# Supplementary material for: Computation and data driven discovery of topological phononic materials
Source: Nat Commun. 2021 Feb 22;12:1204. doi: 10.1038/s41467-021-21293-2 (PMC7900202; doi:10.1038/s41467-021-21293-2)
Supplement: Supplementary file 1 — Supplementary Information [file 41467_2021_21293_MOESM1_ESM.pdf]

Supplementary Materials on "Computation and data driven discovery of topological phononic materials"

Jiangxu Li, Jiaxi Liu, Mingfeng Liu, Lei Wang, and Xing-Qiu Chen*
Shenyang National Laboratory for Materials Science, Institute of Metal Research, Chinese Academy of Science, 110016 Shenyang, Liaoning, People Republic of China and School of Materials Science and Engineering, University of Science and Technology of China, Shenyang 110016, People Republic of China

Stanley A. Baronett and Qiang Zhu†
Department of Physics and Astronomy, University of Nevada, Las Vegas, Nevada 89154, USA

Ronghan Li, Yun Chen, and Dianzhong Li
Shenyang National Laboratory for Materials Science, Institute of Metal Research, Chinese Academy of Science, 110016 Shenyang, Liaoning, People Republic of China
(Dated: January 14, 2021)

Contents

**I**	**Simply summary of supplementary TP materials**	**2**
**II**	**All topological phonon candidates**	**3**
**III**	**Nodal-line (ring) TP materials**	**85**
**IV**	**Single Weyl TP materials**	**96**
**V**	**Mixed TP materials**	**97**
**VI**	**Clean TP materials**	**101**
**VII**	**Hourglass nodal net TP materials**	**103**
**VIII**	**Details of all TP materials**	**104**

*Electronic address: xingqiu.chen@imr.ac.cn
†Electronic address: qiang.zhu@unv.edu

I. SIMPLY SUMMARY OF SUPPLEMENTARY TP MATERIALS

In this supplementary materials, we show the details of each TP material, which contains

(1) 5014 supplementary figures from No. 104 page to No. 14,248 page. They show the calculated phononic dispersions and phonon densities of states from No. 104 page to No. 14,248 page.

and

(2) 11,728 supplementary tables from No. 104 page to No. 14,248 page. In the tables of high-symmetry paths of the BZ of each TP material, the start point and the end point of each separate high symmetry line are specified. In the tables of detailed TPs of each material, we have listed the phononic crossing points on single Weyl nodes, high degenerate Weyl nodes and nodal-line (ring) TPs, together with details of the spatial information of the points (e.g., x, y, z coordinates, frequencies, modes and band paths), and multiplicity, degeneracy, and topological charges to each phononic band crossing points.

II. ALL TOPOLOGICAL PHONON CANDIDATES

Supplementary Table 1: The summary of 5014 topological phonon materials which have been investigated in this work. For each material, they are characterized by materials ID from the online database of Materials Project, chemical formula, number of atoms per unit cell (Z), Space group symbol and number(SP ($No.$)), Prototype, number of nodal lines (NL), nodal rings (NR), Weyl points (WPs), highly-degenerate Weyl points (HDWPs). If the material is defined as the clean materials (i.e.,), the maximum dispersion of nodal points along the high symmetry band path is also shown in the unit of THz·Å.

ID	Formula	Z	SP ($No.$)	Prototype	NL	NR	WPs	HDWPs	Clean	dE/dq
mp-979285	Xe_2	1	Im−3m (229)	A2-BCC	2	0	0	0	False	-
mp-122	Ba_2	1	Im−3m (229)	A2-BCC	2	0	0	0	False	-
mp-127	Na_2	1	Im−3m (229)	A2-BCC	2	0	0	0	False	-
mp-58	K_2	1	Im−3m (229)	A2-BCC	2	0	0	0	False	-
mp-974400	Kr_2	1	Im−3m (229)	A2-BCC	2	0	0	0	False	-
mp-129	Mo_2	1	Im−3m (229)	A2-BCC	2	0	0	0	False	-
mp-39	Tl_2	1	Im−3m (229)	A2-BCC	2	0	0	0	False	-
mp-75	Nb_2	1	Im−3m (229)	A2-BCC	2	0	0	0	False	-
mp-90	Cr_2	1	Im−3m (229)	A2-BCC	3	0	0	0	False	-
mp-91	W_2	1	Im−3m (229)	A2-BCC	2	0	0	0	False	-
mp-126	Pt_4	1	Fm−3m (225)	A1-FCC	1	0	0	0	False	-
mp-2	Pd_4	1	Fm−3m (225)	A1-FCC	1	0	0	0	False	-
mp-51	Li_4	1	Fm−3m (225)	A1-FCC	1	0	0	0	False	-
mp-101	Ir_4	1	Fm−3m (225)	A1-FCC	1	0	0	0	False	-
mp-10157	K_4	1	Fm−3m (225)	A1-FCC	1	0	0	0	False	-
mp-102	Co_4	1	Fm−3m (225)	A1-FCC	1	0	0	0	False	-
mp-10679	Ba_4	1	Fm−3m (225)	A1-FCC	1	0	0	0	False	-
mp-111	Ne_4	1	Fm−3m (225)	A1-FCC	1	0	0	0	False	-
mp-124	Ag_4	1	Fm−3m (225)	A1-FCC	1	0	0	0	False	-
mp-134	Al_4	1	Fm−3m (225)	A1-FCC	1	0	0	0	False	-
mp-28	Ce_4	1	Fm−3m (225)	A1-FCC	1	0	0	0	False	-
mp-30	Cu_4	1	Fm−3m (225)	A1-FCC	1	0	0	0	False	-
mp-45	Ca_4	1	Fm−3m (225)	A1-FCC	1	0	0	0	False	-
mp-611517	Xe_4	1	Fm−3m (225)	A1-FCC	1	0	0	0	False	-
mp-612118	Kr_4	1	Fm−3m (225)	A1-FCC	1	0	0	0	False	-
mp-614456	He_4	1	Fm−3m (225)	A1-FCC	1	0	0	0	False	-
mp-74	Rh_4	1	Fm−3m (225)	A1-FCC	1	0	0	0	False	-
mp-76	Sr_4	1	Fm−3m (225)	A1-FCC	1	0	0	0	False	-
mp-81	Au_4	1	Fm−3m (225)	A1-FCC	1	0	0	0	False	-
mp-974558	Na_4	1	Fm−3m (225)	A1-FCC	1	0	0	0	False	-
mp-23155	Ar_4	1	Fm−3m (225)	A1-FCC	1	1	0	0	False	-
mp-117	Sn_8	2	Fd−3m (227)	A4-diamond	5	4	0	0	False	-
mp-32	Ge_8	2	Fd−3m (227)	A4-diamond	5	7	0	0	False	-
mp-66	C_8	2	Fd−3m (227)	A4-diamond	5	6	0	0	False	-
mp-149	Si_8	2	Fd−3m (227)	A4-diamond	5	6	0	0	False	-
mp-1753	Th_4As_4	2	Fm−3m (225)	B1-NaCl	2	3	0	0	False	-
mp-22926	Bi_4La_4	2	Fm−3m (225)	B1-NaCl	2	8	0	0	False	-
mp-23285	Bi_4Ce_4	2	Fm−3m (225)	B1-NaCl	2	4	0	0	False	-
mp-2637	Y_4Se_4	2	Fm−3m (225)	B1-NaCl	3	5	0	0	False	-
mp-2828	Hf_4N_4	2	Fm−3m (225)	B1-NaCl	2	4	0	0	False	-
mp-2423	U_4S_4	2	Fm−3m (225)	B1-NaCl	3	4	0	0	False	-
mp-571102	Tl_4I_4	2	Fm−3m (225)	B1-NaCl	3	5	0	0	False	-
mp-10192	Lu_4P_4	2	Fm−3m (225)	B1-NaCl	2	5	0	0	False	-
mp-1865	U_4N_4	2	Fm−3m (225)	B1-NaCl	0	4	0	0	False	-
mp-2143	U_4Se_4	2	Fm−3m (225)	B1-NaCl	4	4	0	0	False	-
mp-10108	Np_4P_4	2	Fm−3m (225)	B1-NaCl	2	3	0	0	False	-
mp-10109	Np_4S_4	2	Fm−3m (225)	B1-NaCl	3	5	0	0	False	-
mp-1065	La_4Sb_4	2	Fm−3m (225)	B1-NaCl	3	4	0	0	False	-
mp-1096	Ce_4S_4	2	Fm−3m (225)	B1-NaCl	2	5	0	0	False	-

ID	Formula	Z	SP (*No.*)	Prototype	NL	NR	WPs	HDWPs	Clean	dE/dq
mp-1132	Cd_4O_4	2	$Fm-3m$ (225)	B1-NaCl	2	5	0	0	False	-
mp-1161	La_4Se_4	2	$Fm-3m$ (225)	B1-NaCl	3	3	0	0	False	-
mp-1206874	Co_4H_4	2	$Fm-3m$ (225)	B1-NaCl	2	3	0	0	False	-
mp-1352	Zr_4N_4	2	$Fm-3m$ (225)	B1-NaCl	2	6	0	0	False	-
mp-1674	Pr_4Se_4	2	$Fm-3m$ (225)	B1-NaCl	2	3	0	0	False	-
mp-1751	Th_4Sb_4	2	$Fm-3m$ (225)	B1-NaCl	2	2	0	0	False	-
mp-2052	Sc_4As_4	2	$Fm-3m$ (225)	B1-NaCl	3	3	0	0	False	-
mp-2104	U_4As_4	2	$Fm-3m$ (225)	B1-NaCl	3	5	0	0	False	-
mp-21075	Hf_4C_4	2	$Fm-3m$ (225)	B1-NaCl	2	4	0	0	False	-
mp-215	Y_4Sb_4	2	$Fm-3m$ (225)	B1-NaCl	2	12	0	0	False	-
mp-2182	Sn_4As_4	2	$Fm-3m$ (225)	B1-NaCl	3	5	0	0	False	-
mp-23241	Bi_4Y_4	2	$Fm-3m$ (225)	B1-NaCl	2	3	0	0	False	-
mp-2350	La_4S_4	2	$Fm-3m$ (225)	B1-NaCl	2	4	0	0	False	-
mp-2384	La_4P_4	2	$Fm-3m$ (225)	B1-NaCl	2	3	0	0	False	-
mp-24287	Cr_4H_4	2	$Fm-3m$ (225)	B1-NaCl	3	5	0	0	False	-
mp-24719	Ni_4H_4	2	$Fm-3m$ (225)	B1-NaCl	2	6	0	0	False	-
mp-24722	Rh_4H_4	2	$Fm-3m$ (225)	B1-NaCl	2	4	0	0	False	-
mp-2493	Ce_4N_4	2	$Fm-3m$ (225)	B1-NaCl	2	5	0	0	False	-
mp-2795	Zr_4C_4	2	$Fm-3m$ (225)	B1-NaCl	2	6	0	0	False	-
mp-2807	Sc_4P_4	2	$Fm-3m$ (225)	B1-NaCl	3	3	0	0	False	-
mp-492	Ti_4N_4	2	$Fm-3m$ (225)	B1-NaCl	2	3	0	0	False	-
mp-503	Th_4S_4	2	$Fm-3m$ (225)	B1-NaCl	2	5	0	0	False	-
mp-567580	Pa_4C_4	2	$Fm-3m$ (225)	B1-NaCl	2	5	0	0	False	-
mp-570632	Bi_4Sc_4	2	$Fm-3m$ (225)	B1-NaCl	2	4	0	0	False	-
mp-631	Ti_4C_4	2	$Fm-3m$ (225)	B1-NaCl	2	6	0	0	False	-
mp-931	Th_4P_4	2	$Fm-3m$ (225)	B1-NaCl	2	4	0	0	False	-
mp-933	Y_4As_4	2	$Fm-3m$ (225)	B1-NaCl	3	4	0	0	False	-
mp-994	Y_4P_4	2	$Fm-3m$ (225)	B1-NaCl	2	5	0	0	False	-
mp-1009077	Fe_4H_4	2	$Fm-3m$ (225)	B1-NaCl	2	4	0	0	False	-
mp-1009545	Pa_4N_4	2	$Fm-3m$ (225)	B1-NaCl	2	3	0	0	False	-
mp-1057286	Cs_4H_4	2	$Fm-3m$ (225)	B1-NaCl	2	5	0	0	False	-
mp-10635	Sn_4Sb_4	2	$Fm-3m$ (225)	B1-NaCl	3	9	0	0	False	-
mp-10688	Ce_4O_4	2	$Fm-3m$ (225)	B1-NaCl	2	4	0	0	False	-
mp-1093	Th_4Ge_4	2	$Fm-3m$ (225)	B1-NaCl	2	4	0	0	False	-
mp-1164	Th_4C_4	2	$Fm-3m$ (225)	B1-NaCl	2	5	0	0	False	-
mp-1476	Sc_4S_4	2	$Fm-3m$ (225)	B1-NaCl	3	10	0	0	False	-
mp-1525	Ce_4Te_4	2	$Fm-3m$ (225)	B1-NaCl	2	7	0	0	False	-
mp-1534	Y_4S_4	2	$Fm-3m$ (225)	B1-NaCl	3	3	0	0	False	-
mp-1560	La_4Te_4	2	$Fm-3m$ (225)	B1-NaCl	4	5	0	0	False	-
mp-15694	Pa_4As_4	2	$Fm-3m$ (225)	B1-NaCl	3	4	0	0	False	-
mp-1782	Sc_4Se_4	2	$Fm-3m$ (225)	B1-NaCl	4	4	0	0	False	-
mp-2154	Ce_4P_4	2	$Fm-3m$ (225)	B1-NaCl	2	4	0	0	False	-
mp-2186	Np_4As_4	2	$Fm-3m$ (225)	B1-NaCl	3	3	0	0	False	-
mp-2489	U_4C_4	2	$Fm-3m$ (225)	B1-NaCl	2	6	0	0	False	-
mp-2495	Pr_4S_4	2	$Fm-3m$ (225)	B1-NaCl	2	5	0	0	False	-
mp-2563	Ce_4Se_4	2	$Fm-3m$ (225)	B1-NaCl	3	4	0	0	False	-
mp-2596	Np_4N_4	2	$Fm-3m$ (225)	B1-NaCl	2	3	0	0	False	-
mp-2748	Ce_4As_4	2	$Fm-3m$ (225)	B1-NaCl	2	4	0	0	False	-
mp-343	Pr_4N_4	2	$Fm-3m$ (225)	B1-NaCl	2	4	0	0	False	-
mp-387	Ce_4Sb_4	2	$Fm-3m$ (225)	B1-NaCl	2	8	0	0	False	-
mp-414	Lu_4Se_4	2	$Fm-3m$ (225)	B1-NaCl	2	2	0	0	False	-
mp-549	Sc_4Sb_4	2	$Fm-3m$ (225)	B1-NaCl	2	3	0	0	False	-
mp-627	Np_4Se_4	2	$Fm-3m$ (225)	B1-NaCl	4	4	0	0	False	-
mp-834	Th_4N_4	2	$Fm-3m$ (225)	B1-NaCl	3	5	0	0	False	-
mp-1000	Ba_4Te_4	2	$Fm-3m$ (225)	B1-NaCl	3	6	0	0	False	-
mp-10760	Mg_4Se_4	2	$Fm-3m$ (225)	B1-NaCl	2	7	0	0	False	-
mp-1087	Sr_4S_4	2	$Fm-3m$ (225)	B1-NaCl	2	5	0	0	False	-
mp-1138	Li_4F_4	2	$Fm-3m$ (225)	B1-NaCl	3	7	0	0	False	-
mp-11718	Rb_4F_4	2	$Fm-3m$ (225)	B1-NaCl	2	5	0	0	False	-
mp-1216	Yb_4O_4	2	$Fm-3m$ (225)	B1-NaCl	2	6	0	0	False	-
mp-1253	Ba_4Se_4	2	$Fm-3m$ (225)	B1-NaCl	3	4	0	0	False	-
mp-1265	Mg_4O_4	2	$Fm-3m$ (225)	B1-NaCl	3	6	0	0	False	-

ID	Formula	*Z*	SP (*No.*)	Prototype	NL	NR	WPs	HDWPs	Clean	dE/dq
mp-1315	Mg_4S_4	2	$Fm{-}3m$ (225)	B1-NaCl	3	6	0	0	False	-
mp-1342	Ba_4O_4	2	$Fm{-}3m$ (225)	B1-NaCl	2	7	0	0	False	-
mp-1415	Ca_4Se_4	2	$Fm{-}3m$ (225)	B1-NaCl	2	5	0	0	False	-
mp-1500	Ba_4S_4	2	$Fm{-}3m$ (225)	B1-NaCl	2	4	0	0	False	-
mp-1519	Ca_4Te_4	2	$Fm{-}3m$ (225)	B1-NaCl	2	5	0	0	False	-
mp-1672	Ca_4S_4	2	$Fm{-}3m$ (225)	B1-NaCl	3	7	0	0	False	-
mp-1779	Yb_4Te_4	2	$Fm{-}3m$ (225)	B1-NaCl	3	3	0	0	False	-
mp-1784	Cs_4F_4	2	$Fm{-}3m$ (225)	B1-NaCl	2	7	0	0	False	-
mp-1820	Yb_4S_4	2	$Fm{-}3m$ (225)	B1-NaCl	2	7	0	0	False	-
mp-1958	Sr_4Te_4	2	$Fm{-}3m$ (225)	B1-NaCl	2	12	0	0	False	-
mp-19717	Pb_4Te_4	2	$Fm{-}3m$ (225)	B1-NaCl	2	4	0	0	False	-
mp-22862	Na_4Cl_4	2	$Fm{-}3m$ (225)	B1-NaCl	3	6	0	0	False	-
mp-22867	Rb_4Br_4	2	$Fm{-}3m$ (225)	B1-NaCl	3	5	0	0	False	-
mp-22898	K_4I_4	2	$Fm{-}3m$ (225)	B1-NaCl	2	5	0	0	False	-
mp-22899	Li_4I_4	2	$Fm{-}3m$ (225)	B1-NaCl	2	4	0	0	False	-
mp-22903	Rb_4I_4	2	$Fm{-}3m$ (225)	B1-NaCl	2	9	0	0	False	-
mp-22905	Li_4Cl_4	2	$Fm{-}3m$ (225)	B1-NaCl	2	5	0	0	True	30.55
mp-22916	Na_4Br_4	2	$Fm{-}3m$ (225)	B1-NaCl	2	5	0	0	False	-
mp-22922	Ag_4Cl_4	2	$Fm{-}3m$ (225)	B1-NaCl	3	4	0	0	False	-
mp-23193	K_4Cl_4	2	$Fm{-}3m$ (225)	B1-NaCl	3	4	0	0	False	-
mp-23231	Ag_4Br_4	2	$Fm{-}3m$ (225)	B1-NaCl	3	4	0	0	False	-
mp-23251	K_4Br_4	2	$Fm{-}3m$ (225)	B1-NaCl	2	4	0	0	False	-
mp-23259	Li_4Br_4	2	$Fm{-}3m$ (225)	B1-NaCl	2	5	0	0	True	28.54
mp-23268	Na_4I_4	2	$Fm{-}3m$ (225)	B1-NaCl	2	4	0	0	False	-
mp-23295	Rb_4Cl_4	2	$Fm{-}3m$ (225)	B1-NaCl	2	7	0	0	False	-
mp-23703	Li_4H_4	2	$Fm{-}3m$ (225)	B1-NaCl	2	2	0	0	False	-
mp-23870	Na_4H_4	2	$Fm{-}3m$ (225)	B1-NaCl	2	3	0	0	False	-
mp-24084	K_4H_4	2	$Fm{-}3m$ (225)	B1-NaCl	2	5	0	0	False	-
mp-2472	Sr_4O_4	2	$Fm{-}3m$ (225)	B1-NaCl	2	5	0	0	False	-
mp-24721	Rb_4H_4	2	$Fm{-}3m$ (225)	B1-NaCl	2	5	0	0	False	-
mp-2605	Ca_4O_4	2	$Fm{-}3m$ (225)	B1-NaCl	3	3	0	0	False	-
mp-2758	Sr_4Se_4	2	$Fm{-}3m$ (225)	B1-NaCl	3	4	0	0	False	-
mp-286	Yb_4Se_4	2	$Fm{-}3m$ (225)	B1-NaCl	2	5	0	0	False	-
mp-463	K_4F_4	2	$Fm{-}3m$ (225)	B1-NaCl	3	5	0	0	False	-
mp-568560	Tl_4Br_4	2	$Fm{-}3m$ (225)	B1-NaCl	2	2	0	0	False	-
mp-569639	Tl_4Cl_4	2	$Fm{-}3m$ (225)	B1-NaCl	2	2	0	0	False	-
mp-571222	Cs_4Br_4	2	$Fm{-}3m$ (225)	B1-NaCl	2	11	0	0	False	-
mp-573697	Cs_4Cl_4	2	$Fm{-}3m$ (225)	B1-NaCl	2	5	0	0	False	-
mp-614603	Cs_4I_4	2	$Fm{-}3m$ (225)	B1-NaCl	3	8	0	0	False	-
mp-682	Na_4F_4	2	$Fm{-}3m$ (225)	B1-NaCl	3	7	0	0	False	-
mp-11256	AuSc	2	$Pm{-}3m$ (221)	B2-CsCl	6	2	0	0	False	-
mp-11473	HgTl	2	$Pm{-}3m$ (221)	B2-CsCl	6	7	0	0	False	-
mp-11488	LaTl	2	$Pm{-}3m$ (221)	B2-CsCl	6	6	0	0	False	-
mp-11498	MgTl	2	$Pm{-}3m$ (221)	B2-CsCl	6	2	0	0	False	-
mp-1684	AuZn	2	$Pm{-}3m$ (221)	B2-CsCl	6	2	0	0	False	-
mp-934	LiTl	2	$Pm{-}3m$ (221)	B2-CsCl	6	2	0	0	False	-
mp-11575	TlY	2	$Pm{-}3m$ (221)	B2-CsCl	6	5	0	0	False	-
mp-1885	AlIr	2	$Pm{-}3m$ (221)	B2-CsCl	6	0	0	0	False	-
mp-20582	InLa	2	$Pm{-}3m$ (221)	B2-CsCl	6	5	0	0	False	-
mp-2205	LuPd	2	$Pm{-}3m$ (221)	B2-CsCl	6	1	0	0	False	-
mp-22704	InY	2	$Pm{-}3m$ (221)	B2-CsCl	6	6	0	0	False	-
mp-1014219	HfZn	2	$Pm{-}3m$ (221)	B2-CsCl	6	3	0	0	False	-
mp-1014230	TiZn	2	$Pm{-}3m$ (221)	B2-CsCl	6	3	0	0	False	-
mp-1018029	CuPd	2	$Pm{-}3m$ (221)	B2-CsCl	6	6	0	0	False	-
mp-1033	BeNi	2	$Pm{-}3m$ (221)	B2-CsCl	6	6	0	0	False	-
mp-1039147	CaZn	2	$Pm{-}3m$ (221)	B2-CsCl	5	6	0	0	False	-
mp-1073	CaCd	2	$Pm{-}3m$ (221)	B2-CsCl	6	1	0	0	False	-
mp-1094889	LiMg	2	$Pm{-}3m$ (221)	B2-CsCl	6	4	0	0	False	-
mp-1104	LaMg	2	$Pm{-}3m$ (221)	B2-CsCl	6	2	0	0	False	-
mp-1121	CoGa	2	$Pm{-}3m$ (221)	B2-CsCl	6	11	0	0	False	-
mp-11229	AlY	2	$Pm{-}3m$ (221)	B2-CsCl	6	4	0	0	False	-
mp-11237	AgSc	2	$Pm{-}3m$ (221)	B2-CsCl	6	3	0	0	False	-

ID	Formula	Z	SP (*No.*)	Prototype	NL	NR	WPs	HDWPs	Clean	dE/dq
mp-11274	BePd	2	$Pm-3m$ (221)	B2-CsCl	6	2	0	0	False	-
mp-11276	BeRh	2	$Pm-3m$ (221)	B2-CsCl	7	4	0	0	False	-
mp-11279	BeTi	2	$Pm-3m$ (221)	B2-CsCl	6	6	0	0	False	-
mp-11286	CaHg	2	$Pm-3m$ (221)	B2-CsCl	5	4	0	0	False	-
mp-1129	IrSc	2	$Pm-3m$ (221)	B2-CsCl	5	2	0	0	False	-
mp-11388	GaIr	2	$Pm-3m$ (221)	B2-CsCl	5	2	0	0	False	-
mp-11452	HfOs	2	$Pm-3m$ (221)	B2-CsCl	6	10	0	0	False	-
mp-11457	HfRh	2	$Pm-3m$ (221)	B2-CsCl	7	9	0	0	False	-
mp-11460	HfTc	2	$Pm-3m$ (221)	B2-CsCl	7	3	0	0	False	-
mp-11471	HgSc	2	$Pm-3m$ (221)	B2-CsCl	6	4	0	0	False	-
mp-11496	LuZn	2	$Pm-3m$ (221)	B2-CsCl	6	4	0	0	False	-
mp-11521	NiSc	2	$Pm-3m$ (221)	B2-CsCl	6	6	0	0	False	-
mp-11541	OsZr	2	$Pm-3m$ (221)	B2-CsCl	5	4	0	0	False	-
mp-11566	ScZn	2	$Pm-3m$ (221)	B2-CsCl	5	6	0	0	True	10.01
mp-11572	TaTc	2	$Pm-3m$ (221)	B2-CsCl	5	9	0	0	False	-
mp-11573	TcTi	2	$Pm-3m$ (221)	B2-CsCl	7	2	0	0	False	-
mp-1169	CuSc	2	$Pm-3m$ (221)	B2-CsCl	6	4	0	0	False	-
mp-1172	MgRh	2	$Pm-3m$ (221)	B2-CsCl	6	3	0	0	False	-
mp-1174	MgSc	2	$Pm-3m$ (221)	B2-CsCl	6	4	0	0	False	-
mp-1206794	AuLi	2	$Pm-3m$ (221)	B2-CsCl	5	3	0	0	False	-
mp-1211	CdSc	2	$Pm-3m$ (221)	B2-CsCl	6	4	0	0	False	-
mp-1408	HgMg	2	$Pm-3m$ (221)	B2-CsCl	6	4	0	0	False	-
mp-1487	AlNi	2	$Pm-3m$ (221)	B2-CsCl	5	7	0	0	False	-
mp-1780	RhSc	2	$Pm-3m$ (221)	B2-CsCl	5	2	0	0	False	-
mp-191	RhY	2	$Pm-3m$ (221)	B2-CsCl	6	11	0	0	False	-
mp-1921	ThTe	2	$Pm-3m$ (221)	B2-CsCl	6	8	0	0	False	-
mp-1948	AgLa	2	$Pm-3m$ (221)	B2-CsCl	6	7	0	0	False	-
mp-2012	HgLi	2	$Pm-3m$ (221)	B2-CsCl	6	4	0	0	False	-
mp-20263	CaIn	2	$Pm-3m$ (221)	B2-CsCl	6	1	0	0	False	-
mp-2027	CoHf	2	$Pm-3m$ (221)	B2-CsCl	6	10	0	0	False	-
mp-21215	InPd	2	$Pm-3m$ (221)	B2-CsCl	6	4	0	0	False	-
mp-213	CaPd	2	$Pm-3m$ (221)	B2-CsCl	5	0	0	0	False	-
mp-214	RuZr	2	$Pm-3m$ (221)	B2-CsCl	5	8	0	0	False	-
mp-2179	ReTi	2	$Pm-3m$ (221)	B2-CsCl	6	5	0	0	False	-
mp-2197	BaHg	2	$Pm-3m$ (221)	B2-CsCl	5	5	0	0	False	-
mp-2212	CoSc	2	$Pm-3m$ (221)	B2-CsCl	5	5	0	0	False	-
mp-22320	GaRu	2	$Pm-3m$ (221)	B2-CsCl	6	5	0	0	False	-
mp-2283	CoZr	2	$Pm-3m$ (221)	B2-CsCl	4	8	0	0	False	-
mp-2323	BeCu	2	$Pm-3m$ (221)	B2-CsCl	6	5	0	0	False	-
mp-2399	HgY	2	$Pm-3m$ (221)	B2-CsCl	5	6	0	0	False	-
mp-2426	AgLi	2	$Pm-3m$ (221)	B2-CsCl	6	3	0	0	False	-
mp-2434	SrTl	2	$Pm-3m$ (221)	B2-CsCl	6	4	0	0	False	-
mp-2444	GaRh	2	$Pm-3m$ (221)	B2-CsCl	6	9	0	0	False	-
mp-2474	AgY	2	$Pm-3m$ (221)	B2-CsCl	6	5	0	0	False	-
mp-2516	YZn	2	$Pm-3m$ (221)	B2-CsCl	6	5	0	0	False	-
mp-2540	TcV	2	$Pm-3m$ (221)	B2-CsCl	7	9	0	0	False	-
mp-2564	MgSr	2	$Pm-3m$ (221)	B2-CsCl	6	3	0	0	False	-
mp-2615	LaZn	2	$Pm-3m$ (221)	B2-CsCl	5	12	0	0	False	-
mp-2658	AlFe	2	$Pm-3m$ (221)	B2-CsCl	6	6	0	0	False	-
mp-2696	AgMg	2	$Pm-3m$ (221)	B2-CsCl	6	3	0	0	False	-
mp-2743	LiPd	2	$Pm-3m$ (221)	B2-CsCl	5	3	0	0	False	-
mp-2773	BeCo	2	$Pm-3m$ (221)	B2-CsCl	6	7	0	0	False	-
mp-2781	PdSc	2	$Pm-3m$ (221)	B2-CsCl	6	4	0	0	False	-
mp-2802	HfRu	2	$Pm-3m$ (221)	B2-CsCl	6	0	0	0	False	-
mp-284	AlCo	2	$Pm-3m$ (221)	B2-CsCl	6	4	0	0	False	-
mp-2861	CaTl	2	$Pm-3m$ (221)	B2-CsCl	6	3	0	0	False	-
mp-291	OsTi	2	$Pm-3m$ (221)	B2-CsCl	5	4	0	0	False	-
mp-30373	AuRb	2	$Pm-3m$ (221)	B2-CsCl	6	4	0	0	False	-
mp-30468	BiTh	2	$Pm-3m$ (221)	B2-CsCl	6	1	0	0	False	-
mp-30496	CdSr	2	$Pm-3m$ (221)	B2-CsCl	5	3	0	0	False	-
mp-305	FeTi	2	$Pm-3m$ (221)	B2-CsCl	6	4	0	0	False	-
mp-30746	IrY	2	$Pm-3m$ (221)	B2-CsCl	6	3	0	0	False	-

ID	Formula	Z	SP (No.)	Prototype	NL	NR	WPs	HDWPs	Clean	dE/dq
mp-30867	RuSc	2	$Pm-3m$ (221)	B2-CsCl	6	8	0	0	False	-
mp-316	MnV	2	$Pm-3m$ (221)	B2-CsCl	6	8	0	0	False	-
mp-331	AlSc	2	$Pm-3m$ (221)	B2-CsCl	5	15	0	0	False	-
mp-364	AlRh	2	$Pm-3m$ (221)	B2-CsCl	6	1	0	0	False	-
mp-381	RuSi	2	$Pm-3m$ (221)	B2-CsCl	5	6	0	0	False	-
mp-437	AuMg	2	$Pm-3m$ (221)	B2-CsCl	6	3	0	0	False	-
mp-506	MgPd	2	$Pm-3m$ (221)	B2-CsCl	6	5	0	0	False	-
mp-527	BaCd	2	$Pm-3m$ (221)	B2-CsCl	6	5	0	0	False	-
mp-542	HgSr	2	$Pm-3m$ (221)	B2-CsCl	5	6	0	0	False	-
mp-542569	AlRu	2	$Pm-3m$ (221)	B2-CsCl	6	2	0	0	False	-
mp-570276	ZnZr	2	$Pm-3m$ (221)	B2-CsCl	6	6	0	0	False	-
mp-592	RuTi	2	$Pm-3m$ (221)	B2-CsCl	5	7	0	0	False	-
mp-615	MgY	2	$Pm-3m$ (221)	B2-CsCl	6	4	0	0	False	-
mp-6938	RhZn	2	$Pm-3m$ (221)	B2-CsCl	6	6	0	0	False	-
mp-734	HgLa	2	$Pm-3m$ (221)	B2-CsCl	5	5	0	0	False	-
mp-776	CdLa	2	$Pm-3m$ (221)	B2-CsCl	5	3	0	0	False	-
mp-823	CoTi	2	$Pm-3m$ (221)	B2-CsCl	6	4	0	0	False	-
mp-866199	AcAg	2	$Pm-3m$ (221)	B2-CsCl	6	6	0	0	False	-
mp-866286	AcMg	2	$Pm-3m$ (221)	B2-CsCl	6	1	0	0	False	-
mp-892	PtSc	2	$Pm-3m$ (221)	B2-CsCl	6	2	0	0	False	-
mp-899	InRh	2	$Pm-3m$ (221)	B2-CsCl	6	6	0	0	False	-
mp-902	BaZn	2	$Pm-3m$ (221)	B2-CsCl	6	9	0	0	True	14.35
mp-915	CdY	2	$Pm-3m$ (221)	B2-CsCl	6	2	0	0	False	-
mp-22865	CsCl	2	$Pm-3m$ (221)	B2-CsCl	4	7	0	0	False	-
mp-22875	TlBr	2	$Pm-3m$ (221)	B2-CsCl	3	10	0	0	False	-
mp-22906	CsBr	2	$Pm-3m$ (221)	B2-CsCl	5	0	0	0	False	-
mp-23167	TlCl	2	$Pm-3m$ (221)	B2-CsCl	3	6	0	0	False	-
mp-23302	RbI	2	$Pm-3m$ (221)	B2-CsCl	5	10	0	0	False	-
mp-2667	AuCs	2	$Pm-3m$ (221)	B2-CsCl	5	4	0	0	False	-
mp-632319	CsH	2	$Pm-3m$ (221)	B2-CsCl	5	7	0	0	False	-
mp-1009130	Mn_4N_4	2	$F-43m$ (216)	B3-ZnS	2	1	0	0	False	-
mp-10695	Zn_4S_4	2	$F-43m$ (216)	B3-ZnS	2	1	0	0	False	-
mp-1123	Hg_4S_4	2	$F-43m$ (216)	B3-ZnS	2	0	0	0	False	-
mp-1156	Ga_4Sb_4	2	$F-43m$ (216)	B3-ZnS	3	0	0	0	False	-
mp-1190	Zn_4Se_4	2	$F-43m$ (216)	B3-ZnS	2	2	0	0	False	-
mp-13031	Mg_4Se_4	2	$F-43m$ (216)	B3-ZnS	2	2	0	0	False	-
mp-13032	Mg_4S_4	2	$F-43m$ (216)	B3-ZnS	2	0	0	0	False	-
mp-13033	Mg_4Te_4	2	$F-43m$ (216)	B3-ZnS	2	0	0	0	False	-
mp-1479	B_4P_4	2	$F-43m$ (216)	B3-ZnS	2	0	0	0	False	-
mp-1541	Be_4Se_4	2	$F-43m$ (216)	B3-ZnS	2	1	0	0	False	-
mp-1550	Al_4P_4	2	$F-43m$ (216)	B3-ZnS	3	1	0	0	False	-
mp-1778	Be_4O_4	2	$F-43m$ (216)	B3-ZnS	3	1	0	0	False	-
mp-1986	Zn_4O_4	2	$F-43m$ (216)	B3-ZnS	2	1	0	0	False	-
mp-20012	In_4Sb_4	2	$F-43m$ (216)	B3-ZnS	3	0	0	0	False	-
mp-20305	In_4As_4	2	$F-43m$ (216)	B3-ZnS	3	0	1	0	False	-
mp-20351	In_4P_4	2	$F-43m$ (216)	B3-ZnS	2	1	0	0	False	-
mp-20411	In_4N_4	2	$F-43m$ (216)	B3-ZnS	2	0	0	0	False	-
mp-2172	Al_4As_4	2	$F-43m$ (216)	B3-ZnS	2	0	1	0	False	-
mp-2176	Zn_4Te_4	2	$F-43m$ (216)	B3-ZnS	2	0	0	0	False	-
mp-22895	Cu_4I_4	2	$F-43m$ (216)	B3-ZnS	0	5	1	0	False	-
mp-22913	Cu_4Br_4	2	$F-43m$ (216)	B3-ZnS	2	0	0	0	False	-
mp-22914	Cu_4Cl_4	2	$F-43m$ (216)	B3-ZnS	2	1	0	0	False	-
mp-22925	Ag_4I_4	2	$F-43m$ (216)	B3-ZnS	2	0	0	0	False	-
mp-2469	Cd_4S_4	2	$F-43m$ (216)	B3-ZnS	2	2	0	0	False	-
mp-2490	Ga_4P_4	2	$F-43m$ (216)	B3-ZnS	2	0	0	0	False	-
mp-252	Be_4Te_4	2	$F-43m$ (216)	B3-ZnS	2	1	0	0	False	-
mp-2534	Ga_4As_4	2	$F-43m$ (216)	B3-ZnS	3	0	0	0	False	-
mp-2624	Al_4Sb_4	2	$F-43m$ (216)	B3-ZnS	2	0	0	0	False	-
mp-2691	Cd_4Se_4	2	$F-43m$ (216)	B3-ZnS	2	1	0	0	False	-
mp-2730	Hg_4Te_4	2	$F-43m$ (216)	B3-ZnS	2	0	0	0	False	-
mp-406	Cd_4Te_4	2	$F-43m$ (216)	B3-ZnS	2	2	1	0	False	-
mp-422	Be_4S_4	2	$F-43m$ (216)	B3-ZnS	2	0	0	0	False	-

ID	Formula	Z	SP (*No.*)	Prototype	NL	NR	WPs	HDWPs	Clean	dE/dq
mp-8062	C_4Si_4	2	$F-43$m (216)	B3-ZnS	2	0	0	0	False	-
mp-820	Hg_4Se_4	2	$F-43$m (216)	B3-ZnS	2	1	0	0	False	-
mp-830	Ga_4N_4	2	$F-43$m (216)	B3-ZnS	2	0	0	0	False	-
mp-866291	Ag_4Br_4	2	$F-43$m (216)	B3-ZnS	2	1	0	0	False	-
mp-1700	Al_4N_4	2	$F-43$m (216)	B3-ZnS	2	1	0	0	False	-
mp-23156	He_2	2	$P6_3/mmc$ (194)	A3-HCP	12	6	0	0	False	-
mp-10173	Li_2	2	$P6_3/mmc$ (194)	A3-HCP	11	8	0	0	False	-
mp-1008634	Au_2	2	$P6_3/mmc$ (194)	A3-HCP	12	7	0	0	False	-
mp-10172	Na_2	2	$P6_3/mmc$ (194)	A3-HCP	11	11	0	0	False	-
mp-103	Hf_2	2	$P6_3/mmc$ (194)	A3-HCP	12	10	0	0	False	-
mp-10597	Ag_2	2	$P6_3/mmc$ (194)	A3-HCP	11	14	0	0	False	-
mp-112	Y_2	2	$P6_3/mmc$ (194)	A3-HCP	13	11	0	0	False	-
mp-1184755	K_2	2	$P6_3/mmc$ (194)	A3-HCP	12	7	0	0	False	-
mp-131	Zr_2	2	$P6_3/mmc$ (194)	A3-HCP	12	10	0	0	False	-
mp-132	Ca_2	2	$P6_3/mmc$ (194)	A3-HCP	11	18	0	0	False	-
mp-153	Mg_2	2	$P6_3/mmc$ (194)	A3-HCP	11	11	0	0	False	-
mp-33	Ru_2	2	$P6_3/mmc$ (194)	A3-HCP	11	20	0	0	False	-
mp-46	Ti_2	2	$P6_3/mmc$ (194)	A3-HCP	13	9	0	0	False	-
mp-49	Os_2	2	$P6_3/mmc$ (194)	A3-HCP	11	19	0	0	False	-
mp-56	Ba_2	2	$P6_3/mmc$ (194)	A3-HCP	11	10	0	0	False	-
mp-567365	Kr_2	2	$P6_3/mmc$ (194)	A3-HCP	11	1	0	0	False	-
mp-570510	Xe_2	2	$P6_3/mmc$ (194)	A3-HCP	11	10	0	0	False	-
mp-67	Sc_2	2	$P6_3/mmc$ (194)	A3-HCP	11	11	0	0	False	-
mp-8	Re_2	2	$P6_3/mmc$ (194)	A3-HCP	12	10	0	0	False	-
mp-87	Be_2	2	$P6_3/mmc$ (194)	A3-HCP	12	16	0	0	False	-
mp-94	Cd_2	2	$P6_3/mmc$ (194)	A3-HCP	12	5	0	0	False	-
mp-568145	Ar_2	2	$P6_3/mmc$ (194)	A3-HCP	6	6	0	0	False	-
mp-1217409	IrTc	2	$P-6$m2 (187)	Bh-WC	0	4	3	0	False	-
mp-1219533	IrRe	2	$P-6$m2 (187)	Bh-WC	0	6	0	0	False	-
mp-1220023	OsRu	2	$P-6$m2 (187)	Bh-WC	0	3	0	0	False	-
mp-865430	YTe	2	$P-6$m2 (187)	Bh-WC	0	0	2	0	False	-
mp-867841	GaTc	2	$P-6$m2 (187)	Bh-WC	0	3	4	0	False	-
mp-1018027	VN	2	$P-6$m2 (187)	Bh-WC	0	0	4	0	False	-
mp-1018028	TiS	2	$P-6$m2 (187)	Bh-WC	0	2	5	0	False	-
mp-11807	LiPt	2	$P-6$m2 (187)	Bh-WC	0	1	2	0	False	-
mp-1183040	ZrSe	2	$P-6$m2 (187)	Bh-WC	0	2	6	0	False	-
mp-1183222	AgPd	2	$P-6$m2 (187)	Bh-WC	0	4	1	0	False	-
mp-1183227	AgAu	2	$P-6$m2 (187)	Bh-WC	0	2	2	0	False	-
mp-1187244	TaP	2	$P-6$m2 (187)	Bh-WC	0	0	4	0	False	-
mp-1206743	HfS	2	$P-6$m2 (187)	Bh-WC	0	1	4	0	False	-
mp-1217422	RhTc	2	$P-6$m2 (187)	Bh-WC	0	5	2	0	False	-
mp-1219509	OsRe	2	$P-6$m2 (187)	Bh-WC	0	3	3	0	False	-
mp-1226229	CrIr	2	$P-6$m2 (187)	Bh-WC	0	1	1	0	False	-
mp-1228901	AgAl	2	$P-6$m2 (187)	Bh-WC	0	1	4	0	False	-
mp-1539	ZrTe	2	$P-6$m2 (187)	Bh-WC	0	1	5	0	False	-
mp-1894	WC	2	$P-6$m2 (187)	Bh-WC	0	0	6	0	False	-
mp-219	MoP	2	$P-6$m2 (187)	Bh-WC	0	1	4	0	False	-
mp-2305	MoC	2	$P-6$m2 (187)	Bh-WC	0	1	5	0	False	-
mp-2634	NbN	2	$P-6$m2 (187)	Bh-WC	0	0	4	0	False	-
mp-2744	LiPd	2	$P-6$m2 (187)	Bh-WC	0	1	2	0	False	-
mp-974421	IrRu	2	$P-6$m2 (187)	Bh-WC	0	1	0	0	False	-
mp-983459	HfZr	2	$P-6$m2 (187)	Bh-WC	0	3	4	0	False	-
mp-997617	OsB	2	$P-6$m2 (187)	Bh-WC	0	1	4	0	False	-
mp-1183252	IrB	2	$P-6$m2 (187)	Bh-WC	0	2	4	0	False	-
mp-1183691	CrN	2	$P-6$m2 (187)	Bh-WC	0	0	5	0	False	-
mp-1187974	AgZn	2	$P-6$m2 (187)	Bh-WC	0	1	5	0	False	-
mp-1217363	RuTc	2	$P-6$m2 (187)	Bh-WC	0	4	4	0	False	-
mp-8936	Sn_2Se_2	2	$P4/nmm$ (129)	B1-NaCl	24	40	0	0	False	-
mp-32880	Cu_2Br_2	2	$I-4$m2 (119)	B3-ZnS	0	1	5	0	False	-
mp-684580	Ag_2I_2	2	$I-4$m2 (119)	B3-ZnS	0	0	7	0	False	-
mp-1039148	Ca_2Mg_2	2	$Pmma$ (51)	B2-CsCl	24	28	0	0	False	-
mp-570301	Ag_2Br_2	2	$P2_1/m$ (11)	B1-NaCl	20	38	0	0	False	-

ID	Formula	Z	SP (*No.*)	Prototype	NL	NR	WPs	HDWPs	Clean	dE/dq
mp-673245	Cu_4I_4	2	$P1$ (1)	B3-ZnS	0	23	2	0	False	-
mp-2083	Ir_4Sn_8	3	$Fm{-}3m$ (225)	C1-CaF_2	4	9	0	0	False	-
mp-22095	Ga_8Pt_4	3	$Fm{-}3m$ (225)	C1-CaF_2	5	6	0	0	False	-
mp-2776	Au_4Ga_8	3	$Fm{-}3m$ (225)	C1-CaF_2	5	4	0	0	False	-
mp-24288	Lu_4H_8	3	$Fm{-}3m$ (225)	C1-CaF_2	3	9	0	0	False	-
mp-1008833	Np_4N_8	3	$Fm{-}3m$ (225)	C1-CaF_2	4	4	0	0	False	-
mp-1009733	Ca_8Si_4	3	$Fm{-}3m$ (225)	C1-CaF_2	4	10	0	0	False	-
mp-1009755	Ca_8Ge_4	3	$Fm{-}3m$ (225)	C1-CaF_2	5	13	0	0	False	-
mp-1011695	Cs_8Se_4	3	$Fm{-}3m$ (225)	C1-CaF_2	4	7	0	0	False	-
mp-1062676	K_8Pt_4	3	$Fm{-}3m$ (225)	C1-CaF_2	3	9	0	0	False	-
mp-1367	Mg_8Si_4	3	$Fm{-}3m$ (225)	C1-CaF_2	4	11	0	0	False	-
mp-1502	Al_8Pt_4	3	$Fm{-}3m$ (225)	C1-CaF_2	3	11	0	0	False	-
mp-1597	U_4O_8	3	$Fm{-}3m$ (225)	C1-CaF_2	4	5	0	0	False	-
mp-19962	Pt_4Sn_8	3	$Fm{-}3m$ (225)	C1-CaF_2	4	9	0	0	False	-
mp-20724	Mg_8Pb_4	3	$Fm{-}3m$ (225)	C1-CaF_2	3	10	0	0	False	-
mp-2082	Ir_8P_4	3	$Fm{-}3m$ (225)	C1-CaF_2	4	4	0	0	False	-
mp-22154	Au_4In_8	3	$Fm{-}3m$ (225)	C1-CaF_2	5	9	0	0	False	-
mp-22682	In_8Pt_4	3	$Fm{-}3m$ (225)	C1-CaF_2	4	8	0	0	False	-
mp-2291	Ni_4Si_8	3	$Fm{-}3m$ (225)	C1-CaF_2	4	13	0	0	False	-
mp-2302	Rh_8As_4	3	$Fm{-}3m$ (225)	C1-CaF_2	4	8	0	0	False	-
mp-2343	Mg_8Sn_4	3	$Fm{-}3m$ (225)	C1-CaF_2	4	5	0	0	False	-
mp-2364	Pa_4O_8	3	$Fm{-}3m$ (225)	C1-CaF_2	5	7	0	0	False	-
mp-2379	Co_4Si_8	3	$Fm{-}3m$ (225)	C1-CaF_2	4	13	0	0	False	-
mp-24147	Ac_4H_8	3	$Fm{-}3m$ (225)	C1-CaF_2	4	10	0	0	False	-
mp-24153	La_4H_8	3	$Fm{-}3m$ (225)	C1-CaF_2	4	3	0	0	False	-
mp-24154	Nb_4H_8	3	$Fm{-}3m$ (225)	C1-CaF_2	3	12	0	0	False	-
mp-24208	Cr_4H_8	3	$Fm{-}3m$ (225)	C1-CaF_2	3	10	0	0	False	-
mp-24237	Sc_4H_8	3	$Fm{-}3m$ (225)	C1-CaF_2	4	5	0	0	False	-
mp-24650	Y_4H_8	3	$Fm{-}3m$ (225)	C1-CaF_2	4	6	0	0	False	-
mp-24723	Ta_4H_8	3	$Fm{-}3m$ (225)	C1-CaF_2	4	8	0	0	False	-
mp-24728	V_4H_8	3	$Fm{-}3m$ (225)	C1-CaF_2	3	8	0	0	False	-
mp-2616	Np_4O_8	3	$Fm{-}3m$ (225)	C1-CaF_2	4	8	0	0	False	-
mp-2647	Al_8Au_4	3	$Fm{-}3m$ (225)	C1-CaF_2	5	9	0	0	False	-
mp-2732	Rh_8P_4	3	$Fm{-}3m$ (225)	C1-CaF_2	4	9	0	0	False	-
mp-408	Mg_8Ge_4	3	$Fm{-}3m$ (225)	C1-CaF_2	4	8	0	0	False	-
mp-441	Rb_8Te_4	3	$Fm{-}3m$ (225)	C1-CaF_2	4	13	0	0	False	-
mp-505569	Ce_4H_8	3	$Fm{-}3m$ (225)	C1-CaF_2	3	5	0	0	False	-
mp-985806	Al_8Cu_4	3	$Fm{-}3m$ (225)	C1-CaF_2	5	9	0	0	False	-
mp-1022	K_8S_4	3	$Fm{-}3m$ (225)	C1-CaF_2	4	9	0	0	False	-
mp-1029	Ba_4F_8	3	$Fm{-}3m$ (225)	C1-CaF_2	4	4	0	0	False	-
mp-11327	Rb_8Se_4	3	$Fm{-}3m$ (225)	C1-CaF_2	5	6	0	0	False	-
mp-1153	Li_8S_4	3	$Fm{-}3m$ (225)	C1-CaF_2	3	11	0	0	False	-
mp-1266	Na_8Se_4	3	$Fm{-}3m$ (225)	C1-CaF_2	3	10	0	0	False	-
mp-1394	Rb_8O_4	3	$Fm{-}3m$ (225)	C1-CaF_2	3	13	0	0	False	-
mp-1569	Be_8C_4	3	$Fm{-}3m$ (225)	C1-CaF_2	5	9	0	0	False	-
mp-1747	K_8Te_4	3	$Fm{-}3m$ (225)	C1-CaF_2	3	15	0	0	False	-
mp-1960	Li_8O_4	3	$Fm{-}3m$ (225)	C1-CaF_2	4	8	0	0	False	-
mp-20194	Ce_4O_8	3	$Fm{-}3m$ (225)	C1-CaF_2	5	6	0	0	False	-
mp-2286	Li_8Se_4	3	$Fm{-}3m$ (225)	C1-CaF_2	4	8	0	0	False	-
mp-23209	Sr_4Cl_8	3	$Fm{-}3m$ (225)	C1-CaF_2	4	7	0	0	False	-
mp-2352	Na_8O_4	3	$Fm{-}3m$ (225)	C1-CaF_2	3	14	0	0	False	-
mp-241	Cd_4F_8	3	$Fm{-}3m$ (225)	C1-CaF_2	4	9	0	0	False	-
mp-2530	Li_8Te_4	3	$Fm{-}3m$ (225)	C1-CaF_2	4	7	0	0	False	-
mp-2741	Ca_4F_8	3	$Fm{-}3m$ (225)	C1-CaF_2	4	8	0	0	False	-
mp-2784	Na_8Te_4	3	$Fm{-}3m$ (225)	C1-CaF_2	4	7	0	0	False	-
mp-315	Pb_4F_8	3	$Fm{-}3m$ (225)	C1-CaF_2	4	8	0	0	False	-
mp-568662	Ba_4Cl_8	3	$Fm{-}3m$ (225)	C1-CaF_2	4	9	0	0	False	-
mp-643	Th_4O_8	3	$Fm{-}3m$ (225)	C1-CaF_2	5	7	0	0	False	-
mp-648	Na_8S_4	3	$Fm{-}3m$ (225)	C1-CaF_2	5	4	0	0	False	-
mp-8041	Rb_8S_4	3	$Fm{-}3m$ (225)	C1-CaF_2	4	6	0	0	False	-
mp-8177	Hg_4F_8	3	$Fm{-}3m$ (225)	C1-CaF_2	4	7	0	0	False	-
mp-8426	K_8Se_4	3	$Fm{-}3m$ (225)	C1-CaF_2	4	12	0	0	False	-

ID	Formula	Z	SP (*No.*)	Prototype	NL	NR	WPs	HDWPs	Clean	dE/dq
mp-971	K_8O_4	3	$Fm{-}3m$ (225)	C1-CaF_2	4	7	0	0	False	-
mp-981	Sr_4F_8	3	$Fm{-}3m$ (225)	C1-CaF_2	5	9	0	0	False	-
mp-1216641	$Rh_4Sn_4Ti_4$	3	$F{-}43m$ (216)	C1b-MgAgAs	4	3	1	1	False	-
mp-1018136	$Bi_4La_4Pt_4$	3	$F{-}43m$ (216)	C1b-MgAgAs	4	3	0	1	False	-
mp-10184	$Mg_4Pt_4Sb_4$	3	$F{-}43m$ (216)	C1b-MgAgAs	4	1	0	1	False	-
mp-10755	$Fe_4Ti_4Sb_4$	3	$F{-}43m$ (216)	C1b-MgAgAs	5	4	0	1	False	-
mp-1093991	$Ir_4Zr_4As_4$	3	$F{-}43m$ (216)	C1b-MgAgAs	3	0	0	1	False	-
mp-1094088	$Co_4Nb_4Sn_4$	3	$F{-}43m$ (216)	C1b-MgAgAs	3	1	0	1	False	-
mp-1100408	$Rh_4Sn_4Ta_4$	3	$F{-}43m$ (216)	C1b-MgAgAs	4	1	3	1	False	-
mp-1100415	$Rh_4Zr_4Sb_4$	3	$F{-}43m$ (216)	C1b-MgAgAs	4	1	0	1	False	-
mp-1100430	$Li_4Zn_4Sb_4$	3	$F{-}43m$ (216)	C1b-MgAgAs	3	3	2	1	False	-
mp-11361	$Cu_4Mg_4Sn_4$	3	$F{-}43m$ (216)	C1b-MgAgAs	4	1	1	1	False	-
mp-11520	$Ni_4Y_4Sb_4$	3	$F{-}43m$ (216)	C1b-MgAgAs	4	2	0	1	False	-
mp-11621	$Ni_4Zn_4Sb_4$	3	$F{-}43m$ (216)	C1b-MgAgAs	4	3	0	1	False	-
mp-1206679	$Bi_4Pt_4Y_4$	3	$F{-}43m$ (216)	C1b-MgAgAs	3	5	0	1	False	-
mp-1206717	$Bi_4La_4Pd_4$	3	$F{-}43m$ (216)	C1b-MgAgAs	4	1	0	1	False	-
mp-1207082	$Li_4Mg_4Sb_4$	3	$F{-}43m$ (216)	C1b-MgAgAs	3	2	1	1	False	-
mp-1216635	$Ir_4Sn_4Ti_4$	3	$F{-}43m$ (216)	C1b-MgAgAs	4	2	0	1	False	-
mp-1226720	$Au_4Cd_4Sn_4$	3	$F{-}43m$ (216)	C1b-MgAgAs	5	3	1	1	False	-
mp-15778	$Mg_4Ni_4Sb_4$	3	$F{-}43m$ (216)	C1b-MgAgAs	5	4	0	1	False	-
mp-16246	$Au_4Cd_4Sb_4$	3	$F{-}43m$ (216)	C1b-MgAgAs	4	1	2	1	False	-
mp-16343	$In_4Li_4Ge_4$	3	$F{-}43m$ (216)	C1b-MgAgAs	3	2	0	1	False	-
mp-29733	$Bi_4Mg_4Ni_4$	3	$F{-}43m$ (216)	C1b-MgAgAs	3	5	1	1	False	-
mp-30459	$Bi_4Ni_4Sc_4$	3	$F{-}43m$ (216)	C1b-MgAgAs	3	2	2	1	False	-
mp-30460	$Bi_4Ni_4Y_4$	3	$F{-}43m$ (216)	C1b-MgAgAs	3	4	1	1	False	-
mp-4262	$Al_4Be_4B_4$	3	$F{-}43m$ (216)	C1b-MgAgAs	3	4	1	1	False	-
mp-505297	$Nb_4Ru_4Sb_4$	3	$F{-}43m$ (216)	C1b-MgAgAs	3	0	0	1	False	-
mp-5177	$Au_4Lu_4Sn_4$	3	$F{-}43m$ (216)	C1b-MgAgAs	5	4	0	1	False	-
mp-5318	$Co_4Mn_4Sb_4$	3	$F{-}43m$ (216)	C1b-MgAgAs	4	1	1	1	True	13.58
mp-5549	$Au_4Mg_4Sn_4$	3	$F{-}43m$ (216)	C1b-MgAgAs	4	1	0	1	False	-
mp-570213	$Bi_4Li_4Mg_4$	3	$F{-}43m$ (216)	C1b-MgAgAs	3	1	0	1	False	-
mp-604453	$Bi_4Ce_4Pd_4$	3	$F{-}43m$ (216)	C1b-MgAgAs	5	3	1	1	False	-
mp-961673	$Fe_4Ti_4Te_4$	3	$F{-}43m$ (216)	C1b-MgAgAs	3	4	3	1	False	-
mp-961678	$Co_4Sc_4Te_4$	3	$F{-}43m$ (216)	C1b-MgAgAs	3	1	0	1	False	-
mp-10182	$Li_4Zn_4P_4$	3	$F{-}43m$ (216)	C1b-MgAgAs	3	6	0	1	False	-
mp-10623	$Rh_4Th_4Sb_4$	3	$F{-}43m$ (216)	C1b-MgAgAs	3	2	1	1	False	-
mp-12558	$Li_4Mg_4As_4$	3	$F{-}43m$ (216)	C1b-MgAgAs	3	1	0	1	False	-
mp-16327	$Dy_4Pt_4Sb_4$	3	$F{-}43m$ (216)	C1b-MgAgAs	4	2	0	1	False	-
mp-16329	$Er_4Pt_4Sb_4$	3	$F{-}43m$ (216)	C1b-MgAgAs	4	2	0	1	False	-
mp-16376	$Ho_4Pt_4Sb_4$	3	$F{-}43m$ (216)	C1b-MgAgAs	4	3	1	1	False	-
mp-19886	$Pt_4Sn_4Th_4$	3	$F{-}43m$ (216)	C1b-MgAgAs	4	1	0	1	False	-
mp-30847	$Pt_4Sn_4Ti_4$	3	$F{-}43m$ (216)	C1b-MgAgAs	3	1	0	1	False	-
mp-31451	$Bi_4Co_4Zr_4$	3	$F{-}43m$ (216)	C1b-MgAgAs	3	0	0	1	False	-
mp-31454	$Ru_4Ta_4Sb_4$	3	$F{-}43m$ (216)	C1b-MgAgAs	4	2	2	1	False	-
mp-36111	$Li_4Mg_4P_4$	3	$F{-}43m$ (216)	C1b-MgAgAs	4	2	0	1	False	-
mp-37906	$Li_4Mg_4N_4$	3	$F{-}43m$ (216)	C1b-MgAgAs	4	2	0	1	False	-
mp-567636	$Fe_4V_4Sb_4$	3	$F{-}43m$ (216)	C1b-MgAgAs	3	2	0	1	False	-
mp-5967	$Co_4Ti_4Sb_4$	3	$F{-}43m$ (216)	C1b-MgAgAs	4	2	1	1	False	-
mp-7173	$Pt_4Sc_4Sb_4$	3	$F{-}43m$ (216)	C1b-MgAgAs	4	1	0	1	False	-
mp-7575	$Li_4Zn_4N_4$	3	$F{-}43m$ (216)	C1b-MgAgAs	4	2	1	1	False	-
mp-9124	$Li_4Zn_4As_4$	3	$F{-}43m$ (216)	C1b-MgAgAs	3	5	0	1	False	-
mp-924129	$Ni_4Sn_4Zr_4$	3	$F{-}43m$ (216)	C1b-MgAgAs	4	1	0	1	False	-
mp-924130	$Ni_4Sn_4Ti_4$	3	$F{-}43m$ (216)	C1b-MgAgAs	4	1	0	1	False	-
mp-9437	$Fe_4Nb_4Sb_4$	3	$F{-}43m$ (216)	C1b-MgAgAs	3	3	3	1	False	-
mp-961682	$Pd_4Sn_4Ti_4$	3	$F{-}43m$ (216)	C1b-MgAgAs	4	2	0	1	False	-
mp-961684	$Ca_4Li_4As_4$	3	$F{-}43m$ (216)	C1b-MgAgAs	4	1	1	1	True	3.45
mp-961685	$Ca_4Na_4As_4$	3	$F{-}43m$ (216)	C1b-MgAgAs	4	6	0	1	False	-
mp-961687	$Pd_4Sn_4Zr_4$	3	$F{-}43m$ (216)	C1b-MgAgAs	4	2	1	1	False	-
mp-961693	$Au_4In_4Zr_4$	3	$F{-}43m$ (216)	C1b-MgAgAs	4	1	1	1	False	-
mp-961706	$Pt_4Ti_4Si_4$	3	$F{-}43m$ (216)	C1b-MgAgAs	3	1	0	1	False	-
mp-961711	$Pt_4Zr_4Si_4$	3	$F{-}43m$ (216)	C1b-MgAgAs	3	0	0	1	False	-
mp-961713	$Pt_4Sn_4Zr_4$	3	$F{-}43m$ (216)	C1b-MgAgAs	4	1	0	1	False	-

ID	Formula	*Z*	SP (*No.*)	Prototype	NL	NR	WPs	HDWPs	Clean	dE/dq
mp-1226620	CeCuSi	3	$P-6m2$ (187)	BaLiP	0	2	2	0	False	-
mp-1222888	CuLaSi	3	$P-6m2$ (187)	BaLiP	0	5	2	0	False	-
mp-1061766	CeNiAs	3	$P-6m2$ (187)	BaLiP	0	1	0	0	False	-
mp-1206718	NiYAs	3	$P-6m2$ (187)	BaLiP	0	2	2	0	False	-
mp-1215258	BeZrB	3	$P-6m2$ (187)	BaLiP	0	4	2	0	False	-
mp-1217967	PtSrP	3	$P-6m2$ (187)	BaLiP	0	2	2	0	False	-
mp-1218268	CuSrSi	3	$P-6m2$ (187)	BaLiP	0	7	1	0	False	-
mp-1218273	CuSrGe	3	$P-6m2$ (187)	BaLiP	0	7	5	0	False	-
mp-1222919	CuLaGe	3	$P-6m2$ (187)	BaLiP	0	5	4	0	False	-
mp-1226524	CeNiSi	3	$P-6m2$ (187)	BaLiP	0	0	3	0	False	-
mp-1226602	CeCuGe	3	$P-6m2$ (187)	BaLiP	0	3	1	0	False	-
mp-8606	BaPtSb	3	$P-6m2$ (187)	BaLiP	0	4	4	0	False	-
mp-9744	BaPdAs	3	$P-6m2$ (187)	BaLiP	0	5	1	0	False	-
mp-1215676	YZnSi	3	$P-6m2$ (187)	BaLiP	0	1	1	0	False	-
mp-1226464	CeZnSi	3	$P-6m2$ (187)	BaLiP	0	2	2	0	False	-
mp-1227729	AuBaSi	3	$P-6m2$ (187)	BaLiP	0	3	1	0	False	-
mp-13149	AlBaSi	3	$P-6m2$ (187)	BaLiP	0	0	2	0	False	-
mp-10615	BaLiP	3	$P-6m2$ (187)	BaLiP	0	4	2	0	False	-
mp-10616	BaLiAs	3	$P-6m2$ (187)	BaLiP	0	4	4	0	False	-
mp-24012	HoHSe	3	$P-6m2$ (187)	BaLiP	0	4	3	0	False	-
mp-28797	YHSe	3	$P-6m2$ (187)	BaLiP	0	3	6	0	False	-
mp-22881	Cd_3Cl_6	3	$R-3m$ (166)	$CdCl_2$	2	17	0	0	False	-
mp-23210	Mg_3Cl_6	3	$R-3m$ (166)	$CdCl_2$	2	15	0	0	False	-
mp-568690	Cd_3Br_6	3	$R-3m$ (166)	$CdCl_2$	3	10	0	0	True	6.28
mp-753746	Rb_6O_3	3	$R-3m$ (166)	C1-CaF_2	5	18	0	0	False	-
mp-27922	Ge_3I_6	3	$P-3m1$ (164)	$CdCl_2$	19	101	0	0	False	-
mp-1186	ZrS_2	3	$P-3m1$ (164)	C6-CdI_2	5	14	0	0	False	-
mp-22880	$FeBr_2$	3	$P-3m1$ (164)	C6-CdI_2	6	12	0	0	False	-
mp-22893	PbI_2	3	$P-3m1$ (164)	C6-CdI_2	6	14	0	0	False	-
mp-23205	MgI_2	3	$P-3m1$ (164)	C6-CdI_2	6	17	0	0	False	-
mp-30031	CaI_2	3	$P-3m1$ (164)	C6-CdI_2	6	12	0	0	False	-
mp-30034	$MgBr_2$	3	$P-3m1$ (164)	C6-CdI_2	6	14	0	0	False	-
mp-567259	CdI_2	3	$P-3m1$ (164)	C6-CdI_2	6	19	0	0	False	-
mp-570259	$MgCl_2$	3	$P-3m1$ (164)	C6-CdI_2	6	16	0	0	True	14.69
mp-570418	YbI_2	3	$P-3m1$ (164)	C6-CdI_2	5	13	0	0	False	-
mp-985829	HfS_2	3	$P-3m1$ (164)	C6-CdI_2	4	25	0	0	False	-
mp-985831	$HfSe_2$	3	$P-3m1$ (164)	C6-CdI_2	5	16	0	0	False	-
mp-966	Nb_3S_6	3	$R3m$ (160)	R-MoS_2	2	6	0	0	False	-
mp-1434	Mo_3S_6	3	$R3m$ (160)	R-MoS_2	3	2	0	0	False	-
mp-23162	Zr_3Cl_6	3	$R3m$ (160)	R-MoS_2	2	2	0	0	False	-
mp-7581	Mo_3Se_6	3	$R3m$ (160)	R-MoS_2	3	3	0	0	False	-
mp-9813	W_3S_6	3	$R3m$ (160)	R-MoS_2	3	8	0	0	False	-
mp-38011	$K_3H_3S_3$	3	$R3m$ (160)	KHS	5	5	0	0	False	-
mp-36582	$Na_3H_3S_3$	3	$R3m$ (160)	C1b-MgAgAs	6	5	0	0	False	-
mp-1218912	SnSSe	3	$P3m1$ (156)	BiTeI	3	1	1	0	False	-
mp-1216698	TiSSe	3	$P3m1$ (156)	BiTeI	2	4	0	0	False	-
mp-1220035	NiSeTe	3	$P3m1$ (156)	BiTeI	4	3	3	0	True	5.56
mp-1225994	CoSeTe	3	$P3m1$ (156)	BiTeI	3	4	2	0	False	-
mp-33723	BiBrTe	3	$P3m1$ (156)	BiTeI	3	0	1	0	False	-
mp-22965	BiITe	3	$P3m1$ (156)	BiTeI	3	0	0	0	False	-
mp-1217257	PtSeTe	3	$P3m1$ (156)	BaLiP	4	4	5	0	False	-
mp-1217263	PdSeTe	3	$P3m1$ (156)	BaLiP	4	5	6	0	False	-
mp-1105	Ba_2O_4	3	$I4/mmm$ (139)	BaO_2	0	8	0	0	False	-
mp-22885	F_4Xe_2	3	$I4/mmm$ (139)	BaO_2	0	5	0	0	False	-
mp-30009	F_4Kr_2	3	$I4/mmm$ (139)	BaO_2	0	4	0	0	False	-
mp-685150	Pb_4F_8	3	$Aea2$ (41)	C1-CaF_2	10	31	1	0	False	-
mp-15724	$K_4Na_8Sb_4$	4	$Fm-3m$ (225)	L21-$AlCu_2$	6	13	0	0	False	-
mp-5077	$Li_8Na_4Sb_4$	4	$Fm-3m$ (225)	L21-$AlCu_2$	4	8	0	0	False	-
mp-861937	$Au_4Ba_8As_4$	4	$Fm-3m$ (225)	L21-$AlCu_2$	7	17	0	0	False	-
mp-862631	$Au_4Ba_8Sb_4$	4	$Fm-3m$ (225)	L21-$AlCu_2$	6	14	0	0	False	-
mp-862947	$Au_4Ba_8Bi_4$	4	$Fm-3m$ (225)	L21-$AlCu_2$	6	12	0	0	False	-
mp-865101	$Bi_4Li_8Na_4$	4	$Fm-3m$ (225)	L21-$AlCu_2$	4	5	0	0	False	-

ID	Formula	Z	SP (*No.*)	Prototype	NL	NR	WPs	HDWPs	Clean	dE/dq
mp-865280	$Al_4Fe_8Nb_4$	4	$Fm-3m$ (225)	L21-$AlCu_2$	7	15	0	0	False	-
mp-865648	$Cd_4Pb_4Yb_8$	4	$Fm-3m$ (225)	L21-$AlCu_2$	8	19	0	0	False	-
mp-865681	$Ru_8Ti_4Si_4$	4	$Fm-3m$ (225)	L21-$AlCu_2$	7	7	0	0	False	-
mp-865735	$Li_8Yb_4Ge_4$	4	$Fm-3m$ (225)	L21-$AlCu_2$	4	15	0	0	False	-
mp-865965	$Ca_4Li_8Si_4$	4	$Fm-3m$ (225)	L21-$AlCu_2$	6	13	0	0	False	-
mp-865986	$Ca_4Li_8Ge_4$	4	$Fm-3m$ (225)	L21-$AlCu_2$	6	18	0	0	False	-
mp-866141	$Fe_8Ti_4Si_4$	4	$Fm-3m$ (225)	L21-$AlCu_2$	7	17	0	0	False	-
mp-866154	$Be_4Ir_8Ti_4$	4	$Fm-3m$ (225)	L21-$AlCu_2$	6	15	0	0	False	-
mp-867168	$Au_4Sr_8Sb_4$	4	$Fm-3m$ (225)	L21-$AlCu_2$	6	12	0	0	False	-
mp-867192	$Au_4Sr_8As_4$	4	$Fm-3m$ (225)	L21-$AlCu_2$	5	23	0	0	False	-
mp-984352	$Al_8Co_4Os_4$	4	$Fm-3m$ (225)	L21-$AlCu_2$	5	17	0	0	False	-
mp-30760	$Li_{12}Pb_4$	4	$Fm-3m$ (225)	D03-BiF_3	6	17	0	0	False	-
mp-862559	$Cd_{12}La_4$	4	$Fm-3m$ (225)	D03-BiF_3	6	12	0	0	False	-
mp-865382	Ru_4V_{12}	4	$Fm-3m$ (225)	D03-BiF_3	6	14	0	0	False	-
mp-867343	Cd_4Li_{12}	4	$Fm-3m$ (225)	D03-BiF_3	5	10	0	0	False	-
mp-972071	Mo_4V_{12}	4	$Fm-3m$ (225)	D03-BiF_3	7	12	0	0	False	-
mp-976412	$Li_{12}Zn_4$	4	$Fm-3m$ (225)	D03-BiF_3	5	13	0	0	False	-
mp-1186074	$Na_{12}Pd_4$	4	$Fm-3m$ (225)	D03-BiF_3	6	8	0	0	False	-
mp-2863	$Cd_{12}Ce_4$	4	$Fm-3m$ (225)	D03-BiF_3	6	18	0	0	False	-
mp-867226	In_4Li_{12}	4	$Fm-3m$ (225)	D03-BiF_3	7	12	0	0	False	-
mp-977390	Ru_4Ta_{12}	4	$Fm-3m$ (225)	D03-BiF_3	8	11	0	0	False	-
mp-977434	$Nb_{12}Ru_4$	4	$Fm-3m$ (225)	D03-BiF_3	7	21	0	0	False	-
mp-979289	Ta_4W_{12}	4	$Fm-3m$ (225)	D03-BiF_3	7	22	0	0	False	-
mp-23222	Bi_4Li_{12}	4	$Fm-3m$ (225)	D03-BiF_3	4	14	0	0	False	-
mp-867227	$Li_{12}Pt_4$	4	$Fm-3m$ (225)	D03-BiF_3	5	7	0	0	False	-
mp-976256	$Li_{12}Mg_4$	4	$Fm-3m$ (225)	D03-BiF_3	5	12	0	0	False	-
mp-11247	Au_4Li_{12}	4	$Fm-3m$ (225)	D03-BiF_3	5	8	0	0	False	-
mp-1183324	Ba_4Sr_{12}	4	$Fm-3m$ (225)	D03-BiF_3	8	11	0	0	False	-
mp-1183330	$Ba_{12}Sr_4$	4	$Fm-3m$ (225)	D03-BiF_3	8	7	0	0	False	-
mp-1186024	Cd_4Na_{12}	4	$Fm-3m$ (225)	D03-BiF_3	6	12	0	0	False	-
mp-1187206	$Ta_{12}W_4$	4	$Fm-3m$ (225)	D03-BiF_3	8	17	0	0	False	-
mp-977189	Bi_4Na_{12}	4	$Fm-3m$ (225)	D03-BiF_3	5	2	0	0	False	-
mp-982873	$Na_{12}Tl_4$	4	$Fm-3m$ (225)	D03-BiF_3	5	7	0	0	False	-
mp-1094603	Li_4Mg_{12}	4	$Fm-3m$ (225)	D03-BiF_3	7	13	0	0	False	-
mp-11489	$Li_{12}Pd_4$	4	$Fm-3m$ (225)	D03-BiF_3	5	6	0	0	False	-
mp-1187197	Os_4Ta_{12}	4	$Fm-3m$ (225)	D03-BiF_3	7	18	0	0	False	-
mp-1187203	Re_4Ta_{12}	4	$Fm-3m$ (225)	D03-BiF_3	8	14	0	0	False	-
mp-1798	Ce_4Mg_{12}	4	$Fm-3m$ (225)	D03-BiF_3	6	15	0	0	False	-
mp-2018	Al_4Fe_{12}	4	$Fm-3m$ (225)	D03-BiF_3	7	19	0	0	False	-
mp-2306	La_4Mg_{12}	4	$Fm-3m$ (225)	D03-BiF_3	6	15	0	0	False	-
mp-7396	$Li_{12}Tl_4$	4	$Fm-3m$ (225)	D03-BiF_3	7	9	0	0	False	-
mp-864984	Mn_4V_{12}	4	$Fm-3m$ (225)	D03-BiF_3	8	14	0	0	False	-
mp-865385	Re_4V_{12}	4	$Fm-3m$ (225)	D03-BiF_3	6	13	0	0	False	-
mp-865907	Li_4Zn_{12}	4	$Fm-3m$ (225)	D03-BiF_3	7	6	0	0	False	-
mp-866121	Os_4V_{12}	4	$Fm-3m$ (225)	D03-BiF_3	7	12	0	0	False	-
mp-867342	$Li_{12}Ge_4$	4	$Fm-3m$ (225)	D03-BiF_3	6	10	0	0	False	-
mp-976023	Ga_4Li_{12}	4	$Fm-3m$ (225)	D03-BiF_3	5	13	0	0	False	-
mp-1185265	$Li_{12}Sn_4$	4	$Fm-3m$ (225)	D03-BiF_3	6	14	0	0	False	-
mp-1186218	$Nb_{12}Os_4$	4	$Fm-3m$ (225)	D03-BiF_3	7	12	0	0	False	-
mp-1187196	Ir_4Ta_{12}	4	$Fm-3m$ (225)	D03-BiF_3	7	15	0	0	False	-
mp-1187696	$Cr_{12}V_4$	4	$Fm-3m$ (225)	D03-BiF_3	8	18	0	0	False	-
mp-1187702	V_4W_{12}	4	$Fm-3m$ (225)	D03-BiF_3	7	11	0	0	False	-
mp-2074	$Li_{12}Sb_4$	4	$Fm-3m$ (225)	D03-BiF_3	5	8	0	0	False	-
mp-11231	Al_3	4	$Pm-3m$ (221)	L12-Cu_3	5	25	0	0	False	-
mp-11248	Au_3	4	$Pm-3m$ (221)	L12-Cu_3	7	16	0	0	False	-
mp-11290	$CaSn_3$	4	$Pm-3m$ (221)	L12-Cu_3	7	14	0	0	False	-
mp-11318	$CePt_3$	4	$Pm-3m$ (221)	L12-Cu_3	8	19	0	0	False	-
mp-11474	Hg_3	4	$Pm-3m$ (221)	L12-Cu_3	7	21	0	0	False	-
mp-11501	$MnNi_3$	4	$Pm-3m$ (221)	L12-Cu_3	7	29	0	0	False	-
mp-11504	$MnZn_3$	4	$Pm-3m$ (221)	L12-Cu_3	8	31	0	0	False	-
mp-11517	$NbRu_3$	4	$Pm-3m$ (221)	L12-Cu_3	9	34	0	0	False	-
mp-11522	Ni_3	4	$Pm-3m$ (221)	L12-Cu_3	8	21	0	0	False	-

ID	Formula	*Z*	SP (*No.*)	Prototype	NL	NR	WPs	HDWPs	Clean	dE/dq
mp-1339	Ir_3	4	*Pm*−3m (221)	L12-Cu_3	6	30	0	0	False	-
mp-1911	$CeSn_3$	4	*Pm*−3m (221)	L12-Cu_3	6	27	0	0	False	-
mp-20206	In_3	4	*Pm*−3m (221)	L12-Cu_3	6	18	0	0	False	-
mp-20214	Ga_3	4	*Pm*−3m (221)	L12-Cu_3	6	20	0	0	False	-
mp-20225	$CePb_3$	4	*Pm*−3m (221)	L12-Cu_3	8	24	0	0	False	-
mp-20336	Sn_3	4	*Pm*−3m (221)	L12-Cu_3	6	21	0	0	False	-
mp-20369	$CeIn_3$	4	*Pm*−3m (221)	L12-Cu_3	8	22	0	0	False	-
mp-20603	$CeTl_3$	4	*Pm*−3m (221)	L12-Cu_3	7	16	0	0	False	-
mp-20695	$InZr_3$	4	*Pm*−3m (221)	L12-Cu_3	9	25	0	0	False	-
mp-20821	Ga_3	4	*Pm*−3m (221)	L12-Cu_3	6	17	0	0	False	-
mp-20849	$PbPd_3$	4	*Pm*−3m (221)	L12-Cu_3	7	34	0	0	False	-
mp-20941	In_3	4	*Pm*−3m (221)	L12-Cu_3	6	30	0	0	False	-
mp-21008	Ni_3	4	*Pm*−3m (221)	L12-Cu_3	6	29	0	0	False	-
mp-21289	$TiZn_3$	4	*Pm*−3m (221)	L12-Cu_3	8	13	0	0	False	-
mp-21381	Pb_3	4	*Pm*−3m (221)	L12-Cu_3	7	18	0	0	False	-
mp-21432	$LaPb_3$	4	*Pm*−3m (221)	L12-Cu_3	7	18	0	0	True	8.96
mp-21482	In_3	4	*Pm*−3m (221)	L12-Cu_3	6	28	0	0	False	-
mp-21845	$FePd_3$	4	*Pm*−3m (221)	L12-Cu_3	8	20	0	0	False	-
mp-2393	Tl_3	4	*Pm*−3m (221)	L12-Cu_3	7	18	0	0	False	-
mp-2403	Pt_3	4	*Pm*−3m (221)	L12-Cu_3	8	18	0	0	False	-
mp-2677	Pd_3	4	*Pm*−3m (221)	L12-Cu_3	9	21	0	0	False	-
mp-30741	Ir_3	4	*Pm*−3m (221)	L12-Cu_3	7	21	0	0	False	-
mp-30777	$MgPt_3$	4	*Pm*−3m (221)	L12-Cu_3	7	20	0	0	False	-
mp-481	Pt_3	4	*Pm*−3m (221)	L12-Cu_3	7	17	0	0	False	-
mp-861931	$LiPt_3$	4	*Pm*−3m (221)	L12-Cu_3	7	17	0	0	False	-
mp-861936	$LiPd_3$	4	*Pm*−3m (221)	L12-Cu_3	7	15	0	0	False	-
mp-861939	Ac_3	4	*Pm*−3m (221)	L12-Cu_3	8	19	0	0	False	-
mp-861982	Ga_3	4	*Pm*−3m (221)	L12-Cu_3	5	16	0	0	False	-
mp-861987	In_3	4	*Pm*−3m (221)	L12-Cu_3	6	27	0	0	False	-
mp-862816	$PaGe_3$	4	*Pm*−3m (221)	L12-Cu_3	5	14	0	0	False	-
mp-862820	$PaSi_3$	4	*Pm*−3m (221)	L12-Cu_3	8	21	0	0	False	-
mp-862861	Pa_3	4	*Pm*−3m (221)	L12-Cu_3	9	18	0	0	False	-
mp-862862	Pa_3	4	*Pm*−3m (221)	L12-Cu_3	7	23	0	0	False	-
mp-862863	Pa_3	4	*Pm*−3m (221)	L12-Cu_3	6	32	0	0	False	-
mp-862864	Pa_3	4	*Pm*−3m (221)	L12-Cu_3	8	14	0	0	False	-
mp-1008555	$AlCu_3$	4	*Pm*−3m (221)	L12-Cu_3	7	15	0	0	False	-
mp-1027	$HfRh_3$	4	*Pm*−3m (221)	L12-Cu_3	6	23	0	0	False	-
mp-1089	Ir_3	4	*Pm*−3m (221)	L12-Cu_3	7	24	0	0	False	-
mp-10890	Al_3	4	*Pm*−3m (221)	L12-Cu_3	7	23	0	0	False	-
mp-1094768	$MgZr_3$	4	*Pm*−3m (221)	L12-Cu_3	7	18	0	0	False	-
mp-1094839	Cd_3	4	*Pm*−3m (221)	L12-Cu_3	7	14	0	0	False	-
mp-11358	Co_3	4	*Pm*−3m (221)	L12-Cu_3	6	13	0	0	False	-
mp-1183137	Ag_3	4	*Pm*−3m (221)	L12-Cu_3	7	29	0	0	False	-
mp-1184768	$IrRh_3$	4	*Pm*−3m (221)	L12-Cu_3	5	27	0	0	False	-
mp-1184771	Ir_3	4	*Pm*−3m (221)	L12-Cu_3	6	20	0	0	False	-
mp-1187105	$CaSr_3$	4	*Pm*−3m (221)	L12-Cu_3	7	24	0	0	False	-
mp-1187243	Os_3	4	*Pm*−3m (221)	L12-Cu_3	9	26	0	0	False	-
mp-1187257	$TaZn_3$	4	*Pm*−3m (221)	L12-Cu_3	7	20	0	0	False	-
mp-1187452	Pt_3	4	*Pm*−3m (221)	L12-Cu_3	7	31	0	0	False	-
mp-1263	Ru_3	4	*Pm*−3m (221)	L12-Cu_3	7	23	0	0	False	-
mp-1264	Au_3	4	*Pm*−3m (221)	L12-Cu_3	7	19	0	0	False	-
mp-12798	Ni_3	4	*Pm*−3m (221)	L12-Cu_3	9	14	0	0	False	-
mp-1471	$AlZr_3$	4	*Pm*−3m (221)	L12-Cu_3	7	18	0	0	False	-
mp-1518	$CeRh_3$	4	*Pm*−3m (221)	L12-Cu_3	6	20	0	0	False	-
mp-2001	$LaTl_3$	4	*Pm*−3m (221)	L12-Cu_3	8	20	0	0	False	-
mp-20131	In_3	4	*Pm*−3m (221)	L12-Cu_3	8	14	0	0	False	-
mp-20729	In_3	4	*Pm*−3m (221)	L12-Cu_3	8	19	0	0	False	-
mp-2258	$AuCu_3$	4	*Pm*−3m (221)	L12-Cu_3	7	18	0	0	True	6.61
mp-23159	Bi_3	4	*Pm*−3m (221)	L12-Cu_3	8	16	0	0	False	-
mp-2449	$NbRh_3$	4	*Pm*−3m (221)	L12-Cu_3	7	35	0	0	False	-
mp-265	Ir_3	4	*Pm*−3m (221)	L12-Cu_3	7	36	0	0	False	-
mp-30825	$PaRh_3$	4	*Pm*−3m (221)	L12-Cu_3	7	21	0	0	False	-

ID	Formula	*Z*	SP (*No.*)	Prototype	NL	NR	WPs	HDWPs	Clean	dE/dq
mp-30856	Pt_3	4	*Pm*−3m (221)	L12-Cu_3	7	24	0	0	False	-
mp-435	Sn_3	4	*Pm*−3m (221)	L12-Cu_3	6	20	0	0	False	-
mp-569125	$InMg_3$	4	*Pm*−3m (221)	L12-Cu_3	6	22	0	0	False	-
mp-674	$LaPd_3$	4	*Pm*−3m (221)	L12-Cu_3	7	25	0	0	False	-
mp-1007656	$HfPd_3$	4	*Pm*−3m (221)	L12-Cu_3	8	20	0	0	False	-
mp-1007974	$CrNi_3$	4	*Pm*−3m (221)	L12-Cu_3	6	32	0	0	False	-
mp-1018021	Co_3	4	*Pm*−3m (221)	L12-Cu_3	6	21	0	0	False	-
mp-1018054	$HfPt_3$	4	*Pm*−3m (221)	L12-Cu_3	7	44	0	0	False	-
mp-1044	Ir_3	4	*Pm*−3m (221)	L12-Cu_3	6	21	0	0	False	-
mp-10596	$FeIr_3$	4	*Pm*−3m (221)	L12-Cu_3	7	26	0	0	False	-
mp-1094670	$LiMg_3$	4	*Pm*−3m (221)	L12-Cu_3	7	21	0	0	False	-
mp-1152	Rh_3	4	*Pm*−3m (221)	L12-Cu_3	8	31	0	0	False	-
mp-1186970	Sc_3	4	*Pm*−3m (221)	L12-Cu_3	8	29	0	0	False	-
mp-12083	$CrIr_3$	4	*Pm*−3m (221)	L12-Cu_3	9	16	0	0	False	-
mp-12086	Cu_3	4	*Pm*−3m (221)	L12-Cu_3	6	20	0	0	False	-
mp-12585	$CrRh_3$	4	*Pm*−3m (221)	L12-Cu_3	7	40	0	0	False	-
mp-12593	Ir_3	4	*Pm*−3m (221)	L12-Cu_3	9	20	0	0	False	-
mp-1681	La_3	4	*Pm*−3m (221)	L12-Cu_3	8	28	0	0	False	-
mp-2060	$LuTl_3$	4	*Pm*−3m (221)	L12-Cu_3	7	26	0	0	False	-
mp-2061	$ThTl_3$	4	*Pm*−3m (221)	L12-Cu_3	8	27	0	0	False	-
mp-2092	$CePd_3$	4	*Pm*−3m (221)	L12-Cu_3	8	27	0	0	False	-
mp-2103	Au_3	4	*Pm*−3m (221)	L12-Cu_3	8	22	0	0	False	-
mp-2126	$HfIr_3$	4	*Pm*−3m (221)	L12-Cu_3	7	27	0	0	False	-
mp-2573	Al_3	4	*Pm*−3m (221)	L12-Cu_3	5	13	0	0	False	-
mp-2607	UGe_3	4	*Pm*−3m (221)	L12-Cu_3	6	12	0	0	False	-
mp-2800	USi_3	4	*Pm*−3m (221)	L12-Cu_3	4	9	0	0	False	-
mp-30858	Pt_3	4	*Pm*−3m (221)	L12-Cu_3	7	24	0	0	False	-
mp-580357	Cu_3	4	*Pm*−3m (221)	L12-Cu_3	6	25	0	0	False	-
mp-863750	$AgAu_3$	4	*Pm*−3m (221)	L12-Cu_3	7	11	0	0	False	-
mp-999298	$AuPd_3$	4	*Pm*−3m (221)	L12-Cu_3	10	18	0	0	False	-
mp-20971	Pt_3	4	*Pm*−3m (221)	L12-Cu_3	8	22	0	0	False	-
mp-526	$CaTl_3$	4	*Pm*−3m (221)	L12-Cu_3	7	18	0	0	False	-
mp-976599	Hg_3	4	*Pm*−3m (221)	L12-Cu_3	7	22	0	0	False	-
mp-11536	$NpRh_3$	4	*Pm*−3m (221)	L12-Cu_3	8	34	0	0	False	-
mp-372	Pt_3	4	*Pm*−3m (221)	L12-Cu_3	7	31	0	0	False	-
mp-790	$LuPt_3$	4	*Pm*−3m (221)	L12-Cu_3	8	21	0	0	False	-
mp-976254	Li_3	4	*Pm*−3m (221)	L12-Cu_3	8	25	0	0	False	-
mp-983590	Be_3	4	*Pm*−3m (221)	L12-Cu_3	7	15	0	0	False	-
mp-1017529	Co_3	4	*Pm*−3m (221)	L12-Cu_3	7	36	0	0	False	-
mp-1183655	$CdPd_3$	4	*Pm*−3m (221)	L12-Cu_3	8	28	0	0	False	-
mp-1183995	Cu_3	4	*Pm*−3m (221)	L12-Cu_3	11	29	0	0	False	-
mp-1184703	Hf_3	4	*Pm*−3m (221)	L12-Cu_3	8	26	0	0	False	-
mp-1185846	Au_3	4	*Pm*−3m (221)	L12-Cu_3	8	16	0	0	False	-
mp-1186985	Ni_3	4	*Pm*−3m (221)	L12-Cu_3	8	20	0	0	False	-
mp-1187151	Ca_3	4	*Pm*−3m (221)	L12-Cu_3	6	17	0	0	False	-
mp-1188008	$GaZr_3$	4	*Pm*−3m (221)	L12-Cu_3	7	38	0	0	False	-
mp-1206636	$SrTl_3$	4	*Pm*−3m (221)	L12-Cu_3	6	23	0	0	False	-
mp-229	Rh_3	4	*Pm*−3m (221)	L12-Cu_3	8	19	0	0	False	-
mp-2593	$AlNi_3$	4	*Pm*−3m (221)	L12-Cu_3	7	20	0	0	False	-
mp-376	$PrSn_3$	4	*Pm*−3m (221)	L12-Cu_3	7	25	0	0	False	-
mp-559	Pd_3	4	*Pm*−3m (221)	L12-Cu_3	8	26	0	0	False	-
mp-569775	Al_3	4	*Pm*−3m (221)	L12-Cu_3	7	14	0	0	False	-
mp-570428	$InSc_3$	4	*Pm*−3m (221)	L12-Cu_3	7	29	0	0	False	-
mp-608	Co_3	4	*Pm*−3m (221)	L12-Cu_3	9	38	0	0	False	-
mp-633	$LaSn_3$	4	*Pm*−3m (221)	L12-Cu_3	6	18	0	0	False	-
mp-635	Rh_3	4	*Pm*−3m (221)	L12-Cu_3	7	23	0	0	False	-
mp-718	Pd_3	4	*Pm*−3m (221)	L12-Cu_3	6	33	0	0	False	-
mp-7753	$MgPd_3$	4	*Pm*−3m (221)	L12-Cu_3	8	20	0	0	False	-
mp-815	$GaNi_3$	4	*Pm*−3m (221)	L12-Cu_3	6	33	0	0	False	-
mp-828	Ni_3	4	*Pm*−3m (221)	L12-Cu_3	7	30	0	0	False	-
mp-863257	Ga_3	4	*Pm*−3m (221)	L12-Cu_3	8	27	0	0	False	-
mp-881	$NpSn_3$	4	*Pm*−3m (221)	L12-Cu_3	6	15	0	0	False	-

ID	Formula	Z	SP (*No.*)	Prototype	NL	NR	WPs	HDWPs	Clean	dE/dq
mp-932	Ga_3	4	$Pm-3m$ (221)	L12-Cu_3	8	13	0	0	False	-
mp-973342	Rh_3	4	$Pm-3m$ (221)	L12-Cu_3	6	21	0	0	False	-
mp-973834	Au_3	4	$Pm-3m$ (221)	L12-Cu_3	8	22	0	0	False	-
mp-973865	Al_3	4	$Pm-3m$ (221)	L12-Cu_3	4	15	0	0	False	-
mp-973908	Fe_3	4	$Pm-3m$ (221)	L12-Cu_3	6	25	0	0	False	-
mp-974370	$PtRh_3$	4	$Pm-3m$ (221)	L12-Cu_3	6	17	0	0	False	-
mp-976847	Ni_3	4	$Pm-3m$ (221)	L12-Cu_3	8	36	0	0	False	-
mp-977585	$TlZr_3$	4	$Pm-3m$ (221)	L12-Cu_3	7	20	0	0	False	-
mp-978294	Mg_3	4	$Pm-3m$ (221)	L12-Cu_3	7	21	0	0	False	-
mp-979423	Ga_3	4	$Pm-3m$ (221)	L12-Cu_3	7	19	0	0	False	-
mp-982773	Na_3	4	$Pm-3m$ (221)	L12-Cu_3	6	20	0	0	False	-
mp-1020	Rh_3	4	$Pm-3m$ (221)	L12-Cu_3	8	22	0	0	False	-
mp-11578	VZn_3	4	$Pm-3m$ (221)	L12-Cu_3	9	9	0	0	False	-
mp-1180	$MnPt_3$	4	$Pm-3m$ (221)	L12-Cu_3	7	25	0	0	False	-
mp-1183627	Ag_3	4	$Pm-3m$ (221)	L12-Cu_3	8	29	0	0	False	-
mp-1184558	Hf_3	4	$Pm-3m$ (221)	L12-Cu_3	6	28	0	0	False	-
mp-1185	Rh_3	4	$Pm-3m$ (221)	L12-Cu_3	8	30	0	0	False	-
mp-1185300	Ag_3	4	$Pm-3m$ (221)	L12-Cu_3	7	14	0	0	False	-
mp-1185312	Cu_3	4	$Pm-3m$ (221)	L12-Cu_3	7	19	0	0	False	-
mp-1185427	$AlLu_3$	4	$Pm-3m$ (221)	L12-Cu_3	8	18	0	0	False	-
mp-1185786	Mg_3	4	$Pm-3m$ (221)	L12-Cu_3	6	24	0	0	False	-
mp-1186117	$NiPt_3$	4	$Pm-3m$ (221)	L12-Cu_3	8	27	0	0	False	-
mp-1186137	Hg_3	4	$Pm-3m$ (221)	L12-Cu_3	7	18	0	0	False	-
mp-1187064	$InTh_3$	4	$Pm-3m$ (221)	L12-Cu_3	8	30	0	0	False	-
mp-2509	Rh_3	4	$Pm-3m$ (221)	L12-Cu_3	6	19	0	0	False	-
mp-2515	$CrPt_3$	4	$Pm-3m$ (221)	L12-Cu_3	9	27	0	0	False	-
mp-863027	In_3	4	$Pm-3m$ (221)	L12-Cu_3	7	23	0	0	False	-
mp-974395	Pt_3	4	$Pm-3m$ (221)	L12-Cu_3	8	21	0	0	False	-
mp-976589	In_3	4	$Pm-3m$ (221)	L12-Cu_3	7	15	0	0	False	-
mp-953	$NbZn_3$	4	$Pm-3m$ (221)	L12-Cu_3	8	23	0	0	False	-
mp-30403	$Au_4Li_8Pb_4$	4	$F-43m$ (216)	Li_2	7	2	1	2	False	-
mp-10180	$Li_8Pd_4Sb_4$	4	$F-43m$ (216)	Li_2	7	3	0	2	False	-
mp-1221252	$Hg_4Na_8Tl_4$	4	$F-43m$ (216)	Li_2	6	2	0	2	False	-
mp-1222602	$Au_4Li_8Si_4$	4	$F-43m$ (216)	Li_2	6	2	0	2	False	-
mp-1222604	$Cd_4Li_8Tl_4$	4	$F-43m$ (216)	Li_2	5	3	0	2	False	-
mp-1222607	$Li_8Pt_4Sn_4$	4	$F-43m$ (216)	Li_2	5	2	0	2	False	-
mp-1222608	$Li_8Pd_4Sn_4$	4	$F-43m$ (216)	Li_2	6	0	1	2	False	-
mp-1222610	$Li_8Mg_4Zn_4$	4	$F-43m$ (216)	Li_2	5	3	0	2	False	-
mp-1222611	$Ag_4Li_8Tl_4$	4	$F-43m$ (216)	Li_2	7	3	0	2	False	-
mp-1222612	$Ir_4Li_8Sn_4$	4	$F-43m$ (216)	Li_2	5	1	0	2	False	-
mp-1222613	$Li_8Mg_4Tl_4$	4	$F-43m$ (216)	Li_2	6	2	1	2	False	-
mp-1222615	$Li_8Mg_4Sn_4$	4	$F-43m$ (216)	Li_2	6	4	1	2	False	-
mp-1222646	$Hg_4Li_8Mg_4$	4	$F-43m$ (216)	Li_2	6	2	0	2	False	-
mp-1222647	$Ga_4Li_8Mg_4$	4	$F-43m$ (216)	Li_2	6	1	0	2	False	-
mp-1222648	$Cd_4Li_8Mg_4$	4	$F-43m$ (216)	Li_2	6	2	0	2	False	-
mp-1222650	$Au_4Li_8Mg_4$	4	$F-43m$ (216)	Li_2	4	1	1	2	False	-
mp-1222651	$Li_8Mg_4Ge_4$	4	$F-43m$ (216)	Li_2	6	2	2	2	False	-
mp-1222652	$Li_8Mg_4Pb_4$	4	$F-43m$ (216)	Li_2	6	3	1	2	False	-
mp-1222657	$Li_8Pd_4Ge_4$	4	$F-43m$ (216)	Li_2	6	2	0	2	False	-
mp-1222659	$Ag_4Ga_4Li_8$	4	$F-43m$ (216)	Li_2	6	3	0	2	False	-
mp-1222668	$Al_4Ga_4Li_8$	4	$F-43m$ (216)	Li_2	7	3	0	2	False	-
mp-1222671	$Cd_4In_4Li_8$	4	$F-43m$ (216)	Li_2	5	2	0	2	False	-
mp-20389	$Cd_4Na_8Pb_4$	4	$F-43m$ (216)	Li_2	6	11	0	2	False	-
mp-21026	$In_4Li_8Pt_4$	4	$F-43m$ (216)	Li_2	5	1	0	2	False	-
mp-21029	$In_4Li_8Pd_4$	4	$F-43m$ (216)	Li_2	6	2	1	2	False	-
mp-2935	$Au_4Li_8Ge_4$	4	$F-43m$ (216)	Li_2	5	1	0	2	False	-
mp-2988	$Ga_4Li_8Rh_4$	4	$F-43m$ (216)	Li_2	5	3	0	2	False	-
mp-30344	$Ag_4In_4Li_8$	4	$F-43m$ (216)	Li_2	5	5	1	2	False	-
mp-30349	$Ag_4Li_8Pb_4$	4	$F-43m$ (216)	Li_2	5	1	0	2	False	-
mp-30381	$Au_4Ga_4Li_8$	4	$F-43m$ (216)	Li_2	5	2	0	2	False	-
mp-30392	$Au_4In_4Li_8$	4	$F-43m$ (216)	Li_2	7	2	1	2	True	6.51
mp-30405	$Au_4Li_8Sn_4$	4	$F-43m$ (216)	Li_2	6	8	0	2	False	-

ID	Formula	Z	SP (*No.*)	Prototype	NL	NR	WPs	HDWPs	Clean	dE/dq
mp-30591	$Cu_4Li_8Sn_4$	4	$F-43m$ (216)	Li_2	5	2	0	2	False	-
mp-30816	$Al_4Li_8Pd_4$	4	$F-43m$ (216)	Li_2	5	2	0	2	False	-
mp-30818	$Al_4Li_8Pt_4$	4	$F-43m$ (216)	Li_2	4	3	1	2	False	-
mp-31168	$Ag_4Al_4Li_8$	4	$F-43m$ (216)	Li_2	5	2	0	2	False	-
mp-31441	$Ga_4Ir_4Li_8$	4	$F-43m$ (216)	Li_2	4	1	0	2	False	-
mp-31442	$In_4Li_8Rh_4$	4	$F-43m$ (216)	Li_2	5	1	1	2	False	-
mp-31443	$In_4Ir_4Li_8$	4	$F-43m$ (216)	Li_2	4	2	0	2	False	-
mp-3158	$Li_8Pt_4Sb_4$	4	$F-43m$ (216)	Li_2	6	2	0	2	False	-
mp-3201	$Ga_4Li_8Pd_4$	4	$F-43m$ (216)	Li_2	5	5	1	2	False	-
mp-35841	$Cu_4Li_8Ge_4$	4	$F-43m$ (216)	Li_2	6	2	0	2	False	-
mp-3726	$Ga_4Li_8Pt_4$	4	$F-43m$ (216)	Li_2	6	2	0	2	False	-
mp-567347	$Ag_4Li_8Sn_4$	4	$F-43m$ (216)	Li_2	5	2	0	2	False	-
mp-999576	$Mn_8Ru_4Si_4$	4	$F-43m$ (216)	Li_2	7	4	1	2	False	-
mp-15988	$Cu_4Li_8Sb_4$	4	$F-43m$ (216)	Li_2	6	1	0	2	False	-
mp-165	Si_4	4	$P6_3/mmc$ (194)	Si	10	23	0	0	False	-
mp-984	B_2N_2	4	$P6_3/mmc$ (194)	BN	10	19	0	0	False	-
mp-1066078	Bi_2Pt_2	4	$P6_3/mmc$ (194)	B81-NiAs	22	34	0	0	False	-
mp-999318	Bi_2Ni_2	4	$P6_3/mmc$ (194)	B81-NiAs	24	21	0	0	False	-
mp-10052	Ti_2As_2	4	$P6_3/mmc$ (194)	B81-NiAs	25	24	0	0	False	-
mp-1008488	Bi_2Pd_2	4	$P6_3/mmc$ (194)	B81-NiAs	23	29	0	0	False	-
mp-1066274	Pb_2Pt_2	4	$P6_3/mmc$ (194)	B81-NiAs	23	25	0	0	False	-
mp-1114	V_2P_2	4	$P6_3/mmc$ (194)	B81-NiAs	21	25	0	0	False	-
mp-19856	Pt_2Sn_2	4	$P6_3/mmc$ (194)	B81-NiAs	22	13	0	0	False	-
mp-20033	Ir_2Sn_2	4	$P6_3/mmc$ (194)	B81-NiAs	25	17	0	0	False	-
mp-203	Ni_2Te_2	4	$P6_3/mmc$ (194)	B81-NiAs	23	17	0	0	False	-
mp-2187	Ti_2Sb_2	4	$P6_3/mmc$ (194)	B81-NiAs	25	19	0	0	False	-
mp-2644	Co_2Sb_2	4	$P6_3/mmc$ (194)	B81-NiAs	21	35	0	0	False	-
mp-568565	Cr_2As_2	4	$P6_3/mmc$ (194)	B81-NiAs	22	32	0	0	False	-
mp-590	Ni_2As_2	4	$P6_3/mmc$ (194)	B81-NiAs	22	24	0	0	False	-
mp-810	Ni_2Sb_2	4	$P6_3/mmc$ (194)	B81-NiAs	25	16	0	0	False	-
mp-568273	Li_2I_2	4	$P6_3/mmc$ (194)	B81-NiAs	6	23	0	0	False	-
mp-1007652	In_2As_2	4	$P6_3mc$ (186)	B4-AlN	23	19	0	2	False	-
mp-1007661	In_2Sb_2	4	$P6_3mc$ (186)	B4-AlN	22	23	0	2	False	-
mp-1008559	B_2P_2	4	$P6_3mc$ (186)	B4-AlN	23	20	0	2	False	-
mp-1018040	Mg_2Se_2	4	$P6_3mc$ (186)	B4-AlN	22	19	1	2	False	-
mp-1018059	Ga_2Sb_2	4	$P6_3mc$ (186)	B4-AlN	18	4	4	0	False	-
mp-1018100	Al_2Sb_2	4	$P6_3mc$ (186)	B4-AlN	18	1	6	0	False	-
mp-1178514	Ba_2O_2	4	$P6_3mc$ (186)	B4-AlN	23	14	0	2	False	-
mp-1184046	Cu_2Cl_2	4	$P6_3mc$ (186)	B4-AlN	23	29	0	2	False	-
mp-1184548	Pd_2H_2	4	$P6_3mc$ (186)	B4-AlN	23	14	2	1	False	-
mp-1185301	Li_2F_2	4	$P6_3mc$ (186)	B4-AlN	25	21	0	2	False	-
mp-1185319	Li_2Cl_2	4	$P6_3mc$ (186)	B4-AlN	22	21	1	2	False	-
mp-1185942	Mg_2S_2	4	$P6_3mc$ (186)	B4-AlN	18	6	7	0	False	-
mp-13119	Cd_2O_2	4	$P6_3mc$ (186)	B4-AlN	23	15	0	1	False	-
mp-22205	In_2N_2	4	$P6_3mc$ (186)	B4-AlN	22	18	0	2	False	-
mp-866056	Ac_2N_2	4	$P6_3mc$ (186)	B4-AlN	24	24	0	1	False	-
mp-8883	Ga_2As_2	4	$P6_3mc$ (186)	B4-AlN	22	21	0	2	False	-
mp-973676	Hg_2S_2	4	$P6_3mc$ (186)	B4-AlN	22	32	0	2	False	-
mp-1039	Mg_2Te_2	4	$P6_3mc$ (186)	B4-AlN	5	22	0	0	False	-
mp-1070	Cd_2Se_2	4	$P6_3mc$ (186)	B4-AlN	10	35	0	2	False	-
mp-12779	Cd_2Te_2	4	$P6_3mc$ (186)	B4-AlN	11	23	0	1	False	-
mp-2133	Zn_2O_2	4	$P6_3mc$ (186)	B4-AlN	11	25	0	1	False	-
mp-22894	Ag_2I_2	4	$P6_3mc$ (186)	B4-AlN	5	28	0	0	False	-
mp-2542	Be_2O_2	4	$P6_3mc$ (186)	B4-AlN	11	29	0	1	False	-
mp-380	Zn_2Se_2	4	$P6_3mc$ (186)	B4-AlN	10	27	0	0	False	-
mp-560588	Zn_2S_2	4	$P6_3mc$ (186)	B4-AlN	4	29	0	0	False	-
mp-567290	La_2N_2	4	$P6_3mc$ (186)	B4-AlN	6	19	0	0	False	-
mp-569346	Cu_2I_2	4	$P6_3mc$ (186)	B4-AlN	5	30	0	0	False	-
mp-570935	Li_2I_2	4	$P6_3mc$ (186)	B4-AlN	10	28	1	1	False	-
mp-661	Al_2N_2	4	$P6_3mc$ (186)	B4-AlN	10	20	0	1	False	-
mp-672	Cd_2S_2	4	$P6_3mc$ (186)	B4-AlN	11	25	0	1	False	-
mp-7140	C_2Si_2	4	$P6_3mc$ (186)	B4-AlN	4	20	0	0	False	-

ID	Formula	Z	SP (*No.*)	Prototype	NL	NR	WPs	HDWPs	Clean	dE/dq
mp-804	Ga_2N_2	4	$P6_3mc$ (186)	B4-AlN	11	23	0	0	False	-
mp-8880	Al_2P_2	4	$P6_3mc$ (186)	B4-AlN	4	20	0	0	False	-
mp-8881	Al_2As_2	4	$P6_3mc$ (186)	B4-AlN	11	30	0	1	False	-
mp-8882	Ga_2P_2	4	$P6_3mc$ (186)	B4-AlN	10	21	0	2	False	-
mp-8884	Zn_2Te_2	4	$P6_3mc$ (186)	B4-AlN	10	21	0	0	False	-
mp-559092	Sc_3F_9	4	$R{-}3$m (166)	ScF_3	6	17	0	0	False	-
mp-4636	$Cu_3Sc_3O_6$	4	$R{-}3$m (166)	$CaCN_2$	5	17	0	0	False	-
mp-978857	$Hg_3Sr_3O_6$	4	$R{-}3$m (166)	$CaCN_2$	5	13	0	0	False	-
mp-996981	$Au_3Tl_3O_6$	4	$R{-}3$m (166)	$CaCN_2$	5	16	0	0	True	5.96
mp-753260	$Ag_3La_3O_6$	4	$R{-}3$m (166)	$CaCN_2$	5	13	0	0	False	-
mp-7041	$Ca_3Hg_3O_6$	4	$R{-}3$m (166)	$CaCN_2$	6	12	0	0	False	-
mp-6972	$Cu_3Y_3O_6$	4	$R{-}3$m (166)	$CaCN_2$	5	11	0	0	False	-
mp-10969	$Cd_3C_3N_6$	4	$R{-}3$m (166)	$CaCN_2$	5	16	0	0	False	-
mp-12317	$Sr_3C_3N_6$	4	$R{-}3$m (166)	$CaCN_2$	5	7	0	0	False	-
mp-14116	$Cu_3Rh_3O_6$	4	$R{-}3$m (166)	$CaCN_2$	5	15	0	0	False	-
mp-24199	$Li_3F_6H_3$	4	$R{-}3$m (166)	$CaCN_2$	5	13	0	0	False	-
mp-27837	$Na_3F_6H_3$	4	$R{-}3$m (166)	$CaCN_2$	6	21	0	0	False	-
mp-28688	$Hg_3Sr_3O_6$	4	$R{-}3$m (166)	$CaCN_2$	5	13	0	0	False	-
mp-3915	$Ba_3Hg_3O_6$	4	$R{-}3$m (166)	$CaCN_2$	6	11	0	0	False	-
mp-4124	$Ca_3C_3N_6$	4	$R{-}3$m (166)	$CaCN_2$	5	22	0	0	False	-
mp-10226	$Na_3Y_3S_6$	4	$R{-}3$m (166)	Ca_2	5	17	0	0	False	-
mp-10778	$Pr_3Rb_3Se_6$	4	$R{-}3$m (166)	Ca_2	5	29	0	0	False	-
mp-10779	$Nd_3Rb_3Se_6$	4	$R{-}3$m (166)	Ca_2	5	26	0	0	False	-
mp-10780	$Rb_3Sm_3Se_6$	4	$R{-}3$m (166)	Ca_2	5	23	0	0	False	-
mp-10782	$Rb_3Tb_3Se_6$	4	$R{-}3$m (166)	Ca_2	5	28	0	0	False	-
mp-10783	$Ho_3Rb_3Se_6$	4	$R{-}3$m (166)	Ca_2	5	26	0	0	False	-
mp-10784	$Er_3Rb_3Se_6$	4	$R{-}3$m (166)	Ca_2	5	25	0	0	False	-
mp-10785	$Lu_3Rb_3Se_6$	4	$R{-}3$m (166)	Ca_2	4	24	0	0	False	-
mp-11601	$Er_3Na_3O_6$	4	$R{-}3$m (166)	Ca_2	4	18	0	0	False	-
mp-11739	$K_3La_3Te_6$	4	$R{-}3$m (166)	Ca_2	5	30	0	0	False	-
mp-11740	$K_3Nd_3Te_6$	4	$R{-}3$m (166)	Ca_2	6	28	0	0	False	-
mp-13313	$Sc_3Tl_3Se_6$	4	$R{-}3$m (166)	Ca_2	5	15	0	0	False	-
mp-14115	$Li_3Rh_3O_6$	4	$R{-}3$m (166)	Ca_2	6	19	0	0	False	-
mp-14437	$Rb_3Y_3O_6$	4	$R{-}3$m (166)	Ca_2	5	15	0	0	False	-
mp-15781	$K_3La_3S_6$	4	$R{-}3$m (166)	Ca_2	4	21	0	0	False	-
mp-15782	$K_3Pr_3S_6$	4	$R{-}3$m (166)	Ca_2	4	21	0	0	False	-
mp-15783	$K_3Sm_3S_6$	4	$R{-}3$m (166)	Ca_2	4	25	0	0	False	-
mp-15785	$Dy_3K_3S_6$	4	$R{-}3$m (166)	Ca_2	4	23	0	0	False	-
mp-15786	$Ho_3K_3S_6$	4	$R{-}3$m (166)	Ca_2	4	24	0	0	False	-
mp-15788	$Li_3Y_3S_6$	4	$R{-}3$m (166)	Ca_2	4	28	0	0	False	-
mp-15789	$Dy_3Li_3S_6$	4	$R{-}3$m (166)	Ca_2	5	29	0	0	False	-
mp-15790	$Ho_3Li_3S_6$	4	$R{-}3$m (166)	Ca_2	5	29	0	0	False	-
mp-15791	$Er_3Li_3S_6$	4	$R{-}3$m (166)	Ca_2	5	25	0	0	False	-
mp-15793	$Li_3Tb_3Se_6$	4	$R{-}3$m (166)	Ca_2	5	20	0	0	False	-
mp-15794	$Li_3Y_3Se_6$	4	$R{-}3$m (166)	Ca_2	5	18	0	0	False	-
mp-15795	$Dy_3Li_3Se_6$	4	$R{-}3$m (166)	Ca_2	6	14	0	0	False	-
mp-15796	$Ho_3Li_3Se_6$	4	$R{-}3$m (166)	Ca_2	6	15	0	0	False	-
mp-15797	$Er_3Li_3Se_6$	4	$R{-}3$m (166)	Ca_2	6	19	0	0	False	-
mp-16763	$K_3Y_3Te_6$	4	$R{-}3$m (166)	Ca_2	5	22	0	0	False	-
mp-20289	$In_3Na_3S_6$	4	$R{-}3$m (166)	Ca_2	4	26	0	0	False	-
mp-22473	$In_3Na_3Se_6$	4	$R{-}3$m (166)	Ca_2	4	30	0	0	False	-
mp-22566	$In_3Tl_3S_6$	4	$R{-}3$m (166)	Ca_2	6	18	0	0	False	-
mp-22936	$Ca_6Cl_3N_3$	4	$R{-}3$m (166)	Ca_2	5	28	0	0	False	-
mp-23009	$Ca_6Br_3N_3$	4	$R{-}3$m (166)	Ca_2	5	22	0	0	False	-
mp-23033	$Sr_6Cl_3N_3$	4	$R{-}3$m (166)	Ca_2	4	23	0	0	False	-
mp-23040	$Ca_6I_3P_3$	4	$R{-}3$m (166)	Ca_2	5	17	0	0	False	-
mp-23056	$Sr_6Br_3N_3$	4	$R{-}3$m (166)	Ca_2	5	26	0	0	False	-
mp-27438	$Bi_3Tl_3Te_6$	4	$R{-}3$m (166)	Ca_2	4	51	0	0	False	-
mp-27869	$Ba_6Cl_3P_3$	4	$R{-}3$m (166)	Ca_2	4	24	0	0	False	-
mp-28554	$Ca_6As_3I_3$	4	$R{-}3$m (166)	Ca_2	6	19	0	0	False	-
mp-29678	$Ag_3Bi_3S_6$	4	$R{-}3$m (166)	Ca_2	8	30	0	0	False	-
mp-30041	$Bi_3Rb_3S_6$	4	$R{-}3$m (166)	Ca_2	4	20	0	0	False	-

ID	Formula	Z	SP ($No.$)	Prototype	NL	NR	WPs	HDWPs	Clean	dE/dq
mp-3056	$Na_3Tl_3O_6$	4	R−3m (166)	Ca_2	4	21	0	0	False	-
mp-31166	$Dy_3Tl_3S_6$	4	R−3m (166)	Ca_2	6	25	0	0	False	-
mp-3613	$Er_3Na_3S_6$	4	R−3m (166)	Ca_2	4	19	0	0	False	-
mp-3664	$Nd_3Tl_3S_6$	4	R−3m (166)	Ca_2	7	26	0	0	False	-
mp-4123	$Er_3Tl_3S_6$	4	R−3m (166)	Ca_2	6	24	0	0	False	-
mp-4326	$Er_3K_3S_6$	4	R−3m (166)	Ca_2	4	27	0	0	False	-
mp-4519	$K_3Pr_3O_6$	4	R−3m (166)	Ca_2	5	25	0	0	False	-
mp-505158	$Cs_3Ho_3S_6$	4	R−3m (166)	Ca_2	4	18	0	0	False	-
mp-5175	$In_3Na_3O_6$	4	R−3m (166)	Ca_2	4	20	0	0	False	-
mp-5273	$K_3Pr_3Te_6$	4	R−3m (166)	Ca_2	6	30	0	0	False	-
mp-554310	$Bi_3Tl_3S_6$	4	R−3m (166)	Ca_2	6	16	0	0	False	-
mp-561586	$Cs_3La_3S_6$	4	R−3m (166)	Ca_2	5	19	0	0	False	-
mp-561619	$Cs_3Lu_3S_6$	4	R−3m (166)	Ca_2	4	20	0	0	False	-
mp-561751	$Cs_3Er_3S_6$	4	R−3m (166)	Ca_2	4	25	0	0	False	-
mp-568062	$Dy_3Tl_3Se_6$	4	R−3m (166)	Ca_2	5	22	0	0	False	-
mp-568588	$Nd_3Tl_3Se_6$	4	R−3m (166)	Ca_2	5	16	0	0	False	-
mp-569178	$Ho_3Tl_3Se_6$	4	R−3m (166)	Ca_2	5	19	0	0	False	-
mp-5694	$Ho_3Na_3S_6$	4	R−3m (166)	Ca_2	4	22	0	0	False	-
mp-569507	$Tb_3Tl_3Se_6$	4	R−3m (166)	Ca_2	5	20	0	0	False	-
mp-569677	$Sr_6I_3N_3$	4	R−3m (166)	Ca_2	4	20	0	0	False	-
mp-570117	$Er_3Tl_3Se_6$	4	R−3m (166)	Ca_2	5	21	0	0	False	-
mp-5808	$Nd_3Rb_3Te_6$	4	R−3m (166)	Ca_2	5	24	0	0	False	-
mp-690794	$Sr_6H_3N_3$	4	R−3m (166)	Ca_2	4	19	0	0	False	-
mp-6978	$Ho_3Rb_3O_6$	4	R−3m (166)	Ca_2	6	29	0	0	False	-
mp-7046	$Dy_3Rb_3S_6$	4	R−3m (166)	Ca_2	5	25	0	0	False	-
mp-7176	$La_3Rb_3Se_6$	4	R−3m (166)	Ca_2	4	29	0	0	False	-
mp-7476	$Dy_3Rb_3O_6$	4	R−3m (166)	Ca_2	6	30	0	0	False	-
mp-7477	$Er_3Rb_3O_6$	4	R−3m (166)	Ca_2	6	28	0	0	False	-
mp-7478	$Rb_3Tm_3O_6$	4	R−3m (166)	Ca_2	6	30	0	0	False	-
mp-7480	$Lu_3Rb_3O_6$	4	R−3m (166)	Ca_2	6	28	0	0	False	-
mp-753774	$K_3Tm_3O_6$	4	R−3m (166)	Ca_2	4	27	0	0	False	-
mp-754158	$K_3Nd_3O_6$	4	R−3m (166)	Ca_2	4	23	0	0	False	-
mp-754825	$Na_3Tm_3O_6$	4	R−3m (166)	Ca_2	4	15	0	0	False	-
mp-754883	$Ho_3K_3O_6$	4	R−3m (166)	Ca_2	4	23	0	0	False	-
mp-755311	$Lu_3Na_3O_6$	4	R−3m (166)	Ca_2	3	30	0	0	False	-
mp-756034	$Na_3Sm_3O_6$	4	R−3m (166)	Ca_2	5	23	0	0	True	19.73
mp-7914	$Na_3Sc_3O_6$	4	R−3m (166)	Ca_2	6	30	0	0	False	-
mp-7958	$K_3La_3O_6$	4	R−3m (166)	Ca_2	4	25	0	0	False	-
mp-7959	$Er_3K_3O_6$	4	R−3m (166)	Ca_2	4	24	0	0	False	-
mp-7972	$La_3Rb_3O_6$	4	R−3m (166)	Ca_2	5	18	0	0	False	-
mp-7973	$Nd_3Rb_3O_6$	4	R−3m (166)	Ca_2	6	23	0	0	False	-
mp-7974	$Rb_3Sm_3O_6$	4	R−3m (166)	Ca_2	6	23	0	0	False	-
mp-8001	$Al_3Li_3O_6$	4	R−3m (166)	Ca_2	6	37	0	0	False	-
mp-8145	$Rb_3Sc_3O_6$	4	R−3m (166)	Ca_2	4	27	0	0	False	-
mp-8175	$K_3Tl_3O_6$	4	R−3m (166)	Ca_2	4	17	0	0	False	-
mp-8176	$Rb_3Tl_3O_6$	4	R−3m (166)	Ca_2	5	21	0	0	False	-
mp-8188	$K_3Sc_3O_6$	4	R−3m (166)	Ca_2	5	22	0	0	False	-
mp-8409	$K_3Y_3O_6$	4	R−3m (166)	Ca_2	4	22	0	0	False	-
mp-8584	$Er_3Na_3Se_6$	4	R−3m (166)	Ca_2	5	17	0	0	False	-
mp-8830	$Na_3Rh_3O_6$	4	R−3m (166)	Ca_2	5	32	0	0	False	-
mp-9035	$Lu_3Na_3S_6$	4	R−3m (166)	Ca_2	4	23	0	0	False	-
mp-9036	$K_3Sm_3Te_6$	4	R−3m (166)	Ca_2	6	26	0	0	False	-
mp-9076	$Na_3Tm_3S_6$	4	R−3m (166)	Ca_2	4	21	0	0	False	-
mp-9081	$Cs_3Nd_3S_6$	4	R−3m (166)	Ca_2	4	13	0	0	False	-
mp-9086	$Cs_3Dy_3S_6$	4	R−3m (166)	Ca_2	4	20	0	0	False	-
mp-9089	$Cs_3Tm_3S_6$	4	R−3m (166)	Ca_2	4	22	0	0	False	-
mp-9263	$Er_3K_3Te_6$	4	R−3m (166)	Ca_2	6	27	0	0	False	-
mp-9361	$La_3Rb_3S_6$	4	R−3m (166)	Ca_2	5	23	0	0	False	-
mp-9362	$Pr_3Rb_3S_6$	4	R−3m (166)	Ca_2	5	27	0	0	False	-
mp-9363	$Nd_3Rb_3S_6$	4	R−3m (166)	Ca_2	5	24	0	0	False	-
mp-9364	$Rb_3Sm_3S_6$	4	R−3m (166)	Ca_2	5	25	0	0	False	-
mp-9365	$Rb_3Tb_3S_6$	4	R−3m (166)	Ca_2	5	21	0	0	False	-

ID	Formula	Z	SP (*No.*)	Prototype	NL	NR	WPs	HDWPs	Clean	dE/dq
mp-9366	$Ho_3Rb_3S_6$	4	$R-3m$ (166)	Ca_2	5	27	0	0	False	-
mp-9367	$Er_3Rb_3S_6$	4	$R-3m$ (166)	Ca_2	5	30	0	0	False	-
mp-9368	$Rb_3Tm_3S_6$	4	$R-3m$ (166)	Ca_2	5	25	0	0	False	-
mp-9370	$Lu_3Rb_3S_6$	4	$R-3m$ (166)	Ca_2	5	29	0	0	False	-
mp-19210	$Co_3Pt_3O_6$	4	$R-3m$ (166)	$AlCuO_2$	5	11	0	0	False	-
mp-22660	$Ag_3In_3O_6$	4	$R-3m$ (166)	$AlCuO_2$	5	10	0	0	False	-
mp-997161	$Au_3Co_3O_6$	4	$R-3m$ (166)	$AlCuO_2$	6	24	0	0	False	-
mp-3748	$Al_3Cu_3O_6$	4	$R-3m$ (166)	$AlCuO_2$	6	19	0	0	False	-
mp-4280	$Cu_3Ga_3O_6$	4	$R-3m$ (166)	$AlCuO_2$	5	16	0	0	False	-
mp-753098	$Cu_3Li_3F_6$	4	$R-3m$ (166)	$AlCuO_2$	5	15	0	0	False	-
mp-29803	H_2Si_2	4	$P-3m1$ (164)	SiH	8	16	0	0	False	-
mp-12908	$AgScSe_2$	4	$P-3m1$ (164)	$LiCoS_2$	9	27	0	0	False	-
mp-12953	$AgTmTe_2$	4	$P-3m1$ (164)	$LiCoS_2$	10	39	0	0	False	-
mp-753946	$CoLiS_2$	4	$P-3m1$ (164)	$LiCoS_2$	11	28	0	0	False	-
mp-570081	Cu_2I_2	4	$P-3m1$ (164)	CuI	7	22	0	0	False	-
mp-22691	In_6Se_6	4	$R3m$ (160)	InSe	3	10	0	0	False	-
mp-6980	$CuScS_2$	4	$P3m1$ (156)	$ScCuS_2$	4	3	0	0	False	-
mp-570485	AlSrHSi	4	$P3m1$ (156)	BaAlSiH	4	2	3	0	False	-
mp-571093	AlBaHSi	4	$P3m1$ (156)	BaAlSiH	4	7	1	0	False	-
mp-979137	GaSrHSi	4	$P3m1$ (156)	BaAlSiH	4	5	2	0	False	-
mp-980057	AlSrGeH	4	$P3m1$ (156)	BaAlSiH	5	11	0	0	False	-
mp-22859	Hg_4I_4	4	$I4/mmm$ (139)	HgI	0	12	0	0	False	-
mp-22897	Hg_4Cl_4	4	$I4/mmm$ (139)	HgI	0	8	0	0	False	-
mp-23177	Hg_4Br_4	4	$I4/mmm$ (139)	HgI	0	17	0	0	False	-
mp-706	Hg_4F_4	4	$I4/mmm$ (139)	HgI	0	13	0	0	False	-
mp-19921	Pb_2O_2	4	$P4/nmm$ (129)	CuBr	24	21	0	0	False	-
mp-20311	Fe_2Se_2	4	$P4/nmm$ (129)	CuBr	24	20	0	0	False	-
mp-2097	Sn_2O_2	4	$P4/nmm$ (129)	CuBr	24	23	0	0	False	-
mp-505531	Fe_2S_2	4	$P4/nmm$ (129)	CuBr	24	32	0	0	False	-
mp-604908	Co_2Se_2	4	$P4/nmm$ (129)	CuBr	24	21	0	0	False	-
mp-22917	Cu_2Br_2	4	$P4/nmm$ (129)	CuBr	27	23	0	0	False	-
mp-567809	Ag_2I_2	4	$P4/nmm$ (129)	CuBr	24	22	0	0	False	-
mp-581833	RbN_3	4	$P4/mmm$ (123)	RbN_3	0	19	0	0	False	-
mp-1378	Li_4C_4	4	$Immm$ (71)	LiC	1	23	0	0	False	-
mp-558071	Rb_4S_4	4	$Immm$ (71)	LiC	0	19	0	0	False	-
mp-7895	Rb_4O_4	4	$Immm$ (71)	LiC	0	20	0	0	False	-
mp-22870	In_4Br_4	4	$Cmcm$ (63)	InBr	22	21	0	0	False	-
mp-23202	In_4I_4	4	$Cmcm$ (63)	InBr	19	16	0	0	False	-
mp-27418	$AuKO_2$	4	$Pmmm$ (47)	$KAuO_2$	0	20	0	0	False	-
mp-4541	$Cu_2Na_2O_4$	4	$C2/m$ (12)	$NaCuO_2$	0	24	0	0	False	-
mp-754326	$Ag_2Na_2O_4$	4	$C2/m$ (12)	$NaCuO_2$	1	20	0	0	False	-
mp-10250	$BaLiF_3$	5	$Pm-3m$ (221)	$CsCaH_3$	9	28	0	0	False	-
mp-23737	$KMgH_3$	5	$Pm-3m$ (221)	$CsCaH_3$	9	12	0	0	False	-
mp-23818	$BaLiH_3$	5	$Pm-3m$ (221)	$CsCaH_3$	10	16	0	0	False	-
mp-23949	$CaRbH_3$	5	$Pm-3m$ (221)	$CsCaH_3$	6	25	0	0	False	-
mp-28171	K_3	5	$Pm-3m$ (221)	$CsCaH_3$	8	20	0	0	False	-
mp-28593	Li_3	5	$Pm-3m$ (221)	$CsCaH_3$	9	33	0	0	False	-
mp-3448	$KMgF_3$	5	$Pm-3m$ (221)	$CsCaH_3$	8	22	0	0	False	-
mp-3614	$KTaO_3$	5	$Pm-3m$ (221)	$CsCaH_3$	9	24	0	0	False	-
mp-568544	$CdCsCl_3$	5	$Pm-3m$ (221)	$CsCaH_3$	8	31	0	0	False	-
mp-570223	$CsBr_3$	5	$Pm-3m$ (221)	$CsCaH_3$	7	24	0	0	False	-
mp-5878	$KZnF_3$	5	$Pm-3m$ (221)	$CsCaH_3$	8	36	0	0	False	-
mp-644203	$CaCsH_3$	5	$Pm-3m$ (221)	$CsCaH_3$	6	11	0	0	False	-
mp-7104	$CaCsF_3$	5	$Pm-3m$ (221)	$CsCaH_3$	8	28	0	0	False	-
mp-8399	$CdCsF_3$	5	$Pm-3m$ (221)	$CsCaH_3$	9	19	0	0	False	-
mp-8402	$MgRbF_3$	5	$Pm-3m$ (221)	$CsCaH_3$	8	18	0	0	False	-
mp-862832	$PaTlO_3$	5	$Pm-3m$ (221)	$CsCaH_3$	7	25	0	0	False	-
mp-985586	Na_3	5	$Pm-3m$ (221)	$CsCaH_3$	9	24	0	0	False	-
mp-31406	$Bi_6Se_6Te_3$	5	$R-3m$ (166)	Bi_2TeSe_2	6	30	0	0	False	-
mp-637213	$La_6Ge_3I_6$	5	$R-3m$ (166)	Bi_2TeSe_2	4	36	0	0	False	-
mp-1057	Al_3Ni_2	5	$P-3m1$ (164)	Sm_2O_3	12	32	0	0	False	-
mp-1069815	In_3Pt_2	5	$P-3m1$ (164)	Sm_2O_3	13	30	0	0	False	-

ID	Formula	Z	SP (*No.*)	Prototype	NL	NR	WPs	HDWPs	Clean	dE/dq
mp-10901	Al_3Pd_2	5	$P{-}3m1$ (164)	Sm_2O_3	12	18	0	0	False	-
mp-10905	Al_3Pt_2	5	$P{-}3m1$ (164)	Sm_2O_3	11	33	0	0	False	-
mp-11107	Ac_2O_3	5	$P{-}3m1$ (164)	Sm_2O_3	13	25	0	0	False	-
mp-11397	Ga_3Ni_2	5	$P{-}3m1$ (164)	Sm_2O_3	15	35	0	0	False	-
mp-1207013	Rh_3Si_2	5	$P{-}3m1$ (164)	Sm_2O_3	13	29	0	0	False	-
mp-21385	In_3Ni_2	5	$P{-}3m1$ (164)	Sm_2O_3	17	31	0	0	False	-
mp-21400	Ga_3Pt_2	5	$P{-}3m1$ (164)	Sm_2O_3	13	27	0	0	False	-
mp-2646	Mg_3Sb_2	5	$P{-}3m1$ (164)	Sm_2O_3	13	30	0	0	False	-
mp-510437	In_3Pd_2	5	$P{-}3m1$ (164)	Sm_2O_3	16	42	0	0	False	-
mp-973	U_2N_3	5	$P{-}3m1$ (164)	Sm_2O_3	13	20	0	0	False	-
mp-10733	Sm_2O_3	5	$P{-}3m1$ (164)	Sm_2O_3	11	31	0	0	False	-
mp-1968	La_2O_3	5	$P{-}3m1$ (164)	Sm_2O_3	14	45	0	0	False	-
mp-2063	Pr_2O_3	5	$P{-}3m1$ (164)	Sm_2O_3	11	33	0	0	False	-
mp-2763	Nd_2O_3	5	$P{-}3m1$ (164)	Sm_2O_3	11	35	0	0	False	-
mp-7891	Mg_3As_2	5	$P{-}3m1$ (164)	Sm_2O_3	13	22	0	0	False	-
mp-28479	Sc_2CCl_2	5	$P{-}3m1$ (164)	Sc_2CCl_2	8	35	0	0	True	59.31
mp-10918	$PdRb_2C_2$	5	$P{-}3m1$ (164)	Rb_2PdC_2	17	31	0	0	False	-
mp-10919	$PtRb_2C_2$	5	$P{-}3m1$ (164)	Rb_2PdC_2	14	30	0	0	False	-
mp-4823	Na_2PdC_2	5	$P{-}3m1$ (164)	Rb_2PdC_2	15	28	0	0	False	-
mp-505825	Cs_2PtC_2	5	$P{-}3m1$ (164)	Rb_2PdC_2	15	38	0	0	False	-
mp-976876	K_2PtC_2	5	$P{-}3m1$ (164)	Rb_2PdC_2	14	25	0	0	False	-
mp-1017548	Be_2CaP_2	5	$P{-}3m1$ (164)	Ho_2CF_2	11	30	0	0	False	-
mp-1029337	Zr_2N_2	5	$P{-}3m1$ (164)	Ho_2CF_2	13	29	0	0	True	35.24
mp-1070483	Al_2SrGe_2	5	$P{-}3m1$ (164)	Ho_2CF_2	12	29	0	0	False	-
mp-1206128	Th_2N_2	5	$P{-}3m1$ (164)	Ho_2CF_2	11	22	0	0	False	-
mp-1206998	Al_2LaSi_2	5	$P{-}3m1$ (164)	Ho_2CF_2	11	30	0	0	False	-
mp-1232292	Al_2MgGe_2	5	$P{-}3m1$ (164)	Ho_2CF_2	12	32	0	0	False	-
mp-7429	$CaZn_2Sb_2$	5	$P{-}3m1$ (164)	Ho_2CF_2	14	39	0	0	False	-
mp-9571	$CaZn_2As_2$	5	$P{-}3m1$ (164)	Ho_2CF_2	15	36	0	0	False	-
mp-7431	$SrZn_2Sb_2$	5	$P{-}3m1$ (164)	Ho_2CF_2	11	42	0	0	False	-
mp-1017567	Hf_2N_2	5	$P{-}3m1$ (164)	Ho_2CF_2	12	25	0	0	False	-
mp-1017628	Be_2MgP_2	5	$P{-}3m1$ (164)	Ho_2CF_2	12	32	0	0	False	-
mp-1018630	Be_2CaAs_2	5	$P{-}3m1$ (164)	Ho_2CF_2	11	43	0	0	False	-
mp-1097065	$HfLi_2N_2$	5	$P{-}3m1$ (164)	Ho_2CF_2	13	28	0	0	False	-
mp-15986	Cu_2HfP_2	5	$P{-}3m1$ (164)	Ho_2CF_2	14	38	0	0	False	-
mp-29208	Bi_2CaMg_2	5	$P{-}3m1$ (164)	Ho_2CF_2	11	25	0	0	False	-
mp-29209	$BaBi_2Mg_2$	5	$P{-}3m1$ (164)	Ho_2CF_2	13	29	0	0	False	-
mp-7430	$CaCd_2Sb_2$	5	$P{-}3m1$ (164)	Ho_2CF_2	14	39	0	0	False	-
mp-7432	Cd_2SrSb_2	5	$P{-}3m1$ (164)	Ho_2CF_2	16	39	0	0	False	-
mp-754640	Ce_2O_2	5	$P{-}3m1$ (164)	Ho_2CF_2	12	39	0	0	False	-
mp-7704	Al_2CaSi_2	5	$P{-}3m1$ (164)	Ho_2CF_2	12	39	0	0	False	-
mp-7770	$SrZn_2As_2$	5	$P{-}3m1$ (164)	Ho_2CF_2	13	49	0	0	False	-
mp-7771	Cd_2SrAs_2	5	$P{-}3m1$ (164)	Ho_2CF_2	13	39	0	0	False	-
mp-8150	$BaCd_2Sb_2$	5	$P{-}3m1$ (164)	Ho_2CF_2	13	31	0	0	False	-
mp-8152	$CeLi_2As_2$	5	$P{-}3m1$ (164)	Ho_2CF_2	13	23	0	0	False	-
mp-8219	Cu_2ZrP_2	5	$P{-}3m1$ (164)	Ho_2CF_2	14	31	0	0	False	-
mp-9581	Cu_2ThP_2	5	$P{-}3m1$ (164)	Ho_2CF_2	15	28	0	0	False	-
mp-1029258	$CaZn_2N_2$	5	$P{-}3m1$ (164)	Ho_2CF_2	15	24	0	0	False	-
mp-1029330	Hf_2N_2	5	$P{-}3m1$ (164)	Ho_2CF_2	13	30	0	0	False	-
mp-1029385	Li_2SnN_2	5	$P{-}3m1$ (164)	Ho_2CF_2	12	33	0	0	False	-
mp-10435	Al_2YSi_2	5	$P{-}3m1$ (164)	Ho_2CF_2	11	38	0	0	False	-
mp-10521	Al_2YGe_2	5	$P{-}3m1$ (164)	Ho_2CF_2	12	32	0	0	False	-
mp-10580	Al_2LaGe_2	5	$P{-}3m1$ (164)	Ho_2CF_2	12	30	0	0	False	-
mp-1069558	Ce_2N_2	5	$P{-}3m1$ (164)	Ho_2CF_2	13	26	0	0	False	-
mp-1069695	Bi_2Mg_2	5	$P{-}3m1$ (164)	Ho_2CF_2	11	29	0	0	False	-
mp-3862	Al_2CaGe_2	5	$P{-}3m1$ (164)	Ho_2CF_2	13	31	0	0	False	-
mp-6931	Al_2SrSi_2	5	$P{-}3m1$ (164)	Ho_2CF_2	10	35	0	0	False	-
mp-7067	$CaCd_2As_2$	5	$P{-}3m1$ (164)	Ho_2CF_2	14	42	0	0	False	-
mp-7101	Al_2CeGe_2	5	$P{-}3m1$ (164)	Ho_2CF_2	13	34	0	0	False	-
mp-8281	$BaCd_2As_2$	5	$P{-}3m1$ (164)	Ho_2CF_2	11	27	0	0	False	-
mp-10314	$YbZn_2As_2$	5	$P{-}3m1$ (164)	Ho_2CF_2	12	32	0	0	False	-
mp-10550	Mg_2SrN_2	5	$P{-}3m1$ (164)	Ho_2CF_2	13	26	0	0	False	-

ID	Formula	Z	SP (*No.*)	Prototype	NL	NR	WPs	HDWPs	Clean	dE/dq
mp-10933	Nd_2S_2	5	$P-3m1$ (164)	Ho_2CF_2	13	38	0	0	False	-
mp-10996	Mg_2YbSb_2	5	$P-3m1$ (164)	Ho_2CF_2	15	18	0	0	False	-
mp-11917	Be_2MgN_2	5	$P-3m1$ (164)	Ho_2CF_2	14	45	0	0	True	86
mp-12668	Tb_2O_2	5	$P-3m1$ (164)	Ho_2CF_2	12	16	0	0	False	-
mp-12669	Dy_2O_2	5	$P-3m1$ (164)	Ho_2CF_2	12	17	0	0	False	-
mp-12670	Ho_2O_2	5	$P-3m1$ (164)	Ho_2CF_2	12	21	0	0	False	-
mp-12671	Er_2O_2	5	$P-3m1$ (164)	Ho_2CF_2	12	20	0	0	False	-
mp-12673	Lu_2O_2	5	$P-3m1$ (164)	Ho_2CF_2	12	19	0	0	False	-
mp-12894	Y_2O_2	5	$P-3m1$ (164)	Ho_2CF_2	13	28	0	0	False	-
mp-13971	Nd_2O_2	5	$P-3m1$ (164)	Ho_2CF_2	12	33	0	0	False	-
mp-13972	Sm_2O_2	5	$P-3m1$ (164)	Ho_2CF_2	11	36	0	0	False	-
mp-13974	Ho_2O_2	5	$P-3m1$ (164)	Ho_2CF_2	13	28	0	0	False	-
mp-13975	Er_2O_2	5	$P-3m1$ (164)	Ho_2CF_2	13	31	0	0	False	-
mp-3211	Nd_2O_2	5	$P-3m1$ (164)	Ho_2CF_2	10	22	0	0	False	-
mp-3216	Li_2ZrN_2	5	$P-3m1$ (164)	Ho_2CF_2	13	38	0	0	False	-
mp-3236	Pr_2O_2	5	$P-3m1$ (164)	Ho_2CF_2	10	19	0	0	False	-
mp-3556	Tm_2O_2	5	$P-3m1$ (164)	Ho_2CF_2	12	19	0	0	False	-
mp-4511	La_2O_2	5	$P-3m1$ (164)	Ho_2CF_2	11	29	0	0	False	-
mp-4764	Pr_2O_2	5	$P-3m1$ (164)	Ho_2CF_2	13	30	0	0	False	-
mp-553875	Zr_2N_2	5	$P-3m1$ (164)	Ho_2CF_2	12	30	0	0	False	-
mp-5598	Sm_2O_2	5	$P-3m1$ (164)	Ho_2CF_2	11	27	0	0	False	-
mp-5795	$CaMg_2N_2$	5	$P-3m1$ (164)	Ho_2CF_2	13	41	0	0	False	-
mp-7233	La_2O_2	5	$P-3m1$ (164)	Ho_2CF_2	12	34	0	0	False	-
mp-752658	Y_2O_2	5	$P-3m1$ (164)	Ho_2CF_2	13	37	0	0	False	-
mp-753920	Tm_2O_2	5	$P-3m1$ (164)	Ho_2CF_2	13	29	0	0	False	-
mp-754827	Tm_2O_2	5	$P-3m1$ (164)	Ho_2CF_2	12	35	0	0	False	-
mp-754834	Er_2O_2	5	$P-3m1$ (164)	Ho_2CF_2	12	28	0	0	False	-
mp-755340	Tb_2O_2	5	$P-3m1$ (164)	Ho_2CF_2	12	30	0	0	False	-
mp-768908	Ho_2O_2	5	$P-3m1$ (164)	Ho_2CF_2	12	29	0	0	False	-
mp-769284	Dy_2O_2	5	$P-3m1$ (164)	Ho_2CF_2	12	30	0	0	False	-
mp-7768	Th_2N_2	5	$P-3m1$ (164)	Ho_2CF_2	10	29	0	0	False	-
mp-7769	Th_2N_2	5	$P-3m1$ (164)	Ho_2CF_2	10	18	0	0	False	-
mp-8181	$CeLi_2N_2$	5	$P-3m1$ (164)	Ho_2CF_2	12	23	0	0	False	-
mp-8276	$SrZn_2P_2$	5	$P-3m1$ (164)	Ho_2CF_2	14	38	0	0	False	-
mp-8277	Cd_2SrP_2	5	$P-3m1$ (164)	Ho_2CF_2	12	43	0	0	False	-
mp-8278	$BaMg_2P_2$	5	$P-3m1$ (164)	Ho_2CF_2	12	35	0	0	False	-
mp-8279	$BaCd_2P_2$	5	$P-3m1$ (164)	Ho_2CF_2	13	38	0	0	False	-
mp-8280	$BaMg_2As_2$	5	$P-3m1$ (164)	Ho_2CF_2	11	26	0	0	False	-
mp-8579	Ag_2BaS_2	5	$P-3m1$ (164)	Ho_2CF_2	14	38	0	0	False	-
mp-865185	Be_2MgAs_2	5	$P-3m1$ (164)	Ho_2CF_2	14	31	0	0	False	-
mp-867194	Mg_2SrAs_2	5	$P-3m1$ (164)	Ho_2CF_2	13	31	0	0	False	-
mp-9006	Ho_2CF_2	5	$P-3m1$ (164)	Ho_2CF_2	13	38	0	0	True	58.72
mp-9514	Al_2MgC_2	5	$P-3m1$ (164)	Ho_2CF_2	13	35	0	0	False	-
mp-9564	$CaMg_2As_2$	5	$P-3m1$ (164)	Ho_2CF_2	15	27	0	0	False	-
mp-9565	$CaMg_2Sb_2$	5	$P-3m1$ (164)	Ho_2CF_2	13	27	0	0	False	-
mp-9566	Mg_2SrSb_2	5	$P-3m1$ (164)	Ho_2CF_2	14	30	0	0	False	-
mp-9567	$BaMg_2Sb_2$	5	$P-3m1$ (164)	Ho_2CF_2	11	42	0	0	False	-
mp-9569	$CaZn_2P_2$	5	$P-3m1$ (164)	Ho_2CF_2	13	28	0	0	False	-
mp-9570	$CaCd_2P_2$	5	$P-3m1$ (164)	Ho_2CF_2	13	32	0	0	False	-
mp-9582	$YbZn_2P_2$	5	$P-3m1$ (164)	Ho_2CF_2	11	40	0	0	False	-
mp-9912	$CeLi_2P_2$	5	$P-3m1$ (164)	Ho_2CF_2	14	20	0	0	False	-
mp-29798	$Tl_3Br_3O_9$	5	$R3m$ (160)	$CsCaH_3$	5	11	0	0	False	-
mp-4559	$Ba_3C_3O_9$	5	$R3m$ (160)	$BaCO_3$	7	10	1	0	False	-
mp-2706	Sn_2F_8	5	$I4/mmm$ (139)	SnF_4	0	30	0	0	False	-
mp-341	Pb_2F_8	5	$I4/mmm$ (139)	SnF_4	1	28	0	0	False	-
mp-16032	$Pr_4O_4Te_2$	5	$I4/mmm$ (139)	Pr_2TeO_2	0	18	0	0	False	-
mp-16033	$Sm_4O_4Te_2$	5	$I4/mmm$ (139)	Pr_2TeO_2	0	21	0	0	False	-
mp-16036	$Tb_4O_4Te_2$	5	$I4/mmm$ (139)	Pr_2TeO_2	0	24	0	0	False	-
mp-4255	$Ba_2Cu_4S_4$	5	$I4/mmm$ (139)	Pr_2TeO_2	0	30	0	0	False	-
mp-4547	$La_4O_4Te_2$	5	$I4/mmm$ (139)	Pr_2TeO_2	0	23	0	0	False	-
mp-5459	$Nd_4O_4Te_2$	5	$I4/mmm$ (139)	Pr_2TeO_2	0	22	0	0	False	-
mp-7523	$Th_4N_4Te_2$	5	$I4/mmm$ (139)	Pr_2TeO_2	0	16	0	0	False	-

ID	Formula	Z	SP (*No.*)	Prototype	NL	NR	WPs	HDWPs	Clean	dE/dq
mp-9610	$Li_4C_2N_4$	5	$I4/mmm$ (139)	Li_2CN_2	0	18	0	0	False	-
mp-1029532	$Ca_4Co_2N_4$	5	$I4/mmm$ (139)	Ca_2ZnN_2	0	18	0	0	True	22.08
mp-10343	$Sc_4B_2C_4$	5	$I4/mmm$ (139)	Ca_2ZnN_2	0	16	0	0	False	-
mp-1068685	$Pt_2U_4C_4$	5	$I4/mmm$ (139)	Ca_2ZnN_2	0	17	0	0	False	-
mp-1069004	$Os_2U_4C_4$	5	$I4/mmm$ (139)	Ca_2ZnN_2	0	18	0	0	False	-
mp-1069160	$Ru_2U_4C_4$	5	$I4/mmm$ (139)	Ca_2ZnN_2	0	16	0	0	False	-
mp-1069790	$Rh_2U_4C_4$	5	$I4/mmm$ (139)	Ca_2ZnN_2	0	18	0	0	False	-
mp-1206327	$Ni_2Th_4C_4$	5	$I4/mmm$ (139)	Ca_2ZnN_2	0	19	0	0	False	-
mp-19167	$Ni_2Rb_4O_4$	5	$I4/mmm$ (139)	Ca_2ZnN_2	0	16	0	0	False	-
mp-644389	$Li_4Pd_2H_4$	5	$I4/mmm$ (139)	Ca_2ZnN_2	0	12	0	0	False	-
mp-6940	$Ir_2U_4C_4$	5	$I4/mmm$ (139)	Ca_2ZnN_2	0	21	0	0	False	-
mp-769864	$Cs_4Ni_2O_4$	5	$I4/mmm$ (139)	Ca_2ZnN_2	0	13	0	0	False	-
mp-4961	$Hg_2Na_4O_4$	5	$I4/mmm$ (139)	Ca_2ZnN_2	0	20	0	0	False	-
mp-5072	$Hg_2Rb_4O_4$	5	$I4/mmm$ (139)	Ca_2ZnN_2	0	13	0	0	False	-
mp-5860	$Hg_2K_4O_4$	5	$I4/mmm$ (139)	Ca_2ZnN_2	0	20	0	0	False	-
mp-7232	$Cs_4Hg_2O_4$	5	$I4/mmm$ (139)	Ca_2ZnN_2	0	15	0	0	False	-
mp-8818	$Ca_4Zn_2N_4$	5	$I4/mmm$ (139)	Ca_2ZnN_2	0	19	0	0	False	-
mp-9306	$Sr_4Zn_2N_4$	5	$I4/mmm$ (139)	Ca_2ZnN_2	0	22	0	0	False	-
mp-9307	$Ba_4Zn_2N_4$	5	$I4/mmm$ (139)	Ca_2ZnN_2	1	18	0	0	False	-
mp-774907	$K_2Li_2Zn_2S_4$	5	$I-4m2$ (119)	$KLiZnS_2$	0	3	11	0	False	-
mp-20459	$PbTiO_3$	5	$P4mm$ (99)	$CsCaH_3$	1	36	0	0	False	-
mp-22313	$Na_4Pt_2O_4$	5	$Immm$ (71)	Na_2PtO_2	0	32	0	0	False	-
mp-7608	$Li_4Pd_2O_4$	5	$Immm$ (71)	Na_2PtO_2	0	33	0	0	False	-
mp-540584	$K_4Pd_2O_4$	5	$Immm$ (71)	K_2PdO_2	0	49	0	0	False	-
mp-7928	$K_4Pt_2S_4$	5	$Immm$ (71)	K_2PdO_2	0	35	0	0	False	-
mp-7929	$Pt_2Rb_4S_4$	5	$Immm$ (71)	K_2PdO_2	0	53	0	0	False	-
mp-8621	$K_4Pt_2Se_4$	5	$Immm$ (71)	K_2PdO_2	0	46	0	0	False	-
mp-8622	$Pt_2Rb_4Se_4$	5	$Immm$ (71)	K_2PdO_2	0	44	0	0	False	-
mp-10408	$K_4C_2N_4$	5	$C2/m$ (12)	Ca_2ZnN_2	0	12	0	0	False	-
mp-1189	$Al_{16}Pr_8$	6	$Fd-3m$ (227)	C15-$MgCu_2$	17	23	0	0	False	-
mp-1383	$Co_{16}U_8$	6	$Fd-3m$ (227)	C15-$MgCu_2$	16	29	0	0	True	14.46
mp-1400	Hf_8W_{16}	6	$Fd-3m$ (227)	C15-$MgCu_2$	17	25	0	0	False	-
mp-2028	$Be_{16}Nb_8$	6	$Fd-3m$ (227)	C15-$MgCu_2$	17	18	0	0	False	-
mp-2049	$Mo_{16}Zr_8$	6	$Fd-3m$ (227)	C15-$MgCu_2$	18	30	0	0	False	-
mp-2363	Hf_8Mo_{16}	6	$Fd-3m$ (227)	C15-$MgCu_2$	19	36	0	0	False	-
mp-2708	La_8Ni_{16}	6	$Fd-3m$ (227)	C15-$MgCu_2$	19	27	0	0	False	-
mp-570113	$Au_{16}Bi_8$	6	$Fd-3m$ (227)	C15-$MgCu_2$	18	22	0	0	False	-
mp-582	Ce_8Ir_{16}	6	$Fd-3m$ (227)	C15-$MgCu_2$	19	33	0	0	False	-
mp-675	$W_{16}Zr_8$	6	$Fd-3m$ (227)	C15-$MgCu_2$	18	34	0	0	False	-
mp-819	$Cr_{16}Hf_8$	6	$Fd-3m$ (227)	C15-$MgCu_2$	18	27	0	0	False	-
mp-833	Ca_8Pd_{16}	6	$Fd-3m$ (227)	C15-$MgCu_2$	16	36	0	0	True	12.55
mp-850	$Ni_{16}Sc_8$	6	$Fd-3m$ (227)	C15-$MgCu_2$	17	33	0	0	True	9.67
mp-1425	$Cr_{16}Ti_8$	6	$Fd-3m$ (227)	C15-$MgCu_2$	19	39	0	0	False	-
mp-1638	$Al_{16}Sr_8$	6	$Fd-3m$ (227)	C15-$MgCu_2$	17	25	0	0	False	-
mp-1687	Ba_8Pd_{16}	6	$Fd-3m$ (227)	C15-$MgCu_2$	18	34	0	0	False	-
mp-2098	Ce_8Os_{16}	6	$Fd-3m$ (227)	C15-$MgCu_2$	21	38	0	0	False	-
mp-2295	Ca_8Ni_{16}	6	$Fd-3m$ (227)	C15-$MgCu_2$	18	37	0	0	False	-
mp-253	$Co_{16}Sc_8$	6	$Fd-3m$ (227)	C15-$MgCu_2$	21	35	0	0	True	10.13
mp-2674	$Pt_{16}Y_8$	6	$Fd-3m$ (227)	C15-$MgCu_2$	17	35	0	0	True	6.41
mp-514	Hf_8Zn_{16}	6	$Fd-3m$ (227)	C15-$MgCu_2$	18	23	0	0	False	-
mp-747	Ba_8Pt_{16}	6	$Fd-3m$ (227)	C15-$MgCu_2$	20	30	0	0	False	-
mp-813	$Al_{16}Sc_8$	6	$Fd-3m$ (227)	C15-$MgCu_2$	18	36	0	0	False	-
mp-842	Ca_8Pt_{16}	6	$Fd-3m$ (227)	C15-$MgCu_2$	18	22	0	0	False	-
mp-921	$Rh_{16}Y_8$	6	$Fd-3m$ (227)	C15-$MgCu_2$	17	38	0	0	False	-
mp-1349	$Pt_{16}Sr_8$	6	$Fd-3m$ (227)	C15-$MgCu_2$	18	23	0	0	False	-
mp-2749	$Be_{16}Ti_8$	6	$Fd-3m$ (227)	C15-$MgCu_2$	18	26	0	0	False	-
mp-548	$Cr_{16}Nb_8$	6	$Fd-3m$ (227)	C15-$MgCu_2$	18	36	0	0	False	-
mp-570436	Ca_8Ir_{16}	6	$Fd-3m$ (227)	C15-$MgCu_2$	21	34	0	0	False	-
mp-1038	$Cu_{16}Mg_8$	6	$Fd-3m$ (227)	C15-$MgCu_2$	19	23	0	0	True	8.29
mp-1187198	$Mg_{16}Sr_8$	6	$Fd-3m$ (227)	C15-$MgCu_2$	17	21	0	0	False	-
mp-1187860	$Mg_{16}Y_8$	6	$Fd-3m$ (227)	C15-$MgCu_2$	18	25	0	0	False	-
mp-2206	$Co_{16}Lu_8$	6	$Fd-3m$ (227)	C15-$MgCu_2$	18	27	0	0	False	-

ID	Formula	Z	SP ($No.$)	Prototype	NL	NR	WPs	HDWPs	Clean	dE/dq
mp-540	$Fe_{16}Sc_8$	6	Fd−3m (227)	C15-$MgCu_2$	19	30	0	0	False	-
mp-1909	La_8Mg_{16}	6	Fd−3m (227)	C15-$MgCu_2$	18	35	0	0	False	-
mp-23279	$Bi_{16}K_8$	6	Fd−3m (227)	C15-$MgCu_2$	16	31	0	0	False	-
mp-30352	$Ag_{16}Na_8$	6	Fd−3m (227)	C15-$MgCu_2$	19	29	0	0	True	13.96
mp-333	$Fe_{16}Hf_8$	6	Fd−3m (227)	C15-$MgCu_2$	18	38	0	0	False	-
mp-1183430	Ca_8Mg_{16}	6	Fd−3m (227)	C15-$MgCu_2$	16	28	0	0	True	16.29
mp-1185705	Mg_8Ni_{16}	6	Fd−3m (227)	C15-$MgCu_2$	19	25	0	0	True	13.36
mp-2271	Na_8Pt_{16}	6	Fd−3m (227)	C15-$MgCu_2$	20	29	0	0	False	-
mp-353	Ag_4O_2	6	Pn−3m (224)	C3-Cu_2	16	18	0	0	True	33.87
mp-947	Au_4S_2	6	Pn−3m (224)	C3-Cu_2	16	27	0	0	True	18.89
mp-361	Cu_4O_2	6	Pn−3m (224)	C3-Cu_2	16	34	0	0	True	21.14
mp-976181	$Na_4B_4H_{16}$	6	F−43m (216)	$NaBH_4$	6	9	1	3	False	-
mp-34337	ClH_4	6	P−43m (215)	H_4	9	2	1	5	False	-
mp-36248	BrH_4	6	P−43m (215)	H_4	9	2	0	1	False	-
mp-632667	Yb_2H_4	6	$P6_3/mmc$ (194)	YbH_2	18	36	0	0	False	-
mp-11818	$Ba_2Zn_2Ge_2$	6	$P6_3/mmc$ (194)	KZnSb	34	33	0	0	False	-
mp-1205318	$Ag_2Ba_2Bi_2$	6	$P6_3/mmc$ (194)	KZnSb	36	45	0	0	False	-
mp-20766	$Ce_2Cu_2Ge_2$	6	$P6_3/mmc$ (194)	KZnSb	36	33	0	0	False	-
mp-4120	$Ca_2Cu_2As_2$	6	$P6_3/mmc$ (194)	KZnSb	34	41	0	0	False	-
mp-568895	$Au_2Ba_2Sb_2$	6	$P6_3/mmc$ (194)	KZnSb	34	48	0	0	False	-
mp-10667	$Ag_2Sr_2P_2$	6	$P6_3/mmc$ (194)	KZnSb	33	41	0	0	False	-
mp-10677	$Au_2Ca_2P_2$	6	$P6_3/mmc$ (194)	KZnSb	34	29	0	0	False	-
mp-10749	$Cu_2Sr_2Sb_2$	6	$P6_3/mmc$ (194)	KZnSb	34	33	0	0	False	-
mp-10763	$Hg_2K_2Sb_2$	6	$P6_3/mmc$ (194)	KZnSb	33	32	0	0	False	-
mp-1077361	$Au_2Ba_2As_2$	6	$P6_3/mmc$ (194)	KZnSb	32	45	0	0	False	-
mp-1206169	$Cu_2Sr_2Si_2$	6	$P6_3/mmc$ (194)	KZnSb	37	34	0	0	False	-
mp-1206525	$Au_2Sr_2As_2$	6	$P6_3/mmc$ (194)	KZnSb	34	35	0	0	False	-
mp-12481	$La_2Pt_2P_2$	6	$P6_3/mmc$ (194)	KZnSb	35	31	0	0	False	-
mp-12557	$Cu_2Sr_2As_2$	6	$P6_3/mmc$ (194)	KZnSb	35	44	0	0	True	3.89
mp-16321	$Cu_2Sr_2P_2$	6	$P6_3/mmc$ (194)	KZnSb	34	30	0	0	False	-
mp-22740	$Ce_2Cu_2Si_2$	6	$P6_3/mmc$ (194)	KZnSb	33	17	0	0	False	-
mp-24011	$Ce_2H_2Se_2$	6	$P6_3/mmc$ (194)	KZnSb	35	37	0	0	False	-
mp-31147	$Ba_2Zn_2Si_2$	6	$P6_3/mmc$ (194)	KZnSb	34	32	0	0	False	-
mp-31148	$Ba_2Sn_2Zn_2$	6	$P6_3/mmc$ (194)	KZnSb	32	49	0	0	False	-
mp-4336	$La_2Pd_2P_2$	6	$P6_3/mmc$ (194)	KZnSb	36	33	0	0	False	-
mp-4835	$Cu_2La_2Si_2$	6	$P6_3/mmc$ (194)	KZnSb	34	32	0	0	False	-
mp-985439	$Au_2Ba_2P_2$	6	$P6_3/mmc$ (194)	KZnSb	33	32	0	0	False	-
mp-10200	$Be_2Zr_2Si_2$	6	$P6_3/mmc$ (194)	KZnSb	33	36	0	0	False	-
mp-11700	$Ba_2Cu_2Sb_2$	6	$P6_3/mmc$ (194)	KZnSb	34	48	0	0	False	-
mp-16254	$Ba_2Cu_2P_2$	6	$P6_3/mmc$ (194)	KZnSb	33	26	0	0	False	-
mp-16263	$Ca_2Zn_2Ge_2$	6	$P6_3/mmc$ (194)	KZnSb	34	32	0	0	False	-
mp-16266	$Ca_2Zn_2Si_2$	6	$P6_3/mmc$ (194)	KZnSb	34	32	0	0	False	-
mp-1019279	$Sn_2Sr_2Zn_2$	6	$P6_3/mmc$ (194)	KZnSb	35	38	0	0	True	1.79
mp-3553	$Au_2K_2Te_2$	6	$P6_3/mmc$ (194)	KZnSb	34	33	0	0	False	-
mp-510311	$Ba_2Pb_2Zn_2$	6	$P6_3/mmc$ (194)	KZnSb	35	38	0	0	False	-
mp-11816	$Ba_2Cd_2Ge_2$	6	$P6_3/mmc$ (194)	KZnSb	34	44	0	0	False	-
mp-8948	$Ce_2Pd_2As_2$	6	$P6_3/mmc$ (194)	KZnSb	36	26	0	0	False	-
mp-12571	$Be_2Hf_2Si_2$	6	$P6_3/mmc$ (194)	KZnSb	32	35	0	0	False	-
mp-5910	$Ca_2Cu_2Sb_2$	6	$P6_3/mmc$ (194)	KZnSb	34	37	0	0	True	13.23
mp-7003	$Ce_2Pd_2P_2$	6	$P6_3/mmc$ (194)	KZnSb	33	38	0	0	False	-
mp-7025	$Au_2Rb_2Te_2$	6	$P6_3/mmc$ (194)	KZnSb	33	37	0	0	False	-
mp-7359	$Ag_2Ba_2As_2$	6	$P6_3/mmc$ (194)	KZnSb	34	45	0	0	False	-
mp-7420	$Hg_2K_2As_2$	6	$P6_3/mmc$ (194)	KZnSb	33	42	0	0	False	-
mp-7435	$Cu_2K_2Se_2$	6	$P6_3/mmc$ (194)	KZnSb	33	47	0	0	False	-
mp-8126	$Cu_2Y_2Si_2$	6	$P6_3/mmc$ (194)	KZnSb	35	38	0	0	False	-
mp-8432	$Ca_2Cu_2P_2$	6	$P6_3/mmc$ (194)	KZnSb	33	36	0	0	False	-
mp-8456	$Pt_2Sr_2As_2$	6	$P6_3/mmc$ (194)	KZnSb	34	49	0	0	False	-
mp-9556	$Sr_2Zn_2Si_2$	6	$P6_3/mmc$ (194)	KZnSb	35	37	0	0	False	-
mp-9898	$Ba_2Cu_2As_2$	6	$P6_3/mmc$ (194)	KZnSb	35	33	0	0	False	-
mp-10485	$Ba_2Li_2Sb_2$	6	$P6_3/mmc$ (194)	KZnSb	35	30	0	0	False	-
mp-13276	$Li_2Sr_2P_2$	6	$P6_3/mmc$ (194)	KZnSb	5	23	0	0	False	-
mp-13277	$Ba_2Li_2P_2$	6	$P6_3/mmc$ (194)	KZnSb	6	25	0	0	False	-

ID	Formula	Z	SP (*No.*)	Prototype	NL	NR	WPs	HDWPs	Clean	dE/dq
mp-7436	$Cu_2K_2Te_2$	6	$P6_3/mmc$ (194)	KZnSb	6	29	0	0	False	-
mp-7437	$K_2Zn_2P_2$	6	$P6_3/mmc$ (194)	KZnSb	17	38	0	0	False	-
mp-7438	$K_2Zn_2Sb_2$	6	$P6_3/mmc$ (194)	KZnSb	6	35	0	0	False	-
mp-7738	$La_2F_2Se_2$	6	$P6_3/mmc$ (194)	KZnSb	15	47	0	0	False	-
mp-9244	$Li_2B_2C_2$	6	$P6_3/mmc$ (194)	KZnSb	6	43	0	0	False	-
mp-9573	$Be_2Na_2As_2$	6	$P6_3/mmc$ (194)	KZnSb	10	45	0	0	True	1.4
mp-9574	$Be_2Na_2Sb_2$	6	$P6_3/mmc$ (194)	KZnSb	15	41	0	0	False	-
mp-1013525	V_2S_4	6	$P6_3/mmc$ (194)	C7-H-MoS_2	33	37	0	0	False	-
mp-1077788	Pt_4B_2	6	$P6_3/mmc$ (194)	C7-H-MoS_2	32	47	0	0	False	-
mp-1634	Mo_2Se_4	6	$P6_3/mmc$ (194)	C7-H-MoS_2	33	34	0	0	False	-
mp-2815	Mo_2S_4	6	$P6_3/mmc$ (194)	C7-H-MoS_2	33	25	0	0	False	-
mp-974437	Re_4C_2	6	$P6_3/mmc$ (194)	C7-H-MoS_2	35	31	0	0	False	-
mp-1821	W_2Se_4	6	$P6_3/mmc$ (194)	C7-H-MoS_2	15	36	0	0	False	-
mp-224	W_2S_4	6	$P6_3/mmc$ (194)	C7-H-MoS_2	16	38	0	0	False	-
mp-602	Mo_2Te_4	6	$P6_3/mmc$ (194)	C7-H-MoS_2	16	35	0	0	False	-
mp-1018713	$Cu_2Hf_2Sn_2$	6	$P6_3mc$ (186)	YGeAu	34	35	1	3	False	-
mp-1071260	$Au_2La_2Sn_2$	6	$P6_3mc$ (186)	YGeAu	36	42	1	3	False	-
mp-7874	$Ga_2Li_2Ge_2$	6	$P6_3mc$ (186)	YGeAu	35	49	0	2	False	-
mp-1096880	$Ca_2Pb_2Zn_2$	6	$P6_3mc$ (186)	YGeAu	34	49	0	2	False	-
mp-1096897	$Ce_2Cu_2Pb_2$	6	$P6_3mc$ (186)	YGeAu	33	39	0	2	False	-
mp-20568	$Ag_2La_2Ge_2$	6	$P6_3mc$ (186)	YGeAu	36	34	1	3	False	-
mp-22683	$Ce_2Cu_2Sn_2$	6	$P6_3mc$ (186)	YGeAu	33	36	0	3	False	-
mp-31160	$Ca_2Sn_2Zn_2$	6	$P6_3mc$ (186)	YGeAu	35	32	1	2	False	-
mp-10098	$Au_2Y_2Ge_2$	6	$P6_3mc$ (186)	YGeAu	34	31	0	3	False	-
mp-1019259	$Hg_2Sn_2Sr_2$	6	$P6_3mc$ (186)	YGeAu	36	42	0	3	False	-
mp-1025082	$La_2Pd_2Sb_2$	6	$P6_3mc$ (186)	YGeAu	35	38	0	2	False	-
mp-1025128	$Cu_2La_2Pb_2$	6	$P6_3mc$ (186)	YGeAu	33	43	0	3	False	-
mp-11081	$Au_2Ce_2Sn_2$	6	$P6_3mc$ (186)	YGeAu	33	51	0	1	False	-
mp-19983	$Cu_2Sn_2Ti_2$	6	$P6_3mc$ (186)	YGeAu	34	40	0	3	False	-
mp-5729	$Ag_2Sn_2Y_2$	6	$P6_3mc$ (186)	YGeAu	35	48	1	3	False	-
mp-610631	$Au_2La_2Ge_2$	6	$P6_3mc$ (186)	YGeAu	34	35	1	3	False	-
mp-13406	$Ag_2La_2Pb_2$	6	$P6_3mc$ (186)	YGeAu	33	41	0	2	False	-
mp-7868	Pt_2O_4	6	$P6_3mc$ (186)	PtO_2	14	50	0	0	False	-
mp-27934	Cd_2Br_4	6	$P6_3mc$ (186)	PbI_2	13	44	0	1	False	-
mp-28248	Cd_2I_4	6	$P6_3mc$ (186)	PbI_2	3	50	1	0	False	-
mp-567503	Pb_2I_4	6	$P6_3mc$ (186)	PbI_2	14	59	0	1	False	-
mp-9575	$Be_2Li_2Sb_2$	6	$P6_3mc$ (186)	LiBeSb	9	38	0	0	False	-
mp-9919	$Li_2Zn_2Sb_2$	6	$P6_3mc$ (186)	LiBeSb	7	26	0	0	False	-
mp-4219	$Au_2Ce_2Ge_2$	6	$P6_3mc$ (186)	KZnSb	34	42	0	2	False	-
mp-28944	$Bi_2Cl_2Te_2$	6	$P6_3mc$ (186)	BiTeCl	6	45	0	1	False	-
mp-29529	$Na_2Sn_2P_2$	6	$P6_3mc$ (186)	BiTeCl	5	46	0	0	False	-
mp-14093	$Tb_6F_6O_6$	6	$R{-}3m$ (166)	R-LaOF	8	37	0	0	False	-
mp-3637	$Y_6F_6O_6$	6	$R{-}3m$ (166)	R-LaOF	7	38	0	0	False	-
mp-5634	$Nd_6F_6O_6$	6	$R{-}3m$ (166)	R-LaOF	7	40	0	0	False	-
mp-769173	$Pr_6F_6O_6$	6	$R{-}3m$ (166)	R-LaOF	8	37	0	0	False	-
mp-8111	$La_6F_6O_6$	6	$R{-}3m$ (166)	R-LaOF	8	35	0	0	False	-
mp-8226	$Th_6F_6N_6$	6	$R{-}3m$ (166)	R-LaOF	6	44	0	0	False	-
mp-9488	$Sm_6F_6O_6$	6	$R{-}3m$ (166)	R-LaOF	8	35	0	0	False	-
mp-540789	Pb_6I_{12}	6	$R{-}3m$ (166)	PbI_2	7	57	0	0	False	-
mp-569595	Pb_6I_{12}	6	$R{-}3m$ (166)	PbI_2	8	48	0	0	False	-
mp-555850	$Lu_6F_6S_6$	6	$R{-}3m$ (166)	LuSF	8	43	0	0	True	33.45
mp-29149	$Li_{12}Cl_3N_3$	6	$R{-}3m$ (166)	Li_4	6	35	0	0	False	-
mp-36329	$Sr_3U_3O_{12}$	6	$R{-}3m$ (166)	Li_4	7	26	0	0	False	-
mp-3960	$Ca_3U_3O_{12}$	6	$R{-}3m$ (166)	Li_4	8	29	0	0	False	-
mp-754852	$Ba_3Sr_3I_{12}$	6	$R{-}3m$ (166)	Li_4	11	44	0	0	False	-
mp-30302	$Hf_6Br_6N_6$	6	$R{-}3m$ (166)	HfBrN	5	45	0	0	False	-
mp-541912	$Zr_6Br_6N_6$	6	$R{-}3m$ (166)	HfBrN	3	43	0	0	False	-
mp-567441	$Hf_6I_6N_6$	6	$R{-}3m$ (166)	HfBrN	6	43	0	0	False	-
mp-580886	$Zr_6I_6N_6$	6	$R{-}3m$ (166)	HfBrN	5	36	0	0	False	-
mp-551456	$Ba_6Cu_3Cl_3O_6$	6	$R{-}3m$ (166)	Ba_2CuClO_2	8	27	0	0	False	-
mp-552537	$Cu_3Sr_6Br_3O_6$	6	$R{-}3m$ (166)	Ba_2CuClO_2	7	33	0	0	False	-
mp-552934	$Ba_6Cu_3Br_3O_6$	6	$R{-}3m$ (166)	Ba_2CuClO_2	8	35	0	0	False	-

ID	Formula	Z	SP (*No.*)	Prototype	NL	NR	WPs	HDWPs	Clean	dE/dq
mp-553303	$CsCu_3O_2$	6	$P-3m1$ (164)	$CsCu_3O_2$	12	32	0	0	True	16.69
mp-7786	$CsCu_3S_2$	6	$P-3m1$ (164)	$CsCu_3O_2$	13	31	0	0	True	10.48
mp-9385	Au_3RbSe_2	6	$P-3m1$ (164)	$CsCu_3O_2$	14	23	0	0	False	-
mp-9386	Au_3CsSe_2	6	$P-3m1$ (164)	$CsCu_3O_2$	16	24	0	0	False	-
mp-9252	Hg_3S_3	6	$P3_221$ (154)	HgS	0	0	30	0	True	5.93
mp-634	Hg_3S_3	6	$P3_121$ (152)	HgS	0	0	30	0	True	5.93
mp-7826	Hg_3O_3	6	$P3_121$ (152)	HgS	0	1	33	0	False	-
mp-561243	$Cu_3Sr_6Cl_3O_6$	6	$P3_121$ (152)	Ba_2CuClO_2	21	25	243	2	False	-
mp-147	Se_{18}	6	$R-3$ (148)	Se	12	15	0	0	False	-
mp-7790	Ti_8N_4	6	$I4_1/amd$ (141)	C5-Anatase	27	35	0	0	True	18.81
mp-390	Ti_4O_8	6	$I4_1/amd$ (141)	C5-Anatase	27	39	0	0	False	-
mp-754403	Zr_4O_8	6	$I4_1/amd$ (141)	C5-Anatase	27	37	0	0	False	-
mp-1950	Sr_4S_8	6	$I4/mcm$ (140)	SrS_2	9	41	0	0	False	-
mp-2574	Zr_2O_4	6	$P4_2/nmc$ (137)	ZrO_2	46	48	0	0	False	-
mp-567279	Zn_2Cl_4	6	$P4_2/nmc$ (137)	$ZnCl_2$	47	54	0	0	False	-
mp-1187158	Sr_2Br_4	6	$P4_2/mnm$ (136)	C4-Rutile	45	28	0	0	False	-
mp-1187178	Sr_2I_4	6	$P4_2/mnm$ (136)	C4-Rutile	45	18	0	0	False	-
mp-470	Ge_2O_4	6	$P4_2/mnm$ (136)	C4-Rutile	45	25	0	0	False	-
mp-1014265	Zr_4N_2	6	$P4_2/mnm$ (136)	C4-Rutile	45	23	0	0	False	-
mp-20725	Pb_2O_4	6	$P4_2/mnm$ (136)	C4-Rutile	45	24	0	0	False	-
mp-2723	Ir_2O_4	6	$P4_2/mnm$ (136)	C4-Rutile	45	29	0	0	False	-
mp-556520	Co_2F_4	6	$P4_2/mnm$ (136)	C4-Rutile	45	26	0	0	False	-
mp-559798	Ni_2F_4	6	$P4_2/mnm$ (136)	C4-Rutile	45	33	0	0	False	-
mp-725	Rh_2O_4	6	$P4_2/mnm$ (136)	C4-Rutile	45	25	0	0	False	-
mp-864647	Hf_4N_2	6	$P4_2/mnm$ (136)	C4-Rutile	45	34	0	0	False	-
mp-996	Os_2O_4	6	$P4_2/mnm$ (136)	C4-Rutile	45	13	0	0	False	-
mp-825	Ru_2O_4	6	$P4_2/mnm$ (136)	C4-Rutile	45	17	0	0	False	-
mp-23710	Mg_2H_4	6	$P4_2/mnm$ (136)	C4-Rutile	45	16	0	0	False	-
mp-2657	Ti_2O_4	6	$P4_2/mnm$ (136)	C4-Rutile	45	35	0	0	False	-
mp-776532	Hf_2O_4	6	$P4_2/mnm$ (136)	C4-Rutile	45	34	0	0	False	-
mp-856	Sn_2O_4	6	$P4_2/mnm$ (136)	C4-Rutile	45	25	0	0	False	-
mp-12620	$Nd_2F_2Se_2$	6	$P4/nmm$ (129)	SmIO	37	48	0	0	False	-
mp-12621	$Nd_2F_2Te_2$	6	$P4/nmm$ (129)	SmIO	38	58	0	0	False	-
mp-22939	$Bi_2Cl_2O_2$	6	$P4/nmm$ (129)	SmIO	39	47	0	0	False	-
mp-22969	$Pb_2F_2I_2$	6	$P4/nmm$ (129)	SmIO	38	48	0	0	False	-
mp-22987	$Bi_2I_2O_2$	6	$P4/nmm$ (129)	SmIO	39	52	0	0	False	-
mp-23008	$Pb_2Br_2F_2$	6	$P4/nmm$ (129)	SmIO	37	59	0	0	False	-
mp-23023	$La_2Br_2O_2$	6	$P4/nmm$ (129)	SmIO	39	52	0	0	False	-
mp-23046	$Sr_2F_2I_2$	6	$P4/nmm$ (129)	SmIO	38	51	0	0	False	-
mp-23068	$Nd_2Br_2O_2$	6	$P4/nmm$ (129)	SmIO	38	45	0	0	False	-
mp-23072	$Bi_2Br_2O_2$	6	$P4/nmm$ (129)	SmIO	39	51	0	0	False	-
mp-23859	$Ca_2Cl_2H_2$	6	$P4/nmm$ (129)	SmIO	38	44	0	0	False	-
mp-24204	$Ca_2H_2I_2$	6	$P4/nmm$ (129)	SmIO	37	42	0	0	False	-
mp-24205	$Sr_2H_2I_2$	6	$P4/nmm$ (129)	SmIO	38	46	0	0	False	-
mp-24422	$Ca_2Br_2H_2$	6	$P4/nmm$ (129)	SmIO	38	37	0	0	False	-
mp-27439	$Tm_2I_2O_2$	6	$P4/nmm$ (129)	SmIO	39	48	0	0	False	-
mp-28066	$Th_2Br_2N_2$	6	$P4/nmm$ (129)	SmIO	38	37	0	0	False	-
mp-28067	$Th_2I_2N_2$	6	$P4/nmm$ (129)	SmIO	38	41	0	0	False	-
mp-29254	$Pr_2I_2O_2$	6	$P4/nmm$ (129)	SmIO	39	35	0	0	False	-
mp-29327	$Sm_2Br_2O_2$	6	$P4/nmm$ (129)	SmIO	39	47	0	0	False	-
mp-30993	$La_2I_2O_2$	6	$P4/nmm$ (129)	SmIO	38	43	0	0	False	-
mp-753173	$Ho_2I_2O_2$	6	$P4/nmm$ (129)	SmIO	37	37	0	0	False	-
mp-753712	$Y_2I_2O_2$	6	$P4/nmm$ (129)	SmIO	36	39	0	0	False	-
mp-754217	$Sm_2I_2O_2$	6	$P4/nmm$ (129)	SmIO	36	50	0	0	False	-
mp-754969	$Tm_2Br_2O_2$	6	$P4/nmm$ (129)	SmIO	38	53	0	0	False	-
mp-755336	$Nd_2I_2O_2$	6	$P4/nmm$ (129)	SmIO	36	41	0	0	False	-
mp-768906	$Y_2Br_2O_2$	6	$P4/nmm$ (129)	SmIO	36	39	0	0	False	-
mp-973585	$Lu_2I_2O_2$	6	$P4/nmm$ (129)	SmIO	38	55	0	0	False	-
mp-973981	$Lu_2Br_2O_2$	6	$P4/nmm$ (129)	SmIO	37	40	0	0	False	-
mp-975666	$Pr_2Br_2O_2$	6	$P4/nmm$ (129)	SmIO	37	58	0	0	False	-
mp-10932	$Er_2F_2S_2$	6	$P4/nmm$ (129)	RbNaS	39	59	0	0	False	-
mp-16236	$Ag_2K_2Se_2$	6	$P4/nmm$ (129)	RbNaS	37	56	0	0	False	-

ID	Formula	Z	SP (*No.*)	Prototype	NL	NR	WPs	HDWPs	Clean	dE/dq
mp-22951	$Ba_2F_2I_2$	6	$P4/nmm$ (129)	RbNaS	37	46	0	0	False	-
mp-22957	$Sr_2Cl_2F_2$	6	$P4/nmm$ (129)	RbNaS	39	48	0	0	False	-
mp-22964	$Pb_2Cl_2F_2$	6	$P4/nmm$ (129)	RbNaS	37	59	0	0	False	-
mp-23024	$Sr_2Br_2F_2$	6	$P4/nmm$ (129)	RbNaS	38	58	0	0	False	-
mp-23025	$La_2Cl_2O_2$	6	$P4/nmm$ (129)	RbNaS	39	54	0	0	False	-
mp-23058	$Nd_2Cl_2O_2$	6	$P4/nmm$ (129)	RbNaS	40	48	0	0	False	-
mp-23070	$Ba_2Br_2F_2$	6	$P4/nmm$ (129)	RbNaS	37	48	0	0	False	-
mp-23432	$Ba_2Cl_2F_2$	6	$P4/nmm$ (129)	RbNaS	37	52	0	0	False	-
mp-23860	$Sr_2Cl_2H_2$	6	$P4/nmm$ (129)	RbNaS	40	43	0	0	False	-
mp-23861	$Ba_2Cl_2H_2$	6	$P4/nmm$ (129)	RbNaS	39	41	0	0	False	-
mp-23862	$Ba_2H_2I_2$	6	$P4/nmm$ (129)	RbNaS	38	48	0	0	False	-
mp-24423	$Sr_2Br_2H_2$	6	$P4/nmm$ (129)	RbNaS	38	42	0	0	False	-
mp-24424	$Ba_2Br_2H_2$	6	$P4/nmm$ (129)	RbNaS	38	48	0	0	False	-
mp-27546	$Ca_2Cl_2F_2$	6	$P4/nmm$ (129)	RbNaS	39	52	0	0	False	-
mp-27823	$Sm_2Cl_2O_2$	6	$P4/nmm$ (129)	RbNaS	36	60	0	0	False	-
mp-27984	$Pr_2Cl_2O_2$	6	$P4/nmm$ (129)	RbNaS	39	49	0	0	False	-
mp-27985	$Y_2Cl_2O_2$	6	$P4/nmm$ (129)	RbNaS	37	56	0	0	False	-
mp-28065	$Th_2Cl_2N_2$	6	$P4/nmm$ (129)	RbNaS	39	36	0	0	False	-
mp-29731	$Ho_2Cl_2O_2$	6	$P4/nmm$ (129)	RbNaS	38	51	0	0	False	-
mp-4495	$K_2Li_2Te_2$	6	$P4/nmm$ (129)	RbNaS	38	44	0	0	False	-
mp-4824	$Na_2Zn_2P_2$	6	$P4/nmm$ (129)	RbNaS	36	52	0	0	False	-
mp-5339	$Cs_2Na_2Te_2$	6	$P4/nmm$ (129)	RbNaS	40	48	0	0	False	-
mp-557483	$Yb_2Cl_2F_2$	6	$P4/nmm$ (129)	RbNaS	37	41	0	0	False	-
mp-5962	$Mg_2Na_2As_2$	6	$P4/nmm$ (129)	RbNaS	38	42	0	0	False	-
mp-7089	$K_2Mg_2Sb_2$	6	$P4/nmm$ (129)	RbNaS	37	43	0	0	False	-
mp-7090	$Mg_2Na_2Sb_2$	6	$P4/nmm$ (129)	RbNaS	37	44	0	0	False	-
mp-755323	$Dy_2Cl_2O_2$	6	$P4/nmm$ (129)	RbNaS	37	57	0	0	False	-
mp-773137	$Bi_2Ca_2K_2$	6	$P4/nmm$ (129)	RbNaS	36	40	0	0	False	-
mp-7950	$Th_2O_2Se_2$	6	$P4/nmm$ (129)	RbNaS	38	39	0	0	False	-
mp-8430	$K_2Li_2S_2$	6	$P4/nmm$ (129)	RbNaS	38	51	0	0	False	-
mp-8452	$Li_2Na_2S_2$	6	$P4/nmm$ (129)	RbNaS	38	37	0	0	False	-
mp-8658	$Cs_2Na_2Se_2$	6	$P4/nmm$ (129)	RbNaS	40	31	0	0	False	-
mp-8751	$Li_2Rb_2S_2$	6	$P4/nmm$ (129)	RbNaS	38	41	0	0	False	-
mp-8756	$K_2Li_2Se_2$	6	$P4/nmm$ (129)	RbNaS	37	51	0	0	False	-
mp-8799	$Na_2Rb_2S_2$	6	$P4/nmm$ (129)	RbNaS	38	46	0	0	False	-
mp-9250	$Li_2Rb_2Se_2$	6	$P4/nmm$ (129)	RbNaS	38	40	0	0	False	-
mp-9562	$Be_2Li_2As_2$	6	$P4/nmm$ (129)	RbNaS	41	31	0	0	False	-
mp-9845	$Ca_2Rb_2As_2$	6	$P4/nmm$ (129)	RbNaS	37	45	0	0	False	-
mp-9915	$Be_2Li_2P_2$	6	$P4/nmm$ (129)	RbNaS	38	38	0	0	False	-
mp-10219	$Y_2F_2O_2$	6	$P4/nmm$ (129)	LaOF	38	53	0	0	False	-
mp-7100	$La_2F_2O_2$	6	$P4/nmm$ (129)	LaOF	39	63	0	0	False	-
mp-753594	$Bi_2F_2O_2$	6	$P4/nmm$ (129)	LaOF	37	61	0	0	False	-
mp-10074	Ge_4Se_8	6	$I-42d$ (122)	$GeSe_2$	27	15	13	0	True	16.38
mp-7582	Ge_4S_8	6	$I-42d$ (122)	$GeSe_2$	27	24	14	0	False	-
mp-22993	$Ag_2Cl_2O_8$	6	$I-42m$ (121)	$AgClO_4$	0	12	8	0	False	-
mp-38103	$Na_2Cl_2O_8$	6	$I-42m$ (121)	$AgClO_4$	0	15	13	0	False	-
mp-569055	$CsLi_2I_3$	6	$P-4m2$ (115)	$CsLi_2I_3$	1	37	21	0	False	-
mp-545359	$Cu_2K_2Na_4O_4$	6	$I4mm$ (107)	KNa_2CuO_2	0	18	2	0	False	-
mp-27743	Bi_2F_{10}	6	$I4/m$ (87)	BiF_5	1	26	0	0	False	-
mp-20790	$In_2P_2S_8$	6	$I-4$ (82)	$InPS_4$	0	0	0	0	False	-
mp-29403	$Cu_2Ga_2I_8$	6	$I-4$ (82)	$InPS_4$	0	1	0	0	False	-
mp-570886	Be_4I_8	6	$Ibam$ (72)	BeI_2	18	30	0	0	False	-
mp-569117	$Cs_2Li_4Cl_6$	6	$Immm$ (71)	$CsLi_2Cl_3$	0	44	0	0	False	-
mp-545598	$Cd_2U_2O_8$	6	$Cmmm$ (65)	$UCdO_4$	0	23	0	0	False	-
mp-754478	$Hf_2Zr_2O_8$	6	$Cmmm$ (65)	$UCdO_4$	0	33	0	0	False	-
mp-7077	$Au_4K_4S_4$	6	$Cmcm$ (63)	KAuS	28	14	0	0	False	-
mp-9010	$Au_4Rb_4S_4$	6	$Cmcm$ (63)	KAuS	31	29	0	0	False	-
mp-753826	$Cu_4Li_4S_4$	6	$Pnma$ (62)	RbNaS	174	87	0	0	False	-
mp-27850	$Ti_2Cl_2N_2$	6	$Pmmn$ (59)	SmIO	63	57	0	0	True	94.4
mp-27702	$In_2Cl_2O_2$	6	$Pmmn$ (59)	InClO	64	42	0	0	True	18.16
mp-27703	$In_2Br_2O_2$	6	$Pmmn$ (59)	InClO	63	36	0	0	False	-
mp-28458	$Er_2I_2Se_2$	6	$Pmmn$ (59)	InClO	63	33	0	0	False	-

ID	Formula	Z	SP (No.)	Prototype	NL	NR	WPs	HDWPs	Clean	dE/dq
mp-568346	$Hf_2Br_2N_2$	6	*Pmmn* (59)	InClO	63	49	0	0	False	-
mp-570157	$Zr_2Br_2N_2$	6	*Pmmn* (59)	InClO	63	53	0	0	False	-
mp-571166	Ca_2Br_4	6	*Pnnm* (58)	P_2	81	34	0	0	False	-
mp-1413	Ru_2P_4	6	*Pnnm* (58)	P_2	81	29	0	0	False	-
mp-20027	Fe_2P_4	6	*Pnnm* (58)	P_2	81	34	0	0	False	-
mp-2319	Os_2P_4	6	*Pnnm* (58)	P_2	81	27	0	0	False	-
mp-2455	Os_2As_4	6	*Pnnm* (58)	P_2	82	27	0	0	False	-
mp-766	Ru_2As_4	6	*Pnnm* (58)	P_2	81	35	0	0	False	-
mp-1285	Pt_2O_4	6	*Pnnm* (58)	C4-Rutile	81	15	0	0	False	-
mp-1522	Fe_2S_4	6	*Pnnm* (58)	C4-Rutile	81	30	0	0	False	-
mp-22882	Yb_2Br_4	6	*Pnnm* (58)	C4-Rutile	81	22	0	0	False	-
mp-22888	Ca_2Br_4	6	*Pnnm* (58)	C4-Rutile	81	25	0	0	False	-
mp-23214	Ca_2Cl_4	6	*Pnnm* (58)	C4-Rutile	81	29	0	0	False	-
mp-550172	Sn_2O_4	6	*Pnnm* (58)	C4-Rutile	82	23	0	0	False	-
mp-755072	Na_4O_2	6	*Pnnm* (58)	C4-Rutile	82	34	0	0	False	-
mp-573755	$Au_2Cs_2Te_2$	6	*Pmma* (51)	CsTeAu	36	65	0	0	False	-
mp-9008	$Au_2Rb_2Te_2$	6	*Pmma* (51)	CsTeAu	36	56	0	0	False	-
mp-625136	$Cs_4H_4O_4$	6	$Cmc2_1$ (36)	CsHO	28	29	4	0	False	-
mp-626620	$Rb_4H_4O_4$	6	$Cmc2_1$ (36)	CsHO	31	19	4	0	False	-
mp-626785	$K_4H_4O_4$	6	$Cmc2_1$ (36)	CsHO	29	25	3	0	False	-
mp-28266	Pd_4P_8	6	$C2/c$ (15)	P_2	1	15	0	0	False	-
mp-486	Ni_4P_8	6	$C2/c$ (15)	P_2	2	17	0	0	False	-
mp-684	Ba_4S_8	6	$C2/c$ (15)	BaS_2	1	33	0	0	False	-
mp-7547	Ba_4Se_8	6	$C2/c$ (15)	BaS_2	0	38	0	0	False	-
mp-22945	$Rh_4Cl_4Te_4$	6	$C2/m$ (12)	TeRhCl	0	27	0	0	False	-
mp-28051	$I_4Sb_4Te_4$	6	$C2/m$ (12)	SbTeI	0	23	0	0	False	-
mp-867712	$Ag_2Li_2F_8$	6	$C2/m$ (12)	$LiAgF_4$	0	24	0	0	False	-
mp-27716	$K_4Tl_4O_4$	6	$C2/m$ (12)	KTlO	0	26	0	0	False	-
mp-634657	$Na_2H_2S_2$	6	$P2_1/m$ (11)	NaHS	27	54	0	0	False	-
mp-634676	$K_2H_2S_2$	6	$P2_1/m$ (11)	CsHO	28	48	0	0	False	-
mp-754540	$CaPbI_4$	6	$P2/m$ (10)	$CaPbI_4$	1	46	0	0	False	-
mp-27294	Ca_3AsBr_3	7	$Pm{-}3m$ (221)	Ca_3AsBr_3	13	28	0	0	False	-
mp-28069	Ca_3AsCl_3	7	$Pm{-}3m$ (221)	Ca_3AsBr_3	11	49	0	0	False	-
mp-29342	Ca_3Cl_3	7	$Pm{-}3m$ (221)	Ca_3AsBr_3	12	33	0	0	False	-
mp-7604	Mg_3F_3	7	$Pm{-}3m$ (221)	Ca_3AsBr_3	12	48	0	0	False	-
mp-6867	$CaKCFO_3$	7	$P{-}6m2$ (187)	$KCaCO_3$	0	8	6	0	False	-
mp-867261	$RbSrCFO_3$	7	$P{-}6m2$ (187)	$KCaCO_3$	0	15	9	0	False	-
mp-570097	$Li_{12}Sr_3P_6$	7	$R{-}3m$ (166)	$Sr(Li_2P)_2$	8	43	0	0	False	-
mp-7863	$Rb_6Sn_6O_9$	7	$R{-}3m$ (166)	$Rb_2Sn_2O_3$	8	41	0	0	True	38.01
mp-9479	$Al_6Mg_3Se_{12}$	7	$R{-}3m$ (166)	$Mg(AlSe_2)_2$	6	54	0	0	False	-
mp-11719	$K_{12}Zn_3P_6$	7	$R{-}3m$ (166)	K_4ZnP_2	10	45	0	0	False	-
mp-28302	$Cd_3K_{12}P_6$	7	$R{-}3m$ (166)	K_4ZnP_2	9	57	0	0	False	-
mp-28591	$Hg_3Na_{12}P_6$	7	$R{-}3m$ (166)	K_4ZnP_2	9	61	0	0	False	-
mp-29484	$Hg_3K_{12}As_6$	7	$R{-}3m$ (166)	K_4ZnP_2	8	40	0	0	False	-
mp-29584	$Be_3K_{12}As_6$	7	$R{-}3m$ (166)	K_4ZnP_2	9	39	0	0	False	-
mp-29585	$Cd_3K_{12}As_6$	7	$R{-}3m$ (166)	K_4ZnP_2	10	49	0	0	False	-
mp-571461	$K_{12}Zn_3As_6$	7	$R{-}3m$ (166)	K_4ZnP_2	10	57	0	0	False	-
mp-8753	$Hg_3K_{12}P_6$	7	$R{-}3m$ (166)	K_4ZnP_2	8	40	0	0	False	-
mp-9872	$Be_3K_{12}P_6$	7	$R{-}3m$ (166)	K_4ZnP_2	12	38	0	0	False	-
mp-27948	$Bi_6Ge_3Te_{12}$	7	$R{-}3m$ (166)	$Ge(BiTe_2)_2$	6	77	0	0	False	-
mp-38605	$Bi_6Sn_3Te_{12}$	7	$R{-}3m$ (166)	$Ge(BiTe_2)_2$	7	51	0	0	False	-
mp-675543	$Bi_6Pb_3Se_{12}$	7	$R{-}3m$ (166)	$Ge(BiTe_2)_2$	11	68	0	0	False	-
mp-676250	$Bi_6Pb_3Te_{12}$	7	$R{-}3m$ (166)	$Ge(BiTe_2)_2$	10	58	0	0	False	-
mp-1591	$Al_{12}C_9$	7	$R{-}3m$ (166)	Al_4C_3	10	46	0	0	False	-
mp-13038	$Dy_2CN_2O_2$	7	$P{-}3m1$ (164)	$Dy_2C(NO)_2$	16	39	0	0	False	-
mp-13039	$Tm_2CN_2O_2$	7	$P{-}3m1$ (164)	$Dy_2C(NO)_2$	18	35	0	0	False	-
mp-20374	$Ho_2CN_2O_2$	7	$P{-}3m1$ (164)	$Dy_2C(NO)_2$	16	40	0	0	False	-
mp-546864	$Y_2CN_2O_2$	7	$P{-}3m1$ (164)	$Dy_2C(NO)_2$	17	46	0	0	False	-
mp-6574	$Er_2CN_2O_2$	7	$P{-}3m1$ (164)	$Dy_2C(NO)_2$	17	39	0	0	False	-
mp-9497	$Pr_2CN_2O_2$	7	$P{-}3m1$ (164)	$Dy_2C(NO)_2$	15	42	0	0	False	-
mp-9498	$Nd_2CN_2O_2$	7	$P{-}3m1$ (164)	$Dy_2C(NO)_2$	15	46	0	0	False	-
mp-9499	$Sm_2CN_2O_2$	7	$P{-}3m1$ (164)	$Dy_2C(NO)_2$	14	45	0	0	False	-

ID	Formula	Z	SP ($No.$)	Prototype	NL	NR	WPs	HDWPs	Clean	dE/dq
mp-28483	W_3Br_{18}	7	$R-3$ (148)	WBr_6	9	0	0	0	False	-
mp-22937	$Na_8I_4O_2$	7	$I4/mmm$ (139)	Na_4I_2	0	38	0	0	False	-
mp-23752	$Cs_4Mg_2H_8$	7	$I4/mmm$ (139)	Na_4I_2	0	29	0	0	False	-
mp-23956	$K_4Mg_2H_8$	7	$I4/mmm$ (139)	Na_4I_2	0	32	0	0	False	-
mp-24517	$Ca_2Rb_4H_8$	7	$I4/mmm$ (139)	Na_4I_2	0	28	0	0	False	-
mp-28599	$Na_8Br_4O_2$	7	$I4/mmm$ (139)	Na_4I_2	0	46	0	0	False	-
mp-31212	$K_4Mg_2F_8$	7	$I4/mmm$ (139)	Na_4I_2	0	42	0	0	False	-
mp-3359	$Ba_4Sn_2O_8$	7	$I4/mmm$ (139)	Na_4I_2	0	29	0	0	False	-
mp-3808	$K_4U_2O_8$	7	$I4/mmm$ (139)	Na_4I_2	0	46	0	0	False	-
mp-5532	$Sr_4Ti_2O_8$	7	$I4/mmm$ (139)	Na_4I_2	0	66	0	0	False	-
mp-753192	$Yb_8O_2Sb_4$	7	$I4/mmm$ (139)	Na_4I_2	0	43	0	0	False	-
mp-754363	$Ba_4Hf_2O_8$	7	$I4/mmm$ (139)	Na_4I_2	0	40	0	0	False	-
mp-760417	$Rb_8I_4O_2$	7	$I4/mmm$ (139)	Na_4I_2	0	46	0	0	False	-
mp-768368	$K_8I_4O_2$	7	$I4/mmm$ (139)	Na_4I_2	0	31	0	0	False	-
mp-7726	$Rb_4U_2O_8$	7	$I4/mmm$ (139)	Na_4I_2	0	32	0	0	False	-
mp-8789	$Ca_8As_4O_2$	7	$I4/mmm$ (139)	Na_4I_2	0	62	0	0	False	-
mp-8861	$Mg_2Rb_4F_8$	7	$I4/mmm$ (139)	Na_4I_2	0	40	0	0	False	-
mp-9583	$K_4Zn_2F_8$	7	$I4/mmm$ (139)	Na_4I_2	0	54	0	0	False	-
mp-9799	$Yb_8As_4O_2$	7	$I4/mmm$ (139)	Na_4I_2	0	29	0	0	False	-
mp-643257	$K_4Pd_2H_8$	7	$I4/mmm$ (139)	K_2H_4	0	27	0	0	False	-
mp-643260	$Na_4Pd_2H_8$	7	$I4/mmm$ (139)	K_2H_4	0	23	0	0	False	-
mp-697096	$Na_4Pt_2H_8$	7	$I4/mmm$ (139)	K_2H_4	0	24	0	0	False	-
mp-22934	K_2PtCl_4	7	$P4/mmm$ (123)	K_2PtCl_4	0	29	0	0	False	-
mp-22956	K_2PdCl_4	7	$P4/mmm$ (123)	K_2PtCl_4	0	30	0	0	False	-
mp-27138	K_2PdBr_4	7	$P4/mmm$ (123)	K_2PtCl_4	3	29	0	0	False	-
mp-27243	K_2PtBr_4	7	$P4/mmm$ (123)	K_2PtCl_4	4	31	0	0	False	-
mp-29889	$PdTl_2Cl_4$	7	$P4/mmm$ (123)	K_2PtCl_4	0	19	0	0	False	-
mp-30315	Ca_3BN_3	7	$P4/mmm$ (123)	Ca_3BN_3	6	54	0	0	False	-
mp-35539	$Zn_4Ge_2Se_8$	7	$I-42m$ (121)	Zn_2GeSe_4	0	13	9	0	False	-
mp-568661	$Cd_2In_4Se_8$	7	$I-42m$ (121)	Zn_2GeSe_4	0	21	20	0	False	-
mp-675748	$Zn_4Ge_2S_8$	7	$I-42m$ (121)	Zn_2GeSe_4	0	14	10	0	False	-
mp-557373	$Cu_4W_2S_8$	7	$I-42m$ (121)	Cu_2WS_4	0	14	9	0	False	-
mp-8976	Cu_2WS_4	7	$P-42m$ (111)	Cu_2WS_4	0	13	23	0	False	-
mp-568032	$CdIn_2Se_4$	7	$P-42m$ (111)	$Cd(InSe_2)_2$	0	32	48	0	False	-
mp-570256	Ag_2HgI_4	7	$P-42m$ (111)	$Cd(InSe_2)_2$	0	22	39	0	False	-
mp-33985	$Ba_2In_4Te_8$	7	$I422$ (97)	$Ba(InTe_2)_2$	0	1	43	0	False	-
mp-37091	$Al_4Sr_2Te_8$	7	$I422$ (97)	$Ba(InTe_2)_2$	0	2	43	0	False	-
mp-38117	$Ba_2Ga_4Te_8$	7	$I422$ (97)	$Ba(InTe_2)_2$	0	1	50	0	False	-
mp-38394	$Al_4Ba_2Te_8$	7	$I422$ (97)	$Ba(InTe_2)_2$	0	1	48	0	False	-
mp-2371	Ga_4Te_{10}	7	$I4/m$ (87)	Ga_2Te_5	2	43	0	0	False	-
mp-11175	$Li_2Zn_2P_2S_8$	7	$I-4$ (82)	$LiZnPS_4$	0	0	2	0	False	-
mp-13949	$Cd_2Ga_4Te_8$	7	$I-4$ (82)	$Cd(GaTe_2)_2$	0	5	3	0	False	-
mp-15777	$Ga_4Zn_2Te_8$	7	$I-4$ (82)	$Cd(GaTe_2)_2$	0	4	5	0	False	-
mp-16337	$Ga_4Hg_2Te_8$	7	$I-4$ (82)	$Cd(GaTe_2)_2$	0	4	4	0	False	-
mp-19765	$Hg_2In_4Te_8$	7	$I-4$ (82)	$Cd(GaTe_2)_2$	0	1	6	0	False	-
mp-20731	$Hg_2In_4Se_8$	7	$I-4$ (82)	$Cd(GaTe_2)_2$	0	0	3	0	False	-
mp-20832	$In_4Zn_2Te_8$	7	$I-4$ (82)	$Cd(GaTe_2)_2$	0	1	2	0	False	-
mp-21374	$Cd_2In_4Te_8$	7	$I-4$ (82)	$Cd(GaTe_2)_2$	0	1	1	0	False	-
mp-22304	$Cd_2In_4Se_8$	7	$I-4$ (82)	$Cd(GaTe_2)_2$	0	0	2	0	False	-
mp-22607	$In_4Zn_2Se_8$	7	$I-4$ (82)	$Cd(GaTe_2)_2$	0	0	4	0	False	-
mp-23485	$Ag_4Hg_2I_8$	7	$I-4$ (82)	$Cd(GaTe_2)_2$	0	0	3	0	False	-
mp-3038	$Al_4Hg_2Se_8$	7	$I-4$ (82)	$Cd(GaTe_2)_2$	0	3	1	0	False	-
mp-3159	$Al_4Cd_2Se_8$	7	$I-4$ (82)	$Cd(GaTe_2)_2$	0	0	4	0	False	-
mp-3167	$Hg_4Ge_2Se_8$	7	$I-4$ (82)	$Cd(GaTe_2)_2$	0	0	2	0	False	-
mp-3772	$Cd_2Ga_4Se_8$	7	$I-4$ (82)	$Cd(GaTe_2)_2$	0	1	4	0	False	-
mp-4452	$Cd_2Ga_4S_8$	7	$I-4$ (82)	$Cd(GaTe_2)_2$	0	0	3	0	False	-
mp-4730	$Ga_4Hg_2Se_8$	7	$I-4$ (82)	$Cd(GaTe_2)_2$	0	1	4	0	False	-
mp-4809	$Ga_4Hg_2S_8$	7	$I-4$ (82)	$Cd(GaTe_2)_2$	0	1	5	0	False	-
mp-5928	$Al_4Cd_2S_8$	7	$I-4$ (82)	$Cd(GaTe_2)_2$	0	0	2	0	False	-
mp-7906	$Al_4Hg_2S_8$	7	$I-4$ (82)	$Cd(GaTe_2)_2$	0	0	2	0	False	-
mp-7907	$Al_4Zn_2Se_8$	7	$I-4$ (82)	$Cd(GaTe_2)_2$	0	2	4	0	False	-
mp-7908	$Al_4Zn_2Te_8$	7	$I-4$ (82)	$Cd(GaTe_2)_2$	0	3	2	0	False	-

ID	Formula	Z	SP ($No.$)	Prototype	NL	NR	WPs	HDWPs	Clean	dE/dq
mp-7909	$Al_4Cd_2Te_8$	7	$I-4$ (82)	$Cd(GaTe_2)_2$	0	0	5	0	False	-
mp-7910	$Al_4Hg_2Te_8$	7	$I-4$ (82)	$Cd(GaTe_2)_2$	0	1	3	0	False	-
mp-24148	$La_4Li_2H_2O_6$	7	$Immm$ (71)	$LiLa_2HO_3$	0	52	0	0	False	-
mp-24150	$Li_2Nd_4H_2O_6$	7	$Immm$ (71)	$LiLa_2HO_3$	0	48	0	0	False	-
mp-22967	$Fe_2Li_4Br_8$	7	$Cmmm$ (65)	Li_2FeBr_4	0	36	0	0	False	-
mp-29009	$Li_4Mg_2Br_8$	7	$Cmmm$ (65)	Li_2FeBr_4	0	32	0	0	False	-
mp-29216	$Cs_8Pt_4Cl_{16}$	7	$Cmcm$ (63)	K_2PtCl_4	69	78	0	0	False	-
mp-30314	$Cs_8Pd_4Cl_{16}$	7	$Cmcm$ (63)	K_2PtCl_4	68	78	0	0	False	-
mp-29761	$U_2C_2O_{10}$	7	$Imm2$ (44)	UCO_5	0	15	6	0	False	-
mp-567655	$Sr_4C_2Cl_4N_4$	7	$C2/m$ (12)	$Sr_2C(NCl)_2$	0	24	0	0	False	-
mp-27315	$Ca_4Ti_4F_{24}$	8	$Fm-3m$ (225)	$CaTiF_6$	9	24	0	0	False	-
mp-30871	Sn_2Ta_6	8	$Pm-3n$ (223)	A15-Cr_3	28	39	0	0	False	-
mp-1079399	Fe_2V_6	8	$Pm-3n$ (223)	A15-Cr_3	28	51	0	0	False	-
mp-1079796	Pd_2Ti_6	8	$Pm-3n$ (223)	A15-Cr_3	26	35	0	0	False	-
mp-11263	Au_2Zr_6	8	$Pm-3n$ (223)	A15-Cr_3	25	37	0	0	False	-
mp-1213747	Cr_6As_2	8	$Pm-3n$ (223)	A15-Cr_3	31	40	0	0	False	-
mp-1231	Cr_6Ga_2	8	$Pm-3n$ (223)	A15-Cr_3	28	31	0	0	False	-
mp-1275	Mo_6Si_2	8	$Pm-3n$ (223)	A15-Cr_3	31	45	0	0	False	-
mp-1458	Ir_2Nb_6	8	$Pm-3n$ (223)	A15-Cr_3	28	46	0	0	False	-
mp-1545	Nb_6Rh_2	8	$Pm-3n$ (223)	A15-Cr_3	26	46	0	0	False	-
mp-1555	V_6Sb_2	8	$Pm-3n$ (223)	A15-Cr_3	28	55	0	0	False	-
mp-1578	Rh_2V_6	8	$Pm-3n$ (223)	A15-Cr_3	26	44	0	0	False	-
mp-1585	Co_2V_6	8	$Pm-3n$ (223)	A15-Cr_3	29	46	0	0	False	-
mp-1609	Cr_6Ir_2	8	$Pm-3n$ (223)	A15-Cr_3	30	51	0	0	False	-
mp-1664	Pd_2V_6	8	$Pm-3n$ (223)	A15-Cr_3	30	45	0	0	False	-
mp-1786	Au_2Ti_6	8	$Pm-3n$ (223)	A15-Cr_3	26	42	0	0	False	-
mp-1932	Nb_6Os_2	8	$Pm-3n$ (223)	A15-Cr_3	27	47	0	0	False	-
mp-2006	Ir_2V_6	8	$Pm-3n$ (223)	A15-Cr_3	25	42	0	0	False	-
mp-20685	Cr_6Ge_2	8	$Pm-3n$ (223)	A15-Cr_3	30	32	0	0	False	-
mp-21342	Sn_2V_6	8	$Pm-3n$ (223)	A15-Cr_3	29	60	0	0	False	-
mp-2211	Pt_2V_6	8	$Pm-3n$ (223)	A15-Cr_3	27	50	0	0	False	-
mp-2339	Pt_2Ti_6	8	$Pm-3n$ (223)	A15-Cr_3	26	34	0	0	False	-
mp-2354	Hg_2Ti_6	8	$Pm-3n$ (223)	A15-Cr_3	25	41	0	0	False	-
mp-2377	Ga_2Mo_6	8	$Pm-3n$ (223)	A15-Cr_3	28	58	0	0	False	-
mp-24659	U_2H_6	8	$Pm-3n$ (223)	A15-Cr_3	28	35	0	0	False	-
mp-259	Al_2Mo_6	8	$Pm-3n$ (223)	A15-Cr_3	27	59	0	0	False	-
mp-2663	Nb_6Pt_2	8	$Pm-3n$ (223)	A15-Cr_3	26	46	0	0	False	-
mp-292	V_6As_2	8	$Pm-3n$ (223)	A15-Cr_3	27	38	0	0	False	-
mp-30876	Sn_2Zr_6	8	$Pm-3n$ (223)	A15-Cr_3	27	49	0	0	False	-
mp-416	Cr_6Os_2	8	$Pm-3n$ (223)	A15-Cr_3	31	58	0	0	False	-
mp-494	Mo_6Ge_2	8	$Pm-3n$ (223)	A15-Cr_3	34	52	0	0	False	-
mp-541	Ta_6Sb_2	8	$Pm-3n$ (223)	A15-Cr_3	25	48	0	0	False	-
mp-7226	Ni_2V_6	8	$Pm-3n$ (223)	A15-Cr_3	29	39	0	0	False	-
mp-1630	Hg_2Zr_6	8	$Pm-3n$ (223)	A15-Cr_3	25	34	0	0	False	-
mp-2053	Nb_6Sb_2	8	$Pm-3n$ (223)	A15-Cr_3	27	32	0	0	False	-
mp-729	Cr_6Si_2	8	$Pm-3n$ (223)	A15-Cr_3	29	45	0	0	False	-
mp-978844	Ca_3SrO_4	8	$Pm-3m$ (221)	$SrCa_3O_4$	15	57	0	0	False	-
mp-984729	$BaSr_3O_4$	8	$Pm-3m$ (221)	$SrCa_3O_4$	16	50	0	0	False	-
mp-10644	$Ta_2Tl_6Se_8$	8	$I-43m$ (217)	$TaTl_3Se_4$	21	24	1	10	False	-
mp-981379	$CdZn_3S_4$	8	$P-43m$ (215)	Zn_3CdS_4	12	17	8	3	False	-
mp-10748	Cu_3TaS_4	8	$P-43m$ (215)	$TaCu_3S_4$	10	7	5	4	False	-
mp-21855	Cu_3VSe_4	8	$P-43m$ (215)	$TaCu_3S_4$	10	4	2	6	False	-
mp-3762	Cu_3VS_4	8	$P-43m$ (215)	$TaCu_3S_4$	10	9	4	4	False	-
mp-4043	Cu_3NbSe_4	8	$P-43m$ (215)	$TaCu_3S_4$	11	8	6	4	False	-
mp-4081	Cu_3TaSe_4	8	$P-43m$ (215)	$TaCu_3S_4$	11	8	4	5	False	-
mp-5621	Cu_3NbS_4	8	$P-43m$ (215)	$TaCu_3S_4$	11	8	3	4	False	-
mp-755309	Li_3NbS_4	8	$P-43m$ (215)	$TaCu_3S_4$	9	10	5	7	False	-
mp-760375	Li_3VS_4	8	$P-43m$ (215)	$TaCu_3S_4$	10	9	2	4	False	-
mp-9295	Cu_3TaTe_4	8	$P-43m$ (215)	$TaCu_3S_4$	9	7	5	3	False	-
mp-991652	Cu_3VTe_4	8	$P-43m$ (215)	$TaCu_3S_4$	10	9	2	1	False	-
mp-991676	Cu_3NbTe_4	8	$P-43m$ (215)	$TaCu_3S_4$	9	8	5	4	False	-
mp-2488	Os_4Si_4	8	$P2_13$ (198)	B20-FeSi	48	17	18	10	False	-

ID	Formula	Z	SP (*No.*)	Prototype	NL	NR	WPs	HDWPs	Clean	dE/dq
mp-1025397	Ru_4Ge_4	8	$P2_13$ (198)	B20-FeSi	48	5	43	9	False	-
mp-1025551	Ga_4Pt_4	8	$P2_13$ (198)	B20-FeSi	48	12	34	10	False	-
mp-10692	Co_4Ge_4	8	$P2_13$ (198)	B20-FeSi	48	10	50	10	False	-
mp-1078464	Mn_4Ge_4	8	$P2_13$ (198)	B20-FeSi	48	6	36	10	False	-
mp-1078526	Ga_4Pd_4	8	$P2_13$ (198)	B20-FeSi	48	6	36	10	False	-
mp-1079910	Tc_4Si_4	8	$P2_13$ (198)	B20-FeSi	48	12	32	10	False	-
mp-1079914	Hf_4Sb_4	8	$P2_13$ (198)	B20-FeSi	48	7	37	10	False	-
mp-10904	Al_4Pt_4	8	$P2_13$ (198)	B20-FeSi	48	8	33	10	False	-
mp-12651	Mg_4Pt_4	8	$P2_13$ (198)	B20-FeSi	48	12	24	10	False	-
mp-1431	Mn_4Si_4	8	$P2_13$ (198)	B20-FeSi	49	11	32	10	False	-
mp-189	Ru_4Si_4	8	$P2_13$ (198)	B20-FeSi	48	9	27	10	False	-
mp-30365	Au_4Be_4	8	$P2_13$ (198)	B20-FeSi	48	5	35	11	False	-
mp-317	Rh_4Sn_4	8	$P2_13$ (198)	B20-FeSi	48	11	34	10	False	-
mp-569671	Zr_4Sb_4	8	$P2_13$ (198)	B20-FeSi	48	9	42	10	False	-
mp-7189	Al_4Pd_4	8	$P2_13$ (198)	B20-FeSi	48	8	29	10	False	-
mp-7577	Co_4Si_4	8	$P2_13$ (198)	B20-FeSi	48	6	24	10	False	-
mp-7948	Re_4Si_4	8	$P2_13$ (198)	B20-FeSi	48	7	28	10	False	-
mp-871	Fe_4Si_4	8	$P2_13$ (198)	B20-FeSi	48	11	32	10	False	-
mp-4978	$Al_2Ti_4N_2$	8	$P6_3/mmc$ (194)	Ti_2	45	53	0	0	False	-
mp-1025441	$Al_2Ta_4C_2$	8	$P6_3/mmc$ (194)	Ta_2	44	57	0	0	False	-
mp-1025517	$In_2Ti_4N_2$	8	$P6_3/mmc$ (194)	Ta_2	45	44	0	0	False	-
mp-1084767	$In_2Zr_4N_2$	8	$P6_3/mmc$ (194)	Ta_2	44	47	0	0	False	-
mp-19835	$In_2Nb_4C_2$	8	$P6_3/mmc$ (194)	Ta_2	45	51	0	0	False	-
mp-997099	$Au_2Pd_2O_4$	8	$P6_3/mmc$ (194)	$ScAgO_2$	44	47	0	0	False	-
mp-11021	$Au_2Ga_2O_4$	8	$P6_3/mmc$ (194)	$ScAgO_2$	44	32	0	0	False	-
mp-19723	$Au_2In_2O_4$	8	$P6_3/mmc$ (194)	$ScAgO_2$	47	47	0	0	True	13.58
mp-997008	$Au_2Tl_2O_4$	8	$P6_3/mmc$ (194)	$ScAgO_2$	45	41	0	0	False	-
mp-997010	$Ag_2Tl_2O_4$	8	$P6_3/mmc$ (194)	$ScAgO_2$	46	29	0	0	False	-
mp-997095	$Au_2Ni_2O_4$	8	$P6_3/mmc$ (194)	$ScAgO_2$	45	37	0	0	False	-
mp-997158	$Au_2Co_2O_4$	8	$P6_3/mmc$ (194)	$ScAgO_2$	46	41	0	0	False	-
mp-19178	$Ag_2Co_2O_4$	8	$P6_3/mmc$ (194)	$ScAgO_2$	44	45	0	0	False	-
mp-20329	$Ag_2In_2O_4$	8	$P6_3/mmc$ (194)	$ScAgO_2$	45	39	0	0	False	-
mp-549158	$Cu_2In_2O_4$	8	$P6_3/mmc$ (194)	$ScAgO_2$	45	36	0	0	False	-
mp-11019	$Cu_2Ga_2O_4$	8	$P6_3/mmc$ (194)	$ScAgO_2$	8	33	0	0	False	-
mp-11020	$Ag_2Ga_2O_4$	8	$P6_3/mmc$ (194)	$ScAgO_2$	6	48	0	0	False	-
mp-11022	$Ag_2Sc_2O_4$	8	$P6_3/mmc$ (194)	$ScAgO_2$	11	50	0	0	False	-
mp-11023	$Au_2Sc_2O_4$	8	$P6_3/mmc$ (194)	$ScAgO_2$	8	53	0	0	False	-
mp-16613	$Al_2Au_2O_4$	8	$P6_3/mmc$ (194)	$ScAgO_2$	10	44	0	0	False	-
mp-2918	$Cu_2Y_2O_4$	8	$P6_3/mmc$ (194)	$ScAgO_2$	43	50	0	0	False	-
mp-30250	$Ag_2Y_2O_4$	8	$P6_3/mmc$ (194)	$ScAgO_2$	8	39	0	0	False	-
mp-3098	$Al_2Cu_2O_4$	8	$P6_3/mmc$ (194)	$ScAgO_2$	9	43	0	0	True	26.58
mp-3642	$Cu_2Sc_2O_4$	8	$P6_3/mmc$ (194)	$ScAgO_2$	7	47	0	0	False	-
mp-754110	$Ag_2Sm_2O_4$	8	$P6_3/mmc$ (194)	$ScAgO_2$	19	41	0	0	False	-
mp-754535	$Ag_2La_2O_4$	8	$P6_3/mmc$ (194)	$ScAgO_2$	8	40	0	0	False	-
mp-9631	$Ag_2Al_2O_4$	8	$P6_3/mmc$ (194)	$ScAgO_2$	7	38	0	0	False	-
mp-15668	Na_4Se_4	8	$P6_3/mmc$ (194)	NaS	9	41	0	0	False	-
mp-2072	K_4Te_4	8	$P6_3/mmc$ (194)	NaS	24	44	0	0	False	-
mp-2400	Na_4S_4	8	$P6_3/mmc$ (194)	NaS	21	67	0	0	False	-
mp-841	Li_4O_4	8	$P6_3/mmc$ (194)	NaS	11	49	0	0	False	-
mp-736	Li_6P_2	8	$P6_3/mmc$ (194)	Li_3	23	56	0	0	False	-
mp-757	Li_6As_2	8	$P6_3/mmc$ (194)	Li_3	24	40	0	0	False	-
mp-696944	$La_2Br_4H_2$	8	$P6_3/mmc$ (194)	$LaHBr_2$	20	47	0	0	False	-
mp-10208	$Mo_2Ni_2P_4$	8	$P6_3/mmc$ (194)	$KNbS_2$	45	66	0	0	False	-
mp-1025496	$Li_2Nb_2Se_4$	8	$P6_3/mmc$ (194)	$KNbS_2$	48	50	0	0	False	-
mp-1025557	$Co_2Mo_2P_4$	8	$P6_3/mmc$ (194)	$KNbS_2$	44	51	0	0	False	-
mp-1086669	$Ni_2W_2P_4$	8	$P6_3/mmc$ (194)	$KNbS_2$	44	59	0	0	False	-
mp-1206881	$Li_2Ta_2S_4$	8	$P6_3/mmc$ (194)	$KNbS_2$	44	50	0	0	False	-
mp-7936	$Li_2Nb_2S_4$	8	$P6_3/mmc$ (194)	$KNbS_2$	23	62	0	0	False	-
mp-7937	$Na_2Nb_2S_4$	8	$P6_3/mmc$ (194)	$KNbS_2$	9	53	0	0	False	-
mp-7938	$K_2Nb_2S_4$	8	$P6_3/mmc$ (194)	$KNbS_2$	9	56	0	0	False	-
mp-7939	$Na_2Nb_2Se_4$	8	$P6_3/mmc$ (194)	$KNbS_2$	13	59	0	0	False	-
mp-7940	$K_2Nb_2Se_4$	8	$P6_3/mmc$ (194)	$KNbS_2$	9	59	0	0	False	-

ID	Formula	Z	SP ($No.$)	Prototype	NL	NR	WPs	HDWPs	Clean	dE/dq
mp-10009	Ga_4Te_4	8	$P6_3/mmc$ (194)	GaTe	8	55	0	0	False	-
mp-1943	Ga_4Se_4	8	$P6_3/mmc$ (194)	GaTe	20	69	0	0	False	-
mp-20485	In_4Se_4	8	$P6_3/mmc$ (194)	GaTe	9	61	0	0	False	-
mp-2507	Ga_4S_4	8	$P6_3/mmc$ (194)	GaTe	20	75	0	0	False	-
mp-7955	Li_6Sb_2	8	$P6_3/mmc$ (194)	D03-BiF_3	47	36	0	0	False	-
mp-1078712	$Hf_4Tl_2C_2$	8	$P6_3/mmc$ (194)	$BaCeN_2$	44	58	0	0	False	-
mp-1078811	$Nb_4C_2Ge_2$	8	$P6_3/mmc$ (194)	$BaCeN_2$	43	76	0	0	False	-
mp-1079076	$Hf_4Pb_2C_2$	8	$P6_3/mmc$ (194)	$BaCeN_2$	43	50	0	0	False	-
mp-1079546	$Ga_2Nb_4C_2$	8	$P6_3/mmc$ (194)	$BaCeN_2$	42	60	0	0	False	-
mp-1079635	$Ga_2Mo_4C_2$	8	$P6_3/mmc$ (194)	$BaCeN_2$	44	54	0	0	False	-
mp-1080835	$Ga_2V_4C_2$	8	$P6_3/mmc$ (194)	$BaCeN_2$	44	49	0	0	False	-
mp-1092281	$Ti_4Tl_2C_2$	8	$P6_3/mmc$ (194)	$BaCeN_2$	44	49	0	0	False	-
mp-1208952	$Sc_2Ta_2N_4$	8	$P6_3/mmc$ (194)	$BaCeN_2$	44	44	0	0	False	-
mp-12990	$Al_2Ti_4C_2$	8	$P6_3/mmc$ (194)	$BaCeN_2$	45	54	0	0	False	-
mp-19821	$Cr_4C_2Ge_2$	8	$P6_3/mmc$ (194)	$BaCeN_2$	47	43	0	0	False	-
mp-3886	$Al_2Zr_4C_2$	8	$P6_3/mmc$ (194)	$BaCeN_2$	44	56	0	0	True	1.57
mp-5137	$Zr_4P_2Sb_2$	8	$P6_3/mmc$ (194)	$BaCeN_2$	47	60	0	0	False	-
mp-5443	$Nb_4Sn_2C_2$	8	$P6_3/mmc$ (194)	$BaCeN_2$	44	58	0	0	False	-
mp-567220	$Hf_4P_2Sb_2$	8	$P6_3/mmc$ (194)	$BaCeN_2$	45	51	0	0	False	-
mp-868117	$Co_2Na_2O_4$	8	$P6_3/mmc$ (194)	$BaCeN_2$	44	48	0	0	False	-
mp-971634	$Na_2Ni_2O_4$	8	$P6_3/mmc$ (194)	$BaCeN_2$	44	66	0	0	False	-
mp-1025550	$Ga_2Ti_4N_2$	8	$P6_3/mmc$ (194)	$BaCeN_2$	45	52	0	0	False	-
mp-20197	$Cr_4Ga_2C_2$	8	$P6_3/mmc$ (194)	$BaCeN_2$	45	46	0	0	False	-
mp-22156	$Hf_4In_2C_2$	8	$P6_3/mmc$ (194)	$BaCeN_2$	44	58	0	0	False	-
mp-28553	$Ca_4I_2N_2$	8	$P6_3/mmc$ (194)	$BaCeN_2$	44	49	0	0	False	-
mp-5025	$Zr_4C_2S_2$	8	$P6_3/mmc$ (194)	$BaCeN_2$	43	60	0	0	False	-
mp-996162	$Al_2Nb_4C_2$	8	$P6_3/mmc$ (194)	$BaCeN_2$	45	65	0	0	False	-
mp-1079992	$Pb_2Zr_4C_2$	8	$P6_3/mmc$ (194)	$BaCeN_2$	45	48	0	0	False	-
mp-20004	$In_2Zr_4C_2$	8	$P6_3/mmc$ (194)	$BaCeN_2$	45	49	0	0	False	-
mp-20661	$Pb_2Ti_4C_2$	8	$P6_3/mmc$ (194)	$BaCeN_2$	43	49	0	0	False	-
mp-4893	$Hf_4Sn_2C_2$	8	$P6_3/mmc$ (194)	$BaCeN_2$	44	55	0	0	False	-
mp-4613	$Sn_2Zr_4C_2$	8	$P6_3/mmc$ (194)	$BaCeN_2$	43	40	0	0	False	-
mp-10046	$V_4As_2C_2$	8	$P6_3/mmc$ (194)	$BaCeN_2$	47	67	0	0	False	-
mp-1025427	$Ga_2Ta_4C_2$	8	$P6_3/mmc$ (194)	$BaCeN_2$	45	63	0	0	False	-
mp-1025524	$Tl_2Zr_4C_2$	8	$P6_3/mmc$ (194)	$BaCeN_2$	46	50	0	0	False	-
mp-3732	$Ti_4C_2S_2$	8	$P6_3/mmc$ (194)	$BaCeN_2$	45	56	0	0	False	-
mp-3871	$Sn_2Ti_4C_2$	8	$P6_3/mmc$ (194)	$BaCeN_2$	44	57	0	0	False	-
mp-5244	$Ti_4P_2Sb_2$	8	$P6_3/mmc$ (194)	$BaCeN_2$	45	59	0	0	False	-
mp-8044	$V_4C_2P_2$	8	$P6_3/mmc$ (194)	$BaCeN_2$	45	36	0	0	False	-
mp-9956	$Al_2Cr_4C_2$	8	$P6_3/mmc$ (194)	$BaCeN_2$	44	58	0	0	False	-
mp-9957	$V_4C_2Ge_2$	8	$P6_3/mmc$ (194)	$BaCeN_2$	45	53	0	0	False	-
mp-9958	$Ti_4C_2Ge_2$	8	$P6_3/mmc$ (194)	$BaCeN_2$	45	52	0	0	False	-
mp-9961	$Cd_2Ti_4C_2$	8	$P6_3/mmc$ (194)	$BaCeN_2$	44	51	0	0	False	-
mp-9987	$Nb_4C_2P_2$	8	$P6_3/mmc$ (194)	$BaCeN_2$	45	40	0	0	False	-
mp-9989	$Nb_4As_2C_2$	8	$P6_3/mmc$ (194)	$BaCeN_2$	46	58	0	0	False	-
mp-12537	$Ga_2Ti_4C_2$	8	$P6_3/mmc$ (194)	$BaCeN_2$	44	52	0	0	False	-
mp-13137	$Hf_4C_2S_2$	8	$P6_3/mmc$ (194)	$BaCeN_2$	44	59	0	0	False	-
mp-20983	$In_2V_4C_2$	8	$P6_3/mmc$ (194)	$BaCeN_2$	43	56	0	0	True	1.48
mp-13312	$Sc_2Tl_2S_4$	8	$P6_3/mmc$ (194)	$BaCeN_2$	9	48	0	0	False	-
mp-16764	$Rb_2Y_2Te_4$	8	$P6_3/mmc$ (194)	$BaCeN_2$	10	49	0	0	False	-
mp-20042	$In_2Tl_2S_4$	8	$P6_3/mmc$ (194)	$BaCeN_2$	12	58	0	0	False	-
mp-3744	$Na_2Nb_2O_4$	8	$P6_3/mmc$ (194)	$BaCeN_2$	21	55	0	0	False	-
mp-3924	$Li_2Nb_2O_4$	8	$P6_3/mmc$ (194)	$BaCeN_2$	9	51	0	0	False	-
mp-4887	$Ba_2Ce_2N_4$	8	$P6_3/mmc$ (194)	$BaCeN_2$	20	68	0	0	False	-
mp-541044	$Cs_2Y_2O_4$	8	$P6_3/mmc$ (194)	$BaCeN_2$	7	45	0	0	False	-
mp-553308	$Cs_2Nd_2O_4$	8	$P6_3/mmc$ (194)	$BaCeN_2$	9	43	0	0	False	-
mp-752927	$Ag_2Li_2F_4$	8	$P6_3/mmc$ (194)	$BaCeN_2$	11	34	0	0	False	-
mp-753799	$Cs_2Tm_2O_4$	8	$P6_3/mmc$ (194)	$BaCeN_2$	7	46	0	0	False	-
mp-754055	$Cs_2Er_2O_4$	8	$P6_3/mmc$ (194)	$BaCeN_2$	7	50	0	0	False	-
mp-754138	$Cs_2Dy_2O_4$	8	$P6_3/mmc$ (194)	$BaCeN_2$	20	73	0	0	False	-
mp-754154	$Cs_2Ho_2O_4$	8	$P6_3/mmc$ (194)	$BaCeN_2$	7	64	0	0	False	-
mp-7650	$Rb_2Sc_2O_4$	8	$P6_3/mmc$ (194)	$BaCeN_2$	20	47	0	0	False	-

ID	Formula	Z	SP (*No.*)	Prototype	NL	NR	WPs	HDWPs	Clean	dE/dq
mp-768864	$K_2Nd_2O_4$	8	$P6_3/mmc$ (194)	$BaCeN_2$	20	49	0	0	False	-
mp-7977	$Cs_2Sm_2O_4$	8	$P6_3/mmc$ (194)	$BaCeN_2$	17	44	0	0	False	-
mp-864954	$Mg_2Mo_2N_4$	8	$P6_3/mmc$ (194)	$BaCeN_2$	10	37	0	0	False	-
mp-9037	$Cs_2Pr_2S_4$	8	$P6_3/mmc$ (194)	$BaCeN_2$	44	44	0	0	False	-
mp-972199	$Cs_2Tb_2S_4$	8	$P6_3/mmc$ (194)	$BaCeN_2$	20	63	0	0	False	-
mp-984555	$Cs_2Dy_2S_4$	8	$P6_3/mmc$ (194)	$BaCeN_2$	21	57	0	0	False	-
mp-1025497	$Al_2V_4C_2$	8	$P6_3/mmc$ (194)	AlV_2	46	51	0	0	False	-
mp-20315	$In_2Ti_4C_2$	8	$P6_3/mmc$ (194)	AlV_2	44	39	0	0	False	-
mp-569095	Bi_2Rb_6	8	$P6_3/mmc$ (194)	Al_3	45	48	0	0	False	-
mp-7898	Rb_6As_2	8	$P6_3/mmc$ (194)	Al_3	47	52	0	0	False	-
mp-2294	Al_6Ir_2	8	$P6_3/mmc$ (194)	Al_3	46	50	0	0	False	-
mp-1572	Ga_4Se_4	8	$P{-}6m2$ (187)	GaSe	0	21	2	0	False	-
mp-10281	Zn_4S_4	8	$P6_3mc$ (186)	ZnS	9	76	1	1	False	-
mp-11714	C_4Si_4	8	$P6_3mc$ (186)	ZnS	8	76	0	1	False	-
mp-24066	$Sr_2Cl_2H_2O_2$	8	$P6_3mc$ (186)	SrHClO	9	53	0	1	True	10.14
mp-642725	$Ca_2Cl_2H_2O_2$	8	$P6_3mc$ (186)	SrHClO	19	54	1	2	True	10.1
mp-644222	$Cd_2Cl_2H_2O_2$	8	$P6_3mc$ (186)	SrHClO	20	57	0	1	True	11.89
mp-580941	Ag_4I_4	8	$P6_3mc$ (186)	AgI	20	43	0	1	False	-
mp-22896	La_2Cl_6	8	$P6_3/m$ (176)	$LaCl_3$	50	31	0	0	False	-
mp-23183	Nd_2Cl_6	8	$P6_3/m$ (176)	$LaCl_3$	48	37	0	0	False	-
mp-23211	Pr_2Cl_6	8	$P6_3/m$ (176)	$LaCl_3$	14	44	0	0	False	-
mp-23221	Pr_2Br_6	8	$P6_3/m$ (176)	$LaCl_3$	16	44	0	0	False	-
mp-23263	La_2Br_6	8	$P6_3/m$ (176)	$LaCl_3$	16	56	0	0	False	-
mp-568170	Tb_2Cl_6	8	$P6_3/m$ (176)	$LaCl_3$	49	30	0	0	False	-
mp-2068	Rh_6F_{18}	8	$R{-}3c$ (167)	RhF_3	37	49	0	0	False	-
mp-23933	Al_6H_{18}	8	$R{-}3c$ (167)	RhF_3	38	35	0	0	False	-
mp-2437	Ir_6F_{18}	8	$R{-}3c$ (167)	RhF_3	36	52	0	0	False	-
mp-2552	$O_{18}Te_6$	8	$R{-}3c$ (167)	RhF_3	38	51	0	0	False	-
mp-28044	Tm_6Cl_{18}	8	$R{-}3c$ (167)	RhF_3	37	58	0	0	False	-
mp-468	Al_6F_{18}	8	$R{-}3c$ (167)	RhF_3	38	63	0	0	False	-
mp-588	Ga_6F_{18}	8	$R{-}3c$ (167)	RhF_3	39	54	0	0	False	-
mp-6949	In_6F_{18}	8	$R{-}3c$ (167)	RhF_3	39	51	0	0	False	-
mp-12509	$Y_6Zn_6O_6P_6$	8	$R{-}3m$ (166)	YZnPO	12	60	0	0	False	-
mp-13206	$Pr_6Zn_6O_6P_6$	8	$R{-}3m$ (166)	YZnPO	11	64	0	0	False	-
mp-549728	$Dy_6Zn_6O_6P_6$	8	$R{-}3m$ (166)	YZnPO	11	64	0	0	False	-
mp-550820	$Sm_6Zn_6O_6P_6$	8	$R{-}3m$ (166)	YZnPO	11	64	0	0	False	-
mp-9518	$Nd_6Zn_6O_6P_6$	8	$R{-}3m$ (166)	YZnPO	11	64	0	0	False	-
mp-642807	$Tb_6Br_6H_{12}$	8	$R{-}3m$ (166)	TbH_2	6	40	0	0	False	-
mp-29151	$Li_{15}Cl_6N_3$	8	$R{-}3m$ (166)	Li_5NCl_2	6	63	0	0	False	-
mp-27999	$Cu_3K_{15}Sb_6$	8	$R{-}3m$ (166)	K_5CuSb_2	11	77	0	0	False	-
mp-14205	$Cu_9K_9As_6$	8	$R{-}3m$ (166)	$K_3Cu_3P_2$	9	49	0	0	True	10.25
mp-14206	$Ag_9K_9As_6$	8	$R{-}3m$ (166)	$K_3Cu_3P_2$	9	51	0	0	True	10.53
mp-7439	$Cu_9K_9P_6$	8	$R{-}3m$ (166)	$K_3Cu_3P_2$	12	43	0	0	True	11.02
mp-9273	$Au_9K_9Sb_6$	8	$R{-}3m$ (166)	$K_3Cu_3P_2$	12	39	0	0	True	7.24
mp-9274	$Au_9Rb_9Sb_6$	8	$R{-}3m$ (166)	$K_3Cu_3P_2$	11	40	0	0	True	6.61
mp-32750	$Cu_{12}I_{12}$	8	$R3m$ (160)	CuI	11	36	0	0	False	-
mp-13610	$Pb_3Zn_3F_{18}$	8	$R{-}3$ (148)	$ZnPbF_6$	11	7	0	0	False	-
mp-13903	$Sn_3Zn_3F_{18}$	8	$R{-}3$ (148)	$ZnPbF_6$	11	7	0	0	False	-
mp-13907	$Cd_3Sn_3F_{18}$	8	$R{-}3$ (148)	$ZnPbF_6$	13	3	0	0	False	-
mp-13983	$Pd_3Zn_3F_{18}$	8	$R{-}3$ (148)	$ZnPbF_6$	14	3	0	0	False	-
mp-13984	$Cd_3Pd_3F_{18}$	8	$R{-}3$ (148)	$ZnPbF_6$	9	10	0	0	False	-
mp-19734	$Mg_3Pb_3F_{18}$	8	$R{-}3$ (148)	$ZnPbF_6$	9	7	0	0	False	-
mp-27419	$Bi_3Li_3F_{18}$	8	$R{-}3$ (148)	$ZnPbF_6$	11	11	0	0	False	-
mp-27420	$Bi_3Na_3F_{18}$	8	$R{-}3$ (148)	$ZnPbF_6$	9	7	0	0	False	-
mp-37216	$Cd_3Pb_3F_{18}$	8	$R{-}3$ (148)	$ZnPbF_6$	12	4	0	0	False	-
mp-3980	$Li_3F_{18}Sb_3$	8	$R{-}3$ (148)	$ZnPbF_6$	11	5	0	0	False	-
mp-5858	$Cd_3Pt_3F_{18}$	8	$R{-}3$ (148)	$ZnPbF_6$	10	10	0	0	False	-
mp-623024	$Cd_3Ti_3F_{18}$	8	$R{-}3$ (148)	$ZnPbF_6$	10	0	0	0	False	-
mp-7921	$Mg_3Pd_3F_{18}$	8	$R{-}3$ (148)	$ZnPbF_6$	11	13	0	0	False	-
mp-7922	$Ca_3Pd_3F_{18}$	8	$R{-}3$ (148)	$ZnPbF_6$	9	8	0	0	False	-
mp-8255	$Ca_3Pt_3F_{18}$	8	$R{-}3$ (148)	$ZnPbF_6$	13	6	0	0	False	-
mp-8256	$Pt_3Zn_3F_{18}$	8	$R{-}3$ (148)	$ZnPbF_6$	14	5	0	0	False	-

ID	Formula	Z	SP (*No.*)	Prototype	NL	NR	WPs	HDWPs	Clean	dE/dq
mp-9143	$Li_3F_{18}P_3$	8	$R-3$ (148)	$ZnPbF_6$	12	5	0	0	False	-
mp-9144	$Li_3As_3F_{18}$	8	$R-3$ (148)	$ZnPbF_6$	12	5	0	0	False	-
mp-23218	As_6I_{18}	8	$R-3$ (148)	D05-AsI_3	9	4	0	0	False	-
mp-571442	Y_6I_{18}	8	$R-3$ (148)	D05-AsI_3	8	1	0	0	False	-
mp-10971	$Er_4Li_4O_8$	8	$I4_1/amd$ (141)	$LiErO_2$	36	66	0	0	False	-
mp-33322	$Ho_4Li_4Se_8$	8	$I4_1/amd$ (141)	$LiErO_2$	36	71	0	0	False	-
mp-33363	$Li_4Y_4S_8$	8	$I4_1/amd$ (141)	$LiErO_2$	36	69	0	0	False	-
mp-33526	$Bi_4Li_4S_8$	8	$I4_1/amd$ (141)	$LiErO_2$	36	65	0	0	False	-
mp-33667	$Dy_4Li_4S_8$	8	$I4_1/amd$ (141)	$LiErO_2$	36	48	0	0	False	-
mp-34477	$Li_4Sm_4S_8$	8	$I4_1/amd$ (141)	$LiErO_2$	36	52	0	0	False	-
mp-35015	$Bi_4Na_4Se_8$	8	$I4_1/amd$ (141)	$LiErO_2$	36	85	0	0	False	-
mp-35205	$Er_4Li_4Se_8$	8	$I4_1/amd$ (141)	$LiErO_2$	36	56	0	0	False	-
mp-35388	$Li_4Sm_4Se_8$	8	$I4_1/amd$ (141)	$LiErO_2$	36	66	0	0	False	-
mp-35591	$Er_4Li_4S_8$	8	$I4_1/amd$ (141)	$LiErO_2$	36	45	0	0	False	-
mp-35717	$Dy_4Li_4Se_8$	8	$I4_1/amd$ (141)	$LiErO_2$	36	63	0	0	False	-
mp-36234	$Na_4Tl_4O_8$	8	$I4_1/amd$ (141)	$LiErO_2$	36	53	0	0	False	-
mp-36791	$Li_4Nd_4S_8$	8	$I4_1/amd$ (141)	$LiErO_2$	36	57	0	0	False	-
mp-37605	$Li_4Nd_4Se_8$	8	$I4_1/amd$ (141)	$LiErO_2$	36	61	0	0	False	-
mp-37879	$Li_4Y_4Se_8$	8	$I4_1/amd$ (141)	$LiErO_2$	36	66	0	0	False	-
mp-38695	$Li_4Tb_4Se_8$	8	$I4_1/amd$ (141)	$LiErO_2$	36	72	0	0	False	-
mp-5488	$In_4Li_4O_8$	8	$I4_1/amd$ (141)	$LiErO_2$	35	62	0	0	False	-
mp-5840	$Li_4Sc_4O_8$	8	$I4_1/amd$ (141)	$LiErO_2$	36	64	0	0	False	-
mp-675199	$Na_4Pr_4S_8$	8	$I4_1/amd$ (141)	$LiErO_2$	36	54	0	0	False	-
mp-675230	$La_4Na_4S_8$	8	$I4_1/amd$ (141)	$LiErO_2$	36	73	0	0	False	-
mp-676360	$Na_4Nd_4S_8$	8	$I4_1/amd$ (141)	$LiErO_2$	36	70	0	0	False	-
mp-6964	$Na_4Tb_4O_8$	8	$I4_1/amd$ (141)	$LiErO_2$	36	60	0	0	False	-
mp-754027	$Na_4Sm_4O_8$	8	$I4_1/amd$ (141)	$LiErO_2$	36	61	0	0	False	-
mp-754043	$Lu_4Na_4O_8$	8	$I4_1/amd$ (141)	$LiErO_2$	36	67	0	0	False	-
mp-754334	$Na_4Tm_4O_8$	8	$I4_1/amd$ (141)	$LiErO_2$	35	62	0	0	False	-
mp-754605	$Li_4Lu_4O_8$	8	$I4_1/amd$ (141)	$LiErO_2$	36	51	0	0	False	-
mp-755141	$Ho_4Na_4O_8$	8	$I4_1/amd$ (141)	$LiErO_2$	35	69	0	0	False	-
mp-755586	$La_4Na_4O_8$	8	$I4_1/amd$ (141)	$LiErO_2$	37	54	0	0	False	-
mp-755883	$Dy_4Na_4O_8$	8	$I4_1/amd$ (141)	$LiErO_2$	36	59	0	0	False	-
mp-7589	$Mg_8F_4N_4$	8	$I4_1/amd$ (141)	$LiErO_2$	36	57	0	0	False	-
mp-8128	$Na_4Nd_4O_8$	8	$I4_1/amd$ (141)	$LiErO_2$	36	70	0	0	False	-
mp-8470	$Na_4Pr_4O_8$	8	$I4_1/amd$ (141)	$LiErO_2$	36	69	0	0	False	-
mp-27801	$Ag_4Tl_4I_8$	8	$I4/mcm$ (140)	$TlAgI_2$	13	49	0	0	False	-
mp-10163	$Al_4Na_4Te_8$	8	$I4/mcm$ (140)	$NaAlTe_2$	13	49	0	0	False	-
mp-10165	$Al_4K_4Te_8$	8	$I4/mcm$ (140)	$NaAlTe_2$	12	48	0	0	False	-
mp-10166	$Al_4Na_4Se_8$	8	$I4/mcm$ (140)	$NaAlTe_2$	12	65	0	0	False	-
mp-19851	$In_4K_4Te_8$	8	$I4/mcm$ (140)	$NaAlTe_2$	12	42	0	0	False	-
mp-22255	$In_4Rb_4Te_8$	8	$I4/mcm$ (140)	$NaAlTe_2$	12	40	0	0	False	-
mp-22483	$In_4Na_4Te_8$	8	$I4/mcm$ (140)	$NaAlTe_2$	12	54	0	0	False	-
mp-9579	$Al_4Tl_4Se_8$	8	$I4/mcm$ (140)	$NaAlTe_2$	12	41	0	0	False	-
mp-9580	$Ga_4Tl_4Se_8$	8	$I4/mcm$ (140)	$NaAlTe_2$	12	43	0	0	False	-
mp-23846	$K_4F_8H_4$	8	$I4/mcm$ (140)	KHF_2	12	51	0	0	False	-
mp-24668	$Cs_4F_8H_4$	8	$I4/mcm$ (140)	KHF_2	12	29	0	0	False	-
mp-29764	$Rb_4F_8H_4$	8	$I4/mcm$ (140)	KHF_2	12	34	0	0	False	-
mp-510557	Cs_4N_{12}	8	$I4/mcm$ (140)	CsN_3	12	28	0	0	False	-
mp-743	Rb_4N_{12}	8	$I4/mcm$ (140)	CsN_3	13	36	0	0	False	-
mp-827	K_4N_{12}	8	$I4/mcm$ (140)	CsN_3	12	44	0	0	False	-
mp-27725	Au_4I_4	8	$P4_2/ncm$ (138)	AuBr	63	41	0	0	False	-
mp-1120780	Au_4Cl_4	8	$P4_2/ncm$ (138)	AuBr	65	53	0	0	True	12.01
mp-505366	Au_4Br_4	8	$P4_2/ncm$ (138)	AuBr	62	72	0	0	True	2.78
mp-10322	$Ba_2Hf_2N_4$	8	$P4/nmm$ (129)	$BaHfN_2$	50	62	0	0	False	-
mp-23364	$Cs_2Li_2Cl_4$	8	$P4/nmm$ (129)	$BaHfN_2$	52	66	0	0	False	-
mp-3104	$Ba_2Zr_2N_4$	8	$P4/nmm$ (129)	$BaHfN_2$	51	62	0	0	False	-
mp-9517	$Sr_2Ti_2N_4$	8	$P4/nmm$ (129)	$BaHfN_2$	53	61	0	0	False	-
mp-16742	$Ag_2Ba_2F_2Te_2$	8	$P4/nmm$ (129)	BaAgTeF	53	65	0	0	False	-
mp-21228	$Cu_2Sr_2F_2Se_2$	8	$P4/nmm$ (129)	BaAgTeF	51	55	0	0	False	-
mp-546011	$Y_2Zn_2As_2O_2$	8	$P4/nmm$ (129)	BaAgTeF	51	66	0	0	False	-
mp-549589	$La_2Zn_2As_2O_2$	8	$P4/nmm$ (129)	BaAgTeF	52	65	0	0	False	-

ID	Formula	Z	SP (*No.*)	Prototype	NL	NR	WPs	HDWPs	Clean	dE/dq
mp-552488	$Cu_2La_2O_2Se_2$	8	$P4/nmm$ (129)	BaAgTeF	52	62	0	0	False	-
mp-7060	$La_2Zn_2O_2P_2$	8	$P4/nmm$ (129)	BaAgTeF	53	70	0	0	False	-
mp-7061	$Nd_2Zn_2As_2O_2$	8	$P4/nmm$ (129)	BaAgTeF	54	72	0	0	False	-
mp-9194	$Cu_2Sm_2O_2Se_2$	8	$P4/nmm$ (129)	BaAgTeF	53	62	0	0	False	-
mp-9304	$Cu_2Dy_2O_2Se_2$	8	$P4/nmm$ (129)	BaAgTeF	50	65	0	0	False	-
mp-983603	$Er_2Zn_2As_2O_2$	8	$P4/nmm$ (129)	BaAgTeF	53	67	0	0	False	-
mp-29138	$Au_2K_2Se_4$	8	$P4/mbm$ (127)	$KAuSe_2$	48	28	0	0	False	-
mp-545529	Bi_2NdClO_4	8	$P4/mmm$ (123)	$NdBi_2ClO_4$	4	82	0	0	False	-
mp-545728	Bi_2PrIO_4	8	$P4/mmm$ (123)	$NdBi_2ClO_4$	4	75	0	0	False	-
mp-545847	Bi_2LuBrO_4	8	$P4/mmm$ (123)	$NdBi_2ClO_4$	3	80	0	0	False	-
mp-546082	Bi_2TmClO_4	8	$P4/mmm$ (123)	$NdBi_2ClO_4$	3	77	0	0	False	-
mp-546152	Bi_2SmClO_4	8	$P4/mmm$ (123)	$NdBi_2ClO_4$	3	91	0	0	False	-
mp-546266	Bi_2DyIO_4	8	$P4/mmm$ (123)	$NdBi_2ClO_4$	3	80	0	0	False	-
mp-546350	Bi_2TmBrO_4	8	$P4/mmm$ (123)	$NdBi_2ClO_4$	4	75	0	0	False	-
mp-546621	Bi_2ErBrO_4	8	$P4/mmm$ (123)	$NdBi_2ClO_4$	3	75	0	0	False	-
mp-546625	Bi_2HoBrO_4	8	$P4/mmm$ (123)	$NdBi_2ClO_4$	4	76	0	0	False	-
mp-546672	Bi_2LaIO_4	8	$P4/mmm$ (123)	$NdBi_2ClO_4$	1	94	0	0	False	-
mp-547125	Bi_2TbClO_4	8	$P4/mmm$ (123)	$NdBi_2ClO_4$	3	77	0	0	False	-
mp-549127	Bi_2HoClO_4	8	$P4/mmm$ (123)	$NdBi_2ClO_4$	5	73	0	0	False	-
mp-549333	Bi_2NdIO_4	8	$P4/mmm$ (123)	$NdBi_2ClO_4$	3	82	0	0	False	-
mp-549475	Bi_2TbBrO_4	8	$P4/mmm$ (123)	$NdBi_2ClO_4$	3	82	0	0	False	-
mp-549709	Bi_2ErClO_4	8	$P4/mmm$ (123)	$NdBi_2ClO_4$	4	71	0	0	False	-
mp-550111	Bi_2SmBrO_4	8	$P4/mmm$ (123)	$NdBi_2ClO_4$	4	81	0	0	False	-
mp-550635	Bi_2LuIO_4	8	$P4/mmm$ (123)	$NdBi_2ClO_4$	1	73	0	0	False	-
mp-550805	Bi_2LuClO_4	8	$P4/mmm$ (123)	$NdBi_2ClO_4$	4	79	0	0	False	-
mp-551101	Bi_2HoIO_4	8	$P4/mmm$ (123)	$NdBi_2ClO_4$	3	70	0	0	False	-
mp-551250	Bi_2ErIO_4	8	$P4/mmm$ (123)	$NdBi_2ClO_4$	1	74	0	0	False	-
mp-551816	Bi_2YIO_4	8	$P4/mmm$ (123)	$NdBi_2ClO_4$	5	71	0	0	False	-
mp-551900	Bi_2PrBrO_4	8	$P4/mmm$ (123)	$NdBi_2ClO_4$	2	88	0	0	False	-
mp-552008	Bi_2DyClO_4	8	$P4/mmm$ (123)	$NdBi_2ClO_4$	3	78	0	0	False	-
mp-552292	Bi_2SmIO_4	8	$P4/mmm$ (123)	$NdBi_2ClO_4$	3	80	0	0	False	-
mp-552604	Bi_2YClO_4	8	$P4/mmm$ (123)	$NdBi_2ClO_4$	6	101	0	0	False	-
mp-552738	Bi_2TmIO_4	8	$P4/mmm$ (123)	$NdBi_2ClO_4$	3	75	0	0	False	-
mp-552857	Bi_2TbIO_4	8	$P4/mmm$ (123)	$NdBi_2ClO_4$	3	74	0	0	False	-
mp-552992	Bi_2DyBrO_4	8	$P4/mmm$ (123)	$NdBi_2ClO_4$	4	78	0	0	False	-
mp-553082	Bi_2PrClO_4	8	$P4/mmm$ (123)	$NdBi_2ClO_4$	6	93	0	0	False	-
mp-553242	Bi_2NdBrO_4	8	$P4/mmm$ (123)	$NdBi_2ClO_4$	2	100	0	0	False	-
mp-553243	Bi_2YBrO_4	8	$P4/mmm$ (123)	$NdBi_2ClO_4$	3	84	0	0	False	-
mp-9486	AlK_2F_5	8	$P4/mmm$ (123)	$AlTl_2F_5$	0	22	0	0	False	-
mp-1094098	$Ga_4Na_4Te_8$	8	I−42d (122)	E11-$CuFeS_2$	36	24	26	0	False	-
mp-5518	$Ag_4Ga_4Se_8$	8	I−42d (122)	E11-$CuFeS_2$	36	24	24	0	False	-
mp-10063	$Cd_4Sn_4Sb_8$	8	I−42d (122)	E11-$CuFeS_2$	36	25	32	0	False	-
mp-1009082	$Mg_4Sn_4As_8$	8	I−42d (122)	E11-$CuFeS_2$	36	27	28	0	False	-
mp-1009083	$Mg_4Sn_4P_8$	8	I−42d (122)	E11-$CuFeS_2$	36	17	25	0	False	-
mp-1009084	$Be_4Sn_4As_8$	8	I−42d (122)	E11-$CuFeS_2$	36	27	19	0	True	12.04
mp-1009085	$Be_4P_8Si_4$	8	I−42d (122)	E11-$CuFeS_2$	36	30	30	0	False	-
mp-1009087	$Be_4As_8Si_4$	8	I−42d (122)	E11-$CuFeS_2$	36	27	30	0	False	-
mp-1009088	$Be_4As_8Ge_4$	8	I−42d (122)	E11-$CuFeS_2$	36	32	29	0	False	-
mp-1013527	$Be_4Ge_4P_8$	8	I−42d (122)	E11-$CuFeS_2$	36	24	26	0	False	-
mp-1016197	$Mg_4As_8Si_4$	8	I−42d (122)	E11-$CuFeS_2$	36	22	32	0	False	-
mp-1016200	$Mg_4As_8Ge_4$	8	I−42d (122)	E11-$CuFeS_2$	36	18	27	0	False	-
mp-1232369	$Cd_4Mg_4Te_8$	8	I−42d (122)	E11-$CuFeS_2$	36	23	38	0	False	-
mp-12954	$Cu_4B_4S_8$	8	I−42d (122)	E11-$CuFeS_2$	36	23	20	0	False	-
mp-14089	$Cu_4Tl_4S_8$	8	I−42d (122)	E11-$CuFeS_2$	36	27	33	0	False	-
mp-14090	$Cu_4Tl_4Se_8$	8	I−42d (122)	E11-$CuFeS_2$	36	14	24	0	False	-
mp-14091	$Ag_4Al_4Se_8$	8	I−42d (122)	E11-$CuFeS_2$	36	21	23	0	False	-
mp-14232	$Li_4B_4O_8$	8	I−42d (122)	E11-$CuFeS_2$	36	26	28	0	False	-
mp-15703	$Be_4C_4N_8$	8	I−42d (122)	E11-$CuFeS_2$	36	30	42	0	True	179.6
mp-19833	$Ag_4In_4S_8$	8	I−42d (122)	E11-$CuFeS_2$	36	21	22	0	False	-
mp-20554	$Ag_4In_4Se_8$	8	I−42d (122)	E11-$CuFeS_2$	36	27	20	0	False	-
mp-22261	$Cu_4In_4Te_8$	8	I−42d (122)	E11-$CuFeS_2$	36	26	33	0	False	-
mp-22386	$Ag_4In_4Te_8$	8	I−42d (122)	E11-$CuFeS_2$	36	25	29	0	False	-

ID	Formula	Z	SP (*No.*)	Prototype	NL	NR	WPs	HDWPs	Clean	dE/dq
mp-22736	$Cu_4In_4S_8$	8	$I-42d$ (122)	E11-$CuFeS_2$	36	32	29	0	False	-
mp-22811	$Cu_4In_4Se_8$	8	$I-42d$ (122)	E11-$CuFeS_2$	36	25	32	0	False	-
mp-2961	$Mg_4P_8Si_4$	8	$I-42d$ (122)	E11-$CuFeS_2$	36	28	29	0	False	-
mp-3078	$Cd_4As_8Si_4$	8	$I-42d$ (122)	E11-$CuFeS_2$	36	31	24	0	False	-
mp-34903	$Mg_4Ge_4P_8$	8	$I-42d$ (122)	E11-$CuFeS_2$	36	13	33	0	False	-
mp-3524	$Li_4N_8P_4$	8	$I-42d$ (122)	E11-$CuFeS_2$	36	30	27	0	False	-
mp-3829	$Cd_4Sn_4As_8$	8	$I-42d$ (122)	E11-$CuFeS_2$	36	29	32	0	False	-
mp-3839	$Cu_4Ga_4Te_8$	8	$I-42d$ (122)	E11-$CuFeS_2$	36	29	25	0	False	-
mp-4008	$Zn_4As_8Ge_4$	8	$I-42d$ (122)	E11-$CuFeS_2$	36	29	40	0	False	-
mp-4666	$Cd_4P_8Si_4$	8	$I-42d$ (122)	E11-$CuFeS_2$	36	29	15	0	False	-
mp-4756	$Sn_4Zn_4Sb_8$	8	$I-42d$ (122)	E11-$CuFeS_2$	36	32	29	0	False	-
mp-4840	$Cu_4Ga_4Se_8$	8	$I-42d$ (122)	E11-$CuFeS_2$	36	28	26	0	False	-
mp-4899	$Ag_4Ga_4Te_8$	8	$I-42d$ (122)	E11-$CuFeS_2$	36	20	31	0	False	-
mp-4953	$Cd_4As_8Ge_4$	8	$I-42d$ (122)	E11-$CuFeS_2$	36	19	38	0	False	-
mp-4979	$Al_4Cu_4S_8$	8	$I-42d$ (122)	E11-$CuFeS_2$	36	12	21	0	False	-
mp-5190	$Sn_4Zn_4As_8$	8	$I-42d$ (122)	E11-$CuFeS_2$	36	32	29	0	False	-
mp-5213	$Cd_4Sn_4P_8$	8	$I-42d$ (122)	E11-$CuFeS_2$	36	29	24	0	True	4.26
mp-5342	$Ag_4Ga_4S_8$	8	$I-42d$ (122)	E11-$CuFeS_2$	36	16	20	0	False	-
mp-5782	$Ag_4Al_4S_8$	8	$I-42d$ (122)	E11-$CuFeS_2$	36	26	18	0	False	-
mp-14092	$Ag_4Al_4Te_8$	8	$I-42d$ (122)	E11-$CuFeS_2$	36	23	26	0	False	-
mp-15701	$Mg_4N_8Si_4$	8	$I-42d$ (122)	E11-$CuFeS_2$	36	18	34	0	False	-
mp-15704	$Be_4N_8Si_4$	8	$I-42d$ (122)	E11-$CuFeS_2$	36	23	33	0	False	-
mp-20187	$In_4Li_4Se_8$	8	$I-42d$ (122)	E11-$CuFeS_2$	36	25	25	0	False	-
mp-20782	$In_4Li_4Te_8$	8	$I-42d$ (122)	E11-$CuFeS_2$	36	30	25	0	False	-
mp-3595	$Zn_4As_8Si_4$	8	$I-42d$ (122)	E11-$CuFeS_2$	36	26	27	0	False	-
mp-3668	$Cd_4Ge_4P_8$	8	$I-42d$ (122)	E11-$CuFeS_2$	36	26	11	0	False	-
mp-4175	$Sn_4Zn_4P_8$	8	$I-42d$ (122)	E11-$CuFeS_2$	36	36	35	0	False	-
mp-4524	$Zn_4Ge_4P_8$	8	$I-42d$ (122)	E11-$CuFeS_2$	36	22	25	0	False	-
mp-4586	$Al_4Li_4Te_8$	8	$I-42d$ (122)	E11-$CuFeS_2$	36	17	31	0	False	-
mp-4763	$Zn_4P_8Si_4$	8	$I-42d$ (122)	E11-$CuFeS_2$	36	27	19	0	False	-
mp-5048	$Ga_4Li_4Te_8$	8	$I-42d$ (122)	E11-$CuFeS_2$	36	27	23	0	False	-
mp-5238	$Cu_4Ga_4S_8$	8	$I-42d$ (122)	E11-$CuFeS_2$	35	24	25	0	False	-
mp-550433	$Be_4Zn_4O_8$	8	$I-42d$ (122)	E11-$CuFeS_2$	36	30	21	0	False	-
mp-8016	$Al_4Cu_4Se_8$	8	$I-42d$ (122)	E11-$CuFeS_2$	36	25	25	0	False	-
mp-8017	$Al_4Cu_4Te_8$	8	$I-42d$ (122)	E11-$CuFeS_2$	36	34	19	0	False	-
mp-971837	$Cd_4Zn_4Te_8$	8	$I-42d$ (122)	E11-$CuFeS_2$	36	20	29	0	False	-
mp-983565	$Cu_4B_4Se_8$	8	$I-42d$ (122)	E11-$CuFeS_2$	36	24	22	0	False	-
mp-760415	$Li_6S_8Sb_2$	8	$I-42m$ (121)	Li_3SbS_4	0	26	19	0	False	-
mp-12532	$Ag_4K_2P_2S_8$	8	$I-42m$ (121)	KAg_2PS_4	0	16	14	0	False	-
mp-19896	$Li_4Pb_2Ge_2S_8$	8	$I-42m$ (121)	KAg_2PS_4	0	23	19	0	False	-
mp-568968	$Hg_2K_4Sn_2Se_8$	8	$I-42m$ (121)	$K_2SnHgSe_4$	0	28	8	0	False	-
mp-12902	$Ag_2Er_2Te_4$	8	$P-42_1m$ (113)	$ErAgTe_2$	48	31	15	0	False	-
mp-12903	$Ag_2Y_2Te_4$	8	$P-42_1m$ (113)	$ErAgTe_2$	48	27	16	0	False	-
mp-12904	$Ag_2Ho_2Te_4$	8	$P-42_1m$ (113)	$ErAgTe_2$	48	31	13	0	False	-
mp-3551	$Ag_2Tb_2Te_4$	8	$P-42_1m$ (113)	$ErAgTe_2$	48	30	15	0	False	-
mp-4024	$Ag_2Dy_2Te_4$	8	$P-42_1m$ (113)	$ErAgTe_2$	48	32	14	0	False	-
mp-7548	Ba_2Se_6	8	$P-42_1m$ (113)	$BaSe_3$	48	34	9	0	False	-
mp-239	Ba_2S_6	8	$P-42_1m$ (113)	BaS_3	48	21	8	0	False	-
mp-35152	$Rh_2Sr_4H_{10}$	8	$I4mm$ (107)	Sr_2H_5	0	24	2	0	False	-
mp-29025	$Li_{10}Br_4N_2$	8	$Immm$ (71)	Li_5Br_2	0	59	0	0	False	-
mp-752910	$Bi_2Na_6O_8$	8	$Cmmm$ (65)	Na_3BiO_4	0	37	0	0	False	-
mp-753315	$Mg_6Zn_2O_8$	8	$Cmmm$ (65)	Na_3BiO_4	0	68	0	0	False	-
mp-755287	$Ca_6Cd_2O_8$	8	$Cmmm$ (65)	Na_3BiO_4	0	66	0	0	False	-
mp-2672	K_8O_8	8	$Cmce$ (64)	K_2O_2	51	65	0	0	False	-
mp-23293	Tb_4Cl_{12}	8	$Cmcm$ (63)	$TbCl_3$	37	39	0	0	False	-
mp-27975	Nd_4Br_{12}	8	$Cmcm$ (63)	$TbCl_3$	40	41	0	0	False	-
mp-27976	Sm_4Br_{12}	8	$Cmcm$ (63)	$TbCl_3$	39	41	0	0	False	-
mp-27979	La_4I_{12}	8	$Cmcm$ (63)	$TbCl_3$	39	42	0	0	False	-
mp-28448	Dy_4Cl_{12}	8	$Cmcm$ (63)	$TbCl_3$	40	43	0	0	False	-
mp-28243	$Li_4Rb_4Cl_8$	8	$Cmcm$ (63)	$RbLiCl_2$	39	43	0	0	False	-
mp-7472	$Au_4Rb_4O_8$	8	$Cmcm$ (63)	$NaAuO_2$	41	25	0	0	False	-
mp-7392	$Ag_4Na_8Sb_4$	8	$Cmcm$ (63)	Na_2	41	44	0	0	False	-

ID	Formula	Z	SP (No.)	Prototype	NL	NR	WPs	HDWPs	Clean	dE/dq
mp-8411	$Ag_4Na_8As_4$	8	*Cmcm* (63)	Na_2	37	42	0	0	False	-
mp-15684	$Cu_4K_8As_4$	8	*Cmcm* (63)	K_2	38	47	0	0	False	-
mp-15685	$Cu_4Na_8As_4$	8	*Cmcm* (63)	K_2	39	40	0	0	False	-
mp-27549	$Ag_4Bi_4K_8$	8	*Cmcm* (63)	K_2	39	34	0	0	False	-
mp-7639	$Cu_4Na_8P_4$	8	*Cmcm* (63)	K_2	39	46	0	0	False	-
mp-7642	$Ag_4K_8As_4$	8	*Cmcm* (63)	K_2	37	54	0	0	False	-
mp-7643	$Ag_4K_8Sb_4$	8	*Cmcm* (63)	K_2	37	37	0	0	False	-
mp-7773	$Au_4Na_8As_4$	8	*Cmcm* (63)	K_2	39	43	0	0	False	-
mp-7774	$Au_4Na_8Sb_4$	8	*Cmcm* (63)	K_2	38	40	0	0	False	-
mp-8446	$Cu_4K_8P_4$	8	*Cmcm* (63)	K_2	40	59	0	0	False	-
mp-867335	$Au_4K_8Sb_4$	8	*Cmcm* (63)	K_2	42	32	0	0	False	-
mp-9687	$Au_4K_8P_4$	8	*Cmcm* (63)	K_2	39	37	0	0	False	-
mp-9778	$Ag_4K_8P_4$	8	*Cmcm* (63)	K_2	37	40	0	0	False	-
mp-553310	$Cs_4Cu_4O_8$	8	*Cmcm* (63)	$CsCuO_2$	39	19	0	0	False	-
mp-7467	$Cu_4Rb_4O_8$	8	*Cmcm* (63)	$CsCuO_2$	40	29	0	0	False	-
mp-542772	$Ag_4Cs_4Cl_8$	8	*Cmcm* (63)	$CsAgCl_2$	36	47	0	0	False	-
mp-2231	Sn_4S_4	8	*Pnma* (62)	B29-SnS	117	54	0	0	False	-
mp-2242	Ge_4S_4	8	*Pnma* (62)	B29-SnS	115	47	0	0	False	-
mp-691	Sn_4Se_4	8	*Pnma* (62)	B29-SnS	115	62	0	0	False	-
mp-700	Ge_4Se_4	8	*Pnma* (62)	B29-SnS	117	54	0	0	False	-
mp-28994	$K_2Li_4As_2$	8	*Pmmn* (59)	KLi_2	84	72	0	0	True	21.34
mp-19795	In_4S_4	8	*Pnnm* (58)	InS	108	46	0	0	False	-
mp-8360	Rb_4Te_4	8	*Pbam* (55)	RbTe	84	52	0	0	False	-
mp-772910	$Bi_{12}I_4O_{16}$	8	*Pnna* (52)	Bi_3IO_4	405	290	0	0	True	19.3
mp-675531	$Bi_8Na_8S_{16}$	8	*Fdd*2 (43)	$LiErO_2$	72	45	14	0	False	-
mp-675286	$Ca_{12}U_4N_{16}$	8	*Fmm*2 (42)	Ca_3UN_4	2	48	3	0	False	-
mp-32684	Br_4H_4	8	$P2_12_12_1$ (19)	HBr	112	25	20	9	True	1.38
mp-630528	In_4S_4	8	$P2_1/c$ (14)	InS	60	76	0	0	False	-
mp-569766	I_8Te_8	8	$C2/m$ (12)	TeI	0	41	0	0	False	-
mp-29027	$Ta_4I_8O_4$	8	$C2/m$ (12)	TaI_2	0	36	0	0	False	-
mp-27397	Ir_4Br_{12}	8	$C2/m$ (12)	$RhCl_3$	0	34	0	0	False	-
mp-27666	Ir_4Cl_{12}	8	$C2/m$ (12)	$RhCl_3$	0	29	0	0	False	-
mp-27770	Rh_4Cl_{12}	8	$C2/m$ (12)	$RhCl_3$	0	29	0	0	True	18.08
mp-27871	Rh_4Br_{12}	8	$C2/m$ (12)	$RhCl_3$	0	38	0	0	False	-
mp-643364	$Pb_4Cl_4H_4O_4$	8	$C2/m$ (12)	HPbClO	0	23	0	0	False	-
mp-568388	Bi_8I_8	8	$C2/m$ (12)	BiI	0	49	0	0	False	-
mp-7808	Ba_4P_{12}	8	$C2/m$ (12)	BaP_3	0	15	0	0	False	-
mp-9921	Zr_2S_6	8	$P2_1/m$ (11)	ZrS_3	36	65	0	0	False	-
mp-9922	Hf_2S_6	8	$P2_1/m$ (11)	ZrS_3	36	68	0	0	False	-
mp-24428	$K_2H_4N_2$	8	$P2_1/m$ (11)	KH_2	36	47	0	0	False	-
mp-697144	$Rb_2H_4N_2$	8	$P2_1/m$ (11)	KH_2	36	39	0	0	False	-
mp-549720	$Nb_4I_8O_4$	8	*C*2 (5)	TaI_2	0	0	33	0	False	-
mp-10492	$Rb_8F_{24}Si_4$	9	*Fm*−3m (225)	Cs_2GeCl_6	9	23	0	0	False	-
mp-14088	$Pd_4Rb_8F_{24}$	9	*Fm*−3m (225)	Cs_2GeCl_6	11	30	0	0	False	-
mp-23059	$Rb_8Sn_4Cl_{24}$	9	*Fm*−3m (225)	Cs_2GeCl_6	12	25	0	0	False	-
mp-23060	$Cs_8Pt_4I_{24}$	9	*Fm*−3m (225)	Cs_2GeCl_6	10	18	0	0	False	-
mp-23067	$K_8Pd_4Cl_{24}$	9	*Fm*−3m (225)	Cs_2GeCl_6	13	12	0	0	False	-
mp-23350	$Pt_4Rb_8Cl_{24}$	9	*Fm*−3m (225)	Cs_2GeCl_6	13	16	0	0	False	-
mp-23405	$Cs_8Br_{24}Te_4$	9	*Fm*−3m (225)	Cs_2GeCl_6	12	29	0	0	False	-
mp-23406	$Cs_8Pt_4Cl_{24}$	9	*Fm*−3m (225)	Cs_2GeCl_6	11	19	0	0	False	-
mp-23425	$Cs_8Pb_4Cl_{24}$	9	*Fm*−3m (225)	Cs_2GeCl_6	11	20	0	0	False	-
mp-23465	$Cs_8Cl_{24}Te_4$	9	*Fm*−3m (225)	Cs_2GeCl_6	12	20	0	0	False	-
mp-23804	$Fe_4Mg_8H_{24}$	9	*Fm*−3m (225)	Cs_2GeCl_6	11	16	0	0	False	-
mp-24292	$Ru_4Sr_8H_{24}$	9	*Fm*−3m (225)	Cs_2GeCl_6	10	14	0	0	False	-
mp-27827	$Rb_8Ti_4Cl_{24}$	9	*Fm*−3m (225)	Cs_2GeCl_6	12	23	0	0	False	-
mp-27828	$Cs_8Ti_4Cl_{24}$	9	*Fm*−3m (225)	Cs_2GeCl_6	11	18	0	0	False	-
mp-27829	$Rb_8Cl_{24}Se_4$	9	*Fm*−3m (225)	Cs_2GeCl_6	12	24	0	0	False	-
mp-27830	$Cs_8Cl_{24}Se_4$	9	*Fm*−3m (225)	Cs_2GeCl_6	12	23	0	0	False	-
mp-27831	$Rb_8Zr_4Cl_{24}$	9	*Fm*−3m (225)	Cs_2GeCl_6	12	27	0	0	False	-
mp-27834	$Pt_4Tl_8Cl_{24}$	9	*Fm*−3m (225)	Cs_2GeCl_6	11	21	0	0	False	-
mp-27882	$Cs_8Cl_{24}Ge_4$	9	*Fm*−3m (225)	Cs_2GeCl_6	12	16	0	0	False	-
mp-28145	$Pd_4Rb_8Cl_{24}$	9	*Fm*−3m (225)	Cs_2GeCl_6	13	17	0	0	False	-

ID	Formula	Z	SP (*No.*)	Prototype	NL	NR	WPs	HDWPs	Clean	dE/dq
mp-29398	$Cs_8Hf_4I_{24}$	9	$Fm{-}3m$ (225)	Cs_2GeCl_6	11	23	0	0	False	-
mp-30062	$Cs_8Pt_4Br_{24}$	9	$Fm{-}3m$ (225)	Cs_2GeCl_6	12	14	0	0	False	-
mp-3042	$K_8F_{24}Si_4$	9	$Fm{-}3m$ (225)	Cs_2GeCl_6	11	21	0	0	False	-
mp-4047	$Cs_8F_{24}Si_4$	9	$Fm{-}3m$ (225)	Cs_2GeCl_6	9	20	0	0	False	-
mp-5033	$Tl_8F_{24}Si_4$	9	$Fm{-}3m$ (225)	Cs_2GeCl_6	10	15	0	0	False	-
mp-504729	$Cs_8Pd_4F_{24}$	9	$Fm{-}3m$ (225)	Cs_2GeCl_6	11	34	0	0	False	-
mp-540957	$Cs_8I_{24}Te_4$	9	$Fm{-}3m$ (225)	Cs_2GeCl_6	13	23	0	0	False	-
mp-608555	$Cs_8Sn_4Cl_{24}$	9	$Fm{-}3m$ (225)	Cs_2GeCl_6	11	16	0	0	False	-
mp-638729	$Cs_8Zr_4Cl_{24}$	9	$Fm{-}3m$ (225)	Cs_2GeCl_6	10	25	0	0	False	-
mp-641923	$Cs_8Sn_4Br_{24}$	9	$Fm{-}3m$ (225)	Cs_2GeCl_6	11	20	0	0	False	-
mp-643101	$Pt_4Rb_8H_{24}$	9	$Fm{-}3m$ (225)	Cs_2GeCl_6	10	9	0	0	True	1.32
mp-643243	$Cs_8Pt_4H_{24}$	9	$Fm{-}3m$ (225)	Cs_2GeCl_6	10	11	0	0	False	-
mp-643279	$K_8Pt_4H_{24}$	9	$Fm{-}3m$ (225)	Cs_2GeCl_6	11	14	0	0	False	-
mp-690785	$Na_8Pt_4H_{24}$	9	$Fm{-}3m$ (225)	Cs_2GeCl_6	9	21	0	0	False	-
mp-697038	$Ba_8Ru_4H_{24}$	9	$Fm{-}3m$ (225)	Cs_2GeCl_6	11	13	0	0	False	-
mp-697044	$Ba_8Os_4H_{24}$	9	$Fm{-}3m$ (225)	Cs_2GeCl_6	11	20	0	0	False	-
mp-8217	$Cs_8F_{24}Ge_4$	9	$Fm{-}3m$ (225)	Cs_2GeCl_6	9	26	0	0	False	-
mp-8812	$Rb_8F_{24}Ge_4$	9	$Fm{-}3m$ (225)	Cs_2GeCl_6	10	23	0	0	False	-
mp-9262	$Cs_8Pt_4F_{24}$	9	$Fm{-}3m$ (225)	Cs_2GeCl_6	11	20	0	0	False	-
mp-13978	Rb_2ThF_6	9	$P{-}62m$ (189)	Rb_2ThF_6	14	39	0	0	False	-
mp-14193	K_2ThF_6	9	$P{-}62m$ (189)	Rb_2ThF_6	15	48	0	0	False	-
mp-760418	Ba_2SrI_6	9	$P{-}62m$ (189)	Rb_2ThF_6	15	45	0	0	False	-
mp-13275	$Na_3Sr_3P_3$	9	$P{-}62m$ (189)	NaSrP	13	60	0	0	False	-
mp-9732	$Ba_3Na_3P_3$	9	$P{-}62m$ (189)	NaSrP	14	56	0	0	False	-
mp-9775	$Na_3Sr_3As_3$	9	$P{-}62m$ (189)	NaSrP	12	57	0	0	False	-
mp-567680	Ba_3Cl_6	9	$P{-}62m$ (189)	$BaCl_2$	13	37	0	0	False	-
mp-568536	Ba_3I_6	9	$P{-}62m$ (189)	$BaCl_2$	18	51	0	0	False	-
mp-640058	Pb_9I_{18}	9	$R{-}3m$ (166)	PbI_2	10	94	0	0	False	-
mp-27501	Cs_2ThCl_6	9	$P{-}3m1$ (164)	Rb_2ZrF_6	30	59	0	0	False	-
mp-3821	K_2PtF_6	9	$P{-}3m1$ (164)	Rb_2ZrF_6	29	44	0	0	False	-
mp-643370	Ba_2MgH_6	9	$P{-}3m1$ (164)	Rb_2ZrF_6	27	32	0	0	False	-
mp-567296	Cd_3I_6	9	$P{-}3m1$ (164)	CdI_2	17	94	0	0	False	-
mp-20015	$PbAs_2O_6$	9	$P{-}31m$ (162)	As_2PbO_6	32	59	0	0	False	-
mp-20727	PbO_6Sb_2	9	$P{-}31m$ (162)	As_2PbO_6	34	78	0	0	False	-
mp-3810	$HgAs_2O_6$	9	$P{-}31m$ (162)	As_2PbO_6	33	64	0	0	False	-
mp-4002	Li_2ZrF_6	9	$P{-}31m$ (162)	As_2PbO_6	35	77	0	0	False	-
mp-4555	$CaAs_2O_6$	9	$P{-}31m$ (162)	As_2PbO_6	30	51	0	0	False	-
mp-7128	$CdAs_2O_6$	9	$P{-}31m$ (162)	As_2PbO_6	32	61	0	0	False	-
mp-754065	HgO_6Sb_2	9	$P{-}31m$ (162)	As_2PbO_6	41	83	0	0	False	-
mp-754118	$SrAs_2O_6$	9	$P{-}31m$ (162)	As_2PbO_6	29	53	0	0	False	-
mp-754212	$BaSr_2I_6$	9	$P{-}31m$ (162)	As_2PbO_6	34	78	0	0	False	-
mp-754517	Ca_2SrI_6	9	$P{-}31m$ (162)	As_2PbO_6	36	65	0	0	False	-
mp-754710	$CaSr_2I_6$	9	$P{-}31m$ (162)	As_2PbO_6	35	68	0	0	False	-
mp-757074	$BaCa_2I_6$	9	$P{-}31m$ (162)	As_2PbO_6	37	49	0	0	False	-
mp-8922	CdO_6Sb_2	9	$P{-}31m$ (162)	As_2PbO_6	38	74	0	0	False	-
mp-9125	CaO_6Sb_2	9	$P{-}31m$ (162)	As_2PbO_6	37	65	0	0	False	-
mp-9126	SrO_6Sb_2	9	$P{-}31m$ (162)	As_2PbO_6	33	60	0	0	True	6.2
mp-9127	BaO_6Sb_2	9	$P{-}31m$ (162)	As_2PbO_6	32	65	0	0	False	-
mp-567178	Pb_3I_6	9	$P3m1$ (156)	PbI_2	7	34	6	0	False	-
mp-570437	Cd_3I_6	9	$P3m1$ (156)	CdI_2	8	31	2	0	False	-
mp-553342	$Be_6Cs_3B_3F_6O_9$	9	$R32$ (155)	$CsBe_2BO_3F_2$	2	4	22	0	False	-
mp-6870	$Be_6K_3B_3F_6O_9$	9	$R32$ (155)	$CsBe_2BO_3F_2$	1	3	35	0	False	-
mp-7323	$Be_6Rb_3B_3F_6O_9$	9	$R32$ (155)	$CsBe_2BO_3F_2$	0	6	27	0	False	-
mp-4829	Na_2ThF_6	9	$P321$ (150)	Rb_2ThF_6	0	2	68	0	False	-
mp-867133	$Ba_2Sc_2O_5$	9	$P4/mmm$ (123)	$Ba_2Sc_2O_5$	0	102	0	0	False	-
mp-29091	$Cu_8Ti_2S_8$	9	$I{-}42m$ (121)	$Ti(CuS)_4$	0	22	18	0	False	-
mp-867699	$Li_8Ti_2S_8$	9	$I{-}42m$ (121)	$Ti(CuS)_4$	0	22	18	0	False	-
mp-28698	$Ir_2Na_8O_8$	9	$I4/m$ (87)	Na_4IrO_4	0	60	0	0	False	-
mp-570324	$Rb_4Br_{12}Te_2$	9	$I4/m$ (87)	Cs_2GeCl_6	0	32	0	0	False	-
mp-9523	$Rb_2Th_4Se_{12}$	9	$Immm$ (71)	$Rb(ThSe_3)_2$	0	90	0	0	False	-
mp-27562	$Na_4Pd_6O_8$	9	$Immm$ (71)	$Na_2Pd_3O_4$	0	95	0	0	False	-
mp-11694	$Cs_8Pd_{12}Se_{16}$	9	$Fmmm$ (69)	$Cs_2Pd_3Se_4$	0	33	0	0	False	-

ID	Formula	Z	SP (*No.*)	Prototype	NL	NR	WPs	HDWPs	Clean	dE/dq
mp-11695	$Pd_{12}Rb_8S_{16}$	9	*Fmmm* (69)	$Cs_2Pd_3Se_4$	0	46	0	0	False	-
mp-13992	$Cs_8Pt_{12}S_{16}$	9	*Fmmm* (69)	$Cs_2Pd_3Se_4$	1	44	0	0	False	-
mp-14336	$Cs_8Ni_{12}Se_{16}$	9	*Fmmm* (69)	$Cs_2Pd_3Se_4$	0	23	0	0	False	-
mp-14338	$Cs_8Pt_{12}Se_{16}$	9	*Fmmm* (69)	$Cs_2Pd_3Se_4$	1	48	0	0	False	-
mp-4030	$Pt_{12}Rb_8S_{16}$	9	*Fmmm* (69)	$Cs_2Pd_3Se_4$	0	50	0	0	False	-
mp-510268	$Cs_8Pd_{12}S_{16}$	9	*Fmmm* (69)	$Cs_2Pd_3Se_4$	0	39	0	0	True	31.66
mp-30291	$Tb_8N_4Se_6$	9	$C2/m$ (12)	$Tb_4Se_3N_2$	0	53	0	0	False	-
mp-27194	Sn_6I_{12}	9	$C2/m$ (12)	SnI_2	0	85	0	0	False	-
mp-13800	$Cs_8Na_4Tl_4F_{24}$	10	$Fm-3m$ (225)	Ba_2YTaO_6	12	21	0	0	False	-
mp-13816	$Cs_8Er_4Na_4F_{24}$	10	$Fm-3m$ (225)	Ba_2YTaO_6	14	29	0	0	False	-
mp-13898	$Cs_8In_4Na_4F_{24}$	10	$Fm-3m$ (225)	Ba_2YTaO_6	15	31	0	0	False	-
mp-13925	$Cs_8Na_4Y_4F_{24}$	10	$Fm-3m$ (225)	Ba_2YTaO_6	14	31	0	0	False	-
mp-14037	$Na_4Rh_4Tl_8F_{24}$	10	$Fm-3m$ (225)	Ba_2YTaO_6	13	22	0	0	False	-
mp-14038	$Na_4Rb_8Rh_4F_{24}$	10	$Fm-3m$ (225)	Ba_2YTaO_6	14	29	0	0	False	-
mp-21879	$Ba_8Ce_4Pt_4O_{24}$	10	$Fm-3m$ (225)	Ba_2YTaO_6	15	33	0	0	False	-
mp-24411	$Al_4K_8Li_4H_{24}$	10	$Fm-3m$ (225)	Ba_2YTaO_6	15	27	0	0	False	-
mp-24412	$Al_4K_8Na_4H_{24}$	10	$Fm-3m$ (225)	Ba_2YTaO_6	11	21	0	0	False	-
mp-550685	$Ba_8Ca_4O_{24}Te_4$	10	$Fm-3m$ (225)	Ba_2YTaO_6	16	28	0	0	False	-
mp-560790	$Bi_4Cs_8Na_4F_{24}$	10	$Fm-3m$ (225)	Ba_2YTaO_6	10	45	0	0	False	-
mp-561561	$Cs_8K_4Rh_4F_{24}$	10	$Fm-3m$ (225)	Ba_2YTaO_6	14	25	0	0	False	-
mp-567652	$Cs_8Li_4Y_4Cl_{24}$	10	$Fm-3m$ (225)	Ba_2YTaO_6	13	31	0	0	False	-
mp-570379	$Cs_8Li_4Lu_4Cl_{24}$	10	$Fm-3m$ (225)	Ba_2YTaO_6	14	34	0	0	False	-
mp-580589	$Cs_8Er_4Na_4Cl_{24}$	10	$Fm-3m$ (225)	Ba_2YTaO_6	13	27	0	0	False	-
mp-6204	$Cs_8Na_4Sc_4F_{24}$	10	$Fm-3m$ (225)	Ba_2YTaO_6	12	23	0	0	False	-
mp-6231	$Ba_8Er_4O_{24}Sb_4$	10	$Fm-3m$ (225)	Ba_2YTaO_6	13	28	0	0	False	-
mp-6270	$Ba_8Li_4Re_4O_{24}$	10	$Fm-3m$ (225)	Ba_2YTaO_6	14	30	0	0	False	-
mp-6586	$Al_4K_8Na_4F_{24}$	10	$Fm-3m$ (225)	Ba_2YTaO_6	13	29	0	0	False	-
mp-6687	$Ba_8Ho_4O_{24}Sb_4$	10	$Fm-3m$ (225)	Ba_2YTaO_6	13	29	0	0	False	-
mp-9839	$Al_4K_8Li_4F_{24}$	10	$Fm-3m$ (225)	Ba_2YTaO_6	14	34	0	0	False	-
mp-989400	$Ga_4Na_4Rb_8F_{24}$	10	$Fm-3m$ (225)	Ba_2YTaO_6	12	31	0	0	False	-
mp-989405	$Cs_8In_4Li_4Br_{24}$	10	$Fm-3m$ (225)	Ba_2YTaO_6	13	38	0	0	False	-
mp-989531	$Cs_8Ga_4K_4F_{24}$	10	$Fm-3m$ (225)	Ba_2YTaO_6	13	32	0	0	False	-
mp-989558	$Cs_8Ga_4Tl_4F_{24}$	10	$Fm-3m$ (225)	Ba_2YTaO_6	14	25	0	0	False	-
mp-989561	$Ga_4Na_4Tl_8F_{24}$	10	$Fm-3m$ (225)	Ba_2YTaO_6	13	25	0	0	False	-
mp-989571	$Cs_8In_4Na_4Cl_{24}$	10	$Fm-3m$ (225)	Ba_2YTaO_6	13	29	0	0	False	-
mp-989577	$Ga_4Li_4Tl_8F_{24}$	10	$Fm-3m$ (225)	Ba_2YTaO_6	15	31	0	0	False	-
mp-989578	$In_4Na_4Rb_8F_{24}$	10	$Fm-3m$ (225)	Ba_2YTaO_6	12	25	0	0	False	-
mp-989608	$Cs_8Na_4As_4Cl_{24}$	10	$Fm-3m$ (225)	Ba_2YTaO_6	12	43	0	0	False	-
mp-989610	$Cs_8In_4Na_4H_{24}$	10	$Fm-3m$ (225)	Ba_2YTaO_6	13	34	0	0	False	-
mp-989642	$Al_4Cs_8Na_4H_{24}$	10	$Fm-3m$ (225)	Ba_2YTaO_6	14	26	0	0	False	-
mp-11583	$Zr_4N_4S_2$	10	$P6_3/mmc$ (194)	Zr_2SN_2	11	87	0	0	False	-
mp-754469	$Er_4O_4Te_2$	10	$P6_3/mmc$ (194)	Zr_2SN_2	18	107	0	0	False	-
mp-756922	$Ho_4O_4S_2$	10	$P6_3/mmc$ (194)	Zr_2SN_2	26	67	0	0	False	-
mp-768175	$Dy_4O_4S_2$	10	$P6_3/mmc$ (194)	Zr_2SN_2	25	62	0	0	False	-
mp-6977	Be_6N_4	10	$P6_3/mmc$ (194)	Be_3N_2	14	59	0	0	False	-
mp-12814	$Ba_6N_2Sb_2$	10	$P6_3/mmc$ (194)	Ba_3	19	44	0	0	False	-
mp-29750	$Cs_2Mg_2Br_6$	10	$P6_3/mmc$ (194)	Ba_3	19	43	0	0	False	-
mp-567666	$Ba_6Bi_2N_2$	10	$P6_3/mmc$ (194)	Ba_3	34	51	0	0	False	-
mp-754157	$Al_2In_2O_6$	10	$P6_3/mmc$ (194)	$AlInO_3$	26	60	0	0	False	-
mp-8098	$Ga_2In_2O_6$	10	$P6_3/mmc$ (194)	$AlInO_3$	26	68	0	0	False	-
mp-27366	$K_6Ni_6Cl_{18}$	10	$P6_3cm$ (185)	Ba_3	63	305	1	17	False	-
mp-3953	$Ca_6C_6O_{18}$	10	$R-3c$ (167)	hR10-Calcite	45	82	0	0	False	-
mp-4385	$Cd_6C_6O_{18}$	10	$R-3c$ (167)	hR10-Calcite	43	70	0	0	False	-
mp-4531	$Na_6N_6O_{18}$	10	$R-3c$ (167)	hR10-Calcite	43	77	0	0	False	-
mp-5348	$Mg_6C_6O_{18}$	10	$R-3c$ (167)	hR10-Calcite	45	61	0	0	False	-
mp-7027	$In_6B_6O_{18}$	10	$R-3c$ (167)	hR10-Calcite	44	56	0	0	False	-
mp-754409	$Ho_6B_6O_{18}$	10	$R-3c$ (167)	hR10-Calcite	42	68	0	0	False	-
mp-7560	$Lu_6B_6O_{18}$	10	$R-3c$ (167)	hR10-Calcite	43	64	0	0	False	-
mp-8110	$Al_6B_6O_{18}$	10	$R-3c$ (167)	hR10-Calcite	46	72	0	0	False	-
mp-8180	$Li_6N_6O_{18}$	10	$R-3c$ (167)	hR10-Calcite	42	67	0	0	True	1.2
mp-8697	$Sc_6B_6O_{18}$	10	$R-3c$ (167)	hR10-Calcite	43	80	0	0	False	-
mp-1047	$Ca_{18}N_{12}$	10	$R-3c$ (167)	D51-Al_2O_3	52	76	0	0	False	-

ID	Formula	Z	SP (*No.*)	Prototype	NL	NR	WPs	HDWPs	Clean	dE/dq
mp-1143	$Al_{12}O_{18}$	10	R−3c (167)	D51-Al_2O_3	48	100	0	0	False	-
mp-1243	$Ga_{12}O_{18}$	10	R−3c (167)	D51-Al_2O_3	50	86	0	0	False	-
mp-14787	$Tm_{12}S_{18}$	10	R−3c (167)	D51-Al_2O_3	45	72	0	0	False	-
mp-22323	$In_{12}O_{18}$	10	R−3c (167)	D51-Al_2O_3	45	69	0	0	False	-
mp-2826	$Lu_{12}S_{18}$	10	R−3c (167)	D51-Al_2O_3	42	89	0	0	False	-
mp-542734	$Rh_{12}O_{18}$	10	R−3c (167)	D51-Al_2O_3	50	69	0	0	False	-
mp-755313	$Sc_{12}O_{18}$	10	R−3c (167)	D51-Al_2O_3	45	92	0	0	False	-
mp-861979	$Ir_{12}S_{18}$	10	R−3c (167)	D51-Al_2O_3	46	66	0	0	False	-
mp-974381	$Rh_{12}S_{18}$	10	R−3c (167)	D51-Al_2O_3	46	56	0	0	False	-
mp-978093	$Ho_{12}S_{18}$	10	R−3c (167)	D51-Al_2O_3	45	86	0	0	False	-
mp-3466	$Al_6Pr_6O_{18}$	10	R−3c (167)	$CsPbF_3$	49	103	0	0	False	-
mp-5899	$Al_6Nd_6O_{18}$	10	R−3c (167)	$CsPbF_3$	48	87	0	0	False	-
mp-9890	$Ag_6Ta_6O_{18}$	10	R−3c (167)	$CsPbF_3$	49	93	0	0	False	-
mp-6654	Cs_2GaLiF_6	10	P−3m1 (164)	Cs_2LiGaF_6	33	51	0	0	False	-
mp-30284	$Hg_2As_2O_6$	10	P−31m (162)	$HgAsO_3$	33	64	0	0	False	-
mp-31220	$Al_2Si_2Te_6$	10	P−31m (162)	$AlSiTe_3$	33	71	0	0	False	-
mp-567931	$In_2Si_2Te_6$	10	P−31m (162)	$AlSiTe_3$	36	56	0	0	False	-
mp-13334	$Sn_6Zn_6O_{18}$	10	$R3c$ (161)	hR10-Calcite	48	42	0	0	False	-
mp-23736	$Li_6Mg_6H_{18}$	10	$R3c$ (161)	hR10-Calcite	47	31	1	3	False	-
mp-3666	$Li_6Ta_6O_{18}$	10	$R3c$ (161)	hR10-Calcite	48	52	0	3	False	-
mp-20282	$Cs_6Pb_6F_{18}$	10	$R3c$ (161)	$CsPbF_3$	46	65	0	4	False	-
mp-4360	$Cd_6Na_6F_{18}$	10	$R3c$ (161)	$CsPbF_3$	45	45	0	3	False	-
mp-754009	$Bi_6Sc_6O_{18}$	10	$R3c$ (161)	$CsPbF_3$	47	52	1	4	False	-
mp-754323	$Bi_6Tm_6O_{18}$	10	$R3c$ (161)	$CsPbF_3$	47	61	3	5	False	-
mp-754943	$Bi_6Er_6O_{18}$	10	$R3c$ (161)	$CsPbF_3$	46	61	0	4	False	-
mp-768221	$Bi_6Y_6O_{18}$	10	$R3c$ (161)	$CsPbF_3$	45	67	1	3	False	-
mp-768310	$Bi_6La_6O_{18}$	10	$R3c$ (161)	$CsPbF_3$	47	67	1	4	False	-
mp-35906	$Ag_9Y_3Cl_{18}$	10	R−3 (148)	$Y(AgCl_2)_3$	14	5	0	0	False	-
mp-675104	$Na_9Y_3Cl_{18}$	10	R−3 (148)	$Y(AgCl_2)_3$	14	8	0	0	False	-
mp-676512	$Al_3Na_9F_{18}$	10	R−3 (148)	$Y(AgCl_2)_3$	15	15	0	0	False	-
mp-13641	$Cd_6Ti_6O_{18}$	10	R−3 (148)	$TiCdO_3$	12	9	0	0	False	-
mp-14142	$Ti_6Zn_6O_{18}$	10	R−3 (148)	$TiCdO_3$	16	6	0	0	False	-
mp-23054	$Bi_6Na_6O_{18}$	10	R−3 (148)	$TiCdO_3$	16	1	0	0	False	-
mp-3759	$Mg_6Ge_6O_{18}$	10	R−3 (148)	$TiCdO_3$	16	13	0	0	False	-
mp-3771	$Mg_6Ti_6O_{18}$	10	R−3 (148)	$TiCdO_3$	19	2	0	0	False	-
mp-4190	$Ca_6Sn_6O_{18}$	10	R−3 (148)	$TiCdO_3$	12	6	0	0	False	-
mp-754152	$Bi_6Y_6O_{18}$	10	R−3 (148)	$TiCdO_3$	16	11	0	0	False	-
mp-754329	$Cd_6Sn_6O_{18}$	10	R−3 (148)	$TiCdO_3$	14	2	0	0	False	-
mp-754701	$Ca_6Ti_6O_{18}$	10	R−3 (148)	$TiCdO_3$	15	15	0	0	False	-
mp-8275	$Cd_6Ge_6O_{18}$	10	R−3 (148)	$TiCdO_3$	13	8	0	0	False	-
mp-11715	$Mg_3Sn_3B_6O_{18}$	10	R−3 (148)	$MgSn(BO_3)_2$	11	0	0	0	False	-
mp-14398	$Ca_3Sn_3B_6O_{18}$	10	R−3 (148)	$MgSn(BO_3)_2$	12	1	0	0	False	-
mp-6459	$Ca_3Mg_3C_6O_{18}$	10	R−3 (148)	$MgSn(BO_3)_2$	15	2	0	0	False	-
mp-8000	$Sn_3Sr_3B_6O_{18}$	10	R−3 (148)	$MgSn(BO_3)_2$	12	4	0	0	False	-
mp-30943	$Mg_6P_6Se_{18}$	10	R−3 (148)	$MgPSe_3$	10	2	0	0	False	-
mp-768907	$Sr_9O_{18}Te_3$	10	$R3$ (146)	$Y(AgCl_2)_3$	14	15	0	0	False	-
mp-755478	$Bi_6Cd_6O_{18}$	10	$R3$ (146)	$TiCdO_3$	0	1	14	0	False	-
mp-13900	$Cu_8Sr_4O_8$	10	$I4_1/amd$ (141)	$Sr(CuO)_2$	45	67	0	0	False	-
mp-7374	$Ba_4Cu_8O_8$	10	$I4_1/amd$ (141)	$Sr(CuO)_2$	45	70	0	0	False	-
mp-10289	$Ca_4B_8C_8$	10	$I4/mcm$ (140)	$Ca(BC)_2$	15	57	0	0	False	-
mp-28453	$Au_4Cs_4I_{12}$	10	$I4/mmm$ (139)	$CsPbF_3$	1	62	0	0	False	-
mp-567776	$Ag_2Au_2Cs_4Cl_{12}$	10	$I4/mmm$ (139)	Ba_2YTaO_6	0	49	0	0	False	-
mp-27255	$Ga_8S_8Te_4$	10	$I4_1md$ (109)	Ga_2TeS_2	45	38	20	0	False	-
mp-28423	$Ga_8Se_8Te_4$	10	$I4_1md$ (109)	Ga_2TeS_2	45	27	18	0	True	1.28
mp-567431	Th_4Cl_{16}	10	$I4_1/a$ (88)	$ThCl_4$	15	54	0	0	False	-
mp-570229	Th_4Br_{16}	10	$I4_1/a$ (88)	$ThCl_4$	16	58	0	0	False	-
mp-561623	$Li_8U_2O_{10}$	10	$I4/m$ (87)	Li_4UO_5	0	51	0	0	False	-
mp-7714	$Li_8U_2O_{10}$	10	$I4/m$ (87)	Li_4UO_5	0	46	0	0	False	-
mp-12385	$Ba_4Ta_2Y_2O_{12}$	10	$I4/m$ (87)	Ba_2YTaO_6	0	65	0	0	False	-
mp-13000	$Ba_4Ho_2Ta_2O_{12}$	10	$I4/m$ (87)	Ba_2YTaO_6	0	68	0	0	False	-
mp-15318	$Ho_2Na_2Rb_4F_{12}$	10	$I4/m$ (87)	Ba_2YTaO_6	0	55	0	0	False	-
mp-12535	$K_8Zn_4Te_8$	10	$Ibam$ (72)	K_2ZnTe_2	31	67	0	0	False	-

ID	Formula	Z	SP (*No.*)	Prototype	NL	NR	WPs	HDWPs	Clean	dE/dq
mp-551203	$Sr_8I_8O_4$	10	*Ibam* (72)	K_2ZnTe_2	31	94	0	0	False	-
mp-551835	$Ba_8I_8O_4$	10	*Ibam* (72)	K_2ZnTe_2	30	96	0	0	False	-
mp-6960	$Rb_8As_8Si_4$	10	*Ibam* (72)	K_2ZnTe_2	30	68	0	0	False	-
mp-6984	$K_8As_8Si_4$	10	*Ibam* (72)	K_2ZnTe_2	30	80	0	0	False	-
mp-8187	$K_8Zn_4O_8$	10	*Ibam* (72)	K_2ZnTe_2	30	89	0	0	False	-
mp-8235	$K_8P_8Si_4$	10	*Ibam* (72)	K_2ZnTe_2	31	69	0	0	False	-
mp-8257	$Na_8Zn_4S_8$	10	*Ibam* (72)	K_2ZnTe_2	31	85	0	0	False	-
mp-8716	$K_8Mn_4Se_8$	10	*Ibam* (72)	K_2ZnTe_2	30	93	0	0	False	-
mp-8719	$K_8Mn_4Te_8$	10	*Ibam* (72)	K_2ZnTe_2	30	64	0	0	False	-
mp-8930	$K_8As_8Ge_4$	10	*Ibam* (72)	K_2ZnTe_2	30	79	0	0	False	-
mp-8931	$Rb_8Sn_4As_8$	10	*Ibam* (72)	K_2ZnTe_2	31	94	0	0	False	-
mp-28683	$Ta_4Cl_8I_8$	10	*Immm* (71)	$Ta(ICl)_2$	0	60	0	0	False	-
mp-2079	$Rb_{16}P_{24}$	10	*Fmmm* (69)	Rb_2P_3	0	49	0	0	False	-
mp-8262	$K_{16}P_{24}$	10	*Fmmm* (69)	Rb_2P_3	0	56	0	0	False	-
mp-23104	$Pb_8Cl_4F_4O_4$	10	*Cmme* (67)	Pb_2	15	65	0	0	False	-
mp-989609	$W_4Y_4N_{12}$	10	*Cmcm* (63)	YWN_3	50	53	0	0	False	-
mp-13445	$Pt_4Rb_8As_8$	10	*Cmcm* (63)	Rb_2As_2	52	43	0	0	True	8.09
mp-7505	$K_8Pd_4P_8$	10	*Cmcm* (63)	Rb_2As_2	55	49	0	0	False	-
mp-8147	$K_8Pd_4As_8$	10	*Cmcm* (63)	Rb_2As_2	50	39	0	0	False	-
mp-9672	$K_8Ni_4P_8$	10	*Cmcm* (63)	Rb_2As_2	50	45	0	0	False	-
mp-13326	$Lu_4Pr_4Se_{12}$	10	*Cmcm* (63)	$PrLuSe_3$	51	57	0	0	False	-
mp-13327	$Lu_4Nd_4Se_{12}$	10	*Cmcm* (63)	$PrLuSe_3$	49	58	0	0	False	-
mp-28534	$Ca_4In_4Br_{12}$	10	*Cmcm* (63)	$PrLuSe_3$	50	61	0	0	False	-
mp-4784	$Ca_4Pt_4O_{12}$	10	*Cmcm* (63)	$PrLuSe_3$	53	50	0	0	False	-
mp-556405	$La_4C_4F_4N_8$	10	*Cmcm* (63)	$LaCN_2$	48	43	0	0	True	1.3
mp-23084	$Bi_4Pb_4Cl_4O_8$	10	*Cmcm* (63)	$BiPbClO_2$	51	44	0	0	False	-
mp-23138	$Pb_4Cl_4O_8Sb_4$	10	*Cmcm* (63)	$BiPbClO_2$	50	37	0	0	False	-
mp-545500	$Ba_4Cl_4O_8Sb_4$	10	*Cmcm* (63)	$BiPbClO_2$	48	37	0	0	False	-
mp-547244	$Bi_4Sr_4Cl_4O_8$	10	*Cmcm* (63)	$BiPbClO_2$	49	43	0	0	False	-
mp-551243	$Ba_4Bi_4I_4O_8$	10	*Cmcm* (63)	$BiPbClO_2$	50	39	0	0	False	-
mp-552234	$Bi_4Sr_4Br_4O_8$	10	*Cmcm* (63)	$BiPbClO_2$	48	32	0	0	False	-
mp-552547	$Bi_4Sr_4I_4O_8$	10	*Cmcm* (63)	$BiPbClO_2$	50	32	0	0	False	-
mp-552806	$Ba_4Bi_4Cl_4O_8$	10	*Cmcm* (63)	$BiPbClO_2$	48	31	0	0	False	-
mp-556094	$Ag_4Bi_4Cl_8S_4$	10	*Cmcm* (63)	$AgBiSCl_2$	49	59	0	0	False	-
mp-30528	$Mn_4Tl_4Cl_{12}$	10	*Pnma* (62)	$MnTlCl_3$	285	122	0	0	False	-
mp-27253	$Au_{16}O_{24}$	10	*Fdd*2 (43)	Au_2O_3	60	18	0	0	False	-
mp-558728	$Cs_{12}Li_8F_{20}$	10	*Fmm*2 (42)	$Cs_3Li_2F_5$	0	71	1	0	False	-
mp-989603	$La_4Mo_4N_{12}$	10	*Ama*2 (40)	$LaMoN_3$	0	15	0	0	False	-
mp-989617	$W_4Y_4N_{12}$	10	*Ama*2 (40)	$LaMoN_3$	0	9	0	0	False	-
mp-867203	$Cd_4Sr_8As_8$	10	$Cmc2_1$ (36)	Sr_2CdAs_2	48	57	10	0	False	-
mp-7447	Rb_8Se_{12}	10	$Cmc2_1$ (36)	Rb_2Se_3	49	54	8	0	False	-
mp-7449	Cs_8Se_{12}	10	$Cmc2_1$ (36)	Rb_2Se_3	48	46	3	0	False	-
mp-7667	K_8S_{12}	10	$Cmc2_1$ (36)	Rb_2Se_3	49	64	3	0	False	-
mp-7670	K_8Se_{12}	10	$Cmc2_1$ (36)	Rb_2Se_3	48	51	8	0	False	-
mp-10223	$Na_8Pd_4S_8$	10	$Cmc2_1$ (36)	Na_2PdS_2	51	64	1	0	False	-
mp-10246	$Na_8Pt_4S_8$	10	$Cmc2_1$ (36)	Na_2PdS_2	51	47	3	0	False	-
mp-14588	$Na_8Pt_4Se_8$	10	$Cmc2_1$ (36)	Na_2PdS_2	52	52	4	0	False	-
mp-19885	$Ga_4In_4S_{12}$	10	$Cmc2_1$ (36)	$InGaS_3$	53	36	5	0	False	-
mp-4187	$Ge_8N_8O_4$	10	$Cmc2_1$ (36)	Ge_2N_2	49	61	12	0	False	-
mp-865143	$Ba_4Cd_4Yb_4Sb_8$	10	$Cmc2_1$ (36)	$BaYbCdSb_2$	47	65	5	0	False	-
mp-754401	Al_8O_{12}	10	$Cmc2_1$ (36)	B_2O_3	51	59	8	0	False	-
mp-568328	Fe_4P_{16}	10	$C222_1$ (20)	FeP_4	49	11	57	0	False	-
mp-29396	$Cu_8Pb_4O_8$	10	$C2/c$ (15)	$Sr(CuO)_2$	3	37	0	0	False	-
mp-684690	Sc_8Se_{12}	10	$C2/c$ (15)	Sc_2Se_3	0	70	0	0	False	-
mp-384	Mg_2P_8	10	$P2_1/c$ (14)	MgP_4	75	73	0	0	False	-
mp-7904	Cd_2P_8	10	$P2_1/c$ (14)	MgP_4	79	79	0	0	False	-
mp-9635	$Ag_4Hg_2S_4$	10	$P2_1/c$ (14)	Ag_2HgS_2	77	94	0	0	False	-
mp-27678	Y_8Cl_{12}	10	$C2/m$ (12)	Y_2Cl_3	0	57	0	0	False	-
mp-542180	$Th_4I_8Te_8$	10	$C2/m$ (12)	$Th(TeI)_2$	0	32	0	0	False	-
mp-569623	$Nb_4Br_8Se_8$	10	$C2/m$ (12)	$Th(TeI)_2$	0	38	0	0	False	-
mp-684969	Sc_8S_{12}	10	$C2/m$ (12)	Sc_2S_3	0	59	0	0	False	-
mp-770932	$Li_4O_{12}Sb_4$	10	$C2/m$ (12)	$LiSbO_3$	0	42	0	0	False	-

ID	Formula	Z	SP (*No.*)	Prototype	NL	NR	WPs	HDWPs	Clean	dE/dq
mp-989643	$Co_4La_4N_{12}$	10	$C2/m$ (12)	$LaCoN_3$	0	23	0	0	True	1.69
mp-551092	$K_4Li_4Zn_4O_8$	10	$C2/m$ (12)	$KLiZnO_2$	0	60	0	0	False	-
mp-541785	$Pd_4Ge_4S_{12}$	10	$C2/m$ (12)	$GePdS_3$	0	65	0	0	False	-
mp-886	Ga_8O_{12}	10	$C2/m$ (12)	Ga_2O_3	0	51	0	0	False	-
mp-571409	$Cs_4Li_6Br_{10}$	10	$C2/m$ (12)	$Cs_2Li_3Br_5$	0	60	0	0	True	42.99
mp-570961	$Bi_4Cd_4I_4Se_8$	10	$C2/m$ (12)	$CdBiSe_2$	0	78	0	0	False	-
mp-23127	$Ba_4Bi_2O_{12}Sb_2$	10	$C2/m$ (12)	Ba_2YTaO_6	1	58	0	0	False	-
mp-552191	$Ba_4Bi_2Tb_2O_{12}$	10	$C2/m$ (12)	Ba_2YTaO_6	1	52	0	0	False	-
mp-989645	$W_2Y_2N_6$	10	$P2_1/m$ (11)	YWN_3	45	77	0	0	False	-
mp-24589	$K_4Br_2H_2O_2$	10	$P2_1/m$ (11)	Rb_2	45	96	0	0	True	15.33
mp-867219	$Ga_2Sr_4Ge_2N_2$	10	$P2_1/m$ (11)	Rb_2	45	102	0	0	False	-
mp-545482	$Pd_2O_6Se_2$	10	$P2_1/m$ (11)	$PdSeO_3$	45	44	0	0	False	-
mp-567373	$La_2C_2Cl_2N_4$	10	$P2_1/m$ (11)	$LaCN_2$	45	72	0	0	True	1.44
mp-6989	$Ba_2O_6Se_2$	10	$P2_1/m$ (11)	$BaSeO_3$	46	74	0	0	False	-
mp-569812	$Ba_2Cl_2N_6$	10	$P2_1/m$ (11)	BaN_3	45	62	0	0	False	-
mp-643708	$Li_2H_4I_2O_2$	10	$P2/m$ (10)	LiH_2	0	54	0	0	False	-
mp-539	Ga_8S_{12}	10	Cc (9)	Ga_2S_3	0	29	0	0	False	-
mp-570756	$Cs_6Li_4Cl_{10}$	10	Cm (8)	$Cs_3Li_2Cl_5$	1	61	0	0	False	-
mp-546285	$Nb_4I_{12}O_4$	10	$C2$ (5)	NbI_3	0	2	56	0	True	14.77
mp-608311	$Cs_4Li_6I_{10}$	10	$C2$ (5)	$Cs_2Li_3Br_5$	0	1	39	0	False	-
mp-6494	$Ca_3K_6C_6O_{18}$	11	$R-3$m (166)	$Sr_2Mg(BO_3)_2$	14	76	0	0	False	-
mp-8127	$K_6Mg_3C_6O_{18}$	11	$R-3$m (166)	$Sr_2Mg(BO_3)_2$	14	64	0	0	False	-
mp-9259	$Ba_6Mg_3B_6O_{18}$	11	$R-3$m (166)	$Sr_2Mg(BO_3)_2$	15	48	0	0	False	-
mp-29057	Nb_3Br_7	11	$P3m1$ (156)	Nb_3SBr_7	12	13	3	0	False	-
mp-567713	Nb_3I_7	11	$P3m1$ (156)	Nb_3SBr_7	12	8	5	0	False	-
mp-6026	$Mg_3Na_6C_6O_{18}$	11	$R-3$ (148)	$Sr_2Mg(BO_3)_2$	13	2	0	0	False	-
mp-10776	$K_2SnAs_2S_6$	11	$P-3$ (147)	$K_2Sn(AsS_3)_2$	43	32	0	0	False	-
mp-20352	$Ba_6In_4O_{12}$	11	$I4/mmm$ (139)	$Ba_3In_2O_6$	0	83	0	0	False	-
mp-30192	$Pr_8O_8Se_6$	11	$Amm2$ (38)	$Pr_4Se_3O_4$	0	40	1	0	False	-
mp-4412	$La_8O_8Se_6$	11	$Amm2$ (38)	$Pr_4Se_3O_4$	0	31	0	0	False	-
mp-550117	$Sm_8O_8Se_6$	11	$Amm2$ (38)	$Pr_4Se_3O_4$	0	32	0	0	False	-
mp-553278	$Nd_8O_8Se_6$	11	$Amm2$ (38)	$Pr_4Se_3O_4$	0	28	0	0	False	-
mp-13541	$Mg_2Sr_4B_4O_{12}$	11	$C2/m$ (12)	$Sr_2Mg(BO_3)_2$	0	34	0	0	False	-
mp-549737	$Ba_4Ca_2B_4O_{12}$	11	$C2/m$ (12)	$Sr_2Mg(BO_3)_2$	0	33	0	0	False	-
mp-760400	$Bi_2Na_{10}O_{10}$	11	$C2/m$ (12)	Li_5TaO_5	1	57	0	0	False	-
mp-12803	$Ba_2Er_4F_{16}$	11	$C2/m$ (12)	$BaEr_2F_8$	0	77	0	0	False	-
mp-768240	$Ba_2Y_4F_{16}$	11	$C2/m$ (12)	$BaEr_2F_8$	0	62	0	0	False	-
mp-7693	$Ba_2Tm_4F_{16}$	11	$C2/m$ (12)	$BaEr_2F_8$	0	67	0	0	False	-
mp-759876	$Na_{10}O_{10}Sb_2$	11	$C2$ (5)	Li_5TaO_5	0	0	48	0	False	-
mp-27608	$Be_{16}O_{28}Te_4$	12	$F-43$m (216)	Be_4TeO_7	16	21	2	7	False	-
mp-1095440	Cd_4S_8	12	$Pa-3$ (205)	C2-FeS_2	102	48	0	0	False	-
mp-1102743	Zn_4S_8	12	$Pa-3$ (205)	C2-FeS_2	103	40	0	0	False	-
mp-1103590	Mg_4Se_8	12	$Pa-3$ (205)	C2-FeS_2	103	43	0	0	False	-
mp-1356	Pd_4Sb_8	12	$Pa-3$ (205)	C2-FeS_2	104	49	0	0	False	-
mp-1848	Ru_4Te_8	12	$Pa-3$ (205)	C2-FeS_2	103	50	0	0	False	-
mp-1922	Ru_4Se_8	12	$Pa-3$ (205)	C2-FeS_2	104	58	0	0	False	-
mp-20465	Pd_4As_8	12	$Pa-3$ (205)	C2-FeS_2	101	39	0	0	False	-
mp-2070	Co_4S_8	12	$Pa-3$ (205)	C2-FeS_2	103	42	0	0	False	-
mp-20901	Ni_4Se_8	12	$Pa-3$ (205)	C2-FeS_2	105	48	0	0	False	-
mp-20905	Os_4S_8	12	$Pa-3$ (205)	C2-FeS_2	102	46	0	0	False	-
mp-21065	P_8Si_4	12	$Pa-3$ (205)	C2-FeS_2	103	59	0	0	False	-
mp-2142	Os_4Te_8	12	$Pa-3$ (205)	C2-FeS_2	101	49	0	0	False	-
mp-21873	Ni_4As_8	12	$Pa-3$ (205)	C2-FeS_2	102	48	0	0	False	-
mp-21893	Mn_4Te_8	12	$Pa-3$ (205)	C2-FeS_2	99	52	0	0	False	-
mp-22309	Co_4Se_8	12	$Pa-3$ (205)	C2-FeS_2	102	61	0	0	False	-
mp-22555	Rh_4S_8	12	$Pa-3$ (205)	C2-FeS_2	104	56	0	0	False	-
mp-22619	Ni_4P_8	12	$Pa-3$ (205)	C2-FeS_2	102	44	0	0	False	-
mp-2280	Cu_4Se_8	12	$Pa-3$ (205)	C2-FeS_2	106	47	0	0	False	-
mp-22864	Bi_8Pt_4	12	$Pa-3$ (205)	C2-FeS_2	105	33	0	0	False	-
mp-2480	Os_4Se_8	12	$Pa-3$ (205)	C2-FeS_2	107	42	0	0	False	-
mp-2513	Pt_4As_8	12	$Pa-3$ (205)	C2-FeS_2	98	36	0	0	False	-
mp-562	Pt_4Sb_8	12	$Pa-3$ (205)	C2-FeS_2	102	47	0	0	False	-

ID	Formula	Z	SP (*No.*)	Prototype	NL	NR	WPs	HDWPs	Clean	dE/dq
mp-738	Au_4Sb_8	12	$Pa-3$ (205)	C2-FeS_2	99	45	0	0	False	-
mp-754	Rh_4Te_8	12	$Pa-3$ (205)	C2-FeS_2	104	38	0	0	False	-
mp-2030	Ru_4S_8	12	$Pa-3$ (205)	C2-FeS_2	104	48	0	0	False	-
mp-226	Fe_4S_8	12	$Pa-3$ (205)	C2-FeS_2	105	56	0	0	False	-
mp-2310	Cd_4O_8	12	$Pa-3$ (205)	C2-FeS_2	99	47	0	0	False	-
mp-2589	Mg_4O_8	12	$Pa-3$ (205)	C2-FeS_2	108	46	0	0	False	-
mp-2604	Mg_4Te_8	12	$Pa-3$ (205)	C2-FeS_2	101	33	0	0	False	-
mp-730	Pt_4P_8	12	$Pa-3$ (205)	C2-FeS_2	99	32	0	0	False	-
mp-8484	Zn_4O_8	12	$Pa-3$ (205)	C2-FeS_2	101	37	0	0	False	-
mp-16363	$Co_4As_4S_4$	12	$P2_13$ (198)	CoAsS	72	14	46	16	False	-
mp-756361	$Zr_4O_4Se_4$	12	$P2_13$ (198)	CoAsS	72	14	50	16	False	-
mp-8630	$Ir_4S_4Sb_4$	12	$P2_13$ (198)	CoAsS	72	16	37	16	False	-
mp-1102354	Tc_8Th_4	12	$P6_3/mmc$ (194)	$ThTc_2$	70	85	0	0	False	-
mp-1095643	Cr_8Nb_4	12	$P6_3/mmc$ (194)	C14-$MgZn_2$	67	92	0	0	False	-
mp-1095650	Cs_4Na_8	12	$P6_3/mmc$ (194)	C14-$MgZn_2$	71	70	0	0	False	-
mp-11270	Be_8Mn_4	12	$P6_3/mmc$ (194)	C14-$MgZn_2$	71	67	0	0	True	14.77
mp-11282	Be_8W_4	12	$P6_3/mmc$ (194)	C14-$MgZn_2$	72	53	0	0	True	13.67
mp-11499	Mg_8Y_4	12	$P6_3/mmc$ (194)	C14-$MgZn_2$	70	60	0	0	False	-
mp-1589	Cr_8Ti_4	12	$P6_3/mmc$ (194)	C14-$MgZn_2$	70	120	0	0	False	-
mp-1949	Mn_8Ti_4	12	$P6_3/mmc$ (194)	C14-$MgZn_2$	75	110	0	0	False	-
mp-20868	Fe_8W_4	12	$P6_3/mmc$ (194)	C14-$MgZn_2$	71	85	0	0	False	-
mp-2572	Mg_8Sr_4	12	$P6_3/mmc$ (194)	C14-$MgZn_2$	72	60	0	0	False	-
mp-30577	Cs_4K_8	12	$P6_3/mmc$ (194)	C14-$MgZn_2$	73	90	0	0	False	-
mp-567612	Os_8Sc_4	12	$P6_3/mmc$ (194)	C14-$MgZn_2$	75	93	0	0	False	-
mp-568186	Ru_8Y_4	12	$P6_3/mmc$ (194)	C14-$MgZn_2$	73	103	0	0	False	-
mp-568630	Lu_4Ru_8	12	$P6_3/mmc$ (194)	C14-$MgZn_2$	70	95	0	0	False	-
mp-569624	Cr_8Hf_4	12	$P6_3/mmc$ (194)	C14-$MgZn_2$	68	74	0	0	False	-
mp-570670	Os_8Y_4	12	$P6_3/mmc$ (194)	C14-$MgZn_2$	71	111	0	0	False	-
mp-570684	Os_8Zr_4	12	$P6_3/mmc$ (194)	C14-$MgZn_2$	72	92	0	0	False	-
mp-570786	K_4Na_8	12	$P6_3/mmc$ (194)	C14-$MgZn_2$	69	99	0	0	False	-
mp-1124	Mg_4Zn_8	12	$P6_3/mmc$ (194)	C14-$MgZn_2$	72	83	0	0	False	-
mp-11275	Be_8Re_4	12	$P6_3/mmc$ (194)	C14-$MgZn_2$	72	68	0	0	False	-
mp-11281	Be_8V_4	12	$P6_3/mmc$ (194)	C14-$MgZn_2$	74	66	0	0	False	-
mp-11449	Hf_4Mn_8	12	$P6_3/mmc$ (194)	C14-$MgZn_2$	68	97	0	0	False	-
mp-21170	K_4Pb_8	12	$P6_3/mmc$ (194)	C14-$MgZn_2$	72	74	0	0	False	-
mp-2432	Ca_4Mg_8	12	$P6_3/mmc$ (194)	C14-$MgZn_2$	74	86	0	0	False	-
mp-30438	Be_8Cr_4	12	$P6_3/mmc$ (194)	C14-$MgZn_2$	72	73	0	0	True	15.21
mp-1935	Ba_4Mg_8	12	$P6_3/mmc$ (194)	C14-$MgZn_2$	70	66	0	0	False	-
mp-2225	Be_8Fe_4	12	$P6_3/mmc$ (194)	C14-$MgZn_2$	71	61	0	0	False	-
mp-1078	Ca_4Cd_8	12	$P6_3/mmc$ (194)	C14-$MgZn_2$	73	84	0	0	False	-
mp-1103315	Be_8Ru_4	12	$P6_3/mmc$ (194)	C14-$MgZn_2$	72	81	0	0	False	-
mp-1103373	K_4Sn_8	12	$P6_3/mmc$ (194)	C14-$MgZn_2$	72	104	0	0	False	-
mp-12657	Al_8Hf_4	12	$P6_3/mmc$ (194)	C14-$MgZn_2$	71	47	0	0	False	-
mp-1689	Hf_4Re_8	12	$P6_3/mmc$ (194)	C14-$MgZn_2$	73	110	0	0	False	-
mp-2772	Al_8Zr_4	12	$P6_3/mmc$ (194)	C14-$MgZn_2$	73	74	0	0	False	-
mp-11557	Re_8Sc_4	12	$P6_3/mmc$ (194)	C14-$MgZn_2$	72	100	0	0	False	-
mp-11820	Ba_4Na_8	12	$P6_3/mmc$ (194)	C14-$MgZn_2$	66	76	0	0	False	-
mp-12109	Re_8Zr_4	12	$P6_3/mmc$ (194)	C14-$MgZn_2$	69	111	0	0	False	-
mp-12735	Ag_8K_4	12	$P6_3/mmc$ (194)	C14-$MgZn_2$	76	78	0	0	False	-
mp-13503	Sc_4Zn_8	12	$P6_3/mmc$ (194)	C14-$MgZn_2$	70	122	0	0	False	-
mp-570466	Ca_4Li_8	12	$P6_3/mmc$ (194)	C14-$MgZn_2$	72	63	0	0	False	-
mp-570963	Cr_8Ta_4	12	$P6_3/mmc$ (194)	C14-$MgZn_2$	69	58	0	0	False	-
mp-1677	Be_8Mo_4	12	$P6_3/mmc$ (194)	C14-$MgZn_2$	74	76	0	0	False	-
mp-8723	As_2F_{10}	12	$P6_3/mmc$ (194)	AsF_5	69	41	0	0	False	-
mp-2340	Na_6O_6	12	$P-62m$ (189)	Na_2O_2	19	74	0	0	False	-
mp-409	Na_6S_6	12	$P-62m$ (189)	Na_2O_2	20	61	0	0	False	-
mp-7931	Sr_6P_6	12	$P-62m$ (189)	Na_2O_2	19	57	0	0	False	-
mp-9062	Rb_6S_6	12	$P-62m$ (189)	Na_2O_2	15	66	0	0	False	-
mp-9946	Zn_6S_6	12	$P6_3mc$ (186)	SiC	13	136	1	1	False	-
mp-567542	Pb_4I_8	12	$P6_3mc$ (186)	PbI_2	8	132	0	0	False	-
mp-23794	$F_2H_8N_2$	12	$P6_3mc$ (186)	H_4	14	60	0	4	False	-
mp-15567	$Au_6Na_{18}S_{12}$	12	$R-3c$ (167)	Na_3AuS_2	53	83	0	0	False	-

ID	Formula	Z	SP (*No.*)	Prototype	NL	NR	WPs	HDWPs	Clean	dE/dq
mp-15571	$Au_6K_{18}Se_{12}$	12	$R-3c$ (167)	Na_3AuS_2	55	88	0	0	False	-
mp-779545	$Cu_6Na_{18}O_{12}$	12	$R-3c$ (167)	Na_3AuS_2	57	102	0	0	False	-
mp-867700	$Cu_6Li_{18}S_{12}$	12	$R-3c$ (167)	Na_3AuS_2	54	104	0	0	False	-
mp-14796	$K_6Pt_{12}Se_{18}$	12	$R-3m$ (166)	KPt_2Se_3	15	74	0	0	False	-
mp-14797	$Pt_{12}Rb_6Se_{18}$	12	$R-3m$ (166)	KPt_2Se_3	13	79	0	0	False	-
mp-30533	$K_6Pt_{12}S_{18}$	12	$R-3m$ (166)	KPt_2Se_3	12	67	0	0	False	-
mp-573316	$Cs_6Pt_{12}Se_{18}$	12	$R-3m$ (166)	KPt_2Se_3	14	67	0	0	False	-
mp-23758	$K_3Na_3B_6H_{24}$	12	$R-3m$ (166)	$KNa(BH_4)_2$	13	46	0	0	True	1.17
mp-160	B_{36}	12	$R-3m$ (166)	B	20	92	0	0	False	-
mp-541487	$Pt_4Tl_2Se_6$	12	$P-3m1$ (164)	$TlPt_2Se_3$	30	70	0	0	False	-
mp-9272	$Pt_4Tl_2S_6$	12	$P-3m1$ (164)	$TlPt_2Se_3$	26	79	0	0	False	-
mp-643004	$FeMg_2SrH_8$	12	$P-3m1$ (164)	$SrMg_2FeH_8$	25	52	0	0	False	-
mp-643264	$BaFeMg_2H_8$	12	$P-3m1$ (164)	$SrMg_2FeH_8$	23	56	0	0	False	-
mp-23005	Bi_4PbTe_7	12	$P-3m1$ (164)	Bi_4Te_7	26	155	0	0	False	-
mp-571414	$KNaB_2H_8$	12	$P3m1$ (156)	$KNa(BH_4)_2$	14	10	4	0	False	-
mp-570136	Cu_6I_6	12	$P3m1$ (156)	CuI	10	42	3	0	False	-
mp-4236	$Ba_3Zn_3O_6$	12	$P3_121$ (152)	$BaZnO_2$	0	1	107	0	False	-
mp-756018	$Ba_3Mg_3O_6$	12	$P3_121$ (152)	$BaZnO_2$	0	0	97	0	False	-
mp-568428	$K_3Na_3B_6H_{24}$	12	$R-3$ (148)	$KNa(BH_4)_2$	16	19	0	0	False	-
mp-8724	$K_4O_6Te_2$	12	$P-3$ (147)	K_2TeO_3	50	28	0	0	False	-
mp-554822	CsO_8P_2	12	$P-3$ (147)	$CsSb(PO_4)_2$	38	26	0	0	False	-
mp-12929	$Sm_4As_4O_{16}$	12	$I4_1/amd$ (141)	$SmAsO_4$	36	84	0	0	False	-
mp-12944	$Ho_4As_4O_{16}$	12	$I4_1/amd$ (141)	$SmAsO_4$	36	81	0	0	False	-
mp-34081	$Na_4Y_4F_{16}$	12	$I4_1/amd$ (141)	$NaYF_4$	54	121	0	0	False	-
mp-674459	$Er_4Na_4F_{16}$	12	$I4_1/amd$ (141)	$NaYF_4$	54	112	0	0	False	-
mp-675798	$Ho_4Na_4F_{16}$	12	$I4_1/amd$ (141)	$NaYF_4$	54	120	0	0	False	-
mp-2940	$Lu_4O_{16}P_4$	12	$I4_1/amd$ (141)	$LuPO_4$	36	89	0	0	False	-
mp-3248	$Tb_4As_4O_{16}$	12	$I4_1/amd$ (141)	$LuPO_4$	36	74	0	0	False	-
mp-4006	$Er_4O_{16}P_4$	12	$I4_1/amd$ (141)	$LuPO_4$	36	74	0	0	False	-
mp-4104	$Ho_4O_{16}P_4$	12	$I4_1/amd$ (141)	$LuPO_4$	36	81	0	0	False	-
mp-4200	$Sc_4O_{16}P_4$	12	$I4_1/amd$ (141)	$LuPO_4$	36	96	0	0	False	-
mp-4340	$Tb_4O_{16}P_4$	12	$I4_1/amd$ (141)	$LuPO_4$	36	78	0	0	False	-
mp-4609	$Hf_4O_{16}Si_4$	12	$I4_1/amd$ (141)	$LuPO_4$	36	91	0	0	False	-
mp-4624	$Ta_4B_4O_{16}$	12	$I4_1/amd$ (141)	$LuPO_4$	36	88	0	0	False	-
mp-4790	$Th_4Ge_4O_{16}$	12	$I4_1/amd$ (141)	$LuPO_4$	37	86	0	0	False	-
mp-4820	$Zr_4O_{16}Si_4$	12	$I4_1/amd$ (141)	$LuPO_4$	36	98	0	0	False	-
mp-5132	$Y_4O_{16}P_4$	12	$I4_1/amd$ (141)	$LuPO_4$	36	80	0	0	False	-
mp-546125	$Sc_4As_4O_{16}$	12	$I4_1/amd$ (141)	$LuPO_4$	36	113	0	0	False	-
mp-5632	$Dy_4As_4O_{16}$	12	$I4_1/amd$ (141)	$LuPO_4$	36	76	0	0	False	-
mp-5767	$Dy_4O_{16}P_4$	12	$I4_1/amd$ (141)	$LuPO_4$	36	68	0	0	False	-
mp-5836	$Th_4O_{16}Si_4$	12	$I4_1/amd$ (141)	$LuPO_4$	36	75	0	0	False	-
mp-5884	$Tm_4O_{16}P_4$	12	$I4_1/amd$ (141)	$LuPO_4$	36	88	0	0	False	-
mp-755357	$Nd_4As_4O_{16}$	12	$I4_1/amd$ (141)	$LuPO_4$	36	95	0	0	False	-
mp-7644	$Lu_4As_4O_{16}$	12	$I4_1/amd$ (141)	$LuPO_4$	36	88	0	0	True	12.67
mp-768863	$Pr_4As_4O_{16}$	12	$I4_1/amd$ (141)	$LuPO_4$	36	90	0	0	False	-
mp-771977	$Tm_4As_4O_{16}$	12	$I4_1/amd$ (141)	$LuPO_4$	36	90	0	0	False	-
mp-772156	$Er_4As_4O_{16}$	12	$I4_1/amd$ (141)	$LuPO_4$	36	90	0	0	False	-
mp-772261	$La_4As_4O_{16}$	12	$I4_1/amd$ (141)	$LuPO_4$	36	78	0	0	False	-
mp-8058	$Y_4As_4O_{16}$	12	$I4_1/amd$ (141)	$LuPO_4$	37	89	0	0	False	-
mp-8615	$Nb_4B_4O_{16}$	12	$I4_1/amd$ (141)	$LuPO_4$	36	86	0	0	False	-
mp-29537	$Cl_{16}O_8$	12	$I4_1/amd$ (141)	Cl_2	54	94	0	0	False	-
mp-12622	$Pd_4Sr_4F_{16}$	12	$I4/mcm$ (140)	$SrPdF_4$	18	63	0	0	False	-
mp-12623	$Ba_4Pd_4F_{16}$	12	$I4/mcm$ (140)	$SrPdF_4$	19	84	0	0	False	-
mp-16060	$Ag_4Au_4F_{16}$	12	$I4/mcm$ (140)	$SrPdF_4$	18	68	0	0	False	-
mp-20805	$Pb_4Pd_4F_{16}$	12	$I4/mcm$ (140)	$SrPdF_4$	18	66	0	0	False	-
mp-23550	$K_4Br_4F_{16}$	12	$I4/mcm$ (140)	$SrPdF_4$	18	52	0	0	False	-
mp-3419	$Au_4Rb_4F_{16}$	12	$I4/mcm$ (140)	$SrPdF_4$	18	52	0	0	False	-
mp-5309	$Au_4K_4F_{16}$	12	$I4/mcm$ (140)	$SrPdF_4$	18	53	0	0	True	7.05
mp-7386	$Ag_4Na_4F_{16}$	12	$I4/mcm$ (140)	$SrPdF_4$	18	59	0	0	False	-
mp-7388	$Au_4Na_4F_{16}$	12	$I4/mcm$ (140)	$SrPdF_4$	18	60	0	0	False	-
mp-8161	$Ca_4Pd_4F_{16}$	12	$I4/mcm$ (140)	$SrPdF_4$	18	105	0	0	False	-
mp-10547	$Au_8Rb_8O_8$	12	$I4/mmm$ (139)	RbAuO	0	70	0	0	False	-

ID	Formula	Z	SP ($No.$)	Prototype	NL	NR	WPs	HDWPs	Clean	dE/dq
mp-3074	$Ag_8K_8O_8$	12	$I4/mmm$ (139)	RbAuO	0	66	0	0	False	-
mp-5127	$Cu_8Li_8O_8$	12	$I4/mmm$ (139)	RbAuO	0	88	0	0	False	-
mp-6983	$Ag_8Na_8O_8$	12	$I4/mmm$ (139)	RbAuO	1	81	0	0	False	-
mp-7469	$Cu_8Na_8O_8$	12	$I4/mmm$ (139)	RbAuO	0	83	0	0	False	-
mp-7470	$Cu_8Rb_8O_8$	12	$I4/mmm$ (139)	RbAuO	0	77	0	0	False	-
mp-7911	$Cu_8K_8O_8$	12	$I4/mmm$ (139)	RbAuO	0	74	0	0	False	-
mp-8603	$Ag_8Rb_8O_8$	12	$I4/mmm$ (139)	RbAuO	0	87	0	0	False	-
mp-8666	$Ag_8Cs_8O_8$	12	$I4/mmm$ (139)	RbAuO	0	82	0	0	False	-
mp-27478	$Na_6Pa_2F_{16}$	12	$I4/mmm$ (139)	Na_3PaF_8	0	71	0	0	False	-
mp-756235	$Ba_6Sr_2I_{16}$	12	$I4/mmm$ (139)	Na_3PaF_8	0	146	0	0	False	-
mp-772290	$Ag_8Li_8O_8$	12	$I4/mmm$ (139)	LiAgO	0	87	0	0	False	-
mp-28592	$Li_{14}Br_6O_4$	12	$I4/mmm$ (139)	$Li_7Br_3O_2$	0	91	0	0	False	-
mp-3125	$K_6Zn_4F_{14}$	12	$I4/mmm$ (139)	$Li_7Br_3O_2$	0	105	0	0	False	-
mp-3349	$Sr_6Ti_4O_{14}$	12	$I4/mmm$ (139)	$Li_7Br_3O_2$	0	146	0	0	False	-
mp-568137	$Cs_6Mg_4Cl_{14}$	12	$I4/mmm$ (139)	$Li_7Br_3O_2$	1	83	0	0	False	-
mp-754128	$Ba_6Hf_4O_{14}$	12	$I4/mmm$ (139)	$Li_7Br_3O_2$	0	93	0	0	False	-
mp-755895	$Ba_6Zr_4O_{14}$	12	$I4/mmm$ (139)	$Li_7Br_3O_2$	0	110	0	0	False	-
mp-37722	$K_2B_2H_8$	12	$P4_2/nmc$ (137)	KBH_4	90	70	0	0	True	5.53
mp-38725	$Na_2B_2H_8$	12	$P4_2/nmc$ (137)	KBH_4	90	60	0	0	False	-
mp-24409	$H_8I_2P_2$	12	$P4/nmm$ (129)	PH_4	72	53	0	0	False	-
mp-27689	$Br_2H_8P_2$	12	$P4/nmm$ (129)	PH_4	72	58	0	0	False	-
mp-29456	$Rb_4I_2N_6$	12	$P4/mbm$ (127)	Rb_2IN_3	72	83	0	0	False	-
mp-27888	$Zr_6Ge_2O_{16}$	12	$I{-}42m$ (121)	Na_3PaF_8	0	38	29	0	False	-
mp-561942	$Cs_6Ta_2O_{16}$	12	$I{-}42m$ (121)	Cs_3TaO_8	0	25	14	0	False	-
mp-28020	$Al_2Cu_2Cl_8$	12	$P{-}42c$ (112)	$AlCuCl_4$	18	31	37	0	False	-
mp-29362	$Cu_2Ga_2Cl_8$	12	$P{-}42c$ (112)	$AlCuCl_4$	18	38	61	0	False	-
mp-756638	$Nb_4Rh_4O_{16}$	12	$I4_1md$ (109)	$NbRhO_4$	36	44	31	0	False	-
mp-760402	$Rh_4Ta_4O_{16}$	12	$I4_1md$ (109)	$NbRhO_4$	36	46	22	0	False	-
mp-563025	$Na_{12}Pb_2O_{10}$	12	$I4mm$ (107)	Na_6PbO_5	0	77	18	0	False	-
mp-7812	Ge_4O_8	12	$P4_12_12$ (92)	GeO_2	108	14	40	9	True	27.87
mp-27205	Tl_8Cl_{16}	12	$I4_1/a$ (88)	$TlCl_2$	19	65	0	0	False	-
mp-13530	$Sm_4As_4O_{16}$	12	$I4_1/a$ (88)	$SmAsO_4$	18	70	0	0	False	-
mp-23034	$Sr_4Zn_4Cl_{16}$	12	$I4_1/a$ (88)	$SmAsO_4$	18	84	0	0	False	-
mp-23450	$Rb_4I_4O_{16}$	12	$I4_1/a$ (88)	$SmAsO_4$	18	61	0	0	False	-
mp-23562	$K_4I_4O_{16}$	12	$I4_1/a$ (88)	$SmAsO_4$	18	63	0	0	False	-
mp-23704	$Al_4Na_4H_{16}$	12	$I4_1/a$ (88)	$SmAsO_4$	18	52	0	0	False	-
mp-27385	$K_4Tl_4Cl_{16}$	12	$I4_1/a$ (88)	$SmAsO_4$	18	80	0	0	False	-
mp-27401	$Na_4I_4O_{16}$	12	$I4_1/a$ (88)	$SmAsO_4$	18	54	0	0	False	-
mp-28567	$Bi_4Li_4F_{16}$	12	$I4_1/a$ (88)	$SmAsO_4$	18	65	0	0	False	-
mp-3700	$Li_4Y_4F_{16}$	12	$I4_1/a$ (88)	$SmAsO_4$	18	78	0	0	False	-
mp-4035	$Rb_4Re_4O_{16}$	12	$I4_1/a$ (88)	$SmAsO_4$	18	57	0	0	False	-
mp-4757	$K_4Re_4O_{16}$	12	$I4_1/a$ (88)	$SmAsO_4$	18	55	0	0	False	-
mp-5078	$Sr_4Zn_4F_{16}$	12	$I4_1/a$ (88)	$SmAsO_4$	19	68	0	0	False	-
mp-5558	$Na_4Re_4O_{16}$	12	$I4_1/a$ (88)	$SmAsO_4$	18	52	0	0	False	-
mp-559598	$Li_4Sc_4F_{16}$	12	$I4_1/a$ (88)	$SmAsO_4$	20	107	0	0	False	-
mp-561068	$Bi_4As_4O_{16}$	12	$I4_1/a$ (88)	$SmAsO_4$	18	55	0	0	False	-
mp-644486	$Al_4K_4H_{16}$	12	$I4_1/a$ (88)	$SmAsO_4$	18	57	0	0	False	-
mp-7094	$Ag_4Re_4O_{16}$	12	$I4_1/a$ (88)	$SmAsO_4$	18	48	0	0	False	-
mp-754109	$Pr_4As_4O_{16}$	12	$I4_1/a$ (88)	$SmAsO_4$	19	59	0	0	False	-
mp-754717	$Rb_4Br_4O_{16}$	12	$I4_1/a$ (88)	$SmAsO_4$	18	56	0	0	False	-
mp-754964	$Hf_4Zr_4O_{16}$	12	$I4_1/a$ (88)	$SmAsO_4$	23	110	0	0	False	-
mp-8042	$Zr_4Ge_4O_{16}$	12	$I4_1/a$ (88)	$SmAsO_4$	19	76	0	0	False	-
mp-9755	$Hf_4Ge_4O_{16}$	12	$I4_1/a$ (88)	$SmAsO_4$	20	67	0	0	False	-
mp-9756	$Th_4Ge_4O_{16}$	12	$I4_1/a$ (88)	$SmAsO_4$	18	60	0	0	False	-
mp-976291	$Al_4Li_4H_{16}$	12	$I4_1/a$ (88)	$SmAsO_4$	19	58	0	0	False	-
mp-16577	$Ca_2Hf_2Li_4F_{16}$	12	$I{-}4$ (82)	Li_2CaHfF_8	0	0	9	0	False	-
mp-27227	$Ag_{12}Li_4O_8$	12	$Ibam$ (72)	$LiAg_3O_2$	36	141	0	0	False	-
mp-27303	$Ag_{12}Na_4O_8$	12	$Ibam$ (72)	$LiAg_3O_2$	36	106	0	0	False	-
mp-15999	$Au_4Li_{12}S_8$	12	$Ibam$ (72)	Li_3AuS_2	37	108	0	0	False	-
mp-3527	$Ag_4Na_{12}O_8$	12	$Ibam$ (72)	Li_3AuS_2	37	100	0	0	False	-
mp-754352	$Cu_4Na_{12}O_8$	12	$Ibam$ (72)	Li_3AuS_2	36	112	0	0	False	-
mp-20256	$In_4K_{12}P_8$	12	$Ibam$ (72)	K_3InP_2	37	112	0	0	False	-

ID	Formula	Z	SP (*No.*)	Prototype	NL	NR	WPs	HDWPs	Clean	dE/dq
mp-5122	$Al_4Na_{12}P_8$	12	*Ibam* (72)	K_3InP_2	36	126	0	0	False	-
mp-8543	$Al_4Na_{12}As_8$	12	*Ibam* (72)	K_3InP_2	36	134	0	0	False	-
mp-21510	$In_4K_8Na_4As_8$	12	*Ibam* (72)	$K_2NaInAs_2$	36	115	0	0	False	-
mp-21511	$In_4K_8Na_4P_8$	12	*Ibam* (72)	$K_2NaInAs_2$	36	95	0	0	False	-
mp-6450	$Al_4K_8Li_4P_8$	12	*Ibam* (72)	$K_2NaInAs_2$	36	114	0	0	False	-
mp-9068	$Al_4K_8Na_4P_8$	12	*Ibam* (72)	$K_2NaInAs_2$	36	120	0	0	False	-
mp-9069	$Al_4K_8Na_4As_8$	12	*Ibam* (72)	$K_2NaInAs_2$	37	105	0	0	False	-
mp-9666	$Ga_4K_8Na_4P_8$	12	*Ibam* (72)	$K_2NaInAs_2$	36	117	0	0	False	-
mp-9676	$Ga_4K_8Na_4As_8$	12	*Ibam* (72)	$K_2NaInAs_2$	36	133	0	0	False	-
mp-572767	$Au_4Cs_4F_{16}$	12	*Immm* (71)	$CsAuF_4$	0	82	0	0	False	-
mp-29829	$Au_{16}Li_{16}S_{16}$	12	*Fddd* (70)	LiAuS	108	111	0	0	False	-
mp-567644	$Cu_8Rb_4Br_{12}$	12	*Cmcm* (63)	$RbCu_2Br_3$	60	59	0	0	True	9.97
mp-12630	$Mg_4O_{16}Se_4$	12	*Cmcm* (63)	$MgSeO_4$	55	63	0	0	False	-
mp-13676	$Tl_4O_{16}P_4$	12	*Cmcm* (63)	$MgSeO_4$	58	65	0	0	False	-
mp-754358	$Zn_4O_{16}Se_4$	12	*Cmcm* (63)	$MgSeO_4$	59	53	0	0	False	-
mp-7566	$In_4O_{16}P_4$	12	*Cmcm* (63)	$MgSeO_4$	63	47	0	0	False	-
mp-7572	$Mg_4O_{16}S_4$	12	*Cmcm* (63)	$MgSeO_4$	65	73	0	0	False	-
mp-761036	$Cd_4O_{16}Se_4$	12	*Cmcm* (63)	$MgSeO_4$	60	59	0	0	False	-
mp-8459	$Cd_4O_{16}S_4$	12	*Cmcm* (63)	$MgSeO_4$	58	62	0	0	False	-
mp-770846	$Ba_{12}Sn_8O_{28}$	12	*Cmcm* (63)	$Li_7Br_3O_2$	115	193	0	0	False	-
mp-13133	$K_8Ti_4O_{12}$	12	*Cmcm* (63)	K_2TiO_3	57	53	0	0	False	-
mp-3247	$Cs_8Ti_4S_{12}$	12	*Cmcm* (63)	K_2TiO_3	60	57	0	0	False	-
mp-867730	$Cs_8Sn_4O_{12}$	12	*Cmcm* (63)	K_2TiO_3	58	41	0	0	False	-
mp-8759	$Cs_8Zr_4O_{12}$	12	*Cmcm* (63)	K_2TiO_3	58	43	0	0	False	-
mp-9856	$Cs_8Zr_4Se_{12}$	12	*Cmcm* (63)	K_2TiO_3	58	40	0	0	False	-
mp-560791	$Cs_4Na_4Ti_4O_{12}$	12	*Cmcm* (63)	$CsNaTiO_3$	60	40	0	0	False	-
mp-571390	$Cs_8Li_4Cl_{12}$	12	*Cmcm* (63)	Cs_2LiCl_3	62	62	0	0	False	-
mp-4406	$Ca_4O_{16}S_4$	12	*Cmcm* (63)	$CaSO_4$	61	68	0	0	False	-
mp-10336	$Ba_4Cu_4Y_4Te_{12}$	12	*Cmcm* (63)	$BaYCuTe_3$	59	84	0	0	False	-
mp-10337	$Ag_4Ba_4Y_4Te_{12}$	12	*Cmcm* (63)	$BaYCuTe_3$	58	84	0	0	False	-
mp-11116	$Cd_4Cs_4Y_4Se_{12}$	12	*Cmcm* (63)	$BaYCuTe_3$	56	73	0	0	False	-
mp-11118	$Cd_4Cs_4Pr_4Se_{12}$	12	*Cmcm* (63)	$BaYCuTe_3$	62	63	0	0	False	-
mp-11121	$Cd_4Cs_4Tb_4Se_{12}$	12	*Cmcm* (63)	$BaYCuTe_3$	57	52	0	0	False	-
mp-11122	$Cd_4Cs_4Dy_4Se_{12}$	12	*Cmcm* (63)	$BaYCuTe_3$	57	55	0	0	False	-
mp-11123	$Cs_4Hg_4Y_4Se_{12}$	12	*Cmcm* (63)	$BaYCuTe_3$	58	48	0	0	False	-
mp-12341	$Cs_4Pr_4Zn_4Te_{12}$	12	*Cmcm* (63)	$BaYCuTe_3$	60	62	0	0	False	-
mp-12342	$Cs_4Nd_4Zn_4Te_{12}$	12	*Cmcm* (63)	$BaYCuTe_3$	57	75	0	0	False	-
mp-12343	$Cs_4Sm_4Zn_4Te_{12}$	12	*Cmcm* (63)	$BaYCuTe_3$	59	79	0	0	False	-
mp-12487	$Cd_4Cs_4Dy_4Te_{12}$	12	*Cmcm* (63)	$BaYCuTe_3$	61	66	0	0	False	-
mp-12488	$Cd_4Cs_4Er_4Te_{12}$	12	*Cmcm* (63)	$BaYCuTe_3$	60	55	0	0	False	-
mp-12490	$Cd_4Cs_4Ho_4Te_{12}$	12	*Cmcm* (63)	$BaYCuTe_3$	56	69	0	0	False	-
mp-12491	$Cd_4Cs_4La_4Te_{12}$	12	*Cmcm* (63)	$BaYCuTe_3$	56	70	0	0	False	-
mp-12492	$Cd_4Cs_4Lu_4Te_{12}$	12	*Cmcm* (63)	$BaYCuTe_3$	58	51	0	0	False	-
mp-12494	$Cd_4Cs_4Pr_4Te_{12}$	12	*Cmcm* (63)	$BaYCuTe_3$	57	62	0	0	False	-
mp-12495	$Cd_4Cs_4Tb_4Te_{12}$	12	*Cmcm* (63)	$BaYCuTe_3$	57	60	0	0	False	-
mp-12496	$Cd_4Cs_4Tm_4Te_{12}$	12	*Cmcm* (63)	$BaYCuTe_3$	59	56	0	0	False	-
mp-12497	$Cd_4Cs_4Sm_4Te_{12}$	12	*Cmcm* (63)	$BaYCuTe_3$	63	60	0	0	False	-
mp-13189	$Cu_4Lu_4Sr_4S_{12}$	12	*Cmcm* (63)	$BaYCuTe_3$	60	69	0	0	False	-
mp-14969	$Ba_4Cu_4Er_4S_{12}$	12	*Cmcm* (63)	$BaYCuTe_3$	58	82	0	0	False	-
mp-505709	$Cs_4Sm_4Zn_4Se_{12}$	12	*Cmcm* (63)	$BaYCuTe_3$	60	73	0	0	False	-
mp-505712	$Cs_4Ho_4Zn_4Se_{12}$	12	*Cmcm* (63)	$BaYCuTe_3$	59	65	0	0	False	-
mp-505713	$Cs_4Tm_4Zn_4Se_{12}$	12	*Cmcm* (63)	$BaYCuTe_3$	58	62	0	0	False	-
mp-510062	$Cd_4Cs_4Sm_4Se_{12}$	12	*Cmcm* (63)	$BaYCuTe_3$	61	67	0	0	False	-
mp-510065	$Cs_4Hg_4Nd_4Se_{12}$	12	*Cmcm* (63)	$BaYCuTe_3$	59	62	0	0	False	-
mp-510460	$Cs_4La_4Zn_4Te_{12}$	12	*Cmcm* (63)	$BaYCuTe_3$	61	77	0	0	False	-
mp-510462	$Cs_4Dy_4Zn_4Te_{12}$	12	*Cmcm* (63)	$BaYCuTe_3$	59	65	0	0	False	-
mp-510569	$Ce_4Cs_4Cu_4S_{12}$	12	*Cmcm* (63)	$BaYCuTe_3$	59	62	0	0	False	-
mp-568191	$Cu_4Lu_4Sr_4Se_{12}$	12	*Cmcm* (63)	$BaYCuTe_3$	60	71	0	0	False	-
mp-569107	$Cs_4Er_4Zn_4Te_{12}$	12	*Cmcm* (63)	$BaYCuTe_3$	63	68	0	0	False	-
mp-573710	$Cs_4Tb_4Zn_4Se_{12}$	12	*Cmcm* (63)	$BaYCuTe_3$	60	57	0	0	False	-
mp-574620	$Cs_4Y_4Zn_4Se_{12}$	12	*Cmcm* (63)	$BaYCuTe_3$	58	70	0	0	False	-
mp-579556	$Cs_4Ho_4Zn_4Te_{12}$	12	*Cmcm* (63)	$BaYCuTe_3$	63	61	0	0	False	-

ID	Formula	Z	SP (*No.*)	Prototype	NL	NR	WPs	HDWPs	Clean	dE/dq
mp-638078	$Cs_4Tb_4Zn_4Te_{12}$	12	*Cmcm* (63)	$BaYCuTe_3$	58	65	0	0	False	-
mp-6647	$Ag_4Ba_4Y_4Se_{12}$	12	*Cmcm* (63)	$BaYCuTe_3$	58	76	0	0	False	-
mp-7049	$Cu_4Tl_4Zr_4S_{12}$	12	*Cmcm* (63)	$BaYCuTe_3$	62	63	0	0	False	-
mp-7155	$Cs_4Er_4Zn_4Se_{12}$	12	*Cmcm* (63)	$BaYCuTe_3$	61	68	0	0	False	-
mp-7211	$Cs_4Hg_4Pr_4Se_{12}$	12	*Cmcm* (63)	$BaYCuTe_3$	62	67	0	0	False	-
mp-7212	$Cs_4Hg_4Sm_4Se_{12}$	12	*Cmcm* (63)	$BaYCuTe_3$	61	63	0	0	False	-
mp-9317	$Cu_4K_4Zr_4S_{12}$	12	*Cmcm* (63)	$BaYCuTe_3$	60	67	0	0	False	-
mp-9396	$Cu_4Hf_4Tl_4S_{12}$	12	*Cmcm* (63)	$BaYCuTe_3$	57	59	0	0	False	-
mp-9397	$Cu_4Hf_4Tl_4Se_{12}$	12	*Cmcm* (63)	$BaYCuTe_3$	59	77	0	0	False	-
mp-9503	$Ba_4Cu_4Dy_4Te_{12}$	12	*Cmcm* (63)	$BaYCuTe_3$	59	82	0	0	False	-
mp-9855	$Cu_4Hf_4K_4S_{12}$	12	*Cmcm* (63)	$BaYCuTe_3$	60	67	0	0	False	-
mp-643718	$Ba_4Mg_4H_{16}$	12	*Cmcm* (63)	$BaMgH_4$	56	48	0	0	False	-
mp-755759	Zr_4O_8	12	*Pnma* (62)	ZrO_2	171	66	0	0	False	-
mp-1146	Th_4S_8	12	*Pnma* (62)	ThS_2	173	56	0	0	False	-
mp-23199	Ba_4Cl_8	12	*Pnma* (62)	ThS_2	172	76	0	0	False	-
mp-23260	Ba_4I_8	12	*Pnma* (62)	ThS_2	173	72	0	0	False	-
mp-23291	Pb_4Cl_8	12	*Pnma* (62)	ThS_2	171	73	0	0	False	-
mp-23713	Ca_4H_8	12	*Pnma* (62)	ThS_2	171	72	0	0	False	-
mp-23714	Sr_4H_8	12	*Pnma* (62)	ThS_2	172	69	0	0	False	-
mp-23715	Ba_4H_8	12	*Pnma* (62)	ThS_2	172	68	0	0	False	-
mp-2511	Pb_4F_8	12	*Pnma* (62)	ThS_2	171	72	0	0	False	-
mp-27456	Ba_4Br_8	12	*Pnma* (62)	ThS_2	172	80	0	0	False	-
mp-28077	Pb_4Br_8	12	*Pnma* (62)	ThS_2	171	78	0	0	False	-
mp-540703	Cs_8S_4	12	*Pnma* (62)	ThS_2	171	77	0	0	False	-
mp-555585	Ba_4F_8	12	*Pnma* (62)	ThS_2	169	60	0	0	False	-
mp-556978	Na_8S_4	12	*Pnma* (62)	ThS_2	172	91	0	0	False	-
mp-573763	Cs_8Te_4	12	*Pnma* (62)	ThS_2	172	78	0	0	False	-
mp-755738	Rb_8O_4	12	*Pnma* (62)	ThS_2	172	82	0	0	False	-
mp-7951	Th_4Se_8	12	*Pnma* (62)	ThS_2	174	69	0	0	False	-
mp-864603	Yb_4H_8	12	*Pnma* (62)	ThS_2	170	59	0	0	False	-
mp-568284	Sr_4I_8	12	*Pnma* (62)	SrI_2	172	53	0	0	False	-
mp-12418	$Ca_4Sr_4Ge_4$	12	*Pnma* (62)	SrCaGe	172	78	0	0	True	1.05
mp-16264	$Ca_4Li_4Sb_4$	12	*Pnma* (62)	SrCaGe	172	77	0	0	False	-
mp-22997	$Pb_4Br_4Cl_4$	12	*Pnma* (62)	SrCaGe	171	61	0	0	False	-
mp-28049	$Ba_4Br_4Cl_4$	12	*Pnma* (62)	SrCaGe	173	63	0	0	False	-
mp-28383	$K_4Na_4S_4$	12	*Pnma* (62)	SrCaGe	171	74	0	0	False	-
mp-28545	$Li_4Rb_4O_4$	12	*Pnma* (62)	SrCaGe	172	68	0	0	False	-
mp-28595	$K_4Na_4Se_4$	12	*Pnma* (62)	SrCaGe	171	72	0	0	False	-
mp-28603	$Li_4Na_4Se_4$	12	*Pnma* (62)	SrCaGe	173	75	0	0	False	-
mp-29238	$Ag_4Tl_4Se_4$	12	*Pnma* (62)	SrCaGe	170	75	0	0	False	-
mp-30456	$Bi_4Li_4Sr_4$	12	*Pnma* (62)	SrCaGe	170	65	0	0	False	-
mp-5874	$Ag_4Tl_4Te_4$	12	*Pnma* (62)	SrCaGe	173	81	0	0	False	-
mp-7084	$Ca_4Sr_4Si_4$	12	*Pnma* (62)	SrCaGe	171	69	0	0	False	-
mp-7756	$Li_4Sr_4Sb_4$	12	*Pnma* (62)	SrCaGe	172	71	0	0	False	-
mp-8754	$Li_4Na_4Te_4$	12	*Pnma* (62)	SrCaGe	171	65	0	0	False	-
mp-8755	$K_4Na_4Te_4$	12	*Pnma* (62)	SrCaGe	170	73	0	0	False	-
mp-29862	Sn_4Br_8	12	*Pnma* (62)	$SnBr_2$	171	69	0	0	False	-
mp-22996	$I_4Sb_4Se_4$	12	*Pnma* (62)	SbSI	174	59	0	0	False	-
mp-23041	$I_4S_4Sb_4$	12	*Pnma* (62)	SbSI	172	80	0	0	False	-
mp-28760	$K_4Rb_4S_4$	12	*Pnma* (62)	SbSI	171	69	0	0	False	-
mp-753160	$Bi_4F_4O_4$	12	*Pnma* (62)	SbSI	172	74	0	0	False	-
mp-30156	$Li_4Mg_4N_4$	12	*Pnma* (62)	LiMgN	171	68	0	0	False	-
mp-31468	$Ca_4Li_4N_4$	12	*Pnma* (62)	LiMgN	170	79	0	0	False	-
mp-28270	$Cu_4K_4S_4$	12	*Pnma* (62)	KCuS	173	43	0	0	True	1.32
mp-985782	C_4H_8	12	*Pnma* (62)	H_2	179	40	0	0	True	24.65
mp-5014	$Ag_4Cu_4S_4$	12	*Pnma* (62)	CuAgS	174	73	0	0	False	-
mp-23711	Mg_4H_8	12	*Pbcn* (60)	MgH_2	170	68	0	0	False	-
mp-569051	Mg_4H_8	12	*Pbcn* (60)	MgH_2	171	70	0	0	False	-
mp-571232	Yb_4Br_8	12	*Pbcn* (60)	MgH_2	172	78	0	0	False	-
mp-753737	$Cu_4Li_{12}S_8$	12	*Pbcn* (60)	Li_3CuS_2	345	171	0	0	False	-
mp-555018	$Au_2Na_4Rb_2O_4$	12	*Pnnm* (58)	$RbNa_2AuO_2$	162	49	0	0	False	-
mp-559067	$Au_2K_2Na_4O_4$	12	*Pnnm* (58)	$RbNa_2AuO_2$	162	62	0	0	False	-

ID	Formula	Z	SP (*No.*)	Prototype	NL	NR	WPs	HDWPs	Clean	dE/dq
mp-27449	$Ga_4Cl_4Te_4$	12	$Pnnm$ (58)	GaTeCl	163	48	0	0	False	-
mp-643009	$Mg_4Sr_4H_{16}$	12	$Cmc2_1$ (36)	$SrMgH_4$	62	49	3	0	False	-
mp-14433	$Na_4Ge_8N_{12}$	12	$Cmc2_1$ (36)	$NaGe_2N_3$	58	59	11	0	False	-
mp-5853	$Li_4N_{12}Si_8$	12	$Cmc2_1$ (36)	$NaGe_2N_3$	60	77	15	0	False	-
mp-8973	$Na_4N_{12}Si_8$	12	$Cmc2_1$ (36)	$NaGe_2N_3$	58	70	16	0	False	-
mp-9248	$Cu_8S_{12}Si_4$	12	$Cmc2_1$ (36)	$NaGe_2N_3$	59	59	5	0	False	-
mp-15349	$Li_8Ge_4O_{12}$	12	$Cmc2_1$ (36)	Li_2GeO_3	59	60	19	0	False	-
mp-3933	$Mg_8N_{12}P_4$	12	$Cmc2_1$ (36)	Li_2GeO_3	57	70	11	0	False	-
mp-4533	$Na_8O_{12}Si_4$	12	$Cmc2_1$ (36)	Li_2GeO_3	58	50	15	0	False	-
mp-5012	$Li_8O_{12}Si_4$	12	$Cmc2_1$ (36)	Li_2GeO_3	62	53	21	0	False	-
mp-5784	$Na_8Ge_4O_{12}$	12	$Cmc2_1$ (36)	Li_2GeO_3	61	79	15	0	False	-
mp-21489	$Pb_4Rb_8O_{12}$	12	$Cmc2_1$ (36)	K_2TiO_3	58	45	8	0	False	-
mp-21521	$Cs_8Pb_4O_{12}$	12	$Cmc2_1$ (36)	K_2TiO_3	63	59	9	0	False	-
mp-753798	$Rb_8Sn_4O_{12}$	12	$Cmc2_1$ (36)	K_2TiO_3	57	49	13	0	False	-
mp-756156	$Rb_8Zr_4O_{12}$	12	$Cmc2_1$ (36)	K_2TiO_3	61	53	15	0	False	-
mp-556866	$Ag_8Hg_4I_8S_4$	12	$Cmc2_1$ (36)	Ag_2HgSI_2	61	55	10	0	False	-
mp-22889	Zn_4Cl_8	12	$Pna2_1$ (33)	$ZnCl_2$	146	69	1	8	False	-
mp-402	Cd_4P_8	12	$Pna2_1$ (33)	CdP_2	147	56	0	9	False	-
mp-20817	$Pt_4Ge_4Se_4$	12	$Pca2_1$ (29)	GePtSe	126	86	0	16	False	-
mp-4627	$Co_4As_4S_4$	12	$Pca2_1$ (29)	GePtSe	128	81	0	15	False	-
mp-9270	$Ir_4S_4Sb_4$	12	$Pca2_1$ (29)	GePtSe	129	65	0	18	False	-
mp-28368	$Au_4Tl_4Cl_{16}$	12	$C2/c$ (15)	$TlAuCl_4$	0	61	0	0	False	-
mp-569442	$Au_4Rb_4Cl_{16}$	12	$C2/c$ (15)	$TlAuCl_4$	0	59	0	0	False	-
mp-561536	$Mg_4Na_4B_4O_{12}$	12	$C2/c$ (15)	$NaMgBO_3$	0	100	0	0	False	-
mp-756400	$Bi_4Nd_4O_{16}$	12	$C2/c$ (15)	$LuTaO_4$	0	46	0	0	False	-
mp-768383	$Bi_4Sm_4O_{16}$	12	$C2/c$ (15)	$LuTaO_4$	0	60	0	0	False	-
mp-12263	$Au_4Li_4F_{16}$	12	$C2/c$ (15)	$LiAuF_4$	1	70	0	0	False	-
mp-3054	$Li_8C_4O_{12}$	12	$C2/c$ (15)	Li_2CO_3	0	61	0	0	False	-
mp-573658	$Cs_{12}As_8B_4$	12	$C2/c$ (15)	Cs_3BAs_2	0	44	0	0	False	-
mp-9664	$K_{12}B_4P_8$	12	$C2/c$ (15)	Cs_3BAs_2	4	69	0	0	False	-
mp-9665	$K_{12}As_8B_4$	12	$C2/c$ (15)	Cs_3BAs_2	2	57	0	0	False	-
mp-9675	$Cs_{12}B_4P_8$	12	$C2/c$ (15)	Cs_3BAs_2	0	50	0	0	False	-
mp-9718	$Rb_{12}As_8B_4$	12	$C2/c$ (15)	Cs_3BAs_2	4	61	0	0	False	-
mp-9720	$Rb_{12}B_4P_8$	12	$C2/c$ (15)	Cs_3BAs_2	2	65	0	0	True	1.32
mp-27577	$Cd_8As_{12}I_4$	12	$C2/c$ (15)	Cd_2As_3	0	43	0	0	False	-
mp-28875	$Hg_8Cl_4P_{12}$	12	$C2/c$ (15)	Cd_2As_3	2	49	0	0	False	-
mp-28899	$Hg_8As_{12}Br_4$	12	$C2/c$ (15)	Cd_2As_3	1	62	0	0	False	-
mp-28900	$Cd_8As_{12}Br_4$	12	$C2/c$ (15)	Cd_2As_3	0	52	0	0	False	-
mp-29244	$Cd_8I_4P_{12}$	12	$C2/c$ (15)	Cd_2As_3	1	58	0	0	False	-
mp-29245	$Cd_8Br_4P_{12}$	12	$C2/c$ (15)	Cd_2As_3	0	68	0	0	False	-
mp-644431	$Cd_8Cl_4P_{12}$	12	$C2/c$ (15)	Cd_2As_3	1	72	0	0	False	-
mp-754199	$Ba_4Sr_4I_{16}$	12	$C2/c$ (15)	$BaSrI_4$	0	72	0	0	False	-
mp-570340	$Ag_4Au_4Cl_{16}$	12	$C2/c$ (15)	$AgAuCl_4$	0	48	0	0	False	-
mp-27741	$Au_4I_4Te_4$	12	$P2_1/c$ (14)	TeAuI	90	97	0	0	False	-
mp-30996	$Au_2Rb_2Br_8$	12	$P2_1/c$ (14)	$RbAuBr_4$	92	80	0	0	False	-
mp-27747	Pd_4I_8	12	$P2_1/c$ (14)	PdI_2	92	84	0	0	False	-
mp-28319	Pt_4I_8	12	$P2_1/c$ (14)	PdI_2	90	65	0	0	False	-
mp-10155	Ir_4P_8	12	$P2_1/c$ (14)	P_2	92	81	0	0	False	-
mp-1247	Ir_4Sb_8	12	$P2_1/c$ (14)	P_2	91	110	0	0	False	-
mp-14285	Co_4P_8	12	$P2_1/c$ (14)	P_2	91	98	0	0	False	-
mp-15649	Ir_4As_8	12	$P2_1/c$ (14)	P_2	94	87	0	0	False	-
mp-27180	$Nd_4Br_4S_4$	12	$P2_1/c$ (14)	NdSBr	91	118	0	0	False	-
mp-4165	$Ta_4N_4O_4$	12	$P2_1/c$ (14)	NdSBr	91	112	0	0	False	-
mp-7596	$Nb_4N_4O_4$	12	$P2_1/c$ (14)	NdSBr	90	119	0	0	False	-
mp-13568	Nd_4S_8	12	$P2_1/c$ (14)	NdS_2	92	108	0	0	False	-
mp-753257	$Cu_2Li_2F_8$	12	$P2_1/c$ (14)	$LiCuF_4$	91	86	0	0	False	-
mp-29463	$Be_4Li_4N_4$	12	$P2_1/c$ (14)	LiBeN	90	100	0	0	False	-
mp-753748	$Cu_4Li_{12}S_8$	12	$P2_1/c$ (14)	Li_3CuS_2	184	284	0	0	False	-
mp-642817	$K_4H_4O_4$	12	$P2_1/c$ (14)	KHO	91	108	0	0	False	-
mp-29234	$In_4I_4Te_4$	12	$P2_1/c$ (14)	InTeI	90	81	0	0	True	4.22
mp-29236	$In_4Br_4Te_4$	12	$P2_1/c$ (14)	InTeI	91	77	0	0	False	-
mp-27904	$Fe_4S_4Sb_4$	12	$P2_1/c$ (14)	FeSbS	90	85	0	0	False	-

ID	Formula	Z	SP (*No.*)	Prototype	NL	NR	WPs	HDWPs	Clean	dE/dq
mp-561511	$Fe_4As_4S_4$	12	$P2_1/c$ (14)	FeSbS	91	106	0	0	False	-
mp-8200	Ag_4P_8	12	$P2_1/c$ (14)	AgP_2	91	89	0	0	False	-
mp-927	Cu_4P_8	12	$P2_1/c$ (14)	AgP_2	92	82	0	0	False	-
mp-753809	$Ba_2Ca_2I_8$	12	$P2/c$ (13)	$BaCaI_4$	37	165	0	0	False	-
mp-28580	$Y_8Cl_{12}N_4$	12	$C2/m$ (12)	Y_2NCl_3	0	71	0	0	False	-
mp-14333	$Al_4Ta_4O_{16}$	12	$C2/m$ (12)	$TaAlO_4$	0	68	0	0	False	-
mp-27927	$Al_4Nb_4O_{16}$	12	$C2/m$ (12)	$TaAlO_4$	0	70	0	0	False	-
mp-1863	$As_{12}Si_{12}$	12	$C2/m$ (12)	SiAs	0	51	0	0	False	-
mp-542812	$Ga_{12}Te_{12}$	12	$C2/m$ (12)	SiAs	0	52	0	0	False	-
mp-10985	$Cu_{12}Rb_4S_8$	12	$C2/m$ (12)	$RbCu_3S_2$	1	93	0	0	False	-
mp-9868	$Cu_{12}K_4S_8$	12	$C2/m$ (12)	$RbCu_3S_2$	1	83	0	0	False	-
mp-6660	$Ba_4Na_4B_4O_{12}$	12	$C2/m$ (12)	$NaMgBO_3$	0	66	0	0	False	-
mp-656662	$Na_{10}Re_2O_{12}$	12	$C2/m$ (12)	Na_5ReO_6	1	46	0	0	False	-
mp-8253	$Na_{10}Re_2O_{12}$	12	$C2/m$ (12)	Na_5ReO_6	1	41	0	0	False	-
mp-7219	$Na_8Zr_4Se_{12}$	12	$C2/m$ (12)	Na_2ZrSe_3	2	69	0	0	False	-
mp-754745	$Na_8Sn_4O_{12}$	12	$C2/m$ (12)	Na_2ZrSe_3	2	61	0	0	False	-
mp-760483	$Li_8Pd_4O_{12}$	12	$C2/m$ (12)	Na_2ZrSe_3	1	63	0	0	False	-
mp-5061	$Li_{10}Re_2O_{12}$	12	$C2/m$ (12)	Li_5ReO_6	1	59	0	0	False	-
mp-556475	$K_4La_4Pd_4O_{12}$	12	$C2/m$ (12)	$KLaPdO_3$	0	58	0	0	False	-
mp-557232	$K_4Pd_4Sm_4O_{12}$	12	$C2/m$ (12)	$KLaPdO_3$	0	72	0	0	False	-
mp-558831	$K_4Nd_4Pd_4O_{12}$	12	$C2/m$ (12)	$KLaPdO_3$	0	55	0	0	False	-
mp-560569	$K_4Pd_4Pr_4O_{12}$	12	$C2/m$ (12)	$KLaPdO_3$	0	58	0	0	False	-
mp-567836	$K_8Na_4B_4P_8$	12	$C2/m$ (12)	K_2NaBP_2	0	75	0	0	False	-
mp-9548	$As_{12}Ge_{12}$	12	$C2/m$ (12)	GeAs	0	77	0	0	False	-
mp-559025	$Ag_4Ba_4Er_4S_{12}$	12	$C2/m$ (12)	$BaErAgS_3$	0	103	0	0	False	-
mp-24124	$Nd_2Cl_2H_4O_4$	12	$P2_1/m$ (11)	NdH_2ClO_2	55	78	0	0	False	-
mp-9853	Lu_2P_{10}	12	$P2_1/m$ (11)	LuP_5	54	58	0	0	False	-
mp-976713	$Ho_2Cl_2H_4O_4$	12	$P2_1/m$ (11)	HoH_2ClO_2	55	77	0	0	False	-
mp-579536	$Cs_2Cu_4Cl_4I_2$	12	$P2_1/m$ (11)	$CsCu_2ICl_2$	54	89	0	0	False	-
mp-774736	$Cu_8Li_8S_8$	12	Cc (9)	LiAuS	1	43	0	0	False	-
mp-15895	$Cu_8S_{12}Si_4$	12	Cc (9)	Cu_2SiS_3	2	62	0	0	False	-
mp-29246	$Cd_8Cl_4P_{12}$	12	Cc (9)	Cd_2As_3	2	55	0	0	False	-
mp-8710	$Ca_6Pt_9O_{24}$	13	$R{-}3m$ (166)	$Na_2Ti_3Cl_8$	16	78	0	0	True	24.12
mp-560874	$Ba_3Pb_6C_6F_6O_{18}$	13	$R{-}3m$ (166)	$BaPb_2C_2(O_3F)_2$	17	40	0	0	False	-
mp-3857	$Ba_9O_{24}P_6$	13	$R{-}3m$ (166)	$Ba_3(PO_4)_2$	23	42	0	0	True	2
mp-4632	$Sr_9O_{24}P_6$	13	$R{-}3m$ (166)	$Ba_3(PO_4)_2$	18	42	0	0	False	-
mp-9783	$Ba_9As_6O_{24}$	13	$R{-}3m$ (166)	$Ba_3(PO_4)_2$	19	45	0	0	False	-
mp-3006	$Pd_3P_2S_8$	13	$P{-}3m1$ (164)	$P_2Pd_3S_8$	29	66	0	0	False	-
mp-554959	$Al_2K_2O_7Sb_2$	13	$P{-}3m1$ (164)	$K_2Al_2Sb_2O_7$	27	108	0	0	False	-
mp-556275	$Al_2Rb_2O_7Sb_2$	13	$P{-}3m1$ (164)	$K_2Al_2Sb_2O_7$	30	82	0	0	False	-
mp-557359	$Al_2Cs_2O_7Sb_2$	13	$P{-}3m1$ (164)	$K_2Al_2Sb_2O_7$	29	86	0	0	False	-
mp-27563	$In_4Rb_2O_7$	13	$P{-}31m$ (162)	$Rb_2In_4O_7$	49	88	0	0	False	-
mp-569978	$Na_6Ti_9Cl_{24}$	13	$R3m$ (160)	$Na_2Ti_3Cl_8$	18	29	0	0	False	-
mp-7941	$Li_{18}O_{18}Te_3$	13	$R{-}3$ (148)	Li_6TeO_6	31	25	0	0	False	-
mp-569675	$Hg_4Pd_2Tl_4Cl_{16}$	13	$C2/m$ (12)	$Tl_2Hg_2PdCl_8$	0	67	0	0	False	-
mp-28879	$Ca_{10}P_{16}$	13	$C2/m$ (12)	Ca_5P_8	1	95	0	0	False	-
mp-10811	$Na_2Sr_8B_6N_{12}$	14	$Im{-}3m$ (229)	$NaSr_4(BN_2)_3$	37	81	0	0	False	-
mp-6799	$Ca_8Li_2B_6N_{12}$	14	$Im{-}3m$ (229)	$NaSr_4(BN_2)_3$	37	68	0	0	True	1.62
mp-9723	$Li_2Sr_8B_6N_{12}$	14	$Im{-}3m$ (229)	$NaSr_4(BN_2)_3$	35	42	0	0	False	-
mp-754381	$Zr_{24}N_{32}$	14	$Fd{-}3m$ (227)	Zr_3N_4	44	72	0	0	False	-
mp-755988	$Hf_{24}N_{32}$	14	$Fd{-}3m$ (227)	Zr_3N_4	44	71	0	0	False	-
mp-10191	$Lu_{16}Mg_8Se_{32}$	14	$Fd{-}3m$ (227)	H11-Al_2MgO_4	42	80	0	0	False	-
mp-10953	$Cd_8Sc_{16}S_{32}$	14	$Fd{-}3m$ (227)	H11-Al_2MgO_4	45	76	0	0	False	-
mp-14100	$Cd_8Rh_{16}O_{32}$	14	$Fd{-}3m$ (227)	H11-Al_2MgO_4	38	61	0	0	False	-
mp-14304	$Lu_{16}Mg_8S_{32}$	14	$Fd{-}3m$ (227)	H11-Al_2MgO_4	42	86	0	0	False	-
mp-14307	$Mg_8Sc_{16}S_{32}$	14	$Fd{-}3m$ (227)	H11-Al_2MgO_4	47	84	0	0	False	-
mp-14620	$Cd_8Tm_{16}Se_{32}$	14	$Fd{-}3m$ (227)	H11-Al_2MgO_4	46	89	0	0	False	-
mp-15802	$Mg_8Tm_{16}Se_{32}$	14	$Fd{-}3m$ (227)	H11-Al_2MgO_4	44	86	0	0	False	-
mp-15803	$Mg_8Y_{16}Se_{32}$	14	$Fd{-}3m$ (227)	H11-Al_2MgO_4	43	77	0	0	False	-
mp-16267	$Cd_8Dy_{16}S_{32}$	14	$Fd{-}3m$ (227)	H11-Al_2MgO_4	45	87	0	0	False	-
mp-19803	$Cd_8In_{16}O_{32}$	14	$Fd{-}3m$ (227)	H11-Al_2MgO_4	42	74	0	0	False	-
mp-20493	$In_{16}Mg_8S_{32}$	14	$Fd{-}3m$ (227)	H11-Al_2MgO_4	44	69	0	0	False	-

ID	Formula	Z	SP (*No.*)	Prototype	NL	NR	WPs	HDWPs	Clean	dE/dq
mp-22052	$In_{16}Zn_8S_{32}$	14	$Fd{-}3m$ (227)	H11-Al_2MgO_4	39	90	0	0	False	-
mp-22587	$Cd_8In_{16}Se_{32}$	14	$Fd{-}3m$ (227)	H11-Al_2MgO_4	44	76	0	0	False	-
mp-22766	$Ca_8In_{16}O_{32}$	14	$Fd{-}3m$ (227)	H11-Al_2MgO_4	44	61	0	0	False	-
mp-22961	$Li_{16}Zn_8Cl_{32}$	14	$Fd{-}3m$ (227)	H11-Al_2MgO_4	42	72	0	0	False	-
mp-2908	$Al_{16}Zn_8O_{32}$	14	$Fd{-}3m$ (227)	H11-Al_2MgO_4	44	73	0	0	False	-
mp-3041	$Cd_8Er_{16}S_{32}$	14	$Fd{-}3m$ (227)	H11-Al_2MgO_4	43	85	0	0	False	-
mp-3319	$Mg_8Rh_{16}O_{32}$	14	$Fd{-}3m$ (227)	H11-Al_2MgO_4	41	64	0	0	False	-
mp-3443	$Cd_8Ga_{16}O_{32}$	14	$Fd{-}3m$ (227)	H11-Al_2MgO_4	42	71	0	0	False	-
mp-3536	$Al_{16}Mg_8O_{32}$	14	$Fd{-}3m$ (227)	H11-Al_2MgO_4	45	106	0	0	False	-
mp-35785	$Cd_8Y_{16}S_{32}$	14	$Fd{-}3m$ (227)	H11-Al_2MgO_4	45	70	0	0	False	-
mp-36866	$Al_{16}Cd_8O_{32}$	14	$Fd{-}3m$ (227)	H11-Al_2MgO_4	43	69	0	0	False	-
mp-3807	$Al_{16}Cd_8Se_{32}$	14	$Fd{-}3m$ (227)	H11-Al_2MgO_4	42	76	0	0	False	-
mp-3904	$Mg_{16}Ge_8O_{32}$	14	$Fd{-}3m$ (227)	H11-Al_2MgO_4	42	81	0	0	False	-
mp-3930	$Mn_8Sc_{16}S_{32}$	14	$Fd{-}3m$ (227)	H11-Al_2MgO_4	44	73	0	0	False	-
mp-4284	$Cd_8Ho_{16}Se_{32}$	14	$Fd{-}3m$ (227)	H11-Al_2MgO_4	45	83	0	0	False	-
mp-4324	$Cd_8Tm_{16}S_{32}$	14	$Fd{-}3m$ (227)	H11-Al_2MgO_4	43	73	0	0	False	-
mp-4590	$Ga_{16}Mg_8O_{32}$	14	$Fd{-}3m$ (227)	H11-Al_2MgO_4	43	78	0	0	False	-
mp-4842	$Al_{16}Zn_8S_{32}$	14	$Fd{-}3m$ (227)	H11-Al_2MgO_4	41	77	0	0	False	-
mp-5146	$Rh_{16}Zn_8O_{32}$	14	$Fd{-}3m$ (227)	H11-Al_2MgO_4	43	68	0	0	False	-
mp-559200	$Cd_8In_{16}S_{32}$	14	$Fd{-}3m$ (227)	H11-Al_2MgO_4	44	66	0	0	False	-
mp-570959	$Cd_8Dy_{16}Se_{32}$	14	$Fd{-}3m$ (227)	H11-Al_2MgO_4	45	75	0	0	False	-
mp-5794	$Ga_{16}Zn_8O_{32}$	14	$Fd{-}3m$ (227)	H11-Al_2MgO_4	44	62	0	0	False	-
mp-6942	$Cd_8Ho_{16}S_{32}$	14	$Fd{-}3m$ (227)	H11-Al_2MgO_4	44	94	0	0	False	-
mp-753418	$Nd_{16}Sr_8O_{32}$	14	$Fd{-}3m$ (227)	H11-Al_2MgO_4	47	90	0	0	False	-
mp-754114	$Sc_{16}Sr_8O_{32}$	14	$Fd{-}3m$ (227)	H11-Al_2MgO_4	46	67	0	0	False	-
mp-754211	$La_{16}Sr_8O_{32}$	14	$Fd{-}3m$ (227)	H11-Al_2MgO_4	45	94	0	0	True	20.13
mp-754942	$Sm_{16}Sr_8O_{32}$	14	$Fd{-}3m$ (227)	H11-Al_2MgO_4	46	84	0	0	True	17.15
mp-755044	$Ca_8Tb_{16}O_{32}$	14	$Fd{-}3m$ (227)	H11-Al_2MgO_4	48	66	0	0	False	-
mp-755239	$Ga_{16}Hg_8O_{32}$	14	$Fd{-}3m$ (227)	H11-Al_2MgO_4	43	65	0	0	False	-
mp-755558	$Ba_8La_{16}O_{32}$	14	$Fd{-}3m$ (227)	H11-Al_2MgO_4	45	97	0	0	True	17.74
mp-756297	$In_{16}Zn_8O_{32}$	14	$Fd{-}3m$ (227)	H11-Al_2MgO_4	45	70	0	0	False	-
mp-756646	$Lu_{16}Sr_8O_{32}$	14	$Fd{-}3m$ (227)	H11-Al_2MgO_4	42	67	0	0	False	-
mp-7831	$In_{16}Mg_8O_{32}$	14	$Fd{-}3m$ (227)	H11-Al_2MgO_4	43	71	0	0	False	-
mp-8269	$Cd_8Lu_{16}S_{32}$	14	$Fd{-}3m$ (227)	H11-Al_2MgO_4	43	76	0	0	True	2.48
mp-973261	$Mg_{16}Sn_8O_{32}$	14	$Fd{-}3m$ (227)	H11-Al_2MgO_4	43	74	0	0	False	-
mp-11661	$Zr_{12}N_{16}$	14	$I{-}43d$ (220)	Zr_3N_4	87	54	8	17	False	-
mp-37405	$Al_{20}Cu_4Se_{32}$	14	$F{-}43m$ (216)	Al_5CuSe_8	21	24	4	6	False	-
mp-20694	$K_8Pb_8O_{12}$	14	$I2_13$ (199)	$K_2Pb_2O_3$	1	2	115	19	False	-
mp-558658	$Y_4C_2N_4O_4$	14	$P6_3/mmc$ (194)	$Y_2C(NO)_2$	16	96	0	0	False	-
mp-28989	$Li_{10}BrN_3$	14	$P{-}6m2$ (187)	$Li_{10}BrN_3$	0	26	47	0	False	-
mp-7191	$Ga_2Sr_6N_6$	14	$P6_3/m$ (176)	Sr_3GaN_3	20	48	0	0	False	-
mp-6179	$K_2Li_2O_8S_2$	14	$P6_3$ (173)	$KLiSO_4$	0	9	40	0	True	1.57
mp-6253	$Be_2K_2Li_2F_8$	14	$P6_3$ (173)	$KLiSO_4$	0	6	51	0	False	-
mp-23582	$Bi_3K_3Li_{18}O_{18}$	14	$R{-}3m$ (166)	KLi_6BiO_6	38	85	0	0	False	-
mp-9059	$K_3Li_{18}Ta_3O_{18}$	14	$R{-}3m$ (166)	KLi_6BiO_6	34	90	0	0	False	-
mp-754781	$Ga_{18}N_6O_{18}$	14	$R{-}3m$ (166)	Ga_3NO_3	28	117	0	0	False	-
mp-28395	$B_{36}P_6$	14	$R{-}3m$ (166)	B_6	23	81	0	0	False	-
mp-624	As_6B_{36}	14	$R{-}3m$ (166)	B_6	26	45	0	0	False	-
mp-1346	$B_{36}O_6$	14	$R{-}3m$ (166)	B_6	24	111	0	0	False	-
mp-8564	$CsK_2ScO_8P_2$	14	$P{-}3m1$ (164)	$CsK_2Sc(PO_4)_2$	34	118	0	0	False	-
mp-579560	$Cs_3Br_9Sb_2$	14	$P{-}3m1$ (164)	$Cs_3Sb_2Br_9$	37	108	0	0	False	-
mp-29415	$Cd_6Cl_6P_2$	14	$P{-}3m1$ (164)	Cd_3PCl_3	39	85	0	0	False	-
mp-753677	$Bi_6Li_{18}S_{18}$	14	$R3c$ (161)	Li_3BiS_3	46	46	5	7	False	-
mp-543011	$Zn_{21}S_{21}$	14	$R3m$ (160)	ZnS	83	131	39	0	False	-
mp-9947	$C_{21}Si_{21}$	14	$R3m$ (160)	SiC	78	77	36	0	False	-
mp-9480	$Al_2K_2O_8Si_2$	14	$P31c$ (159)	$KLiSO_4$	84	119	0	5	False	-
mp-861906	$BaMgNa_2O_8P_2$	14	$P{-}3$ (147)	$CsK_2Sc(PO_4)_2$	53	41	0	0	False	-
mp-754060	$Bi_3Li_{21}O_{18}$	14	$R3$ (146)	Li_7BiO_6	0	0	23	0	False	-
mp-15845	$Li_{16}Sr_4N_8$	14	$I4_1/amd$ (141)	$SrLi_4N_2$	62	139	0	0	False	-
mp-753815	$Ca_4Y_8O_{16}$	14	$I4_1/amd$ (141)	H11-Al_2MgO_4	63	125	0	0	False	-
mp-754454	$Ag_8Cd_4O_{16}$	14	$I4_1/amd$ (141)	$Cd(AgO_2)_2$	63	119	0	0	False	-
mp-559446	$Na_4Pd_4P_4S_{16}$	14	$I4/mcm$ (140)	$NaPPdS_4$	22	65	0	0	False	-

ID	Formula	Z	SP (*No.*)	Prototype	NL	NR	WPs	HDWPs	Clean	dE/dq
mp-642735	$Pt_2Rb_4H_8$	14	$P4_2/mnm$ (136)	Rb_2H_4	105	64	0	0	True	3.35
mp-643010	$Cs_4Pt_2H_8$	14	$P4_2/mnm$ (136)	Rb_2H_4	105	64	0	0	False	-
mp-644494	$Pd_2Rb_4H_8$	14	$P4_2/mnm$ (136)	Rb_2H_4	105	72	0	0	False	-
mp-974713	$K_4Pt_2H_8$	14	$P4_2/mnm$ (136)	Rb_2H_4	105	58	0	0	False	-
mp-15844	$Ta_2O_{10}P_2$	14	$P4/nmm$ (129)	$TaPO_5$	84	90	0	0	False	-
mp-28505	$Al_4Ba_2Te_8$	14	$P4/nbm$ (125)	$Ba(AlTe_2)_2$	63	95	0	0	False	-
mp-33406	$La_8Pb_4Se_{16}$	14	I−42d (122)	Pr_2PbS_4	42	51	74	0	False	-
mp-33569	$La_8Sr_4Se_{16}$	14	I−42d (122)	Pr_2PbS_4	53	53	81	0	False	-
mp-33606	$Ba_4Pr_8Se_{16}$	14	I−42d (122)	Pr_2PbS_4	53	52	80	0	False	-
mp-33615	$Ba_4La_8S_{16}$	14	I−42d (122)	Pr_2PbS_4	42	44	58	0	False	-
mp-34011	$Ca_4Ho_8S_{16}$	14	I−42d (122)	Pr_2PbS_4	83	64	60	0	False	-
mp-34141	$La_8Sr_4S_{16}$	14	I−42d (122)	Pr_2PbS_4	42	46	55	0	False	-
mp-34185	$Ca_4Pr_8S_{16}$	14	I−42d (122)	Pr_2PbS_4	58	44	55	0	False	-
mp-34508	$Sm_8Sr_4S_{16}$	14	I−42d (122)	Pr_2PbS_4	53	57	59	0	False	-
mp-35421	$Ca_4La_8S_{16}$	14	I−42d (122)	Pr_2PbS_4	59	49	57	0	False	-
mp-35787	$Ca_4Dy_8S_{16}$	14	I−42d (122)	Pr_2PbS_4	80	53	54	0	False	-
mp-35876	$Ca_4Nd_8S_{16}$	14	I−42d (122)	Pr_2PbS_4	63	47	50	0	False	-
mp-36007	$Ba_4Nd_8Se_{16}$	14	I−42d (122)	Pr_2PbS_4	53	59	74	0	False	-
mp-36031	$Ca_4La_8Te_{16}$	14	I−42d (122)	Pr_2PbS_4	63	52	78	0	False	-
mp-36100	$Ca_4Sm_8S_{16}$	14	I−42d (122)	Pr_2PbS_4	58	53	59	0	False	-
mp-36223	$Ba_4Pr_8S_{16}$	14	I−42d (122)	Pr_2PbS_4	53	59	53	0	False	-
mp-36538	$La_8Pb_4S_{16}$	14	I−42d (122)	Pr_2PbS_4	42	47	55	0	False	-
mp-37108	$Nd_8Sr_4S_{16}$	14	I−42d (122)	Pr_2PbS_4	53	49	67	0	False	-
mp-37263	$Ca_4Nd_8Te_{16}$	14	I−42d (122)	Pr_2PbS_4	63	51	69	0	False	-
mp-37520	$Ca_4Pr_8Te_{16}$	14	I−42d (122)	Pr_2PbS_4	63	66	73	0	False	-
mp-37741	$Ba_4La_8Se_{16}$	14	I−42d (122)	Pr_2PbS_4	42	51	80	0	False	-
mp-37859	$Pr_8Sr_4Se_{16}$	14	I−42d (122)	Pr_2PbS_4	53	69	80	0	False	-
mp-38152	$Sm_8Sr_4Se_{16}$	14	I−42d (122)	Pr_2PbS_4	53	50	69	1	False	-
mp-38240	$Pr_8Sr_4S_{16}$	14	I−42d (122)	Pr_2PbS_4	53	63	69	0	False	-
mp-38327	$Ca_4Tb_8S_{16}$	14	I−42d (122)	Pr_2PbS_4	62	51	59	0	False	-
mp-39033	$Nd_8Sr_4Se_{16}$	14	I−42d (122)	Pr_2PbS_4	53	51	71	0	False	-
mp-673682	$Tb_8Yb_4S_{16}$	14	I−42d (122)	Pr_2PbS_4	53	53	55	0	False	-
mp-674804	$Pb_4Sm_8Se_{16}$	14	I−42d (122)	Pr_2PbS_4	52	50	57	0	False	-
mp-675100	$Nd_8Pb_4Se_{16}$	14	I−42d (122)	Pr_2PbS_4	53	51	56	0	False	-
mp-675146	$Pb_4Pr_8Se_{16}$	14	I−42d (122)	Pr_2PbS_4	53	63	78	0	False	-
mp-675244	$Nd_8Yb_4S_{16}$	14	I−42d (122)	Pr_2PbS_4	62	52	60	0	False	-
mp-675293	$Y_8Yb_4S_{16}$	14	I−42d (122)	Pr_2PbS_4	53	43	64	0	False	-
mp-675406	$Ho_8Yb_4S_{16}$	14	I−42d (122)	Pr_2PbS_4	53	53	66	0	False	-
mp-675458	$La_8Pb_4Te_{16}$	14	I−42d (122)	Pr_2PbS_4	74	40	78	0	False	-
mp-675511	$Pb_4Sm_8S_{16}$	14	I−42d (122)	Pr_2PbS_4	53	45	57	0	True	11.95
mp-675620	$Nd_8Pb_4S_{16}$	14	I−42d (122)	Pr_2PbS_4	53	37	54	0	False	-
mp-675638	$Pb_4Pr_8S_{16}$	14	I−42d (122)	Pr_2PbS_4	53	40	54	0	True	11.31
mp-675668	$Pr_8Yb_4S_{16}$	14	I−42d (122)	Pr_2PbS_4	59	49	58	0	False	-
mp-675677	$Sm_8Yb_4S_{16}$	14	I−42d (122)	Pr_2PbS_4	72	40	63	0	False	-
mp-675767	$La_8Yb_4S_{16}$	14	I−42d (122)	Pr_2PbS_4	62	51	56	0	False	-
mp-676154	$Dy_8Yb_4S_{16}$	14	I−42d (122)	Pr_2PbS_4	53	53	62	0	False	-
mp-673677	$La_4Th_6O_{18}$	14	I−4m2 (119)	$La_2Th_3O_9$	0	37	123	0	False	-
mp-2898	$Au_8Ca_4O_{16}$	14	$I4_1/a$ (88)	$Ca(AuO_2)_2$	21	69	0	0	False	-
mp-752451	$Cu_8Zn_4O_{16}$	14	$I4_1/a$ (88)	$Ca(AuO_2)_2$	23	76	0	0	False	-
mp-9297	$Au_8Ba_4O_{16}$	14	$I4_1/a$ (88)	$Ca(AuO_2)_2$	21	84	0	0	False	-
mp-9298	$Au_8Sr_4O_{16}$	14	$I4_1/a$ (88)	$Ca(AuO_2)_2$	22	74	0	0	False	-
mp-33449	$Ag_2Tb_{10}S_{16}$	14	I−4 (82)	Tb_5AgS_8	0	3	12	0	False	-
mp-34034	$Ba_{16}Br_2P_{10}$	14	I−4 (82)	Tb_5AgS_8	0	1	15	0	False	-
mp-35512	$Ag_2Dy_{10}S_{16}$	14	I−4 (82)	Tb_5AgS_8	0	1	11	0	False	-
mp-35714	$La_{10}Tl_2S_{16}$	14	I−4 (82)	Tb_5AgS_8	0	1	14	0	False	-
mp-35907	$La_{10}Tl_2Te_{16}$	14	I−4 (82)	Tb_5AgS_8	0	0	22	0	False	-
mp-37449	$Ag_2Nd_{10}S_{16}$	14	I−4 (82)	Tb_5AgS_8	0	1	15	0	False	-
mp-37829	$La_{10}Tl_2Se_{16}$	14	I−4 (82)	Tb_5AgS_8	0	1	19	0	False	-
mp-37923	$Ag_2Sm_{10}S_{16}$	14	I−4 (82)	Tb_5AgS_8	0	3	20	0	False	-
mp-38442	$Cs_2Nd_{10}Se_{16}$	14	I−4 (82)	Tb_5AgS_8	0	1	20	0	False	-
mp-38805	$Ag_2Sm_{10}Se_{16}$	14	I−4 (82)	Tb_5AgS_8	0	0	15	0	False	-
mp-33940	$Mg_8Pt_4O_{16}$	14	*Imma* (74)	Mg_2PtO_4	21	152	0	0	False	-

ID	Formula	Z	SP (*No.*)	Prototype	NL	NR	WPs	HDWPs	Clean	dE/dq
mp-34022	$Mg_8Sn_4O_{16}$	14	*Imma* (74)	Mg_2PtO_4	21	183	0	0	False	-
mp-35493	$Sn_4Zn_8O_{16}$	14	*Imma* (74)	Mg_2PtO_4	22	175	0	0	False	-
mp-35647	$Pt_4Zn_8O_{16}$	14	*Imma* (74)	Mg_2PtO_4	21	168	0	0	False	-
mp-38008	$Cd_4Li_8Cl_{16}$	14	*Imma* (74)	Mg_2PtO_4	22	152	0	0	False	-
mp-38307	$Al_8Mg_4O_{16}$	14	*Imma* (74)	Mg_2PtO_4	22	235	0	0	False	-
mp-38684	$Li_8Mg_4Cl_{16}$	14	*Imma* (74)	Mg_2PtO_4	22	168	0	0	False	-
mp-38951	$Ga_8Mg_4O_{16}$	14	*Imma* (74)	Mg_2PtO_4	21	201	0	0	False	-
mp-676338	$In_8Mg_4O_{16}$	14	*Imma* (74)	Mg_2PtO_4	21	198	0	0	False	-
mp-760515	$Ba_6Sr_8O_{14}$	14	*Immm* (71)	$Ba_3Sr_4O_7$	1	270	0	0	False	-
mp-2131	Sr_8N_{48}	14	*Fddd* (70)	SrN_6	84	89	0	0	True	2.3
mp-676	Ca_8N_{48}	14	*Fddd* (70)	SrN_6	84	120	0	0	False	-
mp-13995	$Hg_{16}Ge_8O_{32}$	14	*Fddd* (70)	Hg_2GeO_4	84	105	0	0	False	-
mp-779704	$Hg_{16}O_{32}Si_8$	14	*Fddd* (70)	Hg_2GeO_4	84	76	0	0	False	-
mp-28491	$Cu_{16}O_{32}S_8$	14	*Fddd* (70)	Cu_2SO_4	84	69	0	0	False	-
mp-11641	$Ag_{16}O_{32}Se_8$	14	*Fddd* (70)	Ag_2SeO_4	84	86	0	0	False	-
mp-4530	$Cd_{16}O_{32}Si_8$	14	*Fddd* (70)	Ag_2SeO_4	84	86	0	0	False	-
mp-4770	$Na_{16}O_{32}S_8$	14	*Fddd* (70)	Ag_2SeO_4	84	103	0	0	False	-
mp-5141	$Na_{16}O_{32}Se_8$	14	*Fddd* (70)	Ag_2SeO_4	84	92	0	0	False	-
mp-20397	$In_8Sr_4Te_{16}$	14	*Cccm* (66)	$Sr(InTe_2)_2$	61	99	0	0	False	-
mp-6987	$Ga_8Sr_4Te_{16}$	14	*Cccm* (66)	$Sr(InTe_2)_2$	61	99	0	0	False	-
mp-7841	$Ba_4Ga_8Se_{16}$	14	*Cccm* (66)	$Sr(InTe_2)_2$	62	84	0	0	False	-
mp-8422	$Al_8Sr_4Se_{16}$	14	*Cccm* (66)	$Sr(InTe_2)_2$	63	94	0	0	False	-
mp-560605	$Au_2La_8Li_2O_{16}$	14	*Cmmm* (65)	$LiLa_4AuO_8$	0	122	0	0	False	-
mp-567756	$La_8C_4Cl_4N_{12}$	14	*Cmmm* (65)	La_2CN_3	0	78	0	0	True	3.29
mp-17662	$Lu_8Pd_4O_{16}$	14	*Cmce* (64)	Lu_2PdO_4	91	104	0	0	False	-
mp-8153	$Hg_4Na_4O_{16}P_4$	14	*Cmcm* (63)	$NaHgPO_4$	65	63	0	0	False	-
mp-3143	$Na_8O_{16}S_4$	14	*Cmcm* (63)	Na_2SO_4	70	77	0	0	False	-
mp-643023	$Mg_8Ru_4H_{16}$	14	*Cmcm* (63)	Mg_2H_4	69	62	0	0	False	-
mp-756578	$La_4Mg_4I_{20}$	14	*Cmcm* (63)	$LaMgI_5$	66	81	0	0	False	-
mp-772082	$Ca_4La_4I_{20}$	14	*Cmcm* (63)	$LaMgI_5$	69	78	0	0	False	-
mp-9029	$Ca_{12}V_4N_{12}$	14	*Cmcm* (63)	Ca_3VN_3	72	93	0	0	False	-
mp-752684	$Ba_4Tm_8O_{16}$	14	*Cmcm* (63)	$BaTm_2O_4$	70	88	0	0	False	-
mp-752726	$Ba_4La_8O_{16}$	14	*Cmcm* (63)	$BaTm_2O_4$	67	100	0	0	False	-
mp-756354	$Sc_8Sr_4O_{16}$	14	*Cmcm* (63)	$BaTm_2O_4$	65	96	0	0	False	-
mp-756580	$Lu_8Sr_4O_{16}$	14	*Cmcm* (63)	$BaTm_2O_4$	70	99	0	0	False	-
mp-780535	$Ba_4Lu_8O_{16}$	14	*Cmcm* (63)	$BaTm_2O_4$	69	88	0	0	False	-
mp-8263	$K_2Na_4B_2O_6$	14	*Pmmn* (59)	KNa_2BO_3	147	107	0	0	False	-
mp-8872	$Na_4Rb_2B_2O_6$	14	*Pmmn* (59)	KNa_2BO_3	147	110	0	0	False	-
mp-20944	$Pb_2Sr_4O_8$	14	*Pbam* (55)	Sr_2PbO_4	147	79	0	0	False	-
mp-21137	$Ca_4Pb_2O_8$	14	*Pbam* (55)	Sr_2PbO_4	148	93	0	0	False	-
mp-28657	$Mg_2Na_4Cl_8$	14	*Pbam* (55)	Sr_2PbO_4	149	105	0	0	False	-
mp-28658	$Cd_2Na_4Cl_8$	14	*Pbam* (55)	Sr_2PbO_4	147	124	0	0	False	-
mp-4747	$Ca_4Sn_2O_8$	14	*Pbam* (55)	Sr_2PbO_4	148	79	0	0	False	-
mp-554191	$Na_4U_2O_8$	14	*Pbam* (55)	Sr_2PbO_4	147	67	0	0	False	-
mp-752413	$Ca_4Hf_2O_8$	14	*Pbam* (55)	Sr_2PbO_4	147	97	0	0	False	-
mp-755597	$Ca_4Ce_2O_8$	14	*Pbam* (55)	Sr_2PbO_4	147	111	0	0	False	-
mp-14288	$Sr_{24}P_{32}$	14	*Fdd*2 (43)	Sr_3P_4	84	29	0	0	False	-
mp-14289	$Ba_{24}P_{32}$	14	*Fdd*2 (43)	Sr_3P_4	84	24	0	0	False	-
mp-15339	$Sr_{24}As_{32}$	14	*Fdd*2 (43)	Sr_3P_4	84	45	1	0	False	-
mp-30293	$Sr_8Ge_4Se_{16}$	14	*Ama*2 (40)	Sr_2GeSe_4	0	27	0	0	False	-
mp-30294	$Sn_4Sr_8S_{16}$	14	*Ama*2 (40)	Sr_2GeSe_4	0	25	1	0	False	-
mp-20496	$Ga_8Pb_4O_{16}$	14	*Ama*2 (40)	Ga_2PbO_4	0	15	0	0	False	-
mp-555954	$Ba_4Hg_4Sn_4S_{16}$	14	*Ama*2 (40)	$BaSnHgS_4$	0	27	0	0	False	-
mp-29976	$Na_{12}B_4S_{12}$	14	*C*2/*c* (15)	Na_3BS_3	1	63	0	0	False	-
mp-23720	$Al_4Be_4H_{20}$	14	*C*2/*c* (15)	$BeAlH_5$	1	69	0	0	False	-
mp-756564	$Ba_4Y_4Cl_{20}$	14	*C*2/*c* (15)	$BaYCl_5$	2	118	0	0	False	-
mp-3622	$Na_4Pd_2F_8$	14	$P2_1/c$ (14)	Na_2PdF_4	106	138	0	0	False	-
mp-22999	$Hg_6Cl_4O_4$	14	$P2_1/c$ (14)	$Hg_3(ClO)_2$	106	117	0	0	False	-
mp-29297	$Cd_6Cl_4O_4$	14	$P2_1/c$ (14)	$Hg_3(ClO)_2$	107	125	0	0	False	-
mp-754047	$Rb_{12}O_{12}Sb_4$	14	*C*2/*m* (12)	Rb_3SbO_3	1	59	0	0	False	-
mp-11691	$Hg_4Nd_8O_{16}$	14	*C*2/*m* (12)	Nd_2HgO_4	0	68	0	0	False	-
mp-779287	$Hg_4La_8O_{16}$	14	*C*2/*m* (12)	Nd_2HgO_4	0	77	0	0	False	-

ID	Formula	Z	SP (*No.*)	Prototype	NL	NR	WPs	HDWPs	Clean	dE/dq
mp-14434	$K_{12}Ge_4S_{12}$	14	$C2/m$ (12)	K_3GeS_3	0	87	0	0	False	-
mp-14435	$K_{12}Ge_4Se_{12}$	14	$C2/m$ (12)	K_3GeS_3	0	75	0	0	False	-
mp-7657	$K_{12}Si_4Te_{12}$	14	$C2/m$ (12)	K_3GeS_3	0	66	0	0	False	-
mp-13743	$Ga_4K_{12}O_{12}$	14	$C2/m$ (12)	K_3GaO_3	3	63	0	0	False	-
mp-13744	$Ga_4Rb_{12}O_{12}$	14	$C2/m$ (12)	K_3GaO_3	0	57	0	0	False	-
mp-22303	$In_4Rb_{12}S_{12}$	14	$C2/m$ (12)	K_3GaO_3	0	91	0	0	False	-
mp-554921	$Bi_8Hg_4S_{16}$	14	$C2/m$ (12)	$Hg(BiS_2)_2$	0	99	0	0	False	-
mp-553874	$Ag_{10}Ba_4La_2S_{12}$	14	$C2/m$ (12)	$Ba_2LaAg_5S_6$	1	85	0	0	False	-
mp-28490	$Tl_6B_2Se_6$	14	$P2_1/m$ (11)	Tl_3BSe_3	63	81	0	0	False	-
mp-755393	$La_2Sr_2I_{10}$	14	$P2_1/m$ (11)	$SrLaI_5$	63	162	0	0	False	-
mp-755705	$La_2Sr_2I_{10}$	14	$P2_1/m$ (11)	$SrLaI_5$	65	144	0	0	False	-
mp-11902	$Ba_4Ge_2Se_8$	14	$P2_1/m$ (11)	Ba_2GeSe_4	63	113	0	0	True	1.21
mp-14447	$Ba_4Se_8Si_2$	14	$P2_1/m$ (11)	Ba_2GeSe_4	64	101	0	0	False	-
mp-568987	$Be_2Na_4H_8$	14	$P2_1/m$ (11)	Ba_2GeSe_4	63	61	0	0	False	-
mp-570803	$Ba_4Ge_2Se_4Te_4$	14	$P2_1/m$ (11)	$Ba_2Ge(TeSe)_2$	64	111	0	0	False	-
mp-28967	$Ba_2Pd_4S_8$	14	$P2_1/m$ (11)	$Ba(PdS_2)_2$	63	100	0	0	False	-
mp-674328	$In_4Zn_2S_8$	14	$P2/m$ (10)	$Zn(InS_2)_2$	1	184	0	0	False	-
mp-30126	$Ba_4B_8S_{16}$	14	Cc (9)	$Ba(BS_2)_2$	1	48	0	0	False	-
mp-27821	Ag_6P_{22}	14	Cm (8)	Ag_3P_{11}	0	15	0	0	False	-
mp-11634	$K_2Sm_2Ge_2Se_8$	14	$P2_1$ (4)	$KSmGeSe_4$	63	21	49	0	False	-
mp-12011	$K_2Tb_2Ge_2S_8$	14	$P2_1$ (4)	$KSmGeSe_4$	63	12	40	0	False	-
mp-12012	$K_2Pr_2Ge_2Se_8$	14	$P2_1$ (4)	$KSmGeSe_4$	63	25	52	0	False	-
mp-13538	$K_2Pr_2Se_8Si_2$	14	$P2_1$ (4)	$KSmGeSe_4$	63	18	44	0	False	-
mp-21097	$K_2La_2Ge_2Se_8$	14	$P2_1$ (4)	$KSmGeSe_4$	63	24	50	0	True	1.39
mp-559043	$K_2La_2Ge_2S_8$	14	$P2_1$ (4)	$KSmGeSe_4$	63	19	43	0	False	-
mp-861606	$K_2La_2Se_8Si_2$	14	$P2_1$ (4)	$KSmGeSe_4$	63	20	53	0	False	-
mp-861866	$K_2Nd_2Ge_2S_8$	14	$P2_1$ (4)	$KSmGeSe_4$	63	22	43	0	True	2.74
mp-861938	$K_2La_2S_8Si_2$	14	$P2_1$ (4)	$KSmGeSe_4$	63	22	33	0	False	-
mp-867328	$K_2Y_2S_8Si_2$	14	$P2_1$ (4)	$KSmGeSe_4$	63	23	38	0	False	-
mp-867334	$K_2Y_2Ge_2S_8$	14	$P2_1$ (4)	$KSmGeSe_4$	63	22	56	0	True	2.39
mp-23535	$K_4Zn_2Br_8$	14	$P2_1$ (4)	Ba_2GeSe_4	63	13	42	0	False	-
mp-30532	$Tl_4Zn_2I_8$	14	$P2_1$ (4)	Ba_2GeSe_4	63	20	46	0	False	-
mp-28022	$Cd_4Na_{24}Cl_{32}$	15	$Fm{-}3m$ (225)	Na_6CdCl_8	26	112	0	0	False	-
mp-28828	$Fe_4Li_{24}Cl_{32}$	15	$Fm{-}3m$ (225)	Na_6CdCl_8	23	68	0	0	False	-
mp-29008	$Li_{24}Mg_4Br_{32}$	15	$Fm{-}3m$ (225)	Na_6CdCl_8	23	67	0	0	False	-
mp-755421	$Ca_{24}Hf_4O_{32}$	15	$Fm{-}3m$ (225)	Na_6CdCl_8	25	66	0	0	False	-
mp-755556	$Ba_{24}Zr_4O_{32}$	15	$Fm{-}3m$ (225)	Na_6CdCl_8	25	60	0	0	False	-
mp-23751	$Cs_9Mg_9H_{27}$	15	$R{-}3m$ (166)	$CsMgH_3$	27	44	0	0	True	37.02
mp-696746	$B_{36}C_9$	15	$R{-}3m$ (166)	B_4	29	97	0	0	False	-
mp-6325	$Ba_3MgTa_2O_9$	15	$P{-}3m1$ (164)	$Ba_3MgTa_2O_9$	43	144	0	0	False	-
mp-6474	$Ba_3MgNb_2O_9$	15	$P{-}3m1$ (164)	$Ba_3MgTa_2O_9$	47	140	0	0	False	-
mp-7249	$Ba_3Nb_2ZnO_9$	15	$P{-}3m1$ (164)	$Ba_3MgTa_2O_9$	45	158	0	0	False	-
mp-627401	$Cd_{15}I_{30}$	15	$R3m$ (160)	CdI_2	91	85	39	0	False	-
mp-567199	Pb_5I_{10}	15	$P3m1$ (156)	PbI_2	18	92	3	0	False	-
mp-11168	$Ca_3Ir_6P_6$	15	$P3_221$ (154)	$Ca(PIr)_2$	0	1	112	0	False	-
mp-15074	$Ir_6Sr_3P_6$	15	$P3_221$ (154)	$Ca(PIr)_2$	0	0	130	0	False	-
mp-22538	$Li_{24}Pb_3O_{18}$	15	$R{-}3$ (148)	Li_8PbO_6	26	12	0	0	False	-
mp-752922	$Hf_3Li_{24}O_{18}$	15	$R{-}3$ (148)	Li_8PbO_6	23	25	0	0	False	-
mp-754266	$Li_{24}Ti_3S_{18}$	15	$R{-}3$ (148)	Li_8PbO_6	27	14	0	0	False	-
mp-755225	$Li_{24}Zr_3O_{18}$	15	$R{-}3$ (148)	Li_8PbO_6	25	7	0	0	False	-
mp-632760	$Na_2PtH_6O_6$	15	$P{-}3$ (147)	$Na_2H_6PtO_6$	60	49	0	0	False	-
mp-27487	$Li_6Th_3N_6$	15	$P{-}3$ (147)	Li_2ThN_2	62	26	0	0	False	-
mp-28712	$Hg_{12}O_{14}Si_4$	15	$C2/m$ (12)	$Si_2Hg_6O_7$	0	59	0	0	False	-
mp-567219	$Ca_6Li_8N_{12}Si_4$	15	$C2/m$ (12)	$Li_4Ca_3(SiN_3)_2$	1	74	0	0	False	-
mp-567371	$Li_8Sr_6Ge_4N_{12}$	15	$C2/m$ (12)	$Li_4Ca_3(SiN_3)_2$	2	63	0	0	False	-
mp-11131	$Hg_6K_4Ge_4S_{16}$	15	$C2$ (5)	$K_2Hg_3(GeS_4)_2$	0	0	26	0	False	-
mp-11151	$Na_{16}Pr_{16}Te_{32}$	16	$Fd{-}3m$ (227)	$LiRhO_2$	51	104	0	0	False	-
mp-14476	$Li_{16}Rh_{16}O_{32}$	16	$Fd{-}3m$ (227)	$LiRhO_2$	51	104	0	0	True	11.04
mp-24119	$Ca_{32}H_{16}N_{16}$	16	$Fd{-}3m$ (227)	$LiRhO_2$	49	78	0	0	False	-
mp-5478	$Pd_{24}Tl_8O_{32}$	16	$Fm{-}3m$ (225)	$TlPd_3O_4$	24	64	0	0	True	12.9
mp-7331	$Cs_8Fe_4Mg_4C_{24}N_{24}$	16	$Fm{-}3m$ (225)	$Cs_2MgFe(CN)_6$	18	59	0	0	True	1.99
mp-23287	Cu_8Cl_8	16	$Pa{-}3$ (205)	$CuCl$	133	57	0	0	False	-

ID	Formula	Z	SP (*No.*)	Prototype	NL	NR	WPs	HDWPs	Clean	dE/dq
mp-1106023	Ni_8Sb_{24}	16	$Im-3$ (204)	$NiSb_3$	49	128	0	0	False	-
mp-1239	Ir_8Sb_{24}	16	$Im-3$ (204)	$NiSb_3$	49	130	0	0	False	-
mp-1317	Co_8Sb_{24}	16	$Im-3$ (204)	$NiSb_3$	54	116	0	0	False	-
mp-1357	Rh_8P_{24}	16	$Im-3$ (204)	$NiSb_3$	50	118	0	0	False	-
mp-13853	Ir_8P_{24}	16	$Im-3$ (204)	$NiSb_3$	48	103	0	0	False	-
mp-1944	Co_8P_{24}	16	$Im-3$ (204)	$NiSb_3$	51	117	0	0	False	-
mp-2301	Ni_8P_{24}	16	$Im-3$ (204)	$NiSb_3$	54	152	0	0	False	-
mp-2395	Rh_8Sb_{24}	16	$Im-3$ (204)	$NiSb_3$	51	130	0	0	False	-
mp-452	Co_8As_{24}	16	$Im-3$ (204)	$NiSb_3$	53	146	0	0	False	-
mp-8182	Rh_8As_{24}	16	$Im-3$ (204)	$NiSb_3$	52	138	0	0	False	-
mp-540912	Ir_8As_{24}	16	$Im-3$ (204)	As_3	48	137	0	0	False	-
mp-9702	$Sr_6B_2O_6P_2$	16	$P6_3/mmc$ (194)	Sr_3BPO_3	17	92	0	0	False	-
mp-9712	$Ba_6B_2O_6P_2$	16	$P6_3/mmc$ (194)	Sr_3BPO_3	41	100	0	0	False	-
mp-9793	$Ba_6As_2B_2O_6$	16	$P6_3/mmc$ (194)	Sr_3BPO_3	42	101	0	0	False	-
mp-27791	$Be_6Sr_2O_8$	16	$P-62c$ (190)	$SrBe_3O_4$	92	86	0	2	False	-
mp-558491	$Al_2Ba_2B_2F_4O_6$	16	$P-62c$ (190)	$BaAlBO_3F_2$	89	67	0	5	False	-
mp-561118	Zn_8S_8	16	$P6_3mc$ (186)	ZnS	17	222	5	0	False	-
mp-10509	$Ba_2Ga_2B_2F_4O_6$	16	$P6_3/m$ (176)	$BaGaBO_3F_2$	21	20	0	0	True	1.05
mp-10510	$Al_2Ba_2B_2F_4O_6$	16	$P6_3/m$ (176)	$BaGaBO_3F_2$	20	20	0	0	True	1.14
mp-11166	$Ba_2Zn_2C_2F_4O_6$	16	$P6_3/m$ (176)	$BaGaBO_3F_2$	89	43	0	0	True	2.97
mp-15573	$Tl_6As_2O_8$	16	$P6_3$ (173)	Tl_3AsO_4	72	15	74	0	False	-
mp-5709	$Tl_6O_8P_2$	16	$P6_3$ (173)	Tl_3AsO_4	70	26	85	0	False	-
mp-29331	$K_6F_{24}H_{18}$	16	$R-3c$ (167)	KH_3F_4	68	122	0	0	True	1.84
mp-28815	$Sn_2Th_2I_{12}$	16	$P-31c$ (163)	$ThSnI_6$	90	159	0	0	False	-
mp-505148	$Pb_2Th_2I_{12}$	16	$P-31c$ (163)	$ThSnI_6$	95	172	0	0	False	-
mp-28220	$Fe_2Hf_2Cl_{12}$	16	$P-31c$ (163)	$HfFeCl_6$	69	104	0	0	False	-
mp-8704	$K_{18}Sb_6Se_{24}$	16	$R3c$ (161)	K_3SbSe_4	66	28	6	8	False	-
mp-556468	$Zn_{24}S_{24}$	16	$R3m$ (160)	ZnS	96	138	29	0	False	-
mp-556576	$Zn_{24}S_{24}$	16	$R3m$ (160)	ZnS	94	113	74	1	False	-
mp-557151	$Zn_{24}S_{24}$	16	$R3m$ (160)	ZnS	97	118	49	0	False	-
mp-570641	$C_{24}Si_{24}$	16	$R3m$ (160)	SiC	89	90	43	0	False	-
mp-556005	Zn_8S_8	16	$P3m1$ (156)	ZnS	8	94	4	0	False	-
mp-28080	$K_8Sn_4Te_{20}$	16	$I4/mcm$ (140)	K_2SnTe_5	24	99	0	0	False	-
mp-28520	$Rb_8Sn_4Te_{20}$	16	$I4/mcm$ (140)	K_2SnTe_5	24	121	0	0	False	-
mp-570844	$Ga_{12}Os_4$	16	$P4_2/mnm$ (136)	Ga_3	120	121	0	0	False	-
mp-559893	$Bi_2Li_2Pd_4O_8$	16	$P4/nmm$ (129)	$LiBi(PdO_2)_2$	96	99	0	0	False	-
mp-27824	$Cl_{12}I_2P_2$	16	$P-42_1m$ (113)	$PICl_6$	96	45	12	0	False	-
mp-3427	$Al_4Li_4O_8$	16	$P4_12_12$ (92)	$LiAlO_2$	144	21	82	12	False	-
mp-755633	$K_{20}Se_{12}$	16	$I4/m$ (87)	K_5Se_3	0	151	0	0	False	-
mp-28253	$Bi_8Li_8O_{16}$	16	$Ibam$ (72)	$LiBiO_2$	48	204	0	0	False	-
mp-11923	$Cu_8Rb_{16}Ta_8S_{32}$	16	$Fddd$ (70)	Rb_2TaCuS_4	97	82	0	0	False	-
mp-11924	$Cu_8Rb_{16}Ta_8Se_{32}$	16	$Fddd$ (70)	Rb_2TaCuS_4	97	94	0	0	False	-
mp-14635	$Ag_8Rb_{16}V_8Se_{32}$	16	$Fddd$ (70)	Rb_2TaCuS_4	97	85	0	0	False	-
mp-14636	$Ag_8Nb_8Rb_{16}S_{32}$	16	$Fddd$ (70)	Rb_2TaCuS_4	97	80	0	0	False	-
mp-14637	$Ag_8Cs_{16}Nb_8Se_{32}$	16	$Fddd$ (70)	Rb_2TaCuS_4	96	102	0	0	False	-
mp-15147	$Cu_8K_{16}V_8S_{32}$	16	$Fddd$ (70)	Rb_2TaCuS_4	97	104	0	0	False	-
mp-15217	$Ag_8Rb_{16}Ta_8S_{32}$	16	$Fddd$ (70)	Rb_2TaCuS_4	96	96	0	0	False	-
mp-15218	$Ag_8Cs_{16}Ta_8S_{32}$	16	$Fddd$ (70)	Rb_2TaCuS_4	96	90	0	0	False	-
mp-15221	$Cu_8Nb_8Rb_{16}S_{32}$	16	$Fddd$ (70)	Rb_2TaCuS_4	96	101	0	0	False	-
mp-15222	$Cu_8Nb_8Rb_{16}Se_{32}$	16	$Fddd$ (70)	Rb_2TaCuS_4	97	89	0	0	False	-
mp-623028	$Ag_8Cs_{16}Nb_8S_{32}$	16	$Fddd$ (70)	Rb_2TaCuS_4	96	89	0	0	False	-
mp-8900	$Ag_8K_{16}V_8S_{32}$	16	$Fddd$ (70)	Rb_2TaCuS_4	96	85	0	0	False	-
mp-8972	$Cu_8K_{16}Ta_8Se_{32}$	16	$Fddd$ (70)	Rb_2TaCuS_4	96	114	0	0	False	-
mp-9003	$Cu_8K_{16}Nb_8Se_{32}$	16	$Fddd$ (70)	Rb_2TaCuS_4	96	97	0	0	False	-
mp-9764	$Ag_8Nb_8Rb_{16}Se_{32}$	16	$Fddd$ (70)	Rb_2TaCuS_4	96	100	0	0	False	-
mp-568208	$Ir_{16}Cl_{48}$	16	$Fddd$ (70)	$IrCl_3$	96	80	0	0	False	-
mp-8232	$Ba_4Zr_4F_{24}$	16	$Cmme$ (67)	$BaZrF_6$	24	168	0	0	False	-
mp-3777	$Ba_8N_{16}Si_8$	16	$Cmce$ (64)	$BaSiN_2$	104	84	0	0	False	-
mp-755998	$Zr_{12}N_8O_{12}$	16	$Cmcm$ (63)	$Zr_3N_2O_3$	76	116	0	0	False	-
mp-760007	$Hf_{12}N_8O_{12}$	16	$Cmcm$ (63)	$Zr_3N_2O_3$	77	106	0	0	False	-
mp-10834	$Cu_4Nd_8Rb_4S_{16}$	16	$Cmcm$ (63)	$RbNd_2CuS_4$	82	121	0	0	False	-
mp-10835	$Cu_4Rb_4Sm_8S_{16}$	16	$Cmcm$ (63)	$RbNd_2CuS_4$	76	126	0	0	False	-

ID	Formula	Z	SP (*No.*)	Prototype	NL	NR	WPs	HDWPs	Clean	dE/dq
mp-11602	$Cu_4K_4Y_8S_{16}$	16	*Cmcm* (63)	$RbNd_2CuS_4$	81	100	0	0	False	-
mp-16116	$Cu_4Rb_4Sm_8Se_{16}$	16	*Cmcm* (63)	$RbNd_2CuS_4$	81	102	0	0	False	-
mp-16118	$Cu_4Dy_8Rb_4Se_{16}$	16	*Cmcm* (63)	$RbNd_2CuS_4$	77	102	0	0	False	-
mp-16445	$Cs_4Cu_4Sm_8Se_{16}$	16	*Cmcm* (63)	$RbNd_2CuS_4$	81	108	0	0	False	-
mp-16560	$Cu_4Er_8K_4Se_{16}$	16	*Cmcm* (63)	$RbNd_2CuS_4$	84	126	0	0	False	-
mp-862772	$Cs_4Cu_4Nd_8Se_{16}$	16	*Cmcm* (63)	$RbNd_2CuS_4$	78	110	0	0	False	-
mp-28232	$Mg_4Ti_8O_{20}$	16	*Cmcm* (63)	$MgTi_2O_5$	77	139	0	0	False	-
mp-4930	$Al_8Ti_4O_{20}$	16	*Cmcm* (63)	$MgTi_2O_5$	80	140	0	0	False	-
mp-755537	$Hf_4Y_8O_{20}$	16	*Cmcm* (63)	$MgTi_2O_5$	81	92	0	0	False	-
mp-571666	$Cs_4Li_{12}Cl_{16}$	16	*Cmcm* (63)	$CsLi_3Cl_4$	76	94	0	0	False	-
mp-27758	$Al_4Tl_8F_{20}$	16	*Cmcm* (63)	$AlTl_2F_5$	83	92	0	0	False	-
mp-10533	$Cu_4Y_4S_8$	16	*Pnma* (62)	$YCuS_2$	231	89	0	0	False	-
mp-12453	$Cu_4Ho_4S_8$	16	*Pnma* (62)	$YCuS_2$	233	74	0	0	False	-
mp-12454	$Cu_4Er_4S_8$	16	*Pnma* (62)	$YCuS_2$	231	96	0	0	False	-
mp-12455	$Cu_4Tm_4S_8$	16	*Pnma* (62)	$YCuS_2$	230	83	0	0	False	-
mp-12457	$Cu_4Lu_4S_8$	16	*Pnma* (62)	$YCuS_2$	229	100	0	0	False	-
mp-555044	$Cu_4Dy_4S_8$	16	*Pnma* (62)	$YCuS_2$	230	79	0	0	False	-
mp-7137	$Li_4Tb_4O_8$	16	*Pnma* (62)	$YCuS_2$	231	68	0	0	False	-
mp-976726	$Dy_4Li_4O_8$	16	*Pnma* (62)	$YCuS_2$	232	67	0	0	False	-
mp-11347	Tb_4F_{12}	16	*Pnma* (62)	TbF_3	229	81	0	0	False	-
mp-2416	Y_4F_{12}	16	*Pnma* (62)	TbF_3	228	89	0	0	False	-
mp-2632	Tl_4F_{12}	16	*Pnma* (62)	TbF_3	228	74	0	0	False	-
mp-561877	Ho_4F_{12}	16	*Pnma* (62)	TbF_3	228	86	0	0	False	-
mp-7384	Sm_4F_{12}	16	*Pnma* (62)	TbF_3	227	97	0	0	False	-
mp-9371	Er_4F_{12}	16	*Pnma* (62)	TbF_3	228	100	0	0	False	-
mp-5637	$Sr_4Zn_4O_8$	16	*Pnma* (62)	$SrZnO_2$	230	109	0	0	False	-
mp-9167	$Sr_4C_4N_8$	16	*Pnma* (62)	$SrCN_2$	233	75	0	0	False	-
mp-974682	$Ga_4Rb_4H_8$	16	*Pnma* (62)	$RbGaH_2$	229	76	0	0	False	-
mp-20331	$Cu_4Sb_4Se_8$	16	*Pnma* (62)	$CuSbSe_2$	231	102	0	0	False	-
mp-22982	$Bi_4Cu_4S_8$	16	*Pnma* (62)	$CuSbSe_2$	226	104	0	0	False	-
mp-4468	$Cu_4S_8Sb_4$	16	*Pnma* (62)	$CuSbSe_2$	227	104	0	0	False	-
mp-643036	$Ba_4Br_4H_4O_4$	16	*Pnma* (62)	BaHBrO	230	91	0	0	True	3.05
mp-643384	$Ba_4H_4I_4O_4$	16	*Pnma* (62)	BaHBrO	227	102	0	0	False	-
mp-643385	$Sr_4H_4I_4O_4$	16	*Pnma* (62)	BaHBrO	230	98	0	0	True	8.76
mp-643727	$Pb_4Br_4H_4O_4$	16	*Pnma* (62)	BaHBrO	230	104	0	0	True	10.07
mp-697084	$Pb_4H_4I_4O_4$	16	*Pnma* (62)	BaHBrO	230	91	0	0	True	19.67
mp-654109	$Ba_4Cu_{16}S_{12}$	16	*Pnma* (62)	$BaCu_4S_3$	463	252	0	0	False	-
mp-625404	$Al_4H_4O_8$	16	*Pnma* (62)	$AlHO_2$	230	103	0	0	False	-
mp-17112	$Au_2K_6Ge_8$	16	*Pmmn* (59)	K_3Ge_4	168	95	0	0	False	-
mp-17830	$Au_2Rb_6Ge_8$	16	*Pmmn* (59)	K_3Ge_4	168	112	0	0	False	-
mp-18500	$Au_2K_6Sn_8$	16	*Pmmn* (59)	K_3Ge_4	169	94	0	0	False	-
mp-510341	$Au_2Cs_6Ge_8$	16	*Pmmn* (59)	K_3Ge_4	168	85	0	0	False	-
mp-556872	$Rb_4C_4O_8$	16	*Pbam* (55)	$RbCO_2$	168	94	0	0	False	-
mp-560616	$K_4C_4O_8$	16	*Pbam* (55)	$RbCO_2$	168	105	0	0	False	-
mp-754658	$O_{12}Te_4$	16	*Pnna* (52)	TeO_3	192	96	0	0	False	-
mp-11925	$Cu_8Rb_4Ta_4Se_{16}$	16	*Ama*2 (40)	$RbTa(CuSe_2)_2$	0	30	0	0	True	8.9
mp-6013	$Cu_8K_4Ta_4Se_{16}$	16	*Ama*2 (40)	$RbTa(CuSe_2)_2$	0	23	0	0	False	-
mp-6376	$Cu_8K_4V_4S_{16}$	16	*Ama*2 (40)	$RbTa(CuSe_2)_2$	0	22	0	0	True	50.21
mp-6599	$Cu_8K_4Nb_4Se_{16}$	16	*Ama*2 (40)	$RbTa(CuSe_2)_2$	0	25	0	0	False	-
mp-753153	$Dy_8C_4O_{20}$	16	*Ama*2 (40)	Dy_2CO_5	0	43	0	0	False	-
mp-12364	$Ba_4Cu_8Sn_4Se_{16}$	16	*Ama*2 (40)	$BaCu_2SnSe_4$	0	33	0	0	False	-
mp-16179	$Cu_8Sr_4Ge_4Se_{16}$	16	*Ama*2 (40)	$BaCu_2SnSe_4$	0	34	0	0	False	-
mp-569450	$Li_4B_{24}C_4$	16	*Amm*2 (38)	LiB_6	0	42	0	0	True	3.49
mp-558907	$Bi_8Cs_4Cu_4S_{16}$	16	*Cmc*2_1 (36)	$RbNd_2CuS_4$	77	140	3	0	False	-
mp-9781	$K_{12}S_{16}Sb_4$	16	*Cmc*2_1 (36)	K_3SbSe_4	80	86	17	0	False	-
mp-11582	$Ga_4Li_4Se_8$	16	*Pna*2_1 (33)	$LiGaSe_2$	195	100	0	11	False	-
mp-20310	$In_4Li_4Se_8$	16	*Pna*2_1 (33)	$LiGaSe_2$	196	84	0	11	False	-
mp-21459	$Ag_4In_4S_8$	16	*Pna*2_1 (33)	$LiGaSe_2$	195	93	0	10	False	-
mp-2979	$Zn_4Ge_4N_8$	16	*Pna*2_1 (33)	$LiGaSe_2$	195	95	0	11	False	-
mp-3338	$Ga_4Na_4O_8$	16	*Pna*2_1 (33)	$LiGaSe_2$	194	96	0	11	False	-
mp-3647	$Ga_4Li_4S_8$	16	*Pna*2_1 (33)	$LiGaSe_2$	193	108	0	9	False	-
mp-3677	$Mg_4N_8Si_4$	16	*Pna*2_1 (33)	$LiGaSe_2$	194	118	0	11	False	-

ID	Formula	Z	SP (*No.*)	Prototype	NL	NR	WPs	HDWPs	Clean	dE/dq
mp-5854	$Ga_4Li_4O_8$	16	$Pna2_1$ (33)	$LiGaSe_2$	192	108	0	11	False	-
mp-7117	$Al_4Li_4Se_8$	16	$Pna2_1$ (33)	$LiGaSe_2$	193	96	0	12	False	-
mp-7798	$Mg_4Ge_4N_8$	16	$Pna2_1$ (33)	$LiGaSe_2$	195	102	0	11	False	-
mp-9212	$Al_4Na_4O_8$	16	$Pna2_1$ (33)	$LiGaSe_2$	192	120	0	11	False	-
mp-12023	$Cu_4Mn_2S_8Si_2$	16	$Pmn2_1$ (31)	$MnCu_2SiS_4$	168	79	0	0	False	-
mp-554105	$Ag_4Cd_2Ge_2S_8$	16	$Pmn2_1$ (31)	$MnCu_2SiS_4$	168	83	0	0	False	-
mp-6356	$Ag_2Cd_4Ga_2S_8$	16	$Pmn2_1$ (31)	$MnCu_2SiS_4$	168	85	0	0	False	-
mp-6449	$Cd_2Cu_4S_8Si_2$	16	$Pmn2_1$ (31)	$MnCu_2SiS_4$	168	74	0	0	False	-
mp-7688	$Cd_2Li_4Ge_2O_8$	16	$Pmn2_1$ (31)	$MnCu_2SiS_4$	168	85	0	0	False	-
mp-9066	$Li_4Na_2As_2O_8$	16	$Pmn2_1$ (31)	$MnCu_2SiS_4$	169	87	0	0	False	-
mp-977414	$Cu_4Zn_2S_8Si_2$	16	$Pmn2_1$ (31)	$MnCu_2SiS_4$	168	83	0	0	False	-
mp-13725	$Li_6O_8P_2$	16	$Pmn2_1$ (31)	Li_3PO_4	168	78	0	0	False	-
mp-3934	$Cu_6P_2S_8$	16	$Pmn2_1$ (31)	Cu_3PS_4	168	84	0	0	False	-
mp-755642	$Li_6V_2S_8$	16	$Pmn2_1$ (31)	Cu_3PS_4	169	81	0	0	False	-
mp-756044	$Na_6As_2O_8$	16	$Pmn2_1$ (31)	Cu_3PS_4	168	96	0	0	False	-
mp-756316	$Li_6S_8Sb_2$	16	$Pmn2_1$ (31)	Cu_3PS_4	169	92	0	0	False	-
mp-9197	$Li_6As_2O_8$	16	$Pmn2_1$ (31)	Cu_3PS_4	168	106	0	0	False	-
mp-6955	$K_4Ge_4N_4O_4$	16	$Pca2_1$ (29)	KGeNO	175	125	0	22	False	-
mp-28007	$Ba_4Hg_4S_8$	16	$Pmc2_1$ (26)	$BaHgS_2$	97	107	0	0	False	-
mp-12843	$Cu_4Y_4S_8$	16	$P2_12_12_1$ (19)	$YCuS_2$	219	39	92	13	False	-
mp-554120	$Cu_4Dy_4S_8$	16	$P2_12_12_1$ (19)	$YCuS_2$	220	38	82	12	False	-
mp-559826	$Cu_4Er_4S_8$	16	$P2_12_12_1$ (19)	$YCuS_2$	218	41	84	12	False	-
mp-560611	$Cu_4Ho_4S_8$	16	$P2_12_12_1$ (19)	$YCuS_2$	218	37	78	12	False	-
mp-10802	Rb_8As_8	16	$P2_12_12_1$ (19)	RbAs	216	28	69	12	False	-
mp-713	K_8As_8	16	$P2_12_12_1$ (19)	RbAs	216	32	73	12	False	-
mp-7444	Rb_8Sb_8	16	$P2_12_12_1$ (19)	RbAs	216	24	74	12	False	-
mp-12955	$Ag_4Ho_4Se_8$	16	$P2_12_12_1$ (19)	$HoAgSe_2$	216	28	90	12	False	-
mp-4044	$Ag_4Er_4Se_8$	16	$P2_12_12_1$ (19)	$HoAgSe_2$	216	30	102	12	False	-
mp-556296	Ba_4S_{12}	16	$P2_12_12$ (18)	BaS_3	168	35	95	0	False	-
mp-7593	$Li_8Rb_8F_{16}$	16	$C2/c$ (15)	$RbLiF_2$	0	112	0	0	False	-
mp-14395	$Al_4Na_4As_4F_4O_{16}$	16	$C2/c$ (15)	$NaAlAsO_4$	1	113	0	0	False	-
mp-6873	$Al_4Ca_4F_4O_{16}Si_4$	16	$C2/c$ (15)	$NaAlAsO_4$	2	118	0	0	False	-
mp-8678	$Al_4Na_4F_4O_{16}P_4$	16	$C2/c$ (15)	$NaAlAsO_4$	1	103	0	0	False	-
mp-23421	$Li_4Yb_8Cl_{20}$	16	$C2/c$ (15)	$LiYb_2Cl_5$	0	96	0	0	False	-
mp-621612	$Ag_4Pb_8Br_{20}$	16	$C2/c$ (15)	$LiYb_2Cl_5$	0	159	0	0	False	-
mp-760778	$Ag_4Li_8F_{20}$	16	$C2/c$ (15)	Li_2AgF_5	1	85	0	0	False	-
mp-28136	$Hg_{16}I_8O_8$	16	$C2/c$ (15)	Hg_2	0	120	0	0	False	-
mp-23395	$Hg_{16}Cl_8O_8$	16	$C2/c$ (15)	Hg_2	0	96	0	0	False	-
mp-606510	$Cs_4Ta_4I_{24}$	16	$C2/c$ (15)	$CsTaI_6$	0	121	0	0	False	-
mp-6440	$Ca_4Ti_4O_{20}Si_4$	16	$C2/c$ (15)	$CaTiGeO_5$	1	107	0	0	False	-
mp-6526	$Na_4Ge_4O_{20}Sb_4$	16	$C2/c$ (15)	$CaTiGeO_5$	1	100	0	0	False	-
mp-7594	$Cs_8Li_8F_{16}$	16	$C2/c$ (15)	$BaSiN_2$	0	118	0	0	False	-
mp-768422	$Tm_4H_4O_8$	16	$P2_1/c$ (14)	$TmHO_2$	122	99	0	0	False	-
mp-10487	Rb_8Sb_8	16	$P2_1/c$ (14)	RbSb	121	171	0	0	False	-
mp-1536	K_8Sb_8	16	$P2_1/c$ (14)	RbSb	122	148	0	0	False	-
mp-7943	Li_8As_8	16	$P2_1/c$ (14)	RbSb	121	172	0	0	False	-
mp-7944	Na_8Sb_8	16	$P2_1/c$ (14)	RbSb	122	155	0	0	False	-
mp-9588	Li_8P_8	16	$P2_1/c$ (14)	RbSb	123	209	0	0	False	-
mp-10495	$Cu_4Nd_4S_8$	16	$P2_1/c$ (14)	$NdCuS_2$	121	171	0	0	False	-
mp-11790	$Cu_4La_4Se_8$	16	$P2_1/c$ (14)	$NdCuS_2$	121	198	0	0	False	-
mp-11792	$Cu_4Pr_4Se_8$	16	$P2_1/c$ (14)	$NdCuS_2$	121	180	0	0	False	-
mp-11793	$Cu_4Sm_4Se_8$	16	$P2_1/c$ (14)	$NdCuS_2$	120	175	0	0	False	-
mp-12984	$Cu_4Tb_4Se_8$	16	$P2_1/c$ (14)	$NdCuS_2$	122	158	0	0	False	-
mp-16684	$Cu_4Pr_4S_8$	16	$P2_1/c$ (14)	$NdCuS_2$	121	163	0	0	False	-
mp-3739	$Cu_4Nd_4Se_8$	16	$P2_1/c$ (14)	$NdCuS_2$	120	182	0	0	False	-
mp-4841	$Cu_4La_4S_8$	16	$P2_1/c$ (14)	$NdCuS_2$	121	175	0	0	False	-
mp-5081	$Cu_4Sm_4S_8$	16	$P2_1/c$ (14)	$NdCuS_2$	120	190	0	0	False	-
mp-541378	$Bi_4Cs_4S_8$	16	$P2_1/c$ (14)	$NdCuS_2$	121	128	0	0	False	-
mp-5737	$Cu_4Tb_4S_8$	16	$P2_1/c$ (14)	$NdCuS_2$	120	152	0	0	False	-
mp-29139	$Au_4Na_4Se_8$	16	$P2_1/c$ (14)	$NaAuSe_2$	123	117	0	0	False	-
mp-10620	$Li_4Pr_4O_8$	16	$P2_1/c$ (14)	$LiPrO_2$	121	169	0	0	False	-
mp-7800	$Li_4Sm_4O_8$	16	$P2_1/c$ (14)	$LiPrO_2$	121	183	0	0	False	-

ID	Formula	Z	SP (*No.*)	Prototype	NL	NR	WPs	HDWPs	Clean	dE/dq
mp-10288	$Cu_4La_4S_4Te_4$	16	$P2_1/c$ (14)	LaCuTeS	121	145	0	0	False	-
mp-29567	$Cu_4Br_4Se_8$	16	$P2_1/c$ (14)	$CuSe_2$	125	127	0	0	False	-
mp-30971	$Cu_4Cl_4Te_8$	16	$P2_1/c$ (14)	$CuSe_2$	121	141	0	0	False	-
mp-31037	$Cu_4I_4Te_8$	16	$P2_1/c$ (14)	$CuSe_2$	123	127	0	0	False	-
mp-2969	$Cs_4Sb_4Se_8$	16	$P2_1/c$ (14)	$CsSbSe_2$	120	121	0	0	False	-
mp-561639	$Cs_4S_8Sb_4$	16	$P2_1/c$ (14)	$CsSbSe_2$	121	123	0	0	False	-
mp-12181	$Ba_4Sn_4S_8$	16	$P2_1/c$ (14)	$BaSnS_2$	123	148	0	0	False	-
mp-9576	$K_4Sb_4Se_8$	16	$P2/c$ (13)	$KSbSe_2$	51	193	0	0	False	-
mp-28671	$Zr_4Br_{20}Te_8$	16	$C2/m$ (12)	$ZrTe_2Br_5$	0	35	0	0	False	-
mp-10804	$Na_4Y_4F_4O_{16}P_4$	16	$C2/m$ (12)	$NaYPO_4$	0	81	0	0	False	-
mp-10935	$Er_4Na_4F_4O_{16}P_4$	16	$C2/m$ (12)	$NaYPO_4$	0	58	0	0	False	-
mp-560977	$K_4Ti_4P_4S_{20}$	16	$C2/m$ (12)	$KTiPS_5$	0	57	0	0	True	1.64
mp-559766	$Cs_{12}Li_4F_{16}$	16	$C2/m$ (12)	Cs_3LiF_4	0	68	0	0	False	-
mp-644418	$Ca_4C_4Cl_4H_8O_{12}$	16	$C2/m$ (12)	CaH_2CClO_3	0	43	0	0	False	-
mp-568758	$Bi_{16}Br_{16}$	16	$C2/m$ (12)	BiBr	1	131	0	0	False	-
mp-567318	$Tl_4Sb_4Se_8$	16	$P2_1/m$ (11)	$TlSbSe_2$	72	260	0	0	False	-
mp-11114	$Cu_2K_2Pd_2Se_{10}$	16	$P2_1/m$ (11)	$KCuPdSe_5$	72	109	0	0	False	-
mp-11115	$Cu_2Pd_2Rb_2Se_{10}$	16	$P2_1/m$ (11)	$KCuPdSe_5$	72	133	0	0	False	-
mp-28347	$Al_4K_6As_6$	16	$P2_1/m$ (11)	$K_3Al_2As_3$	72	151	0	0	False	-
mp-619510	$Cs_2Hg_4Br_{10}$	16	$P2_1/m$ (11)	$CsHg_2Br_5$	74	150	0	0	False	-
mp-10555	$Ba_4Li_2Re_2N_8$	16	$P2_1/m$ (11)	Ba_2LiReN_4	72	94	0	0	False	-
mp-10556	$Li_2Re_2Sr_4N_8$	16	$P2_1/m$ (11)	Ba_2LiReN_4	73	102	0	0	False	-
mp-37498	$Li_{10}Cl_4N_2$	16	$P2/m$ (10)	Li_5NCl_2	0	249	0	0	False	-
mp-28261	$Na_{16}Sn_4O_{12}$	16	Cc (9)	Na_4SnO_3	0	133	0	0	False	-
mp-14659	$K_8As_8Se_{16}$	16	Cc (9)	$KAsSe_2$	1	84	0	0	False	-
mp-754668	$Hf_{12}N_8O_{12}$	16	Cm (8)	$Hf_3N_2O_3$	0	15	0	0	False	-
mp-755132	$Zr_{12}N_8O_{12}$	16	Cm (8)	$Hf_3N_2O_3$	0	26	0	0	False	-
mp-555186	$Li_4Sn_2Zn_2S_8$	16	Pc (7)	Li_2ZnSnS_4	49	151	0	0	False	-
mp-6402	$Na_4Zn_2Ge_2O_8$	16	Pc (7)	Li_2ZnSnS_4	48	168	0	0	False	-
mp-6406	$Mg_2Na_4O_8Si_2$	16	Pc (7)	Li_2ZnSnS_4	51	139	0	0	False	-
mp-8184	$Li_4Zn_2Ge_2O_8$	16	Pc (7)	Li_2ZnSnS_4	50	169	0	0	False	-
mp-6109	$Ca_4Ti_4O_{20}Si_4$	16	$P-1$ (2)	$CaTiGeO_5$	1	332	0	0	False	-
mp-16180	$Na_{24}O_{36}S_8$	17	$Fm-3m$ (225)	$Na_6S_2O_9$	23	62	0	0	False	-
mp-21084	$Ga_8In_{24}Pt_4O_{32}$	17	$Fm-3m$ (225)	$In_6Ga_2PtO_8$	27	53	0	0	True	18.26
mp-558992	$In_{28}Ir_4Ge_4O_{32}$	17	$F-43m$ (216)	In_7GeIrO_8	27	16	6	13	False	-
mp-9619	$Fe_8Th_2P_{24}$	17	$Im-3$ (204)	$Th(FeP_3)_4$	56	126	0	0	False	-
mp-643047	$Ba_3Ir_2H_{12}$	17	$P-3m1$ (164)	$Ba_3(H_6Ir)_2$	41	95	0	0	True	6.82
mp-560515	$K_8Ru_4Cl_{20}O_2$	17	$I4/mmm$ (139)	$K_4Ru_2Cl_{10}$	0	62	0	0	False	-
mp-4647	$Cu_{16}K_6Se_{12}$	17	$C2/m$ (12)	$K_3(Cu_4Se_3)_2$	0	118	0	0	False	-
mp-23657	$Na_{24}Cl_4F_4O_{32}S_8$	18	$Fm-3m$ (225)	$Na_6S_2ClO_8$	25	69	0	0	False	-
mp-22186	$In_{24}Pt_4Ge_8O_{36}$	18	$Fm-3m$ (225)	$In_6Ge_2PtO_9$	25	57	0	0	False	-
mp-561543	$Be_{12}F_{24}$	18	$I-43m$ (217)	BeF_2	55	74	1	27	False	-
mp-5496	$Al_{10}C_6N_2$	18	$P6_3/mmc$ (194)	Al_5C_3	19	160	0	0	False	-
mp-24338	$Sr_{10}As_6H_2$	18	$P6_3/mcm$ (193)	Sr_5As_3	30	146	0	0	False	-
mp-983061	$Sr_2As_4O_{12}$	18	$P6_3/mcm$ (193)	$Sr(AsO_3)_2$	25	183	0	0	False	-
mp-13002	$K_4F_{12}Si_2$	18	$P6_3mc$ (186)	K_2SiF_6	47	135	2	6	True	1.52
mp-13954	$Rb_4F_{12}Ge_2$	18	$P6_3mc$ (186)	K_2SiF_6	46	138	1	6	False	-
mp-14168	$K_4F_{12}Ge_2$	18	$P6_3mc$ (186)	K_2SiF_6	22	106	1	8	False	-
mp-567949	$Hg_6As_2Br_2Se_8$	18	$P6_3mc$ (186)	Hg_3AsSe_4	24	100	0	3	True	1.22
mp-570084	$Hg_6As_2I_2Se_8$	18	$P6_3mc$ (186)	Hg_3AsSe_4	22	107	0	4	False	-
mp-37657	$Ca_6O_{10}Si_2$	18	$P6_3mc$ (186)	Ca_3SiO_5	27	96	0	3	True	4.63
mp-570079	$Al_{10}C_6N_2$	18	$P6_3mc$ (186)	Al_5C_3	19	177	1	0	False	-
mp-23110	$Hg_6Na_3I_3O_6$	18	$P6_222$ (180)	$NaHg_2IO_2$	0	4	275	0	True	30.88
mp-31419	$La_3O_{36}Sb_{15}$	18	$R-3m$ (166)	$LaSb_5O_{12}$	25	113	0	0	False	-
mp-13634	$Al_9Cs_6Li_3F_{36}$	18	$R-3m$ (166)	$Cs_2LiAl_3F_{12}$	26	129	0	0	True	14.18
mp-561786	$Cs_6O_{36}Te_{12}$	18	$R-3m$ (166)	$Cs(TeO_3)_2$	27	128	0	0	False	-
mp-10103	$Al_2Li_2Yb_2F_{12}$	18	$P-31c$ (163)	$LiYbAlF_6$	87	149	0	0	False	-
mp-12829	$Ca_2Ga_2Li_2F_{12}$	18	$P-31c$ (163)	$LiYbAlF_6$	96	174	0	0	False	-
mp-6134	$Al_2Ca_2Li_2F_{12}$	18	$P-31c$ (163)	$LiYbAlF_6$	97	155	0	0	False	-
mp-6591	$Al_2Li_2Sr_2F_{12}$	18	$P-31c$ (163)	$LiYbAlF_6$	93	158	0	0	True	6.8
mp-570690	$C_{27}Si_{27}$	18	$R3m$ (160)	SiC	97	107	62	0	False	-
mp-23764	$Mg_3H_4O_9Si_2$	18	$P31m$ (157)	$Mg_3Si_2H_4O_9$	60	93	0	0	False	-

ID	Formula	Z	SP (*No.*)	Prototype	NL	NR	WPs	HDWPs	Clean	dE/dq
mp-29012	$Ag_9Tl_{18}I_{27}$	18	$R-3$ (148)	Tl_2AgI_3	32	17	0	0	False	-
mp-27265	$Au_{18}Cl_{18}O_{18}$	18	$R-3$ (148)	AuClO	30	28	0	0	False	-
mp-556741	$K_2Na_2Th_2F_{12}$	18	$P-3$ (147)	$KNaThF_6$	66	54	0	0	False	-
mp-23571	$Ba_{16}Bi_{12}K_4O_4$	18	$I4/mcm$ (140)	KBa_4Bi_3	27	128	0	0	False	-
mp-557035	$K_{12}F_4O_{16}S_4$	18	$I4/mcm$ (140)	KBa_4Bi_3	27	145	0	0	False	-
mp-559270	$Ba_{16}Rb_4O_4Sb_{12}$	18	$I4/mcm$ (140)	KBa_4Bi_3	28	144	0	0	False	-
mp-17895	$Be_4K_{12}F_{20}$	18	$I4/mcm$ (140)	K_3BeF_5	29	147	0	0	False	-
mp-570262	$Cs_{12}Zn_4Cl_{20}$	18	$I4/mcm$ (140)	K_3BeF_5	27	138	0	0	False	-
mp-643270	$K_{12}Zn_4H_{20}$	18	$I4/mcm$ (140)	K_3BeF_5	27	117	0	0	False	-
mp-643651	$Rb_{12}Zn_4H_{20}$	18	$I4/mcm$ (140)	K_3BeF_5	27	106	0	0	False	-
mp-643702	$Cs_{12}Zn_4H_{20}$	18	$I4/mcm$ (140)	K_3BeF_5	27	116	0	0	False	-
mp-643712	$Mg_4Rb_{12}H_{20}$	18	$I4/mcm$ (140)	K_3BeF_5	27	104	0	0	False	-
mp-643895	$Cs_{12}Mg_4H_{20}$	18	$I4/mcm$ (140)	K_3BeF_5	27	106	0	0	False	-
mp-7765	$Ba_{12}O_{20}Si_4$	18	$I4/mcm$ (140)	K_3BeF_5	27	132	0	0	False	-
mp-9668	$Ba_{12}Ti_4S_{20}$	18	$I4/mcm$ (140)	K_3BeF_5	27	145	0	0	False	-
mp-16124	$Ba_4Mn_4Pr_8S_{20}$	18	$I4/mcm$ (140)	$BaPr_2MnS_5$	27	138	0	0	False	-
mp-16452	$Ba_4La_8Zn_4S_{20}$	18	$I4/mcm$ (140)	$BaPr_2MnS_5$	27	157	0	0	False	-
mp-16454	$Ba_4Pr_8Zn_4S_{20}$	18	$I4/mcm$ (140)	$BaPr_2MnS_5$	27	159	0	0	False	-
mp-16455	$Ba_4Nd_8Zn_4S_{20}$	18	$I4/mcm$ (140)	$BaPr_2MnS_5$	27	155	0	0	False	-
mp-6468	$Ba_4Nd_8Zn_4O_{20}$	18	$I4/mcm$ (140)	$BaPr_2MnS_5$	27	159	0	0	False	-
mp-6770	$Ba_4La_8Mn_4S_{20}$	18	$I4/mcm$ (140)	$BaPr_2MnS_5$	28	118	0	0	False	-
mp-6793	$Ba_4La_8Zn_4O_{20}$	18	$I4/mcm$ (140)	$BaPr_2MnS_5$	27	152	0	0	False	-
mp-557410	$Nd_4Br_4O_{20}Te_8$	18	$I4/mmm$ (139)	$NdTe_2BrO_5$	0	157	0	0	True	9
mp-13836	$Rh_4U_2O_{12}$	18	$P4_2/mnm$ (136)	$U(RhO_3)_2$	135	107	0	0	False	-
mp-13985	$Li_4Pd_2F_{12}$	18	$P4_2/mnm$ (136)	$U(RhO_3)_2$	135	105	0	0	False	-
mp-13986	$Li_4Pt_2F_{12}$	18	$P4_2/mnm$ (136)	$U(RhO_3)_2$	135	98	0	0	False	-
mp-28931	$Ga_4O_{12}Te_2$	18	$P4_2/mnm$ (136)	$U(RhO_3)_2$	135	127	0	0	False	-
mp-756389	$Lu_4O_{12}Te_2$	18	$P4_2/mnm$ (136)	$U(RhO_3)_2$	135	118	0	0	False	-
mp-9650	$Mg_2Ta_4O_{12}$	18	$P4_2/mnm$ (136)	$U(RhO_3)_2$	135	138	0	0	False	-
mp-10488	$Ag_4Cs_4Ti_2S_8$	18	$P4_2/mcm$ (132)	$Cs_2Ti(AgS_2)_2$	54	153	0	0	False	-
mp-10489	$Cs_4Cu_4Ti_2Se_8$	18	$P4_2/mcm$ (132)	$Cs_2Ti(AgS_2)_2$	54	185	0	0	False	-
mp-7129	$Cu_4Rb_4Ti_2S_8$	18	$P4_2/mcm$ (132)	$Cs_2Ti(AgS_2)_2$	54	175	0	0	False	-
mp-23947	$Cs_{12}Mg_4H_{20}$	18	$P4/ncc$ (130)	K_3BeF_5	340	256	0	0	True	1.46
mp-23948	$Mg_4Rb_{12}H_{20}$	18	$P4/ncc$ (130)	K_3BeF_5	337	252	0	0	False	-
mp-6138	$Na_4Ti_2O_{10}Si_2$	18	$P4/nmm$ (129)	Na_2TiSiO_5	109	163	0	0	False	-
mp-6228	$Na_4Ti_2Ge_2O_{10}$	18	$P4/nmm$ (129)	Na_2TiSiO_5	108	156	0	0	False	-
mp-10942	$K_2La_2Na_2Nb_2O_{10}$	18	$P4/nmm$ (129)	$KNaLaNbO_5$	108	148	0	0	False	-
mp-556438	$K_2Na_2Nb_2Sm_2O_{10}$	18	$P4/nmm$ (129)	$KNaLaNbO_5$	108	178	0	0	True	14.88
mp-558212	$K_2Na_2Nb_2Nd_2O_{10}$	18	$P4/nmm$ (129)	$KNaLaNbO_5$	108	155	0	0	True	13.8
mp-558317	$K_2Na_2Nb_2Pr_2O_{10}$	18	$P4/nmm$ (129)	$KNaLaNbO_5$	108	155	0	0	False	-
mp-861607	$K_2Na_2Nd_2Ta_2O_{10}$	18	$P4/nmm$ (129)	$KNaLaNbO_5$	108	156	0	0	False	-
mp-861868	$K_2La_2Na_2Ta_2O_{10}$	18	$P4/nmm$ (129)	$KNaLaNbO_5$	108	146	0	0	True	22.03
mp-861944	$K_2Na_2Pr_2Ta_2O_{10}$	18	$P4/nmm$ (129)	$KNaLaNbO_5$	108	134	0	0	True	17.09
mp-861957	$K_2Na_2Sm_2Ta_2O_{10}$	18	$P4/nmm$ (129)	$KNaLaNbO_5$	108	147	0	0	False	-
mp-13820	$Cd_6O_{10}Si_2$	18	$P4/nmm$ (129)	Cd_3SiO_5	109	130	0	0	False	-
mp-554116	$Ba_2Pb_4Br_2F_{10}$	18	$P4/nmm$ (129)	$BaPb_2BrF_5$	109	199	0	0	False	-
mp-554245	$Ba_2Pb_4F_{10}I_2$	18	$P4/nmm$ (129)	$BaPb_2BrF_5$	108	180	0	0	False	-
mp-28247	$K_4Pt_2I_{12}$	18	$P4/mnc$ (128)	K_2TeI_6	163	85	0	0	False	-
mp-642736	$K_6Pd_2H_{10}$	18	$P4/mbm$ (127)	K_3H_5	108	105	0	0	False	-
mp-642821	$K_6Pt_2H_{10}$	18	$P4/mbm$ (127)	K_3H_5	108	107	0	0	False	-
mp-642845	$Pt_2Rb_6H_{10}$	18	$P4/mbm$ (127)	K_3H_5	109	106	0	0	False	-
mp-643006	$Cs_6Pd_2H_{10}$	18	$P4/mbm$ (127)	K_3H_5	108	95	0	0	False	-
mp-643022	$Pd_2Rb_6H_{10}$	18	$P4/mbm$ (127)	K_3H_5	108	95	0	0	False	-
mp-556525	$Ba_2Nd_4Pt_2O_{10}$	18	$P4/mbm$ (127)	$BaNd_2PtO_5$	108	162	0	0	False	-
mp-8514	$Ba_2Nd_4Pd_2O_{10}$	18	$P4/mbm$ (127)	$BaNd_2PtO_5$	108	169	0	0	False	-
mp-8515	$Ba_2Pd_2Sm_4O_{10}$	18	$P4/mbm$ (127)	$BaNd_2PtO_5$	108	142	0	0	False	-
mp-8959	$Cs_2K_4Pd_2F_{10}$	18	$P4/mbm$ (127)	$BaNd_2PtO_5$	108	113	0	0	False	-
mp-9757	$Ba_2Pt_2Sm_4O_{10}$	18	$P4/mbm$ (127)	$BaNd_2PtO_5$	109	145	0	0	False	-
mp-9759	$Ba_2Pd_2Pr_4O_{10}$	18	$P4/mbm$ (127)	$BaNd_2PtO_5$	108	156	0	0	False	-
mp-557862	$Ag_4Ba_2Hg_4O_8$	18	$P4/nbm$ (125)	$BaAg_2(HgO_2)_2$	81	149	0	0	True	2.08
mp-15472	$Ba_8Cu_2Li_2C_4O_{20}$	18	$I-42m$ (121)	$Ba_4LiCu(CO_5)_2$	0	58	47	0	False	-
mp-6841	$Ba_8Cu_2Na_2C_4O_{20}$	18	$I-42m$ (121)	$Ba_4LiCu(CO_5)_2$	0	55	50	0	False	-

ID	Formula	Z	SP (*No.*)	Prototype	NL	NR	WPs	HDWPs	Clean	dE/dq
mp-28768	$Na_8Sn_2Se_8$	18	$P{-}42_1c$ (114)	Na_4SnSe_4	135	61	38	6	False	-
mp-29628	$Na_8Sn_2S_8$	18	$P{-}42_1c$ (114)	Na_4SnSe_4	135	99	57	6	False	-
mp-14249	$Th_2P_4S_{12}$	18	$P4_2/m$ (84)	$Th(PS_3)_2$	0	20	0	0	False	-
mp-8203	$Zr_2P_4S_{12}$	18	$P4_2/m$ (84)	$Th(PS_3)_2$	0	12	0	0	False	-
mp-560340	$La_4Pd_4O_{10}$	18	$P4_2/m$ (84)	$La_2Pd_2O_5$	0	16	0	0	False	-
mp-18006	$Cu_8Rb_8Sn_4S_{16}$	18	*Ibam* (72)	$Rb_2Cu_2SnS_4$	55	188	0	0	False	-
mp-2884	$Cs_8Mn_{12}S_{16}$	18	*Ibam* (72)	$Cs_2Mn_3S_4$	54	182	0	0	False	-
mp-3704	$Cs_8Mn_{12}Se_{16}$	18	*Ibam* (72)	$Cs_2Mn_3S_4$	54	191	0	0	False	-
mp-4052	$Mn_{12}Rb_8Se_{16}$	18	*Ibam* (72)	$Cs_2Mn_3S_4$	54	199	0	0	False	-
mp-505633	$Cs_8Zn_{12}S_{16}$	18	*Ibam* (72)	$Cs_2Mn_3S_4$	55	175	0	0	False	-
mp-567386	$Cd_{12}Cs_8Te_{16}$	18	*Ibam* (72)	$Cs_2Cd_3Te_4$	54	200	0	0	False	-
mp-768203	$Be_{12}H_{24}$	18	*Ibam* (72)	BeH_2	55	160	0	0	False	-
mp-14339	$K_{16}Pd_{24}Se_{32}$	18	*Fddd* (70)	$K_2Pd_3Se_4$	108	124	0	0	False	-
mp-14340	$Pd_{24}Rb_{16}Se_{32}$	18	*Fddd* (70)	$K_2Pd_3Se_4$	108	124	0	0	False	-
mp-17228	$K_{16}Ni_{24}S_{32}$	18	*Fddd* (70)	$K_2Pd_3Se_4$	109	124	0	0	False	-
mp-9910	$K_{16}Pd_{24}S_{32}$	18	*Fddd* (70)	$K_2Pd_3Se_4$	109	120	0	0	True	34.68
mp-558500	$Ag_{16}K_{16}Ge_8S_{32}$	18	*Fddd* (70)	$K_2Ag_2GeS_4$	108	102	0	0	False	-
mp-561641	$Au_{16}Cs_{16}Sn_8S_{32}$	18	*Fddd* (70)	$K_2Ag_2GeS_4$	106	105	0	0	False	-
mp-29794	$Ba_8Pd_4F_{24}$	18	*Cmce* (64)	Ba_2PdF_6	120	116	0	0	False	-
mp-643253	$Ba_8Pt_4H_{24}$	18	*Cmce* (64)	Ba_2H_6	112	97	0	0	True	13.77
mp-570823	$Ba_4B_8Se_{24}$	18	*Cmce* (64)	$Ba(BSe_3)_2$	117	116	0	0	False	-
mp-22170	$Li_{16}Pb_4O_{16}$	18	*Cmcm* (63)	Li_4PbO_4	91	119	0	0	False	-
mp-756344	$Li_{16}Zr_4O_{16}$	18	*Cmcm* (63)	Li_4PbO_4	88	115	0	0	False	-
mp-9172	$Li_{16}Ti_4O_{16}$	18	*Cmcm* (63)	Li_4PbO_4	83	133	0	0	False	-
mp-4558	$Li_{16}Ge_4O_{16}$	18	*Cmcm* (63)	Li_4GeO_4	92	129	0	0	False	-
mp-29410	$Li_8B_8S_{20}$	18	*Cmcm* (63)	$Li_2B_2S_5$	91	93	0	0	False	-
mp-756624	$Ba_8Sr_4I_{24}$	18	*Pbcn* (60)	Ba_2SrI_6	543	406	0	0	False	-
mp-29898	$Au_8Tl_4S_6$	18	*Pmmn* (59)	$Tl_2Au_4S_3$	192	131	0	0	False	-
mp-28324	$In_4Sr_6P_8$	18	*Pnnm* (58)	$Sr_3(InP_2)_2$	252	84	0	0	False	-
mp-755950	$Ba_4Sc_4O_{10}$	18	*Pbam* (55)	$Ba_2Sc_2O_5$	191	146	0	0	False	-
mp-10444	$Al_8Ca_8O_{20}$	18	*Ima*2 (46)	$Ca_2Al_2O_5$	28	97	81	0	False	-
mp-20546	$Ba_8In_8O_{20}$	18	*Ima*2 (46)	$Ca_2Al_2O_5$	27	100	78	0	False	-
mp-756294	$Li_8Ti_8O_{20}$	18	*Ccc*2 (37)	$Li_2Si_2O_5$	81	88	27	0	False	-
mp-864638	$Ba_8Bi_4In_4S_{20}$	18	$Cmc2_1$ (36)	Ba_2InBiS_5	93	95	9	0	False	-
mp-13703	$Al_{16}C_4O_{16}$	18	$Cmc2_1$ (36)	Al_4CO_4	87	109	10	0	False	-
mp-28744	$Os_4Y_{16}Br_{16}$	18	$C2/c$ (15)	Y_4OsBr_4	0	134	0	0	False	-
mp-7499	$Sn_8Tl_8S_{20}$	18	$C2/c$ (15)	$Tl_2Sn_2S_5$	0	66	0	0	False	-
mp-8965	$K_8Sn_8S_{20}$	18	$C2/c$ (15)	$Tl_2Sn_2S_5$	1	97	0	0	False	-
mp-8966	$K_8Sn_8Se_{20}$	18	$C2/c$ (15)	$Tl_2Sn_2S_5$	1	114	0	0	False	-
mp-9322	$Rb_8Sn_8Se_{20}$	18	$C2/c$ (15)	$Tl_2Sn_2S_5$	0	118	0	0	False	-
mp-25301	$Ti_4O_{24}Si_8$	18	$C2/c$ (15)	$Ti(SiO_3)_2$	1	126	0	0	False	-
mp-11677	$Sr_{12}As_{16}Si_8$	18	$C2/c$ (15)	$Sr_3(SiAs_2)_2$	4	172	0	0	False	-
mp-11692	$Ba_{12}As_{16}Si_8$	18	$C2/c$ (15)	$Sr_3(SiAs_2)_2$	1	150	0	0	False	-
mp-555852	$Ag_8Rb_8Ge_4S_{16}$	18	$C2/c$ (15)	$Rb_2Ag_2GeS_4$	3	90	0	0	False	-
mp-30292	$Nd_{16}N_8Se_{12}$	18	$C2/c$ (15)	$Nd_4Se_3N_2$	2	151	0	0	False	-
mp-31303	$Pr_{16}N_8Se_{12}$	18	$C2/c$ (15)	$Nd_4Se_3N_2$	4	149	0	0	False	-
mp-570258	$Al_8Ca_{12}N_{16}$	18	$C2/c$ (15)	$Nd_4Se_3N_2$	3	111	0	0	False	-
mp-571162	$Ca_{12}Ga_8N_{16}$	18	$C2/c$ (15)	$Nd_4Se_3N_2$	3	138	0	0	False	-
mp-9843	$Al_8Sr_{12}P_{16}$	18	$C2/c$ (15)	$Nd_4Se_3N_2$	0	120	0	0	False	-
mp-23966	$Mg_4H_8O_{20}Se_4$	18	$C2/c$ (15)	MgH_2SeO_5	1	91	0	0	False	-
mp-675800	$Li_{20}Ti_4P_{12}$	18	$C2/c$ (15)	Li_5GeP_3	0	134	0	0	False	-
mp-30100	$Li_8B_8Se_{20}$	18	$C2/c$ (15)	$Li_2B_2Se_5$	1	117	0	0	False	-
mp-28371	$K_8Zn_{12}O_{16}$	18	$C2/c$ (15)	$K_2Zn_3O_4$	0	113	0	0	False	-
mp-675562	$K_8Th_4F_{24}$	18	$C2/c$ (15)	K_2ThF_6	1	111	0	0	False	-
mp-24090	$Ba_4H_{16}I_8O_8$	18	$C2/c$ (15)	$BaH_4(IO)_2$	2	71	0	0	False	-
mp-696806	$Ba_4Br_8H_{16}O_8$	18	$C2/c$ (15)	$BaH_4(IO)_2$	1	66	0	0	False	-
mp-29469	$Pd_2Cl_4Se_{12}$	18	$P2_1/c$ (14)	$Pd(Se_3Cl)_2$	136	125	0	0	False	-
mp-28982	$Pb_4Pd_2Cl_{12}$	18	$P2_1/c$ (14)	$Pd(PbCl_3)_2$	135	146	0	0	False	-
mp-28983	$Pb_4Pd_2Br_{12}$	18	$P2_1/c$ (14)	$Pd(PbCl_3)_2$	135	156	0	0	False	-
mp-27688	$K_4I_{12}Te_2$	18	$P2_1/c$ (14)	K_2TeI_6	139	148	0	0	False	-
mp-574040	$K_4Sn_2Br_{12}$	18	$P2_1/c$ (14)	K_2TeI_6	138	156	0	0	False	-
mp-756202	$Ba_4Sr_2I_{12}$	18	$P2_1/c$ (14)	K_2TeI_6	138	205	0	0	False	-

ID	Formula	Z	SP (*No.*)	Prototype	NL	NR	WPs	HDWPs	Clean	dE/dq
mp-756725	$Ba_4Ca_2I_{12}$	18	$P2_1/c$ (14)	K_2TeI_6	136	184	0	0	False	-
mp-570887	$Ag_4K_4Sn_2Se_8$	18	$P2/c$ (13)	$K_2Ag_2SnSe_4$	63	197	0	0	False	-
mp-975605	$Ir_2Pr_{12}Cl_{22}$	18	$C2/m$ (12)	Pr_6IrCl_{11}	0	77	0	0	False	-
mp-556066	$Li_2Sr_4F_2O_8Si_2$	18	$P2_1/m$ (11)	Sr_2LiSiO_4	81	143	0	0	False	-
mp-760510	$Bi_4Sr_4O_{10}$	18	$P2_1/m$ (11)	$Sr_2Bi_2O_5$	82	130	0	0	False	-
mp-558826	$Be_2Ho_2K_2F_{12}$	18	$P2_1/m$ (11)	$KHoBeF_6$	82	123	0	0	False	-
mp-697181	$Cs_2Li_4H_6O_6$	18	$P2_1/m$ (11)	$CsLi_2(HO)_3$	81	112	0	0	False	-
mp-674531	$Li_{20}Ge_4P_{12}$	18	Cc (9)	Li_5GeP_3	0	37	0	0	False	-
mp-685991	$Li_{20}P_{12}Si_4$	18	Cc (9)	Li_5GeP_3	1	43	1	0	False	-
mp-753048	$Sn_6Ti_6O_{24}$	18	Cm (8)	$TiSnO_4$	0	53	1	0	False	-
mp-753460	$Ba_2Ca_4I_{12}$	18	Pc (7)	$BaCa_2I_6$	56	180	0	0	False	-
mp-31243	Ba_6As_{12}	18	Pc (7)	$BaAs_2$	57	205	0	0	False	-
mp-558102	$K_2Li_2O_{10}Si_4$	18	$P2_1$ (4)	$KLiSi_2O_5$	81	22	100	0	True	34.96
mp-19992	$La_{18}U_3O_{36}$	19	$R-3$ (148)	La_6UO_{12}	46	26	0	0	False	-
mp-20402	$Lu_{18}U_3O_{36}$	19	$R-3$ (148)	La_6UO_{12}	42	50	0	0	False	-
mp-22189	$In_{18}O_{36}Te_3$	19	$R-3$ (148)	La_6UO_{12}	49	19	0	0	False	-
mp-27698	$U_3Y_{18}O_{36}$	19	$R-3$ (148)	La_6UO_{12}	48	37	0	0	False	-
mp-754359	$Y_{18}O_{36}Te_3$	19	$R-3$ (148)	La_6UO_{12}	45	31	0	0	False	-
mp-755804	$Lu_{18}O_{36}Te_3$	19	$R-3$ (148)	La_6UO_{12}	47	26	0	0	False	-
mp-867371	$Cu_8K_4Y_8Se_{18}$	19	$C2/m$ (12)	$K_2Dy_4Cu_4S_9$	0	179	0	0	False	-
mp-680679	$Cu_8Ho_8K_4S_{18}$	19	Cm (8)	$K_2Dy_4Cu_4S_9$	0	62	0	0	False	-
mp-680676	$Cu_8Dy_8K_4S_{18}$	19	$C2$ (5)	$K_2Dy_4Cu_4S_9$	0	10	117	0	False	-
mp-16137	$Ca_2K_8U_6O_{24}$	20	$Im-3m$ (229)	$K_4CaU_3O_{12}$	56	132	0	0	False	-
mp-6273	$Ba_8Na_2O_{24}Sb_6$	20	$Im-3m$ (229)	$K_4CaU_3O_{12}$	64	117	0	0	False	-
mp-16293	$K_{16}O_{48}Sb_{16}$	20	$Fd-3m$ (227)	$KSbO_3$	56	107	0	0	False	-
mp-13654	$Au_{12}Y_{12}Sb_{16}$	20	$I-43d$ (220)	$Y_3Sb_4Au_3$	120	85	7	26	False	-
mp-17926	$Ni_{12}Zr_{12}Sb_{16}$	20	$I-43d$ (220)	$Y_3Sb_4Au_3$	119	59	9	28	False	-
mp-861503	$Au_{12}Pr_{12}Sb_{16}$	20	$I-43d$ (220)	$Y_3Sb_4Au_3$	121	79	14	28	False	-
mp-984350	$Au_{12}Dy_{12}Sb_{16}$	20	$I-43d$ (220)	$Y_3Sb_4Au_3$	124	81	9	27	False	-
mp-569571	$Nb_{12}Sb_8Te_{20}$	20	$I-43m$ (217)	$Nb_3Sb_2Te_5$	68	119	6	35	False	-
mp-561681	$Cs_4O_{12}S_4$	20	$P6_3mc$ (186)	$CsSO_3$	61	114	0	6	True	1.54
mp-554542	$Mg_6B_2F_6O_6$	20	$P6_3/m$ (176)	$Mg_3B(OF)_3$	37	77	0	0	False	-
mp-979264	$Rh_6Cl_{18}Te_{36}$	20	$R-3c$ (167)	Te_6RhCl_3	70	73	0	0	False	-
mp-14638	$Ga_6Li_6Rb_{12}F_{36}$	20	$R-3m$ (166)	Rb_2LiGaF_6	33	117	0	0	False	-
mp-23774	$Al_6K_{12}Li_6H_{36}$	20	$R-3m$ (166)	Rb_2LiGaF_6	27	70	0	0	True	1.46
mp-571527	$Cs_{12}In_6Li_6Cl_{36}$	20	$R-3m$ (166)	Rb_2LiGaF_6	31	98	0	0	False	-
mp-6163	$Cs_{12}Ga_6Na_6F_{36}$	20	$R-3m$ (166)	Rb_2LiGaF_6	29	89	0	0	False	-
mp-6528	$Al_6Cs_{12}Na_6F_{36}$	20	$R-3m$ (166)	Rb_2LiGaF_6	31	79	0	0	False	-
mp-557787	$Tl_4O_{12}Sb_4$	20	$P-31c$ (163)	$TlSbO_3$	89	191	0	0	False	-
mp-13383	$Ag_2Sc_2P_4Se_{12}$	20	$P-31c$ (163)	$ScAg(PSe_3)_2$	91	142	0	0	False	-
mp-13384	$Ag_2Er_2P_4Se_{12}$	20	$P-31c$ (163)	$ScAg(PSe_3)_2$	95	131	0	0	False	-
mp-13385	$Ag_2Tm_2P_4Se_{12}$	20	$P-31c$ (163)	$ScAg(PSe_3)_2$	95	128	0	0	False	-
mp-20902	$Ag_2In_2P_4Se_{12}$	20	$P-31c$ (163)	$ScAg(PSe_3)_2$	93	160	0	0	False	-
mp-7008	$Ag_2Ga_2P_4Se_{12}$	20	$P-31c$ (163)	$ScAg(PSe_3)_2$	93	161	0	0	False	-
mp-8616	$Ag_2Sc_2P_4S_{12}$	20	$P-31c$ (163)	$ScAg(PSe_3)_2$	98	126	0	0	True	5.18
mp-23503	$In_2Na_6Cl_{12}$	20	$P-31c$ (163)	Na_3InCl_6	111	168	0	0	False	-
mp-29417	$Na_6Sc_2Br_{12}$	20	$P-31c$ (163)	Na_3InCl_6	105	188	0	0	False	-
mp-10340	$Na_4Ge_2O_{12}Te_2$	20	$P-31c$ (163)	Na_2GeTeO_6	104	161	0	0	True	1.46
mp-18377	$Zn_{30}S_{30}$	20	$R3m$ (160)	ZnS	100	180	125	0	False	-
mp-555858	$Zn_{30}S_{30}$	20	$R3m$ (160)	ZnS	116	131	95	0	False	-
mp-556105	$Zn_{30}S_{30}$	20	$R3m$ (160)	ZnS	84	190	113	0	False	-
mp-23612	$K_9B_{18}Br_3O_{30}$	20	$R3m$ (160)	$K_3B_6BrO_{10}$	49	29	0	0	False	-
mp-13516	$Al_9Tm_3B_{12}O_{36}$	20	$R32$ (155)	$TmAl_3(BO_3)_4$	0	0	116	0	False	-
mp-6062	$Al_9Y_3B_{12}O_{36}$	20	$R32$ (155)	$TmAl_3(BO_3)_4$	0	2	110	0	False	-
mp-6524	$Ca_3Mg_9C_{12}O_{36}$	20	$R32$ (155)	$TmAl_3(BO_3)_4$	0	0	119	0	True	4.29
mp-7337	$Al_9Nd_3B_{12}O_{36}$	20	$R32$ (155)	$TmAl_3(BO_3)_4$	0	1	101	0	False	-
mp-29699	$Nb_{18}Ti_6O_{36}$	20	$R-3$ (148)	$TiNb_3O_6$	32	12	0	0	False	-
mp-13840	$Ho_{18}Sc_6O_{36}$	20	$R-3$ (148)	Ho_3ScO_6	51	33	0	0	False	-
mp-14252	$Ca_{18}U_6O_{36}$	20	$R-3$ (148)	Ho_3ScO_6	52	28	0	0	False	-
mp-31096	$Al_6Li_{18}H_{36}$	20	$R-3$ (148)	Ho_3ScO_6	55	24	0	0	False	-
mp-3118	$Mg_{18}O_{36}Te_6$	20	$R-3$ (148)	Ho_3ScO_6	47	53	0	0	False	-
mp-752569	$La_{18}Sc_6O_{36}$	20	$R-3$ (148)	Ho_3ScO_6	58	26	0	0	False	-

ID	Formula	Z	SP (*No.*)	Prototype	NL	NR	WPs	HDWPs	Clean	dE/dq
mp-756742	$Al_6Lu_{18}O_{36}$	20	*R*−3 (148)	Ho_3ScO_6	54	15	0	0	False	-
mp-28529	$In_4K_4Br_{12}$	20	*P*−3 (147)	$KInBr_3$	88	43	0	0	False	-
mp-863678	$K_3Zn_{12}O_{36}Sb_9$	20	*R*3 (146)	$KZn_4(SbO_4)_3$	0	0	65	0	False	-
mp-556422	$Nb_4Cl_{12}O_4$	20	*P*−42$_1$m (113)	$NbCl_3$	120	46	8	0	False	-
mp-606393	$Nb_4Br_{12}O_4$	20	*P*−42$_1$m (113)	$NbCl_3$	120	45	9	0	False	-
mp-27809	$Ba_4Ge_8P_8$	20	*P*4$_2$*mc* (105)	$Ba(GeP)_2$	30	164	0	0	False	-
mp-29816	$Ag_8Hg_4O_8$	20	*P*4$_3$2$_1$2 (96)	Ag_2HgO_2	180	37	97	15	False	-
mp-7623	Mg_4As_{16}	20	*P*4$_1$2$_1$2 (92)	$MgAs_4$	180	33	95	15	False	-
mp-644506	$Al_4Na_4C_4H_8O_{20}$	20	*Imma* (74)	$NaAlH_2CO_5$	30	145	0	0	True	3.04
mp-29882	$Cs_8Pd_8Cl_{24}$	20	*Ibam* (72)	$CsPdCl_3$	62	177	0	0	False	-
mp-555140	$Cs_8Ga_4Li_{12}O_{16}$	20	*Ibam* (72)	$Cs_2Li_3GaO_4$	62	205	0	0	False	-
mp-569857	$K_{32}P_{48}$	20	*Fddd* (70)	K_2P_3	121	116	0	0	False	-
mp-209	$Er_{32}Se_{48}$	20	*Fddd* (70)	Er_2Se_3	120	182	0	0	False	-
mp-401	$Sc_{32}S_{48}$	20	*Fddd* (70)	Er_2Se_3	119	223	0	0	False	-
mp-9824	$Ho_{32}Se_{48}$	20	*Fddd* (70)	Er_2Se_3	120	178	0	0	False	-
mp-760489	$H_{24}N_4O_8P_4$	20	*Cmme* (67)	PH_6NO_2	31	88	0	0	False	-
mp-754153	$Li_8Br_8H_{16}O_8$	20	*Cmcm* (63)	LiH_2	101	100	0	0	False	-
mp-17324	$Sn_4Zr_4S_{12}$	20	*Pnma* (62)	$ZrSnS_3$	292	122	0	0	False	-
mp-20244	$Pb_4Zr_4S_{12}$	20	*Pnma* (62)	$ZrSnS_3$	289	114	0	0	False	-
mp-22147	$Hf_4Pb_4S_{12}$	20	*Pnma* (62)	$ZrSnS_3$	287	122	0	0	True	5.51
mp-29483	$In_4Mg_4Br_{12}$	20	*Pnma* (62)	$ZrSnS_3$	293	154	0	0	False	-
mp-505222	$Cd_4In_4Br_{12}$	20	*Pnma* (62)	$ZrSnS_3$	293	141	0	0	False	-
mp-8725	$Hf_4Sn_4S_{12}$	20	*Pnma* (62)	$ZrSnS_3$	292	135	0	0	False	-
mp-3102	$Cu_4Ta_4S_{12}$	20	*Pnma* (62)	$TaCuS_3$	290	104	0	0	False	-
mp-1509	Sn_8S_{12}	20	*Pnma* (62)	Sn_2S_3	289	128	0	0	False	-
mp-1403	Sm_8S_{12}	20	*Pnma* (62)	Sm_2S_3	289	155	0	0	False	-
mp-15179	Pr_8S_{12}	20	*Pnma* (62)	Sm_2S_3	289	156	0	0	False	-
mp-438	Nd_8S_{12}	20	*Pnma* (62)	Sm_2S_3	290	169	0	0	False	-
mp-7475	La_8S_{12}	20	*Pnma* (62)	Sm_2S_3	288	143	0	0	False	-
mp-8850	Dy_8S_{12}	20	*Pnma* (62)	Sm_2S_3	285	150	0	0	False	-
mp-23700	$Rb_4F_4H_8O_4$	20	*Pnma* (62)	RbH_2	285	101	0	0	False	-
mp-23390	$Cd_4Rb_4Cl_{12}$	20	*Pnma* (62)	$RbCdCl_3$	289	129	0	0	False	-
mp-23442	$Cd_4K_4Cl_{12}$	20	*Pnma* (62)	$RbCdCl_3$	292	129	0	0	False	-
mp-23517	$Pb_4Rb_4I_{12}$	20	*Pnma* (62)	$RbCdCl_3$	292	156	0	0	False	-
mp-27252	$Cd_4Tl_4I_{12}$	20	*Pnma* (62)	$RbCdCl_3$	286	161	0	0	False	-
mp-27400	$Cd_4Rb_4Br_{12}$	20	*Pnma* (62)	$RbCdCl_3$	291	148	0	0	False	-
mp-28218	$Cd_4Tl_4Cl_{12}$	20	*Pnma* (62)	$RbCdCl_3$	288	146	0	0	False	-
mp-28219	$Cd_4Tl_4Br_{12}$	20	*Pnma* (62)	$RbCdCl_3$	288	155	0	0	False	-
mp-29288	$Tl_4Ge_4I_{12}$	20	*Pnma* (62)	$RbCdCl_3$	289	161	0	0	False	-
mp-540839	$Cs_4Pb_4I_{12}$	20	*Pnma* (62)	$RbCdCl_3$	291	165	0	0	False	-
mp-558760	$Sr_4Zr_4S_{12}$	20	*Pnma* (62)	$RbCdCl_3$	291	130	0	0	False	-
mp-568796	$Rb_4Yb_4I_{12}$	20	*Pnma* (62)	$RbCdCl_3$	291	140	0	0	False	-
mp-755428	$Rb_{12}Cl_4O_4$	20	*Pnma* (62)	$RbCdCl_3$	288	146	0	0	False	-
mp-643069	$Li_8H_4I_4O_4$	20	*Pnma* (62)	Li_2	287	81	0	0	False	-
mp-11623	$In_4La_4O_{12}$	20	*Pnma* (62)	$LaInO_3$	286	123	0	0	False	-
mp-12271	$Mg_4O_{12}Se_4$	20	*Pnma* (62)	$LaInO_3$	284	117	0	0	False	-
mp-13374	$La_4Tm_4O_{12}$	20	*Pnma* (62)	$LaInO_3$	285	129	0	0	False	-
mp-20489	$Pb_4Sr_4O_{12}$	20	*Pnma* (62)	$LaInO_3$	284	107	0	0	False	-
mp-21872	$Lu_4Pr_4O_{12}$	20	*Pnma* (62)	$LaInO_3$	286	108	0	0	False	-
mp-23730	$Mg_4Na_4H_{12}$	20	*Pnma* (62)	$LaInO_3$	287	95	0	0	False	-
mp-27865	$K_4Mg_4Cl_{12}$	20	*Pnma* (62)	$LaInO_3$	288	137	0	0	False	-
mp-2879	$Sn_4Sr_4O_{12}$	20	*Pnma* (62)	$LaInO_3$	285	125	0	0	False	-
mp-2955	$Mg_4Na_4F_{12}$	20	*Pnma* (62)	$LaInO_3$	285	137	0	0	False	-
mp-30949	$Sr_{12}O_4Si_4$	20	*Pnma* (62)	$LaInO_3$	285	145	0	0	False	-
mp-31117	$Nd_4Sc_4O_{12}$	20	*Pnma* (62)	$LaInO_3$	285	108	0	0	False	-
mp-31118	$Sc_4Sm_4O_{12}$	20	*Pnma* (62)	$LaInO_3$	284	106	0	0	False	-
mp-31119	$Sc_4Tb_4O_{12}$	20	*Pnma* (62)	$LaInO_3$	285	120	0	0	False	-
mp-31120	$Dy_4Sc_4O_{12}$	20	*Pnma* (62)	$LaInO_3$	285	116	0	0	False	-
mp-31121	$Ho_4Sc_4O_{12}$	20	*Pnma* (62)	$LaInO_3$	285	103	0	0	False	-
mp-3196	$Ga_4Nd_4O_{12}$	20	*Pnma* (62)	$LaInO_3$	286	109	0	0	False	-
mp-3317	$Rh_4Sm_4O_{12}$	20	*Pnma* (62)	$LaInO_3$	285	104	0	0	False	-
mp-3378	$Hf_4Sr_4O_{12}$	20	*Pnma* (62)	$LaInO_3$	285	108	0	0	False	-

ID	Formula	Z	SP (*No.*)	Prototype	NL	NR	WPs	HDWPs	Clean	dE/dq
mp-3792	$Al_4Y_4O_{12}$	20	*Pnma* (62)	$LaInO_3$	285	117	0	0	False	-
mp-3795	$Na_4Zn_4F_{12}$	20	*Pnma* (62)	$LaInO_3$	285	144	0	0	False	-
mp-4019	$Ca_4Ti_4O_{12}$	20	*Pnma* (62)	$LaInO_3$	285	147	0	0	False	-
mp-4192	$Ca_{12}As_4N_4$	20	*Pnma* (62)	$LaInO_3$	285	122	0	0	False	-
mp-4387	$Sr_4Zr_4O_{12}$	20	*Pnma* (62)	$LaInO_3$	285	121	0	0	False	-
mp-4434	$Al_4Tb_4O_{12}$	20	*Pnma* (62)	$LaInO_3$	285	132	0	0	False	-
mp-4438	$Ca_4Sn_4O_{12}$	20	*Pnma* (62)	$LaInO_3$	285	129	0	0	False	-
mp-4471	$Al_4Sc_4O_{12}$	20	*Pnma* (62)	$LaInO_3$	285	137	0	0	False	-
mp-4535	$Ga_4Pr_4O_{12}$	20	*Pnma* (62)	$LaInO_3$	284	104	0	0	False	-
mp-4582	$Nd_4Rh_4O_{12}$	20	*Pnma* (62)	$LaInO_3$	285	101	0	0	True	8.13
mp-4789	$Pr_4Rh_4O_{12}$	20	*Pnma* (62)	$LaInO_3$	286	78	0	0	False	-
mp-4891	$Al_4Ho_4O_{12}$	20	*Pnma* (62)	$LaInO_3$	285	138	0	0	False	-
mp-5163	$La_4Rh_4O_{12}$	20	*Pnma* (62)	$LaInO_3$	286	87	0	0	False	-
mp-559756	$Pr_4Sc_4O_{12}$	20	*Pnma* (62)	$LaInO_3$	285	131	0	0	False	-
mp-567629	$Cs_4Pb_4Br_{12}$	20	*Pnma* (62)	$LaInO_3$	284	167	0	0	False	-
mp-5692	$Rh_4Tb_4O_{12}$	20	*Pnma* (62)	$LaInO_3$	285	118	0	0	False	-
mp-571418	$Rb_4Yb_4Br_{12}$	20	*Pnma* (62)	$LaInO_3$	285	158	0	0	False	-
mp-572291	$K_{12}Cl_4O_4$	20	*Pnma* (62)	$LaInO_3$	286	136	0	0	False	-
mp-5926	$Ca_4K_4F_{12}$	20	*Pnma* (62)	$LaInO_3$	285	139	0	0	False	-
mp-752437	$In_4Sm_4O_{12}$	20	*Pnma* (62)	$LaInO_3$	285	131	0	0	False	-
mp-754140	$Ca_4O_{12}Se_4$	20	*Pnma* (62)	$LaInO_3$	286	86	0	0	False	-
mp-754225	$Ti_4Yb_4O_{12}$	20	*Pnma* (62)	$LaInO_3$	285	127	0	0	False	-
mp-754578	$Ga_4Tm_4O_{12}$	20	*Pnma* (62)	$LaInO_3$	284	116	0	0	False	-
mp-754853	$Ca_4Hf_4O_{12}$	20	*Pnma* (62)	$LaInO_3$	285	106	0	0	False	-
mp-755138	$In_4Nd_4O_{12}$	20	*Pnma* (62)	$LaInO_3$	288	124	0	0	False	-
mp-755165	$Sc_4Y_4O_{12}$	20	*Pnma* (62)	$LaInO_3$	286	128	0	0	False	-
mp-755363	$Ba_{12}O_4Si_4$	20	*Pnma* (62)	$LaInO_3$	286	147	0	0	False	-
mp-755416	$Ga_4Ho_4O_{12}$	20	*Pnma* (62)	$LaInO_3$	285	96	0	0	False	-
mp-755574	$Al_4Lu_4O_{12}$	20	*Pnma* (62)	$LaInO_3$	285	135	0	0	False	-
mp-755612	$Dy_4Ga_4O_{12}$	20	*Pnma* (62)	$LaInO_3$	285	111	0	0	False	-
mp-756205	$Bi_4Sc_4O_{12}$	20	*Pnma* (62)	$LaInO_3$	285	116	0	0	False	-
mp-756281	$Al_4Tm_4O_{12}$	20	*Pnma* (62)	$LaInO_3$	285	110	0	0	False	-
mp-756290	$Ga_4Tb_4O_{12}$	20	*Pnma* (62)	$LaInO_3$	287	114	0	0	False	-
mp-756458	$Al_4Er_4O_{12}$	20	*Pnma* (62)	$LaInO_3$	285	119	0	0	False	-
mp-756630	$Al_4Dy_4O_{12}$	20	*Pnma* (62)	$LaInO_3$	285	118	0	0	False	-
mp-7781	$Ca_4Zr_4S_{12}$	20	*Pnma* (62)	$LaInO_3$	287	111	0	0	False	-
mp-7792	$Sc_4Y_4S_{12}$	20	*Pnma* (62)	$LaInO_3$	285	145	0	0	False	-
mp-780387	$Hf_4Yb_4O_{12}$	20	*Pnma* (62)	$LaInO_3$	285	99	0	0	False	-
mp-849371	$Cd_4Sn_4O_{12}$	20	*Pnma* (62)	$LaInO_3$	285	94	0	0	False	-
mp-9186	$Cd_4O_{12}Se_4$	20	*Pnma* (62)	$LaInO_3$	281	81	0	0	False	-
mp-9628	$Cd_4K_4F_{12}$	20	*Pnma* (62)	$LaInO_3$	283	137	0	0	False	-
mp-752418	$La_8O_4Se_8$	20	*Pnma* (62)	La_2Se_2	294	104	0	0	False	-
mp-755184	$Sm_8O_4Se_8$	20	*Pnma* (62)	La_2Se_2	288	113	0	0	False	-
mp-28695	$K_4H_{12}Si_4$	20	*Pnma* (62)	$KSiH_3$	285	81	0	0	False	-
mp-9475	$K_4N_4O_{12}$	20	*Pnma* (62)	KNO_3	289	137	0	0	True	1.83
mp-9228	$Hg_4O_{12}Se_4$	20	*Pnma* (62)	$HgSeO_3$	285	93	0	0	False	-
mp-1270	Dy_8S_{12}	20	*Pnma* (62)	Dy_2S_3	287	137	0	0	False	-
mp-8956	Ho_8S_{12}	20	*Pnma* (62)	Dy_2S_3	289	124	0	0	False	-
mp-9323	Tb_8S_{12}	20	*Pnma* (62)	Dy_2S_3	286	141	0	0	False	-
mp-570590	$Bi_4Cd_4Br_4Se_8$	20	*Pnma* (62)	$CdBiSe_2$	293	124	0	0	False	-
mp-27499	$Ba_4S_{12}Te_4$	20	*Pnma* (62)	$BaTeS_3$	285	124	0	0	False	-
mp-3822	$Sr_4C_4O_{12}$	20	*Pnma* (62)	$BaTeS_3$	298	140	0	0	False	-
mp-7238	$Nd_4B_4O_{12}$	20	*Pnma* (62)	$BaTeS_3$	287	128	0	0	False	-
mp-755213	$Yb_4C_4O_{12}$	20	*Pnma* (62)	$BaTeS_3$	286	109	0	0	False	-
mp-8216	$La_4B_4O_{12}$	20	*Pnma* (62)	$BaTeS_3$	287	124	0	0	False	-
mp-861504	$Pr_4B_4O_{12}$	20	*Pnma* (62)	$BaTeS_3$	292	123	0	0	False	-
mp-30133	$Ba_4Cu_8Te_8$	20	*Pnma* (62)	$Ba(CuTe)_2$	289	126	0	0	False	-
mp-4473	$Ba_4Cu_8Se_8$	20	*Pnma* (62)	$Ba(CuTe)_2$	293	127	0	0	True	6.18
mp-5970	$Ba_4Cu_8S_8$	20	*Pnma* (62)	$Ba(CuTe)_2$	289	131	0	0	False	-
mp-1716	Rh_8O_{12}	20	*Pbcn* (60)	Rh_2O_3	289	125	0	0	False	-
mp-568293	$Ca_{12}N_8$	20	*Pbcn* (60)	Rh_2O_3	286	138	0	0	False	-
mp-27742	$Cd_4K_8O_8$	20	*Pbcn* (60)	K_2CdO_2	288	112	0	0	False	-

ID	Formula	Z	SP (*No.*)	Prototype	NL	NR	WPs	HDWPs	Clean	dE/dq
mp-28364	$Cd_4Rb_8O_8$	20	*Pbcn* (60)	K_2CdO_2	286	118	0	0	False	-
mp-755802	$K_8Mg_4O_8$	20	*Pbcn* (60)	K_2CdO_2	295	99	0	0	False	-
mp-3838	$Li_{10}Re_2N_8$	20	*Pmmn* (59)	Li_5ReN_4	210	159	0	0	False	-
mp-7535	$Al_2Li_{10}O_8$	20	*Pmmn* (59)	Li_5ReN_4	210	165	0	0	False	-
mp-8840	$In_2Na_{10}O_8$	20	*Pmmn* (59)	Li_5ReN_4	210	184	0	0	False	-
mp-28853	$Au_4La_4O_{12}$	20	*Pbcm* (57)	$LaAuO_3$	227	89	0	0	False	-
mp-29920	$Au_6Rb_{10}O_4$	20	*Pbam* (55)	$Rb_5Au_3O_2$	210	122	0	0	False	-
mp-558332	$Au_2K_{10}I_4O_4$	20	*Pbam* (55)	$K_5Au(IO)_2$	210	148	0	0	False	-
mp-558536	$Au_4Cd_4Rb_4S_8$	20	*Pcca* (54)	$RbCdAuS_2$	210	124	0	0	False	-
mp-28741	$Cu_4Br_4Se_{12}$	20	*Pmna* (53)	$CuSe_3$	210	94	0	0	False	-
mp-569052	$Ag_4I_4Se_{12}$	20	*Pmna* (53)	$CuSe_3$	210	89	0	0	False	-
mp-570431	$Ag_4I_4Te_{12}$	20	*Pmna* (53)	$CuSe_3$	210	96	0	0	False	-
mp-4995	$Li_4O_{12}Sb_4$	20	*Pnna* (52)	$LiSbO_3$	240	154	0	0	False	-
mp-11773	$Ga_4Nd_{12}O_{24}$	20	$Cmc2_1$ (36)	Nd_3GaO_6	102	114	20	0	False	-
mp-31110	$Ga_4Sm_{12}O_{24}$	20	$Cmc2_1$ (36)	Nd_3GaO_6	96	107	28	0	False	-
mp-31112	$Ga_4Tb_{12}O_{24}$	20	$Cmc2_1$ (36)	Nd_3GaO_6	94	111	24	0	False	-
mp-31113	$Dy_{12}Ga_4O_{24}$	20	$Cmc2_1$ (36)	Nd_3GaO_6	102	111	28	0	False	-
mp-31114	$Ga_4Ho_{12}O_{24}$	20	$Cmc2_1$ (36)	Nd_3GaO_6	97	122	27	0	False	-
mp-31115	$Er_{12}Ga_4O_{24}$	20	$Cmc2_1$ (36)	Nd_3GaO_6	101	117	26	0	False	-
mp-554722	$Ga_4Y_{12}O_{24}$	20	$Cmc2_1$ (36)	Nd_3GaO_6	99	109	27	0	False	-
mp-7290	$Er_{12}Ga_4S_{24}$	20	$Cmc2_1$ (36)	Nd_3GaO_6	92	107	29	0	False	-
mp-754979	$Al_4Y_{12}O_{24}$	20	$Cmc2_1$ (36)	Nd_3GaO_6	101	111	26	0	False	-
mp-768573	$Al_4Lu_{12}O_{24}$	20	$Cmc2_1$ (36)	Nd_3GaO_6	100	98	16	0	False	-
mp-8673	$Li_4Sn_2O_{12}Te_2$	20	*Pnn*2 (34)	Li_2SnTeO_6	180	177	0	0	False	-
mp-862754	$Pr_4B_4S_{12}$	20	$Pna2_1$ (33)	$PrBS_3$	242	115	0	14	False	-
mp-867286	$Tb_4B_4S_{12}$	20	$Pna2_1$ (33)	$PrBS_3$	243	114	0	13	False	-
mp-972448	$Sm_4B_4S_{12}$	20	$Pna2_1$ (33)	$PrBS_3$	243	105	0	12	False	-
mp-28558	$Rb_4Br_{12}Ge_4$	20	$Pna2_1$ (33)	$LaInO_3$	242	173	0	15	False	-
mp-556892	$Bi_4In_4O_{12}$	20	$Pna2_1$ (33)	$LaInO_3$	242	143	0	14	False	-
mp-697146	$Zn_4H_8O_8$	20	$P2_12_12_1$ (19)	$Zn(HO)_2$	270	27	44	15	False	-
mp-27931	$Rb_8Sn_4O_8$	20	$P2_12_12_1$ (19)	Rb_2SnO_2	270	26	67	15	False	-
mp-781712	$Na_8Sn_4O_8$	20	$P2_12_12_1$ (19)	Rb_2SnO_2	270	37	70	15	True	13.55
mp-11183	$Pr_4B_{12}O_{24}$	20	$C2/c$ (15)	$Pr(BO_2)_3$	0	92	0	0	False	-
mp-11709	$Sm_4B_{12}O_{24}$	20	$C2/c$ (15)	$Pr(BO_2)_3$	0	100	0	0	False	-
mp-3615	$Nd_4B_{12}O_{24}$	20	$C2/c$ (15)	$Pr(BO_2)_3$	0	82	0	0	False	-
mp-6124	$Al_4Na_4O_{24}Si_8$	20	$C2/c$ (15)	$NaAl(SiO_3)_2$	1	153	0	0	False	-
mp-6766	$Ca_4Mg_4O_{24}Si_8$	20	$C2/c$ (15)	$NaAl(SiO_3)_2$	1	142	0	0	False	-
mp-6822	$Ga_4Na_4O_{24}Si_8$	20	$C2/c$ (15)	$NaAl(SiO_3)_2$	1	125	0	0	False	-
mp-7074	$Na_4Sc_4O_{24}Si_8$	20	$C2/c$ (15)	$NaAl(SiO_3)_2$	1	132	0	0	False	-
mp-8709	$In_4K_4Ge_8O_{24}$	20	$C2/c$ (15)	$NaAl(SiO_3)_2$	0	119	0	0	False	-
mp-977298	$Be_8Li_8H_{24}$	20	$C2/c$ (15)	$LiBeH_3$	0	111	0	0	False	-
mp-4426	$Li_{16}O_{20}Se_4$	20	$C2/c$ (15)	Li_4SeO_5	1	150	0	0	False	-
mp-569059	Hf_8I_{32}	20	$C2/c$ (15)	HfI_4	3	68	0	0	False	-
mp-570553	Fe_8P_{32}	20	$C2/c$ (15)	FeP_4	5	166	0	0	False	-
mp-573677	$Ba_8Li_{12}Ta_4N_{16}$	20	$C2/c$ (15)	$Ba_2Li_3NbN_4$	2	105	0	0	False	-
mp-756251	$Ba_8La_4Cl_{28}$	20	$C2/c$ (15)	Ba_2LaCl_7	0	154	0	0	False	-
mp-28532	$Cr_4In_4Br_{12}$	20	$P2_1/c$ (14)	$ZrSnS_3$	152	185	0	0	False	-
mp-28809	$Tl_4B_4S_{12}$	20	$P2_1/c$ (14)	$TlBS_3$	150	116	0	0	True	25.4
mp-27697	Th_4I_{16}	20	$P2_1/c$ (14)	ThI_4	150	179	0	0	False	-
mp-27628	Cl_8Te_{12}	20	$P2_1/c$ (14)	Te_3Cl_2	151	153	0	0	False	-
mp-5045	$Sn_4Ge_4S_{12}$	20	$P2_1/c$ (14)	$SnGeS_3$	153	139	0	0	False	-
mp-624190	$Pb_4Ge_4S_{12}$	20	$P2_1/c$ (14)	$SnGeS_3$	153	158	0	0	False	-
mp-10552	$Rb_4B_4Se_{12}$	20	$P2_1/c$ (14)	$RbBSe_3$	150	118	0	0	False	-
mp-510283	$Cs_4Ga_4Se_{12}$	20	$P2_1/c$ (14)	$RbBSe_3$	150	140	0	0	False	-
mp-9047	$Rb_4B_4S_{12}$	20	$P2_1/c$ (14)	$RbBSe_3$	152	104	0	0	False	-
mp-23705	$Al_2Na_6H_{12}$	20	$P2_1/c$ (14)	Na_3AlH_6	151	180	0	0	False	-
mp-29080	$Na_6Y_2Br_{12}$	20	$P2_1/c$ (14)	Na_3AlH_6	151	199	0	0	False	-
mp-3416	$Al_2Na_6F_{12}$	20	$P2_1/c$ (14)	Na_3AlH_6	152	196	0	0	False	-
mp-5279	$Ca_6O_{12}Te_2$	20	$P2_1/c$ (14)	Na_3AlH_6	152	217	0	0	False	-
mp-768941	$Sr_6O_{12}Te_2$	20	$P2_1/c$ (14)	Na_3AlH_6	152	193	0	0	False	-
mp-10349	$Na_4Sn_2O_{12}Te_2$	20	$P2_1/c$ (14)	Na_2SnTeO_6	153	201	0	0	False	-
mp-11982	$Ca_2Sr_4O_{12}Te_2$	20	$P2_1/c$ (14)	Na_2SnTeO_6	150	211	0	0	False	-

ID	Formula	Z	SP (*No.*)	Prototype	NL	NR	WPs	HDWPs	Clean	dE/dq
mp-12337	$La_4Na_2Os_2O_{12}$	20	$P2_1/c$ (14)	Na_2SnTeO_6	150	142	0	0	False	-
mp-12448	$Li_2Os_2Sm_4O_{12}$	20	$P2_1/c$ (14)	Na_2SnTeO_6	151	151	0	0	False	-
mp-12449	$Li_2Os_2Pr_4O_{12}$	20	$P2_1/c$ (14)	Na_2SnTeO_6	155	145	0	0	False	-
mp-12450	$Li_2Nd_4Os_2O_{12}$	20	$P2_1/c$ (14)	Na_2SnTeO_6	151	164	0	0	True	8.47
mp-12451	$La_4Li_2Os_2O_{12}$	20	$P2_1/c$ (14)	Na_2SnTeO_6	151	176	0	0	False	-
mp-12878	$Sr_4Y_2O_{12}Sb_2$	20	$P2_1/c$ (14)	Na_2SnTeO_6	151	209	0	0	False	-
mp-14551	$Ca_2Sr_4U_2O_{12}$	20	$P2_1/c$ (14)	Na_2SnTeO_6	150	188	0	0	False	-
mp-14552	$Ba_4Sr_2U_2O_{12}$	20	$P2_1/c$ (14)	Na_2SnTeO_6	153	211	0	0	False	-
mp-16605	$Sr_4Ta_2Tm_2O_{12}$	20	$P2_1/c$ (14)	Na_2SnTeO_6	150	209	0	0	False	-
mp-20009	$Na_2Os_2Pr_4O_{12}$	20	$P2_1/c$ (14)	Na_2SnTeO_6	151	163	0	0	False	-
mp-20222	$In_2Nb_2Sr_4O_{12}$	20	$P2_1/c$ (14)	Na_2SnTeO_6	150	192	0	0	False	-
mp-22329	$Na_2Nd_4Os_2O_{12}$	20	$P2_1/c$ (14)	Na_2SnTeO_6	153	162	0	0	False	-
mp-22355	$In_2Sr_4O_{12}Sb_2$	20	$P2_1/c$ (14)	Na_2SnTeO_6	151	209	0	0	False	-
mp-23093	$Bi_2Nd_2Sr_4O_{12}$	20	$P2_1/c$ (14)	Na_2SnTeO_6	152	197	0	0	False	-
mp-23100	$Bi_2Lu_2Sr_4O_{12}$	20	$P2_1/c$ (14)	Na_2SnTeO_6	153	226	0	0	False	-
mp-24413	$Al_2Li_2Na_4H_{12}$	20	$P2_1/c$ (14)	Na_2SnTeO_6	152	166	0	0	False	-
mp-542512	$Na_2Pr_4Ru_2O_{12}$	20	$P2_1/c$ (14)	Na_2SnTeO_6	150	169	0	0	False	-
mp-558039	$Ho_2Sr_4O_{12}Sb_2$	20	$P2_1/c$ (14)	Na_2SnTeO_6	151	207	0	0	False	-
mp-560645	$Ca_4In_2Nb_2O_{12}$	20	$P2_1/c$ (14)	Na_2SnTeO_6	153	209	0	0	False	-
mp-561072	$Dy_2Sr_4O_{12}Sb_2$	20	$P2_1/c$ (14)	Na_2SnTeO_6	150	203	0	0	False	-
mp-6389	$La_4Na_2Ru_2O_{12}$	20	$P2_1/c$ (14)	Na_2SnTeO_6	152	182	0	0	False	-
mp-6604	$Al_2Li_2Na_4F_{12}$	20	$P2_1/c$ (14)	Na_2SnTeO_6	150	184	0	0	False	-
mp-972112	$Mg_2Sr_4U_2O_{12}$	20	$P2_1/c$ (14)	Na_2SnTeO_6	150	198	0	0	False	-
mp-972216	$Sm_2Sr_4O_{12}Sb_2$	20	$P2_1/c$ (14)	Na_2SnTeO_6	152	200	0	0	True	2.68
mp-570948	$Ca_4Ga_4Li_4N_8$	20	$P2_1/c$ (14)	$LiCaGaN_2$	151	179	0	0	False	-
mp-989614	$La_4Re_4N_{12}$	20	$P2_1/c$ (14)	$LaReN_3$	153	134	0	0	False	-
mp-31045	$Au_4K_4I_{12}$	20	$P2_1/c$ (14)	$LaInO_3$	151	182	0	0	False	-
mp-636675	Ga_8I_{12}	20	$P2_1/c$ (14)	Ga_2I_3	152	186	0	0	False	-
mp-31276	$Cd_8Cl_8P_4$	20	$P2_1/c$ (14)	Cd_2PCl_2	155	156	0	0	False	-
mp-643392	$Ca_4H_8O_8$	20	$P2_1/c$ (14)	$Ca(HO)_2$	155	175	0	0	False	-
mp-11006	$Ba_4P_4S_{12}$	20	$P2_1/c$ (14)	$BaPS_3$	154	140	0	0	True	3.8
mp-11007	$Ca_4P_4Se_{12}$	20	$P2_1/c$ (14)	$BaPS_3$	151	166	0	0	False	-
mp-11008	$Ba_4P_4Se_{12}$	20	$P2_1/c$ (14)	$BaPS_3$	152	157	0	0	True	1.23
mp-20199	$Pb_4P_4S_{12}$	20	$P2_1/c$ (14)	$BaPS_3$	150	138	0	0	False	-
mp-20316	$Pb_4P_4Se_{12}$	20	$P2_1/c$ (14)	$BaPS_3$	153	146	0	0	True	1.15
mp-7198	$Sr_4P_4Se_{12}$	20	$P2_1/c$ (14)	$BaPS_3$	153	162	0	0	False	-
mp-9788	$Sr_4P_4S_{12}$	20	$P2_1/c$ (14)	$BaPS_3$	151	138	0	0	False	-
mp-9789	$Ca_4P_4S_{12}$	20	$P2_1/c$ (14)	$BaPS_3$	153	154	0	0	False	-
mp-8194	$Ba_8Ge_4P_8$	20	$P2_1/c$ (14)	Ba_2GeP_2	151	175	0	0	False	-
mp-8195	$Ba_8As_8Ge_4$	20	$P2_1/c$ (14)	Ba_2GeP_2	152	208	0	0	False	-
mp-909	As_8Se_{12}	20	$P2_1/c$ (14)	As_2Se_3	150	190	0	0	False	-
mp-28038	$Nb_4Br_{12}Te_4$	20	$P2/c$ (13)	$NbTeBr_3$	61	131	0	0	False	-
mp-13282	$Er_6Sm_2Se_{12}$	20	$P2_1/m$ (11)	$Sm(ErSe_2)_3$	90	297	0	0	False	-
mp-29376	$In_2Na_{10}S_8$	20	$P2_1/m$ (11)	Na_5InS_4	90	189	0	0	False	-
mp-28327	$Ga_4Li_4Br_{12}$	20	$P2_1/m$ (11)	$LiGaBr_3$	90	173	0	0	False	-
mp-29345	$Ga_4Li_4I_{12}$	20	$P2_1/m$ (11)	$LiGaBr_3$	91	158	0	0	False	-
mp-643749	$Ca_4K_4F_{12}$	20	$P2_1/m$ (11)	$LaInO_3$	0	261	0	0	False	-
mp-569613	$Ba_4Cl_2P_{14}$	20	$P2_1/m$ (11)	Ba_2P_7	90	116	0	0	True	1.21
mp-972120	$Ga_8Sr_4As_8$	20	$P2/m$ (10)	$Sr(GaAs)_2$	1	390	0	0	False	-
mp-570957	$Cs_8Si_8Te_{24}$	20	Cc (9)	$CsSiTe_3$	2	78	1	0	False	-
mp-675941	$Li_{28}I_4N_8$	20	Cm (8)	Li_7IN_2	0	39	1	0	False	-
mp-13923	$Sn_4P_4S_{12}$	20	Pc (7)	$BaPS_3$	66	180	0	0	False	-
mp-570370	$Sn_4P_4Se_{12}$	20	Pc (7)	$BaPS_3$	65	243	0	0	False	-
mp-557728	$Cu_8K_2As_2S_8$	20	$P2_1$ (4)	KCu_4AsS_4	90	26	80	0	False	-
mp-862701	$Ca_3Cs_3C_3F_3O_9$	21	$P{-}62m$ (189)	$CsCaCO_3$	30	105	0	0	False	-
mp-23827	$Sr_7Cl_2H_{12}$	21	$P{-}6$ (174)	$Sr_7(H_6Cl)_2$	0	1	68	0	False	-
mp-23828	$Sr_7Br_2H_{12}$	21	$P{-}6$ (174)	$Sr_7(H_6Cl)_2$	0	9	56	0	False	-
mp-984055	$Ca_7Cl_2H_{12}$	21	$P{-}6$ (174)	$Sr_7(H_6Cl)_2$	0	5	73	0	False	-
mp-756810	$Sr_9Ta_{12}F_6O_{36}$	21	$R{-}3m$ (166)	$Sr_3Ta_4(O_6F)_2$	37	194	0	0	False	-
mp-27377	$K_4Zr_5O_{12}$	21	$P{-}3m1$ (164)	$K_4Zr_5O_{12}$	54	258	0	0	False	-
mp-974482	$Hf_5K_4O_{12}$	21	$P{-}3m1$ (164)	$K_4Zr_5O_{12}$	59	215	0	0	False	-
mp-567246	Pb_7I_{14}	21	$P3m1$ (156)	PbI_2	34	179	4	0	False	-

ID	Formula	Z	SP (*No.*)	Prototype	NL	NR	WPs	HDWPs	Clean	dE/dq
mp-643371	$K_6Sn_3H_{36}N_{18}$	21	$R-3$ (148)	$K_2Sn(H_2N)_6$	23	11	0	0	False	-
mp-555678	$Na_4Ti_5O_{12}$	21	$P-3$ (147)	$K_4Zr_5O_{12}$	90	52	0	0	False	-
mp-560555	$Bi_4Li_{16}Pd_2O_{20}$	21	$C2/m$ (12)	$Li_8Bi_2PdO_{10}$	0	131	0	0	False	-
mp-28167	$Co_2Th_{12}Br_{30}$	22	$Im-3m$ (229)	Th_6CoBr_{15}	64	118	0	0	False	-
mp-28734	$Co_2Zr_{12}Cl_{30}$	22	$Im-3m$ (229)	Th_6CoBr_{15}	56	68	0	0	False	-
mp-17972	$Hg_{16}Zn_{16}F_{48}O_8$	22	$Fd-3m$ (227)	$Zn_2Hg_2OF_6$	68	153	0	0	False	-
mp-12533	$Hf_{16}La_{16}O_{56}$	22	$Fd-3m$ (227)	$La_2Hf_2O_7$	71	127	0	0	False	-
mp-13801	$Pt_{16}Tl_{16}O_{56}$	22	$Fd-3m$ (227)	$La_2Hf_2O_7$	66	105	0	0	False	-
mp-14026	$Ca_{16}Ta_{16}O_{56}$	22	$Fd-3m$ (227)	$La_2Hf_2O_7$	69	148	0	0	False	-
mp-15170	$Lu_{16}Sn_{16}O_{56}$	22	$Fd-3m$ (227)	$La_2Hf_2O_7$	64	116	0	0	False	-
mp-17114	$Nd_{16}Sn_{16}O_{56}$	22	$Fd-3m$ (227)	$La_2Hf_2O_7$	68	105	0	0	False	-
mp-20845	$Dy_{16}Sn_{16}O_{56}$	22	$Fd-3m$ (227)	$La_2Hf_2O_7$	66	109	0	0	False	-
mp-23341	$Bi_{16}Pt_{16}O_{56}$	22	$Fd-3m$ (227)	$La_2Hf_2O_7$	67	110	0	0	False	-
mp-2883	$Sm_{16}Sn_{16}O_{56}$	22	$Fd-3m$ (227)	$La_2Hf_2O_7$	72	94	0	0	False	-
mp-3334	$Sn_{16}Tm_{16}O_{56}$	22	$Fd-3m$ (227)	$La_2Hf_2O_7$	65	117	0	0	False	-
mp-3370	$Sn_{16}Y_{16}O_{56}$	22	$Fd-3m$ (227)	$La_2Hf_2O_7$	67	136	0	0	False	-
mp-35249	$Hf_{16}Sm_{16}O_{56}$	22	$Fd-3m$ (227)	$La_2Hf_2O_7$	71	119	0	0	False	-
mp-3688	$Er_{16}Sn_{16}O_{56}$	22	$Fd-3m$ (227)	$La_2Hf_2O_7$	66	107	0	0	False	-
mp-3884	$Ho_{16}Sn_{16}O_{56}$	22	$Fd-3m$ (227)	$La_2Hf_2O_7$	65	126	0	0	False	-
mp-4086	$La_{16}Sn_{16}O_{56}$	22	$Fd-3m$ (227)	$La_2Hf_2O_7$	69	111	0	0	True	31.96
mp-4394	$Pr_{16}Sn_{16}O_{56}$	22	$Fd-3m$ (227)	$La_2Hf_2O_7$	70	110	0	0	False	-
mp-4408	$Sm_{16}Zr_{16}O_{56}$	22	$Fd-3m$ (227)	$La_2Hf_2O_7$	69	138	0	0	False	-
mp-4974	$La_{16}Zr_{16}O_{56}$	22	$Fd-3m$ (227)	$La_2Hf_2O_7$	73	134	0	0	False	-
mp-4991	$Sn_{16}Tb_{16}O_{56}$	22	$Fd-3m$ (227)	$La_2Hf_2O_7$	68	103	0	0	False	-
mp-5548	$Cd_{16}Ta_{16}O_{56}$	22	$Fd-3m$ (227)	$La_2Hf_2O_7$	72	152	0	0	False	-
mp-559426	$Pr_{16}Zr_{16}O_{56}$	22	$Fd-3m$ (227)	$La_2Hf_2O_7$	70	119	0	0	False	-
mp-5977	$Nd_{16}Zr_{16}O_{56}$	22	$Fd-3m$ (227)	$La_2Hf_2O_7$	69	131	0	0	False	-
mp-752411	$Pb_{16}Tm_{16}O_{56}$	22	$Fd-3m$ (227)	$La_2Hf_2O_7$	69	113	0	0	False	-
mp-752849	$Pb_{16}Y_{16}O_{56}$	22	$Fd-3m$ (227)	$La_2Hf_2O_7$	65	132	0	0	False	-
mp-753414	$Lu_{16}Pt_{16}O_{56}$	22	$Fd-3m$ (227)	$La_2Hf_2O_7$	64	88	0	0	False	-
mp-753692	$Ho_{16}Ge_{16}O_{56}$	22	$Fd-3m$ (227)	$La_2Hf_2O_7$	69	120	0	0	False	-
mp-754302	$Dy_{16}Zr_{16}O_{56}$	22	$Fd-3m$ (227)	$La_2Hf_2O_7$	69	137	0	0	False	-
mp-754646	$Dy_{16}Ge_{16}O_{56}$	22	$Fd-3m$ (227)	$La_2Hf_2O_7$	67	121	0	0	False	-
mp-754768	$Pt_{16}Tb_{16}O_{56}$	22	$Fd-3m$ (227)	$La_2Hf_2O_7$	66	103	0	0	False	-
mp-755026	$La_{16}Pt_{16}O_{56}$	22	$Fd-3m$ (227)	$La_2Hf_2O_7$	68	124	0	0	False	-
mp-755116	$In_{16}Pt_{16}O_{56}$	22	$Fd-3m$ (227)	$La_2Hf_2O_7$	65	105	0	0	False	-
mp-755375	$Lu_{16}Pb_{16}O_{56}$	22	$Fd-3m$ (227)	$La_2Hf_2O_7$	71	102	0	0	False	-
mp-755450	$Ce_{16}La_{16}O_{56}$	22	$Fd-3m$ (227)	$La_2Hf_2O_7$	72	119	0	0	False	-
mp-755635	$Ho_{16}Pt_{16}O_{56}$	22	$Fd-3m$ (227)	$La_2Hf_2O_7$	66	106	0	0	False	-
mp-755723	$Er_{16}Pb_{16}O_{56}$	22	$Fd-3m$ (227)	$La_2Hf_2O_7$	67	129	0	0	False	-
mp-756148	$Pt_{16}Sm_{16}O_{56}$	22	$Fd-3m$ (227)	$La_2Hf_2O_7$	66	123	0	0	True	15.22
mp-756347	$Pt_{16}Y_{16}O_{56}$	22	$Fd-3m$ (227)	$La_2Hf_2O_7$	64	99	0	0	False	-
mp-756440	$Er_{16}Pt_{16}O_{56}$	22	$Fd-3m$ (227)	$La_2Hf_2O_7$	66	91	0	0	False	-
mp-760425	$Tb_{16}Zr_{16}O_{56}$	22	$Fd-3m$ (227)	$La_2Hf_2O_7$	70	137	0	0	False	-
mp-769211	$Pb_{16}Tb_{16}O_{56}$	22	$Fd-3m$ (227)	$La_2Hf_2O_7$	65	102	0	0	False	-
mp-769222	$Dy_{16}Pb_{16}O_{56}$	22	$Fd-3m$ (227)	$La_2Hf_2O_7$	67	114	0	0	False	-
mp-769396	$Y_{16}Ge_{16}O_{56}$	22	$Fd-3m$ (227)	$La_2Hf_2O_7$	73	103	0	0	True	26.55
mp-778114	$Sm_{16}Ge_{16}O_{56}$	22	$Fd-3m$ (227)	$La_2Hf_2O_7$	70	101	0	0	True	44.69
mp-778152	$Pt_{16}Sc_{16}O_{56}$	22	$Fd-3m$ (227)	$La_2Hf_2O_7$	66	122	0	0	False	-
mp-9765	$Nd_{16}Pt_{16}O_{56}$	22	$Fd-3m$ (227)	$La_2Hf_2O_7$	69	97	0	0	False	-
mp-28911	$Ba_2Re_2H_{18}$	22	$P6_3/mmc$ (194)	$BaReH_9$	25	69	0	0	True	1.46
mp-29116	$Ta_6I_{14}Se_2$	22	$P6_3mc$ (186)	Ta_3SeI_7	22	131	0	5	False	-
mp-29117	$Ta_6I_{14}Te_2$	22	$P6_3mc$ (186)	Ta_3SeI_7	23	120	0	0	False	-
mp-29689	$Nb_6I_{14}Te_2$	22	$P6_3mc$ (186)	Ta_3SeI_7	23	118	1	2	False	-
mp-10096	$Ga_2Na_6Sr_6P_8$	22	$P6_3mc$ (186)	$Na_3Sr_3GaP_4$	68	172	3	4	False	-
mp-10097	$Ga_2Na_6Sr_6As_8$	22	$P6_3mc$ (186)	$Na_3Sr_3GaP_4$	38	163	0	9	False	-
mp-15572	$Al_2Ca_6Na_6As_8$	22	$P6_3mc$ (186)	$Na_3Sr_3GaP_4$	40	150	0	8	False	-
mp-12171	$K_{12}Mg_2O_8$	22	$P6_3mc$ (186)	K_6MgO_4	36	149	0	10	False	-
mp-14780	$Mn_2Na_{12}Se_8$	22	$P6_3mc$ (186)	K_6MgO_4	74	149	0	1	False	-
mp-14782	$Mn_2Na_{12}Te_8$	22	$P6_3mc$ (186)	K_6MgO_4	98	152	0	8	False	-
mp-17161	$Cd_2K_{12}O_8$	22	$P6_3mc$ (186)	K_6MgO_4	37	154	1	8	False	-
mp-17698	$Cd_2K_{12}Te_8$	22	$P6_3mc$ (186)	K_6MgO_4	68	139	1	4	False	-

ID	Formula	Z	SP (*No.*)	Prototype	NL	NR	WPs	HDWPs	Clean	dE/dq
mp-18246	$K_{12}Mn_2Te_8$	22	$P6_3mc$ (186)	K_6MgO_4	68	138	0	0	False	-
mp-18481	$K_{12}Mn_2Se_8$	22	$P6_3mc$ (186)	K_6MgO_4	71	154	0	4	False	-
mp-23063	$Ba_8Cl_{12}O_2$	22	$P6_3mc$ (186)	K_6MgO_4	100	169	0	10	False	-
mp-23321	$Sr_8Cl_{12}O_2$	22	$P6_3mc$ (186)	K_6MgO_4	40	171	1	9	False	-
mp-29909	$Ba_8I_{12}O_2$	22	$P6_3mc$ (186)	K_6MgO_4	35	153	1	10	False	-
mp-29910	$Sr_8I_{12}O_2$	22	$P6_3mc$ (186)	K_6MgO_4	32	173	2	8	False	-
mp-5055	$Mn_2Na_{12}S_8$	22	$P6_3mc$ (186)	K_6MgO_4	103	177	1	6	False	-
mp-5114	$Hg_2K_{12}S_8$	22	$P6_3mc$ (186)	K_6MgO_4	35	167	0	8	False	-
mp-554831	$Yb_8Cl_{12}O_2$	22	$P6_3mc$ (186)	K_6MgO_4	66	128	0	3	False	-
mp-555218	$Ba_8Br_{12}O_2$	22	$P6_3mc$ (186)	K_6MgO_4	65	187	0	2	False	-
mp-556049	$Sr_8Br_{12}O_2$	22	$P6_3mc$ (186)	K_6MgO_4	32	194	1	7	False	-
mp-753816	$Mg_2Na_{12}O_8$	22	$P6_3mc$ (186)	K_6MgO_4	36	161	3	5	False	-
mp-755546	$Be_2Na_{12}O_8$	22	$P6_3mc$ (186)	K_6MgO_4	32	181	5	9	False	-
mp-7664	$Na_{12}Zn_2O_8$	22	$P6_3mc$ (186)	K_6MgO_4	40	215	3	8	False	-
mp-8389	$Na_{12}Zn_2S_8$	22	$P6_3mc$ (186)	K_6MgO_4	102	179	0	10	False	-
mp-850213	$Yb_8Br_{12}O_2$	22	$P6_3mc$ (186)	K_6MgO_4	99	149	0	8	False	-
mp-9007	$Na_{12}W_2P_8$	22	$P6_3mc$ (186)	K_6MgO_4	40	148	1	9	False	-
mp-10392	$Cd_6Pt_6Sr_{18}O_{36}$	22	$R-3c$ (167)	Sr_3CdPtO_6	72	125	0	0	False	-
mp-10461	$Na_6Sr_{18}O_{36}Sb_6$	22	$R-3c$ (167)	Sr_3CdPtO_6	95	117	0	0	False	-
mp-12362	$Ag_6Hg_{18}O_{36}Sb_6$	22	$R-3c$ (167)	Sr_3CdPtO_6	92	118	0	0	False	-
mp-13042	$Na_6Sr_{18}Ta_6O_{36}$	22	$R-3c$ (167)	Sr_3CdPtO_6	94	130	0	0	False	-
mp-17149	$Er_6Rh_6Sr_{18}O_{36}$	22	$R-3c$ (167)	Sr_3CdPtO_6	92	99	0	0	False	-
mp-17829	$Rh_6Sm_6Sr_{18}O_{36}$	22	$R-3c$ (167)	Sr_3CdPtO_6	96	114	0	0	True	4.38
mp-18247	$Rh_6Sc_6Sr_{18}O_{36}$	22	$R-3c$ (167)	Sr_3CdPtO_6	88	109	0	0	False	-
mp-18498	$Rh_6Sr_{18}Y_6O_{36}$	22	$R-3c$ (167)	Sr_3CdPtO_6	89	124	0	0	True	5.15
mp-18651	$Dy_6Rh_6Sr_{18}O_{36}$	22	$R-3c$ (167)	Sr_3CdPtO_6	95	86	0	0	False	-
mp-18652	$Ho_6Rh_6Sr_{18}O_{36}$	22	$R-3c$ (167)	Sr_3CdPtO_6	95	105	0	0	False	-
mp-18705	$Rh_6Sr_{18}Tb_6O_{36}$	22	$R-3c$ (167)	Sr_3CdPtO_6	93	92	0	0	False	-
mp-23125	$Fe_6K_{18}Na_6Cl_{36}$	22	$R-3c$ (167)	Sr_3CdPtO_6	89	84	0	0	False	-
mp-555812	$Bi_6Na_6Sr_{18}O_{36}$	22	$R-3c$ (167)	Sr_3CdPtO_6	93	113	0	0	False	-
mp-556316	$Ba_{18}Bi_6Na_6O_{36}$	22	$R-3c$ (167)	Sr_3CdPtO_6	90	100	0	0	False	-
mp-570158	$Cd_6K_6Rb_{18}Cl_{36}$	22	$R-3c$ (167)	Sr_3CdPtO_6	78	108	0	0	False	-
mp-6064	$Mg_6Pt_6Sr_{18}O_{36}$	22	$R-3c$ (167)	Sr_3CdPtO_6	90	112	0	0	False	-
mp-6233	$Na_6Ru_6Sr_{18}O_{36}$	22	$R-3c$ (167)	Sr_3CdPtO_6	77	121	0	0	False	-
mp-6281	$Ca_{18}Na_6Ru_6O_{36}$	22	$R-3c$ (167)	Sr_3CdPtO_6	74	106	0	0	False	-
mp-6361	$Na_6Nb_6Sr_{18}O_{36}$	22	$R-3c$ (167)	Sr_3CdPtO_6	101	99	0	0	False	-
mp-669389	$In_6Rh_6Sr_{18}O_{36}$	22	$R-3c$ (167)	Sr_3CdPtO_6	91	105	0	0	False	-
mp-6730	$Pt_6Sr_{18}Zn_6O_{36}$	22	$R-3c$ (167)	Sr_3CdPtO_6	100	119	0	0	False	-
mp-8960	$Ba_{18}Na_6Nb_6O_{36}$	22	$R-3c$ (167)	Sr_3CdPtO_6	94	120	0	0	False	-
mp-8961	$Ba_{18}Na_6Ta_6O_{36}$	22	$R-3c$ (167)	Sr_3CdPtO_6	95	112	0	0	False	-
mp-9745	$Ba_{18}Na_6Ru_6O_{36}$	22	$R-3c$ (167)	Sr_3CdPtO_6	86	82	0	0	False	-
mp-607712	$Lu_{12}C_{18}N_{36}$	22	$R-3c$ (167)	$Lu_2(CN_2)_3$	100	120	0	0	True	16.07
mp-4710	$Hg_{18}B_{12}O_{36}$	22	$R-3c$ (167)	$Hg_3(BO_3)_2$	92	129	0	0	False	-
mp-10299	$Ca_{24}Pd_6O_{36}$	22	$R-3c$ (167)	Ca_4PdO_6	80	122	0	0	False	-
mp-22930	$Cd_6Rb_{24}Cl_{36}$	22	$R-3c$ (167)	Ca_4PdO_6	87	170	0	0	False	-
mp-23347	$Rb_{24}Yb_6I_{36}$	22	$R-3c$ (167)	Ca_4PdO_6	88	180	0	0	False	-
mp-23392	$Cd_6K_{24}Cl_{36}$	22	$R-3c$ (167)	Ca_4PdO_6	90	183	0	0	False	-
mp-28315	$Cd_6Rb_{24}Br_{36}$	22	$R-3c$ (167)	Ca_4PdO_6	89	169	0	0	False	-
mp-28564	$Pb_6Rb_{24}Br_{36}$	22	$R-3c$ (167)	Ca_4PdO_6	94	181	0	0	False	-
mp-29468	$Rb_{36}Cl_{24}O_6$	22	$R-3c$ (167)	Ca_4PdO_6	84	188	0	0	False	-
mp-29775	$Pd_6Sr_{24}O_{36}$	22	$R-3c$ (167)	Ca_4PdO_6	88	132	0	0	False	-
mp-30005	$Rb_{36}Br_{24}O_6$	22	$R-3c$ (167)	Ca_4PdO_6	96	170	0	0	False	-
mp-4598	$Pt_6Sr_{24}O_{36}$	22	$R-3c$ (167)	Ca_4PdO_6	85	106	0	0	False	-
mp-642847	$Li_{24}Ru_6H_{36}$	22	$R-3c$ (167)	Ca_4PdO_6	89	83	0	0	True	2.58
mp-642996	$Li_{24}Os_6H_{36}$	22	$R-3c$ (167)	Ca_4PdO_6	70	71	0	0	True	9.83
mp-643001	$Na_{24}Ru_6H_{36}$	22	$R-3c$ (167)	Ca_4PdO_6	85	129	0	0	True	10.29
mp-782050	$Ba_{24}Pd_6O_{36}$	22	$R-3c$ (167)	Ca_4PdO_6	93	171	0	0	False	-
mp-8568	$Ca_{24}Pt_6O_{36}$	22	$R-3c$ (167)	Ca_4PdO_6	86	132	0	0	False	-
mp-8727	$Ba_{24}Pt_6O_{36}$	22	$R-3c$ (167)	Ca_4PdO_6	77	162	0	0	False	-
mp-30091	$Mg_6B_{54}N_6$	22	$R-3m$ (166)	MgB_9	29	170	0	0	False	-
mp-28938	$Nb_6Cl_{14}Te_2$	22	$P-3m1$ (164)	Nb_3TeCl_7	36	176	0	0	True	9.51
mp-14495	$Li_{12}Zn_2O_8$	22	$P4_2/nmc$ (137)	Li_6ZnO_4	166	173	0	0	False	-

ID	Formula	Z	SP (*No.*)	Prototype	NL	NR	WPs	HDWPs	Clean	dE/dq
mp-3503	$Li_{12}W_2N_8$	22	$P4_2/nmc$ (137)	Li_6ZnO_4	166	199	0	0	False	-
mp-755084	$Mg_2Na_{12}O_8$	22	$P4_2/nmc$ (137)	Li_6ZnO_4	166	216	0	0	False	-
mp-8804	$Li_{12}Mo_2N_8$	22	$P4_2/nmc$ (137)	Li_6ZnO_4	165	194	0	0	False	-
mp-27375	$Hg_2Tl_8I_{12}$	22	$P4/mnc$ (128)	Tl_4HgI_6	198	181	0	0	False	-
mp-28517	$Tl_{12}I_8Se_2$	22	$P4/mnc$ (128)	Tl_4HgI_6	198	171	0	0	False	-
mp-570339	$Cd_2Tl_8I_{12}$	22	$P4/mnc$ (128)	Tl_4HgI_6	198	163	0	0	True	1.76
mp-616218	$Cd_2In_8I_{12}$	22	$P4/mnc$ (128)	Tl_4HgI_6	199	193	0	0	False	-
mp-29169	$Au_4Cd_2F_{16}$	22	$P4/mcc$ (124)	$Cd(AuF_4)_2$	99	153	0	0	False	-
mp-29170	$Au_4Hg_2F_{16}$	22	$P4/mcc$ (124)	$Cd(AuF_4)_2$	99	139	0	0	False	-
mp-973793	$Li_{32}N_8Se_4$	22	$I4_1md$ (109)	Li_8SeN_2	99	112	54	0	False	-
mp-984766	$Ca_2B_4H_{16}$	22	$P4_2$ (77)	$Ca(BH_4)_2$	0	13	61	0	False	-
mp-697263	$Li_4As_4F_{24}H_8O_4$	22	$Imma$ (74)	$LiAsH_2OF_6$	34	175	0	0	False	-
mp-16280	$Ca_8O_{28}Sb_8$	22	$Imma$ (74)	$Ca_2Sb_2O_7$	35	315	0	0	False	-
mp-20254	$Na_8O_{28}Te_8$	22	$Imma$ (74)	$Ca_2Sb_2O_7$	34	296	0	0	False	-
mp-4103	$Sr_8O_{28}Sb_8$	22	$Imma$ (74)	$Ca_2Sb_2O_7$	33	287	0	0	False	-
mp-752404	$Ba_8O_{28}Sb_8$	22	$Imma$ (74)	$Ca_2Sb_2O_7$	34	257	0	0	False	-
mp-555070	$Ba_8Be_{16}B_{16}O_{48}$	22	$Fddd$ (70)	$BaBe_2(BO_3)_2$	132	115	0	0	False	-
mp-769366	$Nd_{12}O_{28}Sb_4$	22	$Cmcm$ (63)	Tb_3NbO_7	111	156	0	0	False	-
mp-981511	$Y_{12}O_{28}Sb_4$	22	$Cmcm$ (63)	Tb_3NbO_7	108	160	0	0	False	-
mp-16884	$Cu_{12}Er_8Rb_4Se_{20}$	22	$Cmcm$ (63)	$RbEr_2Cu_3Se_5$	114	183	0	0	False	-
mp-17344	$Cu_{12}Er_8Rb_4S_{20}$	22	$Cmcm$ (63)	$RbEr_2Cu_3Se_5$	106	169	0	0	False	-
mp-17929	$Cu_{12}Ho_8Rb_4S_{20}$	22	$Cmcm$ (63)	$RbEr_2Cu_3Se_5$	106	164	0	0	False	-
mp-18710	$Ag_{12}Rb_4Sm_8Se_{20}$	22	$Cmcm$ (63)	$RbEr_2Cu_3Se_5$	107	163	0	0	False	-
mp-542164	$Ag_{12}Cs_4Tb_8Se_{20}$	22	$Cmcm$ (63)	$RbEr_2Cu_3Se_5$	107	189	0	0	False	-
mp-581696	$Cs_4Cu_{12}Lu_8Se_{20}$	22	$Cmcm$ (63)	$RbEr_2Cu_3Se_5$	104	182	0	0	False	-
mp-581749	$Cs_4Cu_{12}Tm_8Se_{20}$	22	$Cmcm$ (63)	$RbEr_2Cu_3Se_5$	109	156	0	0	False	-
mp-865972	$Cu_{12}K_4Tm_8Se_{20}$	22	$Cmcm$ (63)	$RbEr_2Cu_3Se_5$	106	172	0	0	False	-
mp-5005	$Mg_6B_4O_{12}$	22	$Pnnm$ (58)	$Mg_3(BO_3)_2$	297	127	0	0	False	-
mp-5560	$Cd_6B_4O_{12}$	22	$Pnnm$ (58)	$Mg_3(BO_3)_2$	297	108	0	0	False	-
mp-29212	$Pb_2Tl_8I_{12}$	22	$Pbam$ (55)	Tl_4PbI_6	231	152	0	0	False	-
mp-28035	Ba_4P_{40}	22	$Cmc2_1$ (36)	BaP_{10}	106	115	8	0	False	-
mp-510346	$In_{10}Br_2Se_{10}$	22	$Pmn2_1$ (31)	In_5Se_5	231	196	0	0	False	-
mp-756591	$Nb_4Sm_{12}O_{28}$	22	$C222_1$ (20)	Tb_3NbO_7	109	22	191	0	False	-
mp-555318	$K_8Zn_4O_{24}Si_8$	22	$C222_1$ (20)	$K_2Zn(SiO_3)_2$	109	27	96	0	True	11.77
mp-560398	$K_8Zn_4Ge_8O_{24}$	22	$C222_1$ (20)	$K_2Zn(SiO_3)_2$	109	29	111	0	True	1.54
mp-16183	$Tl_8B_8Se_{28}$	22	$C2/c$ (15)	$Tl_2B_2Se_7$	0	69	0	0	False	-
mp-16184	$Rb_8B_8Se_{28}$	22	$C2/c$ (15)	$Tl_2B_2Se_7$	0	71	0	0	False	-
mp-4351	$K_8B_8S_{28}$	22	$C2/c$ (15)	$Tl_2B_2Se_7$	0	97	0	0	True	1.13
mp-5004	$Na_8B_8Se_{28}$	22	$C2/c$ (15)	$Tl_2B_2Se_7$	0	105	0	0	False	-
mp-542637	$K_8B_8Se_{28}$	22	$C2/c$ (15)	$Tl_2B_2Se_7$	0	79	0	0	True	5.48
mp-30239	$Sr_{12}B_8S_{24}$	22	$C2/c$ (15)	$Sr_3(BS_3)_2$	0	183	0	0	False	-
mp-5477	$Na_{20}Nb_4O_{20}$	22	$C2/c$ (15)	Na_5NbO_5	3	158	0	0	False	-
mp-8957	$Na_{20}Ta_4O_{20}$	22	$C2/c$ (15)	Na_5NbO_5	3	142	0	0	False	-
mp-755043	$Al_8O_{28}Si_8$	22	$C2/c$ (15)	$Al_2Si_2O_7$	0	145	0	0	False	-
mp-9751	$Al_8Ge_8O_{28}$	22	$C2/c$ (15)	$Al_2Si_2O_7$	3	118	0	0	False	-
mp-643346	$Al_8Ge_4H_8O_{24}$	22	$C2/c$ (15)	$Al_2Ge(HO_3)_2$	1	146	0	0	False	-
mp-17512	$Au_4Zn_2F_{16}$	22	$P2_1/c$ (14)	$Zn(AuF_4)_2$	167	195	0	0	False	-
mp-541897	$Cd_2Rb_4P_4Se_{12}$	22	$P2_1/c$ (14)	$Rb_2Cd(PSe_3)_2$	168	143	0	0	True	2.03
mp-11643	$K_4Mg_2P_4Se_{12}$	22	$P2_1/c$ (14)	$K_2Mg(PSe_3)_2$	166	146	0	0	True	1.54
mp-771981	$Ba_2La_4Br_{16}$	22	$P2/c$ (13)	$BaLa_2Br_8$	70	321	0	0	False	-
mp-29849	$Ag_6Sn_2P_{14}$	22	$P2_1/m$ (11)	Ag_3SnP_7	99	113	0	0	False	-
mp-505607	$Ag_8In_8Ge_4Se_{24}$	22	Cc (9)	$In_2Ag_2GeSe_6$	0	40	0	0	False	-
mp-558407	$Ag_8In_8S_{24}Si_4$	22	Cc (9)	$In_2Ag_2GeSe_6$	0	30	0	0	False	-
mp-560386	$Ag_8In_8Ge_4S_{24}$	22	Cc (9)	$In_2Ag_2GeSe_6$	0	29	0	0	False	-
mp-640614	$Ag_8In_8Se_{24}Si_4$	22	Cc (9)	$In_2Ag_2GeSe_6$	0	38	0	0	False	-
mp-17791	$Cd_{16}Se_{24}Si_4$	22	Cc (9)	Cd_4SiSe_6	0	36	1	0	False	-
mp-17948	$Hg_{16}S_{24}Si_4$	22	Cc (9)	Cd_4SiSe_6	0	44	0	0	False	-
mp-18163	$Cd_{16}Ge_4Se_{24}$	22	Cc (9)	Cd_4SiSe_6	2	37	0	0	False	-
mp-18179	$Cd_{16}S_{24}Si_4$	22	Cc (9)	Cd_4SiSe_6	0	33	0	0	False	-
mp-5151	$Cd_{16}Ge_4S_{24}$	22	Cc (9)	Cd_4SiSe_6	0	41	0	0	False	-
mp-556806	$Ba_2Na_4O_{12}Si_4$	22	$P2_1$ (4)	$BaNa_2(SiO_3)_2$	99	34	114	0	False	-
mp-14921	$Zn_8B_{12}O_{24}Se_2$	23	$I{-}43m$ (217)	$Zn_4B_6SeO_{12}$	74	87	6	31	False	-

ID	Formula	Z	SP (*No.*)	Prototype	NL	NR	WPs	HDWPs	Clean	dE/dq
mp-15833	$Zn_8N_{24}P_{12}S_2$	23	$I-43m$ (217)	$Zn_4B_6SeO_{12}$	63	71	1	35	False	-
mp-557537	$Zn_8N_{24}O_2P_{12}$	23	$I-43m$ (217)	$Zn_4B_6SeO_{12}$	60	78	1	36	True	9.51
mp-6137	$Mg_8N_{24}P_{12}S_2$	23	$I-43m$ (217)	$Zn_4B_6SeO_{12}$	62	109	6	32	False	-
mp-8876	$Al_{12}Ca_8O_{24}S_2$	23	$I-43m$ (217)	$Zn_4B_6SeO_{12}$	65	110	5	34	False	-
mp-8921	$Cd_8N_{24}P_{12}S_2$	23	$I-43m$ (217)	$Zn_4B_6SeO_{12}$	63	64	4	30	True	2
mp-9203	$Al_{12}Cd_8O_{24}S_2$	23	$I-43m$ (217)	$Zn_4B_6SeO_{12}$	67	73	0	28	False	-
mp-9535	$Al_{12}Cd_8O_{24}Te_2$	23	$I-43m$ (217)	$Zn_4B_6SeO_{12}$	65	67	1	33	False	-
mp-4812	$Zn_8B_{12}O_{26}$	23	$I-43m$ (217)	$Zn_4B_6O_{13}$	73	89	1	35	False	-
mp-7531	$Al_{12}Ca_8O_{26}$	23	$I-43m$ (217)	$Zn_4B_6O_{13}$	67	113	1	38	False	-
mp-755260	$Cd_8B_{12}O_{26}$	23	$I-43m$ (217)	$Zn_4B_6O_{13}$	70	68	4	37	True	22.2
mp-18062	$Na_{20}Sn_{24}Yb_2$	23	$I-43m$ (217)	$Na_{10}YbSn_{12}$	68	144	3	36	False	-
mp-30252	$Ca_2Na_{20}Sn_{24}$	23	$I-43m$ (217)	$Na_{10}YbSn_{12}$	70	120	3	33	False	-
mp-30253	$Na_{20}Sn_{24}Sr_2$	23	$I-43m$ (217)	$Na_{10}YbSn_{12}$	68	111	5	31	False	-
mp-39215	$Ga_3Zn_9B_{18}O_{36}P_3$	23	$R3m$ (160)	$Zn_3GaB_6PO_{12}$	41	75	1	0	False	-
mp-985697	$Ba_3N_2O_{12}Si_6$	23	$P-3$ (147)	$Ba_3Si_6(NO_6)_2$	90	54	0	0	False	-
mp-672339	$Ag_4Hg_{14}I_{12}P_{16}$	23	$C2/m$ (12)	$Ag_2Hg_7(P_4I_3)_2$	0	117	0	0	False	-
mp-581857	$Bi_{16}K_{16}F_{64}$	24	$Fd-3m$ (227)	$KBiF_4$	85	195	0	0	False	-
mp-555740	$O_{32}Si_{16}$	24	$Ia-3$ (206)	SiO_2	113	151	0	0	False	-
mp-866635	$Cu_8Cl_8H_{24}N_8$	24	$I2_13$ (199)	CuH_3	0	10	171	33	True	6.29
mp-561277	$K_8Pb_4O_{12}$	24	$P6_3/mcm$ (193)	K_2PbO_3	31	179	0	0	False	-
mp-755486	$Na_8Sn_4O_{12}$	24	$P6_3/mcm$ (193)	K_2PbO_3	32	183	0	0	False	-
mp-28987	$Na_4Pt_8Se_{12}$	24	$P6_3mc$ (186)	$NaPt_2Se_3$	20	172	5	2	False	-
mp-568916	$In_2K_{12}Cl_2Te_8$	24	$P6_3mc$ (186)	K_6InTe_4	39	161	1	9	False	-
mp-942	Au_6F_{18}	24	$P6_122$ (178)	AuF_3	0	8	150	0	False	-
mp-12485	$Na_2Sm_6S_{14}Si_2$	24	$P6_3$ (173)	$NaSm_3SiS_7$	0	19	165	0	False	-
mp-504891	$Ga_2La_6Mn_2S_{14}$	24	$P6_3$ (173)	$NaSm_3SiS_7$	1	19	155	0	False	-
mp-866696	$Be_2La_6Sc_2S_{14}$	24	$P6_3$ (173)	$NaSm_3SiS_7$	0	18	175	0	False	-
mp-17155	$Ag_2La_6Sn_2Se_{14}$	24	$P6_3$ (173)	$La_3AgSnSe_7$	0	13	154	0	False	-
mp-17243	$Cu_2Pr_6Sn_2Se_{14}$	24	$P6_3$ (173)	$La_3AgSnSe_7$	0	19	128	0	False	-
mp-17389	$Ag_2Pr_6Se_{14}Si_2$	24	$P6_3$ (173)	$La_3AgSnSe_7$	1	17	181	0	False	-
mp-17719	$Ag_2La_6S_{14}Si_2$	24	$P6_3$ (173)	$La_3AgSnSe_7$	0	12	158	0	False	-
mp-17747	$Cu_2Sn_2Y_6S_{14}$	24	$P6_3$ (173)	$La_3AgSnSe_7$	0	13	141	0	False	-
mp-17827	$Ag_2Nd_6Se_{14}Si_2$	24	$P6_3$ (173)	$La_3AgSnSe_7$	1	22	177	0	False	-
mp-17916	$Cu_2Y_6Ge_2Se_{14}$	24	$P6_3$ (173)	$La_3AgSnSe_7$	0	19	158	0	True	1.73
mp-18126	$Cu_2Sn_2Tb_6Se_{14}$	24	$P6_3$ (173)	$La_3AgSnSe_7$	0	16	151	0	False	-
mp-18303	$Cu_2Nd_6Sn_2Se_{14}$	24	$P6_3$ (173)	$La_3AgSnSe_7$	0	19	139	0	False	-
mp-505558	$Cu_2Dy_6Ge_2Se_{14}$	24	$P6_3$ (173)	$La_3AgSnSe_7$	0	14	151	0	False	-
mp-510566	$Cu_2La_6Sn_2S_{14}$	24	$P6_3$ (173)	$La_3AgSnSe_7$	0	14	140	0	True	2.05
mp-542888	$Ag_2La_6Sn_2S_{14}$	24	$P6_3$ (173)	$La_3AgSnSe_7$	0	17	160	1	False	-
mp-554097	$Cu_2Sm_6S_{14}Si_2$	24	$P6_3$ (173)	$La_3AgSnSe_7$	0	11	123	0	True	1.88
mp-554150	$Cu_2Nd_6Ge_2S_{14}$	24	$P6_3$ (173)	$La_3AgSnSe_7$	0	7	120	0	False	-
mp-554781	$Cu_2Sn_2Tb_6S_{14}$	24	$P6_3$ (173)	$La_3AgSnSe_7$	0	9	125	0	False	-
mp-555081	$Cu_2Er_6Ge_2S_{14}$	24	$P6_3$ (173)	$La_3AgSnSe_7$	0	12	133	0	False	-
mp-555893	$Cu_2Pr_6S_{14}Si_2$	24	$P6_3$ (173)	$La_3AgSnSe_7$	0	9	131	0	True	2.71
mp-555978	$Cu_2Sm_6Ge_2S_{14}$	24	$P6_3$ (173)	$La_3AgSnSe_7$	0	9	137	0	True	1.8
mp-556781	$Cu_2Y_6Ge_2S_{14}$	24	$P6_3$ (173)	$La_3AgSnSe_7$	0	14	145	0	True	1.57
mp-556962	$Cu_2Pr_6Ge_2S_{14}$	24	$P6_3$ (173)	$La_3AgSnSe_7$	0	9	134	0	False	-
mp-556975	$Cu_2Nd_6S_{14}Si_2$	24	$P6_3$ (173)	$La_3AgSnSe_7$	0	12	134	0	True	1.05
mp-557517	$Cu_2Tb_6Ge_2S_{14}$	24	$P6_3$ (173)	$La_3AgSnSe_7$	0	15	141	0	False	-
mp-557998	$Cu_2Dy_6S_{14}Si_2$	24	$P6_3$ (173)	$La_3AgSnSe_7$	0	14	135	0	False	-
mp-558042	$Cu_2Sm_6Sn_2S_{14}$	24	$P6_3$ (173)	$La_3AgSnSe_7$	0	9	129	0	False	-
mp-558980	$Cu_2Er_6S_{14}Si_2$	24	$P6_3$ (173)	$La_3AgSnSe_7$	0	19	142	0	False	-
mp-560014	$Cu_2Pr_6Sn_2S_{14}$	24	$P6_3$ (173)	$La_3AgSnSe_7$	0	20	144	0	False	-
mp-560300	$Cu_2Nd_6Sn_2S_{14}$	24	$P6_3$ (173)	$La_3AgSnSe_7$	0	14	135	0	False	-
mp-560501	$Cu_2Tb_6S_{14}Si_2$	24	$P6_3$ (173)	$La_3AgSnSe_7$	0	16	137	0	True	2.3
mp-561173	$Cu_2Y_6S_{14}Si_2$	24	$P6_3$ (173)	$La_3AgSnSe_7$	0	11	144	0	False	-
mp-567428	$Cu_2Tb_6Ge_2Se_{14}$	24	$P6_3$ (173)	$La_3AgSnSe_7$	0	11	149	0	False	-
mp-567753	$Cu_2La_6Sn_2Se_{14}$	24	$P6_3$ (173)	$La_3AgSnSe_7$	0	19	138	0	True	1.12
mp-568638	$Cu_2Y_6Se_{14}Si_2$	24	$P6_3$ (173)	$La_3AgSnSe_7$	1	13	179	0	False	-
mp-568779	$Cu_2Tb_6Se_{14}Si_2$	24	$P6_3$ (173)	$La_3AgSnSe_7$	1	13	155	0	False	-
mp-568801	$Cu_2Pr_6Se_{14}Si_2$	24	$P6_3$ (173)	$La_3AgSnSe_7$	2	14	165	0	True	1.4
mp-568954	$Cu_2Nd_6Ge_2Se_{14}$	24	$P6_3$ (173)	$La_3AgSnSe_7$	0	20	137	0	False	-

ID	Formula	Z	SP (*No.*)	Prototype	NL	NR	WPs	HDWPs	Clean	dE/dq
mp-569557	$Cu_2Dy_6Se_{14}Si_2$	24	$P6_3$ (173)	$La_3AgSnSe_7$	1	9	143	0	False	-
mp-570045	$Cu_2Sm_6Se_{14}Si_2$	24	$P6_3$ (173)	$La_3AgSnSe_7$	1	8	168	0	False	-
mp-570226	$Cu_2Sm_6Ge_2Se_{14}$	24	$P6_3$ (173)	$La_3AgSnSe_7$	0	19	144	0	False	-
mp-570594	$Cu_2Sm_6Sn_2Se_{14}$	24	$P6_3$ (173)	$La_3AgSnSe_7$	0	12	130	0	False	-
mp-571347	$Cu_2Pr_6Ge_2Se_{14}$	24	$P6_3$ (173)	$La_3AgSnSe_7$	0	11	141	0	False	-
mp-571468	$Cu_2Ho_6Se_{14}Si_2$	24	$P6_3$ (173)	$La_3AgSnSe_7$	1	10	138	0	False	-
mp-617632	$Ag_2La_6Ge_2S_{14}$	24	$P6_3$ (173)	$La_3AgSnSe_7$	0	14	153	0	True	1.28
mp-641534	$Cu_2Nd_6Se_{14}Si_2$	24	$P6_3$ (173)	$La_3AgSnSe_7$	2	9	159	0	False	-
mp-6485	$Ag_2La_6Se_{14}Si_2$	24	$P6_3$ (173)	$La_3AgSnSe_7$	1	14	165	0	False	-
mp-862792	$Ag_2Pr_6Ge_2S_{14}$	24	$P6_3$ (173)	$La_3AgSnSe_7$	0	16	157	0	True	2.43
mp-864666	$Ag_2Nd_6S_{14}Si_2$	24	$P6_3$ (173)	$La_3AgSnSe_7$	0	12	165	0	False	-
mp-867322	$Ag_2Pr_6S_{14}Si_2$	24	$P6_3$ (173)	$La_3AgSnSe_7$	0	11	162	0	True	1.76
mp-867929	$Ag_2Sm_6S_{14}Si_2$	24	$P6_3$ (173)	$La_3AgSnSe_7$	0	14	156	0	False	-
mp-31358	$Rb_{18}B_{18}O_{36}$	24	R−3c (167)	$NaBS_2$	82	115	0	0	True	2.22
mp-3889	$Na_{18}B_{18}O_{36}$	24	R−3c (167)	$NaBS_2$	87	124	0	0	False	-
mp-3919	$K_{18}B_{18}O_{36}$	24	R−3c (167)	$NaBS_2$	84	85	0	0	True	2.46
mp-561725	$Cs_{18}B_{18}O_{36}$	24	R−3c (167)	$NaBS_2$	83	94	0	0	False	-
mp-905	La_6F_{18}	24	P−3c1 (165)	LaF_3	139	231	0	0	False	-
mp-3563	$Ba_5Nb_4O_{15}$	24	P−3m1 (164)	$Ba_5Nb_4O_{15}$	58	245	0	0	False	-
mp-504554	$Ba_5Ta_4O_{15}$	24	P−3m1 (164)	$Ba_5Nb_4O_{15}$	64	275	0	0	False	-
mp-581258	$Zn_{36}S_{36}$	24	$R3m$ (160)	ZnS	113	199	124	0	False	-
mp-581476	$Zn_{36}S_{36}$	24	$R3m$ (160)	ZnS	134	198	98	0	False	-
mp-581601	$Zn_{36}S_{36}$	24	$R3m$ (160)	ZnS	129	197	135	1	False	-
mp-17756	$Ag_6Rb_3S_{12}Sb_3$	24	$P3_221$ (154)	$RbAg_2SbS_4$	0	5	209	0	True	3.12
mp-18685	$Cu_6Sr_3Ge_3S_{12}$	24	$P3_221$ (154)	$RbAg_2SbS_4$	0	6	208	0	False	-
mp-555818	$Cu_6Pb_3S_{12}Si_3$	24	$P3_221$ (154)	$RbAg_2SbS_4$	0	3	191	0	False	-
mp-17947	$Ba_3Cu_6Ge_3S_{12}$	24	$P3_121$ (152)	$RbAg_2SbS_4$	0	2	195	0	True	4.37
mp-13740	$Ag_{18}As_{18}S_{36}$	24	R−3 (148)	$AgAsS_2$	43	35	0	0	False	-
mp-14591	$Li_{18}S_{36}Sb_{18}$	24	R−3 (148)	$AgAsS_2$	50	32	0	0	False	-
mp-5914	$Li_{24}B_8N_{16}$	24	$I4_1/amd$ (141)	Li_3BN_2	108	249	0	0	True	3.21
mp-582259	$Li_{14}V_2N_8$	24	$P4_2/nmc$ (137)	Li_7VN_4	184	184	0	0	False	-
mp-568742	Hg_8I_{16}	24	$P4_2/nmc$ (137)	HgI_2	181	210	0	0	False	-
mp-28365	$Au_4Na_{12}O_8$	24	$P4_2/mnm$ (136)	Na_3AuO_2	180	146	0	0	False	-
mp-755401	$Cu_4Na_{12}O_8$	24	$P4_2/mnm$ (136)	Na_3AuO_2	180	167	0	0	False	-
mp-643248	$Al_2Na_4Cl_2H_8O_8$	24	$P4/nmm$ (129)	$Na_2AlH_4ClO_4$	144	155	0	0	True	21
mp-690684	$Na_4B_2Cl_2H_8O_8$	24	$P4/nmm$ (129)	$Na_2AlH_4ClO_4$	145	162	0	0	True	15.13
mp-28255	$La_8H_8O_8$	24	$P4/nmm$ (129)	LaHO	144	211	0	0	False	-
mp-3269	$Na_4Th_8F_{36}$	24	I−42m (121)	$NaTh_2F_9$	0	119	81	0	False	-
mp-14415	$Be_4Pr_4Ge_2O_{14}$	24	P−42$_1$m (113)	$Pr_2Be_2GeO_7$	144	98	35	0	False	-
mp-14416	$Be_4Sm_4Ge_2O_{14}$	24	P−42$_1$m (113)	$Pr_2Be_2GeO_7$	144	106	35	0	False	-
mp-14420	$Be_4Er_4Ge_2O_{14}$	24	P−42$_1$m (113)	$Pr_2Be_2GeO_7$	144	97	33	0	False	-
mp-19926	$Be_4La_4Ge_2O_{14}$	24	P−42$_1$m (113)	$Pr_2Be_2GeO_7$	144	105	39	0	False	-
mp-20778	$Be_4Dy_4Ge_2O_{14}$	24	P−42$_1$m (113)	$Pr_2Be_2GeO_7$	144	90	33	0	True	2.67
mp-541040	$Be_4Y_4Ge_2O_{14}$	24	P−42$_1$m (113)	$Pr_2Be_2GeO_7$	144	99	38	0	False	-
mp-559691	$Al_4Ca_4O_{14}Si_2$	24	P−42$_1$m (113)	$Pr_2Be_2GeO_7$	144	122	50	0	False	-
mp-6655	$Be_4Y_4O_{14}Si_2$	24	P−42$_1$m (113)	$Pr_2Be_2GeO_7$	144	114	42	0	False	-
mp-7013	$Be_4Ho_4O_{14}Si_2$	24	P−42$_1$m (113)	$Pr_2Be_2GeO_7$	144	105	45	0	False	-
mp-9077	$Be_4Nd_4O_{14}Si_2$	24	P−42$_1$m (113)	$Pr_2Be_2GeO_7$	144	114	45	0	False	-
mp-9338	$Ba_4Mg_2O_{14}Si_4$	24	P−42$_1$m (113)	$Pr_2Be_2GeO_7$	144	105	29	0	False	-
mp-17244	$Ba_4Zn_2Ge_4O_2S_{12}$	24	P−42$_1$m (113)	$Ba_2ZnGe_2S_6$	144	83	35	0	False	-
mp-18129	$Ga_4La_4Zn_2O_2S_{12}$	24	P−42$_1$m (113)	$Ba_2ZnGe_2S_6$	144	100	34	0	True	1.08
mp-11025	Zn_8P_{16}	24	$P4_32_12$ (96)	ZnP_2	216	39	125	18	False	-
mp-913	Cd_8P_{16}	24	$P4_32_12$ (96)	ZnP_2	216	30	107	18	False	-
mp-12112	Cd_8P_{16}	24	$P4_12_12$ (92)	ZnP_2	216	45	109	18	False	-
mp-2782	Zn_8P_{16}	24	$P4_12_12$ (92)	ZnP_2	216	32	122	18	True	1
mp-29289	$Ba_4Pt_8S_{12}$	24	$P4_12_12$ (92)	$BaPt_2S_3$	216	32	128	18	False	-
mp-616572	$Li_8Pd_4O_{28}P_8$	24	$Imma$ (74)	$Li_2P_2PdO_7$	36	219	0	0	False	-
mp-14532	$Na_4Ta_{12}O_{32}$	24	$Ibam$ (72)	$NaTa_3O_8$	72	308	0	0	False	-
mp-18480	$Ca_{16}Na_{24}Ta_8O_{48}$	24	$Fddd$ (70)	$Na_3Ca_2TaO_6$	144	177	0	0	False	-
mp-557506	$Bi_8Ca_{16}Na_{24}O_{48}$	24	$Fddd$ (70)	$Na_3Ca_2TaO_6$	145	201	0	0	False	-
mp-560860	$Na_{32}Pt_{16}O_{48}$	24	$Fddd$ (70)	Na_2PtO_3	144	204	0	0	False	-
mp-754848	$Na_{32}Sn_{16}O_{48}$	24	$Fddd$ (70)	Na_2PtO_3	143	227	0	0	False	-

ID	Formula	*Z*	SP (*No.*)	Prototype	NL	NR	WPs	HDWPs	Clean	dE/dq
mp-17380	$Ba_{16}Ca_8Pd_{24}O_{48}$	24	*Fmmm* (69)	$Ba_2Ca(PdO_2)_3$	0	172	0	0	False	-
mp-556532	$Al_4Ca_4B_{12}O_{28}$	24	*Cmme* (67)	$CaAlB_3O_7$	36	252	0	0	False	-
mp-17696	$Ba_{16}V_8N_{24}$	24	*Cmce* (64)	Sr_2VN_3	156	176	0	0	False	-
mp-8977	$Ca_{16}N_{24}P_8$	24	*Cmce* (64)	Sr_2VN_3	153	188	0	0	False	-
mp-17123	$Os_{16}Si_{32}$	24	*Cmce* (64)	Si_2	152	226	0	0	False	-
mp-1714	$Fe_{16}Si_{32}$	24	*Cmce* (64)	Si_2	154	307	0	0	False	-
mp-569815	$Ru_{16}Si_{32}$	24	*Cmce* (64)	Si_2	155	238	0	0	False	-
mp-29835	$K_{16}Sn_8Te_{24}$	24	*Cmce* (64)	K_2SnTe_3	158	156	0	0	False	-
mp-505431	$In_8K_{16}Li_8As_{16}$	24	*Cmce* (64)	$K_2LiInAs_2$	151	157	0	0	False	-
mp-505767	$In_8K_{16}Na_8Sb_{16}$	24	*Cmce* (64)	$K_2LiInAs_2$	160	178	0	0	False	-
mp-9703	$Ga_8K_{16}Li_8As_{16}$	24	*Cmce* (64)	$K_2LiInAs_2$	152	210	0	0	False	-
mp-9719	$Al_8Li_8Na_{16}P_{16}$	24	*Cmce* (64)	$K_2LiInAs_2$	157	181	0	0	False	-
mp-556046	$Ga_8Sr_4B_8O_{28}$	24	*Cmcm* (63)	$SrGa_2B_2O_7$	119	123	0	0	False	-
mp-560870	$Ba_4Ga_8B_8O_{28}$	24	*Cmcm* (63)	$SrGa_2B_2O_7$	117	90	0	0	False	-
mp-557401	$Na_{24}Pb_4O_{20}$	24	*Cmcm* (63)	Na_6PbO_5	123	216	0	0	False	-
mp-556896	$Ag_4Au_4Ba_{16}O_{24}$	24	*Cmcm* (63)	Ba_4AgAuO_6	116	186	0	0	False	-
mp-554903	$Tb_4Cl_4O_{12}Se_4$	24	*Pnma* (62)	$TbSeClO_3$	351	148	0	0	True	4.44
mp-1244	Sr_8Ge_{16}	24	*Pnma* (62)	$SrGe_2$	340	162	0	0	True	1.28
mp-1477	Ba_8Si_{16}	24	*Pnma* (62)	$SrGe_2$	344	135	0	0	False	-
mp-2139	Ba_8Ge_{16}	24	*Pnma* (62)	$SrGe_2$	341	170	0	0	False	-
mp-23632	$Ca_8B_4Cl_4N_8$	24	*Pnma* (62)	Sr_2BN_2	345	150	0	0	False	-
mp-757145	$Sm_4Cl_4H_8O_8$	24	*Pnma* (62)	SmH_2ClO_2	351	130	0	0	True	2.31
mp-976349	$Lu_4Cl_4H_8O_8$	24	*Pnma* (62)	SmH_2ClO_2	355	117	0	0	False	-
mp-569764	$Cu_8Rb_4I_{12}$	24	*Pnma* (62)	$RbCu_2I_3$	349	142	0	0	False	-
mp-29454	$Na_{12}Tl_4O_8$	24	*Pnma* (62)	Na_3TlO_2	342	126	0	0	False	-
mp-31086	Na_4P_{20}	24	*Pnma* (62)	LiP_5	345	133	0	0	False	-
mp-28679	$Ga_4Na_4Br_{16}$	24	*Pnma* (62)	$LiGaBr_4$	347	111	0	0	False	-
mp-29395	$Al_4Na_4I_{16}$	24	*Pnma* (62)	$LiGaBr_4$	348	86	0	0	True	1.12
mp-30209	$Li_4B_4H_{16}$	24	*Pnma* (62)	$LiBH_4$	342	99	0	0	True	1.93
mp-1508	La_8S_{16}	24	*Pnma* (62)	LaS_2	341	146	0	0	False	-
mp-17697	$K_4Sm_4F_{16}$	24	*Pnma* (62)	$KSmF_4$	346	192	0	0	False	-
mp-18040	$K_4Pr_4F_{16}$	24	*Pnma* (62)	$KSmF_4$	349	178	0	0	False	-
mp-31097	$Al_4K_4H_{16}$	24	*Pnma* (62)	$KAlH_4$	342	104	0	0	False	-
mp-3164	$Ba_4O_{16}S_4$	24	*Pnma* (62)	$KAlH_4$	341	100	0	0	True	3.38
mp-696983	$Ga_4K_4H_{16}$	24	*Pnma* (62)	$KAlH_4$	345	118	0	0	False	-
mp-17730	$K_8Sn_4O_{12}$	24	*Pnma* (62)	K_2SnO_3	342	127	0	0	True	6.39
mp-21461	$Pb_4Rb_8O_{12}$	24	*Pnma* (62)	K_2SnO_3	352	130	0	0	False	-
mp-2833	Ir_8S_{16}	24	*Pnma* (62)	IrS_2	352	172	0	0	False	-
mp-22569	$In_4O_{16}P_4$	24	*Pnma* (62)	$InPO_4$	342	65	0	0	False	-
mp-4967	$Mg_4O_{16}S_4$	24	*Pnma* (62)	$InPO_4$	343	98	0	0	False	-
mp-560670	$Mg_4O_{16}Se_4$	24	*Pnma* (62)	$InPO_4$	342	108	0	0	False	-
mp-778924	$Zn_4O_{16}Se_4$	24	*Pnma* (62)	$InPO_4$	342	100	0	0	False	-
mp-779156	$Cd_4O_{16}Se_4$	24	*Pnma* (62)	$InPO_4$	342	109	0	0	False	-
mp-557807	$Au_4Cs_4K_8O_8$	24	*Pnma* (62)	CsK_2AuO_2	344	117	0	0	False	-
mp-540881	$Ag_4Cs_8I_{12}$	24	*Pnma* (62)	Cs_2AgI_3	343	220	0	0	True	1.69
mp-5587	$Ba_8Zn_4S_{12}$	24	*Pnma* (62)	Cs_2AgI_3	344	141	0	0	False	-
mp-569531	$Cu_4Rb_8Br_{12}$	24	*Pnma* (62)	Cs_2AgI_3	346	226	0	0	False	-
mp-569952	$Cu_4K_8Cl_{12}$	24	*Pnma* (62)	Cs_2AgI_3	342	195	0	0	False	-
mp-571440	$Cu_4K_8Br_{12}$	24	*Pnma* (62)	Cs_2AgI_3	345	183	0	0	False	-
mp-640053	$Cu_4Rb_8Cl_{12}$	24	*Pnma* (62)	Cs_2AgI_3	348	157	0	0	False	-
mp-10233	$Ca_8B_4F_4N_8$	24	*Pnma* (62)	Ca_2BN_2	346	185	0	0	False	-
mp-10234	$Sr_8B_4F_4N_8$	24	*Pnma* (62)	Ca_2BN_2	349	171	0	0	True	12.11
mp-24266	$Ca_8B_4H_4N_8$	24	*Pnma* (62)	Ca_2BN_2	346	149	0	0	False	-
mp-17063	$Ba_4Cu_4La_4Te_{12}$	24	*Pnma* (62)	$BaLaCuTe_3$	345	189	0	0	False	-
mp-17660	$Cu_4Pr_4Sr_4Se_{12}$	24	*Pnma* (62)	$BaLaCuTe_3$	344	199	0	0	False	-
mp-541430	$Ba_4Cu_4La_4Se_{12}$	24	*Pnma* (62)	$BaLaCuTe_3$	343	202	0	0	False	-
mp-542802	$Cu_4Pb_4Y_4S_{12}$	24	*Pnma* (62)	$BaLaCuTe_3$	343	155	0	0	False	-
mp-567983	$Cu_4Er_4Pb_4Se_{12}$	24	*Pnma* (62)	$BaLaCuTe_3$	347	152	0	0	False	-
mp-601257	$Cu_4Dy_4Pb_4Se_{12}$	24	*Pnma* (62)	$BaLaCuTe_3$	344	164	0	0	False	-
mp-601286	$Cu_4Pb_4Tb_4Se_{12}$	24	*Pnma* (62)	$BaLaCuTe_3$	345	166	0	0	False	-
mp-6244	$Cu_4Ho_4Pb_4Se_{12}$	24	*Pnma* (62)	$BaLaCuTe_3$	348	175	0	0	False	-
mp-640324	$Cu_4Pb_4Tm_4Se_{12}$	24	*Pnma* (62)	$BaLaCuTe_3$	347	186	0	0	False	-

ID	Formula	Z	SP (*No.*)	Prototype	NL	NR	WPs	HDWPs	Clean	dE/dq
mp-16870	$Ba_8Cd_4Se_{12}$	24	*Pnma* (62)	Ba_2CdSe_3	349	203	0	0	False	-
mp-16904	$Ba_8Cd_4Te_{12}$	24	*Pnma* (62)	Ba_2CdSe_3	345	208	0	0	False	-
mp-16905	$Ba_8Mn_4Te_{12}$	24	*Pnma* (62)	Ba_2CdSe_3	344	229	0	0	False	-
mp-18008	$Ba_8Hg_4S_{12}$	24	*Pnma* (62)	Ba_2CdSe_3	349	144	0	0	False	-
mp-18309	$Ba_8Cd_4S_{12}$	24	*Pnma* (62)	Ba_2CdSe_3	347	143	0	0	False	-
mp-18445	$Ba_8Mn_4S_{12}$	24	*Pnma* (62)	Ba_2CdSe_3	341	157	0	0	False	-
mp-18513	$Ba_8Mn_4Se_{12}$	24	*Pnma* (62)	Ba_2CdSe_3	348	214	0	0	False	-
mp-23399	$Ag_4Rb_8I_{12}$	24	*Pnma* (62)	Ba_2CdSe_3	341	204	0	0	False	-
mp-29693	$Ag_4Rb_8Cl_{12}$	24	*Pnma* (62)	Ba_2CdSe_3	346	171	0	0	True	1.56
mp-567714	$Ag_4Rb_8Br_{12}$	24	*Pnma* (62)	Ba_2CdSe_3	342	212	0	0	False	-
mp-569943	$Ag_4K_8I_{12}$	24	*Pnma* (62)	Ba_2CdSe_3	343	201	0	0	False	-
mp-27199	$Au_8Br_8Se_8$	24	*Pnma* (62)	AuSeBr	341	121	0	0	False	-
mp-776890	$Rb_{16}O_8$	24	*Pbca* (61)	Rb_2	378	137	0	0	False	-
mp-10368	$Co_8P_8Se_8$	24	*Pbca* (61)	CoPSe	379	160	0	0	False	-
mp-1858	Hf_8O_{16}	24	*Pbca* (61)	C21-Brookite	376	145	0	0	False	-
mp-23181	Sr_8I_{16}	24	*Pbca* (61)	C21-Brookite	378	148	0	0	False	-
mp-23220	Yb_8Cl_{16}	24	*Pbca* (61)	C21-Brookite	375	147	0	0	False	-
mp-568441	Mg_8H_{16}	24	*Pbca* (61)	C21-Brookite	378	130	0	0	False	-
mp-775757	Hf_8O_{16}	24	*Pbca* (61)	C21-Brookite	375	199	0	0	False	-
mp-776404	Zr_8O_{16}	24	*Pbca* (61)	C21-Brookite	379	202	0	0	False	-
mp-3123	$Pd_8P_8Se_8$	24	*Pbcn* (60)	PPdSe	373	129	0	0	False	-
mp-7280	$Pd_8P_8S_8$	24	*Pbcn* (60)	PPdSe	371	96	0	0	False	-
mp-28970	$Nd_8Cl_{12}N_4$	24	*Pbcn* (60)	Nd_2NCl_3	353	138	0	0	False	-
mp-28874	$Hg_8Br_4P_{12}$	24	*Pbcn* (60)	Hg_2P_3	347	150	0	0	False	-
mp-12221	$Ca_4O_{16}Te_4$	24	*Pbcn* (60)	$CaTeO_4$	341	104	0	0	False	-
mp-29001	$Lu_4Na_4Cl_{16}$	24	*Pbcn* (60)	$CaTeO_4$	340	161	0	0	False	-
mp-29432	$Na_4Sc_4Cl_{16}$	24	*Pbcn* (60)	$CaTeO_4$	343	114	0	0	False	-
mp-754885	$Ca_4Sr_4I_{16}$	24	*Pbcn* (60)	$CaTeO_4$	345	175	0	0	False	-
mp-8892	$In_4Li_4F_{16}$	24	*Pbcn* (60)	$CaTeO_4$	343	158	0	0	False	-
mp-8898	$Al_4Ta_4O_{16}$	24	*Pbcn* (60)	$CaTeO_4$	344	150	0	0	False	-
mp-23522	$Ba_4Zn_4Cl_{16}$	24	*Pbcn* (60)	$BaZnCl_4$	346	161	0	0	False	-
mp-28584	$Cu_4Tl_8I_{12}$	24	*Pnnm* (58)	Tl_2CuI_3	329	161	0	0	False	-
mp-555495	$K_6Y_2B_4O_{12}$	24	*Pnnm* (58)	$K_3Y(BO_3)_2$	324	126	0	0	False	-
mp-3311	$Sr_4U_4O_{16}$	24	*Pbcm* (57)	$SrUO_4$	270	172	0	0	False	-
mp-5611	$Ba_4U_4O_{16}$	24	*Pbcm* (57)	$SrUO_4$	270	152	0	0	False	-
mp-978553	$As_{16}Si_8$	24	*Pbam* (55)	$SiAs_2$	252	142	0	0	False	-
mp-29249	$As_8Ge_8Se_8$	24	*Pnna* (52)	GeAsSe	290	220	0	0	False	-
mp-28384	Ga_8Br_{16}	24	*Pnna* (52)	$GaBr_2$	289	206	0	0	False	-
mp-29312	In_8I_{16}	24	*Pnna* (52)	$GaBr_2$	288	229	0	0	False	-
mp-23373	$Ba_4Zn_4Cl_{16}$	24	*Pnna* (52)	$BaZnCl_4$	290	164	0	0	False	-
mp-780656	$Bi_{16}Na_{32}O_{48}$	24	*Fdd*2 (43)	Na_2PtO_3	145	85	17	0	False	-
mp-2798	$P_{24}Si_{24}$	24	*Cmc*2$_1$ (36)	SiP	119	150	3	0	False	-
mp-230	$O_{16}Sb_8$	24	*Pna*2$_1$ (33)	SbO_2	288	193	0	16	False	-
mp-2412	Li_4P_{20}	24	*Pna*2$_1$ (33)	LiP_5	295	119	0	16	False	-
mp-558085	$K_4Na_4Ge_4O_{12}$	24	*Pna*2$_1$ (33)	$KNaGeO_3$	296	141	1	17	True	2.69
mp-18440	$Nb_2Rb_{14}As_8$	24	*Pmn*2$_1$ (31)	K_7TaAs_4	252	132	0	0	False	-
mp-8258	$Al_8Ba_2S_{14}$	24	*Pmn*2$_1$ (31)	CaB_4O_7	252	113	0	0	False	-
mp-27411	Tl_4P_{20}	24	*Pmc*2$_1$ (26)	TlP_5	144	104	0	0	False	-
mp-17095	$Cs_4Cu_4Se_{16}$	24	*P*2$_1$2$_1$2$_1$ (19)	$CsCuSe_4$	325	48	96	18	False	-
mp-18003	$Cs_4Cu_4S_{16}$	24	*P*2$_1$2$_1$2$_1$ (19)	$CsCuSe_4$	324	44	71	18	True	1.69
mp-18105	$Ag_4Cs_4Se_{16}$	24	*P*2$_1$2$_1$2$_1$ (19)	$CsCuSe_4$	324	44	84	18	False	-
mp-18365	$Cu_4Rb_4Se_{16}$	24	*P*2$_1$2$_1$2$_1$ (19)	$CsCuSe_4$	324	67	103	18	False	-
mp-18585	$Ag_4Rb_4Se_{16}$	24	*P*2$_1$2$_1$2$_1$ (19)	$CsCuSe_4$	324	37	87	18	False	-
mp-983589	$Ag_8Ge_4O_{12}$	24	*P*2$_1$2$_1$2$_1$ (19)	Ag_2GeO_3	325	34	109	18	True	1.62
mp-17012	$Sr_{16}V_8N_{24}$	24	*C*2/*c* (15)	Sr_2VN_3	4	203	0	0	False	-
mp-568036	$Ga_8La_{16}N_{24}$	24	*C*2/*c* (15)	Sr_2VN_3	7	196	0	0	False	-
mp-779511	$Ba_{16}Mg_8O_{24}$	24	*C*2/*c* (15)	Sr_2VN_3	5	182	0	0	False	-
mp-573581	$Na_{16}Se_{24}Te_8$	24	*C*2/*c* (15)	Na_2TeSe_3	0	208	0	0	False	-
mp-557450	$Na_{16}Pt_8O_{24}$	24	*C*2/*c* (15)	Na_2PtO_3	7	183	0	0	False	-
mp-673833	$Li_{28}V_4As_{16}$	24	*C*2/*c* (15)	Li_7VAs_4	1	202	0	0	False	-
mp-27231	$Li_{16}O_{24}Te_8$	24	*C*2/*c* (15)	Li_2TeO_3	6	179	0	0	True	15.5
mp-6678	$Hg_8K_8S_{24}Sb_8$	24	*C*2/*c* (15)	$KHgSbS_3$	2	131	0	0	True	2.36

ID	Formula	Z	SP (*No.*)	Prototype	NL	NR	WPs	HDWPs	Clean	dE/dq
mp-28158	$Be_{12}K_{16}O_{20}$	24	$C2/c$ (15)	$K_4Be_3O_5$	0	170	0	0	False	-
mp-17914	$Ca_4Ga_{16}O_{28}$	24	$C2/c$ (15)	$CaGa_4O_7$	4	165	0	0	False	-
mp-29598	$Na_4N_{28}P_{16}$	24	$C2/c$ (15)	$CaGa_4O_7$	1	156	0	0	False	-
mp-3788	$Al_{16}Sr_4O_{28}$	24	$C2/c$ (15)	$CaGa_4O_7$	3	162	0	0	False	-
mp-769971	$Ba_4Ga_{16}O_{28}$	24	$C2/c$ (15)	$CaGa_4O_7$	3	145	0	0	False	-
mp-27148	$Be_{16}P_{32}$	24	$C2/c$ (15)	BeP_2	4	135	0	0	False	-
mp-558629	$Ba_8Zn_4O_{28}Si_8$	24	$C2/c$ (15)	$Ba_2ZnSi_2O_7$	0	148	0	0	False	-
mp-560493	$Ba_8Cu_{12}V_4S_{24}$	24	$C2/c$ (15)	$Ba_2V(CuS_2)_3$	0	97	0	0	True	2.68
mp-1392	Zn_8P_{16}	24	$P2_1/c$ (14)	ZnP_2	183	205	0	0	False	-
mp-569129	$Cu_4Tl_4P_4Se_{12}$	24	$P2_1/c$ (14)	$TlCuPSe_3$	183	181	0	0	False	-
mp-10814	$Li_4Sr_4B_4O_{12}$	24	$P2_1/c$ (14)	$SrLiBO_3$	184	200	0	0	False	-
mp-3831	$Sm_4O_{16}P_4$	24	$P2_1/c$ (14)	$SmPO_4$	184	210	0	0	False	-
mp-6300	$Hg_4Rb_4Sb_4Se_{12}$	24	$P2_1/c$ (14)	$RbHgSbSe_3$	180	196	0	0	False	-
mp-28568	$Cu_4Rb_{12}O_8$	24	$P2_1/c$ (14)	Rb_3CuO_2	185	240	0	0	False	-
mp-28630	$Na_{12}B_4N_8$	24	$P2_1/c$ (14)	Rb_3CuO_2	181	250	0	0	True	1.79
mp-755530	$Cu_4Na_{12}O_8$	24	$P2_1/c$ (14)	Rb_3CuO_2	183	265	0	0	False	-
mp-756241	$Bi_4Pr_4O_{16}$	24	$P2_1/c$ (14)	$PrBiO_4$	182	187	0	0	False	-
mp-31487	Nb_4I_{20}	24	$P2_1/c$ (14)	NbI_5	181	218	0	0	False	-
mp-556695	$Na_4Sr_4B_4O_{12}$	24	$P2_1/c$ (14)	$NaSrBO_3$	181	219	0	0	True	1.33
mp-561190	$Au_2Na_2O_{16}S_4$	24	$P2_1/c$ (14)	$NaAu(SO_4)_2$	183	134	0	0	True	1.95
mp-5416	$Na_8O_{12}Se_4$	24	$P2_1/c$ (14)	Na_2SeO_3	182	242	0	0	False	-
mp-541619	$Na_8O_{12}Te_4$	24	$P2_1/c$ (14)	Na_2SeO_3	180	226	0	0	False	-
mp-558731	$Li_8S_{12}Te_4$	24	$P2_1/c$ (14)	Na_2SeO_3	183	241	0	0	False	-
mp-570080	$Li_8Se_{12}Te_4$	24	$P2_1/c$ (14)	Na_2SeO_3	181	233	0	0	False	-
mp-28278	$Na_8Ge_4Se_{12}$	24	$P2_1/c$ (14)	Na_2GeSe_3	181	191	0	0	False	-
mp-4068	$Na_8Ge_4S_{12}$	24	$P2_1/c$ (14)	Na_2GeSe_3	182	202	0	0	False	-
mp-568543	$Na_8Sn_4Se_{12}$	24	$P2_1/c$ (14)	Na_2GeSe_3	181	199	0	0	False	-
mp-555022	$Lu_4F_4O_{12}Se_4$	24	$P2_1/c$ (14)	$LuSeO_3$	180	165	0	0	False	-
mp-556999	$Er_4F_4O_{12}Se_4$	24	$P2_1/c$ (14)	$LuSeO_3$	181	163	0	0	False	-
mp-557457	$Tm_4F_4O_{12}Se_4$	24	$P2_1/c$ (14)	$LuSeO_3$	182	164	0	0	False	-
mp-558538	$Ho_4F_4O_{12}Se_4$	24	$P2_1/c$ (14)	$LuSeO_3$	182	163	0	0	False	-
mp-28326	$Ga_4Li_4Br_{16}$	24	$P2_1/c$ (14)	$LiGaBr_4$	183	223	0	0	False	-
mp-28570	$In_4Na_4I_{16}$	24	$P2_1/c$ (14)	$LiGaBr_4$	183	210	0	0	False	-
mp-541001	$In_4Li_4I_{16}$	24	$P2_1/c$ (14)	$LiGaBr_4$	183	222	0	0	False	-
mp-567967	$Ga_4Li_4I_{16}$	24	$P2_1/c$ (14)	$LiGaBr_4$	181	207	0	0	False	-
mp-557012	$Li_6Sc_2B_4O_{12}$	24	$P2_1/c$ (14)	$Li_3Sc(BO_3)_2$	181	204	0	0	False	-
mp-769287	$Li_8Ti_4O_4S_8$	24	$P2_1/c$ (14)	Li_2TiS_2	181	228	0	0	True	3.31
mp-568986	$Au_4K_4Cl_{16}$	24	$P2_1/c$ (14)	$KAuCl_4$	182	203	0	0	True	1.49
mp-9726	$Be_4Sr_4F_{16}$	24	$P2_1/c$ (14)	$KAlH_4$	182	237	0	0	False	-
mp-29602	$K_8Zr_4Te_{12}$	24	$P2_1/c$ (14)	K_2ZrTe_3	182	186	0	0	False	-
mp-504630	$In_8Cl_8Te_8$	24	$P2_1/c$ (14)	InTeCl	180	176	0	0	True	5.9
mp-865980	$Ag_4Cs_4P_4Se_{12}$	24	$P2_1/c$ (14)	$CsAgPSe_3$	180	178	0	0	False	-
mp-574138	$Cs_8As_4Te_{12}$	24	$P2_1/c$ (14)	Cs_2Te_3	182	250	0	0	True	1.78
mp-755980	$Ca_4Sr_4I_{16}$	24	$P2_1/c$ (14)	$CaTeO_4$	181	320	0	0	False	-
mp-22665	$Ag_4Pb_4As_4S_{12}$	24	$P2_1/c$ (14)	$AgAsPbS_3$	180	183	0	0	False	-
mp-560848	$Ag_4Pb_4S_{12}Sb_4$	24	$P2_1/c$ (14)	$AgAsPbS_3$	181	213	0	0	False	-
mp-861513	$Rb_{20}H_4O_{20}Si_4$	24	$C2/m$ (12)	Rb_5SiHO_5	1	134	0	0	False	-
mp-505747	$In_{10}Tl_2S_{12}$	24	$P2_1/m$ (11)	$TlIn_5S_6$	108	339	0	0	False	-
mp-558195	$Ba_4Ho_2B_4Cl_2O_{12}$	24	$P2_1/m$ (11)	$Ba_2HoB_2ClO_6$	108	197	0	0	False	-
mp-866310	$Ba_4Y_2B_4Cl_2O_{12}$	24	$P2_1/m$ (11)	$Ba_2HoB_2ClO_6$	108	183	0	0	False	-
mp-561179	$Ba_8Cu_4I_4O_8$	24	$P2_1/m$ (11)	Ba_2CuIO_2	108	298	0	0	True	4.82
mp-571260	$Al_4In_4I_{16}$	24	$P2_1/m$ (11)	$AlInI_4$	111	160	0	0	False	-
mp-2804	$La_{16}P_{32}$	24	Cc (9)	LaP_2	1	74	0	0	False	-
mp-29815	$La_{16}As_{32}$	24	Cc (9)	LaP_2	1	95	0	0	False	-
mp-27181	$Au_4K_4Cl_{16}$	24	Pc (7)	$KAuCl_4$	75	205	0	0	False	-
mp-27559	$Cd_2Na_{14}O_9$	25	$P-3$ (147)	$Na_{14}Cd_2O_9$	112	87	0	0	False	-
mp-31408	$Al_{12}B_{18}O_{45}$	25	$R3$ (146)	$Al_4(B_2O_5)_3$	0	1	88	0	False	-
mp-9071	$Na_{64}Ti_8As_{32}$	26	$Fd-3$m (227)	Na_8TiAs_4	85	177	0	0	False	-
mp-23691	$Tl_8B_{48}H_{48}$	26	$Fm-3$ (202)	$Tl(BH)_6$	32	58	0	0	True	3
mp-24123	$Rb_8B_{48}H_{48}$	26	$Fm-3$ (202)	$Tl(BH)_6$	36	71	0	0	False	-
mp-24136	$Cs_8B_{48}H_{48}$	26	$Fm-3$ (202)	$Tl(BH)_6$	35	65	0	0	True	3.09
mp-24239	$K_8B_{48}H_{48}$	26	$Fm-3$ (202)	$Tl(BH)_6$	37	101	0	0	True	2.45

ID	Formula	*Z*	SP (*No.*)	Prototype	NL	NR	WPs	HDWPs	Clean	dE/dq
mp-557577	$K_6Sc_2O_{14}Si_4$	26	$P6_3/mmc$ (194)	$K_3LuSi_2O_7$	34	184	0	0	False	-
mp-11670	$Ba_2Y_2N_{14}Si_8$	26	$P6_3mc$ (186)	$BaYSi_4N_7$	33	142	2	10	False	-
mp-8075	$Be_8Na_2O_{14}Sb_2$	26	$P6_3mc$ (186)	$BaYSi_4N_7$	75	232	6	4	False	-
mp-554719	$Mg_6B_2H_6O_{12}$	26	$P6_3/m$ (176)	$Mg_3B(HO_2)_3$	163	101	0	0	False	-
mp-554303	$La_6U_2Cl_6O_{12}$	26	$P6_3/m$ (176)	$La_3U(ClO_2)_3$	159	81	0	0	False	-
mp-556183	$Pr_6U_2Cl_6O_{12}$	26	$P6_3/m$ (176)	$La_3U(ClO_2)_3$	43	103	0	0	False	-
mp-558533	$Nd_6U_2Cl_6O_{12}$	26	$P6_3/m$ (176)	$La_3U(ClO_2)_3$	46	97	0	0	False	-
mp-570804	$C_{39}Si_{39}$	26	$R3m$ (160)	SiC	138	155	103	8	False	-
mp-14324	$Be_4K_4Na_8O_{10}$	26	$P4_2/mnm$ (136)	$K_2Na_4Be_2O_5$	195	242	0	0	False	-
mp-30311	$Zn_6P_4S_{16}$	26	$P-4n2$ (118)	$Zn_3(PS_4)_2$	117	110	64	0	True	1.27
mp-6081	$Ba_4Ti_2O_{16}Si_4$	26	$P4bm$ (100)	$Ba_2Ti(SiO_4)_2$	117	189	0	0	False	-
mp-28995	$Cu_{16}P_{32}Si_4$	26	$I4_1/a$ (88)	Cu_4SiP_8	42	151	0	0	False	-
mp-568105	$Ba_4Zr_{12}B_2Cl_{34}$	26	$I4/m$ (87)	$Ba_2Zr_6BCl_{17}$	2	126	0	0	False	-
mp-23768	$Al_4Zn_4F_{20}H_{16}O_8$	26	$Imma$ (74)	$AlZnH_4O_2F_5$	40	290	0	0	False	-
mp-24142	$Al_4Mg_4F_{20}H_{16}O_8$	26	$Imma$ (74)	$AlZnH_4O_2F_5$	39	341	0	0	False	-
mp-769381	$Ca_{12}Ta_8O_{32}$	26	$Cmce$ (64)	$Ca_3Ta_2O_8$	163	188	0	0	False	-
mp-770741	$Mg_{12}As_8O_{32}$	26	$Cmce$ (64)	$Ca_3Ta_2O_8$	173	190	0	0	False	-
mp-31418	$La_4O_{36}Sb_{12}$	26	$Cmcm$ (63)	$La(SbO_3)_3$	128	147	0	0	False	-
mp-560303	$Ba_4Ca_8C_8F_8O_{24}$	26	$Cmcm$ (63)	$BaCa_2C_2(O_3F)_2$	126	195	0	0	False	-
mp-557467	$Ca_2Li_8B_4O_{12}$	26	$Pnnm$ (58)	$Li_4Ca(BO_3)_2$	351	131	0	0	False	-
mp-18010	$Pd_6Ta_4Se_{16}$	26	$Pbam$ (55)	$Ta_2Pd_3Se_8$	278	141	0	0	False	-
mp-4425	$Pt_6Ta_4Se_{16}$	26	$Pbam$ (55)	$Ta_2Pd_3Se_8$	274	125	0	0	False	-
mp-504898	$Nb_4Pd_6Se_{16}$	26	$Pbam$ (55)	$Ta_2Pd_3Se_8$	273	127	0	0	False	-
mp-560046	$Pt_6Ta_4S_{16}$	26	$Pbam$ (55)	$Ta_2Pd_3Se_8$	273	124	0	0	False	-
mp-27482	$Ag_{16}P_8S_{28}$	26	$C2/c$ (15)	$Ag_4P_2S_7$	4	126	0	0	False	-
mp-28805	$Pt_{10}Tl_4S_{12}$	26	$P2_1/c$ (14)	$Tl_2Pt_5S_6$	197	233	0	0	False	-
mp-766217	$Cu_4Sr_8O_{14}$	26	$P2_1/c$ (14)	$Sr_4Cu_2O_7$	201	248	0	0	True	10.67
mp-6637	$Ag_8Mn_2S_{12}Sb_4$	26	$P2_1/c$ (14)	$MnAg_4(SbS_3)_2$	196	210	0	0	False	-
mp-560623	$Mg_6O_{16}P_4$	26	$P2_1/c$ (14)	$Mg_3(PO_4)_2$	197	222	0	0	False	-
mp-756919	$Mg_6As_4O_{16}$	26	$P2_1/c$ (14)	$Mg_3(AsO_4)_2$	199	235	0	0	False	-
mp-557698	$K_8Na_4O_{12}Te_2$	26	$P2_1/c$ (14)	$K_4Na_2TeO_6$	196	226	0	0	False	-
mp-554627	$Cu_{24}Br_4P_4S_{20}$	26	Cc (9)	Cu_6PS_5	1	69	1	0	False	-
mp-504908	$In_6O_{18}Te_3$	27	$P321$ (150)	In_2TeO_6	0	11	265	0	False	-
mp-752695	$Ca_6Sr_3I_{18}$	27	$P321$ (150)	In_2TeO_6	0	9	388	0	False	-
mp-14871	$Li_{48}Ta_6N_{24}O_3$	27	$R-3$ (148)	$Li_{16}Ta_2N_8$	40	26	0	0	False	-
mp-6031	$Li_{48}Nb_6N_{24}O_3$	27	$R-3$ (148)	$Li_{16}Ta_2N_8$	42	23	0	0	False	-
mp-569413	$K_6Nb_{18}Sr_3Cl_{54}$	27	$R-3$ (148)	$K_2Sr(NbCl_3)_6$	40	20	0	0	False	-
mp-569465	$Cs_6Nb_{18}Pb_3Cl_{54}$	27	$R-3$ (148)	$K_2Sr(NbCl_3)_6$	34	12	0	0	False	-
mp-984696	$Hf_9Tl_{36}Te_{36}$	27	$R-3$ (148)	$Hf(TlTe)_4$	63	27	0	0	False	-
mp-667	$Tl_{54}S_{27}$	27	$R3$ (146)	Tl_2	0	3	77	1	False	-
mp-756639	$Li_6Nb_3F_{15}O_3$	27	$P3_1$ (144)	Li_2NbOF_5	0	0	126	0	False	-
mp-541097	$Pb_8Zr_{24}F_{48}O_{32}$	28	$Fm-3m$ (225)	$Zr_3Pb(O_2F_3)_2$	46	157	0	0	False	-
mp-17838	$K_8Tb_{24}F_{80}$	28	$Fm-3m$ (225)	KTb_3F_{10}	42	161	0	0	False	-
mp-18451	$Er_{24}K_8F_{80}$	28	$Fm-3m$ (225)	KTb_3F_{10}	42	159	0	0	False	-
mp-2943	$K_8Y_{24}F_{80}$	28	$Fm-3m$ (225)	KTb_3F_{10}	47	149	0	0	False	-
mp-27914	$Bi_8Na_{24}O_{24}$	28	$I-43m$ (217)	Na_3BiO_3	88	167	2	45	False	-
mp-8076	$Na_{24}O_{24}Sb_8$	28	$I-43m$ (217)	Na_3BiO_3	95	121	4	46	False	-
mp-17862	$Ag_{12}Ge_{20}P_{24}$	28	$I-43m$ (217)	$Ag_3Ge_5P_6$	84	167	4	49	False	-
mp-27272	$Ca_{24}I_{24}P_8$	28	$I4_132$ (214)	Ca_3PI_3	1	11	409	40	False	-
mp-675142	$Dy_4Yb_{12}Sb_{12}$	28	$P2_13$ (198)	Yb_3DySb_3	168	31	203	40	False	-
mp-8686	$Na_{12}As_4Se_{12}$	28	$P2_13$ (198)	Yb_3DySb_3	168	36	112	39	False	-
mp-9191	$Na_{12}Sb_4Te_{12}$	28	$P2_13$ (198)	Yb_3DySb_3	168	38	158	40	False	-
mp-17250	$K_{12}O_{12}Sb_4$	28	$P2_13$ (198)	K_3SbO_3	168	48	128	40	True	6.54
mp-17538	$K_{12}Sb_4Se_{12}$	28	$P2_13$ (198)	K_3SbO_3	168	27	107	40	False	-
mp-28980	$Bi_4K_{12}Se_{12}$	28	$P2_13$ (198)	K_3SbO_3	168	24	110	40	False	-
mp-29168	$Bi_4Rb_{12}Se_{12}$	28	$P2_13$ (198)	K_3SbO_3	168	27	120	39	False	-
mp-29379	$Bi_4K_{12}Te_{12}$	28	$P2_13$ (198)	K_3SbO_3	0	12	98	39	False	-
mp-29525	$Bi_4Rb_{12}O_{12}$	28	$P2_13$ (198)	K_3SbO_3	168	26	156	40	False	-
mp-5626	$K_{12}Sb_4Te_{12}$	28	$P2_13$ (198)	K_3SbO_3	168	27	94	40	True	1.09
mp-768232	$Rb_{12}O_{12}Sb_4$	28	$P2_13$ (198)	K_3SbO_3	168	29	135	40	False	-
mp-621964	$Bi_4K_8Rb_4Se_{12}$	28	$P2_13$ (198)	$K_2RbBiSe_3$	168	41	116	40	False	-
mp-24052	$Ca_4Mg_4Ni_4H_{16}$	28	$P2_13$ (198)	$CaMgNiH_4$	168	32	89	41	False	-

ID	Formula	Z	SP (*No.*)	Prototype	NL	NR	WPs	HDWPs	Clean	dE/dq
mp-643246	$Mg_4Ni_4Yb_4H_{16}$	28	$P2_13$ (198)	$CaMgNiH_4$	168	43	81	41	True	2.16
mp-643293	$Mg_4Ni_4Sr_4H_{16}$	28	$P2_13$ (198)	$CaMgNiH_4$	168	33	78	41	False	-
mp-6872	$K_6F_{12}N_2O_6Si_2$	28	$P6_3/mmc$ (194)	$K_3SiN(OF_2)_3$	34	235	0	0	True	1.43
mp-6185	$Co_2Er_2C_{12}N_{12}$	28	$P6_3/mmc$ (194)	$ErCo(CN)_6$	44	160	0	0	True	2.79
mp-17120	$Al_2Cs_{12}K_6Sb_8$	28	$P6_3/mmc$ (194)	$Cs_6K_3AlSb_4$	163	179	0	0	False	-
mp-541707	$Al_2K_{12}Na_6Sb_8$	28	$P6_3/mmc$ (194)	$Cs_6K_3AlSb_4$	170	207	0	0	False	-
mp-541811	$Cs_{12}Ga_2K_6Sb_8$	28	$P6_3/mmc$ (194)	$Cs_6K_3AlSb_4$	40	176	0	0	False	-
mp-6112	$Ga_2K_{12}Na_6Sb_8$	28	$P6_3/mmc$ (194)	$Cs_6K_3AlSb_4$	44	195	0	0	False	-
mp-23015	$Cs_6Mo_4Br_{18}$	28	$P6_3/mmc$ (194)	$Cs_3Mo_2Br_9$	30	159	0	0	True	2.37
mp-23451	$Cr_4Cs_6Br_{18}$	28	$P6_3/mmc$ (194)	$Cs_3Mo_2Br_9$	31	155	0	0	False	-
mp-30167	$Os_4Rb_6Br_{18}$	28	$P6_3/mmc$ (194)	$Cs_3Mo_2Br_9$	76	142	0	0	False	-
mp-541646	$Cs_6Ru_4Cl_{18}$	28	$P6_3/mmc$ (194)	$Cs_3Mo_2Br_9$	69	123	0	0	False	-
mp-541742	$Cs_6Mo_4Cl_{18}$	28	$P6_3/mmc$ (194)	$Cs_3Mo_2Br_9$	65	145	0	0	False	-
mp-17009	$Ba_6O_{18}Te_4$	28	$P6_3/mmc$ (194)	$Ba_3Te_2O_9$	33	142	0	0	False	-
mp-11625	$Ca_2K_2O_{18}P_6$	28	$P{-}6c2$ (188)	$KCa(PO_3)_3$	126	81	45	9	False	-
mp-17229	$Be_6Rb_2Zn_2F_{18}$	28	$P{-}6c2$ (188)	$KCa(PO_3)_3$	125	61	62	2	False	-
mp-18502	$Ba_2Sn_2O_{18}Si_6$	28	$P{-}6c2$ (188)	$KCa(PO_3)_3$	125	48	59	6	False	-
mp-18509	$Be_6K_2Zn_2F_{18}$	28	$P{-}6c2$ (188)	$KCa(PO_3)_3$	126	89	54	9	False	-
mp-540631	$K_2Ta_2Ge_6O_{18}$	28	$P{-}6c2$ (188)	$KCa(PO_3)_3$	125	82	55	9	False	-
mp-540634	$Ta_2Tl_2Ge_6O_{18}$	28	$P{-}6c2$ (188)	$KCa(PO_3)_3$	126	88	51	7	False	-
mp-6661	$Ba_2Ti_2O_{18}Si_6$	28	$P{-}6c2$ (188)	$KCa(PO_3)_3$	125	46	68	6	True	30.17
mp-9478	$Ba_2O_{18}Si_8$	28	$P{-}6c2$ (188)	$BaSi_4O_9$	126	83	70	11	False	-
mp-626161	$Al_{10}H_2O_{16}$	28	$P6_3mc$ (186)	Al_5HO_8	34	323	5	4	False	-
mp-29218	$Bi_4Ge_6O_{18}$	28	$P6_3/m$ (176)	$Ge_3Bi_2O_9$	35	121	0	0	False	-
mp-558672	$Bi_4O_{18}Si_6$	28	$P6_3/m$ (176)	$Ge_3Bi_2O_9$	40	39	0	0	False	-
mp-756888	$La_4O_{18}Si_6$	28	$P6_3/m$ (176)	$Ge_3Bi_2O_9$	36	81	0	0	False	-
mp-27506	$K_6W_4Cl_{18}$	28	$P6_3/m$ (176)	$Cs_3Mo_2Br_9$	35	59	0	0	True	1.02
mp-541738	$Cr_{12}Cs_{18}I_{54}$	28	$R{-}3c$ (167)	$Cs_3Cr_2I_9$	124	155	0	0	False	-
mp-568937	$Cs_{18}Y_{12}Br_{54}$	28	$R{-}3c$ (167)	$Cs_3Cr_2I_9$	124	207	0	0	False	-
mp-637194	$Cs_{18}Sc_{12}Cl_{54}$	28	$R{-}3c$ (167)	$Cs_3Cr_2I_9$	122	191	0	0	True	1.09
mp-669304	$Cs_{18}Tb_{12}Br_{54}$	28	$R{-}3c$ (167)	$Cs_3Cr_2I_9$	128	243	0	0	False	-
mp-24241	$Rb_9B_{36}H_{36}I_3$	28	$R{-}3m$ (166)	$Rb_3B_{12}H_{12}$	62	34	0	0	True	1.81
mp-24796	$Rb_9B_{36}Br_3H_{36}$	28	$R{-}3m$ (166)	$Rb_3B_{12}H_{12}$	65	54	0	0	False	-
mp-24798	$Rb_9B_{36}Cl_3H_{36}$	28	$R{-}3m$ (166)	$Rb_3B_{12}H_{12}$	64	45	0	0	False	-
mp-24799	$Cs_9B_{36}Cl_3H_{36}$	28	$R{-}3m$ (166)	$Rb_3B_{12}H_{12}$	61	57	0	0	True	2.39
mp-510549	$K_9B_{36}Br_3H_{36}$	28	$R{-}3m$ (166)	$Rb_3B_{12}H_{12}$	65	60	0	0	True	1.55
mp-542819	$Cs_9B_{36}Br_3H_{36}$	28	$R{-}3m$ (166)	$Rb_3B_{12}H_{12}$	64	39	0	0	True	1.85
mp-556301	$Ca_2Ce_2Li_{12}O_{12}$	28	$P{-}31c$ (163)	Li_6CaCeO_6	154	241	0	0	False	-
mp-27417	$In_4Li_{12}O_{12}$	28	$P{-}31c$ (163)	Li_3InO_3	158	257	0	0	False	-
mp-2245	$N_{16}Si_{12}$	28	$P31c$ (159)	Si_3N_4	187	255	0	10	False	-
mp-672289	$Ge_{12}N_{16}$	28	$P31c$ (159)	Si_3N_4	188	244	0	9	False	-
mp-29890	$Au_4Th_8F_{44}$	28	$I4/mcm$ (140)	Th_2AuF_{11}	42	259	0	0	False	-
mp-4287	$Sn_4Sr_8O_{16}$	28	$P4_2/ncm$ (138)	Sr_2SnO_4	210	322	0	0	False	-
mp-754761	$Hf_4Sr_8O_{16}$	28	$P4_2/ncm$ (138)	Sr_2SnO_4	213	327	0	0	False	-
mp-19970	$Cu_4Li_{12}O_{12}$	28	$P4_2/mnm$ (136)	Li_3CuO_3	210	231	0	0	False	-
mp-662530	$K_4Ni_4P_4S_{16}$	28	$P4_2/mnm$ (136)	$KNiPS_4$	212	188	0	0	False	-
mp-866637	$K_4Pd_4P_4S_{16}$	28	$P4_2/mnm$ (136)	$KNiPS_4$	210	176	0	0	False	-
mp-29359	$Na_8Pd_4Cl_{16}$	28	$P4/ncc$ (130)	Na_2PdCl_4	258	144	0	0	False	-
mp-555736	$Ba_4Hg_8Cl_8O_8$	28	$P4/mbm$ (127)	$BaHg_2(ClO)_2$	168	180	0	0	False	-
mp-556712	$Bi_4Ga_8S_{16}$	28	$P4/nnc$ (126)	Ga_2BiS_4	169	180	0	0	False	-
mp-557737	$Al_8Bi_4S_{16}$	28	$P4/nnc$ (126)	Ga_2BiS_4	169	182	0	0	False	-
mp-571085	$Bi_4Ga_8Se_{16}$	28	$P4/nnc$ (126)	Ga_2BiS_4	168	177	0	0	False	-
mp-571623	$Al_8Bi_4Se_{16}$	28	$P4/nnc$ (126)	Ga_2BiS_4	169	193	0	0	False	-
mp-29136	$Cu_6Sr_{12}N_{10}$	28	$P4_2mc$ (105)	$Sr_6Cu_3N_5$	43	245	0	0	False	-
mp-771976	$Be_8Rb_8O_{12}$	28	$P4_32_12$ (96)	$Rb_2Be_2O_3$	252	46	158	21	False	-
mp-554845	$Na_8Zn_8O_{12}$	28	$P4_32_12$ (96)	$Na_2Zn_2O_3$	252	40	179	21	True	12.47
mp-769025	$K_8Mg_8O_{12}$	28	$P4_32_12$ (96)	$Na_2Zn_2O_3$	252	38	167	21	False	-
mp-13843	$Li_8O_{16}Te_4$	28	$P4_122$ (91)	Li_2TeO_4	126	19	225	0	False	-
mp-542737	$Ti_4Zn_8O_{16}$	28	$P4_122$ (91)	Li_2TeO_4	126	26	299	0	False	-
mp-757031	$Mg_8Ti_4O_{16}$	28	$P4_122$ (91)	Li_2TeO_4	126	29	378	0	False	-
mp-3257	$Au_8K_8Se_{40}$	28	$Ibam$ (72)	$KAuSe_5$	85	220	0	0	False	-
mp-3592	$Au_8K_8S_{40}$	28	$Ibam$ (72)	$KAuSe_5$	86	239	0	0	False	-

ID	Formula	Z	SP (*No.*)	Prototype	NL	NR	WPs	HDWPs	Clean	dE/dq
mp-17667	$Al_8Sr_{24}Sb_{24}$	28	*Cmce* (64)	Sr_3AlSb_3	182	242	0	0	False	-
mp-18439	$Al_8Ba_{24}Sb_{24}$	28	*Cmce* (64)	Sr_3AlSb_3	185	252	0	0	False	-
mp-541715	$Ba_{24}Ga_8P_{24}$	28	*Cmce* (64)	Sr_3AlSb_3	183	187	0	0	False	-
mp-277	$Zr_{12}N_{16}$	28	*Pnma* (62)	Zr_3N_4	399	188	0	0	False	-
mp-17769	$Y_4F_4O_{16}S_4$	28	*Pnma* (62)	YSO_4	410	132	0	0	False	-
mp-17043	$Tm_8Zn_4S_{16}$	28	*Pnma* (62)	Tm_2ZnS_4	401	151	0	0	False	-
mp-17441	$Mg_8Ge_4S_{16}$	28	*Pnma* (62)	Tm_2ZnS_4	401	136	0	0	False	-
mp-18332	$Lu_8Zn_4S_{16}$	28	*Pnma* (62)	Tm_2ZnS_4	401	170	0	0	False	-
mp-23416	$Li_8Zn_4Cl_{16}$	28	*Pnma* (62)	Tm_2ZnS_4	400	155	0	0	False	-
mp-23497	$Li_8Zn_4I_{16}$	28	*Pnma* (62)	Tm_2ZnS_4	402	147	0	0	False	-
mp-28829	$Li_8Zn_4Br_{16}$	28	*Pnma* (62)	Tm_2ZnS_4	403	156	0	0	False	-
mp-28830	$Na_8Zn_4Br_{16}$	28	*Pnma* (62)	Tm_2ZnS_4	401	140	0	0	False	-
mp-2895	$Mg_8O_{16}Si_4$	28	*Pnma* (62)	Tm_2ZnS_4	400	132	0	0	False	-
mp-3051	$Mg_8Ge_4O_{16}$	28	*Pnma* (62)	Tm_2ZnS_4	399	152	0	0	False	-
mp-3081	$Al_8Be_4O_{16}$	28	*Pnma* (62)	Tm_2ZnS_4	398	159	0	0	False	-
mp-3318	$Be_4Na_8F_{16}$	28	*Pnma* (62)	Tm_2ZnS_4	400	151	0	0	False	-
mp-3917	$Cd_8Ge_4O_{16}$	28	*Pnma* (62)	Tm_2ZnS_4	401	125	0	0	False	-
mp-4481	$Ca_8O_{16}Si_4$	28	*Pnma* (62)	Tm_2ZnS_4	397	147	0	0	False	-
mp-540773	$Ca_8Ge_4S_{16}$	28	*Pnma* (62)	Tm_2ZnS_4	401	173	0	0	False	-
mp-560647	$Ca_8Ge_4O_{16}$	28	*Pnma* (62)	Tm_2ZnS_4	404	151	0	0	True	2.65
mp-616461	$Na_8Zn_4Cl_{16}$	28	*Pnma* (62)	Tm_2ZnS_4	400	169	0	0	False	-
mp-752410	$Ba_8Ge_4O_{16}$	28	*Pnma* (62)	Tm_2ZnS_4	400	123	0	0	False	-
mp-866503	$Ca_8Sn_4S_{16}$	28	*Pnma* (62)	Tm_2ZnS_4	399	172	0	0	False	-
mp-17832	$Ba_4Rb_4O_{16}P_4$	28	*Pnma* (62)	$RbBaPO_4$	398	167	0	0	False	-
mp-6812	$Ba_4K_4O_{16}P_4$	28	*Pnma* (62)	$RbBaPO_4$	426	217	0	0	False	-
mp-17048	$Rb_8W_4S_{16}$	28	*Pnma* (62)	Rb_2WS_4	406	204	0	0	False	-
mp-17361	$Cs_8W_4S_{16}$	28	*Pnma* (62)	Rb_2WS_4	400	167	0	0	False	-
mp-17612	$Ba_8O_{16}Si_4$	28	*Pnma* (62)	Rb_2WS_4	400	178	0	0	True	15.58
mp-17908	$Ba_8Ti_4S_{16}$	28	*Pnma* (62)	Rb_2WS_4	406	189	0	0	False	-
mp-17980	$K_8Mo_4S_{16}$	28	*Pnma* (62)	Rb_2WS_4	415	192	0	0	False	-
mp-18138	$K_8W_4Se_{16}$	28	*Pnma* (62)	Rb_2WS_4	417	168	0	0	True	2.49
mp-18612	$Mo_4Rb_8S_{16}$	28	*Pnma* (62)	Rb_2WS_4	397	178	0	0	False	-
mp-3009	$Be_4Rb_8F_{16}$	28	*Pnma* (62)	Rb_2WS_4	399	204	0	0	False	-
mp-3372	$Rb_8O_{16}S_4$	28	*Pnma* (62)	Rb_2WS_4	400	181	0	0	False	-
mp-5013	$Be_4Cs_8F_{16}$	28	*Pnma* (62)	Rb_2WS_4	404	174	0	0	False	-
mp-5185	$Be_4K_8F_{16}$	28	*Pnma* (62)	Rb_2WS_4	405	187	0	0	False	-
mp-540578	$Cs_8Zn_4Cl_{16}$	28	*Pnma* (62)	Rb_2WS_4	401	173	0	0	False	-
mp-540805	$Ba_8Ge_4S_{16}$	28	*Pnma* (62)	Rb_2WS_4	407	166	0	0	False	-
mp-562442	$Cs_8O_{16}Se_4$	28	*Pnma* (62)	Rb_2WS_4	416	169	0	0	True	2.08
mp-5838	$Ba_8S_{16}Si_4$	28	*Pnma* (62)	Rb_2WS_4	413	166	0	0	True	1.95
mp-644236	$Cs_8Mg_4H_{16}$	28	*Pnma* (62)	Rb_2WS_4	404	159	0	0	True	3.11
mp-695998	$K_8Zn_4H_{16}$	28	*Pnma* (62)	Rb_2WS_4	409	161	0	0	False	-
mp-697575	$Rb_8Zn_4H_{16}$	28	*Pnma* (62)	Rb_2WS_4	414	158	0	0	False	-
mp-505284	$Pb_{12}Br_8O_8$	28	*Pnma* (62)	$Pb_3(BrO)_2$	410	166	0	0	True	34.66
mp-605821	$Nd_{12}Br_4N_8Se_4$	28	*Pnma* (62)	Nd_3SeBrN_2	420	206	0	0	False	-
mp-16927	$Na_4Tb_4Ge_4O_{16}$	28	*Pnma* (62)	$NaTbGeO_4$	400	132	0	0	False	-
mp-17394	$Na_4Y_4Ge_4O_{16}$	28	*Pnma* (62)	$NaTbGeO_4$	399	142	0	0	False	-
mp-17487	$Er_4Na_4Ge_4O_{16}$	28	*Pnma* (62)	$NaTbGeO_4$	399	116	0	0	False	-
mp-17854	$In_4Li_4Ge_4O_{16}$	28	*Pnma* (62)	$NaTbGeO_4$	398	126	0	0	False	-
mp-17891	$Na_4Y_4O_{16}Si_4$	28	*Pnma* (62)	$NaTbGeO_4$	403	152	0	0	False	-
mp-18334	$Ho_4Na_4Ge_4O_{16}$	28	*Pnma* (62)	$NaTbGeO_4$	399	146	0	0	False	-
mp-18629	$Na_4Tm_4Ge_4O_{16}$	28	*Pnma* (62)	$NaTbGeO_4$	399	159	0	0	False	-
mp-504948	$Li_4Sc_4Ge_4O_{16}$	28	*Pnma* (62)	$NaTbGeO_4$	398	143	0	0	False	-
mp-555904	$Na_4Sm_4Ge_4O_{16}$	28	*Pnma* (62)	$NaTbGeO_4$	398	125	0	0	False	-
mp-558362	$Ca_4Mg_4Ge_4O_{16}$	28	*Pnma* (62)	$NaTbGeO_4$	399	144	0	0	False	-
mp-559969	$Ca_4Ga_4La_4O_{16}$	28	*Pnma* (62)	$NaTbGeO_4$	401	153	0	0	False	-
mp-6224	$Li_4Sc_4O_{16}Si_4$	28	*Pnma* (62)	$NaTbGeO_4$	402	157	0	0	False	-
mp-6493	$Ca_4Mg_4O_{16}Si_4$	28	*Pnma* (62)	$NaTbGeO_4$	399	110	0	0	False	-
mp-6684	$Cd_4Na_4O_{16}P_4$	28	*Pnma* (62)	$NaTbGeO_4$	399	117	0	0	True	1.94
mp-6712	$Lu_4Na_4Ge_4O_{16}$	28	*Pnma* (62)	$NaTbGeO_4$	403	164	0	0	False	-
mp-7205	$In_4Li_4O_{16}Si_4$	28	*Pnma* (62)	$NaTbGeO_4$	400	127	0	0	False	-
mp-8376	$Al_4Mg_4B_4O_{16}$	28	*Pnma* (62)	$NaTbGeO_4$	400	151	0	0	False	-

ID	Formula	Z	SP (*No.*)	Prototype	NL	NR	WPs	HDWPs	Clean	dE/dq
mp-8870	$Li_4Mg_4As_4O_{16}$	28	*Pnma* (62)	$NaTbGeO_4$	400	137	0	0	False	-
mp-9625	$Li_4Mg_4O_{16}P_4$	28	*Pnma* (62)	$NaTbGeO_4$	399	113	0	0	False	-
mp-28127	Na_8S_{20}	28	*Pnma* (62)	Na_2S_5	406	177	0	0	False	-
mp-556125	$Na_8B_4H_4O_{12}$	28	*Pnma* (62)	Na_2BHO_3	413	175	0	0	False	-
mp-3872	$Al_8Mg_4S_{16}$	28	*Pnma* (62)	$Mg(AlS_2)_2$	402	171	0	0	False	-
mp-9607	$Li_8U_4O_{16}$	28	*Pnma* (62)	$Mg(AlS_2)_2$	401	137	0	0	False	-
mp-5614	$Li_{12}B_4S_{12}$	28	*Pnma* (62)	Li_3BS_3	398	121	0	0	False	-
mp-17785	$Ga_4K_4Na_8O_{12}$	28	*Pnma* (62)	KNa_2GaO_3	402	231	0	0	True	1.51
mp-17088	$Ba_4K_4P_4S_{16}$	28	*Pnma* (62)	$KBaPS_4$	411	174	0	0	True	2.99
mp-17090	$Sr_4Tl_4P_4S_{16}$	28	*Pnma* (62)	$KBaPS_4$	400	150	0	0	False	-
mp-776470	$Hf_{12}N_{16}$	28	*Pnma* (62)	Hf_3N_4	397	170	0	0	False	-
mp-28608	$Ga_{12}I_4Te_{12}$	28	*Pnma* (62)	Ga_3Te_3	410	178	0	0	False	-
mp-616327	$In_{12}I_4Te_{12}$	28	*Pnma* (62)	Ga_3Te_3	416	201	0	0	False	-
mp-29476	$Cu_{12}Sb_4Se_{12}$	28	*Pnma* (62)	Cu_3SbSe_3	400	164	0	0	False	-
mp-28877	$Cs_8Sn_4Te_{16}$	28	*Pnma* (62)	Cs_2SnTe_4	417	203	0	0	False	-
mp-27899	$Cd_{12}As_4Cl_{12}$	28	*Pnma* (62)	Cd_3AsCl_3	396	145	0	0	False	-
mp-17186	$Al_4Ca_{12}As_{12}$	28	*Pnma* (62)	Ca_3AlAs_3	404	243	0	0	False	-
mp-567391	$Dy_{12}N_4Se_{12}$	28	*Pnma* (62)	Ca_3AlAs_3	408	208	0	0	False	-
mp-614572	$Ca_{12}In_4P_{12}$	28	*Pnma* (62)	Ca_3AlAs_3	405	203	0	0	False	-
mp-616026	$In_4Sr_{12}P_{12}$	28	*Pnma* (62)	Ca_3AlAs_3	406	190	0	0	False	-
mp-620346	$Ba_{12}In_4As_{12}$	28	*Pnma* (62)	Ca_3AlAs_3	406	231	0	0	False	-
mp-18642	$Ca_4Y_8S_{16}$	28	*Pnma* (62)	$Ca(YS_2)_2$	408	213	0	0	False	-
mp-505362	$Ca_4Lu_8S_{16}$	28	*Pnma* (62)	$Ca(YS_2)_2$	406	215	0	0	False	-
mp-865865	$Er_8Yb_4S_{16}$	28	*Pnma* (62)	$Ca(YS_2)_2$	410	172	0	0	False	-
mp-559186	$Ba_4Bi_4B_4O_{16}$	28	*Pnma* (62)	$BaBiBO_4$	408	134	0	0	False	-
mp-866301	$Ba_4B_4S_{16}Sb_4$	28	*Pnma* (62)	$BaBiBO_4$	407	144	0	0	True	2.69
mp-31275	$Ba_4P_{16}Te_8$	28	*Pnma* (62)	$Ba(TeP_2)_2$	419	172	0	0	True	37.6
mp-17153	$Ba_4Tb_8Te_{16}$	28	*Pnma* (62)	$Ba(TbTe_2)_2$	406	256	0	0	False	-
mp-17273	$Ba_4Ho_8Te_{16}$	28	*Pnma* (62)	$Ba(TbTe_2)_2$	404	251	0	0	False	-
mp-17275	$Sr_4Y_8O_{16}$	28	*Pnma* (62)	$Ba(TbTe_2)_2$	404	203	0	0	False	-
mp-17363	$Ba_4Ho_8O_{16}$	28	*Pnma* (62)	$Ba(TbTe_2)_2$	401	187	0	0	False	-
mp-17412	$Ba_4Pr_8O_{16}$	28	*Pnma* (62)	$Ba(TbTe_2)_2$	403	211	0	0	False	-
mp-17501	$Ba_4Sc_8Te_{16}$	28	*Pnma* (62)	$Ba(TbTe_2)_2$	404	251	0	0	False	-
mp-17597	$Ca_4Sc_8S_{16}$	28	*Pnma* (62)	$Ba(TbTe_2)_2$	403	238	0	0	False	-
mp-17678	$Sc_8Sr_4S_{16}$	28	*Pnma* (62)	$Ba(TbTe_2)_2$	404	209	0	0	False	-
mp-17786	$Ba_4Sm_8Te_{16}$	28	*Pnma* (62)	$Ba(TbTe_2)_2$	405	267	0	0	False	-
mp-17872	$Ba_4Y_8Te_{16}$	28	*Pnma* (62)	$Ba(TbTe_2)_2$	409	221	0	0	False	-
mp-17927	$Ba_4Dy_8Te_{16}$	28	*Pnma* (62)	$Ba(TbTe_2)_2$	406	229	0	0	False	-
mp-17928	$Ba_4Er_8Te_{16}$	28	*Pnma* (62)	$Ba(TbTe_2)_2$	406	250	0	0	False	-
mp-17960	$Ba_4Sm_8O_{16}$	28	*Pnma* (62)	$Ba(TbTe_2)_2$	403	216	0	0	False	-
mp-17998	$Ca_4Rh_8O_{16}$	28	*Pnma* (62)	$Ba(TbTe_2)_2$	399	216	0	0	False	-
mp-18042	$Sr_4Tb_8O_{16}$	28	*Pnma* (62)	$Ba(TbTe_2)_2$	404	178	0	0	False	-
mp-18196	$Ba_4Tm_8Te_{16}$	28	*Pnma* (62)	$Ba(TbTe_2)_2$	403	224	0	0	False	-
mp-18258	$Ba_4Tb_8O_{16}$	28	*Pnma* (62)	$Ba(TbTe_2)_2$	404	195	0	0	False	-
mp-18660	$Sc_8Sr_4Te_{16}$	28	*Pnma* (62)	$Ba(TbTe_2)_2$	404	224	0	0	False	-
mp-22630	$Pb_4Sc_8S_{16}$	28	*Pnma* (62)	$Ba(TbTe_2)_2$	403	218	0	0	False	-
mp-2892	$Ba_4Nd_8O_{16}$	28	*Pnma* (62)	$Ba(TbTe_2)_2$	404	227	0	0	False	-
mp-29035	$Sr_4Y_8S_{16}$	28	*Pnma* (62)	$Ba(TbTe_2)_2$	402	215	0	0	False	-
mp-29036	$Ba_4Y_8S_{16}$	28	*Pnma* (62)	$Ba(TbTe_2)_2$	399	231	0	0	False	-
mp-29605	$Pr_8Sr_4O_{16}$	28	*Pnma* (62)	$Ba(TbTe_2)_2$	408	187	0	0	False	-
mp-3446	$Sr_4Tm_8O_{16}$	28	*Pnma* (62)	$Ba(TbTe_2)_2$	401	204	0	0	False	-
mp-3952	$Ba_4Y_8O_{16}$	28	*Pnma* (62)	$Ba(TbTe_2)_2$	408	213	0	0	False	-
mp-4056	$Ca_4Sc_8O_{16}$	28	*Pnma* (62)	$Ba(TbTe_2)_2$	406	246	0	0	False	-
mp-4959	$Ca_4Ga_8O_{16}$	28	*Pnma* (62)	$Ba(TbTe_2)_2$	404	203	0	0	False	-
mp-504539	$Ca_4Lu_8O_{16}$	28	*Pnma* (62)	$Ba(TbTe_2)_2$	404	206	0	0	False	-
mp-540688	$In_8Sr_4O_{16}$	28	*Pnma* (62)	$Ba(TbTe_2)_2$	400	203	0	0	False	-
mp-542826	$Pb_4Sc_8Se_{16}$	28	*Pnma* (62)	$Ba(TbTe_2)_2$	407	222	0	0	False	-
mp-556075	$Dy_8Sr_4O_{16}$	28	*Pnma* (62)	$Ba(TbTe_2)_2$	406	195	0	0	False	-
mp-559385	$Ho_8Sr_4O_{16}$	28	*Pnma* (62)	$Ba(TbTe_2)_2$	406	185	0	0	False	-
mp-560445	$Er_8Sr_4O_{16}$	28	*Pnma* (62)	$Ba(TbTe_2)_2$	406	209	0	0	False	-
mp-569786	$Er_8Pb_4Se_{16}$	28	*Pnma* (62)	$Ba(TbTe_2)_2$	404	206	0	0	False	-
mp-619279	$In_8Pb_4S_{16}$	28	*Pnma* (62)	$Ba(TbTe_2)_2$	403	194	0	0	False	-

ID	Formula	Z	SP (*No.*)	Prototype	NL	NR	WPs	HDWPs	Clean	dE/dq
mp-752442	$Ba_4Lu_8O_{16}$	28	*Pnma* (62)	$Ba(TbTe_2)_2$	407	187	0	0	False	-
mp-752656	$Ba_4La_8O_{16}$	28	*Pnma* (62)	$Ba(TbTe_2)_2$	404	212	0	0	False	-
mp-754410	$Ca_4Tm_8O_{16}$	28	*Pnma* (62)	$Ba(TbTe_2)_2$	403	211	0	0	False	-
mp-754414	$Sm_8Sr_4O_{16}$	28	*Pnma* (62)	$Ba(TbTe_2)_2$	408	209	0	0	False	-
mp-754599	$Ba_4Tm_8O_{16}$	28	*Pnma* (62)	$Ba(TbTe_2)_2$	410	196	0	0	False	-
mp-755405	$Nd_8Sr_4O_{16}$	28	*Pnma* (62)	$Ba(TbTe_2)_2$	404	216	0	0	False	-
mp-770771	$Sc_8Sr_4O_{16}$	28	*Pnma* (62)	$Ba(TbTe_2)_2$	402	205	0	0	False	-
mp-772820	$Lu_8Sr_4O_{16}$	28	*Pnma* (62)	$Ba(TbTe_2)_2$	404	214	0	0	False	-
mp-862729	$Dy_8Sr_4Se_{16}$	28	*Pnma* (62)	$Ba(TbTe_2)_2$	401	243	0	0	False	-
mp-866306	$Ba_4Nd_8S_{16}$	28	*Pnma* (62)	$Ba(TbTe_2)_2$	406	201	0	0	False	-
mp-980666	$Dy_8Sr_4S_{16}$	28	*Pnma* (62)	$Ba(TbTe_2)_2$	407	193	0	0	False	-
mp-984052	$Ba_4Lu_8S_{16}$	28	*Pnma* (62)	$Ba(TbTe_2)_2$	404	183	0	0	False	-
mp-675601	$Al_{12}N_4O_{12}$	28	*Pnma* (62)	Al_3NO_3	399	204	0	0	False	-
mp-1666	Th_8S_{20}	28	*Pbcn* (60)	Th_2S_5	399	171	0	0	False	-
mp-27537	$Na_8O_{16}Te_4$	28	*Pbcn* (60)	Na_2TeO_4	403	129	0	0	False	-
mp-556814	$Li_4Nd_4Ge_4O_{16}$	28	*Pbcn* (60)	$LiNdGeO_4$	405	166	0	0	False	-
mp-3417	$Ca_4B_8O_{16}$	28	*Pbcn* (60)	$Ca(BO_2)_2$	410	160	0	0	True	47.46
mp-766173	$Rh_8Sr_4O_{16}$	28	*Pmmn* (59)	$Sr(RhO_2)_2$	295	209	0	0	False	-
mp-29792	$Ca_4Nb_8O_{16}$	28	*Pbcm* (57)	$CaNb_2O_4$	317	170	0	0	False	-
mp-768342	$Nb_8Sr_4O_{16}$	28	*Pbcm* (57)	$CaNb_2O_4$	332	153	0	0	False	-
mp-18099	$Ba_4F_{20}Sb_4$	28	*Pbcm* (57)	$BaSbF_5$	316	150	0	0	False	-
mp-669528	$Na_8U_4O_{16}$	28	*Pccn* (56)	Na_2UO_4	383	152	0	0	False	-
mp-531523	$Ga_{10}Li_2O_{16}$	28	*Pmna* (53)	$LiGa_5O_8$	295	202	0	0	False	-
mp-752573	$Ba_8Na_{16}Si_{32}$	28	*Ima*2 (46)	$Ba(NaSi_2)_2$	43	122	87	0	False	-
mp-558807	$Ag_4Zn_4P_4S_{16}$	28	$Pna2_1$ (33)	$ZnAgPS_4$	339	111	0	19	False	-
mp-28471	$Li_{12}As_4S_{12}$	28	$Pna2_1$ (33)	Li_3AsS_3	337	179	0	17	False	-
mp-753520	$Bi_4Li_{12}S_{12}$	28	$Pna2_1$ (33)	Li_3AsS_3	338	182	0	18	False	-
mp-6057	$Sn_4Tl_4P_4S_{16}$	28	$Pna2_1$ (33)	$KBaPS_4$	342	151	0	18	False	-
mp-644097	$Al_4Ba_4H_{20}$	28	$Pna2_1$ (33)	$BaAlH_5$	339	150	0	19	False	-
mp-568450	$Al_4Sr_4H_{20}$	28	$P2_12_12_1$ (19)	$SrAlH_5$	378	43	83	21	False	-
mp-16911	Rb_8S_{20}	28	$P2_12_12_1$ (19)	Rb_2S_5	378	77	109	21	True	1.16
mp-620372	Rb_8Se_{20}	28	$P2_12_12_1$ (19)	Rb_2S_5	378	45	112	21	False	-
mp-17691	$Cu_{12}S_{12}Sb_4$	28	$P2_12_12_1$ (19)	Cu_3SbS_3	378	34	109	21	False	-
mp-16972	$Cs_4Er_4S_{16}Si_4$	28	$P2_12_12_1$ (19)	$CsErSiS_4$	380	61	114	21	True	1.89
mp-556979	$Cs_4Sm_4Ge_4S_{16}$	28	$P2_12_12_1$ (19)	$CsErSiS_4$	378	69	104	21	False	-
mp-561635	$Cs_4Sm_4S_{16}Si_4$	28	$P2_12_12_1$ (19)	$CsErSiS_4$	379	61	111	21	True	1.53
mp-567873	$Rb_4Sm_4Ge_4Se_{16}$	28	$P2_12_12_1$ (19)	$CsErSiS_4$	379	49	127	21	False	-
mp-16934	$Al_4Ba_4La_4O_{16}$	28	$P2_12_12_1$ (19)	$BaLaAlO_4$	378	34	137	21	False	-
mp-541280	$Ba_4Ga_4La_4O_{16}$	28	$P2_12_12_1$ (19)	$BaLaAlO_4$	378	27	151	21	False	-
mp-541281	$Ba_4Ga_4Nd_4O_{16}$	28	$P2_12_12_1$ (19)	$BaLaAlO_4$	378	40	143	21	False	-
mp-985293	$Al_4H_{12}O_{12}$	28	$P2_12_12_1$ (19)	$Al(HO)_3$	378	38	127	21	True	34.2
mp-561467	$Ag_{12}Y_4P_8S_{32}$	28	$C2/c$ (15)	$YAg_3(PS_4)_2$	0	154	0	0	False	-
mp-554641	$Mg_4Sr_{12}O_{32}Si_8$	28	$C2/c$ (15)	$Sr_3Mg(SiO_4)_2$	3	243	0	0	False	-
mp-570536	$Ag_4Au_{12}Rb_8I_{32}$	28	$C2/c$ (15)	$Rb_2AgAu_3I_8$	1	202	0	0	False	-
mp-28079	$Mn_{16}Na_{16}S_{24}$	28	$C2/c$ (15)	$Na_2Mn_2S_3$	1	193	0	0	False	-
mp-697331	$Mg_{16}Ni_8H_{32}$	28	$C2/c$ (15)	Mg_2NiH_4	2	159	0	0	False	-
mp-23090	$La_{12}I_4S_{32}Si_8$	28	$C2/c$ (15)	$La_3Si_2S_8$	1	156	0	0	False	-
mp-554826	$Pr_{12}Cl_4O_{32}Si_8$	28	$C2/c$ (15)	$La_3Si_2S_8$	2	170	0	0	False	-
mp-555527	$Sm_{12}Br_4S_{32}Si_8$	28	$C2/c$ (15)	$La_3Si_2S_8$	0	180	0	0	False	-
mp-558259	$Pr_{12}I_4S_{32}Si_8$	28	$C2/c$ (15)	$La_3Si_2S_8$	0	163	0	0	False	-
mp-558985	$La_{12}Cl_4O_{32}Si_8$	28	$C2/c$ (15)	$La_3Si_2S_8$	1	188	0	0	False	-
mp-560356	$Sm_{12}I_4S_{32}Si_8$	28	$C2/c$ (15)	$La_3Si_2S_8$	0	156	0	0	False	-
mp-560830	$Nd_{12}Cl_4O_{32}Si_8$	28	$C2/c$ (15)	$La_3Si_2S_8$	3	166	0	0	False	-
mp-560853	$Tb_{12}I_4S_{32}Si_8$	28	$C2/c$ (15)	$La_3Si_2S_8$	0	183	0	0	False	-
mp-561126	$Nd_{12}I_4S_{32}Si_8$	28	$C2/c$ (15)	$La_3Si_2S_8$	2	150	0	0	False	-
mp-27331	$K_{24}Ge_8Te_{24}$	28	$C2/c$ (15)	K_3GeTe_3	2	207	0	0	False	-
mp-607806	$Ho_{24}N_8Se_{24}$	28	$C2/c$ (15)	Ho_3Se_3	2	223	0	0	False	-
mp-28579	$Hg_{24}I_{16}Te_{16}$	28	$C2/c$ (15)	$Hg_3(TeI)_2$	1	147	0	0	False	-
mp-555082	$Cs_4Li_{20}B_8O_{24}$	28	$C2/c$ (15)	$CsLi_5(BO_3)_2$	1	125	0	0	True	3.59
mp-561620	$Ag_{24}As_8S_{24}$	28	$C2/c$ (15)	Ag_3AsS_3	1	175	0	0	False	-
mp-554351	$Hg_8Sr_4Cl_8O_8$	28	$P2_1/c$ (14)	$SrHg_2(ClO)_2$	212	247	0	0	False	-
mp-643460	$Hg_8Sn_4Br_8S_8$	28	$P2_1/c$ (14)	$SrHg_2(ClO)_2$	213	258	0	0	False	-

ID	Formula	Z	SP (*No.*)	Prototype	NL	NR	WPs	HDWPs	Clean	dE/dq
mp-8947	$Sr_4B_8S_{16}$	28	$P2_1/c$ (14)	$Sr(BS_2)_2$	210	186	0	0	True	2.6
mp-560051	$Bi_4Rb_4S_{16}Si_4$	28	$P2_1/c$ (14)	$RbGeBiS_4$	217	247	0	0	False	-
mp-866651	$Bi_4K_4S_{16}Si_4$	28	$P2_1/c$ (14)	$RbGeBiS_4$	217	266	0	0	False	-
mp-849527	$In_4Rb_{12}O_{12}$	28	$P2_1/c$ (14)	Rb_3InO_3	214	292	0	0	False	-
mp-29977	$Rb_{12}B_4S_{12}$	28	$P2_1/c$ (14)	Rb_3BS_3	213	288	0	0	True	2.04
mp-30222	$Cs_{12}B_4S_{12}$	28	$P2_1/c$ (14)	Rb_3BS_3	213	273	0	0	False	-
mp-18510	$Sr_8O_{16}Si_4$	28	$P2_1/c$ (14)	Rb_2WS_4	211	295	0	0	True	2.04
mp-752730	$Ba_8Ge_4O_{16}$	28	$P2_1/c$ (14)	Rb_2WS_4	213	310	0	0	True	1.1
mp-28860	P_8Se_{20}	28	$P2_1/c$ (14)	P_2Se_5	214	192	0	0	False	-
mp-16930	$Na_{12}Ge_4Te_{12}$	28	$P2_1/c$ (14)	Na_3GeTe_3	214	237	0	0	False	-
mp-17291	$Na_{12}Si_4Te_{12}$	28	$P2_1/c$ (14)	Na_3GeTe_3	215	218	0	0	True	1.94
mp-18411	$Na_{12}Ge_4Se_{12}$	28	$P2_1/c$ (14)	Na_3GeTe_3	210	259	0	0	False	-
mp-540619	$K_{12}Sn_4Te_{12}$	28	$P2_1/c$ (14)	Na_3GeTe_3	214	259	0	0	False	-
mp-540923	$Ba_{12}Sn_4P_{12}$	28	$P2_1/c$ (14)	Na_3GeTe_3	215	240	0	0	False	-
mp-541875	$K_{12}Sn_4Se_{12}$	28	$P2_1/c$ (14)	Na_3GeTe_3	214	280	0	0	False	-
mp-18165	$Ga_4Na_{12}S_{12}$	28	$P2_1/c$ (14)	Na_3GaS_3	214	346	0	0	False	-
mp-9680	$Al_4Na_{12}Te_{12}$	28	$P2_1/c$ (14)	Na_3GaS_3	215	267	0	0	False	-
mp-30975	$Na_{12}B_4O_{12}$	28	$P2_1/c$ (14)	Na_3BO_3	210	340	0	0	False	-
mp-3855	$Be_4Na_8F_{16}$	28	$P2_1/c$ (14)	Na_2BeF_4	212	252	0	0	False	-
mp-558941	$Li_4Y_4O_{16}Si_4$	28	$P2_1/c$ (14)	$LiYSiO_4$	215	271	0	0	False	-
mp-559820	$La_4Cl_4O_{16}S_4$	28	$P2_1/c$ (14)	$LaSClO_4$	213	223	0	0	False	-
mp-18217	$Al_4K_{12}Se_{12}$	28	$P2_1/c$ (14)	K_3AlSe_3	215	269	0	0	False	-
mp-18378	$Al_4K_{12}Te_{12}$	28	$P2_1/c$ (14)	K_3AlSe_3	211	239	0	0	False	-
mp-541686	$Ga_4K_{12}S_{12}$	28	$P2_1/c$ (14)	K_3AlSe_3	211	238	0	0	False	-
mp-541687	$Ga_4K_{12}Se_{12}$	28	$P2_1/c$ (14)	K_3AlSe_3	211	238	0	0	False	-
mp-28126	$K_8Ge_4Te_{16}$	28	$P2_1/c$ (14)	K_2GeTe_4	87	193	0	0	True	3.16
mp-554343	$Cu_4K_8S_{12}Sb_4$	28	$P2_1/c$ (14)	K_2CuSbS_3	212	257	0	0	False	-
mp-555871	$Cu_4Na_8S_{12}Sb_4$	28	$P2_1/c$ (14)	K_2CuSbS_3	213	237	0	0	False	-
mp-560370	$Pb_8Ge_4S_{16}$	28	$P2_1/c$ (14)	$Ge(PbS_2)_2$	213	227	0	0	False	-
mp-866615	$Ag_8Cs_4As_4S_{12}$	28	$P2_1/c$ (14)	$CsAg_2AsS_3$	211	182	0	0	False	-
mp-16795	$Al_4Ca_4F_{20}$	28	$P2_1/c$ (14)	$CaAlF_5$	213	293	0	0	False	-
mp-554263	$Ca_8B_4Cl_4O_{12}$	28	$P2_1/c$ (14)	Ca_2BClO_3	213	291	0	0	True	1.61
mp-3397	$Ba_8Ti_4O_{16}$	28	$P2_1/c$ (14)	Ba_2TiO_4	210	313	0	0	False	-
mp-4180	$Ca_8O_{16}Si_4$	28	$P2_1/c$ (14)	Ba_2TiO_4	211	322	0	0	False	-
mp-542085	$Sr_8Ge_4O_{16}$	28	$P2_1/c$ (14)	Ba_2TiO_4	212	258	0	0	False	-
mp-555269	$Ag_{12}S_{12}Sb_4$	28	$P2_1/c$ (14)	Ag_3SbS_3	212	237	0	0	False	-
mp-16839	$Na_{24}W_8N_{24}$	28	Cc (9)	Na_3WN_3	0	112	0	0	False	-
mp-542200	$Ag_{16}Cd_4Ge_8S_{28}$	28	Cc (9)	$CdAg_4Ge_2S_7$	1	46	0	0	False	-
mp-3926	$Ag_{14}Nb_2S_{12}$	28	Pc (7)	$NbAg_7S_6$	93	190	0	0	False	-
mp-560847	$Nb_4Tl_2P_2S_{20}$	28	Pc (7)	Nb_2TlPS_{10}	87	133	0	0	True	2.67
mp-674465	$K_8Sr_4Cl_{16}$	28	Pc (7)	K_2SrCl_4	91	376	0	0	False	-
mp-29187	$Co_4P_{12}Si_{12}$	28	$P2_1$ (4)	$Co(SiP)_3$	126	34	137	0	False	-
mp-23738	$Mg_6Rb_6H_{18}$	30	$P6_3/mmc$ (194)	$RbMgH_3$	37	173	0	0	False	-
mp-29019	$Rb_{18}I_6O_6$	30	$P6_3/mmc$ (194)	$RbMgH_3$	44	193	0	0	False	-
mp-4975	$Rb_6Zn_6F_{18}$	30	$P6_3/mmc$ (194)	$RbMgH_3$	38	263	0	0	False	-
mp-541813	$Mg_6Rb_6F_{18}$	30	$P6_3/mmc$ (194)	$RbMgH_3$	41	228	0	0	False	-
mp-619015	$Cd_6Cs_6Cl_{18}$	30	$P6_3/mmc$ (194)	$RbMgH_3$	38	265	0	0	False	-
mp-769398	$Na_{18}I_6O_6$	30	$P6_3/mmc$ (194)	$RbMgH_3$	38	222	0	0	False	-
mp-16978	$Ba_6Mg_2O_{18}Sb_4$	30	$P6_3/mmc$ (194)	$Ba_3MgSb_2O_9$	36	224	0	0	False	-
mp-29090	$Au_{12}Rb_8S_{10}$	30	P−62c (190)	$Rb_4Au_6S_5$	179	101	0	2	False	-
mp-29341	$Au_{12}K_8S_{10}$	30	P−62c (190)	$Rb_4Au_6S_5$	170	129	0	7	False	-
mp-17853	$Ga_6Y_6O_{18}$	30	$P6_3cm$ (185)	$YGaO_3$	49	309	1	17	False	-
mp-504607	$In_6Y_6O_{18}$	30	$P6_3cm$ (185)	$YGaO_3$	47	310	2	24	False	-
mp-768149	$Dy_6In_6O_{18}$	30	$P6_3cm$ (185)	$YGaO_3$	45	310	2	25	False	-
mp-768267	$Ho_6In_6O_{18}$	30	$P6_3cm$ (185)	$YGaO_3$	47	289	1	17	False	-
mp-768469	$Ga_6Tm_6O_{18}$	30	$P6_3cm$ (185)	$YGaO_3$	49	328	1	26	False	-
mp-768505	$Ga_6Lu_6O_{18}$	30	$P6_3cm$ (185)	$YGaO_3$	51	325	1	14	False	-
mp-769007	$Sc_6Y_6O_{18}$	30	$P6_3cm$ (185)	$YGaO_3$	41	345	4	14	False	-
mp-769015	$Dy_6Ga_6O_{18}$	30	$P6_3cm$ (185)	$YGaO_3$	47	314	3	26	False	-
mp-769045	$Ga_6Ho_6O_{18}$	30	$P6_3cm$ (185)	$YGaO_3$	49	306	2	23	False	-
mp-769079	$Ga_6Sc_6O_{18}$	30	$P6_3cm$ (185)	$YGaO_3$	47	368	0	20	False	-
mp-772180	$Lu_6Sc_6O_{18}$	30	$P6_3cm$ (185)	$YGaO_3$	42	311	1	20	False	-

ID	Formula	Z	SP (*No.*)	Prototype	NL	NR	WPs	HDWPs	Clean	dE/dq
mp-772217	$Er_6In_6O_{18}$	30	$P6_3cm$ (185)	$YGaO_3$	135	346	0	29	False	-
mp-28548	$Li_{16}Ge_2O_{12}$	30	$P6_3cm$ (185)	Li_8GeO_6	44	293	4	24	False	-
mp-28549	$Li_{16}O_{12}Si_2$	30	$P6_3cm$ (185)	Li_8GeO_6	40	293	4	17	True	2.82
mp-753546	$Li_{16}Ti_2S_{12}$	30	$P6_3cm$ (185)	Li_8GeO_6	36	267	2	25	False	-
mp-761931	$Na_{16}Sn_2O_{12}$	30	$P6_3cm$ (185)	Li_8GeO_6	44	325	8	22	False	-
mp-769294	$Rb_{16}Sn_2O_{12}$	30	$P6_3cm$ (185)	Li_8GeO_6	36	307	1	19	False	-
mp-772521	$Li_{16}Ti_2O_{12}$	30	$P6_3cm$ (185)	Li_8GeO_6	49	289	1	20	False	-
mp-17037	$Rb_4Ti_2O_{18}Si_6$	30	$P6_3/m$ (176)	$Rb_2Ti(SiO_3)_3$	43	57	0	0	False	-
mp-17382	$Rb_4Sn_2O_{18}Si_6$	30	$P6_3/m$ (176)	$Rb_2Ti(SiO_3)_3$	41	51	0	0	True	1.3
mp-540707	$Cs_4Sn_2Ge_6O_{18}$	30	$P6_3/m$ (176)	$Rb_2Ti(SiO_3)_3$	48	47	0	0	False	-
mp-6694	$Cs_4Zr_2O_{18}Si_6$	30	$P6_3/m$ (176)	$Rb_2Ti(SiO_3)_3$	36	52	0	0	False	-
mp-8380	$K_4O_{18}Si_8$	30	$P6_3/m$ (176)	$K_2Si_4O_9$	178	81	0	0	False	-
mp-540949	$Ba_6Nb_4Sr_2O_{18}$	30	$P6_3/m$ (176)	$Ba_3MgSb_2O_9$	36	46	0	0	False	-
mp-540950	$Ba_6Sr_2Ta_4O_{18}$	30	$P6_3/m$ (176)	$Ba_3MgSb_2O_9$	37	59	0	0	False	-
mp-504482	$Al_6In_6S_{18}$	30	$P6_1$ (169)	$AlInS_3$	133	28	77	0	True	1.17
mp-504951	$Ga_6In_6S_{18}$	30	$P6_1$ (169)	$AlInS_3$	135	17	93	0	False	-
mp-504952	$Ga_6In_6Se_{18}$	30	$P6_1$ (169)	$AlInS_3$	135	22	90	0	False	-
mp-862787	$Al_6In_6Se_{18}$	30	$P6_1$ (169)	$AlInS_3$	133	30	90	1	False	-
mp-2654	$Al_{12}S_{18}$	30	$P6_1$ (169)	Al_2S_3	134	18	104	0	False	-
mp-612740	$In_{12}Se_{18}$	30	$P6_1$ (169)	Al_2S_3	133	26	90	0	False	-
mp-29450	$Cu_{18}I_{18}Se_{54}$	30	$R{-}3m$ (166)	$CuSe_3$	65	140	0	0	False	-
mp-17481	$Mg_8Ta_4O_{18}$	30	$P{-}3c1$ (165)	$Mg_4Ta_2O_9$	169	295	0	0	False	-
mp-17808	$Nb_4Zn_8O_{18}$	30	$P{-}3c1$ (165)	$Mg_4Ta_2O_9$	171	288	0	0	False	-
mp-17841	$Mg_8Nb_4O_{18}$	30	$P{-}3c1$ (165)	$Mg_4Ta_2O_9$	168	325	0	0	False	-
mp-762258	$Na_4Sn_8O_{18}$	30	$P{-}3c1$ (165)	$Mg_4Ta_2O_9$	171	260	0	0	False	-
mp-769268	$Sr_8Ta_4O_{18}$	30	$P{-}3c1$ (165)	$Mg_4Ta_2O_9$	175	243	0	0	False	-
mp-769307	$Ca_8Ta_4O_{18}$	30	$P{-}3c1$ (165)	$Mg_4Ta_2O_9$	174	243	0	0	False	-
mp-772151	$Li_4Ti_8O_{18}$	30	$P{-}3c1$ (165)	$Mg_4Ta_2O_9$	174	320	0	0	False	-
mp-556996	$Al_3K_6Li_3F_{18}$	30	$P{-}3m1$ (164)	K_2LiAlF_6	81	355	0	0	False	-
mp-580202	$Pb_{10}I_{20}$	30	$P3m1$ (156)	PbI_2	50	329	6	0	False	-
mp-556458	$Ba_3Ca_3C_6O_{18}$	30	$P321$ (150)	$BaCa(CO_3)_2$	1	11	258	0	False	-
mp-571164	$Ag_2Rb_6Sn_6Se_{16}$	30	$P4/nbm$ (125)	$Rb_3AgSn_3Se_8$	135	295	0	0	False	-
mp-571594	$Ag_2K_6Sn_6Se_{16}$	30	$P4/nbm$ (125)	$Rb_3AgSn_3Se_8$	135	271	0	0	True	1.31
mp-628185	$K_6Na_2Sn_6Se_{16}$	30	$P4/nbm$ (125)	$Rb_3AgSn_3Se_8$	135	292	0	0	False	-
mp-17905	$Cs_4Hg_{12}S_{14}$	30	$P4_2nm$ (102)	$Cs_2Hg_6S_7$	136	186	0	0	False	-
mp-28021	$Sr_{10}Br_{16}Cl_4$	30	$P4/n$ (85)	$Sr_5(Br_4Cl)_2$	47	94	0	0	False	-
mp-556228	$Ca_8Na_{10}F_2O_{32}P_8$	30	$I{-}4$ (82)	$Na_5Ca_4P_4O_{16}$	0	2	71	0	False	-
mp-557696	$Na_{10}Y_8F_2O_{32}Si_8$	30	$I{-}4$ (82)	$Na_5Ca_4P_4O_{16}$	0	0	79	0	False	-
mp-866662	$K_{10}La_8F_2O_{32}Si_8$	30	$I{-}4$ (82)	$Na_5Ca_4P_4O_{16}$	0	3	60	0	False	-
mp-568803	$Mg_4B_{48}C_8$	30	$Imma$ (74)	$Mg(B_6C)_2$	46	509	0	0	False	-
mp-558121	$Ba_4K_{16}V_8S_{32}$	30	$Ibam$ (72)	$K_4Ba(VS_4)_2$	91	318	0	0	False	-
mp-4971	$K_{16}Sn_{12}Se_{32}$	30	$Ccce$ (68)	$K_4Sn_3Se_8$	179	237	0	0	False	-
mp-765970	$Sn_{14}Ti_6O_{40}$	30	$Cmmm$ (65)	$Ti_3Sn_7O_{20}$	0	554	0	0	False	-
mp-29205	$Y_{12}B_{12}O_{36}$	30	$Cmcm$ (63)	YBO_3	151	186	0	0	True	1.59
mp-752713	$Ho_{12}B_{12}O_{36}$	30	$Cmcm$ (63)	YBO_3	145	166	0	0	False	-
mp-768518	$Al_{12}La_{12}O_{36}$	30	$Cmcm$ (63)	YBO_3	144	216	0	0	False	-
mp-977148	$Be_{12}Li_{12}H_{36}$	30	$Cmcm$ (63)	YBO_3	151	191	0	0	True	1.13
mp-571020	$Cs_6Mg_6H_{18}$	30	$Pmmn$ (59)	$CsMgH_3$	315	199	0	0	True	50.16
mp-23426	$Al_8Bi_4O_{18}$	30	$Pbam$ (55)	$Al_4Bi_2O_9$	318	189	0	0	False	-
mp-17792	$Hg_{12}K_8Ge_8S_{32}$	30	$Aea2$ (41)	$K_2Hg_3(GeS_4)_2$	1	93	0	0	False	-
mp-757375	$Sn_{12}Ti_8O_{40}$	30	$Cmm2$ (35)	$Ti_2Sn_3O_{10}$	0	639	36	0	False	-
mp-13872	$Cu_{16}S_{12}Si_2$	30	$Pmn2_1$ (31)	Cu_8SiS_6	315	166	0	0	False	-
mp-9711	$Ba_4N_{16}Si_{10}$	30	$Pmn2_1$ (31)	$Ba_2Si_5N_8$	315	206	0	0	False	-
mp-17464	$Sr_{12}Ge_{12}O_{36}$	30	$C2/c$ (15)	$SrGeO_3$	2	244	0	0	False	-
mp-17552	$Ca_{12}O_{36}Si_{12}$	30	$C2/c$ (15)	$SrGeO_3$	2	240	0	0	False	-
mp-3978	$Sr_{12}O_{36}Si_{12}$	30	$C2/c$ (15)	$SrGeO_3$	2	207	0	0	False	-
mp-776074	$Ca_{12}Ge_{12}O_{36}$	30	$C2/c$ (15)	$SrGeO_3$	1	258	0	0	False	-
mp-776084	$Ba_{12}O_{36}Si_{12}$	30	$C2/c$ (15)	$SrGeO_3$	0	164	0	0	False	-
mp-16961	$Na_{24}Se_{28}Si_8$	30	$C2/c$ (15)	$Na_6Si_2Se_7$	2	181	0	0	False	-
mp-18100	$Na_{24}Ge_8Se_{28}$	30	$C2/c$ (15)	$Na_6Si_2Se_7$	6	203	0	0	False	-
mp-27752	$Na_{24}Ge_8S_{28}$	30	$C2/c$ (15)	$Na_6Si_2Se_7$	3	192	0	0	False	-
mp-31033	$Hf_{12}F_{48}$	30	$C2/c$ (15)	HfF_4	1	178	0	0	False	-

ID	Formula	Z	SP (*No.*)	Prototype	NL	NR	WPs	HDWPs	Clean	dE/dq
mp-27164	Fe_6P_{24}	30	$P2_1/c$ (14)	FeP_4	226	297	0	0	False	-
mp-18315	$Cs_{12}O_{14}Si_4$	30	$P2_1/c$ (14)	$Cs_6Si_2O_7$	229	260	0	0	False	-
mp-18483	$Rb_{12}O_{14}Si_4$	30	$P2_1/c$ (14)	$Cs_6Si_2O_7$	230	287	0	0	True	1.43
mp-30990	$K_{12}O_{14}Si_4$	30	$P2_1/c$ (14)	$Cs_6Si_2O_7$	227	262	0	0	False	-
mp-770408	$Rb_{12}Ti_4O_{14}$	30	$P2_1/c$ (14)	$Cs_6Si_2O_7$	228	296	0	0	False	-
mp-866688	$Cs_8Pd_2P_4Se_{16}$	30	$P2_1/c$ (14)	$Cs_4P_2PdSe_8$	226	216	0	0	False	-
mp-571235	Zr_6I_{24}	30	$P2/c$ (13)	ZrI_4	96	225	0	0	False	-
mp-778364	$Na_{12}Ti_4O_{14}$	30	$P2/c$ (13)	$Na_6Ti_2O_7$	95	494	0	0	False	-
mp-733604	$Ba_8Mg_{12}H_{40}$	30	$C2/m$ (12)	$Ba_2Mg_3H_{10}$	1	127	0	0	False	-
mp-28922	$Ca_8N_{32}Si_{20}$	30	Cc (9)	$Ca_2Si_5N_8$	1	75	0	0	False	-
mp-555191	$La_6Cl_6O_{14}Si_4$	30	$P2_1$ (4)	$La_3Si_2Cl_3O_7$	135	42	51	0	False	-
mp-559391	$Pr_6Cl_6O_{14}Si_4$	30	$P2_1$ (4)	$La_3Si_2Cl_3O_7$	135	38	47	0	False	-
mp-558539	$Ba_8Cs_8C_{12}O_{36}$	32	$I2_13$ (199)	$Cs_2Ba_2(CO_3)_3$	0	9	291	45	True	5.27
mp-866617	$Cs_8Sr_8C_{12}O_{36}$	32	$I2_13$ (199)	$Cs_2Ba_2(CO_3)_3$	3	8	275	45	True	1.6
mp-984332	$Rb_8Sr_8C_{12}O_{36}$	32	$I2_13$ (199)	$Cs_2Ba_2(CO_3)_3$	1	7	298	45	False	-
mp-643655	$Al_4Li_2Cl_2H_{12}O_{12}$	32	$P6_3/mcm$ (193)	$LiAl_2H_6ClO_6$	41	209	0	0	True	12.73
mp-3079	$Ca_2Ta_8O_{22}$	32	$P6_322$ (182)	$CaTa_4O_{11}$	0	14	300	0	False	-
mp-2956	$Co_{24}Ge_{36}S_{36}$	32	$R{-}3$ (148)	$Co_2(GeS)_3$	63	40	0	0	False	-
mp-2994	$Co_{24}Ge_{36}Te_{36}$	32	$R{-}3$ (148)	$Co_2(GeS)_3$	65	71	0	0	False	-
mp-5094	$Co_{24}Ge_{36}Se_{36}$	32	$R{-}3$ (148)	$Co_2(GeS)_3$	64	52	0	0	False	-
mp-866481	$Co_{24}Sn_{36}Te_{36}$	32	$R{-}3$ (148)	$Co_2(GeS)_3$	64	85	0	0	False	-
mp-975924	$Ir_{24}Ge_{36}Se_{36}$	32	$R{-}3$ (148)	$Co_2(GeS)_3$	54	76	0	0	False	-
mp-976401	$Rh_{24}Ge_{36}Se_{36}$	32	$R{-}3$ (148)	$Co_2(GeS)_3$	64	71	0	0	False	-
mp-866313	$Ba_{18}In_6B_{18}O_{54}$	32	$R{-}3$ (148)	$Ba_3In(BO_3)_3$	61	65	0	0	False	-
mp-11054	$Rb_{32}Sn_{32}$	32	$I4_1/acd$ (142)	RbSn	195	288	0	0	False	-
mp-11055	$Cs_{32}Sn_{32}$	32	$I4_1/acd$ (142)	RbSn	193	331	0	0	False	-
mp-21525	$Pb_{32}Rb_{32}$	32	$I4_1/acd$ (142)	RbSn	192	290	0	0	False	-
mp-542374	$K_{32}Sn_{32}$	32	$I4_1/acd$ (142)	RbSn	192	287	0	0	False	-
mp-866482	$Cs_{32}Si_{32}$	32	$I4_1/acd$ (142)	RbSn	193	299	0	0	True	1.31
mp-28920	$Tl_{20}Cl_4Se_8$	32	$P4/ncc$ (130)	Tl_5Se_2	293	296	0	0	False	-
mp-557304	$Sr_{16}Cl_{16}O_{24}Si_8$	32	$I4/m$ (87)	$Ca_2SiCl_2O_3$	0	241	0	0	False	-
mp-556364	$Ca_{16}Cl_{16}O_{24}Si_8$	32	$I4$ (79)	$Ca_2SiCl_2O_3$	2	13	86	0	False	-
mp-569128	$B_{48}Si_{16}$	32	$Imma$ (74)	SiB_3	50	458	0	0	False	-
mp-978275	Mg_8B_{56}	32	$Imma$ (74)	MgB_7	48	506	0	0	False	-
mp-655591	$Li_4B_{52}C_8$	32	$Imma$ (74)	$LiB_{13}C_2$	48	513	0	0	False	-
mp-8204	$Al_4Li_4B_{56}$	32	$Imma$ (74)	$LiAlB_{14}$	48	525	0	0	False	-
mp-30129	$Li_{32}Ta_8N_{24}$	32	$Ibca$ (73)	Li_4TaN_3	144	452	0	0	False	-
mp-973391	$Li_8B_{48}Si_8$	32	$Cmce$ (64)	$LiSiB_6$	210	226	0	0	False	-
mp-583615	$In_{16}K_{24}As_{24}$	32	$Cmce$ (64)	$K_3In_2As_3$	206	280	0	0	False	-
mp-752579	$Ti_4Tm_8O_{20}$	32	$Pnma$ (62)	Y_2TiO_5	463	216	0	0	False	-
mp-768865	$Lu_8Ge_4O_{20}$	32	$Pnma$ (62)	Y_2TiO_5	463	230	0	0	False	-
mp-770379	$Tm_8Ge_4O_{20}$	32	$Pnma$ (62)	Y_2TiO_5	461	246	0	0	False	-
mp-771031	$Lu_8Ti_4O_{20}$	32	$Pnma$ (62)	Y_2TiO_5	463	216	0	0	False	-
mp-772833	$Hf_4Y_8O_{20}$	32	$Pnma$ (62)	Y_2TiO_5	460	261	0	0	False	-
mp-16919	$Hf_4Y_8S_{20}$	32	$Pnma$ (62)	Y_2HfS_5	457	192	0	0	False	-
mp-28275	$Pr_4Rb_8Cl_{20}$	32	$Pnma$ (62)	Y_2HfS_5	458	159	0	0	False	-
mp-28276	$K_8Pr_4Br_{20}$	32	$Pnma$ (62)	Y_2HfS_5	458	224	0	0	False	-
mp-28277	$K_8Pr_4I_{20}$	32	$Pnma$ (62)	Y_2HfS_5	460	203	0	0	False	-
mp-569967	$K_8Pr_4Cl_{20}$	32	$Pnma$ (62)	Y_2HfS_5	459	203	0	0	False	-
mp-3075	$Tl_8Ge_8S_{16}$	32	$Pnma$ (62)	$TlGeS_2$	458	152	0	0	False	-
mp-16848	$Tl_{12}P_4S_{16}$	32	$Pnma$ (62)	Tl_3PS_4	458	150	0	0	False	-
mp-16863	$Rb_{12}P_4S_{16}$	32	$Pnma$ (62)	Tl_3PS_4	463	213	0	0	False	-
mp-17154	$Rb_{12}S_{16}Sb_4$	32	$Pnma$ (62)	Tl_3PS_4	454	164	0	0	False	-
mp-17220	$Rb_{12}Ta_4S_{16}$	32	$Pnma$ (62)	Tl_3PS_4	459	223	0	0	False	-
mp-17283	$K_{12}Nb_4Se_{16}$	32	$Pnma$ (62)	Tl_3PS_4	457	182	0	0	False	-
mp-17638	$Rb_{12}Sb_4Se_{16}$	32	$Pnma$ (62)	Tl_3PS_4	454	194	0	0	False	-
mp-17989	$K_{12}P_4S_{16}$	32	$Pnma$ (62)	Tl_3PS_4	457	193	0	0	False	-
mp-18148	$K_{12}Ta_4S_{16}$	32	$Pnma$ (62)	Tl_3PS_4	453	137	0	0	True	2.44
mp-18305	$Rb_{12}As_4Se_{16}$	32	$Pnma$ (62)	Tl_3PS_4	457	191	0	0	True	5.55
mp-18383	$K_{12}Nb_4S_{16}$	32	$Pnma$ (62)	Tl_3PS_4	457	192	0	0	False	-
mp-31313	$K_{12}P_4Se_{16}$	32	$Pnma$ (62)	Tl_3PS_4	457	234	0	0	False	-
mp-3529	$K_{12}V_4S_{16}$	32	$Pnma$ (62)	Tl_3PS_4	456	188	0	0	True	1.41

ID	Formula	Z	SP (*No.*)	Prototype	NL	NR	WPs	HDWPs	Clean	dE/dq
mp-3719	$Nb_4Rb_{12}S_{16}$	32	*Pnma* (62)	Tl_3PS_4	458	199	0	0	False	-
mp-3797	$K_{12}As_4S_{16}$	32	*Pnma* (62)	Tl_3PS_4	458	172	0	0	False	-
mp-4160	$Tl_{12}P_4Se_{16}$	32	*Pnma* (62)	Tl_3PS_4	460	192	0	0	False	-
mp-505721	$Rb_{12}V_4S_{16}$	32	*Pnma* (62)	Tl_3PS_4	455	187	0	0	False	-
mp-5704	$Tl_{12}As_4S_{16}$	32	*Pnma* (62)	Tl_3PS_4	462	149	0	0	False	-
mp-583193	$Cs_{12}P_4Se_{16}$	32	*Pnma* (62)	Tl_3PS_4	464	200	0	0	False	-
mp-614491	$Tl_{12}P_4Se_{16}$	32	*Pnma* (62)	Tl_3PS_4	458	171	0	0	False	-
mp-17057	$Sn_4Sr_8F_8Se_{12}$	32	*Pnma* (62)	$Sr_2SnSe_3F_2$	461	205	0	0	False	-
mp-17805	$Ba_8Sn_4F_8Se_{12}$	32	*Pnma* (62)	$Sr_2SnSe_3F_2$	459	188	0	0	False	-
mp-558170	$Rb_{12}O_{16}P_4$	32	*Pnma* (62)	Rb_3PO_4	462	180	0	0	False	-
mp-558160	$Na_4Y_4C_4F_8O_{12}$	32	*Pnma* (62)	$NaYCO_3F_2$	474	204	0	0	False	-
mp-6113	$Li_4Ti_4As_4O_{20}$	32	*Pnma* (62)	$LiTiAsO_5$	456	188	0	0	False	-
mp-6456	$Li_4Nb_4Ge_4O_{20}$	32	*Pnma* (62)	$LiTiAsO_5$	457	180	0	0	False	-
mp-2878	$Li_{12}O_{16}P_4$	32	*Pnma* (62)	Li_3PO_4	456	154	0	0	False	-
mp-557293	$Cd_4Li_8O_{16}Si_4$	32	*Pnma* (62)	Li_2CdSiO_4	456	126	0	0	False	-
mp-28316	$K_{12}Li_4Si_{16}$	32	*Pnma* (62)	K_3LiSi_4	456	186	0	0	True	3.52
mp-18177	$Ba_{12}Nb_4As_{12}O_4$	32	*Pnma* (62)	Ba_3NbAs_3	462	219	0	0	False	-
mp-18199	$Sr_{12}Ta_4As_{12}O_4$	32	*Pnma* (62)	Ba_3NbAs_3	459	174	0	0	False	-
mp-4934	$Al_8O_{20}Si_4$	32	*Pnma* (62)	Al_2SiO_5	457	214	0	0	False	-
mp-4753	$Al_8O_{20}Si_4$	32	*Pnnm* (58)	Al_2SiO_5	432	169	0	0	False	-
mp-17633	$Er_8O_{16}Se_4Si_4$	32	*Pbcm* (57)	Er_2SiSeO_4	362	224	0	0	False	-
mp-17750	$Pr_8O_{16}Si_4Te_4$	32	*Pbcm* (57)	Er_2SiSeO_4	359	207	0	0	False	-
mp-17834	$La_8O_{16}Se_4Si_4$	32	*Pbcm* (57)	Er_2SiSeO_4	360	187	0	0	False	-
mp-17913	$Tb_8O_{16}Se_4Si_4$	32	*Pbcm* (57)	Er_2SiSeO_4	360	220	0	0	False	-
mp-18219	$Dy_8O_{16}Se_4Si_4$	32	*Pbcm* (57)	Er_2SiSeO_4	360	232	0	0	False	-
mp-18466	$Nd_8O_{16}Se_4Si_4$	32	*Pbcm* (57)	Er_2SiSeO_4	360	209	0	0	False	-
mp-18542	$Nd_8O_{16}Si_4Te_4$	32	*Pbcm* (57)	Er_2SiSeO_4	360	213	0	0	False	-
mp-18584	$Ho_8O_{16}Se_4Si_4$	32	*Pbcm* (57)	Er_2SiSeO_4	361	222	0	0	False	-
mp-18610	$Sm_8O_{16}Se_4Si_4$	32	*Pbcm* (57)	Er_2SiSeO_4	357	221	0	0	True	4.2
mp-560595	$Ca_8As_4Cl_4O_{16}$	32	*Pbcm* (57)	Er_2SiSeO_4	362	303	0	0	False	-
mp-6055	$Sm_8O_{16}Si_4Te_4$	32	*Pbcm* (57)	Er_2SiSeO_4	360	211	0	0	False	-
mp-4279	$Ca_4Ge_8O_{20}$	32	*Pbam* (55)	$CaGe_2O_5$	338	262	0	0	False	-
mp-558188	$Ag_4Hg_8As_4O_{16}$	32	*Pbam* (55)	$AgHg_2AsO_4$	339	214	0	0	False	-
mp-30107	$Nb_8Rb_2Cl_{22}$	32	*Pmma* (51)	$RbNb_4Cl_{11}$	192	308	0	0	False	-
mp-582146	$Cs_2Nb_8Cl_{22}$	32	*Pmma* (51)	$RbNb_4Cl_{11}$	196	292	0	0	False	-
mp-638070	$Li_4B_{52}C_8$	32	*Imm2* (44)	$LiB_{13}C_2$	0	294	97	0	False	-
mp-505322	$Cs_4Cu_8Ta_4Te_{16}$	32	$Pna2_1$ (33)	$CsTa(CuTe_2)_2$	393	189	0	23	False	-
mp-17945	$Rb_4P_4Se_{24}$	32	$Pca2_1$ (29)	$RbPSe_6$	344	187	0	44	True	1.11
mp-18625	$K_4P_4Se_{24}$	32	$Pca2_1$ (29)	$RbPSe_6$	346	202	0	47	False	-
mp-16969	$Lu_{16}O_{40}Si_8$	32	$C2/c$ (15)	Lu_2SiO_5	0	201	0	0	False	-
mp-28351	$Dy_{16}Ge_8O_{40}$	32	$C2/c$ (15)	Lu_2SiO_5	0	200	0	0	False	-
mp-555983	$Er_{16}O_{40}Si_8$	32	$C2/c$ (15)	Lu_2SiO_5	1	182	0	0	False	-
mp-768388	$Lu_{16}Ge_8O_{40}$	32	$C2/c$ (15)	Lu_2SiO_5	0	186	0	0	False	-
mp-770409	$Tm_{16}Ge_8O_{40}$	32	$C2/c$ (15)	Lu_2SiO_5	0	195	0	0	False	-
mp-561095	$Ho_{12}Li_4F_8Ge_8O_{32}$	32	$C2/c$ (15)	$LiHo_3Ge_2(O_4F)_2$	3	232	0	0	False	-
mp-582161	$Ag_{12}K_{12}Ta_8Se_{32}$	32	$C2/c$ (15)	$K_3Ta_2Ag_3Se_8$	0	208	0	0	False	-
mp-557910	$Cd_{16}F_8O_{32}P_8$	32	$C2/c$ (15)	Cd_2PO_4	1	184	0	0	True	2.54
mp-553884	$Ba_{20}Br_{24}O_{16}Si_4$	32	$C2/c$ (15)	$Ba_5Si(Br_3O_2)_2$	2	214	0	0	True	3.66
mp-555087	$Ba_{20}Cl_{24}O_{16}Si_4$	32	$C2/c$ (15)	$Ba_5Si(Br_3O_2)_2$	3	223	0	0	True	2.1
mp-559571	$Zr_4B_8O_{20}$	32	$P2_1/c$ (14)	ZrB_2O_5	243	278	0	0	False	-
mp-559617	$Hf_4B_8O_{20}$	32	$P2_1/c$ (14)	ZrB_2O_5	244	298	0	0	False	-
mp-554420	$Y_8O_{20}Si_4$	32	$P2_1/c$ (14)	Y_2SiO_5	242	301	0	0	True	29.75
mp-752405	$Dy_8O_{20}Si_4$	32	$P2_1/c$ (14)	Y_2SiO_5	243	296	0	0	True	1.94
mp-4988	$Tl_8As_8S_{16}$	32	$P2_1/c$ (14)	$TlAsS_2$	241	238	0	0	False	-
mp-653561	$Pb_4Tl_8Ge_4S_{16}$	32	$P2_1/c$ (14)	Tl_2GePbS_4	241	258	0	0	False	-
mp-17282	$Sr_4Th_8Se_{20}$	32	$P2_1/c$ (14)	$SrTh_2Se_5$	245	409	0	0	False	-
mp-541722	$Ba_8Cs_4Br_{20}$	32	$P2_1/c$ (14)	$SrTh_2Se_5$	243	384	0	0	False	-
mp-568044	$K_4Sm_8Cl_{20}$	32	$P2_1/c$ (14)	$SrTh_2Se_5$	100	345	0	0	False	-
mp-677055	$Cu_8Sc_8Se_{16}$	32	$P2_1/c$ (14)	$ScCuSe_2$	243	420	0	0	False	-
mp-624200	$Re_{12}Br_4Se_{16}$	32	$P2_1/c$ (14)	Re_3Se_4	243	236	0	0	False	-
mp-652046	$Re_{12}Br_4S_{16}$	32	$P2_1/c$ (14)	Re_3Se_4	241	196	0	0	False	-
mp-557540	$Ag_4Rb_8S_{16}Sb_4$	32	$P2_1/c$ (14)	Rb_2AgSbS_4	241	273	0	0	False	-

ID	Formula	Z	SP (*No.*)	Prototype	NL	NR	WPs	HDWPs	Clean	dE/dq
mp-558241	$Ag_4Ta_4Tl_8S_{16}$	32	$P2_1/c$ (14)	Rb_2AgSbS_4	241	254	0	0	True	2.13
mp-29657	$Na_{16}Ge_{16}$	32	$P2_1/c$ (14)	NaGe	244	313	0	0	False	-
mp-18197	$Be_4Na_8Ge_4O_{16}$	32	$P2_1/c$ (14)	Na_2BeGeO_4	244	318	0	0	False	-
mp-556156	$Li_4U_4B_4O_{20}$	32	$P2_1/c$ (14)	$LiUBO_5$	242	233	0	0	True	2.63
mp-698205	$Li_4B_4H_8O_{16}$	32	$P2_1/c$ (14)	$LiB(HO_2)_2$	244	216	0	0	True	3.69
mp-11189	$Li_8Mg_4O_{16}Si_4$	32	$P2_1/c$ (14)	Li_2MgSiO_4	245	335	0	0	False	-
mp-17288	$Li_8Zn_4O_{16}Si_4$	32	$P2_1/c$ (14)	Li_2MgSiO_4	244	318	0	0	False	-
mp-200	La_4P_{28}	32	$P2_1/c$ (14)	LaP_7	242	247	0	0	False	-
mp-558724	$La_8S_{20}Si_4$	32	$P2_1/c$ (14)	La_2SiS_5	247	276	0	0	False	-
mp-622086	$La_8Ge_4S_{20}$	32	$P2_1/c$ (14)	La_2SiS_5	243	279	0	0	False	-
mp-556237	$K_4Li_4Y_4F_{20}$	32	$P2_1/c$ (14)	$KLiYF_5$	243	304	0	0	False	-
mp-31104	$Bi_{16}K_{16}$	32	$P2_1/c$ (14)	KBi	242	353	0	0	False	-
mp-554950	$Hg_{12}As_4S_{12}Sb_4$	32	$P2_1/c$ (14)	Hg_3SbAsS_3	103	247	0	0	False	-
mp-29566	$Cs_8Na_8Ge_{16}$	32	$P2_1/c$ (14)	$CsNaGe_2$	242	274	0	0	False	-
mp-581864	$Cs_8As_8Se_{16}$	32	$P2_1/c$ (14)	$CsAsSe_2$	246	291	0	0	False	-
mp-559957	$Sr_4Ti_4Ge_4O_{20}$	32	$P2_1/c$ (14)	$CaTiGeO_5$	244	400	0	0	False	-
mp-6899	$Be_4Ca_4F_4O_{16}P_4$	32	$P2_1/c$ (14)	$CaBePO_4$	242	298	0	0	False	-
mp-17956	$Ca_8U_4O_{20}$	32	$P2_1/c$ (14)	Ca_2UO_5	243	342	0	0	False	-
mp-18471	$Sr_8U_4O_{20}$	32	$P2_1/c$ (14)	Ca_2UO_5	243	298	0	0	False	-
mp-555414	$Ba_{12}O_4Sb_{16}$	32	$P2_1/c$ (14)	Ba_3Sb_4	246	301	0	0	False	-
mp-28456	$Os_6Sn_{30}O_{28}$	32	Cm (8)	$Sn_{15}Os_3O_{14}$	0	45	1	0	False	-
mp-643071	$Fe_3Mg_4Yb_4H_{22}$	33	P−43m (215)	$Yb_4Mg_4Fe_3H_{22}$	44	36	12	17	False	-
mp-644264	$Ca_4Fe_3Mg_4H_{22}$	33	P−43m (215)	$Yb_4Mg_4Fe_3H_{22}$	43	49	16	15	False	-
mp-17208	$K_4Li_{28}Zr_6O_{28}$	33	$Immm$ (71)	$K_2Li_{14}Zr_3O_{14}$	3	488	0	0	False	-
mp-504806	$K_4Li_{28}Pb_6O_{28}$	33	$Immm$ (71)	$K_2Li_{14}Zr_3O_{14}$	0	403	0	0	False	-
mp-504810	$Li_{28}Pb_6Rb_4O_{28}$	33	$Immm$ (71)	$K_2Li_{14}Zr_3O_{14}$	0	385	0	0	False	-
mp-770481	$Ta_{18}O_{50}P_2$	35	I−4 (82)	Ta_9PO_{25}	0	7	150	2	False	-
mp-20820	$Pb_4N_8O_{24}$	36	Pa−3 (205)	$Pb(NO_3)_2$	304	122	0	0	True	2.4
mp-4157	$Sr_4N_8O_{24}$	36	Pa−3 (205)	$Pb(NO_3)_2$	302	166	0	0	False	-
mp-4396	$Ba_4N_8O_{24}$	36	Pa−3 (205)	$Pb(NO_3)_2$	306	117	0	0	True	1.39
mp-771046	$Mg_4N_8O_{24}$	36	Pa−3 (205)	$Pb(NO_3)_2$	305	117	0	0	False	-
mp-772617	$Zn_4N_8O_{24}$	36	Pa−3 (205)	$Pb(NO_3)_2$	301	140	0	0	False	-
mp-541995	$La_6F_6O_{18}Si_6$	36	P−62c (190)	$LaSiO_3$	208	137	0	1	False	-
mp-29877	$K_6Re_3H_{27}$	36	P−62m (189)	K_2ReH_9	48	125	0	0	True	2.09
mp-560641	$Ba_{12}Cl_5F_{19}$	36	P−62m (189)	$Ba_{12}Cl_5F_{19}$	64	198	0	0	False	-
mp-24097	$Mg_6H_8O_{18}Si_4$	36	$P6_3cm$ (185)	$Mg_3Si_2H_4O_9$	45	465	5	16	True	11.91
mp-29462	$Ga_{36}Na_{12}Te_{60}$	36	$R32$ (155)	$NaGa_3Te_5$	0	1	247	0	False	-
mp-554540	$Li_{15}Re_3O_{18}$	36	$P3_112$ (151)	Li_5ReO_6	0	14	374	0	False	-
mp-3273	$O_{75}P_{18}Si_{15}$	36	R−3 (148)	$Si_5P_6O_{25}$	57	23	0	0	False	-
mp-18203	$K_6Ge_{12}O_{72}P_{18}$	36	R−3 (148)	$KGe_2(PO_4)_3$	65	30	0	0	False	-
mp-23583	$Bi_4Cs_{16}Cl_{48}Sb_4$	36	$I4_1/amd$ (141)	$Cs_4BiSbCl_{12}$	163	301	0	0	True	1.44
mp-650007	$Cs_{16}Tl_4Cl_{48}Sb_4$	36	$I4_1/amd$ (141)	$Cs_4BiSbCl_{12}$	162	252	0	0	True	1.16
mp-541923	$Cu_{20}Tl_4Se_{12}$	36	$P4_2/mnm$ (136)	$TlCu_5Se_3$	270	213	0	0	False	-
mp-5487	$Sr_{12}O_{20}Si_4$	36	$P4/ncc$ (130)	Sr_3SiO_5	326	270	0	0	False	-
mp-778602	$Ba_{12}Ge_4O_{20}$	36	$P4/ncc$ (130)	Sr_3SiO_5	326	277	0	0	False	-
mp-772861	$Ba_4Sr_8I_{24}$	36	P−4b2 (117)	$BaSr_2I_6$	165	545	500	1	False	-
mp-772875	$Ba_8Sr_4I_{24}$	36	P−4b2 (117)	$BaSr_2I_6$	166	566	505	1	False	-
mp-625541	$Sn_{12}H_8O_{16}$	36	P−4$2_1$c (114)	$Sn_3(HO_2)_2$	271	148	74	13	True	6.06
mp-30519	$Pb_4Tl_{12}Cl_{20}$	36	$P4_1$ (76)	Tl_3PbCl_5	162	24	128	1	False	-
mp-28870	$Rh_{12}O_{48}P_{12}$	36	$Imma$ (74)	$PRhO_4$	58	380	0	0	False	-
mp-29498	$W_{24}Br_{48}$	36	$Cmce$ (64)	WBr_2	230	151	0	0	False	-
mp-29555	$Mo_{24}Br_{48}$	36	$Cmce$ (64)	WBr_2	229	159	0	0	False	-
mp-620029	$Mo_{24}Cl_{48}$	36	$Cmce$ (64)	WBr_2	234	168	0	0	False	-
mp-16847	$K_{24}Na_{16}Sn_8P_{24}$	36	$Cmce$ (64)	$K_3Na_2SnP_3$	229	287	0	0	False	-
mp-18447	$K_{24}Na_{16}Sn_8As_{24}$	36	$Cmce$ (64)	$K_3Na_2SnP_3$	228	313	0	0	False	-
mp-17715	$Sr_4Ta_8O_{24}$	36	$Pnma$ (62)	$SrTa_2O_6$	524	249	0	0	False	-
mp-570623	$Pr_{16}N_8Te_{12}$	36	$Pnma$ (62)	$Sr_3(GaN_2)_2$	512	244	0	0	False	-
mp-570646	$Nd_{16}N_8Te_{12}$	36	$Pnma$ (62)	$Sr_3(GaN_2)_2$	512	222	0	0	False	-
mp-17978	$Rb_{20}Ge_4P_{12}$	36	$Pnma$ (62)	Rb_5GeP_3	521	341	0	0	False	-
mp-29883	$Pb_4Rb_{12}Cl_{20}$	36	$Pnma$ (62)	Rb_3PbCl_5	512	179	0	0	False	-
mp-29936	$Pd_4Rb_8I_{24}$	36	$Pnma$ (62)	Rb_2PdI_6	513	220	0	0	True	1.3
mp-16818	$Cd_{12}Rb_8Se_{16}$	36	$Pnma$ (62)	$Rb_2Cd_3Se_4$	516	213	0	0	False	-

ID	Formula	Z	SP (*No.*)	Prototype	NL	NR	WPs	HDWPs	Clean	dE/dq
mp-18101	$Cd_{12}K_8S_{16}$	36	*Pnma* (62)	$Rb_2Cd_3Se_4$	518	163	0	0	False	-
mp-18531	$Cd_{12}K_8Te_{16}$	36	*Pnma* (62)	$Rb_2Cd_3Se_4$	528	239	0	0	False	-
mp-18556	$Cd_{12}Rb_8S_{16}$	36	*Pnma* (62)	$Rb_2Cd_3Se_4$	515	214	0	0	False	-
mp-608106	$I_8P_{16}S_{12}$	36	*Pnma* (62)	$P_4S_3I_2$	510	141	0	0	False	-
mp-653559	$I_8P_{16}Se_{12}$	36	*Pnma* (62)	$P_4S_3I_2$	515	115	0	0	True	2.04
mp-29411	$Na_8B_8S_{20}$	36	*Pnma* (62)	$Na_2B_2S_5$	519	133	0	0	True	1.53
mp-23740	$Mg_8H_4O_{20}P_4$	36	*Pnma* (62)	Mg_2PHO_5	522	225	0	0	True	11.41
mp-30249	$Li_{16}Ge_4S_{16}$	36	*Pnma* (62)	Li_4GeS_4	530	195	0	0	False	-
mp-766540	$Li_{16}Ti_4S_{16}$	36	*Pnma* (62)	Li_4GeS_4	511	185	0	0	False	-
mp-641010	$La_{16}N_8Te_{12}$	36	*Pnma* (62)	$La_4Te_3N_2$	512	219	0	0	False	-
mp-21390	$Cu_4In_4La_8Se_{20}$	36	*Pnma* (62)	$La_2InCuSe_5$	515	319	0	0	False	-
mp-18132	$K_{16}Sn_4Se_{16}$	36	*Pnma* (62)	K_4SnSe_4	513	195	0	0	False	-
mp-569427	$K_{16}Sn_4Se_8Te_8$	36	*Pnma* (62)	$K_4Sn(TeSe)_2$	515	194	0	0	False	-
mp-556347	$K_8Re_4Cl_{20}O_4$	36	*Pnma* (62)	K_2ReCl_5	514	141	0	0	True	2.29
mp-541694	$K_8Na_{12}P_{12}Si_4$	36	*Pnma* (62)	$K_2Na_3SiP_3$	519	280	0	0	False	-
mp-27354	$Cs_{12}Hg_4Cl_{20}$	36	*Pnma* (62)	Cs_3HgCl_5	516	233	0	0	False	-
mp-505001	$Rb_{12}Zn_4Br_{20}$	36	*Pnma* (62)	Cs_3HgCl_5	515	230	0	0	False	-
mp-24216	$Hg_4Zn_4As_4H_4O_{20}$	36	*Pnma* (62)	$CaZnAsHO_5$	515	162	0	0	True	6
mp-18084	$Ba_4Tb_8Zn_4O_{20}$	36	*Pnma* (62)	$BaTb_2ZnO_5$	549	256	0	0	False	-
mp-6121	$Ba_4Y_8Zn_4O_{20}$	36	*Pnma* (62)	$BaTb_2ZnO_5$	512	243	0	0	False	-
mp-6250	$Ba_4Tm_8Zn_4O_{20}$	36	*Pnma* (62)	$BaTb_2ZnO_5$	523	287	0	0	False	-
mp-6452	$Ba_4Sm_8Zn_4O_{20}$	36	*Pnma* (62)	$BaTb_2ZnO_5$	533	229	0	0	False	-
mp-6495	$Ba_4Er_8Zn_4O_{20}$	36	*Pnma* (62)	$BaTb_2ZnO_5$	546	241	0	0	False	-
mp-6501	$Ba_4Ho_8Zn_4O_{20}$	36	*Pnma* (62)	$BaTb_2ZnO_5$	537	245	0	0	False	-
mp-6643	$Ba_4Dy_8Zn_4O_{20}$	36	*Pnma* (62)	$BaTb_2ZnO_5$	541	240	0	0	False	-
mp-27805	$Ba_{12}S_{20}Si_4$	36	*Pnma* (62)	Ba_3SiS_5	514	201	0	0	False	-
mp-707218	$Al_4Ba_{12}H_4O_{16}$	36	*Pnma* (62)	Ba_3AlHO_4	513	201	0	0	False	-
mp-18327	$Ba_8Tl_8O_{20}$	36	*Pnma* (62)	$Ba_2Tl_2O_5$	522	269	0	0	False	-
mp-770936	$Ba_8Sc_8O_{20}$	36	*Pnma* (62)	$Ba_2Tl_2O_5$	515	232	0	0	False	-
mp-18335	$Ba_8Ge_8Se_{20}$	36	*Pnma* (62)	$Ba_2Ge_2Se_5$	523	229	0	0	False	-
mp-561437	$Al_8F_8Ge_4O_{16}$	36	*Pnma* (62)	$Al_2Ge(O_2F)_2$	512	222	0	0	False	-
mp-6280	$Al_8F_8O_{16}Si_4$	36	*Pnma* (62)	$Al_2Ge(O_2F)_2$	514	196	0	0	False	-
mp-505121	$Hg_{12}Na_8S_{16}$	36	*Pbca* (61)	$Na_2Hg_3S_4$	564	205	0	0	False	-
mp-756798	$Ca_4Sr_8I_{24}$	36	*Pbcn* (60)	Sr_2CaI_6	518	302	0	0	False	-
mp-780396	$Ca_8Sr_4I_{24}$	36	*Pbcn* (60)	Sr_2CaI_6	519	297	0	0	False	-
mp-555487	$Sn_{12}O_{24}$	36	*Pbcn* (60)	SnO_2	515	364	0	0	False	-
mp-29107	$Hg_{12}Rb_8Te_{16}$	36	*Pbcn* (60)	$Rb_2Hg_3Te_4$	515	261	0	0	False	-
mp-772049	$Li_8Ti_8O_{20}$	36	*Pbcn* (60)	$Li_2Ti_2O_5$	515	294	0	0	False	-
mp-3552	$Li_8O_{20}Si_8$	36	*Pbcn* (60)	$Li_2Si_2O_5$	519	303	0	0	False	-
mp-571582	$Hg_{12}Tl_4Br_{12}Sb_8$	36	*Pbcm* (57)	$TlHg_3Sb_2Br_3$	412	308	0	0	False	-
mp-628647	$Hg_{12}Tl_4As_8Cl_{12}$	36	*Pbcm* (57)	$TlHg_3Sb_2Br_3$	412	327	0	0	False	-
mp-567398	$Mg_4Sr_{12}Ge_4N_{16}$	36	*Pnna* (52)	Sr_3MgGeN_4	434	264	0	0	False	-
mp-16945	$Ga_8Sr_{12}N_{16}$	36	*Pnna* (52)	$Sr_3(GaN_2)_2$	433	283	0	0	False	-
mp-17129	$Al_8Sr_{12}N_{16}$	36	*Pnna* (52)	$Sr_3(GaN_2)_2$	435	327	0	0	False	-
mp-17133	$Al_8Ba_{12}N_{16}$	36	*Pnna* (52)	$Sr_3(GaN_2)_2$	435	307	0	0	False	-
mp-18297	$Ba_{12}Ga_8N_{16}$	36	*Pnna* (52)	$Sr_3(GaN_2)_2$	433	294	0	0	False	-
mp-27522	$K_8Zr_8O_{20}$	36	*Pnna* (52)	$K_2Zr_2O_5$	432	290	0	0	False	-
mp-568854	$Cs_{12}Re_{12}Br_{12}Cl_{36}$	36	*Ama*2 (40)	$CsReBrCl_3$	0	26	0	0	False	-
mp-20205	$Cu_8Yb_8O_{20}$	36	*Pna*2$_1$ (33)	$Yb_2Cu_2O_5$	433	234	0	22	False	-
mp-772788	$Ba_8Cu_8O_{20}$	36	*Pna*2$_1$ (33)	$Yb_2Cu_2O_5$	434	196	2	25	True	2.59
mp-772813	$Ca_8Cu_8O_{20}$	36	*Pna*2$_1$ (33)	$Yb_2Cu_2O_5$	432	219	0	24	False	-
mp-554383	$Na_8O_{20}Si_8$	36	*Pna*2$_1$ (33)	$Na_2Si_2O_5$	444	194	0	24	False	-
mp-17964	$Na_8Ge_8Se_{20}$	36	*Pna*2$_1$ (33)	$Na_2Ge_2Se_5$	438	209	0	26	False	-
mp-771248	$Na_8Ge_8O_{20}$	36	*Pna*2$_1$ (33)	$Na_2Ge_2O_5$	442	219	0	25	False	-
mp-555076	$K_4Na_4Sn_4F_{24}$	36	*Pna*2$_1$ (33)	$KNaSnF_6$	435	235	0	25	False	-
mp-14280	$Ga_8Sn_8S_{20}$	36	*Pna*2$_1$ (33)	$Ga_2Sn_2S_5$	435	222	0	25	False	-
mp-17885	$Ba_8Ge_8Te_{20}$	36	*Pna*2$_1$ (33)	$Ba_2Ge_2Te_5$	437	241	0	28	False	-
mp-554856	$Na_8O_{20}Si_8$	36	*Pca*2$_1$ (29)	$Na_2Si_2O_5$	382	241	0	46	False	-
mp-555203	$Sr_{20}B_{12}Cl_4O_{36}$	36	*C*222$_1$ (20)	$Sr_5B_3ClO_9$	180	41	288	0	False	-
mp-17066	$Y_8O_{24}Te_4$	36	*P*2$_1$2$_1$2$_1$ (19)	Y_2TeO_6	486	50	176	27	False	-
mp-31318	$Nd_8O_{24}Te_4$	36	*P*2$_1$2$_1$2$_1$ (19)	Y_2TeO_6	486	50	151	27	False	-
mp-752526	$Lu_8O_{24}Te_4$	36	*P*2$_1$2$_1$2$_1$ (19)	Y_2TeO_6	486	55	144	27	False	-

ID	Formula	Z	SP (*No.*)	Prototype	NL	NR	WPs	HDWPs	Clean	dE/dq
mp-768313	$Dy_8O_{24}Te_4$	36	$P2_12_12_1$ (19)	Y_2TeO_6	486	47	150	27	False	-
mp-768762	$Ho_8O_{24}Te_4$	36	$P2_12_12_1$ (19)	Y_2TeO_6	486	52	146	27	False	-
mp-23380	$Pb_4Tl_{12}I_{20}$	36	$P2_12_12_1$ (19)	Tl_3PbI_5	486	70	256	27	False	-
mp-27451	$Pb_4Tl_{12}Br_{20}$	36	$P2_12_12_1$ (19)	Tl_3PbI_5	486	61	254	27	False	-
mp-554390	$Al_8Sr_4N_8O_{12}Si_4$	36	$P2_12_12_1$ (19)	$SrAl_2SiN_2O_3$	486	61	183	27	False	-
mp-772900	$Sm_8O_{24}Te_4$	36	$P2_12_12_1$ (19)	Sm_2TeO_6	486	60	142	27	False	-
mp-774557	$Er_8O_{24}Te_4$	36	$P2_12_12_1$ (19)	Sm_2TeO_6	486	59	144	27	False	-
mp-780172	$Tm_8O_{24}Te_4$	36	$P2_12_12_1$ (19)	Sm_2TeO_6	486	54	141	27	False	-
mp-28108	$Na_{16}Sn_4Te_{16}$	36	$P2_12_12_1$ (19)	Na_4SnTe_4	486	66	177	27	False	-
mp-24018	$Ca_4Zn_4As_4H_4O_{20}$	36	$P2_12_12_1$ (19)	$CaZnAsHO_5$	486	52	160	27	True	6.5
mp-24402	$Al_4Ca_4H_4O_{20}Si_4$	36	$P2_12_12_1$ (19)	$CaZnAsHO_5$	487	63	199	27	False	-
mp-510279	$Pb_4Zn_4As_4H_4O_{20}$	36	$P2_12_12_1$ (19)	$CaZnAsHO_5$	486	41	163	27	True	3.04
mp-29901	$Cd_{24}Rb_{16}Te_{32}$	36	$C2/c$ (15)	$Rb_2Cd_3Te_4$	1	236	0	0	False	-
mp-571490	$Pr_{24}C_8Cl_{40}$	36	$C2/c$ (15)	Pr_3CCl_5	1	276	0	0	False	-
mp-554395	$Li_{12}Na_{20}Ti_8S_{32}$	36	$C2/c$ (15)	$Na_5Li_3(TiS_4)_2$	1	285	0	0	False	-
mp-556679	$Ca_{12}Na_8O_{40}Si_{12}$	36	$C2/c$ (15)	$Na_2Ca_3Si_3O_{10}$	1	284	0	0	False	-
mp-6078	$Cd_{12}Na_8O_{40}Si_{12}$	36	$C2/c$ (15)	$Na_2Ca_3Si_3O_{10}$	2	282	0	0	False	-
mp-557104	$Al_8Li_8B_{16}O_{40}$	36	$C2/c$ (15)	$LiAlB_2O_5$	0	167	0	0	False	-
mp-568484	$La_{24}Br_{40}C_8$	36	$C2/c$ (15)	La_3CBr_5	0	321	0	0	False	-
mp-540939	$Ti_4Tl_{16}S_{16}$	36	$P2_1/c$ (14)	$Ti(TlS)_4$	274	377	0	0	False	-
mp-540940	$Ti_4Tl_{16}Se_{16}$	36	$P2_1/c$ (14)	$Ti(TlS)_4$	275	383	0	0	False	-
mp-5818	$Sn_4Tl_{16}S_{16}$	36	$P2_1/c$ (14)	$Ti(TlS)_4$	273	411	0	0	False	-
mp-757407	$Sr_8Tl_8O_{20}$	36	$P2_1/c$ (14)	$Sr_2Tl_2O_5$	271	352	0	0	False	-
mp-867615	$Ca_8Tl_8O_{20}$	36	$P2_1/c$ (14)	$Sr_2Tl_2O_5$	274	402	0	0	False	-
mp-18112	$Sr_8Ge_8Se_{20}$	36	$P2_1/c$ (14)	$Sr_2Ge_2Se_5$	273	347	0	0	False	-
mp-18464	$Rb_{16}Ge_4O_{16}$	36	$P2_1/c$ (14)	Rb_4GeO_4	273	368	0	0	False	-
mp-554584	$Na_4Pb_4Rb_{12}O_{16}$	36	$P2_1/c$ (14)	Rb_3NaPbO_4	273	369	0	0	False	-
mp-560476	$Na_4Rb_{12}Ti_4O_{16}$	36	$P2_1/c$ (14)	Rb_3NaPbO_4	274	327	0	0	False	-
mp-28745	$Nb_8I_4Te_{24}$	36	$P2_1/c$ (14)	Nb_2Te_6	277	256	0	0	False	-
mp-16861	$Na_{20}As_{12}Ge_4$	36	$P2_1/c$ (14)	Na_5GeAs_3	276	436	0	0	False	-
mp-16989	$K_{20}Sn_4As_{12}$	36	$P2_1/c$ (14)	Na_5GeAs_3	274	423	0	0	True	1.42
mp-18139	$Na_{20}As_{12}Si_4$	36	$P2_1/c$ (14)	Na_5GeAs_3	276	414	0	0	False	-
mp-18317	$Na_{20}Sn_4P_{12}$	36	$P2_1/c$ (14)	Na_5GeAs_3	277	412	0	0	False	-
mp-29387	$Bi_{12}K_{20}Sn_4$	36	$P2_1/c$ (14)	Na_5GeAs_3	272	353	0	0	False	-
mp-4172	$Na_{20}Ge_4P_{12}$	36	$P2_1/c$ (14)	Na_5GeAs_3	274	428	0	0	True	1.01
mp-5367	$K_{20}Sn_4Sb_{12}$	36	$P2_1/c$ (14)	Na_5GeAs_3	278	414	0	0	False	-
mp-541236	$Hf_4Na_{20}As_{12}$	36	$P2_1/c$ (14)	Na_5GeAs_3	275	383	0	0	False	-
mp-5929	$Na_{20}P_{12}Si_4$	36	$P2_1/c$ (14)	Na_5GeAs_3	273	453	0	0	False	-
mp-623030	$Cu_4Na_{12}Sn_4Se_{16}$	36	$P2_1/c$ (14)	$Na_3CuSnSe_4$	273	359	0	0	False	-
mp-3193	$Na_8O_{20}Si_8$	36	$P2_1/c$ (14)	$Na_2Si_2O_5$	275	408	0	0	False	-
mp-772811	$Na_8Ge_8O_{20}$	36	$P2_1/c$ (14)	$Na_2Ge_2O_5$	276	399	0	0	False	-
mp-696528	$K_4H_{12}N_4O_{12}P_4$	36	$P2_1/c$ (14)	KPH_3NO_3	273	284	0	0	True	6.6
mp-6758	$K_4Na_{16}Sn_4Sb_{12}$	36	$P2_1/c$ (14)	KNa_4SnSb_3	273	376	0	0	False	-
mp-762240	$K_8Li_8Ti_4O_{16}$	36	$P2_1/c$ (14)	$K_2Li_2TiO_4$	273	315	0	0	False	-
mp-568493	$In_{12}Sn_4I_{20}$	36	$P2_1/c$ (14)	In_3SnI_5	273	486	0	0	False	-
mp-568522	$In_{12}Sn_4I_{20}$	36	$P2_1/c$ (14)	In_3SnI_5	275	492	0	0	False	-
mp-573145	$Cs_4Ga_{12}S_{20}$	36	$P2_1/c$ (14)	$CsGa_3S_5$	270	263	0	0	False	-
mp-29808	$Ca_{16}Ge_4N_{16}$	36	$P2_1/c$ (14)	Ca_4GeN_4	274	403	0	0	False	-
mp-570505	$Al_8Ca_{12}N_{16}$	36	$P2_1/c$ (14)	$Ca_3(AlN_2)_2$	273	396	0	0	False	-
mp-29284	$Ca_8S_{20}Sb_8$	36	$P2_1/c$ (14)	$Ca_2Sb_2S_5$	273	434	0	0	False	-
mp-759686	$Al_4Be_4H_4O_{20}Si_4$	36	$P2_1/c$ (14)	$BeAlSiHO_5$	273	346	0	0	False	-
mp-2911	$Ba_{12}Sn_8P_{16}$	36	$P2_1/c$ (14)	$Ba_3(SnP_2)_2$	275	374	0	0	False	-
mp-17470	$Ba_{12}Sn_8As_{16}$	36	$P2_1/c$ (14)	$Ba_3(SnAs_2)_2$	273	397	0	0	False	-
mp-17504	$Sr_{12}As_{16}Ge_8$	36	$P2_1/c$ (14)	$Ba_3(SnAs_2)_2$	272	465	0	0	False	-
mp-17817	$Ca_{12}Ge_8P_{16}$	36	$P2_1/c$ (14)	$Ba_3(SnAs_2)_2$	273	384	0	0	False	-
mp-18351	$Sr_{12}Ge_8P_{16}$	36	$P2_1/c$ (14)	$Ba_3(SnAs_2)_2$	273	360	0	0	False	-
mp-18504	$Ca_{12}As_{16}Ge_8$	36	$P2_1/c$ (14)	$Ba_3(SnAs_2)_2$	274	401	0	0	False	-
mp-540696	$Ca_{12}As_{16}Si_8$	36	$P2_1/c$ (14)	$Ba_3(SnAs_2)_2$	272	404	0	0	False	-
mp-601867	$Ba_{12}Sn_8P_{16}$	36	$P2_1/c$ (14)	$Ba_3(SnAs_2)_2$	274	363	0	0	False	-
mp-768735	$Ba_8Sr_4I_{24}$	36	$P2_1/c$ (14)	Ba_2SrI_6	275	504	0	0	False	-
mp-771238	$Y_8O_{24}Te_4$	36	$P2/c$ (13)	Y_2TeO_6	111	460	0	0	False	-
mp-28034	$Cu_{16}P_{56}$	36	$C2/m$ (12)	Cu_2P_7	0	206	0	0	False	-

ID	Formula	Z	SP (*No.*)	Prototype	NL	NR	WPs	HDWPs	Clean	dE/dq
mp-6075	$Ba_4Li_2B_{10}O_{20}$	36	$P2_1/m$ (11)	$Ba_2Li(BO_2)_5$	162	171	0	0	False	-
mp-29198	$Au_2Na_{10}Se_{24}$	36	$P2_1$ (4)	Na_5AuSe_{12}	162	41	62	0	False	-
mp-16773	$K_2Tb_2O_{24}P_8$	36	$P2_1$ (4)	$KTb(PO_3)_4$	162	30	79	0	True	1.56
mp-18661	$K_2Y_2O_{24}P_8$	36	$P2_1$ (4)	$KTb(PO_3)_4$	162	47	81	0	False	-
mp-28348	$Ba_8As_8Se_{20}$	36	$P2_1$ (4)	$Ba_2As_2Se_5$	162	39	136	0	False	-

III. NODAL-LINE (RING) TP MATERIALS

Supplementary Table 2: The summary of 4068 nodal line(NDL) and nodal ring(NDR) phonon materials categorized by their space group number and symbol(*No.* (SP)). The true or false in the column of Clean denotes whether the material holds clean topological phonons.

No. (SP)	Clean	NDL and NDR (NDL(R)) candidates
2 ($P-1$)	False	$Ca_4Ti_4O_{20}Si_4$
7 (Pc)	False	$Li_4Sn_2Zn_2S_8$, $Na_4Zn_2Ge_2O_8$, $Mg_2Na_4O_8Si_2$, $Li_4Zn_2Ge_2O_8$, $Ba_2Ca_4I_{12}$, Ba_6As_{12}, $Sn_4P_4S_{12}$, $Sn_4P_4Se_{12}$, $Au_4K_4Cl_{16}$, $Ag_{14}Nb_2S_{12}$, $K_8Sr_4Cl_{16}$
7 (Pc)	True	$Nb_4Tl_2P_2S_{20}$
8 (Cm)	False	$Cs_6Li_4Cl_{10}$, Ag_6P_{22}, $Hf_{12}N_8O_{12}$, $Zr_{12}N_8O_{12}$, $Cu_8Ho_8K_4S_{18}$
9 (Cc)	False	Ga_8S_{12}, $Cu_8Li_8S_8$, $Cu_8S_{12}Si_4$, $Cd_8Cl_4P_{12}$, $Ba_4B_8S_{16}$, $Na_{16}Sn_4O_{12}$, $K_8As_8Se_{16}$, $Li_{20}Ge_4P_{12}$, $Ag_8In_8Ge_4Se_{24}$, $Ag_8In_8S_{24}Si_4$, $Ag_8In_8Ge_4S_{24}$, $Ag_8In_8Se_{24}Si_4$, $Hg_{16}S_{24}Si_4$, $Cd_{16}Ge_4Se_{24}$, $Cd_{16}S_{24}Si_4$, $Cd_{16}Ge_4S_{24}$, $La_{16}P_{32}$, $La_{16}As_{32}$, $Na_{24}W_8N_{24}$, $Ag_{16}Cd_4Ge_8S_{28}$, $Ca_8N_{32}Si_{20}$
10 ($P2/m$)	False	$CaPbI_4$, $Li_2H_4I_2O_2$, $In_4Zn_2S_8$, $Li_{10}Cl_4N_2$, $Ga_8Sr_4As_8$
11 ($P2_1/m$)	False	Ag_2Br_2, $Na_2H_2S_2$, $K_2H_2S_2$, Zr_2S_6, Hf_2S_6, $K_2H_4N_2$, $Rb_2H_4N_2$, $W_2Y_2N_6$, $Ga_2Sr_4Ge_2N_2$, $Pd_2O_6Se_2$, $Ba_2O_6Se_2$, $Ba_2Cl_2N_6$, $Nd_2Cl_2H_4O_4$, Lu_2P_{10}, $Ho_2Cl_2H_4O_4$, $Cs_2Cu_4Cl_4I_2$, $Tl_6B_2Se_6$, $La_2Sr_2I_{10}$, $La_2Sr_2I_{10}$, $Ba_4Se_8Si_2$, $Be_2Na_4H_8$, $Ba_4Ge_2Se_4Te_4$, $Ba_2Pd_4S_8$, $Tl_4Sb_4Se_8$, $Cu_2K_2Pd_2Se_{10}$, $Cu_2Pd_2Rb_2Se_{10}$, $Al_4K_6As_6$, $Cs_2Hg_4Br_{10}$, $Ba_4Li_2Re_2N_8$, $Li_2Re_2Sr_4N_8$, $Li_2Sr_4F_2O_8Si_2$, $Bi_4Sr_4O_{10}$, $Be_2Ho_2K_2F_{12}$, $Cs_2Li_4H_6O_6$, $Er_6Sm_2Se_{12}$, $In_2Na_{10}S_8$, $Ga_4Li_4Br_{12}$, $Ga_4Li_4I_{12}$, $Ca_4K_4F_{12}$, $Ag_6Sn_2P_{14}$, $In_{10}Tl_2S_{12}$, $Ba_4Ho_2B_4Cl_2O_{12}$, $Ba_4Y_2B_4Cl_2O_{12}$, $Al_4In_4I_{16}$, $Ba_4Li_2B_{10}O_{20}$
11 ($P2_1/m$)	True	$K_4Br_2H_2O_2$, $La_2C_2Cl_2N_4$, $Ba_4Ge_2Se_8$, $Ba_4Cl_2P_{14}$, $Ba_8Cu_4I_4O_8$
12 ($C2/m$)	False	$Cu_2Na_2O_4$, $Ag_2Na_2O_4$, $K_4C_2N_4$, $Rh_4Cl_4Te_4$, $I_4Sb_4Te_4$, $Ag_2Li_2F_8$, $K_4Tl_4O_4$, $Sr_4C_2Cl_4N_4$, I_8Te_8, $Ta_4I_8O_4$, Ir_4Br_{12}, Ir_4Cl_{12}, Rh_4Br_{12}, $Pb_4Cl_4H_4O_4$, Bi_8I_8, Ba_4P_{12}, $Tb_8N_4Se_6$, Sn_6I_{12}, Y_8Cl_{12}, $Th_4I_8Te_8$, $Nb_4Br_8Se_8$, Sc_8S_{12}, $Li_4O_{12}Sb_4$, $K_4Li_4Zn_4O_8$, $Pd_4Ge_4S_{12}$, Ga_8O_{12}, $Bi_4Cd_4I_4Se_8$, $Ba_4Bi_2O_{12}Sb_2$, $Ba_4Bi_2Tb_2O_{12}$, $Mg_2Sr_4B_4O_{12}$, $Ba_4Ca_2B_4O_{12}$, $Bi_2Na_{10}O_{10}$, $Ba_2Er_4F_{16}$, $Ba_2Y_4F_{16}$, $Ba_2Tm_4F_{16}$, $Y_8Cl_{12}N_4$, $Al_4Ta_4O_{16}$, $Al_4Nb_4O_{16}$, $As_{12}Si_{12}$, $Ga_{12}Te_{12}$, $Cu_{12}Rb_4S_8$, $Cu_{12}K_4S_8$, $Ba_4Na_4B_4O_{12}$, $Na_{10}Re_2O_{12}$, $Na_{10}Re_2O_{12}$, $Na_8Zr_4Se_{12}$, $Na_8Sn_4O_{12}$, $Li_8Pd_4O_{12}$, $Li_{10}Re_2O_{12}$, $K_4La_4Pd_4O_{12}$, $K_4Pd_4Sm_4O_{12}$, $K_4Nd_4Pd_4O_{12}$, $K_4Pd_4Pr_4O_{12}$, $K_8Na_4B_4P_8$, $As_{12}Ge_{12}$, $Ag_4Ba_4Er_4S_{12}$, $Hg_4Pd_2Tl_4Cl_{16}$, $Ca_{10}P_{16}$, $Rb_{12}O_{12}Sb_4$, $Hg_4Nd_8O_{16}$, $Hg_4La_8O_{16}$, $K_{12}Ge_4S_{12}$, $K_{12}Ge_4Se_{12}$, $K_{12}Si_4Te_{12}$, $Ga_4K_{12}O_{12}$, $Ga_4Rb_{12}O_{12}$, $In_4Rb_{12}S_{12}$, $Bi_8Hg_4S_{16}$, $Ag_{10}Ba_4La_2S_{12}$, $Hg_{12}O_{14}Si_4$, $Ca_6Li_8N_{12}Si_4$, $Li_8Sr_6Ge_4N_{12}$, $Zr_4Br_{20}Te_8$, $Na_4Y_4F_4O_{16}P_4$, $Er_4Na_4F_4O_{16}P_4$, $Cs_{12}Li_4F_{16}$, $Ca_4C_4Cl_4H_8O_{12}$, $Bi_{16}Br_{16}$, $Cu_{16}K_6Se_{12}$, $Ir_2Pr_{12}Cl_{22}$, $Cu_8K_4Y_8Se_{18}$, $Bi_4Li_{16}Pd_2O_{20}$, $Ag_4Hg_{14}I_{12}P_{16}$, $Rb_{20}H_4O_{20}Si_4$, $Ba_8Mg_{12}H_{40}$, $Cu_{16}P_{56}$
12 ($C2/m$)	True	Rh_4Cl_{12}, $Co_4La_4N_{12}$, $Cs_4Li_6Br_{10}$, $K_4Ti_4P_4S_{20}$
13 ($P2/c$)	False	$Ba_2Ca_2I_8$, $K_4Sb_4Se_8$, $Ag_4K_4Sn_2Se_8$, $Nb_4Br_{12}Te_4$, $Ba_2La_4Br_{16}$, Zr_6I_{24}, $Na_{12}Ti_4O_{14}$, $Y_8O_{24}Te_4$

No. (SP)	Clean	NDL and NDR (NDL(R)) candidates
14 ($P2_1/c$)	False	In_4S_4, Mg_2P_8, Cd_2P_8, $Ag_4Hg_2S_4$, $Au_4I_4Te_4$, $Au_2Rb_2Br_8$, Pd_4I_8, Pt_4I_8, Ir_4P_8, Ir_4Sb_8, Co_4P_8, Ir_4As_8, $Nd_4Br_4S_4$, $Ta_4N_4O_4$, $Nb_4N_4O_4$, Nd_4S_8, $Cu_2Li_2F_8$, $Be_4Li_4N_4$, $Cu_4Li_{12}S_8$, $K_4H_4O_4$, $In_4Br_4Te_4$, $Fe_4S_4Sb_4$, $Fe_4As_4S_4$, Ag_4P_8, Cu_4P_8, $Na_4Pd_2F_8$, $Hg_6Cl_4O_4$, $Cd_6Cl_4O_4$, $Tm_4H_4O_8$, Rb_8Sb_8, K_8Sb_8, Li_8As_8, Na_8Sb_8, Li_8P_8, $Cu_4Nd_4S_8$, $Cu_4La_4Se_8$, $Cu_4Pr_4Se_8$, $Cu_4Sm_4Se_8$, $Cu_4Tb_4Se_8$, $Cu_4Pr_4S_8$, $Cu_4Nd_4Se_8$, $Cu_4La_4S_8$, $Cu_4Sm_4S_8$, $Bi_4Cs_4S_8$, $Cu_4Tb_4S_8$, $Au_4Na_4Se_8$, $Li_4Pr_4O_8$, $Li_4Sm_4O_8$, $Cu_4La_4S_4Te_4$, $Cu_4Br_4Se_8$, $Cu_4Cl_4Te_8$, $Cu_4I_4Te_8$, $Cs_4Sb_4Se_8$, $Cs_4S_8Sb_4$, $Ba_4Sn_4S_8$, $Pd_2Cl_4Se_{12}$, $Pb_4Pd_2Cl_{12}$, $Pb_4Pd_2Br_{12}$, $K_4I_{12}Te_2$, $K_4Sn_2Br_{12}$, $Ba_4Sr_2I_{12}$, $Ba_4Ca_2I_{12}$, $Cr_4In_4Br_{12}$, Th_4I_{16}, Cl_8Te_{12}, $Sn_4Ge_4S_{12}$, $Pb_4Ge_4S_{12}$, $Rb_4B_4Se_{12}$, $Cs_4Ga_4Se_{12}$, $Rb_4B_4S_{12}$, $Al_2Na_6H_{12}$, $Na_6Y_2Br_{12}$, $Al_2Na_6F_{12}$, $Ca_6O_{12}Te_2$, $Sr_6O_{12}Te_2$, $Na_4Sn_2O_{12}Te_2$, $Ca_2Sr_4O_{12}Te_2$, $La_4Na_2Os_2O_{12}$, $Li_2Os_2Sm_4O_{12}$, $Li_2Os_2Pr_4O_{12}$, $La_4Li_2Os_2O_{12}$, $Sr_4Y_2O_{12}Sb_2$, $Ca_2Sr_4U_2O_{12}$, $Ba_4Sr_2U_2O_{12}$, $Sr_4Ta_2Tm_2O_{12}$, $Na_2Os_2Pr_4O_{12}$, $In_2Nb_2Sr_4O_{12}$, $Na_2Nd_4Os_2O_{12}$, $In_2Sr_4O_{12}Sb_2$, $Bi_2Nd_2Sr_4O_{12}$, $Bi_2Lu_2Sr_4O_{12}$, $Al_2Li_2Na_4H_{12}$, $Na_2Pr_4Ru_2O_{12}$, $Ho_2Sr_4O_{12}Sb_2$, $Ca_4In_2Nb_2O_{12}$, $Dy_2Sr_4O_{12}Sb_2$, $La_4Na_2Ru_2O_{12}$, $Al_2Li_2Na_4F_{12}$, $Mg_2Sr_4U_2O_{12}$, $Ca_4Ga_4Li_4N_8$, $La_4Re_4N_{12}$, $Au_4K_4I_{12}$, Ga_8I_{12}, $Cd_8Cl_8P_4$, $Ca_4H_8O_8$, $Ca_4P_4Se_{12}$, $Pb_4P_4S_{12}$, $Sr_4P_4Se_{12}$, $Sr_4P_4S_{12}$, $Ca_4P_4S_{12}$, $Ba_8Ge_4P_8$, $Ba_8As_8Ge_4$, As_8Se_{12}, $Au_4Zn_2F_{16}$, Zn_8P_{16}, $Cu_4Tl_4P_4Se_{12}$, $Li_4Sr_4B_4O_{12}$, $Sm_4O_{16}P_4$, $Hg_4Rb_4Sb_4Se_{12}$, $Cu_4Rb_{12}O_8$, $Cu_4Na_{12}O_8$, $Bi_4Pr_4O_{16}$, Nb_4I_{20}, $Na_8O_{12}Se_4$, $Na_8O_{12}Te_4$, $Li_8S_{12}Te_4$, $Li_8Se_{12}Te_4$, $Na_8Ge_4Se_{12}$, $Na_8Ge_4S_{12}$, $Na_8Sn_4Se_{12}$, $Lu_4F_4O_{12}Se_4$, $Er_4F_4O_{12}Se_4$, $Tm_4F_4O_{12}Se_4$, $Ho_4F_4O_{12}Se_4$, $Ga_4Li_4Br_{16}$, $In_4Na_4I_{16}$, $In_4Li_4I_{16}$, $Ga_4Li_4I_{16}$, $Li_6Sc_2B_4O_{12}$, $Be_4Sr_4F_{16}$, $K_8Zr_4Te_{12}$, $Ag_4Cs_4P_4Se_{12}$, $Ca_4Sr_4I_{16}$, $Ag_4Pb_4As_4S_{12}$, $Ag_4Pb_4S_{12}Sb_4$, $Pt_{10}Tl_4S_{12}$, $Ag_8Mn_2S_{12}Sb_4$, $Mg_6O_{16}P_4$, $Mg_6As_4O_{16}$, $K_8Na_4O_{12}Te_2$, $Hg_8Sr_4Cl_8O_8$, $Hg_8Sn_4Br_8S_8$, $Bi_4Rb_4S_{16}Si_4$, $Bi_4K_4S_{16}Si_4$, $In_4Rb_{12}O_{12}$, $Cs_{12}B_4S_{12}$, P_8Se_{20}, $Na_{12}Ge_4Te_{12}$, $Na_{12}Ge_4Se_{12}$, $K_{12}Sn_4Te_{12}$, $Ba_{12}Sn_4P_{12}$, $K_{12}Sn_4Se_{12}$, $Ga_4Na_{12}S_{12}$, $Al_4Na_{12}Te_{12}$, $Na_{12}B_4O_{12}$, $Be_4Na_8F_{16}$, $Li_4Y_4O_{16}Si_4$, $La_4Cl_4O_{16}S_4$, $Al_4K_{12}Se_{12}$, $Al_4K_{12}Te_{12}$, $Ga_4K_{12}S_{12}$, $Ga_4K_{12}Se_{12}$, $Cu_4K_8S_{12}Sb_4$, $Cu_4Na_8S_{12}Sb_4$, $Pb_8Ge_4S_{16}$, $Ag_8Cs_4As_4S_{12}$, $Al_4Ca_4F_{20}$, $Ba_8Ti_4O_{16}$, $Ca_8O_{16}Si_4$, $Sr_8Ge_4O_{16}$, $Ag_{12}S_{12}Sb_4$, Fe_6P_{24}, $Cs_{12}O_{14}Si_4$, $K_{12}O_{14}Si_4$, $Rb_{12}Ti_4O_{14}$, $Cs_8Pd_2P_4Se_{16}$, $Zr_4B_8O_{20}$, $Hf_4B_8O_{20}$, $Tl_8As_8S_{16}$, $Pb_4Tl_8Ge_4S_{16}$, $Sr_4Th_8Se_{20}$, $Ba_8Cs_4Br_{20}$, $K_4Sm_8Cl_{20}$, $Cu_8Sc_8Se_{16}$, $Re_{12}Br_4Se_{16}$, $Re_{12}Br_4S_{16}$, $Ag_4Rb_8S_{16}Sb_4$, $Na_{16}Ge_{16}$, $Be_4Na_8Ge_4O_{16}$, $Li_8Mg_4O_{16}Si_4$, $Li_8Zn_4O_{16}Si_4$, La_4P_{28}, $La_8S_{20}Si_4$, $La_8Ge_4S_{20}$, $K_4Li_4Y_4F_{20}$, $Bi_{16}K_{16}$, $Hg_{12}As_4S_{12}Sb_4$, $Cs_8Na_8Ge_{16}$, $Cs_8As_8Se_{16}$, $Sr_4Ti_4Ge_4O_{20}$, $Be_4Ca_4F_4O_{16}P_4$, $Ca_8U_4O_{20}$, $Sr_8U_4O_{20}$, $Ba_{12}O_4Sb_{16}$, $Ti_4Tl_{16}S_{16}$, $Ti_4Tl_{16}Se_{16}$, $Sn_4Tl_{16}S_{16}$, $Sr_8Tl_8O_{20}$, $Ca_8Tl_8O_{20}$, $Sr_8Ge_8Se_{20}$, $Rb_{16}Ge_4O_{16}$, $Na_4Pb_4Rb_{12}O_{16}$, $Na_4Rb_{12}Ti_4O_{16}$, $Nb_8I_4Te_{24}$, $Na_{20}As_{12}Ge_4$, $Na_{20}As_{12}Si_4$, $Na_{20}Sn_4P_{12}$, $Bi_{12}K_{20}Sn_4$, $K_{20}Sn_4Sb_{12}$, $Hf_4Na_{20}As_{12}$, $Na_{20}P_{12}Si_4$, $Cu_4Na_{12}Sn_4Se_{16}$, $Na_8O_{20}Si_8$, $Na_8Ge_8O_{20}$, $K_4Na_{16}Sn_4Sb_{12}$, $K_8Li_8Ti_4O_{16}$, $In_{12}Sn_4I_{20}$, $In_{12}Sn_4I_{20}$, $Cs_4Ga_{12}S_{20}$, $Ca_{16}Ge_4N_{16}$, $Al_8Ca_{12}N_{16}$, $Ca_8S_{20}Sb_8$, $Al_4Be_4H_4O_{20}Si_4$, $Ba_{12}Sn_8P_{16}$, $Ba_{12}Sn_8As_{16}$, $Sr_{12}As_{16}Ge_8$, $Ca_{12}Ge_8P_{16}$, $Sr_{12}Ge_8P_{16}$, $Ca_{12}As_{16}Ge_8$, $Ca_{12}As_{16}Si_8$, $Ba_{12}Sn_8P_{16}$, $Ba_8Sr_4I_{24}$
14 ($P2_1/c$)	True	$In_4I_4Te_4$, $Tl_4B_4S_{12}$, $Li_2Nd_4Os_2O_{12}$, $Sm_2Sr_4O_{12}Sb_2$, $Ba_4P_4S_{12}$, $Ba_4P_4Se_{12}$, $Pb_4P_4Se_{12}$, $Cd_2Rb_4P_4Se_{12}$, $K_4Mg_2P_4Se_{12}$, $Na_{12}B_4N_8$, $Na_4Sr_4B_4O_{12}$, $Au_2Na_2O_{16}S_4$, $Li_8Ti_4O_4S_8$, $Au_4K_4Cl_{16}$, $In_8Cl_8Te_8$, $Cs_8As_4Te_{12}$, $Cu_4Sr_8O_{14}$, $Sr_4B_8S_{16}$, $Rb_{12}B_4S_{12}$, $Sr_8O_{16}Si_4$, $Ba_8Ge_4O_{16}$, $Na_{12}Si_4Te_{12}$, $K_8Ge_4Te_{16}$, $Ca_8B_4Cl_4O_{12}$, $Rb_{12}O_{14}Si_4$, $Y_8O_{20}Si_4$, $Dy_8O_{20}Si_4$, $Ag_4Ta_4Tl_8S_{16}$, $Li_4U_4B_4O_{20}$, $Li_4B_4H_8O_{16}$, $K_{20}Sn_4As_{12}$, $Na_{20}Ge_4P_{12}$, $K_4H_{12}N_4O_{12}P_4$
15 ($C2/c$)	False	Pd_4P_8, Ni_4P_8, Ba_4S_8, Ba_4Se_8, $Cu_8Pb_4O_8$, Sc_8Se_{12}, $Au_4Tl_4Cl_{16}$, $Au_4Rb_4Cl_{16}$, $Mg_4Na_4B_4O_{12}$, $Bi_4Nd_4O_{16}$, $Bi_4Sm_4O_{16}$, $Au_4Li_4F_{16}$, $Li_8C_4O_{12}$, $Cs_{12}As_8B_4$, $K_{12}B_4P_8$, $K_{12}As_8B_4$, $Cs_{12}B_4P_8$, $Rb_{12}As_8B_4$, $Cd_8As_{12}I_4$, $Hg_8Cl_4P_{12}$, $Hg_8As_{12}Br_4$, $Cd_8As_{12}Br_4$, $Cd_8I_4P_{12}$, $Cd_8Br_4P_{12}$, $Cd_8Cl_4P_{12}$, $Ba_4Sr_4I_{16}$, $Ag_4Au_4Cl_{16}$, $Na_{12}B_4S_{12}$, $Al_4Be_4H_{20}$, $Ba_4Y_4Cl_{20}$, $Li_8Rb_8F_{16}$, $Al_4Na_4As_4F_4O_{16}$, $Al_4Ca_4F_4O_{16}Si_4$, $Al_4Na_4F_4O_{16}P_4$, $Li_4Yb_8Cl_{20}$, $Ag_4Pb_8Br_{20}$, $Ag_4Li_8F_{20}$, $Hg_{16}I_8O_8$, $Hg_{16}Cl_8O_8$, $Cs_4Ta_4I_{24}$, $Ca_4Ti_4O_{20}Si_4$, $Na_4Ge_4O_{20}Sb_4$, $Cs_8Li_8F_{16}$, $Os_4Y_{16}Br_{16}$, $Sn_8Tl_8S_{20}$, $K_8Sn_8S_{20}$, $K_8Sn_8Se_{20}$, $Rb_8Sn_8Se_{20}$, $Ti_4O_{24}Si_8$, $Sr_{12}As_{16}Si_8$, $Ba_{12}As_{16}Si_8$, $Ag_8Rb_8Ge_4S_{16}$, $Nd_{16}N_8Se_{12}$, $Pr_{16}N_8Se_{12}$, $Al_8Ca_{12}N_{16}$, $Ca_{12}Ga_8N_{16}$, $Al_8Sr_{12}P_{16}$, $Mg_4H_8O_{20}Se_4$, $Li_{20}Ti_4P_{12}$, $Li_8B_8Se_{20}$, $K_8Zn_{12}O_{16}$, $K_8Th_4F_{24}$, $Ba_4H_{16}I_8O_8$, $Ba_4Br_8H_{16}O_8$, $Pr_4B_{12}O_{24}$, $Sm_4B_{12}O_{24}$, $Nd_4B_{12}O_{24}$, $Al_4Na_4O_{24}Si_8$, $Ca_4Mg_4O_{24}Si_8$, $Ga_4Na_4O_{24}Si_8$, $Na_4Sc_4O_{24}Si_8$, $In_4K_4Ge_8O_{24}$, $Be_8Li_8H_{24}$, $Li_{16}O_{20}Se_4$, Hf_8I_{32}, Fe_8P_{32}, $Ba_8Li_{12}Ta_4N_{16}$, $Ba_8La_4Cl_{28}$, $Tl_8B_8Se_{28}$, $Rb_8B_8Se_{28}$, $Na_8B_8Se_{28}$, $Sr_{12}B_8S_{24}$, $Na_{20}Nb_4O_{20}$, $Na_{20}Ta_4O_{20}$, $Al_8O_{28}Si_8$, $Al_8Ge_8O_{28}$, $Al_8Ge_4H_8O_{24}$, $Sr_{16}V_8N_{24}$, $Ga_8La_{16}N_{24}$, $Ba_{16}Mg_8O_{24}$, $Na_{16}Se_{24}Te_8$, $Na_{16}Pt_8O_{24}$, $Li_{28}V_4As_{16}$, $Be_{12}K_{16}O_{20}$, $Ca_4Ga_{16}O_{28}$, $Na_4N_{28}P_{16}$, $Al_{16}Sr_4O_{28}$, $Ba_4Ga_{16}O_{28}$, $Be_{16}P_{32}$, $Ba_8Zn_4O_{28}Si_8$, $Ag_{16}P_8S_{28}$, $Ag_{12}Y_4P_8S_{32}$, $Mg_4Sr_{12}O_{32}Si_8$, $Ag_4Au_{12}Rb_8I_{32}$, $Mn_{16}Na_{16}S_{24}$, $Mg_{16}Ni_8H_{32}$, $La_{12}I_4S_{32}Si_8$, $Pr_{12}Cl_4O_{32}Si_8$, $Sm_{12}Br_4S_{32}Si_8$, $Pr_{12}I_4S_{32}Si_8$, $La_{12}Cl_4O_{32}Si_8$, $Sm_{12}I_4S_{32}Si_8$, $Nd_{12}Cl_4O_{32}Si_8$, $Tb_{12}I_4S_{32}Si_8$, $Nd_{12}I_4S_{32}Si_8$, $K_{24}Ge_8Te_{24}$, $Ho_{24}N_8Se_{24}$, $Hg_{24}I_{16}Te_{16}$, $Ag_{24}As_8S_{24}$, $Sr_{12}Ge_{12}O_{36}$, $Ca_{12}O_{36}Si_{12}$, $Sr_{12}O_{36}Si_{12}$, $Ca_{12}Ge_{12}O_{36}$, $Ba_{12}O_{36}Si_{12}$, $Na_{24}Se_{28}Si_8$, $Na_{24}Ge_8Se_{28}$, $Na_{24}Ge_8S_{28}$, $Hf_{12}F_{48}$, $Lu_{16}O_{40}Si_8$, $Dy_{16}Ge_8O_{40}$, $Er_{16}O_{40}Si_8$, $Lu_{16}Ge_8O_{40}$, $Tm_{16}Ge_8O_{40}$, $Ho_{12}Li_4F_8Ge_8O_{32}$, $Ag_{12}K_{12}Ta_8Se_{32}$, $Cd_{24}Rb_{16}Te_{32}$, $Pr_{24}C_8Cl_{40}$, $Li_{12}Na_{20}Ti_8S_{32}$, $Ca_{12}Na_8O_{40}Si_{12}$, $Cd_{12}Na_8O_{40}Si_{12}$, $Al_8Li_8B_{16}O_{40}$, $La_{24}Br_{40}C_8$
15 ($C2/c$)	True	$Rb_{12}B_4P_8$, $K_8B_8S_{28}$, $K_8B_8Se_{28}$, $Li_{16}O_{24}Te_8$, $Hg_8K_8S_{24}Sb_8$, $Ba_8Cu_{12}V_4S_{24}$, $Cs_4Li_{20}B_8O_{24}$, $Cd_{16}F_8O_{32}P_8$, $Ba_{20}Br_{24}O_{16}Si_4$, $Ba_{20}Cl_{24}O_{16}Si_4$

No. (SP)	Clean	NDL and NDR (NDL(R)) candidates
26 ($Pmc2_1$)	False	$Ba_4Hg_4S_8$, Tl_4P_{20}
31 ($Pmn2_1$)	False	$Cu_4Mn_2S_8Si_2$, $Ag_4Cd_2Ge_2S_8$, $Ag_2Cd_4Ga_2S_8$, $Cd_2Cu_4S_8Si_2$, $Cd_2Li_4Ge_2O_8$, $Li_4Na_2As_2O_8$, $Cu_4Zn_2S_8Si_2$, $Li_6O_8P_2$, $Cu_6P_2S_8$, $Li_6V_2S_8$, $Na_6As_2O_8$, $Li_6S_8Sb_2$, $Li_6As_2O_8$, $In_{10}Br_2Se_{10}$, $Nb_2Rb_{14}As_8$, $Al_8Ba_2S_{14}$, $Cu_{16}S_{12}Si_2$, $Ba_4N_{16}Si_{10}$
34 ($Pnn2$)	False	$Li_4Sn_2O_{12}Te_2$
38 ($Amm2$)	False	$La_8O_8Se_6$, $Sm_8O_8Se_6$, $Nd_8O_8Se_6$
38 ($Amm2$)	True	$Li_4B_{24}C_4$
40 ($Ama2$)	False	$La_4Mo_4N_{12}$, $W_4Y_4N_{12}$, $Sr_8Ge_4Se_{16}$, $Ga_8Pb_4O_{16}$, $Ba_4Hg_4Sn_4S_{16}$, $Cu_8K_4Ta_4Se_{16}$, $Cu_8K_4Nb_4Se_{16}$, $Dy_8C_4O_{20}$, $Ba_4Cu_8Sn_4Se_{16}$, $Cu_8Sr_4Ge_4Se_{16}$, $Cs_{12}Re_{12}Br_{12}Cl_{36}$
40 ($Ama2$)	True	$Cu_8Rb_4Ta_4Se_{16}$, $Cu_8K_4V_4S_{16}$
41 ($Aea2$)	False	$Hg_{12}K_8Ge_8S_{32}$
43 ($Fdd2$)	False	$Au_{16}O_{24}$, $Sr_{24}P_{32}$, $Ba_{24}P_{32}$
47 ($Pmmm$)	False	$AuKO_2$
51 ($Pmma$)	False	Ca_2Mg_2, $Au_2Cs_2Te_2$, $Au_2Rb_2Te_2$, $Nb_8Rb_2Cl_{22}$, $Cs_2Nb_8Cl_{22}$
52 ($Pnna$)	False	$O_{12}Te_4$, $Li_4O_{12}Sb_4$, $As_8Ge_8Se_8$, Ga_8Br_{16}, In_8I_{16}, $Ba_4Zn_4Cl_{16}$, $Mg_4Sr_{12}Ge_4N_{16}$, $Ga_8Sr_{12}N_{16}$, $Al_8Sr_{12}N_{16}$, $Al_8Ba_{12}N_{16}$, $Ba_{12}Ga_8N_{16}$, $K_8Zr_8O_{20}$
52 ($Pnna$)	True	$Bi_{12}I_4O_{16}$
53 ($Pmna$)	False	$Cu_4Br_4Se_{12}$, $Ag_4I_4Se_{12}$, $Ag_4I_4Te_{12}$, $Ga_{10}Li_2O_{16}$
54 ($Pcca$)	False	$Au_4Cd_4Rb_4S_8$
55 ($Pbam$)	False	Rb_4Te_4, $Pb_2Sr_4O_8$, $Ca_4Pb_2O_8$, $Mg_2Na_4Cl_8$, $Cd_2Na_4Cl_8$, $Ca_4Sn_2O_8$, $Na_4U_2O_8$, $Ca_4Hf_2O_8$, $Ca_4Ce_2O_8$, $Rb_4C_4O_8$, $K_4C_4O_8$, $Ba_4Sc_4O_{10}$, $Au_6Rb_{10}O_4$, $Au_2K_{10}I_4O_4$, $Pb_2Tl_8I_{12}$, $As_{16}Si_8$, $Pd_6Ta_4Se_{16}$, $Pt_6Ta_4Se_{16}$, $Nb_4Pd_6Se_{16}$, $Pt_6Ta_4S_{16}$, $Al_8Bi_4O_{18}$, $Ca_4Ge_8O_{20}$, $Ag_4Hg_8As_4O_{16}$
56 ($Pccn$)	False	$Na_8U_4O_{16}$
57 ($Pbcm$)	False	$Au_4La_4O_{12}$, $Sr_4U_4O_{16}$, $Ba_4U_4O_{16}$, $Ca_4Nb_8O_{16}$, $Nb_8Sr_4O_{16}$, $Ba_4F_{20}Sb_4$, $Er_8O_{16}Se_4Si_4$, $Pr_8O_{16}Si_4Te_4$, $La_8O_{16}Se_4Si_4$, $Tb_8O_{16}Se_4Si_4$, $Dy_8O_{16}Se_4Si_4$, $Nd_8O_{16}Se_4Si_4$, $Nd_8O_{16}Si_4Te_4$, $Ho_8O_{16}Se_4Si_4$, $Ca_8As_4Cl_4O_{16}$, $Sm_8O_{16}Si_4Te_4$, $Hg_{12}Tl_4Br_{12}Sb_8$, $Hg_{12}Tl_4As_8Cl_{12}$
57 ($Pbcm$)	True	$Sm_8O_{16}Se_4Si_4$
58 ($Pnnm$)	False	Ca_2Br_4, Ru_2P_4, Fe_2P_4, Os_2P_4, Os_2As_4, Ru_2As_4, Pt_2O_4, Fe_2S_4, Yb_2Br_4, Ca_2Br_4, Ca_2Cl_4, Sn_2O_4, Na_4O_2, In_4S_4, $Au_2Na_4Rb_2O_4$, $Au_2K_2Na_4O_4$, $Ga_4Cl_4Te_4$, $In_4Sr_6P_8$, $Mg_6B_4O_{12}$, $Cd_6B_4O_{12}$, $Cu_4Tl_8I_{12}$, $K_6Y_2B_4O_{12}$, $Ca_2Li_8B_4O_{12}$, $Al_8O_{20}Si_4$
59 ($Pmmn$)	False	$In_2Br_2O_2$, $Er_2I_2Se_2$, $Hf_2Br_2N_2$, $Zr_2Br_2N_2$, $K_2Na_4B_2O_6$, $Na_4Rb_2B_2O_6$, $Au_2K_6Ge_8$, $Au_2Rb_6Ge_8$, $Au_2K_6Sn_8$, $Au_2Cs_6Ge_8$, $Au_8Tl_4S_6$, $Li_{10}Re_2N_8$, $Al_2Li_{10}O_8$, $In_2Na_{10}O_8$, $Rh_8Sr_4O_{16}$
59 ($Pmmn$)	True	$Ti_2Cl_2N_2$, $In_2Cl_2O_2$, $K_2Li_4As_2$, $Cs_6Mg_6H_{18}$
60 ($Pbcn$)	False	Mg_4H_8, Mg_4H_8, Yb_4Br_8, $Cu_4Li_{12}S_8$, $Ba_8Sr_4I_{24}$, Rh_8O_{12}, $Ca_{12}N_8$, $Cd_4K_8O_8$, $Cd_4Rb_8O_8$, $K_8Mg_4O_8$, $Pd_8P_8Se_8$, $Pd_8P_8S_8$, $Nd_8Cl_{12}N_4$, $Hg_8Br_4P_{12}$, $Ca_4O_{16}Te_4$, $Lu_4Na_4Cl_{16}$, $Na_4Sc_4Cl_{16}$, $Ca_4Sr_4I_{16}$, $In_4Li_4F_{16}$, $Al_4Ta_4O_{16}$, $Ba_4Zn_4Cl_{16}$, Th_8S_{20}, $Na_8O_{16}Te_4$, $Li_4Nd_4Ge_4O_{16}$, $Ca_4Sr_8I_{24}$, $Ca_8Sr_4I_{24}$, $Sn_{12}O_{24}$, $Hg_{12}Rb_8Te_{16}$, $Li_8Ti_8O_{20}$, $Li_8O_{20}Si_8$
60 ($Pbcn$)	True	$Ca_4B_8O_{16}$
61 ($Pbca$)	False	$Rb_{16}O_8$, $Co_8P_8Se_8$, Hf_8O_{16}, Sr_8I_{16}, Yb_8Cl_{16}, Mg_8H_{16}, Hf_8O_{16}, Zr_8O_{16}, $Hg_{12}Na_8S_{16}$

No. (SP)	Clean	NDL and NDR (NDL(R)) candidates
62 (*Pnma*)	False	$Cu_4Li_4S_4$, Sn_4S_4, Ge_4S_4, Sn_4Se_4, Ge_4Se_4, $Mn_4Tl_4Cl_{12}$, Zr_4O_8, Th_4S_8, Ba_4Cl_8, Ba_4I_8, Pb_4Cl_8, Ca_4H_8, Sr_4H_8, Ba_4H_8, Pb_4F_8, Ba_4Br_8, Pb_4Br_8, Cs_8S_4, Ba_4F_8, Na_8S_4, Cs_8Te_4, Rb_8O_4, Th_4Se_8, Yb_4H_8, Sr_4I_8, $Ca_4Li_4Sb_4$, $Pb_4Br_4Cl_4$, $Ba_4Br_4Cl_4$, $K_4Na_4S_4$, $Li_4Rb_4O_4$, $K_4Na_4Se_4$, $Li_4Na_4Se_4$, $Ag_4Tl_4Se_4$, $Bi_4Li_4Sr_4$, $Ag_4Tl_4Te_4$, $Ca_4Sr_4Si_4$, $Li_4Sr_4Sb_4$, $Li_4Na_4Te_4$, $K_4Na_4Te_4$, Sn_4Br_8, $I_4Sb_4Se_4$, $I_4S_4Sb_4$, $K_4Rb_4S_4$, $Bi_4F_4O_4$, $Li_4Mg_4N_4$, $Ca_4Li_4N_4$, $Ag_4Cu_4S_4$, $Cu_4Y_4S_8$, $Cu_4Ho_4S_8$, $Cu_4Er_4S_8$, $Cu_4Tm_4S_8$, $Cu_4Lu_4S_8$, $Cu_4Dy_4S_8$, $Li_4Tb_4O_8$, $Dy_4Li_4O_8$, Tb_4F_{12}, Y_4F_{12}, Tl_4F_{12}, Ho_4F_{12}, Sm_4F_{12}, Er_4F_{12}, $Sr_4Zn_4O_8$, $Sr_4C_4N_8$, $Ga_4Rb_4H_8$, $Cu_4Sb_4Se_8$, $Bi_4Cu_4S_8$, $Cu_4S_8Sb_4$, $Ba_4H_4I_4O_4$, $Ba_4Cu_{16}S_{12}$, $Al_4H_4O_8$, $Sn_4Zr_4S_{12}$, $Pb_4Zr_4S_{12}$, $In_4Mg_4Br_{12}$, $Cd_4In_4Br_{12}$, $Hf_4Sn_4S_{12}$, $Cu_4Ta_4S_{12}$, Sn_8S_{12}, Sm_8S_{12}, Pr_8S_{12}, Nd_8S_{12}, La_8S_{12}, Dy_8S_{12}, $Rb_4F_4H_8O_4$, $Cd_4Rb_4Cl_{12}$, $Cd_4K_4Cl_{12}$, $Pb_4Rb_4I_{12}$, $Cd_4Tl_4I_{12}$, $Cd_4Rb_4Br_{12}$, $Cd_4Tl_4Cl_{12}$, $Cd_4Tl_4Br_{12}$, $Tl_4Ge_4I_{12}$, $Cs_4Pb_4I_{12}$, $Sr_4Zr_4S_{12}$, $Rb_4Yb_4I_{12}$, $Rb_{12}Cl_4O_4$, $Li_8H_4I_4O_4$, $In_4La_4O_{12}$, $Mg_4O_{12}Se_4$, $La_4Tm_4O_{12}$, $Pb_4Sr_4O_{12}$, $Lu_4Pr_4O_{12}$, $Mg_4Na_4H_{12}$, $K_4Mg_4Cl_{12}$, $Sn_4Sr_4O_{12}$, $Mg_4Na_4F_{12}$, $Sr_{12}O_4Si_4$, $Nd_4Sc_4O_{12}$, $Sc_4Sm_4O_{12}$, $Sc_4Tb_4O_{12}$, $Dy_4Sc_4O_{12}$, $Ho_4Sc_4O_{12}$, $Ga_4Nd_4O_{12}$, $Rh_4Sm_4O_{12}$, $Hf_4Sr_4O_{12}$, $Al_4Y_4O_{12}$, $Na_4Zn_4F_{12}$, $Ca_4Ti_4O_{12}$, $Ca_{12}As_4N_4$, $Sr_4Zr_4O_{12}$, $Al_4Tb_4O_{12}$, $Ca_4Sn_4O_{12}$, $Al_4Sc_4O_{12}$, $Ga_4Pr_4O_{12}$, $Pr_4Rh_4O_{12}$, $Al_4Ho_4O_{12}$, $La_4Rh_4O_{12}$, $Pr_4Sc_4O_{12}$, $Cs_4Pb_4Br_{12}$, $Rh_4Tb_4O_{12}$, $Rb_4Yb_4Br_{12}$, $K_{12}Cl_4O_4$, $Ca_4K_4F_{12}$, $In_4Sm_4O_{12}$, $Ca_4O_{12}Se_4$, $Ti_4Yb_4O_{12}$, $Ga_4Tm_4O_{12}$, $Ca_4Hf_4O_{12}$, $In_4Nd_4O_{12}$, $Sc_4Y_4O_{12}$, $Ba_{12}O_4Si_4$, $Ga_4Ho_4O_{12}$, $Al_4Lu_4O_{12}$, $Dy_4Ga_4O_{12}$, $Bi_4Sc_4O_{12}$, $Al_4Tm_4O_{12}$, $Ga_4Tb_4O_{12}$, $Al_4Er_4O_{12}$, $Al_4Dy_4O_{12}$, $Ca_4Zr_4S_{12}$, $Sc_4Y_4S_{12}$, $Hf_4Yb_4O_{12}$, $Cd_4Sn_4O_{12}$, $Cd_4O_{12}Se_4$, $Cd_4K_4F_{12}$, $La_8O_4Se_8$, $Sm_8O_4Se_8$, $K_4H_{12}Si_4$, $Hg_4O_{12}Se_4$, Dy_8S_{12}, Ho_8S_{12}, Tb_8S_{12}, $Bi_4Cd_4Br_4Se_8$, $Ba_4S_{12}Te_4$, $Sr_4C_4O_{12}$, $Nd_4B_4O_{12}$, $Yb_4C_4O_{12}$, $La_4B_4O_{12}$, $Pr_4B_4O_{12}$, $Ba_4Cu_8Te_8$, $Ba_4Cu_8S_8$, Ba_8Si_{16}, Ba_8Ge_{16}, $Ca_8B_4Cl_4N_8$, $Lu_4Cl_4H_8O_8$, $Cu_8Rb_4I_{12}$, $Na_{12}Tl_4O_8$, Na_4P_{20}, $Ga_4Na_4Br_{16}$, La_8S_{16}, $K_4Sm_4F_{16}$, $K_4Pr_4F_{16}$, $Al_4K_4H_{16}$, $Ga_4K_4H_{16}$, $Pb_4Rb_8O_{12}$, Ir_8S_{16}, $In_4O_{16}P_4$, $Mg_4O_{16}S_4$, $Mg_4O_{16}Se_4$, $Zn_4O_{16}Se_4$, $Cd_4O_{16}Se_4$, $Au_4Cs_4K_8O_8$, $Ba_8Zn_4S_{12}$, $Cu_4Rb_8Br_{12}$, $Cu_4K_8Cl_{12}$, $Cu_4K_8Br_{12}$, $Cu_4Rb_8Cl_{12}$, $Ca_8B_4F_4N_8$, $Ca_8B_4H_4N_8$, $Ba_4Cu_4La_4Te_{12}$, $Cu_4Pr_4Sr_4Se_{12}$, $Ba_4Cu_4La_4Se_{12}$, $Cu_4Pb_4Y_4S_{12}$, $Cu_4Er_4Pb_4Se_{12}$, $Cu_4Dy_4Pb_4Se_{12}$, $Cu_4Pb_4Tb_4Se_{12}$, $Cu_4Ho_4Pb_4Se_{12}$, $Cu_4Pb_4Tm_4Se_{12}$, $Ba_8Cd_4Se_{12}$, $Ba_8Cd_4Te_{12}$, $Ba_8Mn_4Te_{12}$, $Ba_8Hg_4S_{12}$, $Ba_8Cd_4S_{12}$, $Ba_8Mn_4S_{12}$, $Ba_8Mn_4Se_{12}$, $Ag_4Rb_8I_{12}$, $Ag_4Rb_8Br_{12}$, $Ag_4K_8I_{12}$, $Au_8Br_8Se_8$, $Zr_{12}N_{16}$, $Y_4F_4O_{16}S_4$, $Tm_8Zn_4S_{16}$, $Mg_8Ge_4S_{16}$, $Lu_8Zn_4S_{16}$, $Li_8Zn_4Cl_{16}$, $Li_8Zn_4I_{16}$, $Li_8Zn_4Br_{16}$, $Na_8Zn_4Br_{16}$, $Mg_8O_{16}Si_4$, $Mg_8Ge_4O_{16}$, $Al_8Be_4O_{16}$, $Be_4Na_8F_{16}$, $Cd_8Ge_4O_{16}$, $Ca_8O_{16}Si_4$, $Ca_8Ge_4S_{16}$, $Na_8Zn_4Cl_{16}$, $Ba_8Ge_4O_{16}$, $Ca_8Sn_4S_{16}$, $Ba_4Rb_4O_{16}P_4$, $Ba_4K_4O_{16}P_4$, $Rb_8W_4S_{16}$, $Cs_8W_4S_{16}$, $Ba_8Ti_4S_{16}$, $K_8Mo_4S_{16}$, $Mo_4Rb_8S_{16}$, $Be_4Rb_8F_{16}$, $Rb_8O_{16}S_4$, $Be_4Cs_8F_{16}$, $Be_4K_8F_{16}$, $Cs_8Zn_4Cl_{16}$, $Ba_8Ge_4S_{16}$, $K_8Zn_4H_{16}$, $Rb_8Zn_4H_{16}$, $Nd_{12}Br_4N_8Se_4$, $Na_4Tb_4Ge_4O_{16}$, $Na_4Y_4Ge_4O_{16}$, $Er_4Na_4Ge_4O_{16}$, $In_4Li_4Ge_4O_{16}$, $Na_4Y_4O_{16}Si_4$, $Ho_4Na_4Ge_4O_{16}$, $Na_4Tm_4Ge_4O_{16}$, $Li_4Sc_4Ge_4O_{16}$, $Na_4Sm_4Ge_4O_{16}$, $Ca_4Mg_4Ge_4O_{16}$, $Ca_4Ga_4La_4O_{16}$, $Li_4Sc_4O_{16}Si_4$, $Ca_4Mg_4O_{16}Si_4$, $Lu_4Na_4Ge_4O_{16}$, $In_4Li_4O_{16}Si_4$, $Al_4Mg_4B_4O_{16}$, $Li_4Mg_4As_4O_{16}$, $Li_4Mg_4O_{16}P_4$, Na_8S_{20}, $Na_8B_4H_4O_{12}$, $Al_8Mg_4S_{16}$, $Li_8U_4O_{16}$, $Li_{12}B_4S_{12}$, $Sr_4Tl_4P_4S_{16}$, $Hf_{12}N_{16}$, $Ga_{12}I_4Te_{12}$, $In_{12}I_4Te_{12}$, $Cu_{12}Sb_4Se_{12}$, $Cs_8Sn_4Te_{16}$, $Cd_{12}As_4Cl_{12}$, $Al_4Ca_{12}As_{12}$, $Dy_{12}N_4Se_{12}$, $Ca_{12}In_4P_{12}$, $In_4Sr_{12}P_{12}$, $Ba_{12}In_4As_{12}$, $Ca_4Y_8S_{16}$, $Ca_4Lu_8S_{16}$, $Er_8Yb_4S_{16}$, $Ba_4Bi_4B_4O_{16}$, $Ba_4Tb_8Te_{16}$, $Ba_4Ho_8Te_{16}$, $Sr_4Y_8O_{16}$, $Ba_4Ho_8O_{16}$, $Ba_4Pr_8O_{16}$, $Ba_4Sc_8Te_{16}$, $Ca_4Sc_8S_{16}$, $Sc_8Sr_4S_{16}$, $Ba_4Sm_8Te_{16}$, $Ba_4Y_8Te_{16}$, $Ba_4Dy_8Te_{16}$, $Ba_4Er_8Te_{16}$, $Ba_4Sm_8O_{16}$, $Ca_4Rh_8O_{16}$, $Sr_4Tb_8O_{16}$, $Ba_4Tm_8Te_{16}$, $Ba_4Tb_8O_{16}$, $Sc_8Sr_4Te_{16}$, $Pb_4Sc_8S_{16}$, $Ba_4Nd_8O_{16}$, $Sr_4Y_8S_{16}$, $Ba_4Y_8S_{16}$, $Pr_8Sr_4O_{16}$, $Sr_4Tm_8O_{16}$, $Ba_4Y_8O_{16}$, $Ca_4Sc_8O_{16}$, $Ca_4Ga_8O_{16}$, $Ca_4Lu_8O_{16}$, $In_8Sr_4O_{16}$, $Pb_4Sc_8Se_{16}$, $Dy_8Sr_4O_{16}$, $Ho_8Sr_4O_{16}$, $Er_8Sr_4O_{16}$, $Er_8Pb_4Se_{16}$, $In_8Pb_4S_{16}$, $Ba_4Lu_8O_{16}$, $Ba_4La_8O_{16}$, $Ca_4Tm_8O_{16}$, $Sm_8Sr_4O_{16}$, $Ba_4Tm_8O_{16}$, $Nd_8Sr_4O_{16}$, $Sc_8Sr_4O_{16}$, $Lu_8Sr_4O_{16}$, $Dy_8Sr_4Se_{16}$, $Ba_4Nd_8S_{16}$, $Dy_8Sr_4S_{16}$, $Ba_4Lu_8S_{16}$, $Al_{12}N_4O_{12}$, $Ti_4Tm_8O_{20}$, $Lu_8Ge_4O_{20}$, $Tm_8Ge_4O_{20}$, $Lu_8Ti_4O_{20}$, $Hf_4Y_8O_{20}$, $Hf_4Y_8S_{20}$, $Pr_4Rb_8Cl_{20}$, $K_8Pr_4Br_{20}$, $K_8Pr_4I_{20}$, $K_8Pr_4Cl_{20}$, $Tl_8Ge_8S_{16}$, $Tl_{12}P_4S_{16}$, $Rb_{12}P_4S_{16}$, $Rb_{12}S_{16}Sb_4$, $Rb_{12}Ta_4S_{16}$, $K_{12}Nb_4Se_{16}$, $Rb_{12}Sb_4Se_{16}$, $K_{12}P_4S_{16}$, $K_{12}Nb_4S_{16}$, $K_{12}P_4Se_{16}$, $Nb_4Rb_{12}S_{16}$, $K_{12}As_4S_{16}$, $Tl_{12}P_4Se_{16}$, $Rb_{12}V_4S_{16}$, $Tl_{12}As_4S_{16}$, $Cs_{12}P_4Se_{16}$, $Tl_{12}P_4Se_{16}$, $Sn_4Sr_8F_8Se_{12}$, $Ba_8Sn_4F_8Se_{12}$, $Rb_{12}O_{16}P_4$, $Na_4Y_4C_4F_8O_{12}$, $Li_4Ti_4As_4O_{20}$, $Li_4Nb_4Ge_4O_{20}$, $Li_{12}O_{16}P_4$, $Cd_4Li_8O_{16}Si_4$, $Ba_{12}Nb_4As_{12}O_4$, $Sr_{12}Ta_4As_{12}O_4$, $Al_8O_{20}Si_4$, $Sr_4Ta_8O_{24}$, $Pr_{16}N_8Te_{12}$, $Nd_{16}N_8Te_{12}$, $Rb_{20}Ge_4P_{12}$, $Pb_4Rb_{12}Cl_{20}$, $Cd_{12}Rb_8Se_{16}$, $Cd_{12}K_8S$
62 (*Pnma*)	True	$Ca_4Sr_4Ge_4$, $Cu_4K_4S_4$, C_4H_8, $Ba_4Br_4H_4O_4$, $Sr_4H_4I_4O_4$, $Pb_4Br_4H_4O_4$, $Pb_4H_4I_4O_4$, $Hf_4Pb_4S_{12}$, $Nd_4Rh_4O_{12}$, $K_4N_4O_{12}$, $Ba_4Cu_8Se_8$, $Tb_4Cl_4O_{12}Se_4$, Sr_8Ge_{16}, $Sm_4Cl_4H_8O_8$, $Al_4Na_4I_{16}$, $Li_4B_4H_{16}$, $Ba_4O_{16}S_4$, $K_8Sn_4O_{12}$, $Ag_4Cs_8I_{12}$, $Sr_8B_4F_4N_8$, $Ag_4Rb_8Cl_{12}$, $Ca_8Ge_4O_{16}$, $Ba_8O_{16}Si_4$, $K_8W_4Se_{16}$, $Cs_8O_{16}Se_4$, $Ba_8S_{16}Si_4$, $Cs_8Mg_4H_{16}$, $Pb_{12}Br_8O_8$, $Cd_4Na_4O_{16}P_4$, $Ga_4K_4Na_8O_{12}$, $Ba_4K_4P_4S_{16}$, $Ba_4B_4S_{16}Sb_4$, $Ba_4P_{16}Te_8$, $K_{12}Ta_4S_{16}$, $Rb_{12}As_4Se_{16}$, $K_{12}V_4S_{16}$, $K_{12}Li_4Si_{16}$, $Pd_4Rb_8I_{24}$, $I_8P_{16}Se_{12}$, $Na_8B_8S_{20}$, $Mg_8H_4O_{20}P_4$, $K_8Re_4Cl_{20}O_4$, $Hg_4Zn_4As_4H_4O_{20}$

No. (SP)	Clean	NDL and NDR (NDL(R)) candidates
63 (*Cmcm*)	False	In_4Br_4, In_4I_4, $Au_4K_4S_4$, $Au_4Rb_4S_4$, $Cs_8Pt_4Cl_{16}$, $Cs_8Pd_4Cl_{16}$, Tb_4Cl_{12}, Nd_4Br_{12}, Sm_4Br_{12}, La_4I_{12}, Dy_4Cl_{12}, $Li_4Rb_4Cl_8$, $Au_4Rb_4O_8$, $Ag_4Na_8Sb_4$, $Ag_4Na_8As_4$, $Cu_4K_8As_4$, $Cu_4Na_8As_4$, $Ag_4Bi_4K_8$, $Cu_4Na_8P_4$, $Ag_4K_8As_4$, $Ag_4K_8Sb_4$, $Au_4Na_8As_4$, $Au_4Na_8Sb_4$, $Cu_4K_8P_4$, $Au_4K_8Sb_4$, $Au_4K_8P_4$, $Ag_4K_8P_4$, $Cs_4Cu_4O_8$, $Cu_4Rb_4O_8$, $Ag_4Cs_4Cl_8$, $W_4Y_4N_{12}$, $K_8Pd_4P_8$, $K_8Pd_4As_8$, $K_8Ni_4P_8$, $Lu_4Pr_4Se_{12}$, $Lu_4Nd_4Se_{12}$, $Ca_4In_4Br_{12}$, $Ca_4Pt_4O_{12}$, $Bi_4Pb_4Cl_4O_8$, $Pb_4Cl_4O_8Sb_4$, $Ba_4Cl_4O_8Sb_4$, $Bi_4Sr_4Cl_4O_8$, $Ba_4Bi_4I_4O_8$, $Bi_4Sr_4Br_4O_8$, $Bi_4Sr_4I_4O_8$, $Ba_4Bi_4Cl_4O_8$, $Ag_4Bi_4Cl_8S_4$, $Mg_4O_{16}Se_4$, $Tl_4O_{16}P_4$, $Zn_4O_{16}Se_4$, $In_4O_{16}P_4$, $Mg_4O_{16}S_4$, $Cd_4O_{16}Se_4$, $Cd_4O_{16}S_4$, $Ba_{12}Sn_8O_{28}$, $K_8Ti_4O_{12}$, $Cs_8Ti_4S_{12}$, $Cs_8Sn_4O_{12}$, $Cs_8Zr_4O_{12}$, $Cs_8Zr_4Se_{12}$, $Cs_4Na_4Ti_4O_{12}$, $Cs_8Li_4Cl_{12}$, $Ca_4O_{16}S_4$, $Ba_4Cu_4Y_4Te_{12}$, $Ag_4Ba_4Y_4Te_{12}$, $Cd_4Cs_4Y_4Se_{12}$, $Cd_4Cs_4Pr_4Se_{12}$, $Cd_4Cs_4Tb_4Se_{12}$, $Cd_4Cs_4Dy_4Se_{12}$, $Cs_4Hg_4Y_4Se_{12}$, $Cs_4Pr_4Zn_4Te_{12}$, $Cs_4Nd_4Zn_4Te_{12}$, $Cs_4Sm_4Zn_4Te_{12}$, $Cd_4Cs_4Dy_4Te_{12}$, $Cd_4Cs_4Er_4Te_{12}$, $Cd_4Cs_4Ho_4Te_{12}$, $Cd_4Cs_4La_4Te_{12}$, $Cd_4Cs_4Lu_4Te_{12}$, $Cd_4Cs_4Pr_4Te_{12}$, $Cd_4Cs_4Tb_4Te_{12}$, $Cd_4Cs_4Tm_4Te_{12}$, $Cd_4Cs_4Sm_4Te_{12}$, $Cu_4Lu_4Sr_4S_{12}$, $Ba_4Cu_4Er_4S_{12}$, $Cs_4Sm_4Zn_4Se_{12}$, $Cs_4Ho_4Zn_4Se_{12}$, $Cs_4Tm_4Zn_4Se_{12}$, $Cd_4Cs_4Sm_4Se_{12}$, $Cs_4Hg_4Nd_4Se_{12}$, $Cs_4La_4Zn_4Te_{12}$, $Cs_4Dy_4Zn_4Te_{12}$, $Ce_4Cs_4Cu_4S_{12}$, $Cu_4Lu_4Sr_4Se_{12}$, $Cs_4Er_4Zn_4Te_{12}$, $Cs_4Tb_4Zn_4Se_{12}$, $Cs_4Y_4Zn_4Se_{12}$, $Cs_4Ho_4Zn_4Te_{12}$, $Cs_4Tb_4Zn_4Te_{12}$, $Ag_4Ba_4Y_4Se_{12}$, $Cu_4Tl_4Zr_4S_{12}$, $Cs_4Er_4Zn_4Se_{12}$, $Cs_4Hg_4Pr_4Se_{12}$, $Cs_4Hg_4Sm_4Se_{12}$, $Cu_4K_4Zr_4S_{12}$, $Cu_4Hf_4Tl_4S_{12}$, $Cu_4Hf_4Tl_4Se_{12}$, $Ba_4Cu_4Dy_4Te_{12}$, $Cu_4Hf_4K_4S_{12}$, $Ba_4Mg_4H_{16}$, $Hg_4Na_4O_{16}P_4$, $Na_8O_{16}S_4$, $Mg_8Ru_4H_{16}$, $La_4Mg_4I_{20}$, $Ca_4La_4I_{20}$, $Ca_{12}V_4N_{12}$, $Ba_4Tm_8O_{16}$, $Ba_4La_8O_{16}$, $Sc_8Sr_4O_{16}$, $Lu_8Sr_4O_{16}$, $Ba_4Lu_8O_{16}$, $Zr_{12}N_8O_{12}$, $Hf_{12}N_8O_{12}$, $Cu_4Nd_8Rb_4S_{16}$, $Cu_4Rb_4Sm_8S_{16}$, $Cu_4K_4Y_8S_{16}$, $Cu_4Rb_4Sm_8Se_{16}$, $Cu_4Dy_8Rb_4Se_{16}$, $Cs_4Cu_4Sm_8Se_{16}$, $Cu_4Er_8K_4Se_{16}$, $Cs_4Cu_4Nd_8Se_{16}$, $Mg_4Ti_8O_{20}$, $Al_8Ti_4O_{20}$, $Hf_4Y_8O_{20}$, $Cs_4Li_{12}Cl_{16}$, $Al_4Tl_8F_{20}$, $Li_{16}Pb_4O_{16}$, $Li_{16}Zr_4O_{16}$, $Li_{16}Ti_4O_{16}$, $Li_{16}Ge_4O_{16}$, $Li_8B_8S_{20}$, $Li_8Br_8H_{16}O_8$, $Nd_{12}O_{28}Sb_4$, $Y_{12}O_{28}Sb_4$, $Cu_{12}Er_8Rb_4Se_{20}$, $Cu_{12}Er_8Rb_4S_{20}$, $Cu_{12}Ho_8Rb_4S_{20}$, $Ag_{12}Rb_4Sm_8Se_{20}$, $Ag_{12}Cs_4Tb_8Se_{20}$, $Cs_4Cu_{12}Lu_8Se_{20}$, $Cs_4Cu_{12}Tm_8Se_{20}$, $Cu_{12}K_4Tm_8Se_{20}$, $Ga_8Sr_4B_8O_{28}$, $Ba_4Ga_8B_8O_{28}$, $Na_{24}Pb_4O_{20}$, $Ag_4Au_4Ba_{16}O_{24}$, $La_4O_{36}Sb_{12}$, $Ba_4Ca_8C_8F_8O_{24}$, $Ho_{12}B_{12}O_{36}$, $Al_{12}La_{12}O_{36}$
63 (*Cmcm*)	True	$Pt_4Rb_8As_8$, $La_4C_4F_4N_8$, $Cu_8Rb_4Br_{12}$, $Y_{12}B_{12}O_{36}$, $Be_{12}Li_{12}H_{36}$
64 (*Cmce*)	False	K_8O_8, $Lu_8Pd_4O_{16}$, $Ba_8N_{16}Si_8$, $Ba_8Pd_4F_{24}$, $Ba_4B_8Se_{24}$, $Ba_{16}V_8N_{24}$, $Ca_{16}N_{24}P_8$, $Os_{16}Si_{32}$, $Fe_{16}Si_{32}$, $Ru_{16}Si_{32}$, $K_{16}Sn_8Te_{24}$, $In_8K_{16}Li_8As_{16}$, $In_8K_{16}Na_8Sb_{16}$, $Ga_8K_{16}Li_8As_{16}$, $Al_8Li_8Na_{16}P_{16}$, $Ca_{12}Ta_8O_{32}$, $Mg_{12}As_8O_{32}$, $Al_8Sr_{24}Sb_{24}$, $Al_8Ba_{24}Sb_{24}$, $Ba_{24}Ga_8P_{24}$, $Li_8B_{48}Si_8$, $In_{16}K_{24}As_{24}$, $W_{24}Br_{48}$, $Mo_{24}Br_{48}$, $Mo_{24}Cl_{48}$, $K_{24}Na_{16}Sn_8P_{24}$, $K_{24}Na_{16}Sn_8As_{24}$
64 (*Cmce*)	True	$Ba_8Pt_4H_{24}$
65 (*Cmmm*)	False	$Cd_2U_2O_8$, $Hf_2Zr_2O_8$, $Fe_2Li_4Br_8$, $Li_4Mg_2Br_8$, $Bi_2Na_6O_8$, $Mg_6Zn_2O_8$, $Ca_6Cd_2O_8$, $Au_2La_8Li_2O_{16}$, $Sn_{14}Ti_6O_{40}$
65 (*Cmmm*)	True	$La_8C_4Cl_4N_{12}$
66 (*Cccm*)	False	$In_8Sr_4Te_{16}$, $Ga_8Sr_4Te_{16}$, $Ba_4Ga_8Se_{16}$, $Al_8Sr_4Se_{16}$
67 (*Cmme*)	False	$Pb_8Cl_4F_4O_4$, $Ba_4Zr_4F_{24}$, $H_{24}N_4O_8P_4$, $Al_4Ca_4B_{12}O_{28}$
68 (*Ccce*)	False	$K_{16}Sn_{12}Se_{32}$
69 (*Fmmm*)	False	$Cs_8Pd_{12}Se_{16}$, $Pd_{12}Rb_8S_{16}$, $Cs_8Pt_{12}S_{16}$, $Cs_8Ni_{12}Se_{16}$, $Cs_8Pt_{12}Se_{16}$, $Pt_{12}Rb_8S_{16}$, $Rb_{16}P_{24}$, $K_{16}P_{24}$, $Ba_{16}Ca_8Pd_{24}O_{48}$
69 (*Fmmm*)	True	$Cs_8Pd_{12}S_{16}$
70 (*Fddd*)	False	$Au_{16}Li_{16}S_{16}$, Ca_8N_{48}, $Hg_{16}Ge_8O_{32}$, $Hg_{16}O_{32}Si_8$, $Cu_{16}O_{32}S_8$, $Ag_{16}O_{32}Se_8$, $Cd_{16}O_{32}Si_8$, $Na_{16}O_{32}S_8$, $Na_{16}O_{32}Se_8$, $Cu_8Rb_{16}Ta_8S_{32}$, $Cu_8Rb_{16}Ta_8Se_{32}$, $Ag_8Rb_{16}V_8Se_{32}$, $Ag_8Nb_8Rb_{16}S_{32}$, $Ag_8Cs_{16}Nb_8Se_{32}$, $Cu_8K_{16}V_8S_{32}$, $Ag_8Rb_{16}Ta_8S_{32}$, $Ag_8Cs_{16}Ta_8S_{32}$, $Cu_8Nb_8Rb_{16}S_{32}$, $Cu_8Nb_8Rb_{16}Se_{32}$, $Ag_8Cs_{16}Nb_8S_{32}$, $Ag_8K_{16}V_8S_{32}$, $Cu_8K_{16}Ta_8Se_{32}$, $Cu_8K_{16}Nb_8Se_{32}$, $Ag_8Nb_8Rb_{16}Se_{32}$, $Ir_{16}Cl_{48}$, $K_{16}Pd_{24}Se_{32}$, $Pd_{24}Rb_{16}Se_{32}$, $K_{16}Ni_{24}S_{32}$, $Ag_{16}K_{16}Ge_8S_{32}$, $Au_{16}Cs_{16}Sn_8S_{32}$, $K_{32}P_{48}$, $Er_{32}Se_{48}$, $Sc_{32}S_{48}$, $Ho_{32}Se_{48}$, $Ba_8Be_{16}B_{16}O_{48}$, $Ca_{16}Na_{24}Ta_8O_{48}$, $Bi_8Ca_{16}Na_{24}O_{48}$, $Na_{32}Pt_{16}O_{48}$, $Na_{32}Sn_{16}O_{48}$
70 (*Fddd*)	True	Sr_8N_{48}, $K_{16}Pd_{24}S_{32}$
71 (*Immm*)	False	Li_4C_4, Rb_4S_4, Rb_4O_4, $Na_4Pt_2O_4$, $Li_4Pd_2O_4$, $K_4Pd_2O_4$, $K_4Pt_2S_4$, $Pt_2Rb_4S_4$, $K_4Pt_2Se_4$, $Pt_2Rb_4Se_4$, $Cs_2Li_4Cl_6$, $La_4Li_2H_2O_6$, $Li_2Nd_4H_2O_6$, $Li_{10}Br_4N_2$, $Rb_2Th_4Se_{12}$, $Na_4Pd_6O_8$, $Ta_4Cl_8I_8$, $Au_4Cs_4F_{16}$, $Ba_6Sr_8O_{14}$, $K_4Li_{28}Zr_6O_{28}$, $K_4Li_{28}Pb_6O_{28}$, $Li_{28}Pb_6Rb_4O_{28}$
72 (*Ibam*)	False	Be_4I_8, $K_8Zn_4Te_8$, $Sr_8I_8O_4$, $Ba_8I_8O_4$, $Rb_8As_8Si_4$, $K_8As_8Si_4$, $K_8Zn_4O_8$, $K_8P_8Si_4$, $Na_8Zn_4S_8$, $K_8Mn_4Se_8$, $K_8Mn_4Te_8$, $K_8As_8Ge_4$, $Rb_8Sn_4As_8$, $Ag_{12}Li_4O_8$, $Ag_{12}Na_4O_8$, $Au_4Li_{12}S_8$, $Ag_4Na_{12}O_8$, $Cu_4Na_{12}O_8$, $In_4K_{12}P_8$, $Al_4Na_{12}P_8$, $Al_4Na_{12}As_8$, $In_4K_8Na_4As_8$, $In_4K_8Na_4P_8$, $Al_4K_8Li_4P_8$, $Al_4K_8Na_4P_8$, $Al_4K_8Na_4As_8$, $Ga_4K_8Na_4P_8$, $Ga_4K_8Na_4As_8$, $Bi_8Li_8O_{16}$, $Cu_8Rb_8Sn_4S_{16}$, $Cs_8Mn_{12}S_{16}$, $Cs_8Mn_{12}Se_{16}$, $Mn_{12}Rb_8Se_{16}$, $Cs_8Zn_{12}S_{16}$, $Cd_{12}Cs_8Te_{16}$, $Be_{12}H_{24}$, $Cs_8Pd_8Cl_{24}$, $Cs_8Ga_4Li_{12}O_{16}$, $Na_4Ta_{12}O_{32}$, $Au_8K_8Se_{40}$, $Au_8K_8S_{40}$, $Ba_4K_{16}V_8S_{32}$
73 (*Ibca*)	False	$Li_{32}Ta_8N_{24}$
74 (*Imma*)	False	$Mg_8Pt_4O_{16}$, $Mg_8Sn_4O_{16}$, $Sn_4Zn_8O_{16}$, $Pt_4Zn_8O_{16}$, $Cd_4Li_8Cl_{16}$, $Al_8Mg_4O_{16}$, $Li_8Mg_4Cl_{16}$, $Ga_8Mg_4O_{16}$, $In_8Mg_4O_{16}$, $Li_4As_4F_{24}H_8O_4$, $Ca_8O_{28}Sb_8$, $Na_8O_{28}Te_8$, $Sr_8O_{28}Sb_8$, $Ba_8O_{28}Sb_8$, $Li_8Pd_4O_{28}P_8$, $Al_4Zn_4F_{20}H_{16}O_8$, $Al_4Mg_4F_{20}H_{16}O_8$, $Mg_4B_{48}C_8$, $B_{48}Si_{16}$, Mg_8B_{56}, $Li_4B_{52}C_8$, $Al_4Li_4B_{56}$, $Rh_{12}O_{48}P_{12}$
74 (*Imma*)	True	$Al_4Na_4C_4H_8O_{20}$

No. (SP)	Clean	NDL and NDR (NDL(R)) candidates
82 ($I-4$)	False	$In_2P_2S_8, Cu_2Ga_2I_8$
84 ($P4_2/m$)	False	$Th_2P_4S_{12}, Zr_2P_4S_{12}, La_4Pd_4O_{10}$
85 ($P4/n$)	False	$Sr_{10}Br_{16}Cl_4$
87 ($I4/m$)	False	$Bi_2F_{10}, Ga_4Te_{10}, Ir_2Na_8O_8, Rb_4Br_{12}Te_2, Li_8U_2O_{10}, Li_8U_2O_{10}, Ba_4Ta_2Y_2O_{12}, Ba_4Ho_2Ta_2O_{12}, Ho_2Na_2Rb_4F_{12}, K_{20}Se_{12}, Ba_4Zr_{12}B_2Cl_{34}, Sr_{16}Cl_{16}O_{24}Si_8$
88 ($I4_1/a$)	False	$Th_4Cl_{16}, Th_4Br_{16}, Tl_8Cl_{16}, Sm_4As_4O_{16}, Sr_4Zn_4Cl_{16}, Rb_4I_4O_{16}, K_4I_4O_{16}, Al_4Na_4H_{16}, K_4Tl_4Cl_{16}, Na_4I_4O_{16}, Bi_4Li_4F_{16}, Li_4Y_4F_{16}, Rb_4Re_4O_{16}, K_4Re_4O_{16}, Sr_4Zn_4F_{16}, Na_4Re_4O_{16}, Li_4Sc_4F_{16}, Bi_4As_4O_{16}, Al_4K_4H_{16}, Ag_4Re_4O_{16}, Pr_4As_4O_{16}, Rb_4Br_4O_{16}, Hf_4Zr_4O_{16}, Zr_4Ge_4O_{16}, Hf_4Ge_4O_{16}, Th_4Ge_4O_{16}, Al_4Li_4H_{16}, Au_8Ca_4O_{16}, Cu_8Zn_4O_{16}, Au_8Ba_4O_{16}, Au_8Sr_4O_{16}, Cu_{16}P_{32}Si_4$
99 ($P4mm$)	False	$PbTiO_3$
100 ($P4bm$)	False	$Ba_4Ti_2O_{16}Si_4$
102 ($P4_2nm$)	False	$Cs_4Hg_{12}S_{14}$
105 ($P4_2mc$)	False	$Ba_4Ge_8P_8, Cu_6Sr_{12}N_{10}$
123 ($P4/mmm$)	False	$RbN_3, K_2PtCl_4, K_2PdCl_4, K_2PdBr_4, K_2PtBr_4, PdTl_2Cl_4, Ca_3BN_3, Bi_2NdClO_4, Bi_2PrIO_4, Bi_2LuBrO_4, Bi_2TmClO_4, Bi_2SmClO_4, Bi_2DyIO_4, Bi_2TmBrO_4, Bi_2ErBrO_4, Bi_2HoBrO_4, Bi_2LaIO_4, Bi_2TbClO_4, Bi_2HoClO_4, Bi_2NdIO_4, Bi_2TbBrO_4, Bi_2ErClO_4, Bi_2SmBrO_4, Bi_2LuIO_4, Bi_2LuClO_4, Bi_2HoIO_4, Bi_2ErIO_4, Bi_2YIO_4, Bi_2PrBrO_4, Bi_2DyClO_4, Bi_2SmIO_4, Bi_2YClO_4, Bi_2TmIO_4, Bi_2TbIO_4, Bi_2DyBrO_4, Bi_2PrClO_4, Bi_2NdBrO_4, Bi_2YBrO_4, AlK_2F_5, Ba_2Sc_2O_5$
124 ($P4/mcc$)	False	$Au_4Cd_2F_{16}, Au_4Hg_2F_{16}$
125 ($P4/nbm$)	False	$Al_4Ba_2Te_8, Ag_2Rb_6Sn_6Se_{16}, K_6Na_2Sn_6Se_{16}$
125 ($P4/nbm$)	True	$Ag_4Ba_2Hg_4O_8, Ag_2K_6Sn_6Se_{16}$
126 ($P4/nnc$)	False	$Bi_4Ga_8S_{16}, Al_8Bi_4S_{16}, Bi_4Ga_8Se_{16}, Al_8Bi_4Se_{16}$
127 ($P4/mbm$)	False	$Au_2K_2Se_4, Rb_4I_2N_6, K_6Pd_2H_{10}, K_6Pt_2H_{10}, Pt_2Rb_6H_{10}, Cs_6Pd_2H_{10}, Pd_2Rb_6H_{10}, Ba_2Nd_4Pt_2O_{10}, Ba_2Nd_4Pd_2O_{10}, Ba_2Pd_2Sm_4O_{10}, Cs_2K_4Pd_2F_{10}, Ba_2Pt_2Sm_4O_{10}, Ba_2Pd_2Pr_4O_{10}, Ba_4Hg_8Cl_8O_8$
128 ($P4/mnc$)	False	$K_4Pt_2I_{12}, Hg_2Tl_8I_{12}, Tl_{12}I_8Se_2, Cd_2In_8I_{12}$
128 ($P4/mnc$)	True	$Cd_2Tl_8I_{12}$
129 ($P4/nmm$)	False	$Sn_2Se_2, Pb_2O_2, Fe_2Se_2, Sn_2O_2, Fe_2S_2, Co_2Se_2, Cu_2Br_2, Ag_2I_2, Nd_2F_2Se_2, Nd_2F_2Te_2, Bi_2Cl_2O_2, Pb_2F_2I_2, Bi_2I_2O_2, Pb_2Br_2F_2, La_2Br_2O_2, Sr_2F_2I_2, Nd_2Br_2O_2, Bi_2Br_2O_2, Ca_2Cl_2H_2, Ca_2H_2I_2, Sr_2H_2I_2, Ca_2Br_2H_2, Tm_2I_2O_2, Th_2Br_2N_2, Th_2I_2N_2, Pr_2I_2O_2, Sm_2Br_2O_2, La_2I_2O_2, Ho_2I_2O_2, Y_2I_2O_2, Sm_2I_2O_2, Tm_2Br_2O_2, Nd_2I_2O_2, Y_2Br_2O_2, Lu_2I_2O_2, Lu_2Br_2O_2, Pr_2Br_2O_2, Er_2F_2S_2, Ag_2K_2Se_2, Ba_2F_2I_2, Sr_2Cl_2F_2, Pb_2Cl_2F_2, Sr_2Br_2F_2, La_2Cl_2O_2, Nd_2Cl_2O_2, Ba_2Br_2F_2, Ba_2Cl_2F_2, Sr_2Cl_2H_2, Ba_2Cl_2H_2, Ba_2H_2I_2, Sr_2Br_2H_2, Ba_2Br_2H_2, Ca_2Cl_2F_2, Sm_2Cl_2O_2, Pr_2Cl_2O_2, Y_2Cl_2O_2, Th_2Cl_2N_2, Ho_2Cl_2O_2, K_2Li_2Te_2, Na_2Zn_2P_2, Cs_2Na_2Te_2, Yb_2Cl_2F_2, Mg_2Na_2As_2, K_2Mg_2Sb_2, Mg_2Na_2Sb_2, Dy_2Cl_2O_2, Bi_2Ca_2K_2, Th_2O_2Se_2, K_2Li_2S_2, Li_2Na_2S_2, Cs_2Na_2Se_2, Li_2Rb_2S_2, K_2Li_2Se_2, Na_2Rb_2S_2, Li_2Rb_2Se_2, Be_2Li_2As_2, Ca_2Rb_2As_2, Be_2Li_2P_2, Y_2F_2O_2, La_2F_2O_2, Bi_2F_2O_2, Ba_2Hf_2N_4, Cs_2Li_2Cl_4, Ba_2Zr_2N_4, Sr_2Ti_2N_4, Ag_2Ba_2F_2Te_2, Cu_2Sr_2F_2Se_2, Y_2Zn_2As_2O_2, La_2Zn_2As_2O_2, Cu_2La_2O_2Se_2, La_2Zn_2O_2P_2, Nd_2Zn_2As_2O_2, Cu_2Sm_2O_2Se_2, Cu_2Dy_2O_2Se_2, Er_2Zn_2As_2O_2, H_8I_2P_2, Br_2H_8P_2, Ta_2O_{10}P_2, Bi_2Li_2Pd_4O_8, Na_4Ti_2O_{10}Si_2, Na_4Ti_2Ge_2O_{10}, K_2La_2Na_2Nb_2O_{10}, K_2Na_2Nb_2Pr_2O_{10}, K_2Na_2Nd_2Ta_2O_{10}, K_2Na_2Sm_2Ta_2O_{10}, Cd_6O_{10}Si_2, Ba_2Pb_4Br_2F_{10}, Ba_2Pb_4F_{10}I_2, La_8H_8O_8$
129 ($P4/nmm$)	True	$K_2Na_2Nb_2Sm_2O_{10}, K_2Na_2Nb_2Nd_2O_{10}, K_2La_2Na_2Ta_2O_{10}, K_2Na_2Pr_2Ta_2O_{10}, Al_2Na_4Cl_2H_8O_8, Na_4B_2Cl_2H_8O_8$
130 ($P4/ncc$)	False	$Mg_4Rb_{12}H_{20}, Na_8Pd_4Cl_{16}, Tl_{20}Cl_4Se_8, Sr_{12}O_{20}Si_4, Ba_{12}Ge_4O_{20}$
130 ($P4/ncc$)	True	$Cs_{12}Mg_4H_{20}$
132 ($P4_2/mcm$)	False	$Ag_4Cs_4Ti_2S_8, Cs_4Cu_4Ti_2Se_8, Cu_4Rb_4Ti_2S_8$
136 ($P4_2/mnm$)	False	$Sr_2Br_4, Sr_2I_4, Ge_2O_4, Zr_4N_2, Pb_2O_4, Ir_2O_4, Co_2F_4, Ni_2F_4, Rh_2O_4, Hf_4N_2, Os_2O_4, Ru_2O_4, Mg_2H_4, Ti_2O_4, Hf_2O_4, Sn_2O_4, Cs_4Pt_2H_8, Pd_2Rb_4H_8, K_4Pt_2H_8, Ga_{12}Os_4, Rh_4U_2O_{12}, Li_4Pd_2F_{12}, Li_4Pt_2F_{12}, Ga_4O_{12}Te_2, Lu_4O_{12}Te_2, Mg_2Ta_4O_{12}, Au_4Na_{12}O_8, Cu_4Na_{12}O_8, Be_4K_4Na_8O_{10}, Cu_4Li_{12}O_{12}, K_4Ni_4P_4S_{16}, K_4Pd_4P_4S_{16}, Cu_{20}Tl_4Se_{12}$
136 ($P4_2/mnm$)	True	$Pt_2Rb_4H_8$
137 ($P4_2/nmc$)	False	$Zr_2O_4, Zn_2Cl_4, Na_2B_2H_8, Li_{12}Zn_2O_8, Li_{12}W_2N_8, Mg_2Na_{12}O_8, Li_{12}Mo_2N_8, Li_{14}V_2N_8, Hg_8I_{16}$
137 ($P4_2/nmc$)	True	$K_2B_2H_8$
138 ($P4_2/ncm$)	False	$Au_4I_4, Sn_4Sr_8O_{16}, Hf_4Sr_8O_{16}$
138 ($P4_2/ncm$)	True	Au_4Cl_4, Au_4Br_4
139 ($I4/mmm$)	False	$Ba_2O_4, F_4Xe_2, F_4Kr_2, Hg_4I_4, Hg_4Cl_4, Hg_4Br_4, Hg_4F_4, Sn_2F_8, Pb_2F_8, Pr_4O_4Te_2, Sm_4O_4Te_2, Tb_4O_4Te_2, Ba_2Cu_4S_4, La_4O_4Te_2, Nd_4O_4Te_2, Th_4N_4Te_2, Li_4C_2N_4, Sc_4B_2C_4, Pt_2U_4C_4, Os_2U_4C_4, Ru_2U_4C_4, Rh_2U_4C_4, Ni_2Th_4C_4, Ni_2Rb_4O_4, Li_4Pd_2H_4, Ir_2U_4C_4, Cs_4Ni_2O_4, Hg_2Na_4O_4, Hg_2Rb_4O_4, Hg_2K_4O_4, Cs_4Hg_2O_4, Ca_4Zn_2N_4, Sr_4Zn_2N_4, Ba_4Zn_2N_4, Na_8I_4O_2, Cs_4Mg_2H_8, K_4Mg_2H_8, Ca_2Rb_4H_8, Na_8Br_4O_2, K_4Mg_2F_8, Ba_4Sn_2O_8, K_4U_2O_8, Sr_4Ti_2O_8, Yb_8O_2Sb_4, Ba_4Hf_2O_8, Rb_8I_4O_2, K_8I_4O_2, Rb_4U_2O_8, Ca_8As_4O_2, Mg_2Rb_4F_8, K_4Zn_2F_8, Yb_8As_4O_2, K_4Pd_2H_8, Na_4Pd_2H_8, Na_4Pt_2H_8, Au_4Cs_4I_{12}, Ag_2Au_2Cs_4Cl_{12}, Ba_6In_4O_{12}, Au_8Rb_8O_8, Ag_8K_8O_8, Cu_8Li_8O_8, Ag_8Na_8O_8, Cu_8Na_8O_8, Cu_8Rb_8O_8, Cu_8K_8O_8, Ag_8Rb_8O_8, Ag_8Cs_8O_8, Na_6Pa_2F_{16}, Ba_6Sr_2I_{16}, Ag_8Li_8O_8, Li_{14}Br_6O_4, K_6Zn_4F_{14}, Sr_6Ti_4O_{14}, Cs_6Mg_4Cl_{14}, Ba_6Hf_4O_{14}, Ba_6Zr_4O_{14}, K_8Ru_4Cl_{20}O_2$

No. (SP)	Clean	NDL and NDR (NDL(R)) candidates
139 ($I4/mmm$)	True	$Ca_4Co_2N_4$, $Nd_4Br_4O_{20}Te_8$
140 ($I4/mcm$)	False	Sr_4S_8, $Ag_4Tl_4I_8$, $Al_4Na_4Te_8$, $Al_4K_4Te_8$, $Al_4Na_4Se_8$, $In_4K_4Te_8$, $In_4Rb_4Te_8$, $In_4Na_4Te_8$, $Al_4Tl_4Se_8$, $Ga_4Tl_4Se_8$, $K_4F_8H_4$, $Cs_4F_8H_4$, $Rb_4F_8H_4$, Cs_4N_{12}, Rb_4N_{12}, K_4N_{12}, $Ca_4B_8C_8$, $Pd_4Sr_4F_{16}$, $Ba_4Pd_4F_{16}$, $Ag_4Au_4F_{16}$, $Pb_4Pd_4F_{16}$, $K_4Br_4F_{16}$, $Au_4Rb_4F_{16}$, $Ag_4Na_4F_{16}$, $Au_4Na_4F_{16}$, $Ca_4Pd_4F_{16}$, $Na_4Pd_4P_4S_{16}$, $K_8Sn_4Te_{20}$, $Rb_8Sn_4Te_{20}$, $Ba_{16}Bi_{12}K_4O_4$, $K_{12}F_4O_{16}S_4$, $Ba_{16}Rb_4O_4Sb_{12}$, $Be_4K_{12}F_{20}$, $Cs_{12}Zn_4Cl_{20}$, $K_{12}Zn_4H_{20}$, $Rb_{12}Zn_4H_{20}$, $Cs_{12}Zn_4H_{20}$, $Mg_4Rb_{12}H_{20}$, $Cs_{12}Mg_4H_{20}$, $Ba_{12}O_{20}Si_4$, $Ba_{12}Ti_4S_{20}$, $Ba_4Mn_4Pr_8S_{20}$, $Ba_4La_8Zn_4S_{20}$, $Ba_4Pr_8Zn_4S_{20}$, $Ba_4Nd_8Zn_4S_{20}$, $Ba_4Nd_8Zn_4O_{20}$, $Ba_4La_8Mn_4S_{20}$, $Ba_4La_8Zn_4O_{20}$, $Au_4Th_8F_{44}$
140 ($I4/mcm$)	True	$Au_4K_4F_{16}$
141 ($I4_1/amd$)	False	Ti_4O_8, Zr_4O_8, $Er_4Li_4O_8$, $Ho_4Li_4Se_8$, $Li_4Y_4S_8$, $Bi_4Li_4S_8$, $Dy_4Li_4S_8$, $Li_4Sm_4S_8$, $Bi_4Na_4Se_8$, $Er_4Li_4Se_8$, $Li_4Sm_4Se_8$, $Er_4Li_4S_8$, $Dy_4Li_4Se_8$, $Na_4Tl_4O_8$, $Li_4Nd_4S_8$, $Li_4Nd_4Se_8$, $Li_4Y_4Se_8$, $Li_4Tb_4Se_8$, $In_4Li_4O_8$, $Li_4Sc_4O_8$, $Na_4Pr_4S_8$, $La_4Na_4S_8$, $Na_4Nd_4S_8$, $Na_4Tb_4O_8$, $Na_4Sm_4O_8$, $Lu_4Na_4O_8$, $Na_4Tm_4O_8$, $Li_4Lu_4O_8$, $Ho_4Na_4O_8$, $La_4Na_4O_8$, $Dy_4Na_4O_8$, $Mg_8F_4N_4$, $Na_4Nd_4O_8$, $Na_4Pr_4O_8$, $Cu_8Sr_4O_8$, $Ba_4Cu_8O_8$, $Sm_4As_4O_{16}$, $Ho_4As_4O_{16}$, $Na_4Y_4F_{16}$, $Er_4Na_4F_{16}$, $Ho_4Na_4F_{16}$, $Lu_4O_{16}P_4$, $Tb_4As_4O_{16}$, $Er_4O_{16}P_4$, $Ho_4O_{16}P_4$, $Sc_4O_{16}P_4$, $Tb_4O_{16}P_4$, $Hf_4O_{16}Si_4$, $Ta_4B_4O_{16}$, $Th_4Ge_4O_{16}$, $Zr_4O_{16}Si_4$, $Y_4O_{16}P_4$, $Sc_4As_4O_{16}$, $Dy_4As_4O_{16}$, $Dy_4O_{16}P_4$, $Th_4O_{16}Si_4$, $Tm_4O_{16}P_4$, $Nd_4As_4O_{16}$, $Pr_4As_4O_{16}$, $Tm_4As_4O_{16}$, $Er_4As_4O_{16}$, $La_4As_4O_{16}$, $Y_4As_4O_{16}$, $Nb_4B_4O_{16}$, $Cl_{16}O_8$, $Li_{16}Sr_4N_8$, $Ca_4Y_8O_{16}$, $Ag_8Cd_4O_{16}$
141 ($I4_1/amd$)	True	Ti_8N_4, $Lu_4As_4O_{16}$, $Li_{24}B_8N_{16}$, $Bi_4Cs_{16}Cl_{48}Sb_4$, $Cs_{16}Tl_4Cl_{48}Sb_4$
142 ($I4_1/acd$)	False	$Rb_{32}Sn_{32}$, $Cs_{32}Sn_{32}$, $Pb_{32}Rb_{32}$, $K_{32}Sn_{32}$
142 ($I4_1/acd$)	True	$Cs_{32}Si_{32}$
146 ($R3$)	False	$Sr_9O_{18}Te_3$
147 ($P-3$)	False	$K_2SnAs_2S_6$, $K_4O_6Te_2$, CsO_8P_2, $BaMgNa_2O_8P_2$, $Na_2PtH_6O_6$, $Li_6Th_3N_6$, $K_2Na_2Th_2F_{12}$, $In_4K_4Br_{12}$, $Na_4Ti_5O_{12}$, $Ba_3N_2O_{12}Si_6$, $Cd_2Na_{14}O_9$
148 ($R-3$)	False	Se_{18}, W_3Br_{18}, $Pb_3Zn_3F_{18}$, $Sn_3Zn_3F_{18}$, $Cd_3Sn_3F_{18}$, $Pd_3Zn_3F_{18}$, $Cd_3Pd_3F_{18}$, $Mg_3Pb_3F_{18}$, $Bi_3Li_3F_{18}$, $Bi_3Na_3F_{18}$, $Cd_3Pb_3F_{18}$, $Li_3F_{18}Sb_3$, $Cd_3Pt_3F_{18}$, $Cd_3Ti_3F_{18}$, $Mg_3Pd_3F_{18}$, $Ca_3Pd_3F_{18}$, $Ca_3Pt_3F_{18}$, $Pt_3Zn_3F_{18}$, $Li_3F_{18}P_3$, $Li_3As_3F_{18}$, As_6I_{18}, Y_6I_{18}, $Ag_9Y_3Cl_{18}$, $Na_9Y_3Cl_{18}$, $Al_3Na_9F_{18}$, $Cd_6Ti_6O_{18}$, $Ti_6Zn_6O_{18}$, $Bi_6Na_6O_{18}$, $Mg_6Ge_6O_{18}$, $Mg_6Ti_6O_{18}$, $Ca_6Sn_6O_{18}$, $Bi_6Y_6O_{18}$, $Cd_6Sn_6O_{18}$, $Ca_6Ti_6O_{18}$, $Cd_6Ge_6O_{18}$, $Mg_3Sn_3B_6O_{18}$, $Ca_3Sn_3B_6O_{18}$, $Ca_3Mg_3C_6O_{18}$, $Sn_3Sr_3B_6O_{18}$, $Mg_6P_6Se_{18}$, $Mg_3Na_6C_6O_{18}$, $K_3Na_3B_6H_{24}$, $Li_{18}O_{18}Te_3$, $Li_{24}Pb_3O_{18}$, $Hf_3Li_{24}O_{18}$, $Li_{24}Ti_3S_{18}$, $Li_{24}Zr_3O_{18}$, $Ag_9Tl_{18}I_{27}$, $Au_{18}Cl_{18}O_{18}$, $La_{18}U_3O_{36}$, $Lu_{18}U_3O_{36}$, $In_{18}O_{36}Te_3$, $U_3Y_{18}O_{36}$, $Y_{18}O_{36}Te_3$, $Lu_{18}O_{36}Te_3$, $Nb_{18}Ti_6O_{36}$, $Ho_{18}Sc_6O_{36}$, $Ca_{18}U_6O_{36}$, $Al_6Li_{18}H_{36}$, $Mg_{18}O_{36}Te_6$, $La_{18}Sc_6O_{36}$, $Al_6Lu_{18}O_{36}$, $K_6Sn_3H_{36}N_{18}$, $Ag_{18}As_{18}S_{36}$, $Li_{18}S_{36}Sb_{18}$, $Li_{48}Ta_6N_{24}O_3$, $Li_{48}Nb_6N_{24}O_3$, $K_6Nb_{18}Sr_3Cl_{54}$, $Cs_6Nb_{18}Pb_3Cl_{54}$, $Hf_9Tl_{36}Te_{36}$, $Co_{24}Ge_{36}S_{36}$, $Co_{24}Ge_{36}Te_{36}$, $Co_{24}Ge_{36}Se_{36}$, $Co_{24}Sn_{36}Te_{36}$, $Ir_{24}Ge_{36}Se_{36}$, $Rh_{24}Ge_{36}Se_{36}$, $Ba_{18}In_6B_{18}O_{54}$, $O_{75}P_{18}Si_{15}$, $K_6Ge_{12}O_{72}P_{18}$
156 ($P3m1$)	False	TiSSe, BiITe, $CuScS_2$, AlSrGeH
157 ($P31m$)	False	$Mg_3H_4O_9Si_2$
160 ($R3m$)	False	Nb_3S_6, Mo_3S_6, Zr_3Cl_6, Mo_3Se_6, W_3S_6, $K_3H_3S_3$, $Na_3H_3S_3$, In_6Se_6, $Tl_3Br_3O_9$, $Cu_{12}I_{12}$, $Na_6Ti_9Cl_{24}$, $K_9B_{18}Br_3O_{30}$
161 ($R3c$)	False	$Sn_6Zn_6O_{18}$
162 ($P-31$m)	False	$PbAs_2O_6$, PbO_6Sb_2, $HgAs_2O_6$, Li_2ZrF_6, $CaAs_2O_6$, $CdAs_2O_6$, HgO_6Sb_2, $SrAs_2O_6$, $BaSr_2I_6$, Ca_2SrI_6, $CaSr_2I_6$, $BaCa_2I_6$, CdO_6Sb_2, CaO_6Sb_2, BaO_6Sb_2, $Hg_2As_2O_6$, $Al_2Si_2Te_6$, $In_2Si_2Te_6$, $In_4Rb_2O_7$
162 ($P-31$m)	True	SrO_6Sb_2
163 ($P-31$c)	False	$Sn_2Th_2I_{12}$, $Pb_2Th_2I_{12}$, $Fe_2Hf_2Cl_{12}$, $Al_2Li_2Yb_2F_{12}$, $Ca_2Ga_2Li_2F_{12}$, $Al_2Ca_2Li_2F_{12}$, $Tl_4O_{12}Sb_4$, $Ag_2Sc_2P_4Se_{12}$, $Ag_2Er_2P_4Se_{12}$, $Ag_2Tm_2P_4Se_{12}$, $Ag_2In_2P_4Se_{12}$, $Ag_2Ga_2P_4Se_{12}$, $In_2Na_6Cl_{12}$, $Na_6Sc_2Br_{12}$, $Ca_2Ce_2Li_{12}O_{12}$, $In_4Li_{12}O_{12}$
163 ($P-31$c)	True	$Al_2Li_2Sr_2F_{12}$, $Ag_2Sc_2P_4S_{12}$, $Na_4Ge_2O_{12}Te_2$

No. (SP)	Clean	NDL and NDR (NDL(R)) candidates
164 (*P*−3m1)	False	Ge_3I_6, ZrS_2, $FeBr_2$, PbI_2, MgI_2, CaI_2, $MgBr_2$, CdI_2, YbI_2, HfS_2, $HfSe_2$, H_2Si_2, $AgScSe_2$, $AgTmTe_2$, $CoLiS_2$, Cu_2I_2, Al_3Ni_2, In_3Pt_2, Al_3Pd_2, Al_3Pt_2, Ac_2O_3, Ga_3Ni_2, Rh_3Si_2, In_3Ni_2, Ga_3Pt_2, Mg_3Sb_2, In_3Pd_2, U_2N_3, Sm_2O_3, La_2O_3, Pr_2O_3, Nd_2O_3, Mg_3As_2, $PdRb_2C_2$, $PtRb_2C_2$, Na_2PdC_2, Cs_2PtC_2, K_2PtC_2, Be_2CaP_2, Al_2SrGe_2, Th_2N_2, Al_2LaSi_2, Al_2MgGe_2, $CaZn_2Sb_2$, $CaZn_2As_2$, $SrZn_2Sb_2$, Hf_2N_2, Be_2MgP_2, Be_2CaAs_2, $HfLi_2N_2$, Cu_2HfP_2, Bi_2CaMg_2, $BaBi_2Mg_2$, $CaCd_2Sb_2$, Cd_2SrSb_2, Ce_2O_2, Al_2CaSi_2, $SrZn_2As_2$, Cd_2SrAs_2, $BaCd_2Sb_2$, $CeLi_2As_2$, Cu_2ZrP_2, Cu_2ThP_2, $CaZn_2N_2$, Hf_2N_2, Li_2SnN_2, Al_2YSi_2, Al_2YGe_2, Al_2LaGe_2, Ce_2N_2, Bi_2Mg_2, Al_2CaGe_2, Al_2SrSi_2, $CaCd_2As_2$, Al_2CeGe_2, $BaCd_2As_2$, $YbZn_2As_2$, Mg_2SrN_2, Nd_2S_2, Mg_2YbSb_2, Tb_2O_2, Dy_2O_2, Ho_2O_2, Er_2O_2, Lu_2O_2, Y_2O_2, Nd_2O_2, Sm_2O_2, Ho_2O_2, Er_2O_2, Nd_2O_2, Li_2ZrN_2, Pr_2O_2, Tm_2O_2, La_2O_2, Pr_2O_2, Zr_2N_2, Sm_2O_2, $CaMg_2N_2$, La_2O_2, Y_2O_2, Tm_2O_2, Tm_2O_2, Er_2O_2, Tb_2O_2, Ho_2O_2, Dy_2O_2, Th_2N_2, Th_2N_2, $CeLi_2N_2$, $SrZn_2P_2$, Cd_2SrP_2, $BaMg_2P_2$, $BaCd_2P_2$, $BaMg_2As_2$, Ag_2BaS_2, Be_2MgAs_2, Mg_2SrAs_2, Al_2MgC_2, $CaMg_2As_2$, $CaMg_2Sb_2$, Mg_2SrSb_2, $BaMg_2Sb_2$, $CaZn_2P_2$, $CaCd_2P_2$, $YbZn_2P_2$, $CeLi_2P_2$, Au_3RbSe_2, Au_3CsSe_2, $Dy_2CN_2O_2$, $Tm_2CN_2O_2$, $Ho_2CN_2O_2$, $Y_2CN_2O_2$, $Er_2CN_2O_2$, $Pr_2CN_2O_2$, $Nd_2CN_2O_2$, $Sm_2CN_2O_2$, Cs_2ThCl_6, K_2PtF_6, Ba_2MgH_6, Cd_3I_6, Cs_2GaLiF_6, $Pt_4Tl_2Se_6$, $Pt_4Tl_2S_6$, $FeMg_2SrH_8$, $BaFeMg_2H_8$, Bi_4PbTe_7, $Pd_3P_2S_8$, $Al_2K_2O_7Sb_2$, $Al_2Rb_2O_7Sb_2$, $Al_2Cs_2O_7Sb_2$, $CsK_2ScO_8P_2$, $Cs_3Br_9Sb_2$, $Cd_6Cl_6P_2$, $Ba_3MgTa_2O_9$, $Ba_3MgNb_2O_9$, $Ba_3Nb_2ZnO_9$, $K_4Zr_5O_{12}$, $Hf_5K_4O_{12}$, $Ba_5Nb_4O_{15}$, $Ba_5Ta_4O_{15}$, $Al_3K_6Li_3F_{18}$
164 (*P*−3m1)	True	$MgCl_2$, Sc_2CCl_2, Zr_2N_2, Be_2MgN_2, Ho_2CF_2, $CsCu_3O_2$, $CsCu_3S_2$, $Ba_3Ir_2H_{12}$, $Nb_6Cl_{14}Te_2$
165 (*P*−3c1)	False	La_6F_{18}, $Mg_8Ta_4O_{18}$, $Nb_4Zn_8O_{18}$, $Mg_8Nb_4O_{18}$, $Na_4Sn_8O_{18}$, $Sr_8Ta_4O_{18}$, $Ca_8Ta_4O_{18}$, $Li_4Ti_8O_{18}$
166 (*R*−3m)	False	Cd_3Cl_6, Mg_3Cl_6, Rb_6O_3, Sc_3F_9, $Cu_3Sc_3O_6$, $Hg_3Sr_3O_6$, $Ag_3La_3O_6$, $Ca_3Hg_3O_6$, $Cu_3Y_3O_6$, $Cd_3C_3N_6$, $Sr_3C_3N_6$, $Cu_3Rh_3O_6$, $Li_3F_6H_3$, $Na_3F_6H_3$, $Hg_3Sr_3O_6$, $Ba_3Hg_3O_6$, $Ca_3C_3N_6$, $Na_3Y_3S_6$, $Pr_3Rb_3Se_6$, $Nd_3Rb_3Se_6$, $Rb_3Sm_3Se_6$, $Rb_3Tb_3Se_6$, $Ho_3Rb_3Se_6$, $Er_3Rb_3Se_6$, $Lu_3Rb_3Se_6$, $Er_3Na_3O_6$, $K_3La_3Te_6$, $K_3Nd_3Te_6$, $Sc_3Tl_3Se_6$, $Li_3Rh_3O_6$, $Rb_3Y_3O_6$, $K_3La_3S_6$, $K_3Pr_3S_6$, $K_3Sm_3S_6$, $Dy_3K_3S_6$, $Ho_3K_3S_6$, $Li_3Y_3S_6$, $Dy_3Li_3S_6$, $Ho_3Li_3S_6$, $Er_3Li_3S_6$, $Li_3Tb_3Se_6$, $Li_3Y_3Se_6$, $Dy_3Li_3Se_6$, $Ho_3Li_3Se_6$, $Er_3Li_3Se_6$, $K_3Y_3Te_6$, $In_3Na_3S_6$, $In_3Na_3Se_6$, $In_3Tl_3S_6$, $Ca_6Cl_3N_3$, $Ca_6Br_3N_3$, $Sr_6Cl_3N_3$, $Ca_6I_3P_3$, $Sr_6Br_3N_3$, $Bi_3Tl_3Te_6$, $Ba_6Cl_3P_3$, $Ca_6As_3I_3$, $Ag_3Bi_3S_6$, $Bi_3Rb_3S_6$, $Na_3Tl_3O_6$, $Dy_3Tl_3S_6$, $Er_3Na_3S_6$, $Nd_3Tl_3S_6$, $Er_3Tl_3S_6$, $Er_3K_3S_6$, $K_3Pr_3O_6$, $Cs_3Ho_3S_6$, $In_3Na_3O_6$, $K_3Pr_3Te_6$, $Bi_3Tl_3S_6$, $Cs_3La_3S_6$, $Cs_3Lu_3S_6$, $Cs_3Er_3S_6$, $Dy_3Tl_3Se_6$, $Nd_3Tl_3Se_6$, $Ho_3Tl_3Se_6$, $Ho_3Na_3S_6$, $Tb_3Tl_3Se_6$, $Sr_6I_3N_3$, $Er_3Tl_3Se_6$, $Nd_3Rb_3Te_6$, $Sr_6H_3N_3$, $Ho_3Rb_3O_6$, $Dy_3Rb_3S_6$, $La_3Rb_3Se_6$, $Dy_3Rb_3O_6$, $Er_3Rb_3O_6$, $Rb_3Tm_3O_6$, $Lu_3Rb_3O_6$, $K_3Tm_3O_6$, $K_3Nd_3O_6$, $Na_3Tm_3O_6$, $Ho_3K_3O_6$, $Lu_3Na_3O_6$, $Na_3Sc_3O_6$, $K_3La_3O_6$, $Er_3K_3O_6$, $La_3Rb_3O_6$, $Nd_3Rb_3O_6$, $Rb_3Sm_3O_6$, $Al_3Li_3O_6$, $Rb_3Sc_3O_6$, $K_3Tl_3O_6$, $Rb_3Tl_3O_6$, $K_3Sc_3O_6$, $K_3Y_3O_6$, $Er_3Na_3Se_6$, $Na_3Rh_3O_6$, $Lu_3Na_3S_6$, $K_3Sm_3Te_6$, $Na_3Tm_3S_6$, $Cs_3Nd_3S_6$, $Cs_3Dy_3S_6$, $Cs_3Tm_3S_6$, $Er_3K_3Te_6$, $La_3Rb_3S_6$, $Pr_3Rb_3S_6$, $Nd_3Rb_3S_6$, $Rb_3Sm_3S_6$, $Rb_3Tb_3S_6$, $Ho_3Rb_3S_6$, $Er_3Rb_3S_6$, $Rb_3Tm_3S_6$, $Lu_3Rb_3S_6$, $Co_3Pt_3O_6$, $Ag_3In_3O_6$, $Au_3Co_3O_6$, $Al_3Cu_3O_6$, $Cu_3Ga_3O_6$, $Cu_3Li_3F_6$, $Bi_6Se_6Te_3$, $La_6Ge_3I_6$, $Tb_6F_6O_6$, $Y_6F_6O_6$, $Nd_6F_6O_6$, $Pr_6F_6O_6$, $La_6F_6O_6$, $Th_6F_6N_6$, $Sm_6F_6O_6$, Pb_6I_{12}, Pb_6I_{12}, $Li_{12}Cl_3N_3$, $Sr_3U_3O_{12}$, $Ca_3U_3O_{12}$, $Ba_3Sr_3I_{12}$, $Hf_6Br_6N_6$, $Zr_6Br_6N_6$, $Hf_6I_6N_6$, $Zr_6I_6N_6$, $Ba_6Cu_3Cl_3O_6$, $Cu_3Sr_6Br_3O_6$, $Ba_6Cu_3Br_3O_6$, $Li_{12}Sr_3P_6$, $Al_6Mg_3Se_{12}$, $K_{12}Zn_3P_6$, $Cd_3K_{12}P_6$, $Hg_3Na_{12}P_6$, $Hg_3K_{12}As_6$, $Be_3K_{12}As_6$, $Cd_3K_{12}As_6$, $K_{12}Zn_3As_6$, $Hg_3K_{12}P_6$, $Be_3K_{12}P_6$, $Bi_6Ge_3Te_{12}$, $Bi_6Sn_3Te_{12}$, $Bi_6Pb_3Se_{12}$, $Bi_6Pb_3Te_{12}$, $Al_{12}C_9$, $Y_6Zn_6O_6P_6$, $Pr_6Zn_6O_6P_6$, $Dy_6Zn_6O_6P_6$, $Sm_6Zn_6O_6P_6$, $Nd_6Zn_6O_6P_6$, $Tb_6Br_6H_{12}$, $Li_{15}Cl_6N_3$, $Cu_3K_{15}Sb_6$, Pb_9I_{18}, $Ca_3K_6C_6O_{18}$, $K_6Mg_3C_6O_{18}$, $Ba_6Mg_3B_6O_{18}$, $K_6Pt_{12}Se_{18}$, $Pt_{12}Rb_6Se_{18}$, $K_6Pt_{12}S_{18}$, $Cs_6Pt_{12}Se_{18}$, B_{36}, $Ba_3Pb_6C_6F_6O_{18}$, $Sr_9O_{24}P_6$, $Ba_9As_6O_{24}$, $Bi_3K_3Li_{18}O_{18}$, $K_3Li_{18}Ta_3O_{18}$, $Ga_{18}N_6O_{18}$, $B_{36}P_6$, As_6B_{36}, $B_{36}O_6$, $B_{36}C_9$, $La_3O_{36}Sb_{15}$, $Cs_6O_{36}Te_{12}$, $Ga_6Li_6Rb_{12}F_{36}$, $Cs_{12}In_6Li_6Cl_{36}$, $Cs_{12}Ga_6Na_6F_{36}$, $Al_6Cs_{12}Na_6F_{36}$, $Sr_9Ta_{12}F_6O_{36}$, $Mg_6B_{54}N_6$, $Rb_9B_{36}Br_3H_{36}$, $Rb_9B_{36}Cl_3H_{36}$, $Cu_{18}I_{18}Se_{54}$
166 (*R*−3m)	True	Cd_3Br_6, $Au_3Tl_3O_6$, $Na_3Sm_3O_6$, $Lu_6F_6S_6$, $Rb_6Sn_6O_9$, $Cu_9K_9As_6$, $Ag_9K_9As_6$, $Cu_9K_9P_6$, $Au_9K_9Sb_6$, $Au_9Rb_9Sb_6$, $K_3Na_3B_6H_{24}$, $Ca_6Pt_9O_{24}$, $Ba_9O_{24}P_6$, $Cs_9Mg_9H_{27}$, $Al_9Cs_6Li_3F_{36}$, $Al_6K_{12}Li_6H_{36}$, $Rb_9B_{36}H_{36}I_3$, $Cs_9B_{36}Cl_3H_{36}$, $K_9B_{36}Br_3H_{36}$, $Cs_9B_{36}Br_3H_{36}$
167 (*R*−3c)	False	Rh_6F_{18}, Al_6H_{18}, Ir_6F_{18}, $O_{18}Te_6$, Tm_6Cl_{18}, Al_6F_{18}, Ga_6F_{18}, In_6F_{18}, $Ca_6C_6O_{18}$, $Cd_6C_6O_{18}$, $Na_6N_6O_{18}$, $Mg_6C_6O_{18}$, $In_6B_6O_{18}$, $Ho_6B_6O_{18}$, $Lu_6B_6O_{18}$, $Al_6B_6O_{18}$, $Sc_6B_6O_{18}$, $Ca_{18}N_{12}$, $Al_{12}O_{18}$, $Ga_{12}O_{18}$, $Tm_{12}S_{18}$, $In_{12}O_{18}$, $Lu_{12}S_{18}$, $Rh_{12}O_{18}$, $Sc_{12}O_{18}$, $Ir_{12}S_{18}$, $Rh_{12}S_{18}$, $Ho_{12}S_{18}$, $Al_6Pr_6O_{18}$, $Al_6Nd_6O_{18}$, $Ag_6Ta_6O_{18}$, $Au_6Na_{18}S_{12}$, $Au_6K_{18}Se_{12}$, $Cu_6Na_{18}O_{12}$, $Cu_6Li_{18}S_{12}$, $Rh_6Cl_{18}Te_{36}$, $Cd_6Pt_6Sr_{18}O_{36}$, $Na_6Sr_{18}O_{36}Sb_6$, $Ag_6Hg_{18}O_{36}Sb_6$, $Na_6Sr_{18}Ta_6O_{36}$, $Er_6Rh_6Sr_{18}O_{36}$, $Rh_6Sc_6Sr_{18}O_{36}$, $Dy_6Rh_6Sr_{18}O_{36}$, $Ho_6Rh_6Sr_{18}O_{36}$, $Rh_6Sr_{18}Tb_6O_{36}$, $Fe_6K_{18}Na_6Cl_{36}$, $Bi_6Na_6Sr_{18}O_{36}$, $Ba_{18}Bi_6Na_6O_{36}$, $Cd_6K_6Rb_{18}Cl_{36}$, $Mg_6Pt_6Sr_{18}O_{36}$, $Na_6Ru_6Sr_{18}O_{36}$, $Ca_{18}Na_6Ru_6O_{36}$, $Na_6Nb_6Sr_{18}O_{36}$, $In_6Rh_6Sr_{18}O_{36}$, $Pt_6Sr_{18}Zn_6O_{36}$, $Ba_{18}Na_6Nb_6O_{36}$, $Ba_{18}Na_6Ta_6O_{36}$, $Ba_{18}Na_6Ru_6O_{36}$, $Hg_{18}B_{12}O_{36}$, $Ca_{24}Pd_6O_{36}$, $Cd_6Rb_{24}Cl_{36}$, $Rb_{24}Yb_6I_{36}$, $Cd_6K_{24}Cl_{36}$, $Cd_6Rb_{24}Br_{36}$, $Pb_6Rb_{24}Br_{36}$, $Rb_{36}Cl_{24}O_6$, $Pd_6Sr_{24}O_{36}$, $Rb_{36}Br_{24}O_6$, $Pt_6Sr_{24}O_{36}$, $Ba_{24}Pd_6O_{36}$, $Ca_{24}Pt_6O_{36}$, $Ba_{24}Pt_6O_{36}$, $Na_{18}B_{18}O_{36}$, $Cs_{18}B_{18}O_{36}$, $Cr_{12}Cs_{18}I_{54}$, $Cs_{18}Y_{12}Br_{54}$, $Cs_{18}Tb_{12}Br_{54}$
167 (*R*−3c)	True	$Li_6N_6O_{18}$, $K_6F_{24}H_{18}$, $Rh_6Sm_6Sr_{18}O_{36}$, $Rh_6Sr_{18}Y_6O_{36}$, $Lu_{12}C_{18}N_{36}$, $Li_{24}Ru_6H_{36}$, $Li_{24}Os_6H_{36}$, $Na_{24}Ru_6H_{36}$, $Rb_{18}B_{18}O_{36}$, $K_{18}B_{18}O_{36}$, $Cs_{18}Sc_{12}Cl_{54}$

No. (SP)	Clean	NDL and NDR (NDL(R)) candidates
176 ($P6_3/m$)	False	La_2Cl_6, Nd_2Cl_6, Pr_2Cl_6, Pr_2Br_6, La_2Br_6, Tb_2Cl_6, $Ga_2Sr_6N_6$, $Mg_6B_2F_6O_6$, $Mg_6B_2H_6O_{12}$, $La_6U_2Cl_6O_{12}$, $Pr_6U_2Cl_6O_{12}$, $Nd_6U_2Cl_6O_{12}$, $Bi_4Ge_6O_{18}$, $Bi_4O_{18}Si_6$, $La_4O_{18}Si_6$, $Rb_4Ti_2O_{18}Si_6$, $Cs_4Sn_2Ge_6O_{18}$, $Cs_4Zr_2O_{18}Si_6$, $K_4O_{18}Si_8$, $Ba_6Nb_4Sr_2O_{18}$, $Ba_6Sr_2Ta_4O_{18}$
176 ($P6_3/m$)	True	$Ba_2Ga_2B_2F_4O_6$, $Al_2Ba_2B_2F_4O_6$, $Ba_2Zn_2C_2F_4O_6$, $K_6W_4Cl_{18}$, $Rb_4Sn_2O_{18}Si_6$
186 ($P6_3mc$)	False	Mg_2Te_2, Ag_2I_2, Zn_2Se_2, Zn_2S_2, La_2N_2, Cu_2I_2, C_2Si_2, Ga_2N_2, Al_2P_2, Zn_2Te_2, Pt_2O_4, $Be_2Li_2Sb_2$, $Li_2Zn_2Sb_2$, $Na_2Sn_2P_2$, Pb_4I_8, $Ta_6I_{14}Te_2$, $K_{12}Mn_2Te_8$
187 (P−6m2)	False	IrRe, OsRu, IrRu, CeNiAs
189 (P−62m)	False	Rb_2ThF_6, K_2ThF_6, Ba_2SrI_6, $Na_3Sr_3P_3$, $Ba_3Na_3P_3$, $Na_3Sr_3As_3$, Ba_3Cl_6, Ba_3I_6, Na_6O_6, Na_6S_6, Sr_6P_6, Rb_6S_6, $Ca_3Cs_3C_3F_3O_9$, $Ba_{12}Cl_5F_{19}$
189 (P−62m)	True	$K_6Re_3H_{27}$
193 ($P6_3/mcm$)	False	$Sr_{10}As_6H_2$, $Sr_2As_4O_{12}$, $K_8Pb_4O_{12}$, $Na_8Sn_4O_{12}$
193 ($P6_3/mcm$)	True	$Al_4Li_2Cl_2H_{12}O_{12}$
194 ($P6_3/mmc$)	False	He_2, Li_2, Au_2, Na_2, Hf_2, Ag_2, Y_2, K_2, Zr_2, Ca_2, Mg_2, Ru_2, Ti_2, Os_2, Ba_2, Kr_2, Xe_2, Sc_2, Re_2, Be_2, Cd_2, Ar_2, Si_4, B_2N_2, Bi_2Pt_2, Bi_2Ni_2, Ti_2As_2, Bi_2Pd_2, Pb_2Pt_2, V_2P_2, Pt_2Sn_2, Ir_2Sn_2, Ni_2Te_2, Ti_2Sb_2, Co_2Sb_2, Cr_2As_2, Ni_2As_2, Ni_2Sb_2, Li_2I_2, Yb_2H_4, $Ba_2Zn_2Ge_2$, $Ag_2Ba_2Bi_2$, $Ce_2Cu_2Ge_2$, $Ca_2Cu_2As_2$, $Au_2Ba_2Sb_2$, $Ag_2Sr_2P_2$, $Au_2Ca_2P_2$, $Cu_2Sr_2Sb_2$, $Hg_2K_2Sb_2$, $Au_2Ba_2As_2$, $Cu_2Sr_2Si_2$, $Au_2Sr_2As_2$, $La_2Pt_2P_2$, $Cu_2Sr_2P_2$, $Ce_2Cu_2Si_2$, $Ce_2H_2Se_2$, $Ba_2Zn_2Si_2$, $Ba_2Sn_2Zn_2$, $La_2Pd_2P_2$, $Cu_2La_2Si_2$, $Au_2Ba_2P_2$, $Be_2Zr_2Si_2$, $Ba_2Cu_2Sb_2$, $Ba_2Cu_2P_2$, $Ca_2Zn_2Ge_2$, $Ca_2Zn_2Si_2$, $Au_2K_2Te_2$, $Ba_2Pb_2Zn_2$, $Ba_2Cd_2Ge_2$, $Ce_2Pd_2As_2$, $Be_2Hf_2Si_2$, $Ce_2Pd_2P_2$, $Au_2Rb_2Te_2$, $Ag_2Ba_2As_2$, $Hg_2K_2As_2$, $Cu_2K_2Se_2$, $Cu_2Y_2Si_2$, $Ca_2Cu_2P_2$, $Pt_2Sr_2As_2$, $Sr_2Zn_2Si_2$, $Ba_2Cu_2As_2$, $Ba_2Li_2Sb_2$, $Li_2Sr_2P_2$, $Ba_2Li_2P_2$, $Cu_2K_2Te_2$, $K_2Zn_2P_2$, $K_2Zn_2Sb_2$, $La_2F_2Se_2$, $Li_2B_2C_2$, $Be_2Na_2Sb_2$, V_2S_4, Pt_4B_2, Mo_2Se_4, Mo_2S_4, Re_4C_2, W_2Se_4, W_2S_4, Mo_2Te_4, $Al_2Ti_4N_2$, $Al_2Ta_4C_2$, $In_2Ti_4N_2$, $In_2Zr_4N_2$, $In_2Nb_4C_2$, $Au_2Pd_2O_4$, $Au_2Ga_2O_4$, $Au_2Tl_2O_4$, $Ag_2Tl_2O_4$, $Au_2Ni_2O_4$, $Au_2Co_2O_4$, $Ag_2Co_2O_4$, $Ag_2In_2O_4$, $Cu_2In_2O_4$, $Cu_2Ga_2O_4$, $Ag_2Ga_2O_4$, $Ag_2Sc_2O_4$, $Au_2Sc_2O_4$, $Al_2Au_2O_4$, $Cu_2Y_2O_4$, $Ag_2Y_2O_4$, $Cu_2Sc_2O_4$, $Ag_2Sm_2O_4$, $Ag_2La_2O_4$, $Ag_2Al_2O_4$, Na_4Se_4, K_4Te_4, Na_4S_4, Li_4O_4, Li_6P_2, Li_6As_2, $La_2Br_4H_2$, $Mo_2Ni_2P_4$, $Li_2Nb_2Se_4$, $Co_2Mo_2P_4$, $Ni_2W_2P_4$, $Li_2Ta_2S_4$, $Li_2Nb_2S_4$, $Na_2Nb_2S_4$, $K_2Nb_2S_4$, $Na_2Nb_2Se_4$, $K_2Nb_2Se_4$, Ga_4Te_4, Ga_4Se_4, In_4Se_4, Ga_4S_4, Li_6Sb_2, $Hf_4Tl_2C_2$, $Nb_4C_2Ge_2$, $Hf_4Pb_2C_2$, $Ga_2Nb_4C_2$, $Ga_2Mo_4C_2$, $Ga_2V_4C_2$, $Ti_4Tl_2C_2$, $Sc_2Ta_2N_4$, $Al_2Ti_4C_2$, $Cr_4C_2Ge_2$, $Zr_4P_2Sb_2$, $Nb_4Sn_2C_2$, $Hf_4P_2Sb_2$, $Co_2Na_2O_4$, $Na_2Ni_2O_4$, $Ga_2Ti_4N_2$, $Cr_4Ga_2C_2$, $Hf_4In_2C_2$, $Ca_4I_2N_2$, $Zr_4C_2S_2$, $Al_2Nb_4C_2$, $Pb_2Zr_4C_2$, $In_2Zr_4C_2$, $Pb_2Ti_4C_2$, $Hf_4Sn_2C_2$, $Sn_2Zr_4C_2$, $V_4As_2C_2$, $Ga_2Ta_4C_2$, $Tl_2Zr_4C_2$, $Ti_4C_2S_2$, $Sn_2Ti_4C_2$, $Ti_4P_2Sb_2$, $V_4C_2P_2$, $Al_2Cr_4C_2$, $V_4C_2Ge_2$, $Ti_4C_2Ge_2$, $Cd_2Ti_4C_2$, $Nb_4C_2P_2$, $Nb_4As_2C_2$, $Ga_2Ti_4C_2$, $Hf_4C_2S_2$, $Sc_2Tl_2S_4$, $Rb_2Y_2Te_4$, $In_2Tl_2S_4$, $Na_2Nb_2O_4$, $Li_2Nb_2O_4$, $Ba_2Ce_2N_4$, $Cs_2Y_2O_4$, $Cs_2Nd_2O_4$, $Ag_2Li_2F_4$, $Cs_2Tm_2O_4$, $Cs_2Er_2O_4$, $Cs_2Dy_2O_4$, $Cs_2Ho_2O_4$, $Rb_2Sc_2O_4$, $K_2Nd_2O_4$, $Cs_2Sm_2O_4$, $Mg_2Mo_2N_4$, $Cs_2Pr_2S_4$, $Cs_2Tb_2S_4$, $Cs_2Dy_2S_4$, $Al_2V_4C_2$, $In_2Ti_4C_2$, Bi_2Rb_6, Rb_6As_2, Al_6Ir_2, $Zr_4N_4S_2$, $Er_4O_4Te_2$, $Ho_4O_4S_2$, $Dy_4O_4S_2$, Be_6N_4, $Ba_6N_2Sb_2$, $Cs_2Mg_2Br_6$, $Ba_6Bi_2N_2$, $Al_2In_2O_6$, $Ga_2In_2O_6$, Tc_8Th_4, Cr_8Nb_4, Cs_4Na_8, Mg_8Y_4, Cr_8Ti_4, Mn_8Ti_4, Fe_8W_4, Mg_8Sr_4, Cs_4K_8, Os_8Sc_4, Ru_8Y_4, Lu_4Ru_8, Cr_8Hf_4, Os_8Y_4, Os_8Zr_4, K_4Na_8, Mg_4Zn_8, Be_8Re_4, Be_8V_4, Hf_4Mn_8, K_4Pb_8, Ca_4Mg_8, Ba_4Mg_8, Be_8Fe_4, Ca_4Cd_8, Be_8Ru_4, K_4Sn_8, Al_8Hf_4, Hf_4Re_8, Al_8Zr_4, Re_8Sc_4, Ba_4Na_8, Re_8Zr_4, Ag_8K_4, Sc_4Zn_8, Ca_4Li_8, Cr_8Ta_4, Be_8Mo_4, As_2F_{10}, $Y_4C_2N_4O_4$, $Sr_6B_2O_6P_2$, $Ba_6B_2O_6P_2$, $Ba_6As_2B_2O_6$, $Al_{10}C_6N_2$, $K_6Sc_2O_{14}Si_4$, $Al_2Cs_{12}K_6Sb_8$, $Al_2K_{12}Na_6Sb_8$, $Cs_{12}Ga_2K_6Sb_8$, $Ga_2K_{12}Na_6Sb_8$, $Cr_4Cs_6Br_{18}$, $Os_4Rb_6Br_{18}$, $Cs_6Ru_4Cl_{18}$, $Cs_6Mo_4Cl_{18}$, $Ba_6O_{18}Te_4$, $Mg_6Rb_6H_{18}$, $Rb_{18}I_6O_6$, $Rb_6Zn_6F_{18}$, $Mg_6Rb_6F_{18}$, $Cd_6Cs_6Cl_{18}$, $Na_{18}I_6O_6$, $Ba_6Mg_2O_{18}Sb_4$
194 ($P6_3/mmc$)	True	$Cu_2Sr_2As_2$, $Sn_2Sr_2Zn_2$, $Ca_2Cu_2Sb_2$, $Be_2Na_2As_2$, $Au_2In_2O_4$, $Al_2Cu_2O_4$, $Al_2Zr_4C_2$, $In_2V_4C_2$, Be_8Mn_4, Be_8W_4, Be_8Cr_4, $Ba_2Re_2H_{18}$, $K_6F_{12}N_2O_6Si_2$, $Co_2Er_2C_{12}N_{12}$, $Cs_6Mo_4Br_{18}$
202 (Fm−3)	False	$Rb_8B_{48}H_{48}$
202 (Fm−3)	True	$Tl_8B_{48}H_{48}$, $Cs_8B_{48}H_{48}$, $K_8B_{48}H_{48}$
204 (Im−3)	False	Ni_8Sb_{24}, Ir_8Sb_{24}, Co_8Sb_{24}, Rh_8P_{24}, Ir_8P_{24}, Co_8P_{24}, Ni_8P_{24}, Rh_8Sb_{24}, Co_8As_{24}, Rh_8As_{24}, Ir_8As_{24}, $Fe_8Th_2P_{24}$
205 (Pa−3)	False	Cd_4S_8, Zn_4S_8, Mg_4Se_8, Pd_4Sb_8, Ru_4Te_8, Ru_4Se_8, Pd_4As_8, Co_4S_8, Ni_4Se_8, Os_4S_8, P_8Si_4, Os_4Te_8, Ni_4As_8, Mn_4Te_8, Co_4Se_8, Rh_4S_8, Ni_4P_8, Cu_4Se_8, Bi_8Pt_4, Os_4Se_8, Pt_4As_8, Pt_4Sb_8, Au_4Sb_8, Rh_4Te_8, Ru_4S_8, Fe_4S_8, Cd_4O_8, Mg_4O_8, Mg_4Te_8, Pt_4P_8, Zn_4O_8, Cu_8Cl_8, $Sr_4N_8O_{24}$, $Mg_4N_8O_{24}$, $Zn_4N_8O_{24}$
205 (Pa−3)	True	$Pb_4N_8O_{24}$, $Ba_4N_8O_{24}$
206 (Ia−3)	False	$O_{32}Si_{16}$
216 (F−43m)	False	Mn_4N_4, Zn_4S_4, Hg_4S_4, Ga_4Sb_4, Zn_4Se_4, Mg_4Se_4, Mg_4S_4, Mg_4Te_4, B_4P_4, Be_4Se_4, Al_4P_4, Be_4O_4, Zn_4O_4, In_4Sb_4, In_4P_4, In_4N_4, Zn_4Te_4, Cu_4Br_4, Cu_4Cl_4, Ag_4I_4, Cd_4S_4, Ga_4P_4, Be_4Te_4, Ga_4As_4, Al_4Sb_4, Cd_4Se_4, Hg_4Te_4, Be_4S_4, C_4Si_4, Hg_4Se_4, Ga_4N_4, Ag_4Br_4, Al_4N_4

No. (SP)	Clean	NDL and NDR (NDL(R)) candidates
221 (*Pm*−3m)	False	AuSc, HgTl, LaTl, MgTl, AuZn, LiTl, TlY, AlIr, InLa, LuPd, InY, HfZn, TiZn, CuPd, BeNi, CaZn, CaCd, LiMg, LaMg, CoGa, AlY, AgSc, BePd, BeRh, BeTi, CaHg, IrSc, GaIr, HfOs, HfRh, HfTc, HgSc, LuZn, NiSc, OsZr, TaTc, TcTi, CuSc, MgRh, MgSc, AuLi, CdSc, HgMg, AlNi, RhSc, RhY, ThTe, AgLa, HgLi, CaIn, CoHf, InPd, CaPd, RuZr, ReTi, BaHg, CoSc, GaRu, CoZr, BeCu, HgY, AgLi, SrTl, GaRh, AgY, YZn, TcV, MgSr, LaZn, AlFe, AgMg, LiPd, BeCo, PdSc, HfRu, AlCo, CaTl, OsTi, AuRb, BiTh, CdSr, FeTi, IrY, RuSc, MnV, AlSc, AlRh, RuSi, AuMg, MgPd, BaCd, HgSr, AlRu, ZnZr, RuTi, MgY, RhZn, HgLa, CdLa, CoTi, AcAg, AcMg, PtSc, InRh, CdY, CsCl, TlBr, CsBr, TlCl, RbI, AuCs, CsH, Al_3, Au_3, $CaSn_3$, $CePt_3$, Hg_3, $MnNi_3$, $MnZn_3$, $NbRu_3$, Ni_3, Ir_3, $CeSn_3$, In_3, Ga_3, $CePb_3$, Sn_3, $CeIn_3$, $CeTl_3$, $InZr_3$, Ga_3, $PbPd_3$, In_3, Ni_3, $TiZn_3$, Pb_3, In_3, $FePd_3$, Tl_3, Pt_3, Pd_3, Ir_3, $MgPt_3$, Pt_3, $LiPt_3$, $LiPd_3$, Ac_3, Ga_3, In_3, $PaGe_3$, $PaSi_3$, Pa_3, Pa_3, Pa_3, Pa_3, $AlCu_3$, $HfRh_3$, Ir_3, Al_3, $MgZr_3$, Cd_3, Co_3, Ag_3, $IrRh_3$, Ir_3, $CaSr_3$, Os_3, $TaZn_3$, Pt_3, Ru_3, Au_3, Ni_3, $AlZr_3$, $CeRh_3$, $LaTl_3$, In_3, In_3, Bi_3, $NbRh_3$, Ir_3, $PaRh_3$, Pt_3, Sn_3, $InMg_3$, $LaPd_3$, $HfPd_3$, $CrNi_3$, Co_3, $HfPt_3$, Ir_3, $FeIr_3$, $LiMg_3$, Rh_3, Sc_3, $CrIr_3$, Cu_3, $CrRh_3$, Ir_3, La_3, $LuTl_3$, $ThTl_3$, $CePd_3$, Au_3, $HfIr_3$, Al_3, UGe_3, USi_3, Pt_3, Cu_3, $AgAu_3$, $AuPd_3$, Pt_3, $CaTl_3$, Hg_3, $NpRh_3$, Pt_3, $LuPt_3$, Li_3, Be_3, Co_3, $CdPd_3$, Cu_3, Hf_3, Au_3, Ni_3, Ca_3, $GaZr_3$, $SrTl_3$, Rh_3, $AlNi_3$, $PrSn_3$, Pd_3, Al_3, $InSc_3$, Co_3, $LaSn_3$, Rh_3, Pd_3, $MgPd_3$, $GaNi_3$, Ni_3, Ga_3, $NpSn_3$, Ga_3, Rh_3, Au_3, Al_3, Fe_3, $PtRh_3$, Ni_3, $TlZr_3$, Mg_3, Ga_3, Na_3, Rh_3, VZn_3, $MnPt_3$, Ag_3, Hf_3, Rh_3, Ag_3, Cu_3, $AlLu_3$, Mg_3, $NiPt_3$, Hg_3, $InTh_3$, Rh_3, $CrPt_3$, In_3, Pt_3, In_3, $NbZn_3$, $BaLiF_3$, $KMgH_3$, $BaLiH_3$, $CaRbH_3$, K_3, Li_3, $KMgF_3$, $KTaO_3$, $CdCsCl_3$, $CsBr_3$, $KZnF_3$, $CaCsH_3$, $CaCsF_3$, $CdCsF_3$, $MgRbF_3$, $PaTlO_3$, Na_3, Ca_3AsBr_3, Ca_3AsCl_3, Ca_3Cl_3, Mg_3F_3, Ca_3SrO_4, $BaSr_3O_4$
221 (*Pm*−3m)	True	ScZn, BaZn, $LaPb_3$, $AuCu_3$
223 (*Pm*−3n)	False	Sn_2Ta_6, Fe_2V_6, Pd_2Ti_6, Au_2Zr_6, Cr_6As_2, Cr_6Ga_2, Mo_6Si_2, Ir_2Nb_6, Nb_6Rh_2, V_6Sb_2, Rh_2V_6, Co_2V_6, Cr_6Ir_2, Pd_2V_6, Au_2Ti_6, Nb_6Os_2, Ir_2V_6, Cr_6Ge_2, Sn_2V_6, Pt_2V_6, Pt_2Ti_6, Hg_2Ti_6, Ga_2Mo_6, U_2H_6, Al_2Mo_6, Nb_6Pt_2, V_6As_2, Sn_2Zr_6, Cr_6Os_2, Mo_6Ge_2, Ta_6Sb_2, Ni_2V_6, Hg_2Zr_6, Nb_6Sb_2, Cr_6Si_2
224 (*Pn*−3m)	True	Ag_4O_2, Au_4S_2, Cu_4O_2
225 (*Fm*−3m)	False	Pt_4, Pd_4, Li_4, Ir_4, K_4, Co_4, Ba_4, Ne_4, Ag_4, Al_4, Ce_4, Cu_4, Ca_4, Xe_4, Kr_4, He_4, Rh_4, Sr_4, Au_4, Na_4, Ar_4, Th_4As_4, Bi_4La_4, Bi_4Ce_4, Y_4Se_4, Hf_4N_4, U_4S_4, Tl_4I_4, Lu_4P_4, U_4N_4, U_4Se_4, Np_4P_4, Np_4S_4, La_4Sb_4, Ce_4S_4, Cd_4O_4, La_4Se_4, Co_4H_4, Zr_4N_4, Pr_4Se_4, Th_4Sb_4, Sc_4As_4, U_4As_4, Hf_4C_4, Y_4Sb_4, Sn_4As_4, Bi_4Y_4, La_4S_4, La_4P_4, Cr_4H_4, Ni_4H_4, Rh_4H_4, Ce_4N_4, Zr_4C_4, Sc_4P_4, Ti_4N_4, Th_4S_4, Pa_4C_4, Bi_4Sc_4, Ti_4C_4, Th_4P_4, Y_4As_4, Y_4P_4, Fe_4H_4, Pa_4N_4, Cs_4H_4, Sn_4Sb_4, Ce_4O_4, Th_4Ge_4, Th_4C_4, Sc_4S_4, Ce_4Te_4, Y_4S_4, La_4Te_4, Pa_4As_4, Sc_4Se_4, Ce_4P_4, Np_4As_4, U_4C_4, Pr_4S_4, Ce_4Se_4, Np_4N_4, Ce_4As_4, Pr_4N_4, Ce_4Sb_4, Lu_4Se_4, Sc_4Sb_4, Np_4Se_4, Th_4N_4, Ba_4Te_4, Mg_4Se_4, Sr_4S_4, Li_4F_4, Rb_4F_4, Yb_4O_4, Ba_4Se_4, Mg_4O_4, Mg_4S_4, Ba_4O_4, Ca_4Se_4, Ba_4S_4, Ca_4Te_4, Ca_4S_4, Yb_4Te_4, Cs_4F_4, Yb_4S_4, Sr_4Te_4, Pb_4Te_4, Na_4Cl_4, Rb_4Br_4, K_4I_4, Li_4I_4, Rb_4I_4, Na_4Br_4, Ag_4Cl_4, K_4Cl_4, Ag_4Br_4, K_4Br_4, Na_4I_4, Rb_4Cl_4, Li_4H_4, Na_4H_4, K_4H_4, Sr_4O_4, Rb_4H_4, Ca_4O_4, Sr_4Se_4, Yb_4Se_4, K_4F_4, Tl_4Br_4, Tl_4Cl_4, Cs_4Br_4, Cs_4Cl_4, Cs_4I_4, Na_4F_4, Ir_4Sn_8, Ga_8Pt_4, Au_4Ga_8, Lu_4H_8, Np_4N_8, Ca_8Si_4, Ca_8Ge_4, Cs_8Se_4, K_8Pt_4, Mg_8Si_4, Al_8Pt_4, U_4O_8, Pt_4Sn_8, Mg_8Pb_4, Ir_8P_4, Au_4In_8, In_8Pt_4, Ni_4Si_8, Rh_8As_4, Mg_8Sn_4, Pa_4O_8, Co_4Si_8, Ac_4H_8, La_4H_8, Nb_4H_8, Cr_4H_8, Sc_4H_8, Y_4H_8, Ta_4H_8, V_4H_8, Np_4O_8, Al_8Au_4, Rh_8P_4, Mg_8Ge_4, Rb_8Te_4, Ce_4H_8, Al_8Cu_4, K_8S_4, Ba_4F_8, Rb_8Se_4, Li_8S_4, Na_8Se_4, Rb_8O_4, Be_8C_4, K_8Te_4, Li_8O_4, Ce_4O_8, Li_8Se_4, Sr_4Cl_8, Na_8O_4, Cd_4F_8, Li_8Te_4, Ca_4F_8, Na_8Te_4, Pb_4F_8, Ba_4Cl_8, Th_4O_8, Na_8S_4, Rb_8S_4, Hg_4F_8, K_8Se_4, K_8O_4, Sr_4F_8, $K_4Na_8Sb_4$, $Li_8Na_4Sb_4$, $Au_4Ba_8As_4$, $Au_4Ba_8Sb_4$, $Au_4Ba_8Bi_4$, $Bi_4Li_8Na_4$, $Al_4Fe_8Nb_4$, $Cd_4Pb_4Yb_8$, $Ru_8Ti_4Si_4$, $Li_8Yb_4Ge_4$, $Ca_4Li_8Si_4$, $Ca_4Li_8Ge_4$, $Fe_8Ti_4Si_4$, $Be_4Ir_8Ti_4$, $Au_4Sr_8Sb_4$, $Au_4Sr_8As_4$, $Al_8Co_4Os_4$, $Li_{12}Pb_4$, $Cd_{12}La_4$, Ru_4V_{12}, Cd_4Li_{12}, Mo_4V_{12}, $Li_{12}Zn_4$, $Na_{12}Pd_4$, $Cd_{12}Ce_4$, In_4Li_{12}, Ru_4Ta_{12}, $Nb_{12}Ru_4$, Ta_4W_{12}, Bi_4Li_{12}, $Li_{12}Pt_4$, $Li_{12}Mg_4$, Au_4Li_{12}, Ba_4Sr_{12}, $Ba_{12}Sr_4$, Cd_4Na_{12}, $Ta_{12}W_4$, Bi_4Na_{12}, $Na_{12}Tl_4$, Li_4Mg_{12}, $Li_{12}Pd_4$, Os_4Ta_{12}, Re_4Ta_{12}, Ce_4Mg_{12}, Al_4Fe_{12}, La_4Mg_{12}, $Li_{12}Tl_4$, Mn_4V_{12}, Re_4V_{12}, Li_4Zn_{12}, Os_4V_{12}, $Li_{12}Ge_4$, Ga_4Li_{12}, $Li_{12}Sn_4$, $Nb_{12}Os_4$, Ir_4Ta_{12}, $Cr_{12}V_4$, V_4W_{12}, $Li_{12}Sb_4$, $Ca_4Ti_4F_{24}$, $Rb_8F_{24}Si_4$, $Pd_4Rb_8F_{24}$, $Rb_8Sn_4Cl_{24}$, $Cs_8Pt_4I_{24}$, $K_8Pd_4Cl_{24}$, $Pt_4Rb_8Cl_{24}$, $Cs_8Br_{24}Te_4$, $Cs_8Pt_4Cl_{24}$, $Cs_8Pb_4Cl_{24}$, $Cs_8Cl_{24}Te_4$, $Fe_4Mg_8H_{24}$, $Ru_4Sr_8H_{24}$, $Rb_8Ti_4Cl_{24}$, $Cs_8Ti_4Cl_{24}$, $Rb_8Cl_{24}Se_4$, $Cs_8Cl_{24}Se_4$, $Rb_8Zr_4Cl_{24}$, $Pt_4Tl_8Cl_{24}$, $Cs_8Cl_{24}Ge_4$, $Pd_4Rb_8Cl_{24}$, $Cs_8Hf_4I_{24}$, $Cs_8Pt_4Br_{24}$, $K_8F_{24}Si_4$, $Cs_8F_{24}Si_4$, $Tl_8F_{24}Si_4$, $Cs_8Pd_4F_{24}$, $Cs_8I_{24}Te_4$, $Cs_8Sn_4Cl_{24}$, $Cs_8Zr_4Cl_{24}$, $Cs_8Sn_4Br_{24}$, $Cs_8Pt_4H_{24}$, $K_8Pt_4H_{24}$, $Na_8Pt_4H_{24}$, $Ba_8Ru_4H_{24}$, $Ba_8Os_4H_{24}$, $Cs_8F_{24}Ge_4$, $Rb_8F_{24}Ge_4$, $Cs_8Pt_4F_{24}$, $Cs_8Na_4Tl_4F_{24}$, $Cs_8Er_4Na_4F_{24}$, $Cs_8In_4Na_4F_{24}$, $Cs_8Na_4Y_4F_{24}$, $Na_4Rh_4Tl_8F_{24}$, $Na_4Rb_8Rh_4F_{24}$, $Ba_8Ce_4Pt_4O_{24}$, $Al_4K_8Li_4H_{24}$, $Al_4K_8Na_4H_{24}$, $Ba_8Ca_4O_{24}Te_4$, $Bi_4Cs_8Na_4F_{24}$, $Cs_8K_4Rh_4F_{24}$, $Cs_8Li_4Y_4Cl_{24}$, $Cs_8Li_4Lu_4Cl_{24}$, $Cs_8Er_4Na_4Cl_{24}$, $Cs_8Na_4Sc_4F_{24}$, $Ba_8Er_4O_{24}Sb_4$, $Ba_8Li_4Re_4O_{24}$, $Al_4K_8Na_4F_{24}$, $Ba_8Ho_4O_{24}Sb_4$, $Al_4K_8Li_4F_{24}$, $Ga_4Na_4Rb_8F_{24}$, $Cs_8In_4Li_4Br_{24}$, $Cs_8Ga_4K_4F_{24}$, $Cs_8Ga_4Tl_4F_{24}$, $Ga_4Na_4Tl_8F_{24}$, $Cs_8In_4Na_4Cl_{24}$, $Ga_4Li_4Tl_8F_{24}$, $In_4Na_4Rb_8F_{24}$, $Cs_8Na_4As_4Cl_{24}$, $Cs_8In_4Na_4H_{24}$, $Al_4Cs_8Na_4H_{24}$, $Cd_4Na_{24}Cl_{32}$, $Fe_4Li_{24}Cl_{32}$, $Li_{24}Mg_4Br_{32}$, $Ca_{24}Hf_4O_{32}$, $Ba_{24}Zr_4O_{32}$, $Na_{24}O_{36}S_8$, $Na_{24}Cl_4F_4O_{32}S_8$, $In_{24}Pt_4Ge_8O_{36}$, $Pb_8Zr_{24}F_{48}O_{32}$, $K_8Tb_{24}F_{80}$, $Er_{24}K_8F_{80}$, $K_8Y_{24}F_{80}$
225 (*Fm*−3m)	True	Li_4Cl_4, Li_4Br_4, $Pt_4Rb_8H_{24}$, $Pd_{24}Tl_8O_{32}$, $Cs_8Fe_4Mg_4C_{24}N_{24}$, $Ga_8In_{24}Pt_4O_{32}$

No. (SP)	Clean	NDL and NDR (NDL(R)) candidates
227 ($Fd-3m$)	False	Sn_8, Ge_8, C_8, Si_8, $Al_{16}Pr_8$, Hf_8W_{16}, $Be_{16}Nb_8$, $Mo_{16}Zr_8$, Hf_8Mo_{16}, La_8Ni_{16}, $Au_{16}Bi_8$, Ce_8Ir_{16}, $W_{16}Zr_8$, $Cr_{16}Hf_8$, $Cr_{16}Ti_8$, $Al_{16}Sr_8$, Ba_8Pd_{16}, Ce_8Os_{16}, Ca_8Ni_{16}, Hf_8Zn_{16}, Ba_8Pt_{16}, $Al_{16}Sc_8$, Ca_8Pt_{16}, $Rh_{16}Y_8$, $Pt_{16}Sr_8$, $Be_{16}Ti_8$, $Cr_{16}Nb_8$, Ca_8Ir_{16}, $Mg_{16}Sr_8$, $Mg_{16}Y_8$, $Co_{16}Lu_8$, $Fe_{16}Sc_8$, La_8Mg_{16}, $Bi_{16}K_8$, $Fe_{16}Hf_8$, Na_8Pt_{16}, $Zr_{24}N_{32}$, $Hf_{24}N_{32}$, $Lu_{16}Mg_8Se_{32}$, $Cd_8Sc_{16}S_{32}$, $Cd_8Rh_{16}O_{32}$, $Lu_{16}Mg_8S_{32}$, $Mg_8Sc_{16}S_{32}$, $Cd_8Tm_{16}Se_{32}$, $Mg_8Tm_{16}Se_{32}$, $Mg_8Y_{16}Se_{32}$, $Cd_8Dy_{16}S_{32}$, $Cd_8In_{16}O_{32}$, $In_{16}Mg_8S_{32}$, $In_{16}Zn_8S_{32}$, $Cd_8In_{16}Se_{32}$, $Ca_8In_{16}O_{32}$, $Li_{16}Zn_8Cl_{32}$, $Al_{16}Zn_8O_{32}$, $Cd_8Er_{16}S_{32}$, $Mg_8Rh_{16}O_{32}$, $Cd_8Ga_{16}O_{32}$, $Al_{16}Mg_8O_{32}$, $Cd_8Y_{16}S_{32}$, $Al_{16}Cd_8O_{32}$, $Al_{16}Cd_8Se_{32}$, $Mg_{16}Ge_8O_{32}$, $Mn_8Sc_{16}S_{32}$, $Cd_8Ho_{16}Se_{32}$, $Cd_8Tm_{16}S_{32}$, $Ga_{16}Mg_8O_{32}$, $Al_{16}Zn_8S_{32}$, $Rh_{16}Zn_8O_{32}$, $Cd_8In_{16}S_{32}$, $Cd_8Dy_{16}Se_{32}$, $Ga_{16}Zn_8O_{32}$, $Cd_8Ho_{16}S_{32}$, $Nd_{16}Sr_8O_{32}$, $Sc_{16}Sr_8O_{32}$, $Ca_8Tb_{16}O_{32}$, $Ga_{16}Hg_8O_{32}$, $In_{16}Zn_8O_{32}$, $Lu_{16}Sr_8O_{32}$, $In_{16}Mg_8O_{32}$, $Mg_{16}Sn_8O_{32}$, $Na_{16}Pr_{16}Te_{32}$, $Ca_{32}H_{16}N_{16}$, $K_{16}O_{48}Sb_{16}$, $Hg_{16}Zn_{16}F_{48}O_8$, $Hf_{16}La_{16}O_{56}$, $Pt_{16}Tl_{16}O_{56}$, $Ca_{16}Ta_{16}O_{56}$, $Lu_{16}Sn_{16}O_{56}$, $Nd_{16}Sn_{16}O_{56}$, $Dy_{16}Sn_{16}O_{56}$, $Bi_{16}Pt_{16}O_{56}$, $Sm_{16}Sn_{16}O_{56}$, $Sn_{16}Tm_{16}O_{56}$, $Sn_{16}Y_{16}O_{56}$, $Hf_{16}Sm_{16}O_{56}$, $Er_{16}Sn_{16}O_{56}$, $Ho_{16}Sn_{16}O_{56}$, $Pr_{16}Sn_{16}O_{56}$, $Sm_{16}Zr_{16}O_{56}$, $La_{16}Zr_{16}O_{56}$, $Sn_{16}Tb_{16}O_{56}$, $Cd_{16}Ta_{16}O_{56}$, $Pr_{16}Zr_{16}O_{56}$, $Nd_{16}Zr_{16}O_{56}$, $Pb_{16}Tm_{16}O_{56}$, $Pb_{16}Y_{16}O_{56}$, $Lu_{16}Pt_{16}O_{56}$, $Ho_{16}Ge_{16}O_{56}$, $Dy_{16}Zr_{16}O_{56}$, $Dy_{16}Ge_{16}O_{56}$, $Pt_{16}Tb_{16}O_{56}$, $La_{16}Pt_{16}O_{56}$, $In_{16}Pt_{16}O_{56}$, $Lu_{16}Pb_{16}O_{56}$, $Ce_{16}La_{16}O_{56}$, $Ho_{16}Pt_{16}O_{56}$, $Er_{16}Pb_{16}O_{56}$, $Pt_{16}Y_{16}O_{56}$, $Er_{16}Pt_{16}O_{56}$, $Tb_{16}Zr_{16}O_{56}$, $Pb_{16}Tb_{16}O_{56}$, $Dy_{16}Pb_{16}O_{56}$, $Pt_{16}Sc_{16}O_{56}$, $Nd_{16}Pt_{16}O_{56}$, $Bi_{16}K_{16}F_{64}$, $Na_{64}Ti_8As_{32}$
227 ($Fd-3m$)	True	$Co_{16}U_8$, Ca_8Pd_{16}, $Ni_{16}Sc_8$, $Co_{16}Sc_8$, $Pt_{16}Y_8$, $Cu_{16}Mg_8$, $Ag_{16}Na_8$, Ca_8Mg_{16}, Mg_8Ni_{16}, $La_{16}Sr_8O_{32}$, $Sm_{16}Sr_8O_{32}$, $Ba_8La_{16}O_{32}$, $Cd_8Lu_{16}S_{32}$, $Li_{16}Rh_{16}O_{32}$, $La_{16}Sn_{16}O_{56}$, $Pt_{16}Sm_{16}O_{56}$, $Y_{16}Ge_{16}O_{56}$, $Sm_{16}Ge_{16}O_{56}$
229 ($Im-3m$)	False	Xe_2, Ba_2, Na_2, K_2, Kr_2, Mo_2, Tl_2, Nb_2, Cr_2, W_2, $Na_2Sr_8B_6N_{12}$, $Li_2Sr_8B_6N_{12}$, $Ca_2K_8U_6O_{24}$, $Ba_8Na_2O_{24}Sb_6$, $Co_2Th_{12}Br_{30}$, $Co_2Zr_{12}Cl_{30}$
229 ($Im-3m$)	True	$Ca_8Li_2B_6N_{12}$

IV. SINGLE WEYL TP MATERIALS

Supplementary Table 3: The summary of 36 single Weyl phonon materials categorized by their space group number and symbol(*No.* (SP)). The true or false in the column of Clean denotes whether the material holds clean WPs

No. (SP)	Clean	Single WPs candidates
5 ($C2$)	False	$Nb_4I_8O_4$, $Na_{10}O_{10}Sb_2$, $Hg_6K_4Ge_4S_{16}$
82 ($I-4$)	False	$Li_2Zn_2P_2S_8$, $Hg_2In_4Se_8$, $Cd_2In_4Se_8$, $In_4Zn_2Se_8$, $Ag_4Hg_2I_8$, $Al_4Cd_2Se_8$, $Hg_4Ge_2Se_8$, $Cd_2Ga_4S_8$, $Al_4Cd_2S_8$, $Al_4Hg_2S_8$, $Al_4Cd_2Te_8$, $Ca_2Hf_2Li_4F_{16}$, $La_{10}Tl_2Te_{16}$, $Ag_2Sm_{10}Se_{16}$, $Na_{10}Y_8F_2O_{32}Si_8$
119 ($I-4m2$)	False	Ag_2I_2
144 ($P3_1$)	False	$Li_6Nb_3F_{15}O_3$
146 ($R3$)	False	$Bi_3Li_{21}O_{18}$, $K_3Zn_{12}O_{36}Sb_9$
152 ($P3_121$)	False	$Ba_3Mg_3O_6$
152 ($P3_121$)	True	Hg_3S_3
154 ($P3_221$)	False	$Ir_6Sr_3P_6$
154 ($P3_221$)	True	Hg_3S_3
155 ($R32$)	False	$Al_9Tm_3B_{12}O_{36}$
155 ($R32$)	True	$Ca_3Mg_9C_{12}O_{36}$
187 ($P-6m2$)	False	YTe, VN, TaP, WC, NbN, CrN, CeNiSi, AlBaSi

V. MIXED TP MATERIALS

Supplementary Table 4: The summary of 910 mixed topological phonon(TPs) materials categorized by their space group number and symbol(*No.* (SP)). The true or false in the column of Clean denotes whether the material holds clean topological phonons Those mixed TPs are classified into A, B and C. 463 TPs in block A holds both single Weyl points and NDL(R). The block B represents 266 topological phonon materials have single WPs, high degenerate WPs, and NDL(R). 181 TPs in block C mixed by high degenerate WPs and DNL(R).

Type	*No.* (SP)	Clean	mixed TPs candidates
	1 ($P1$)	False	Cu_4I_4
	4 ($P2_1$)	False	$K_2Sm_2Ge_2Se_8$, $K_2Tb_2Ge_2S_8$, $K_2Pr_2Ge_2Se_8$, $K_2Pr_2Se_8Si_2$, $K_2La_2Ge_2S_8$, $K_2La_2Se_8Si_2$, $K_2La_2S_8Si_2$, $K_2Y_2S_8Si_2$, $K_4Zn_2Br_8$, $Tl_4Zn_2I_8$, $Cu_8K_2As_2S_8$, $Ba_2Na_4O_{12}Si_4$, $Co_4P_{12}Si_{12}$, $La_6Cl_6O_{14}Si_4$, $Pr_6Cl_6O_{14}Si_4$, $Au_2Na_{10}Se_{24}$, $K_2Y_2O_{24}P_8$, $Ba_8As_8Se_{20}$
	4 ($P2_1$)	True	$K_2La_2Ge_2Se_8$, $K_2Nd_2Ge_2S_8$, $K_2Y_2Ge_2S_8$, $K_2Li_2O_{10}Si_4$, $K_2Tb_2O_{24}P_8$
	5 ($C2$)	False	$Cs_4Li_6I_{10}$, $Cu_8Dy_8K_4S_{18}$
	5 ($C2$)	True	$Nb_4I_{12}O_4$
	8 (Cm)	False	$Sn_6Ti_6O_{24}$, $Li_{28}I_4N_8$, $Os_6Sn_{30}O_{28}$
	9 (Cc)	False	$Li_{20}P_{12}Si_4$, $Cs_8Si_8Te_{24}$, $Cd_{16}Se_{24}Si_4$, $Cu_{24}Br_4P_4S_{20}$
	18 ($P2_12_12$)	False	Ba_4S_{12}
	20 ($C222_1$)	False	Fe_4P_{16}, $Nb_4Sm_{12}O_{28}$, $Sr_{20}B_{12}Cl_4O_{36}$
	20 ($C222_1$)	True	$K_8Zn_4O_{24}Si_8$, $K_8Zn_4Ge_8O_{24}$
	35 ($Cmm2$)	False	$Sn_{12}Ti_8O_{40}$
	36 ($Cmc2_1$)	False	$Cs_4H_4O_4$, $Rb_4H_4O_4$, $K_4H_4O_4$, $Cd_4Sr_8As_8$, Rb_8Se_{12}, Cs_8Se_{12}, K_8S_{12}, K_8Se_{12}, $Na_8Pd_4S_8$, $Na_8Pt_4S_8$, $Na_8Pt_4Se_8$, $Ga_4In_4S_{12}$, $Ge_8N_8O_4$, $Ba_4Cd_4Yb_4Sb_8$, Al_8O_{12}, $Mg_4Sr_4H_{16}$, $Na_4Ge_8N_{12}$, $Li_4N_{12}Si_8$, $Na_4N_{12}Si_8$, $Cu_8S_{12}Si_4$, $Li_8Ge_4O_{12}$, $Mg_8N_{12}P_4$, $Na_8O_{12}Si_4$, $Li_8O_{12}Si_4$, $Na_8Ge_4O_{12}$, $Pb_4Rb_8O_{12}$, $Cs_8Pb_4O_{12}$, $Rb_8Sn_4O_{12}$, $Rb_8Zr_4O_{12}$, $Ag_8Hg_4I_8S_4$, $Bi_8Cs_4Cu_4S_{16}$, $K_{12}S_{16}Sb_4$, $Ba_8Bi_4In_4S_{20}$, $Al_{16}C_4O_{16}$, $Ga_4Nd_{12}O_{24}$, $Ga_4Sm_{12}O_{24}$, $Ga_4Tb_{12}O_{24}$, $Dy_{12}Ga_4O_{24}$, $Ga_4Ho_{12}O_{24}$, $Er_{12}Ga_4O_{24}$, $Ga_4Y_{12}O_{24}$, $Er_{12}Ga_4S_{24}$, $Al_4Y_{12}O_{24}$, $Al_4Lu_{12}O_{24}$, Ba_4P_{40}, $P_{24}Si_{24}$
	37 ($Ccc2$)	False	$Li_8Ti_8O_{20}$
	38 ($Amm2$)	False	$Pr_8O_8Se_6$
	40 ($Ama2$)	False	$Sn_4Sr_8S_{16}$
	41 ($Aea2$)	False	Pb_4F_8
	42 ($Fmm2$)	False	$Ca_{12}U_4N_{16}$, $Cs_{12}Li_8F_{20}$
	43 ($Fdd2$)	False	$Bi_8Na_8S_{16}$, $Sr_{24}As_{32}$, $Bi_{16}Na_{32}O_{48}$
A	44 ($Imm2$)	False	$U_2C_2O_{10}$, $Li_4B_{52}C_8$
	46 ($Ima2$)	False	$Al_8Ca_8O_{20}$, $Ba_8In_8O_{20}$, $Ba_8Na_{16}Si_{32}$
	77 ($P4_2$)	False	$Ca_2B_4H_{16}$
	79 ($I4$)	False	$Ca_{16}Cl_{16}O_{24}Si_8$
	82 ($I-4$)	False	$Cd_2Ga_4Te_8$, $Ga_4Zn_2Te_8$, $Ga_4Hg_2Te_8$, $Hg_2In_4Te_8$, $In_4Zn_2Te_8$, $Cd_2In_4Te_8$, $Al_4Hg_2Se_8$, $Cd_2Ga_4Se_8$, $Ga_4Hg_2Se_8$, $Ga_4Hg_2S_8$, $Al_4Zn_2Se_8$, $Al_4Zn_2Te_8$, $Al_4Hg_2Te_8$, $Ag_2Tb_{10}S_{16}$, $Ba_{16}Br_2P_{10}$, $Ag_2Dy_{10}S_{16}$, $La_{10}Tl_2S_{16}$, $Ag_2Nd_{10}S_{16}$, $La_{10}Tl_2Se_{16}$, $Ag_2Sm_{10}S_{16}$, $Cs_2Nd_{10}Se_{16}$, $Ca_8Na_{10}F_2O_{32}P_8$, $K_{10}La_8F_2O_{32}Si_8$
	91 ($P4_122$)	False	$Li_8O_{16}Te_4$, $Ti_4Zn_8O_{16}$, $Mg_8Ti_4O_{16}$
	97 ($I422$)	False	$Ba_2In_4Te_8$, $Al_4Sr_2Te_8$, $Ba_2Ga_4Te_8$, $Al_4Ba_2Te_8$
	107 ($I4mm$)	False	$Cu_2K_2Na_4O_4$, $Rh_2Sr_4H_{10}$, $Na_{12}Pb_2O_{10}$
	109 ($I4_1md$)	False	$Ga_8S_8Te_4$, $Nb_4Rh_4O_{16}$, $Rh_4Ta_4O_{16}$, $Li_{32}N_8Se_4$
	109 ($I4_1md$)	True	$Ga_8Se_8Te_4$
	111 ($P-42$m)	False	Cu_2WS_4, $CdIn_2Se_4$, Ag_2HgI_4
	112 ($P-42$c)	False	$Al_2Cu_2Cl_8$, $Cu_2Ga_2Cl_8$
	113 ($P-42_1$m)	False	$Ag_2Er_2Te_4$, $Ag_2Y_2Te_4$, $Ag_2Ho_2Te_4$, $Ag_2Tb_2Te_4$, $Ag_2Dy_2Te_4$, Ba_2Se_6, Ba_2S_6, $Cl_{12}I_2P_2$, $Nb_4Cl_{12}O_4$, $Nb_4Br_{12}O_4$, $Be_4Pr_4Ge_2O_{14}$, $Be_4Sm_4Ge_2O_{14}$, $Be_4Er_4Ge_2O_{14}$, $Be_4La_4Ge_2O_{14}$, $Be_4Y_4Ge_2O_{14}$, $Al_4Ca_4O_{14}Si_2$, $Be_4Y_4O_{14}Si_2$, $Be_4Ho_4O_{14}Si_2$, $Be_4Nd_4O_{14}Si_2$, $Ba_4Mg_2O_{14}Si_4$, $Ba_4Zn_2Ge_4O_2S_{12}$
	113 ($P-42_1$m)	True	$Be_4Dy_4Ge_2O_{14}$, $Ga_4La_4Zn_2O_2S_{12}$
	115 ($P-4$m2)	False	$CsLi_2I_3$
	118 ($P-4$n2)	True	$Zn_6P_4S_{16}$
	119 ($I-4$m2)	False	Cu_2Br_2, $K_2Li_2Zn_2S_4$, $La_4Th_6O_{18}$
	121 ($I-42$m)	False	$Ag_2Cl_2O_8$, $Na_2Cl_2O_8$, $Zn_4Ge_2Se_8$, $Cd_2In_4Se_8$, $Zn_4Ge_2S_8$, $Cu_4W_2S_8$, $Li_6S_8Sb_2$, $Ag_4K_2P_2S_8$, $Li_4Pb_2Ge_2S_8$, $Hg_2K_4Sn_2Se_8$, $Cu_8Ti_2S_8$, $Li_8Ti_2S_8$, $Zr_6Ge_2O_{16}$, $Cs_6Ta_2O_{16}$, $Ba_8Cu_2Li_2C_4O_{20}$, $Ba_8Cu_2Na_2C_4O_{20}$, $Na_4Th_8F_{36}$

Type	*No.* (SP)	Clean	mixed TPs candidates
	122 (*I*−42d)	False	Ge_4S_8, $Ga_4Na_4Te_8$, $Ag_4Ga_4Se_8$, $Cd_4Sn_4Sb_8$, $Mg_4Sn_4As_8$, $Mg_4Sn_4P_8$, $Be_4P_8Si_4$, $Be_4As_8Si_4$, $Be_4As_8Ge_4$, $Be_4Ge_4P_8$, $Mg_4As_8Si_4$, $Mg_4As_8Ge_4$, $Cd_4Mg_4Te_8$, $Cu_4B_4S_8$, $Cu_4Tl_4S_8$, $Cu_4Tl_4Se_8$, $Ag_4Al_4Se_8$, $Li_4B_4O_8$, $Ag_4In_4S_8$, $Ag_4In_4Se_8$, $Cu_4In_4Te_8$, $Ag_4In_4Te_8$, $Cu_4In_4S_8$, $Cu_4In_4Se_8$, $Mg_4P_8Si_4$, $Cd_4As_8Si_4$, $Mg_4Ge_4P_8$, $Li_4N_8P_4$, $Cd_4Sn_4As_8$, $Cu_4Ga_4Te_8$, $Zn_4As_8Ge_4$, $Cd_4P_8Si_4$, $Sn_4Zn_4Sb_8$, $Cu_4Ga_4Se_8$, $Ag_4Ga_4Te_8$, $Cd_4As_8Ge_4$, $Al_4Cu_4S_8$, $Sn_4Zn_4As_8$, $Ag_4Ga_4S_8$, $Ag_4Al_4S_8$, $Ag_4Al_4Te_8$, $Mg_4N_8Si_4$, $Be_4N_8Si_4$, $In_4Li_4Se_8$, $In_4Li_4Te_8$, $Zn_4As_8Si_4$, $Cd_4Ge_4P_8$, $Sn_4Zn_4P_8$, $Zn_4Ge_4P_8$, $Al_4Li_4Te_8$, $Zn_4P_8Si_4$, $Ga_4Li_4Te_8$, $Cu_4Ga_4S_8$, $Be_4Zn_4O_8$, $Al_4Cu_4Se_8$, $Al_4Cu_4Te_8$, $Cd_4Zn_4Te_8$, $Cu_4B_4Se_8$, $La_8Pb_4Se_{16}$, $La_8Sr_4Se_{16}$, $Ba_4Pr_8Se_{16}$, $Ba_4La_8S_{16}$, $Ca_4Ho_8S_{16}$, $La_8Sr_4S_{16}$, $Ca_4Pr_8S_{16}$, $Sm_8Sr_4S_{16}$, $Ca_4La_8S_{16}$, $Ca_4Dy_8S_{16}$, $Ca_4Nd_8S_{16}$, $Ba_4Nd_8Se_{16}$, $Ca_4La_8Te_{16}$, $Ca_4Sm_8S_{16}$, $Ba_4Pr_8S_{16}$, $La_8Pb_4S_{16}$, $Nd_8Sr_4S_{16}$, $Ca_4Nd_8Te_{16}$, $Ca_4Pr_8Te_{16}$, $Ba_4La_8Se_{16}$, $Pr_8Sr_4Se_{16}$, $Pr_8Sr_4S_{16}$, $Ca_4Tb_8S_{16}$, $Nd_8Sr_4Se_{16}$, $Tb_8Yb_4S_{16}$, $Pb_4Sm_8Se_{16}$, $Nd_8Pb_4Se_{16}$, $Pb_4Pr_8Se_{16}$, $Nd_8Yb_4S_{16}$, $Y_8Yb_4S_{16}$, $Ho_8Yb_4S_{16}$, $La_8Pb_4Te_{16}$, $Nd_8Pb_4S_{16}$, $Pr_8Yb_4S_{16}$, $Sm_8Yb_4S_{16}$, $La_8Yb_4S_{16}$, $Dy_8Yb_4S_{16}$
	122 (*I*−42d)	True	Ge_4Se_8, $Be_4Sn_4As_8$, $Be_4C_4N_8$, $Cd_4Sn_4P_8$, $Pb_4Sm_8S_{16}$, $Pb_4Pr_8S_{16}$
	146 (*R*3)	False	$Bi_6Cd_6O_{18}$, $Al_{12}B_{18}O_{45}$
	150 (*P*321)	False	Na_2ThF_6, $In_6O_{18}Te_3$, $Ca_6Sr_3I_{18}$, $Ba_3Ca_3C_6O_{18}$
	151 ($P3_112$)	False	$Li_{15}Re_3O_{18}$
	152 ($P3_121$)	False	Hg_3O_3, $Ba_3Zn_3O_6$
	152 ($P3_121$)	True	$Ba_3Cu_6Ge_3S_{12}$
	154 ($P3_221$)	False	$Ca_3Ir_6P_6$, $Cu_6Sr_3Ge_3S_{12}$, $Cu_6Pb_3S_{12}Si_3$
	154 ($P3_221$)	True	$Ag_6Rb_3S_{12}Sb_3$
	155 (*R*32)	False	$Be_6Cs_3B_3F_6O_9$, $Be_6K_3B_3F_6O_9$, $Be_6Rb_3B_3F_6O_9$, $Al_9Y_3B_{12}O_{36}$, $Al_9Nd_3B_{12}O_{36}$, $Ga_{36}Na_{12}Te_{60}$
A	156 (*P*3*m*1)	False	SnSSe, CoSeTe, BiBrTe, PtSeTe, PdSeTe, AlSrHSi, AlBaHSi, GaSrHSi, Pb_3I_6, Cd_3I_6, Nb_3Br_7, Nb_3I_7, $KNaB_2H_8$, Cu_6I_6, Pb_5I_{10}, Zn_8S_8, Pb_7I_{14}, $Pb_{10}I_{20}$
	156 (*P*3*m*1)	True	NiSeTe
	160 (*R*3*m*)	False	$Ba_3C_3O_9$, $Zn_{21}S_{21}$, $C_{21}Si_{21}$, $Cd_{15}I_{30}$, $Zn_{24}S_{24}$, $Zn_{24}S_{24}$, $C_{24}Si_{24}$, $C_{27}Si_{27}$, $Zn_{30}S_{30}$, $Zn_{30}S_{30}$, $Zn_{30}S_{30}$, $Ga_3Zn_9B_{18}O_{36}P_3$, $Zn_{36}S_{36}$, $Zn_{36}S_{36}$
	169 ($P6_1$)	False	$Ga_6In_6S_{18}$, $Ga_6In_6Se_{18}$, $Al_{12}S_{18}$, $In_{12}Se_{18}$
	169 ($P6_1$)	True	$Al_6In_6S_{18}$
	173 ($P6_3$)	False	$Be_2K_2Li_2F_8$, $Tl_6As_2O_8$, $Tl_6O_8P_2$, $Na_2Sm_6S_{14}Si_2$, $Ga_2La_6Mn_2S_{14}$, $Be_2La_6Sc_2S_{14}$, $Ag_2La_6Sn_2Se_{14}$, $Cu_2Pr_6Sn_2Se_{14}$, $Ag_2Pr_6Se_{14}Si_2$, $Ag_2La_6S_{14}Si_2$, $Cu_2Sn_2Y_6S_{14}$, $Ag_2Nd_6Se_{14}Si_2$, $Cu_2Sn_2Tb_6Se_{14}$, $Cu_2Nd_6Sn_2Se_{14}$, $Cu_2Dy_6Ge_2Se_{14}$, $Cu_2Nd_6Ge_2S_{14}$, $Cu_2Sn_2Tb_6S_{14}$, $Cu_2Er_6Ge_2S_{14}$, $Cu_2Pr_6Ge_2S_{14}$, $Cu_2Tb_6Ge_2S_{14}$, $Cu_2Dy_6S_{14}Si_2$, $Cu_2Sm_6Sn_2S_{14}$, $Cu_2Er_6S_{14}Si_2$, $Cu_2Pr_6Sn_2S_{14}$, $Cu_2Nd_6Sn_2S_{14}$, $Cu_2Y_6S_{14}Si_2$, $Cu_2Tb_6Ge_2Se_{14}$, $Cu_2Y_6Se_{14}Si_2$, $Cu_2Tb_6Se_{14}Si_2$, $Cu_2Nd_6Ge_2Se_{14}$, $Cu_2Dy_6Se_{14}Si_2$, $Cu_2Sm_6Se_{14}Si_2$, $Cu_2Sm_6Ge_2Se_{14}$, $Cu_2Sm_6Sn_2Se_{14}$, $Cu_2Pr_6Ge_2Se_{14}$, $Cu_2Ho_6Se_{14}Si_2$, $Cu_2Nd_6Se_{14}Si_2$, $Ag_2La_6Se_{14}Si_2$, $Ag_2Nd_6S_{14}Si_2$, $Ag_2Sm_6S_{14}Si_2$
	173 ($P6_3$)	True	$K_2Li_2O_8S_2$, $Cu_2Y_6Ge_2Se_{14}$, $Cu_2La_6Sn_2S_{14}$, $Cu_2Sm_6S_{14}Si_2$, $Cu_2Pr_6S_{14}Si_2$, $Cu_2Sm_6Ge_2S_{14}$, $Cu_2Y_6Ge_2S_{14}$, $Cu_2Nd_6S_{14}Si_2$, $Cu_2Tb_6S_{14}Si_2$, $Cu_2La_6Sn_2Se_{14}$, $Cu_2Pr_6Se_{14}Si_2$, $Ag_2La_6Ge_2S_{14}$, $Ag_2Pr_6Ge_2S_{14}$, $Ag_2Pr_6S_{14}Si_2$
	174 (*P*−6)	False	$Sr_7Cl_2H_{12}$, $Sr_7Br_2H_{12}$, $Ca_7Cl_2H_{12}$
	178 ($P6_122$)	False	Au_6F_{18}
	180 ($P6_222$)	True	$Hg_6Na_3I_3O_6$
	182 ($P6_322$)	False	$Ca_2Ta_8O_{22}$
	186 ($P6_3mc$)	False	Ga_2Sb_2, Al_2Sb_2, Mg_2S_2, Cd_2I_4, Zn_8S_8, $Al_{10}C_6N_2$
	187 (*P*−6m2)	False	IrTc, GaTc, TiS, LiPt, ZrSe, AgPd, AgAu, HfS, RhTc, OsRe, CrIr, AgAl, ZrTe, MoP, MoC, LiPd, HfZr, OsB, IrB, AgZn, RuTc, CeCuSi, CuLaSi, NiYAs, BeZrB, PtSrP, CuSrSi, CuSrGe, CuLaGe, CeCuGe, BaPtSb, BaPdAs, YZnSi, CeZnSi, AuBaSi, BaLiP, BaLiAs, HoHSe, YHSe, $CaKCFO_3$, $RbSrCFO_3$, Ga_4Se_4, $Li_{10}BrN_3$
	216 (*F*−43m)	False	In_4As_4, Al_4As_4, Cu_4I_4, Cd_4Te_4
	19 ($P2_12_12_1$)	False	$Cu_4Y_4S_8$, $Cu_4Dy_4S_8$, $Cu_4Er_4S_8$, $Cu_4Ho_4S_8$, Rb_8As_8, K_8As_8, Rb_8Sb_8, $Ag_4Ho_4Se_8$, $Ag_4Er_4Se_8$, $Zn_4H_8O_8$, $Rb_8Sn_4O_8$, $Cs_4Cu_4Se_{16}$, $Ag_4Cs_4Se_{16}$, $Cu_4Rb_4Se_{16}$, $Ag_4Rb_4Se_{16}$, $Al_4Sr_4H_{20}$, Rb_8Se_{20}, $Cu_{12}S_{12}Sb_4$, $Cs_4Sm_4Ge_4S_{16}$, $Rb_4Sm_4Ge_4Se_{16}$, $Al_4Ba_4La_4O_{16}$, $Ba_4Ga_4La_4O_{16}$, $Ba_4Ga_4Nd_4O_{16}$, $Y_8O_{24}Te_4$, $Nd_8O_{24}Te_4$, $Lu_8O_{24}Te_4$, $Dy_8O_{24}Te_4$, $Ho_8O_{24}Te_4$, $Pb_4Tl_{12}I_{20}$, $Pb_4Tl_{12}Br_{20}$, $Al_8Sr_4N_8O_{12}Si_4$, $Sm_8O_{24}Te_4$, $Er_8O_{24}Te_4$, $Tm_8O_{24}Te_4$, $Na_{16}Sn_4Te_{16}$, $Al_4Ca_4H_4O_{20}Si_4$
	19 ($P2_12_12_1$)	True	Br_4H_4, $Na_8Sn_4O_8$, $Cs_4Cu_4S_{16}$, $Ag_8Ge_4O_{12}$, Rb_8S_{20}, $Cs_4Er_4S_{16}Si_4$, $Cs_4Sm_4S_{16}Si_4$, $Al_4H_{12}O_{12}$, $Ca_4Zn_4As_4H_4O_{20}$, $Pb_4Zn_4As_4H_4O_{20}$
	33 ($Pna2_1$)	False	Zn_4Cl_8
B	33 ($Pna2_1$)	True	$K_4Na_4Ge_4O_{12}$, $Ba_8Cu_8O_{20}$
	76 ($P4_1$)	False	$Pb_4Tl_{12}Cl_{20}$
	82 (*I*−4)	False	$Ta_{18}O_{50}P_2$
	92 ($P4_12_12$)	False	$Al_4Li_4O_8$, Mg_4As_{16}, Cd_8P_{16}, $Ba_4Pt_8S_{12}$
	92 ($P4_12_12$)	True	Ge_4O_8, Zn_8P_{16}
	96 ($P4_32_12$)	False	$Ag_8Hg_4O_8$, Zn_8P_{16}, Cd_8P_{16}, $Be_8Rb_8O_{12}$, $K_8Mg_8O_{12}$

Type	*No.* (SP)	Clean	mixed TPs candidates
	96 ($P4_32_12$)	True	$Na_8Zn_8O_{12}$
	114 ($P{-}42_1c$)	False	$Na_8Sn_2Se_8$, $Na_8Sn_2S_8$
	114 ($P{-}42_1c$)	True	$Sn_{12}H_8O_{16}$
	117 ($P{-}4b2$)	False	$Ba_4Sr_8I_{24}$, $Ba_8Sr_4I_{24}$
	122 ($I{-}42d$)	False	$Sm_8Sr_4Se_{16}$
	146 ($R3$)	False	$Tl_{54}S_{27}$
	152 ($P3_121$)	False	$Cu_3Sr_6Cl_3O_6$
	160 ($R3m$)	False	$Zn_{24}S_{24}$, $Zn_{36}S_{36}$, $C_{39}Si_{39}$
	161 ($R3c$)	False	$Li_6Mg_6H_{18}$, $Bi_6Sc_6O_{18}$, $Bi_6Tm_6O_{18}$, $Bi_6Y_6O_{18}$, $Bi_6La_6O_{18}$, $Bi_6Li_{18}S_{18}$, $K_{18}Sb_6Se_{24}$
	169 ($P6_1$)	False	$Al_6In_6Se_{18}$
	173 ($P6_3$)	False	$Ag_2La_6Sn_2S_{14}$
	185 ($P6_3cm$)	False	$K_6Ni_6Cl_{18}$, $Ga_6Y_6O_{18}$, $In_6Y_6O_{18}$, $Dy_6In_6O_{18}$, $Ho_6In_6O_{18}$, $Ga_6Tm_6O_{18}$, $Ga_6Lu_6O_{18}$, $Sc_6Y_6O_{18}$, $Dy_6Ga_6O_{18}$, $Ga_6Ho_6O_{18}$, $Lu_6Sc_6O_{18}$, $Li_{16}Ge_2O_{12}$, $Li_{16}Ti_2S_{12}$, $Na_{16}Sn_2O_{12}$, $Rb_{16}Sn_2O_{12}$, $Li_{16}Ti_2O_{12}$
	185 ($P6_3cm$)	True	$Li_{16}O_{12}Si_2$, $Mg_6H_8O_{18}Si_4$
	186 ($P6_3mc$)	False	Mg_2Se_2, Pd_2H_2, Li_2Cl_2, Li_2I_2, $Cu_2Hf_2Sn_2$, $Au_2La_2Sn_2$, $Ag_2La_2Ge_2$, $Ca_2Sn_2Zn_2$, $Ag_2Sn_2Y_2$, $Au_2La_2Ge_2$, Zn_4S_4, Zn_6S_6, $Rb_4F_{12}Ge_2$, $K_4F_{12}Ge_2$, $Nb_6I_{14}Te_2$, $Ga_2Na_6Sr_6P_8$, $Cd_2K_{12}O_8$, $Cd_2K_{12}Te_8$, $Sr_8Cl_{12}O_2$, $Ba_8I_{12}O_2$, $Sr_8I_{12}O_2$, $Mn_2Na_{12}S_8$, $Sr_8Br_{12}O_2$, $Mg_2Na_{12}O_8$, $Be_2Na_{12}O_8$, $Na_{12}Zn_2O_8$, $Na_{12}W_2P_8$, $Na_4Pt_8Se_{12}$, $In_2K_{12}Cl_2Te_8$, $Ba_2Y_2N_{14}Si_8$, $Be_8Na_2O_{14}Sb_2$, $Al_{10}H_2O_{16}$
	186 ($P6_3mc$)	True	$Ca_2Cl_2H_2O_2$, $K_4F_{12}Si_2$
	188 ($P{-}6c2$)	False	$Ca_2K_2O_{18}P_6$, $Be_6Rb_2Zn_2F_{18}$, $Ba_2Sn_2O_{18}Si_6$, $Be_6K_2Zn_2F_{18}$, $K_2Ta_2Ge_6O_{18}$, $Ta_2Tl_2Ge_6O_{18}$, $Ba_2O_{18}Si_8$
B	188 ($P{-}6c2$)	True	$Ba_2Ti_2O_{18}Si_6$
	198 ($P2_13$)	False	Os_4Si_4, Ru_4Ge_4, Ga_4Pt_4, Co_4Ge_4, Mn_4Ge_4, Ga_4Pd_4, Tc_4Si_4, Hf_4Sb_4, Al_4Pt_4, Mg_4Pt_4, Mn_4Si_4, Ru_4Si_4, Au_4Be_4, Rh_4Sn_4, Zr_4Sb_4, Al_4Pd_4, Co_4Si_4, Re_4Si_4, Fe_4Si_4, $Co_4As_4S_4$, $Zr_4O_4Se_4$, $Ir_4S_4Sb_4$, $Dy_4Yb_{12}Sb_{12}$, $Na_{12}As_4Se_{12}$, $Na_{12}Sb_4Te_{12}$, $K_{12}Sb_4Se_{12}$, $Bi_4K_{12}Se_{12}$, $Bi_4Rb_{12}Se_{12}$, $Bi_4K_{12}Te_{12}$, $Bi_4Rb_{12}O_{12}$, $Rb_{12}O_{12}Sb_4$, $Bi_4K_8Rb_4Se_{12}$, $Ca_4Mg_4Ni_4H_{16}$, $Mg_4Ni_4Sr_4H_{16}$
	198 ($P2_13$)	True	$K_{12}O_{12}Sb_4$, $K_{12}Sb_4Te_{12}$, $Mg_4Ni_4Yb_4H_{16}$
	199 ($I2_13$)	False	$K_8Pb_8O_{12}$, $Rb_8Sr_8C_{12}O_{36}$
	199 ($I2_13$)	True	$Cu_8Cl_8H_{24}N_8$, $Ba_8Cs_8C_{12}O_{36}$, $Cs_8Sr_8C_{12}O_{36}$
	214 ($I4_132$)	False	$Ca_{24}I_{24}P_8$
	215 ($P{-}43m$)	False	ClH_4, $CdZn_3S_4$, Cu_3TaS_4, Cu_3VSe_4, Cu_3VS_4, Cu_3NbSe_4, Cu_3TaSe_4, Cu_3NbS_4, Li_3NbS_4, Li_3VS_4, Cu_3TaTe_4, Cu_3VTe_4, Cu_3NbTe_4, $Fe_3Mg_4Yb_4H_{22}$, $Ca_4Fe_3Mg_4H_{22}$
	216 ($F{-}43m$)	False	$Rh_4Sn_4Ti_4$, $Rh_4Sn_4Ta_4$, $Li_4Zn_4Sb_4$, $Cu_4Mg_4Sn_4$, $Li_4Mg_4Sb_4$, $Au_4Cd_4Sn_4$, $Au_4Cd_4Sb_4$, $Bi_4Mg_4Ni_4$, $Bi_4Ni_4Sc_4$, $Bi_4Ni_4Y_4$, $Al_4Be_4B_4$, $Bi_4Ce_4Pd_4$, $Fe_4Ti_4Te_4$, $Rh_4Th_4Sb_4$, $Ho_4Pt_4Sb_4$, $Ru_4Ta_4Sb_4$, $Co_4Ti_4Sb_4$, $Li_4Zn_4N_4$, $Fe_4Nb_4Sb_4$, $Pd_4Sn_4Zr_4$, $Au_4In_4Zr_4$, $Au_4Li_8Pb_4$, $Li_8Pd_4Sn_4$, $Li_8Mg_4Tl_4$, $Li_8Mg_4Sn_4$, $Au_4Li_8Mg_4$, $Li_8Mg_4Ge_4$, $Li_8Mg_4Pb_4$, $In_4Li_8Pd_4$, $Ag_4In_4Li_8$, $Al_4Li_8Pt_4$, $In_4Li_8Rh_4$, $Ga_4Li_8Pd_4$, $Mn_8Ru_4Si_4$, $Na_4B_4H_{16}$, $Be_{16}O_{28}Te_4$, $Al_{20}Cu_4Se_{32}$, $In_{28}Ir_4Ge_4O_{32}$
	216 ($F{-}43m$)	True	$Co_4Mn_4Sb_4$, $Ca_4Li_4As_4$, $Au_4In_4Li_8$
	217 ($I{-}43m$)	False	$Ta_2Tl_6Se_8$, $Be_{12}F_{24}$, $Nb_{12}Sb_8Te_{20}$, $Zn_8B_{12}O_{24}Se_2$, $Zn_8N_{24}P_{12}S_2$, $Mg_8N_{24}P_{12}S_2$, $Al_{12}Ca_8O_{24}S_2$, $Al_{12}Cd_8O_{24}Te_2$, $Zn_8B_{12}O_{26}$, $Al_{12}Ca_8O_{26}$, $Na_{20}Sn_{24}Yb_2$, $Ca_2Na_{20}Sn_{24}$, $Na_{20}Sn_{24}Sr_2$, $Bi_8Na_{24}O_{24}$, $Na_{24}O_{24}Sb_8$, $Ag_{12}Ge_{20}P_{24}$
	217 ($I{-}43m$)	True	$Zn_8N_{24}O_2P_{12}$, $Cd_8N_{24}P_{12}S_2$, $Cd_8B_{12}O_{26}$
	220 ($I{-}43d$)	False	$Zr_{12}N_{16}$, $Au_{12}Y_{12}Sb_{16}$, $Ni_{12}Zr_{12}Sb_{16}$, $Au_{12}Pr_{12}Sb_{16}$, $Au_{12}Dy_{12}Sb_{16}$
	29 ($Pca2_1$)	False	$Pt_4Ge_4Se_4$, $Co_4As_4S_4$, $Ir_4S_4Sb_4$, $K_4Ge_4N_4O_4$, $K_4P_4Se_{24}$, $Na_8O_{20}Si_8$
	29 ($Pca2_1$)	True	$Rb_4P_4Se_{24}$
	33 ($Pna2_1$)	False	Cd_4P_8, $Ga_4Li_4Se_8$, $In_4Li_4Se_8$, $Ag_4In_4S_8$, $Zn_4Ge_4N_8$, $Ga_4Na_4O_8$, $Ga_4Li_4S_8$, $Mg_4N_8Si_4$, $Ga_4Li_4O_8$, $Al_4Li_4Se_8$, $Mg_4Ge_4N_8$, $Al_4Na_4O_8$, $Pr_4B_4S_{12}$, $Tb_4B_4S_{12}$, $Sm_4B_4S_{12}$, $Rb_4Br_{12}Ge_4$, $Bi_4In_4O_{12}$, $O_{16}Sb_8$, Li_4P_{20}, $Ag_4Zn_4P_4S_{16}$, $Li_{12}As_4S_{12}$, $Bi_4Li_{12}S_{12}$, $Sn_4Tl_4P_4S_{16}$, $Al_4Ba_4H_{20}$, $Cs_4Cu_8Ta_4Te_{16}$, $Cu_8Yb_8O_{20}$, $Ca_8Cu_8O_{20}$, $Na_8O_{20}Si_8$, $Na_8Ge_8Se_{20}$, $Na_8Ge_8O_{20}$, $K_4Na_4Sn_4F_{24}$, $Ga_8Sn_8S_{20}$, $Ba_8Ge_8Te_{20}$
	159 ($P31c$)	False	$Al_2K_2O_8Si_2$, $N_{16}Si_{12}$, $Ge_{12}N_{16}$
	161 ($R3c$)	False	$Li_6Ta_6O_{18}$, $Cs_6Pb_6F_{18}$, $Cd_6Na_6F_{18}$, $Bi_6Er_6O_{18}$
C	185 ($P6_3cm$)	False	$Ga_6Sc_6O_{18}$, $Er_6In_6O_{18}$
	186 ($P6_3mc$)	False	In_2As_2, In_2Sb_2, B_2P_2, Ba_2O_2, Cu_2Cl_2, Li_2F_2, Cd_2O_2, In_2N_2, Ac_2N_2, Ga_2As_2, Hg_2S_2, Cd_2Se_2, Cd_2Te_2, Zn_2O_2, Be_2O_2, Al_2N_2, Cd_2S_2, Al_2As_2, Ga_2P_2, $Ga_2Li_2Ge_2$, $Ca_2Pb_2Zn_2$, $Ce_2Cu_2Pb_2$, $Ce_2Cu_2Sn_2$, $Au_2Y_2Ge_2$, $Hg_2Sn_2Sr_2$, $La_2Pd_2Sb_2$, $Cu_2La_2Pb_2$, $Au_2Ce_2Sn_2$, $Cu_2Sn_2Ti_2$, $Ag_2La_2Pb_2$, Cd_2Br_4, Pb_2I_4, $Au_2Ce_2Ge_2$, $Bi_2Cl_2Te_2$, C_4Si_4, Ag_4I_4, $F_2H_8N_2$, $Hg_6As_2I_2Se_8$, $Ta_6I_{14}Se_2$, $Ga_2Na_6Sr_6As_8$, $Al_2Ca_6Na_6As_8$, $K_{12}Mg_2O_8$, $Mn_2Na_{12}Se_8$, $Mn_2Na_{12}Te_8$, $K_{12}Mn_2Se_8$, $Ba_8Cl_{12}O_2$, $Hg_2K_{12}S_8$, $Yb_8Cl_{12}O_2$, $Ba_8Br_{12}O_2$, $Na_{12}Zn_2S_8$, $Yb_8Br_{12}O_2$
	186 ($P6_3mc$)	True	$Sr_2Cl_2H_2O_2$, $Cd_2Cl_2H_2O_2$, $Hg_6As_2Br_2Se_8$, $Ca_6O_{10}Si_2$, $Cs_4O_{12}S_4$
	190 ($P{-}62c$)	False	$Be_6Sr_2O_8$, $Al_2Ba_2B_2F_4O_6$, $Au_{12}Rb_8S_{10}$, $Au_{12}K_8S_{10}$, $La_6F_6O_{18}Si_6$
	215 ($P{-}43m$)	False	BrH_4

Type	*No.* (SP)	Clean	mixed TPs candidates
C	216 ($F-43$m)	False	$Bi_4La_4Pt_4$, $Mg_4Pt_4Sb_4$, $Fe_4Ti_4Sb_4$, $Ir_4Zr_4As_4$, $Co_4Nb_4Sn_4$, $Rh_4Zr_4Sb_4$, $Ni_4Y_4Sb_4$, $Ni_4Zn_4Sb_4$, $Bi_4Pt_4Y_4$, $Bi_4La_4Pd_4$, $Ir_4Sn_4Ti_4$, $Mg_4Ni_4Sb_4$, $In_4Li_4Ge_4$, $Nb_4Ru_4Sb_4$, $Au_4Lu_4Sn_4$, $Au_4Mg_4Sn_4$, $Bi_4Li_4Mg_4$, $Co_4Sc_4Te_4$, $Li_4Zn_4P_4$, $Li_4Mg_4As_4$, $Dy_4Pt_4Sb_4$, $Er_4Pt_4Sb_4$, $Pt_4Sn_4Th_4$, $Pt_4Sn_4Ti_4$, $Bi_4Co_4Zr_4$, $Li_4Mg_4P_4$, $Li_4Mg_4N_4$, $Fe_4V_4Sb_4$, $Pt_4Sc_4Sb_4$, $Li_4Zn_4As_4$, $Ni_4Sn_4Zr_4$, $Ni_4Sn_4Ti_4$, $Pd_4Sn_4Ti_4$, $Ca_4Na_4As_4$, $Pt_4Ti_4Si_4$, $Pt_4Zr_4Si_4$, $Pt_4Sn_4Zr_4$, $Li_8Pd_4Sb_4$, $Hg_4Na_8Tl_4$, $Au_4Li_8Si_4$, $Cd_4Li_8Tl_4$, $Li_8Pt_4Sn_4$, $Li_8Mg_4Zn_4$, $Ag_4Li_8Tl_4$, $Ir_4Li_8Sn_4$, $Hg_4Li_8Mg_4$, $Ga_4Li_8Mg_4$, $Cd_4Li_8Mg_4$, $Li_8Pd_4Ge_4$, $Ag_4Ga_4Li_8$, $Al_4Ga_4Li_8$, $Cd_4In_4Li_8$, $Cd_4Na_8Pb_4$, $In_4Li_8Pt_4$, $Au_4Li_8Ge_4$, $Ga_4Li_8Rh_4$, $Ag_4Li_8Pb_4$, $Au_4Ga_4Li_8$, $Au_4Li_8Sn_4$, $Cu_4Li_8Sn_4$, $Al_4Li_8Pd_4$, $Ag_4Al_4Li_8$, $Ga_4Ir_4Li_8$, $In_4Ir_4Li_8$, $Li_8Pt_4Sb_4$, $Cu_4Li_8Ge_4$, $Ga_4Li_8Pt_4$, $Ag_4Li_8Sn_4$, $Cu_4Li_8Sb_4$
	217 ($I-43$m)	False	$Al_{12}Cd_8O_{24}S_2$

VI. CLEAN TP MATERIALS

Supplementary Table 5: The summary of 322 clean TP materials categorized by their space group number and symbol(*No.* (SP)). Those mixed TPs are classified into s1, n1, A, B and C. s1 and n1 are topological phonon materials with single WPs and NDL(R), respectively. A, B and C are the same as A, B and C in Table S3.

Type	*No.* (SP)	mixed TPs candidates
	152 ($P3_121$)	$\mathrm{Hg_3S_3}$
s1	154 ($P3_221$)	$\mathrm{Hg_3S_3}$
	155 ($R32$)	$\mathrm{Ca_3Mg_9C_{12}O_{36}}$
	7 (Pc)	$\mathrm{Nb_4Tl_2P_2S_{20}}$
	11 ($P2_1/m$)	$\mathrm{K_4Br_2H_2O_2}$, $\mathrm{La_2C_2Cl_2N_4}$, $\mathrm{Ba_4Ge_2Se_8}$, $\mathrm{Ba_4Cl_2P_{14}}$, $\mathrm{Ba_8Cu_4I_4O_8}$
	12 ($C2/m$)	$\mathrm{Rh_4Cl_{12}}$, $\mathrm{Co_4La_4N_{12}}$, $\mathrm{Cs_4Li_6Br_{10}}$, $\mathrm{K_4Ti_4P_4S_{20}}$
	14 ($P2_1/c$)	$\mathrm{In_4I_4Te_4}$, $\mathrm{Tl_4B_4S_{12}}$, $\mathrm{Li_2Nd_4Os_2O_{12}}$, $\mathrm{Sm_2Sr_4O_{12}Sb_2}$, $\mathrm{Ba_4P_4S_{12}}$, $\mathrm{Ba_4P_4Se_{12}}$, $\mathrm{Pb_4P_4Se_{12}}$, $\mathrm{Cd_2Rb_4P_4Se_{12}}$, $\mathrm{K_4Mg_2P_4Se_{12}}$, $\mathrm{Na_{12}B_4N_8}$, $\mathrm{Na_4Sr_4B_4O_{12}}$, $\mathrm{Au_2Na_2O_{16}S_4}$, $\mathrm{Li_8Ti_4O_4S_8}$, $\mathrm{Au_4K_4Cl_{16}}$, $\mathrm{In_8Cl_8Te_8}$, $\mathrm{Cs_8As_4Te_{12}}$, $\mathrm{Cu_4Sr_8O_{14}}$, $\mathrm{Sr_4B_8S_{16}}$, $\mathrm{Rb_{12}B_4S_{12}}$, $\mathrm{Sr_8O_{16}Si_4}$, $\mathrm{Ba_8Ge_4O_{16}}$, $\mathrm{Na_{12}Si_4Te_{12}}$, $\mathrm{K_8Ge_4Te_{16}}$, $\mathrm{Ca_8B_4Cl_4O_{12}}$, $\mathrm{Rb_{12}O_{14}Si_4}$, $\mathrm{Y_8O_{20}Si_4}$, $\mathrm{Dy_8O_{20}Si_4}$, $\mathrm{Ag_4Ta_4Tl_8S_{16}}$, $\mathrm{Li_4U_4B_4O_{20}}$, $\mathrm{Li_4B_4H_8O_{16}}$, $\mathrm{K_{20}Sn_4As_{12}}$, $\mathrm{Na_{20}Ge_4P_{12}}$, $\mathrm{K_4H_{12}N_4O_{12}P_4}$
	15 ($C2/c$)	$\mathrm{Rb_{12}B_4P_8}$, $\mathrm{K_8B_8S_{28}}$, $\mathrm{K_8B_8Se_{28}}$, $\mathrm{Li_{16}O_{24}Te_8}$, $\mathrm{Hg_8K_8S_{24}Sb_8}$, $\mathrm{Ba_8Cu_{12}V_4S_{24}}$, $\mathrm{Cs_4Li_{20}B_8O_{24}}$, $\mathrm{Cd_{16}F_8O_{32}P_8}$, $\mathrm{Ba_{20}Br_{24}O_{16}Si_4}$, $\mathrm{Ba_{20}Cl_{24}O_{16}Si_4}$
	38 ($Amm2$)	$\mathrm{Li_4B_{24}C_4}$
	40 ($Ama2$)	$\mathrm{Cu_8Rb_4Ta_4Se_{16}}$, $\mathrm{Cu_8K_4V_4S_{16}}$
	52 ($Pnna$)	$\mathrm{Bi_{12}I_4O_{16}}$
	57 ($Pbcm$)	$\mathrm{Sm_8O_{16}Se_4Si_4}$
	59 ($Pmmn$)	$\mathrm{Ti_2Cl_2N_2}$, $\mathrm{In_2Cl_2O_2}$, $\mathrm{K_2Li_4As_2}$, $\mathrm{Cs_6Mg_6H_{18}}$
	60 ($Pbcn$)	$\mathrm{Ca_4B_8O_{16}}$
	62 ($Pnma$)	$\mathrm{Ca_4Sr_4Ge_4}$, $\mathrm{Cu_4K_4S_4}$, $\mathrm{C_4H_8}$, $\mathrm{Ba_4Br_4H_4O_4}$, $\mathrm{Sr_4H_4I_4O_4}$, $\mathrm{Pb_4Br_4H_4O_4}$, $\mathrm{Pb_4H_4I_4O_4}$, $\mathrm{Hf_4Pb_4S_{12}}$, $\mathrm{Nd_4Rh_4O_{12}}$, $\mathrm{K_4N_4O_{12}}$, $\mathrm{Ba_4Cu_8Se_8}$, $\mathrm{Tb_4Cl_4O_{12}Se_4}$, $\mathrm{Sr_8Ge_{16}}$, $\mathrm{Sm_4Cl_4H_8O_8}$, $\mathrm{Al_4Na_4I_{16}}$, $\mathrm{Li_4B_4H_{16}}$, $\mathrm{Ba_4O_{16}S_4}$, $\mathrm{K_8Sn_4O_{12}}$, $\mathrm{Ag_4Cs_8I_{12}}$, $\mathrm{Sr_8B_4F_4N_8}$, $\mathrm{Ag_4Rb_8Cl_{12}}$, $\mathrm{Ca_8Ge_4O_{16}}$, $\mathrm{Ba_8O_{16}Si_4}$, $\mathrm{K_8W_4Se_{16}}$, $\mathrm{Cs_8O_{16}Se_4}$, $\mathrm{Ba_8S_{16}Si_4}$, $\mathrm{Cs_8Mg_4H_{16}}$, $\mathrm{Pb_{12}Br_8O_8}$, $\mathrm{Cd_4Na_4O_{16}P_4}$, $\mathrm{Ga_4K_4Na_8O_{12}}$, $\mathrm{Ba_4K_4P_4S_{16}}$, $\mathrm{Ba_4B_4S_{16}Sb_4}$, $\mathrm{Ba_4P_{16}Te_8}$, $\mathrm{K_{12}Ta_4S_{16}}$, $\mathrm{Rb_{12}As_4Se_{16}}$, $\mathrm{K_{12}V_4S_{16}}$, $\mathrm{K_{12}Li_4Si_{16}}$, $\mathrm{Pd_4Rb_8I_{24}}$, $\mathrm{I_8P_{16}Se_{12}}$, $\mathrm{Na_8B_8S_{20}}$, $\mathrm{Mg_8H_4O_{20}P_4}$, $\mathrm{K_8Re_4Cl_{20}O_4}$, $\mathrm{Hg_4Zn_4As_4H_4O_{20}}$
n1	63 ($Cmcm$)	$\mathrm{Pt_4Rb_8As_8}$, $\mathrm{La_4C_4F_4N_8}$, $\mathrm{Cu_8Rb_4Br_{12}}$, $\mathrm{Y_{12}B_{12}O_{36}}$, $\mathrm{Be_{12}Li_{12}H_{36}}$
	64 ($Cmce$)	$\mathrm{Ba_8Pt_4H_{24}}$
	65 ($Cmmm$)	$\mathrm{La_8C_4Cl_4N_{12}}$
	69 ($Fmmm$)	$\mathrm{Cs_8Pd_{12}S_{16}}$
	70 ($Fddd$)	$\mathrm{Sr_8N_{48}}$, $\mathrm{K_{16}Pd_{24}S_{32}}$
	74 ($Imma$)	$\mathrm{Al_4Na_4C_4H_8O_{20}}$
	125 ($P4/nbm$)	$\mathrm{Ag_4Ba_2Hg_4O_8}$, $\mathrm{Ag_2K_6Sn_6Se_{16}}$
	128 ($P4/mnc$)	$\mathrm{Cd_2Tl_8I_{12}}$
	129 ($P4/nmm$)	$\mathrm{K_2Na_2Nb_2Sm_2O_{10}}$, $\mathrm{K_2Na_2Nb_2Nd_2O_{10}}$, $\mathrm{K_2La_2Na_2Ta_2O_{10}}$, $\mathrm{K_2Na_2Pr_2Ta_2O_{10}}$, $\mathrm{Al_2Na_4Cl_2H_8O_8}$, $\mathrm{Na_4B_2Cl_2H_8O_8}$
	130 ($P4/ncc$)	$\mathrm{Cs_{12}Mg_4H_{20}}$
	136 ($P4_2/mnm$)	$\mathrm{Pt_2Rb_4H_8}$
	137 ($P4_2/nmc$)	$\mathrm{K_2B_2H_8}$
	138 ($P4_2/ncm$)	$\mathrm{Au_4Cl_4}$, $\mathrm{Au_4Br_4}$
	139 ($I4/mmm$)	$\mathrm{Ca_4Co_2N_4}$, $\mathrm{Nd_4Br_4O_{20}Te_8}$
	140 ($I4/mcm$)	$\mathrm{Au_4K_4F_{16}}$
	141 ($I4_1/amd$)	$\mathrm{Ti_8N_4}$, $\mathrm{Lu_4As_4O_{16}}$, $\mathrm{Li_{24}B_8N_{16}}$, $\mathrm{Bi_4Cs_{16}Cl_{48}Sb_4}$, $\mathrm{Cs_{16}Tl_4Cl_{48}Sb_4}$
	142 ($I4_1/acd$)	$\mathrm{Cs_{32}Si_{32}}$
	162 (P−31m)	$\mathrm{SrO_6Sb_2}$
	163 (P−31c)	$\mathrm{Al_2Li_2Sr_2F_{12}}$, $\mathrm{Ag_2Sc_2P_4S_{12}}$, $\mathrm{Na_4Ge_2O_{12}Te_2}$
	164 (P−3m1)	$\mathrm{MgCl_2}$, $\mathrm{Sc_2CCl_2}$, $\mathrm{Zr_2N_2}$, $\mathrm{Be_2MgN_2}$, $\mathrm{Ho_2CF_2}$, $\mathrm{CsCu_3O_2}$, $\mathrm{CsCu_3S_2}$, $\mathrm{Ba_3Ir_2H_{12}}$, $\mathrm{Nb_6Cl_{14}Te_2}$
	166 (R−3m)	$\mathrm{Cd_3Br_6}$, $\mathrm{Au_3Tl_3O_6}$, $\mathrm{Na_3Sm_3O_6}$, $\mathrm{Lu_6F_6S_6}$, $\mathrm{Rb_6Sn_6O_9}$, $\mathrm{Cu_9K_9As_6}$, $\mathrm{Ag_9K_9As_6}$, $\mathrm{Cu_9K_9P_6}$, $\mathrm{Au_9K_9Sb_6}$, $\mathrm{Au_9Rb_9Sb_6}$, $\mathrm{K_3Na_3B_6H_{24}}$, $\mathrm{Ca_6Pt_9O_{24}}$, $\mathrm{Ba_9O_{24}P_6}$, $\mathrm{Cs_9Mg_9H_{27}}$, $\mathrm{Al_9Cs_6Li_3F_{36}}$, $\mathrm{Al_6K_{12}Li_6H_{36}}$, $\mathrm{Rb_9B_{36}H_{36}I_3}$, $\mathrm{Cs_9B_{36}Cl_3H_{36}}$, $\mathrm{K_9B_{36}Br_3H_{36}}$, $\mathrm{Cs_9B_{36}Br_3H_{36}}$
	167 (R−3c)	$\mathrm{Li_6N_6O_{18}}$, $\mathrm{K_6F_{24}H_{18}}$, $\mathrm{Rh_6Sm_6Sr_{18}O_{36}}$, $\mathrm{Rh_6Sr_{18}Y_6O_{36}}$, $\mathrm{Lu_{12}C_{18}N_{36}}$, $\mathrm{Li_{24}Ru_6H_{36}}$, $\mathrm{Li_{24}Os_6H_{36}}$, $\mathrm{Na_{24}Ru_6H_{36}}$, $\mathrm{Rb_{18}B_{18}O_{36}}$, $\mathrm{K_{18}B_{18}O_{36}}$, $\mathrm{Cs_{18}Sc_{12}Cl_{54}}$
	176 ($P6_3/m$)	$\mathrm{Ba_2Ga_2B_2F_4O_6}$, $\mathrm{Al_2Ba_2B_2F_4O_6}$, $\mathrm{Ba_2Zn_2C_2F_4O_6}$, $\mathrm{K_6W_4Cl_{18}}$, $\mathrm{Rb_4Sn_2O_{18}Si_6}$
	189 (P−62m)	$\mathrm{K_6Re_3H_{27}}$
	193 ($P6_3/mcm$)	$\mathrm{Al_4Li_2Cl_2H_{12}O_{12}}$

Type	*No.* (SP)	mixed TPs candidates
	194 ($P6_3/mmc$)	$Cu_2Sr_2As_2$, $Sn_2Sr_2Zn_2$, $Ca_2Cu_2Sb_2$, $Be_2Na_2As_2$, $Au_2In_2O_4$, $Al_2Cu_2O_4$, $Al_2Zr_4C_2$, $In_2V_4C_2$, Be_8Mn_4, Be_8W_4, Be_8Cr_4, $Ba_2Re_2H_{18}$, $K_6F_{12}N_2O_6Si_2$, $Co_2Er_2C_{12}N_{12}$, $Cs_6Mo_4Br_{18}$
	202 ($Fm-3$)	$Tl_8B_{48}H_{48}$, $Cs_8B_{48}H_{48}$, $K_8B_{48}H_{48}$
	205 ($Pa-3$)	$Pb_4N_8O_{24}$, $Ba_4N_8O_{24}$
	221 ($Pm-3$m)	ScZn, BaZn, $LaPb_3$, $AuCu_3$
	224 ($Pn-3$m)	Ag_4O_2, Au_4S_2, Cu_4O_2
	225 ($Fm-3$m)	Li_4Cl_4, Li_4Br_4, $Pt_4Rb_8H_{24}$, $Pd_{24}Tl_8O_{32}$, $Cs_8Fe_4Mg_4C_{24}N_{24}$, $Ga_8In_{24}Pt_4O_{32}$
	227 ($Fd-3$m)	$Co_{16}U_8$, Ca_8Pd_{16}, $Ni_{16}Sc_8$, $Co_{16}Sc_8$, $Pt_{16}Y_8$, $Cu_{16}Mg_8$, $Ag_{16}Na_8$, Ca_8Mg_{16}, Mg_8Ni_{16}, $La_{16}Sr_8O_{32}$, $Sm_{16}Sr_8O_{32}$, $Ba_8La_{16}O_{32}$, $Cd_8Lu_{16}S_{32}$, $Li_{16}Rh_{16}O_{32}$, $La_{16}Sn_{16}O_{56}$, $Pt_{16}Sm_{16}O_{56}$, $Y_{16}Ge_{16}O_{56}$, $Sm_{16}Ge_{16}O_{56}$
	229 ($Im-3$m)	$Ca_8Li_2B_6N_{12}$
A	4 ($P2_1$)	$K_2La_2Ge_2Se_8$, $K_2Nd_2Ge_2S_8$, $K_2Y_2Ge_2S_8$, $K_2Li_2O_{10}Si_4$, $K_2Tb_2O_{24}P_8$
	5 ($C2$)	$Nb_4I_{12}O_4$
	20 ($C222_1$)	$K_8Zn_4O_{24}Si_8$, $K_8Zn_4Ge_8O_{24}$
	109 ($I4_1md$)	$Ga_8Se_8Te_4$
	113 ($P-42_1$m)	$Be_4Dy_4Ge_2O_{14}$, $Ga_4La_4Zn_2O_2S_{12}$
	118 ($P-4$n2)	$Zn_6P_4S_{16}$
	122 ($I-42$d)	Ge_4Se_8, $Be_4Sn_4As_8$, $Be_4C_4N_8$, $Cd_4Sn_4P_8$, $Pb_4Sm_8S_{16}$, $Pb_4Pr_8S_{16}$
	152 ($P3_121$)	$Ba_3Cu_6Ge_3S_{12}$
	154 ($P3_221$)	$Ag_6Rb_3S_{12}Sb_3$
	156 ($P3m1$)	NiSeTe
	169 ($P6_1$)	$Al_6In_6S_{18}$
	173 ($P6_3$)	$K_2Li_2O_8S_2$, $Cu_2Y_6Ge_2Se_{14}$, $Cu_2La_6Sn_2S_{14}$, $Cu_2Sm_6S_{14}Si_2$, $Cu_2Pr_6S_{14}Si_2$, $Cu_2Sm_6Ge_2S_{14}$, $Cu_2Y_6Ge_2S_{14}$, $Cu_2Nd_6S_{14}Si_2$, $Cu_2Tb_6S_{14}Si_2$, $Cu_2La_6Sn_2Se_{14}$, $Cu_2Pr_6Se_{14}Si_2$, $Ag_2La_6Ge_2S_{14}$, $Ag_2Pr_6Ge_2S_{14}$, $Ag_2Pr_6S_{14}Si_2$
	180 ($P6_222$)	$Hg_6Na_3I_3O_6$
B	19 ($P2_12_12_1$)	Br_4H_4, $Na_8Sn_4O_8$, $Cs_4Cu_4S_{16}$, $Ag_8Ge_4O_{12}$, Rb_8S_{20}, $Cs_4Er_4S_{16}Si_4$, $Cs_4Sm_4S_{16}Si_4$, $Al_4H_{12}O_{12}$, $Ca_4Zn_4As_4H_4O_{20}$, $Pb_4Zn_4As_4H_4O_{20}$
	33 ($Pna2_1$)	$K_4Na_4Ge_4O_{12}$, $Ba_8Cu_8O_{20}$
	92 ($P4_12_12$)	Ge_4O_8, Zn_8P_{16}
	96 ($P4_32_12$)	$Na_8Zn_8O_{12}$
	114 ($P-42_1$c)	$Sn_{12}H_8O_{16}$
	185 ($P6_3cm$)	$Li_{16}O_{12}Si_2$, $Mg_6H_8O_{18}Si_4$
	186 ($P6_3mc$)	$Ca_2Cl_2H_2O_2$, $K_4F_{12}Si_2$
	188 ($P-6$c2)	$Ba_2Ti_2O_{18}Si_6$
	198 ($P2_13$)	$K_{12}O_{12}Sb_4$, $K_{12}Sb_4Te_{12}$, $Mg_4Ni_4Yb_4H_{16}$
	199 ($I2_13$)	$Cu_8Cl_8H_{24}N_8$, $Ba_8Cs_8C_{12}O_{36}$, $Cs_8Sr_8C_{12}O_{36}$
	216 ($F-43$m)	$Co_4Mn_4Sb_4$, $Ca_4Li_4As_4$, $Au_4In_4Li_8$
	217 ($I-43$m)	$Zn_8N_{24}O_2P_{12}$, $Cd_8N_{24}P_{12}S_2$, $Cd_8B_{12}O_{26}$
C	29 ($Pca2_1$)	$Rb_4P_4Se_{24}$
	186 ($P6_3mc$)	$Sr_2Cl_2H_2O_2$, $Cd_2Cl_2H_2O_2$, $Hg_6As_2Br_2Se_8$, $Ca_6O_{10}Si_2$, $Cs_4O_{12}S_4$

VII. HOURGLASS NODAL NET TP MATERIALS

Supplementary Table 6: The summary of 15 hourglass nodal net materials categorized by their space group number and symbol(*No.* (SP)). The true or false in the column of Clean denotes whether the material holds clean WPs

No. (SP)	Clean	Hourglass nodal net candidates
52 (*Pnna*)	False	$Li_4O_{12}Sb_4$, $Ba_{12}Ga_8N_{16}$, $K_8Zr_8O_{20}$, $Mg_4Sr_{12}Ge_4N_{16}$, $Ag_8Ge_8Se_8$
53 (*Pmna*)	False	$Cu_4Br_4Se_{12}$, $Ag_4I_4Se_{12}$
54 (*Pcca*)	False	$Au_4Cd_4Rb_4S_8$
55 (*Pbam*)	False	$Ba_2Sc_2O_5$, $Ag_4Hg_8As_4O_{16}$
57 (*Pbcm*)	False	$Hg_{12}Tl_4As_8Cl_{12}$, $Er_8O_{16}Se_4Si_4$
60 (*Pbcn*)	False	$Cd_4K_8O_8$, $Li_8O_{20}Si_8$
61 (*Pbca*)	False	$Hg_{12}Na_8S_{16}$

VIII. DETAILS OF ALL TP MATERIALS

The followings are details for Pt_4 and it only has 1 topological nodal line along high symmetry line as shown in Fig 1.

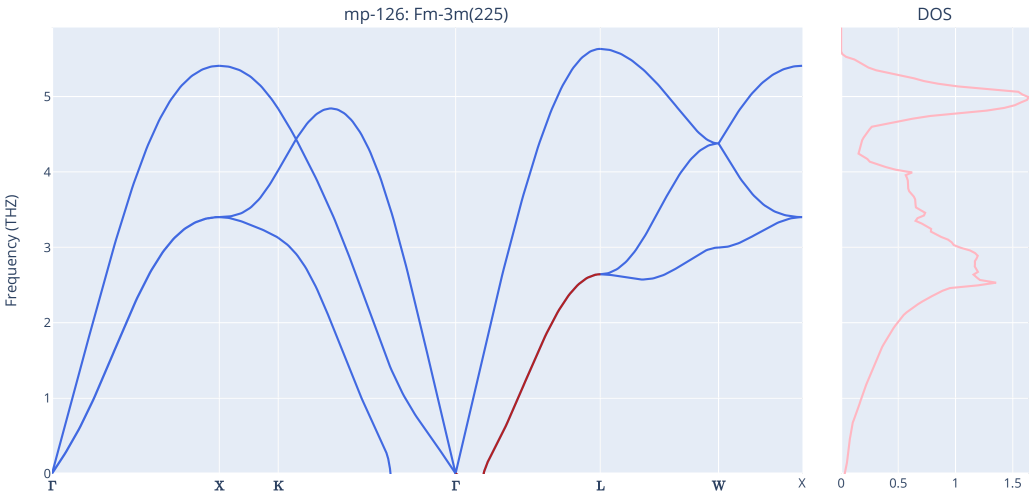

Supplementary Figure 1: The phonon band and DOS of Pt4.

Supplementary Table 7: High symmetry lines of mp-126-Pt4

	Start	End
0	Γ (0.000, 0.000, 0.000)	X (0.500, 0.000, 0.500)
1	X (0.500, 0.000, 0.500)	U (0.625, 0.250, 0.625)
2	K (0.375, 0.375, 0.750)	Γ (0.000, 0.000, 0.000)
3	Γ (0.000, 0.000, 0.000)	L (0.500, 0.500, 0.500)
4	L (0.500, 0.500, 0.500)	W (0.500, 0.250, 0.750)
5	W (0.500, 0.250, 0.750)	X (0.500, 0.000, 0.500)

The followings are details for Pd_4 and it only has 1 topological nodal line along high symmetry line as shown in Fig 2.

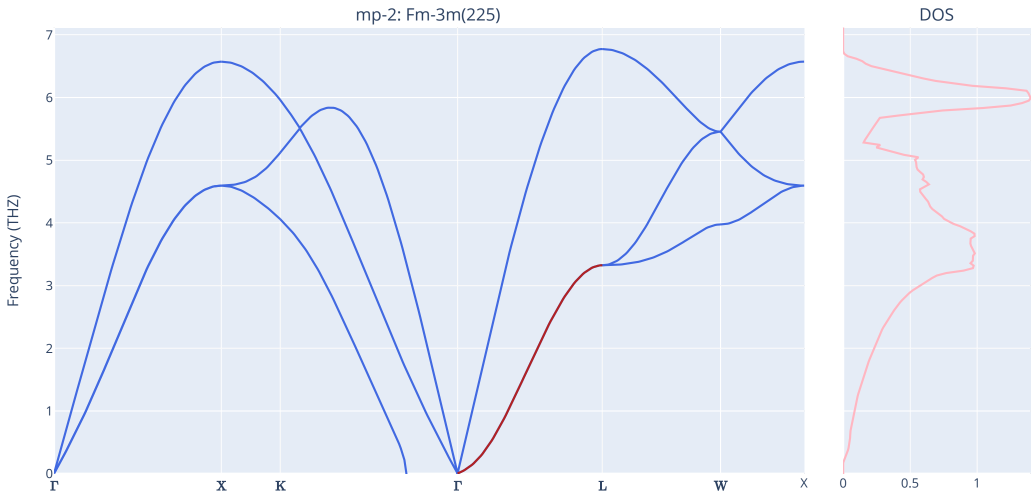

Supplementary Figure 2: The phonon band and DOS of Pd4.

Supplementary Table 8: High symmetry lines of mp-2-Pd4

	Start	End
0	Γ (0.000, 0.000, 0.000)	X (0.500, 0.000, 0.500)
1	X (0.500, 0.000, 0.500)	U (0.625, 0.250, 0.625)
2	K (0.375, 0.375, 0.750)	Γ (0.000, 0.000, 0.000)
3	Γ (0.000, 0.000, 0.000)	L (0.500, 0.500, 0.500)
4	L (0.500, 0.500, 0.500)	W (0.500, 0.250, 0.750)
5	W (0.500, 0.250, 0.750)	X (0.500, 0.000, 0.500)

The followings are details for Xe_2 and it only has 2 topological nodal lines along high symmetry line as shown in Fig **3**.

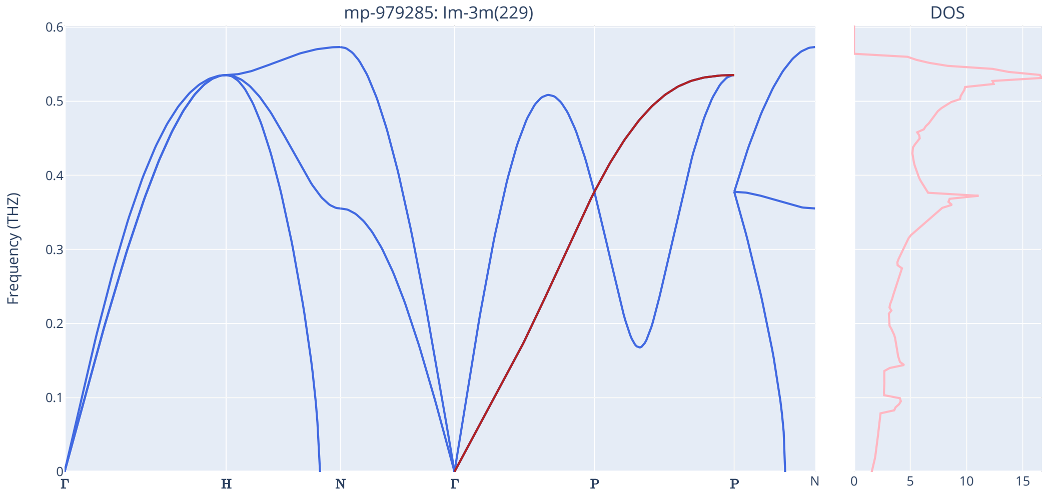

Supplementary Figure 3: The phonon band and DOS of Xe2.

Supplementary Table 9: High symmetry lines of mp-979285-Xe2

	Start	End
0	Γ (0.000, 0.000, 0.000)	H (0.500, -0.500, 0.500)
1	H (0.500, -0.500, 0.500)	N (0.000, 0.000, 0.500)
2	N (0.000, 0.000, 0.500)	Γ (0.000, 0.000, 0.000)
3	Γ (0.000, 0.000, 0.000)	P (0.250, 0.250, 0.250)
4	P (0.250, 0.250, 0.250)	H (0.500, -0.500, 0.500)
5	P (0.250, 0.250, 0.250)	N (0.000, 0.000, 0.500)

The followings are details for Ba_2 and it only has 2 topological nodal lines along high symmetry line as shown in Fig 4.

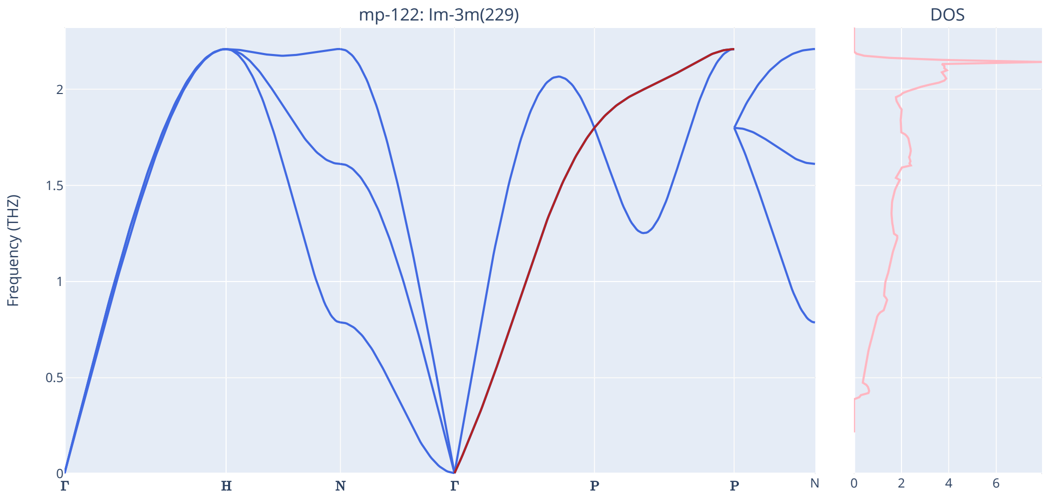

Supplementary Figure 4: The phonon band and DOS of Ba2.

Supplementary Table 10: High symmetry lines of mp-122-Ba2

	Start	End
0	Γ (0.000, 0.000, 0.000)	H (0.500, -0.500, 0.500)
1	H (0.500, -0.500, 0.500)	N (0.000, 0.000, 0.500)
2	N (0.000, 0.000, 0.500)	Γ (0.000, 0.000, 0.000)
3	Γ (0.000, 0.000, 0.000)	P (0.250, 0.250, 0.250)
4	P (0.250, 0.250, 0.250)	H (0.500, -0.500, 0.500)
5	P (0.250, 0.250, 0.250)	N (0.000, 0.000, 0.500)

The followings are details for Na_2 and it only has 2 topological nodal lines along high symmetry line as shown in Fig 5.

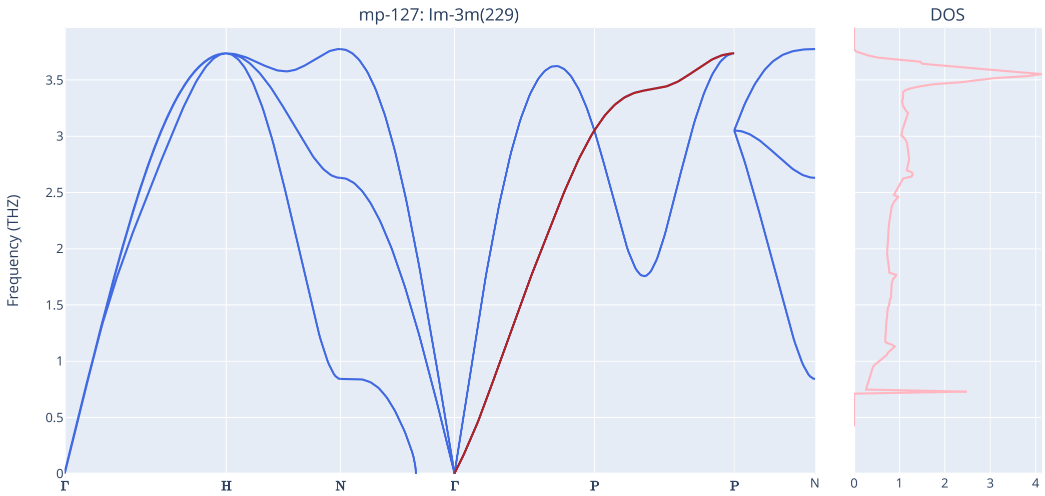

Supplementary Figure 5: The phonon band and DOS of Na2.

Supplementary Table 11: High symmetry lines of mp-127-Na2

	Start	End
0	Γ (0.000, 0.000, 0.000)	H (0.500, -0.500, 0.500)
1	H (0.500, -0.500, 0.500)	N (0.000, 0.000, 0.500)
2	N (0.000, 0.000, 0.500)	Γ (0.000, 0.000, 0.000)
3	Γ (0.000, 0.000, 0.000)	P (0.250, 0.250, 0.250)
4	P (0.250, 0.250, 0.250)	H (0.500, -0.500, 0.500)
5	P (0.250, 0.250, 0.250)	N (0.000, 0.000, 0.500)

The followings are details for Li_4 and it only has 1 topological nodal line along high symmetry line as shown in Fig **6**.

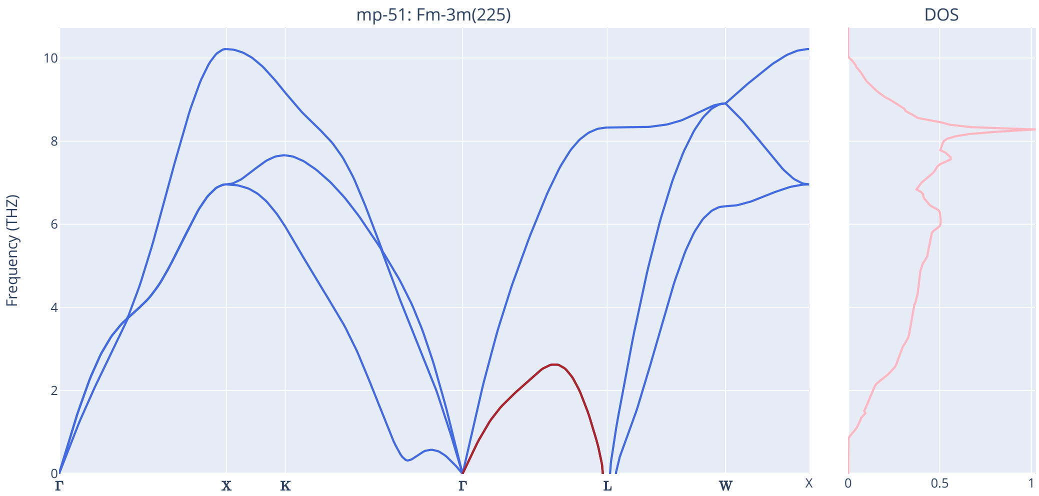

Supplementary Figure 6: The phonon band and DOS of Li4.

Supplementary Table 12: High symmetry lines of mp-51-Li4

	Start	End
0	Γ (0.000, 0.000, 0.000)	X (0.500, 0.000, 0.500)
1	X (0.500, 0.000, 0.500)	U (0.625, 0.250, 0.625)
2	K (0.375, 0.375, 0.750)	Γ (0.000, 0.000, 0.000)
3	Γ (0.000, 0.000, 0.000)	L (0.500, 0.500, 0.500)
4	L (0.500, 0.500, 0.500)	W (0.500, 0.250, 0.750)
5	W (0.500, 0.250, 0.750)	X (0.500, 0.000, 0.500)

The followings are details for K_2 and it only has 2 topological nodal lines along high symmetry line as shown in Fig 7.

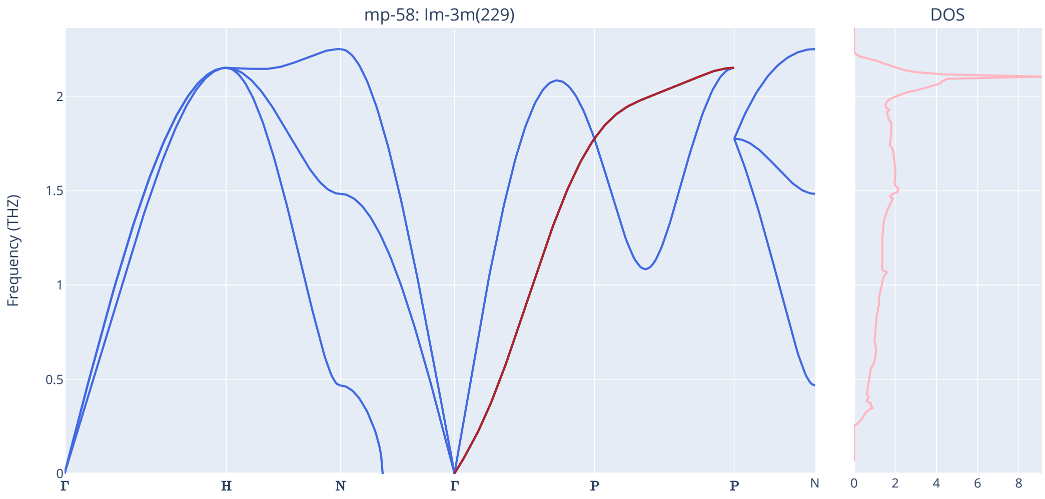

Supplementary Figure 7: The phonon band and DOS of K2.

Supplementary Table 13: High symmetry lines of mp-58-K2

	Start	End
0	Γ (0.000, 0.000, 0.000)	H (0.500, -0.500, 0.500)
1	H (0.500, -0.500, 0.500)	N (0.000, 0.000, 0.500)
2	N (0.000, 0.000, 0.500)	Γ (0.000, 0.000, 0.000)
3	Γ (0.000, 0.000, 0.000)	P (0.250, 0.250, 0.250)
4	P (0.250, 0.250, 0.250)	H (0.500, -0.500, 0.500)
5	P (0.250, 0.250, 0.250)	N (0.000, 0.000, 0.500)

The followings are details for Kr_2 and it only has 2 topological nodal lines along high symmetry line as shown in Fig **8**.

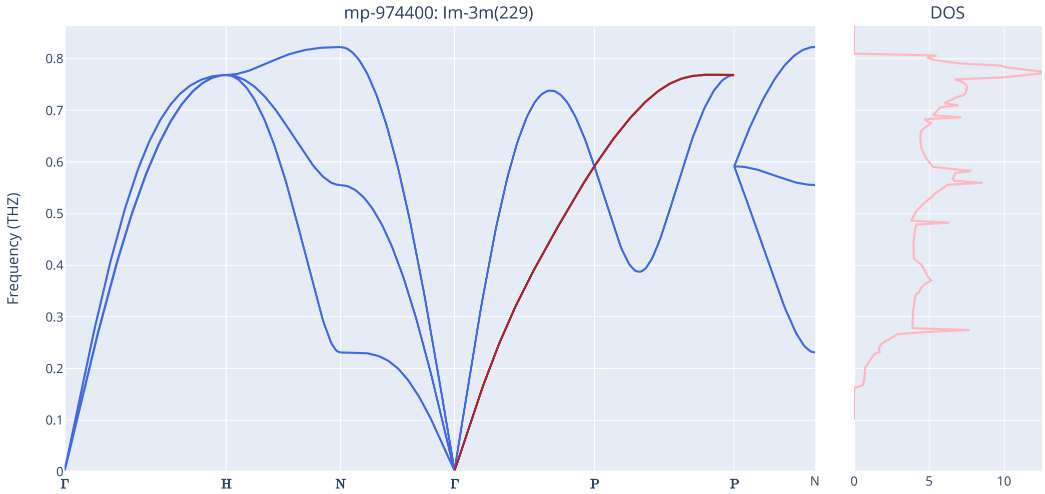

Supplementary Figure 8: The phonon band and DOS of Kr2.

Supplementary Table 14: High symmetry lines of mp-974400-Kr2

	Start	End
0	Γ (0.000, 0.000, 0.000)	H (0.500, -0.500, 0.500)
1	H (0.500, -0.500, 0.500)	N (0.000, 0.000, 0.500)
2	N (0.000, 0.000, 0.500)	Γ (0.000, 0.000, 0.000)
3	Γ (0.000, 0.000, 0.000)	P (0.250, 0.250, 0.250)
4	P (0.250, 0.250, 0.250)	H (0.500, -0.500, 0.500)
5	P (0.250, 0.250, 0.250)	N (0.000, 0.000, 0.500)

The followings are details for Ir_4 and it only has 1 topological nodal line along high symmetry line as shown in Fig **9**.

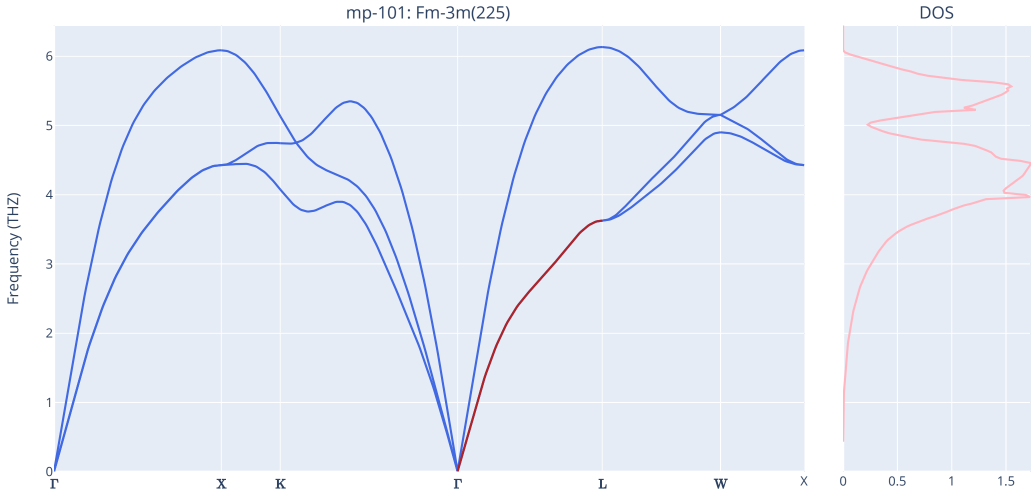

Supplementary Figure 9: The phonon band and DOS of Ir4.

Supplementary Table 15: High symmetry lines of mp-101-Ir4

	Start	End
0	Γ (0.000, 0.000, 0.000)	X (0.500, 0.000, 0.500)
1	X (0.500, 0.000, 0.500)	U (0.625, 0.250, 0.625)
2	K (0.375, 0.375, 0.750)	Γ (0.000, 0.000, 0.000)
3	Γ (0.000, 0.000, 0.000)	L (0.500, 0.500, 0.500)
4	L (0.500, 0.500, 0.500)	W (0.500, 0.250, 0.750)
5	W (0.500, 0.250, 0.750)	X (0.500, 0.000, 0.500)

The followings are details for K_4 and it only has 1 topological nodal line along high symmetry line as shown in Fig **10**.

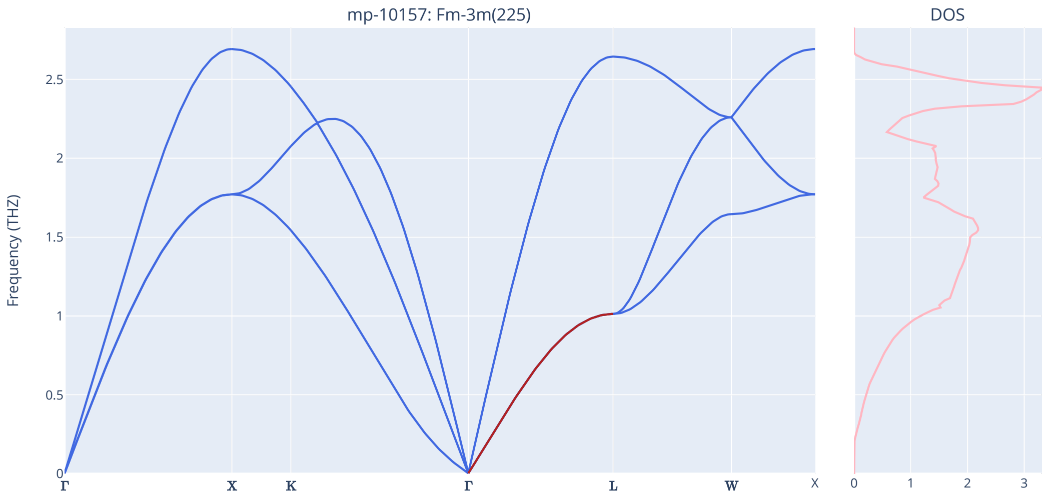

Supplementary Figure 10: The phonon band and DOS of K4.

Supplementary Table 16: High symmetry lines of mp-10157-K4

	Start	End
0	Γ (0.000, 0.000, 0.000)	X (0.500, 0.000, 0.500)
1	X (0.500, 0.000, 0.500)	U (0.625, 0.250, 0.625)
2	K (0.375, 0.375, 0.750)	Γ (0.000, 0.000, 0.000)
3	Γ (0.000, 0.000, 0.000)	L (0.500, 0.500, 0.500)
4	L (0.500, 0.500, 0.500)	W (0.500, 0.250, 0.750)
5	W (0.500, 0.250, 0.750)	X (0.500, 0.000, 0.500)

The followings are details for Co_4 and it only has 1 topological nodal line along high symmetry line as shown in Fig **11**.

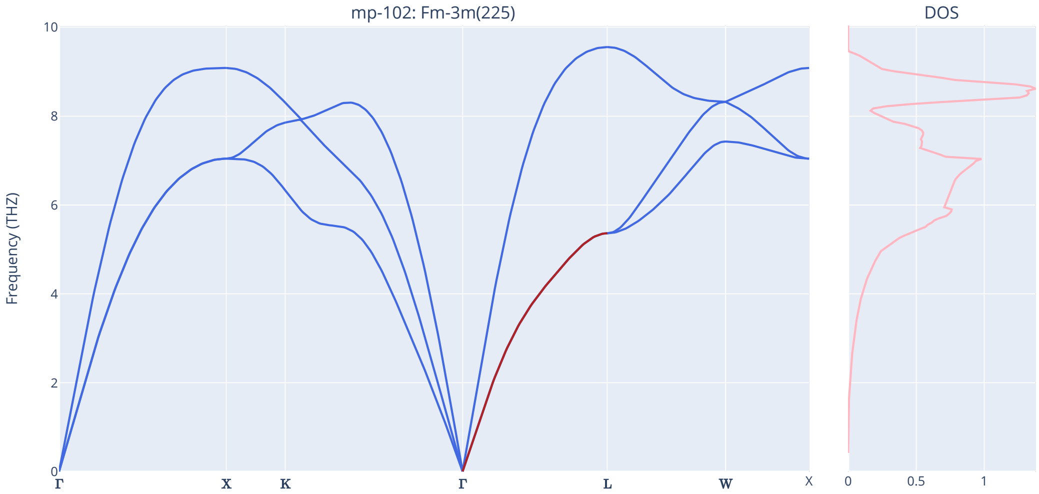

Supplementary Figure 11: The phonon band and DOS of Co4.

Supplementary Table 17: High symmetry lines of mp-102-Co4

	Start	End
0	Γ (0.000, 0.000, 0.000)	X (0.500, 0.000, 0.500)
1	X (0.500, 0.000, 0.500)	U (0.625, 0.250, 0.625)
2	K (0.375, 0.375, 0.750)	Γ (0.000, 0.000, 0.000)
3	Γ (0.000, 0.000, 0.000)	L (0.500, 0.500, 0.500)
4	L (0.500, 0.500, 0.500)	W (0.500, 0.250, 0.750)
5	W (0.500, 0.250, 0.750)	X (0.500, 0.000, 0.500)

The followings are details for Ba$_4$ and it only has 1 topological nodal line along high symmetry line as shown in Fig **12**.

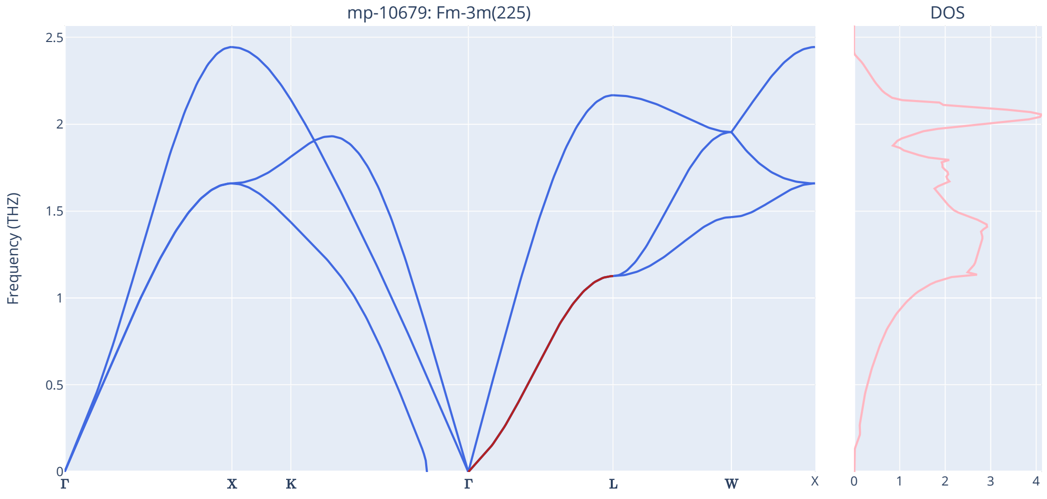

Supplementary Figure 12: The phonon band and DOS of Ba4.

Supplementary Table 18: High symmetry lines of mp-10679-Ba4

	Start	End
0	Γ (0.000, 0.000, 0.000)	X (0.500, 0.000, 0.500)
1	X (0.500, 0.000, 0.500)	U (0.625, 0.250, 0.625)
2	K (0.375, 0.375, 0.750)	Γ (0.000, 0.000, 0.000)
3	Γ (0.000, 0.000, 0.000)	L (0.500, 0.500, 0.500)
4	L (0.500, 0.500, 0.500)	W (0.500, 0.250, 0.750)
5	W (0.500, 0.250, 0.750)	X (0.500, 0.000, 0.500)

The followings are details for Ne_4 and it only has 1 topological nodal line along high symmetry line as shown in Fig **13**.

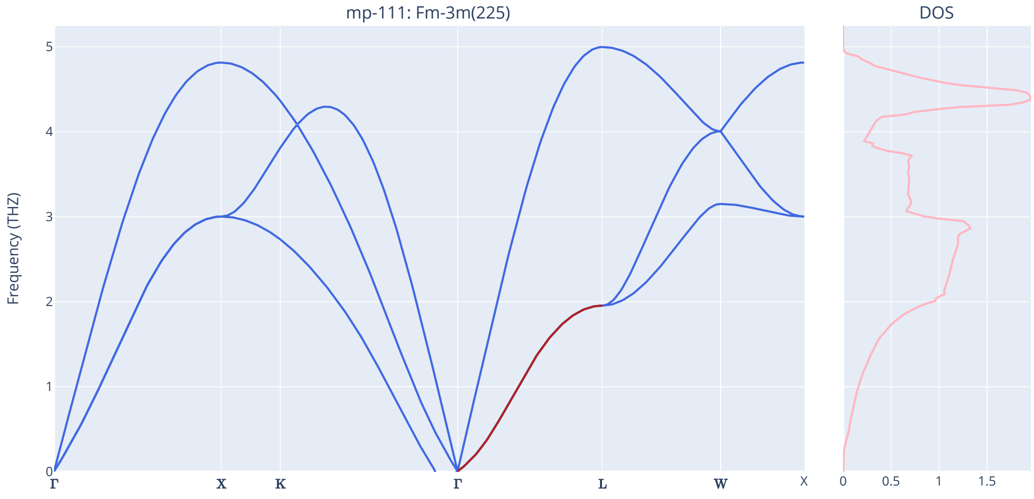

Supplementary Figure 13: The phonon band and DOS of Ne4.

Supplementary Table 19: High symmetry lines of mp-111-Ne4

	Start	End
0	Γ (0.000, 0.000, 0.000)	X (0.500, 0.000, 0.500)
1	X (0.500, 0.000, 0.500)	U (0.625, 0.250, 0.625)
2	K (0.375, 0.375, 0.750)	Γ (0.000, 0.000, 0.000)
3	Γ (0.000, 0.000, 0.000)	L (0.500, 0.500, 0.500)
4	L (0.500, 0.500, 0.500)	W (0.500, 0.250, 0.750)
5	W (0.500, 0.250, 0.750)	X (0.500, 0.000, 0.500)

The followings are details for Ag_4 and it only has 1 topological nodal line along high symmetry line as shown in Fig **14**.

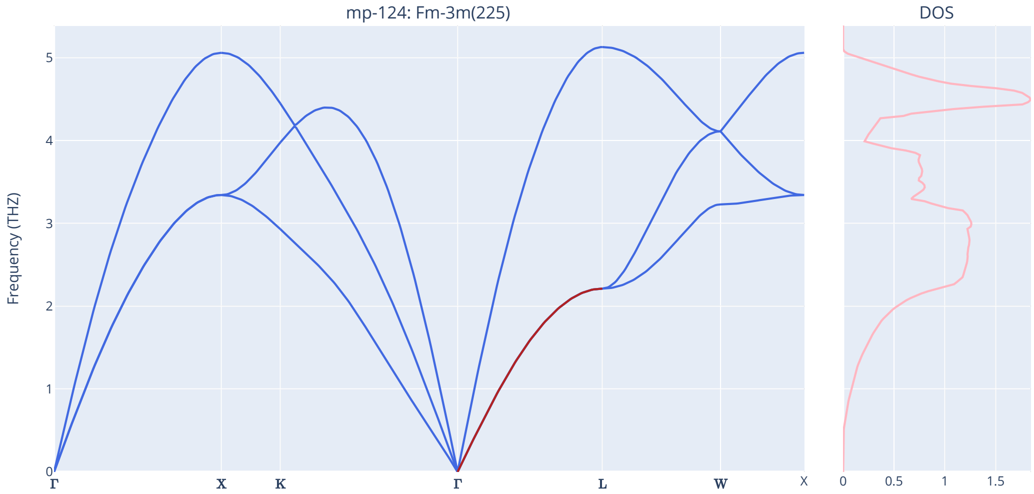

Supplementary Figure 14: The phonon band and DOS of Ag4.

Supplementary Table 20: High symmetry lines of mp-124-Ag4

	Start	End
0	Γ (0.000, 0.000, 0.000)	X (0.500, 0.000, 0.500)
1	X (0.500, 0.000, 0.500)	U (0.625, 0.250, 0.625)
2	K (0.375, 0.375, 0.750)	Γ (0.000, 0.000, 0.000)
3	Γ (0.000, 0.000, 0.000)	L (0.500, 0.500, 0.500)
4	L (0.500, 0.500, 0.500)	W (0.500, 0.250, 0.750)
5	W (0.500, 0.250, 0.750)	X (0.500, 0.000, 0.500)

The followings are details for Mo_2 and it only has 2 topological nodal lines along high symmetry line as shown in Fig **15**.

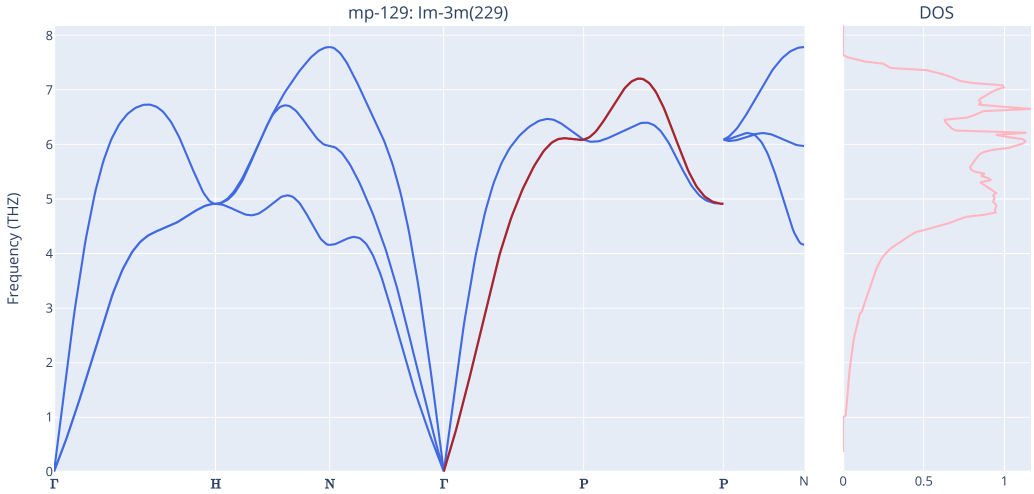

Supplementary Figure 15: The phonon band and DOS of Mo2.

Supplementary Table 21: High symmetry lines of mp-129-Mo2

	Start	End
0	Γ (0.000, 0.000, 0.000)	H (0.500, -0.500, 0.500)
1	H (0.500, -0.500, 0.500)	N (0.000, 0.000, 0.500)
2	N (0.000, 0.000, 0.500)	Γ (0.000, 0.000, 0.000)
3	Γ (0.000, 0.000, 0.000)	P (0.250, 0.250, 0.250)
4	P (0.250, 0.250, 0.250)	H (0.500, -0.500, 0.500)
5	P (0.250, 0.250, 0.250)	N (0.000, 0.000, 0.500)

The followings are details for Al$_4$ and it only has 1 topological nodal line along high symmetry line as shown in Fig **16**.

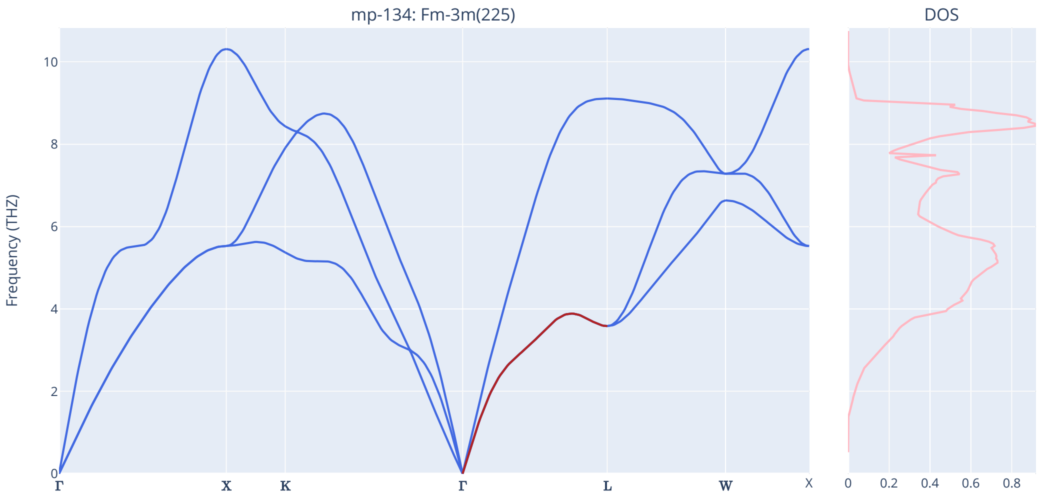

Supplementary Figure 16: The phonon band and DOS of Al4.

Supplementary Table 22: High symmetry lines of mp-134-Al4

	Start	End
0	Γ (0.000, 0.000, 0.000)	X (0.500, 0.000, 0.500)
1	X (0.500, 0.000, 0.500)	U (0.625, 0.250, 0.625)
2	K (0.375, 0.375, 0.750)	Γ (0.000, 0.000, 0.000)
3	Γ (0.000, 0.000, 0.000)	L (0.500, 0.500, 0.500)
4	L (0.500, 0.500, 0.500)	W (0.500, 0.250, 0.750)
5	W (0.500, 0.250, 0.750)	X (0.500, 0.000, 0.500)

The followings are details for Ce$_4$ and it only has 1 topological nodal line along high symmetry line as shown in Fig **17**.

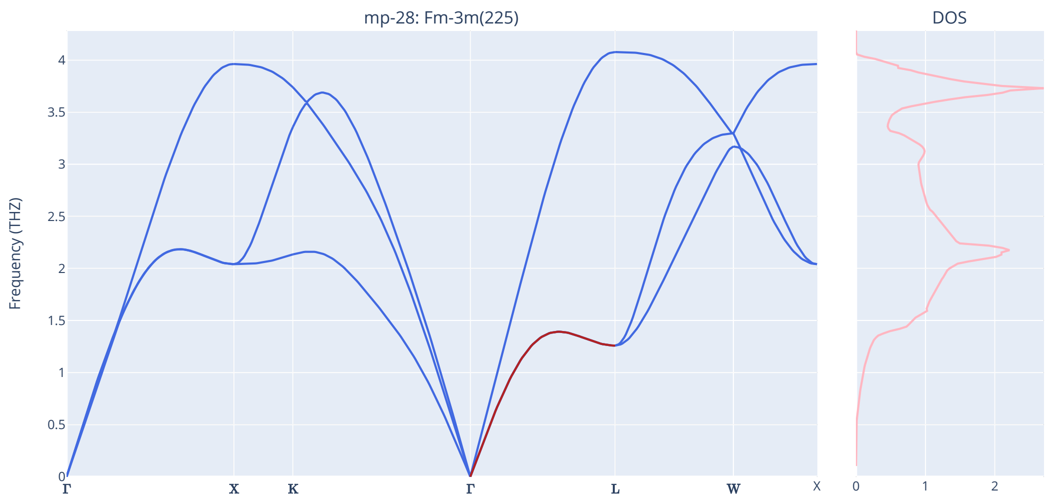

Supplementary Figure 17: The phonon band and DOS of Ce4.

Supplementary Table 23: High symmetry lines of mp-28-Ce4

	Start	End
0	Γ (0.000, 0.000, 0.000)	X (0.500, 0.000, 0.500)
1	X (0.500, 0.000, 0.500)	U (0.625, 0.250, 0.625)
2	K (0.375, 0.375, 0.750)	Γ (0.000, 0.000, 0.000)
3	Γ (0.000, 0.000, 0.000)	L (0.500, 0.500, 0.500)
4	L (0.500, 0.500, 0.500)	W (0.500, 0.250, 0.750)
5	W (0.500, 0.250, 0.750)	X (0.500, 0.000, 0.500)

The followings are details for Cu_4 and it only has 1 topological nodal line along high symmetry line as shown in Fig **18**.

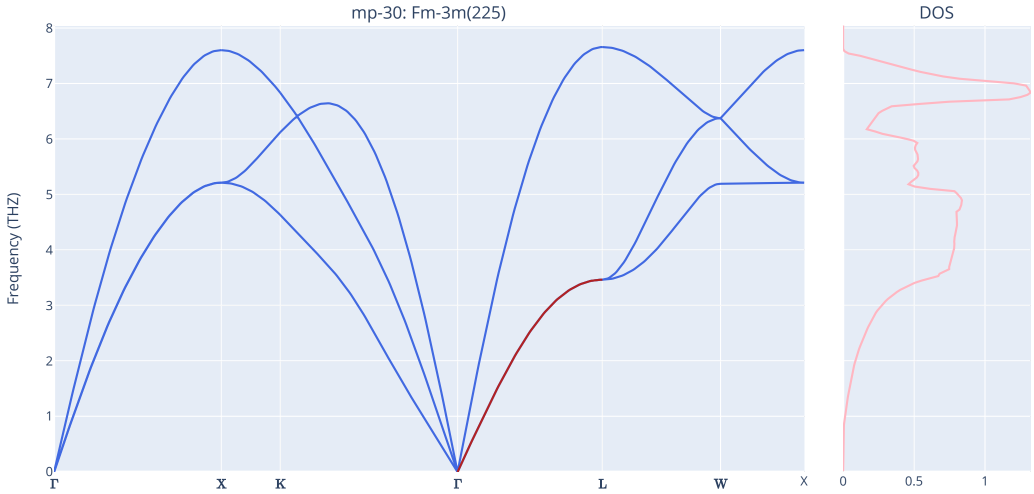

Supplementary Figure 18: The phonon band and DOS of Cu4.

Supplementary Table 24: High symmetry lines of mp-30-Cu4

	Start	End
0	Γ (0.000, 0.000, 0.000)	X (0.500, 0.000, 0.500)
1	X (0.500, 0.000, 0.500)	U (0.625, 0.250, 0.625)
2	K (0.375, 0.375, 0.750)	Γ (0.000, 0.000, 0.000)
3	Γ (0.000, 0.000, 0.000)	L (0.500, 0.500, 0.500)
4	L (0.500, 0.500, 0.500)	W (0.500, 0.250, 0.750)
5	W (0.500, 0.250, 0.750)	X (0.500, 0.000, 0.500)

The followings are details for Tl_2 and it only has 2 topological nodal lines along high symmetry line as shown in Fig **19**.

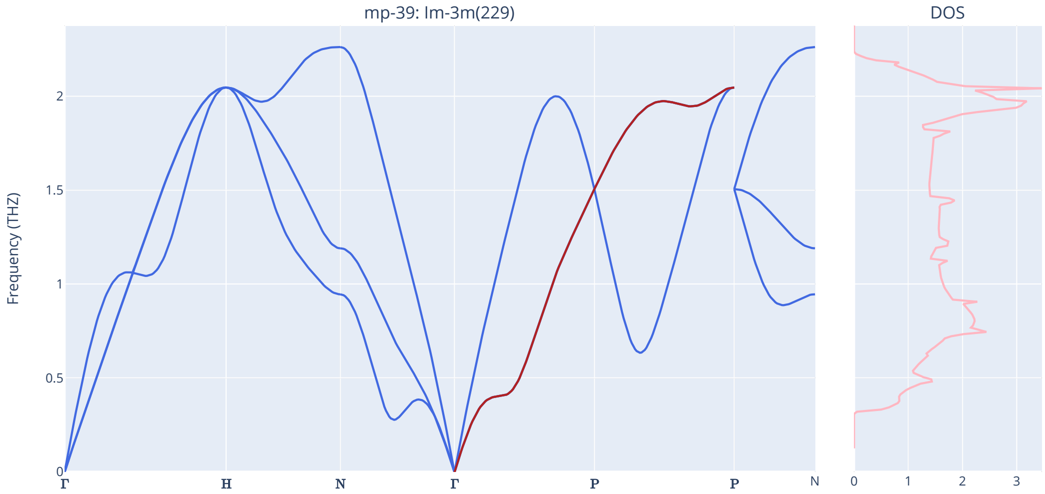

Supplementary Figure 19: The phonon band and DOS of Tl2.

Supplementary Table 25: High symmetry lines of mp-39-Tl2

	Start	End
0	Γ (0.000, 0.000, 0.000)	H (0.500, -0.500, 0.500)
1	H (0.500, -0.500, 0.500)	N (0.000, 0.000, 0.500)
2	N (0.000, 0.000, 0.500)	Γ (0.000, 0.000, 0.000)
3	Γ (0.000, 0.000, 0.000)	P (0.250, 0.250, 0.250)
4	P (0.250, 0.250, 0.250)	H (0.500, -0.500, 0.500)
5	P (0.250, 0.250, 0.250)	N (0.000, 0.000, 0.500)

The followings are details for Ca_4 and it only has 1 topological nodal line along high symmetry line as shown in Fig **20**.

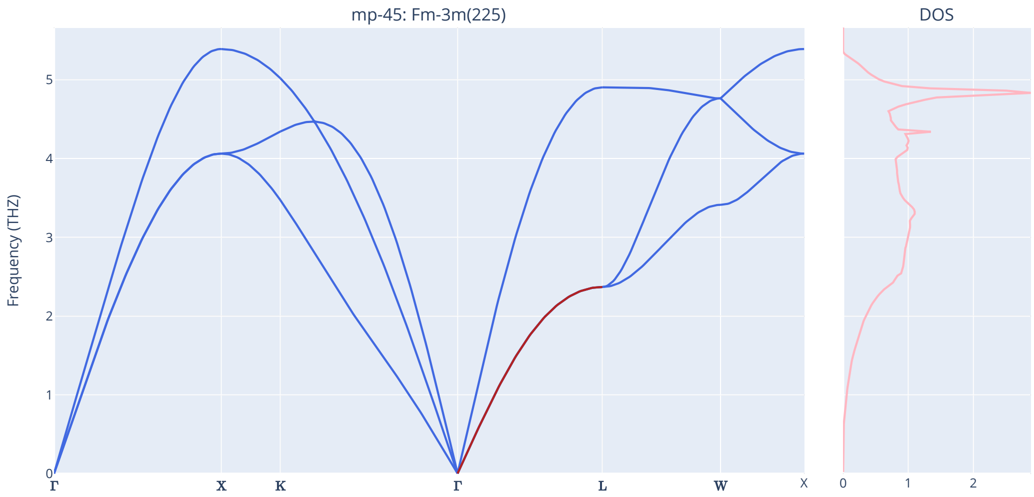

Supplementary Figure 20: The phonon band and DOS of Ca4.

Supplementary Table 26: High symmetry lines of mp-45-Ca4

	Start	End
0	Γ (0.000, 0.000, 0.000)	X (0.500, 0.000, 0.500)
1	X (0.500, 0.000, 0.500)	U (0.625, 0.250, 0.625)
2	K (0.375, 0.375, 0.750)	Γ (0.000, 0.000, 0.000)
3	Γ (0.000, 0.000, 0.000)	L (0.500, 0.500, 0.500)
4	L (0.500, 0.500, 0.500)	W (0.500, 0.250, 0.750)
5	W (0.500, 0.250, 0.750)	X (0.500, 0.000, 0.500)

The followings are details for Xe_4 and it only has 1 topological nodal line along high symmetry line as shown in Fig **21**.

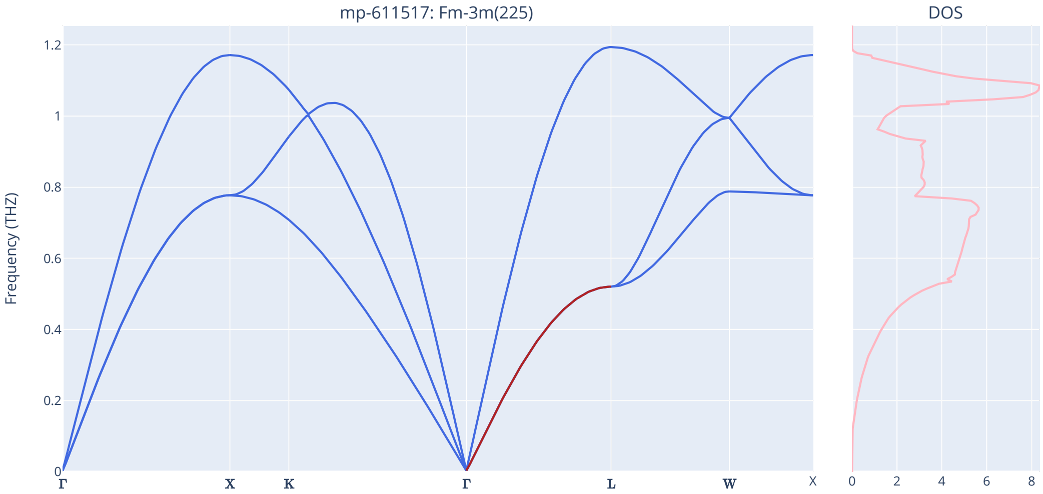

Supplementary Figure 21: The phonon band and DOS of Xe4.

Supplementary Table 27: High symmetry lines of mp-611517-Xe4

	Start	End
0	Γ (0.000, 0.000, 0.000)	X (0.500, 0.000, 0.500)
1	X (0.500, 0.000, 0.500)	U (0.625, 0.250, 0.625)
2	K (0.375, 0.375, 0.750)	Γ (0.000, 0.000, 0.000)
3	Γ (0.000, 0.000, 0.000)	L (0.500, 0.500, 0.500)
4	L (0.500, 0.500, 0.500)	W (0.500, 0.250, 0.750)
5	W (0.500, 0.250, 0.750)	X (0.500, 0.000, 0.500)

The followings are details for Kr_4 and it only has 1 topological nodal line along high symmetry line as shown in Fig 22.

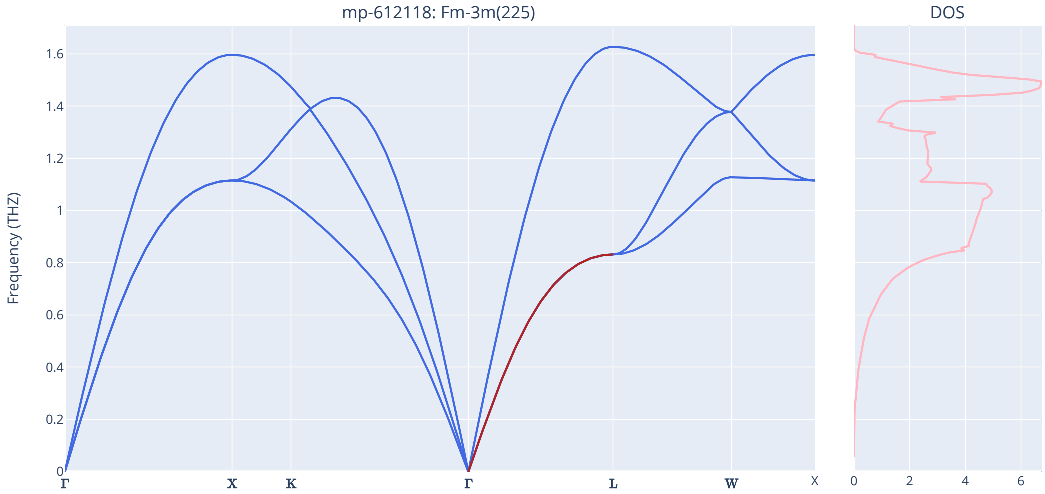

Supplementary Figure 22: The phonon band and DOS of Kr4.

Supplementary Table 28: High symmetry lines of mp-612118-Kr4

	Start	End
0	Γ (0.000, 0.000, 0.000)	X (0.500, 0.000, 0.500)
1	X (0.500, 0.000, 0.500)	U (0.625, 0.250, 0.625)
2	K (0.375, 0.375, 0.750)	Γ (0.000, 0.000, 0.000)
3	Γ (0.000, 0.000, 0.000)	L (0.500, 0.500, 0.500)
4	L (0.500, 0.500, 0.500)	W (0.500, 0.250, 0.750)
5	W (0.500, 0.250, 0.750)	X (0.500, 0.000, 0.500)

The followings are details for He_4 and it only has 1 topological nodal line along high symmetry line as shown in Fig **23**.

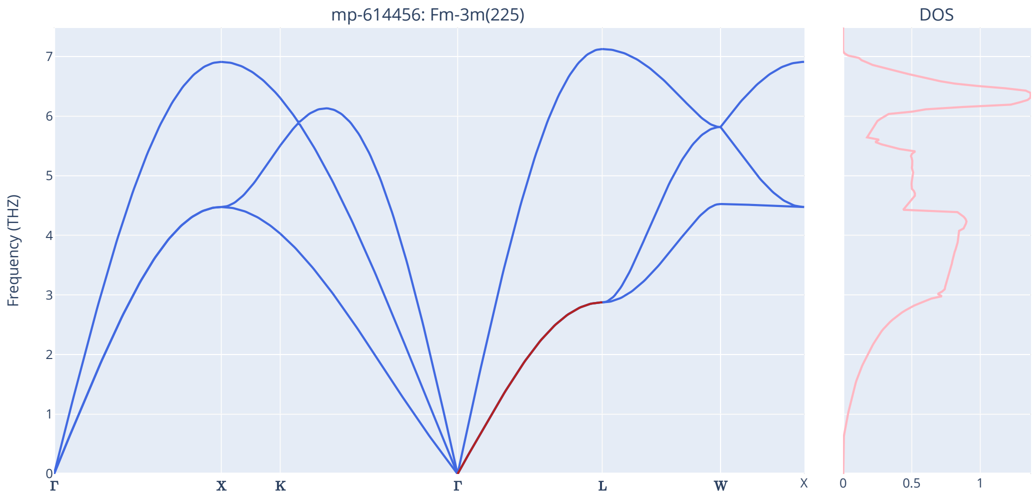

Supplementary Figure 23: The phonon band and DOS of He4.

Supplementary Table 29: High symmetry lines of mp-614456-He4

	Start	End
0	Γ (0.000, 0.000, 0.000)	X (0.500, 0.000, 0.500)
1	X (0.500, 0.000, 0.500)	U (0.625, 0.250, 0.625)
2	K (0.375, 0.375, 0.750)	Γ (0.000, 0.000, 0.000)
3	Γ (0.000, 0.000, 0.000)	L (0.500, 0.500, 0.500)
4	L (0.500, 0.500, 0.500)	W (0.500, 0.250, 0.750)
5	W (0.500, 0.250, 0.750)	X (0.500, 0.000, 0.500)

The followings are details for Rh$_4$ and it only has 1 topological nodal line along high symmetry line as shown in Fig 24.

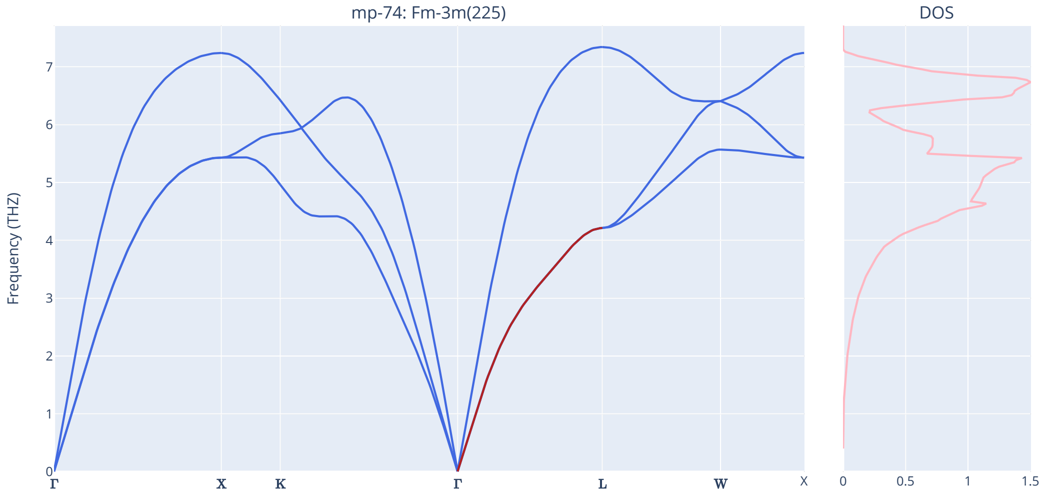

Supplementary Figure 24: The phonon band and DOS of Rh4.

Supplementary Table 30: High symmetry lines of mp-74-Rh4

	Start	End
0	Γ (0.000, 0.000, 0.000)	X (0.500, 0.000, 0.500)
1	X (0.500, 0.000, 0.500)	U (0.625, 0.250, 0.625)
2	K (0.375, 0.375, 0.750)	Γ (0.000, 0.000, 0.000)
3	Γ (0.000, 0.000, 0.000)	L (0.500, 0.500, 0.500)
4	L (0.500, 0.500, 0.500)	W (0.500, 0.250, 0.750)
5	W (0.500, 0.250, 0.750)	X (0.500, 0.000, 0.500)

The followings are details for Nb_2 and it only has 2 topological nodal lines along high symmetry line as shown in Fig **25**.

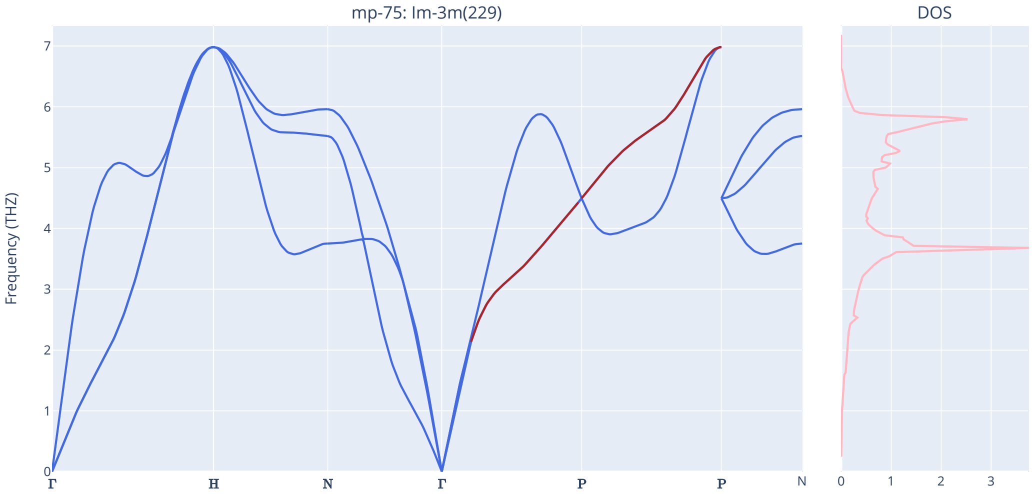

Supplementary Figure 25: The phonon band and DOS of Nb2.

Supplementary Table 31: High symmetry lines of mp-75-Nb2

	Start	End
0	Γ (0.000, 0.000, 0.000)	H (0.500, -0.500, 0.500)
1	H (0.500, -0.500, 0.500)	N (0.000, 0.000, 0.500)
2	N (0.000, 0.000, 0.500)	Γ (0.000, 0.000, 0.000)
3	Γ (0.000, 0.000, 0.000)	P (0.250, 0.250, 0.250)
4	P (0.250, 0.250, 0.250)	H (0.500, -0.500, 0.500)
5	P (0.250, 0.250, 0.250)	N (0.000, 0.000, 0.500)

The followings are details for Sr_4 and it only has 1 topological nodal line along high symmetry line as shown in Fig **26**.

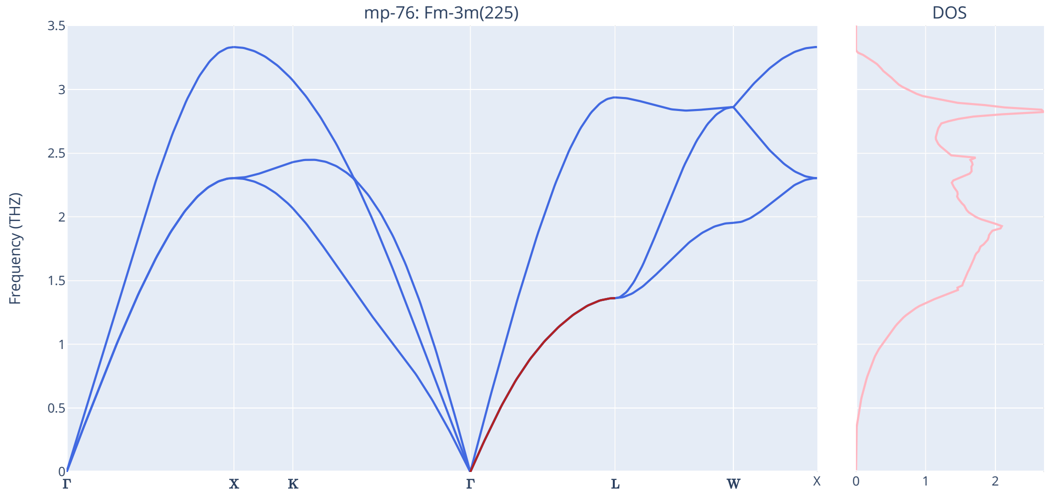

Supplementary Figure 26: The phonon band and DOS of Sr4.

Supplementary Table 32: High symmetry lines of mp-76-Sr4

	Start	End
0	Γ (0.000, 0.000, 0.000)	X (0.500, 0.000, 0.500)
1	X (0.500, 0.000, 0.500)	U (0.625, 0.250, 0.625)
2	K (0.375, 0.375, 0.750)	Γ (0.000, 0.000, 0.000)
3	Γ (0.000, 0.000, 0.000)	L (0.500, 0.500, 0.500)
4	L (0.500, 0.500, 0.500)	W (0.500, 0.250, 0.750)
5	W (0.500, 0.250, 0.750)	X (0.500, 0.000, 0.500)

The followings are details for Au_4 and it only has 1 topological nodal line along high symmetry line as shown in Fig **27**.

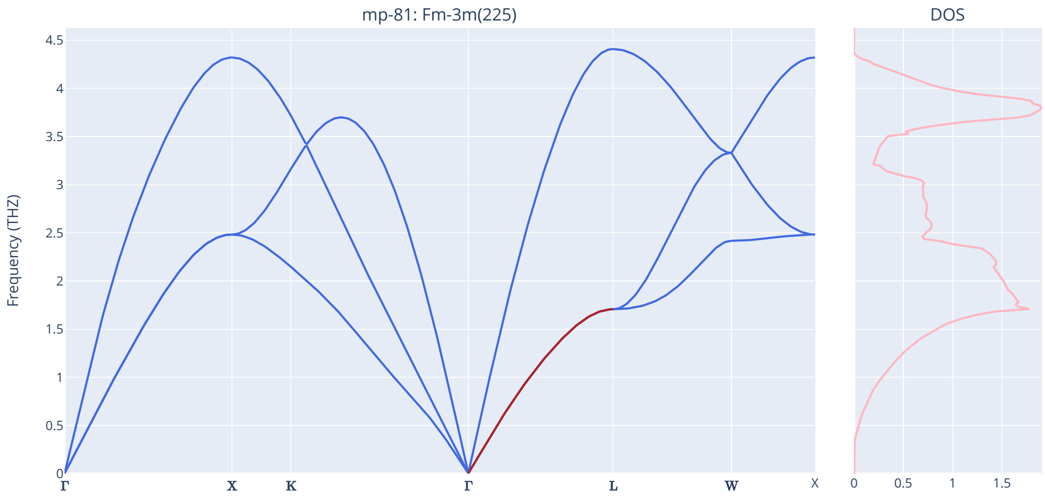

Supplementary Figure 27: The phonon band and DOS of Au4.

Supplementary Table 33: High symmetry lines of mp-81-Au4

	Start	End
0	Γ (0.000, 0.000, 0.000)	X (0.500, 0.000, 0.500)
1	X (0.500, 0.000, 0.500)	U (0.625, 0.250, 0.625)
2	K (0.375, 0.375, 0.750)	Γ (0.000, 0.000, 0.000)
3	Γ (0.000, 0.000, 0.000)	L (0.500, 0.500, 0.500)
4	L (0.500, 0.500, 0.500)	W (0.500, 0.250, 0.750)
5	W (0.500, 0.250, 0.750)	X (0.500, 0.000, 0.500)

The followings are details for Cr_2 and it only has 3 topological nodal lines along high symmetry line as shown in Fig **28**.

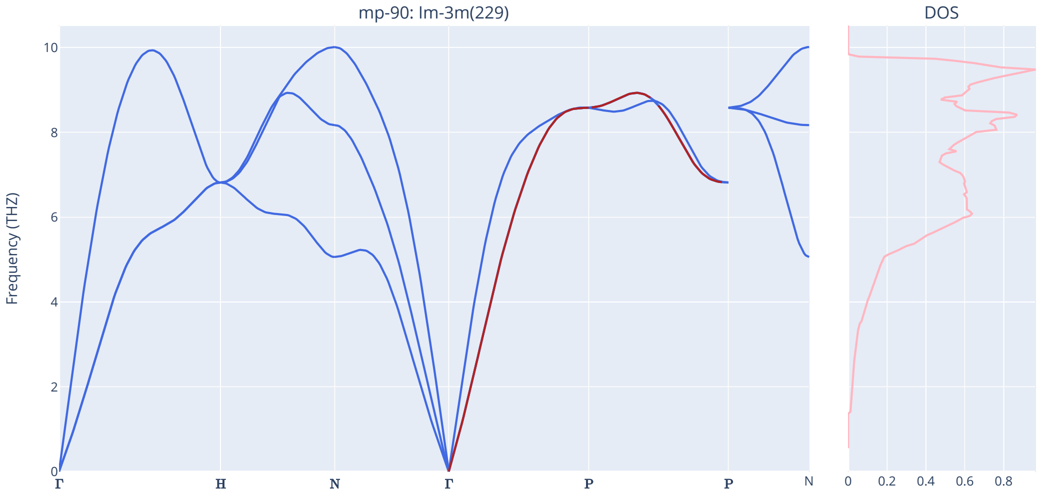

Supplementary Figure 28: The phonon band and DOS of Cr2.

Supplementary Table 34: High symmetry lines of mp-90-Cr2

	Start	End
0	Γ (0.000, 0.000, 0.000)	H (0.500, -0.500, 0.500)
1	H (0.500, -0.500, 0.500)	N (0.000, 0.000, 0.500)
2	N (0.000, 0.000, 0.500)	Γ (0.000, 0.000, 0.000)
3	Γ (0.000, 0.000, 0.000)	P (0.250, 0.250, 0.250)
4	P (0.250, 0.250, 0.250)	H (0.500, -0.500, 0.500)
5	P (0.250, 0.250, 0.250)	N (0.000, 0.000, 0.500)

The followings are details for W_2 and it only has 2 topological nodal lines along high symmetry line as shown in Fig **29**.

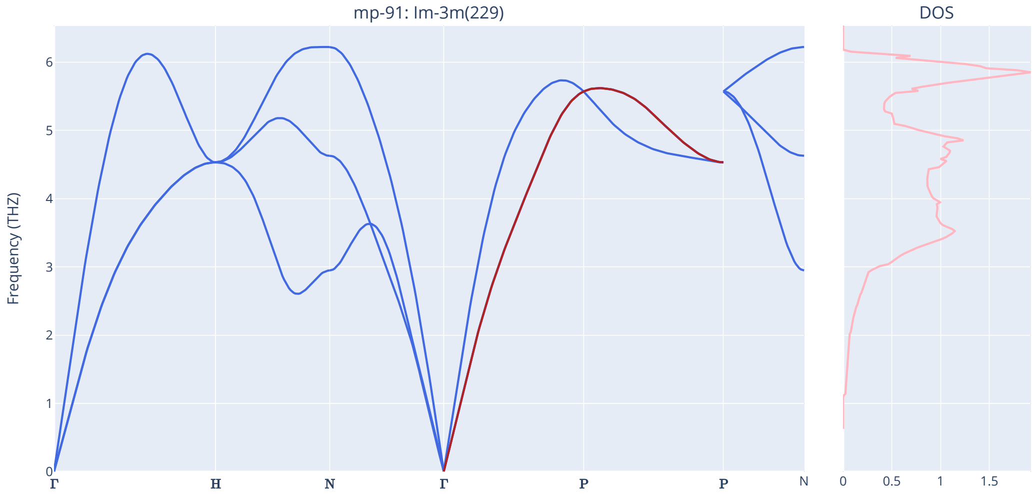

Supplementary Figure 29: The phonon band and DOS of W2.

Supplementary Table 35: High symmetry lines of mp-91-W2

	Start	End
0	Γ (0.000, 0.000, 0.000)	H (0.500, -0.500, 0.500)
1	H (0.500, -0.500, 0.500)	N (0.000, 0.000, 0.500)
2	N (0.000, 0.000, 0.500)	Γ (0.000, 0.000, 0.000)
3	Γ (0.000, 0.000, 0.000)	P (0.250, 0.250, 0.250)
4	P (0.250, 0.250, 0.250)	H (0.500, -0.500, 0.500)
5	P (0.250, 0.250, 0.250)	N (0.000, 0.000, 0.500)

The followings are details for Na_4 and it only has 1 topological nodal line along high symmetry line as shown in Fig **30**.

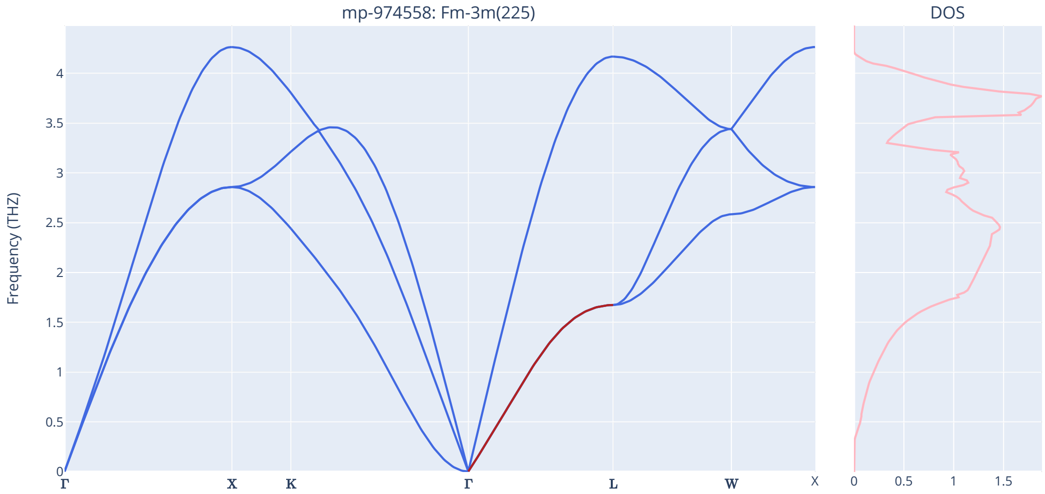

Supplementary Figure 30: The phonon band and DOS of Na4.

Supplementary Table 36: High symmetry lines of mp-974558-Na4

	Start	End
0	Γ (0.000, 0.000, 0.000)	X (0.500, 0.000, 0.500)
1	X (0.500, 0.000, 0.500)	U (0.625, 0.250, 0.625)
2	K (0.375, 0.375, 0.750)	Γ (0.000, 0.000, 0.000)
3	Γ (0.000, 0.000, 0.000)	L (0.500, 0.500, 0.500)
4	L (0.500, 0.500, 0.500)	W (0.500, 0.250, 0.750)
5	W (0.500, 0.250, 0.750)	X (0.500, 0.000, 0.500)

The followings are details for Ar$_4$. It has 1 topological nodal line and 1 nodal ring point along high symmetry line as shown in Fig **31**.

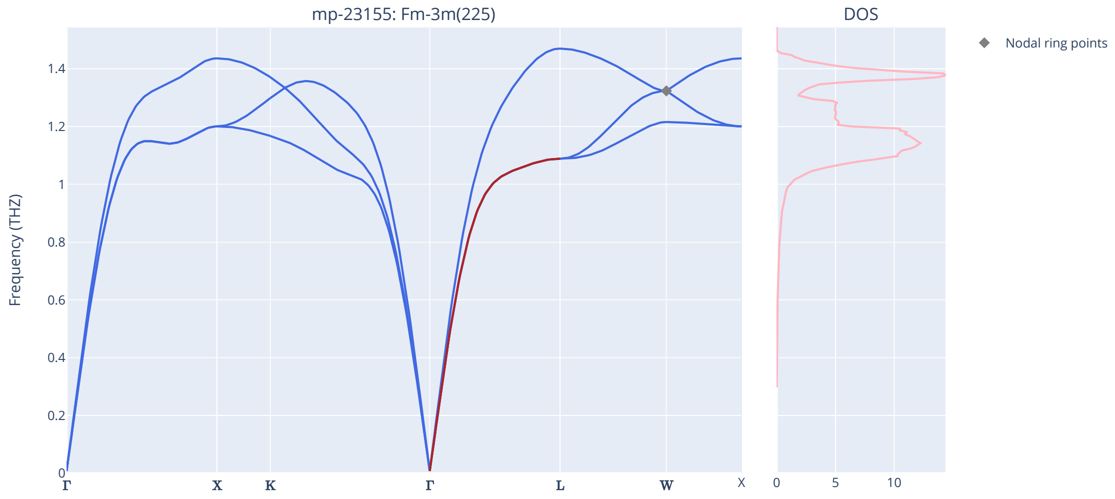

Supplementary Figure 31: The phonon band and DOS of Ar4.

Supplementary Table 37: High symmetry lines of mp-23155-Ar4

	Start	End
0	Γ (0.000, 0.000, 0.000)	X (0.500, 0.000, 0.500)
1	X (0.500, 0.000, 0.500)	U (0.625, 0.250, 0.625)
2	K (0.375, 0.375, 0.750)	Γ (0.000, 0.000, 0.000)
3	Γ (0.000, 0.000, 0.000)	L (0.500, 0.500, 0.500)
4	L (0.500, 0.500, 0.500)	W (0.500, 0.250, 0.750)
5	W (0.500, 0.250, 0.750)	X (0.500, 0.000, 0.500)

Supplementary Table 38: Nodal ring points along high symmetry lines of mp-23155-Ar4.

	Coordinates	Frequency	Mode 1	Mode 2	Multi.	Deg.	Path	Ratio
0	(0.500, 0.250, 0.750)	1.323	3	2	6	2	5	1.000

The followings are details for IrTc. It has 4 nodal ring points and 3 single Weyl points along high symmetry line as shown in Fig 32.

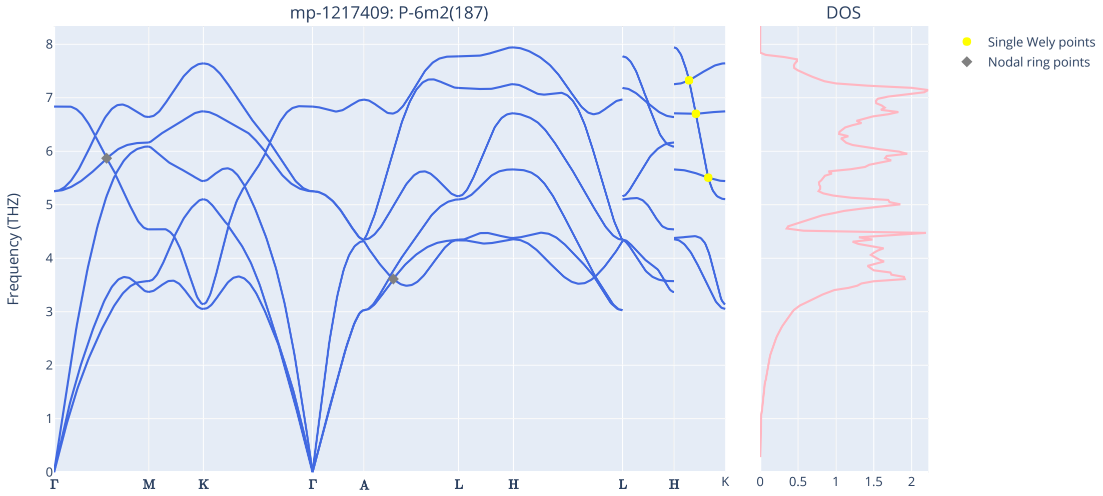

Supplementary Figure 32: The phonon band and DOS of IrTc.

Supplementary Table 39: High symmetry lines of mp-1217409-IrTc

	Start	End
0	Γ (0.000, 0.000, 0.000)	M (0.500, 0.000, 0.000)
1	M (0.500, 0.000, 0.000)	K (0.333, 0.333, 0.000)
2	K (0.333, 0.333, 0.000)	Γ (0.000, 0.000, 0.000)
3	Γ (0.000, 0.000, 0.000)	A (0.000, 0.000, 0.500)
4	A (0.000, 0.000, 0.500)	L (0.500, 0.000, 0.500)
5	L (0.500, 0.000, 0.500)	H (0.333, 0.333, 0.500)
6	H (0.333, 0.333, 0.500)	A (0.000, 0.000, 0.500)
7	L (0.500, 0.000, 0.500)	M (0.500, 0.000, 0.000)
8	H (0.333, 0.333, 0.500)	K (0.333, 0.333, 0.000)

Supplementary Table 40: Single Weyl points along high symmetry lines of mp-1217409-IrTc.

	Coordinates	Frequency	Mode 1	Mode 2	Multi.	Deg.	Path	Ratio
0	(0.333, 0.333, 0.166)	5.504	4	3	4	2	9	0.668
1	(0.333, 0.333, 0.286)	6.698	5	4	4	2	9	0.427
2	(0.333, 0.333, 0.353)	7.324	6	5	4	2	9	0.293

Supplementary Table 41: Nodal ring points along high symmetry lines of mp-1217409-IrTc.

	Coordinates	Frequency	Mode 1	Mode 2	Multi.	Deg.	Path	Ratio
0	(0.155, -0.001, 0.500)	3.610	2	1	10	2	5	0.310

Supplementary Table 41: Nodal ring points along high symmetry lines of mp-1217409-IrTc.

	Coordinates	Frequency	Mode 1	Mode 2	Multi.	Deg.	Path	Ratio
1	(0.278, 0.001, -0.000)	5.866	5	4	12	2	1	0.556

The followings are details for IrRe and it only has 6 topological nodal ring points along high symmetry line as shown in Fig 33.

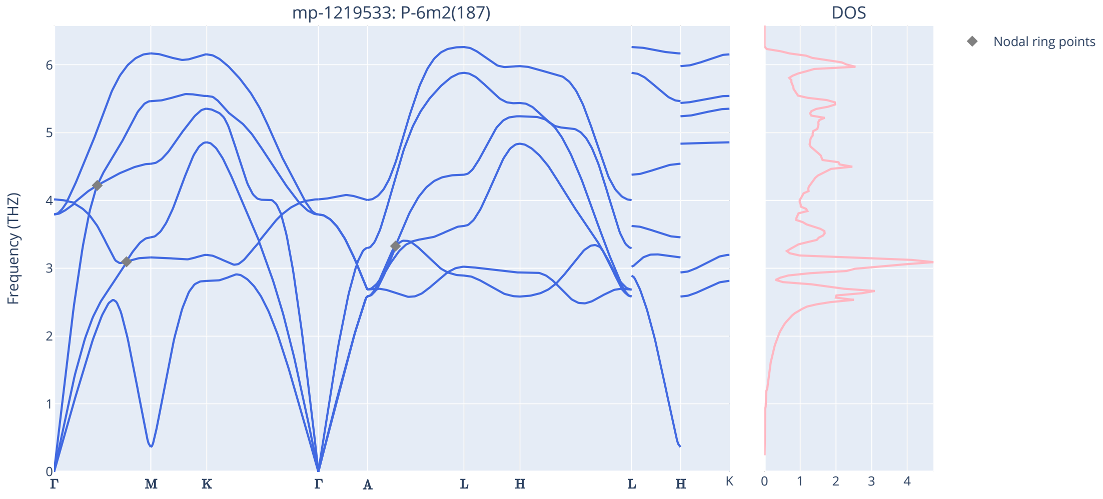

Supplementary Figure 33: The phonon band and DOS of IrRe.

Supplementary Table 42: High symmetry lines of mp-1219533-IrRe

	Start	End
0	Γ (0.000, 0.000, 0.000)	M (0.500, 0.000, 0.000)
1	M (0.500, 0.000, 0.000)	K (0.333, 0.333, 0.000)
2	K (0.333, 0.333, 0.000)	Γ (0.000, 0.000, 0.000)
3	Γ (0.000, 0.000, 0.000)	A (0.000, 0.000, 0.500)
4	A (0.000, 0.000, 0.500)	L (0.500, 0.000, 0.500)
5	L (0.500, 0.000, 0.500)	H (0.333, 0.333, 0.500)
6	H (0.333, 0.333, 0.500)	A (0.000, 0.000, 0.500)
7	L (0.500, 0.000, 0.500)	M (0.500, 0.000, 0.000)
8	H (0.333, 0.333, 0.500)	K (0.333, 0.333, 0.000)

Supplementary Table 43: Nodal ring points along high symmetry lines of mp-1219533-IrRe.

	Coordinates	Frequency	Mode 1	Mode 2	Multi.	Deg.	Path	Ratio
0	(0.374, -0.002, 0.000)	3.096	3	2	12	2	1	0.749
1	(0.146, -0.000, 0.500)	3.327	4	3	6	2	5	0.292
2	(0.223, -0.000, 0.000)	4.221	5	4	6	2	1	0.446

The followings are details for OsRu and it only has 3 topological nodal ring points along high symmetry line as shown in Fig 34.

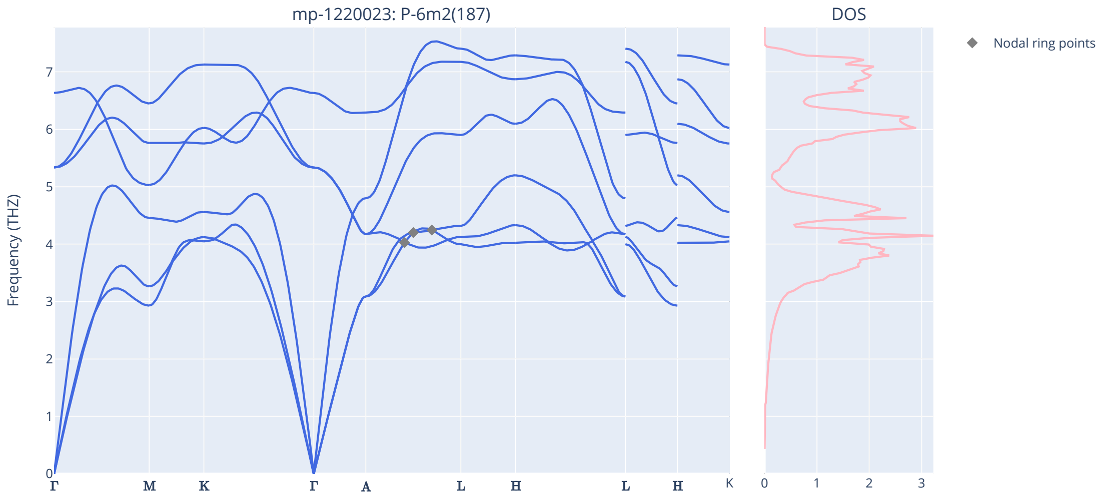

Supplementary Figure 34: The phonon band and DOS of OsRu.

Supplementary Table 44: High symmetry lines of mp-1220023-OsRu

	Start	End
0	Γ (0.000, 0.000, 0.000)	M (0.500, 0.000, 0.000)
1	M (0.500, 0.000, 0.000)	K (0.333, 0.333, 0.000)
2	K (0.333, 0.333, 0.000)	Γ (0.000, 0.000, 0.000)
3	Γ (0.000, 0.000, 0.000)	A (0.000, 0.000, 0.500)
4	A (0.000, 0.000, 0.500)	L (0.500, 0.000, 0.500)
5	L (0.500, 0.000, 0.500)	H (0.333, 0.333, 0.500)
6	H (0.333, 0.333, 0.500)	A (0.000, 0.000, 0.500)
7	L (0.500, 0.000, 0.500)	M (0.500, 0.000, 0.000)
8	H (0.333, 0.333, 0.500)	K (0.333, 0.333, 0.000)

Supplementary Table 45: Nodal ring points along high symmetry lines of mp-1220023-OsRu.

	Coordinates	Frequency	Mode 1	Mode 2	Multi.	Deg.	Path	Ratio
0	(0.204, 0.000, 0.497)	4.026	2	1	12	2	5	0.408
1	(0.250, 0.000, 0.500)	4.201	3	2	6	2	5	0.501
2	(0.348, -0.000, 0.500)	4.246	3	2	6	2	5	0.695

The followings are details for Th_4As_4. It has 2 topological nodal lines and 3 nodal ring points along high symmetry line as shown in Fig **35**.

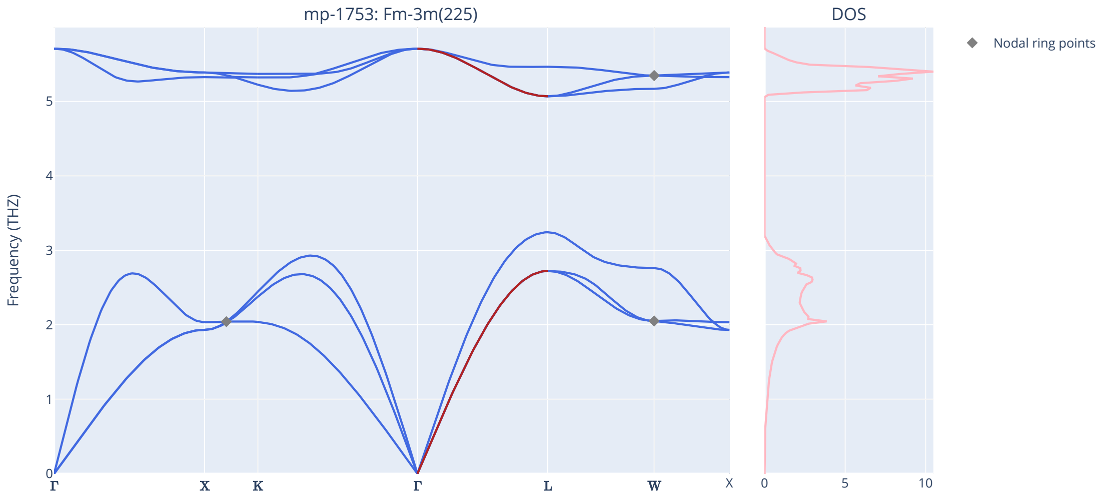

Supplementary Figure 35: The phonon band and DOS of Th4As4.

Supplementary Table 46: High symmetry lines of mp-1753-Th4As4

	Start	End
0	Γ (0.000, 0.000, 0.000)	X (0.500, 0.000, 0.500)
1	X (0.500, 0.000, 0.500)	U (0.625, 0.250, 0.625)
2	K (0.375, 0.375, 0.750)	Γ (0.000, 0.000, 0.000)
3	Γ (0.000, 0.000, 0.000)	L (0.500, 0.500, 0.500)
4	L (0.500, 0.500, 0.500)	W (0.500, 0.250, 0.750)
5	W (0.500, 0.250, 0.750)	X (0.500, 0.000, 0.500)

Supplementary Table 47: Nodal ring points along high symmetry lines of mp-1753-Th4As4.

	Coordinates	Frequency	Mode 1	Mode 2	Multi.	Deg.	Path	Ratio
0	(0.500, 0.250, 0.750)	2.051	2	1	6	2	5	1.000
1	(0.551, 0.102, 0.551)	2.041	3	2	18	2	2	0.410
2	(0.500, 0.250, 0.750)	5.345	6	5	6	2	5	1.000

The followings are details for Bi_4La_4. It has 2 topological nodal lines and 8 nodal ring points along high symmetry line as shown in Fig 36.

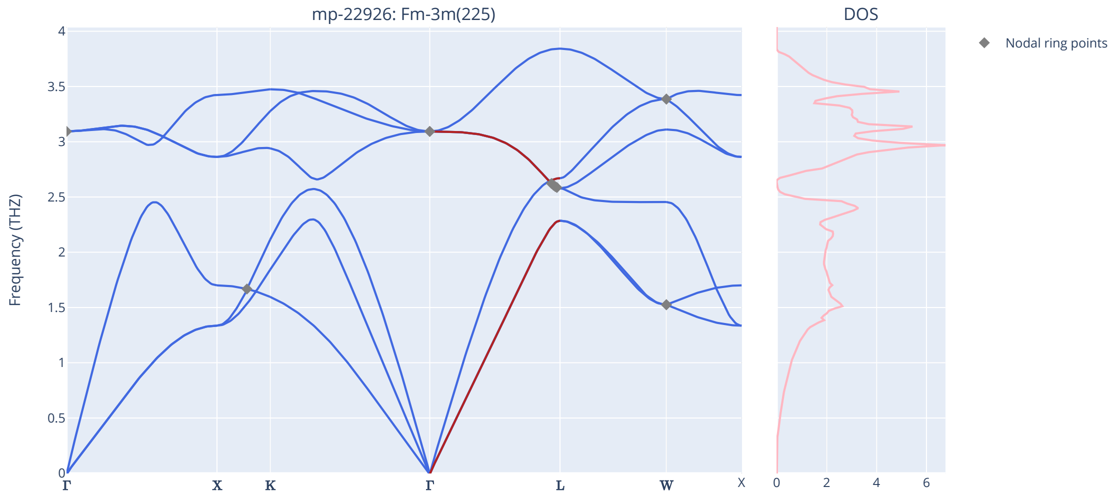

Supplementary Figure 36: The phonon band and DOS of Bi4La4.

Supplementary Table 48: High symmetry lines of mp-22926-Bi4La4

	Start	End
0	Γ (0.000, 0.000, 0.000)	X (0.500, 0.000, 0.500)
1	X (0.500, 0.000, 0.500)	U (0.625, 0.250, 0.625)
2	K (0.375, 0.375, 0.750)	Γ (0.000, 0.000, 0.000)
3	Γ (0.000, 0.000, 0.000)	L (0.500, 0.500, 0.500)
4	L (0.500, 0.500, 0.500)	W (0.500, 0.250, 0.750)
5	W (0.500, 0.250, 0.750)	X (0.500, 0.000, 0.500)

Supplementary Table 49: Nodal ring points along high symmetry lines of mp-22926-Bi4La4.

	Coordinates	Frequency	Mode 1	Mode 2	Multi.	Deg.	Path	Ratio
0	(0.500, 0.250, 0.750)	1.525	2	1	6	2	5	1.000
1	(0.570, 0.141, 0.570)	1.668	3	2	12	2	2	0.563
2	(0.488, 0.488, 0.488)	2.586	4	3	8	2	4	0.976
3	(0.467, 0.467, 0.467)	2.623	4	3	8	2	4	0.934
4	(0.482, 0.482, 0.482)	2.594	4	3	8	2	4	0.964
5	(0.475, 0.475, 0.475)	2.606	4	3	8	2	4	0.951
6	(0.000, 0.000, 0.000)	3.093	5	4	1	3	1	0.000
7	(0.500, 0.250, 0.750)	3.386	6	5	6	2	5	1.000

The followings are details for Bi_4Ce_4. It has 2 topological nodal lines and 4 nodal ring points along high symmetry line as shown in Fig **37**.

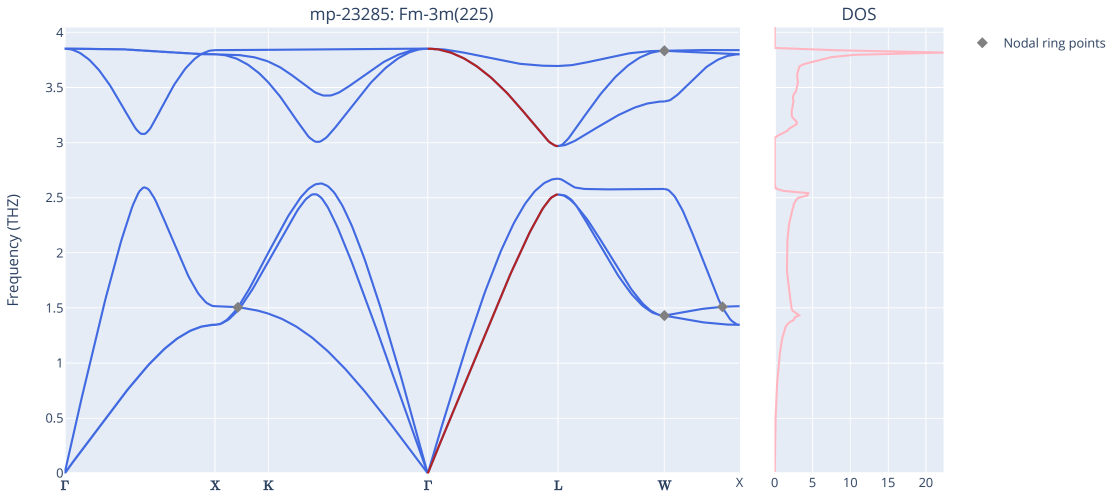

Supplementary Figure 37: The phonon band and DOS of Bi4Ce4.

Supplementary Table 50: High symmetry lines of mp-23285-Bi4Ce4

	Start	End
0	Γ (0.000, 0.000, 0.000)	X (0.500, 0.000, 0.500)
1	X (0.500, 0.000, 0.500)	U (0.625, 0.250, 0.625)
2	K (0.375, 0.375, 0.750)	Γ (0.000, 0.000, 0.000)
3	Γ (0.000, 0.000, 0.000)	L (0.500, 0.500, 0.500)
4	L (0.500, 0.500, 0.500)	W (0.500, 0.250, 0.750)
5	W (0.500, 0.250, 0.750)	X (0.500, 0.000, 0.500)

Supplementary Table 51: Nodal ring points along high symmetry lines of mp-23285-Bi4Ce4.

	Coordinates	Frequency	Mode 1	Mode 2	Multi.	Deg.	Path	Ratio
0	(0.500, 0.250, 0.750)	1.431	2	1	6	2	5	1.000
1	(0.554, 0.107, 0.554)	1.506	3	2	12	2	2	0.430
2	(0.498, 0.056, 0.558)	1.510	3	2	24	2	6	0.772
3	(0.500, 0.250, 0.750)	3.833	6	5	6	2	5	1.000

The followings are details for Y_4Se_4. It has 3 topological nodal lines and 5 nodal ring points along high symmetry line as shown in Fig 38.

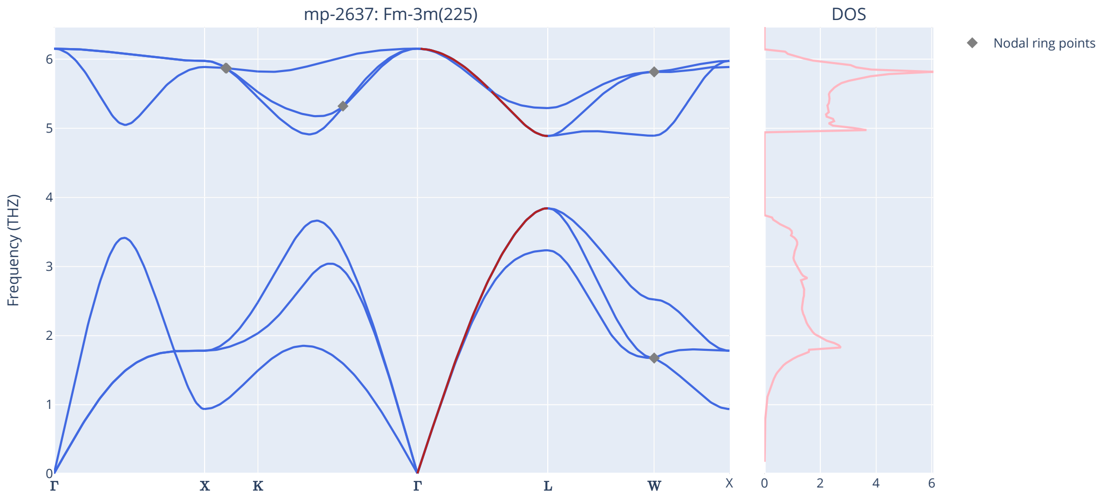

Supplementary Figure 38: The phonon band and DOS of Y4Se4.

Supplementary Table 52: High symmetry lines of mp-2637-Y4Se4

	Start	End
0	Γ (0.000, 0.000, 0.000)	X (0.500, 0.000, 0.500)
1	X (0.500, 0.000, 0.500)	U (0.625, 0.250, 0.625)
2	K (0.375, 0.375, 0.750)	Γ (0.000, 0.000, 0.000)
3	Γ (0.000, 0.000, 0.000)	L (0.500, 0.500, 0.500)
4	L (0.500, 0.500, 0.500)	W (0.500, 0.250, 0.750)
5	W (0.500, 0.250, 0.750)	X (0.500, 0.000, 0.500)

Supplementary Table 53: Nodal ring points along high symmetry lines of mp-2637-Y4Se4.

	Coordinates	Frequency	Mode 1	Mode 2	Multi.	Deg.	Path	Ratio
0	(0.500, 0.250, 0.750)	1.674	2	1	6	2	5	1.000
1	(0.550, 0.098, 0.548)	5.870	5	4	24	2	2	0.393
2	(0.176, 0.176, 0.349)	5.320	5	4	24	2	3	0.534
3	(0.552, 0.104, 0.552)	5.868	6	5	12	2	2	0.415
4	(0.500, 0.250, 0.750)	5.815	6	5	6	2	5	1.000

The followings are details for Hf_4N_4. It has 2 topological nodal lines and 4 nodal ring points along high symmetry line as shown in Fig 39.

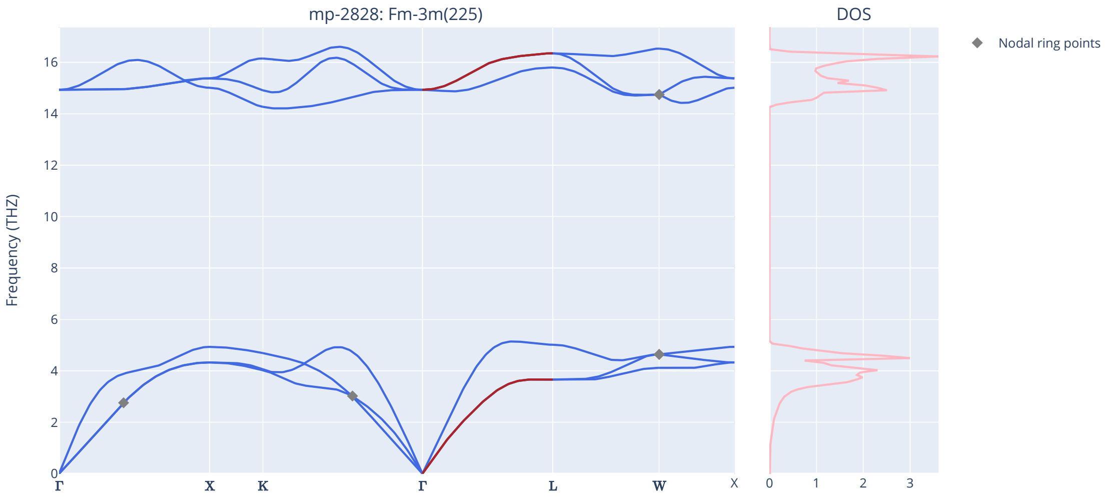

Supplementary Figure 39: The phonon band and DOS of Hf4N4.

Supplementary Table 54: High symmetry lines of mp-2828-Hf4N4

	Start	End
0	Γ (0.000, 0.000, 0.000)	X (0.500, 0.000, 0.500)
1	X (0.500, 0.000, 0.500)	U (0.625, 0.250, 0.625)
2	K (0.375, 0.375, 0.750)	Γ (0.000, 0.000, 0.000)
3	Γ (0.000, 0.000, 0.000)	L (0.500, 0.500, 0.500)
4	L (0.500, 0.500, 0.500)	W (0.500, 0.250, 0.750)
5	W (0.500, 0.250, 0.750)	X (0.500, 0.000, 0.500)

Supplementary Table 55: Nodal ring points along high symmetry lines of mp-2828-Hf4N4.

	Coordinates	Frequency	Mode 1	Mode 2	Multi.	Deg.	Path	Ratio
0	(0.214, 0.000, 0.214)	2.760	2	1	6	2	1	0.429
1	(0.161, 0.161, 0.332)	3.024	2	1	24	2	3	0.562
2	(0.500, 0.250, 0.750)	4.642	3	2	6	2	5	1.000
3	(0.500, 0.250, 0.750)	14.747	5	4	6	2	5	1.000

The followings are details for YTe and it only has 2 single Weyl point along high symmetry line as shown in Fig 40.

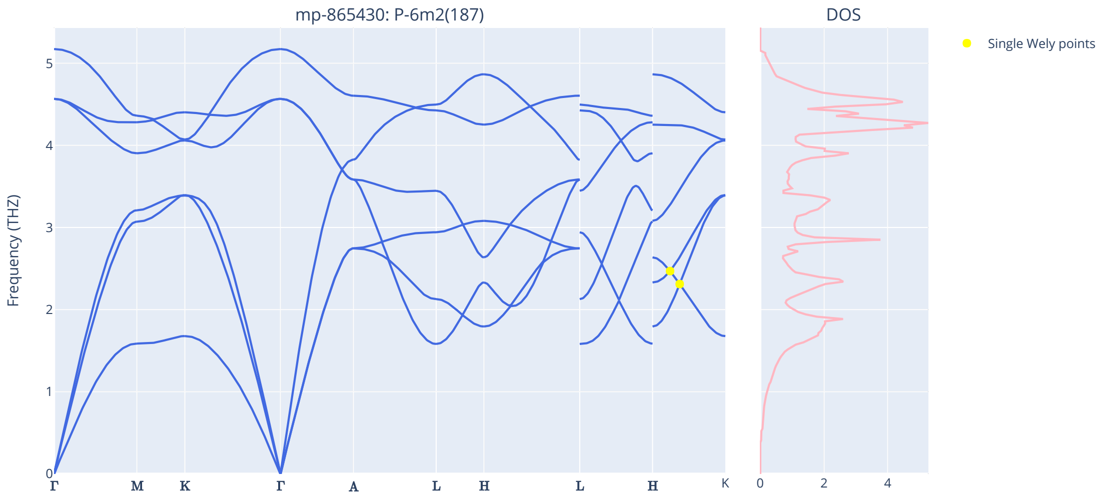

Supplementary Figure 40: The phonon band and DOS of YTe.

Supplementary Table 56: High symmetry lines of mp-865430-YTe

	Start	End
0	Γ (0.000, 0.000, 0.000)	M (0.500, 0.000, 0.000)
1	M (0.500, 0.000, 0.000)	K (0.333, 0.333, 0.000)
2	K (0.333, 0.333, 0.000)	Γ (0.000, 0.000, 0.000)
3	Γ (0.000, 0.000, 0.000)	A (0.000, 0.000, 0.500)
4	A (0.000, 0.000, 0.500)	L (0.500, 0.000, 0.500)
5	L (0.500, 0.000, 0.500)	H (0.333, 0.333, 0.500)
6	H (0.333, 0.333, 0.500)	A (0.000, 0.000, 0.500)
7	L (0.500, 0.000, 0.500)	M (0.500, 0.000, 0.000)
8	H (0.333, 0.333, 0.500)	K (0.333, 0.333, 0.000)

Supplementary Table 57: Single Weyl points along high symmetry lines of mp-865430-YTe.

	Coordinates	Frequency	Mode 1	Mode 2	Multi.	Deg.	Path	Ratio
0	(0.333, 0.333, 0.314)	2.311	2	1	4	2	9	0.373
1	(0.333, 0.333, 0.380)	2.469	3	2	4	2	9	0.241

The followings are details for GaTc. It has 3 nodal ring points and 4 single Weyl points along high symmetry line as shown in Fig **41**.

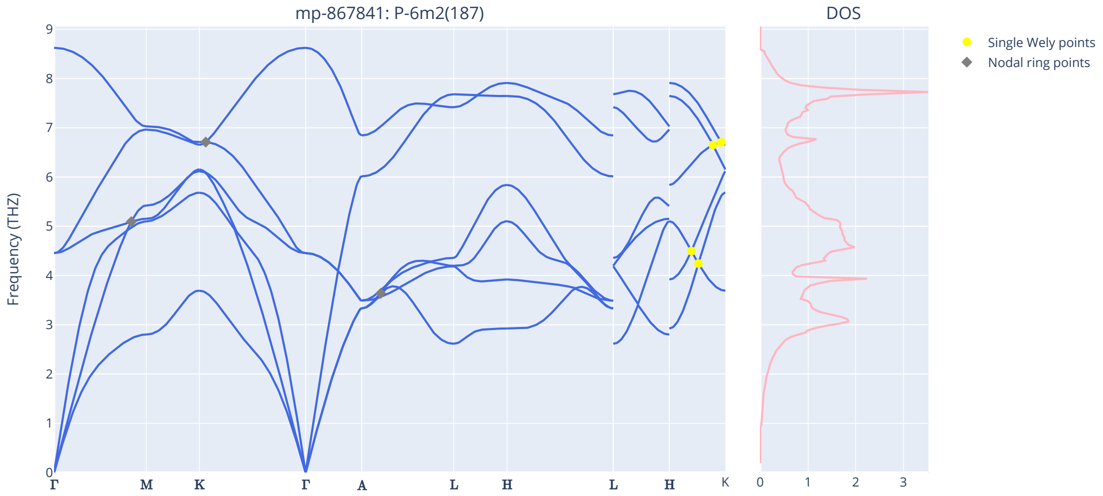

Supplementary Figure 41: The phonon band and DOS of GaTc.

Supplementary Table 58: High symmetry lines of mp-867841-GaTc

	Start	End
0	Γ (0.000, 0.000, 0.000)	M (0.500, 0.000, 0.000)
1	M (0.500, 0.000, 0.000)	K (0.333, 0.333, 0.000)
2	K (0.333, 0.333, 0.000)	Γ (0.000, 0.000, 0.000)
3	Γ (0.000, 0.000, 0.000)	A (0.000, 0.000, 0.500)
4	A (0.000, 0.000, 0.500)	L (0.500, 0.000, 0.500)
5	L (0.500, 0.000, 0.500)	H (0.333, 0.333, 0.500)
6	H (0.333, 0.333, 0.500)	A (0.000, 0.000, 0.500)
7	L (0.500, 0.000, 0.500)	M (0.500, 0.000, 0.000)
8	H (0.333, 0.333, 0.500)	K (0.333, 0.333, 0.000)

Supplementary Table 59: Single Weyl points along high symmetry lines of mp-867841-GaTc.

	Coordinates	Frequency	Mode 1	Mode 2	Multi.	Deg.	Path	Ratio
0	(0.333, 0.333, 0.240)	4.242	2	1	4	2	9	0.520
1	(0.333, 0.333, 0.303)	4.496	3	2	4	2	9	0.395
2	(0.333, 0.333, 0.111)	6.650	5	4	4	2	9	0.779
3	(0.333, 0.333, 0.037)	6.705	6	5	4	2	9	0.927

Supplementary Table 60: Nodal ring points along high symmetry lines of mp-867841-GaTc.

	Coordinates	Frequency	Mode 1	Mode 2	Multi.	Deg.	Path	Ratio
0	(0.103, -0.000, 0.500)	3.638	3	2	6	2	5	0.207

Supplementary Table 60: Nodal ring points along high symmetry lines of mp-867841-GaTc.

	Coordinates	Frequency	Mode 1	Mode 2	Multi.	Deg.	Path	Ratio
1	(0.419, 0.000, 0.000)	5.093	4	3	6	2	1	0.838
2	(0.314, 0.313, 0.000)	6.710	6	5	12	2	3	0.060

The followings are details for AuSc. It has 6 topological nodal lines and 2 nodal ring points along high symmetry line as shown in Fig 42.

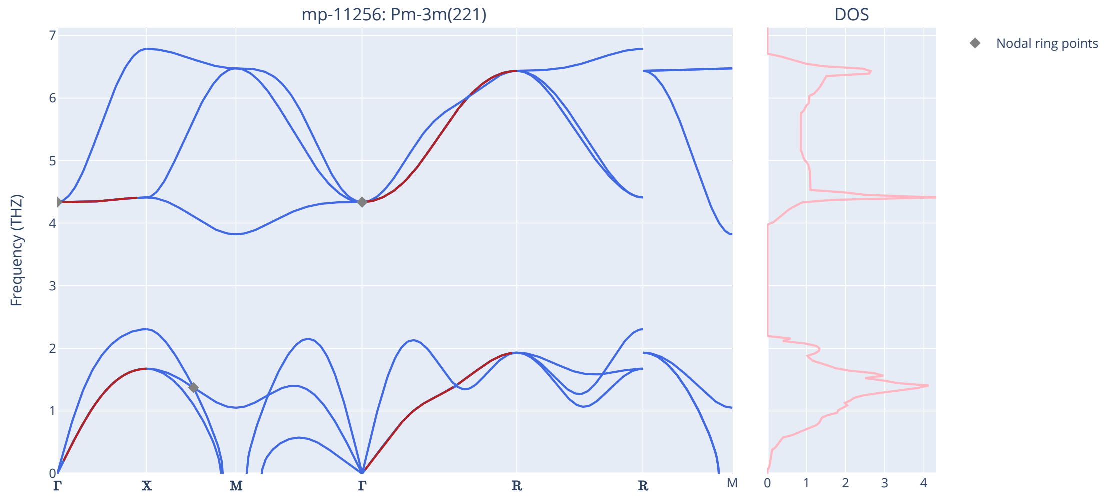

Supplementary Figure 42: The phonon band and DOS of AuSc.

Supplementary Table 61: High symmetry lines of mp-11256-AuSc

	Start	End
0	Γ (0.000, 0.000, 0.000)	X (0.000, 0.500, 0.000)
1	X (0.000, 0.500, 0.000)	M (0.500, 0.500, 0.000)
2	M (0.500, 0.500, 0.000)	Γ (0.000, 0.000, 0.000)
3	Γ (0.000, 0.000, 0.000)	R (0.500, 0.500, 0.500)
4	R (0.500, 0.500, 0.500)	X (0.000, 0.500, 0.000)
5	R (0.500, 0.500, 0.500)	M (0.500, 0.500, 0.000)

Supplementary Table 62: Nodal ring points along high symmetry lines of mp-11256-AuSc.

	Coordinates	Frequency	Mode 1	Mode 2	Multi.	Deg.	Path	Ratio
0	(0.263, 0.500, 0.000)	1.374	3	2	12	2	2	0.525
1	(0.000, 0.000, 0.000)	4.336	6	5	1	3	1	0.000

The followings are details for HgTl. It has 6 topological nodal lines and 7 nodal ring points along high symmetry line as shown in Fig 43.

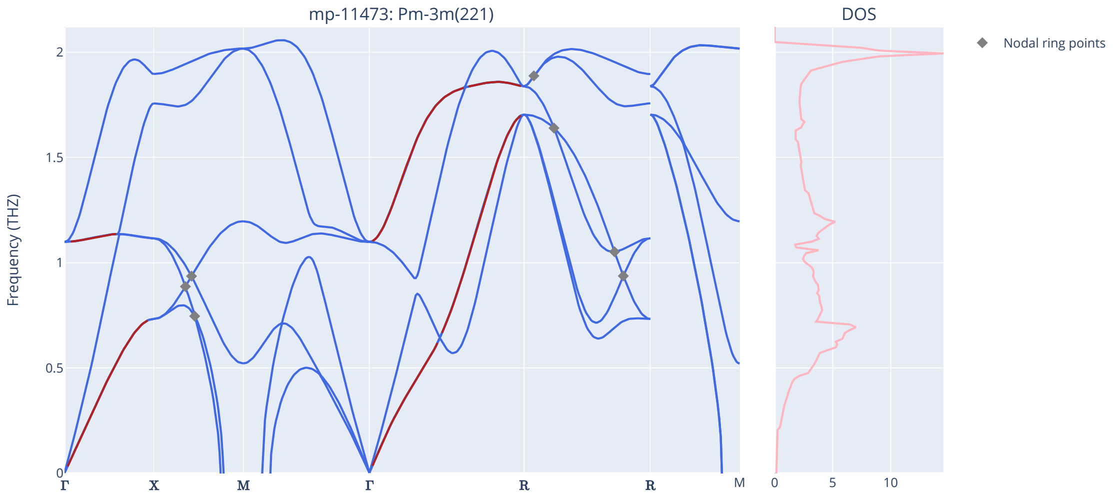

Supplementary Figure 43: The phonon band and DOS of HgTl.

Supplementary Table 63: High symmetry lines of mp-11473-HgTl

	Start	End
0	Γ (0.000, 0.000, 0.000)	X (0.000, 0.500, 0.000)
1	X (0.000, 0.500, 0.000)	M (0.500, 0.500, 0.000)
2	M (0.500, 0.500, 0.000)	Γ (0.000, 0.000, 0.000)
3	Γ (0.000, 0.000, 0.000)	R (0.500, 0.500, 0.500)
4	R (0.500, 0.500, 0.500)	X (0.000, 0.500, 0.000)
5	R (0.500, 0.500, 0.500)	M (0.500, 0.500, 0.000)

Supplementary Table 64: Nodal ring points along high symmetry lines of mp-11473-HgTl.

	Coordinates	Frequency	Mode 1	Mode 2	Multi.	Deg.	Path	Ratio
0	(0.229, 0.500, 0.000)	0.746	2	1	12	2	2	0.457
1	(0.177, 0.500, -0.000)	0.887	3	2	12	2	2	0.354
2	(0.107, 0.500, 0.107)	0.937	3	2	12	2	5	0.786
3	(0.211, 0.500, -0.000)	0.936	4	3	12	2	2	0.422
4	(0.385, 0.500, 0.378)	1.639	4	3	24	2	5	0.237
5	(0.142, 0.500, 0.142)	1.052	4	3	12	2	5	0.717
6	(0.461, 0.500, 0.461)	1.887	6	5	12	2	5	0.078

The followings are details for LaTl. It has 6 topological nodal lines and 6 nodal ring points along high symmetry line as shown in Fig **44**.

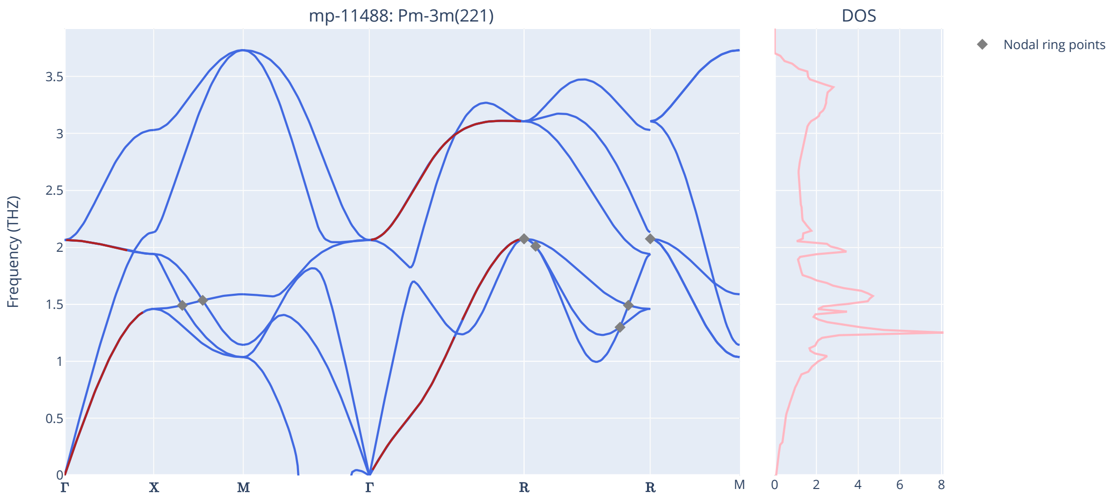

Supplementary Figure 44: The phonon band and DOS of LaTl.

Supplementary Table 65: High symmetry lines of mp-11488-LaTl

	Start	End
0	Γ (0.000, 0.000, 0.000)	X (0.000, 0.500, 0.000)
1	X (0.000, 0.500, 0.000)	M (0.500, 0.500, 0.000)
2	M (0.500, 0.500, 0.000)	Γ (0.000, 0.000, 0.000)
3	Γ (0.000, 0.000, 0.000)	R (0.500, 0.500, 0.500)
4	R (0.500, 0.500, 0.500)	X (0.000, 0.500, 0.000)
5	R (0.500, 0.500, 0.500)	M (0.500, 0.500, 0.000)

Supplementary Table 66: Nodal ring points along high symmetry lines of mp-11488-LaTl.

	Coordinates	Frequency	Mode 1	Mode 2	Multi.	Deg.	Path	Ratio
0	(0.500, 0.500, 0.500)	2.075	2	1	1	3	4	1.000
1	(0.454, 0.500, 0.454)	2.010	2	1	12	2	5	0.092
2	(0.120, 0.500, 0.120)	1.299	2	1	12	2	5	0.760
3	(0.160, 0.500, 0.001)	1.491	3	2	24	2	2	0.319
4	(0.087, 0.500, 0.087)	1.492	3	2	12	2	5	0.827
5	(0.274, 0.500, -0.000)	1.535	4	3	12	2	2	0.548

The followings are details for MgTl. It has 6 topological nodal lines and 2 nodal ring points along high symmetry line as shown in Fig **45**.

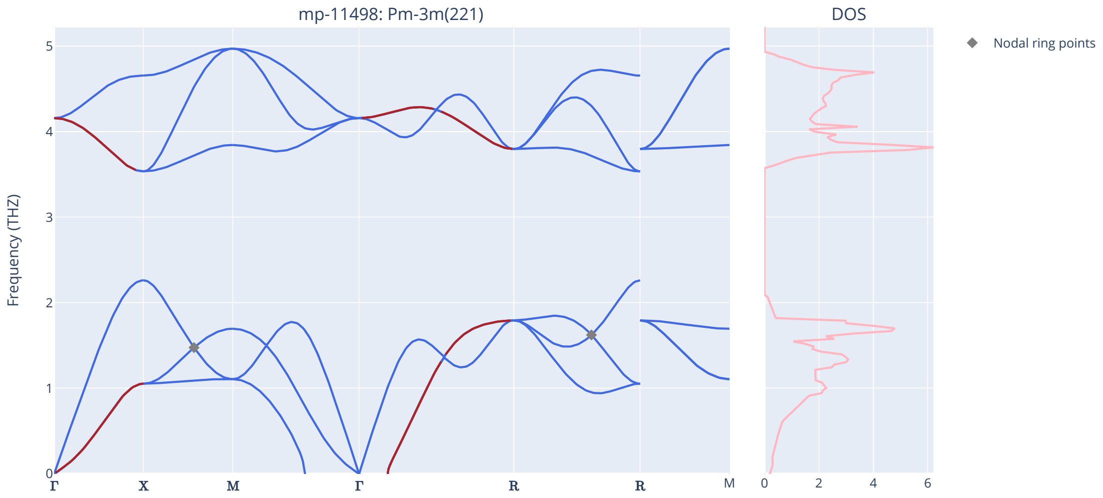

Supplementary Figure 45: The phonon band and DOS of MgTl.

Supplementary Table 67: High symmetry lines of mp-11498-MgTl

	Start	End
0	Γ (0.000, 0.000, 0.000)	X (0.000, 0.500, 0.000)
1	X (0.000, 0.500, 0.000)	M (0.500, 0.500, 0.000)
2	M (0.500, 0.500, 0.000)	Γ (0.000, 0.000, 0.000)
3	Γ (0.000, 0.000, 0.000)	R (0.500, 0.500, 0.500)
4	R (0.500, 0.500, 0.500)	X (0.000, 0.500, 0.000)
5	R (0.500, 0.500, 0.500)	M (0.500, 0.500, 0.000)

Supplementary Table 68: Nodal ring points along high symmetry lines of mp-11498-MgTl.

	Coordinates	Frequency	Mode 1	Mode 2	Multi.	Deg.	Path	Ratio
0	(0.283, 0.500, -0.000)	1.475	3	2	12	2	2	0.567
1	(0.192, 0.500, 0.192)	1.623	3	2	12	2	5	0.615

The followings are details for AuZn. It has 6 topological nodal lines and 2 nodal ring points along high symmetry line as shown in Fig 46.

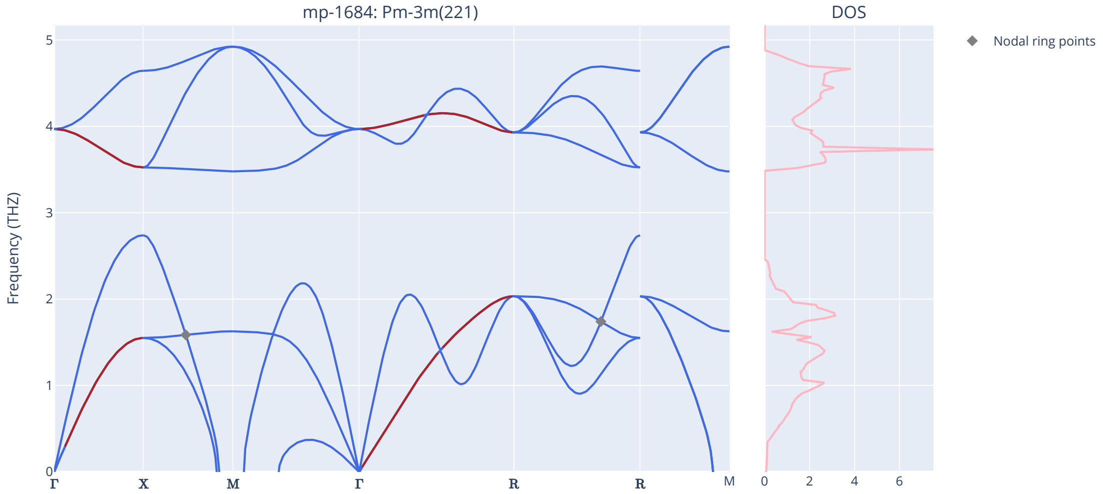

Supplementary Figure 46: The phonon band and DOS of AuZn.

Supplementary Table 69: High symmetry lines of mp-1684-AuZn

	Start	End
0	Γ (0.000, 0.000, 0.000)	X (0.000, 0.500, 0.000)
1	X (0.000, 0.500, 0.000)	M (0.500, 0.500, 0.000)
2	M (0.500, 0.500, 0.000)	Γ (0.000, 0.000, 0.000)
3	Γ (0.000, 0.000, 0.000)	R (0.500, 0.500, 0.500)
4	R (0.500, 0.500, 0.500)	X (0.000, 0.500, 0.000)
5	R (0.500, 0.500, 0.500)	M (0.500, 0.500, 0.000)

Supplementary Table 70: Nodal ring points along high symmetry lines of mp-1684-AuZn.

	Coordinates	Frequency	Mode 1	Mode 2	Multi.	Deg.	Path	Ratio
0	(0.237, 0.500, -0.000)	1.585	3	2	12	2	2	0.475
1	(0.155, 0.500, 0.155)	1.740	3	2	12	2	5	0.690

The followings are details for He_2. It has 12 topological nodal lines and 6 nodal ring points along high symmetry line as shown in Fig **47**.

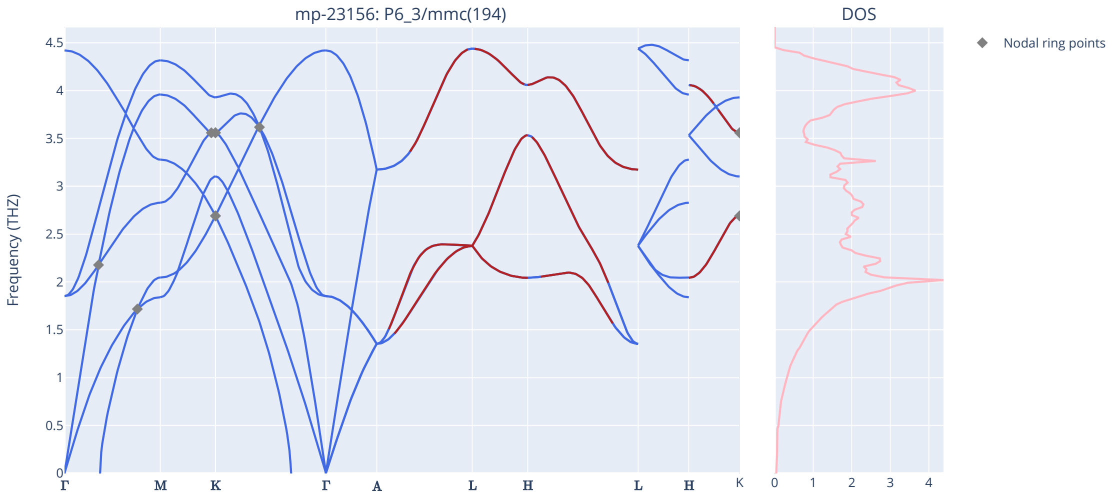

Supplementary Figure 47: The phonon band and DOS of He2.

Supplementary Table 71: High symmetry lines of mp-23156-He2

	Start	End
0	Γ (0.000, 0.000, 0.000)	M (0.500, 0.000, 0.000)
1	M (0.500, 0.000, 0.000)	K (0.333, 0.333, 0.000)
2	K (0.333, 0.333, 0.000)	Γ (0.000, 0.000, 0.000)
3	Γ (0.000, 0.000, 0.000)	A (0.000, 0.000, 0.500)
4	A (0.000, 0.000, 0.500)	L (0.500, 0.000, 0.500)
5	L (0.500, 0.000, 0.500)	H (0.333, 0.333, 0.500)
6	H (0.333, 0.333, 0.500)	A (0.000, 0.000, 0.500)
7	L (0.500, 0.000, 0.500)	M (0.500, 0.000, 0.000)
8	H (0.333, 0.333, 0.500)	K (0.333, 0.333, 0.000)

Supplementary Table 72: Nodal ring points along high symmetry lines of mp-23156-He2.

	Coordinates	Frequency	Mode 1	Mode 2	Multi.	Deg.	Path	Ratio
0	(0.380, 0.001, 0.000)	1.716	2	1	12	2	1	0.761
1	(0.333, 0.333, 0.000)	2.690	2	1	2	2	2	1.000
2	(0.178, 0.000, 0.000)	2.177	4	3	6	2	1	0.355
3	(0.201, 0.201, 0.000)	3.617	5	4	6	2	3	0.398
4	(0.346, 0.308, 0.000)	3.559	5	4	6	2	2	0.925
5	(0.333, 0.333, 0.000)	3.559	5	4	2	2	2	1.000

The followings are details for U_4S_4. It has 3 topological nodal lines and 4 nodal ring points along high symmetry line as shown in Fig **48**.

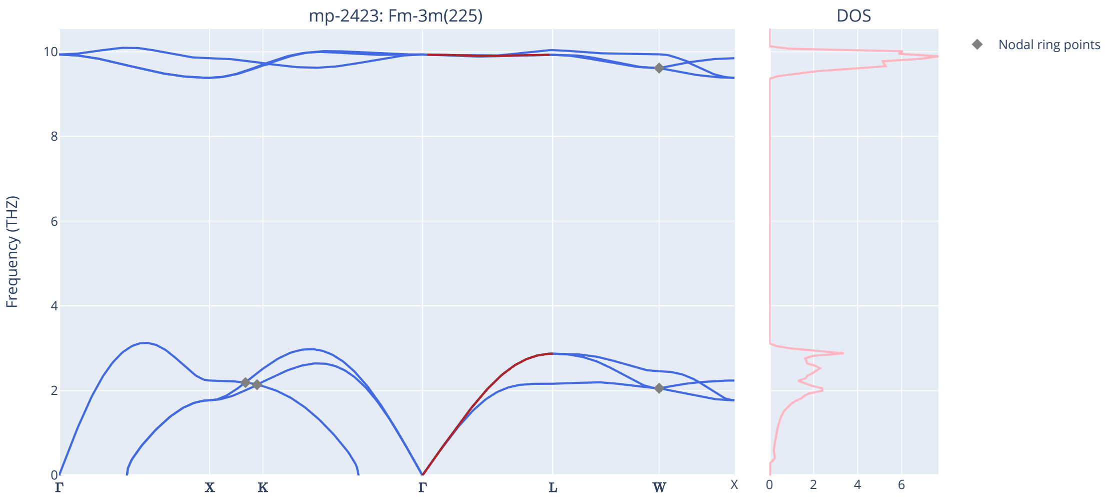

Supplementary Figure 48: The phonon band and DOS of U4S4.

Supplementary Table 73: High symmetry lines of mp-2423-U4S4

	Start	End
0	Γ (0.000, 0.000, 0.000)	X (0.500, 0.000, 0.500)
1	X (0.500, 0.000, 0.500)	U (0.625, 0.250, 0.625)
2	K (0.375, 0.375, 0.750)	Γ (0.000, 0.000, 0.000)
3	Γ (0.000, 0.000, 0.000)	L (0.500, 0.500, 0.500)
4	L (0.500, 0.500, 0.500)	W (0.500, 0.250, 0.750)
5	W (0.500, 0.250, 0.750)	X (0.500, 0.000, 0.500)

Supplementary Table 74: Nodal ring points along high symmetry lines of mp-2423-U4S4.

	Coordinates	Frequency	Mode 1	Mode 2	Multi.	Deg.	Path	Ratio
0	(0.612, 0.223, 0.612)	2.138	2	1	12	2	2	0.892
1	(0.500, 0.250, 0.750)	2.054	2	1	6	2	5	1.000
2	(0.586, 0.167, 0.586)	2.188	3	2	24	2	2	0.675
3	(0.500, 0.250, 0.750)	9.614	5	4	6	2	5	1.000

The followings are details for Tl_4I_4. It has 3 topological nodal lines and 5 nodal ring points along high symmetry line as shown in Fig **49**.

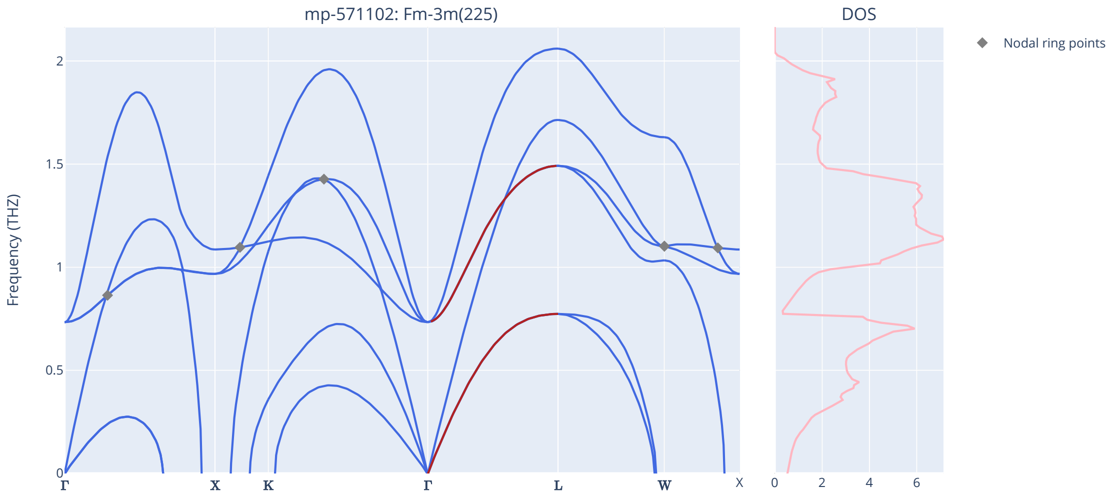

Supplementary Figure 49: The phonon band and DOS of Tl4I4.

Supplementary Table 75: High symmetry lines of mp-571102-Tl4I4

	Start	End
0	Γ (0.000, 0.000, 0.000)	X (0.500, 0.000, 0.500)
1	X (0.500, 0.000, 0.500)	U (0.625, 0.250, 0.625)
2	K (0.375, 0.375, 0.750)	Γ (0.000, 0.000, 0.000)
3	Γ (0.000, 0.000, 0.000)	L (0.500, 0.500, 0.500)
4	L (0.500, 0.500, 0.500)	W (0.500, 0.250, 0.750)
5	W (0.500, 0.250, 0.750)	X (0.500, 0.000, 0.500)

Supplementary Table 76: Nodal ring points along high symmetry lines of mp-571102-Tl4I4.

	Coordinates	Frequency	Mode 1	Mode 2	Multi.	Deg.	Path	Ratio
0	(0.143, 0.000, 0.143)	0.863	4	3	6	2	1	0.286
1	(0.241, 0.241, 0.491)	1.427	5	4	24	2	3	0.350
2	(0.500, 0.250, 0.750)	1.102	5	4	6	2	5	1.000
3	(0.559, 0.117, 0.559)	1.096	6	5	12	2	2	0.470
4	(0.498, 0.071, 0.573)	1.093	6	5	24	2	6	0.710

The followings are details for LiTl. It has 6 topological nodal lines and 2 nodal ring points along high symmetry line as shown in Fig **50**.

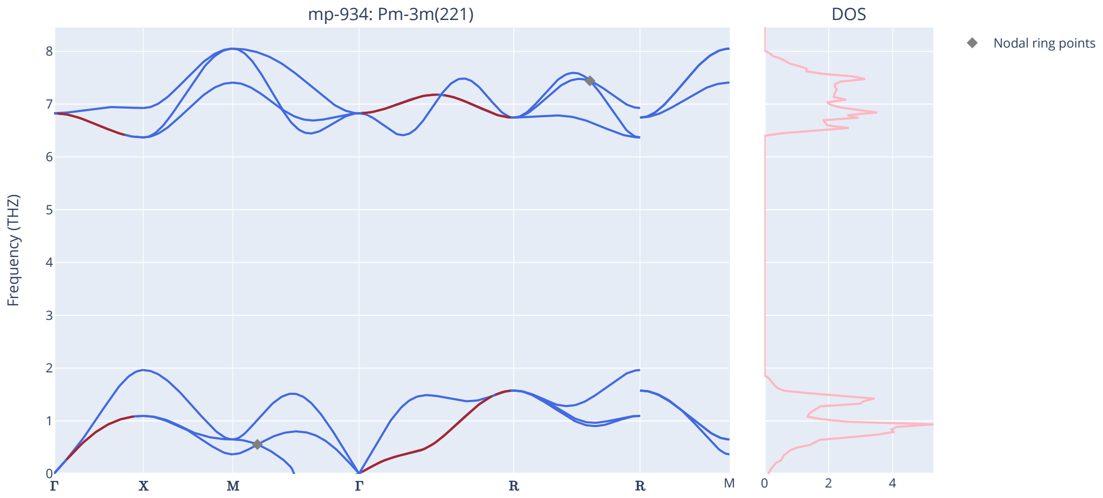

Supplementary Figure 50: The phonon band and DOS of LiTl.

Supplementary Table 77: High symmetry lines of mp-934-LiTl

	Start	End
0	Γ (0.000, 0.000, 0.000)	X (0.000, 0.500, 0.000)
1	X (0.000, 0.500, 0.000)	M (0.500, 0.500, 0.000)
2	M (0.500, 0.500, 0.000)	Γ (0.000, 0.000, 0.000)
3	Γ (0.000, 0.000, 0.000)	R (0.500, 0.500, 0.500)
4	R (0.500, 0.500, 0.500)	X (0.000, 0.500, 0.000)
5	R (0.500, 0.500, 0.500)	M (0.500, 0.500, 0.000)

Supplementary Table 78: Nodal ring points along high symmetry lines of mp-934-LiTl.

	Coordinates	Frequency	Mode 1	Mode 2	Multi.	Deg.	Path	Ratio
0	(0.402, 0.402, -0.000)	0.555	2	1	12	2	3	0.195
1	(0.199, 0.500, 0.199)	7.440	6	5	12	2	5	0.603

The followings are details for Li_2. It has 11 topological nodal lines and 8 nodal ring points along high symmetry line as shown in Fig **51**.

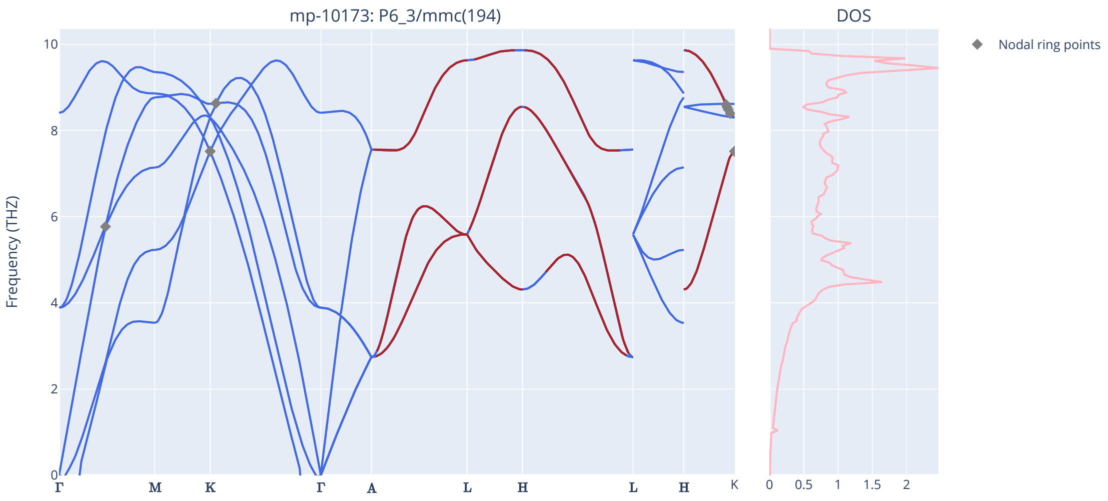

Supplementary Figure 51: The phonon band and DOS of Li2.

Supplementary Table 79: High symmetry lines of mp-10173-Li2

	Start	End
0	Γ (0.000, 0.000, 0.000)	M (0.500, 0.000, 0.000)
1	M (0.500, 0.000, 0.000)	K (0.333, 0.333, 0.000)
2	K (0.333, 0.333, 0.000)	Γ (0.000, 0.000, 0.000)
3	Γ (0.000, 0.000, 0.000)	A (0.000, 0.000, 0.500)
4	A (0.000, 0.000, 0.500)	L (0.500, 0.000, 0.500)
5	L (0.500, 0.000, 0.500)	H (0.333, 0.333, 0.500)
6	H (0.333, 0.333, 0.500)	A (0.000, 0.000, 0.500)
7	L (0.500, 0.000, 0.500)	M (0.500, 0.000, 0.000)
8	H (0.333, 0.333, 0.500)	K (0.333, 0.333, 0.000)

Supplementary Table 80: Nodal ring points along high symmetry lines of mp-10173-Li2.

	Coordinates	Frequency	Mode 1	Mode 2	Multi.	Deg.	Path	Ratio
0	(0.333, 0.333, 0.000)	7.516	2	1	2	2	2	1.000
1	(0.242, 0.000, 0.000)	5.769	4	3	6	2	1	0.485
2	(0.333, 0.333, 0.083)	8.598	5	4	4	2	9	0.834
3	(0.333, 0.333, 0.061)	8.482	5	4	4	2	9	0.879
4	(0.333, 0.333, 0.071)	8.537	5	4	4	2	9	0.857
5	(0.333, 0.333, 0.041)	8.392	5	4	4	2	9	0.918
6	(0.316, 0.316, -0.000)	8.629	6	5	6	2	3	0.052

The followings are details for Lu_4P_4. It has 2 topological nodal lines and 5 nodal ring points along high symmetry line as shown in Fig 52.

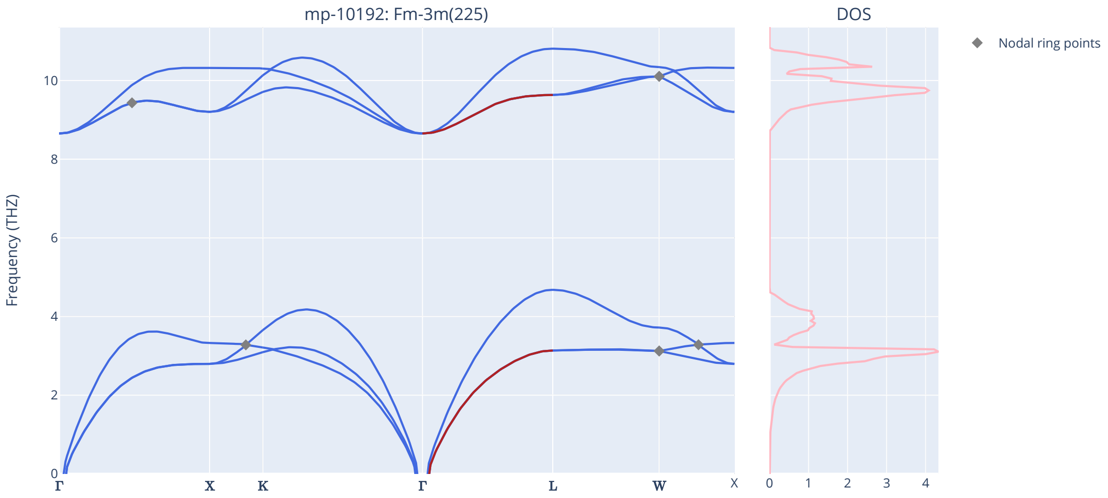

Supplementary Figure 52: The phonon band and DOS of Lu4P4.

Supplementary Table 81: High symmetry lines of mp-10192-Lu4P4

	Start	End
0	Γ (0.000, 0.000, 0.000)	X (0.500, 0.000, 0.500)
1	X (0.500, 0.000, 0.500)	U (0.625, 0.250, 0.625)
2	K (0.375, 0.375, 0.750)	Γ (0.000, 0.000, 0.000)
3	Γ (0.000, 0.000, 0.000)	L (0.500, 0.500, 0.500)
4	L (0.500, 0.500, 0.500)	W (0.500, 0.250, 0.750)
5	W (0.500, 0.250, 0.750)	X (0.500, 0.000, 0.500)

Supplementary Table 82: Nodal ring points along high symmetry lines of mp-10192-Lu4P4.

	Coordinates	Frequency	Mode 1	Mode 2	Multi.	Deg.	Path	Ratio
0	(0.500, 0.250, 0.750)	3.122	2	1	6	2	5	1.000
1	(0.585, 0.170, 0.585)	3.277	3	2	12	2	2	0.681
2	(0.497, 0.118, 0.621)	3.281	3	2	24	2	6	0.522
3	(0.237, -0.000, 0.248)	9.434	5	4	24	2	1	0.484
4	(0.500, 0.250, 0.750)	10.106	5	4	6	2	5	1.000

The followings are details for TlY. It has 6 topological nodal lines and 5 nodal ring points along high symmetry line as shown in Fig 53.

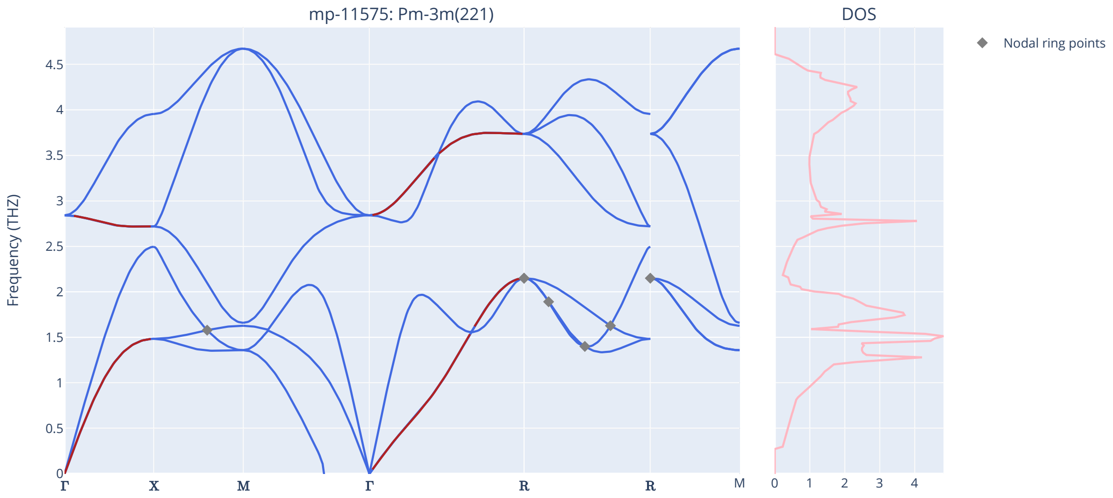

Supplementary Figure 53: The phonon band and DOS of TlY.

Supplementary Table 83: High symmetry lines of mp-11575-TlY

	Start	End
0	Γ (0.000, 0.000, 0.000)	X (0.000, 0.500, 0.000)
1	X (0.000, 0.500, 0.000)	M (0.500, 0.500, 0.000)
2	M (0.500, 0.500, 0.000)	Γ (0.000, 0.000, 0.000)
3	Γ (0.000, 0.000, 0.000)	R (0.500, 0.500, 0.500)
4	R (0.500, 0.500, 0.500)	X (0.000, 0.500, 0.000)
5	R (0.500, 0.500, 0.500)	M (0.500, 0.500, 0.000)

Supplementary Table 84: Nodal ring points along high symmetry lines of mp-11575-TlY.

	Coordinates	Frequency	Mode 1	Mode 2	Multi.	Deg.	Path	Ratio
0	(0.500, 0.500, 0.500)	2.150	2	1	1	3	4	1.000
1	(0.403, 0.500, 0.403)	1.891	2	1	12	2	5	0.195
2	(0.259, 0.500, 0.259)	1.400	2	1	12	2	5	0.482
3	(0.299, 0.500, -0.000)	1.578	3	2	12	2	2	0.597
4	(0.158, 0.500, 0.158)	1.627	3	2	12	2	5	0.684

The followings are details for U_4N_4 and it only has 4 topological nodal ring points along high symmetry line as shown in Fig **54**.

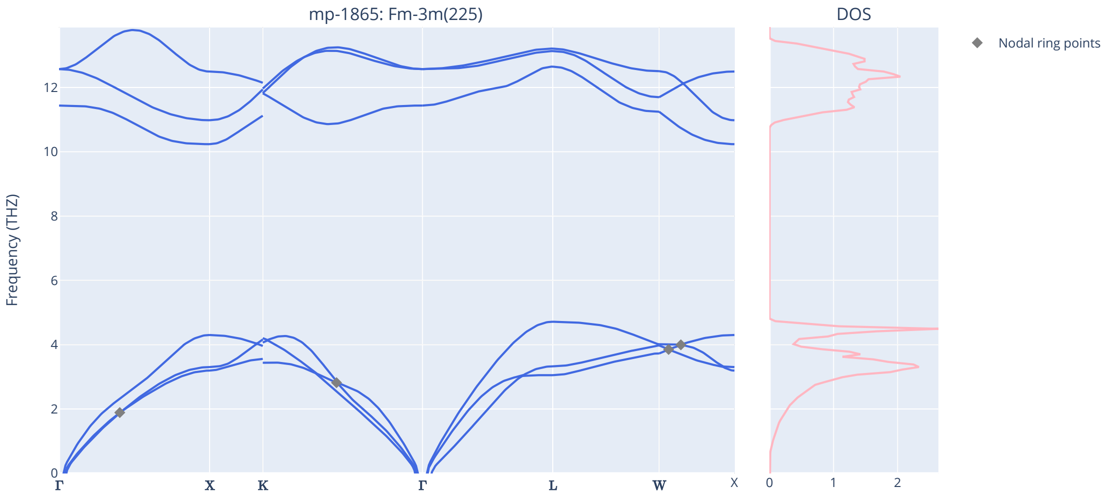

Supplementary Figure 54: The phonon band and DOS of U4N4.

Supplementary Table 85: High symmetry lines of mp-1865-U4N4

	Start	End
0	Γ (0.000, 0.000, 0.000)	X (0.500, 0.000, 0.500)
1	X (0.500, 0.000, 0.500)	U (0.625, 0.250, 0.625)
2	K (0.375, 0.375, 0.750)	Γ (0.000, 0.000, 0.000)
3	Γ (0.000, 0.000, 0.000)	L (0.500, 0.500, 0.500)
4	L (0.500, 0.500, 0.500)	W (0.500, 0.250, 0.750)
5	W (0.500, 0.250, 0.750)	X (0.500, 0.000, 0.500)

Supplementary Table 86: Nodal ring points along high symmetry lines of mp-1865-U4N4.

	Coordinates	Frequency	Mode 1	Mode 2	Multi.	Deg.	Path	Ratio
0	(0.202, -0.002, 0.201)	1.888	2	1	24	2	1	0.403
1	(0.500, 0.219, 0.719)	3.851	2	1	12	2	6	0.125
2	(0.499, 0.177, 0.679)	3.996	3	2	24	2	6	0.288
3	(0.207, 0.207, 0.399)	2.818	3	2	24	2	3	0.462

The followings are details for AlIr and it only has 6 topological nodal lines along high symmetry line as shown in Fig **55**.

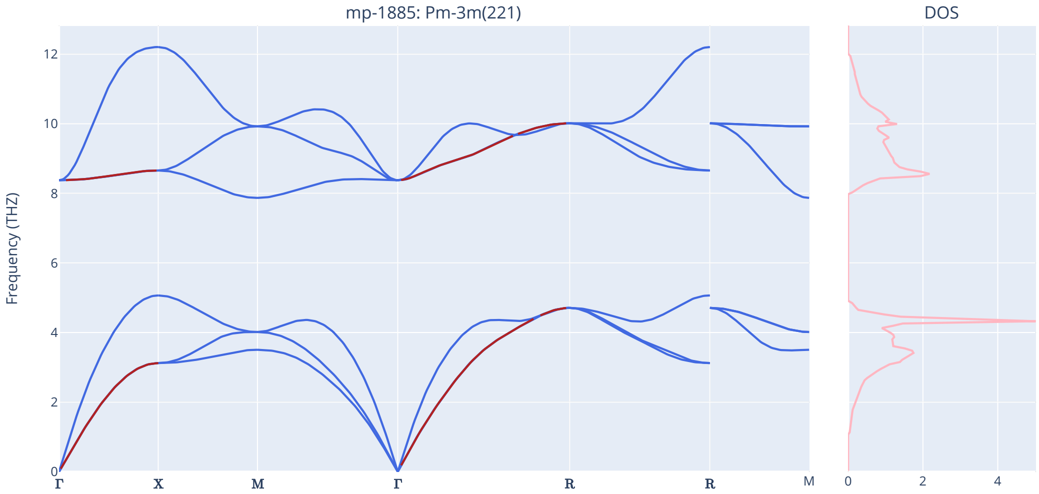

Supplementary Figure 55: The phonon band and DOS of AlIr.

Supplementary Table 87: High symmetry lines of mp-1885-AlIr

	Start	End
0	Γ (0.000, 0.000, 0.000)	X (0.000, 0.500, 0.000)
1	X (0.000, 0.500, 0.000)	M (0.500, 0.500, 0.000)
2	M (0.500, 0.500, 0.000)	Γ (0.000, 0.000, 0.000)
3	Γ (0.000, 0.000, 0.000)	R (0.500, 0.500, 0.500)
4	R (0.500, 0.500, 0.500)	X (0.000, 0.500, 0.000)
5	R (0.500, 0.500, 0.500)	M (0.500, 0.500, 0.000)

The followings are details for InLa. It has 6 topological nodal lines and 5 nodal ring points along high symmetry line as shown in Fig 56.

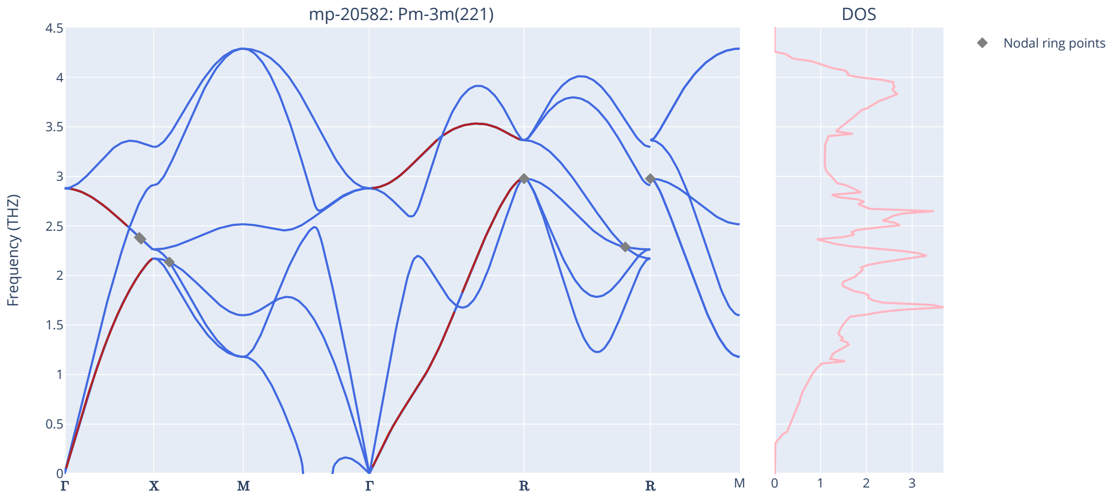

Supplementary Figure 56: The phonon band and DOS of InLa.

Supplementary Table 88: High symmetry lines of mp-20582-InLa

	Start	End
0	Γ (0.000, 0.000, 0.000)	X (0.000, 0.500, 0.000)
1	X (0.000, 0.500, 0.000)	M (0.500, 0.500, 0.000)
2	M (0.500, 0.500, 0.000)	Γ (0.000, 0.000, 0.000)
3	Γ (0.000, 0.000, 0.000)	R (0.500, 0.500, 0.500)
4	R (0.500, 0.500, 0.500)	X (0.000, 0.500, 0.000)
5	R (0.500, 0.500, 0.500)	M (0.500, 0.500, 0.000)

Supplementary Table 89: Nodal ring points along high symmetry lines of mp-20582-InLa.

	Coordinates	Frequency	Mode 1	Mode 2	Multi.	Deg.	Path	Ratio
0	(0.500, 0.500, 0.500)	2.978	2	1	1	3	4	1.000
1	(0.087, 0.500, 0.000)	2.136	3	2	12	2	2	0.174
2	(0.000, 0.418, 0.000)	2.385	4	3	6	2	1	0.837
3	(0.000, 0.429, 0.000)	2.367	4	3	6	2	1	0.857
4	(0.099, 0.500, 0.099)	2.288	4	3	12	2	5	0.802

The followings are details for U_4Se_4. It has 4 topological nodal lines and 4 nodal ring points along high symmetry line as shown in Fig 57.

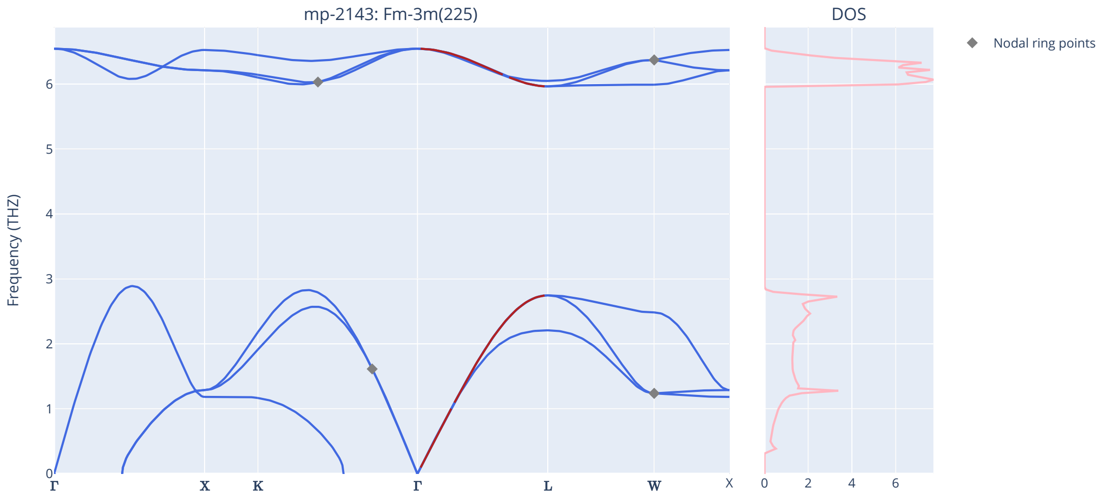

Supplementary Figure 57: The phonon band and DOS of U4Se4.

Supplementary Table 90: High symmetry lines of mp-2143-U4Se4

	Start	End
0	Γ (0.000, 0.000, 0.000)	X (0.500, 0.000, 0.500)
1	X (0.500, 0.000, 0.500)	U (0.625, 0.250, 0.625)
2	K (0.375, 0.375, 0.750)	Γ (0.000, 0.000, 0.000)
3	Γ (0.000, 0.000, 0.000)	L (0.500, 0.500, 0.500)
4	L (0.500, 0.500, 0.500)	W (0.500, 0.250, 0.750)
5	W (0.500, 0.250, 0.750)	X (0.500, 0.000, 0.500)

Supplementary Table 91: Nodal ring points along high symmetry lines of mp-2143-U4Se4.

	Coordinates	Frequency	Mode 1	Mode 2	Multi.	Deg.	Path	Ratio
0	(0.500, 0.250, 0.750)	1.238	2	1	6	2	5	1.000
1	(0.107, 0.107, 0.212)	1.613	3	2	24	2	3	0.717
2	(0.237, 0.237, 0.464)	6.032	5	4	24	2	3	0.377
3	(0.500, 0.250, 0.750)	6.371	6	5	6	2	5	1.000

The followings are details for LuPd. It has 6 topological nodal lines and 1 nodal ring point along high symmetry line as shown in Fig **58**.

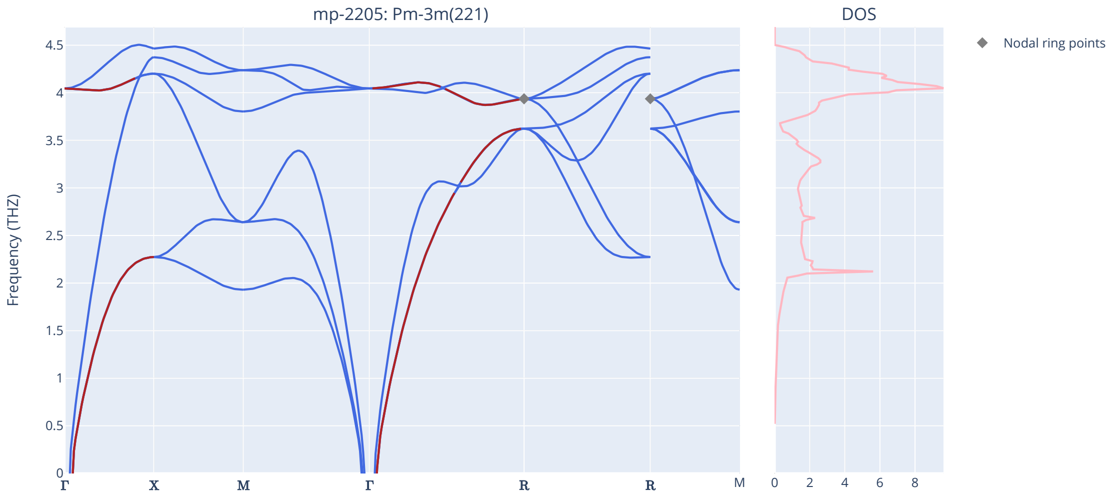

Supplementary Figure 58: The phonon band and DOS of LuPd.

Supplementary Table 92: High symmetry lines of mp-2205-LuPd

	Start	End
0	Γ (0.000, 0.000, 0.000)	X (0.000, 0.500, 0.000)
1	X (0.000, 0.500, 0.000)	M (0.500, 0.500, 0.000)
2	M (0.500, 0.500, 0.000)	Γ (0.000, 0.000, 0.000)
3	Γ (0.000, 0.000, 0.000)	R (0.500, 0.500, 0.500)
4	R (0.500, 0.500, 0.500)	X (0.000, 0.500, 0.000)
5	R (0.500, 0.500, 0.500)	M (0.500, 0.500, 0.000)

Supplementary Table 93: Nodal ring points along high symmetry lines of mp-2205-LuPd.

	Coordinates	Frequency	Mode 1	Mode 2	Multi.	Deg.	Path	Ratio
0	(0.500, 0.500, 0.500)	3.937	6	5	1	3	4	1.000

The followings are details for InY. It has 6 topological nodal lines and 6 nodal ring points along high symmetry line as shown in Fig 59.

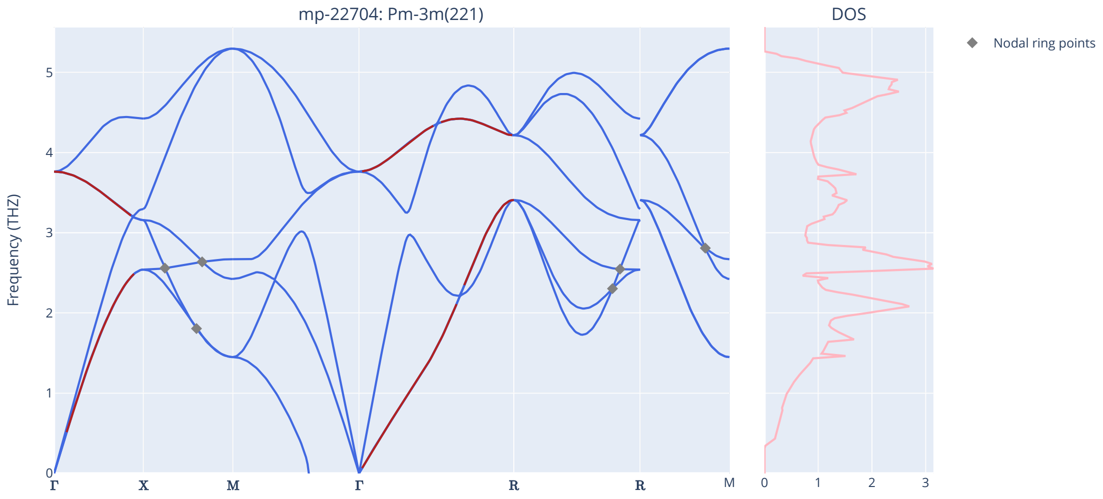

Supplementary Figure 59: The phonon band and DOS of InY.

Supplementary Table 94: High symmetry lines of mp-22704-InY

	Start	End
0	Γ (0.000, 0.000, 0.000)	X (0.000, 0.500, 0.000)
1	X (0.000, 0.500, 0.000)	M (0.500, 0.500, 0.000)
2	M (0.500, 0.500, 0.000)	Γ (0.000, 0.000, 0.000)
3	Γ (0.000, 0.000, 0.000)	R (0.500, 0.500, 0.500)
4	R (0.500, 0.500, 0.500)	X (0.000, 0.500, 0.000)
5	R (0.500, 0.500, 0.500)	M (0.500, 0.500, 0.000)

Supplementary Table 95: Nodal ring points along high symmetry lines of mp-22704-InY.

	Coordinates	Frequency	Mode 1	Mode 2	Multi.	Deg.	Path	Ratio
0	(0.297, 0.500, -0.000)	1.805	2	1	12	2	2	0.594
1	(0.109, 0.500, 0.109)	2.303	2	1	12	2	5	0.781
2	(0.120, 0.500, 0.000)	2.558	3	2	12	2	2	0.241
3	(0.080, 0.500, 0.080)	2.546	3	2	12	2	5	0.841
4	(0.329, 0.500, -0.000)	2.637	4	3	12	2	2	0.658
5	(0.498, 0.500, 0.135)	2.808	4	3	24	2	6	0.730

The followings are details for Au_2. It has 12 topological nodal lines and 7 nodal ring points along high symmetry line as shown in Fig **60**.

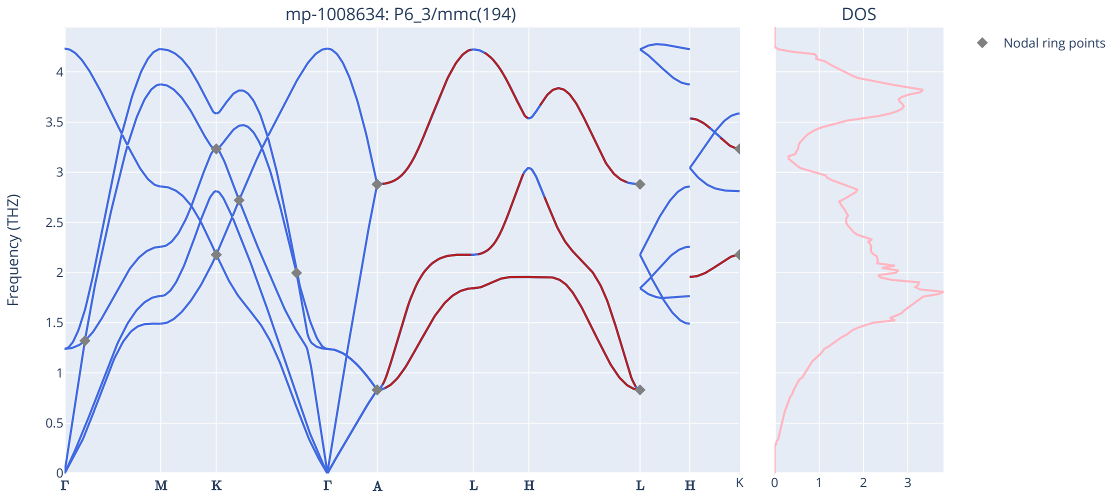

Supplementary Figure 60: The phonon band and DOS of Au2.

Supplementary Table 96: High symmetry lines of mp-1008634-Au2

	Start	End
0	Γ (0.000, 0.000, 0.000)	M (0.500, 0.000, 0.000)
1	M (0.500, 0.000, 0.000)	K (0.333, 0.333, 0.000)
2	K (0.333, 0.333, 0.000)	Γ (0.000, 0.000, 0.000)
3	Γ (0.000, 0.000, 0.000)	A (0.000, 0.000, 0.500)
4	A (0.000, 0.000, 0.500)	L (0.500, 0.000, 0.500)
5	L (0.500, 0.000, 0.500)	H (0.333, 0.333, 0.500)
6	H (0.333, 0.333, 0.500)	A (0.000, 0.000, 0.500)
7	L (0.500, 0.000, 0.500)	M (0.500, 0.000, 0.000)
8	H (0.333, 0.333, 0.500)	K (0.333, 0.333, 0.000)

Supplementary Table 97: Nodal ring points along high symmetry lines of mp-1008634-Au2.

	Coordinates	Frequency	Mode 1	Mode 2	Multi.	Deg.	Path	Ratio
0	(0.333, 0.333, 0.000)	2.178	2	1	2	2	2	1.000
1	(0.000, 0.000, 0.500)	0.831	2	1	1	4	4	1.000
2	(0.106, 0.000, 0.000)	1.320	4	3	6	2	1	0.212
3	(0.265, 0.265, 0.000)	2.721	4	3	6	2	3	0.205
4	(0.093, 0.093, -0.000)	1.997	5	4	6	2	3	0.722
5	(0.333, 0.333, 0.000)	3.233	5	4	2	2	2	1.000
6	(0.000, 0.000, 0.500)	2.880	6	5	1	2	4	1.000

The followings are details for Mn_4N_4. It has 2 topological nodal lines and 1 nodal ring point along high symmetry line as shown in Fig **61**.

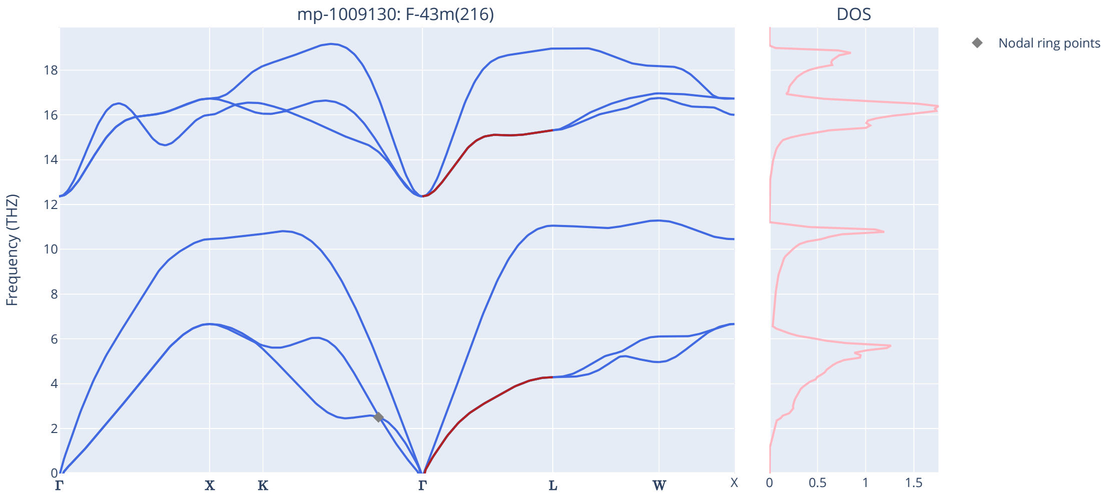

Supplementary Figure 61: The phonon band and DOS of Mn4N4.

Supplementary Table 98: High symmetry lines of mp-1009130-Mn4N4

	Start	End
0	Γ (0.000, 0.000, 0.000)	X (0.500, 0.000, 0.500)
1	X (0.500, 0.000, 0.500)	U (0.625, 0.250, 0.625)
2	K (0.375, 0.375, 0.750)	Γ (0.000, 0.000, 0.000)
3	Γ (0.000, 0.000, 0.000)	L (0.500, 0.500, 0.500)
4	L (0.500, 0.500, 0.500)	W (0.500, 0.250, 0.750)
5	W (0.500, 0.250, 0.750)	X (0.500, 0.000, 0.500)

Supplementary Table 99: Nodal ring points along high symmetry lines of mp-1009130-Mn4N4.

	Coordinates	Frequency	Mode 1	Mode 2	Multi.	Deg.	Path	Ratio
0	(0.107, 0.107, 0.202)	2.504	2	1	24	2	3	0.725

The followings are details for Np_4P_4. It has 2 topological nodal lines and 3 nodal ring points along high symmetry line as shown in Fig 62.

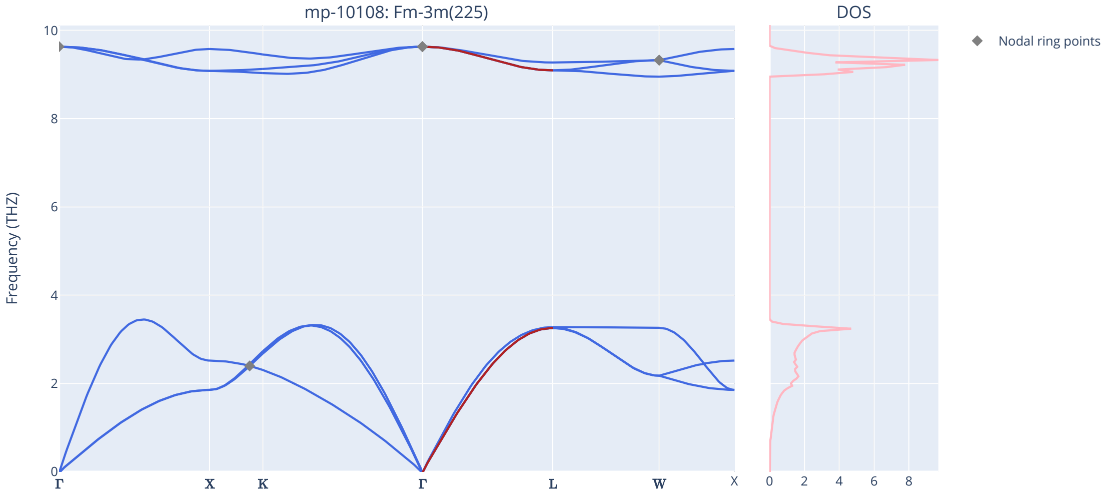

Supplementary Figure 62: The phonon band and DOS of Np4P4.

Supplementary Table 100: High symmetry lines of mp-10108-Np4P4

	Start	End
0	Γ (0.000, 0.000, 0.000)	X (0.500, 0.000, 0.500)
1	X (0.500, 0.000, 0.500)	U (0.625, 0.250, 0.625)
2	K (0.375, 0.375, 0.750)	Γ (0.000, 0.000, 0.000)
3	Γ (0.000, 0.000, 0.000)	L (0.500, 0.500, 0.500)
4	L (0.500, 0.500, 0.500)	W (0.500, 0.250, 0.750)
5	W (0.500, 0.250, 0.750)	X (0.500, 0.000, 0.500)

Supplementary Table 101: Nodal ring points along high symmetry lines of mp-10108-Np4P4.

	Coordinates	Frequency	Mode 1	Mode 2	Multi.	Deg.	Path	Ratio
0	(0.594, 0.188, 0.594)	2.397	2	1	12	2	2	0.753
1	(0.000, 0.000, 0.000)	9.631	6	5	1	3	1	0.000
2	(0.500, 0.250, 0.750)	9.324	6	5	6	2	5	1.000

The followings are details for Np_4S_4. It has 3 topological nodal lines and 5 nodal ring points along high symmetry line as shown in Fig 63.

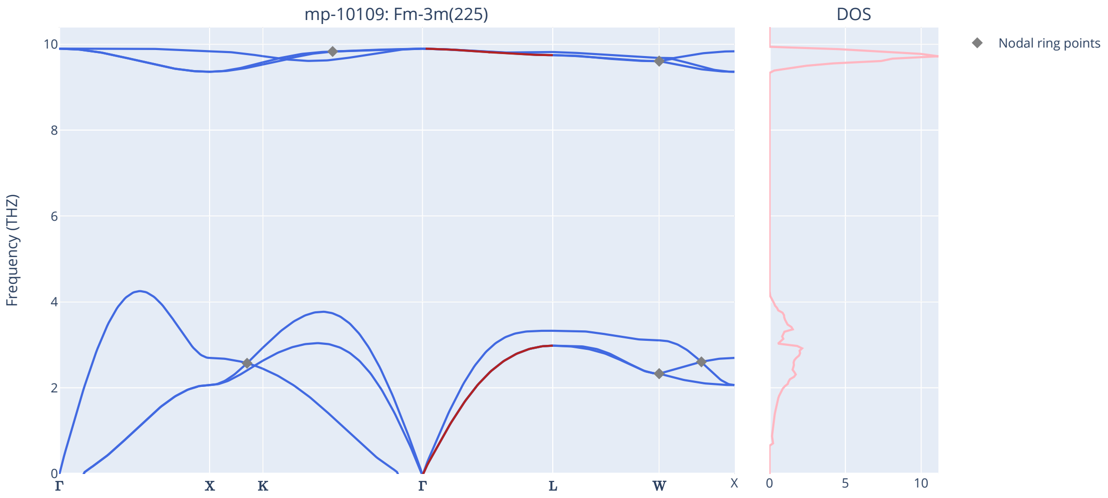

Supplementary Figure 63: The phonon band and DOS of Np4S4.

Supplementary Table 102: High symmetry lines of mp-10109-Np4S4

	Start	End
0	Γ (0.000, 0.000, 0.000)	X (0.500, 0.000, 0.500)
1	X (0.500, 0.000, 0.500)	U (0.625, 0.250, 0.625)
2	K (0.375, 0.375, 0.750)	Γ (0.000, 0.000, 0.000)
3	Γ (0.000, 0.000, 0.000)	L (0.500, 0.500, 0.500)
4	L (0.500, 0.500, 0.500)	W (0.500, 0.250, 0.750)
5	W (0.500, 0.250, 0.750)	X (0.500, 0.000, 0.500)

Supplementary Table 103: Nodal ring points along high symmetry lines of mp-10109-Np4S4.

	Coordinates	Frequency	Mode 1	Mode 2	Multi.	Deg.	Path	Ratio
0	(0.500, 0.250, 0.750)	2.330	2	1	6	2	5	1.000
1	(0.588, 0.176, 0.588)	2.573	3	2	12	2	2	0.704
2	(0.498, 0.109, 0.611)	2.604	3	2	24	2	6	0.561
3	(0.500, 0.250, 0.750)	9.605	5	4	6	2	5	1.000
4	(0.213, 0.213, 0.419)	9.829	6	5	24	2	3	0.437

The followings are details for HfZn. It has 6 topological nodal lines and 3 nodal ring points along high symmetry line as shown in Fig **64**.

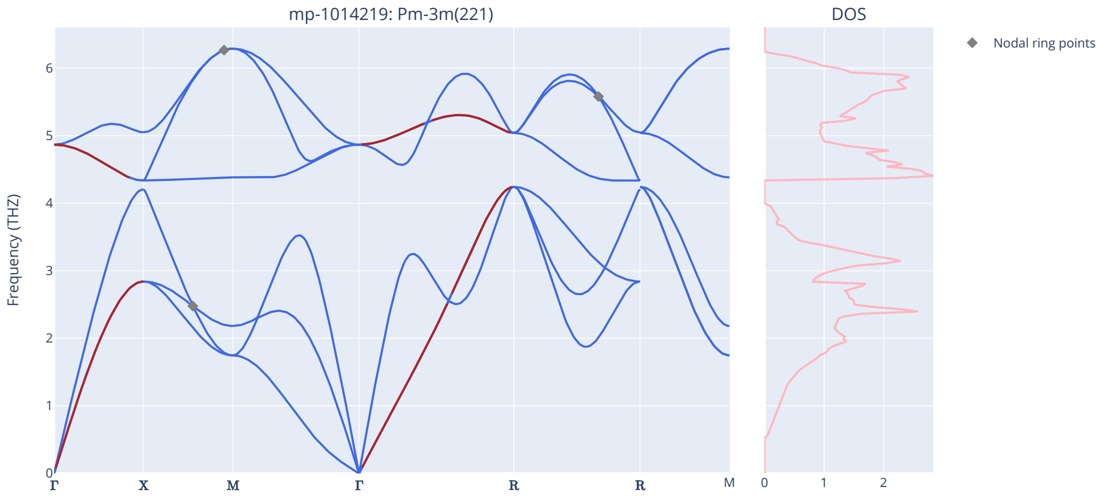

Supplementary Figure 64: The phonon band and DOS of HfZn.

Supplementary Table 104: High symmetry lines of mp-1014219-HfZn

	Start	End
0	Γ (0.000, 0.000, 0.000)	X (0.000, 0.500, 0.000)
1	X (0.000, 0.500, 0.000)	M (0.500, 0.500, 0.000)
2	M (0.500, 0.500, 0.000)	Γ (0.000, 0.000, 0.000)
3	Γ (0.000, 0.000, 0.000)	R (0.500, 0.500, 0.500)
4	R (0.500, 0.500, 0.500)	X (0.000, 0.500, 0.000)
5	R (0.500, 0.500, 0.500)	M (0.500, 0.500, 0.000)

Supplementary Table 105: Nodal ring points along high symmetry lines of mp-1014219-HfZn.

	Coordinates	Frequency	Mode 1	Mode 2	Multi.	Deg.	Path	Ratio
0	(0.277, 0.500, -0.000)	2.477	3	2	12	2	2	0.553
1	(0.165, 0.500, 0.165)	5.578	6	5	12	2	5	0.671
2	(0.451, 0.500, 0.000)	6.266	6	5	12	2	2	0.903

The followings are details for TiZn. It has 6 topological nodal lines and 3 nodal ring points along high symmetry line as shown in Fig 65.

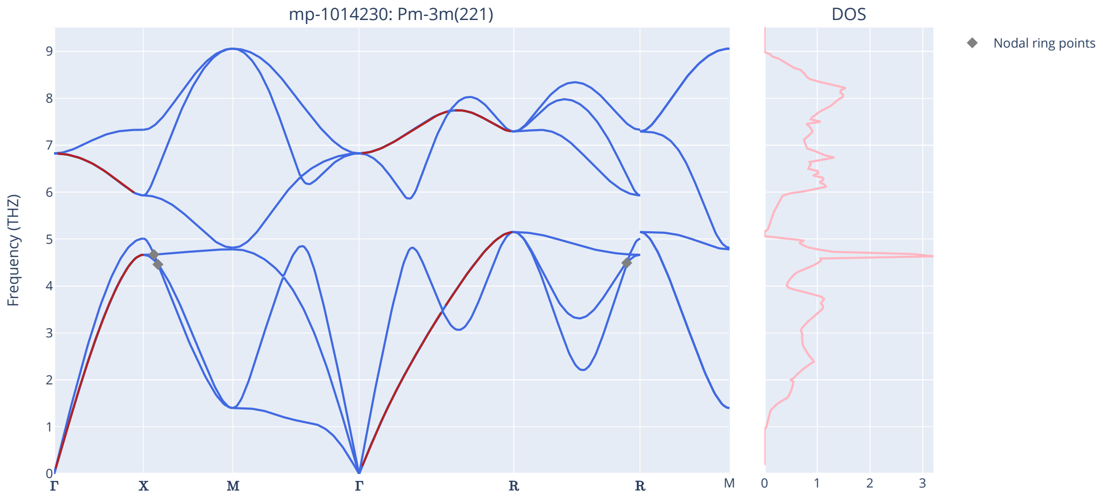

Supplementary Figure 65: The phonon band and DOS of TiZn.

Supplementary Table 106: High symmetry lines of mp-1014230-TiZn

	Start	End
0	Γ (0.000, 0.000, 0.000)	X (0.000, 0.500, 0.000)
1	X (0.000, 0.500, 0.000)	M (0.500, 0.500, 0.000)
2	M (0.500, 0.500, 0.000)	Γ (0.000, 0.000, 0.000)
3	Γ (0.000, 0.000, 0.000)	R (0.500, 0.500, 0.500)
4	R (0.500, 0.500, 0.500)	X (0.000, 0.500, 0.000)
5	R (0.500, 0.500, 0.500)	M (0.500, 0.500, 0.000)

Supplementary Table 107: Nodal ring points along high symmetry lines of mp-1014230-TiZn.

	Coordinates	Frequency	Mode 1	Mode 2	Multi.	Deg.	Path	Ratio
0	(0.081, 0.500, -0.000)	4.460	2	1	12	2	2	0.163
1	(0.053, 0.500, 0.053)	4.492	2	1	12	2	5	0.894
2	(0.059, 0.500, -0.000)	4.670	3	2	12	2	2	0.118

The followings are details for Na_2. It has 11 topological nodal lines and 11 nodal ring points along high symmetry line as shown in Fig 66.

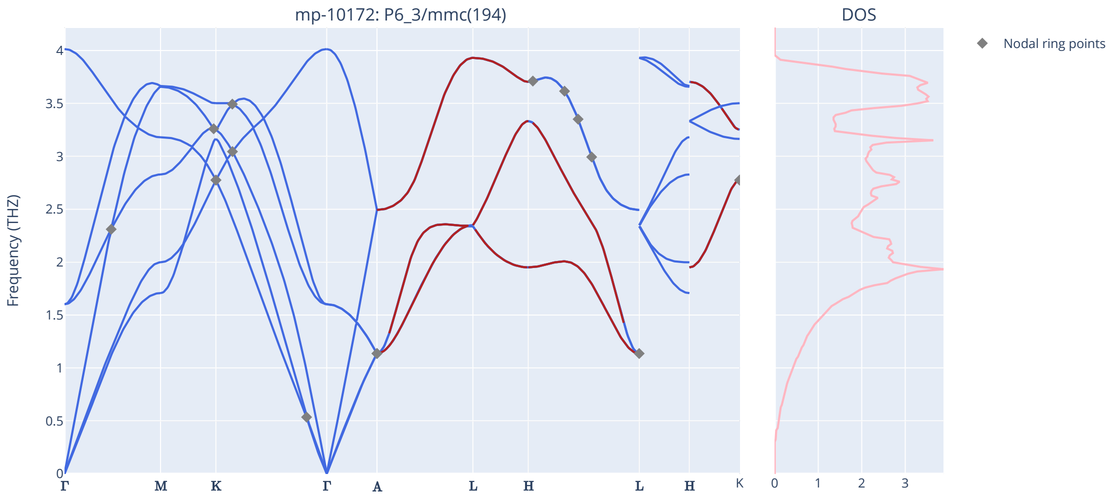

Supplementary Figure 66: The phonon band and DOS of Na2.

Supplementary Table 108: High symmetry lines of mp-10172-Na2

	Start	End
0	Γ (0.000, 0.000, 0.000)	M (0.500, 0.000, 0.000)
1	M (0.500, 0.000, 0.000)	K (0.333, 0.333, 0.000)
2	K (0.333, 0.333, 0.000)	Γ (0.000, 0.000, 0.000)
3	Γ (0.000, 0.000, 0.000)	A (0.000, 0.000, 0.500)
4	A (0.000, 0.000, 0.500)	L (0.500, 0.000, 0.500)
5	L (0.500, 0.000, 0.500)	H (0.333, 0.333, 0.500)
6	H (0.333, 0.333, 0.500)	A (0.000, 0.000, 0.500)
7	L (0.500, 0.000, 0.500)	M (0.500, 0.000, 0.000)
8	H (0.333, 0.333, 0.500)	K (0.333, 0.333, 0.000)

Supplementary Table 109: Nodal ring points along high symmetry lines of mp-10172-Na2.

	Coordinates	Frequency	Mode 1	Mode 2	Multi.	Deg.	Path	Ratio
0	(0.333, 0.333, 0.000)	2.775	2	1	2	2	2	1.000
1	(0.061, 0.061, 0.000)	0.535	2	1	6	2	3	0.818
2	(0.243, -0.000, -0.000)	2.311	4	3	6	2	1	0.486
3	(0.284, 0.284, 0.000)	3.045	4	3	6	2	3	0.149
4	(0.000, 0.000, 0.500)	1.136	4	3	1	4	4	1.000
5	(0.340, 0.320, 0.000)	3.260	5	4	6	2	2	0.959
6	(0.284, 0.284, -0.000)	3.493	6	5	6	2	3	0.148
7	(0.320, 0.320, 0.500)	3.711	6	5	6	2	7	0.041
8	(0.224, 0.224, 0.500)	3.616	6	5	6	2	7	0.327
9	(0.184, 0.184, 0.500)	3.351	6	5	6	2	7	0.449
10	(0.143, 0.143, 0.500)	2.994	6	5	6	2	7	0.571

The followings are details for VN and it only has 4 single Weyl point along high symmetry line as shown in Fig 67.

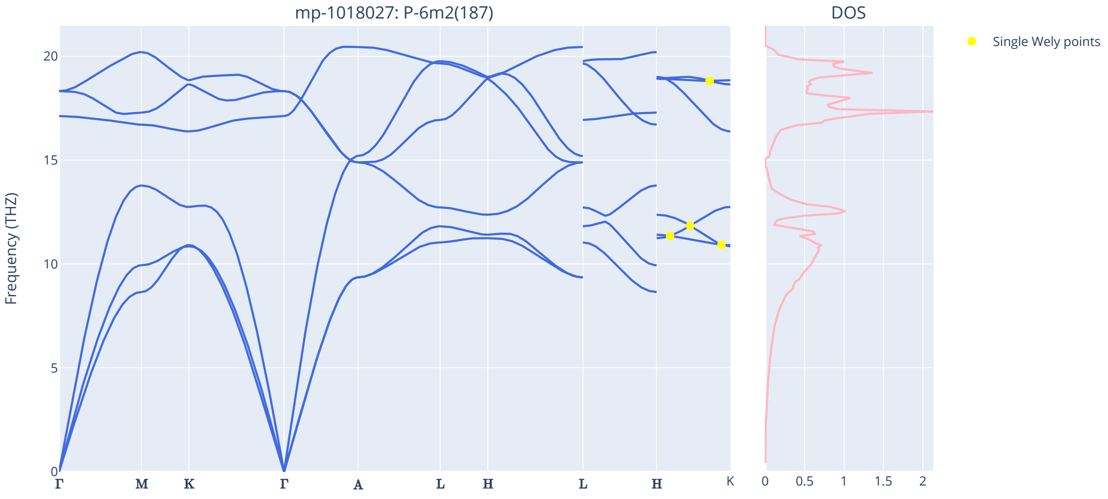

Supplementary Figure 67: The phonon band and DOS of VN.

Supplementary Table 110: High symmetry lines of mp-1018027-VN

	Start	End
0	Γ (0.000, 0.000, 0.000)	M (0.500, 0.000, 0.000)
1	M (0.500, 0.000, 0.000)	K (0.333, 0.333, 0.000)
2	K (0.333, 0.333, 0.000)	Γ (0.000, 0.000, 0.000)
3	Γ (0.000, 0.000, 0.000)	A (0.000, 0.000, 0.500)
4	A (0.000, 0.000, 0.500)	L (0.500, 0.000, 0.500)
5	L (0.500, 0.000, 0.500)	H (0.333, 0.333, 0.500)
6	H (0.333, 0.333, 0.500)	A (0.000, 0.000, 0.500)
7	L (0.500, 0.000, 0.500)	M (0.500, 0.000, 0.000)
8	H (0.333, 0.333, 0.500)	K (0.333, 0.333, 0.000)

Supplementary Table 111: Single Weyl points along high symmetry lines of mp-1018027-VN.

	Coordinates	Frequency	Mode 1	Mode 2	Multi.	Deg.	Path	Ratio
0	(0.333, 0.333, 0.409)	11.354	2	1	4	2	9	0.183
1	(0.333, 0.333, 0.060)	10.924	2	1	4	2	9	0.879
2	(0.333, 0.333, 0.273)	11.843	3	2	4	2	9	0.455
3	(0.333, 0.333, 0.141)	18.811	6	5	4	2	9	0.718

The followings are details for TiS. It has 2 nodal ring points and 5 single Weyl points along high symmetry line as shown in Fig 68.

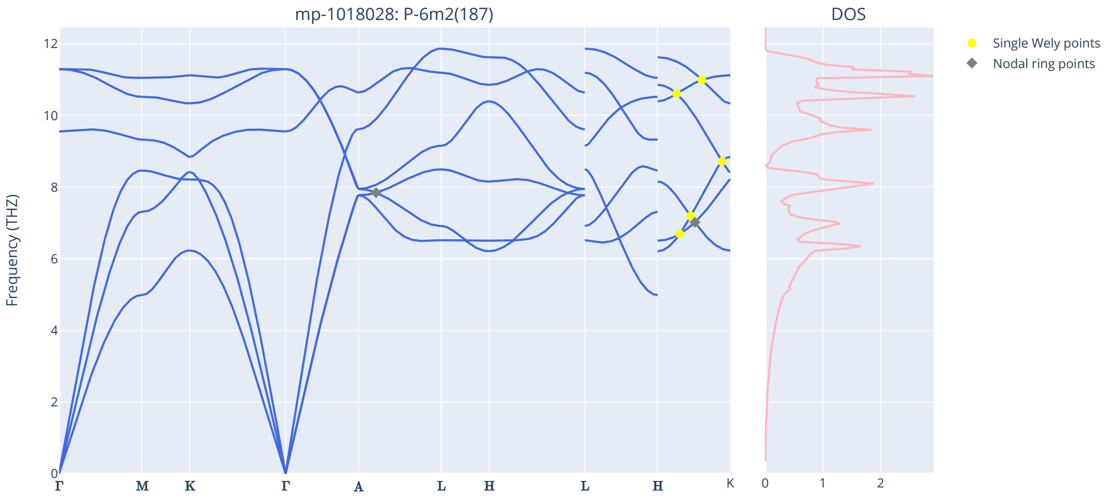

Supplementary Figure 68: The phonon band and DOS of TiS.

Supplementary Table 112: High symmetry lines of mp-1018028-TiS

	Start	End
0	Γ (0.000, 0.000, 0.000)	M (0.500, 0.000, 0.000)
1	M (0.500, 0.000, 0.000)	K (0.333, 0.333, 0.000)
2	K (0.333, 0.333, 0.000)	Γ (0.000, 0.000, 0.000)
3	Γ (0.000, 0.000, 0.000)	A (0.000, 0.000, 0.500)
4	A (0.000, 0.000, 0.500)	L (0.500, 0.000, 0.500)
5	L (0.500, 0.000, 0.500)	H (0.333, 0.333, 0.500)
6	H (0.333, 0.333, 0.500)	A (0.000, 0.000, 0.500)
7	L (0.500, 0.000, 0.500)	M (0.500, 0.000, 0.000)
8	H (0.333, 0.333, 0.500)	K (0.333, 0.333, 0.000)

Supplementary Table 113: Single Weyl points along high symmetry lines of mp-1018028-TiS.

	Coordinates	Frequency	Mode 1	Mode 2	Multi.	Deg.	Path	Ratio
0	(0.333, 0.333, 0.347)	6.693	2	1	4	2	9	0.306
1	(0.333, 0.333, 0.273)	7.189	3	2	4	2	9	0.454
2	(0.333, 0.333, 0.056)	8.713	4	3	4	2	9	0.887
3	(0.333, 0.333, 0.369)	10.609	5	4	4	2	9	0.261
4	(0.333, 0.333, 0.196)	10.990	6	5	4	2	9	0.609

Supplementary Table 114: Nodal ring points along high symmetry lines of mp-1018028-TiS.

	Coordinates	Frequency	Mode 1	Mode 2	Multi.	Deg.	Path	Ratio
0	(0.333, 0.333, 0.244)	7.019	2	1	4	2	9	0.513
1	(0.107, -0.002, 0.500)	7.844	3	2	12	2	5	0.214

The followings are details for CuPd. It has 6 topological nodal lines and 6 nodal ring points along high symmetry line as shown in Fig 69.

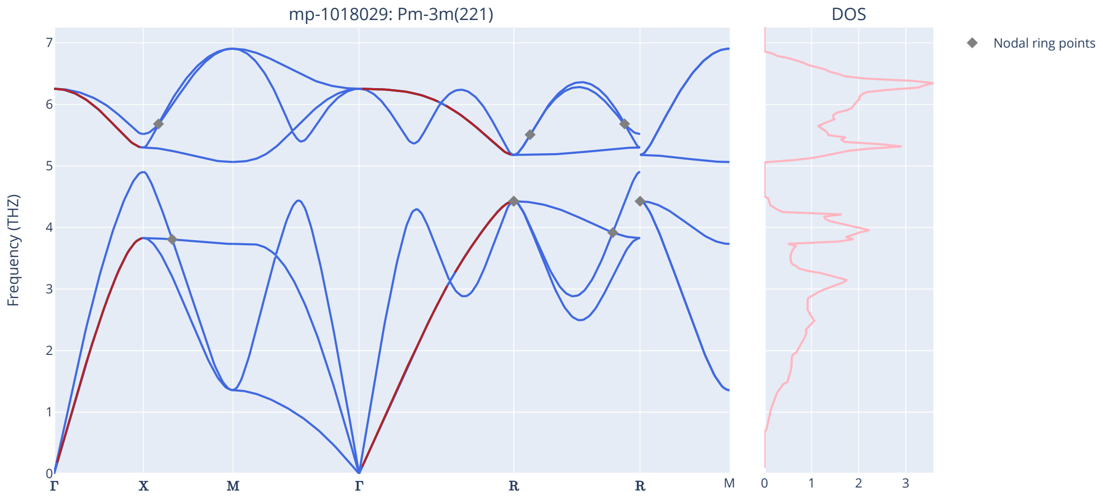

Supplementary Figure 69: The phonon band and DOS of CuPd.

Supplementary Table 115: High symmetry lines of mp-1018029-CuPd

	Start	End
0	Γ (0.000, 0.000, 0.000)	X (0.000, 0.500, 0.000)
1	X (0.000, 0.500, 0.000)	M (0.500, 0.500, 0.000)
2	M (0.500, 0.500, 0.000)	Γ (0.000, 0.000, 0.000)
3	Γ (0.000, 0.000, 0.000)	R (0.500, 0.500, 0.500)
4	R (0.500, 0.500, 0.500)	X (0.000, 0.500, 0.000)
5	R (0.500, 0.500, 0.500)	M (0.500, 0.500, 0.000)

Supplementary Table 116: Nodal ring points along high symmetry lines of mp-1018029-CuPd.

	Coordinates	Frequency	Mode 1	Mode 2	Multi.	Deg.	Path	Ratio
0	(0.500, 0.500, 0.500)	4.426	2	1	1	3	4	1.000
1	(0.161, 0.500, -0.000)	3.806	3	2	12	2	2	0.323
2	(0.108, 0.500, 0.108)	3.916	3	2	12	2	5	0.783
3	(0.061, 0.500, 0.061)	5.682	6	5	12	2	5	0.877
4	(0.084, 0.500, 0.000)	5.680	6	5	12	2	2	0.169
5	(0.435, 0.500, 0.435)	5.507	6	5	12	2	5	0.129

The followings are details for Hf_2. It has 12 topological nodal lines and 10 nodal ring points along high symmetry line as shown in Fig 70.

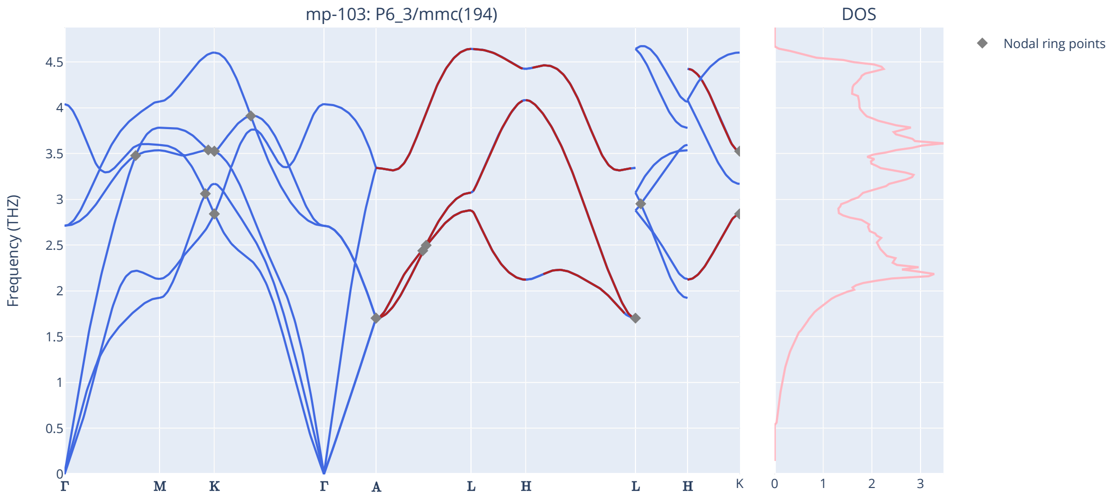

Supplementary Figure 70: The phonon band and DOS of Hf2.

Supplementary Table 117: High symmetry lines of mp-103-Hf2

	Start	End
0	Γ (0.000, 0.000, 0.000)	M (0.500, 0.000, 0.000)
1	M (0.500, 0.000, 0.000)	K (0.333, 0.333, 0.000)
2	K (0.333, 0.333, 0.000)	Γ (0.000, 0.000, 0.000)
3	Γ (0.000, 0.000, 0.000)	A (0.000, 0.000, 0.500)
4	A (0.000, 0.000, 0.500)	L (0.500, 0.000, 0.500)
5	L (0.500, 0.000, 0.500)	H (0.333, 0.333, 0.500)
6	H (0.333, 0.333, 0.500)	A (0.000, 0.000, 0.500)
7	L (0.500, 0.000, 0.500)	M (0.500, 0.000, 0.000)
8	H (0.333, 0.333, 0.500)	K (0.333, 0.333, 0.000)

Supplementary Table 118: Nodal ring points along high symmetry lines of mp-103-Hf2.

	Coordinates	Frequency	Mode 1	Mode 2	Multi.	Deg.	Path	Ratio
0	(0.333, 0.333, 0.000)	2.841	2	1	2	2	2	1.000
1	(0.000, 0.000, 0.500)	1.702	2	1	1	4	4	1.000
2	(0.362, 0.279, 0.000)	3.062	3	2	12	2	2	0.836
3	(0.246, -0.000, 0.497)	2.439	3	2	12	2	5	0.492
4	(0.264, -0.000, 0.496)	2.498	3	2	12	2	5	0.528
5	(0.499, -0.000, 0.450)	2.951	3	2	12	2	8	0.101
6	(0.374, 0.000, -0.000)	3.478	4	3	6	2	1	0.748
7	(0.333, 0.333, 0.000)	3.526	5	4	2	2	2	1.000
8	(0.352, 0.296, 0.000)	3.539	5	4	6	2	2	0.889
9	(0.222, 0.222, 0.000)	3.911	6	5	6	2	3	0.333

The followings are details for BeNi. It has 6 topological nodal lines and 6 nodal ring points along high symmetry line as shown in Fig **71**.

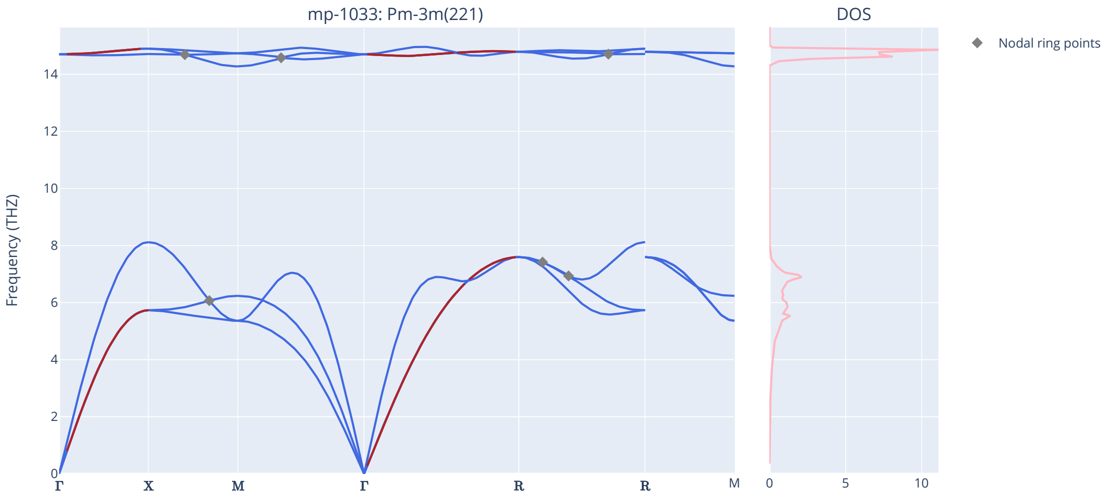

Supplementary Figure 71: The phonon band and DOS of BeNi.

Supplementary Table 119: High symmetry lines of mp-1033-BeNi

	Start	End
0	Γ (0.000, 0.000, 0.000)	X (0.000, 0.500, 0.000)
1	X (0.000, 0.500, 0.000)	M (0.500, 0.500, 0.000)
2	M (0.500, 0.500, 0.000)	Γ (0.000, 0.000, 0.000)
3	Γ (0.000, 0.000, 0.000)	R (0.500, 0.500, 0.500)
4	R (0.500, 0.500, 0.500)	X (0.000, 0.500, 0.000)
5	R (0.500, 0.500, 0.500)	M (0.500, 0.500, 0.000)

Supplementary Table 120: Nodal ring points along high symmetry lines of mp-1033-BeNi.

	Coordinates	Frequency	Mode 1	Mode 2	Multi.	Deg.	Path	Ratio
0	(0.341, 0.500, 0.000)	6.064	3	2	12	2	2	0.682
1	(0.406, 0.500, 0.406)	7.414	3	2	12	2	5	0.188
2	(0.303, 0.500, 0.303)	6.931	3	2	12	2	5	0.394
3	(0.204, 0.500, -0.000)	14.683	5	4	12	2	2	0.408
4	(0.328, 0.328, 0.000)	14.573	5	4	12	2	3	0.343
5	(0.145, 0.500, 0.145)	14.701	5	4	12	2	5	0.710

The followings are details for CaZn. It has 5 topological nodal lines and 6 nodal ring points along high symmetry line as shown in Fig 72.

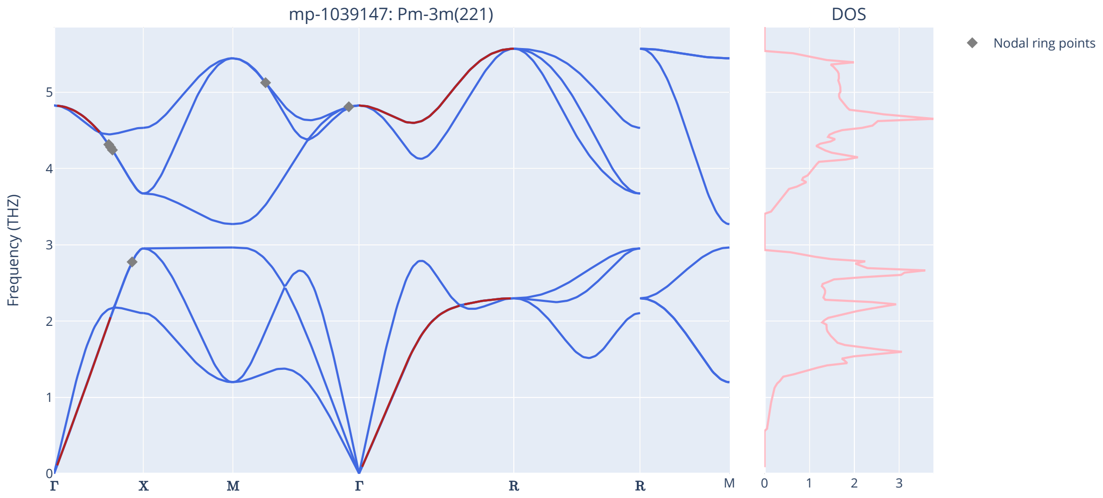

Supplementary Figure 72: The phonon band and DOS of CaZn.

Supplementary Table 121: High symmetry lines of mp-1039147-CaZn

	Start	End
0	Γ (0.000, 0.000, 0.000)	X (0.000, 0.500, 0.000)
1	X (0.000, 0.500, 0.000)	M (0.500, 0.500, 0.000)
2	M (0.500, 0.500, 0.000)	Γ (0.000, 0.000, 0.000)
3	Γ (0.000, 0.000, 0.000)	R (0.500, 0.500, 0.500)
4	R (0.500, 0.500, 0.500)	X (0.000, 0.500, 0.000)
5	R (0.500, 0.500, 0.500)	M (0.500, 0.500, 0.000)

Supplementary Table 122: Nodal ring points along high symmetry lines of mp-1039147-CaZn.

	Coordinates	Frequency	Mode 1	Mode 2	Multi.	Deg.	Path	Ratio
0	(0.000, 0.437, 0.000)	2.776	3	2	6	2	1	0.874
1	(0.000, 0.306, 0.000)	4.314	5	4	6	2	1	0.612
2	(0.000, 0.316, 0.000)	4.279	5	4	6	2	1	0.633
3	(0.000, 0.327, 0.000)	4.243	5	4	6	2	1	0.653
4	(0.370, 0.370, 0.000)	5.125	6	5	12	2	3	0.260
5	(0.040, 0.040, -0.000)	4.809	6	5	12	2	3	0.919

The followings are details for Ca_2Mg_2. It has 24 topological nodal lines and 28 nodal ring points along high symmetry line as shown in Fig 73.

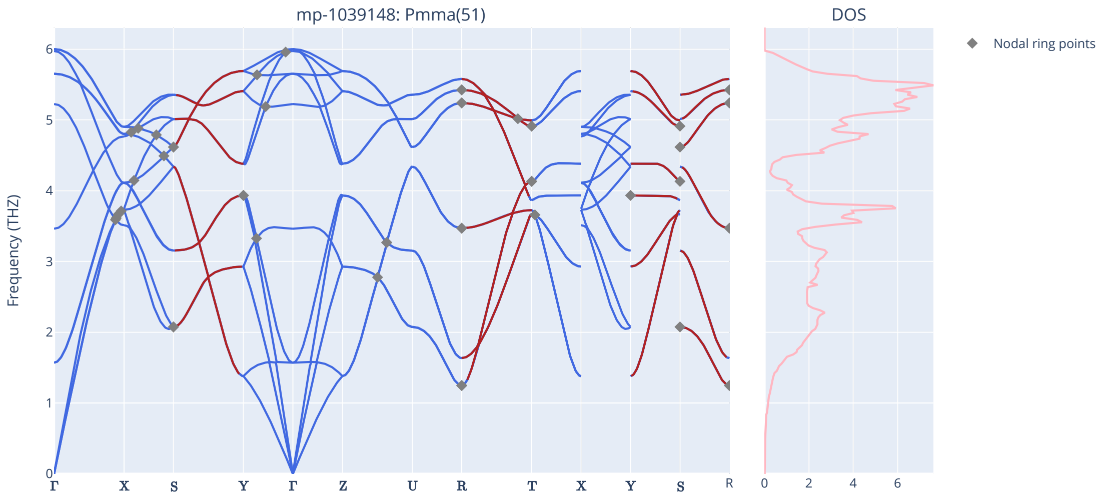

Supplementary Figure 73: The phonon band and DOS of Ca2Mg2.

Supplementary Table 123: High symmetry lines of mp-1039148-Ca2Mg2

	Start	End
0	Γ (0.000, 0.000, 0.000)	X (0.500, 0.000, 0.000)
1	X (0.500, 0.000, 0.000)	S (0.500, 0.500, 0.000)
2	S (0.500, 0.500, 0.000)	Y (0.000, 0.500, 0.000)
3	Y (0.000, 0.500, 0.000)	Γ (0.000, 0.000, 0.000)
4	Γ (0.000, 0.000, 0.000)	Z (0.000, 0.000, 0.500)
5	Z (0.000, 0.000, 0.500)	U (0.500, 0.000, 0.500)
6	U (0.500, 0.000, 0.500)	R (0.500, 0.500, 0.500)
7	R (0.500, 0.500, 0.500)	T (0.000, 0.500, 0.500)
8	T (0.000, 0.500, 0.500)	Z (0.000, 0.000, 0.500)
9	X (0.500, 0.000, 0.000)	U (0.500, 0.000, 0.500)
10	Y (0.000, 0.500, 0.000)	T (0.000, 0.500, 0.500)
11	S (0.500, 0.500, 0.000)	R (0.500, 0.500, 0.500)

Supplementary Table 124: Nodal ring points along high symmetry lines of mp-1039148-Ca2Mg2.

	Coordinates	Frequency	Mode 1	Mode 2	Multi.	Deg.	Path	Ratio
0	(0.439, 0.000, 0.000)	3.589	2	1	2	2	1	0.878
1	(0.500, 0.500, 0.000)	2.075	2	1	1	2	2	1.000
2	(0.252, 0.000, 0.500)	2.776	2	1	2	2	6	0.504
3	(0.500, 0.500, 0.500)	1.246	2	1	1	2	7	1.000
4	(0.479, 0.000, -0.000)	3.712	3	2	2	2	1	0.959
5	(0.445, 0.000, 0.000)	3.613	3	2	2	2	1	0.889
6	(0.460, 0.000, 0.000)	3.667	3	2	2	2	1	0.920
7	(0.317, -0.000, 0.500)	3.267	4	3	2	2	6	0.634

Supplementary Table 124: Nodal ring points along high symmetry lines of mp-1039148-Ca2Mg2.

	Coordinates	Frequency	Mode 1	Mode 2	Multi.	Deg.	Path	Ratio
8	(-0.000, 0.467, 0.500)	3.658	4	3	2	2	9	0.066
9	(0.317, 0.000, 0.500)	3.267	5	4	2	2	6	0.634
10	(0.000, 0.500, 0.000)	3.933	6	5	1	2	3	1.000
11	(-0.000, 0.367, 0.000)	3.326	6	5	2	2	4	0.265
12	(0.500, 0.500, 0.500)	3.470	6	5	1	2	7	1.000
13	(0.500, 0.403, 0.000)	4.491	7	6	2	2	2	0.806
14	(0.500, 0.101, -0.000)	4.145	7	6	2	2	2	0.201
15	(0.500, 0.500, 0.000)	4.616	8	7	1	2	2	1.000
16	(-0.000, 0.275, -0.000)	5.188	8	7	2	2	4	0.449
17	(0.500, 0.500, 0.500)	5.240	8	7	1	2	7	1.000
18	(0.000, 0.500, 0.500)	4.132	8	7	1	2	8	1.000
19	(0.500, 0.327, -0.000)	4.788	9	8	2	2	2	0.654
20	(0.500, 0.143, 0.000)	4.882	10	9	2	2	2	0.286
21	(0.500, 0.072, 0.000)	4.826	10	9	2	2	2	0.144
22	(0.500, 0.500, 0.500)	5.425	10	9	1	2	7	1.000
23	(0.000, 0.500, 0.500)	4.912	10	9	1	2	8	1.000
24	(0.000, 0.073, -0.000)	5.959	11	10	2	2	4	0.854
25	(0.000, 0.362, 0.000)	5.637	11	10	2	2	4	0.275
26	(0.099, 0.500, 0.500)	5.017	11	10	2	2	8	0.803

The followings are details for Ag_2. It has 11 topological nodal lines and 14 nodal ring points along high symmetry line as shown in Fig 74.

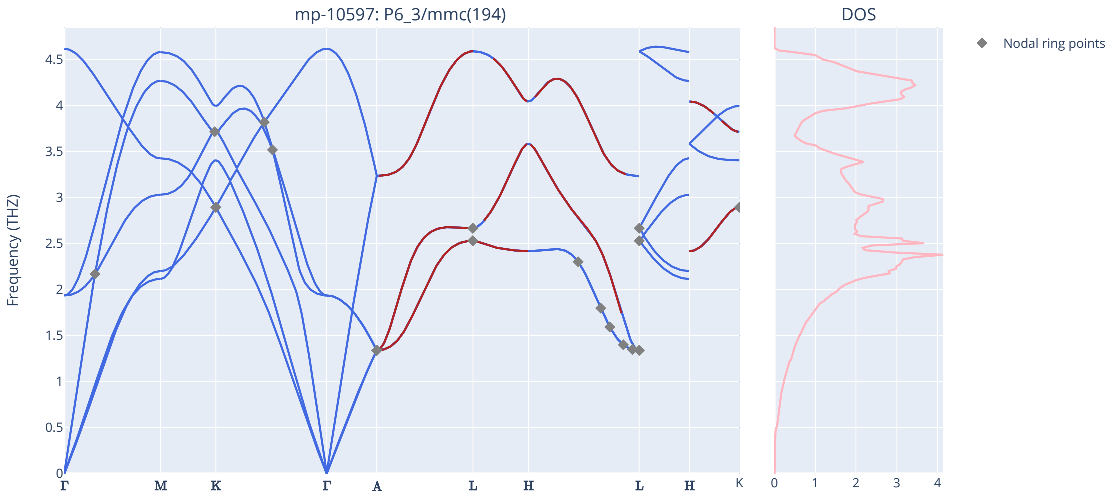

Supplementary Figure 74: The phonon band and DOS of Ag2.

Supplementary Table 125: High symmetry lines of mp-10597-Ag2

	Start	End
0	Γ (0.000, 0.000, 0.000)	M (0.500, 0.000, 0.000)
1	M (0.500, 0.000, 0.000)	K (0.333, 0.333, 0.000)
2	K (0.333, 0.333, 0.000)	Γ (0.000, 0.000, 0.000)
3	Γ (0.000, 0.000, 0.000)	A (0.000, 0.000, 0.500)
4	A (0.000, 0.000, 0.500)	L (0.500, 0.000, 0.500)
5	L (0.500, 0.000, 0.500)	H (0.333, 0.333, 0.500)
6	H (0.333, 0.333, 0.500)	A (0.000, 0.000, 0.500)
7	L (0.500, 0.000, 0.500)	M (0.500, 0.000, 0.000)
8	H (0.333, 0.333, 0.500)	K (0.333, 0.333, 0.000)

Supplementary Table 126: Nodal ring points along high symmetry lines of mp-10597-Ag2.

	Coordinates	Frequency	Mode 1	Mode 2	Multi.	Deg.	Path	Ratio
0	(0.333, 0.333, 0.000)	2.893	2	1	2	2	2	1.000
1	(0.000, 0.000, 0.500)	1.338	2	1	1	4	4	1.000
2	(0.500, 0.000, 0.500)	2.530	2	1	3	2	5	1.000
3	(0.184, 0.184, 0.500)	2.302	2	1	6	2	7	0.449
4	(0.116, 0.116, 0.500)	1.798	2	1	6	2	7	0.653
5	(0.088, 0.088, 0.500)	1.592	2	1	6	2	7	0.735
6	(0.048, 0.048, 0.500)	1.399	2	1	6	2	7	0.857
7	(0.020, 0.020, 0.500)	1.348	2	1	6	2	7	0.939
8	(0.160, -0.000, 0.000)	2.168	4	3	6	2	1	0.320
9	(0.000, 0.000, 0.500)	1.339	4	3	1	4	4	1.000
10	(0.500, 0.000, 0.500)	2.665	4	3	3	2	5	1.000

Supplementary Table 126: Nodal ring points along high symmetry lines of mp-10597-Ag2.

	Coordinates	Frequency	Mode 1	Mode 2	Multi.	Deg.	Path	Ratio
11	(0.163, 0.163, 0.000)	3.517	5	4	6	2	3	0.511
12	(0.337, 0.326, 0.000)	3.714	5	4	6	2	2	0.979
13	(0.188, 0.188, 0.000)	3.819	6	5	6	2	3	0.437

The followings are details for La_4Sb_4. It has 3 topological nodal lines and 4 nodal ring points along high symmetry line as shown in Fig **75**.

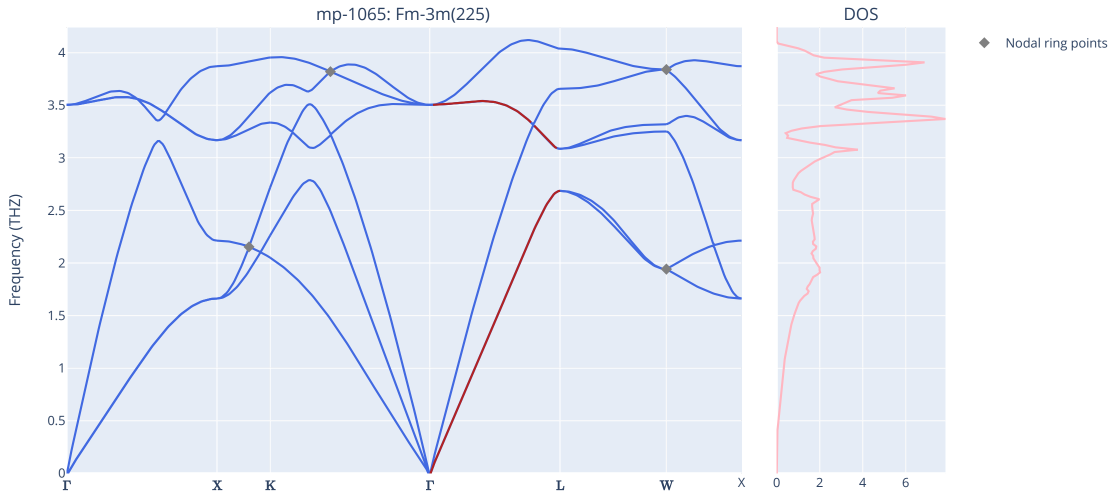

Supplementary Figure 75: The phonon band and DOS of La4Sb4.

Supplementary Table 127: High symmetry lines of mp-1065-La4Sb4

	Start	End
0	Γ (0.000, 0.000, 0.000)	X (0.500, 0.000, 0.500)
1	X (0.500, 0.000, 0.500)	U (0.625, 0.250, 0.625)
2	K (0.375, 0.375, 0.750)	Γ (0.000, 0.000, 0.000)
3	Γ (0.000, 0.000, 0.000)	L (0.500, 0.500, 0.500)
4	L (0.500, 0.500, 0.500)	W (0.500, 0.250, 0.750)
5	W (0.500, 0.250, 0.750)	X (0.500, 0.000, 0.500)

Supplementary Table 128: Nodal ring points along high symmetry lines of mp-1065-La4Sb4.

	Coordinates	Frequency	Mode 1	Mode 2	Multi.	Deg.	Path	Ratio
0	(0.500, 0.250, 0.750)	1.942	2	1	6	2	5	1.000
1	(0.575, 0.151, 0.575)	2.153	3	2	12	2	2	0.602
2	(0.500, 0.250, 0.750)	3.840	6	5	6	2	5	1.000
3	(0.229, 0.238, 0.467)	3.820	6	5	24	2	3	0.378

The followings are details for Zn_4S_4. It has 2 topological nodal lines and 1 nodal ring point along high symmetry line as shown in Fig **76**.

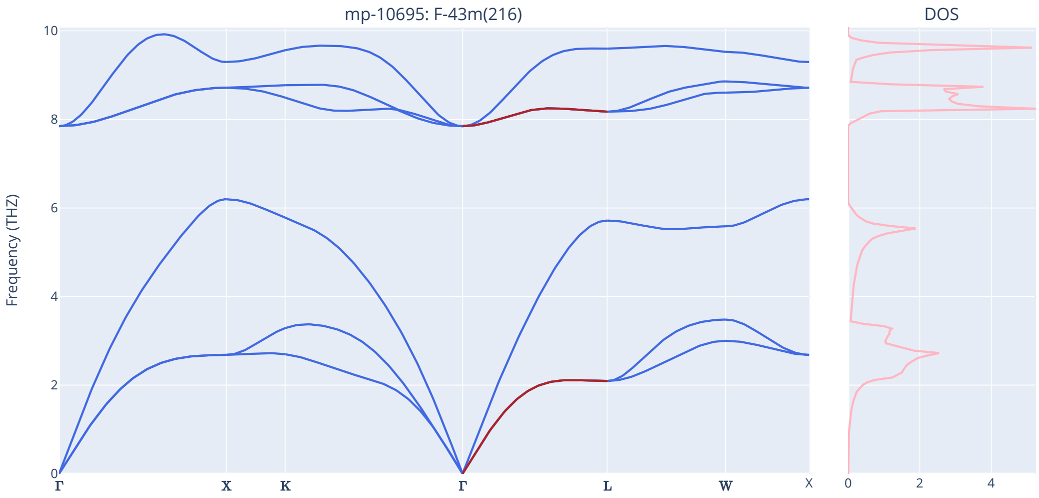

Supplementary Figure 76: The phonon band and DOS of Zn4S4.

Supplementary Table 129: High symmetry lines of mp-10695-Zn4S4

	Start	End
0	Γ (0.000, 0.000, 0.000)	X (0.500, 0.000, 0.500)
1	X (0.500, 0.000, 0.500)	U (0.625, 0.250, 0.625)
2	K (0.375, 0.375, 0.750)	Γ (0.000, 0.000, 0.000)
3	Γ (0.000, 0.000, 0.000)	L (0.500, 0.500, 0.500)
4	L (0.500, 0.500, 0.500)	W (0.500, 0.250, 0.750)
5	W (0.500, 0.250, 0.750)	X (0.500, 0.000, 0.500)

The followings are details for CaCd. It has 6 topological nodal lines and 1 nodal ring point along high symmetry line as shown in Fig 77.

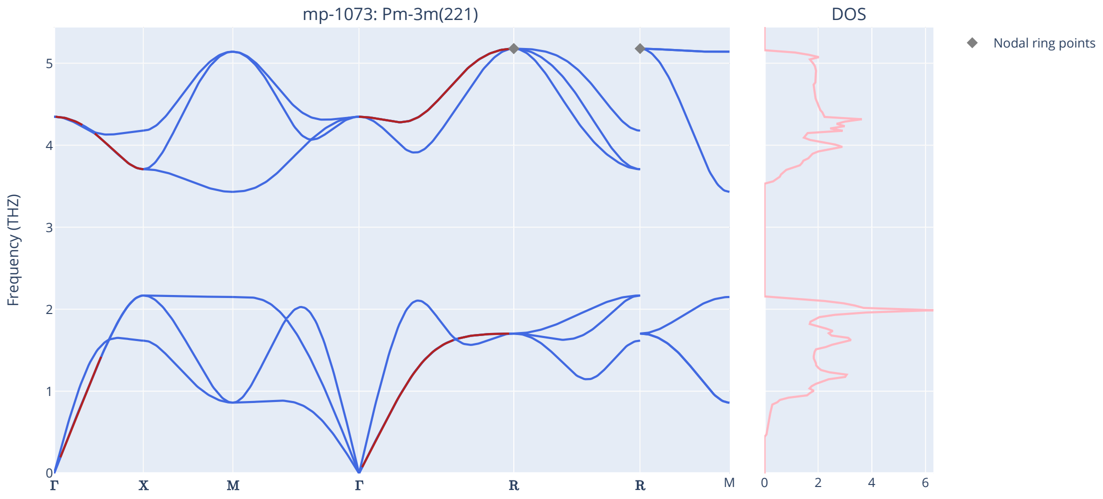

Supplementary Figure 77: The phonon band and DOS of CaCd.

Supplementary Table 130: High symmetry lines of mp-1073-CaCd

	Start	End
0	Γ (0.000, 0.000, 0.000)	X (0.000, 0.500, 0.000)
1	X (0.000, 0.500, 0.000)	M (0.500, 0.500, 0.000)
2	M (0.500, 0.500, 0.000)	Γ (0.000, 0.000, 0.000)
3	Γ (0.000, 0.000, 0.000)	R (0.500, 0.500, 0.500)
4	R (0.500, 0.500, 0.500)	X (0.000, 0.500, 0.000)
5	R (0.500, 0.500, 0.500)	M (0.500, 0.500, 0.000)

Supplementary Table 131: Nodal ring points along high symmetry lines of mp-1073-CaCd.

	Coordinates	Frequency	Mode 1	Mode 2	Multi.	Deg.	Path	Ratio
0	(0.500, 0.500, 0.500)	5.180	6	5	1	3	4	1.000

The followings are details for LiMg. It has 6 topological nodal lines and 4 nodal ring points along high symmetry line as shown in Fig 78.

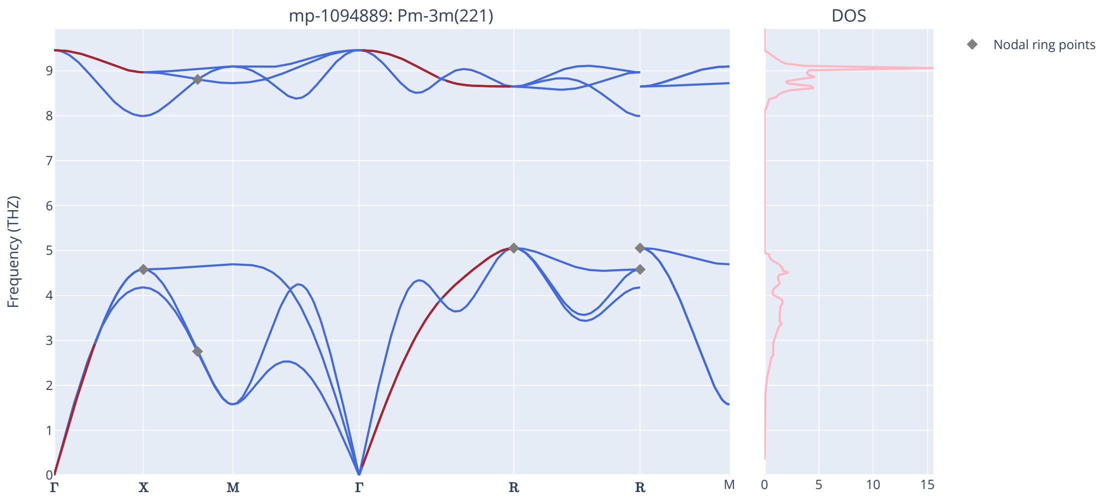

Supplementary Figure 78: The phonon band and DOS of LiMg.

Supplementary Table 132: High symmetry lines of mp-1094889-LiMg

	Start	End
0	Γ (0.000, 0.000, 0.000)	X (0.000, 0.500, 0.000)
1	X (0.000, 0.500, 0.000)	M (0.500, 0.500, 0.000)
2	M (0.500, 0.500, 0.000)	Γ (0.000, 0.000, 0.000)
3	Γ (0.000, 0.000, 0.000)	R (0.500, 0.500, 0.500)
4	R (0.500, 0.500, 0.500)	X (0.000, 0.500, 0.000)
5	R (0.500, 0.500, 0.500)	M (0.500, 0.500, 0.000)

Supplementary Table 133: Nodal ring points along high symmetry lines of mp-1094889-LiMg.

	Coordinates	Frequency	Mode 1	Mode 2	Multi.	Deg.	Path	Ratio
0	(0.303, 0.500, 0.000)	2.755	2	1	12	2	2	0.605
1	(0.500, 0.500, 0.500)	5.055	2	1	1	3	4	1.000
2	(0.000, 0.500, 0.000)	4.579	3	2	3	2	1	1.000
3	(0.304, 0.500, 0.000)	8.807	5	4	12	2	2	0.607

The followings are details for Ce_4S_4. It has 2 topological nodal lines and 5 nodal ring points along high symmetry line as shown in Fig 79.

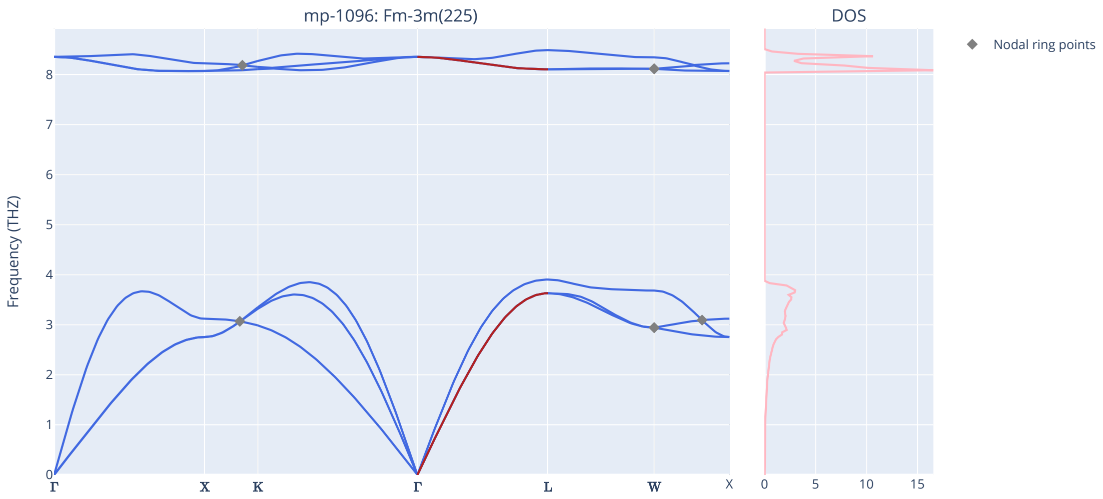

Supplementary Figure 79: The phonon band and DOS of Ce4S4.

Supplementary Table 134: High symmetry lines of mp-1096-Ce4S4

	Start	End
0	Γ (0.000, 0.000, 0.000)	X (0.500, 0.000, 0.500)
1	X (0.500, 0.000, 0.500)	U (0.625, 0.250, 0.625)
2	K (0.375, 0.375, 0.750)	Γ (0.000, 0.000, 0.000)
3	Γ (0.000, 0.000, 0.000)	L (0.500, 0.500, 0.500)
4	L (0.500, 0.500, 0.500)	W (0.500, 0.250, 0.750)
5	W (0.500, 0.250, 0.750)	X (0.500, 0.000, 0.500)

Supplementary Table 135: Nodal ring points along high symmetry lines of mp-1096-Ce4S4.

	Coordinates	Frequency	Mode 1	Mode 2	Multi.	Deg.	Path	Ratio
0	(0.500, 0.250, 0.750)	2.941	2	1	6	2	5	1.000
1	(0.584, 0.165, 0.582)	3.067	3	2	24	2	2	0.662
2	(0.499, 0.090, 0.591)	3.092	3	2	24	2	6	0.637
3	(0.500, 0.250, 0.750)	8.114	5	4	6	2	5	1.000
4	(0.589, 0.178, 0.589)	8.184	6	5	12	2	2	0.710

The followings are details for LaMg. It has 6 topological nodal lines and 2 nodal ring points along high symmetry line as shown in Fig 80.

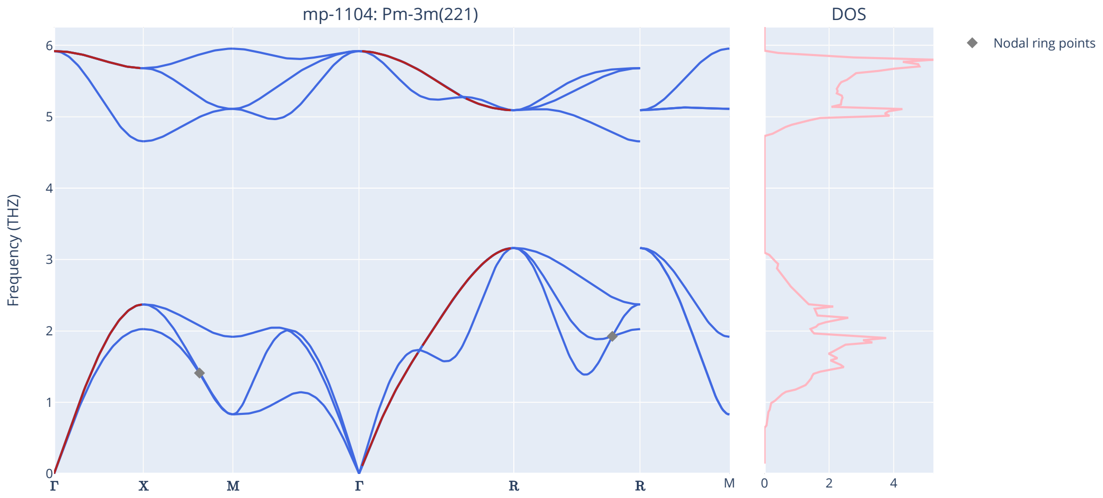

Supplementary Figure 80: The phonon band and DOS of LaMg.

Supplementary Table 136: High symmetry lines of mp-1104-LaMg

	Start	End
0	Γ (0.000, 0.000, 0.000)	X (0.000, 0.500, 0.000)
1	X (0.000, 0.500, 0.000)	M (0.500, 0.500, 0.000)
2	M (0.500, 0.500, 0.000)	Γ (0.000, 0.000, 0.000)
3	Γ (0.000, 0.000, 0.000)	R (0.500, 0.500, 0.500)
4	R (0.500, 0.500, 0.500)	X (0.000, 0.500, 0.000)
5	R (0.500, 0.500, 0.500)	M (0.500, 0.500, 0.000)

Supplementary Table 137: Nodal ring points along high symmetry lines of mp-1104-LaMg.

	Coordinates	Frequency	Mode 1	Mode 2	Multi.	Deg.	Path	Ratio
0	(0.314, 0.500, -0.000)	1.411	2	1	12	2	2	0.628
1	(0.110, 0.500, 0.110)	1.925	2	1	12	2	5	0.780

The followings are details for Y$_2$. It has 13 topological nodal lines and 11 nodal ring points along high symmetry line as shown in Fig 81.

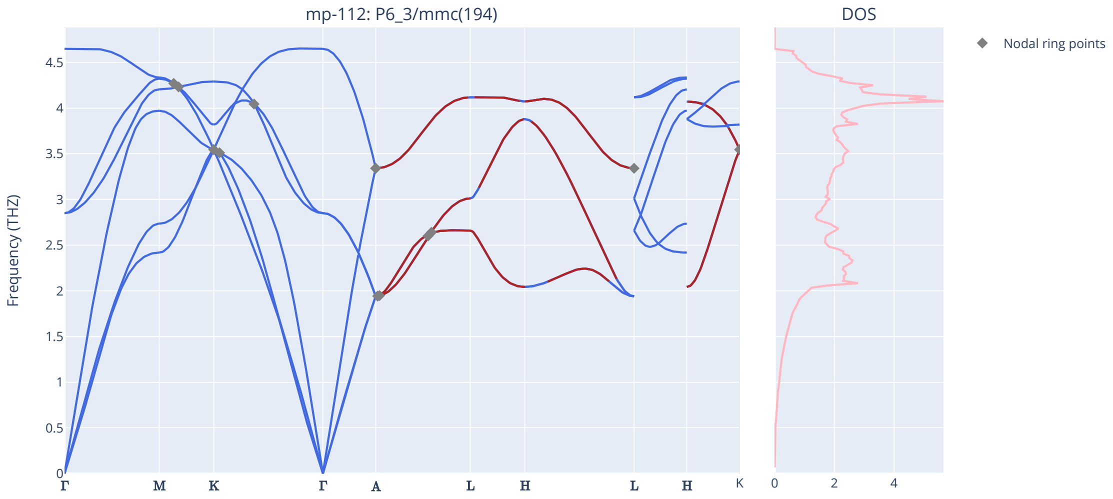

Supplementary Figure 81: The phonon band and DOS of Y2.

Supplementary Table 138: High symmetry lines of mp-112-Y2

	Start	End
0	Γ (0.000, 0.000, 0.000)	M (0.500, 0.000, 0.000)
1	M (0.500, 0.000, 0.000)	K (0.333, 0.333, 0.000)
2	K (0.333, 0.333, 0.000)	Γ (0.000, 0.000, 0.000)
3	Γ (0.000, 0.000, 0.000)	A (0.000, 0.000, 0.500)
4	A (0.000, 0.000, 0.500)	L (0.500, 0.000, 0.500)
5	L (0.500, 0.000, 0.500)	H (0.333, 0.333, 0.500)
6	H (0.333, 0.333, 0.500)	A (0.000, 0.000, 0.500)
7	L (0.500, 0.000, 0.500)	M (0.500, 0.000, 0.000)
8	H (0.333, 0.333, 0.500)	K (0.333, 0.333, 0.000)

Supplementary Table 139: Nodal ring points along high symmetry lines of mp-112-Y2.

	Coordinates	Frequency	Mode 1	Mode 2	Multi.	Deg.	Path	Ratio
0	(0.316, 0.316, 0.000)	3.511	3	2	6	2	3	0.053
1	(0.010, 0.000, 0.500)	1.942	3	2	6	2	5	0.020
2	(0.020, -0.000, 0.499)	1.948	3	2	12	2	5	0.041
3	(0.277, -0.000, 0.496)	2.601	3	2	12	2	5	0.554
4	(0.295, -0.000, 0.497)	2.641	3	2	12	2	5	0.590
5	(0.333, 0.333, 0.000)	3.546	4	3	2	2	2	1.000
6	(0.210, 0.210, 0.000)	4.044	5	4	6	2	3	0.369
7	(0.456, 0.089, -0.000)	4.270	6	5	6	2	2	0.267
8	(0.441, 0.119, 0.000)	4.232	6	5	6	2	2	0.356
9	(0.000, 0.000, 0.500)	3.341	6	5	1	2	4	1.000

The followings are details for CoGa. It has 6 topological nodal lines and 11 nodal ring points along high symmetry line as shown in Fig 82.

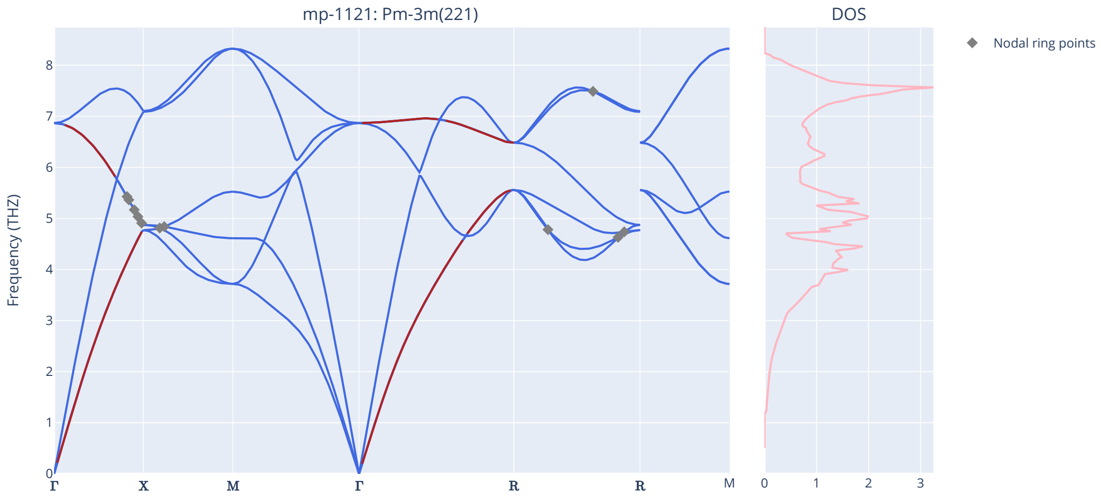

Supplementary Figure 82: The phonon band and DOS of CoGa.

Supplementary Table 140: High symmetry lines of mp-1121-CoGa

	Start	End
0	Γ (0.000, 0.000, 0.000)	X (0.000, 0.500, 0.000)
1	X (0.000, 0.500, 0.000)	M (0.500, 0.500, 0.000)
2	M (0.500, 0.500, 0.000)	Γ (0.000, 0.000, 0.000)
3	Γ (0.000, 0.000, 0.000)	R (0.500, 0.500, 0.500)
4	R (0.500, 0.500, 0.500)	X (0.000, 0.500, 0.000)
5	R (0.500, 0.500, 0.500)	M (0.500, 0.500, 0.000)

Supplementary Table 141: Nodal ring points along high symmetry lines of mp-1121-CoGa.

	Coordinates	Frequency	Mode 1	Mode 2	Multi.	Deg.	Path	Ratio
0	(0.365, 0.500, 0.365)	4.779	2	1	12	2	5	0.271
1	(0.087, 0.500, 0.087)	4.629	2	1	12	2	5	0.826
2	(0.091, 0.499, -0.000)	4.811	3	2	24	2	2	0.183
3	(0.063, 0.500, 0.063)	4.737	3	2	12	2	5	0.875
4	(0.000, 0.408, 0.000)	5.427	4	3	6	2	1	0.816
5	(0.000, 0.418, 0.000)	5.363	4	3	6	2	1	0.837
6	(0.000, 0.449, 0.000)	5.167	4	3	6	2	1	0.898
7	(0.000, 0.469, 0.000)	5.034	4	3	6	2	1	0.939
8	(0.000, 0.490, 0.000)	4.907	4	3	6	2	1	0.980
9	(0.117, 0.500, 0.000)	4.838	4	3	12	2	2	0.233
10	(0.186, 0.500, 0.186)	7.487	6	5	12	2	5	0.627

The followings are details for AlY. It has 6 topological nodal lines and 4 nodal ring points along high symmetry line as shown in Fig 83.

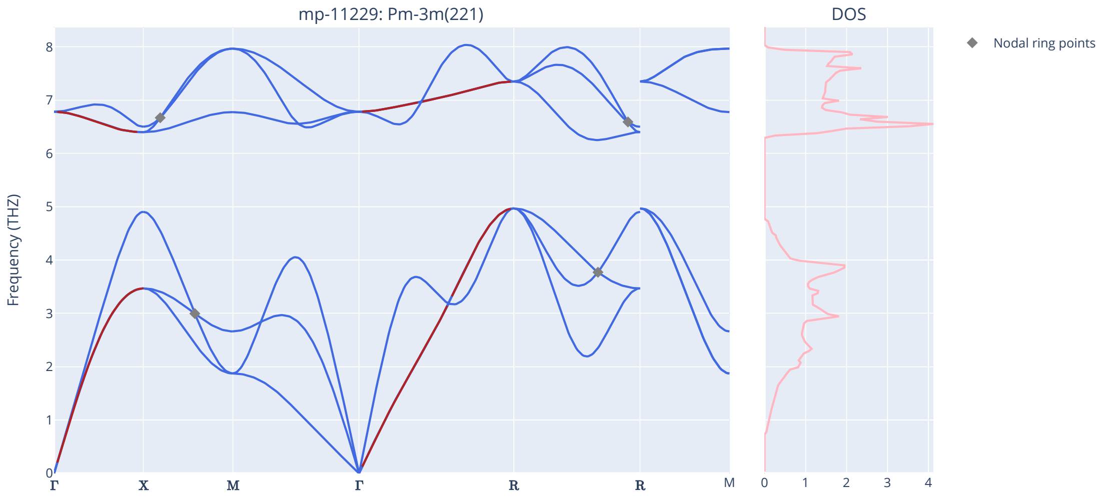

Supplementary Figure 83: The phonon band and DOS of AlY.

Supplementary Table 142: High symmetry lines of mp-11229-AlY

	Start	End
0	Γ (0.000, 0.000, 0.000)	X (0.000, 0.500, 0.000)
1	X (0.000, 0.500, 0.000)	M (0.500, 0.500, 0.000)
2	M (0.500, 0.500, 0.000)	Γ (0.000, 0.000, 0.000)
3	Γ (0.000, 0.000, 0.000)	R (0.500, 0.500, 0.500)
4	R (0.500, 0.500, 0.500)	X (0.000, 0.500, 0.000)
5	R (0.500, 0.500, 0.500)	M (0.500, 0.500, 0.000)

Supplementary Table 143: Nodal ring points along high symmetry lines of mp-11229-AlY.

	Coordinates	Frequency	Mode 1	Mode 2	Multi.	Deg.	Path	Ratio
0	(0.288, 0.500, 0.000)	2.995	3	2	12	2	2	0.576
1	(0.167, 0.500, 0.167)	3.771	3	2	12	2	5	0.667
2	(0.048, 0.500, 0.048)	6.590	6	5	12	2	5	0.904
3	(0.094, 0.500, -0.000)	6.669	6	5	12	2	2	0.188

The followings are details for Hg_4S_4 and it only has 2 topological nodal lines along high symmetry line as shown in Fig **84**.

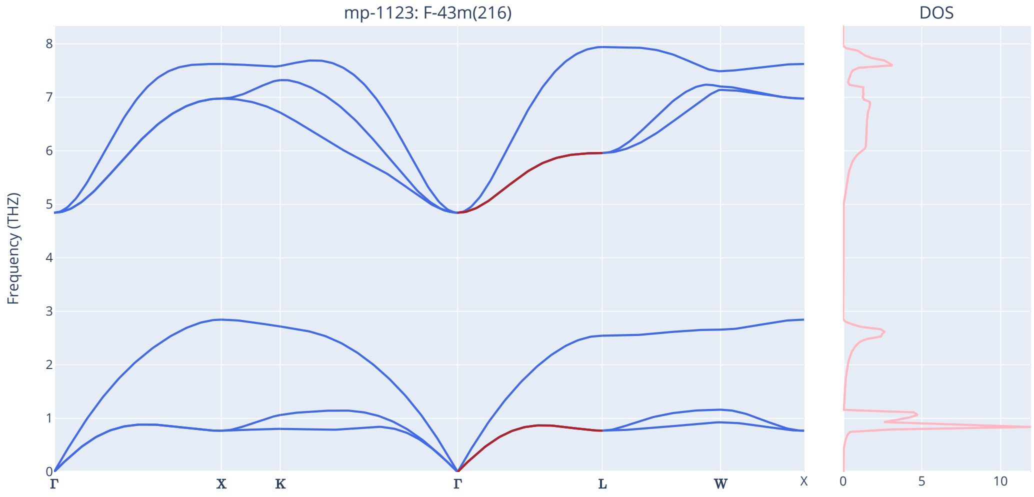

Supplementary Figure 84: The phonon band and DOS of Hg4S4.

Supplementary Table 144: High symmetry lines of mp-1123-Hg4S4

	Start	End
0	Γ (0.000, 0.000, 0.000)	X (0.500, 0.000, 0.500)
1	X (0.500, 0.000, 0.500)	U (0.625, 0.250, 0.625)
2	K (0.375, 0.375, 0.750)	Γ (0.000, 0.000, 0.000)
3	Γ (0.000, 0.000, 0.000)	L (0.500, 0.500, 0.500)
4	L (0.500, 0.500, 0.500)	W (0.500, 0.250, 0.750)
5	W (0.500, 0.250, 0.750)	X (0.500, 0.000, 0.500)

The followings are details for AgSc. It has 6 topological nodal lines and 3 nodal ring points along high symmetry line as shown in Fig 85.

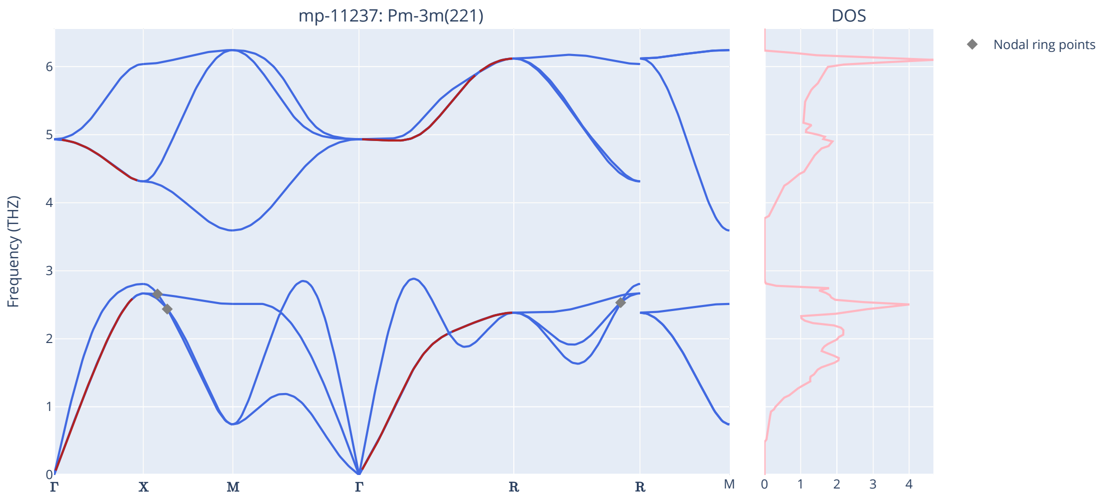

Supplementary Figure 85: The phonon band and DOS of AgSc.

Supplementary Table 145: High symmetry lines of mp-11237-AgSc

	Start	End
0	Γ (0.000, 0.000, 0.000)	X (0.000, 0.500, 0.000)
1	X (0.000, 0.500, 0.000)	M (0.500, 0.500, 0.000)
2	M (0.500, 0.500, 0.000)	Γ (0.000, 0.000, 0.000)
3	Γ (0.000, 0.000, 0.000)	R (0.500, 0.500, 0.500)
4	R (0.500, 0.500, 0.500)	X (0.000, 0.500, 0.000)
5	R (0.500, 0.500, 0.500)	M (0.500, 0.500, 0.000)

Supplementary Table 146: Nodal ring points along high symmetry lines of mp-11237-AgSc.

	Coordinates	Frequency	Mode 1	Mode 2	Multi.	Deg.	Path	Ratio
0	(0.133, 0.500, -0.000)	2.436	2	1	12	2	2	0.267
1	(0.077, 0.500, 0.077)	2.530	2	1	12	2	5	0.846
2	(0.079, 0.500, -0.000)	2.657	3	2	12	2	2	0.158

The followings are details for BePd. It has 6 topological nodal lines and 2 nodal ring points along high symmetry line as shown in Fig 86.

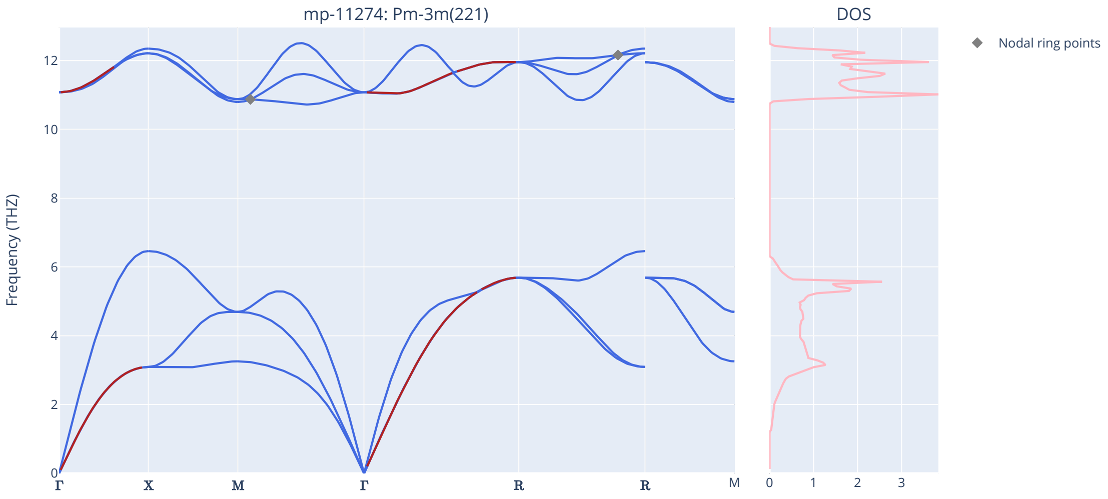

Supplementary Figure 86: The phonon band and DOS of BePd.

Supplementary Table 147: High symmetry lines of mp-11274-BePd

	Start	End
0	Γ (0.000, 0.000, 0.000)	X (0.000, 0.500, 0.000)
1	X (0.000, 0.500, 0.000)	M (0.500, 0.500, 0.000)
2	M (0.500, 0.500, 0.000)	Γ (0.000, 0.000, 0.000)
3	Γ (0.000, 0.000, 0.000)	R (0.500, 0.500, 0.500)
4	R (0.500, 0.500, 0.500)	X (0.000, 0.500, 0.000)
5	R (0.500, 0.500, 0.500)	M (0.500, 0.500, 0.000)

Supplementary Table 148: Nodal ring points along high symmetry lines of mp-11274-BePd.

	Coordinates	Frequency	Mode 1	Mode 2	Multi.	Deg.	Path	Ratio
0	(0.450, 0.450, -0.000)	10.872	5	4	12	2	3	0.100
1	(0.107, 0.500, 0.107)	12.161	6	5	12	2	5	0.785

The followings are details for BeRh. It has 7 topological nodal lines and 4 nodal ring points along high symmetry line as shown in Fig **87**.

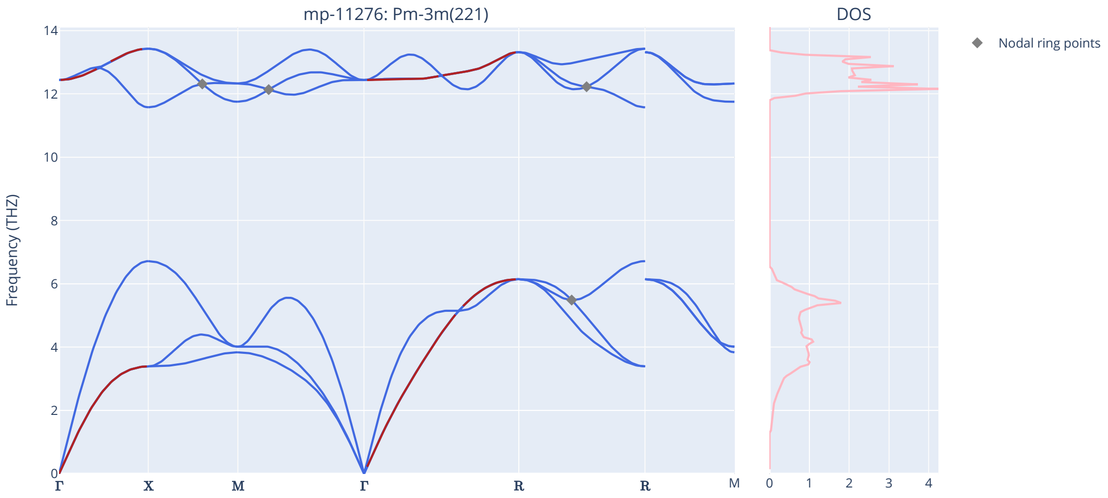

Supplementary Figure 87: The phonon band and DOS of BeRh.

Supplementary Table 149: High symmetry lines of mp-11276-BeRh

	Start	End
0	Γ (0.000, 0.000, 0.000)	X (0.000, 0.500, 0.000)
1	X (0.000, 0.500, 0.000)	M (0.500, 0.500, 0.000)
2	M (0.500, 0.500, 0.000)	Γ (0.000, 0.000, 0.000)
3	Γ (0.000, 0.000, 0.000)	R (0.500, 0.500, 0.500)
4	R (0.500, 0.500, 0.500)	X (0.000, 0.500, 0.000)
5	R (0.500, 0.500, 0.500)	M (0.500, 0.500, 0.000)

Supplementary Table 150: Nodal ring points along high symmetry lines of mp-11276-BeRh.

	Coordinates	Frequency	Mode 1	Mode 2	Multi.	Deg.	Path	Ratio
0	(0.291, 0.500, 0.291)	5.488	3	2	12	2	5	0.419
1	(0.302, 0.500, -0.000)	12.315	5	4	12	2	2	0.604
2	(0.377, 0.377, 0.000)	12.136	5	4	12	2	3	0.245
3	(0.232, 0.500, 0.232)	12.227	5	4	12	2	5	0.537

The followings are details for BeTi. It has 6 topological nodal lines and 6 nodal ring points along high symmetry line as shown in Fig 88.

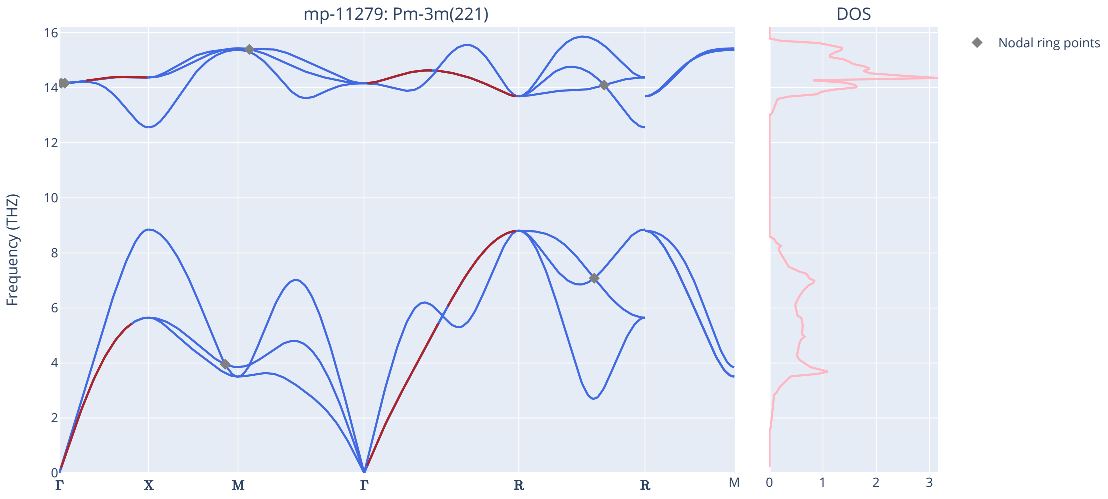

Supplementary Figure 88: The phonon band and DOS of BeTi.

Supplementary Table 151: High symmetry lines of mp-11279-BeTi

	Start	End
0	Γ (0.000, 0.000, 0.000)	X (0.000, 0.500, 0.000)
1	X (0.000, 0.500, 0.000)	M (0.500, 0.500, 0.000)
2	M (0.500, 0.500, 0.000)	Γ (0.000, 0.000, 0.000)
3	Γ (0.000, 0.000, 0.000)	R (0.500, 0.500, 0.500)
4	R (0.500, 0.500, 0.500)	X (0.000, 0.500, 0.000)
5	R (0.500, 0.500, 0.500)	M (0.500, 0.500, 0.000)

Supplementary Table 152: Nodal ring points along high symmetry lines of mp-11279-BeTi.

	Coordinates	Frequency	Mode 1	Mode 2	Multi.	Deg.	Path	Ratio
0	(0.429, 0.500, 0.000)	3.952	3	2	12	2	2	0.858
1	(0.201, 0.500, 0.201)	7.077	3	2	12	2	5	0.597
2	(0.000, 0.010, 0.000)	14.162	5	4	6	3	1	0.020
3	(0.000, 0.031, 0.000)	14.167	5	4	6	2	1	0.061
4	(0.162, 0.500, 0.162)	14.096	5	4	12	2	5	0.676
5	(0.455, 0.455, -0.000)	15.395	6	5	12	2	3	0.091

The followings are details for CaHg. It has 5 topological nodal lines and 4 nodal ring points along high symmetry line as shown in Fig 89.

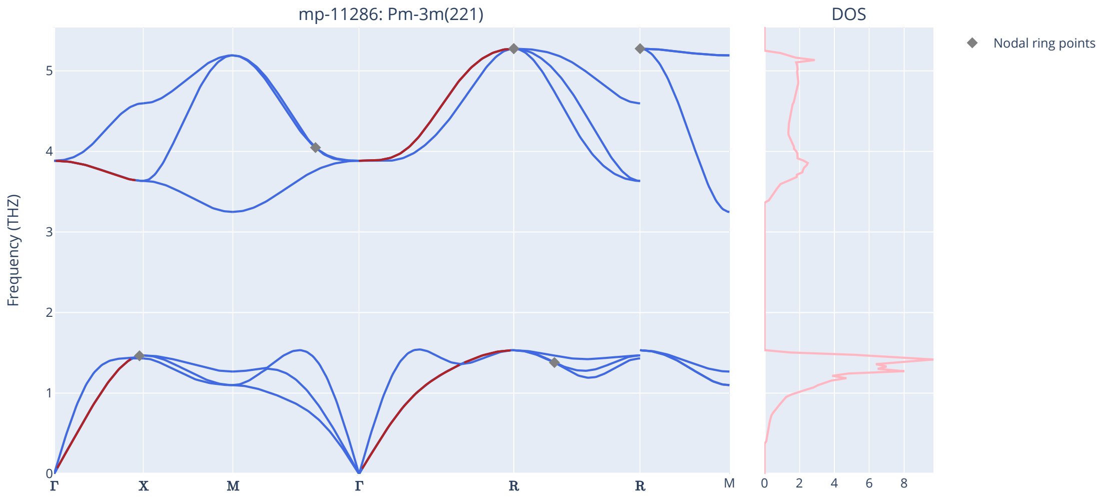

Supplementary Figure 89: The phonon band and DOS of CaHg.

Supplementary Table 153: High symmetry lines of mp-11286-CaHg

	Start	End
0	Γ (0.000, 0.000, 0.000)	X (0.000, 0.500, 0.000)
1	X (0.000, 0.500, 0.000)	M (0.500, 0.500, 0.000)
2	M (0.500, 0.500, 0.000)	Γ (0.000, 0.000, 0.000)
3	Γ (0.000, 0.000, 0.000)	R (0.500, 0.500, 0.500)
4	R (0.500, 0.500, 0.500)	X (0.000, 0.500, 0.000)
5	R (0.500, 0.500, 0.500)	M (0.500, 0.500, 0.000)

Supplementary Table 154: Nodal ring points along high symmetry lines of mp-11286-CaHg.

	Coordinates	Frequency	Mode 1	Mode 2	Multi.	Deg.	Path	Ratio
0	(0.339, 0.500, 0.339)	1.380	2	1	12	2	5	0.322
1	(-0.000, 0.478, -0.000)	1.463	3	2	6	2	1	0.956
2	(0.172, 0.172, -0.000)	4.047	6	5	12	2	3	0.655
3	(0.500, 0.500, 0.500)	5.275	6	5	1	3	4	1.000

The followings are details for IrSc. It has 5 topological nodal lines and 2 nodal ring points along high symmetry line as shown in Fig 90.

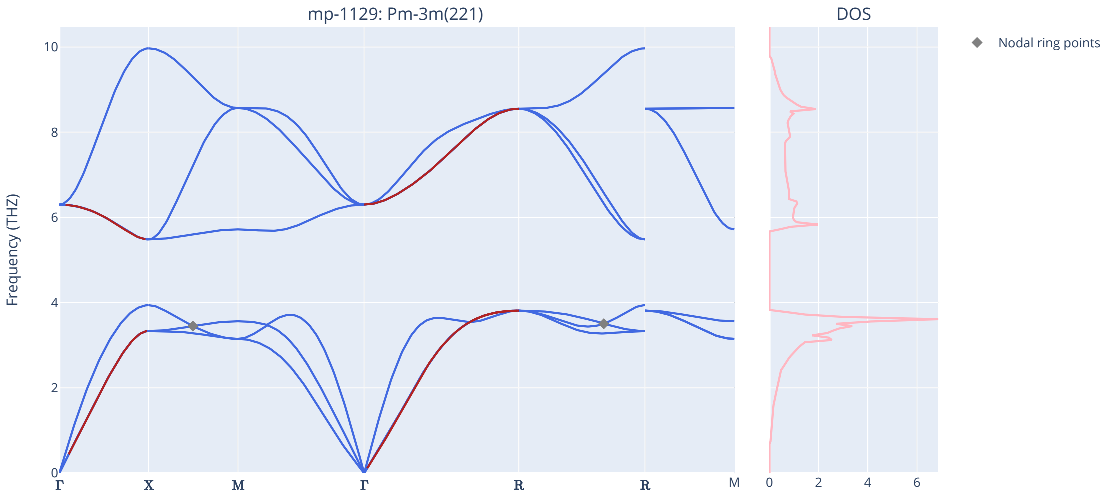

Supplementary Figure 90: The phonon band and DOS of IrSc.

Supplementary Table 155: High symmetry lines of mp-1129-IrSc

	Start	End
0	Γ (0.000, 0.000, 0.000)	X (0.000, 0.500, 0.000)
1	X (0.000, 0.500, 0.000)	M (0.500, 0.500, 0.000)
2	M (0.500, 0.500, 0.000)	Γ (0.000, 0.000, 0.000)
3	Γ (0.000, 0.000, 0.000)	R (0.500, 0.500, 0.500)
4	R (0.500, 0.500, 0.500)	X (0.000, 0.500, 0.000)
5	R (0.500, 0.500, 0.500)	M (0.500, 0.500, 0.000)

Supplementary Table 156: Nodal ring points along high symmetry lines of mp-1129-IrSc.

	Coordinates	Frequency	Mode 1	Mode 2	Multi.	Deg.	Path	Ratio
0	(0.248, 0.500, -0.000)	3.448	3	2	12	2	2	0.496
1	(0.163, 0.500, 0.163)	3.504	3	2	12	2	5	0.673

The followings are details for Cd_4O_4. It has 2 topological nodal lines and 5 nodal ring points along high symmetry line as shown in Fig 91.

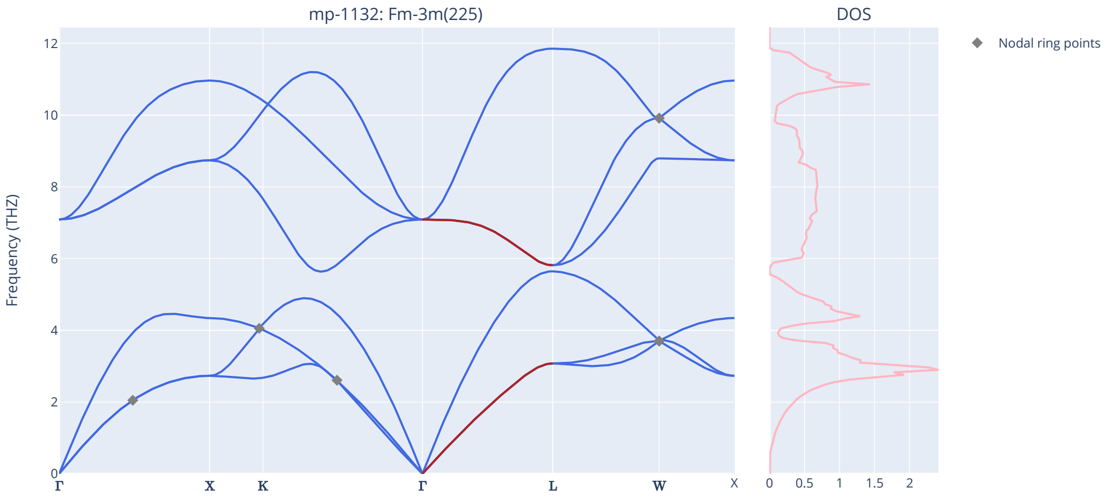

Supplementary Figure 91: The phonon band and DOS of Cd4O4.

Supplementary Table 157: High symmetry lines of mp-1132-Cd4O4

	Start	End
0	Γ (0.000, 0.000, 0.000)	X (0.500, 0.000, 0.500)
1	X (0.500, 0.000, 0.500)	U (0.625, 0.250, 0.625)
2	K (0.375, 0.375, 0.750)	Γ (0.000, 0.000, 0.000)
3	Γ (0.000, 0.000, 0.000)	L (0.500, 0.500, 0.500)
4	L (0.500, 0.500, 0.500)	W (0.500, 0.250, 0.750)
5	W (0.500, 0.250, 0.750)	X (0.500, 0.000, 0.500)

Supplementary Table 158: Nodal ring points along high symmetry lines of mp-1132-Cd4O4.

	Coordinates	Frequency	Mode 1	Mode 2	Multi.	Deg.	Path	Ratio
0	(0.245, 0.000, 0.245)	2.049	2	1	6	2	1	0.490
1	(0.199, 0.199, 0.403)	2.608	2	1	24	2	3	0.465
2	(0.500, 0.250, 0.750)	3.700	2	1	6	2	5	1.000
3	(0.617, 0.233, 0.617)	4.054	3	2	12	2	2	0.933
4	(0.500, 0.250, 0.750)	9.916	6	5	6	2	5	1.000

The followings are details for GaIr. It has 5 topological nodal lines and 2 nodal ring points along high symmetry line as shown in Fig 92.

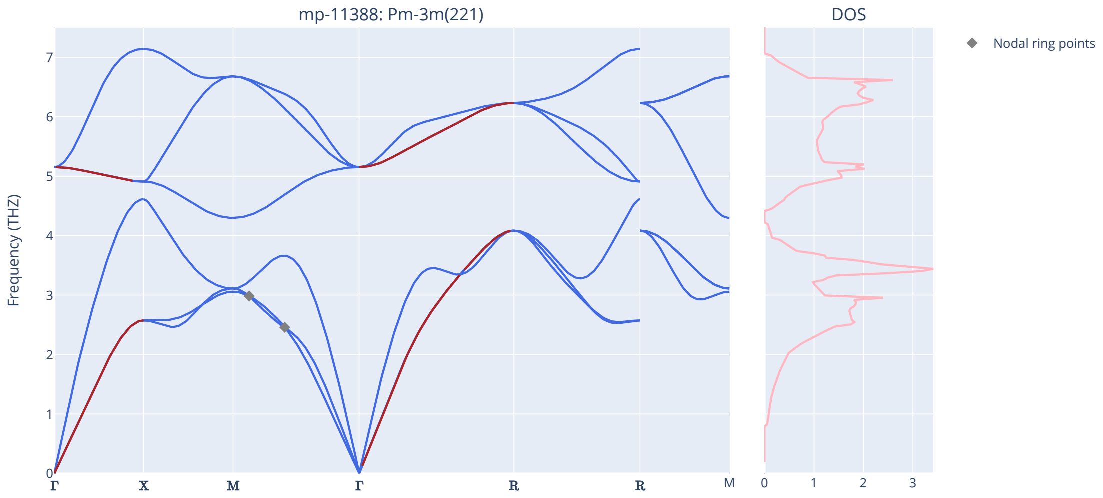

Supplementary Figure 92: The phonon band and DOS of GaIr.

Supplementary Table 159: High symmetry lines of mp-11388-GaIr

	Start	End
0	Γ (0.000, 0.000, 0.000)	X (0.000, 0.500, 0.000)
1	X (0.000, 0.500, 0.000)	M (0.500, 0.500, 0.000)
2	M (0.500, 0.500, 0.000)	Γ (0.000, 0.000, 0.000)
3	Γ (0.000, 0.000, 0.000)	R (0.500, 0.500, 0.500)
4	R (0.500, 0.500, 0.500)	X (0.000, 0.500, 0.000)
5	R (0.500, 0.500, 0.500)	M (0.500, 0.500, 0.000)

Supplementary Table 160: Nodal ring points along high symmetry lines of mp-11388-GaIr.

	Coordinates	Frequency	Mode 1	Mode 2	Multi.	Deg.	Path	Ratio
0	(0.436, 0.436, -0.000)	2.986	2	1	12	2	3	0.128
1	(0.295, 0.295, 0.000)	2.460	2	1	12	2	3	0.410

The followings are details for HfOs. It has 6 topological nodal lines and 10 nodal ring points along high symmetry line as shown in Fig 93.

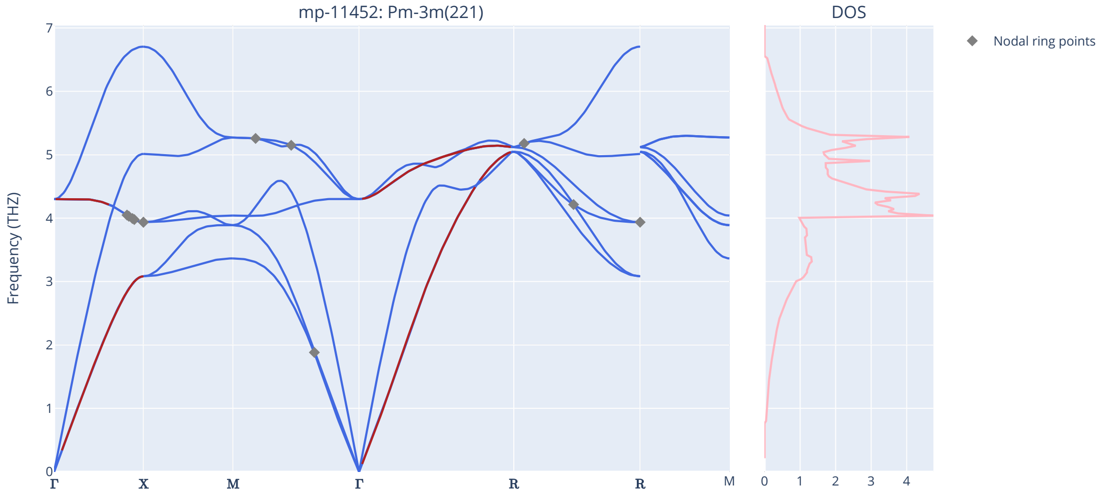

Supplementary Figure 93: The phonon band and DOS of HfOs.

Supplementary Table 161: High symmetry lines of mp-11452-HfOs

	Start	End
0	Γ (0.000, 0.000, 0.000)	X (0.000, 0.500, 0.000)
1	X (0.000, 0.500, 0.000)	M (0.500, 0.500, 0.000)
2	M (0.500, 0.500, 0.000)	Γ (0.000, 0.000, 0.000)
3	Γ (0.000, 0.000, 0.000)	R (0.500, 0.500, 0.500)
4	R (0.500, 0.500, 0.500)	X (0.000, 0.500, 0.000)
5	R (0.500, 0.500, 0.500)	M (0.500, 0.500, 0.000)

Supplementary Table 162: Nodal ring points along high symmetry lines of mp-11452-HfOs.

	Coordinates	Frequency	Mode 1	Mode 2	Multi.	Deg.	Path	Ratio
0	(0.177, 0.177, -0.000)	1.882	2	1	12	2	3	0.646
1	(0.263, 0.500, 0.263)	4.215	3	2	12	2	5	0.473
2	(0.000, 0.408, 0.000)	4.049	4	3	6	2	1	0.816
3	(0.000, 0.418, 0.000)	4.030	4	3	6	2	1	0.837
4	(0.000, 0.439, 0.000)	3.994	4	3	6	2	1	0.878
5	(0.000, 0.449, 0.000)	3.978	4	3	6	2	1	0.898
6	(0.000, 0.500, 0.000)	3.936	4	3	3	2	1	1.000
7	(0.410, 0.410, -0.000)	5.258	6	5	12	2	3	0.181
8	(0.269, 0.269, 0.000)	5.150	6	5	12	2	3	0.463
9	(0.459, 0.500, 0.459)	5.179	6	5	12	2	5	0.082

The followings are details for HfRh. It has 7 topological nodal lines and 9 nodal ring points along high symmetry line as shown in Fig **94**.

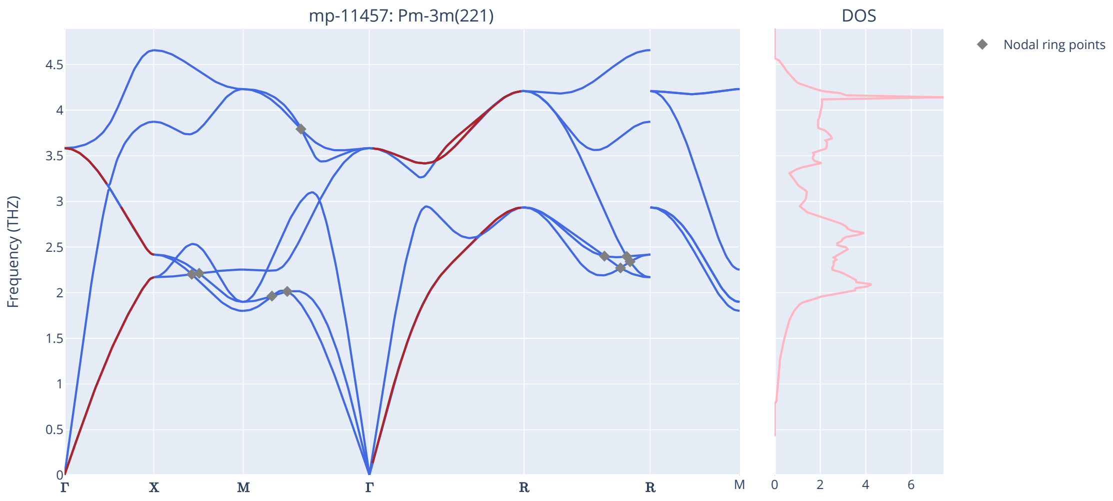

Supplementary Figure 94: The phonon band and DOS of HfRh.

Supplementary Table 163: High symmetry lines of mp-11457-HfRh

	Start	End
0	Γ (0.000, 0.000, 0.000)	X (0.000, 0.500, 0.000)
1	X (0.000, 0.500, 0.000)	M (0.500, 0.500, 0.000)
2	M (0.500, 0.500, 0.000)	Γ (0.000, 0.000, 0.000)
3	Γ (0.000, 0.000, 0.000)	R (0.500, 0.500, 0.500)
4	R (0.500, 0.500, 0.500)	X (0.000, 0.500, 0.000)
5	R (0.500, 0.500, 0.500)	M (0.500, 0.500, 0.000)

Supplementary Table 164: Nodal ring points along high symmetry lines of mp-11457-HfRh.

	Coordinates	Frequency	Mode 1	Mode 2	Multi.	Deg.	Path	Ratio
0	(0.212, 0.500, 0.000)	2.202	2	1	12	2	2	0.425
1	(0.386, 0.386, 0.000)	1.963	2	1	12	2	3	0.228
2	(0.325, 0.325, 0.000)	2.014	2	1	12	2	3	0.349
3	(0.118, 0.500, 0.118)	2.271	2	1	12	2	5	0.764
4	(0.254, 0.500, 0.000)	2.213	3	2	12	2	2	0.508
5	(0.182, 0.500, 0.182)	2.400	3	2	12	2	5	0.637
6	(0.080, 0.500, 0.080)	2.340	3	2	12	2	5	0.840
7	(0.093, 0.500, 0.093)	2.397	4	3	12	2	5	0.813
8	(0.271, 0.271, 0.000)	3.793	6	5	12	2	3	0.458

The followings are details for HfTc. It has 7 topological nodal lines and 3 nodal ring points along high symmetry line as shown in Fig 95.

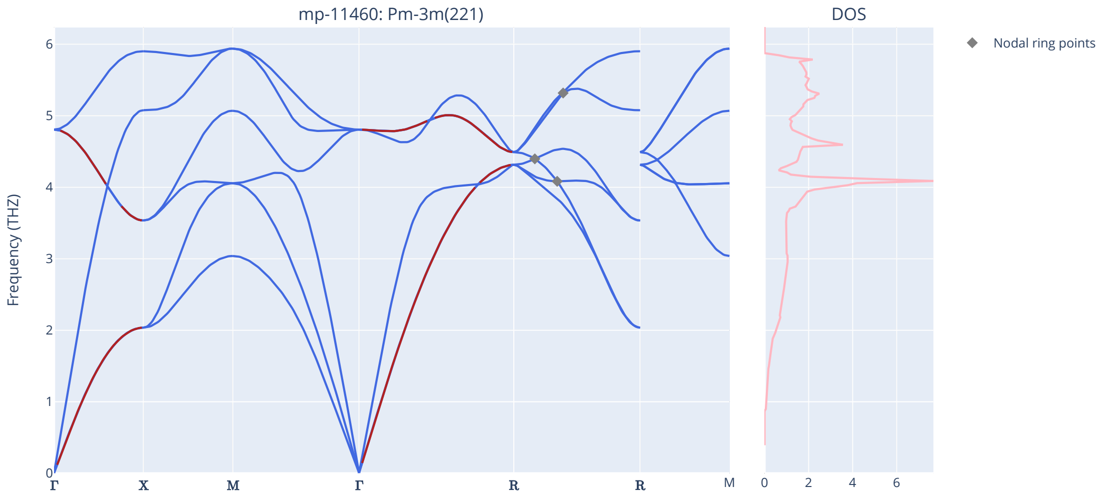

Supplementary Figure 95: The phonon band and DOS of HfTc.

Supplementary Table 165: High symmetry lines of mp-11460-HfTc

	Start	End
0	Γ (0.000, 0.000, 0.000)	X (0.000, 0.500, 0.000)
1	X (0.000, 0.500, 0.000)	M (0.500, 0.500, 0.000)
2	M (0.500, 0.500, 0.000)	Γ (0.000, 0.000, 0.000)
3	Γ (0.000, 0.000, 0.000)	R (0.500, 0.500, 0.500)
4	R (0.500, 0.500, 0.500)	X (0.000, 0.500, 0.000)
5	R (0.500, 0.500, 0.500)	M (0.500, 0.500, 0.000)

Supplementary Table 166: Nodal ring points along high symmetry lines of mp-11460-HfTc.

	Coordinates	Frequency	Mode 1	Mode 2	Multi.	Deg.	Path	Ratio
0	(0.328, 0.500, 0.328)	4.082	3	2	12	2	5	0.344
1	(0.417, 0.500, 0.417)	4.396	4	3	12	2	5	0.167
2	(0.305, 0.500, 0.305)	5.316	6	5	12	2	5	0.391

The followings are details for HgSc. It has 6 topological nodal lines and 4 nodal ring points along high symmetry line as shown in Fig **96**.

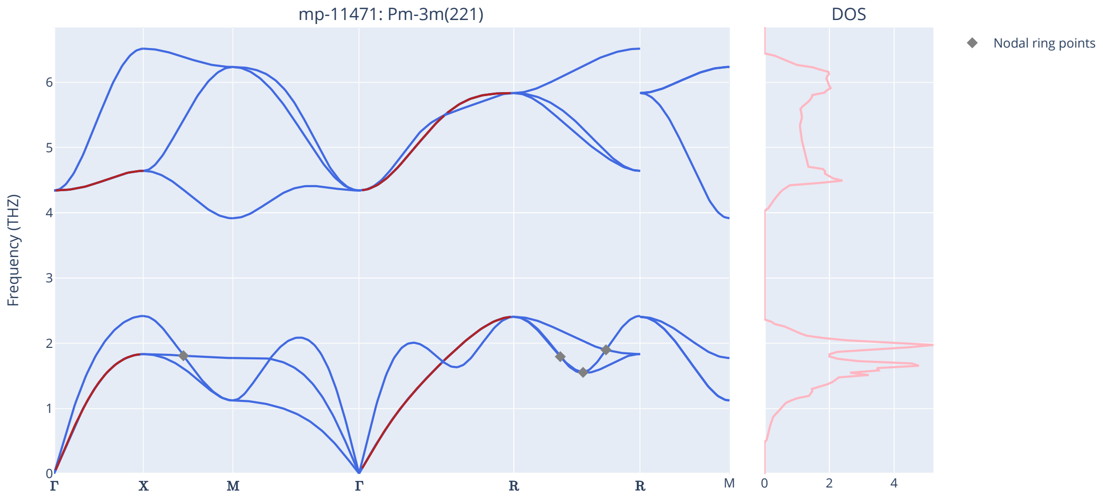

Supplementary Figure 96: The phonon band and DOS of HgSc.

Supplementary Table 167: High symmetry lines of mp-11471-HgSc

	Start	End
0	Γ (0.000, 0.000, 0.000)	X (0.000, 0.500, 0.000)
1	X (0.000, 0.500, 0.000)	M (0.500, 0.500, 0.000)
2	M (0.500, 0.500, 0.000)	Γ (0.000, 0.000, 0.000)
3	Γ (0.000, 0.000, 0.000)	R (0.500, 0.500, 0.500)
4	R (0.500, 0.500, 0.500)	X (0.000, 0.500, 0.000)
5	R (0.500, 0.500, 0.500)	M (0.500, 0.500, 0.000)

Supplementary Table 168: Nodal ring points along high symmetry lines of mp-11471-HgSc.

	Coordinates	Frequency	Mode 1	Mode 2	Multi.	Deg.	Path	Ratio
0	(0.316, 0.500, 0.316)	1.795	2	1	12	2	5	0.368
1	(0.226, 0.500, 0.226)	1.554	2	1	12	2	5	0.549
2	(0.224, 0.500, -0.000)	1.809	3	2	12	2	2	0.448
3	(0.135, 0.500, 0.135)	1.899	3	2	12	2	5	0.730

The followings are details for LuZn. It has 6 topological nodal lines and 4 nodal ring points along high symmetry line as shown in Fig **97**.

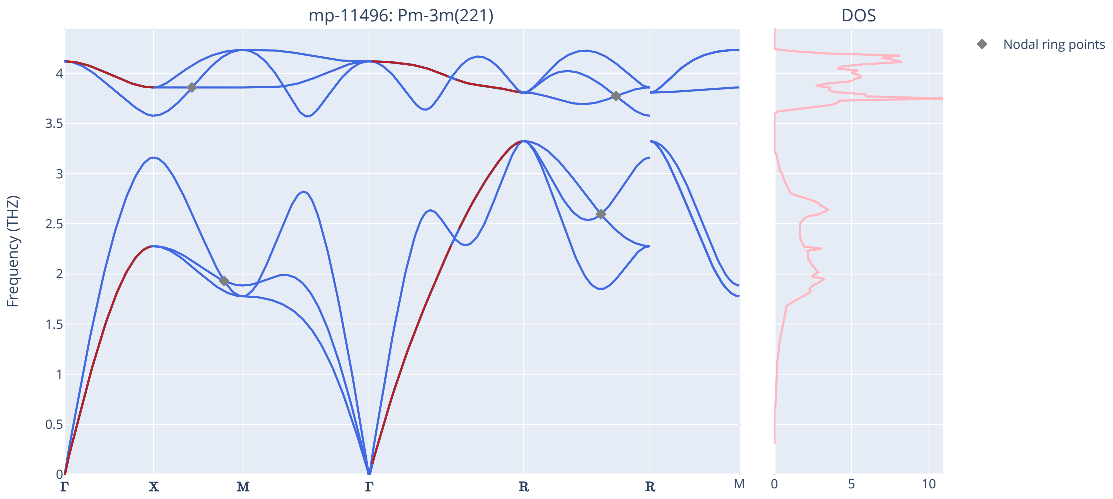

Supplementary Figure 97: The phonon band and DOS of LuZn.

Supplementary Table 169: High symmetry lines of mp-11496-LuZn

	Start	End
0	Γ (0.000, 0.000, 0.000)	X (0.000, 0.500, 0.000)
1	X (0.000, 0.500, 0.000)	M (0.500, 0.500, 0.000)
2	M (0.500, 0.500, 0.000)	Γ (0.000, 0.000, 0.000)
3	Γ (0.000, 0.000, 0.000)	R (0.500, 0.500, 0.500)
4	R (0.500, 0.500, 0.500)	X (0.000, 0.500, 0.000)
5	R (0.500, 0.500, 0.500)	M (0.500, 0.500, 0.000)

Supplementary Table 170: Nodal ring points along high symmetry lines of mp-11496-LuZn.

	Coordinates	Frequency	Mode 1	Mode 2	Multi.	Deg.	Path	Ratio
0	(0.396, 0.500, -0.001)	1.929	3	2	24	2	2	0.791
1	(0.194, 0.500, 0.194)	2.594	3	2	12	2	5	0.611
2	(0.215, 0.500, -0.000)	3.859	5	4	12	2	2	0.429
3	(0.135, 0.500, 0.135)	3.773	5	4	12	2	5	0.730

The followings are details for NiSc. It has 6 topological nodal lines and 6 nodal ring points along high symmetry line as shown in Fig 98.

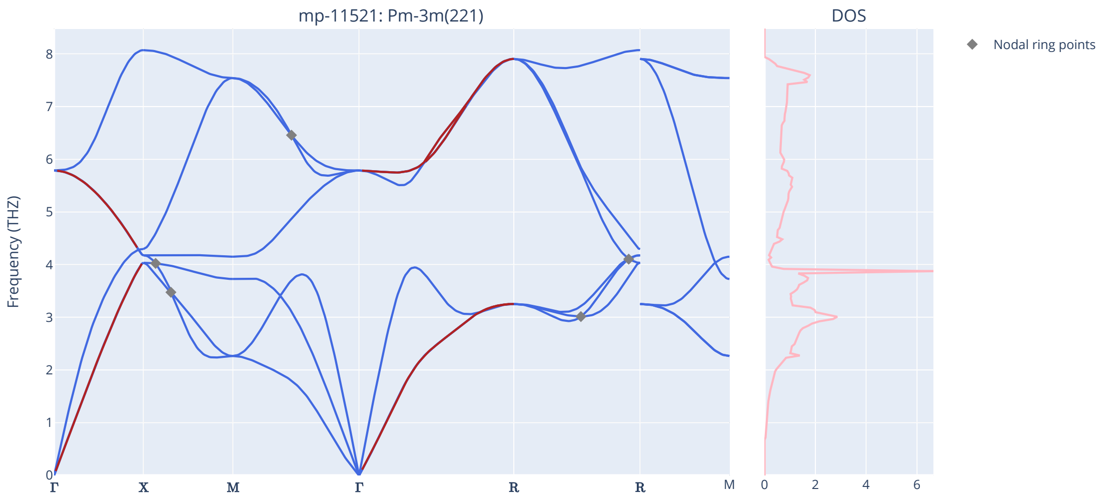

Supplementary Figure 98: The phonon band and DOS of NiSc.

Supplementary Table 171: High symmetry lines of mp-11521-NiSc

	Start	End
0	Γ (0.000, 0.000, 0.000)	X (0.000, 0.500, 0.000)
1	X (0.000, 0.500, 0.000)	M (0.500, 0.500, 0.000)
2	M (0.500, 0.500, 0.000)	Γ (0.000, 0.000, 0.000)
3	Γ (0.000, 0.000, 0.000)	R (0.500, 0.500, 0.500)
4	R (0.500, 0.500, 0.500)	X (0.000, 0.500, 0.000)
5	R (0.500, 0.500, 0.500)	M (0.500, 0.500, 0.000)

Supplementary Table 172: Nodal ring points along high symmetry lines of mp-11521-NiSc.

	Coordinates	Frequency	Mode 1	Mode 2	Multi.	Deg.	Path	Ratio
0	(0.154, 0.500, 0.000)	3.474	2	1	12	2	2	0.308
1	(0.234, 0.500, 0.234)	3.013	2	1	12	2	5	0.531
2	(0.068, 0.500, -0.000)	4.023	3	2	12	2	2	0.137
3	(0.045, 0.498, 0.045)	4.103	3	2	24	2	5	0.910
4	(0.267, 0.267, 0.000)	6.457	6	5	12	2	3	0.465

The followings are details for OsZr. It has 5 topological nodal lines and 4 nodal ring points along high symmetry line as shown in Fig **99**.

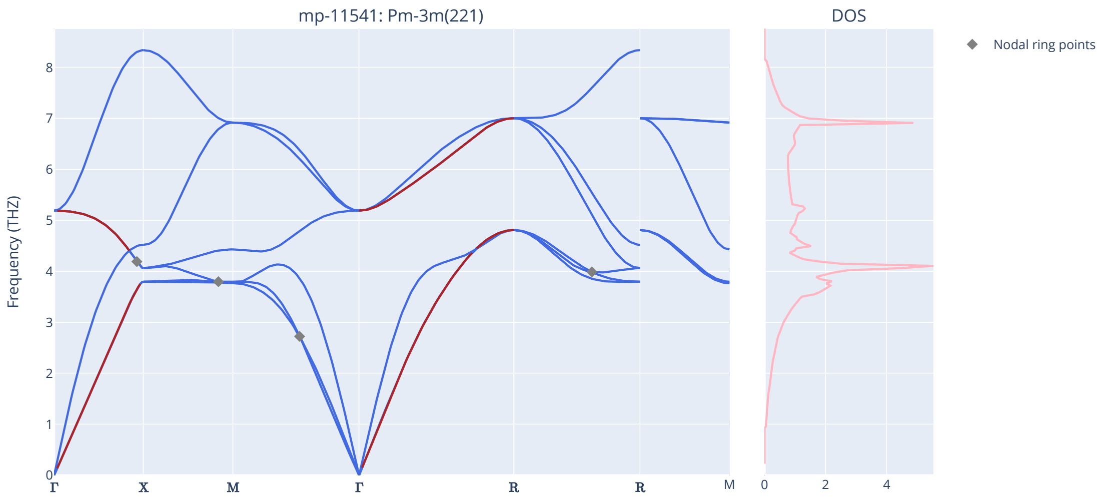

Supplementary Figure 99: The phonon band and DOS of OsZr.

Supplementary Table 173: High symmetry lines of mp-11541-OsZr

	Start	End
0	Γ (0.000, 0.000, 0.000)	X (0.000, 0.500, 0.000)
1	X (0.000, 0.500, 0.000)	M (0.500, 0.500, 0.000)
2	M (0.500, 0.500, 0.000)	Γ (0.000, 0.000, 0.000)
3	Γ (0.000, 0.000, 0.000)	R (0.500, 0.500, 0.500)
4	R (0.500, 0.500, 0.500)	X (0.000, 0.500, 0.000)
5	R (0.500, 0.500, 0.500)	M (0.500, 0.500, 0.000)

Supplementary Table 174: Nodal ring points along high symmetry lines of mp-11541-OsZr.

	Coordinates	Frequency	Mode 1	Mode 2	Multi.	Deg.	Path	Ratio
0	(0.235, 0.235, -0.000)	2.723	2	1	12	2	3	0.529
1	(0.420, 0.500, 0.000)	3.797	3	2	12	2	2	0.840
2	(0.191, 0.500, 0.191)	3.988	3	2	12	2	5	0.618
3	(-0.000, 0.463, -0.000)	4.191	4	3	6	2	1	0.927

The followings are details for Ga_4Sb_4 and it only has 3 topological nodal lines along high symmetry line as shown in Fig **100**.

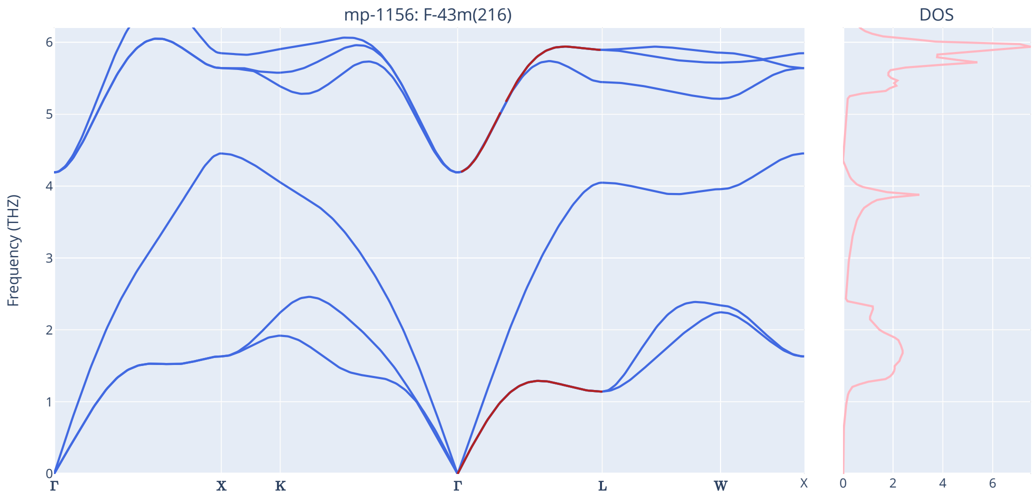

Supplementary Figure 100: The phonon band and DOS of Ga4Sb4.

Supplementary Table 175: High symmetry lines of mp-1156-Ga4Sb4

	Start	End
0	Γ (0.000, 0.000, 0.000)	X (0.500, 0.000, 0.500)
1	X (0.500, 0.000, 0.500)	U (0.625, 0.250, 0.625)
2	K (0.375, 0.375, 0.750)	Γ (0.000, 0.000, 0.000)
3	Γ (0.000, 0.000, 0.000)	L (0.500, 0.500, 0.500)
4	L (0.500, 0.500, 0.500)	W (0.500, 0.250, 0.750)
5	W (0.500, 0.250, 0.750)	X (0.500, 0.000, 0.500)

The followings are details for ScZn. It has 5 topological nodal lines and 6 nodal ring points along high symmetry line as shown in Fig 101.

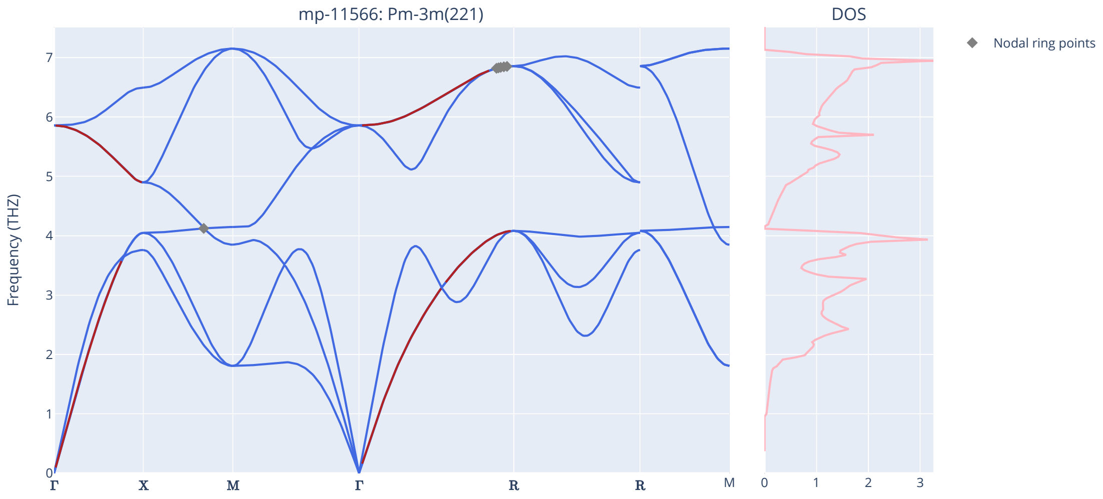

Supplementary Figure 101: The phonon band and DOS of ScZn.

Supplementary Table 176: High symmetry lines of mp-11566-ScZn

	Start	End
0	Γ (0.000, 0.000, 0.000)	X (0.000, 0.500, 0.000)
1	X (0.000, 0.500, 0.000)	M (0.500, 0.500, 0.000)
2	M (0.500, 0.500, 0.000)	Γ (0.000, 0.000, 0.000)
3	Γ (0.000, 0.000, 0.000)	R (0.500, 0.500, 0.500)
4	R (0.500, 0.500, 0.500)	X (0.000, 0.500, 0.000)
5	R (0.500, 0.500, 0.500)	M (0.500, 0.500, 0.000)

Supplementary Table 177: Nodal ring points along high symmetry lines of mp-11566-ScZn.

	Coordinates	Frequency	Mode 1	Mode 2	Multi.	Deg.	Path	Ratio
0	(0.338, 0.500, 0.000)	4.123	4	3	12	2	2	0.675
1	(0.444, 0.444, 0.444)	6.819	5	4	8	2	4	0.888
2	(0.458, 0.458, 0.458)	6.835	5	4	8	2	4	0.915
3	(0.468, 0.468, 0.468)	6.844	5	4	8	2	4	0.936
4	(0.478, 0.478, 0.478)	6.850	5	4	8	2	4	0.956
5	(0.450, 0.450, 0.450)	6.827	5	4	8	2	4	0.901

The followings are details for TaTc. It has 5 topological nodal lines and 9 nodal ring points along high symmetry line as shown in Fig **102**.

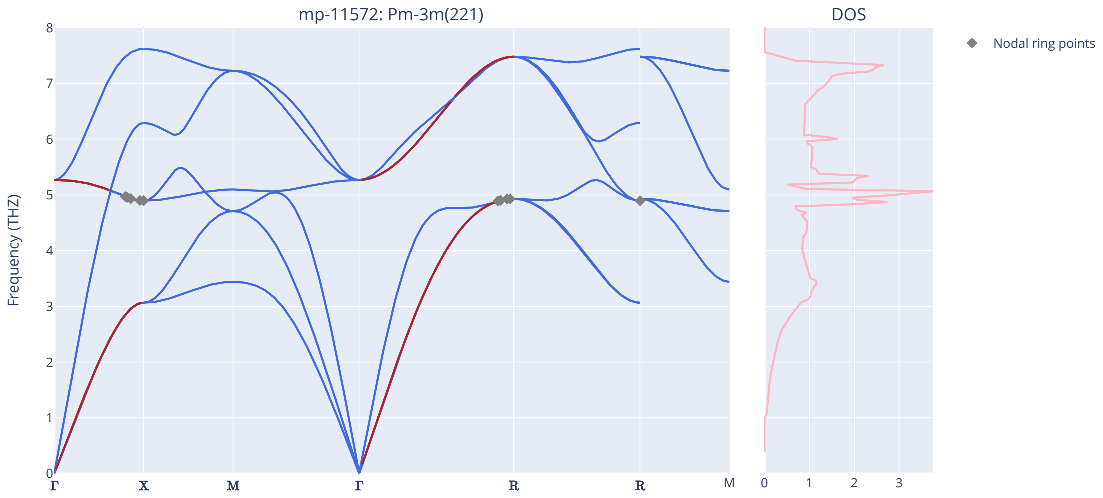

Supplementary Figure 102: The phonon band and DOS of TaTc.

Supplementary Table 178: High symmetry lines of mp-11572-TaTc

	Start	End
0	Γ (0.000, 0.000, 0.000)	X (0.000, 0.500, 0.000)
1	X (0.000, 0.500, 0.000)	M (0.500, 0.500, 0.000)
2	M (0.500, 0.500, 0.000)	Γ (0.000, 0.000, 0.000)
3	Γ (0.000, 0.000, 0.000)	R (0.500, 0.500, 0.500)
4	R (0.500, 0.500, 0.500)	X (0.000, 0.500, 0.000)
5	R (0.500, 0.500, 0.500)	M (0.500, 0.500, 0.000)

Supplementary Table 179: Nodal ring points along high symmetry lines of mp-11572-TaTc.

	Coordinates	Frequency	Mode 1	Mode 2	Multi.	Deg.	Path	Ratio
0	(0.449, 0.448, 0.449)	4.888	3	2	8	2	4	0.898
1	(0.458, 0.458, 0.458)	4.902	3	2	8	2	4	0.915
2	(0.478, 0.478, 0.478)	4.923	3	2	8	2	4	0.956
3	(0.488, 0.488, 0.488)	4.929	3	2	8	2	4	0.977
4	(0.000, 0.398, 0.000)	4.967	4	3	6	2	1	0.796
5	(0.000, 0.408, 0.000)	4.955	4	3	6	2	1	0.816
6	(0.000, 0.429, 0.000)	4.934	4	3	6	2	1	0.857
7	(0.000, 0.480, 0.000)	4.899	4	3	6	2	1	0.959
8	(0.000, 0.500, 0.000)	4.896	4	3	3	2	1	1.000

The followings are details for TcTi. It has 7 topological nodal lines and 2 nodal ring points along high symmetry line as shown in Fig **103**.

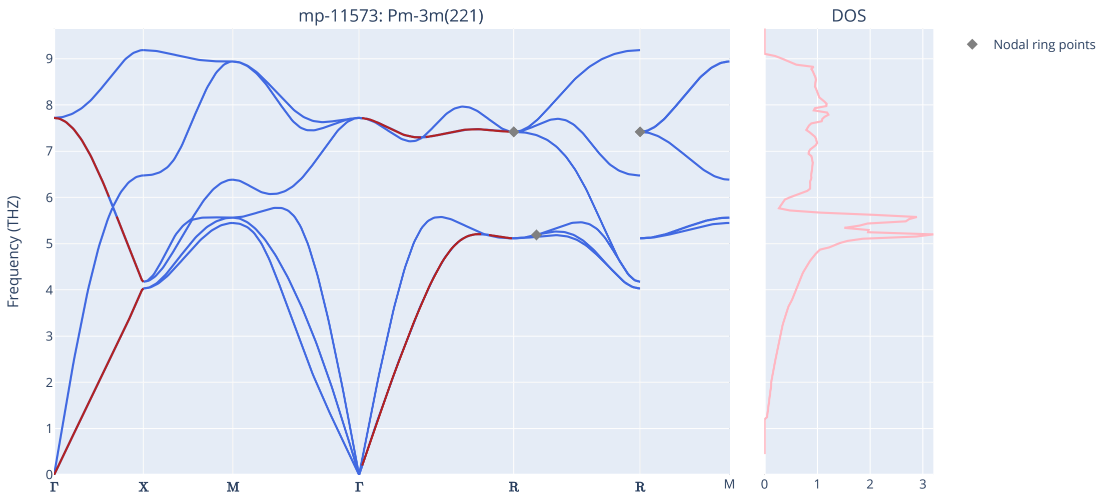

Supplementary Figure 103: The phonon band and DOS of TcTi.

Supplementary Table 180: High symmetry lines of mp-11573-TcTi

	Start	End
0	Γ (0.000, 0.000, 0.000)	X (0.000, 0.500, 0.000)
1	X (0.000, 0.500, 0.000)	M (0.500, 0.500, 0.000)
2	M (0.500, 0.500, 0.000)	Γ (0.000, 0.000, 0.000)
3	Γ (0.000, 0.000, 0.000)	R (0.500, 0.500, 0.500)
4	R (0.500, 0.500, 0.500)	X (0.000, 0.500, 0.000)
5	R (0.500, 0.500, 0.500)	M (0.500, 0.500, 0.000)

Supplementary Table 181: Nodal ring points along high symmetry lines of mp-11573-TcTi.

	Coordinates	Frequency	Mode 1	Mode 2	Multi.	Deg.	Path	Ratio
0	(0.410, 0.500, 0.410)	5.188	3	2	12	2	5	0.180
1	(0.500, 0.500, 0.500)	7.418	6	5	1	3	4	1.000

The followings are details for La_4Se_4. It has 3 topological nodal lines and 3 nodal ring points along high symmetry line as shown in Fig **104**.

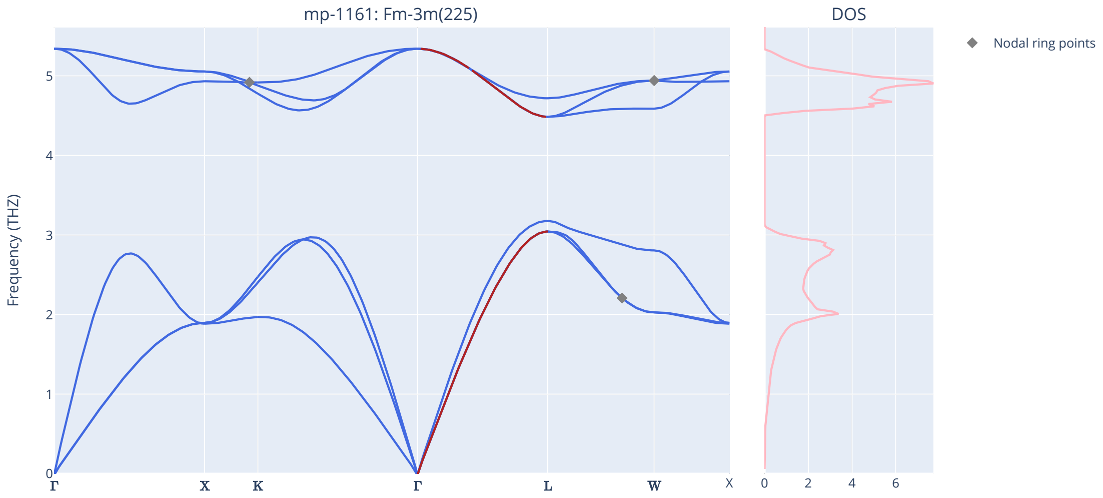

Supplementary Figure 104: The phonon band and DOS of La4Se4.

Supplementary Table 182: High symmetry lines of mp-1161-La4Se4

	Start	End
0	Γ (0.000, 0.000, 0.000)	X (0.500, 0.000, 0.500)
1	X (0.500, 0.000, 0.500)	U (0.625, 0.250, 0.625)
2	K (0.375, 0.375, 0.750)	Γ (0.000, 0.000, 0.000)
3	Γ (0.000, 0.000, 0.000)	L (0.500, 0.500, 0.500)
4	L (0.500, 0.500, 0.500)	W (0.500, 0.250, 0.750)
5	W (0.500, 0.250, 0.750)	X (0.500, 0.000, 0.500)

Supplementary Table 183: Nodal ring points along high symmetry lines of mp-1161-La4Se4.

	Coordinates	Frequency	Mode 1	Mode 2	Multi.	Deg.	Path	Ratio
0	(0.500, 0.325, 0.675)	2.208	2	1	24	2	5	0.699
1	(0.605, 0.211, 0.605)	4.920	6	5	12	2	2	0.843
2	(0.500, 0.250, 0.750)	4.942	6	5	6	2	5	1.000

The followings are details for CuSc. It has 6 topological nodal lines and 4 nodal ring points along high symmetry line as shown in Fig 105.

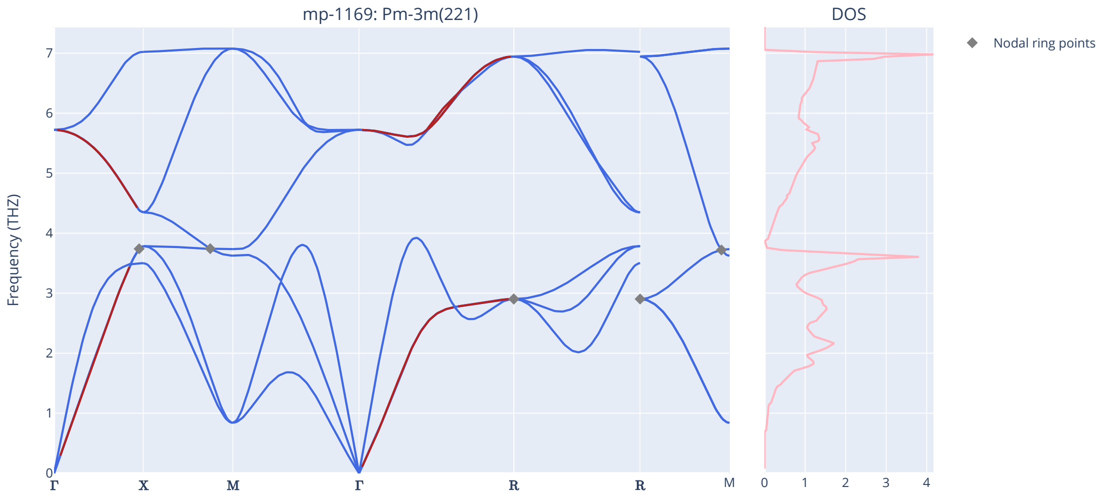

Supplementary Figure 105: The phonon band and DOS of CuSc.

Supplementary Table 184: High symmetry lines of mp-1169-CuSc

	Start	End
0	Γ (0.000, 0.000, 0.000)	X (0.000, 0.500, 0.000)
1	X (0.000, 0.500, 0.000)	M (0.500, 0.500, 0.000)
2	M (0.500, 0.500, 0.000)	Γ (0.000, 0.000, 0.000)
3	Γ (0.000, 0.000, 0.000)	R (0.500, 0.500, 0.500)
4	R (0.500, 0.500, 0.500)	X (0.000, 0.500, 0.000)
5	R (0.500, 0.500, 0.500)	M (0.500, 0.500, 0.000)

Supplementary Table 185: Nodal ring points along high symmetry lines of mp-1169-CuSc.

	Coordinates	Frequency	Mode 1	Mode 2	Multi.	Deg.	Path	Ratio
0	(0.500, 0.500, 0.500)	2.904	2	1	1	3	4	1.000
1	(-0.000, 0.476, -0.000)	3.741	3	2	6	2	1	0.952
2	(0.374, 0.500, -0.000)	3.743	4	3	12	2	2	0.748
3	(0.498, 0.500, 0.045)	3.721	4	3	24	2	6	0.910

The followings are details for Sn$_8$. It has 5 topological nodal lines and 4 nodal ring points along high symmetry line as shown in Fig **106**.

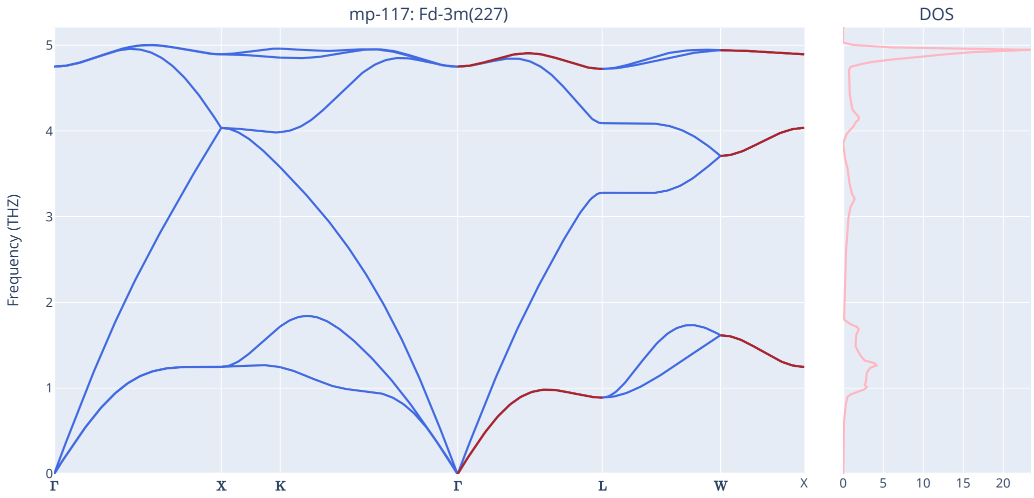

Supplementary Figure 106: The phonon band and DOS of Sn8.

Supplementary Table 186: High symmetry lines of mp-117-Sn8

	Start	End
0	Γ (0.000, 0.000, 0.000)	X (0.500, 0.000, 0.500)
1	X (0.500, 0.000, 0.500)	U (0.625, 0.250, 0.625)
2	K (0.375, 0.375, 0.750)	Γ (0.000, 0.000, 0.000)
3	Γ (0.000, 0.000, 0.000)	L (0.500, 0.500, 0.500)
4	L (0.500, 0.500, 0.500)	W (0.500, 0.250, 0.750)
5	W (0.500, 0.250, 0.750)	X (0.500, 0.000, 0.500)

The followings are details for MgRh. It has 6 topological nodal lines and 3 nodal ring points along high symmetry line as shown in Fig 107.

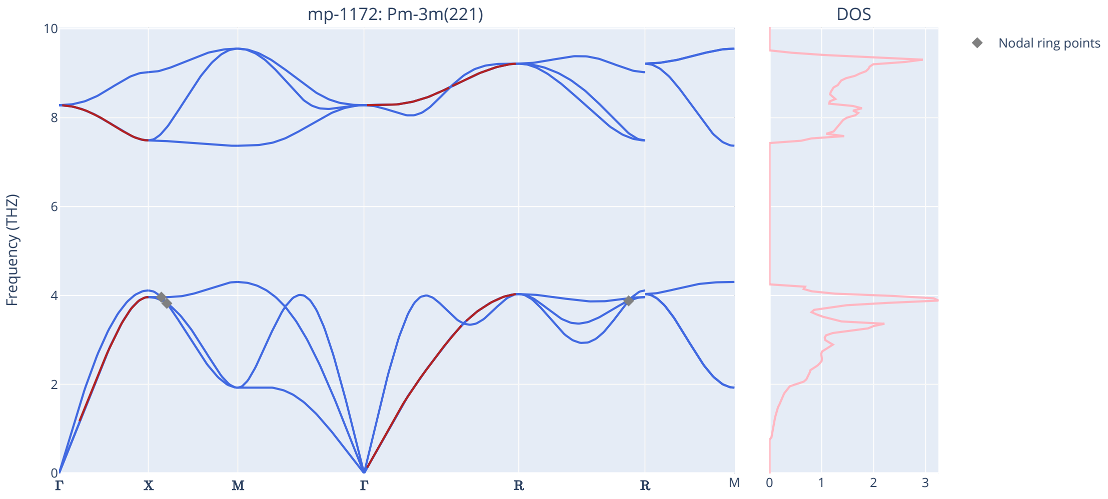

Supplementary Figure 107: The phonon band and DOS of MgRh.

Supplementary Table 187: High symmetry lines of mp-1172-MgRh

	Start	End
0	Γ (0.000, 0.000, 0.000)	X (0.000, 0.500, 0.000)
1	X (0.000, 0.500, 0.000)	M (0.500, 0.500, 0.000)
2	M (0.500, 0.500, 0.000)	Γ (0.000, 0.000, 0.000)
3	Γ (0.000, 0.000, 0.000)	R (0.500, 0.500, 0.500)
4	R (0.500, 0.500, 0.500)	X (0.000, 0.500, 0.000)
5	R (0.500, 0.500, 0.500)	M (0.500, 0.500, 0.000)

Supplementary Table 188: Nodal ring points along high symmetry lines of mp-1172-MgRh.

	Coordinates	Frequency	Mode 1	Mode 2	Multi.	Deg.	Path	Ratio
0	(0.103, 0.500, -0.000)	3.822	2	1	12	2	2	0.206
1	(0.065, 0.500, 0.065)	3.879	2	1	12	2	5	0.870
2	(0.072, 0.500, -0.000)	3.962	3	2	12	2	2	0.145

The followings are details for MgSc. It has 6 topological nodal lines and 4 nodal ring points along high symmetry line as shown in Fig **108**.

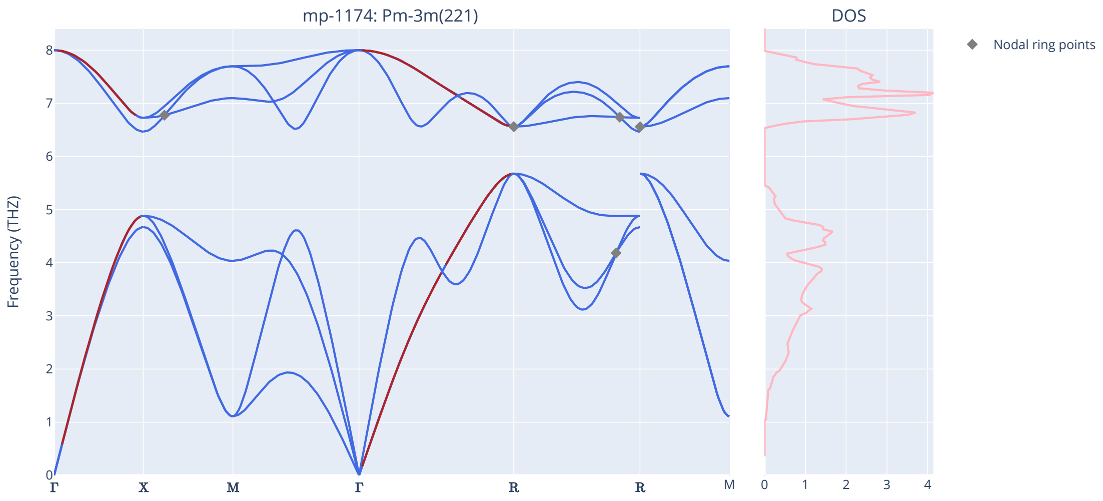

Supplementary Figure 108: The phonon band and DOS of MgSc.

Supplementary Table 189: High symmetry lines of mp-1174-MgSc

	Start	End
0	Γ (0.000, 0.000, 0.000)	X (0.000, 0.500, 0.000)
1	X (0.000, 0.500, 0.000)	M (0.500, 0.500, 0.000)
2	M (0.500, 0.500, 0.000)	Γ (0.000, 0.000, 0.000)
3	Γ (0.000, 0.000, 0.000)	R (0.500, 0.500, 0.500)
4	R (0.500, 0.500, 0.500)	X (0.000, 0.500, 0.000)
5	R (0.500, 0.500, 0.500)	M (0.500, 0.500, 0.000)

Supplementary Table 190: Nodal ring points along high symmetry lines of mp-1174-MgSc.

	Coordinates	Frequency	Mode 1	Mode 2	Multi.	Deg.	Path	Ratio
0	(0.095, 0.500, 0.095)	4.182	2	1	12	2	5	0.810
1	(0.118, 0.500, -0.000)	6.775	5	4	12	2	2	0.235
2	(0.081, 0.500, 0.081)	6.738	5	4	12	2	5	0.839
3	(0.500, 0.500, 0.500)	6.559	6	5	1	3	4	1.000

The followings are details for LiPt. It has 1 nodal ring points and 2 single Weyl point along high symmetry line as shown in Fig 109.

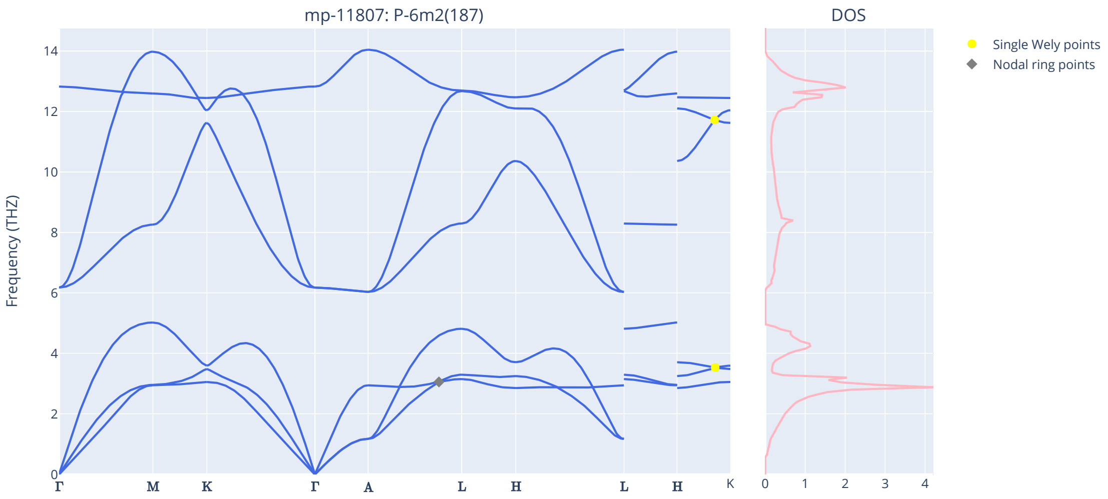

Supplementary Figure 109: The phonon band and DOS of LiPt.

Supplementary Table 191: High symmetry lines of mp-11807-LiPt

	Start	End
0	Γ (0.000, 0.000, 0.000)	M (0.500, 0.000, 0.000)
1	M (0.500, 0.000, 0.000)	K (0.333, 0.333, 0.000)
2	K (0.333, 0.333, 0.000)	Γ (0.000, 0.000, 0.000)
3	Γ (0.000, 0.000, 0.000)	A (0.000, 0.000, 0.500)
4	A (0.000, 0.000, 0.500)	L (0.500, 0.000, 0.500)
5	L (0.500, 0.000, 0.500)	H (0.333, 0.333, 0.500)
6	H (0.333, 0.333, 0.500)	A (0.000, 0.000, 0.500)
7	L (0.500, 0.000, 0.500)	M (0.500, 0.000, 0.000)
8	H (0.333, 0.333, 0.500)	K (0.333, 0.333, 0.000)

Supplementary Table 192: Single Weyl points along high symmetry lines of mp-11807-LiPt.

	Coordinates	Frequency	Mode 1	Mode 2	Multi.	Deg.	Path	Ratio
0	(0.333, 0.333, 0.143)	3.532	3	2	4	2	9	0.715
1	(0.333, 0.333, 0.148)	11.720	5	4	4	2	9	0.704

Supplementary Table 193: Nodal ring points along high symmetry lines of mp-11807-LiPt.

	Coordinates	Frequency	Mode 1	Mode 2	Multi.	Deg.	Path	Ratio
0	(0.378, -0.000, 0.500)	3.056	2	1	6	2	5	0.757

The followings are details for ZrSe. It has 2 nodal ring points and 6 single Weyl points along high symmetry line as shown in Fig 110.

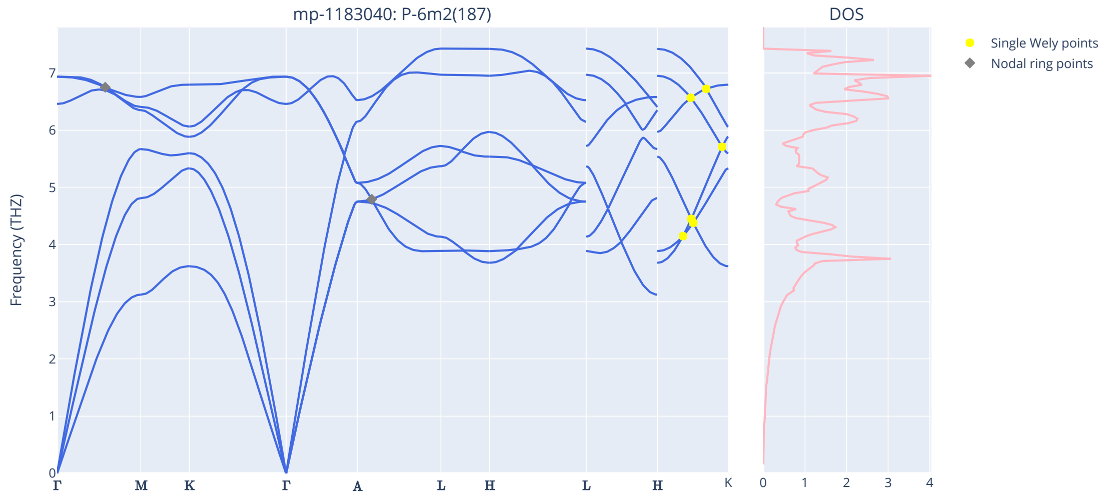

Supplementary Figure 110: The phonon band and DOS of ZrSe.

Supplementary Table 194: High symmetry lines of mp-1183040-ZrSe

	Start	End
0	Γ (0.000, 0.000, 0.000)	M (0.500, 0.000, 0.000)
1	M (0.500, 0.000, 0.000)	K (0.333, 0.333, 0.000)
2	K (0.333, 0.333, 0.000)	Γ (0.000, 0.000, 0.000)
3	Γ (0.000, 0.000, 0.000)	A (0.000, 0.000, 0.500)
4	A (0.000, 0.000, 0.500)	L (0.500, 0.000, 0.500)
5	L (0.500, 0.000, 0.500)	H (0.333, 0.333, 0.500)
6	H (0.333, 0.333, 0.500)	A (0.000, 0.000, 0.500)
7	L (0.500, 0.000, 0.500)	M (0.500, 0.000, 0.000)
8	H (0.333, 0.333, 0.500)	K (0.333, 0.333, 0.000)

Supplementary Table 195: Single Weyl points along high symmetry lines of mp-1183040-ZrSe.

	Coordinates	Frequency	Mode 1	Mode 2	Multi.	Deg.	Path	Ratio
0	(0.333, 0.333, 0.319)	4.147	2	1	4	2	9	0.362
1	(0.333, 0.333, 0.247)	4.379	2	1	4	2	9	0.507
2	(0.333, 0.333, 0.260)	4.449	3	2	4	2	9	0.480
3	(0.333, 0.333, 0.044)	5.710	4	3	4	2	9	0.912
4	(0.333, 0.333, 0.264)	6.566	5	4	4	2	9	0.471
5	(0.333, 0.333, 0.156)	6.723	6	5	4	2	9	0.688

Supplementary Table 196: Nodal ring points along high symmetry lines of mp-1183040-ZrSe.

	Coordinates	Frequency	Mode 1	Mode 2	Multi.	Deg.	Path	Ratio
0	(0.087, -0.002, 0.500)	4.792	3	2	12	2	5	0.174
1	(0.287, -0.000, 0.000)	6.751	6	5	6	2	1	0.574

The followings are details for AgPd. It has 4 nodal ring points and 1 single Weyl point along high symmetry line as shown in Fig 111.

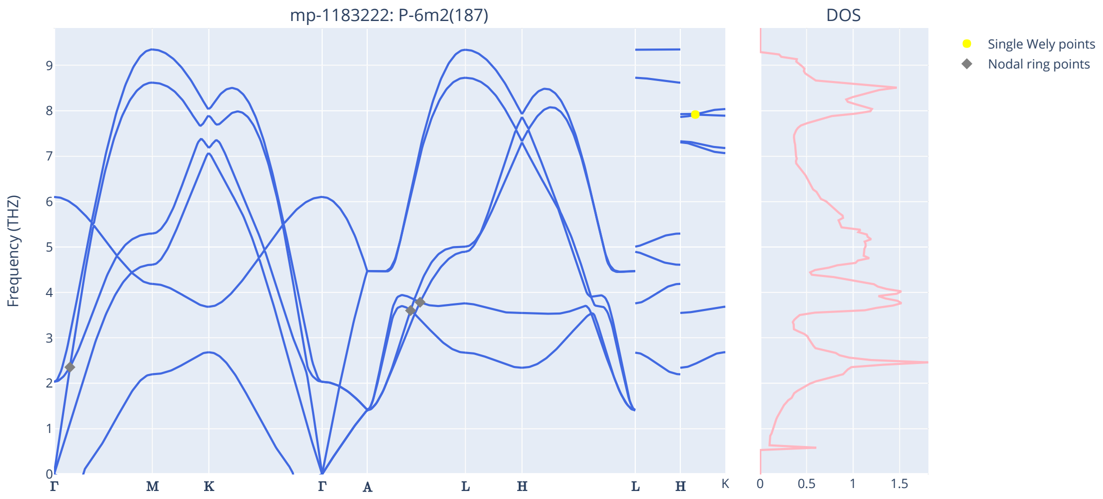

Supplementary Figure 111: The phonon band and DOS of AgPd.

Supplementary Table 197: High symmetry lines of mp-1183222-AgPd

	Start	End
0	Γ (0.000, 0.000, 0.000)	M (0.500, 0.000, 0.000)
1	M (0.500, 0.000, 0.000)	K (0.333, 0.333, 0.000)
2	K (0.333, 0.333, 0.000)	Γ (0.000, 0.000, 0.000)
3	Γ (0.000, 0.000, 0.000)	A (0.000, 0.000, 0.500)
4	A (0.000, 0.000, 0.500)	L (0.500, 0.000, 0.500)
5	L (0.500, 0.000, 0.500)	H (0.333, 0.333, 0.500)
6	H (0.333, 0.333, 0.500)	A (0.000, 0.000, 0.500)
7	L (0.500, 0.000, 0.500)	M (0.500, 0.000, 0.000)
8	H (0.333, 0.333, 0.500)	K (0.333, 0.333, 0.000)

Supplementary Table 198: Single Weyl points along high symmetry lines of mp-1183222-AgPd.

	Coordinates	Frequency	Mode 1	Mode 2	Multi.	Deg.	Path	Ratio
0	(0.333, 0.333, 0.334)	7.917	6	5	4	2	9	0.333

Supplementary Table 199: Nodal ring points along high symmetry lines of mp-1183222-AgPd.

	Coordinates	Frequency	Mode 1	Mode 2	Multi.	Deg.	Path	Ratio
0	(0.270, -0.000, 0.500)	3.785	3	2	6	2	5	0.539
1	(0.221, -0.000, 0.496)	3.602	3	2	12	2	5	0.442
2	(0.081, 0.000, 0.000)	2.354	4	3	6	2	1	0.161

Supplementary Table 199: Nodal ring points along high symmetry lines of mp-1183222-AgPd.

Coordinates	Frequency	Mode 1	Mode 2	Multi.	Deg.	Path	Ratio

The followings are details for AgAu. It has 2 nodal ring points and 2 single Weyl points along high symmetry line as shown in Fig 112.

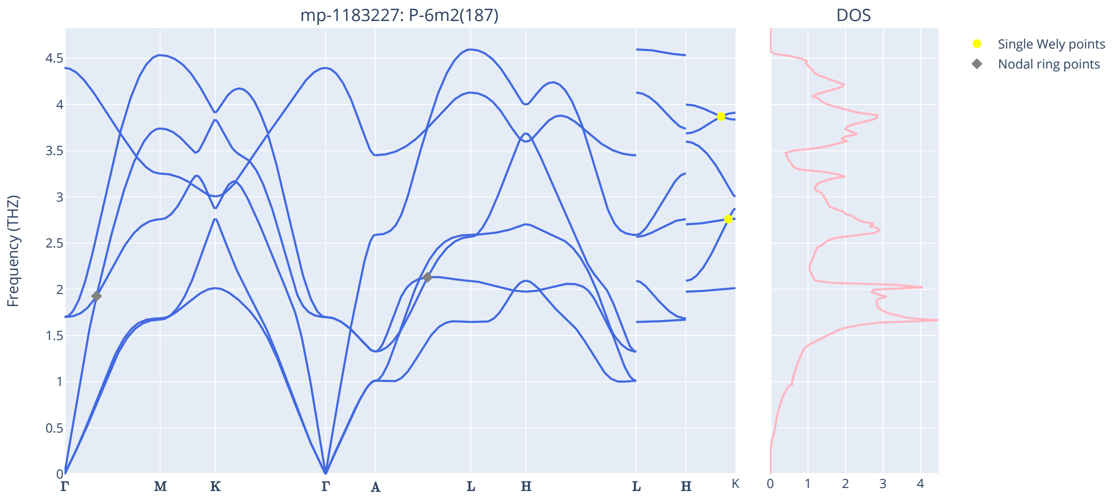

Supplementary Figure 112: The phonon band and DOS of AgAu.

Supplementary Table 200: High symmetry lines of mp-1183227-AgAu

	Start	End
0	Γ (0.000, 0.000, 0.000)	M (0.500, 0.000, 0.000)
1	M (0.500, 0.000, 0.000)	K (0.333, 0.333, 0.000)
2	K (0.333, 0.333, 0.000)	Γ (0.000, 0.000, 0.000)
3	Γ (0.000, 0.000, 0.000)	A (0.000, 0.000, 0.500)
4	A (0.000, 0.000, 0.500)	L (0.500, 0.000, 0.500)
5	L (0.500, 0.000, 0.500)	H (0.333, 0.333, 0.500)
6	H (0.333, 0.333, 0.500)	A (0.000, 0.000, 0.500)
7	L (0.500, 0.000, 0.500)	M (0.500, 0.000, 0.000)
8	H (0.333, 0.333, 0.500)	K (0.333, 0.333, 0.000)

Supplementary Table 201: Single Weyl points along high symmetry lines of mp-1183227-AgAu.

	Coordinates	Frequency	Mode 1	Mode 2	Multi.	Deg.	Path	Ratio
0	(0.333, 0.333, 0.072)	2.759	3	2	4	2	9	0.856
1	(0.333, 0.333, 0.146)	3.869	6	5	4	2	9	0.709

Supplementary Table 202: Nodal ring points along high symmetry lines of mp-1183227-AgAu.

	Coordinates	Frequency	Mode 1	Mode 2	Multi.	Deg.	Path	Ratio
0	(0.274, -0.000, 0.500)	2.129	3	2	6	2	5	0.547
1	(0.167, 0.000, 0.000)	1.926	4	3	6	2	1	0.335

Supplementary Table 202: Nodal ring points along high symmetry lines of mp-1183227-AgAu.

Coordinates	Frequency	Mode 1	Mode 2	Multi.	Deg.	Path	Ratio

The followings are details for K_2. It has 12 topological nodal lines and 7 nodal ring points along high symmetry line as shown in Fig **113**.

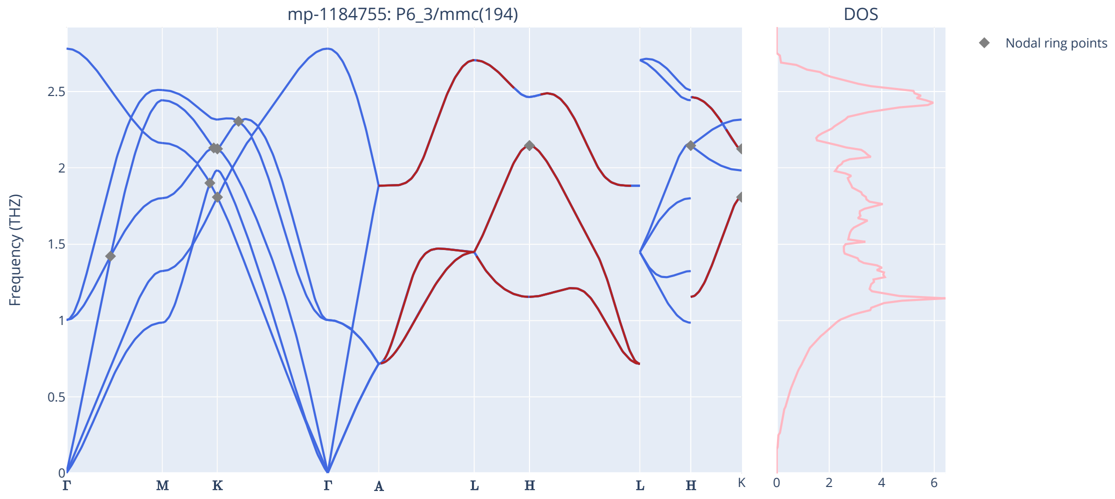

Supplementary Figure 113: The phonon band and DOS of K2.

Supplementary Table 203: High symmetry lines of mp-1184755-K2

	Start	End
0	Γ (0.000, 0.000, 0.000)	M (0.500, 0.000, 0.000)
1	M (0.500, 0.000, 0.000)	K (0.333, 0.333, 0.000)
2	K (0.333, 0.333, 0.000)	Γ (0.000, 0.000, 0.000)
3	Γ (0.000, 0.000, 0.000)	A (0.000, 0.000, 0.500)
4	A (0.000, 0.000, 0.500)	L (0.500, 0.000, 0.500)
5	L (0.500, 0.000, 0.500)	H (0.333, 0.333, 0.500)
6	H (0.333, 0.333, 0.500)	A (0.000, 0.000, 0.500)
7	L (0.500, 0.000, 0.500)	M (0.500, 0.000, 0.000)
8	H (0.333, 0.333, 0.500)	K (0.333, 0.333, 0.000)

Supplementary Table 204: Nodal ring points along high symmetry lines of mp-1184755-K2.

	Coordinates	Frequency	Mode 1	Mode 2	Multi.	Deg.	Path	Ratio
0	(0.333, 0.333, 0.000)	1.808	2	1	2	2	2	1.000
1	(0.357, 0.288, -0.000)	1.901	3	2	12	2	2	0.864
2	(0.230, 0.000, -0.000)	1.422	4	3	6	2	1	0.459
3	(0.333, 0.333, 0.500)	2.146	4	3	2	2	6	1.000
4	(0.344, 0.311, 0.000)	2.130	5	4	6	2	2	0.934
5	(0.333, 0.333, 0.000)	2.124	5	4	2	2	2	1.000
6	(0.269, 0.269, -0.000)	2.305	6	5	6	2	3	0.192

The followings are details for TaP and it only has 4 single Weyl point along high symmetry line as shown in Fig **114**.

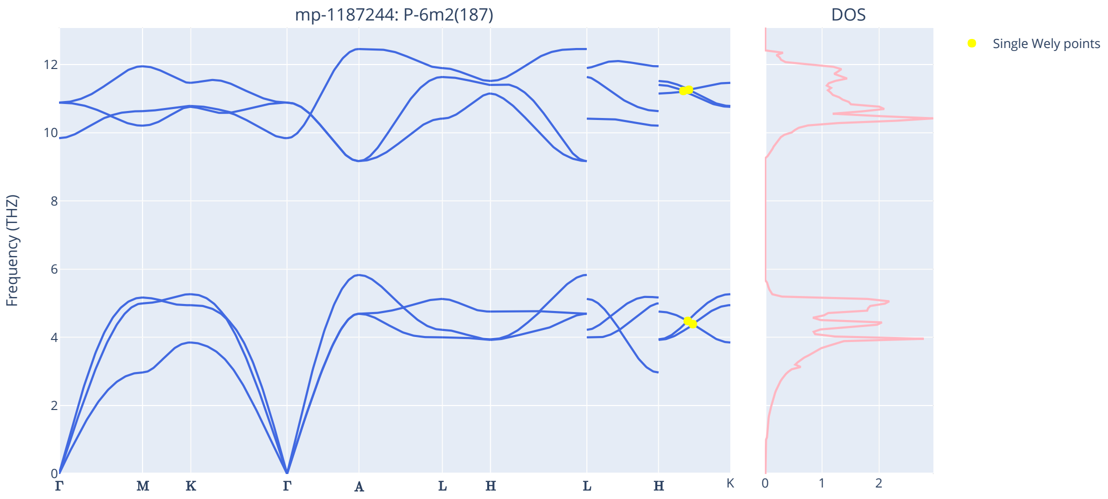

Supplementary Figure 114: The phonon band and DOS of TaP.

Supplementary Table 205: High symmetry lines of mp-1187244-TaP

	Start	End
0	Γ (0.000, 0.000, 0.000)	M (0.500, 0.000, 0.000)
1	M (0.500, 0.000, 0.000)	K (0.333, 0.333, 0.000)
2	K (0.333, 0.333, 0.000)	Γ (0.000, 0.000, 0.000)
3	Γ (0.000, 0.000, 0.000)	A (0.000, 0.000, 0.500)
4	A (0.000, 0.000, 0.500)	L (0.500, 0.000, 0.500)
5	L (0.500, 0.000, 0.500)	H (0.333, 0.333, 0.500)
6	H (0.333, 0.333, 0.500)	A (0.000, 0.000, 0.500)
7	L (0.500, 0.000, 0.500)	M (0.500, 0.000, 0.000)
8	H (0.333, 0.333, 0.500)	K (0.333, 0.333, 0.000)

Supplementary Table 206: Single Weyl points along high symmetry lines of mp-1187244-TaP.

	Coordinates	Frequency	Mode 1	Mode 2	Multi.	Deg.	Path	Ratio
0	(0.333, 0.333, 0.261)	4.387	2	1	4	2	9	0.477
1	(0.333, 0.333, 0.297)	4.480	3	2	4	2	9	0.407
2	(0.333, 0.333, 0.327)	11.225	5	4	4	2	9	0.345
3	(0.333, 0.333, 0.289)	11.259	6	5	4	2	9	0.423

The followings are details for Zn_4Se_4. It has 2 topological nodal lines and 2 nodal ring points along high symmetry line as shown in Fig 115.

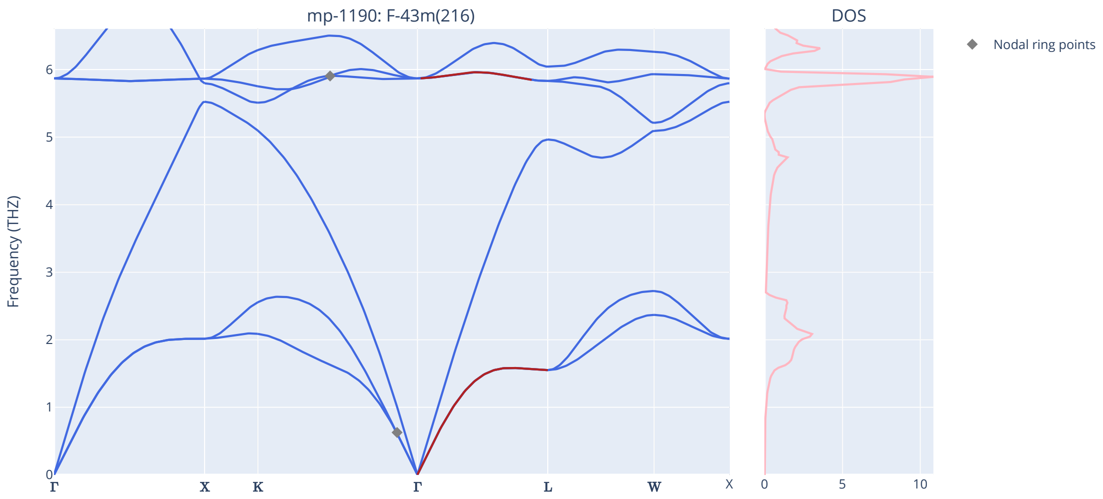

Supplementary Figure 115: The phonon band and DOS of Zn4Se4.

Supplementary Table 207: High symmetry lines of mp-1190-Zn4Se4

	Start	End
0	Γ (0.000, 0.000, 0.000)	X (0.500, 0.000, 0.500)
1	X (0.500, 0.000, 0.500)	U (0.625, 0.250, 0.625)
2	K (0.375, 0.375, 0.750)	Γ (0.000, 0.000, 0.000)
3	Γ (0.000, 0.000, 0.000)	L (0.500, 0.500, 0.500)
4	L (0.500, 0.500, 0.500)	W (0.500, 0.250, 0.750)
5	W (0.500, 0.250, 0.750)	X (0.500, 0.000, 0.500)

Supplementary Table 208: Nodal ring points along high symmetry lines of mp-1190-Zn4Se4.

	Coordinates	Frequency	Mode 1	Mode 2	Multi.	Deg.	Path	Ratio
0	(0.046, 0.046, 0.097)	0.626	2	1	24	2	3	0.873
1	(0.207, 0.207, 0.409)	5.904	5	4	24	2	3	0.453

The followings are details for HfS. It has 1 nodal ring points and 4 single Weyl point along high symmetry line as shown in Fig **116**.

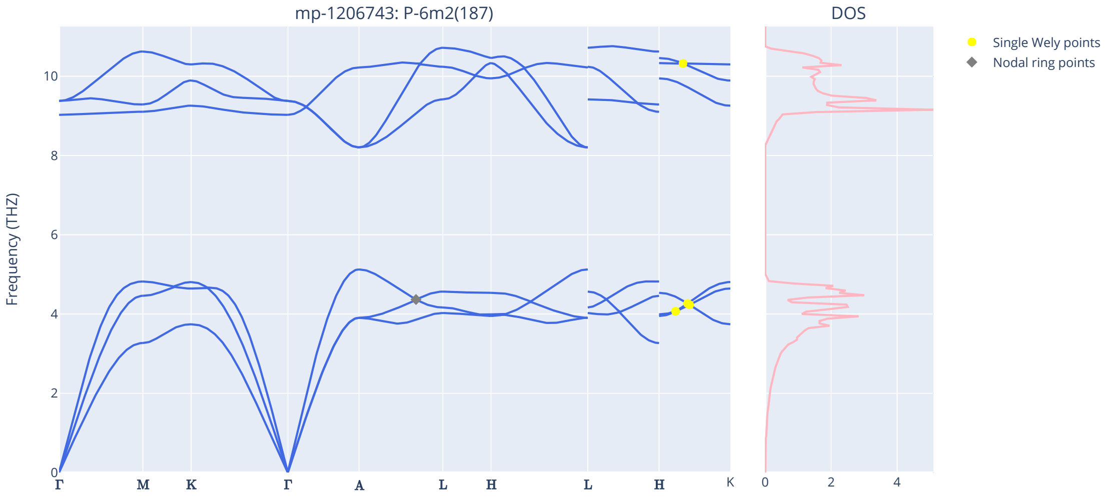

Supplementary Figure 116: The phonon band and DOS of HfS.

Supplementary Table 209: High symmetry lines of mp-1206743-HfS

	Start	End
0	Γ (0.000, 0.000, 0.000)	M (0.500, 0.000, 0.000)
1	M (0.500, 0.000, 0.000)	K (0.333, 0.333, 0.000)
2	K (0.333, 0.333, 0.000)	Γ (0.000, 0.000, 0.000)
3	Γ (0.000, 0.000, 0.000)	A (0.000, 0.000, 0.500)
4	A (0.000, 0.000, 0.500)	L (0.500, 0.000, 0.500)
5	L (0.500, 0.000, 0.500)	H (0.333, 0.333, 0.500)
6	H (0.333, 0.333, 0.500)	A (0.000, 0.000, 0.500)
7	L (0.500, 0.000, 0.500)	M (0.500, 0.000, 0.000)
8	H (0.333, 0.333, 0.500)	K (0.333, 0.333, 0.000)

Supplementary Table 210: Single Weyl points along high symmetry lines of mp-1206743-HfS.

	Coordinates	Frequency	Mode 1	Mode 2	Multi.	Deg.	Path	Ratio
0	(0.333, 0.333, 0.384)	4.067	2	1	4	2	9	0.232
1	(0.333, 0.333, 0.286)	4.231	2	1	4	2	9	0.428
2	(0.333, 0.333, 0.300)	4.264	3	2	4	2	9	0.400
3	(0.333, 0.333, 0.332)	10.323	6	5	4	2	9	0.336

Supplementary Table 211: Nodal ring points along high symmetry lines of mp-1206743-HfS.

	Coordinates	Frequency	Mode 1	Mode 2	Multi.	Deg.	Path	Ratio
0	(0.340, -0.001, 0.500)	4.365	3	2	12	2	5	0.680

Supplementary Table 211: Nodal ring points along high symmetry lines of mp-1206743-HfS.

Coordinates	Frequency	Mode 1	Mode 2	Multi.	Deg.	Path	Ratio

The followings are details for AuLi. It has 5 topological nodal lines and 3 nodal ring points along high symmetry line as shown in Fig **117**.

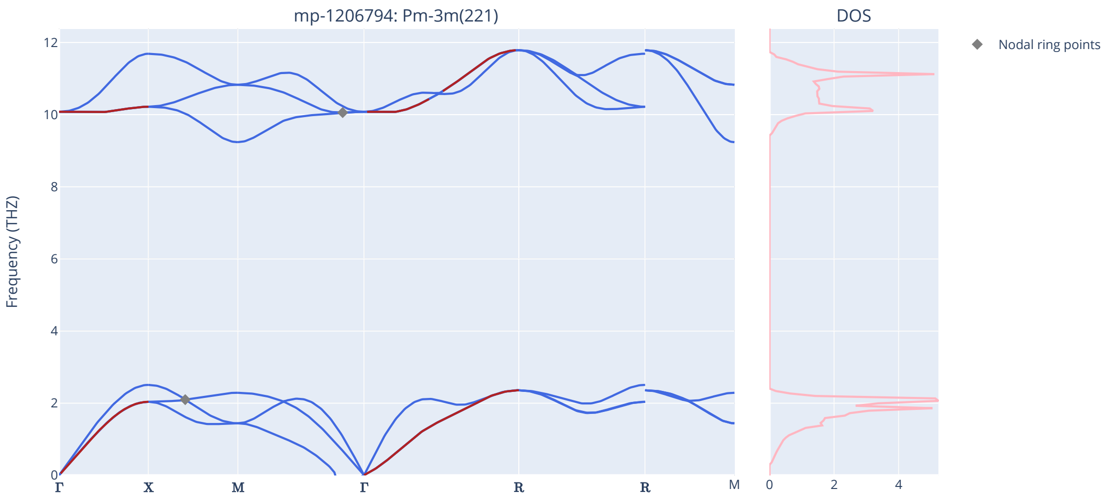

Supplementary Figure 117: The phonon band and DOS of AuLi.

Supplementary Table 212: High symmetry lines of mp-1206794-AuLi

	Start	End
0	Γ (0.000, 0.000, 0.000)	X (0.000, 0.500, 0.000)
1	X (0.000, 0.500, 0.000)	M (0.500, 0.500, 0.000)
2	M (0.500, 0.500, 0.000)	Γ (0.000, 0.000, 0.000)
3	Γ (0.000, 0.000, 0.000)	R (0.500, 0.500, 0.500)
4	R (0.500, 0.500, 0.500)	X (0.000, 0.500, 0.000)
5	R (0.500, 0.500, 0.500)	M (0.500, 0.500, 0.000)

Supplementary Table 213: Nodal ring points along high symmetry lines of mp-1206794-AuLi.

	Coordinates	Frequency	Mode 1	Mode 2	Multi.	Deg.	Path	Ratio
0	(0.206, 0.500, -0.000)	2.096	3	2	12	2	2	0.412
1	(0.085, 0.085, 0.000)	10.057	5	4	12	2	3	0.831

The followings are details for Co_4H_4. It has 2 topological nodal lines and 3 nodal ring points along high symmetry line as shown in Fig **118**.

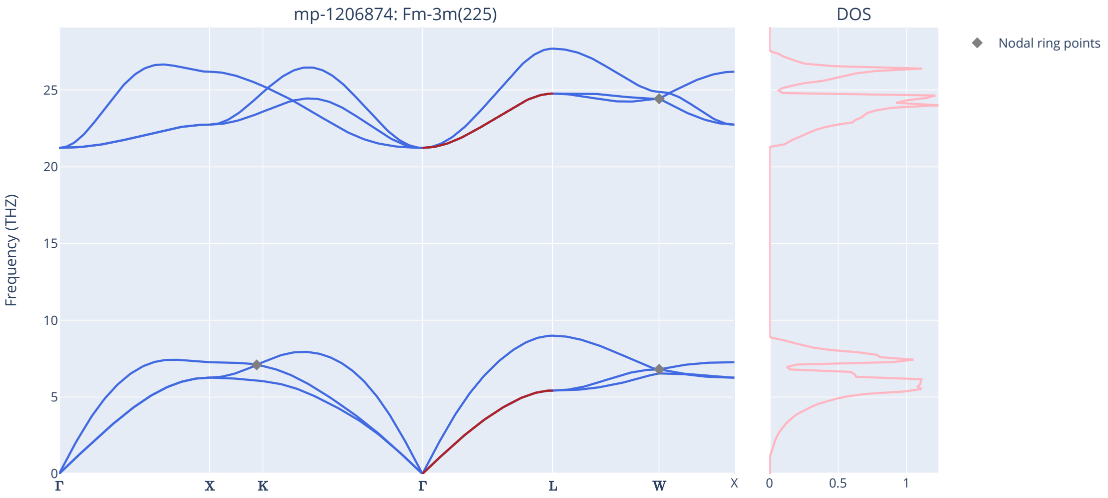

Supplementary Figure 118: The phonon band and DOS of Co4H4.

Supplementary Table 214: High symmetry lines of mp-1206874-Co4H4

	Start	End
0	Γ (0.000, 0.000, 0.000)	X (0.500, 0.000, 0.500)
1	X (0.500, 0.000, 0.500)	U (0.625, 0.250, 0.625)
2	K (0.375, 0.375, 0.750)	Γ (0.000, 0.000, 0.000)
3	Γ (0.000, 0.000, 0.000)	L (0.500, 0.500, 0.500)
4	L (0.500, 0.500, 0.500)	W (0.500, 0.250, 0.750)
5	W (0.500, 0.250, 0.750)	X (0.500, 0.000, 0.500)

Supplementary Table 215: Nodal ring points along high symmetry lines of mp-1206874-Co4H4.

	Coordinates	Frequency	Mode 1	Mode 2	Multi.	Deg.	Path	Ratio
0	(0.611, 0.221, 0.611)	7.091	3	2	12	2	2	0.885
1	(0.500, 0.250, 0.750)	6.801	3	2	6	2	5	1.000
2	(0.500, 0.250, 0.750)	24.432	5	4	6	2	5	1.000

The followings are details for CdSc. It has 6 topological nodal lines and 4 nodal ring points along high symmetry line as shown in Fig **119**.

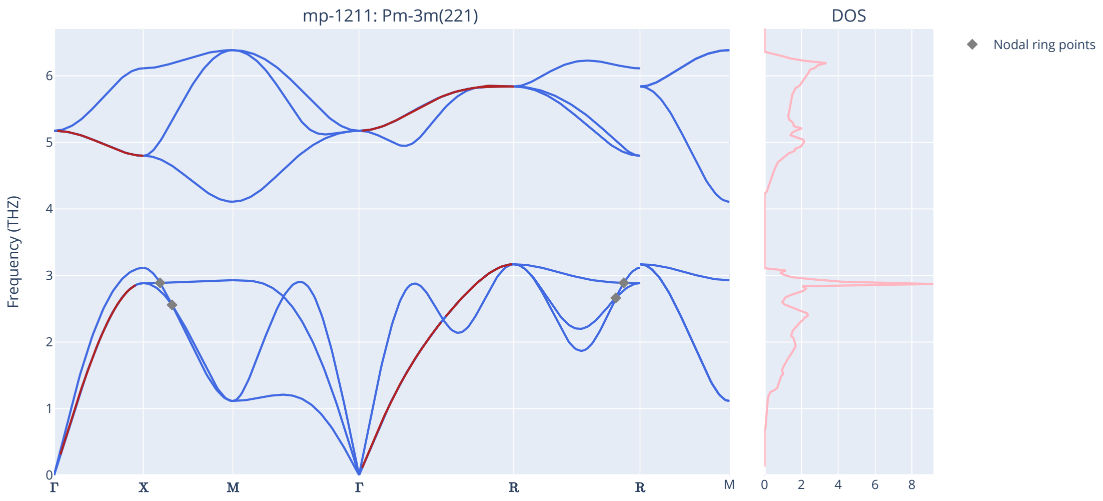

Supplementary Figure 119: The phonon band and DOS of CdSc.

Supplementary Table 216: High symmetry lines of mp-1211-CdSc

	Start	End
0	Γ (0.000, 0.000, 0.000)	X (0.000, 0.500, 0.000)
1	X (0.000, 0.500, 0.000)	M (0.500, 0.500, 0.000)
2	M (0.500, 0.500, 0.000)	Γ (0.000, 0.000, 0.000)
3	Γ (0.000, 0.000, 0.000)	R (0.500, 0.500, 0.500)
4	R (0.500, 0.500, 0.500)	X (0.000, 0.500, 0.000)
5	R (0.500, 0.500, 0.500)	M (0.500, 0.500, 0.000)

Supplementary Table 217: Nodal ring points along high symmetry lines of mp-1211-CdSc.

	Coordinates	Frequency	Mode 1	Mode 2	Multi.	Deg.	Path	Ratio
0	(0.160, 0.500, -0.000)	2.559	2	1	12	2	2	0.321
1	(0.096, 0.500, 0.096)	2.663	2	1	12	2	5	0.807
2	(0.093, 0.500, -0.000)	2.890	3	2	12	2	2	0.186
3	(0.065, 0.500, 0.065)	2.889	3	2	12	2	5	0.871

The followings are details for RhTc. It has 5 nodal ring points and 2 single Weyl points along high symmetry line as shown in Fig 120.

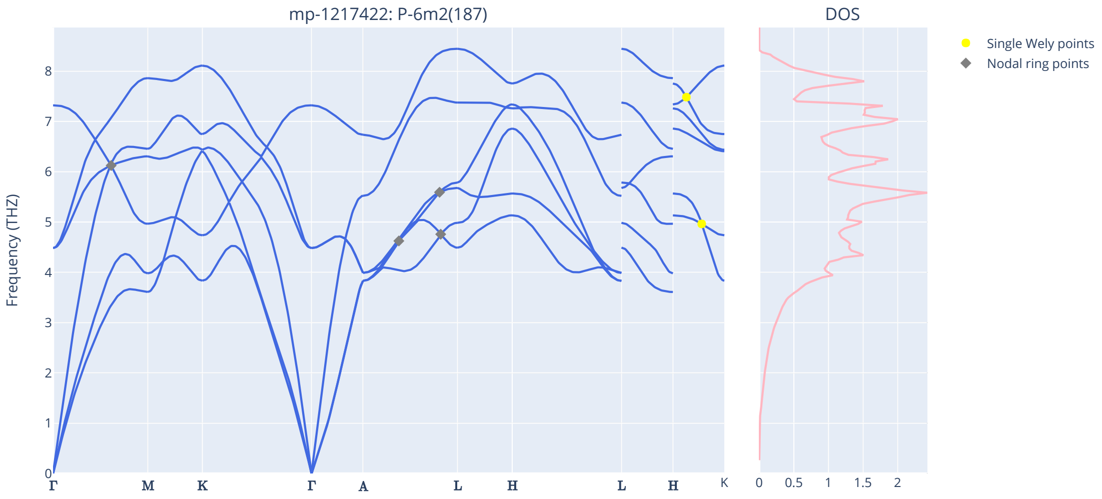

Supplementary Figure 120: The phonon band and DOS of RhTc.

Supplementary Table 218: High symmetry lines of mp-1217422-RhTc

	Start	End
0	Γ (0.000, 0.000, 0.000)	M (0.500, 0.000, 0.000)
1	M (0.500, 0.000, 0.000)	K (0.333, 0.333, 0.000)
2	K (0.333, 0.333, 0.000)	Γ (0.000, 0.000, 0.000)
3	Γ (0.000, 0.000, 0.000)	A (0.000, 0.000, 0.500)
4	A (0.000, 0.000, 0.500)	L (0.500, 0.000, 0.500)
5	L (0.500, 0.000, 0.500)	H (0.333, 0.333, 0.500)
6	H (0.333, 0.333, 0.500)	A (0.000, 0.000, 0.500)
7	L (0.500, 0.000, 0.500)	M (0.500, 0.000, 0.000)
8	H (0.333, 0.333, 0.500)	K (0.333, 0.333, 0.000)

Supplementary Table 219: Single Weyl points along high symmetry lines of mp-1217422-RhTc.

	Coordinates	Frequency	Mode 1	Mode 2	Multi.	Deg.	Path	Ratio
0	(0.333, 0.333, 0.220)	4.963	2	1	4	2	9	0.560
1	(0.333, 0.333, 0.369)	7.483	6	5	4	2	9	0.262

Supplementary Table 220: Nodal ring points along high symmetry lines of mp-1217422-RhTc.

	Coordinates	Frequency	Mode 1	Mode 2	Multi.	Deg.	Path	Ratio
0	(0.411, 0.000, 0.500)	4.757	2	1	6	2	5	0.821

Supplementary Table 220: Nodal ring points along high symmetry lines of mp-1217422-RhTc.

	Coordinates	Frequency	Mode 1	Mode 2	Multi.	Deg.	Path	Ratio
1	(0.189, -0.000, 0.500)	4.624	3	2	6	2	5	0.379
2	(0.307, -0.001, 0.000)	6.124	4	3	10	2	1	0.614
3	(0.405, -0.000, 0.500)	5.592	4	3	6	2	5	0.809

The followings are details for OsRe. It has 3 nodal ring points and 3 single Weyl points along high symmetry line as shown in Fig 121.

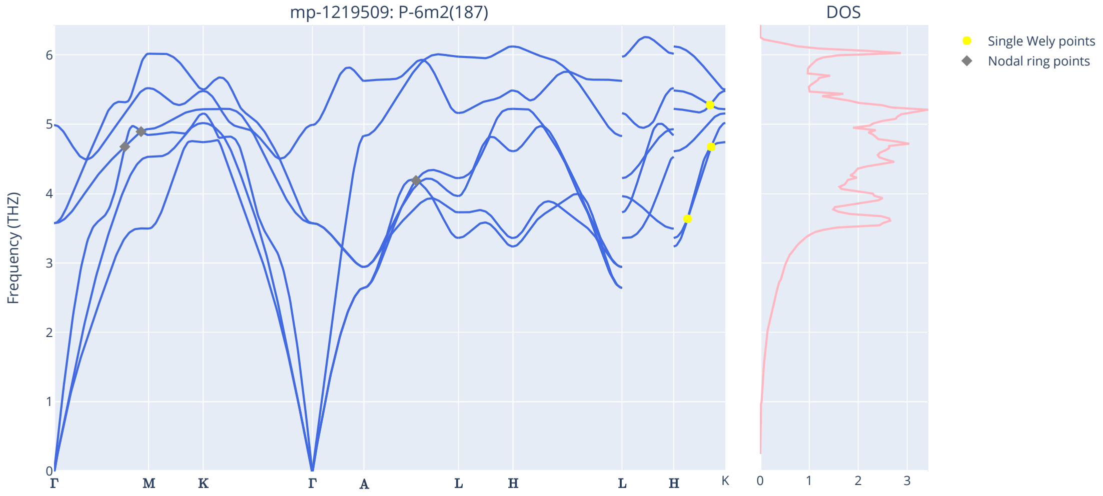

Supplementary Figure 121: The phonon band and DOS of OsRe.

Supplementary Table 221: High symmetry lines of mp-1219509-OsRe

	Start	End
0	Γ (0.000, 0.000, 0.000)	M (0.500, 0.000, 0.000)
1	M (0.500, 0.000, 0.000)	K (0.333, 0.333, 0.000)
2	K (0.333, 0.333, 0.000)	Γ (0.000, 0.000, 0.000)
3	Γ (0.000, 0.000, 0.000)	A (0.000, 0.000, 0.500)
4	A (0.000, 0.000, 0.500)	L (0.500, 0.000, 0.500)
5	L (0.500, 0.000, 0.500)	H (0.333, 0.333, 0.500)
6	H (0.333, 0.333, 0.500)	A (0.000, 0.000, 0.500)
7	L (0.500, 0.000, 0.500)	M (0.500, 0.000, 0.000)
8	H (0.333, 0.333, 0.500)	K (0.333, 0.333, 0.000)

Supplementary Table 222: Single Weyl points along high symmetry lines of mp-1219509-OsRe.

	Coordinates	Frequency	Mode 1	Mode 2	Multi.	Deg.	Path	Ratio
0	(0.333, 0.333, 0.366)	3.638	2	1	4	2	9	0.268
1	(0.333, 0.333, 0.137)	4.675	2	1	4	2	9	0.726
2	(0.333, 0.333, 0.149)	5.278	5	4	4	2	9	0.703

Supplementary Table 223: Nodal ring points along high symmetry lines of mp-1219509-OsRe.

	Coordinates	Frequency	Mode 1	Mode 2	Multi.	Deg.	Path	Ratio
0	(0.460, 0.000, 0.000)	4.894	4	3	6	2	1	0.920

Supplementary Table 223: Nodal ring points along high symmetry lines of mp-1219509-OsRe.

	Coordinates	Frequency	Mode 1	Mode 2	Multi.	Deg.	Path	Ratio
1	(0.374, -0.000, -0.000)	4.677	4	3	6	2	1	0.748
2	(0.275, -0.000, 0.500)	4.190	4	3	6	2	5	0.551

The followings are details for CrIr. It has 1 nodal ring point and 1 single Weyl point along high symmetry line as shown in Fig 122.

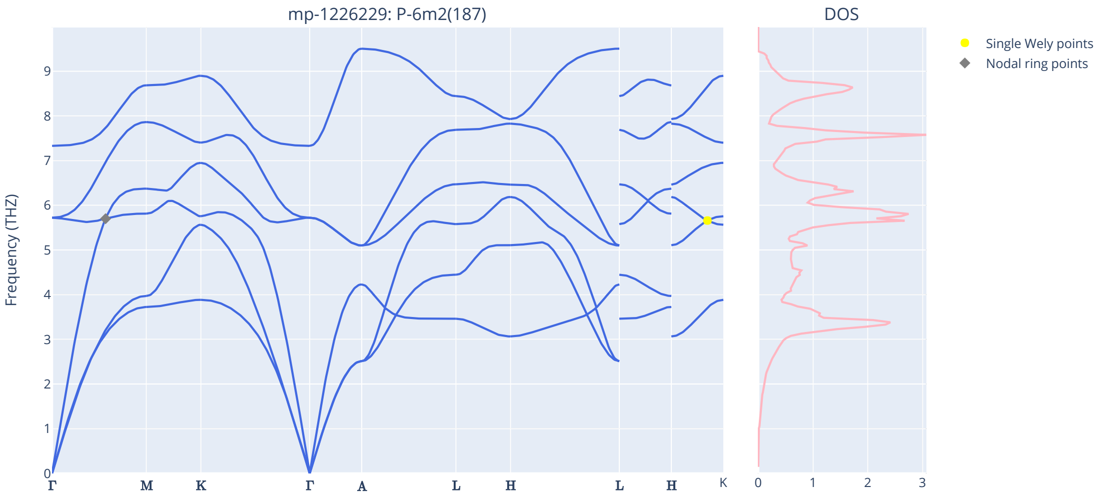

Supplementary Figure 122: The phonon band and DOS of CrIr.

Supplementary Table 224: High symmetry lines of mp-1226229-CrIr

	Start	End
0	Γ (0.000, 0.000, 0.000)	M (0.500, 0.000, 0.000)
1	M (0.500, 0.000, 0.000)	K (0.333, 0.333, 0.000)
2	K (0.333, 0.333, 0.000)	Γ (0.000, 0.000, 0.000)
3	Γ (0.000, 0.000, 0.000)	A (0.000, 0.000, 0.500)
4	A (0.000, 0.000, 0.500)	L (0.500, 0.000, 0.500)
5	L (0.500, 0.000, 0.500)	H (0.333, 0.333, 0.500)
6	H (0.333, 0.333, 0.500)	A (0.000, 0.000, 0.500)
7	L (0.500, 0.000, 0.500)	M (0.500, 0.000, 0.000)
8	H (0.333, 0.333, 0.500)	K (0.333, 0.333, 0.000)

Supplementary Table 225: Single Weyl points along high symmetry lines of mp-1226229-CrIr.

	Coordinates	Frequency	Mode 1	Mode 2	Multi.	Deg.	Path	Ratio
0	(0.333, 0.333, 0.153)	5.658	3	2	4	2	9	0.694

Supplementary Table 226: Nodal ring points along high symmetry lines of mp-1226229-CrIr.

	Coordinates	Frequency	Mode 1	Mode 2	Multi.	Deg.	Path	Ratio
0	(0.284, 0.000, -0.000)	5.699	4	3	6	2	1	0.567

The followings are details for AgAl. It has 1 nodal ring points and 4 single Weyl point along high symmetry line as shown in Fig 123.

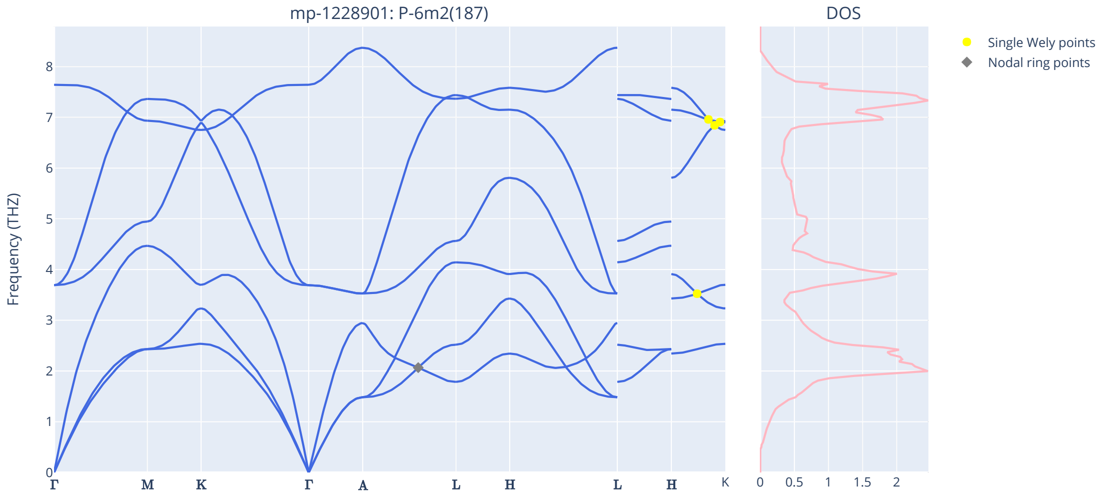

Supplementary Figure 123: The phonon band and DOS of AgAl.

Supplementary Table 227: High symmetry lines of mp-1228901-AgAl

	Start	End
0	Γ (0.000, 0.000, 0.000)	M (0.500, 0.000, 0.000)
1	M (0.500, 0.000, 0.000)	K (0.333, 0.333, 0.000)
2	K (0.333, 0.333, 0.000)	Γ (0.000, 0.000, 0.000)
3	Γ (0.000, 0.000, 0.000)	A (0.000, 0.000, 0.500)
4	A (0.000, 0.000, 0.500)	L (0.500, 0.000, 0.500)
5	L (0.500, 0.000, 0.500)	H (0.333, 0.333, 0.500)
6	H (0.333, 0.333, 0.500)	A (0.000, 0.000, 0.500)
7	L (0.500, 0.000, 0.500)	M (0.500, 0.000, 0.000)
8	H (0.333, 0.333, 0.500)	K (0.333, 0.333, 0.000)

Supplementary Table 228: Single Weyl points along high symmetry lines of mp-1228901-AgAl.

	Coordinates	Frequency	Mode 1	Mode 2	Multi.	Deg.	Path	Ratio
0	(0.333, 0.333, 0.261)	3.523	3	2	4	2	9	0.477
1	(0.333, 0.333, 0.100)	6.842	5	4	4	2	9	0.800
2	(0.333, 0.333, 0.049)	6.907	6	5	4	2	9	0.901
3	(0.333, 0.333, 0.156)	6.959	6	5	4	2	9	0.687

Supplementary Table 229: Nodal ring points along high symmetry lines of mp-1228901-AgAl.

	Coordinates	Frequency	Mode 1	Mode 2	Multi.	Deg.	Path	Ratio
0	(0.298, -0.001, 0.500)	2.066	2	1	10	2	5	0.596

Supplementary Table 229: Nodal ring points along high symmetry lines of mp-1228901-AgAl.

Coordinates	Frequency	Mode 1	Mode 2	Multi.	Deg.	Path	Ratio

The followings are details for Mg_4Se_4. It has 2 topological nodal lines and 2 nodal ring points along high symmetry line as shown in Fig 124.

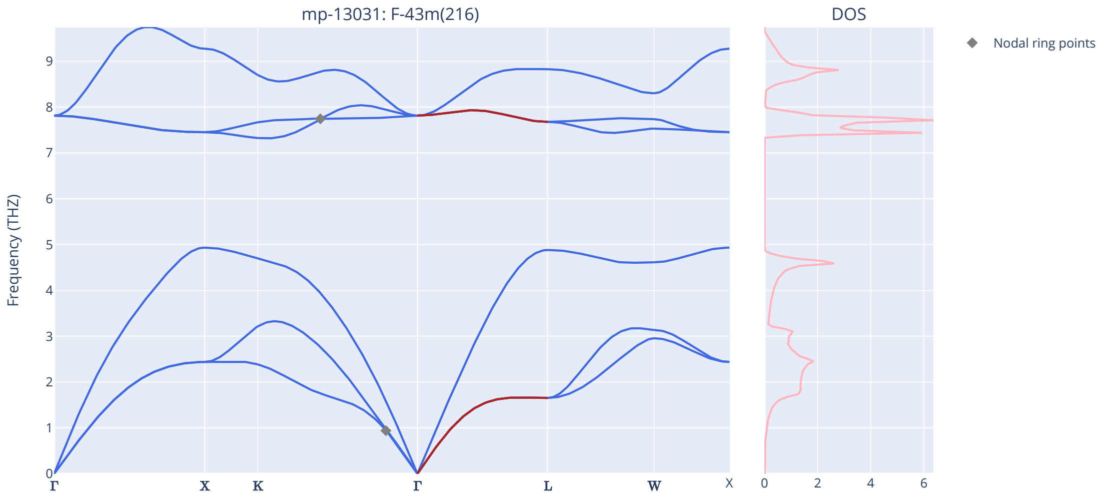

Supplementary Figure 124: The phonon band and DOS of Mg4Se4.

Supplementary Table 230: High symmetry lines of mp-13031-Mg4Se4

	Start	End
0	Γ (0.000, 0.000, 0.000)	X (0.500, 0.000, 0.500)
1	X (0.500, 0.000, 0.500)	U (0.625, 0.250, 0.625)
2	K (0.375, 0.375, 0.750)	Γ (0.000, 0.000, 0.000)
3	Γ (0.000, 0.000, 0.000)	L (0.500, 0.500, 0.500)
4	L (0.500, 0.500, 0.500)	W (0.500, 0.250, 0.750)
5	W (0.500, 0.250, 0.750)	X (0.500, 0.000, 0.500)

Supplementary Table 231: Nodal ring points along high symmetry lines of mp-13031-Mg4Se4.

	Coordinates	Frequency	Mode 1	Mode 2	Multi.	Deg.	Path	Ratio
0	(0.077, 0.077, 0.145)	0.940	2	1	24	2	3	0.803
1	(0.230, 0.230, 0.454)	7.743	5	4	24	2	3	0.392

The followings are details for Mg_4S_4 and it only has 2 topological nodal lines along high symmetry line as shown in Fig **125**.

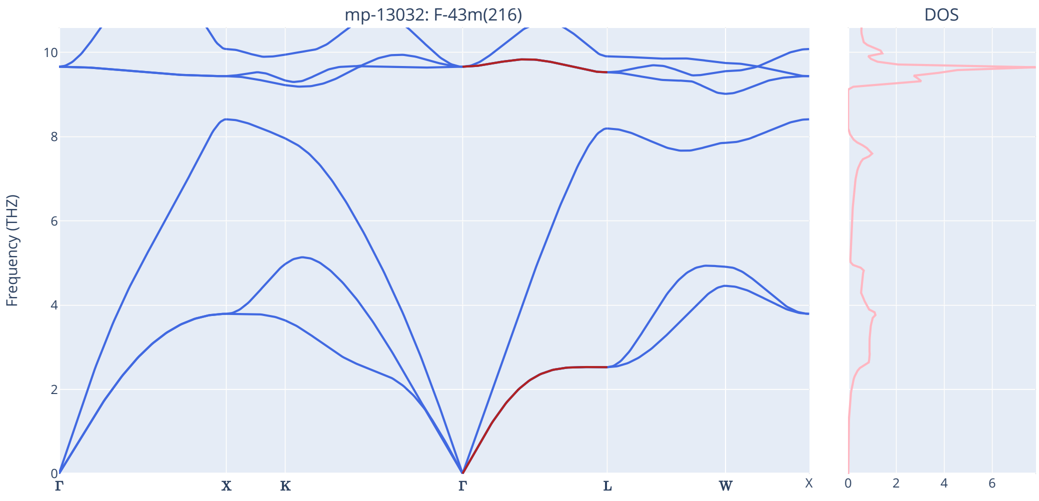

Supplementary Figure 125: The phonon band and DOS of Mg4S4.

Supplementary Table 232: High symmetry lines of mp-13032-Mg4S4

	Start	End
0	Γ (0.000, 0.000, 0.000)	X (0.500, 0.000, 0.500)
1	X (0.500, 0.000, 0.500)	U (0.625, 0.250, 0.625)
2	K (0.375, 0.375, 0.750)	Γ (0.000, 0.000, 0.000)
3	Γ (0.000, 0.000, 0.000)	L (0.500, 0.500, 0.500)
4	L (0.500, 0.500, 0.500)	W (0.500, 0.250, 0.750)
5	W (0.500, 0.250, 0.750)	X (0.500, 0.000, 0.500)

The followings are details for Mg_4Te_4 and it only has 2 topological nodal lines along high symmetry line as shown in Fig **126**.

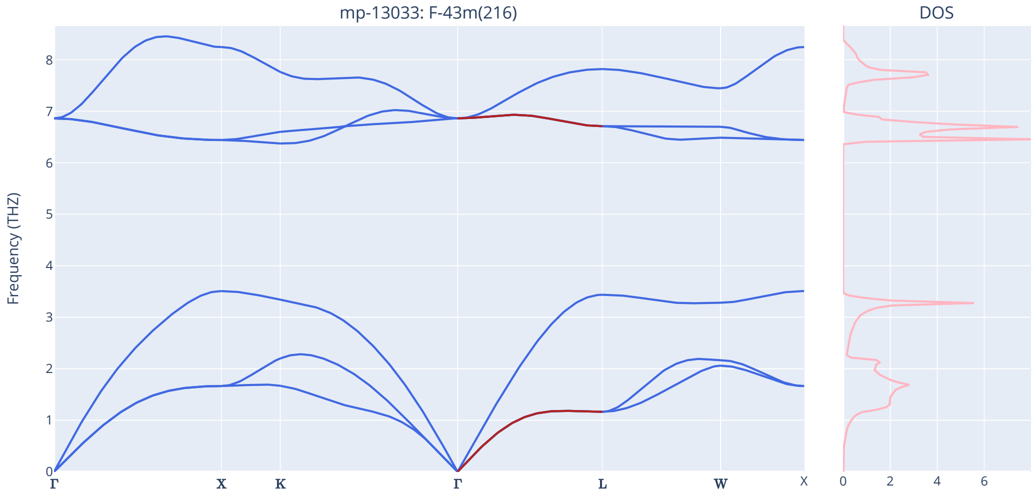

Supplementary Figure 126: The phonon band and DOS of Mg4Te4.

Supplementary Table 233: High symmetry lines of mp-13033-Mg4Te4

	Start	End
0	Γ (0.000, 0.000, 0.000)	X (0.500, 0.000, 0.500)
1	X (0.500, 0.000, 0.500)	U (0.625, 0.250, 0.625)
2	K (0.375, 0.375, 0.750)	Γ (0.000, 0.000, 0.000)
3	Γ (0.000, 0.000, 0.000)	L (0.500, 0.500, 0.500)
4	L (0.500, 0.500, 0.500)	W (0.500, 0.250, 0.750)
5	W (0.500, 0.250, 0.750)	X (0.500, 0.000, 0.500)

The followings are details for Zr_2. It has 12 topological nodal lines and 10 nodal ring points along high symmetry line as shown in Fig **127**.

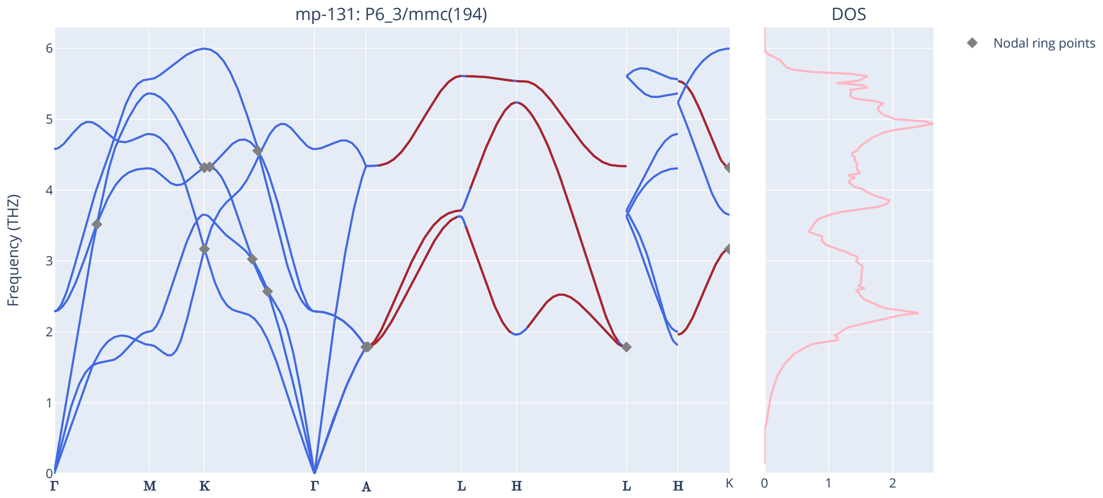

Supplementary Figure 127: The phonon band and DOS of Zr2.

Supplementary Table 234: High symmetry lines of mp-131-Zr2

	Start	End
0	Γ (0.000, 0.000, 0.000)	M (0.500, 0.000, 0.000)
1	M (0.500, 0.000, 0.000)	K (0.333, 0.333, 0.000)
2	K (0.333, 0.333, 0.000)	Γ (0.000, 0.000, 0.000)
3	Γ (0.000, 0.000, 0.000)	A (0.000, 0.000, 0.500)
4	A (0.000, 0.000, 0.500)	L (0.500, 0.000, 0.500)
5	L (0.500, 0.000, 0.500)	H (0.333, 0.333, 0.500)
6	H (0.333, 0.333, 0.500)	A (0.000, 0.000, 0.500)
7	L (0.500, 0.000, 0.500)	M (0.500, 0.000, 0.000)
8	H (0.333, 0.333, 0.500)	K (0.333, 0.333, 0.000)

Supplementary Table 235: Nodal ring points along high symmetry lines of mp-131-Zr2.

	Coordinates	Frequency	Mode 1	Mode 2	Multi.	Deg.	Path	Ratio
0	(0.333, 0.333, 0.000)	3.169	2	1	2	2	2	1.000
1	(0.189, 0.189, 0.000)	3.026	3	2	6	2	3	0.434
2	(0.142, 0.142, 0.000)	2.572	3	2	6	2	3	0.573
3	(0.010, 0.000, 0.500)	1.790	3	2	6	2	5	0.020
4	(0.225, -0.000, 0.000)	3.518	4	3	6	2	1	0.449
5	(0.000, 0.000, 0.500)	1.787	4	3	1	4	4	1.000
6	(0.318, 0.318, 0.000)	4.327	5	4	6	2	3	0.047
7	(0.333, 0.333, 0.000)	4.318	5	4	2	2	2	1.000
8	(0.172, 0.172, -0.000)	4.556	6	5	6	2	3	0.485

The followings are details for Ca_2. It has 11 topological nodal lines and 18 nodal ring points along high symmetry line as shown in Fig **128**.

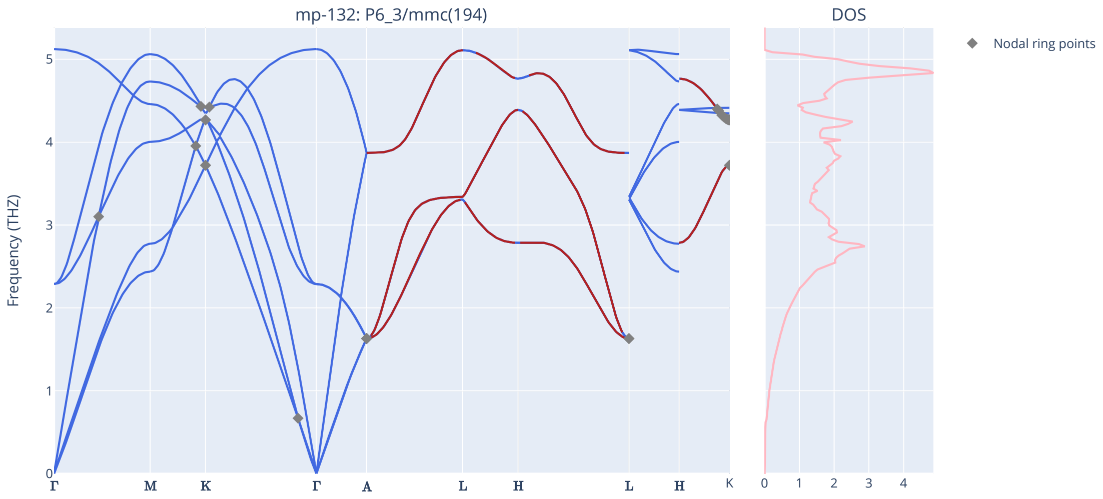

Supplementary Figure 128: The phonon band and DOS of Ca2.

Supplementary Table 236: High symmetry lines of mp-132-Ca2

	Start	End
0	Γ (0.000, 0.000, 0.000)	M (0.500, 0.000, 0.000)
1	M (0.500, 0.000, 0.000)	K (0.333, 0.333, 0.000)
2	K (0.333, 0.333, 0.000)	Γ (0.000, 0.000, 0.000)
3	Γ (0.000, 0.000, 0.000)	A (0.000, 0.000, 0.500)
4	A (0.000, 0.000, 0.500)	L (0.500, 0.000, 0.500)
5	L (0.500, 0.000, 0.500)	H (0.333, 0.333, 0.500)
6	H (0.333, 0.333, 0.500)	A (0.000, 0.000, 0.500)
7	L (0.500, 0.000, 0.500)	M (0.500, 0.000, 0.000)
8	H (0.333, 0.333, 0.500)	K (0.333, 0.333, 0.000)

Supplementary Table 237: Nodal ring points along high symmetry lines of mp-132-Ca2.

	Coordinates	Frequency	Mode 1	Mode 2	Multi.	Deg.	Path	Ratio
0	(0.057, 0.054, 0.000)	0.670	2	1	12	2	3	0.834
1	(0.333, 0.333, 0.000)	3.721	2	1	2	2	2	1.000
2	(0.000, 0.000, 0.500)	1.630	2	1	1	4	4	1.000
3	(0.362, 0.274, 0.000)	3.954	3	2	12	2	2	0.824
4	(0.000, 0.000, 0.500)	1.630	3	2	1	4	4	1.000
5	(0.232, -0.000, 0.000)	3.101	4	3	6	2	1	0.464
6	(0.333, 0.333, 0.000)	4.268	4	3	2	2	2	1.000
7	(0.000, 0.000, 0.500)	1.630	4	3	1	4	4	1.000
8	(0.333, 0.333, 0.066)	4.316	4	3	4	2	9	0.868
9	(0.333, 0.333, 0.034)	4.282	4	3	4	2	9	0.932
10	(0.332, 0.332, 0.076)	4.326	4	3	12	2	9	0.848

Supplementary Table 237: Nodal ring points along high symmetry lines of mp-132-Ca2.

	Coordinates	Frequency	Mode 1	Mode 2	Multi.	Deg.	Path	Ratio
11	(0.333, 0.333, 0.051)	4.298	4	3	4	2	9	0.898
12	(0.333, 0.333, 0.020)	4.273	4	3	4	2	9	0.959
13	(0.333, 0.333, 0.010)	4.270	4	3	4	2	9	0.980
14	(0.333, 0.333, 0.121)	4.400	5	4	4	2	9	0.758
15	(0.335, 0.335, 0.108)	4.382	5	4	12	2	9	0.783
16	(0.322, 0.322, 0.000)	4.422	6	5	6	2	3	0.034
17	(0.347, 0.305, -0.000)	4.431	6	5	6	2	2	0.916

The followings are details for Zr_4N_4. It has 2 topological nodal lines and 6 nodal ring points along high symmetry line as shown in Fig 129.

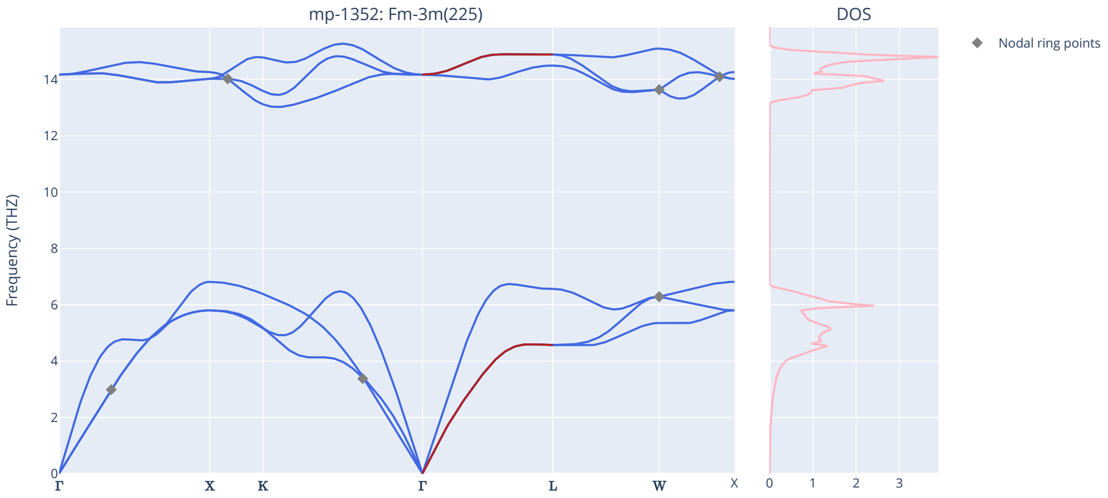

Supplementary Figure 129: The phonon band and DOS of Zr4N4.

Supplementary Table 238: High symmetry lines of mp-1352-Zr4N4

	Start	End
0	Γ (0.000, 0.000, 0.000)	X (0.500, 0.000, 0.500)
1	X (0.500, 0.000, 0.500)	U (0.625, 0.250, 0.625)
2	K (0.375, 0.375, 0.750)	Γ (0.000, 0.000, 0.000)
3	Γ (0.000, 0.000, 0.000)	L (0.500, 0.500, 0.500)
4	L (0.500, 0.500, 0.500)	W (0.500, 0.250, 0.750)
5	W (0.500, 0.250, 0.750)	X (0.500, 0.000, 0.500)

Supplementary Table 239: Nodal ring points along high symmetry lines of mp-1352-Zr4N4.

	Coordinates	Frequency	Mode 1	Mode 2	Multi.	Deg.	Path	Ratio
0	(0.173, 0.000, 0.173)	2.988	2	1	6	2	1	0.347
1	(0.145, 0.145, 0.275)	3.376	2	1	24	2	3	0.626
2	(0.500, 0.250, 0.750)	6.287	3	2	6	2	5	1.000
3	(0.543, 0.085, 0.543)	14.021	5	4	12	2	2	0.341
4	(0.500, 0.250, 0.750)	13.637	5	4	6	2	5	1.000
5	(0.500, 0.049, 0.549)	14.097	5	4	12	2	6	0.803

The followings are details for HgMg. It has 6 topological nodal lines and 4 nodal ring points along high symmetry line as shown in Fig **130**.

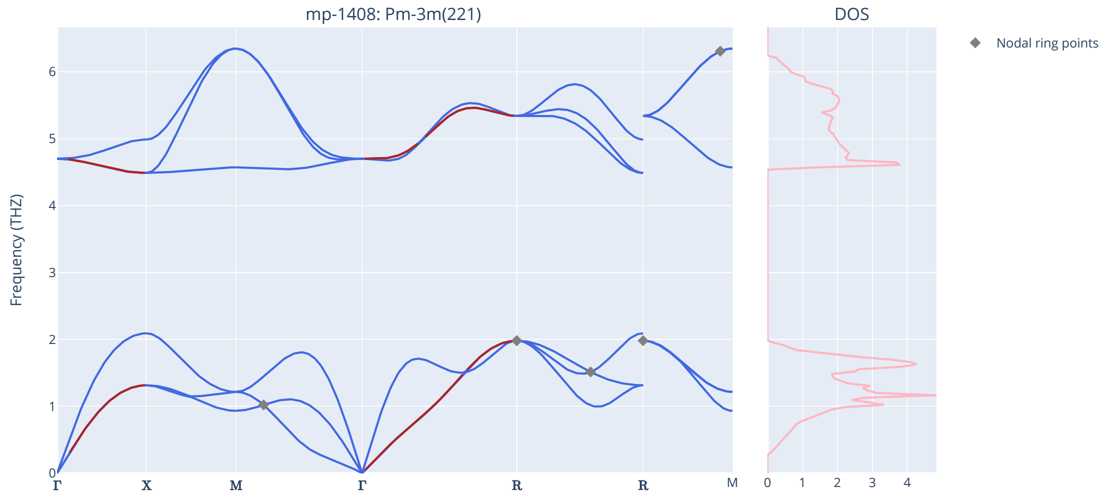

Supplementary Figure 130: The phonon band and DOS of HgMg.

Supplementary Table 240: High symmetry lines of mp-1408-HgMg

	Start	End
0	Γ (0.000, 0.000, 0.000)	X (0.000, 0.500, 0.000)
1	X (0.000, 0.500, 0.000)	M (0.500, 0.500, 0.000)
2	M (0.500, 0.500, 0.000)	Γ (0.000, 0.000, 0.000)
3	Γ (0.000, 0.000, 0.000)	R (0.500, 0.500, 0.500)
4	R (0.500, 0.500, 0.500)	X (0.000, 0.500, 0.000)
5	R (0.500, 0.500, 0.500)	M (0.500, 0.500, 0.000)

Supplementary Table 241: Nodal ring points along high symmetry lines of mp-1408-HgMg.

	Coordinates	Frequency	Mode 1	Mode 2	Multi.	Deg.	Path	Ratio
0	(0.390, 0.390, 0.000)	1.022	2	1	12	2	3	0.221
1	(0.500, 0.500, 0.500)	1.981	2	1	1	3	4	1.000
2	(0.208, 0.500, 0.208)	1.513	3	2	12	2	5	0.584
3	(0.499, 0.499, 0.068)	6.306	6	5	24	2	6	0.864

The followings are details for B_4P_4 and it only has 2 topological nodal lines along high symmetry line as shown in Fig **131**.

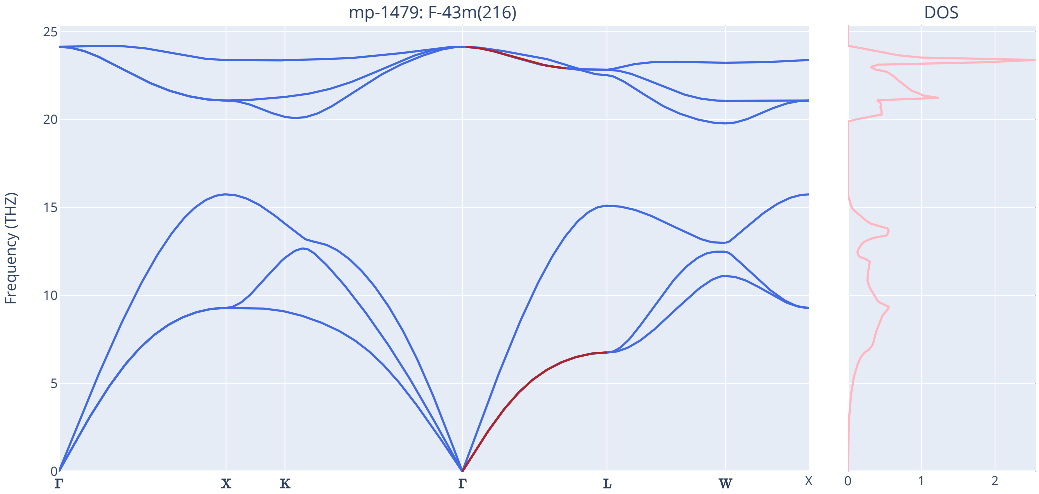

Supplementary Figure 131: The phonon band and DOS of B4P4.

Supplementary Table 242: High symmetry lines of mp-1479-B4P4

	Start	End
0	Γ (0.000, 0.000, 0.000)	X (0.500, 0.000, 0.500)
1	X (0.500, 0.000, 0.500)	U (0.625, 0.250, 0.625)
2	K (0.375, 0.375, 0.750)	Γ (0.000, 0.000, 0.000)
3	Γ (0.000, 0.000, 0.000)	L (0.500, 0.500, 0.500)
4	L (0.500, 0.500, 0.500)	W (0.500, 0.250, 0.750)
5	W (0.500, 0.250, 0.750)	X (0.500, 0.000, 0.500)

The followings are details for AlNi. It has 5 topological nodal lines and 7 nodal ring points along high symmetry line as shown in Fig 132.

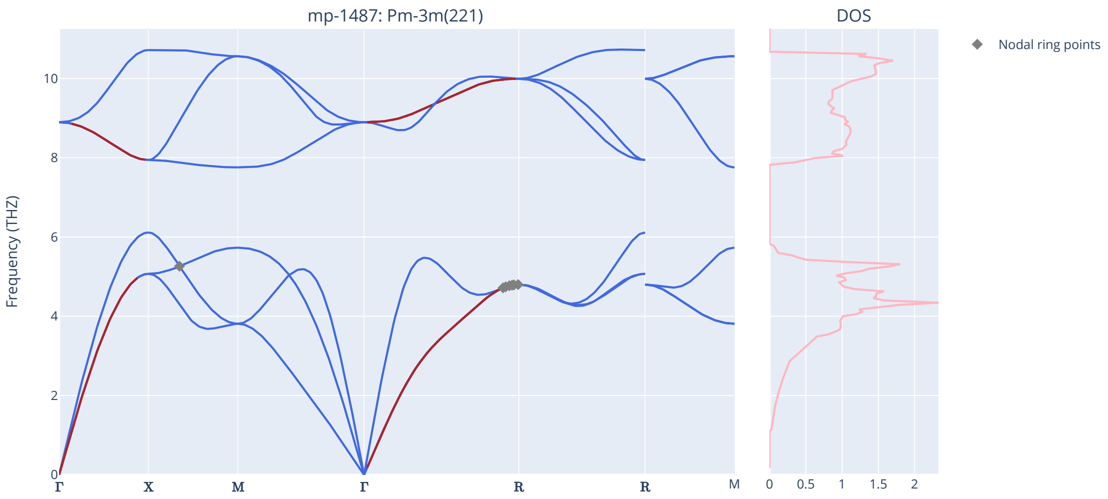

Supplementary Figure 132: The phonon band and DOS of AlNi.

Supplementary Table 243: High symmetry lines of mp-1487-AlNi

	Start	End
0	Γ (0.000, 0.000, 0.000)	X (0.000, 0.500, 0.000)
1	X (0.000, 0.500, 0.000)	M (0.500, 0.500, 0.000)
2	M (0.500, 0.500, 0.000)	Γ (0.000, 0.000, 0.000)
3	Γ (0.000, 0.000, 0.000)	R (0.500, 0.500, 0.500)
4	R (0.500, 0.500, 0.500)	X (0.000, 0.500, 0.000)
5	R (0.500, 0.500, 0.500)	M (0.500, 0.500, 0.000)

Supplementary Table 244: Nodal ring points along high symmetry lines of mp-1487-AlNi.

	Coordinates	Frequency	Mode 1	Mode 2	Multi.	Deg.	Path	Ratio
0	(0.174, 0.500, 0.000)	5.263	3	2	12	2	2	0.348
1	(0.498, 0.498, 0.498)	4.800	3	2	8	3	4	0.996
2	(0.449, 0.449, 0.449)	4.718	3	2	8	2	4	0.898
3	(0.478, 0.478, 0.478)	4.785	3	2	8	2	4	0.957
4	(0.458, 0.458, 0.458)	4.743	3	2	8	2	4	0.915
5	(0.484, 0.484, 0.484)	4.792	3	2	8	2	4	0.968
6	(0.469, 0.469, 0.469)	4.770	3	2	8	2	4	0.939

The followings are details for Mg_2. It has 11 topological nodal lines and 11 nodal ring points along high symmetry line as shown in Fig 133.

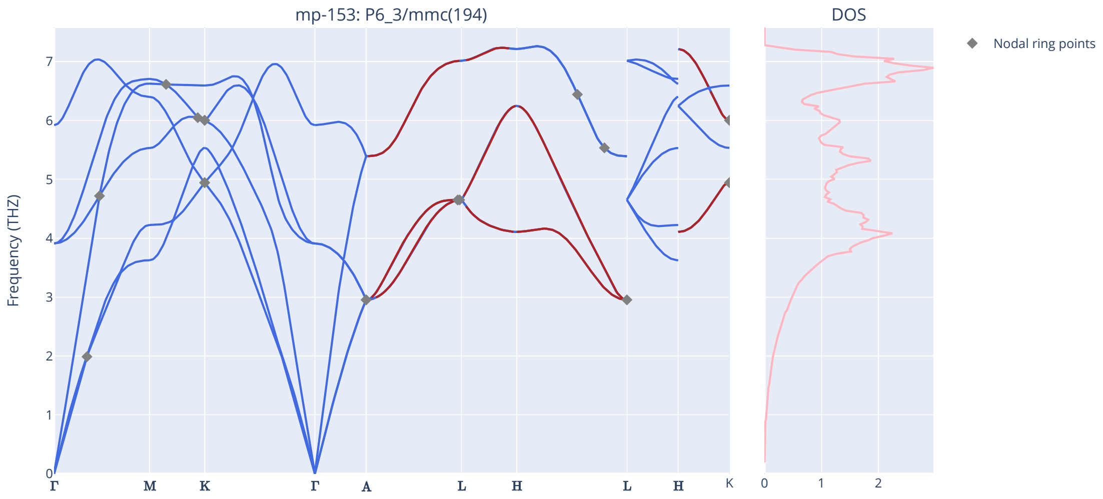

Supplementary Figure 133: The phonon band and DOS of Mg2.

Supplementary Table 245: High symmetry lines of mp-153-Mg2

	Start	End
0	Γ (0.000, 0.000, 0.000)	M (0.500, 0.000, 0.000)
1	M (0.500, 0.000, 0.000)	K (0.333, 0.333, 0.000)
2	K (0.333, 0.333, 0.000)	Γ (0.000, 0.000, 0.000)
3	Γ (0.000, 0.000, 0.000)	A (0.000, 0.000, 0.500)
4	A (0.000, 0.000, 0.500)	L (0.500, 0.000, 0.500)
5	L (0.500, 0.000, 0.500)	H (0.333, 0.333, 0.500)
6	H (0.333, 0.333, 0.500)	A (0.000, 0.000, 0.500)
7	L (0.500, 0.000, 0.500)	M (0.500, 0.000, 0.000)
8	H (0.333, 0.333, 0.500)	K (0.333, 0.333, 0.000)

Supplementary Table 246: Nodal ring points along high symmetry lines of mp-153-Mg2.

	Coordinates	Frequency	Mode 1	Mode 2	Multi.	Deg.	Path	Ratio
0	(0.172, -0.001, 0.000)	1.988	2	1	10	2	1	0.344
1	(0.333, 0.333, 0.000)	4.943	2	1	2	2	2	1.000
2	(0.000, 0.000, 0.500)	2.953	2	1	1	4	4	1.000
3	(0.480, 0.000, 0.499)	4.649	3	2	12	2	5	0.959
4	(0.490, 0.000, 0.500)	4.651	3	2	6	2	5	0.980
5	(0.238, 0.000, 0.000)	4.718	4	3	6	2	1	0.476
6	(0.354, 0.292, -0.000)	6.047	5	4	6	2	2	0.875
7	(0.333, 0.333, 0.000)	6.002	5	4	2	2	2	1.000
8	(0.450, 0.100, -0.000)	6.612	6	5	6	2	2	0.300
9	(0.150, 0.150, 0.500)	6.441	6	5	6	2	7	0.551
10	(0.068, 0.068, 0.500)	5.534	6	5	6	2	7	0.796

The followings are details for ZrTe. It has 1 nodal ring points and 5 single Weyl point along high symmetry line as shown in Fig 134.

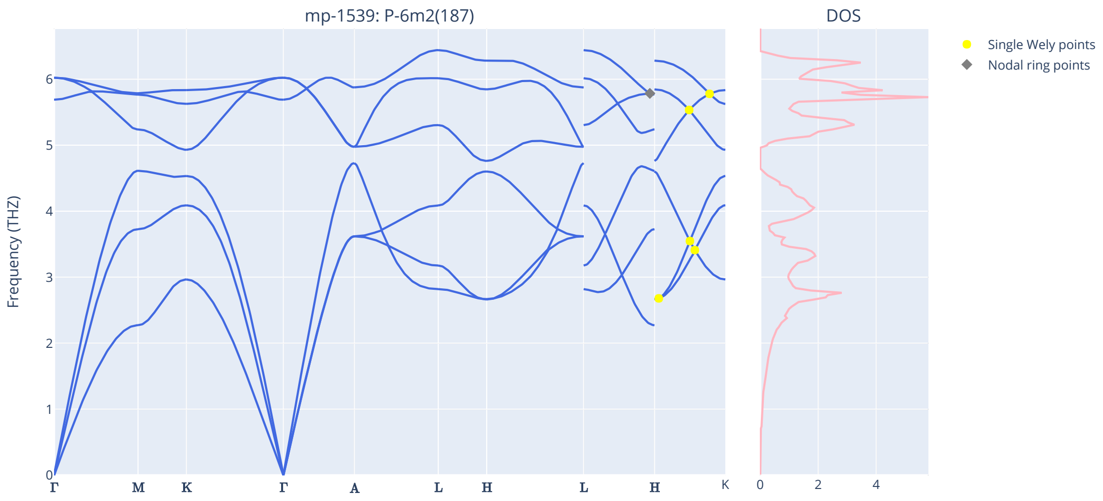

Supplementary Figure 134: The phonon band and DOS of ZrTe.

Supplementary Table 247: High symmetry lines of mp-1539-ZrTe

	Start	End
0	Γ (0.000, 0.000, 0.000)	M (0.500, 0.000, 0.000)
1	M (0.500, 0.000, 0.000)	K (0.333, 0.333, 0.000)
2	K (0.333, 0.333, 0.000)	Γ (0.000, 0.000, 0.000)
3	Γ (0.000, 0.000, 0.000)	A (0.000, 0.000, 0.500)
4	A (0.000, 0.000, 0.500)	L (0.500, 0.000, 0.500)
5	L (0.500, 0.000, 0.500)	H (0.333, 0.333, 0.500)
6	H (0.333, 0.333, 0.500)	A (0.000, 0.000, 0.500)
7	L (0.500, 0.000, 0.500)	M (0.500, 0.000, 0.000)
8	H (0.333, 0.333, 0.500)	K (0.333, 0.333, 0.000)

Supplementary Table 248: Single Weyl points along high symmetry lines of mp-1539-ZrTe.

	Coordinates	Frequency	Mode 1	Mode 2	Multi.	Deg.	Path	Ratio
0	(0.333, 0.333, 0.470)	2.679	2	1	4	2	9	0.061
1	(0.333, 0.333, 0.215)	3.411	2	1	4	2	9	0.569
2	(0.333, 0.333, 0.250)	3.548	3	2	4	2	9	0.500
3	(0.333, 0.333, 0.255)	5.533	5	4	4	2	9	0.489
4	(0.333, 0.333, 0.112)	5.777	6	5	4	2	9	0.777

Supplementary Table 249: Nodal ring points along high symmetry lines of mp-1539-ZrTe.

	Coordinates	Frequency	Mode 1	Mode 2	Multi.	Deg.	Path	Ratio
0	(0.499, 0.000, 0.033)	5.784	6	5	12	2	8	0.935

The followings are details for Be_4Se_4. It has 2 topological nodal lines and 1 nodal ring point along high symmetry line as shown in Fig **135**.

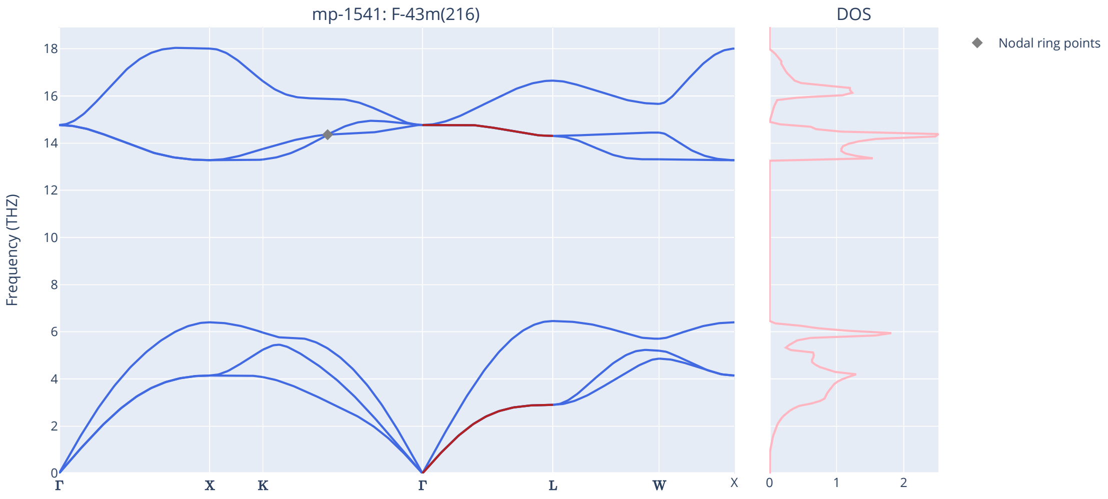

Supplementary Figure 135: The phonon band and DOS of Be4Se4.

Supplementary Table 250: High symmetry lines of mp-1541-Be4Se4

	Start	End
0	Γ (0.000, 0.000, 0.000)	X (0.500, 0.000, 0.500)
1	X (0.500, 0.000, 0.500)	U (0.625, 0.250, 0.625)
2	K (0.375, 0.375, 0.750)	Γ (0.000, 0.000, 0.000)
3	Γ (0.000, 0.000, 0.000)	L (0.500, 0.500, 0.500)
4	L (0.500, 0.500, 0.500)	W (0.500, 0.250, 0.750)
5	W (0.500, 0.250, 0.750)	X (0.500, 0.000, 0.500)

Supplementary Table 251: Nodal ring points along high symmetry lines of mp-1541-Be4Se4.

	Coordinates	Frequency	Mode 1	Mode 2	Multi.	Deg.	Path	Ratio
0	(0.222, 0.222, 0.446)	14.357	5	4	24	2	3	0.406

The followings are details for Al_4P_4. It has 3 topological nodal lines and 1 nodal ring point along high symmetry line as shown in Fig **136**.

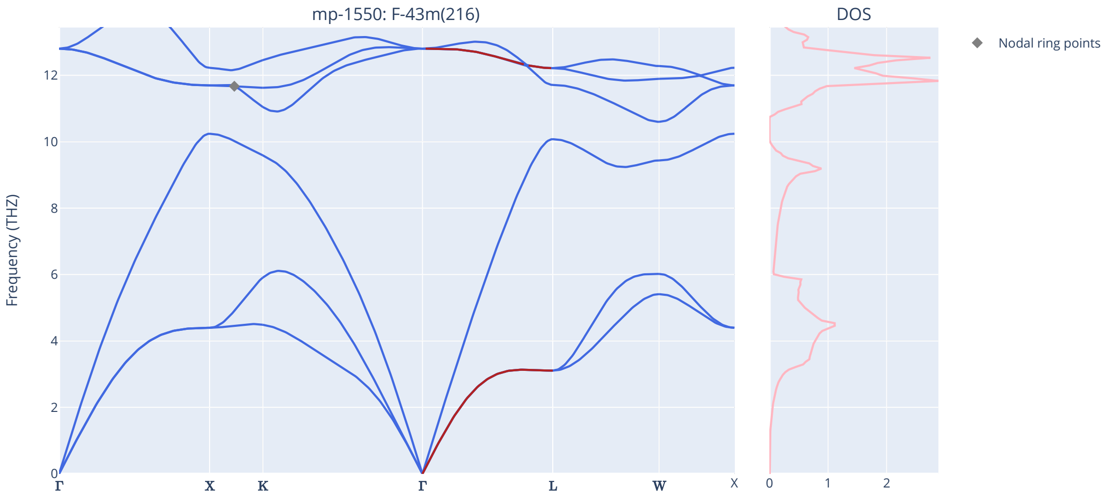

Supplementary Figure 136: The phonon band and DOS of Al4P4.

Supplementary Table 252: High symmetry lines of mp-1550-Al4P4

	Start	End
0	Γ (0.000, 0.000, 0.000)	X (0.500, 0.000, 0.500)
1	X (0.500, 0.000, 0.500)	U (0.625, 0.250, 0.625)
2	K (0.375, 0.375, 0.750)	Γ (0.000, 0.000, 0.000)
3	Γ (0.000, 0.000, 0.000)	L (0.500, 0.500, 0.500)
4	L (0.500, 0.500, 0.500)	W (0.500, 0.250, 0.750)
5	W (0.500, 0.250, 0.750)	X (0.500, 0.000, 0.500)

Supplementary Table 253: Nodal ring points along high symmetry lines of mp-1550-Al4P4.

	Coordinates	Frequency	Mode 1	Mode 2	Multi.	Deg.	Path	Ratio
0	(0.559, 0.115, 0.559)	11.671	5	4	24	2	2	0.465

The followings are details for Pr_4Se_4. It has 2 topological nodal lines and 3 nodal ring points along high symmetry line as shown in Fig 137.

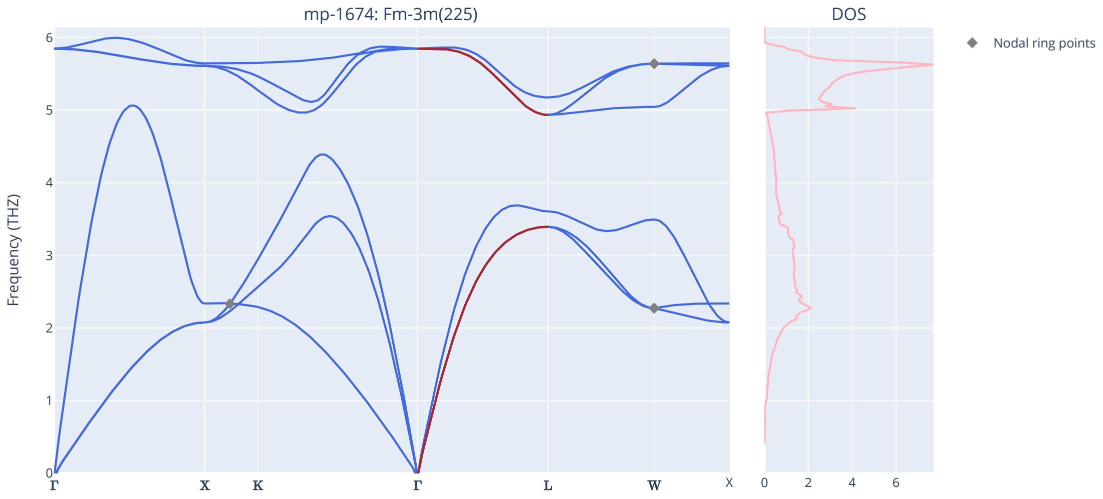

Supplementary Figure 137: The phonon band and DOS of Pr4Se4.

Supplementary Table 254: High symmetry lines of mp-1674-Pr4Se4

	Start	End
0	Γ (0.000, 0.000, 0.000)	X (0.500, 0.000, 0.500)
1	X (0.500, 0.000, 0.500)	U (0.625, 0.250, 0.625)
2	K (0.375, 0.375, 0.750)	Γ (0.000, 0.000, 0.000)
3	Γ (0.000, 0.000, 0.000)	L (0.500, 0.500, 0.500)
4	L (0.500, 0.500, 0.500)	W (0.500, 0.250, 0.750)
5	W (0.500, 0.250, 0.750)	X (0.500, 0.000, 0.500)

Supplementary Table 255: Nodal ring points along high symmetry lines of mp-1674-Pr4Se4.

	Coordinates	Frequency	Mode 1	Mode 2	Multi.	Deg.	Path	Ratio
0	(0.500, 0.250, 0.750)	2.272	2	1	6	2	5	1.000
1	(0.559, 0.119, 0.559)	2.334	3	2	12	2	2	0.476
2	(0.500, 0.250, 0.750)	5.638	6	5	6	2	5	1.000

The followings are details for Th_4Sb_4. It has 2 topological nodal lines and 2 nodal ring points along high symmetry line as shown in Fig **138**.

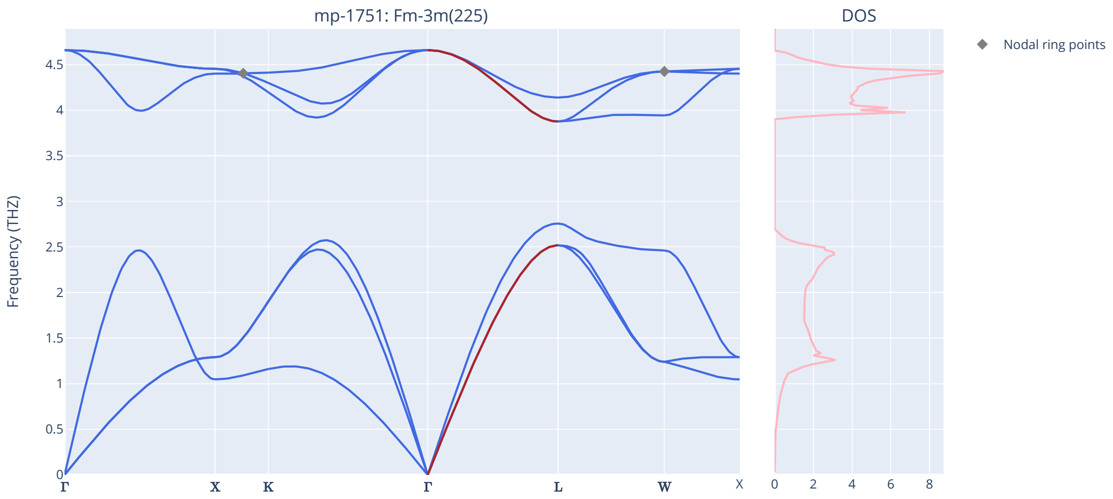

Supplementary Figure 138: The phonon band and DOS of Th4Sb4.

Supplementary Table 256: High symmetry lines of mp-1751-Th4Sb4

	Start	End
0	Γ (0.000, 0.000, 0.000)	X (0.500, 0.000, 0.500)
1	X (0.500, 0.000, 0.500)	U (0.625, 0.250, 0.625)
2	K (0.375, 0.375, 0.750)	Γ (0.000, 0.000, 0.000)
3	Γ (0.000, 0.000, 0.000)	L (0.500, 0.500, 0.500)
4	L (0.500, 0.500, 0.500)	W (0.500, 0.250, 0.750)
5	W (0.500, 0.250, 0.750)	X (0.500, 0.000, 0.500)

Supplementary Table 257: Nodal ring points along high symmetry lines of mp-1751-Th4Sb4.

	Coordinates	Frequency	Mode 1	Mode 2	Multi.	Deg.	Path	Ratio
0	(0.566, 0.133, 0.566)	4.405	6	5	12	2	2	0.531
1	(0.500, 0.250, 0.750)	4.425	6	5	6	2	5	1.000

The followings are details for Be_4O_4. It has 3 topological nodal lines and 1 nodal ring point along high symmetry line as shown in Fig **139**.

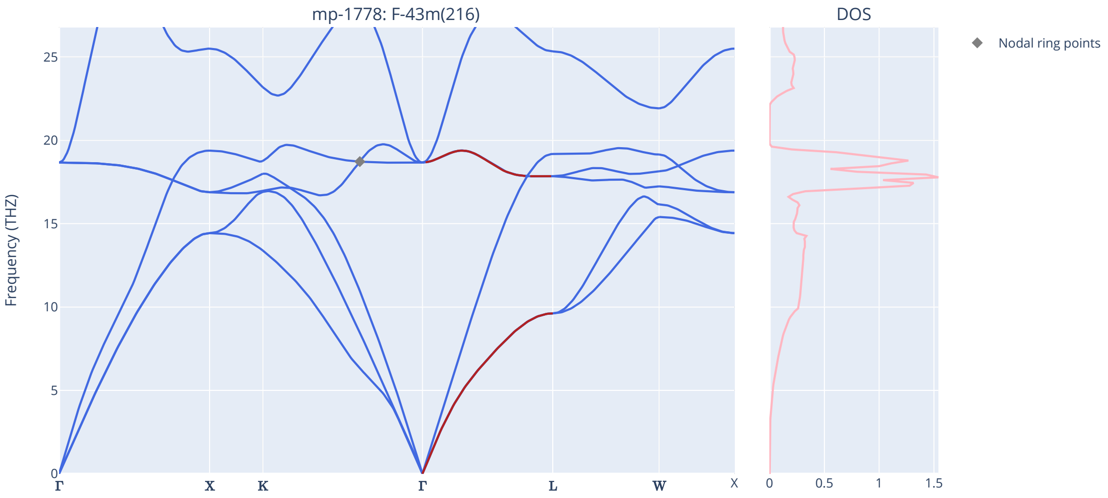

Supplementary Figure 139: The phonon band and DOS of Be4O4.

Supplementary Table 258: High symmetry lines of mp-1778-Be4O4

	Start	End
0	Γ (0.000, 0.000, 0.000)	X (0.500, 0.000, 0.500)
1	X (0.500, 0.000, 0.500)	U (0.625, 0.250, 0.625)
2	K (0.375, 0.375, 0.750)	Γ (0.000, 0.000, 0.000)
3	Γ (0.000, 0.000, 0.000)	L (0.500, 0.500, 0.500)
4	L (0.500, 0.500, 0.500)	W (0.500, 0.250, 0.750)
5	W (0.500, 0.250, 0.750)	X (0.500, 0.000, 0.500)

Supplementary Table 259: Nodal ring points along high symmetry lines of mp-1778-Be4O4.

	Coordinates	Frequency	Mode 1	Mode 2	Multi.	Deg.	Path	Ratio
0	(0.145, 0.145, 0.296)	18.723	5	4	24	2	3	0.608

The followings are details for RhSc. It has 5 topological nodal lines and 2 nodal ring points along high symmetry line as shown in Fig **140**.

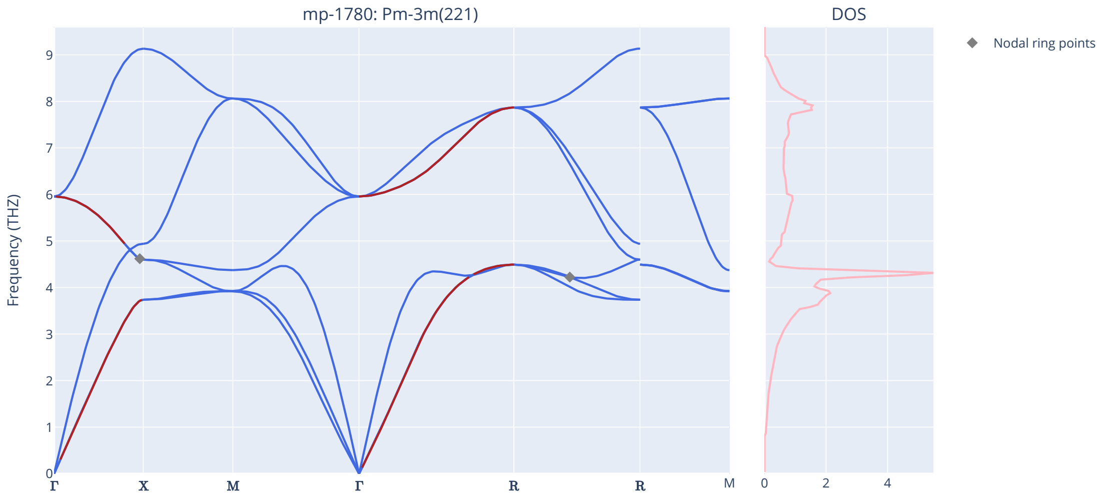

Supplementary Figure 140: The phonon band and DOS of RhSc.

Supplementary Table 260: High symmetry lines of mp-1780-RhSc

	Start	End
0	Γ (0.000, 0.000, 0.000)	X (0.000, 0.500, 0.000)
1	X (0.000, 0.500, 0.000)	M (0.500, 0.500, 0.000)
2	M (0.500, 0.500, 0.000)	Γ (0.000, 0.000, 0.000)
3	Γ (0.000, 0.000, 0.000)	R (0.500, 0.500, 0.500)
4	R (0.500, 0.500, 0.500)	X (0.000, 0.500, 0.000)
5	R (0.500, 0.500, 0.500)	M (0.500, 0.500, 0.000)

Supplementary Table 261: Nodal ring points along high symmetry lines of mp-1780-RhSc.

	Coordinates	Frequency	Mode 1	Mode 2	Multi.	Deg.	Path	Ratio
0	(0.278, 0.500, 0.278)	4.226	3	2	12	2	5	0.443
1	(0.000, 0.480, 0.000)	4.621	4	3	6	2	1	0.959

The followings are details for WC and it only has 6 single Weyl point along high symmetry line as shown in Fig **141**.

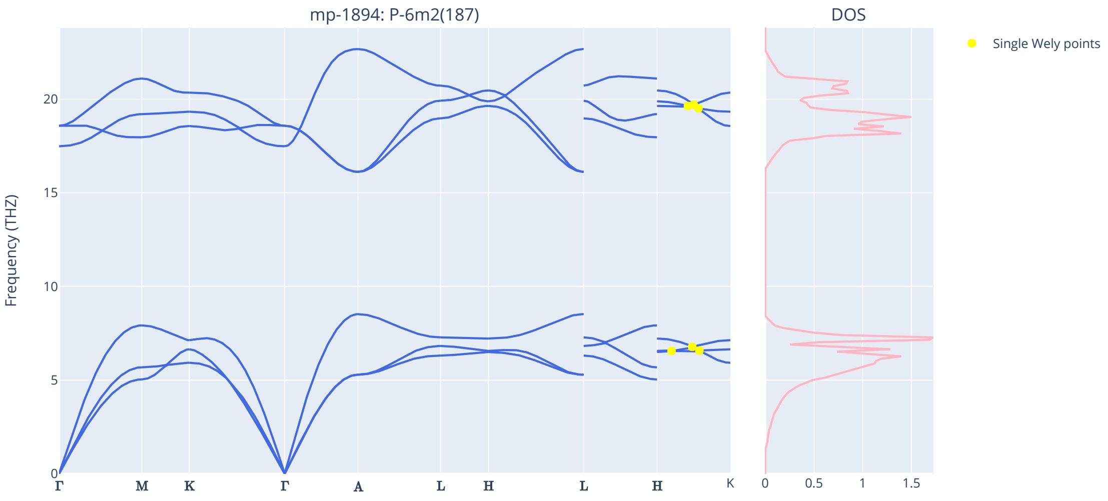

Supplementary Figure 141: The phonon band and DOS of WC.

Supplementary Table 262: High symmetry lines of mp-1894-WC

	Start	End
0	Γ (0.000, 0.000, 0.000)	M (0.500, 0.000, 0.000)
1	M (0.500, 0.000, 0.000)	K (0.333, 0.333, 0.000)
2	K (0.333, 0.333, 0.000)	Γ (0.000, 0.000, 0.000)
3	Γ (0.000, 0.000, 0.000)	A (0.000, 0.000, 0.500)
4	A (0.000, 0.000, 0.500)	L (0.500, 0.000, 0.500)
5	L (0.500, 0.000, 0.500)	H (0.333, 0.333, 0.500)
6	H (0.333, 0.333, 0.500)	A (0.000, 0.000, 0.500)
7	L (0.500, 0.000, 0.500)	M (0.500, 0.000, 0.000)
8	H (0.333, 0.333, 0.500)	K (0.333, 0.333, 0.000)

Supplementary Table 263: Single Weyl points along high symmetry lines of mp-1894-WC.

	Coordinates	Frequency	Mode 1	Mode 2	Multi.	Deg.	Path	Ratio
0	(0.333, 0.333, 0.401)	6.551	2	1	4	2	9	0.198
1	(0.333, 0.333, 0.211)	6.576	2	1	4	2	9	0.578
2	(0.333, 0.333, 0.260)	6.775	3	2	4	2	9	0.479
3	(0.333, 0.333, 0.217)	19.508	5	4	4	2	9	0.565
4	(0.333, 0.333, 0.289)	19.634	5	4	4	2	9	0.422
5	(0.333, 0.333, 0.249)	19.705	6	5	4	2	9	0.501

The followings are details for RhY. It has 6 topological nodal lines and 11 nodal ring points along high symmetry line as shown in Fig 142.

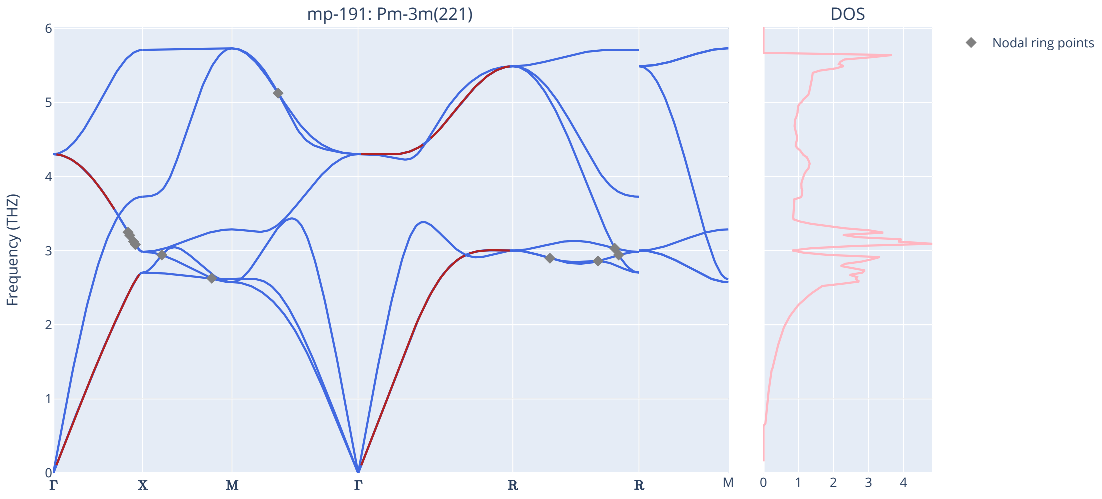

Supplementary Figure 142: The phonon band and DOS of RhY.

Supplementary Table 264: High symmetry lines of mp-191-RhY

	Start	End
0	Γ (0.000, 0.000, 0.000)	X (0.000, 0.500, 0.000)
1	X (0.000, 0.500, 0.000)	M (0.500, 0.500, 0.000)
2	M (0.500, 0.500, 0.000)	Γ (0.000, 0.000, 0.000)
3	Γ (0.000, 0.000, 0.000)	R (0.500, 0.500, 0.500)
4	R (0.500, 0.500, 0.500)	X (0.000, 0.500, 0.000)
5	R (0.500, 0.500, 0.500)	M (0.500, 0.500, 0.000)

Supplementary Table 265: Nodal ring points along high symmetry lines of mp-191-RhY.

	Coordinates	Frequency	Mode 1	Mode 2	Multi.	Deg.	Path	Ratio
0	(0.387, 0.500, -0.000)	2.628	2	1	12	2	2	0.775
1	(0.353, 0.500, 0.353)	2.897	2	1	12	2	5	0.294
2	(0.163, 0.500, 0.163)	2.859	2	1	12	2	5	0.675
3	(0.108, 0.500, -0.000)	2.942	3	2	12	2	2	0.215
4	(0.081, 0.500, 0.081)	2.942	3	2	12	2	5	0.838
5	(0.000, 0.418, 0.000)	3.248	4	3	6	2	1	0.837
6	(0.000, 0.429, 0.000)	3.206	4	3	6	2	1	0.857
7	(0.000, 0.449, 0.000)	3.122	4	3	6	2	1	0.898
8	(0.000, 0.459, 0.000)	3.083	4	3	6	2	1	0.918
9	(0.096, 0.500, 0.096)	3.031	4	3	12	2	5	0.808
10	(0.317, 0.317, -0.000)	5.124	6	5	12	2	3	0.366

The followings are details for ThTe. It has 6 topological nodal lines and 8 nodal ring points along high symmetry line as shown in Fig 143.

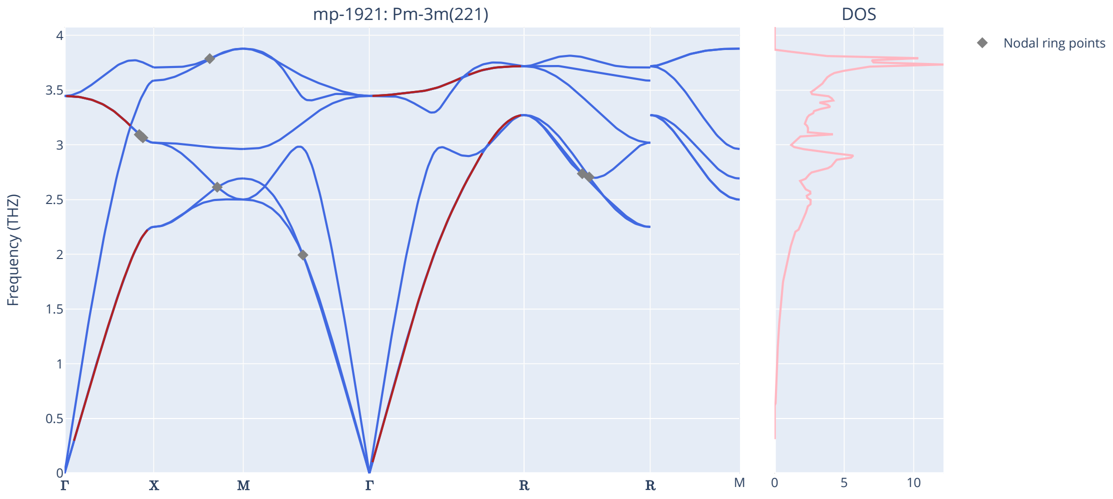

Supplementary Figure 143: The phonon band and DOS of ThTe.

Supplementary Table 266: High symmetry lines of mp-1921-ThTe

	Start	End
0	Γ (0.000, 0.000, 0.000)	X (0.000, 0.500, 0.000)
1	X (0.000, 0.500, 0.000)	M (0.500, 0.500, 0.000)
2	M (0.500, 0.500, 0.000)	Γ (0.000, 0.000, 0.000)
3	Γ (0.000, 0.000, 0.000)	R (0.500, 0.500, 0.500)
4	R (0.500, 0.500, 0.500)	X (0.000, 0.500, 0.000)
5	R (0.500, 0.500, 0.500)	M (0.500, 0.500, 0.000)

Supplementary Table 267: Nodal ring points along high symmetry lines of mp-1921-ThTe.

	Coordinates	Frequency	Mode 1	Mode 2	Multi.	Deg.	Path	Ratio
0	(0.263, 0.263, -0.000)	1.994	2	1	12	2	3	0.474
1	(0.269, 0.500, 0.269)	2.737	2	1	12	2	5	0.461
2	(0.355, 0.500, -0.000)	2.613	3	2	12	2	2	0.710
3	(0.242, 0.500, 0.242)	2.706	3	2	12	2	5	0.516
4	(0.000, 0.418, 0.000)	3.094	4	3	6	2	1	0.837
5	(0.000, 0.429, 0.000)	3.079	4	3	6	2	1	0.857
6	(0.000, 0.439, 0.000)	3.065	4	3	6	2	1	0.878
7	(0.313, 0.500, 0.000)	3.787	6	5	12	2	2	0.626

The followings are details for AgLa. It has 6 topological nodal lines and 7 nodal ring points along high symmetry line as shown in Fig **144**.

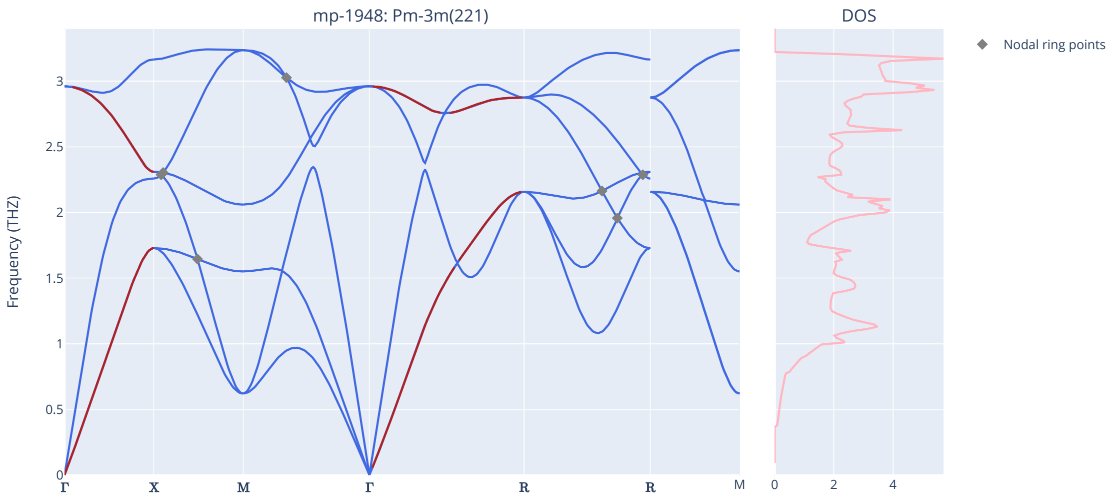

Supplementary Figure 144: The phonon band and DOS of AgLa.

Supplementary Table 268: High symmetry lines of mp-1948-AgLa

	Start	End
0	Γ (0.000, 0.000, 0.000)	X (0.000, 0.500, 0.000)
1	X (0.000, 0.500, 0.000)	M (0.500, 0.500, 0.000)
2	M (0.500, 0.500, 0.000)	Γ (0.000, 0.000, 0.000)
3	Γ (0.000, 0.000, 0.000)	R (0.500, 0.500, 0.500)
4	R (0.500, 0.500, 0.500)	X (0.000, 0.500, 0.000)
5	R (0.500, 0.500, 0.500)	M (0.500, 0.500, 0.000)

Supplementary Table 269: Nodal ring points along high symmetry lines of mp-1948-AgLa.

	Coordinates	Frequency	Mode 1	Mode 2	Multi.	Deg.	Path	Ratio
0	(0.244, 0.500, -0.000)	1.645	3	2	12	2	2	0.489
1	(0.131, 0.500, 0.131)	1.957	3	2	12	2	5	0.739
2	(0.030, 0.500, 0.029)	2.287	4	3	24	2	5	0.941
3	(0.041, 0.500, -0.000)	2.287	4	3	12	2	2	0.082
4	(0.191, 0.500, 0.191)	2.163	4	3	12	2	5	0.617
5	(0.052, 0.500, -0.000)	2.303	5	4	12	2	2	0.104
6	(0.328, 0.328, 0.000)	3.026	6	5	12	2	3	0.343

The followings are details for Zn_4O_4. It has 2 topological nodal lines and 1 nodal ring point along high symmetry line as shown in Fig **145**.

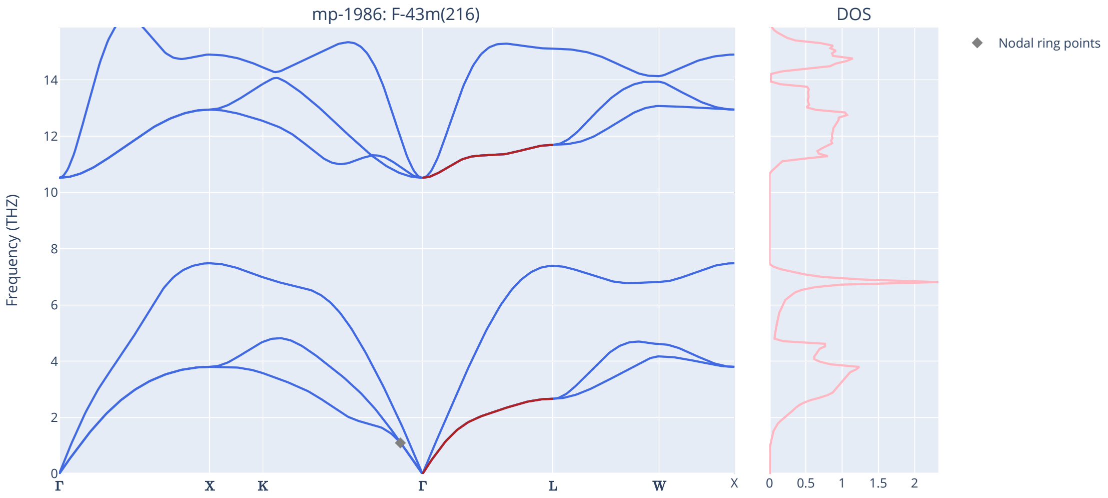

Supplementary Figure 145: The phonon band and DOS of Zn4O4.

Supplementary Table 270: High symmetry lines of mp-1986-Zn4O4

	Start	End
0	Γ (0.000, 0.000, 0.000)	X (0.500, 0.000, 0.500)
1	X (0.500, 0.000, 0.500)	U (0.625, 0.250, 0.625)
2	K (0.375, 0.375, 0.750)	Γ (0.000, 0.000, 0.000)
3	Γ (0.000, 0.000, 0.000)	L (0.500, 0.500, 0.500)
4	L (0.500, 0.500, 0.500)	W (0.500, 0.250, 0.750)
5	W (0.500, 0.250, 0.750)	X (0.500, 0.000, 0.500)

Supplementary Table 271: Nodal ring points along high symmetry lines of mp-1986-Zn4O4.

	Coordinates	Frequency	Mode 1	Mode 2	Multi.	Deg.	Path	Ratio
0	(0.054, 0.054, 0.102)	1.097	2	1	24	2	3	0.862

The followings are details for In_4Sb_4 and it only has 3 topological nodal lines along high symmetry line as shown in Fig **146**.

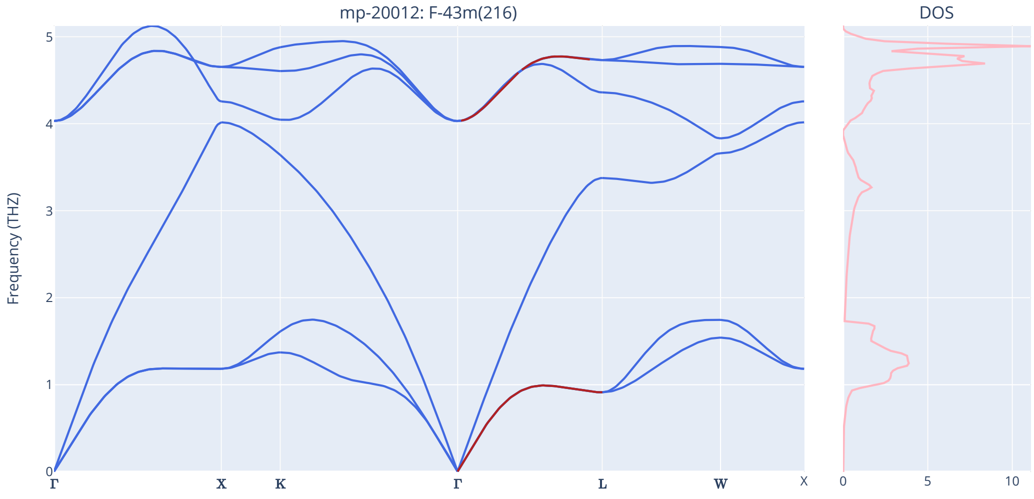

Supplementary Figure 146: The phonon band and DOS of In4Sb4.

Supplementary Table 272: High symmetry lines of mp-20012-In4Sb4

	Start	End
0	Γ (0.000, 0.000, 0.000)	X (0.500, 0.000, 0.500)
1	X (0.500, 0.000, 0.500)	U (0.625, 0.250, 0.625)
2	K (0.375, 0.375, 0.750)	Γ (0.000, 0.000, 0.000)
3	Γ (0.000, 0.000, 0.000)	L (0.500, 0.500, 0.500)
4	L (0.500, 0.500, 0.500)	W (0.500, 0.250, 0.750)
5	W (0.500, 0.250, 0.750)	X (0.500, 0.000, 0.500)

The followings are details for HgLi. It has 6 topological nodal lines and 4 nodal ring points along high symmetry line as shown in Fig 147.

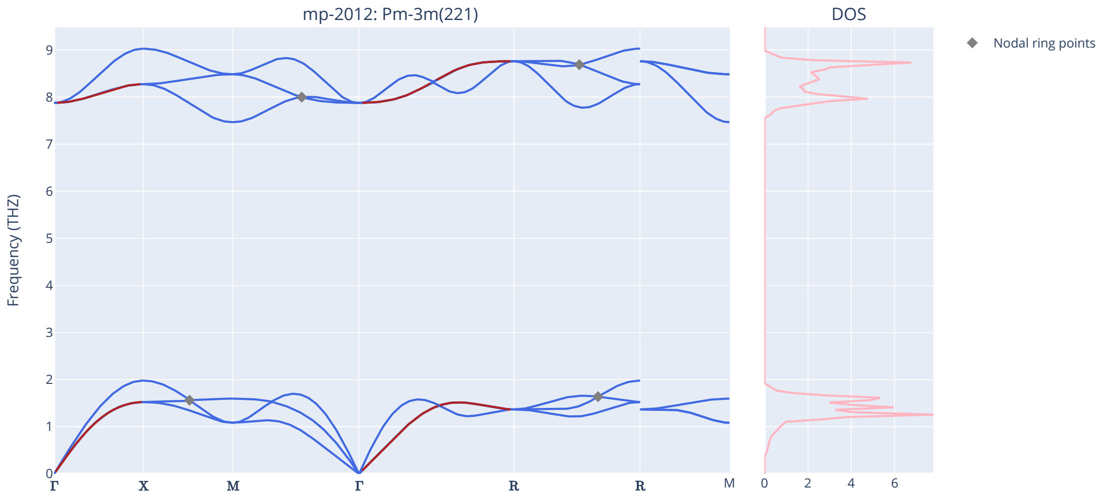

Supplementary Figure 147: The phonon band and DOS of HgLi.

Supplementary Table 273: High symmetry lines of mp-2012-HgLi

	Start	End
0	Γ (0.000, 0.000, 0.000)	X (0.000, 0.500, 0.000)
1	X (0.000, 0.500, 0.000)	M (0.500, 0.500, 0.000)
2	M (0.500, 0.500, 0.000)	Γ (0.000, 0.000, 0.000)
3	Γ (0.000, 0.000, 0.000)	R (0.500, 0.500, 0.500)
4	R (0.500, 0.500, 0.500)	X (0.000, 0.500, 0.000)
5	R (0.500, 0.500, 0.500)	M (0.500, 0.500, 0.000)

Supplementary Table 274: Nodal ring points along high symmetry lines of mp-2012-HgLi.

	Coordinates	Frequency	Mode 1	Mode 2	Multi.	Deg.	Path	Ratio
0	(0.258, 0.500, -0.000)	1.562	3	2	12	2	2	0.516
1	(0.166, 0.500, 0.166)	1.637	3	2	12	2	5	0.667
2	(0.227, 0.227, -0.000)	7.999	5	4	12	2	3	0.546
3	(0.240, 0.500, 0.240)	8.687	6	5	12	2	5	0.519

The followings are details for CaIn. It has 6 topological nodal lines and 1 nodal ring point along high symmetry line as shown in Fig 148.

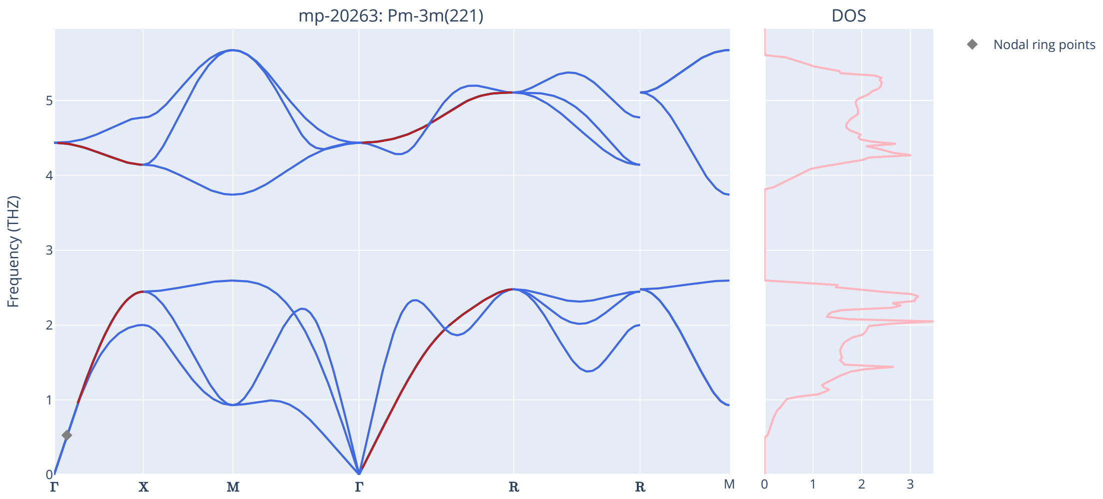

Supplementary Figure 148: The phonon band and DOS of CaIn.

Supplementary Table 275: High symmetry lines of mp-20263-CaIn

	Start	End
0	Γ (0.000, 0.000, 0.000)	X (0.000, 0.500, 0.000)
1	X (0.000, 0.500, 0.000)	M (0.500, 0.500, 0.000)
2	M (0.500, 0.500, 0.000)	Γ (0.000, 0.000, 0.000)
3	Γ (0.000, 0.000, 0.000)	R (0.500, 0.500, 0.500)
4	R (0.500, 0.500, 0.500)	X (0.000, 0.500, 0.000)
5	R (0.500, 0.500, 0.500)	M (0.500, 0.500, 0.000)

Supplementary Table 276: Nodal ring points along high symmetry lines of mp-20263-CaIn.

	Coordinates	Frequency	Mode 1	Mode 2	Multi.	Deg.	Path	Ratio
0	(0.000, 0.071, 0.000)	0.528	2	1	6	2	1	0.143

The followings are details for CoHf. It has 6 topological nodal lines and 10 nodal ring points along high symmetry line as shown in Fig 149.

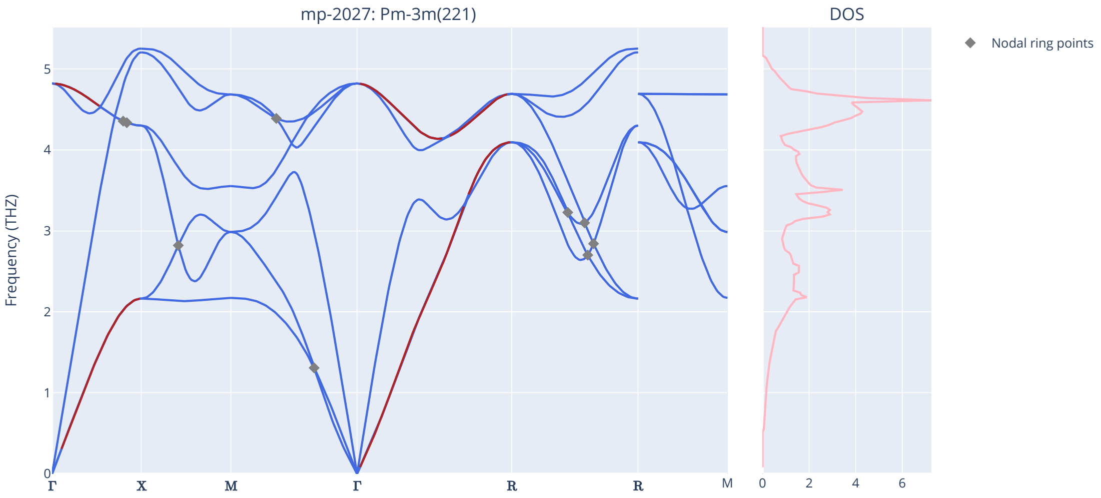

Supplementary Figure 149: The phonon band and DOS of CoHf.

Supplementary Table 277: High symmetry lines of mp-2027-CoHf

	Start	End
0	Γ (0.000, 0.000, 0.000)	X (0.000, 0.500, 0.000)
1	X (0.000, 0.500, 0.000)	M (0.500, 0.500, 0.000)
2	M (0.500, 0.500, 0.000)	Γ (0.000, 0.000, 0.000)
3	Γ (0.000, 0.000, 0.000)	R (0.500, 0.500, 0.500)
4	R (0.500, 0.500, 0.500)	X (0.000, 0.500, 0.000)
5	R (0.500, 0.500, 0.500)	M (0.500, 0.500, 0.000)

Supplementary Table 278: Nodal ring points along high symmetry lines of mp-2027-CoHf.

	Coordinates	Frequency	Mode 1	Mode 2	Multi.	Deg.	Path	Ratio
0	(0.170, 0.170, 0.000)	1.308	2	1	12	2	3	0.660
1	(0.199, 0.500, 0.199)	2.701	2	1	12	2	5	0.602
2	(0.206, 0.500, -0.000)	2.820	3	2	12	2	2	0.413
3	(0.278, 0.500, 0.278)	3.228	3	2	12	2	5	0.443
4	(0.176, 0.500, 0.176)	2.842	3	2	12	2	5	0.647
5	(0.000, 0.398, 0.000)	4.355	4	3	6	2	1	0.796
6	(0.000, 0.418, 0.000)	4.336	4	3	6	2	1	0.837
7	(0.212, 0.500, 0.212)	3.100	4	3	12	2	5	0.576
8	(0.320, 0.320, -0.000)	4.389	6	5	12	2	3	0.361

The followings are details for In_4As_4. It has 3 topological nodal lines and 1 single Weyl point along high symmetry line as shown in Fig **150**.

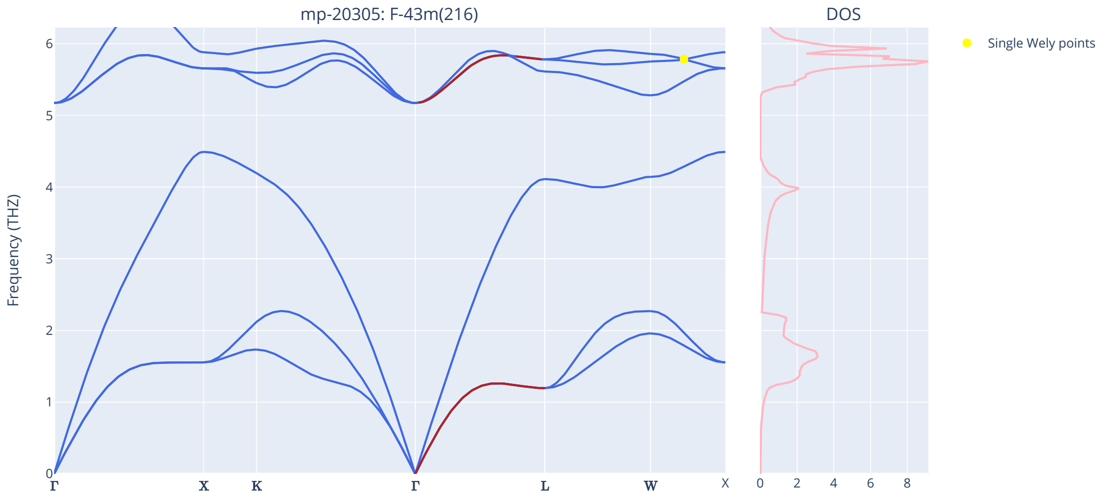

Supplementary Figure 150: The phonon band and DOS of In4As4.

Supplementary Table 279: High symmetry lines of mp-20305-In4As4

	Start	End
0	Γ (0.000, 0.000, 0.000)	X (0.500, 0.000, 0.500)
1	X (0.500, 0.000, 0.500)	U (0.625, 0.250, 0.625)
2	K (0.375, 0.375, 0.750)	Γ (0.000, 0.000, 0.000)
3	Γ (0.000, 0.000, 0.000)	L (0.500, 0.500, 0.500)
4	L (0.500, 0.500, 0.500)	W (0.500, 0.250, 0.750)
5	W (0.500, 0.250, 0.750)	X (0.500, 0.000, 0.500)

Supplementary Table 280: Single Weyl points along high symmetry lines of mp-20305-In4As4.

	Coordinates	Frequency	Mode 1	Mode 2	Multi.	Deg.	Path	Ratio
0	(0.500, 0.138, 0.638)	5.784	6	5	12	2	6	0.447

The followings are details for In_4P_4. It has 2 topological nodal lines and 1 nodal ring point along high symmetry line as shown in Fig **151**.

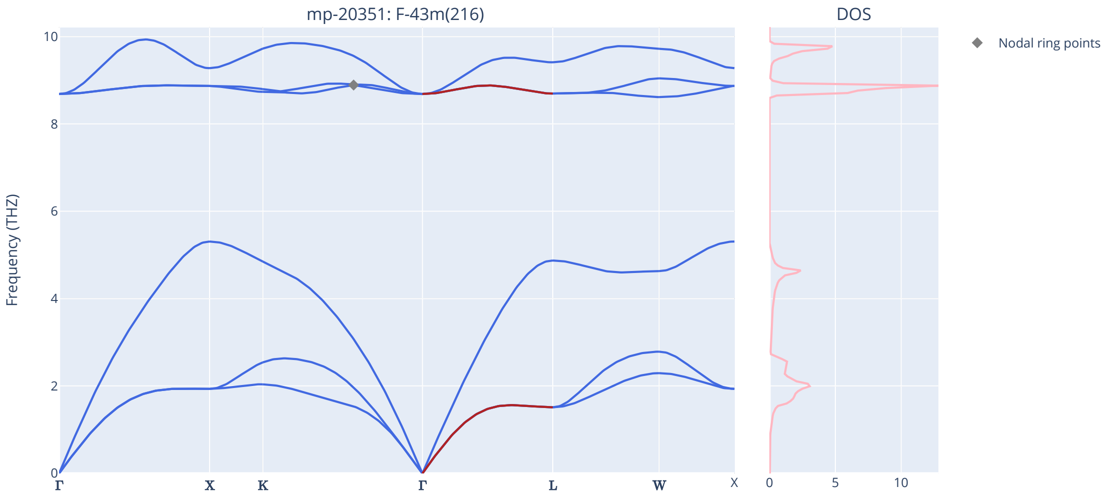

Supplementary Figure 151: The phonon band and DOS of In4P4.

Supplementary Table 281: High symmetry lines of mp-20351-In4P4

	Start	End
0	Γ (0.000, 0.000, 0.000)	X (0.500, 0.000, 0.500)
1	X (0.500, 0.000, 0.500)	U (0.625, 0.250, 0.625)
2	K (0.375, 0.375, 0.750)	Γ (0.000, 0.000, 0.000)
3	Γ (0.000, 0.000, 0.000)	L (0.500, 0.500, 0.500)
4	L (0.500, 0.500, 0.500)	W (0.500, 0.250, 0.750)
5	W (0.500, 0.250, 0.750)	X (0.500, 0.000, 0.500)

Supplementary Table 282: Nodal ring points along high symmetry lines of mp-20351-In4P4.

	Coordinates	Frequency	Mode 1	Mode 2	Multi.	Deg.	Path	Ratio
0	(0.161, 0.161, 0.324)	8.890	5	4	24	2	3	0.569

The followings are details for In_4N_4 and it only has 2 topological nodal lines along high symmetry line as shown in Fig **152**.

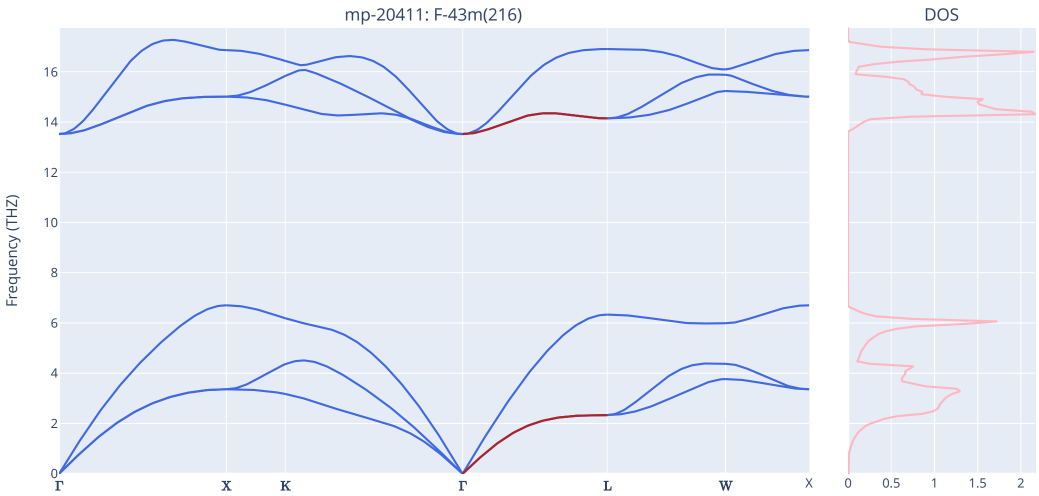

Supplementary Figure 152: The phonon band and DOS of In4N4.

Supplementary Table 283: High symmetry lines of mp-20411-In4N4

	Start	End
0	Γ (0.000, 0.000, 0.000)	X (0.500, 0.000, 0.500)
1	X (0.500, 0.000, 0.500)	U (0.625, 0.250, 0.625)
2	K (0.375, 0.375, 0.750)	Γ (0.000, 0.000, 0.000)
3	Γ (0.000, 0.000, 0.000)	L (0.500, 0.500, 0.500)
4	L (0.500, 0.500, 0.500)	W (0.500, 0.250, 0.750)
5	W (0.500, 0.250, 0.750)	X (0.500, 0.000, 0.500)

The followings are details for Sc_4As_4. It has 3 topological nodal lines and 3 nodal ring points along high symmetry line as shown in Fig **153**.

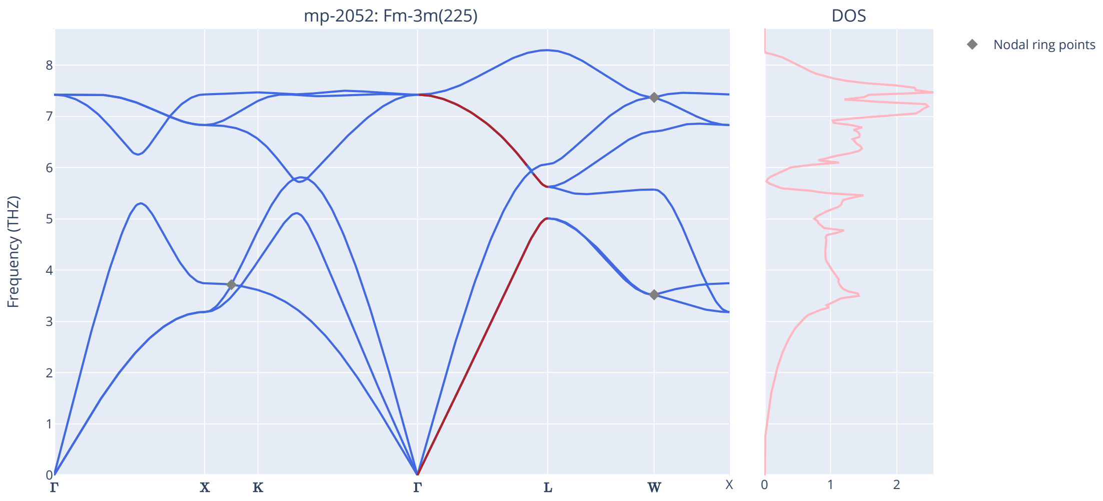

Supplementary Figure 153: The phonon band and DOS of Sc4As4.

Supplementary Table 284: High symmetry lines of mp-2052-Sc4As4

	Start	End
0	Γ (0.000, 0.000, 0.000)	X (0.500, 0.000, 0.500)
1	X (0.500, 0.000, 0.500)	U (0.625, 0.250, 0.625)
2	K (0.375, 0.375, 0.750)	Γ (0.000, 0.000, 0.000)
3	Γ (0.000, 0.000, 0.000)	L (0.500, 0.500, 0.500)
4	L (0.500, 0.500, 0.500)	W (0.500, 0.250, 0.750)
5	W (0.500, 0.250, 0.750)	X (0.500, 0.000, 0.500)

Supplementary Table 285: Nodal ring points along high symmetry lines of mp-2052-Sc4As4.

	Coordinates	Frequency	Mode 1	Mode 2	Multi.	Deg.	Path	Ratio
0	(0.500, 0.250, 0.750)	3.521	2	1	6	2	5	1.000
1	(0.563, 0.126, 0.563)	3.716	3	2	12	2	2	0.503
2	(0.500, 0.250, 0.750)	7.369	6	5	6	2	5	1.000

The followings are details for U_4As_4. It has 3 topological nodal lines and 5 nodal ring points along high symmetry line as shown in Fig **154**.

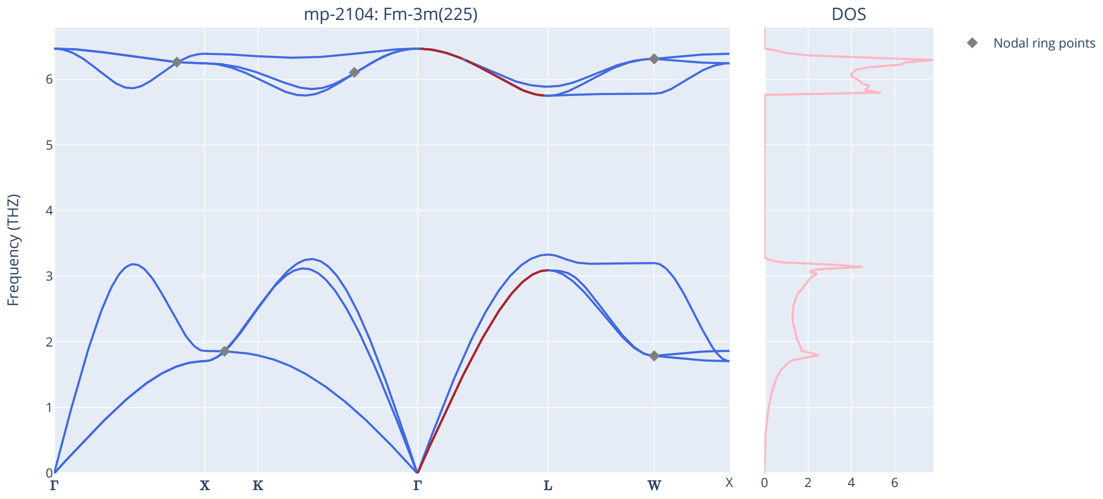

Supplementary Figure 154: The phonon band and DOS of U4As4.

Supplementary Table 286: High symmetry lines of mp-2104-U4As4

	Start	End
0	Γ (0.000, 0.000, 0.000)	X (0.500, 0.000, 0.500)
1	X (0.500, 0.000, 0.500)	U (0.625, 0.250, 0.625)
2	K (0.375, 0.375, 0.750)	Γ (0.000, 0.000, 0.000)
3	Γ (0.000, 0.000, 0.000)	L (0.500, 0.500, 0.500)
4	L (0.500, 0.500, 0.500)	W (0.500, 0.250, 0.750)
5	W (0.500, 0.250, 0.750)	X (0.500, 0.000, 0.500)

Supplementary Table 287: Nodal ring points along high symmetry lines of mp-2104-U4As4.

	Coordinates	Frequency	Mode 1	Mode 2	Multi.	Deg.	Path	Ratio
0	(0.547, 0.094, 0.547)	1.856	2	1	12	2	2	0.375
1	(0.500, 0.250, 0.750)	1.787	2	1	6	2	5	1.000
2	(0.153, 0.153, 0.291)	6.103	5	4	24	2	3	0.605
3	(0.408, 0.000, 0.408)	6.259	6	5	6	2	1	0.816
4	(0.500, 0.250, 0.750)	6.309	6	5	6	2	5	1.000

The followings are details for Hf_4C_4. It has 2 topological nodal lines and 4 nodal ring points along high symmetry line as shown in Fig 155.

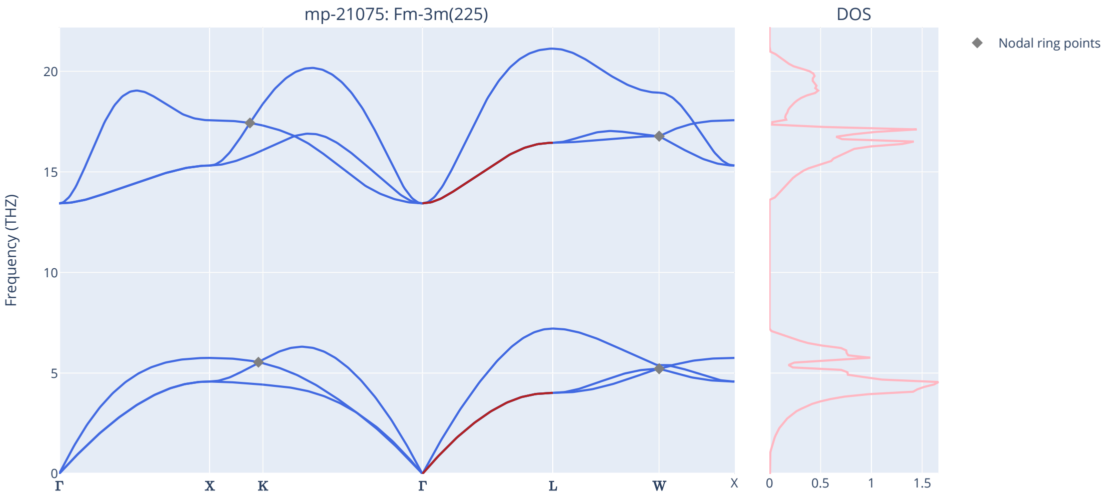

Supplementary Figure 155: The phonon band and DOS of Hf4C4.

Supplementary Table 288: High symmetry lines of mp-21075-Hf4C4

	Start	End
0	Γ (0.000, 0.000, 0.000)	X (0.500, 0.000, 0.500)
1	X (0.500, 0.000, 0.500)	U (0.625, 0.250, 0.625)
2	K (0.375, 0.375, 0.750)	Γ (0.000, 0.000, 0.000)
3	Γ (0.000, 0.000, 0.000)	L (0.500, 0.500, 0.500)
4	L (0.500, 0.500, 0.500)	W (0.500, 0.250, 0.750)
5	W (0.500, 0.250, 0.750)	X (0.500, 0.000, 0.500)

Supplementary Table 289: Nodal ring points along high symmetry lines of mp-21075-Hf4C4.

	Coordinates	Frequency	Mode 1	Mode 2	Multi.	Deg.	Path	Ratio
0	(0.500, 0.250, 0.750)	5.220	2	1	6	2	5	1.000
1	(0.615, 0.229, 0.615)	5.545	3	2	12	2	2	0.918
2	(0.500, 0.250, 0.750)	16.781	5	4	6	2	5	1.000
3	(0.595, 0.190, 0.595)	17.438	6	5	12	2	2	0.759

The followings are details for InPd. It has 6 topological nodal lines and 4 nodal ring points along high symmetry line as shown in Fig 156.

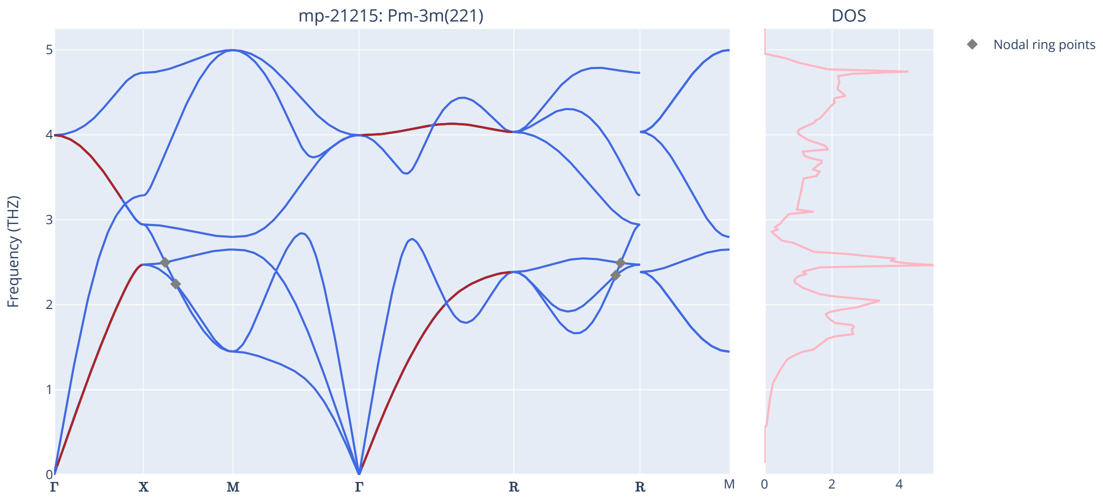

Supplementary Figure 156: The phonon band and DOS of InPd.

Supplementary Table 290: High symmetry lines of mp-21215-InPd

	Start	End
0	Γ (0.000, 0.000, 0.000)	X (0.000, 0.500, 0.000)
1	X (0.000, 0.500, 0.000)	M (0.500, 0.500, 0.000)
2	M (0.500, 0.500, 0.000)	Γ (0.000, 0.000, 0.000)
3	Γ (0.000, 0.000, 0.000)	R (0.500, 0.500, 0.500)
4	R (0.500, 0.500, 0.500)	X (0.000, 0.500, 0.000)
5	R (0.500, 0.500, 0.500)	M (0.500, 0.500, 0.000)

Supplementary Table 291: Nodal ring points along high symmetry lines of mp-21215-InPd.

	Coordinates	Frequency	Mode 1	Mode 2	Multi.	Deg.	Path	Ratio
0	(0.181, 0.500, -0.000)	2.244	2	1	12	2	2	0.361
1	(0.097, 0.500, 0.097)	2.346	2	1	12	2	5	0.806
2	(0.121, 0.500, 0.000)	2.498	3	2	12	2	2	0.243
3	(0.077, 0.500, 0.077)	2.492	3	2	12	2	5	0.845

The followings are details for CaPd and it only has 5 topological nodal lines along high symmetry line as shown in Fig **157**.

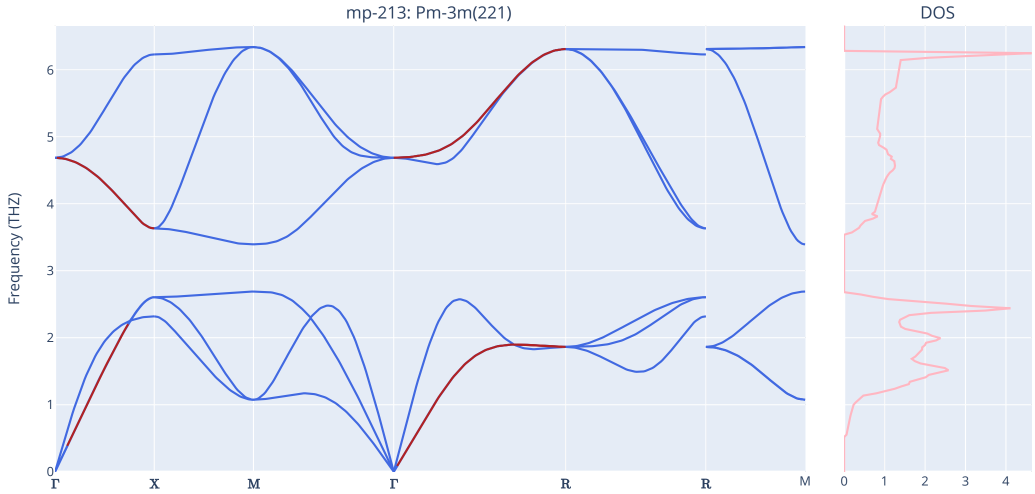

Supplementary Figure 157: The phonon band and DOS of CaPd.

Supplementary Table 292: High symmetry lines of mp-213-CaPd

	Start	End
0	Γ (0.000, 0.000, 0.000)	X (0.000, 0.500, 0.000)
1	X (0.000, 0.500, 0.000)	M (0.500, 0.500, 0.000)
2	M (0.500, 0.500, 0.000)	Γ (0.000, 0.000, 0.000)
3	Γ (0.000, 0.000, 0.000)	R (0.500, 0.500, 0.500)
4	R (0.500, 0.500, 0.500)	X (0.000, 0.500, 0.000)
5	R (0.500, 0.500, 0.500)	M (0.500, 0.500, 0.000)

The followings are details for RuZr. It has 5 topological nodal lines and 8 nodal ring points along high symmetry line as shown in Fig 158.

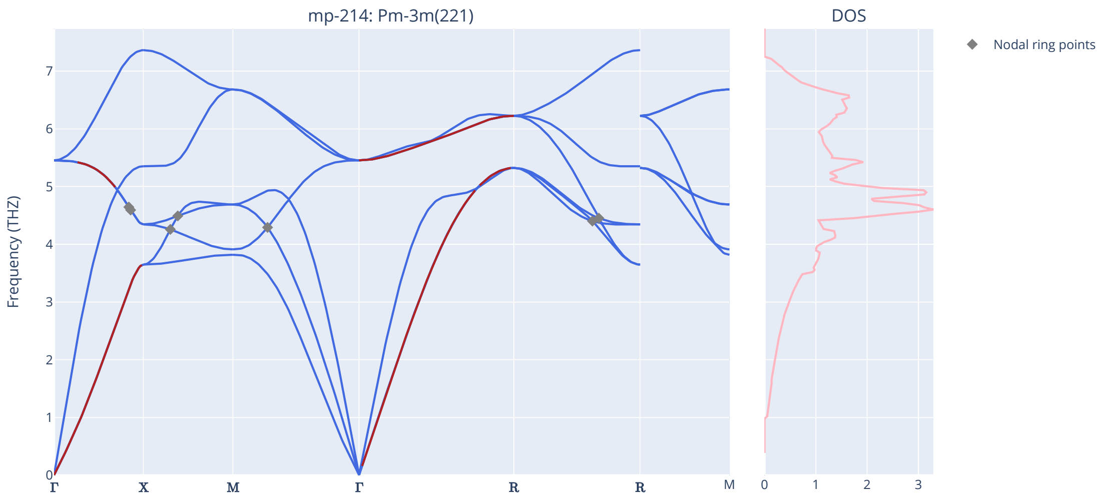

Supplementary Figure 158: The phonon band and DOS of RuZr.

Supplementary Table 293: High symmetry lines of mp-214-RuZr

	Start	End
0	Γ (0.000, 0.000, 0.000)	X (0.000, 0.500, 0.000)
1	X (0.000, 0.500, 0.000)	M (0.500, 0.500, 0.000)
2	M (0.500, 0.500, 0.000)	Γ (0.000, 0.000, 0.000)
3	Γ (0.000, 0.000, 0.000)	R (0.500, 0.500, 0.500)
4	R (0.500, 0.500, 0.500)	X (0.000, 0.500, 0.000)
5	R (0.500, 0.500, 0.500)	M (0.500, 0.500, 0.000)

Supplementary Table 294: Nodal ring points along high symmetry lines of mp-214-RuZr.

	Coordinates	Frequency	Mode 1	Mode 2	Multi.	Deg.	Path	Ratio
0	(0.189, 0.500, 0.189)	4.402	2	1	12	2	5	0.623
1	(0.151, 0.500, 0.000)	4.258	3	2	12	2	2	0.302
2	(0.362, 0.362, 0.000)	4.292	3	2	12	2	3	0.276
3	(0.000, 0.418, 0.000)	4.644	4	3	6	2	1	0.837
4	(0.000, 0.429, 0.000)	4.593	4	3	6	2	1	0.857
5	(0.192, 0.500, -0.000)	4.491	4	3	12	2	2	0.385
6	(0.163, 0.500, 0.163)	4.452	4	3	12	2	5	0.673

The followings are details for Y_4Sb_4. It has 2 topological nodal lines and 12 nodal ring points along high symmetry line as shown in Fig **159**.

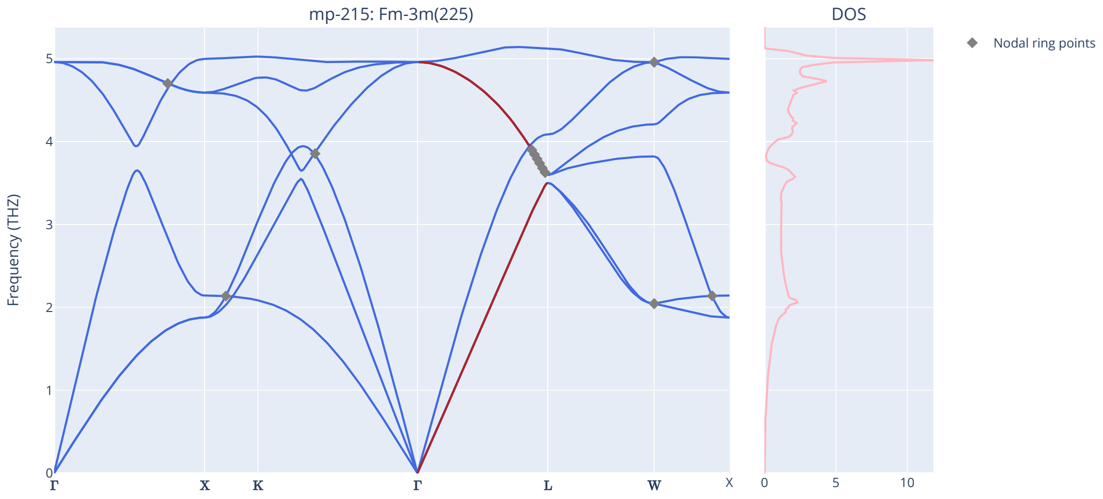

Supplementary Figure 159: The phonon band and DOS of Y4Sb4.

Supplementary Table 295: High symmetry lines of mp-215-Y4Sb4

	Start	End
0	Γ (0.000, 0.000, 0.000)	X (0.500, 0.000, 0.500)
1	X (0.500, 0.000, 0.500)	U (0.625, 0.250, 0.625)
2	K (0.375, 0.375, 0.750)	Γ (0.000, 0.000, 0.000)
3	Γ (0.000, 0.000, 0.000)	L (0.500, 0.500, 0.500)
4	L (0.500, 0.500, 0.500)	W (0.500, 0.250, 0.750)
5	W (0.500, 0.250, 0.750)	X (0.500, 0.000, 0.500)

Supplementary Table 296: Nodal ring points along high symmetry lines of mp-215-Y4Sb4.

	Coordinates	Frequency	Mode 1	Mode 2	Multi.	Deg.	Path	Ratio
0	(0.500, 0.250, 0.750)	2.045	2	1	6	2	5	1.000
1	(0.550, 0.100, 0.550)	2.136	3	2	12	2	2	0.399
2	(0.498, 0.056, 0.558)	2.139	3	2	24	2	6	0.771
3	(0.237, 0.237, 0.485)	3.854	4	3	24	2	3	0.359
4	(0.469, 0.469, 0.469)	3.739	4	3	8	2	4	0.937
5	(0.439, 0.439, 0.439)	3.898	4	3	8	2	4	0.878
6	(0.449, 0.449, 0.449)	3.845	4	3	8	2	4	0.898
7	(0.459, 0.459, 0.459)	3.790	4	3	8	2	4	0.918
8	(0.480, 0.480, 0.480)	3.679	4	3	8	2	4	0.959
9	(0.490, 0.490, 0.490)	3.626	4	3	8	2	4	0.980
10	(0.378, 0.000, 0.378)	4.700	6	5	6	2	1	0.755
11	(0.500, 0.250, 0.750)	4.956	6	5	6	2	5	1.000

The followings are details for Al_4As_4. It has 2 topological nodal lines and 1 single Weyl point along high symmetry line as shown in Fig **160**.

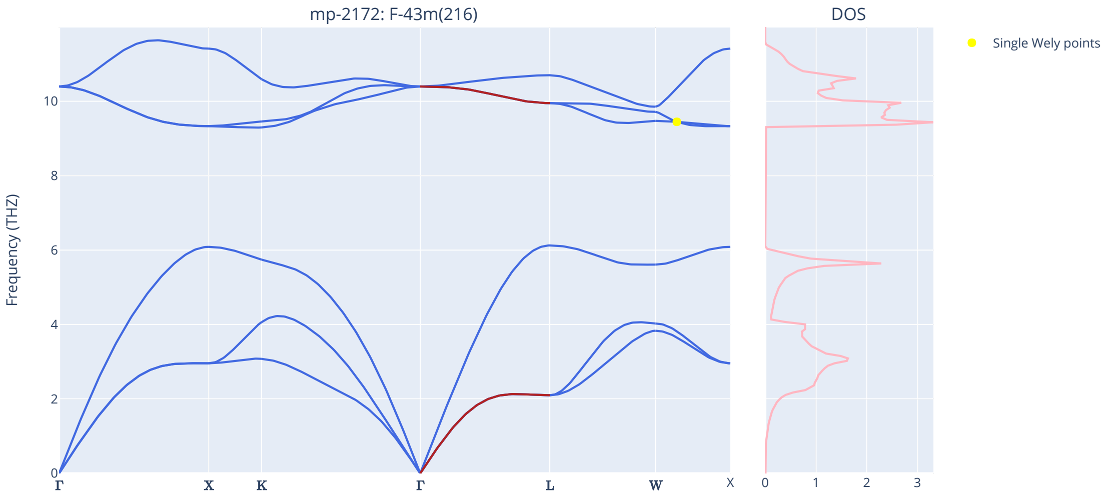

Supplementary Figure 160: The phonon band and DOS of Al4As4.

Supplementary Table 297: High symmetry lines of mp-2172-Al4As4

	Start	End
0	Γ (0.000, 0.000, 0.000)	X (0.500, 0.000, 0.500)
1	X (0.500, 0.000, 0.500)	U (0.625, 0.250, 0.625)
2	K (0.375, 0.375, 0.750)	Γ (0.000, 0.000, 0.000)
3	Γ (0.000, 0.000, 0.000)	L (0.500, 0.500, 0.500)
4	L (0.500, 0.500, 0.500)	W (0.500, 0.250, 0.750)
5	W (0.500, 0.250, 0.750)	X (0.500, 0.000, 0.500)

Supplementary Table 298: Single Weyl points along high symmetry lines of mp-2172-Al4As4.

	Coordinates	Frequency	Mode 1	Mode 2	Multi.	Deg.	Path	Ratio
0	(0.500, 0.179, 0.679)	9.447	5	4	12	2	6	0.284

The followings are details for Zn_4Te_4 and it only has 2 topological nodal lines along high symmetry line as shown in Fig **161**.

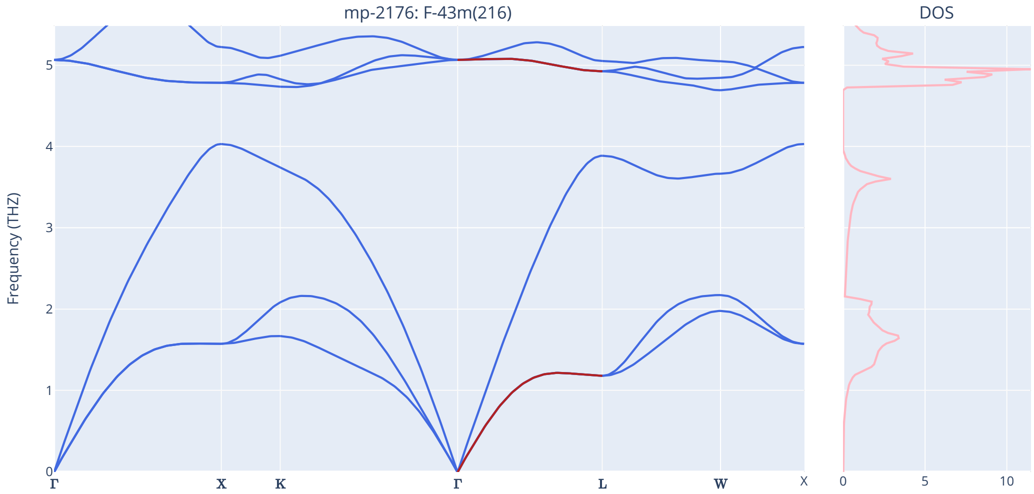

Supplementary Figure 161: The phonon band and DOS of Zn4Te4.

Supplementary Table 299: High symmetry lines of mp-2176-Zn4Te4

	Start	End
0	Γ (0.000, 0.000, 0.000)	X (0.500, 0.000, 0.500)
1	X (0.500, 0.000, 0.500)	U (0.625, 0.250, 0.625)
2	K (0.375, 0.375, 0.750)	Γ (0.000, 0.000, 0.000)
3	Γ (0.000, 0.000, 0.000)	L (0.500, 0.500, 0.500)
4	L (0.500, 0.500, 0.500)	W (0.500, 0.250, 0.750)
5	W (0.500, 0.250, 0.750)	X (0.500, 0.000, 0.500)

The followings are details for ReTi. It has 6 topological nodal lines and 5 nodal ring points along high symmetry line as shown in Fig 162.

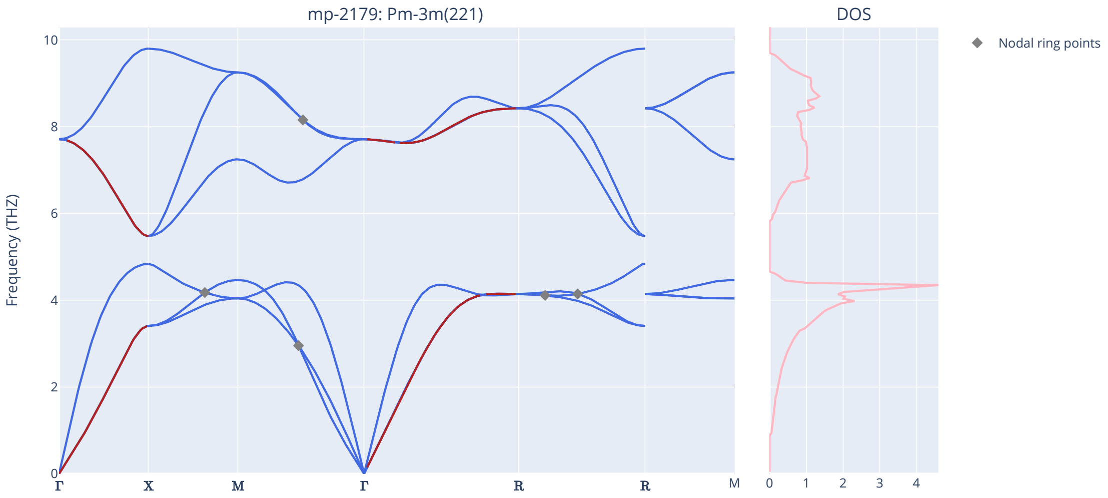

Supplementary Figure 162: The phonon band and DOS of ReTi.

Supplementary Table 300: High symmetry lines of mp-2179-ReTi

	Start	End
0	Γ (0.000, 0.000, 0.000)	X (0.000, 0.500, 0.000)
1	X (0.000, 0.500, 0.000)	M (0.500, 0.500, 0.000)
2	M (0.500, 0.500, 0.000)	Γ (0.000, 0.000, 0.000)
3	Γ (0.000, 0.000, 0.000)	R (0.500, 0.500, 0.500)
4	R (0.500, 0.500, 0.500)	X (0.000, 0.500, 0.000)
5	R (0.500, 0.500, 0.500)	M (0.500, 0.500, 0.000)

Supplementary Table 301: Nodal ring points along high symmetry lines of mp-2179-ReTi.

	Coordinates	Frequency	Mode 1	Mode 2	Multi.	Deg.	Path	Ratio
0	(0.259, 0.259, 0.000)	2.954	2	1	12	2	3	0.481
1	(0.396, 0.500, 0.396)	4.112	2	1	12	2	5	0.209
2	(0.316, 0.500, -0.000)	4.178	3	2	12	2	2	0.631
3	(0.267, 0.500, 0.267)	4.146	3	2	12	2	5	0.466
4	(0.242, 0.242, -0.000)	8.156	6	5	12	2	3	0.516

The followings are details for Sn_4As_4. It has 3 topological nodal lines and 5 nodal ring points along high symmetry line as shown in Fig **163**.

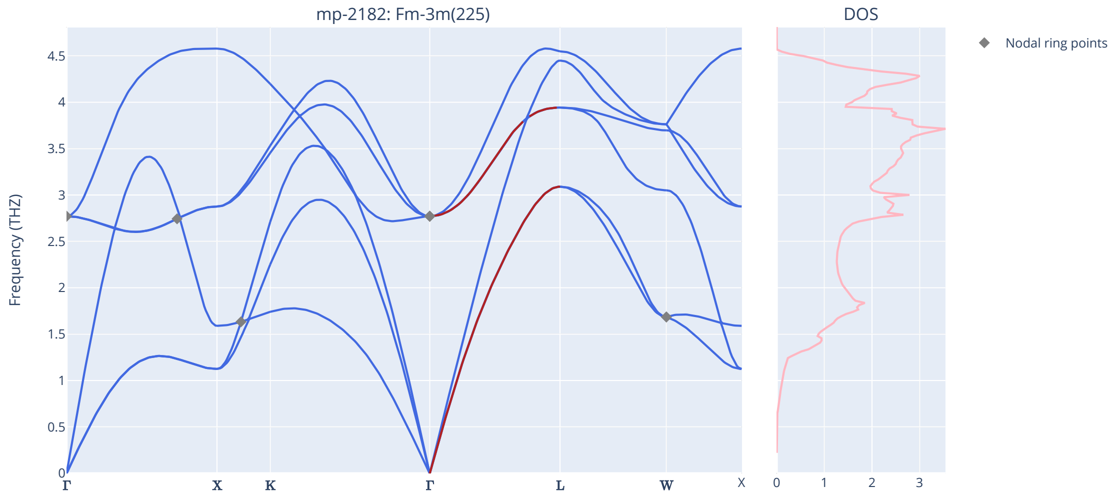

Supplementary Figure 163: The phonon band and DOS of Sn4As4.

Supplementary Table 302: High symmetry lines of mp-2182-Sn4As4

	Start	End
0	Γ (0.000, 0.000, 0.000)	X (0.500, 0.000, 0.500)
1	X (0.500, 0.000, 0.500)	U (0.625, 0.250, 0.625)
2	K (0.375, 0.375, 0.750)	Γ (0.000, 0.000, 0.000)
3	Γ (0.000, 0.000, 0.000)	L (0.500, 0.500, 0.500)
4	L (0.500, 0.500, 0.500)	W (0.500, 0.250, 0.750)
5	W (0.500, 0.250, 0.750)	X (0.500, 0.000, 0.500)

Supplementary Table 303: Nodal ring points along high symmetry lines of mp-2182-Sn4As4.

	Coordinates	Frequency	Mode 1	Mode 2	Multi.	Deg.	Path	Ratio
0	(0.500, 0.250, 0.750)	1.685	2	1	6	2	5	1.000
1	(0.556, 0.113, 0.556)	1.633	3	2	12	2	2	0.451
2	(0.367, 0.000, 0.367)	2.743	4	3	6	2	1	0.735
3	(0.000, 0.000, 0.000)	2.771	5	4	1	3	1	0.000
4	(0.000, 0.000, 0.000)	2.771	6	5	1	3	1	0.000

The followings are details for MoP. It has 1 nodal ring points and 4 single Weyl point along high symmetry line as shown in Fig 164.

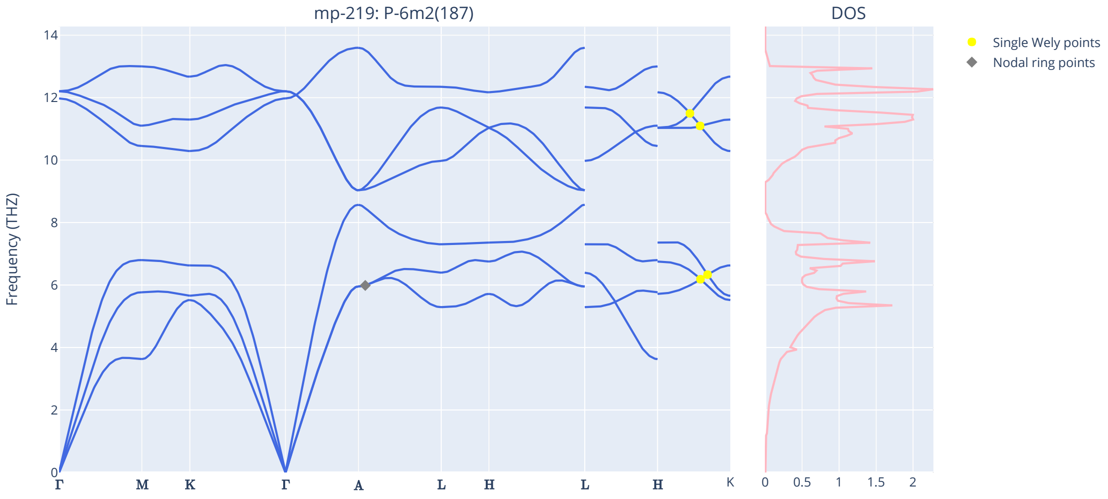

Supplementary Figure 164: The phonon band and DOS of MoP.

Supplementary Table 304: High symmetry lines of mp-219-MoP

	Start	End
0	Γ (0.000, 0.000, 0.000)	M (0.500, 0.000, 0.000)
1	M (0.500, 0.000, 0.000)	K (0.333, 0.333, 0.000)
2	K (0.333, 0.333, 0.000)	Γ (0.000, 0.000, 0.000)
3	Γ (0.000, 0.000, 0.000)	A (0.000, 0.000, 0.500)
4	A (0.000, 0.000, 0.500)	L (0.500, 0.000, 0.500)
5	L (0.500, 0.000, 0.500)	H (0.333, 0.333, 0.500)
6	H (0.333, 0.333, 0.500)	A (0.000, 0.000, 0.500)
7	L (0.500, 0.000, 0.500)	M (0.500, 0.000, 0.000)
8	H (0.333, 0.333, 0.500)	K (0.333, 0.333, 0.000)

Supplementary Table 305: Single Weyl points along high symmetry lines of mp-219-MoP.

	Coordinates	Frequency	Mode 1	Mode 2	Multi.	Deg.	Path	Ratio
0	(0.333, 0.333, 0.205)	6.186	2	1	4	2	9	0.589
1	(0.333, 0.333, 0.157)	6.332	3	2	4	2	9	0.687
2	(0.333, 0.333, 0.208)	11.092	5	4	4	2	9	0.585
3	(0.333, 0.333, 0.278)	11.495	6	5	4	2	9	0.444

Supplementary Table 306: Nodal ring points along high symmetry lines of mp-219-MoP.

	Coordinates	Frequency	Mode 1	Mode 2	Multi.	Deg.	Path	Ratio
0	(0.042, 0.000, 0.500)	5.987	2	1	6	2	5	0.084

Supplementary Table 306: Nodal ring points along high symmetry lines of mp-219-MoP.

Coordinates	Frequency	Mode 1	Mode 2	Multi.	Deg.	Path	Ratio

The followings are details for BaHg. It has 5 topological nodal lines and 5 nodal ring points along high symmetry line as shown in Fig 165.

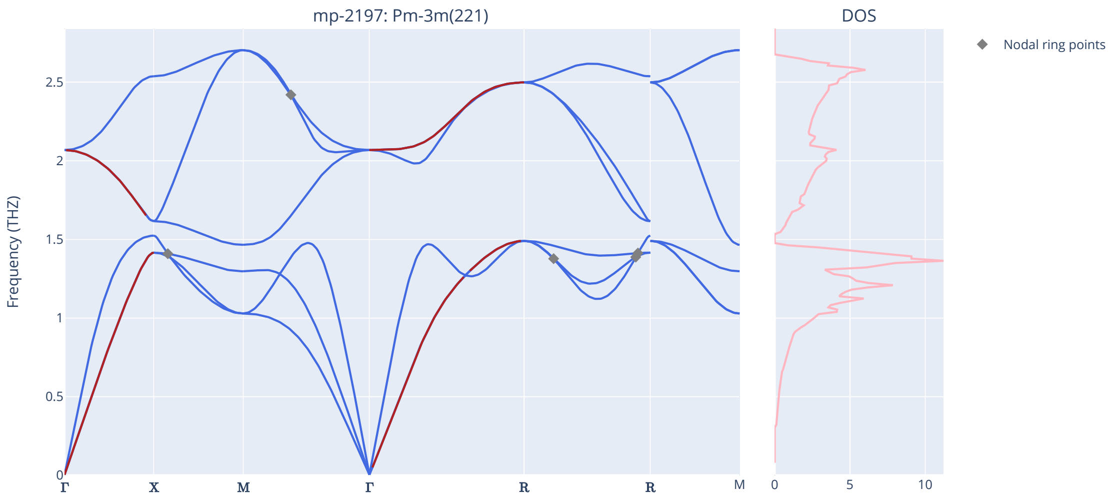

Supplementary Figure 165: The phonon band and DOS of BaHg.

Supplementary Table 307: High symmetry lines of mp-2197-BaHg

	Start	End
0	Γ (0.000, 0.000, 0.000)	X (0.000, 0.500, 0.000)
1	X (0.000, 0.500, 0.000)	M (0.500, 0.500, 0.000)
2	M (0.500, 0.500, 0.000)	Γ (0.000, 0.000, 0.000)
3	Γ (0.000, 0.000, 0.000)	R (0.500, 0.500, 0.500)
4	R (0.500, 0.500, 0.500)	X (0.000, 0.500, 0.000)
5	R (0.500, 0.500, 0.500)	M (0.500, 0.500, 0.000)

Supplementary Table 308: Nodal ring points along high symmetry lines of mp-2197-BaHg.

	Coordinates	Frequency	Mode 1	Mode 2	Multi.	Deg.	Path	Ratio
0	(0.383, 0.500, 0.383)	1.377	2	1	12	2	5	0.234
1	(0.058, 0.500, 0.058)	1.387	2	1	12	2	5	0.885
2	(0.078, 0.500, -0.000)	1.409	3	2	12	2	2	0.157
3	(0.049, 0.500, 0.049)	1.413	3	2	12	2	5	0.902
4	(0.311, 0.311, -0.000)	2.419	6	5	12	2	3	0.378

The followings are details for CoSc. It has 5 topological nodal lines and 5 nodal ring points along high symmetry line as shown in Fig 166.

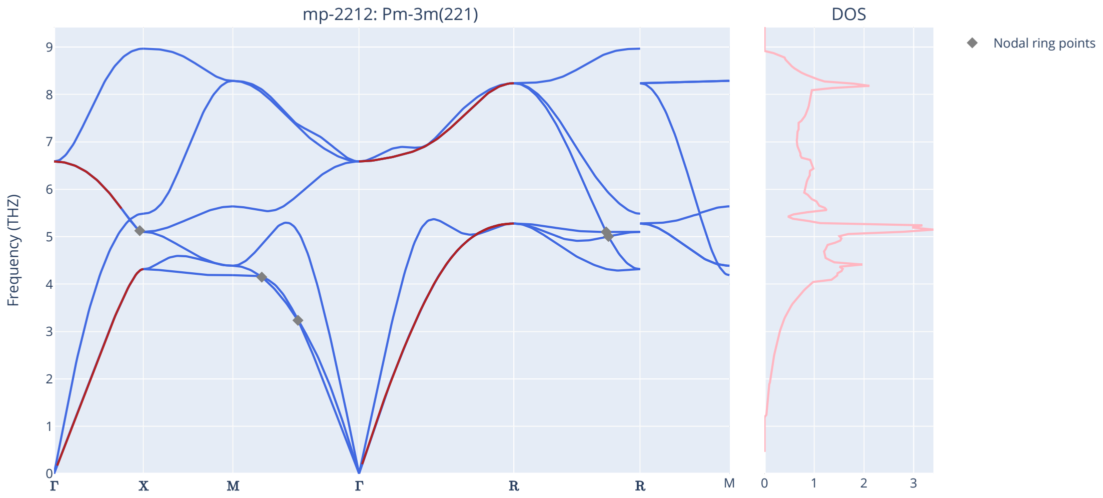

Supplementary Figure 166: The phonon band and DOS of CoSc.

Supplementary Table 309: High symmetry lines of mp-2212-CoSc

	Start	End
0	Γ (0.000, 0.000, 0.000)	X (0.000, 0.500, 0.000)
1	X (0.000, 0.500, 0.000)	M (0.500, 0.500, 0.000)
2	M (0.500, 0.500, 0.000)	Γ (0.000, 0.000, 0.000)
3	Γ (0.000, 0.000, 0.000)	R (0.500, 0.500, 0.500)
4	R (0.500, 0.500, 0.500)	X (0.000, 0.500, 0.000)
5	R (0.500, 0.500, 0.500)	M (0.500, 0.500, 0.000)

Supplementary Table 310: Nodal ring points along high symmetry lines of mp-2212-CoSc.

	Coordinates	Frequency	Mode 1	Mode 2	Multi.	Deg.	Path	Ratio
0	(0.242, 0.242, 0.000)	3.236	2	1	12	2	3	0.516
1	(0.385, 0.385, 0.000)	4.146	2	1	12	2	3	0.230
2	(0.125, 0.500, 0.125)	5.005	3	2	12	2	5	0.750
3	(0.000, 0.480, 0.000)	5.126	4	3	6	2	1	0.959
4	(0.134, 0.500, 0.134)	5.100	4	3	12	2	5	0.731

The followings are details for GaRu. It has 6 topological nodal lines and 5 nodal ring points along high symmetry line as shown in Fig 167.

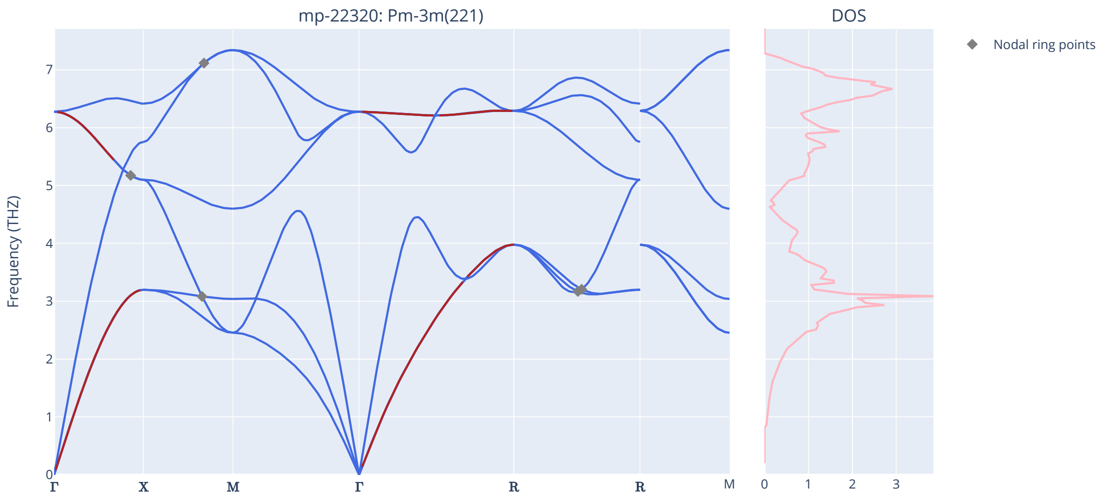

Supplementary Figure 167: The phonon band and DOS of GaRu.

Supplementary Table 311: High symmetry lines of mp-22320-GaRu

	Start	End
0	Γ (0.000, 0.000, 0.000)	X (0.000, 0.500, 0.000)
1	X (0.000, 0.500, 0.000)	M (0.500, 0.500, 0.000)
2	M (0.500, 0.500, 0.000)	Γ (0.000, 0.000, 0.000)
3	Γ (0.000, 0.000, 0.000)	R (0.500, 0.500, 0.500)
4	R (0.500, 0.500, 0.500)	X (0.000, 0.500, 0.000)
5	R (0.500, 0.500, 0.500)	M (0.500, 0.500, 0.000)

Supplementary Table 312: Nodal ring points along high symmetry lines of mp-22320-GaRu.

	Coordinates	Frequency	Mode 1	Mode 2	Multi.	Deg.	Path	Ratio
0	(0.246, 0.500, 0.246)	3.168	2	1	12	2	5	0.508
1	(0.328, 0.500, 0.000)	3.082	3	2	12	2	2	0.657
2	(0.231, 0.500, 0.231)	3.205	3	2	12	2	5	0.538
3	(0.000, 0.429, 0.000)	5.173	4	3	6	2	1	0.857
4	(0.339, 0.500, -0.000)	7.114	6	5	12	2	2	0.678

The followings are details for CoZr. It has 4 topological nodal lines and 8 nodal ring points along high symmetry line as shown in Fig **168**.

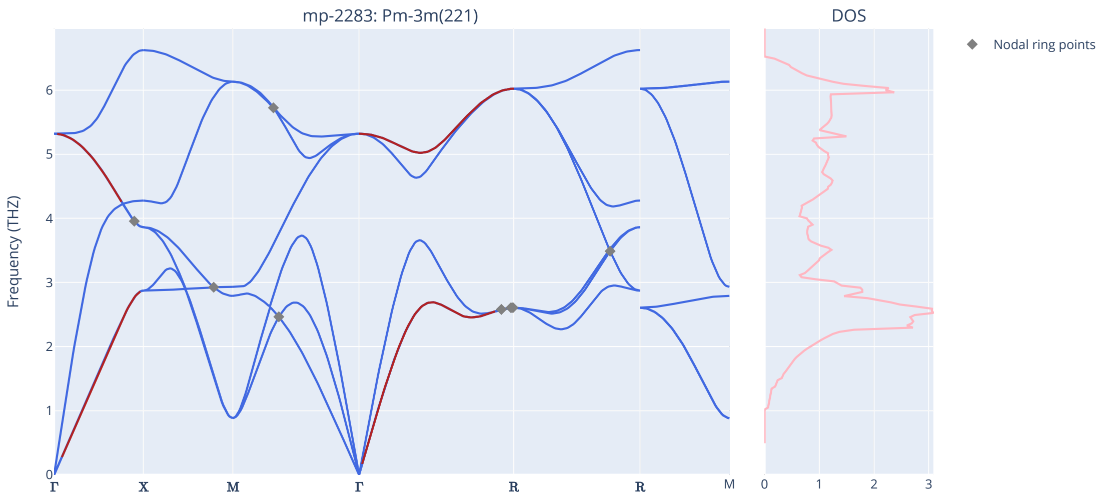

Supplementary Figure 168: The phonon band and DOS of CoZr.

Supplementary Table 313: High symmetry lines of mp-2283-CoZr

	Start	End
0	Γ (0.000, 0.000, 0.000)	X (0.000, 0.500, 0.000)
1	X (0.000, 0.500, 0.000)	M (0.500, 0.500, 0.000)
2	M (0.500, 0.500, 0.000)	Γ (0.000, 0.000, 0.000)
3	Γ (0.000, 0.000, 0.000)	R (0.500, 0.500, 0.500)
4	R (0.500, 0.500, 0.500)	X (0.000, 0.500, 0.000)
5	R (0.500, 0.500, 0.500)	M (0.500, 0.500, 0.000)

Supplementary Table 314: Nodal ring points along high symmetry lines of mp-2283-CoZr.

	Coordinates	Frequency	Mode 1	Mode 2	Multi.	Deg.	Path	Ratio
0	(0.317, 0.317, 0.000)	2.464	2	1	12	2	3	0.365
1	(0.497, 0.497, 0.497)	2.606	3	2	8	3	4	0.995
2	(0.491, 0.491, 0.491)	2.605	3	2	8	2	4	0.982
3	(0.460, 0.460, 0.460)	2.579	3	2	8	2	4	0.920
4	(0.119, 0.500, 0.119)	3.487	3	2	12	2	5	0.762
5	(0.000, 0.449, 0.000)	3.955	4	3	6	2	1	0.898
6	(0.393, 0.500, 0.000)	2.925	4	3	12	2	2	0.787
7	(0.339, 0.339, 0.000)	5.724	6	5	12	2	3	0.322

The followings are details for Cu_4I_4. It has 5 nodal ring points and 1 single Weyl point along high symmetry line as shown in Fig 169.

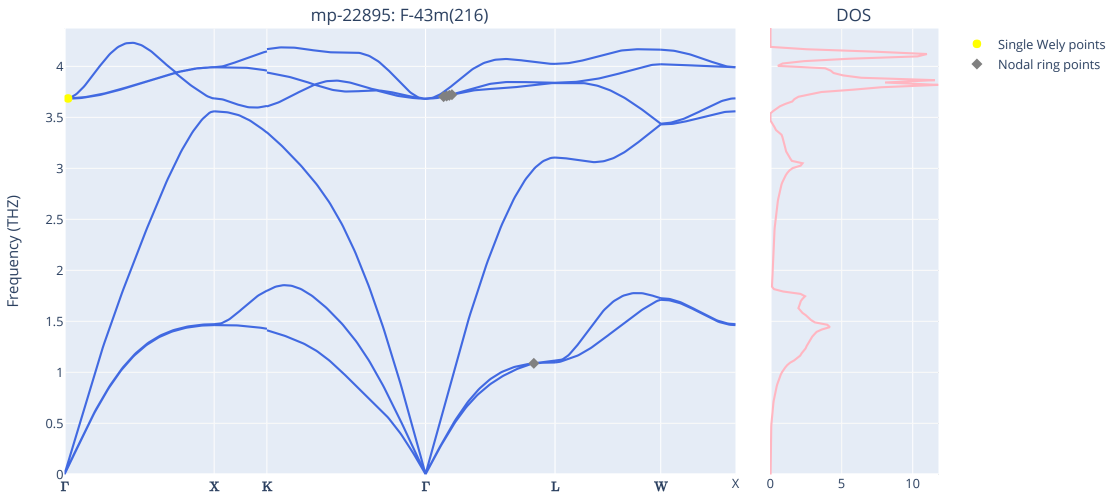

Supplementary Figure 169: The phonon band and DOS of Cu4I4.

Supplementary Table 315: High symmetry lines of mp-22895-Cu4I4

	Start	End
0	Γ (0.000, 0.000, 0.000)	X (0.500, 0.000, 0.500)
1	X (0.500, 0.000, 0.500)	U (0.625, 0.250, 0.625)
2	K (0.375, 0.375, 0.750)	Γ (0.000, 0.000, 0.000)
3	Γ (0.000, 0.000, 0.000)	L (0.500, 0.500, 0.500)
4	L (0.500, 0.500, 0.500)	W (0.500, 0.250, 0.750)
5	W (0.500, 0.250, 0.750)	X (0.500, 0.000, 0.500)

Supplementary Table 316: Single Weyl points along high symmetry lines of mp-22895-Cu4I4.

	Coordinates	Frequency	Mode 1	Mode 2	Multi.	Deg.	Path	Ratio
0	(0.012, -0.000, 0.012)	3.686	6	5	6	2	1	0.023

Supplementary Table 317: Nodal ring points along high symmetry lines of mp-22895-Cu4I4.

	Coordinates	Frequency	Mode 1	Mode 2	Multi.	Deg.	Path	Ratio
0	(0.418, 0.418, 0.419)	1.089	2	1	24	2	4	0.836
1	(0.068, 0.068, 0.073)	3.703	5	4	24	2	4	0.140
2	(0.080, 0.080, 0.085)	3.710	5	4	24	2	4	0.163
3	(0.090, 0.090, 0.096)	3.716	5	4	24	2	4	0.183
4	(0.100, 0.100, 0.106)	3.723	5	4	24	2	4	0.204

The followings are details for Cu_4Br_4 and it only has 2 topological nodal lines along high symmetry line as shown in Fig **170**.

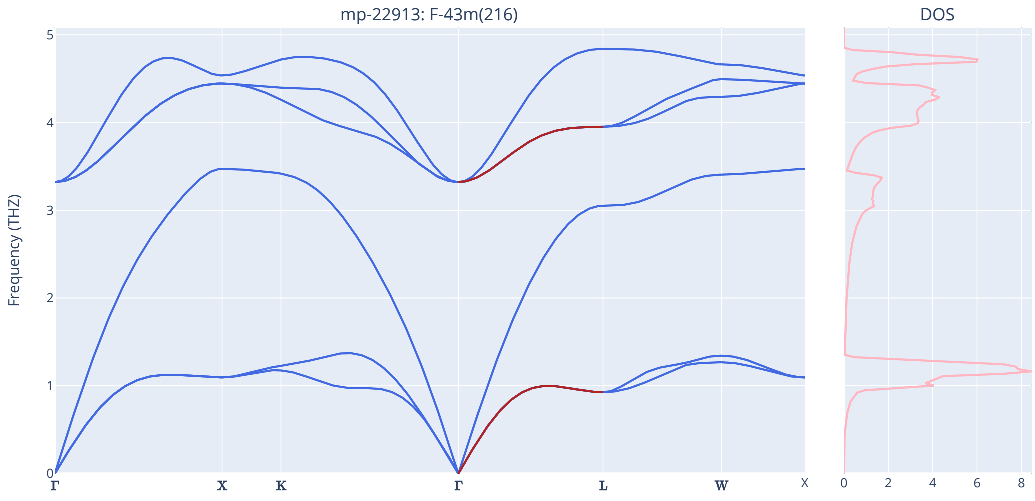

Supplementary Figure 170: The phonon band and DOS of Cu4Br4.

Supplementary Table 318: High symmetry lines of mp-22913-Cu4Br4

	Start	End
0	Γ (0.000, 0.000, 0.000)	X (0.500, 0.000, 0.500)
1	X (0.500, 0.000, 0.500)	U (0.625, 0.250, 0.625)
2	K (0.375, 0.375, 0.750)	Γ (0.000, 0.000, 0.000)
3	Γ (0.000, 0.000, 0.000)	L (0.500, 0.500, 0.500)
4	L (0.500, 0.500, 0.500)	W (0.500, 0.250, 0.750)
5	W (0.500, 0.250, 0.750)	X (0.500, 0.000, 0.500)

The followings are details for Cu_4Cl_4. It has 2 topological nodal lines and 1 nodal ring point along high symmetry line as shown in Fig **171**.

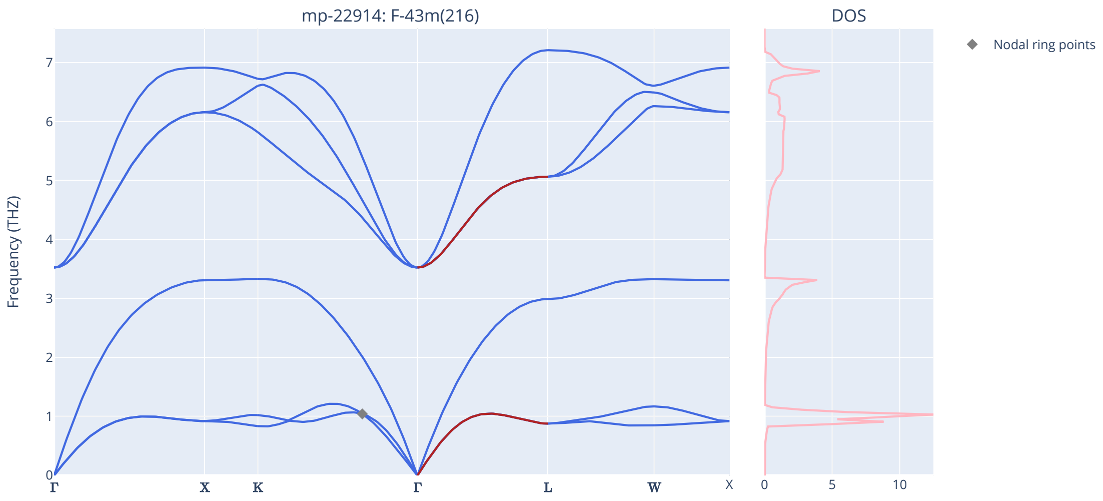

Supplementary Figure 171: The phonon band and DOS of Cu4Cl4.

Supplementary Table 319: High symmetry lines of mp-22914-Cu4Cl4

	Start	End
0	Γ (0.000, 0.000, 0.000)	X (0.500, 0.000, 0.500)
1	X (0.500, 0.000, 0.500)	U (0.625, 0.250, 0.625)
2	K (0.375, 0.375, 0.750)	Γ (0.000, 0.000, 0.000)
3	Γ (0.000, 0.000, 0.000)	L (0.500, 0.500, 0.500)
4	L (0.500, 0.500, 0.500)	W (0.500, 0.250, 0.750)
5	W (0.500, 0.250, 0.750)	X (0.500, 0.000, 0.500)

Supplementary Table 320: Nodal ring points along high symmetry lines of mp-22914-Cu4Cl4.

	Coordinates	Frequency	Mode 1	Mode 2	Multi.	Deg.	Path	Ratio
0	(0.130, 0.130, 0.258)	1.040	2	1	24	2	3	0.655

The followings are details for Ag_4I_4 and it only has 2 topological nodal lines along high symmetry line as shown in Fig **172**.

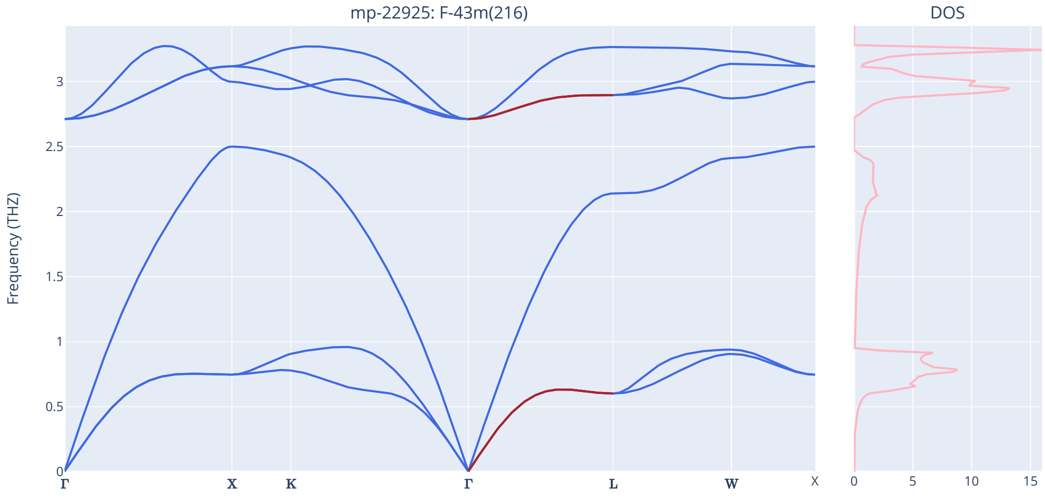

Supplementary Figure 172: The phonon band and DOS of Ag4I4.

Supplementary Table 321: High symmetry lines of mp-22925-Ag4I4

	Start	End
0	Γ (0.000, 0.000, 0.000)	X (0.500, 0.000, 0.500)
1	X (0.500, 0.000, 0.500)	U (0.625, 0.250, 0.625)
2	K (0.375, 0.375, 0.750)	Γ (0.000, 0.000, 0.000)
3	Γ (0.000, 0.000, 0.000)	L (0.500, 0.500, 0.500)
4	L (0.500, 0.500, 0.500)	W (0.500, 0.250, 0.750)
5	W (0.500, 0.250, 0.750)	X (0.500, 0.000, 0.500)

The followings are details for MoC. It has 1 nodal ring points and 5 single Weyl point along high symmetry line as shown in Fig 173.

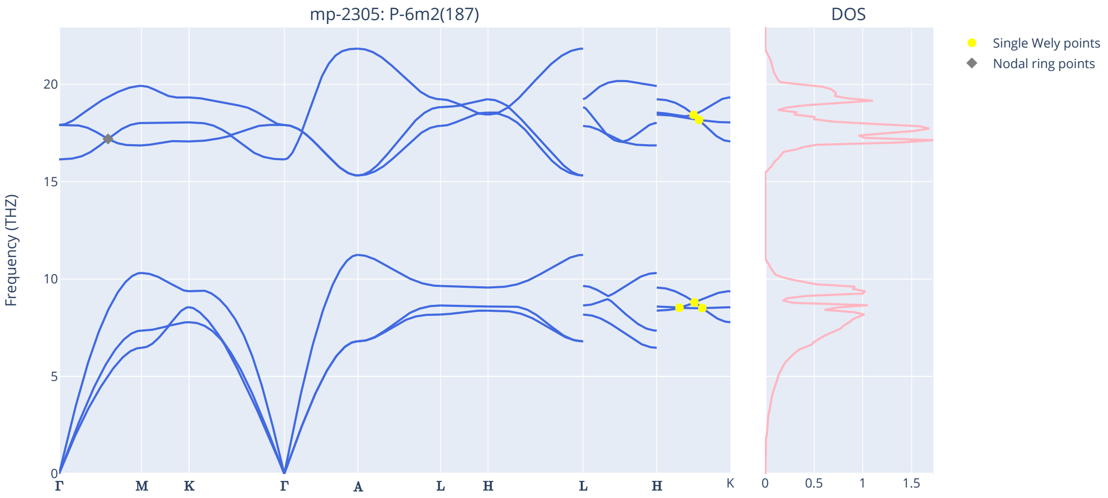

Supplementary Figure 173: The phonon band and DOS of MoC.

Supplementary Table 322: High symmetry lines of mp-2305-MoC

	Start	End
0	Γ (0.000, 0.000, 0.000)	M (0.500, 0.000, 0.000)
1	M (0.500, 0.000, 0.000)	K (0.333, 0.333, 0.000)
2	K (0.333, 0.333, 0.000)	Γ (0.000, 0.000, 0.000)
3	Γ (0.000, 0.000, 0.000)	A (0.000, 0.000, 0.500)
4	A (0.000, 0.000, 0.500)	L (0.500, 0.000, 0.500)
5	L (0.500, 0.000, 0.500)	H (0.333, 0.333, 0.500)
6	H (0.333, 0.333, 0.500)	A (0.000, 0.000, 0.500)
7	L (0.500, 0.000, 0.500)	M (0.500, 0.000, 0.000)
8	H (0.333, 0.333, 0.500)	K (0.333, 0.333, 0.000)

Supplementary Table 323: Single Weyl points along high symmetry lines of mp-2305-MoC.

	Coordinates	Frequency	Mode 1	Mode 2	Multi.	Deg.	Path	Ratio
0	(0.333, 0.333, 0.346)	8.527	2	1	4	2	9	0.309
1	(0.333, 0.333, 0.190)	8.503	2	1	4	2	9	0.619
2	(0.333, 0.333, 0.243)	8.802	3	2	4	2	9	0.513
3	(0.333, 0.333, 0.213)	18.167	5	4	4	2	9	0.575
4	(0.333, 0.333, 0.250)	18.430	6	5	4	2	9	0.500

Supplementary Table 324: Nodal ring points along high symmetry lines of mp-2305-MoC.

	Coordinates	Frequency	Mode 1	Mode 2	Multi.	Deg.	Path	Ratio
0	(0.297, 0.000, 0.000)	17.192	5	4	10	2	1	0.594

The followings are details for BeCu. It has 6 topological nodal lines and 5 nodal ring points along high symmetry line as shown in Fig 174.

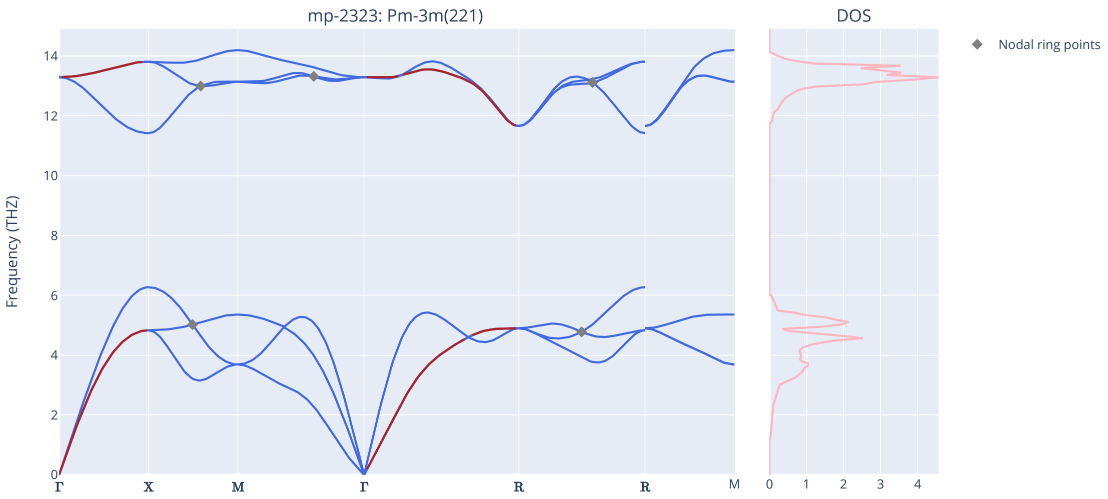

Supplementary Figure 174: The phonon band and DOS of BeCu.

Supplementary Table 325: High symmetry lines of mp-2323-BeCu

	Start	End
0	Γ (0.000, 0.000, 0.000)	X (0.000, 0.500, 0.000)
1	X (0.000, 0.500, 0.000)	M (0.500, 0.500, 0.000)
2	M (0.500, 0.500, 0.000)	Γ (0.000, 0.000, 0.000)
3	Γ (0.000, 0.000, 0.000)	R (0.500, 0.500, 0.500)
4	R (0.500, 0.500, 0.500)	X (0.000, 0.500, 0.000)
5	R (0.500, 0.500, 0.500)	M (0.500, 0.500, 0.000)

Supplementary Table 326: Nodal ring points along high symmetry lines of mp-2323-BeCu.

	Coordinates	Frequency	Mode 1	Mode 2	Multi.	Deg.	Path	Ratio
0	(0.247, 0.500, 0.000)	5.021	3	2	12	2	2	0.495
1	(0.251, 0.500, 0.251)	4.777	3	2	12	2	5	0.498
2	(0.293, 0.500, 0.000)	12.996	5	4	12	2	2	0.585
3	(0.199, 0.199, 0.000)	13.319	5	4	12	2	3	0.602
4	(0.208, 0.500, 0.208)	13.112	5	4	12	2	5	0.584

The followings are details for Bi_4Y_4. It has 2 topological nodal lines and 3 nodal ring points along high symmetry line as shown in Fig **175**.

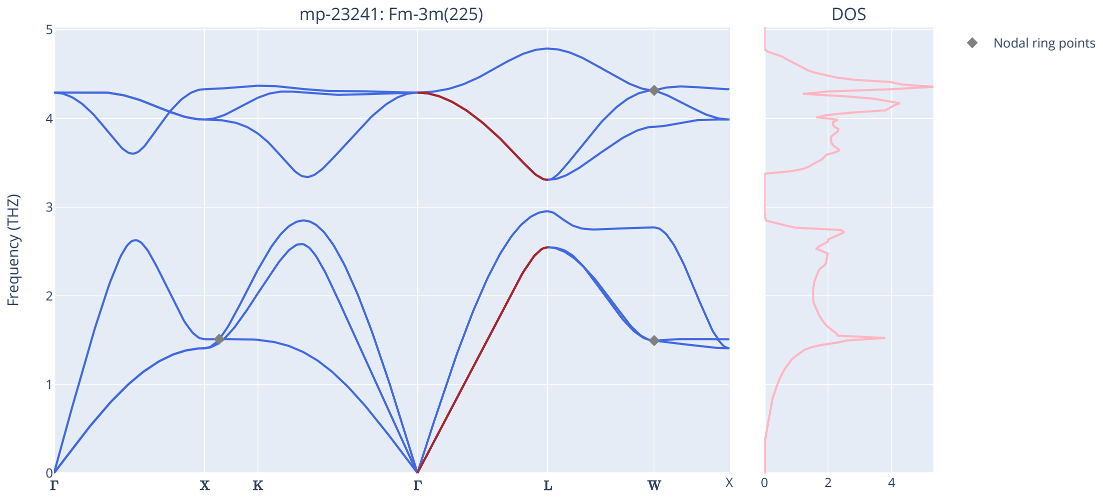

Supplementary Figure 175: The phonon band and DOS of Bi4Y4.

Supplementary Table 327: High symmetry lines of mp-23241-Bi4Y4

	Start	End
0	Γ (0.000, 0.000, 0.000)	X (0.500, 0.000, 0.500)
1	X (0.500, 0.000, 0.500)	U (0.625, 0.250, 0.625)
2	K (0.375, 0.375, 0.750)	Γ (0.000, 0.000, 0.000)
3	Γ (0.000, 0.000, 0.000)	L (0.500, 0.500, 0.500)
4	L (0.500, 0.500, 0.500)	W (0.500, 0.250, 0.750)
5	W (0.500, 0.250, 0.750)	X (0.500, 0.000, 0.500)

Supplementary Table 328: Nodal ring points along high symmetry lines of mp-23241-Bi4Y4.

	Coordinates	Frequency	Mode 1	Mode 2	Multi.	Deg.	Path	Ratio
0	(0.500, 0.250, 0.750)	1.496	2	1	6	2	5	1.000
1	(0.534, 0.069, 0.534)	1.512	3	2	12	2	2	0.274
2	(0.500, 0.250, 0.750)	4.316	6	5	6	2	5	1.000

The followings are details for La_4S_4. It has 2 topological nodal lines and 4 nodal ring points along high symmetry line as shown in Fig 176.

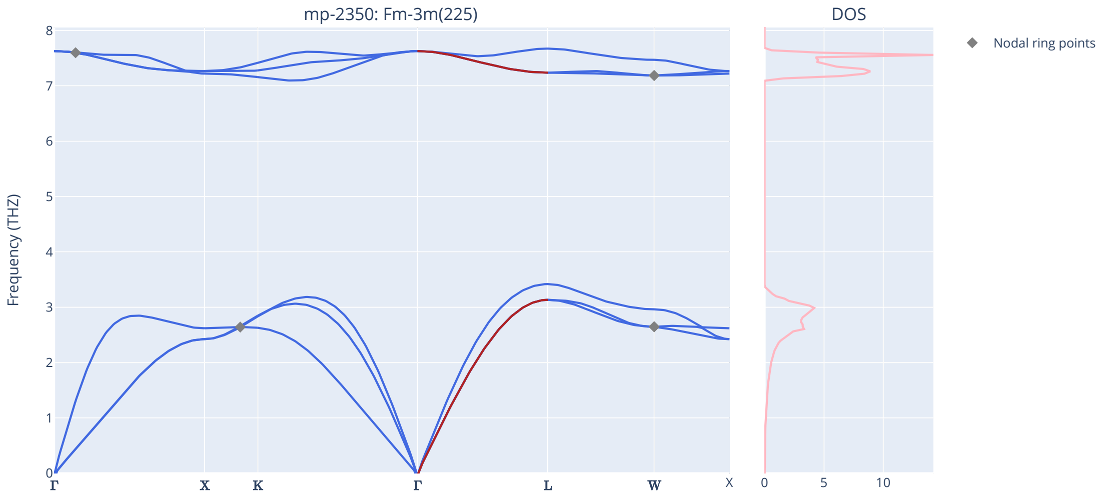

Supplementary Figure 176: The phonon band and DOS of La4S4.

Supplementary Table 329: High symmetry lines of mp-2350-La4S4

	Start	End
0	Γ (0.000, 0.000, 0.000)	X (0.500, 0.000, 0.500)
1	X (0.500, 0.000, 0.500)	U (0.625, 0.250, 0.625)
2	K (0.375, 0.375, 0.750)	Γ (0.000, 0.000, 0.000)
3	Γ (0.000, 0.000, 0.000)	L (0.500, 0.500, 0.500)
4	L (0.500, 0.500, 0.500)	W (0.500, 0.250, 0.750)
5	W (0.500, 0.250, 0.750)	X (0.500, 0.000, 0.500)

Supplementary Table 330: Nodal ring points along high symmetry lines of mp-2350-La4S4.

	Coordinates	Frequency	Mode 1	Mode 2	Multi.	Deg.	Path	Ratio
0	(0.584, 0.168, 0.584)	2.641	2	1	12	2	2	0.670
1	(0.500, 0.250, 0.750)	2.647	2	1	6	2	5	1.000
2	(0.071, 0.000, 0.071)	7.598	5	4	6	2	1	0.143
3	(0.500, 0.250, 0.750)	7.186	5	4	6	2	5	1.000

The followings are details for La_4P_4. It has 2 topological nodal lines and 3 nodal ring points along high symmetry line as shown in Fig **177**.

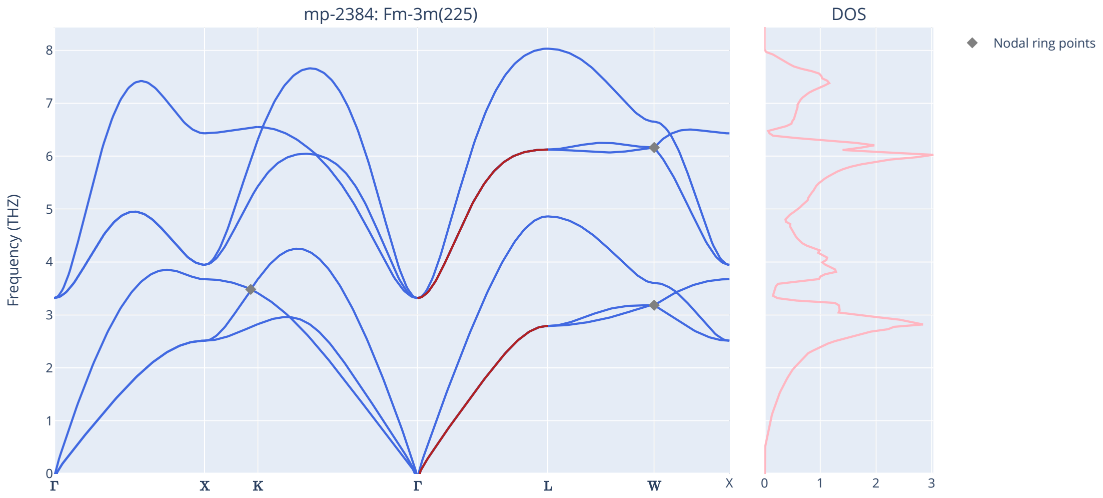

Supplementary Figure 177: The phonon band and DOS of La4P4.

Supplementary Table 331: High symmetry lines of mp-2384-La4P4

	Start	End
0	Γ (0.000, 0.000, 0.000)	X (0.500, 0.000, 0.500)
1	X (0.500, 0.000, 0.500)	U (0.625, 0.250, 0.625)
2	K (0.375, 0.375, 0.750)	Γ (0.000, 0.000, 0.000)
3	Γ (0.000, 0.000, 0.000)	L (0.500, 0.500, 0.500)
4	L (0.500, 0.500, 0.500)	W (0.500, 0.250, 0.750)
5	W (0.500, 0.250, 0.750)	X (0.500, 0.000, 0.500)

Supplementary Table 332: Nodal ring points along high symmetry lines of mp-2384-La4P4.

	Coordinates	Frequency	Mode 1	Mode 2	Multi.	Deg.	Path	Ratio
0	(0.500, 0.250, 0.750)	3.184	2	1	6	2	5	1.000
1	(0.608, 0.217, 0.608)	3.482	3	2	12	2	2	0.867
2	(0.500, 0.250, 0.750)	6.162	5	4	6	2	5	1.000

The followings are details for HgY. It has 5 topological nodal lines and 6 nodal ring points along high symmetry line as shown in Fig **178**.

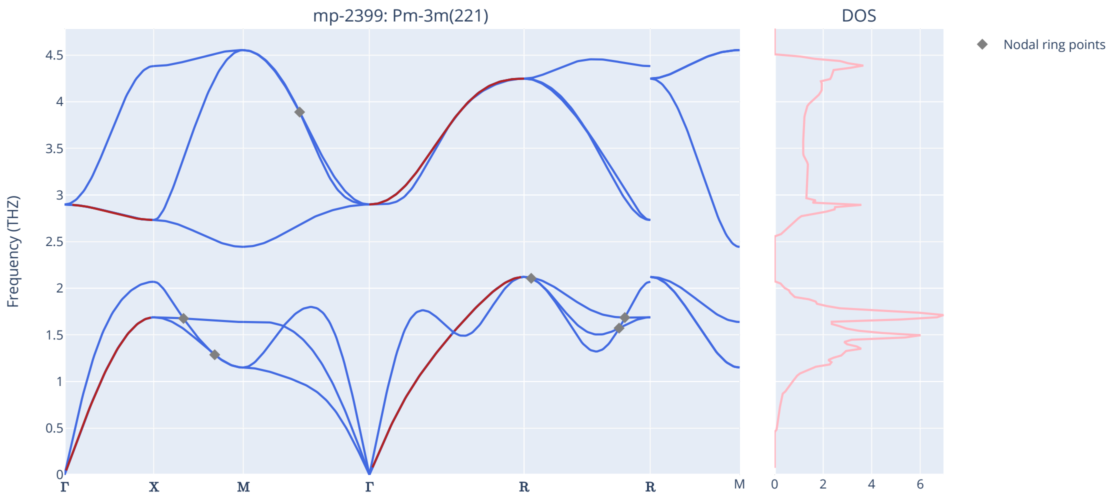

Supplementary Figure 178: The phonon band and DOS of HgY.

Supplementary Table 333: High symmetry lines of mp-2399-HgY

	Start	End
0	Γ (0.000, 0.000, 0.000)	X (0.000, 0.500, 0.000)
1	X (0.000, 0.500, 0.000)	M (0.500, 0.500, 0.000)
2	M (0.500, 0.500, 0.000)	Γ (0.000, 0.000, 0.000)
3	Γ (0.000, 0.000, 0.000)	R (0.500, 0.500, 0.500)
4	R (0.500, 0.500, 0.500)	X (0.000, 0.500, 0.000)
5	R (0.500, 0.500, 0.500)	M (0.500, 0.500, 0.000)

Supplementary Table 334: Nodal ring points along high symmetry lines of mp-2399-HgY.

	Coordinates	Frequency	Mode 1	Mode 2	Multi.	Deg.	Path	Ratio
0	(0.341, 0.500, -0.000)	1.287	2	1	12	2	2	0.682
1	(0.472, 0.500, 0.472)	2.106	2	1	12	2	5	0.057
2	(0.124, 0.500, 0.124)	1.572	2	1	12	2	5	0.753
3	(0.166, 0.500, -0.000)	1.676	3	2	12	2	2	0.332
4	(0.101, 0.500, 0.101)	1.686	3	2	12	2	5	0.798
5	(0.276, 0.276, 0.000)	3.889	6	5	12	2	3	0.447

The followings are details for AgLi. It has 6 topological nodal lines and 3 nodal ring points along high symmetry line as shown in Fig **179**.

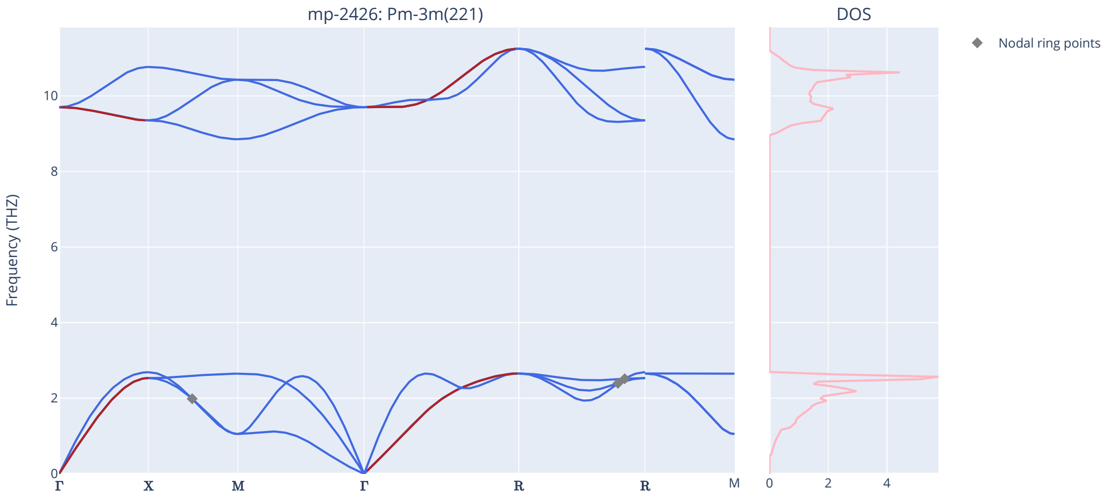

Supplementary Figure 179: The phonon band and DOS of AgLi.

Supplementary Table 335: High symmetry lines of mp-2426-AgLi

	Start	End
0	Γ (0.000, 0.000, 0.000)	X (0.000, 0.500, 0.000)
1	X (0.000, 0.500, 0.000)	M (0.500, 0.500, 0.000)
2	M (0.500, 0.500, 0.000)	Γ (0.000, 0.000, 0.000)
3	Γ (0.000, 0.000, 0.000)	R (0.500, 0.500, 0.500)
4	R (0.500, 0.500, 0.500)	X (0.000, 0.500, 0.000)
5	R (0.500, 0.500, 0.500)	M (0.500, 0.500, 0.000)

Supplementary Table 336: Nodal ring points along high symmetry lines of mp-2426-AgLi.

	Coordinates	Frequency	Mode 1	Mode 2	Multi.	Deg.	Path	Ratio
0	(0.245, 0.500, -0.000)	1.984	2	1	12	2	2	0.490
1	(0.108, 0.500, 0.108)	2.393	2	1	12	2	5	0.785
2	(0.081, 0.500, 0.081)	2.513	3	2	12	2	5	0.838

The followings are details for Cr_4H_4. It has 3 topological nodal lines and 5 nodal ring points along high symmetry line as shown in Fig **180**.

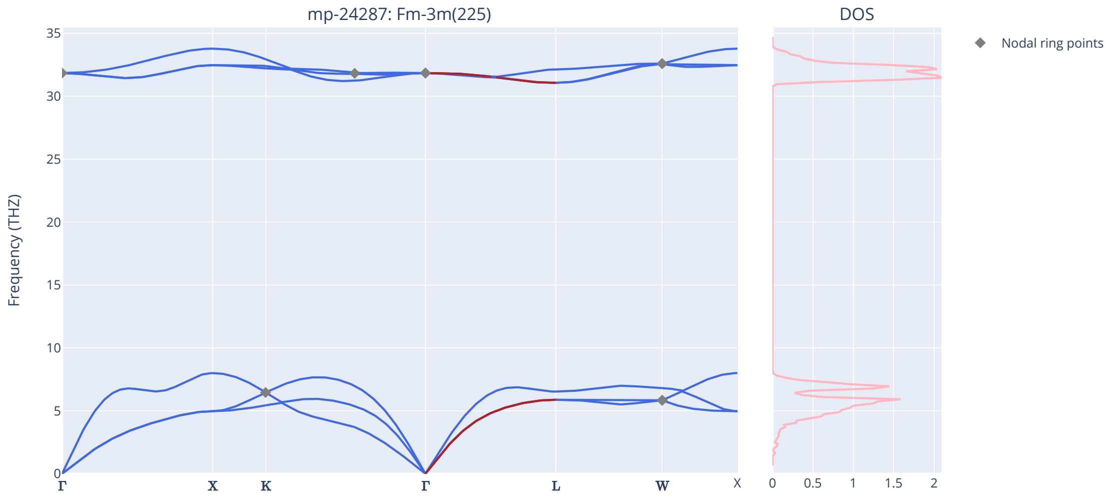

Supplementary Figure 180: The phonon band and DOS of Cr4H4.

Supplementary Table 337: High symmetry lines of mp-24287-Cr4H4

	Start	End
0	Γ (0.000, 0.000, 0.000)	X (0.500, 0.000, 0.500)
1	X (0.500, 0.000, 0.500)	U (0.625, 0.250, 0.625)
2	K (0.375, 0.375, 0.750)	Γ (0.000, 0.000, 0.000)
3	Γ (0.000, 0.000, 0.000)	L (0.500, 0.500, 0.500)
4	L (0.500, 0.500, 0.500)	W (0.500, 0.250, 0.750)
5	W (0.500, 0.250, 0.750)	X (0.500, 0.000, 0.500)

Supplementary Table 338: Nodal ring points along high symmetry lines of mp-24287-Cr4H4.

	Coordinates	Frequency	Mode 1	Mode 2	Multi.	Deg.	Path	Ratio
0	(0.500, 0.250, 0.750)	5.836	2	1	6	2	5	1.000
1	(0.624, 0.249, 0.624)	6.457	3	2	12	2	2	0.996
2	(0.000, 0.000, 0.000)	31.837	6	5	1	3	1	0.000
3	(0.168, 0.168, 0.330)	31.816	6	5	24	2	3	0.557
4	(0.500, 0.250, 0.750)	32.587	6	5	6	2	5	1.000

The followings are details for SrTl. It has 6 topological nodal lines and 4 nodal ring points along high symmetry line as shown in Fig **181**.

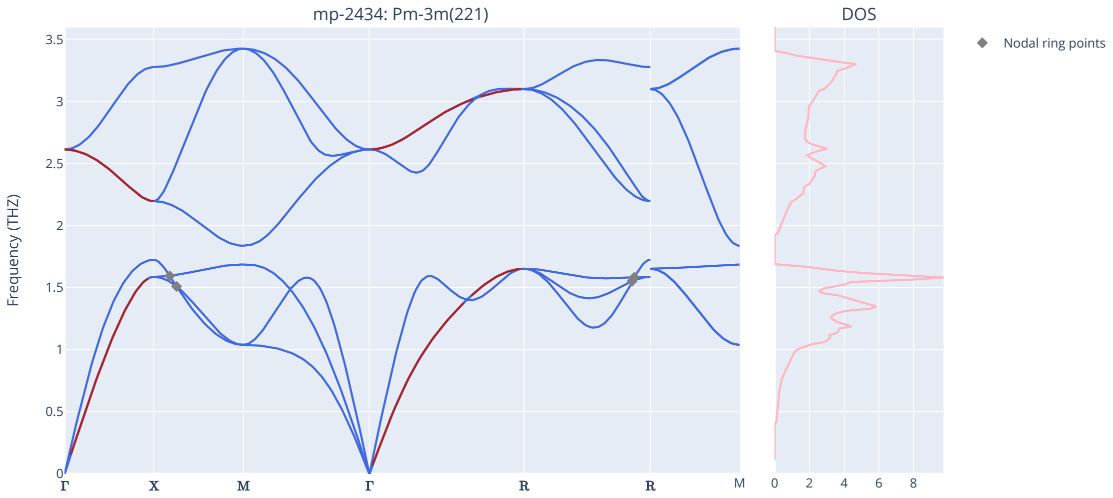

Supplementary Figure 181: The phonon band and DOS of SrTl.

Supplementary Table 339: High symmetry lines of mp-2434-SrTl

	Start	End
0	Γ (0.000, 0.000, 0.000)	X (0.000, 0.500, 0.000)
1	X (0.000, 0.500, 0.000)	M (0.500, 0.500, 0.000)
2	M (0.500, 0.500, 0.000)	Γ (0.000, 0.000, 0.000)
3	Γ (0.000, 0.000, 0.000)	R (0.500, 0.500, 0.500)
4	R (0.500, 0.500, 0.500)	X (0.000, 0.500, 0.000)
5	R (0.500, 0.500, 0.500)	M (0.500, 0.500, 0.000)

Supplementary Table 340: Nodal ring points along high symmetry lines of mp-2434-SrTl.

	Coordinates	Frequency	Mode 1	Mode 2	Multi.	Deg.	Path	Ratio
0	(0.127, 0.500, -0.000)	1.509	2	1	12	2	2	0.255
1	(0.072, 0.500, 0.072)	1.552	2	1	12	2	5	0.856
2	(0.089, 0.500, -0.000)	1.594	3	2	12	2	2	0.178
3	(0.063, 0.500, 0.063)	1.583	3	2	12	2	5	0.873

The followings are details for GaRh. It has 6 topological nodal lines and 9 nodal ring points along high symmetry line as shown in Fig 182.

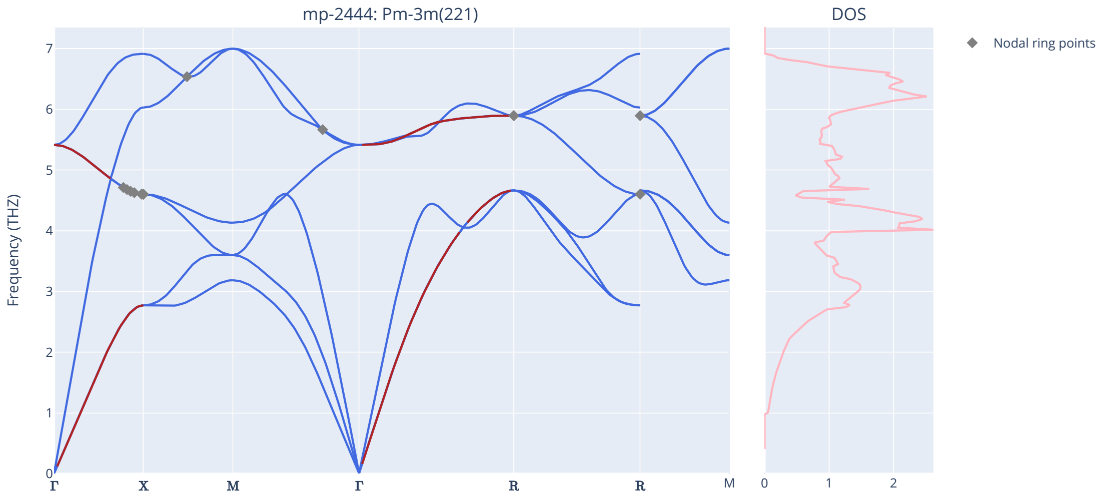

Supplementary Figure 182: The phonon band and DOS of GaRh.

Supplementary Table 341: High symmetry lines of mp-2444-GaRh

	Start	End
0	Γ (0.000, 0.000, 0.000)	X (0.000, 0.500, 0.000)
1	X (0.000, 0.500, 0.000)	M (0.500, 0.500, 0.000)
2	M (0.500, 0.500, 0.000)	Γ (0.000, 0.000, 0.000)
3	Γ (0.000, 0.000, 0.000)	R (0.500, 0.500, 0.500)
4	R (0.500, 0.500, 0.500)	X (0.000, 0.500, 0.000)
5	R (0.500, 0.500, 0.500)	M (0.500, 0.500, 0.000)

Supplementary Table 342: Nodal ring points along high symmetry lines of mp-2444-GaRh.

	Coordinates	Frequency	Mode 1	Mode 2	Multi.	Deg.	Path	Ratio
0	(0.000, 0.388, 0.000)	4.713	4	3	6	2	1	0.776
1	(0.000, 0.408, 0.000)	4.679	4	3	6	2	1	0.816
2	(0.000, 0.429, 0.000)	4.650	4	3	6	2	1	0.857
3	(0.000, 0.449, 0.000)	4.627	4	3	6	2	1	0.898
4	(0.000, 0.490, 0.000)	4.602	4	3	6	2	1	0.980
5	(0.000, 0.500, 0.000)	4.601	4	3	3	2	1	1.000
6	(0.244, 0.500, 0.000)	6.537	6	5	12	2	2	0.488
7	(0.144, 0.144, 0.000)	5.665	6	5	12	2	3	0.712
8	(0.500, 0.500, 0.500)	5.895	6	5	1	3	4	1.000

The followings are details for Cd_4S_4. It has 2 topological nodal lines and 2 nodal ring points along high symmetry line as shown in Fig **183**.

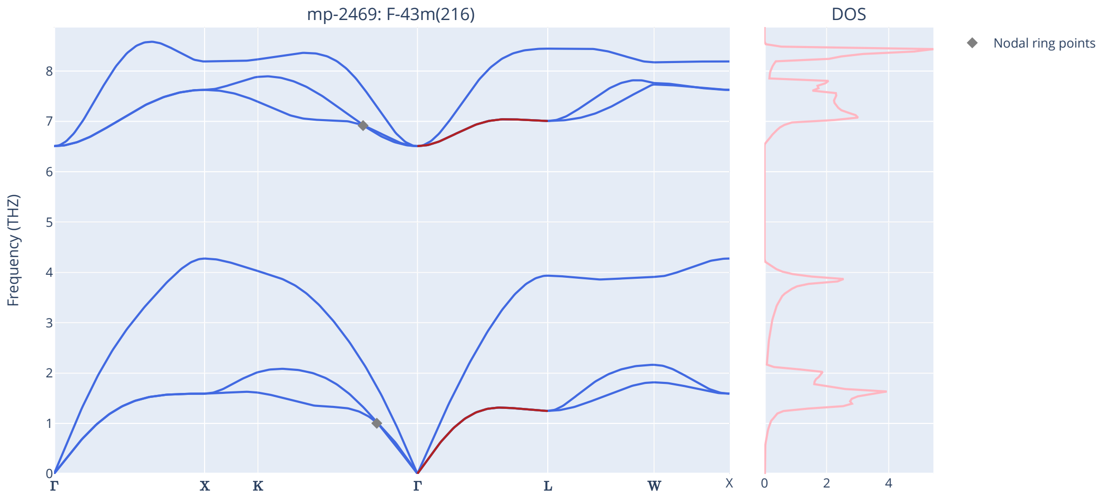

Supplementary Figure 183: The phonon band and DOS of Cd4S4.

Supplementary Table 343: High symmetry lines of mp-2469-Cd4S4

	Start	End
0	Γ (0.000, 0.000, 0.000)	X (0.500, 0.000, 0.500)
1	X (0.500, 0.000, 0.500)	U (0.625, 0.250, 0.625)
2	K (0.375, 0.375, 0.750)	Γ (0.000, 0.000, 0.000)
3	Γ (0.000, 0.000, 0.000)	L (0.500, 0.500, 0.500)
4	L (0.500, 0.500, 0.500)	W (0.500, 0.250, 0.750)
5	W (0.500, 0.250, 0.750)	X (0.500, 0.000, 0.500)

Supplementary Table 344: Nodal ring points along high symmetry lines of mp-2469-Cd4S4.

	Coordinates	Frequency	Mode 1	Mode 2	Multi.	Deg.	Path	Ratio
0	(0.099, 0.099, 0.187)	1.003	2	1	24	2	3	0.745
1	(0.130, 0.130, 0.253)	6.916	5	4	24	2	3	0.660

The followings are details for Ni_4H_4. It has 2 topological nodal lines and 6 nodal ring points along high symmetry line as shown in Fig **184**.

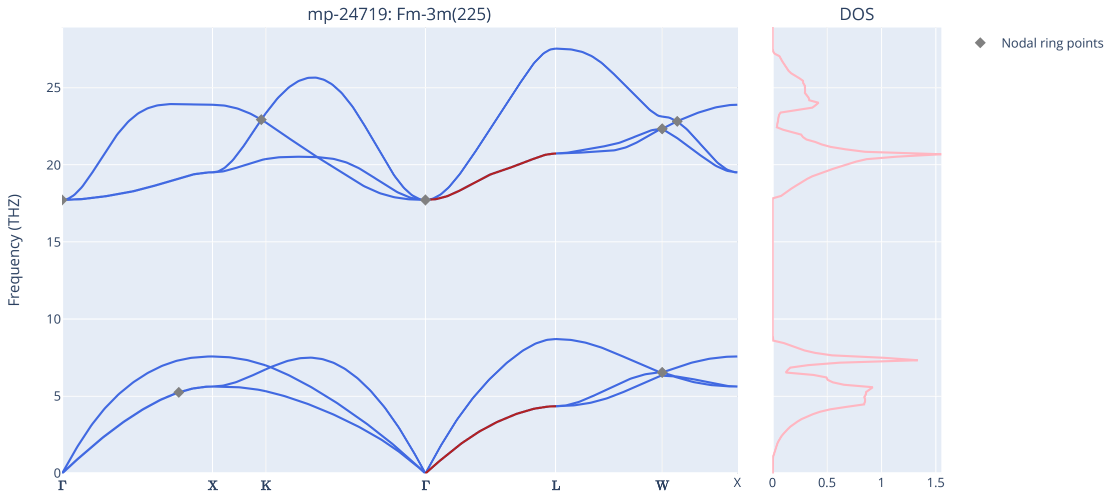

Supplementary Figure 184: The phonon band and DOS of Ni4H4.

Supplementary Table 345: High symmetry lines of mp-24719-Ni4H4

	Start	End
0	Γ (0.000, 0.000, 0.000)	X (0.500, 0.000, 0.500)
1	X (0.500, 0.000, 0.500)	U (0.625, 0.250, 0.625)
2	K (0.375, 0.375, 0.750)	Γ (0.000, 0.000, 0.000)
3	Γ (0.000, 0.000, 0.000)	L (0.500, 0.500, 0.500)
4	L (0.500, 0.500, 0.500)	W (0.500, 0.250, 0.750)
5	W (0.500, 0.250, 0.750)	X (0.500, 0.000, 0.500)

Supplementary Table 346: Nodal ring points along high symmetry lines of mp-24719-Ni4H4.

	Coordinates	Frequency	Mode 1	Mode 2	Multi.	Deg.	Path	Ratio
0	(0.388, 0.000, 0.388)	5.247	2	1	6	2	1	0.776
1	(0.500, 0.250, 0.750)	6.537	3	2	6	2	5	1.000
2	(0.000, 0.000, 0.000)	17.720	5	4	1	3	1	0.000
3	(0.500, 0.250, 0.750)	22.332	5	4	6	2	5	1.000
4	(0.614, 0.229, 0.614)	22.927	6	5	12	2	2	0.916
5	(0.498, 0.199, 0.701)	22.816	6	5	24	2	6	0.200

The followings are details for Rh_4H_4. It has 2 topological nodal lines and 4 nodal ring points along high symmetry line as shown in Fig **185**.

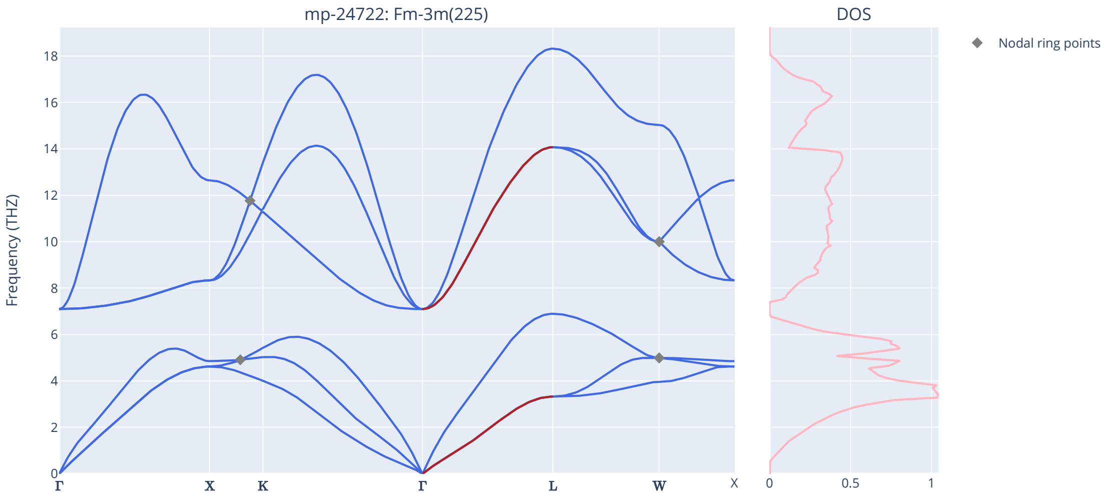

Supplementary Figure 185: The phonon band and DOS of Rh4H4.

Supplementary Table 347: High symmetry lines of mp-24722-Rh4H4

	Start	End
0	Γ (0.000, 0.000, 0.000)	X (0.500, 0.000, 0.500)
1	X (0.500, 0.000, 0.500)	U (0.625, 0.250, 0.625)
2	K (0.375, 0.375, 0.750)	Γ (0.000, 0.000, 0.000)
3	Γ (0.000, 0.000, 0.000)	L (0.500, 0.500, 0.500)
4	L (0.500, 0.500, 0.500)	W (0.500, 0.250, 0.750)
5	W (0.500, 0.250, 0.750)	X (0.500, 0.000, 0.500)

Supplementary Table 348: Nodal ring points along high symmetry lines of mp-24722-Rh4H4.

	Coordinates	Frequency	Mode 1	Mode 2	Multi.	Deg.	Path	Ratio
0	(0.572, 0.144, 0.572)	4.911	3	2	12	2	2	0.578
1	(0.500, 0.250, 0.750)	4.990	3	2	6	2	5	1.000
2	(0.500, 0.250, 0.750)	10.000	5	4	6	2	5	1.000
3	(0.595, 0.190, 0.595)	11.765	6	5	12	2	2	0.760

The followings are details for AgY. It has 6 topological nodal lines and 5 nodal ring points along high symmetry line as shown in Fig **186**.

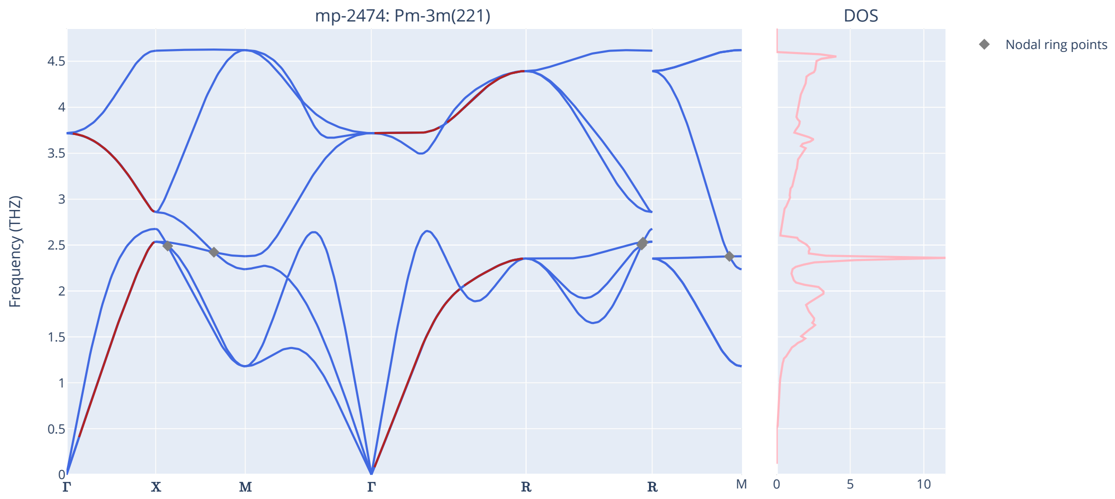

Supplementary Figure 186: The phonon band and DOS of AgY.

Supplementary Table 349: High symmetry lines of mp-2474-AgY

	Start	End
0	Γ (0.000, 0.000, 0.000)	X (0.000, 0.500, 0.000)
1	X (0.000, 0.500, 0.000)	M (0.500, 0.500, 0.000)
2	M (0.500, 0.500, 0.000)	Γ (0.000, 0.000, 0.000)
3	Γ (0.000, 0.000, 0.000)	R (0.500, 0.500, 0.500)
4	R (0.500, 0.500, 0.500)	X (0.000, 0.500, 0.000)
5	R (0.500, 0.500, 0.500)	M (0.500, 0.500, 0.000)

Supplementary Table 350: Nodal ring points along high symmetry lines of mp-2474-AgY.

	Coordinates	Frequency	Mode 1	Mode 2	Multi.	Deg.	Path	Ratio
0	(0.066, 0.500, 0.000)	2.490	2	1	12	2	2	0.133
1	(0.042, 0.500, 0.042)	2.501	2	1	12	2	5	0.916
2	(0.037, 0.499, 0.037)	2.531	3	2	24	2	5	0.926
3	(0.325, 0.500, 0.000)	2.422	4	3	12	2	2	0.651
4	(0.499, 0.500, 0.068)	2.377	4	3	24	2	6	0.864

The followings are details for Ga_4P_4 and it only has 2 topological nodal lines along high symmetry line as shown in Fig **187**.

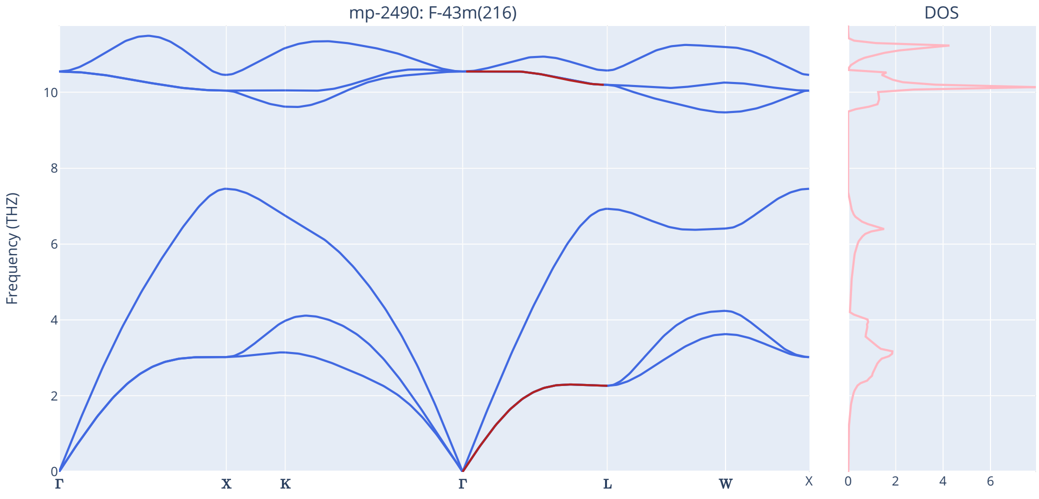

Supplementary Figure 187: The phonon band and DOS of Ga4P4.

Supplementary Table 351: High symmetry lines of mp-2490-Ga4P4

	Start	End
0	Γ (0.000, 0.000, 0.000)	X (0.500, 0.000, 0.500)
1	X (0.500, 0.000, 0.500)	U (0.625, 0.250, 0.625)
2	K (0.375, 0.375, 0.750)	Γ (0.000, 0.000, 0.000)
3	Γ (0.000, 0.000, 0.000)	L (0.500, 0.500, 0.500)
4	L (0.500, 0.500, 0.500)	W (0.500, 0.250, 0.750)
5	W (0.500, 0.250, 0.750)	X (0.500, 0.000, 0.500)

The followings are details for Ce_4N_4. It has 2 topological nodal lines and 5 nodal ring points along high symmetry line as shown in Fig **188**.

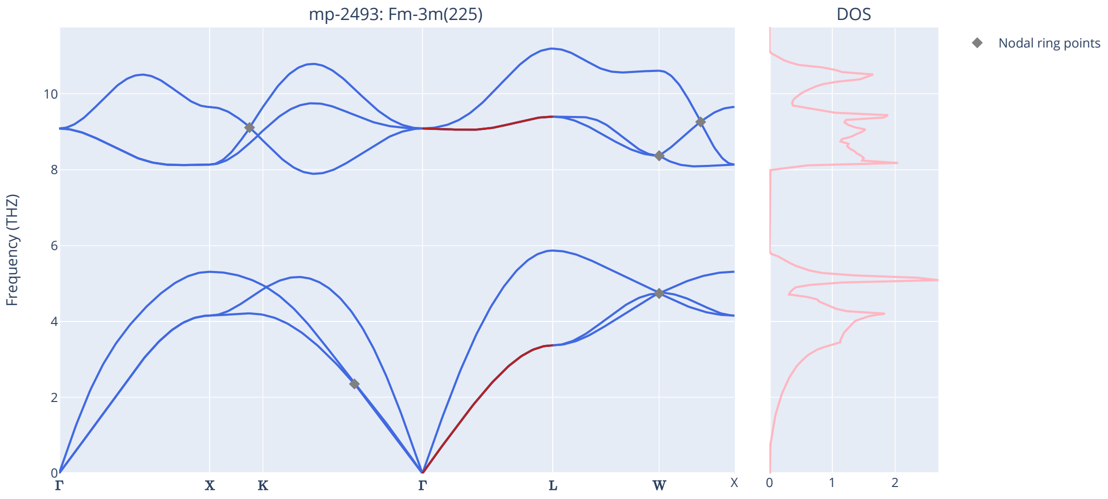

Supplementary Figure 188: The phonon band and DOS of Ce4N4.

Supplementary Table 352: High symmetry lines of mp-2493-Ce4N4

	Start	End
0	Γ (0.000, 0.000, 0.000)	X (0.500, 0.000, 0.500)
1	X (0.500, 0.000, 0.500)	U (0.625, 0.250, 0.625)
2	K (0.375, 0.375, 0.750)	Γ (0.000, 0.000, 0.000)
3	Γ (0.000, 0.000, 0.000)	L (0.500, 0.500, 0.500)
4	L (0.500, 0.500, 0.500)	W (0.500, 0.250, 0.750)
5	W (0.500, 0.250, 0.750)	X (0.500, 0.000, 0.500)

Supplementary Table 353: Nodal ring points along high symmetry lines of mp-2493-Ce4N4.

	Coordinates	Frequency	Mode 1	Mode 2	Multi.	Deg.	Path	Ratio
0	(0.161, 0.161, 0.318)	2.354	2	1	24	2	3	0.574
1	(0.500, 0.250, 0.750)	4.739	2	1	6	2	5	1.000
2	(0.500, 0.250, 0.750)	8.368	5	4	6	2	5	1.000
3	(0.594, 0.188, 0.594)	9.112	6	5	12	2	2	0.751
4	(0.498, 0.111, 0.613)	9.259	6	5	24	2	6	0.551

The followings are details for YZn. It has 6 topological nodal lines and 5 nodal ring points along high symmetry line as shown in Fig 189.

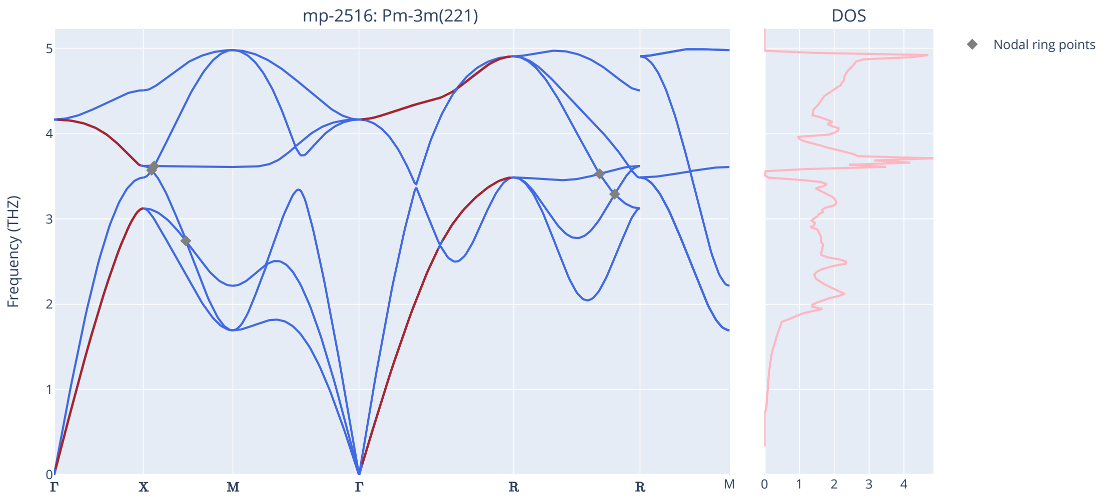

Supplementary Figure 189: The phonon band and DOS of YZn.

Supplementary Table 354: High symmetry lines of mp-2516-YZn

	Start	End
0	Γ (0.000, 0.000, 0.000)	X (0.000, 0.500, 0.000)
1	X (0.000, 0.500, 0.000)	M (0.500, 0.500, 0.000)
2	M (0.500, 0.500, 0.000)	Γ (0.000, 0.000, 0.000)
3	Γ (0.000, 0.000, 0.000)	R (0.500, 0.500, 0.500)
4	R (0.500, 0.500, 0.500)	X (0.000, 0.500, 0.000)
5	R (0.500, 0.500, 0.500)	M (0.500, 0.500, 0.000)

Supplementary Table 355: Nodal ring points along high symmetry lines of mp-2516-YZn.

	Coordinates	Frequency	Mode 1	Mode 2	Multi.	Deg.	Path	Ratio
0	(0.237, 0.500, -0.000)	2.743	3	2	12	2	2	0.475
1	(0.101, 0.500, 0.101)	3.291	3	2	12	2	5	0.799
2	(0.046, 0.500, -0.000)	3.572	4	3	12	2	2	0.092
3	(0.160, 0.500, 0.160)	3.529	4	3	12	2	5	0.680
4	(0.060, 0.500, -0.000)	3.620	5	4	12	2	2	0.120

The followings are details for Be_4Te_4. It has 2 topological nodal lines and 1 nodal ring point along high symmetry line as shown in Fig **190**.

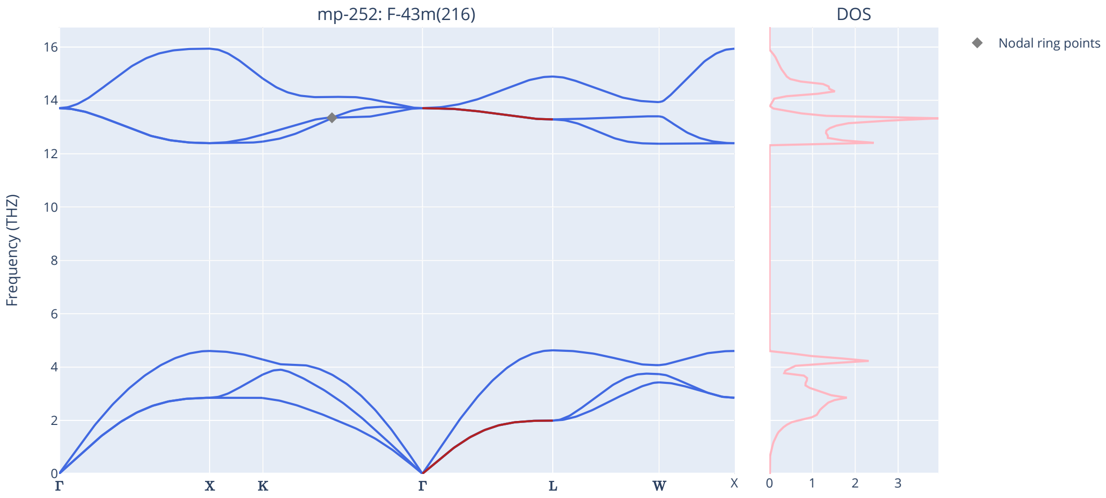

Supplementary Figure 190: The phonon band and DOS of Be4Te4.

Supplementary Table 356: High symmetry lines of mp-252-Be4Te4

	Start	End
0	Γ (0.000, 0.000, 0.000)	X (0.500, 0.000, 0.500)
1	X (0.500, 0.000, 0.500)	U (0.625, 0.250, 0.625)
2	K (0.375, 0.375, 0.750)	Γ (0.000, 0.000, 0.000)
3	Γ (0.000, 0.000, 0.000)	L (0.500, 0.500, 0.500)
4	L (0.500, 0.500, 0.500)	W (0.500, 0.250, 0.750)
5	W (0.500, 0.250, 0.750)	X (0.500, 0.000, 0.500)

Supplementary Table 357: Nodal ring points along high symmetry lines of mp-252-Be4Te4.

	Coordinates	Frequency	Mode 1	Mode 2	Multi.	Deg.	Path	Ratio
0	(0.214, 0.214, 0.423)	13.349	5	4	24	2	3	0.433

The followings are details for Ga_4As_4 and it only has 3 topological nodal lines along high symmetry line as shown in Fig **191**.

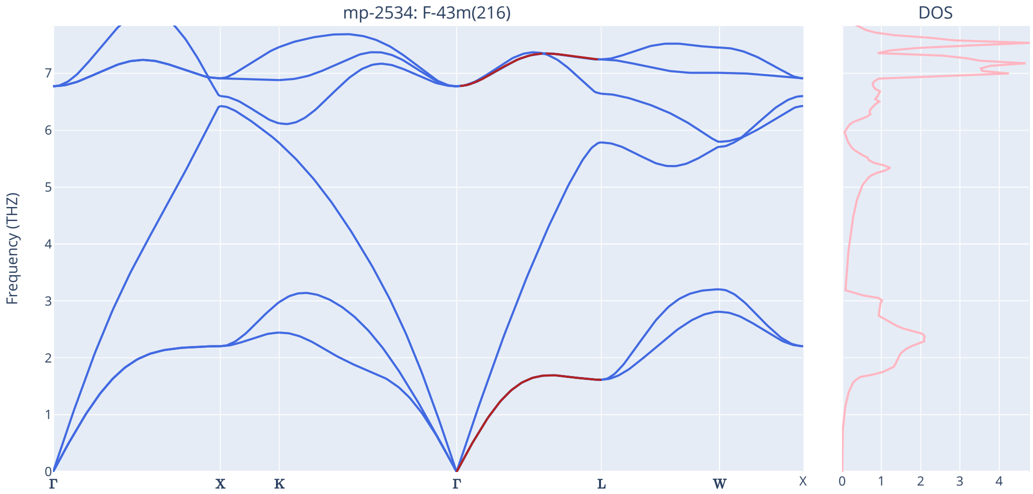

Supplementary Figure 191: The phonon band and DOS of Ga4As4.

Supplementary Table 358: High symmetry lines of mp-2534-Ga4As4

	Start	End
0	Γ (0.000, 0.000, 0.000)	X (0.500, 0.000, 0.500)
1	X (0.500, 0.000, 0.500)	U (0.625, 0.250, 0.625)
2	K (0.375, 0.375, 0.750)	Γ (0.000, 0.000, 0.000)
3	Γ (0.000, 0.000, 0.000)	L (0.500, 0.500, 0.500)
4	L (0.500, 0.500, 0.500)	W (0.500, 0.250, 0.750)
5	W (0.500, 0.250, 0.750)	X (0.500, 0.000, 0.500)

The followings are details for TcV. It has 7 topological nodal lines and 9 nodal ring points along high symmetry line as shown in Fig 192.

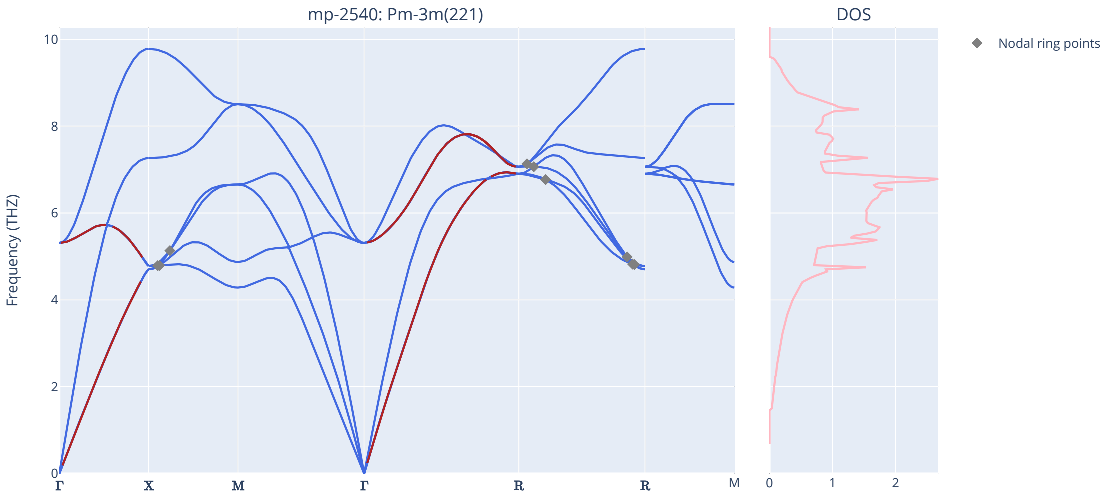

Supplementary Figure 192: The phonon band and DOS of TcV.

Supplementary Table 359: High symmetry lines of mp-2540-TcV

	Start	End
0	Γ (0.000, 0.000, 0.000)	X (0.000, 0.500, 0.000)
1	X (0.000, 0.500, 0.000)	M (0.500, 0.500, 0.000)
2	M (0.500, 0.500, 0.000)	Γ (0.000, 0.000, 0.000)
3	Γ (0.000, 0.000, 0.000)	R (0.500, 0.500, 0.500)
4	R (0.500, 0.500, 0.500)	X (0.000, 0.500, 0.000)
5	R (0.500, 0.500, 0.500)	M (0.500, 0.500, 0.000)

Supplementary Table 360: Nodal ring points along high symmetry lines of mp-2540-TcV.

	Coordinates	Frequency	Mode 1	Mode 2	Multi.	Deg.	Path	Ratio
0	(0.062, 0.500, 0.000)	4.792	2	1	12	2	2	0.123
1	(0.394, 0.500, 0.394)	6.769	2	1	12	2	5	0.213
2	(0.050, 0.500, 0.050)	4.823	2	1	12	2	5	0.901
3	(0.051, 0.500, -0.000)	4.788	3	2	12	2	2	0.103
4	(0.042, 0.500, 0.042)	4.811	3	2	12	2	5	0.915
5	(0.119, 0.500, -0.000)	5.134	4	3	12	2	2	0.239
6	(0.440, 0.500, 0.440)	7.067	4	3	12	2	5	0.119
7	(0.071, 0.500, 0.071)	4.988	4	3	12	2	5	0.858
8	(0.467, 0.500, 0.467)	7.133	6	5	12	2	5	0.065

The followings are details for MgSr. It has 6 topological nodal lines and 3 nodal ring points along high symmetry line as shown in Fig 193.

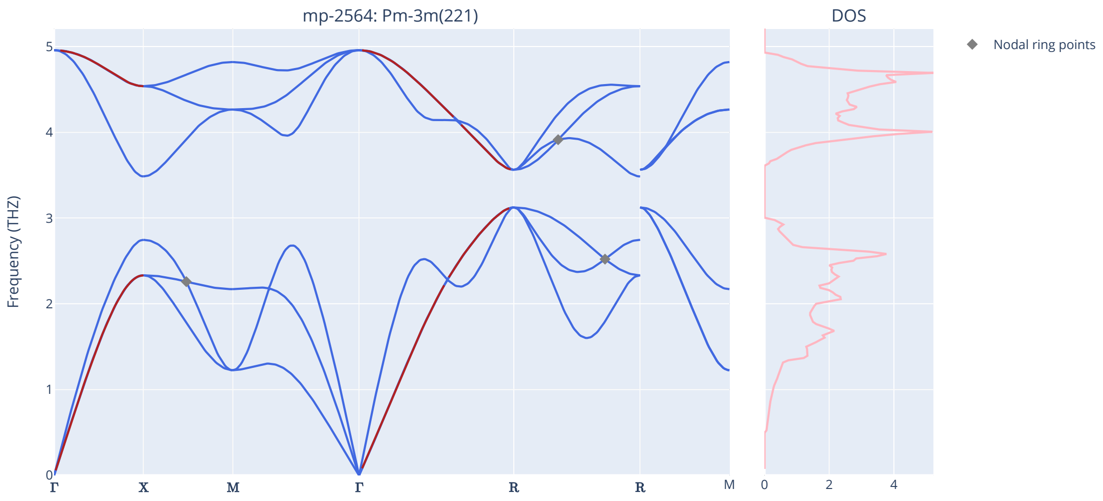

Supplementary Figure 193: The phonon band and DOS of MgSr.

Supplementary Table 361: High symmetry lines of mp-2564-MgSr

	Start	End
0	Γ (0.000, 0.000, 0.000)	X (0.000, 0.500, 0.000)
1	X (0.000, 0.500, 0.000)	M (0.500, 0.500, 0.000)
2	M (0.500, 0.500, 0.000)	Γ (0.000, 0.000, 0.000)
3	Γ (0.000, 0.000, 0.000)	R (0.500, 0.500, 0.500)
4	R (0.500, 0.500, 0.500)	X (0.000, 0.500, 0.000)
5	R (0.500, 0.500, 0.500)	M (0.500, 0.500, 0.000)

Supplementary Table 362: Nodal ring points along high symmetry lines of mp-2564-MgSr.

	Coordinates	Frequency	Mode 1	Mode 2	Multi.	Deg.	Path	Ratio
0	(0.240, 0.500, 0.000)	2.258	3	2	12	2	2	0.481
1	(0.139, 0.500, 0.139)	2.520	3	2	12	2	5	0.722
2	(0.324, 0.500, 0.324)	3.914	5	4	12	2	5	0.351

The followings are details for LaZn. It has 5 topological nodal lines and 12 nodal ring points along high symmetry line as shown in Fig 194.

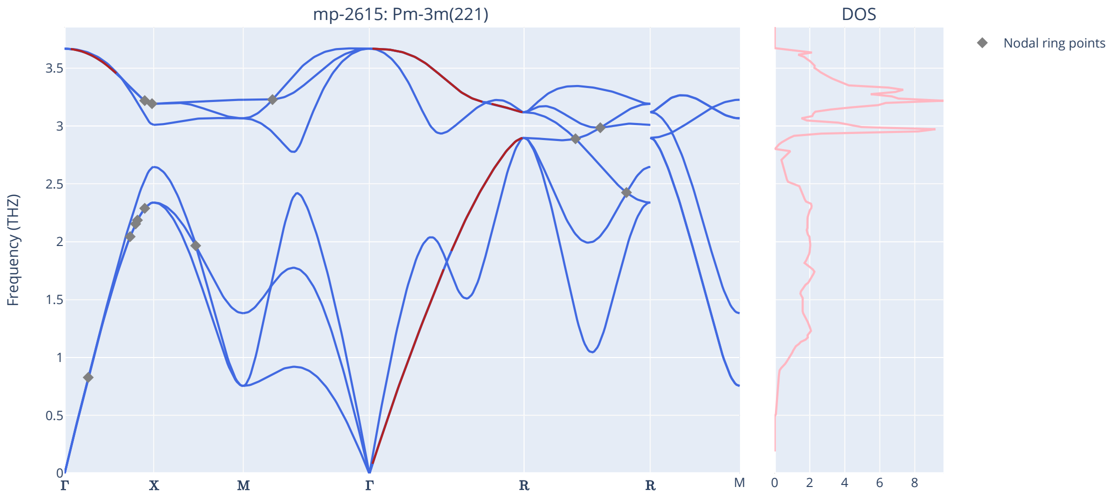

Supplementary Figure 194: The phonon band and DOS of LaZn.

Supplementary Table 363: High symmetry lines of mp-2615-LaZn

	Start	End
0	Γ (0.000, 0.000, 0.000)	X (0.000, 0.500, 0.000)
1	X (0.000, 0.500, 0.000)	M (0.500, 0.500, 0.000)
2	M (0.500, 0.500, 0.000)	Γ (0.000, 0.000, 0.000)
3	Γ (0.000, 0.000, 0.000)	R (0.500, 0.500, 0.500)
4	R (0.500, 0.500, 0.500)	X (0.000, 0.500, 0.000)
5	R (0.500, 0.500, 0.500)	M (0.500, 0.500, 0.000)

Supplementary Table 364: Nodal ring points along high symmetry lines of mp-2615-LaZn.

	Coordinates	Frequency	Mode 1	Mode 2	Multi.	Deg.	Path	Ratio
0	(0.000, 0.367, 0.000)	2.044	2	1	6	2	1	0.735
1	(0.000, 0.398, 0.000)	2.154	2	1	6	2	1	0.796
2	(0.000, 0.408, 0.000)	2.186	2	1	6	2	1	0.816
3	(0.000, 0.449, 0.000)	2.289	2	1	6	2	1	0.898
4	(0.000, 0.133, 0.000)	0.828	3	2	6	2	1	0.265
5	(0.234, 0.500, -0.000)	1.966	3	2	12	2	2	0.469
6	(0.094, 0.500, 0.094)	2.425	3	2	12	2	5	0.811
7	(0.296, 0.500, 0.296)	2.889	4	3	12	2	5	0.407
8	(0.198, 0.500, 0.198)	2.985	5	4	12	2	5	0.604
9	(0.000, 0.449, 0.000)	3.219	6	5	6	2	1	0.898
10	(0.000, 0.490, 0.000)	3.193	6	5	6	2	1	0.980
11	(0.384, 0.384, 0.000)	3.228	6	5	12	2	3	0.233

The followings are details for Al_4Sb_4 and it only has 2 topological nodal lines along high symmetry line as shown in Fig **195**.

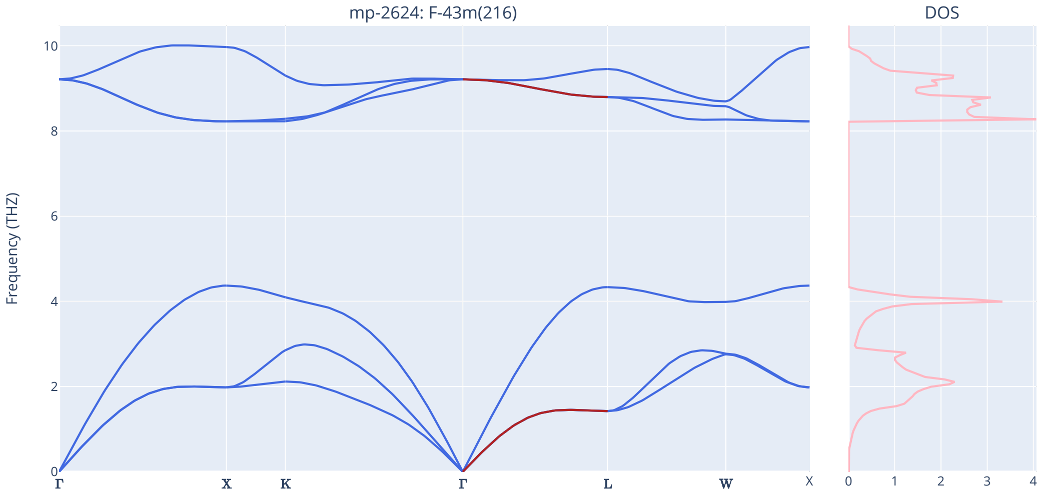

Supplementary Figure 195: The phonon band and DOS of Al4Sb4.

Supplementary Table 365: High symmetry lines of mp-2624-Al4Sb4

	Start	End
0	Γ (0.000, 0.000, 0.000)	X (0.500, 0.000, 0.500)
1	X (0.500, 0.000, 0.500)	U (0.625, 0.250, 0.625)
2	K (0.375, 0.375, 0.750)	Γ (0.000, 0.000, 0.000)
3	Γ (0.000, 0.000, 0.000)	L (0.500, 0.500, 0.500)
4	L (0.500, 0.500, 0.500)	W (0.500, 0.250, 0.750)
5	W (0.500, 0.250, 0.750)	X (0.500, 0.000, 0.500)

The followings are details for NbN and it only has 4 single Weyl point along high symmetry line as shown in Fig **196**.

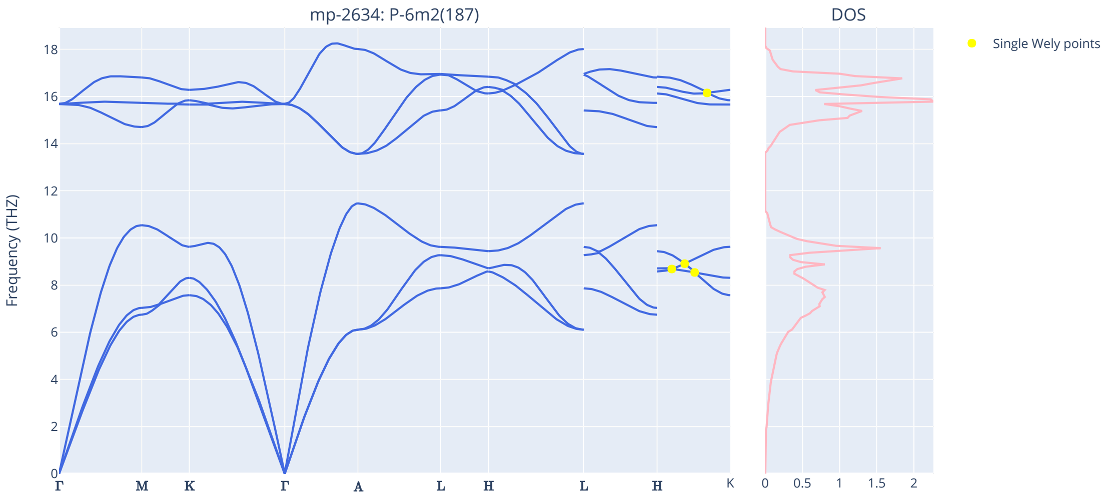

Supplementary Figure 196: The phonon band and DOS of NbN.

Supplementary Table 366: High symmetry lines of mp-2634-NbN

	Start	End
0	Γ (0.000, 0.000, 0.000)	M (0.500, 0.000, 0.000)
1	M (0.500, 0.000, 0.000)	K (0.333, 0.333, 0.000)
2	K (0.333, 0.333, 0.000)	Γ (0.000, 0.000, 0.000)
3	Γ (0.000, 0.000, 0.000)	A (0.000, 0.000, 0.500)
4	A (0.000, 0.000, 0.500)	L (0.500, 0.000, 0.500)
5	L (0.500, 0.000, 0.500)	H (0.333, 0.333, 0.500)
6	H (0.333, 0.333, 0.500)	A (0.000, 0.000, 0.500)
7	L (0.500, 0.000, 0.500)	M (0.500, 0.000, 0.000)
8	H (0.333, 0.333, 0.500)	K (0.333, 0.333, 0.000)

Supplementary Table 367: Single Weyl points along high symmetry lines of mp-2634-NbN.

	Coordinates	Frequency	Mode 1	Mode 2	Multi.	Deg.	Path	Ratio
0	(0.333, 0.333, 0.399)	8.683	2	1	4	2	9	0.201
1	(0.333, 0.333, 0.243)	8.537	2	1	4	2	9	0.513
2	(0.333, 0.333, 0.311)	8.910	3	2	4	2	9	0.377
3	(0.333, 0.333, 0.158)	16.153	6	5	4	2	9	0.683

The followings are details for AlFe. It has 6 topological nodal lines and 6 nodal ring points along high symmetry line as shown in Fig 197.

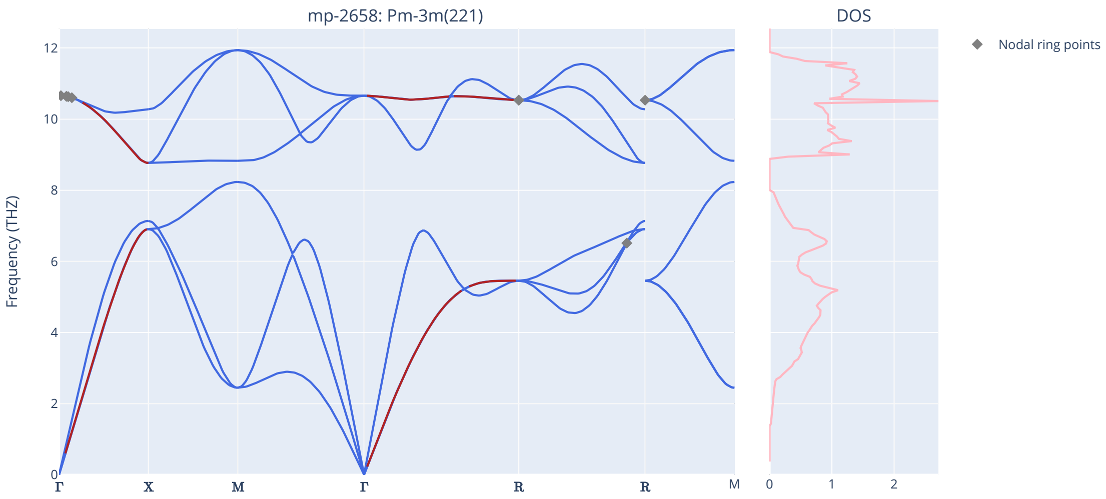

Supplementary Figure 197: The phonon band and DOS of AlFe.

Supplementary Table 368: High symmetry lines of mp-2658-AlFe

	Start	End
0	Γ (0.000, 0.000, 0.000)	X (0.000, 0.500, 0.000)
1	X (0.000, 0.500, 0.000)	M (0.500, 0.500, 0.000)
2	M (0.500, 0.500, 0.000)	Γ (0.000, 0.000, 0.000)
3	Γ (0.000, 0.000, 0.000)	R (0.500, 0.500, 0.500)
4	R (0.500, 0.500, 0.500)	X (0.000, 0.500, 0.000)
5	R (0.500, 0.500, 0.500)	M (0.500, 0.500, 0.000)

Supplementary Table 369: Nodal ring points along high symmetry lines of mp-2658-AlFe.

	Coordinates	Frequency	Mode 1	Mode 2	Multi.	Deg.	Path	Ratio
0	(0.072, 0.500, 0.072)	6.513	2	1	12	2	5	0.856
1	(0.000, 0.010, 0.000)	10.658	6	5	6	3	1	0.020
2	(0.000, 0.041, 0.000)	10.640	6	5	6	2	1	0.082
3	(0.000, 0.051, 0.000)	10.629	6	5	6	2	1	0.102
4	(0.000, 0.071, 0.000)	10.601	6	5	6	2	1	0.143
5	(0.500, 0.500, 0.500)	10.531	6	5	1	3	4	1.000

The followings are details for Cd_4Se_4. It has 2 topological nodal lines and 1 nodal ring point along high symmetry line as shown in Fig **198**.

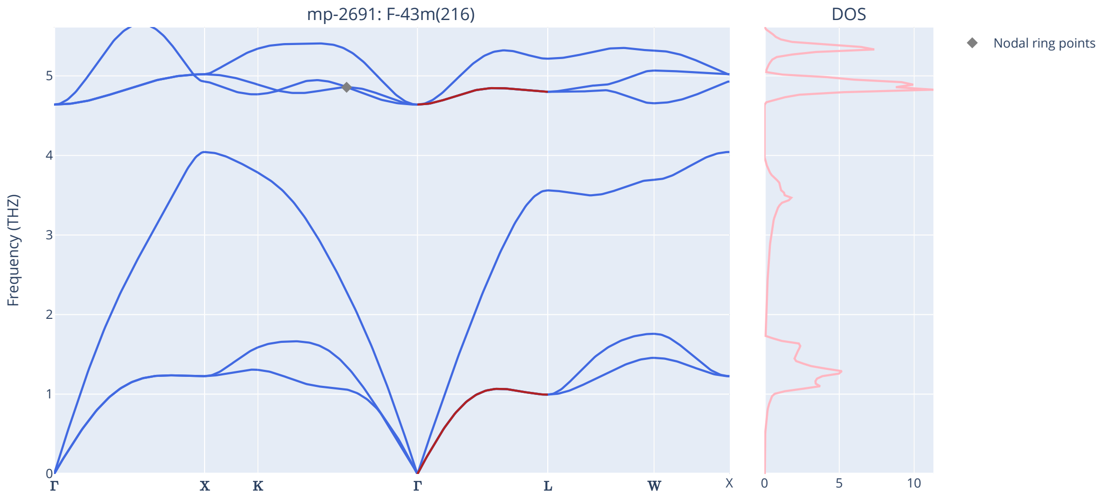

Supplementary Figure 198: The phonon band and DOS of Cd4Se4.

Supplementary Table 370: High symmetry lines of mp-2691-Cd4Se4

	Start	End
0	Γ (0.000, 0.000, 0.000)	X (0.500, 0.000, 0.500)
1	X (0.500, 0.000, 0.500)	U (0.625, 0.250, 0.625)
2	K (0.375, 0.375, 0.750)	Γ (0.000, 0.000, 0.000)
3	Γ (0.000, 0.000, 0.000)	L (0.500, 0.500, 0.500)
4	L (0.500, 0.500, 0.500)	W (0.500, 0.250, 0.750)
5	W (0.500, 0.250, 0.750)	X (0.500, 0.000, 0.500)

Supplementary Table 371: Nodal ring points along high symmetry lines of mp-2691-Cd4Se4.

	Coordinates	Frequency	Mode 1	Mode 2	Multi.	Deg.	Path	Ratio
0	(0.168, 0.168, 0.331)	4.856	5	4	24	2	3	0.556

The followings are details for AgMg. It has 6 topological nodal lines and 3 nodal ring points along high symmetry line as shown in Fig 199.

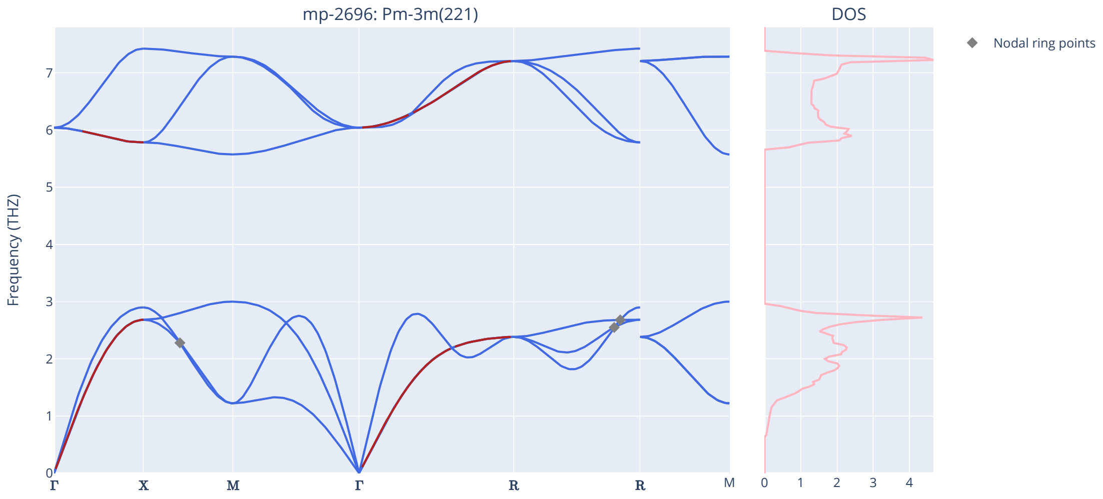

Supplementary Figure 199: The phonon band and DOS of AgMg.

Supplementary Table 372: High symmetry lines of mp-2696-AgMg

	Start	End
0	Γ (0.000, 0.000, 0.000)	X (0.000, 0.500, 0.000)
1	X (0.000, 0.500, 0.000)	M (0.500, 0.500, 0.000)
2	M (0.500, 0.500, 0.000)	Γ (0.000, 0.000, 0.000)
3	Γ (0.000, 0.000, 0.000)	R (0.500, 0.500, 0.500)
4	R (0.500, 0.500, 0.500)	X (0.000, 0.500, 0.000)
5	R (0.500, 0.500, 0.500)	M (0.500, 0.500, 0.000)

Supplementary Table 373: Nodal ring points along high symmetry lines of mp-2696-AgMg.

	Coordinates	Frequency	Mode 1	Mode 2	Multi.	Deg.	Path	Ratio
0	(0.204, 0.500, -0.000)	2.280	2	1	12	2	2	0.409
1	(0.103, 0.500, 0.103)	2.549	2	1	12	2	5	0.795
2	(0.079, 0.500, 0.079)	2.681	3	2	12	2	5	0.843

The followings are details for Hg_4Te_4 and it only has 2 topological nodal lines along high symmetry line as shown in Fig **200**.

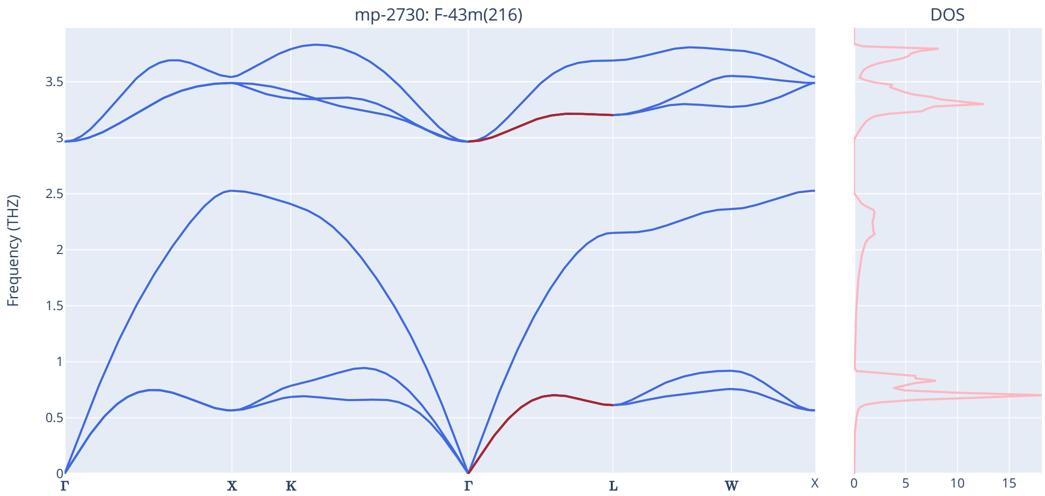

Supplementary Figure 200: The phonon band and DOS of Hg4Te4.

Supplementary Table 374: High symmetry lines of mp-2730-Hg4Te4

	Start	End
0	Γ (0.000, 0.000, 0.000)	X (0.500, 0.000, 0.500)
1	X (0.500, 0.000, 0.500)	U (0.625, 0.250, 0.625)
2	K (0.375, 0.375, 0.750)	Γ (0.000, 0.000, 0.000)
3	Γ (0.000, 0.000, 0.000)	L (0.500, 0.500, 0.500)
4	L (0.500, 0.500, 0.500)	W (0.500, 0.250, 0.750)
5	W (0.500, 0.250, 0.750)	X (0.500, 0.000, 0.500)

The followings are details for LiPd. It has 5 topological nodal lines and 3 nodal ring points along high symmetry line as shown in Fig 201.

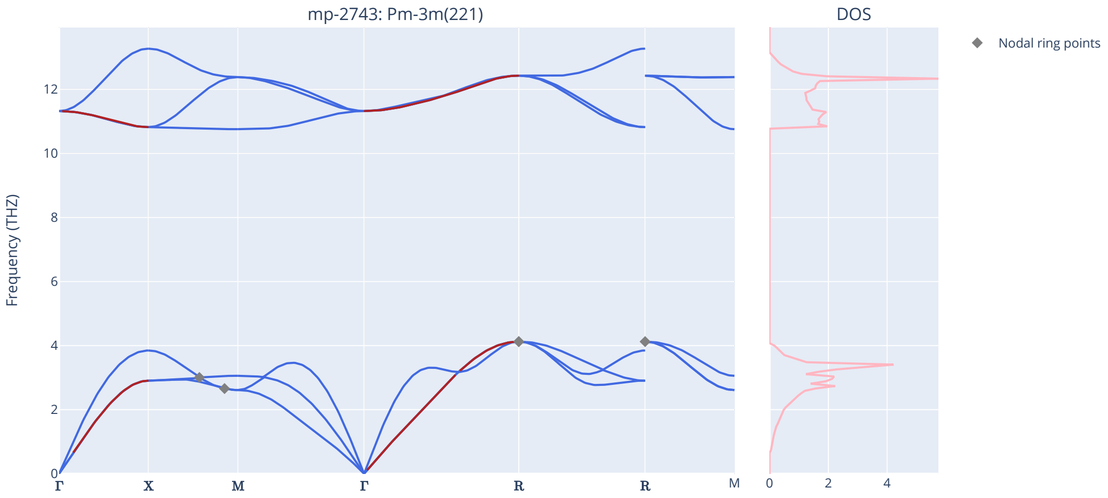

Supplementary Figure 201: The phonon band and DOS of LiPd.

Supplementary Table 375: High symmetry lines of mp-2743-LiPd

	Start	End
0	Γ (0.000, 0.000, 0.000)	X (0.000, 0.500, 0.000)
1	X (0.000, 0.500, 0.000)	M (0.500, 0.500, 0.000)
2	M (0.500, 0.500, 0.000)	Γ (0.000, 0.000, 0.000)
3	Γ (0.000, 0.000, 0.000)	R (0.500, 0.500, 0.500)
4	R (0.500, 0.500, 0.500)	X (0.000, 0.500, 0.000)
5	R (0.500, 0.500, 0.500)	M (0.500, 0.500, 0.000)

Supplementary Table 376: Nodal ring points along high symmetry lines of mp-2743-LiPd.

	Coordinates	Frequency	Mode 1	Mode 2	Multi.	Deg.	Path	Ratio
0	(0.426, 0.500, 0.000)	2.659	2	1	12	2	2	0.852
1	(0.500, 0.500, 0.500)	4.124	2	1	1	3	4	1.000
2	(0.285, 0.500, -0.000)	3.004	3	2	12	2	2	0.571

The followings are details for LiPd. It has 1 nodal ring points and 2 single Weyl point along high symmetry line as shown in Fig 202.

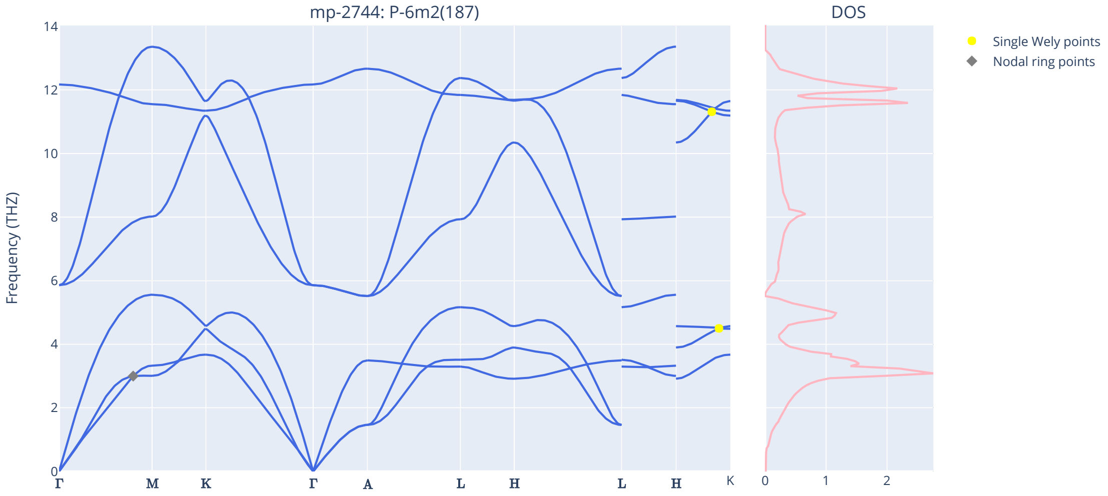

Supplementary Figure 202: The phonon band and DOS of LiPd.

Supplementary Table 377: High symmetry lines of mp-2744-LiPd

	Start	End
0	Γ (0.000, 0.000, 0.000)	M (0.500, 0.000, 0.000)
1	M (0.500, 0.000, 0.000)	K (0.333, 0.333, 0.000)
2	K (0.333, 0.333, 0.000)	Γ (0.000, 0.000, 0.000)
3	Γ (0.000, 0.000, 0.000)	A (0.000, 0.000, 0.500)
4	A (0.000, 0.000, 0.500)	L (0.500, 0.000, 0.500)
5	L (0.500, 0.000, 0.500)	H (0.333, 0.333, 0.500)
6	H (0.333, 0.333, 0.500)	A (0.000, 0.000, 0.500)
7	L (0.500, 0.000, 0.500)	M (0.500, 0.000, 0.000)
8	H (0.333, 0.333, 0.500)	K (0.333, 0.333, 0.000)

Supplementary Table 378: Single Weyl points along high symmetry lines of mp-2744-LiPd.

	Coordinates	Frequency	Mode 1	Mode 2	Multi.	Deg.	Path	Ratio
0	(0.333, 0.333, 0.105)	4.498	3	2	4	2	9	0.790
1	(0.333, 0.333, 0.171)	11.313	5	4	4	2	9	0.658

Supplementary Table 379: Nodal ring points along high symmetry lines of mp-2744-LiPd.

	Coordinates	Frequency	Mode 1	Mode 2	Multi.	Deg.	Path	Ratio
0	(0.398, 0.000, -0.000)	2.997	2	1	6	2	1	0.797

The followings are details for BeCo. It has 6 topological nodal lines and 7 nodal ring points along high symmetry line as shown in Fig 203.

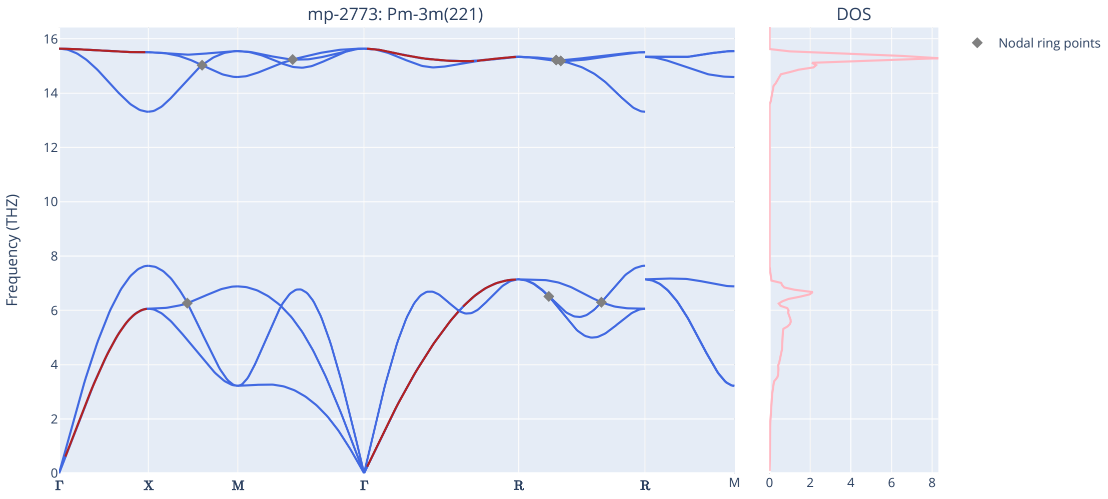

Supplementary Figure 203: The phonon band and DOS of BeCo.

Supplementary Table 380: High symmetry lines of mp-2773-BeCo

	Start	End
0	Γ (0.000, 0.000, 0.000)	X (0.000, 0.500, 0.000)
1	X (0.000, 0.500, 0.000)	M (0.500, 0.500, 0.000)
2	M (0.500, 0.500, 0.000)	Γ (0.000, 0.000, 0.000)
3	Γ (0.000, 0.000, 0.000)	R (0.500, 0.500, 0.500)
4	R (0.500, 0.500, 0.500)	X (0.000, 0.500, 0.000)
5	R (0.500, 0.500, 0.500)	M (0.500, 0.500, 0.000)

Supplementary Table 381: Nodal ring points along high symmetry lines of mp-2773-BeCo.

	Coordinates	Frequency	Mode 1	Mode 2	Multi.	Deg.	Path	Ratio
0	(0.381, 0.500, 0.381)	6.512	2	1	12	2	5	0.238
1	(0.217, 0.500, -0.000)	6.266	3	2	12	2	2	0.435
2	(0.173, 0.500, 0.173)	6.298	3	2	12	2	5	0.654
3	(0.300, 0.500, 0.000)	15.027	5	4	12	2	2	0.601
4	(0.334, 0.500, 0.334)	15.185	5	4	12	2	5	0.333
5	(0.283, 0.283, -0.000)	15.237	6	5	12	2	3	0.435
6	(0.352, 0.500, 0.352)	15.218	6	5	12	2	5	0.296

The followings are details for PdSc. It has 6 topological nodal lines and 4 nodal ring points along high symmetry line as shown in Fig 204.

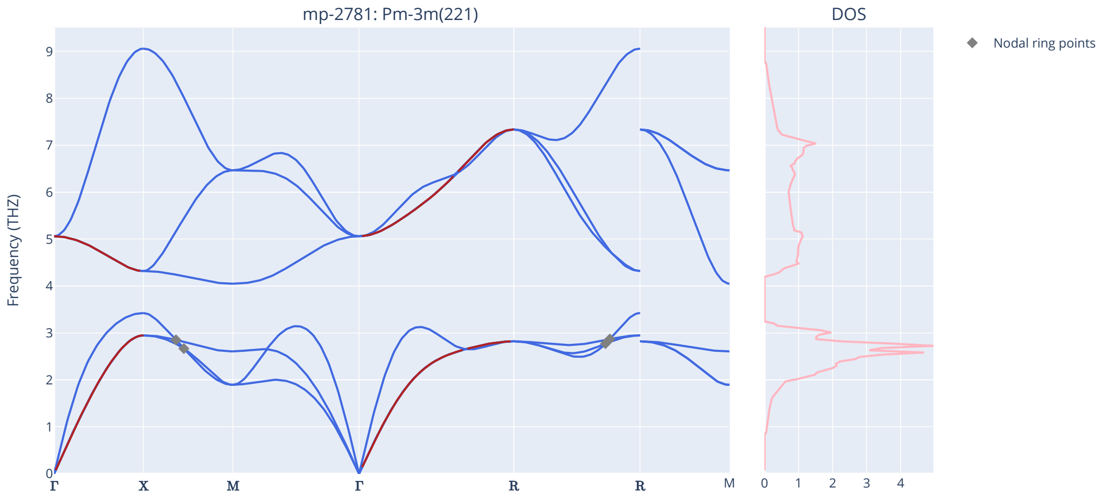

Supplementary Figure 204: The phonon band and DOS of PdSc.

Supplementary Table 382: High symmetry lines of mp-2781-PdSc

	Start	End
0	Γ (0.000, 0.000, 0.000)	X (0.000, 0.500, 0.000)
1	X (0.000, 0.500, 0.000)	M (0.500, 0.500, 0.000)
2	M (0.500, 0.500, 0.000)	Γ (0.000, 0.000, 0.000)
3	Γ (0.000, 0.000, 0.000)	R (0.500, 0.500, 0.500)
4	R (0.500, 0.500, 0.500)	X (0.000, 0.500, 0.000)
5	R (0.500, 0.500, 0.500)	M (0.500, 0.500, 0.000)

Supplementary Table 383: Nodal ring points along high symmetry lines of mp-2781-PdSc.

	Coordinates	Frequency	Mode 1	Mode 2	Multi.	Deg.	Path	Ratio
0	(0.227, 0.500, 0.000)	2.667	2	1	12	2	2	0.453
1	(0.137, 0.500, 0.137)	2.773	2	1	12	2	5	0.726
2	(0.182, 0.500, 0.003)	2.853	3	2	24	2	2	0.364
3	(0.120, 0.500, 0.120)	2.870	3	2	12	2	5	0.761

The followings are details for Zr_4C_4. It has 2 topological nodal lines and 6 nodal ring points along high symmetry line as shown in Fig **205**.

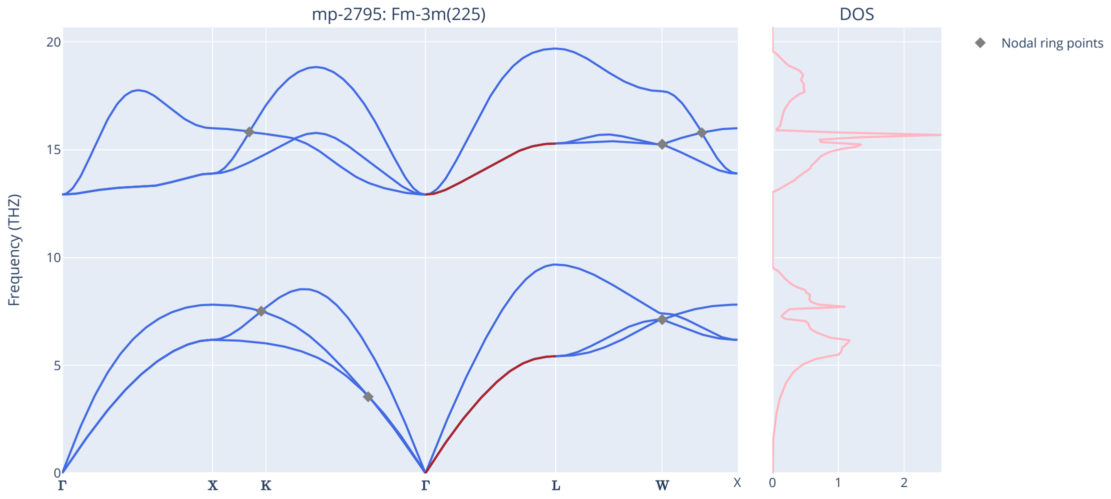

Supplementary Figure 205: The phonon band and DOS of Zr4C4.

Supplementary Table 384: High symmetry lines of mp-2795-Zr4C4

	Start	End
0	Γ (0.000, 0.000, 0.000)	X (0.500, 0.000, 0.500)
1	X (0.500, 0.000, 0.500)	U (0.625, 0.250, 0.625)
2	K (0.375, 0.375, 0.750)	Γ (0.000, 0.000, 0.000)
3	Γ (0.000, 0.000, 0.000)	L (0.500, 0.500, 0.500)
4	L (0.500, 0.500, 0.500)	W (0.500, 0.250, 0.750)
5	W (0.500, 0.250, 0.750)	X (0.500, 0.000, 0.500)

Supplementary Table 385: Nodal ring points along high symmetry lines of mp-2795-Zr4C4.

	Coordinates	Frequency	Mode 1	Mode 2	Multi.	Deg.	Path	Ratio
0	(0.138, 0.138, 0.266)	3.546	2	1	24	2	3	0.641
1	(0.500, 0.250, 0.750)	7.126	2	1	6	2	5	1.000
2	(0.614, 0.229, 0.614)	7.513	3	2	12	2	2	0.914
3	(0.500, 0.250, 0.750)	15.253	5	4	6	2	5	1.000
4	(0.586, 0.173, 0.586)	15.824	6	5	12	2	2	0.690
5	(0.498, 0.117, 0.619)	15.790	6	5	24	2	6	0.527

The followings are details for HfRu and it only has 6 topological nodal lines along high symmetry line as shown in Fig **206**.

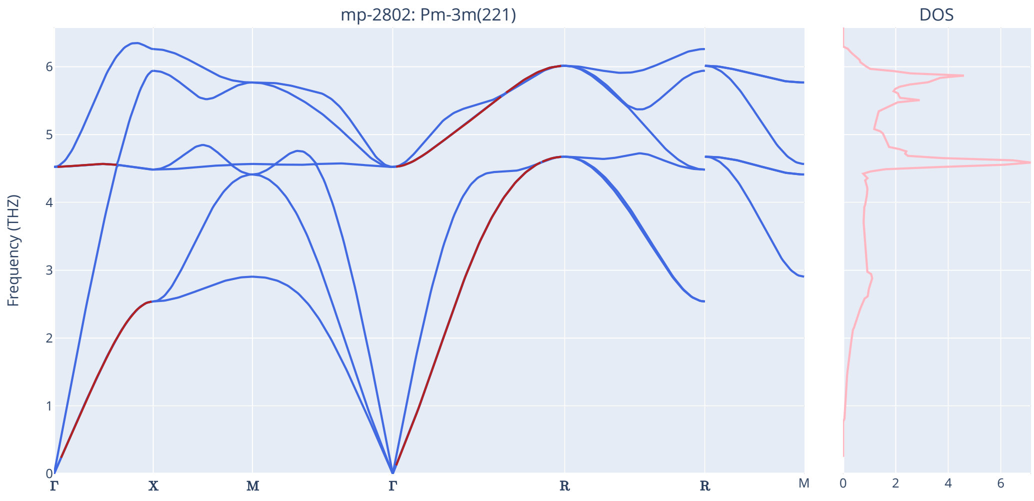

Supplementary Figure 206: The phonon band and DOS of HfRu.

Supplementary Table 386: High symmetry lines of mp-2802-HfRu

	Start	End
0	Γ (0.000, 0.000, 0.000)	X (0.000, 0.500, 0.000)
1	X (0.000, 0.500, 0.000)	M (0.500, 0.500, 0.000)
2	M (0.500, 0.500, 0.000)	Γ (0.000, 0.000, 0.000)
3	Γ (0.000, 0.000, 0.000)	R (0.500, 0.500, 0.500)
4	R (0.500, 0.500, 0.500)	X (0.000, 0.500, 0.000)
5	R (0.500, 0.500, 0.500)	M (0.500, 0.500, 0.000)

The followings are details for Sc_4P_4. It has 3 topological nodal lines and 3 nodal ring points along high symmetry line as shown in Fig **207**.

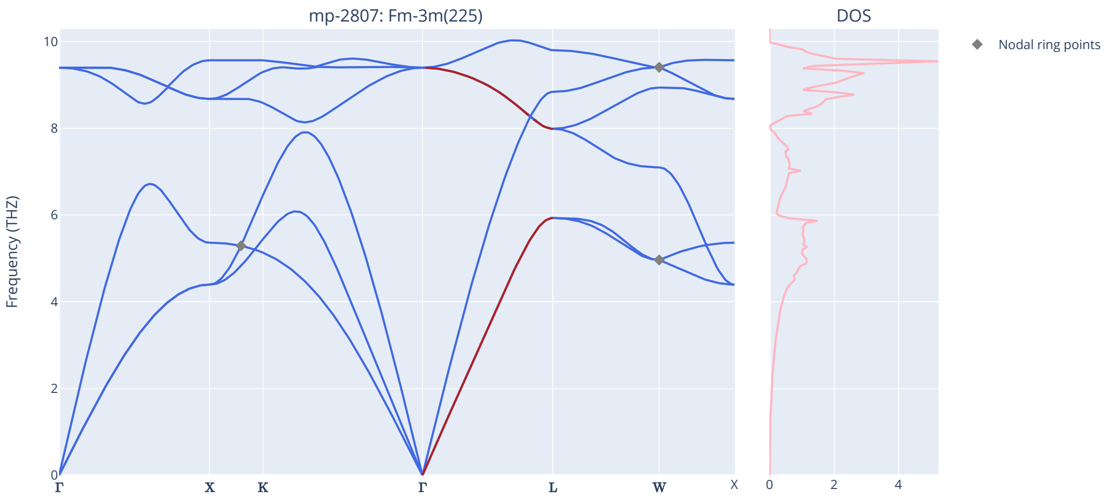

Supplementary Figure 207: The phonon band and DOS of Sc4P4.

Supplementary Table 387: High symmetry lines of mp-2807-Sc4P4

	Start	End
0	Γ (0.000, 0.000, 0.000)	X (0.500, 0.000, 0.500)
1	X (0.500, 0.000, 0.500)	U (0.625, 0.250, 0.625)
2	K (0.375, 0.375, 0.750)	Γ (0.000, 0.000, 0.000)
3	Γ (0.000, 0.000, 0.000)	L (0.500, 0.500, 0.500)
4	L (0.500, 0.500, 0.500)	W (0.500, 0.250, 0.750)
5	W (0.500, 0.250, 0.750)	X (0.500, 0.000, 0.500)

Supplementary Table 388: Nodal ring points along high symmetry lines of mp-2807-Sc4P4.

	Coordinates	Frequency	Mode 1	Mode 2	Multi.	Deg.	Path	Ratio
0	(0.500, 0.250, 0.750)	4.964	2	1	6	2	5	1.000
1	(0.574, 0.147, 0.574)	5.290	3	2	12	2	2	0.590
2	(0.500, 0.250, 0.750)	9.405	6	5	6	2	5	1.000

The followings are details for AlCo. It has 6 topological nodal lines and 4 nodal ring points along high symmetry line as shown in Fig 208.

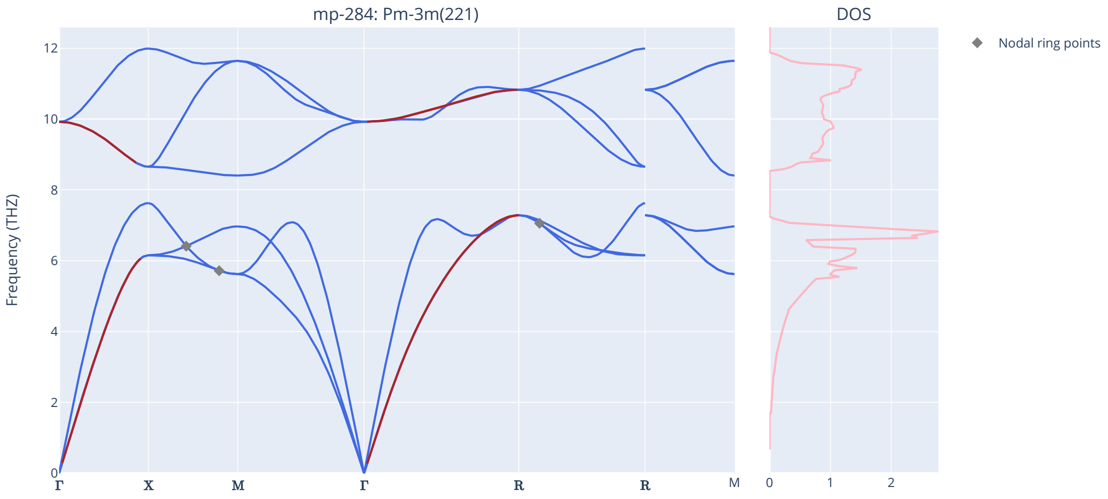

Supplementary Figure 208: The phonon band and DOS of AlCo.

Supplementary Table 389: High symmetry lines of mp-284-AlCo

	Start	End
0	Γ (0.000, 0.000, 0.000)	X (0.000, 0.500, 0.000)
1	X (0.000, 0.500, 0.000)	M (0.500, 0.500, 0.000)
2	M (0.500, 0.500, 0.000)	Γ (0.000, 0.000, 0.000)
3	Γ (0.000, 0.000, 0.000)	R (0.500, 0.500, 0.500)
4	R (0.500, 0.500, 0.500)	X (0.000, 0.500, 0.000)
5	R (0.500, 0.500, 0.500)	M (0.500, 0.500, 0.000)

Supplementary Table 390: Nodal ring points along high symmetry lines of mp-284-AlCo.

	Coordinates	Frequency	Mode 1	Mode 2	Multi.	Deg.	Path	Ratio
0	(0.396, 0.500, -0.000)	5.717	2	1	12	2	2	0.792
1	(0.418, 0.500, 0.418)	7.056	2	1	12	2	5	0.165
2	(0.212, 0.500, -0.000)	6.411	3	2	12	2	2	0.423

The followings are details for CaTl. It has 6 topological nodal lines and 3 nodal ring points along high symmetry line as shown in Fig 209.

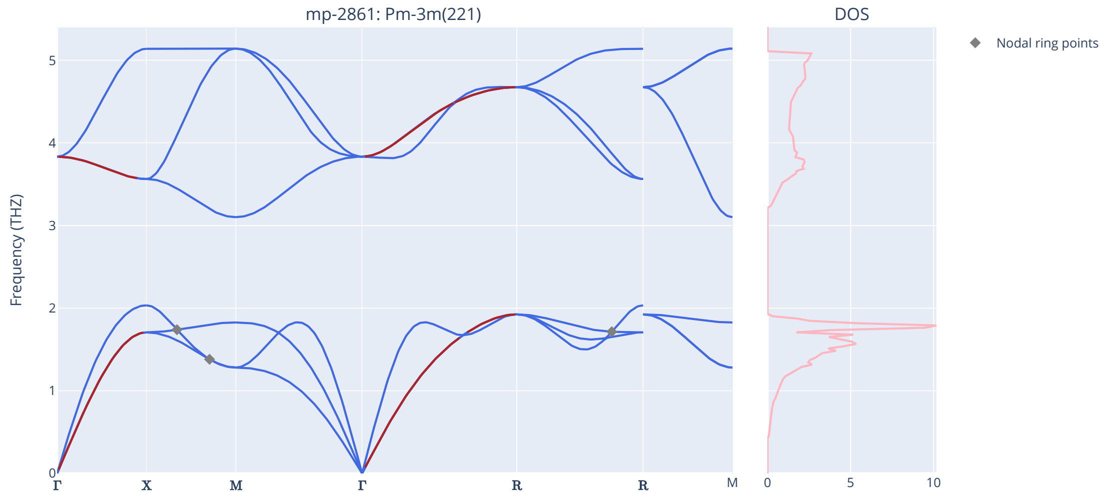

Supplementary Figure 209: The phonon band and DOS of CaTl.

Supplementary Table 391: High symmetry lines of mp-2861-CaTl

	Start	End
0	Γ (0.000, 0.000, 0.000)	X (0.000, 0.500, 0.000)
1	X (0.000, 0.500, 0.000)	M (0.500, 0.500, 0.000)
2	M (0.500, 0.500, 0.000)	Γ (0.000, 0.000, 0.000)
3	Γ (0.000, 0.000, 0.000)	R (0.500, 0.500, 0.500)
4	R (0.500, 0.500, 0.500)	X (0.000, 0.500, 0.000)
5	R (0.500, 0.500, 0.500)	M (0.500, 0.500, 0.000)

Supplementary Table 392: Nodal ring points along high symmetry lines of mp-2861-CaTl.

	Coordinates	Frequency	Mode 1	Mode 2	Multi.	Deg.	Path	Ratio
0	(0.353, 0.500, -0.000)	1.380	2	1	12	2	2	0.707
1	(0.171, 0.500, -0.000)	1.738	3	2	12	2	2	0.342
2	(0.124, 0.500, 0.124)	1.714	3	2	12	2	5	0.752

The followings are details for OsTi. It has 5 topological nodal lines and 4 nodal ring points along high symmetry line as shown in Fig **210**.

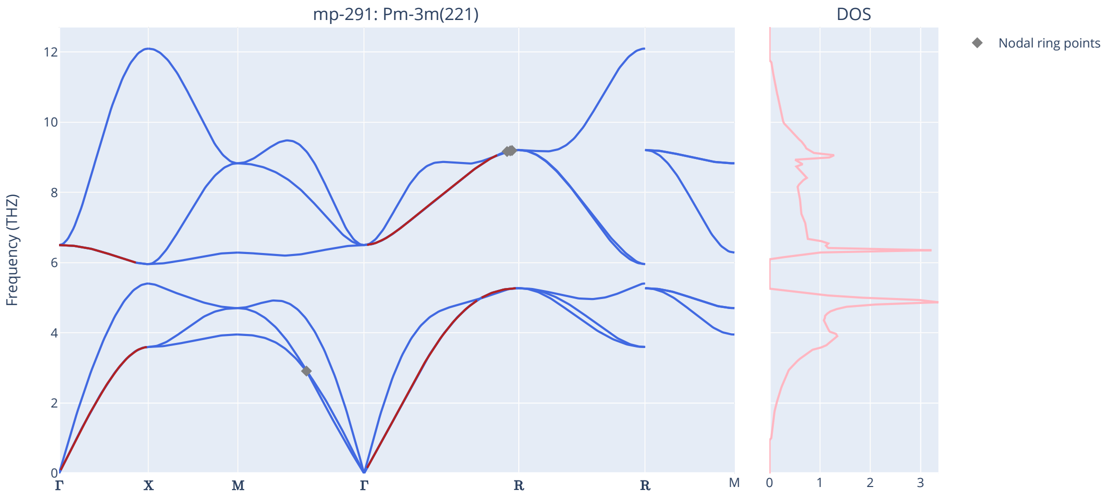

Supplementary Figure 210: The phonon band and DOS of OsTi.

Supplementary Table 393: High symmetry lines of mp-291-OsTi

	Start	End
0	Γ (0.000, 0.000, 0.000)	X (0.000, 0.500, 0.000)
1	X (0.000, 0.500, 0.000)	M (0.500, 0.500, 0.000)
2	M (0.500, 0.500, 0.000)	Γ (0.000, 0.000, 0.000)
3	Γ (0.000, 0.000, 0.000)	R (0.500, 0.500, 0.500)
4	R (0.500, 0.500, 0.500)	X (0.000, 0.500, 0.000)
5	R (0.500, 0.500, 0.500)	M (0.500, 0.500, 0.000)

Supplementary Table 394: Nodal ring points along high symmetry lines of mp-291-OsTi.

	Coordinates	Frequency	Mode 1	Mode 2	Multi.	Deg.	Path	Ratio
0	(0.228, 0.228, -0.000)	2.911	2	1	12	2	3	0.544
1	(0.462, 0.462, 0.462)	9.163	6	5	8	2	4	0.924
2	(0.478, 0.478, 0.478)	9.193	6	5	8	2	4	0.956
3	(0.472, 0.472, 0.472)	9.184	6	5	8	2	4	0.945

The followings are details for AuRb. It has 6 topological nodal lines and 4 nodal ring points along high symmetry line as shown in Fig **211**.

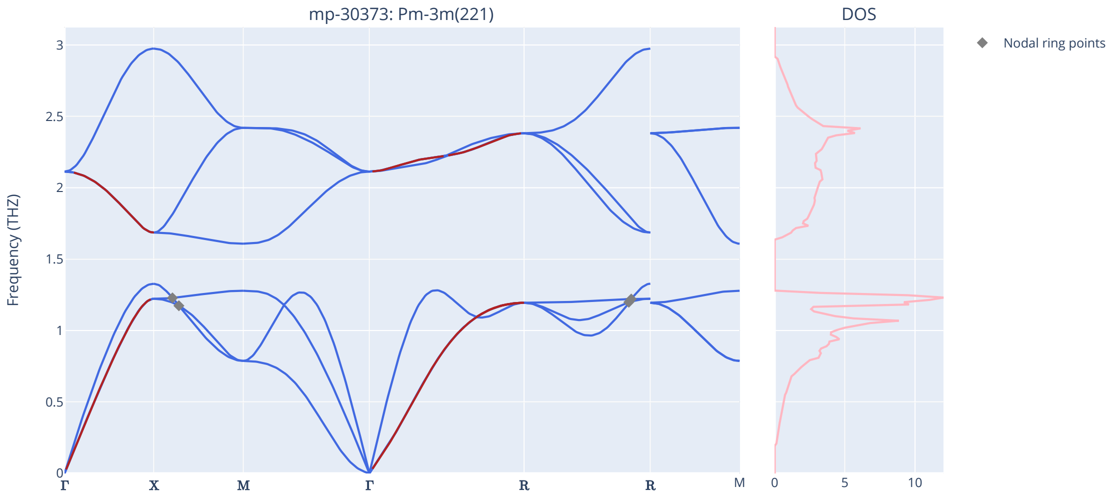

Supplementary Figure 211: The phonon band and DOS of AuRb.

Supplementary Table 395: High symmetry lines of mp-30373-AuRb

	Start	End
0	Γ (0.000, 0.000, 0.000)	X (0.000, 0.500, 0.000)
1	X (0.000, 0.500, 0.000)	M (0.500, 0.500, 0.000)
2	M (0.500, 0.500, 0.000)	Γ (0.000, 0.000, 0.000)
3	Γ (0.000, 0.000, 0.000)	R (0.500, 0.500, 0.500)
4	R (0.500, 0.500, 0.500)	X (0.000, 0.500, 0.000)
5	R (0.500, 0.500, 0.500)	M (0.500, 0.500, 0.000)

Supplementary Table 396: Nodal ring points along high symmetry lines of mp-30373-AuRb.

	Coordinates	Frequency	Mode 1	Mode 2	Multi.	Deg.	Path	Ratio
0	(0.140, 0.500, 0.000)	1.174	2	1	12	2	2	0.281
1	(0.085, 0.500, 0.085)	1.198	2	1	12	2	5	0.830
2	(0.104, 0.500, -0.000)	1.228	3	2	12	2	2	0.209
3	(0.075, 0.500, 0.075)	1.220	3	2	12	2	5	0.849

The followings are details for BiTh. It has 6 topological nodal lines and 1 nodal ring point along high symmetry line as shown in Fig **212**.

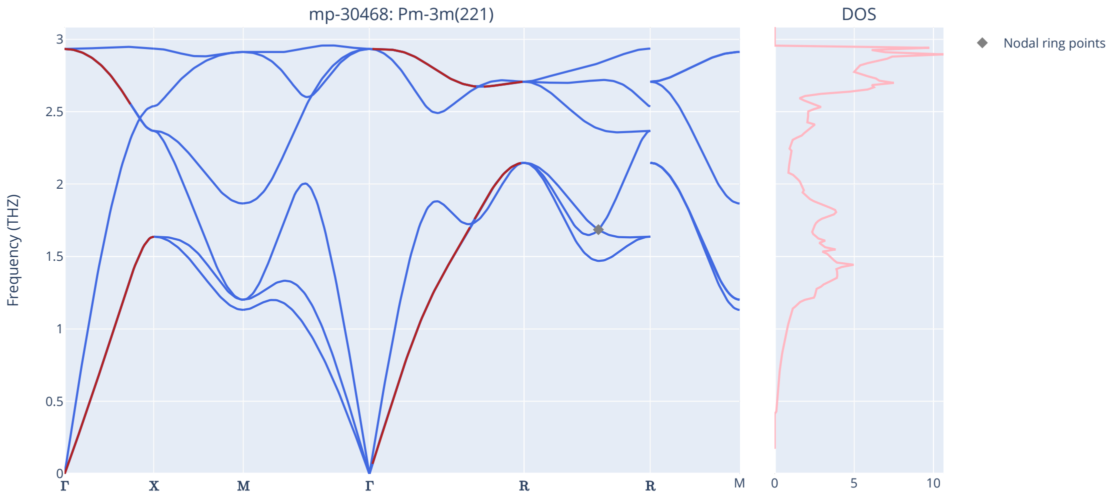

Supplementary Figure 212: The phonon band and DOS of BiTh.

Supplementary Table 397: High symmetry lines of mp-30468-BiTh

	Start	End
0	Γ (0.000, 0.000, 0.000)	X (0.000, 0.500, 0.000)
1	X (0.000, 0.500, 0.000)	M (0.500, 0.500, 0.000)
2	M (0.500, 0.500, 0.000)	Γ (0.000, 0.000, 0.000)
3	Γ (0.000, 0.000, 0.000)	R (0.500, 0.500, 0.500)
4	R (0.500, 0.500, 0.500)	X (0.000, 0.500, 0.000)
5	R (0.500, 0.500, 0.500)	M (0.500, 0.500, 0.000)

Supplementary Table 398: Nodal ring points along high symmetry lines of mp-30468-BiTh.

	Coordinates	Frequency	Mode 1	Mode 2	Multi.	Deg.	Path	Ratio
0	(0.206, 0.500, 0.206)	1.685	3	2	12	2	5	0.589

The followings are details for CdSr. It has 5 topological nodal lines and 3 nodal ring points along high symmetry line as shown in Fig 213.

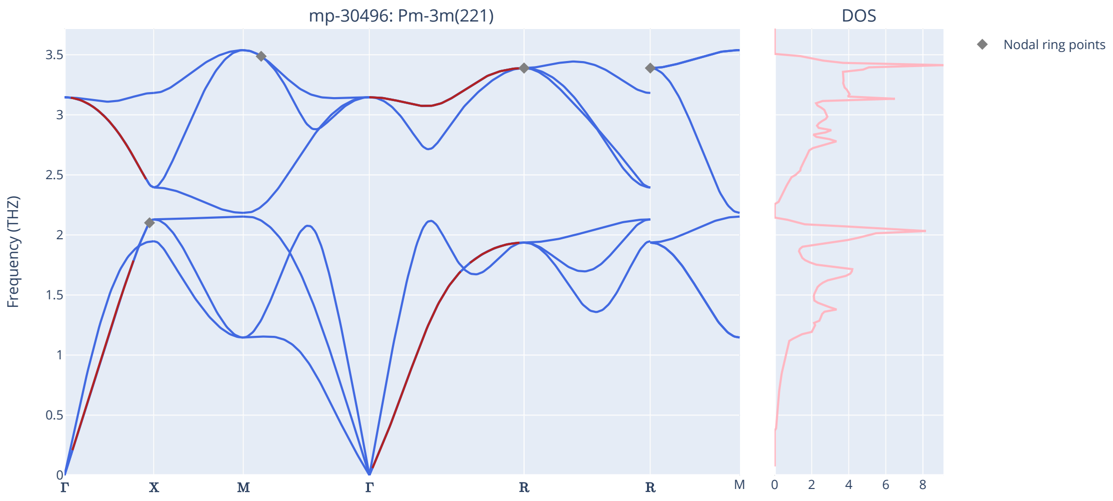

Supplementary Figure 213: The phonon band and DOS of CdSr.

Supplementary Table 399: High symmetry lines of mp-30496-CdSr

	Start	End
0	Γ (0.000, 0.000, 0.000)	X (0.000, 0.500, 0.000)
1	X (0.000, 0.500, 0.000)	M (0.500, 0.500, 0.000)
2	M (0.500, 0.500, 0.000)	Γ (0.000, 0.000, 0.000)
3	Γ (0.000, 0.000, 0.000)	R (0.500, 0.500, 0.500)
4	R (0.500, 0.500, 0.500)	X (0.000, 0.500, 0.000)
5	R (0.500, 0.500, 0.500)	M (0.500, 0.500, 0.000)

Supplementary Table 400: Nodal ring points along high symmetry lines of mp-30496-CdSr.

	Coordinates	Frequency	Mode 1	Mode 2	Multi.	Deg.	Path	Ratio
0	(0.000, 0.476, 0.000)	2.102	3	2	6	2	1	0.952
1	(0.429, 0.429, -0.000)	3.488	6	5	12	2	3	0.143
2	(0.500, 0.500, 0.500)	3.390	6	5	1	3	4	1.000

The followings are details for FeTi. It has 6 topological nodal lines and 4 nodal ring points along high symmetry line as shown in Fig **214**.

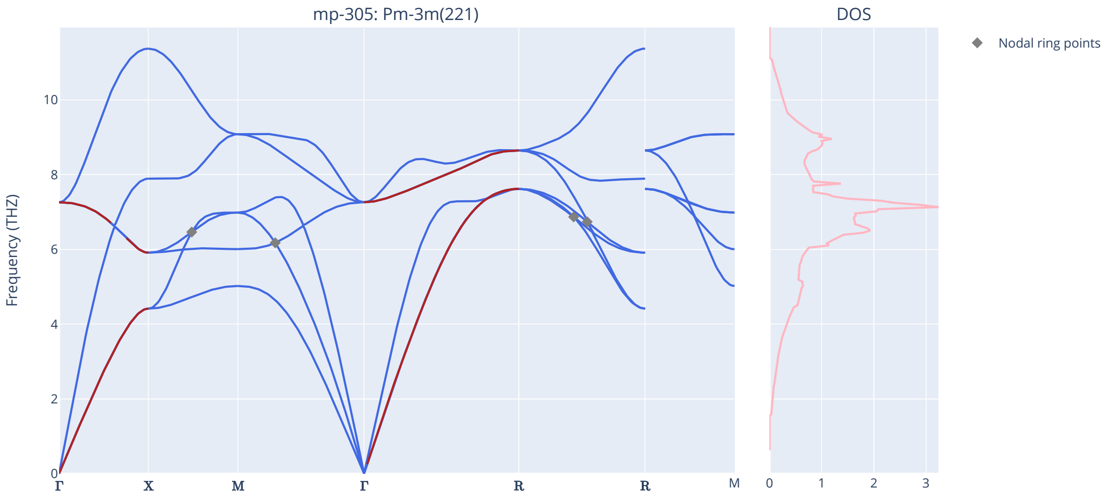

Supplementary Figure 214: The phonon band and DOS of FeTi.

Supplementary Table 401: High symmetry lines of mp-305-FeTi

	Start	End
0	Γ (0.000, 0.000, 0.000)	X (0.000, 0.500, 0.000)
1	X (0.000, 0.500, 0.000)	M (0.500, 0.500, 0.000)
2	M (0.500, 0.500, 0.000)	Γ (0.000, 0.000, 0.000)
3	Γ (0.000, 0.000, 0.000)	R (0.500, 0.500, 0.500)
4	R (0.500, 0.500, 0.500)	X (0.000, 0.500, 0.000)
5	R (0.500, 0.500, 0.500)	M (0.500, 0.500, 0.000)

Supplementary Table 402: Nodal ring points along high symmetry lines of mp-305-FeTi.

	Coordinates	Frequency	Mode 1	Mode 2	Multi.	Deg.	Path	Ratio
0	(0.283, 0.500, 0.283)	6.867	2	1	12	2	5	0.434
1	(0.352, 0.352, 0.000)	6.175	3	2	12	2	3	0.297
2	(0.242, 0.500, -0.000)	6.463	4	3	12	2	2	0.485
3	(0.229, 0.500, 0.229)	6.736	4	3	12	2	5	0.542

The followings are details for IrY. It has 6 topological nodal lines and 3 nodal ring points along high symmetry line as shown in Fig 215.

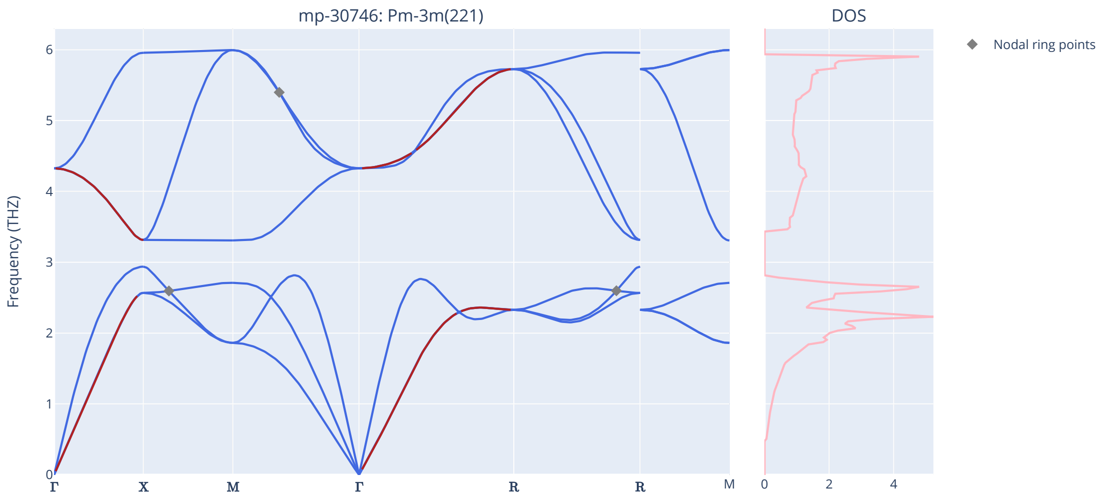

Supplementary Figure 215: The phonon band and DOS of IrY.

Supplementary Table 403: High symmetry lines of mp-30746-IrY

	Start	End
0	Γ (0.000, 0.000, 0.000)	X (0.000, 0.500, 0.000)
1	X (0.000, 0.500, 0.000)	M (0.500, 0.500, 0.000)
2	M (0.500, 0.500, 0.000)	Γ (0.000, 0.000, 0.000)
3	Γ (0.000, 0.000, 0.000)	R (0.500, 0.500, 0.500)
4	R (0.500, 0.500, 0.500)	X (0.000, 0.500, 0.000)
5	R (0.500, 0.500, 0.500)	M (0.500, 0.500, 0.000)

Supplementary Table 404: Nodal ring points along high symmetry lines of mp-30746-IrY.

	Coordinates	Frequency	Mode 1	Mode 2	Multi.	Deg.	Path	Ratio
0	(0.143, 0.500, -0.000)	2.595	3	2	12	2	2	0.286
1	(0.094, 0.500, 0.094)	2.598	3	2	12	2	5	0.812
2	(0.316, 0.316, 0.000)	5.398	6	5	12	2	3	0.368

The followings are details for RuSc. It has 6 topological nodal lines and 8 nodal ring points along high symmetry line as shown in Fig **216**.

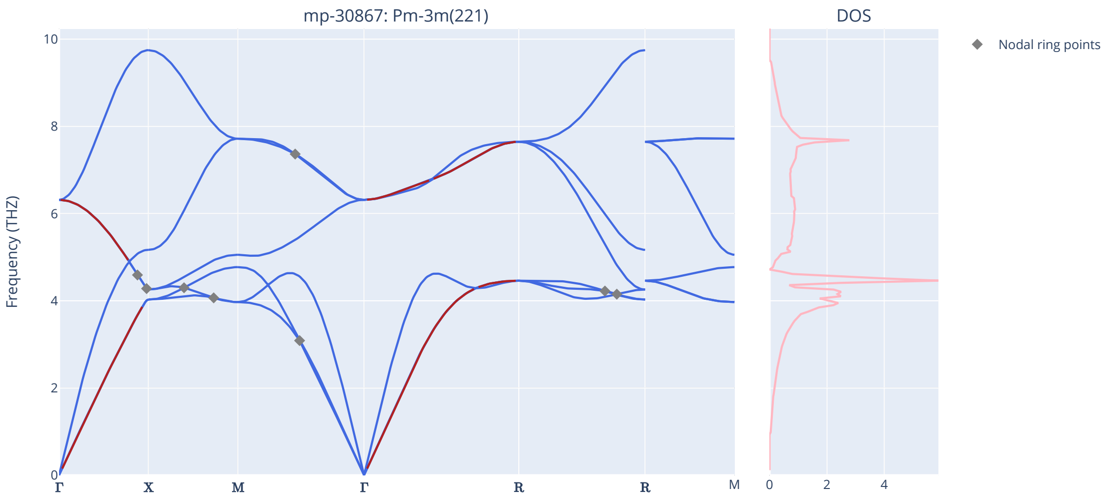

Supplementary Figure 216: The phonon band and DOS of RuSc.

Supplementary Table 405: High symmetry lines of mp-30867-RuSc

	Start	End
0	Γ (0.000, 0.000, 0.000)	X (0.000, 0.500, 0.000)
1	X (0.000, 0.500, 0.000)	M (0.500, 0.500, 0.000)
2	M (0.500, 0.500, 0.000)	Γ (0.000, 0.000, 0.000)
3	Γ (0.000, 0.000, 0.000)	R (0.500, 0.500, 0.500)
4	R (0.500, 0.500, 0.500)	X (0.000, 0.500, 0.000)
5	R (0.500, 0.500, 0.500)	M (0.500, 0.500, 0.000)

Supplementary Table 406: Nodal ring points along high symmetry lines of mp-30867-RuSc.

	Coordinates	Frequency	Mode 1	Mode 2	Multi.	Deg.	Path	Ratio
0	(0.365, 0.500, -0.000)	4.067	2	1	12	2	2	0.731
1	(0.255, 0.255, 0.000)	3.087	2	1	12	2	3	0.490
2	(0.159, 0.496, 0.159)	4.223	3	2	24	2	5	0.682
3	(0.199, 0.500, 0.000)	4.298	3	2	12	2	2	0.399
4	(0.112, 0.500, 0.112)	4.151	3	2	12	2	5	0.776
5	(0.000, 0.439, 0.000)	4.592	4	3	6	2	1	0.878
6	(0.000, 0.490, 0.000)	4.280	4	3	6	2	1	0.980
7	(0.273, 0.273, 0.000)	7.364	6	5	12	2	3	0.455

The followings are details for MnV. It has 6 topological nodal lines and 8 nodal ring points along high symmetry line as shown in Fig 217.

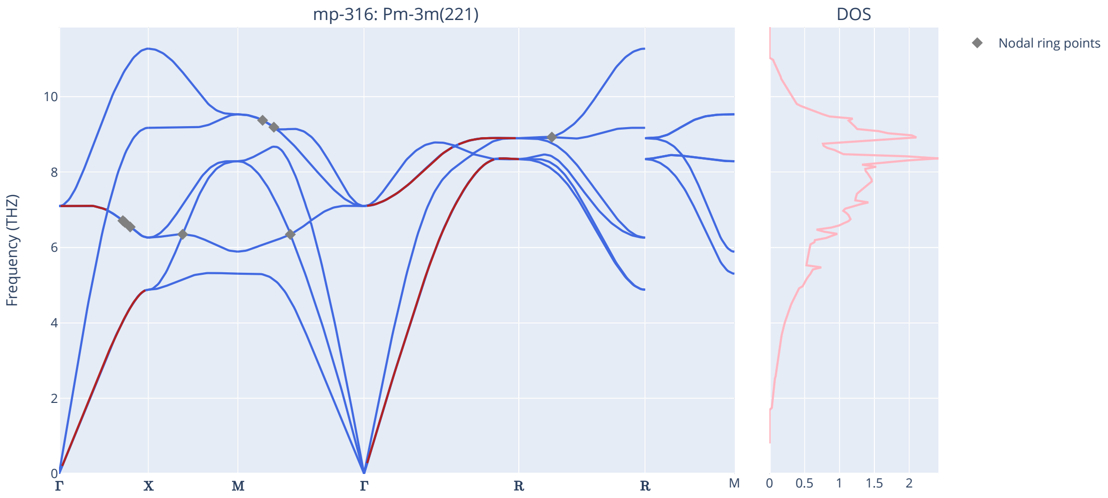

Supplementary Figure 217: The phonon band and DOS of MnV.

Supplementary Table 407: High symmetry lines of mp-316-MnV

	Start	End
0	Γ (0.000, 0.000, 0.000)	X (0.000, 0.500, 0.000)
1	X (0.000, 0.500, 0.000)	M (0.500, 0.500, 0.000)
2	M (0.500, 0.500, 0.000)	Γ (0.000, 0.000, 0.000)
3	Γ (0.000, 0.000, 0.000)	R (0.500, 0.500, 0.500)
4	R (0.500, 0.500, 0.500)	X (0.000, 0.500, 0.000)
5	R (0.500, 0.500, 0.500)	M (0.500, 0.500, 0.000)

Supplementary Table 408: Nodal ring points along high symmetry lines of mp-316-MnV.

	Coordinates	Frequency	Mode 1	Mode 2	Multi.	Deg.	Path	Ratio
0	(0.192, 0.500, -0.000)	6.349	3	2	12	2	2	0.384
1	(0.292, 0.292, 0.000)	6.347	3	2	12	2	3	0.416
2	(0.000, 0.357, 0.000)	6.710	4	3	6	2	1	0.714
3	(0.000, 0.367, 0.000)	6.670	4	3	6	2	1	0.735
4	(0.000, 0.398, 0.000)	6.544	4	3	6	2	1	0.796
5	(0.402, 0.402, -0.000)	9.373	6	5	12	2	3	0.197
6	(0.357, 0.357, 0.000)	9.190	6	5	12	2	3	0.286
7	(0.369, 0.500, 0.369)	8.927	6	5	12	2	5	0.262

The followings are details for Ge_8. It has 5 topological nodal lines and 7 nodal ring points along high symmetry line as shown in Fig **218**.

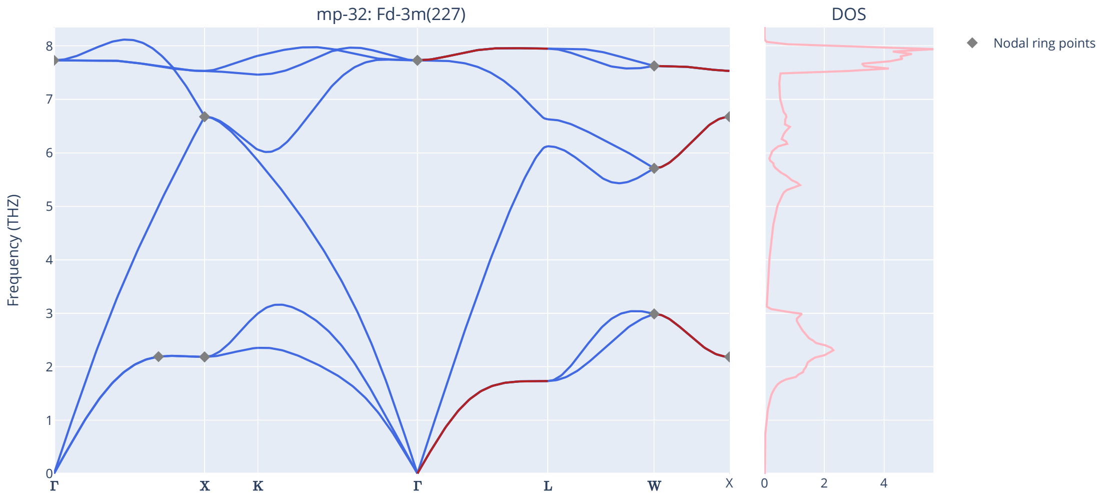

Supplementary Figure 218: The phonon band and DOS of Ge8.

Supplementary Table 409: High symmetry lines of mp-32-Ge8

	Start	End
0	Γ (0.000, 0.000, 0.000)	X (0.500, 0.000, 0.500)
1	X (0.500, 0.000, 0.500)	U (0.625, 0.250, 0.625)
2	K (0.375, 0.375, 0.750)	Γ (0.000, 0.000, 0.000)
3	Γ (0.000, 0.000, 0.000)	L (0.500, 0.500, 0.500)
4	L (0.500, 0.500, 0.500)	W (0.500, 0.250, 0.750)
5	W (0.500, 0.250, 0.750)	X (0.500, 0.000, 0.500)

Supplementary Table 410: Nodal ring points along high symmetry lines of mp-32-Ge8.

	Coordinates	Frequency	Mode 1	Mode 2	Multi.	Deg.	Path	Ratio
0	(0.347, 0.000, 0.347)	2.190	2	1	6	2	1	0.694
1	(0.500, 0.000, 0.500)	2.184	2	1	3	2	1	1.000
2	(0.500, 0.250, 0.750)	2.988	2	1	6	2	5	1.000
3	(0.500, 0.000, 0.500)	6.675	4	3	3	2	1	1.000
4	(0.500, 0.250, 0.750)	5.711	4	3	6	2	5	1.000
5	(0.000, 0.000, 0.000)	7.730	6	5	1	3	1	0.000
6	(0.500, 0.250, 0.750)	7.625	6	5	6	2	5	1.000

The followings are details for Ru_2. It has 11 topological nodal lines and 20 nodal ring points along high symmetry line as shown in Fig **219**.

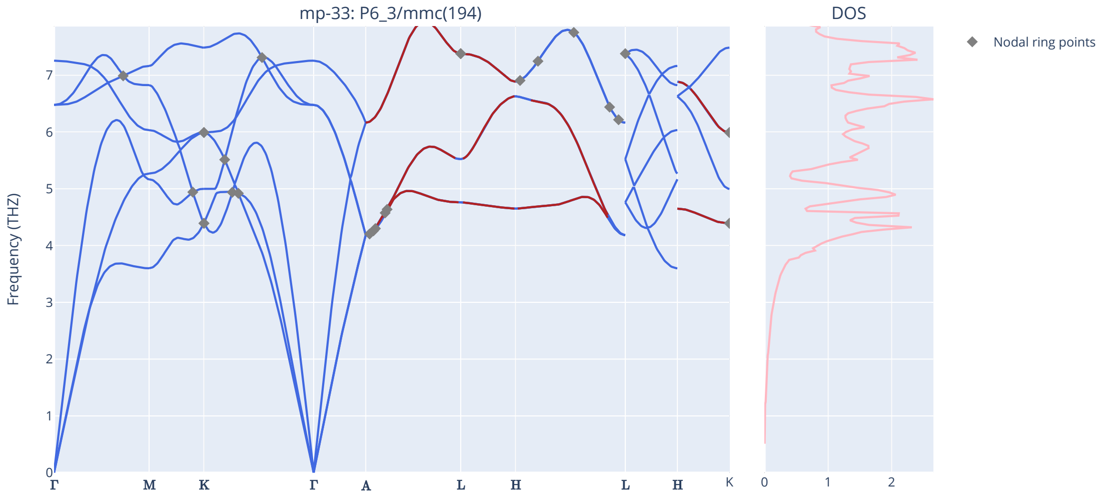

Supplementary Figure 219: The phonon band and DOS of Ru2.

Supplementary Table 411: High symmetry lines of mp-33-Ru2

	Start	End
0	Γ (0.000, 0.000, 0.000)	M (0.500, 0.000, 0.000)
1	M (0.500, 0.000, 0.000)	K (0.333, 0.333, 0.000)
2	K (0.333, 0.333, 0.000)	Γ (0.000, 0.000, 0.000)
3	Γ (0.000, 0.000, 0.000)	A (0.000, 0.000, 0.500)
4	A (0.000, 0.000, 0.500)	L (0.500, 0.000, 0.500)
5	L (0.500, 0.000, 0.500)	H (0.333, 0.333, 0.500)
6	H (0.333, 0.333, 0.500)	A (0.000, 0.000, 0.500)
7	L (0.500, 0.000, 0.500)	M (0.500, 0.000, 0.000)
8	H (0.333, 0.333, 0.500)	K (0.333, 0.333, 0.000)

Supplementary Table 412: Nodal ring points along high symmetry lines of mp-33-Ru2.

	Coordinates	Frequency	Mode 1	Mode 2	Multi.	Deg.	Path	Ratio
0	(0.247, 0.247, -0.000)	4.938	2	1	6	2	3	0.258
1	(0.333, 0.333, 0.000)	4.391	2	1	2	2	2	1.000
2	(0.229, 0.229, -0.000)	4.919	2	1	6	2	3	0.314
3	(0.367, 0.266, -0.000)	4.941	3	2	6	2	2	0.797
4	(0.020, -0.000, 0.500)	4.204	3	2	6	2	5	0.041
5	(0.041, -0.000, 0.499)	4.260	3	2	12	2	5	0.082
6	(0.051, -0.000, 0.498)	4.299	3	2	12	2	5	0.102
7	(0.102, -0.000, 0.497)	4.575	3	2	12	2	5	0.205
8	(0.112, -0.000, 0.497)	4.637	3	2	12	2	5	0.225
9	(0.270, 0.270, 0.000)	5.512	4	3	6	2	3	0.189
10	(0.333, 0.333, 0.000)	5.990	5	4	2	2	2	1.000

Supplementary Table 412: Nodal ring points along high symmetry lines of mp-33-Ru2.

	Coordinates	Frequency	Mode 1	Mode 2	Multi.	Deg.	Path	Ratio
11	(0.364, -0.000, 0.000)	6.988	6	5	6	2	1	0.727
12	(0.157, 0.157, -0.000)	7.310	6	5	6	2	3	0.530
13	(0.500, 0.000, 0.500)	7.379	6	5	3	2	5	1.000
14	(0.320, 0.320, 0.500)	6.906	6	5	6	2	7	0.041
15	(0.265, 0.265, 0.500)	7.246	6	5	6	2	7	0.204
16	(0.156, 0.156, 0.500)	7.752	6	5	6	2	7	0.531
17	(0.048, 0.048, 0.500)	6.438	6	5	6	2	7	0.857
18	(0.020, 0.020, 0.500)	6.215	6	5	6	2	7	0.939

The followings are details for AlSc. It has 5 topological nodal lines and 15 nodal ring points along high symmetry line as shown in Fig 220.

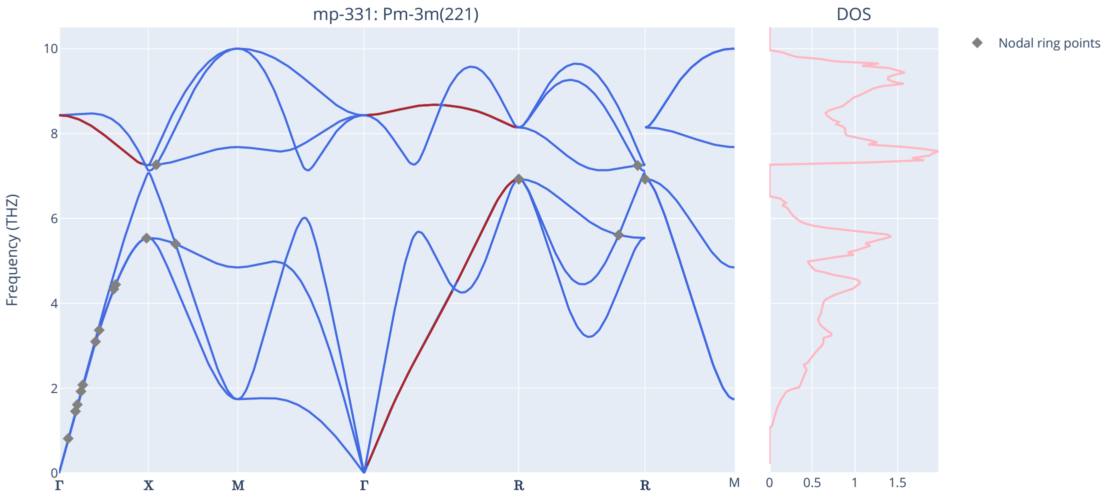

Supplementary Figure 220: The phonon band and DOS of AlSc.

Supplementary Table 413: High symmetry lines of mp-331-AlSc

	Start	End
0	Γ (0.000, 0.000, 0.000)	X (0.000, 0.500, 0.000)
1	X (0.000, 0.500, 0.000)	M (0.500, 0.500, 0.000)
2	M (0.500, 0.500, 0.000)	Γ (0.000, 0.000, 0.000)
3	Γ (0.000, 0.000, 0.000)	R (0.500, 0.500, 0.500)
4	R (0.500, 0.500, 0.500)	X (0.000, 0.500, 0.000)
5	R (0.500, 0.500, 0.500)	M (0.500, 0.500, 0.000)

Supplementary Table 414: Nodal ring points along high symmetry lines of mp-331-AlSc.

	Coordinates	Frequency	Mode 1	Mode 2	Multi.	Deg.	Path	Ratio
0	(0.000, 0.204, 0.000)	3.100	2	1	6	2	1	0.408
1	(0.000, 0.224, 0.000)	3.370	2	1	6	2	1	0.449
2	(0.000, 0.306, 0.000)	4.337	2	1	6	2	1	0.612
3	(0.000, 0.316, 0.000)	4.444	2	1	6	2	1	0.633
4	(0.000, 0.490, 0.000)	5.538	2	1	6	2	1	0.980
5	(0.500, 0.500, 0.500)	6.928	2	1	1	3	4	1.000
6	(0.000, 0.051, 0.000)	0.817	3	2	6	2	1	0.102
7	(0.000, 0.092, 0.000)	1.459	3	2	6	2	1	0.184
8	(0.000, 0.102, 0.000)	1.616	3	2	6	2	1	0.204
9	(0.000, 0.122, 0.000)	1.928	3	2	6	2	1	0.245
10	(0.000, 0.133, 0.000)	2.081	3	2	6	2	1	0.265
11	(0.152, 0.500, -0.000)	5.403	3	2	12	2	2	0.303
12	(0.105, 0.500, 0.105)	5.612	3	2	12	2	5	0.791
13	(0.044, 0.500, -0.000)	7.266	5	4	12	2	2	0.089
14	(0.029, 0.500, 0.029)	7.249	5	4	12	2	5	0.941

Supplementary Table 414: Nodal ring points along high symmetry lines of mp-331-AlSc.

Coordinates	Frequency	Mode 1	Mode 2	Multi.	Deg.	Path	Ratio

The followings are details for AlRh. It has 6 topological nodal lines and 1 nodal ring point along high symmetry line as shown in Fig **221**.

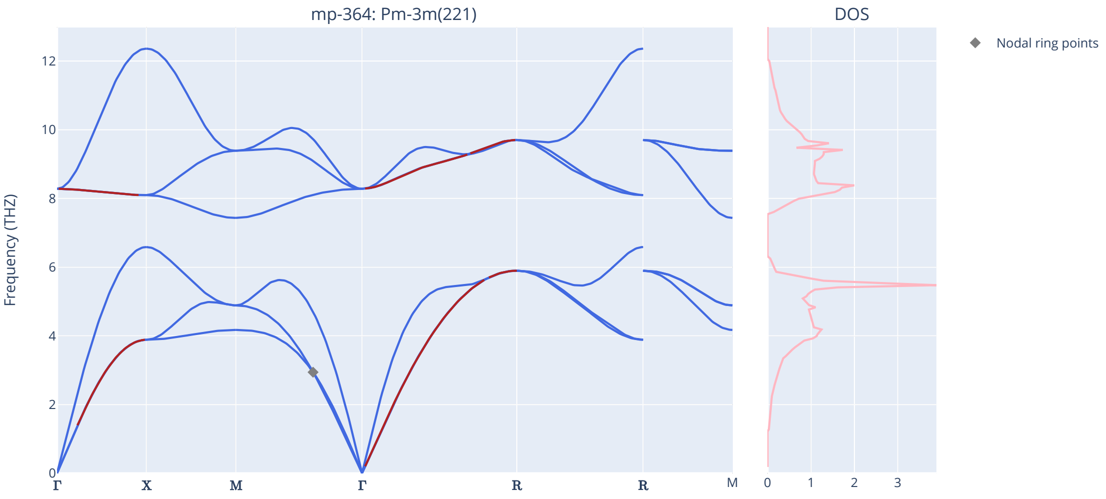

Supplementary Figure 221: The phonon band and DOS of AlRh.

Supplementary Table 415: High symmetry lines of mp-364-AlRh

	Start	End
0	Γ (0.000, 0.000, 0.000)	X (0.000, 0.500, 0.000)
1	X (0.000, 0.500, 0.000)	M (0.500, 0.500, 0.000)
2	M (0.500, 0.500, 0.000)	Γ (0.000, 0.000, 0.000)
3	Γ (0.000, 0.000, 0.000)	R (0.500, 0.500, 0.500)
4	R (0.500, 0.500, 0.500)	X (0.000, 0.500, 0.000)
5	R (0.500, 0.500, 0.500)	M (0.500, 0.500, 0.000)

Supplementary Table 416: Nodal ring points along high symmetry lines of mp-364-AlRh.

	Coordinates	Frequency	Mode 1	Mode 2	Multi.	Deg.	Path	Ratio
0	(0.194, 0.194, 0.000)	2.942	2	1	12	2	3	0.612

The followings are details for RuSi. It has 5 topological nodal lines and 6 nodal ring points along high symmetry line as shown in Fig 222.

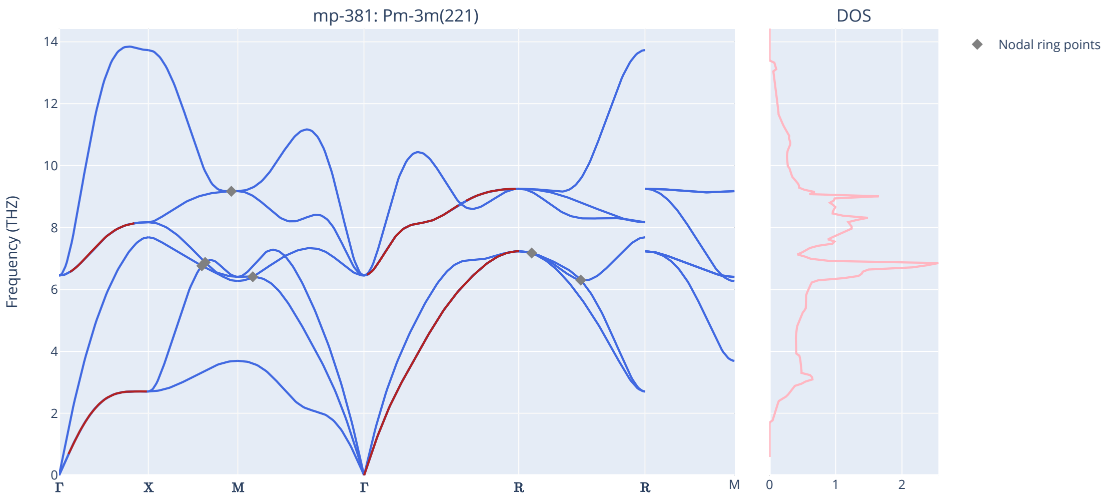

Supplementary Figure 222: The phonon band and DOS of RuSi.

Supplementary Table 417: High symmetry lines of mp-381-RuSi

	Start	End
0	Γ (0.000, 0.000, 0.000)	X (0.000, 0.500, 0.000)
1	X (0.000, 0.500, 0.000)	M (0.500, 0.500, 0.000)
2	M (0.500, 0.500, 0.000)	Γ (0.000, 0.000, 0.000)
3	Γ (0.000, 0.000, 0.000)	R (0.500, 0.500, 0.500)
4	R (0.500, 0.500, 0.500)	X (0.000, 0.500, 0.000)
5	R (0.500, 0.500, 0.500)	M (0.500, 0.500, 0.000)

Supplementary Table 418: Nodal ring points along high symmetry lines of mp-381-RuSi.

	Coordinates	Frequency	Mode 1	Mode 2	Multi.	Deg.	Path	Ratio
0	(0.299, 0.500, -0.000)	6.764	3	2	12	2	2	0.598
1	(0.440, 0.440, 0.000)	6.411	3	2	12	2	3	0.119
2	(0.448, 0.500, 0.450)	7.177	3	2	24	2	5	0.102
3	(0.255, 0.500, 0.255)	6.302	3	2	12	2	5	0.490
4	(0.318, 0.500, -0.000)	6.875	4	3	12	2	2	0.635
5	(0.464, 0.500, 0.000)	9.171	6	5	12	2	2	0.928

The followings are details for Cd_4Te_4. As shown in Fig **223**, it has 2 topological nodal lines, 2 nodal ring points and 1 single Weyl point.

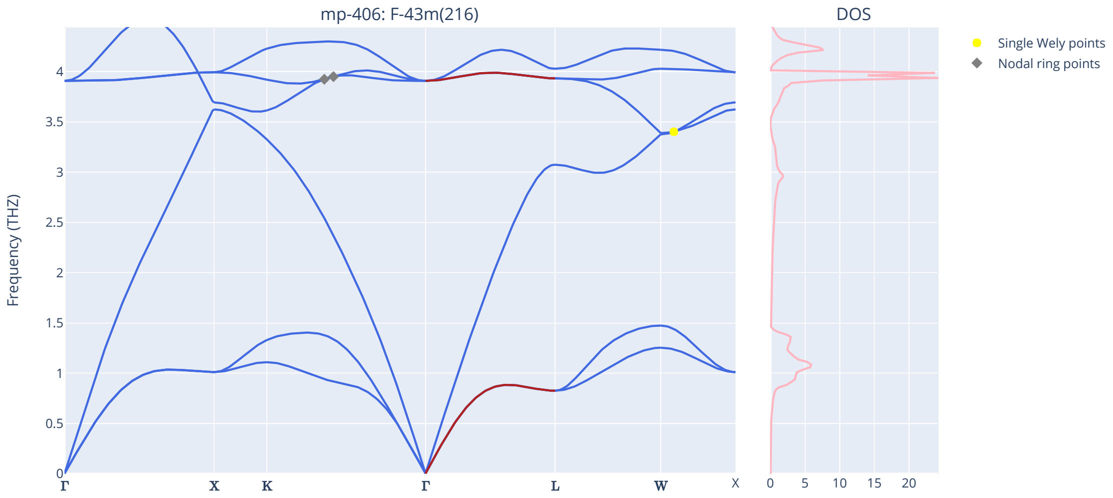

Supplementary Figure 223: The phonon band and DOS of Cd4Te4.

Supplementary Table 419: High symmetry lines of mp-406-Cd4Te4

	Start	End
0	Γ (0.000, 0.000, 0.000)	X (0.500, 0.000, 0.500)
1	X (0.500, 0.000, 0.500)	U (0.625, 0.250, 0.625)
2	K (0.375, 0.375, 0.750)	Γ (0.000, 0.000, 0.000)
3	Γ (0.000, 0.000, 0.000)	L (0.500, 0.500, 0.500)
4	L (0.500, 0.500, 0.500)	W (0.500, 0.250, 0.750)
5	W (0.500, 0.250, 0.750)	X (0.500, 0.000, 0.500)

Supplementary Table 420: Single Weyl points along high symmetry lines of mp-406-Cd4Te4.

	Coordinates	Frequency	Mode 1	Mode 2	Multi.	Deg.	Path	Ratio
0	(0.500, 0.206, 0.706)	3.402	4	3	12	2	6	0.175

Supplementary Table 421: Nodal ring points along high symmetry lines of mp-406-Cd4Te4.

	Coordinates	Frequency	Mode 1	Mode 2	Multi.	Deg.	Path	Ratio
0	(0.237, 0.237, 0.481)	3.923	5	4	24	2	3	0.362
1	(0.214, 0.214, 0.439)	3.950	5	4	24	2	3	0.419

The followings are details for Be_4S_4 and it only has 2 topological nodal lines along high symmetry line as shown in Fig **224**.

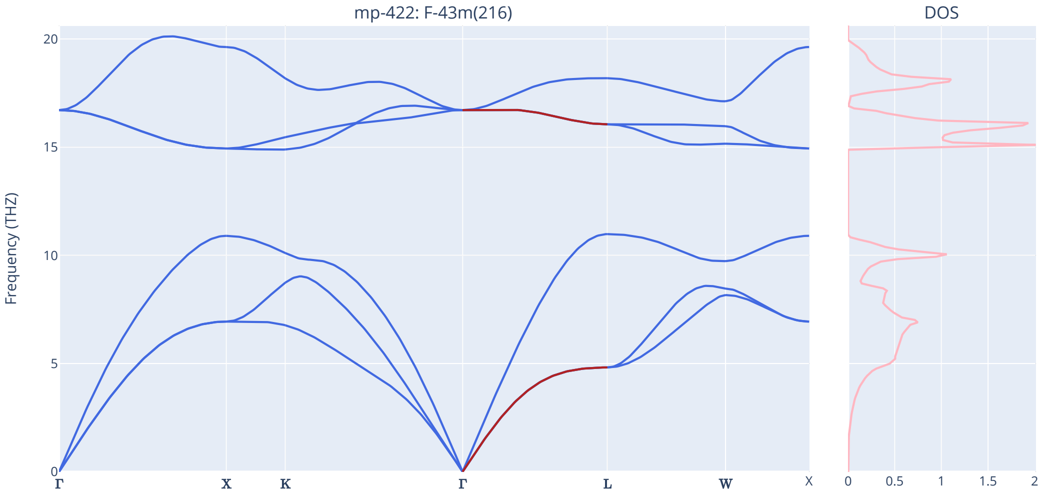

Supplementary Figure 224: The phonon band and DOS of Be4S4.

Supplementary Table 422: High symmetry lines of mp-422-Be4S4

	Start	End
0	Γ (0.000, 0.000, 0.000)	X (0.500, 0.000, 0.500)
1	X (0.500, 0.000, 0.500)	U (0.625, 0.250, 0.625)
2	K (0.375, 0.375, 0.750)	Γ (0.000, 0.000, 0.000)
3	Γ (0.000, 0.000, 0.000)	L (0.500, 0.500, 0.500)
4	L (0.500, 0.500, 0.500)	W (0.500, 0.250, 0.750)
5	W (0.500, 0.250, 0.750)	X (0.500, 0.000, 0.500)

The followings are details for AuMg. It has 6 topological nodal lines and 3 nodal ring points along high symmetry line as shown in Fig **225**.

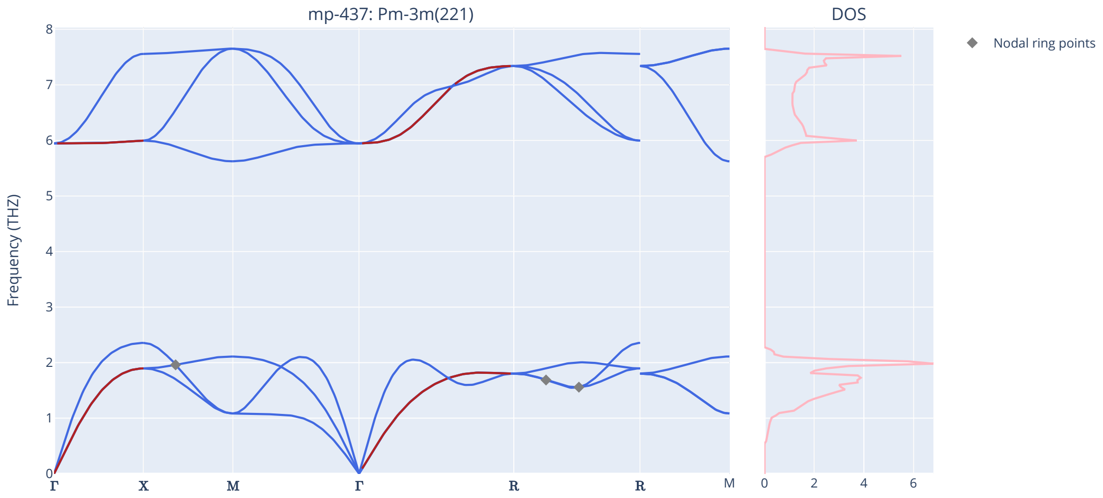

Supplementary Figure 225: The phonon band and DOS of AuMg.

Supplementary Table 423: High symmetry lines of mp-437-AuMg

	Start	End
0	Γ (0.000, 0.000, 0.000)	X (0.000, 0.500, 0.000)
1	X (0.000, 0.500, 0.000)	M (0.500, 0.500, 0.000)
2	M (0.500, 0.500, 0.000)	Γ (0.000, 0.000, 0.000)
3	Γ (0.000, 0.000, 0.000)	R (0.500, 0.500, 0.500)
4	R (0.500, 0.500, 0.500)	X (0.000, 0.500, 0.000)
5	R (0.500, 0.500, 0.500)	M (0.500, 0.500, 0.000)

Supplementary Table 424: Nodal ring points along high symmetry lines of mp-437-AuMg.

	Coordinates	Frequency	Mode 1	Mode 2	Multi.	Deg.	Path	Ratio
0	(0.372, 0.500, 0.372)	1.687	2	1	12	2	5	0.255
1	(0.242, 0.500, 0.242)	1.558	2	1	12	2	5	0.516
2	(0.180, 0.500, 0.000)	1.960	3	2	12	2	2	0.360

The followings are details for Ti_2. It has 13 topological nodal lines and 9 nodal ring points along high symmetry line as shown in Fig **226**.

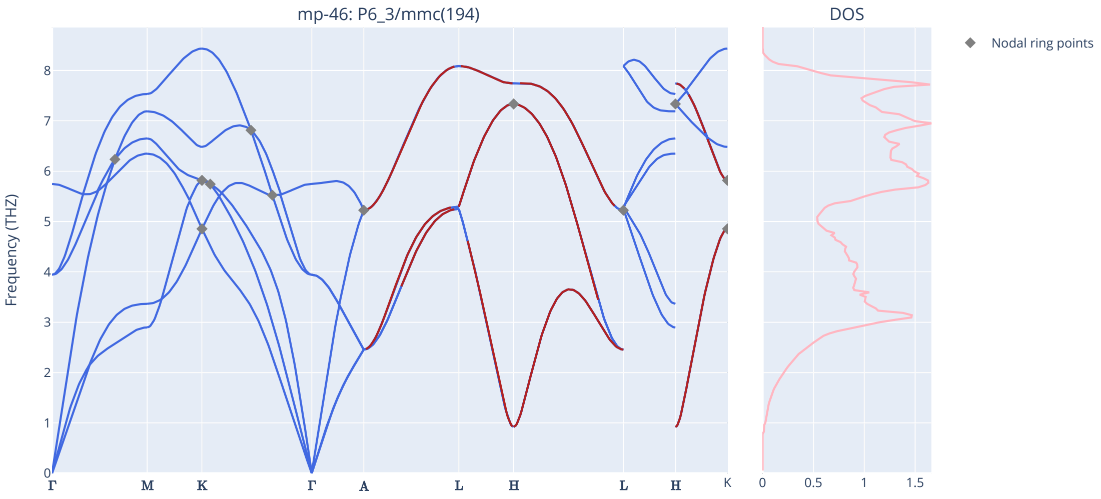

Supplementary Figure 226: The phonon band and DOS of Ti2.

Supplementary Table 425: High symmetry lines of mp-46-Ti2

	Start	End
0	Γ (0.000, 0.000, 0.000)	M (0.500, 0.000, 0.000)
1	M (0.500, 0.000, 0.000)	K (0.333, 0.333, 0.000)
2	K (0.333, 0.333, 0.000)	Γ (0.000, 0.000, 0.000)
3	Γ (0.000, 0.000, 0.000)	A (0.000, 0.000, 0.500)
4	A (0.000, 0.000, 0.500)	L (0.500, 0.000, 0.500)
5	L (0.500, 0.000, 0.500)	H (0.333, 0.333, 0.500)
6	H (0.333, 0.333, 0.500)	A (0.000, 0.000, 0.500)
7	L (0.500, 0.000, 0.500)	M (0.500, 0.000, 0.000)
8	H (0.333, 0.333, 0.500)	K (0.333, 0.333, 0.000)

Supplementary Table 426: Nodal ring points along high symmetry lines of mp-46-Ti2.

	Coordinates	Frequency	Mode 1	Mode 2	Multi.	Deg.	Path	Ratio
0	(0.333, 0.333, 0.000)	4.855	2	1	2	2	2	1.000
1	(0.333, 0.333, 0.000)	5.817	4	3	2	2	2	1.000
2	(0.308, 0.308, -0.000)	5.742	4	3	6	2	3	0.075
3	(0.333, 0.333, 0.500)	7.335	4	3	2	2	6	1.000
4	(0.331, -0.000, 0.000)	6.235	5	4	6	2	1	0.661
5	(0.119, 0.119, -0.000)	5.522	5	4	6	2	3	0.642
6	(0.185, 0.185, -0.000)	6.813	6	5	6	2	3	0.446
7	(0.000, 0.000, 0.500)	5.224	6	5	1	2	4	1.000

The followings are details for Os_2. It has 11 topological nodal lines and 19 nodal ring points along high symmetry line as shown in Fig 227.

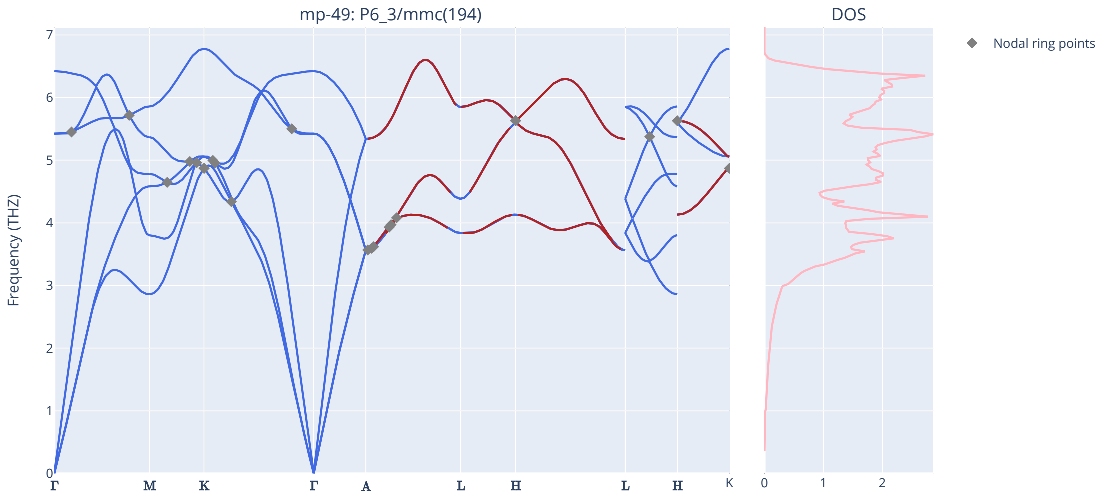

Supplementary Figure 227: The phonon band and DOS of Os2.

Supplementary Table 427: High symmetry lines of mp-49-Os2

	Start	End
0	Γ (0.000, 0.000, 0.000)	M (0.500, 0.000, 0.000)
1	M (0.500, 0.000, 0.000)	K (0.333, 0.333, 0.000)
2	K (0.333, 0.333, 0.000)	Γ (0.000, 0.000, 0.000)
3	Γ (0.000, 0.000, 0.000)	A (0.000, 0.000, 0.500)
4	A (0.000, 0.000, 0.500)	L (0.500, 0.000, 0.500)
5	L (0.500, 0.000, 0.500)	H (0.333, 0.333, 0.500)
6	H (0.333, 0.333, 0.500)	A (0.000, 0.000, 0.500)
7	L (0.500, 0.000, 0.500)	M (0.500, 0.000, 0.000)
8	H (0.333, 0.333, 0.500)	K (0.333, 0.333, 0.000)

Supplementary Table 428: Nodal ring points along high symmetry lines of mp-49-Os2.

	Coordinates	Frequency	Mode 1	Mode 2	Multi.	Deg.	Path	Ratio
0	(0.251, 0.251, 0.000)	4.338	2	1	6	2	3	0.248
1	(0.333, 0.333, 0.000)	4.870	2	1	2	2	2	1.000
2	(0.356, 0.288, -0.000)	4.959	3	2	6	2	2	0.865
3	(0.010, 0.000, 0.500)	3.567	3	2	6	2	5	0.020
4	(0.031, 0.000, 0.499)	3.595	3	2	12	2	5	0.061
5	(0.041, 0.000, 0.499)	3.618	3	2	12	2	5	0.081
6	(0.123, -0.000, 0.496)	3.930	3	2	12	2	5	0.246
7	(0.133, 0.000, 0.498)	3.971	3	2	12	2	5	0.266
8	(0.162, -0.000, 0.495)	4.079	3	2	12	2	5	0.324
9	(0.446, 0.109, -0.000)	4.646	4	3	6	2	2	0.326
10	(0.300, 0.300, -0.000)	4.956	4	3	6	2	3	0.099

Supplementary Table 428: Nodal ring points along high symmetry lines of mp-49-Os2.

	Coordinates	Frequency	Mode 1	Mode 2	Multi.	Deg.	Path	Ratio
11	(0.091, -0.000, 0.000)	5.450	5	4	6	2	1	0.181
12	(0.376, 0.246, -0.000)	4.979	5	4	12	2	2	0.740
13	(0.067, 0.067, -0.000)	5.500	5	4	6	2	3	0.799
14	(0.307, 0.307, -0.000)	4.996	5	4	6	2	3	0.080
15	(0.497, 0.000, 0.265)	5.375	5	4	12	2	8	0.470
16	(0.394, -0.000, -0.000)	5.716	6	5	6	2	1	0.788
17	(0.333, 0.333, 0.500)	5.629	6	5	2	2	6	1.000

The followings are details for Ti_4N_4. It has 2 topological nodal lines and 3 nodal ring points along high symmetry line as shown in Fig **228**.

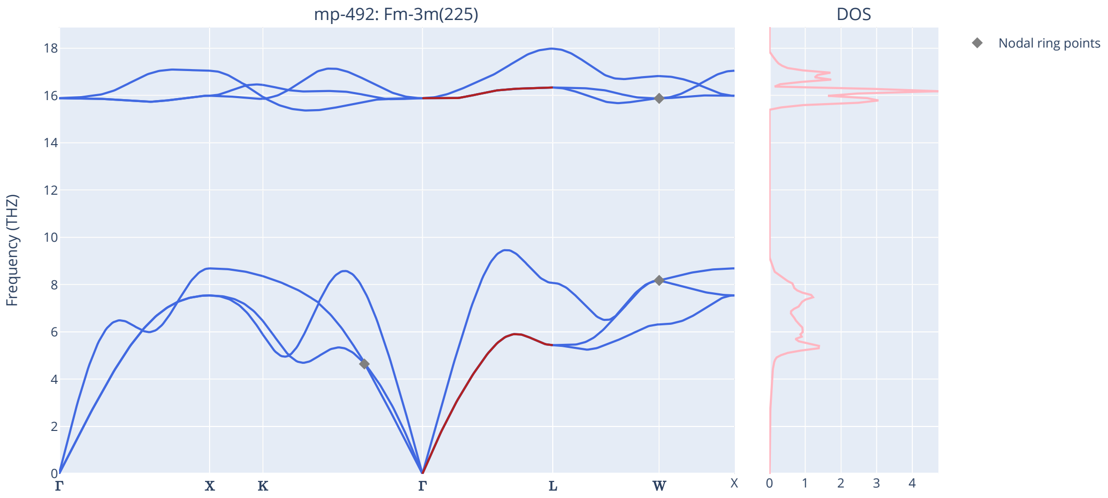

Supplementary Figure 228: The phonon band and DOS of Ti4N4.

Supplementary Table 429: High symmetry lines of mp-492-Ti4N4

	Start	End
0	Γ (0.000, 0.000, 0.000)	X (0.500, 0.000, 0.500)
1	X (0.500, 0.000, 0.500)	U (0.625, 0.250, 0.625)
2	K (0.375, 0.375, 0.750)	Γ (0.000, 0.000, 0.000)
3	Γ (0.000, 0.000, 0.000)	L (0.500, 0.500, 0.500)
4	L (0.500, 0.500, 0.500)	W (0.500, 0.250, 0.750)
5	W (0.500, 0.250, 0.750)	X (0.500, 0.000, 0.500)

Supplementary Table 430: Nodal ring points along high symmetry lines of mp-492-Ti4N4.

	Coordinates	Frequency	Mode 1	Mode 2	Multi.	Deg.	Path	Ratio
0	(0.138, 0.138, 0.271)	4.643	2	1	24	2	3	0.636
1	(0.500, 0.250, 0.750)	8.176	3	2	6	2	5	1.000
2	(0.500, 0.250, 0.750)	15.876	5	4	6	2	5	1.000

The followings are details for Th_4S_4. It has 2 topological nodal lines and 5 nodal ring points along high symmetry line as shown in Fig 229.

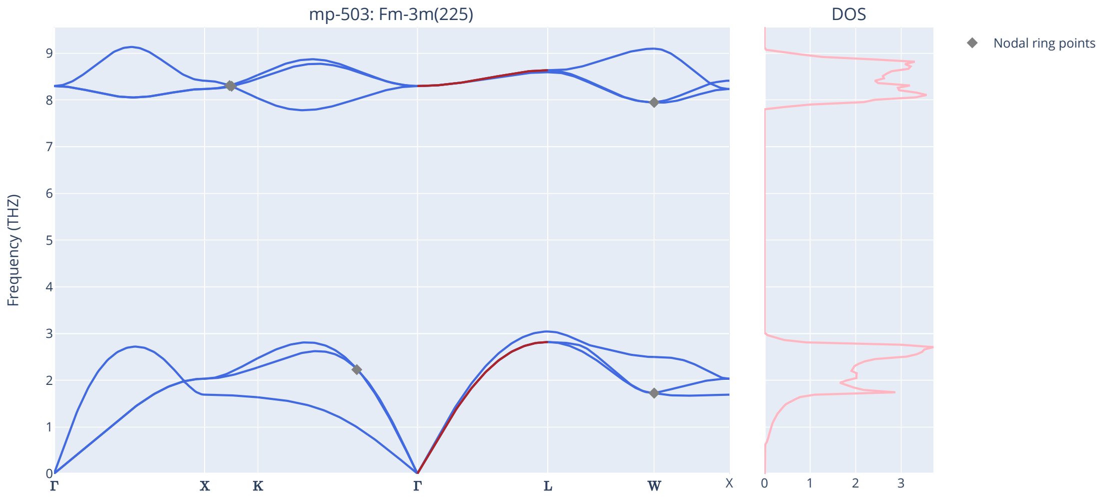

Supplementary Figure 229: The phonon band and DOS of Th4S4.

Supplementary Table 431: High symmetry lines of mp-503-Th4S4

	Start	End
0	Γ (0.000, 0.000, 0.000)	X (0.500, 0.000, 0.500)
1	X (0.500, 0.000, 0.500)	U (0.625, 0.250, 0.625)
2	K (0.375, 0.375, 0.750)	Γ (0.000, 0.000, 0.000)
3	Γ (0.000, 0.000, 0.000)	L (0.500, 0.500, 0.500)
4	L (0.500, 0.500, 0.500)	W (0.500, 0.250, 0.750)
5	W (0.500, 0.250, 0.750)	X (0.500, 0.000, 0.500)

Supplementary Table 432: Nodal ring points along high symmetry lines of mp-503-Th4S4.

	Coordinates	Frequency	Mode 1	Mode 2	Multi.	Deg.	Path	Ratio
0	(0.500, 0.250, 0.750)	1.725	2	1	6	2	5	1.000
1	(0.145, 0.145, 0.282)	2.229	3	2	24	2	3	0.620
2	(0.565, 0.126, 0.565)	8.293	5	4	24	2	2	0.508
3	(0.500, 0.250, 0.750)	7.948	5	4	6	2	5	1.000
4	(0.559, 0.117, 0.559)	8.308	6	5	12	2	2	0.469

The followings are details for MgPd. It has 6 topological nodal lines and 5 nodal ring points along high symmetry line as shown in Fig 230.

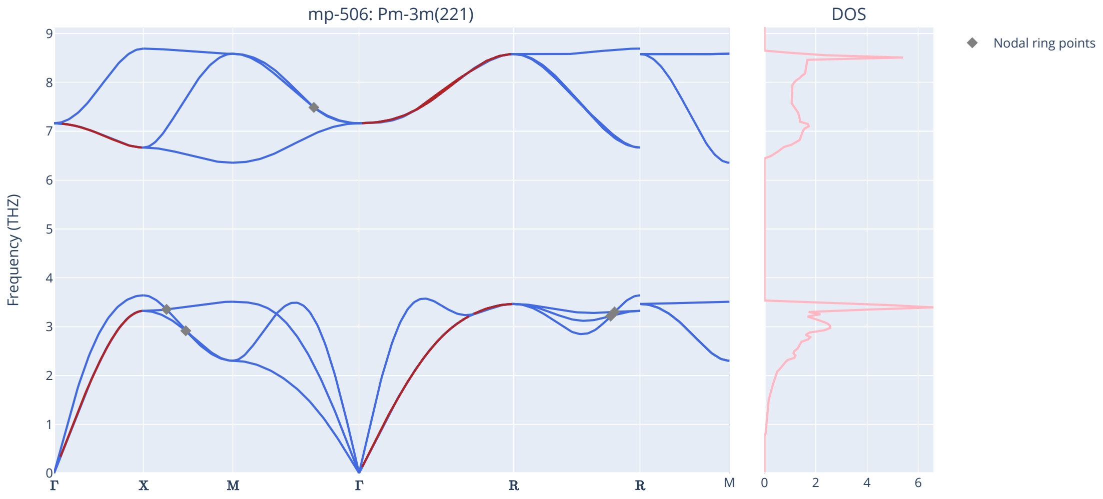

Supplementary Figure 230: The phonon band and DOS of MgPd.

Supplementary Table 433: High symmetry lines of mp-506-MgPd

	Start	End
0	Γ (0.000, 0.000, 0.000)	X (0.000, 0.500, 0.000)
1	X (0.000, 0.500, 0.000)	M (0.500, 0.500, 0.000)
2	M (0.500, 0.500, 0.000)	Γ (0.000, 0.000, 0.000)
3	Γ (0.000, 0.000, 0.000)	R (0.500, 0.500, 0.500)
4	R (0.500, 0.500, 0.500)	X (0.000, 0.500, 0.000)
5	R (0.500, 0.500, 0.500)	M (0.500, 0.500, 0.000)

Supplementary Table 434: Nodal ring points along high symmetry lines of mp-506-MgPd.

	Coordinates	Frequency	Mode 1	Mode 2	Multi.	Deg.	Path	Ratio
0	(0.237, 0.500, 0.000)	2.916	2	1	12	2	2	0.474
1	(0.117, 0.500, 0.117)	3.218	2	1	12	2	5	0.767
2	(0.130, 0.500, -0.000)	3.354	3	2	12	2	2	0.259
3	(0.101, 0.500, 0.101)	3.303	3	2	12	2	5	0.798
4	(0.179, 0.179, 0.000)	7.488	6	5	12	2	3	0.642

The followings are details for BaCd. It has 6 topological nodal lines and 5 nodal ring points along high symmetry line as shown in Fig **231**.

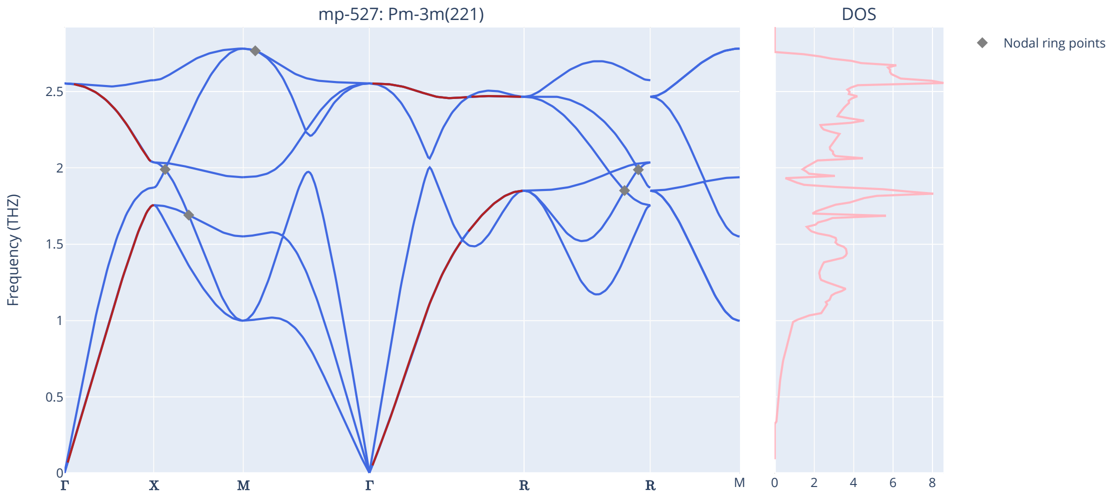

Supplementary Figure 231: The phonon band and DOS of BaCd.

Supplementary Table 435: High symmetry lines of mp-527-BaCd

	Start	End
0	Γ (0.000, 0.000, 0.000)	X (0.000, 0.500, 0.000)
1	X (0.000, 0.500, 0.000)	M (0.500, 0.500, 0.000)
2	M (0.500, 0.500, 0.000)	Γ (0.000, 0.000, 0.000)
3	Γ (0.000, 0.000, 0.000)	R (0.500, 0.500, 0.500)
4	R (0.500, 0.500, 0.500)	X (0.000, 0.500, 0.000)
5	R (0.500, 0.500, 0.500)	M (0.500, 0.500, 0.000)

Supplementary Table 436: Nodal ring points along high symmetry lines of mp-527-BaCd.

	Coordinates	Frequency	Mode 1	Mode 2	Multi.	Deg.	Path	Ratio
0	(0.195, 0.500, -0.000)	1.691	3	2	12	2	2	0.391
1	(0.102, 0.500, 0.102)	1.852	3	2	12	2	5	0.796
2	(0.047, 0.500, 0.047)	1.988	4	3	12	2	5	0.905
3	(0.063, 0.500, -0.000)	1.990	4	3	12	2	2	0.127
4	(0.452, 0.452, -0.000)	2.767	6	5	12	2	3	0.096

The followings are details for HgSr. It has 5 topological nodal lines and 6 nodal ring points along high symmetry line as shown in Fig 232.

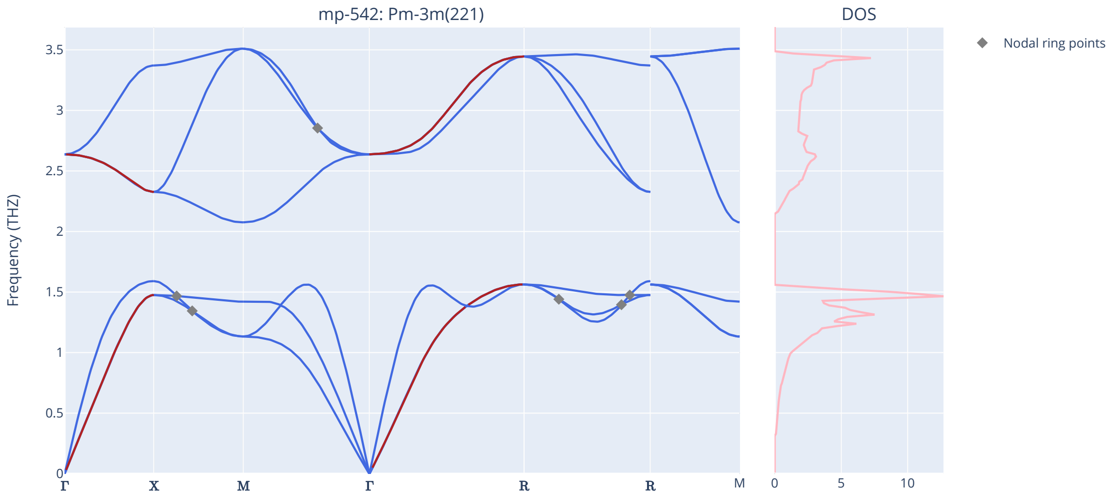

Supplementary Figure 232: The phonon band and DOS of HgSr.

Supplementary Table 437: High symmetry lines of mp-542-HgSr

	Start	End
0	Γ (0.000, 0.000, 0.000)	X (0.000, 0.500, 0.000)
1	X (0.000, 0.500, 0.000)	M (0.500, 0.500, 0.000)
2	M (0.500, 0.500, 0.000)	Γ (0.000, 0.000, 0.000)
3	Γ (0.000, 0.000, 0.000)	R (0.500, 0.500, 0.500)
4	R (0.500, 0.500, 0.500)	X (0.000, 0.500, 0.000)
5	R (0.500, 0.500, 0.500)	M (0.500, 0.500, 0.000)

Supplementary Table 438: Nodal ring points along high symmetry lines of mp-542-HgSr.

	Coordinates	Frequency	Mode 1	Mode 2	Multi.	Deg.	Path	Ratio
0	(0.215, 0.500, -0.000)	1.344	2	1	12	2	2	0.430
1	(0.362, 0.500, 0.362)	1.441	2	1	12	2	5	0.276
2	(0.114, 0.500, 0.114)	1.395	2	1	12	2	5	0.771
3	(0.128, 0.500, 0.000)	1.467	3	2	12	2	2	0.255
4	(0.081, 0.500, 0.081)	1.475	3	2	12	2	5	0.837
5	(0.205, 0.205, 0.000)	2.854	6	5	12	2	3	0.590

The followings are details for AlRu. It has 6 topological nodal lines and 2 nodal ring points along high symmetry line as shown in Fig 233.

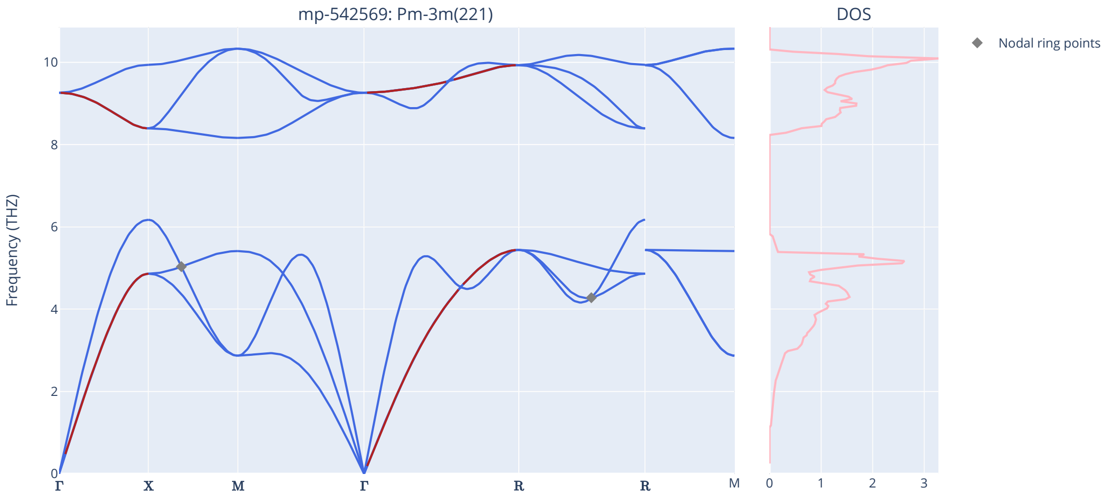

Supplementary Figure 233: The phonon band and DOS of AlRu.

Supplementary Table 439: High symmetry lines of mp-542569-AlRu

	Start	End
0	Γ (0.000, 0.000, 0.000)	X (0.000, 0.500, 0.000)
1	X (0.000, 0.500, 0.000)	M (0.500, 0.500, 0.000)
2	M (0.500, 0.500, 0.000)	Γ (0.000, 0.000, 0.000)
3	Γ (0.000, 0.000, 0.000)	R (0.500, 0.500, 0.500)
4	R (0.500, 0.500, 0.500)	X (0.000, 0.500, 0.000)
5	R (0.500, 0.500, 0.500)	M (0.500, 0.500, 0.000)

Supplementary Table 440: Nodal ring points along high symmetry lines of mp-542569-AlRu.

	Coordinates	Frequency	Mode 1	Mode 2	Multi.	Deg.	Path	Ratio
0	(0.213, 0.500, 0.213)	4.279	2	1	12	2	5	0.574
1	(0.184, 0.500, -0.000)	5.034	3	2	12	2	2	0.369

The followings are details for Ba$_2$. It has 11 topological nodal lines and 10 nodal ring points along high symmetry line as shown in Fig 234.

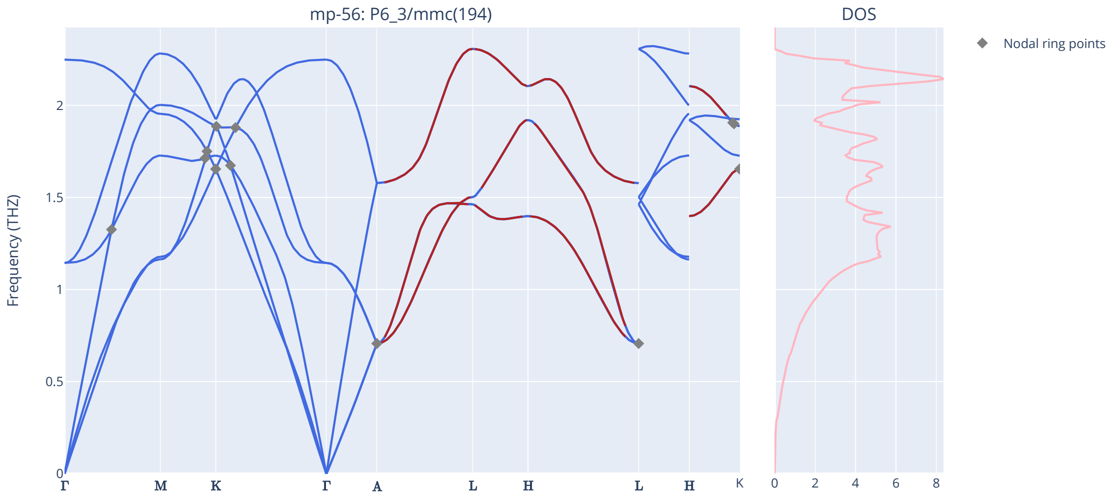

Supplementary Figure 234: The phonon band and DOS of Ba2.

Supplementary Table 441: High symmetry lines of mp-56-Ba2

	Start	End
0	Γ (0.000, 0.000, 0.000)	M (0.500, 0.000, 0.000)
1	M (0.500, 0.000, 0.000)	K (0.333, 0.333, 0.000)
2	K (0.333, 0.333, 0.000)	Γ (0.000, 0.000, 0.000)
3	Γ (0.000, 0.000, 0.000)	A (0.000, 0.000, 0.500)
4	A (0.000, 0.000, 0.500)	L (0.500, 0.000, 0.500)
5	L (0.500, 0.000, 0.500)	H (0.333, 0.333, 0.500)
6	H (0.333, 0.333, 0.500)	A (0.000, 0.000, 0.500)
7	L (0.500, 0.000, 0.500)	M (0.500, 0.000, 0.000)
8	H (0.333, 0.333, 0.500)	K (0.333, 0.333, 0.000)

Supplementary Table 442: Nodal ring points along high symmetry lines of mp-56-Ba2.

	Coordinates	Frequency	Mode 1	Mode 2	Multi.	Deg.	Path	Ratio
0	(0.333, 0.333, 0.000)	1.654	2	1	2	2	2	1.000
1	(0.366, 0.269, 0.000)	1.713	3	2	6	2	2	0.807
2	(0.289, 0.289, -0.000)	1.674	3	2	6	2	3	0.133
3	(0.245, -0.000, 0.000)	1.326	4	3	6	2	1	0.490
4	(0.361, 0.281, 0.000)	1.751	4	3	12	2	2	0.842
5	(0.000, 0.000, 0.500)	0.706	4	3	1	4	4	1.000
6	(0.331, 0.331, -0.000)	1.886	5	4	6	2	3	0.006
7	(0.274, 0.274, -0.000)	1.880	5	4	6	2	3	0.178
8	(0.333, 0.333, 0.055)	1.900	5	4	4	2	9	0.890
9	(0.333, 0.333, 0.069)	1.906	5	4	4	2	9	0.863

The followings are details for Kr_2. It has 11 topological nodal lines and 1 nodal ring point along high symmetry line as shown in Fig **235**.

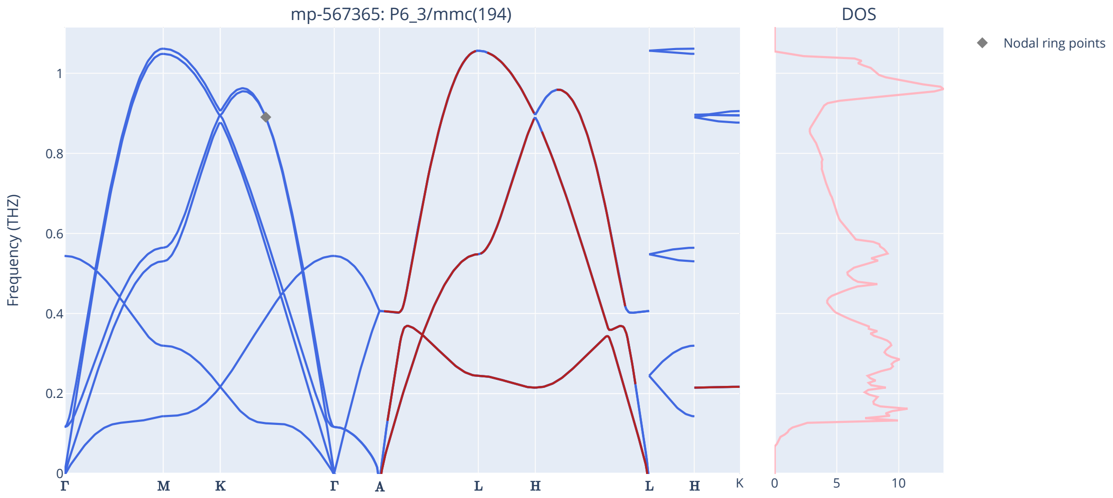

Supplementary Figure 235: The phonon band and DOS of Kr2.

Supplementary Table 443: High symmetry lines of mp-567365-Kr2

	Start	End
0	Γ (0.000, 0.000, 0.000)	M (0.500, 0.000, 0.000)
1	M (0.500, 0.000, 0.000)	K (0.333, 0.333, 0.000)
2	K (0.333, 0.333, 0.000)	Γ (0.000, 0.000, 0.000)
3	Γ (0.000, 0.000, 0.000)	A (0.000, 0.000, 0.500)
4	A (0.000, 0.000, 0.500)	L (0.500, 0.000, 0.500)
5	L (0.500, 0.000, 0.500)	H (0.333, 0.333, 0.500)
6	H (0.333, 0.333, 0.500)	A (0.000, 0.000, 0.500)
7	L (0.500, 0.000, 0.500)	M (0.500, 0.000, 0.000)
8	H (0.333, 0.333, 0.500)	K (0.333, 0.333, 0.000)

Supplementary Table 444: Nodal ring points along high symmetry lines of mp-567365-Kr2.

	Coordinates	Frequency	Mode 1	Mode 2	Multi.	Deg.	Path	Ratio
0	(0.200, 0.200, 0.000)	0.891	6	5	6	2	3	0.399

The followings are details for Pa_4C_4. It has 2 topological nodal lines and 5 nodal ring points along high symmetry line as shown in Fig 236.

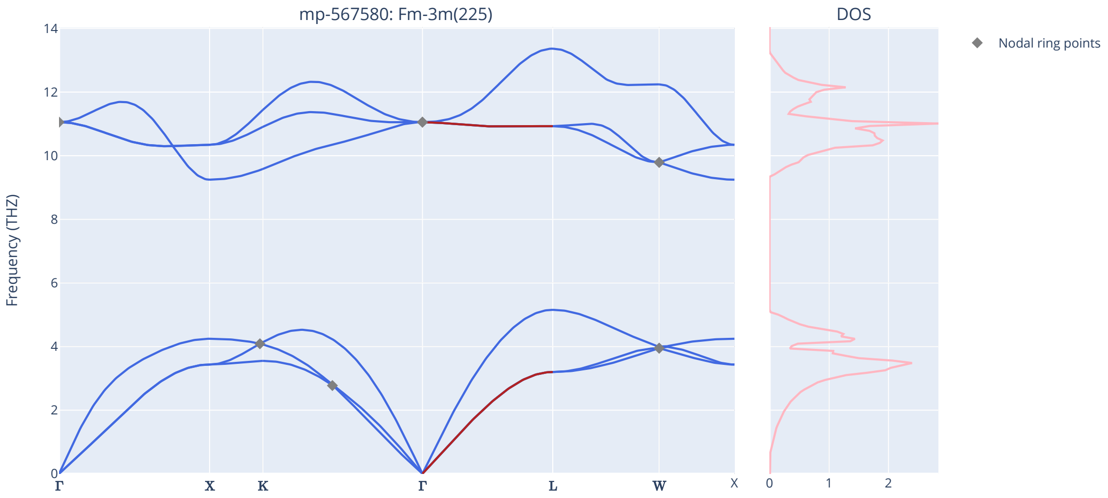

Supplementary Figure 236: The phonon band and DOS of Pa4C4.

Supplementary Table 445: High symmetry lines of mp-567580-Pa4C4

	Start	End
0	Γ (0.000, 0.000, 0.000)	X (0.500, 0.000, 0.500)
1	X (0.500, 0.000, 0.500)	U (0.625, 0.250, 0.625)
2	K (0.375, 0.375, 0.750)	Γ (0.000, 0.000, 0.000)
3	Γ (0.000, 0.000, 0.000)	L (0.500, 0.500, 0.500)
4	L (0.500, 0.500, 0.500)	W (0.500, 0.250, 0.750)
5	W (0.500, 0.250, 0.750)	X (0.500, 0.000, 0.500)

Supplementary Table 446: Nodal ring points along high symmetry lines of mp-567580-Pa4C4.

	Coordinates	Frequency	Mode 1	Mode 2	Multi.	Deg.	Path	Ratio
0	(0.214, 0.214, 0.420)	2.773	2	1	24	2	3	0.436
1	(0.500, 0.250, 0.750)	3.948	2	1	6	2	5	1.000
2	(0.618, 0.237, 0.618)	4.087	3	2	12	2	2	0.946
3	(0.500, 0.250, 0.750)	9.790	5	4	6	2	5	1.000
4	(0.000, 0.000, 0.000)	11.055	6	5	1	3	1	0.000

The followings are details for ZnZr. It has 6 topological nodal lines and 6 nodal ring points along high symmetry line as shown in Fig 237.

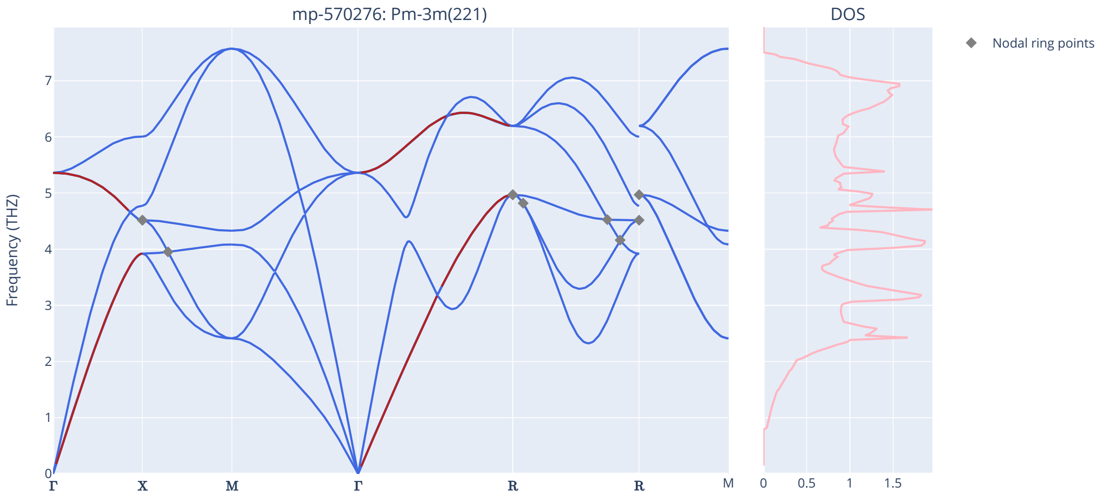

Supplementary Figure 237: The phonon band and DOS of ZnZr.

Supplementary Table 447: High symmetry lines of mp-570276-ZnZr

	Start	End
0	Γ (0.000, 0.000, 0.000)	X (0.000, 0.500, 0.000)
1	X (0.000, 0.500, 0.000)	M (0.500, 0.500, 0.000)
2	M (0.500, 0.500, 0.000)	Γ (0.000, 0.000, 0.000)
3	Γ (0.000, 0.000, 0.000)	R (0.500, 0.500, 0.500)
4	R (0.500, 0.500, 0.500)	X (0.000, 0.500, 0.000)
5	R (0.500, 0.500, 0.500)	M (0.500, 0.500, 0.000)

Supplementary Table 448: Nodal ring points along high symmetry lines of mp-570276-ZnZr.

	Coordinates	Frequency	Mode 1	Mode 2	Multi.	Deg.	Path	Ratio
0	(0.500, 0.500, 0.500)	4.970	2	1	1	3	4	1.000
1	(0.459, 0.500, 0.459)	4.820	2	1	12	2	5	0.082
2	(0.143, 0.500, -0.000)	3.953	3	2	12	2	2	0.287
3	(0.075, 0.500, 0.075)	4.163	3	2	12	2	5	0.850
4	(0.000, 0.500, 0.000)	4.517	4	3	3	2	1	1.000
5	(0.126, 0.500, 0.126)	4.527	4	3	12	2	5	0.748

The followings are details for Xe_2. It has 11 topological nodal lines and 10 nodal ring points along high symmetry line as shown in Fig 238.

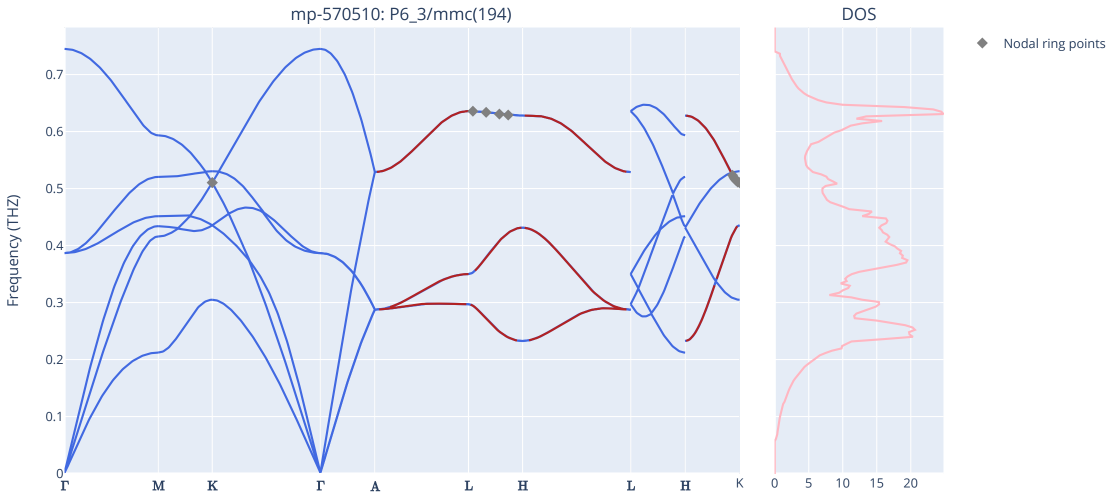

Supplementary Figure 238: The phonon band and DOS of Xe2.

Supplementary Table 449: High symmetry lines of mp-570510-Xe2

	Start	End
0	Γ (0.000, 0.000, 0.000)	M (0.500, 0.000, 0.000)
1	M (0.500, 0.000, 0.000)	K (0.333, 0.333, 0.000)
2	K (0.333, 0.333, 0.000)	Γ (0.000, 0.000, 0.000)
3	Γ (0.000, 0.000, 0.000)	A (0.000, 0.000, 0.500)
4	A (0.000, 0.000, 0.500)	L (0.500, 0.000, 0.500)
5	L (0.500, 0.000, 0.500)	H (0.333, 0.333, 0.500)
6	H (0.333, 0.333, 0.500)	A (0.000, 0.000, 0.500)
7	L (0.500, 0.000, 0.500)	M (0.500, 0.000, 0.000)
8	H (0.333, 0.333, 0.500)	K (0.333, 0.333, 0.000)

Supplementary Table 450: Nodal ring points along high symmetry lines of mp-570510-Xe2.

	Coordinates	Frequency	Mode 1	Mode 2	Multi.	Deg.	Path	Ratio
0	(0.333, 0.333, 0.000)	0.510	5	4	2	2	2	1.000
1	(0.333, 0.333, 0.017)	0.511	5	4	4	2	9	0.966
2	(0.333, 0.333, 0.065)	0.523	5	4	4	2	9	0.871
3	(0.333, 0.333, 0.051)	0.519	5	4	4	2	9	0.898
4	(0.333, 0.333, 0.041)	0.516	5	4	4	2	9	0.918
5	(0.333, 0.333, 0.031)	0.514	5	4	4	2	9	0.939
6	(0.486, 0.027, 0.500)	0.636	6	5	6	2	6	0.082
7	(0.446, 0.109, 0.500)	0.634	6	5	6	2	6	0.327
8	(0.405, 0.190, 0.500)	0.631	6	5	6	2	6	0.571
9	(0.378, 0.245, 0.500)	0.629	6	5	6	2	6	0.735

The followings are details for Bi_4Sc_4. It has 2 topological nodal lines and 4 nodal ring points along high symmetry line as shown in Fig 239.

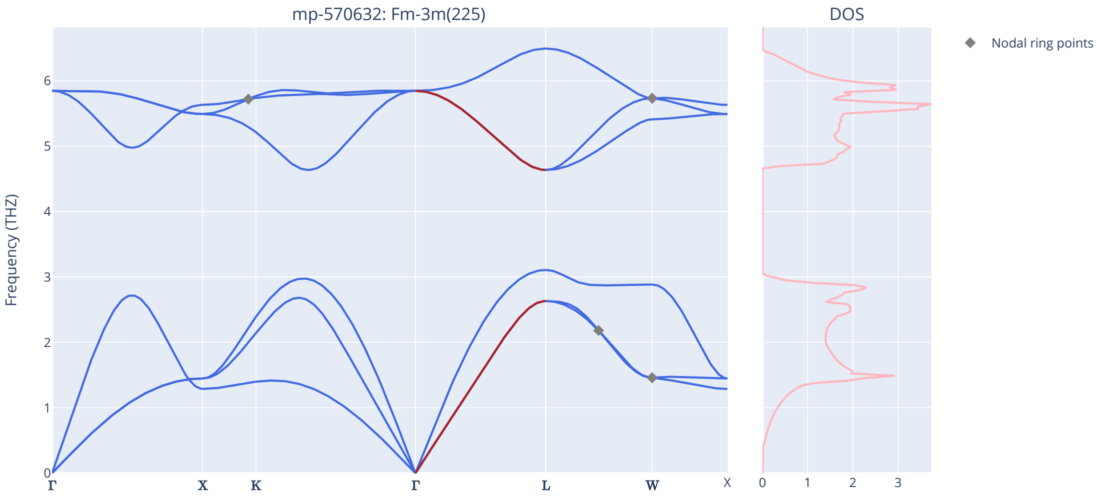

Supplementary Figure 239: The phonon band and DOS of Bi4Sc4.

Supplementary Table 451: High symmetry lines of mp-570632-Bi4Sc4

	Start	End
0	Γ (0.000, 0.000, 0.000)	X (0.500, 0.000, 0.500)
1	X (0.500, 0.000, 0.500)	U (0.625, 0.250, 0.625)
2	K (0.375, 0.375, 0.750)	Γ (0.000, 0.000, 0.000)
3	Γ (0.000, 0.000, 0.000)	L (0.500, 0.500, 0.500)
4	L (0.500, 0.500, 0.500)	W (0.500, 0.250, 0.750)
5	W (0.500, 0.250, 0.750)	X (0.500, 0.000, 0.500)

Supplementary Table 452: Nodal ring points along high symmetry lines of mp-570632-Bi4Sc4.

	Coordinates	Frequency	Mode 1	Mode 2	Multi.	Deg.	Path	Ratio
0	(0.500, 0.250, 0.750)	1.460	2	1	6	2	5	1.000
1	(0.500, 0.376, 0.624)	2.182	2	1	24	2	5	0.496
2	(0.607, 0.215, 0.607)	5.717	6	5	12	2	2	0.859
3	(0.500, 0.250, 0.750)	5.731	6	5	6	2	5	1.000

The followings are details for RuTi. It has 5 topological nodal lines and 7 nodal ring points along high symmetry line as shown in Fig 240.

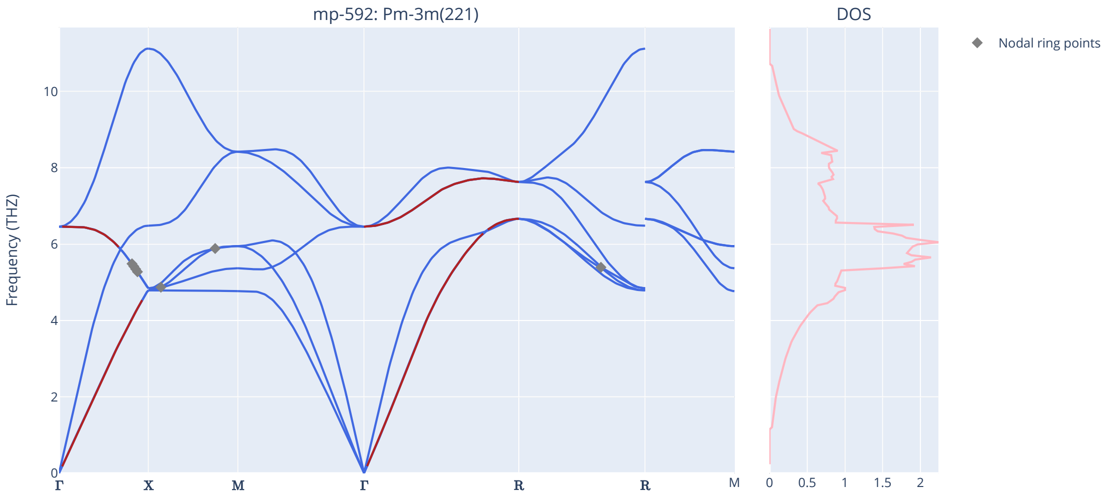

Supplementary Figure 240: The phonon band and DOS of RuTi.

Supplementary Table 453: High symmetry lines of mp-592-RuTi

	Start	End
0	Γ (0.000, 0.000, 0.000)	X (0.000, 0.500, 0.000)
1	X (0.000, 0.500, 0.000)	M (0.500, 0.500, 0.000)
2	M (0.500, 0.500, 0.000)	Γ (0.000, 0.000, 0.000)
3	Γ (0.000, 0.000, 0.000)	R (0.500, 0.500, 0.500)
4	R (0.500, 0.500, 0.500)	X (0.000, 0.500, 0.000)
5	R (0.500, 0.500, 0.500)	M (0.500, 0.500, 0.000)

Supplementary Table 454: Nodal ring points along high symmetry lines of mp-592-RuTi.

	Coordinates	Frequency	Mode 1	Mode 2	Multi.	Deg.	Path	Ratio
0	(0.070, 0.500, -0.000)	4.872	3	2	12	2	2	0.140
1	(0.175, 0.500, 0.175)	5.394	3	2	12	2	5	0.650
2	(0.000, 0.408, 0.000)	5.486	4	3	6	2	1	0.816
3	(0.000, 0.418, 0.000)	5.418	4	3	6	2	1	0.837
4	(0.000, 0.429, 0.000)	5.348	4	3	6	2	1	0.857
5	(0.000, 0.439, 0.000)	5.276	4	3	6	2	1	0.878
6	(0.374, 0.500, 0.000)	5.886	4	3	12	2	2	0.747

The followings are details for MgY. It has 6 topological nodal lines and 4 nodal ring points along high symmetry line as shown in Fig 241.

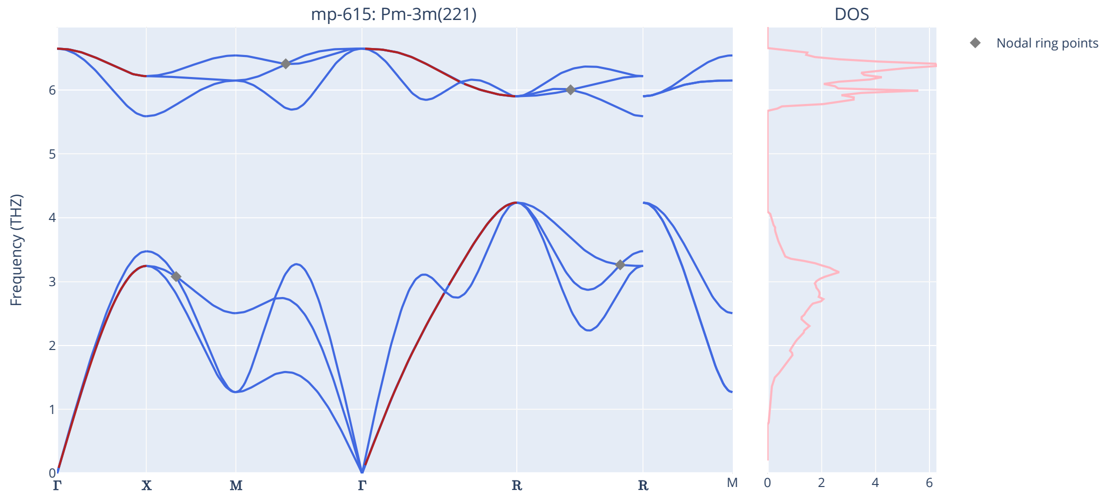

Supplementary Figure 241: The phonon band and DOS of MgY.

Supplementary Table 455: High symmetry lines of mp-615-MgY

	Start	End
0	Γ (0.000, 0.000, 0.000)	X (0.000, 0.500, 0.000)
1	X (0.000, 0.500, 0.000)	M (0.500, 0.500, 0.000)
2	M (0.500, 0.500, 0.000)	Γ (0.000, 0.000, 0.000)
3	Γ (0.000, 0.000, 0.000)	R (0.500, 0.500, 0.500)
4	R (0.500, 0.500, 0.500)	X (0.000, 0.500, 0.000)
5	R (0.500, 0.500, 0.500)	M (0.500, 0.500, 0.000)

Supplementary Table 456: Nodal ring points along high symmetry lines of mp-615-MgY.

	Coordinates	Frequency	Mode 1	Mode 2	Multi.	Deg.	Path	Ratio
0	(0.166, 0.500, -0.000)	3.079	3	2	12	2	2	0.333
1	(0.091, 0.500, 0.091)	3.263	3	2	12	2	5	0.819
2	(0.287, 0.500, 0.287)	6.003	5	4	12	2	5	0.426
3	(0.302, 0.302, 0.000)	6.410	6	5	12	2	3	0.396

The followings are details for Ti_4C_4. It has 2 topological nodal lines and 6 nodal ring points along high symmetry line as shown in Fig **242**.

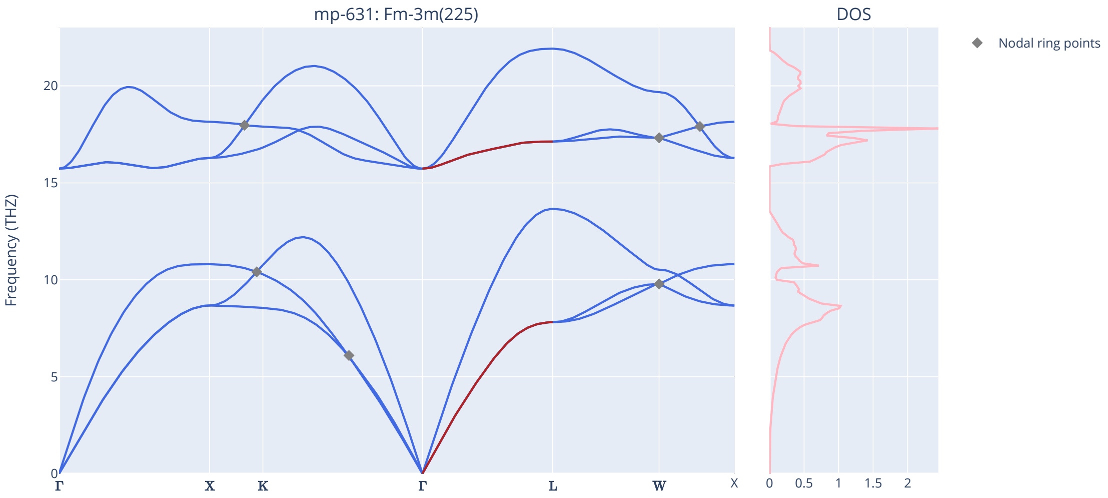

Supplementary Figure 242: The phonon band and DOS of Ti4C4.

Supplementary Table 457: High symmetry lines of mp-631-Ti4C4

	Start	End
0	Γ (0.000, 0.000, 0.000)	X (0.500, 0.000, 0.500)
1	X (0.500, 0.000, 0.500)	U (0.625, 0.250, 0.625)
2	K (0.375, 0.375, 0.750)	Γ (0.000, 0.000, 0.000)
3	Γ (0.000, 0.000, 0.000)	L (0.500, 0.500, 0.500)
4	L (0.500, 0.500, 0.500)	W (0.500, 0.250, 0.750)
5	W (0.500, 0.250, 0.750)	X (0.500, 0.000, 0.500)

Supplementary Table 458: Nodal ring points along high symmetry lines of mp-631-Ti4C4.

	Coordinates	Frequency	Mode 1	Mode 2	Multi.	Deg.	Path	Ratio
0	(0.168, 0.168, 0.349)	6.098	2	1	24	2	3	0.540
1	(0.500, 0.250, 0.750)	9.785	2	1	6	2	5	1.000
2	(0.611, 0.222, 0.611)	10.408	3	2	12	2	2	0.886
3	(0.500, 0.250, 0.750)	17.319	5	4	6	2	5	1.000
4	(0.582, 0.164, 0.582)	17.967	6	5	12	2	2	0.657
5	(0.497, 0.113, 0.616)	17.905	6	5	24	2	6	0.541

The followings are details for C_8. It has 5 topological nodal lines and 6 nodal ring points along high symmetry line as shown in Fig **243**.

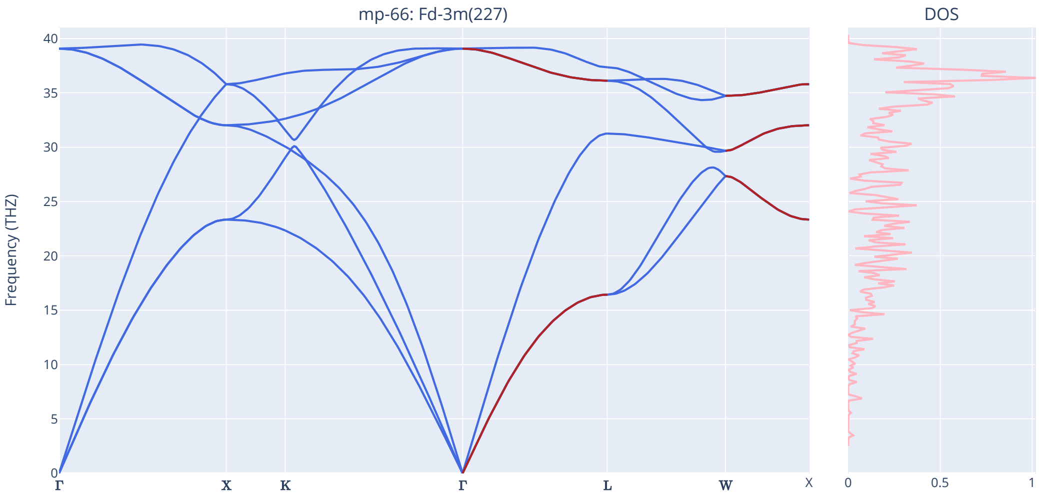

Supplementary Figure 243: The phonon band and DOS of C8.

Supplementary Table 459: High symmetry lines of mp-66-C8

	Start	End
0	Γ (0.000, 0.000, 0.000)	X (0.500, 0.000, 0.500)
1	X (0.500, 0.000, 0.500)	U (0.625, 0.250, 0.625)
2	K (0.375, 0.375, 0.750)	Γ (0.000, 0.000, 0.000)
3	Γ (0.000, 0.000, 0.000)	L (0.500, 0.500, 0.500)
4	L (0.500, 0.500, 0.500)	W (0.500, 0.250, 0.750)
5	W (0.500, 0.250, 0.750)	X (0.500, 0.000, 0.500)

The followings are details for Sc_2. It has 11 topological nodal lines and 11 nodal ring points along high symmetry line as shown in Fig 244.

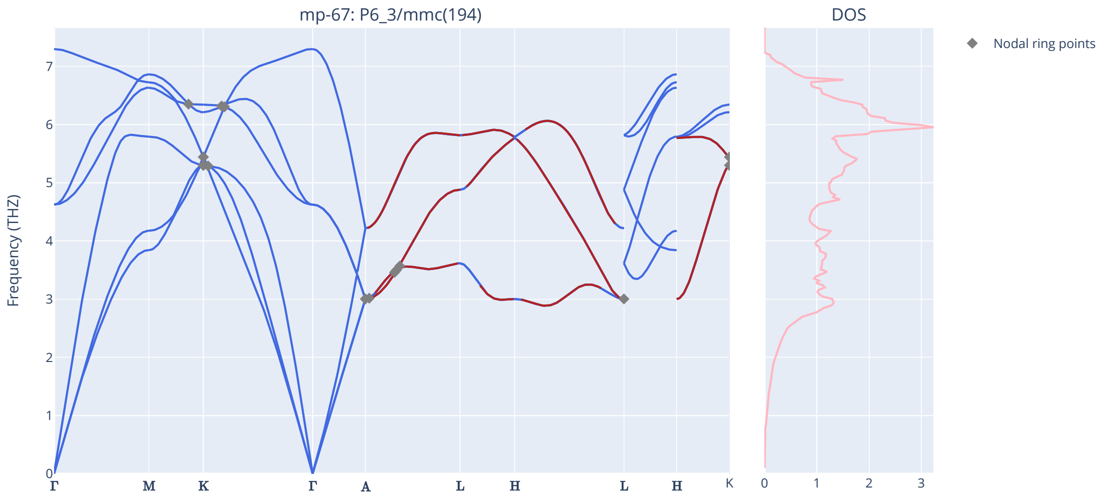

Supplementary Figure 244: The phonon band and DOS of Sc2.

Supplementary Table 460: High symmetry lines of mp-67-Sc2

	Start	End
0	Γ (0.000, 0.000, 0.000)	M (0.500, 0.000, 0.000)
1	M (0.500, 0.000, 0.000)	K (0.333, 0.333, 0.000)
2	K (0.333, 0.333, 0.000)	Γ (0.000, 0.000, 0.000)
3	Γ (0.000, 0.000, 0.000)	A (0.000, 0.000, 0.500)
4	A (0.000, 0.000, 0.500)	L (0.500, 0.000, 0.500)
5	L (0.500, 0.000, 0.500)	H (0.333, 0.333, 0.500)
6	H (0.333, 0.333, 0.500)	A (0.000, 0.000, 0.500)
7	L (0.500, 0.000, 0.500)	M (0.500, 0.000, 0.000)
8	H (0.333, 0.333, 0.500)	K (0.333, 0.333, 0.000)

Supplementary Table 461: Nodal ring points along high symmetry lines of mp-67-Sc2.

	Coordinates	Frequency	Mode 1	Mode 2	Multi.	Deg.	Path	Ratio
0	(0.333, 0.333, 0.000)	5.299	2	1	2	2	2	1.000
1	(0.319, 0.319, 0.000)	5.289	3	2	6	2	3	0.044
2	(0.000, 0.000, 0.500)	3.003	3	2	1	4	4	1.000
3	(0.020, 0.000, 0.499)	3.016	3	2	12	2	5	0.041
4	(0.154, 0.000, 0.496)	3.459	3	2	12	2	5	0.308
5	(0.164, 0.000, 0.498)	3.498	3	2	12	2	5	0.327
6	(0.182, 0.000, 0.496)	3.570	3	2	12	2	5	0.364
7	(0.333, 0.333, 0.000)	5.441	4	3	2	2	2	1.000
8	(0.269, 0.269, 0.000)	6.305	5	4	6	2	3	0.194
9	(0.278, 0.278, 0.000)	6.319	6	5	6	2	3	0.167
10	(0.379, 0.243, 0.000)	6.351	6	5	6	2	2	0.729

The followings are details for RhZn. It has 6 topological nodal lines and 6 nodal ring points along high symmetry line as shown in Fig 245.

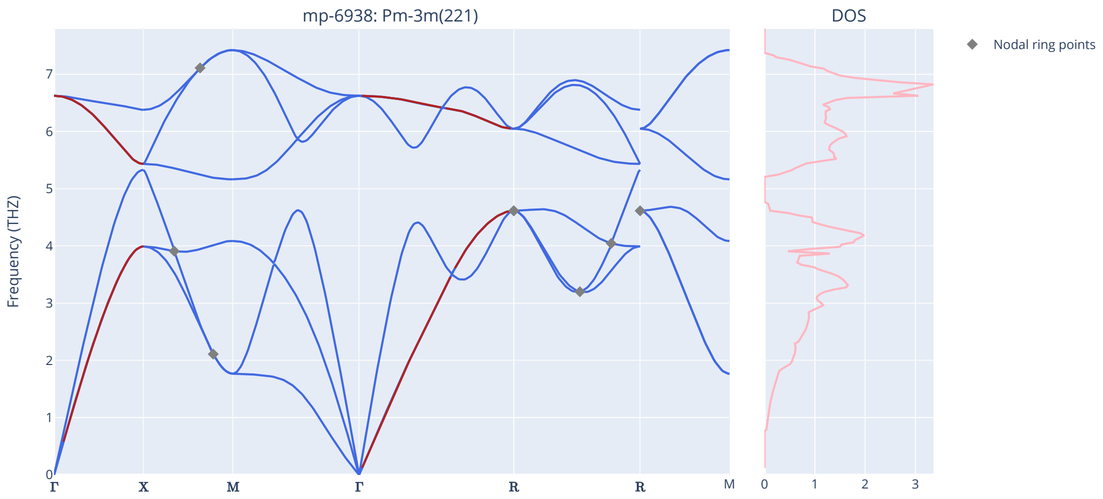

Supplementary Figure 245: The phonon band and DOS of RhZn.

Supplementary Table 462: High symmetry lines of mp-6938-RhZn

	Start	End
0	Γ (0.000, 0.000, 0.000)	X (0.000, 0.500, 0.000)
1	X (0.000, 0.500, 0.000)	M (0.500, 0.500, 0.000)
2	M (0.500, 0.500, 0.000)	Γ (0.000, 0.000, 0.000)
3	Γ (0.000, 0.000, 0.000)	R (0.500, 0.500, 0.500)
4	R (0.500, 0.500, 0.500)	X (0.000, 0.500, 0.000)
5	R (0.500, 0.500, 0.500)	M (0.500, 0.500, 0.000)

Supplementary Table 463: Nodal ring points along high symmetry lines of mp-6938-RhZn.

	Coordinates	Frequency	Mode 1	Mode 2	Multi.	Deg.	Path	Ratio
0	(0.391, 0.500, -0.000)	2.108	2	1	12	2	2	0.782
1	(0.500, 0.500, 0.500)	4.613	2	1	1	3	4	1.000
2	(0.238, 0.500, 0.238)	3.199	2	1	12	2	5	0.523
3	(0.173, 0.500, -0.000)	3.908	3	2	12	2	2	0.345
4	(0.114, 0.500, 0.114)	4.044	3	2	12	2	5	0.771
5	(0.317, 0.500, 0.000)	7.110	6	5	12	2	2	0.635

The followings are details for HgLa. It has 5 topological nodal lines and 5 nodal ring points along high symmetry line as shown in Fig **246**.

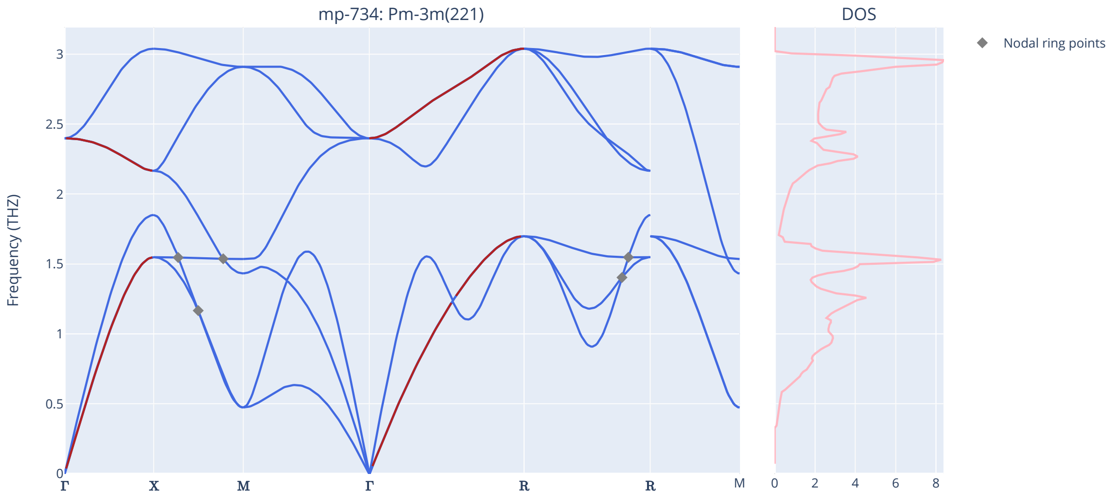

Supplementary Figure 246: The phonon band and DOS of HgLa.

Supplementary Table 464: High symmetry lines of mp-734-HgLa

	Start	End
0	Γ (0.000, 0.000, 0.000)	X (0.000, 0.500, 0.000)
1	X (0.000, 0.500, 0.000)	M (0.500, 0.500, 0.000)
2	M (0.500, 0.500, 0.000)	Γ (0.000, 0.000, 0.000)
3	Γ (0.000, 0.000, 0.000)	R (0.500, 0.500, 0.500)
4	R (0.500, 0.500, 0.500)	X (0.000, 0.500, 0.000)
5	R (0.500, 0.500, 0.500)	M (0.500, 0.500, 0.000)

Supplementary Table 465: Nodal ring points along high symmetry lines of mp-734-HgLa.

	Coordinates	Frequency	Mode 1	Mode 2	Multi.	Deg.	Path	Ratio
0	(0.249, 0.500, -0.000)	1.166	2	1	12	2	2	0.497
1	(0.112, 0.500, 0.112)	1.403	2	1	12	2	5	0.776
2	(0.136, 0.500, -0.000)	1.546	3	2	12	2	2	0.273
3	(0.087, 0.500, 0.087)	1.548	3	2	12	2	5	0.827
4	(0.388, 0.500, -0.000)	1.537	4	3	12	2	2	0.777

The followings are details for CdLa. It has 5 topological nodal lines and 3 nodal ring points along high symmetry line as shown in Fig 247.

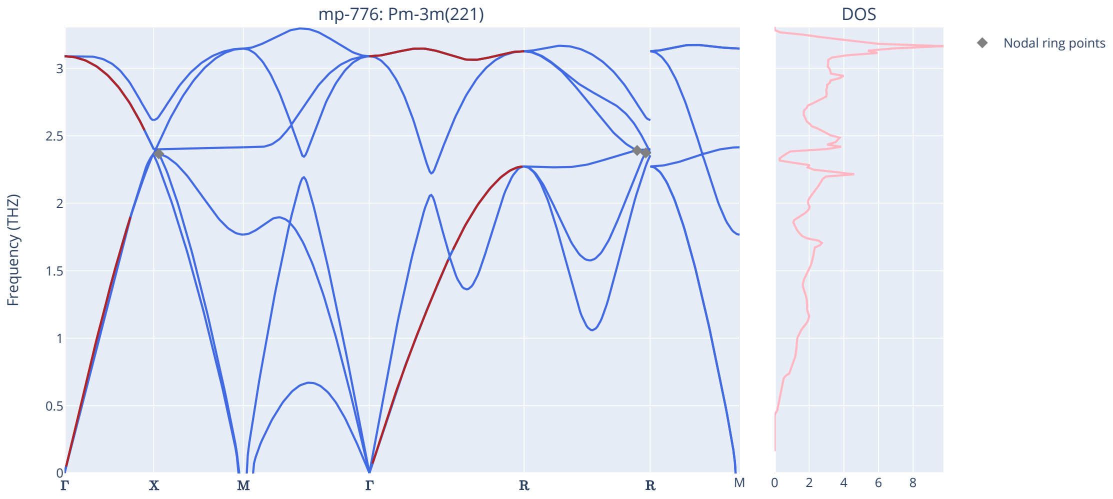

Supplementary Figure 247: The phonon band and DOS of CdLa.

Supplementary Table 466: High symmetry lines of mp-776-CdLa

	Start	End
0	Γ (0.000, 0.000, 0.000)	X (0.000, 0.500, 0.000)
1	X (0.000, 0.500, 0.000)	M (0.500, 0.500, 0.000)
2	M (0.500, 0.500, 0.000)	Γ (0.000, 0.000, 0.000)
3	Γ (0.000, 0.000, 0.000)	R (0.500, 0.500, 0.500)
4	R (0.500, 0.500, 0.500)	X (0.000, 0.500, 0.000)
5	R (0.500, 0.500, 0.500)	M (0.500, 0.500, 0.000)

Supplementary Table 467: Nodal ring points along high symmetry lines of mp-776-CdLa.

	Coordinates	Frequency	Mode 1	Mode 2	Multi.	Deg.	Path	Ratio
0	(0.028, 0.500, 0.000)	2.367	3	2	12	2	2	0.056
1	(0.018, 0.500, 0.018)	2.374	3	2	12	2	5	0.964
2	(0.052, 0.500, 0.052)	2.392	4	3	12	2	5	0.896

The followings are details for Re_2. It has 12 topological nodal lines and 10 nodal ring points along high symmetry line as shown in Fig **248**.

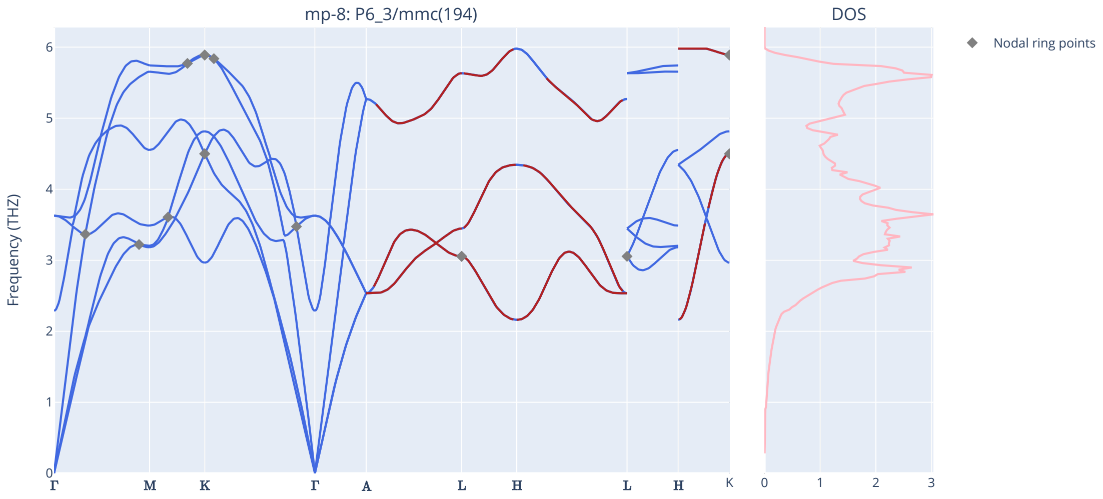

Supplementary Figure 248: The phonon band and DOS of Re2.

Supplementary Table 468: High symmetry lines of mp-8-Re2

	Start	End
0	Γ (0.000, 0.000, 0.000)	M (0.500, 0.000, 0.000)
1	M (0.500, 0.000, 0.000)	K (0.333, 0.333, 0.000)
2	K (0.333, 0.333, 0.000)	Γ (0.000, 0.000, 0.000)
3	Γ (0.000, 0.000, 0.000)	A (0.000, 0.000, 0.500)
4	A (0.000, 0.000, 0.500)	L (0.500, 0.000, 0.500)
5	L (0.500, 0.000, 0.500)	H (0.333, 0.333, 0.500)
6	H (0.333, 0.333, 0.500)	A (0.000, 0.000, 0.500)
7	L (0.500, 0.000, 0.500)	M (0.500, 0.000, 0.000)
8	H (0.333, 0.333, 0.500)	K (0.333, 0.333, 0.000)

Supplementary Table 469: Nodal ring points along high symmetry lines of mp-8-Re2.

	Coordinates	Frequency	Mode 1	Mode 2	Multi.	Deg.	Path	Ratio
0	(0.445, -0.001, 0.000)	3.220	2	1	10	2	1	0.890
1	(0.500, 0.000, 0.500)	3.054	2	1	3	2	5	1.000
2	(0.444, 0.111, 0.000)	3.606	3	2	6	2	2	0.334
3	(0.333, 0.333, 0.000)	4.497	3	2	2	2	2	1.000
4	(0.164, 0.000, 0.000)	3.365	4	3	6	2	1	0.328
5	(0.056, 0.056, -0.000)	3.473	5	4	6	2	3	0.832
6	(0.306, 0.306, -0.000)	5.836	6	5	6	2	3	0.083
7	(0.385, 0.229, 0.000)	5.770	6	5	6	2	2	0.687
8	(0.333, 0.333, 0.000)	5.888	6	5	2	2	2	1.000

The followings are details for C_4Si_4 and it only has 2 topological nodal lines along high symmetry line as shown in Fig **249**.

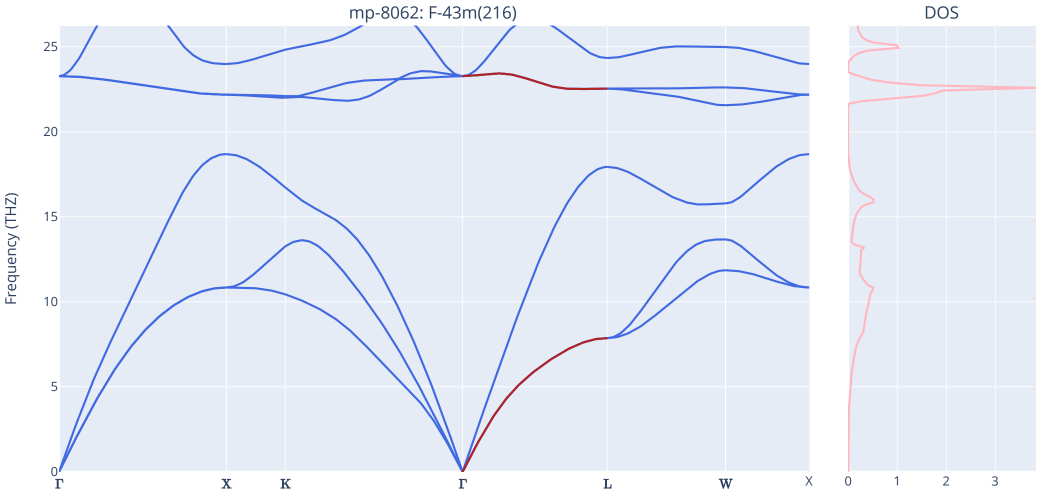

Supplementary Figure 249: The phonon band and DOS of C4Si4.

Supplementary Table 470: High symmetry lines of mp-8062-C4Si4

	Start	End
0	Γ (0.000, 0.000, 0.000)	X (0.500, 0.000, 0.500)
1	X (0.500, 0.000, 0.500)	U (0.625, 0.250, 0.625)
2	K (0.375, 0.375, 0.750)	Γ (0.000, 0.000, 0.000)
3	Γ (0.000, 0.000, 0.000)	L (0.500, 0.500, 0.500)
4	L (0.500, 0.500, 0.500)	W (0.500, 0.250, 0.750)
5	W (0.500, 0.250, 0.750)	X (0.500, 0.000, 0.500)

The followings are details for Hg_4Se_4. It has 2 topological nodal lines and 1 nodal ring point along high symmetry line as shown in Fig **250**.

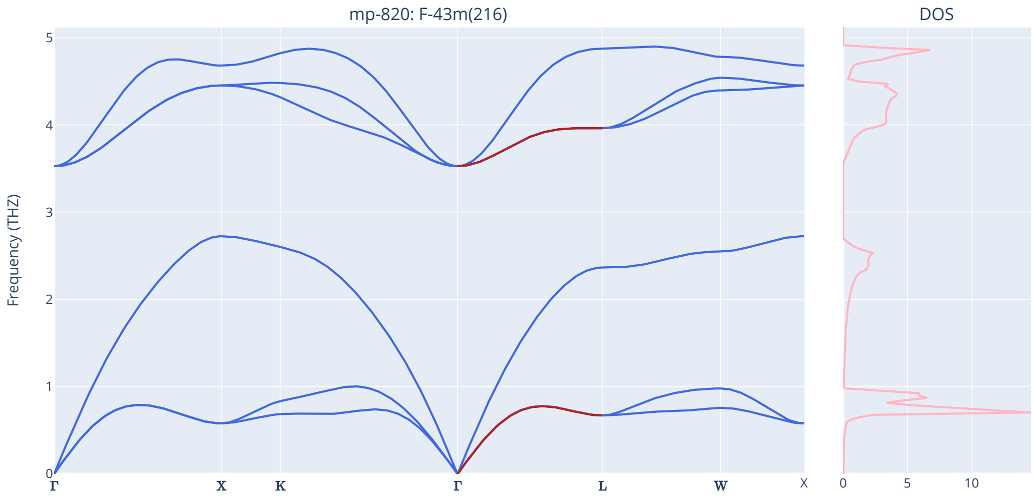

Supplementary Figure 250: The phonon band and DOS of Hg4Se4.

Supplementary Table 471: High symmetry lines of mp-820-Hg4Se4

	Start	End
0	Γ (0.000, 0.000, 0.000)	X (0.500, 0.000, 0.500)
1	X (0.500, 0.000, 0.500)	U (0.625, 0.250, 0.625)
2	K (0.375, 0.375, 0.750)	Γ (0.000, 0.000, 0.000)
3	Γ (0.000, 0.000, 0.000)	L (0.500, 0.500, 0.500)
4	L (0.500, 0.500, 0.500)	W (0.500, 0.250, 0.750)
5	W (0.500, 0.250, 0.750)	X (0.500, 0.000, 0.500)

The followings are details for CoTi. It has 6 topological nodal lines and 4 nodal ring points along high symmetry line as shown in Fig 251.

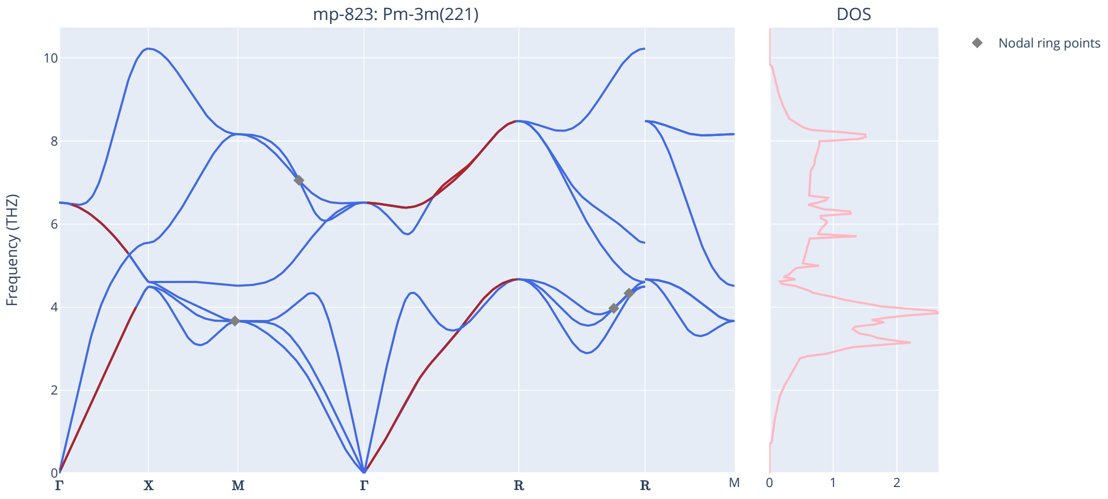

Supplementary Figure 251: The phonon band and DOS of CoTi.

Supplementary Table 472: High symmetry lines of mp-823-CoTi

	Start	End
0	Γ (0.000, 0.000, 0.000)	X (0.000, 0.500, 0.000)
1	X (0.000, 0.500, 0.000)	M (0.500, 0.500, 0.000)
2	M (0.500, 0.500, 0.000)	Γ (0.000, 0.000, 0.000)
3	Γ (0.000, 0.000, 0.000)	R (0.500, 0.500, 0.500)
4	R (0.500, 0.500, 0.500)	X (0.000, 0.500, 0.000)
5	R (0.500, 0.500, 0.500)	M (0.500, 0.500, 0.000)

Supplementary Table 473: Nodal ring points along high symmetry lines of mp-823-CoTi.

	Coordinates	Frequency	Mode 1	Mode 2	Multi.	Deg.	Path	Ratio
0	(0.484, 0.500, -0.000)	3.666	3	2	12	2	2	0.968
1	(0.064, 0.500, 0.063)	4.334	3	2	24	2	5	0.873
2	(0.124, 0.500, 0.124)	3.972	3	2	12	2	5	0.752
3	(0.258, 0.258, -0.000)	7.053	6	5	12	2	3	0.485

The followings are details for Ga_4N_4 and it only has 2 topological nodal lines along high symmetry line as shown in Fig **252**.

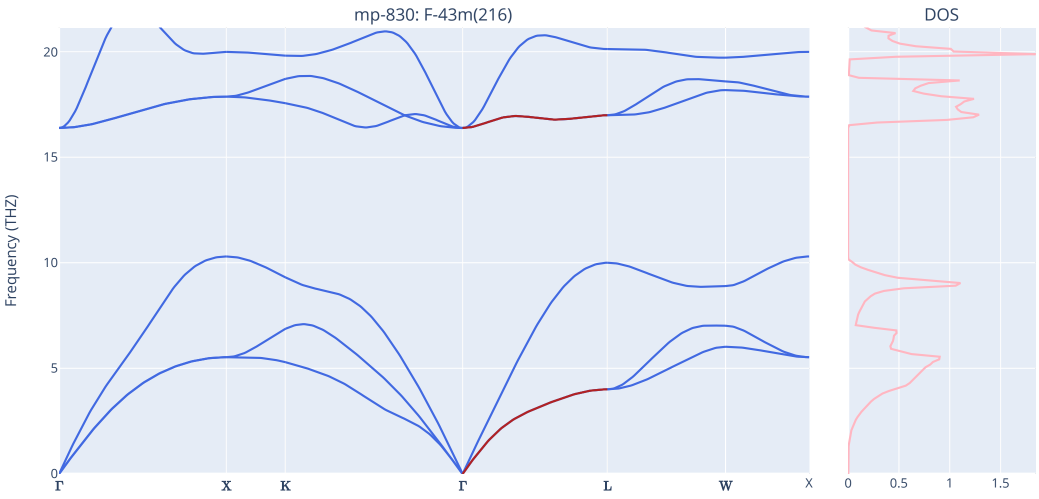

Supplementary Figure 252: The phonon band and DOS of Ga4N4.

Supplementary Table 474: High symmetry lines of mp-830-Ga4N4

	Start	End
0	Γ (0.000, 0.000, 0.000)	X (0.500, 0.000, 0.500)
1	X (0.500, 0.000, 0.500)	U (0.625, 0.250, 0.625)
2	K (0.375, 0.375, 0.750)	Γ (0.000, 0.000, 0.000)
3	Γ (0.000, 0.000, 0.000)	L (0.500, 0.500, 0.500)
4	L (0.500, 0.500, 0.500)	W (0.500, 0.250, 0.750)
5	W (0.500, 0.250, 0.750)	X (0.500, 0.000, 0.500)

The followings are details for AcAg. It has 6 topological nodal lines and 6 nodal ring points along high symmetry line as shown in Fig 253.

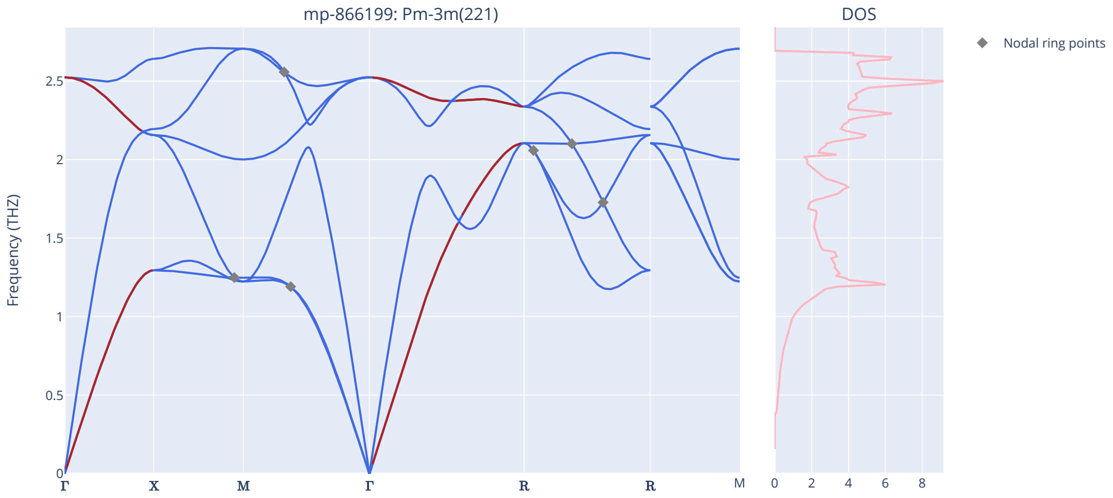

Supplementary Figure 253: The phonon band and DOS of AcAg.

Supplementary Table 475: High symmetry lines of mp-866199-AcAg

	Start	End
0	Γ (0.000, 0.000, 0.000)	X (0.000, 0.500, 0.000)
1	X (0.000, 0.500, 0.000)	M (0.500, 0.500, 0.000)
2	M (0.500, 0.500, 0.000)	Γ (0.000, 0.000, 0.000)
3	Γ (0.000, 0.000, 0.000)	R (0.500, 0.500, 0.500)
4	R (0.500, 0.500, 0.500)	X (0.000, 0.500, 0.000)
5	R (0.500, 0.500, 0.500)	M (0.500, 0.500, 0.000)

Supplementary Table 476: Nodal ring points along high symmetry lines of mp-866199-AcAg.

	Coordinates	Frequency	Mode 1	Mode 2	Multi.	Deg.	Path	Ratio
0	(0.312, 0.312, -0.000)	1.191	2	1	12	2	3	0.377
1	(0.463, 0.500, 0.463)	2.058	2	1	12	2	5	0.075
2	(0.451, 0.500, -0.000)	1.249	3	2	12	2	2	0.901
3	(0.187, 0.500, 0.187)	1.727	3	2	12	2	5	0.626
4	(0.314, 0.500, 0.307)	2.101	4	3	24	2	5	0.379
5	(0.338, 0.338, -0.000)	2.558	6	5	12	2	3	0.325

The followings are details for AcMg. It has 6 topological nodal lines and 1 nodal ring point along high symmetry line as shown in Fig 254.

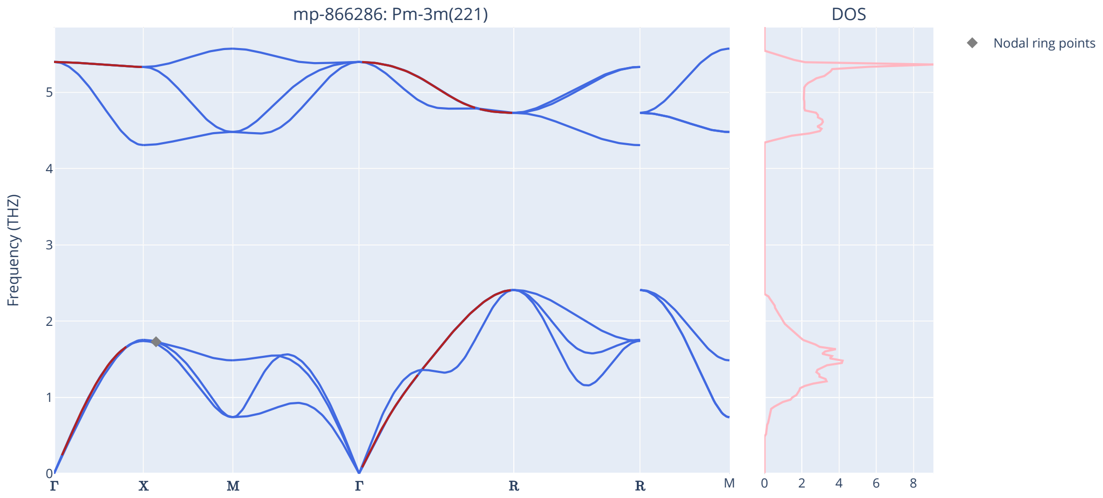

Supplementary Figure 254: The phonon band and DOS of AcMg.

Supplementary Table 477: High symmetry lines of mp-866286-AcMg

	Start	End
0	Γ (0.000, 0.000, 0.000)	X (0.000, 0.500, 0.000)
1	X (0.000, 0.500, 0.000)	M (0.500, 0.500, 0.000)
2	M (0.500, 0.500, 0.000)	Γ (0.000, 0.000, 0.000)
3	Γ (0.000, 0.000, 0.000)	R (0.500, 0.500, 0.500)
4	R (0.500, 0.500, 0.500)	X (0.000, 0.500, 0.000)
5	R (0.500, 0.500, 0.500)	M (0.500, 0.500, 0.000)

Supplementary Table 478: Nodal ring points along high symmetry lines of mp-866286-AcMg.

	Coordinates	Frequency	Mode 1	Mode 2	Multi.	Deg.	Path	Ratio
0	(0.071, 0.500, -0.000)	1.730	3	2	12	2	2	0.141

The followings are details for Ag_4Br_4. It has 2 topological nodal lines and 1 nodal ring point along high symmetry line as shown in Fig **255**.

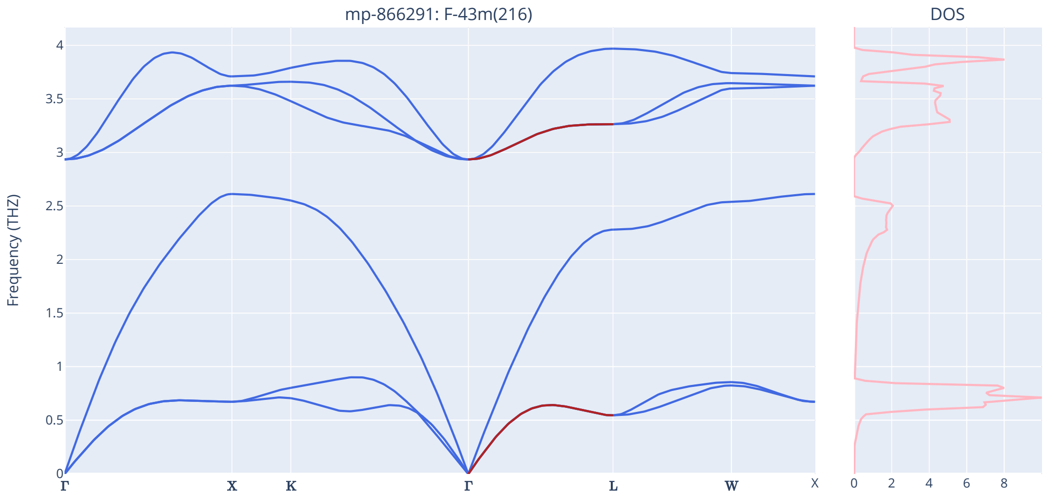

Supplementary Figure 255: The phonon band and DOS of Ag4Br4.

Supplementary Table 479: High symmetry lines of mp-866291-Ag4Br4

	Start	End
0	Γ (0.000, 0.000, 0.000)	X (0.500, 0.000, 0.500)
1	X (0.500, 0.000, 0.500)	U (0.625, 0.250, 0.625)
2	K (0.375, 0.375, 0.750)	Γ (0.000, 0.000, 0.000)
3	Γ (0.000, 0.000, 0.000)	L (0.500, 0.500, 0.500)
4	L (0.500, 0.500, 0.500)	W (0.500, 0.250, 0.750)
5	W (0.500, 0.250, 0.750)	X (0.500, 0.000, 0.500)

The followings are details for Be_2. It has 12 topological nodal lines and 16 nodal ring points along high symmetry line as shown in Fig 256.

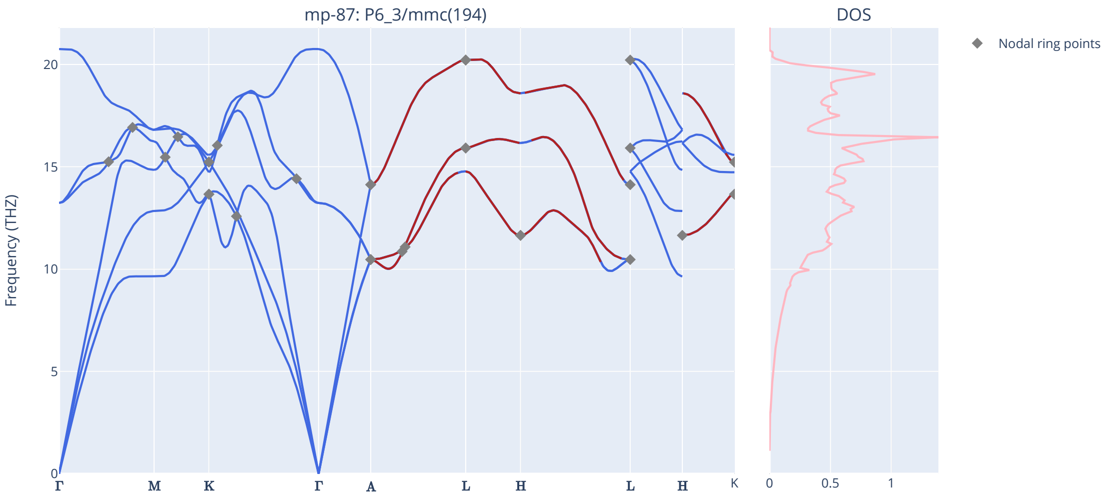

Supplementary Figure 256: The phonon band and DOS of Be2.

Supplementary Table 480: High symmetry lines of mp-87-Be2

	Start	End
0	Γ (0.000, 0.000, 0.000)	M (0.500, 0.000, 0.000)
1	M (0.500, 0.000, 0.000)	K (0.333, 0.333, 0.000)
2	K (0.333, 0.333, 0.000)	Γ (0.000, 0.000, 0.000)
3	Γ (0.000, 0.000, 0.000)	A (0.000, 0.000, 0.500)
4	A (0.000, 0.000, 0.500)	L (0.500, 0.000, 0.500)
5	L (0.500, 0.000, 0.500)	H (0.333, 0.333, 0.500)
6	H (0.333, 0.333, 0.500)	A (0.000, 0.000, 0.500)
7	L (0.500, 0.000, 0.500)	M (0.500, 0.000, 0.000)
8	H (0.333, 0.333, 0.500)	K (0.333, 0.333, 0.000)

Supplementary Table 481: Nodal ring points along high symmetry lines of mp-87-Be2.

	Coordinates	Frequency	Mode 1	Mode 2	Multi.	Deg.	Path	Ratio
0	(0.250, 0.250, -0.000)	12.582	2	1	6	2	3	0.250
1	(0.333, 0.333, 0.000)	13.660	2	1	2	2	2	1.000
2	(0.000, 0.000, 0.500)	10.468	2	1	1	4	4	1.000
3	(0.333, 0.333, 0.500)	11.649	2	1	2	2	6	1.000
4	(0.165, 0.000, 0.496)	10.860	3	2	12	2	5	0.331
5	(0.181, 0.000, 0.496)	11.085	3	2	12	2	5	0.362
6	(0.466, 0.068, 0.000)	15.470	4	3	6	2	2	0.203
7	(0.500, 0.000, 0.500)	15.918	4	3	3	2	5	1.000
8	(0.067, 0.067, 0.000)	14.417	5	4	6	2	3	0.798
9	(0.261, 0.000, 0.000)	15.247	5	4	6	2	1	0.521
10	(0.428, 0.145, -0.000)	16.464	5	4	6	2	2	0.434

Supplementary Table 481: Nodal ring points along high symmetry lines of mp-87-Be2.

	Coordinates	Frequency	Mode 1	Mode 2	Multi.	Deg.	Path	Ratio
11	(0.387, -0.000, -0.000)	16.924	5	4	6	2	1	0.773
12	(0.333, 0.333, 0.000)	15.231	5	4	2	2	2	1.000
13	(0.308, 0.308, 0.000)	16.045	6	5	6	2	3	0.076
14	(0.000, 0.000, 0.500)	14.126	6	5	1	2	4	1.000
15	(0.500, 0.000, 0.500)	20.217	6	5	3	2	5	1.000

The followings are details for PtSc. It has 6 topological nodal lines and 2 nodal ring points along high symmetry line as shown in Fig 257.

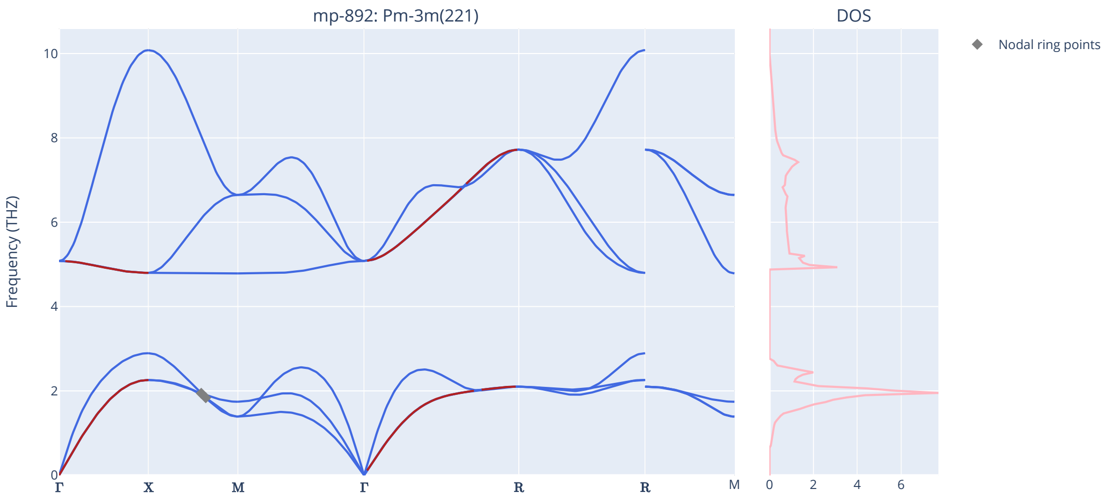

Supplementary Figure 257: The phonon band and DOS of PtSc.

Supplementary Table 482: High symmetry lines of mp-892-PtSc

	Start	End
0	Γ (0.000, 0.000, 0.000)	X (0.000, 0.500, 0.000)
1	X (0.000, 0.500, 0.000)	M (0.500, 0.500, 0.000)
2	M (0.500, 0.500, 0.000)	Γ (0.000, 0.000, 0.000)
3	Γ (0.000, 0.000, 0.000)	R (0.500, 0.500, 0.500)
4	R (0.500, 0.500, 0.500)	X (0.000, 0.500, 0.000)
5	R (0.500, 0.500, 0.500)	M (0.500, 0.500, 0.000)

Supplementary Table 483: Nodal ring points along high symmetry lines of mp-892-PtSc.

	Coordinates	Frequency	Mode 1	Mode 2	Multi.	Deg.	Path	Ratio
0	(0.321, 0.500, 0.000)	1.838	2	1	12	2	2	0.642
1	(0.296, 0.500, -0.000)	1.944	3	2	12	2	2	0.591

The followings are details for InRh. It has 6 topological nodal lines and 6 nodal ring points along high symmetry line as shown in Fig 258.

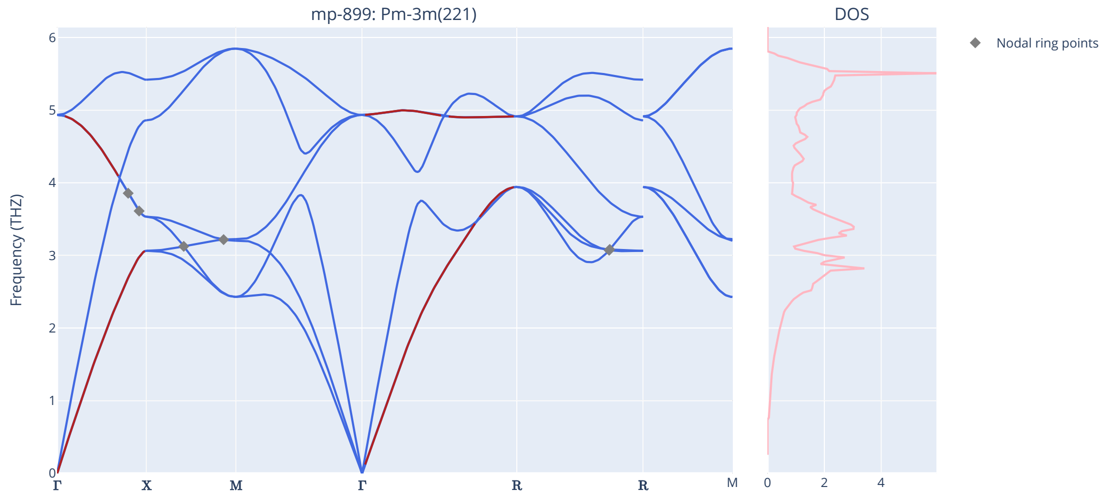

Supplementary Figure 258: The phonon band and DOS of InRh.

Supplementary Table 484: High symmetry lines of mp-899-InRh

	Start	End
0	Γ (0.000, 0.000, 0.000)	X (0.000, 0.500, 0.000)
1	X (0.000, 0.500, 0.000)	M (0.500, 0.500, 0.000)
2	M (0.500, 0.500, 0.000)	Γ (0.000, 0.000, 0.000)
3	Γ (0.000, 0.000, 0.000)	R (0.500, 0.500, 0.500)
4	R (0.500, 0.500, 0.500)	X (0.000, 0.500, 0.000)
5	R (0.500, 0.500, 0.500)	M (0.500, 0.500, 0.000)

Supplementary Table 485: Nodal ring points along high symmetry lines of mp-899-InRh.

	Coordinates	Frequency	Mode 1	Mode 2	Multi.	Deg.	Path	Ratio
0	(0.135, 0.500, 0.135)	3.071	2	1	12	2	5	0.731
1	(0.209, 0.500, -0.000)	3.124	3	2	12	2	2	0.418
2	(0.132, 0.500, 0.132)	3.081	3	2	12	2	5	0.736
3	(0.000, 0.398, 0.000)	3.855	4	3	6	2	1	0.796
4	(0.000, 0.459, 0.000)	3.611	4	3	6	2	1	0.918
5	(0.432, 0.500, 0.000)	3.217	4	3	12	2	2	0.864

The followings are details for BaZn. It has 6 topological nodal lines and 9 nodal ring points along high symmetry line as shown in Fig 259.

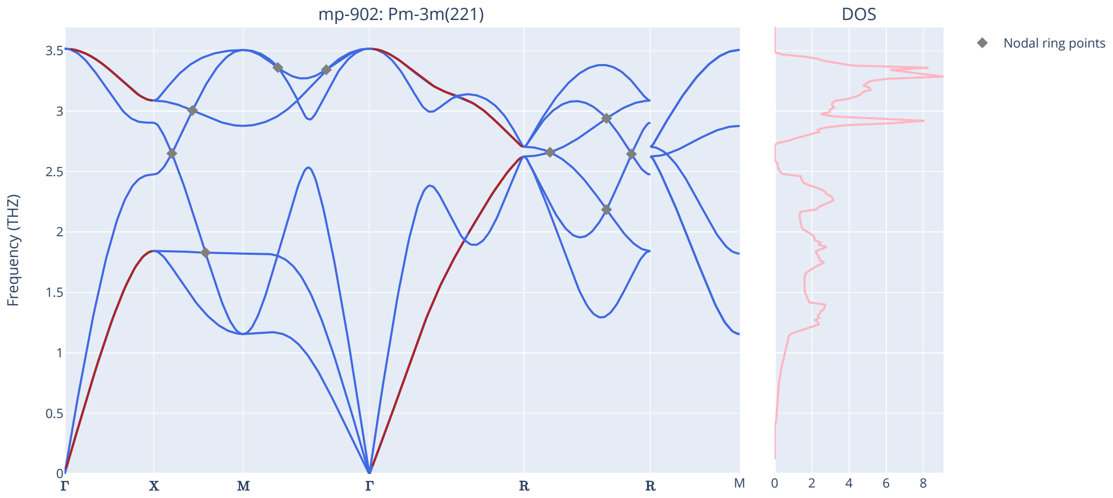

Supplementary Figure 259: The phonon band and DOS of BaZn.

Supplementary Table 486: High symmetry lines of mp-902-BaZn

	Start	End
0	Γ (0.000, 0.000, 0.000)	X (0.000, 0.500, 0.000)
1	X (0.000, 0.500, 0.000)	M (0.500, 0.500, 0.000)
2	M (0.500, 0.500, 0.000)	Γ (0.000, 0.000, 0.000)
3	Γ (0.000, 0.000, 0.000)	R (0.500, 0.500, 0.500)
4	R (0.500, 0.500, 0.500)	X (0.000, 0.500, 0.000)
5	R (0.500, 0.500, 0.500)	M (0.500, 0.500, 0.000)

Supplementary Table 487: Nodal ring points along high symmetry lines of mp-902-BaZn.

	Coordinates	Frequency	Mode 1	Mode 2	Multi.	Deg.	Path	Ratio
0	(0.289, 0.500, 0.000)	1.830	3	2	12	2	2	0.578
1	(0.173, 0.500, 0.173)	2.185	3	2	12	2	5	0.653
2	(0.072, 0.500, 0.077)	2.645	4	3	24	2	5	0.851
3	(0.101, 0.500, -0.000)	2.650	4	3	12	2	2	0.202
4	(0.397, 0.500, 0.397)	2.659	4	3	12	2	5	0.206
5	(0.217, 0.500, -0.000)	3.006	5	4	12	2	2	0.433
6	(0.173, 0.500, 0.173)	2.940	5	4	12	2	5	0.653
7	(0.363, 0.363, 0.000)	3.360	6	5	12	2	3	0.275
8	(0.171, 0.171, 0.000)	3.342	6	5	12	2	3	0.659

The followings are details for CdY. It has 6 topological nodal lines and 2 nodal ring points along high symmetry line as shown in Fig 260.

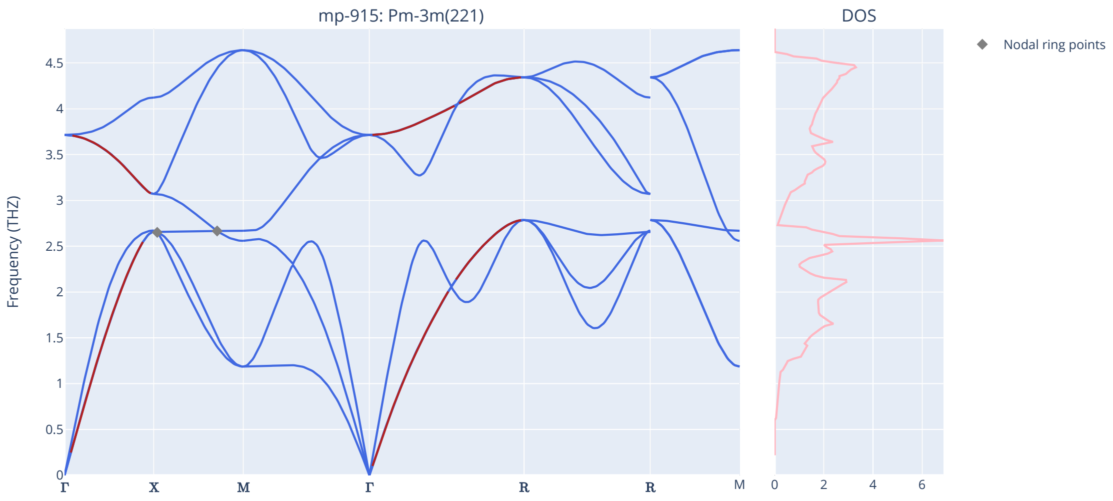

Supplementary Figure 260: The phonon band and DOS of CdY.

Supplementary Table 488: High symmetry lines of mp-915-CdY

	Start	End
0	Γ (0.000, 0.000, 0.000)	X (0.000, 0.500, 0.000)
1	X (0.000, 0.500, 0.000)	M (0.500, 0.500, 0.000)
2	M (0.500, 0.500, 0.000)	Γ (0.000, 0.000, 0.000)
3	Γ (0.000, 0.000, 0.000)	R (0.500, 0.500, 0.500)
4	R (0.500, 0.500, 0.500)	X (0.000, 0.500, 0.000)
5	R (0.500, 0.500, 0.500)	M (0.500, 0.500, 0.000)

Supplementary Table 489: Nodal ring points along high symmetry lines of mp-915-CdY.

	Coordinates	Frequency	Mode 1	Mode 2	Multi.	Deg.	Path	Ratio
0	(0.019, 0.500, 0.000)	2.651	2	1	12	2	2	0.038
1	(0.355, 0.500, 0.000)	2.666	4	3	12	2	2	0.709

The followings are details for Th_4P_4. It has 2 topological nodal lines and 4 nodal ring points along high symmetry line as shown in Fig **261**.

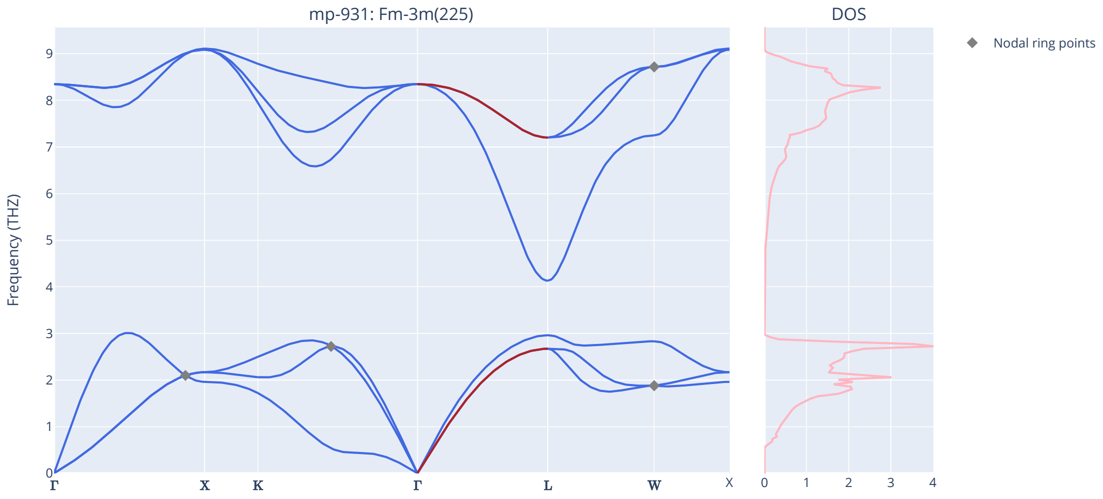

Supplementary Figure 261: The phonon band and DOS of Th4P4.

Supplementary Table 490: High symmetry lines of mp-931-Th4P4

	Start	End
0	Γ (0.000, 0.000, 0.000)	X (0.500, 0.000, 0.500)
1	X (0.500, 0.000, 0.500)	U (0.625, 0.250, 0.625)
2	K (0.375, 0.375, 0.750)	Γ (0.000, 0.000, 0.000)
3	Γ (0.000, 0.000, 0.000)	L (0.500, 0.500, 0.500)
4	L (0.500, 0.500, 0.500)	W (0.500, 0.250, 0.750)
5	W (0.500, 0.250, 0.750)	X (0.500, 0.000, 0.500)

Supplementary Table 491: Nodal ring points along high symmetry lines of mp-931-Th4P4.

	Coordinates	Frequency	Mode 1	Mode 2	Multi.	Deg.	Path	Ratio
0	(0.436, -0.000, 0.436)	2.098	2	1	6	2	1	0.873
1	(0.500, 0.250, 0.750)	1.879	2	1	6	2	5	1.000
2	(0.207, 0.207, 0.402)	2.721	3	2	24	2	3	0.459
3	(0.500, 0.250, 0.750)	8.719	6	5	6	2	5	1.000

The followings are details for Y_4As_4. It has 3 topological nodal lines and 4 nodal ring points along high symmetry line as shown in Fig **262**.

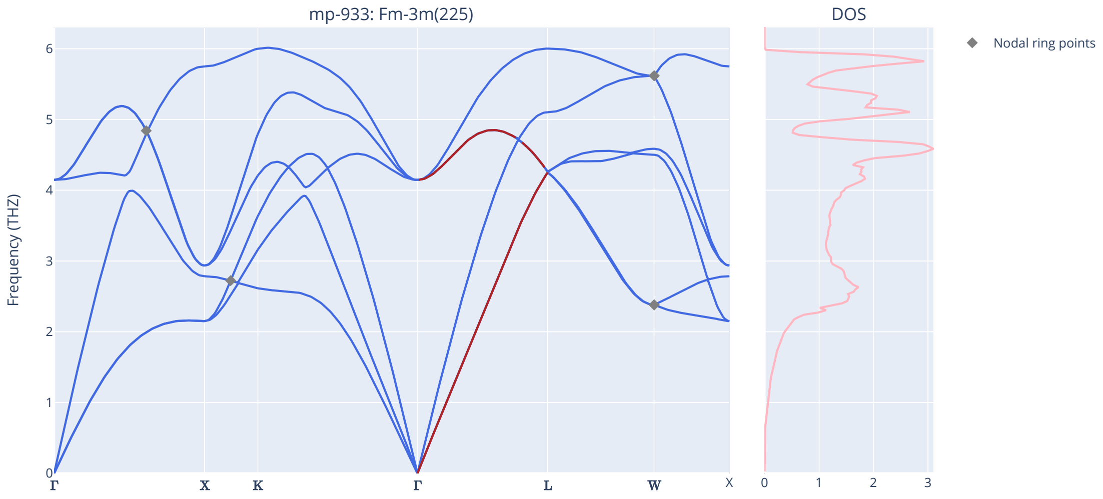

Supplementary Figure 262: The phonon band and DOS of Y4As4.

Supplementary Table 492: High symmetry lines of mp-933-Y4As4

	Start	End
0	Γ (0.000, 0.000, 0.000)	X (0.500, 0.000, 0.500)
1	X (0.500, 0.000, 0.500)	U (0.625, 0.250, 0.625)
2	K (0.375, 0.375, 0.750)	Γ (0.000, 0.000, 0.000)
3	Γ (0.000, 0.000, 0.000)	L (0.500, 0.500, 0.500)
4	L (0.500, 0.500, 0.500)	W (0.500, 0.250, 0.750)
5	W (0.500, 0.250, 0.750)	X (0.500, 0.000, 0.500)

Supplementary Table 493: Nodal ring points along high symmetry lines of mp-933-Y4As4.

	Coordinates	Frequency	Mode 1	Mode 2	Multi.	Deg.	Path	Ratio
0	(0.500, 0.250, 0.750)	2.380	2	1	6	2	5	1.000
1	(0.561, 0.123, 0.561)	2.723	3	2	12	2	2	0.490
2	(0.306, 0.000, 0.306)	4.842	6	5	6	2	1	0.612
3	(0.500, 0.250, 0.750)	5.618	6	5	6	2	5	1.000

The followings are details for Cd_2. It has 12 topological nodal lines and 5 nodal ring points along high symmetry line as shown in Fig **263**.

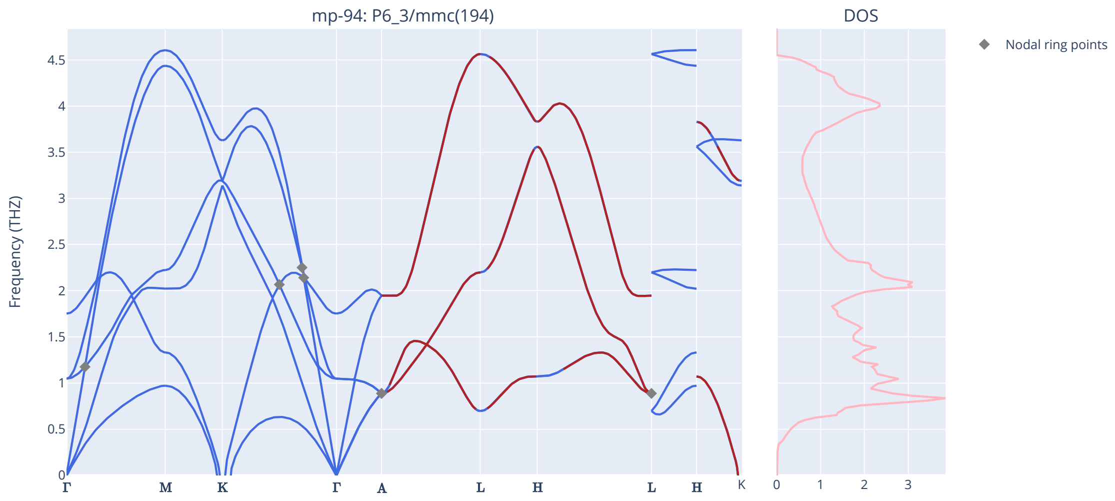

Supplementary Figure 263: The phonon band and DOS of Cd2.

Supplementary Table 494: High symmetry lines of mp-94-Cd2

	Start	End
0	Γ (0.000, 0.000, 0.000)	M (0.500, 0.000, 0.000)
1	M (0.500, 0.000, 0.000)	K (0.333, 0.333, 0.000)
2	K (0.333, 0.333, 0.000)	Γ (0.000, 0.000, 0.000)
3	Γ (0.000, 0.000, 0.000)	A (0.000, 0.000, 0.500)
4	A (0.000, 0.000, 0.500)	L (0.500, 0.000, 0.500)
5	L (0.500, 0.000, 0.500)	H (0.333, 0.333, 0.500)
6	H (0.333, 0.333, 0.500)	A (0.000, 0.000, 0.500)
7	L (0.500, 0.000, 0.500)	M (0.500, 0.000, 0.000)
8	H (0.333, 0.333, 0.500)	K (0.333, 0.333, 0.000)

Supplementary Table 495: Nodal ring points along high symmetry lines of mp-94-Cd2.

	Coordinates	Frequency	Mode 1	Mode 2	Multi.	Deg.	Path	Ratio
0	(0.000, 0.000, 0.500)	0.887	2	1	1	4	4	1.000
1	(0.093, 0.000, 0.000)	1.175	4	3	6	2	1	0.187
2	(0.167, 0.167, -0.000)	2.066	4	3	6	2	3	0.499
3	(0.096, 0.096, 0.000)	2.141	5	4	6	2	3	0.713
4	(0.101, 0.101, 0.000)	2.251	6	5	6	2	3	0.698

The followings are details for IrRu and it only has 1 topological nodal ring point along high symmetry line as shown in Fig 264.

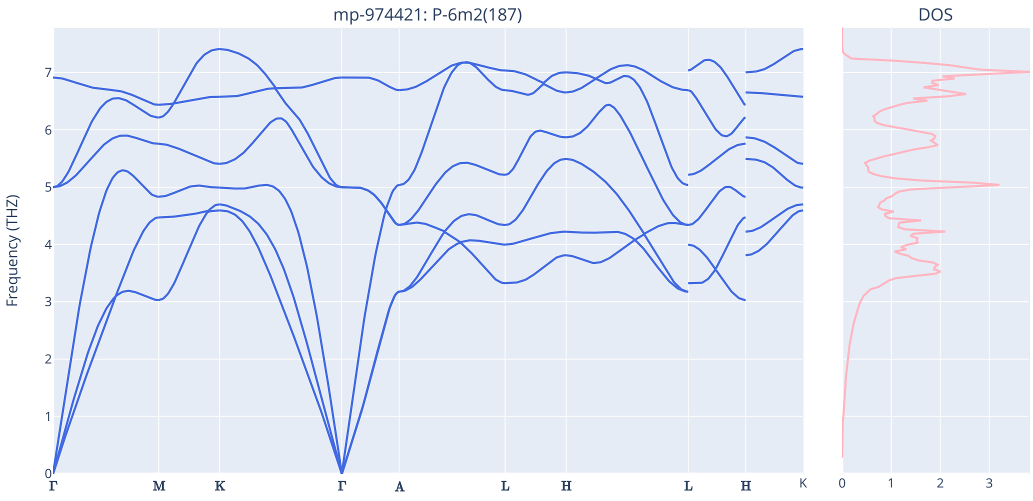

Supplementary Figure 264: The phonon band and DOS of IrRu.

Supplementary Table 496: High symmetry lines of mp-974421-IrRu

	Start	End
0	Γ (0.000, 0.000, 0.000)	M (0.500, 0.000, 0.000)
1	M (0.500, 0.000, 0.000)	K (0.333, 0.333, 0.000)
2	K (0.333, 0.333, 0.000)	Γ (0.000, 0.000, 0.000)
3	Γ (0.000, 0.000, 0.000)	A (0.000, 0.000, 0.500)
4	A (0.000, 0.000, 0.500)	L (0.500, 0.000, 0.500)
5	L (0.500, 0.000, 0.500)	H (0.333, 0.333, 0.500)
6	H (0.333, 0.333, 0.500)	A (0.000, 0.000, 0.500)
7	L (0.500, 0.000, 0.500)	M (0.500, 0.000, 0.000)
8	H (0.333, 0.333, 0.500)	K (0.333, 0.333, 0.000)

The followings are details for HfZr. It has 3 nodal ring points and 4 single Weyl points along high symmetry line as shown in Fig **265**.

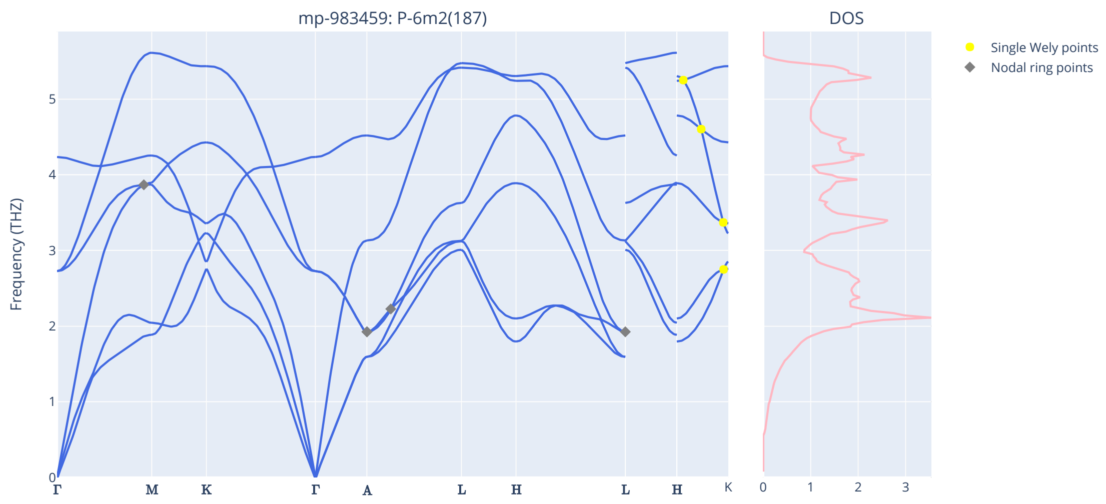

Supplementary Figure 265: The phonon band and DOS of HfZr.

Supplementary Table 497: High symmetry lines of mp-983459-HfZr

	Start	End
0	Γ (0.000, 0.000, 0.000)	M (0.500, 0.000, 0.000)
1	M (0.500, 0.000, 0.000)	K (0.333, 0.333, 0.000)
2	K (0.333, 0.333, 0.000)	Γ (0.000, 0.000, 0.000)
3	Γ (0.000, 0.000, 0.000)	A (0.000, 0.000, 0.500)
4	A (0.000, 0.000, 0.500)	L (0.500, 0.000, 0.500)
5	L (0.500, 0.000, 0.500)	H (0.333, 0.333, 0.500)
6	H (0.333, 0.333, 0.500)	A (0.000, 0.000, 0.500)
7	L (0.500, 0.000, 0.500)	M (0.500, 0.000, 0.000)
8	H (0.333, 0.333, 0.500)	K (0.333, 0.333, 0.000)

Supplementary Table 498: Single Weyl points along high symmetry lines of mp-983459-HfZr.

	Coordinates	Frequency	Mode 1	Mode 2	Multi.	Deg.	Path	Ratio
0	(0.333, 0.333, 0.046)	2.749	2	1	4	2	9	0.908
1	(0.333, 0.333, 0.049)	3.370	4	3	4	2	9	0.901
2	(0.333, 0.333, 0.263)	4.603	5	4	4	2	9	0.473
3	(0.333, 0.333, 0.437)	5.251	6	5	4	2	9	0.125

Supplementary Table 499: Nodal ring points along high symmetry lines of mp-983459-HfZr.

	Coordinates	Frequency	Mode 1	Mode 2	Multi.	Deg.	Path	Ratio
0	(0.459, 0.000, 0.000)	3.867	4	3	6	2	1	0.917

Supplementary Table 499: Nodal ring points along high symmetry lines of mp-983459-HfZr.

	Coordinates	Frequency	Mode 1	Mode 2	Multi.	Deg.	Path	Ratio
1	(0.000, 0.000, 0.500)	1.925	4	3	1	2	4	1.000
2	(0.126, -0.000, 0.500)	2.227	4	3	6	2	5	0.252

The followings are details for Y_4P_4. It has 2 topological nodal lines and 5 nodal ring points along high symmetry line as shown in Fig **266**.

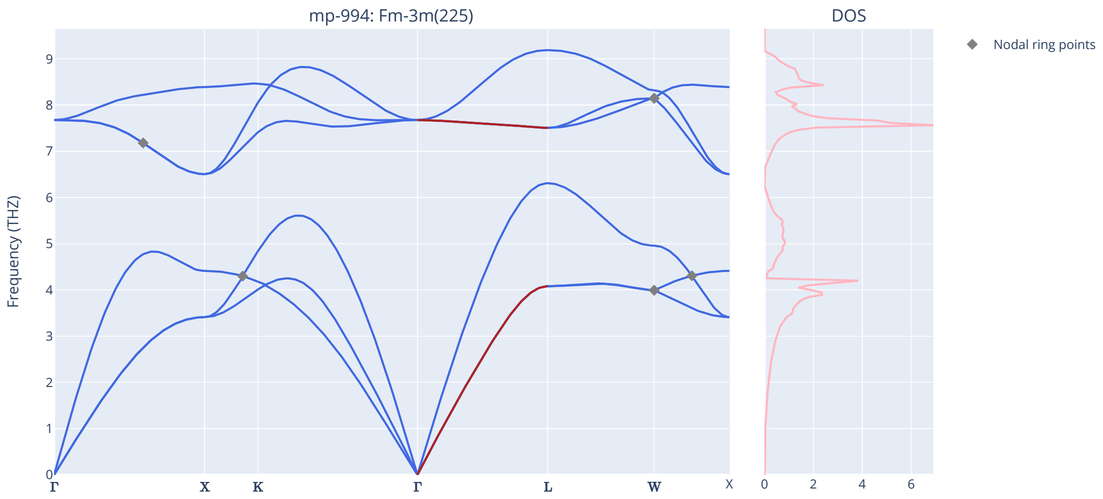

Supplementary Figure 266: The phonon band and DOS of Y4P4.

Supplementary Table 500: High symmetry lines of mp-994-Y4P4

	Start	End
0	Γ (0.000, 0.000, 0.000)	X (0.500, 0.000, 0.500)
1	X (0.500, 0.000, 0.500)	U (0.625, 0.250, 0.625)
2	K (0.375, 0.375, 0.750)	Γ (0.000, 0.000, 0.000)
3	Γ (0.000, 0.000, 0.000)	L (0.500, 0.500, 0.500)
4	L (0.500, 0.500, 0.500)	W (0.500, 0.250, 0.750)
5	W (0.500, 0.250, 0.750)	X (0.500, 0.000, 0.500)

Supplementary Table 501: Nodal ring points along high symmetry lines of mp-994-Y4P4.

	Coordinates	Frequency	Mode 1	Mode 2	Multi.	Deg.	Path	Ratio
0	(0.500, 0.250, 0.750)	3.992	2	1	6	2	5	1.000
1	(0.589, 0.179, 0.589)	4.299	3	2	12	2	2	0.716
2	(0.499, 0.124, 0.625)	4.306	3	2	24	2	6	0.501
3	(0.296, 0.000, 0.296)	7.179	5	4	6	2	1	0.592
4	(0.500, 0.250, 0.750)	8.146	5	4	6	2	5	1.000

The followings are details for OsB. It has 1 nodal ring points and 4 single Weyl point along high symmetry line as shown in Fig 267.

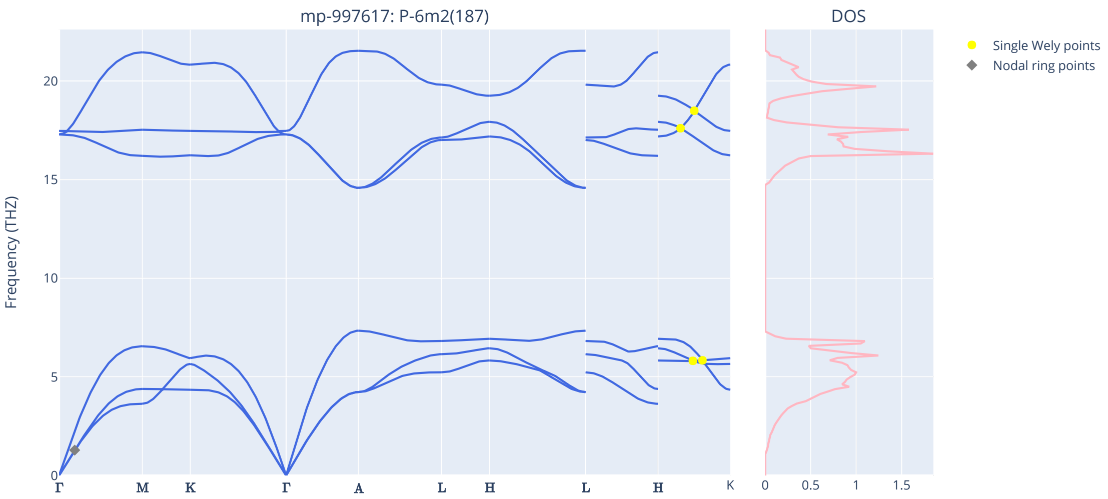

Supplementary Figure 267: The phonon band and DOS of OsB.

Supplementary Table 502: High symmetry lines of mp-997617-OsB

	Start	End
0	Γ (0.000, 0.000, 0.000)	M (0.500, 0.000, 0.000)
1	M (0.500, 0.000, 0.000)	K (0.333, 0.333, 0.000)
2	K (0.333, 0.333, 0.000)	Γ (0.000, 0.000, 0.000)
3	Γ (0.000, 0.000, 0.000)	A (0.000, 0.000, 0.500)
4	A (0.000, 0.000, 0.500)	L (0.500, 0.000, 0.500)
5	L (0.500, 0.000, 0.500)	H (0.333, 0.333, 0.500)
6	H (0.333, 0.333, 0.500)	A (0.000, 0.000, 0.500)
7	L (0.500, 0.000, 0.500)	M (0.500, 0.000, 0.000)
8	H (0.333, 0.333, 0.500)	K (0.333, 0.333, 0.000)

Supplementary Table 503: Single Weyl points along high symmetry lines of mp-997617-OsB.

	Coordinates	Frequency	Mode 1	Mode 2	Multi.	Deg.	Path	Ratio
0	(0.333, 0.333, 0.259)	5.811	2	1	4	2	9	0.482
1	(0.333, 0.333, 0.193)	5.837	3	2	4	2	9	0.615
2	(0.333, 0.333, 0.344)	17.603	5	4	4	2	9	0.312
3	(0.333, 0.333, 0.249)	18.490	6	5	4	2	9	0.502

Supplementary Table 504: Nodal ring points along high symmetry lines of mp-997617-OsB.

	Coordinates	Frequency	Mode 1	Mode 2	Multi.	Deg.	Path	Ratio
0	(0.094, 0.001, 0.000)	1.284	2	1	12	2	1	0.188

Supplementary Table 504: Nodal ring points along high symmetry lines of mp-997617-OsB.

Coordinates	Frequency	Mode 1	Mode 2	Multi.	Deg.	Path	Ratio

The followings are details for Fe_4H_4. It has 2 topological nodal lines and 4 nodal ring points along high symmetry line as shown in Fig 268.

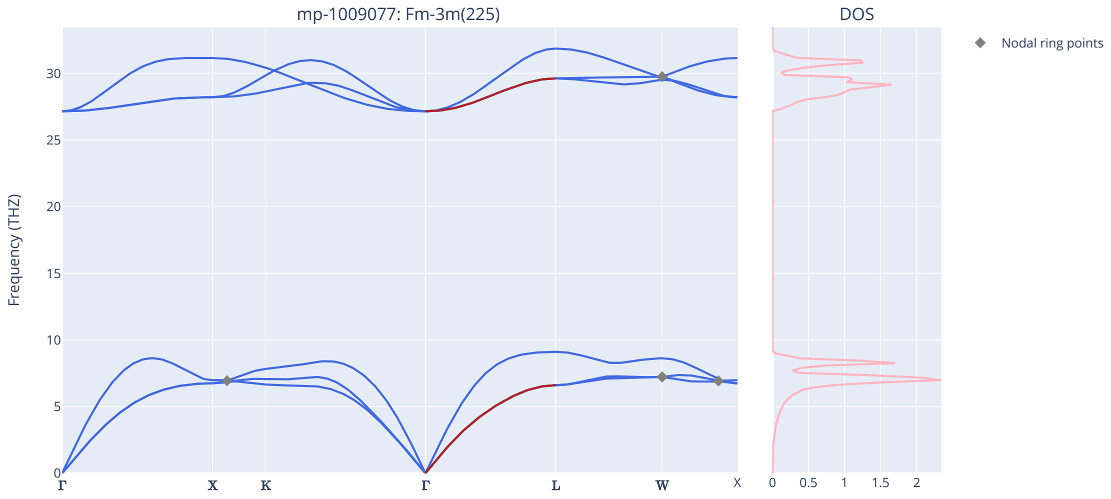

Supplementary Figure 268: The phonon band and DOS of Fe4H4.

Supplementary Table 505: High symmetry lines of mp-1009077-Fe4H4

	Start	End
0	Γ (0.000, 0.000, 0.000)	X (0.500, 0.000, 0.500)
1	X (0.500, 0.000, 0.500)	U (0.625, 0.250, 0.625)
2	K (0.375, 0.375, 0.750)	Γ (0.000, 0.000, 0.000)
3	Γ (0.000, 0.000, 0.000)	L (0.500, 0.500, 0.500)
4	L (0.500, 0.500, 0.500)	W (0.500, 0.250, 0.750)
5	W (0.500, 0.250, 0.750)	X (0.500, 0.000, 0.500)

Supplementary Table 506: Nodal ring points along high symmetry lines of mp-1009077-Fe4H4.

	Coordinates	Frequency	Mode 1	Mode 2	Multi.	Deg.	Path	Ratio
0	(0.500, 0.250, 0.750)	7.226	2	1	6	2	5	1.000
1	(0.498, 0.063, 0.563)	6.926	2	1	48	2	6	0.748
2	(0.534, 0.068, 0.534)	6.945	3	2	12	2	2	0.272
3	(0.500, 0.250, 0.750)	29.747	6	5	6	2	5	1.000

The followings are details for Pa_4N_4. It has 2 topological nodal lines and 3 nodal ring points along high symmetry line as shown in Fig **269**.

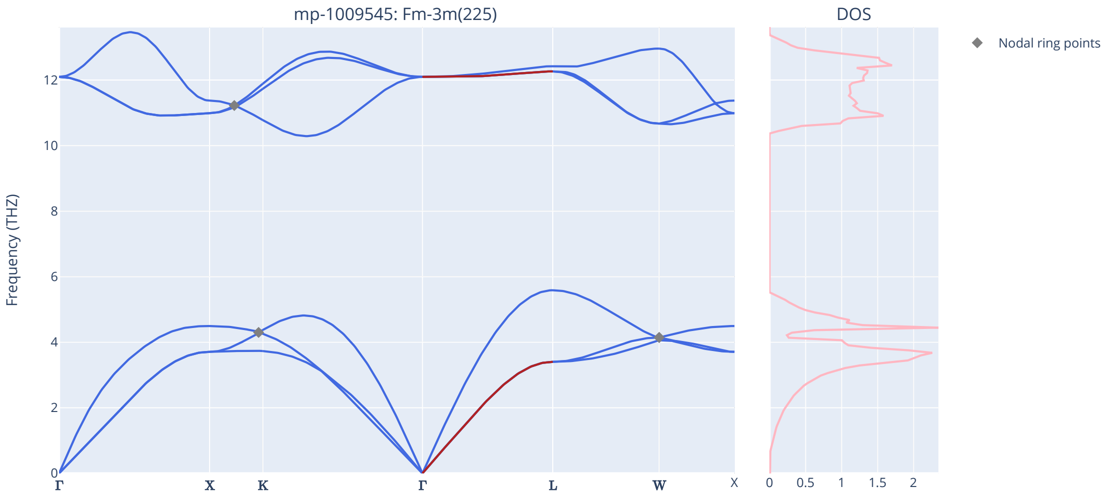

Supplementary Figure 269: The phonon band and DOS of Pa4N4.

Supplementary Table 507: High symmetry lines of mp-1009545-Pa4N4

	Start	End
0	Γ (0.000, 0.000, 0.000)	X (0.500, 0.000, 0.500)
1	X (0.500, 0.000, 0.500)	U (0.625, 0.250, 0.625)
2	K (0.375, 0.375, 0.750)	Γ (0.000, 0.000, 0.000)
3	Γ (0.000, 0.000, 0.000)	L (0.500, 0.500, 0.500)
4	L (0.500, 0.500, 0.500)	W (0.500, 0.250, 0.750)
5	W (0.500, 0.250, 0.750)	X (0.500, 0.000, 0.500)

Supplementary Table 508: Nodal ring points along high symmetry lines of mp-1009545-Pa4N4.

	Coordinates	Frequency	Mode 1	Mode 2	Multi.	Deg.	Path	Ratio
0	(0.615, 0.230, 0.615)	4.298	3	2	12	2	2	0.921
1	(0.500, 0.250, 0.750)	4.144	3	2	6	2	5	1.000
2	(0.558, 0.116, 0.558)	11.222	6	5	12	2	2	0.465

The followings are details for Cs_4H_4. It has 2 topological nodal lines and 5 nodal ring points along high symmetry line as shown in Fig 270.

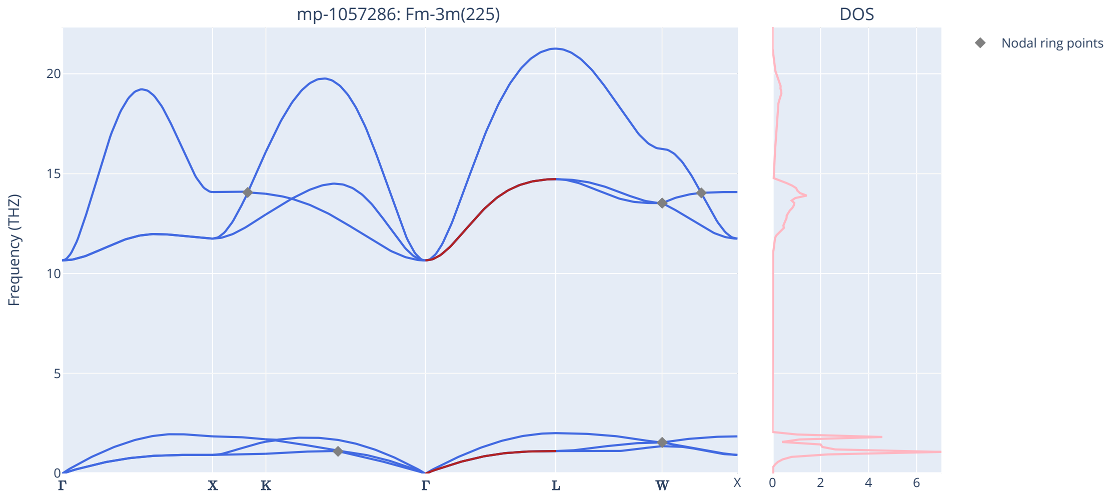

Supplementary Figure 270: The phonon band and DOS of Cs4H4.

Supplementary Table 509: High symmetry lines of mp-1057286-Cs4H4

	Start	End
0	Γ (0.000, 0.000, 0.000)	X (0.500, 0.000, 0.500)
1	X (0.500, 0.000, 0.500)	U (0.625, 0.250, 0.625)
2	K (0.375, 0.375, 0.750)	Γ (0.000, 0.000, 0.000)
3	Γ (0.000, 0.000, 0.000)	L (0.500, 0.500, 0.500)
4	L (0.500, 0.500, 0.500)	W (0.500, 0.250, 0.750)
5	W (0.500, 0.250, 0.750)	X (0.500, 0.000, 0.500)

Supplementary Table 510: Nodal ring points along high symmetry lines of mp-1057286-Cs4H4.

	Coordinates	Frequency	Mode 1	Mode 2	Multi.	Deg.	Path	Ratio
0	(0.207, 0.207, 0.409)	1.096	2	1	24	2	3	0.453
1	(0.500, 0.250, 0.750)	1.543	3	2	6	2	5	1.000
2	(0.500, 0.250, 0.750)	13.525	5	4	6	2	5	1.000
3	(0.583, 0.165, 0.583)	14.059	6	5	12	2	2	0.660
4	(0.499, 0.119, 0.620)	14.036	6	5	24	2	6	0.521

The followings are details for Sn_4Sb_4. It has 3 topological nodal lines and 9 nodal ring points along high symmetry line as shown in Fig 271.

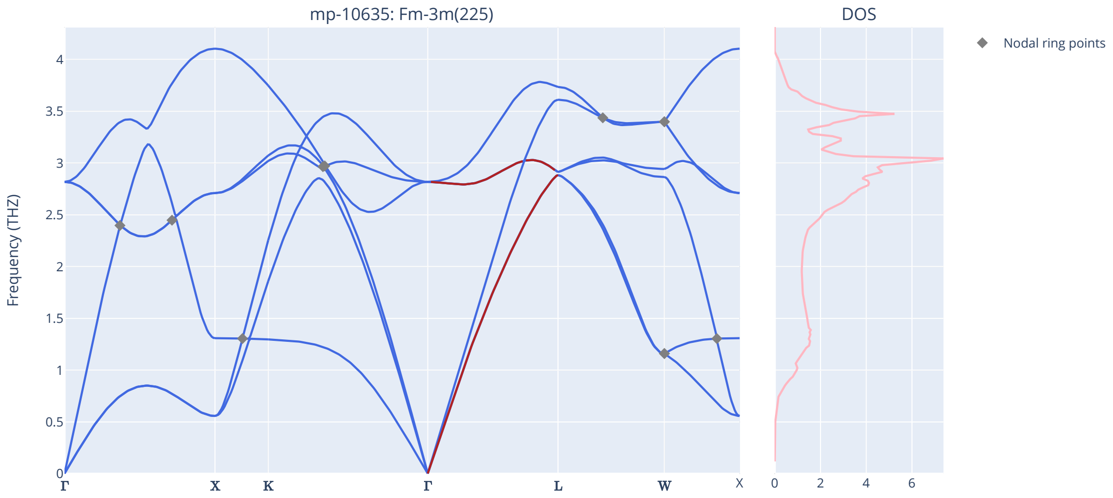

Supplementary Figure 271: The phonon band and DOS of Sn4Sb4.

Supplementary Table 511: High symmetry lines of mp-10635-Sn4Sb4

	Start	End
0	Γ (0.000, 0.000, 0.000)	X (0.500, 0.000, 0.500)
1	X (0.500, 0.000, 0.500)	U (0.625, 0.250, 0.625)
2	K (0.375, 0.375, 0.750)	Γ (0.000, 0.000, 0.000)
3	Γ (0.000, 0.000, 0.000)	L (0.500, 0.500, 0.500)
4	L (0.500, 0.500, 0.500)	W (0.500, 0.250, 0.750)
5	W (0.500, 0.250, 0.750)	X (0.500, 0.000, 0.500)

Supplementary Table 512: Nodal ring points along high symmetry lines of mp-10635-Sn4Sb4.

	Coordinates	Frequency	Mode 1	Mode 2	Multi.	Deg.	Path	Ratio
0	(0.500, 0.250, 0.750)	1.160	2	1	6	2	5	1.000
1	(0.565, 0.129, 0.565)	1.305	3	2	12	2	2	0.517
2	(0.498, 0.074, 0.577)	1.305	3	2	24	2	6	0.698
3	(0.357, 0.000, 0.357)	2.448	4	3	6	2	1	0.714
4	(0.249, 0.249, 0.490)	2.965	4	3	24	2	3	0.343
5	(0.184, -0.003, 0.184)	2.397	4	3	24	2	1	0.368
6	(0.245, 0.245, 0.481)	2.972	5	4	24	2	3	0.355
7	(0.500, 0.395, 0.605)	3.436	6	5	24	2	5	0.421
8	(0.500, 0.250, 0.750)	3.398	6	5	6	2	5	1.000

The followings are details for Ce_4O_4. It has 2 topological nodal lines and 4 nodal ring points along high symmetry line as shown in Fig 272.

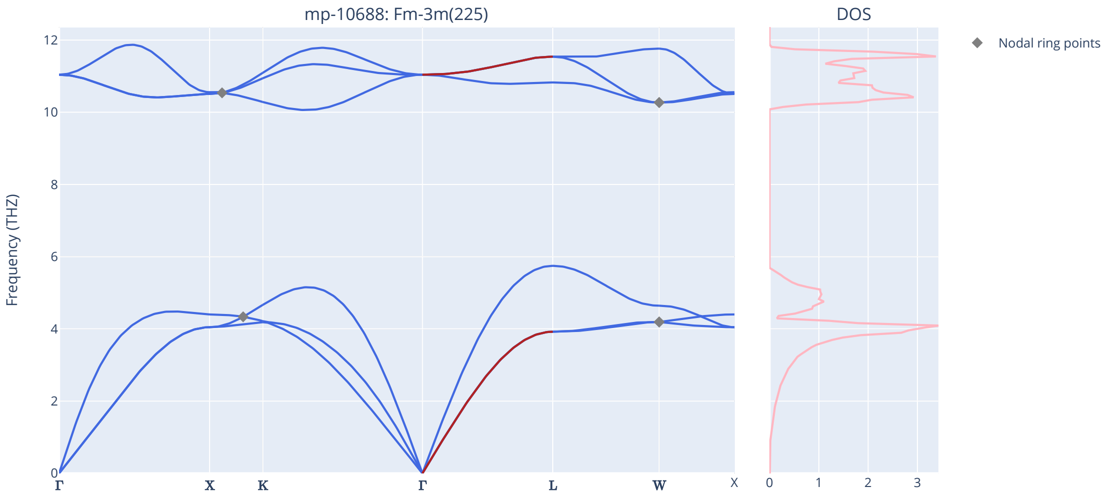

Supplementary Figure 272: The phonon band and DOS of Ce4O4.

Supplementary Table 513: High symmetry lines of mp-10688-Ce4O4

	Start	End
0	Γ (0.000, 0.000, 0.000)	X (0.500, 0.000, 0.500)
1	X (0.500, 0.000, 0.500)	U (0.625, 0.250, 0.625)
2	K (0.375, 0.375, 0.750)	Γ (0.000, 0.000, 0.000)
3	Γ (0.000, 0.000, 0.000)	L (0.500, 0.500, 0.500)
4	L (0.500, 0.500, 0.500)	W (0.500, 0.250, 0.750)
5	W (0.500, 0.250, 0.750)	X (0.500, 0.000, 0.500)

Supplementary Table 514: Nodal ring points along high symmetry lines of mp-10688-Ce4O4.

	Coordinates	Frequency	Mode 1	Mode 2	Multi.	Deg.	Path	Ratio
0	(0.500, 0.250, 0.750)	4.190	2	1	6	2	5	1.000
1	(0.579, 0.158, 0.579)	4.334	3	2	12	2	2	0.631
2	(0.529, 0.059, 0.529)	10.538	5	4	12	2	2	0.234
3	(0.500, 0.250, 0.750)	10.269	5	4	6	2	5	1.000

The followings are details for Th_4Ge_4. It has 2 topological nodal lines and 4 nodal ring points along high symmetry line as shown in Fig 273.

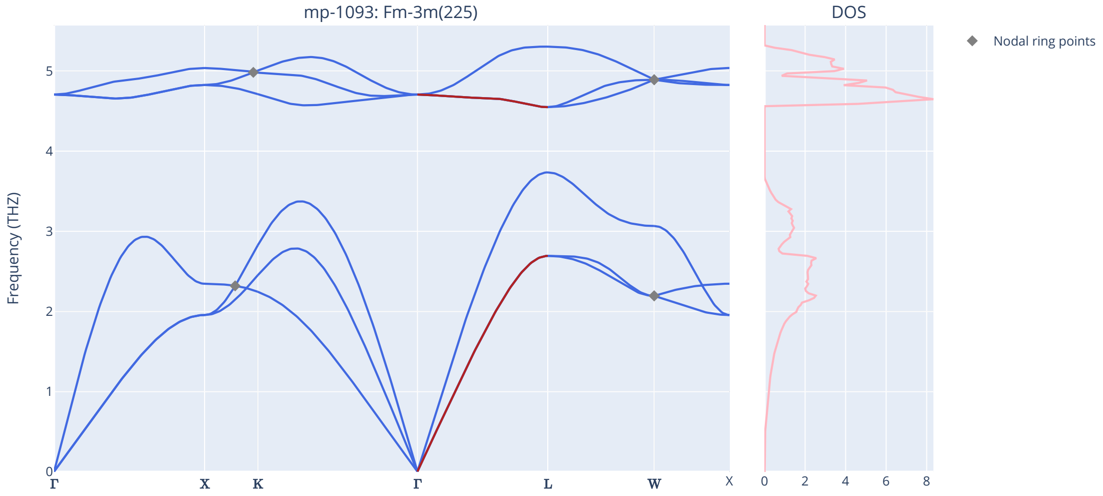

Supplementary Figure 273: The phonon band and DOS of Th4Ge4.

Supplementary Table 515: High symmetry lines of mp-1093-Th4Ge4

	Start	End
0	Γ (0.000, 0.000, 0.000)	X (0.500, 0.000, 0.500)
1	X (0.500, 0.000, 0.500)	U (0.625, 0.250, 0.625)
2	K (0.375, 0.375, 0.750)	Γ (0.000, 0.000, 0.000)
3	Γ (0.000, 0.000, 0.000)	L (0.500, 0.500, 0.500)
4	L (0.500, 0.500, 0.500)	W (0.500, 0.250, 0.750)
5	W (0.500, 0.250, 0.750)	X (0.500, 0.000, 0.500)

Supplementary Table 516: Nodal ring points along high symmetry lines of mp-1093-Th4Ge4.

	Coordinates	Frequency	Mode 1	Mode 2	Multi.	Deg.	Path	Ratio
0	(0.500, 0.250, 0.750)	2.194	2	1	6	2	5	1.000
1	(0.572, 0.144, 0.572)	2.319	3	2	12	2	2	0.574
2	(0.500, 0.250, 0.750)	4.891	5	4	6	2	5	1.000
3	(0.615, 0.229, 0.615)	4.983	6	5	12	2	2	0.916

The followings are details for Th_4C_4. It has 2 topological nodal lines and 5 nodal ring points along high symmetry line as shown in Fig **274**.

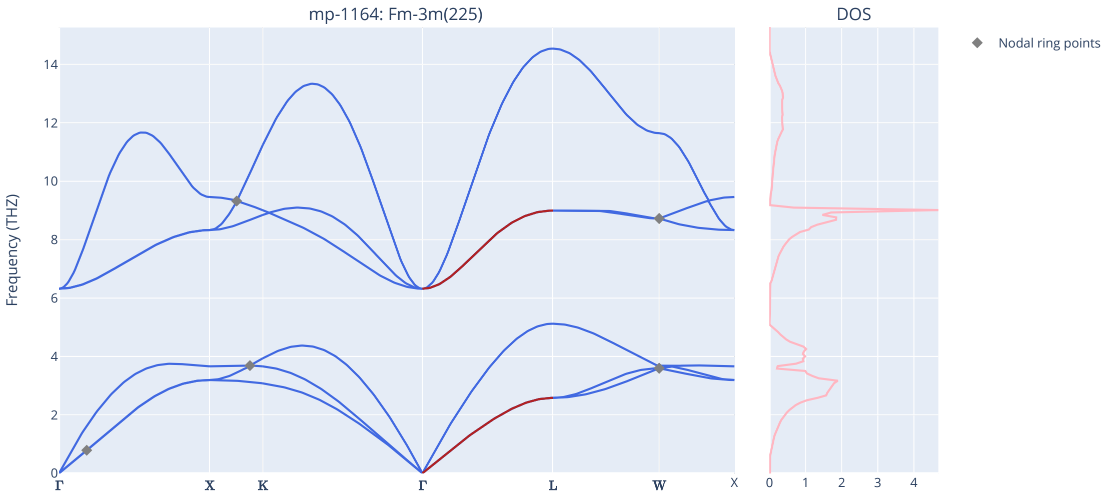

Supplementary Figure 274: The phonon band and DOS of Th4C4.

Supplementary Table 517: High symmetry lines of mp-1164-Th4C4

	Start	End
0	Γ (0.000, 0.000, 0.000)	X (0.500, 0.000, 0.500)
1	X (0.500, 0.000, 0.500)	U (0.625, 0.250, 0.625)
2	K (0.375, 0.375, 0.750)	Γ (0.000, 0.000, 0.000)
3	Γ (0.000, 0.000, 0.000)	L (0.500, 0.500, 0.500)
4	L (0.500, 0.500, 0.500)	W (0.500, 0.250, 0.750)
5	W (0.500, 0.250, 0.750)	X (0.500, 0.000, 0.500)

Supplementary Table 518: Nodal ring points along high symmetry lines of mp-1164-Th4C4.

	Coordinates	Frequency	Mode 1	Mode 2	Multi.	Deg.	Path	Ratio
0	(0.092, 0.000, 0.092)	0.788	2	1	6	2	1	0.184
1	(0.500, 0.250, 0.750)	3.596	2	1	6	2	5	1.000
2	(0.595, 0.190, 0.595)	3.687	3	2	12	2	2	0.758
3	(0.500, 0.250, 0.750)	8.724	5	4	6	2	5	1.000
4	(0.564, 0.127, 0.564)	9.322	6	5	12	2	2	0.509

The followings are details for IrB. It has 2 nodal ring points and 4 single Weyl points along high symmetry line as shown in Fig 275.

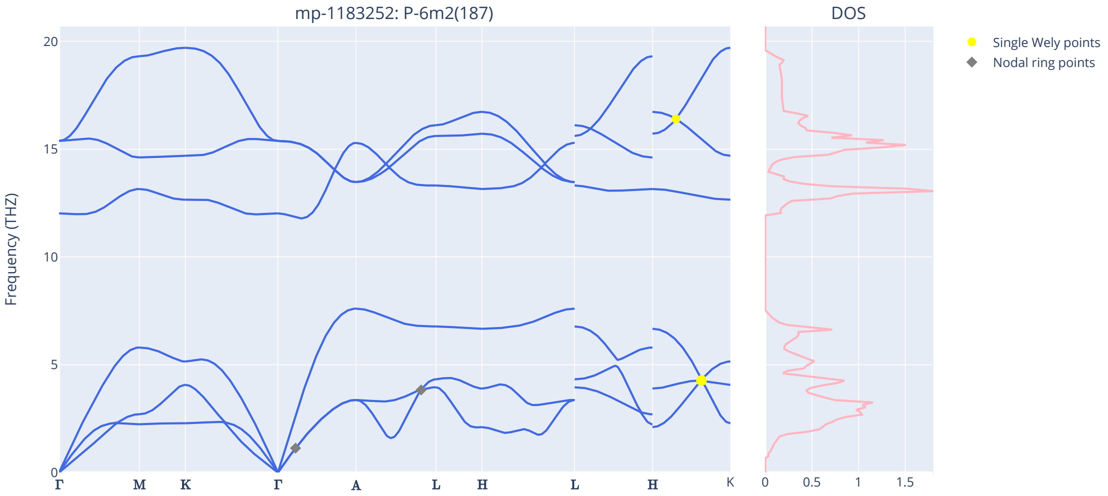

Supplementary Figure 275: The phonon band and DOS of IrB.

Supplementary Table 519: High symmetry lines of mp-1183252-IrB

	Start	End
0	Γ (0.000, 0.000, 0.000)	M (0.500, 0.000, 0.000)
1	M (0.500, 0.000, 0.000)	K (0.333, 0.333, 0.000)
2	K (0.333, 0.333, 0.000)	Γ (0.000, 0.000, 0.000)
3	Γ (0.000, 0.000, 0.000)	A (0.000, 0.000, 0.500)
4	A (0.000, 0.000, 0.500)	L (0.500, 0.000, 0.500)
5	L (0.500, 0.000, 0.500)	H (0.333, 0.333, 0.500)
6	H (0.333, 0.333, 0.500)	A (0.000, 0.000, 0.500)
7	L (0.500, 0.000, 0.500)	M (0.500, 0.000, 0.000)
8	H (0.333, 0.333, 0.500)	K (0.333, 0.333, 0.000)

Supplementary Table 520: Single Weyl points along high symmetry lines of mp-1183252-IrB.

	Coordinates	Frequency	Mode 1	Mode 2	Multi.	Deg.	Path	Ratio
0	(0.333, 0.333, 0.192)	4.250	2	1	4	2	9	0.616
1	(0.333, 0.333, 0.179)	4.243	2	1	4	2	9	0.642
2	(0.333, 0.333, 0.184)	4.315	3	2	4	2	9	0.632
3	(0.333, 0.333, 0.349)	16.404	6	5	4	2	9	0.301

Supplementary Table 521: Nodal ring points along high symmetry lines of mp-1183252-IrB.

	Coordinates	Frequency	Mode 1	Mode 2	Multi.	Deg.	Path	Ratio
0	(0.002, 0.000, 0.113)	1.128	2	1	12	2	4	0.226

Supplementary Table 521: Nodal ring points along high symmetry lines of mp-1183252-IrB.

	Coordinates	Frequency	Mode 1	Mode 2	Multi.	Deg.	Path	Ratio
1	(0.408, 0.000, 0.500)	3.826	2	1	6	2	5	0.815

The followings are details for CrN and it only has 5 single Weyl point along high symmetry line as shown in Fig **276**.

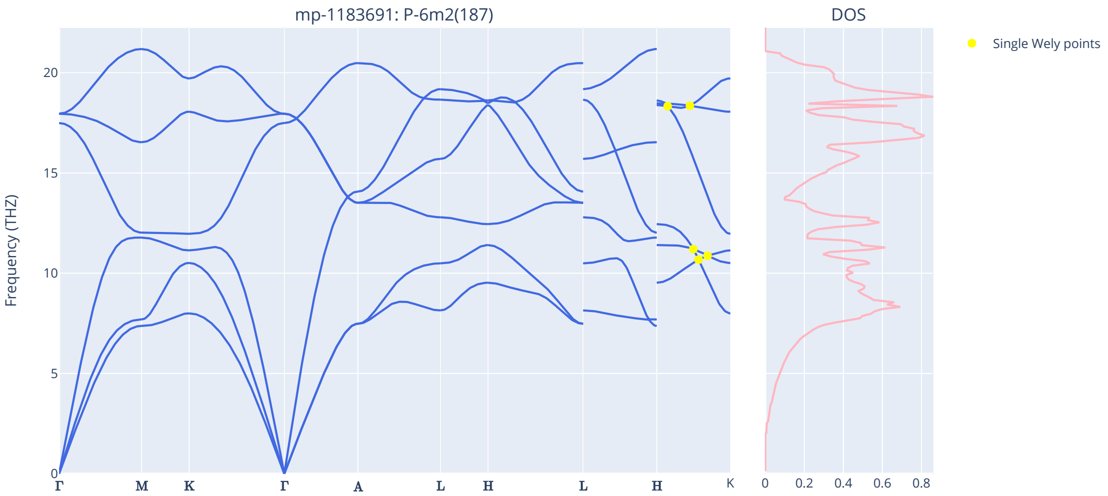

Supplementary Figure 276: The phonon band and DOS of CrN.

Supplementary Table 522: High symmetry lines of mp-1183691-CrN

	Start	End
0	Γ (0.000, 0.000, 0.000)	M (0.500, 0.000, 0.000)
1	M (0.500, 0.000, 0.000)	K (0.333, 0.333, 0.000)
2	K (0.333, 0.333, 0.000)	Γ (0.000, 0.000, 0.000)
3	Γ (0.000, 0.000, 0.000)	A (0.000, 0.000, 0.500)
4	A (0.000, 0.000, 0.500)	L (0.500, 0.000, 0.500)
5	L (0.500, 0.000, 0.500)	H (0.333, 0.333, 0.500)
6	H (0.333, 0.333, 0.500)	A (0.000, 0.000, 0.500)
7	L (0.500, 0.000, 0.500)	M (0.500, 0.000, 0.000)
8	H (0.333, 0.333, 0.500)	K (0.333, 0.333, 0.000)

Supplementary Table 523: Single Weyl points along high symmetry lines of mp-1183691-CrN.

	Coordinates	Frequency	Mode 1	Mode 2	Multi.	Deg.	Path	Ratio
0	(0.333, 0.333, 0.216)	10.680	2	1	4	2	9	0.569
1	(0.333, 0.333, 0.155)	10.871	3	2	4	2	9	0.690
2	(0.333, 0.333, 0.252)	11.201	3	2	4	2	9	0.496
3	(0.333, 0.333, 0.426)	18.332	5	4	4	2	9	0.149
4	(0.333, 0.333, 0.275)	18.357	6	5	4	2	9	0.450

The followings are details for AgZn. It has 1 nodal ring points and 5 single Weyl point along high symmetry line as shown in Fig 277.

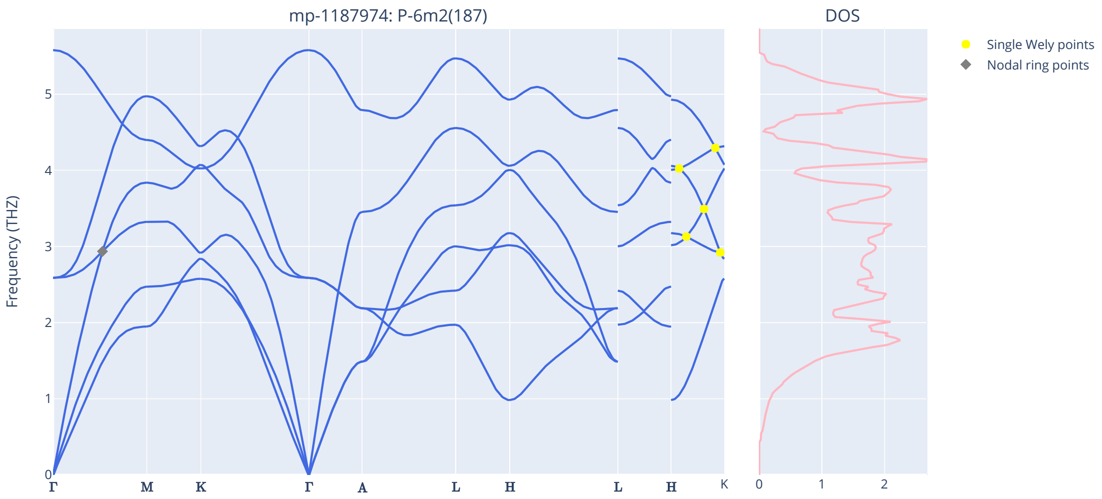

Supplementary Figure 277: The phonon band and DOS of AgZn.

Supplementary Table 524: High symmetry lines of mp-1187974-AgZn

	Start	End
0	Γ (0.000, 0.000, 0.000)	M (0.500, 0.000, 0.000)
1	M (0.500, 0.000, 0.000)	K (0.333, 0.333, 0.000)
2	K (0.333, 0.333, 0.000)	Γ (0.000, 0.000, 0.000)
3	Γ (0.000, 0.000, 0.000)	A (0.000, 0.000, 0.500)
4	A (0.000, 0.000, 0.500)	L (0.500, 0.000, 0.500)
5	L (0.500, 0.000, 0.500)	H (0.333, 0.333, 0.500)
6	H (0.333, 0.333, 0.500)	A (0.000, 0.000, 0.500)
7	L (0.500, 0.000, 0.500)	M (0.500, 0.000, 0.000)
8	H (0.333, 0.333, 0.500)	K (0.333, 0.333, 0.000)

Supplementary Table 525: Single Weyl points along high symmetry lines of mp-1187974-AgZn.

	Coordinates	Frequency	Mode 1	Mode 2	Multi.	Deg.	Path	Ratio
0	(0.333, 0.333, 0.038)	2.920	3	2	4	2	9	0.924
1	(0.333, 0.333, 0.357)	3.129	3	2	4	2	9	0.286
2	(0.333, 0.333, 0.191)	3.493	4	3	4	2	9	0.618
3	(0.333, 0.333, 0.425)	4.024	5	4	4	2	9	0.151
4	(0.333, 0.333, 0.086)	4.294	6	5	4	2	9	0.829

Supplementary Table 526: Nodal ring points along high symmetry lines of mp-1187974-AgZn.

	Coordinates	Frequency	Mode 1	Mode 2	Multi.	Deg.	Path	Ratio
0	(0.264, -0.000, -0.000)	2.935	4	3	6	2	1	0.528

The followings are details for RuTc. It has 4 nodal ring points and 4 single Weyl points along high symmetry line as shown in Fig 278.

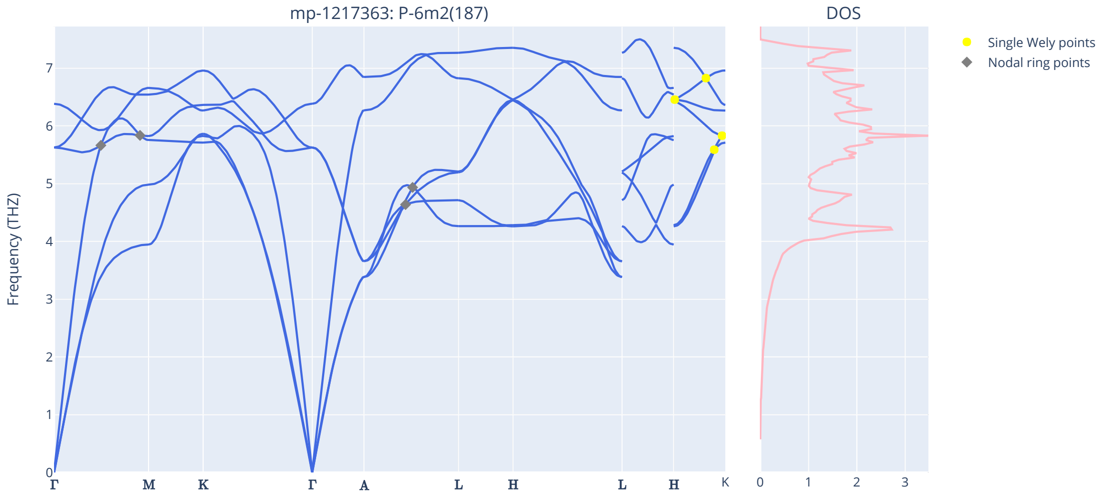

Supplementary Figure 278: The phonon band and DOS of RuTc.

Supplementary Table 527: High symmetry lines of mp-1217363-RuTc

	Start	End
0	Γ (0.000, 0.000, 0.000)	M (0.500, 0.000, 0.000)
1	M (0.500, 0.000, 0.000)	K (0.333, 0.333, 0.000)
2	K (0.333, 0.333, 0.000)	Γ (0.000, 0.000, 0.000)
3	Γ (0.000, 0.000, 0.000)	A (0.000, 0.000, 0.500)
4	A (0.000, 0.000, 0.500)	L (0.500, 0.000, 0.500)
5	L (0.500, 0.000, 0.500)	H (0.333, 0.333, 0.500)
6	H (0.333, 0.333, 0.500)	A (0.000, 0.000, 0.500)
7	L (0.500, 0.000, 0.500)	M (0.500, 0.000, 0.000)
8	H (0.333, 0.333, 0.500)	K (0.333, 0.333, 0.000)

Supplementary Table 528: Single Weyl points along high symmetry lines of mp-1217363-RuTc.

	Coordinates	Frequency	Mode 1	Mode 2	Multi.	Deg.	Path	Ratio
0	(0.333, 0.333, 0.108)	5.588	2	1	4	2	9	0.785
1	(0.333, 0.333, 0.032)	5.831	3	2	4	2	9	0.935
2	(0.333, 0.333, 0.490)	6.455	5	4	4	2	9	0.021
3	(0.333, 0.333, 0.188)	6.827	6	5	4	2	9	0.623

Supplementary Table 529: Nodal ring points along high symmetry lines of mp-1217363-RuTc.

	Coordinates	Frequency	Mode 1	Mode 2	Multi.	Deg.	Path	Ratio
0	(0.221, 0.000, 0.500)	4.639	2	1	6	2	5	0.441

Supplementary Table 529: Nodal ring points along high symmetry lines of mp-1217363-RuTc.

	Coordinates	Frequency	Mode 1	Mode 2	Multi.	Deg.	Path	Ratio
1	(0.248, -0.000, 0.000)	5.663	4	3	6	2	1	0.495
2	(0.455, 0.000, 0.000)	5.836	4	3	6	2	1	0.910
3	(0.258, -0.000, 0.500)	4.935	4	3	6	2	5	0.516

The followings are details for Sc_4S_4. It has 3 topological nodal lines and 10 nodal ring points along high symmetry line as shown in Fig **279**.

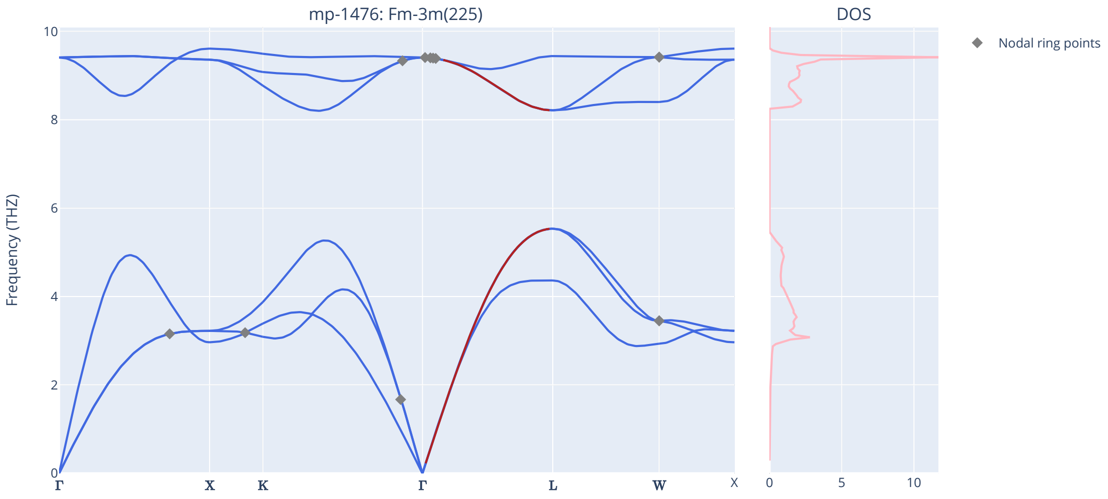

Supplementary Figure 279: The phonon band and DOS of Sc4S4.

Supplementary Table 530: High symmetry lines of mp-1476-Sc4S4

	Start	End
0	Γ (0.000, 0.000, 0.000)	X (0.500, 0.000, 0.500)
1	X (0.500, 0.000, 0.500)	U (0.625, 0.250, 0.625)
2	K (0.375, 0.375, 0.750)	Γ (0.000, 0.000, 0.000)
3	Γ (0.000, 0.000, 0.000)	L (0.500, 0.500, 0.500)
4	L (0.500, 0.500, 0.500)	W (0.500, 0.250, 0.750)
5	W (0.500, 0.250, 0.750)	X (0.500, 0.000, 0.500)

Supplementary Table 531: Nodal ring points along high symmetry lines of mp-1476-Sc4S4.

	Coordinates	Frequency	Mode 1	Mode 2	Multi.	Deg.	Path	Ratio
0	(0.367, 0.000, 0.367)	3.155	2	1	6	2	1	0.735
1	(0.583, 0.167, 0.583)	3.181	2	1	12	2	2	0.668
2	(0.054, 0.054, 0.100)	1.669	3	2	24	2	3	0.863
3	(0.500, 0.250, 0.750)	3.450	3	2	6	2	5	1.000
4	(0.048, 0.048, 0.092)	9.334	5	4	24	2	3	0.875
5	(0.010, 0.010, 0.010)	9.408	6	5	8	3	4	0.020
6	(0.031, 0.031, 0.031)	9.401	6	5	8	2	4	0.061
7	(0.041, 0.041, 0.041)	9.394	6	5	8	2	4	0.082
8	(0.051, 0.051, 0.051)	9.387	6	5	8	2	4	0.102
9	(0.500, 0.250, 0.750)	9.417	6	5	6	2	5	1.000

The followings are details for Ce_4Te_4. It has 2 topological nodal lines and 7 nodal ring points along high symmetry line as shown in Fig **280**.

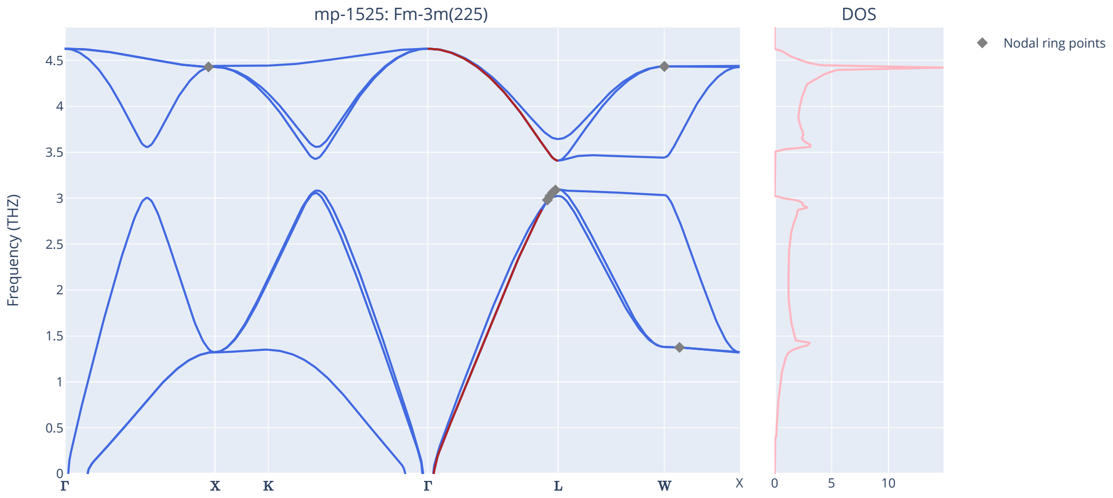

Supplementary Figure 280: The phonon band and DOS of Ce4Te4.

Supplementary Table 532: High symmetry lines of mp-1525-Ce4Te4

	Start	End
0	Γ (0.000, 0.000, 0.000)	X (0.500, 0.000, 0.500)
1	X (0.500, 0.000, 0.500)	U (0.625, 0.250, 0.625)
2	K (0.375, 0.375, 0.750)	Γ (0.000, 0.000, 0.000)
3	Γ (0.000, 0.000, 0.000)	L (0.500, 0.500, 0.500)
4	L (0.500, 0.500, 0.500)	W (0.500, 0.250, 0.750)
5	W (0.500, 0.250, 0.750)	X (0.500, 0.000, 0.500)

Supplementary Table 533: Nodal ring points along high symmetry lines of mp-1525-Ce4Te4.

	Coordinates	Frequency	Mode 1	Mode 2	Multi.	Deg.	Path	Ratio
0	(0.499, 0.199, 0.700)	1.375	2	1	24	2	6	0.201
1	(0.490, 0.490, 0.490)	3.088	3	2	8	2	4	0.981
2	(0.459, 0.459, 0.459)	2.980	3	2	8	2	4	0.918
3	(0.469, 0.469, 0.469)	3.024	3	2	8	2	4	0.939
4	(0.480, 0.480, 0.480)	3.061	3	2	8	2	4	0.959
5	(0.478, -0.000, 0.478)	4.429	6	5	6	2	1	0.957
6	(0.500, 0.250, 0.750)	4.434	6	5	6	2	5	1.000

The followings are details for Y_4S_4. It has 3 topological nodal lines and 3 nodal ring points along high symmetry line as shown in Fig **281**.

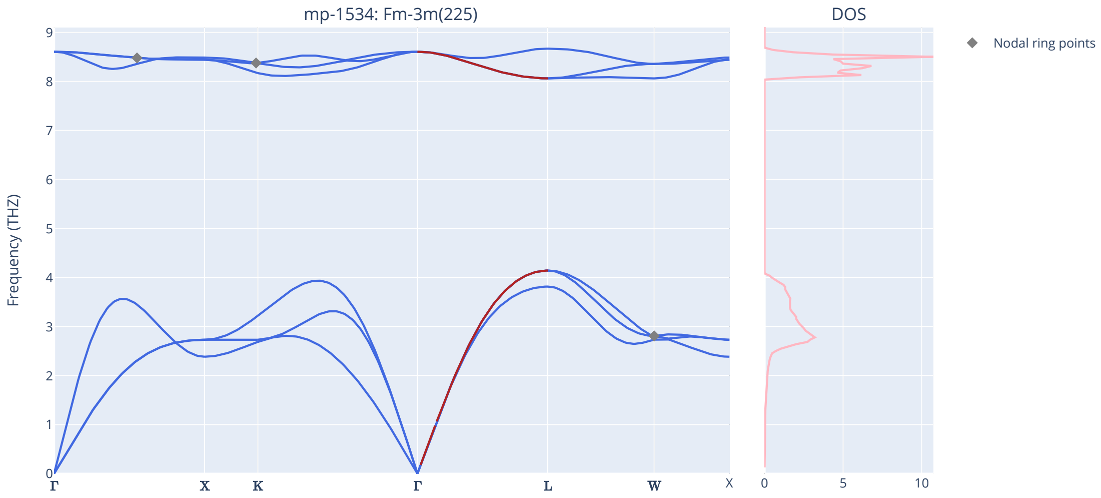

Supplementary Figure 281: The phonon band and DOS of Y4S4.

Supplementary Table 534: High symmetry lines of mp-1534-Y4S4

	Start	End
0	Γ (0.000, 0.000, 0.000)	X (0.500, 0.000, 0.500)
1	X (0.500, 0.000, 0.500)	U (0.625, 0.250, 0.625)
2	K (0.375, 0.375, 0.750)	Γ (0.000, 0.000, 0.000)
3	Γ (0.000, 0.000, 0.000)	L (0.500, 0.500, 0.500)
4	L (0.500, 0.500, 0.500)	W (0.500, 0.250, 0.750)
5	W (0.500, 0.250, 0.750)	X (0.500, 0.000, 0.500)

Supplementary Table 535: Nodal ring points along high symmetry lines of mp-1534-Y4S4.

	Coordinates	Frequency	Mode 1	Mode 2	Multi.	Deg.	Path	Ratio
0	(0.500, 0.250, 0.750)	2.810	3	2	6	2	5	1.000
1	(0.276, 0.000, 0.276)	8.480	6	5	6	2	1	0.551
2	(0.621, 0.242, 0.621)	8.377	6	5	12	2	2	0.968

The followings are details for La_4Te_4. It has 4 topological nodal lines and 5 nodal ring points along high symmetry line as shown in Fig 282.

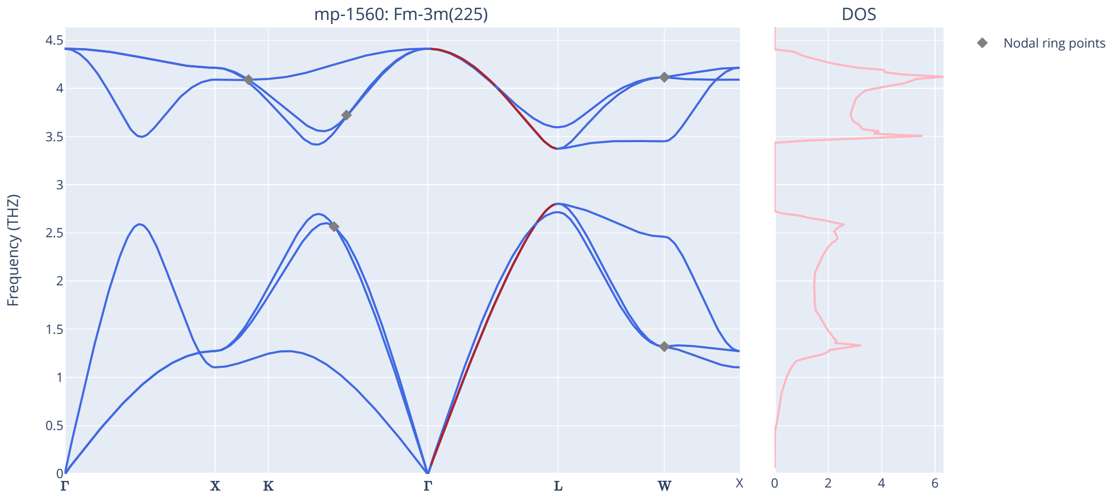

Supplementary Figure 282: The phonon band and DOS of La4Te4.

Supplementary Table 536: High symmetry lines of mp-1560-La4Te4

	Start	End
0	Γ (0.000, 0.000, 0.000)	X (0.500, 0.000, 0.500)
1	X (0.500, 0.000, 0.500)	U (0.625, 0.250, 0.625)
2	K (0.375, 0.375, 0.750)	Γ (0.000, 0.000, 0.000)
3	Γ (0.000, 0.000, 0.000)	L (0.500, 0.500, 0.500)
4	L (0.500, 0.500, 0.500)	W (0.500, 0.250, 0.750)
5	W (0.500, 0.250, 0.750)	X (0.500, 0.000, 0.500)

Supplementary Table 537: Nodal ring points along high symmetry lines of mp-1560-La4Te4.

	Coordinates	Frequency	Mode 1	Mode 2	Multi.	Deg.	Path	Ratio
0	(0.500, 0.250, 0.750)	1.320	2	1	6	2	5	1.000
1	(0.222, 0.222, 0.438)	2.564	3	2	24	2	3	0.413
2	(0.196, 0.196, 0.377)	3.722	5	4	24	2	3	0.491
3	(0.579, 0.158, 0.579)	4.090	6	5	12	2	2	0.631
4	(0.500, 0.250, 0.750)	4.116	6	5	6	2	5	1.000

The followings are details for Pa_4As_4. It has 3 topological nodal lines and 4 nodal ring points along high symmetry line as shown in Fig **283**.

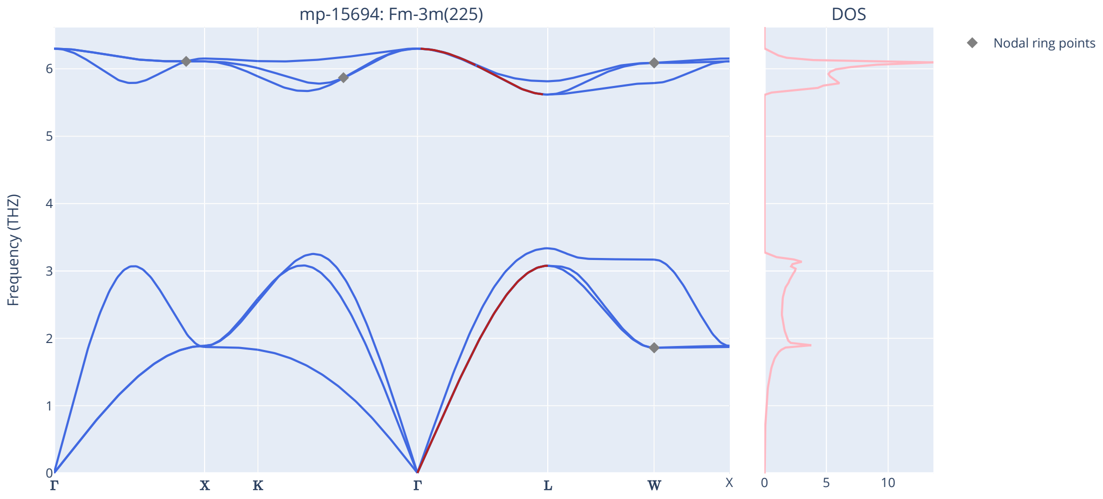

Supplementary Figure 283: The phonon band and DOS of Pa4As4.

Supplementary Table 538: High symmetry lines of mp-15694-Pa4As4

	Start	End
0	Γ (0.000, 0.000, 0.000)	X (0.500, 0.000, 0.500)
1	X (0.500, 0.000, 0.500)	U (0.625, 0.250, 0.625)
2	K (0.375, 0.375, 0.750)	Γ (0.000, 0.000, 0.000)
3	Γ (0.000, 0.000, 0.000)	L (0.500, 0.500, 0.500)
4	L (0.500, 0.500, 0.500)	W (0.500, 0.250, 0.750)
5	W (0.500, 0.250, 0.750)	X (0.500, 0.000, 0.500)

Supplementary Table 539: Nodal ring points along high symmetry lines of mp-15694-Pa4As4.

	Coordinates	Frequency	Mode 1	Mode 2	Multi.	Deg.	Path	Ratio
0	(0.500, 0.250, 0.750)	1.862	2	1	6	2	5	1.000
1	(0.176, 0.176, 0.345)	5.867	5	4	24	2	3	0.537
2	(0.439, 0.000, 0.439)	6.108	6	5	6	2	1	0.878
3	(0.500, 0.250, 0.750)	6.087	6	5	6	2	5	1.000

The followings are details for Sc_4Se_4. It has 4 topological nodal lines and 4 nodal ring points along high symmetry line as shown in Fig **284**.

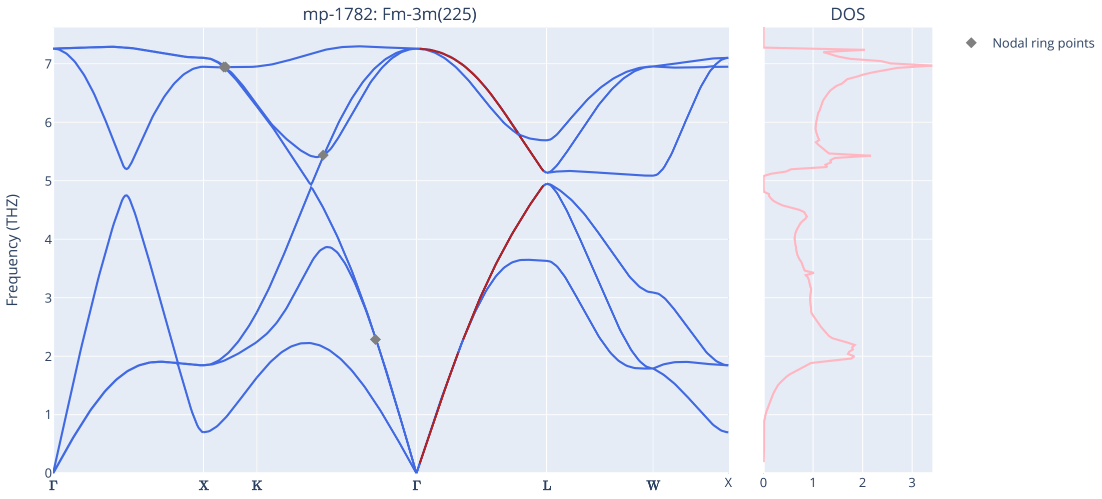

Supplementary Figure 284: The phonon band and DOS of Sc4Se4.

Supplementary Table 540: High symmetry lines of mp-1782-Sc4Se4

	Start	End
0	Γ (0.000, 0.000, 0.000)	X (0.500, 0.000, 0.500)
1	X (0.500, 0.000, 0.500)	U (0.625, 0.250, 0.625)
2	K (0.375, 0.375, 0.750)	Γ (0.000, 0.000, 0.000)
3	Γ (0.000, 0.000, 0.000)	L (0.500, 0.500, 0.500)
4	L (0.500, 0.500, 0.500)	W (0.500, 0.250, 0.750)
5	W (0.500, 0.250, 0.750)	X (0.500, 0.000, 0.500)

Supplementary Table 541: Nodal ring points along high symmetry lines of mp-1782-Sc4Se4.

	Coordinates	Frequency	Mode 1	Mode 2	Multi.	Deg.	Path	Ratio
0	(0.099, 0.099, 0.189)	2.288	3	2	24	2	3	0.743
1	(0.549, 0.095, 0.546)	6.946	5	4	24	2	2	0.379
2	(0.222, 0.222, 0.435)	5.440	5	4	24	2	3	0.416
3	(0.553, 0.102, 0.553)	6.945	6	5	24	2	2	0.414

The followings are details for Ce_4P_4. It has 2 topological nodal lines and 4 nodal ring points along high symmetry line as shown in Fig 285.

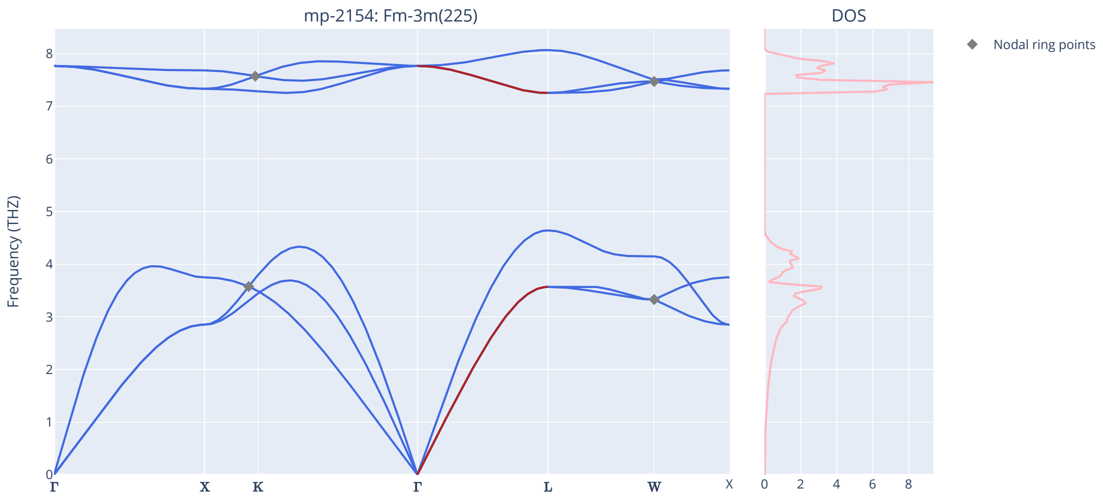

Supplementary Figure 285: The phonon band and DOS of Ce4P4.

Supplementary Table 542: High symmetry lines of mp-2154-Ce4P4

	Start	End
0	Γ (0.000, 0.000, 0.000)	X (0.500, 0.000, 0.500)
1	X (0.500, 0.000, 0.500)	U (0.625, 0.250, 0.625)
2	K (0.375, 0.375, 0.750)	Γ (0.000, 0.000, 0.000)
3	Γ (0.000, 0.000, 0.000)	L (0.500, 0.500, 0.500)
4	L (0.500, 0.500, 0.500)	W (0.500, 0.250, 0.750)
5	W (0.500, 0.250, 0.750)	X (0.500, 0.000, 0.500)

Supplementary Table 543: Nodal ring points along high symmetry lines of mp-2154-Ce4P4.

	Coordinates	Frequency	Mode 1	Mode 2	Multi.	Deg.	Path	Ratio
0	(0.500, 0.250, 0.750)	3.329	2	1	6	2	5	1.000
1	(0.604, 0.207, 0.604)	3.575	3	2	12	2	2	0.829
2	(0.500, 0.250, 0.750)	7.470	5	4	6	2	5	1.000
3	(0.619, 0.238, 0.619)	7.571	6	5	12	2	2	0.953

The followings are details for Np_4As_4. It has 3 topological nodal lines and 3 nodal ring points along high symmetry line as shown in Fig 286.

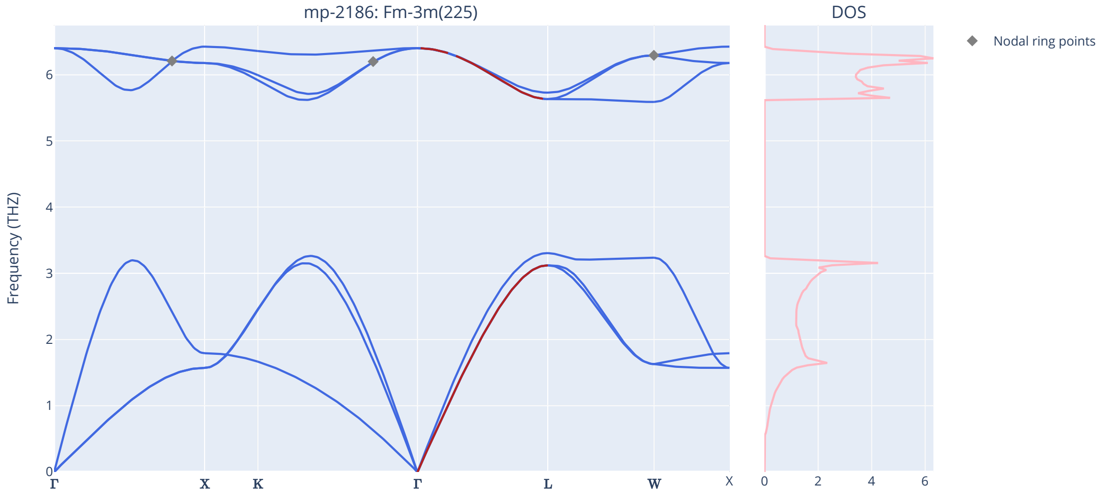

Supplementary Figure 286: The phonon band and DOS of Np4As4.

Supplementary Table 544: High symmetry lines of mp-2186-Np4As4

	Start	End
0	Γ (0.000, 0.000, 0.000)	X (0.500, 0.000, 0.500)
1	X (0.500, 0.000, 0.500)	U (0.625, 0.250, 0.625)
2	K (0.375, 0.375, 0.750)	Γ (0.000, 0.000, 0.000)
3	Γ (0.000, 0.000, 0.000)	L (0.500, 0.500, 0.500)
4	L (0.500, 0.500, 0.500)	W (0.500, 0.250, 0.750)
5	W (0.500, 0.250, 0.750)	X (0.500, 0.000, 0.500)

Supplementary Table 545: Nodal ring points along high symmetry lines of mp-2186-Np4As4.

	Coordinates	Frequency	Mode 1	Mode 2	Multi.	Deg.	Path	Ratio
0	(0.396, 0.000, 0.388)	6.209	5	4	24	2	1	0.784
1	(0.107, 0.107, 0.205)	6.200	5	4	24	2	3	0.723
2	(0.500, 0.251, 0.749)	6.295	6	5	24	2	5	0.997

The followings are details for U_4C_4. It has 2 topological nodal lines and 6 nodal ring points along high symmetry line as shown in Fig **287**.

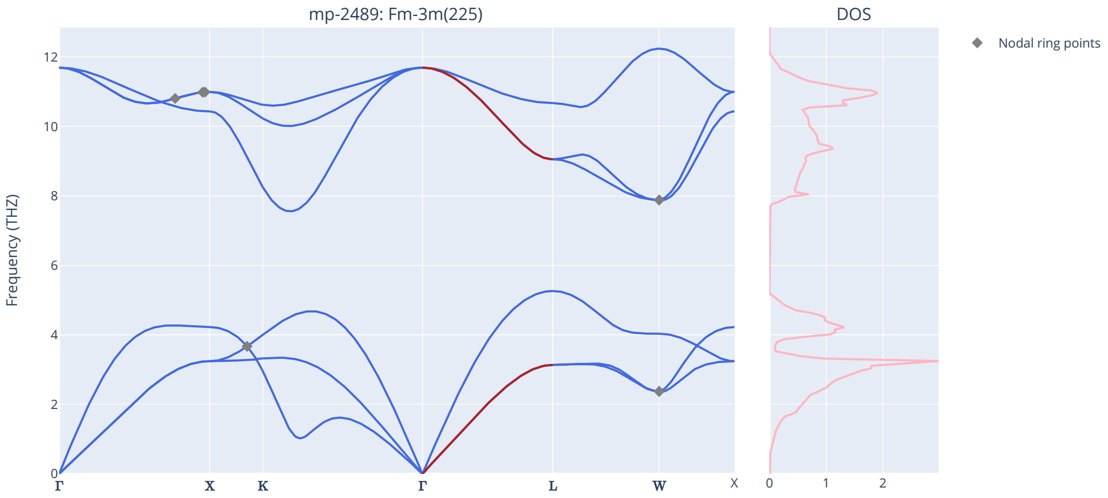

Supplementary Figure 287: The phonon band and DOS of U4C4.

Supplementary Table 546: High symmetry lines of mp-2489-U4C4

	Start	End
0	Γ (0.000, 0.000, 0.000)	X (0.500, 0.000, 0.500)
1	X (0.500, 0.000, 0.500)	U (0.625, 0.250, 0.625)
2	K (0.375, 0.375, 0.750)	Γ (0.000, 0.000, 0.000)
3	Γ (0.000, 0.000, 0.000)	L (0.500, 0.500, 0.500)
4	L (0.500, 0.500, 0.500)	W (0.500, 0.250, 0.750)
5	W (0.500, 0.250, 0.750)	X (0.500, 0.000, 0.500)

Supplementary Table 547: Nodal ring points along high symmetry lines of mp-2489-U4C4.

	Coordinates	Frequency	Mode 1	Mode 2	Multi.	Deg.	Path	Ratio
0	(0.500, 0.250, 0.750)	2.370	2	1	6	2	5	1.000
1	(0.588, 0.176, 0.588)	3.668	3	2	12	2	2	0.704
2	(0.500, 0.250, 0.750)	7.881	5	4	6	2	5	1.000
3	(0.386, -0.000, 0.386)	10.805	6	5	6	2	1	0.771
4	(0.477, -0.000, 0.477)	10.984	6	5	6	2	1	0.954
5	(0.485, -0.000, 0.485)	10.989	6	5	6	2	1	0.969

The followings are details for Pr_4S_4. It has 2 topological nodal lines and 5 nodal ring points along high symmetry line as shown in Fig **288**.

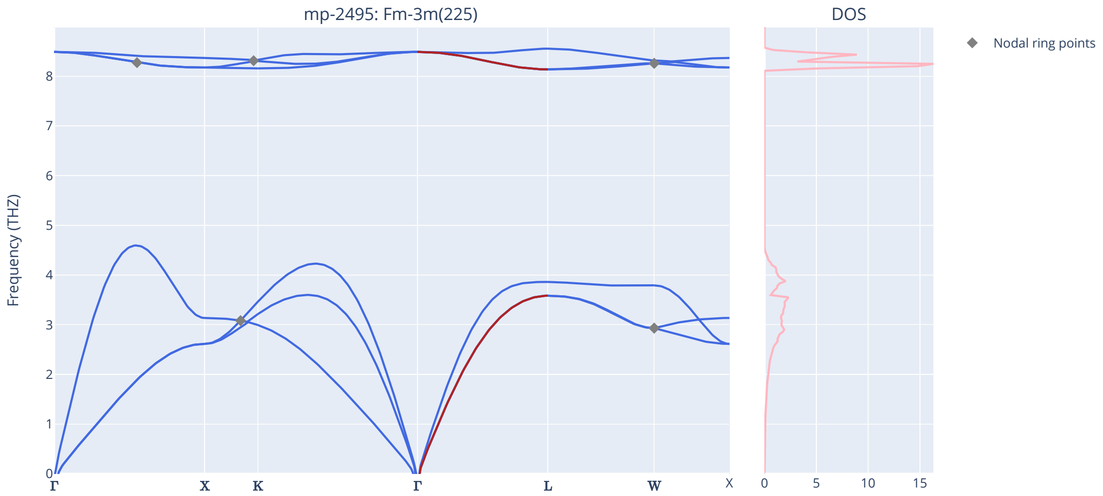

Supplementary Figure 288: The phonon band and DOS of Pr4S4.

Supplementary Table 548: High symmetry lines of mp-2495-Pr4S4

	Start	End
0	Γ (0.000, 0.000, 0.000)	X (0.500, 0.000, 0.500)
1	X (0.500, 0.000, 0.500)	U (0.625, 0.250, 0.625)
2	K (0.375, 0.375, 0.750)	Γ (0.000, 0.000, 0.000)
3	Γ (0.000, 0.000, 0.000)	L (0.500, 0.500, 0.500)
4	L (0.500, 0.500, 0.500)	W (0.500, 0.250, 0.750)
5	W (0.500, 0.250, 0.750)	X (0.500, 0.000, 0.500)

Supplementary Table 549: Nodal ring points along high symmetry lines of mp-2495-Pr4S4.

	Coordinates	Frequency	Mode 1	Mode 2	Multi.	Deg.	Path	Ratio
0	(0.500, 0.250, 0.750)	2.930	2	1	6	2	5	1.000
1	(0.584, 0.169, 0.584)	3.081	3	2	12	2	2	0.676
2	(0.276, 0.000, 0.276)	8.273	5	4	6	2	1	0.551
3	(0.500, 0.250, 0.750)	8.259	5	4	6	2	5	1.000
4	(0.615, 0.230, 0.615)	8.312	6	5	12	2	2	0.921

The followings are details for Ce_4Se_4. It has 3 topological nodal lines and 4 nodal ring points along high symmetry line as shown in Fig **289**.

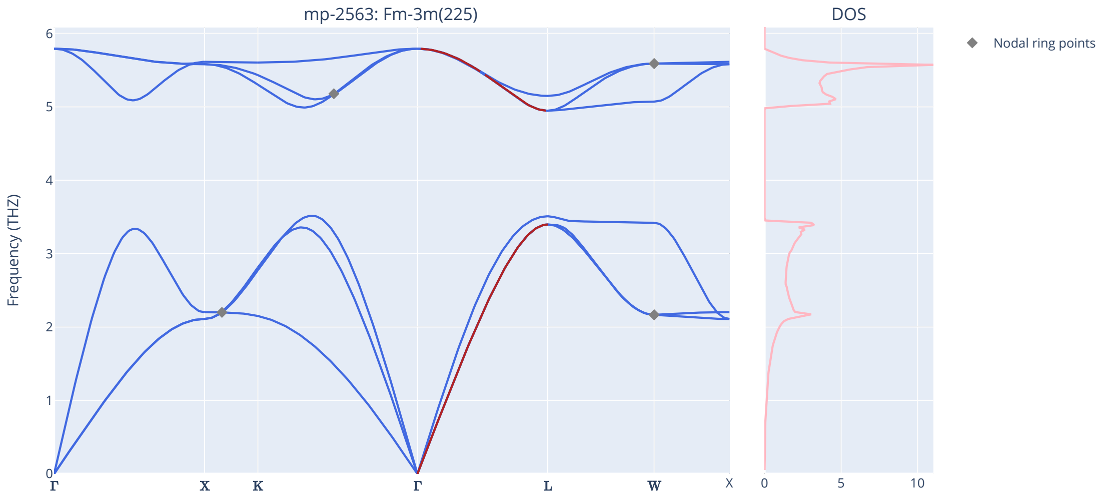

Supplementary Figure 289: The phonon band and DOS of Ce4Se4.

Supplementary Table 550: High symmetry lines of mp-2563-Ce4Se4

	Start	End
0	Γ (0.000, 0.000, 0.000)	X (0.500, 0.000, 0.500)
1	X (0.500, 0.000, 0.500)	U (0.625, 0.250, 0.625)
2	K (0.375, 0.375, 0.750)	Γ (0.000, 0.000, 0.000)
3	Γ (0.000, 0.000, 0.000)	L (0.500, 0.500, 0.500)
4	L (0.500, 0.500, 0.500)	W (0.500, 0.250, 0.750)
5	W (0.500, 0.250, 0.750)	X (0.500, 0.000, 0.500)

Supplementary Table 551: Nodal ring points along high symmetry lines of mp-2563-Ce4Se4.

	Coordinates	Frequency	Mode 1	Mode 2	Multi.	Deg.	Path	Ratio
0	(0.541, 0.082, 0.541)	2.199	2	1	12	2	2	0.326
1	(0.500, 0.250, 0.750)	2.166	2	1	6	2	5	1.000
2	(0.199, 0.199, 0.390)	5.178	5	4	24	2	3	0.477
3	(0.500, 0.250, 0.750)	5.589	6	5	6	2	5	1.000

The followings are details for Np_4N_4. It has 2 topological nodal lines and 3 nodal ring points along high symmetry line as shown in Fig 290.

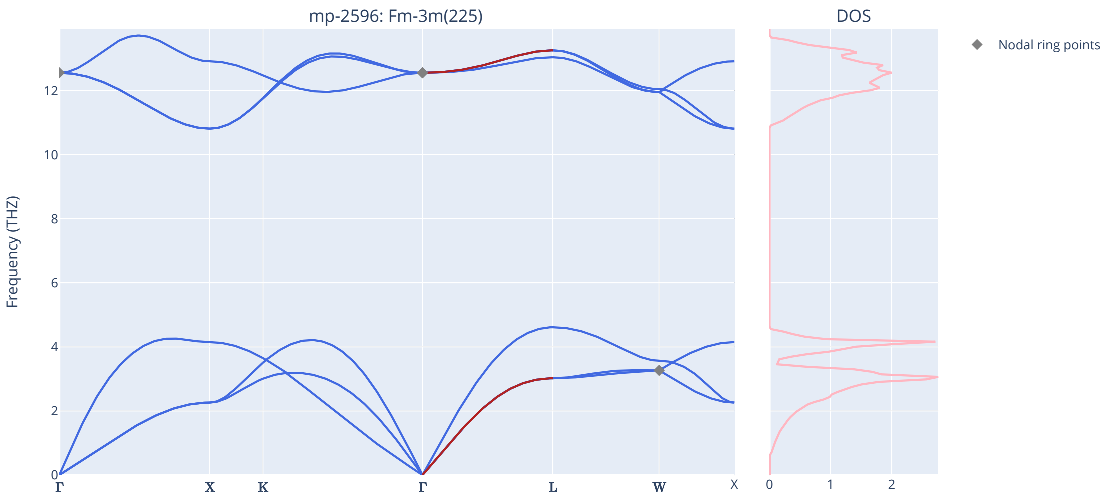

Supplementary Figure 290: The phonon band and DOS of Np4N4.

Supplementary Table 552: High symmetry lines of mp-2596-Np4N4

	Start	End
0	Γ (0.000, 0.000, 0.000)	X (0.500, 0.000, 0.500)
1	X (0.500, 0.000, 0.500)	U (0.625, 0.250, 0.625)
2	K (0.375, 0.375, 0.750)	Γ (0.000, 0.000, 0.000)
3	Γ (0.000, 0.000, 0.000)	L (0.500, 0.500, 0.500)
4	L (0.500, 0.500, 0.500)	W (0.500, 0.250, 0.750)
5	W (0.500, 0.250, 0.750)	X (0.500, 0.000, 0.500)

Supplementary Table 553: Nodal ring points along high symmetry lines of mp-2596-Np4N4.

	Coordinates	Frequency	Mode 1	Mode 2	Multi.	Deg.	Path	Ratio
0	(0.500, 0.250, 0.750)	3.265	2	1	6	2	5	1.000
1	(0.000, 0.000, 0.000)	12.553	5	4	1	3	1	0.000
2	(0.000, 0.000, 0.000)	12.553	6	5	1	3	1	0.000

The followings are details for Ce_4As_4. It has 2 topological nodal lines and 4 nodal ring points along high symmetry line as shown in Fig **291**.

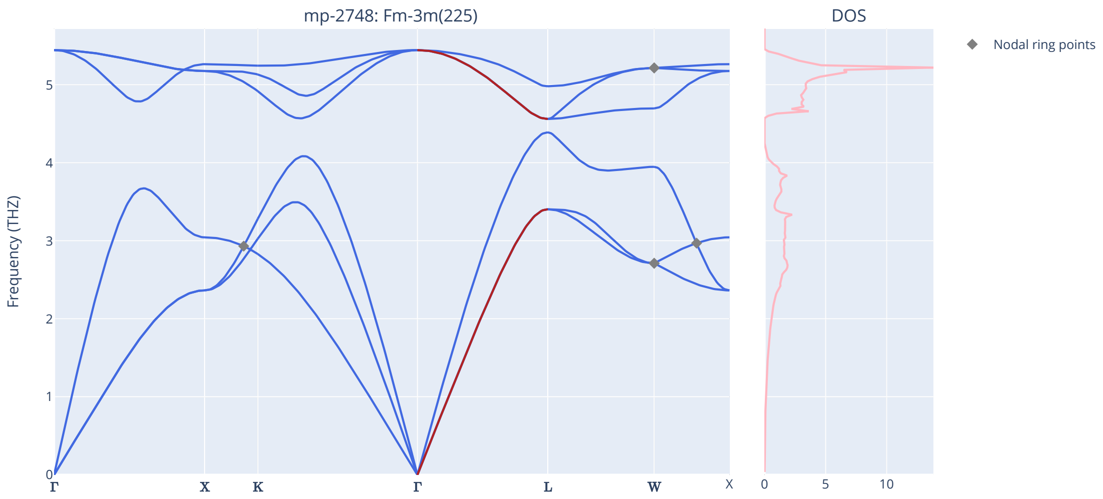

Supplementary Figure 291: The phonon band and DOS of Ce4As4.

Supplementary Table 554: High symmetry lines of mp-2748-Ce4As4

	Start	End
0	Γ (0.000, 0.000, 0.000)	X (0.500, 0.000, 0.500)
1	X (0.500, 0.000, 0.500)	U (0.625, 0.250, 0.625)
2	K (0.375, 0.375, 0.750)	Γ (0.000, 0.000, 0.000)
3	Γ (0.000, 0.000, 0.000)	L (0.500, 0.500, 0.500)
4	L (0.500, 0.500, 0.500)	W (0.500, 0.250, 0.750)
5	W (0.500, 0.250, 0.750)	X (0.500, 0.000, 0.500)

Supplementary Table 555: Nodal ring points along high symmetry lines of mp-2748-Ce4As4.

	Coordinates	Frequency	Mode 1	Mode 2	Multi.	Deg.	Path	Ratio
0	(0.500, 0.250, 0.750)	2.712	2	1	6	2	5	1.000
1	(0.592, 0.184, 0.592)	2.932	3	2	12	2	2	0.735
2	(0.499, 0.108, 0.610)	2.970	3	2	24	2	6	0.564
3	(0.500, 0.250, 0.750)	5.217	6	5	6	2	5	1.000

The followings are details for Pr_4N_4. It has 2 topological nodal lines and 4 nodal ring points along high symmetry line as shown in Fig 292.

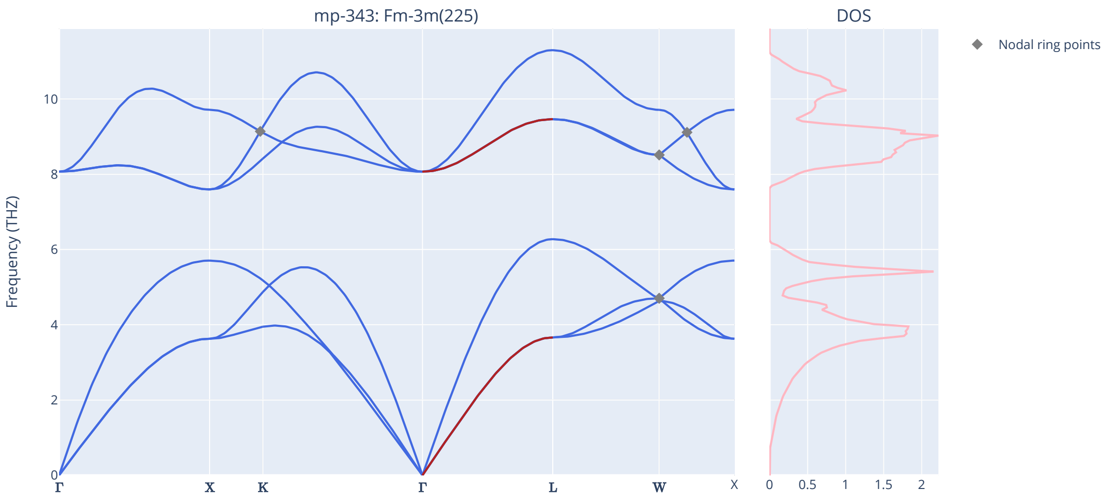

Supplementary Figure 292: The phonon band and DOS of Pr4N4.

Supplementary Table 556: High symmetry lines of mp-343-Pr4N4

	Start	End
0	Γ (0.000, 0.000, 0.000)	X (0.500, 0.000, 0.500)
1	X (0.500, 0.000, 0.500)	U (0.625, 0.250, 0.625)
2	K (0.375, 0.375, 0.750)	Γ (0.000, 0.000, 0.000)
3	Γ (0.000, 0.000, 0.000)	L (0.500, 0.500, 0.500)
4	L (0.500, 0.500, 0.500)	W (0.500, 0.250, 0.750)
5	W (0.500, 0.250, 0.750)	X (0.500, 0.000, 0.500)

Supplementary Table 557: Nodal ring points along high symmetry lines of mp-343-Pr4N4.

	Coordinates	Frequency	Mode 1	Mode 2	Multi.	Deg.	Path	Ratio
0	(0.500, 0.250, 0.750)	4.704	3	2	6	2	5	1.000
1	(0.500, 0.250, 0.750)	8.514	5	4	6	2	5	1.000
2	(0.619, 0.238, 0.619)	9.140	6	5	12	2	2	0.952
3	(0.498, 0.156, 0.659)	9.115	6	5	24	2	6	0.370

The followings are details for Ce_4Sb_4. It has 2 topological nodal lines and 8 nodal ring points along high symmetry line as shown in Fig **293**.

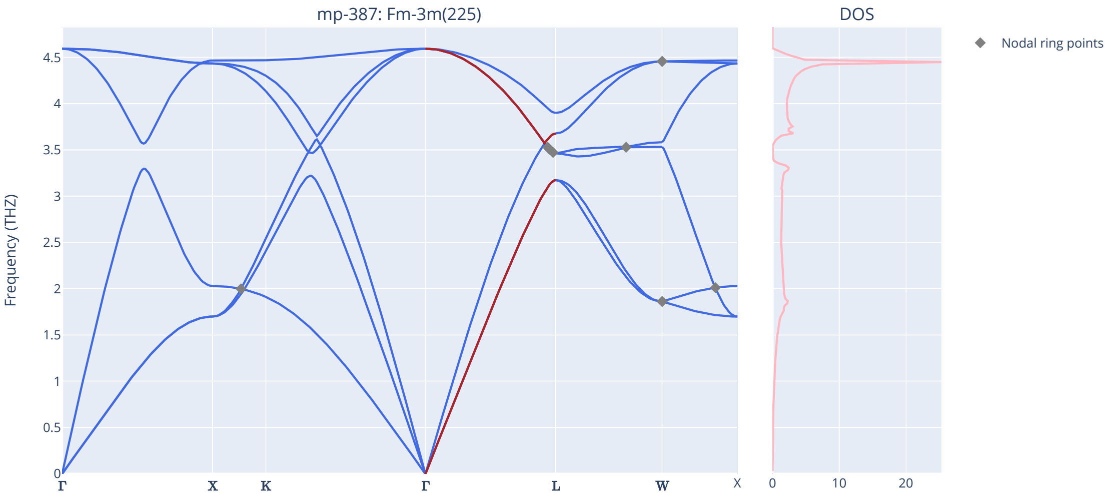

Supplementary Figure 293: The phonon band and DOS of Ce4Sb4.

Supplementary Table 558: High symmetry lines of mp-387-Ce4Sb4

	Start	End
0	Γ (0.000, 0.000, 0.000)	X (0.500, 0.000, 0.500)
1	X (0.500, 0.000, 0.500)	U (0.625, 0.250, 0.625)
2	K (0.375, 0.375, 0.750)	Γ (0.000, 0.000, 0.000)
3	Γ (0.000, 0.000, 0.000)	L (0.500, 0.500, 0.500)
4	L (0.500, 0.500, 0.500)	W (0.500, 0.250, 0.750)
5	W (0.500, 0.250, 0.750)	X (0.500, 0.000, 0.500)

Supplementary Table 559: Nodal ring points along high symmetry lines of mp-387-Ce4Sb4.

	Coordinates	Frequency	Mode 1	Mode 2	Multi.	Deg.	Path	Ratio
0	(0.500, 0.250, 0.750)	1.862	2	1	6	2	5	1.000
1	(0.567, 0.133, 0.567)	1.998	3	2	12	2	2	0.534
2	(0.498, 0.072, 0.574)	2.011	3	2	24	2	6	0.708
3	(0.476, 0.476, 0.476)	3.507	4	3	8	2	4	0.951
4	(0.469, 0.469, 0.469)	3.527	4	3	8	2	4	0.939
5	(0.490, 0.490, 0.490)	3.472	4	3	8	2	4	0.980
6	(0.500, 0.335, 0.665)	3.529	4	3	24	2	5	0.661
7	(0.500, 0.250, 0.750)	4.457	6	5	6	2	5	1.000

The followings are details for Lu_4Se_4. It has 2 topological nodal lines and 2 nodal ring points along high symmetry line as shown in Fig **294**.

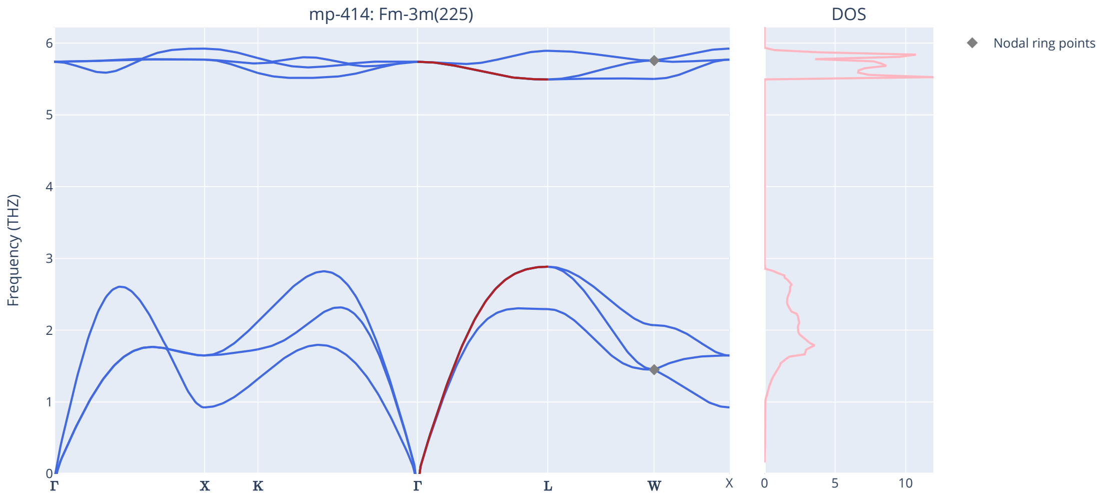

Supplementary Figure 294: The phonon band and DOS of Lu4Se4.

Supplementary Table 560: High symmetry lines of mp-414-Lu4Se4

	Start	End
0	Γ (0.000, 0.000, 0.000)	X (0.500, 0.000, 0.500)
1	X (0.500, 0.000, 0.500)	U (0.625, 0.250, 0.625)
2	K (0.375, 0.375, 0.750)	Γ (0.000, 0.000, 0.000)
3	Γ (0.000, 0.000, 0.000)	L (0.500, 0.500, 0.500)
4	L (0.500, 0.500, 0.500)	W (0.500, 0.250, 0.750)
5	W (0.500, 0.250, 0.750)	X (0.500, 0.000, 0.500)

Supplementary Table 561: Nodal ring points along high symmetry lines of mp-414-Lu4Se4.

	Coordinates	Frequency	Mode 1	Mode 2	Multi.	Deg.	Path	Ratio
0	(0.500, 0.250, 0.750)	1.450	2	1	6	2	5	1.000
1	(0.500, 0.250, 0.750)	5.757	6	5	6	2	5	1.000

The followings are details for Sc_4Sb_4. It has 2 topological nodal lines and 3 nodal ring points along high symmetry line as shown in Fig **295**.

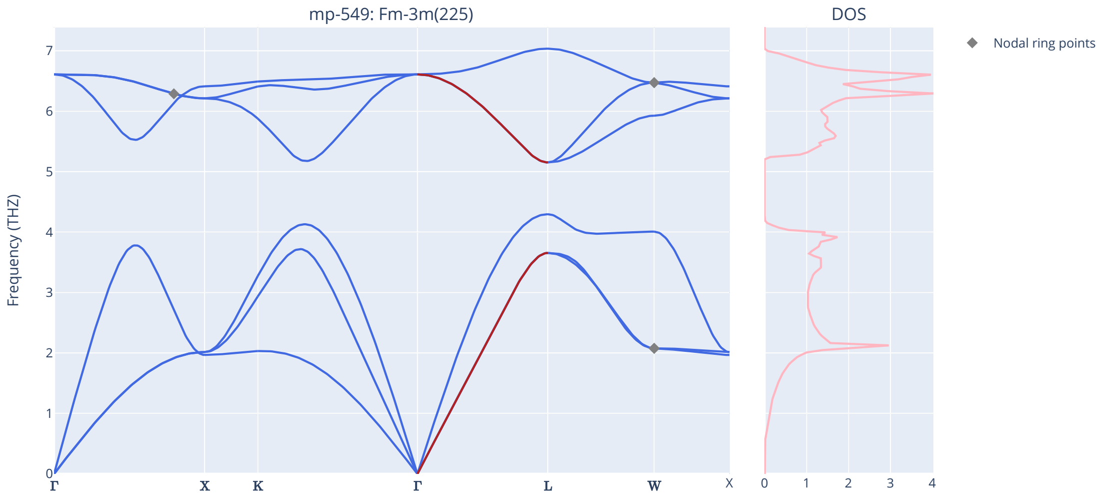

Supplementary Figure 295: The phonon band and DOS of Sc4Sb4.

Supplementary Table 562: High symmetry lines of mp-549-Sc4Sb4

	Start	End
0	Γ (0.000, 0.000, 0.000)	X (0.500, 0.000, 0.500)
1	X (0.500, 0.000, 0.500)	U (0.625, 0.250, 0.625)
2	K (0.375, 0.375, 0.750)	Γ (0.000, 0.000, 0.000)
3	Γ (0.000, 0.000, 0.000)	L (0.500, 0.500, 0.500)
4	L (0.500, 0.500, 0.500)	W (0.500, 0.250, 0.750)
5	W (0.500, 0.250, 0.750)	X (0.500, 0.000, 0.500)

Supplementary Table 563: Nodal ring points along high symmetry lines of mp-549-Sc4Sb4.

	Coordinates	Frequency	Mode 1	Mode 2	Multi.	Deg.	Path	Ratio
0	(0.500, 0.250, 0.750)	2.075	2	1	6	2	5	1.000
1	(0.398, 0.000, 0.398)	6.289	6	5	6	2	1	0.796
2	(0.500, 0.250, 0.750)	6.473	6	5	6	2	5	1.000

The followings are details for Np_4Se_4. It has 4 topological nodal lines and 4 nodal ring points along high symmetry line as shown in Fig **296**.

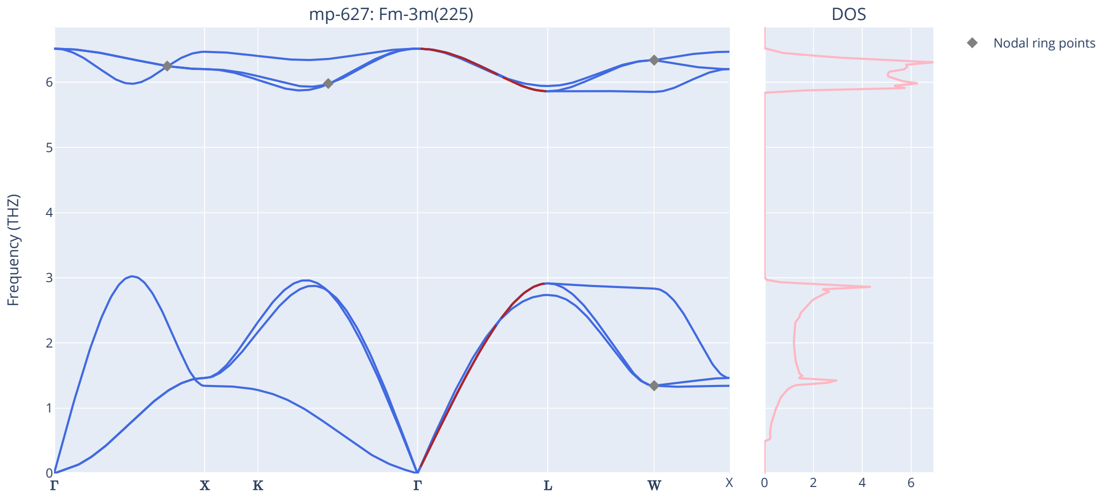

Supplementary Figure 296: The phonon band and DOS of Np4Se4.

Supplementary Table 564: High symmetry lines of mp-627-Np4Se4

	Start	End
0	Γ (0.000, 0.000, 0.000)	X (0.500, 0.000, 0.500)
1	X (0.500, 0.000, 0.500)	U (0.625, 0.250, 0.625)
2	K (0.375, 0.375, 0.750)	Γ (0.000, 0.000, 0.000)
3	Γ (0.000, 0.000, 0.000)	L (0.500, 0.500, 0.500)
4	L (0.500, 0.500, 0.500)	W (0.500, 0.250, 0.750)
5	W (0.500, 0.250, 0.750)	X (0.500, 0.000, 0.500)

Supplementary Table 565: Nodal ring points along high symmetry lines of mp-627-Np4Se4.

	Coordinates	Frequency	Mode 1	Mode 2	Multi.	Deg.	Path	Ratio
0	(0.500, 0.250, 0.750)	1.344	2	1	6	2	5	1.000
1	(0.214, 0.214, 0.414)	5.980	5	4	24	2	3	0.441
2	(0.376, 0.000, 0.376)	6.249	6	5	6	2	1	0.752
3	(0.500, 0.250, 0.750)	6.339	6	5	6	2	5	1.000

The followings are details for Th_4N_4. It has 3 topological nodal lines and 5 nodal ring points along high symmetry line as shown in Fig **297**.

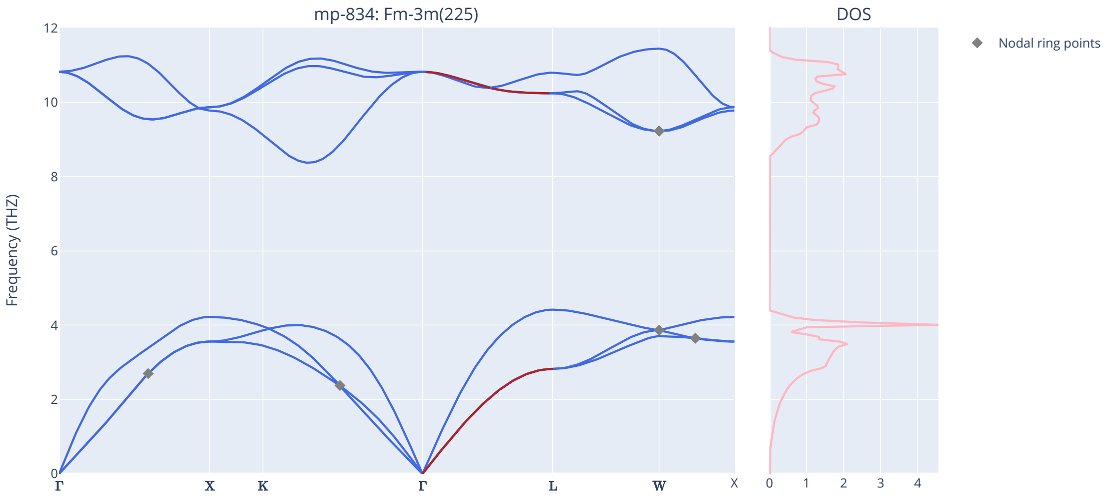

Supplementary Figure 297: The phonon band and DOS of Th4N4.

Supplementary Table 566: High symmetry lines of mp-834-Th4N4

	Start	End
0	Γ (0.000, 0.000, 0.000)	X (0.500, 0.000, 0.500)
1	X (0.500, 0.000, 0.500)	U (0.625, 0.250, 0.625)
2	K (0.375, 0.375, 0.750)	Γ (0.000, 0.000, 0.000)
3	Γ (0.000, 0.000, 0.000)	L (0.500, 0.500, 0.500)
4	L (0.500, 0.500, 0.500)	W (0.500, 0.250, 0.750)
5	W (0.500, 0.250, 0.750)	X (0.500, 0.000, 0.500)

Supplementary Table 567: Nodal ring points along high symmetry lines of mp-834-Th4N4.

	Coordinates	Frequency	Mode 1	Mode 2	Multi.	Deg.	Path	Ratio
0	(0.296, 0.000, 0.296)	2.697	2	1	6	2	1	0.592
1	(0.191, 0.191, 0.390)	2.375	2	1	24	2	3	0.483
2	(0.499, 0.130, 0.629)	3.649	2	1	24	2	6	0.482
3	(0.500, 0.250, 0.750)	3.864	3	2	6	2	5	1.000
4	(0.500, 0.250, 0.750)	9.224	5	4	6	2	5	1.000

The followings are details for Ag_2Br_2. It has 20 topological nodal lines and 38 nodal ring points along high symmetry line as shown in Fig **298**.

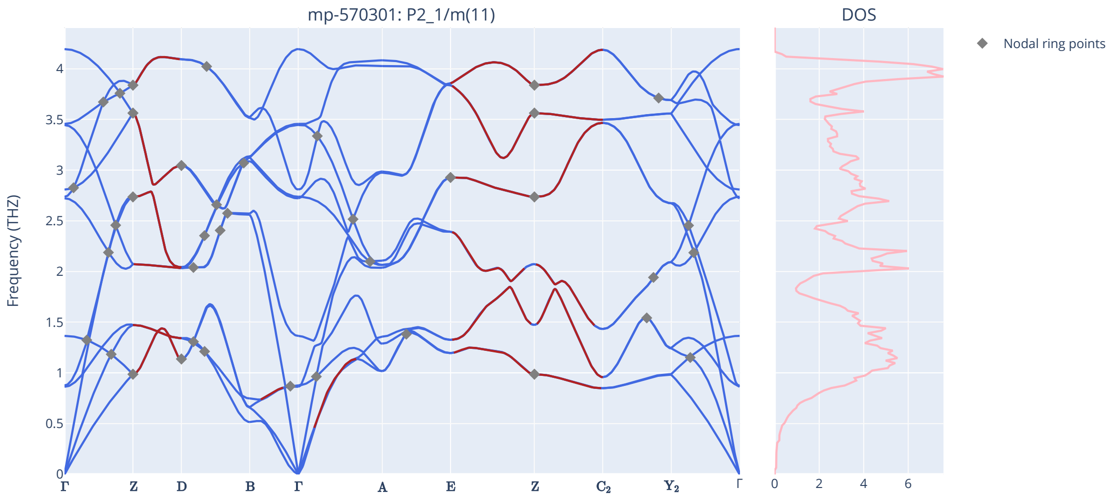

Supplementary Figure 298: The phonon band and DOS of Ag2Br2.

Supplementary Table 568: High symmetry lines of mp-570301-Ag2Br2

	Start	End
0	Γ (0.000, 0.000, 0.000)	Z (0.000, 0.500, 0.000)
1	Z (0.000, 0.500, 0.000)	D (0.000, 0.500, 0.500)
2	D (0.000, 0.500, 0.500)	B (0.000, 0.000, 0.500)
3	B (0.000, 0.000, 0.500)	Γ (0.000, 0.000, 0.000)
4	Γ (0.000, 0.000, 0.000)	A (-0.500, 0.000, 0.500)
5	A (-0.500, 0.000, 0.500)	E (-0.500, 0.500, 0.500)
6	E (-0.500, 0.500, 0.500)	Z (0.000, 0.500, 0.000)
7	Z (0.000, 0.500, 0.000)	C_2 (-0.500, 0.500, 0.000)
8	C_2 (-0.500, 0.500, 0.000)	Y_2 (-0.500, 0.000, 0.000)
9	Y_2 (-0.500, 0.000, 0.000)	Γ (0.000, 0.000, 0.000)

Supplementary Table 569: Nodal ring points along high symmetry lines of mp-570301-Ag2Br2.

	Coordinates	Frequency	Mode 1	Mode 2	Multi.	Deg.	Path	Ratio
0	(0.000, 0.500, 0.000)	0.986	2	1	1	2	1	1.000
1	(0.000, 0.500, 0.500)	1.137	2	1	1	2	2	1.000
2	(-0.000, 0.331, 0.500)	1.211	2	1	2	2	3	0.339
3	(-0.500, 0.177, 0.500)	1.382	2	1	2	2	6	0.354
4	(-0.000, 0.341, -0.000)	1.183	3	2	2	2	1	0.682
5	(-0.003, 0.412, 0.500)	1.307	3	2	4	2	3	0.176
6	(-0.360, 0.000, 0.004)	1.151	3	2	2	2	10	0.279
7	(-0.108, -0.000, 0.108)	0.963	4	3	2	2	5	0.215
8	(-0.500, 0.179, -0.000)	1.542	4	3	2	2	9	0.643
9	(-0.003, -0.000, 0.082)	0.869	5	4	2	2	4	0.836

Supplementary Table 569: Nodal ring points along high symmetry lines of mp-570301-Ag2Br2.

	Coordinates	Frequency	Mode 1	Mode 2	Multi.	Deg.	Path	Ratio
10	(0.000, 0.321, 0.000)	2.188	6	5	2	2	1	0.642
11	(-0.000, 0.162, 0.000)	1.323	6	5	2	2	1	0.325
12	(-0.000, 0.412, 0.500)	2.040	6	5	2	2	3	0.176
13	(-0.000, 0.217, 0.500)	2.405	6	5	2	2	3	0.566
14	(-0.000, 0.163, 0.500)	2.574	6	5	2	2	3	0.674
15	(-0.500, 0.130, -0.000)	1.941	6	5	2	2	9	0.741
16	(-0.335, 0.000, 0.000)	2.186	6	5	2	2	10	0.330
17	(-0.428, -0.000, 0.429)	2.098	7	6	2	2	5	0.857
18	(0.000, 0.500, 0.000)	2.736	8	7	1	2	1	1.000
19	(0.000, 0.374, -0.000)	2.456	8	7	2	2	1	0.748
20	(0.000, 0.045, 0.500)	3.070	8	7	2	2	3	0.910
21	(-0.000, 0.331, 0.500)	2.353	8	7	2	2	3	0.338
22	(-0.500, 0.500, 0.500)	2.928	8	7	1	2	6	1.000
23	(-0.373, 0.000, 0.000)	2.454	8	7	2	2	10	0.254
24	(0.000, 0.066, 0.000)	2.826	9	8	2	2	1	0.132
25	(-0.326, 0.000, 0.327)	2.516	9	8	2	2	5	0.653
26	(0.000, 0.500, 0.000)	3.563	10	9	1	2	1	1.000
27	(0.000, 0.500, 0.500)	3.046	10	9	1	2	2	1.000
28	(-0.000, 0.243, 0.500)	2.658	10	9	2	2	3	0.513
29	(-0.114, 0.000, 0.113)	3.336	10	9	2	2	5	0.227
30	(0.000, 0.284, 0.000)	3.672	11	10	2	2	1	0.567
31	(-0.000, 0.405, 0.000)	3.758	11	10	2	2	1	0.810
32	(0.000, 0.500, 0.000)	3.838	12	11	1	2	1	1.000
33	(0.000, 0.316, 0.500)	4.023	12	11	2	2	3	0.369
34	(-0.499, 0.092, 0.001)	3.711	12	11	4	2	9	0.817

The followings are details for Sn_2Se_2. It has 24 topological nodal lines and 40 nodal ring points along high symmetry line as shown in Fig 299.

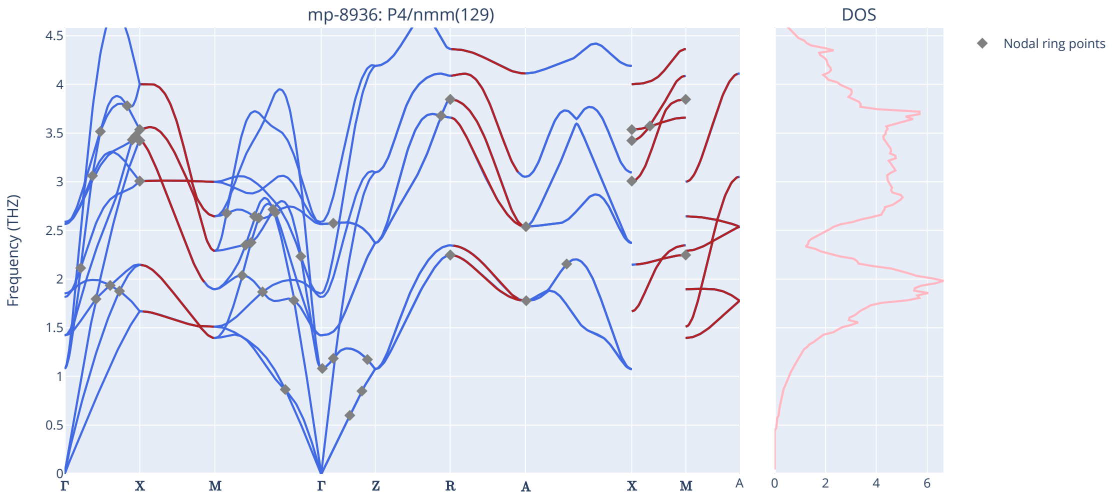

Supplementary Figure 299: The phonon band and DOS of Sn2Se2.

Supplementary Table 570: High symmetry lines of mp-8936-Sn2Se2

	Start	End
0	Γ (0.000, 0.000, 0.000)	X (0.000, 0.500, 0.000)
1	X (0.000, 0.500, 0.000)	M (0.500, 0.500, 0.000)
2	M (0.500, 0.500, 0.000)	Γ (0.000, 0.000, 0.000)
3	Γ (0.000, 0.000, 0.000)	Z (0.000, 0.000, 0.500)
4	Z (0.000, 0.000, 0.500)	R (0.000, 0.500, 0.500)
5	R (0.000, 0.500, 0.500)	A (0.500, 0.500, 0.500)
6	A (0.500, 0.500, 0.500)	Z (0.000, 0.000, 0.500)
7	X (0.000, 0.500, 0.000)	R (0.000, 0.500, 0.500)
8	M (0.500, 0.500, 0.000)	A (0.500, 0.500, 0.500)

Supplementary Table 571: Nodal ring points along high symmetry lines of mp-8936-Sn2Se2.

	Coordinates	Frequency	Mode 1	Mode 2	Multi.	Deg.	Path	Ratio
0	(0.169, 0.169, -0.000)	0.865	2	1	4	2	3	0.662
1	(0.000, 0.000, 0.265)	0.600	2	1	2	2	4	0.531
2	(0.000, 0.000, 0.378)	0.850	2	1	2	2	4	0.755
3	(0.000, 0.500, 0.500)	2.247	2	1	2	2	5	1.000
4	(-0.000, 0.365, -0.000)	1.876	3	2	4	2	1	0.731
5	(0.496, 0.496, 0.500)	1.778	3	2	4	2	7	0.007
6	(0.000, 0.304, 0.000)	1.934	4	3	4	2	1	0.607
7	(-0.000, 0.209, -0.000)	1.795	4	3	4	2	1	0.418
8	(0.276, 0.276, 0.000)	1.868	4	3	4	2	3	0.448
9	(0.128, 0.128, -0.000)	1.781	4	3	4	2	3	0.744
10	(0.000, 0.000, 0.429)	1.173	4	3	2	2	4	0.857

Supplementary Table 571: Nodal ring points along high symmetry lines of mp-8936-Sn2Se2.

	Coordinates	Frequency	Mode 1	Mode 2	Multi.	Deg.	Path	Ratio
11	(0.306, 0.306, 0.500)	2.155	4	3	4	2	7	0.388
12	(0.372, 0.372, -0.000)	2.037	5	4	4	2	3	0.257
13	(0.000, 0.000, 0.010)	1.081	5	4	2	2	4	0.020
14	(0.000, 0.000, 0.112)	1.184	5	4	2	2	4	0.224
15	(0.000, 0.500, 0.000)	3.007	6	5	2	2	1	1.000
16	(0.330, 0.330, 0.000)	2.376	6	5	4	2	3	0.340
17	(0.216, 0.216, -0.000)	2.683	7	6	4	2	3	0.567
18	(0.356, 0.356, -0.000)	2.350	7	6	4	2	3	0.289
19	(0.294, 0.294, -0.000)	2.628	7	6	4	2	3	0.412
20	(0.227, 0.227, -0.000)	2.715	7	6	4	2	3	0.546
21	(0.498, 0.498, 0.500)	2.538	7	6	4	2	7	0.004
22	(-0.000, 0.107, -0.000)	2.113	8	7	4	2	1	0.214
23	(0.000, 0.500, 0.000)	3.423	8	7	2	2	1	1.000
24	(0.313, 0.313, 0.000)	2.644	8	7	4	2	3	0.373
25	(0.096, 0.096, 0.000)	2.233	8	7	4	2	3	0.808
26	(0.000, 0.439, 0.500)	3.678	8	7	4	2	5	0.878
27	(0.000, 0.500, 0.500)	3.846	8	7	2	2	5	1.000
28	(0.000, 0.451, -0.000)	3.431	9	8	4	2	1	0.901
29	(-0.000, 0.471, -0.000)	3.468	9	8	4	2	1	0.943
30	(0.445, 0.445, 0.000)	2.676	9	8	4	2	3	0.110
31	(0.000, 0.498, 0.169)	3.573	9	8	8	2	8	0.337
32	(-0.000, 0.186, -0.000)	3.061	10	9	4	2	1	0.371
33	(0.000, 0.500, 0.000)	3.535	10	9	2	2	1	1.000
34	(0.001, 0.238, -0.000)	3.516	11	10	8	2	1	0.477
35	(0.000, 0.416, 0.000)	3.780	11	10	4	2	1	0.833
36	(0.000, 0.000, 0.112)	2.573	11	10	2	2	4	0.224

The followings are details for Ba_4Te_4. It has 3 topological nodal lines and 6 nodal ring points along high symmetry line as shown in Fig 300.

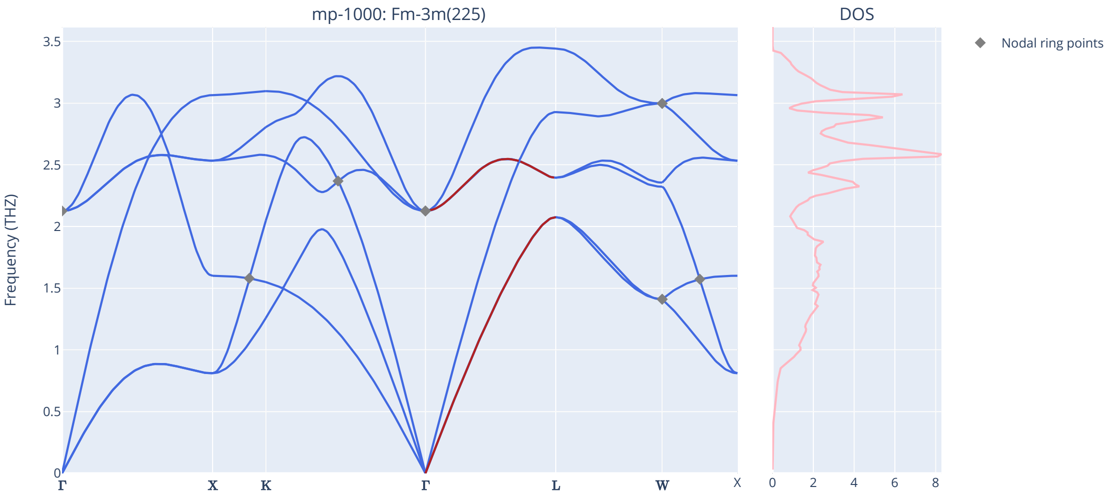

Supplementary Figure 300: The phonon band and DOS of Ba4Te4.

Supplementary Table 572: High symmetry lines of mp-1000-Ba4Te4

	Start	End
0	Γ (0.000, 0.000, 0.000)	X (0.500, 0.000, 0.500)
1	X (0.500, 0.000, 0.500)	U (0.625, 0.250, 0.625)
2	K (0.375, 0.375, 0.750)	Γ (0.000, 0.000, 0.000)
3	Γ (0.000, 0.000, 0.000)	L (0.500, 0.500, 0.500)
4	L (0.500, 0.500, 0.500)	W (0.500, 0.250, 0.750)
5	W (0.500, 0.250, 0.750)	X (0.500, 0.000, 0.500)

Supplementary Table 573: Nodal ring points along high symmetry lines of mp-1000-Ba4Te4.

	Coordinates	Frequency	Mode 1	Mode 2	Multi.	Deg.	Path	Ratio
0	(0.500, 0.250, 0.750)	1.410	2	1	6	2	5	1.000
1	(0.586, 0.172, 0.586)	1.580	3	2	12	2	2	0.689
2	(0.499, 0.124, 0.625)	1.573	3	2	24	2	6	0.501
3	(0.207, 0.207, 0.409)	2.368	4	3	24	2	3	0.453
4	(0.000, 0.000, 0.000)	2.126	5	4	1	3	1	0.000
5	(0.500, 0.250, 0.750)	2.997	6	5	6	2	5	1.000

The followings are details for Mg_4Se_4. It has 2 topological nodal lines and 7 nodal ring points along high symmetry line as shown in Fig 301.

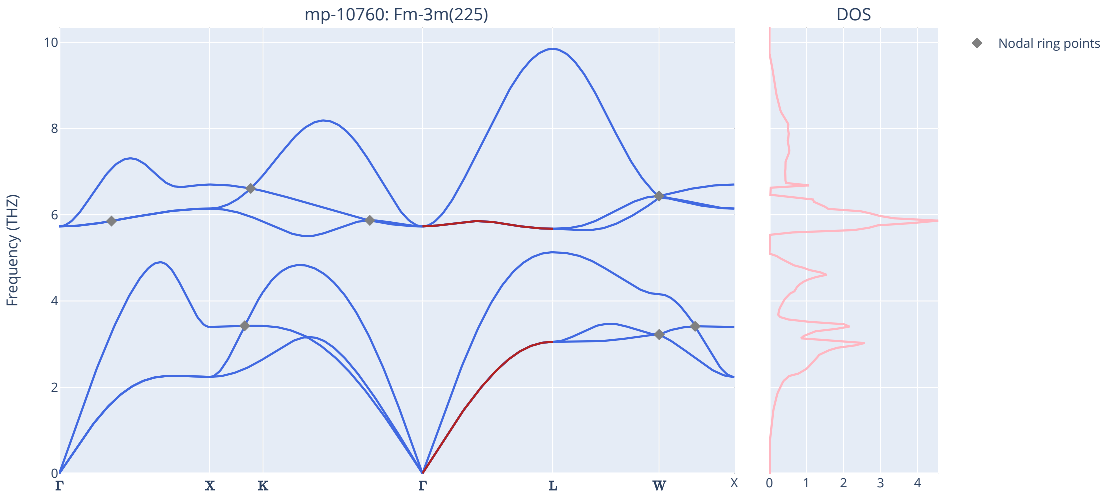

Supplementary Figure 301: The phonon band and DOS of Mg4Se4.

Supplementary Table 574: High symmetry lines of mp-10760-Mg4Se4

	Start	End
0	Γ (0.000, 0.000, 0.000)	X (0.500, 0.000, 0.500)
1	X (0.500, 0.000, 0.500)	U (0.625, 0.250, 0.625)
2	K (0.375, 0.375, 0.750)	Γ (0.000, 0.000, 0.000)
3	Γ (0.000, 0.000, 0.000)	L (0.500, 0.500, 0.500)
4	L (0.500, 0.500, 0.500)	W (0.500, 0.250, 0.750)
5	W (0.500, 0.250, 0.750)	X (0.500, 0.000, 0.500)

Supplementary Table 575: Nodal ring points along high symmetry lines of mp-10760-Mg4Se4.

	Coordinates	Frequency	Mode 1	Mode 2	Multi.	Deg.	Path	Ratio
0	(0.500, 0.250, 0.750)	3.223	2	1	6	2	5	1.000
1	(0.582, 0.164, 0.582)	3.425	3	2	12	2	2	0.657
2	(0.499, 0.130, 0.631)	3.412	3	2	24	2	6	0.478
3	(0.173, 0.000, 0.173)	5.854	5	4	6	2	1	0.347
4	(0.122, 0.122, 0.249)	5.869	5	4	24	2	3	0.670
5	(0.596, 0.193, 0.596)	6.610	6	5	12	2	2	0.771
6	(0.500, 0.250, 0.750)	6.435	6	5	6	2	5	1.000

The followings are details for Sr_4S_4. It has 2 topological nodal lines and 5 nodal ring points along high symmetry line as shown in Fig **302**.

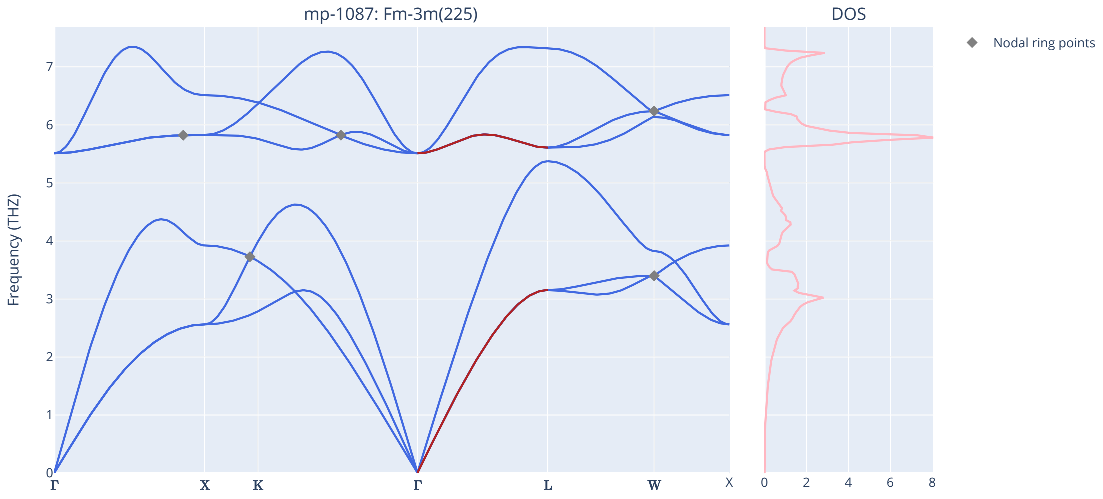

Supplementary Figure 302: The phonon band and DOS of Sr4S4.

Supplementary Table 576: High symmetry lines of mp-1087-Sr4S4

	Start	End
0	Γ (0.000, 0.000, 0.000)	X (0.500, 0.000, 0.500)
1	X (0.500, 0.000, 0.500)	U (0.625, 0.250, 0.625)
2	K (0.375, 0.375, 0.750)	Γ (0.000, 0.000, 0.000)
3	Γ (0.000, 0.000, 0.000)	L (0.500, 0.500, 0.500)
4	L (0.500, 0.500, 0.500)	W (0.500, 0.250, 0.750)
5	W (0.500, 0.250, 0.750)	X (0.500, 0.000, 0.500)

Supplementary Table 577: Nodal ring points along high symmetry lines of mp-1087-Sr4S4.

	Coordinates	Frequency	Mode 1	Mode 2	Multi.	Deg.	Path	Ratio
0	(0.500, 0.250, 0.750)	3.400	2	1	6	2	5	1.000
1	(0.606, 0.213, 0.606)	3.728	3	2	12	2	2	0.850
2	(0.429, 0.000, 0.429)	5.821	5	4	6	2	1	0.857
3	(0.176, 0.176, 0.364)	5.821	5	4	24	2	3	0.520
4	(0.500, 0.250, 0.750)	6.237	6	5	6	2	5	1.000

The followings are details for Li_4F_4. It has 3 topological nodal lines and 7 nodal ring points along high symmetry line as shown in Fig 303.

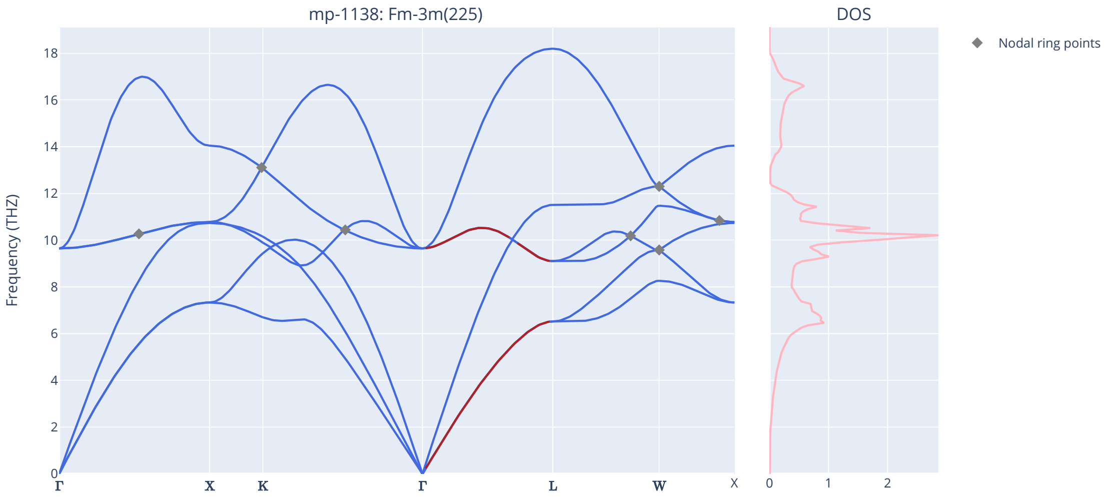

Supplementary Figure 303: The phonon band and DOS of Li4F4.

Supplementary Table 578: High symmetry lines of mp-1138-Li4F4

	Start	End
0	Γ (0.000, 0.000, 0.000)	X (0.500, 0.000, 0.500)
1	X (0.500, 0.000, 0.500)	U (0.625, 0.250, 0.625)
2	K (0.375, 0.375, 0.750)	Γ (0.000, 0.000, 0.000)
3	Γ (0.000, 0.000, 0.000)	L (0.500, 0.500, 0.500)
4	L (0.500, 0.500, 0.500)	W (0.500, 0.250, 0.750)
5	W (0.500, 0.250, 0.750)	X (0.500, 0.000, 0.500)

Supplementary Table 579: Nodal ring points along high symmetry lines of mp-1138-Li4F4.

	Coordinates	Frequency	Mode 1	Mode 2	Multi.	Deg.	Path	Ratio
0	(0.500, 0.250, 0.750)	9.580	3	2	6	2	5	1.000
1	(0.500, 0.317, 0.683)	10.183	4	3	24	2	5	0.732
2	(0.265, 0.000, 0.265)	10.268	5	4	6	2	1	0.531
3	(0.176, 0.176, 0.368)	10.438	5	4	24	2	3	0.517
4	(0.500, 0.050, 0.550)	10.840	5	4	24	2	6	0.800
5	(0.622, 0.245, 0.622)	13.107	6	5	12	2	2	0.978
6	(0.500, 0.250, 0.750)	12.300	6	5	6	2	5	1.000

The followings are details for Rb_4F_4. It has 2 topological nodal lines and 5 nodal ring points along high symmetry line as shown in Fig **304**.

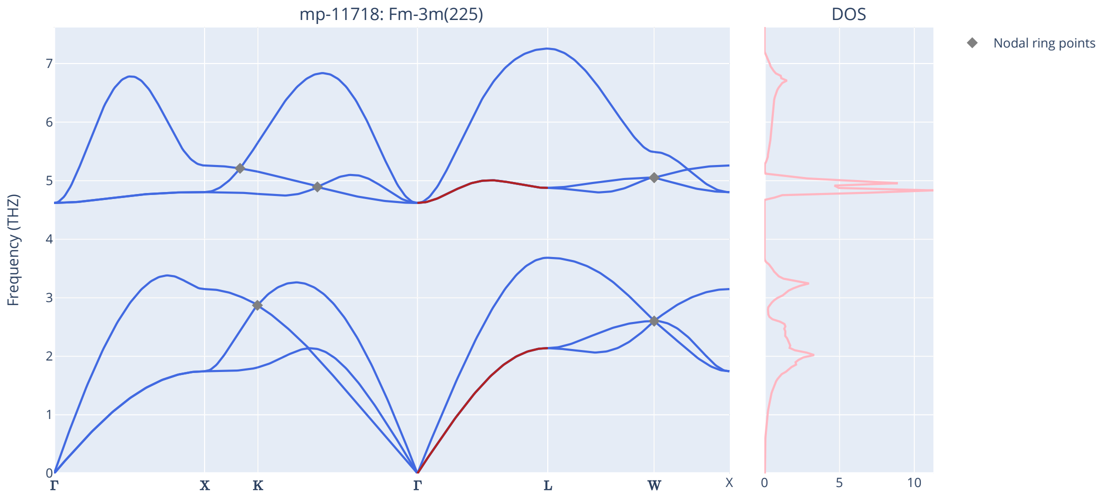

Supplementary Figure 304: The phonon band and DOS of Rb4F4.

Supplementary Table 580: High symmetry lines of mp-11718-Rb4F4

	Start	End
0	Γ (0.000, 0.000, 0.000)	X (0.500, 0.000, 0.500)
1	X (0.500, 0.000, 0.500)	U (0.625, 0.250, 0.625)
2	K (0.375, 0.375, 0.750)	Γ (0.000, 0.000, 0.000)
3	Γ (0.000, 0.000, 0.000)	L (0.500, 0.500, 0.500)
4	L (0.500, 0.500, 0.500)	W (0.500, 0.250, 0.750)
5	W (0.500, 0.250, 0.750)	X (0.500, 0.000, 0.500)

Supplementary Table 581: Nodal ring points along high symmetry lines of mp-11718-Rb4F4.

	Coordinates	Frequency	Mode 1	Mode 2	Multi.	Deg.	Path	Ratio
0	(0.500, 0.250, 0.750)	2.600	2	1	6	2	5	1.000
1	(0.624, 0.248, 0.624)	2.869	3	2	12	2	2	0.993
2	(0.500, 0.250, 0.750)	5.050	5	4	6	2	5	1.000
3	(0.238, 0.233, 0.470)	4.891	5	4	24	2	3	0.373
4	(0.583, 0.167, 0.583)	5.209	6	5	12	2	2	0.667

The followings are details for Yb_4O_4. It has 2 topological nodal lines and 6 nodal ring points along high symmetry line as shown in Fig **305**.

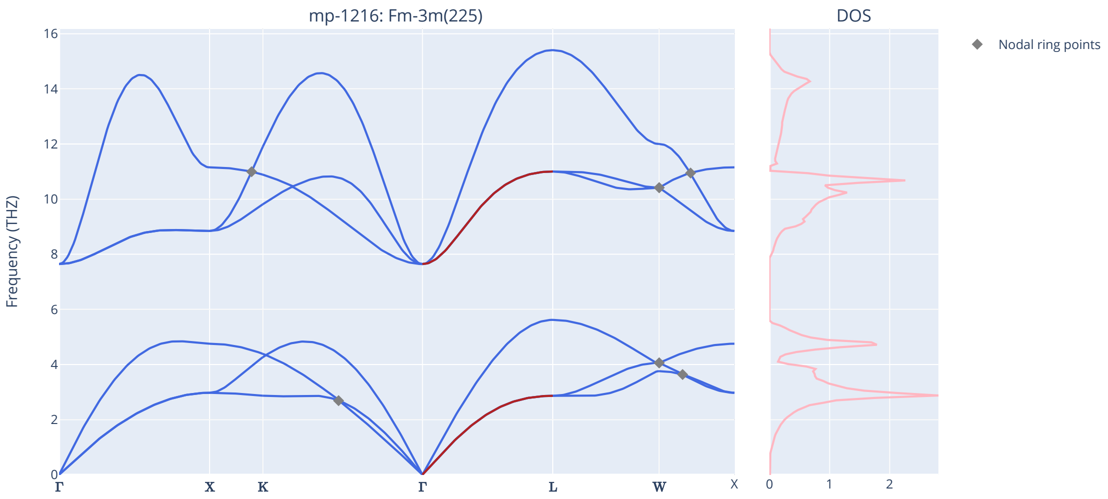

Supplementary Figure 305: The phonon band and DOS of Yb4O4.

Supplementary Table 582: High symmetry lines of mp-1216-Yb4O4

	Start	End
0	Γ (0.000, 0.000, 0.000)	X (0.500, 0.000, 0.500)
1	X (0.500, 0.000, 0.500)	U (0.625, 0.250, 0.625)
2	K (0.375, 0.375, 0.750)	Γ (0.000, 0.000, 0.000)
3	Γ (0.000, 0.000, 0.000)	L (0.500, 0.500, 0.500)
4	L (0.500, 0.500, 0.500)	W (0.500, 0.250, 0.750)
5	W (0.500, 0.250, 0.750)	X (0.500, 0.000, 0.500)

Supplementary Table 583: Nodal ring points along high symmetry lines of mp-1216-Yb4O4.

	Coordinates	Frequency	Mode 1	Mode 2	Multi.	Deg.	Path	Ratio
0	(0.199, 0.199, 0.392)	2.688	2	1	24	2	3	0.475
1	(0.497, 0.173, 0.671)	3.637	2	1	24	2	6	0.311
2	(0.500, 0.250, 0.750)	4.059	3	2	6	2	5	1.000
3	(0.500, 0.250, 0.750)	10.415	5	4	6	2	5	1.000
4	(0.599, 0.197, 0.599)	10.995	6	5	12	2	2	0.790
5	(0.497, 0.144, 0.647)	10.947	6	5	24	2	6	0.417

The followings are details for Ba_4Se_4. It has 3 topological nodal lines and 4 nodal ring points along high symmetry line as shown in Fig 306.

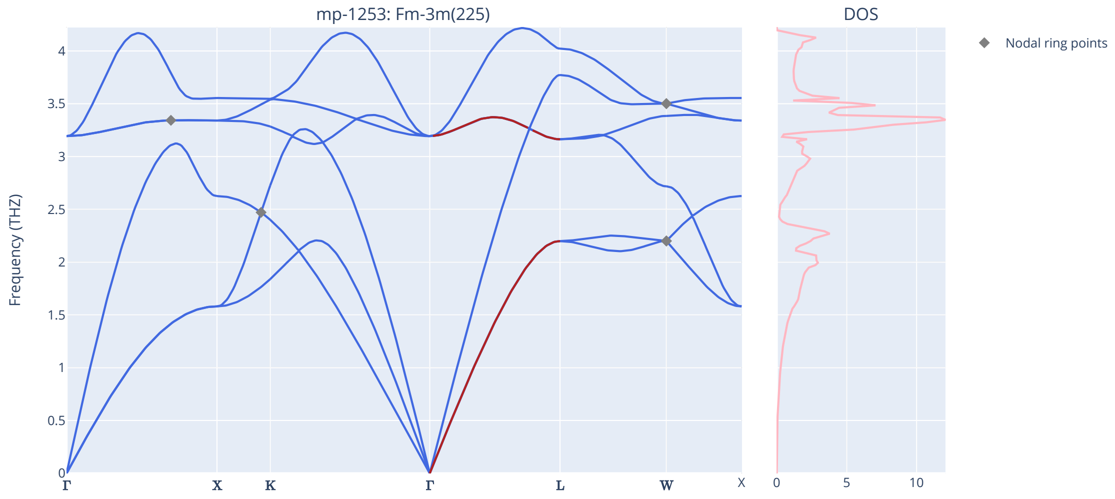

Supplementary Figure 306: The phonon band and DOS of Ba4Se4.

Supplementary Table 584: High symmetry lines of mp-1253-Ba4Se4

	Start	End
0	Γ (0.000, 0.000, 0.000)	X (0.500, 0.000, 0.500)
1	X (0.500, 0.000, 0.500)	U (0.625, 0.250, 0.625)
2	K (0.375, 0.375, 0.750)	Γ (0.000, 0.000, 0.000)
3	Γ (0.000, 0.000, 0.000)	L (0.500, 0.500, 0.500)
4	L (0.500, 0.500, 0.500)	W (0.500, 0.250, 0.750)
5	W (0.500, 0.250, 0.750)	X (0.500, 0.000, 0.500)

Supplementary Table 585: Nodal ring points along high symmetry lines of mp-1253-Ba4Se4.

	Coordinates	Frequency	Mode 1	Mode 2	Multi.	Deg.	Path	Ratio
0	(0.500, 0.250, 0.750)	2.200	2	1	6	2	5	1.000
1	(0.604, 0.207, 0.604)	2.471	3	2	12	2	2	0.829
2	(0.347, 0.000, 0.347)	3.343	5	4	6	2	1	0.694
3	(0.500, 0.250, 0.750)	3.501	6	5	6	2	5	1.000

The followings are details for Mg_4O_4. It has 3 topological nodal lines and 6 nodal ring points along high symmetry line as shown in Fig **307**.

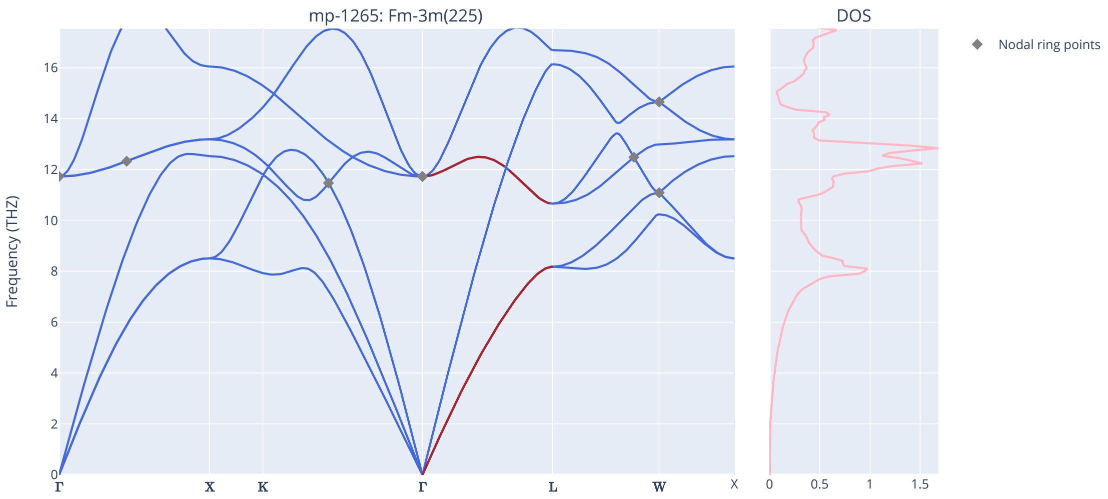

Supplementary Figure 307: The phonon band and DOS of Mg4O4.

Supplementary Table 586: High symmetry lines of mp-1265-Mg4O4

	Start	End
0	Γ (0.000, 0.000, 0.000)	X (0.500, 0.000, 0.500)
1	X (0.500, 0.000, 0.500)	U (0.625, 0.250, 0.625)
2	K (0.375, 0.375, 0.750)	Γ (0.000, 0.000, 0.000)
3	Γ (0.000, 0.000, 0.000)	L (0.500, 0.500, 0.500)
4	L (0.500, 0.500, 0.500)	W (0.500, 0.250, 0.750)
5	W (0.500, 0.250, 0.750)	X (0.500, 0.000, 0.500)

Supplementary Table 587: Nodal ring points along high symmetry lines of mp-1265-Mg4O4.

	Coordinates	Frequency	Mode 1	Mode 2	Multi.	Deg.	Path	Ratio
0	(0.500, 0.250, 0.750)	11.086	3	2	6	2	5	1.000
1	(0.222, 0.222, 0.440)	11.468	4	3	24	2	3	0.411
2	(0.500, 0.309, 0.691)	12.483	4	3	24	2	5	0.762
3	(0.000, 0.000, 0.000)	11.724	5	4	1	3	1	0.000
4	(0.224, 0.000, 0.224)	12.325	5	4	6	2	1	0.449
5	(0.500, 0.250, 0.750)	14.657	6	5	6	2	5	1.000

The followings are details for Mg_4S_4. It has 3 topological nodal lines and 6 nodal ring points along high symmetry line as shown in Fig 308.

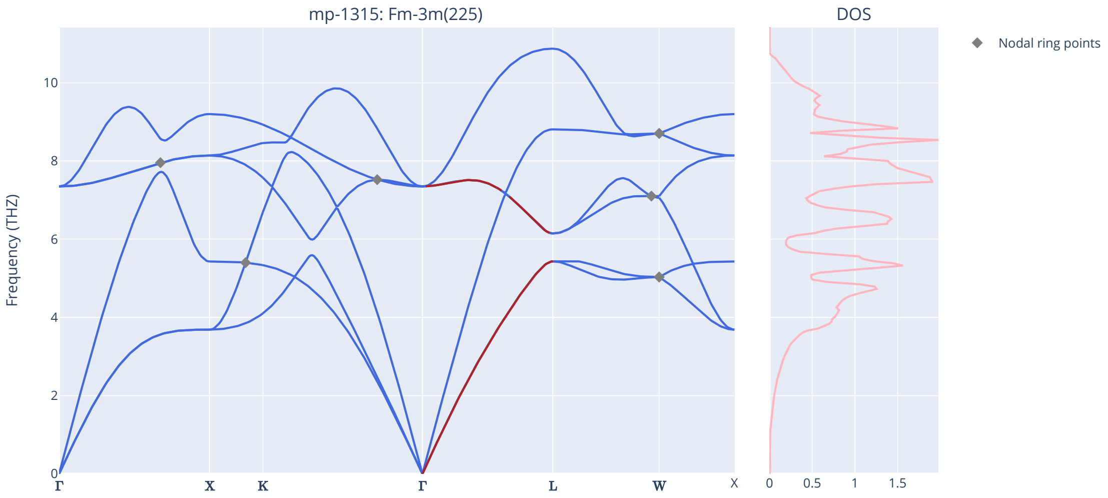

Supplementary Figure 308: The phonon band and DOS of Mg4S4.

Supplementary Table 588: High symmetry lines of mp-1315-Mg4S4

	Start	End
0	Γ (0.000, 0.000, 0.000)	X (0.500, 0.000, 0.500)
1	X (0.500, 0.000, 0.500)	U (0.625, 0.250, 0.625)
2	K (0.375, 0.375, 0.750)	Γ (0.000, 0.000, 0.000)
3	Γ (0.000, 0.000, 0.000)	L (0.500, 0.500, 0.500)
4	L (0.500, 0.500, 0.500)	W (0.500, 0.250, 0.750)
5	W (0.500, 0.250, 0.750)	X (0.500, 0.000, 0.500)

Supplementary Table 589: Nodal ring points along high symmetry lines of mp-1315-Mg4S4.

	Coordinates	Frequency	Mode 1	Mode 2	Multi.	Deg.	Path	Ratio
0	(0.500, 0.250, 0.750)	5.031	2	1	6	2	5	1.000
1	(0.585, 0.169, 0.585)	5.399	3	2	12	2	2	0.677
2	(0.500, 0.268, 0.732)	7.102	4	3	24	2	5	0.927
3	(0.337, 0.000, 0.337)	7.955	5	4	6	2	1	0.673
4	(0.107, 0.107, 0.211)	7.522	5	4	24	2	3	0.717
5	(0.500, 0.250, 0.750)	8.703	6	5	6	2	5	1.000

The followings are details for Ba_4O_4. It has 2 topological nodal lines and 7 nodal ring points along high symmetry line as shown in Fig **309**.

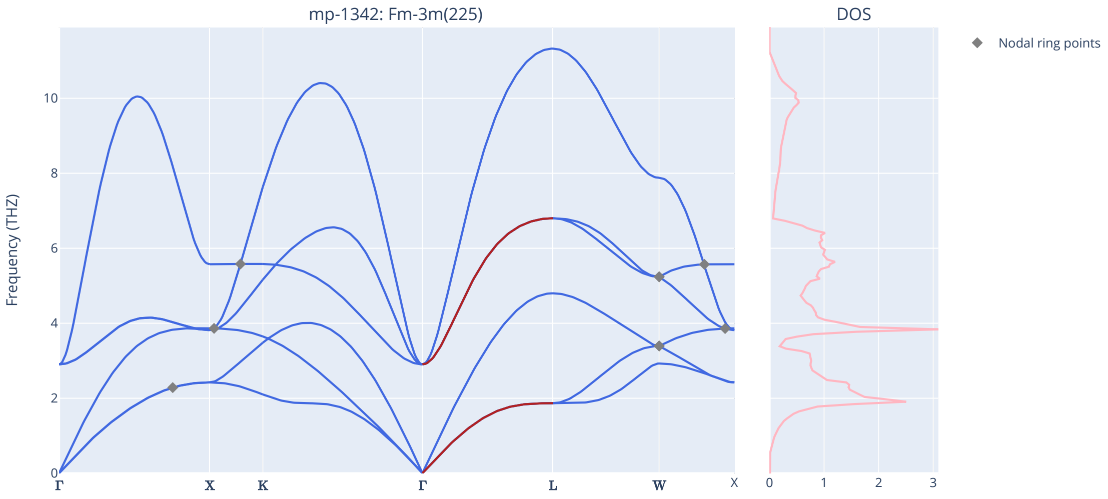

Supplementary Figure 309: The phonon band and DOS of Ba4O4.

Supplementary Table 590: High symmetry lines of mp-1342-Ba4O4

	Start	End
0	Γ (0.000, 0.000, 0.000)	X (0.500, 0.000, 0.500)
1	X (0.500, 0.000, 0.500)	U (0.625, 0.250, 0.625)
2	K (0.375, 0.375, 0.750)	Γ (0.000, 0.000, 0.000)
3	Γ (0.000, 0.000, 0.000)	L (0.500, 0.500, 0.500)
4	L (0.500, 0.500, 0.500)	W (0.500, 0.250, 0.750)
5	W (0.500, 0.250, 0.750)	X (0.500, 0.000, 0.500)

Supplementary Table 591: Nodal ring points along high symmetry lines of mp-1342-Ba4O4.

	Coordinates	Frequency	Mode 1	Mode 2	Multi.	Deg.	Path	Ratio
0	(0.378, 0.000, 0.378)	2.285	2	1	6	2	1	0.755
1	(0.500, 0.250, 0.750)	3.395	3	2	6	2	5	1.000
2	(0.499, 0.031, 0.531)	3.860	4	3	48	2	6	0.876
3	(0.511, 0.021, 0.511)	3.863	5	4	12	2	2	0.084
4	(0.500, 0.250, 0.750)	5.239	5	4	6	2	5	1.000
5	(0.573, 0.145, 0.573)	5.581	6	5	12	2	2	0.580
6	(0.497, 0.098, 0.601)	5.570	6	5	24	2	6	0.601

The followings are details for Ca_4Se_4. It has 2 topological nodal lines and 5 nodal ring points along high symmetry line as shown in Fig **310**.

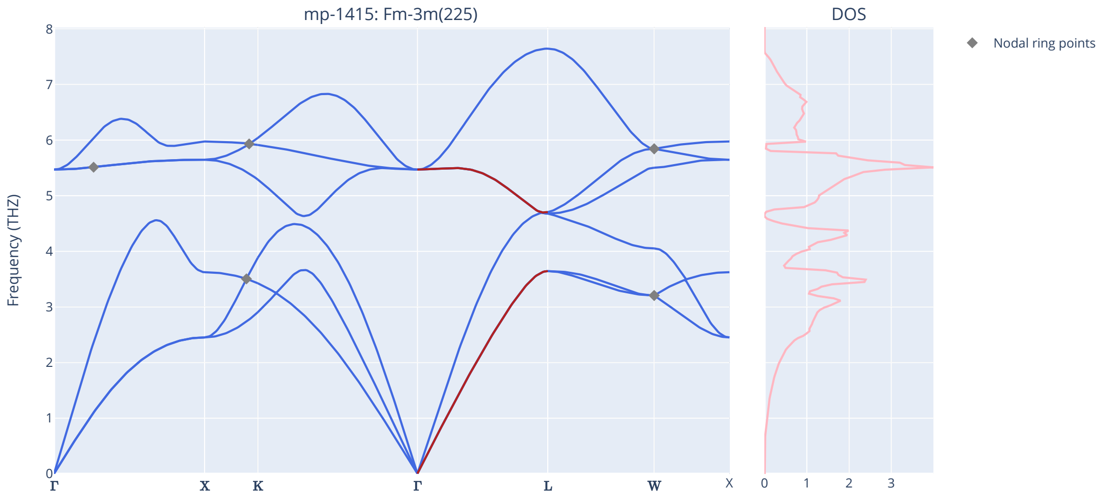

Supplementary Figure 310: The phonon band and DOS of Ca4Se4.

Supplementary Table 592: High symmetry lines of mp-1415-Ca4Se4

	Start	End
0	Γ (0.000, 0.000, 0.000)	X (0.500, 0.000, 0.500)
1	X (0.500, 0.000, 0.500)	U (0.625, 0.250, 0.625)
2	K (0.375, 0.375, 0.750)	Γ (0.000, 0.000, 0.000)
3	Γ (0.000, 0.000, 0.000)	L (0.500, 0.500, 0.500)
4	L (0.500, 0.500, 0.500)	W (0.500, 0.250, 0.750)
5	W (0.500, 0.250, 0.750)	X (0.500, 0.000, 0.500)

Supplementary Table 593: Nodal ring points along high symmetry lines of mp-1415-Ca4Se4.

	Coordinates	Frequency	Mode 1	Mode 2	Multi.	Deg.	Path	Ratio
0	(0.500, 0.250, 0.750)	3.205	2	1	6	2	5	1.000
1	(0.598, 0.197, 0.598)	3.504	3	2	12	2	2	0.787
2	(0.127, 0.000, 0.136)	5.513	5	4	24	2	1	0.263
3	(0.605, 0.209, 0.605)	5.931	6	5	12	2	2	0.838
4	(0.500, 0.250, 0.750)	5.843	6	5	6	2	5	1.000

The followings are details for Si_8. It has 5 topological nodal lines and 6 nodal ring points along high symmetry line as shown in Fig **311**.

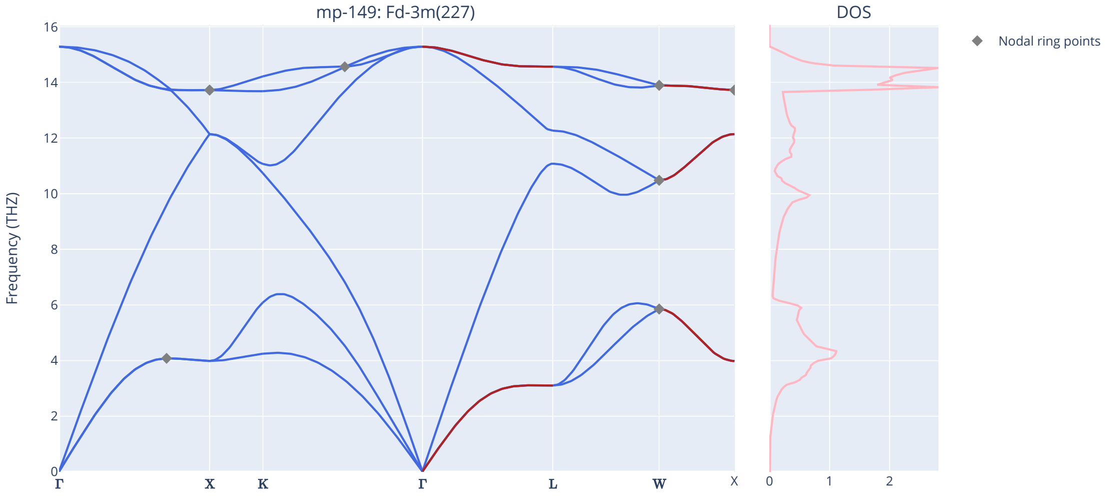

Supplementary Figure 311: The phonon band and DOS of Si8.

Supplementary Table 594: High symmetry lines of mp-149-Si8

	Start	End
0	Γ (0.000, 0.000, 0.000)	X (0.500, 0.000, 0.500)
1	X (0.500, 0.000, 0.500)	U (0.625, 0.250, 0.625)
2	K (0.375, 0.375, 0.750)	Γ (0.000, 0.000, 0.000)
3	Γ (0.000, 0.000, 0.000)	L (0.500, 0.500, 0.500)
4	L (0.500, 0.500, 0.500)	W (0.500, 0.250, 0.750)
5	W (0.500, 0.250, 0.750)	X (0.500, 0.000, 0.500)

Supplementary Table 595: Nodal ring points along high symmetry lines of mp-149-Si8.

	Coordinates	Frequency	Mode 1	Mode 2	Multi.	Deg.	Path	Ratio
0	(0.357, 0.000, 0.357)	4.079	2	1	6	2	1	0.714
1	(0.500, 0.250, 0.750)	5.851	2	1	6	2	5	1.000
2	(0.500, 0.250, 0.750)	10.480	4	3	6	2	5	1.000
3	(0.500, 0.000, 0.500)	13.725	6	5	3	2	1	1.000
4	(0.184, 0.184, 0.364)	14.563	6	5	24	2	3	0.514
5	(0.500, 0.250, 0.750)	13.896	6	5	6	2	5	1.000

The followings are details for Ba_4S_4. It has 2 topological nodal lines and 4 nodal ring points along high symmetry line as shown in Fig 312.

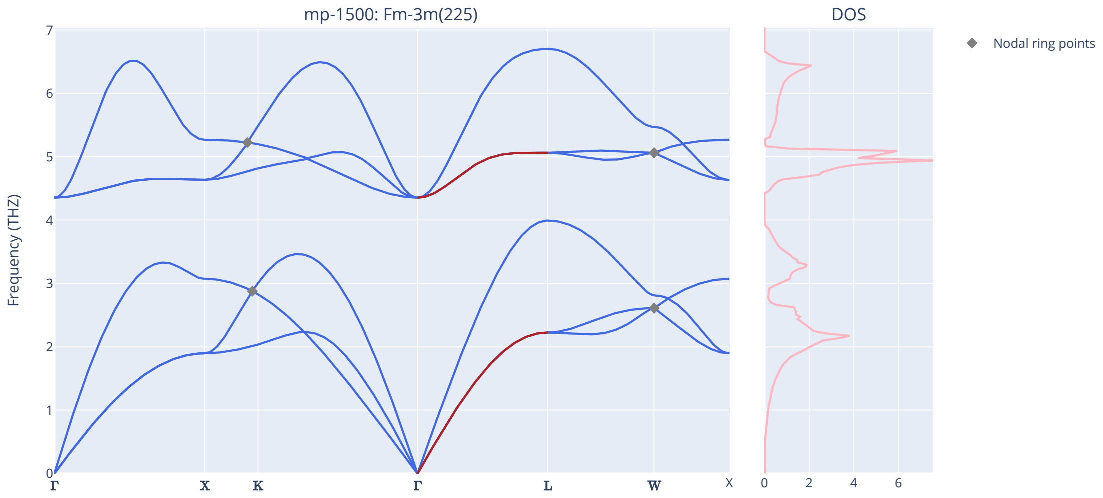

Supplementary Figure 312: The phonon band and DOS of Ba4S4.

Supplementary Table 596: High symmetry lines of mp-1500-Ba4S4

	Start	End
0	Γ (0.000, 0.000, 0.000)	X (0.500, 0.000, 0.500)
1	X (0.500, 0.000, 0.500)	U (0.625, 0.250, 0.625)
2	K (0.375, 0.375, 0.750)	Γ (0.000, 0.000, 0.000)
3	Γ (0.000, 0.000, 0.000)	L (0.500, 0.500, 0.500)
4	L (0.500, 0.500, 0.500)	W (0.500, 0.250, 0.750)
5	W (0.500, 0.250, 0.750)	X (0.500, 0.000, 0.500)

Supplementary Table 597: Nodal ring points along high symmetry lines of mp-1500-Ba4S4.

	Coordinates	Frequency	Mode 1	Mode 2	Multi.	Deg.	Path	Ratio
0	(0.500, 0.250, 0.750)	2.611	2	1	6	2	5	1.000
1	(0.612, 0.224, 0.612)	2.878	3	2	12	2	2	0.895
2	(0.500, 0.250, 0.750)	5.062	5	4	6	2	5	1.000
3	(0.600, 0.201, 0.600)	5.226	6	5	12	2	2	0.802

The followings are details for Ca_4Te_4. It has 2 topological nodal lines and 5 nodal ring points along high symmetry line as shown in Fig **313**.

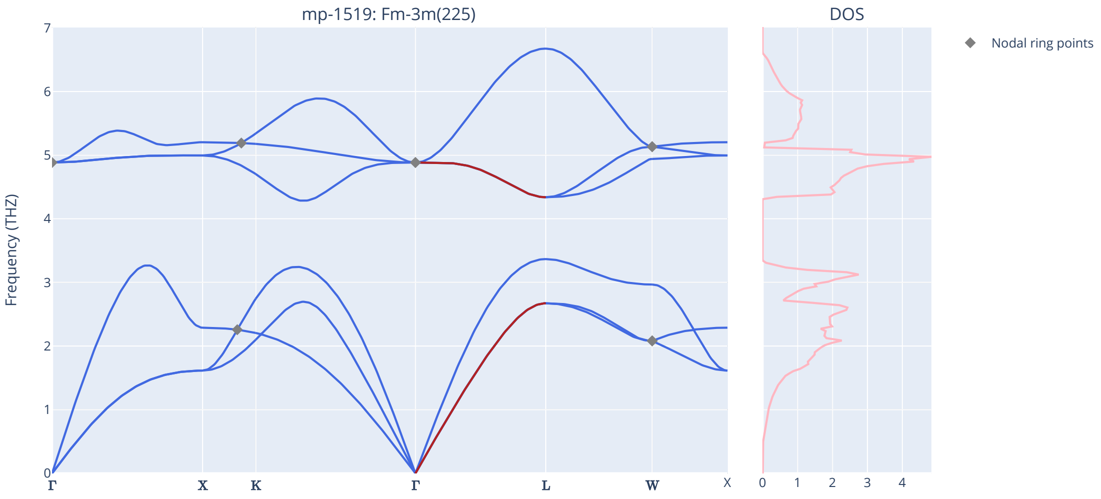

Supplementary Figure 313: The phonon band and DOS of Ca4Te4.

Supplementary Table 598: High symmetry lines of mp-1519-Ca4Te4

	Start	End
0	Γ (0.000, 0.000, 0.000)	X (0.500, 0.000, 0.500)
1	X (0.500, 0.000, 0.500)	U (0.625, 0.250, 0.625)
2	K (0.375, 0.375, 0.750)	Γ (0.000, 0.000, 0.000)
3	Γ (0.000, 0.000, 0.000)	L (0.500, 0.500, 0.500)
4	L (0.500, 0.500, 0.500)	W (0.500, 0.250, 0.750)
5	W (0.500, 0.250, 0.750)	X (0.500, 0.000, 0.500)

Supplementary Table 599: Nodal ring points along high symmetry lines of mp-1519-Ca4Te4.

	Coordinates	Frequency	Mode 1	Mode 2	Multi.	Deg.	Path	Ratio
0	(0.500, 0.250, 0.750)	2.081	2	1	6	2	5	1.000
1	(0.582, 0.163, 0.582)	2.256	3	2	12	2	2	0.652
2	(0.000, 0.000, 0.000)	4.884	5	4	1	3	1	0.000
3	(0.591, 0.182, 0.591)	5.190	6	5	12	2	2	0.728
4	(0.500, 0.250, 0.750)	5.133	6	5	6	2	5	1.000

The followings are details for Ca_4S_4. It has 3 topological nodal lines and 7 nodal ring points along high symmetry line as shown in Fig 314.

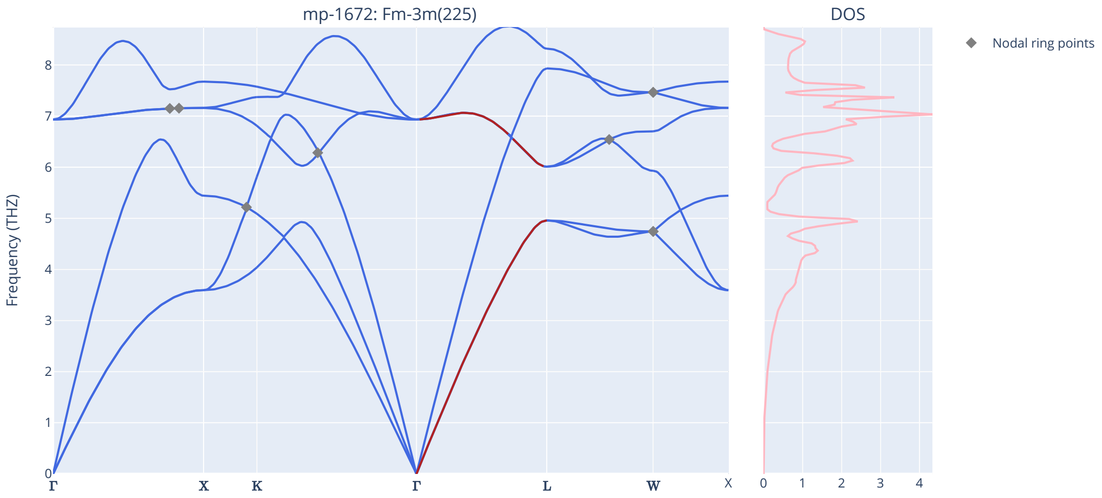

Supplementary Figure 314: The phonon band and DOS of Ca4S4.

Supplementary Table 600: High symmetry lines of mp-1672-Ca4S4

	Start	End
0	Γ (0.000, 0.000, 0.000)	X (0.500, 0.000, 0.500)
1	X (0.500, 0.000, 0.500)	U (0.625, 0.250, 0.625)
2	K (0.375, 0.375, 0.750)	Γ (0.000, 0.000, 0.000)
3	Γ (0.000, 0.000, 0.000)	L (0.500, 0.500, 0.500)
4	L (0.500, 0.500, 0.500)	W (0.500, 0.250, 0.750)
5	W (0.500, 0.250, 0.750)	X (0.500, 0.000, 0.500)

Supplementary Table 601: Nodal ring points along high symmetry lines of mp-1672-Ca4S4.

	Coordinates	Frequency	Mode 1	Mode 2	Multi.	Deg.	Path	Ratio
0	(0.500, 0.250, 0.750)	4.742	2	1	6	2	5	1.000
1	(0.601, 0.201, 0.601)	5.217	3	2	12	2	2	0.806
2	(0.237, 0.237, 0.458)	6.283	4	3	24	2	3	0.382
3	(0.500, 0.353, 0.647)	6.542	4	3	24	2	5	0.587
4	(0.388, 0.000, 0.388)	7.149	5	4	6	2	1	0.776
5	(0.418, 0.000, 0.418)	7.155	5	4	6	2	1	0.837
6	(0.500, 0.250, 0.750)	7.466	6	5	6	2	5	1.000

The followings are details for Al_4N_4. It has 2 topological nodal lines and 1 nodal ring point along high symmetry line as shown in Fig **315**.

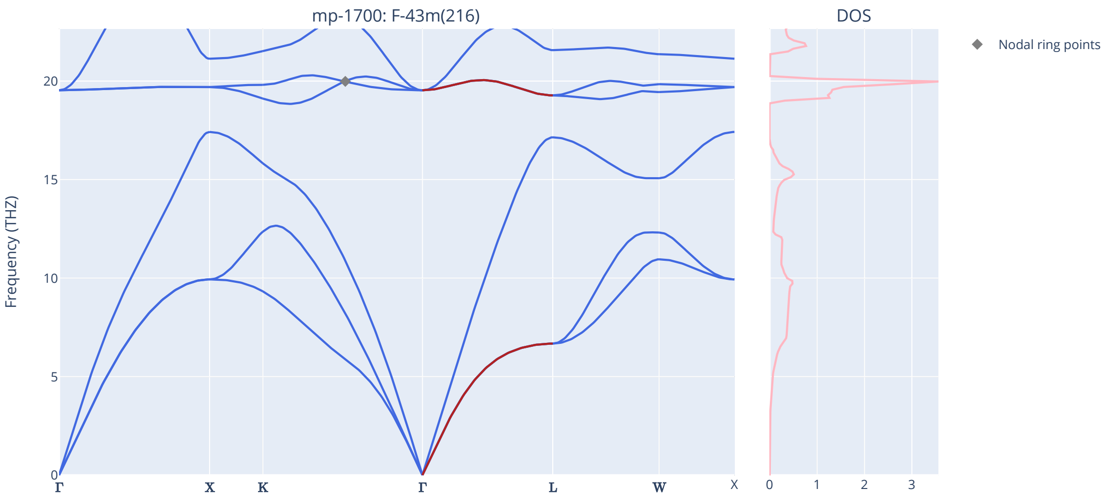

Supplementary Figure 315: The phonon band and DOS of Al4N4.

Supplementary Table 602: High symmetry lines of mp-1700-Al4N4

	Start	End
0	Γ (0.000, 0.000, 0.000)	X (0.500, 0.000, 0.500)
1	X (0.500, 0.000, 0.500)	U (0.625, 0.250, 0.625)
2	K (0.375, 0.375, 0.750)	Γ (0.000, 0.000, 0.000)
3	Γ (0.000, 0.000, 0.000)	L (0.500, 0.500, 0.500)
4	L (0.500, 0.500, 0.500)	W (0.500, 0.250, 0.750)
5	W (0.500, 0.250, 0.750)	X (0.500, 0.000, 0.500)

Supplementary Table 603: Nodal ring points along high symmetry lines of mp-1700-Al4N4.

	Coordinates	Frequency	Mode 1	Mode 2	Multi.	Deg.	Path	Ratio
0	(0.176, 0.176, 0.367)	19.984	5	4	24	2	3	0.517

The followings are details for Yb_4Te_4. It has 3 topological nodal lines and 3 nodal ring points along high symmetry line as shown in Fig **316**.

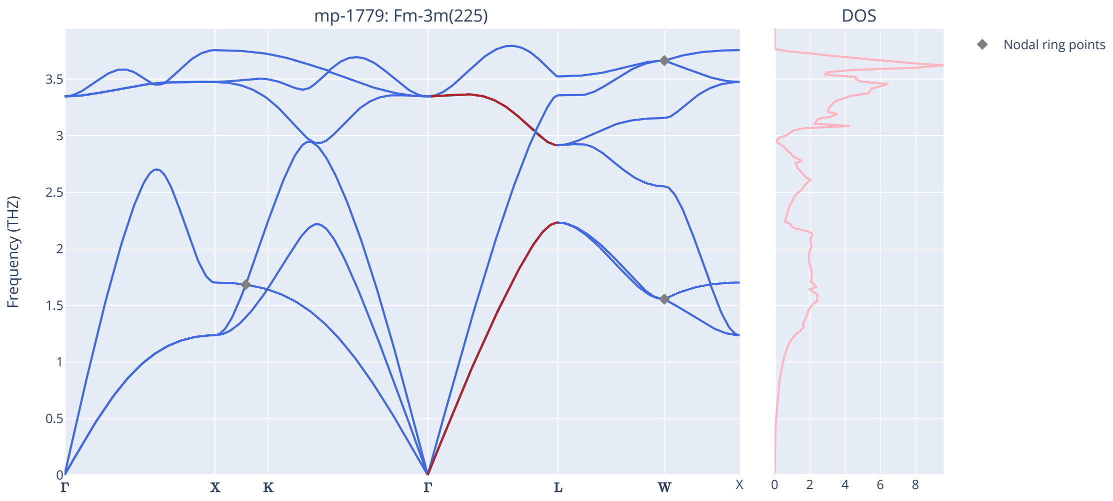

Supplementary Figure 316: The phonon band and DOS of Yb4Te4.

Supplementary Table 604: High symmetry lines of mp-1779-Yb4Te4

	Start	End
0	Γ (0.000, 0.000, 0.000)	X (0.500, 0.000, 0.500)
1	X (0.500, 0.000, 0.500)	U (0.625, 0.250, 0.625)
2	K (0.375, 0.375, 0.750)	Γ (0.000, 0.000, 0.000)
3	Γ (0.000, 0.000, 0.000)	L (0.500, 0.500, 0.500)
4	L (0.500, 0.500, 0.500)	W (0.500, 0.250, 0.750)
5	W (0.500, 0.250, 0.750)	X (0.500, 0.000, 0.500)

Supplementary Table 605: Nodal ring points along high symmetry lines of mp-1779-Yb4Te4.

	Coordinates	Frequency	Mode 1	Mode 2	Multi.	Deg.	Path	Ratio
0	(0.500, 0.250, 0.750)	1.556	2	1	6	2	5	1.000
1	(0.572, 0.144, 0.572)	1.684	3	2	12	2	2	0.578
2	(0.500, 0.250, 0.750)	3.663	6	5	6	2	5	1.000

The followings are details for Cs_4F_4. It has 2 topological nodal lines and 7 nodal ring points along high symmetry line as shown in Fig 317.

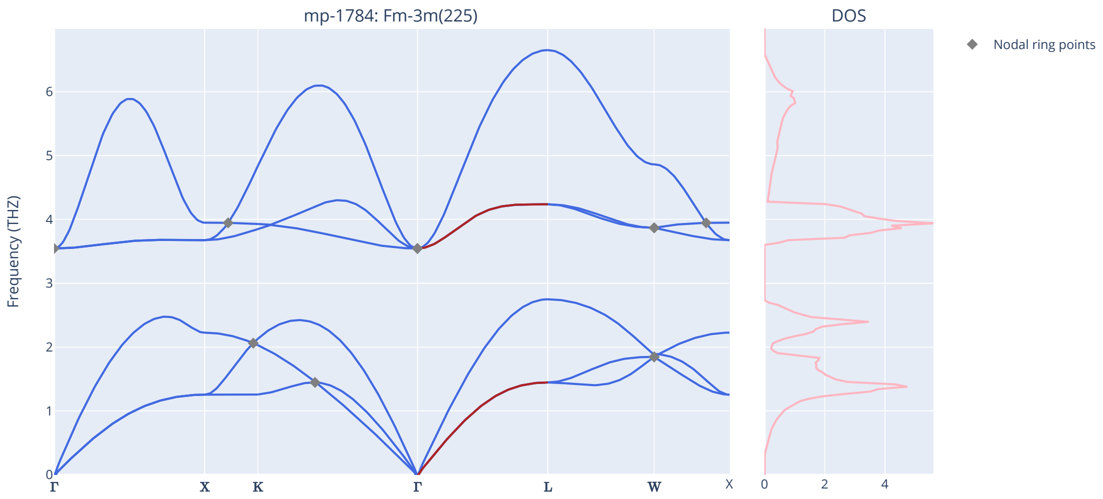

Supplementary Figure 317: The phonon band and DOS of Cs4F4.

Supplementary Table 606: High symmetry lines of mp-1784-Cs4F4

	Start	End
0	Γ (0.000, 0.000, 0.000)	X (0.500, 0.000, 0.500)
1	X (0.500, 0.000, 0.500)	U (0.625, 0.250, 0.625)
2	K (0.375, 0.375, 0.750)	Γ (0.000, 0.000, 0.000)
3	Γ (0.000, 0.000, 0.000)	L (0.500, 0.500, 0.500)
4	L (0.500, 0.500, 0.500)	W (0.500, 0.250, 0.750)
5	W (0.500, 0.250, 0.750)	X (0.500, 0.000, 0.500)

Supplementary Table 607: Nodal ring points along high symmetry lines of mp-1784-Cs4F4.

	Coordinates	Frequency	Mode 1	Mode 2	Multi.	Deg.	Path	Ratio
0	(0.245, 0.245, 0.477)	1.446	2	1	24	2	3	0.358
1	(0.500, 0.250, 0.750)	1.846	2	1	6	2	5	1.000
2	(0.614, 0.229, 0.614)	2.062	3	2	12	2	2	0.915
3	(0.000, 0.000, 0.000)	3.544	5	4	1	3	1	0.000
4	(0.500, 0.250, 0.750)	3.868	5	4	6	2	5	1.000
5	(0.555, 0.111, 0.555)	3.946	6	5	12	2	2	0.443
6	(0.498, 0.076, 0.578)	3.945	6	5	24	2	6	0.691

The followings are details for Yb_4S_4. It has 2 topological nodal lines and 7 nodal ring points along high symmetry line as shown in Fig 318.

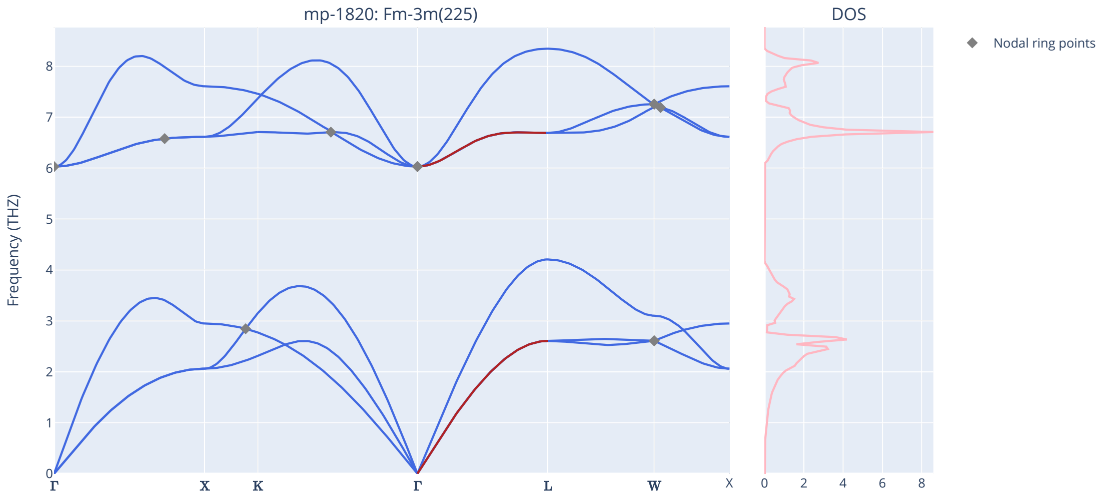

Supplementary Figure 318: The phonon band and DOS of Yb4S4.

Supplementary Table 608: High symmetry lines of mp-1820-Yb4S4

	Start	End
0	Γ (0.000, 0.000, 0.000)	X (0.500, 0.000, 0.500)
1	X (0.500, 0.000, 0.500)	U (0.625, 0.250, 0.625)
2	K (0.375, 0.375, 0.750)	Γ (0.000, 0.000, 0.000)
3	Γ (0.000, 0.000, 0.000)	L (0.500, 0.500, 0.500)
4	L (0.500, 0.500, 0.500)	W (0.500, 0.250, 0.750)
5	W (0.500, 0.250, 0.750)	X (0.500, 0.000, 0.500)

Supplementary Table 609: Nodal ring points along high symmetry lines of mp-1820-Yb4S4.

	Coordinates	Frequency	Mode 1	Mode 2	Multi.	Deg.	Path	Ratio
0	(0.500, 0.250, 0.750)	2.610	2	1	6	2	5	1.000
1	(0.596, 0.193, 0.596)	2.846	3	2	12	2	2	0.772
2	(0.000, 0.000, 0.000)	6.029	5	4	1	3	1	0.000
3	(0.367, 0.000, 0.367)	6.578	5	4	6	2	1	0.735
4	(0.207, 0.207, 0.403)	6.705	5	4	24	2	3	0.458
5	(0.500, 0.229, 0.729)	7.188	5	4	12	2	6	0.084
6	(0.500, 0.250, 0.750)	7.255	6	5	6	2	5	1.000

The followings are details for Sr_4Te_4. It has 2 topological nodal lines and 12 nodal ring points along high symmetry line as shown in Fig 319.

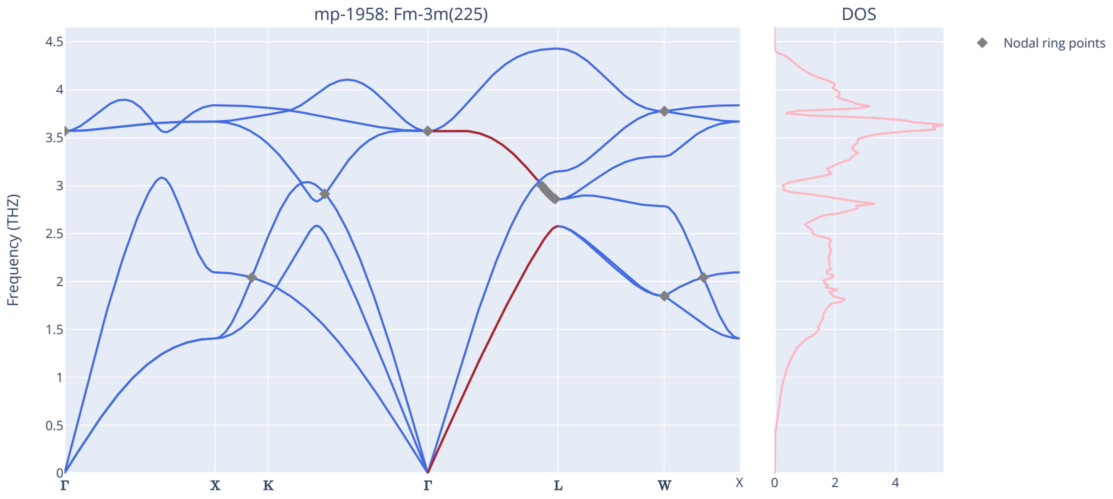

Supplementary Figure 319: The phonon band and DOS of Sr4Te4.

Supplementary Table 610: High symmetry lines of mp-1958-Sr4Te4

	Start	End
0	Γ (0.000, 0.000, 0.000)	X (0.500, 0.000, 0.500)
1	X (0.500, 0.000, 0.500)	U (0.625, 0.250, 0.625)
2	K (0.375, 0.375, 0.750)	Γ (0.000, 0.000, 0.000)
3	Γ (0.000, 0.000, 0.000)	L (0.500, 0.500, 0.500)
4	L (0.500, 0.500, 0.500)	W (0.500, 0.250, 0.750)
5	W (0.500, 0.250, 0.750)	X (0.500, 0.000, 0.500)

Supplementary Table 611: Nodal ring points along high symmetry lines of mp-1958-Sr4Te4.

	Coordinates	Frequency	Mode 1	Mode 2	Multi.	Deg.	Path	Ratio
0	(0.500, 0.250, 0.750)	1.849	2	1	6	2	5	1.000
1	(0.586, 0.173, 0.586)	2.041	3	2	12	2	2	0.692
2	(0.499, 0.119, 0.620)	2.042	3	2	24	2	6	0.520
3	(0.482, 0.482, 0.482)	2.872	4	3	8	2	4	0.964
4	(0.452, 0.452, 0.452)	2.955	4	3	8	2	4	0.903
5	(0.245, 0.245, 0.481)	2.914	4	3	24	2	3	0.354
6	(0.489, 0.489, 0.489)	2.861	4	3	8	2	4	0.979
7	(0.439, 0.439, 0.439)	2.996	4	3	8	2	4	0.878
8	(0.459, 0.459, 0.459)	2.931	4	3	8	2	4	0.918
9	(0.469, 0.469, 0.469)	2.902	4	3	8	2	4	0.939
10	(0.000, 0.000, 0.000)	3.568	5	4	1	3	1	0.000
11	(0.500, 0.250, 0.750)	3.776	6	5	6	2	5	1.000

The followings are details for Pb_4Te_4. It has 2 topological nodal lines and 4 nodal ring points along high symmetry line as shown in Fig **320**.

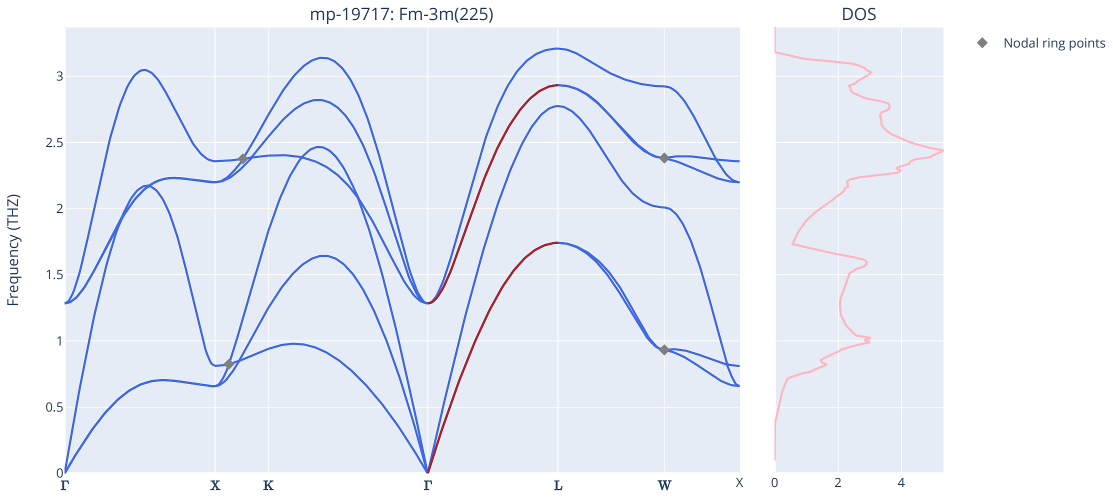

Supplementary Figure 320: The phonon band and DOS of Pb4Te4.

Supplementary Table 612: High symmetry lines of mp-19717-Pb4Te4

	Start	End
0	Γ (0.000, 0.000, 0.000)	X (0.500, 0.000, 0.500)
1	X (0.500, 0.000, 0.500)	U (0.625, 0.250, 0.625)
2	K (0.375, 0.375, 0.750)	Γ (0.000, 0.000, 0.000)
3	Γ (0.000, 0.000, 0.000)	L (0.500, 0.500, 0.500)
4	L (0.500, 0.500, 0.500)	W (0.500, 0.250, 0.750)
5	W (0.500, 0.250, 0.750)	X (0.500, 0.000, 0.500)

Supplementary Table 613: Nodal ring points along high symmetry lines of mp-19717-Pb4Te4.

	Coordinates	Frequency	Mode 1	Mode 2	Multi.	Deg.	Path	Ratio
0	(0.500, 0.250, 0.750)	0.932	2	1	6	2	5	1.000
1	(0.533, 0.066, 0.533)	0.825	3	2	12	2	2	0.262
2	(0.500, 0.250, 0.750)	2.382	5	4	6	2	5	1.000
3	(0.566, 0.131, 0.566)	2.376	6	5	12	2	2	0.525

The followings are details for Na_4Cl_4. It has 3 topological nodal lines and 6 nodal ring points along high symmetry line as shown in Fig 321.

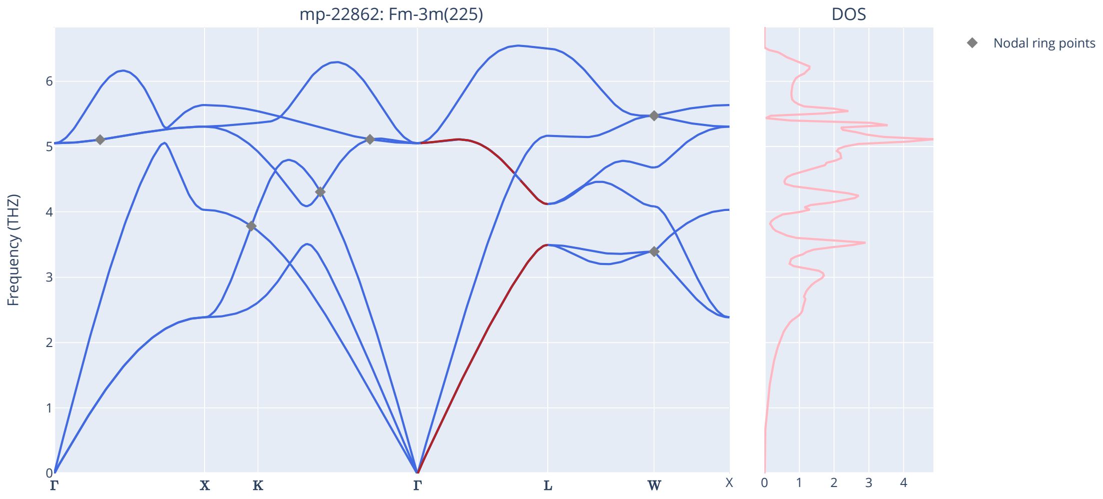

Supplementary Figure 321: The phonon band and DOS of Na4Cl4.

Supplementary Table 614: High symmetry lines of mp-22862-Na4Cl4

	Start	End
0	Γ (0.000, 0.000, 0.000)	X (0.500, 0.000, 0.500)
1	X (0.500, 0.000, 0.500)	U (0.625, 0.250, 0.625)
2	K (0.375, 0.375, 0.750)	Γ (0.000, 0.000, 0.000)
3	Γ (0.000, 0.000, 0.000)	L (0.500, 0.500, 0.500)
4	L (0.500, 0.500, 0.500)	W (0.500, 0.250, 0.750)
5	W (0.500, 0.250, 0.750)	X (0.500, 0.000, 0.500)

Supplementary Table 615: Nodal ring points along high symmetry lines of mp-22862-Na4Cl4.

	Coordinates	Frequency	Mode 1	Mode 2	Multi.	Deg.	Path	Ratio
0	(0.500, 0.250, 0.750)	3.393	2	1	6	2	5	1.000
1	(0.610, 0.220, 0.610)	3.783	3	2	12	2	2	0.878
2	(0.230, 0.230, 0.455)	4.306	4	3	24	2	3	0.392
3	(0.153, 0.000, 0.153)	5.104	5	4	6	2	1	0.306
4	(0.115, 0.115, 0.219)	5.109	5	4	24	2	3	0.703
5	(0.500, 0.250, 0.750)	5.471	6	5	6	2	5	1.000

The followings are details for CsCl. It has 4 topological nodal lines and 7 nodal ring points along high symmetry line as shown in Fig 322.

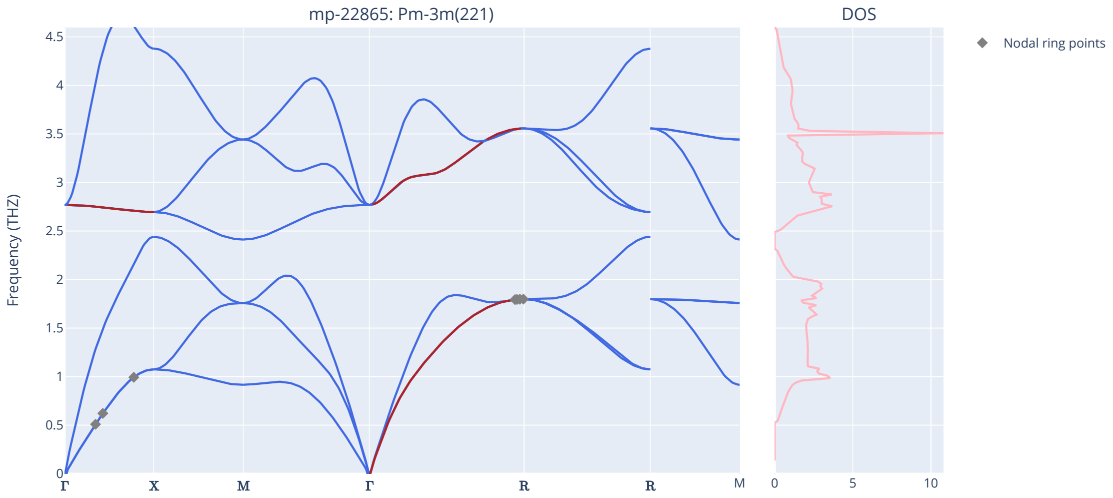

Supplementary Figure 322: The phonon band and DOS of CsCl.

Supplementary Table 616: High symmetry lines of mp-22865-CsCl

	Start	End
0	Γ (0.000, 0.000, 0.000)	X (0.000, 0.500, 0.000)
1	X (0.000, 0.500, 0.000)	M (0.500, 0.500, 0.000)
2	M (0.500, 0.500, 0.000)	Γ (0.000, 0.000, 0.000)
3	Γ (0.000, 0.000, 0.000)	R (0.500, 0.500, 0.500)
4	R (0.500, 0.500, 0.500)	X (0.000, 0.500, 0.000)
5	R (0.500, 0.500, 0.500)	M (0.500, 0.500, 0.000)

Supplementary Table 617: Nodal ring points along high symmetry lines of mp-22865-CsCl.

	Coordinates	Frequency	Mode 1	Mode 2	Multi.	Deg.	Path	Ratio
0	(0.000, 0.173, 0.000)	0.510	2	1	6	2	1	0.347
1	(0.000, 0.214, 0.000)	0.622	2	1	6	2	1	0.429
2	(0.000, 0.388, 0.000)	0.993	2	1	6	2	1	0.776
3	(0.498, 0.498, 0.498)	1.798	3	2	8	3	4	0.996
4	(0.487, 0.487, 0.487)	1.797	3	2	8	2	4	0.973
5	(0.471, 0.471, 0.471)	1.793	3	2	8	2	4	0.942
6	(0.477, 0.477, 0.477)	1.795	3	2	8	2	4	0.954

The followings are details for Rb_4Br_4. It has 3 topological nodal lines and 5 nodal ring points along high symmetry line as shown in Fig 323.

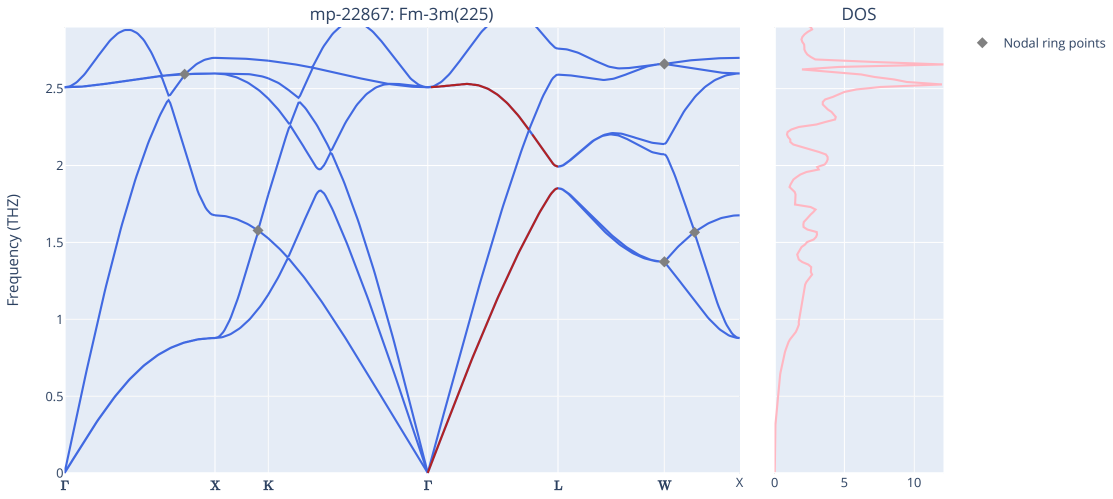

Supplementary Figure 323: The phonon band and DOS of Rb4Br4.

Supplementary Table 618: High symmetry lines of mp-22867-Rb4Br4

	Start	End
0	Γ (0.000, 0.000, 0.000)	X (0.500, 0.000, 0.500)
1	X (0.500, 0.000, 0.500)	U (0.625, 0.250, 0.625)
2	K (0.375, 0.375, 0.750)	Γ (0.000, 0.000, 0.000)
3	Γ (0.000, 0.000, 0.000)	L (0.500, 0.500, 0.500)
4	L (0.500, 0.500, 0.500)	W (0.500, 0.250, 0.750)
5	W (0.500, 0.250, 0.750)	X (0.500, 0.000, 0.500)

Supplementary Table 619: Nodal ring points along high symmetry lines of mp-22867-Rb4Br4.

	Coordinates	Frequency	Mode 1	Mode 2	Multi.	Deg.	Path	Ratio
0	(0.500, 0.250, 0.750)	1.374	2	1	6	2	5	1.000
1	(0.601, 0.202, 0.601)	1.578	3	2	12	2	2	0.809
2	(0.499, 0.149, 0.650)	1.566	3	2	24	2	6	0.401
3	(0.399, -0.000, 0.399)	2.593	6	5	6	2	1	0.798
4	(0.500, 0.250, 0.750)	2.660	6	5	6	2	5	1.000

The followings are details for TlBr. It has 3 topological nodal lines and 10 nodal ring points along high symmetry line as shown in Fig **324**.

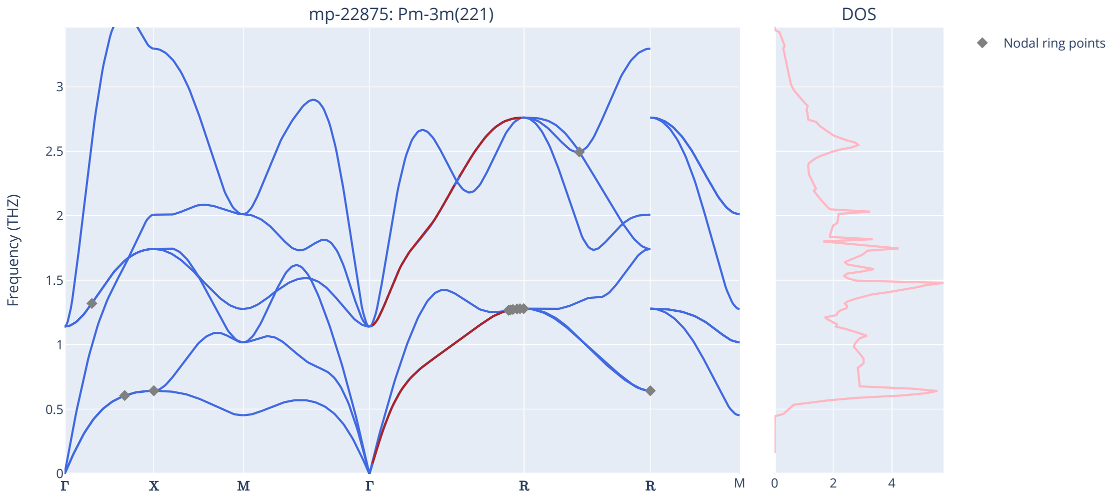

Supplementary Figure 324: The phonon band and DOS of TlBr.

Supplementary Table 620: High symmetry lines of mp-22875-TlBr

	Start	End
0	Γ (0.000, 0.000, 0.000)	X (0.000, 0.500, 0.000)
1	X (0.000, 0.500, 0.000)	M (0.500, 0.500, 0.000)
2	M (0.500, 0.500, 0.000)	Γ (0.000, 0.000, 0.000)
3	Γ (0.000, 0.000, 0.000)	R (0.500, 0.500, 0.500)
4	R (0.500, 0.500, 0.500)	X (0.000, 0.500, 0.000)
5	R (0.500, 0.500, 0.500)	M (0.500, 0.500, 0.000)

Supplementary Table 621: Nodal ring points along high symmetry lines of mp-22875-TlBr.

	Coordinates	Frequency	Mode 1	Mode 2	Multi.	Deg.	Path	Ratio
0	(0.000, 0.337, 0.000)	0.606	2	1	6	2	1	0.673
1	(0.000, 0.500, 0.000)	0.643	2	1	3	2	1	1.000
2	(0.499, 0.499, 0.499)	1.280	3	2	8	3	4	0.998
3	(0.487, 0.487, 0.487)	1.279	3	2	8	2	4	0.974
4	(0.450, 0.450, 0.450)	1.267	3	2	8	2	4	0.900
5	(0.477, 0.477, 0.477)	1.277	3	2	8	2	4	0.955
6	(0.456, 0.456, 0.456)	1.270	3	2	8	2	4	0.912
7	(0.464, 0.464, 0.464)	1.273	3	2	8	2	4	0.928
8	(0.000, 0.153, 0.000)	1.320	5	4	6	2	1	0.306
9	(0.281, 0.500, 0.281)	2.494	6	5	12	2	5	0.438

The followings are details for K_4I_4. It has 2 topological nodal lines and 5 nodal ring points along high symmetry line as shown in Fig **325**.

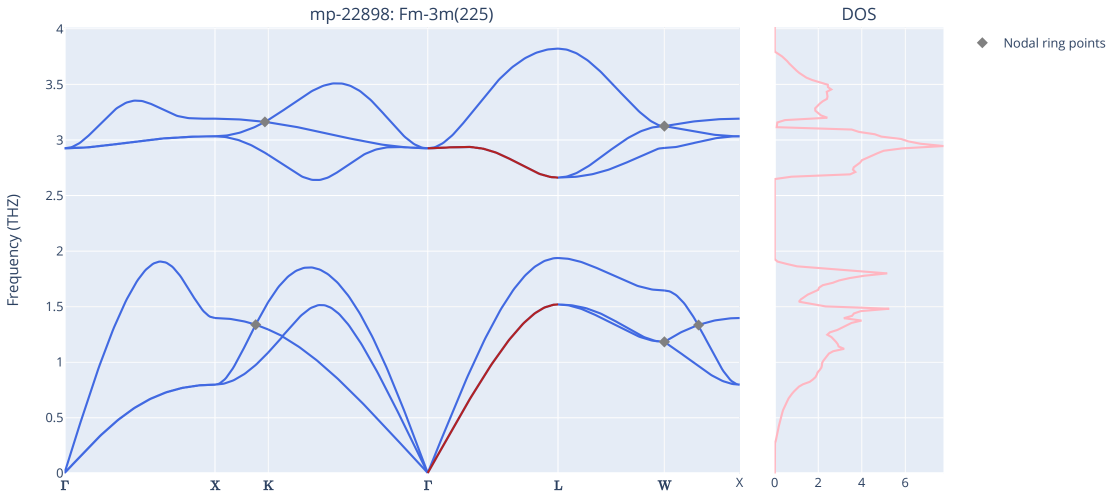

Supplementary Figure 325: The phonon band and DOS of K4I4.

Supplementary Table 622: High symmetry lines of mp-22898-K4I4

	Start	End
0	Γ (0.000, 0.000, 0.000)	X (0.500, 0.000, 0.500)
1	X (0.500, 0.000, 0.500)	U (0.625, 0.250, 0.625)
2	K (0.375, 0.375, 0.750)	Γ (0.000, 0.000, 0.000)
3	Γ (0.000, 0.000, 0.000)	L (0.500, 0.500, 0.500)
4	L (0.500, 0.500, 0.500)	W (0.500, 0.250, 0.750)
5	W (0.500, 0.250, 0.750)	X (0.500, 0.000, 0.500)

Supplementary Table 623: Nodal ring points along high symmetry lines of mp-22898-K4I4.

	Coordinates	Frequency	Mode 1	Mode 2	Multi.	Deg.	Path	Ratio
0	(0.500, 0.250, 0.750)	1.183	2	1	6	2	5	1.000
1	(0.596, 0.191, 0.596)	1.337	3	2	12	2	2	0.765
2	(0.497, 0.135, 0.638)	1.334	3	2	24	2	6	0.454
3	(0.617, 0.235, 0.617)	3.163	6	5	12	2	2	0.938
4	(0.500, 0.250, 0.750)	3.125	6	5	6	2	5	1.000

The followings are details for Li_4I_4. It has 2 topological nodal lines and 4 nodal ring points along high symmetry line as shown in Fig **326**.

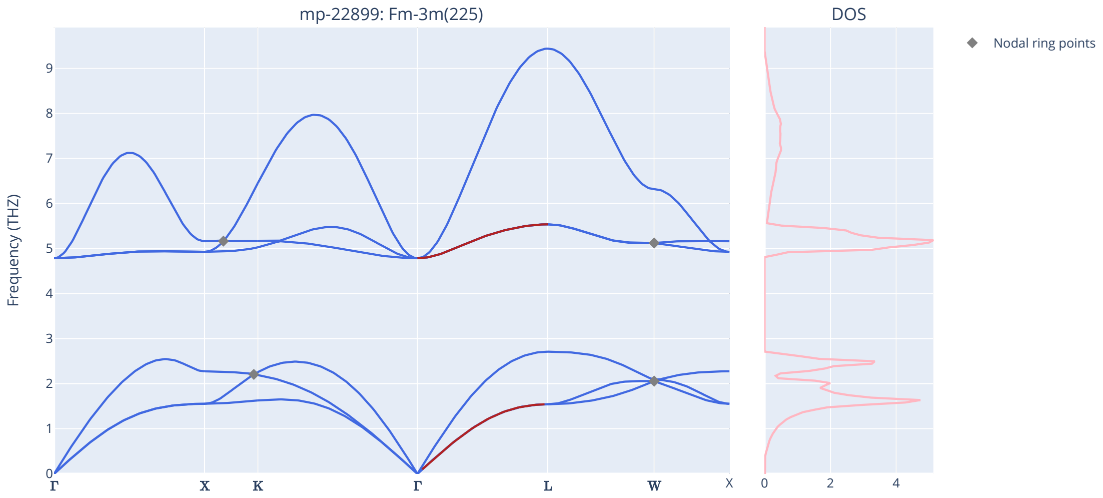

Supplementary Figure 326: The phonon band and DOS of Li4I4.

Supplementary Table 624: High symmetry lines of mp-22899-Li4I4

	Start	End
0	Γ (0.000, 0.000, 0.000)	X (0.500, 0.000, 0.500)
1	X (0.500, 0.000, 0.500)	U (0.625, 0.250, 0.625)
2	K (0.375, 0.375, 0.750)	Γ (0.000, 0.000, 0.000)
3	Γ (0.000, 0.000, 0.000)	L (0.500, 0.500, 0.500)
4	L (0.500, 0.500, 0.500)	W (0.500, 0.250, 0.750)
5	W (0.500, 0.250, 0.750)	X (0.500, 0.000, 0.500)

Supplementary Table 625: Nodal ring points along high symmetry lines of mp-22899-Li4I4.

	Coordinates	Frequency	Mode 1	Mode 2	Multi.	Deg.	Path	Ratio
0	(0.500, 0.250, 0.750)	2.056	2	1	6	2	5	1.000
1	(0.616, 0.231, 0.616)	2.205	3	2	12	2	2	0.926
2	(0.500, 0.250, 0.750)	5.121	5	4	6	2	5	1.000
3	(0.544, 0.088, 0.544)	5.163	6	5	12	2	2	0.354

The followings are details for Rb_4I_4. It has 2 topological nodal lines and 9 nodal ring points along high symmetry line as shown in Fig **327**.

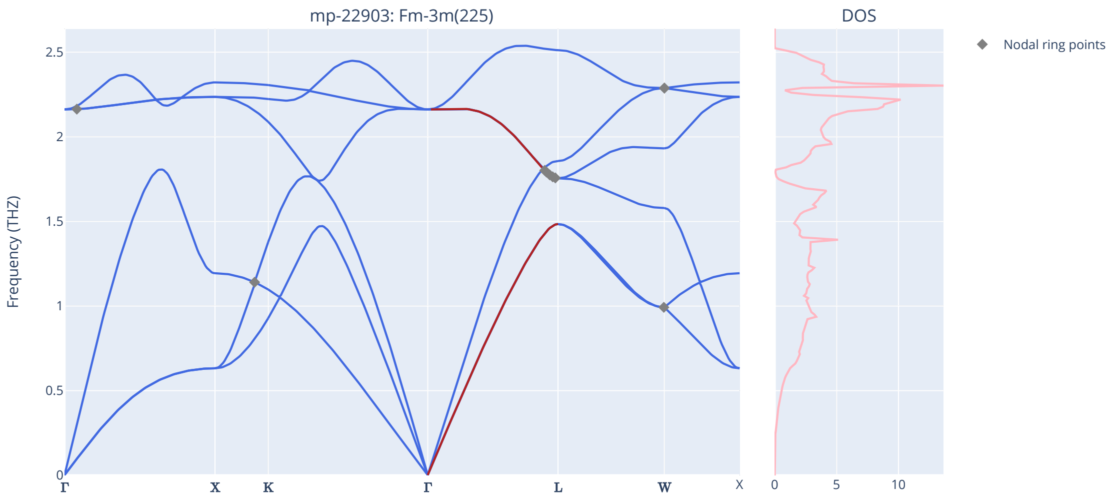

Supplementary Figure 327: The phonon band and DOS of Rb4I4.

Supplementary Table 626: High symmetry lines of mp-22903-Rb4I4

	Start	End
0	Γ (0.000, 0.000, 0.000)	X (0.500, 0.000, 0.500)
1	X (0.500, 0.000, 0.500)	U (0.625, 0.250, 0.625)
2	K (0.375, 0.375, 0.750)	Γ (0.000, 0.000, 0.000)
3	Γ (0.000, 0.000, 0.000)	L (0.500, 0.500, 0.500)
4	L (0.500, 0.500, 0.500)	W (0.500, 0.250, 0.750)
5	W (0.500, 0.250, 0.750)	X (0.500, 0.000, 0.500)

Supplementary Table 627: Nodal ring points along high symmetry lines of mp-22903-Rb4I4.

	Coordinates	Frequency	Mode 1	Mode 2	Multi.	Deg.	Path	Ratio
0	(0.500, 0.251, 0.749)	0.992	2	1	24	2	5	0.995
1	(0.593, 0.186, 0.593)	1.141	3	2	12	2	2	0.745
2	(0.456, 0.456, 0.456)	1.791	4	3	8	2	4	0.911
3	(0.468, 0.468, 0.468)	1.775	4	3	8	2	4	0.936
4	(0.449, 0.449, 0.449)	1.801	4	3	8	2	4	0.898
5	(0.480, 0.480, 0.480)	1.763	4	3	8	2	4	0.959
6	(0.490, 0.490, 0.490)	1.757	4	3	8	2	4	0.980
7	(0.041, 0.000, 0.041)	2.163	5	4	6	2	1	0.082
8	(0.500, 0.250, 0.750)	2.287	6	5	6	2	5	1.000

The followings are details for Li_4Cl_4. It has 2 topological nodal lines and 5 nodal ring points along high symmetry line as shown in Fig 328.

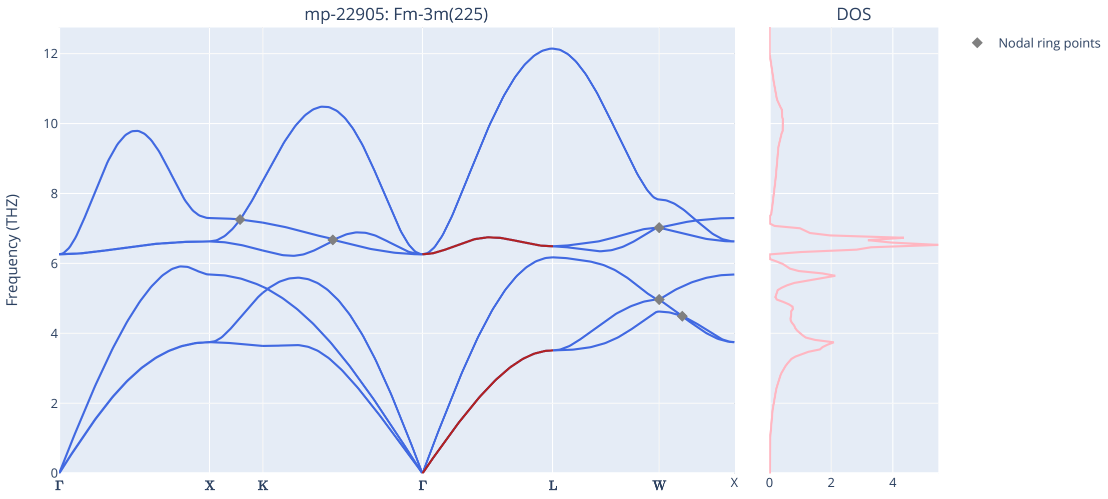

Supplementary Figure 328: The phonon band and DOS of Li4Cl4.

Supplementary Table 628: High symmetry lines of mp-22905-Li4Cl4

	Start	End
0	Γ (0.000, 0.000, 0.000)	X (0.500, 0.000, 0.500)
1	X (0.500, 0.000, 0.500)	U (0.625, 0.250, 0.625)
2	K (0.375, 0.375, 0.750)	Γ (0.000, 0.000, 0.000)
3	Γ (0.000, 0.000, 0.000)	L (0.500, 0.500, 0.500)
4	L (0.500, 0.500, 0.500)	W (0.500, 0.250, 0.750)
5	W (0.500, 0.250, 0.750)	X (0.500, 0.000, 0.500)

Supplementary Table 629: Nodal ring points along high symmetry lines of mp-22905-Li4Cl4.

	Coordinates	Frequency	Mode 1	Mode 2	Multi.	Deg.	Path	Ratio
0	(0.500, 0.173, 0.673)	4.490	2	1	12	2	6	0.307
1	(0.500, 0.250, 0.750)	4.968	3	2	6	2	5	1.000
2	(0.214, 0.214, 0.417)	6.673	5	4	24	2	3	0.439
3	(0.500, 0.250, 0.750)	7.022	5	4	6	2	5	1.000
4	(0.572, 0.143, 0.572)	7.255	6	5	12	2	2	0.573

The followings are details for CsBr and it only has 5 topological nodal lines along high symmetry line as shown in Fig **329**.

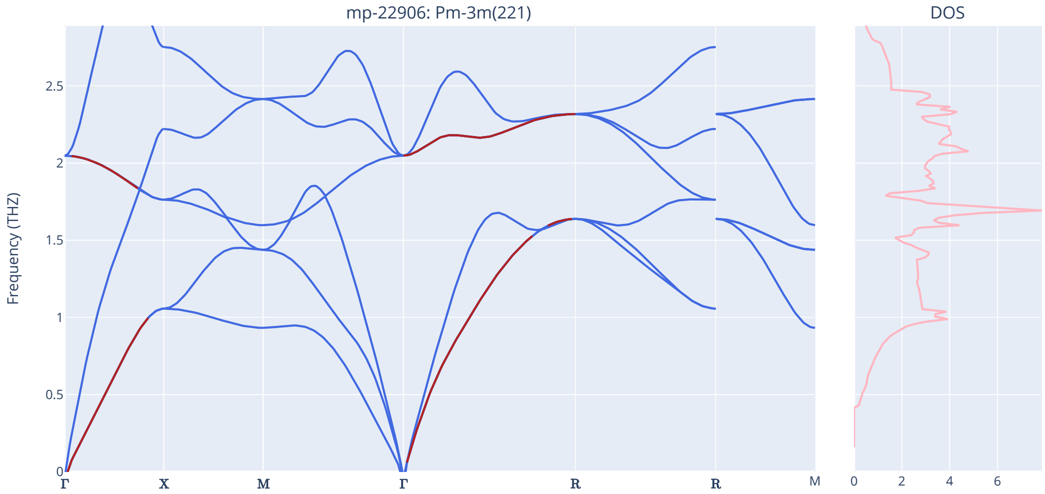

Supplementary Figure 329: The phonon band and DOS of CsBr.

Supplementary Table 630: High symmetry lines of mp-22906-CsBr

	Start	End
0	Γ (0.000, 0.000, 0.000)	X (0.000, 0.500, 0.000)
1	X (0.000, 0.500, 0.000)	M (0.500, 0.500, 0.000)
2	M (0.500, 0.500, 0.000)	Γ (0.000, 0.000, 0.000)
3	Γ (0.000, 0.000, 0.000)	R (0.500, 0.500, 0.500)
4	R (0.500, 0.500, 0.500)	X (0.000, 0.500, 0.000)
5	R (0.500, 0.500, 0.500)	M (0.500, 0.500, 0.000)

The followings are details for Na_4Br_4. It has 2 topological nodal lines and 5 nodal ring points along high symmetry line as shown in Fig 330.

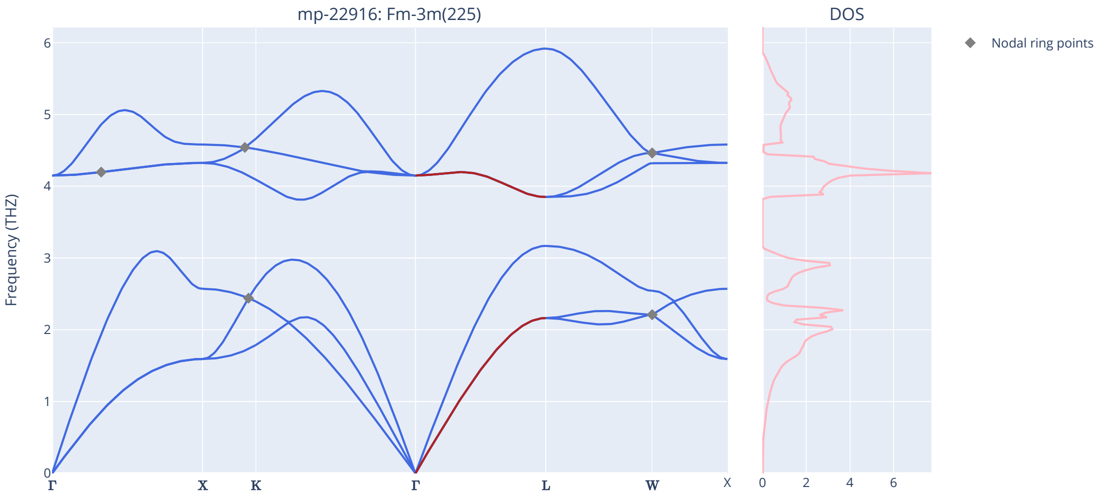

Supplementary Figure 330: The phonon band and DOS of Na4Br4.

Supplementary Table 631: High symmetry lines of mp-22916-Na4Br4

	Start	End
0	Γ (0.000, 0.000, 0.000)	X (0.500, 0.000, 0.500)
1	X (0.500, 0.000, 0.500)	U (0.625, 0.250, 0.625)
2	K (0.375, 0.375, 0.750)	Γ (0.000, 0.000, 0.000)
3	Γ (0.000, 0.000, 0.000)	L (0.500, 0.500, 0.500)
4	L (0.500, 0.500, 0.500)	W (0.500, 0.250, 0.750)
5	W (0.500, 0.250, 0.750)	X (0.500, 0.000, 0.500)

Supplementary Table 632: Nodal ring points along high symmetry lines of mp-22916-Na4Br4.

	Coordinates	Frequency	Mode 1	Mode 2	Multi.	Deg.	Path	Ratio
0	(0.500, 0.250, 0.750)	2.210	2	1	6	2	5	1.000
1	(0.608, 0.216, 0.608)	2.441	3	2	12	2	2	0.864
2	(0.163, 0.000, 0.163)	4.197	5	4	6	2	1	0.327
3	(0.599, 0.199, 0.599)	4.542	6	5	12	2	2	0.795
4	(0.500, 0.250, 0.750)	4.464	6	5	6	2	5	1.000

The followings are details for Ag_4Cl_4. It has 3 topological nodal lines and 4 nodal ring points along high symmetry line as shown in Fig 331.

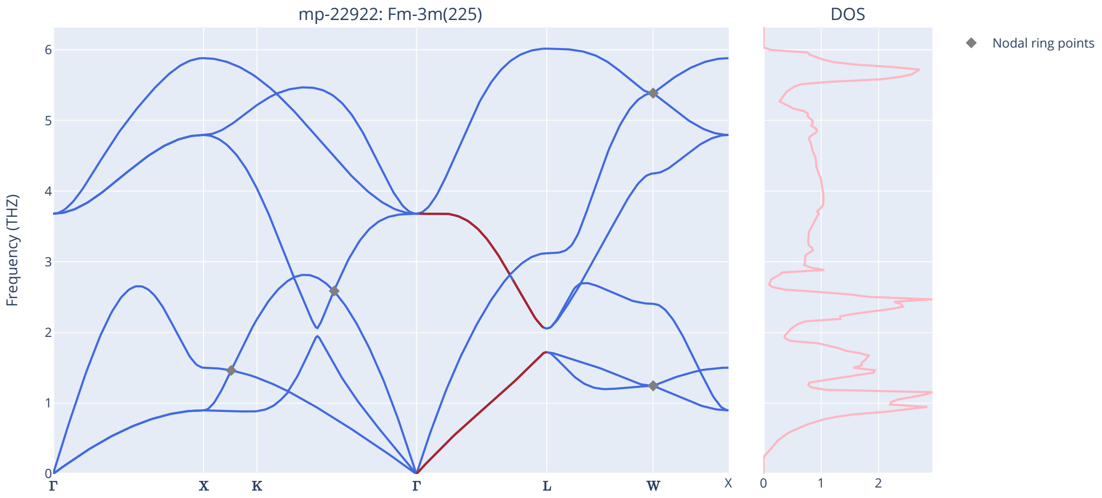

Supplementary Figure 331: The phonon band and DOS of Ag4Cl4.

Supplementary Table 633: High symmetry lines of mp-22922-Ag4Cl4

	Start	End
0	Γ (0.000, 0.000, 0.000)	X (0.500, 0.000, 0.500)
1	X (0.500, 0.000, 0.500)	U (0.625, 0.250, 0.625)
2	K (0.375, 0.375, 0.750)	Γ (0.000, 0.000, 0.000)
3	Γ (0.000, 0.000, 0.000)	L (0.500, 0.500, 0.500)
4	L (0.500, 0.500, 0.500)	W (0.500, 0.250, 0.750)
5	W (0.500, 0.250, 0.750)	X (0.500, 0.000, 0.500)

Supplementary Table 634: Nodal ring points along high symmetry lines of mp-22922-Ag4Cl4.

	Coordinates	Frequency	Mode 1	Mode 2	Multi.	Deg.	Path	Ratio
0	(0.500, 0.250, 0.750)	1.245	2	1	6	2	5	1.000
1	(0.565, 0.130, 0.565)	1.462	3	2	12	2	2	0.519
2	(0.191, 0.191, 0.388)	2.586	4	3	24	2	3	0.485
3	(0.500, 0.250, 0.750)	5.387	6	5	6	2	5	1.000

The followings are details for TlCl. It has 3 topological nodal lines and 6 nodal ring points along high symmetry line as shown in Fig 332.

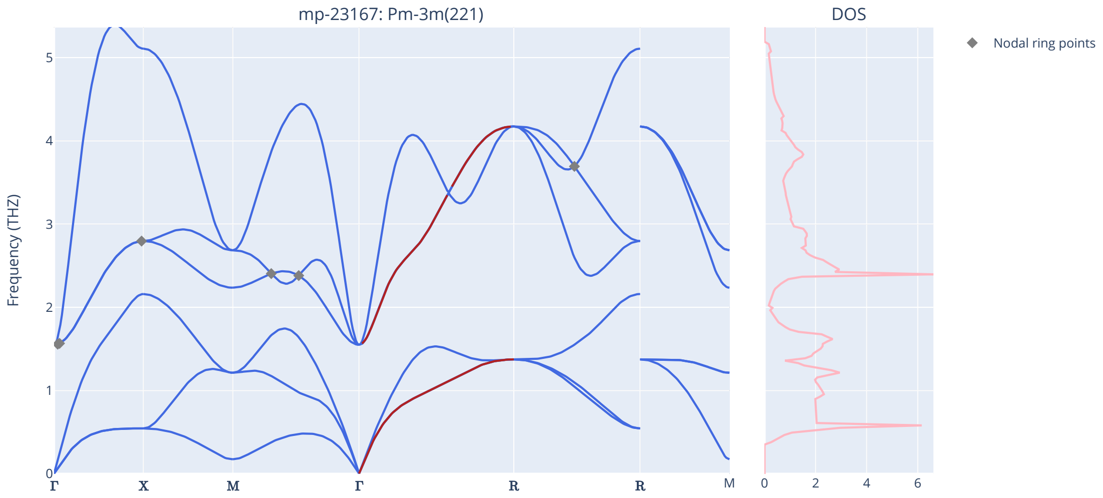

Supplementary Figure 332: The phonon band and DOS of TlCl.

Supplementary Table 635: High symmetry lines of mp-23167-TlCl

	Start	End
0	Γ (0.000, 0.000, 0.000)	X (0.000, 0.500, 0.000)
1	X (0.000, 0.500, 0.000)	M (0.500, 0.500, 0.000)
2	M (0.500, 0.500, 0.000)	Γ (0.000, 0.000, 0.000)
3	Γ (0.000, 0.000, 0.000)	R (0.500, 0.500, 0.500)
4	R (0.500, 0.500, 0.500)	X (0.000, 0.500, 0.000)
5	R (0.500, 0.500, 0.500)	M (0.500, 0.500, 0.000)

Supplementary Table 636: Nodal ring points along high symmetry lines of mp-23167-TlCl.

	Coordinates	Frequency	Mode 1	Mode 2	Multi.	Deg.	Path	Ratio
0	(0.000, 0.020, 0.000)	1.557	5	4	6	2	1	0.041
1	(0.000, 0.031, 0.000)	1.566	5	4	6	2	1	0.061
2	(0.000, 0.490, 0.000)	2.796	5	4	6	2	1	0.980
3	(0.239, 0.239, 0.000)	2.381	5	4	12	2	3	0.523
4	(0.347, 0.347, -0.000)	2.405	5	4	12	2	3	0.305
5	(0.260, 0.500, 0.260)	3.693	6	5	12	2	5	0.480

The followings are details for K_4Cl_4. It has 3 topological nodal lines and 4 nodal ring points along high symmetry line as shown in Fig 333.

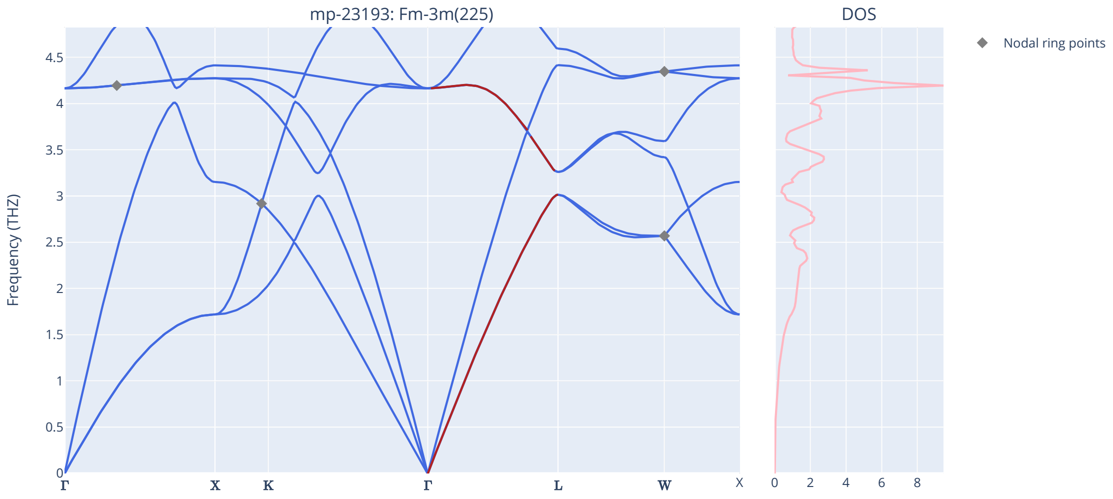

Supplementary Figure 333: The phonon band and DOS of K4Cl4.

Supplementary Table 637: High symmetry lines of mp-23193-K4Cl4

	Start	End
0	Γ (0.000, 0.000, 0.000)	X (0.500, 0.000, 0.500)
1	X (0.500, 0.000, 0.500)	U (0.625, 0.250, 0.625)
2	K (0.375, 0.375, 0.750)	Γ (0.000, 0.000, 0.000)
3	Γ (0.000, 0.000, 0.000)	L (0.500, 0.500, 0.500)
4	L (0.500, 0.500, 0.500)	W (0.500, 0.250, 0.750)
5	W (0.500, 0.250, 0.750)	X (0.500, 0.000, 0.500)

Supplementary Table 638: Nodal ring points along high symmetry lines of mp-23193-K4Cl4.

	Coordinates	Frequency	Mode 1	Mode 2	Multi.	Deg.	Path	Ratio
0	(0.500, 0.250, 0.750)	2.569	2	1	6	2	5	1.000
1	(0.609, 0.219, 0.609)	2.918	3	2	12	2	2	0.876
2	(0.169, -0.000, 0.178)	4.196	5	4	24	2	1	0.347
3	(0.500, 0.250, 0.750)	4.347	6	5	6	2	5	1.000

The followings are details for Ag_4Br_4. It has 3 topological nodal lines and 4 nodal ring points along high symmetry line as shown in Fig 334.

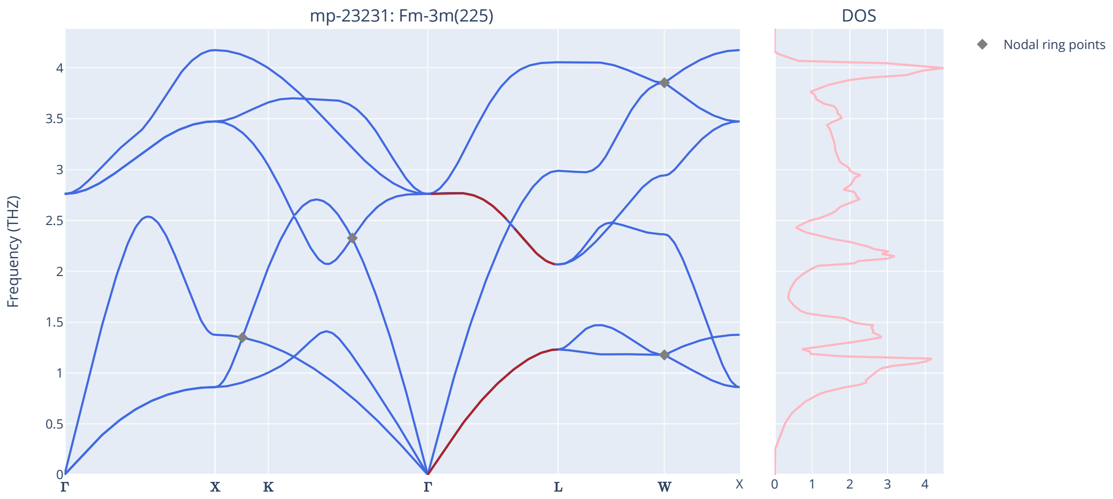

Supplementary Figure 334: The phonon band and DOS of Ag4Br4.

Supplementary Table 639: High symmetry lines of mp-23231-Ag4Br4

	Start	End
0	Γ (0.000, 0.000, 0.000)	X (0.500, 0.000, 0.500)
1	X (0.500, 0.000, 0.500)	U (0.625, 0.250, 0.625)
2	K (0.375, 0.375, 0.750)	Γ (0.000, 0.000, 0.000)
3	Γ (0.000, 0.000, 0.000)	L (0.500, 0.500, 0.500)
4	L (0.500, 0.500, 0.500)	W (0.500, 0.250, 0.750)
5	W (0.500, 0.250, 0.750)	X (0.500, 0.000, 0.500)

Supplementary Table 640: Nodal ring points along high symmetry lines of mp-23231-Ag4Br4.

	Coordinates	Frequency	Mode 1	Mode 2	Multi.	Deg.	Path	Ratio
0	(0.500, 0.250, 0.750)	1.178	2	1	6	2	5	1.000
1	(0.564, 0.129, 0.564)	1.349	3	2	12	2	2	0.516
2	(0.176, 0.176, 0.356)	2.327	4	3	24	2	3	0.527
3	(0.500, 0.250, 0.750)	3.854	6	5	6	2	5	1.000

The followings are details for K_4Br_4. It has 2 topological nodal lines and 4 nodal ring points along high symmetry line as shown in Fig 335.

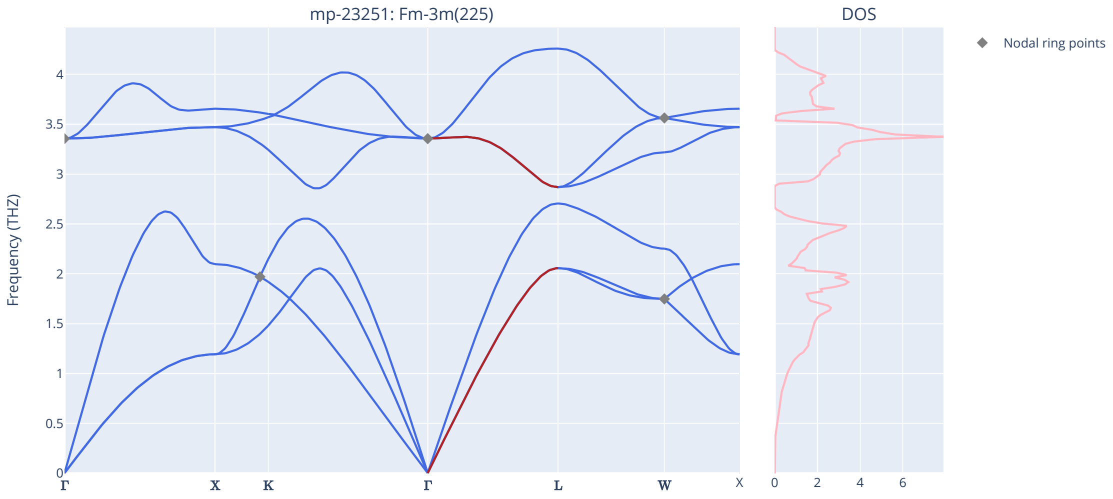

Supplementary Figure 335: The phonon band and DOS of K4Br4.

Supplementary Table 641: High symmetry lines of mp-23251-K4Br4

	Start	End
0	Γ (0.000, 0.000, 0.000)	X (0.500, 0.000, 0.500)
1	X (0.500, 0.000, 0.500)	U (0.625, 0.250, 0.625)
2	K (0.375, 0.375, 0.750)	Γ (0.000, 0.000, 0.000)
3	Γ (0.000, 0.000, 0.000)	L (0.500, 0.500, 0.500)
4	L (0.500, 0.500, 0.500)	W (0.500, 0.250, 0.750)
5	W (0.500, 0.250, 0.750)	X (0.500, 0.000, 0.500)

Supplementary Table 642: Nodal ring points along high symmetry lines of mp-23251-K4Br4.

	Coordinates	Frequency	Mode 1	Mode 2	Multi.	Deg.	Path	Ratio
0	(0.500, 0.250, 0.750)	1.748	2	1	6	2	5	1.000
1	(0.606, 0.212, 0.606)	1.970	3	2	12	2	2	0.847
2	(0.000, 0.000, 0.000)	3.356	5	4	1	3	1	0.000
3	(0.500, 0.250, 0.750)	3.563	6	5	6	2	5	1.000

The followings are details for Li_4Br_4. It has 2 topological nodal lines and 5 nodal ring points along high symmetry line as shown in Fig 336.

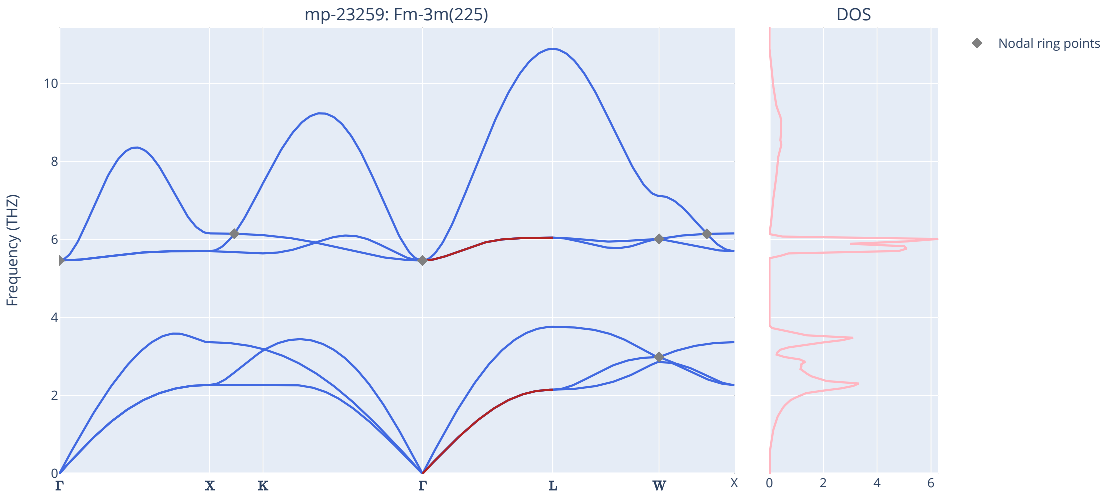

Supplementary Figure 336: The phonon band and DOS of Li4Br4.

Supplementary Table 643: High symmetry lines of mp-23259-Li4Br4

	Start	End
0	Γ (0.000, 0.000, 0.000)	X (0.500, 0.000, 0.500)
1	X (0.500, 0.000, 0.500)	U (0.625, 0.250, 0.625)
2	K (0.375, 0.375, 0.750)	Γ (0.000, 0.000, 0.000)
3	Γ (0.000, 0.000, 0.000)	L (0.500, 0.500, 0.500)
4	L (0.500, 0.500, 0.500)	W (0.500, 0.250, 0.750)
5	W (0.500, 0.250, 0.750)	X (0.500, 0.000, 0.500)

Supplementary Table 644: Nodal ring points along high symmetry lines of mp-23259-Li4Br4.

	Coordinates	Frequency	Mode 1	Mode 2	Multi.	Deg.	Path	Ratio
0	(0.500, 0.250, 0.750)	2.985	3	2	6	2	5	1.000
1	(0.000, 0.000, 0.000)	5.460	5	4	1	3	1	0.000
2	(0.500, 0.250, 0.750)	6.012	5	4	6	2	5	1.000
3	(0.558, 0.115, 0.558)	6.146	6	5	12	2	2	0.461
4	(0.498, 0.090, 0.593)	6.142	6	5	24	2	6	0.634

The followings are details for Na_4I_4. It has 2 topological nodal lines and 4 nodal ring points along high symmetry line as shown in Fig **337**.

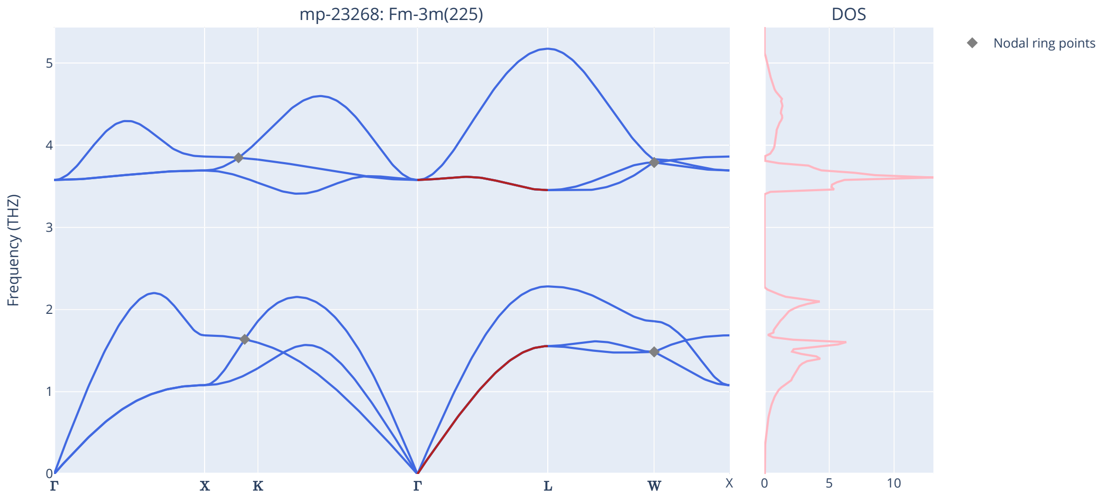

Supplementary Figure 337: The phonon band and DOS of Na4I4.

Supplementary Table 645: High symmetry lines of mp-23268-Na4I4

	Start	End
0	Γ (0.000, 0.000, 0.000)	X (0.500, 0.000, 0.500)
1	X (0.500, 0.000, 0.500)	U (0.625, 0.250, 0.625)
2	K (0.375, 0.375, 0.750)	Γ (0.000, 0.000, 0.000)
3	Γ (0.000, 0.000, 0.000)	L (0.500, 0.500, 0.500)
4	L (0.500, 0.500, 0.500)	W (0.500, 0.250, 0.750)
5	W (0.500, 0.250, 0.750)	X (0.500, 0.000, 0.500)

Supplementary Table 646: Nodal ring points along high symmetry lines of mp-23268-Na4I4.

	Coordinates	Frequency	Mode 1	Mode 2	Multi.	Deg.	Path	Ratio
0	(0.500, 0.250, 0.750)	1.483	2	1	6	2	5	1.000
1	(0.594, 0.188, 0.594)	1.636	3	2	12	2	2	0.754
2	(0.500, 0.250, 0.750)	3.789	5	4	6	2	5	1.000
3	(0.580, 0.160, 0.580)	3.847	6	5	12	2	2	0.638

The followings are details for Rb_4Cl_4. It has 2 topological nodal lines and 7 nodal ring points along high symmetry line as shown in Fig 338.

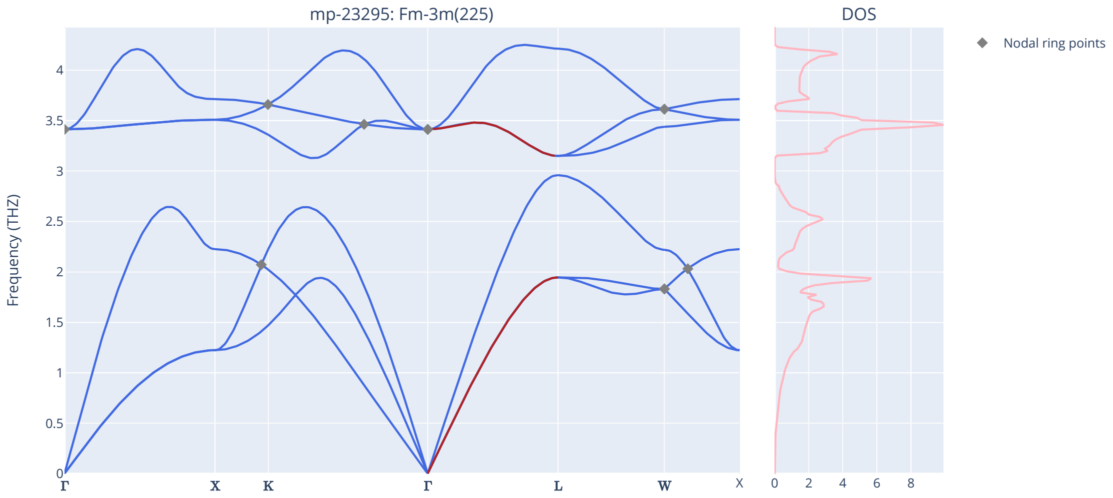

Supplementary Figure 338: The phonon band and DOS of Rb4Cl4.

Supplementary Table 647: High symmetry lines of mp-23295-Rb4Cl4

	Start	End
0	Γ (0.000, 0.000, 0.000)	X (0.500, 0.000, 0.500)
1	X (0.500, 0.000, 0.500)	U (0.625, 0.250, 0.625)
2	K (0.375, 0.375, 0.750)	Γ (0.000, 0.000, 0.000)
3	Γ (0.000, 0.000, 0.000)	L (0.500, 0.500, 0.500)
4	L (0.500, 0.500, 0.500)	W (0.500, 0.250, 0.750)
5	W (0.500, 0.250, 0.750)	X (0.500, 0.000, 0.500)

Supplementary Table 648: Nodal ring points along high symmetry lines of mp-23295-Rb4Cl4.

	Coordinates	Frequency	Mode 1	Mode 2	Multi.	Deg.	Path	Ratio
0	(0.500, 0.250, 0.750)	1.832	2	1	6	2	5	1.000
1	(0.609, 0.218, 0.609)	2.072	3	2	12	2	2	0.871
2	(0.497, 0.171, 0.673)	2.031	3	2	24	2	6	0.312
3	(0.000, 0.000, 0.000)	3.413	5	4	1	3	1	0.000
4	(0.153, 0.153, 0.296)	3.463	5	4	24	2	3	0.601
5	(0.625, 0.249, 0.625)	3.659	6	5	12	2	2	0.996
6	(0.500, 0.250, 0.750)	3.613	6	5	6	2	5	1.000

The followings are details for RbI. It has 5 topological nodal lines and 10 nodal ring points along high symmetry line as shown in Fig 339.

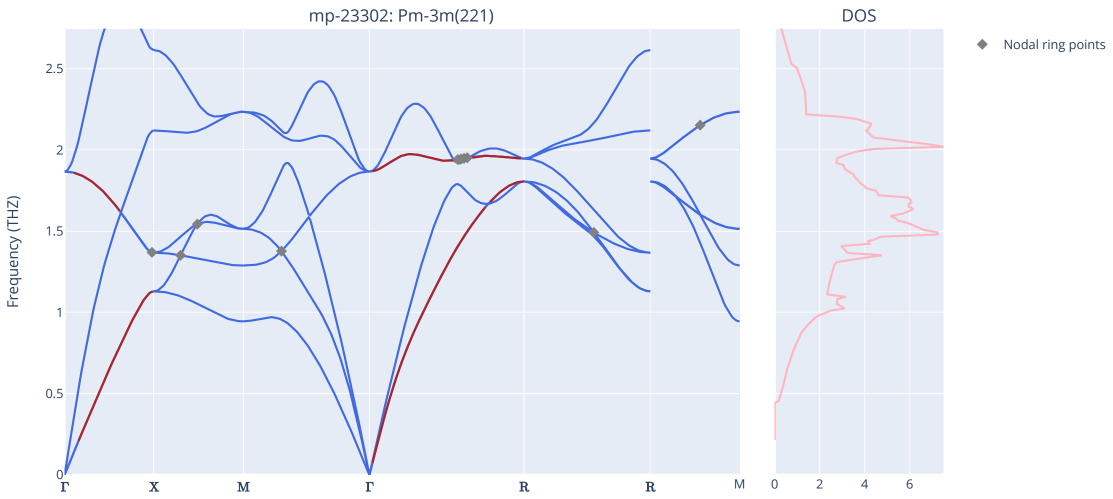

Supplementary Figure 339: The phonon band and DOS of RbI.

Supplementary Table 649: High symmetry lines of mp-23302-RbI

	Start	End
0	Γ (0.000, 0.000, 0.000)	X (0.000, 0.500, 0.000)
1	X (0.000, 0.500, 0.000)	M (0.500, 0.500, 0.000)
2	M (0.500, 0.500, 0.000)	Γ (0.000, 0.000, 0.000)
3	Γ (0.000, 0.000, 0.000)	R (0.500, 0.500, 0.500)
4	R (0.500, 0.500, 0.500)	X (0.000, 0.500, 0.000)
5	R (0.500, 0.500, 0.500)	M (0.500, 0.500, 0.000)

Supplementary Table 650: Nodal ring points along high symmetry lines of mp-23302-RbI.

	Coordinates	Frequency	Mode 1	Mode 2	Multi.	Deg.	Path	Ratio
0	(0.150, 0.500, -0.000)	1.351	3	2	12	2	2	0.300
1	(0.348, 0.348, 0.000)	1.377	3	2	12	2	3	0.304
2	(0.224, 0.500, 0.224)	1.492	3	2	12	2	5	0.553
3	(0.000, 0.490, 0.000)	1.369	4	3	6	2	1	0.980
4	(0.244, 0.500, -0.000)	1.543	4	3	12	2	2	0.487
5	(0.499, 0.499, 0.221)	2.152	6	5	24	2	6	0.559
6	(0.286, 0.286, 0.286)	1.941	6	5	8	2	4	0.571
7	(0.296, 0.296, 0.296)	1.944	6	5	8	2	4	0.592
8	(0.306, 0.306, 0.306)	1.948	6	5	8	2	4	0.612
9	(0.316, 0.316, 0.316)	1.952	6	5	8	2	4	0.633

The followings are details for Li_4H_4. It has 2 topological nodal lines and 2 nodal ring points along high symmetry line as shown in Fig **340**.

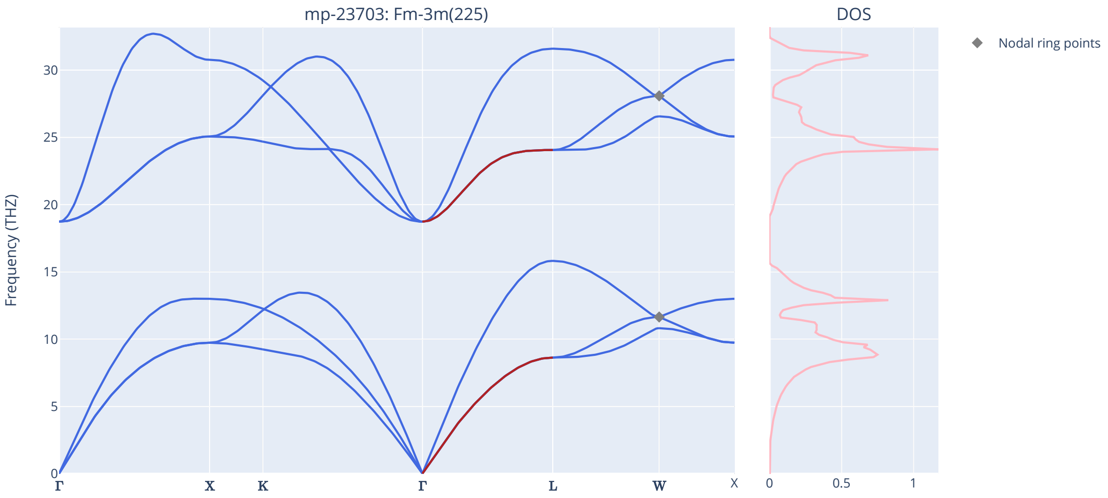

Supplementary Figure 340: The phonon band and DOS of Li4H4.

Supplementary Table 651: High symmetry lines of mp-23703-Li4H4

	Start	End
0	Γ (0.000, 0.000, 0.000)	X (0.500, 0.000, 0.500)
1	X (0.500, 0.000, 0.500)	U (0.625, 0.250, 0.625)
2	K (0.375, 0.375, 0.750)	Γ (0.000, 0.000, 0.000)
3	Γ (0.000, 0.000, 0.000)	L (0.500, 0.500, 0.500)
4	L (0.500, 0.500, 0.500)	W (0.500, 0.250, 0.750)
5	W (0.500, 0.250, 0.750)	X (0.500, 0.000, 0.500)

Supplementary Table 652: Nodal ring points along high symmetry lines of mp-23703-Li4H4.

	Coordinates	Frequency	Mode 1	Mode 2	Multi.	Deg.	Path	Ratio
0	(0.500, 0.250, 0.750)	11.650	3	2	6	2	5	1.000
1	(0.500, 0.250, 0.750)	28.082	6	5	6	2	5	1.000

The followings are details for Na_4H_4. It has 2 topological nodal lines and 3 nodal ring points along high symmetry line as shown in Fig **341**.

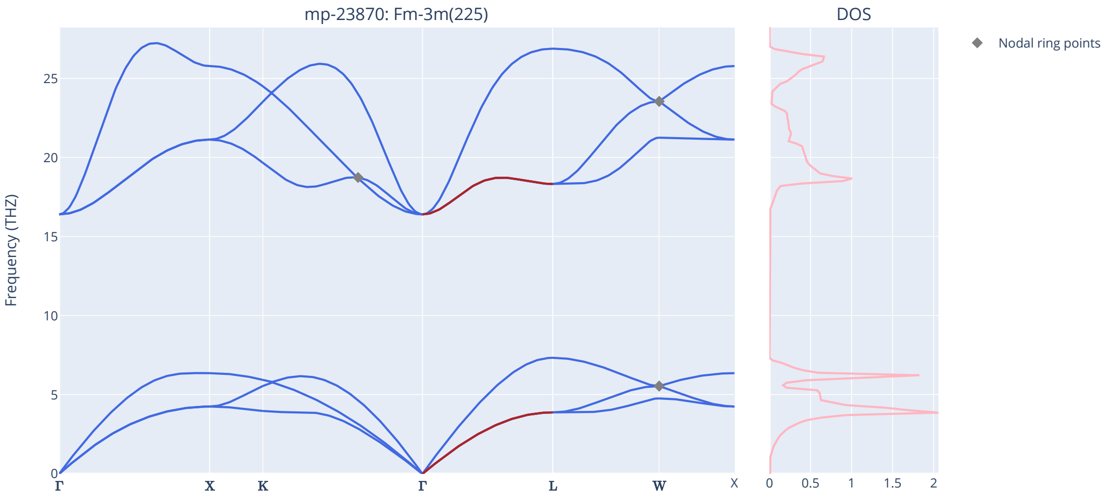

Supplementary Figure 341: The phonon band and DOS of Na4H4.

Supplementary Table 653: High symmetry lines of mp-23870-Na4H4

	Start	End
0	Γ (0.000, 0.000, 0.000)	X (0.500, 0.000, 0.500)
1	X (0.500, 0.000, 0.500)	U (0.625, 0.250, 0.625)
2	K (0.375, 0.375, 0.750)	Γ (0.000, 0.000, 0.000)
3	Γ (0.000, 0.000, 0.000)	L (0.500, 0.500, 0.500)
4	L (0.500, 0.500, 0.500)	W (0.500, 0.250, 0.750)
5	W (0.500, 0.250, 0.750)	X (0.500, 0.000, 0.500)

Supplementary Table 654: Nodal ring points along high symmetry lines of mp-23870-Na4H4.

	Coordinates	Frequency	Mode 1	Mode 2	Multi.	Deg.	Path	Ratio
0	(0.500, 0.250, 0.750)	5.545	3	2	6	2	5	1.000
1	(0.146, 0.146, 0.307)	18.740	5	4	24	2	3	0.597
2	(0.500, 0.250, 0.750)	23.551	6	5	6	2	5	1.000

The followings are details for K_4H_4. It has 2 topological nodal lines and 5 nodal ring points along high symmetry line as shown in Fig **342**.

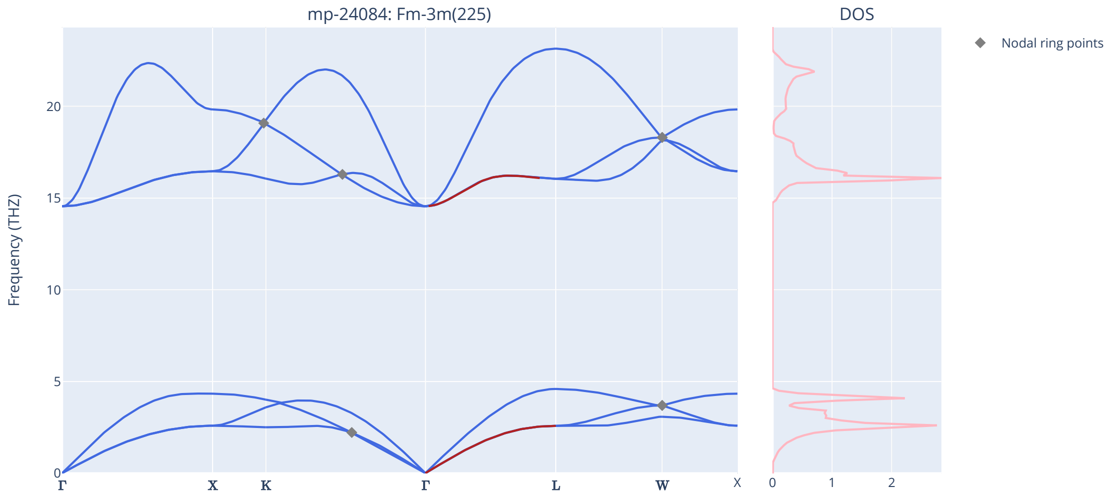

Supplementary Figure 342: The phonon band and DOS of K4H4.

Supplementary Table 655: High symmetry lines of mp-24084-K4H4

	Start	End
0	Γ (0.000, 0.000, 0.000)	X (0.500, 0.000, 0.500)
1	X (0.500, 0.000, 0.500)	U (0.625, 0.250, 0.625)
2	K (0.375, 0.375, 0.750)	Γ (0.000, 0.000, 0.000)
3	Γ (0.000, 0.000, 0.000)	L (0.500, 0.500, 0.500)
4	L (0.500, 0.500, 0.500)	W (0.500, 0.250, 0.750)
5	W (0.500, 0.250, 0.750)	X (0.500, 0.000, 0.500)

Supplementary Table 656: Nodal ring points along high symmetry lines of mp-24084-K4H4.

	Coordinates	Frequency	Mode 1	Mode 2	Multi.	Deg.	Path	Ratio
0	(0.168, 0.168, 0.351)	2.223	2	1	24	2	3	0.539
1	(0.500, 0.250, 0.750)	3.699	3	2	6	2	5	1.000
2	(0.191, 0.191, 0.393)	16.291	5	4	24	2	3	0.480
3	(0.620, 0.240, 0.620)	19.085	6	5	12	2	2	0.961
4	(0.500, 0.250, 0.750)	18.307	6	5	6	2	5	1.000

The followings are details for Sr_4O_4. It has 2 topological nodal lines and 5 nodal ring points along high symmetry line as shown in Fig 343.

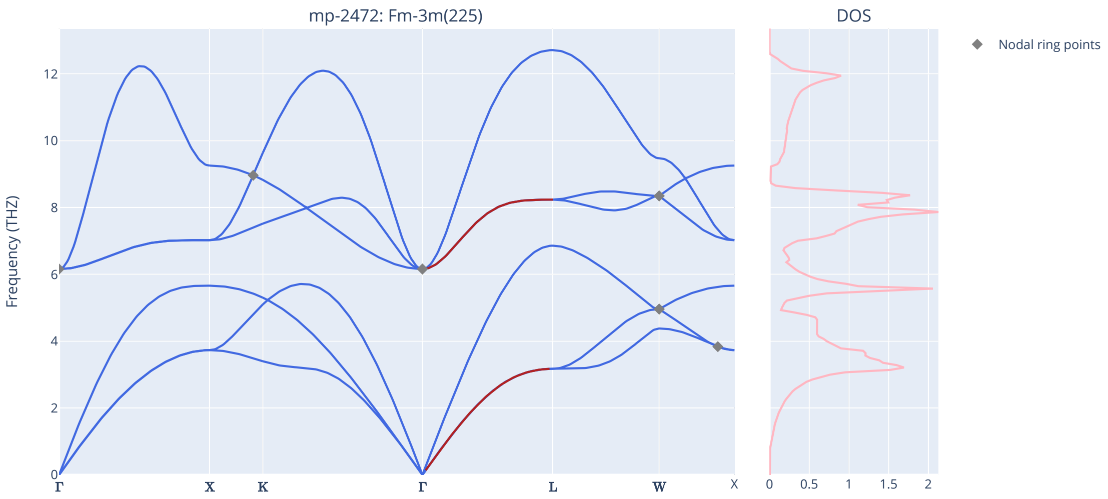

Supplementary Figure 343: The phonon band and DOS of Sr4O4.

Supplementary Table 657: High symmetry lines of mp-2472-Sr4O4

	Start	End
0	Γ (0.000, 0.000, 0.000)	X (0.500, 0.000, 0.500)
1	X (0.500, 0.000, 0.500)	U (0.625, 0.250, 0.625)
2	K (0.375, 0.375, 0.750)	Γ (0.000, 0.000, 0.000)
3	Γ (0.000, 0.000, 0.000)	L (0.500, 0.500, 0.500)
4	L (0.500, 0.500, 0.500)	W (0.500, 0.250, 0.750)
5	W (0.500, 0.250, 0.750)	X (0.500, 0.000, 0.500)

Supplementary Table 658: Nodal ring points along high symmetry lines of mp-2472-Sr4O4.

	Coordinates	Frequency	Mode 1	Mode 2	Multi.	Deg.	Path	Ratio
0	(0.500, 0.055, 0.555)	3.835	2	1	24	2	6	0.779
1	(0.500, 0.250, 0.750)	4.959	3	2	6	2	5	1.000
2	(0.000, 0.000, 0.000)	6.157	5	4	1	3	1	0.000
3	(0.500, 0.250, 0.750)	8.346	5	4	6	2	5	1.000
4	(0.603, 0.205, 0.603)	8.963	6	5	12	2	2	0.820

The followings are details for Rb_4H_4. It has 2 topological nodal lines and 5 nodal ring points along high symmetry line as shown in Fig 344.

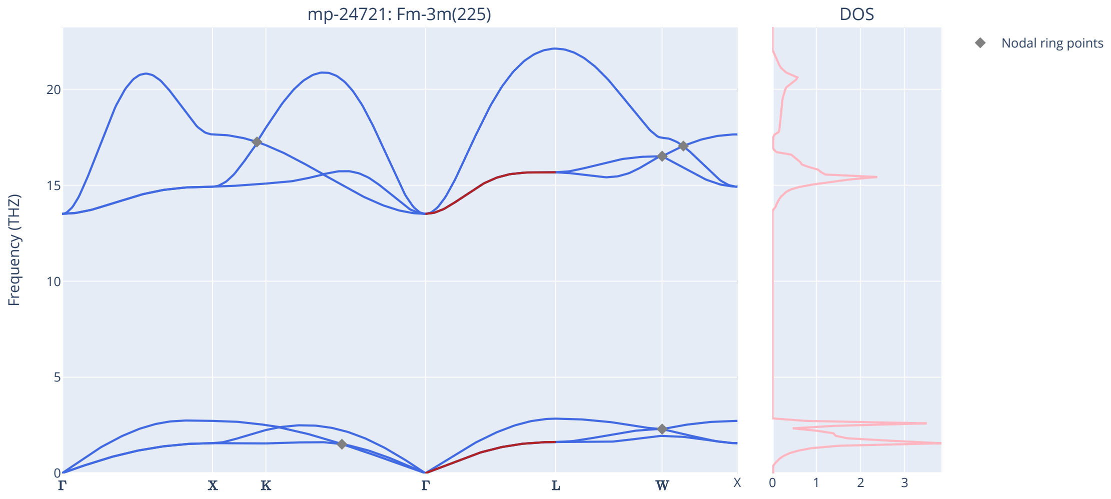

Supplementary Figure 344: The phonon band and DOS of Rb4H4.

Supplementary Table 659: High symmetry lines of mp-24721-Rb4H4

	Start	End
0	Γ (0.000, 0.000, 0.000)	X (0.500, 0.000, 0.500)
1	X (0.500, 0.000, 0.500)	U (0.625, 0.250, 0.625)
2	K (0.375, 0.375, 0.750)	Γ (0.000, 0.000, 0.000)
3	Γ (0.000, 0.000, 0.000)	L (0.500, 0.500, 0.500)
4	L (0.500, 0.500, 0.500)	W (0.500, 0.250, 0.750)
5	W (0.500, 0.250, 0.750)	X (0.500, 0.000, 0.500)

Supplementary Table 660: Nodal ring points along high symmetry lines of mp-24721-Rb4H4.

	Coordinates	Frequency	Mode 1	Mode 2	Multi.	Deg.	Path	Ratio
0	(0.199, 0.199, 0.390)	1.512	2	1	24	2	3	0.477
1	(0.500, 0.250, 0.750)	2.301	3	2	6	2	5	1.000
2	(0.500, 0.250, 0.750)	16.515	5	4	6	2	5	1.000
3	(0.604, 0.208, 0.604)	17.259	6	5	12	2	2	0.834
4	(0.498, 0.179, 0.680)	17.046	6	5	24	2	6	0.282

The followings are details for Ca_4O_4. It has 3 topological nodal lines and 3 nodal ring points along high symmetry line as shown in Fig **345**.

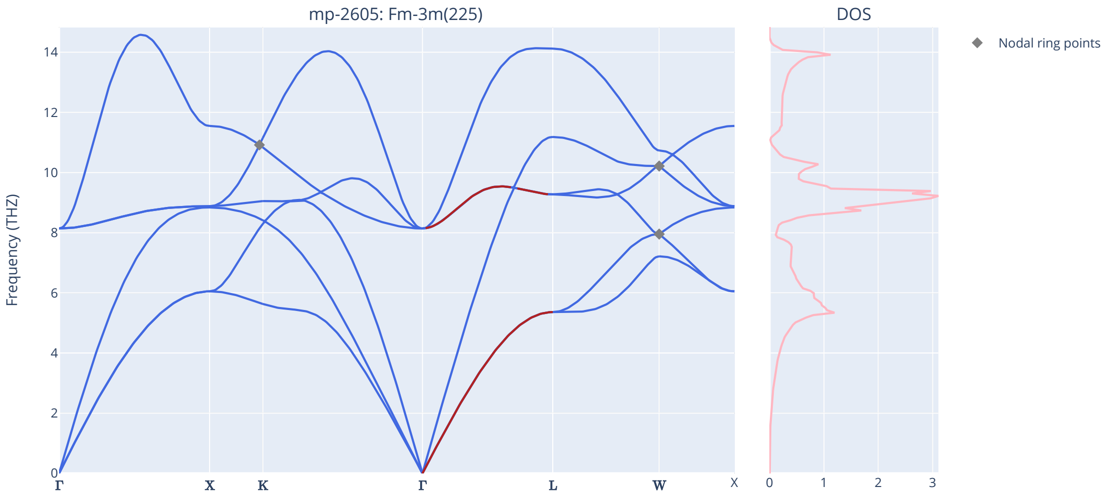

Supplementary Figure 345: The phonon band and DOS of Ca4O4.

Supplementary Table 661: High symmetry lines of mp-2605-Ca4O4

	Start	End
0	Γ (0.000, 0.000, 0.000)	X (0.500, 0.000, 0.500)
1	X (0.500, 0.000, 0.500)	U (0.625, 0.250, 0.625)
2	K (0.375, 0.375, 0.750)	Γ (0.000, 0.000, 0.000)
3	Γ (0.000, 0.000, 0.000)	L (0.500, 0.500, 0.500)
4	L (0.500, 0.500, 0.500)	W (0.500, 0.250, 0.750)
5	W (0.500, 0.250, 0.750)	X (0.500, 0.000, 0.500)

Supplementary Table 662: Nodal ring points along high symmetry lines of mp-2605-Ca4O4.

	Coordinates	Frequency	Mode 1	Mode 2	Multi.	Deg.	Path	Ratio
0	(0.500, 0.250, 0.750)	7.954	3	2	6	2	5	1.000
1	(0.500, 0.250, 0.750)	10.216	5	4	6	2	5	1.000
2	(0.617, 0.234, 0.617)	10.919	6	5	12	2	2	0.937

The followings are details for AuCs. It has 5 topological nodal lines and 4 nodal ring points along high symmetry line as shown in Fig 346.

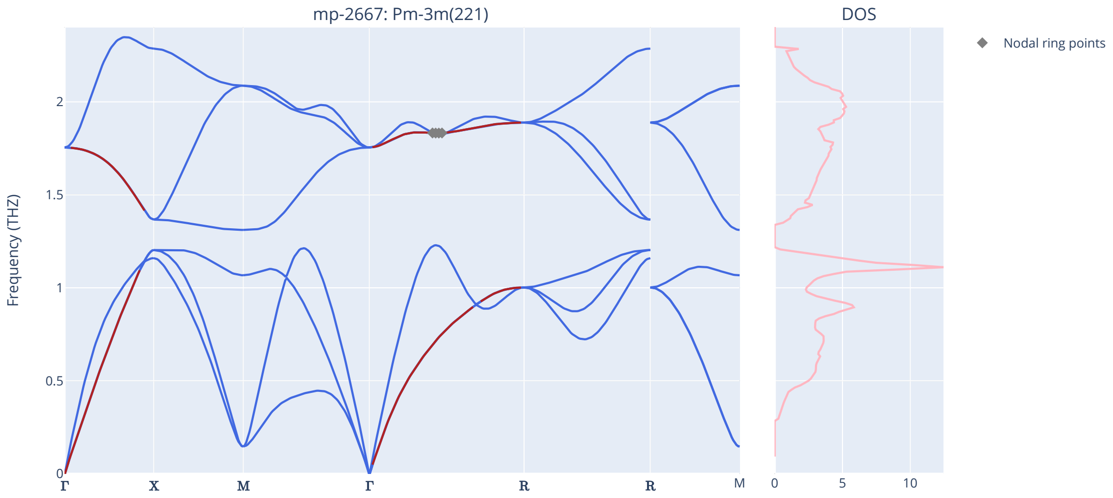

Supplementary Figure 346: The phonon band and DOS of AuCs.

Supplementary Table 663: High symmetry lines of mp-2667-AuCs

	Start	End
0	Γ (0.000, 0.000, 0.000)	X (0.000, 0.500, 0.000)
1	X (0.000, 0.500, 0.000)	M (0.500, 0.500, 0.000)
2	M (0.500, 0.500, 0.000)	Γ (0.000, 0.000, 0.000)
3	Γ (0.000, 0.000, 0.000)	R (0.500, 0.500, 0.500)
4	R (0.500, 0.500, 0.500)	X (0.000, 0.500, 0.000)
5	R (0.500, 0.500, 0.500)	M (0.500, 0.500, 0.000)

Supplementary Table 664: Nodal ring points along high symmetry lines of mp-2667-AuCs.

	Coordinates	Frequency	Mode 1	Mode 2	Multi.	Deg.	Path	Ratio
0	(0.204, 0.204, 0.204)	1.833	6	5	8	2	4	0.408
1	(0.214, 0.214, 0.214)	1.832	6	5	8	2	4	0.429
2	(0.224, 0.224, 0.224)	1.832	6	5	8	2	4	0.449
3	(0.235, 0.235, 0.235)	1.833	6	5	8	2	4	0.469

The followings are details for Sr_4Se_4. It has 3 topological nodal lines and 4 nodal ring points along high symmetry line as shown in Fig 347.

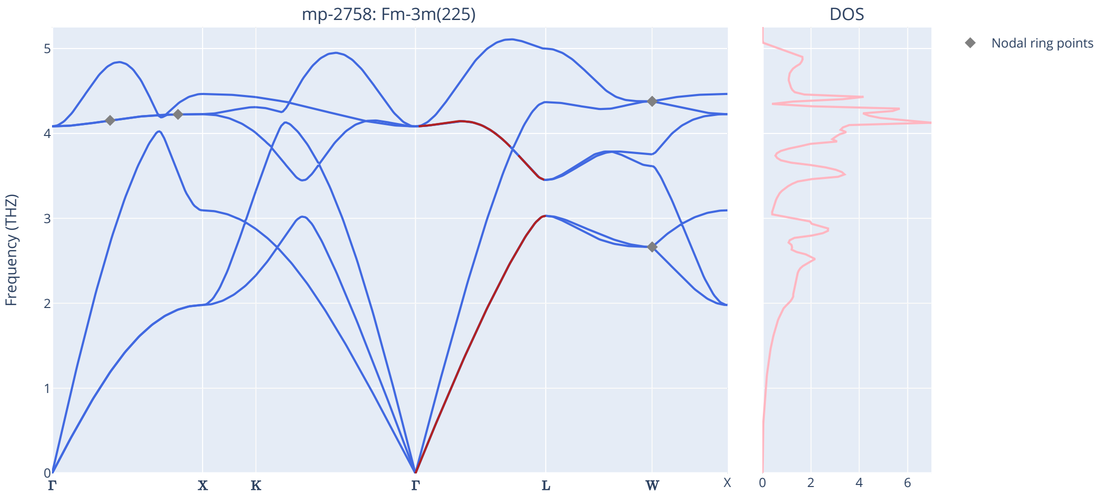

Supplementary Figure 347: The phonon band and DOS of Sr4Se4.

Supplementary Table 665: High symmetry lines of mp-2758-Sr4Se4

	Start	End
0	Γ (0.000, 0.000, 0.000)	X (0.500, 0.000, 0.500)
1	X (0.500, 0.000, 0.500)	U (0.625, 0.250, 0.625)
2	K (0.375, 0.375, 0.750)	Γ (0.000, 0.000, 0.000)
3	Γ (0.000, 0.000, 0.000)	L (0.500, 0.500, 0.500)
4	L (0.500, 0.500, 0.500)	W (0.500, 0.250, 0.750)
5	W (0.500, 0.250, 0.750)	X (0.500, 0.000, 0.500)

Supplementary Table 666: Nodal ring points along high symmetry lines of mp-2758-Sr4Se4.

	Coordinates	Frequency	Mode 1	Mode 2	Multi.	Deg.	Path	Ratio
0	(0.500, 0.250, 0.750)	2.664	2	1	6	2	5	1.000
1	(0.418, 0.000, 0.418)	4.225	5	4	6	2	1	0.837
2	(0.189, 0.000, 0.197)	4.154	5	4	24	2	1	0.386
3	(0.500, 0.250, 0.750)	4.379	6	5	6	2	5	1.000

The followings are details for Yb_4Se_4. It has 2 topological nodal lines and 5 nodal ring points along high symmetry line as shown in Fig **348**.

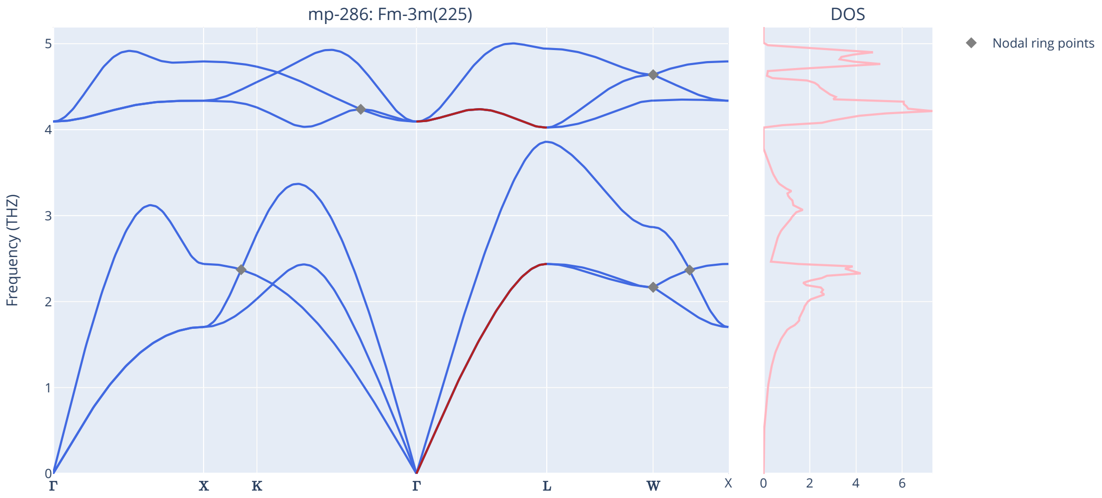

Supplementary Figure 348: The phonon band and DOS of Yb4Se4.

Supplementary Table 667: High symmetry lines of mp-286-Yb4Se4

	Start	End
0	Γ (0.000, 0.000, 0.000)	X (0.500, 0.000, 0.500)
1	X (0.500, 0.000, 0.500)	U (0.625, 0.250, 0.625)
2	K (0.375, 0.375, 0.750)	Γ (0.000, 0.000, 0.000)
3	Γ (0.000, 0.000, 0.000)	L (0.500, 0.500, 0.500)
4	L (0.500, 0.500, 0.500)	W (0.500, 0.250, 0.750)
5	W (0.500, 0.250, 0.750)	X (0.500, 0.000, 0.500)

Supplementary Table 668: Nodal ring points along high symmetry lines of mp-286-Yb4Se4.

	Coordinates	Frequency	Mode 1	Mode 2	Multi.	Deg.	Path	Ratio
0	(0.500, 0.250, 0.750)	2.168	2	1	6	2	5	1.000
1	(0.588, 0.177, 0.588)	2.373	3	2	12	2	2	0.706
2	(0.498, 0.128, 0.630)	2.368	3	2	24	2	6	0.485
3	(0.130, 0.130, 0.262)	4.237	5	4	24	2	3	0.651
4	(0.500, 0.250, 0.750)	4.638	6	5	6	2	5	1.000

The followings are details for Cu_2Br_2. It has 1 nodal ring points and 5 single Weyl point along high symmetry line as shown in Fig **349**.

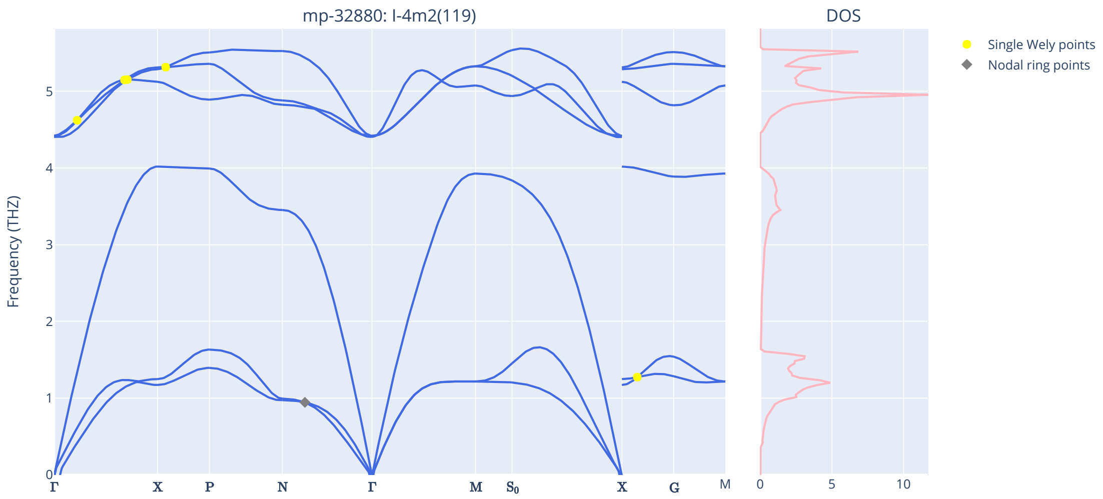

Supplementary Figure 349: The phonon band and DOS of Cu2Br2.

Supplementary Table 669: High symmetry lines of mp-32880-Cu2Br2

	Start	End
0	Γ (0.000, 0.000, 0.000)	X (0.000, 0.000, 0.500)
1	X (0.000, 0.000, 0.500)	P (0.250, 0.250, 0.250)
2	P (0.250, 0.250, 0.250)	N (0.000, 0.500, 0.000)
3	N (0.000, 0.500, 0.000)	Γ (0.000, 0.000, 0.000)
4	Γ (0.000, 0.000, 0.000)	M (0.500, 0.500, -0.500)
5	M (0.500, 0.500, -0.500)	S (0.375, 0.625, -0.375)
6	S_0 (-0.375, 0.375, 0.375)	Γ (0.000, 0.000, 0.000)
7	X (0.000, 0.000, 0.500)	R (-0.250, 0.250, 0.500)
8	G (0.500, 0.500, -0.250)	M (0.500, 0.500, -0.500)

Supplementary Table 670: Single Weyl points along high symmetry lines of mp-32880-Cu2Br2.

	Coordinates	Frequency	Mode 1	Mode 2	Multi.	Deg.	Path	Ratio
0	(-0.074, 0.074, 0.500)	1.273	2	1	4	2	8	0.296
1	(-0.000, -0.000, 0.354)	5.153	5	4	4	2	1	0.709
2	(-0.000, -0.000, 0.112)	4.623	6	5	4	2	1	0.223
3	(0.039, 0.039, 0.461)	5.314	6	5	4	2	2	0.157
4	(-0.000, -0.000, 0.340)	5.148	6	5	4	2	1	0.681

Supplementary Table 671: Nodal ring points along high symmetry lines of mp-32880-Cu2Br2.

	Coordinates	Frequency	Mode 1	Mode 2	Multi.	Deg.	Path	Ratio
0	(-0.003, 0.375, 0.003)	0.944	2	1	8	2	4	0.251

The followings are details for K_4F_4. It has 3 topological nodal lines and 5 nodal ring points along high symmetry line as shown in Fig **350**.

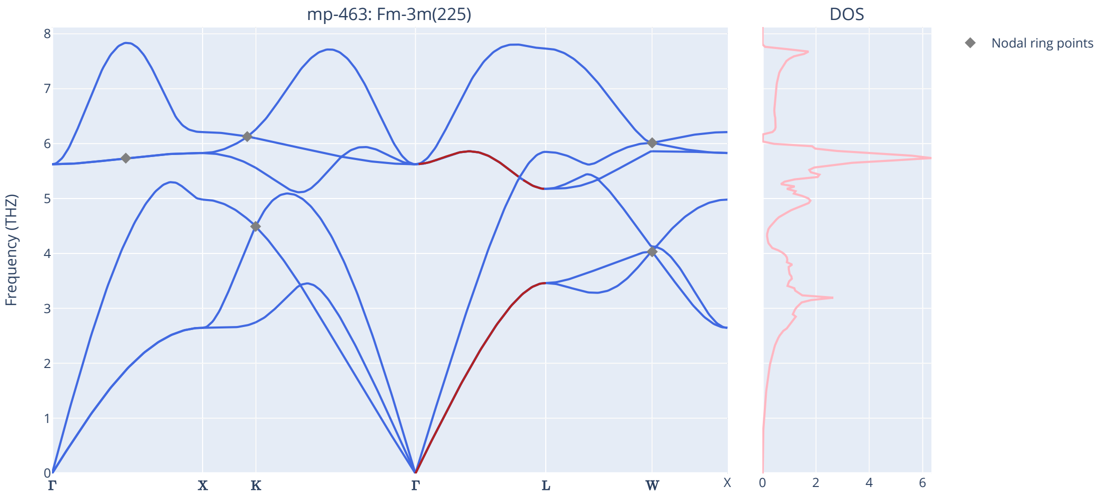

Supplementary Figure 350: The phonon band and DOS of K4F4.

Supplementary Table 672: High symmetry lines of mp-463-K4F4

	Start	End
0	Γ (0.000, 0.000, 0.000)	X (0.500, 0.000, 0.500)
1	X (0.500, 0.000, 0.500)	U (0.625, 0.250, 0.625)
2	K (0.375, 0.375, 0.750)	Γ (0.000, 0.000, 0.000)
3	Γ (0.000, 0.000, 0.000)	L (0.500, 0.500, 0.500)
4	L (0.500, 0.500, 0.500)	W (0.500, 0.250, 0.750)
5	W (0.500, 0.250, 0.750)	X (0.500, 0.000, 0.500)

Supplementary Table 673: Nodal ring points along high symmetry lines of mp-463-K4F4.

	Coordinates	Frequency	Mode 1	Mode 2	Multi.	Deg.	Path	Ratio
0	(0.500, 0.250, 0.750)	4.032	2	1	6	2	5	1.000
1	(0.625, 0.249, 0.625)	4.492	3	2	12	2	2	0.996
2	(0.245, 0.000, 0.245)	5.734	5	4	6	2	1	0.490
3	(0.605, 0.210, 0.605)	6.127	6	5	12	2	2	0.839
4	(0.500, 0.250, 0.750)	6.013	6	5	6	2	5	1.000

The followings are details for Ar_2. It has 6 topological nodal lines and 6 nodal ring points along high symmetry line as shown in Fig **351**.

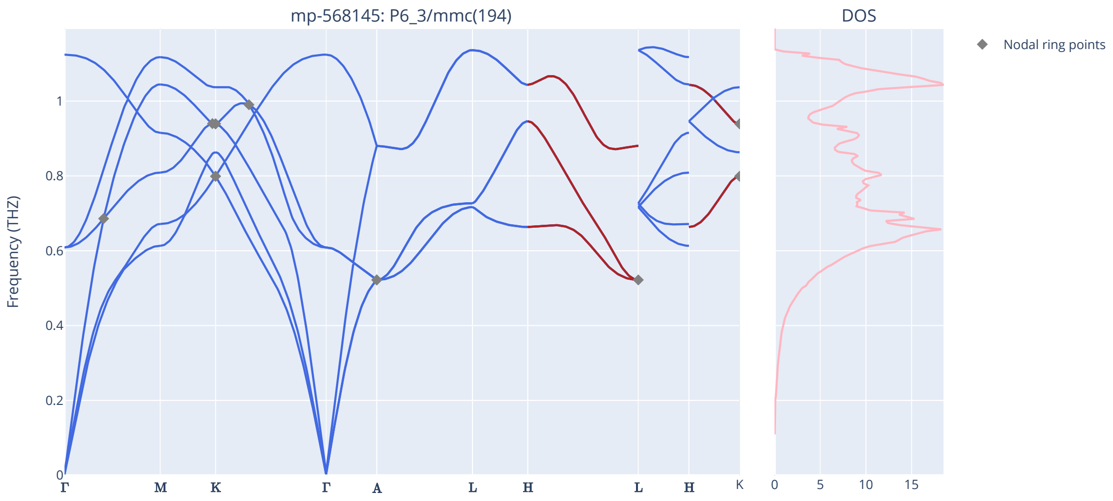

Supplementary Figure 351: The phonon band and DOS of Ar2.

Supplementary Table 674: High symmetry lines of mp-568145-Ar2

	Start	End
0	Γ (0.000, 0.000, 0.000)	M (0.500, 0.000, 0.000)
1	M (0.500, 0.000, 0.000)	K (0.333, 0.333, 0.000)
2	K (0.333, 0.333, 0.000)	Γ (0.000, 0.000, 0.000)
3	Γ (0.000, 0.000, 0.000)	A (0.000, 0.000, 0.500)
4	A (0.000, 0.000, 0.500)	L (0.500, 0.000, 0.500)
5	L (0.500, 0.000, 0.500)	H (0.333, 0.333, 0.500)
6	H (0.333, 0.333, 0.500)	A (0.000, 0.000, 0.500)
7	L (0.500, 0.000, 0.500)	M (0.500, 0.000, 0.000)
8	H (0.333, 0.333, 0.500)	K (0.333, 0.333, 0.000)

Supplementary Table 675: Nodal ring points along high symmetry lines of mp-568145-Ar2.

	Coordinates	Frequency	Mode 1	Mode 2	Multi.	Deg.	Path	Ratio
0	(0.333, 0.333, 0.000)	0.799	2	1	2	2	2	1.000
1	(0.000, 0.000, 0.500)	0.522	2	1	1	4	4	1.000
2	(0.204, -0.000, -0.000)	0.686	4	3	6	2	1	0.408
3	(0.333, 0.333, 0.000)	0.939	5	4	2	2	2	1.000
4	(0.343, 0.315, -0.000)	0.940	5	4	6	2	2	0.944
5	(0.233, 0.233, 0.000)	0.990	6	5	6	2	3	0.302

The followings are details for Tl_4Br_4. It has 2 topological nodal lines and 2 nodal ring points along high symmetry line as shown in Fig **352**.

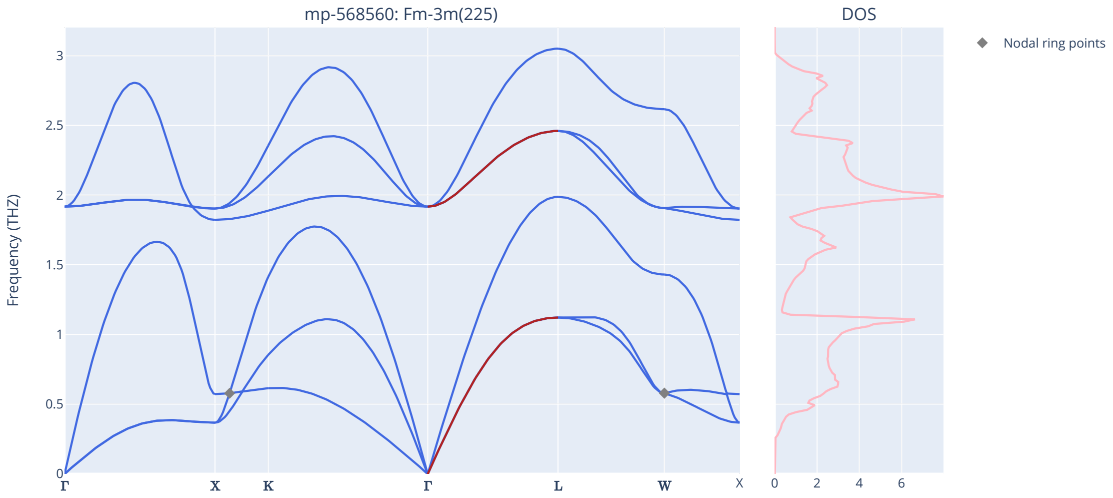

Supplementary Figure 352: The phonon band and DOS of Tl4Br4.

Supplementary Table 676: High symmetry lines of mp-568560-Tl4Br4

	Start	End
0	Γ (0.000, 0.000, 0.000)	X (0.500, 0.000, 0.500)
1	X (0.500, 0.000, 0.500)	U (0.625, 0.250, 0.625)
2	K (0.375, 0.375, 0.750)	Γ (0.000, 0.000, 0.000)
3	Γ (0.000, 0.000, 0.000)	L (0.500, 0.500, 0.500)
4	L (0.500, 0.500, 0.500)	W (0.500, 0.250, 0.750)
5	W (0.500, 0.250, 0.750)	X (0.500, 0.000, 0.500)

Supplementary Table 677: Nodal ring points along high symmetry lines of mp-568560-Tl4Br4.

	Coordinates	Frequency	Mode 1	Mode 2	Multi.	Deg.	Path	Ratio
0	(0.500, 0.250, 0.750)	0.578	2	1	6	2	5	1.000
1	(0.534, 0.069, 0.534)	0.577	3	2	12	2	2	0.275

The followings are details for Tl_4Cl_4. It has 2 topological nodal lines and 2 nodal ring points along high symmetry line as shown in Fig **353**.

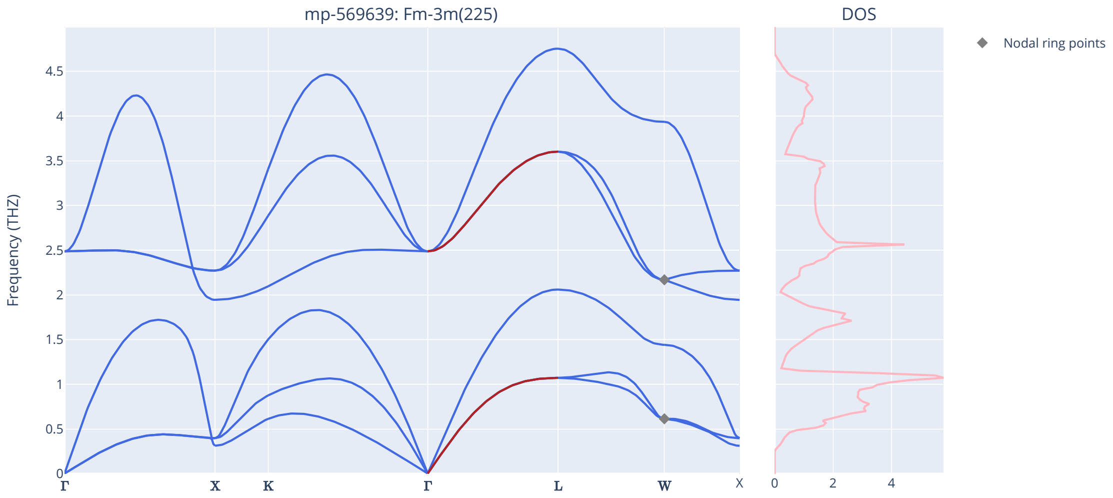

Supplementary Figure 353: The phonon band and DOS of Tl4Cl4.

Supplementary Table 678: High symmetry lines of mp-569639-Tl4Cl4

	Start	End
0	Γ (0.000, 0.000, 0.000)	X (0.500, 0.000, 0.500)
1	X (0.500, 0.000, 0.500)	U (0.625, 0.250, 0.625)
2	K (0.375, 0.375, 0.750)	Γ (0.000, 0.000, 0.000)
3	Γ (0.000, 0.000, 0.000)	L (0.500, 0.500, 0.500)
4	L (0.500, 0.500, 0.500)	W (0.500, 0.250, 0.750)
5	W (0.500, 0.250, 0.750)	X (0.500, 0.000, 0.500)

Supplementary Table 679: Nodal ring points along high symmetry lines of mp-569639-Tl4Cl4.

	Coordinates	Frequency	Mode 1	Mode 2	Multi.	Deg.	Path	Ratio
0	(0.500, 0.250, 0.750)	0.615	2	1	6	2	5	1.000
1	(0.500, 0.250, 0.750)	2.169	5	4	6	2	5	1.000

The followings are details for Cs_4Br_4. It has 2 topological nodal lines and 11 nodal ring points along high symmetry line as shown in Fig **354**.

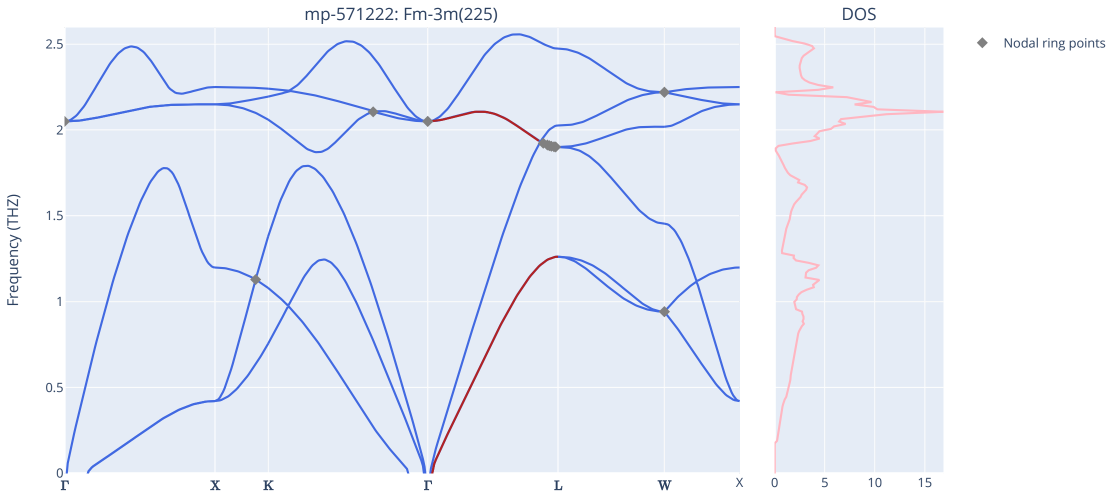

Supplementary Figure 354: The phonon band and DOS of Cs4Br4.

Supplementary Table 680: High symmetry lines of mp-571222-Cs4Br4

	Start	End
0	Γ (0.000, 0.000, 0.000)	X (0.500, 0.000, 0.500)
1	X (0.500, 0.000, 0.500)	U (0.625, 0.250, 0.625)
2	K (0.375, 0.375, 0.750)	Γ (0.000, 0.000, 0.000)
3	Γ (0.000, 0.000, 0.000)	L (0.500, 0.500, 0.500)
4	L (0.500, 0.500, 0.500)	W (0.500, 0.250, 0.750)
5	W (0.500, 0.250, 0.750)	X (0.500, 0.000, 0.500)

Supplementary Table 681: Nodal ring points along high symmetry lines of mp-571222-Cs4Br4.

	Coordinates	Frequency	Mode 1	Mode 2	Multi.	Deg.	Path	Ratio
0	(0.500, 0.250, 0.750)	0.941	2	1	6	2	5	1.000
1	(0.596, 0.191, 0.596)	1.128	3	2	12	2	2	0.765
2	(0.474, 0.474, 0.474)	1.905	4	3	8	2	4	0.949
3	(0.443, 0.443, 0.443)	1.923	4	3	8	2	4	0.887
4	(0.491, 0.491, 0.491)	1.901	4	3	8	2	4	0.982
5	(0.485, 0.485, 0.485)	1.902	4	3	8	2	4	0.969
6	(0.467, 0.467, 0.467)	1.908	4	3	8	2	4	0.934
7	(0.459, 0.459, 0.459)	1.912	4	3	8	2	4	0.918
8	(0.000, 0.000, 0.000)	2.050	5	4	1	3	1	0.000
9	(0.130, 0.130, 0.255)	2.105	5	4	24	2	3	0.658
10	(0.500, 0.250, 0.750)	2.219	6	5	6	2	5	1.000

The followings are details for Cs_4Cl_4. It has 2 topological nodal lines and 5 nodal ring points along high symmetry line as shown in Fig **355**.

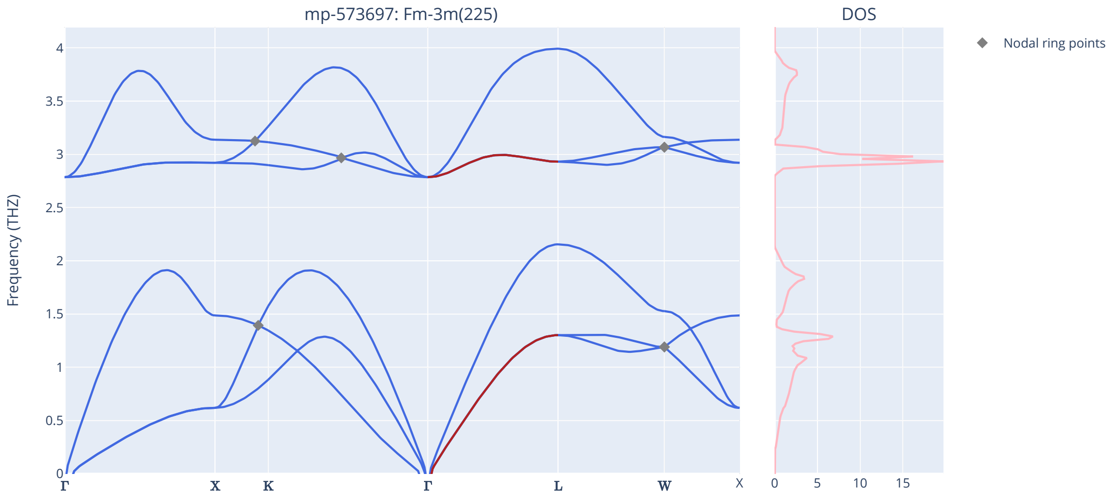

Supplementary Figure 355: The phonon band and DOS of Cs4Cl4.

Supplementary Table 682: High symmetry lines of mp-573697-Cs4Cl4

	Start	End
0	Γ (0.000, 0.000, 0.000)	X (0.500, 0.000, 0.500)
1	X (0.500, 0.000, 0.500)	U (0.625, 0.250, 0.625)
2	K (0.375, 0.375, 0.750)	Γ (0.000, 0.000, 0.000)
3	Γ (0.000, 0.000, 0.000)	L (0.500, 0.500, 0.500)
4	L (0.500, 0.500, 0.500)	W (0.500, 0.250, 0.750)
5	W (0.500, 0.250, 0.750)	X (0.500, 0.000, 0.500)

Supplementary Table 683: Nodal ring points along high symmetry lines of mp-573697-Cs4Cl4.

	Coordinates	Frequency	Mode 1	Mode 2	Multi.	Deg.	Path	Ratio
0	(0.500, 0.250, 0.750)	1.191	2	1	6	2	5	1.000
1	(0.602, 0.203, 0.602)	1.393	3	2	12	2	2	0.813
2	(0.207, 0.207, 0.403)	2.967	5	4	24	2	3	0.458
3	(0.500, 0.250, 0.750)	3.067	5	4	6	2	5	1.000
4	(0.594, 0.188, 0.594)	3.125	6	5	12	2	2	0.753

The followings are details for Cs_4I_4. It has 3 topological nodal lines and 8 nodal ring points along high symmetry line as shown in Fig **356**.

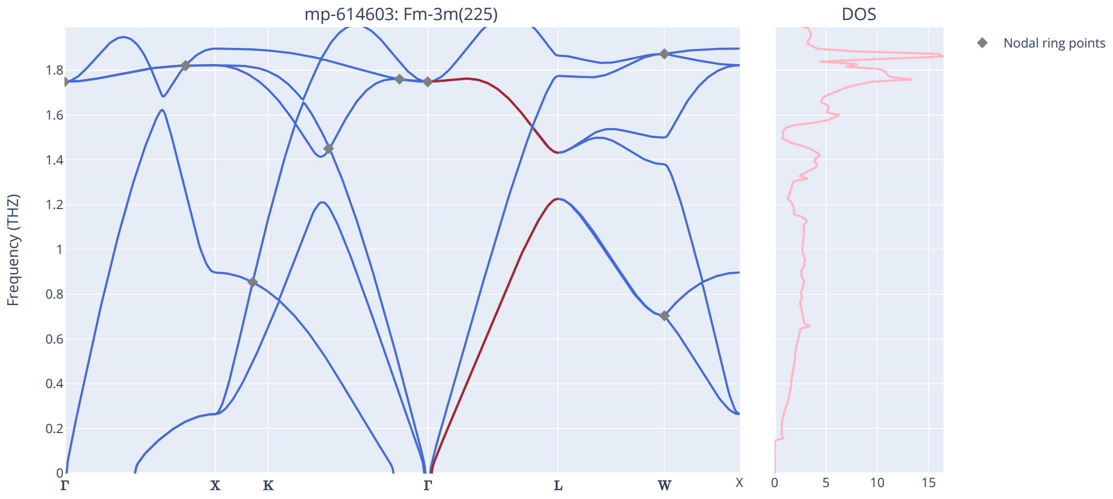

Supplementary Figure 356: The phonon band and DOS of Cs4I4.

Supplementary Table 684: High symmetry lines of mp-614603-Cs4I4

	Start	End
0	Γ (0.000, 0.000, 0.000)	X (0.500, 0.000, 0.500)
1	X (0.500, 0.000, 0.500)	U (0.625, 0.250, 0.625)
2	K (0.375, 0.375, 0.750)	Γ (0.000, 0.000, 0.000)
3	Γ (0.000, 0.000, 0.000)	L (0.500, 0.500, 0.500)
4	L (0.500, 0.500, 0.500)	W (0.500, 0.250, 0.750)
5	W (0.500, 0.250, 0.750)	X (0.500, 0.000, 0.500)

Supplementary Table 685: Nodal ring points along high symmetry lines of mp-614603-Cs4I4.

	Coordinates	Frequency	Mode 1	Mode 2	Multi.	Deg.	Path	Ratio
0	(0.500, 0.250, 0.750)	0.704	2	1	6	2	5	1.000
1	(0.589, 0.177, 0.589)	0.854	3	2	12	2	2	0.709
2	(0.238, 0.238, 0.461)	1.450	4	3	24	2	3	0.379
3	(0.000, 0.000, 0.000)	1.749	5	4	1	3	1	0.000
4	(0.406, -0.000, 0.400)	1.821	5	4	24	2	1	0.806
5	(0.065, 0.065, 0.134)	1.760	5	4	24	2	3	0.823
6	(0.401, 0.000, 0.401)	1.821	6	5	6	2	1	0.803
7	(0.500, 0.250, 0.750)	1.873	6	5	6	2	5	1.000

The followings are details for CsH. It has 5 topological nodal lines and 7 nodal ring points along high symmetry line as shown in Fig 357.

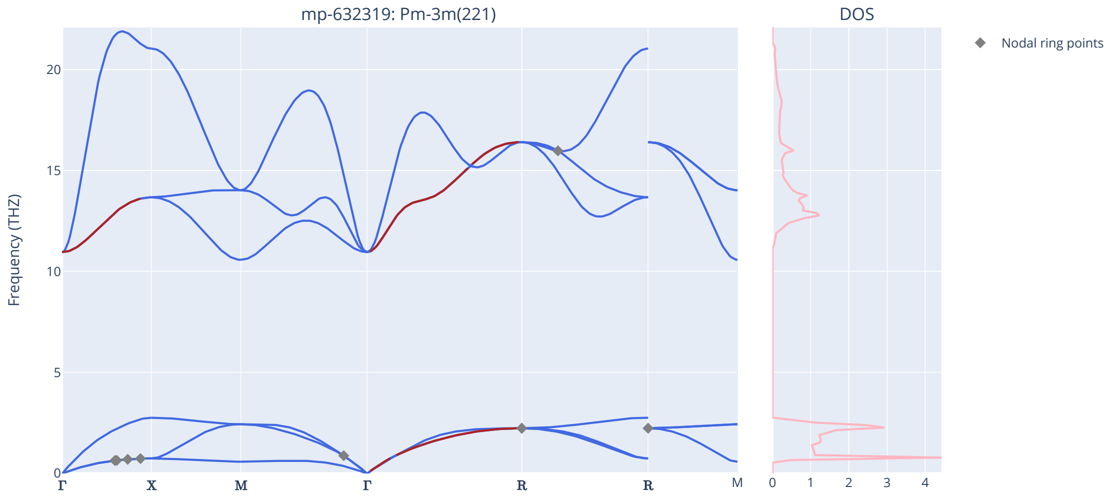

Supplementary Figure 357: The phonon band and DOS of CsH.

Supplementary Table 686: High symmetry lines of mp-632319-CsH

	Start	End
0	Γ (0.000, 0.000, 0.000)	X (0.000, 0.500, 0.000)
1	X (0.000, 0.500, 0.000)	M (0.500, 0.500, 0.000)
2	M (0.500, 0.500, 0.000)	Γ (0.000, 0.000, 0.000)
3	Γ (0.000, 0.000, 0.000)	R (0.500, 0.500, 0.500)
4	R (0.500, 0.500, 0.500)	X (0.000, 0.500, 0.000)
5	R (0.500, 0.500, 0.500)	M (0.500, 0.500, 0.000)

Supplementary Table 687: Nodal ring points along high symmetry lines of mp-632319-CsH.

	Coordinates	Frequency	Mode 1	Mode 2	Multi.	Deg.	Path	Ratio
0	(0.000, 0.296, 0.000)	0.631	2	1	6	2	1	0.592
1	(0.000, 0.306, 0.000)	0.641	2	1	6	2	1	0.612
2	(0.000, 0.367, 0.000)	0.692	2	1	6	2	1	0.735
3	(0.000, 0.439, 0.000)	0.727	2	1	6	2	1	0.878
4	(0.092, 0.092, -0.000)	0.872	3	2	12	2	3	0.815
5	(0.500, 0.500, 0.500)	2.231	3	2	1	3	4	1.000
6	(0.357, 0.500, 0.357)	15.974	6	5	12	2	5	0.286

The followings are details for Na_4F_4. It has 3 topological nodal lines and 7 nodal ring points along high symmetry line as shown in Fig 358.

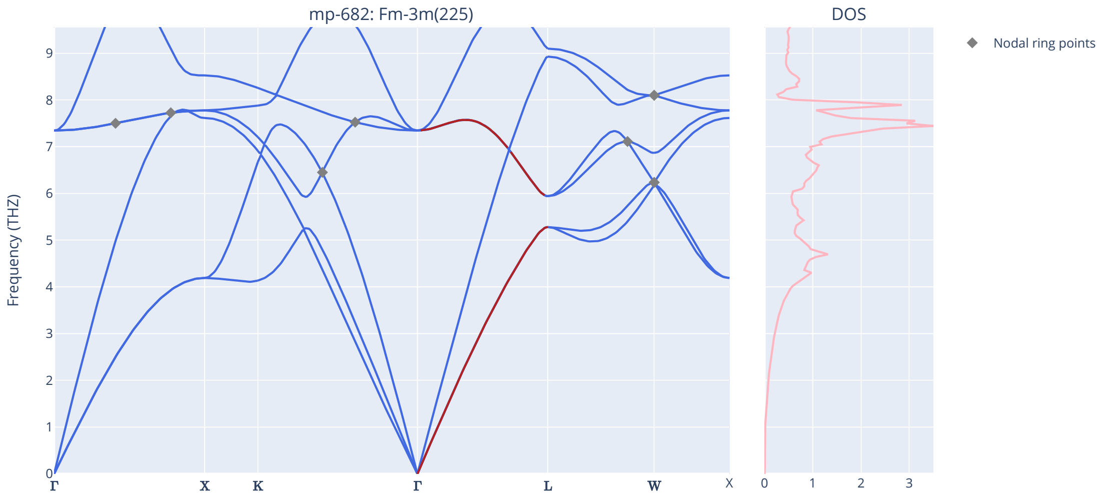

Supplementary Figure 358: The phonon band and DOS of Na4F4.

Supplementary Table 688: High symmetry lines of mp-682-Na4F4

	Start	End
0	Γ (0.000, 0.000, 0.000)	X (0.500, 0.000, 0.500)
1	X (0.500, 0.000, 0.500)	U (0.625, 0.250, 0.625)
2	K (0.375, 0.375, 0.750)	Γ (0.000, 0.000, 0.000)
3	Γ (0.000, 0.000, 0.000)	L (0.500, 0.500, 0.500)
4	L (0.500, 0.500, 0.500)	W (0.500, 0.250, 0.750)
5	W (0.500, 0.250, 0.750)	X (0.500, 0.000, 0.500)

Supplementary Table 689: Nodal ring points along high symmetry lines of mp-682-Na4F4.

	Coordinates	Frequency	Mode 1	Mode 2	Multi.	Deg.	Path	Ratio
0	(0.500, 0.250, 0.750)	6.235	3	2	6	2	5	1.000
1	(0.222, 0.222, 0.448)	6.450	4	3	24	2	3	0.405
2	(0.500, 0.313, 0.687)	7.110	4	3	24	2	5	0.750
3	(0.204, 0.000, 0.204)	7.500	5	4	6	2	1	0.408
4	(0.388, 0.000, 0.388)	7.725	5	4	6	2	1	0.776
5	(0.145, 0.145, 0.293)	7.524	5	4	24	2	3	0.610
6	(0.500, 0.250, 0.750)	8.099	6	5	6	2	5	1.000

The followings are details for Ag_2I_2 and it only has 7 single Weyl point along high symmetry line as shown in Fig **359**.

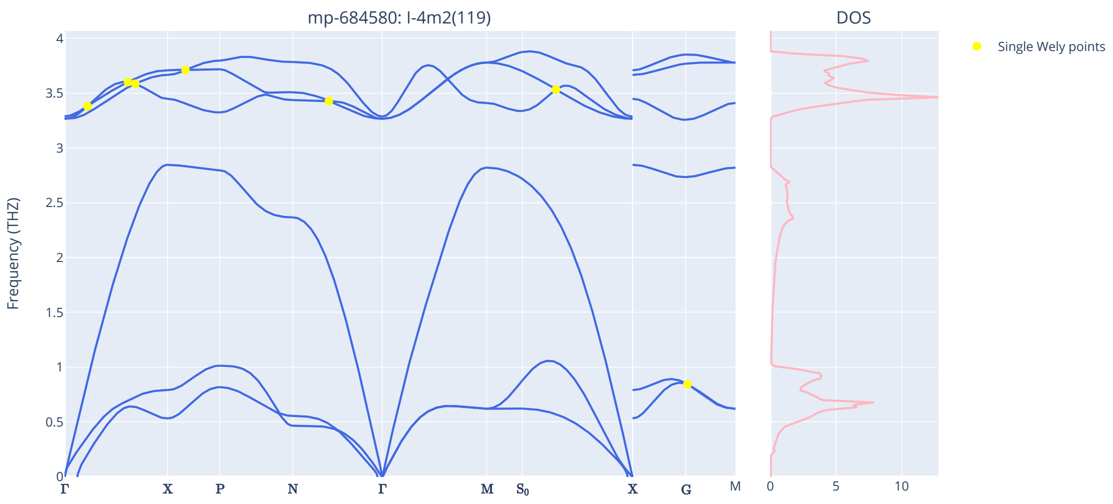

Supplementary Figure 359: The phonon band and DOS of Ag2I2.

Supplementary Table 690: High symmetry lines of mp-684580-Ag2I2

	Start	End
0	Γ (0.000, 0.000, 0.000)	X (0.000, 0.000, 0.500)
1	X (0.000, 0.000, 0.500)	P (0.250, 0.250, 0.250)
2	P (0.250, 0.250, 0.250)	N (0.000, 0.500, 0.000)
3	N (0.000, 0.500, 0.000)	Γ (0.000, 0.000, 0.000)
4	Γ (0.000, 0.000, 0.000)	M (0.500, 0.500, -0.500)
5	M (0.500, 0.500, -0.500)	S (0.379, 0.621, -0.379)
6	S_0 (-0.379, 0.379, 0.379)	Γ (0.000, 0.000, 0.000)
7	X (0.000, 0.000, 0.500)	R (-0.258, 0.258, 0.500)
8	G (0.500, 0.500, -0.258)	M (0.500, 0.500, -0.500)

Supplementary Table 691: Single Weyl points along high symmetry lines of mp-684580-Ag2I2.

	Coordinates	Frequency	Mode 1	Mode 2	Multi.	Deg.	Path	Ratio
0	(0.500, 0.500, -0.267)	0.843	2	1	4	2	9	0.038
1	(-0.000, -0.000, 0.343)	3.586	5	4	4	2	1	0.687
2	(-0.262, 0.264, 0.262)	3.532	5	4	8	2	7	0.306
3	(-0.002, 0.298, 0.002)	3.427	5	4	8	2	4	0.404
4	(-0.000, -0.000, 0.111)	3.380	6	5	4	2	1	0.222
5	(0.086, 0.086, 0.414)	3.711	6	5	4	2	2	0.343
6	(-0.000, -0.000, 0.308)	3.601	6	5	4	2	1	0.616

The followings are details for Cu_4I_4. It has 23 nodal ring points and 2 single Weyl points along high symmetry line as shown in Fig 360.

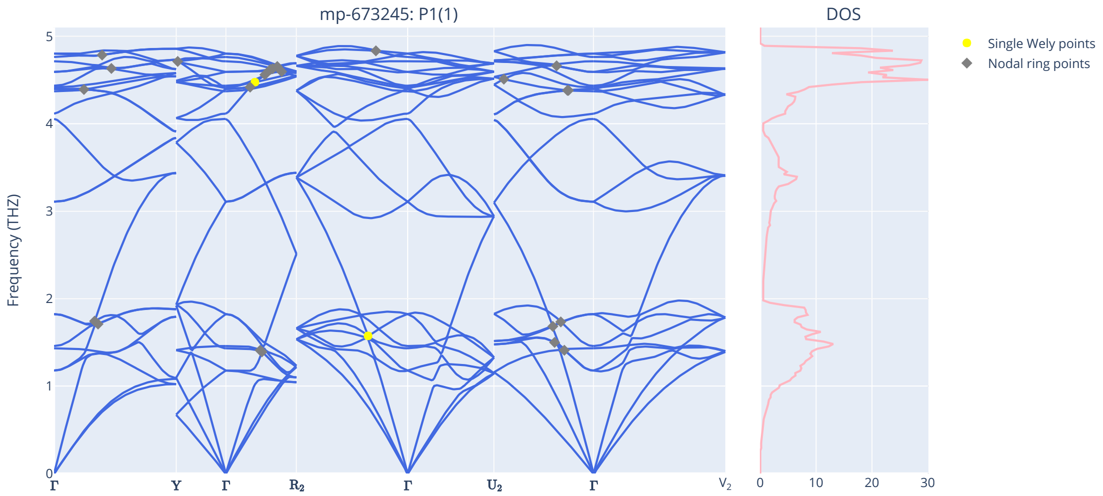

Supplementary Figure 360: The phonon band and DOS of Cu4I4.

Supplementary Table 692: High symmetry lines of mp-673245-Cu4I4

	Start	End
0	Γ (0.000, 0.000, 0.000)	X (0.500, 0.000, 0.000)
1	Y (0.000, 0.500, 0.000)	Γ (0.000, 0.000, 0.000)
2	Γ (0.000, 0.000, 0.000)	Z (0.000, 0.000, 0.500)
3	R_2 (-0.500, -0.500, 0.500)	Γ (0.000, 0.000, 0.000)
4	Γ (0.000, 0.000, 0.000)	T_2 (0.000, -0.500, 0.500)
5	U_2 (-0.500, 0.000, 0.500)	Γ (0.000, 0.000, 0.000)
6	Γ (0.000, 0.000, 0.000)	V_2 (0.500, -0.500, 0.000)

Supplementary Table 693: Single Weyl points along high symmetry lines of mp-673245-Cu4I4.

	Coordinates	Frequency	Mode 1	Mode 2	Multi.	Deg.	Path	Ratio
0	(-0.177, -0.175, 0.182)	1.574	7	6	2	2	4	0.644
1	(0.004, -0.000, 0.206)	4.476	17	16	2	2	3	0.412

Supplementary Table 694: Nodal ring points along high symmetry lines of mp-673245-Cu4I4.

	Coordinates	Frequency	Mode 1	Mode 2	Multi.	Deg.	Path	Ratio
0	(-0.197, -0.000, 0.195)	1.499	5	4	2	2	6	0.608
1	(-0.145, -0.000, 0.148)	1.409	5	4	2	2	6	0.706
2	(0.001, -0.003, 0.256)	1.391	6	5	2	2	3	0.512

Supplementary Table 694: Nodal ring points along high symmetry lines of mp-673245-Cu4I4.

	Coordinates	Frequency	Mode 1	Mode 2	Multi.	Deg.	Path	Ratio
3	(0.180, 0.000, 0.004)	1.709	7	6	2	2	1	0.360
4	(-0.002, -0.000, 0.243)	1.408	7	6	2	2	3	0.486
5	(-0.207, -0.000, 0.202)	1.682	7	6	2	2	6	0.591
6	(0.166, -0.000, 0.001)	1.743	9	8	2	2	1	0.332
7	(-0.163, -0.004, 0.166)	1.733	9	8	2	2	6	0.671
8	(0.122, 0.000, 0.001)	4.392	15	14	2	2	1	0.245
9	(0.000, -0.002, 0.172)	4.419	15	14	2	2	3	0.344
10	(-0.130, -0.000, 0.133)	4.382	15	14	2	2	6	0.737
11	(-0.130, -0.000, 0.122)	4.382	15	14	2	2	6	0.748
12	(-0.452, 0.000, 0.449)	4.510	17	16	2	2	6	0.099
13	(0.004, -0.000, 0.277)	4.561	18	17	2	2	3	0.553
14	(0.001, 0.000, 0.395)	4.597	19	18	2	2	3	0.790
15	(0.235, -0.001, 0.000)	4.633	20	19	2	2	1	0.469
16	(-0.001, 0.000, 0.315)	4.621	20	19	2	2	3	0.630
17	(-0.184, -0.000, 0.188)	4.663	20	19	2	2	6	0.629
18	(0.000, 0.484, 0.000)	4.713	21	20	2	2	2	0.031
19	(-0.003, 0.000, 0.331)	4.630	21	20	2	2	3	0.663
20	(0.197, 0.000, 0.004)	4.786	23	22	2	2	1	0.394
21	(0.002, 0.000, 0.364)	4.655	23	22	2	2	3	0.729
22	(-0.143, -0.144, 0.142)	4.834	24	23	2	2	4	0.714

The followings are details for CeCuSi. It has 2 nodal ring points and 2 single Weyl points along high symmetry line as shown in Fig **361**.

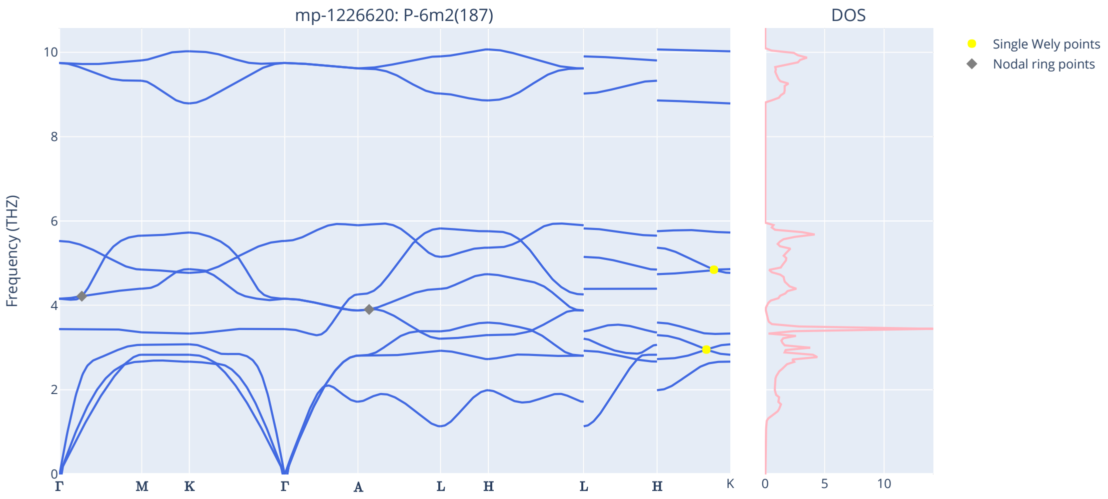

Supplementary Figure 361: The phonon band and DOS of CeCuSi.

Supplementary Table 695: High symmetry lines of mp-1226620-CeCuSi

	Start	End
0	Γ (0.000, 0.000, 0.000)	M (0.500, 0.000, 0.000)
1	M (0.500, 0.000, 0.000)	K (0.333, 0.333, 0.000)
2	K (0.333, 0.333, 0.000)	Γ (0.000, 0.000, 0.000)
3	Γ (0.000, 0.000, 0.000)	A (0.000, 0.000, 0.500)
4	A (0.000, 0.000, 0.500)	L (0.500, 0.000, 0.500)
5	L (0.500, 0.000, 0.500)	H (0.333, 0.333, 0.500)
6	H (0.333, 0.333, 0.500)	A (0.000, 0.000, 0.500)
7	L (0.500, 0.000, 0.500)	M (0.500, 0.000, 0.000)
8	H (0.333, 0.333, 0.500)	K (0.333, 0.333, 0.000)

Supplementary Table 696: Single Weyl points along high symmetry lines of mp-1226620-CeCuSi.

	Coordinates	Frequency	Mode 1	Mode 2	Multi.	Deg.	Path	Ratio
0	(0.333, 0.333, 0.163)	2.952	3	2	4	2	9	0.673
1	(0.333, 0.333, 0.112)	4.845	6	5	4	2	9	0.776

Supplementary Table 697: Nodal ring points along high symmetry lines of mp-1226620-CeCuSi.

	Coordinates	Frequency	Mode 1	Mode 2	Multi.	Deg.	Path	Ratio
0	(0.067, -0.000, 0.500)	3.903	5	4	6	2	5	0.134
1	(0.138, -0.000, 0.000)	4.217	6	5	6	2	1	0.276

Supplementary Table 697: Nodal ring points along high symmetry lines of mp-1226620-CeCuSi.

Coordinates	Frequency	Mode 1	Mode 2	Multi.	Deg.	Path	Ratio

The followings are details for $Rh_4Sn_4Ti_4$. As shown in Fig 362, it has 4 topological nodal lines and 3 nodal ring points. At the same time, it has 1 single Weyl point and 1 high degenerate Weyl point whose degeneracy is more than 2 and charge is bigger than 1.

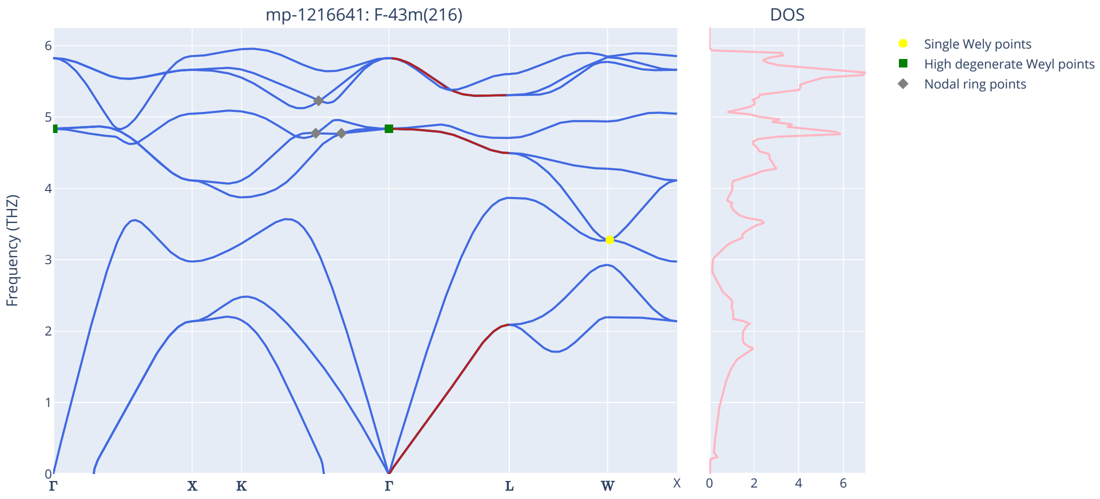

Supplementary Figure 362: The phonon band and DOS of Rh4Sn4Ti4.

Supplementary Table 698: High symmetry lines of mp-1216641-Rh4Sn4Ti4

	Start	End
0	Γ (0.000, 0.000, 0.000)	X (0.500, 0.000, 0.500)
1	X (0.500, 0.000, 0.500)	U (0.625, 0.250, 0.625)
2	K (0.375, 0.375, 0.750)	Γ (0.000, 0.000, 0.000)
3	Γ (0.000, 0.000, 0.000)	L (0.500, 0.500, 0.500)
4	L (0.500, 0.500, 0.500)	W (0.500, 0.250, 0.750)
5	W (0.500, 0.250, 0.750)	X (0.500, 0.000, 0.500)

Supplementary Table 699: Single Weyl points along high symmetry lines of mp-1216641-Rh4Sn4Ti4.

	Coordinates	Frequency	Mode 1	Mode 2	Multi.	Deg.	Path	Ratio
0	(0.500, 0.242, 0.742)	3.278	4	3	12	2	6	0.033

Supplementary Table 700: High degenerate Weyl points along high symmetry lines of mp-1216641-Rh4Sn4Ti4.

	Coordinates	Frequency	Mode 1	Mode 2	Multi.	Deg.	Path	Ratio
0	(0.000, 0.000, 0.000)	4.836	6	4	1	3	1	0.000

Supplementary Table 701: Nodal ring points along high symmetry lines of mp-1216641-Rh4Sn4Ti4.

	Coordinates	Frequency	Mode 1	Mode 2	Multi.	Deg.	Path	Ratio
0	(0.122, 0.122, 0.238)	4.773	5	4	24	2	3	0.679
1	(0.191, 0.191, 0.366)	4.773	6	5	24	2	3	0.505
2	(0.184, 0.184, 0.352)	5.228	8	7	24	2	3	0.524

The followings are details for SnSSe. As shown in Fig 363, it has 3 topological nodal lines, 1 nodal ring point and 1 single Weyl point.

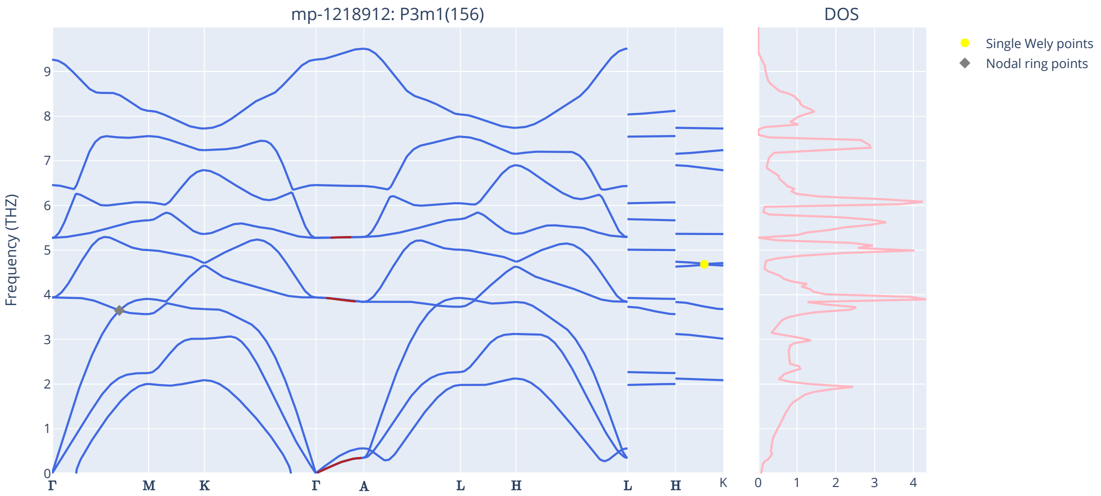

Supplementary Figure 363: The phonon band and DOS of SnSSe.

Supplementary Table 702: High symmetry lines of mp-1218912-SnSSe

	Start	End
0	Γ (0.000, 0.000, 0.000)	M (0.500, 0.000, 0.000)
1	M (0.500, 0.000, 0.000)	K (0.333, 0.333, 0.000)
2	K (0.333, 0.333, 0.000)	Γ (0.000, 0.000, 0.000)
3	Γ (0.000, 0.000, 0.000)	A (0.000, 0.000, 0.500)
4	A (0.000, 0.000, 0.500)	L (0.500, 0.000, 0.500)
5	L (0.500, 0.000, 0.500)	H (0.333, 0.333, 0.500)
6	H (0.333, 0.333, 0.500)	A (0.000, 0.000, 0.500)
7	L (0.500, 0.000, 0.500)	M (0.500, 0.000, 0.000)
8	H (0.333, 0.333, 0.500)	K (0.333, 0.333, 0.000)

Supplementary Table 703: Single Weyl points along high symmetry lines of mp-1218912-SnSSe.

	Coordinates	Frequency	Mode 1	Mode 2	Multi.	Deg.	Path	Ratio
0	(0.333, 0.333, 0.198)	4.683	5	4	4	2	9	0.604

Supplementary Table 704: Nodal ring points along high symmetry lines of mp-1218912-SnSSe.

	Coordinates	Frequency	Mode 1	Mode 2	Multi.	Deg.	Path	Ratio
0	(0.348, -0.000, -0.000)	3.648	4	3	6	2	1	0.697

The followings are details for Ir_4Sn_8. It has 4 topological nodal lines and 9 nodal ring points along high symmetry line as shown in Fig **364**.

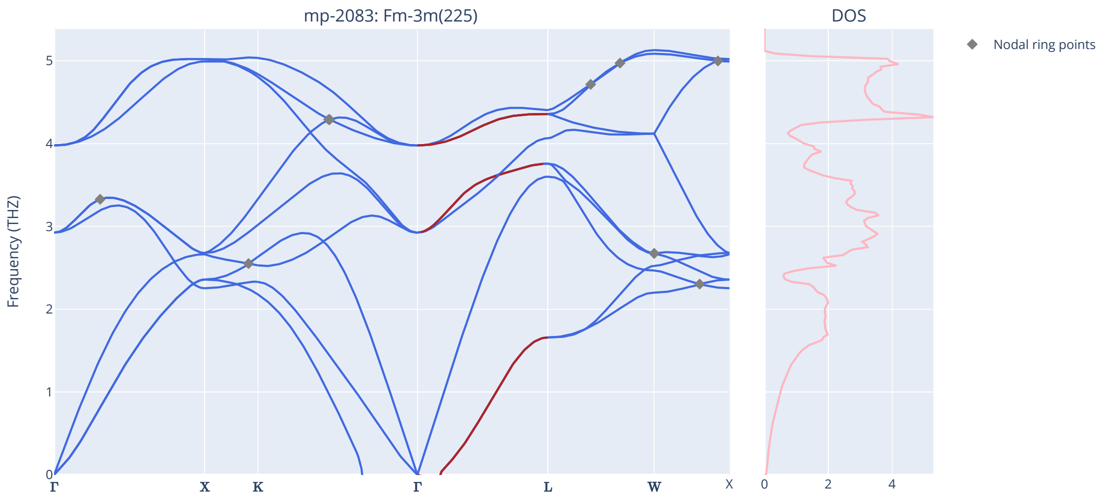

Supplementary Figure 364: The phonon band and DOS of Ir4Sn8.

Supplementary Table 705: High symmetry lines of mp-2083-Ir4Sn8

	Start	End
0	Γ (0.000, 0.000, 0.000)	X (0.500, 0.000, 0.500)
1	X (0.500, 0.000, 0.500)	U (0.625, 0.250, 0.625)
2	K (0.375, 0.375, 0.750)	Γ (0.000, 0.000, 0.000)
3	Γ (0.000, 0.000, 0.000)	L (0.500, 0.500, 0.500)
4	L (0.500, 0.500, 0.500)	W (0.500, 0.250, 0.750)
5	W (0.500, 0.250, 0.750)	X (0.500, 0.000, 0.500)

Supplementary Table 706: Nodal ring points along high symmetry lines of mp-2083-Ir4Sn8.

	Coordinates	Frequency	Mode 1	Mode 2	Multi.	Deg.	Path	Ratio
0	(0.499, 0.099, 0.599)	2.303	2	1	48	2	6	0.605
1	(0.603, 0.206, 0.603)	2.551	4	3	12	2	2	0.826
2	(0.500, 0.250, 0.750)	2.673	5	4	6	2	5	1.000
3	(0.153, 0.000, 0.153)	3.327	6	5	6	2	1	0.306
4	(0.214, 0.214, 0.412)	4.290	8	7	24	2	3	0.444
5	(0.207, 0.207, 0.412)	4.290	8	7	24	2	3	0.450
6	(0.499, 0.039, 0.538)	4.998	8	7	24	2	6	0.847
7	(0.500, 0.330, 0.670)	4.972	9	8	24	2	5	0.679
8	(0.500, 0.399, 0.601)	4.715	9	8	24	2	5	0.404

The followings are details for Ga_8Pt_4. It has 5 topological nodal lines and 6 nodal ring points along high symmetry line as shown in Fig **365**.

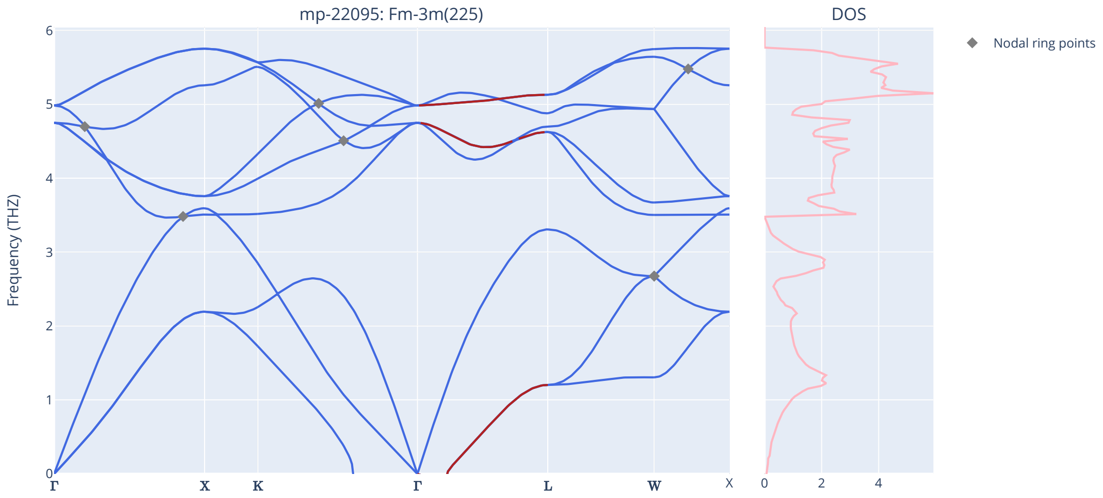

Supplementary Figure 365: The phonon band and DOS of Ga8Pt4.

Supplementary Table 707: High symmetry lines of mp-22095-Ga8Pt4

	Start	End
0	Γ (0.000, 0.000, 0.000)	X (0.500, 0.000, 0.500)
1	X (0.500, 0.000, 0.500)	U (0.625, 0.250, 0.625)
2	K (0.375, 0.375, 0.750)	Γ (0.000, 0.000, 0.000)
3	Γ (0.000, 0.000, 0.000)	L (0.500, 0.500, 0.500)
4	L (0.500, 0.500, 0.500)	W (0.500, 0.250, 0.750)
5	W (0.500, 0.250, 0.750)	X (0.500, 0.000, 0.500)

Supplementary Table 708: Nodal ring points along high symmetry lines of mp-22095-Ga8Pt4.

	Coordinates	Frequency	Mode 1	Mode 2	Multi.	Deg.	Path	Ratio
0	(0.500, 0.250, 0.750)	2.675	3	2	6	2	5	1.000
1	(0.428, -0.001, 0.428)	3.482	4	3	10	2	1	0.857
2	(0.176, 0.176, 0.344)	4.507	6	5	24	2	3	0.537
3	(0.102, -0.000, 0.102)	4.698	7	6	6	2	1	0.204
4	(0.230, 0.230, 0.466)	5.012	8	7	24	2	3	0.381
5	(0.498, 0.138, 0.636)	5.478	8	7	24	2	6	0.452

The followings are details for Au_4Ga_8. It has 5 topological nodal lines and 4 nodal ring points along high symmetry line as shown in Fig **366**.

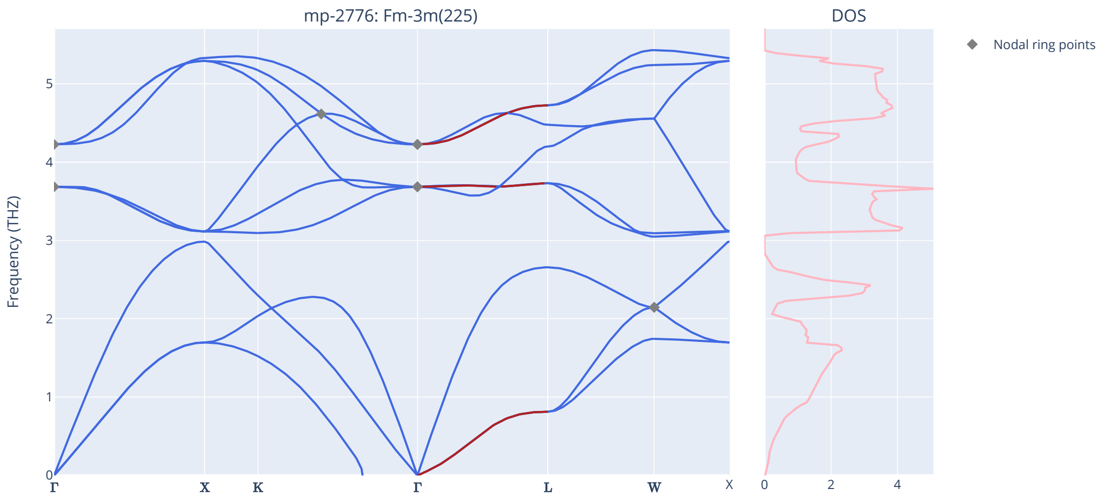

Supplementary Figure 366: The phonon band and DOS of Au4Ga8.

Supplementary Table 709: High symmetry lines of mp-2776-Au4Ga8

	Start	End
0	Γ (0.000, 0.000, 0.000)	X (0.500, 0.000, 0.500)
1	X (0.500, 0.000, 0.500)	U (0.625, 0.250, 0.625)
2	K (0.375, 0.375, 0.750)	Γ (0.000, 0.000, 0.000)
3	Γ (0.000, 0.000, 0.000)	L (0.500, 0.500, 0.500)
4	L (0.500, 0.500, 0.500)	W (0.500, 0.250, 0.750)
5	W (0.500, 0.250, 0.750)	X (0.500, 0.000, 0.500)

Supplementary Table 710: Nodal ring points along high symmetry lines of mp-2776-Au4Ga8.

	Coordinates	Frequency	Mode 1	Mode 2	Multi.	Deg.	Path	Ratio
0	(0.500, 0.250, 0.750)	2.145	3	2	6	2	5	1.000
1	(0.000, 0.000, 0.000)	3.685	6	5	1	3	1	0.000
2	(0.230, 0.230, 0.448)	4.615	8	7	24	2	3	0.397
3	(0.000, 0.000, 0.000)	4.226	9	8	1	3	1	0.000

The followings are details for CuLaSi. It has 5 nodal ring points and 2 single Weyl points along high symmetry line as shown in Fig 367.

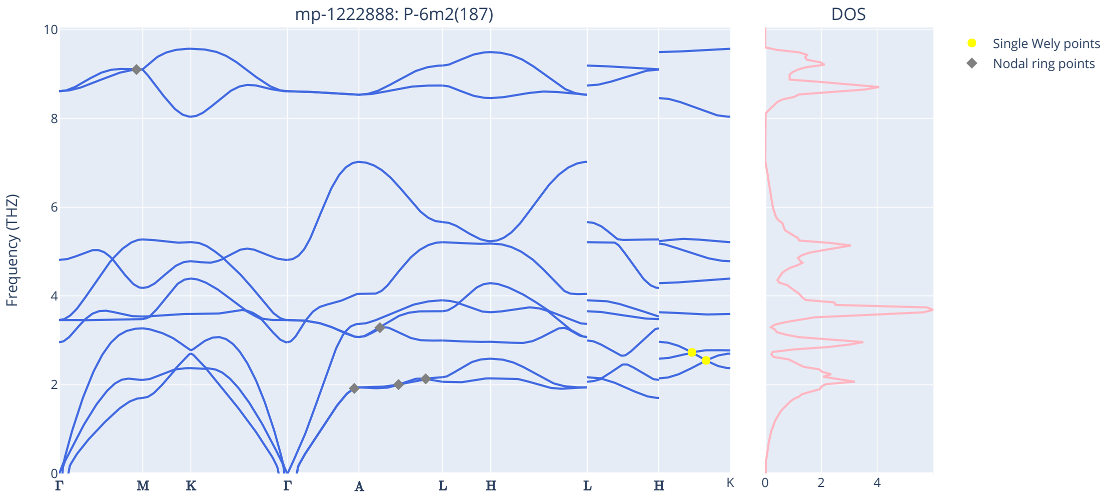

Supplementary Figure 367: The phonon band and DOS of CuLaSi.

Supplementary Table 711: High symmetry lines of mp-1222888-CuLaSi

	Start	End
0	Γ (0.000, 0.000, 0.000)	M (0.500, 0.000, 0.000)
1	M (0.500, 0.000, 0.000)	K (0.333, 0.333, 0.000)
2	K (0.333, 0.333, 0.000)	Γ (0.000, 0.000, 0.000)
3	Γ (0.000, 0.000, 0.000)	A (0.000, 0.000, 0.500)
4	A (0.000, 0.000, 0.500)	L (0.500, 0.000, 0.500)
5	L (0.500, 0.000, 0.500)	H (0.333, 0.333, 0.500)
6	H (0.333, 0.333, 0.500)	A (0.000, 0.000, 0.500)
7	L (0.500, 0.000, 0.500)	M (0.500, 0.000, 0.000)
8	H (0.333, 0.333, 0.500)	K (0.333, 0.333, 0.000)

Supplementary Table 712: Single Weyl points along high symmetry lines of mp-1222888-CuLaSi.

	Coordinates	Frequency	Mode 1	Mode 2	Multi.	Deg.	Path	Ratio
0	(0.333, 0.333, 0.170)	2.546	2	1	4	2	9	0.660
1	(0.333, 0.333, 0.269)	2.727	3	2	4	2	9	0.463

Supplementary Table 713: Nodal ring points along high symmetry lines of mp-1222888-CuLaSi.

	Coordinates	Frequency	Mode 1	Mode 2	Multi.	Deg.	Path	Ratio
0	(-0.001, 0.000, 0.468)	1.916	2	1	12	2	4	0.935

Supplementary Table 713: Nodal ring points along high symmetry lines of mp-1222888-CuLaSi.

	Coordinates	Frequency	Mode 1	Mode 2	Multi.	Deg.	Path	Ratio
1	(0.237, 0.000, 0.500)	2.002	2	1	6	2	5	0.474
2	(0.399, 0.000, 0.500)	2.132	2	1	6	2	5	0.799
3	(0.125, -0.000, 0.500)	3.284	4	3	6	2	5	0.251
4	(0.463, 0.000, -0.000)	9.101	9	8	6	2	1	0.926

The followings are details for Lu_4H_8. It has 3 topological nodal lines and 9 nodal ring points along high symmetry line as shown in Fig 368.

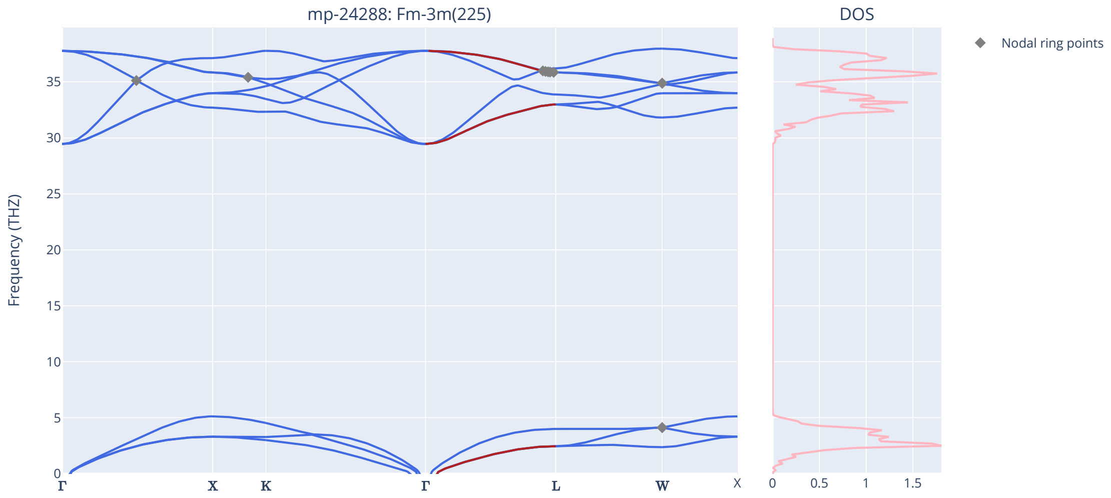

Supplementary Figure 368: The phonon band and DOS of Lu4H8.

Supplementary Table 714: High symmetry lines of mp-24288-Lu4H8

	Start	End
0	Γ (0.000, 0.000, 0.000)	X (0.500, 0.000, 0.500)
1	X (0.500, 0.000, 0.500)	U (0.625, 0.250, 0.625)
2	K (0.375, 0.375, 0.750)	Γ (0.000, 0.000, 0.000)
3	Γ (0.000, 0.000, 0.000)	L (0.500, 0.500, 0.500)
4	L (0.500, 0.500, 0.500)	W (0.500, 0.250, 0.750)
5	W (0.500, 0.250, 0.750)	X (0.500, 0.000, 0.500)

Supplementary Table 715: Nodal ring points along high symmetry lines of mp-24288-Lu4H8.

	Coordinates	Frequency	Mode 1	Mode 2	Multi.	Deg.	Path	Ratio
0	(0.500, 0.250, 0.750)	4.121	3	2	6	2	5	1.000
1	(0.247, -0.002, 0.247)	35.117	7	6	24	2	1	0.494
2	(0.492, 0.492, 0.492)	35.852	8	7	8	2	4	0.984
3	(0.584, 0.166, 0.584)	35.404	8	7	24	2	2	0.667
4	(0.470, 0.470, 0.470)	35.896	8	7	8	2	4	0.941
5	(0.478, 0.478, 0.478)	35.875	8	7	8	2	4	0.956
6	(0.461, 0.461, 0.461)	35.931	8	7	8	2	4	0.921
7	(0.450, 0.450, 0.450)	35.976	8	7	8	2	4	0.901
8	(0.500, 0.250, 0.750)	34.881	8	7	6	2	5	1.000

The followings are details for Np_4N_8. It has 4 topological nodal lines and 4 nodal ring points along high symmetry line as shown in Fig **369**.

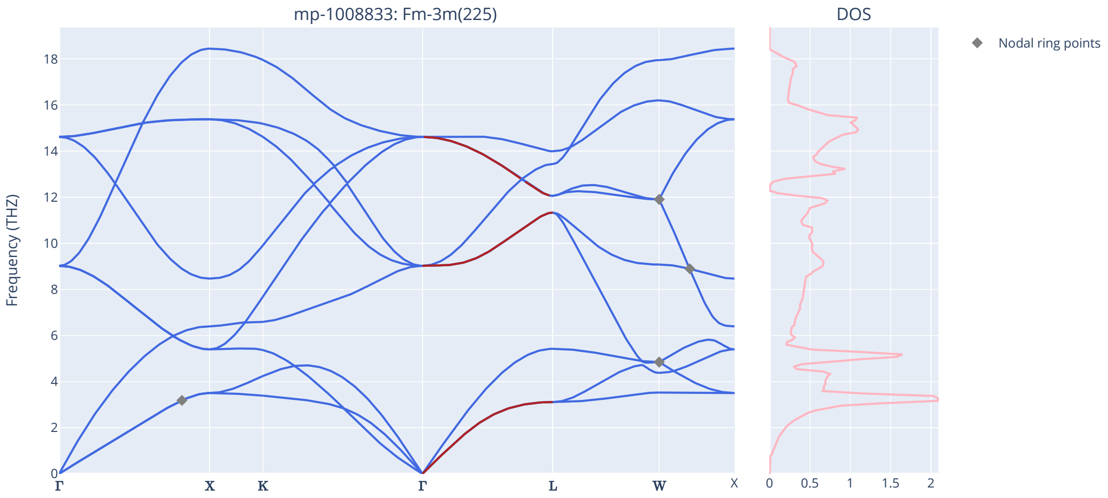

Supplementary Figure 369: The phonon band and DOS of Np4N8.

Supplementary Table 716: High symmetry lines of mp-1008833-Np4N8

	Start	End
0	Γ (0.000, 0.000, 0.000)	X (0.500, 0.000, 0.500)
1	X (0.500, 0.000, 0.500)	U (0.625, 0.250, 0.625)
2	K (0.375, 0.375, 0.750)	Γ (0.000, 0.000, 0.000)
3	Γ (0.000, 0.000, 0.000)	L (0.500, 0.500, 0.500)
4	L (0.500, 0.500, 0.500)	W (0.500, 0.250, 0.750)
5	W (0.500, 0.250, 0.750)	X (0.500, 0.000, 0.500)

Supplementary Table 717: Nodal ring points along high symmetry lines of mp-1008833-Np4N8.

	Coordinates	Frequency	Mode 1	Mode 2	Multi.	Deg.	Path	Ratio
0	(0.408, 0.000, 0.408)	3.182	2	1	6	2	1	0.816
1	(0.500, 0.250, 0.750)	4.844	4	3	6	2	5	1.000
2	(0.498, 0.149, 0.647)	8.893	6	5	24	2	6	0.407
3	(0.500, 0.250, 0.750)	11.904	7	6	6	2	5	1.000

The followings are details for Ca_8Si_4. It has 4 topological nodal lines and 10 nodal ring points along high symmetry line as shown in Fig 370.

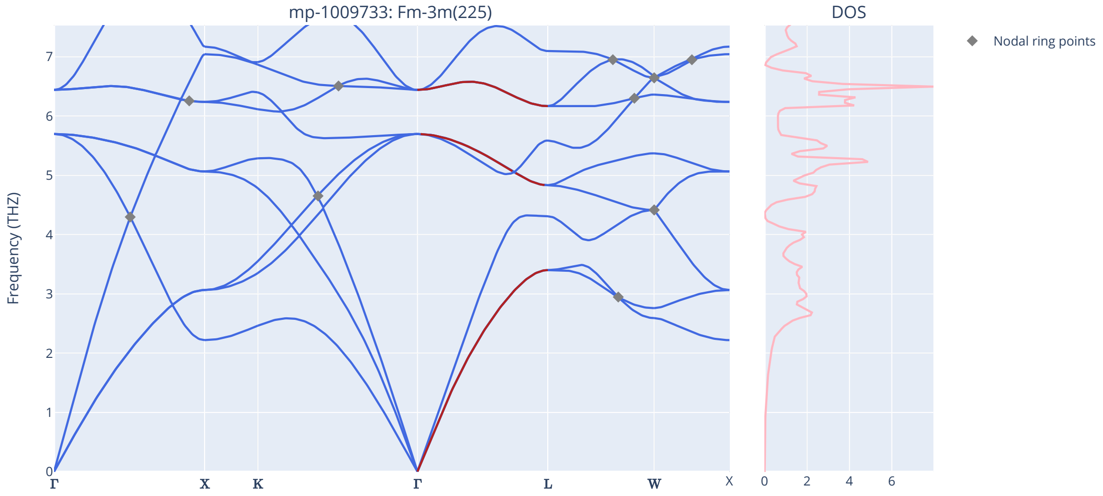

Supplementary Figure 370: The phonon band and DOS of Ca8Si4.

Supplementary Table 718: High symmetry lines of mp-1009733-Ca8Si4

	Start	End
0	Γ (0.000, 0.000, 0.000)	X (0.500, 0.000, 0.500)
1	X (0.500, 0.000, 0.500)	U (0.625, 0.250, 0.625)
2	K (0.375, 0.375, 0.750)	Γ (0.000, 0.000, 0.000)
3	Γ (0.000, 0.000, 0.000)	L (0.500, 0.500, 0.500)
4	L (0.500, 0.500, 0.500)	W (0.500, 0.250, 0.750)
5	W (0.500, 0.250, 0.750)	X (0.500, 0.000, 0.500)

Supplementary Table 719: Nodal ring points along high symmetry lines of mp-1009733-Ca8Si4.

	Coordinates	Frequency	Mode 1	Mode 2	Multi.	Deg.	Path	Ratio
0	(0.500, 0.334, 0.666)	2.947	2	1	24	2	5	0.663
1	(0.253, -0.001, 0.253)	4.297	4	3	10	2	1	0.506
2	(0.500, 0.250, 0.750)	4.416	4	3	6	2	5	1.000
3	(0.230, 0.230, 0.471)	4.652	5	4	24	2	3	0.377
4	(0.449, 0.000, 0.449)	6.256	7	6	6	2	1	0.898
5	(0.500, 0.297, 0.703)	6.300	7	6	24	2	5	0.814
6	(0.184, 0.184, 0.372)	6.506	8	7	24	2	3	0.506
7	(0.500, 0.250, 0.750)	6.644	8	7	6	2	5	1.000
8	(0.500, 0.347, 0.653)	6.950	9	8	24	2	5	0.612
9	(0.499, 0.124, 0.625)	6.949	9	8	24	2	6	0.501

The followings are details for Ca_8Ge_4. It has 5 topological nodal lines and 13 nodal ring points along high symmetry line as shown in Fig **371**.

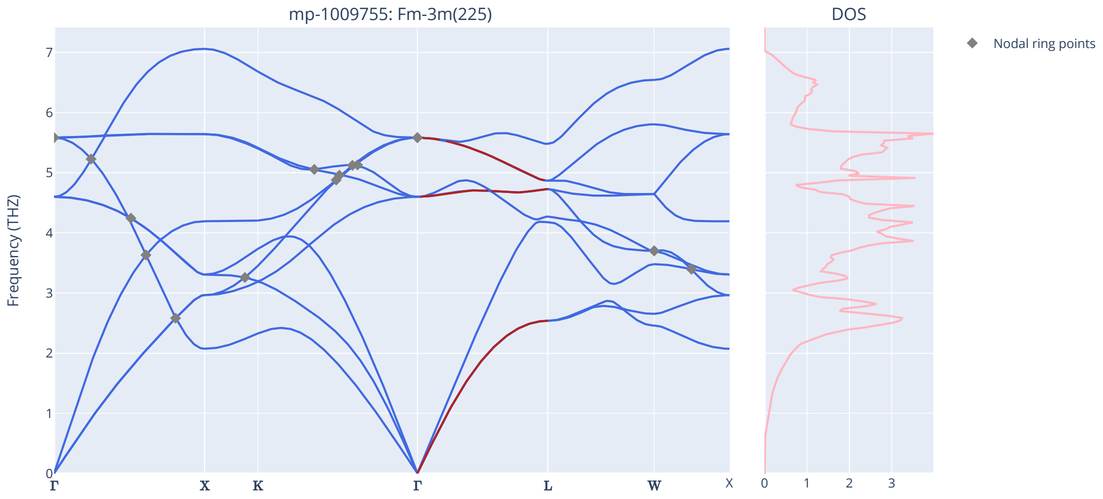

Supplementary Figure 371: The phonon band and DOS of Ca8Ge4.

Supplementary Table 720: High symmetry lines of mp-1009755-Ca8Ge4

	Start	End
0	Γ (0.000, 0.000, 0.000)	X (0.500, 0.000, 0.500)
1	X (0.500, 0.000, 0.500)	U (0.625, 0.250, 0.625)
2	K (0.375, 0.375, 0.750)	Γ (0.000, 0.000, 0.000)
3	Γ (0.000, 0.000, 0.000)	L (0.500, 0.500, 0.500)
4	L (0.500, 0.500, 0.500)	W (0.500, 0.250, 0.750)
5	W (0.500, 0.250, 0.750)	X (0.500, 0.000, 0.500)

Supplementary Table 721: Nodal ring points along high symmetry lines of mp-1009755-Ca8Ge4.

	Coordinates	Frequency	Mode 1	Mode 2	Multi.	Deg.	Path	Ratio
0	(0.406, -0.000, 0.401)	2.583	2	1	24	2	1	0.807
1	(0.305, -0.001, 0.305)	3.633	4	3	24	2	1	0.610
2	(0.595, 0.189, 0.595)	3.259	4	3	12	2	2	0.758
3	(0.498, 0.125, 0.627)	3.396	4	3	24	2	6	0.496
4	(0.500, 0.250, 0.750)	3.702	5	4	6	2	5	1.000
5	(0.255, 0.000, 0.255)	4.245	6	5	6	2	1	0.510
6	(0.191, 0.191, 0.381)	4.879	6	5	24	2	3	0.491
7	(0.123, -0.002, 0.123)	5.228	7	6	24	2	1	0.246
8	(0.188, 0.179, 0.367)	4.959	7	6	24	2	3	0.511
9	(0.147, 0.134, 0.281)	5.130	7	6	24	2	3	0.625
10	(0.237, 0.237, 0.490)	5.055	8	7	24	2	3	0.354
11	(0.153, 0.153, 0.305)	5.123	8	7	24	2	3	0.593
12	(0.000, 0.000, 0.000)	5.584	9	8	1	3	1	0.000

The followings are details for Cs_8Se_4. It has 4 topological nodal lines and 7 nodal ring points along high symmetry line as shown in Fig 372.

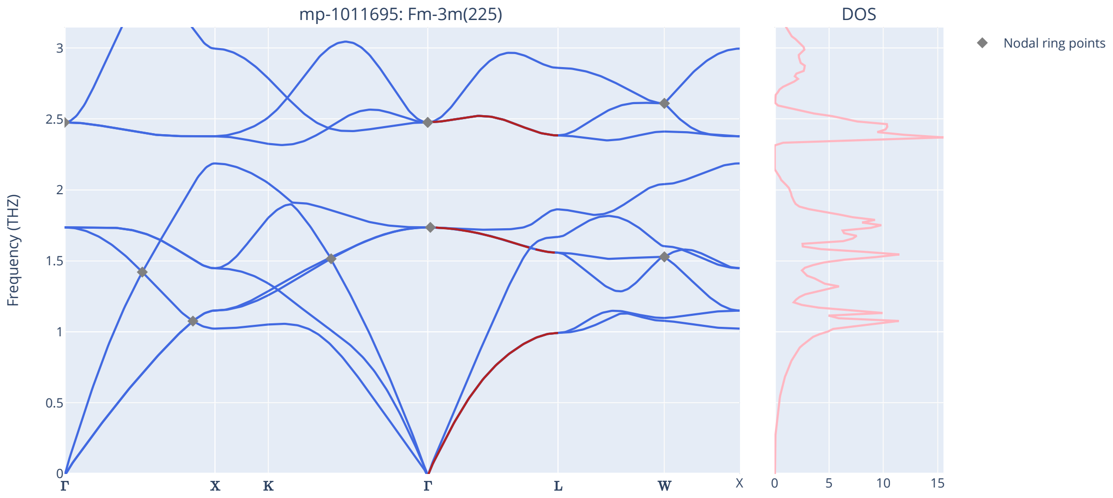

Supplementary Figure 372: The phonon band and DOS of Cs8Se4.

Supplementary Table 722: High symmetry lines of mp-1011695-Cs8Se4

	Start	End
0	Γ (0.000, 0.000, 0.000)	X (0.500, 0.000, 0.500)
1	X (0.500, 0.000, 0.500)	U (0.625, 0.250, 0.625)
2	K (0.375, 0.375, 0.750)	Γ (0.000, 0.000, 0.000)
3	Γ (0.000, 0.000, 0.000)	L (0.500, 0.500, 0.500)
4	L (0.500, 0.500, 0.500)	W (0.500, 0.250, 0.750)
5	W (0.500, 0.250, 0.750)	X (0.500, 0.000, 0.500)

Supplementary Table 723: Nodal ring points along high symmetry lines of mp-1011695-Cs8Se4.

	Coordinates	Frequency	Mode 1	Mode 2	Multi.	Deg.	Path	Ratio
0	(0.427, -0.000, 0.427)	1.076	3	2	6	2	1	0.853
1	(0.225, 0.225, 0.456)	1.515	4	3	24	2	3	0.395
2	(0.258, -0.000, 0.258)	1.421	4	3	6	2	1	0.517
3	(0.500, 0.250, 0.750)	1.528	4	3	6	2	5	1.000
4	(0.010, 0.010, 0.010)	1.736	6	5	8	3	4	0.020
5	(0.000, 0.000, 0.000)	2.476	8	7	1	3	1	0.000
6	(0.500, 0.250, 0.750)	2.610	9	8	6	2	5	1.000

The followings are details for $Bi_4La_4Pt_4$. As shown in Fig 373, it has 4 topological nodal lines, 3 nodal ring points and 1 high degenerate Weyl point whose degeneracy is more than 2 and charge is bigger than 1.

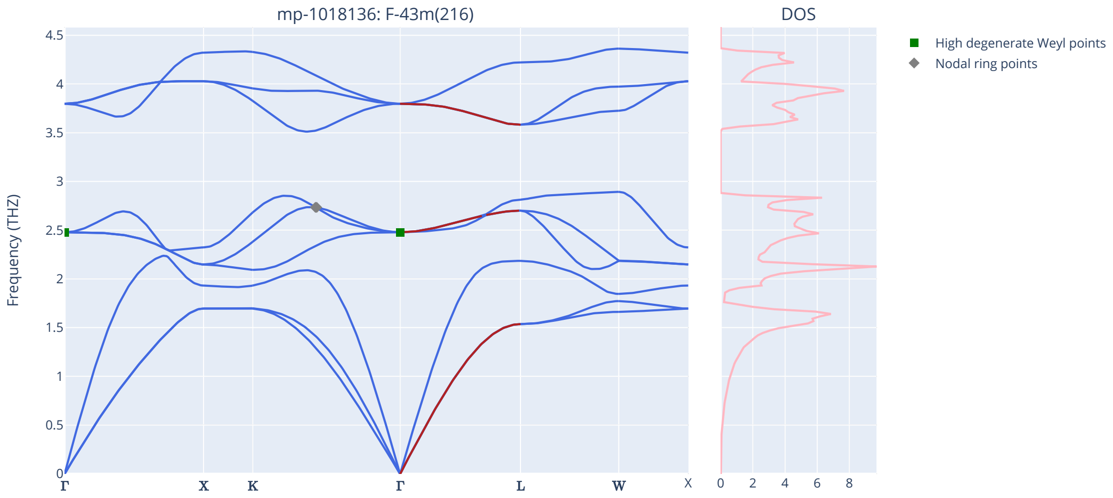

Supplementary Figure 373: The phonon band and DOS of Bi4La4Pt4.

Supplementary Table 724: High symmetry lines of mp-1018136-Bi4La4Pt4

	Start	End
0	Γ (0.000, 0.000, 0.000)	X (0.500, 0.000, 0.500)
1	X (0.500, 0.000, 0.500)	U (0.625, 0.250, 0.625)
2	K (0.375, 0.375, 0.750)	Γ (0.000, 0.000, 0.000)
3	Γ (0.000, 0.000, 0.000)	L (0.500, 0.500, 0.500)
4	L (0.500, 0.500, 0.500)	W (0.500, 0.250, 0.750)
5	W (0.500, 0.250, 0.750)	X (0.500, 0.000, 0.500)

Supplementary Table 725: High degenerate Weyl points along high symmetry lines of mp-1018136-Bi4La4Pt4.

	Coordinates	Frequency	Mode 1	Mode 2	Multi.	Deg.	Path	Ratio
0	(0.000, 0.000, 0.000)	2.478	6	4	1	3	1	0.000

Supplementary Table 726: Nodal ring points along high symmetry lines of mp-1018136-Bi4La4Pt4.

	Coordinates	Frequency	Mode 1	Mode 2	Multi.	Deg.	Path	Ratio
0	(0.206, 0.206, 0.430)	2.734	6	5	24	2	3	0.434
1	(0.216, 0.216, 0.430)	2.734	6	5	24	2	3	0.426

The followings are details for $Mg_4Pt_4Sb_4$. As shown in Fig 374, it has 4 topological nodal lines, 1 nodal ring point and 1 high degenerate Weyl point whose degeneracy is more than 2 and charge is bigger than 1.

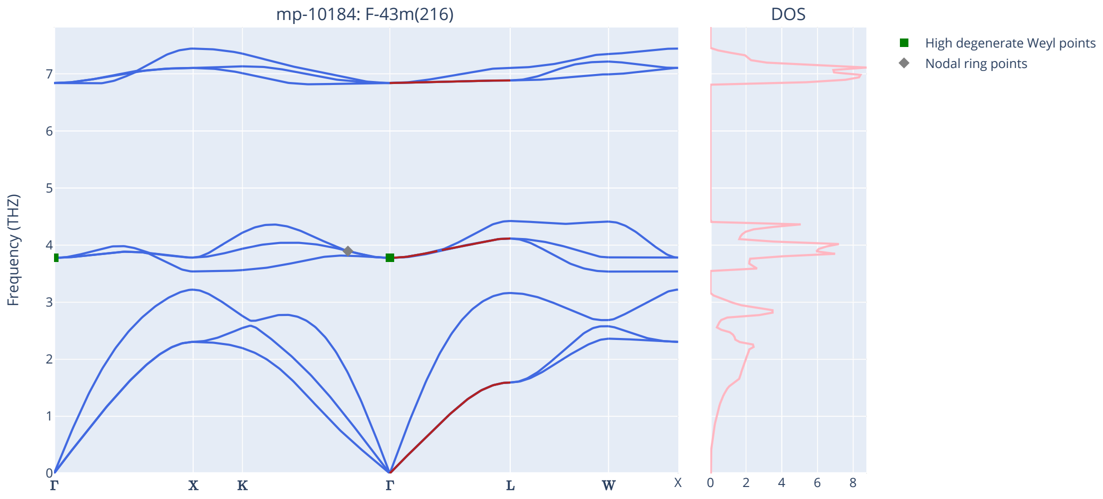

Supplementary Figure 374: The phonon band and DOS of Mg4Pt4Sb4.

Supplementary Table 727: High symmetry lines of mp-10184-Mg4Pt4Sb4

	Start	End
0	Γ (0.000, 0.000, 0.000)	X (0.500, 0.000, 0.500)
1	X (0.500, 0.000, 0.500)	U (0.625, 0.250, 0.625)
2	K (0.375, 0.375, 0.750)	Γ (0.000, 0.000, 0.000)
3	Γ (0.000, 0.000, 0.000)	L (0.500, 0.500, 0.500)
4	L (0.500, 0.500, 0.500)	W (0.500, 0.250, 0.750)
5	W (0.500, 0.250, 0.750)	X (0.500, 0.000, 0.500)

Supplementary Table 728: High degenerate Weyl points along high symmetry lines of mp-10184-Mg4Pt4Sb4.

	Coordinates	Frequency	Mode 1	Mode 2	Multi.	Deg.	Path	Ratio
0	(0.000, 0.000, 0.000)	3.774	6	4	1	3	1	0.000

Supplementary Table 729: Nodal ring points along high symmetry lines of mp-10184-Mg4Pt4Sb4.

	Coordinates	Frequency	Mode 1	Mode 2	Multi.	Deg.	Path	Ratio
0	(0.107, 0.107, 0.212)	3.894	6	5	24	2	3	0.716

The followings are details for CeNiAs and it only has 1 topological nodal ring point along high symmetry line as shown in Fig **375**.

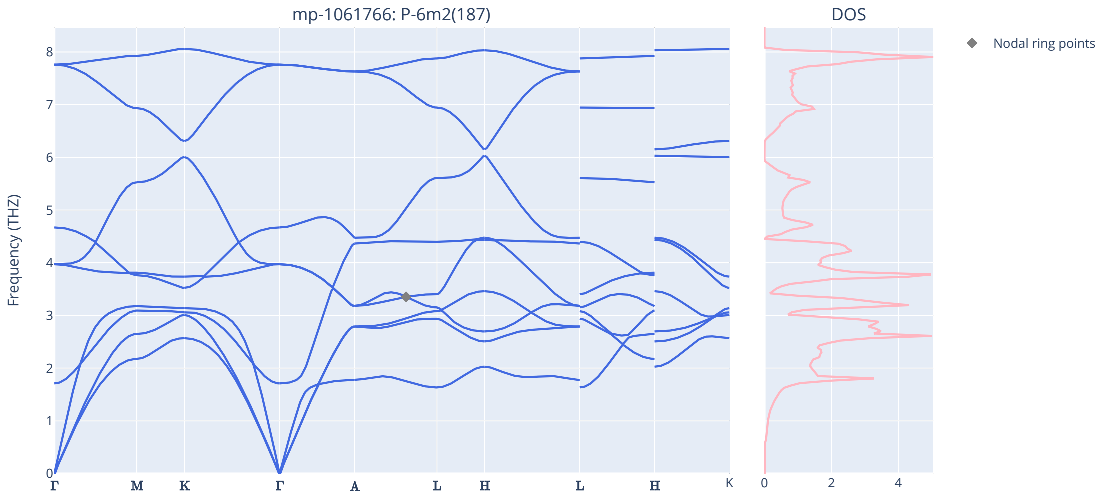

Supplementary Figure 375: The phonon band and DOS of CeNiAs.

Supplementary Table 730: High symmetry lines of mp-1061766-CeNiAs

	Start	End
0	Γ (0.000, 0.000, 0.000)	M (0.500, 0.000, 0.000)
1	M (0.500, 0.000, 0.000)	K (0.333, 0.333, 0.000)
2	K (0.333, 0.333, 0.000)	Γ (0.000, 0.000, 0.000)
3	Γ (0.000, 0.000, 0.000)	A (0.000, 0.000, 0.500)
4	A (0.000, 0.000, 0.500)	L (0.500, 0.000, 0.500)
5	L (0.500, 0.000, 0.500)	H (0.333, 0.333, 0.500)
6	H (0.333, 0.333, 0.500)	A (0.000, 0.000, 0.500)
7	L (0.500, 0.000, 0.500)	M (0.500, 0.000, 0.000)
8	H (0.333, 0.333, 0.500)	K (0.333, 0.333, 0.000)

Supplementary Table 731: Nodal ring points along high symmetry lines of mp-1061766-CeNiAs.

	Coordinates	Frequency	Mode 1	Mode 2	Multi.	Deg.	Path	Ratio
0	(0.313, 0.000, 0.500)	3.354	5	4	6	2	5	0.626

The followings are details for K_8Pt_4. It has 3 topological nodal lines and 9 nodal ring points along high symmetry line as shown in Fig **376**.

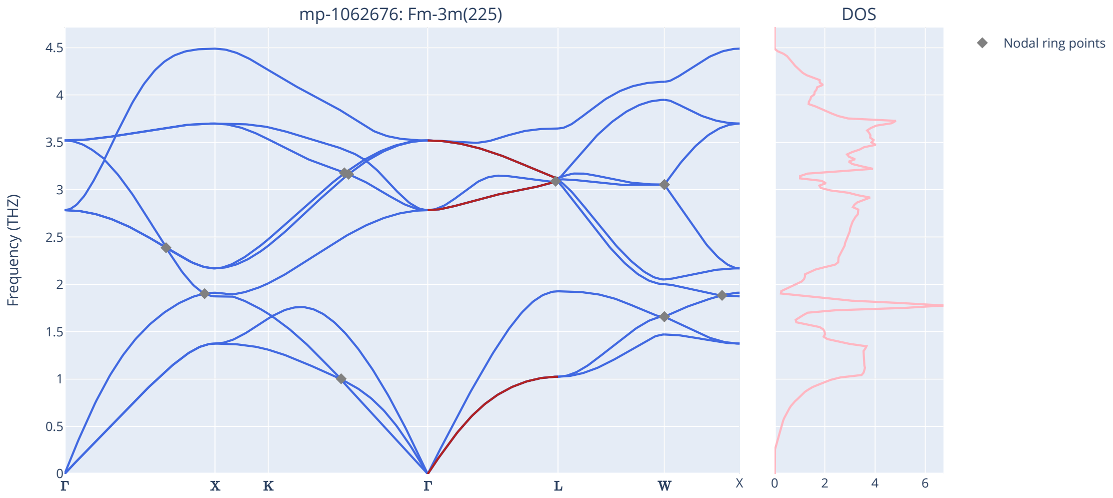

Supplementary Figure 376: The phonon band and DOS of K8Pt4.

Supplementary Table 732: High symmetry lines of mp-1062676-K8Pt4

	Start	End
0	Γ (0.000, 0.000, 0.000)	X (0.500, 0.000, 0.500)
1	X (0.500, 0.000, 0.500)	U (0.625, 0.250, 0.625)
2	K (0.375, 0.375, 0.750)	Γ (0.000, 0.000, 0.000)
3	Γ (0.000, 0.000, 0.000)	L (0.500, 0.500, 0.500)
4	L (0.500, 0.500, 0.500)	W (0.500, 0.250, 0.750)
5	W (0.500, 0.250, 0.750)	X (0.500, 0.000, 0.500)

Supplementary Table 733: Nodal ring points along high symmetry lines of mp-1062676-K8Pt4.

	Coordinates	Frequency	Mode 1	Mode 2	Multi.	Deg.	Path	Ratio
0	(0.199, 0.199, 0.412)	1.003	2	1	24	2	3	0.457
1	(0.500, 0.250, 0.750)	1.658	3	2	6	2	5	1.000
2	(0.466, 0.000, 0.466)	1.902	4	3	6	2	1	0.931
3	(0.499, 0.059, 0.558)	1.884	4	3	24	2	6	0.767
4	(0.187, 0.185, 0.372)	3.163	6	5	24	2	3	0.504
5	(0.337, 0.000, 0.337)	2.387	6	5	6	2	1	0.675
6	(0.491, 0.491, 0.491)	3.089	6	5	8	2	4	0.981
7	(0.199, 0.199, 0.389)	3.178	7	6	24	2	3	0.477
8	(0.500, 0.250, 0.750)	3.054	7	6	6	2	5	1.000

The followings are details for $Fe_4Ti_4Sb_4$. As shown in Fig **377**, it has 5 topological nodal lines, 4 nodal ring points and 1 high degenerate Weyl point whose degeneracy is more than 2 and charge is bigger than 1.

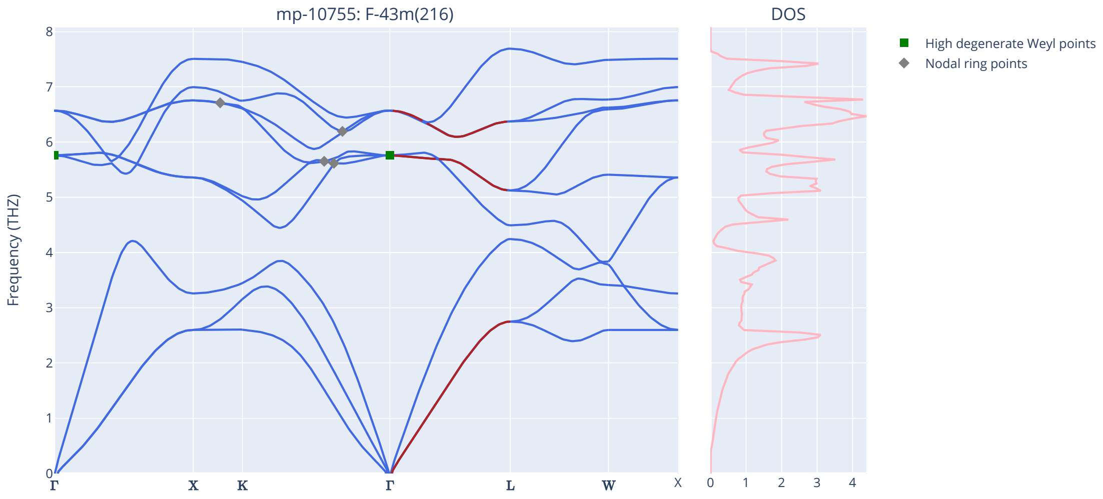

Supplementary Figure 377: The phonon band and DOS of Fe4Ti4Sb4.

Supplementary Table 734: High symmetry lines of mp-10755-Fe4Ti4Sb4

	Start	End
0	Γ (0.000, 0.000, 0.000)	X (0.500, 0.000, 0.500)
1	X (0.500, 0.000, 0.500)	U (0.625, 0.250, 0.625)
2	K (0.375, 0.375, 0.750)	Γ (0.000, 0.000, 0.000)
3	Γ (0.000, 0.000, 0.000)	L (0.500, 0.500, 0.500)
4	L (0.500, 0.500, 0.500)	W (0.500, 0.250, 0.750)
5	W (0.500, 0.250, 0.750)	X (0.500, 0.000, 0.500)

Supplementary Table 735: High degenerate Weyl points along high symmetry lines of mp-10755-Fe4Ti4Sb4.

	Coordinates	Frequency	Mode 1	Mode 2	Multi.	Deg.	Path	Ratio
0	(0.000, 0.000, 0.000)	5.758	6	4	1	3	1	0.000

Supplementary Table 736: Nodal ring points along high symmetry lines of mp-10755-Fe4Ti4Sb4.

	Coordinates	Frequency	Mode 1	Mode 2	Multi.	Deg.	Path	Ratio
0	(0.145, 0.145, 0.281)	5.616	5	4	24	2	3	0.621
1	(0.168, 0.168, 0.332)	5.651	6	5	24	2	3	0.555
2	(0.570, 0.137, 0.570)	6.710	7	6	24	2	2	0.550
3	(0.122, 0.122, 0.239)	6.193	8	7	24	2	3	0.679

The followings are details for $Ir_4Zr_4As_4$. It has 3 topological nodal lines and 1 high degenerate Weyl point whose degeneracy is more than 2 and charge is bigger than 1 along high symmetry line as shown in Fig 378.

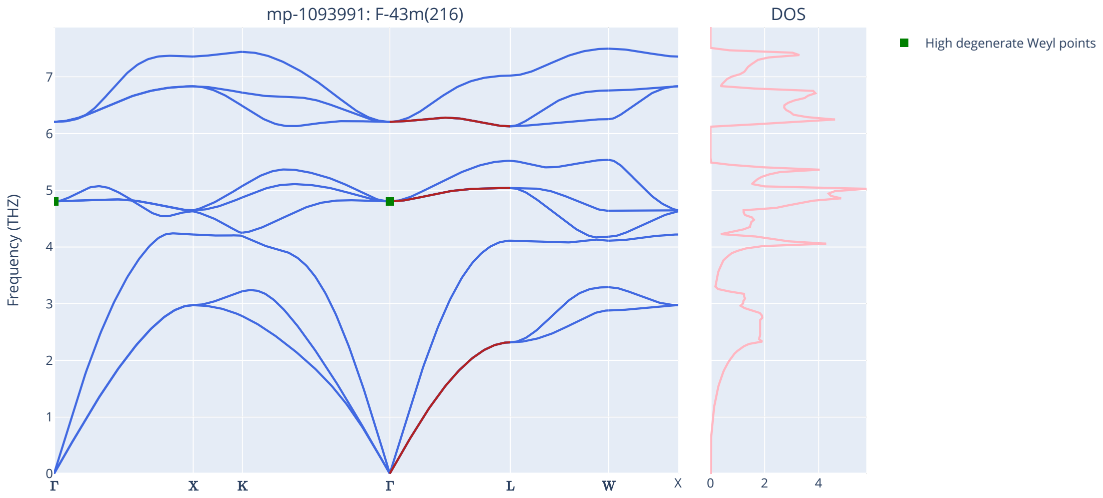

Supplementary Figure 378: The phonon band and DOS of Ir4Zr4As4.

Supplementary Table 737: High symmetry lines of mp-1093991-Ir4Zr4As4

	Start	End
0	Γ (0.000, 0.000, 0.000)	X (0.500, 0.000, 0.500)
1	X (0.500, 0.000, 0.500)	U (0.625, 0.250, 0.625)
2	K (0.375, 0.375, 0.750)	Γ (0.000, 0.000, 0.000)
3	Γ (0.000, 0.000, 0.000)	L (0.500, 0.500, 0.500)
4	L (0.500, 0.500, 0.500)	W (0.500, 0.250, 0.750)
5	W (0.500, 0.250, 0.750)	X (0.500, 0.000, 0.500)

Supplementary Table 738: High degenerate Weyl points along high symmetry lines of mp-1093991-Ir4Zr4As4.

	Coordinates	Frequency	Mode 1	Mode 2	Multi.	Deg.	Path	Ratio
0	(0.000, 0.000, 0.000)	4.805	6	4	1	3	1	0.000

The followings are details for $Co_4Nb_4Sn_4$. As shown in Fig **379**, it has 3 topological nodal lines, 1 nodal ring point and 1 high degenerate Weyl point whose degeneracy is more than 2 and charge is bigger than 1.

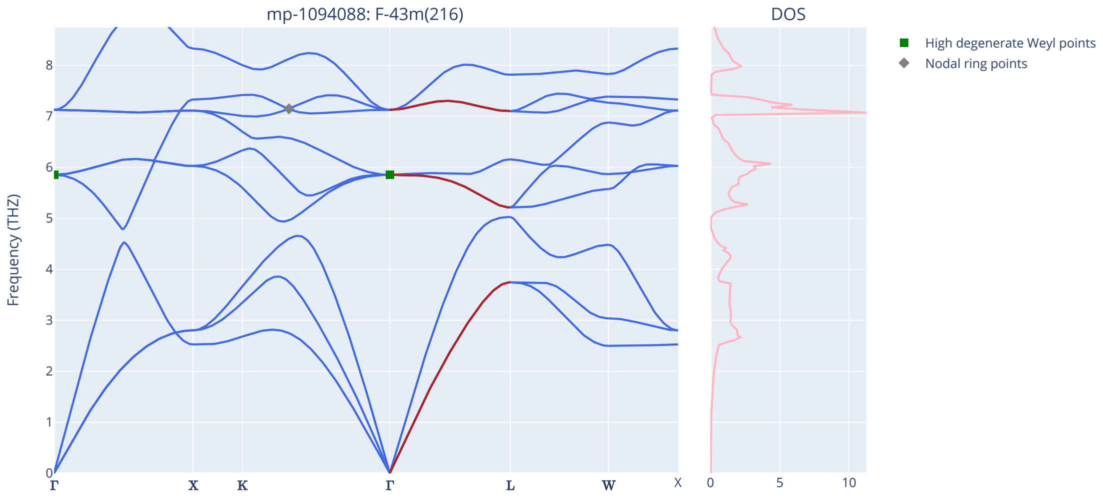

Supplementary Figure 379: The phonon band and DOS of Co4Nb4Sn4.

Supplementary Table 739: High symmetry lines of mp-1094088-Co4Nb4Sn4

	Start	End
0	Γ (0.000, 0.000, 0.000)	X (0.500, 0.000, 0.500)
1	X (0.500, 0.000, 0.500)	U (0.625, 0.250, 0.625)
2	K (0.375, 0.375, 0.750)	Γ (0.000, 0.000, 0.000)
3	Γ (0.000, 0.000, 0.000)	L (0.500, 0.500, 0.500)
4	L (0.500, 0.500, 0.500)	W (0.500, 0.250, 0.750)
5	W (0.500, 0.250, 0.750)	X (0.500, 0.000, 0.500)

Supplementary Table 740: High degenerate Weyl points along high symmetry lines of mp-1094088-Co4Nb4Sn4.

	Coordinates	Frequency	Mode 1	Mode 2	Multi.	Deg.	Path	Ratio
0	(0.000, 0.000, 0.000)	5.850	6	4	1	3	1	0.000

Supplementary Table 741: Nodal ring points along high symmetry lines of mp-1094088-Co4Nb4Sn4.

	Coordinates	Frequency	Mode 1	Mode 2	Multi.	Deg.	Path	Ratio
0	(0.252, 0.252, 0.517)	7.142	8	7	24	2	3	0.317

The followings are details for $Rh_4Sn_4Ta_4$. As shown in Fig **380**, it has 4 topological nodal lines and 1 nodal ring point. At the same time, it has 3 single Weyl points and 1 high degenerate Weyl point whose degeneracy is more than 2 and charge is bigger than 1.

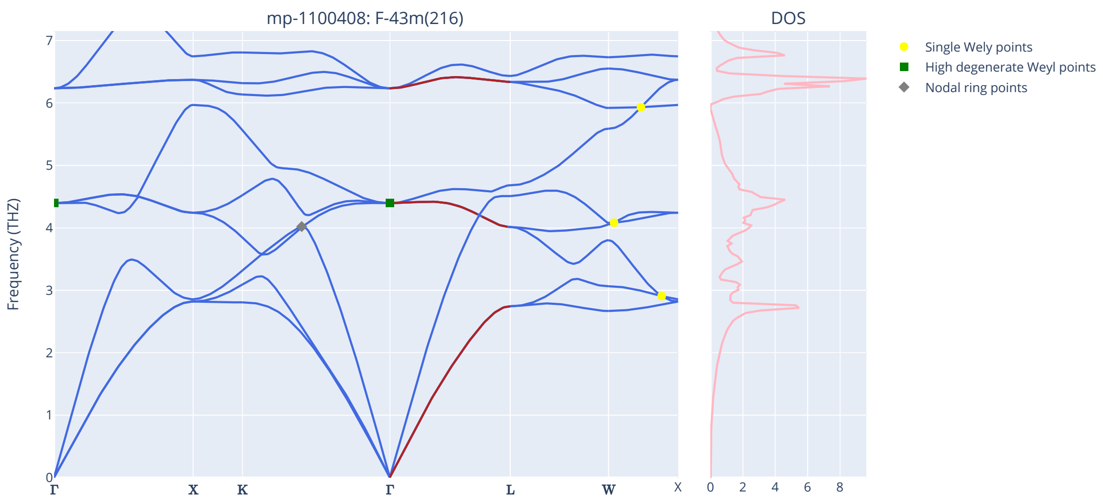

Supplementary Figure 380: The phonon band and DOS of Rh4Sn4Ta4.

Supplementary Table 742: High symmetry lines of mp-1100408-Rh4Sn4Ta4

	Start	End
0	Γ (0.000, 0.000, 0.000)	X (0.500, 0.000, 0.500)
1	X (0.500, 0.000, 0.500)	U (0.625, 0.250, 0.625)
2	K (0.375, 0.375, 0.750)	Γ (0.000, 0.000, 0.000)
3	Γ (0.000, 0.000, 0.000)	L (0.500, 0.500, 0.500)
4	L (0.500, 0.500, 0.500)	W (0.500, 0.250, 0.750)
5	W (0.500, 0.250, 0.750)	X (0.500, 0.000, 0.500)

Supplementary Table 743: Single Weyl points along high symmetry lines of mp-1100408-Rh4Sn4Ta4.

	Coordinates	Frequency	Mode 1	Mode 2	Multi.	Deg.	Path	Ratio
0	(0.500, 0.059, 0.559)	2.908	3	2	12	2	6	0.765
1	(0.500, 0.230, 0.730)	4.082	5	4	12	2	6	0.080
2	(0.500, 0.133, 0.633)	5.932	7	6	12	2	6	0.467

Supplementary Table 744: High degenerate Weyl points along high symmetry lines of mp-1100408-Rh4Sn4Ta4.

	Coordinates	Frequency	Mode 1	Mode 2	Multi.	Deg.	Path	Ratio
0	(0.000, 0.000, 0.000)	4.395	6	4	1	3	1	0.000

Supplementary Table 745: Nodal ring points along high symmetry lines of mp-1100408-Rh4Sn4Ta4.

	Coordinates	Frequency	Mode 1	Mode 2	Multi.	Deg.	Path	Ratio
0	(0.230, 0.230, 0.442)	4.020	4	3	24	2	3	0.403

The followings are details for $Rh_4Zr_4Sb_4$. As shown in Fig **381**, it has 4 topological nodal lines, 1 nodal ring point and 1 high degenerate Weyl point whose degeneracy is more than 2 and charge is bigger than 1.

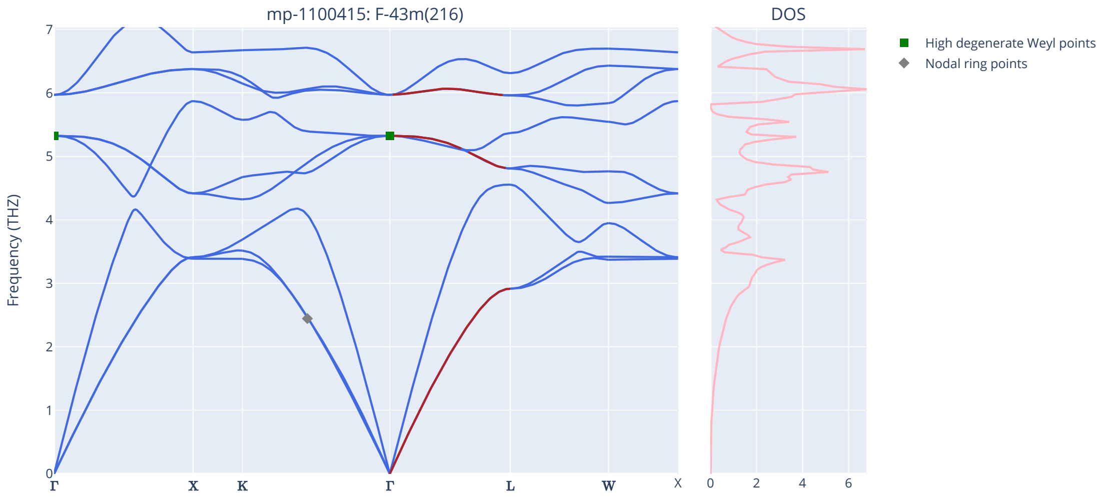

Supplementary Figure 381: The phonon band and DOS of Rh4Zr4Sb4.

Supplementary Table 746: High symmetry lines of mp-1100415-Rh4Zr4Sb4

	Start	End
0	Γ (0.000, 0.000, 0.000)	X (0.500, 0.000, 0.500)
1	X (0.500, 0.000, 0.500)	U (0.625, 0.250, 0.625)
2	K (0.375, 0.375, 0.750)	Γ (0.000, 0.000, 0.000)
3	Γ (0.000, 0.000, 0.000)	L (0.500, 0.500, 0.500)
4	L (0.500, 0.500, 0.500)	W (0.500, 0.250, 0.750)
5	W (0.500, 0.250, 0.750)	X (0.500, 0.000, 0.500)

Supplementary Table 747: High degenerate Weyl points along high symmetry lines of mp-1100415-Rh4Zr4Sb4.

	Coordinates	Frequency	Mode 1	Mode 2	Multi.	Deg.	Path	Ratio
0	(0.000, 0.000, 0.000)	5.325	6	4	1	3	1	0.000

Supplementary Table 748: Nodal ring points along high symmetry lines of mp-1100415-Rh4Zr4Sb4.

	Coordinates	Frequency	Mode 1	Mode 2	Multi.	Deg.	Path	Ratio
0	(0.214, 0.214, 0.414)	2.445	2	1	24	2	3	0.441

The followings are details for $Li_4Zn_4Sb_4$. As shown in Fig **382**, it has 3 topological nodal lines and 3 nodal ring points. At the same time, it has 2 single Weyl points and 1 high degenerate Weyl point whose degeneracy is more than 2 and charge is bigger than 1.

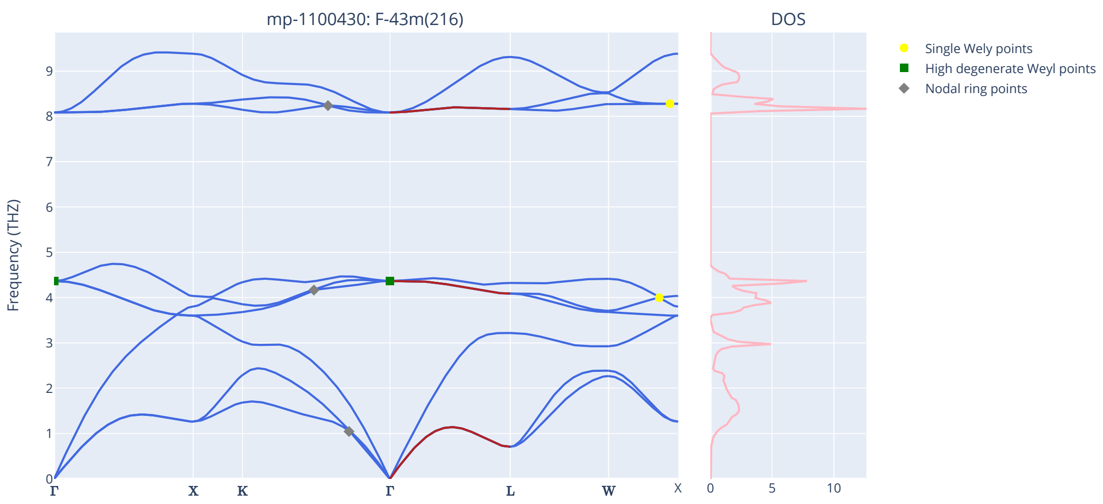

Supplementary Figure 382: The phonon band and DOS of Li4Zn4Sb4.

Supplementary Table 749: High symmetry lines of mp-1100430-Li4Zn4Sb4

	Start	End
0	Γ (0.000, 0.000, 0.000)	X (0.500, 0.000, 0.500)
1	X (0.500, 0.000, 0.500)	U (0.625, 0.250, 0.625)
2	K (0.375, 0.375, 0.750)	Γ (0.000, 0.000, 0.000)
3	Γ (0.000, 0.000, 0.000)	L (0.500, 0.500, 0.500)
4	L (0.500, 0.500, 0.500)	W (0.500, 0.250, 0.750)
5	W (0.500, 0.250, 0.750)	X (0.500, 0.000, 0.500)

Supplementary Table 750: Single Weyl points along high symmetry lines of mp-1100430-Li4Zn4Sb4.

	Coordinates	Frequency	Mode 1	Mode 2	Multi.	Deg.	Path	Ratio
0	(0.500, 0.067, 0.567)	3.999	6	5	12	2	6	0.731
1	(0.500, 0.029, 0.529)	8.280	8	7	12	2	6	0.885

Supplementary Table 751: High degenerate Weyl points along high symmetry lines of mp-1100430-Li4Zn4Sb4.

	Coordinates	Frequency	Mode 1	Mode 2	Multi.	Deg.	Path	Ratio
0	(0.000, 0.000, 0.000)	4.366	6	4	1	3	1	0.000

Supplementary Table 752: Nodal ring points along high symmetry lines of mp-1100430-Li4Zn4Sb4.

	Coordinates	Frequency	Mode 1	Mode 2	Multi.	Deg.	Path	Ratio
0	(0.107, 0.107, 0.204)	1.045	2	1	24	2	3	0.724
1	(0.191, 0.191, 0.387)	4.166	5	4	24	2	3	0.486
2	(0.161, 0.161, 0.311)	8.238	8	7	24	2	3	0.581

The followings are details for $Cu_4Mg_4Sn_4$. As shown in Fig **383**, it has 4 topological nodal lines and 1 nodal ring point. At the same time, it has 1 single Weyl point and 1 high degenerate Weyl point whose degeneracy is more than 2 and charge is bigger than 1.

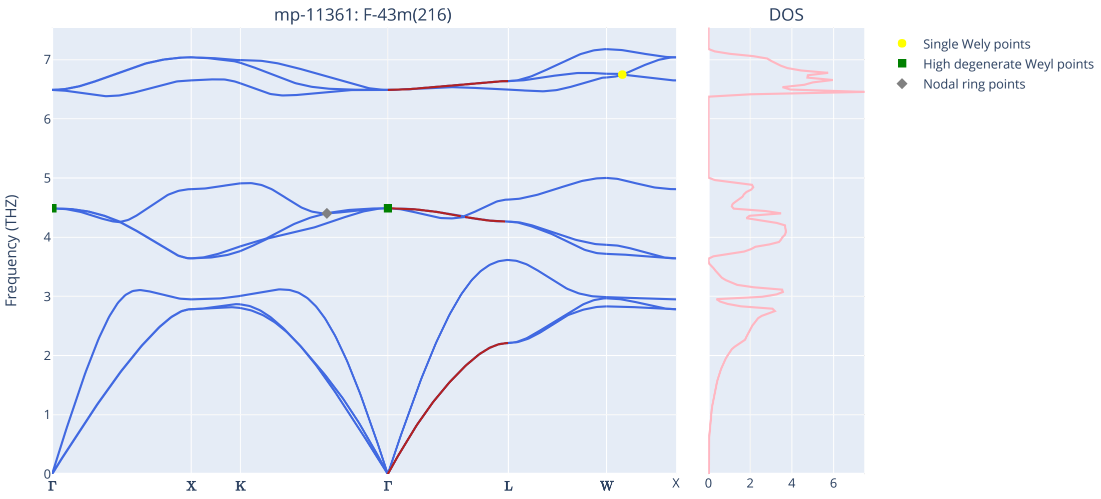

Supplementary Figure 383: The phonon band and DOS of Cu4Mg4Sn4.

Supplementary Table 753: High symmetry lines of mp-11361-Cu4Mg4Sn4

	Start	End
0	Γ (0.000, 0.000, 0.000)	X (0.500, 0.000, 0.500)
1	X (0.500, 0.000, 0.500)	U (0.625, 0.250, 0.625)
2	K (0.375, 0.375, 0.750)	Γ (0.000, 0.000, 0.000)
3	Γ (0.000, 0.000, 0.000)	L (0.500, 0.500, 0.500)
4	L (0.500, 0.500, 0.500)	W (0.500, 0.250, 0.750)
5	W (0.500, 0.250, 0.750)	X (0.500, 0.000, 0.500)

Supplementary Table 754: Single Weyl points along high symmetry lines of mp-11361-Cu4Mg4Sn4.

	Coordinates	Frequency	Mode 1	Mode 2	Multi.	Deg.	Path	Ratio
0	(0.500, 0.193, 0.693)	6.748	8	7	12	2	6	0.228

Supplementary Table 755: High degenerate Weyl points along high symmetry lines of mp-11361-Cu4Mg4Sn4.

	Coordinates	Frequency	Mode 1	Mode 2	Multi.	Deg.	Path	Ratio
0	(0.000, 0.000, 0.000)	4.488	6	4	1	3	1	0.000

Supplementary Table 756: Nodal ring points along high symmetry lines of mp-11361-Cu4Mg4Sn4.

	Coordinates	Frequency	Mode 1	Mode 2	Multi.	Deg.	Path	Ratio
0	(0.153, 0.153, 0.311)	4.402	6	5	24	2	3	0.587

The followings are details for $Ni_4Y_4Sb_4$. As shown in Fig **384**, it has 4 topological nodal lines, 2 nodal ring points and 1 high degenerate Weyl point whose degeneracy is more than 2 and charge is bigger than 1.

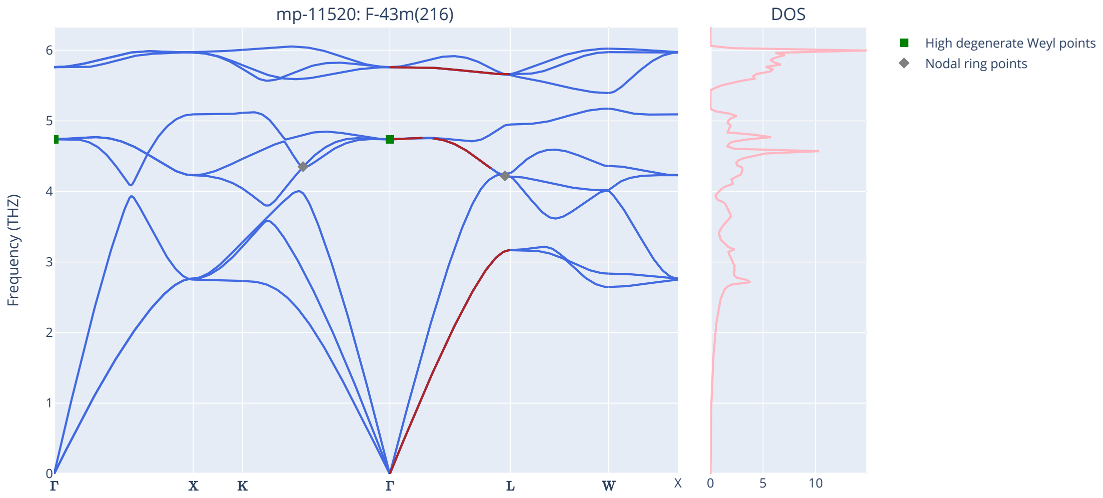

Supplementary Figure 384: The phonon band and DOS of Ni4Y4Sb4.

Supplementary Table 757: High symmetry lines of mp-11520-Ni4Y4Sb4

	Start	End
0	Γ (0.000, 0.000, 0.000)	X (0.500, 0.000, 0.500)
1	X (0.500, 0.000, 0.500)	U (0.625, 0.250, 0.625)
2	K (0.375, 0.375, 0.750)	Γ (0.000, 0.000, 0.000)
3	Γ (0.000, 0.000, 0.000)	L (0.500, 0.500, 0.500)
4	L (0.500, 0.500, 0.500)	W (0.500, 0.250, 0.750)
5	W (0.500, 0.250, 0.750)	X (0.500, 0.000, 0.500)

Supplementary Table 758: High degenerate Weyl points along high symmetry lines of mp-11520-Ni4Y4Sb4.

	Coordinates	Frequency	Mode 1	Mode 2	Multi.	Deg.	Path	Ratio
0	(0.000, 0.000, 0.000)	4.737	6	4	1	3	1	0.000

Supplementary Table 759: Nodal ring points along high symmetry lines of mp-11520-Ni4Y4Sb4.

	Coordinates	Frequency	Mode 1	Mode 2	Multi.	Deg.	Path	Ratio
0	(0.479, 0.478, 0.478)	4.218	4	3	24	2	4	0.957
1	(0.222, 0.222, 0.440)	4.350	5	4	24	2	3	0.412

The followings are details for $Ni_4Zn_4Sb_4$. As shown in Fig **385**, it has 4 topological nodal lines, 3 nodal ring points and 1 high degenerate Weyl point whose degeneracy is more than 2 and charge is bigger than 1.

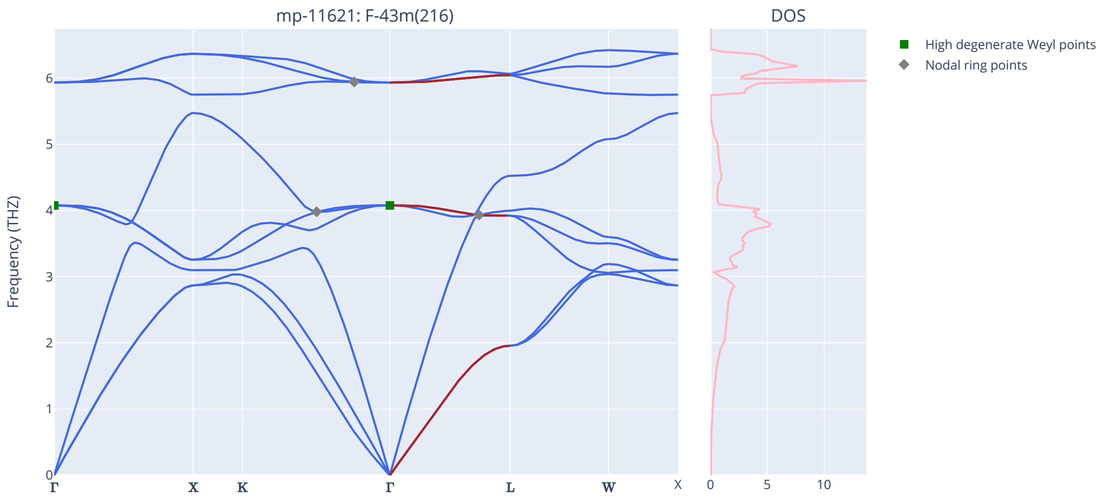

Supplementary Figure 385: The phonon band and DOS of Ni4Zn4Sb4.

Supplementary Table 760: High symmetry lines of mp-11621-Ni4Zn4Sb4

	Start	End
0	Γ (0.000, 0.000, 0.000)	X (0.500, 0.000, 0.500)
1	X (0.500, 0.000, 0.500)	U (0.625, 0.250, 0.625)
2	K (0.375, 0.375, 0.750)	Γ (0.000, 0.000, 0.000)
3	Γ (0.000, 0.000, 0.000)	L (0.500, 0.500, 0.500)
4	L (0.500, 0.500, 0.500)	W (0.500, 0.250, 0.750)
5	W (0.500, 0.250, 0.750)	X (0.500, 0.000, 0.500)

Supplementary Table 761: High degenerate Weyl points along high symmetry lines of mp-11621-Ni4Zn4Sb4.

	Coordinates	Frequency	Mode 1	Mode 2	Multi.	Deg.	Path	Ratio
0	(0.000, 0.000, 0.000)	4.078	6	4	1	3	1	0.000

Supplementary Table 762: Nodal ring points along high symmetry lines of mp-11621-Ni4Zn4Sb4.

	Coordinates	Frequency	Mode 1	Mode 2	Multi.	Deg.	Path	Ratio
0	(0.369, 0.375, 0.369)	3.931	5	4	24	2	4	0.742
1	(0.191, 0.191, 0.366)	3.979	6	5	24	2	3	0.504
2	(0.092, 0.092, 0.179)	5.940	8	7	24	2	3	0.759

The followings are details for $Bi_4Pt_4Y_4$. As shown in Fig **386**, it has 3 topological nodal lines, 5 nodal ring points and 1 high degenerate Weyl point whose degeneracy is more than 2 and charge is bigger than 1.

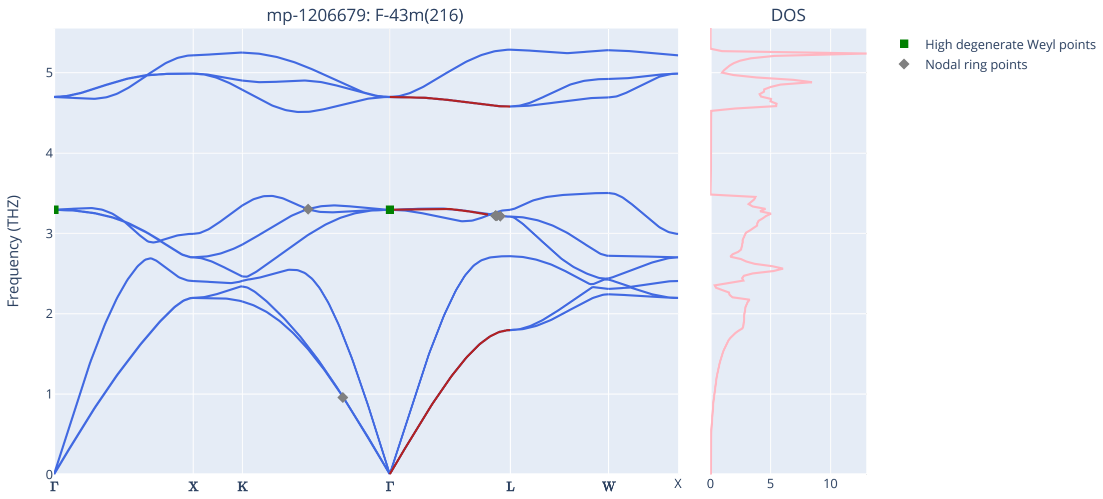

Supplementary Figure 386: The phonon band and DOS of Bi4Pt4Y4.

Supplementary Table 763: High symmetry lines of mp-1206679-Bi4Pt4Y4

	Start	End
0	Γ (0.000, 0.000, 0.000)	X (0.500, 0.000, 0.500)
1	X (0.500, 0.000, 0.500)	U (0.625, 0.250, 0.625)
2	K (0.375, 0.375, 0.750)	Γ (0.000, 0.000, 0.000)
3	Γ (0.000, 0.000, 0.000)	L (0.500, 0.500, 0.500)
4	L (0.500, 0.500, 0.500)	W (0.500, 0.250, 0.750)
5	W (0.500, 0.250, 0.750)	X (0.500, 0.000, 0.500)

Supplementary Table 764: High degenerate Weyl points along high symmetry lines of mp-1206679-Bi4Pt4Y4.

	Coordinates	Frequency	Mode 1	Mode 2	Multi.	Deg.	Path	Ratio
0	(0.000, 0.000, 0.000)	3.295	6	4	1	3	1	0.000

Supplementary Table 765: Nodal ring points along high symmetry lines of mp-1206679-Bi4Pt4Y4.

	Coordinates	Frequency	Mode 1	Mode 2	Multi.	Deg.	Path	Ratio
0	(0.122, 0.122, 0.236)	0.957	2	1	24	2	3	0.682
1	(0.459, 0.459, 0.459)	3.215	5	4	8	2	4	0.918
2	(0.446, 0.442, 0.446)	3.218	5	4	24	2	4	0.889
3	(0.439, 0.439, 0.439)	3.221	5	4	8	2	4	0.878
4	(0.207, 0.207, 0.416)	3.302	6	5	24	2	3	0.446

The followings are details for $Bi_4La_4Pd_4$. As shown in Fig **387**, it has 4 topological nodal lines, 1 nodal ring point and 1 high degenerate Weyl point whose degeneracy is more than 2 and charge is bigger than 1.

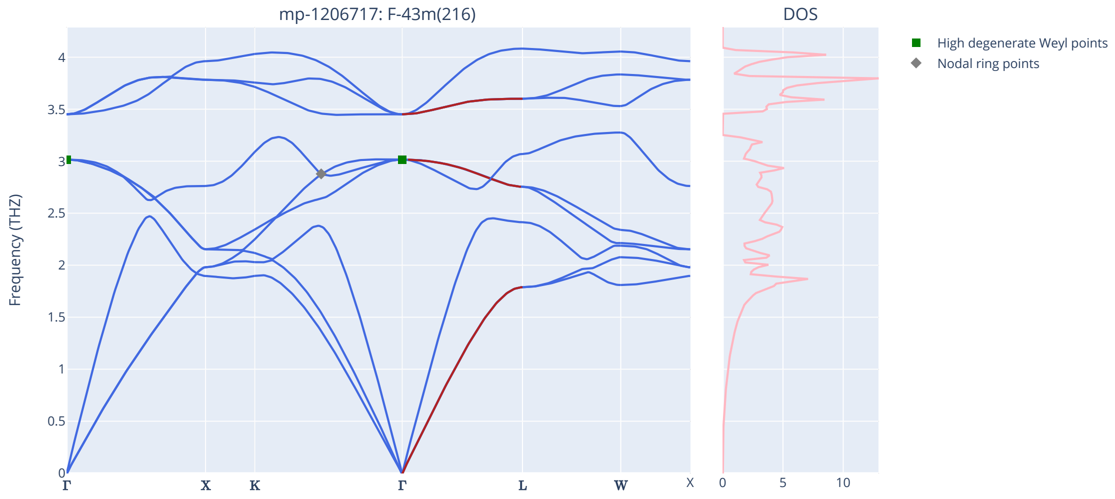

Supplementary Figure 387: The phonon band and DOS of Bi4La4Pd4.

Supplementary Table 766: High symmetry lines of mp-1206717-Bi4La4Pd4

	Start	End
0	Γ (0.000, 0.000, 0.000)	X (0.500, 0.000, 0.500)
1	X (0.500, 0.000, 0.500)	U (0.625, 0.250, 0.625)
2	K (0.375, 0.375, 0.750)	Γ (0.000, 0.000, 0.000)
3	Γ (0.000, 0.000, 0.000)	L (0.500, 0.500, 0.500)
4	L (0.500, 0.500, 0.500)	W (0.500, 0.250, 0.750)
5	W (0.500, 0.250, 0.750)	X (0.500, 0.000, 0.500)

Supplementary Table 767: High degenerate Weyl points along high symmetry lines of mp-1206717-Bi4La4Pd4.

	Coordinates	Frequency	Mode 1	Mode 2	Multi.	Deg.	Path	Ratio
0	(0.000, 0.000, 0.000)	3.017	6	4	1	3	1	0.000

Supplementary Table 768: Nodal ring points along high symmetry lines of mp-1206717-Bi4La4Pd4.

	Coordinates	Frequency	Mode 1	Mode 2	Multi.	Deg.	Path	Ratio
0	(0.211, 0.211, 0.407)	2.879	6	5	24	2	3	0.451

The followings are details for NiYAs. It has 2 nodal ring points and 2 single Weyl points along high symmetry line as shown in Fig 388.

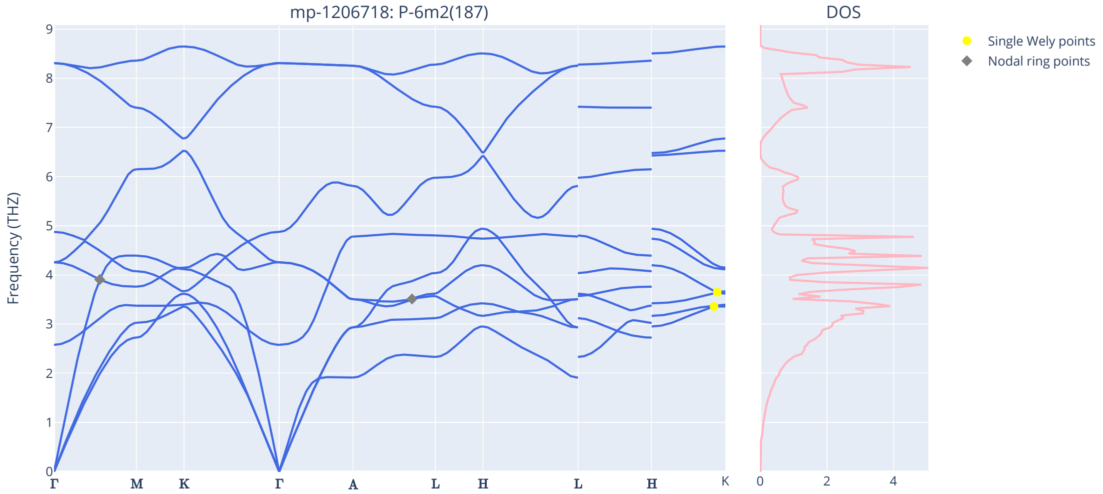

Supplementary Figure 388: The phonon band and DOS of NiYAs.

Supplementary Table 769: High symmetry lines of mp-1206718-NiYAs

	Start	End
0	Γ (0.000, 0.000, 0.000)	M (0.500, 0.000, 0.000)
1	M (0.500, 0.000, 0.000)	K (0.333, 0.333, 0.000)
2	K (0.333, 0.333, 0.000)	Γ (0.000, 0.000, 0.000)
3	Γ (0.000, 0.000, 0.000)	A (0.000, 0.000, 0.500)
4	A (0.000, 0.000, 0.500)	L (0.500, 0.000, 0.500)
5	L (0.500, 0.000, 0.500)	H (0.333, 0.333, 0.500)
6	H (0.333, 0.333, 0.500)	A (0.000, 0.000, 0.500)
7	L (0.500, 0.000, 0.500)	M (0.500, 0.000, 0.000)
8	H (0.333, 0.333, 0.500)	K (0.333, 0.333, 0.000)

Supplementary Table 770: Single Weyl points along high symmetry lines of mp-1206718-NiYAs.

	Coordinates	Frequency	Mode 1	Mode 2	Multi.	Deg.	Path	Ratio
0	(0.333, 0.333, 0.077)	3.358	2	1	4	2	9	0.847
1	(0.333, 0.333, 0.055)	3.648	4	3	4	2	9	0.890

Supplementary Table 771: Nodal ring points along high symmetry lines of mp-1206718-NiYAs.

	Coordinates	Frequency	Mode 1	Mode 2	Multi.	Deg.	Path	Ratio
0	(0.359, 0.000, 0.500)	3.511	4	3	6	2	5	0.718
1	(0.277, 0.000, 0.000)	3.906	5	4	6	2	1	0.554

Supplementary Table 771: Nodal ring points along high symmetry lines of mp-1206718-NiYAs.

Coordinates	Frequency	Mode 1	Mode 2	Multi.	Deg.	Path	Ratio

The followings are details for $Li_4Mg_4Sb_4$. As shown in Fig 389, it has 3 topological nodal lines and 2 nodal ring points. At the same time, it has 1 single Weyl point and 1 high degenerate Weyl point whose degeneracy is more than 2 and charge is bigger than 1.

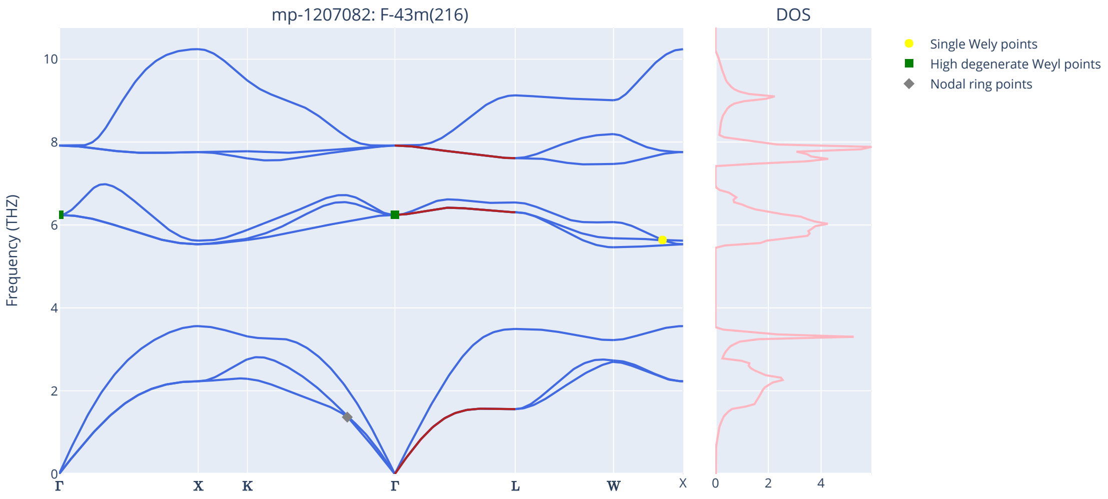

Supplementary Figure 389: The phonon band and DOS of Li4Mg4Sb4.

Supplementary Table 772: High symmetry lines of mp-1207082-Li4Mg4Sb4

	Start	End
0	Γ (0.000, 0.000, 0.000)	X (0.500, 0.000, 0.500)
1	X (0.500, 0.000, 0.500)	U (0.625, 0.250, 0.625)
2	K (0.375, 0.375, 0.750)	Γ (0.000, 0.000, 0.000)
3	Γ (0.000, 0.000, 0.000)	L (0.500, 0.500, 0.500)
4	L (0.500, 0.500, 0.500)	W (0.500, 0.250, 0.750)
5	W (0.500, 0.250, 0.750)	X (0.500, 0.000, 0.500)

Supplementary Table 773: Single Weyl points along high symmetry lines of mp-1207082-Li4Mg4Sb4.

	Coordinates	Frequency	Mode 1	Mode 2	Multi.	Deg.	Path	Ratio
0	(0.500, 0.074, 0.574)	5.639	6	5	12	2	6	0.704

Supplementary Table 774: High degenerate Weyl points along high symmetry lines of mp-1207082-Li4Mg4Sb4.

	Coordinates	Frequency	Mode 1	Mode 2	Multi.	Deg.	Path	Ratio
0	(0.000, 0.000, 0.000)	6.242	6	4	1	3	1	0.000

Supplementary Table 775: Nodal ring points along high symmetry lines of mp-1207082-Li4Mg4Sb4.

	Coordinates	Frequency	Mode 1	Mode 2	Multi.	Deg.	Path	Ratio
0	(0.122, 0.122, 0.240)	1.367	2	1	24	2	3	0.678

The followings are details for BeZrB. It has 4 nodal ring points and 2 single Weyl points along high symmetry line as shown in Fig 390.

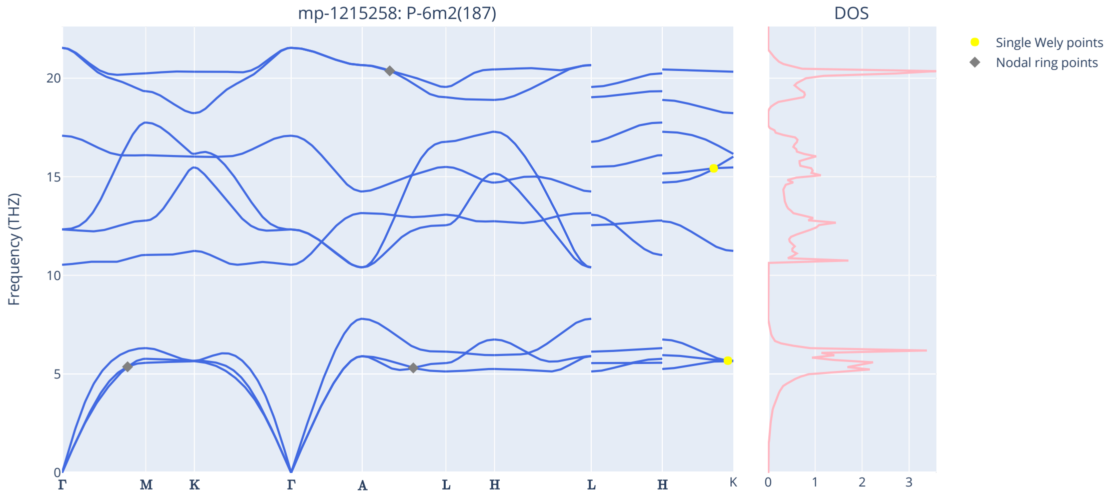

Supplementary Figure 390: The phonon band and DOS of BeZrB.

Supplementary Table 776: High symmetry lines of mp-1215258-BeZrB

	Start	End
0	Γ (0.000, 0.000, 0.000)	M (0.500, 0.000, 0.000)
1	M (0.500, 0.000, 0.000)	K (0.333, 0.333, 0.000)
2	K (0.333, 0.333, 0.000)	Γ (0.000, 0.000, 0.000)
3	Γ (0.000, 0.000, 0.000)	A (0.000, 0.000, 0.500)
4	A (0.000, 0.000, 0.500)	L (0.500, 0.000, 0.500)
5	L (0.500, 0.000, 0.500)	H (0.333, 0.333, 0.500)
6	H (0.333, 0.333, 0.500)	A (0.000, 0.000, 0.500)
7	L (0.500, 0.000, 0.500)	M (0.500, 0.000, 0.000)
8	H (0.333, 0.333, 0.500)	K (0.333, 0.333, 0.000)

Supplementary Table 777: Single Weyl points along high symmetry lines of mp-1215258-BeZrB.

	Coordinates	Frequency	Mode 1	Mode 2	Multi.	Deg.	Path	Ratio
0	(0.333, 0.333, 0.038)	5.675	3	2	4	2	9	0.924
1	(0.333, 0.333, 0.138)	15.430	6	5	4	2	9	0.724

Supplementary Table 778: Nodal ring points along high symmetry lines of mp-1215258-BeZrB.

	Coordinates	Frequency	Mode 1	Mode 2	Multi.	Deg.	Path	Ratio
0	(0.391, 0.000, -0.000)	5.364	2	1	6	2	1	0.781

Supplementary Table 778: Nodal ring points along high symmetry lines of mp-1215258-BeZrB.

	Coordinates	Frequency	Mode 1	Mode 2	Multi.	Deg.	Path	Ratio
1	(0.305, 0.000, 0.500)	5.306	2	1	6	2	5	0.610
2	(0.164, 0.000, 0.500)	20.376	9	8	6	2	5	0.329

The followings are details for $Ir_4Sn_4Ti_4$. As shown in Fig **391**, it has 4 topological nodal lines, 2 nodal ring points and 1 high degenerate Weyl point whose degeneracy is more than 2 and charge is bigger than 1.

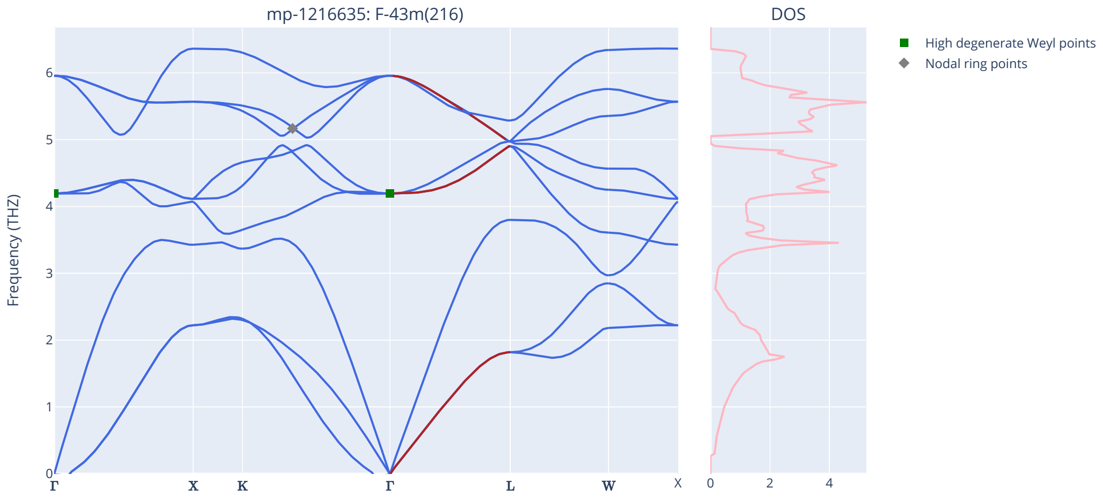

Supplementary Figure 391: The phonon band and DOS of Ir4Sn4Ti4.

Supplementary Table 779: High symmetry lines of mp-1216635-Ir4Sn4Ti4

	Start	End
0	Γ (0.000, 0.000, 0.000)	X (0.500, 0.000, 0.500)
1	X (0.500, 0.000, 0.500)	U (0.625, 0.250, 0.625)
2	K (0.375, 0.375, 0.750)	Γ (0.000, 0.000, 0.000)
3	Γ (0.000, 0.000, 0.000)	L (0.500, 0.500, 0.500)
4	L (0.500, 0.500, 0.500)	W (0.500, 0.250, 0.750)
5	W (0.500, 0.250, 0.750)	X (0.500, 0.000, 0.500)

Supplementary Table 780: High degenerate Weyl points along high symmetry lines of mp-1216635-Ir4Sn4Ti4.

	Coordinates	Frequency	Mode 1	Mode 2	Multi.	Deg.	Path	Ratio
0	(0.000, 0.000, 0.000)	4.192	6	4	1	3	1	0.000

Supplementary Table 781: Nodal ring points along high symmetry lines of mp-1216635-Ir4Sn4Ti4.

	Coordinates	Frequency	Mode 1	Mode 2	Multi.	Deg.	Path	Ratio
0	(0.253, 0.253, 0.489)	5.166	8	7	24	2	3	0.341

The followings are details for TiSSe. It has 2 topological nodal lines and 4 nodal ring points along high symmetry line as shown in Fig 392.

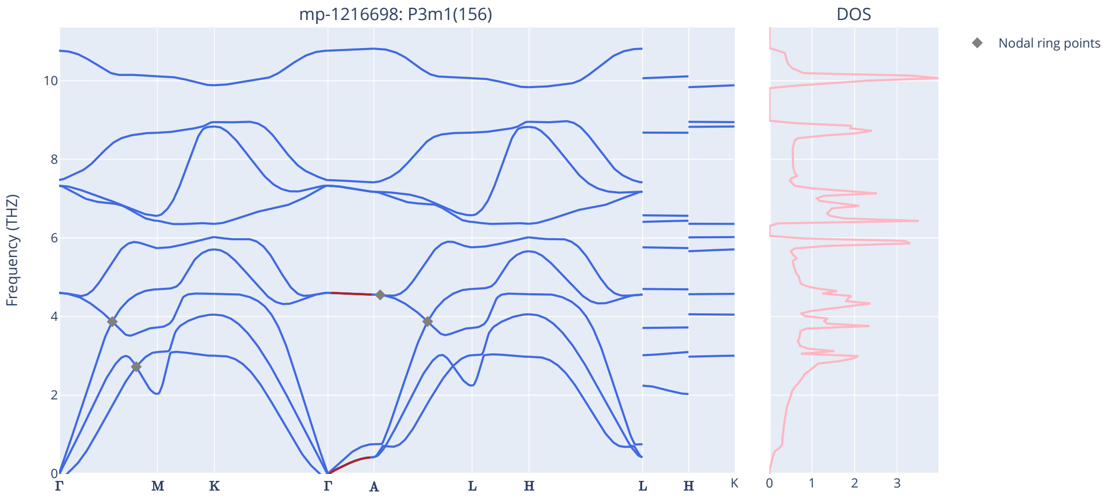

Supplementary Figure 392: The phonon band and DOS of TiSSe.

Supplementary Table 782: High symmetry lines of mp-1216698-TiSSe

	Start	End
0	Γ (0.000, 0.000, 0.000)	M (0.500, 0.000, 0.000)
1	M (0.500, 0.000, 0.000)	K (0.333, 0.333, 0.000)
2	K (0.333, 0.333, 0.000)	Γ (0.000, 0.000, 0.000)
3	Γ (0.000, 0.000, 0.000)	A (0.000, 0.000, 0.500)
4	A (0.000, 0.000, 0.500)	L (0.500, 0.000, 0.500)
5	L (0.500, 0.000, 0.500)	H (0.333, 0.333, 0.500)
6	H (0.333, 0.333, 0.500)	A (0.000, 0.000, 0.500)
7	L (0.500, 0.000, 0.500)	M (0.500, 0.000, 0.000)
8	H (0.333, 0.333, 0.500)	K (0.333, 0.333, 0.000)

Supplementary Table 783: Nodal ring points along high symmetry lines of mp-1216698-TiSSe.

	Coordinates	Frequency	Mode 1	Mode 2	Multi.	Deg.	Path	Ratio
0	(0.392, -0.000, -0.003)	2.724	2	1	6	2	1	0.784
1	(0.270, -0.000, 0.000)	3.871	4	3	6	2	1	0.541
2	(0.274, -0.000, 0.500)	3.875	4	3	6	2	5	0.548
3	(0.033, -0.000, 0.499)	4.551	5	4	6	2	5	0.065

The followings are details for PtSeTe. As shown in Fig 393, it has 4 topological nodal lines, 4 nodal ring points and 5 single Weyl points.

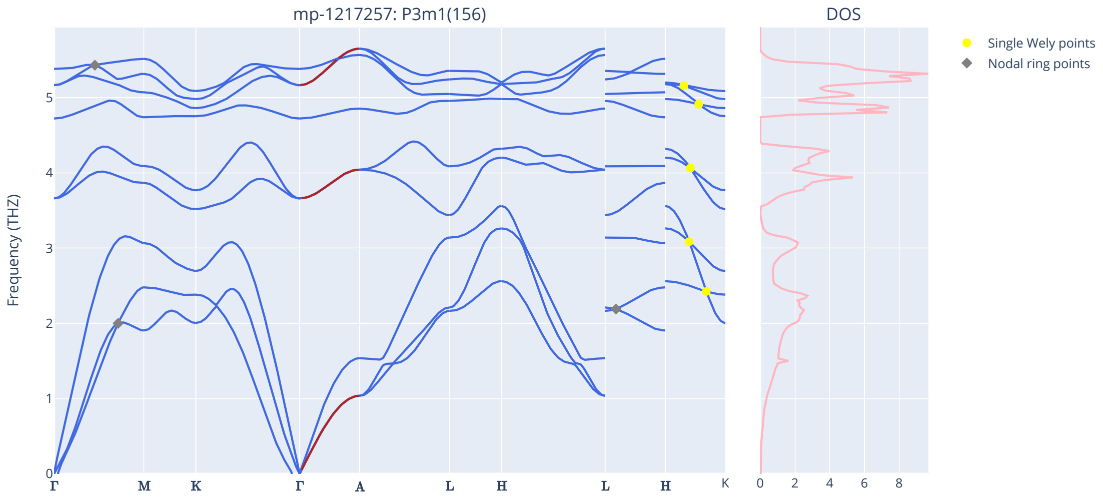

Supplementary Figure 393: The phonon band and DOS of PtSeTe.

Supplementary Table 784: High symmetry lines of mp-1217257-PtSeTe

	Start	End
0	Γ (0.000, 0.000, 0.000)	M (0.500, 0.000, 0.000)
1	M (0.500, 0.000, 0.000)	K (0.333, 0.333, 0.000)
2	K (0.333, 0.333, 0.000)	Γ (0.000, 0.000, 0.000)
3	Γ (0.000, 0.000, 0.000)	A (0.000, 0.000, 0.500)
4	A (0.000, 0.000, 0.500)	L (0.500, 0.000, 0.500)
5	L (0.500, 0.000, 0.500)	H (0.333, 0.333, 0.500)
6	H (0.333, 0.333, 0.500)	A (0.000, 0.000, 0.500)
7	L (0.500, 0.000, 0.500)	M (0.500, 0.000, 0.000)
8	H (0.333, 0.333, 0.500)	K (0.333, 0.333, 0.000)

Supplementary Table 785: Single Weyl points along high symmetry lines of mp-1217257-PtSeTe.

	Coordinates	Frequency	Mode 1	Mode 2	Multi.	Deg.	Path	Ratio
0	(0.333, 0.333, 0.159)	2.424	2	1	4	2	9	0.683
1	(0.333, 0.333, 0.301)	3.086	3	2	4	2	9	0.397
2	(0.333, 0.333, 0.291)	4.063	5	4	4	2	9	0.417
3	(0.333, 0.333, 0.222)	4.912	7	6	4	2	9	0.556
4	(0.333, 0.333, 0.346)	5.159	9	8	4	2	9	0.309

Supplementary Table 786: Nodal ring points along high symmetry lines of mp-1217257-PtSeTe.

	Coordinates	Frequency	Mode 1	Mode 2	Multi.	Deg.	Path	Ratio
0	(0.356, 0.000, 0.000)	1.999	2	1	6	2	1	0.712
1	(0.498, 0.000, 0.411)	2.190	2	1	6	2	8	0.178
2	(0.227, 0.000, 0.002)	5.431	9	8	6	2	1	0.454

The followings are details for PdSeTe. As shown in Fig 394, it has 4 topological nodal lines, 5 nodal ring points and 6 single Weyl points.

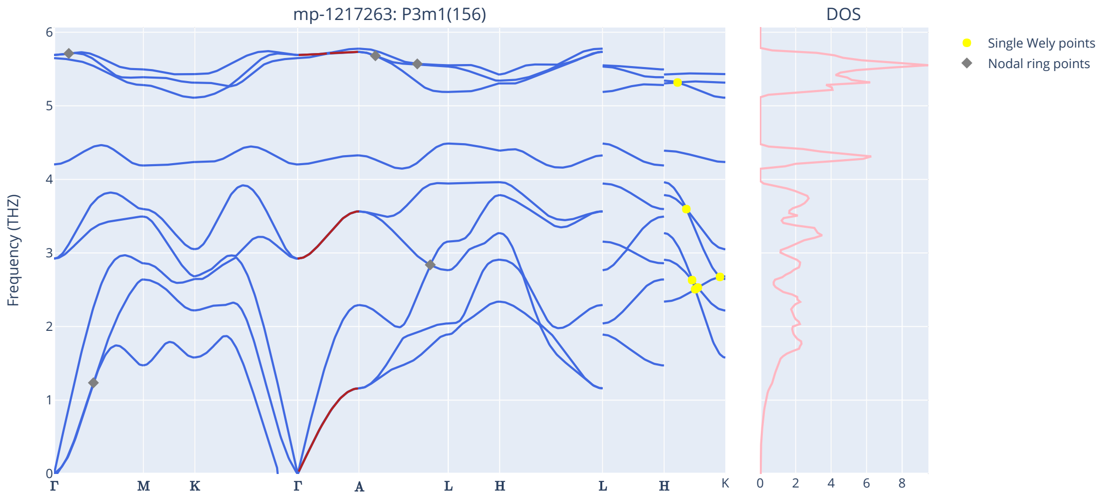

Supplementary Figure 394: The phonon band and DOS of PdSeTe.

Supplementary Table 787: High symmetry lines of mp-1217263-PdSeTe

	Start	End
0	Γ (0.000, 0.000, 0.000)	M (0.500, 0.000, 0.000)
1	M (0.500, 0.000, 0.000)	K (0.333, 0.333, 0.000)
2	K (0.333, 0.333, 0.000)	Γ (0.000, 0.000, 0.000)
3	Γ (0.000, 0.000, 0.000)	A (0.000, 0.000, 0.500)
4	A (0.000, 0.000, 0.500)	L (0.500, 0.000, 0.500)
5	L (0.500, 0.000, 0.500)	H (0.333, 0.333, 0.500)
6	H (0.333, 0.333, 0.500)	A (0.000, 0.000, 0.500)
7	L (0.500, 0.000, 0.500)	M (0.500, 0.000, 0.000)
8	H (0.333, 0.333, 0.500)	K (0.333, 0.333, 0.000)

Supplementary Table 788: Single Weyl points along high symmetry lines of mp-1217263-PdSeTe.

	Coordinates	Frequency	Mode 1	Mode 2	Multi.	Deg.	Path	Ratio
0	(0.333, 0.333, 0.246)	2.508	2	1	4	2	9	0.508
1	(0.333, 0.333, 0.224)	2.531	3	2	4	2	9	0.552
2	(0.333, 0.333, 0.271)	2.632	3	2	4	2	9	0.458
3	(0.333, 0.333, 0.044)	2.676	4	3	4	2	9	0.912
4	(0.333, 0.333, 0.318)	3.597	5	4	4	2	9	0.364
5	(0.333, 0.333, 0.389)	5.318	8	7	4	2	9	0.222

Supplementary Table 789: Nodal ring points along high symmetry lines of mp-1217263-PdSeTe.

	Coordinates	Frequency	Mode 1	Mode 2	Multi.	Deg.	Path	Ratio
0	(0.220, -0.000, 0.002)	1.236	2	1	6	2	1	0.441
1	(0.399, -0.000, 0.498)	2.840	4	3	6	2	5	0.799
2	(0.092, -0.000, 0.497)	5.681	8	7	6	2	5	0.184
3	(0.083, -0.000, -0.002)	5.713	9	8	6	2	1	0.165
4	(0.327, 0.000, 0.500)	5.571	9	8	6	2	5	0.653

The followings are details for PtSrP. It has 2 nodal ring points and 2 single Weyl points along high symmetry line as shown in Fig 395.

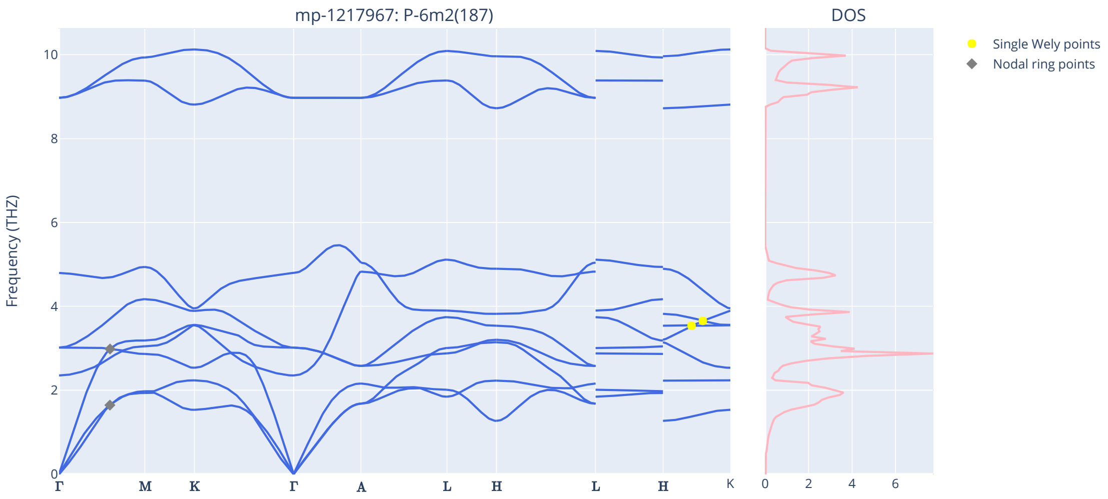

Supplementary Figure 395: The phonon band and DOS of PtSrP.

Supplementary Table 790: High symmetry lines of mp-1217967-PtSrP

	Start	End
0	Γ (0.000, 0.000, 0.000)	M (0.500, 0.000, 0.000)
1	M (0.500, 0.000, 0.000)	K (0.333, 0.333, 0.000)
2	K (0.333, 0.333, 0.000)	Γ (0.000, 0.000, 0.000)
3	Γ (0.000, 0.000, 0.000)	A (0.000, 0.000, 0.500)
4	A (0.000, 0.000, 0.500)	L (0.500, 0.000, 0.500)
5	L (0.500, 0.000, 0.500)	H (0.333, 0.333, 0.500)
6	H (0.333, 0.333, 0.500)	A (0.000, 0.000, 0.500)
7	L (0.500, 0.000, 0.500)	M (0.500, 0.000, 0.000)
8	H (0.333, 0.333, 0.500)	K (0.333, 0.333, 0.000)

Supplementary Table 791: Single Weyl points along high symmetry lines of mp-1217967-PtSrP.

	Coordinates	Frequency	Mode 1	Mode 2	Multi.	Deg.	Path	Ratio
0	(0.333, 0.333, 0.288)	3.538	5	4	4	2	9	0.425
1	(0.333, 0.333, 0.204)	3.654	6	5	4	2	9	0.591

Supplementary Table 792: Nodal ring points along high symmetry lines of mp-1217967-PtSrP.

	Coordinates	Frequency	Mode 1	Mode 2	Multi.	Deg.	Path	Ratio
0	(0.296, 0.000, 0.000)	1.645	2	1	6	2	1	0.592
1	(0.297, 0.000, -0.000)	2.984	5	4	6	2	1	0.593

Supplementary Table 792: Nodal ring points along high symmetry lines of mp-1217967-PtSrP.

Coordinates	Frequency	Mode 1	Mode 2	Multi.	Deg.	Path	Ratio

The followings are details for CuSrSi. It has 7 nodal ring points and 1 single Weyl point along high symmetry line as shown in Fig **396**.

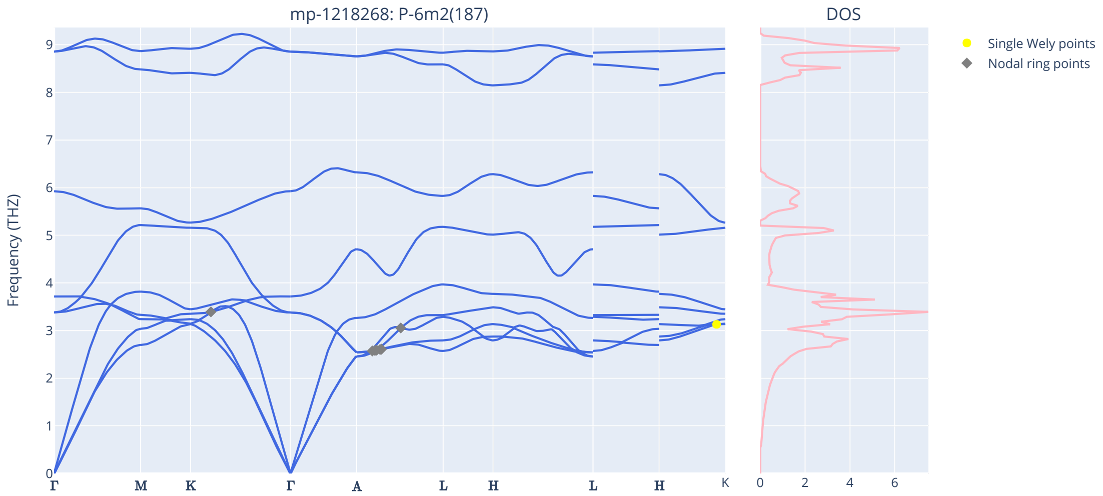

Supplementary Figure 396: The phonon band and DOS of CuSrSi.

Supplementary Table 793: High symmetry lines of mp-1218268-CuSrSi

	Start	End
0	Γ (0.000, 0.000, 0.000)	M (0.500, 0.000, 0.000)
1	M (0.500, 0.000, 0.000)	K (0.333, 0.333, 0.000)
2	K (0.333, 0.333, 0.000)	Γ (0.000, 0.000, 0.000)
3	Γ (0.000, 0.000, 0.000)	A (0.000, 0.000, 0.500)
4	A (0.000, 0.000, 0.500)	L (0.500, 0.000, 0.500)
5	L (0.500, 0.000, 0.500)	H (0.333, 0.333, 0.500)
6	H (0.333, 0.333, 0.500)	A (0.000, 0.000, 0.500)
7	L (0.500, 0.000, 0.500)	M (0.500, 0.000, 0.000)
8	H (0.333, 0.333, 0.500)	K (0.333, 0.333, 0.000)

Supplementary Table 794: Single Weyl points along high symmetry lines of mp-1218268-CuSrSi.

	Coordinates	Frequency	Mode 1	Mode 2	Multi.	Deg.	Path	Ratio
0	(0.333, 0.333, 0.066)	3.129	2	1	4	2	9	0.868

Supplementary Table 795: Nodal ring points along high symmetry lines of mp-1218268-CuSrSi.

	Coordinates	Frequency	Mode 1	Mode 2	Multi.	Deg.	Path	Ratio
0	(0.116, -0.001, 0.500)	2.588	2	1	12	2	5	0.231
1	(0.136, -0.000, 0.498)	2.603	2	1	12	2	5	0.271

Supplementary Table 795: Nodal ring points along high symmetry lines of mp-1218268-CuSrSi.

	Coordinates	Frequency	Mode 1	Mode 2	Multi.	Deg.	Path	Ratio
2	(0.144, 0.000, 0.496)	2.609	2	1	12	2	5	0.287
3	(0.106, 0.000, 0.500)	2.582	3	2	6	2	5	0.211
4	(0.090, 0.000, 0.499)	2.572	3	2	12	2	5	0.181
5	(0.265, 0.265, 0.000)	3.393	4	3	6	2	3	0.204
6	(0.255, 0.000, 0.500)	3.054	4	3	6	2	5	0.510

The followings are details for CuSrGe. It has 7 nodal ring points and 5 single Weyl points along high symmetry line as shown in Fig 397.

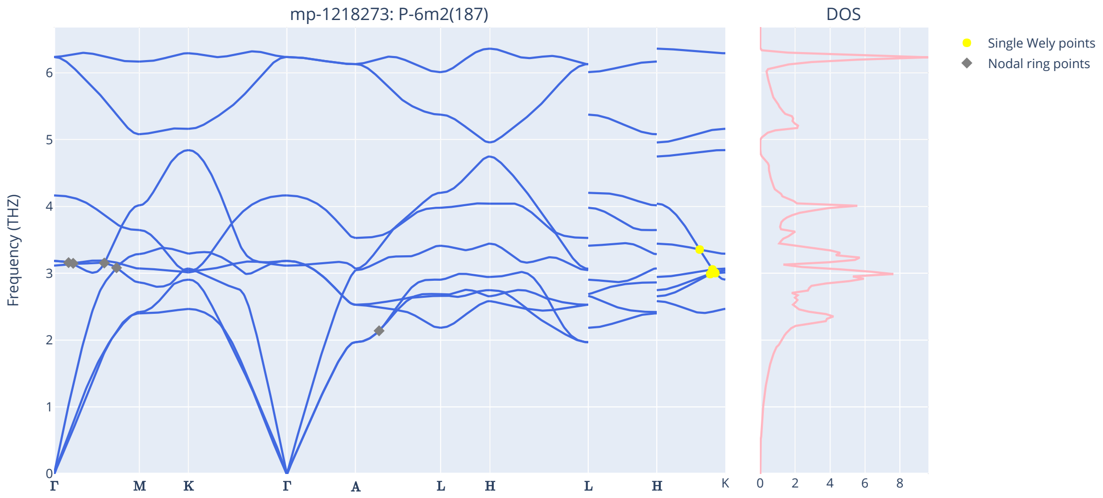

Supplementary Figure 397: The phonon band and DOS of CuSrGe.

Supplementary Table 796: High symmetry lines of mp-1218273-CuSrGe

	Start	End
0	Γ (0.000, 0.000, 0.000)	M (0.500, 0.000, 0.000)
1	M (0.500, 0.000, 0.000)	K (0.333, 0.333, 0.000)
2	K (0.333, 0.333, 0.000)	Γ (0.000, 0.000, 0.000)
3	Γ (0.000, 0.000, 0.000)	A (0.000, 0.000, 0.500)
4	A (0.000, 0.000, 0.500)	L (0.500, 0.000, 0.500)
5	L (0.500, 0.000, 0.500)	H (0.333, 0.333, 0.500)
6	H (0.333, 0.333, 0.500)	A (0.000, 0.000, 0.500)
7	L (0.500, 0.000, 0.500)	M (0.500, 0.000, 0.000)
8	H (0.333, 0.333, 0.500)	K (0.333, 0.333, 0.000)

Supplementary Table 797: Single Weyl points along high symmetry lines of mp-1218273-CuSrGe.

	Coordinates	Frequency	Mode 1	Mode 2	Multi.	Deg.	Path	Ratio
0	(0.333, 0.333, 0.111)	2.987	3	2	4	2	9	0.778
1	(0.333, 0.333, 0.073)	3.001	3	2	4	2	9	0.854
2	(0.333, 0.333, 0.078)	3.013	4	3	4	2	9	0.844
3	(0.333, 0.333, 0.095)	3.057	5	4	4	2	9	0.811
4	(0.333, 0.333, 0.187)	3.354	6	5	4	2	9	0.625

Supplementary Table 798: Nodal ring points along high symmetry lines of mp-1218273-CuSrGe.

	Coordinates	Frequency	Mode 1	Mode 2	Multi.	Deg.	Path	Ratio
0	(0.139, -0.000, 0.500)	2.139	2	1	6	2	5	0.278
1	(0.367, 0.000, 0.000)	3.081	4	3	6	2	1	0.733
2	(0.295, -0.000, -0.000)	3.153	5	4	6	2	1	0.589
3	(0.085, -0.000, -0.000)	3.159	6	5	6	2	1	0.170
4	(0.112, -0.000, -0.000)	3.148	6	5	6	2	1	0.224

The followings are details for NiSeTe. As shown in Fig 398, it has 4 topological nodal lines, 3 nodal ring points and 3 single Weyl points.

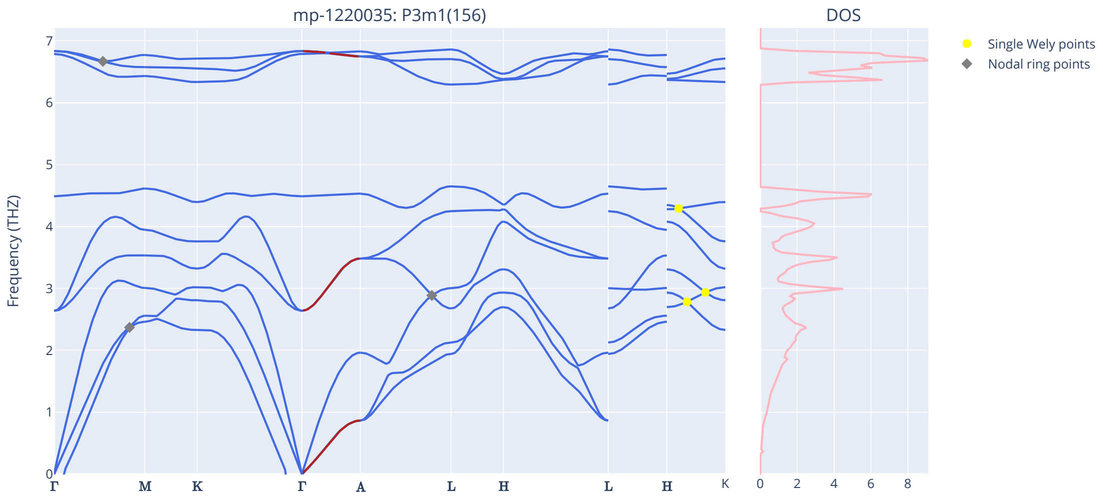

Supplementary Figure 398: The phonon band and DOS of NiSeTe.

Supplementary Table 799: High symmetry lines of mp-1220035-NiSeTe

	Start	End
0	Γ (0.000, 0.000, 0.000)	M (0.500, 0.000, 0.000)
1	M (0.500, 0.000, 0.000)	K (0.333, 0.333, 0.000)
2	K (0.333, 0.333, 0.000)	Γ (0.000, 0.000, 0.000)
3	Γ (0.000, 0.000, 0.000)	A (0.000, 0.000, 0.500)
4	A (0.000, 0.000, 0.500)	L (0.500, 0.000, 0.500)
5	L (0.500, 0.000, 0.500)	H (0.333, 0.333, 0.500)
6	H (0.333, 0.333, 0.500)	A (0.000, 0.000, 0.500)
7	L (0.500, 0.000, 0.500)	M (0.500, 0.000, 0.000)
8	H (0.333, 0.333, 0.500)	K (0.333, 0.333, 0.000)

Supplementary Table 800: Single Weyl points along high symmetry lines of mp-1220035-NiSeTe.

	Coordinates	Frequency	Mode 1	Mode 2	Multi.	Deg.	Path	Ratio
0	(0.333, 0.333, 0.328)	2.784	2	1	4	2	9	0.344
1	(0.333, 0.333, 0.170)	2.936	3	2	4	2	9	0.659
2	(0.333, 0.333, 0.399)	4.293	6	5	4	2	9	0.202

Supplementary Table 801: Nodal ring points along high symmetry lines of mp-1220035-NiSeTe.

	Coordinates	Frequency	Mode 1	Mode 2	Multi.	Deg.	Path	Ratio
0	(0.416, 0.000, 0.002)	2.370	2	1	6	2	1	0.832

Supplementary Table 801: Nodal ring points along high symmetry lines of mp-1220035-NiSeTe.

	Coordinates	Frequency	Mode 1	Mode 2	Multi.	Deg.	Path	Ratio
1	(0.394, 0.000, 0.499)	2.887	4	3	6	2	5	0.787
2	(0.269, -0.000, 0.005)	6.669	9	8	6	2	1	0.538

The followings are details for CuLaGe. It has 5 nodal ring points and 4 single Weyl points along high symmetry line as shown in Fig 399.

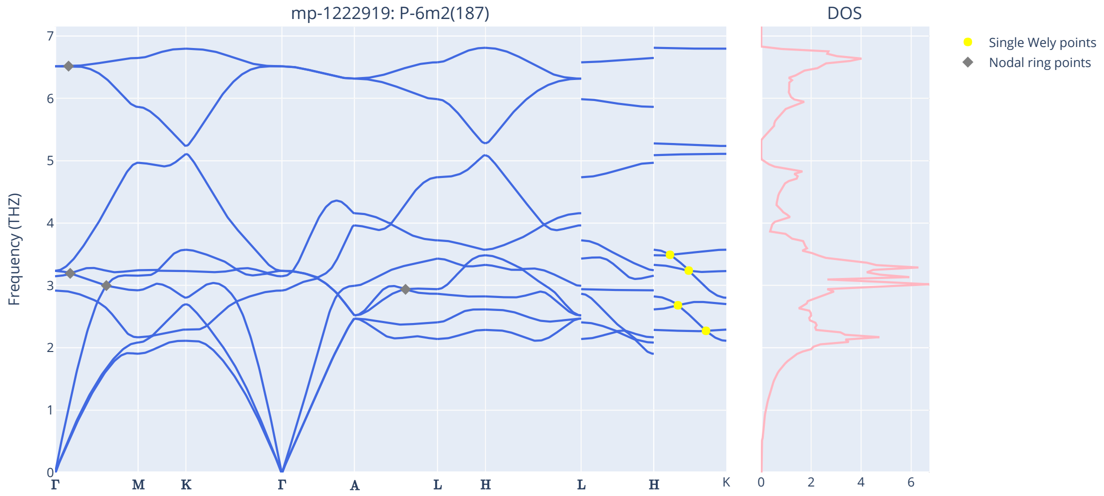

Supplementary Figure 399: The phonon band and DOS of CuLaGe.

Supplementary Table 802: High symmetry lines of mp-1222919-CuLaGe

	Start	End
0	Γ (0.000, 0.000, 0.000)	M (0.500, 0.000, 0.000)
1	M (0.500, 0.000, 0.000)	K (0.333, 0.333, 0.000)
2	K (0.333, 0.333, 0.000)	Γ (0.000, 0.000, 0.000)
3	Γ (0.000, 0.000, 0.000)	A (0.000, 0.000, 0.500)
4	A (0.000, 0.000, 0.500)	L (0.500, 0.000, 0.500)
5	L (0.500, 0.000, 0.500)	H (0.333, 0.333, 0.500)
6	H (0.333, 0.333, 0.500)	A (0.000, 0.000, 0.500)
7	L (0.500, 0.000, 0.500)	M (0.500, 0.000, 0.000)
8	H (0.333, 0.333, 0.500)	K (0.333, 0.333, 0.000)

Supplementary Table 803: Single Weyl points along high symmetry lines of mp-1222919-CuLaGe.

	Coordinates	Frequency	Mode 1	Mode 2	Multi.	Deg.	Path	Ratio
0	(0.333, 0.333, 0.140)	2.272	2	1	4	2	9	0.719
1	(0.333, 0.333, 0.334)	2.681	3	2	4	2	9	0.333
2	(0.333, 0.333, 0.259)	3.238	5	4	4	2	9	0.482
3	(0.333, 0.333, 0.388)	3.491	6	5	4	2	9	0.223

Supplementary Table 804: Nodal ring points along high symmetry lines of mp-1222919-CuLaGe.

	Coordinates	Frequency	Mode 1	Mode 2	Multi.	Deg.	Path	Ratio
0	(0.308, -0.000, 0.500)	2.937	4	3	6	2	5	0.616

Supplementary Table 804: Nodal ring points along high symmetry lines of mp-1222919-CuLaGe.

	Coordinates	Frequency	Mode 1	Mode 2	Multi.	Deg.	Path	Ratio
1	(0.309, 0.000, -0.000)	2.997	5	4	6	2	1	0.618
2	(0.091, -0.000, -0.000)	3.193	6	5	6	2	1	0.182
3	(0.081, -0.000, 0.000)	6.517	9	8	6	2	1	0.162

The followings are details for CoSeTe. As shown in Fig **400**, it has 3 topological nodal lines, 4 nodal ring points and 2 single Weyl points.

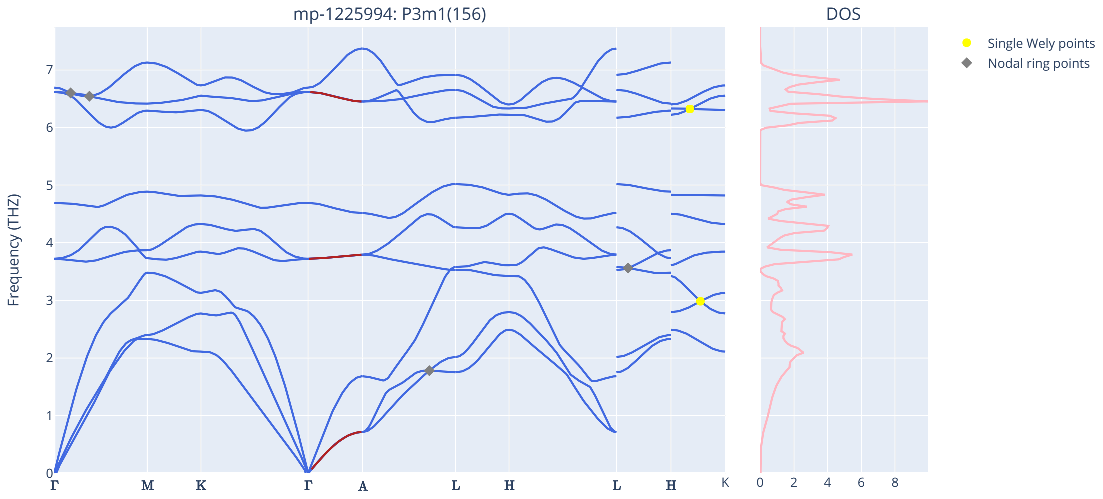

Supplementary Figure 400: The phonon band and DOS of CoSeTe.

Supplementary Table 805: High symmetry lines of mp-1225994-CoSeTe

	Start	End
0	Γ (0.000, 0.000, 0.000)	M (0.500, 0.000, 0.000)
1	M (0.500, 0.000, 0.000)	K (0.333, 0.333, 0.000)
2	K (0.333, 0.333, 0.000)	Γ (0.000, 0.000, 0.000)
3	Γ (0.000, 0.000, 0.000)	A (0.000, 0.000, 0.500)
4	A (0.000, 0.000, 0.500)	L (0.500, 0.000, 0.500)
5	L (0.500, 0.000, 0.500)	H (0.333, 0.333, 0.500)
6	H (0.333, 0.333, 0.500)	A (0.000, 0.000, 0.500)
7	L (0.500, 0.000, 0.500)	M (0.500, 0.000, 0.000)
8	H (0.333, 0.333, 0.500)	K (0.333, 0.333, 0.000)

Supplementary Table 806: Single Weyl points along high symmetry lines of mp-1225994-CoSeTe.

	Coordinates	Frequency	Mode 1	Mode 2	Multi.	Deg.	Path	Ratio
0	(0.333, 0.333, 0.228)	2.984	3	2	4	2	9	0.543
1	(0.333, 0.333, 0.325)	6.322	8	7	4	2	9	0.350

Supplementary Table 807: Nodal ring points along high symmetry lines of mp-1225994-CoSeTe.

	Coordinates	Frequency	Mode 1	Mode 2	Multi.	Deg.	Path	Ratio
0	(0.359, 0.000, 0.499)	1.780	2	1	6	2	5	0.718

Supplementary Table 807: Nodal ring points along high symmetry lines of mp-1225994-CoSeTe.

	Coordinates	Frequency	Mode 1	Mode 2	Multi.	Deg.	Path	Ratio
1	(0.500, 0.000, 0.393)	3.561	4	3	6	2	8	0.215
2	(0.088, -0.000, 0.003)	6.600	9	8	6	2	1	0.175
3	(0.189, 0.000, 0.003)	6.544	9	8	6	2	1	0.378

The followings are details for CeNiSi and it only has 3 single Weyl point along high symmetry line as shown in Fig 401.

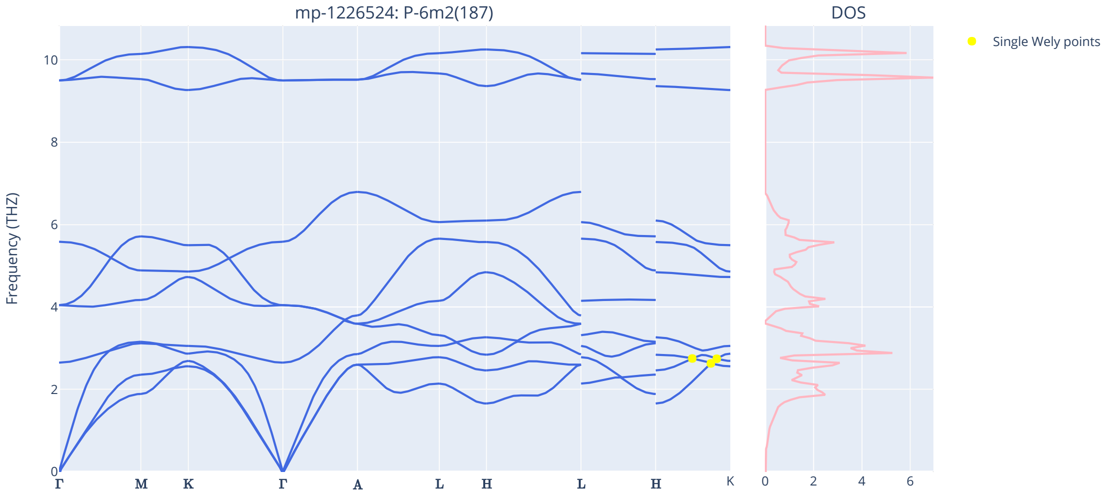

Supplementary Figure 401: The phonon band and DOS of CeNiSi.

Supplementary Table 808: High symmetry lines of mp-1226524-CeNiSi

	Start	End
0	Γ (0.000, 0.000, 0.000)	M (0.500, 0.000, 0.000)
1	M (0.500, 0.000, 0.000)	K (0.333, 0.333, 0.000)
2	K (0.333, 0.333, 0.000)	Γ (0.000, 0.000, 0.000)
3	Γ (0.000, 0.000, 0.000)	A (0.000, 0.000, 0.500)
4	A (0.000, 0.000, 0.500)	L (0.500, 0.000, 0.500)
5	L (0.500, 0.000, 0.500)	H (0.333, 0.333, 0.500)
6	H (0.333, 0.333, 0.500)	A (0.000, 0.000, 0.500)
7	L (0.500, 0.000, 0.500)	M (0.500, 0.000, 0.000)
8	H (0.333, 0.333, 0.500)	K (0.333, 0.333, 0.000)

Supplementary Table 809: Single Weyl points along high symmetry lines of mp-1226524-CeNiSi.

	Coordinates	Frequency	Mode 1	Mode 2	Multi.	Deg.	Path	Ratio
0	(0.333, 0.333, 0.131)	2.629	2	1	4	2	9	0.739
1	(0.333, 0.333, 0.255)	2.749	3	2	4	2	9	0.490
2	(0.333, 0.333, 0.091)	2.743	3	2	4	2	9	0.818

The followings are details for CeCuGe. It has 3 nodal ring points and 1 single Weyl point along high symmetry line as shown in Fig **402**.

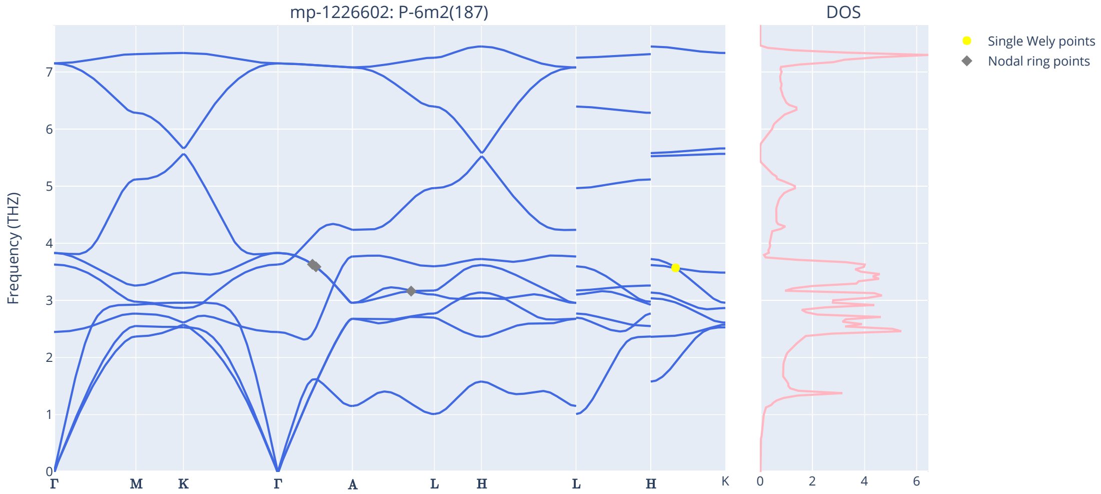

Supplementary Figure 402: The phonon band and DOS of CeCuGe.

Supplementary Table 810: High symmetry lines of mp-1226602-CeCuGe

	Start	End
0	Γ (0.000, 0.000, 0.000)	M (0.500, 0.000, 0.000)
1	M (0.500, 0.000, 0.000)	K (0.333, 0.333, 0.000)
2	K (0.333, 0.333, 0.000)	Γ (0.000, 0.000, 0.000)
3	Γ (0.000, 0.000, 0.000)	A (0.000, 0.000, 0.500)
4	A (0.000, 0.000, 0.500)	L (0.500, 0.000, 0.500)
5	L (0.500, 0.000, 0.500)	H (0.333, 0.333, 0.500)
6	H (0.333, 0.333, 0.500)	A (0.000, 0.000, 0.500)
7	L (0.500, 0.000, 0.500)	M (0.500, 0.000, 0.000)
8	H (0.333, 0.333, 0.500)	K (0.333, 0.333, 0.000)

Supplementary Table 811: Single Weyl points along high symmetry lines of mp-1226602-CeCuGe.

	Coordinates	Frequency	Mode 1	Mode 2	Multi.	Deg.	Path	Ratio
0	(0.333, 0.333, 0.334)	3.569	6	5	4	2	9	0.333

Supplementary Table 812: Nodal ring points along high symmetry lines of mp-1226602-CeCuGe.

	Coordinates	Frequency	Mode 1	Mode 2	Multi.	Deg.	Path	Ratio
0	(0.359, 0.000, 0.500)	3.162	5	4	6	2	5	0.717
1	(0.001, 0.000, 0.256)	3.588	6	5	12	2	4	0.511
2	(0.001, -0.000, 0.232)	3.631	6	5	6	2	4	0.464

Supplementary Table 812: Nodal ring points along high symmetry lines of mp-1226602-CeCuGe.

Coordinates	Frequency	Mode 1	Mode 2	Multi.	Deg.	Path	Ratio

The followings are details for $Au_4Cd_4Sn_4$. As shown in Fig **403**, it has 5 topological nodal lines and 3 nodal ring points. At the same time, it has 1 single Weyl point and 1 high degenerate Weyl point whose degeneracy is more than 2 and charge is bigger than 1.

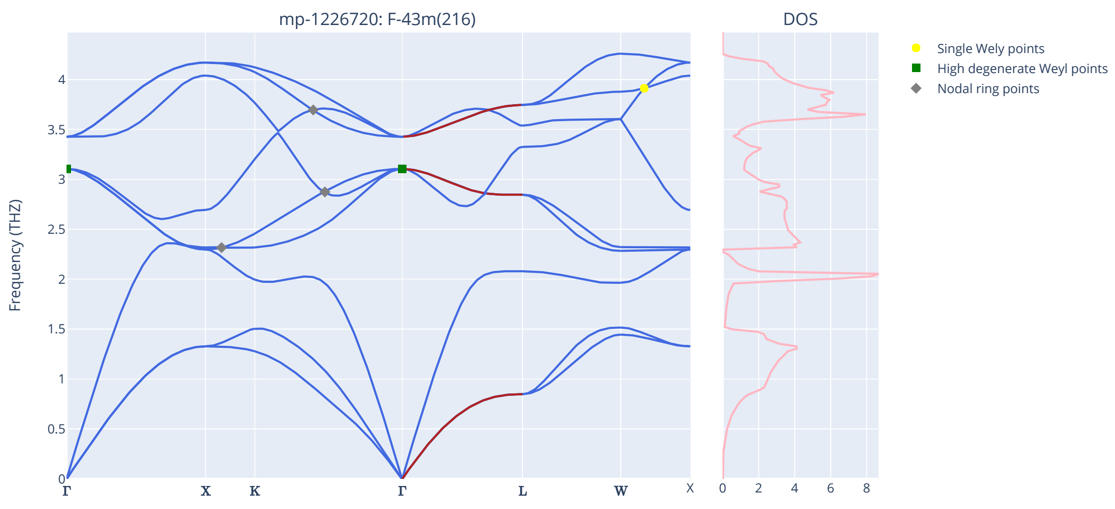

Supplementary Figure 403: The phonon band and DOS of Au4Cd4Sn4.

Supplementary Table 813: High symmetry lines of mp-1226720-Au4Cd4Sn4

	Start	End
0	Γ (0.000, 0.000, 0.000)	X (0.500, 0.000, 0.500)
1	X (0.500, 0.000, 0.500)	U (0.625, 0.250, 0.625)
2	K (0.375, 0.375, 0.750)	Γ (0.000, 0.000, 0.000)
3	Γ (0.000, 0.000, 0.000)	L (0.500, 0.500, 0.500)
4	L (0.500, 0.500, 0.500)	W (0.500, 0.250, 0.750)
5	W (0.500, 0.250, 0.750)	X (0.500, 0.000, 0.500)

Supplementary Table 814: Single Weyl points along high symmetry lines of mp-1226720-Au4Cd4Sn4.

	Coordinates	Frequency	Mode 1	Mode 2	Multi.	Deg.	Path	Ratio
0	(0.500, 0.165, 0.665)	3.912	8	7	12	2	6	0.338

Supplementary Table 815: High degenerate Weyl points along high symmetry lines of mp-1226720-Au4Cd4Sn4.

	Coordinates	Frequency	Mode 1	Mode 2	Multi.	Deg.	Path	Ratio
0	(0.000, 0.000, 0.000)	3.105	6	4	1	3	1	0.000

Supplementary Table 816: Nodal ring points along high symmetry lines of mp-1226720-Au4Cd4Sn4.

	Coordinates	Frequency	Mode 1	Mode 2	Multi.	Deg.	Path	Ratio
0	(0.541, 0.081, 0.541)	2.317	5	4	12	2	2	0.326
1	(0.191, 0.191, 0.398)	2.874	6	5	24	2	3	0.476
2	(0.222, 0.222, 0.456)	3.695	8	7	24	2	3	0.397

The followings are details for Mg_8Si_4. It has 4 topological nodal lines and 11 nodal ring points along high symmetry line as shown in Fig **404**.

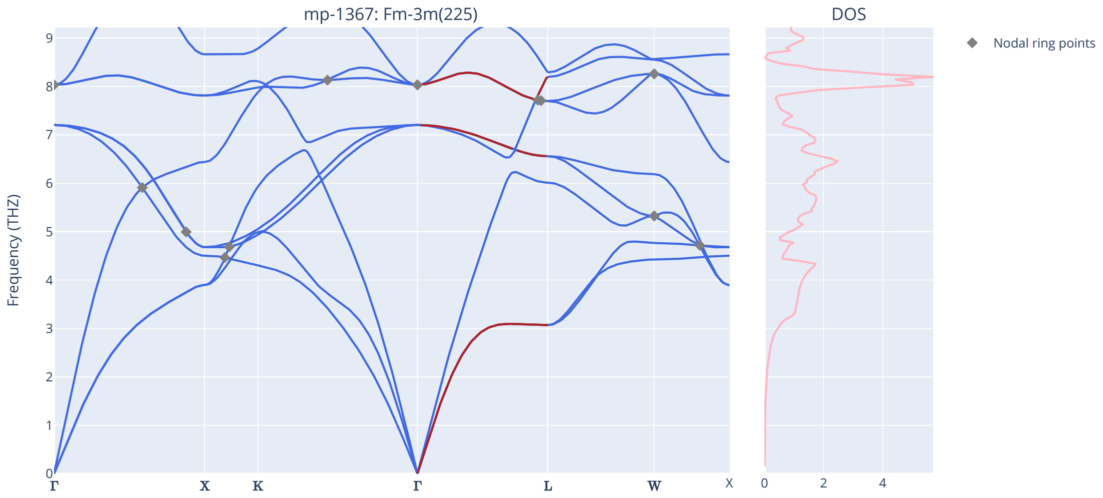

Supplementary Figure 404: The phonon band and DOS of Mg8Si4.

Supplementary Table 817: High symmetry lines of mp-1367-Mg8Si4

	Start	End
0	Γ (0.000, 0.000, 0.000)	X (0.500, 0.000, 0.500)
1	X (0.500, 0.000, 0.500)	U (0.625, 0.250, 0.625)
2	K (0.375, 0.375, 0.750)	Γ (0.000, 0.000, 0.000)
3	Γ (0.000, 0.000, 0.000)	L (0.500, 0.500, 0.500)
4	L (0.500, 0.500, 0.500)	W (0.500, 0.250, 0.750)
5	W (0.500, 0.250, 0.750)	X (0.500, 0.000, 0.500)

Supplementary Table 818: Nodal ring points along high symmetry lines of mp-1367-Mg8Si4.

	Coordinates	Frequency	Mode 1	Mode 2	Multi.	Deg.	Path	Ratio
0	(0.548, 0.095, 0.548)	4.466	3	2	12	2	2	0.380
1	(0.498, 0.097, 0.599)	4.717	3	2	24	2	6	0.608
2	(0.558, 0.116, 0.558)	4.688	4	3	12	2	2	0.464
3	(0.293, 0.000, 0.293)	5.911	4	3	6	2	1	0.586
4	(0.500, 0.250, 0.750)	5.325	4	3	6	2	5	1.000
5	(0.439, 0.000, 0.439)	4.995	5	4	6	2	1	0.878
6	(0.474, 0.474, 0.474)	7.707	7	6	8	2	4	0.949
7	(0.462, 0.462, 0.462)	7.718	7	6	8	2	4	0.924
8	(0.500, 0.250, 0.750)	8.260	7	6	6	2	5	1.000
9	(0.000, 0.000, 0.000)	8.033	8	7	1	3	1	0.000
10	(0.207, 0.207, 0.427)	8.131	8	7	24	2	3	0.437

The followings are details for Al_8Pt_4. It has 3 topological nodal lines and 11 nodal ring points along high symmetry line as shown in Fig **405**.

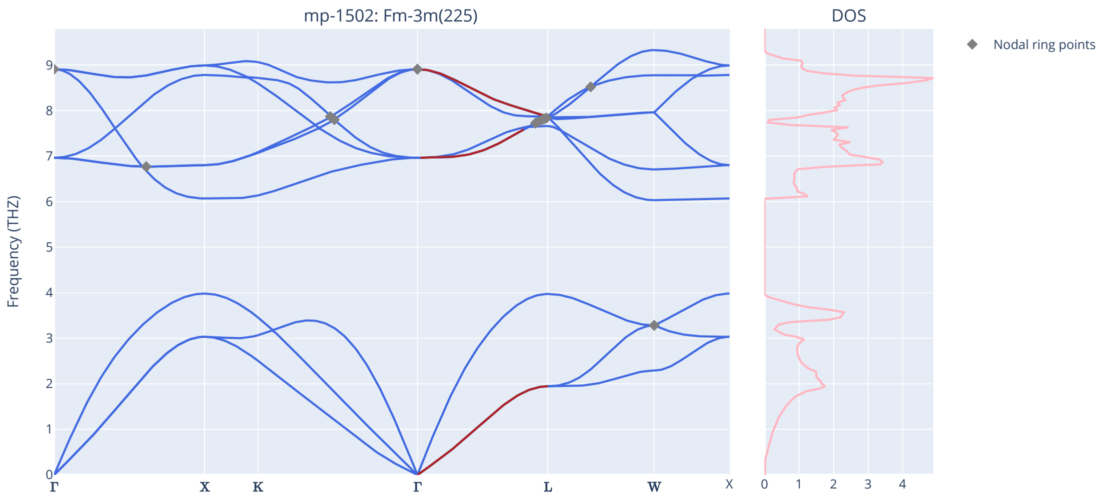

Supplementary Figure 405: The phonon band and DOS of Al8Pt4.

Supplementary Table 819: High symmetry lines of mp-1502-Al8Pt4

	Start	End
0	Γ (0.000, 0.000, 0.000)	X (0.500, 0.000, 0.500)
1	X (0.500, 0.000, 0.500)	U (0.625, 0.250, 0.625)
2	K (0.375, 0.375, 0.750)	Γ (0.000, 0.000, 0.000)
3	Γ (0.000, 0.000, 0.000)	L (0.500, 0.500, 0.500)
4	L (0.500, 0.500, 0.500)	W (0.500, 0.250, 0.750)
5	W (0.500, 0.250, 0.750)	X (0.500, 0.000, 0.500)

Supplementary Table 820: Nodal ring points along high symmetry lines of mp-1502-Al8Pt4.

	Coordinates	Frequency	Mode 1	Mode 2	Multi.	Deg.	Path	Ratio
0	(0.500, 0.250, 0.750)	3.280	3	2	6	2	5	1.000
1	(0.453, 0.453, 0.453)	7.723	6	5	8	2	4	0.906
2	(0.306, 0.000, 0.306)	6.767	6	5	6	2	1	0.612
3	(0.495, 0.495, 0.495)	7.836	6	5	8	2	4	0.991
4	(0.464, 0.464, 0.464)	7.754	6	5	8	2	4	0.928
5	(0.488, 0.488, 0.488)	7.818	6	5	8	2	4	0.976
6	(0.479, 0.479, 0.479)	7.794	6	5	8	2	4	0.957
7	(0.192, 0.199, 0.390)	7.797	7	6	24	2	3	0.480
8	(0.207, 0.207, 0.407)	7.868	8	7	24	2	3	0.455
9	(0.000, 0.000, 0.000)	8.907	9	8	1	3	1	0.000
10	(0.500, 0.399, 0.601)	8.519	9	8	24	2	5	0.403

The followings are details for $Mg_4Ni_4Sb_4$. As shown in Fig **406**, it has 5 topological nodal lines, 4 nodal ring points and 1 high degenerate Weyl point whose degeneracy is more than 2 and charge is bigger than 1.

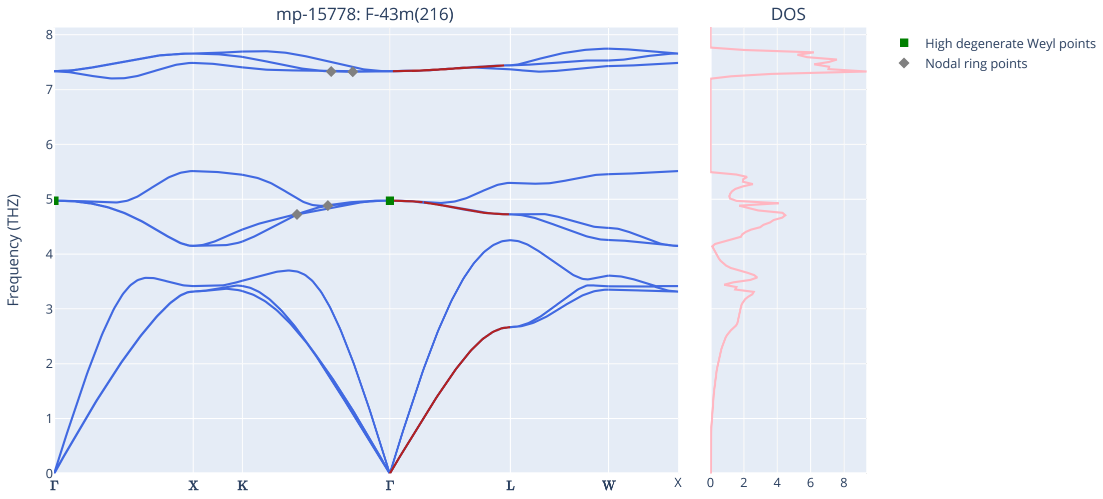

Supplementary Figure 406: The phonon band and DOS of Mg4Ni4Sb4.

Supplementary Table 821: High symmetry lines of mp-15778-Mg4Ni4Sb4

	Start	End
0	Γ (0.000, 0.000, 0.000)	X (0.500, 0.000, 0.500)
1	X (0.500, 0.000, 0.500)	U (0.625, 0.250, 0.625)
2	K (0.375, 0.375, 0.750)	Γ (0.000, 0.000, 0.000)
3	Γ (0.000, 0.000, 0.000)	L (0.500, 0.500, 0.500)
4	L (0.500, 0.500, 0.500)	W (0.500, 0.250, 0.750)
5	W (0.500, 0.250, 0.750)	X (0.500, 0.000, 0.500)

Supplementary Table 822: High degenerate Weyl points along high symmetry lines of mp-15778-Mg4Ni4Sb4.

	Coordinates	Frequency	Mode 1	Mode 2	Multi.	Deg.	Path	Ratio
0	(0.000, 0.000, 0.000)	4.977	6	4	1	3	1	0.000

Supplementary Table 823: Nodal ring points along high symmetry lines of mp-15778-Mg4Ni4Sb4.

	Coordinates	Frequency	Mode 1	Mode 2	Multi.	Deg.	Path	Ratio
0	(0.237, 0.237, 0.471)	4.723	5	4	24	2	3	0.371
1	(0.153, 0.153, 0.319)	4.884	6	5	24	2	3	0.580
2	(0.152, 0.152, 0.294)	7.330	8	7	24	2	3	0.603
3	(0.090, 0.090, 0.193)	7.325	8	7	24	2	3	0.749

The followings are details for U_4O_8. It has 4 topological nodal lines and 5 nodal ring points along high symmetry line as shown in Fig **407**.

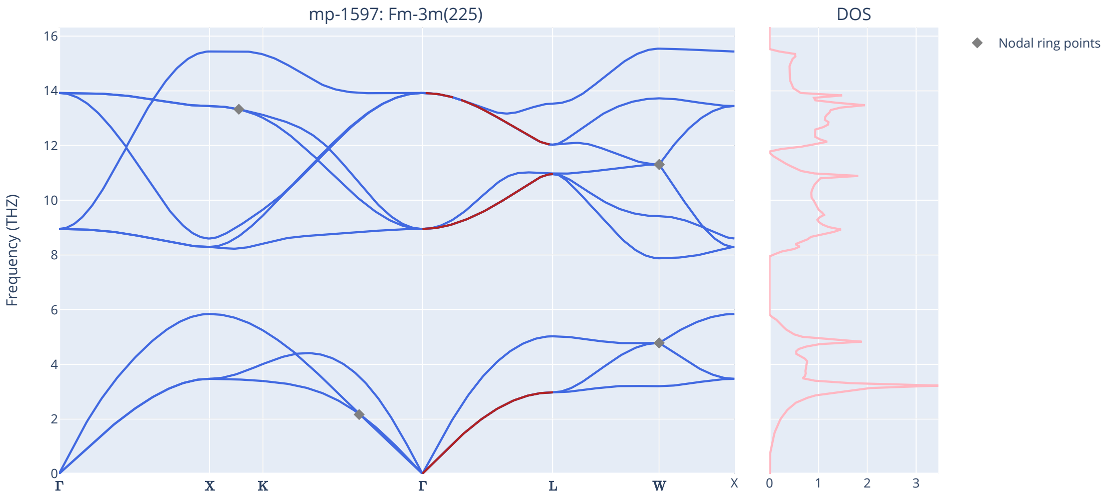

Supplementary Figure 407: The phonon band and DOS of U4O8.

Supplementary Table 824: High symmetry lines of mp-1597-U4O8

	Start	End
0	Γ (0.000, 0.000, 0.000)	X (0.500, 0.000, 0.500)
1	X (0.500, 0.000, 0.500)	U (0.625, 0.250, 0.625)
2	K (0.375, 0.375, 0.750)	Γ (0.000, 0.000, 0.000)
3	Γ (0.000, 0.000, 0.000)	L (0.500, 0.500, 0.500)
4	L (0.500, 0.500, 0.500)	W (0.500, 0.250, 0.750)
5	W (0.500, 0.250, 0.750)	X (0.500, 0.000, 0.500)

Supplementary Table 825: Nodal ring points along high symmetry lines of mp-1597-U4O8.

	Coordinates	Frequency	Mode 1	Mode 2	Multi.	Deg.	Path	Ratio
0	(0.153, 0.153, 0.293)	2.165	2	1	24	2	3	0.604
1	(0.500, 0.250, 0.750)	4.783	3	2	6	2	5	1.000
2	(0.500, 0.250, 0.750)	11.306	7	6	6	2	5	1.000
3	(0.569, 0.138, 0.569)	13.324	8	7	12	2	2	0.550

The followings are details for $Au_4Cd_4Sb_4$. As shown in Fig **408**, it has 4 topological nodal lines and 1 nodal ring point. At the same time, it has 2 single Weyl points and 1 high degenerate Weyl point whose degeneracy is more than 2 and charge is bigger than 1.

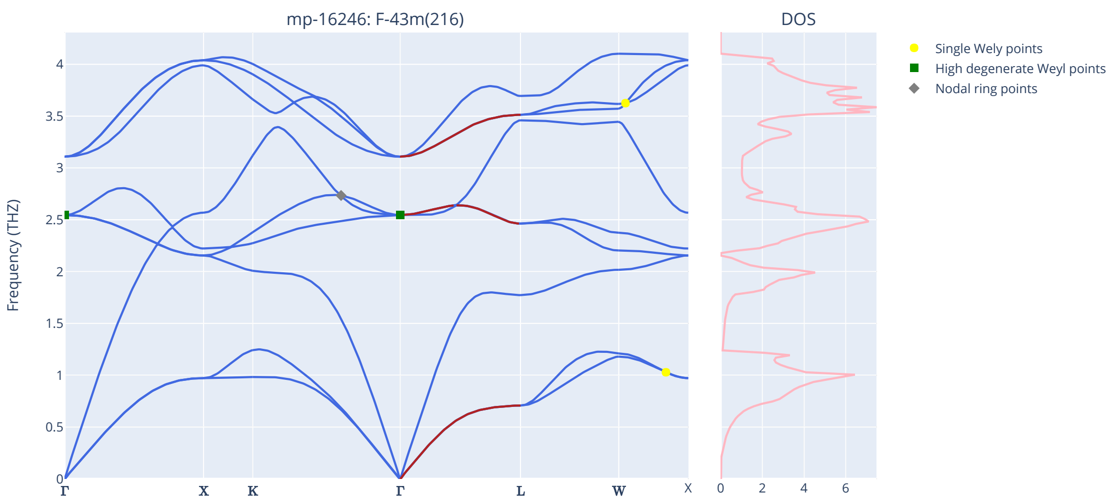

Supplementary Figure 408: The phonon band and DOS of Au4Cd4Sb4.

Supplementary Table 826: High symmetry lines of mp-16246-Au4Cd4Sb4

	Start	End
0	Γ (0.000, 0.000, 0.000)	X (0.500, 0.000, 0.500)
1	X (0.500, 0.000, 0.500)	U (0.625, 0.250, 0.625)
2	K (0.375, 0.375, 0.750)	Γ (0.000, 0.000, 0.000)
3	Γ (0.000, 0.000, 0.000)	L (0.500, 0.500, 0.500)
4	L (0.500, 0.500, 0.500)	W (0.500, 0.250, 0.750)
5	W (0.500, 0.250, 0.750)	X (0.500, 0.000, 0.500)

Supplementary Table 827: Single Weyl points along high symmetry lines of mp-16246-Au4Cd4Sb4.

	Coordinates	Frequency	Mode 1	Mode 2	Multi.	Deg.	Path	Ratio
0	(0.500, 0.080, 0.580)	1.028	2	1	12	2	6	0.680
1	(0.500, 0.226, 0.726)	3.625	8	7	12	2	6	0.097

Supplementary Table 828: High degenerate Weyl points along high symmetry lines of mp-16246-Au4Cd4Sb4.

	Coordinates	Frequency	Mode 1	Mode 2	Multi.	Deg.	Path	Ratio
0	(0.000, 0.000, 0.000)	2.545	6	4	1	3	1	0.000

Supplementary Table 829: Nodal ring points along high symmetry lines of mp-16246-Au4Cd4Sb4.

	Coordinates	Frequency	Mode 1	Mode 2	Multi.	Deg.	Path	Ratio
0	(0.147, 0.147, 0.304)	2.734	6	5	24	2	3	0.600

The followings are details for $In_4Li_4Ge_4$. As shown in Fig **409**, it has 3 topological nodal lines, 2 nodal ring points and 1 high degenerate Weyl point whose degeneracy is more than 2 and charge is bigger than 1.

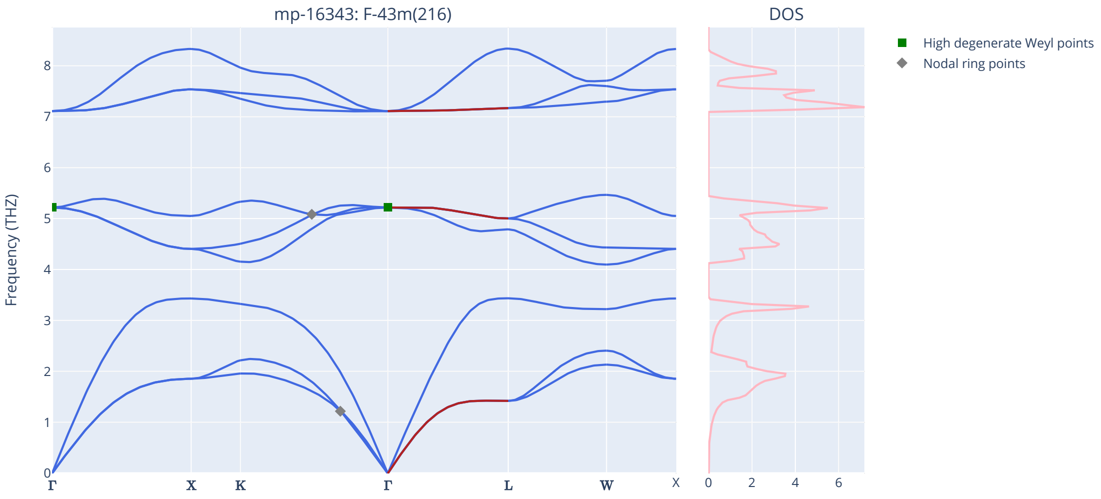

Supplementary Figure 409: The phonon band and DOS of In4Li4Ge4.

Supplementary Table 830: High symmetry lines of mp-16343-In4Li4Ge4

	Start	End
0	Γ (0.000, 0.000, 0.000)	X (0.500, 0.000, 0.500)
1	X (0.500, 0.000, 0.500)	U (0.625, 0.250, 0.625)
2	K (0.375, 0.375, 0.750)	Γ (0.000, 0.000, 0.000)
3	Γ (0.000, 0.000, 0.000)	L (0.500, 0.500, 0.500)
4	L (0.500, 0.500, 0.500)	W (0.500, 0.250, 0.750)
5	W (0.500, 0.250, 0.750)	X (0.500, 0.000, 0.500)

Supplementary Table 831: High degenerate Weyl points along high symmetry lines of mp-16343-In4Li4Ge4.

	Coordinates	Frequency	Mode 1	Mode 2	Multi.	Deg.	Path	Ratio
0	(0.000, 0.000, 0.000)	5.217	6	4	1	3	1	0.000

Supplementary Table 832: Nodal ring points along high symmetry lines of mp-16343-In4Li4Ge4.

	Coordinates	Frequency	Mode 1	Mode 2	Multi.	Deg.	Path	Ratio
0	(0.122, 0.122, 0.239)	1.218	2	1	24	2	3	0.678
1	(0.199, 0.199, 0.381)	5.083	6	5	24	2	3	0.485

The followings are details for Pt_4Sn_8. It has 4 topological nodal lines and 9 nodal ring points along high symmetry line as shown in Fig **410**.

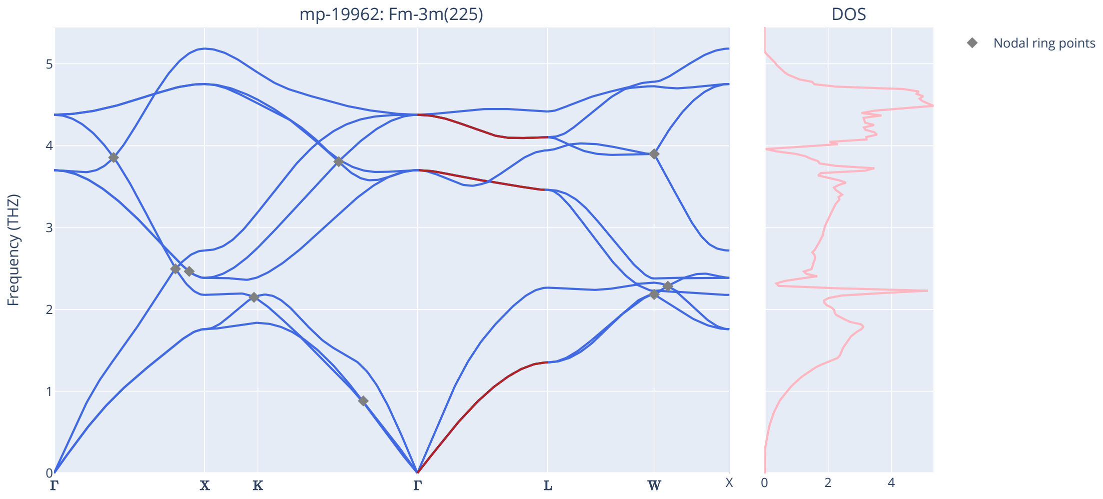

Supplementary Figure 410: The phonon band and DOS of Pt4Sn8.

Supplementary Table 833: High symmetry lines of mp-19962-Pt4Sn8

	Start	End
0	Γ (0.000, 0.000, 0.000)	X (0.500, 0.000, 0.500)
1	X (0.500, 0.000, 0.500)	U (0.625, 0.250, 0.625)
2	K (0.375, 0.375, 0.750)	Γ (0.000, 0.000, 0.000)
3	Γ (0.000, 0.000, 0.000)	L (0.500, 0.500, 0.500)
4	L (0.500, 0.500, 0.500)	W (0.500, 0.250, 0.750)
5	W (0.500, 0.250, 0.750)	X (0.500, 0.000, 0.500)

Supplementary Table 834: Nodal ring points along high symmetry lines of mp-19962-Pt4Sn8.

	Coordinates	Frequency	Mode 1	Mode 2	Multi.	Deg.	Path	Ratio
0	(0.122, 0.122, 0.259)	0.884	2	1	24	2	3	0.661
1	(0.500, 0.250, 0.750)	2.184	2	1	6	2	5	1.000
2	(0.616, 0.232, 0.616)	2.148	3	2	12	2	2	0.929
3	(0.403, -0.002, 0.403)	2.496	4	3	24	2	1	0.806
4	(0.498, 0.204, 0.705)	2.284	4	3	24	2	6	0.182
5	(0.449, 0.000, 0.449)	2.465	5	4	6	2	1	0.898
6	(0.184, 0.184, 0.371)	3.805	6	5	24	2	3	0.507
7	(0.198, -0.001, 0.198)	3.856	7	6	10	2	1	0.395
8	(0.500, 0.250, 0.750)	3.899	7	6	6	2	5	1.000

The followings are details for Mg_8Pb_4. It has 3 topological nodal lines and 10 nodal ring points along high symmetry line as shown in Fig **411**.

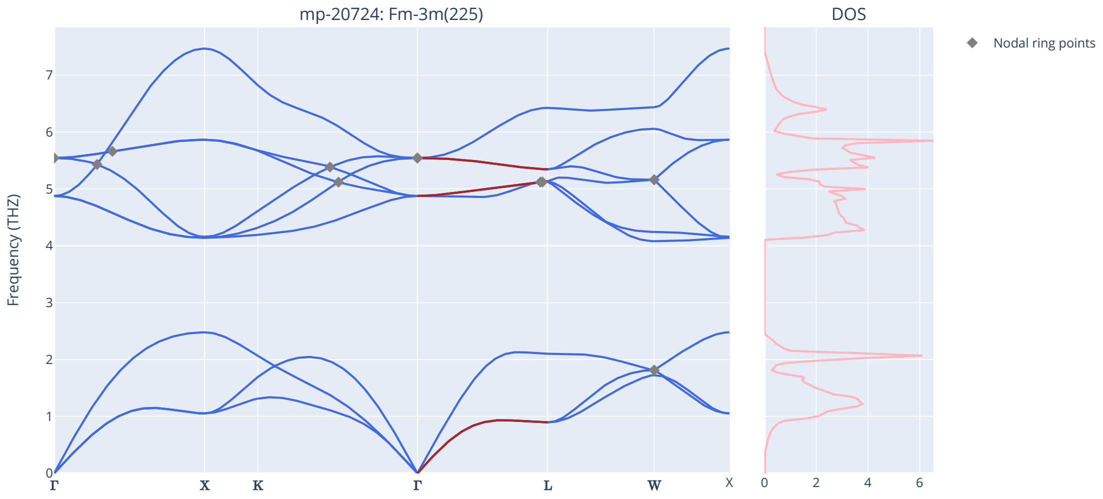

Supplementary Figure 411: The phonon band and DOS of Mg8Pb4.

Supplementary Table 835: High symmetry lines of mp-20724-Mg8Pb4

	Start	End
0	Γ (0.000, 0.000, 0.000)	X (0.500, 0.000, 0.500)
1	X (0.500, 0.000, 0.500)	U (0.625, 0.250, 0.625)
2	K (0.375, 0.375, 0.750)	Γ (0.000, 0.000, 0.000)
3	Γ (0.000, 0.000, 0.000)	L (0.500, 0.500, 0.500)
4	L (0.500, 0.500, 0.500)	W (0.500, 0.250, 0.750)
5	W (0.500, 0.250, 0.750)	X (0.500, 0.000, 0.500)

Supplementary Table 836: Nodal ring points along high symmetry lines of mp-20724-Mg8Pb4.

	Coordinates	Frequency	Mode 1	Mode 2	Multi.	Deg.	Path	Ratio
0	(0.500, 0.250, 0.750)	1.813	3	2	6	2	5	1.000
1	(0.472, 0.472, 0.472)	5.119	5	4	8	2	4	0.944
2	(0.480, 0.480, 0.480)	5.121	5	4	8	2	4	0.959
3	(0.184, 0.184, 0.373)	5.118	6	5	24	2	3	0.505
4	(0.143, 0.001, 0.143)	5.429	7	6	24	2	1	0.286
5	(0.500, 0.250, 0.750)	5.161	7	6	6	2	5	1.000
6	(0.000, 0.000, 0.000)	5.542	8	7	1	3	1	0.000
7	(0.194, 0.000, 0.194)	5.660	8	7	6	2	1	0.388
8	(0.207, 0.207, 0.410)	5.384	8	7	24	2	3	0.452
9	(0.000, 0.000, 0.000)	5.542	9	8	1	3	1	0.000

The followings are details for Ir_8P_4. It has 4 topological nodal lines and 4 nodal ring points along high symmetry line as shown in Fig **412**.

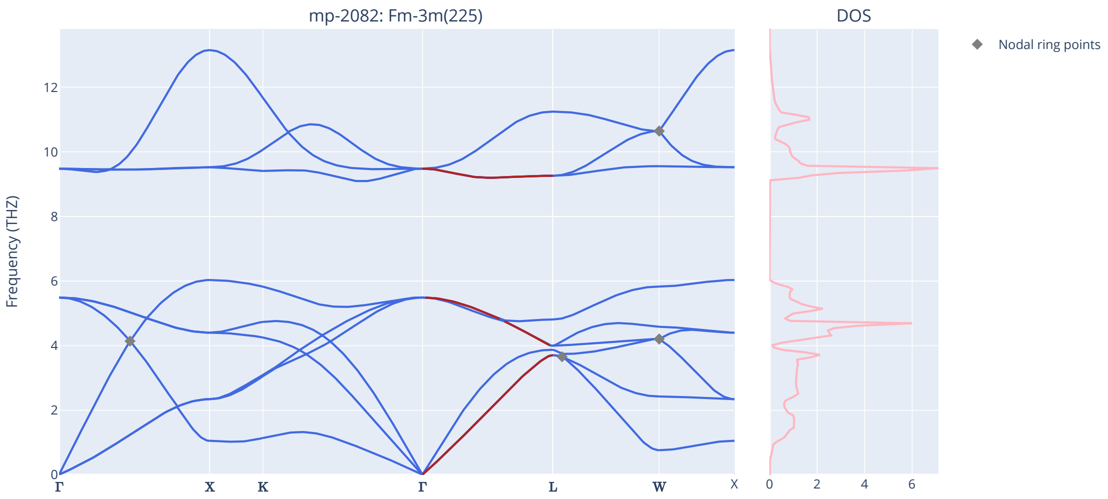

Supplementary Figure 412: The phonon band and DOS of Ir8P4.

Supplementary Table 837: High symmetry lines of mp-2082-Ir8P4

	Start	End
0	Γ (0.000, 0.000, 0.000)	X (0.500, 0.000, 0.500)
1	X (0.500, 0.000, 0.500)	U (0.625, 0.250, 0.625)
2	K (0.375, 0.375, 0.750)	Γ (0.000, 0.000, 0.000)
3	Γ (0.000, 0.000, 0.000)	L (0.500, 0.500, 0.500)
4	L (0.500, 0.500, 0.500)	W (0.500, 0.250, 0.750)
5	W (0.500, 0.250, 0.750)	X (0.500, 0.000, 0.500)

Supplementary Table 838: Nodal ring points along high symmetry lines of mp-2082-Ir8P4.

	Coordinates	Frequency	Mode 1	Mode 2	Multi.	Deg.	Path	Ratio
0	(0.500, 0.478, 0.522)	3.655	2	1	24	2	5	0.088
1	(0.236, -0.001, 0.236)	4.140	4	3	24	2	1	0.471
2	(0.500, 0.250, 0.750)	4.205	4	3	6	2	5	1.000
3	(0.500, 0.250, 0.750)	10.645	9	8	6	2	5	1.000

The followings are details for Au_4In_8. It has 5 topological nodal lines and 9 nodal ring points along high symmetry line as shown in Fig **413**.

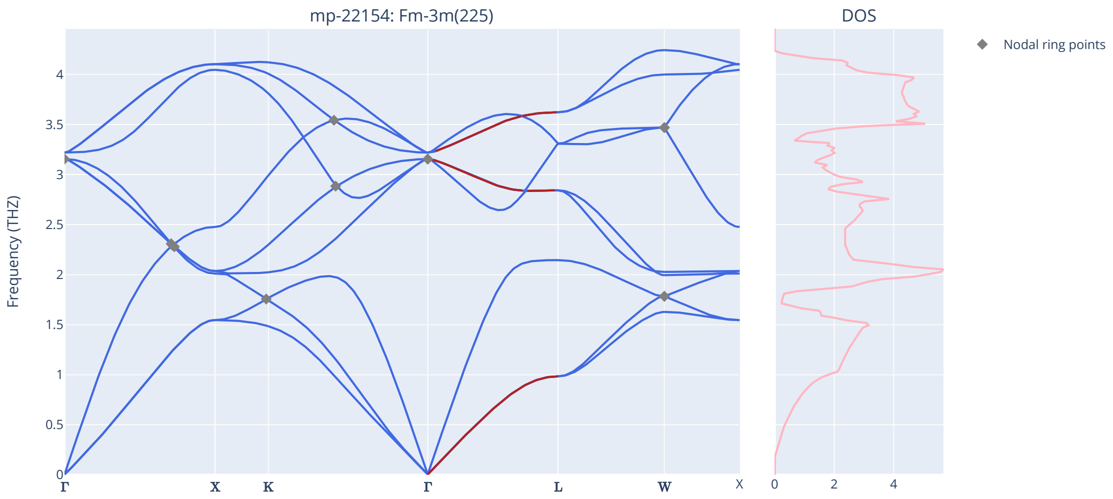

Supplementary Figure 413: The phonon band and DOS of Au4In8.

Supplementary Table 839: High symmetry lines of mp-22154-Au4In8

	Start	End
0	Γ (0.000, 0.000, 0.000)	X (0.500, 0.000, 0.500)
1	X (0.500, 0.000, 0.500)	U (0.625, 0.250, 0.625)
2	K (0.375, 0.375, 0.750)	Γ (0.000, 0.000, 0.000)
3	Γ (0.000, 0.000, 0.000)	L (0.500, 0.500, 0.500)
4	L (0.500, 0.500, 0.500)	W (0.500, 0.250, 0.750)
5	W (0.500, 0.250, 0.750)	X (0.500, 0.000, 0.500)

Supplementary Table 840: Nodal ring points along high symmetry lines of mp-22154-Au4In8.

	Coordinates	Frequency	Mode 1	Mode 2	Multi.	Deg.	Path	Ratio
0	(0.620, 0.241, 0.620)	1.756	3	2	12	2	2	0.964
1	(0.500, 0.250, 0.750)	1.783	3	2	6	2	5	1.000
2	(0.359, 0.000, 0.359)	2.292	4	3	6	2	1	0.718
3	(0.363, 0.005, 0.367)	2.278	4	3	24	2	1	0.730
4	(0.000, 0.000, 0.000)	3.154	6	5	1	3	1	0.000
5	(0.355, -0.001, 0.354)	2.309	6	5	12	2	1	0.708
6	(0.222, 0.222, 0.427)	2.884	6	5	24	2	3	0.423
7	(0.500, 0.250, 0.750)	3.469	7	6	6	2	5	1.000
8	(0.222, 0.222, 0.439)	3.542	8	7	24	2	3	0.412

The followings are details for In_8Pt_4. It has 4 topological nodal lines and 8 nodal ring points along high symmetry line as shown in Fig **414**.

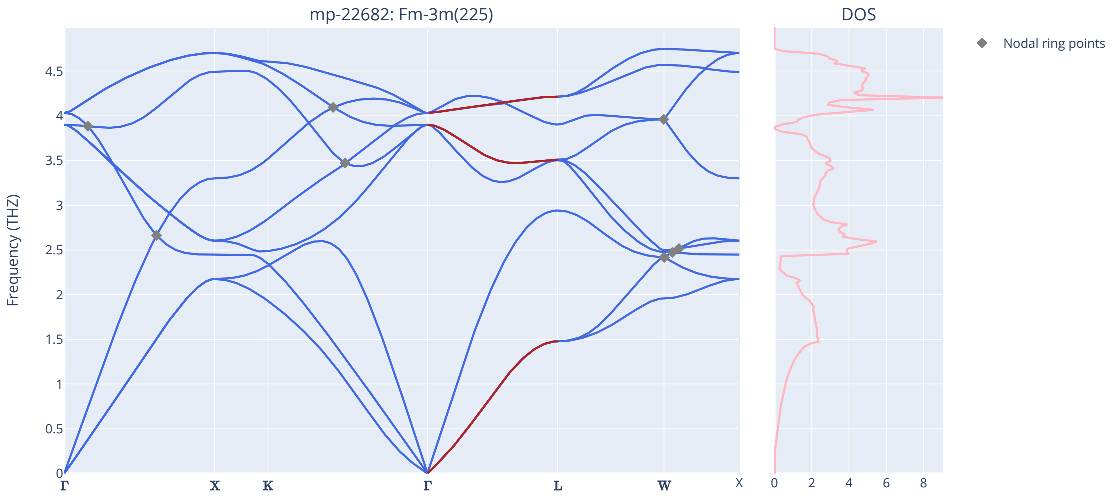

Supplementary Figure 414: The phonon band and DOS of In8Pt4.

Supplementary Table 841: High symmetry lines of mp-22682-In8Pt4

	Start	End
0	Γ (0.000, 0.000, 0.000)	X (0.500, 0.000, 0.500)
1	X (0.500, 0.000, 0.500)	U (0.625, 0.250, 0.625)
2	K (0.375, 0.375, 0.750)	Γ (0.000, 0.000, 0.000)
3	Γ (0.000, 0.000, 0.000)	L (0.500, 0.500, 0.500)
4	L (0.500, 0.500, 0.500)	W (0.500, 0.250, 0.750)
5	W (0.500, 0.250, 0.750)	X (0.500, 0.000, 0.500)

Supplementary Table 842: Nodal ring points along high symmetry lines of mp-22682-In8Pt4.

	Coordinates	Frequency	Mode 1	Mode 2	Multi.	Deg.	Path	Ratio
0	(0.500, 0.250, 0.750)	2.417	3	2	6	2	5	1.000
1	(0.307, -0.000, 0.307)	2.664	4	3	6	2	1	0.613
2	(0.499, 0.223, 0.722)	2.473	4	3	24	2	6	0.110
3	(0.499, 0.201, 0.700)	2.516	5	4	24	2	6	0.197
4	(0.196, 0.196, 0.385)	3.473	6	5	24	2	3	0.483
5	(0.079, 0.000, 0.079)	3.883	7	6	6	2	1	0.157
6	(0.500, 0.251, 0.749)	3.960	7	6	24	2	5	0.997
7	(0.224, 0.224, 0.442)	4.094	8	7	24	2	3	0.408

The followings are details for Ni_4Si_8. It has 4 topological nodal lines and 13 nodal ring points along high symmetry line as shown in Fig **415**.

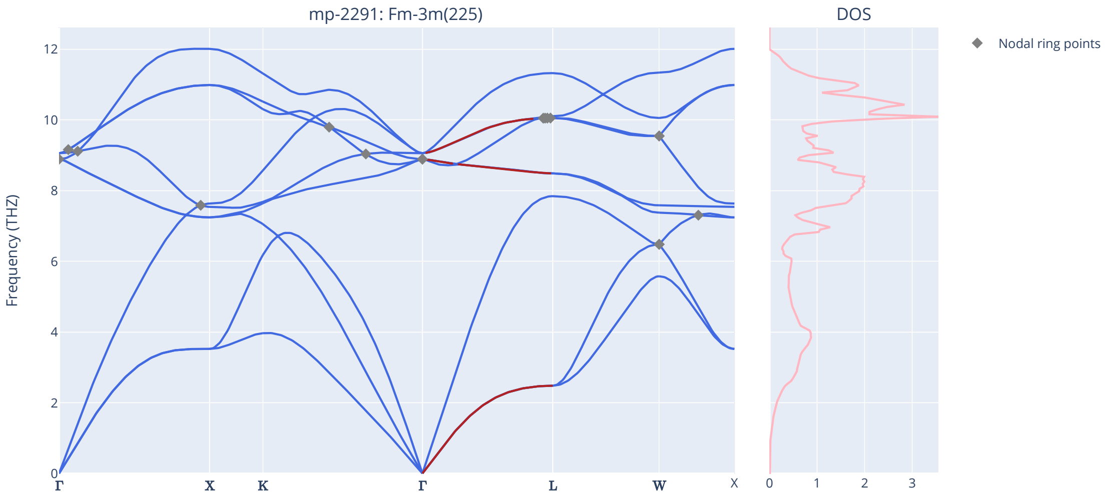

Supplementary Figure 415: The phonon band and DOS of Ni4Si8.

Supplementary Table 843: High symmetry lines of mp-2291-Ni4Si8

	Start	End
0	Γ (0.000, 0.000, 0.000)	X (0.500, 0.000, 0.500)
1	X (0.500, 0.000, 0.500)	U (0.625, 0.250, 0.625)
2	K (0.375, 0.375, 0.750)	Γ (0.000, 0.000, 0.000)
3	Γ (0.000, 0.000, 0.000)	L (0.500, 0.500, 0.500)
4	L (0.500, 0.500, 0.500)	W (0.500, 0.250, 0.750)
5	W (0.500, 0.250, 0.750)	X (0.500, 0.000, 0.500)

Supplementary Table 844: Nodal ring points along high symmetry lines of mp-2291-Ni4Si8.

	Coordinates	Frequency	Mode 1	Mode 2	Multi.	Deg.	Path	Ratio
0	(0.500, 0.250, 0.750)	6.481	3	2	6	2	5	1.000
1	(0.497, 0.121, 0.618)	7.305	4	3	24	2	6	0.521
2	(0.000, 0.000, 0.000)	8.890	6	5	1	3	1	0.000
3	(0.471, 0.000, 0.471)	7.586	6	5	6	2	1	0.941
4	(0.130, 0.130, 0.269)	9.037	6	5	24	2	3	0.646
5	(0.062, 0.000, 0.062)	9.113	7	6	6	2	1	0.124
6	(0.222, 0.222, 0.435)	9.795	7	6	24	2	3	0.416
7	(0.479, 0.479, 0.479)	10.051	7	6	8	2	4	0.959
8	(0.491, 0.491, 0.491)	10.052	7	6	8	2	4	0.982
9	(0.464, 0.464, 0.464)	10.048	7	6	8	2	4	0.927
10	(0.471, 0.471, 0.471)	10.049	7	6	8	2	4	0.941
11	(0.500, 0.250, 0.750)	9.544	7	6	6	2	5	1.000
12	(0.031, 0.000, 0.031)	9.160	9	8	6	2	1	0.061

The followings are details for Rh_8As_4. It has 4 topological nodal lines and 8 nodal ring points along high symmetry line as shown in Fig **416**.

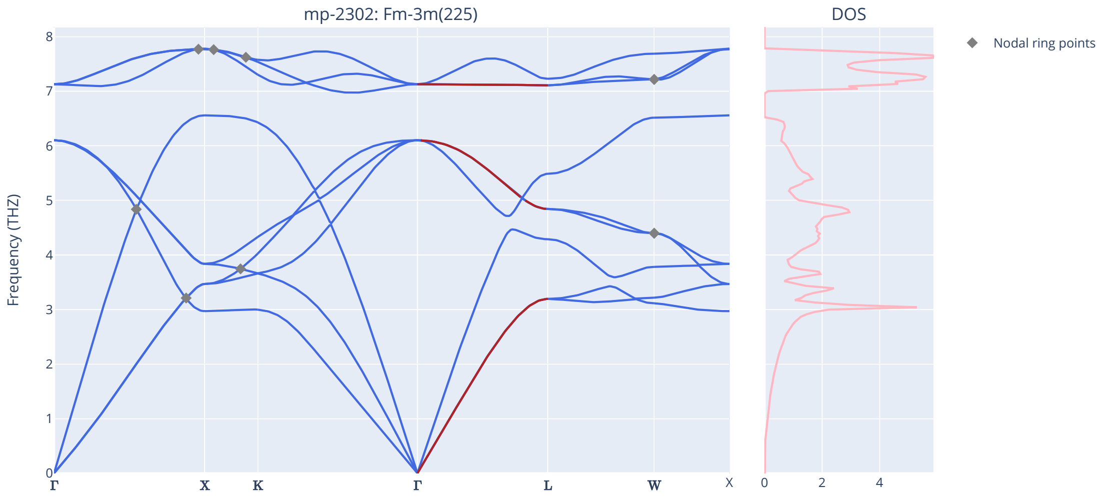

Supplementary Figure 416: The phonon band and DOS of Rh8As4.

Supplementary Table 845: High symmetry lines of mp-2302-Rh8As4

	Start	End
0	Γ (0.000, 0.000, 0.000)	X (0.500, 0.000, 0.500)
1	X (0.500, 0.000, 0.500)	U (0.625, 0.250, 0.625)
2	K (0.375, 0.375, 0.750)	Γ (0.000, 0.000, 0.000)
3	Γ (0.000, 0.000, 0.000)	L (0.500, 0.500, 0.500)
4	L (0.500, 0.500, 0.500)	W (0.500, 0.250, 0.750)
5	W (0.500, 0.250, 0.750)	X (0.500, 0.000, 0.500)

Supplementary Table 846: Nodal ring points along high symmetry lines of mp-2302-Rh8As4.

	Coordinates	Frequency	Mode 1	Mode 2	Multi.	Deg.	Path	Ratio
0	(0.439, 0.000, 0.439)	3.211	3	2	6	2	1	0.878
1	(0.273, -0.001, 0.273)	4.837	4	3	24	2	1	0.546
2	(0.584, 0.169, 0.584)	3.745	4	3	12	2	2	0.676
3	(0.500, 0.250, 0.750)	4.398	5	4	6	2	5	1.000
4	(0.480, 0.000, 0.480)	7.771	8	7	6	2	1	0.959
5	(0.521, 0.043, 0.521)	7.762	8	7	12	2	2	0.171
6	(0.500, 0.250, 0.750)	7.217	8	7	6	2	5	1.000
7	(0.597, 0.194, 0.597)	7.623	9	8	12	2	2	0.776

The followings are details for Mg_8Sn_4. It has 4 topological nodal lines and 5 nodal ring points along high symmetry line as shown in Fig **417**.

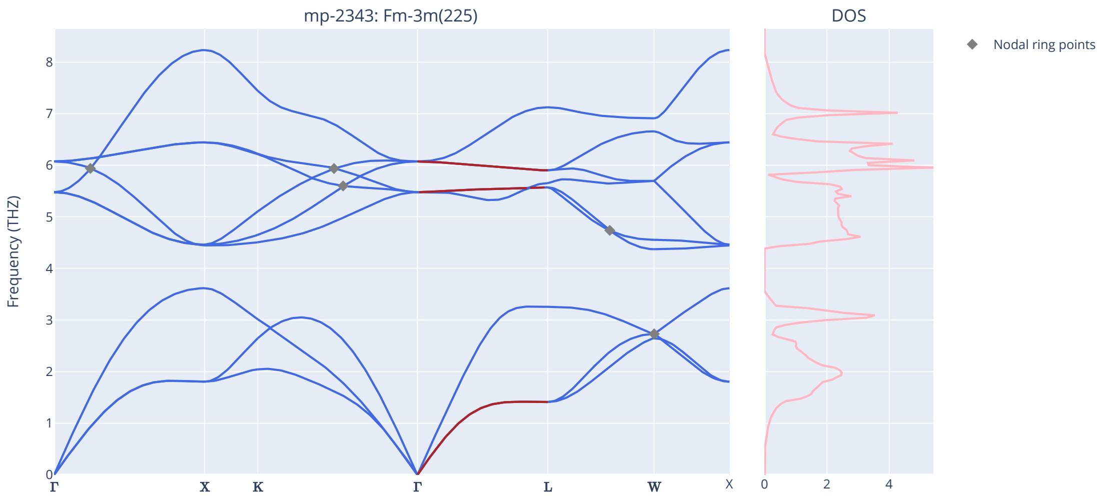

Supplementary Figure 417: The phonon band and DOS of Mg8Sn4.

Supplementary Table 847: High symmetry lines of mp-2343-Mg8Sn4

	Start	End
0	Γ (0.000, 0.000, 0.000)	X (0.500, 0.000, 0.500)
1	X (0.500, 0.000, 0.500)	U (0.625, 0.250, 0.625)
2	K (0.375, 0.375, 0.750)	Γ (0.000, 0.000, 0.000)
3	Γ (0.000, 0.000, 0.000)	L (0.500, 0.500, 0.500)
4	L (0.500, 0.500, 0.500)	W (0.500, 0.250, 0.750)
5	W (0.500, 0.250, 0.750)	X (0.500, 0.000, 0.500)

Supplementary Table 848: Nodal ring points along high symmetry lines of mp-2343-Mg8Sn4.

	Coordinates	Frequency	Mode 1	Mode 2	Multi.	Deg.	Path	Ratio
0	(0.500, 0.250, 0.750)	2.730	3	2	6	2	5	1.000
1	(0.500, 0.354, 0.646)	4.737	5	4	24	2	5	0.583
2	(0.176, 0.176, 0.348)	5.599	6	5	24	2	3	0.535
3	(0.121, -0.000, 0.121)	5.939	7	6	10	2	1	0.241
4	(0.199, 0.199, 0.389)	5.936	8	7	24	2	3	0.478

The followings are details for Pa_4O_8. It has 5 topological nodal lines and 7 nodal ring points along high symmetry line as shown in Fig **418**.

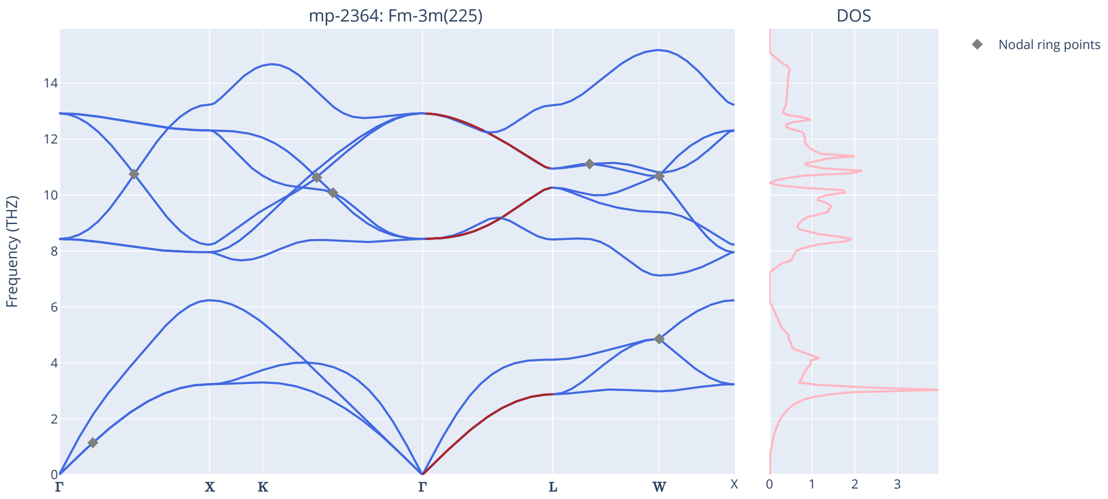

Supplementary Figure 418: The phonon band and DOS of Pa4O8.

Supplementary Table 849: High symmetry lines of mp-2364-Pa4O8

	Start	End
0	Γ (0.000, 0.000, 0.000)	X (0.500, 0.000, 0.500)
1	X (0.500, 0.000, 0.500)	U (0.625, 0.250, 0.625)
2	K (0.375, 0.375, 0.750)	Γ (0.000, 0.000, 0.000)
3	Γ (0.000, 0.000, 0.000)	L (0.500, 0.500, 0.500)
4	L (0.500, 0.500, 0.500)	W (0.500, 0.250, 0.750)
5	W (0.500, 0.250, 0.750)	X (0.500, 0.000, 0.500)

Supplementary Table 850: Nodal ring points along high symmetry lines of mp-2364-Pa4O8.

	Coordinates	Frequency	Mode 1	Mode 2	Multi.	Deg.	Path	Ratio
0	(0.112, 0.000, 0.112)	1.145	2	1	6	2	1	0.224
1	(0.500, 0.250, 0.750)	4.855	3	2	6	2	5	1.000
2	(0.204, 0.204, 0.426)	10.084	6	5	24	2	3	0.439
3	(0.249, -0.001, 0.249)	10.748	7	6	24	2	1	0.498
4	(0.242, 0.254, 0.496)	10.629	7	6	24	2	3	0.338
5	(0.500, 0.250, 0.750)	10.676	7	6	6	2	5	1.000
6	(0.500, 0.413, 0.587)	11.110	8	7	24	2	5	0.347

The followings are details for Co_4Si_8. It has 4 topological nodal lines and 13 nodal ring points along high symmetry line as shown in Fig **419**.

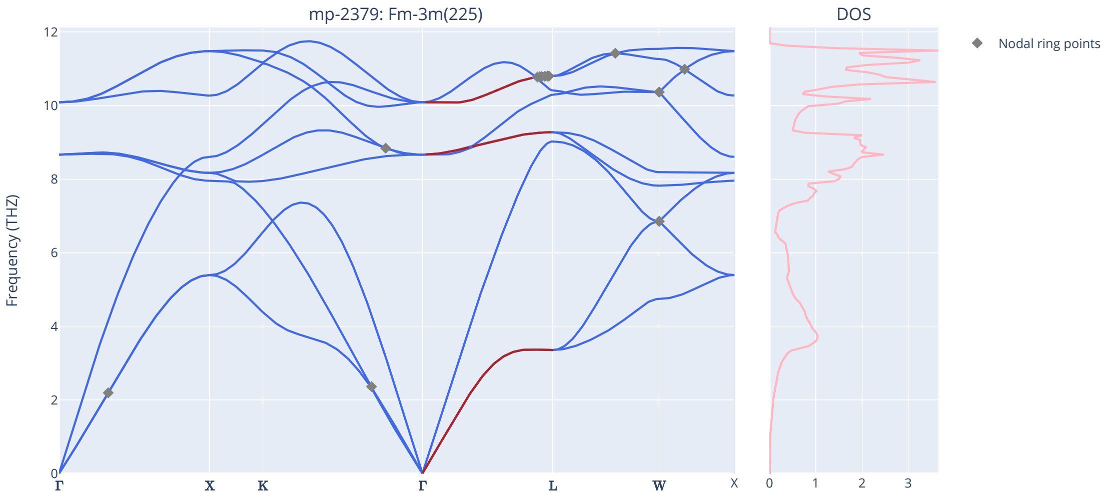

Supplementary Figure 419: The phonon band and DOS of Co4Si8.

Supplementary Table 851: High symmetry lines of mp-2379-Co4Si8

	Start	End
0	Γ (0.000, 0.000, 0.000)	X (0.500, 0.000, 0.500)
1	X (0.500, 0.000, 0.500)	U (0.625, 0.250, 0.625)
2	K (0.375, 0.375, 0.750)	Γ (0.000, 0.000, 0.000)
3	Γ (0.000, 0.000, 0.000)	L (0.500, 0.500, 0.500)
4	L (0.500, 0.500, 0.500)	W (0.500, 0.250, 0.750)
5	W (0.500, 0.250, 0.750)	X (0.500, 0.000, 0.500)

Supplementary Table 852: Nodal ring points along high symmetry lines of mp-2379-Co4Si8.

	Coordinates	Frequency	Mode 1	Mode 2	Multi.	Deg.	Path	Ratio
0	(0.163, 0.000, 0.163)	2.194	2	1	6	2	1	0.327
1	(0.115, 0.115, 0.243)	2.361	2	1	24	2	3	0.681
2	(0.500, 0.250, 0.750)	6.852	3	2	6	2	5	1.000
3	(0.085, 0.085, 0.174)	8.841	6	5	24	2	3	0.770
4	(0.500, 0.250, 0.750)	10.366	7	6	6	2	5	1.000
5	(0.499, 0.166, 0.665)	10.986	8	7	24	2	6	0.338
6	(0.479, 0.479, 0.479)	10.799	9	8	8	2	4	0.959
7	(0.485, 0.485, 0.485)	10.801	9	8	8	2	4	0.971
8	(0.441, 0.441, 0.441)	10.773	9	8	8	2	4	0.882
9	(0.450, 0.450, 0.450)	10.781	9	8	8	2	4	0.900
10	(0.457, 0.457, 0.457)	10.787	9	8	8	2	4	0.915
11	(0.469, 0.469, 0.469)	10.795	9	8	8	2	4	0.939
12	(0.500, 0.353, 0.647)	11.420	9	8	24	2	5	0.588

The followings are details for Ac_4H_8. It has 4 topological nodal lines and 10 nodal ring points along high symmetry line as shown in Fig **420**.

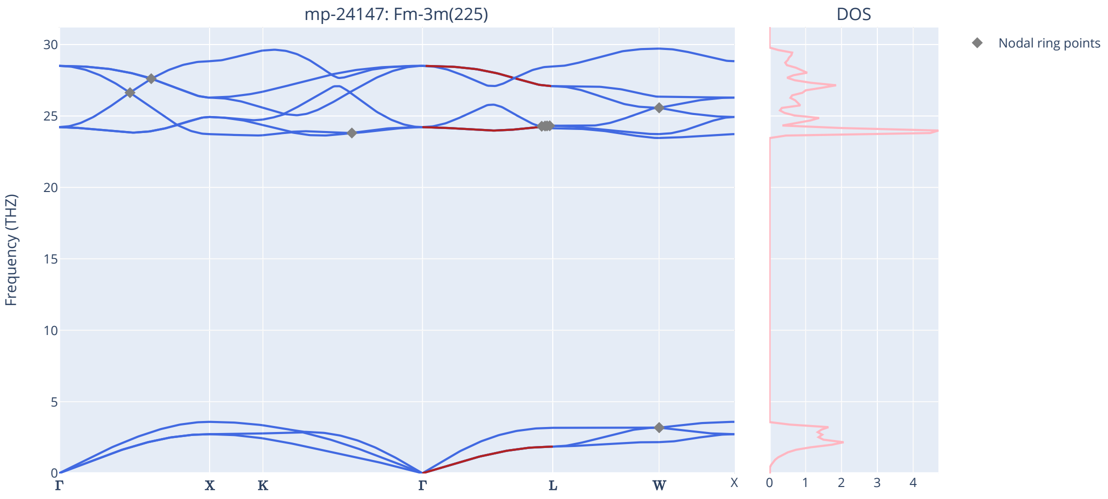

Supplementary Figure 420: The phonon band and DOS of Ac4H8.

Supplementary Table 853: High symmetry lines of mp-24147-Ac4H8

	Start	End
0	Γ (0.000, 0.000, 0.000)	X (0.500, 0.000, 0.500)
1	X (0.500, 0.000, 0.500)	U (0.625, 0.250, 0.625)
2	K (0.375, 0.375, 0.750)	Γ (0.000, 0.000, 0.000)
3	Γ (0.000, 0.000, 0.000)	L (0.500, 0.500, 0.500)
4	L (0.500, 0.500, 0.500)	W (0.500, 0.250, 0.750)
5	W (0.500, 0.250, 0.750)	X (0.500, 0.000, 0.500)

Supplementary Table 854: Nodal ring points along high symmetry lines of mp-24147-Ac4H8.

	Coordinates	Frequency	Mode 1	Mode 2	Multi.	Deg.	Path	Ratio
0	(0.500, 0.250, 0.750)	3.193	3	2	6	2	5	1.000
1	(0.161, 0.161, 0.337)	23.808	5	4	24	2	3	0.558
2	(0.470, 0.470, 0.470)	24.297	6	5	8	2	4	0.941
3	(0.458, 0.458, 0.458)	24.280	6	5	8	2	4	0.915
4	(0.478, 0.478, 0.478)	24.306	6	5	8	2	4	0.956
5	(0.488, 0.488, 0.488)	24.313	6	5	8	2	4	0.977
6	(0.236, 0.001, 0.236)	26.631	7	6	24	2	1	0.471
7	(0.500, 0.250, 0.750)	25.572	7	6	6	2	5	1.000
8	(0.306, 0.000, 0.306)	27.609	9	8	6	2	1	0.612

The followings are details for La_4H_8. It has 4 topological nodal lines and 3 nodal ring points along high symmetry line as shown in Fig **421**.

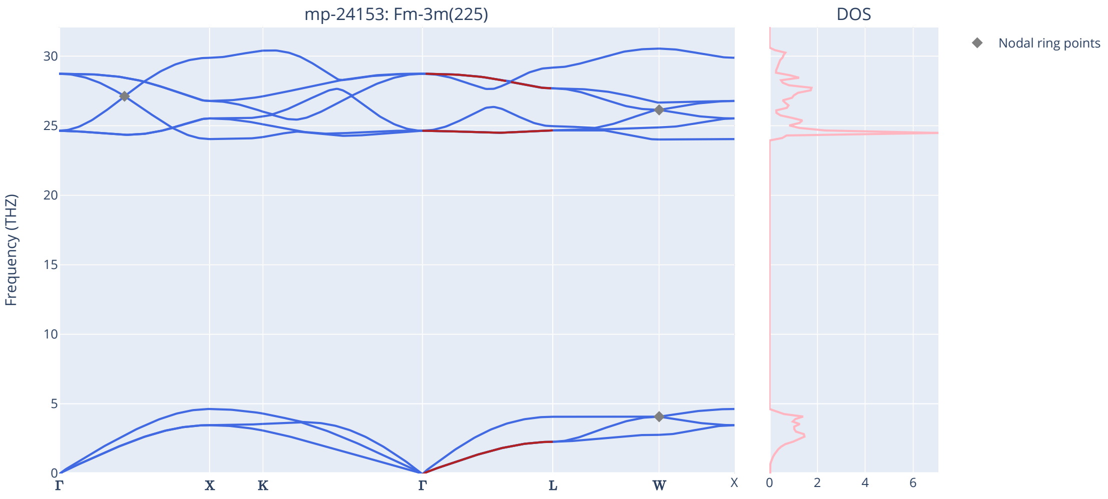

Supplementary Figure 421: The phonon band and DOS of La4H8.

Supplementary Table 855: High symmetry lines of mp-24153-La4H8

	Start	End
0	Γ (0.000, 0.000, 0.000)	X (0.500, 0.000, 0.500)
1	X (0.500, 0.000, 0.500)	U (0.625, 0.250, 0.625)
2	K (0.375, 0.375, 0.750)	Γ (0.000, 0.000, 0.000)
3	Γ (0.000, 0.000, 0.000)	L (0.500, 0.500, 0.500)
4	L (0.500, 0.500, 0.500)	W (0.500, 0.250, 0.750)
5	W (0.500, 0.250, 0.750)	X (0.500, 0.000, 0.500)

Supplementary Table 856: Nodal ring points along high symmetry lines of mp-24153-La4H8.

	Coordinates	Frequency	Mode 1	Mode 2	Multi.	Deg.	Path	Ratio
0	(0.500, 0.250, 0.750)	4.070	3	2	6	2	5	1.000
1	(0.217, -0.000, 0.217)	27.110	7	6	6	2	1	0.435
2	(0.500, 0.250, 0.750)	26.130	7	6	6	2	5	1.000

The followings are details for Nb_4H_8. It has 3 topological nodal lines and 12 nodal ring points along high symmetry line as shown in Fig 422.

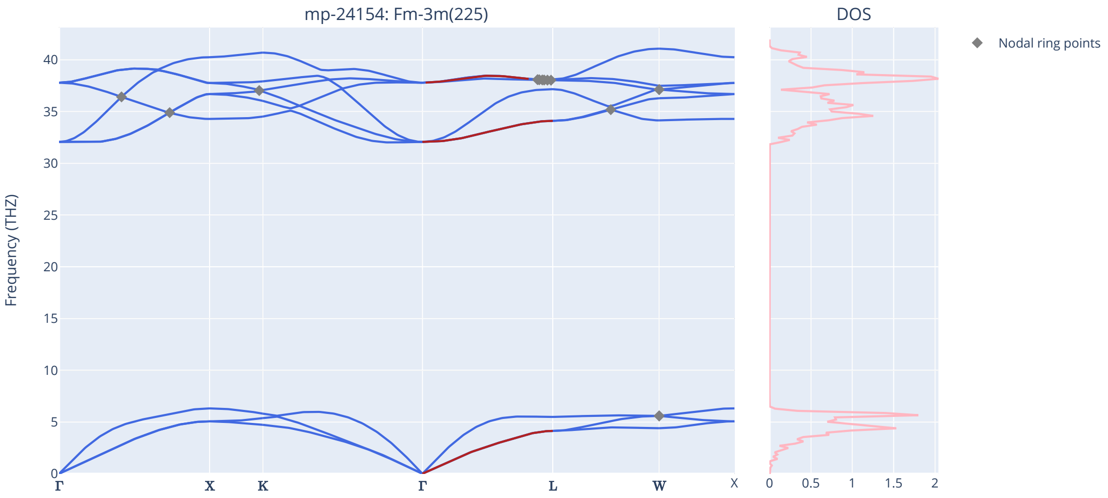

Supplementary Figure 422: The phonon band and DOS of Nb4H8.

Supplementary Table 857: High symmetry lines of mp-24154-Nb4H8

	Start	End
0	Γ (0.000, 0.000, 0.000)	X (0.500, 0.000, 0.500)
1	X (0.500, 0.000, 0.500)	U (0.625, 0.250, 0.625)
2	K (0.375, 0.375, 0.750)	Γ (0.000, 0.000, 0.000)
3	Γ (0.000, 0.000, 0.000)	L (0.500, 0.500, 0.500)
4	L (0.500, 0.500, 0.500)	W (0.500, 0.250, 0.750)
5	W (0.500, 0.250, 0.750)	X (0.500, 0.000, 0.500)

Supplementary Table 858: Nodal ring points along high symmetry lines of mp-24154-Nb4H8.

	Coordinates	Frequency	Mode 1	Mode 2	Multi.	Deg.	Path	Ratio
0	(0.500, 0.250, 0.750)	5.590	3	2	6	2	5	1.000
1	(0.500, 0.364, 0.636)	35.179	5	4	24	2	5	0.545
2	(0.367, 0.000, 0.367)	34.892	6	5	6	2	1	0.735
3	(0.207, 0.000, 0.207)	36.415	7	6	6	2	1	0.415
4	(0.617, 0.233, 0.617)	37.026	7	6	12	2	2	0.933
5	(0.500, 0.250, 0.750)	37.119	7	6	6	2	5	1.000
6	(0.493, 0.493, 0.493)	38.027	8	7	8	2	4	0.987
7	(0.467, 0.467, 0.467)	38.044	8	7	8	2	4	0.934
8	(0.442, 0.442, 0.442)	38.080	8	7	8	2	4	0.883
9	(0.448, 0.448, 0.448)	38.069	8	7	8	2	4	0.897
10	(0.460, 0.460, 0.460)	38.052	8	7	8	2	4	0.920
11	(0.480, 0.480, 0.480)	38.033	8	7	8	2	4	0.959

The followings are details for Cr_4H_8. It has 3 topological nodal lines and 10 nodal ring points along high symmetry line as shown in Fig **423**.

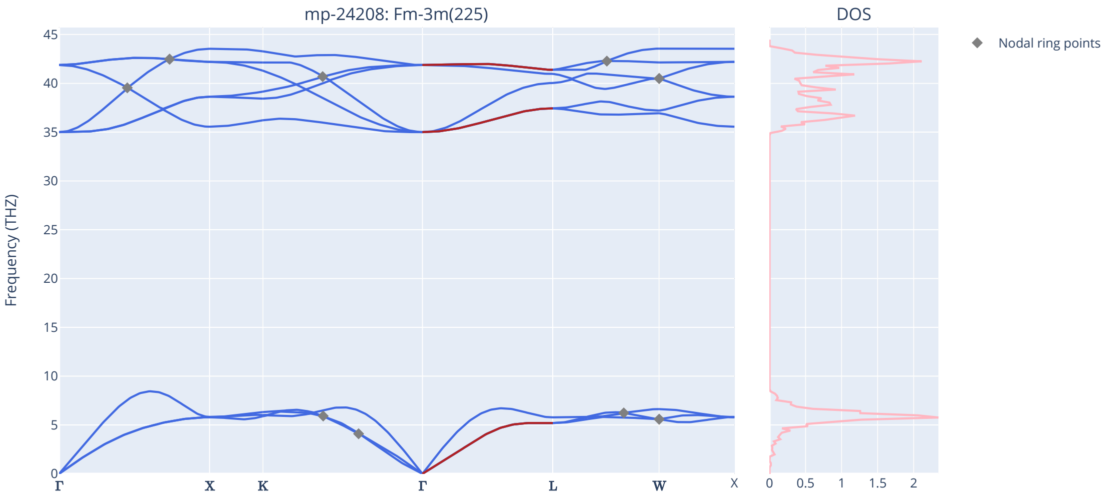

Supplementary Figure 423: The phonon band and DOS of Cr4H8.

Supplementary Table 859: High symmetry lines of mp-24208-Cr4H8

	Start	End
0	Γ (0.000, 0.000, 0.000)	X (0.500, 0.000, 0.500)
1	X (0.500, 0.000, 0.500)	U (0.625, 0.250, 0.625)
2	K (0.375, 0.375, 0.750)	Γ (0.000, 0.000, 0.000)
3	Γ (0.000, 0.000, 0.000)	L (0.500, 0.500, 0.500)
4	L (0.500, 0.500, 0.500)	W (0.500, 0.250, 0.750)
5	W (0.500, 0.250, 0.750)	X (0.500, 0.000, 0.500)

Supplementary Table 860: Nodal ring points along high symmetry lines of mp-24208-Cr4H8.

	Coordinates	Frequency	Mode 1	Mode 2	Multi.	Deg.	Path	Ratio
0	(0.230, 0.230, 0.470)	5.915	2	1	24	2	3	0.378
1	(0.153, 0.153, 0.297)	4.096	2	1	24	2	3	0.600
2	(0.500, 0.250, 0.750)	5.586	2	1	6	2	5	1.000
3	(0.500, 0.333, 0.667)	6.230	3	2	24	2	5	0.667
4	(0.227, -0.002, 0.227)	39.536	7	6	24	2	1	0.453
5	(0.500, 0.250, 0.750)	40.488	7	6	6	2	5	1.000
6	(0.367, 0.000, 0.367)	42.468	8	7	6	2	1	0.735
7	(0.363, -0.000, 0.371)	42.469	8	7	24	2	1	0.734
8	(0.237, 0.237, 0.466)	40.683	8	7	24	2	3	0.375
9	(0.500, 0.373, 0.627)	42.272	9	8	24	2	5	0.508

The followings are details for Sc_4H_8. It has 4 topological nodal lines and 5 nodal ring points along high symmetry line as shown in Fig 424.

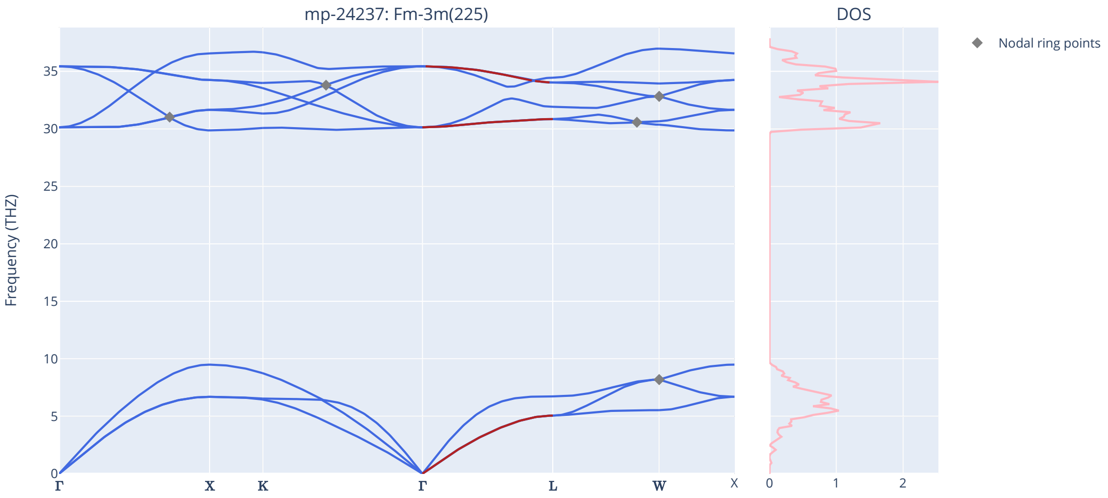

Supplementary Figure 424: The phonon band and DOS of Sc4H8.

Supplementary Table 861: High symmetry lines of mp-24237-Sc4H8

	Start	End
0	Γ (0.000, 0.000, 0.000)	X (0.500, 0.000, 0.500)
1	X (0.500, 0.000, 0.500)	U (0.625, 0.250, 0.625)
2	K (0.375, 0.375, 0.750)	Γ (0.000, 0.000, 0.000)
3	Γ (0.000, 0.000, 0.000)	L (0.500, 0.500, 0.500)
4	L (0.500, 0.500, 0.500)	W (0.500, 0.250, 0.750)
5	W (0.500, 0.250, 0.750)	X (0.500, 0.000, 0.500)

Supplementary Table 862: Nodal ring points along high symmetry lines of mp-24237-Sc4H8.

	Coordinates	Frequency	Mode 1	Mode 2	Multi.	Deg.	Path	Ratio
0	(0.500, 0.250, 0.750)	8.189	3	2	6	2	5	1.000
1	(0.500, 0.302, 0.698)	30.572	5	4	24	2	5	0.791
2	(0.367, 0.000, 0.367)	31.019	6	5	6	2	1	0.735
3	(0.500, 0.250, 0.750)	32.822	7	6	6	2	5	1.000
4	(0.222, 0.222, 0.457)	33.795	8	7	24	2	3	0.396

The followings are details for Y_4H_8. It has 4 topological nodal lines and 6 nodal ring points along high symmetry line as shown in Fig **425**.

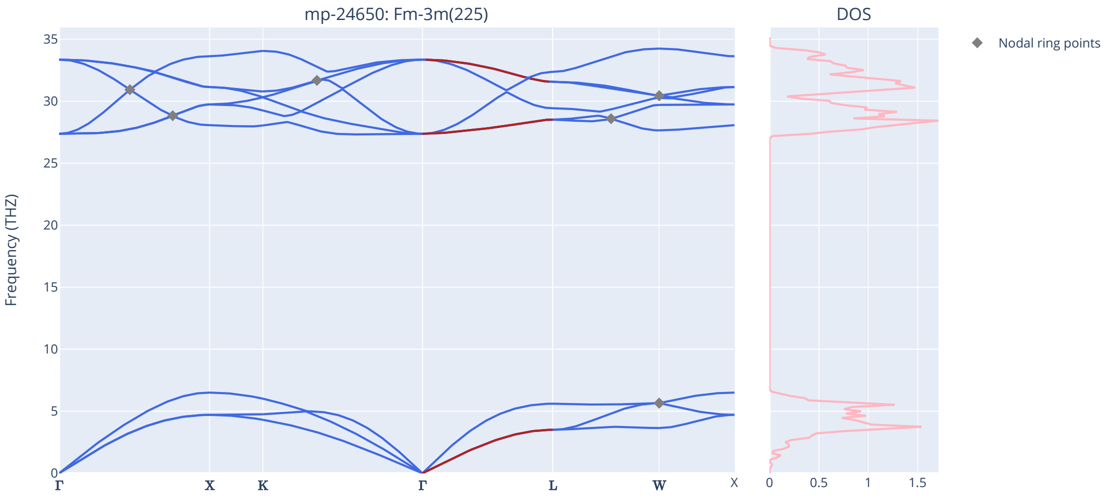

Supplementary Figure 425: The phonon band and DOS of Y4H8.

Supplementary Table 863: High symmetry lines of mp-24650-Y4H8

	Start	End
0	Γ (0.000, 0.000, 0.000)	X (0.500, 0.000, 0.500)
1	X (0.500, 0.000, 0.500)	U (0.625, 0.250, 0.625)
2	K (0.375, 0.375, 0.750)	Γ (0.000, 0.000, 0.000)
3	Γ (0.000, 0.000, 0.000)	L (0.500, 0.500, 0.500)
4	L (0.500, 0.500, 0.500)	W (0.500, 0.250, 0.750)
5	W (0.500, 0.250, 0.750)	X (0.500, 0.000, 0.500)

Supplementary Table 864: Nodal ring points along high symmetry lines of mp-24650-Y4H8.

	Coordinates	Frequency	Mode 1	Mode 2	Multi.	Deg.	Path	Ratio
0	(0.500, 0.250, 0.750)	5.656	3	2	6	2	5	1.000
1	(0.500, 0.363, 0.637)	28.592	5	4	24	2	5	0.548
2	(0.378, 0.000, 0.378)	28.833	6	5	6	2	1	0.755
3	(0.235, -0.001, 0.235)	30.930	7	6	10	2	1	0.470
4	(0.253, 0.253, 0.491)	31.678	8	7	24	2	3	0.339
5	(0.500, 0.250, 0.750)	30.442	8	7	6	2	5	1.000

The followings are details for Ta_4H_8. It has 4 topological nodal lines and 8 nodal ring points along high symmetry line as shown in Fig **426**.

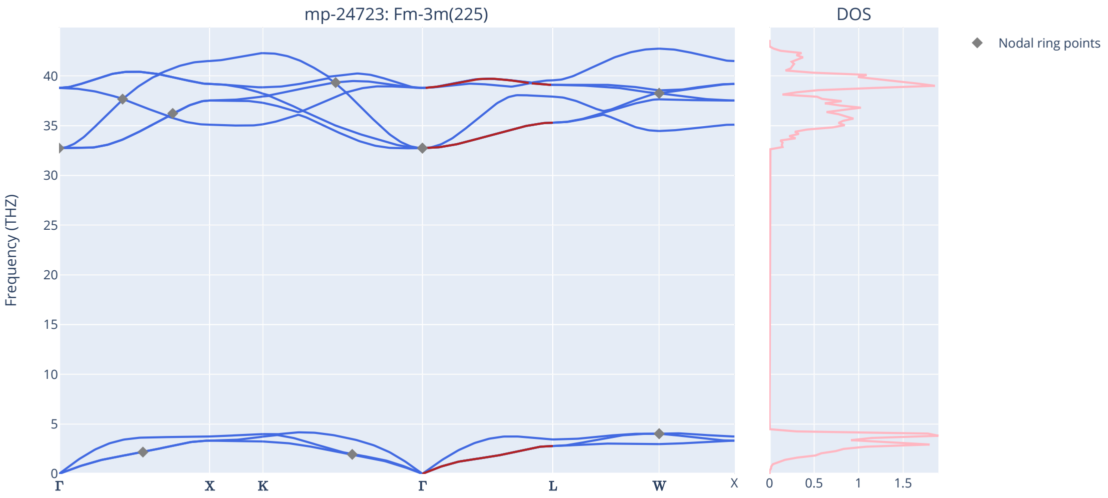

Supplementary Figure 426: The phonon band and DOS of Ta4H8.

Supplementary Table 865: High symmetry lines of mp-24723-Ta4H8

	Start	End
0	Γ (0.000, 0.000, 0.000)	X (0.500, 0.000, 0.500)
1	X (0.500, 0.000, 0.500)	U (0.625, 0.250, 0.625)
2	K (0.375, 0.375, 0.750)	Γ (0.000, 0.000, 0.000)
3	Γ (0.000, 0.000, 0.000)	L (0.500, 0.500, 0.500)
4	L (0.500, 0.500, 0.500)	W (0.500, 0.250, 0.750)
5	W (0.500, 0.250, 0.750)	X (0.500, 0.000, 0.500)

Supplementary Table 866: Nodal ring points along high symmetry lines of mp-24723-Ta4H8.

	Coordinates	Frequency	Mode 1	Mode 2	Multi.	Deg.	Path	Ratio
0	(0.284, 0.000, 0.274)	2.179	2	1	24	2	1	0.557
1	(0.168, 0.168, 0.326)	1.947	2	1	24	2	3	0.560
2	(0.500, 0.250, 0.750)	4.027	3	2	6	2	5	1.000
3	(0.000, 0.000, 0.000)	32.742	5	4	1	3	1	0.000
4	(0.378, 0.000, 0.378)	36.235	6	5	6	2	1	0.755
5	(0.211, -0.001, 0.211)	37.674	7	6	24	2	1	0.422
6	(0.500, 0.250, 0.750)	38.257	7	6	6	2	5	1.000
7	(0.199, 0.199, 0.413)	39.335	8	7	24	2	3	0.456

The followings are details for V_4H_8. It has 3 topological nodal lines and 8 nodal ring points along high symmetry line as shown in Fig 427.

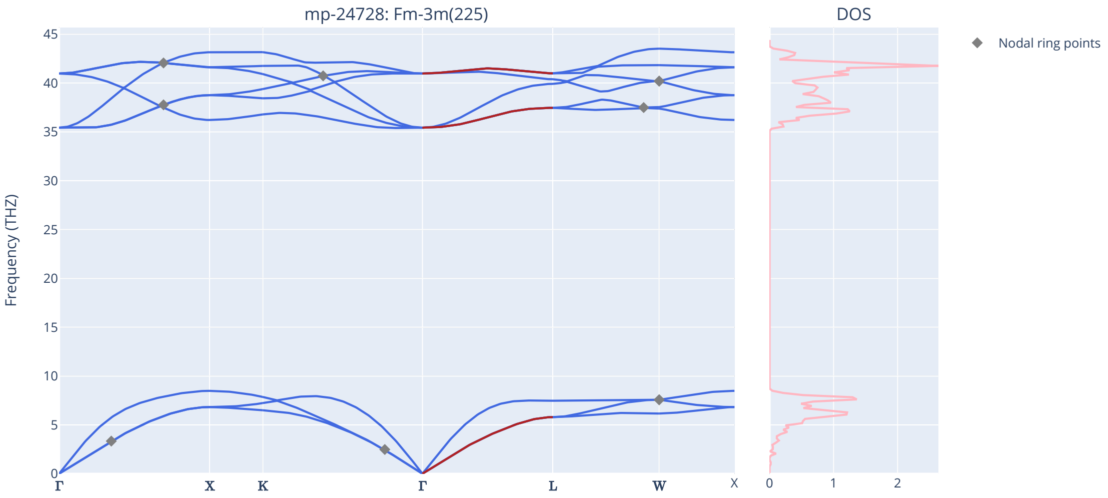

Supplementary Figure 427: The phonon band and DOS of V4H8.

Supplementary Table 867: High symmetry lines of mp-24728-V4H8

	Start	End
0	Γ (0.000, 0.000, 0.000)	X (0.500, 0.000, 0.500)
1	X (0.500, 0.000, 0.500)	U (0.625, 0.250, 0.625)
2	K (0.375, 0.375, 0.750)	Γ (0.000, 0.000, 0.000)
3	Γ (0.000, 0.000, 0.000)	L (0.500, 0.500, 0.500)
4	L (0.500, 0.500, 0.500)	W (0.500, 0.250, 0.750)
5	W (0.500, 0.250, 0.750)	X (0.500, 0.000, 0.500)

Supplementary Table 868: Nodal ring points along high symmetry lines of mp-24728-V4H8.

	Coordinates	Frequency	Mode 1	Mode 2	Multi.	Deg.	Path	Ratio
0	(0.173, 0.000, 0.173)	3.342	2	1	6	2	1	0.347
1	(0.084, 0.084, 0.181)	2.484	2	1	24	2	3	0.764
2	(0.500, 0.250, 0.750)	7.575	3	2	6	2	5	1.000
3	(0.500, 0.286, 0.714)	37.489	5	4	24	2	5	0.854
4	(0.347, 0.000, 0.347)	37.767	6	5	6	2	1	0.694
5	(0.500, 0.250, 0.750)	40.194	7	6	6	2	5	1.000
6	(0.347, 0.000, 0.347)	42.055	8	7	6	2	1	0.694
7	(0.237, 0.237, 0.462)	40.735	8	7	24	2	3	0.378

The followings are details for Np_4O_8. It has 4 topological nodal lines and 8 nodal ring points along high symmetry line as shown in Fig **428**.

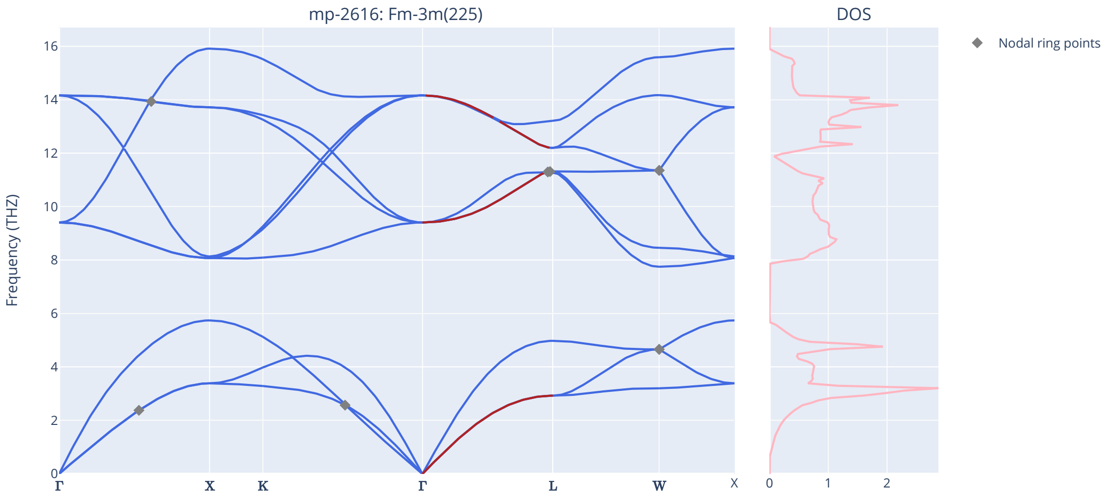

Supplementary Figure 428: The phonon band and DOS of Np4O8.

Supplementary Table 869: High symmetry lines of mp-2616-Np4O8

	Start	End
0	Γ (0.000, 0.000, 0.000)	X (0.500, 0.000, 0.500)
1	X (0.500, 0.000, 0.500)	U (0.625, 0.250, 0.625)
2	K (0.375, 0.375, 0.750)	Γ (0.000, 0.000, 0.000)
3	Γ (0.000, 0.000, 0.000)	L (0.500, 0.500, 0.500)
4	L (0.500, 0.500, 0.500)	W (0.500, 0.250, 0.750)
5	W (0.500, 0.250, 0.750)	X (0.500, 0.000, 0.500)

Supplementary Table 870: Nodal ring points along high symmetry lines of mp-2616-Np4O8.

	Coordinates	Frequency	Mode 1	Mode 2	Multi.	Deg.	Path	Ratio
0	(0.265, 0.000, 0.265)	2.374	2	1	6	2	1	0.531
1	(0.184, 0.184, 0.361)	2.568	2	1	24	2	3	0.515
2	(0.500, 0.250, 0.750)	4.656	3	2	6	2	5	1.000
3	(0.490, 0.490, 0.490)	11.311	6	5	8	2	4	0.979
4	(0.481, 0.481, 0.481)	11.296	6	5	8	2	4	0.962
5	(0.500, 0.250, 0.750)	11.354	7	6	6	2	5	1.000
6	(0.306, 0.000, 0.306)	13.934	8	7	6	2	1	0.612

The followings are details for Al_8Au_4. It has 5 topological nodal lines and 9 nodal ring points along high symmetry line as shown in Fig **429**.

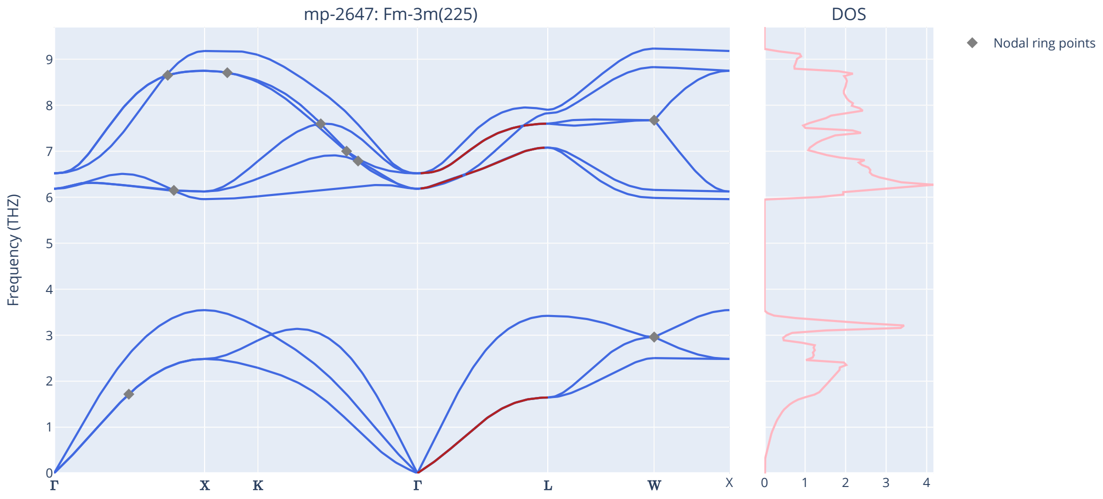

Supplementary Figure 429: The phonon band and DOS of Al8Au4.

Supplementary Table 871: High symmetry lines of mp-2647-Al8Au4

	Start	End
0	Γ (0.000, 0.000, 0.000)	X (0.500, 0.000, 0.500)
1	X (0.500, 0.000, 0.500)	U (0.625, 0.250, 0.625)
2	K (0.375, 0.375, 0.750)	Γ (0.000, 0.000, 0.000)
3	Γ (0.000, 0.000, 0.000)	L (0.500, 0.500, 0.500)
4	L (0.500, 0.500, 0.500)	W (0.500, 0.250, 0.750)
5	W (0.500, 0.250, 0.750)	X (0.500, 0.000, 0.500)

Supplementary Table 872: Nodal ring points along high symmetry lines of mp-2647-Al8Au4.

	Coordinates	Frequency	Mode 1	Mode 2	Multi.	Deg.	Path	Ratio
0	(0.243, 0.000, 0.254)	1.720	2	1	24	2	1	0.497
1	(0.500, 0.250, 0.750)	2.962	3	2	6	2	5	1.000
2	(0.398, 0.000, 0.398)	6.151	6	5	6	2	1	0.796
3	(0.138, 0.138, 0.280)	6.790	6	5	24	2	3	0.628
4	(0.168, 0.168, 0.331)	7.003	7	6	24	2	3	0.556
5	(0.500, 0.250, 0.750)	7.676	7	6	6	2	5	1.000
6	(0.229, 0.225, 0.454)	7.598	8	7	24	2	3	0.395
7	(0.378, 0.000, 0.378)	8.654	8	7	6	2	1	0.755
8	(0.553, 0.107, 0.553)	8.710	8	7	12	2	2	0.427

The followings are details for Rh_8P_4. It has 4 topological nodal lines and 9 nodal ring points along high symmetry line as shown in Fig 430.

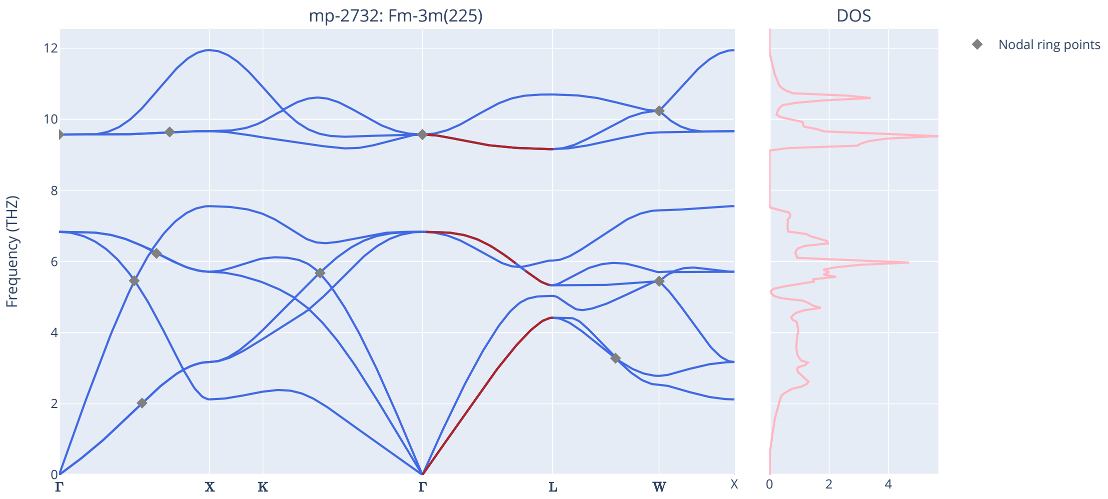

Supplementary Figure 430: The phonon band and DOS of Rh8P4.

Supplementary Table 873: High symmetry lines of mp-2732-Rh8P4

	Start	End
0	Γ (0.000, 0.000, 0.000)	X (0.500, 0.000, 0.500)
1	X (0.500, 0.000, 0.500)	U (0.625, 0.250, 0.625)
2	K (0.375, 0.375, 0.750)	Γ (0.000, 0.000, 0.000)
3	Γ (0.000, 0.000, 0.000)	L (0.500, 0.500, 0.500)
4	L (0.500, 0.500, 0.500)	W (0.500, 0.250, 0.750)
5	W (0.500, 0.250, 0.750)	X (0.500, 0.000, 0.500)

Supplementary Table 874: Nodal ring points along high symmetry lines of mp-2732-Rh8P4.

	Coordinates	Frequency	Mode 1	Mode 2	Multi.	Deg.	Path	Ratio
0	(0.276, 0.000, 0.276)	2.017	2	1	6	2	1	0.551
1	(0.500, 0.352, 0.648)	3.278	2	1	24	2	5	0.590
2	(0.250, -0.002, 0.250)	5.458	4	3	24	2	1	0.499
3	(0.500, 0.250, 0.750)	5.444	4	3	6	2	5	1.000
4	(0.324, -0.004, 0.324)	6.227	5	4	24	2	1	0.647
5	(0.237, 0.237, 0.484)	5.674	5	4	24	2	3	0.359
6	(0.000, 0.000, 0.000)	9.569	8	7	1	3	1	0.000
7	(0.367, 0.000, 0.367)	9.642	8	7	6	2	1	0.735
8	(0.500, 0.250, 0.750)	10.235	9	8	6	2	5	1.000

The followings are details for $Bi_4Mg_4Ni_4$. As shown in Fig **431**, it has 3 topological nodal lines and 5 nodal ring points. At the same time, it has 1 single Weyl point and 1 high degenerate Weyl point whose degeneracy is more than 2 and charge is bigger than 1.

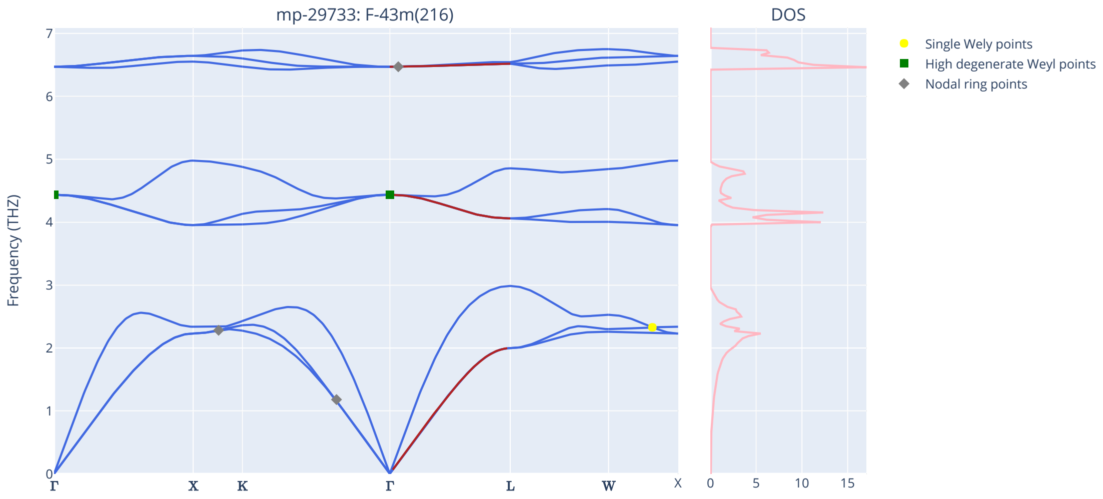

Supplementary Figure 431: The phonon band and DOS of Bi4Mg4Ni4.

Supplementary Table 875: High symmetry lines of mp-29733-Bi4Mg4Ni4

	Start	End
0	Γ (0.000, 0.000, 0.000)	X (0.500, 0.000, 0.500)
1	X (0.500, 0.000, 0.500)	U (0.625, 0.250, 0.625)
2	K (0.375, 0.375, 0.750)	Γ (0.000, 0.000, 0.000)
3	Γ (0.000, 0.000, 0.000)	L (0.500, 0.500, 0.500)
4	L (0.500, 0.500, 0.500)	W (0.500, 0.250, 0.750)
5	W (0.500, 0.250, 0.750)	X (0.500, 0.000, 0.500)

Supplementary Table 876: Single Weyl points along high symmetry lines of mp-29733-Bi4Mg4Ni4.

	Coordinates	Frequency	Mode 1	Mode 2	Multi.	Deg.	Path	Ratio
0	(0.500, 0.092, 0.592)	2.329	3	2	12	2	6	0.631

Supplementary Table 877: High degenerate Weyl points along high symmetry lines of mp-29733-Bi4Mg4Ni4.

	Coordinates	Frequency	Mode 1	Mode 2	Multi.	Deg.	Path	Ratio
0	(0.000, 0.000, 0.000)	4.437	6	4	1	3	1	0.000

Supplementary Table 878: Nodal ring points along high symmetry lines of mp-29733-Bi4Mg4Ni4.

	Coordinates	Frequency	Mode 1	Mode 2	Multi.	Deg.	Path	Ratio
0	(0.567, 0.128, 0.567)	2.282	2	1	24	2	2	0.518
1	(0.130, 0.130, 0.276)	1.180	2	1	24	2	3	0.639
2	(0.036, 0.036, 0.034)	6.471	9	8	24	2	4	0.071

The followings are details for $Bi_4Ni_4Sc_4$. As shown in Fig **432**, it has 3 topological nodal lines and 2 nodal ring points. At the same time, it has 2 single Weyl points and 1 high degenerate Weyl point whose degeneracy is more than 2 and charge is bigger than 1.

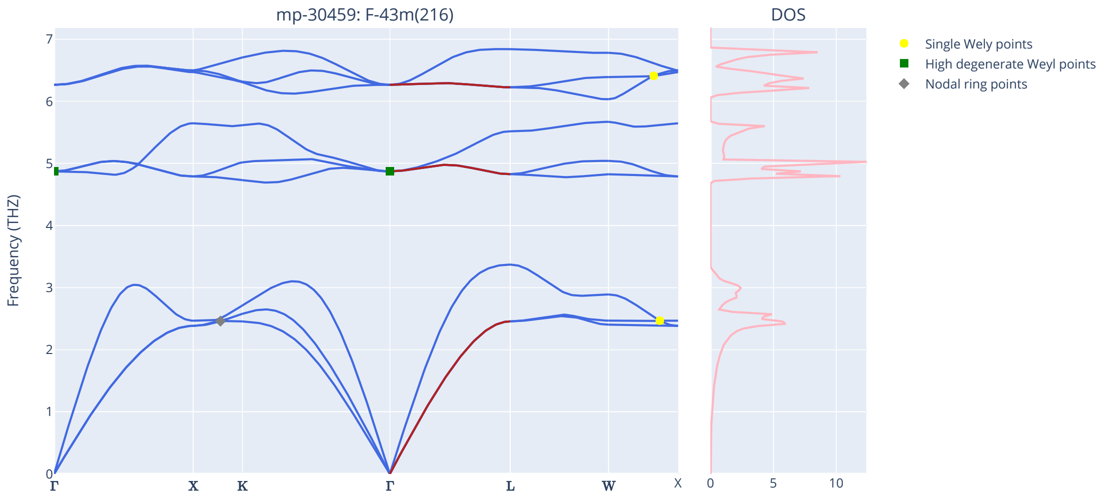

Supplementary Figure 432: The phonon band and DOS of Bi4Ni4Sc4.

Supplementary Table 879: High symmetry lines of mp-30459-Bi4Ni4Sc4

	Start	End
0	Γ (0.000, 0.000, 0.000)	X (0.500, 0.000, 0.500)
1	X (0.500, 0.000, 0.500)	U (0.625, 0.250, 0.625)
2	K (0.375, 0.375, 0.750)	Γ (0.000, 0.000, 0.000)
3	Γ (0.000, 0.000, 0.000)	L (0.500, 0.500, 0.500)
4	L (0.500, 0.500, 0.500)	W (0.500, 0.250, 0.750)
5	W (0.500, 0.250, 0.750)	X (0.500, 0.000, 0.500)

Supplementary Table 880: Single Weyl points along high symmetry lines of mp-30459-Bi4Ni4Sc4.

	Coordinates	Frequency	Mode 1	Mode 2	Multi.	Deg.	Path	Ratio
0	(0.500, 0.066, 0.566)	2.467	3	2	12	2	6	0.738
1	(0.500, 0.087, 0.587)	6.411	8	7	12	2	6	0.650

Supplementary Table 881: High degenerate Weyl points along high symmetry lines of mp-30459-Bi4Ni4Sc4.

	Coordinates	Frequency	Mode 1	Mode 2	Multi.	Deg.	Path	Ratio
0	(0.000, 0.000, 0.000)	4.872	6	4	1	3	1	0.000

Supplementary Table 882: Nodal ring points along high symmetry lines of mp-30459-Bi4Ni4Sc4.

	Coordinates	Frequency	Mode 1	Mode 2	Multi.	Deg.	Path	Ratio
0	(0.569, 0.140, 0.569)	2.460	2	1	24	2	2	0.556

The followings are details for $Bi_4Ni_4Y_4$. As shown in Fig 433, it has 3 topological nodal lines and 4 nodal ring points. At the same time, it has 1 single Weyl point and 1 high degenerate Weyl point whose degeneracy is more than 2 and charge is bigger than 1.

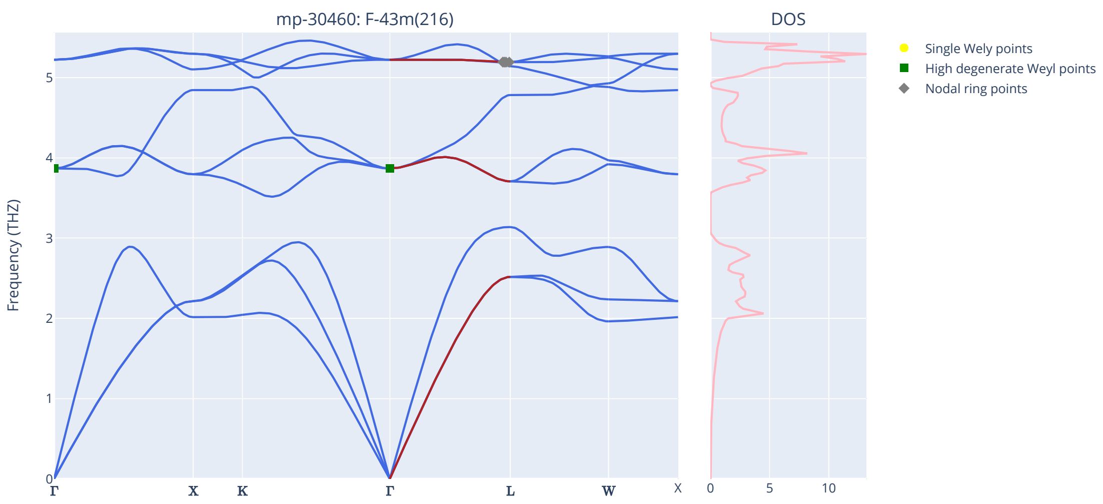

Supplementary Figure 433: The phonon band and DOS of Bi4Ni4Y4.

Supplementary Table 883: High symmetry lines of mp-30460-Bi4Ni4Y4

	Start	End
0	Γ (0.000, 0.000, 0.000)	X (0.500, 0.000, 0.500)
1	X (0.500, 0.000, 0.500)	U (0.625, 0.250, 0.625)
2	K (0.375, 0.375, 0.750)	Γ (0.000, 0.000, 0.000)
3	Γ (0.000, 0.000, 0.000)	L (0.500, 0.500, 0.500)
4	L (0.500, 0.500, 0.500)	W (0.500, 0.250, 0.750)
5	W (0.500, 0.250, 0.750)	X (0.500, 0.000, 0.500)

Supplementary Table 884: Single Weyl points along high symmetry lines of mp-30460-Bi4Ni4Y4.

	Coordinates	Frequency	Mode 1	Mode 2	Multi.	Deg.	Path	Ratio
0	(0.490, 0.490, 0.490)	5.192	9	8	8	2	4	0.980

Supplementary Table 885: High degenerate Weyl points along high symmetry lines of mp-30460-Bi4Ni4Y4.

	Coordinates	Frequency	Mode 1	Mode 2	Multi.	Deg.	Path	Ratio
0	(0.000, 0.000, 0.000)	3.868	6	4	1	3	1	0.000

Supplementary Table 886: Nodal ring points along high symmetry lines of mp-30460-Bi4Ni4Y4.

	Coordinates	Frequency	Mode 1	Mode 2	Multi.	Deg.	Path	Ratio
0	(0.476, 0.476, 0.476)	5.193	9	8	8	2	4	0.951
1	(0.482, 0.482, 0.482)	5.192	9	8	8	2	4	0.965
2	(0.496, 0.496, 0.496)	5.192	9	8	8	2	4	0.992
3	(0.469, 0.469, 0.469)	5.193	9	8	8	2	4	0.939

The followings are details for BiBrTe. It has 3 topological nodal lines and 1 single Weyl point along high symmetry line as shown in Fig **434**.

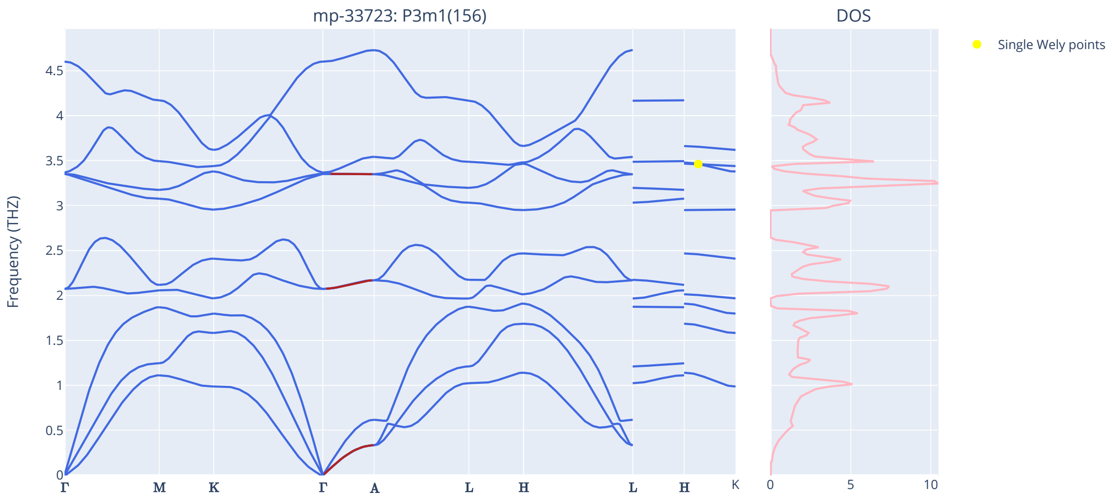

Supplementary Figure 434: The phonon band and DOS of BiBrTe.

Supplementary Table 887: High symmetry lines of mp-33723-BiBrTe

	Start	End
0	Γ (0.000, 0.000, 0.000)	M (0.500, 0.000, 0.000)
1	M (0.500, 0.000, 0.000)	K (0.333, 0.333, 0.000)
2	K (0.333, 0.333, 0.000)	Γ (0.000, 0.000, 0.000)
3	Γ (0.000, 0.000, 0.000)	A (0.000, 0.000, 0.500)
4	A (0.000, 0.000, 0.500)	L (0.500, 0.000, 0.500)
5	L (0.500, 0.000, 0.500)	H (0.333, 0.333, 0.500)
6	H (0.333, 0.333, 0.500)	A (0.000, 0.000, 0.500)
7	L (0.500, 0.000, 0.500)	M (0.500, 0.000, 0.000)
8	H (0.333, 0.333, 0.500)	K (0.333, 0.333, 0.000)

Supplementary Table 888: Single Weyl points along high symmetry lines of mp-33723-BiBrTe.

	Coordinates	Frequency	Mode 1	Mode 2	Multi.	Deg.	Path	Ratio
0	(0.333, 0.333, 0.363)	3.461	8	7	4	2	9	0.274

The followings are details for Mg_8Ge_4. It has 4 topological nodal lines and 8 nodal ring points along high symmetry line as shown in Fig **435**.

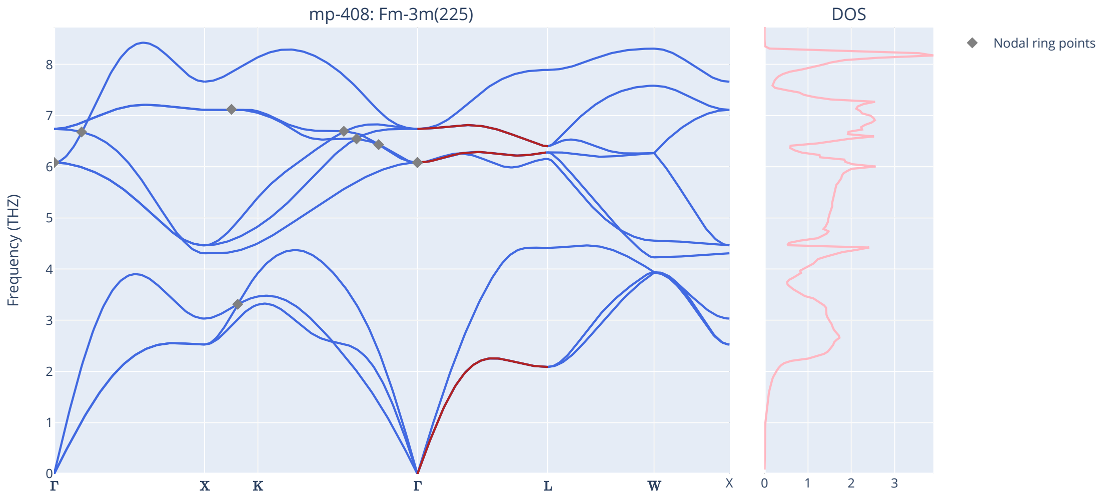

Supplementary Figure 435: The phonon band and DOS of Mg8Ge4.

Supplementary Table 889: High symmetry lines of mp-408-Mg8Ge4

	Start	End
0	Γ (0.000, 0.000, 0.000)	X (0.500, 0.000, 0.500)
1	X (0.500, 0.000, 0.500)	U (0.625, 0.250, 0.625)
2	K (0.375, 0.375, 0.750)	Γ (0.000, 0.000, 0.000)
3	Γ (0.000, 0.000, 0.000)	L (0.500, 0.500, 0.500)
4	L (0.500, 0.500, 0.500)	W (0.500, 0.250, 0.750)
5	W (0.500, 0.250, 0.750)	X (0.500, 0.000, 0.500)

Supplementary Table 890: Nodal ring points along high symmetry lines of mp-408-Mg8Ge4.

	Coordinates	Frequency	Mode 1	Mode 2	Multi.	Deg.	Path	Ratio
0	(0.578, 0.156, 0.578)	3.312	3	2	12	2	2	0.623
1	(0.000, 0.000, 0.000)	6.081	5	4	1	3	1	0.000
2	(0.000, 0.000, 0.000)	6.081	6	5	1	3	1	0.000
3	(0.140, 0.140, 0.289)	6.544	6	5	24	2	3	0.619
4	(0.092, 0.092, 0.182)	6.431	6	5	24	2	3	0.757
5	(0.091, -0.003, 0.091)	6.679	7	6	24	2	1	0.183
6	(0.564, 0.126, 0.564)	7.121	8	7	24	2	2	0.506
7	(0.176, 0.176, 0.342)	6.692	8	7	24	2	3	0.539

The followings are details for $Al_4Be_4B_4$. As shown in Fig **436**, it has 3 topological nodal lines and 4 nodal ring points. At the same time, it has 1 single Weyl point and 1 high degenerate Weyl point whose degeneracy is more than 2 and charge is bigger than 1.

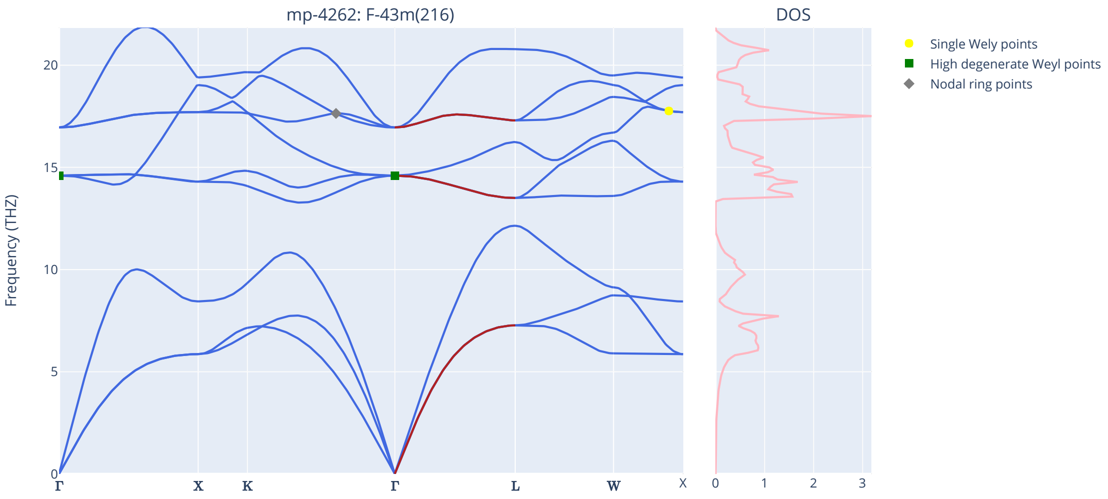

Supplementary Figure 436: The phonon band and DOS of Al4Be4B4.

Supplementary Table 891: High symmetry lines of mp-4262-Al4Be4B4

	Start	End
0	Γ (0.000, 0.000, 0.000)	X (0.500, 0.000, 0.500)
1	X (0.500, 0.000, 0.500)	U (0.625, 0.250, 0.625)
2	K (0.375, 0.375, 0.750)	Γ (0.000, 0.000, 0.000)
3	Γ (0.000, 0.000, 0.000)	L (0.500, 0.500, 0.500)
4	L (0.500, 0.500, 0.500)	W (0.500, 0.250, 0.750)
5	W (0.500, 0.250, 0.750)	X (0.500, 0.000, 0.500)

Supplementary Table 892: Single Weyl points along high symmetry lines of mp-4262-Al4Be4B4.

	Coordinates	Frequency	Mode 1	Mode 2	Multi.	Deg.	Path	Ratio
0	(0.500, 0.051, 0.551)	17.763	7	6	12	2	6	0.796

Supplementary Table 893: High degenerate Weyl points along high symmetry lines of mp-4262-Al4Be4B4.

	Coordinates	Frequency	Mode 1	Mode 2	Multi.	Deg.	Path	Ratio
0	(0.000, 0.000, 0.000)	14.598	6	4	1	3	1	0.000

Supplementary Table 894: Nodal ring points along high symmetry lines of mp-4262-Al4Be4B4.

	Coordinates	Frequency	Mode 1	Mode 2	Multi.	Deg.	Path	Ratio
0	(0.145, 0.145, 0.303)	17.642	8	7	24	2	3	0.602

The followings are details for Rb_8Te_4. It has 4 topological nodal lines and 13 nodal ring points along high symmetry line as shown in Fig **437**.

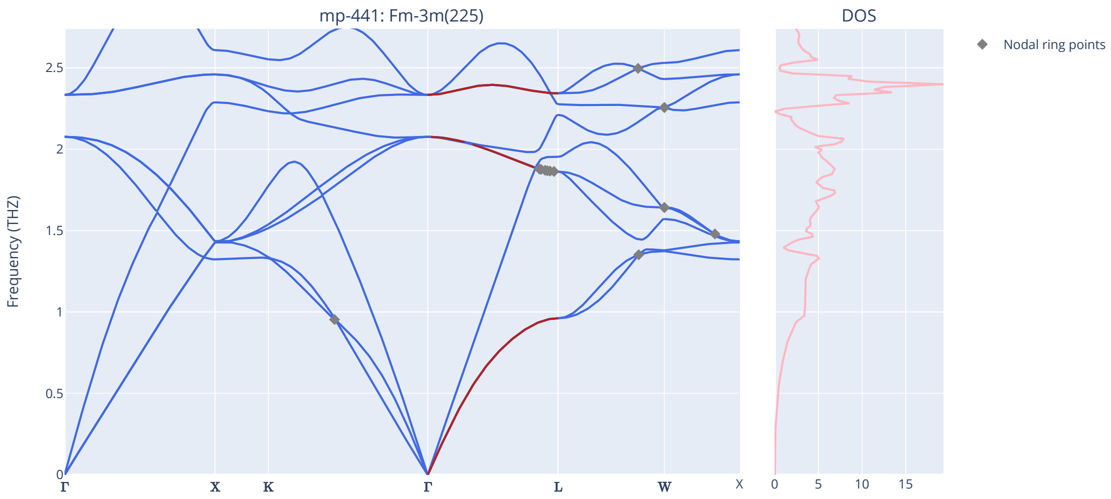

Supplementary Figure 437: The phonon band and DOS of Rb8Te4.

Supplementary Table 895: High symmetry lines of mp-441-Rb8Te4

	Start	End
0	Γ (0.000, 0.000, 0.000)	X (0.500, 0.000, 0.500)
1	X (0.500, 0.000, 0.500)	U (0.625, 0.250, 0.625)
2	K (0.375, 0.375, 0.750)	Γ (0.000, 0.000, 0.000)
3	Γ (0.000, 0.000, 0.000)	L (0.500, 0.500, 0.500)
4	L (0.500, 0.500, 0.500)	W (0.500, 0.250, 0.750)
5	W (0.500, 0.250, 0.750)	X (0.500, 0.000, 0.500)

Supplementary Table 896: Nodal ring points along high symmetry lines of mp-441-Rb8Te4.

	Coordinates	Frequency	Mode 1	Mode 2	Multi.	Deg.	Path	Ratio
0	(0.222, 0.222, 0.435)	0.953	2	1	24	2	3	0.416
1	(0.500, 0.310, 0.690)	1.352	2	1	24	2	5	0.759
2	(0.435, 0.435, 0.435)	1.876	4	3	8	2	4	0.869
3	(0.450, 0.450, 0.450)	1.870	4	3	8	2	4	0.901
4	(0.485, 0.485, 0.485)	1.863	4	3	8	2	4	0.969
5	(0.429, 0.429, 0.429)	1.878	4	3	8	2	4	0.857
6	(0.459, 0.459, 0.459)	1.868	4	3	8	2	4	0.918
7	(0.469, 0.469, 0.469)	1.865	4	3	8	2	4	0.939
8	(0.500, 0.250, 0.750)	1.641	5	4	6	2	5	1.000
9	(0.498, 0.081, 0.583)	1.479	5	4	24	2	6	0.672
10	(0.500, 0.250, 0.750)	2.256	7	6	6	2	5	1.000
11	(0.500, 0.312, 0.688)	2.496	9	8	24	2	5	0.752

The followings are details for $Nb_4Ru_4Sb_4$. It has 3 topological nodal lines and 1 high degenerate Weyl point whose degeneracy is more than 2 and charge is bigger than 1 along high symmetry line as shown in Fig **438**.

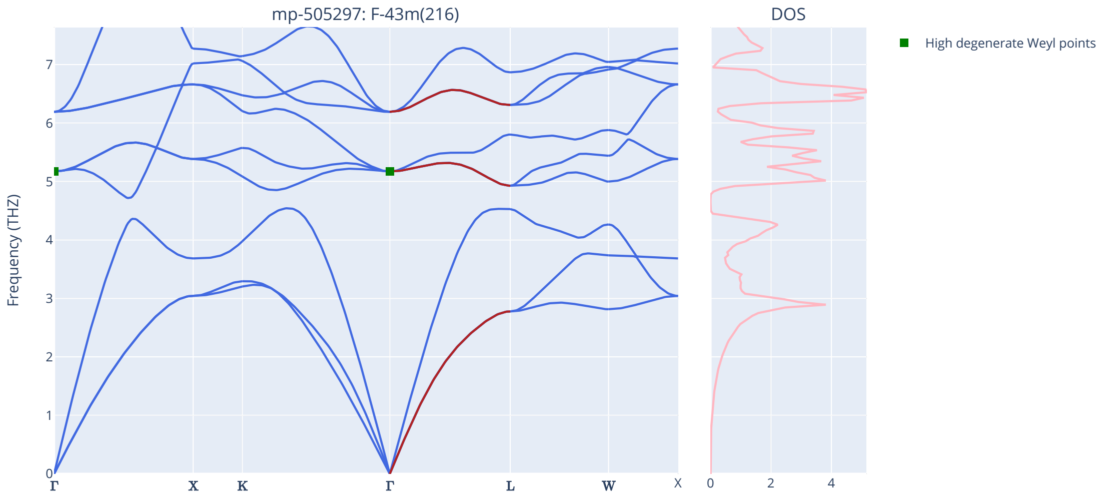

Supplementary Figure 438: The phonon band and DOS of Nb4Ru4Sb4.

Supplementary Table 897: High symmetry lines of mp-505297-Nb4Ru4Sb4

	Start	End
0	Γ (0.000, 0.000, 0.000)	X (0.500, 0.000, 0.500)
1	X (0.500, 0.000, 0.500)	U (0.625, 0.250, 0.625)
2	K (0.375, 0.375, 0.750)	Γ (0.000, 0.000, 0.000)
3	Γ (0.000, 0.000, 0.000)	L (0.500, 0.500, 0.500)
4	L (0.500, 0.500, 0.500)	W (0.500, 0.250, 0.750)
5	W (0.500, 0.250, 0.750)	X (0.500, 0.000, 0.500)

Supplementary Table 898: High degenerate Weyl points along high symmetry lines of mp-505297-Nb4Ru4Sb4.

	Coordinates	Frequency	Mode 1	Mode 2	Multi.	Deg.	Path	Ratio
0	(0.000, 0.000, 0.000)	5.172	6	4	1	3	1	0.000

The followings are details for Ce_4H_8. It has 3 topological nodal lines and 5 nodal ring points along high symmetry line as shown in Fig **439**.

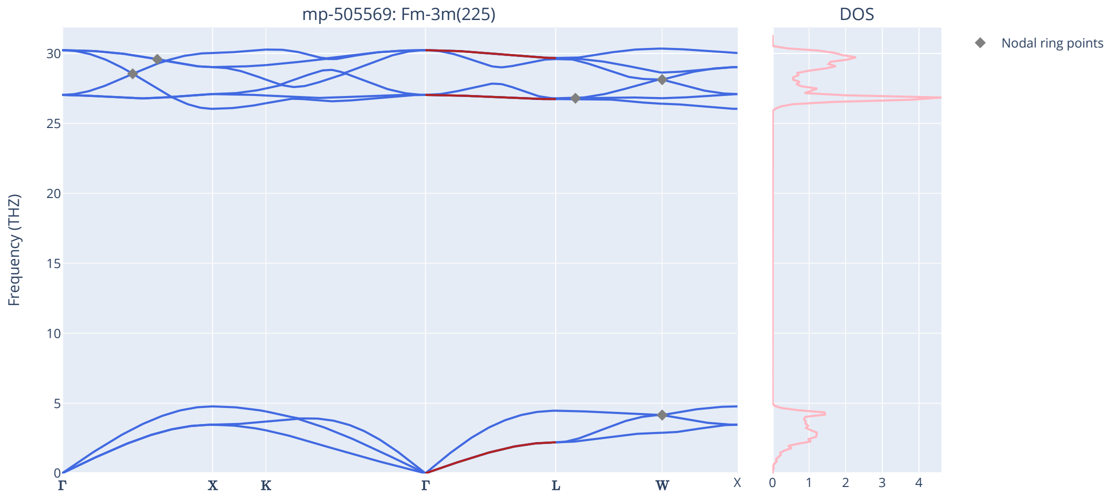

Supplementary Figure 439: The phonon band and DOS of Ce4H8.

Supplementary Table 899: High symmetry lines of mp-505569-Ce4H8

	Start	End
0	Γ (0.000, 0.000, 0.000)	X (0.500, 0.000, 0.500)
1	X (0.500, 0.000, 0.500)	U (0.625, 0.250, 0.625)
2	K (0.375, 0.375, 0.750)	Γ (0.000, 0.000, 0.000)
3	Γ (0.000, 0.000, 0.000)	L (0.500, 0.500, 0.500)
4	L (0.500, 0.500, 0.500)	W (0.500, 0.250, 0.750)
5	W (0.500, 0.250, 0.750)	X (0.500, 0.000, 0.500)

Supplementary Table 900: Nodal ring points along high symmetry lines of mp-505569-Ce4H8.

	Coordinates	Frequency	Mode 1	Mode 2	Multi.	Deg.	Path	Ratio
0	(0.500, 0.250, 0.750)	4.160	3	2	6	2	5	1.000
1	(0.500, 0.454, 0.546)	26.799	6	5	24	2	5	0.185
2	(0.235, -0.001, 0.235)	28.547	7	6	24	2	1	0.469
3	(0.500, 0.250, 0.750)	28.131	7	6	6	2	5	1.000
4	(0.316, 0.000, 0.316)	29.584	9	8	6	2	1	0.633

The followings are details for $Au_4Lu_4Sn_4$. As shown in Fig **440**, it has 5 topological nodal lines, 4 nodal ring points and 1 high degenerate Weyl point whose degeneracy is more than 2 and charge is bigger than 1.

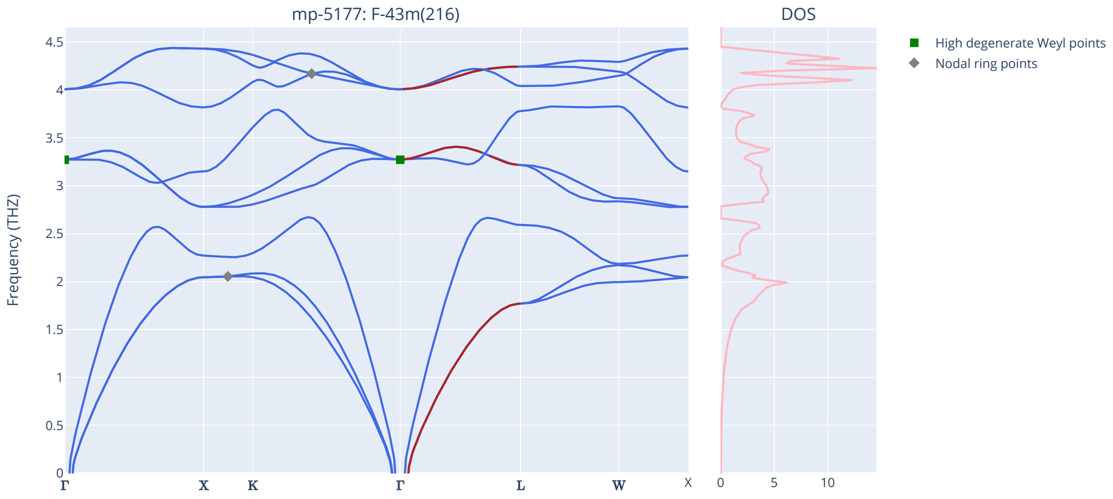

Supplementary Figure 440: The phonon band and DOS of Au4Lu4Sn4.

Supplementary Table 901: High symmetry lines of mp-5177-Au4Lu4Sn4

	Start	End
0	Γ (0.000, 0.000, 0.000)	X (0.500, 0.000, 0.500)
1	X (0.500, 0.000, 0.500)	U (0.625, 0.250, 0.625)
2	K (0.375, 0.375, 0.750)	Γ (0.000, 0.000, 0.000)
3	Γ (0.000, 0.000, 0.000)	L (0.500, 0.500, 0.500)
4	L (0.500, 0.500, 0.500)	W (0.500, 0.250, 0.750)
5	W (0.500, 0.250, 0.750)	X (0.500, 0.000, 0.500)

Supplementary Table 902: High degenerate Weyl points along high symmetry lines of mp-5177-Au4Lu4Sn4.

	Coordinates	Frequency	Mode 1	Mode 2	Multi.	Deg.	Path	Ratio
0	(0.000, 0.000, 0.000)	3.274	6	4	1	3	1	0.000

Supplementary Table 903: Nodal ring points along high symmetry lines of mp-5177-Au4Lu4Sn4.

	Coordinates	Frequency	Mode 1	Mode 2	Multi.	Deg.	Path	Ratio
0	(0.562, 0.123, 0.562)	2.054	2	1	24	2	2	0.494
1	(0.230, 0.230, 0.446)	4.169	8	7	24	2	3	0.400

The followings are details for $Co_4Mn_4Sb_4$. As shown in Fig **441**, it has 4 topological nodal lines and 1 nodal ring point. At the same time, it has 1 single Weyl point and 1 high degenerate Weyl point whose degeneracy is more than 2 and charge is bigger than 1.

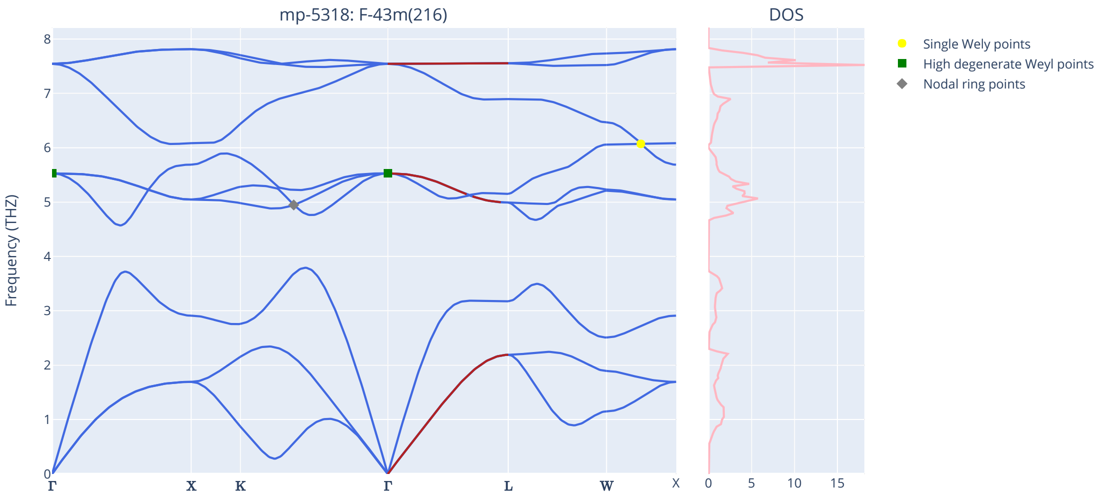

Supplementary Figure 441: The phonon band and DOS of Co4Mn4Sb4.

Supplementary Table 904: High symmetry lines of mp-5318-Co4Mn4Sb4

	Start	End
0	Γ (0.000, 0.000, 0.000)	X (0.500, 0.000, 0.500)
1	X (0.500, 0.000, 0.500)	U (0.625, 0.250, 0.625)
2	K (0.375, 0.375, 0.750)	Γ (0.000, 0.000, 0.000)
3	Γ (0.000, 0.000, 0.000)	L (0.500, 0.500, 0.500)
4	L (0.500, 0.500, 0.500)	W (0.500, 0.250, 0.750)
5	W (0.500, 0.250, 0.750)	X (0.500, 0.000, 0.500)

Supplementary Table 905: Single Weyl points along high symmetry lines of mp-5318-Co4Mn4Sb4.

	Coordinates	Frequency	Mode 1	Mode 2	Multi.	Deg.	Path	Ratio
0	(0.500, 0.127, 0.627)	6.071	7	6	12	2	6	0.494

Supplementary Table 906: High degenerate Weyl points along high symmetry lines of mp-5318-Co4Mn4Sb4.

	Coordinates	Frequency	Mode 1	Mode 2	Multi.	Deg.	Path	Ratio
0	(0.000, 0.000, 0.000)	5.529	6	4	1	3	1	0.000

Supplementary Table 907: Nodal ring points along high symmetry lines of mp-5318-Co4Mn4Sb4.

	Coordinates	Frequency	Mode 1	Mode 2	Multi.	Deg.	Path	Ratio
0	(0.240, 0.240, 0.477)	4.947	5	4	24	2	3	0.362

The followings are details for $Au_4Mg_4Sn_4$. As shown in Fig **442**, it has 4 topological nodal lines, 1 nodal ring point and 1 high degenerate Weyl point whose degeneracy is more than 2 and charge is bigger than 1.

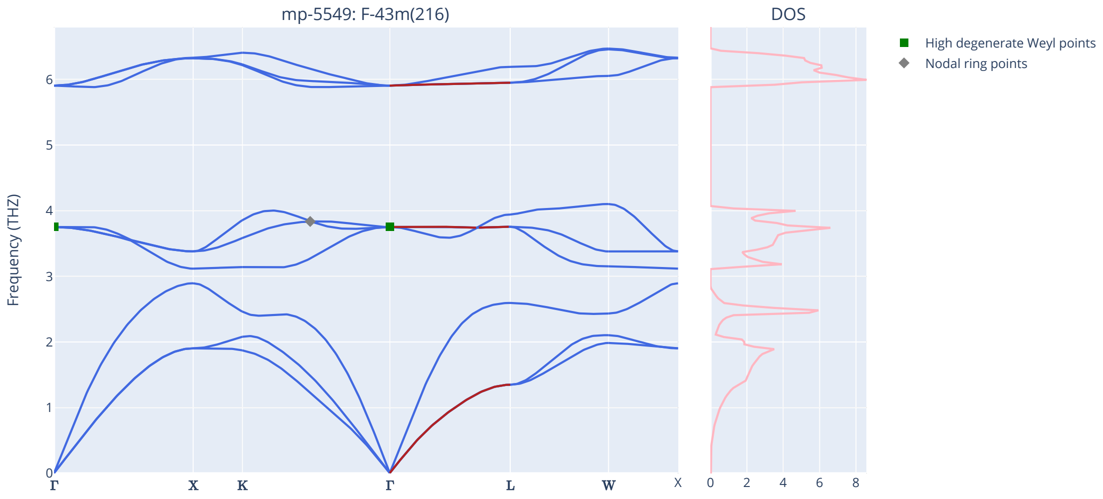

Supplementary Figure 442: The phonon band and DOS of Au4Mg4Sn4.

Supplementary Table 908: High symmetry lines of mp-5549-Au4Mg4Sn4

	Start	End
0	Γ (0.000, 0.000, 0.000)	X (0.500, 0.000, 0.500)
1	X (0.500, 0.000, 0.500)	U (0.625, 0.250, 0.625)
2	K (0.375, 0.375, 0.750)	Γ (0.000, 0.000, 0.000)
3	Γ (0.000, 0.000, 0.000)	L (0.500, 0.500, 0.500)
4	L (0.500, 0.500, 0.500)	W (0.500, 0.250, 0.750)
5	W (0.500, 0.250, 0.750)	X (0.500, 0.000, 0.500)

Supplementary Table 909: High degenerate Weyl points along high symmetry lines of mp-5549-Au4Mg4Sn4.

	Coordinates	Frequency	Mode 1	Mode 2	Multi.	Deg.	Path	Ratio
0	(0.000, 0.000, 0.000)	3.751	6	4	1	3	1	0.000

Supplementary Table 910: Nodal ring points along high symmetry lines of mp-5549-Au4Mg4Sn4.

	Coordinates	Frequency	Mode 1	Mode 2	Multi.	Deg.	Path	Ratio
0	(0.207, 0.207, 0.400)	3.835	6	5	24	2	3	0.460

The followings are details for $Bi_4Li_4Mg_4$. As shown in Fig **443**, it has 3 topological nodal lines, 1 nodal ring point and 1 high degenerate Weyl point whose degeneracy is more than 2 and charge is bigger than 1.

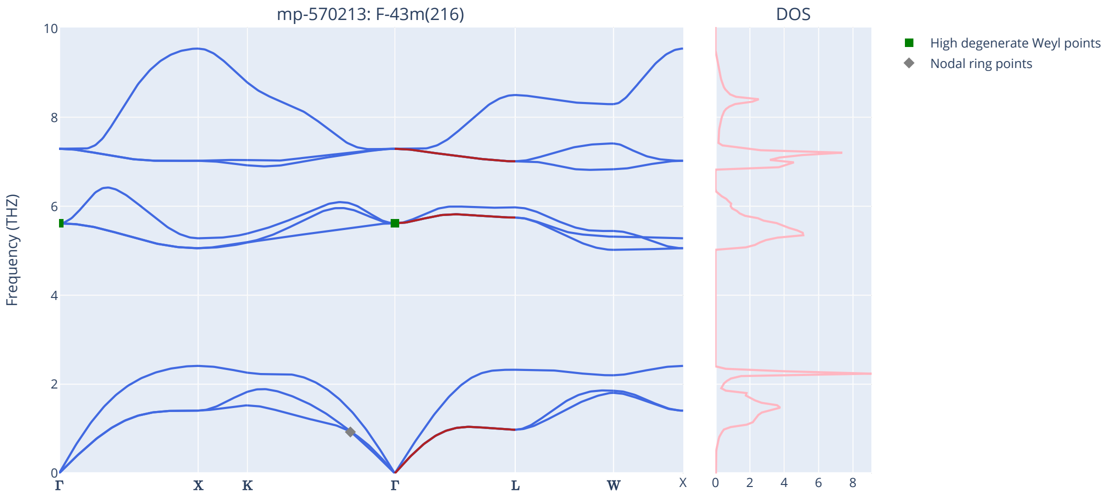

Supplementary Figure 443: The phonon band and DOS of Bi4Li4Mg4.

Supplementary Table 911: High symmetry lines of mp-570213-Bi4Li4Mg4

	Start	End
0	Γ (0.000, 0.000, 0.000)	X (0.500, 0.000, 0.500)
1	X (0.500, 0.000, 0.500)	U (0.625, 0.250, 0.625)
2	K (0.375, 0.375, 0.750)	Γ (0.000, 0.000, 0.000)
3	Γ (0.000, 0.000, 0.000)	L (0.500, 0.500, 0.500)
4	L (0.500, 0.500, 0.500)	W (0.500, 0.250, 0.750)
5	W (0.500, 0.250, 0.750)	X (0.500, 0.000, 0.500)

Supplementary Table 912: High degenerate Weyl points along high symmetry lines of mp-570213-Bi4Li4Mg4.

	Coordinates	Frequency	Mode 1	Mode 2	Multi.	Deg.	Path	Ratio
0	(0.000, 0.000, 0.000)	5.617	6	4	1	3	1	0.000

Supplementary Table 913: Nodal ring points along high symmetry lines of mp-570213-Bi4Li4Mg4.

	Coordinates	Frequency	Mode 1	Mode 2	Multi.	Deg.	Path	Ratio
0	(0.115, 0.115, 0.224)	0.928	2	1	24	2	3	0.699

The followings are details for $Bi_4Ce_4Pd_4$. As shown in Fig **444**, it has 5 topological nodal lines and 3 nodal ring points. At the same time, it has 1 single Weyl point and 1 high degenerate Weyl point whose degeneracy is more than 2 and charge is bigger than 1.

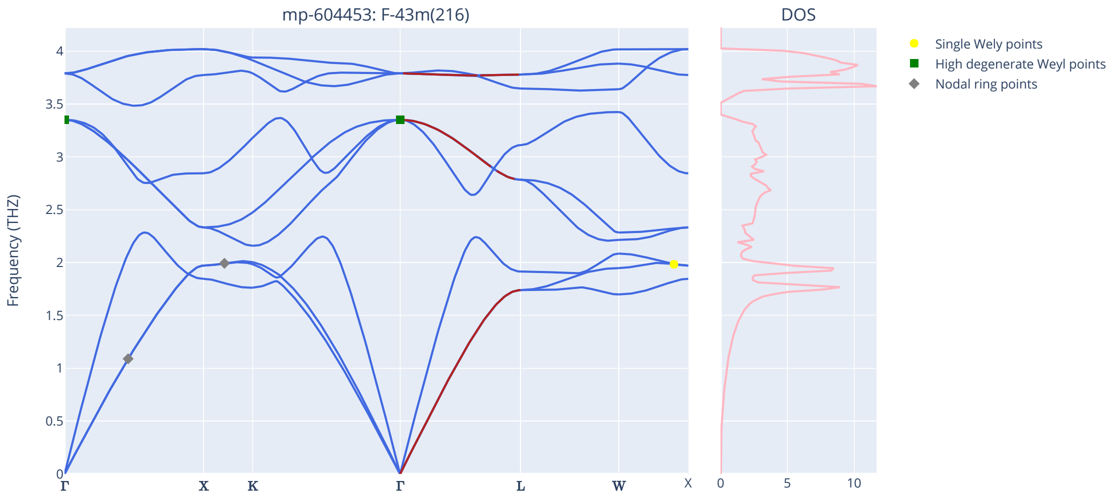

Supplementary Figure 444: The phonon band and DOS of Bi4Ce4Pd4.

Supplementary Table 914: High symmetry lines of mp-604453-Bi4Ce4Pd4

	Start	End
0	Γ (0.000, 0.000, 0.000)	X (0.500, 0.000, 0.500)
1	X (0.500, 0.000, 0.500)	U (0.625, 0.250, 0.625)
2	K (0.375, 0.375, 0.750)	Γ (0.000, 0.000, 0.000)
3	Γ (0.000, 0.000, 0.000)	L (0.500, 0.500, 0.500)
4	L (0.500, 0.500, 0.500)	W (0.500, 0.250, 0.750)
5	W (0.500, 0.250, 0.750)	X (0.500, 0.000, 0.500)

Supplementary Table 915: Single Weyl points along high symmetry lines of mp-604453-Bi4Ce4Pd4.

	Coordinates	Frequency	Mode 1	Mode 2	Multi.	Deg.	Path	Ratio
0	(0.500, 0.051, 0.551)	1.983	3	2	12	2	6	0.795

Supplementary Table 916: High degenerate Weyl points along high symmetry lines of mp-604453-Bi4Ce4Pd4.

	Coordinates	Frequency	Mode 1	Mode 2	Multi.	Deg.	Path	Ratio
0	(0.000, 0.000, 0.000)	3.351	6	4	1	3	1	0.000

Supplementary Table 917: Nodal ring points along high symmetry lines of mp-604453-Bi4Ce4Pd4.

	Coordinates	Frequency	Mode 1	Mode 2	Multi.	Deg.	Path	Ratio
0	(0.234, -0.000, 0.223)	1.089	2	1	24	2	1	0.457
1	(0.554, 0.105, 0.554)	1.992	3	2	24	2	2	0.425

The followings are details for BaPtSb. It has 4 nodal ring points and 4 single Weyl points along high symmetry line as shown in Fig **445**.

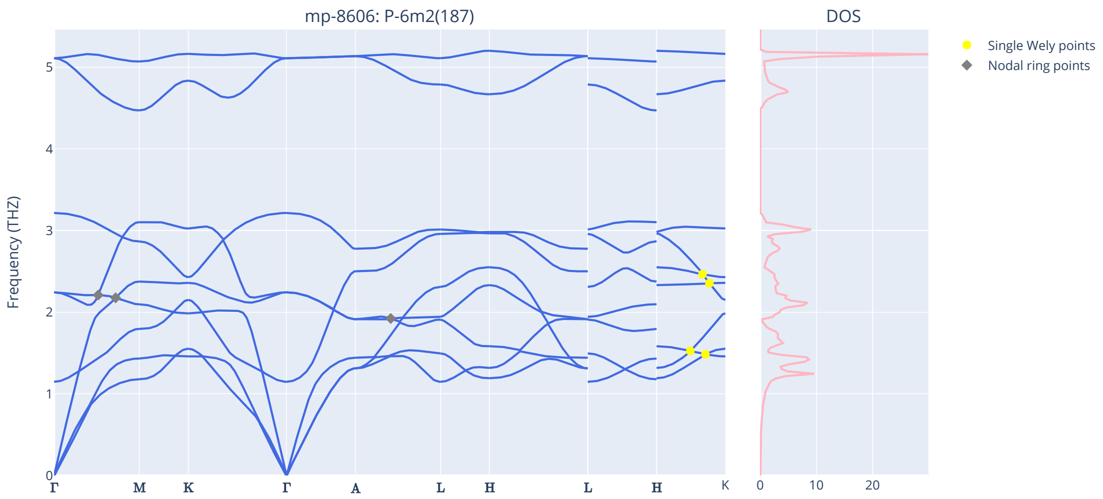

Supplementary Figure 445: The phonon band and DOS of BaPtSb.

Supplementary Table 918: High symmetry lines of mp-8606-BaPtSb

	Start	End
0	Γ (0.000, 0.000, 0.000)	M (0.500, 0.000, 0.000)
1	M (0.500, 0.000, 0.000)	K (0.333, 0.333, 0.000)
2	K (0.333, 0.333, 0.000)	Γ (0.000, 0.000, 0.000)
3	Γ (0.000, 0.000, 0.000)	A (0.000, 0.000, 0.500)
4	A (0.000, 0.000, 0.500)	L (0.500, 0.000, 0.500)
5	L (0.500, 0.000, 0.500)	H (0.333, 0.333, 0.500)
6	H (0.333, 0.333, 0.500)	A (0.000, 0.000, 0.500)
7	L (0.500, 0.000, 0.500)	M (0.500, 0.000, 0.000)
8	H (0.333, 0.333, 0.500)	K (0.333, 0.333, 0.000)

Supplementary Table 919: Single Weyl points along high symmetry lines of mp-8606-BaPtSb.

	Coordinates	Frequency	Mode 1	Mode 2	Multi.	Deg.	Path	Ratio
0	(0.333, 0.333, 0.145)	1.484	2	1	4	2	9	0.710
1	(0.333, 0.333, 0.254)	1.524	3	2	4	2	9	0.491
2	(0.333, 0.333, 0.116)	2.355	5	4	4	2	9	0.768
3	(0.333, 0.333, 0.166)	2.465	6	5	4	2	9	0.668

Supplementary Table 920: Nodal ring points along high symmetry lines of mp-8606-BaPtSb.

	Coordinates	Frequency	Mode 1	Mode 2	Multi.	Deg.	Path	Ratio
0	(0.362, -0.000, -0.000)	2.176	5	4	6	2	1	0.723

Supplementary Table 920: Nodal ring points along high symmetry lines of mp-8606-BaPtSb.

	Coordinates	Frequency	Mode 1	Mode 2	Multi.	Deg.	Path	Ratio
1	(0.208, 0.000, 0.500)	1.922	5	4	6	2	5	0.416
2	(0.259, -0.000, -0.000)	2.210	6	5	6	2	1	0.518

The followings are details for $Fe_4Ti_4Te_4$. As shown in Fig **446**, it has 3 topological nodal lines and 4 nodal ring points. At the same time, it has 3 single Weyl points and 1 high degenerate Weyl point whose degeneracy is more than 2 and charge is bigger than 1.

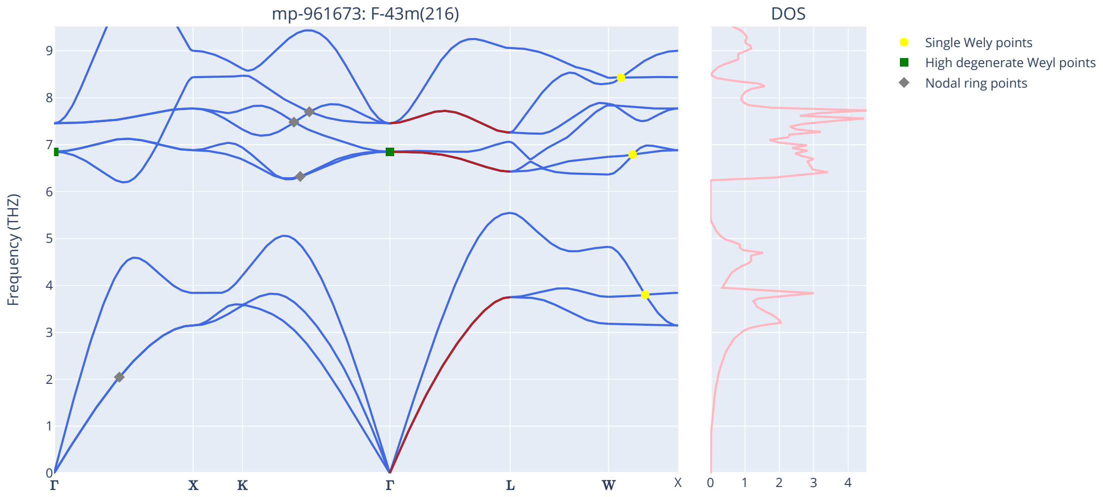

Supplementary Figure 446: The phonon band and DOS of Fe4Ti4Te4.

Supplementary Table 921: High symmetry lines of mp-961673-Fe4Ti4Te4

	Start	End
0	Γ (0.000, 0.000, 0.000)	X (0.500, 0.000, 0.500)
1	X (0.500, 0.000, 0.500)	U (0.625, 0.250, 0.625)
2	K (0.375, 0.375, 0.750)	Γ (0.000, 0.000, 0.000)
3	Γ (0.000, 0.000, 0.000)	L (0.500, 0.500, 0.500)
4	L (0.500, 0.500, 0.500)	W (0.500, 0.250, 0.750)
5	W (0.500, 0.250, 0.750)	X (0.500, 0.000, 0.500)

Supplementary Table 922: Single Weyl points along high symmetry lines of mp-961673-Fe4Ti4Te4.

	Coordinates	Frequency	Mode 1	Mode 2	Multi.	Deg.	Path	Ratio
0	(0.500, 0.118, 0.618)	3.802	3	2	12	2	6	0.528
1	(0.500, 0.163, 0.663)	6.788	5	4	12	2	6	0.347
2	(0.500, 0.205, 0.705)	8.428	9	8	12	2	6	0.181

Supplementary Table 923: High degenerate Weyl points along high symmetry lines of mp-961673-Fe4Ti4Te4.

	Coordinates	Frequency	Mode 1	Mode 2	Multi.	Deg.	Path	Ratio
0	(0.000, 0.000, 0.000)	6.850	6	4	1	3	1	0.000

Supplementary Table 924: Nodal ring points along high symmetry lines of mp-961673-Fe4Ti4Te4.

	Coordinates	Frequency	Mode 1	Mode 2	Multi.	Deg.	Path	Ratio
0	(0.235, 0.000, 0.235)	2.045	2	1	6	2	1	0.469
1	(0.230, 0.230, 0.454)	6.322	5	4	24	2	3	0.393
2	(0.246, 0.246, 0.485)	7.480	7	6	24	2	3	0.350
3	(0.207, 0.207, 0.407)	7.701	8	7	24	2	3	0.455

The followings are details for $Co_4Sc_4Te_4$. As shown in Fig **447**, it has 3 topological nodal lines, 1 nodal ring point and 1 high degenerate Weyl point whose degeneracy is more than 2 and charge is bigger than 1.

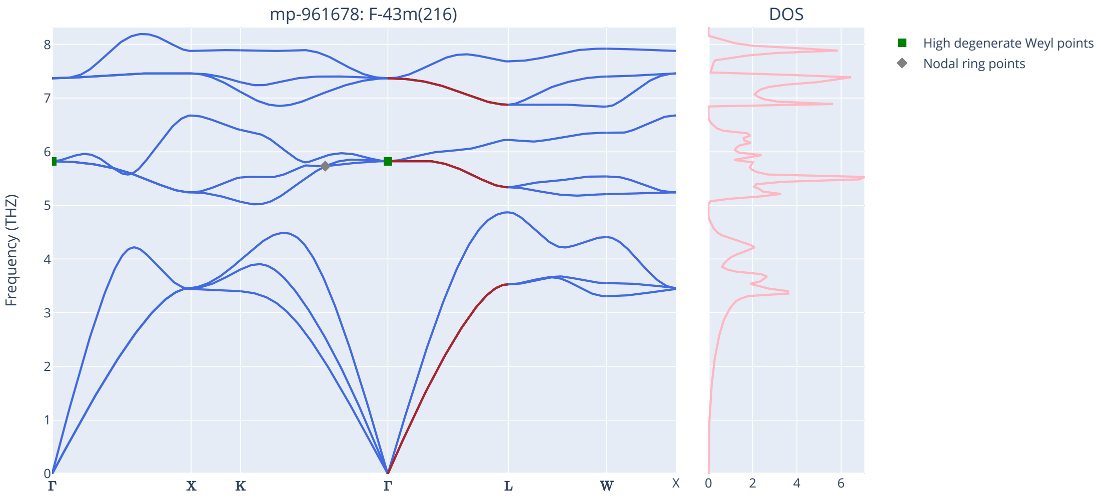

Supplementary Figure 447: The phonon band and DOS of Co4Sc4Te4.

Supplementary Table 925: High symmetry lines of mp-961678-Co4Sc4Te4

	Start	End
0	Γ (0.000, 0.000, 0.000)	X (0.500, 0.000, 0.500)
1	X (0.500, 0.000, 0.500)	U (0.625, 0.250, 0.625)
2	K (0.375, 0.375, 0.750)	Γ (0.000, 0.000, 0.000)
3	Γ (0.000, 0.000, 0.000)	L (0.500, 0.500, 0.500)
4	L (0.500, 0.500, 0.500)	W (0.500, 0.250, 0.750)
5	W (0.500, 0.250, 0.750)	X (0.500, 0.000, 0.500)

Supplementary Table 926: High degenerate Weyl points along high symmetry lines of mp-961678-Co4Sc4Te4.

	Coordinates	Frequency	Mode 1	Mode 2	Multi.	Deg.	Path	Ratio
0	(0.000, 0.000, 0.000)	5.823	6	4	1	3	1	0.000

Supplementary Table 927: Nodal ring points along high symmetry lines of mp-961678-Co4Sc4Te4.

	Coordinates	Frequency	Mode 1	Mode 2	Multi.	Deg.	Path	Ratio
0	(0.161, 0.161, 0.316)	5.732	5	4	24	2	3	0.576

The followings are details for Nb_3S_6. It has 2 topological nodal lines and 6 nodal ring points along high symmetry line as shown in Fig **448**.

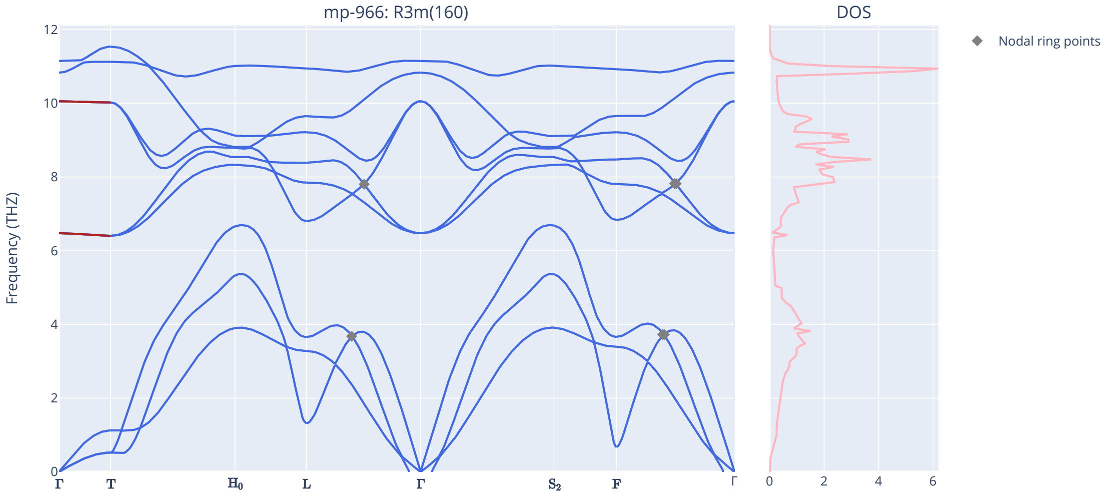

Supplementary Figure 448: The phonon band and DOS of Nb3S6.

Supplementary Table 928: High symmetry lines of mp-966-Nb3S6

	Start	End
0	Γ (0.000, 0.000, 0.000)	T (0.500, 0.500, 0.500)
1	T (0.500, 0.500, 0.500)	H_2 (0.818, 0.182, 0.500)
2	H_0 (0.500, -0.182, 0.182)	L (0.500, 0.000, 0.000)
3	L (0.500, 0.000, 0.000)	Γ (0.000, 0.000, 0.000)
4	Γ (0.000, 0.000, 0.000)	S_0 (0.341, -0.341, 0.000)
5	S_2 (0.659, 0.000, 0.341)	F (0.500, 0.000, 0.500)
6	F (0.500, 0.000, 0.500)	Γ (0.000, 0.000, 0.000)

Supplementary Table 929: Nodal ring points along high symmetry lines of mp-966-Nb3S6.

	Coordinates	Frequency	Mode 1	Mode 2	Multi.	Deg.	Path	Ratio
0	(0.301, 0.002, 0.002)	3.676	3	2	6	2	4	0.397
1	(0.304, 0.005, 0.304)	3.723	3	2	6	2	7	0.393
2	(0.297, -0.002, 0.297)	3.722	3	2	6	2	7	0.406
3	(0.247, -0.001, -0.001)	7.801	6	5	6	2	4	0.506
4	(0.253, 0.004, 0.253)	7.816	6	5	6	2	7	0.494
5	(0.246, -0.003, 0.246)	7.816	6	5	6	2	7	0.507

The followings are details for BaPdAs. It has 5 nodal ring points and 1 single Weyl point along high symmetry line as shown in Fig 449.

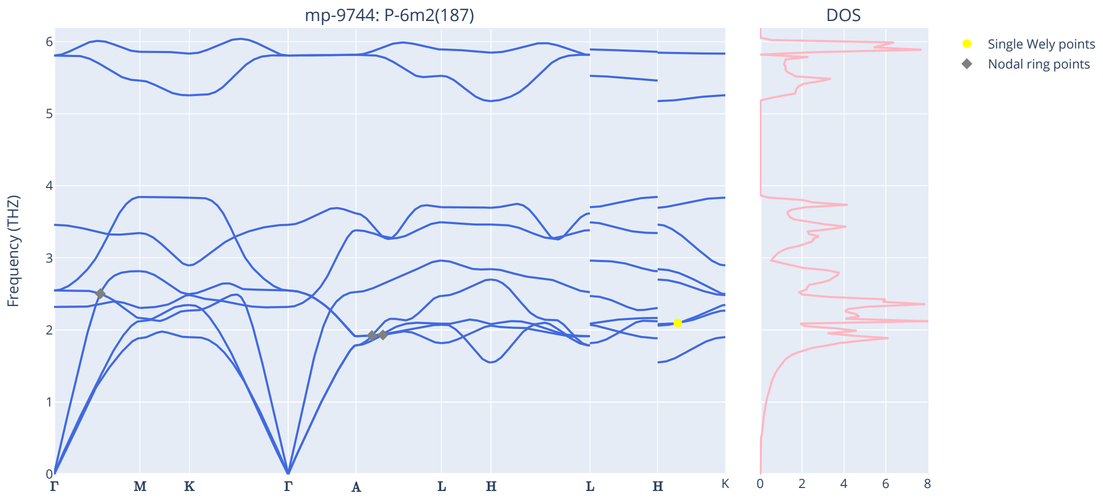

Supplementary Figure 449: The phonon band and DOS of BaPdAs.

Supplementary Table 930: High symmetry lines of mp-9744-BaPdAs

	Start	End
0	Γ (0.000, 0.000, 0.000)	M (0.500, 0.000, 0.000)
1	M (0.500, 0.000, 0.000)	K (0.333, 0.333, 0.000)
2	K (0.333, 0.333, 0.000)	Γ (0.000, 0.000, 0.000)
3	Γ (0.000, 0.000, 0.000)	A (0.000, 0.000, 0.500)
4	A (0.000, 0.000, 0.500)	L (0.500, 0.000, 0.500)
5	L (0.500, 0.000, 0.500)	H (0.333, 0.333, 0.500)
6	H (0.333, 0.333, 0.500)	A (0.000, 0.000, 0.500)
7	L (0.500, 0.000, 0.500)	M (0.500, 0.000, 0.000)
8	H (0.333, 0.333, 0.500)	K (0.333, 0.333, 0.000)

Supplementary Table 931: Single Weyl points along high symmetry lines of mp-9744-BaPdAs.

	Coordinates	Frequency	Mode 1	Mode 2	Multi.	Deg.	Path	Ratio
0	(0.333, 0.333, 0.351)	2.097	3	2	4	2	9	0.298

Supplementary Table 932: Nodal ring points along high symmetry lines of mp-9744-BaPdAs.

	Coordinates	Frequency	Mode 1	Mode 2	Multi.	Deg.	Path	Ratio
0	(0.158, 0.000, 0.495)	1.931	2	1	12	2	5	0.316
1	(0.094, -0.001, 0.500)	1.922	4	3	10	2	5	0.188
2	(0.270, 0.000, -0.000)	2.503	5	4	6	2	1	0.540

Supplementary Table 932: Nodal ring points along high symmetry lines of mp-9744-BaPdAs.

Coordinates	Frequency	Mode 1	Mode 2	Multi.	Deg.	Path	Ratio

The followings are details for Al_8Cu_4. It has 5 topological nodal lines and 9 nodal ring points along high symmetry line as shown in Fig 450.

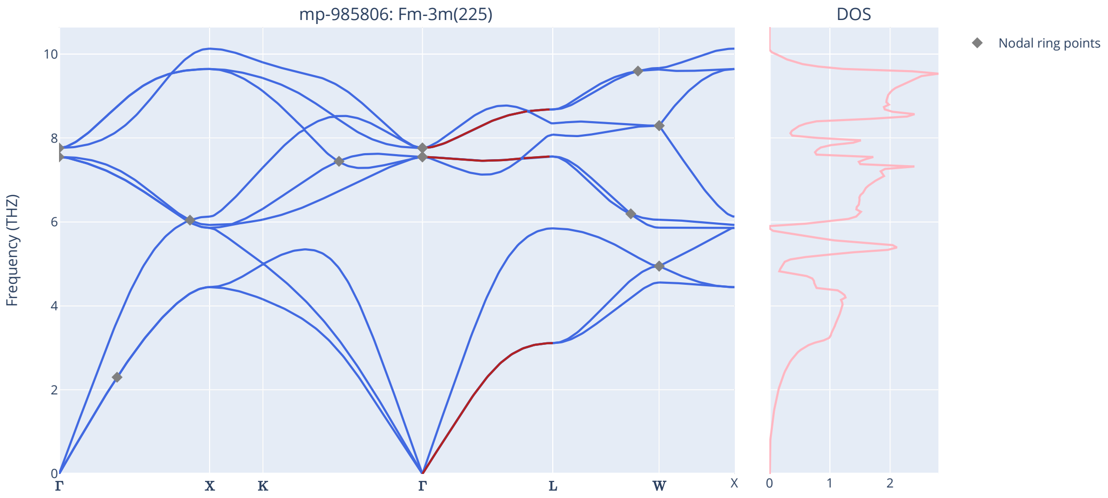

Supplementary Figure 450: The phonon band and DOS of Al8Cu4.

Supplementary Table 933: High symmetry lines of mp-985806-Al8Cu4

	Start	End
0	Γ (0.000, 0.000, 0.000)	X (0.500, 0.000, 0.500)
1	X (0.500, 0.000, 0.500)	U (0.625, 0.250, 0.625)
2	K (0.375, 0.375, 0.750)	Γ (0.000, 0.000, 0.000)
3	Γ (0.000, 0.000, 0.000)	L (0.500, 0.500, 0.500)
4	L (0.500, 0.500, 0.500)	W (0.500, 0.250, 0.750)
5	W (0.500, 0.250, 0.750)	X (0.500, 0.000, 0.500)

Supplementary Table 934: Nodal ring points along high symmetry lines of mp-985806-Al8Cu4.

	Coordinates	Frequency	Mode 1	Mode 2	Multi.	Deg.	Path	Ratio
0	(0.189, -0.000, 0.197)	2.300	2	1	24	2	1	0.385
1	(0.500, 0.250, 0.750)	4.947	3	2	6	2	5	1.000
2	(0.500, 0.317, 0.683)	6.194	5	4	24	2	5	0.733
3	(0.000, 0.000, 0.000)	7.551	6	5	1	3	1	0.000
4	(0.435, -0.001, 0.435)	6.043	6	5	24	2	1	0.870
5	(0.191, 0.191, 0.397)	7.445	6	5	24	2	3	0.477
6	(0.500, 0.250, 0.750)	8.295	7	6	6	2	5	1.000
7	(0.000, 0.000, 0.000)	7.762	8	7	1	3	1	0.000
8	(0.500, 0.300, 0.700)	9.595	9	8	24	2	5	0.801

The followings are details for YZnSi. It has 1 nodal ring point and 1 single Weyl point along high symmetry line as shown in Fig **451**.

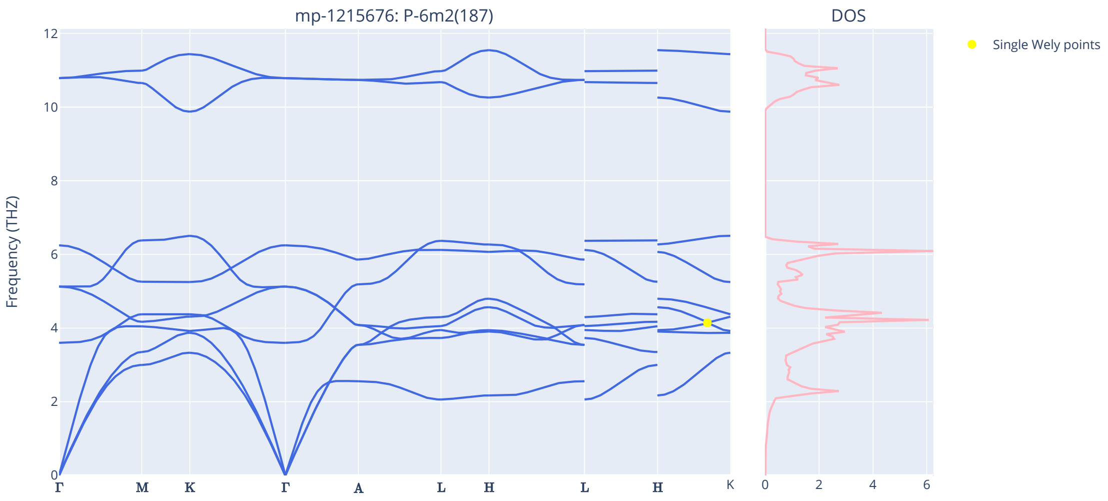

Supplementary Figure 451: The phonon band and DOS of YZnSi.

Supplementary Table 935: High symmetry lines of mp-1215676-YZnSi

	Start	End
0	Γ (0.000, 0.000, 0.000)	M (0.500, 0.000, 0.000)
1	M (0.500, 0.000, 0.000)	K (0.333, 0.333, 0.000)
2	K (0.333, 0.333, 0.000)	Γ (0.000, 0.000, 0.000)
3	Γ (0.000, 0.000, 0.000)	A (0.000, 0.000, 0.500)
4	A (0.000, 0.000, 0.500)	L (0.500, 0.000, 0.500)
5	L (0.500, 0.000, 0.500)	H (0.333, 0.333, 0.500)
6	H (0.333, 0.333, 0.500)	A (0.000, 0.000, 0.500)
7	L (0.500, 0.000, 0.500)	M (0.500, 0.000, 0.000)
8	H (0.333, 0.333, 0.500)	K (0.333, 0.333, 0.000)

Supplementary Table 936: Single Weyl points along high symmetry lines of mp-1215676-YZnSi.

	Coordinates	Frequency	Mode 1	Mode 2	Multi.	Deg.	Path	Ratio
0	(0.333, 0.333, 0.157)	4.142	4	3	4	2	9	0.685

The followings are details for CeZnSi. It has 2 nodal ring points and 2 single Weyl points along high symmetry line as shown in Fig 452.

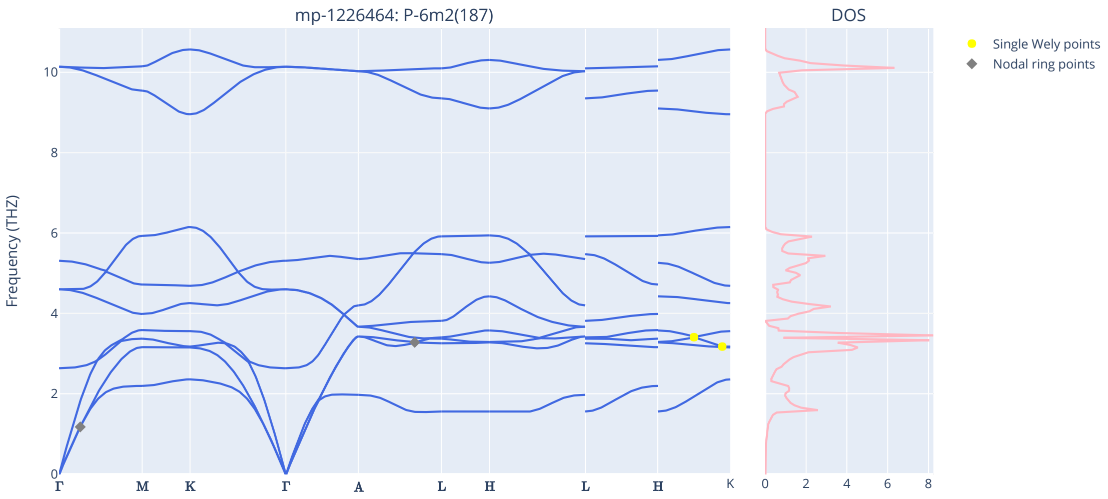

Supplementary Figure 452: The phonon band and DOS of CeZnSi.

Supplementary Table 937: High symmetry lines of mp-1226464-CeZnSi

	Start	End
0	Γ (0.000, 0.000, 0.000)	M (0.500, 0.000, 0.000)
1	M (0.500, 0.000, 0.000)	K (0.333, 0.333, 0.000)
2	K (0.333, 0.333, 0.000)	Γ (0.000, 0.000, 0.000)
3	Γ (0.000, 0.000, 0.000)	A (0.000, 0.000, 0.500)
4	A (0.000, 0.000, 0.500)	L (0.500, 0.000, 0.500)
5	L (0.500, 0.000, 0.500)	H (0.333, 0.333, 0.500)
6	H (0.333, 0.333, 0.500)	A (0.000, 0.000, 0.500)
7	L (0.500, 0.000, 0.500)	M (0.500, 0.000, 0.000)
8	H (0.333, 0.333, 0.500)	K (0.333, 0.333, 0.000)

Supplementary Table 938: Single Weyl points along high symmetry lines of mp-1226464-CeZnSi.

	Coordinates	Frequency	Mode 1	Mode 2	Multi.	Deg.	Path	Ratio
0	(0.333, 0.333, 0.056)	3.173	3	2	4	2	9	0.888
1	(0.333, 0.333, 0.251)	3.408	4	3	4	2	9	0.498

Supplementary Table 939: Nodal ring points along high symmetry lines of mp-1226464-CeZnSi.

	Coordinates	Frequency	Mode 1	Mode 2	Multi.	Deg.	Path	Ratio
0	(0.127, -0.001, 0.000)	1.175	2	1	10	2	1	0.255
1	(0.339, -0.000, 0.500)	3.279	3	2	6	2	5	0.678

Supplementary Table 939: Nodal ring points along high symmetry lines of mp-1226464-CeZnSi.

Coordinates	Frequency	Mode 1	Mode 2	Multi.	Deg.	Path	Ratio

The followings are details for AuBaSi. It has 3 nodal ring points and 1 single Weyl point along high symmetry line as shown in Fig 453.

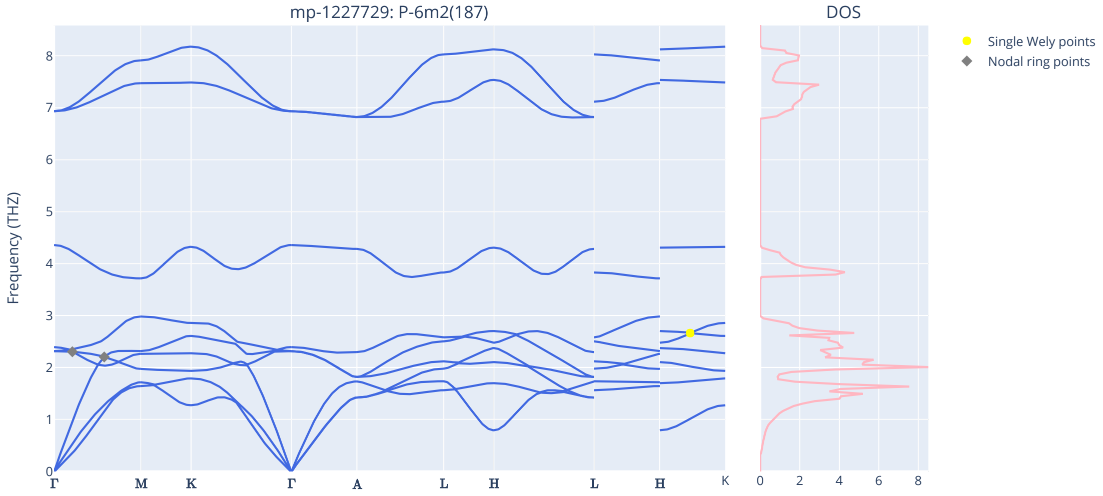

Supplementary Figure 453: The phonon band and DOS of AuBaSi.

Supplementary Table 940: High symmetry lines of mp-1227729-AuBaSi

	Start	End
0	Γ (0.000, 0.000, 0.000)	M (0.500, 0.000, 0.000)
1	M (0.500, 0.000, 0.000)	K (0.333, 0.333, 0.000)
2	K (0.333, 0.333, 0.000)	Γ (0.000, 0.000, 0.000)
3	Γ (0.000, 0.000, 0.000)	A (0.000, 0.000, 0.500)
4	A (0.000, 0.000, 0.500)	L (0.500, 0.000, 0.500)
5	L (0.500, 0.000, 0.500)	H (0.333, 0.333, 0.500)
6	H (0.333, 0.333, 0.500)	A (0.000, 0.000, 0.500)
7	L (0.500, 0.000, 0.500)	M (0.500, 0.000, 0.000)
8	H (0.333, 0.333, 0.500)	K (0.333, 0.333, 0.000)

Supplementary Table 941: Single Weyl points along high symmetry lines of mp-1227729-AuBaSi.

	Coordinates	Frequency	Mode 1	Mode 2	Multi.	Deg.	Path	Ratio
0	(0.333, 0.333, 0.268)	2.662	6	5	4	2	9	0.464

Supplementary Table 942: Nodal ring points along high symmetry lines of mp-1227729-AuBaSi.

	Coordinates	Frequency	Mode 1	Mode 2	Multi.	Deg.	Path	Ratio
0	(0.105, 0.002, 0.000)	2.303	5	4	12	2	1	0.210
1	(0.289, 0.000, 0.000)	2.202	5	4	6	2	1	0.578

The followings are details for AlBaSi and it only has 2 single Weyl point along high symmetry line as shown in Fig 454.

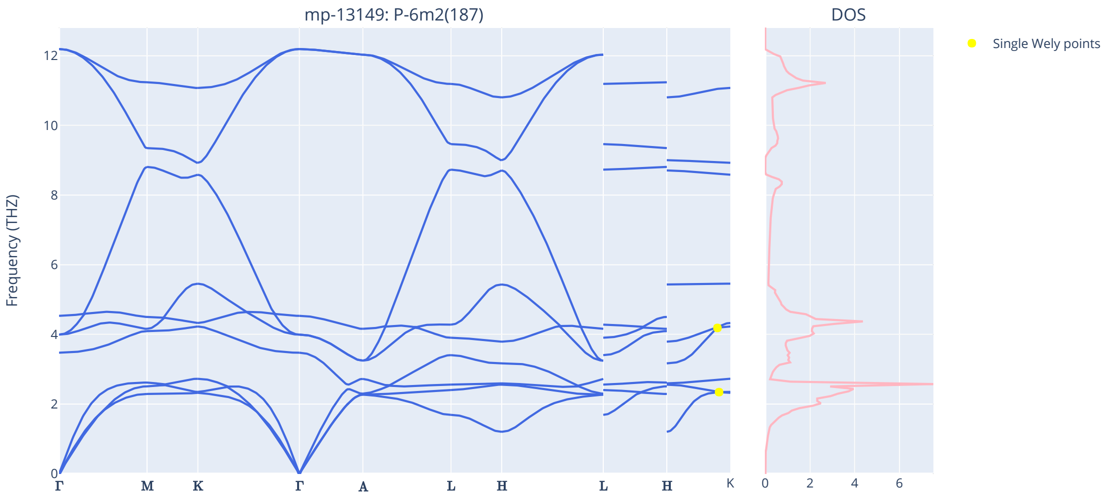

Supplementary Figure 454: The phonon band and DOS of AlBaSi.

Supplementary Table 943: High symmetry lines of mp-13149-AlBaSi

	Start	End
0	Γ (0.000, 0.000, 0.000)	M (0.500, 0.000, 0.000)
1	M (0.500, 0.000, 0.000)	K (0.333, 0.333, 0.000)
2	K (0.333, 0.333, 0.000)	Γ (0.000, 0.000, 0.000)
3	Γ (0.000, 0.000, 0.000)	A (0.000, 0.000, 0.500)
4	A (0.000, 0.000, 0.500)	L (0.500, 0.000, 0.500)
5	L (0.500, 0.000, 0.500)	H (0.333, 0.333, 0.500)
6	H (0.333, 0.333, 0.500)	A (0.000, 0.000, 0.500)
7	L (0.500, 0.000, 0.500)	M (0.500, 0.000, 0.000)
8	H (0.333, 0.333, 0.500)	K (0.333, 0.333, 0.000)

Supplementary Table 944: Single Weyl points along high symmetry lines of mp-13149-AlBaSi.

	Coordinates	Frequency	Mode 1	Mode 2	Multi.	Deg.	Path	Ratio
0	(0.333, 0.333, 0.089)	2.342	2	1	4	2	9	0.822
1	(0.333, 0.333, 0.101)	4.185	5	4	4	2	9	0.797

The followings are details for Ge_3I_6. It has 19 topological nodal lines and 101 nodal ring points along high symmetry line as shown in Fig **455**.

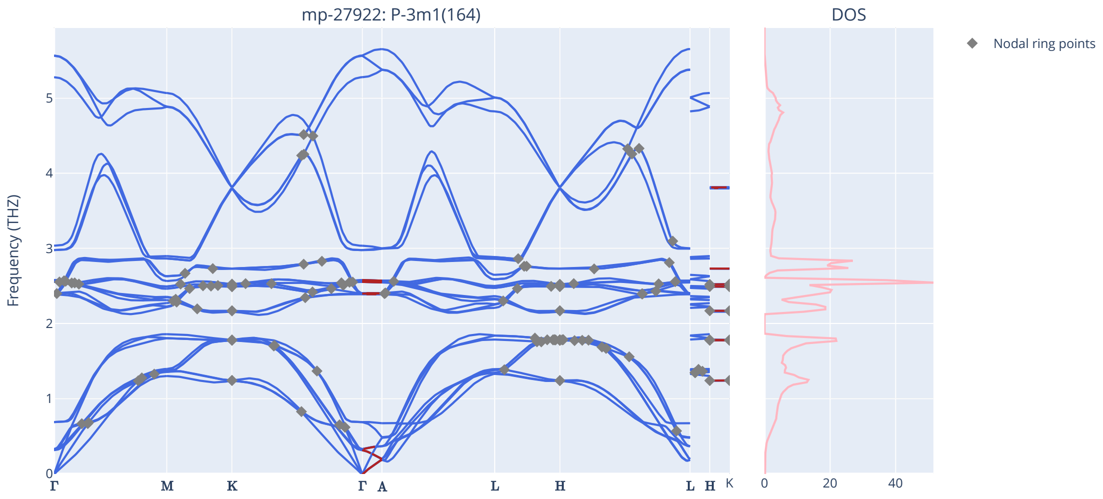

Supplementary Figure 455: The phonon band and DOS of Ge3I6.

Supplementary Table 945: High symmetry lines of mp-27922-Ge3I6

	Start	End
0	Γ (0.000, 0.000, 0.000)	M (0.500, 0.000, 0.000)
1	M (0.500, 0.000, 0.000)	K (0.333, 0.333, 0.000)
2	K (0.333, 0.333, 0.000)	Γ (0.000, 0.000, 0.000)
3	Γ (0.000, 0.000, 0.000)	A (0.000, 0.000, 0.500)
4	A (0.000, 0.000, 0.500)	L (0.500, 0.000, 0.500)
5	L (0.500, 0.000, 0.500)	H (0.333, 0.333, 0.500)
6	H (0.333, 0.333, 0.500)	A (0.000, 0.000, 0.500)
7	L (0.500, 0.000, 0.500)	M (0.500, 0.000, 0.000)
8	H (0.333, 0.333, 0.500)	K (0.333, 0.333, 0.000)

Supplementary Table 946: Nodal ring points along high symmetry lines of mp-27922-Ge3I6.

	Coordinates	Frequency	Mode 1	Mode 2	Multi.	Deg.	Path	Ratio
0	(0.444, 0.000, 0.001)	1.326	3	2	6	2	1	0.888
1	(0.333, 0.333, 0.000)	1.242	3	2	2	2	2	1.000
2	(0.156, 0.156, 0.000)	0.827	3	2	6	2	3	0.533
3	(0.149, -0.000, 0.003)	0.668	3	2	6	2	1	0.299
4	(0.333, 0.333, 0.500)	1.240	3	2	2	2	6	1.000
5	(0.497, -0.000, 0.377)	1.346	3	2	6	2	8	0.246
6	(0.498, 0.000, 0.367)	1.346	3	2	6	2	8	0.266
7	(0.153, 0.000, -0.000)	0.682	4	3	6	2	1	0.306
8	(0.373, 0.000, -0.000)	1.237	4	3	6	2	1	0.746
9	(0.497, 0.000, 0.185)	1.364	4	3	6	2	8	0.631
10	(0.475, 0.049, 0.500)	1.384	4	3	6	2	6	0.148

Supplementary Table 946: Nodal ring points along high symmetry lines of mp-27922-Ge3I6.

	Coordinates	Frequency	Mode 1	Mode 2	Multi.	Deg.	Path	Ratio
11	(0.389, -0.000, -0.000)	1.276	5	4	6	2	1	0.778
12	(0.333, 0.333, 0.000)	1.777	5	4	2	2	2	1.000
13	(0.226, 0.226, 0.500)	1.687	5	4	6	2	7	0.323
14	(0.381, 0.239, 0.498)	1.756	5	4	12	2	6	0.715
15	(0.333, 0.333, 0.500)	1.776	5	4	2	2	6	1.000
16	(0.123, -0.000, -0.000)	0.666	6	5	6	2	1	0.246
17	(0.226, 0.226, -0.002)	1.701	6	5	12	2	3	0.321
18	(0.058, 0.058, 0.000)	0.651	6	5	6	2	3	0.826
19	(0.391, 0.216, 0.498)	1.757	6	5	12	2	6	0.649
20	(0.344, 0.311, 0.499)	1.781	6	5	12	2	6	0.934
21	(0.351, 0.298, 0.499)	1.782	6	5	12	2	6	0.894
22	(0.276, 0.276, 0.500)	1.776	6	5	6	2	7	0.172
23	(0.296, 0.296, 0.500)	1.772	6	5	6	2	7	0.113
24	(0.499, -0.000, 0.286)	1.389	6	5	6	2	8	0.427
25	(0.044, 0.044, 0.000)	0.619	7	6	6	2	3	0.868
26	(0.215, 0.215, 0.500)	1.668	7	6	6	2	7	0.355
27	(0.366, 0.269, 0.499)	1.783	7	6	12	2	6	0.806
28	(0.349, 0.306, 0.500)	1.783	7	6	12	2	6	0.915
29	(0.327, 0.327, 0.500)	1.782	7	6	6	2	7	0.018
30	(0.035, 0.035, 0.500)	0.570	7	6	6	2	7	0.896
31	(0.333, 0.333, 0.000)	1.780	8	7	2	2	2	1.000
32	(0.225, 0.225, -0.000)	1.709	8	7	6	2	3	0.325
33	(0.333, 0.333, 0.500)	1.783	8	7	2	2	6	1.000
34	(0.260, 0.260, 0.500)	1.778	8	7	6	2	7	0.220
35	(0.116, 0.116, 0.000)	1.367	9	8	6	2	3	0.652
36	(0.397, 0.206, 0.499)	1.806	9	8	12	2	6	0.617
37	(0.325, 0.325, 0.500)	1.785	9	8	6	2	7	0.026
38	(0.347, 0.305, 0.500)	1.785	9	8	6	2	6	0.916
39	(0.156, 0.156, 0.500)	1.557	9	8	6	2	7	0.532
40	(0.475, 0.050, 0.002)	2.286	11	10	12	2	2	0.150
41	(0.012, 0.000, -0.000)	2.399	12	11	6	2	1	0.024
42	(0.422, 0.155, -0.001)	2.195	12	11	12	2	2	0.466
43	(0.333, 0.333, 0.000)	2.169	12	11	2	2	2	1.000
44	(0.146, 0.146, -0.000)	2.344	12	11	6	2	3	0.562
45	(0.011, 0.000, 0.500)	2.398	12	11	6	2	5	0.023
46	(0.478, 0.043, 0.498)	2.300	12	11	12	2	6	0.130
47	(0.333, 0.333, 0.500)	2.169	12	11	2	2	6	1.000
48	(0.088, 0.088, 0.500)	2.430	12	11	6	2	7	0.737
49	(0.122, 0.122, 0.500)	2.394	12	11	6	2	7	0.635
50	(0.484, 0.033, -0.000)	2.299	13	12	6	2	2	0.099
51	(0.016, -0.000, 0.500)	2.406	13	12	6	2	5	0.031
52	(0.121, 0.126, 0.499)	2.394	13	12	12	2	7	0.630
53	(0.477, 0.045, 0.001)	2.323	14	13	12	2	2	0.135
54	(0.387, 0.226, 0.001)	2.498	14	13	12	2	2	0.677
55	(0.333, 0.333, 0.000)	2.490	14	13	2	2	2	1.000
56	(0.127, 0.127, 0.000)	2.420	14	13	6	2	3	0.618
57	(0.049, 0.049, 0.000)	2.509	14	13	6	2	3	0.854
58	(0.079, 0.079, 0.000)	2.464	14	13	6	2	3	0.762
59	(0.356, 0.289, 0.499)	2.494	14	13	12	2	6	0.866
60	(0.333, 0.333, 0.500)	2.492	14	13	2	2	6	1.000
61	(0.406, 0.184, 0.003)	2.502	15	14	12	2	2	0.555
62	(0.442, 0.116, -0.000)	2.465	15	14	6	2	2	0.348
63	(0.442, 0.116, 0.500)	2.465	15	14	6	2	6	0.348
64	(0.078, 0.000, 0.000)	2.539	16	15	6	2	1	0.156
65	(0.110, 0.000, 0.000)	2.524	16	15	6	2	1	0.220
66	(0.369, 0.261, 0.001)	2.505	16	15	12	2	2	0.784
67	(0.232, 0.232, -0.000)	2.530	16	15	6	2	3	0.303
68	(0.033, 0.033, 0.000)	2.549	16	15	6	2	3	0.902

Supplementary Table 946: Nodal ring points along high symmetry lines of mp-27922-Ge3I6.

	Coordinates	Frequency	Mode 1	Mode 2	Multi.	Deg.	Path	Ratio
69	(0.299, 0.299, 0.000)	2.531	17	16	6	2	3	0.104
70	(0.024, 0.024, 0.000)	2.551	17	16	6	2	3	0.927
71	(0.024, 0.000, -0.000)	2.553	18	17	6	2	1	0.048
72	(0.051, -0.000, 0.000)	2.563	18	17	6	2	1	0.102
73	(0.091, -0.000, 0.000)	2.540	18	17	6	2	1	0.183
74	(0.053, 0.053, 0.001)	2.541	18	17	12	2	3	0.840
75	(0.465, 0.070, -0.000)	2.525	18	17	8	2	2	0.211
76	(0.333, 0.333, 0.000)	2.523	18	17	2	2	2	1.000
77	(0.052, 0.000, 0.500)	2.561	18	17	6	2	5	0.105
78	(0.080, 0.080, 0.500)	2.528	18	17	6	2	7	0.759
79	(0.298, 0.298, 0.500)	2.531	18	17	6	2	7	0.107
80	(0.333, 0.333, 0.500)	2.520	18	17	2	2	6	1.000
81	(0.038, 0.038, 0.500)	2.557	18	17	6	2	7	0.886
82	(0.046, 0.000, -0.001)	2.566	19	18	6	2	1	0.092
83	(0.043, -0.000, -0.000)	2.567	20	19	6	2	1	0.087
84	(0.103, 0.103, -0.000)	2.828	20	19	12	2	3	0.690
85	(0.383, 0.235, 0.001)	2.731	20	19	12	2	2	0.705
86	(0.150, 0.150, 0.000)	2.789	20	19	6	2	3	0.550
87	(0.243, 0.248, 0.498)	2.727	20	19	12	2	7	0.263
88	(0.452, 0.092, 0.002)	2.668	21	20	12	2	2	0.279
89	(0.053, 0.054, 0.498)	2.811	21	20	12	2	7	0.840
90	(0.425, 0.151, 0.497)	2.762	21	20	12	2	6	0.452
91	(0.419, 0.163, 0.497)	2.762	21	20	12	2	6	0.489
92	(0.149, 0.149, -0.002)	4.253	23	22	12	2	3	0.552
93	(0.149, 0.149, 0.500)	4.257	23	22	6	2	7	0.554
94	(0.155, 0.155, 0.000)	4.237	24	23	6	2	3	0.535
95	(0.440, 0.120, 0.499)	2.863	24	23	12	2	6	0.360
96	(0.044, 0.045, 0.499)	3.097	24	23	12	2	7	0.867
97	(0.131, 0.131, 0.500)	4.331	24	23	6	2	7	0.608
98	(0.126, 0.126, -0.000)	4.496	25	24	6	2	3	0.621
99	(0.160, 0.160, 0.500)	4.325	25	24	6	2	7	0.520
100	(0.150, 0.150, 0.000)	4.515	26	25	6	2	3	0.550

The followings are details for $Li_4Zn_4P_4$. As shown in Fig **456**, it has 3 topological nodal lines, 6 nodal ring points and 1 high degenerate Weyl point whose degeneracy is more than 2 and charge is bigger than 1.

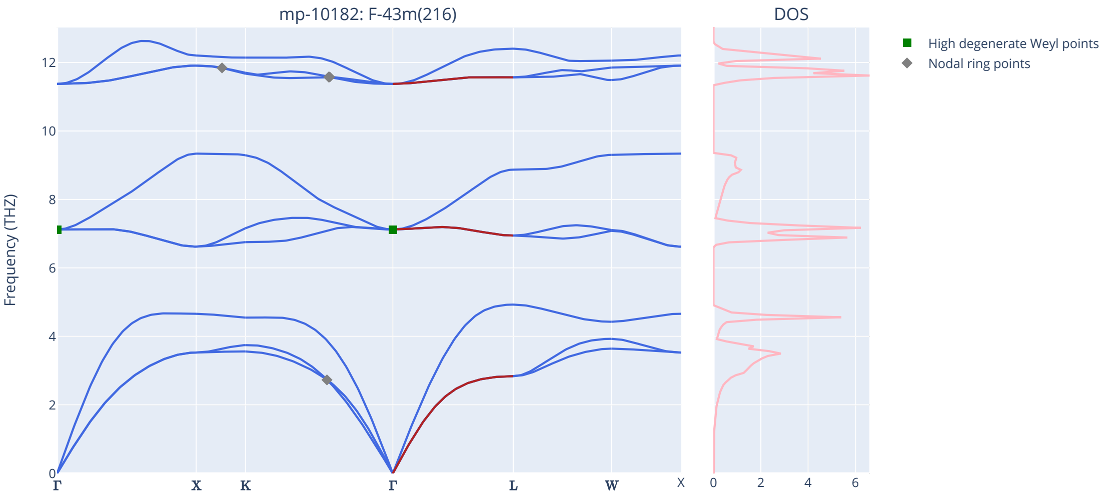

Supplementary Figure 456: The phonon band and DOS of Li4Zn4P4.

Supplementary Table 947: High symmetry lines of mp-10182-Li4Zn4P4

	Start	End
0	Γ (0.000, 0.000, 0.000)	X (0.500, 0.000, 0.500)
1	X (0.500, 0.000, 0.500)	U (0.625, 0.250, 0.625)
2	K (0.375, 0.375, 0.750)	Γ (0.000, 0.000, 0.000)
3	Γ (0.000, 0.000, 0.000)	L (0.500, 0.500, 0.500)
4	L (0.500, 0.500, 0.500)	W (0.500, 0.250, 0.750)
5	W (0.500, 0.250, 0.750)	X (0.500, 0.000, 0.500)

Supplementary Table 948: High degenerate Weyl points along high symmetry lines of mp-10182-Li4Zn4P4.

	Coordinates	Frequency	Mode 1	Mode 2	Multi.	Deg.	Path	Ratio
0	(0.000, 0.000, 0.000)	7.123	6	4	1	3	1	0.000

Supplementary Table 949: Nodal ring points along high symmetry lines of mp-10182-Li4Zn4P4.

	Coordinates	Frequency	Mode 1	Mode 2	Multi.	Deg.	Path	Ratio
0	(0.168, 0.168, 0.334)	2.730	2	1	24	2	3	0.554
1	(0.567, 0.131, 0.567)	11.843	8	7	24	2	2	0.527
2	(0.168, 0.168, 0.320)	11.576	8	7	24	2	3	0.565
3	(0.161, 0.161, 0.320)	11.576	8	7	24	2	3	0.572

The followings are details for K_8S_4. It has 4 topological nodal lines and 9 nodal ring points along high symmetry line as shown in Fig **457**.

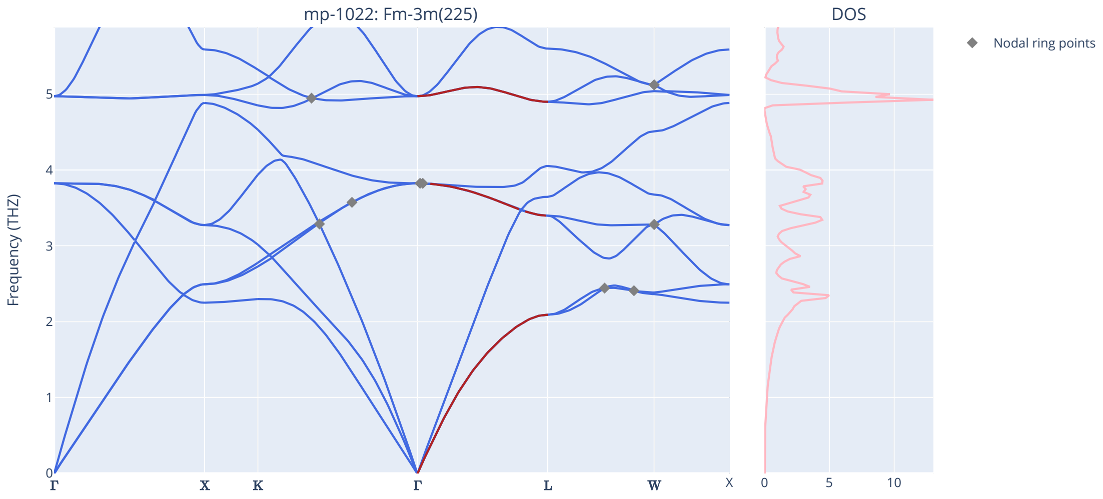

Supplementary Figure 457: The phonon band and DOS of K8S4.

Supplementary Table 950: High symmetry lines of mp-1022-K8S4

	Start	End
0	Γ (0.000, 0.000, 0.000)	X (0.500, 0.000, 0.500)
1	X (0.500, 0.000, 0.500)	U (0.625, 0.250, 0.625)
2	K (0.375, 0.375, 0.750)	Γ (0.000, 0.000, 0.000)
3	Γ (0.000, 0.000, 0.000)	L (0.500, 0.500, 0.500)
4	L (0.500, 0.500, 0.500)	W (0.500, 0.250, 0.750)
5	W (0.500, 0.250, 0.750)	X (0.500, 0.000, 0.500)

Supplementary Table 951: Nodal ring points along high symmetry lines of mp-1022-K8S4.

	Coordinates	Frequency	Mode 1	Mode 2	Multi.	Deg.	Path	Ratio
0	(0.500, 0.297, 0.703)	2.408	2	1	24	2	5	0.810
1	(0.500, 0.366, 0.634)	2.442	2	1	24	2	5	0.534
2	(0.500, 0.250, 0.750)	3.281	4	3	6	2	5	1.000
3	(0.225, 0.235, 0.460)	3.288	4	3	24	2	3	0.386
4	(0.153, 0.153, 0.308)	3.573	5	4	24	2	3	0.590
5	(0.010, 0.010, 0.010)	3.824	6	5	8	3	4	0.020
6	(0.020, 0.020, 0.020)	3.824	6	5	8	3	4	0.041
7	(0.253, 0.253, 0.494)	4.947	8	7	24	2	3	0.337
8	(0.500, 0.250, 0.750)	5.122	9	8	6	2	5	1.000

The followings are details for Ba_4F_8. It has 4 topological nodal lines and 4 nodal ring points along high symmetry line as shown in Fig 458.

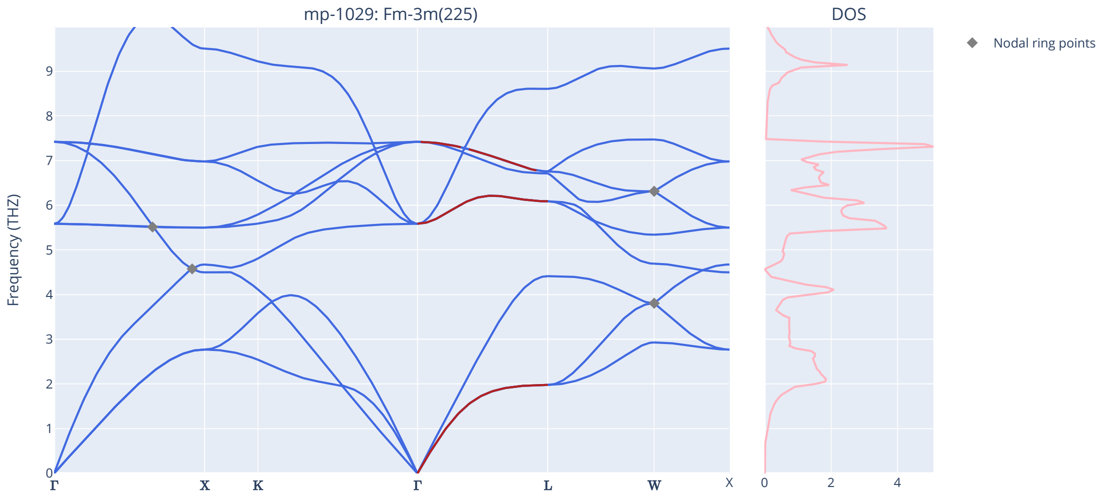

Supplementary Figure 458: The phonon band and DOS of Ba4F8.

Supplementary Table 952: High symmetry lines of mp-1029-Ba4F8

	Start	End
0	Γ (0.000, 0.000, 0.000)	X (0.500, 0.000, 0.500)
1	X (0.500, 0.000, 0.500)	U (0.625, 0.250, 0.625)
2	K (0.375, 0.375, 0.750)	Γ (0.000, 0.000, 0.000)
3	Γ (0.000, 0.000, 0.000)	L (0.500, 0.500, 0.500)
4	L (0.500, 0.500, 0.500)	W (0.500, 0.250, 0.750)
5	W (0.500, 0.250, 0.750)	X (0.500, 0.000, 0.500)

Supplementary Table 953: Nodal ring points along high symmetry lines of mp-1029-Ba4F8.

	Coordinates	Frequency	Mode 1	Mode 2	Multi.	Deg.	Path	Ratio
0	(0.500, 0.250, 0.750)	3.806	3	2	6	2	5	1.000
1	(0.459, 0.001, 0.459)	4.575	4	3	24	2	1	0.918
2	(0.332, 0.000, 0.323)	5.512	5	4	24	2	1	0.654
3	(0.500, 0.250, 0.750)	6.312	7	6	6	2	5	1.000

The followings are details for BaLiP. It has 4 nodal ring points and 2 single Weyl points along high symmetry line as shown in Fig 459.

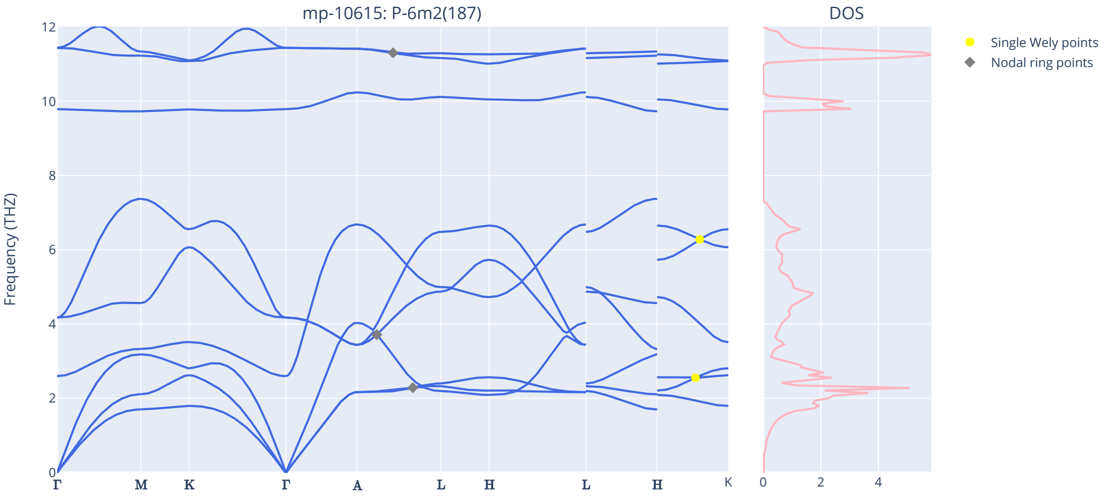

Supplementary Figure 459: The phonon band and DOS of BaLiP.

Supplementary Table 954: High symmetry lines of mp-10615-BaLiP

	Start	End
0	Γ (0.000, 0.000, 0.000)	M (0.500, 0.000, 0.000)
1	M (0.500, 0.000, 0.000)	K (0.333, 0.333, 0.000)
2	K (0.333, 0.333, 0.000)	Γ (0.000, 0.000, 0.000)
3	Γ (0.000, 0.000, 0.000)	A (0.000, 0.000, 0.500)
4	A (0.000, 0.000, 0.500)	L (0.500, 0.000, 0.500)
5	L (0.500, 0.000, 0.500)	H (0.333, 0.333, 0.500)
6	H (0.333, 0.333, 0.500)	A (0.000, 0.000, 0.500)
7	L (0.500, 0.000, 0.500)	M (0.500, 0.000, 0.000)
8	H (0.333, 0.333, 0.500)	K (0.333, 0.333, 0.000)

Supplementary Table 955: Single Weyl points along high symmetry lines of mp-10615-BaLiP.

	Coordinates	Frequency	Mode 1	Mode 2	Multi.	Deg.	Path	Ratio
0	(0.333, 0.333, 0.231)	2.554	3	2	4	2	9	0.539
1	(0.333, 0.333, 0.201)	6.280	6	5	4	2	9	0.598

Supplementary Table 956: Nodal ring points along high symmetry lines of mp-10615-BaLiP.

	Coordinates	Frequency	Mode 1	Mode 2	Multi.	Deg.	Path	Ratio
0	(0.333, 0.000, 0.500)	2.285	2	1	6	2	5	0.666

Supplementary Table 956: Nodal ring points along high symmetry lines of mp-10615-BaLiP.

	Coordinates	Frequency	Mode 1	Mode 2	Multi.	Deg.	Path	Ratio
1	(0.118, 0.000, 0.500)	3.714	4	3	6	2	5	0.236
2	(0.215, 0.000, 0.500)	11.305	9	8	6	2	5	0.430

The followings are details for BaLiAs. It has 4 nodal ring points and 4 single Weyl points along high symmetry line as shown in Fig 460.

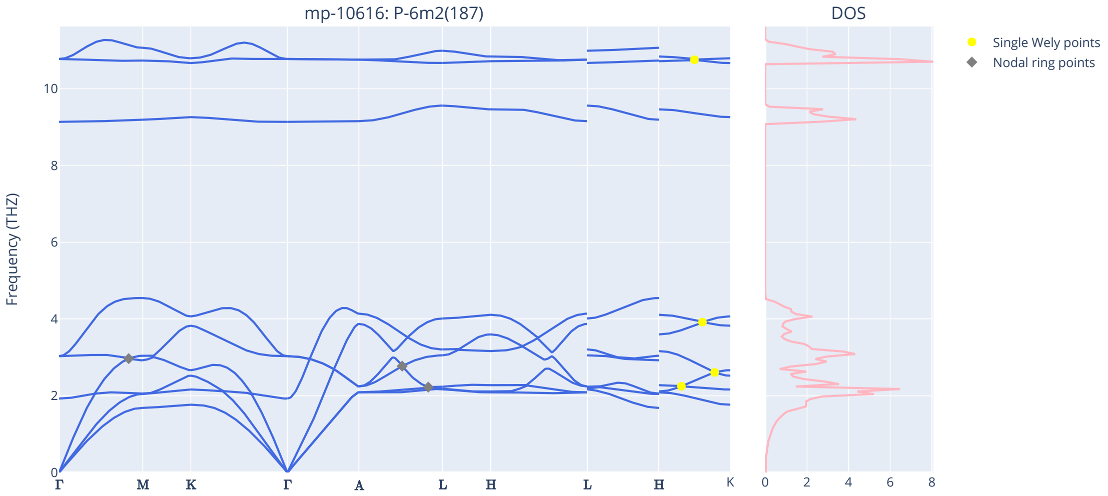

Supplementary Figure 460: The phonon band and DOS of BaLiAs.

Supplementary Table 957: High symmetry lines of mp-10616-BaLiAs

	Start	End
0	Γ (0.000, 0.000, 0.000)	M (0.500, 0.000, 0.000)
1	M (0.500, 0.000, 0.000)	K (0.333, 0.333, 0.000)
2	K (0.333, 0.333, 0.000)	Γ (0.000, 0.000, 0.000)
3	Γ (0.000, 0.000, 0.000)	A (0.000, 0.000, 0.500)
4	A (0.000, 0.000, 0.500)	L (0.500, 0.000, 0.500)
5	L (0.500, 0.000, 0.500)	H (0.333, 0.333, 0.500)
6	H (0.333, 0.333, 0.500)	A (0.000, 0.000, 0.500)
7	L (0.500, 0.000, 0.500)	M (0.500, 0.000, 0.000)
8	H (0.333, 0.333, 0.500)	K (0.333, 0.333, 0.000)

Supplementary Table 958: Single Weyl points along high symmetry lines of mp-10616-BaLiAs.

	Coordinates	Frequency	Mode 1	Mode 2	Multi.	Deg.	Path	Ratio
0	(0.333, 0.333, 0.342)	2.246	3	2	4	2	9	0.317
1	(0.333, 0.333, 0.110)	2.606	4	3	4	2	9	0.780
2	(0.333, 0.333, 0.195)	3.917	6	5	4	2	9	0.611
3	(0.333, 0.333, 0.251)	10.753	9	8	4	2	9	0.498

Supplementary Table 959: Nodal ring points along high symmetry lines of mp-10616-BaLiAs.

	Coordinates	Frequency	Mode 1	Mode 2	Multi.	Deg.	Path	Ratio
0	(0.415, 0.002, 0.500)	2.223	3	2	12	2	5	0.829

Supplementary Table 959: Nodal ring points along high symmetry lines of mp-10616-BaLiAs.

	Coordinates	Frequency	Mode 1	Mode 2	Multi.	Deg.	Path	Ratio
1	(0.259, 0.000, 0.500)	2.770	4	3	6	2	5	0.517
2	(0.417, 0.000, -0.000)	2.966	5	4	6	2	1	0.833

The followings are details for $Rh_4Th_4Sb_4$. As shown in Fig 461, it has 3 topological nodal lines and 2 nodal ring points. At the same time, it has 1 single Weyl point and 1 high degenerate Weyl point whose degeneracy is more than 2 and charge is bigger than 1.

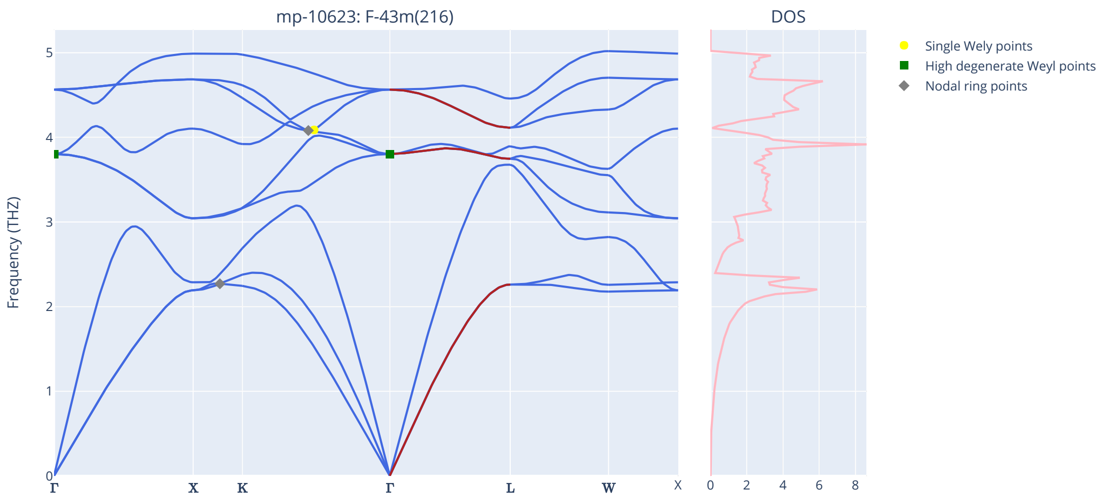

Supplementary Figure 461: The phonon band and DOS of Rh4Th4Sb4.

Supplementary Table 960: High symmetry lines of mp-10623-Rh4Th4Sb4

	Start	End
0	Γ (0.000, 0.000, 0.000)	X (0.500, 0.000, 0.500)
1	X (0.500, 0.000, 0.500)	U (0.625, 0.250, 0.625)
2	K (0.375, 0.375, 0.750)	Γ (0.000, 0.000, 0.000)
3	Γ (0.000, 0.000, 0.000)	L (0.500, 0.500, 0.500)
4	L (0.500, 0.500, 0.500)	W (0.500, 0.250, 0.750)
5	W (0.500, 0.250, 0.750)	X (0.500, 0.000, 0.500)

Supplementary Table 961: Single Weyl points along high symmetry lines of mp-10623-Rh4Th4Sb4.

	Coordinates	Frequency	Mode 1	Mode 2	Multi.	Deg.	Path	Ratio
0	(0.185, 0.199, 0.385)	4.088	7	6	24	2	3	0.487

Supplementary Table 962: High degenerate Weyl points along high symmetry lines of mp-10623-Rh4Th4Sb4.

	Coordinates	Frequency	Mode 1	Mode 2	Multi.	Deg.	Path	Ratio
0	(0.000, 0.000, 0.000)	3.803	6	4	1	3	1	0.000

Supplementary Table 963: Nodal ring points along high symmetry lines of mp-10623-Rh4Th4Sb4.

	Coordinates	Frequency	Mode 1	Mode 2	Multi.	Deg.	Path	Ratio
0	(0.569, 0.135, 0.569)	2.270	2	1	24	2	2	0.544
1	(0.207, 0.207, 0.415)	4.081	7	6	24	2	3	0.448

The followings are details for Ba_2O_4 and it only has 8 topological nodal ring points along high symmetry line as shown in Fig 462.

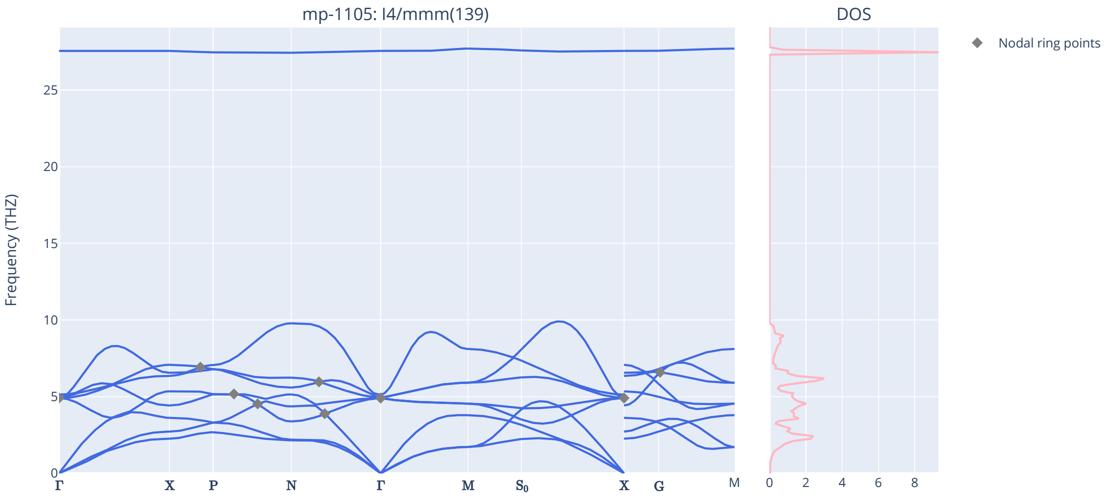

Supplementary Figure 462: The phonon band and DOS of Ba2O4.

Supplementary Table 964: High symmetry lines of mp-1105-Ba2O4

	Start	End
0	Γ (0.000, 0.000, 0.000)	X (0.000, 0.000, 0.500)
1	X (0.000, 0.000, 0.500)	P (0.250, 0.250, 0.250)
2	P (0.250, 0.250, 0.250)	N (0.000, 0.500, 0.000)
3	N (0.000, 0.500, 0.000)	Γ (0.000, 0.000, 0.000)
4	Γ (0.000, 0.000, 0.000)	M (0.500, 0.500, -0.500)
5	M (0.500, 0.500, -0.500)	S (0.328, 0.672, -0.328)
6	S_0 (-0.328, 0.328, 0.328)	Γ (0.000, 0.000, 0.000)
7	X (0.000, 0.000, 0.500)	R (-0.156, 0.156, 0.500)
8	G (0.500, 0.500, -0.156)	M (0.500, 0.500, -0.500)

Supplementary Table 965: Nodal ring points along high symmetry lines of mp-1105-Ba2O4.

	Coordinates	Frequency	Mode 1	Mode 2	Multi.	Deg.	Path	Ratio
0	(0.108, 0.393, 0.107)	4.524	4	3	16	2	3	0.570
1	(0.001, 0.312, -0.001)	3.886	4	3	8	2	4	0.375
2	(0.000, 0.000, 0.000)	4.907	5	4	1	2	1	0.000
3	(0.183, 0.317, 0.183)	5.174	5	4	8	2	3	0.267
4	(0.001, 0.345, -0.001)	5.963	7	6	8	2	4	0.310
5	(0.500, 0.500, -0.163)	6.585	7	6	4	2	9	0.022
6	(0.177, 0.177, 0.323)	6.923	8	7	4	2	2	0.708

The followings are details for Rb_8Se_4. It has 5 topological nodal lines and 6 nodal ring points along high symmetry line as shown in Fig 463.

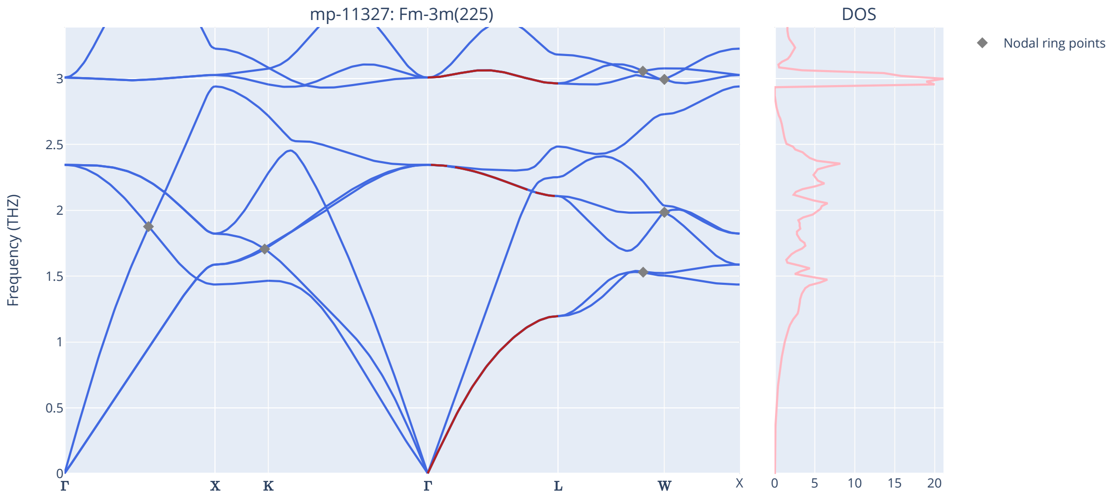

Supplementary Figure 463: The phonon band and DOS of Rb8Se4.

Supplementary Table 966: High symmetry lines of mp-11327-Rb8Se4

	Start	End
0	Γ (0.000, 0.000, 0.000)	X (0.500, 0.000, 0.500)
1	X (0.500, 0.000, 0.500)	U (0.625, 0.250, 0.625)
2	K (0.375, 0.375, 0.750)	Γ (0.000, 0.000, 0.000)
3	Γ (0.000, 0.000, 0.000)	L (0.500, 0.500, 0.500)
4	L (0.500, 0.500, 0.500)	W (0.500, 0.250, 0.750)
5	W (0.500, 0.250, 0.750)	X (0.500, 0.000, 0.500)

Supplementary Table 967: Nodal ring points along high symmetry lines of mp-11327-Rb8Se4.

	Coordinates	Frequency	Mode 1	Mode 2	Multi.	Deg.	Path	Ratio
0	(0.500, 0.300, 0.700)	1.530	2	1	24	2	5	0.801
1	(0.617, 0.234, 0.617)	1.706	3	2	12	2	2	0.934
2	(0.279, -0.002, 0.279)	1.876	4	3	24	2	1	0.558
3	(0.500, 0.250, 0.750)	1.985	4	3	6	2	5	1.000
4	(0.500, 0.250, 0.750)	2.994	8	7	6	2	5	1.000
5	(0.500, 0.301, 0.699)	3.056	9	8	24	2	5	0.798

The followings are details for Li_8S_4. It has 3 topological nodal lines and 11 nodal ring points along high symmetry line as shown in Fig **464**.

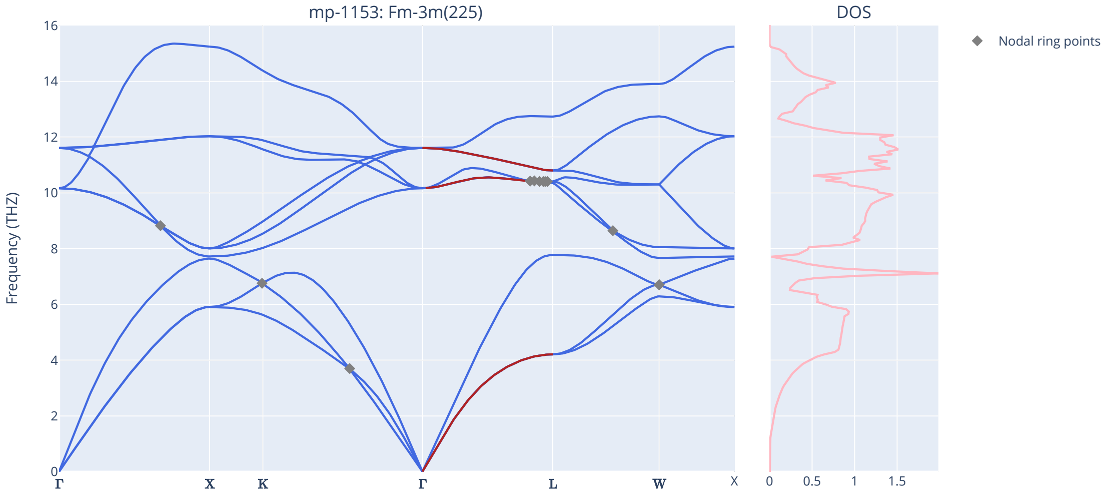

Supplementary Figure 464: The phonon band and DOS of Li8S4.

Supplementary Table 968: High symmetry lines of mp-1153-Li8S4

	Start	End
0	Γ (0.000, 0.000, 0.000)	X (0.500, 0.000, 0.500)
1	X (0.500, 0.000, 0.500)	U (0.625, 0.250, 0.625)
2	K (0.375, 0.375, 0.750)	Γ (0.000, 0.000, 0.000)
3	Γ (0.000, 0.000, 0.000)	L (0.500, 0.500, 0.500)
4	L (0.500, 0.500, 0.500)	W (0.500, 0.250, 0.750)
5	W (0.500, 0.250, 0.750)	X (0.500, 0.000, 0.500)

Supplementary Table 969: Nodal ring points along high symmetry lines of mp-1153-Li8S4.

	Coordinates	Frequency	Mode 1	Mode 2	Multi.	Deg.	Path	Ratio
0	(0.168, 0.168, 0.344)	3.700	2	1	24	2	3	0.544
1	(0.623, 0.247, 0.623)	6.757	3	2	12	2	2	0.986
2	(0.500, 0.250, 0.750)	6.704	3	2	6	2	5	1.000
3	(0.500, 0.359, 0.641)	8.643	5	4	24	2	5	0.565
4	(0.464, 0.464, 0.464)	10.405	6	5	8	2	4	0.927
5	(0.480, 0.480, 0.480)	10.404	6	5	8	2	4	0.959
6	(0.337, -0.000, 0.337)	8.821	6	5	6	2	1	0.674
7	(0.433, 0.428, 0.428)	10.427	6	5	24	2	4	0.859
8	(0.449, 0.449, 0.449)	10.407	6	5	8	2	4	0.898
9	(0.413, 0.413, 0.413)	10.420	6	5	8	2	4	0.827
10	(0.471, 0.471, 0.471)	10.404	6	5	8	2	4	0.942

The followings are details for ZrS_2. It has 5 topological nodal lines and 14 nodal ring points along high symmetry line as shown in Fig **465**.

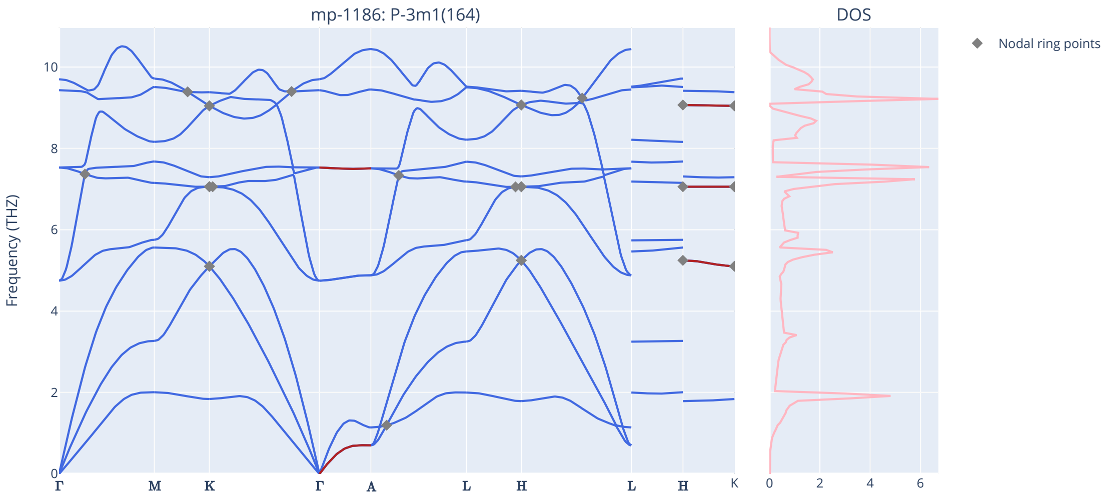

Supplementary Figure 465: The phonon band and DOS of ZrS2.

Supplementary Table 970: High symmetry lines of mp-1186-ZrS2

	Start	End
0	Γ (0.000, 0.000, 0.000)	M (0.500, 0.000, 0.000)
1	M (0.500, 0.000, 0.000)	K (0.333, 0.333, 0.000)
2	K (0.333, 0.333, 0.000)	Γ (0.000, 0.000, 0.000)
3	Γ (0.000, 0.000, 0.000)	A (0.000, 0.000, 0.500)
4	A (0.000, 0.000, 0.500)	L (0.500, 0.000, 0.500)
5	L (0.500, 0.000, 0.500)	H (0.333, 0.333, 0.500)
6	H (0.333, 0.333, 0.500)	A (0.000, 0.000, 0.500)
7	L (0.500, 0.000, 0.500)	M (0.500, 0.000, 0.000)
8	H (0.333, 0.333, 0.500)	K (0.333, 0.333, 0.000)

Supplementary Table 971: Nodal ring points along high symmetry lines of mp-1186-ZrS2.

	Coordinates	Frequency	Mode 1	Mode 2	Multi.	Deg.	Path	Ratio
0	(0.082, -0.000, 0.499)	1.193	2	1	6	2	5	0.164
1	(0.333, 0.333, 0.000)	5.100	3	2	2	2	2	1.000
2	(0.333, 0.333, 0.500)	5.247	3	2	2	2	6	1.000
3	(0.333, 0.333, 0.000)	7.058	5	4	2	2	2	1.000
4	(0.323, 0.323, 0.000)	7.057	5	4	6	2	3	0.030
5	(0.351, 0.298, 0.499)	7.054	5	4	12	2	6	0.894
6	(0.333, 0.333, 0.500)	7.057	5	4	2	2	6	1.000
7	(0.136, -0.000, -0.000)	7.368	6	5	6	2	1	0.271
8	(0.146, -0.000, 0.500)	7.335	6	5	6	2	5	0.292
9	(0.333, 0.333, 0.000)	9.046	8	7	2	2	2	1.000
10	(0.333, 0.333, 0.500)	9.067	8	7	2	2	6	1.000

Supplementary Table 971: Nodal ring points along high symmetry lines of mp-1186-ZrS2.

	Coordinates	Frequency	Mode 1	Mode 2	Multi.	Deg.	Path	Ratio
11	(0.399, 0.202, -0.000)	9.390	9	8	6	2	2	0.605
12	(0.084, 0.084, -0.000)	9.398	9	8	6	2	3	0.747
13	(0.149, 0.149, 0.500)	9.236	9	8	6	2	7	0.553

The followings are details for $Li_4Mg_4As_4$. As shown in Fig **466**, it has 3 topological nodal lines, 1 nodal ring point and 1 high degenerate Weyl point whose degeneracy is more than 2 and charge is bigger than 1.

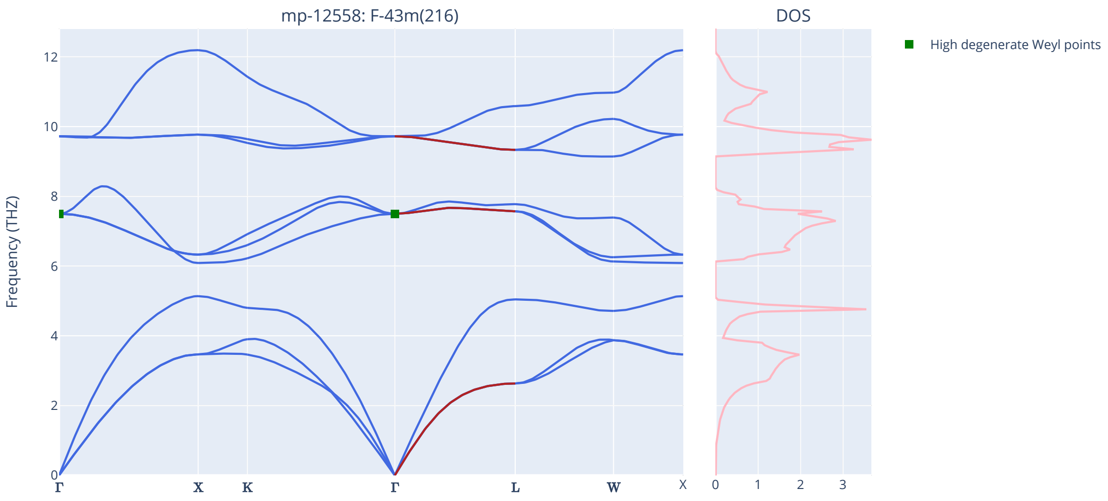

Supplementary Figure 466: The phonon band and DOS of Li4Mg4As4.

Supplementary Table 972: High symmetry lines of mp-12558-Li4Mg4As4

	Start	End
0	Γ (0.000, 0.000, 0.000)	X (0.500, 0.000, 0.500)
1	X (0.500, 0.000, 0.500)	U (0.625, 0.250, 0.625)
2	K (0.375, 0.375, 0.750)	Γ (0.000, 0.000, 0.000)
3	Γ (0.000, 0.000, 0.000)	L (0.500, 0.500, 0.500)
4	L (0.500, 0.500, 0.500)	W (0.500, 0.250, 0.750)
5	W (0.500, 0.250, 0.750)	X (0.500, 0.000, 0.500)

Supplementary Table 973: High degenerate Weyl points along high symmetry lines of mp-12558-Li4Mg4As4.

	Coordinates	Frequency	Mode 1	Mode 2	Multi.	Deg.	Path	Ratio
0	(0.000, 0.000, 0.000)	7.498	6	4	1	3	1	0.000

The followings are details for Na_8Se_4. It has 3 topological nodal lines and 10 nodal ring points along high symmetry line as shown in Fig **467**.

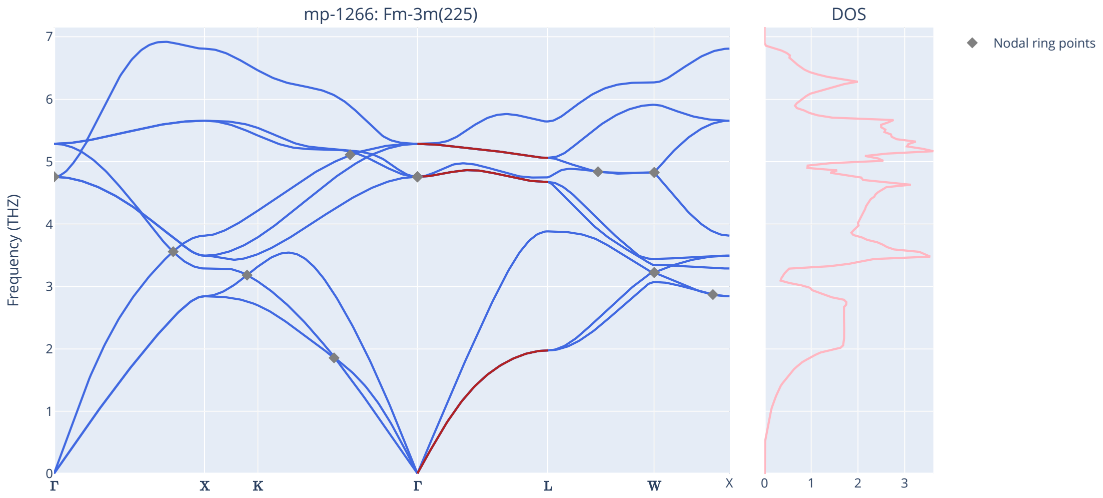

Supplementary Figure 467: The phonon band and DOS of Na8Se4.

Supplementary Table 974: High symmetry lines of mp-1266-Na8Se4

	Start	End
0	Γ (0.000, 0.000, 0.000)	X (0.500, 0.000, 0.500)
1	X (0.500, 0.000, 0.500)	U (0.625, 0.250, 0.625)
2	K (0.375, 0.375, 0.750)	Γ (0.000, 0.000, 0.000)
3	Γ (0.000, 0.000, 0.000)	L (0.500, 0.500, 0.500)
4	L (0.500, 0.500, 0.500)	W (0.500, 0.250, 0.750)
5	W (0.500, 0.250, 0.750)	X (0.500, 0.000, 0.500)

Supplementary Table 975: Nodal ring points along high symmetry lines of mp-1266-Na8Se4.

	Coordinates	Frequency	Mode 1	Mode 2	Multi.	Deg.	Path	Ratio
0	(0.199, 0.199, 0.389)	1.857	2	1	24	2	3	0.478
1	(0.498, 0.056, 0.554)	2.870	2	1	24	2	6	0.781
2	(0.600, 0.200, 0.600)	3.182	3	2	12	2	2	0.799
3	(0.500, 0.250, 0.750)	3.224	3	2	6	2	5	1.000
4	(0.396, 0.002, 0.396)	3.557	4	3	24	2	1	0.791
5	(0.000, 0.000, 0.000)	4.757	5	4	1	3	1	0.000
6	(0.000, 0.000, 0.000)	4.757	6	5	1	3	1	0.000
7	(0.152, 0.164, 0.316)	5.110	7	6	24	2	3	0.579
8	(0.500, 0.250, 0.750)	4.827	7	6	6	2	5	1.000
9	(0.500, 0.382, 0.618)	4.839	7	6	24	2	5	0.472

The followings are details for Rb_8O_4. It has 3 topological nodal lines and 13 nodal ring points along high symmetry line as shown in Fig **468**.

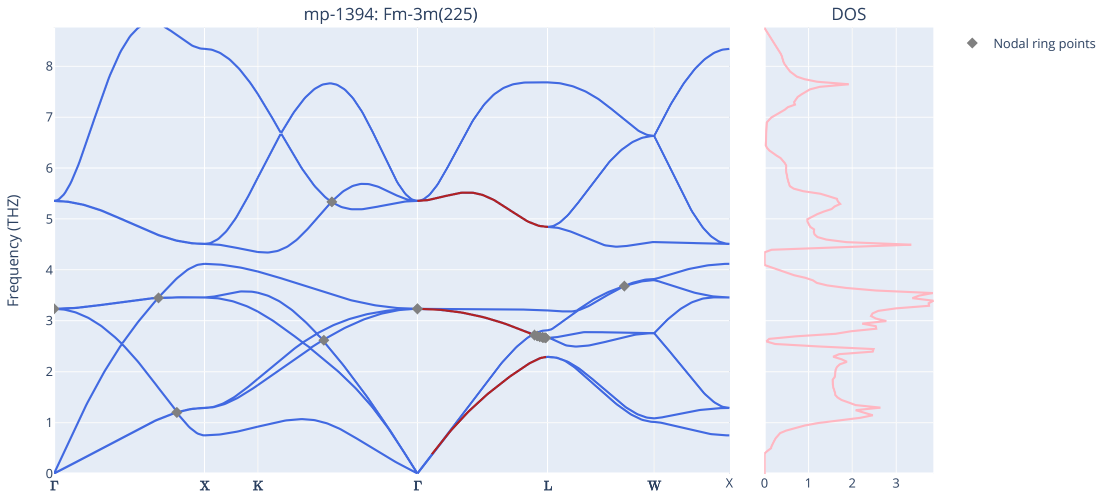

Supplementary Figure 468: The phonon band and DOS of Rb8O4.

Supplementary Table 976: High symmetry lines of mp-1394-Rb8O4

	Start	End
0	Γ (0.000, 0.000, 0.000)	X (0.500, 0.000, 0.500)
1	X (0.500, 0.000, 0.500)	U (0.625, 0.250, 0.625)
2	K (0.375, 0.375, 0.750)	Γ (0.000, 0.000, 0.000)
3	Γ (0.000, 0.000, 0.000)	L (0.500, 0.500, 0.500)
4	L (0.500, 0.500, 0.500)	W (0.500, 0.250, 0.750)
5	W (0.500, 0.250, 0.750)	X (0.500, 0.000, 0.500)

Supplementary Table 977: Nodal ring points along high symmetry lines of mp-1394-Rb8O4.

	Coordinates	Frequency	Mode 1	Mode 2	Multi.	Deg.	Path	Ratio
0	(0.408, 0.003, 0.408)	1.199	2	1	24	2	1	0.816
1	(0.408, 0.000, 0.408)	1.201	3	2	6	2	1	0.815
2	(0.492, 0.492, 0.492)	2.664	4	3	8	2	4	0.985
3	(0.214, 0.214, 0.445)	2.618	4	3	24	2	3	0.414
4	(0.485, 0.485, 0.485)	2.668	4	3	8	2	4	0.971
5	(0.449, 0.449, 0.449)	2.719	4	3	8	2	4	0.898
6	(0.459, 0.459, 0.459)	2.701	4	3	8	2	4	0.918
7	(0.469, 0.469, 0.469)	2.686	4	3	8	2	4	0.939
8	(0.480, 0.480, 0.480)	2.674	4	3	8	2	4	0.959
9	(0.000, 0.000, 0.000)	3.235	5	4	1	3	1	0.000
10	(0.347, 0.000, 0.347)	3.449	5	4	6	2	1	0.694
11	(0.500, 0.320, 0.680)	3.681	6	5	24	2	5	0.720
12	(0.199, 0.199, 0.404)	5.335	8	7	24	2	3	0.464

The followings are details for Mo_3S_6. It has 3 topological nodal lines and 2 nodal ring points along high symmetry line as shown in Fig **469**.

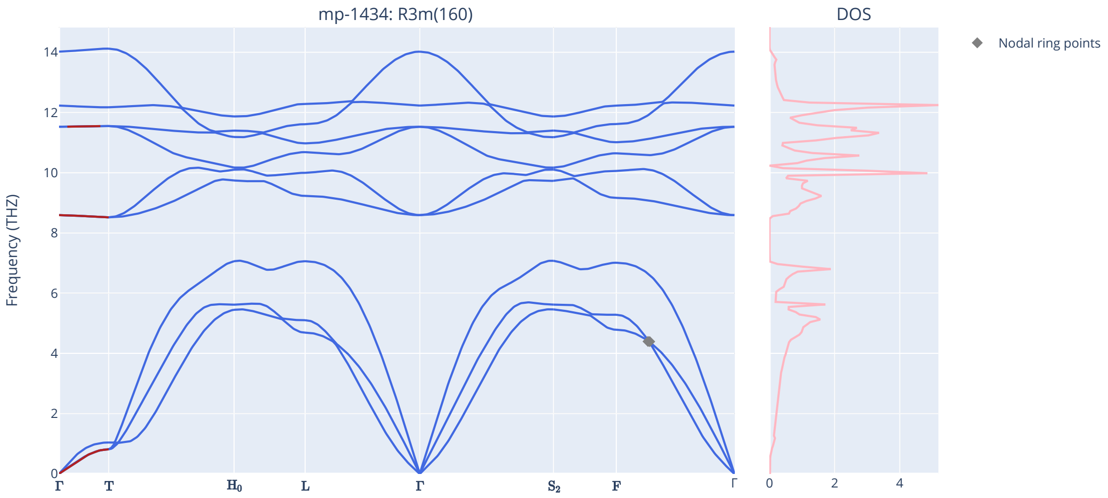

Supplementary Figure 469: The phonon band and DOS of Mo3S6.

Supplementary Table 978: High symmetry lines of mp-1434-Mo3S6

	Start	End
0	Γ (0.000, 0.000, 0.000)	T (0.500, 0.500, 0.500)
1	T (0.500, 0.500, 0.500)	H_2 (0.819, 0.181, 0.500)
2	H_0 (0.500, -0.181, 0.181)	L (0.500, 0.000, 0.000)
3	L (0.500, 0.000, 0.000)	Γ (0.000, 0.000, 0.000)
4	Γ (0.000, 0.000, 0.000)	S_0 (0.340, -0.340, 0.000)
5	S_2 (0.660, 0.000, 0.340)	F (0.500, 0.000, 0.500)
6	F (0.500, 0.000, 0.500)	Γ (0.000, 0.000, 0.000)

Supplementary Table 979: Nodal ring points along high symmetry lines of mp-1434-Mo3S6.

	Coordinates	Frequency	Mode 1	Mode 2	Multi.	Deg.	Path	Ratio
0	(0.366, 0.004, 0.366)	4.395	2	1	6	2	7	0.269
1	(0.359, -0.003, 0.359)	4.390	2	1	6	2	7	0.283

The followings are details for Be_8C_4. It has 5 topological nodal lines and 9 nodal ring points along high symmetry line as shown in Fig **470**.

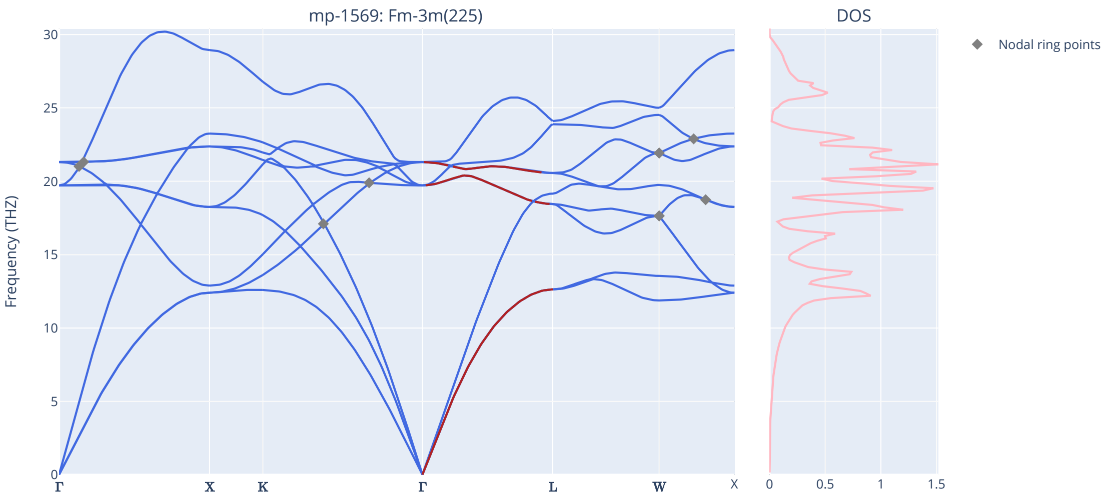

Supplementary Figure 470: The phonon band and DOS of Be8C4.

Supplementary Table 980: High symmetry lines of mp-1569-Be8C4

	Start	End
0	Γ (0.000, 0.000, 0.000)	X (0.500, 0.000, 0.500)
1	X (0.500, 0.000, 0.500)	U (0.625, 0.250, 0.625)
2	K (0.375, 0.375, 0.750)	Γ (0.000, 0.000, 0.000)
3	Γ (0.000, 0.000, 0.000)	L (0.500, 0.500, 0.500)
4	L (0.500, 0.500, 0.500)	W (0.500, 0.250, 0.750)
5	W (0.500, 0.250, 0.750)	X (0.500, 0.000, 0.500)

Supplementary Table 981: Nodal ring points along high symmetry lines of mp-1569-Be8C4.

	Coordinates	Frequency	Mode 1	Mode 2	Multi.	Deg.	Path	Ratio
0	(0.237, 0.237, 0.461)	17.102	4	3	24	2	3	0.379
1	(0.500, 0.250, 0.750)	17.642	4	3	6	2	5	1.000
2	(0.130, 0.130, 0.246)	19.898	5	4	24	2	3	0.666
3	(0.499, 0.096, 0.595)	18.744	5	4	24	2	6	0.617
4	(0.066, -0.002, 0.066)	21.014	7	6	24	2	1	0.133
5	(0.500, 0.250, 0.750)	21.926	7	6	6	2	5	1.000
6	(0.082, 0.000, 0.082)	21.320	8	7	6	2	1	0.163
7	(0.497, 0.134, 0.637)	22.891	8	7	24	2	6	0.458

The followings are details for $Dy_4Pt_4Sb_4$. As shown in Fig **471**, it has 4 topological nodal lines, 2 nodal ring points and 1 high degenerate Weyl point whose degeneracy is more than 2 and charge is bigger than 1.

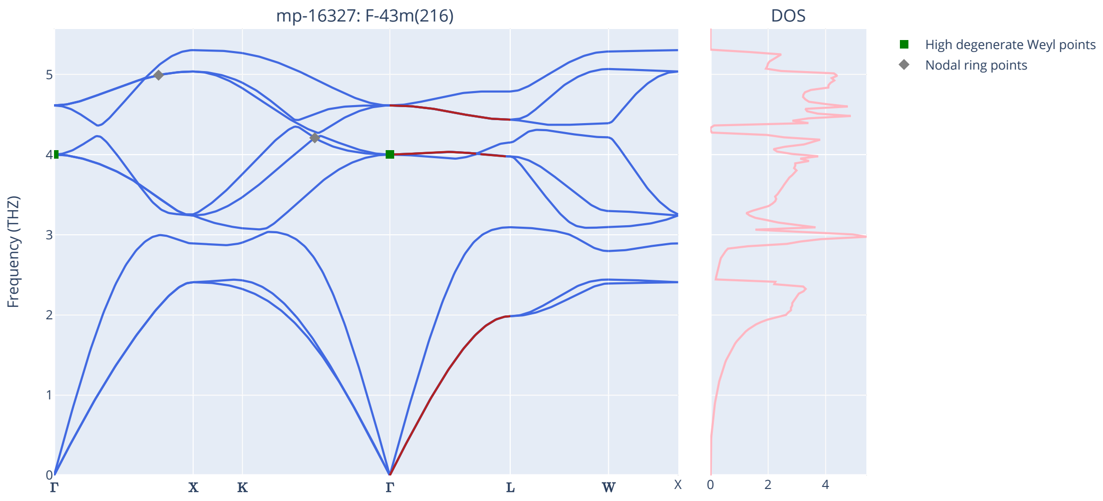

Supplementary Figure 471: The phonon band and DOS of Dy4Pt4Sb4.

Supplementary Table 982: High symmetry lines of mp-16327-Dy4Pt4Sb4

	Start	End
0	Γ (0.000, 0.000, 0.000)	X (0.500, 0.000, 0.500)
1	X (0.500, 0.000, 0.500)	U (0.625, 0.250, 0.625)
2	K (0.375, 0.375, 0.750)	Γ (0.000, 0.000, 0.000)
3	Γ (0.000, 0.000, 0.000)	L (0.500, 0.500, 0.500)
4	L (0.500, 0.500, 0.500)	W (0.500, 0.250, 0.750)
5	W (0.500, 0.250, 0.750)	X (0.500, 0.000, 0.500)

Supplementary Table 983: High degenerate Weyl points along high symmetry lines of mp-16327-Dy4Pt4Sb4.

	Coordinates	Frequency	Mode 1	Mode 2	Multi.	Deg.	Path	Ratio
0	(0.000, 0.000, 0.000)	4.001	6	4	1	3	1	0.000

Supplementary Table 984: Nodal ring points along high symmetry lines of mp-16327-Dy4Pt4Sb4.

	Coordinates	Frequency	Mode 1	Mode 2	Multi.	Deg.	Path	Ratio
0	(0.191, 0.191, 0.381)	4.209	6	5	24	2	3	0.491
1	(0.372, 0.000, 0.380)	4.991	8	7	24	2	1	0.751

The followings are details for $Er_4Pt_4Sb_4$. As shown in Fig **472**, it has 4 topological nodal lines, 2 nodal ring points and 1 high degenerate Weyl point whose degeneracy is more than 2 and charge is bigger than 1.

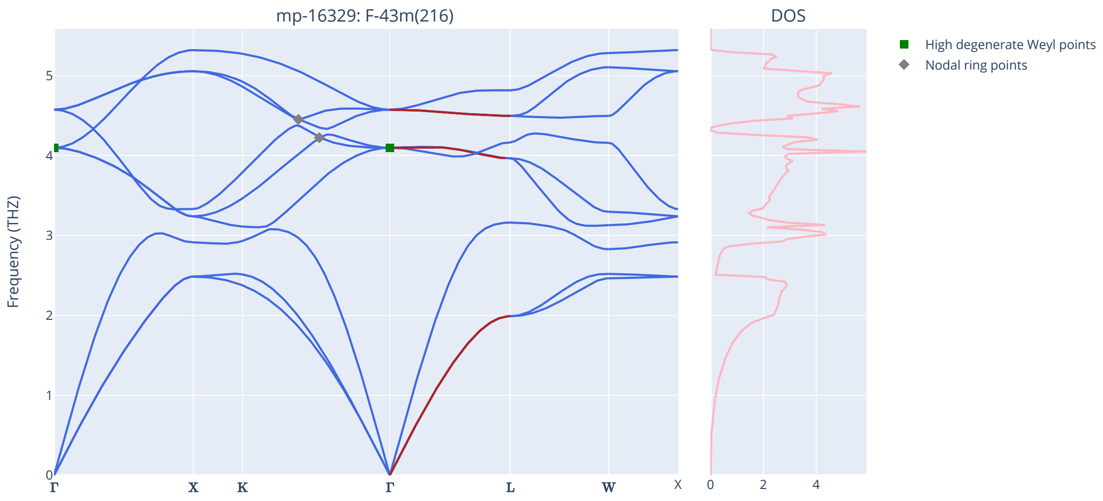

Supplementary Figure 472: The phonon band and DOS of Er4Pt4Sb4.

Supplementary Table 985: High symmetry lines of mp-16329-Er4Pt4Sb4

	Start	End
0	Γ (0.000, 0.000, 0.000)	X (0.500, 0.000, 0.500)
1	X (0.500, 0.000, 0.500)	U (0.625, 0.250, 0.625)
2	K (0.375, 0.375, 0.750)	Γ (0.000, 0.000, 0.000)
3	Γ (0.000, 0.000, 0.000)	L (0.500, 0.500, 0.500)
4	L (0.500, 0.500, 0.500)	W (0.500, 0.250, 0.750)
5	W (0.500, 0.250, 0.750)	X (0.500, 0.000, 0.500)

Supplementary Table 986: High degenerate Weyl points along high symmetry lines of mp-16329-Er4Pt4Sb4.

	Coordinates	Frequency	Mode 1	Mode 2	Multi.	Deg.	Path	Ratio
0	(0.000, 0.000, 0.000)	4.098	6	4	1	3	1	0.000

Supplementary Table 987: Nodal ring points along high symmetry lines of mp-16329-Er4Pt4Sb4.

	Coordinates	Frequency	Mode 1	Mode 2	Multi.	Deg.	Path	Ratio
0	(0.184, 0.184, 0.354)	4.224	6	5	24	2	3	0.522
1	(0.237, 0.237, 0.460)	4.453	8	7	24	2	3	0.380

The followings are details for $Ho_4Pt_4Sb_4$. As shown in Fig **473**, it has 4 topological nodal lines and 3 nodal ring points. At the same time, it has 1 single Weyl point and 1 high degenerate Weyl point whose degeneracy is more than 2 and charge is bigger than 1.

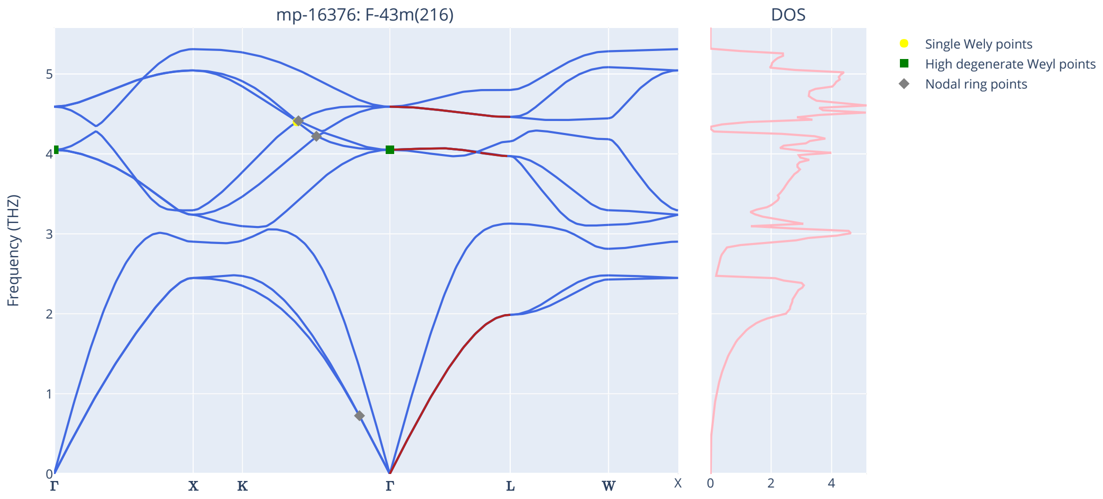

Supplementary Figure 473: The phonon band and DOS of Ho4Pt4Sb4.

Supplementary Table 988: High symmetry lines of mp-16376-Ho4Pt4Sb4

	Start	End
0	Γ (0.000, 0.000, 0.000)	X (0.500, 0.000, 0.500)
1	X (0.500, 0.000, 0.500)	U (0.625, 0.250, 0.625)
2	K (0.375, 0.375, 0.750)	Γ (0.000, 0.000, 0.000)
3	Γ (0.000, 0.000, 0.000)	L (0.500, 0.500, 0.500)
4	L (0.500, 0.500, 0.500)	W (0.500, 0.250, 0.750)
5	W (0.500, 0.250, 0.750)	X (0.500, 0.000, 0.500)

Supplementary Table 989: Single Weyl points along high symmetry lines of mp-16376-Ho4Pt4Sb4.

	Coordinates	Frequency	Mode 1	Mode 2	Multi.	Deg.	Path	Ratio
0	(0.237, 0.235, 0.472)	4.401	7	6	24	2	3	0.371

Supplementary Table 990: High degenerate Weyl points along high symmetry lines of mp-16376-Ho4Pt4Sb4.

	Coordinates	Frequency	Mode 1	Mode 2	Multi.	Deg.	Path	Ratio
0	(0.000, 0.000, 0.000)	4.052	6	4	1	3	1	0.000

Supplementary Table 991: Nodal ring points along high symmetry lines of mp-16376-Ho4Pt4Sb4.

	Coordinates	Frequency	Mode 1	Mode 2	Multi.	Deg.	Path	Ratio
0	(0.077, 0.077, 0.154)	0.727	2	1	24	2	3	0.795
1	(0.191, 0.191, 0.368)	4.221	6	5	24	2	3	0.502
2	(0.234, 0.234, 0.463)	4.415	8	7	24	2	3	0.381

The followings are details for K_8Te_4. It has 3 topological nodal lines and 15 nodal ring points along high symmetry line as shown in Fig **474**.

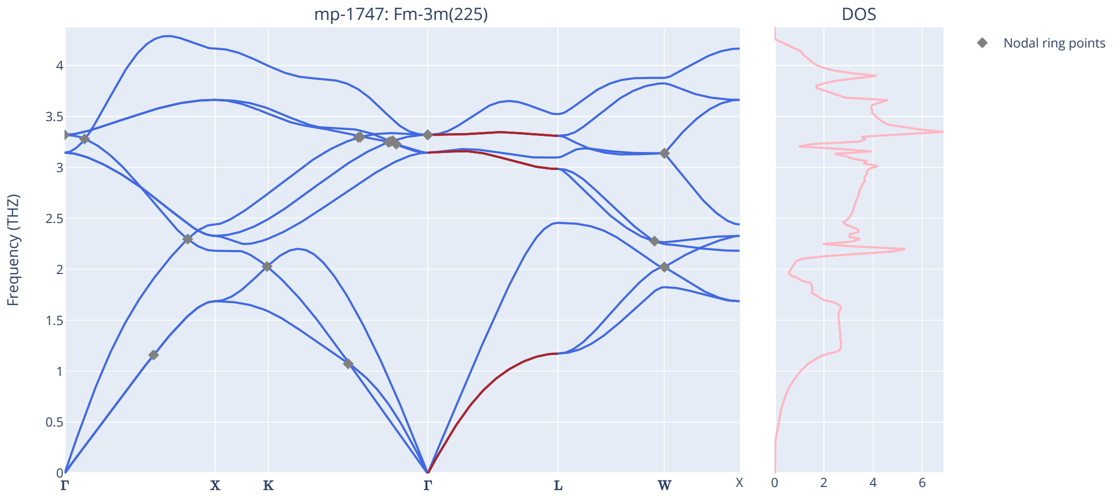

Supplementary Figure 474: The phonon band and DOS of K8Te4.

Supplementary Table 992: High symmetry lines of mp-1747-K8Te4

	Start	End
0	Γ (0.000, 0.000, 0.000)	X (0.500, 0.000, 0.500)
1	X (0.500, 0.000, 0.500)	U (0.625, 0.250, 0.625)
2	K (0.375, 0.375, 0.750)	Γ (0.000, 0.000, 0.000)
3	Γ (0.000, 0.000, 0.000)	L (0.500, 0.500, 0.500)
4	L (0.500, 0.500, 0.500)	W (0.500, 0.250, 0.750)
5	W (0.500, 0.250, 0.750)	X (0.500, 0.000, 0.500)

Supplementary Table 993: Nodal ring points along high symmetry lines of mp-1747-K8Te4.

	Coordinates	Frequency	Mode 1	Mode 2	Multi.	Deg.	Path	Ratio
0	(0.296, 0.000, 0.296)	1.160	2	1	6	2	1	0.592
1	(0.191, 0.191, 0.369)	1.074	2	1	24	2	3	0.502
2	(0.622, 0.245, 0.622)	2.028	3	2	12	2	2	0.979
3	(0.500, 0.250, 0.750)	2.023	3	2	6	2	5	1.000
4	(0.409, 0.001, 0.409)	2.297	4	3	24	2	1	0.818
5	(0.500, 0.273, 0.727)	2.277	5	4	24	2	5	0.907
6	(0.072, 0.072, 0.150)	3.229	6	5	24	2	3	0.803
7	(0.092, 0.092, 0.185)	3.247	6	5	24	2	3	0.754
8	(0.067, -0.001, 0.067)	3.279	7	6	24	2	1	0.133
9	(0.088, 0.086, 0.174)	3.255	7	6	24	2	3	0.768
10	(0.154, 0.162, 0.317)	3.299	7	6	24	2	3	0.578
11	(0.087, 0.077, 0.164)	3.261	7	6	24	2	3	0.782
12	(0.157, 0.169, 0.326)	3.295	7	6	24	2	3	0.565
13	(0.500, 0.250, 0.750)	3.139	7	6	6	2	5	1.000
14	(0.000, 0.000, 0.000)	3.320	8	7	1	3	1	0.000

Supplementary Table 993: Nodal ring points along high symmetry lines of mp-1747-K8Te4.

Coordinates	Frequency	Mode 1	Mode 2	Multi.	Deg.	Path	Ratio

The followings are details for Li_8O_4. It has 4 topological nodal lines and 8 nodal ring points along high symmetry line as shown in Fig **475**.

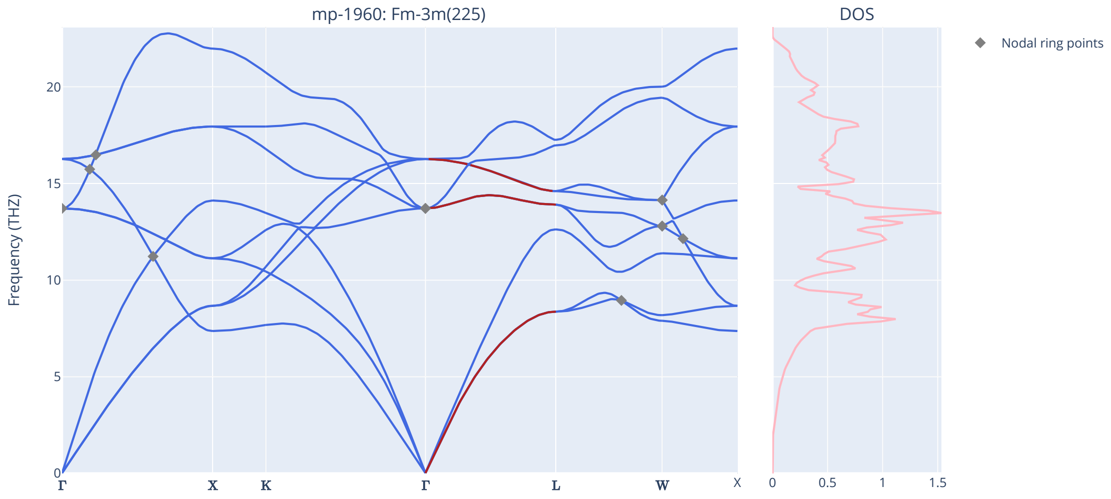

Supplementary Figure 475: The phonon band and DOS of Li8O4.

Supplementary Table 994: High symmetry lines of mp-1960-Li8O4

	Start	End
0	Γ (0.000, 0.000, 0.000)	X (0.500, 0.000, 0.500)
1	X (0.500, 0.000, 0.500)	U (0.625, 0.250, 0.625)
2	K (0.375, 0.375, 0.750)	Γ (0.000, 0.000, 0.000)
3	Γ (0.000, 0.000, 0.000)	L (0.500, 0.500, 0.500)
4	L (0.500, 0.500, 0.500)	W (0.500, 0.250, 0.750)
5	W (0.500, 0.250, 0.750)	X (0.500, 0.000, 0.500)

Supplementary Table 995: Nodal ring points along high symmetry lines of mp-1960-Li8O4.

	Coordinates	Frequency	Mode 1	Mode 2	Multi.	Deg.	Path	Ratio
0	(0.500, 0.346, 0.654)	8.947	2	1	24	2	5	0.617
1	(0.302, -0.001, 0.302)	11.225	4	3	10	2	1	0.605
2	(0.000, 0.000, 0.000)	13.711	5	4	1	3	1	0.000
3	(0.500, 0.250, 0.750)	12.795	5	4	6	2	5	1.000
4	(0.500, 0.181, 0.681)	12.144	5	4	12	2	6	0.277
5	(0.092, -0.001, 0.092)	15.737	7	6	24	2	1	0.184
6	(0.500, 0.250, 0.750)	14.142	7	6	6	2	5	1.000
7	(0.112, 0.000, 0.112)	16.480	8	7	6	2	1	0.224

The followings are details for $Pt_4Sn_4Th_4$. As shown in Fig **476**, it has 4 topological nodal lines, 1 nodal ring point and 1 high degenerate Weyl point whose degeneracy is more than 2 and charge is bigger than 1.

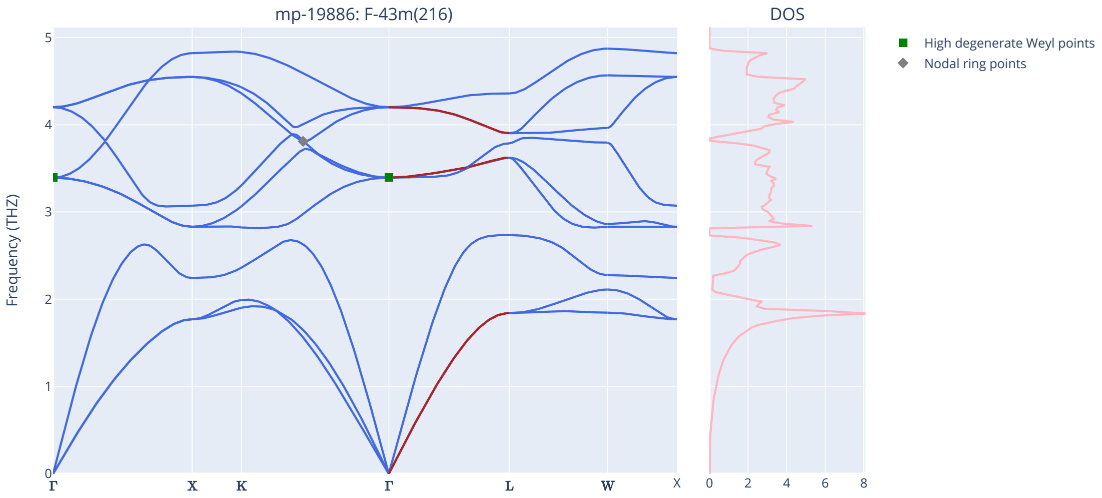

Supplementary Figure 476: The phonon band and DOS of Pt4Sn4Th4.

Supplementary Table 996: High symmetry lines of mp-19886-Pt4Sn4Th4

	Start	End
0	Γ (0.000, 0.000, 0.000)	X (0.500, 0.000, 0.500)
1	X (0.500, 0.000, 0.500)	U (0.625, 0.250, 0.625)
2	K (0.375, 0.375, 0.750)	Γ (0.000, 0.000, 0.000)
3	Γ (0.000, 0.000, 0.000)	L (0.500, 0.500, 0.500)
4	L (0.500, 0.500, 0.500)	W (0.500, 0.250, 0.750)
5	W (0.500, 0.250, 0.750)	X (0.500, 0.000, 0.500)

Supplementary Table 997: High degenerate Weyl points along high symmetry lines of mp-19886-Pt4Sn4Th4.

	Coordinates	Frequency	Mode 1	Mode 2	Multi.	Deg.	Path	Ratio
0	(0.000, 0.000, 0.000)	3.393	6	4	1	3	1	0.000

Supplementary Table 998: Nodal ring points along high symmetry lines of mp-19886-Pt4Sn4Th4.

	Coordinates	Frequency	Mode 1	Mode 2	Multi.	Deg.	Path	Ratio
0	(0.222, 0.222, 0.431)	3.809	7	6	24	2	3	0.419

The followings are details for Ce_4O_8. It has 5 topological nodal lines and 6 nodal ring points along high symmetry line as shown in Fig 477.

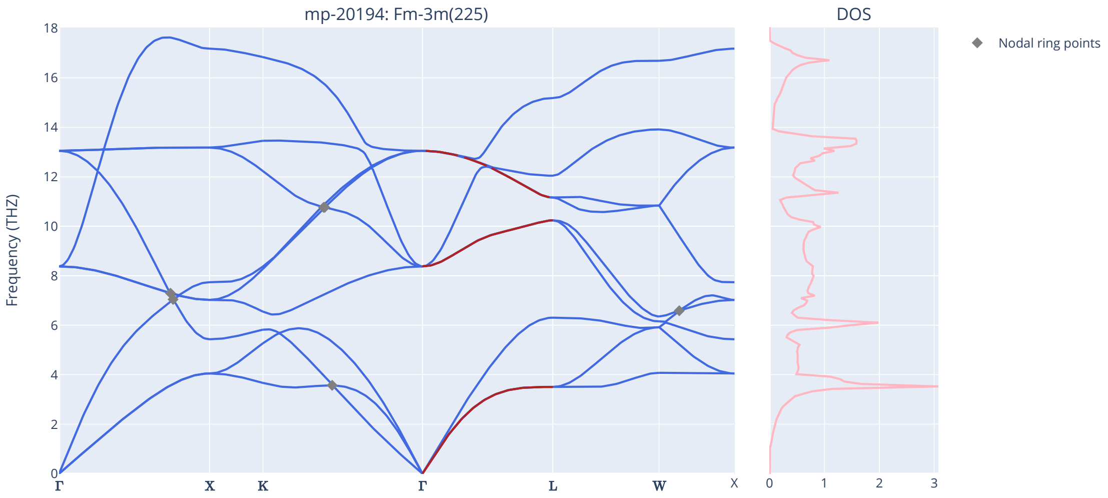

Supplementary Figure 477: The phonon band and DOS of Ce4O8.

Supplementary Table 999: High symmetry lines of mp-20194-Ce4O8

	Start	End
0	Γ (0.000, 0.000, 0.000)	X (0.500, 0.000, 0.500)
1	X (0.500, 0.000, 0.500)	U (0.625, 0.250, 0.625)
2	K (0.375, 0.375, 0.750)	Γ (0.000, 0.000, 0.000)
3	Γ (0.000, 0.000, 0.000)	L (0.500, 0.500, 0.500)
4	L (0.500, 0.500, 0.500)	W (0.500, 0.250, 0.750)
5	W (0.500, 0.250, 0.750)	X (0.500, 0.000, 0.500)

Supplementary Table 1000: Nodal ring points along high symmetry lines of mp-20194-Ce4O8.

	Coordinates	Frequency	Mode 1	Mode 2	Multi.	Deg.	Path	Ratio
0	(0.214, 0.214, 0.422)	3.572	2	1	24	2	3	0.435
1	(0.379, -0.002, 0.379)	7.044	4	3	24	2	1	0.758
2	(0.500, 0.183, 0.683)	6.587	5	4	12	2	6	0.267
3	(0.370, -0.000, 0.370)	7.294	6	5	6	2	1	0.740
4	(0.237, 0.237, 0.459)	10.764	6	5	24	2	3	0.381
5	(0.230, 0.230, 0.459)	10.766	6	5	12	2	3	0.388

The followings are details for Li_8Se_4. It has 4 topological nodal lines and 8 nodal ring points along high symmetry line as shown in Fig **478**.

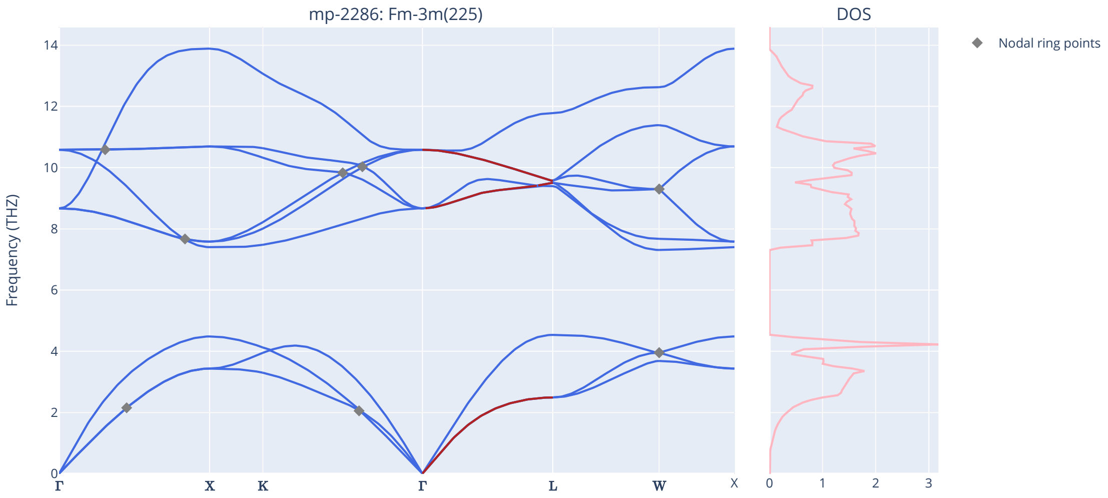

Supplementary Figure 478: The phonon band and DOS of Li8Se4.

Supplementary Table 1001: High symmetry lines of mp-2286-Li8Se4

	Start	End
0	Γ (0.000, 0.000, 0.000)	X (0.500, 0.000, 0.500)
1	X (0.500, 0.000, 0.500)	U (0.625, 0.250, 0.625)
2	K (0.375, 0.375, 0.750)	Γ (0.000, 0.000, 0.000)
3	Γ (0.000, 0.000, 0.000)	L (0.500, 0.500, 0.500)
4	L (0.500, 0.500, 0.500)	W (0.500, 0.250, 0.750)
5	W (0.500, 0.250, 0.750)	X (0.500, 0.000, 0.500)

Supplementary Table 1002: Nodal ring points along high symmetry lines of mp-2286-Li8Se4.

	Coordinates	Frequency	Mode 1	Mode 2	Multi.	Deg.	Path	Ratio
0	(0.224, 0.000, 0.224)	2.154	2	1	6	2	1	0.449
1	(0.153, 0.153, 0.293)	2.054	2	1	24	2	3	0.603
2	(0.500, 0.250, 0.750)	3.955	3	2	6	2	5	1.000
3	(0.418, 0.000, 0.418)	7.669	6	5	6	2	1	0.837
4	(0.184, 0.184, 0.378)	9.829	7	6	24	2	3	0.501
5	(0.145, 0.145, 0.277)	10.028	7	6	24	2	3	0.624
6	(0.500, 0.250, 0.750)	9.299	7	6	6	2	5	1.000
7	(0.153, 0.000, 0.153)	10.585	8	7	6	2	1	0.306

The followings are details for $FeBr_2$. It has 6 topological nodal lines and 12 nodal ring points along high symmetry line as shown in Fig **479**.

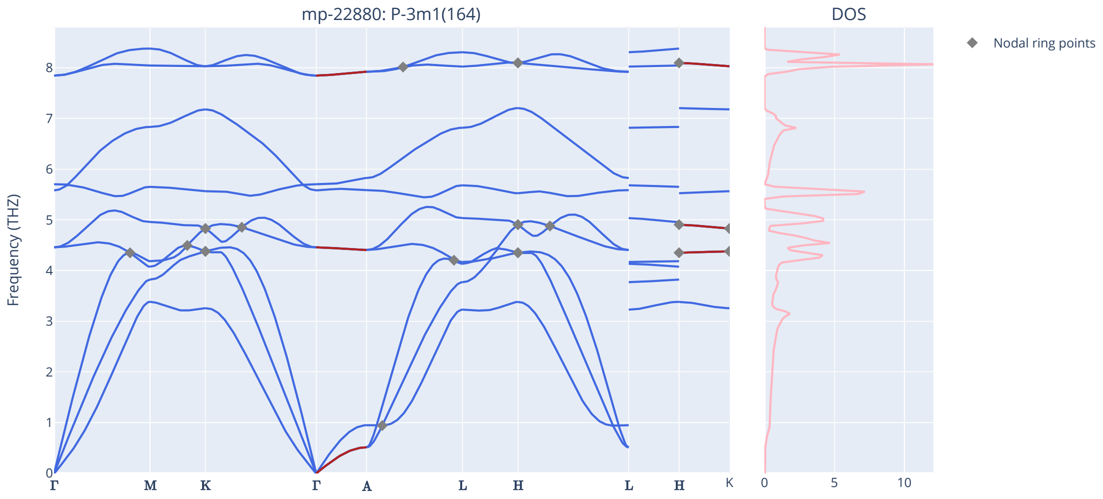

Supplementary Figure 479: The phonon band and DOS of FeBr2.

Supplementary Table 1003: High symmetry lines of mp-22880-FeBr2

	Start	End
0	Γ (0.000, 0.000, 0.000)	M (0.500, 0.000, 0.000)
1	M (0.500, 0.000, 0.000)	K (0.333, 0.333, 0.000)
2	K (0.333, 0.333, 0.000)	Γ (0.000, 0.000, 0.000)
3	Γ (0.000, 0.000, 0.000)	A (0.000, 0.000, 0.500)
4	A (0.000, 0.000, 0.500)	L (0.500, 0.000, 0.500)
5	L (0.500, 0.000, 0.500)	H (0.333, 0.333, 0.500)
6	H (0.333, 0.333, 0.500)	A (0.000, 0.000, 0.500)
7	L (0.500, 0.000, 0.500)	M (0.500, 0.000, 0.000)
8	H (0.333, 0.333, 0.500)	K (0.333, 0.333, 0.000)

Supplementary Table 1004: Nodal ring points along high symmetry lines of mp-22880-FeBr2.

	Coordinates	Frequency	Mode 1	Mode 2	Multi.	Deg.	Path	Ratio
0	(0.082, -0.000, 0.500)	0.941	2	1	6	2	5	0.164
1	(0.333, 0.333, 0.000)	4.378	3	2	2	2	2	1.000
2	(0.333, 0.333, 0.500)	4.350	3	2	2	2	6	1.000
3	(0.396, 0.000, 0.001)	4.348	4	3	6	2	1	0.792
4	(0.388, 0.224, -0.002)	4.493	4	3	12	2	2	0.672
5	(0.455, 0.000, 0.500)	4.202	4	3	6	2	5	0.911
6	(0.224, 0.224, -0.001)	4.852	5	4	12	2	3	0.328
7	(0.333, 0.333, 0.000)	4.827	5	4	2	2	2	1.000
8	(0.237, 0.237, 0.500)	4.877	5	4	6	2	7	0.289
9	(0.333, 0.333, 0.500)	4.903	5	4	2	2	6	1.000
10	(0.190, 0.000, 0.497)	8.014	9	8	6	2	5	0.380
11	(0.333, 0.333, 0.500)	8.095	9	8	2	2	6	1.000

Supplementary Table 1004: Nodal ring points along high symmetry lines of mp-22880-FeBr2.

Coordinates	Frequency	Mode 1	Mode 2	Multi.	Deg.	Path	Ratio

The followings are details for Cd_3Cl_6. It has 2 topological nodal lines and 17 nodal ring points along high symmetry line as shown in Fig **480**.

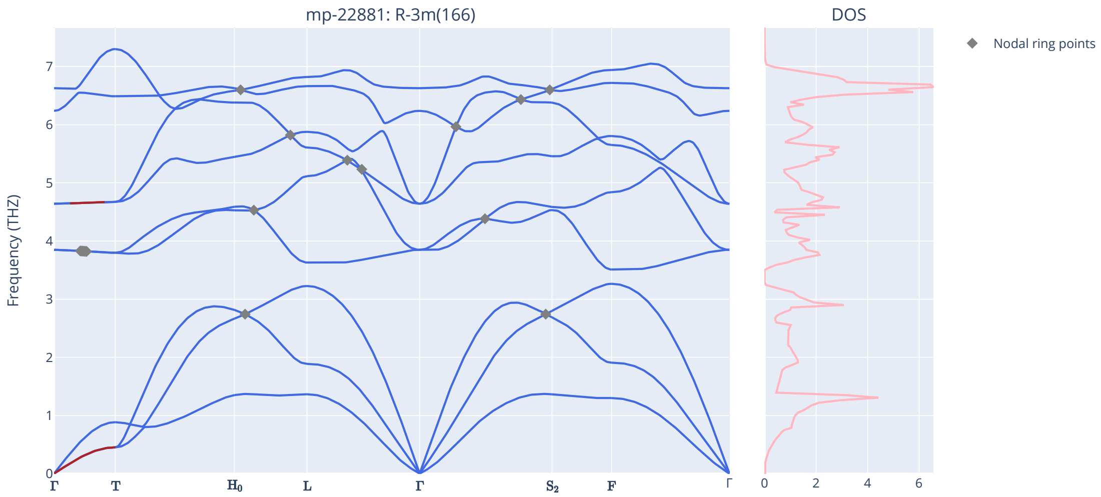

Supplementary Figure 480: The phonon band and DOS of Cd3Cl6.

Supplementary Table 1005: High symmetry lines of mp-22881-Cd3Cl6

	Start	End
0	Γ (0.000, 0.000, 0.000)	T (0.500, 0.500, 0.500)
1	T (0.500, 0.500, 0.500)	H_2 (0.811, 0.189, 0.500)
2	H_0 (0.500, -0.189, 0.189)	L (0.500, 0.000, 0.000)
3	L (0.500, 0.000, 0.000)	Γ (0.000, 0.000, 0.000)
4	Γ (0.000, 0.000, 0.000)	S_0 (0.345, -0.345, 0.000)
5	S_2 (0.655, 0.000, 0.345)	F (0.500, 0.000, 0.500)
6	F (0.500, 0.000, 0.500)	Γ (0.000, 0.000, 0.000)

Supplementary Table 1006: Nodal ring points along high symmetry lines of mp-22881-Cd3Cl6.

	Coordinates	Frequency	Mode 1	Mode 2	Multi.	Deg.	Path	Ratio
0	(0.500, -0.162, 0.162)	2.743	3	2	6	2	3	0.146
1	(0.329, -0.329, -0.000)	2.743	3	2	6	2	5	0.954
2	(0.214, 0.214, 0.214)	3.830	5	4	2	2	1	0.429
3	(0.224, 0.224, 0.224)	3.828	5	4	2	2	1	0.449
4	(0.235, 0.235, 0.235)	3.827	5	4	2	2	1	0.469
5	(0.245, 0.245, 0.245)	3.825	5	4	2	2	1	0.490
6	(0.255, 0.255, 0.255)	3.823	5	4	2	2	1	0.510
7	(0.265, 0.265, 0.265)	3.822	5	4	2	2	1	0.531
8	(0.500, -0.139, 0.139)	4.531	5	4	6	2	3	0.266
9	(0.170, -0.170, 0.000)	4.383	5	4	6	2	5	0.494
10	(0.257, -0.004, -0.004)	5.233	6	5	6	2	4	0.486
11	(0.321, -0.003, -0.003)	5.390	6	5	6	2	4	0.357
12	(0.500, -0.044, 0.044)	5.821	7	6	6	2	3	0.770

Supplementary Table 1006: Nodal ring points along high symmetry lines of mp-22881-Cd3Cl6.

	Coordinates	Frequency	Mode 1	Mode 2	Multi.	Deg.	Path	Ratio
13	(0.264, -0.264, 0.000)	6.432	8	7	6	2	5	0.766
14	(0.094, -0.094, 0.000)	5.965	8	7	6	2	5	0.274
15	(0.500, -0.173, 0.173)	6.599	9	8	6	2	3	0.084
16	(0.339, -0.339, -0.000)	6.599	9	8	6	2	5	0.984

The followings are details for F_4Xe_2 and it only has 5 topological nodal ring points along high symmetry line as shown in Fig **481**.

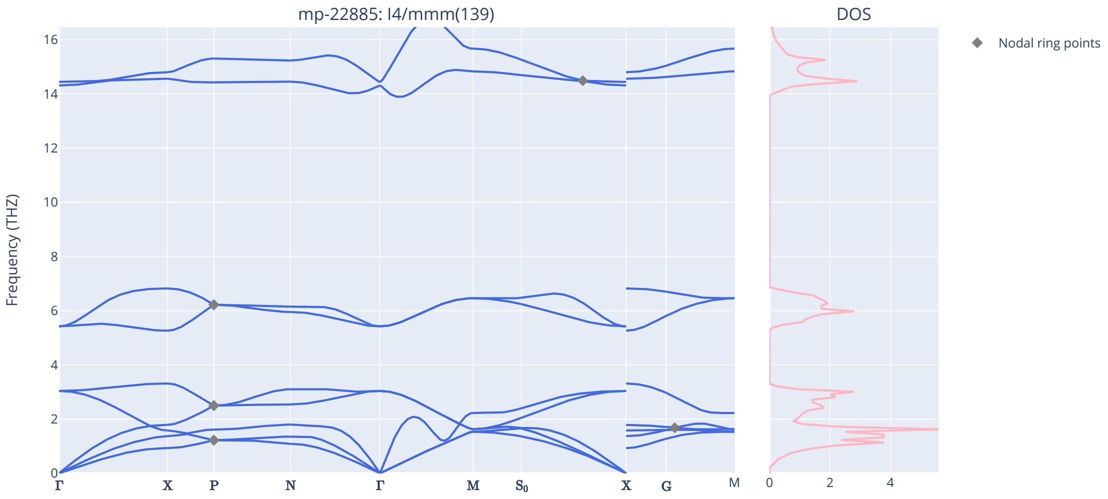

Supplementary Figure 481: The phonon band and DOS of F4Xe2.

Supplementary Table 1007: High symmetry lines of mp-22885-F4Xe2

	Start	End
0	Γ (0.000, 0.000, 0.000)	X (0.000, 0.000, 0.500)
1	X (0.000, 0.000, 0.500)	P (0.250, 0.250, 0.250)
2	P (0.250, 0.250, 0.250)	N (0.000, 0.500, 0.000)
3	N (0.000, 0.500, 0.000)	Γ (0.000, 0.000, 0.000)
4	Γ (0.000, 0.000, 0.000)	M (0.500, 0.500, -0.500)
5	M (0.500, 0.500, -0.500)	S (0.342, 0.658, -0.342)
6	S_0 (-0.342, 0.342, 0.342)	Γ (0.000, 0.000, 0.000)
7	X (0.000, 0.000, 0.500)	R (-0.183, 0.183, 0.500)
8	G (0.500, 0.500, -0.183)	M (0.500, 0.500, -0.500)

Supplementary Table 1008: Nodal ring points along high symmetry lines of mp-22885-F4Xe2.

	Coordinates	Frequency	Mode 1	Mode 2	Multi.	Deg.	Path	Ratio
0	(0.250, 0.250, 0.250)	1.220	2	1	2	2	2	1.000
1	(0.500, 0.500, -0.224)	1.677	4	3	8	2	9	0.130
2	(0.250, 0.250, 0.250)	2.497	5	4	2	2	2	1.000
3	(0.250, 0.250, 0.250)	6.217	7	6	2	2	2	1.000
4	(-0.141, 0.141, 0.141)	14.483	9	8	8	2	7	0.587

The followings are details for PbI_2. It has 6 topological nodal lines and 14 nodal ring points along high symmetry line as shown in Fig 482.

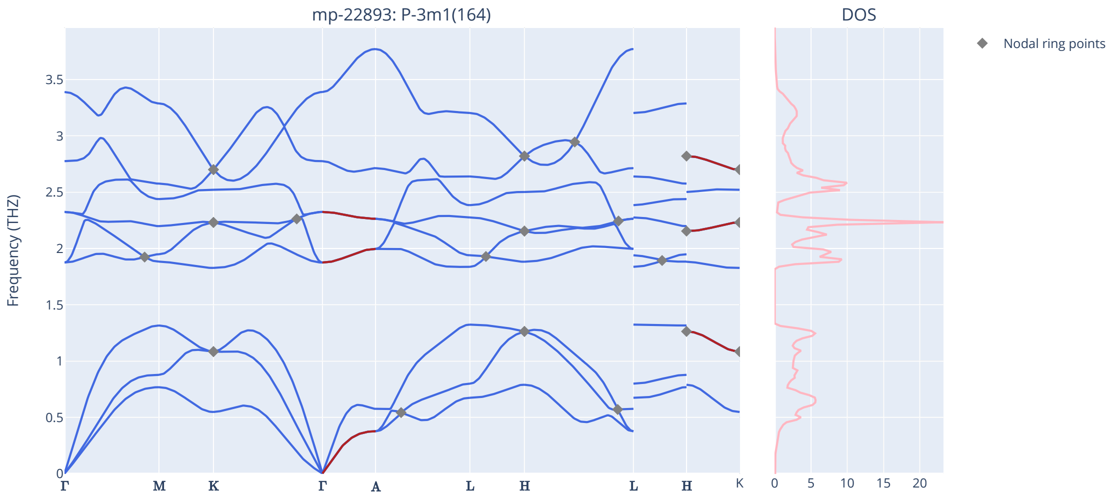

Supplementary Figure 482: The phonon band and DOS of PbI2.

Supplementary Table 1009: High symmetry lines of mp-22893-PbI2

	Start	End
0	Γ (0.000, 0.000, 0.000)	M (0.500, 0.000, 0.000)
1	M (0.500, 0.000, 0.000)	K (0.333, 0.333, 0.000)
2	K (0.333, 0.333, 0.000)	Γ (0.000, 0.000, 0.000)
3	Γ (0.000, 0.000, 0.000)	A (0.000, 0.000, 0.500)
4	A (0.000, 0.000, 0.500)	L (0.500, 0.000, 0.500)
5	L (0.500, 0.000, 0.500)	H (0.333, 0.333, 0.500)
6	H (0.333, 0.333, 0.500)	A (0.000, 0.000, 0.500)
7	L (0.500, 0.000, 0.500)	M (0.500, 0.000, 0.000)
8	H (0.333, 0.333, 0.500)	K (0.333, 0.333, 0.000)

Supplementary Table 1010: Nodal ring points along high symmetry lines of mp-22893-PbI2.

	Coordinates	Frequency	Mode 1	Mode 2	Multi.	Deg.	Path	Ratio
0	(0.135, 0.000, 0.497)	0.543	2	1	6	2	5	0.271
1	(0.333, 0.333, 0.000)	1.084	3	2	2	2	2	1.000
2	(0.333, 0.333, 0.500)	1.263	3	2	2	2	6	1.000
3	(0.048, 0.048, 0.500)	0.571	3	2	6	2	7	0.856
4	(0.424, 0.000, 0.001)	1.924	5	4	6	2	1	0.848
5	(0.451, 0.098, 0.499)	1.929	5	4	12	2	6	0.293
6	(0.495, 0.000, 0.230)	1.893	5	4	6	2	8	0.540
7	(0.080, 0.077, 0.001)	2.262	6	5	12	2	3	0.764
8	(0.333, 0.333, 0.000)	2.231	6	5	2	2	2	1.000
9	(0.333, 0.333, 0.500)	2.154	6	5	2	2	6	1.000
10	(0.046, 0.046, 0.500)	2.244	7	6	6	2	7	0.863

Supplementary Table 1010: Nodal ring points along high symmetry lines of mp-22893-PbI2.

	Coordinates	Frequency	Mode 1	Mode 2	Multi.	Deg.	Path	Ratio
11	(0.179, 0.180, 0.497)	2.946	9	8	12	2	7	0.461
12	(0.333, 0.333, 0.000)	2.700	9	8	2	2	2	1.000
13	(0.333, 0.333, 0.500)	2.820	9	8	2	2	6	1.000

The followings are details for BiITe and it only has 3 topological nodal lines along high symmetry line as shown in Fig **483**.

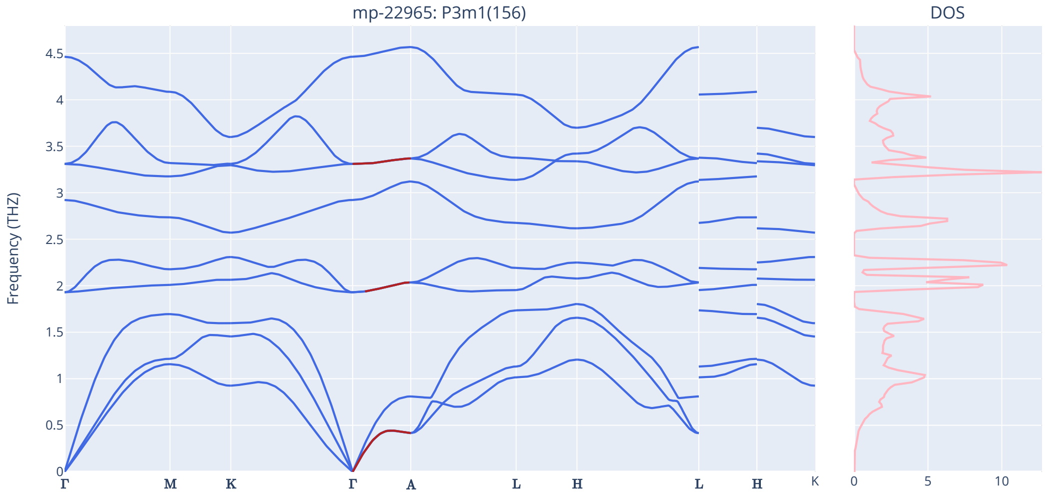

Supplementary Figure 483: The phonon band and DOS of BiITe.

Supplementary Table 1011: High symmetry lines of mp-22965-BiITe

	Start	End
0	Γ (0.000, 0.000, 0.000)	M (0.500, 0.000, 0.000)
1	M (0.500, 0.000, 0.000)	K (0.333, 0.333, 0.000)
2	K (0.333, 0.333, 0.000)	Γ (0.000, 0.000, 0.000)
3	Γ (0.000, 0.000, 0.000)	A (0.000, 0.000, 0.500)
4	A (0.000, 0.000, 0.500)	L (0.500, 0.000, 0.500)
5	L (0.500, 0.000, 0.500)	H (0.333, 0.333, 0.500)
6	H (0.333, 0.333, 0.500)	A (0.000, 0.000, 0.500)
7	L (0.500, 0.000, 0.500)	M (0.500, 0.000, 0.000)
8	H (0.333, 0.333, 0.500)	K (0.333, 0.333, 0.000)

The followings are details for Zr_3Cl_6. It has 2 topological nodal lines and 2 nodal ring points along high symmetry line as shown in Fig 484.

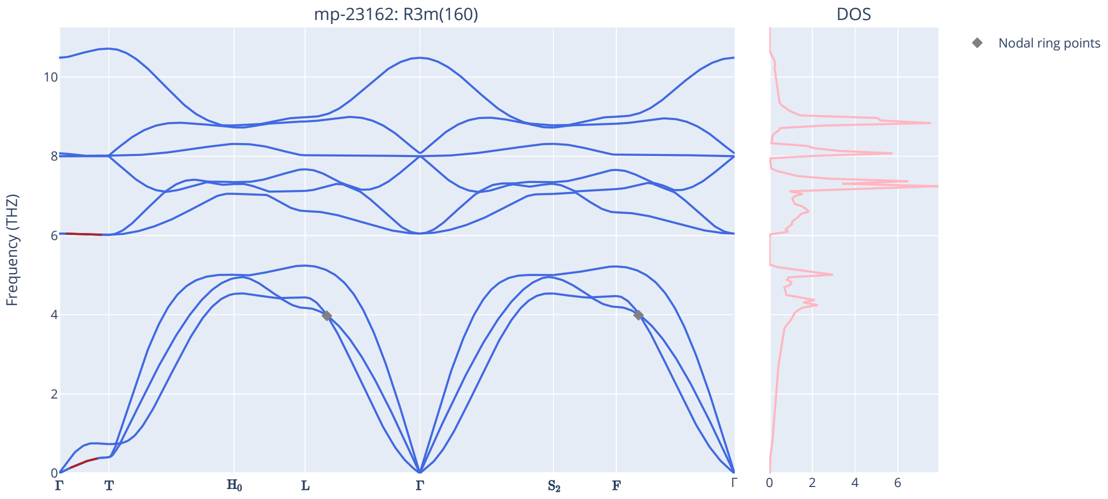

Supplementary Figure 484: The phonon band and DOS of Zr3Cl6.

Supplementary Table 1012: High symmetry lines of mp-23162-Zr3Cl6

	Start	End
0	Γ (0.000, 0.000, 0.000)	T (0.500, 0.500, 0.500)
1	T (0.500, 0.500, 0.500)	H_2 (0.819, 0.181, 0.500)
2	H_0 (0.500, -0.181, 0.181)	L (0.500, 0.000, 0.000)
3	L (0.500, 0.000, 0.000)	Γ (0.000, 0.000, 0.000)
4	Γ (0.000, 0.000, 0.000)	S_0 (0.340, -0.340, 0.000)
5	S_2 (0.660, 0.000, 0.340)	F (0.500, 0.000, 0.500)
6	F (0.500, 0.000, 0.500)	Γ (0.000, 0.000, 0.000)

Supplementary Table 1013: Nodal ring points along high symmetry lines of mp-23162-Zr3Cl6.

	Coordinates	Frequency	Mode 1	Mode 2	Multi.	Deg.	Path	Ratio
0	(0.405, 0.001, 0.001)	3.974	2	1	6	2	4	0.189
1	(0.406, 0.005, 0.406)	3.989	2	1	6	2	7	0.188

The followings are details for MgI_2. It has 6 topological nodal lines and 17 nodal ring points along high symmetry line as shown in Fig **485**.

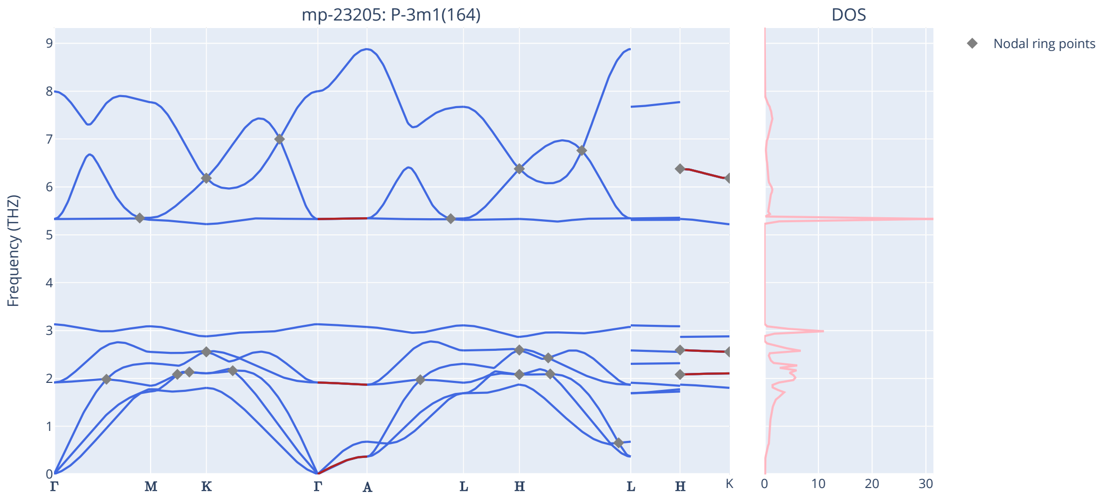

Supplementary Figure 485: The phonon band and DOS of MgI2.

Supplementary Table 1014: High symmetry lines of mp-23205-MgI2

	Start	End
0	Γ (0.000, 0.000, 0.000)	M (0.500, 0.000, 0.000)
1	M (0.500, 0.000, 0.000)	K (0.333, 0.333, 0.000)
2	K (0.333, 0.333, 0.000)	Γ (0.000, 0.000, 0.000)
3	Γ (0.000, 0.000, 0.000)	A (0.000, 0.000, 0.500)
4	A (0.000, 0.000, 0.500)	L (0.500, 0.000, 0.500)
5	L (0.500, 0.000, 0.500)	H (0.333, 0.333, 0.500)
6	H (0.333, 0.333, 0.500)	A (0.000, 0.000, 0.500)
7	L (0.500, 0.000, 0.500)	M (0.500, 0.000, 0.000)
8	H (0.333, 0.333, 0.500)	K (0.333, 0.333, 0.000)

Supplementary Table 1015: Nodal ring points along high symmetry lines of mp-23205-MgI2.

	Coordinates	Frequency	Mode 1	Mode 2	Multi.	Deg.	Path	Ratio
0	(0.254, 0.254, 0.000)	2.161	3	2	6	2	3	0.237
1	(0.420, 0.160, -0.001)	2.085	3	2	12	2	2	0.481
2	(0.385, 0.232, -0.003)	2.134	3	2	12	2	2	0.695
3	(0.333, 0.333, 0.500)	2.082	3	2	2	2	6	1.000
4	(0.241, 0.241, 0.500)	2.094	3	2	6	2	7	0.277
5	(0.036, 0.036, 0.500)	0.655	3	2	6	2	7	0.892
6	(0.271, -0.000, -0.004)	1.983	4	3	6	2	1	0.543
7	(0.276, -0.000, 0.498)	1.972	4	3	6	2	5	0.552
8	(0.333, 0.333, 0.000)	2.556	5	4	2	2	2	1.000
9	(0.333, 0.333, 0.500)	2.593	5	4	2	2	6	1.000
10	(0.247, 0.247, 0.500)	2.429	5	4	6	2	7	0.259

Supplementary Table 1015: Nodal ring points along high symmetry lines of mp-23205-MgI2.

	Coordinates	Frequency	Mode 1	Mode 2	Multi.	Deg.	Path	Ratio
11	(0.444, 0.000, 0.001)	5.352	8	7	6	2	1	0.887
12	(0.433, 0.000, 0.498)	5.337	8	7	6	2	5	0.867
13	(0.333, 0.333, 0.000)	6.182	9	8	2	2	2	1.000
14	(0.114, 0.114, 0.000)	6.999	9	8	6	2	3	0.657
15	(0.333, 0.333, 0.500)	6.379	9	8	2	2	6	1.000
16	(0.147, 0.146, 0.498)	6.762	9	8	12	2	7	0.561

The followings are details for Sr_4Cl_8. It has 4 topological nodal lines and 7 nodal ring points along high symmetry line as shown in Fig **486**.

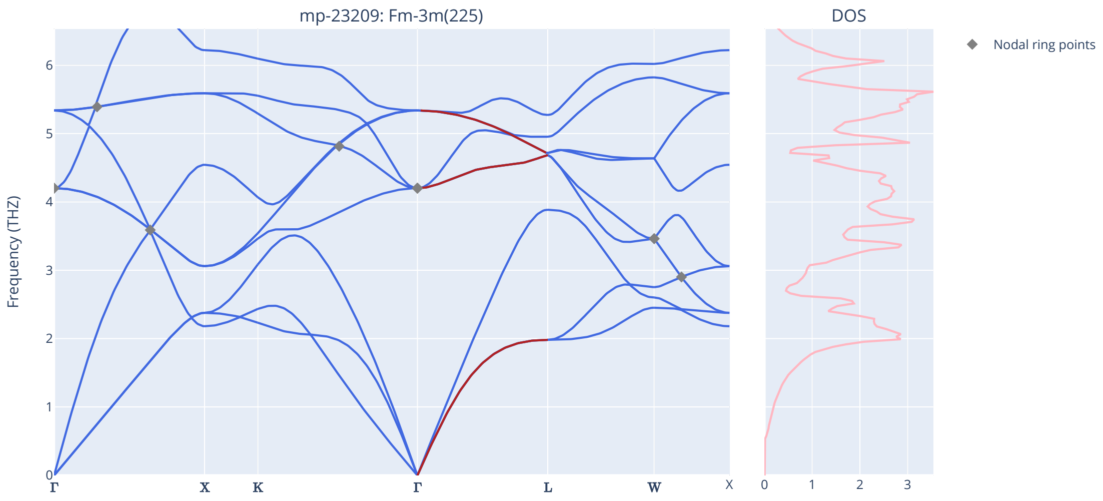

Supplementary Figure 486: The phonon band and DOS of Sr4Cl8.

Supplementary Table 1016: High symmetry lines of mp-23209-Sr4Cl8

	Start	End
0	Γ (0.000, 0.000, 0.000)	X (0.500, 0.000, 0.500)
1	X (0.500, 0.000, 0.500)	U (0.625, 0.250, 0.625)
2	K (0.375, 0.375, 0.750)	Γ (0.000, 0.000, 0.000)
3	Γ (0.000, 0.000, 0.000)	L (0.500, 0.500, 0.500)
4	L (0.500, 0.500, 0.500)	W (0.500, 0.250, 0.750)
5	W (0.500, 0.250, 0.750)	X (0.500, 0.000, 0.500)

Supplementary Table 1017: Nodal ring points along high symmetry lines of mp-23209-Sr4Cl8.

	Coordinates	Frequency	Mode 1	Mode 2	Multi.	Deg.	Path	Ratio
0	(0.319, 0.002, 0.320)	3.589	4	3	24	2	1	0.639
1	(0.498, 0.158, 0.660)	2.902	4	3	24	2	6	0.363
2	(0.000, 0.000, 0.000)	4.201	5	4	1	3	1	0.000
3	(0.500, 0.250, 0.750)	3.462	5	4	6	2	5	1.000
4	(0.000, 0.000, 0.000)	4.201	6	5	1	3	1	0.000
5	(0.178, 0.189, 0.368)	4.814	6	5	24	2	3	0.510
6	(0.143, 0.000, 0.143)	5.392	8	7	6	2	1	0.286

The followings are details for Mg_3Cl_6. It has 2 topological nodal lines and 15 nodal ring points along high symmetry line as shown in Fig **487**.

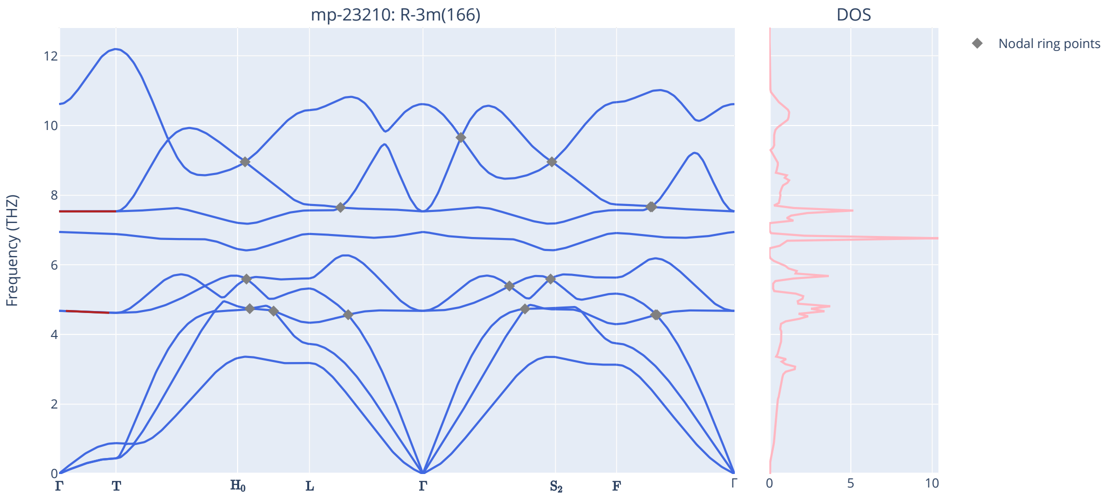

Supplementary Figure 487: The phonon band and DOS of Mg3Cl6.

Supplementary Table 1018: High symmetry lines of mp-23210-Mg3Cl6

	Start	End
0	Γ (0.000, 0.000, 0.000)	T (0.500, 0.500, 0.500)
1	T (0.500, 0.500, 0.500)	H_2 (0.814, 0.186, 0.500)
2	H_0 (0.500, -0.186, 0.186)	L (0.500, 0.000, 0.000)
3	L (0.500, 0.000, 0.000)	Γ (0.000, 0.000, 0.000)
4	Γ (0.000, 0.000, 0.000)	S_0 (0.343, -0.343, 0.000)
5	S_2 (0.657, 0.000, 0.343)	F (0.500, 0.000, 0.500)
6	F (0.500, 0.000, 0.500)	Γ (0.000, 0.000, 0.000)

Supplementary Table 1019: Nodal ring points along high symmetry lines of mp-23210-Mg3Cl6.

	Coordinates	Frequency	Mode 1	Mode 2	Multi.	Deg.	Path	Ratio
0	(0.500, -0.155, 0.155)	4.741	3	2	6	2	3	0.169
1	(0.500, -0.093, 0.093)	4.671	3	2	6	2	3	0.500
2	(0.264, -0.264, 0.000)	4.732	3	2	6	2	5	0.769
3	(0.330, -0.002, -0.002)	4.566	4	3	6	2	4	0.341
4	(0.335, 0.004, 0.335)	4.564	4	3	6	2	7	0.330
5	(0.328, -0.003, 0.328)	4.564	4	3	6	2	7	0.344
6	(0.500, -0.163, 0.163)	5.591	5	4	6	2	3	0.124
7	(0.330, -0.330, 0.000)	5.591	5	4	6	2	5	0.962
8	(0.224, -0.224, -0.000)	5.390	5	4	6	2	5	0.652
9	(0.363, -0.001, -0.001)	7.647	8	7	6	2	4	0.273
10	(0.355, 0.003, 0.355)	7.665	8	7	6	2	7	0.289
11	(0.349, -0.004, 0.349)	7.664	8	7	6	2	7	0.302
12	(0.500, -0.167, 0.167)	8.956	9	8	6	2	3	0.104

Supplementary Table 1019: Nodal ring points along high symmetry lines of mp-23210-Mg3Cl6.

	Coordinates	Frequency	Mode 1	Mode 2	Multi.	Deg.	Path	Ratio
13	(0.333, -0.333, 0.000)	8.956	9	8	6	2	5	0.972
14	(0.098, -0.098, 0.000)	9.656	9	8	6	2	5	0.287

The followings are details for Na_8O_4. It has 3 topological nodal lines and 14 nodal ring points along high symmetry line as shown in Fig **488**.

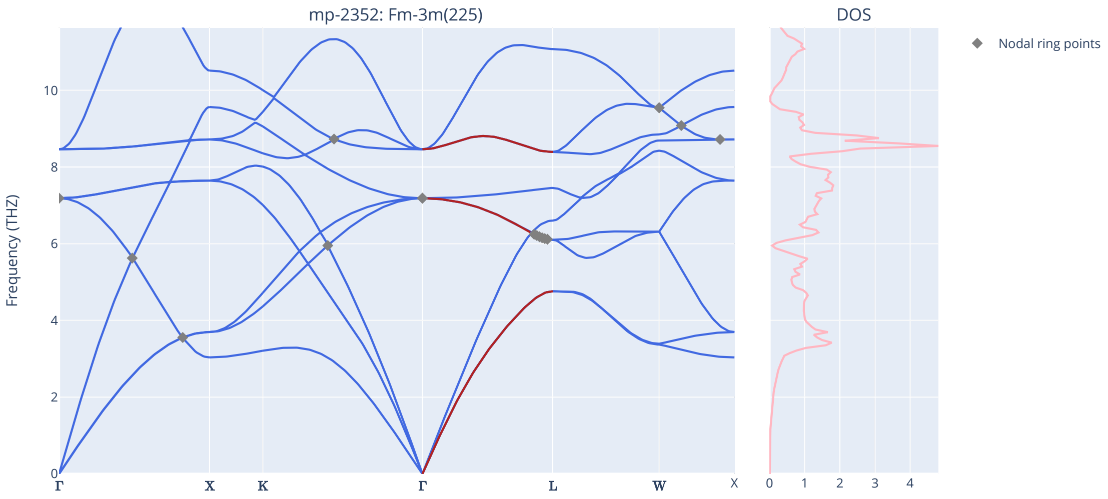

Supplementary Figure 488: The phonon band and DOS of Na8O4.

Supplementary Table 1020: High symmetry lines of mp-2352-Na8O4

	Start	End
0	Γ (0.000, 0.000, 0.000)	X (0.500, 0.000, 0.500)
1	X (0.500, 0.000, 0.500)	U (0.625, 0.250, 0.625)
2	K (0.375, 0.375, 0.750)	Γ (0.000, 0.000, 0.000)
3	Γ (0.000, 0.000, 0.000)	L (0.500, 0.500, 0.500)
4	L (0.500, 0.500, 0.500)	W (0.500, 0.250, 0.750)
5	W (0.500, 0.250, 0.750)	X (0.500, 0.000, 0.500)

Supplementary Table 1021: Nodal ring points along high symmetry lines of mp-2352-Na8O4.

	Coordinates	Frequency	Mode 1	Mode 2	Multi.	Deg.	Path	Ratio
0	(0.411, 0.000, 0.411)	3.554	3	2	6	2	1	0.822
1	(0.243, -0.002, 0.243)	5.625	4	3	24	2	1	0.486
2	(0.222, 0.222, 0.445)	5.952	4	3	24	2	3	0.407
3	(0.429, 0.429, 0.429)	6.246	4	3	8	2	4	0.857
4	(0.439, 0.439, 0.439)	6.212	4	3	8	2	4	0.878
5	(0.449, 0.449, 0.449)	6.181	4	3	8	2	4	0.898
6	(0.459, 0.459, 0.459)	6.154	4	3	8	2	4	0.918
7	(0.469, 0.469, 0.469)	6.132	4	3	8	2	4	0.939
8	(0.480, 0.480, 0.480)	6.116	4	3	8	2	4	0.959
9	(0.000, 0.000, 0.000)	7.186	5	4	1	3	1	0.000
10	(0.499, 0.048, 0.547)	8.718	7	6	24	2	6	0.809
11	(0.207, 0.207, 0.416)	8.729	8	7	24	2	3	0.446
12	(0.499, 0.177, 0.676)	9.081	8	7	24	2	6	0.294
13	(0.500, 0.250, 0.750)	9.545	9	8	6	2	5	1.000

The followings are details for HoHSe. It has 4 nodal ring points and 3 single Weyl points along high symmetry line as shown in Fig 489.

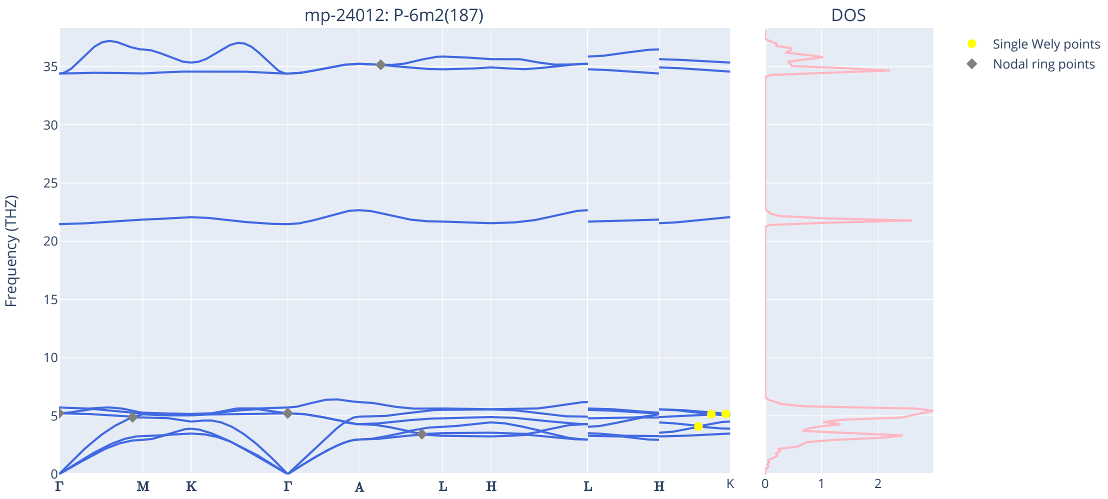

Supplementary Figure 489: The phonon band and DOS of HoHSe.

Supplementary Table 1022: High symmetry lines of mp-24012-HoHSe

	Start	End
0	Γ (0.000, 0.000, 0.000)	M (0.500, 0.000, 0.000)
1	M (0.500, 0.000, 0.000)	K (0.333, 0.333, 0.000)
2	K (0.333, 0.333, 0.000)	Γ (0.000, 0.000, 0.000)
3	Γ (0.000, 0.000, 0.000)	A (0.000, 0.000, 0.500)
4	A (0.000, 0.000, 0.500)	L (0.500, 0.000, 0.500)
5	L (0.500, 0.000, 0.500)	H (0.333, 0.333, 0.500)
6	H (0.333, 0.333, 0.500)	A (0.000, 0.000, 0.500)
7	L (0.500, 0.000, 0.500)	M (0.500, 0.000, 0.000)
8	H (0.333, 0.333, 0.500)	K (0.333, 0.333, 0.000)

Supplementary Table 1023: Single Weyl points along high symmetry lines of mp-24012-HoHSe.

	Coordinates	Frequency	Mode 1	Mode 2	Multi.	Deg.	Path	Ratio
0	(0.333, 0.333, 0.223)	4.093	3	2	4	2	9	0.553
1	(0.333, 0.333, 0.133)	5.141	5	4	4	2	9	0.734
2	(0.333, 0.333, 0.032)	5.165	6	5	4	2	9	0.935

Supplementary Table 1024: Nodal ring points along high symmetry lines of mp-24012-HoHSe.

	Coordinates	Frequency	Mode 1	Mode 2	Multi.	Deg.	Path	Ratio
0	(0.375, 0.001, 0.500)	3.432	2	1	10	2	5	0.751

Supplementary Table 1024: Nodal ring points along high symmetry lines of mp-24012-HoHSe.

	Coordinates	Frequency	Mode 1	Mode 2	Multi.	Deg.	Path	Ratio
1	(0.439, 0.000, 0.000)	4.883	4	3	6	2	1	0.879
2	(0.000, 0.000, 0.000)	5.225	5	4	1	2	1	0.000
3	(0.130, 0.000, 0.500)	35.154	9	8	6	2	5	0.260

The followings are details for Cd_4F_8. It has 4 topological nodal lines and 9 nodal ring points along high symmetry line as shown in Fig **490**.

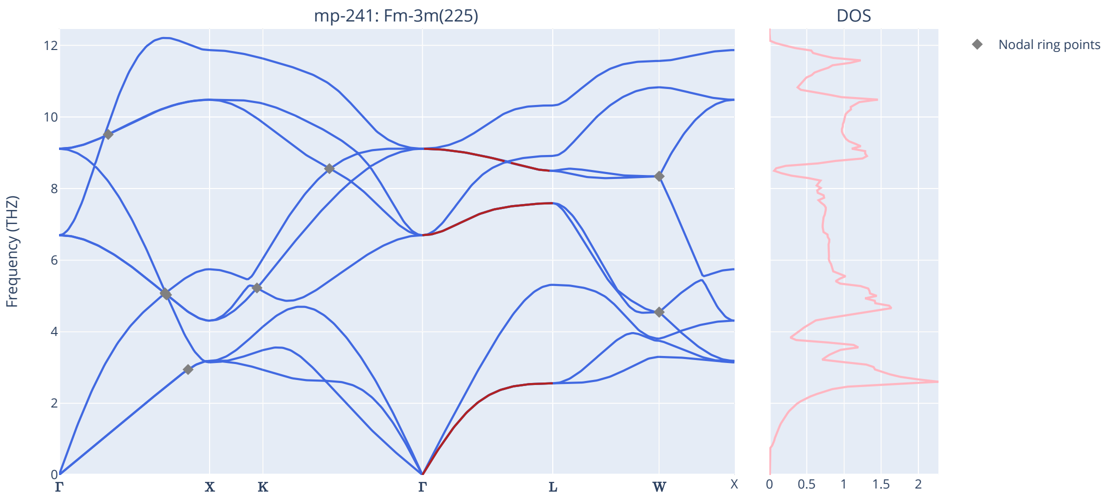

Supplementary Figure 490: The phonon band and DOS of Cd4F8.

Supplementary Table 1025: High symmetry lines of mp-241-Cd4F8

	Start	End
0	Γ (0.000, 0.000, 0.000)	X (0.500, 0.000, 0.500)
1	X (0.500, 0.000, 0.500)	U (0.625, 0.250, 0.625)
2	K (0.375, 0.375, 0.750)	Γ (0.000, 0.000, 0.000)
3	Γ (0.000, 0.000, 0.000)	L (0.500, 0.500, 0.500)
4	L (0.500, 0.500, 0.500)	W (0.500, 0.250, 0.750)
5	W (0.500, 0.250, 0.750)	X (0.500, 0.000, 0.500)

Supplementary Table 1026: Nodal ring points along high symmetry lines of mp-241-Cd4F8.

	Coordinates	Frequency	Mode 1	Mode 2	Multi.	Deg.	Path	Ratio
0	(0.429, 0.000, 0.429)	2.942	2	1	6	2	1	0.857
1	(0.353, 0.000, 0.353)	5.056	4	3	6	2	1	0.706
2	(0.357, 0.000, 0.357)	5.028	5	4	6	2	1	0.714
3	(0.611, 0.223, 0.611)	5.222	5	4	24	2	2	0.890
4	(0.500, 0.250, 0.750)	4.546	5	4	6	2	5	1.000
5	(0.354, -0.003, 0.350)	5.083	6	5	24	2	1	0.704
6	(0.222, 0.222, 0.434)	8.556	7	6	24	2	3	0.417
7	(0.500, 0.250, 0.750)	8.342	7	6	6	2	5	1.000
8	(0.163, 0.000, 0.163)	9.511	8	7	6	2	1	0.327

The followings are details for Li_8Te_4. It has 4 topological nodal lines and 7 nodal ring points along high symmetry line as shown in Fig **491**.

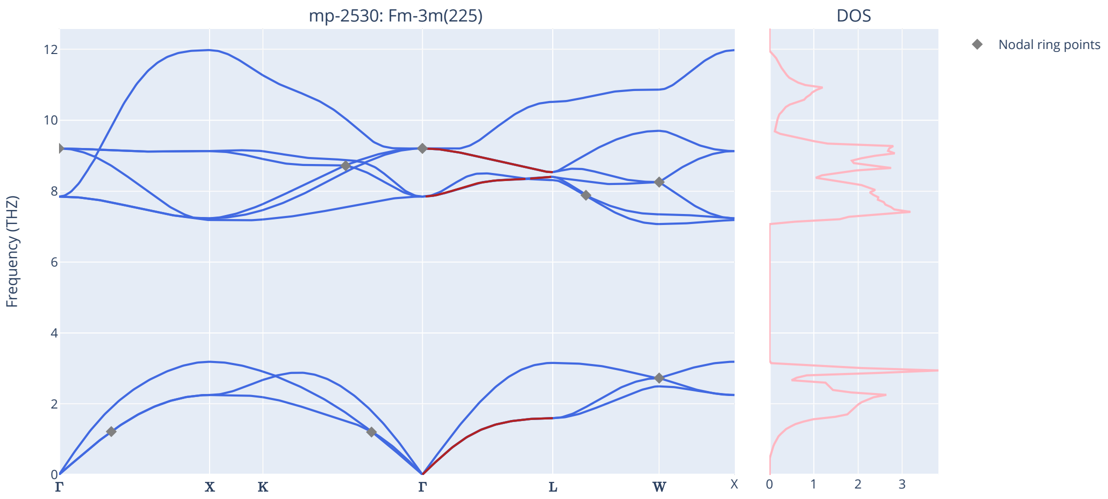

Supplementary Figure 491: The phonon band and DOS of Li8Te4.

Supplementary Table 1027: High symmetry lines of mp-2530-Li8Te4

	Start	End
0	Γ (0.000, 0.000, 0.000)	X (0.500, 0.000, 0.500)
1	X (0.500, 0.000, 0.500)	U (0.625, 0.250, 0.625)
2	K (0.375, 0.375, 0.750)	Γ (0.000, 0.000, 0.000)
3	Γ (0.000, 0.000, 0.000)	L (0.500, 0.500, 0.500)
4	L (0.500, 0.500, 0.500)	W (0.500, 0.250, 0.750)
5	W (0.500, 0.250, 0.750)	X (0.500, 0.000, 0.500)

Supplementary Table 1028: Nodal ring points along high symmetry lines of mp-2530-Li8Te4.

	Coordinates	Frequency	Mode 1	Mode 2	Multi.	Deg.	Path	Ratio
0	(0.173, 0.000, 0.173)	1.218	2	1	6	2	1	0.347
1	(0.122, 0.122, 0.236)	1.205	2	1	24	2	3	0.681
2	(0.500, 0.250, 0.750)	2.727	3	2	6	2	5	1.000
3	(0.500, 0.422, 0.578)	7.884	5	4	24	2	5	0.312
4	(0.176, 0.176, 0.365)	8.716	7	6	24	2	3	0.519
5	(0.500, 0.250, 0.750)	8.256	7	6	6	2	5	1.000
6	(0.000, 0.000, 0.000)	9.206	8	7	1	3	1	0.000

The followings are details for Ca_4F_8. It has 4 topological nodal lines and 8 nodal ring points along high symmetry line as shown in Fig 492.

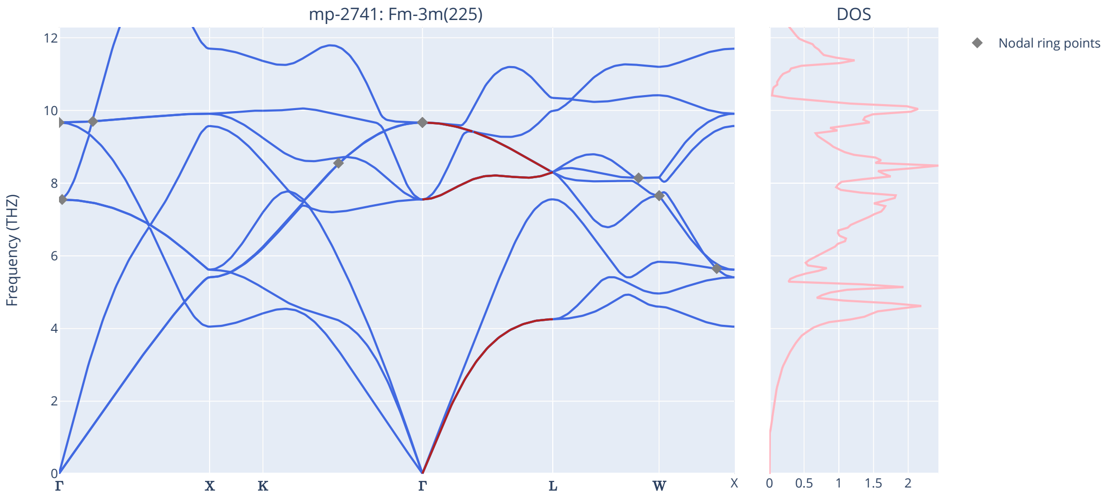

Supplementary Figure 492: The phonon band and DOS of Ca4F8.

Supplementary Table 1029: High symmetry lines of mp-2741-Ca4F8

	Start	End
0	Γ (0.000, 0.000, 0.000)	X (0.500, 0.000, 0.500)
1	X (0.500, 0.000, 0.500)	U (0.625, 0.250, 0.625)
2	K (0.375, 0.375, 0.750)	Γ (0.000, 0.000, 0.000)
3	Γ (0.000, 0.000, 0.000)	L (0.500, 0.500, 0.500)
4	L (0.500, 0.500, 0.500)	W (0.500, 0.250, 0.750)
5	W (0.500, 0.250, 0.750)	X (0.500, 0.000, 0.500)

Supplementary Table 1030: Nodal ring points along high symmetry lines of mp-2741-Ca4F8.

	Coordinates	Frequency	Mode 1	Mode 2	Multi.	Deg.	Path	Ratio
0	(0.499, 0.058, 0.559)	5.648	4	3	24	2	6	0.765
1	(0.010, 0.000, 0.010)	7.551	5	4	6	2	1	0.020
2	(0.500, 0.250, 0.750)	7.656	5	4	6	2	5	1.000
3	(0.201, 0.201, 0.390)	8.552	6	5	24	2	3	0.474
4	(0.499, 0.298, 0.701)	8.145	7	6	48	2	5	0.806
5	(0.000, 0.000, 0.000)	9.671	8	7	1	3	1	0.000
6	(0.112, 0.000, 0.112)	9.702	8	7	6	2	1	0.224
7	(0.000, 0.000, 0.000)	9.671	9	8	1	3	1	0.000

The followings are details for Na_8Te_4. It has 4 topological nodal lines and 7 nodal ring points along high symmetry line as shown in Fig **493**.

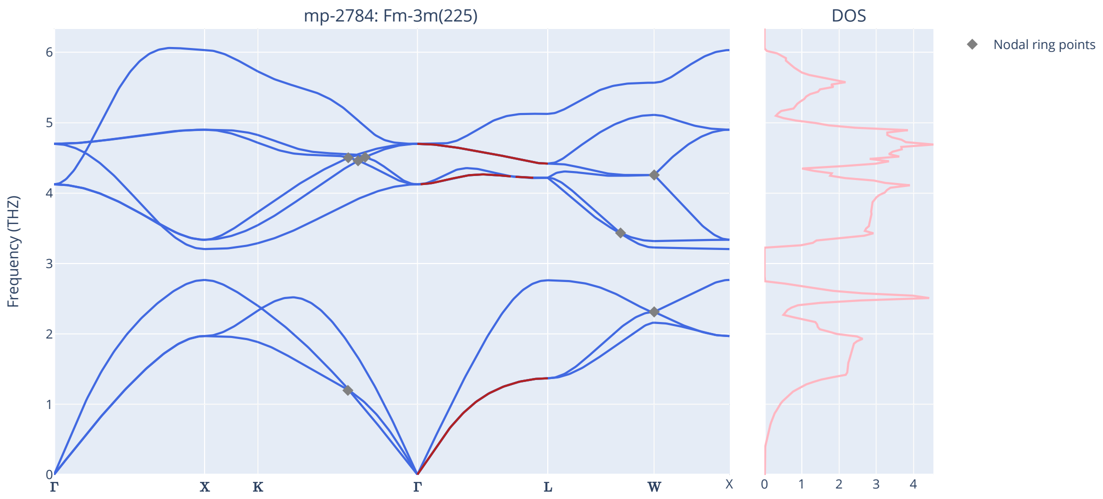

Supplementary Figure 493: The phonon band and DOS of Na8Te4.

Supplementary Table 1031: High symmetry lines of mp-2784-Na8Te4

	Start	End
0	Γ (0.000, 0.000, 0.000)	X (0.500, 0.000, 0.500)
1	X (0.500, 0.000, 0.500)	U (0.625, 0.250, 0.625)
2	K (0.375, 0.375, 0.750)	Γ (0.000, 0.000, 0.000)
3	Γ (0.000, 0.000, 0.000)	L (0.500, 0.500, 0.500)
4	L (0.500, 0.500, 0.500)	W (0.500, 0.250, 0.750)
5	W (0.500, 0.250, 0.750)	X (0.500, 0.000, 0.500)

Supplementary Table 1032: Nodal ring points along high symmetry lines of mp-2784-Na8Te4.

	Coordinates	Frequency	Mode 1	Mode 2	Multi.	Deg.	Path	Ratio
0	(0.168, 0.168, 0.322)	1.198	2	1	24	2	3	0.564
1	(0.500, 0.250, 0.750)	2.313	3	2	6	2	5	1.000
2	(0.500, 0.329, 0.671)	3.434	5	4	24	2	5	0.684
3	(0.142, 0.137, 0.279)	4.458	6	5	24	2	3	0.628
4	(0.166, 0.166, 0.321)	4.502	7	6	24	2	3	0.567
5	(0.122, 0.122, 0.246)	4.510	7	6	24	2	3	0.673
6	(0.500, 0.250, 0.750)	4.257	7	6	6	2	5	1.000

The followings are details for YHSe. It has 3 nodal ring points and 6 single Weyl points along high symmetry line as shown in Fig 494.

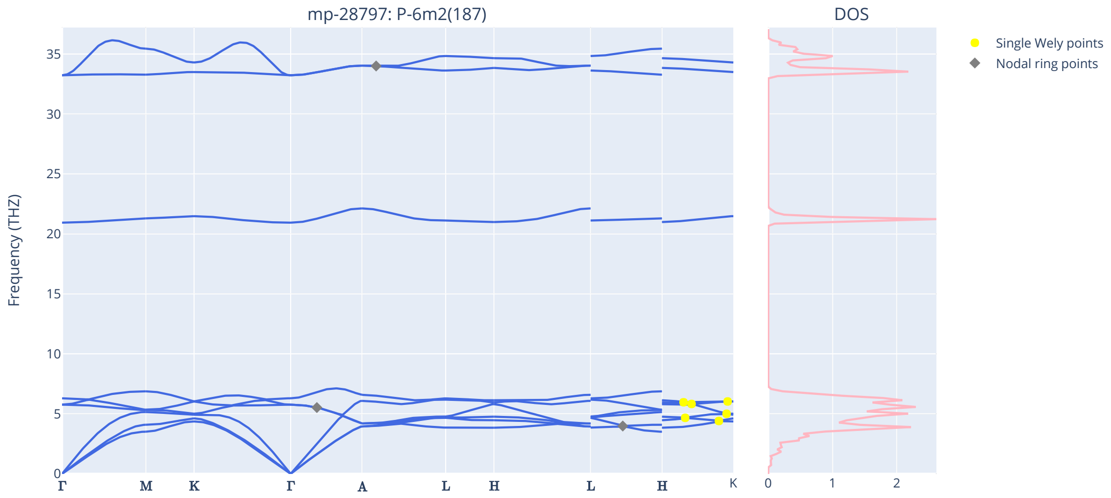

Supplementary Figure 494: The phonon band and DOS of YHSe.

Supplementary Table 1033: High symmetry lines of mp-28797-YHSe

	Start	End
0	Γ (0.000, 0.000, 0.000)	M (0.500, 0.000, 0.000)
1	M (0.500, 0.000, 0.000)	K (0.333, 0.333, 0.000)
2	K (0.333, 0.333, 0.000)	Γ (0.000, 0.000, 0.000)
3	Γ (0.000, 0.000, 0.000)	A (0.000, 0.000, 0.500)
4	A (0.000, 0.000, 0.500)	L (0.500, 0.000, 0.500)
5	L (0.500, 0.000, 0.500)	H (0.333, 0.333, 0.500)
6	H (0.333, 0.333, 0.500)	A (0.000, 0.000, 0.500)
7	L (0.500, 0.000, 0.500)	M (0.500, 0.000, 0.000)
8	H (0.333, 0.333, 0.500)	K (0.333, 0.333, 0.000)

Supplementary Table 1034: Single Weyl points along high symmetry lines of mp-28797-YHSe.

	Coordinates	Frequency	Mode 1	Mode 2	Multi.	Deg.	Path	Ratio
0	(0.333, 0.333, 0.101)	4.401	2	1	4	2	9	0.797
1	(0.333, 0.333, 0.338)	4.663	3	2	4	2	9	0.325
2	(0.333, 0.333, 0.047)	5.002	4	3	4	2	9	0.905
3	(0.333, 0.333, 0.293)	5.821	5	4	4	2	9	0.414
4	(0.333, 0.333, 0.349)	5.960	6	5	4	2	9	0.302
5	(0.333, 0.333, 0.040)	6.027	6	5	4	2	9	0.920

Supplementary Table 1035: Nodal ring points along high symmetry lines of mp-28797-YHSe.

	Coordinates	Frequency	Mode 1	Mode 2	Multi.	Deg.	Path	Ratio
0	(0.499, -0.000, 0.275)	3.980	2	1	12	2	8	0.451
1	(0.000, 0.000, 0.184)	5.520	5	4	2	2	4	0.367
2	(0.084, 0.000, 0.500)	34.003	9	8	6	2	5	0.169

The followings are details for F_4Kr_2 and it only has 4 topological nodal ring points along high symmetry line as shown in Fig 495.

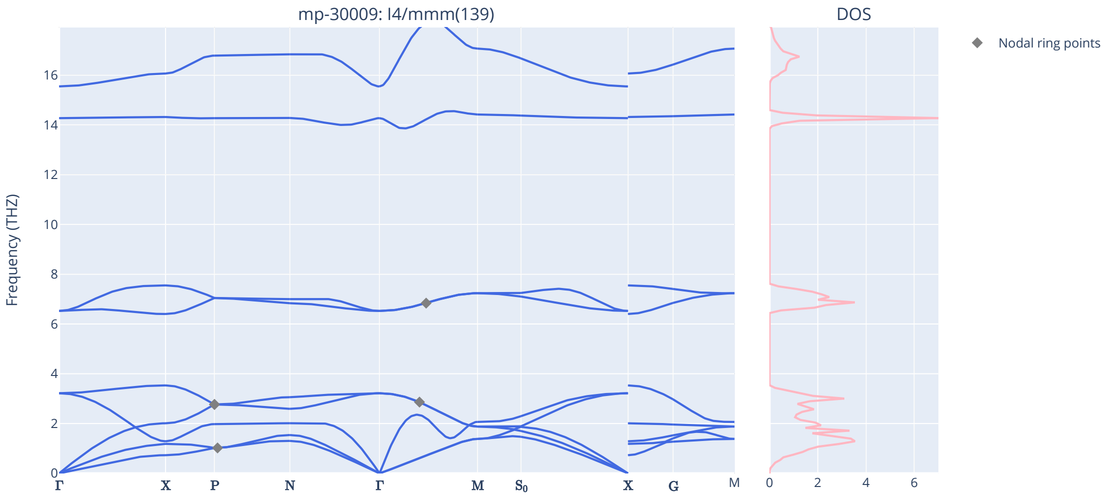

Supplementary Figure 495: The phonon band and DOS of F4Kr2.

Supplementary Table 1036: High symmetry lines of mp-30009-F4Kr2

	Start	End
0	Γ (0.000, 0.000, 0.000)	X (0.000, 0.000, 0.500)
1	X (0.000, 0.000, 0.500)	P (0.250, 0.250, 0.250)
2	P (0.250, 0.250, 0.250)	N (0.000, 0.500, 0.000)
3	N (0.000, 0.500, 0.000)	Γ (0.000, 0.000, 0.000)
4	Γ (0.000, 0.000, 0.000)	M (0.500, 0.500, -0.500)
5	M (0.500, 0.500, -0.500)	S (0.356, 0.644, -0.356)
6	S_0 (-0.356, 0.356, 0.356)	Γ (0.000, 0.000, 0.000)
7	X (0.000, 0.000, 0.500)	R (-0.211, 0.211, 0.500)
8	G (0.500, 0.500, -0.211)	M (0.500, 0.500, -0.500)

Supplementary Table 1037: Nodal ring points along high symmetry lines of mp-30009-F4Kr2.

	Coordinates	Frequency	Mode 1	Mode 2	Multi.	Deg.	Path	Ratio
0	(0.240, 0.260, 0.240)	1.018	2	1	8	2	3	0.041
1	(0.250, 0.250, 0.250)	2.766	5	4	2	2	2	1.000
2	(0.204, 0.204, -0.204)	2.861	5	4	2	2	5	0.408
3	(0.236, 0.236, -0.242)	6.838	7	6	8	2	5	0.476

The followings are details for CaI_2. It has 6 topological nodal lines and 12 nodal ring points along high symmetry line as shown in Fig **496**.

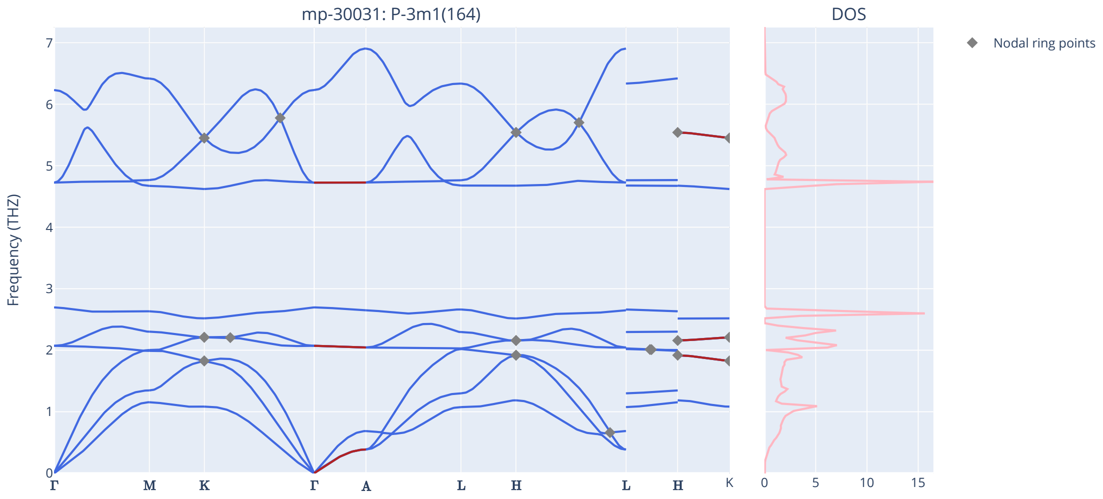

Supplementary Figure 496: The phonon band and DOS of CaI2.

Supplementary Table 1038: High symmetry lines of mp-30031-CaI2

	Start	End
0	Γ (0.000, 0.000, 0.000)	M (0.500, 0.000, 0.000)
1	M (0.500, 0.000, 0.000)	K (0.333, 0.333, 0.000)
2	K (0.333, 0.333, 0.000)	Γ (0.000, 0.000, 0.000)
3	Γ (0.000, 0.000, 0.000)	A (0.000, 0.000, 0.500)
4	A (0.000, 0.000, 0.500)	L (0.500, 0.000, 0.500)
5	L (0.500, 0.000, 0.500)	H (0.333, 0.333, 0.500)
6	H (0.333, 0.333, 0.500)	A (0.000, 0.000, 0.500)
7	L (0.500, 0.000, 0.500)	M (0.500, 0.000, 0.000)
8	H (0.333, 0.333, 0.500)	K (0.333, 0.333, 0.000)

Supplementary Table 1039: Nodal ring points along high symmetry lines of mp-30031-CaI2.

	Coordinates	Frequency	Mode 1	Mode 2	Multi.	Deg.	Path	Ratio
0	(0.333, 0.333, 0.000)	1.827	3	2	2	2	2	1.000
1	(0.048, 0.049, 0.500)	0.661	3	2	8	2	7	0.855
2	(0.333, 0.333, 0.500)	1.919	3	2	2	2	6	1.000
3	(0.498, 0.000, 0.253)	2.011	4	3	6	2	8	0.493
4	(0.496, 0.000, 0.268)	2.012	4	3	6	2	8	0.465
5	(0.255, 0.254, 0.002)	2.206	5	4	12	2	3	0.237
6	(0.333, 0.333, 0.000)	2.211	5	4	2	2	2	1.000
7	(0.333, 0.333, 0.500)	2.159	5	4	2	2	6	1.000
8	(0.333, 0.333, 0.000)	5.452	9	8	2	2	2	1.000
9	(0.103, 0.103, -0.000)	5.779	9	8	6	2	3	0.691
10	(0.333, 0.333, 0.500)	5.543	9	8	2	2	6	1.000
11	(0.142, 0.143, 0.499)	5.703	9	8	12	2	7	0.573

Supplementary Table 1039: Nodal ring points along high symmetry lines of mp-30031-CaI2.

Coordinates	Frequency	Mode 1	Mode 2	Multi.	Deg.	Path	Ratio

The followings are details for MgBr$_2$. It has 6 topological nodal lines and 14 nodal ring points along high symmetry line as shown in Fig 497.

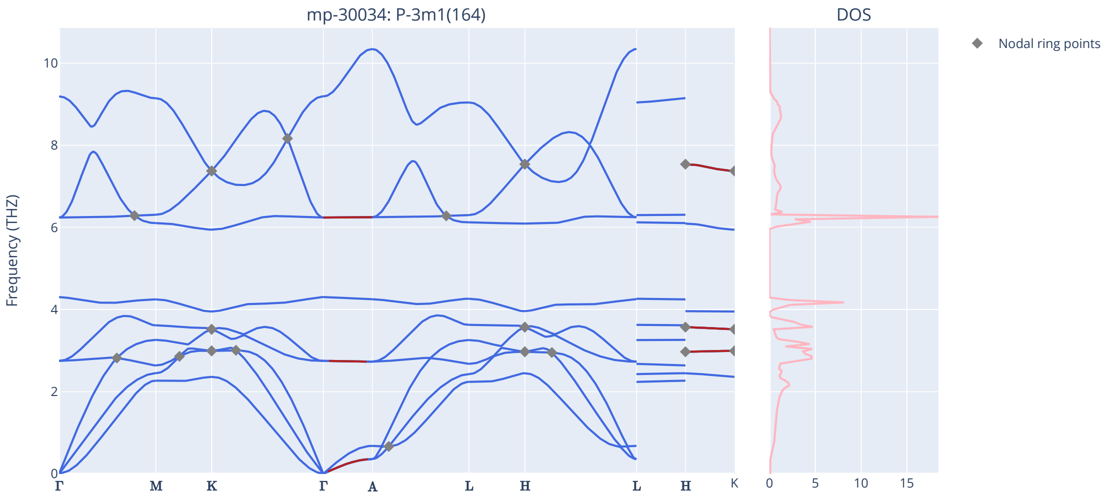

Supplementary Figure 497: The phonon band and DOS of MgBr2.

Supplementary Table 1040: High symmetry lines of mp-30034-MgBr2

	Start	End
0	Γ (0.000, 0.000, 0.000)	M (0.500, 0.000, 0.000)
1	M (0.500, 0.000, 0.000)	K (0.333, 0.333, 0.000)
2	K (0.333, 0.333, 0.000)	Γ (0.000, 0.000, 0.000)
3	Γ (0.000, 0.000, 0.000)	A (0.000, 0.000, 0.500)
4	A (0.000, 0.000, 0.500)	L (0.500, 0.000, 0.500)
5	L (0.500, 0.000, 0.500)	H (0.333, 0.333, 0.500)
6	H (0.333, 0.333, 0.500)	A (0.000, 0.000, 0.500)
7	L (0.500, 0.000, 0.500)	M (0.500, 0.000, 0.000)
8	H (0.333, 0.333, 0.500)	K (0.333, 0.333, 0.000)

Supplementary Table 1041: Nodal ring points along high symmetry lines of mp-30034-MgBr2.

	Coordinates	Frequency	Mode 1	Mode 2	Multi.	Deg.	Path	Ratio
0	(0.085, -0.000, 0.498)	0.664	2	1	6	2	5	0.171
1	(0.261, 0.261, 0.000)	3.006	3	2	6	2	3	0.218
2	(0.429, 0.141, 0.001)	2.858	3	2	12	2	2	0.424
3	(0.333, 0.333, 0.000)	2.991	3	2	2	2	2	1.000
4	(0.253, 0.253, 0.500)	2.951	3	2	6	2	7	0.242
5	(0.333, 0.333, 0.500)	2.970	3	2	2	2	6	1.000
6	(0.299, -0.000, -0.005)	2.818	4	3	6	2	1	0.597
7	(0.333, 0.333, 0.000)	3.517	5	4	2	2	2	1.000
8	(0.333, 0.333, 0.500)	3.570	5	4	2	2	6	1.000
9	(0.390, 0.000, 0.003)	6.285	8	7	6	2	1	0.780
10	(0.382, 0.000, 0.496)	6.280	8	7	6	2	5	0.765

Supplementary Table 1041: Nodal ring points along high symmetry lines of mp-30034-MgBr2.

	Coordinates	Frequency	Mode 1	Mode 2	Multi.	Deg.	Path	Ratio
11	(0.333, 0.333, 0.000)	7.373	9	8	2	2	2	1.000
12	(0.107, 0.107, -0.000)	8.163	9	8	6	2	3	0.679
13	(0.333, 0.333, 0.500)	7.538	9	8	2	2	6	1.000

The followings are details for $Pt_4Sn_4Ti_4$. As shown in Fig **498**, it has 3 topological nodal lines, 1 nodal ring point and 1 high degenerate Weyl point whose degeneracy is more than 2 and charge is bigger than 1.

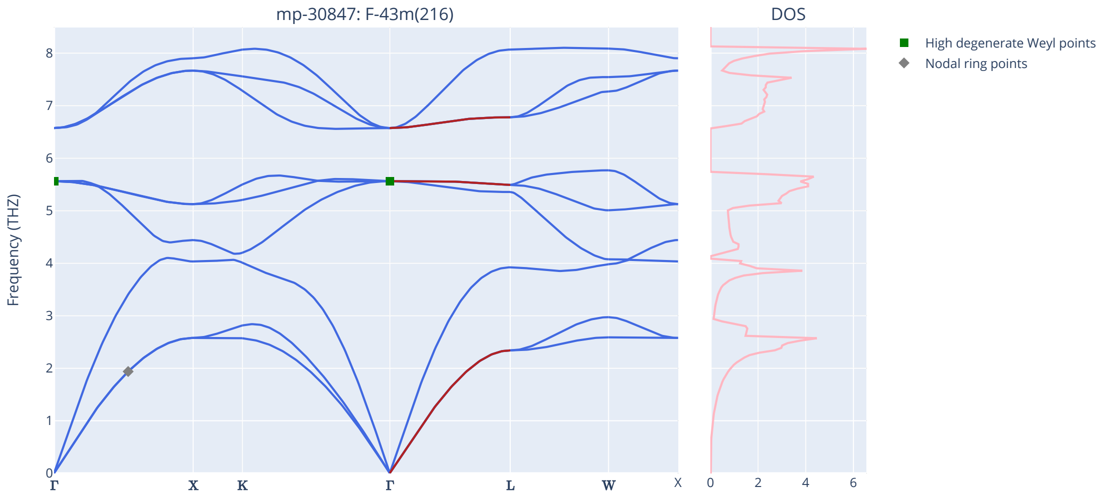

Supplementary Figure 498: The phonon band and DOS of Pt4Sn4Ti4.

Supplementary Table 1042: High symmetry lines of mp-30847-Pt4Sn4Ti4

	Start	End
0	Γ (0.000, 0.000, 0.000)	X (0.500, 0.000, 0.500)
1	X (0.500, 0.000, 0.500)	U (0.625, 0.250, 0.625)
2	K (0.375, 0.375, 0.750)	Γ (0.000, 0.000, 0.000)
3	Γ (0.000, 0.000, 0.000)	L (0.500, 0.500, 0.500)
4	L (0.500, 0.500, 0.500)	W (0.500, 0.250, 0.750)
5	W (0.500, 0.250, 0.750)	X (0.500, 0.000, 0.500)

Supplementary Table 1043: High degenerate Weyl points along high symmetry lines of mp-30847-Pt4Sn4Ti4.

	Coordinates	Frequency	Mode 1	Mode 2	Multi.	Deg.	Path	Ratio
0	(0.000, 0.000, 0.000)	5.561	6	4	1	3	1	0.000

Supplementary Table 1044: Nodal ring points along high symmetry lines of mp-30847-Pt4Sn4Ti4.

	Coordinates	Frequency	Mode 1	Mode 2	Multi.	Deg.	Path	Ratio
0	(0.261, -0.000, 0.272)	1.937	2	1	24	2	1	0.533

The followings are details for $Bi_4Co_4Zr_4$. It has 3 topological nodal lines and 1 high degenerate Weyl point whose degeneracy is more than 2 and charge is bigger than 1 along high symmetry line as shown in Fig **499**.

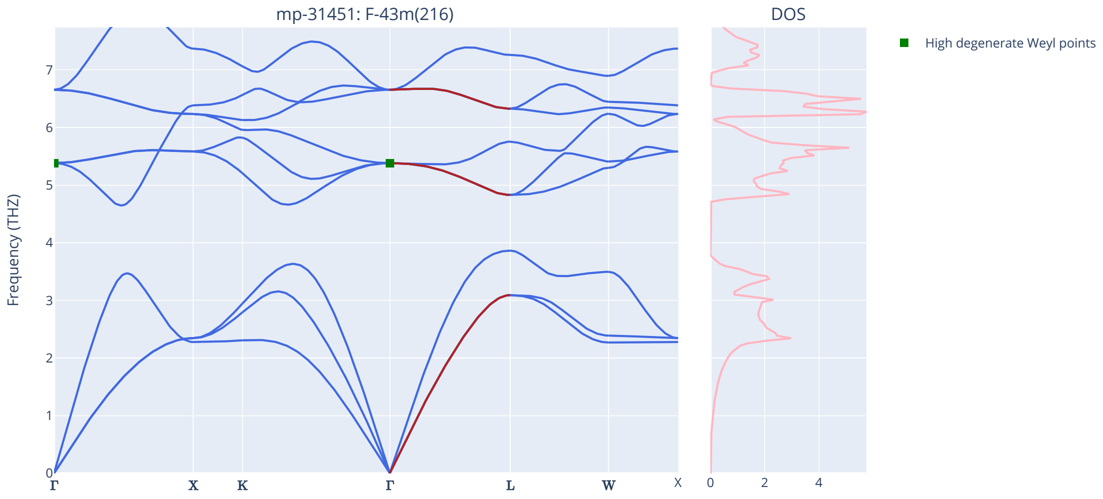

Supplementary Figure 499: The phonon band and DOS of Bi4Co4Zr4.

Supplementary Table 1045: High symmetry lines of mp-31451-Bi4Co4Zr4

	Start	End
0	Γ (0.000, 0.000, 0.000)	X (0.500, 0.000, 0.500)
1	X (0.500, 0.000, 0.500)	U (0.625, 0.250, 0.625)
2	K (0.375, 0.375, 0.750)	Γ (0.000, 0.000, 0.000)
3	Γ (0.000, 0.000, 0.000)	L (0.500, 0.500, 0.500)
4	L (0.500, 0.500, 0.500)	W (0.500, 0.250, 0.750)
5	W (0.500, 0.250, 0.750)	X (0.500, 0.000, 0.500)

Supplementary Table 1046: High degenerate Weyl points along high symmetry lines of mp-31451-Bi4Co4Zr4.

	Coordinates	Frequency	Mode 1	Mode 2	Multi.	Deg.	Path	Ratio
0	(0.000, 0.000, 0.000)	5.382	6	4	1	3	1	0.000

The followings are details for $Ru_4Ta_4Sb_4$. As shown in Fig **500**, it has 4 topological nodal lines and 2 nodal ring points. At the same time, it has 2 single Weyl points and 1 high degenerate Weyl point whose degeneracy is more than 2 and charge is bigger than 1.

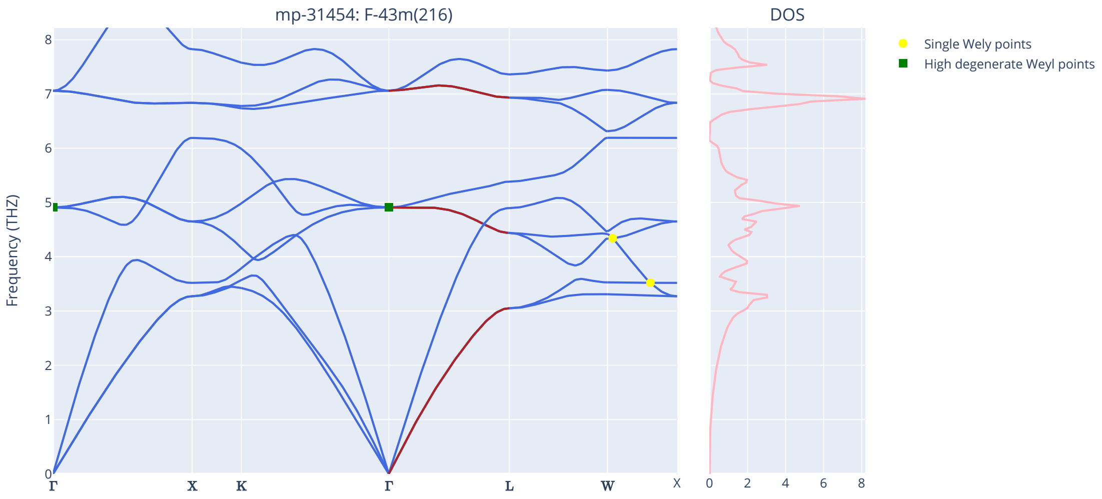

Supplementary Figure 500: The phonon band and DOS of Ru4Ta4Sb4.

Supplementary Table 1047: High symmetry lines of mp-31454-Ru4Ta4Sb4

	Start	End
0	Γ (0.000, 0.000, 0.000)	X (0.500, 0.000, 0.500)
1	X (0.500, 0.000, 0.500)	U (0.625, 0.250, 0.625)
2	K (0.375, 0.375, 0.750)	Γ (0.000, 0.000, 0.000)
3	Γ (0.000, 0.000, 0.000)	L (0.500, 0.500, 0.500)
4	L (0.500, 0.500, 0.500)	W (0.500, 0.250, 0.750)
5	W (0.500, 0.250, 0.750)	X (0.500, 0.000, 0.500)

Supplementary Table 1048: Single Weyl points along high symmetry lines of mp-31454-Ru4Ta4Sb4.

	Coordinates	Frequency	Mode 1	Mode 2	Multi.	Deg.	Path	Ratio
0	(0.500, 0.096, 0.596)	3.520	3	2	12	2	6	0.617
1	(0.500, 0.231, 0.731)	4.340	4	3	12	2	6	0.075

Supplementary Table 1049: High degenerate Weyl points along high symmetry lines of mp-31454-Ru4Ta4Sb4.

	Coordinates	Frequency	Mode 1	Mode 2	Multi.	Deg.	Path	Ratio
0	(0.000, 0.000, 0.000)	4.908	6	4	1	3	1	0.000

The followings are details for Pb_4F_8. It has 4 topological nodal lines and 8 nodal ring points along high symmetry line as shown in Fig **501**.

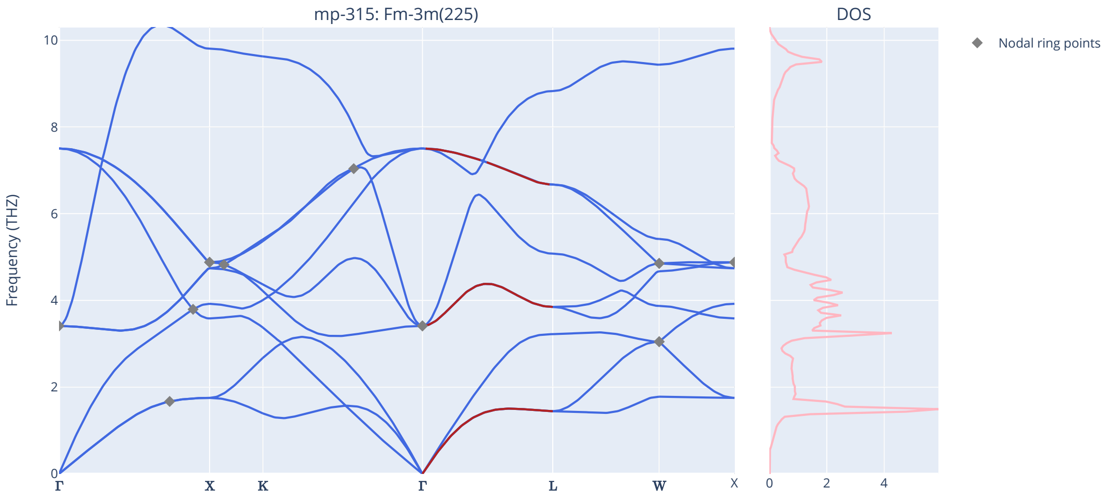

Supplementary Figure 501: The phonon band and DOS of Pb4F8.

Supplementary Table 1050: High symmetry lines of mp-315-Pb4F8

	Start	End
0	Γ (0.000, 0.000, 0.000)	X (0.500, 0.000, 0.500)
1	X (0.500, 0.000, 0.500)	U (0.625, 0.250, 0.625)
2	K (0.375, 0.375, 0.750)	Γ (0.000, 0.000, 0.000)
3	Γ (0.000, 0.000, 0.000)	L (0.500, 0.500, 0.500)
4	L (0.500, 0.500, 0.500)	W (0.500, 0.250, 0.750)
5	W (0.500, 0.250, 0.750)	X (0.500, 0.000, 0.500)

Supplementary Table 1051: Nodal ring points along high symmetry lines of mp-315-Pb4F8.

	Coordinates	Frequency	Mode 1	Mode 2	Multi.	Deg.	Path	Ratio
0	(0.367, 0.000, 0.367)	1.668	2	1	6	2	1	0.735
1	(0.500, 0.250, 0.750)	3.046	3	2	6	2	5	1.000
2	(0.445, 0.000, 0.445)	3.793	4	3	10	2	1	0.890
3	(0.000, 0.000, 0.000)	3.411	5	4	1	3	1	0.000
4	(0.534, 0.067, 0.534)	4.825	7	6	12	2	2	0.270
5	(0.500, 0.250, 0.750)	4.853	7	6	6	2	5	1.000
6	(0.500, 0.000, 0.500)	4.878	8	7	3	2	1	1.000
7	(0.161, 0.161, 0.323)	7.038	8	7	24	2	3	0.570

The followings are details for $Li_4Mg_4P_4$. As shown in Fig 502, it has 4 topological nodal lines, 2 nodal ring points and 1 high degenerate Weyl point whose degeneracy is more than 2 and charge is bigger than 1.

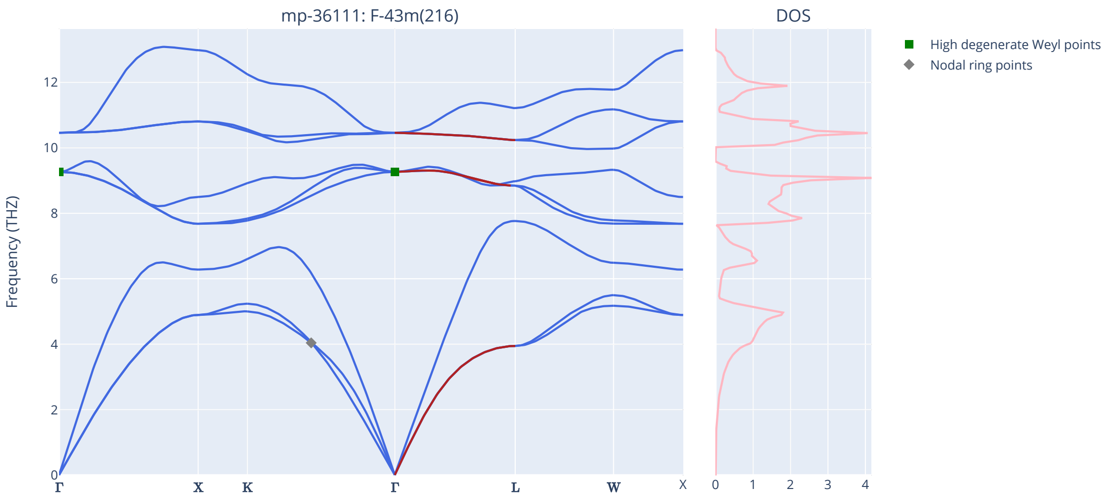

Supplementary Figure 502: The phonon band and DOS of Li4Mg4P4.

Supplementary Table 1052: High symmetry lines of mp-36111-Li4Mg4P4

	Start	End
0	Γ (0.000, 0.000, 0.000)	X (0.500, 0.000, 0.500)
1	X (0.500, 0.000, 0.500)	U (0.625, 0.250, 0.625)
2	K (0.375, 0.375, 0.750)	Γ (0.000, 0.000, 0.000)
3	Γ (0.000, 0.000, 0.000)	L (0.500, 0.500, 0.500)
4	L (0.500, 0.500, 0.500)	W (0.500, 0.250, 0.750)
5	W (0.500, 0.250, 0.750)	X (0.500, 0.000, 0.500)

Supplementary Table 1053: High degenerate Weyl points along high symmetry lines of mp-36111-Li4Mg4P4.

	Coordinates	Frequency	Mode 1	Mode 2	Multi.	Deg.	Path	Ratio
0	(0.000, 0.000, 0.000)	9.263	6	4	1	3	1	0.000

Supplementary Table 1054: Nodal ring points along high symmetry lines of mp-36111-Li4Mg4P4.

	Coordinates	Frequency	Mode 1	Mode 2	Multi.	Deg.	Path	Ratio
0	(0.214, 0.214, 0.423)	4.042	2	1	24	2	3	0.433

The followings are details for $Na_3H_3S_3$. It has 6 topological nodal lines and 5 nodal ring points along high symmetry line as shown in Fig **503**.

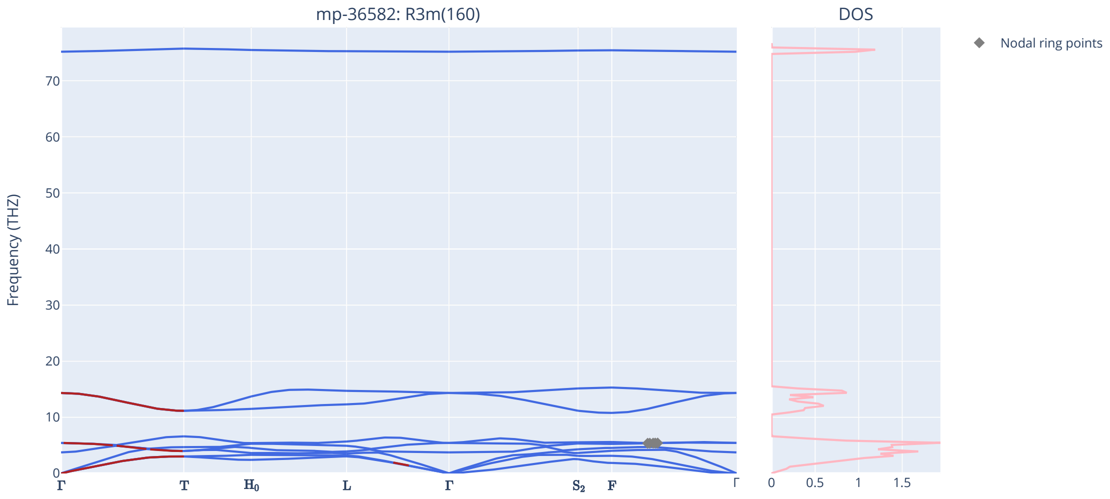

Supplementary Figure 503: The phonon band and DOS of Na3H3S3.

Supplementary Table 1055: High symmetry lines of mp-36582-Na3H3S3

	Start	End
0	Γ (0.000, 0.000, 0.000)	T (0.500, 0.500, 0.500)
1	T (0.500, 0.500, 0.500)	H_2 (0.707, 0.293, 0.500)
2	H_0 (0.500, -0.293, 0.293)	L (0.500, 0.000, 0.000)
3	L (0.500, 0.000, 0.000)	Γ (0.000, 0.000, 0.000)
4	Γ (0.000, 0.000, 0.000)	S_0 (0.397, -0.397, 0.000)
5	S_2 (0.603, 0.000, 0.397)	F (0.500, 0.000, 0.500)
6	F (0.500, 0.000, 0.500)	Γ (0.000, 0.000, 0.000)

Supplementary Table 1056: Nodal ring points along high symmetry lines of mp-36582-Na3H3S3.

	Coordinates	Frequency	Mode 1	Mode 2	Multi.	Deg.	Path	Ratio
0	(0.356, 0.003, 0.356)	5.351	6	5	6	2	7	0.287
1	(0.334, -0.006, 0.334)	5.378	6	5	6	2	7	0.332
2	(0.348, -0.003, 0.348)	5.363	6	5	6	2	7	0.305
3	(0.326, -0.005, 0.326)	5.388	6	5	6	2	7	0.348
4	(0.316, -0.001, 0.316)	5.399	6	5	6	2	7	0.368

The followings are details for $Li_4Mg_4N_4$. As shown in Fig **504**, it has 4 topological nodal lines, 2 nodal ring points and 1 high degenerate Weyl point whose degeneracy is more than 2 and charge is bigger than 1.

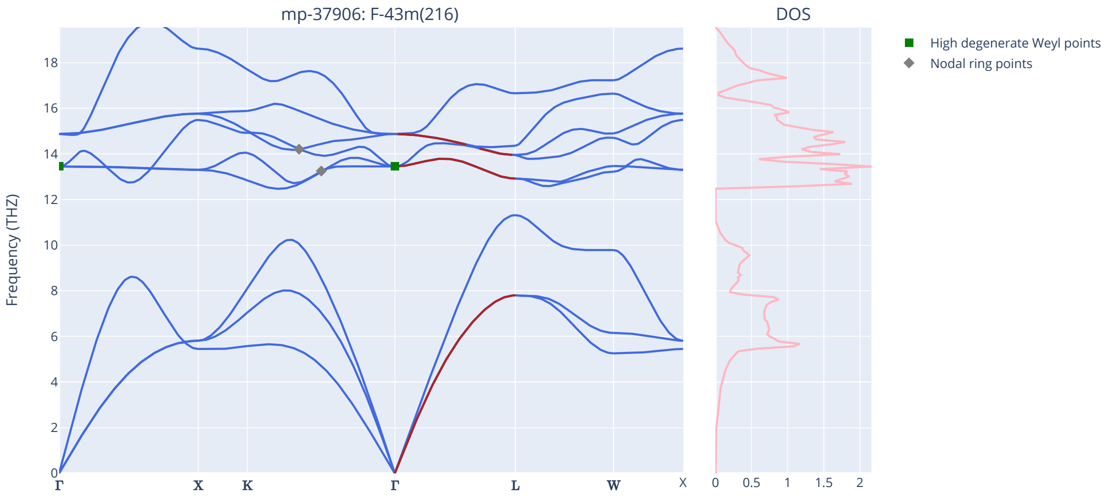

Supplementary Figure 504: The phonon band and DOS of Li4Mg4N4.

Supplementary Table 1057: High symmetry lines of mp-37906-Li4Mg4N4

	Start	End
0	Γ (0.000, 0.000, 0.000)	X (0.500, 0.000, 0.500)
1	X (0.500, 0.000, 0.500)	U (0.625, 0.250, 0.625)
2	K (0.375, 0.375, 0.750)	Γ (0.000, 0.000, 0.000)
3	Γ (0.000, 0.000, 0.000)	L (0.500, 0.500, 0.500)
4	L (0.500, 0.500, 0.500)	W (0.500, 0.250, 0.750)
5	W (0.500, 0.250, 0.750)	X (0.500, 0.000, 0.500)

Supplementary Table 1058: High degenerate Weyl points along high symmetry lines of mp-37906-Li4Mg4N4.

	Coordinates	Frequency	Mode 1	Mode 2	Multi.	Deg.	Path	Ratio
0	(0.000, 0.000, 0.000)	13.450	6	4	1	3	1	0.000

Supplementary Table 1059: Nodal ring points along high symmetry lines of mp-37906-Li4Mg4N4.

	Coordinates	Frequency	Mode 1	Mode 2	Multi.	Deg.	Path	Ratio
0	(0.189, 0.189, 0.371)	13.250	5	4	24	2	3	0.502
1	(0.245, 0.245, 0.485)	14.205	7	6	24	2	3	0.351

The followings are details for $K_3H_3S_3$. It has 5 topological nodal lines and 5 nodal ring points along high symmetry line as shown in Fig **505**.

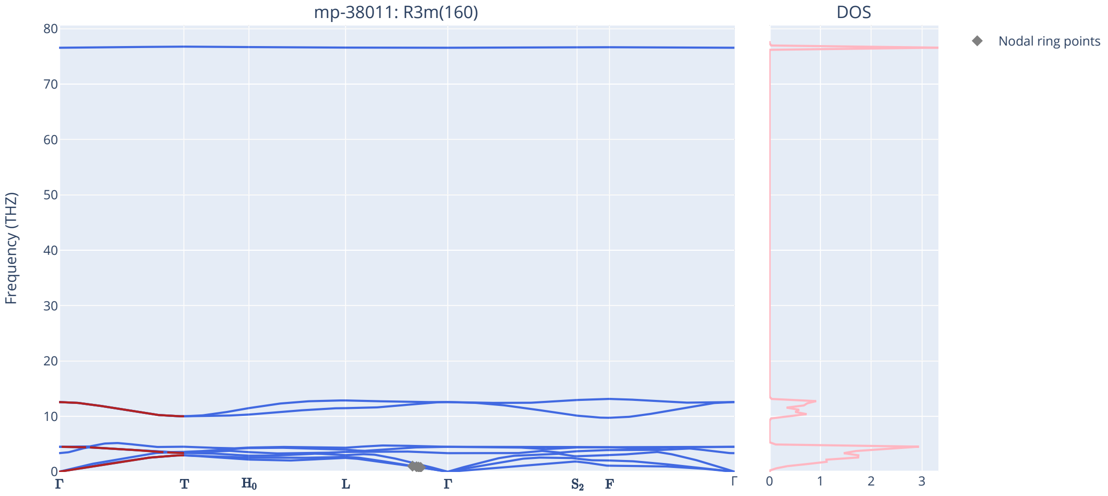

Supplementary Figure 505: The phonon band and DOS of K3H3S3.

Supplementary Table 1060: High symmetry lines of mp-38011-K3H3S3

	Start	End
0	Γ (0.000, 0.000, 0.000)	T (0.500, 0.500, 0.500)
1	T (0.500, 0.500, 0.500)	H_2 (0.701, 0.299, 0.500)
2	H_0 (0.500, -0.299, 0.299)	L (0.500, 0.000, 0.000)
3	L (0.500, 0.000, 0.000)	Γ (0.000, 0.000, 0.000)
4	Γ (0.000, 0.000, 0.000)	S_0 (0.400, -0.400, 0.000)
5	S_2 (0.600, 0.000, 0.400)	F (0.500, 0.000, 0.500)
6	F (0.500, 0.000, 0.500)	Γ (0.000, 0.000, 0.000)

Supplementary Table 1061: Nodal ring points along high symmetry lines of mp-38011-K3H3S3.

	Coordinates	Frequency	Mode 1	Mode 2	Multi.	Deg.	Path	Ratio
0	(0.172, -0.003, -0.003)	1.023	2	1	6	2	4	0.655
1	(0.153, -0.000, -0.000)	0.906	2	1	6	2	4	0.694
2	(0.143, 0.001, 0.001)	0.847	2	1	6	2	4	0.714
3	(0.133, 0.002, 0.002)	0.787	2	1	6	2	4	0.734

The followings are details for CdI_2. It has 6 topological nodal lines and 19 nodal ring points along high symmetry line as shown in Fig **506**.

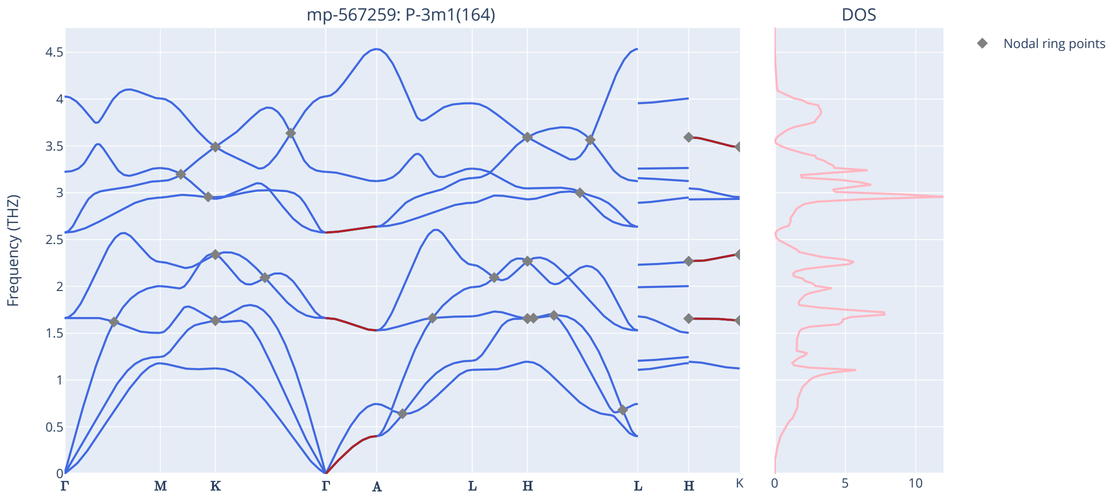

Supplementary Figure 506: The phonon band and DOS of CdI2.

Supplementary Table 1062: High symmetry lines of mp-567259-CdI2

	Start	End
0	Γ (0.000, 0.000, 0.000)	M (0.500, 0.000, 0.000)
1	M (0.500, 0.000, 0.000)	K (0.333, 0.333, 0.000)
2	K (0.333, 0.333, 0.000)	Γ (0.000, 0.000, 0.000)
3	Γ (0.000, 0.000, 0.000)	A (0.000, 0.000, 0.500)
4	A (0.000, 0.000, 0.500)	L (0.500, 0.000, 0.500)
5	L (0.500, 0.000, 0.500)	H (0.333, 0.333, 0.500)
6	H (0.333, 0.333, 0.500)	A (0.000, 0.000, 0.500)
7	L (0.500, 0.000, 0.500)	M (0.500, 0.000, 0.000)
8	H (0.333, 0.333, 0.500)	K (0.333, 0.333, 0.000)

Supplementary Table 1063: Nodal ring points along high symmetry lines of mp-567259-CdI2.

	Coordinates	Frequency	Mode 1	Mode 2	Multi.	Deg.	Path	Ratio
0	(0.136, 0.000, 0.498)	0.641	2	1	6	2	5	0.271
1	(0.333, 0.333, 0.000)	1.635	3	2	2	2	2	1.000
2	(0.253, 0.254, 0.498)	1.690	3	2	12	2	7	0.240
3	(0.315, 0.315, 0.500)	1.661	3	2	6	2	7	0.055
4	(0.333, 0.333, 0.500)	1.657	3	2	2	2	6	1.000
5	(0.046, 0.046, 0.500)	0.681	3	2	6	2	7	0.863
6	(0.259, -0.000, -0.000)	1.621	4	3	6	2	1	0.517
7	(0.292, 0.000, 0.498)	1.662	4	3	6	2	5	0.584
8	(0.333, 0.333, 0.000)	2.340	5	4	2	2	2	1.000
9	(0.184, 0.184, -0.000)	2.094	5	4	6	2	3	0.449
10	(0.433, 0.133, 0.498)	2.094	5	4	12	2	6	0.398

Supplementary Table 1063: Nodal ring points along high symmetry lines of mp-567259-CdI2.

	Coordinates	Frequency	Mode 1	Mode 2	Multi.	Deg.	Path	Ratio
11	(0.333, 0.333, 0.500)	2.269	5	4	2	2	6	1.000
12	(0.355, 0.290, -0.000)	2.956	7	6	6	2	2	0.869
13	(0.176, 0.173, 0.500)	2.999	7	6	12	2	7	0.475
14	(0.438, 0.125, -0.000)	3.197	8	7	6	2	2	0.374
15	(0.333, 0.333, 0.000)	3.489	9	8	2	2	2	1.000
16	(0.106, 0.106, 0.000)	3.636	9	8	6	2	3	0.683
17	(0.333, 0.333, 0.500)	3.592	9	8	2	2	6	1.000
18	(0.144, 0.144, 0.500)	3.565	9	8	6	2	7	0.569

The followings are details for $Fe_4V_4Sb_4$. As shown in Fig **507**, it has 3 topological nodal lines, 2 nodal ring points and 1 high degenerate Weyl point whose degeneracy is more than 2 and charge is bigger than 1.

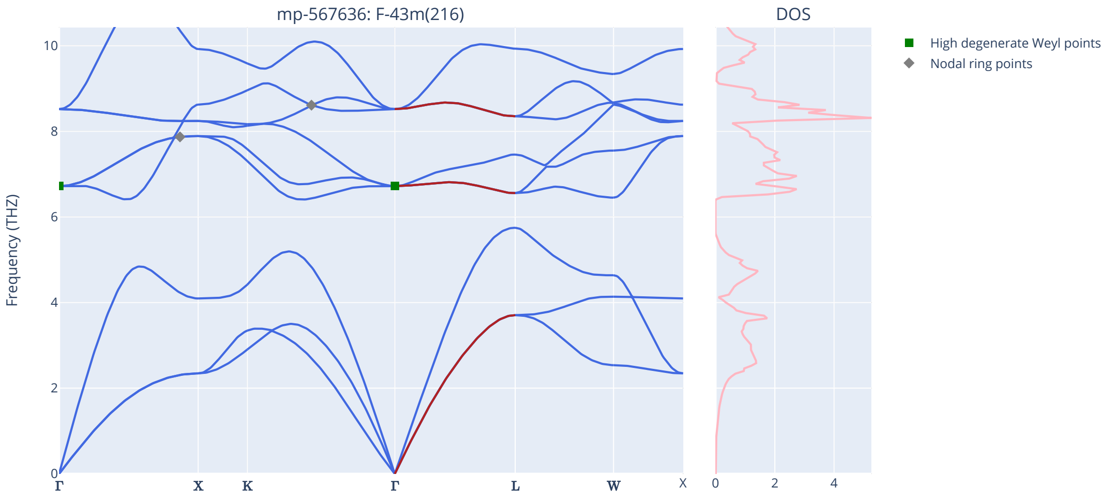

Supplementary Figure 507: The phonon band and DOS of Fe4V4Sb4.

Supplementary Table 1064: High symmetry lines of mp-567636-Fe4V4Sb4

	Start	End
0	Γ (0.000, 0.000, 0.000)	X (0.500, 0.000, 0.500)
1	X (0.500, 0.000, 0.500)	U (0.625, 0.250, 0.625)
2	K (0.375, 0.375, 0.750)	Γ (0.000, 0.000, 0.000)
3	Γ (0.000, 0.000, 0.000)	L (0.500, 0.500, 0.500)
4	L (0.500, 0.500, 0.500)	W (0.500, 0.250, 0.750)
5	W (0.500, 0.250, 0.750)	X (0.500, 0.000, 0.500)

Supplementary Table 1065: High degenerate Weyl points along high symmetry lines of mp-567636-Fe4V4Sb4.

	Coordinates	Frequency	Mode 1	Mode 2	Multi.	Deg.	Path	Ratio
0	(0.000, 0.000, 0.000)	6.718	6	4	1	3	1	0.000

Supplementary Table 1066: Nodal ring points along high symmetry lines of mp-567636-Fe4V4Sb4.

	Coordinates	Frequency	Mode 1	Mode 2	Multi.	Deg.	Path	Ratio
0	(0.435, -0.001, 0.435)	7.869	5	4	24	2	1	0.869
1	(0.214, 0.214, 0.421)	8.610	8	7	24	2	3	0.435

The followings are details for Ba_4Cl_8. It has 4 topological nodal lines and 9 nodal ring points along high symmetry line as shown in Fig **508**.

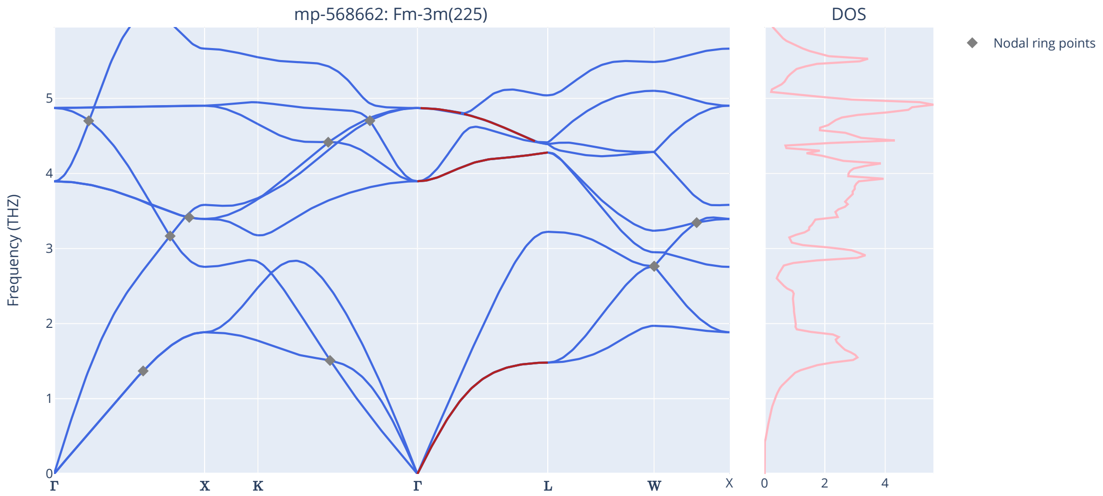

Supplementary Figure 508: The phonon band and DOS of Ba4Cl8.

Supplementary Table 1067: High symmetry lines of mp-568662-Ba4Cl8

	Start	End
0	Γ (0.000, 0.000, 0.000)	X (0.500, 0.000, 0.500)
1	X (0.500, 0.000, 0.500)	U (0.625, 0.250, 0.625)
2	K (0.375, 0.375, 0.750)	Γ (0.000, 0.000, 0.000)
3	Γ (0.000, 0.000, 0.000)	L (0.500, 0.500, 0.500)
4	L (0.500, 0.500, 0.500)	W (0.500, 0.250, 0.750)
5	W (0.500, 0.250, 0.750)	X (0.500, 0.000, 0.500)

Supplementary Table 1068: Nodal ring points along high symmetry lines of mp-568662-Ba4Cl8.

	Coordinates	Frequency	Mode 1	Mode 2	Multi.	Deg.	Path	Ratio
0	(0.296, 0.000, 0.296)	1.369	2	1	6	2	1	0.592
1	(0.207, 0.207, 0.409)	1.508	2	1	24	2	3	0.452
2	(0.500, 0.250, 0.750)	2.763	3	2	6	2	5	1.000
3	(0.386, 0.001, 0.386)	3.165	4	3	24	2	1	0.771
4	(0.449, 0.000, 0.449)	3.414	5	4	6	2	1	0.898
5	(0.499, 0.110, 0.608)	3.343	5	4	24	2	6	0.565
6	(0.110, 0.113, 0.223)	4.701	7	6	24	2	3	0.702
7	(0.115, -0.003, 0.115)	4.699	7	6	24	2	1	0.230
8	(0.205, 0.214, 0.419)	4.414	7	6	24	2	3	0.441

The followings are details for Cd_3Br_6. It has 3 topological nodal lines and 10 nodal ring points along high symmetry line as shown in Fig **509**.

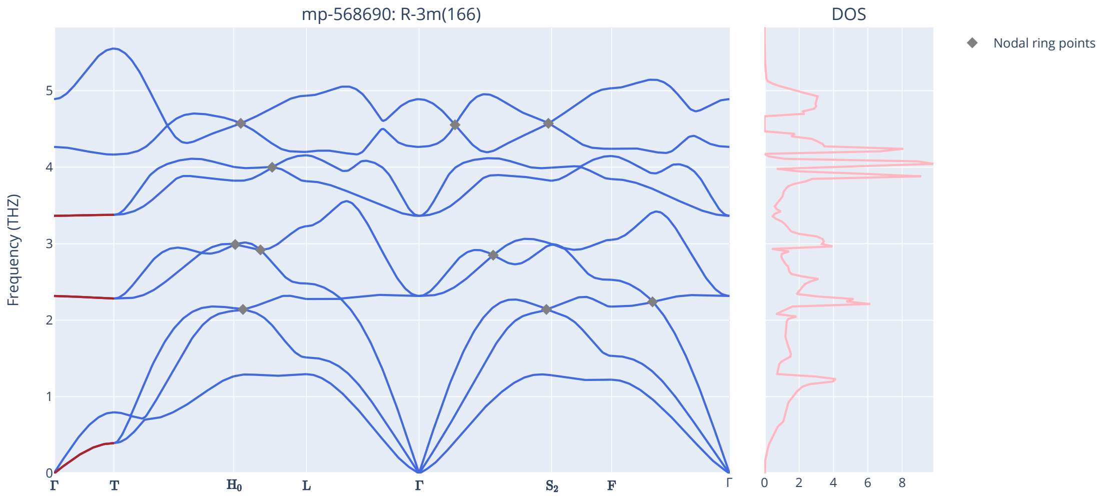

Supplementary Figure 509: The phonon band and DOS of Cd3Br6.

Supplementary Table 1069: High symmetry lines of mp-568690-Cd3Br6

	Start	End
0	Γ (0.000, 0.000, 0.000)	T (0.500, 0.500, 0.500)
1	T (0.500, 0.500, 0.500)	H_2 (0.812, 0.188, 0.500)
2	H_0 (0.500, -0.188, 0.188)	L (0.500, 0.000, 0.000)
3	L (0.500, 0.000, 0.000)	Γ (0.000, 0.000, 0.000)
4	Γ (0.000, 0.000, 0.000)	S_0 (0.344, -0.344, 0.000)
5	S_2 (0.656, 0.000, 0.344)	F (0.500, 0.000, 0.500)
6	F (0.500, 0.000, 0.500)	Γ (0.000, 0.000, 0.000)

Supplementary Table 1070: Nodal ring points along high symmetry lines of mp-568690-Cd3Br6.

	Coordinates	Frequency	Mode 1	Mode 2	Multi.	Deg.	Path	Ratio
0	(0.500, -0.165, 0.165)	2.142	3	2	6	2	3	0.125
1	(0.331, -0.331, 0.000)	2.142	3	2	6	2	5	0.962
2	(0.326, 0.002, 0.326)	2.241	4	3	6	2	7	0.348
3	(0.500, -0.185, 0.185)	2.990	5	4	6	2	3	0.018
4	(0.500, -0.119, 0.119)	2.920	5	4	6	2	3	0.367
5	(0.193, -0.193, 0.000)	2.851	5	4	6	2	5	0.560
6	(0.500, -0.089, 0.089)	4.000	7	6	6	2	3	0.529
7	(0.500, -0.170, 0.170)	4.574	9	8	6	2	3	0.095
8	(0.336, -0.336, -0.000)	4.574	9	8	6	2	5	0.977
9	(0.093, -0.093, 0.000)	4.555	9	8	6	2	5	0.271

The followings are details for $MgCl_2$. It has 6 topological nodal lines and 16 nodal ring points along high symmetry line as shown in Fig **510**.

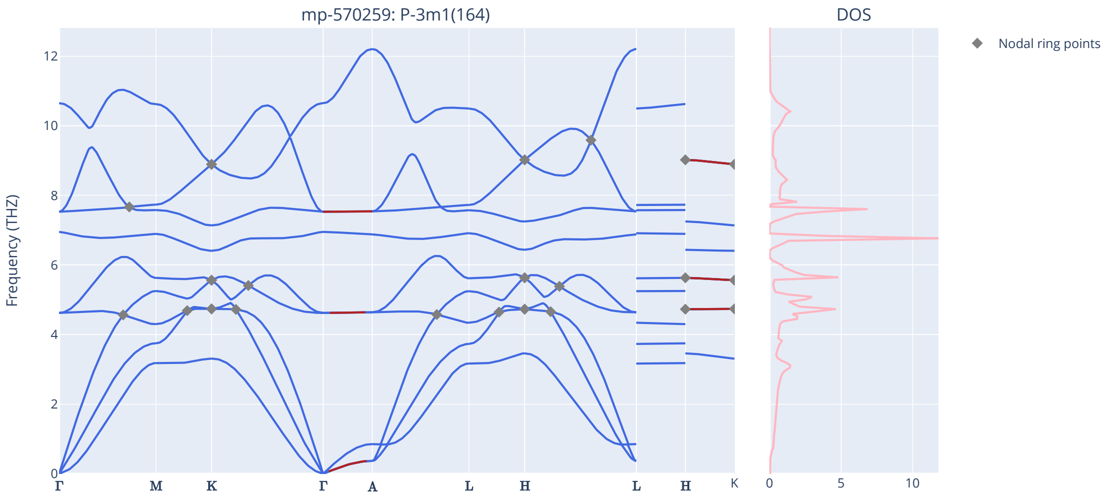

Supplementary Figure 510: The phonon band and DOS of MgCl2.

Supplementary Table 1071: High symmetry lines of mp-570259-MgCl2

	Start	End
0	Γ (0.000, 0.000, 0.000)	M (0.500, 0.000, 0.000)
1	M (0.500, 0.000, 0.000)	K (0.333, 0.333, 0.000)
2	K (0.333, 0.333, 0.000)	Γ (0.000, 0.000, 0.000)
3	Γ (0.000, 0.000, 0.000)	A (0.000, 0.000, 0.500)
4	A (0.000, 0.000, 0.500)	L (0.500, 0.000, 0.500)
5	L (0.500, 0.000, 0.500)	H (0.333, 0.333, 0.500)
6	H (0.333, 0.333, 0.500)	A (0.000, 0.000, 0.500)
7	L (0.500, 0.000, 0.500)	M (0.500, 0.000, 0.000)
8	H (0.333, 0.333, 0.500)	K (0.333, 0.333, 0.000)

Supplementary Table 1072: Nodal ring points along high symmetry lines of mp-570259-MgCl2.

	Coordinates	Frequency	Mode 1	Mode 2	Multi.	Deg.	Path	Ratio
0	(0.260, 0.260, 0.000)	4.722	3	2	6	2	3	0.219
1	(0.333, 0.333, 0.000)	4.737	3	2	2	2	2	1.000
2	(0.406, 0.188, -0.000)	4.687	3	2	6	2	2	0.564
3	(0.256, 0.256, 0.500)	4.656	3	2	6	2	7	0.233
4	(0.333, 0.333, 0.500)	4.728	3	2	2	2	6	1.000
5	(0.410, 0.180, 0.500)	4.645	3	2	6	2	6	0.540
6	(0.332, -0.000, 0.000)	4.564	4	3	6	2	1	0.663
7	(0.334, 0.000, 0.499)	4.575	4	3	6	2	5	0.668
8	(0.223, 0.223, -0.003)	5.408	5	4	12	2	3	0.330
9	(0.333, 0.333, 0.000)	5.561	5	4	2	2	2	1.000
10	(0.230, 0.230, 0.497)	5.385	5	4	12	2	7	0.311

Supplementary Table 1072: Nodal ring points along high symmetry lines of mp-570259-MgCl2.

	Coordinates	Frequency	Mode 1	Mode 2	Multi.	Deg.	Path	Ratio
11	(0.333, 0.333, 0.500)	5.631	5	4	2	2	6	1.000
12	(0.363, 0.000, 0.001)	7.667	8	7	6	2	1	0.727
13	(0.333, 0.333, 0.000)	8.891	9	8	2	2	2	1.000
14	(0.333, 0.333, 0.500)	9.020	9	8	2	2	6	1.000
15	(0.135, 0.135, 0.500)	9.587	9	8	6	2	7	0.594

The followings are details for YbI_2. It has 5 topological nodal lines and 13 nodal ring points along high symmetry line as shown in Fig **511**.

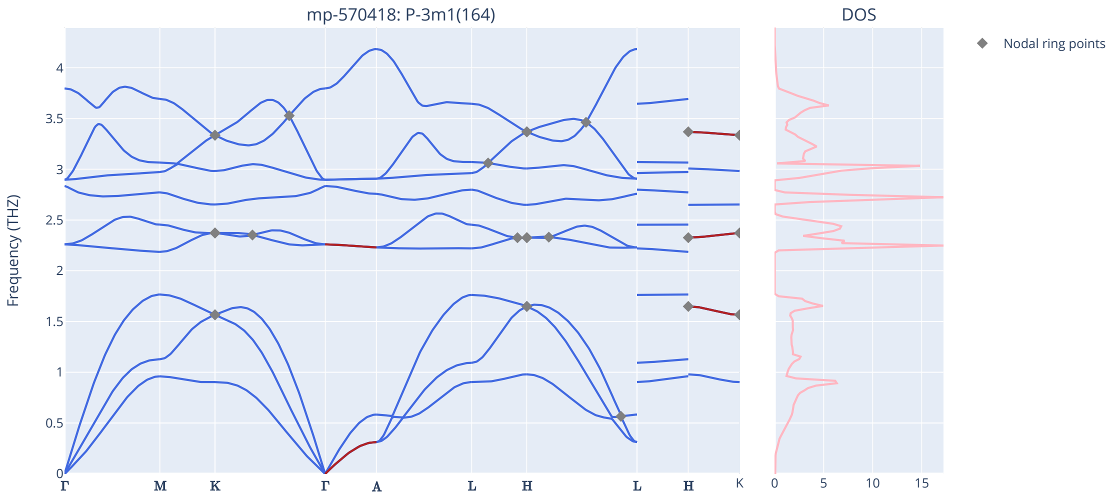

Supplementary Figure 511: The phonon band and DOS of YbI2.

Supplementary Table 1073: High symmetry lines of mp-570418-YbI2

	Start	End
0	Γ (0.000, 0.000, 0.000)	M (0.500, 0.000, 0.000)
1	M (0.500, 0.000, 0.000)	K (0.333, 0.333, 0.000)
2	K (0.333, 0.333, 0.000)	Γ (0.000, 0.000, 0.000)
3	Γ (0.000, 0.000, 0.000)	A (0.000, 0.000, 0.500)
4	A (0.000, 0.000, 0.500)	L (0.500, 0.000, 0.500)
5	L (0.500, 0.000, 0.500)	H (0.333, 0.333, 0.500)
6	H (0.333, 0.333, 0.500)	A (0.000, 0.000, 0.500)
7	L (0.500, 0.000, 0.500)	M (0.500, 0.000, 0.000)
8	H (0.333, 0.333, 0.500)	K (0.333, 0.333, 0.000)

Supplementary Table 1074: Nodal ring points along high symmetry lines of mp-570418-YbI2.

	Coordinates	Frequency	Mode 1	Mode 2	Multi.	Deg.	Path	Ratio
0	(0.333, 0.333, 0.000)	1.566	3	2	2	2	2	1.000
1	(0.333, 0.333, 0.500)	1.649	3	2	2	2	6	1.000
2	(0.049, 0.049, 0.500)	0.565	3	2	6	2	7	0.854
3	(0.220, 0.220, 0.004)	2.352	5	4	12	2	3	0.340
4	(0.333, 0.333, 0.000)	2.373	5	4	2	2	2	1.000
5	(0.333, 0.333, 0.500)	2.326	5	4	2	2	6	1.000
6	(0.267, 0.267, 0.499)	2.331	5	4	12	2	7	0.199
7	(0.361, 0.276, 0.498)	2.326	5	4	12	2	6	0.829
8	(0.451, 0.099, 0.499)	3.062	8	7	12	2	6	0.296
9	(0.333, 0.333, 0.000)	3.336	9	8	2	2	2	1.000
10	(0.109, 0.109, 0.000)	3.528	9	8	6	2	3	0.674

Supplementary Table 1074: Nodal ring points along high symmetry lines of mp-570418-YbI2.

	Coordinates	Frequency	Mode 1	Mode 2	Multi.	Deg.	Path	Ratio
11	(0.333, 0.333, 0.500)	3.369	9	8	2	2	6	1.000
12	(0.154, 0.154, 0.500)	3.463	9	8	6	2	7	0.537

The followings are details for $Co_4Ti_4Sb_4$. As shown in Fig **512**, it has 4 topological nodal lines and 2 nodal ring points. At the same time, it has 1 single Weyl point and 1 high degenerate Weyl point whose degeneracy is more than 2 and charge is bigger than 1.

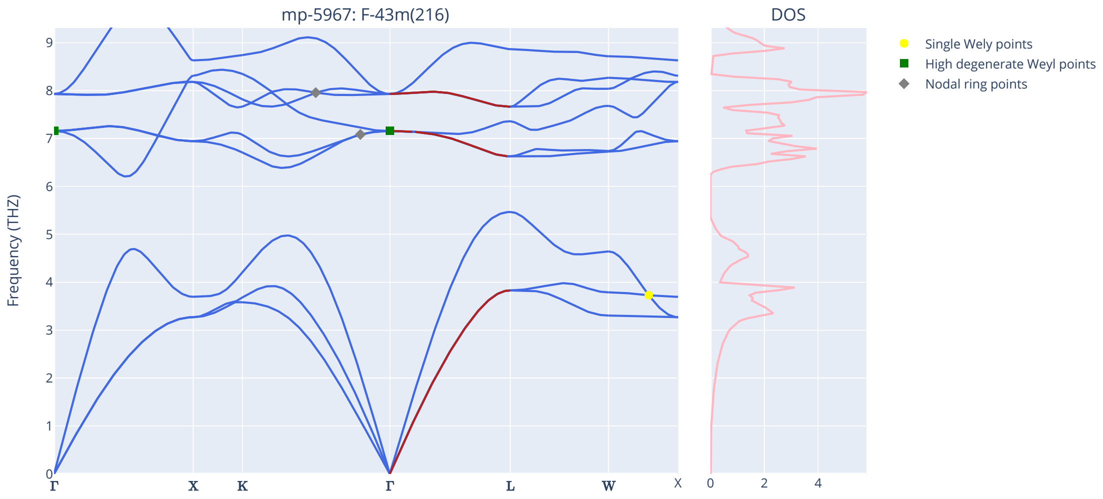

Supplementary Figure 512: The phonon band and DOS of Co4Ti4Sb4.

Supplementary Table 1075: High symmetry lines of mp-5967-Co4Ti4Sb4

	Start	End
0	Γ (0.000, 0.000, 0.000)	X (0.500, 0.000, 0.500)
1	X (0.500, 0.000, 0.500)	U (0.625, 0.250, 0.625)
2	K (0.375, 0.375, 0.750)	Γ (0.000, 0.000, 0.000)
3	Γ (0.000, 0.000, 0.000)	L (0.500, 0.500, 0.500)
4	L (0.500, 0.500, 0.500)	W (0.500, 0.250, 0.750)
5	W (0.500, 0.250, 0.750)	X (0.500, 0.000, 0.500)

Supplementary Table 1076: Single Weyl points along high symmetry lines of mp-5967-Co4Ti4Sb4.

	Coordinates	Frequency	Mode 1	Mode 2	Multi.	Deg.	Path	Ratio
0	(0.500, 0.105, 0.605)	3.728	3	2	12	2	6	0.579

Supplementary Table 1077: High degenerate Weyl points along high symmetry lines of mp-5967-Co4Ti4Sb4.

	Coordinates	Frequency	Mode 1	Mode 2	Multi.	Deg.	Path	Ratio
0	(0.000, 0.000, 0.000)	7.156	6	4	1	3	1	0.000

Supplementary Table 1078: Nodal ring points along high symmetry lines of mp-5967-Co4Ti4Sb4.

	Coordinates	Frequency	Mode 1	Mode 2	Multi.	Deg.	Path	Ratio
0	(0.077, 0.077, 0.148)	7.083	5	4	24	2	3	0.801
1	(0.191, 0.191, 0.374)	7.956	8	7	24	2	3	0.498

The followings are details for Th_4O_8. It has 5 topological nodal lines and 7 nodal ring points along high symmetry line as shown in Fig 513.

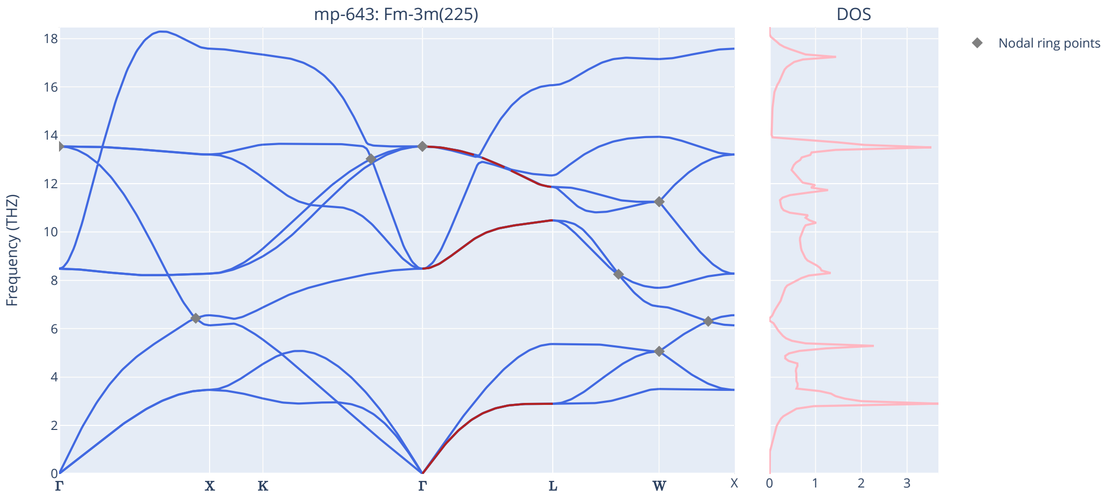

Supplementary Figure 513: The phonon band and DOS of Th4O8.

Supplementary Table 1079: High symmetry lines of mp-643-Th4O8

	Start	End
0	Γ (0.000, 0.000, 0.000)	X (0.500, 0.000, 0.500)
1	X (0.500, 0.000, 0.500)	U (0.625, 0.250, 0.625)
2	K (0.375, 0.375, 0.750)	Γ (0.000, 0.000, 0.000)
3	Γ (0.000, 0.000, 0.000)	L (0.500, 0.500, 0.500)
4	L (0.500, 0.500, 0.500)	W (0.500, 0.250, 0.750)
5	W (0.500, 0.250, 0.750)	X (0.500, 0.000, 0.500)

Supplementary Table 1080: Nodal ring points along high symmetry lines of mp-643-Th4O8.

	Coordinates	Frequency	Mode 1	Mode 2	Multi.	Deg.	Path	Ratio
0	(0.500, 0.250, 0.750)	5.064	3	2	6	2	5	1.000
1	(0.454, 0.001, 0.454)	6.434	4	3	24	2	1	0.908
2	(0.498, 0.088, 0.586)	6.306	4	3	24	2	6	0.652
3	(0.500, 0.345, 0.655)	8.252	5	4	24	2	5	0.618
4	(0.500, 0.250, 0.750)	11.254	7	6	6	2	5	1.000
5	(0.122, 0.122, 0.240)	13.025	8	7	24	2	3	0.678
6	(0.000, 0.000, 0.000)	13.542	9	8	1	3	1	0.000

The followings are details for Na_8S_4. It has 5 topological nodal lines and 4 nodal ring points along high symmetry line as shown in Fig **514**.

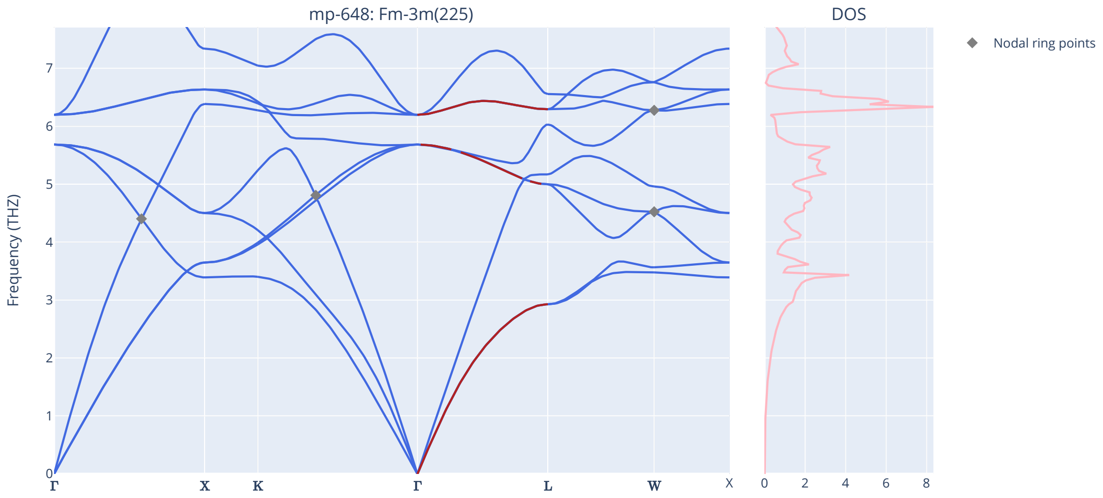

Supplementary Figure 514: The phonon band and DOS of Na8S4.

Supplementary Table 1081: High symmetry lines of mp-648-Na8S4

	Start	End
0	Γ (0.000, 0.000, 0.000)	X (0.500, 0.000, 0.500)
1	X (0.500, 0.000, 0.500)	U (0.625, 0.250, 0.625)
2	K (0.375, 0.375, 0.750)	Γ (0.000, 0.000, 0.000)
3	Γ (0.000, 0.000, 0.000)	L (0.500, 0.500, 0.500)
4	L (0.500, 0.500, 0.500)	W (0.500, 0.250, 0.750)
5	W (0.500, 0.250, 0.750)	X (0.500, 0.000, 0.500)

Supplementary Table 1082: Nodal ring points along high symmetry lines of mp-648-Na8S4.

	Coordinates	Frequency	Mode 1	Mode 2	Multi.	Deg.	Path	Ratio
0	(0.290, -0.001, 0.290)	4.402	4	3	24	2	1	0.581
1	(0.500, 0.250, 0.750)	4.523	4	3	6	2	5	1.000
2	(0.237, 0.237, 0.479)	4.809	5	4	24	2	3	0.363
3	(0.500, 0.250, 0.750)	6.275	7	6	6	2	5	1.000

The followings are details for $Pt_4Sc_4Sb_4$. As shown in Fig **515**, it has 4 topological nodal lines, 1 nodal ring point and 1 high degenerate Weyl point whose degeneracy is more than 2 and charge is bigger than 1.

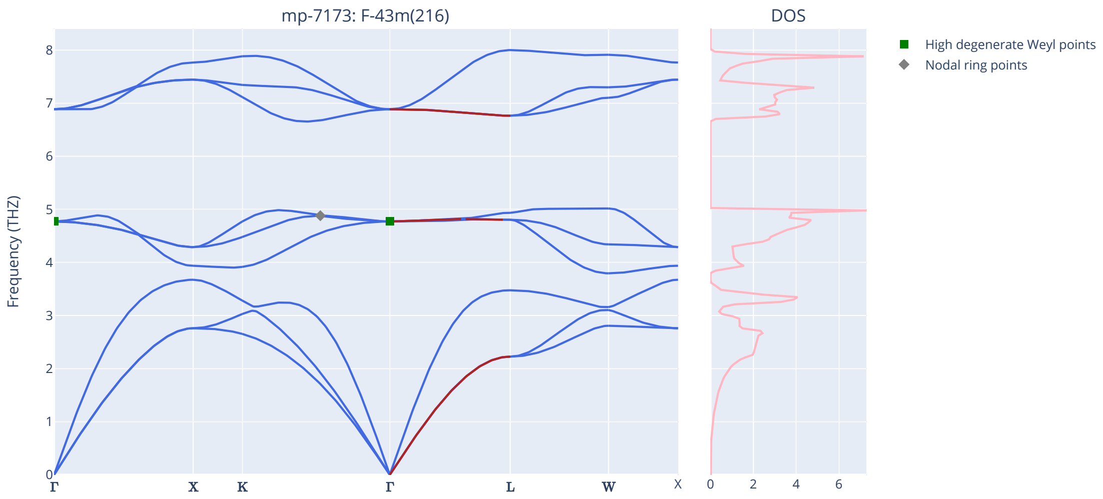

Supplementary Figure 515: The phonon band and DOS of Pt4Sc4Sb4.

Supplementary Table 1083: High symmetry lines of mp-7173-Pt4Sc4Sb4

	Start	End
0	Γ (0.000, 0.000, 0.000)	X (0.500, 0.000, 0.500)
1	X (0.500, 0.000, 0.500)	U (0.625, 0.250, 0.625)
2	K (0.375, 0.375, 0.750)	Γ (0.000, 0.000, 0.000)
3	Γ (0.000, 0.000, 0.000)	L (0.500, 0.500, 0.500)
4	L (0.500, 0.500, 0.500)	W (0.500, 0.250, 0.750)
5	W (0.500, 0.250, 0.750)	X (0.500, 0.000, 0.500)

Supplementary Table 1084: High degenerate Weyl points along high symmetry lines of mp-7173-Pt4Sc4Sb4.

	Coordinates	Frequency	Mode 1	Mode 2	Multi.	Deg.	Path	Ratio
0	(0.000, 0.000, 0.000)	4.771	6	4	1	3	1	0.000

Supplementary Table 1085: Nodal ring points along high symmetry lines of mp-7173-Pt4Sc4Sb4.

	Coordinates	Frequency	Mode 1	Mode 2	Multi.	Deg.	Path	Ratio
0	(0.171, 0.171, 0.359)	4.880	6	5	24	2	3	0.529

The followings are details for Rb_6O_3. It has 5 topological nodal lines and 18 nodal ring points along high symmetry line as shown in Fig 516.

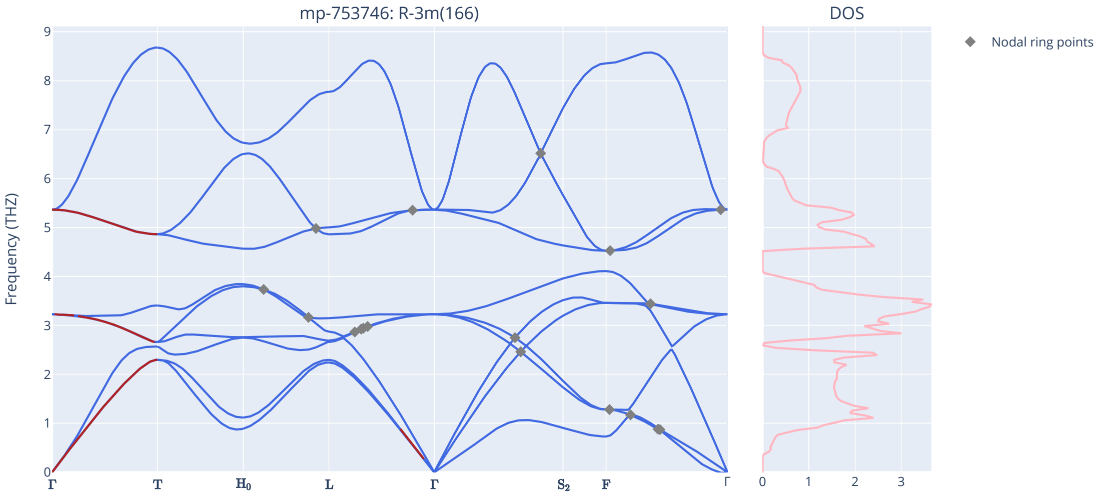

Supplementary Figure 516: The phonon band and DOS of Rb6O3.

Supplementary Table 1086: High symmetry lines of mp-753746-Rb6O3

	Start	End
0	Γ (0.000, 0.000, 0.000)	T (0.500, 0.500, 0.500)
1	T (0.500, 0.500, 0.500)	H_2 (0.750, 0.250, 0.500)
2	H_0 (0.500, -0.250, 0.250)	L (0.500, 0.000, 0.000)
3	L (0.500, 0.000, 0.000)	Γ (0.000, 0.000, 0.000)
4	Γ (0.000, 0.000, 0.000)	S_0 (0.375, -0.375, 0.000)
5	S_2 (0.625, 0.000, 0.375)	F (0.500, 0.000, 0.500)
6	F (0.500, 0.000, 0.500)	Γ (0.000, 0.000, 0.000)

Supplementary Table 1087: Nodal ring points along high symmetry lines of mp-753746-Rb6O3.

	Coordinates	Frequency	Mode 1	Mode 2	Multi.	Deg.	Path	Ratio
0	(0.286, 0.007, 0.286)	0.880	2	1	6	2	7	0.429
1	(0.399, -0.001, 0.399)	1.173	2	1	6	2	7	0.202
2	(0.278, -0.004, 0.278)	0.871	2	1	6	2	7	0.444
3	(0.252, -0.252, -0.000)	2.461	3	2	6	2	5	0.672
4	(0.486, -0.001, 0.485)	1.280	3	2	6	2	7	0.029
5	(0.377, -0.001, -0.001)	2.866	5	4	6	2	4	0.245
6	(0.347, 0.001, 0.001)	2.924	5	4	6	2	4	0.306
7	(0.337, 0.001, 0.001)	2.943	5	4	6	2	4	0.326
8	(0.316, 0.002, 0.002)	2.978	5	4	6	2	4	0.367
9	(0.235, -0.235, 0.000)	2.749	5	4	6	2	5	0.627
10	(0.500, -0.190, 0.190)	3.734	6	5	6	2	3	0.239
11	(0.500, -0.060, 0.060)	3.165	6	5	6	2	3	0.761
12	(0.317, -0.002, 0.317)	3.442	6	5	6	2	7	0.366

Supplementary Table 1087: Nodal ring points along high symmetry lines of mp-753746-Rb6O3.

	Coordinates	Frequency	Mode 1	Mode 2	Multi.	Deg.	Path	Ratio
13	(0.500, -0.038, 0.038)	4.981	8	7	6	2	3	0.848
14	(0.028, -0.000, 0.028)	5.365	8	7	6	2	7	0.945
15	(0.103, 0.002, 0.002)	5.354	8	7	6	2	4	0.795
16	(0.483, 0.000, 0.483)	4.528	8	7	6	2	7	0.034
17	(0.310, -0.310, 0.000)	6.517	9	8	6	2	5	0.827

The followings are details for $Li_4Zn_4N_4$. As shown in Fig 517, it has 4 topological nodal lines and 2 nodal ring points. At the same time, it has 1 single Weyl point and 1 high degenerate Weyl point whose degeneracy is more than 2 and charge is bigger than 1.

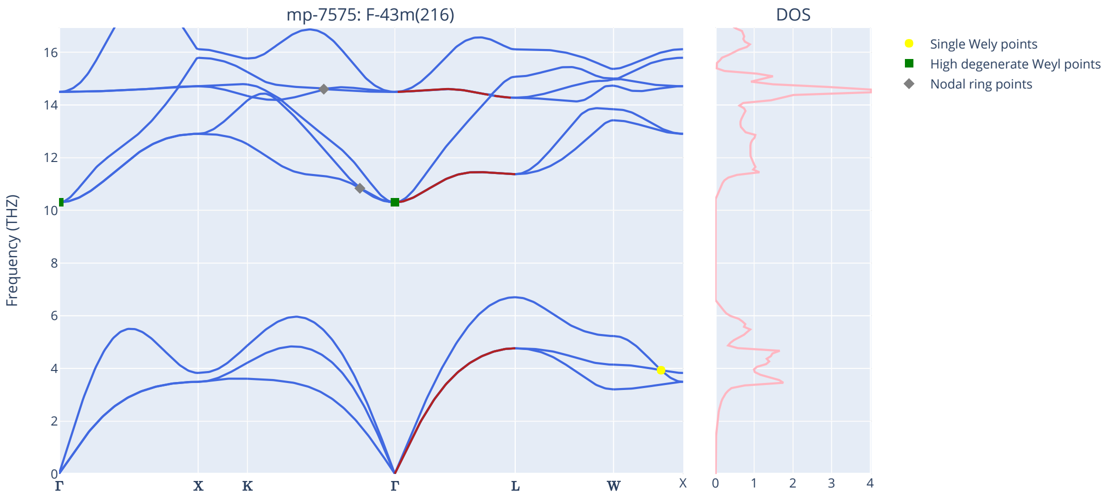

Supplementary Figure 517: The phonon band and DOS of Li4Zn4N4.

Supplementary Table 1088: High symmetry lines of mp-7575-Li4Zn4N4

	Start	End
0	Γ (0.000, 0.000, 0.000)	X (0.500, 0.000, 0.500)
1	X (0.500, 0.000, 0.500)	U (0.625, 0.250, 0.625)
2	K (0.375, 0.375, 0.750)	Γ (0.000, 0.000, 0.000)
3	Γ (0.000, 0.000, 0.000)	L (0.500, 0.500, 0.500)
4	L (0.500, 0.500, 0.500)	W (0.500, 0.250, 0.750)
5	W (0.500, 0.250, 0.750)	X (0.500, 0.000, 0.500)

Supplementary Table 1089: Single Weyl points along high symmetry lines of mp-7575-Li4Zn4N4.

	Coordinates	Frequency	Mode 1	Mode 2	Multi.	Deg.	Path	Ratio
0	(0.500, 0.079, 0.579)	3.933	3	2	12	2	6	0.684

Supplementary Table 1090: High degenerate Weyl points along high symmetry lines of mp-7575-Li4Zn4N4.

	Coordinates	Frequency	Mode 1	Mode 2	Multi.	Deg.	Path	Ratio
0	(0.000, 0.000, 0.000)	10.305	6	4	1	3	1	0.000

Supplementary Table 1091: Nodal ring points along high symmetry lines of mp-7575-Li4Zn4N4.

	Coordinates	Frequency	Mode 1	Mode 2	Multi.	Deg.	Path	Ratio
0	(0.092, 0.092, 0.174)	10.838	5	4	24	2	3	0.763
1	(0.184, 0.184, 0.358)	14.601	8	7	24	2	3	0.519

The followings are details for Mo_3Se_6. It has 3 topological nodal lines and 3 nodal ring points along high symmetry line as shown in Fig **518**.

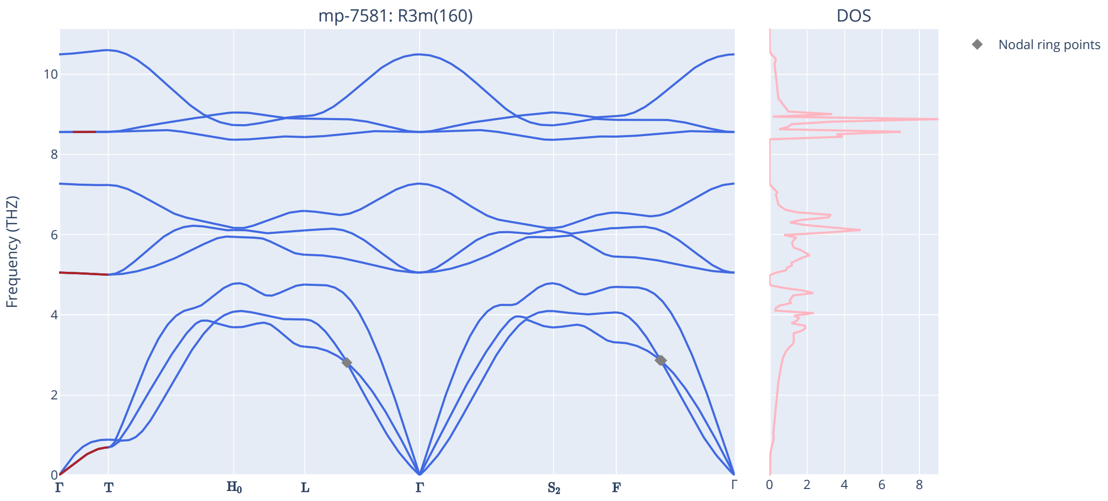

Supplementary Figure 518: The phonon band and DOS of Mo3Se6.

Supplementary Table 1092: High symmetry lines of mp-7581-Mo3Se6

	Start	End
0	Γ (0.000, 0.000, 0.000)	T (0.500, 0.500, 0.500)
1	T (0.500, 0.500, 0.500)	H_2 (0.819, 0.181, 0.500)
2	H_0 (0.500, -0.181, 0.181)	L (0.500, 0.000, 0.000)
3	L (0.500, 0.000, 0.000)	Γ (0.000, 0.000, 0.000)
4	Γ (0.000, 0.000, 0.000)	S_0 (0.340, -0.340, 0.000)
5	S_2 (0.660, 0.000, 0.340)	F (0.500, 0.000, 0.500)
6	F (0.500, 0.000, 0.500)	Γ (0.000, 0.000, 0.000)

Supplementary Table 1093: Nodal ring points along high symmetry lines of mp-7581-Mo3Se6.

	Coordinates	Frequency	Mode 1	Mode 2	Multi.	Deg.	Path	Ratio
0	(0.317, -0.001, -0.001)	2.808	2	1	6	2	4	0.365
1	(0.315, 0.003, 0.315)	2.867	2	1	6	2	7	0.370
2	(0.308, -0.004, 0.308)	2.861	2	1	6	2	7	0.384

The followings are details for Rb_8S_4. It has 4 topological nodal lines and 6 nodal ring points along high symmetry line as shown in Fig 519.

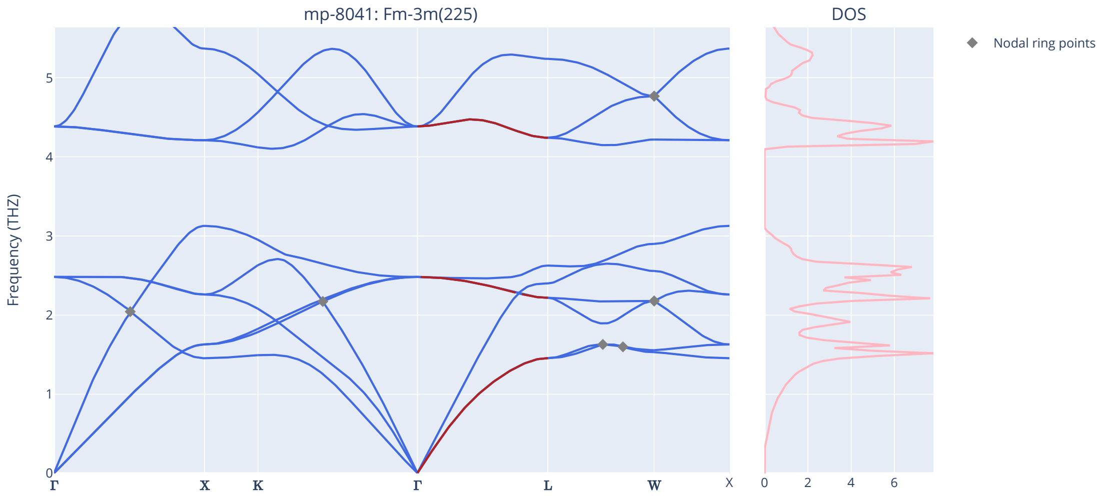

Supplementary Figure 519: The phonon band and DOS of Rb8S4.

Supplementary Table 1094: High symmetry lines of mp-8041-Rb8S4

	Start	End
0	Γ (0.000, 0.000, 0.000)	X (0.500, 0.000, 0.500)
1	X (0.500, 0.000, 0.500)	U (0.625, 0.250, 0.625)
2	K (0.375, 0.375, 0.750)	Γ (0.000, 0.000, 0.000)
3	Γ (0.000, 0.000, 0.000)	L (0.500, 0.500, 0.500)
4	L (0.500, 0.500, 0.500)	W (0.500, 0.250, 0.750)
5	W (0.500, 0.250, 0.750)	X (0.500, 0.000, 0.500)

Supplementary Table 1095: Nodal ring points along high symmetry lines of mp-8041-Rb8S4.

	Coordinates	Frequency	Mode 1	Mode 2	Multi.	Deg.	Path	Ratio
0	(0.500, 0.323, 0.677)	1.599	2	1	24	2	5	0.707
1	(0.500, 0.371, 0.629)	1.629	2	1	24	2	5	0.517
2	(0.253, -0.002, 0.253)	2.044	4	3	24	2	1	0.506
3	(0.222, 0.222, 0.445)	2.172	4	3	24	2	3	0.407
4	(0.500, 0.250, 0.750)	2.178	4	3	6	2	5	1.000
5	(0.500, 0.250, 0.750)	4.768	9	8	6	2	5	1.000

The followings are details for Hg_4F_8. It has 4 topological nodal lines and 7 nodal ring points along high symmetry line as shown in Fig 520.

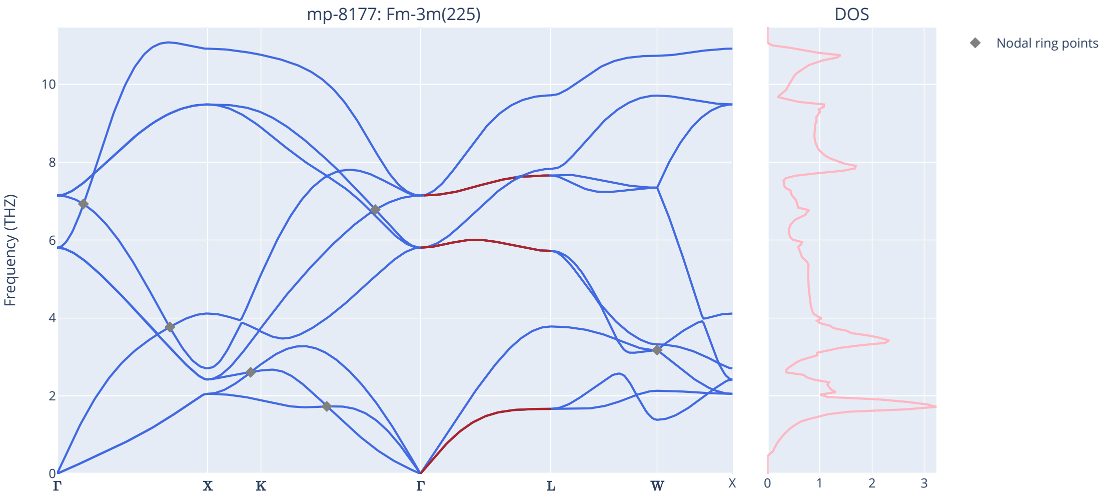

Supplementary Figure 520: The phonon band and DOS of Hg4F8.

Supplementary Table 1096: High symmetry lines of mp-8177-Hg4F8

	Start	End
0	Γ (0.000, 0.000, 0.000)	X (0.500, 0.000, 0.500)
1	X (0.500, 0.000, 0.500)	U (0.625, 0.250, 0.625)
2	K (0.375, 0.375, 0.750)	Γ (0.000, 0.000, 0.000)
3	Γ (0.000, 0.000, 0.000)	L (0.500, 0.500, 0.500)
4	L (0.500, 0.500, 0.500)	W (0.500, 0.250, 0.750)
5	W (0.500, 0.250, 0.750)	X (0.500, 0.000, 0.500)

Supplementary Table 1097: Nodal ring points along high symmetry lines of mp-8177-Hg4F8.

	Coordinates	Frequency	Mode 1	Mode 2	Multi.	Deg.	Path	Ratio
0	(0.222, 0.222, 0.438)	1.728	2	1	24	2	3	0.414
1	(0.601, 0.202, 0.601)	2.605	3	2	12	2	2	0.808
2	(0.500, 0.250, 0.750)	3.178	4	3	6	2	5	1.000
3	(0.375, 0.002, 0.375)	3.767	6	5	24	2	1	0.750
4	(0.087, -0.001, 0.087)	6.928	7	6	24	2	1	0.175
5	(0.107, 0.107, 0.212)	6.784	7	6	24	2	3	0.717

The followings are details for K_8Se_4. It has 4 topological nodal lines and 12 nodal ring points along high symmetry line as shown in Fig 521.

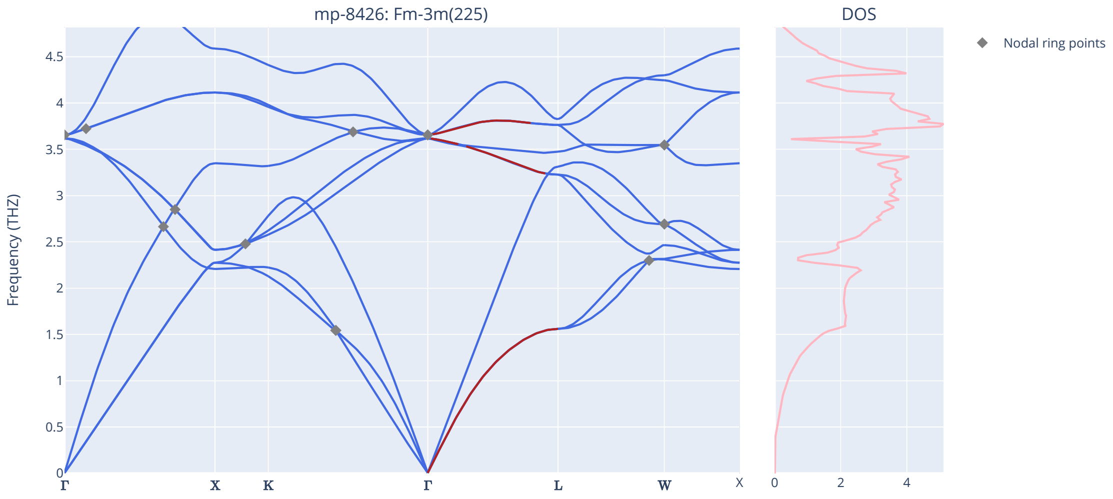

Supplementary Figure 521: The phonon band and DOS of K8Se4.

Supplementary Table 1098: High symmetry lines of mp-8426-K8Se4

	Start	End
0	Γ (0.000, 0.000, 0.000)	X (0.500, 0.000, 0.500)
1	X (0.500, 0.000, 0.500)	U (0.625, 0.250, 0.625)
2	K (0.375, 0.375, 0.750)	Γ (0.000, 0.000, 0.000)
3	Γ (0.000, 0.000, 0.000)	L (0.500, 0.500, 0.500)
4	L (0.500, 0.500, 0.500)	W (0.500, 0.250, 0.750)
5	W (0.500, 0.250, 0.750)	X (0.500, 0.000, 0.500)

Supplementary Table 1099: Nodal ring points along high symmetry lines of mp-8426-K8Se4.

	Coordinates	Frequency	Mode 1	Mode 2	Multi.	Deg.	Path	Ratio
0	(0.214, 0.214, 0.433)	1.543	2	1	24	2	3	0.425
1	(0.500, 0.286, 0.714)	2.299	2	1	24	2	5	0.856
2	(0.211, 0.221, 0.433)	1.544	2	1	24	2	3	0.423
3	(0.329, -0.000, 0.329)	2.664	4	3	6	2	1	0.657
4	(0.573, 0.143, 0.571)	2.478	4	3	24	2	2	0.573
5	(0.367, -0.000, 0.367)	2.849	5	4	6	2	1	0.735
6	(0.500, 0.250, 0.750)	2.692	5	4	6	2	5	1.000
7	(0.182, 0.169, 0.351)	3.688	7	6	24	2	3	0.532
8	(0.500, 0.250, 0.750)	3.547	7	6	6	2	5	1.000
9	(0.000, 0.000, 0.000)	3.656	8	7	1	3	1	0.000
10	(0.071, 0.000, 0.071)	3.723	8	7	6	2	1	0.143

The followings are details for $Li_4Zn_4As_4$. As shown in Fig 522, it has 3 topological nodal lines, 5 nodal ring points and 1 high degenerate Weyl point whose degeneracy is more than 2 and charge is bigger than 1.

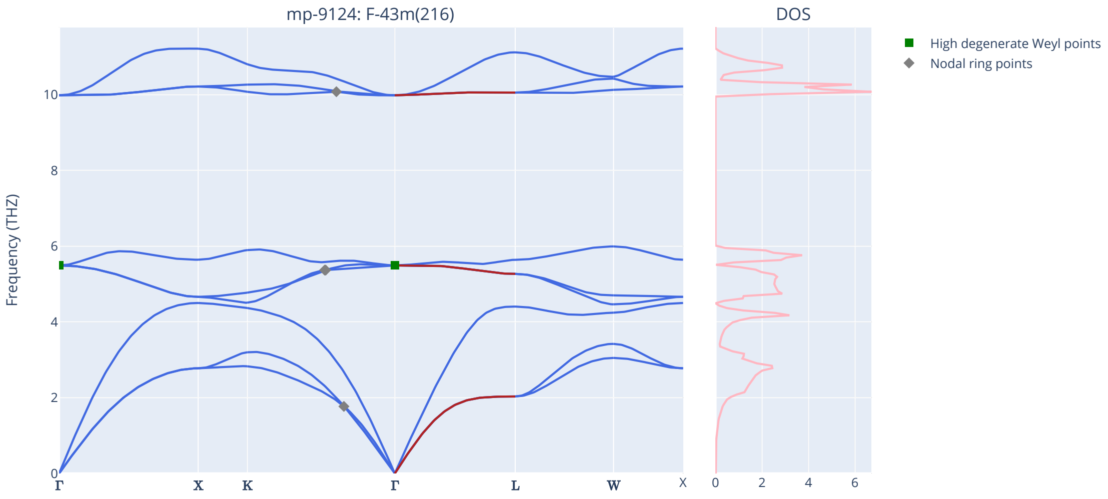

Supplementary Figure 522: The phonon band and DOS of Li4Zn4As4.

Supplementary Table 1100: High symmetry lines of mp-9124-Li4Zn4As4

	Start	End
0	Γ (0.000, 0.000, 0.000)	X (0.500, 0.000, 0.500)
1	X (0.500, 0.000, 0.500)	U (0.625, 0.250, 0.625)
2	K (0.375, 0.375, 0.750)	Γ (0.000, 0.000, 0.000)
3	Γ (0.000, 0.000, 0.000)	L (0.500, 0.500, 0.500)
4	L (0.500, 0.500, 0.500)	W (0.500, 0.250, 0.750)
5	W (0.500, 0.250, 0.750)	X (0.500, 0.000, 0.500)

Supplementary Table 1101: High degenerate Weyl points along high symmetry lines of mp-9124-Li4Zn4As4.

	Coordinates	Frequency	Mode 1	Mode 2	Multi.	Deg.	Path	Ratio
0	(0.000, 0.000, 0.000)	5.490	6	4	1	3	1	0.000

Supplementary Table 1102: Nodal ring points along high symmetry lines of mp-9124-Li4Zn4As4.

	Coordinates	Frequency	Mode 1	Mode 2	Multi.	Deg.	Path	Ratio
0	(0.130, 0.130, 0.258)	1.767	2	1	24	2	3	0.655
1	(0.184, 0.184, 0.351)	5.367	5	4	24	2	3	0.525
2	(0.176, 0.176, 0.351)	5.367	5	4	24	2	3	0.532
3	(0.153, 0.153, 0.292)	10.078	8	7	24	2	3	0.604

The followings are details for $Ni_4Sn_4Zr_4$. As shown in Fig **523**, it has 4 topological nodal lines, 1 nodal ring point and 1 high degenerate Weyl point whose degeneracy is more than 2 and charge is bigger than 1.

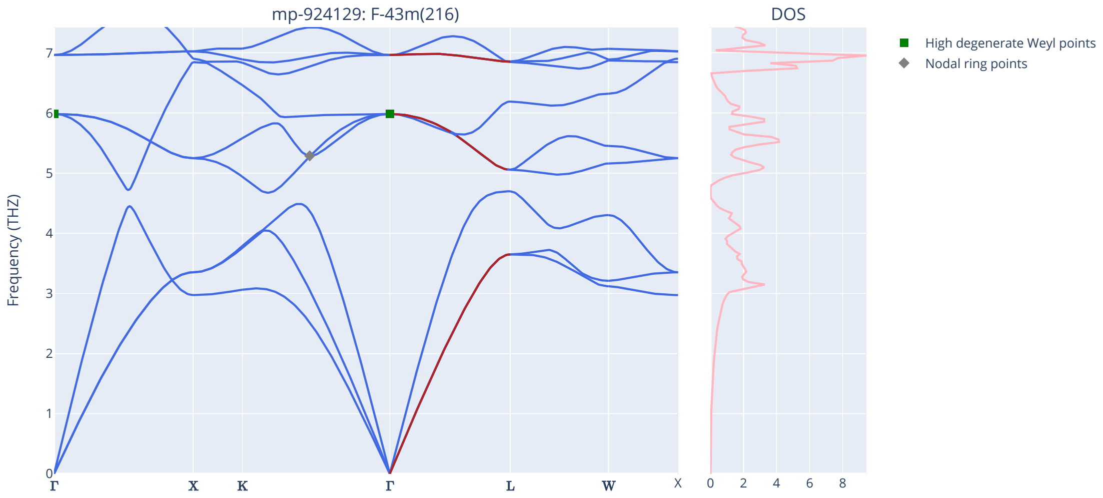

Supplementary Figure 523: The phonon band and DOS of Ni4Sn4Zr4.

Supplementary Table 1103: High symmetry lines of mp-924129-Ni4Sn4Zr4

	Start	End
0	Γ (0.000, 0.000, 0.000)	X (0.500, 0.000, 0.500)
1	X (0.500, 0.000, 0.500)	U (0.625, 0.250, 0.625)
2	K (0.375, 0.375, 0.750)	Γ (0.000, 0.000, 0.000)
3	Γ (0.000, 0.000, 0.000)	L (0.500, 0.500, 0.500)
4	L (0.500, 0.500, 0.500)	W (0.500, 0.250, 0.750)
5	W (0.500, 0.250, 0.750)	X (0.500, 0.000, 0.500)

Supplementary Table 1104: High degenerate Weyl points along high symmetry lines of mp-924129-Ni4Sn4Zr4.

	Coordinates	Frequency	Mode 1	Mode 2	Multi.	Deg.	Path	Ratio
0	(0.000, 0.000, 0.000)	5.985	6	4	1	3	1	0.000

Supplementary Table 1105: Nodal ring points along high symmetry lines of mp-924129-Ni4Sn4Zr4.

	Coordinates	Frequency	Mode 1	Mode 2	Multi.	Deg.	Path	Ratio
0	(0.207, 0.207, 0.404)	5.287	5	4	24	2	3	0.457

The followings are details for $Ni_4Sn_4Ti_4$. As shown in Fig **524**, it has 4 topological nodal lines, 1 nodal ring point and 1 high degenerate Weyl point whose degeneracy is more than 2 and charge is bigger than 1.

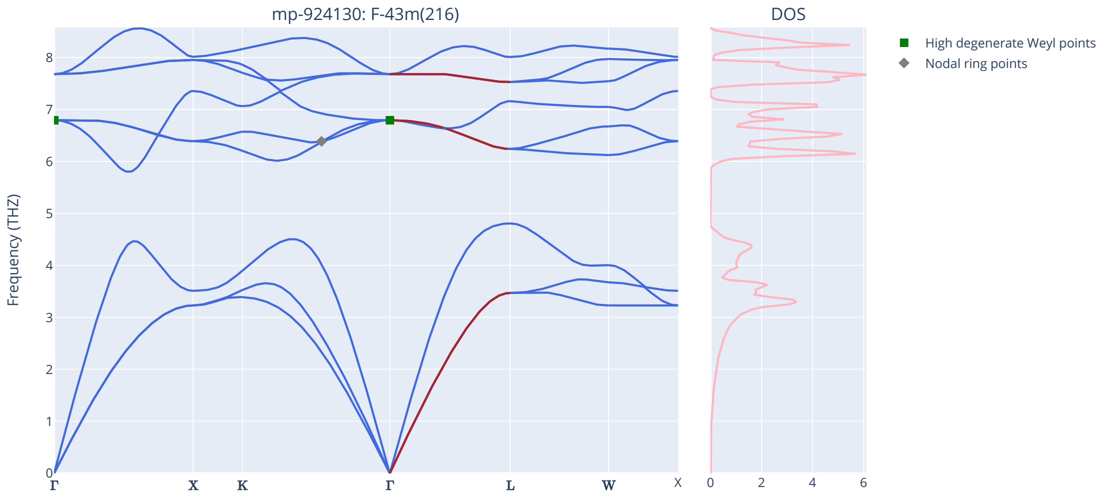

Supplementary Figure 524: The phonon band and DOS of Ni4Sn4Ti4.

Supplementary Table 1106: High symmetry lines of mp-924130-Ni4Sn4Ti4

	Start	End
0	Γ (0.000, 0.000, 0.000)	X (0.500, 0.000, 0.500)
1	X (0.500, 0.000, 0.500)	U (0.625, 0.250, 0.625)
2	K (0.375, 0.375, 0.750)	Γ (0.000, 0.000, 0.000)
3	Γ (0.000, 0.000, 0.000)	L (0.500, 0.500, 0.500)
4	L (0.500, 0.500, 0.500)	W (0.500, 0.250, 0.750)
5	W (0.500, 0.250, 0.750)	X (0.500, 0.000, 0.500)

Supplementary Table 1107: High degenerate Weyl points along high symmetry lines of mp-924130-Ni4Sn4Ti4.

	Coordinates	Frequency	Mode 1	Mode 2	Multi.	Deg.	Path	Ratio
0	(0.000, 0.000, 0.000)	6.795	6	4	1	3	1	0.000

Supplementary Table 1108: Nodal ring points along high symmetry lines of mp-924130-Ni4Sn4Ti4.

	Coordinates	Frequency	Mode 1	Mode 2	Multi.	Deg.	Path	Ratio
0	(0.176, 0.176, 0.344)	6.388	5	4	24	2	3	0.537

The followings are details for $Fe_4Nb_4Sb_4$. As shown in Fig **525**, it has 3 topological nodal lines and 3 nodal ring points. At the same time, it has 3 single Weyl points and 1 high degenerate Weyl point whose degeneracy is more than 2 and charge is bigger than 1.

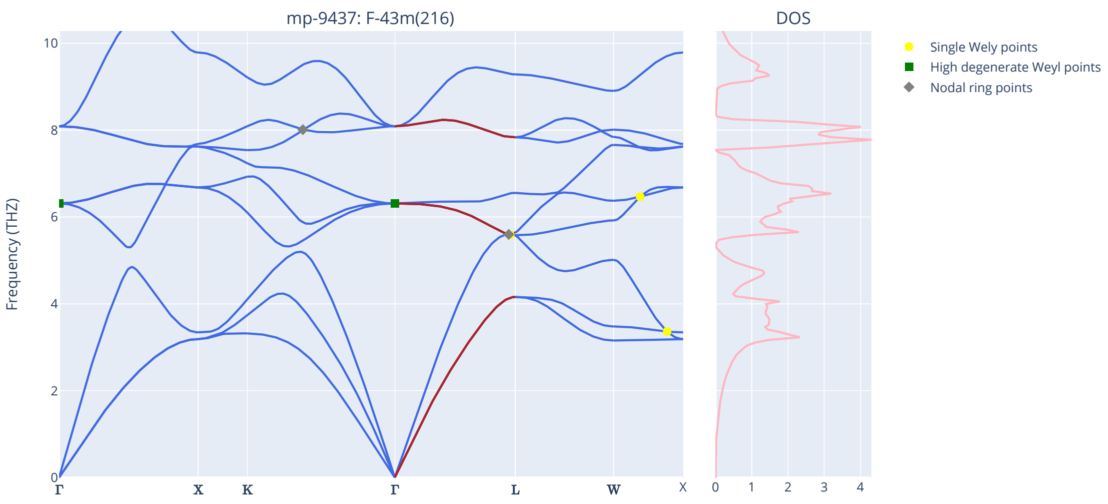

Supplementary Figure 525: The phonon band and DOS of Fe4Nb4Sb4.

Supplementary Table 1109: High symmetry lines of mp-9437-Fe4Nb4Sb4

	Start	End
0	Γ (0.000, 0.000, 0.000)	X (0.500, 0.000, 0.500)
1	X (0.500, 0.000, 0.500)	U (0.625, 0.250, 0.625)
2	K (0.375, 0.375, 0.750)	Γ (0.000, 0.000, 0.000)
3	Γ (0.000, 0.000, 0.000)	L (0.500, 0.500, 0.500)
4	L (0.500, 0.500, 0.500)	W (0.500, 0.250, 0.750)
5	W (0.500, 0.250, 0.750)	X (0.500, 0.000, 0.500)

Supplementary Table 1110: Single Weyl points along high symmetry lines of mp-9437-Fe4Nb4Sb4.

	Coordinates	Frequency	Mode 1	Mode 2	Multi.	Deg.	Path	Ratio
0	(0.500, 0.057, 0.557)	3.363	3	2	12	2	6	0.771
1	(0.480, 0.480, 0.480)	5.591	4	3	8	2	4	0.959
2	(0.500, 0.154, 0.654)	6.468	5	4	12	2	6	0.383

Supplementary Table 1111: High degenerate Weyl points along high symmetry lines of mp-9437-Fe4Nb4Sb4.

	Coordinates	Frequency	Mode 1	Mode 2	Multi.	Deg.	Path	Ratio
0	(0.000, 0.000, 0.000)	6.313	6	4	1	3	1	0.000

Supplementary Table 1112: Nodal ring points along high symmetry lines of mp-9437-Fe4Nb4Sb4.

	Coordinates	Frequency	Mode 1	Mode 2	Multi.	Deg.	Path	Ratio
0	(0.474, 0.473, 0.473)	5.598	4	3	24	2	4	0.946
1	(0.237, 0.237, 0.464)	8.011	8	7	24	2	3	0.377

The followings are details for $Pd_4Sn_4Ti_4$. As shown in Fig **526**, it has 4 topological nodal lines, 2 nodal ring points and 1 high degenerate Weyl point whose degeneracy is more than 2 and charge is bigger than 1.

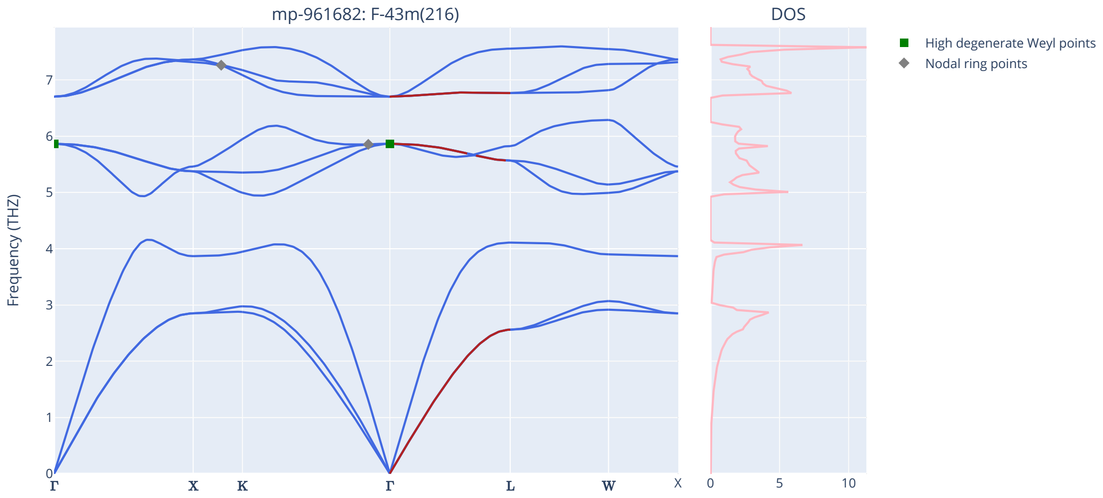

Supplementary Figure 526: The phonon band and DOS of Pd4Sn4Ti4.

Supplementary Table 1113: High symmetry lines of mp-961682-Pd4Sn4Ti4

	Start	End
0	Γ (0.000, 0.000, 0.000)	X (0.500, 0.000, 0.500)
1	X (0.500, 0.000, 0.500)	U (0.625, 0.250, 0.625)
2	K (0.375, 0.375, 0.750)	Γ (0.000, 0.000, 0.000)
3	Γ (0.000, 0.000, 0.000)	L (0.500, 0.500, 0.500)
4	L (0.500, 0.500, 0.500)	W (0.500, 0.250, 0.750)
5	W (0.500, 0.250, 0.750)	X (0.500, 0.000, 0.500)

Supplementary Table 1114: High degenerate Weyl points along high symmetry lines of mp-961682-Pd4Sn4Ti4.

	Coordinates	Frequency	Mode 1	Mode 2	Multi.	Deg.	Path	Ratio
0	(0.000, 0.000, 0.000)	5.866	6	4	1	3	1	0.000

Supplementary Table 1115: Nodal ring points along high symmetry lines of mp-961682-Pd4Sn4Ti4.

	Coordinates	Frequency	Mode 1	Mode 2	Multi.	Deg.	Path	Ratio
0	(0.054, 0.054, 0.110)	5.855	6	5	24	2	3	0.854
1	(0.571, 0.143, 0.571)	7.262	8	7	12	2	2	0.571

The followings are details for $Ca_4Li_4As_4$. As shown in Fig 527, it has 4 topological nodal lines and 1 nodal ring point. At the same time, it has 1 single Weyl point and 1 high degenerate Weyl point whose degeneracy is more than 2 and charge is bigger than 1.

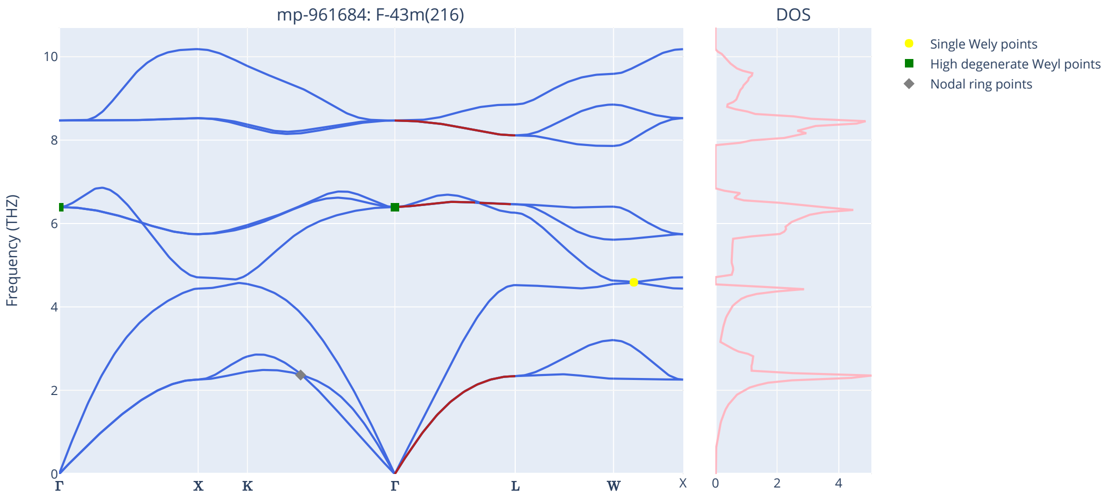

Supplementary Figure 527: The phonon band and DOS of Ca4Li4As4.

Supplementary Table 1116: High symmetry lines of mp-961684-Ca4Li4As4

	Start	End
0	Γ (0.000, 0.000, 0.000)	X (0.500, 0.000, 0.500)
1	X (0.500, 0.000, 0.500)	U (0.625, 0.250, 0.625)
2	K (0.375, 0.375, 0.750)	Γ (0.000, 0.000, 0.000)
3	Γ (0.000, 0.000, 0.000)	L (0.500, 0.500, 0.500)
4	L (0.500, 0.500, 0.500)	W (0.500, 0.250, 0.750)
5	W (0.500, 0.250, 0.750)	X (0.500, 0.000, 0.500)

Supplementary Table 1117: Single Weyl points along high symmetry lines of mp-961684-Ca4Li4As4.

	Coordinates	Frequency	Mode 1	Mode 2	Multi.	Deg.	Path	Ratio
0	(0.500, 0.177, 0.677)	4.590	4	3	12	2	6	0.293

Supplementary Table 1118: High degenerate Weyl points along high symmetry lines of mp-961684-Ca4Li4As4.

	Coordinates	Frequency	Mode 1	Mode 2	Multi.	Deg.	Path	Ratio
0	(0.000, 0.000, 0.000)	6.395	6	4	1	3	1	0.000

Supplementary Table 1119: Nodal ring points along high symmetry lines of mp-961684-Ca4Li4As4.

	Coordinates	Frequency	Mode 1	Mode 2	Multi.	Deg.	Path	Ratio
0	(0.245, 0.245, 0.473)	2.368	2	1	24	2	3	0.362

The followings are details for $Ca_4Na_4As_4$. As shown in Fig **528**, it has 4 topological nodal lines, 6 nodal ring points and 1 high degenerate Weyl point whose degeneracy is more than 2 and charge is bigger than 1.

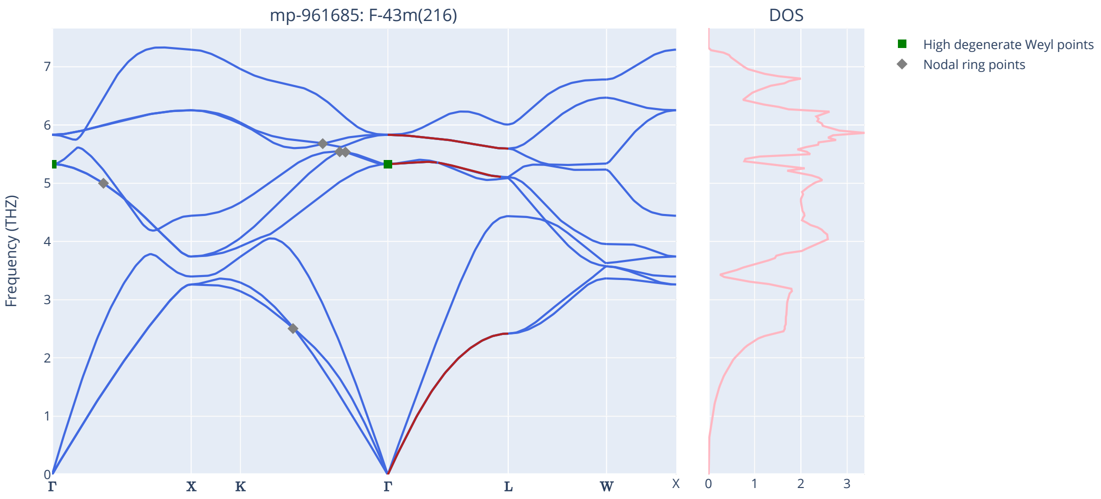

Supplementary Figure 528: The phonon band and DOS of Ca4Na4As4.

Supplementary Table 1120: High symmetry lines of mp-961685-Ca4Na4As4

	Start	End
0	Γ (0.000, 0.000, 0.000)	X (0.500, 0.000, 0.500)
1	X (0.500, 0.000, 0.500)	U (0.625, 0.250, 0.625)
2	K (0.375, 0.375, 0.750)	Γ (0.000, 0.000, 0.000)
3	Γ (0.000, 0.000, 0.000)	L (0.500, 0.500, 0.500)
4	L (0.500, 0.500, 0.500)	W (0.500, 0.250, 0.750)
5	W (0.500, 0.250, 0.750)	X (0.500, 0.000, 0.500)

Supplementary Table 1121: High degenerate Weyl points along high symmetry lines of mp-961685-Ca4Na4As4.

	Coordinates	Frequency	Mode 1	Mode 2	Multi.	Deg.	Path	Ratio
0	(0.000, 0.000, 0.000)	5.328	6	4	1	3	1	0.000

Supplementary Table 1122: Nodal ring points along high symmetry lines of mp-961685-Ca4Na4As4.

	Coordinates	Frequency	Mode 1	Mode 2	Multi.	Deg.	Path	Ratio
0	(0.245, 0.245, 0.478)	2.503	2	1	24	2	3	0.357
1	(0.190, -0.000, 0.180)	4.999	5	4	24	2	1	0.370
2	(0.127, 0.127, 0.239)	5.540	6	5	24	2	3	0.674
3	(0.107, 0.107, 0.216)	5.534	6	5	24	2	3	0.713
4	(0.168, 0.168, 0.328)	5.679	8	7	24	2	3	0.559

The followings are details for $Pd_4Sn_4Zr_4$. As shown in Fig **529**, it has 4 topological nodal lines and 2 nodal ring points. At the same time, it has 1 single Weyl point and 1 high degenerate Weyl point whose degeneracy is more than 2 and charge is bigger than 1.

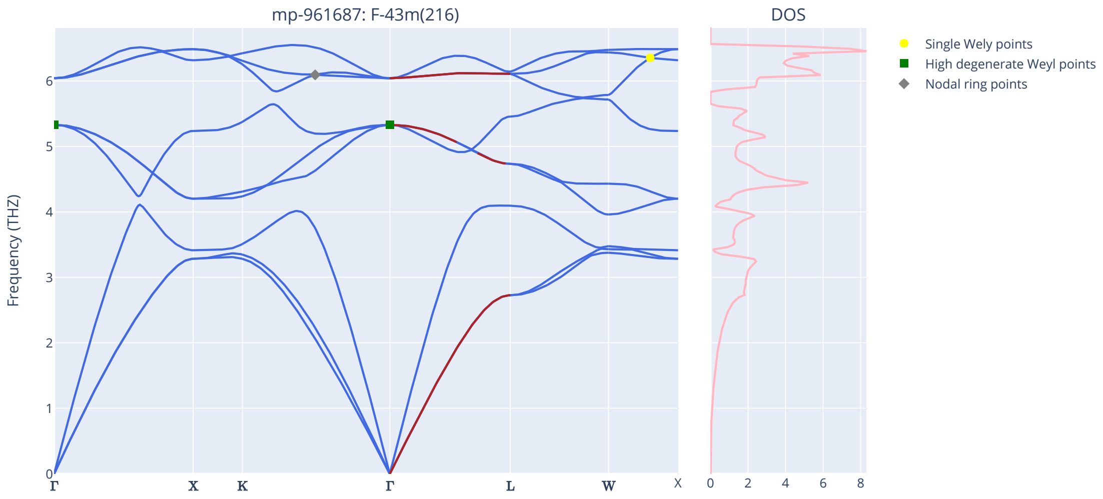

Supplementary Figure 529: The phonon band and DOS of Pd4Sn4Zr4.

Supplementary Table 1123: High symmetry lines of mp-961687-Pd4Sn4Zr4

	Start	End
0	Γ (0.000, 0.000, 0.000)	X (0.500, 0.000, 0.500)
1	X (0.500, 0.000, 0.500)	U (0.625, 0.250, 0.625)
2	K (0.375, 0.375, 0.750)	Γ (0.000, 0.000, 0.000)
3	Γ (0.000, 0.000, 0.000)	L (0.500, 0.500, 0.500)
4	L (0.500, 0.500, 0.500)	W (0.500, 0.250, 0.750)
5	W (0.500, 0.250, 0.750)	X (0.500, 0.000, 0.500)

Supplementary Table 1124: Single Weyl points along high symmetry lines of mp-961687-Pd4Sn4Zr4.

	Coordinates	Frequency	Mode 1	Mode 2	Multi.	Deg.	Path	Ratio
0	(0.499, 0.100, 0.600)	6.354	8	7	24	2	6	0.600

Supplementary Table 1125: High degenerate Weyl points along high symmetry lines of mp-961687-Pd4Sn4Zr4.

	Coordinates	Frequency	Mode 1	Mode 2	Multi.	Deg.	Path	Ratio
0	(0.000, 0.000, 0.000)	5.330	6	4	1	3	1	0.000

Supplementary Table 1126: Nodal ring points along high symmetry lines of mp-961687-Pd4Sn4Zr4.

	Coordinates	Frequency	Mode 1	Mode 2	Multi.	Deg.	Path	Ratio
0	(0.191, 0.191, 0.378)	6.094	8	7	24	2	3	0.494

The followings are details for $Au_4In_4Zr_4$. As shown in Fig **530**, it has 4 topological nodal lines and 1 nodal ring point. At the same time, it has 1 single Weyl point and 1 high degenerate Weyl point whose degeneracy is more than 2 and charge is bigger than 1.

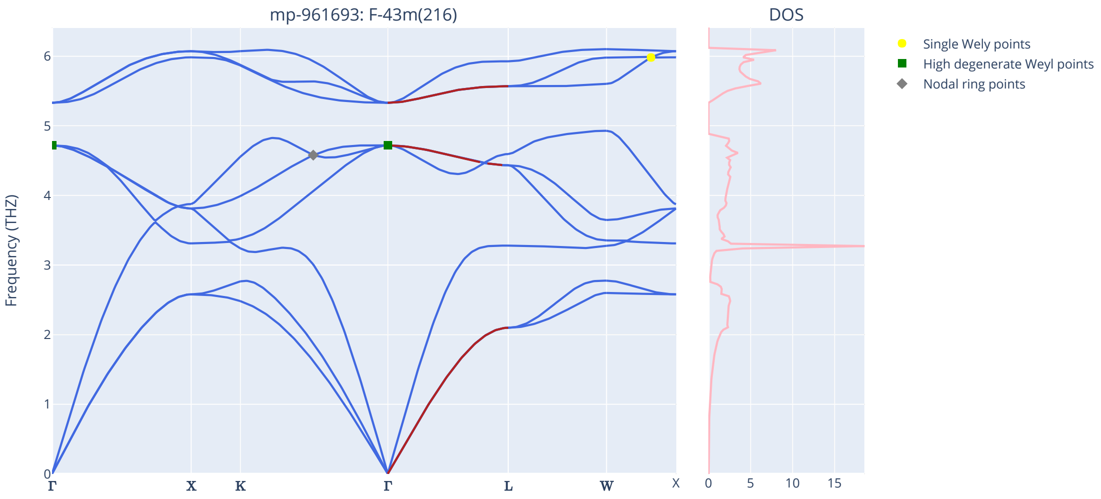

Supplementary Figure 530: The phonon band and DOS of Au4In4Zr4.

Supplementary Table 1127: High symmetry lines of mp-961693-Au4In4Zr4

	Start	End
0	Γ (0.000, 0.000, 0.000)	X (0.500, 0.000, 0.500)
1	X (0.500, 0.000, 0.500)	U (0.625, 0.250, 0.625)
2	K (0.375, 0.375, 0.750)	Γ (0.000, 0.000, 0.000)
3	Γ (0.000, 0.000, 0.000)	L (0.500, 0.500, 0.500)
4	L (0.500, 0.500, 0.500)	W (0.500, 0.250, 0.750)
5	W (0.500, 0.250, 0.750)	X (0.500, 0.000, 0.500)

Supplementary Table 1128: Single Weyl points along high symmetry lines of mp-961693-Au4In4Zr4.

	Coordinates	Frequency	Mode 1	Mode 2	Multi.	Deg.	Path	Ratio
0	(0.500, 0.090, 0.590)	5.982	8	7	12	2	6	0.640

Supplementary Table 1129: High degenerate Weyl points along high symmetry lines of mp-961693-Au4In4Zr4.

	Coordinates	Frequency	Mode 1	Mode 2	Multi.	Deg.	Path	Ratio
0	(0.000, 0.000, 0.000)	4.719	6	4	1	3	1	0.000

Supplementary Table 1130: Nodal ring points along high symmetry lines of mp-961693-Au4In4Zr4.

	Coordinates	Frequency	Mode 1	Mode 2	Multi.	Deg.	Path	Ratio
0	(0.191, 0.191, 0.377)	4.579	6	5	24	2	3	0.495

The followings are details for $Pt_4Ti_4Si_4$. As shown in Fig 531, it has 3 topological nodal lines, 1 nodal ring point and 1 high degenerate Weyl point whose degeneracy is more than 2 and charge is bigger than 1.

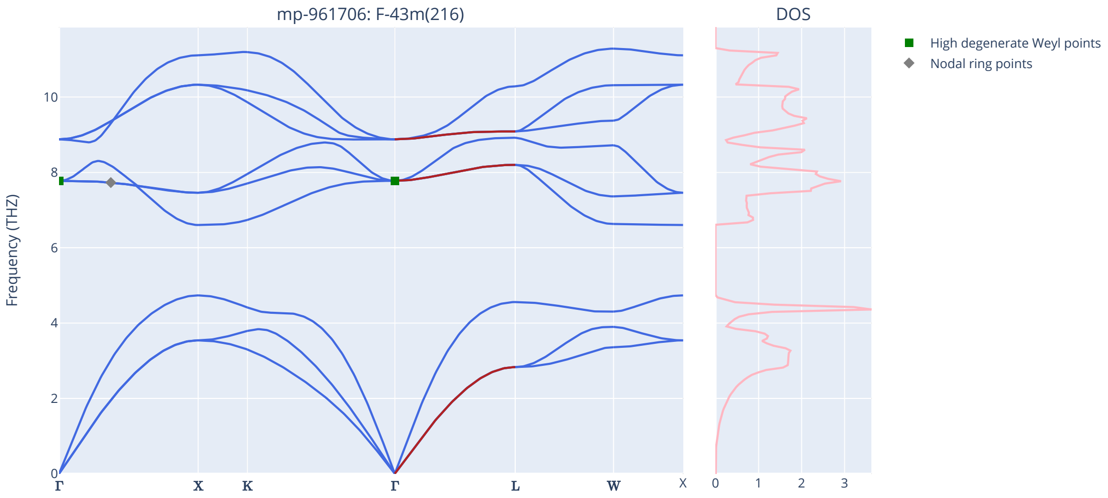

Supplementary Figure 531: The phonon band and DOS of Pt4Ti4Si4.

Supplementary Table 1131: High symmetry lines of mp-961706-Pt4Ti4Si4

	Start	End
0	Γ (0.000, 0.000, 0.000)	X (0.500, 0.000, 0.500)
1	X (0.500, 0.000, 0.500)	U (0.625, 0.250, 0.625)
2	K (0.375, 0.375, 0.750)	Γ (0.000, 0.000, 0.000)
3	Γ (0.000, 0.000, 0.000)	L (0.500, 0.500, 0.500)
4	L (0.500, 0.500, 0.500)	W (0.500, 0.250, 0.750)
5	W (0.500, 0.250, 0.750)	X (0.500, 0.000, 0.500)

Supplementary Table 1132: High degenerate Weyl points along high symmetry lines of mp-961706-Pt4Ti4Si4.

	Coordinates	Frequency	Mode 1	Mode 2	Multi.	Deg.	Path	Ratio
0	(0.000, 0.000, 0.000)	7.775	6	4	1	3	1	0.000

Supplementary Table 1133: Nodal ring points along high symmetry lines of mp-961706-Pt4Ti4Si4.

	Coordinates	Frequency	Mode 1	Mode 2	Multi.	Deg.	Path	Ratio
0	(0.191, -0.000, 0.182)	7.730	5	4	24	2	1	0.372

The followings are details for $Pt_4Zr_4Si_4$. It has 3 topological nodal lines and 1 high degenerate Weyl point whose degeneracy is more than 2 and charge is bigger than 1 along high symmetry line as shown in Fig **532**.

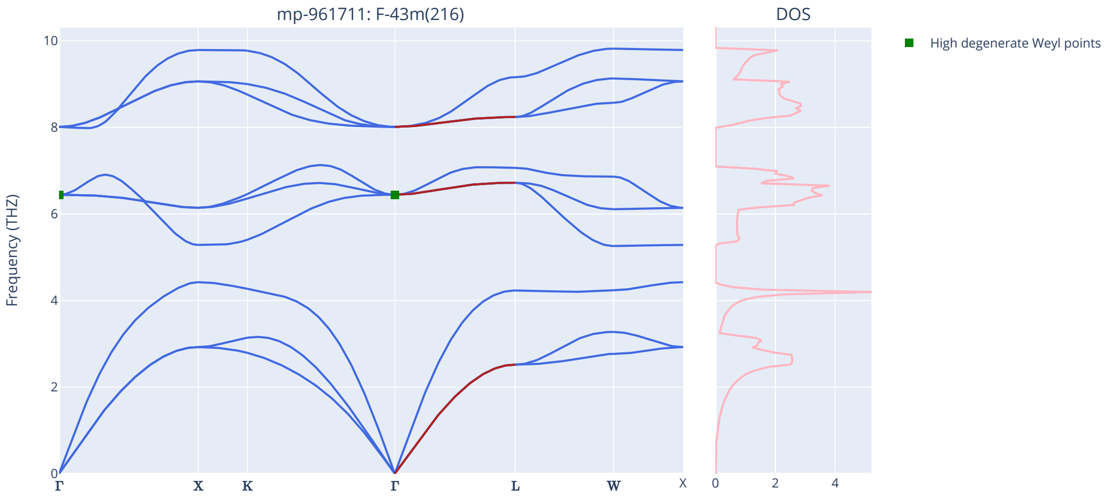

Supplementary Figure 532: The phonon band and DOS of Pt4Zr4Si4.

Supplementary Table 1134: High symmetry lines of mp-961711-Pt4Zr4Si4

	Start	End
0	Γ (0.000, 0.000, 0.000)	X (0.500, 0.000, 0.500)
1	X (0.500, 0.000, 0.500)	U (0.625, 0.250, 0.625)
2	K (0.375, 0.375, 0.750)	Γ (0.000, 0.000, 0.000)
3	Γ (0.000, 0.000, 0.000)	L (0.500, 0.500, 0.500)
4	L (0.500, 0.500, 0.500)	W (0.500, 0.250, 0.750)
5	W (0.500, 0.250, 0.750)	X (0.500, 0.000, 0.500)

Supplementary Table 1135: High degenerate Weyl points along high symmetry lines of mp-961711-Pt4Zr4Si4.

	Coordinates	Frequency	Mode 1	Mode 2	Multi.	Deg.	Path	Ratio
0	(0.000, 0.000, 0.000)	6.444	6	4	1	3	1	0.000

The followings are details for $Pt_4Sn_4Zr_4$. As shown in Fig 533, it has 4 topological nodal lines, 1 nodal ring point and 1 high degenerate Weyl point whose degeneracy is more than 2 and charge is bigger than 1.

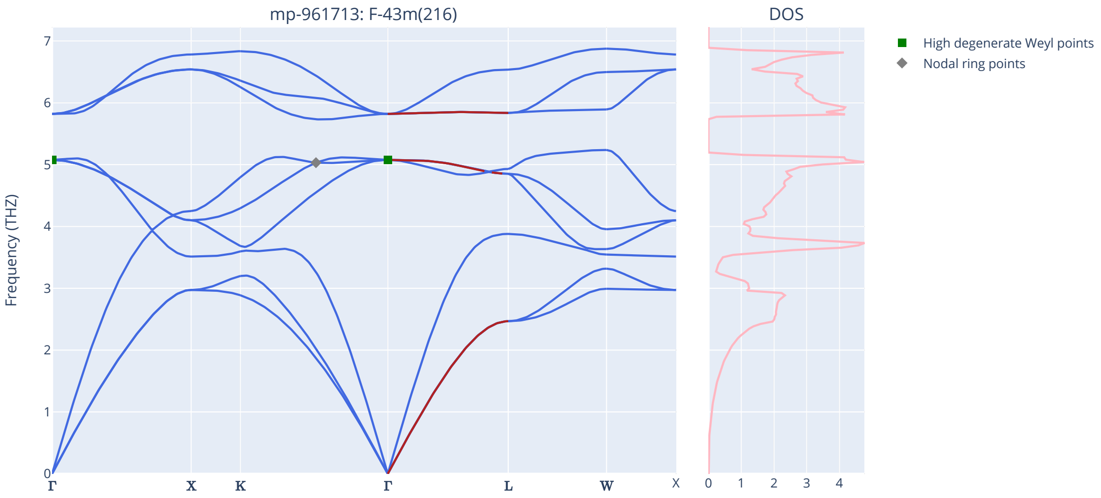

Supplementary Figure 533: The phonon band and DOS of Pt4Sn4Zr4.

Supplementary Table 1136: High symmetry lines of mp-961713-Pt4Sn4Zr4

	Start	End
0	Γ (0.000, 0.000, 0.000)	X (0.500, 0.000, 0.500)
1	X (0.500, 0.000, 0.500)	U (0.625, 0.250, 0.625)
2	K (0.375, 0.375, 0.750)	Γ (0.000, 0.000, 0.000)
3	Γ (0.000, 0.000, 0.000)	L (0.500, 0.500, 0.500)
4	L (0.500, 0.500, 0.500)	W (0.500, 0.250, 0.750)
5	W (0.500, 0.250, 0.750)	X (0.500, 0.000, 0.500)

Supplementary Table 1137: High degenerate Weyl points along high symmetry lines of mp-961713-Pt4Sn4Zr4.

	Coordinates	Frequency	Mode 1	Mode 2	Multi.	Deg.	Path	Ratio
0	(0.000, 0.000, 0.000)	5.077	6	4	1	3	1	0.000

Supplementary Table 1138: Nodal ring points along high symmetry lines of mp-961713-Pt4Sn4Zr4.

	Coordinates	Frequency	Mode 1	Mode 2	Multi.	Deg.	Path	Ratio
0	(0.184, 0.184, 0.364)	5.032	6	5	24	2	3	0.513

The followings are details for K_8O_4. It has 4 topological nodal lines and 7 nodal ring points along high symmetry line as shown in Fig **534**.

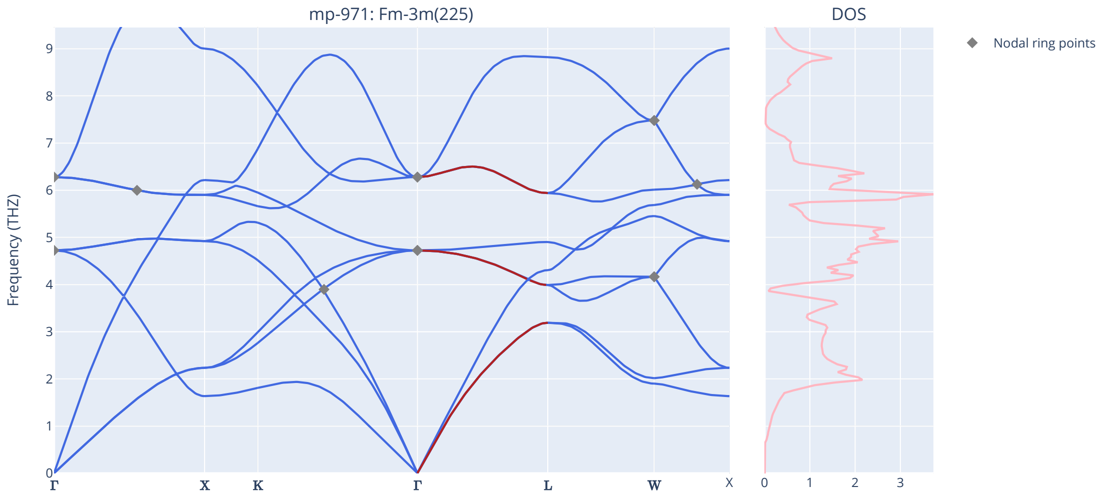

Supplementary Figure 534: The phonon band and DOS of K8O4.

Supplementary Table 1139: High symmetry lines of mp-971-K8O4

	Start	End
0	Γ (0.000, 0.000, 0.000)	X (0.500, 0.000, 0.500)
1	X (0.500, 0.000, 0.500)	U (0.625, 0.250, 0.625)
2	K (0.375, 0.375, 0.750)	Γ (0.000, 0.000, 0.000)
3	Γ (0.000, 0.000, 0.000)	L (0.500, 0.500, 0.500)
4	L (0.500, 0.500, 0.500)	W (0.500, 0.250, 0.750)
5	W (0.500, 0.250, 0.750)	X (0.500, 0.000, 0.500)

Supplementary Table 1140: Nodal ring points along high symmetry lines of mp-971-K8O4.

	Coordinates	Frequency	Mode 1	Mode 2	Multi.	Deg.	Path	Ratio
0	(0.214, 0.214, 0.444)	3.898	4	3	24	2	3	0.415
1	(0.500, 0.250, 0.750)	4.166	4	3	6	2	5	1.000
2	(0.000, 0.000, 0.000)	4.725	5	4	1	3	1	0.000
3	(0.000, 0.000, 0.000)	6.279	8	7	1	3	1	0.000
4	(0.276, 0.000, 0.276)	5.998	8	7	6	2	1	0.551
5	(0.498, 0.108, 0.607)	6.126	8	7	24	2	6	0.570
6	(0.500, 0.250, 0.750)	7.479	9	8	6	2	5	1.000

The followings are details for Sr_4F_8. It has 5 topological nodal lines and 9 nodal ring points along high symmetry line as shown in Fig **535**.

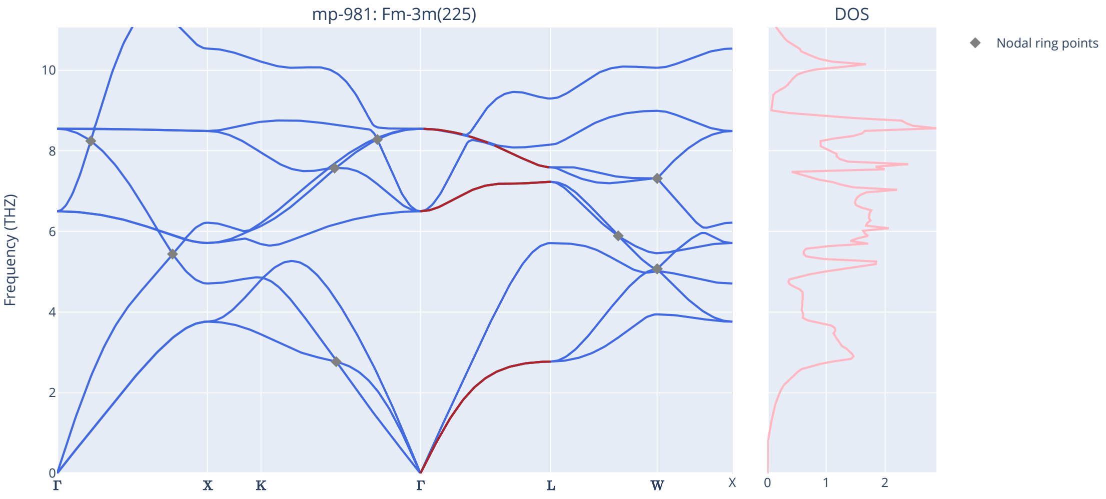

Supplementary Figure 535: The phonon band and DOS of Sr4F8.

Supplementary Table 1141: High symmetry lines of mp-981-Sr4F8

	Start	End
0	Γ (0.000, 0.000, 0.000)	X (0.500, 0.000, 0.500)
1	X (0.500, 0.000, 0.500)	U (0.625, 0.250, 0.625)
2	K (0.375, 0.375, 0.750)	Γ (0.000, 0.000, 0.000)
3	Γ (0.000, 0.000, 0.000)	L (0.500, 0.500, 0.500)
4	L (0.500, 0.500, 0.500)	W (0.500, 0.250, 0.750)
5	W (0.500, 0.250, 0.750)	X (0.500, 0.000, 0.500)

Supplementary Table 1142: Nodal ring points along high symmetry lines of mp-981-Sr4F8.

	Coordinates	Frequency	Mode 1	Mode 2	Multi.	Deg.	Path	Ratio
0	(0.199, 0.199, 0.395)	2.769	2	1	24	2	3	0.472
1	(0.384, -0.002, 0.384)	5.439	4	3	24	2	1	0.767
2	(0.500, 0.250, 0.750)	5.071	4	3	6	2	5	1.000
3	(0.500, 0.341, 0.659)	5.890	5	4	24	2	5	0.634
4	(0.207, 0.207, 0.398)	7.571	6	5	24	2	3	0.462
5	(0.112, 0.000, 0.112)	8.245	7	6	6	2	1	0.224
6	(0.099, 0.103, 0.202)	8.281	7	6	24	2	3	0.730
7	(0.500, 0.250, 0.750)	7.315	7	6	6	2	5	1.000

The followings are details for W_3S_6. It has 3 topological nodal lines and 8 nodal ring points along high symmetry line as shown in Fig **536**.

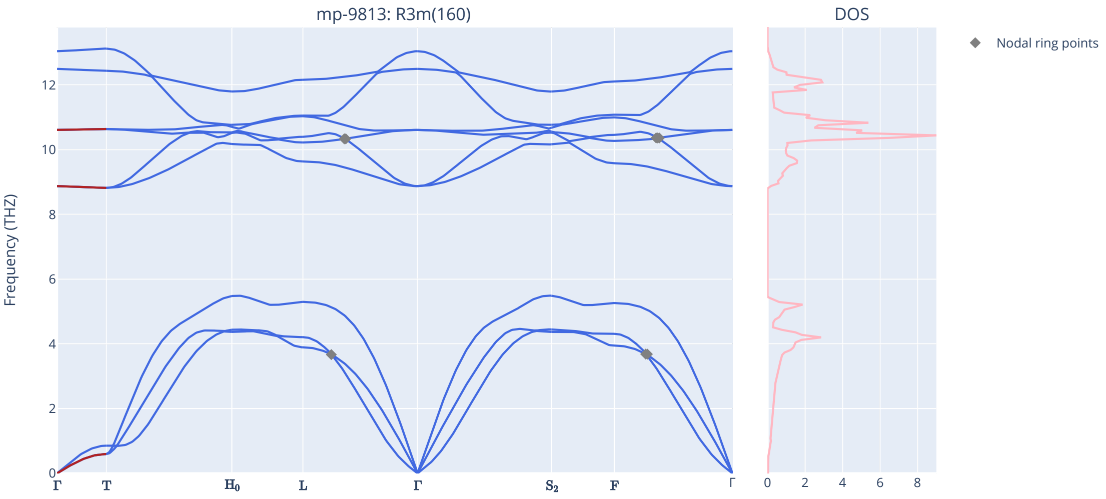

Supplementary Figure 536: The phonon band and DOS of W3S6.

Supplementary Table 1143: High symmetry lines of mp-9813-W3S6

	Start	End
0	Γ (0.000, 0.000, 0.000)	T (0.500, 0.500, 0.500)
1	T (0.500, 0.500, 0.500)	H_2 (0.819, 0.181, 0.500)
2	H_0 (0.500, -0.181, 0.181)	L (0.500, 0.000, 0.000)
3	L (0.500, 0.000, 0.000)	Γ (0.000, 0.000, 0.000)
4	Γ (0.000, 0.000, 0.000)	S_0 (0.340, -0.340, 0.000)
5	S_2 (0.660, 0.000, 0.340)	F (0.500, 0.000, 0.500)
6	F (0.500, 0.000, 0.500)	Γ (0.000, 0.000, 0.000)

Supplementary Table 1144: Nodal ring points along high symmetry lines of mp-9813-W3S6.

	Coordinates	Frequency	Mode 1	Mode 2	Multi.	Deg.	Path	Ratio
0	(0.376, 0.001, 0.001)	3.666	2	1	6	2	4	0.248
1	(0.367, 0.001, 0.367)	3.683	2	1	6	2	7	0.266
2	(0.360, -0.006, 0.360)	3.680	2	1	6	2	7	0.280
3	(0.319, 0.003, 0.003)	10.333	6	5	6	2	4	0.361
4	(0.313, -0.003, -0.003)	10.332	6	5	6	2	4	0.374
5	(0.323, 0.007, 0.323)	10.361	6	5	6	2	7	0.354
6	(0.316, 0.000, 0.316)	10.360	6	5	6	2	7	0.367
7	(0.310, -0.007, 0.310)	10.359	6	5	6	2	7	0.381

The followings are details for HfS_2. It has 4 topological nodal lines and 25 nodal ring points along high symmetry line as shown in Fig 537.

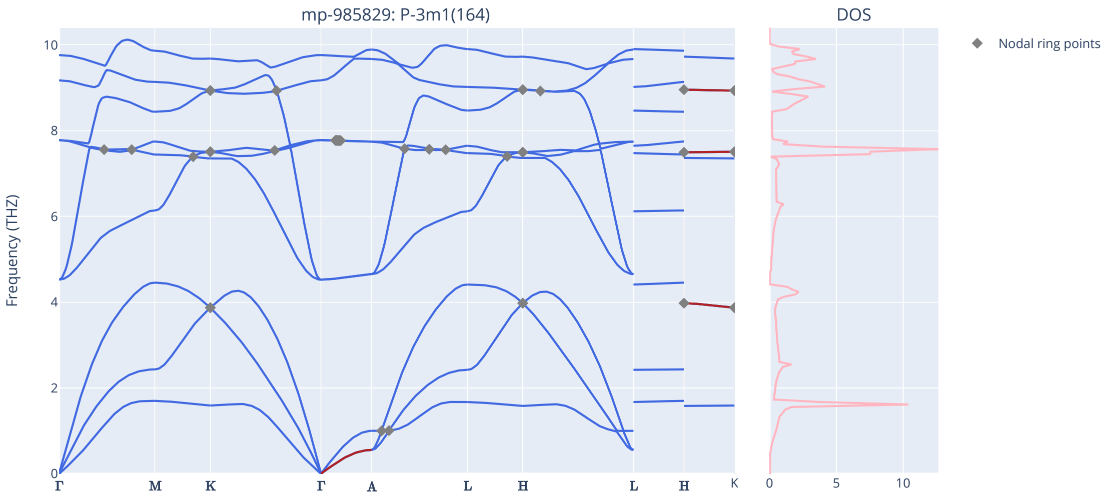

Supplementary Figure 537: The phonon band and DOS of HfS2.

Supplementary Table 1145: High symmetry lines of mp-985829-HfS2

	Start	End
0	Γ (0.000, 0.000, 0.000)	M (0.500, 0.000, 0.000)
1	M (0.500, 0.000, 0.000)	K (0.333, 0.333, 0.000)
2	K (0.333, 0.333, 0.000)	Γ (0.000, 0.000, 0.000)
3	Γ (0.000, 0.000, 0.000)	A (0.000, 0.000, 0.500)
4	A (0.000, 0.000, 0.500)	L (0.500, 0.000, 0.500)
5	L (0.500, 0.000, 0.500)	H (0.333, 0.333, 0.500)
6	H (0.333, 0.333, 0.500)	A (0.000, 0.000, 0.500)
7	L (0.500, 0.000, 0.500)	M (0.500, 0.000, 0.000)
8	H (0.333, 0.333, 0.500)	K (0.333, 0.333, 0.000)

Supplementary Table 1146: Nodal ring points along high symmetry lines of mp-985829-HfS2.

	Coordinates	Frequency	Mode 1	Mode 2	Multi.	Deg.	Path	Ratio
0	(0.091, 0.000, 0.497)	1.006	2	1	6	2	5	0.182
1	(0.333, 0.333, 0.000)	3.871	3	2	2	2	2	1.000
2	(0.053, -0.001, 0.498)	1.000	3	2	12	2	5	0.105
3	(0.333, 0.333, 0.500)	3.977	3	2	2	2	6	1.000
4	(0.385, 0.230, 0.002)	7.392	5	4	12	2	2	0.690
5	(0.380, 0.239, 0.497)	7.399	5	4	12	2	6	0.718
6	(0.235, 0.000, -0.003)	7.557	6	5	6	2	1	0.470
7	(0.379, 0.000, -0.003)	7.556	6	5	6	2	1	0.757
8	(0.333, 0.333, 0.000)	7.507	6	5	2	2	2	1.000
9	(0.139, 0.139, -0.000)	7.538	6	5	6	2	3	0.582
10	(0.173, 0.000, 0.500)	7.578	6	5	6	2	5	0.345

Supplementary Table 1146: Nodal ring points along high symmetry lines of mp-985829-HfS2.

	Coordinates	Frequency	Mode 1	Mode 2	Multi.	Deg.	Path	Ratio
11	(0.301, -0.000, 0.498)	7.565	6	5	6	2	5	0.602
12	(0.387, -0.000, 0.500)	7.554	6	5	6	2	5	0.773
13	(0.333, 0.333, 0.500)	7.495	6	5	2	2	6	1.000
14	(0.000, -0.000, 0.143)	7.772	7	6	2	2	4	0.286
15	(0.000, -0.000, 0.153)	7.771	7	6	2	2	4	0.306
16	(0.000, -0.000, 0.163)	7.770	7	6	2	2	4	0.327
17	(0.000, -0.000, 0.173)	7.769	7	6	2	2	4	0.347
18	(0.000, -0.000, 0.184)	7.768	7	6	2	2	4	0.367
19	(0.000, -0.000, 0.194)	7.767	7	6	2	2	4	0.388
20	(0.333, 0.333, 0.000)	8.934	8	7	2	2	2	1.000
21	(0.134, 0.134, -0.000)	8.932	8	7	6	2	3	0.598
22	(0.280, 0.280, 0.500)	8.922	8	7	6	2	7	0.159
23	(0.333, 0.333, 0.500)	8.954	8	7	2	2	6	1.000

The followings are details for $HfSe_2$. It has 5 topological nodal lines and 16 nodal ring points along high symmetry line as shown in Fig **538**.

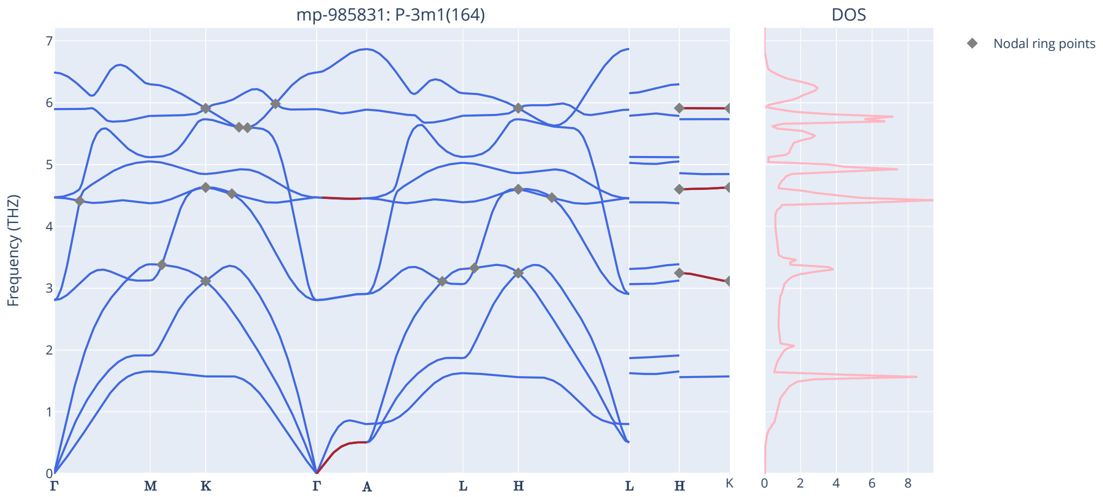

Supplementary Figure 538: The phonon band and DOS of HfSe2.

Supplementary Table 1147: High symmetry lines of mp-985831-HfSe2

	Start	End
0	Γ (0.000, 0.000, 0.000)	M (0.500, 0.000, 0.000)
1	M (0.500, 0.000, 0.000)	K (0.333, 0.333, 0.000)
2	K (0.333, 0.333, 0.000)	Γ (0.000, 0.000, 0.000)
3	Γ (0.000, 0.000, 0.000)	A (0.000, 0.000, 0.500)
4	A (0.000, 0.000, 0.500)	L (0.500, 0.000, 0.500)
5	L (0.500, 0.000, 0.500)	H (0.333, 0.333, 0.500)
6	H (0.333, 0.333, 0.500)	A (0.000, 0.000, 0.500)
7	L (0.500, 0.000, 0.500)	M (0.500, 0.000, 0.000)
8	H (0.333, 0.333, 0.500)	K (0.333, 0.333, 0.000)

Supplementary Table 1148: Nodal ring points along high symmetry lines of mp-985831-HfSe2.

	Coordinates	Frequency	Mode 1	Mode 2	Multi.	Deg.	Path	Ratio
0	(0.333, 0.333, 0.000)	3.116	3	2	2	2	2	1.000
1	(0.333, 0.333, 0.500)	3.246	3	2	2	2	6	1.000
2	(0.465, 0.070, -0.001)	3.380	4	3	12	2	2	0.211
3	(0.393, 0.000, 0.500)	3.111	4	3	6	2	5	0.787
4	(0.466, 0.070, 0.498)	3.327	4	3	12	2	6	0.209
5	(0.333, 0.333, 0.000)	4.630	5	4	2	2	2	1.000
6	(0.255, 0.255, -0.000)	4.529	5	4	6	2	3	0.236
7	(0.333, 0.333, 0.500)	4.601	5	4	2	2	6	1.000
8	(0.233, 0.233, 0.500)	4.466	5	4	6	2	7	0.302
9	(0.133, 0.000, -0.000)	4.411	6	5	6	2	1	0.267
10	(0.208, 0.208, -0.002)	5.596	8	7	12	2	3	0.377

Supplementary Table 1148: Nodal ring points along high symmetry lines of mp-985831-HfSe2.

	Coordinates	Frequency	Mode 1	Mode 2	Multi.	Deg.	Path	Ratio
11	(0.233, 0.233, -0.000)	5.604	8	7	6	2	3	0.300
12	(0.122, 0.125, -0.004)	5.982	9	8	12	2	3	0.629
13	(0.333, 0.333, 0.000)	5.909	9	8	2	2	2	1.000
14	(0.333, 0.333, 0.500)	5.913	9	8	2	2	6	1.000

The followings are details for Pb_4F_8. As shown in Fig **539**, it has 10 topological nodal lines, 31 nodal ring points and 1 single Weyl point.

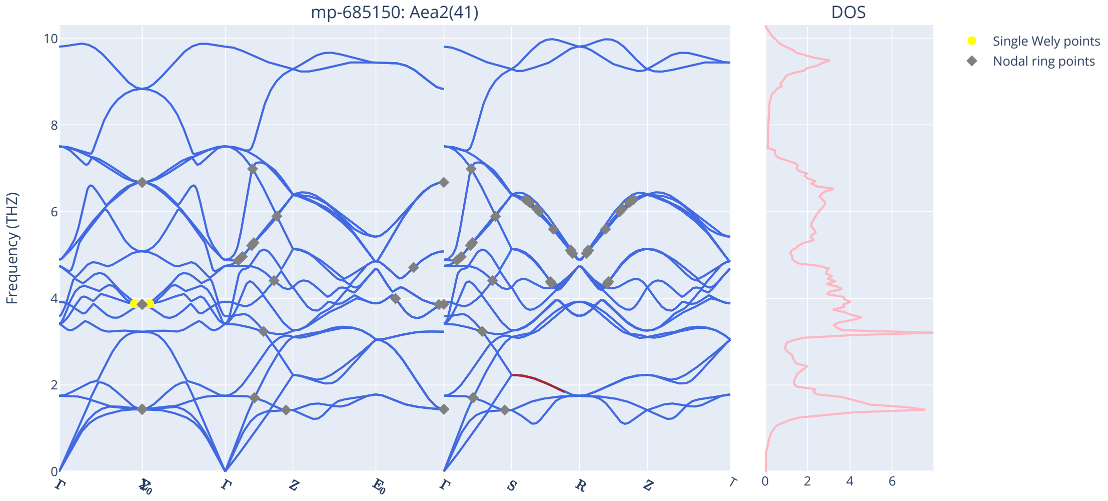

Supplementary Figure 539: The phonon band and DOS of Pb4F8.

Supplementary Table 1149: High symmetry lines of mp-685150-Pb4F8

	Start	End
0	Γ (0.000, 0.000, 0.000)	Y (-0.500, 0.500, 0.000)
1	Y (-0.500, 0.500, 0.000)	C_0 (-0.500, 0.500, 0.000)
2	Σ_0 (0.500, 0.500, 0.000)	Γ (0.000, 0.000, 0.000)
3	Γ (0.000, 0.000, 0.000)	Z (0.000, 0.000, 0.500)
4	Z (0.000, 0.000, 0.500)	A_0 (0.500, 0.500, 0.500)
5	E_0 (-0.500, 0.500, 0.500)	T (-0.500, 0.500, 0.500)
6	T (-0.500, 0.500, 0.500)	Y (-0.500, 0.500, 0.000)
7	Γ (0.000, 0.000, 0.000)	S (0.000, 0.500, 0.000)
8	S (0.000, 0.500, 0.000)	R (0.000, 0.500, 0.500)
9	R (0.000, 0.500, 0.500)	Z (0.000, 0.000, 0.500)
10	Z (0.000, 0.000, 0.500)	T (-0.500, 0.500, 0.500)

Supplementary Table 1150: Single Weyl points along high symmetry lines of mp-685150-Pb4F8.

	Coordinates	Frequency	Mode 1	Mode 2	Multi.	Deg.	Path	Ratio
0	(0.455, 0.451, 0.001)	3.872	9	8	8	2	1	0.906

Supplementary Table 1151: Nodal ring points along high symmetry lines of mp-685150-Pb4F8.

	Coordinates	Frequency	Mode 1	Mode 2	Multi.	Deg.	Path	Ratio
0	(-0.500, 0.500, 0.000)	1.435	2	1	2	2	1	1.000

Supplementary Table 1151: Nodal ring points along high symmetry lines of mp-685150-Pb4F8.

	Coordinates	Frequency	Mode 1	Mode 2	Multi.	Deg.	Path	Ratio
1	(-0.000, 0.449, 0.001)	1.422	2	1	4	2	4	0.898
2	(-0.500, 0.500, 0.000)	1.442	4	3	2	2	1	1.000
3	(0.000, 0.215, 0.005)	1.707	4	3	4	2	4	0.429
4	(-0.000, 0.217, -0.005)	1.701	4	3	4	2	4	0.433
5	(0.000, 0.283, -0.001)	3.236	7	6	4	2	4	0.566
6	(-0.500, 0.496, 0.356)	3.994	8	7	4	2	7	0.287
7	(-0.500, 0.500, 0.000)	3.859	10	9	2	2	1	1.000
8	(-0.000, 0.360, -0.001)	4.409	10	9	4	2	4	0.720
9	(-0.500, 0.499, 0.036)	3.855	10	9	4	2	7	0.927
10	(-0.500, 0.495, 0.222)	4.711	12	11	4	2	7	0.556
11	(0.000, 0.286, 0.500)	4.383	12	11	4	2	9	0.571
12	(0.000, 0.296, 0.499)	4.339	12	11	4	2	9	0.592
13	(0.000, 0.097, 0.002)	4.873	13	12	4	2	4	0.193
14	(0.000, 0.127, -0.000)	4.961	13	12	4	2	4	0.254
15	(0.000, 0.196, -0.005)	5.217	13	12	4	2	4	0.391
16	(0.000, 0.212, 0.004)	5.283	13	12	4	2	4	0.424
17	(0.000, 0.381, -0.001)	5.893	13	12	4	2	4	0.762
18	(-0.500, 0.500, 0.000)	6.674	14	13	2	2	1	1.000
19	(0.000, 0.308, 0.495)	5.597	14	13	4	2	9	0.616
20	(0.000, 0.433, 0.498)	5.102	14	13	4	2	9	0.866
21	(0.000, 0.449, 0.499)	5.038	14	13	4	2	9	0.898
22	(0.000, 0.202, 0.001)	6.987	15	14	4	2	4	0.404
23	(0.000, 0.102, 0.497)	6.278	15	14	4	2	9	0.204
24	(0.000, 0.122, 0.497)	6.234	15	14	4	2	9	0.245
25	(0.000, 0.133, 0.497)	6.209	15	14	4	2	9	0.265
26	(0.000, 0.184, 0.497)	6.064	15	14	4	2	9	0.367
27	(0.000, 0.204, 0.497)	5.997	15	14	4	2	9	0.408
28	(-0.000, 0.435, 0.498)	5.114	16	15	4	2	9	0.869

The followings are details for $Co_3Pt_3O_6$. It has 5 topological nodal lines and 11 nodal ring points along high symmetry line as shown in Fig **540**.

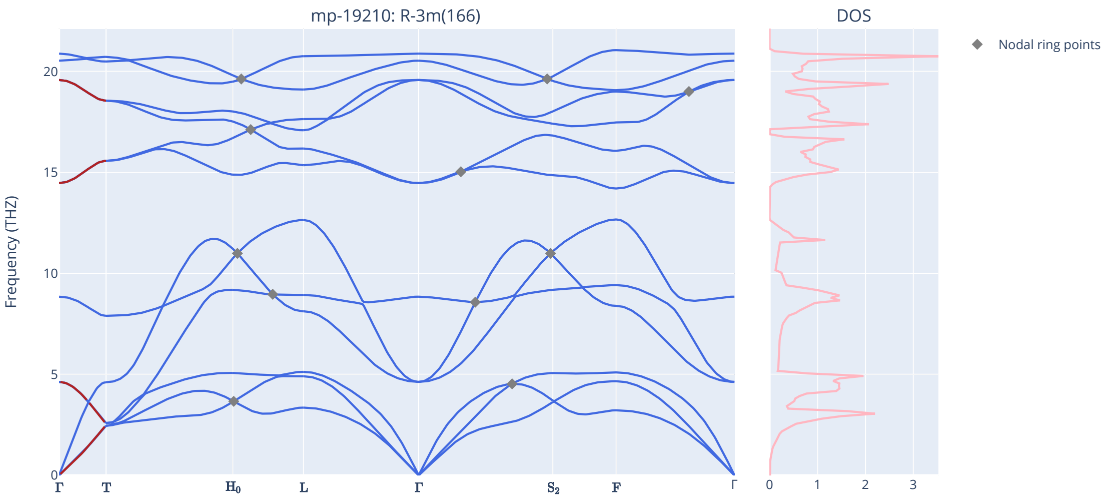

Supplementary Figure 540: The phonon band and DOS of Co3Pt3O6.

Supplementary Table 1152: High symmetry lines of mp-19210-Co3Pt3O6

	Start	End
0	Γ (0.000, 0.000, 0.000)	T (0.500, 0.500, 0.500)
1	T (0.500, 0.500, 0.500)	H_2 (0.821, 0.179, 0.500)
2	H_0 (0.500, -0.179, 0.179)	L (0.500, 0.000, 0.000)
3	L (0.500, 0.000, 0.000)	Γ (0.000, 0.000, 0.000)
4	Γ (0.000, 0.000, 0.000)	S_0 (0.340, -0.340, 0.000)
5	S_2 (0.660, 0.000, 0.340)	F (0.500, 0.000, 0.500)
6	F (0.500, 0.000, 0.500)	Γ (0.000, 0.000, 0.000)

Supplementary Table 1153: Nodal ring points along high symmetry lines of mp-19210-Co3Pt3O6.

	Coordinates	Frequency	Mode 1	Mode 2	Multi.	Deg.	Path	Ratio
0	(0.500, -0.177, 0.177)	3.659	2	1	6	2	3	0.014
1	(0.237, -0.237, -0.000)	4.526	3	2	6	2	5	0.697
2	(0.500, -0.078, 0.078)	8.960	5	4	6	2	3	0.565
3	(0.500, -0.167, 0.167)	10.989	6	5	6	2	3	0.066
4	(0.334, -0.334, 0.000)	10.989	6	5	6	2	5	0.983
5	(0.143, -0.143, -0.000)	8.571	6	5	6	2	5	0.422
6	(0.107, -0.107, 0.000)	15.030	8	7	6	2	5	0.315
7	(0.500, -0.134, 0.134)	17.118	9	8	6	2	3	0.254
8	(0.192, 0.003, 0.192)	19.003	10	9	6	2	7	0.615
9	(0.500, -0.158, 0.158)	19.624	12	11	6	2	3	0.121
10	(0.325, -0.325, 0.000)	19.625	12	11	6	2	5	0.958

The followings are details for $Ag_3In_3O_6$. It has 5 topological nodal lines and 10 nodal ring points along high symmetry line as shown in Fig **541**.

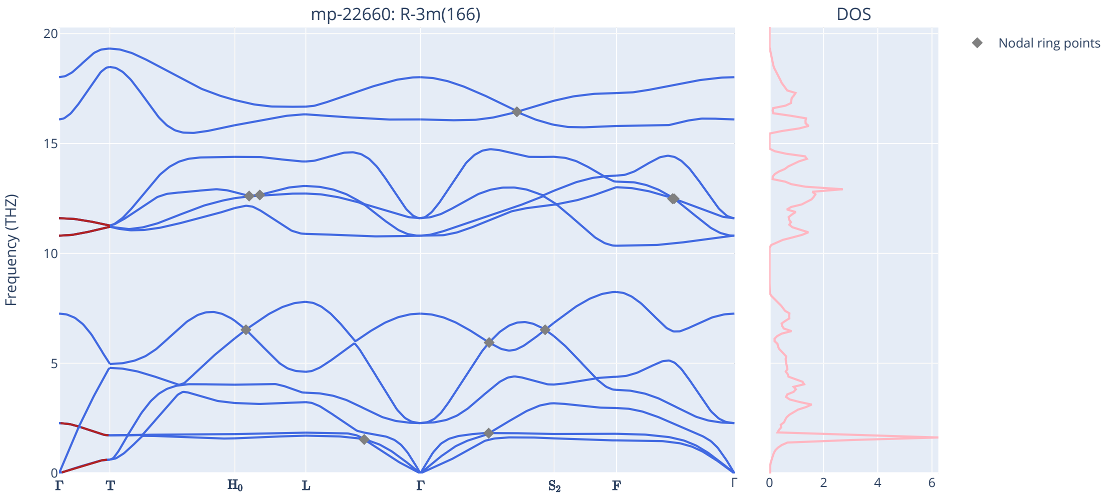

Supplementary Figure 541: The phonon band and DOS of Ag3In3O6.

Supplementary Table 1154: High symmetry lines of mp-22660-Ag3In3O6

	Start	End
0	Γ (0.000, 0.000, 0.000)	T (0.500, 0.500, 0.500)
1	T (0.500, 0.500, 0.500)	H_2 (0.818, 0.182, 0.500)
2	H_0 (0.500, -0.182, 0.182)	L (0.500, 0.000, 0.000)
3	L (0.500, 0.000, 0.000)	Γ (0.000, 0.000, 0.000)
4	Γ (0.000, 0.000, 0.000)	S_0 (0.341, -0.341, 0.000)
5	S_2 (0.659, 0.000, 0.341)	F (0.500, 0.000, 0.500)
6	F (0.500, 0.000, 0.500)	Γ (0.000, 0.000, 0.000)

Supplementary Table 1155: Nodal ring points along high symmetry lines of mp-22660-Ag3In3O6.

	Coordinates	Frequency	Mode 1	Mode 2	Multi.	Deg.	Path	Ratio
0	(0.245, -0.000, -0.000)	1.536	2	1	6	2	4	0.510
1	(0.174, -0.174, -0.000)	1.824	3	2	6	2	5	0.512
2	(0.500, -0.153, 0.153)	6.528	6	5	6	2	3	0.158
3	(0.176, -0.176, 0.000)	5.946	6	5	6	2	5	0.515
4	(0.318, -0.318, -0.000)	6.527	6	5	6	2	5	0.934
5	(0.500, -0.118, 0.118)	12.658	9	8	6	2	3	0.352
6	(0.500, -0.145, 0.145)	12.610	9	8	6	2	3	0.203
7	(0.262, 0.007, 0.262)	12.488	9	8	6	2	7	0.476
8	(0.255, -0.000, 0.255)	12.487	9	8	6	2	7	0.489
9	(0.246, -0.246, -0.000)	16.442	12	11	6	2	5	0.723

The followings are details for $Cu_3Sc_3O_6$. It has 5 topological nodal lines and 17 nodal ring points along high symmetry line as shown in Fig **542**.

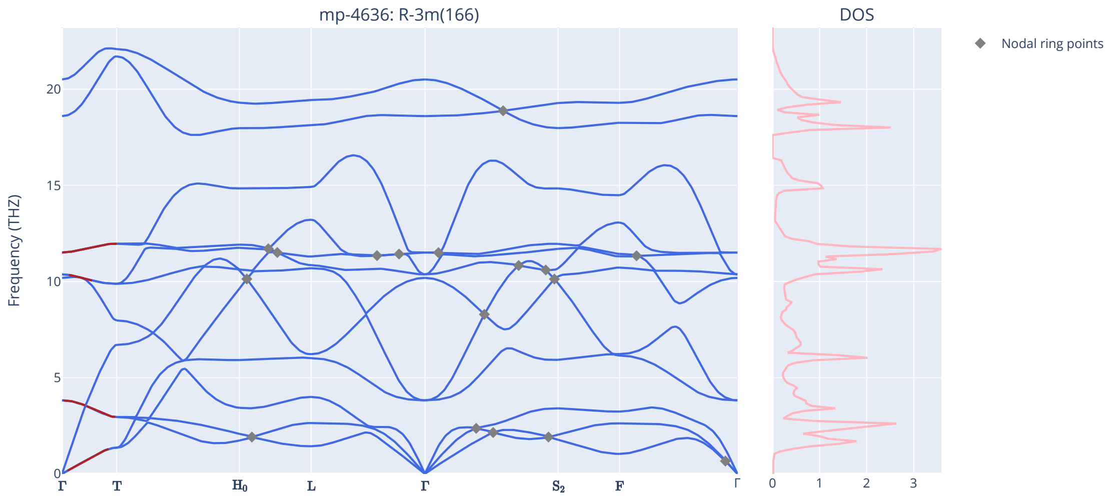

Supplementary Figure 542: The phonon band and DOS of Cu3Sc3O6.

Supplementary Table 1156: High symmetry lines of mp-4636-Cu3Sc3O6

	Start	End
0	Γ (0.000, 0.000, 0.000)	T (0.500, 0.500, 0.500)
1	T (0.500, 0.500, 0.500)	H_2 (0.816, 0.184, 0.500)
2	H_0 (0.500, -0.184, 0.184)	L (0.500, 0.000, 0.000)
3	L (0.500, 0.000, 0.000)	Γ (0.000, 0.000, 0.000)
4	Γ (0.000, 0.000, 0.000)	S_0 (0.342, -0.342, 0.000)
5	S_2 (0.658, 0.000, 0.342)	F (0.500, 0.000, 0.500)
6	F (0.500, 0.000, 0.500)	Γ (0.000, 0.000, 0.000)

Supplementary Table 1157: Nodal ring points along high symmetry lines of mp-4636-Cu3Sc3O6.

	Coordinates	Frequency	Mode 1	Mode 2	Multi.	Deg.	Path	Ratio
0	(0.500, -0.152, 0.152)	1.908	2	1	6	2	3	0.173
1	(0.318, -0.318, -0.000)	1.910	2	1	6	2	5	0.929
2	(0.175, -0.175, -0.000)	2.145	2	1	6	2	5	0.513
3	(0.051, 0.003, 0.051)	0.659	2	1	6	2	7	0.898
4	(0.132, -0.132, -0.000)	2.360	3	2	6	2	5	0.385
5	(0.500, -0.165, 0.165)	10.132	6	5	6	2	3	0.102
6	(0.153, -0.153, -0.000)	8.289	6	5	6	2	5	0.447
7	(0.333, -0.333, 0.000)	10.126	6	5	6	2	5	0.973
8	(0.311, -0.311, 0.000)	10.599	7	6	6	2	5	0.908
9	(0.241, -0.241, -0.000)	10.831	7	6	6	2	5	0.705
10	(0.500, -0.087, 0.087)	11.508	8	7	6	2	3	0.528
11	(0.427, 0.003, 0.427)	11.339	8	7	6	2	7	0.145
12	(0.500, -0.110, 0.110)	11.705	9	8	6	2	3	0.402

Supplementary Table 1157: Nodal ring points along high symmetry lines of mp-4636-Cu3Sc3O6.

	Coordinates	Frequency	Mode 1	Mode 2	Multi.	Deg.	Path	Ratio
13	(0.210, 0.002, 0.002)	11.347	9	8	6	2	4	0.579
14	(0.113, -0.001, -0.001)	11.428	9	8	6	2	4	0.773
15	(0.035, -0.035, -0.000)	11.501	10	9	6	2	5	0.102
16	(0.201, -0.201, 0.000)	18.880	12	11	6	2	5	0.588

The followings are details for $Hg_3Sr_3O_6$. It has 5 topological nodal lines and 13 nodal ring points along high symmetry line as shown in Fig 543.

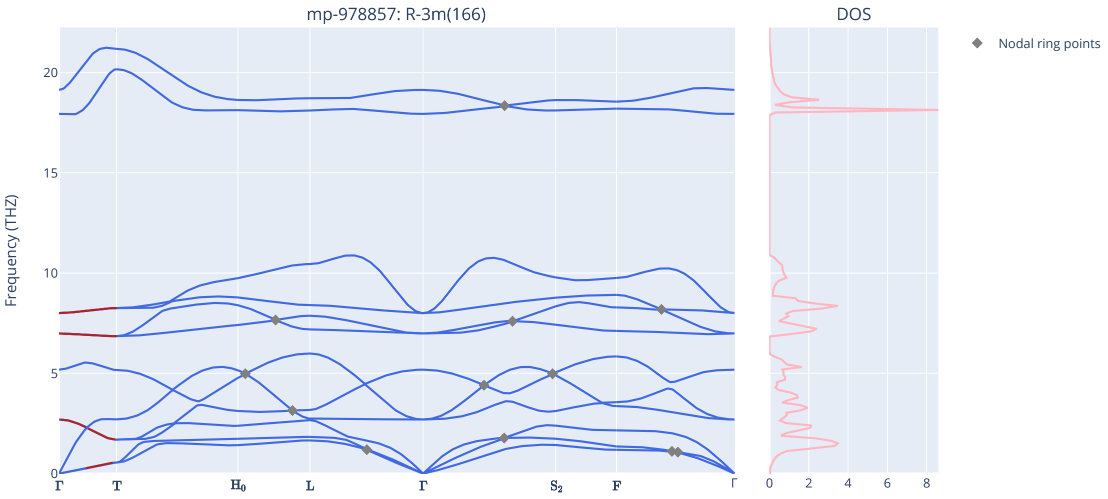

Supplementary Figure 543: The phonon band and DOS of Hg3Sr3O6.

Supplementary Table 1158: High symmetry lines of mp-978857-Hg3Sr3O6

	Start	End
0	Γ (0.000, 0.000, 0.000)	T (0.500, 0.500, 0.500)
1	T (0.500, 0.500, 0.500)	H_2 (0.814, 0.186, 0.500)
2	H_0 (0.500, -0.186, 0.186)	L (0.500, 0.000, 0.000)
3	L (0.500, 0.000, 0.000)	Γ (0.000, 0.000, 0.000)
4	Γ (0.000, 0.000, 0.000)	S_0 (0.343, -0.343, 0.000)
5	S_2 (0.657, 0.000, 0.343)	F (0.500, 0.000, 0.500)
6	F (0.500, 0.000, 0.500)	Γ (0.000, 0.000, 0.000)

Supplementary Table 1159: Nodal ring points along high symmetry lines of mp-978857-Hg3Sr3O6.

	Coordinates	Frequency	Mode 1	Mode 2	Multi.	Deg.	Path	Ratio
0	(0.249, 0.002, 0.002)	1.190	2	1	6	2	4	0.503
1	(0.240, -0.008, 0.240)	1.068	2	1	6	2	7	0.521
2	(0.265, 0.003, 0.265)	1.108	2	1	6	2	7	0.471
3	(0.210, -0.210, 0.000)	1.771	3	2	6	2	5	0.611
4	(0.500, -0.045, 0.045)	3.143	5	4	6	2	3	0.758
5	(0.500, -0.167, 0.167)	4.976	6	5	6	2	3	0.104
6	(0.158, -0.158, 0.000)	4.404	6	5	6	2	5	0.459
7	(0.335, -0.335, 0.000)	4.976	6	5	6	2	5	0.975
8	(0.500, -0.089, 0.089)	7.658	8	7	6	2	3	0.524
9	(0.231, -0.231, -0.000)	7.595	8	7	6	2	5	0.674
10	(0.309, -0.003, 0.309)	8.188	9	8	6	2	7	0.381
11	(0.211, -0.211, -0.000)	18.349	12	11	6	2	5	0.614

The followings are details for $Au_3Tl_3O_6$. It has 5 topological nodal lines and 16 nodal ring points along high symmetry line as shown in Fig **544**.

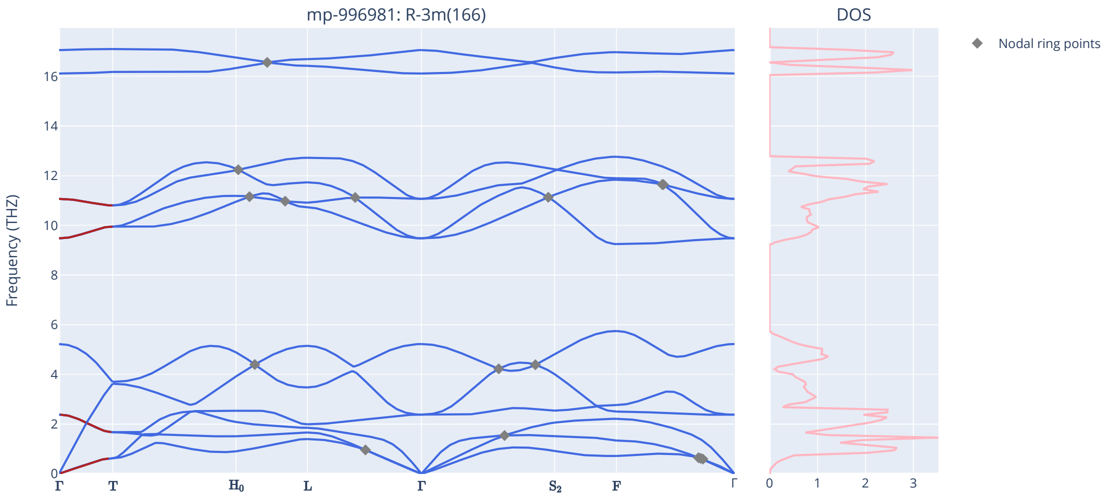

Supplementary Figure 544: The phonon band and DOS of Au3Tl3O6.

Supplementary Table 1160: High symmetry lines of mp-996981-Au3Tl3O6

	Start	End
0	Γ (0.000, 0.000, 0.000)	T (0.500, 0.500, 0.500)
1	T (0.500, 0.500, 0.500)	H_2 (0.817, 0.183, 0.500)
2	H_0 (0.500, -0.183, 0.183)	L (0.500, 0.000, 0.000)
3	L (0.500, 0.000, 0.000)	Γ (0.000, 0.000, 0.000)
4	Γ (0.000, 0.000, 0.000)	S_0 (0.342, -0.342, 0.000)
5	S_2 (0.658, 0.000, 0.342)	F (0.500, 0.000, 0.500)
6	F (0.500, 0.000, 0.500)	Γ (0.000, 0.000, 0.000)

Supplementary Table 1161: Nodal ring points along high symmetry lines of mp-996981-Au3Tl3O6.

	Coordinates	Frequency	Mode 1	Mode 2	Multi.	Deg.	Path	Ratio
0	(0.246, -0.000, -0.000)	0.959	2	1	6	2	4	0.509
1	(0.152, -0.000, 0.152)	0.645	2	1	6	2	7	0.696
2	(0.143, -0.003, 0.143)	0.620	2	1	6	2	7	0.713
3	(0.133, -0.006, 0.133)	0.590	2	1	6	2	7	0.733
4	(0.213, -0.213, -0.000)	1.539	3	2	6	2	5	0.624
5	(0.500, -0.135, 0.135)	4.397	6	5	6	2	3	0.262
6	(0.292, -0.292, 0.000)	4.388	6	5	6	2	5	0.855
7	(0.198, -0.198, 0.000)	4.223	6	5	6	2	5	0.580
8	(0.500, -0.149, 0.149)	11.155	8	7	6	2	3	0.188
9	(0.500, -0.057, 0.057)	10.974	8	7	6	2	3	0.687
10	(0.325, -0.325, 0.000)	11.131	8	7	6	2	5	0.952
11	(0.291, -0.002, -0.002)	11.121	9	8	6	2	4	0.418
12	(0.306, 0.001, 0.306)	11.643	9	8	6	2	7	0.389

Supplementary Table 1161: Nodal ring points along high symmetry lines of mp-996981-Au3Tl3O6.

	Coordinates	Frequency	Mode 1	Mode 2	Multi.	Deg.	Path	Ratio
13	(0.301, -0.007, 0.301)	11.642	9	8	6	2	7	0.399
14	(0.500, -0.178, 0.178)	12.238	10	9	6	2	3	0.031
15	(0.500, -0.104, 0.103)	16.560	12	11	6	2	3	0.436

The followings are details for $Au_3Co_3O_6$. It has 6 topological nodal lines and 24 nodal ring points along high symmetry line as shown in Fig 545.

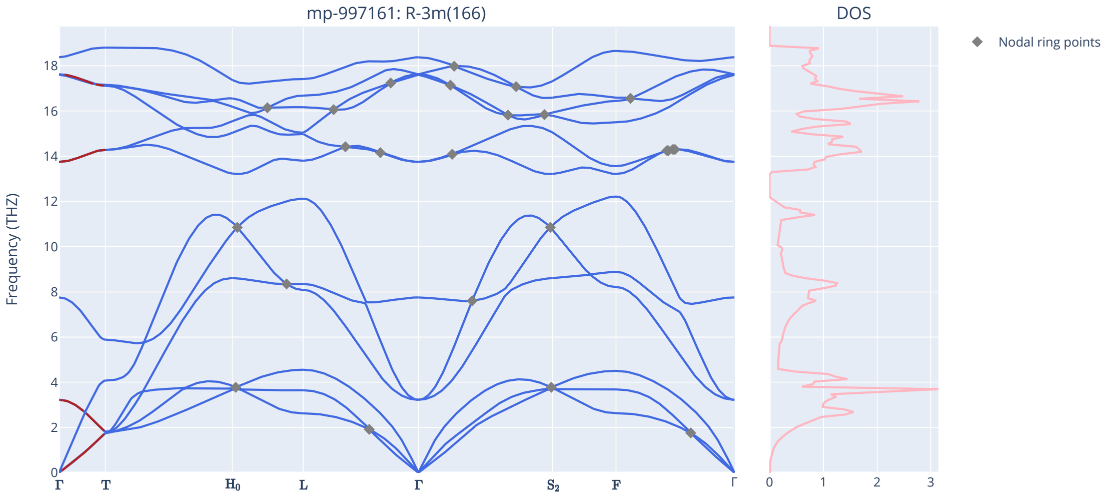

Supplementary Figure 545: The phonon band and DOS of Au3Co3O6.

Supplementary Table 1162: High symmetry lines of mp-997161-Au3Co3O6

	Start	End
0	Γ (0.000, 0.000, 0.000)	T (0.500, 0.500, 0.500)
1	T (0.500, 0.500, 0.500)	H_2 (0.821, 0.179, 0.500)
2	H_0 (0.500, -0.179, 0.179)	L (0.500, 0.000, 0.000)
3	L (0.500, 0.000, 0.000)	Γ (0.000, 0.000, 0.000)
4	Γ (0.000, 0.000, 0.000)	S_0 (0.339, -0.339, 0.000)
5	S_2 (0.661, 0.000, 0.339)	F (0.500, 0.000, 0.500)
6	F (0.500, 0.000, 0.500)	Γ (0.000, 0.000, 0.000)

Supplementary Table 1163: Nodal ring points along high symmetry lines of mp-997161-Au3Co3O6.

	Coordinates	Frequency	Mode 1	Mode 2	Multi.	Deg.	Path	Ratio
0	(0.213, 0.000, 0.000)	1.919	2	1	6	2	4	0.574
1	(0.185, -0.001, 0.185)	1.761	2	1	6	2	7	0.631
2	(0.500, -0.170, 0.170)	3.778	3	2	6	2	3	0.047
3	(0.336, -0.336, -0.000)	3.778	3	2	6	2	5	0.990
4	(0.500, -0.042, 0.042)	8.350	5	4	6	2	3	0.765
5	(0.500, -0.167, 0.167)	10.853	6	5	6	2	3	0.069
6	(0.136, -0.136, 0.000)	7.605	6	5	6	2	5	0.400
7	(0.333, -0.333, -0.000)	10.853	6	5	6	2	5	0.982
8	(0.317, -0.000, -0.000)	14.418	8	7	6	2	4	0.366
9	(0.165, 0.002, 0.002)	14.157	8	7	6	2	4	0.670
10	(0.085, -0.085, -0.000)	14.087	8	7	6	2	5	0.252
11	(0.252, -0.002, 0.252)	14.301	8	7	6	2	7	0.497
12	(0.260, 0.004, 0.260)	14.301	8	7	6	2	7	0.480

Supplementary Table 1163: Nodal ring points along high symmetry lines of mp-997161-Au3Co3O6.

	Coordinates	Frequency	Mode 1	Mode 2	Multi.	Deg.	Path	Ratio
13	(0.285, 0.001, 0.285)	14.252	8	7	6	2	7	0.430
14	(0.280, -0.006, 0.280)	14.245	8	7	6	2	7	0.441
15	(0.368, -0.000, -0.000)	16.061	10	9	6	2	4	0.264
16	(0.120, 0.001, 0.001)	17.241	10	9	6	2	4	0.760
17	(0.319, -0.319, 0.000)	15.839	10	9	6	2	5	0.939
18	(0.081, -0.081, 0.000)	17.144	10	9	6	2	5	0.238
19	(0.227, -0.227, 0.000)	15.818	10	9	6	2	5	0.667
20	(0.500, -0.090, 0.090)	16.145	11	10	6	2	3	0.494
21	(0.439, -0.001, 0.439)	16.559	11	10	6	2	7	0.121
22	(0.247, -0.247, -0.000)	17.082	12	11	6	2	5	0.728
23	(0.090, -0.090, 0.000)	17.982	12	11	6	2	5	0.267

The followings are details for Al3. It has 5 topological nodal lines and 25 nodal ring points along high symmetry line as shown in Fig **546**.

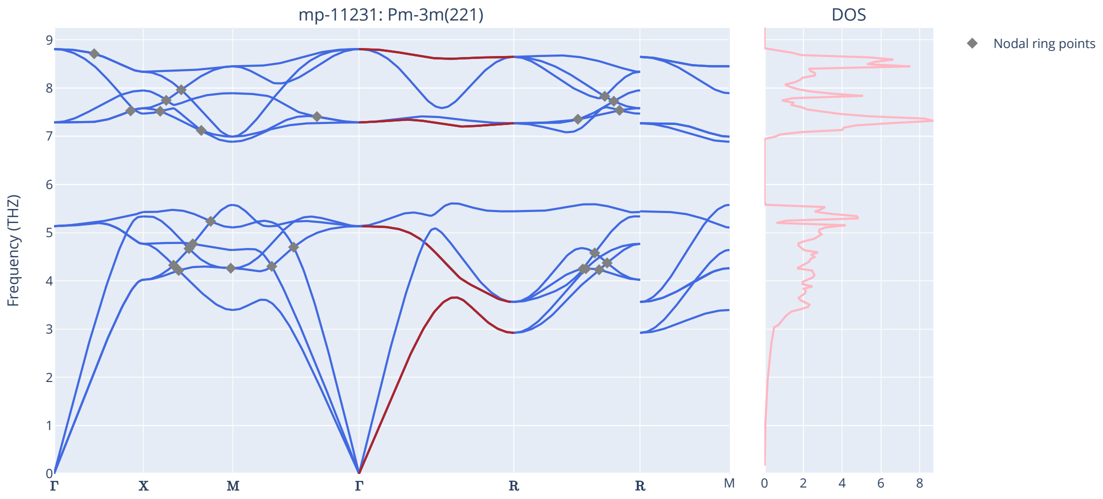

Supplementary Figure 546: The phonon band and DOS of Al3Y.

Supplementary Table 1164: High symmetry lines of mp-11231-Al3Y

	Start	End
0	Γ (0.000, 0.000, 0.000)	X (0.000, 0.500, 0.000)
1	X (0.000, 0.500, 0.000)	M (0.500, 0.500, 0.000)
2	M (0.500, 0.500, 0.000)	Γ (0.000, 0.000, 0.000)
3	Γ (0.000, 0.000, 0.000)	R (0.500, 0.500, 0.500)
4	R (0.500, 0.500, 0.500)	X (0.000, 0.500, 0.000)
5	R (0.500, 0.500, 0.500)	M (0.500, 0.500, 0.000)

Supplementary Table 1165: Nodal ring points along high symmetry lines of mp-11231-Al3Y.

	Coordinates	Frequency	Mode 1	Mode 2	Multi.	Deg.	Path	Ratio
0	(0.198, 0.500, -0.000)	4.216	2	1	12	2	2	0.395
1	(0.162, 0.500, 0.162)	4.231	2	1	12	2	5	0.676
2	(0.489, 0.500, 0.000)	4.263	3	2	12	2	2	0.978
3	(0.169, 0.500, -0.000)	4.325	3	2	12	2	2	0.337
4	(0.345, 0.345, -0.000)	4.302	3	2	12	2	3	0.309
5	(0.131, 0.500, 0.131)	4.373	3	2	12	2	5	0.739
6	(0.215, 0.500, 0.215)	4.253	3	2	12	2	5	0.571
7	(0.255, 0.500, 0.000)	4.669	4	3	12	2	2	0.510
8	(0.259, 0.259, 0.000)	4.700	4	3	12	2	3	0.482
9	(0.227, 0.500, 0.227)	4.246	4	3	12	2	5	0.546
10	(0.277, 0.500, 0.000)	4.767	5	4	12	2	2	0.553
11	(0.180, 0.500, 0.180)	4.580	5	4	12	2	5	0.641
12	(0.376, 0.500, 0.000)	5.235	6	5	12	2	2	0.753
13	(0.000, 0.429, 0.000)	7.526	8	7	6	2	1	0.857

Supplementary Table 1165: Nodal ring points along high symmetry lines of mp-11231-Al3Y.

	Coordinates	Frequency	Mode 1	Mode 2	Multi.	Deg.	Path	Ratio
14	(0.324, 0.500, -0.000)	7.120	8	7	12	2	2	0.648
15	(0.094, 0.500, -0.000)	7.519	8	7	12	2	2	0.189
16	(0.082, 0.500, 0.082)	7.538	8	7	12	2	5	0.837
17	(0.167, 0.167, 0.000)	7.407	9	8	12	2	3	0.667
18	(0.246, 0.500, 0.246)	7.353	9	8	12	2	5	0.507
19	(0.128, 0.500, -0.000)	7.745	10	9	12	2	2	0.255
20	(0.104, 0.500, 0.104)	7.727	10	9	12	2	5	0.792
21	(0.212, 0.500, -0.000)	7.963	11	10	12	2	2	0.423
22	(0.140, 0.500, 0.140)	7.828	11	10	12	2	5	0.720
23	(0.000, 0.224, 0.000)	8.711	12	11	6	2	1	0.449

The followings are details for Au3. It has 7 topological nodal lines and 16 nodal ring points along high symmetry line as shown in Fig 547.

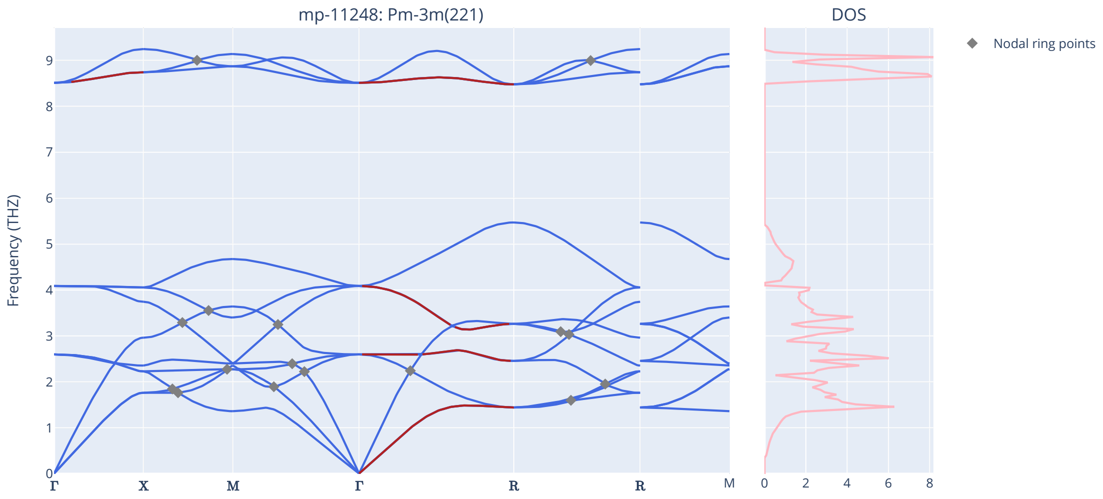

Supplementary Figure 547: The phonon band and DOS of Au3Li.

Supplementary Table 1166: High symmetry lines of mp-11248-Au3Li

	Start	End
0	Γ (0.000, 0.000, 0.000)	X (0.000, 0.500, 0.000)
1	X (0.000, 0.500, 0.000)	M (0.500, 0.500, 0.000)
2	M (0.500, 0.500, 0.000)	Γ (0.000, 0.000, 0.000)
3	Γ (0.000, 0.000, 0.000)	R (0.500, 0.500, 0.500)
4	R (0.500, 0.500, 0.500)	X (0.000, 0.500, 0.000)
5	R (0.500, 0.500, 0.500)	M (0.500, 0.500, 0.000)

Supplementary Table 1167: Nodal ring points along high symmetry lines of mp-11248-Au3Li.

	Coordinates	Frequency	Mode 1	Mode 2	Multi.	Deg.	Path	Ratio
0	(0.194, 0.500, 0.000)	1.763	2	1	12	2	2	0.388
1	(0.274, 0.500, 0.274)	1.598	2	1	12	2	5	0.453
2	(0.162, 0.500, -0.000)	1.846	3	2	12	2	2	0.325
3	(0.337, 0.337, 0.000)	1.894	3	2	12	2	3	0.325
4	(0.468, 0.500, -0.000)	2.275	4	3	12	2	2	0.937
5	(0.216, 0.216, -0.000)	2.223	4	3	12	2	3	0.567
6	(0.166, 0.166, 0.166)	2.242	4	3	8	2	4	0.331
7	(0.138, 0.500, 0.138)	1.952	4	3	12	2	5	0.724
8	(0.265, 0.265, -0.000)	2.395	5	4	12	2	3	0.471
9	(0.281, 0.500, 0.281)	3.032	6	5	12	2	5	0.437
10	(0.218, 0.500, 0.000)	3.291	7	6	12	2	2	0.436
11	(0.321, 0.321, -0.000)	3.249	7	6	12	2	3	0.358
12	(0.314, 0.500, 0.314)	3.093	7	6	12	2	5	0.371
13	(0.365, 0.500, 0.000)	3.554	8	7	12	2	2	0.730

Supplementary Table 1167: Nodal ring points along high symmetry lines of mp-11248-Au3Li.

	Coordinates	Frequency	Mode 1	Mode 2	Multi.	Deg.	Path	Ratio
14	(0.300, 0.500, -0.000)	9.002	12	11	12	2	2	0.600
15	(0.196, 0.500, 0.196)	8.995	12	11	12	2	5	0.608

The followings are details for $CaSn_3$. It has 7 topological nodal lines and 14 nodal ring points along high symmetry line as shown in Fig **548**.

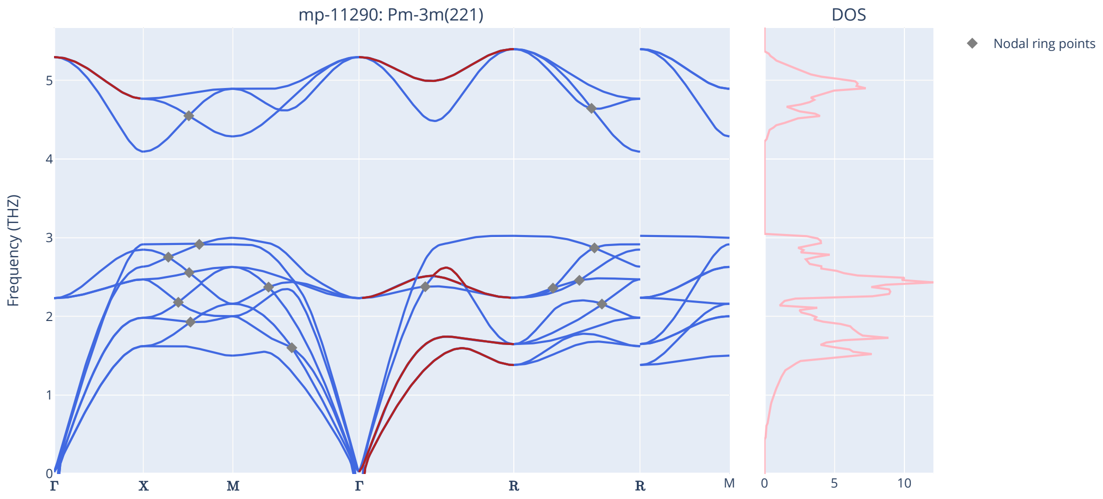

Supplementary Figure 548: The phonon band and DOS of CaSn3.

Supplementary Table 1168: High symmetry lines of mp-11290-CaSn3

	Start	End
0	Γ (0.000, 0.000, 0.000)	X (0.000, 0.500, 0.000)
1	X (0.000, 0.500, 0.000)	M (0.500, 0.500, 0.000)
2	M (0.500, 0.500, 0.000)	Γ (0.000, 0.000, 0.000)
3	Γ (0.000, 0.000, 0.000)	R (0.500, 0.500, 0.500)
4	R (0.500, 0.500, 0.500)	X (0.000, 0.500, 0.000)
5	R (0.500, 0.500, 0.500)	M (0.500, 0.500, 0.000)

Supplementary Table 1169: Nodal ring points along high symmetry lines of mp-11290-CaSn3.

	Coordinates	Frequency	Mode 1	Mode 2	Multi.	Deg.	Path	Ratio
0	(0.264, 0.500, 0.000)	1.928	3	2	12	2	2	0.527
1	(0.266, 0.266, 0.000)	1.601	3	2	12	2	3	0.468
2	(0.195, 0.500, -0.000)	2.179	5	4	12	2	2	0.391
3	(0.151, 0.500, 0.151)	2.157	5	4	12	2	5	0.698
4	(0.358, 0.358, -0.000)	2.372	6	5	12	2	3	0.284
5	(0.214, 0.214, 0.214)	2.377	6	5	8	2	4	0.428
6	(0.256, 0.500, 0.000)	2.557	7	6	12	2	2	0.512
7	(0.240, 0.500, 0.240)	2.458	7	6	12	2	5	0.520
8	(0.140, 0.500, -0.000)	2.753	8	7	12	2	2	0.279
9	(0.345, 0.500, 0.345)	2.359	8	7	12	2	5	0.310
10	(0.313, 0.500, -0.000)	2.915	9	8	12	2	2	0.626
11	(0.181, 0.500, 0.181)	2.870	9	8	12	2	5	0.639
12	(0.254, 0.500, 0.000)	4.550	11	10	12	2	2	0.508
13	(0.192, 0.500, 0.192)	4.644	11	10	12	2	5	0.616

The followings are details for CePt$_3$. It has 8 topological nodal lines and 19 nodal ring points along high symmetry line as shown in Fig **549**.

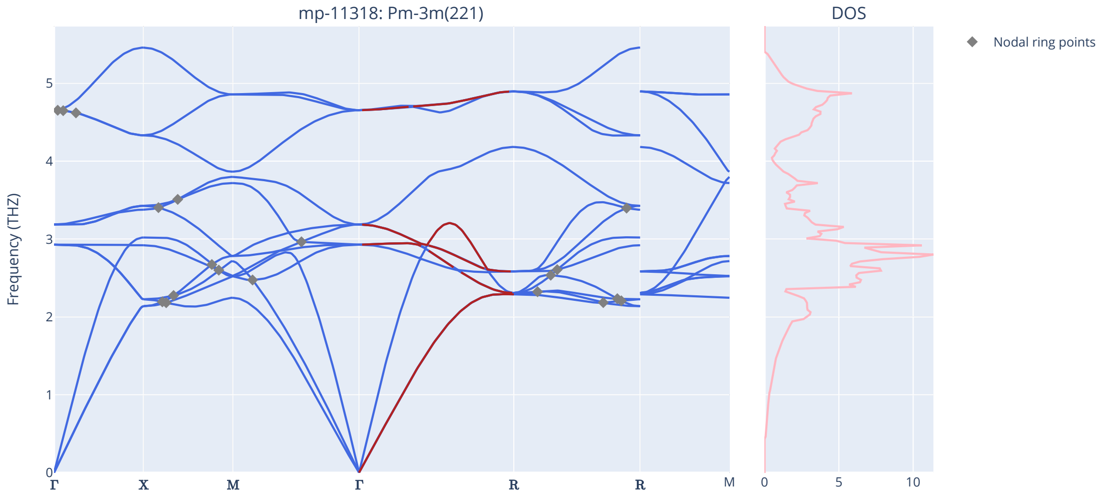

Supplementary Figure 549: The phonon band and DOS of CePt3.

Supplementary Table 1170: High symmetry lines of mp-11318-CePt3

	Start	End
0	Γ (0.000, 0.000, 0.000)	X (0.000, 0.500, 0.000)
1	X (0.000, 0.500, 0.000)	M (0.500, 0.500, 0.000)
2	M (0.500, 0.500, 0.000)	Γ (0.000, 0.000, 0.000)
3	Γ (0.000, 0.000, 0.000)	R (0.500, 0.500, 0.500)
4	R (0.500, 0.500, 0.500)	X (0.000, 0.500, 0.000)
5	R (0.500, 0.500, 0.500)	M (0.500, 0.500, 0.000)

Supplementary Table 1171: Nodal ring points along high symmetry lines of mp-11318-CePt3.

	Coordinates	Frequency	Mode 1	Mode 2	Multi.	Deg.	Path	Ratio
0	(0.128, 0.500, -0.000)	2.182	2	1	12	2	2	0.255
1	(0.146, 0.500, 0.146)	2.184	2	1	12	2	5	0.709
2	(0.106, 0.500, -0.000)	2.194	3	2	12	2	2	0.212
3	(0.422, 0.422, 0.000)	2.475	3	2	12	2	3	0.157
4	(0.406, 0.500, 0.406)	2.324	3	2	12	2	5	0.188
5	(0.074, 0.500, 0.074)	2.207	3	2	12	2	5	0.851
6	(0.169, 0.500, -0.000)	2.275	4	3	12	2	2	0.338
7	(0.090, 0.500, 0.090)	2.234	4	3	12	2	5	0.820
8	(0.422, 0.500, 0.000)	2.601	5	4	12	2	2	0.844
9	(0.354, 0.500, 0.354)	2.536	5	4	12	2	5	0.292
10	(0.383, 0.500, -0.000)	2.673	6	5	12	2	2	0.766
11	(0.328, 0.500, 0.328)	2.607	6	5	12	2	5	0.344
12	(0.228, 0.228, -0.000)	2.964	7	6	12	2	3	0.544
13	(0.084, 0.500, -0.000)	3.403	8	7	12	2	2	0.168

Supplementary Table 1171: Nodal ring points along high symmetry lines of mp-11318-CePt3.

	Coordinates	Frequency	Mode 1	Mode 2	Multi.	Deg.	Path	Ratio
14	(0.054, 0.500, 0.054)	3.397	8	7	12	2	5	0.893
15	(0.192, 0.500, -0.000)	3.509	9	8	12	2	2	0.384
16	(0.000, 0.020, 0.000)	4.653	11	10	6	2	1	0.041
17	(0.000, 0.051, 0.000)	4.648	11	10	6	2	1	0.102
18	(0.000, 0.122, 0.000)	4.620	11	10	6	2	1	0.245

The followings are details for Hg_3. It has 7 topological nodal lines and 21 nodal ring points along high symmetry line as shown in Fig **550**.

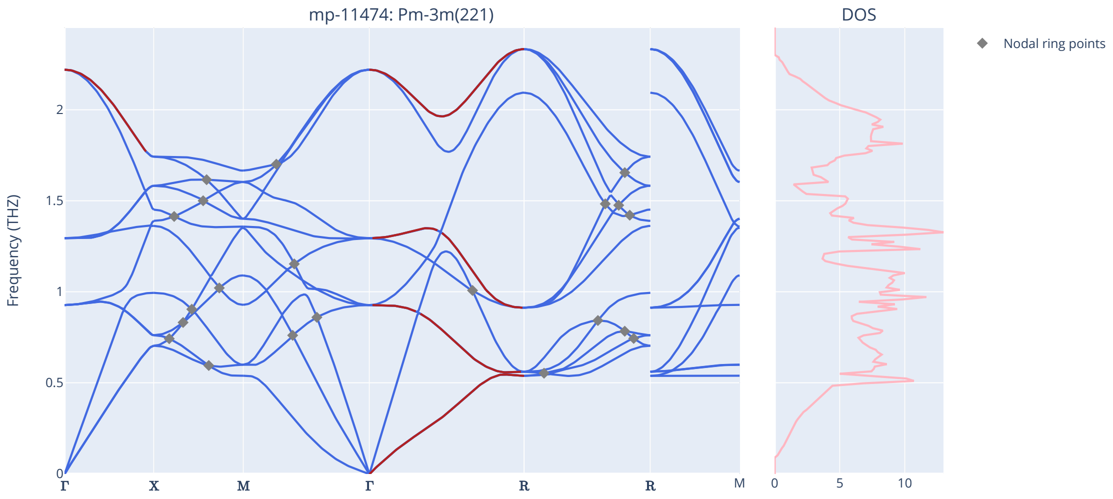

Supplementary Figure 550: The phonon band and DOS of Hg3Tl.

Supplementary Table 1172: High symmetry lines of mp-11474-Hg3Tl

	Start	End
0	Γ (0.000, 0.000, 0.000)	X (0.000, 0.500, 0.000)
1	X (0.000, 0.500, 0.000)	M (0.500, 0.500, 0.000)
2	M (0.500, 0.500, 0.000)	Γ (0.000, 0.000, 0.000)
3	Γ (0.000, 0.000, 0.000)	R (0.500, 0.500, 0.500)
4	R (0.500, 0.500, 0.500)	X (0.000, 0.500, 0.000)
5	R (0.500, 0.500, 0.500)	M (0.500, 0.500, 0.000)

Supplementary Table 1173: Nodal ring points along high symmetry lines of mp-11474-Hg3Tl.

	Coordinates	Frequency	Mode 1	Mode 2	Multi.	Deg.	Path	Ratio
0	(0.308, 0.500, 0.000)	0.595	2	1	12	2	2	0.615
1	(0.086, 0.500, -0.000)	0.742	3	2	12	2	2	0.172
2	(0.304, 0.304, 0.000)	0.760	3	2	12	2	3	0.392
3	(0.421, 0.500, 0.421)	0.552	3	2	12	2	5	0.158
4	(0.066, 0.500, 0.066)	0.743	3	2	12	2	5	0.868
5	(0.164, 0.500, -0.000)	0.831	4	3	12	2	2	0.327
6	(0.208, 0.208, 0.000)	0.859	4	3	12	2	3	0.584
7	(0.101, 0.500, 0.101)	0.783	4	3	12	2	5	0.798
8	(0.367, 0.500, -0.000)	1.021	5	4	12	2	2	0.733
9	(0.212, 0.500, 0.000)	0.904	5	4	12	2	2	0.425
10	(0.207, 0.500, 0.207)	0.842	5	4	12	2	5	0.585
11	(0.333, 0.333, 0.333)	1.007	6	5	8	2	4	0.667
12	(0.297, 0.297, -0.000)	1.153	7	6	12	2	3	0.405
13	(0.114, 0.500, -0.000)	1.414	8	7	12	2	2	0.228

Supplementary Table 1173: Nodal ring points along high symmetry lines of mp-11474-Hg3Tl.

	Coordinates	Frequency	Mode 1	Mode 2	Multi.	Deg.	Path	Ratio
14	(0.081, 0.500, 0.081)	1.421	8	7	12	2	5	0.837
15	(0.276, 0.500, 0.000)	1.500	9	8	12	2	2	0.552
16	(0.178, 0.500, 0.178)	1.483	9	8	12	2	5	0.643
17	(0.125, 0.500, 0.125)	1.475	9	8	12	2	5	0.750
18	(0.296, 0.500, -0.000)	1.616	11	10	12	2	2	0.591
19	(0.101, 0.500, 0.101)	1.654	11	10	12	2	5	0.798
20	(0.368, 0.368, -0.000)	1.701	12	11	12	2	3	0.263

The followings are details for MnNi$_3$. It has 7 topological nodal lines and 29 nodal ring points along high symmetry line as shown in Fig **551**.

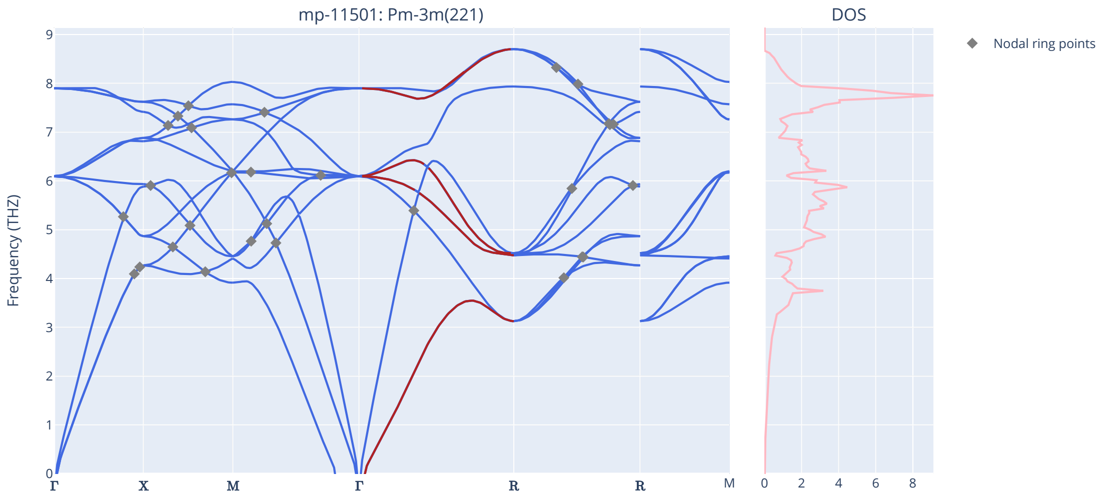

Supplementary Figure 551: The phonon band and DOS of MnNi3.

Supplementary Table 1174: High symmetry lines of mp-11501-MnNi3

	Start	End
0	Γ (0.000, 0.000, 0.000)	X (0.000, 0.500, 0.000)
1	X (0.000, 0.500, 0.000)	M (0.500, 0.500, 0.000)
2	M (0.500, 0.500, 0.000)	Γ (0.000, 0.000, 0.000)
3	Γ (0.000, 0.000, 0.000)	R (0.500, 0.500, 0.500)
4	R (0.500, 0.500, 0.500)	X (0.000, 0.500, 0.000)
5	R (0.500, 0.500, 0.500)	M (0.500, 0.500, 0.000)

Supplementary Table 1175: Nodal ring points along high symmetry lines of mp-11501-MnNi3.

	Coordinates	Frequency	Mode 1	Mode 2	Multi.	Deg.	Path	Ratio
0	(0.000, 0.449, 0.000)	4.095	2	1	6	2	1	0.898
1	(0.000, 0.480, 0.000)	4.236	2	1	6	2	1	0.959
2	(0.346, 0.500, 0.000)	4.139	2	1	12	2	2	0.693
3	(0.165, 0.500, -0.000)	4.647	3	2	12	2	2	0.330
4	(0.329, 0.329, 0.000)	4.729	3	2	12	2	3	0.342
5	(0.302, 0.500, 0.302)	4.016	3	2	12	2	5	0.396
6	(0.225, 0.500, 0.225)	4.441	3	2	12	2	5	0.550
7	(0.261, 0.500, 0.000)	5.087	4	3	12	2	2	0.522
8	(0.427, 0.427, 0.000)	4.766	4	3	12	2	3	0.146
9	(0.365, 0.365, 0.000)	5.125	4	3	12	2	3	0.269
10	(0.174, 0.184, 0.174)	5.392	4	3	24	2	4	0.354
11	(0.229, 0.500, 0.229)	4.445	4	3	12	2	5	0.542
12	(0.000, 0.388, 0.000)	5.267	5	4	6	2	1	0.776
13	(0.040, 0.500, -0.000)	5.906	6	5	12	2	2	0.080

Supplementary Table 1175: Nodal ring points along high symmetry lines of mp-11501-MnNi3.

	Coordinates	Frequency	Mode 1	Mode 2	Multi.	Deg.	Path	Ratio
14	(0.493, 0.500, 0.000)	6.167	6	5	12	2	2	0.987
15	(0.028, 0.500, 0.028)	5.906	6	5	12	2	5	0.943
16	(0.428, 0.428, 0.000)	6.181	7	6	12	2	3	0.144
17	(0.152, 0.152, 0.000)	6.116	7	6	12	2	3	0.696
18	(0.270, 0.500, 0.270)	5.845	8	7	12	2	5	0.460
19	(0.270, 0.499, -0.000)	7.089	9	8	24	2	2	0.539
20	(0.120, 0.500, 0.120)	7.152	9	8	12	2	5	0.761
21	(0.107, 0.500, 0.104)	7.160	10	9	24	2	5	0.789
22	(0.138, 0.500, -0.000)	7.137	10	9	12	2	2	0.276
23	(0.124, 0.500, 0.124)	7.175	10	9	12	2	5	0.753
24	(0.193, 0.500, -0.000)	7.329	11	10	12	2	2	0.387
25	(0.374, 0.374, -0.000)	7.407	11	10	12	2	3	0.251
26	(0.331, 0.500, 0.331)	8.323	11	10	12	2	5	0.337
27	(0.252, 0.500, 0.000)	7.540	12	11	12	2	2	0.503
28	(0.246, 0.500, 0.246)	7.984	12	11	12	2	5	0.508

The followings are details for MnZn$_3$. It has 8 topological nodal lines and 31 nodal ring points along high symmetry line as shown in Fig 552.

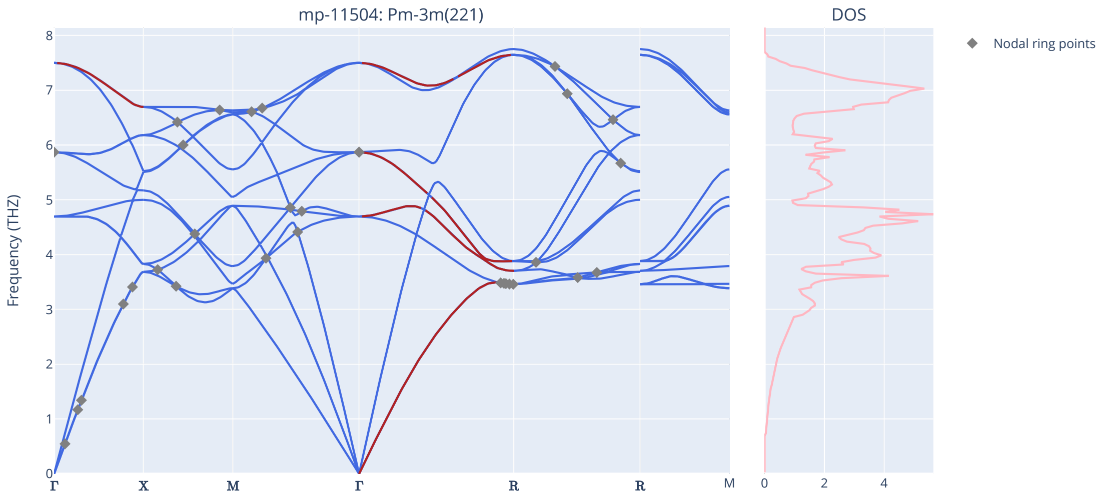

Supplementary Figure 552: The phonon band and DOS of MnZn3.

Supplementary Table 1176: High symmetry lines of mp-11504-MnZn3

	Start	End
0	Γ (0.000, 0.000, 0.000)	X (0.000, 0.500, 0.000)
1	X (0.000, 0.500, 0.000)	M (0.500, 0.500, 0.000)
2	M (0.500, 0.500, 0.000)	Γ (0.000, 0.000, 0.000)
3	Γ (0.000, 0.000, 0.000)	R (0.500, 0.500, 0.500)
4	R (0.500, 0.500, 0.500)	X (0.000, 0.500, 0.000)
5	R (0.500, 0.500, 0.500)	M (0.500, 0.500, 0.000)

Supplementary Table 1177: Nodal ring points along high symmetry lines of mp-11504-MnZn3.

	Coordinates	Frequency	Mode 1	Mode 2	Multi.	Deg.	Path	Ratio
0	(0.000, 0.061, 0.000)	0.545	2	1	6	2	1	0.122
1	(0.000, 0.133, 0.000)	1.168	2	1	6	2	1	0.265
2	(0.000, 0.153, 0.000)	1.341	2	1	6	2	1	0.306
3	(0.000, 0.388, 0.000)	3.098	2	1	6	2	1	0.776
4	(0.000, 0.439, 0.000)	3.408	2	1	6	2	1	0.878
5	(0.183, 0.500, 0.000)	3.423	2	1	12	2	2	0.366
6	(0.080, 0.500, 0.000)	3.725	3	2	12	2	2	0.160
7	(0.368, 0.368, -0.000)	3.934	3	2	12	2	3	0.263
8	(0.486, 0.486, 0.486)	3.462	3	2	8	2	4	0.972
9	(0.468, 0.468, 0.468)	3.475	3	2	8	2	4	0.937
10	(0.474, 0.474, 0.474)	3.470	3	2	8	2	4	0.949
11	(0.458, 0.458, 0.458)	3.484	3	2	8	2	4	0.915
12	(0.498, 0.498, 0.498)	3.458	3	2	8	3	4	0.997
13	(0.247, 0.500, 0.247)	3.581	3	2	12	2	5	0.506

Supplementary Table 1177: Nodal ring points along high symmetry lines of mp-11504-MnZn3.

	Coordinates	Frequency	Mode 1	Mode 2	Multi.	Deg.	Path	Ratio
14	(0.243, 0.243, -0.000)	4.412	4	3	12	2	3	0.514
15	(0.171, 0.500, 0.171)	3.674	4	3	12	2	5	0.657
16	(0.288, 0.500, -0.000)	4.377	6	5	12	2	2	0.575
17	(0.227, 0.227, -0.000)	4.791	6	5	12	2	3	0.547
18	(0.412, 0.500, 0.412)	3.859	6	5	12	2	5	0.176
19	(0.272, 0.272, -0.000)	4.857	7	6	12	2	3	0.456
20	(0.000, 0.000, 0.000)	5.870	8	7	1	3	1	0.000
21	(0.077, 0.500, 0.077)	5.668	8	7	12	2	5	0.846
22	(0.223, 0.500, -0.000)	6.000	9	8	12	2	2	0.446
23	(0.288, 0.500, 0.288)	6.936	10	9	12	2	5	0.423
24	(0.190, 0.500, -0.000)	6.419	11	10	12	2	2	0.380
25	(0.425, 0.425, -0.000)	6.607	11	10	12	2	3	0.149
26	(0.107, 0.500, 0.107)	6.462	11	10	12	2	5	0.786
27	(0.427, 0.500, 0.000)	6.639	12	11	12	2	2	0.854
28	(0.383, 0.383, -0.000)	6.674	12	11	12	2	3	0.233
29	(0.337, 0.500, 0.337)	7.434	12	11	12	2	5	0.326

The followings are details for NbRu$_3$. It has 9 topological nodal lines and 34 nodal ring points along high symmetry line as shown in Fig 553.

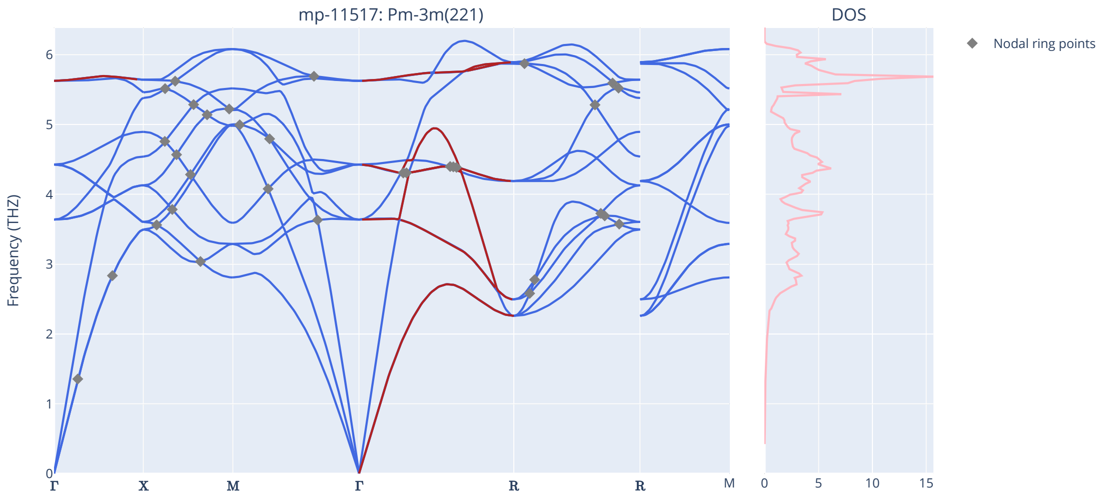

Supplementary Figure 553: The phonon band and DOS of NbRu3.

Supplementary Table 1178: High symmetry lines of mp-11517-NbRu3

	Start	End
0	Γ (0.000, 0.000, 0.000)	X (0.000, 0.500, 0.000)
1	X (0.000, 0.500, 0.000)	M (0.500, 0.500, 0.000)
2	M (0.500, 0.500, 0.000)	Γ (0.000, 0.000, 0.000)
3	Γ (0.000, 0.000, 0.000)	R (0.500, 0.500, 0.500)
4	R (0.500, 0.500, 0.500)	X (0.000, 0.500, 0.000)
5	R (0.500, 0.500, 0.500)	M (0.500, 0.500, 0.000)

Supplementary Table 1179: Nodal ring points along high symmetry lines of mp-11517-NbRu3.

	Coordinates	Frequency	Mode 1	Mode 2	Multi.	Deg.	Path	Ratio
0	(0.000, 0.133, 0.000)	1.356	2	1	6	2	1	0.265
1	(0.000, 0.327, 0.000)	2.836	2	1	6	2	1	0.653
2	(0.319, 0.500, -0.000)	3.038	2	1	12	2	2	0.639
3	(0.074, 0.500, 0.000)	3.561	3	2	12	2	2	0.149
4	(0.083, 0.500, 0.083)	3.574	3	2	12	2	5	0.835
5	(0.162, 0.500, 0.000)	3.783	4	3	12	2	2	0.323
6	(0.164, 0.164, -0.000)	3.632	4	3	12	2	3	0.672
7	(0.437, 0.500, 0.437)	2.581	4	3	12	2	5	0.126
8	(0.140, 0.500, 0.140)	3.691	4	3	12	2	5	0.720
9	(0.360, 0.360, 0.000)	4.078	5	4	12	2	3	0.280
10	(0.156, 0.500, 0.156)	3.727	5	4	12	2	5	0.689
11	(0.417, 0.500, 0.417)	2.778	5	4	12	2	5	0.165
12	(0.264, 0.499, 0.000)	4.283	6	5	24	2	2	0.527
13	(0.186, 0.500, -0.000)	4.568	7	6	12	2	2	0.371

Supplementary Table 1179: Nodal ring points along high symmetry lines of mp-11517-NbRu3.

	Coordinates	Frequency	Mode 1	Mode 2	Multi.	Deg.	Path	Ratio
14	(0.354, 0.354, -0.000)	4.794	7	6	12	2	3	0.292
15	(0.472, 0.472, -0.000)	4.994	7	6	12	2	3	0.055
16	(0.315, 0.315, 0.315)	4.386	7	6	8	2	4	0.630
17	(0.305, 0.305, 0.305)	4.393	7	6	8	2	4	0.609
18	(0.294, 0.294, 0.294)	4.397	7	6	8	2	4	0.589
19	(0.143, 0.143, 0.143)	4.306	7	6	8	2	4	0.286
20	(0.153, 0.153, 0.153)	4.307	7	6	8	2	4	0.306
21	(0.357, 0.500, -0.000)	5.138	8	7	12	2	2	0.713
22	(0.120, 0.500, -0.000)	4.757	8	7	12	2	2	0.240
23	(0.480, 0.500, -0.000)	5.221	9	8	12	2	2	0.960
24	(0.281, 0.500, -0.000)	5.284	9	8	12	2	2	0.562
25	(0.179, 0.500, 0.179)	5.280	9	8	12	2	5	0.643
26	(0.121, 0.500, 0.000)	5.513	10	9	12	2	2	0.243
27	(0.458, 0.498, 0.458)	5.870	10	9	24	2	5	0.085
28	(0.085, 0.500, 0.085)	5.520	10	9	12	2	5	0.829
29	(0.178, 0.500, -0.000)	5.620	11	10	12	2	2	0.355
30	(0.109, 0.500, 0.109)	5.592	11	10	12	2	5	0.782
31	(0.178, 0.178, -0.000)	5.689	12	11	12	2	3	0.643

The followings are details for Ni3. It has 8 topological nodal lines and 21 nodal ring points along high symmetry line as shown in Fig 554.

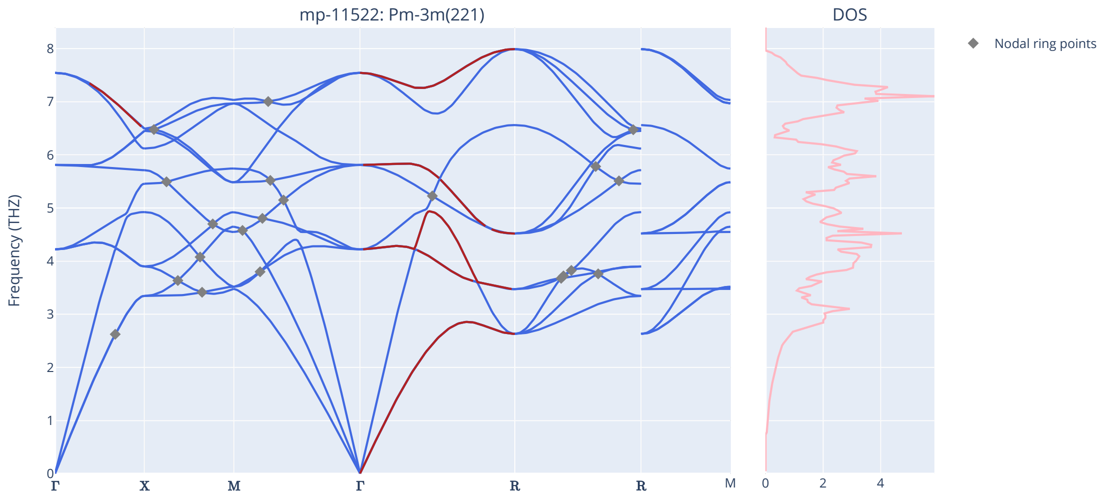

Supplementary Figure 554: The phonon band and DOS of Ni3Sn.

Supplementary Table 1180: High symmetry lines of mp-11522-Ni3Sn

	Start	End
0	Γ (0.000, 0.000, 0.000)	X (0.000, 0.500, 0.000)
1	X (0.000, 0.500, 0.000)	M (0.500, 0.500, 0.000)
2	M (0.500, 0.500, 0.000)	Γ (0.000, 0.000, 0.000)
3	Γ (0.000, 0.000, 0.000)	R (0.500, 0.500, 0.500)
4	R (0.500, 0.500, 0.500)	X (0.000, 0.500, 0.000)
5	R (0.500, 0.500, 0.500)	M (0.500, 0.500, 0.000)

Supplementary Table 1181: Nodal ring points along high symmetry lines of mp-11522-Ni3Sn.

	Coordinates	Frequency	Mode 1	Mode 2	Multi.	Deg.	Path	Ratio
0	(0.000, 0.337, 0.000)	2.624	2	1	6	2	1	0.673
1	(0.323, 0.500, 0.000)	3.414	2	1	12	2	2	0.646
2	(0.187, 0.500, -0.000)	3.635	3	2	12	2	2	0.374
3	(0.396, 0.396, -0.000)	3.800	3	2	12	2	3	0.209
4	(0.170, 0.500, 0.170)	3.761	3	2	12	2	5	0.660
5	(0.312, 0.500, 0.000)	4.077	4	3	12	2	2	0.623
6	(0.316, 0.500, 0.316)	3.677	4	3	12	2	5	0.367
7	(0.465, 0.465, 0.000)	4.578	5	4	12	2	3	0.069
8	(0.307, 0.500, 0.307)	3.722	5	4	12	2	5	0.386
9	(0.276, 0.500, 0.276)	3.825	5	4	12	2	5	0.448
10	(0.383, 0.500, -0.000)	4.700	6	5	12	2	2	0.765
11	(0.387, 0.387, 0.000)	4.805	6	5	12	2	3	0.227
12	(0.124, 0.500, -0.000)	5.493	7	6	12	2	2	0.247
13	(0.303, 0.303, -0.000)	5.149	7	6	12	2	3	0.394

Supplementary Table 1181: Nodal ring points along high symmetry lines of mp-11522-Ni3Sn.

	Coordinates	Frequency	Mode 1	Mode 2	Multi.	Deg.	Path	Ratio
14	(0.234, 0.234, 0.234)	5.226	7	6	8	2	4	0.467
15	(0.088, 0.500, 0.088)	5.510	7	6	12	2	5	0.825
16	(0.355, 0.355, -0.000)	5.519	8	7	12	2	3	0.290
17	(0.180, 0.500, 0.180)	5.781	8	7	12	2	5	0.640
18	(0.053, 0.500, -0.000)	6.476	11	10	12	2	2	0.106
19	(0.031, 0.500, 0.031)	6.472	11	10	12	2	5	0.938
20	(0.364, 0.364, -0.000)	7.003	12	11	12	2	3	0.273

The followings are details for Ir$_3$. It has 6 topological nodal lines and 30 nodal ring points along high symmetry line as shown in Fig 555.

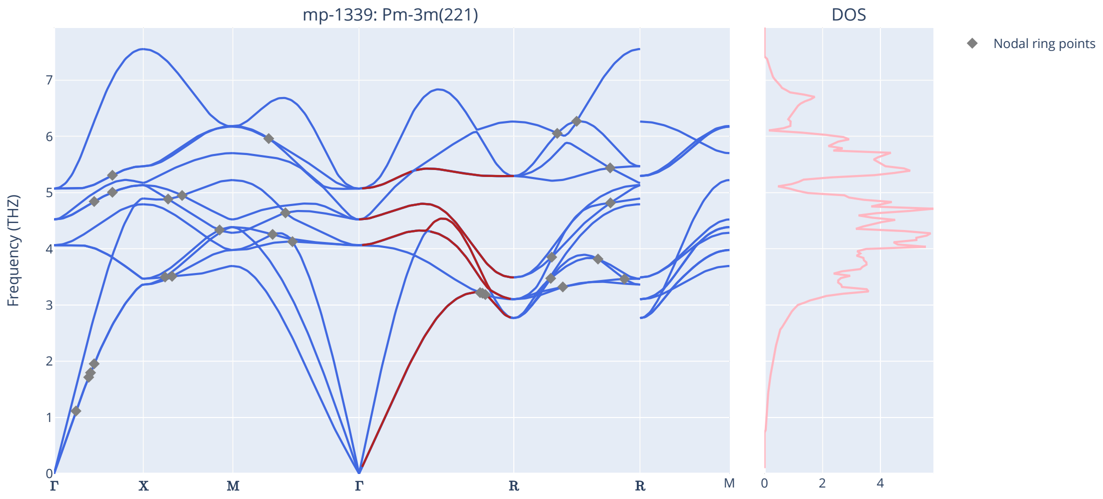

Supplementary Figure 555: The phonon band and DOS of Ir3Nb.

Supplementary Table 1182: High symmetry lines of mp-1339-Ir3Nb

	Start	End
0	Γ (0.000, 0.000, 0.000)	X (0.000, 0.500, 0.000)
1	X (0.000, 0.500, 0.000)	M (0.500, 0.500, 0.000)
2	M (0.500, 0.500, 0.000)	Γ (0.000, 0.000, 0.000)
3	Γ (0.000, 0.000, 0.000)	R (0.500, 0.500, 0.500)
4	R (0.500, 0.500, 0.500)	X (0.000, 0.500, 0.000)
5	R (0.500, 0.500, 0.500)	M (0.500, 0.500, 0.000)

Supplementary Table 1183: Nodal ring points along high symmetry lines of mp-1339-Ir3Nb.

	Coordinates	Frequency	Mode 1	Mode 2	Multi.	Deg.	Path	Ratio
0	(0.000, 0.122, 0.000)	1.115	2	1	6	2	1	0.245
1	(0.000, 0.194, 0.000)	1.716	2	1	6	2	1	0.388
2	(0.000, 0.204, 0.000)	1.797	2	1	6	2	1	0.408
3	(0.000, 0.224, 0.000)	1.955	2	1	6	2	1	0.449
4	(0.160, 0.500, -0.000)	3.511	2	1	12	2	2	0.321
5	(0.306, 0.500, 0.306)	3.322	2	1	12	2	5	0.388
6	(0.121, 0.500, -0.000)	3.499	3	2	12	2	2	0.243
7	(0.399, 0.399, 0.399)	3.210	3	2	8	2	4	0.798
8	(0.391, 0.391, 0.391)	3.221	3	2	8	2	4	0.782
9	(0.408, 0.408, 0.408)	3.186	3	2	8	2	4	0.816
10	(0.062, 0.499, 0.062)	3.463	3	2	24	2	5	0.876
11	(0.264, 0.264, -0.000)	4.128	4	3	12	2	3	0.473
12	(0.167, 0.500, 0.167)	3.818	4	3	12	2	5	0.667
13	(0.354, 0.500, 0.354)	3.474	4	3	12	2	5	0.292

Supplementary Table 1183: Nodal ring points along high symmetry lines of mp-1339-Ir3Nb.

	Coordinates	Frequency	Mode 1	Mode 2	Multi.	Deg.	Path	Ratio
14	(0.342, 0.342, -0.000)	4.256	5	4	12	2	3	0.316
15	(0.427, 0.500, -0.000)	4.335	6	5	12	2	2	0.853
16	(0.138, 0.500, 0.000)	4.885	7	6	12	2	2	0.276
17	(0.291, 0.291, -0.000)	4.639	7	6	12	2	3	0.417
18	(0.350, 0.500, 0.350)	3.852	7	6	12	2	5	0.301
19	(0.117, 0.500, 0.117)	4.817	7	6	12	2	5	0.765
20	(0.000, 0.224, 0.000)	4.841	8	7	6	2	1	0.449
21	(0.000, 0.327, 0.000)	5.006	8	7	6	2	1	0.653
22	(0.217, 0.500, -0.000)	4.948	8	7	12	2	2	0.433
23	(0.114, 0.500, 0.122)	5.439	10	9	24	2	5	0.763
24	(0.000, 0.327, 0.000)	5.308	11	10	6	2	1	0.653
25	(0.358, 0.358, -0.000)	5.960	11	10	12	2	3	0.285
26	(0.328, 0.500, 0.328)	6.053	12	11	12	2	5	0.344
27	(0.251, 0.500, 0.251)	6.268	12	11	12	2	5	0.497

The followings are details for $CeSn_3$. It has 6 topological nodal lines and 27 nodal ring points along high symmetry line as shown in Fig **556**.

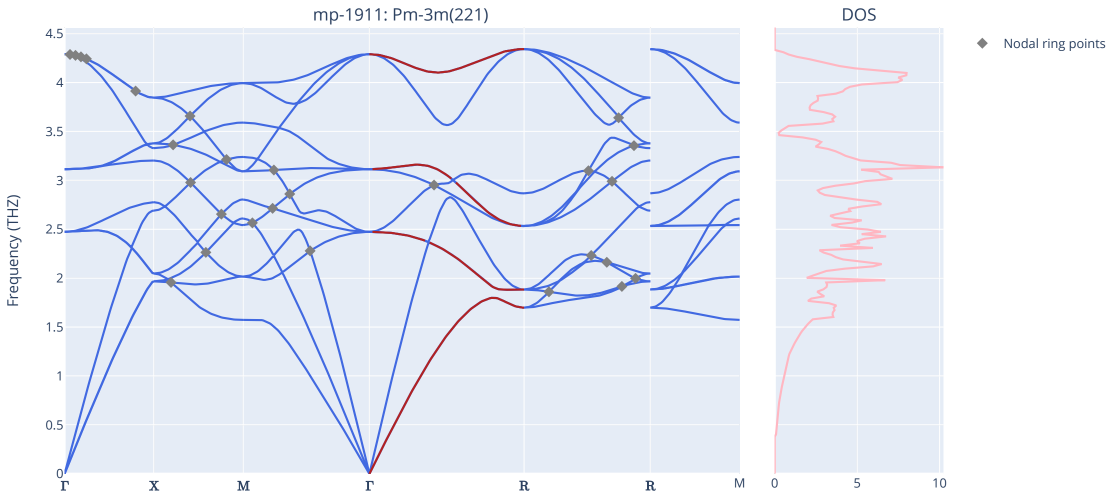

Supplementary Figure 556: The phonon band and DOS of CeSn3.

Supplementary Table 1184: High symmetry lines of mp-1911-CeSn3

	Start	End
0	Γ (0.000, 0.000, 0.000)	X (0.000, 0.500, 0.000)
1	X (0.000, 0.500, 0.000)	M (0.500, 0.500, 0.000)
2	M (0.500, 0.500, 0.000)	Γ (0.000, 0.000, 0.000)
3	Γ (0.000, 0.000, 0.000)	R (0.500, 0.500, 0.500)
4	R (0.500, 0.500, 0.500)	X (0.000, 0.500, 0.000)
5	R (0.500, 0.500, 0.500)	M (0.500, 0.500, 0.000)

Supplementary Table 1185: Nodal ring points along high symmetry lines of mp-1911-CeSn3.

	Coordinates	Frequency	Mode 1	Mode 2	Multi.	Deg.	Path	Ratio
0	(0.096, 0.500, 0.000)	1.956	2	1	12	2	2	0.193
1	(0.113, 0.500, 0.113)	1.915	2	1	12	2	5	0.775
2	(0.399, 0.500, 0.404)	1.860	3	2	24	2	5	0.197
3	(0.059, 0.500, 0.059)	1.998	3	2	12	2	5	0.883
4	(0.291, 0.500, -0.000)	2.263	4	3	12	2	2	0.583
5	(0.235, 0.235, -0.000)	2.279	4	3	12	2	3	0.530
6	(0.172, 0.500, 0.172)	2.162	4	3	12	2	5	0.656
7	(0.463, 0.463, 0.000)	2.565	5	4	12	2	3	0.073
8	(0.234, 0.500, 0.234)	2.232	5	4	12	2	5	0.532
9	(0.378, 0.500, 0.000)	2.655	6	5	12	2	2	0.757
10	(0.383, 0.383, 0.000)	2.714	6	5	12	2	3	0.234
11	(0.206, 0.500, -0.000)	2.978	7	6	12	2	2	0.411
12	(0.315, 0.315, 0.000)	2.860	7	6	12	2	3	0.369
13	(0.209, 0.209, 0.209)	2.952	7	6	8	2	4	0.418

Supplementary Table 1185: Nodal ring points along high symmetry lines of mp-1911-CeSn3.

	Coordinates	Frequency	Mode 1	Mode 2	Multi.	Deg.	Path	Ratio
14	(0.152, 0.500, 0.152)	2.990	7	6	12	2	5	0.697
15	(0.378, 0.378, -0.000)	3.105	8	7	12	2	3	0.244
16	(0.108, 0.500, -0.000)	3.363	9	8	12	2	2	0.216
17	(0.407, 0.499, -0.000)	3.213	9	8	24	2	2	0.814
18	(0.245, 0.500, 0.245)	3.098	9	8	12	2	5	0.510
19	(0.065, 0.500, 0.065)	3.355	9	8	12	2	5	0.871
20	(0.203, 0.500, 0.000)	3.658	11	10	12	2	2	0.406
21	(0.125, 0.500, 0.125)	3.640	11	10	12	2	5	0.749
22	(0.000, 0.031, 0.000)	4.287	12	11	6	2	1	0.061
23	(0.000, 0.061, 0.000)	4.278	12	11	6	2	1	0.122
24	(0.000, 0.092, 0.000)	4.263	12	11	6	2	1	0.184
25	(0.000, 0.122, 0.000)	4.242	12	11	6	2	1	0.245
26	(0.000, 0.398, 0.000)	3.913	12	11	6	2	1	0.796

The followings are details for In$_3$. It has 6 topological nodal lines and 18 nodal ring points along high symmetry line as shown in Fig 557.

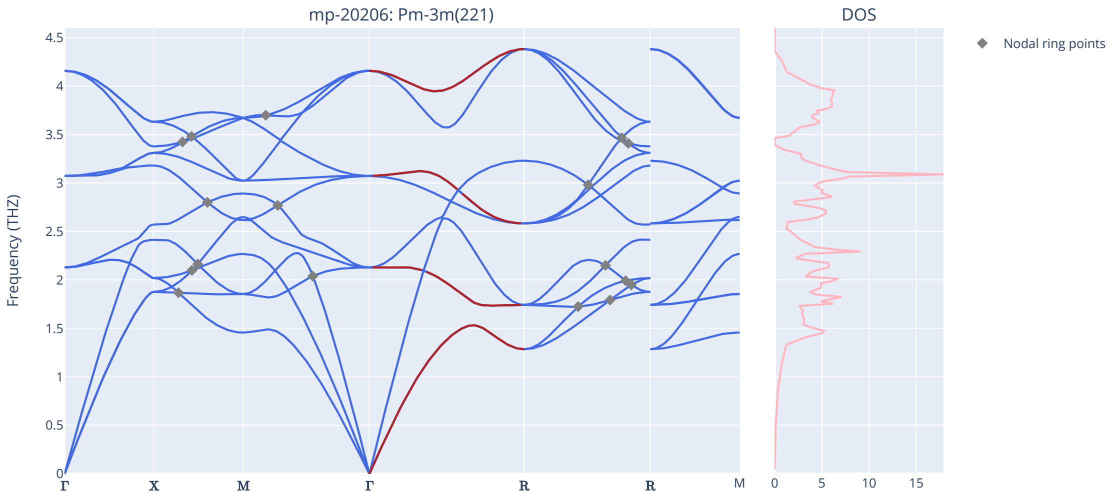

Supplementary Figure 557: The phonon band and DOS of In3Th.

Supplementary Table 1186: High symmetry lines of mp-20206-In3Th

	Start	End
0	Γ (0.000, 0.000, 0.000)	X (0.000, 0.500, 0.000)
1	X (0.000, 0.500, 0.000)	M (0.500, 0.500, 0.000)
2	M (0.500, 0.500, 0.000)	Γ (0.000, 0.000, 0.000)
3	Γ (0.000, 0.000, 0.000)	R (0.500, 0.500, 0.500)
4	R (0.500, 0.500, 0.500)	X (0.000, 0.500, 0.000)
5	R (0.500, 0.500, 0.500)	M (0.500, 0.500, 0.000)

Supplementary Table 1187: Nodal ring points along high symmetry lines of mp-20206-In3Th.

	Coordinates	Frequency	Mode 1	Mode 2	Multi.	Deg.	Path	Ratio
0	(0.138, 0.500, -0.000)	1.868	2	1	12	2	2	0.276
1	(0.159, 0.500, 0.159)	1.793	2	1	12	2	5	0.682
2	(0.286, 0.500, 0.286)	1.725	3	2	12	2	5	0.429
3	(0.075, 0.500, 0.075)	1.949	3	2	12	2	5	0.850
4	(0.213, 0.500, 0.000)	2.099	4	3	12	2	2	0.427
5	(0.224, 0.224, -0.000)	2.039	4	3	12	2	3	0.552
6	(0.097, 0.500, 0.097)	1.991	4	3	12	2	5	0.805
7	(0.246, 0.500, 0.000)	2.164	5	4	12	2	2	0.491
8	(0.177, 0.500, 0.177)	2.150	5	4	12	2	5	0.645
9	(0.300, 0.500, -0.000)	2.800	7	6	12	2	2	0.600
10	(0.363, 0.363, -0.000)	2.769	7	6	12	2	3	0.274
11	(0.246, 0.500, 0.246)	2.981	9	8	12	2	5	0.508
12	(0.161, 0.500, -0.000)	3.424	10	9	12	2	2	0.322
13	(0.087, 0.500, 0.087)	3.409	10	9	12	2	5	0.826

Supplementary Table 1187: Nodal ring points along high symmetry lines of mp-20206-In3Th.

	Coordinates	Frequency	Mode 1	Mode 2	Multi.	Deg.	Path	Ratio
14	(0.211, 0.500, -0.000)	3.481	11	10	12	2	2	0.422
15	(0.113, 0.500, 0.113)	3.467	11	10	12	2	5	0.774
16	(0.410, 0.410, 0.000)	3.699	12	11	12	2	3	0.179

The followings are details for Ga$_3$. It has 6 topological nodal lines and 20 nodal ring points along high symmetry line as shown in Fig **558**.

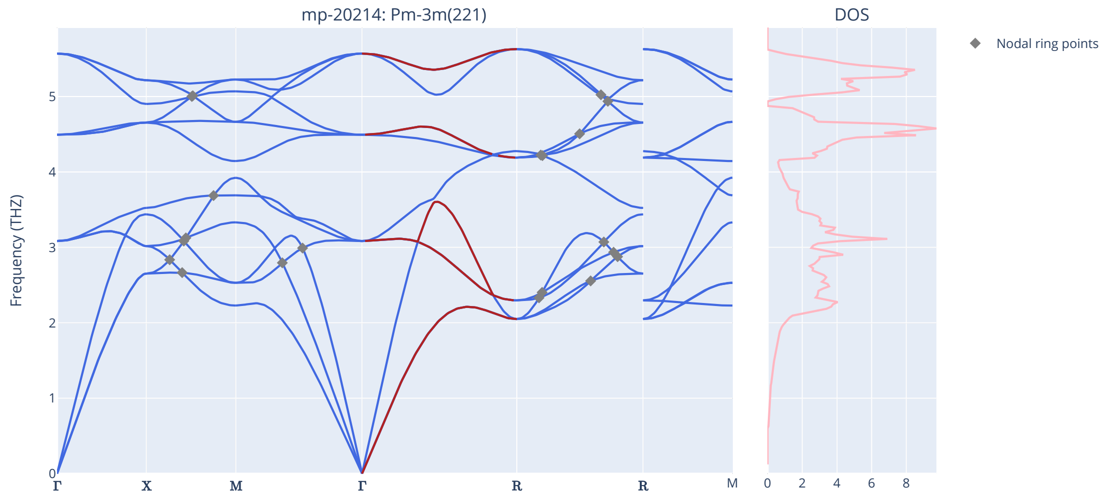

Supplementary Figure 558: The phonon band and DOS of Ga3U.

Supplementary Table 1188: High symmetry lines of mp-20214-Ga3U

	Start	End
0	Γ (0.000, 0.000, 0.000)	X (0.000, 0.500, 0.000)
1	X (0.000, 0.500, 0.000)	M (0.500, 0.500, 0.000)
2	M (0.500, 0.500, 0.000)	Γ (0.000, 0.000, 0.000)
3	Γ (0.000, 0.000, 0.000)	R (0.500, 0.500, 0.500)
4	R (0.500, 0.500, 0.500)	X (0.000, 0.500, 0.000)
5	R (0.500, 0.500, 0.500)	M (0.500, 0.500, 0.000)

Supplementary Table 1189: Nodal ring points along high symmetry lines of mp-20214-Ga3U.

	Coordinates	Frequency	Mode 1	Mode 2	Multi.	Deg.	Path	Ratio
0	(0.200, 0.500, 0.000)	2.666	2	1	12	2	2	0.400
1	(0.208, 0.500, 0.208)	2.556	2	1	12	2	5	0.585
2	(0.130, 0.500, 0.000)	2.837	3	2	12	2	2	0.260
3	(0.316, 0.316, 0.000)	2.796	3	2	12	2	3	0.369
4	(0.099, 0.500, 0.103)	2.877	3	2	24	2	5	0.798
5	(0.209, 0.500, -0.000)	3.085	4	3	12	2	2	0.419
6	(0.235, 0.235, -0.000)	2.992	4	3	12	2	3	0.529
7	(0.412, 0.500, 0.412)	2.332	4	3	12	2	5	0.176
8	(0.117, 0.500, 0.117)	2.936	4	3	12	2	5	0.766
9	(0.221, 0.500, -0.000)	3.126	5	4	12	2	2	0.442
10	(0.400, 0.500, 0.400)	2.404	5	4	12	2	5	0.201
11	(0.155, 0.500, 0.155)	3.072	5	4	12	2	5	0.689
12	(0.376, 0.500, 0.000)	3.687	6	5	12	2	2	0.752
13	(0.398, 0.500, 0.398)	4.217	7	6	12	2	5	0.203

Supplementary Table 1189: Nodal ring points along high symmetry lines of mp-20214-Ga3U.

	Coordinates	Frequency	Mode 1	Mode 2	Multi.	Deg.	Path	Ratio
14	(0.405, 0.500, 0.405)	4.224	8	7	12	2	5	0.190
15	(0.251, 0.500, 0.251)	4.506	9	8	12	2	5	0.498
16	(0.254, 0.500, 0.000)	5.001	10	9	12	2	2	0.509
17	(0.139, 0.500, 0.139)	4.937	10	9	12	2	5	0.721
18	(0.260, 0.500, 0.001)	5.010	11	10	24	2	2	0.520
19	(0.167, 0.500, 0.167)	5.023	11	10	12	2	5	0.666

The followings are details for $CePb_3$. It has 8 topological nodal lines and 24 nodal ring points along high symmetry line as shown in Fig **559**.

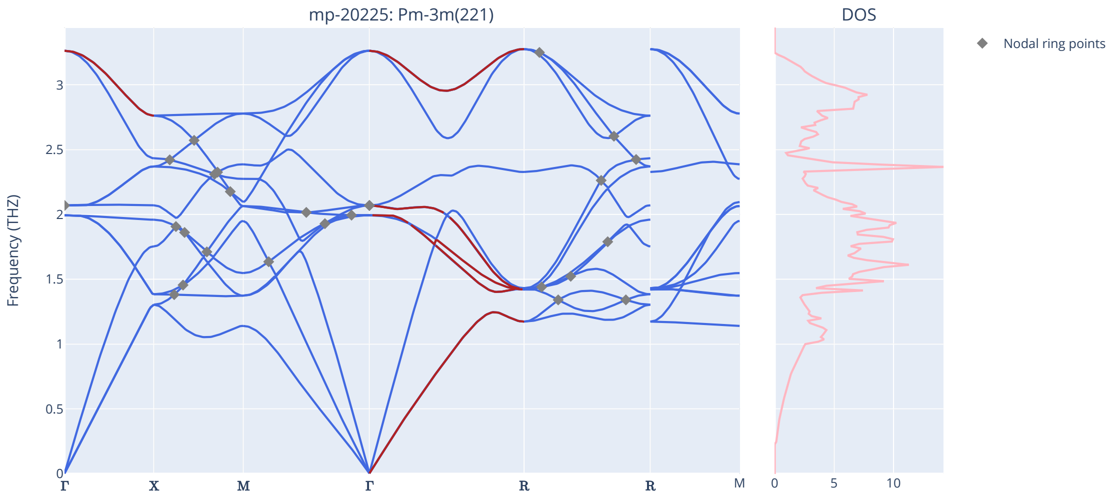

Supplementary Figure 559: The phonon band and DOS of CePb3.

Supplementary Table 1190: High symmetry lines of mp-20225-CePb3

	Start	End
0	Γ (0.000, 0.000, 0.000)	X (0.000, 0.500, 0.000)
1	X (0.000, 0.500, 0.000)	M (0.500, 0.500, 0.000)
2	M (0.500, 0.500, 0.000)	Γ (0.000, 0.000, 0.000)
3	Γ (0.000, 0.000, 0.000)	R (0.500, 0.500, 0.500)
4	R (0.500, 0.500, 0.500)	X (0.000, 0.500, 0.000)
5	R (0.500, 0.500, 0.500)	M (0.500, 0.500, 0.000)

Supplementary Table 1191: Nodal ring points along high symmetry lines of mp-20225-CePb3.

	Coordinates	Frequency	Mode 1	Mode 2	Multi.	Deg.	Path	Ratio
0	(0.114, 0.500, -0.000)	1.382	3	2	12	2	2	0.228
1	(0.365, 0.500, 0.365)	1.341	3	2	12	2	5	0.271
2	(0.097, 0.500, 0.097)	1.341	3	2	12	2	5	0.806
3	(0.164, 0.500, -0.000)	1.455	4	3	12	2	2	0.327
4	(0.399, 0.399, -0.000)	1.635	5	4	12	2	3	0.202
5	(0.315, 0.500, 0.315)	1.523	5	4	12	2	5	0.370
6	(0.172, 0.500, -0.000)	1.861	6	5	12	2	2	0.344
7	(0.124, 0.500, -0.000)	1.907	6	5	12	2	2	0.247
8	(0.297, 0.500, 0.000)	1.711	6	5	12	2	2	0.594
9	(0.176, 0.176, 0.000)	1.929	6	5	12	2	3	0.648
10	(0.071, 0.071, 0.000)	1.995	6	5	12	2	3	0.858
11	(0.430, 0.498, 0.430)	1.440	6	5	24	2	5	0.140
12	(0.169, 0.500, 0.169)	1.789	6	5	12	2	5	0.663
13	(0.000, 0.000, 0.000)	2.069	8	7	1	3	1	0.000

Supplementary Table 1191: Nodal ring points along high symmetry lines of mp-20225-CePb3.

	Coordinates	Frequency	Mode 1	Mode 2	Multi.	Deg.	Path	Ratio
14	(0.428, 0.500, -0.000)	2.176	8	7	12	2	2	0.856
15	(0.250, 0.250, -0.000)	2.016	8	7	12	2	3	0.500
16	(0.340, 0.500, 0.000)	2.310	9	8	12	2	2	0.680
17	(0.195, 0.500, 0.195)	2.263	9	8	12	2	5	0.611
18	(0.089, 0.500, -0.000)	2.421	10	9	12	2	2	0.178
19	(0.357, 0.500, -0.001)	2.326	10	9	24	2	2	0.713
20	(0.056, 0.500, 0.056)	2.424	10	9	12	2	5	0.888
21	(0.226, 0.500, -0.000)	2.571	11	10	12	2	2	0.451
22	(0.144, 0.500, 0.144)	2.603	11	10	12	2	5	0.712
23	(0.439, 0.500, 0.439)	3.249	12	11	12	2	5	0.122

The followings are details for Sn$_3$. It has 6 topological nodal lines and 21 nodal ring points along high symmetry line as shown in Fig 560.

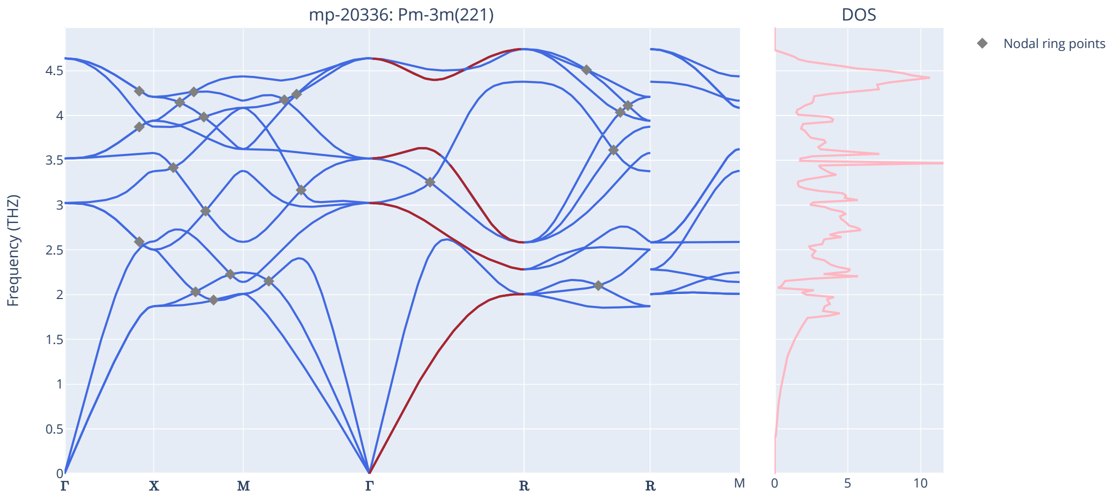

Supplementary Figure 560: The phonon band and DOS of Sn3U.

Supplementary Table 1192: High symmetry lines of mp-20336-Sn3U

	Start	End
0	Γ (0.000, 0.000, 0.000)	X (0.000, 0.500, 0.000)
1	X (0.000, 0.500, 0.000)	M (0.500, 0.500, 0.000)
2	M (0.500, 0.500, 0.000)	Γ (0.000, 0.000, 0.000)
3	Γ (0.000, 0.000, 0.000)	R (0.500, 0.500, 0.500)
4	R (0.500, 0.500, 0.500)	X (0.000, 0.500, 0.000)
5	R (0.500, 0.500, 0.500)	M (0.500, 0.500, 0.000)

Supplementary Table 1193: Nodal ring points along high symmetry lines of mp-20336-Sn3U.

	Coordinates	Frequency	Mode 1	Mode 2	Multi.	Deg.	Path	Ratio
0	(0.334, 0.500, -0.000)	1.940	2	1	12	2	2	0.669
1	(0.234, 0.499, 0.000)	2.031	3	2	24	2	2	0.467
2	(0.398, 0.398, 0.000)	2.150	3	2	12	2	3	0.203
3	(0.206, 0.500, 0.206)	2.101	3	2	12	2	5	0.588
4	(0.429, 0.500, -0.000)	2.227	4	3	12	2	2	0.857
5	(0.000, 0.418, 0.000)	2.590	5	4	6	2	1	0.837
6	(0.290, 0.500, 0.000)	2.933	6	5	12	2	2	0.579
7	(0.109, 0.500, -0.000)	3.418	7	6	12	2	2	0.218
8	(0.270, 0.270, -0.000)	3.168	7	6	12	2	3	0.460
9	(0.196, 0.196, 0.196)	3.255	7	6	8	2	4	0.393
10	(0.146, 0.500, 0.146)	3.614	8	7	12	2	5	0.708
11	(0.000, 0.418, 0.000)	3.872	9	8	6	2	1	0.837
12	(0.280, 0.500, 0.000)	3.983	10	9	12	2	2	0.560
13	(0.119, 0.500, 0.119)	4.035	10	9	12	2	5	0.762

Supplementary Table 1193: Nodal ring points along high symmetry lines of mp-20336-Sn3U.

	Coordinates	Frequency	Mode 1	Mode 2	Multi.	Deg.	Path	Ratio
14	(0.145, 0.500, -0.000)	4.146	11	10	12	2	2	0.290
15	(0.337, 0.337, -0.000)	4.174	11	10	12	2	3	0.326
16	(0.288, 0.288, -0.000)	4.237	11	10	12	2	3	0.423
17	(0.089, 0.500, 0.089)	4.111	11	10	12	2	5	0.823
18	(0.000, 0.418, 0.000)	4.272	12	11	6	2	1	0.837
19	(0.224, 0.500, 0.000)	4.263	12	11	12	2	2	0.448
20	(0.253, 0.500, 0.253)	4.509	12	11	12	2	5	0.495

The followings are details for CeIn$_3$. It has 8 topological nodal lines and 22 nodal ring points along high symmetry line as shown in Fig **561**.

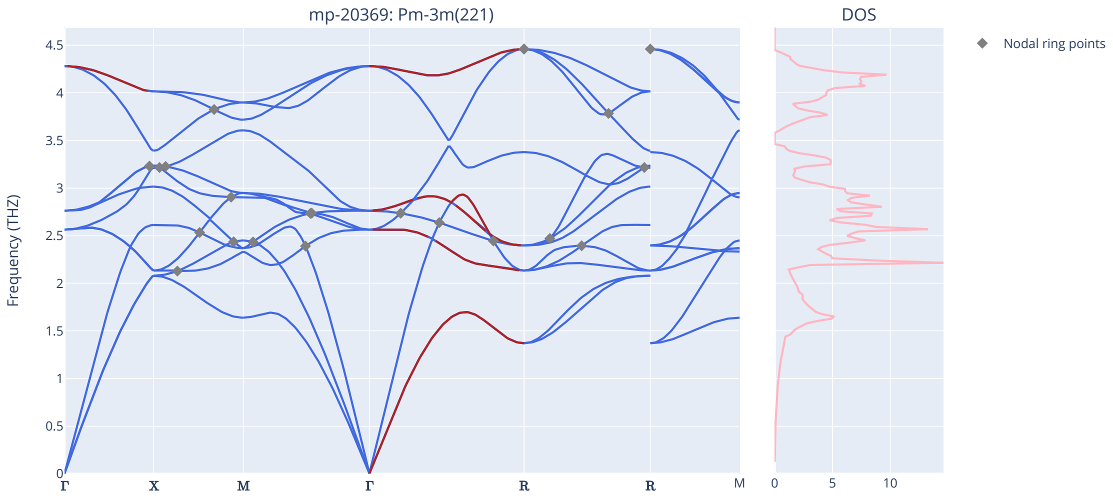

Supplementary Figure 561: The phonon band and DOS of CeIn3.

Supplementary Table 1194: High symmetry lines of mp-20369-CeIn3

	Start	End
0	Γ (0.000, 0.000, 0.000)	X (0.000, 0.500, 0.000)
1	X (0.000, 0.500, 0.000)	M (0.500, 0.500, 0.000)
2	M (0.500, 0.500, 0.000)	Γ (0.000, 0.000, 0.000)
3	Γ (0.000, 0.000, 0.000)	R (0.500, 0.500, 0.500)
4	R (0.500, 0.500, 0.500)	X (0.000, 0.500, 0.000)
5	R (0.500, 0.500, 0.500)	M (0.500, 0.500, 0.000)

Supplementary Table 1195: Nodal ring points along high symmetry lines of mp-20369-CeIn3.

	Coordinates	Frequency	Mode 1	Mode 2	Multi.	Deg.	Path	Ratio
0	(0.132, 0.500, 0.000)	2.128	3	2	12	2	2	0.263
1	(0.253, 0.253, -0.000)	2.391	4	3	12	2	3	0.494
2	(0.447, 0.500, 0.000)	2.436	5	4	12	2	2	0.894
3	(0.257, 0.500, 0.000)	2.533	5	4	12	2	2	0.513
4	(0.461, 0.461, -0.000)	2.434	5	4	12	2	3	0.078
5	(0.272, 0.500, 0.272)	2.394	5	4	12	2	5	0.456
6	(0.233, 0.233, 0.000)	2.734	6	5	12	2	3	0.533
7	(0.401, 0.401, 0.401)	2.445	6	5	8	2	4	0.802
8	(0.226, 0.226, 0.226)	2.637	6	5	8	2	4	0.452
9	(0.433, 0.500, 0.000)	2.905	7	6	12	2	2	0.866
10	(0.228, 0.228, -0.000)	2.736	7	6	12	2	3	0.543
11	(0.101, 0.101, 0.101)	2.736	7	6	8	2	4	0.202
12	(0.032, 0.500, -0.000)	3.216	8	7	12	2	2	0.063
13	(0.398, 0.500, 0.398)	2.471	8	7	12	2	5	0.205

Supplementary Table 1195: Nodal ring points along high symmetry lines of mp-20369-CeIn3.

	Coordinates	Frequency	Mode 1	Mode 2	Multi.	Deg.	Path	Ratio
14	(0.024, 0.500, 0.024)	3.216	8	7	12	2	5	0.953
15	(0.000, 0.475, 0.000)	3.231	9	8	6	2	1	0.951
16	(0.066, 0.500, -0.000)	3.227	9	8	12	2	2	0.131
17	(0.338, 0.500, -0.000)	3.825	11	10	12	2	2	0.675
18	(0.500, 0.500, 0.500)	4.460	11	10	1	3	4	1.000
19	(0.165, 0.500, 0.165)	3.784	11	10	12	2	5	0.669

The followings are details for $CeTl_3$. It has 7 topological nodal lines and 16 nodal ring points along high symmetry line as shown in Fig **562**.

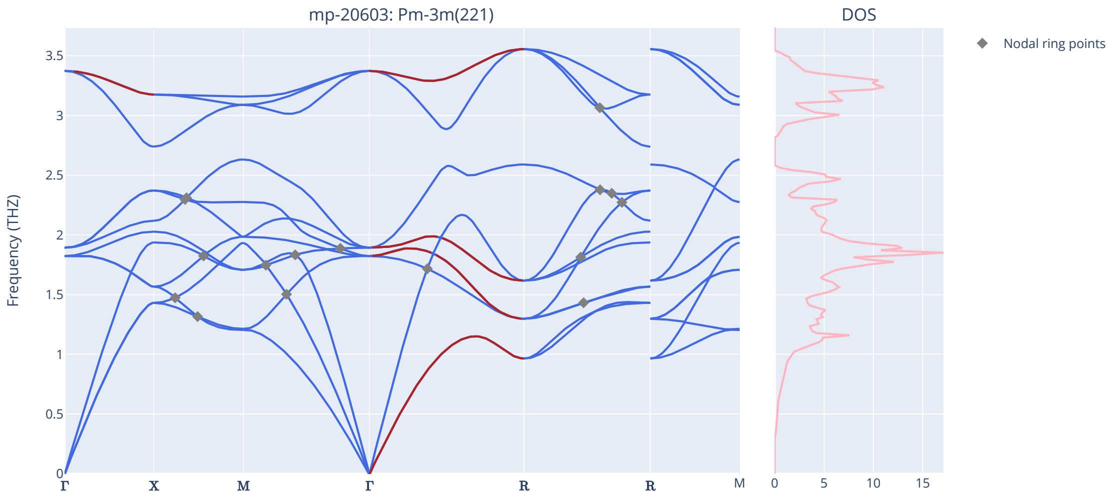

Supplementary Figure 562: The phonon band and DOS of CeTl3.

Supplementary Table 1196: High symmetry lines of mp-20603-CeTl3

	Start	End
0	Γ (0.000, 0.000, 0.000)	X (0.000, 0.500, 0.000)
1	X (0.000, 0.500, 0.000)	M (0.500, 0.500, 0.000)
2	M (0.500, 0.500, 0.000)	Γ (0.000, 0.000, 0.000)
3	Γ (0.000, 0.000, 0.000)	R (0.500, 0.500, 0.500)
4	R (0.500, 0.500, 0.500)	X (0.000, 0.500, 0.000)
5	R (0.500, 0.500, 0.500)	M (0.500, 0.500, 0.000)

Supplementary Table 1197: Nodal ring points along high symmetry lines of mp-20603-CeTl3.

	Coordinates	Frequency	Mode 1	Mode 2	Multi.	Deg.	Path	Ratio
0	(0.245, 0.500, 0.000)	1.316	2	1	12	2	2	0.490
1	(0.119, 0.500, 0.000)	1.473	3	2	12	2	2	0.238
2	(0.328, 0.328, -0.000)	1.503	3	2	12	2	3	0.344
3	(0.410, 0.410, 0.000)	1.746	4	3	12	2	3	0.180
4	(0.264, 0.498, 0.264)	1.432	4	3	24	2	5	0.472
5	(0.187, 0.187, 0.187)	1.717	4	3	8	2	4	0.375
6	(0.278, 0.500, 0.000)	1.824	5	4	12	2	2	0.557
7	(0.294, 0.294, -0.000)	1.833	5	4	12	2	3	0.413
8	(0.115, 0.115, 0.002)	1.887	7	6	24	2	3	0.770
9	(0.275, 0.500, 0.275)	1.814	7	6	12	2	5	0.449
10	(0.174, 0.500, -0.000)	2.297	8	7	12	2	2	0.349
11	(0.113, 0.500, 0.112)	2.273	8	7	12	2	5	0.775
12	(0.183, 0.500, 0.000)	2.311	9	8	12	2	2	0.366
13	(0.153, 0.500, 0.153)	2.348	9	8	12	2	5	0.695

Supplementary Table 1197: Nodal ring points along high symmetry lines of mp-20603-CeTl3.

	Coordinates	Frequency	Mode 1	Mode 2	Multi.	Deg.	Path	Ratio
14	(0.199, 0.500, 0.199)	2.378	9	8	12	2	5	0.602
15	(0.200, 0.500, 0.200)	3.065	11	10	12	2	5	0.600

The followings are details for InZr$_3$. It has 9 topological nodal lines and 25 nodal ring points along high symmetry line as shown in Fig **563**.

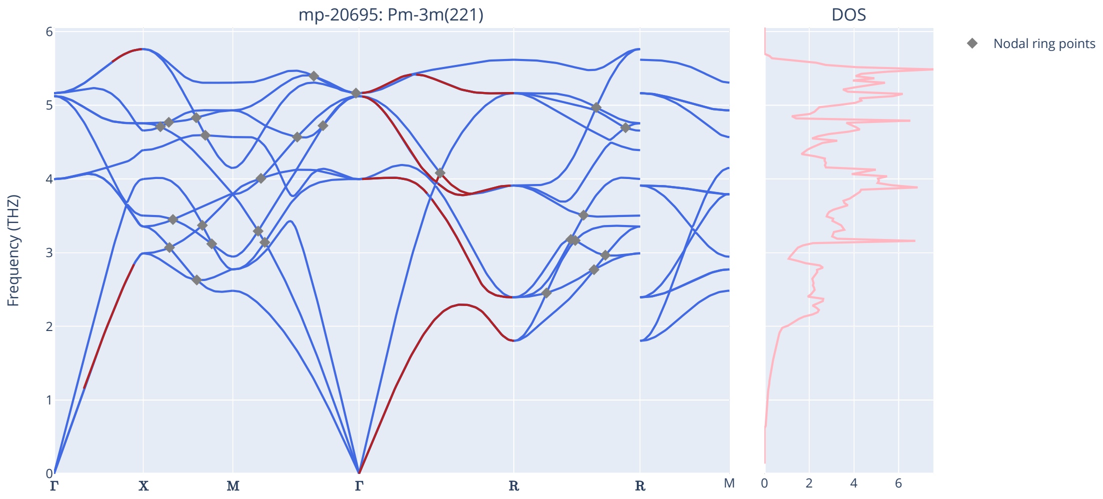

Supplementary Figure 563: The phonon band and DOS of InZr3.

Supplementary Table 1198: High symmetry lines of mp-20695-InZr3

	Start	End
0	Γ (0.000, 0.000, 0.000)	X (0.000, 0.500, 0.000)
1	X (0.000, 0.500, 0.000)	M (0.500, 0.500, 0.000)
2	M (0.500, 0.500, 0.000)	Γ (0.000, 0.000, 0.000)
3	Γ (0.000, 0.000, 0.000)	R (0.500, 0.500, 0.500)
4	R (0.500, 0.500, 0.500)	X (0.000, 0.500, 0.000)
5	R (0.500, 0.500, 0.500)	M (0.500, 0.500, 0.000)

Supplementary Table 1199: Nodal ring points along high symmetry lines of mp-20695-InZr3.

	Coordinates	Frequency	Mode 1	Mode 2	Multi.	Deg.	Path	Ratio
0	(0.299, 0.500, 0.000)	2.629	2	1	12	2	2	0.598
1	(0.182, 0.500, 0.182)	2.769	2	1	12	2	5	0.635
2	(0.146, 0.500, 0.000)	3.069	3	2	12	2	2	0.292
3	(0.371, 0.500, 0.371)	2.452	3	2	12	2	5	0.259
4	(0.137, 0.500, 0.137)	2.965	3	2	12	2	5	0.725
5	(0.383, 0.500, -0.000)	3.119	4	3	12	2	2	0.765
6	(0.373, 0.373, 0.000)	3.140	4	3	12	2	3	0.254
7	(0.257, 0.500, 0.257)	3.165	4	3	12	2	5	0.487
8	(0.165, 0.500, 0.000)	3.450	5	4	12	2	2	0.331
9	(0.330, 0.500, -0.000)	3.371	5	4	12	2	2	0.660
10	(0.400, 0.400, 0.000)	3.292	5	4	12	2	3	0.201
11	(0.274, 0.500, 0.274)	3.179	5	4	12	2	5	0.452
12	(0.224, 0.500, 0.224)	3.508	6	5	12	2	5	0.553
13	(0.388, 0.388, -0.000)	4.008	7	6	12	2	3	0.223

Supplementary Table 1199: Nodal ring points along high symmetry lines of mp-20695-InZr3.

	Coordinates	Frequency	Mode 1	Mode 2	Multi.	Deg.	Path	Ratio
14	(0.143, 0.143, -0.000)	4.723	8	7	12	2	3	0.715
15	(0.095, 0.500, -0.000)	4.708	9	8	12	2	2	0.190
16	(0.346, 0.500, -0.000)	4.593	9	8	12	2	2	0.693
17	(0.245, 0.245, 0.000)	4.568	9	8	12	2	3	0.511
18	(0.262, 0.262, 0.262)	4.081	9	8	8	2	4	0.524
19	(0.058, 0.500, 0.058)	4.695	9	8	12	2	5	0.885
20	(0.142, 0.500, 0.000)	4.769	10	9	12	2	2	0.284
21	(0.296, 0.500, 0.000)	4.831	10	9	12	2	2	0.591
22	(0.174, 0.500, 0.174)	4.970	11	10	12	2	5	0.653
23	(0.179, 0.179, 0.000)	5.395	12	11	12	2	3	0.642
24	(0.012, 0.012, -0.000)	5.165	12	11	12	2	3	0.975

The followings are details for Ga$_3$. It has 6 topological nodal lines and 17 nodal ring points along high symmetry line as shown in Fig **564**.

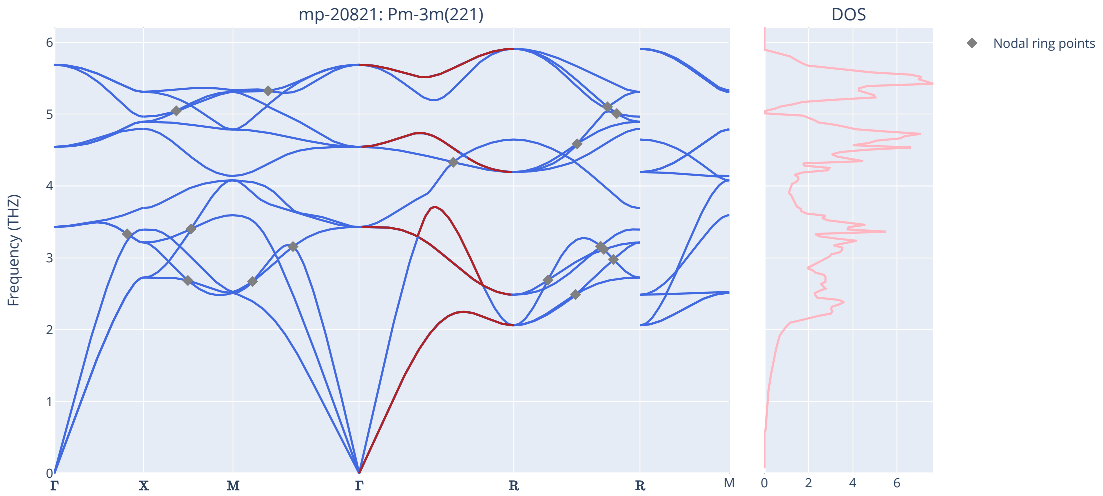

Supplementary Figure 564: The phonon band and DOS of Ga3Np.

Supplementary Table 1200: High symmetry lines of mp-20821-Ga3Np

	Start	End
0	Γ (0.000, 0.000, 0.000)	X (0.000, 0.500, 0.000)
1	X (0.000, 0.500, 0.000)	M (0.500, 0.500, 0.000)
2	M (0.500, 0.500, 0.000)	Γ (0.000, 0.000, 0.000)
3	Γ (0.000, 0.000, 0.000)	R (0.500, 0.500, 0.500)
4	R (0.500, 0.500, 0.500)	X (0.000, 0.500, 0.000)
5	R (0.500, 0.500, 0.500)	M (0.500, 0.500, 0.000)

Supplementary Table 1201: Nodal ring points along high symmetry lines of mp-20821-Ga3Np.

	Coordinates	Frequency	Mode 1	Mode 2	Multi.	Deg.	Path	Ratio
0	(0.248, 0.500, 0.000)	2.685	2	1	12	2	2	0.496
1	(0.256, 0.500, 0.256)	2.489	2	1	12	2	5	0.488
2	(0.422, 0.422, 0.000)	2.672	3	2	12	2	3	0.155
3	(0.106, 0.500, 0.106)	2.978	3	2	12	2	5	0.788
4	(0.262, 0.262, -0.000)	3.158	4	3	12	2	3	0.476
5	(0.143, 0.500, 0.143)	3.119	4	3	12	2	5	0.713
6	(0.000, 0.408, 0.000)	3.334	5	4	6	2	1	0.816
7	(0.265, 0.500, 0.000)	3.402	5	4	12	2	2	0.530
8	(0.365, 0.500, 0.365)	2.690	5	4	12	2	5	0.270
9	(0.156, 0.500, 0.156)	3.162	5	4	12	2	5	0.687
10	(0.304, 0.304, 0.304)	4.331	7	6	8	2	4	0.609
11	(0.249, 0.500, 0.249)	4.586	9	8	12	2	5	0.502
12	(0.092, 0.500, 0.092)	5.008	10	9	12	2	5	0.816
13	(0.184, 0.500, -0.000)	5.045	10	9	12	2	2	0.367

Supplementary Table 1201: Nodal ring points along high symmetry lines of mp-20821-Ga3Np.

	Coordinates	Frequency	Mode 1	Mode 2	Multi.	Deg.	Path	Ratio
14	(0.129, 0.500, 0.129)	5.101	11	10	12	2	5	0.742
15	(0.361, 0.361, 0.000)	5.325	12	11	12	2	3	0.279

The followings are details for $PbPd_3$. It has 7 topological nodal lines and 34 nodal ring points along high symmetry line as shown in Fig **565**.

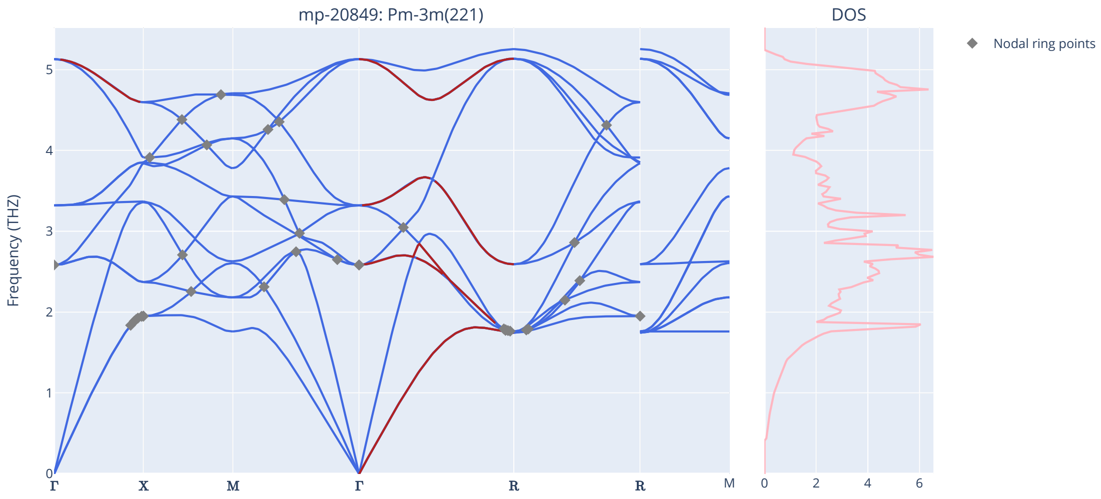

Supplementary Figure 565: The phonon band and DOS of PbPd3.

Supplementary Table 1202: High symmetry lines of mp-20849-PbPd3

	Start	End
0	Γ (0.000, 0.000, 0.000)	X (0.000, 0.500, 0.000)
1	X (0.000, 0.500, 0.000)	M (0.500, 0.500, 0.000)
2	M (0.500, 0.500, 0.000)	Γ (0.000, 0.000, 0.000)
3	Γ (0.000, 0.000, 0.000)	R (0.500, 0.500, 0.500)
4	R (0.500, 0.500, 0.500)	X (0.000, 0.500, 0.000)
5	R (0.500, 0.500, 0.500)	M (0.500, 0.500, 0.000)

Supplementary Table 1203: Nodal ring points along high symmetry lines of mp-20849-PbPd3.

	Coordinates	Frequency	Mode 1	Mode 2	Multi.	Deg.	Path	Ratio
0	(0.000, 0.429, 0.000)	1.837	2	1	6	2	1	0.857
1	(0.000, 0.449, 0.000)	1.888	2	1	6	2	1	0.898
2	(0.000, 0.459, 0.000)	1.909	2	1	6	2	1	0.918
3	(0.000, 0.469, 0.000)	1.926	2	1	6	2	1	0.939
4	(0.000, 0.490, 0.000)	1.947	2	1	6	2	1	0.980
5	(0.000, 0.500, 0.000)	1.950	2	1	3	2	1	1.000
6	(0.267, 0.500, -0.000)	2.254	3	2	12	2	2	0.534
7	(0.376, 0.376, 0.000)	2.312	3	2	12	2	3	0.248
8	(0.450, 0.500, 0.450)	1.779	3	2	12	2	5	0.099
9	(0.249, 0.249, 0.000)	2.746	4	3	12	2	3	0.501
10	(0.473, 0.473, 0.473)	1.773	4	3	8	2	4	0.947
11	(0.442, 0.500, 0.442)	1.791	4	3	12	2	5	0.115
12	(0.297, 0.500, 0.297)	2.148	4	3	12	2	5	0.405
13	(0.000, 0.000, 0.000)	2.583	5	4	1	3	1	0.000

Supplementary Table 1203: Nodal ring points along high symmetry lines of mp-20849-PbPd3.

	Coordinates	Frequency	Mode 1	Mode 2	Multi.	Deg.	Path	Ratio
14	(0.219, 0.500, -0.000)	2.708	5	4	12	2	2	0.437
15	(0.086, 0.086, 0.000)	2.649	5	4	12	2	3	0.828
16	(0.239, 0.500, 0.239)	2.389	5	4	12	2	5	0.523
17	(0.481, 0.481, 0.481)	1.771	6	5	8	2	4	0.962
18	(0.468, 0.468, 0.468)	1.792	6	5	8	2	4	0.936
19	(0.488, 0.488, 0.488)	1.765	6	5	8	2	4	0.977
20	(0.474, 0.474, 0.474)	1.781	6	5	8	2	4	0.948
21	(0.260, 0.500, 0.259)	2.860	7	6	24	2	5	0.480
22	(0.236, 0.236, 0.000)	2.976	7	6	12	2	3	0.528
23	(0.143, 0.143, 0.143)	3.047	7	6	8	2	4	0.286
24	(0.296, 0.296, 0.000)	3.391	8	7	12	2	3	0.409
25	(0.355, 0.500, -0.000)	4.068	9	8	12	2	2	0.709
26	(0.035, 0.500, -0.000)	3.913	10	9	12	2	2	0.071
27	(0.316, 0.316, 0.000)	4.354	10	9	12	2	3	0.368
28	(0.360, 0.360, -0.000)	4.259	10	9	12	2	3	0.279
29	(0.216, 0.500, 0.000)	4.382	11	10	12	2	2	0.431
30	(0.133, 0.500, 0.133)	4.313	11	10	12	2	5	0.734
31	(0.434, 0.500, -0.000)	4.690	12	11	12	2	2	0.868

The followings are details for In$_3$. It has 6 topological nodal lines and 30 nodal ring points along high symmetry line as shown in Fig **566**.

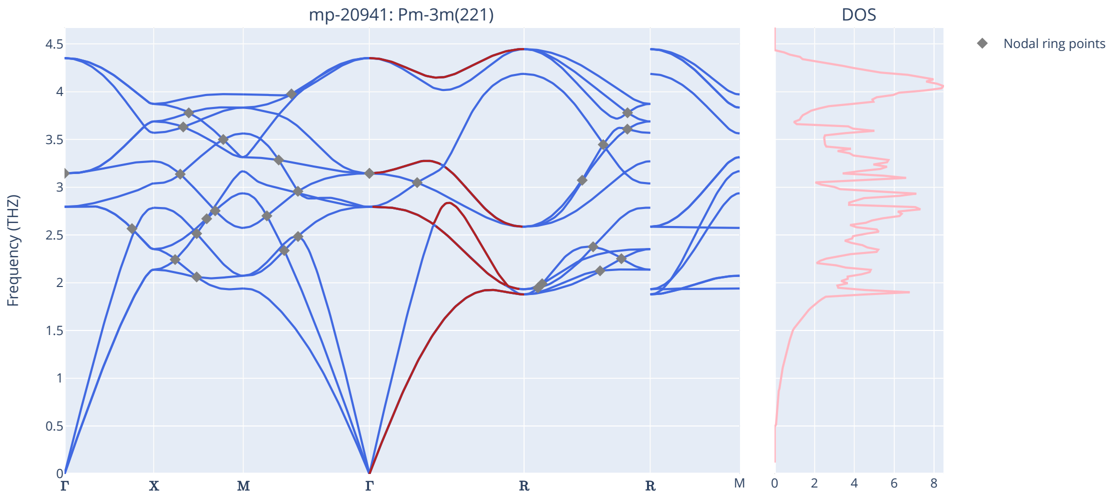

Supplementary Figure 566: The phonon band and DOS of In3U.

Supplementary Table 1204: High symmetry lines of mp-20941-In3U

	Start	End
0	Γ (0.000, 0.000, 0.000)	X (0.000, 0.500, 0.000)
1	X (0.000, 0.500, 0.000)	M (0.500, 0.500, 0.000)
2	M (0.500, 0.500, 0.000)	Γ (0.000, 0.000, 0.000)
3	Γ (0.000, 0.000, 0.000)	R (0.500, 0.500, 0.500)
4	R (0.500, 0.500, 0.500)	X (0.000, 0.500, 0.000)
5	R (0.500, 0.500, 0.500)	M (0.500, 0.500, 0.000)

Supplementary Table 1205: Nodal ring points along high symmetry lines of mp-20941-In3U.

	Coordinates	Frequency	Mode 1	Mode 2	Multi.	Deg.	Path	Ratio
0	(0.240, 0.500, -0.000)	2.060	2	1	12	2	2	0.480
1	(0.199, 0.500, 0.199)	2.124	2	1	12	2	5	0.602
2	(0.119, 0.500, -0.000)	2.242	3	2	12	2	2	0.238
3	(0.337, 0.337, -0.000)	2.337	3	2	12	2	3	0.326
4	(0.115, 0.500, 0.115)	2.251	3	2	12	2	5	0.771
5	(0.240, 0.500, -0.000)	2.514	4	3	12	2	2	0.480
6	(0.283, 0.283, -0.000)	2.483	4	3	12	2	3	0.435
7	(0.445, 0.500, 0.445)	1.945	4	3	12	2	5	0.110
8	(0.000, 0.378, 0.000)	2.566	5	4	6	2	1	0.755
9	(0.296, 0.500, 0.000)	2.669	5	4	12	2	2	0.592
10	(0.344, 0.500, -0.000)	2.753	5	4	12	2	2	0.687
11	(0.406, 0.406, 0.000)	2.700	5	4	12	2	3	0.188
12	(0.428, 0.500, 0.428)	1.989	5	4	12	2	5	0.143
13	(0.226, 0.500, 0.226)	2.376	5	4	12	2	5	0.547

Supplementary Table 1205: Nodal ring points along high symmetry lines of mp-20941-In3U.

	Coordinates	Frequency	Mode 1	Mode 2	Multi.	Deg.	Path	Ratio
14	(0.148, 0.500, -0.000)	3.137	7	6	12	2	2	0.296
15	(0.283, 0.283, 0.000)	2.957	7	6	12	2	3	0.434
16	(0.154, 0.154, 0.154)	3.048	7	6	8	2	4	0.309
17	(0.000, 0.000, 0.000)	3.145	8	7	1	3	1	0.000
18	(0.359, 0.359, -0.000)	3.285	8	7	12	2	3	0.282
19	(0.270, 0.500, 0.270)	3.073	8	7	12	2	5	0.461
20	(0.165, 0.500, -0.000)	3.631	9	8	12	2	2	0.329
21	(0.389, 0.500, -0.000)	3.500	9	8	12	2	2	0.778
22	(0.091, 0.500, 0.091)	3.608	9	8	12	2	5	0.818
23	(0.186, 0.500, 0.186)	3.446	9	8	12	2	5	0.627
24	(0.196, 0.500, -0.000)	3.778	11	10	12	2	2	0.391
25	(0.090, 0.500, 0.090)	3.779	11	10	12	2	5	0.820
26	(0.309, 0.309, 0.000)	3.976	12	11	12	2	3	0.383

The followings are details for Ni_3. It has 6 topological nodal lines and 29 nodal ring points along high symmetry line as shown in Fig 567.

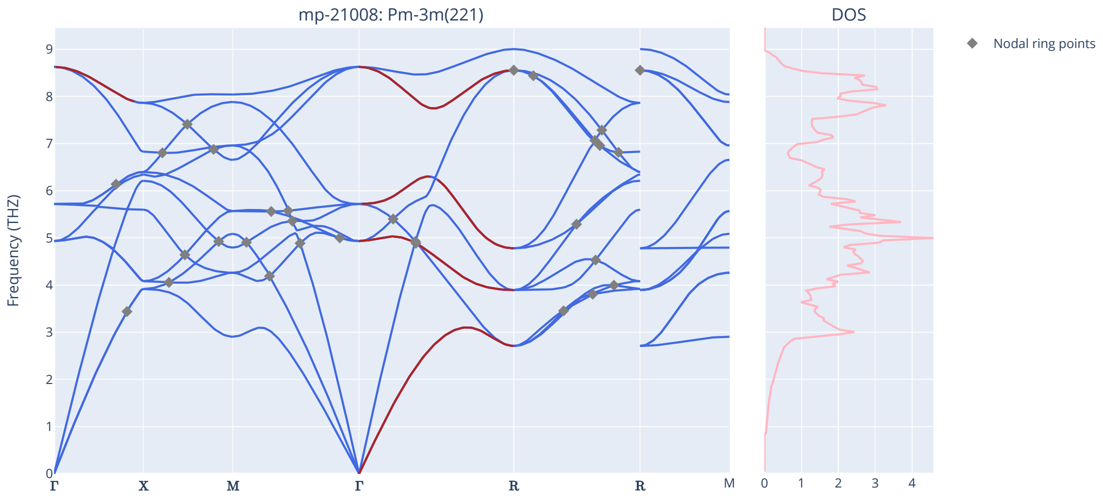

Supplementary Figure 567: The phonon band and DOS of Ni3Ge.

Supplementary Table 1206: High symmetry lines of mp-21008-Ni3Ge

	Start	End
0	Γ (0.000, 0.000, 0.000)	X (0.000, 0.500, 0.000)
1	X (0.000, 0.500, 0.000)	M (0.500, 0.500, 0.000)
2	M (0.500, 0.500, 0.000)	Γ (0.000, 0.000, 0.000)
3	Γ (0.000, 0.000, 0.000)	R (0.500, 0.500, 0.500)
4	R (0.500, 0.500, 0.500)	X (0.000, 0.500, 0.000)
5	R (0.500, 0.500, 0.500)	M (0.500, 0.500, 0.000)

Supplementary Table 1207: Nodal ring points along high symmetry lines of mp-21008-Ni3Ge.

	Coordinates	Frequency	Mode 1	Mode 2	Multi.	Deg.	Path	Ratio
0	(0.000, 0.408, 0.000)	3.439	2	1	6	2	1	0.816
1	(0.188, 0.500, 0.188)	3.807	2	1	12	2	5	0.625
2	(0.143, 0.500, -0.000)	4.056	3	2	12	2	2	0.286
3	(0.354, 0.354, -0.000)	4.190	3	2	12	2	3	0.291
4	(0.303, 0.500, 0.303)	3.454	3	2	12	2	5	0.394
5	(0.104, 0.500, 0.104)	3.995	3	2	12	2	5	0.793
6	(0.233, 0.233, -0.000)	4.886	4	3	12	2	3	0.533
7	(0.184, 0.184, 0.184)	4.886	4	3	8	2	4	0.367
8	(0.232, 0.500, -0.000)	4.641	5	4	12	2	2	0.465
9	(0.421, 0.500, 0.000)	4.925	5	4	12	2	2	0.842
10	(0.446, 0.446, 0.000)	4.907	5	4	12	2	3	0.109
11	(0.076, 0.076, -0.000)	5.000	5	4	12	2	3	0.848
12	(0.176, 0.500, 0.176)	4.530	5	4	12	2	5	0.647
13	(0.264, 0.264, -0.000)	5.358	6	5	12	2	3	0.472

Supplementary Table 1207: Nodal ring points along high symmetry lines of mp-21008-Ni3Ge.

	Coordinates	Frequency	Mode 1	Mode 2	Multi.	Deg.	Path	Ratio
14	(0.182, 0.182, 0.182)	4.924	6	5	8	2	4	0.364
15	(0.348, 0.348, -0.000)	5.560	7	6	12	2	3	0.305
16	(0.251, 0.500, 0.251)	5.290	7	6	12	2	5	0.497
17	(0.110, 0.110, 0.110)	5.400	7	6	8	2	4	0.220
18	(0.281, 0.281, -0.000)	5.576	8	7	12	2	3	0.439
19	(0.000, 0.347, 0.000)	6.138	9	8	6	2	1	0.694
20	(0.393, 0.500, -0.000)	6.877	9	8	12	2	2	0.786
21	(0.159, 0.500, 0.159)	6.958	9	8	12	2	5	0.681
22	(0.106, 0.500, 0.000)	6.803	10	9	12	2	2	0.212
23	(0.500, 0.500, 0.500)	8.555	10	9	1	3	4	1.000
24	(0.422, 0.500, 0.422)	8.438	10	9	12	2	5	0.155
25	(0.179, 0.500, 0.179)	7.067	10	9	12	2	5	0.642
26	(0.085, 0.500, 0.085)	6.813	10	9	12	2	5	0.829
27	(0.246, 0.500, -0.000)	7.408	11	10	12	2	2	0.491
28	(0.151, 0.500, 0.151)	7.285	11	10	12	2	5	0.698

The followings are details for $TiZn_3$. It has 8 topological nodal lines and 13 nodal ring points along high symmetry line as shown in Fig **568**.

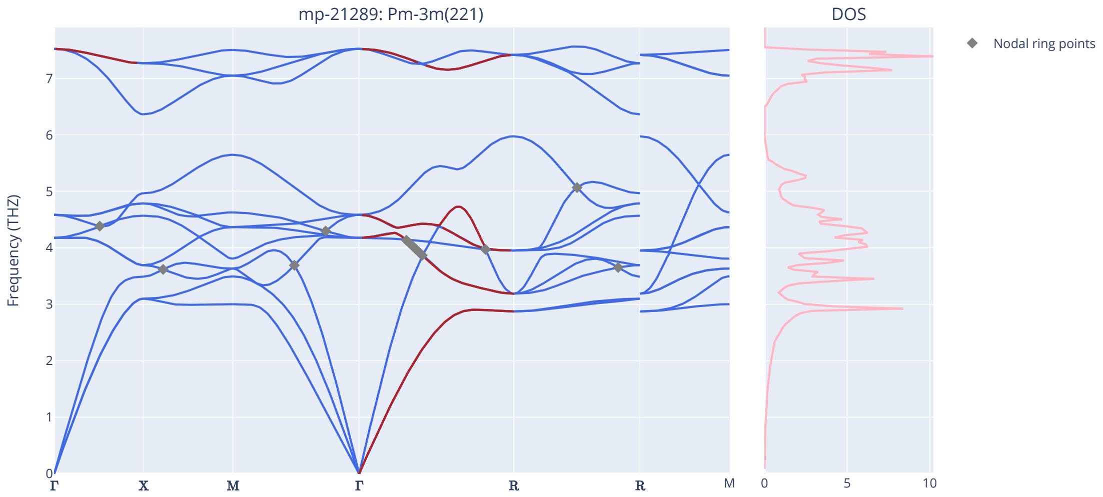

Supplementary Figure 568: The phonon band and DOS of TiZn3.

Supplementary Table 1208: High symmetry lines of mp-21289-TiZn3

	Start	End
0	Γ (0.000, 0.000, 0.000)	X (0.000, 0.500, 0.000)
1	X (0.000, 0.500, 0.000)	M (0.500, 0.500, 0.000)
2	M (0.500, 0.500, 0.000)	Γ (0.000, 0.000, 0.000)
3	Γ (0.000, 0.000, 0.000)	R (0.500, 0.500, 0.500)
4	R (0.500, 0.500, 0.500)	X (0.000, 0.500, 0.000)
5	R (0.500, 0.500, 0.500)	M (0.500, 0.500, 0.000)

Supplementary Table 1209: Nodal ring points along high symmetry lines of mp-21289-TiZn3.

	Coordinates	Frequency	Mode 1	Mode 2	Multi.	Deg.	Path	Ratio
0	(0.110, 0.500, 0.000)	3.616	4	3	12	2	2	0.221
1	(0.256, 0.256, 0.000)	3.689	4	3	12	2	3	0.488
2	(0.087, 0.500, 0.087)	3.651	4	3	12	2	5	0.826
3	(0.173, 0.173, 0.173)	4.032	5	4	8	2	4	0.346
4	(0.153, 0.153, 0.153)	4.132	5	4	8	2	4	0.306
5	(0.163, 0.163, 0.163)	4.082	5	4	8	2	4	0.327
6	(0.184, 0.184, 0.184)	3.976	5	4	8	2	4	0.367
7	(0.194, 0.194, 0.194)	3.921	5	4	8	2	4	0.388
8	(0.204, 0.204, 0.204)	3.867	5	4	8	2	4	0.408
9	(0.410, 0.408, 0.409)	3.970	6	5	24	2	4	0.818
10	(-0.001, 0.255, -0.001)	4.383	7	6	24	2	1	0.511
11	(0.132, 0.132, -0.000)	4.296	7	6	12	2	3	0.736
12	(0.249, 0.500, 0.249)	5.066	9	8	12	2	5	0.501

The followings are details for Pb$_3$. It has 7 topological nodal lines and 18 nodal ring points along high symmetry line as shown in Fig **569**.

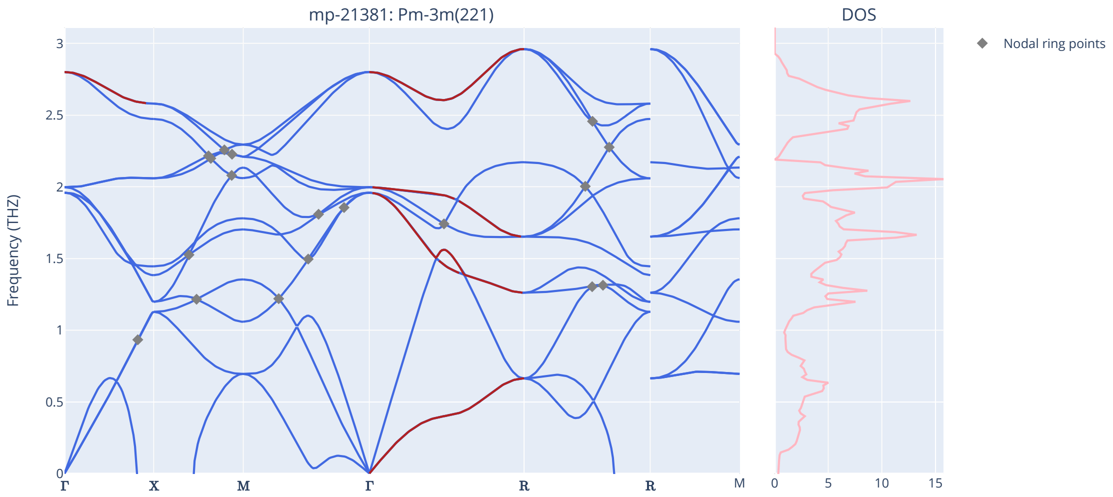

Supplementary Figure 569: The phonon band and DOS of Pb3Th.

Supplementary Table 1210: High symmetry lines of mp-21381-Pb3Th

	Start	End
0	Γ (0.000, 0.000, 0.000)	X (0.000, 0.500, 0.000)
1	X (0.000, 0.500, 0.000)	M (0.500, 0.500, 0.000)
2	M (0.500, 0.500, 0.000)	Γ (0.000, 0.000, 0.000)
3	Γ (0.000, 0.000, 0.000)	R (0.500, 0.500, 0.500)
4	R (0.500, 0.500, 0.500)	X (0.000, 0.500, 0.000)
5	R (0.500, 0.500, 0.500)	M (0.500, 0.500, 0.000)

Supplementary Table 1211: Nodal ring points along high symmetry lines of mp-21381-Pb3Th.

	Coordinates	Frequency	Mode 1	Mode 2	Multi.	Deg.	Path	Ratio
0	(0.000, 0.410, 0.000)	0.934	3	2	6	2	1	0.820
1	(0.239, 0.500, -0.000)	1.216	4	3	12	2	2	0.479
2	(0.359, 0.359, 0.000)	1.220	4	3	12	2	3	0.282
3	(0.187, 0.500, 0.187)	1.314	4	3	12	2	5	0.625
4	(0.230, 0.500, 0.230)	1.304	4	3	12	2	5	0.540
5	(0.100, 0.100, 0.000)	1.856	5	4	12	2	3	0.799
6	(0.242, 0.242, 0.000)	1.496	5	4	12	2	3	0.515
7	(0.197, 0.500, 0.000)	1.526	6	5	12	2	2	0.394
8	(0.201, 0.201, 0.000)	1.809	7	6	12	2	3	0.597
9	(0.241, 0.241, 0.241)	1.742	7	6	8	2	4	0.482
10	(0.436, 0.500, 0.000)	2.080	8	7	12	2	2	0.872
11	(0.319, 0.500, 0.000)	2.198	9	8	12	2	2	0.638
12	(0.257, 0.500, 0.257)	2.004	9	8	12	2	5	0.485
13	(0.163, 0.500, 0.163)	2.276	10	9	12	2	5	0.675

Supplementary Table 1211: Nodal ring points along high symmetry lines of mp-21381-Pb3Th.

	Coordinates	Frequency	Mode 1	Mode 2	Multi.	Deg.	Path	Ratio
14	(0.438, 0.500, -0.000)	2.227	10	9	12	2	2	0.876
15	(0.306, 0.500, -0.000)	2.216	10	9	12	2	2	0.612
16	(0.395, 0.500, -0.001)	2.258	11	10	24	2	2	0.790
17	(0.229, 0.500, 0.229)	2.457	11	10	12	2	5	0.542

The followings are details for LaPb$_3$. It has 7 topological nodal lines and 18 nodal ring points along high symmetry line as shown in Fig **570**.

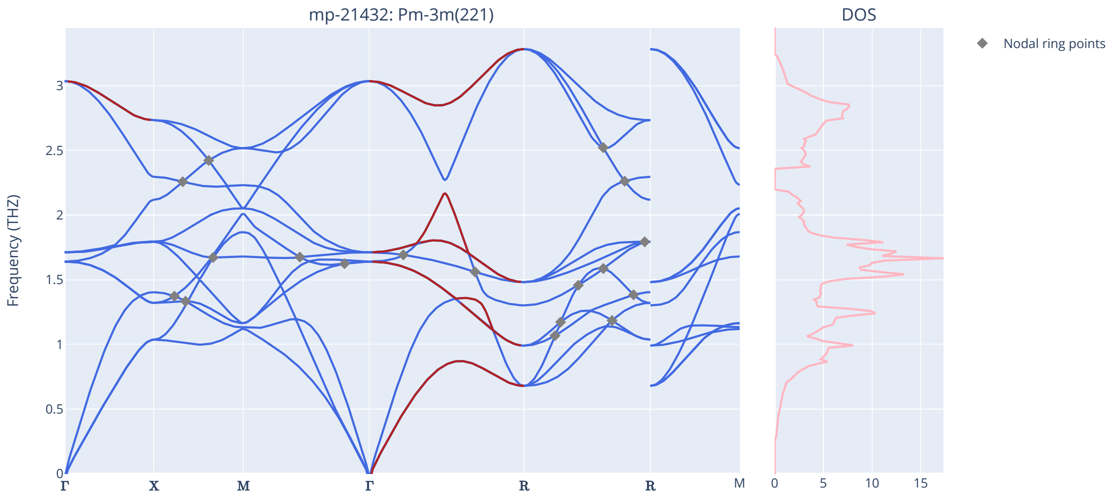

Supplementary Figure 570: The phonon band and DOS of LaPb3.

Supplementary Table 1212: High symmetry lines of mp-21432-LaPb3

	Start	End
0	Γ (0.000, 0.000, 0.000)	X (0.000, 0.500, 0.000)
1	X (0.000, 0.500, 0.000)	M (0.500, 0.500, 0.000)
2	M (0.500, 0.500, 0.000)	Γ (0.000, 0.000, 0.000)
3	Γ (0.000, 0.000, 0.000)	R (0.500, 0.500, 0.500)
4	R (0.500, 0.500, 0.500)	X (0.000, 0.500, 0.000)
5	R (0.500, 0.500, 0.500)	M (0.500, 0.500, 0.000)

Supplementary Table 1213: Nodal ring points along high symmetry lines of mp-21432-LaPb3.

	Coordinates	Frequency	Mode 1	Mode 2	Multi.	Deg.	Path	Ratio
0	(0.151, 0.500, 0.151)	1.184	3	2	12	2	5	0.697
1	(0.177, 0.500, 0.000)	1.333	4	3	12	2	2	0.355
2	(0.377, 0.500, 0.377)	1.067	4	3	12	2	5	0.245
3	(0.115, 0.500, -0.000)	1.372	5	4	12	2	2	0.230
4	(0.099, 0.099, -0.000)	1.624	5	4	12	2	3	0.803
5	(0.355, 0.500, 0.355)	1.172	5	4	12	2	5	0.289
6	(0.066, 0.500, 0.066)	1.383	5	4	12	2	5	0.868
7	(0.332, 0.500, -0.000)	1.671	6	5	12	2	2	0.664
8	(0.285, 0.500, 0.285)	1.457	6	5	12	2	5	0.430
9	(0.186, 0.500, 0.186)	1.586	6	5	12	2	5	0.628
10	(0.276, 0.276, 0.000)	1.675	7	6	12	2	3	0.449
11	(0.110, 0.110, 0.110)	1.692	7	6	8	2	4	0.220
12	(0.341, 0.341, 0.341)	1.560	7	6	8	2	4	0.683
13	(0.022, 0.500, 0.022)	1.792	8	7	12	2	5	0.956

Supplementary Table 1213: Nodal ring points along high symmetry lines of mp-21432-LaPb3.

	Coordinates	Frequency	Mode 1	Mode 2	Multi.	Deg.	Path	Ratio
14	(0.162, 0.500, -0.000)	2.257	10	9	12	2	2	0.324
15	(0.101, 0.500, 0.101)	2.261	10	9	12	2	5	0.797
16	(0.307, 0.500, 0.001)	2.422	11	10	24	2	2	0.614
17	(0.186, 0.500, 0.186)	2.521	11	10	12	2	5	0.627

The followings are details for In3. It has 6 topological nodal lines and 28 nodal ring points along high symmetry line as shown in Fig **571**.

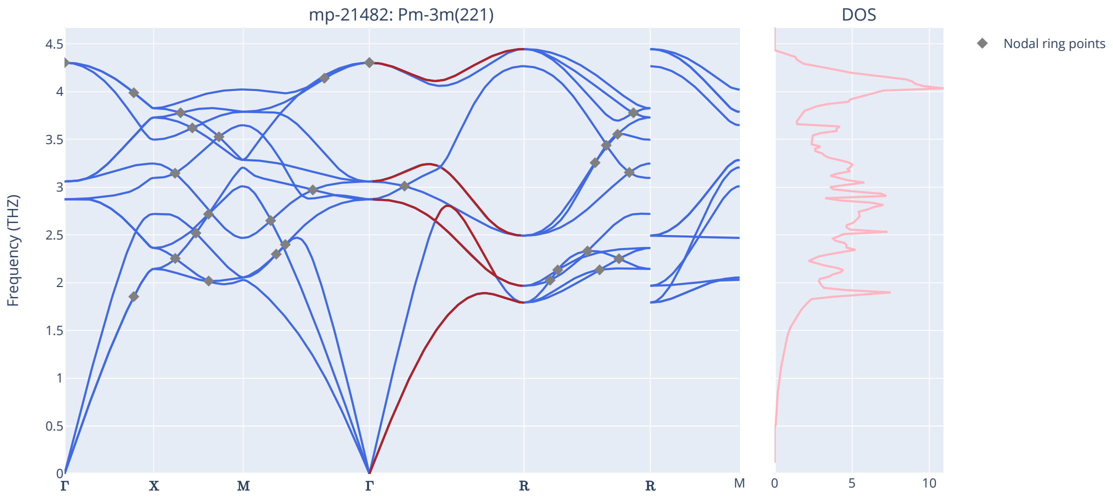

Supplementary Figure 571: The phonon band and DOS of In3Np.

Supplementary Table 1214: High symmetry lines of mp-21482-In3Np

	Start	End
0	Γ (0.000, 0.000, 0.000)	X (0.000, 0.500, 0.000)
1	X (0.000, 0.500, 0.000)	M (0.500, 0.500, 0.000)
2	M (0.500, 0.500, 0.000)	Γ (0.000, 0.000, 0.000)
3	Γ (0.000, 0.000, 0.000)	R (0.500, 0.500, 0.500)
4	R (0.500, 0.500, 0.500)	X (0.000, 0.500, 0.000)
5	R (0.500, 0.500, 0.500)	M (0.500, 0.500, 0.000)

Supplementary Table 1215: Nodal ring points along high symmetry lines of mp-21482-In3Np.

	Coordinates	Frequency	Mode 1	Mode 2	Multi.	Deg.	Path	Ratio
0	(0.000, 0.388, 0.000)	1.854	2	1	6	2	1	0.776
1	(0.307, 0.500, 0.000)	2.017	2	1	12	2	2	0.614
2	(0.201, 0.500, 0.201)	2.134	2	1	12	2	5	0.598
3	(0.120, 0.500, 0.000)	2.254	3	2	12	2	2	0.240
4	(0.369, 0.369, -0.000)	2.299	3	2	12	2	3	0.262
5	(0.124, 0.500, 0.124)	2.251	3	2	12	2	5	0.752
6	(0.236, 0.500, -0.000)	2.519	4	3	12	2	2	0.471
7	(0.333, 0.333, -0.000)	2.399	4	3	12	2	3	0.334
8	(0.397, 0.500, 0.397)	2.026	4	3	12	2	5	0.206
9	(0.306, 0.500, 0.000)	2.717	5	4	12	2	2	0.613
10	(0.391, 0.391, -0.000)	2.649	5	4	12	2	3	0.218
11	(0.249, 0.500, 0.249)	2.331	5	4	12	2	5	0.503
12	(0.367, 0.500, 0.367)	2.133	5	4	12	2	5	0.267
13	(0.085, 0.500, 0.081)	3.154	7	6	24	2	5	0.834

Supplementary Table 1215: Nodal ring points along high symmetry lines of mp-21482-In3Np.

	Coordinates	Frequency	Mode 1	Mode 2	Multi.	Deg.	Path	Ratio
14	(0.119, 0.500, -0.000)	3.146	7	6	12	2	2	0.238
15	(0.224, 0.224, -0.000)	2.971	7	6	12	2	3	0.552
16	(0.114, 0.114, 0.114)	3.011	7	6	8	2	4	0.227
17	(0.219, 0.500, 0.219)	3.256	8	7	12	2	5	0.563
18	(0.365, 0.500, -0.000)	3.527	9	8	12	2	2	0.730
19	(0.216, 0.500, 0.000)	3.620	9	8	12	2	2	0.431
20	(0.174, 0.500, 0.174)	3.438	9	8	12	2	5	0.651
21	(0.129, 0.500, 0.129)	3.553	9	8	12	2	5	0.741
22	(0.150, 0.500, -0.000)	3.778	11	10	12	2	2	0.299
23	(0.178, 0.178, -0.000)	4.140	11	10	12	2	3	0.644
24	(0.067, 0.500, 0.067)	3.778	11	10	12	2	5	0.866
25	(0.000, 0.000, 0.000)	4.303	12	11	1	3	1	0.000
26	(0.000, 0.388, 0.000)	3.988	12	11	6	2	1	0.776

The followings are details for $FePd_3$. It has 8 topological nodal lines and 20 nodal ring points along high symmetry line as shown in Fig **572**.

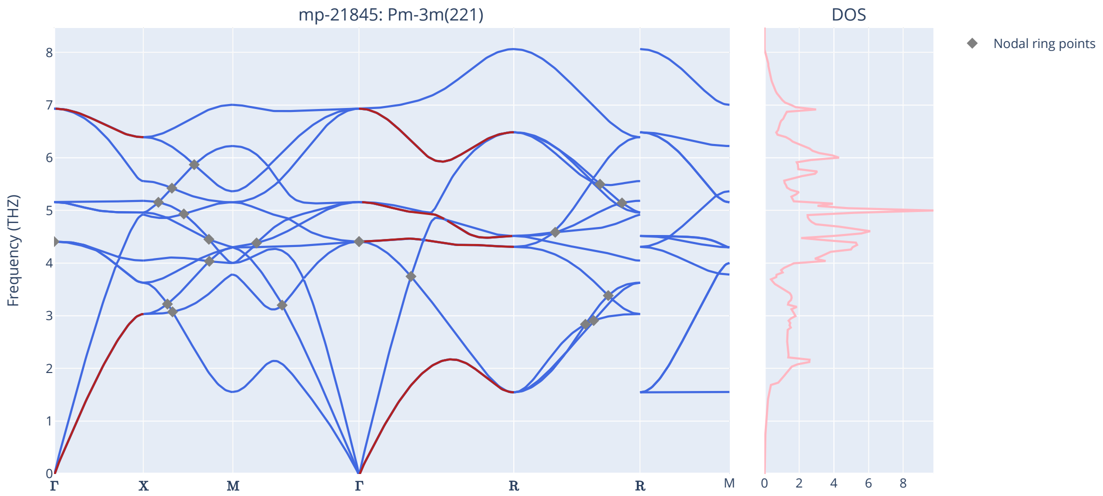

Supplementary Figure 572: The phonon band and DOS of FePd3.

Supplementary Table 1216: High symmetry lines of mp-21845-FePd3

	Start	End
0	Γ (0.000, 0.000, 0.000)	X (0.000, 0.500, 0.000)
1	X (0.000, 0.500, 0.000)	M (0.500, 0.500, 0.000)
2	M (0.500, 0.500, 0.000)	Γ (0.000, 0.000, 0.000)
3	Γ (0.000, 0.000, 0.000)	R (0.500, 0.500, 0.500)
4	R (0.500, 0.500, 0.500)	X (0.000, 0.500, 0.000)
5	R (0.500, 0.500, 0.500)	M (0.500, 0.500, 0.000)

Supplementary Table 1217: Nodal ring points along high symmetry lines of mp-21845-FePd3.

	Coordinates	Frequency	Mode 1	Mode 2	Multi.	Deg.	Path	Ratio
0	(0.163, 0.500, -0.000)	3.073	2	1	12	2	2	0.326
1	(0.184, 0.500, 0.184)	2.909	2	1	12	2	5	0.632
2	(0.134, 0.500, -0.000)	3.222	3	2	12	2	2	0.269
3	(0.304, 0.304, -0.000)	3.201	3	2	12	2	3	0.391
4	(0.216, 0.500, 0.216)	2.838	3	2	12	2	5	0.567
5	(0.369, 0.500, -0.000)	4.034	4	3	12	2	2	0.738
6	(0.168, 0.168, 0.168)	3.747	4	3	8	2	4	0.336
7	(0.126, 0.500, 0.126)	3.383	4	3	12	2	5	0.748
8	(0.000, 0.000, 0.000)	4.407	6	5	1	3	1	0.000
9	(0.367, 0.499, 0.000)	4.451	7	6	24	2	2	0.733
10	(0.406, 0.406, 0.000)	4.382	7	6	12	2	3	0.189
11	(0.336, 0.500, 0.336)	4.584	7	6	12	2	5	0.328
12	(0.226, 0.500, 0.000)	4.933	8	7	12	2	2	0.453
13	(0.084, 0.500, -0.000)	5.154	9	8	12	2	2	0.168

Supplementary Table 1217: Nodal ring points along high symmetry lines of mp-21845-FePd3.

	Coordinates	Frequency	Mode 1	Mode 2	Multi.	Deg.	Path	Ratio
14	(0.072, 0.500, 0.072)	5.141	9	8	12	2	5	0.857
15	(0.160, 0.500, -0.000)	5.422	10	9	12	2	2	0.319
16	(0.159, 0.500, 0.159)	5.497	10	9	12	2	5	0.683
17	(0.284, 0.500, 0.000)	5.868	11	10	12	2	2	0.569

The followings are details for Tl_3. It has 7 topological nodal lines and 18 nodal ring points along high symmetry line as shown in Fig **573**.

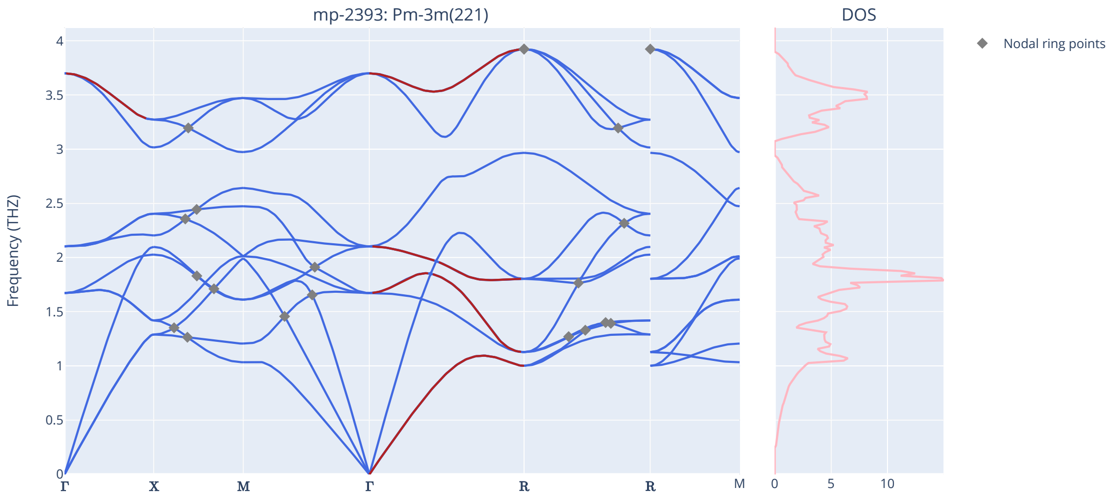

Supplementary Figure 573: The phonon band and DOS of Tl3Y.

Supplementary Table 1218: High symmetry lines of mp-2393-Tl3Y

	Start	End
0	Γ (0.000, 0.000, 0.000)	X (0.000, 0.500, 0.000)
1	X (0.000, 0.500, 0.000)	M (0.500, 0.500, 0.000)
2	M (0.500, 0.500, 0.000)	Γ (0.000, 0.000, 0.000)
3	Γ (0.000, 0.000, 0.000)	R (0.500, 0.500, 0.500)
4	R (0.500, 0.500, 0.500)	X (0.000, 0.500, 0.000)
5	R (0.500, 0.500, 0.500)	M (0.500, 0.500, 0.000)

Supplementary Table 1219: Nodal ring points along high symmetry lines of mp-2393-Tl3Y.

	Coordinates	Frequency	Mode 1	Mode 2	Multi.	Deg.	Path	Ratio
0	(0.188, 0.500, -0.000)	1.264	2	1	12	2	2	0.376
1	(0.113, 0.500, -0.000)	1.351	3	2	12	2	2	0.226
2	(0.336, 0.336, -0.000)	1.457	3	2	12	2	3	0.328
3	(0.156, 0.500, 0.156)	1.393	3	2	12	2	5	0.687
4	(0.257, 0.500, 0.257)	1.329	3	2	12	2	5	0.485
5	(0.226, 0.226, -0.000)	1.655	4	3	12	2	3	0.547
6	(0.177, 0.497, 0.177)	1.400	4	3	24	2	5	0.646
7	(0.323, 0.494, 0.323)	1.270	4	3	24	2	5	0.353
8	(0.337, 0.500, -0.000)	1.711	5	4	12	2	2	0.673
9	(0.241, 0.500, -0.000)	1.830	6	5	12	2	2	0.482
10	(0.285, 0.500, 0.285)	1.765	6	5	12	2	5	0.431
11	(0.216, 0.216, -0.000)	1.911	7	6	12	2	3	0.568
12	(0.176, 0.500, -0.000)	2.356	8	7	12	2	2	0.352
13	(0.103, 0.500, 0.103)	2.316	8	7	12	2	5	0.793

Supplementary Table 1219: Nodal ring points along high symmetry lines of mp-2393-Tl3Y.

	Coordinates	Frequency	Mode 1	Mode 2	Multi.	Deg.	Path	Ratio
14	(0.240, 0.500, 0.000)	2.444	9	8	12	2	2	0.480
15	(0.193, 0.500, 0.000)	3.196	11	10	12	2	2	0.386
16	(0.127, 0.500, 0.127)	3.195	11	10	12	2	5	0.745
17	(0.500, 0.500, 0.500)	3.924	12	11	1	3	4	1.000

The followings are details for Pt_3. It has 8 topological nodal lines and 18 nodal ring points along high symmetry line as shown in Fig **574**.

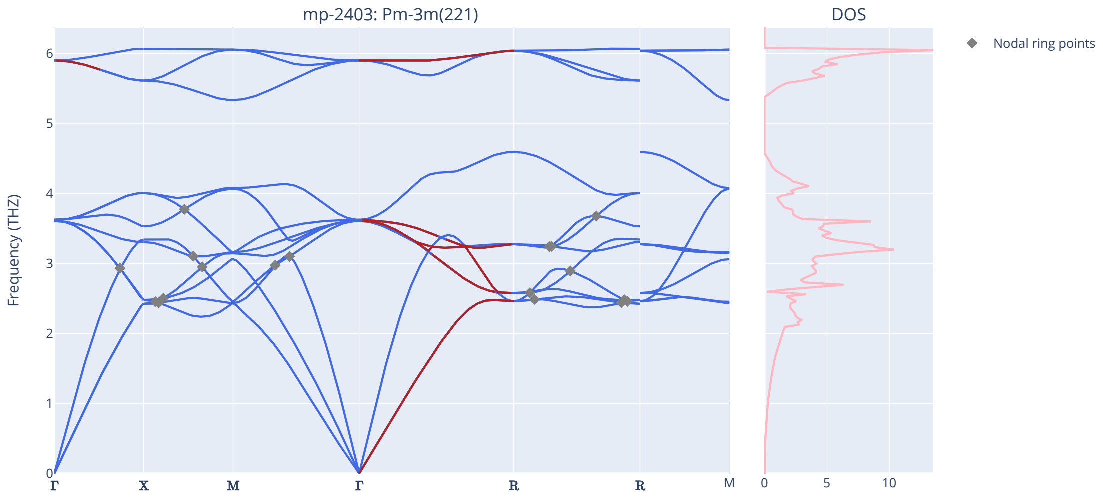

Supplementary Figure 574: The phonon band and DOS of Pt3Y.

Supplementary Table 1220: High symmetry lines of mp-2403-Pt3Y

	Start	End
0	Γ (0.000, 0.000, 0.000)	X (0.000, 0.500, 0.000)
1	X (0.000, 0.500, 0.000)	M (0.500, 0.500, 0.000)
2	M (0.500, 0.500, 0.000)	Γ (0.000, 0.000, 0.000)
3	Γ (0.000, 0.000, 0.000)	R (0.500, 0.500, 0.500)
4	R (0.500, 0.500, 0.500)	X (0.000, 0.500, 0.000)
5	R (0.500, 0.500, 0.500)	M (0.500, 0.500, 0.000)

Supplementary Table 1221: Nodal ring points along high symmetry lines of mp-2403-Pt3Y.

	Coordinates	Frequency	Mode 1	Mode 2	Multi.	Deg.	Path	Ratio
0	(0.084, 0.500, 0.000)	2.440	2	1	12	2	2	0.168
1	(0.418, 0.500, 0.418)	2.487	2	1	12	2	5	0.163
2	(0.074, 0.500, 0.074)	2.441	2	1	12	2	5	0.851
3	(0.066, 0.500, 0.000)	2.454	3	2	12	2	2	0.132
4	(0.050, 0.500, 0.050)	2.460	3	2	12	2	5	0.900
5	(0.111, 0.500, -0.000)	2.504	4	3	12	2	2	0.221
6	(0.276, 0.276, -0.000)	3.103	4	3	12	2	3	0.448
7	(0.333, 0.333, -0.000)	2.974	4	3	12	2	3	0.334
8	(0.436, 0.500, 0.436)	2.583	4	3	12	2	5	0.128
9	(0.062, 0.497, 0.062)	2.483	4	3	24	2	5	0.875
10	(0.000, 0.367, 0.000)	2.933	5	4	6	2	1	0.735
11	(0.329, 0.500, -0.000)	2.952	5	4	12	2	2	0.657
12	(0.276, 0.500, 0.276)	2.893	5	4	12	2	5	0.448
13	(0.278, 0.500, -0.000)	3.105	6	5	12	2	2	0.555

Supplementary Table 1221: Nodal ring points along high symmetry lines of mp-2403-Pt3Y.

	Coordinates	Frequency	Mode 1	Mode 2	Multi.	Deg.	Path	Ratio
14	(0.360, 0.500, 0.360)	3.242	7	6	12	2	5	0.281
15	(0.351, 0.500, 0.351)	3.246	7	6	12	2	5	0.298
16	(0.228, 0.500, 0.000)	3.775	8	7	12	2	2	0.456
17	(0.173, 0.500, 0.173)	3.680	8	7	12	2	5	0.653

The followings are details for Pd3. It has 9 topological nodal lines and 21 nodal ring points along high symmetry line as shown in Fig **575**.

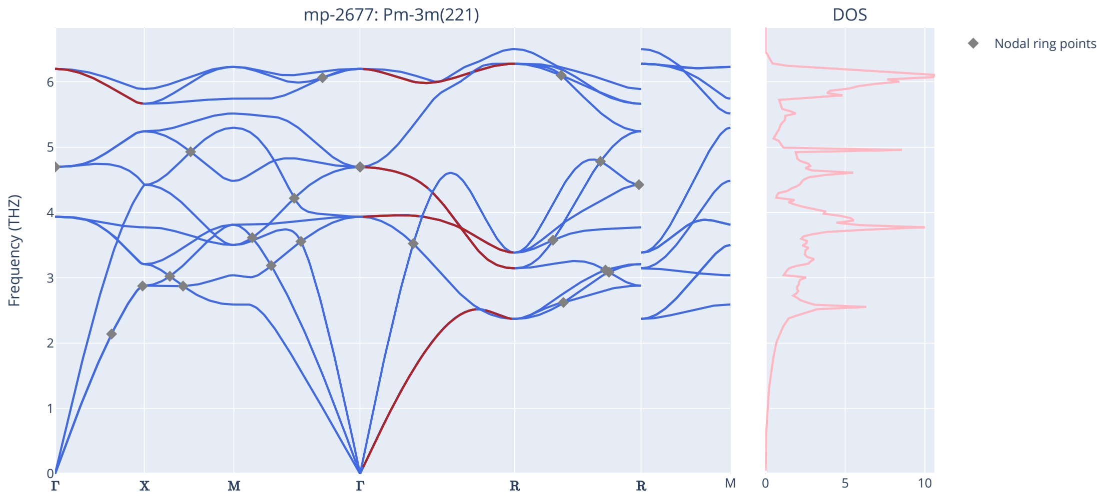

Supplementary Figure 575: The phonon band and DOS of Pd3Sc.

Supplementary Table 1222: High symmetry lines of mp-2677-Pd3Sc

	Start	End
0	Γ (0.000, 0.000, 0.000)	X (0.000, 0.500, 0.000)
1	X (0.000, 0.500, 0.000)	M (0.500, 0.500, 0.000)
2	M (0.500, 0.500, 0.000)	Γ (0.000, 0.000, 0.000)
3	Γ (0.000, 0.000, 0.000)	R (0.500, 0.500, 0.500)
4	R (0.500, 0.500, 0.500)	X (0.000, 0.500, 0.000)
5	R (0.500, 0.500, 0.500)	M (0.500, 0.500, 0.000)

Supplementary Table 1223: Nodal ring points along high symmetry lines of mp-2677-Pd3Sc.

	Coordinates	Frequency	Mode 1	Mode 2	Multi.	Deg.	Path	Ratio
0	(0.000, 0.316, 0.000)	2.140	2	1	6	2	1	0.633
1	(0.000, 0.490, 0.000)	2.874	2	1	6	2	1	0.980
2	(0.216, 0.500, -0.000)	2.873	2	1	12	2	2	0.433
3	(0.307, 0.500, 0.307)	2.622	2	1	12	2	5	0.385
4	(0.142, 0.500, -0.000)	3.023	3	2	12	2	2	0.285
5	(0.352, 0.352, 0.000)	3.188	3	2	12	2	3	0.297
6	(0.127, 0.500, 0.127)	3.086	3	2	12	2	5	0.746
7	(0.234, 0.234, 0.000)	3.556	4	3	12	2	3	0.532
8	(0.172, 0.172, 0.172)	3.524	4	3	8	2	4	0.344
9	(0.141, 0.500, 0.141)	3.118	4	3	12	2	5	0.718
10	(0.427, 0.427, -0.000)	3.614	5	4	12	2	3	0.147
11	(0.349, 0.500, 0.349)	3.576	6	5	12	2	5	0.302
12	(0.009, 0.500, 0.009)	4.425	7	6	12	2	5	0.983
13	(0.261, 0.261, -0.000)	4.218	7	6	12	2	3	0.479

Supplementary Table 1223: Nodal ring points along high symmetry lines of mp-2677-Pd3Sc.

	Coordinates	Frequency	Mode 1	Mode 2	Multi.	Deg.	Path	Ratio
14	(0.000, 0.000, 0.000)	4.697	8	7	1	3	1	0.000
15	(0.259, 0.500, 0.000)	4.930	8	7	12	2	2	0.518
16	(0.161, 0.500, 0.161)	4.782	8	7	12	2	5	0.678
17	(0.000, 0.000, 0.000)	4.697	9	8	1	3	1	0.000
18	(0.316, 0.500, 0.316)	6.100	10	9	12	2	5	0.368
19	(0.148, 0.148, -0.000)	6.064	11	10	12	2	3	0.703

The followings are details for Ir$_3$. It has 7 topological nodal lines and 21 nodal ring points along high symmetry line as shown in Fig **576**.

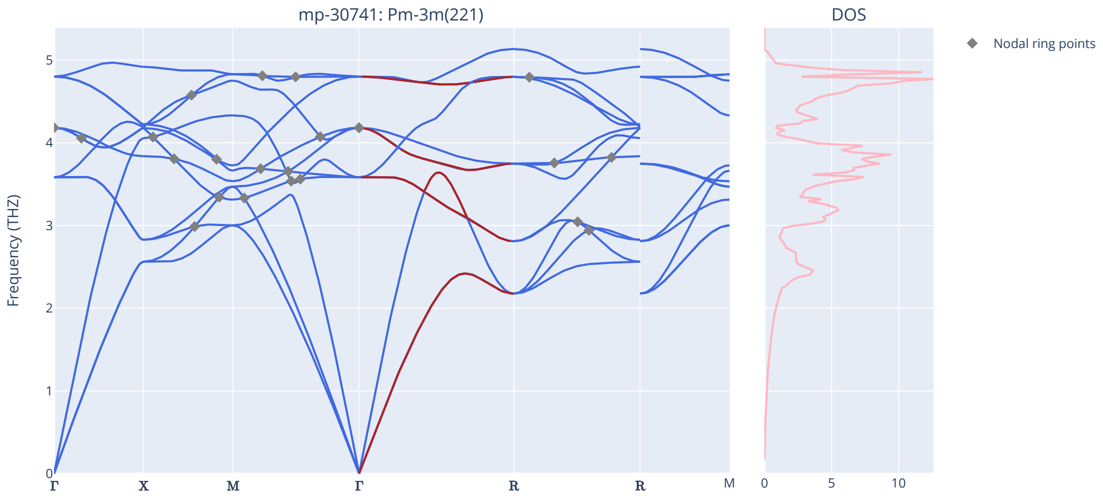

Supplementary Figure 576: The phonon band and DOS of Ir3Pa.

Supplementary Table 1224: High symmetry lines of mp-30741-Ir3Pa

	Start	End
0	Γ (0.000, 0.000, 0.000)	X (0.000, 0.500, 0.000)
1	X (0.000, 0.500, 0.000)	M (0.500, 0.500, 0.000)
2	M (0.500, 0.500, 0.000)	Γ (0.000, 0.000, 0.000)
3	Γ (0.000, 0.000, 0.000)	R (0.500, 0.500, 0.500)
4	R (0.500, 0.500, 0.500)	X (0.000, 0.500, 0.000)
5	R (0.500, 0.500, 0.500)	M (0.500, 0.500, 0.000)

Supplementary Table 1225: Nodal ring points along high symmetry lines of mp-30741-Ir3Pa.

	Coordinates	Frequency	Mode 1	Mode 2	Multi.	Deg.	Path	Ratio
0	(0.287, 0.500, 0.000)	2.988	3	2	12	2	2	0.574
1	(0.202, 0.500, 0.202)	2.942	3	2	12	2	5	0.595
2	(0.424, 0.500, -0.000)	3.343	4	3	12	2	2	0.848
3	(0.454, 0.454, -0.000)	3.332	4	3	12	2	3	0.091
4	(0.247, 0.500, 0.247)	3.044	4	3	12	2	5	0.506
5	(0.268, 0.268, 0.000)	3.536	5	4	12	2	3	0.463
6	(0.232, 0.232, -0.000)	3.561	5	4	12	2	3	0.535
7	(0.172, 0.500, -0.000)	3.806	6	5	12	2	2	0.343
8	(0.113, 0.500, 0.113)	3.823	6	5	12	2	5	0.774
9	(0.054, 0.500, -0.000)	4.069	7	6	12	2	2	0.107
10	(0.390, 0.390, -0.000)	3.686	7	6	12	2	3	0.221
11	(0.281, 0.281, 0.000)	3.654	7	6	12	2	3	0.439
12	(0.000, 0.153, 0.000)	4.056	8	7	6	2	1	0.306
13	(0.409, 0.500, -0.000)	3.801	8	7	12	2	2	0.818

Supplementary Table 1225: Nodal ring points along high symmetry lines of mp-30741-Ir3Pa.

	Coordinates	Frequency	Mode 1	Mode 2	Multi.	Deg.	Path	Ratio
14	(0.340, 0.500, 0.340)	3.756	8	7	12	2	5	0.321
15	(0.000, 0.000, 0.000)	4.181	9	8	1	3	1	0.000
16	(0.153, 0.153, 0.000)	4.072	9	8	12	2	3	0.693
17	(0.269, 0.500, 0.000)	4.575	11	10	12	2	2	0.539
18	(0.438, 0.500, 0.438)	4.792	11	10	12	2	5	0.123
19	(0.383, 0.383, -0.000)	4.809	12	11	12	2	3	0.235
20	(0.251, 0.251, -0.000)	4.796	12	11	12	2	3	0.498

The followings are details for $Li_{12}Pb_4$. It has 6 topological nodal lines and 17 nodal ring points along high symmetry line as shown in Fig **577**.

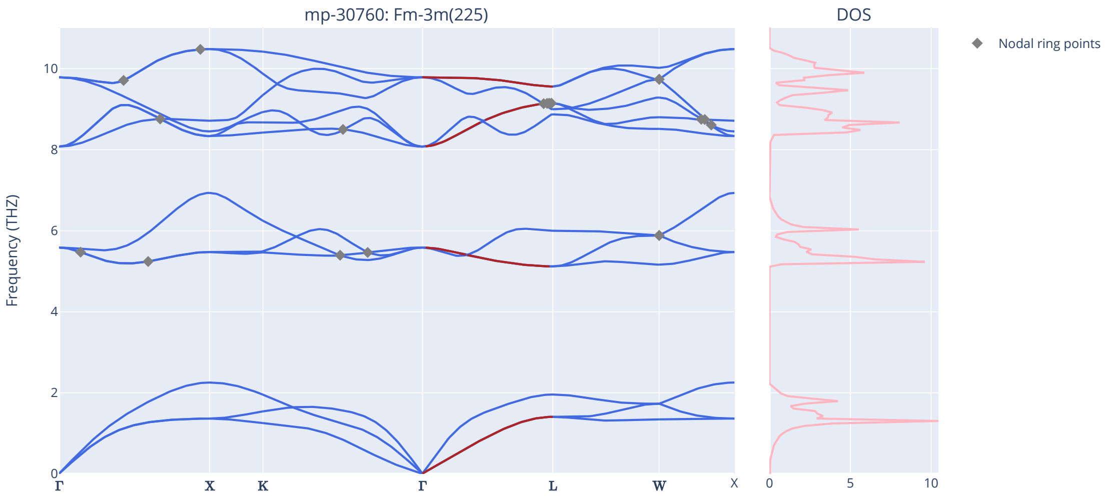

Supplementary Figure 577: The phonon band and DOS of Li12Pb4.

Supplementary Table 1226: High symmetry lines of mp-30760-Li12Pb4

	Start	End
0	Γ (0.000, 0.000, 0.000)	X (0.500, 0.000, 0.500)
1	X (0.500, 0.000, 0.500)	U (0.625, 0.250, 0.625)
2	K (0.375, 0.375, 0.750)	Γ (0.000, 0.000, 0.000)
3	Γ (0.000, 0.000, 0.000)	L (0.500, 0.500, 0.500)
4	L (0.500, 0.500, 0.500)	W (0.500, 0.250, 0.750)
5	W (0.500, 0.250, 0.750)	X (0.500, 0.000, 0.500)

Supplementary Table 1227: Nodal ring points along high symmetry lines of mp-30760-Li12Pb4.

	Coordinates	Frequency	Mode 1	Mode 2	Multi.	Deg.	Path	Ratio
0	(0.071, 0.000, 0.071)	5.469	5	4	6	2	1	0.143
1	(0.296, 0.000, 0.296)	5.238	5	4	6	2	1	0.592
2	(0.191, 0.191, 0.389)	5.392	5	4	24	2	3	0.484
3	(0.132, 0.132, 0.254)	5.462	6	5	24	2	3	0.657
4	(0.500, 0.250, 0.750)	5.881	6	5	6	2	5	1.000
5	(0.191, 0.191, 0.369)	8.498	8	7	24	2	3	0.502
6	(0.332, 0.000, 0.340)	8.756	9	8	24	2	1	0.672
7	(0.499, 0.111, 0.610)	8.749	9	8	24	2	6	0.559
8	(0.498, 0.076, 0.578)	8.607	9	8	24	2	6	0.693
9	(0.495, 0.495, 0.495)	9.145	10	9	8	2	4	0.990
10	(0.486, 0.486, 0.486)	9.144	10	9	8	2	4	0.972
11	(0.465, 0.465, 0.465)	9.134	10	9	8	2	4	0.930
12	(0.478, 0.478, 0.478)	9.141	10	9	8	2	4	0.956
13	(0.499, 0.099, 0.598)	8.743	10	9	24	2	6	0.606

Supplementary Table 1227: Nodal ring points along high symmetry lines of mp-30760-Li12Pb4.

	Coordinates	Frequency	Mode 1	Mode 2	Multi.	Deg.	Path	Ratio
14	(0.500, 0.250, 0.750)	9.736	11	10	6	2	5	1.000
15	(0.214, 0.000, 0.214)	9.708	12	11	6	2	1	0.429
16	(0.469, 0.000, 0.469)	10.474	12	11	6	2	1	0.939

The followings are details for MgPt$_3$. It has 7 topological nodal lines and 20 nodal ring points along high symmetry line as shown in Fig **578**.

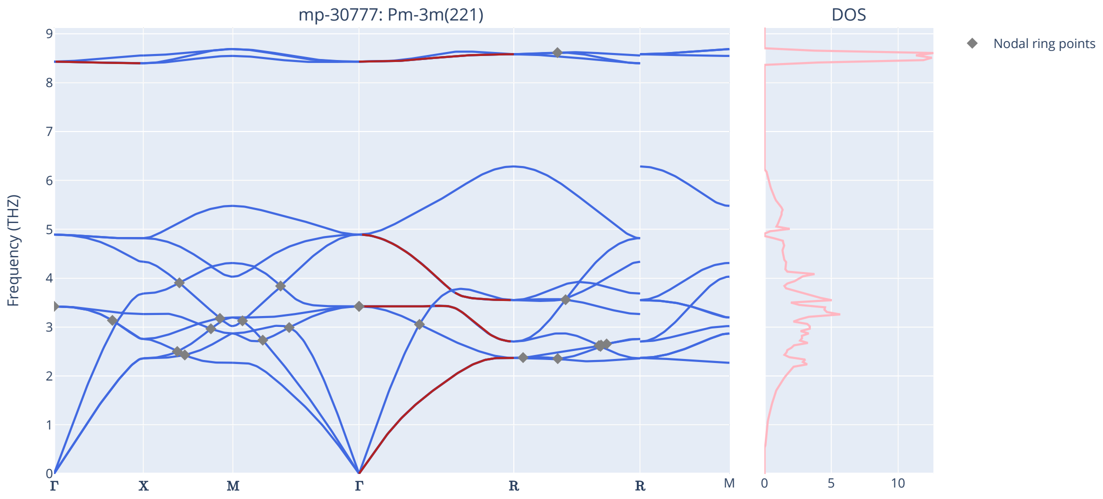

Supplementary Figure 578: The phonon band and DOS of MgPt3.

Supplementary Table 1228: High symmetry lines of mp-30777-MgPt3

	Start	End
0	Γ (0.000, 0.000, 0.000)	X (0.000, 0.500, 0.000)
1	X (0.000, 0.500, 0.000)	M (0.500, 0.500, 0.000)
2	M (0.500, 0.500, 0.000)	Γ (0.000, 0.000, 0.000)
3	Γ (0.000, 0.000, 0.000)	R (0.500, 0.500, 0.500)
4	R (0.500, 0.500, 0.500)	X (0.000, 0.500, 0.000)
5	R (0.500, 0.500, 0.500)	M (0.500, 0.500, 0.000)

Supplementary Table 1229: Nodal ring points along high symmetry lines of mp-30777-MgPt3.

	Coordinates	Frequency	Mode 1	Mode 2	Multi.	Deg.	Path	Ratio
0	(0.232, 0.500, 0.000)	2.429	2	1	12	2	2	0.463
1	(0.326, 0.500, 0.326)	2.354	2	1	12	2	5	0.348
2	(0.190, 0.500, -0.000)	2.500	3	2	12	2	2	0.379
3	(0.381, 0.381, 0.000)	2.732	3	2	12	2	3	0.238
4	(0.462, 0.500, 0.464)	2.376	3	2	24	2	5	0.074
5	(0.157, 0.500, 0.157)	2.606	3	2	12	2	5	0.686
6	(0.378, 0.500, 0.000)	2.965	4	3	12	2	2	0.756
7	(0.277, 0.277, 0.000)	2.990	4	3	12	2	3	0.447
8	(0.196, 0.195, 0.195)	3.055	4	3	24	2	4	0.390
9	(0.155, 0.494, 0.155)	2.631	4	3	24	2	5	0.691
10	(0.132, 0.495, 0.132)	2.654	4	3	24	2	5	0.735
11	(0.163, 0.500, 0.163)	2.621	4	3	12	2	5	0.674
12	(0.000, 0.000, 0.000)	3.423	5	4	1	3	1	0.000
13	(0.000, 0.327, 0.000)	3.137	5	4	6	2	1	0.653

Supplementary Table 1229: Nodal ring points along high symmetry lines of mp-30777-MgPt3.

	Coordinates	Frequency	Mode 1	Mode 2	Multi.	Deg.	Path	Ratio
14	(0.461, 0.461, -0.000)	3.128	5	4	12	2	3	0.077
15	(0.428, 0.500, -0.000)	3.177	6	5	12	2	2	0.857
16	(0.295, 0.500, 0.295)	3.560	6	5	12	2	5	0.410
17	(0.201, 0.500, -0.000)	3.905	7	6	12	2	2	0.403
18	(0.311, 0.311, -0.000)	3.840	7	6	12	2	3	0.379
19	(0.327, 0.500, 0.327)	8.614	12	11	12	2	5	0.346

The followings are details for Pt$_3$. It has 7 topological nodal lines and 17 nodal ring points along high symmetry line as shown in Fig **579**.

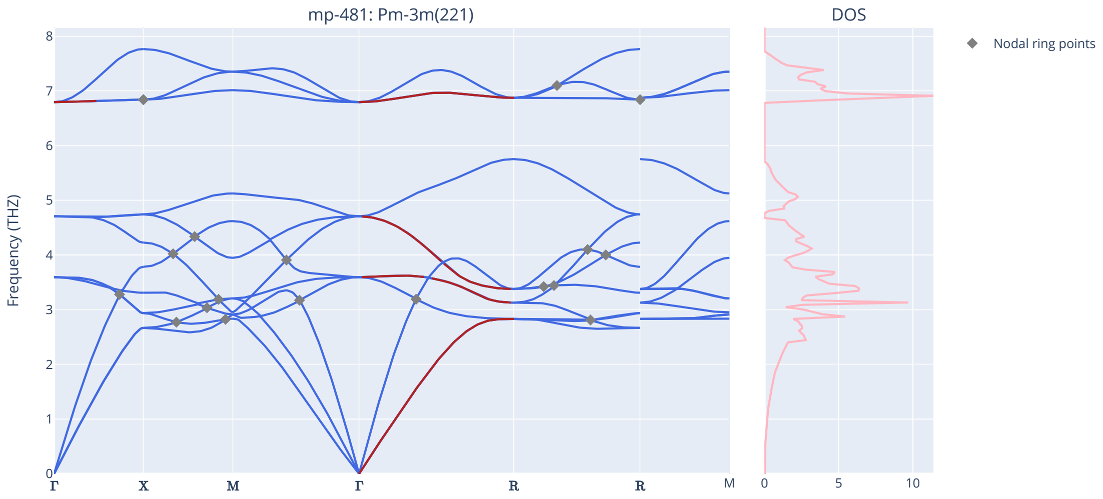

Supplementary Figure 579: The phonon band and DOS of Pt3Sc.

Supplementary Table 1230: High symmetry lines of mp-481-Pt3Sc

	Start	End
0	Γ (0.000, 0.000, 0.000)	X (0.000, 0.500, 0.000)
1	X (0.000, 0.500, 0.000)	M (0.500, 0.500, 0.000)
2	M (0.500, 0.500, 0.000)	Γ (0.000, 0.000, 0.000)
3	Γ (0.000, 0.000, 0.000)	R (0.500, 0.500, 0.500)
4	R (0.500, 0.500, 0.500)	X (0.000, 0.500, 0.000)
5	R (0.500, 0.500, 0.500)	M (0.500, 0.500, 0.000)

Supplementary Table 1231: Nodal ring points along high symmetry lines of mp-481-Pt3Sc.

	Coordinates	Frequency	Mode 1	Mode 2	Multi.	Deg.	Path	Ratio
0	(0.460, 0.500, 0.000)	2.823	2	1	12	2	2	0.919
1	(0.185, 0.500, 0.000)	2.772	3	2	12	2	2	0.369
2	(0.196, 0.500, 0.196)	2.811	3	2	12	2	5	0.607
3	(0.356, 0.500, 0.000)	3.035	4	3	12	2	2	0.712
4	(0.236, 0.236, 0.000)	3.172	4	3	12	2	3	0.529
5	(0.184, 0.184, 0.184)	3.191	4	3	8	2	4	0.369
6	(-0.000, 0.366, -0.000)	3.279	5	4	6	2	1	0.733
7	(0.421, 0.500, -0.000)	3.188	6	5	12	2	2	0.841
8	(0.341, 0.500, 0.341)	3.441	6	5	12	2	5	0.318
9	(0.166, 0.500, -0.000)	4.021	7	6	12	2	2	0.332
10	(0.287, 0.287, -0.000)	3.908	7	6	12	2	3	0.425
11	(0.136, 0.500, 0.136)	4.000	7	6	12	2	5	0.728
12	(0.382, 0.500, 0.382)	3.421	7	6	12	2	5	0.237
13	(0.287, 0.500, 0.000)	4.336	8	7	12	2	2	0.574

Supplementary Table 1231: Nodal ring points along high symmetry lines of mp-481-Pt3Sc.

	Coordinates	Frequency	Mode 1	Mode 2	Multi.	Deg.	Path	Ratio
14	(0.208, 0.500, 0.208)	4.099	8	7	12	2	5	0.584
15	(0.000, 0.500, 0.000)	6.839	11	10	3	2	1	1.000
16	(0.329, 0.500, 0.329)	7.098	12	11	12	2	5	0.342

The followings are details for $LiPt_3$. It has 7 topological nodal lines and 17 nodal ring points along high symmetry line as shown in Fig **580**.

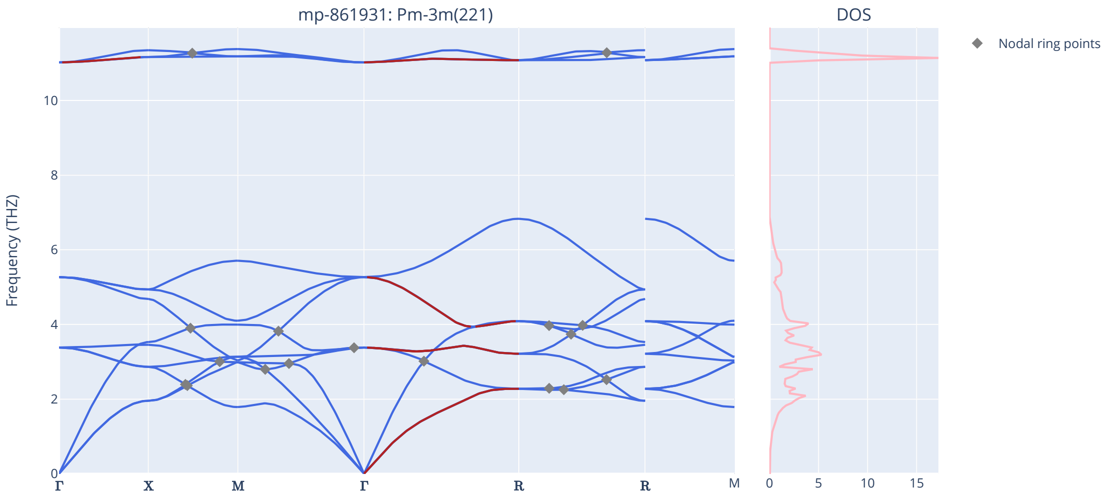

Supplementary Figure 580: The phonon band and DOS of LiPt3.

Supplementary Table 1232: High symmetry lines of mp-861931-LiPt3

	Start	End
0	Γ (0.000, 0.000, 0.000)	X (0.000, 0.500, 0.000)
1	X (0.000, 0.500, 0.000)	M (0.500, 0.500, 0.000)
2	M (0.500, 0.500, 0.000)	Γ (0.000, 0.000, 0.000)
3	Γ (0.000, 0.000, 0.000)	R (0.500, 0.500, 0.500)
4	R (0.500, 0.500, 0.500)	X (0.000, 0.500, 0.000)
5	R (0.500, 0.500, 0.500)	M (0.500, 0.500, 0.000)

Supplementary Table 1233: Nodal ring points along high symmetry lines of mp-861931-LiPt3.

	Coordinates	Frequency	Mode 1	Mode 2	Multi.	Deg.	Path	Ratio
0	(0.218, 0.500, 0.000)	2.361	2	1	12	2	2	0.436
1	(0.322, 0.500, 0.322)	2.252	2	1	12	2	5	0.357
2	(0.207, 0.500, -0.000)	2.393	3	2	12	2	2	0.414
3	(0.391, 0.391, 0.000)	2.796	3	2	12	2	3	0.218
4	(0.380, 0.500, 0.380)	2.289	3	2	12	2	5	0.241
5	(0.153, 0.500, 0.153)	2.520	3	2	12	2	5	0.695
6	(0.399, 0.500, -0.000)	3.003	4	3	12	2	2	0.798
7	(0.297, 0.297, -0.000)	2.953	4	3	12	2	3	0.406
8	(0.193, 0.193, 0.193)	3.016	4	3	8	2	4	0.387
9	(0.040, 0.040, -0.000)	3.376	6	5	12	2	3	0.921
10	(0.293, 0.500, 0.293)	3.739	6	5	12	2	5	0.414
11	(0.236, 0.500, -0.000)	3.900	7	6	12	2	2	0.471
12	(0.339, 0.339, 0.000)	3.820	7	6	12	2	3	0.322
13	(0.380, 0.500, 0.380)	3.969	7	6	12	2	5	0.240

Supplementary Table 1233: Nodal ring points along high symmetry lines of mp-861931-LiPt3.

	Coordinates	Frequency	Mode 1	Mode 2	Multi.	Deg.	Path	Ratio
14	(0.247, 0.500, 0.247)	3.975	8	7	12	2	5	0.506
15	(0.246, 0.500, -0.000)	11.268	12	11	12	2	2	0.491
16	(0.152, 0.500, 0.152)	11.280	12	11	12	2	5	0.696

The followings are details for $LiPd_3$. It has 7 topological nodal lines and 15 nodal ring points along high symmetry line as shown in Fig **581**.

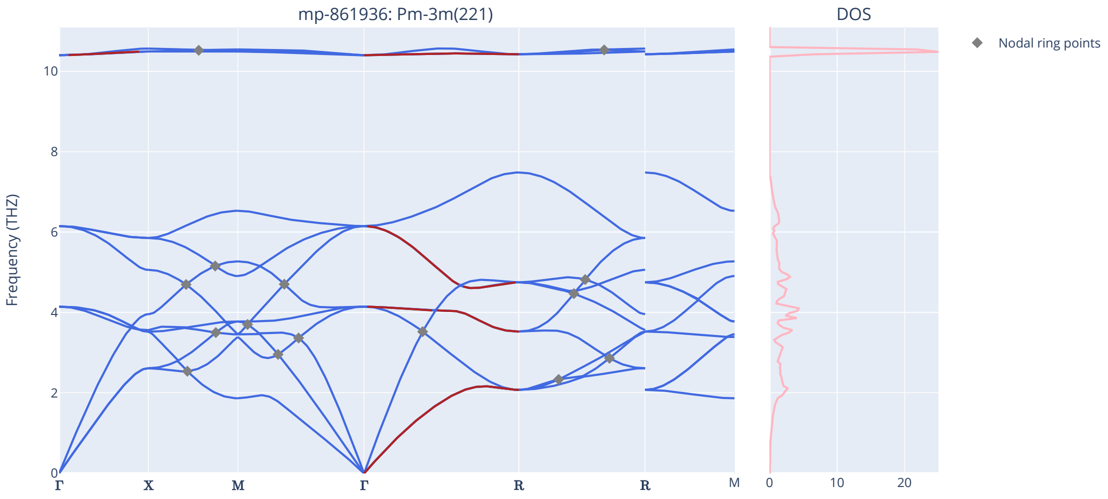

Supplementary Figure 581: The phonon band and DOS of LiPd3.

Supplementary Table 1234: High symmetry lines of mp-861936-LiPd3

	Start	End
0	Γ (0.000, 0.000, 0.000)	X (0.000, 0.500, 0.000)
1	X (0.000, 0.500, 0.000)	M (0.500, 0.500, 0.000)
2	M (0.500, 0.500, 0.000)	Γ (0.000, 0.000, 0.000)
3	Γ (0.000, 0.000, 0.000)	R (0.500, 0.500, 0.500)
4	R (0.500, 0.500, 0.500)	X (0.000, 0.500, 0.000)
5	R (0.500, 0.500, 0.500)	M (0.500, 0.500, 0.000)

Supplementary Table 1235: Nodal ring points along high symmetry lines of mp-861936-LiPd3.

	Coordinates	Frequency	Mode 1	Mode 2	Multi.	Deg.	Path	Ratio
0	(0.218, 0.500, -0.000)	2.537	2	1	12	2	2	0.436
1	(0.340, 0.340, -0.000)	2.956	3	2	12	2	3	0.319
2	(0.342, 0.500, 0.342)	2.333	3	2	12	2	5	0.316
3	(0.141, 0.500, 0.141)	2.861	3	2	12	2	5	0.718
4	(0.377, 0.500, -0.000)	3.499	4	3	12	2	2	0.755
5	(0.259, 0.259, 0.000)	3.364	4	3	12	2	3	0.482
6	(0.189, 0.189, 0.189)	3.521	4	3	8	2	4	0.379
7	(0.461, 0.461, -0.000)	3.701	5	4	12	2	3	0.078
8	(0.282, 0.500, 0.282)	4.468	6	5	12	2	5	0.436
9	(0.211, 0.500, -0.000)	4.695	7	6	12	2	2	0.422
10	(0.316, 0.316, -0.000)	4.702	7	6	12	2	3	0.368
11	(0.374, 0.500, 0.000)	5.155	8	7	12	2	2	0.748
12	(0.237, 0.500, 0.237)	4.818	8	7	12	2	5	0.526
13	(0.282, 0.500, 0.000)	10.521	12	11	12	2	2	0.563
14	(0.162, 0.500, 0.162)	10.530	12	11	12	2	5	0.676

Supplementary Table 1235: Nodal ring points along high symmetry lines of mp-861936-LiPd3.

Coordinates	Frequency	Mode 1	Mode 2	Multi.	Deg.	Path	Ratio

The followings are details for Ac$_3$. It has 8 topological nodal lines and 19 nodal ring points along high symmetry line as shown in Fig **582**.

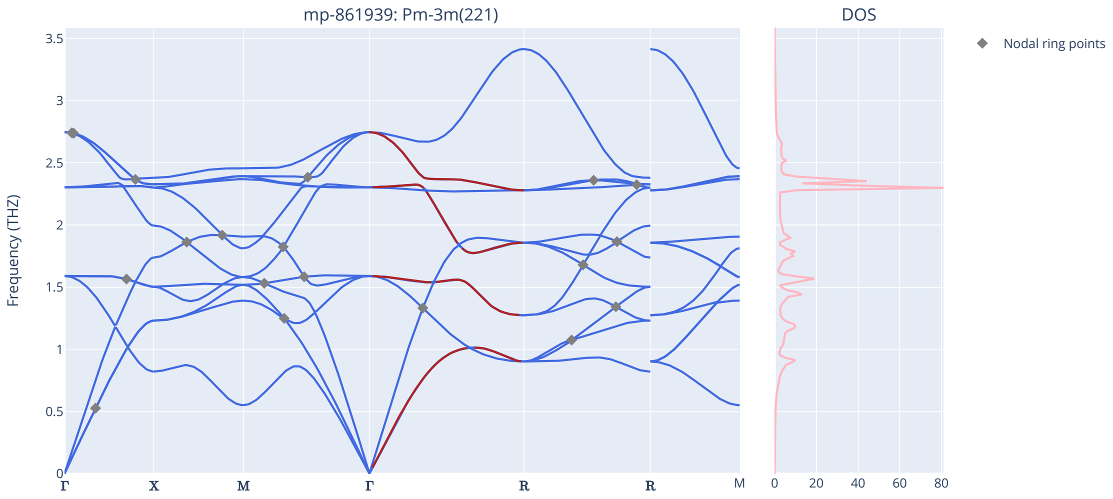

Supplementary Figure 582: The phonon band and DOS of Ac3Sn.

Supplementary Table 1236: High symmetry lines of mp-861939-Ac3Sn

	Start	End
0	Γ (0.000, 0.000, 0.000)	X (0.000, 0.500, 0.000)
1	X (0.000, 0.500, 0.000)	M (0.500, 0.500, 0.000)
2	M (0.500, 0.500, 0.000)	Γ (0.000, 0.000, 0.000)
3	Γ (0.000, 0.000, 0.000)	R (0.500, 0.500, 0.500)
4	R (0.500, 0.500, 0.500)	X (0.000, 0.500, 0.000)
5	R (0.500, 0.500, 0.500)	M (0.500, 0.500, 0.000)

Supplementary Table 1237: Nodal ring points along high symmetry lines of mp-861939-Ac3Sn.

	Coordinates	Frequency	Mode 1	Mode 2	Multi.	Deg.	Path	Ratio
0	(0.000, 0.173, 0.000)	0.527	2	1	6	2	1	0.347
1	(0.337, 0.337, 0.000)	1.249	3	2	12	2	3	0.325
2	(0.312, 0.500, 0.312)	1.075	3	2	12	2	5	0.377
3	(0.173, 0.173, 0.173)	1.332	4	3	8	2	4	0.346
4	(0.136, 0.500, 0.136)	1.343	4	3	12	2	5	0.727
5	(0.416, 0.416, -0.000)	1.532	5	4	12	2	3	0.168
6	(0.000, 0.347, 0.000)	1.565	6	5	6	2	1	0.694
7	(0.258, 0.258, 0.000)	1.584	6	5	12	2	3	0.484
8	(0.266, 0.500, 0.266)	1.679	6	5	12	2	5	0.468
9	(0.184, 0.500, -0.000)	1.863	7	6	12	2	2	0.368
10	(0.341, 0.341, -0.000)	1.824	7	6	12	2	3	0.317
11	(0.132, 0.500, 0.132)	1.866	7	6	12	2	5	0.736
12	(0.383, 0.500, -0.000)	1.918	8	7	12	2	2	0.765
13	(0.054, 0.500, 0.053)	2.324	10	9	24	2	5	0.892

Supplementary Table 1237: Nodal ring points along high symmetry lines of mp-861939-Ac3Sn.

	Coordinates	Frequency	Mode 1	Mode 2	Multi.	Deg.	Path	Ratio
14	(0.000, 0.397, 0.000)	2.367	11	10	6	2	1	0.794
15	(0.244, 0.244, 0.000)	2.384	11	10	12	2	3	0.512
16	(0.224, 0.500, 0.224)	2.360	11	10	12	2	5	0.551
17	(0.000, 0.041, 0.000)	2.741	12	11	6	2	1	0.082
18	(0.000, 0.051, 0.000)	2.738	12	11	6	2	1	0.102

The followings are details for Ga$_3$. It has 5 topological nodal lines and 16 nodal ring points along high symmetry line as shown in Fig **583**.

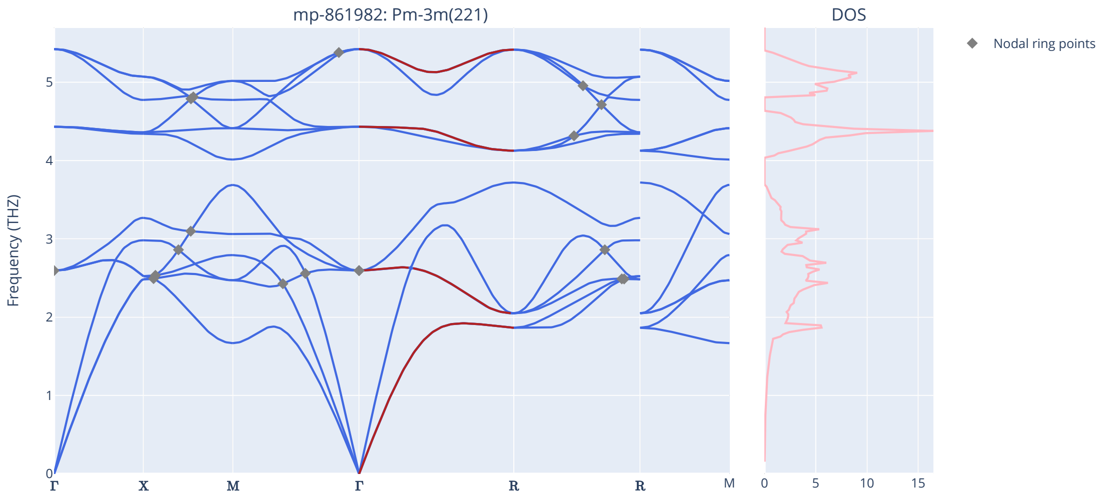

Supplementary Figure 583: The phonon band and DOS of Ga3Pa.

Supplementary Table 1238: High symmetry lines of mp-861982-Ga3Pa

	Start	End
0	Γ (0.000, 0.000, 0.000)	X (0.000, 0.500, 0.000)
1	X (0.000, 0.500, 0.000)	M (0.500, 0.500, 0.000)
2	M (0.500, 0.500, 0.000)	Γ (0.000, 0.000, 0.000)
3	Γ (0.000, 0.000, 0.000)	R (0.500, 0.500, 0.500)
4	R (0.500, 0.500, 0.500)	X (0.000, 0.500, 0.000)
5	R (0.500, 0.500, 0.500)	M (0.500, 0.500, 0.000)

Supplementary Table 1239: Nodal ring points along high symmetry lines of mp-861982-Ga3Pa.

	Coordinates	Frequency	Mode 1	Mode 2	Multi.	Deg.	Path	Ratio
0	(0.057, 0.500, -0.000)	2.492	2	1	12	2	2	0.114
1	(0.063, 0.500, 0.063)	2.488	2	1	12	2	5	0.874
2	(0.302, 0.302, -0.000)	2.427	3	2	12	2	3	0.397
3	(0.072, 0.500, 0.072)	2.489	3	2	12	2	5	0.855
4	(0.067, 0.500, -0.000)	2.534	4	3	12	2	2	0.134
5	(0.213, 0.213, -0.000)	2.558	4	3	12	2	3	0.575
6	(0.000, 0.000, 0.000)	2.594	5	4	1	3	1	0.000
7	(0.196, 0.500, -0.000)	2.860	5	4	12	2	2	0.392
8	(0.140, 0.500, 0.140)	2.861	5	4	12	2	5	0.721
9	(0.265, 0.500, 0.000)	3.098	6	5	12	2	2	0.530
10	(0.262, 0.500, 0.262)	4.316	9	8	12	2	5	0.477
11	(0.266, 0.500, -0.000)	4.788	10	9	12	2	2	0.532
12	(0.153, 0.500, 0.153)	4.714	10	9	12	2	5	0.694
13	(0.280, 0.500, -0.000)	4.813	11	10	12	2	2	0.559

Supplementary Table 1239: Nodal ring points along high symmetry lines of mp-861982-Ga3Pa.

	Coordinates	Frequency	Mode 1	Mode 2	Multi.	Deg.	Path	Ratio
14	(0.226, 0.500, 0.226)	4.955	11	10	12	2	5	0.548
15	(0.080, 0.080, 0.000)	5.380	12	11	12	2	3	0.840

The followings are details for In$_3$. It has 6 topological nodal lines and 27 nodal ring points along high symmetry line as shown in Fig 584.

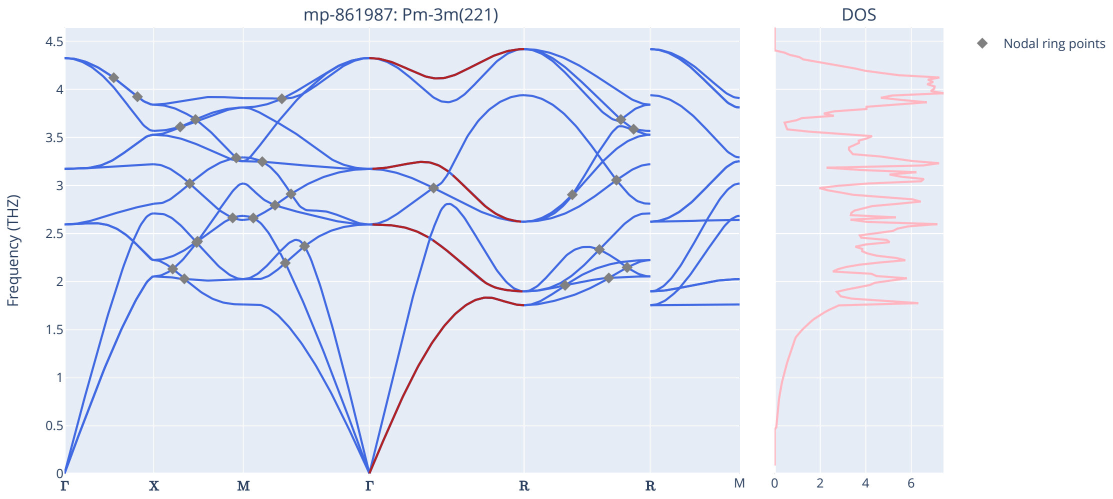

Supplementary Figure 584: The phonon band and DOS of In3Pa.

Supplementary Table 1240: High symmetry lines of mp-861987-In3Pa

	Start	End
0	Γ (0.000, 0.000, 0.000)	X (0.000, 0.500, 0.000)
1	X (0.000, 0.500, 0.000)	M (0.500, 0.500, 0.000)
2	M (0.500, 0.500, 0.000)	Γ (0.000, 0.000, 0.000)
3	Γ (0.000, 0.000, 0.000)	R (0.500, 0.500, 0.500)
4	R (0.500, 0.500, 0.500)	X (0.000, 0.500, 0.000)
5	R (0.500, 0.500, 0.500)	M (0.500, 0.500, 0.000)

Supplementary Table 1241: Nodal ring points along high symmetry lines of mp-861987-In3Pa.

	Coordinates	Frequency	Mode 1	Mode 2	Multi.	Deg.	Path	Ratio
0	(0.171, 0.500, 0.000)	2.029	2	1	12	2	2	0.342
1	(0.164, 0.500, 0.164)	2.037	2	1	12	2	5	0.671
2	(0.106, 0.500, -0.000)	2.131	3	2	12	2	2	0.212
3	(0.333, 0.333, 0.000)	2.193	3	2	12	2	3	0.334
4	(0.337, 0.500, 0.337)	1.961	3	2	12	2	5	0.325
5	(0.092, 0.500, 0.092)	2.147	3	2	12	2	5	0.817
6	(0.240, 0.500, 0.000)	2.407	4	3	12	2	2	0.480
7	(0.257, 0.257, 0.000)	2.368	4	3	12	2	3	0.486
8	(0.245, 0.500, -0.000)	2.419	5	4	12	2	2	0.489
9	(0.442, 0.500, 0.000)	2.663	5	4	12	2	2	0.883
10	(0.460, 0.460, 0.000)	2.661	5	4	12	2	3	0.080
11	(0.202, 0.500, 0.202)	2.333	5	4	12	2	5	0.597
12	(0.374, 0.374, 0.000)	2.795	6	5	12	2	3	0.253
13	(0.201, 0.500, -0.000)	3.020	7	6	12	2	2	0.402

Supplementary Table 1241: Nodal ring points along high symmetry lines of mp-861987-In3Pa.

	Coordinates	Frequency	Mode 1	Mode 2	Multi.	Deg.	Path	Ratio
14	(0.310, 0.310, 0.000)	2.911	7	6	12	2	3	0.380
15	(0.208, 0.208, 0.208)	2.974	7	6	8	2	4	0.415
16	(0.134, 0.500, 0.134)	3.056	7	6	12	2	5	0.732
17	(0.424, 0.424, -0.000)	3.247	8	7	12	2	3	0.152
18	(0.309, 0.500, 0.309)	2.904	8	7	12	2	5	0.383
19	(0.462, 0.499, 0.000)	3.287	9	8	24	2	2	0.924
20	(0.148, 0.500, -0.000)	3.610	10	9	12	2	2	0.295
21	(0.066, 0.500, 0.066)	3.586	10	9	12	2	5	0.868
22	(0.233, 0.500, -0.000)	3.686	11	10	12	2	2	0.467
23	(0.117, 0.500, 0.117)	3.686	11	10	12	2	5	0.767
24	(0.000, 0.276, 0.000)	4.122	12	11	6	2	1	0.551
25	(0.000, 0.408, 0.000)	3.924	12	11	6	2	1	0.816
26	(0.347, 0.347, 0.000)	3.902	12	11	12	2	3	0.307

The followings are details for $Cd_{12}La_4$. It has 6 topological nodal lines and 12 nodal ring points along high symmetry line as shown in Fig **585**.

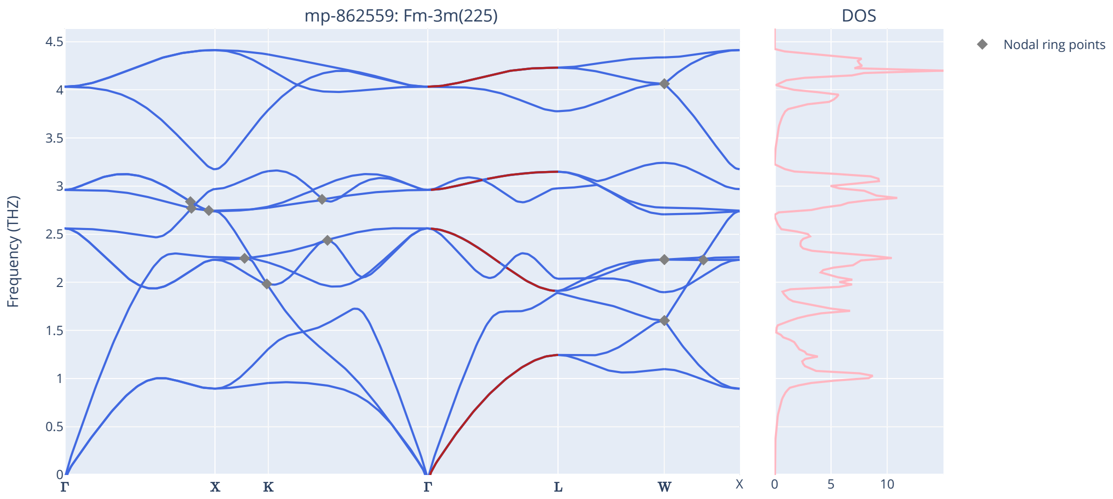

Supplementary Figure 585: The phonon band and DOS of Cd12La4.

Supplementary Table 1242: High symmetry lines of mp-862559-Cd12La4

	Start	End
0	Γ (0.000, 0.000, 0.000)	X (0.500, 0.000, 0.500)
1	X (0.500, 0.000, 0.500)	U (0.625, 0.250, 0.625)
2	K (0.375, 0.375, 0.750)	Γ (0.000, 0.000, 0.000)
3	Γ (0.000, 0.000, 0.000)	L (0.500, 0.500, 0.500)
4	L (0.500, 0.500, 0.500)	W (0.500, 0.250, 0.750)
5	W (0.500, 0.250, 0.750)	X (0.500, 0.000, 0.500)

Supplementary Table 1243: Nodal ring points along high symmetry lines of mp-862559-Cd12La4.

	Coordinates	Frequency	Mode 1	Mode 2	Multi.	Deg.	Path	Ratio
0	(0.500, 0.250, 0.750)	1.603	3	2	6	2	5	1.000
1	(0.622, 0.244, 0.622)	1.983	4	3	12	2	2	0.975
2	(0.571, 0.137, 0.571)	2.249	5	4	24	2	2	0.556
3	(0.499, 0.120, 0.620)	2.232	5	4	24	2	6	0.520
4	(0.241, 0.241, 0.466)	2.436	6	5	24	2	3	0.372
5	(0.500, 0.250, 0.750)	2.236	6	5	6	2	5	1.000
6	(0.422, -0.001, 0.422)	2.769	7	6	24	2	1	0.844
7	(0.480, 0.000, 0.480)	2.746	7	6	6	2	1	0.959
8	(0.253, 0.253, 0.492)	2.862	8	7	24	2	3	0.338
9	(0.418, 0.000, 0.418)	2.838	9	8	6	2	1	0.837
10	(0.500, 0.250, 0.750)	4.063	11	10	6	2	5	1.000

The followings are details for $PaGe_3$. It has 5 topological nodal lines and 14 nodal ring points along high symmetry line as shown in Fig **586**.

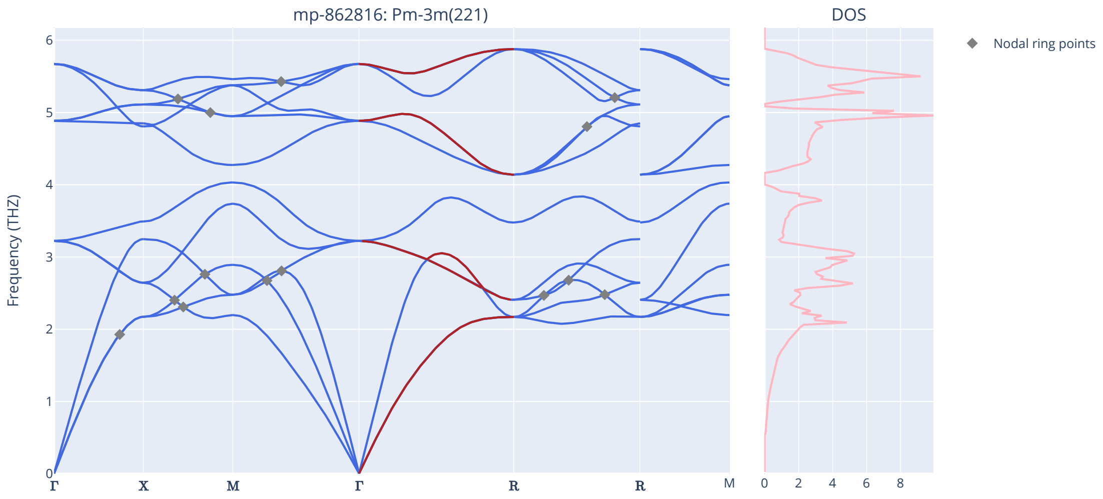

Supplementary Figure 586: The phonon band and DOS of PaGe3.

Supplementary Table 1244: High symmetry lines of mp-862816-PaGe3

	Start	End
0	Γ (0.000, 0.000, 0.000)	X (0.000, 0.500, 0.000)
1	X (0.000, 0.500, 0.000)	M (0.500, 0.500, 0.000)
2	M (0.500, 0.500, 0.000)	Γ (0.000, 0.000, 0.000)
3	Γ (0.000, 0.000, 0.000)	R (0.500, 0.500, 0.500)
4	R (0.500, 0.500, 0.500)	X (0.000, 0.500, 0.000)
5	R (0.500, 0.500, 0.500)	M (0.500, 0.500, 0.000)

Supplementary Table 1245: Nodal ring points along high symmetry lines of mp-862816-PaGe3.

	Coordinates	Frequency	Mode 1	Mode 2	Multi.	Deg.	Path	Ratio
0	(0.000, 0.367, 0.000)	1.928	2	1	6	2	1	0.735
1	(0.223, 0.500, 0.000)	2.309	2	1	12	2	2	0.446
2	(0.174, 0.500, -0.000)	2.402	3	2	12	2	2	0.349
3	(0.364, 0.364, 0.000)	2.673	3	2	12	2	3	0.271
4	(0.140, 0.500, 0.140)	2.479	3	2	12	2	5	0.720
5	(0.344, 0.500, -0.000)	2.761	4	3	12	2	2	0.688
6	(0.307, 0.307, 0.000)	2.808	4	3	12	2	3	0.386
7	(0.283, 0.500, 0.283)	2.679	4	3	12	2	5	0.435
8	(0.381, 0.500, 0.381)	2.469	4	3	12	2	5	0.238
9	(0.375, 0.500, -0.000)	4.999	9	8	12	2	2	0.749
10	(0.210, 0.500, 0.210)	4.805	9	8	12	2	5	0.579
11	(0.193, 0.500, 0.000)	5.187	11	10	12	2	2	0.387
12	(0.100, 0.500, 0.100)	5.206	11	10	12	2	5	0.799
13	(0.308, 0.308, -0.000)	5.428	12	11	12	2	3	0.384

The followings are details for $PaSi_3$. It has 8 topological nodal lines and 21 nodal ring points along high symmetry line as shown in Fig **587**.

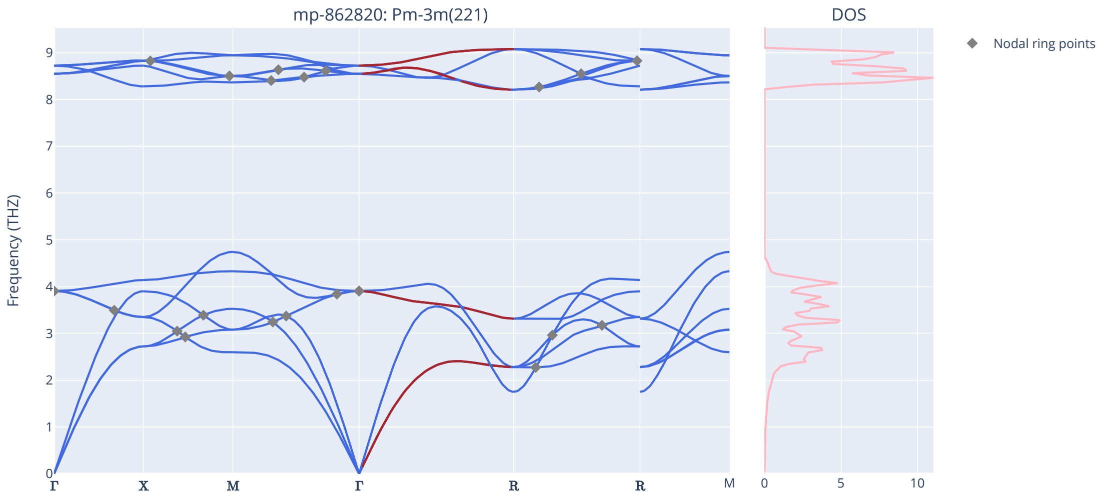

Supplementary Figure 587: The phonon band and DOS of PaSi3.

Supplementary Table 1246: High symmetry lines of mp-862820-PaSi3

	Start	End
0	Γ (0.000, 0.000, 0.000)	X (0.000, 0.500, 0.000)
1	X (0.000, 0.500, 0.000)	M (0.500, 0.500, 0.000)
2	M (0.500, 0.500, 0.000)	Γ (0.000, 0.000, 0.000)
3	Γ (0.000, 0.000, 0.000)	R (0.500, 0.500, 0.500)
4	R (0.500, 0.500, 0.500)	X (0.000, 0.500, 0.000)
5	R (0.500, 0.500, 0.500)	M (0.500, 0.500, 0.000)

Supplementary Table 1247: Nodal ring points along high symmetry lines of mp-862820-PaSi3.

	Coordinates	Frequency	Mode 1	Mode 2	Multi.	Deg.	Path	Ratio
0	(0.234, 0.500, 0.000)	2.925	2	1	12	2	2	0.469
1	(0.413, 0.500, 0.413)	2.273	2	1	12	2	5	0.173
2	(0.189, 0.500, -0.000)	3.048	3	2	12	2	2	0.378
3	(0.341, 0.341, 0.000)	3.244	3	2	12	2	3	0.318
4	(0.151, 0.500, 0.151)	3.172	3	2	12	2	5	0.698
5	(0.336, 0.500, -0.000)	3.389	4	3	12	2	2	0.672
6	(0.288, 0.288, -0.000)	3.371	4	3	12	2	3	0.423
7	(0.347, 0.500, 0.347)	2.962	4	3	12	2	5	0.305
8	(0.000, 0.000, 0.000)	3.906	5	4	1	3	1	0.000
9	(0.000, 0.337, 0.000)	3.494	5	4	6	2	1	0.673
10	(0.088, 0.088, 0.000)	3.840	5	4	12	2	3	0.825
11	(0.348, 0.348, 0.000)	8.404	8	7	12	2	3	0.304
12	(0.217, 0.217, -0.000)	8.478	8	7	12	2	3	0.565
13	(0.132, 0.132, 0.000)	8.611	9	8	12	2	3	0.735

Supplementary Table 1247: Nodal ring points along high symmetry lines of mp-862820-PaSi3.

	Coordinates	Frequency	Mode 1	Mode 2	Multi.	Deg.	Path	Ratio
14	(0.400, 0.500, 0.400)	8.266	9	8	12	2	5	0.201
15	(0.039, 0.500, -0.000)	8.823	10	9	12	2	2	0.077
16	(0.481, 0.500, 0.000)	8.503	10	9	12	2	2	0.963
17	(0.319, 0.319, -0.000)	8.636	10	9	12	2	3	0.362
18	(0.233, 0.500, 0.233)	8.549	10	9	12	2	5	0.533
19	(0.012, 0.500, 0.012)	8.827	11	10	12	2	5	0.976

The followings are details for Pa$_3$. It has 9 topological nodal lines and 18 nodal ring points along high symmetry line as shown in Fig **588**.

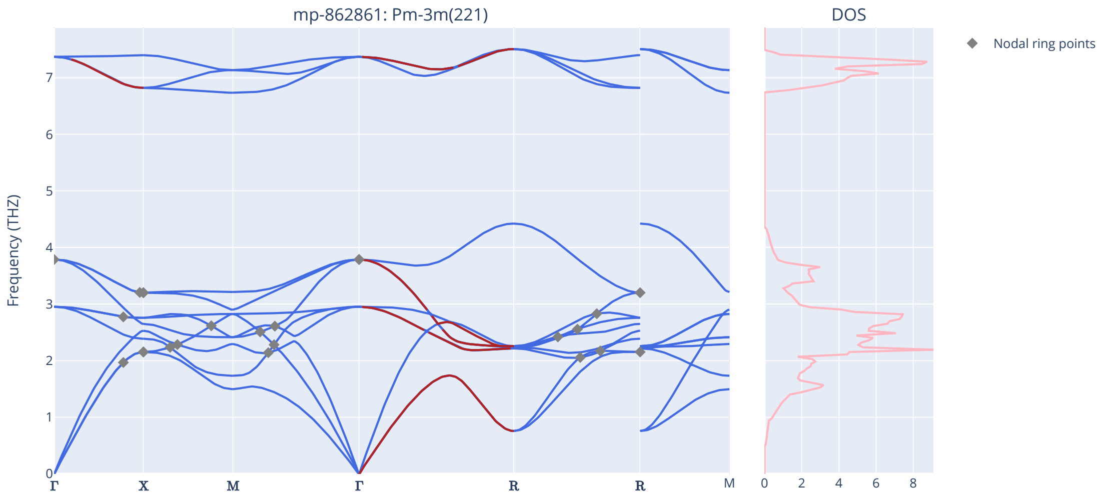

Supplementary Figure 588: The phonon band and DOS of Pa3Si.

Supplementary Table 1248: High symmetry lines of mp-862861-Pa3Si

	Start	End
0	Γ (0.000, 0.000, 0.000)	X (0.000, 0.500, 0.000)
1	X (0.000, 0.500, 0.000)	M (0.500, 0.500, 0.000)
2	M (0.500, 0.500, 0.000)	Γ (0.000, 0.000, 0.000)
3	Γ (0.000, 0.000, 0.000)	R (0.500, 0.500, 0.500)
4	R (0.500, 0.500, 0.500)	X (0.000, 0.500, 0.000)
5	R (0.500, 0.500, 0.500)	M (0.500, 0.500, 0.000)

Supplementary Table 1249: Nodal ring points along high symmetry lines of mp-862861-Pa3Si.

	Coordinates	Frequency	Mode 1	Mode 2	Multi.	Deg.	Path	Ratio
0	(0.000, 0.388, 0.000)	1.966	2	1	6	2	1	0.776
1	(0.000, 0.500, 0.000)	2.152	2	1	3	2	1	1.000
2	(0.149, 0.500, -0.000)	2.234	3	2	12	2	2	0.299
3	(0.359, 0.359, -0.000)	2.140	3	2	12	2	3	0.282
4	(0.157, 0.500, 0.157)	2.170	3	2	12	2	5	0.685
5	(0.237, 0.500, 0.237)	2.056	3	2	12	2	5	0.526
6	(0.190, 0.500, -0.000)	2.283	4	3	12	2	2	0.381
7	(0.337, 0.337, 0.000)	2.280	4	3	12	2	3	0.327
8	(0.391, 0.391, 0.000)	2.508	5	4	12	2	3	0.217
9	(0.000, 0.388, 0.000)	2.772	6	5	6	2	1	0.776
10	(0.380, 0.500, -0.000)	2.616	6	5	12	2	2	0.760
11	(0.333, 0.333, -0.000)	2.608	6	5	12	2	3	0.334
12	(0.248, 0.500, 0.248)	2.554	7	6	12	2	5	0.504
13	(0.326, 0.500, 0.326)	2.419	7	6	12	2	5	0.349

Supplementary Table 1249: Nodal ring points along high symmetry lines of mp-862861-Pa3Si.

	Coordinates	Frequency	Mode 1	Mode 2	Multi.	Deg.	Path	Ratio
14	(0.171, 0.500, 0.171)	2.829	8	7	12	2	5	0.657
15	(0.000, 0.000, 0.000)	3.787	9	8	1	3	1	0.000
16	(0.000, 0.480, 0.000)	3.205	9	8	6	2	1	0.959
17	(0.000, 0.500, 0.000)	3.200	9	8	3	2	1	1.000

The followings are details for Pa$_3$. It has 7 topological nodal lines and 23 nodal ring points along high symmetry line as shown in Fig **589**.

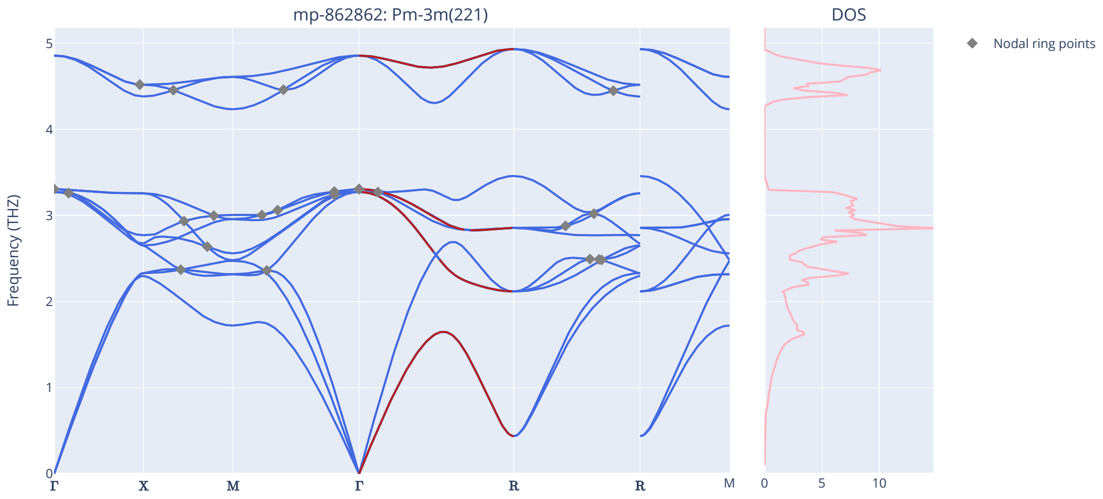

Supplementary Figure 589: The phonon band and DOS of Pa3Sb.

Supplementary Table 1250: High symmetry lines of mp-862862-Pa3Sb

	Start	End
0	Γ (0.000, 0.000, 0.000)	X (0.000, 0.500, 0.000)
1	X (0.000, 0.500, 0.000)	M (0.500, 0.500, 0.000)
2	M (0.500, 0.500, 0.000)	Γ (0.000, 0.000, 0.000)
3	Γ (0.000, 0.000, 0.000)	R (0.500, 0.500, 0.500)
4	R (0.500, 0.500, 0.500)	X (0.000, 0.500, 0.000)
5	R (0.500, 0.500, 0.500)	M (0.500, 0.500, 0.000)

Supplementary Table 1251: Nodal ring points along high symmetry lines of mp-862862-Pa3Sb.

	Coordinates	Frequency	Mode 1	Mode 2	Multi.	Deg.	Path	Ratio
0	(0.209, 0.500, 0.000)	2.370	3	2	12	2	2	0.419
1	(0.365, 0.365, 0.000)	2.361	4	3	12	2	3	0.270
2	(0.152, 0.500, 0.152)	2.483	4	3	12	2	5	0.695
3	(0.199, 0.500, 0.199)	2.494	5	4	12	2	5	0.603
4	(0.163, 0.500, 0.163)	2.490	5	4	12	2	5	0.675
5	(0.000, 0.082, 0.000)	3.260	6	5	6	2	1	0.163
6	(0.357, 0.500, -0.000)	2.638	6	5	12	2	2	0.715
7	(0.097, 0.097, 0.000)	3.240	7	6	12	2	3	0.805
8	(0.061, 0.061, 0.061)	3.271	7	6	8	2	4	0.122
9	(0.227, 0.500, -0.000)	2.935	8	7	12	2	2	0.455
10	(0.295, 0.500, 0.295)	2.879	8	7	12	2	5	0.410
11	(0.183, 0.500, 0.183)	3.020	8	7	12	2	5	0.635
12	(0.000, 0.000, 0.000)	3.307	9	8	1	3	1	0.000
13	(0.394, 0.500, 0.000)	2.998	9	8	12	2	2	0.788

Supplementary Table 1251: Nodal ring points along high symmetry lines of mp-862862-Pa3Sb.

	Coordinates	Frequency	Mode 1	Mode 2	Multi.	Deg.	Path	Ratio
14	(0.385, 0.385, 0.000)	3.005	9	8	12	2	3	0.231
15	(0.098, 0.098, 0.000)	3.277	9	8	12	2	3	0.804
16	(0.322, 0.322, -0.000)	3.061	9	8	12	2	3	0.355
17	(0.168, 0.500, -0.000)	4.456	11	10	12	2	2	0.335
18	(0.299, 0.299, 0.000)	4.461	11	10	12	2	3	0.401
19	(0.106, 0.500, 0.106)	4.449	11	10	12	2	5	0.788
20	(0.000, 0.480, 0.000)	4.521	12	11	6	2	1	0.959

The followings are details for Pa$_3$. It has 6 topological nodal lines and 32 nodal ring points along high symmetry line as shown in Fig **590**.

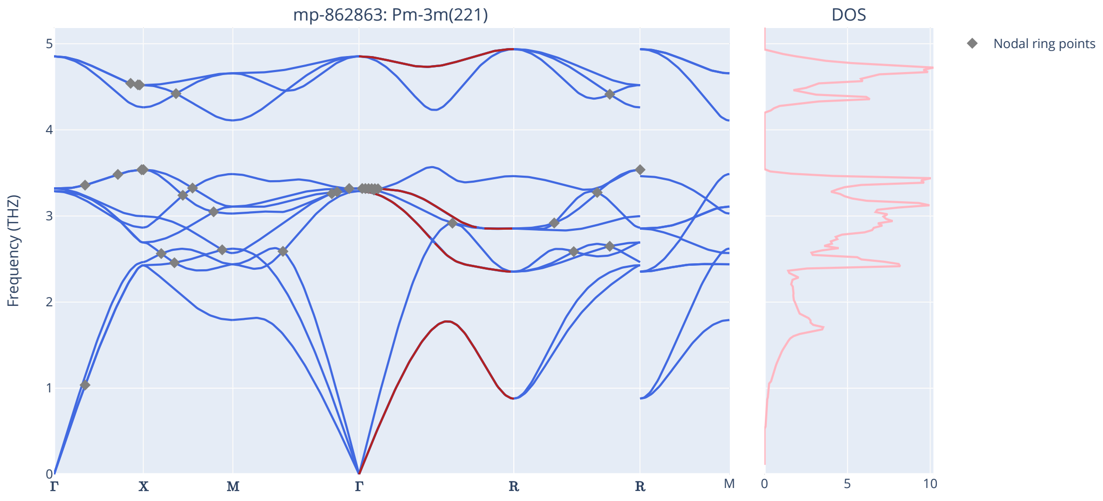

Supplementary Figure 590: The phonon band and DOS of Pa3Te.

Supplementary Table 1252: High symmetry lines of mp-862863-Pa3Te

	Start	End
0	Γ (0.000, 0.000, 0.000)	X (0.000, 0.500, 0.000)
1	X (0.000, 0.500, 0.000)	M (0.500, 0.500, 0.000)
2	M (0.500, 0.500, 0.000)	Γ (0.000, 0.000, 0.000)
3	Γ (0.000, 0.000, 0.000)	R (0.500, 0.500, 0.500)
4	R (0.500, 0.500, 0.500)	X (0.000, 0.500, 0.000)
5	R (0.500, 0.500, 0.500)	M (0.500, 0.500, 0.000)

Supplementary Table 1253: Nodal ring points along high symmetry lines of mp-862863-Pa3Te.

	Coordinates	Frequency	Mode 1	Mode 2	Multi.	Deg.	Path	Ratio
0	(0.000, 0.173, 0.000)	1.036	2	1	6	2	1	0.347
1	(0.174, 0.500, 0.000)	2.456	3	2	12	2	2	0.347
2	(0.100, 0.500, 0.000)	2.562	4	3	12	2	2	0.200
3	(0.301, 0.301, 0.000)	2.588	4	3	12	2	3	0.398
4	(0.441, 0.500, 0.000)	2.606	5	4	12	2	2	0.882
5	(0.120, 0.500, 0.120)	2.647	5	4	12	2	5	0.760
6	(0.263, 0.500, 0.263)	2.584	5	4	12	2	5	0.475
7	(0.108, 0.108, 0.000)	3.258	6	5	12	2	3	0.784
8	(0.302, 0.302, 0.302)	2.915	6	5	8	2	4	0.603
9	(0.393, 0.500, 0.000)	3.047	7	6	12	2	2	0.786
10	(0.087, 0.087, -0.000)	3.279	7	6	12	2	3	0.826
11	(0.048, 0.048, 0.048)	3.301	7	6	8	2	4	0.096
12	(0.221, 0.500, -0.000)	3.239	8	7	12	2	2	0.441
13	(0.340, 0.500, 0.340)	2.917	8	7	12	2	5	0.320

Supplementary Table 1253: Nodal ring points along high symmetry lines of mp-862863-Pa3Te.

	Coordinates	Frequency	Mode 1	Mode 2	Multi.	Deg.	Path	Ratio
14	(0.170, 0.500, 0.170)	3.272	8	7	12	2	5	0.660
15	(0.000, 0.173, 0.000)	3.357	9	8	6	2	1	0.347
16	(0.000, 0.357, 0.000)	3.481	9	8	6	2	1	0.714
17	(0.000, 0.490, 0.000)	3.536	9	8	6	2	1	0.980
18	(0.000, 0.500, 0.000)	3.536	9	8	3	2	1	1.000
19	(0.275, 0.500, -0.000)	3.323	9	8	12	2	2	0.550
20	(0.039, 0.039, -0.000)	3.317	9	8	12	2	3	0.922
21	(0.010, 0.010, 0.010)	3.318	9	8	8	2	4	0.020
22	(0.020, 0.020, 0.020)	3.317	9	8	8	2	4	0.041
23	(0.031, 0.031, 0.031)	3.317	9	8	8	2	4	0.061
24	(0.041, 0.041, 0.041)	3.316	9	8	8	2	4	0.082
25	(0.051, 0.051, 0.051)	3.315	9	8	8	2	4	0.102
26	(0.061, 0.061, 0.061)	3.314	9	8	8	2	4	0.122
27	(0.182, 0.500, -0.000)	4.419	11	10	12	2	2	0.365
28	(0.120, 0.500, 0.120)	4.412	11	10	12	2	5	0.760
29	(0.000, 0.429, 0.000)	4.539	12	11	6	2	1	0.857
30	(0.000, 0.469, 0.000)	4.522	12	11	6	2	1	0.939
31	(0.000, 0.480, 0.000)	4.520	12	11	6	2	1	0.959

The followings are details for Pa$_3$. It has 8 topological nodal lines and 14 nodal ring points along high symmetry line as shown in Fig **591**.

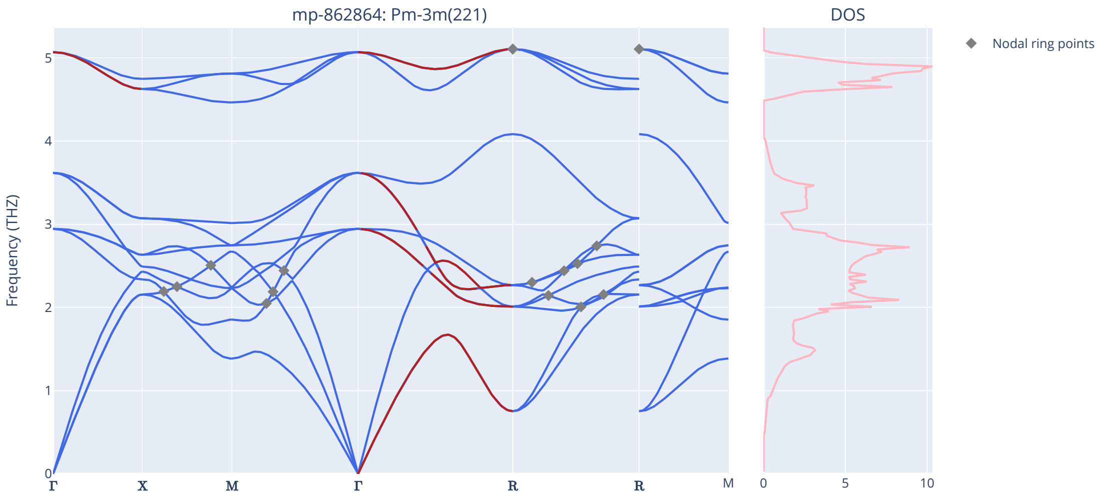

Supplementary Figure 591: The phonon band and DOS of Pa3Ge.

Supplementary Table 1254: High symmetry lines of mp-862864-Pa3Ge

	Start	End
0	Γ (0.000, 0.000, 0.000)	X (0.000, 0.500, 0.000)
1	X (0.000, 0.500, 0.000)	M (0.500, 0.500, 0.000)
2	M (0.500, 0.500, 0.000)	Γ (0.000, 0.000, 0.000)
3	Γ (0.000, 0.000, 0.000)	R (0.500, 0.500, 0.500)
4	R (0.500, 0.500, 0.500)	X (0.000, 0.500, 0.000)
5	R (0.500, 0.500, 0.500)	M (0.500, 0.500, 0.000)

Supplementary Table 1255: Nodal ring points along high symmetry lines of mp-862864-Pa3Ge.

	Coordinates	Frequency	Mode 1	Mode 2	Multi.	Deg.	Path	Ratio
0	(0.120, 0.500, 0.000)	2.192	3	2	12	2	2	0.239
1	(0.363, 0.363, 0.000)	2.048	3	2	12	2	3	0.275
2	(0.230, 0.500, 0.230)	2.006	3	2	12	2	5	0.540
3	(0.193, 0.500, -0.000)	2.250	4	3	12	2	2	0.387
4	(0.336, 0.336, -0.000)	2.188	4	3	12	2	3	0.327
5	(0.140, 0.500, 0.140)	2.155	4	3	12	2	5	0.719
6	(0.293, 0.293, -0.000)	2.442	5	4	12	2	3	0.413
7	(0.359, 0.500, 0.359)	2.143	5	4	12	2	5	0.282
8	(0.383, 0.500, 0.000)	2.506	6	5	12	2	2	0.766
9	(0.244, 0.500, 0.244)	2.526	7	6	12	2	5	0.512
10	(0.297, 0.500, 0.297)	2.440	7	6	12	2	5	0.406
11	(0.424, 0.500, 0.424)	2.301	8	7	12	2	5	0.153
12	(0.168, 0.500, 0.168)	2.740	8	7	12	2	5	0.665
13	(0.500, 0.500, 0.500)	5.106	12	11	1	3	4	1.000

The followings are details for Ru_4V_{12}. It has 6 topological nodal lines and 14 nodal ring points along high symmetry line as shown in Fig **592**.

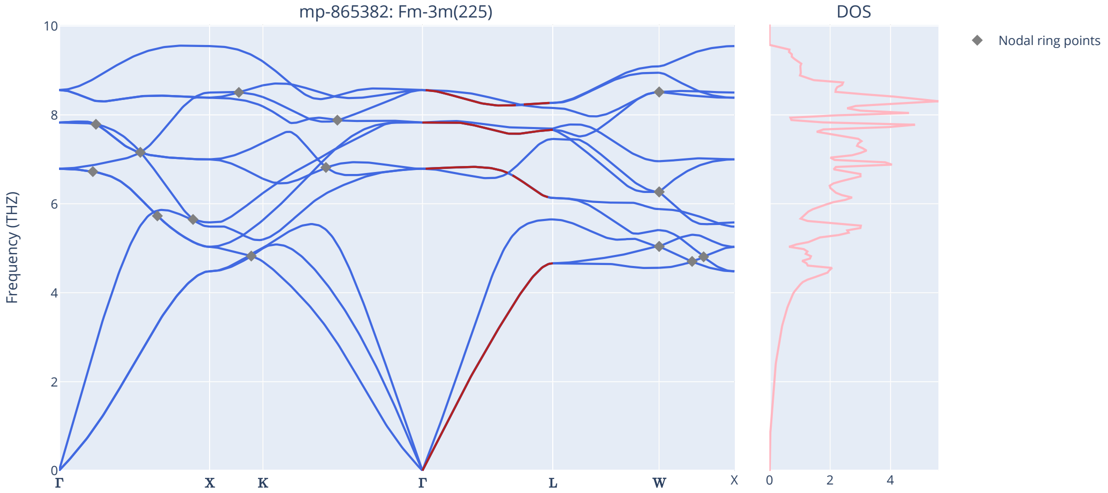

Supplementary Figure 592: The phonon band and DOS of Ru4V12.

Supplementary Table 1256: High symmetry lines of mp-865382-Ru4V12

	Start	End
0	Γ (0.000, 0.000, 0.000)	X (0.500, 0.000, 0.500)
1	X (0.500, 0.000, 0.500)	U (0.625, 0.250, 0.625)
2	K (0.375, 0.375, 0.750)	Γ (0.000, 0.000, 0.000)
3	Γ (0.000, 0.000, 0.000)	L (0.500, 0.500, 0.500)
4	L (0.500, 0.500, 0.500)	W (0.500, 0.250, 0.750)
5	W (0.500, 0.250, 0.750)	X (0.500, 0.000, 0.500)

Supplementary Table 1257: Nodal ring points along high symmetry lines of mp-865382-Ru4V12.

	Coordinates	Frequency	Mode 1	Mode 2	Multi.	Deg.	Path	Ratio
0	(0.598, 0.196, 0.598)	4.827	2	1	12	2	2	0.783
1	(0.497, 0.139, 0.642)	4.706	2	1	24	2	6	0.438
2	(0.500, 0.250, 0.750)	5.043	3	2	6	2	5	1.000
3	(0.498, 0.103, 0.602)	4.811	3	2	48	2	6	0.590
4	(0.327, 0.000, 0.327)	5.730	4	3	6	2	1	0.653
5	(0.112, 0.000, 0.112)	6.726	5	4	6	2	1	0.224
6	(0.445, -0.000, 0.445)	5.650	6	5	6	2	1	0.890
7	(0.229, 0.229, 0.451)	6.819	6	5	24	2	3	0.396
8	(0.500, 0.250, 0.750)	6.270	7	6	6	2	5	1.000
9	(0.122, 0.000, 0.122)	7.790	9	8	6	2	1	0.245
10	(0.270, -0.002, 0.270)	7.159	9	8	24	2	1	0.540
11	(0.195, 0.204, 0.400)	7.882	10	9	24	2	3	0.467
12	(0.500, 0.250, 0.750)	8.516	10	9	6	2	5	1.000
13	(0.570, 0.136, 0.570)	8.506	11	10	24	2	2	0.551

The followings are details for Cd_4Li_{12}. It has 5 topological nodal lines and 10 nodal ring points along high symmetry line as shown in Fig **593**.

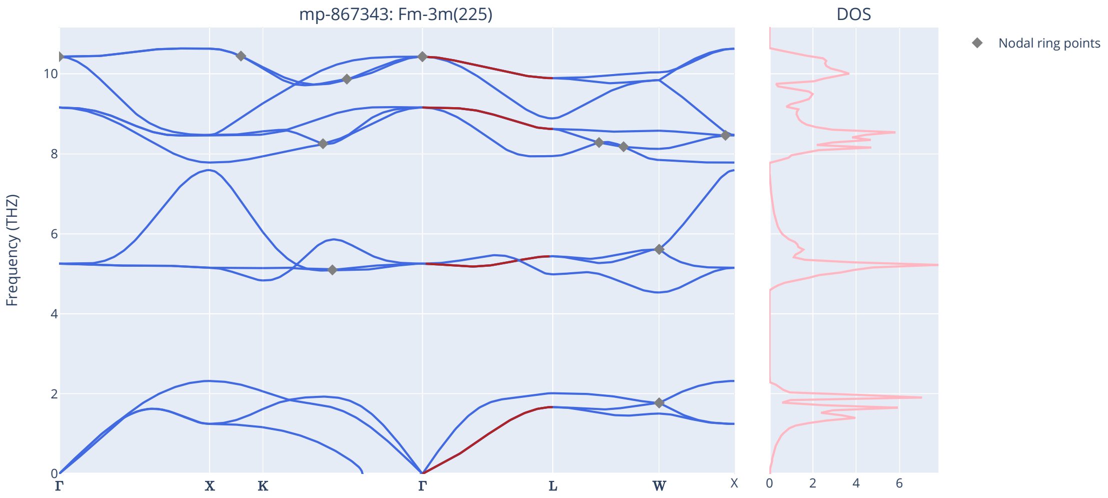

Supplementary Figure 593: The phonon band and DOS of Cd4Li12.

Supplementary Table 1258: High symmetry lines of mp-867343-Cd4Li12

	Start	End
0	Γ (0.000, 0.000, 0.000)	X (0.500, 0.000, 0.500)
1	X (0.500, 0.000, 0.500)	U (0.625, 0.250, 0.625)
2	K (0.375, 0.375, 0.750)	Γ (0.000, 0.000, 0.000)
3	Γ (0.000, 0.000, 0.000)	L (0.500, 0.500, 0.500)
4	L (0.500, 0.500, 0.500)	W (0.500, 0.250, 0.750)
5	W (0.500, 0.250, 0.750)	X (0.500, 0.000, 0.500)

Supplementary Table 1259: Nodal ring points along high symmetry lines of mp-867343-Cd4Li12.

	Coordinates	Frequency	Mode 1	Mode 2	Multi.	Deg.	Path	Ratio
0	(0.500, 0.250, 0.750)	1.771	3	2	6	2	5	1.000
1	(0.214, 0.214, 0.420)	5.097	5	4	24	2	3	0.436
2	(0.500, 0.250, 0.750)	5.611	6	5	6	2	5	1.000
3	(0.500, 0.334, 0.666)	8.177	8	7	24	2	5	0.666
4	(0.500, 0.391, 0.609)	8.282	8	7	24	2	5	0.435
5	(0.228, 0.239, 0.467)	8.252	8	7	24	2	3	0.377
6	(0.499, 0.029, 0.530)	8.462	9	8	24	2	6	0.881
7	(0.182, 0.182, 0.350)	9.869	11	10	24	2	3	0.527
8	(0.000, 0.000, 0.000)	10.429	12	11	1	3	1	0.000
9	(0.575, 0.146, 0.575)	10.445	12	11	24	2	2	0.590

The followings are details for Mo_4V_{12}. It has 7 topological nodal lines and 12 nodal ring points along high symmetry line as shown in Fig **594**.

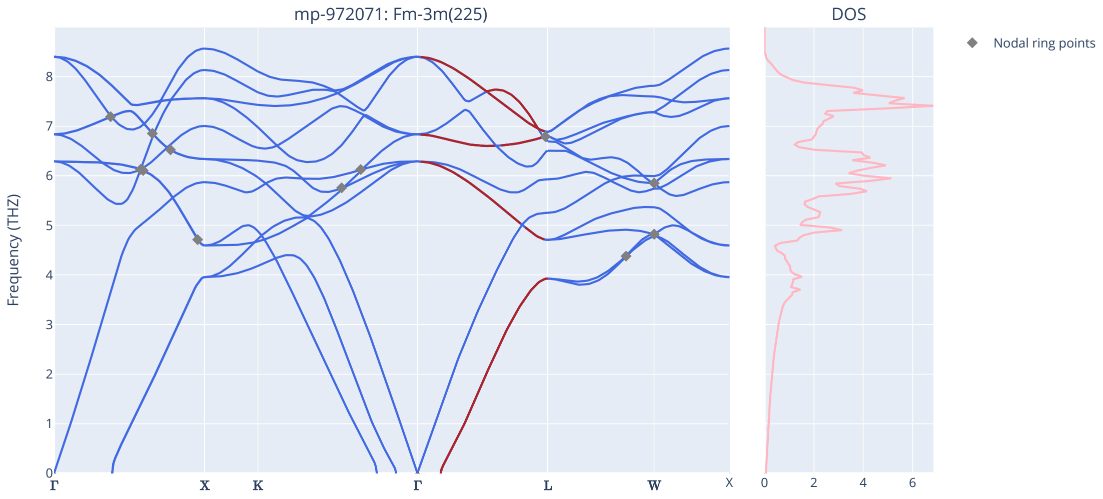

Supplementary Figure 594: The phonon band and DOS of Mo4V12.

Supplementary Table 1260: High symmetry lines of mp-972071-Mo4V12

	Start	End
0	Γ (0.000, 0.000, 0.000)	X (0.500, 0.000, 0.500)
1	X (0.500, 0.000, 0.500)	U (0.625, 0.250, 0.625)
2	K (0.375, 0.375, 0.750)	Γ (0.000, 0.000, 0.000)
3	Γ (0.000, 0.000, 0.000)	L (0.500, 0.500, 0.500)
4	L (0.500, 0.500, 0.500)	W (0.500, 0.250, 0.750)
5	W (0.500, 0.250, 0.750)	X (0.500, 0.000, 0.500)

Supplementary Table 1261: Nodal ring points along high symmetry lines of mp-972071-Mo4V12.

	Coordinates	Frequency	Mode 1	Mode 2	Multi.	Deg.	Path	Ratio
0	(0.500, 0.316, 0.684)	4.380	2	1	24	2	5	0.736
1	(0.500, 0.250, 0.750)	4.821	3	2	6	2	5	1.000
2	(0.477, -0.000, 0.477)	4.712	4	3	6	2	1	0.954
3	(0.296, 0.000, 0.296)	6.106	5	4	6	2	1	0.592
4	(0.184, 0.184, 0.350)	5.757	6	5	24	2	3	0.525
5	(0.287, -0.000, 0.287)	6.127	7	6	6	2	1	0.575
6	(0.383, 0.000, 0.390)	6.523	7	6	24	2	1	0.773
7	(0.138, 0.138, 0.262)	6.126	7	6	24	2	3	0.645
8	(0.500, 0.250, 0.750)	5.851	7	6	6	2	5	1.000
9	(0.327, 0.000, 0.327)	6.853	9	8	6	2	1	0.653
10	(0.192, -0.000, 0.184)	7.191	9	8	24	2	1	0.376
11	(0.491, 0.491, 0.491)	6.791	10	9	8	2	4	0.982

The followings are details for $Li_{12}Zn_4$. It has 5 topological nodal lines and 13 nodal ring points along high symmetry line as shown in Fig **595**.

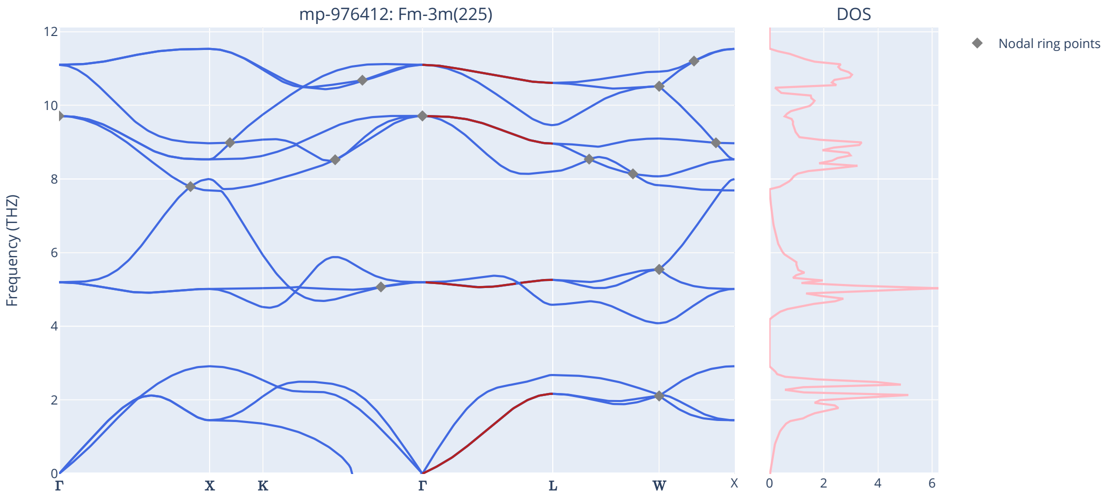

Supplementary Figure 595: The phonon band and DOS of Li12Zn4.

Supplementary Table 1262: High symmetry lines of mp-976412-Li12Zn4

	Start	End
0	Γ (0.000, 0.000, 0.000)	X (0.500, 0.000, 0.500)
1	X (0.500, 0.000, 0.500)	U (0.625, 0.250, 0.625)
2	K (0.375, 0.375, 0.750)	Γ (0.000, 0.000, 0.000)
3	Γ (0.000, 0.000, 0.000)	L (0.500, 0.500, 0.500)
4	L (0.500, 0.500, 0.500)	W (0.500, 0.250, 0.750)
5	W (0.500, 0.250, 0.750)	X (0.500, 0.000, 0.500)

Supplementary Table 1263: Nodal ring points along high symmetry lines of mp-976412-Li12Zn4.

	Coordinates	Frequency	Mode 1	Mode 2	Multi.	Deg.	Path	Ratio
0	(0.500, 0.250, 0.750)	2.107	2	1	6	2	5	1.000
1	(0.099, 0.099, 0.193)	5.068	5	4	24	2	3	0.740
2	(0.500, 0.250, 0.750)	5.545	6	5	6	2	5	1.000
3	(0.437, 0.000, 0.437)	7.794	7	6	6	2	1	0.873
4	(0.500, 0.312, 0.688)	8.140	8	7	24	2	5	0.754
5	(0.500, 0.414, 0.586)	8.539	8	7	24	2	5	0.342
6	(0.210, 0.200, 0.410)	8.522	8	7	24	2	3	0.453
7	(0.000, 0.000, 0.000)	9.713	9	8	1	3	1	0.000
8	(0.548, 0.096, 0.548)	8.986	10	9	12	2	2	0.383
9	(0.498, 0.060, 0.562)	8.983	10	9	24	2	6	0.754
10	(0.145, 0.145, 0.278)	10.683	11	10	24	2	3	0.624
11	(0.500, 0.250, 0.750)	10.518	11	10	6	2	5	1.000
12	(0.500, 0.134, 0.634)	11.203	12	11	12	2	6	0.463

The followings are details for AlCu$_3$. It has 7 topological nodal lines and 15 nodal ring points along high symmetry line as shown in Fig **596**.

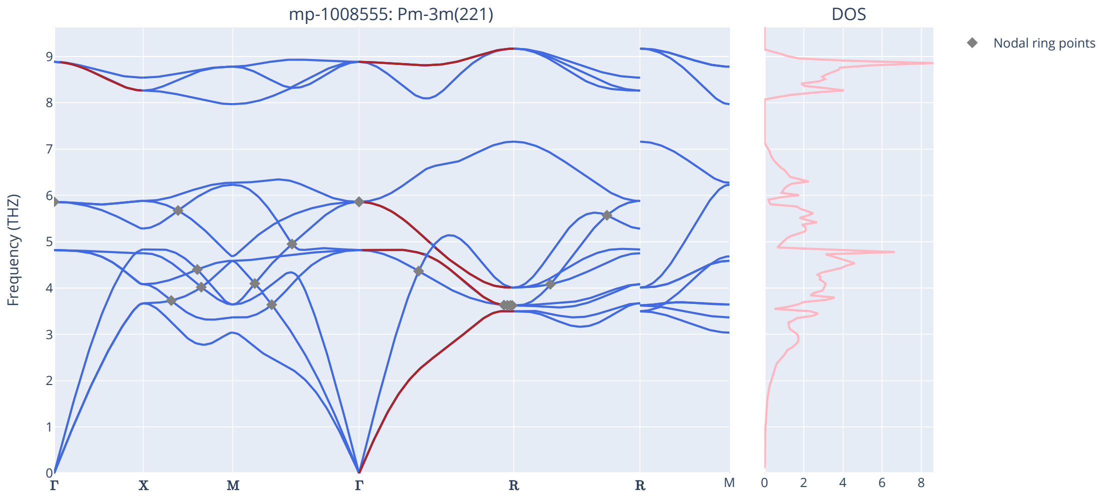

Supplementary Figure 596: The phonon band and DOS of AlCu3.

Supplementary Table 1264: High symmetry lines of mp-1008555-AlCu3

	Start	End
0	Γ (0.000, 0.000, 0.000)	X (0.000, 0.500, 0.000)
1	X (0.000, 0.500, 0.000)	M (0.500, 0.500, 0.000)
2	M (0.500, 0.500, 0.000)	Γ (0.000, 0.000, 0.000)
3	Γ (0.000, 0.000, 0.000)	R (0.500, 0.500, 0.500)
4	R (0.500, 0.500, 0.500)	X (0.000, 0.500, 0.000)
5	R (0.500, 0.500, 0.500)	M (0.500, 0.500, 0.000)

Supplementary Table 1265: Nodal ring points along high symmetry lines of mp-1008555-AlCu3.

	Coordinates	Frequency	Mode 1	Mode 2	Multi.	Deg.	Path	Ratio
0	(0.156, 0.500, 0.000)	3.729	3	2	12	2	2	0.312
1	(0.346, 0.346, 0.000)	3.640	3	2	12	2	3	0.309
2	(0.324, 0.500, 0.000)	4.019	4	3	12	2	2	0.648
3	(0.193, 0.193, 0.193)	4.365	4	3	8	2	4	0.385
4	(0.495, 0.495, 0.495)	3.623	4	3	8	3	4	0.990
5	(0.468, 0.468, 0.468)	3.630	4	3	8	2	4	0.937
6	(0.489, 0.489, 0.489)	3.624	4	3	8	2	4	0.978
7	(0.480, 0.480, 0.480)	3.625	4	3	8	2	4	0.959
8	(0.413, 0.413, 0.000)	4.096	5	4	12	2	3	0.175
9	(0.302, 0.500, 0.000)	4.399	6	5	12	2	2	0.604
10	(0.355, 0.500, 0.355)	4.080	6	5	12	2	5	0.289
11	(0.265, 0.265, 0.000)	4.950	7	6	12	2	3	0.469
12	(0.194, 0.500, -0.000)	5.674	8	7	12	2	2	0.388
13	(0.131, 0.500, 0.131)	5.570	8	7	12	2	5	0.738
14	(0.000, 0.000, 0.000)	5.861	9	8	1	3	1	0.000

Supplementary Table 1265: Nodal ring points along high symmetry lines of mp-1008555-AlCu3.

Coordinates	Frequency	Mode 1	Mode 2	Multi.	Deg.	Path	Ratio

The followings are details for HfRh$_3$. It has 6 topological nodal lines and 23 nodal ring points along high symmetry line as shown in Fig 597.

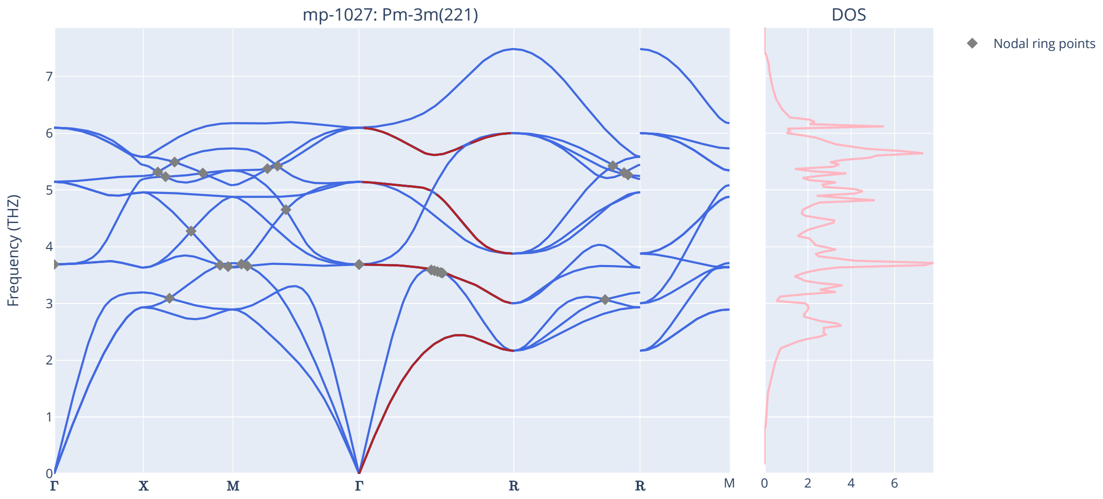

Supplementary Figure 597: The phonon band and DOS of HfRh3.

Supplementary Table 1266: High symmetry lines of mp-1027-HfRh3

	Start	End
0	Γ (0.000, 0.000, 0.000)	X (0.000, 0.500, 0.000)
1	X (0.000, 0.500, 0.000)	M (0.500, 0.500, 0.000)
2	M (0.500, 0.500, 0.000)	Γ (0.000, 0.000, 0.000)
3	Γ (0.000, 0.000, 0.000)	R (0.500, 0.500, 0.500)
4	R (0.500, 0.500, 0.500)	X (0.000, 0.500, 0.000)
5	R (0.500, 0.500, 0.500)	M (0.500, 0.500, 0.000)

Supplementary Table 1267: Nodal ring points along high symmetry lines of mp-1027-HfRh3.

	Coordinates	Frequency	Mode 1	Mode 2	Multi.	Deg.	Path	Ratio
0	(0.146, 0.500, -0.000)	3.092	3	2	12	2	2	0.292
1	(0.138, 0.500, 0.138)	3.068	3	2	12	2	5	0.723
2	(0.474, 0.500, 0.000)	3.649	4	3	12	2	2	0.948
3	(0.429, 0.500, 0.000)	3.674	4	3	12	2	2	0.859
4	(0.442, 0.442, 0.000)	3.659	4	3	12	2	3	0.116
5	(0.270, 0.270, 0.268)	3.535	4	3	24	2	4	0.539
6	(0.264, 0.264, 0.264)	3.545	4	3	8	2	4	0.528
7	(0.254, 0.254, 0.254)	3.561	4	3	8	2	4	0.507
8	(0.243, 0.243, 0.243)	3.577	4	3	8	2	4	0.487
9	(0.233, 0.233, 0.233)	3.591	4	3	8	2	4	0.466
10	(0.465, 0.465, -0.000)	3.693	5	4	12	2	3	0.069
11	(0.000, 0.000, 0.000)	3.687	6	5	1	3	1	0.000
12	(0.267, 0.500, 0.000)	4.275	6	5	12	2	2	0.533
13	(0.289, 0.289, -0.000)	4.652	7	6	12	2	3	0.421

Supplementary Table 1267: Nodal ring points along high symmetry lines of mp-1027-HfRh3.

	Coordinates	Frequency	Mode 1	Mode 2	Multi.	Deg.	Path	Ratio
14	(0.124, 0.500, -0.000)	5.233	9	8	12	2	2	0.248
15	(0.048, 0.498, 0.048)	5.265	9	8	24	2	5	0.904
16	(0.063, 0.500, 0.063)	5.305	10	9	12	2	5	0.873
17	(0.081, 0.500, -0.000)	5.315	10	9	12	2	2	0.161
18	(0.332, 0.500, -0.000)	5.297	10	9	12	2	2	0.665
19	(0.323, 0.323, 0.000)	5.422	10	9	12	2	3	0.354
20	(0.363, 0.363, -0.000)	5.375	10	9	12	2	3	0.275
21	(0.175, 0.500, 0.000)	5.491	11	10	12	2	2	0.349
22	(0.108, 0.500, 0.108)	5.425	11	10	12	2	5	0.784

The followings are details for Ir$_3$. It has 7 topological nodal lines and 24 nodal ring points along high symmetry line as shown in Fig **598**.

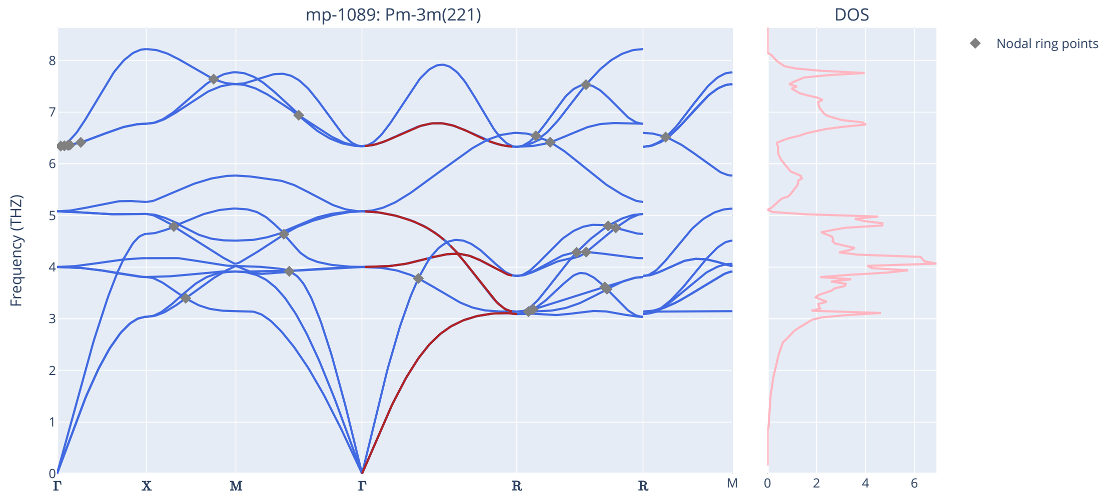

Supplementary Figure 598: The phonon band and DOS of Ir3Ti.

Supplementary Table 1268: High symmetry lines of mp-1089-Ir3Ti

	Start	End
0	Γ (0.000, 0.000, 0.000)	X (0.000, 0.500, 0.000)
1	X (0.000, 0.500, 0.000)	M (0.500, 0.500, 0.000)
2	M (0.500, 0.500, 0.000)	Γ (0.000, 0.000, 0.000)
3	Γ (0.000, 0.000, 0.000)	R (0.500, 0.500, 0.500)
4	R (0.500, 0.500, 0.500)	X (0.000, 0.500, 0.000)
5	R (0.500, 0.500, 0.500)	M (0.500, 0.500, 0.000)

Supplementary Table 1269: Nodal ring points along high symmetry lines of mp-1089-Ir3Ti.

	Coordinates	Frequency	Mode 1	Mode 2	Multi.	Deg.	Path	Ratio
0	(0.220, 0.500, -0.002)	3.396	2	1	24	2	2	0.440
1	(0.454, 0.500, 0.454)	3.138	3	2	24	2	5	0.093
2	(0.143, 0.500, 0.143)	3.572	3	2	12	2	5	0.714
3	(0.288, 0.288, 0.000)	3.918	4	3	12	2	3	0.424
4	(0.182, 0.182, 0.182)	3.780	4	3	8	2	4	0.364
5	(0.435, 0.500, 0.435)	3.182	4	3	12	2	5	0.130
6	(0.151, 0.500, 0.151)	3.614	4	3	12	2	5	0.697
7	(0.225, 0.500, 0.225)	4.291	6	5	12	2	5	0.549
8	(0.154, 0.500, -0.000)	4.785	7	6	12	2	2	0.307
9	(0.310, 0.310, 0.000)	4.636	7	6	12	2	3	0.381
10	(0.108, 0.500, 0.108)	4.759	7	6	12	2	5	0.783
11	(0.263, 0.500, 0.263)	4.285	7	6	12	2	5	0.474
12	(0.139, 0.500, 0.139)	4.796	8	7	12	2	5	0.723
13	(0.368, 0.500, 0.368)	6.416	10	9	12	2	5	0.265

Supplementary Table 1269: Nodal ring points along high symmetry lines of mp-1089-Ir3Ti.

	Coordinates	Frequency	Mode 1	Mode 2	Multi.	Deg.	Path	Ratio
14	(0.000, 0.020, 0.000)	6.342	11	10	6	2	1	0.041
15	(0.000, 0.041, 0.000)	6.347	11	10	6	2	1	0.082
16	(0.000, 0.061, 0.000)	6.356	11	10	6	2	1	0.122
17	(0.000, 0.071, 0.000)	6.362	11	10	6	2	1	0.143
18	(0.000, 0.133, 0.000)	6.413	11	10	6	2	1	0.265
19	(0.251, 0.251, -0.000)	6.939	11	10	12	2	3	0.499
20	(0.376, 0.500, 0.000)	7.638	12	11	12	2	2	0.753
21	(0.424, 0.500, 0.424)	6.540	12	11	12	2	5	0.151
22	(0.226, 0.500, 0.226)	7.530	12	11	12	2	5	0.548
23	(0.497, 0.500, 0.374)	6.516	12	11	24	2	6	0.252

The followings are details for Al$_3$. It has 7 topological nodal lines and 23 nodal ring points along high symmetry line as shown in Fig **599**.

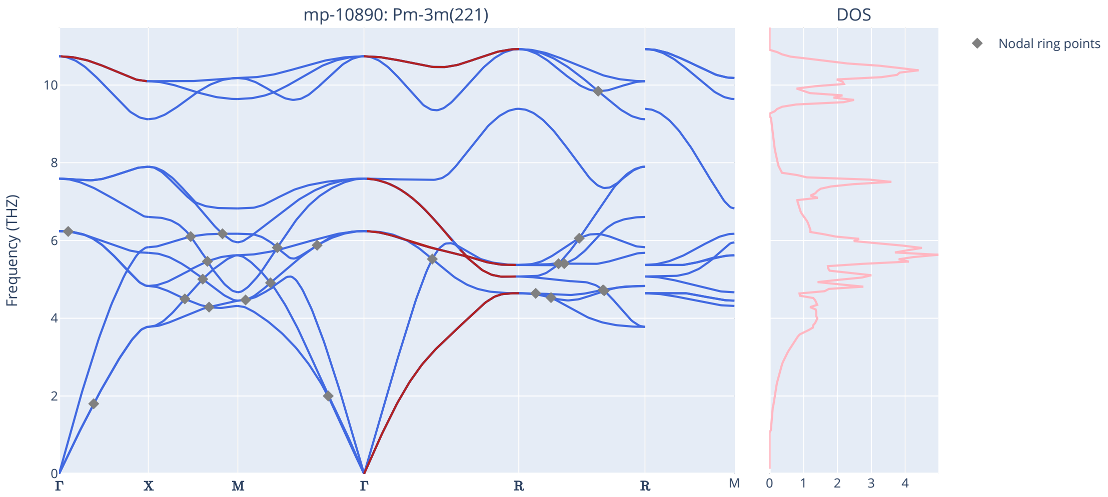

Supplementary Figure 599: The phonon band and DOS of Al3Li.

Supplementary Table 1270: High symmetry lines of mp-10890-Al3Li

	Start	End
0	Γ (0.000, 0.000, 0.000)	X (0.000, 0.500, 0.000)
1	X (0.000, 0.500, 0.000)	M (0.500, 0.500, 0.000)
2	M (0.500, 0.500, 0.000)	Γ (0.000, 0.000, 0.000)
3	Γ (0.000, 0.000, 0.000)	R (0.500, 0.500, 0.500)
4	R (0.500, 0.500, 0.500)	X (0.000, 0.500, 0.000)
5	R (0.500, 0.500, 0.500)	M (0.500, 0.500, 0.000)

Supplementary Table 1271: Nodal ring points along high symmetry lines of mp-10890-Al3Li.

	Coordinates	Frequency	Mode 1	Mode 2	Multi.	Deg.	Path	Ratio
0	(0.000, 0.194, 0.000)	1.801	2	1	6	2	1	0.388
1	(0.340, 0.500, 0.000)	4.286	2	1	12	2	2	0.679
2	(0.142, 0.142, -0.000)	2.000	2	1	12	2	3	0.716
3	(0.372, 0.500, 0.372)	4.531	2	1	12	2	5	0.255
4	(0.204, 0.500, -0.000)	4.498	3	2	12	2	2	0.408
5	(0.469, 0.469, 0.000)	4.471	3	2	12	2	3	0.062
6	(0.433, 0.500, 0.433)	4.640	3	2	12	2	5	0.134
7	(0.162, 0.500, 0.162)	4.707	3	2	12	2	5	0.676
8	(0.304, 0.500, -0.000)	5.006	4	3	12	2	2	0.608
9	(0.370, 0.370, -0.000)	4.911	4	3	12	2	3	0.260
10	(0.221, 0.221, 0.221)	5.523	4	3	8	2	4	0.441
11	(0.166, 0.500, 0.166)	4.731	4	3	12	2	5	0.667
12	(0.186, 0.186, -0.000)	5.878	5	4	12	2	3	0.628
13	(0.000, 0.051, 0.000)	6.233	6	5	6	2	1	0.102

Supplementary Table 1271: Nodal ring points along high symmetry lines of mp-10890-Al3Li.

	Coordinates	Frequency	Mode 1	Mode 2	Multi.	Deg.	Path	Ratio
14	(0.330, 0.500, -0.000)	5.464	6	5	12	2	2	0.661
15	(0.320, 0.500, 0.320)	5.406	6	5	12	2	5	0.360
16	(0.237, 0.500, -0.000)	6.102	7	6	12	2	2	0.473
17	(0.343, 0.343, 0.000)	5.817	7	6	12	2	3	0.313
18	(0.343, 0.500, 0.343)	5.403	7	6	12	2	5	0.315
19	(0.415, 0.500, -0.000)	6.173	8	7	12	2	2	0.829
20	(0.261, 0.500, 0.261)	6.058	8	7	12	2	5	0.479
21	(0.186, 0.500, 0.186)	9.842	11	10	12	2	5	0.629

The followings are details for $MgZr_3$. It has 7 topological nodal lines and 18 nodal ring points along high symmetry line as shown in Fig **600**.

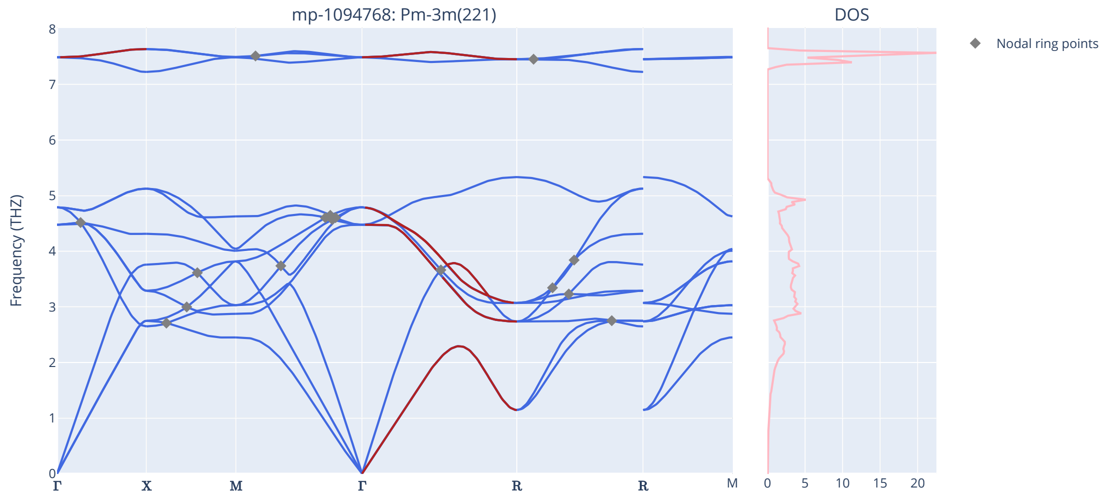

Supplementary Figure 600: The phonon band and DOS of MgZr3.

Supplementary Table 1272: High symmetry lines of mp-1094768-MgZr3

	Start	End
0	Γ (0.000, 0.000, 0.000)	X (0.000, 0.500, 0.000)
1	X (0.000, 0.500, 0.000)	M (0.500, 0.500, 0.000)
2	M (0.500, 0.500, 0.000)	Γ (0.000, 0.000, 0.000)
3	Γ (0.000, 0.000, 0.000)	R (0.500, 0.500, 0.500)
4	R (0.500, 0.500, 0.500)	X (0.000, 0.500, 0.000)
5	R (0.500, 0.500, 0.500)	M (0.500, 0.500, 0.000)

Supplementary Table 1273: Nodal ring points along high symmetry lines of mp-1094768-MgZr3.

	Coordinates	Frequency	Mode 1	Mode 2	Multi.	Deg.	Path	Ratio
0	(0.112, 0.500, -0.000)	2.708	2	1	12	2	2	0.224
1	(0.124, 0.500, 0.124)	2.752	3	2	12	2	5	0.752
2	(0.226, 0.500, 0.000)	2.999	4	3	12	2	2	0.451
3	(0.285, 0.500, -0.000)	3.614	6	5	12	2	2	0.570
4	(0.321, 0.321, -0.000)	3.738	6	5	12	2	3	0.357
5	(0.255, 0.255, 0.255)	3.664	6	5	8	2	4	0.509
6	(0.294, 0.500, 0.294)	3.231	6	5	12	2	5	0.412
7	(0.000, 0.132, 0.000)	4.516	7	6	6	2	1	0.263
8	(0.118, 0.118, -0.000)	4.571	7	6	12	2	3	0.765
9	(0.145, 0.145, 0.000)	4.603	8	7	12	2	3	0.710
10	(0.105, 0.105, 0.000)	4.605	8	7	12	2	3	0.790
11	(0.359, 0.500, 0.359)	3.344	8	7	12	2	5	0.283
12	(0.273, 0.500, 0.273)	3.842	8	7	12	2	5	0.454
13	(0.126, 0.126, 0.000)	4.646	9	8	12	2	3	0.749

Supplementary Table 1273: Nodal ring points along high symmetry lines of mp-1094768-MgZr3.

	Coordinates	Frequency	Mode 1	Mode 2	Multi.	Deg.	Path	Ratio
14	(0.434, 0.500, 0.434)	7.453	11	10	12	2	5	0.133
15	(0.422, 0.422, 0.000)	7.512	12	11	12	2	3	0.157

The followings are details for Cd$_3$. It has 7 topological nodal lines and 14 nodal ring points along high symmetry line as shown in Fig **601**.

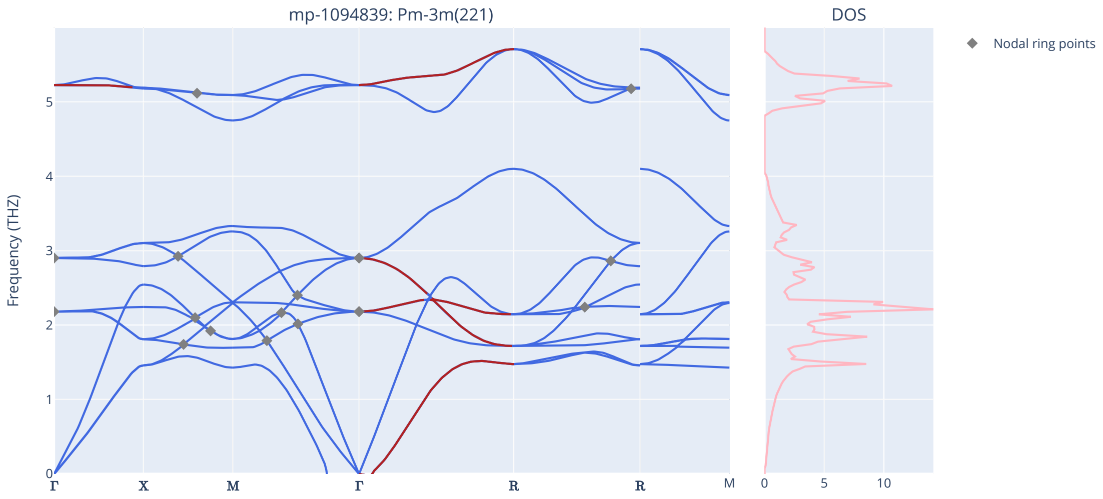

Supplementary Figure 601: The phonon band and DOS of Cd3Mg.

Supplementary Table 1274: High symmetry lines of mp-1094839-Cd3Mg

	Start	End
0	Γ (0.000, 0.000, 0.000)	X (0.000, 0.500, 0.000)
1	X (0.000, 0.500, 0.000)	M (0.500, 0.500, 0.000)
2	M (0.500, 0.500, 0.000)	Γ (0.000, 0.000, 0.000)
3	Γ (0.000, 0.000, 0.000)	R (0.500, 0.500, 0.500)
4	R (0.500, 0.500, 0.500)	X (0.000, 0.500, 0.000)
5	R (0.500, 0.500, 0.500)	M (0.500, 0.500, 0.000)

Supplementary Table 1275: Nodal ring points along high symmetry lines of mp-1094839-Cd3Mg.

	Coordinates	Frequency	Mode 1	Mode 2	Multi.	Deg.	Path	Ratio
0	(0.225, 0.500, 0.000)	1.739	3	2	12	2	2	0.450
1	(0.365, 0.365, 0.000)	1.790	3	2	12	2	3	0.270
2	(0.376, 0.500, -0.000)	1.921	4	3	12	2	2	0.752
3	(0.242, 0.242, -0.000)	2.015	4	3	12	2	3	0.516
4	(0.307, 0.307, 0.000)	2.167	5	4	12	2	3	0.386
5	(0.000, 0.000, 0.000)	2.180	6	5	1	3	1	0.000
6	(0.289, 0.500, 0.000)	2.099	6	5	12	2	2	0.579
7	(0.219, 0.500, 0.219)	2.241	6	5	12	2	5	0.562
8	(0.244, 0.244, 0.000)	2.401	7	6	12	2	3	0.512
9	(0.194, 0.500, 0.000)	2.924	8	7	12	2	2	0.388
10	(0.116, 0.500, 0.116)	2.862	8	7	12	2	5	0.768
11	(0.000, 0.000, 0.000)	2.901	9	8	1	3	1	0.000
12	(0.035, 0.500, 0.035)	5.177	11	10	12	2	5	0.929
13	(0.300, 0.500, 0.000)	5.117	12	11	12	2	2	0.600

The followings are details for Co_3. It has 6 topological nodal lines and 13 nodal ring points along high symmetry line as shown in Fig **602**.

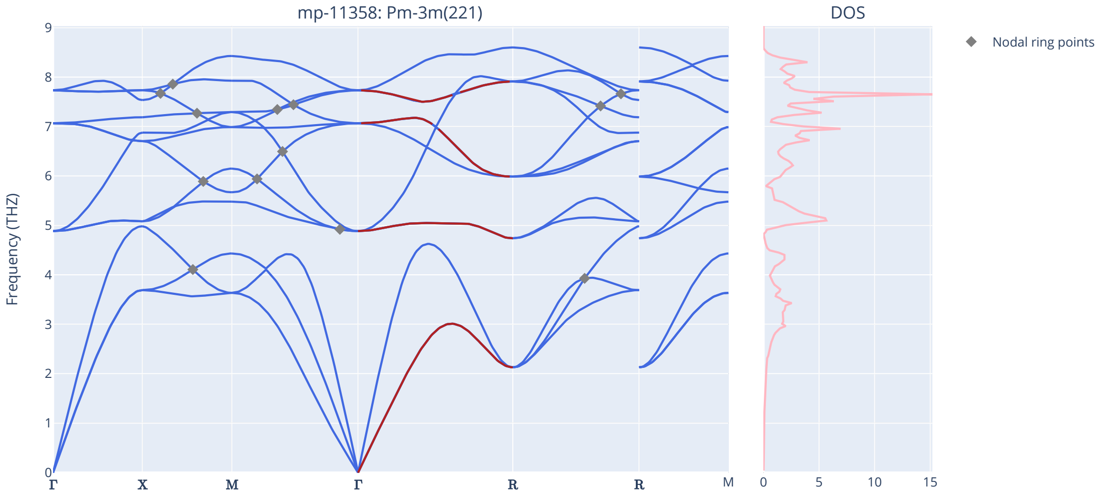

Supplementary Figure 602: The phonon band and DOS of Co3Ta.

Supplementary Table 1276: High symmetry lines of mp-11358-Co3Ta

	Start	End
0	Γ (0.000, 0.000, 0.000)	X (0.000, 0.500, 0.000)
1	X (0.000, 0.500, 0.000)	M (0.500, 0.500, 0.000)
2	M (0.500, 0.500, 0.000)	Γ (0.000, 0.000, 0.000)
3	Γ (0.000, 0.000, 0.000)	R (0.500, 0.500, 0.500)
4	R (0.500, 0.500, 0.500)	X (0.000, 0.500, 0.000)
5	R (0.500, 0.500, 0.500)	M (0.500, 0.500, 0.000)

Supplementary Table 1277: Nodal ring points along high symmetry lines of mp-11358-Co3Ta.

	Coordinates	Frequency	Mode 1	Mode 2	Multi.	Deg.	Path	Ratio
0	(0.282, 0.500, 0.000)	4.107	3	2	12	2	2	0.565
1	(0.216, 0.500, 0.216)	3.928	3	2	12	2	5	0.569
2	(0.072, 0.072, 0.000)	4.920	5	4	12	2	3	0.856
3	(0.341, 0.500, 0.000)	5.889	6	5	12	2	2	0.682
4	(0.400, 0.400, 0.000)	5.940	6	5	12	2	3	0.200
5	(0.299, 0.299, -0.000)	6.495	7	6	12	2	3	0.402
6	(0.306, 0.500, -0.000)	7.269	10	9	12	2	2	0.611
7	(0.320, 0.320, 0.000)	7.344	10	9	12	2	3	0.361
8	(0.256, 0.256, -0.000)	7.441	10	9	12	2	3	0.489
9	(0.153, 0.500, 0.153)	7.416	10	9	12	2	5	0.695
10	(0.101, 0.500, -0.000)	7.670	11	10	12	2	2	0.203
11	(0.072, 0.500, 0.072)	7.662	11	10	12	2	5	0.856
12	(0.170, 0.500, -0.000)	7.857	12	11	12	2	2	0.340

The followings are details for Ag$_3$. It has 7 topological nodal lines and 29 nodal ring points along high symmetry line as shown in Fig **603**.

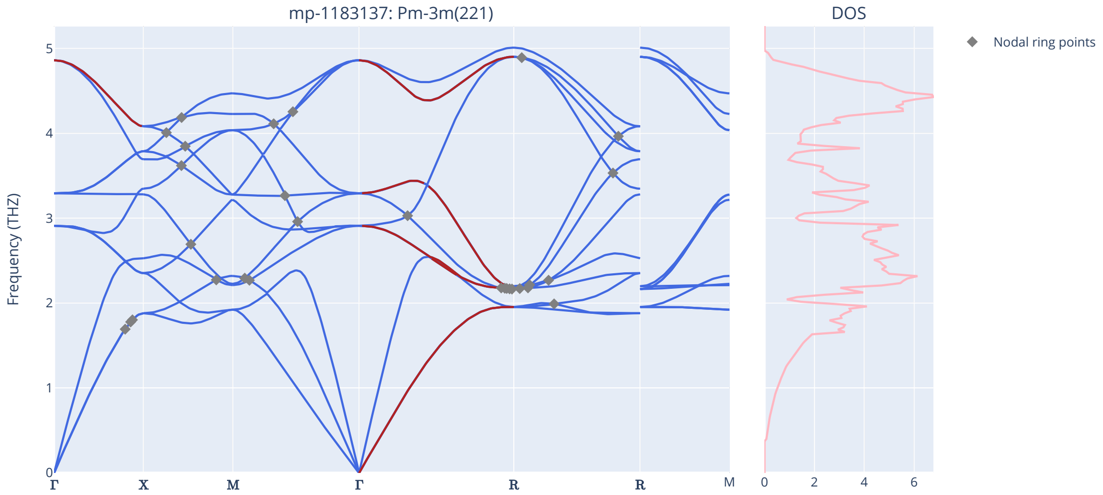

Supplementary Figure 603: The phonon band and DOS of Ag3Au.

Supplementary Table 1278: High symmetry lines of mp-1183137-Ag3Au

	Start	End
0	Γ (0.000, 0.000, 0.000)	X (0.000, 0.500, 0.000)
1	X (0.000, 0.500, 0.000)	M (0.500, 0.500, 0.000)
2	M (0.500, 0.500, 0.000)	Γ (0.000, 0.000, 0.000)
3	Γ (0.000, 0.000, 0.000)	R (0.500, 0.500, 0.500)
4	R (0.500, 0.500, 0.500)	X (0.000, 0.500, 0.000)
5	R (0.500, 0.500, 0.500)	M (0.500, 0.500, 0.000)

Supplementary Table 1279: Nodal ring points along high symmetry lines of mp-1183137-Ag3Au.

	Coordinates	Frequency	Mode 1	Mode 2	Multi.	Deg.	Path	Ratio
0	(0.000, 0.398, 0.000)	1.692	2	1	6	2	1	0.796
1	(0.000, 0.429, 0.000)	1.777	2	1	6	2	1	0.857
2	(0.000, 0.439, 0.000)	1.801	2	1	6	2	1	0.878
3	(0.340, 0.500, 0.340)	1.989	3	2	12	2	5	0.319
4	(0.408, 0.500, 0.000)	2.273	4	3	12	2	2	0.816
5	(0.434, 0.434, 0.000)	2.268	4	3	12	2	3	0.132
6	(0.452, 0.452, 0.000)	2.293	5	4	12	2	3	0.095
7	(0.444, 0.500, 0.444)	2.179	5	4	12	2	5	0.112
8	(0.266, 0.500, -0.000)	2.692	6	5	12	2	2	0.531
9	(0.459, 0.459, 0.459)	2.178	6	5	8	2	4	0.919
10	(0.477, 0.477, 0.477)	2.171	6	5	8	2	4	0.954
11	(0.471, 0.471, 0.471)	2.174	6	5	8	2	4	0.942
12	(0.494, 0.494, 0.494)	2.164	6	5	8	2	4	0.988
13	(0.486, 0.486, 0.486)	2.167	6	5	8	2	4	0.973

Supplementary Table 1279: Nodal ring points along high symmetry lines of mp-1183137-Ag3Au.

	Coordinates	Frequency	Mode 1	Mode 2	Multi.	Deg.	Path	Ratio
14	(0.476, 0.500, 0.476)	2.170	6	5	12	2	5	0.047
15	(0.362, 0.500, 0.362)	2.268	6	5	12	2	5	0.276
16	(0.243, 0.243, -0.000)	2.959	7	6	12	2	3	0.513
17	(0.157, 0.157, 0.157)	3.029	7	6	8	2	4	0.314
18	(0.437, 0.500, 0.437)	2.214	7	6	12	2	5	0.126
19	(0.213, 0.500, 0.000)	3.619	8	7	12	2	2	0.425
20	(0.293, 0.293, -0.000)	3.266	8	7	12	2	3	0.413
21	(0.107, 0.500, 0.107)	3.532	8	7	12	2	5	0.785
22	(0.234, 0.500, 0.000)	3.848	10	9	12	2	2	0.468
23	(0.468, 0.500, 0.468)	4.891	10	9	12	2	5	0.063
24	(0.129, 0.500, -0.000)	4.008	11	10	12	2	2	0.257
25	(0.338, 0.338, 0.000)	4.112	11	10	12	2	3	0.324
26	(0.262, 0.262, -0.000)	4.254	11	10	12	2	3	0.476
27	(0.086, 0.500, 0.086)	3.966	11	10	12	2	5	0.828
28	(0.214, 0.500, 0.000)	4.186	12	11	12	2	2	0.427

The followings are details for IrRh$_3$. It has 5 topological nodal lines and 27 nodal ring points along high symmetry line as shown in Fig 604.

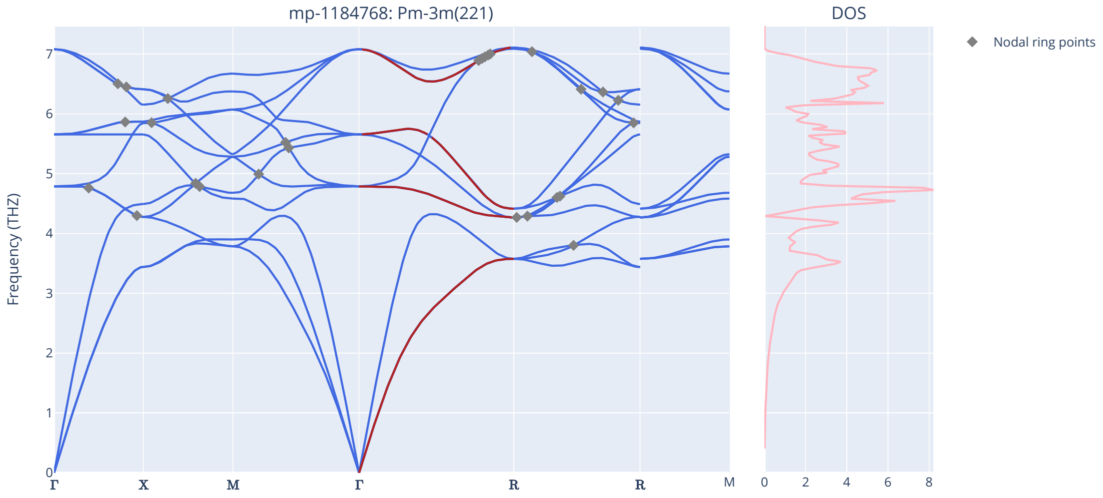

Supplementary Figure 604: The phonon band and DOS of IrRh3.

Supplementary Table 1280: High symmetry lines of mp-1184768-IrRh3

	Start	End
0	Γ (0.000, 0.000, 0.000)	X (0.000, 0.500, 0.000)
1	X (0.000, 0.500, 0.000)	M (0.500, 0.500, 0.000)
2	M (0.500, 0.500, 0.000)	Γ (0.000, 0.000, 0.000)
3	Γ (0.000, 0.000, 0.000)	R (0.500, 0.500, 0.500)
4	R (0.500, 0.500, 0.500)	X (0.000, 0.500, 0.000)
5	R (0.500, 0.500, 0.500)	M (0.500, 0.500, 0.000)

Supplementary Table 1281: Nodal ring points along high symmetry lines of mp-1184768-IrRh3.

	Coordinates	Frequency	Mode 1	Mode 2	Multi.	Deg.	Path	Ratio
0	(0.263, 0.500, 0.263)	3.800	3	2	12	2	5	0.473
1	(0.000, 0.463, -0.000)	4.298	4	3	6	2	1	0.927
2	(0.000, 0.194, 0.000)	4.760	5	4	6	2	1	0.388
3	(0.315, 0.500, -0.000)	4.783	5	4	12	2	2	0.630
4	(0.446, 0.500, 0.446)	4.289	5	4	12	2	5	0.108
5	(0.292, 0.500, -0.000)	4.838	6	5	12	2	2	0.584
6	(0.397, 0.397, 0.000)	4.991	6	5	12	2	3	0.205
7	(0.488, 0.500, 0.488)	4.268	6	5	12	2	5	0.023
8	(0.317, 0.500, 0.317)	4.623	6	5	12	2	5	0.366
9	(0.278, 0.278, -0.000)	5.430	7	6	12	2	3	0.443
10	(0.329, 0.498, 0.329)	4.602	7	6	24	2	5	0.342
11	(0.046, 0.500, -0.000)	5.851	8	7	12	2	2	0.092
12	(0.291, 0.291, 0.000)	5.521	8	7	12	2	3	0.418
13	(0.025, 0.500, 0.025)	5.848	8	7	12	2	5	0.949

Supplementary Table 1281: Nodal ring points along high symmetry lines of mp-1184768-IrRh3.

	Coordinates	Frequency	Mode 1	Mode 2	Multi.	Deg.	Path	Ratio
14	(0.000, 0.398, 0.000)	5.863	9	8	6	2	1	0.796
15	(0.407, 0.407, 0.407)	6.953	10	9	8	2	4	0.814
16	(0.398, 0.398, 0.398)	6.925	10	9	8	2	4	0.795
17	(0.387, 0.387, 0.387)	6.890	10	9	8	2	4	0.773
18	(0.417, 0.417, 0.417)	6.981	10	9	8	2	4	0.834
19	(0.424, 0.424, 0.424)	7.000	10	9	8	2	4	0.848
20	(0.428, 0.500, 0.428)	7.038	10	9	12	2	5	0.144
21	(0.234, 0.500, 0.234)	6.410	10	9	12	2	5	0.533
22	(0.000, 0.357, 0.000)	6.501	11	10	6	2	1	0.714
23	(0.137, 0.500, -0.000)	6.255	11	10	12	2	2	0.273
24	(0.086, 0.500, 0.086)	6.225	11	10	12	2	5	0.827
25	(0.000, 0.404, 0.000)	6.449	12	11	6	2	1	0.809
26	(0.147, 0.500, 0.147)	6.364	12	11	12	2	5	0.706

The followings are details for Ir_3. It has 6 topological nodal lines and 20 nodal ring points along high symmetry line as shown in Fig **605**.

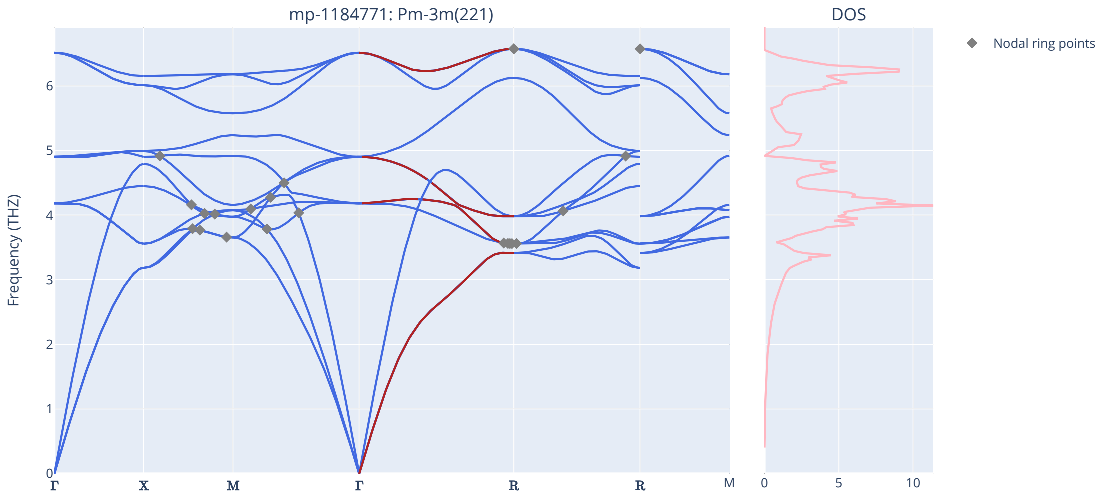

Supplementary Figure 605: The phonon band and DOS of Ir3Rh.

Supplementary Table 1282: High symmetry lines of mp-1184771-Ir3Rh

	Start	End
0	Γ (0.000, 0.000, 0.000)	X (0.000, 0.500, 0.000)
1	X (0.000, 0.500, 0.000)	M (0.500, 0.500, 0.000)
2	M (0.500, 0.500, 0.000)	Γ (0.000, 0.000, 0.000)
3	Γ (0.000, 0.000, 0.000)	R (0.500, 0.500, 0.500)
4	R (0.500, 0.500, 0.500)	X (0.000, 0.500, 0.000)
5	R (0.500, 0.500, 0.500)	M (0.500, 0.500, 0.000)

Supplementary Table 1283: Nodal ring points along high symmetry lines of mp-1184771-Ir3Rh.

	Coordinates	Frequency	Mode 1	Mode 2	Multi.	Deg.	Path	Ratio
0	(0.465, 0.500, -0.000)	3.660	2	1	12	2	2	0.929
1	(0.315, 0.500, -0.000)	3.768	2	1	12	2	2	0.630
2	(0.274, 0.500, 0.000)	3.788	3	2	12	2	2	0.548
3	(0.365, 0.365, 0.000)	3.785	3	2	12	2	3	0.270
4	(0.239, 0.239, -0.000)	4.034	4	3	12	2	3	0.522
5	(0.494, 0.494, 0.494)	3.559	4	3	8	2	4	0.988
6	(0.467, 0.467, 0.467)	3.568	4	3	8	2	4	0.935
7	(0.487, 0.487, 0.487)	3.560	4	3	8	2	4	0.975
8	(0.481, 0.481, 0.481)	3.561	4	3	8	2	4	0.962
9	(0.399, 0.500, -0.000)	4.015	5	4	12	2	2	0.797
10	(0.341, 0.500, 0.000)	4.026	5	4	12	2	2	0.683
11	(0.489, 0.500, 0.489)	3.560	5	4	12	2	5	0.022
12	(0.268, 0.500, -0.000)	4.159	6	5	12	2	2	0.536
13	(0.429, 0.429, -0.000)	4.095	6	5	12	2	3	0.142

Supplementary Table 1283: Nodal ring points along high symmetry lines of mp-1184771-Ir3Rh.

	Coordinates	Frequency	Mode 1	Mode 2	Multi.	Deg.	Path	Ratio
14	(0.351, 0.351, -0.000)	4.276	6	5	12	2	3	0.299
15	(0.304, 0.500, 0.304)	4.065	6	5	12	2	5	0.391
16	(0.297, 0.297, 0.000)	4.500	7	6	12	2	3	0.406
17	(0.091, 0.500, -0.000)	4.916	8	7	12	2	2	0.182
18	(0.057, 0.500, 0.057)	4.914	8	7	12	2	5	0.885
19	(0.500, 0.500, 0.500)	6.574	12	11	1	3	4	1.000

The followings are details for $Na_{12}Pd_4$. It has 6 topological nodal lines and 8 nodal ring points along high symmetry line as shown in Fig **606**.

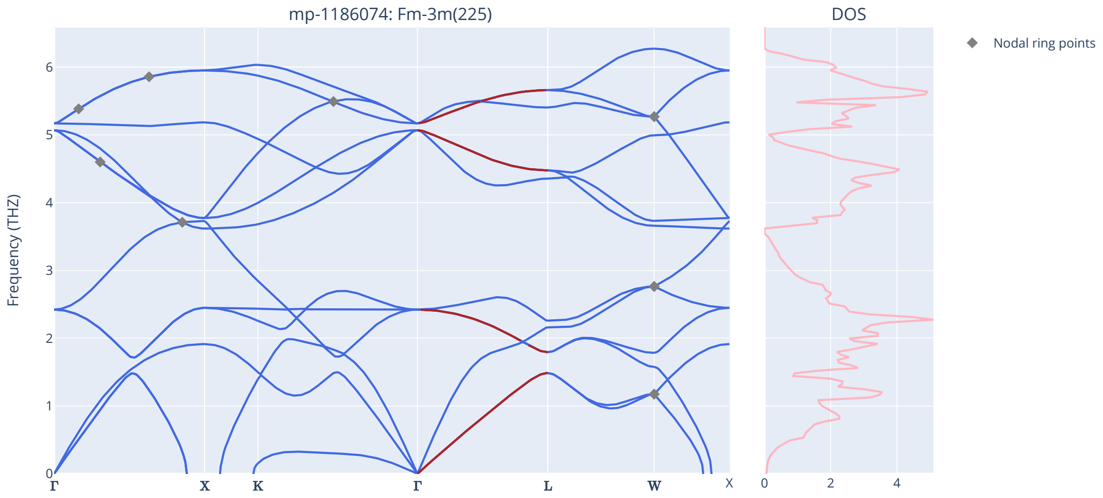

Supplementary Figure 606: The phonon band and DOS of Na12Pd4.

Supplementary Table 1284: High symmetry lines of mp-1186074-Na12Pd4

	Start	End
0	Γ (0.000, 0.000, 0.000)	X (0.500, 0.000, 0.500)
1	X (0.500, 0.000, 0.500)	U (0.625, 0.250, 0.625)
2	K (0.375, 0.375, 0.750)	Γ (0.000, 0.000, 0.000)
3	Γ (0.000, 0.000, 0.000)	L (0.500, 0.500, 0.500)
4	L (0.500, 0.500, 0.500)	W (0.500, 0.250, 0.750)
5	W (0.500, 0.250, 0.750)	X (0.500, 0.000, 0.500)

Supplementary Table 1285: Nodal ring points along high symmetry lines of mp-1186074-Na12Pd4.

	Coordinates	Frequency	Mode 1	Mode 2	Multi.	Deg.	Path	Ratio
0	(0.500, 0.250, 0.750)	1.175	2	1	6	2	5	1.000
1	(0.500, 0.250, 0.750)	2.763	6	5	6	2	5	1.000
2	(0.426, -0.001, 0.426)	3.710	7	6	10	2	1	0.852
3	(0.153, 0.000, 0.153)	4.600	8	7	6	2	1	0.306
4	(0.194, 0.194, 0.398)	5.492	11	10	24	2	3	0.474
5	(0.500, 0.250, 0.750)	5.269	11	10	6	2	5	1.000
6	(0.082, 0.000, 0.082)	5.384	12	11	6	2	1	0.163
7	(0.320, -0.000, 0.311)	5.857	12	11	24	2	1	0.632

The followings are details for $CaSr_3$. It has 7 topological nodal lines and 24 nodal ring points along high symmetry line as shown in Fig 607.

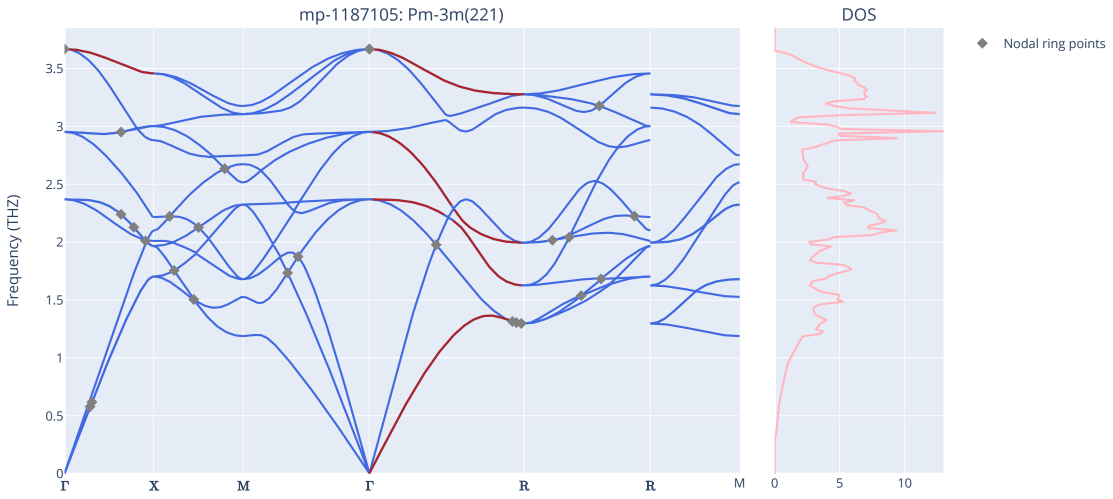

Supplementary Figure 607: The phonon band and DOS of CaSr3.

Supplementary Table 1286: High symmetry lines of mp-1187105-CaSr3

	Start	End
0	Γ (0.000, 0.000, 0.000)	X (0.000, 0.500, 0.000)
1	X (0.000, 0.500, 0.000)	M (0.500, 0.500, 0.000)
2	M (0.500, 0.500, 0.000)	Γ (0.000, 0.000, 0.000)
3	Γ (0.000, 0.000, 0.000)	R (0.500, 0.500, 0.500)
4	R (0.500, 0.500, 0.500)	X (0.000, 0.500, 0.000)
5	R (0.500, 0.500, 0.500)	M (0.500, 0.500, 0.000)

Supplementary Table 1287: Nodal ring points along high symmetry lines of mp-1187105-CaSr3.

	Coordinates	Frequency	Mode 1	Mode 2	Multi.	Deg.	Path	Ratio
0	(0.000, 0.143, 0.000)	0.579	2	1	6	2	1	0.286
1	(0.000, 0.153, 0.000)	0.619	2	1	6	2	1	0.306
2	(0.223, 0.500, -0.000)	1.505	2	1	12	2	2	0.447
3	(0.114, 0.500, 0.000)	1.755	3	2	12	2	2	0.228
4	(0.324, 0.324, 0.000)	1.735	3	2	12	2	3	0.352
5	(0.463, 0.463, 0.463)	1.314	3	2	8	2	4	0.925
6	(0.475, 0.475, 0.475)	1.305	3	2	8	2	4	0.951
7	(0.491, 0.491, 0.491)	1.298	3	2	8	3	4	0.982
8	(0.274, 0.500, 0.274)	1.538	3	2	12	2	5	0.453
9	(0.282, 0.282, -0.000)	1.875	4	3	12	2	3	0.435
10	(0.216, 0.216, 0.216)	1.978	4	3	8	2	4	0.432
11	(0.195, 0.500, 0.195)	1.683	4	3	12	2	5	0.610
12	(0.000, 0.454, 0.000)	2.011	5	4	6	2	1	0.907
13	(0.000, 0.316, 0.000)	2.241	6	5	6	2	1	0.633

Supplementary Table 1287: Nodal ring points along high symmetry lines of mp-1187105-CaSr3.

	Coordinates	Frequency	Mode 1	Mode 2	Multi.	Deg.	Path	Ratio
14	(0.000, 0.388, 0.000)	2.129	6	5	6	2	1	0.776
15	(0.250, 0.500, 0.000)	2.128	6	5	12	2	2	0.501
16	(0.321, 0.500, 0.321)	2.043	6	5	12	2	5	0.357
17	(0.088, 0.500, -0.000)	2.223	7	6	12	2	2	0.177
18	(0.063, 0.500, 0.063)	2.225	7	6	12	2	5	0.874
19	(0.388, 0.500, 0.388)	2.017	7	6	12	2	5	0.224
20	(0.397, 0.500, 0.000)	2.635	8	7	12	2	2	0.794
21	(0.000, 0.316, 0.000)	2.952	9	8	6	2	1	0.633
22	(0.000, 0.000, 0.000)	3.668	11	10	1	3	1	0.000
23	(0.202, 0.500, 0.202)	3.177	11	10	12	2	5	0.596

The followings are details for Os$_3$. It has 9 topological nodal lines and 26 nodal ring points along high symmetry line as shown in Fig **608**.

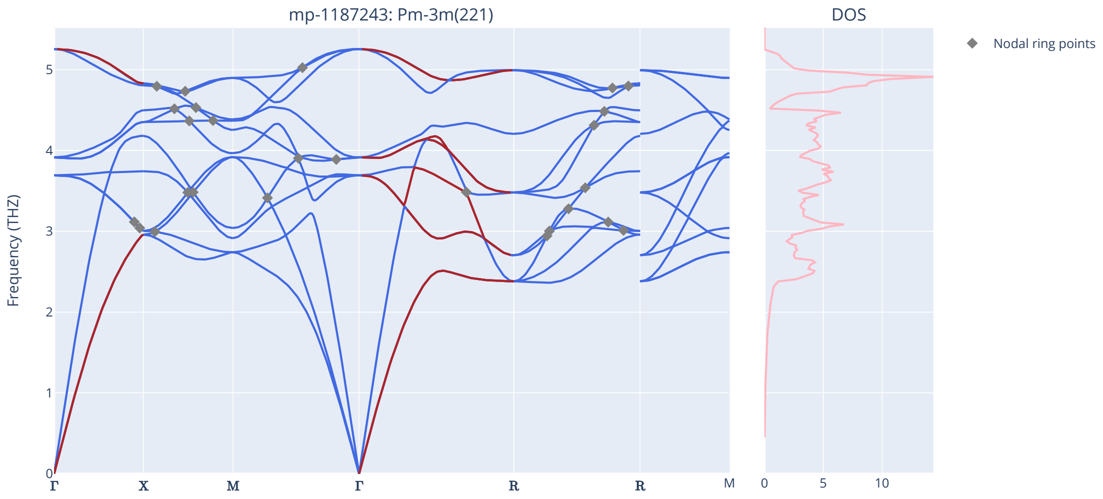

Supplementary Figure 608: The phonon band and DOS of Os3Ta.

Supplementary Table 1288: High symmetry lines of mp-1187243-Os3Ta

	Start	End
0	Γ (0.000, 0.000, 0.000)	X (0.000, 0.500, 0.000)
1	X (0.000, 0.500, 0.000)	M (0.500, 0.500, 0.000)
2	M (0.500, 0.500, 0.000)	Γ (0.000, 0.000, 0.000)
3	Γ (0.000, 0.000, 0.000)	R (0.500, 0.500, 0.500)
4	R (0.500, 0.500, 0.500)	X (0.000, 0.500, 0.000)
5	R (0.500, 0.500, 0.500)	M (0.500, 0.500, 0.000)

Supplementary Table 1289: Nodal ring points along high symmetry lines of mp-1187243-Os3Ta.

	Coordinates	Frequency	Mode 1	Mode 2	Multi.	Deg.	Path	Ratio
0	(0.064, 0.500, 0.000)	2.995	3	2	12	2	2	0.128
1	(0.066, 0.499, 0.066)	3.011	3	2	24	2	5	0.869
2	(0.000, 0.449, 0.000)	3.116	4	3	6	2	1	0.898
3	(0.000, 0.480, 0.000)	3.040	4	3	6	2	1	0.959
4	(0.369, 0.500, 0.369)	2.943	4	3	12	2	5	0.263
5	(0.126, 0.500, 0.126)	3.114	4	3	12	2	5	0.747
6	(0.248, 0.499, -0.000)	3.480	5	4	24	2	2	0.497
7	(0.279, 0.500, -0.000)	3.483	5	4	12	2	2	0.557
8	(0.363, 0.363, 0.000)	3.413	5	4	12	2	3	0.275
9	(0.359, 0.500, 0.359)	3.003	5	4	12	2	5	0.282
10	(0.283, 0.500, 0.283)	3.278	5	4	12	2	5	0.433
11	(0.347, 0.347, 0.347)	3.484	6	5	8	2	4	0.693
12	(0.217, 0.500, 0.217)	3.538	6	5	12	2	5	0.566
13	(0.240, 0.240, -0.000)	3.903	7	6	12	2	3	0.520

Supplementary Table 1289: Nodal ring points along high symmetry lines of mp-1187243-Os3Ta.

	Coordinates	Frequency	Mode 1	Mode 2	Multi.	Deg.	Path	Ratio
14	(0.257, 0.499, -0.000)	4.365	8	7	24	2	2	0.514
15	(0.090, 0.090, -0.000)	3.889	8	7	12	2	3	0.820
16	(0.182, 0.500, 0.182)	4.312	8	7	12	2	5	0.636
17	(0.173, 0.500, -0.000)	4.515	9	8	12	2	2	0.347
18	(0.390, 0.500, -0.000)	4.370	9	8	12	2	2	0.779
19	(0.142, 0.500, 0.142)	4.483	9	8	12	2	5	0.717
20	(0.294, 0.500, -0.000)	4.530	10	9	12	2	2	0.588
21	(0.075, 0.500, -0.000)	4.794	11	10	12	2	2	0.150
22	(0.046, 0.500, 0.046)	4.797	11	10	12	2	5	0.907
23	(0.234, 0.500, 0.000)	4.732	12	11	12	2	2	0.468
24	(0.224, 0.224, 0.000)	5.024	12	11	12	2	3	0.552
25	(0.109, 0.500, 0.109)	4.773	12	11	12	2	5	0.781

The followings are details for $TaZn_3$. It has 7 topological nodal lines and 20 nodal ring points along high symmetry line as shown in Fig **609**.

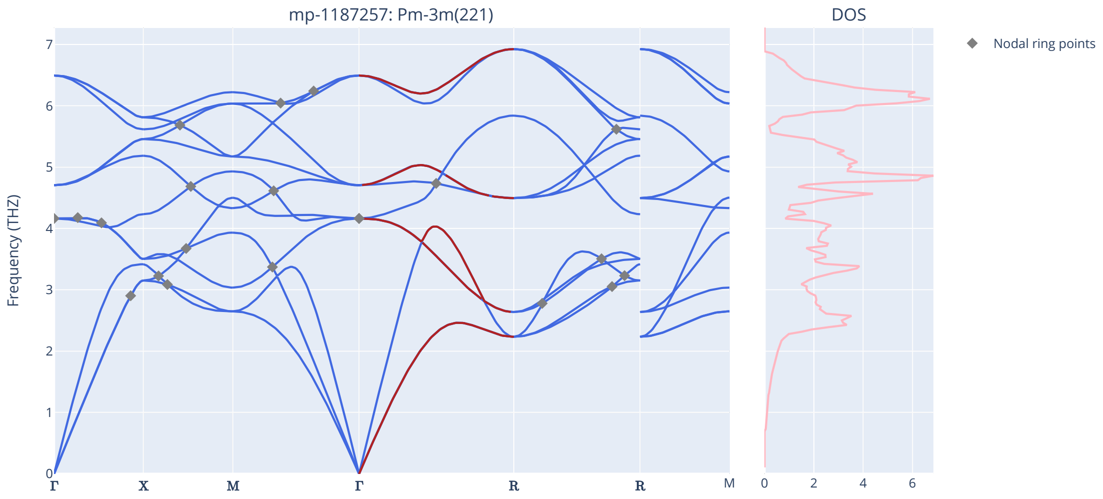

Supplementary Figure 609: The phonon band and DOS of TaZn3.

Supplementary Table 1290: High symmetry lines of mp-1187257-TaZn3

	Start	End
0	Γ (0.000, 0.000, 0.000)	X (0.000, 0.500, 0.000)
1	X (0.000, 0.500, 0.000)	M (0.500, 0.500, 0.000)
2	M (0.500, 0.500, 0.000)	Γ (0.000, 0.000, 0.000)
3	Γ (0.000, 0.000, 0.000)	R (0.500, 0.500, 0.500)
4	R (0.500, 0.500, 0.500)	X (0.000, 0.500, 0.000)
5	R (0.500, 0.500, 0.500)	M (0.500, 0.500, 0.000)

Supplementary Table 1291: Nodal ring points along high symmetry lines of mp-1187257-TaZn3.

	Coordinates	Frequency	Mode 1	Mode 2	Multi.	Deg.	Path	Ratio
0	(0.000, 0.429, 0.000)	2.902	2	1	6	2	1	0.857
1	(0.134, 0.500, -0.000)	3.086	2	1	12	2	2	0.269
2	(0.111, 0.500, 0.111)	3.052	2	1	12	2	5	0.778
3	(0.085, 0.500, -0.000)	3.225	3	2	12	2	2	0.169
4	(0.062, 0.500, 0.062)	3.230	3	2	12	2	5	0.877
5	(0.343, 0.343, -0.000)	3.371	4	3	12	2	3	0.314
6	(0.387, 0.500, 0.387)	2.778	4	3	12	2	5	0.225
7	(0.239, 0.500, 0.000)	3.673	5	4	12	2	2	0.478
8	(0.153, 0.500, 0.153)	3.505	5	4	12	2	5	0.695
9	(0.000, 0.000, 0.000)	4.163	6	5	1	3	1	0.000
10	(0.000, 0.133, 0.000)	4.176	6	5	6	2	1	0.265
11	(0.000, 0.265, 0.000)	4.091	6	5	6	2	1	0.531
12	(0.265, 0.500, -0.000)	4.685	7	6	12	2	2	0.531
13	(0.338, 0.338, -0.000)	4.613	7	6	12	2	3	0.324

Supplementary Table 1291: Nodal ring points along high symmetry lines of mp-1187257-TaZn3.

	Coordinates	Frequency	Mode 1	Mode 2	Multi.	Deg.	Path	Ratio
14	(0.249, 0.249, 0.249)	4.734	7	6	8	2	4	0.498
15	(0.205, 0.500, -0.000)	5.685	10	9	12	2	2	0.409
16	(0.094, 0.500, 0.094)	5.618	10	9	12	2	5	0.812
17	(0.180, 0.180, -0.000)	6.241	12	11	12	2	3	0.640
18	(0.310, 0.310, -0.000)	6.044	12	11	12	2	3	0.379

The followings are details for Pt$_3$. It has 7 topological nodal lines and 31 nodal ring points along high symmetry line as shown in Fig **610**.

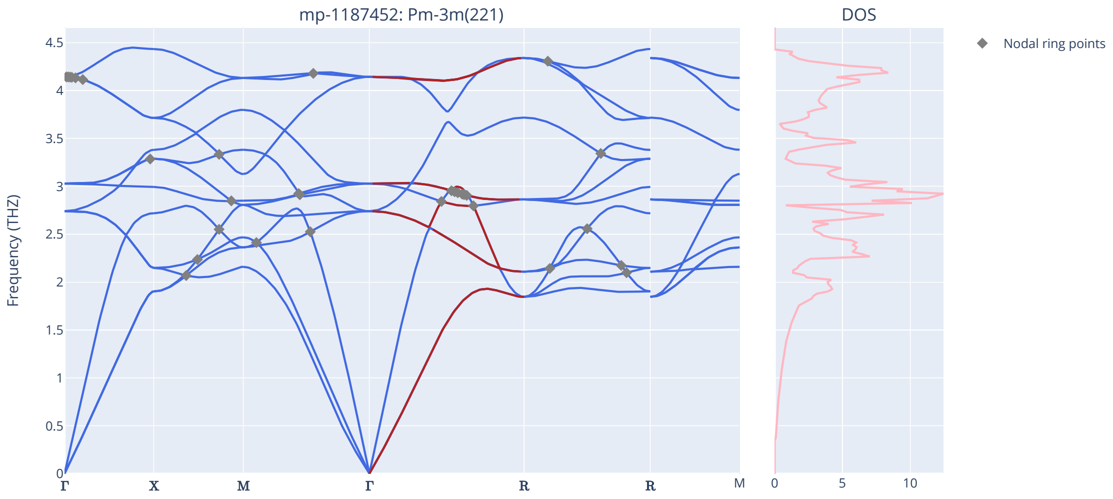

Supplementary Figure 610: The phonon band and DOS of Pt3Th.

Supplementary Table 1292: High symmetry lines of mp-1187452-Pt3Th

	Start	End
0	Γ (0.000, 0.000, 0.000)	X (0.000, 0.500, 0.000)
1	X (0.000, 0.500, 0.000)	M (0.500, 0.500, 0.000)
2	M (0.500, 0.500, 0.000)	Γ (0.000, 0.000, 0.000)
3	Γ (0.000, 0.000, 0.000)	R (0.500, 0.500, 0.500)
4	R (0.500, 0.500, 0.500)	X (0.000, 0.500, 0.000)
5	R (0.500, 0.500, 0.500)	M (0.500, 0.500, 0.000)

Supplementary Table 1293: Nodal ring points along high symmetry lines of mp-1187452-Pt3Th.

	Coordinates	Frequency	Mode 1	Mode 2	Multi.	Deg.	Path	Ratio
0	(0.181, 0.500, -0.000)	2.069	2	1	12	2	2	0.362
1	(0.094, 0.500, 0.094)	2.098	3	2	12	2	5	0.812
2	(0.244, 0.500, 0.000)	2.236	4	3	12	2	2	0.488
3	(0.234, 0.234, -0.000)	2.526	4	3	12	2	3	0.532
4	(0.447, 0.447, 0.000)	2.413	4	3	12	2	3	0.105
5	(0.398, 0.500, 0.398)	2.142	4	3	12	2	5	0.204
6	(0.115, 0.500, 0.115)	2.174	4	3	12	2	5	0.770
7	(0.365, 0.500, -0.000)	2.551	5	4	12	2	2	0.731
8	(0.250, 0.500, 0.250)	2.557	5	4	12	2	5	0.500
9	(0.233, 0.233, 0.233)	2.842	6	5	8	2	4	0.466
10	(0.335, 0.338, 0.336)	2.796	6	5	48	2	4	0.673
11	(0.434, 0.500, 0.000)	2.848	7	6	12	2	2	0.868
12	(0.275, 0.275, -0.000)	2.912	7	6	12	2	3	0.449
13	(0.300, 0.300, 0.300)	2.919	7	6	8	2	4	0.600

Supplementary Table 1293: Nodal ring points along high symmetry lines of mp-1187452-Pt3Th.

	Coordinates	Frequency	Mode 1	Mode 2	Multi.	Deg.	Path	Ratio
14	(0.315, 0.315, 0.315)	2.905	7	6	8	2	4	0.630
15	(0.265, 0.265, 0.265)	2.954	7	6	8	2	4	0.531
16	(0.276, 0.276, 0.276)	2.943	7	6	8	2	4	0.551
17	(0.286, 0.286, 0.286)	2.933	7	6	8	2	4	0.571
18	(0.306, 0.306, 0.306)	2.913	7	6	8	2	4	0.612
19	(0.000, 0.480, 0.000)	3.285	8	7	6	2	1	0.959
20	(0.280, 0.280, -0.000)	2.924	8	7	12	2	3	0.440
21	(0.366, 0.500, -0.002)	3.334	9	8	24	2	2	0.731
22	(0.196, 0.500, 0.196)	3.343	9	8	12	2	5	0.608
23	(0.000, 0.010, 0.000)	4.142	11	10	6	2	1	0.020
24	(0.000, 0.020, 0.000)	4.141	11	10	6	2	1	0.041
25	(0.000, 0.031, 0.000)	4.140	11	10	6	2	1	0.061
26	(0.000, 0.041, 0.000)	4.138	11	10	6	2	1	0.082
27	(0.000, 0.061, 0.000)	4.132	11	10	6	2	1	0.122
28	(0.000, 0.102, 0.000)	4.114	11	10	6	2	1	0.204
29	(0.222, 0.222, -0.000)	4.179	12	11	12	2	3	0.556
30	(0.406, 0.500, 0.406)	4.306	12	11	12	2	5	0.189

The followings are details for Ru3. It has 7 topological nodal lines and 23 nodal ring points along high symmetry line as shown in Fig **611**.

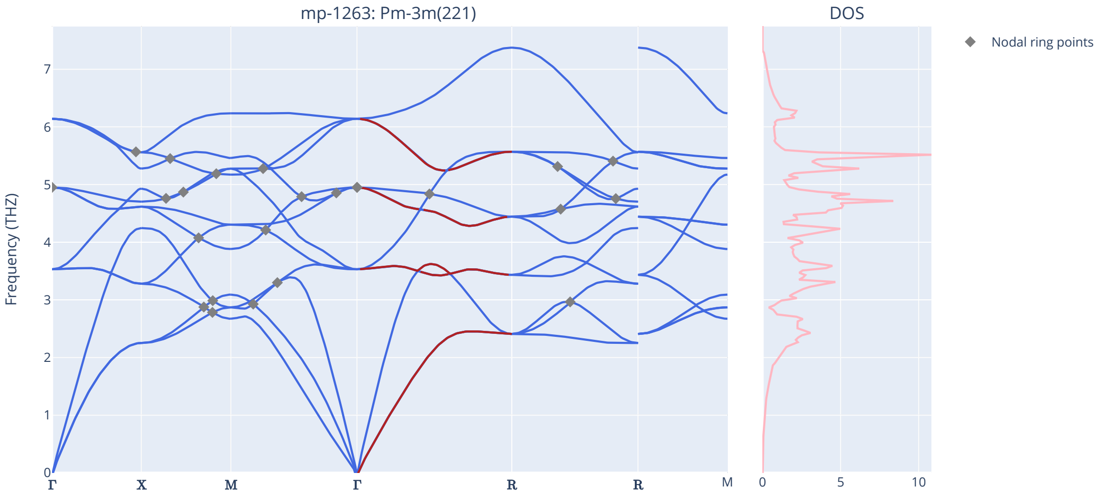

Supplementary Figure 611: The phonon band and DOS of Ru3U.

Supplementary Table 1294: High symmetry lines of mp-1263-Ru3U

	Start	End
0	Γ (0.000, 0.000, 0.000)	X (0.000, 0.500, 0.000)
1	X (0.000, 0.500, 0.000)	M (0.500, 0.500, 0.000)
2	M (0.500, 0.500, 0.000)	Γ (0.000, 0.000, 0.000)
3	Γ (0.000, 0.000, 0.000)	R (0.500, 0.500, 0.500)
4	R (0.500, 0.500, 0.500)	X (0.000, 0.500, 0.000)
5	R (0.500, 0.500, 0.500)	M (0.500, 0.500, 0.000)

Supplementary Table 1295: Nodal ring points along high symmetry lines of mp-1263-Ru3U.

	Coordinates	Frequency	Mode 1	Mode 2	Multi.	Deg.	Path	Ratio
0	(0.398, 0.500, -0.000)	2.782	2	1	12	2	2	0.795
1	(0.349, 0.500, 0.000)	2.875	3	2	12	2	2	0.699
2	(0.411, 0.411, 0.000)	2.926	3	2	12	2	3	0.178
3	(0.269, 0.500, 0.269)	2.964	3	2	12	2	5	0.462
4	(0.399, 0.500, 0.000)	2.986	4	3	12	2	2	0.799
5	(0.315, 0.315, -0.000)	3.299	4	3	12	2	3	0.369
6	(0.320, 0.500, -0.000)	4.076	6	5	12	2	2	0.640
7	(0.362, 0.362, 0.000)	4.211	6	5	12	2	3	0.277
8	(0.000, 0.000, 0.000)	4.952	8	7	1	3	1	0.000
9	(0.082, 0.082, 0.000)	4.856	8	7	12	2	3	0.836
10	(0.306, 0.500, 0.306)	4.574	8	7	12	2	5	0.387
11	(0.235, 0.500, 0.000)	4.870	9	8	12	2	2	0.470
12	(0.138, 0.500, -0.000)	4.761	9	8	12	2	2	0.276
13	(0.419, 0.500, -0.000)	5.189	9	8	12	2	2	0.838

Supplementary Table 1295: Nodal ring points along high symmetry lines of mp-1263-Ru3U.

	Coordinates	Frequency	Mode 1	Mode 2	Multi.	Deg.	Path	Ratio
14	(0.220, 0.220, 0.000)	4.790	9	8	12	2	3	0.561
15	(0.234, 0.234, 0.234)	4.837	9	8	8	2	4	0.468
16	(0.088, 0.500, 0.088)	4.757	9	8	12	2	5	0.824
17	(0.372, 0.372, -0.000)	5.278	10	9	12	2	3	0.256
18	(0.319, 0.500, 0.319)	5.314	10	9	12	2	5	0.363
19	(0.161, 0.500, -0.000)	5.451	11	10	12	2	2	0.322
20	(0.099, 0.500, 0.099)	5.407	11	10	12	2	5	0.802
21	(0.000, 0.469, 0.000)	5.569	12	11	6	2	1	0.939

The followings are details for Au$_3$. It has 7 topological nodal lines and 19 nodal ring points along high symmetry line as shown in Fig **612**.

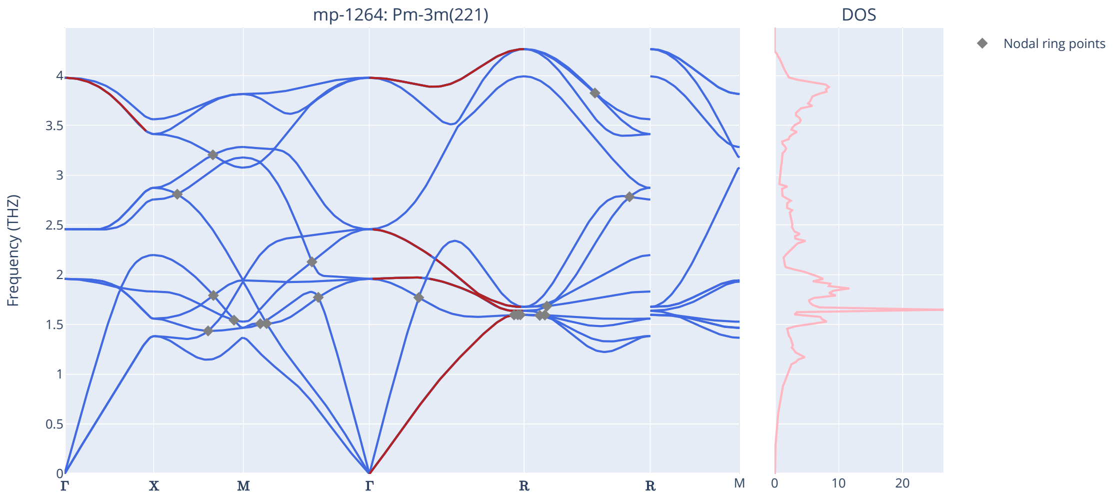

Supplementary Figure 612: The phonon band and DOS of Au3Cd.

Supplementary Table 1296: High symmetry lines of mp-1264-Au3Cd

	Start	End
0	Γ (0.000, 0.000, 0.000)	X (0.000, 0.500, 0.000)
1	X (0.000, 0.500, 0.000)	M (0.500, 0.500, 0.000)
2	M (0.500, 0.500, 0.000)	Γ (0.000, 0.000, 0.000)
3	Γ (0.000, 0.000, 0.000)	R (0.500, 0.500, 0.500)
4	R (0.500, 0.500, 0.500)	X (0.000, 0.500, 0.000)
5	R (0.500, 0.500, 0.500)	M (0.500, 0.500, 0.000)

Supplementary Table 1297: Nodal ring points along high symmetry lines of mp-1264-Au3Cd.

	Coordinates	Frequency	Mode 1	Mode 2	Multi.	Deg.	Path	Ratio
0	(0.438, 0.500, 0.438)	1.591	2	1	12	2	5	0.124
1	(0.304, 0.500, -0.000)	1.435	3	2	12	2	2	0.608
2	(0.432, 0.432, -0.000)	1.508	3	2	12	2	3	0.136
3	(0.407, 0.407, 0.000)	1.509	3	2	12	2	3	0.186
4	(0.480, 0.480, 0.480)	1.597	3	2	8	2	4	0.960
5	(0.468, 0.468, 0.468)	1.595	3	2	8	2	4	0.936
6	(0.488, 0.488, 0.488)	1.597	3	2	8	2	4	0.977
7	(0.449, 0.500, 0.000)	1.543	4	3	12	2	2	0.898
8	(0.202, 0.202, -0.000)	1.772	4	3	12	2	3	0.595
9	(0.418, 0.500, 0.418)	1.594	4	3	12	2	5	0.165
10	(0.160, 0.160, 0.160)	1.770	4	3	8	2	4	0.319
11	(0.334, 0.500, -0.000)	1.791	6	5	12	2	2	0.667
12	(0.410, 0.500, 0.410)	1.684	6	5	12	2	5	0.181
13	(0.227, 0.227, -0.000)	2.128	7	6	12	2	3	0.545

Supplementary Table 1297: Nodal ring points along high symmetry lines of mp-1264-Au3Cd.

	Coordinates	Frequency	Mode 1	Mode 2	Multi.	Deg.	Path	Ratio
14	(0.131, 0.500, -0.000)	2.808	8	7	12	2	2	0.263
15	(0.082, 0.500, 0.082)	2.782	8	7	12	2	5	0.835
16	(0.331, 0.500, 0.005)	3.204	10	9	24	2	2	0.661
17	(0.219, 0.500, 0.219)	3.825	12	11	12	2	5	0.562

The followings are details for Ni3. It has 9 topological nodal lines and 14 nodal ring points along high symmetry line as shown in Fig 613.

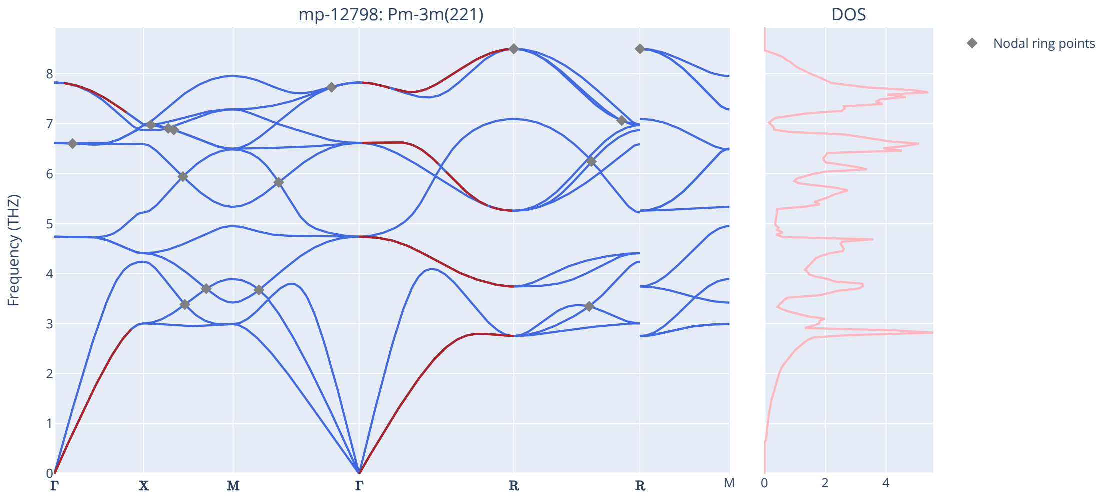

Supplementary Figure 613: The phonon band and DOS of Ni3Pt.

Supplementary Table 1298: High symmetry lines of mp-12798-Ni3Pt

	Start	End
0	Γ (0.000, 0.000, 0.000)	X (0.000, 0.500, 0.000)
1	X (0.000, 0.500, 0.000)	M (0.500, 0.500, 0.000)
2	M (0.500, 0.500, 0.000)	Γ (0.000, 0.000, 0.000)
3	Γ (0.000, 0.000, 0.000)	R (0.500, 0.500, 0.500)
4	R (0.500, 0.500, 0.500)	X (0.000, 0.500, 0.000)
5	R (0.500, 0.500, 0.500)	M (0.500, 0.500, 0.000)

Supplementary Table 1299: Nodal ring points along high symmetry lines of mp-12798-Ni3Pt.

	Coordinates	Frequency	Mode 1	Mode 2	Multi.	Deg.	Path	Ratio
0	(0.231, 0.500, -0.000)	3.381	3	2	12	2	2	0.462
1	(0.202, 0.500, 0.202)	3.345	3	2	12	2	5	0.597
2	(0.351, 0.500, -0.000)	3.699	4	3	12	2	2	0.702
3	(0.397, 0.397, -0.000)	3.673	4	3	12	2	3	0.206
4	(0.220, 0.500, -0.000)	5.940	7	6	12	2	2	0.440
5	(0.319, 0.319, -0.000)	5.827	7	6	12	2	3	0.363
6	(0.000, 0.102, 0.000)	6.601	8	7	6	2	1	0.204
7	(0.193, 0.500, 0.193)	6.244	8	7	12	2	5	0.615
8	(0.169, 0.500, 0.000)	6.877	9	8	12	2	2	0.338
9	(0.138, 0.500, -0.002)	6.907	9	8	24	2	2	0.276
10	(0.041, 0.500, -0.000)	6.975	11	10	12	2	2	0.081
11	(0.109, 0.109, -0.000)	7.729	11	10	12	2	3	0.782
12	(0.500, 0.500, 0.500)	8.498	11	10	1	3	4	1.000
13	(0.073, 0.500, 0.073)	7.063	11	10	12	2	5	0.855

The followings are details for $AlZr_3$. It has 7 topological nodal lines and 18 nodal ring points along high symmetry line as shown in Fig **614**.

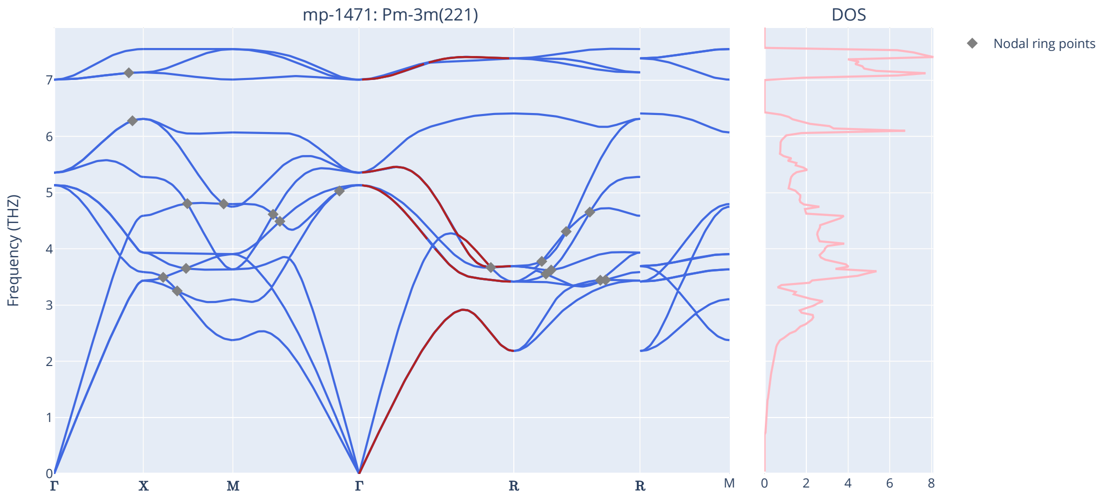

Supplementary Figure 614: The phonon band and DOS of AlZr3.

Supplementary Table 1300: High symmetry lines of mp-1471-AlZr3

	Start	End
0	Γ (0.000, 0.000, 0.000)	X (0.000, 0.500, 0.000)
1	X (0.000, 0.500, 0.000)	M (0.500, 0.500, 0.000)
2	M (0.500, 0.500, 0.000)	Γ (0.000, 0.000, 0.000)
3	Γ (0.000, 0.000, 0.000)	R (0.500, 0.500, 0.500)
4	R (0.500, 0.500, 0.500)	X (0.000, 0.500, 0.000)
5	R (0.500, 0.500, 0.500)	M (0.500, 0.500, 0.000)

Supplementary Table 1301: Nodal ring points along high symmetry lines of mp-1471-AlZr3.

	Coordinates	Frequency	Mode 1	Mode 2	Multi.	Deg.	Path	Ratio
0	(0.189, 0.500, -0.000)	3.251	2	1	12	2	2	0.378
1	(0.111, 0.500, 0.000)	3.494	3	2	12	2	2	0.221
2	(0.137, 0.500, 0.137)	3.446	3	2	12	2	5	0.727
3	(0.239, 0.500, -0.000)	3.652	4	3	12	2	2	0.478
4	(0.157, 0.500, 0.157)	3.442	4	3	12	2	5	0.685
5	(0.078, 0.078, 0.000)	5.029	5	4	12	2	3	0.844
6	(0.373, 0.500, 0.373)	3.553	5	4	12	2	5	0.255
7	(0.313, 0.313, -0.000)	4.490	6	5	12	2	3	0.373
8	(0.426, 0.426, 0.426)	3.668	6	5	8	2	4	0.852
9	(0.352, 0.500, 0.352)	3.623	6	5	12	2	5	0.296
10	(0.246, 0.500, -0.000)	4.805	7	6	12	2	2	0.492
11	(0.340, 0.340, -0.000)	4.614	7	6	12	2	3	0.320
12	(0.199, 0.500, 0.199)	4.654	7	6	12	2	5	0.602
13	(0.449, 0.500, -0.000)	4.800	8	7	12	2	2	0.899

Supplementary Table 1301: Nodal ring points along high symmetry lines of mp-1471-AlZr3.

	Coordinates	Frequency	Mode 1	Mode 2	Multi.	Deg.	Path	Ratio
14	(0.389, 0.500, 0.389)	3.777	8	7	12	2	5	0.222
15	(0.292, 0.500, 0.292)	4.308	8	7	12	2	5	0.415
16	(0.000, 0.439, 0.000)	6.278	9	8	6	2	1	0.878
17	(0.000, 0.418, 0.000)	7.131	11	10	6	2	1	0.837

The followings are details for $CeRh_3$. It has 6 topological nodal lines and 20 nodal ring points along high symmetry line as shown in Fig **615**.

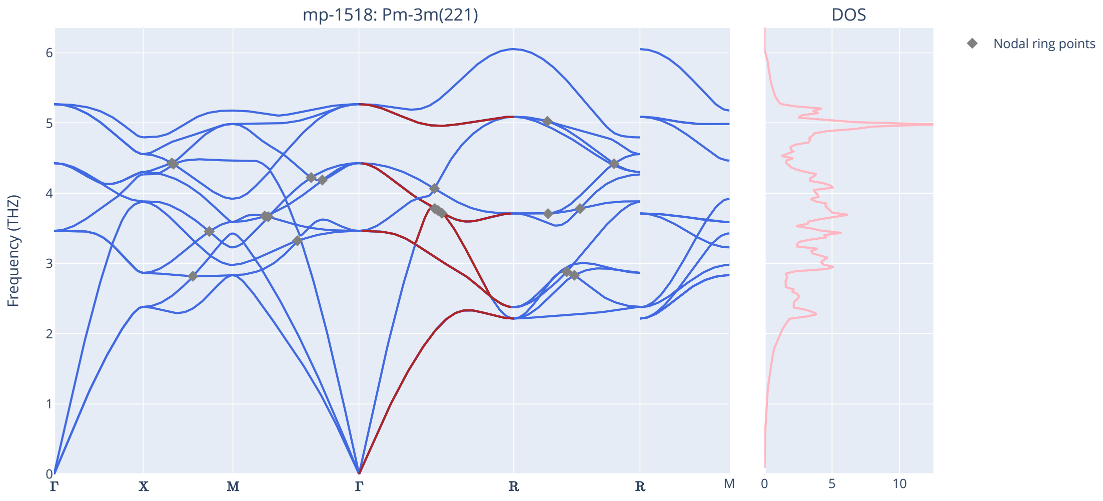

Supplementary Figure 615: The phonon band and DOS of CeRh3.

Supplementary Table 1302: High symmetry lines of mp-1518-CeRh3

	Start	End
0	Γ (0.000, 0.000, 0.000)	X (0.000, 0.500, 0.000)
1	X (0.000, 0.500, 0.000)	M (0.500, 0.500, 0.000)
2	M (0.500, 0.500, 0.000)	Γ (0.000, 0.000, 0.000)
3	Γ (0.000, 0.000, 0.000)	R (0.500, 0.500, 0.500)
4	R (0.500, 0.500, 0.500)	X (0.000, 0.500, 0.000)
5	R (0.500, 0.500, 0.500)	M (0.500, 0.500, 0.000)

Supplementary Table 1303: Nodal ring points along high symmetry lines of mp-1518-CeRh3.

	Coordinates	Frequency	Mode 1	Mode 2	Multi.	Deg.	Path	Ratio
0	(0.277, 0.500, -0.000)	2.817	3	2	12	2	2	0.553
1	(0.260, 0.500, 0.260)	2.832	3	2	12	2	5	0.480
2	(0.244, 0.244, 0.000)	3.322	4	3	12	2	3	0.512
3	(0.290, 0.500, 0.290)	2.883	4	3	12	2	5	0.420
4	(0.369, 0.500, 0.000)	3.455	6	5	12	2	2	0.737
5	(0.359, 0.359, -0.000)	3.664	6	5	12	2	3	0.281
6	(0.263, 0.271, 0.271)	3.713	6	5	24	2	4	0.536
7	(0.245, 0.245, 0.245)	3.781	6	5	8	2	4	0.490
8	(0.255, 0.255, 0.255)	3.754	6	5	8	2	4	0.510
9	(0.238, 0.498, 0.238)	3.781	7	6	24	2	5	0.524
10	(0.373, 0.373, -0.000)	3.675	7	6	12	2	3	0.253
11	(0.145, 0.145, 0.000)	4.187	8	7	12	2	3	0.709
12	(0.364, 0.500, 0.364)	3.709	8	7	12	2	5	0.271
13	(0.169, 0.500, -0.000)	4.414	9	8	12	2	2	0.337

Supplementary Table 1303: Nodal ring points along high symmetry lines of mp-1518-CeRh3.

	Coordinates	Frequency	Mode 1	Mode 2	Multi.	Deg.	Path	Ratio
14	(0.190, 0.190, 0.000)	4.222	9	8	12	2	3	0.621
15	(0.244, 0.244, 0.244)	4.064	9	8	8	2	4	0.487
16	(0.159, 0.500, -0.000)	4.429	10	9	12	2	2	0.317
17	(0.104, 0.500, 0.102)	4.420	10	9	24	2	5	0.793
18	(0.367, 0.500, 0.367)	5.022	11	10	12	2	5	0.266

The followings are details for $LaTl_3$. It has 8 topological nodal lines and 20 nodal ring points along high symmetry line as shown in Fig **616**.

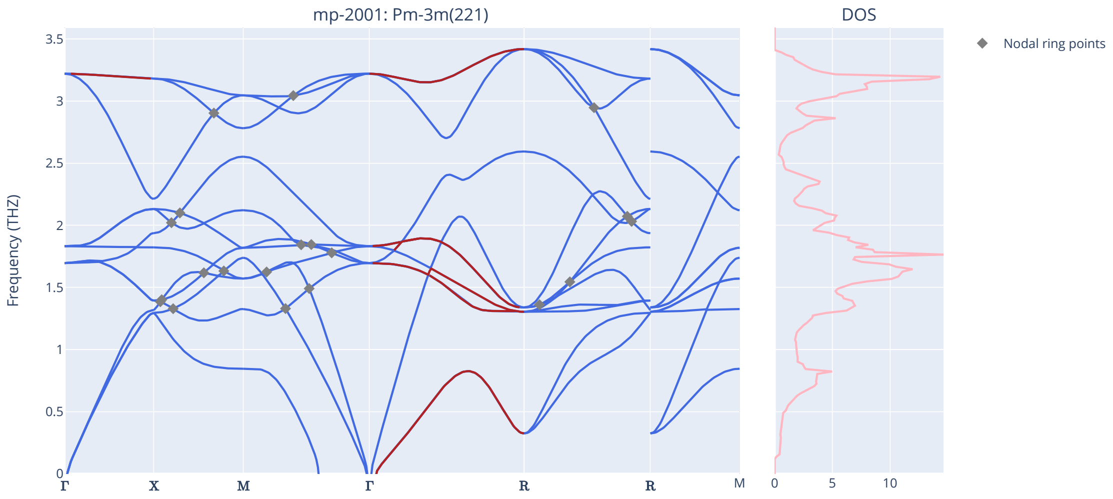

Supplementary Figure 616: The phonon band and DOS of LaTl3.

Supplementary Table 1304: High symmetry lines of mp-2001-LaTl3

	Start	End
0	Γ (0.000, 0.000, 0.000)	X (0.000, 0.500, 0.000)
1	X (0.000, 0.500, 0.000)	M (0.500, 0.500, 0.000)
2	M (0.500, 0.500, 0.000)	Γ (0.000, 0.000, 0.000)
3	Γ (0.000, 0.000, 0.000)	R (0.500, 0.500, 0.500)
4	R (0.500, 0.500, 0.500)	X (0.000, 0.500, 0.000)
5	R (0.500, 0.500, 0.500)	M (0.500, 0.500, 0.000)

Supplementary Table 1305: Nodal ring points along high symmetry lines of mp-2001-LaTl3.

	Coordinates	Frequency	Mode 1	Mode 2	Multi.	Deg.	Path	Ratio
0	(0.108, 0.500, 0.000)	1.329	3	2	12	2	2	0.216
1	(0.332, 0.332, -0.000)	1.330	3	2	12	2	3	0.336
2	(0.036, 0.500, -0.000)	1.385	4	3	12	2	2	0.073
3	(0.238, 0.238, 0.000)	1.491	4	3	12	2	3	0.523
4	(0.044, 0.500, -0.000)	1.401	5	4	12	2	2	0.088
5	(0.280, 0.500, -0.000)	1.617	5	4	12	2	2	0.560
6	(0.392, 0.500, 0.000)	1.632	5	4	12	2	2	0.784
7	(0.407, 0.407, 0.000)	1.624	5	4	12	2	3	0.186
8	(0.149, 0.149, -0.000)	1.778	6	5	12	2	3	0.701
9	(0.270, 0.270, -0.000)	1.843	7	6	12	2	3	0.460
10	(0.439, 0.500, 0.439)	1.361	7	6	12	2	5	0.122
11	(0.319, 0.500, 0.319)	1.545	7	6	12	2	5	0.363
12	(0.099, 0.500, 0.000)	2.021	8	7	12	2	2	0.197
13	(0.230, 0.230, 0.000)	1.845	8	7	12	2	3	0.540

Supplementary Table 1305: Nodal ring points along high symmetry lines of mp-2001-LaTl3.

	Coordinates	Frequency	Mode 1	Mode 2	Multi.	Deg.	Path	Ratio
14	(0.074, 0.500, 0.074)	2.031	8	7	12	2	5	0.852
15	(0.146, 0.500, -0.000)	2.100	9	8	12	2	2	0.292
16	(0.091, 0.500, 0.091)	2.072	9	8	12	2	5	0.818
17	(0.336, 0.500, 0.003)	2.903	11	10	24	2	2	0.673
18	(0.223, 0.500, 0.223)	2.947	11	10	12	2	5	0.554
19	(0.301, 0.301, 0.000)	3.044	12	11	12	2	3	0.397

The followings are details for In$_3$. It has 8 topological nodal lines and 14 nodal ring points along high symmetry line as shown in Fig **617**.

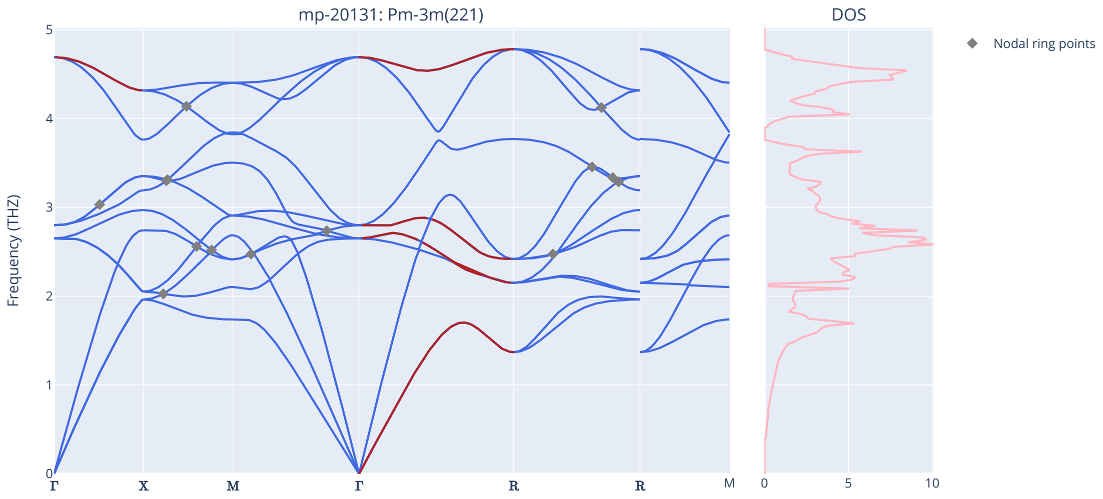

Supplementary Figure 617: The phonon band and DOS of In3Y.

Supplementary Table 1306: High symmetry lines of mp-20131-In3Y

	Start	End
0	Γ (0.000, 0.000, 0.000)	X (0.000, 0.500, 0.000)
1	X (0.000, 0.500, 0.000)	M (0.500, 0.500, 0.000)
2	M (0.500, 0.500, 0.000)	Γ (0.000, 0.000, 0.000)
3	Γ (0.000, 0.000, 0.000)	R (0.500, 0.500, 0.500)
4	R (0.500, 0.500, 0.500)	X (0.000, 0.500, 0.000)
5	R (0.500, 0.500, 0.500)	M (0.500, 0.500, 0.000)

Supplementary Table 1307: Nodal ring points along high symmetry lines of mp-20131-In3Y.

	Coordinates	Frequency	Mode 1	Mode 2	Multi.	Deg.	Path	Ratio
0	(0.111, 0.500, 0.000)	2.025	3	2	12	2	2	0.223
1	(0.428, 0.428, -0.000)	2.472	4	3	12	2	3	0.144
2	(0.382, 0.500, 0.000)	2.518	5	4	12	2	2	0.764
3	(0.298, 0.500, -0.000)	2.558	5	4	12	2	2	0.596
4	(0.128, 0.128, 0.000)	2.736	7	6	12	2	3	0.744
5	(0.345, 0.500, 0.345)	2.474	7	6	12	2	5	0.310
6	(0.128, 0.500, -0.000)	3.299	8	7	12	2	2	0.256
7	(0.085, 0.500, 0.085)	3.286	8	7	12	2	5	0.829
8	(0.000, 0.255, 0.000)	3.031	9	8	6	2	1	0.510
9	(0.135, 0.500, -0.000)	3.311	9	8	12	2	2	0.270
10	(0.108, 0.500, 0.108)	3.334	9	8	12	2	5	0.784
11	(0.190, 0.500, 0.190)	3.453	9	8	12	2	5	0.620
12	(0.241, 0.500, -0.000)	4.137	11	10	12	2	2	0.482
13	(0.153, 0.500, 0.153)	4.122	11	10	12	2	5	0.694

The followings are details for In$_3$. It has 8 topological nodal lines and 19 nodal ring points along high symmetry line as shown in Fig 618.

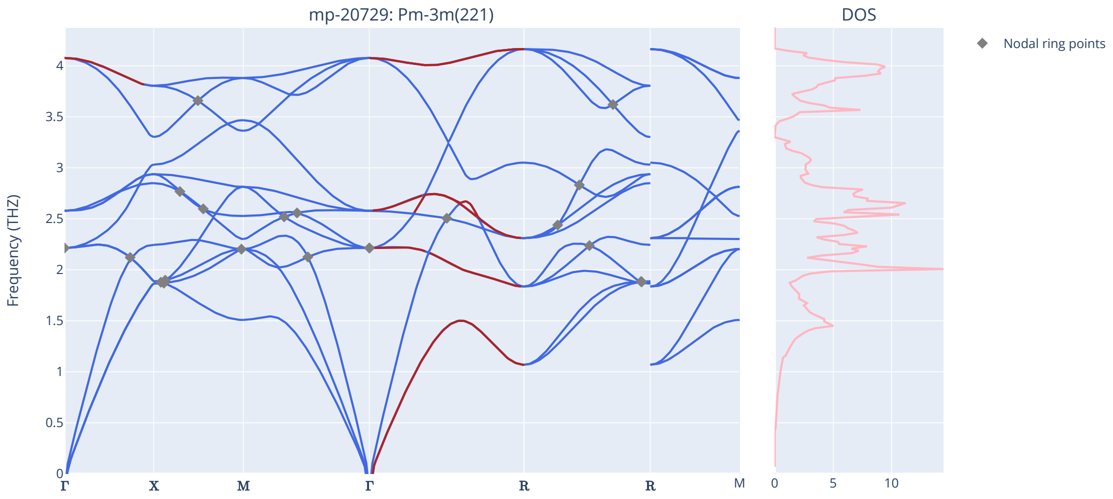

Supplementary Figure 618: The phonon band and DOS of In3La.

Supplementary Table 1308: High symmetry lines of mp-20729-In3La

	Start	End
0	Γ (0.000, 0.000, 0.000)	X (0.000, 0.500, 0.000)
1	X (0.000, 0.500, 0.000)	M (0.500, 0.500, 0.000)
2	M (0.500, 0.500, 0.000)	Γ (0.000, 0.000, 0.000)
3	Γ (0.000, 0.000, 0.000)	R (0.500, 0.500, 0.500)
4	R (0.500, 0.500, 0.500)	X (0.000, 0.500, 0.000)
5	R (0.500, 0.500, 0.500)	M (0.500, 0.500, 0.000)

Supplementary Table 1309: Nodal ring points along high symmetry lines of mp-20729-In3La.

	Coordinates	Frequency	Mode 1	Mode 2	Multi.	Deg.	Path	Ratio
0	(0.057, 0.500, 0.000)	1.869	2	1	12	2	2	0.113
1	(0.040, 0.500, 0.000)	1.877	3	2	12	2	2	0.079
2	(0.490, 0.500, -0.000)	2.201	3	2	12	2	2	0.981
3	(0.034, 0.500, 0.034)	1.881	3	2	12	2	5	0.932
4	(0.064, 0.500, -0.000)	1.897	4	3	12	2	2	0.127
5	(0.244, 0.244, 0.000)	2.125	4	3	12	2	3	0.513
6	(0.037, 0.500, 0.037)	1.884	4	3	12	2	5	0.926
7	(0.000, 0.000, 0.000)	2.212	5	4	1	3	1	0.000
8	(0.000, 0.367, 0.000)	2.120	5	4	6	2	1	0.735
9	(0.241, 0.500, 0.241)	2.236	5	4	12	2	5	0.518
10	(0.338, 0.338, -0.000)	2.518	6	5	12	2	3	0.325
11	(0.250, 0.250, 0.250)	2.504	6	5	8	2	4	0.500
12	(0.146, 0.500, 0.000)	2.766	7	6	12	2	2	0.292
13	(0.277, 0.500, 0.000)	2.596	7	6	12	2	2	0.553

Supplementary Table 1309: Nodal ring points along high symmetry lines of mp-20729-In3La.

	Coordinates	Frequency	Mode 1	Mode 2	Multi.	Deg.	Path	Ratio
14	(0.287, 0.287, 0.000)	2.556	7	6	12	2	3	0.427
15	(0.367, 0.500, 0.367)	2.438	8	7	12	2	5	0.266
16	(0.281, 0.500, 0.281)	2.830	9	8	12	2	5	0.438
17	(0.247, 0.500, -0.000)	3.657	11	10	12	2	2	0.494
18	(0.148, 0.500, 0.148)	3.619	11	10	12	2	5	0.704

The followings are details for $AuCu_3$. It has 7 topological nodal lines and 18 nodal ring points along high symmetry line as shown in Fig **619**.

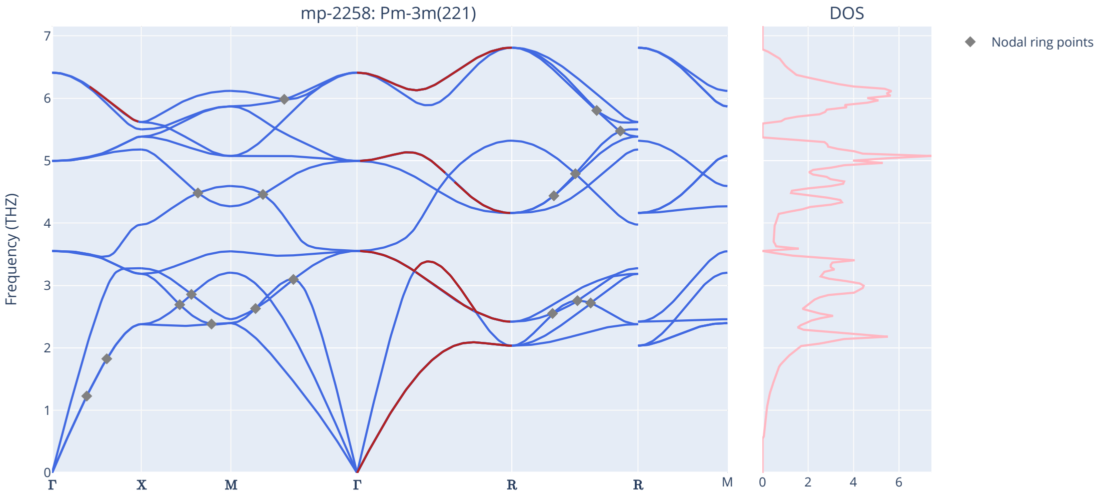

Supplementary Figure 619: The phonon band and DOS of AuCu3.

Supplementary Table 1310: High symmetry lines of mp-2258-AuCu3

	Start	End
0	Γ (0.000, 0.000, 0.000)	X (0.000, 0.500, 0.000)
1	X (0.000, 0.500, 0.000)	M (0.500, 0.500, 0.000)
2	M (0.500, 0.500, 0.000)	Γ (0.000, 0.000, 0.000)
3	Γ (0.000, 0.000, 0.000)	R (0.500, 0.500, 0.500)
4	R (0.500, 0.500, 0.500)	X (0.000, 0.500, 0.000)
5	R (0.500, 0.500, 0.500)	M (0.500, 0.500, 0.000)

Supplementary Table 1311: Nodal ring points along high symmetry lines of mp-2258-AuCu3.

	Coordinates	Frequency	Mode 1	Mode 2	Multi.	Deg.	Path	Ratio
0	(0.000, 0.194, 0.000)	1.228	2	1	6	2	1	0.388
1	(0.000, 0.306, 0.000)	1.825	2	1	6	2	1	0.612
2	(0.392, 0.500, 0.000)	2.382	2	1	12	2	2	0.785
3	(0.214, 0.500, -0.000)	2.692	3	2	12	2	2	0.429
4	(0.402, 0.402, 0.000)	2.631	3	2	12	2	3	0.196
5	(0.188, 0.500, 0.188)	2.719	3	2	12	2	5	0.624
6	(0.280, 0.500, -0.000)	2.857	4	3	12	2	2	0.560
7	(0.252, 0.252, 0.000)	3.097	4	3	12	2	3	0.496
8	(0.240, 0.500, 0.240)	2.757	4	3	12	2	5	0.520
9	(0.339, 0.500, 0.339)	2.551	4	3	12	2	5	0.323
10	(0.316, 0.500, -0.000)	4.482	7	6	12	2	2	0.633
11	(0.372, 0.372, -0.000)	4.459	7	6	12	2	3	0.255
12	(0.333, 0.500, 0.333)	4.437	8	7	12	2	5	0.333
13	(0.248, 0.500, 0.248)	4.791	9	8	12	2	5	0.504

Supplementary Table 1311: Nodal ring points along high symmetry lines of mp-2258-AuCu3.

	Coordinates	Frequency	Mode 1	Mode 2	Multi.	Deg.	Path	Ratio
14	(0.070, 0.500, 0.070)	5.475	10	9	12	2	5	0.860
15	(0.164, 0.500, 0.164)	5.804	11	10	12	2	5	0.672
16	(0.288, 0.288, -0.004)	5.984	12	11	24	2	3	0.424

The followings are details for Bi3. It has 8 topological nodal lines and 16 nodal ring points along high symmetry line as shown in Fig 620.

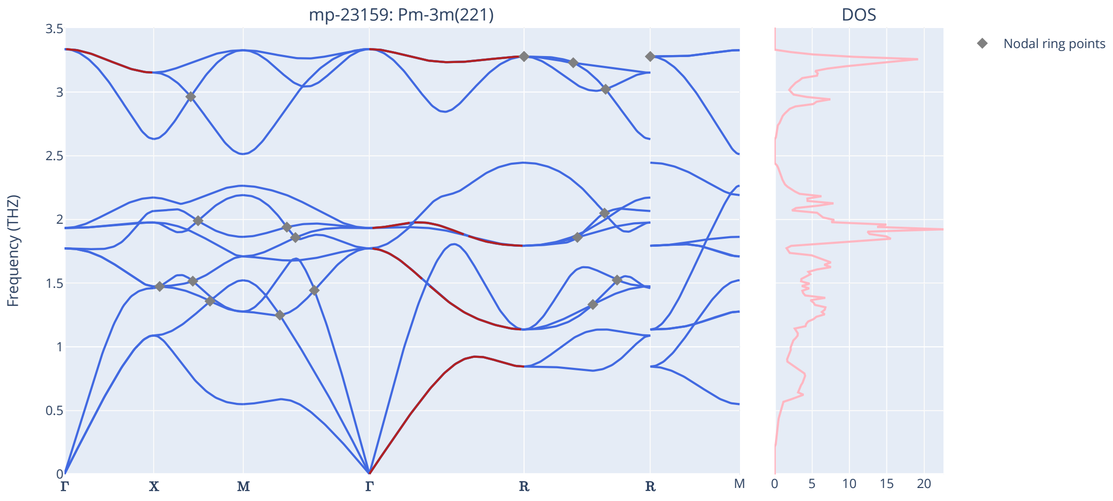

Supplementary Figure 620: The phonon band and DOS of Bi3Sr.

Supplementary Table 1312: High symmetry lines of mp-23159-Bi3Sr

	Start	End
0	Γ (0.000, 0.000, 0.000)	X (0.000, 0.500, 0.000)
1	X (0.000, 0.500, 0.000)	M (0.500, 0.500, 0.000)
2	M (0.500, 0.500, 0.000)	Γ (0.000, 0.000, 0.000)
3	Γ (0.000, 0.000, 0.000)	R (0.500, 0.500, 0.500)
4	R (0.500, 0.500, 0.500)	X (0.000, 0.500, 0.000)
5	R (0.500, 0.500, 0.500)	M (0.500, 0.500, 0.000)

Supplementary Table 1313: Nodal ring points along high symmetry lines of mp-23159-Bi3Sr.

	Coordinates	Frequency	Mode 1	Mode 2	Multi.	Deg.	Path	Ratio
0	(0.315, 0.500, 0.000)	1.359	3	2	12	2	2	0.629
1	(0.355, 0.355, 0.000)	1.249	3	2	12	2	3	0.291
2	(0.033, 0.500, -0.000)	1.474	4	3	12	2	2	0.065
3	(0.218, 0.218, 0.000)	1.443	4	3	12	2	3	0.564
4	(0.227, 0.500, 0.227)	1.332	4	3	12	2	5	0.545
5	(0.218, 0.500, 0.000)	1.516	5	4	12	2	2	0.437
6	(0.131, 0.500, 0.131)	1.524	5	4	12	2	5	0.737
7	(0.293, 0.293, 0.000)	1.858	7	6	12	2	3	0.414
8	(0.289, 0.500, 0.289)	1.859	7	6	12	2	5	0.423
9	(0.248, 0.500, 0.000)	1.990	8	7	12	2	2	0.496
10	(0.327, 0.327, -0.000)	1.940	8	7	12	2	3	0.346
11	(0.181, 0.500, 0.181)	2.049	9	8	12	2	5	0.637
12	(0.207, 0.500, -0.000)	2.964	11	10	12	2	2	0.413
13	(0.177, 0.500, 0.177)	3.022	11	10	12	2	5	0.646

Supplementary Table 1313: Nodal ring points along high symmetry lines of mp-23159-Bi3Sr.

	Coordinates	Frequency	Mode 1	Mode 2	Multi.	Deg.	Path	Ratio
14	(0.500, 0.500, 0.500)	3.279	12	11	1	3	4	1.000
15	(0.305, 0.500, 0.305)	3.230	12	11	12	2	5	0.390

The followings are details for NbRh$_3$. It has 7 topological nodal lines and 35 nodal ring points along high symmetry line as shown in Fig 621.

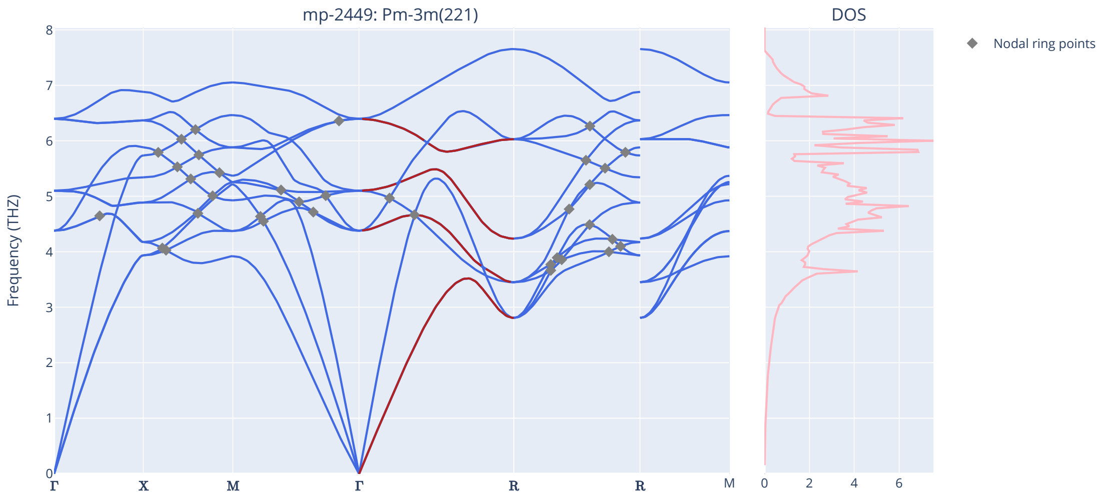

Supplementary Figure 621: The phonon band and DOS of NbRh3.

Supplementary Table 1314: High symmetry lines of mp-2449-NbRh3

	Start	End
0	Γ (0.000, 0.000, 0.000)	X (0.000, 0.500, 0.000)
1	X (0.000, 0.500, 0.000)	M (0.500, 0.500, 0.000)
2	M (0.500, 0.500, 0.000)	Γ (0.000, 0.000, 0.000)
3	Γ (0.000, 0.000, 0.000)	R (0.500, 0.500, 0.500)
4	R (0.500, 0.500, 0.500)	X (0.000, 0.500, 0.000)
5	R (0.500, 0.500, 0.500)	M (0.500, 0.500, 0.000)

Supplementary Table 1315: Nodal ring points along high symmetry lines of mp-2449-NbRh3.

	Coordinates	Frequency	Mode 1	Mode 2	Multi.	Deg.	Path	Ratio
0	(0.127, 0.500, 0.000)	4.029	2	1	12	2	2	0.253
1	(0.123, 0.500, 0.123)	4.001	2	1	12	2	5	0.753
2	(0.106, 0.500, -0.000)	4.068	3	2	12	2	2	0.212
3	(0.379, 0.379, 0.000)	4.552	3	2	12	2	3	0.243
4	(0.353, 0.500, 0.353)	3.665	3	2	12	2	5	0.293
5	(0.310, 0.500, 0.310)	3.859	3	2	12	2	5	0.379
6	(0.077, 0.500, 0.077)	4.099	3	2	12	2	5	0.845
7	(0.305, 0.500, -0.000)	4.695	4	3	12	2	2	0.610
8	(0.390, 0.390, 0.000)	4.637	4	3	12	2	3	0.220
9	(0.110, 0.500, 0.110)	4.227	4	3	12	2	5	0.781
10	(0.000, 0.255, 0.000)	4.648	5	4	6	2	1	0.510
11	(0.181, 0.181, 0.000)	4.717	5	4	12	2	3	0.638
12	(0.328, 0.500, 0.328)	3.894	5	4	12	2	5	0.343
13	(0.200, 0.500, 0.200)	4.489	5	4	12	2	5	0.600

Supplementary Table 1315: Nodal ring points along high symmetry lines of mp-2449-NbRh3.

	Coordinates	Frequency	Mode 1	Mode 2	Multi.	Deg.	Path	Ratio
14	(0.354, 0.500, 0.354)	3.771	5	4	12	2	5	0.293
15	(0.388, 0.500, 0.000)	5.009	6	5	12	2	2	0.775
16	(0.237, 0.237, -0.000)	4.901	6	5	12	2	3	0.526
17	(0.179, 0.179, 0.179)	4.667	6	5	8	2	4	0.358
18	(0.265, 0.500, -0.000)	5.314	7	6	12	2	2	0.530
19	(0.309, 0.309, 0.000)	5.115	7	6	12	2	3	0.382
20	(0.132, 0.132, -0.000)	5.012	7	6	12	2	3	0.736
21	(0.098, 0.098, 0.098)	4.969	7	6	8	2	4	0.197
22	(0.199, 0.500, 0.199)	5.212	7	6	12	2	5	0.602
23	(0.281, 0.500, 0.281)	4.771	7	6	12	2	5	0.438
24	(0.190, 0.500, -0.000)	5.531	8	7	12	2	2	0.380
25	(0.426, 0.500, 0.000)	5.427	8	7	12	2	2	0.851
26	(0.139, 0.500, 0.139)	5.508	8	7	12	2	5	0.723
27	(0.083, 0.500, -0.000)	5.793	9	8	12	2	2	0.166
28	(0.310, 0.500, 0.000)	5.748	9	8	12	2	2	0.621
29	(0.214, 0.500, 0.214)	5.651	9	8	12	2	5	0.571
30	(0.058, 0.500, 0.058)	5.794	9	8	12	2	5	0.884
31	(0.213, 0.500, -0.000)	6.030	10	9	12	2	2	0.426
32	(0.291, 0.500, 0.000)	6.203	11	10	12	2	2	0.583
33	(0.079, 0.079, 0.000)	6.360	11	10	12	2	3	0.841
34	(0.199, 0.500, 0.199)	6.266	11	10	12	2	5	0.603

The followings are details for Ir$_3$. It has 7 topological nodal lines and 36 nodal ring points along high symmetry line as shown in Fig **622**.

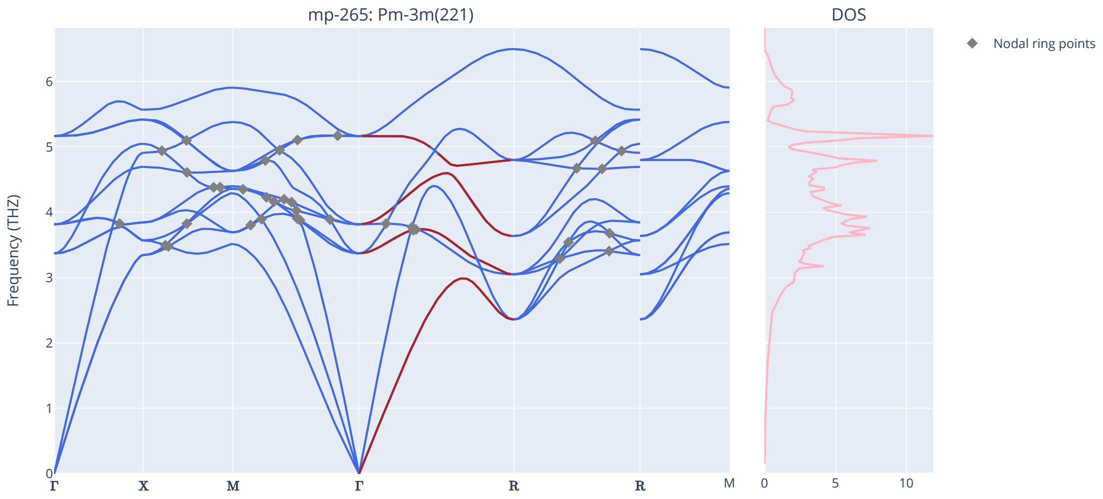

Supplementary Figure 622: The phonon band and DOS of Ir3Ta.

Supplementary Table 1316: High symmetry lines of mp-265-Ir3Ta

	Start	End
0	Γ (0.000, 0.000, 0.000)	X (0.000, 0.500, 0.000)
1	X (0.000, 0.500, 0.000)	M (0.500, 0.500, 0.000)
2	M (0.500, 0.500, 0.000)	Γ (0.000, 0.000, 0.000)
3	Γ (0.000, 0.000, 0.000)	R (0.500, 0.500, 0.500)
4	R (0.500, 0.500, 0.500)	X (0.000, 0.500, 0.000)
5	R (0.500, 0.500, 0.500)	M (0.500, 0.500, 0.000)

Supplementary Table 1317: Nodal ring points along high symmetry lines of mp-265-Ir3Ta.

	Coordinates	Frequency	Mode 1	Mode 2	Multi.	Deg.	Path	Ratio
0	(0.140, 0.500, -0.000)	3.477	2	1	12	2	2	0.279
1	(0.123, 0.500, 0.123)	3.404	2	1	12	2	5	0.755
2	(0.123, 0.500, 0.000)	3.494	3	2	12	2	2	0.245
3	(0.429, 0.429, 0.000)	3.802	3	2	12	2	3	0.142
4	(0.386, 0.386, -0.000)	3.894	3	2	12	2	3	0.228
5	(0.318, 0.500, 0.318)	3.288	3	2	12	2	5	0.365
6	(0.243, 0.500, -0.000)	3.824	4	3	12	2	2	0.487
7	(0.244, 0.244, -0.000)	3.903	4	3	12	2	3	0.511
8	(0.175, 0.175, 0.175)	3.727	4	3	8	2	4	0.350
9	(0.184, 0.184, 0.184)	3.735	4	3	8	2	4	0.367
10	(0.121, 0.500, 0.121)	3.678	4	3	12	2	5	0.757
11	(0.234, 0.234, 0.000)	3.880	5	4	12	2	3	0.533
12	(0.283, 0.500, 0.283)	3.540	5	4	12	2	5	0.434
13	(0.000, 0.366, -0.000)	3.825	6	5	6	2	1	0.733

Supplementary Table 1317: Nodal ring points along high symmetry lines of mp-265-Ir3Ta.

	Coordinates	Frequency	Mode 1	Mode 2	Multi.	Deg.	Path	Ratio
14	(0.459, 0.459, 0.000)	4.350	6	5	12	2	3	0.082
15	(0.337, 0.337, -0.000)	4.162	6	5	12	2	3	0.326
16	(0.366, 0.366, 0.000)	4.227	6	5	12	2	3	0.267
17	(0.248, 0.248, -0.000)	4.024	6	5	12	2	3	0.503
18	(0.171, 0.171, 0.171)	3.773	6	5	8	2	4	0.343
19	(0.429, 0.500, -0.000)	4.382	7	6	12	2	2	0.859
20	(0.393, 0.500, 0.000)	4.380	7	6	12	2	2	0.787
21	(0.114, 0.114, -0.000)	3.886	7	6	12	2	3	0.771
22	(0.297, 0.297, -0.000)	4.197	7	6	12	2	3	0.407
23	(0.267, 0.267, -0.000)	4.154	7	6	12	2	3	0.465
24	(0.087, 0.087, 0.087)	3.825	7	6	8	2	4	0.174
25	(0.244, 0.500, 0.000)	4.607	8	7	12	2	2	0.488
26	(0.151, 0.500, 0.151)	4.662	8	7	12	2	5	0.699
27	(0.103, 0.500, 0.000)	4.939	9	8	12	2	2	0.207
28	(0.371, 0.371, -0.000)	4.791	9	8	12	2	3	0.258
29	(0.073, 0.500, 0.073)	4.936	9	8	12	2	5	0.853
30	(0.251, 0.500, 0.251)	4.672	9	8	12	2	5	0.498
31	(0.315, 0.315, 0.000)	4.947	10	9	12	2	3	0.371
32	(0.241, 0.500, -0.000)	5.097	11	10	12	2	2	0.482
33	(0.085, 0.085, 0.000)	5.169	11	10	12	2	3	0.831
34	(0.244, 0.244, -0.000)	5.104	11	10	12	2	3	0.513
35	(0.177, 0.500, 0.177)	5.091	11	10	12	2	5	0.646

The followings are details for $Cd_{12}Ce_4$. It has 6 topological nodal lines and 18 nodal ring points along high symmetry line as shown in Fig **623**.

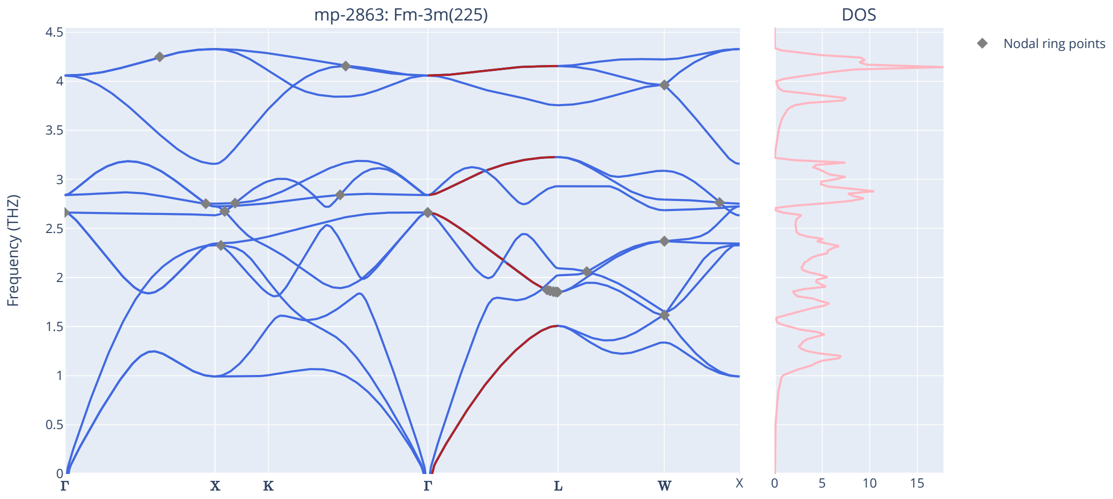

Supplementary Figure 623: The phonon band and DOS of Cd12Ce4.

Supplementary Table 1318: High symmetry lines of mp-2863-Cd12Ce4

	Start	End
0	Γ (0.000, 0.000, 0.000)	X (0.500, 0.000, 0.500)
1	X (0.500, 0.000, 0.500)	U (0.625, 0.250, 0.625)
2	K (0.375, 0.375, 0.750)	Γ (0.000, 0.000, 0.000)
3	Γ (0.000, 0.000, 0.000)	L (0.500, 0.500, 0.500)
4	L (0.500, 0.500, 0.500)	W (0.500, 0.250, 0.750)
5	W (0.500, 0.250, 0.750)	X (0.500, 0.000, 0.500)

Supplementary Table 1319: Nodal ring points along high symmetry lines of mp-2863-Cd12Ce4.

	Coordinates	Frequency	Mode 1	Mode 2	Multi.	Deg.	Path	Ratio
0	(0.500, 0.250, 0.750)	1.617	3	2	6	2	5	1.000
1	(0.499, 0.499, 0.499)	1.851	4	3	8	2	4	0.997
2	(0.514, 0.028, 0.514)	2.326	4	3	12	2	2	0.112
3	(0.458, 0.458, 0.458)	1.874	4	3	8	2	4	0.916
4	(0.482, 0.482, 0.482)	1.856	4	3	8	2	4	0.964
5	(0.492, 0.492, 0.492)	1.852	4	3	8	2	4	0.983
6	(0.469, 0.469, 0.469)	1.864	4	3	8	2	4	0.939
7	(0.000, 0.000, 0.000)	2.662	6	5	1	3	1	0.000
8	(0.500, 0.250, 0.750)	2.370	6	5	6	2	5	1.000
9	(0.500, 0.432, 0.568)	2.059	6	5	24	2	5	0.270
10	(0.523, 0.046, 0.523)	2.673	7	6	12	2	2	0.183
11	(0.469, 0.000, 0.469)	2.752	8	7	6	2	1	0.939
12	(0.207, 0.207, 0.411)	2.842	8	7	24	2	3	0.451
13	(0.547, 0.095, 0.547)	2.758	9	8	12	2	2	0.378

Supplementary Table 1319: Nodal ring points along high symmetry lines of mp-2863-Cd12Ce4.

	Coordinates	Frequency	Mode 1	Mode 2	Multi.	Deg.	Path	Ratio
14	(0.498, 0.067, 0.567)	2.764	9	8	48	2	6	0.732
15	(0.500, 0.250, 0.750)	3.961	11	10	6	2	5	1.000
16	(0.316, 0.000, 0.316)	4.248	12	11	6	2	1	0.633
17	(0.191, 0.191, 0.387)	4.154	12	11	24	2	3	0.486

The followings are details for PaRh$_3$. It has 7 topological nodal lines and 21 nodal ring points along high symmetry line as shown in Fig 624.

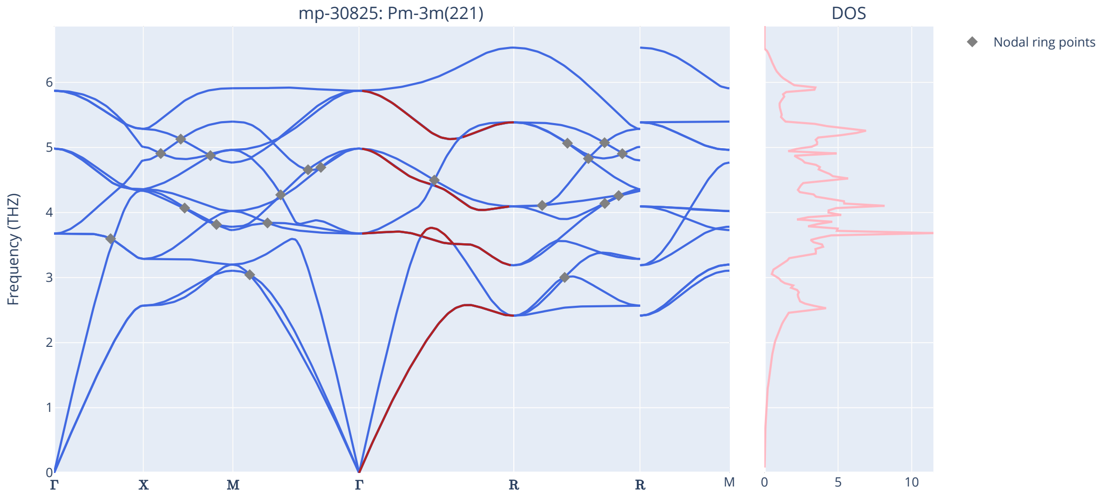

Supplementary Figure 624: The phonon band and DOS of PaRh3.

Supplementary Table 1320: High symmetry lines of mp-30825-PaRh3

	Start	End
0	Γ (0.000, 0.000, 0.000)	X (0.000, 0.500, 0.000)
1	X (0.000, 0.500, 0.000)	M (0.500, 0.500, 0.000)
2	M (0.500, 0.500, 0.000)	Γ (0.000, 0.000, 0.000)
3	Γ (0.000, 0.000, 0.000)	R (0.500, 0.500, 0.500)
4	R (0.500, 0.500, 0.500)	X (0.000, 0.500, 0.000)
5	R (0.500, 0.500, 0.500)	M (0.500, 0.500, 0.000)

Supplementary Table 1321: Nodal ring points along high symmetry lines of mp-30825-PaRh3.

	Coordinates	Frequency	Mode 1	Mode 2	Multi.	Deg.	Path	Ratio
0	(0.433, 0.433, -0.000)	3.042	2	1	12	2	3	0.134
1	(0.299, 0.500, 0.299)	3.001	3	2	12	2	5	0.403
2	(0.000, 0.316, 0.000)	3.595	5	4	6	2	1	0.633
3	(0.409, 0.500, -0.000)	3.814	5	4	12	2	2	0.818
4	(0.362, 0.362, 0.000)	3.838	5	4	12	2	3	0.275
5	(0.231, 0.500, 0.000)	4.067	6	5	12	2	2	0.462
6	(0.140, 0.500, 0.140)	4.138	6	5	12	2	5	0.721
7	(0.085, 0.500, 0.085)	4.257	7	6	12	2	5	0.830
8	(0.310, 0.310, -0.000)	4.269	8	7	12	2	3	0.380
9	(0.151, 0.151, 0.000)	4.692	8	7	12	2	3	0.698
10	(0.388, 0.500, 0.388)	4.107	8	7	12	2	5	0.225
11	(0.375, 0.500, 0.000)	4.872	9	8	12	2	2	0.750
12	(0.202, 0.202, -0.000)	4.655	9	8	12	2	3	0.596
13	(0.243, 0.243, 0.243)	4.495	9	8	8	2	4	0.486

Supplementary Table 1321: Nodal ring points along high symmetry lines of mp-30825-PaRh3.

	Coordinates	Frequency	Mode 1	Mode 2	Multi.	Deg.	Path	Ratio
14	(0.205, 0.500, 0.205)	4.827	9	8	12	2	5	0.591
15	(0.070, 0.500, 0.070)	4.902	10	9	12	2	5	0.859
16	(0.097, 0.500, 0.000)	4.904	10	9	12	2	2	0.194
17	(0.288, 0.500, 0.288)	5.063	10	9	12	2	5	0.425
18	(0.209, 0.500, -0.000)	5.126	11	10	12	2	2	0.418
19	(0.141, 0.500, 0.141)	5.071	11	10	12	2	5	0.719

The followings are details for Pt$_3$. It has 7 topological nodal lines and 24 nodal ring points along high symmetry line as shown in Fig 625.

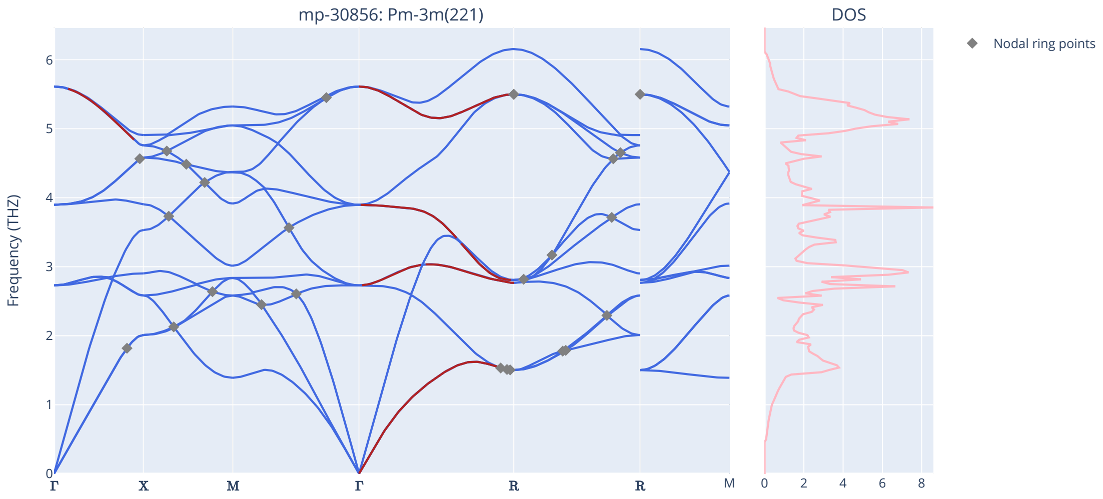

Supplementary Figure 625: The phonon band and DOS of Pt3Zn.

Supplementary Table 1322: High symmetry lines of mp-30856-Pt3Zn

	Start	End
0	Γ (0.000, 0.000, 0.000)	X (0.000, 0.500, 0.000)
1	X (0.000, 0.500, 0.000)	M (0.500, 0.500, 0.000)
2	M (0.500, 0.500, 0.000)	Γ (0.000, 0.000, 0.000)
3	Γ (0.000, 0.000, 0.000)	R (0.500, 0.500, 0.500)
4	R (0.500, 0.500, 0.500)	X (0.000, 0.500, 0.000)
5	R (0.500, 0.500, 0.500)	M (0.500, 0.500, 0.000)

Supplementary Table 1323: Nodal ring points along high symmetry lines of mp-30856-Pt3Zn.

	Coordinates	Frequency	Mode 1	Mode 2	Multi.	Deg.	Path	Ratio
0	(0.000, 0.408, 0.000)	1.819	2	1	6	2	1	0.816
1	(0.294, 0.500, 0.294)	1.788	2	1	12	2	5	0.411
2	(0.170, 0.500, 0.000)	2.129	3	2	12	2	2	0.339
3	(0.386, 0.386, 0.000)	2.451	3	2	12	2	3	0.228
4	(0.458, 0.458, 0.458)	1.533	3	2	8	2	4	0.915
5	(0.478, 0.478, 0.478)	1.511	3	2	8	2	4	0.956
6	(0.488, 0.488, 0.488)	1.505	3	2	8	2	4	0.977
7	(0.306, 0.500, 0.306)	1.777	3	2	12	2	5	0.388
8	(0.387, 0.500, -0.000)	2.639	4	3	12	2	2	0.773
9	(0.249, 0.249, 0.000)	2.607	4	3	12	2	3	0.503
10	(0.132, 0.500, 0.132)	2.295	4	3	12	2	5	0.737
11	(0.142, 0.500, -0.000)	3.732	7	6	12	2	2	0.284
12	(0.277, 0.277, -0.000)	3.566	7	6	12	2	3	0.445
13	(0.461, 0.500, 0.461)	2.819	7	6	12	2	5	0.078

Supplementary Table 1323: Nodal ring points along high symmetry lines of mp-30856-Pt3Zn.

	Coordinates	Frequency	Mode 1	Mode 2	Multi.	Deg.	Path	Ratio
14	(0.112, 0.500, 0.112)	3.715	7	6	12	2	5	0.776
15	(0.343, 0.499, 0.000)	4.222	8	7	24	2	2	0.686
16	(0.349, 0.500, 0.349)	3.170	8	7	12	2	5	0.302
17	(0.000, 0.480, 0.000)	4.566	9	8	6	2	1	0.959
18	(0.240, 0.500, -0.000)	4.486	9	8	12	2	2	0.480
19	(0.106, 0.500, 0.106)	4.565	9	8	12	2	5	0.788
20	(0.131, 0.500, -0.000)	4.677	10	9	12	2	2	0.262
21	(0.078, 0.500, 0.078)	4.654	10	9	12	2	5	0.844
22	(0.129, 0.129, -0.000)	5.451	11	10	12	2	3	0.741
23	(0.500, 0.500, 0.500)	5.500	11	10	1	3	4	1.000

The followings are details for Sn_3. It has 6 topological nodal lines and 20 nodal ring points along high symmetry line as shown in Fig **626**.

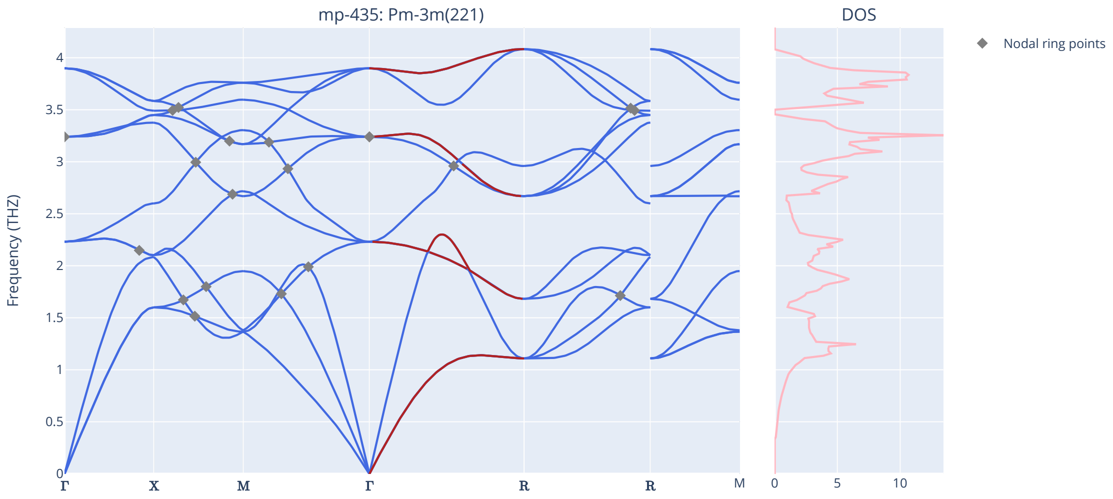

Supplementary Figure 626: The phonon band and DOS of Sn3Th.

Supplementary Table 1324: High symmetry lines of mp-435-Sn3Th

	Start	End
0	Γ (0.000, 0.000, 0.000)	X (0.000, 0.500, 0.000)
1	X (0.000, 0.500, 0.000)	M (0.500, 0.500, 0.000)
2	M (0.500, 0.500, 0.000)	Γ (0.000, 0.000, 0.000)
3	Γ (0.000, 0.000, 0.000)	R (0.500, 0.500, 0.500)
4	R (0.500, 0.500, 0.500)	X (0.000, 0.500, 0.000)
5	R (0.500, 0.500, 0.500)	M (0.500, 0.500, 0.000)

Supplementary Table 1325: Nodal ring points along high symmetry lines of mp-435-Sn3Th.

	Coordinates	Frequency	Mode 1	Mode 2	Multi.	Deg.	Path	Ratio
0	(0.229, 0.500, 0.000)	1.515	2	1	12	2	2	0.459
1	(0.165, 0.500, -0.000)	1.672	3	2	12	2	2	0.331
2	(0.348, 0.348, 0.000)	1.730	3	2	12	2	3	0.303
3	(0.119, 0.500, 0.119)	1.713	3	2	12	2	5	0.762
4	(0.293, 0.500, -0.000)	1.799	4	3	12	2	2	0.586
5	(0.242, 0.242, -0.000)	1.990	4	3	12	2	3	0.517
6	(0.000, 0.418, 0.000)	2.148	5	4	6	2	1	0.837
7	(0.441, 0.500, 0.000)	2.688	6	5	12	2	2	0.881
8	(0.234, 0.500, -0.000)	2.995	7	6	12	2	2	0.469
9	(0.323, 0.323, 0.000)	2.933	7	6	12	2	3	0.355
10	(0.272, 0.272, 0.272)	2.960	7	6	8	2	4	0.545
11	(0.424, 0.500, 0.000)	3.199	8	7	12	2	2	0.847
12	(0.398, 0.398, -0.000)	3.189	8	7	12	2	3	0.205
13	(0.000, 0.000, 0.000)	3.239	9	8	1	3	1	0.000

Supplementary Table 1325: Nodal ring points along high symmetry lines of mp-435-Sn3Th.

	Coordinates	Frequency	Mode 1	Mode 2	Multi.	Deg.	Path	Ratio
14	(0.105, 0.500, -0.000)	3.494	10	9	12	2	2	0.210
15	(0.062, 0.500, 0.062)	3.493	10	9	12	2	5	0.875
16	(0.138, 0.500, -0.000)	3.522	11	10	12	2	2	0.276
17	(0.078, 0.500, 0.078)	3.515	11	10	12	2	5	0.844

The followings are details for InMg$_3$. It has 6 topological nodal lines and 22 nodal ring points along high symmetry line as shown in Fig 627.

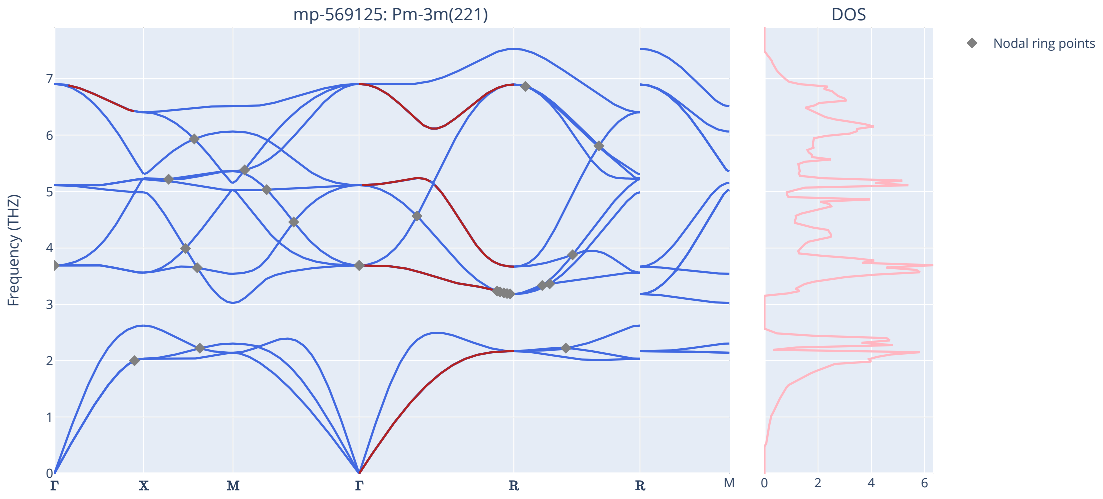

Supplementary Figure 627: The phonon band and DOS of InMg3.

Supplementary Table 1326: High symmetry lines of mp-569125-InMg3

	Start	End
0	Γ (0.000, 0.000, 0.000)	X (0.000, 0.500, 0.000)
1	X (0.000, 0.500, 0.000)	M (0.500, 0.500, 0.000)
2	M (0.500, 0.500, 0.000)	Γ (0.000, 0.000, 0.000)
3	Γ (0.000, 0.000, 0.000)	R (0.500, 0.500, 0.500)
4	R (0.500, 0.500, 0.500)	X (0.000, 0.500, 0.000)
5	R (0.500, 0.500, 0.500)	M (0.500, 0.500, 0.000)

Supplementary Table 1327: Nodal ring points along high symmetry lines of mp-569125-InMg3.

	Coordinates	Frequency	Mode 1	Mode 2	Multi.	Deg.	Path	Ratio
0	(0.000, 0.449, 0.000)	1.999	2	1	6	2	1	0.898
1	(0.315, 0.500, -0.000)	2.221	3	2	12	2	2	0.630
2	(0.294, 0.500, 0.294)	2.224	3	2	12	2	5	0.412
3	(0.000, 0.000, 0.000)	3.690	5	4	1	3	1	0.000
4	(0.301, 0.500, -0.000)	3.648	5	4	12	2	2	0.601
5	(0.358, 0.500, 0.358)	3.363	5	4	12	2	5	0.284
6	(0.235, 0.500, -0.000)	3.992	6	5	12	2	2	0.469
7	(0.456, 0.456, 0.456)	3.219	6	5	8	2	4	0.912
8	(0.468, 0.468, 0.468)	3.203	6	5	8	2	4	0.936
9	(0.478, 0.478, 0.478)	3.192	6	5	8	2	4	0.956
10	(0.488, 0.488, 0.488)	3.184	6	5	8	2	4	0.977
11	(0.447, 0.447, 0.445)	3.235	6	5	24	2	4	0.892
12	(0.387, 0.500, 0.387)	3.334	6	5	12	2	5	0.225
13	(0.259, 0.259, -0.000)	4.460	7	6	12	2	3	0.482

Supplementary Table 1327: Nodal ring points along high symmetry lines of mp-569125-InMg3.

	Coordinates	Frequency	Mode 1	Mode 2	Multi.	Deg.	Path	Ratio
14	(0.270, 0.500, 0.264)	3.877	7	6	24	2	5	0.466
15	(0.187, 0.187, 0.187)	4.566	7	6	8	2	4	0.374
16	(0.366, 0.366, -0.000)	5.034	8	7	12	2	3	0.268
17	(0.140, 0.500, -0.000)	5.219	9	8	12	2	2	0.280
18	(0.453, 0.453, 0.000)	5.383	10	9	12	2	3	0.093
19	(0.454, 0.500, 0.454)	6.865	10	9	12	2	5	0.092
20	(0.284, 0.500, -0.000)	5.932	11	10	12	2	2	0.569
21	(0.163, 0.500, 0.163)	5.812	11	10	12	2	5	0.674

The followings are details for LaPd$_3$. It has 7 topological nodal lines and 25 nodal ring points along high symmetry line as shown in Fig 628.

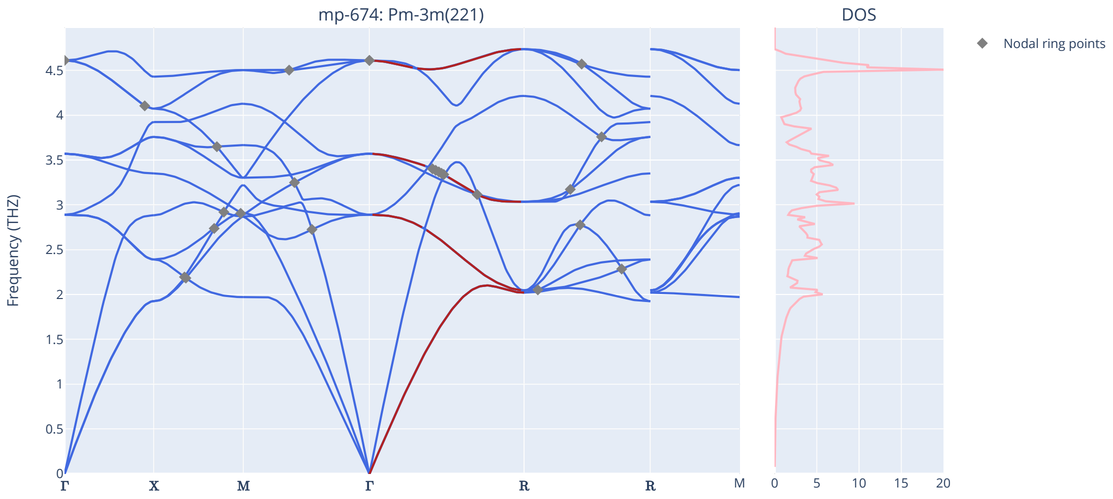

Supplementary Figure 628: The phonon band and DOS of LaPd3.

Supplementary Table 1328: High symmetry lines of mp-674-LaPd3

	Start	End
0	Γ (0.000, 0.000, 0.000)	X (0.000, 0.500, 0.000)
1	X (0.000, 0.500, 0.000)	M (0.500, 0.500, 0.000)
2	M (0.500, 0.500, 0.000)	Γ (0.000, 0.000, 0.000)
3	Γ (0.000, 0.000, 0.000)	R (0.500, 0.500, 0.500)
4	R (0.500, 0.500, 0.500)	X (0.000, 0.500, 0.000)
5	R (0.500, 0.500, 0.500)	M (0.500, 0.500, 0.000)

Supplementary Table 1329: Nodal ring points along high symmetry lines of mp-674-LaPd3.

	Coordinates	Frequency	Mode 1	Mode 2	Multi.	Deg.	Path	Ratio
0	(0.179, 0.500, -0.000)	2.183	2	1	12	2	2	0.359
1	(0.171, 0.500, -0.000)	2.195	3	2	12	2	2	0.343
2	(0.447, 0.500, 0.444)	2.052	3	2	24	2	5	0.109
3	(0.113, 0.500, 0.113)	2.284	3	2	12	2	5	0.773
4	(0.338, 0.500, 0.000)	2.737	4	3	12	2	2	0.676
5	(0.227, 0.227, -0.000)	2.725	4	3	12	2	3	0.545
6	(0.486, 0.500, 0.000)	2.905	5	4	12	2	2	0.973
7	(0.392, 0.500, -0.000)	2.919	5	4	12	2	2	0.784
8	(0.278, 0.500, 0.278)	2.776	5	4	12	2	5	0.444
9	(0.348, 0.348, 0.348)	3.115	6	5	8	2	4	0.697
10	(0.297, 0.297, 0.000)	3.249	7	6	12	2	3	0.407
11	(0.240, 0.240, 0.240)	3.339	8	7	8	2	4	0.481
12	(0.233, 0.233, 0.233)	3.352	8	7	8	2	4	0.466
13	(0.204, 0.204, 0.204)	3.402	8	7	8	2	4	0.408

Supplementary Table 1329: Nodal ring points along high symmetry lines of mp-674-LaPd3.

	Coordinates	Frequency	Mode 1	Mode 2	Multi.	Deg.	Path	Ratio
14	(0.214, 0.214, 0.214)	3.385	8	7	8	2	4	0.429
15	(0.224, 0.224, 0.224)	3.368	8	7	8	2	4	0.449
16	(0.317, 0.500, 0.317)	3.173	8	7	12	2	5	0.366
17	(0.353, 0.500, -0.000)	3.649	9	8	12	2	2	0.706
18	(0.193, 0.500, 0.193)	3.759	9	8	12	2	5	0.614
19	(0.000, 0.000, 0.000)	4.611	11	10	1	3	1	0.000
20	(0.000, 0.449, 0.000)	4.104	11	10	6	2	1	0.898
21	(0.318, 0.318, 0.000)	4.503	12	11	12	2	3	0.364
22	(0.272, 0.500, 0.272)	4.571	12	11	12	2	5	0.456

The followings are details for In_4Li_{12}. It has 7 topological nodal lines and 12 nodal ring points along high symmetry line as shown in Fig 629.

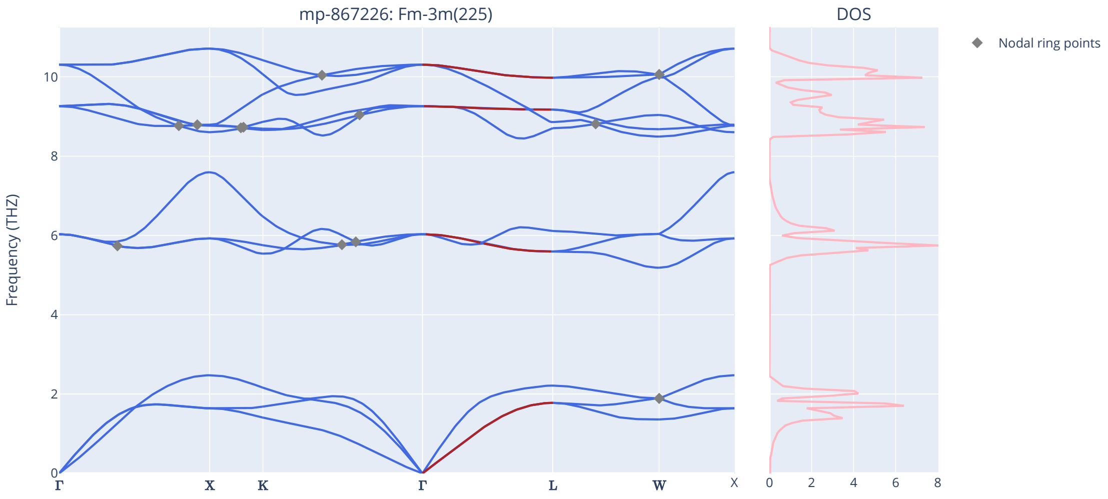

Supplementary Figure 629: The phonon band and DOS of In4Li12.

Supplementary Table 1330: High symmetry lines of mp-867226-In4Li12

	Start	End
0	Γ (0.000, 0.000, 0.000)	X (0.500, 0.000, 0.500)
1	X (0.500, 0.000, 0.500)	U (0.625, 0.250, 0.625)
2	K (0.375, 0.375, 0.750)	Γ (0.000, 0.000, 0.000)
3	Γ (0.000, 0.000, 0.000)	L (0.500, 0.500, 0.500)
4	L (0.500, 0.500, 0.500)	W (0.500, 0.250, 0.750)
5	W (0.500, 0.250, 0.750)	X (0.500, 0.000, 0.500)

Supplementary Table 1331: Nodal ring points along high symmetry lines of mp-867226-In4Li12.

	Coordinates	Frequency	Mode 1	Mode 2	Multi.	Deg.	Path	Ratio
0	(0.500, 0.250, 0.750)	1.888	3	2	6	2	5	1.000
1	(0.194, 0.000, 0.194)	5.737	5	4	6	2	1	0.388
2	(0.184, 0.184, 0.384)	5.766	5	4	24	2	3	0.496
3	(0.152, 0.152, 0.317)	5.838	6	5	24	2	3	0.583
4	(0.398, -0.000, 0.398)	8.767	8	7	12	2	1	0.795
5	(0.573, 0.147, 0.573)	8.715	8	7	12	2	2	0.588
6	(0.153, 0.153, 0.290)	9.043	8	7	24	2	3	0.606
7	(0.500, 0.399, 0.601)	8.814	8	7	24	2	5	0.403
8	(0.581, 0.158, 0.581)	8.730	9	8	24	2	2	0.637
9	(0.459, 0.000, 0.459)	8.791	10	9	6	2	1	0.918
10	(0.500, 0.250, 0.750)	10.061	12	11	6	2	5	1.000
11	(0.241, 0.231, 0.472)	10.042	12	11	24	2	3	0.371

The followings are details for Ru_4Ta_{12}. It has 8 topological nodal lines and 11 nodal ring points along high symmetry line as shown in Fig **630**.

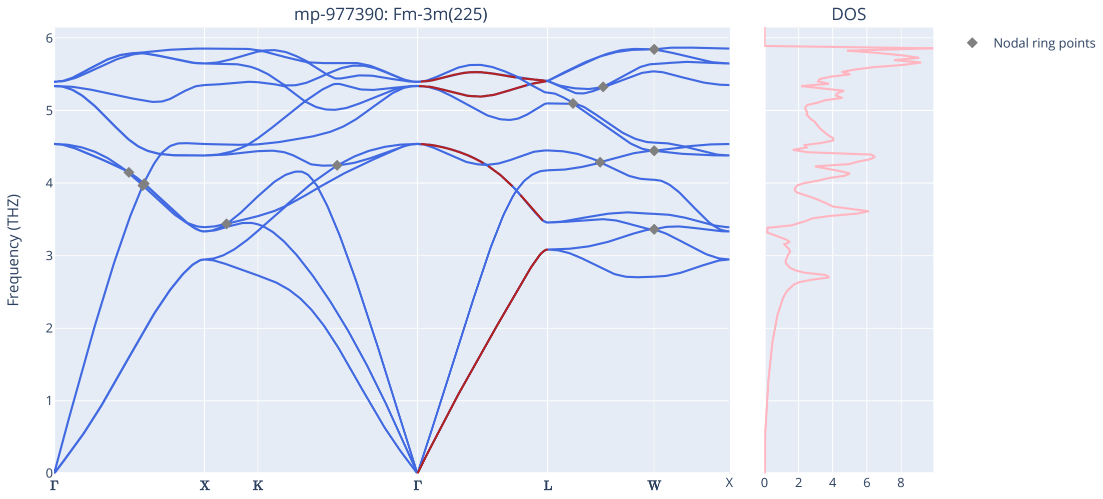

Supplementary Figure 630: The phonon band and DOS of Ru4Ta12.

Supplementary Table 1332: High symmetry lines of mp-977390-Ru4Ta12

	Start	End
0	Γ (0.000, 0.000, 0.000)	X (0.500, 0.000, 0.500)
1	X (0.500, 0.000, 0.500)	U (0.625, 0.250, 0.625)
2	K (0.375, 0.375, 0.750)	Γ (0.000, 0.000, 0.000)
3	Γ (0.000, 0.000, 0.000)	L (0.500, 0.500, 0.500)
4	L (0.500, 0.500, 0.500)	W (0.500, 0.250, 0.750)
5	W (0.500, 0.250, 0.750)	X (0.500, 0.000, 0.500)

Supplementary Table 1333: Nodal ring points along high symmetry lines of mp-977390-Ru4Ta12.

	Coordinates	Frequency	Mode 1	Mode 2	Multi.	Deg.	Path	Ratio
0	(0.500, 0.250, 0.750)	3.362	3	2	6	2	5	1.000
1	(0.295, 0.001, 0.295)	3.966	4	3	24	2	1	0.590
2	(0.304, -0.000, 0.295)	3.992	5	4	24	2	1	0.599
3	(0.552, 0.103, 0.551)	3.437	5	4	12	2	2	0.413
4	(0.249, -0.000, 0.249)	4.147	6	5	6	2	1	0.497
5	(0.191, 0.191, 0.375)	4.244	6	5	24	2	3	0.497
6	(0.500, 0.377, 0.623)	4.287	6	5	24	2	5	0.492
7	(0.500, 0.250, 0.750)	4.445	7	6	6	2	5	1.000
8	(0.500, 0.441, 0.559)	5.097	8	7	24	2	5	0.237
9	(0.500, 0.370, 0.630)	5.325	10	9	24	2	5	0.520
10	(0.500, 0.250, 0.750)	5.842	12	11	6	2	5	1.000

The followings are details for $Nb_{12}Ru_4$. It has 7 topological nodal lines and 21 nodal ring points along high symmetry line as shown in Fig **631**.

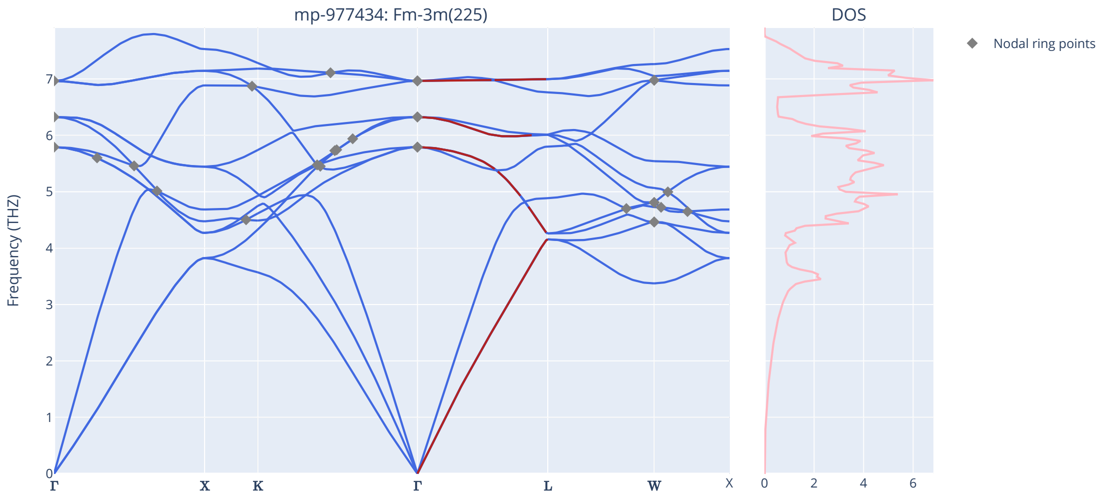

Supplementary Figure 631: The phonon band and DOS of Nb12Ru4.

Supplementary Table 1334: High symmetry lines of mp-977434-Nb12Ru4

	Start	End
0	Γ (0.000, 0.000, 0.000)	X (0.500, 0.000, 0.500)
1	X (0.500, 0.000, 0.500)	U (0.625, 0.250, 0.625)
2	K (0.375, 0.375, 0.750)	Γ (0.000, 0.000, 0.000)
3	Γ (0.000, 0.000, 0.000)	L (0.500, 0.500, 0.500)
4	L (0.500, 0.500, 0.500)	W (0.500, 0.250, 0.750)
5	W (0.500, 0.250, 0.750)	X (0.500, 0.000, 0.500)

Supplementary Table 1335: Nodal ring points along high symmetry lines of mp-977434-Nb12Ru4.

	Coordinates	Frequency	Mode 1	Mode 2	Multi.	Deg.	Path	Ratio
0	(0.500, 0.250, 0.750)	4.464	3	2	6	2	5	1.000
1	(0.598, 0.195, 0.598)	4.503	4	3	12	2	2	0.780
2	(0.143, 0.000, 0.143)	5.601	5	4	6	2	1	0.286
3	(0.500, 0.315, 0.685)	4.702	5	4	24	2	5	0.739
4	(0.499, 0.228, 0.727)	4.724	5	4	24	2	6	0.092
5	(0.499, 0.140, 0.638)	4.649	5	4	24	2	6	0.443
6	(0.000, 0.000, 0.000)	5.791	6	5	1	3	1	0.000
7	(0.223, 0.223, 0.461)	5.452	6	5	24	2	3	0.392
8	(0.342, 0.000, 0.342)	5.013	6	5	6	2	1	0.684
9	(0.500, 0.250, 0.750)	4.810	6	5	6	2	5	1.000
10	(0.266, -0.000, 0.266)	5.459	7	6	10	2	1	0.532
11	(0.237, 0.237, 0.469)	5.476	7	6	24	2	3	0.372
12	(0.498, 0.205, 0.704)	4.997	7	6	24	2	6	0.182
13	(0.000, 0.000, 0.000)	6.326	8	7	1	3	1	0.000

Supplementary Table 1335: Nodal ring points along high symmetry lines of mp-977434-Nb12Ru4.

	Coordinates	Frequency	Mode 1	Mode 2	Multi.	Deg.	Path	Ratio
14	(0.199, 0.199, 0.384)	5.729	8	7	24	2	3	0.482
15	(0.191, 0.191, 0.378)	5.743	8	7	24	2	3	0.494
16	(0.153, 0.153, 0.303)	5.939	8	7	24	2	3	0.594
17	(0.611, 0.223, 0.611)	6.871	10	9	12	2	2	0.892
18	(0.500, 0.250, 0.750)	6.977	10	9	6	2	5	1.000
19	(0.000, 0.000, 0.000)	6.966	11	10	1	3	1	0.000
20	(0.207, 0.207, 0.406)	7.110	12	11	24	2	3	0.455

The followings are details for Ta_4W_{12}. It has 7 topological nodal lines and 22 nodal ring points along high symmetry line as shown in Fig **632**.

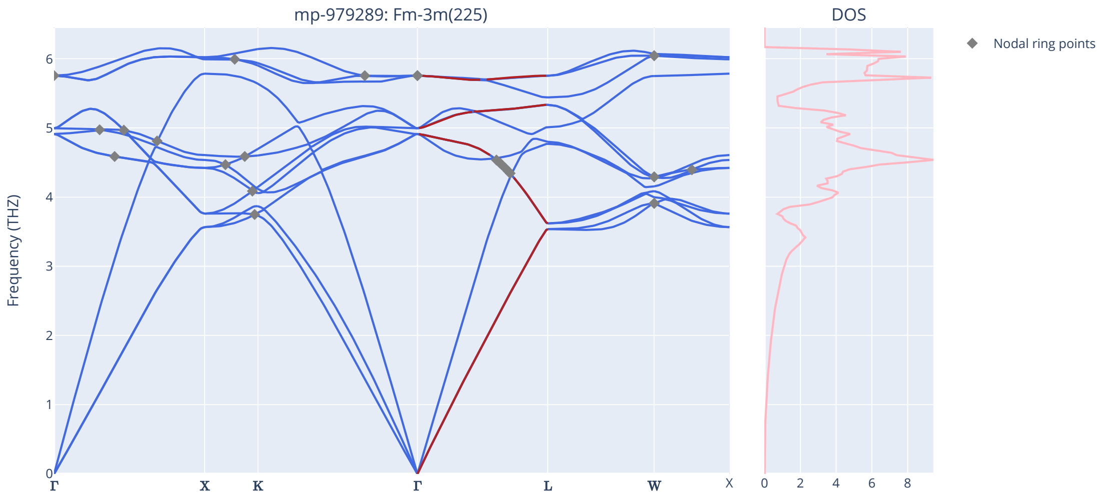

Supplementary Figure 632: The phonon band and DOS of Ta4W12.

Supplementary Table 1336: High symmetry lines of mp-979289-Ta4W12

	Start	End
0	Γ (0.000, 0.000, 0.000)	X (0.500, 0.000, 0.500)
1	X (0.500, 0.000, 0.500)	U (0.625, 0.250, 0.625)
2	K (0.375, 0.375, 0.750)	Γ (0.000, 0.000, 0.000)
3	Γ (0.000, 0.000, 0.000)	L (0.500, 0.500, 0.500)
4	L (0.500, 0.500, 0.500)	W (0.500, 0.250, 0.750)
5	W (0.500, 0.250, 0.750)	X (0.500, 0.000, 0.500)

Supplementary Table 1337: Nodal ring points along high symmetry lines of mp-979289-Ta4W12.

	Coordinates	Frequency	Mode 1	Mode 2	Multi.	Deg.	Path	Ratio
0	(0.618, 0.235, 0.618)	3.748	2	1	12	2	2	0.941
1	(0.500, 0.250, 0.750)	3.910	2	1	6	2	5	1.000
2	(0.206, 0.000, 0.196)	4.587	5	4	24	2	1	0.402
3	(0.612, 0.225, 0.612)	4.087	5	4	12	2	2	0.898
4	(0.337, 0.337, 0.337)	4.420	5	4	8	2	4	0.673
5	(0.303, 0.303, 0.303)	4.536	5	4	8	2	4	0.606
6	(0.322, 0.322, 0.322)	4.472	5	4	8	2	4	0.645
7	(0.315, 0.315, 0.315)	4.497	5	4	8	2	4	0.631
8	(0.356, 0.356, 0.356)	4.344	5	4	8	2	4	0.711
9	(0.347, 0.347, 0.347)	4.380	5	4	8	2	4	0.694
10	(0.151, -0.000, 0.151)	4.974	7	6	6	2	1	0.303
11	(0.549, 0.098, 0.549)	4.469	7	6	12	2	2	0.391
12	(0.500, 0.125, 0.625)	4.395	7	6	24	2	6	0.501
13	(0.594, 0.189, 0.594)	4.588	8	7	12	2	2	0.755

Supplementary Table 1337: Nodal ring points along high symmetry lines of mp-979289-Ta4W12.

	Coordinates	Frequency	Mode 1	Mode 2	Multi.	Deg.	Path	Ratio
14	(0.500, 0.250, 0.750)	4.293	8	7	6	2	5	1.000
15	(0.342, -0.003, 0.342)	4.811	9	8	24	2	1	0.684
16	(0.237, 0.000, 0.226)	4.968	9	8	24	2	1	0.463
17	(0.000, 0.000, 0.000)	5.757	11	10	1	3	1	0.000
18	(0.571, 0.142, 0.571)	5.993	11	10	12	2	2	0.566
19	(0.500, 0.250, 0.750)	6.045	11	10	6	2	5	1.000
20	(0.126, 0.126, 0.245)	5.756	12	11	24	2	3	0.670

The followings are details for HfPd$_3$. It has 8 topological nodal lines and 20 nodal ring points along high symmetry line as shown in Fig 633.

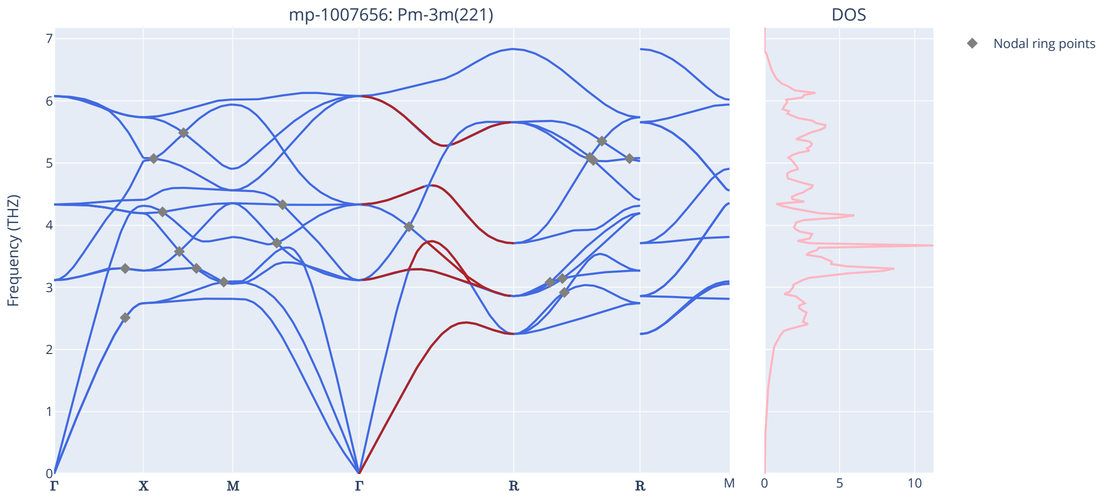

Supplementary Figure 633: The phonon band and DOS of HfPd3.

Supplementary Table 1338: High symmetry lines of mp-1007656-HfPd3

	Start	End
0	Γ (0.000, 0.000, 0.000)	X (0.000, 0.500, 0.000)
1	X (0.000, 0.500, 0.000)	M (0.500, 0.500, 0.000)
2	M (0.500, 0.500, 0.000)	Γ (0.000, 0.000, 0.000)
3	Γ (0.000, 0.000, 0.000)	R (0.500, 0.500, 0.500)
4	R (0.500, 0.500, 0.500)	X (0.000, 0.500, 0.000)
5	R (0.500, 0.500, 0.500)	M (0.500, 0.500, 0.000)

Supplementary Table 1339: Nodal ring points along high symmetry lines of mp-1007656-HfPd3.

	Coordinates	Frequency	Mode 1	Mode 2	Multi.	Deg.	Path	Ratio
0	(0.000, 0.398, 0.000)	2.511	2	1	6	2	1	0.796
1	(0.300, 0.500, 0.300)	2.918	3	2	12	2	5	0.401
2	(0.000, 0.398, 0.000)	3.303	4	3	6	2	1	0.796
3	(0.297, 0.500, -0.000)	3.305	4	3	12	2	2	0.593
4	(0.450, 0.500, -0.000)	3.085	4	3	12	2	2	0.899
5	(0.201, 0.500, 0.001)	3.577	5	4	24	2	2	0.402
6	(0.308, 0.500, 0.308)	3.142	5	4	12	2	5	0.385
7	(0.326, 0.326, 0.000)	3.714	6	5	12	2	3	0.348
8	(0.357, 0.500, 0.357)	3.081	6	5	12	2	5	0.287
9	(0.107, 0.500, 0.000)	4.213	7	6	12	2	2	0.214
10	(0.161, 0.161, 0.161)	3.975	7	6	8	2	4	0.323
11	(0.303, 0.303, -0.000)	4.329	8	7	12	2	3	0.395
12	(0.185, 0.500, 0.185)	5.043	9	8	12	2	5	0.630
13	(0.058, 0.500, -0.000)	5.072	10	9	12	2	2	0.115

Supplementary Table 1339: Nodal ring points along high symmetry lines of mp-1007656-HfPd3.

	Coordinates	Frequency	Mode 1	Mode 2	Multi.	Deg.	Path	Ratio
14	(0.042, 0.500, 0.042)	5.072	10	9	12	2	5	0.916
15	(0.199, 0.500, 0.199)	5.091	10	9	12	2	5	0.601
16	(0.225, 0.500, -0.000)	5.486	11	10	12	2	2	0.449
17	(0.151, 0.500, 0.151)	5.353	11	10	12	2	5	0.698

The followings are details for $CrNi_3$. It has 6 topological nodal lines and 32 nodal ring points along high symmetry line as shown in Fig **634**.

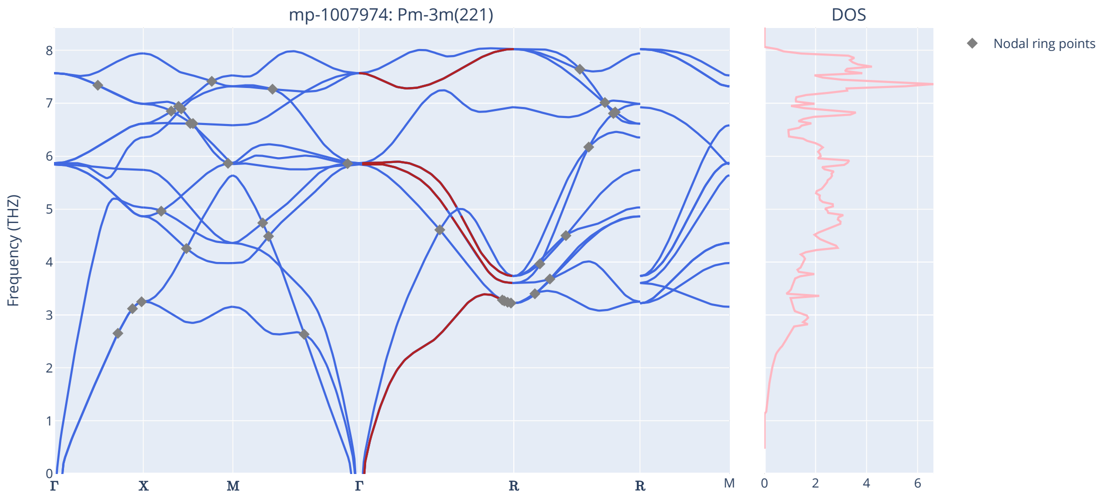

Supplementary Figure 634: The phonon band and DOS of CrNi3.

Supplementary Table 1340: High symmetry lines of mp-1007974-CrNi3

	Start	End
0	Γ (0.000, 0.000, 0.000)	X (0.000, 0.500, 0.000)
1	X (0.000, 0.500, 0.000)	M (0.500, 0.500, 0.000)
2	M (0.500, 0.500, 0.000)	Γ (0.000, 0.000, 0.000)
3	Γ (0.000, 0.000, 0.000)	R (0.500, 0.500, 0.500)
4	R (0.500, 0.500, 0.500)	X (0.000, 0.500, 0.000)
5	R (0.500, 0.500, 0.500)	M (0.500, 0.500, 0.000)

Supplementary Table 1341: Nodal ring points along high symmetry lines of mp-1007974-CrNi3.

	Coordinates	Frequency	Mode 1	Mode 2	Multi.	Deg.	Path	Ratio
0	(0.000, 0.357, 0.000)	2.653	2	1	6	2	1	0.714
1	(0.000, 0.439, 0.000)	3.119	2	1	6	2	1	0.878
2	(0.000, 0.490, 0.000)	3.249	2	1	6	2	1	0.980
3	(0.217, 0.217, -0.000)	2.635	2	1	12	2	3	0.565
4	(0.241, 0.500, -0.000)	4.255	3	2	12	2	2	0.481
5	(0.463, 0.463, 0.463)	3.282	3	2	8	2	4	0.926
6	(0.491, 0.491, 0.491)	3.225	3	2	8	2	4	0.982
7	(0.480, 0.480, 0.480)	3.240	3	2	8	2	4	0.960
8	(0.469, 0.469, 0.469)	3.264	3	2	8	2	4	0.939
9	(0.416, 0.500, 0.416)	3.400	3	2	12	2	5	0.167
10	(0.358, 0.358, 0.000)	4.486	4	3	12	2	3	0.284
11	(0.261, 0.261, 0.261)	4.608	4	3	8	2	4	0.522
12	(0.353, 0.500, 0.361)	3.679	4	3	24	2	5	0.286
13	(0.100, 0.500, 0.000)	4.961	5	4	12	2	2	0.200

Supplementary Table 1341: Nodal ring points along high symmetry lines of mp-1007974-CrNi3.

	Coordinates	Frequency	Mode 1	Mode 2	Multi.	Deg.	Path	Ratio
14	(0.381, 0.381, -0.000)	4.739	5	4	12	2	3	0.237
15	(0.294, 0.500, 0.294)	4.500	6	5	12	2	5	0.413
16	(0.397, 0.500, 0.397)	3.965	6	5	12	2	5	0.206
17	(0.045, 0.045, -0.002)	5.860	7	6	24	2	3	0.909
18	(0.474, 0.500, -0.000)	5.866	7	6	12	2	2	0.947
19	(0.262, 0.500, -0.000)	6.618	8	7	12	2	2	0.525
20	(0.203, 0.500, 0.203)	6.172	8	7	12	2	5	0.593
21	(0.278, 0.499, -0.000)	6.617	9	8	24	2	2	0.555
22	(0.106, 0.500, 0.106)	6.807	9	8	12	2	5	0.789
23	(0.156, 0.500, -0.000)	6.852	10	9	12	2	2	0.311
24	(0.212, 0.500, -0.000)	6.894	10	9	12	2	2	0.424
25	(0.099, 0.500, 0.099)	6.834	10	9	12	2	5	0.803
26	(0.000, 0.245, 0.000)	7.340	11	10	6	2	1	0.490
27	(0.197, 0.500, 0.000)	6.941	11	10	12	2	2	0.393
28	(0.343, 0.343, 0.000)	7.266	11	10	12	2	3	0.315
29	(0.139, 0.500, 0.139)	7.014	11	10	12	2	5	0.721
30	(0.383, 0.500, -0.000)	7.415	12	11	12	2	2	0.765
31	(0.238, 0.500, 0.238)	7.643	12	11	12	2	5	0.523

The followings are details for Co$_3$. It has 6 topological nodal lines and 21 nodal ring points along high symmetry line as shown in Fig 635.

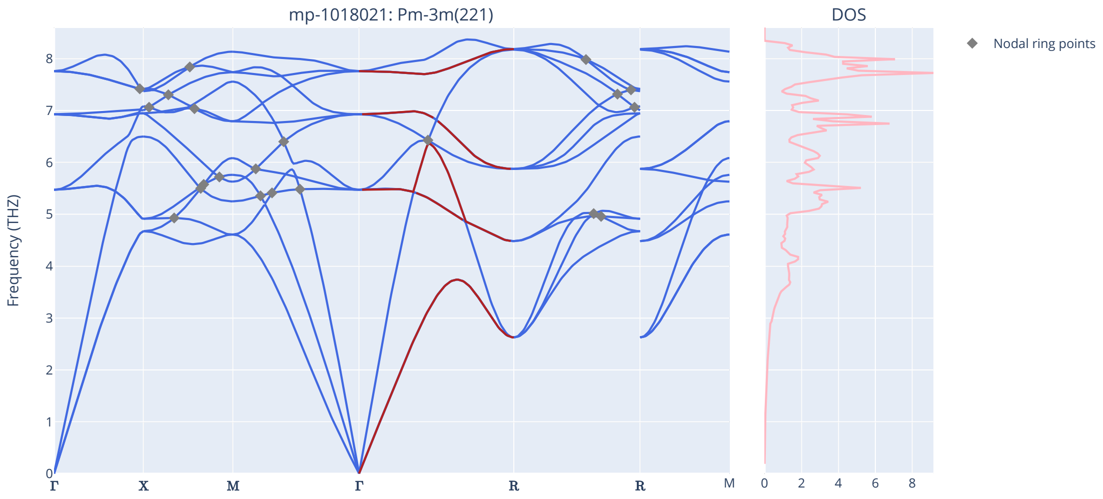

Supplementary Figure 635: The phonon band and DOS of Co3Nb.

Supplementary Table 1342: High symmetry lines of mp-1018021-Co3Nb

	Start	End
0	Γ (0.000, 0.000, 0.000)	X (0.000, 0.500, 0.000)
1	X (0.000, 0.500, 0.000)	M (0.500, 0.500, 0.000)
2	M (0.500, 0.500, 0.000)	Γ (0.000, 0.000, 0.000)
3	Γ (0.000, 0.000, 0.000)	R (0.500, 0.500, 0.500)
4	R (0.500, 0.500, 0.500)	X (0.000, 0.500, 0.000)
5	R (0.500, 0.500, 0.500)	M (0.500, 0.500, 0.000)

Supplementary Table 1343: Nodal ring points along high symmetry lines of mp-1018021-Co3Nb.

	Coordinates	Frequency	Mode 1	Mode 2	Multi.	Deg.	Path	Ratio
0	(0.172, 0.500, -0.000)	4.929	3	2	12	2	2	0.344
1	(0.154, 0.500, 0.154)	4.959	3	2	12	2	5	0.691
2	(0.321, 0.500, -0.000)	5.500	4	3	12	2	2	0.641
3	(0.344, 0.344, 0.000)	5.412	4	3	12	2	3	0.311
4	(0.234, 0.234, 0.000)	5.483	4	3	12	2	3	0.532
5	(0.390, 0.390, 0.000)	5.355	4	3	12	2	3	0.220
6	(0.184, 0.500, 0.184)	5.012	4	3	12	2	5	0.633
7	(0.338, 0.500, -0.000)	5.575	5	4	12	2	2	0.675
8	(0.425, 0.500, -0.000)	5.719	5	4	12	2	2	0.851
9	(0.409, 0.409, 0.000)	5.875	6	5	12	2	3	0.182
10	(0.298, 0.298, -0.000)	6.399	7	6	12	2	3	0.403
11	(0.222, 0.222, 0.222)	6.428	7	6	8	2	4	0.445
12	(0.286, 0.500, -0.000)	7.035	8	7	12	2	2	0.572
13	(0.032, 0.500, -0.000)	7.062	9	8	12	2	2	0.063

Supplementary Table 1343: Nodal ring points along high symmetry lines of mp-1018021-Co3Nb.

	Coordinates	Frequency	Mode 1	Mode 2	Multi.	Deg.	Path	Ratio
14	(0.022, 0.500, 0.022)	7.062	9	8	12	2	5	0.956
15	(0.140, 0.499, -0.000)	7.302	10	9	24	2	2	0.279
16	(0.090, 0.500, 0.090)	7.316	10	9	12	2	5	0.821
17	(0.000, 0.480, 0.000)	7.419	11	10	6	2	1	0.959
18	(0.036, 0.500, 0.036)	7.399	11	10	12	2	5	0.928
19	(0.260, 0.500, -0.000)	7.839	12	11	12	2	2	0.519
20	(0.214, 0.500, 0.214)	7.982	12	11	12	2	5	0.571

The followings are details for HfPt$_3$. It has 7 topological nodal lines and 44 nodal ring points along high symmetry line as shown in Fig 636.

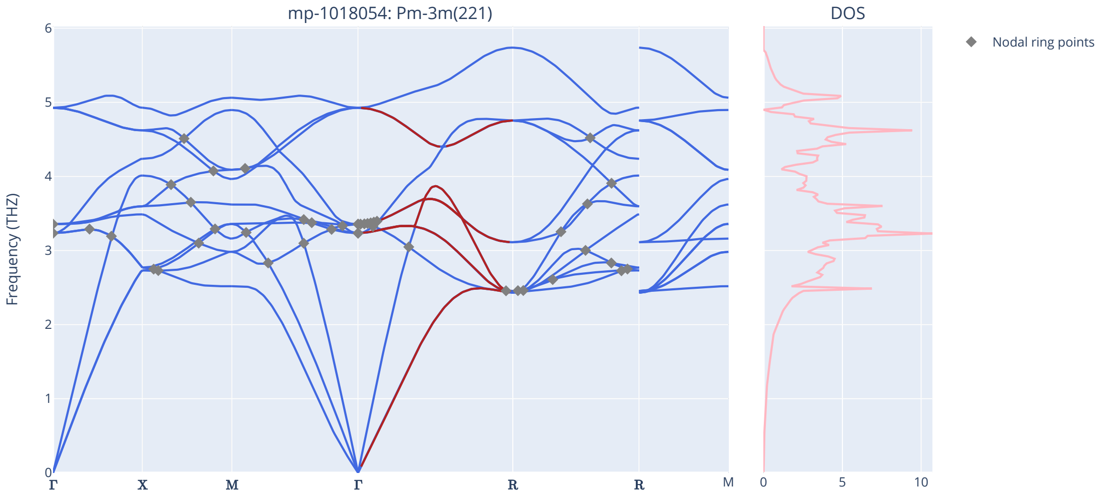

Supplementary Figure 636: The phonon band and DOS of HfPt3.

Supplementary Table 1344: High symmetry lines of mp-1018054-HfPt3

	Start	End
0	Γ (0.000, 0.000, 0.000)	X (0.000, 0.500, 0.000)
1	X (0.000, 0.500, 0.000)	M (0.500, 0.500, 0.000)
2	M (0.500, 0.500, 0.000)	Γ (0.000, 0.000, 0.000)
3	Γ (0.000, 0.000, 0.000)	R (0.500, 0.500, 0.500)
4	R (0.500, 0.500, 0.500)	X (0.000, 0.500, 0.000)
5	R (0.500, 0.500, 0.500)	M (0.500, 0.500, 0.000)

Supplementary Table 1345: Nodal ring points along high symmetry lines of mp-1018054-HfPt3.

	Coordinates	Frequency	Mode 1	Mode 2	Multi.	Deg.	Path	Ratio
0	(0.089, 0.500, 0.000)	2.728	2	1	12	2	2	0.178
1	(0.070, 0.500, 0.070)	2.725	2	1	12	2	5	0.860
2	(0.063, 0.500, -0.000)	2.747	3	2	12	2	2	0.126
3	(0.356, 0.356, 0.000)	2.831	3	2	12	2	3	0.288
4	(0.458, 0.500, 0.458)	2.459	3	2	12	2	5	0.083
5	(0.342, 0.500, 0.342)	2.607	3	2	12	2	5	0.316
6	(0.046, 0.500, 0.046)	2.749	3	2	12	2	5	0.908
7	(0.315, 0.500, -0.000)	3.098	4	3	12	2	2	0.630
8	(0.215, 0.215, -0.000)	3.096	4	3	12	2	3	0.570
9	(0.164, 0.164, 0.164)	3.047	4	3	8	2	4	0.328
10	(0.478, 0.478, 0.478)	2.455	4	3	8	2	4	0.956
11	(0.110, 0.500, 0.110)	2.830	4	3	12	2	5	0.780
12	(0.000, 0.000, 0.000)	3.233	5	4	1	3	1	0.000
13	(0.000, 0.204, 0.000)	3.288	5	4	6	2	1	0.408

Supplementary Table 1345: Nodal ring points along high symmetry lines of mp-1018054-HfPt3.

	Coordinates	Frequency	Mode 1	Mode 2	Multi.	Deg.	Path	Ratio
14	(0.000, 0.327, 0.000)	3.192	5	4	6	2	1	0.653
15	(0.105, 0.105, 0.000)	3.284	5	4	12	2	3	0.791
16	(0.443, 0.443, 0.000)	3.242	5	4	12	2	3	0.114
17	(0.212, 0.500, 0.212)	3.000	5	4	12	2	5	0.576
18	(0.407, 0.500, -0.000)	3.290	6	5	12	2	2	0.813
19	(0.183, 0.183, 0.000)	3.374	6	5	12	2	3	0.633
20	(0.480, 0.500, 0.480)	2.457	6	5	12	2	5	0.041
21	(0.271, 0.500, -0.000)	3.650	7	6	12	2	2	0.542
22	(0.062, 0.062, -0.000)	3.331	7	6	12	2	3	0.877
23	(0.215, 0.215, -0.000)	3.416	7	6	12	2	3	0.570
24	(0.048, 0.048, 0.048)	3.323	7	6	8	2	4	0.096
25	(0.204, 0.500, 0.204)	3.628	7	6	12	2	5	0.591
26	(0.309, 0.500, 0.309)	3.252	7	6	12	2	5	0.382
27	(0.160, 0.500, 0.000)	3.888	8	7	12	2	2	0.321
28	(0.109, 0.500, 0.109)	3.906	8	7	12	2	5	0.782
29	(0.000, 0.000, 0.000)	3.354	9	8	1	3	1	0.000
30	(0.008, 0.008, 0.008)	3.355	9	8	8	2	4	0.016
31	(0.397, 0.500, 0.000)	4.072	9	8	12	2	2	0.795
32	(0.020, 0.020, 0.020)	3.359	9	8	8	2	4	0.041
33	(0.031, 0.031, 0.031)	3.364	9	8	8	2	4	0.061
34	(0.041, 0.041, 0.041)	3.372	9	8	8	2	4	0.082
35	(0.051, 0.051, 0.051)	3.382	9	8	8	2	4	0.102
36	(0.061, 0.061, 0.061)	3.394	9	8	8	2	4	0.122
37	(0.447, 0.447, -0.000)	4.106	10	9	12	2	3	0.106
38	(0.233, 0.500, -0.000)	4.509	11	10	12	2	2	0.466
39	(0.194, 0.500, 0.194)	4.519	11	10	12	2	5	0.613

The followings are details for Ir$_3$. It has 6 topological nodal lines and 21 nodal ring points along high symmetry line as shown in Fig 637.

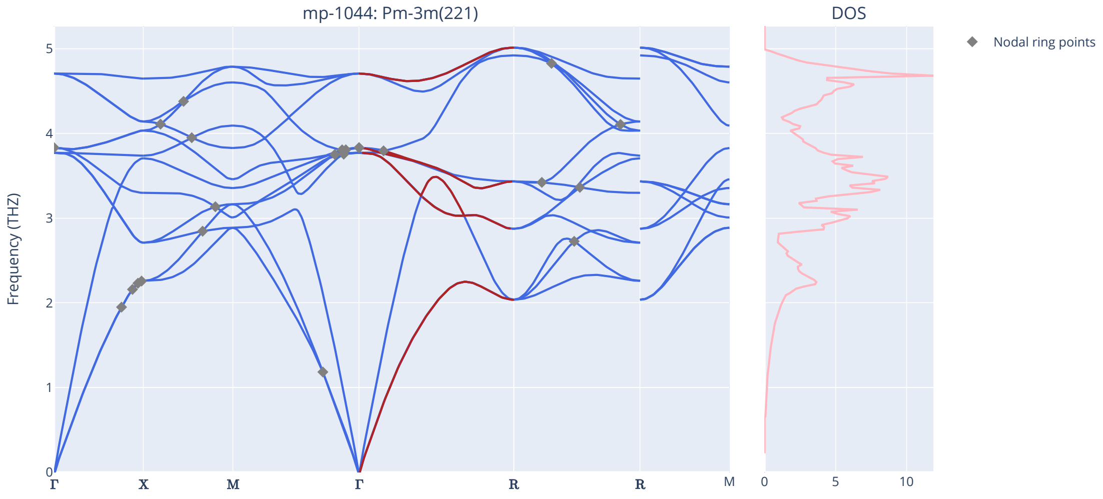

Supplementary Figure 637: The phonon band and DOS of Ir3U.

Supplementary Table 1346: High symmetry lines of mp-1044-Ir3U

	Start	End
0	Γ (0.000, 0.000, 0.000)	X (0.000, 0.500, 0.000)
1	X (0.000, 0.500, 0.000)	M (0.500, 0.500, 0.000)
2	M (0.500, 0.500, 0.000)	Γ (0.000, 0.000, 0.000)
3	Γ (0.000, 0.000, 0.000)	R (0.500, 0.500, 0.500)
4	R (0.500, 0.500, 0.500)	X (0.000, 0.500, 0.000)
5	R (0.500, 0.500, 0.500)	M (0.500, 0.500, 0.000)

Supplementary Table 1347: Nodal ring points along high symmetry lines of mp-1044-Ir3U.

	Coordinates	Frequency	Mode 1	Mode 2	Multi.	Deg.	Path	Ratio
0	(0.000, 0.378, 0.000)	1.949	2	1	6	2	1	0.755
1	(0.000, 0.439, 0.000)	2.158	2	1	6	2	1	0.878
2	(0.000, 0.469, 0.000)	2.231	2	1	6	2	1	0.939
3	(0.000, 0.490, 0.000)	2.257	2	1	6	2	1	0.980
4	(0.143, 0.143, 0.000)	1.183	2	1	12	2	3	0.713
5	(0.331, 0.500, -0.000)	2.846	3	2	12	2	2	0.663
6	(0.261, 0.500, 0.261)	2.726	3	2	12	2	5	0.478
7	(0.403, 0.500, -0.000)	3.135	5	4	12	2	2	0.806
8	(0.061, 0.061, -0.000)	3.755	5	4	12	2	3	0.879
9	(0.238, 0.500, 0.238)	3.363	6	5	12	2	5	0.523
10	(0.096, 0.096, -0.000)	3.752	7	6	12	2	3	0.809
11	(0.000, 0.000, 0.000)	3.832	8	7	1	3	1	0.000
12	(0.052, 0.052, -0.000)	3.807	8	7	12	2	3	0.895
13	(0.389, 0.500, 0.389)	3.422	8	7	12	2	5	0.222

Supplementary Table 1347: Nodal ring points along high symmetry lines of mp-1044-Ir3U.

	Coordinates	Frequency	Mode 1	Mode 2	Multi.	Deg.	Path	Ratio
14	(0.271, 0.500, -0.000)	3.950	9	8	12	2	2	0.541
15	(0.067, 0.067, 0.000)	3.807	9	8	12	2	3	0.866
16	(0.079, 0.079, 0.079)	3.794	9	8	8	2	4	0.158
17	(0.096, 0.500, 0.000)	4.110	10	9	12	2	2	0.192
18	(0.351, 0.500, 0.351)	4.827	10	9	12	2	5	0.299
19	(0.078, 0.500, 0.078)	4.106	10	9	12	2	5	0.845
20	(0.225, 0.500, 0.000)	4.379	11	10	12	2	2	0.450

The followings are details for $FeIr_3$. It has 7 topological nodal lines and 26 nodal ring points along high symmetry line as shown in Fig 638.

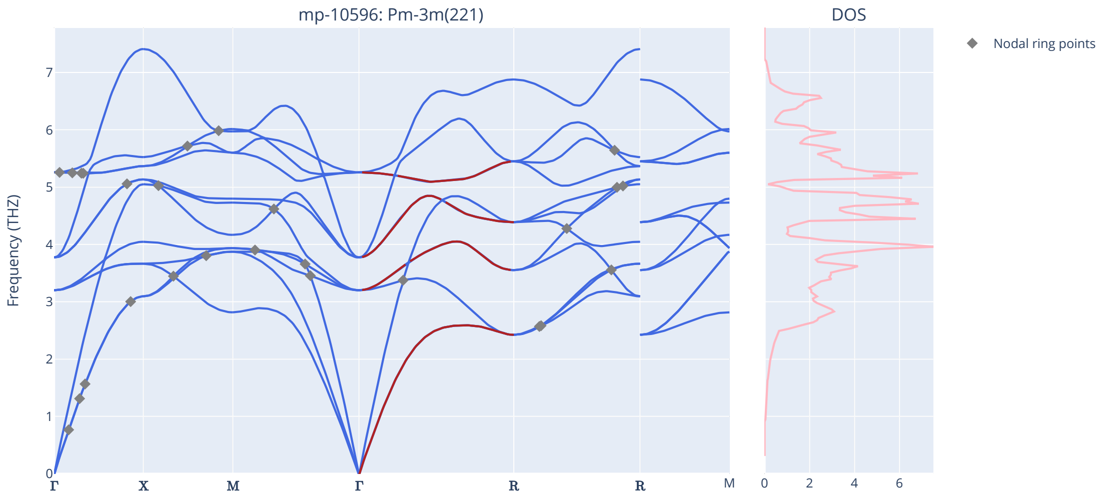

Supplementary Figure 638: The phonon band and DOS of FeIr3.

Supplementary Table 1348: High symmetry lines of mp-10596-FeIr3

	Start	End
0	Γ (0.000, 0.000, 0.000)	X (0.000, 0.500, 0.000)
1	X (0.000, 0.500, 0.000)	M (0.500, 0.500, 0.000)
2	M (0.500, 0.500, 0.000)	Γ (0.000, 0.000, 0.000)
3	Γ (0.000, 0.000, 0.000)	R (0.500, 0.500, 0.500)
4	R (0.500, 0.500, 0.500)	X (0.000, 0.500, 0.000)
5	R (0.500, 0.500, 0.500)	M (0.500, 0.500, 0.000)

Supplementary Table 1349: Nodal ring points along high symmetry lines of mp-10596-FeIr3.

	Coordinates	Frequency	Mode 1	Mode 2	Multi.	Deg.	Path	Ratio
0	(0.000, 0.082, 0.000)	0.766	2	1	6	2	1	0.163
1	(0.000, 0.143, 0.000)	1.310	2	1	6	2	1	0.286
2	(0.000, 0.173, 0.000)	1.566	2	1	6	2	1	0.347
3	(0.000, 0.429, 0.000)	3.003	2	1	6	2	1	0.857
4	(0.392, 0.500, 0.392)	2.583	2	1	12	2	5	0.216
5	(0.352, 0.500, 0.002)	3.803	3	2	24	2	2	0.703
6	(0.168, 0.500, -0.002)	3.447	3	2	24	2	2	0.336
7	(0.399, 0.500, 0.399)	2.569	3	2	12	2	5	0.203
8	(0.114, 0.500, 0.114)	3.556	3	2	12	2	5	0.773
9	(0.193, 0.193, 0.000)	3.455	4	3	12	2	3	0.614
10	(0.142, 0.142, 0.142)	3.375	4	3	8	2	4	0.285
11	(0.412, 0.412, -0.000)	3.903	5	4	12	2	3	0.176
12	(0.214, 0.214, -0.000)	3.658	5	4	12	2	3	0.573
13	(0.290, 0.500, 0.290)	4.278	6	5	12	2	5	0.419

Supplementary Table 1349: Nodal ring points along high symmetry lines of mp-10596-FeIr3.

	Coordinates	Frequency	Mode 1	Mode 2	Multi.	Deg.	Path	Ratio
14	(0.084, 0.500, 0.000)	5.025	7	6	12	2	2	0.169
15	(0.337, 0.337, -0.000)	4.618	7	6	12	2	3	0.326
16	(0.068, 0.500, 0.068)	5.020	7	6	12	2	5	0.864
17	(0.000, 0.408, 0.000)	5.057	8	7	6	2	1	0.816
18	(0.091, 0.500, 0.091)	4.997	8	7	12	2	5	0.817
19	(0.000, 0.031, 0.000)	5.257	11	10	6	2	1	0.061
20	(0.000, 0.102, 0.000)	5.249	11	10	6	2	1	0.204
21	(0.000, 0.153, 0.000)	5.243	11	10	6	2	1	0.306
22	(0.000, 0.163, 0.000)	5.242	11	10	6	2	1	0.327
23	(0.247, 0.500, -0.000)	5.717	11	10	12	2	2	0.493
24	(0.101, 0.500, 0.101)	5.643	11	10	12	2	5	0.798
25	(0.421, 0.500, 0.000)	5.984	12	11	12	2	2	0.842

The followings are details for $LiMg_3$. It has 7 topological nodal lines and 21 nodal ring points along high symmetry line as shown in Fig 639.

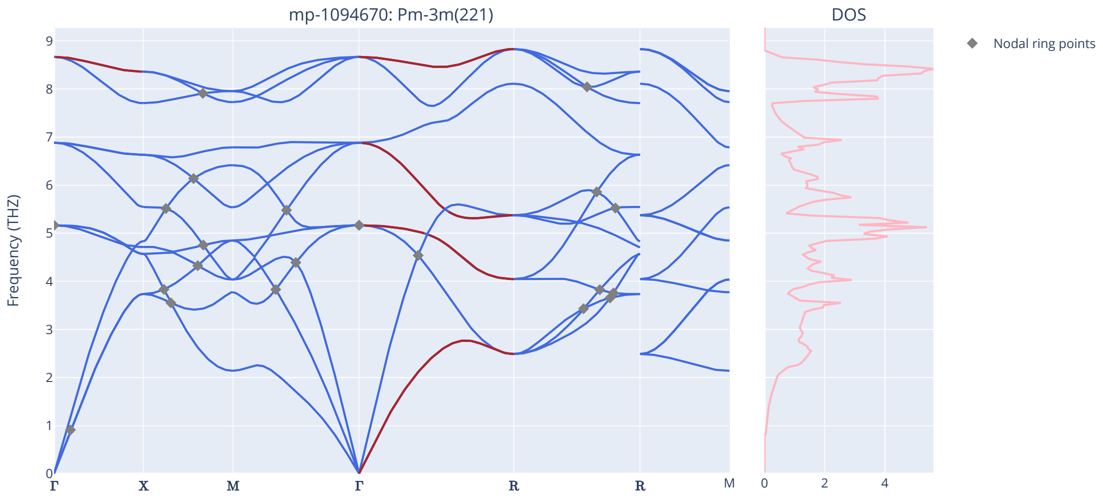

Supplementary Figure 639: The phonon band and DOS of LiMg3.

Supplementary Table 1350: High symmetry lines of mp-1094670-LiMg3

	Start	End
0	Γ (0.000, 0.000, 0.000)	X (0.000, 0.500, 0.000)
1	X (0.000, 0.500, 0.000)	M (0.500, 0.500, 0.000)
2	M (0.500, 0.500, 0.000)	Γ (0.000, 0.000, 0.000)
3	Γ (0.000, 0.000, 0.000)	R (0.500, 0.500, 0.500)
4	R (0.500, 0.500, 0.500)	X (0.000, 0.500, 0.000)
5	R (0.500, 0.500, 0.500)	M (0.500, 0.500, 0.000)

Supplementary Table 1351: Nodal ring points along high symmetry lines of mp-1094670-LiMg3.

	Coordinates	Frequency	Mode 1	Mode 2	Multi.	Deg.	Path	Ratio
0	(0.000, 0.092, 0.000)	0.910	2	1	6	2	1	0.184
1	(0.154, 0.500, 0.000)	3.552	2	1	12	2	2	0.308
2	(0.119, 0.500, 0.119)	3.655	2	1	12	2	5	0.761
3	(0.115, 0.500, -0.000)	3.832	3	2	12	2	2	0.230
4	(0.330, 0.330, -0.000)	3.830	3	2	12	2	3	0.341
5	(0.224, 0.500, 0.224)	3.432	3	2	12	2	5	0.553
6	(0.105, 0.500, 0.105)	3.757	3	2	12	2	5	0.789
7	(0.304, 0.500, -0.000)	4.326	4	3	12	2	2	0.608
8	(0.250, 0.250, 0.000)	4.391	4	3	12	2	3	0.500
9	(0.191, 0.191, 0.191)	4.540	4	3	8	2	4	0.382
10	(0.159, 0.500, 0.159)	3.826	4	3	12	2	5	0.681
11	(0.000, 0.000, 0.000)	5.166	5	4	1	3	1	0.000
12	(0.336, 0.500, -0.000)	4.754	6	5	12	2	2	0.671
13	(0.126, 0.500, -0.000)	5.513	7	6	12	2	2	0.252

Supplementary Table 1351: Nodal ring points along high symmetry lines of mp-1094670-LiMg3.

	Coordinates	Frequency	Mode 1	Mode 2	Multi.	Deg.	Path	Ratio
14	(0.287, 0.287, 0.000)	5.480	7	6	12	2	3	0.426
15	(0.098, 0.500, 0.098)	5.524	7	6	12	2	5	0.805
16	(0.281, 0.500, 0.000)	6.137	8	7	12	2	2	0.562
17	(0.172, 0.500, 0.172)	5.858	8	7	12	2	5	0.656
18	(0.333, 0.500, -0.000)	7.909	11	10	12	2	2	0.665
19	(0.210, 0.500, 0.210)	8.042	11	10	12	2	5	0.580

The followings are details for Rh$_3$. It has 8 topological nodal lines and 31 nodal ring points along high symmetry line as shown in Fig **640**.

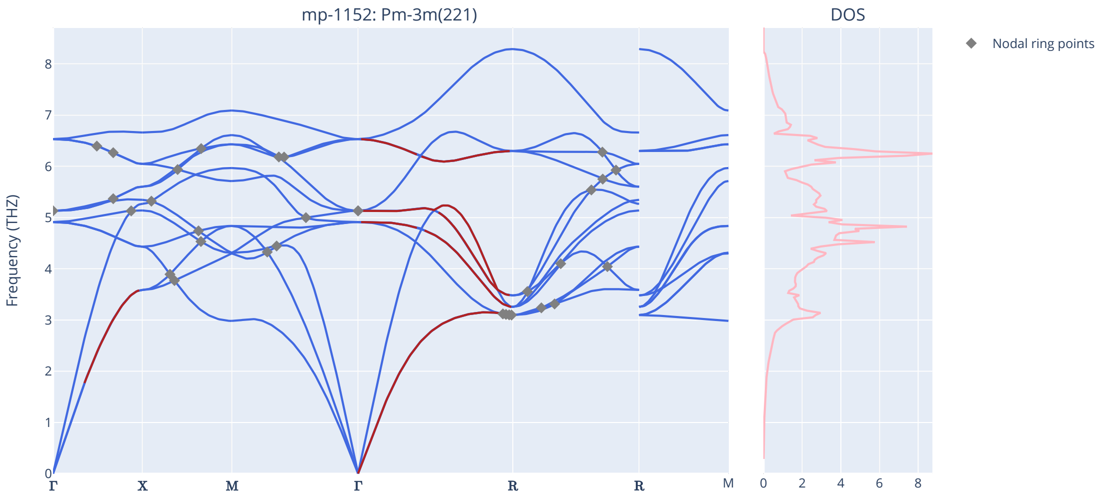

Supplementary Figure 640: The phonon band and DOS of Rh3Ti.

Supplementary Table 1352: High symmetry lines of mp-1152-Rh3Ti

	Start	End
0	Γ (0.000, 0.000, 0.000)	X (0.000, 0.500, 0.000)
1	X (0.000, 0.500, 0.000)	M (0.500, 0.500, 0.000)
2	M (0.500, 0.500, 0.000)	Γ (0.000, 0.000, 0.000)
3	Γ (0.000, 0.000, 0.000)	R (0.500, 0.500, 0.500)
4	R (0.500, 0.500, 0.500)	X (0.000, 0.500, 0.000)
5	R (0.500, 0.500, 0.500)	M (0.500, 0.500, 0.000)

Supplementary Table 1353: Nodal ring points along high symmetry lines of mp-1152-Rh3Ti.

	Coordinates	Frequency	Mode 1	Mode 2	Multi.	Deg.	Path	Ratio
0	(0.180, 0.500, -0.000)	3.771	2	1	12	2	2	0.359
1	(0.334, 0.500, 0.334)	3.314	2	1	12	2	5	0.331
2	(0.154, 0.500, -0.000)	3.893	3	2	12	2	2	0.309
3	(0.359, 0.359, -0.000)	4.328	3	2	12	2	3	0.281
4	(0.468, 0.468, 0.468)	3.120	3	2	8	2	4	0.936
5	(0.478, 0.478, 0.478)	3.109	3	2	8	2	4	0.956
6	(0.488, 0.488, 0.488)	3.101	3	2	8	2	4	0.977
7	(0.496, 0.496, 0.496)	3.098	3	2	8	2	4	0.992
8	(0.387, 0.500, 0.387)	3.238	3	2	12	2	5	0.226
9	(0.125, 0.500, 0.125)	4.043	3	2	12	2	5	0.749
10	(0.328, 0.500, -0.000)	4.533	4	3	12	2	2	0.655
11	(0.322, 0.322, 0.000)	4.445	4	3	12	2	3	0.355
12	(0.310, 0.500, 0.310)	4.100	5	4	12	2	5	0.380
13	(-0.001, 0.437, -0.001)	5.131	6	5	24	2	1	0.875

Supplementary Table 1353: Nodal ring points along high symmetry lines of mp-1152-Rh3Ti.

	Coordinates	Frequency	Mode 1	Mode 2	Multi.	Deg.	Path	Ratio
14	(0.314, 0.500, -0.000)	4.740	6	5	12	2	2	0.628
15	(0.050, 0.500, -0.000)	5.320	7	6	12	2	2	0.101
16	(0.206, 0.206, -0.000)	4.999	7	6	12	2	3	0.587
17	(0.442, 0.500, 0.442)	3.556	7	6	12	2	5	0.116
18	(0.000, 0.000, 0.000)	5.132	8	7	1	3	1	0.000
19	(0.189, 0.500, 0.189)	5.539	8	7	12	2	5	0.622
20	(0.000, 0.337, 0.000)	5.368	9	8	6	2	1	0.673
21	(0.197, 0.500, 0.000)	5.938	9	8	12	2	2	0.394
22	(0.144, 0.500, 0.144)	5.751	9	8	12	2	5	0.712
23	(0.091, 0.500, 0.091)	5.927	10	9	12	2	5	0.817
24	(0.000, 0.245, 0.000)	6.395	11	10	6	2	1	0.490
25	(0.000, 0.337, 0.000)	6.266	11	10	6	2	1	0.673
26	(0.329, 0.500, -0.000)	6.347	11	10	12	2	2	0.658
27	(0.293, 0.293, 0.000)	6.181	11	10	12	2	3	0.414
28	(0.313, 0.313, 0.000)	6.183	11	10	12	2	3	0.373
29	(0.145, 0.500, 0.145)	6.278	11	10	12	2	5	0.709

The followings are details for Sc$_3$. It has 8 topological nodal lines and 29 nodal ring points along high symmetry line as shown in Fig **641**.

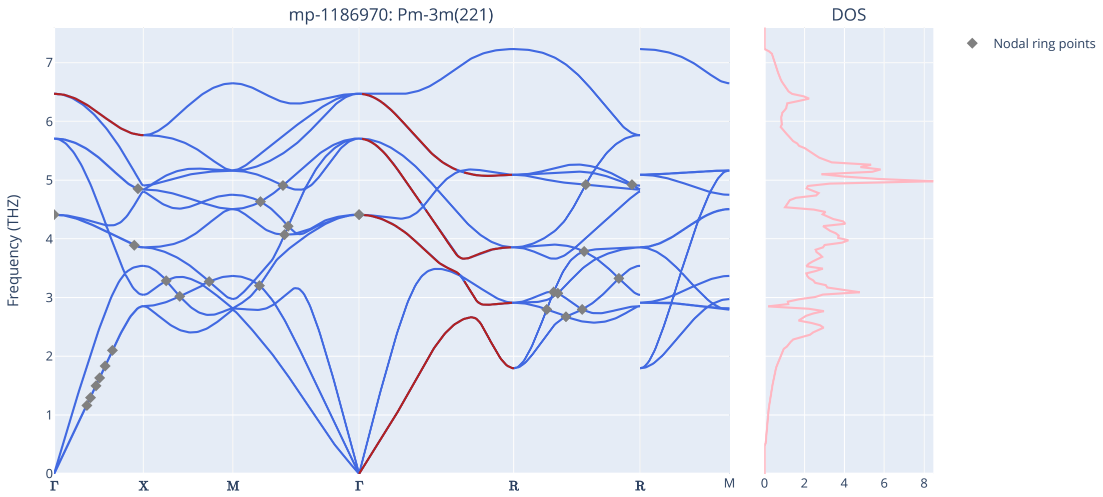

Supplementary Figure 641: The phonon band and DOS of Sc3Sn.

Supplementary Table 1354: High symmetry lines of mp-1186970-Sc3Sn

	Start	End
0	Γ (0.000, 0.000, 0.000)	X (0.000, 0.500, 0.000)
1	X (0.000, 0.500, 0.000)	M (0.500, 0.500, 0.000)
2	M (0.500, 0.500, 0.000)	Γ (0.000, 0.000, 0.000)
3	Γ (0.000, 0.000, 0.000)	R (0.500, 0.500, 0.500)
4	R (0.500, 0.500, 0.500)	X (0.000, 0.500, 0.000)
5	R (0.500, 0.500, 0.500)	M (0.500, 0.500, 0.000)

Supplementary Table 1355: Nodal ring points along high symmetry lines of mp-1186970-Sc3Sn.

	Coordinates	Frequency	Mode 1	Mode 2	Multi.	Deg.	Path	Ratio
0	(0.000, 0.184, 0.000)	1.163	2	1	6	2	1	0.367
1	(0.000, 0.204, 0.000)	1.296	2	1	6	2	1	0.408
2	(0.000, 0.235, 0.000)	1.497	2	1	6	2	1	0.469
3	(0.000, 0.255, 0.000)	1.632	2	1	6	2	1	0.510
4	(0.000, 0.286, 0.000)	1.834	2	1	6	2	1	0.571
5	(0.000, 0.327, 0.000)	2.101	2	1	6	2	1	0.653
6	(0.293, 0.500, 0.293)	2.673	2	1	12	2	5	0.413
7	(0.203, 0.500, 0.000)	3.020	3	2	12	2	2	0.407
8	(0.369, 0.500, 0.369)	2.797	3	2	12	2	5	0.261
9	(0.229, 0.500, 0.229)	2.798	3	2	12	2	5	0.541
10	(0.128, 0.500, -0.000)	3.285	4	3	12	2	2	0.255
11	(0.394, 0.394, 0.000)	3.202	4	3	12	2	3	0.211
12	(0.325, 0.500, 0.325)	3.073	4	3	12	2	5	0.349
13	(0.084, 0.500, 0.084)	3.326	4	3	12	2	5	0.832

Supplementary Table 1355: Nodal ring points along high symmetry lines of mp-1186970-Sc3Sn.

	Coordinates	Frequency	Mode 1	Mode 2	Multi.	Deg.	Path	Ratio
14	(0.000, 0.000, 0.000)	4.411	5	4	1	3	1	0.000
15	(0.368, 0.500, 0.000)	3.271	5	4	12	2	2	0.736
16	(0.342, 0.500, 0.342)	3.094	5	4	12	2	5	0.316
17	(0.000, 0.449, 0.000)	3.890	6	5	6	2	1	0.898
18	(0.295, 0.295, 0.000)	4.071	6	5	12	2	3	0.410
19	(0.221, 0.500, 0.221)	3.784	6	5	12	2	5	0.557
20	(0.281, 0.281, 0.000)	4.214	7	6	12	2	3	0.439
21	(0.390, 0.390, -0.000)	4.632	8	7	12	2	3	0.220
22	(0.000, 0.469, 0.000)	4.853	9	8	6	2	1	0.939
23	(0.301, 0.301, 0.000)	4.908	9	8	12	2	3	0.398
24	(0.215, 0.500, 0.215)	4.922	9	8	12	2	5	0.570
25	(0.031, 0.500, 0.034)	4.922	10	9	24	2	5	0.936

The followings are details for CrIr$_3$. It has 9 topological nodal lines and 16 nodal ring points along high symmetry line as shown in Fig 642.

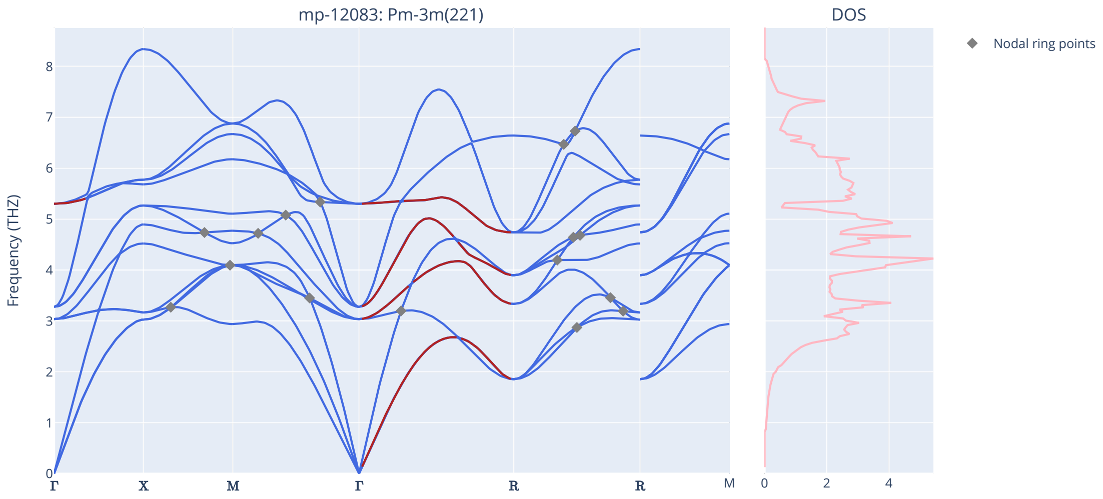

Supplementary Figure 642: The phonon band and DOS of CrIr3.

Supplementary Table 1356: High symmetry lines of mp-12083-CrIr3

	Start	End
0	Γ (0.000, 0.000, 0.000)	X (0.000, 0.500, 0.000)
1	X (0.000, 0.500, 0.000)	M (0.500, 0.500, 0.000)
2	M (0.500, 0.500, 0.000)	Γ (0.000, 0.000, 0.000)
3	Γ (0.000, 0.000, 0.000)	R (0.500, 0.500, 0.500)
4	R (0.500, 0.500, 0.500)	X (0.000, 0.500, 0.000)
5	R (0.500, 0.500, 0.500)	M (0.500, 0.500, 0.000)

Supplementary Table 1357: Nodal ring points along high symmetry lines of mp-12083-CrIr3.

	Coordinates	Frequency	Mode 1	Mode 2	Multi.	Deg.	Path	Ratio
0	(0.250, 0.500, 0.250)	2.872	2	1	12	2	5	0.500
1	(0.153, 0.500, -0.003)	3.269	3	2	24	2	2	0.306
2	(0.067, 0.500, 0.067)	3.192	3	2	12	2	5	0.866
3	(0.485, 0.500, -0.000)	4.095	4	3	12	2	2	0.970
4	(0.136, 0.136, 0.136)	3.198	4	3	8	2	4	0.272
5	(0.117, 0.500, 0.117)	3.457	4	3	12	2	5	0.765
6	(0.196, 0.196, -0.000)	3.451	5	4	12	2	3	0.608
7	(0.328, 0.500, 0.328)	4.196	6	5	12	2	5	0.345
8	(0.342, 0.500, 0.000)	4.737	7	6	12	2	2	0.684
9	(0.399, 0.399, -0.000)	4.720	7	6	12	2	3	0.201
10	(0.238, 0.500, 0.238)	4.681	7	6	12	2	5	0.525
11	(0.290, 0.290, 0.000)	5.079	8	7	12	2	3	0.420
12	(0.264, 0.500, 0.264)	4.643	8	7	12	2	5	0.472
13	(0.154, 0.154, -0.000)	5.335	10	9	12	2	3	0.692

Supplementary Table 1357: Nodal ring points along high symmetry lines of mp-12083-CrIr3.

	Coordinates	Frequency	Mode 1	Mode 2	Multi.	Deg.	Path	Ratio
14	(0.302, 0.500, 0.302)	6.467	12	11	12	2	5	0.396
15	(0.257, 0.500, 0.257)	6.728	12	11	12	2	5	0.485

The followings are details for Cu$_3$. It has 6 topological nodal lines and 20 nodal ring points along high symmetry line as shown in Fig **643**.

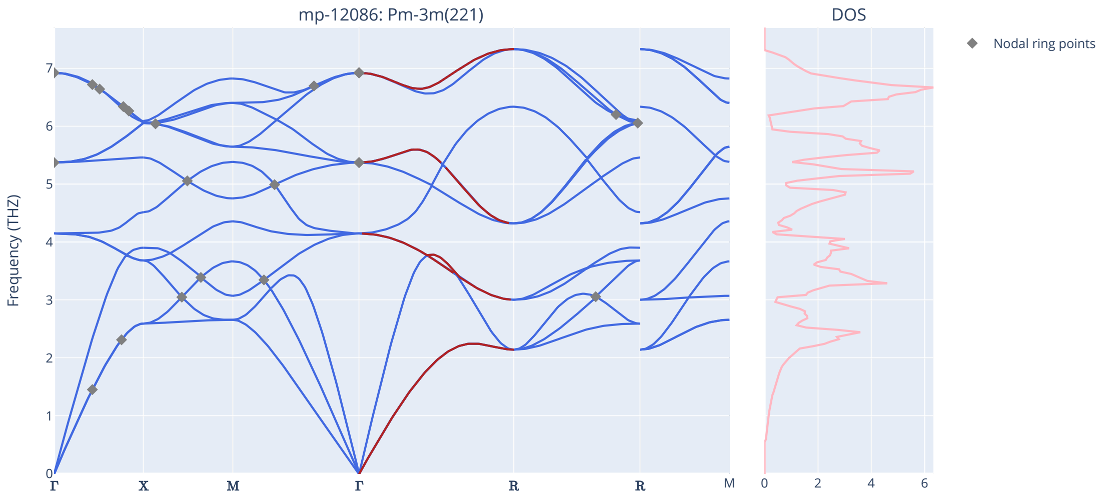

Supplementary Figure 643: The phonon band and DOS of Cu3Pt.

Supplementary Table 1358: High symmetry lines of mp-12086-Cu3Pt

	Start	End
0	Γ (0.000, 0.000, 0.000)	X (0.000, 0.500, 0.000)
1	X (0.000, 0.500, 0.000)	M (0.500, 0.500, 0.000)
2	M (0.500, 0.500, 0.000)	Γ (0.000, 0.000, 0.000)
3	Γ (0.000, 0.000, 0.000)	R (0.500, 0.500, 0.500)
4	R (0.500, 0.500, 0.500)	X (0.000, 0.500, 0.000)
5	R (0.500, 0.500, 0.500)	M (0.500, 0.500, 0.000)

Supplementary Table 1359: Nodal ring points along high symmetry lines of mp-12086-Cu3Pt.

	Coordinates	Frequency	Mode 1	Mode 2	Multi.	Deg.	Path	Ratio
0	(0.000, 0.214, 0.000)	1.452	2	1	6	2	1	0.429
1	(0.000, 0.378, 0.000)	2.312	2	1	6	2	1	0.755
2	(0.215, 0.499, -0.000)	3.048	3	2	24	2	2	0.430
3	(0.176, 0.500, 0.176)	3.055	3	2	12	2	5	0.649
4	(0.322, 0.500, 0.000)	3.388	4	3	12	2	2	0.643
5	(0.376, 0.376, -0.000)	3.346	4	3	12	2	3	0.247
6	(0.246, 0.500, 0.000)	5.054	7	6	12	2	2	0.492
7	(0.334, 0.334, 0.000)	4.989	7	6	12	2	3	0.331
8	(0.000, 0.000, 0.000)	5.371	9	8	1	3	1	0.000
9	(0.009, 0.500, 0.009)	6.052	9	8	12	2	5	0.981
10	(0.068, 0.500, 0.000)	6.040	9	8	12	2	2	0.135
11	(0.000, 0.000, 0.000)	6.920	11	10	1	3	1	0.000
12	(0.000, 0.214, 0.000)	6.716	11	10	6	2	1	0.429
13	(0.000, 0.255, 0.000)	6.639	11	10	6	2	1	0.510

Supplementary Table 1359: Nodal ring points along high symmetry lines of mp-12086-Cu3Pt.

	Coordinates	Frequency	Mode 1	Mode 2	Multi.	Deg.	Path	Ratio
14	(0.095, 0.500, 0.095)	6.199	11	10	12	2	5	0.810
15	(0.000, 0.000, 0.000)	6.920	12	11	1	3	1	0.000
16	(0.000, 0.388, 0.000)	6.337	12	11	6	2	1	0.776
17	(0.000, 0.418, 0.000)	6.261	12	11	6	2	1	0.837
18	(0.178, 0.178, -0.000)	6.693	12	11	12	2	3	0.643

The followings are details for $CrRh_3$. It has 7 topological nodal lines and 40 nodal ring points along high symmetry line as shown in Fig **644**.

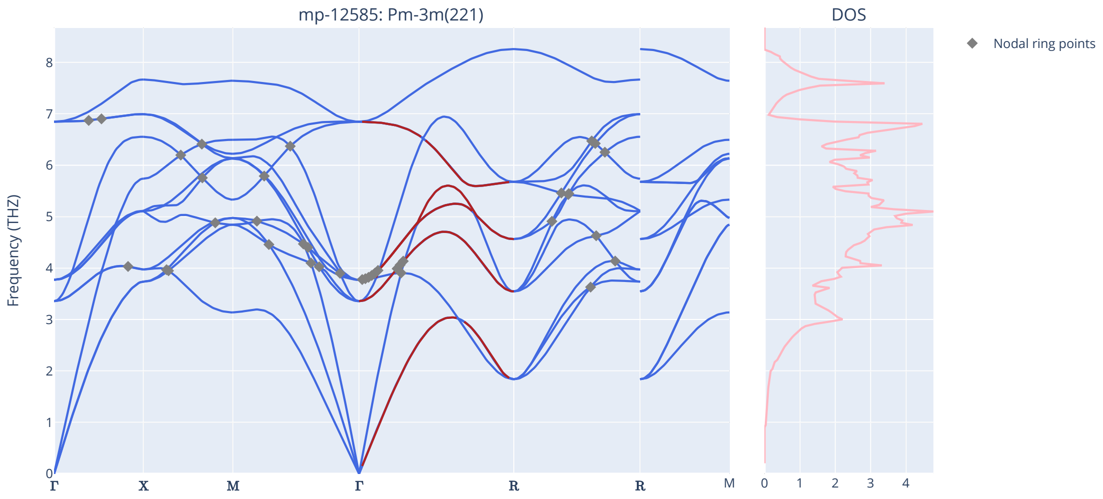

Supplementary Figure 644: The phonon band and DOS of CrRh3.

Supplementary Table 1360: High symmetry lines of mp-12585-CrRh3

	Start	End
0	Γ (0.000, 0.000, 0.000)	X (0.000, 0.500, 0.000)
1	X (0.000, 0.500, 0.000)	M (0.500, 0.500, 0.000)
2	M (0.500, 0.500, 0.000)	Γ (0.000, 0.000, 0.000)
3	Γ (0.000, 0.000, 0.000)	R (0.500, 0.500, 0.500)
4	R (0.500, 0.500, 0.500)	X (0.000, 0.500, 0.000)
5	R (0.500, 0.500, 0.500)	M (0.500, 0.500, 0.000)

Supplementary Table 1361: Nodal ring points along high symmetry lines of mp-12585-CrRh3.

	Coordinates	Frequency	Mode 1	Mode 2	Multi.	Deg.	Path	Ratio
0	(0.141, 0.500, -0.000)	3.948	2	1	12	2	2	0.281
1	(0.196, 0.500, 0.196)	3.630	2	1	12	2	5	0.608
2	(0.129, 0.500, -0.000)	3.968	3	2	12	2	2	0.259
3	(0.357, 0.357, -0.000)	4.458	3	2	12	2	3	0.286
4	(0.000, 0.414, 0.000)	4.031	4	3	6	2	1	0.828
5	(0.402, 0.500, -0.001)	4.881	4	3	24	2	2	0.804
6	(0.190, 0.190, 0.000)	4.099	4	3	12	2	3	0.620
7	(0.136, 0.136, 0.136)	3.906	4	3	8	2	4	0.272
8	(0.099, 0.500, 0.099)	4.136	4	3	12	2	5	0.803
9	(0.404, 0.404, 0.000)	4.913	5	4	12	2	3	0.191
10	(0.158, 0.158, 0.000)	4.024	5	4	12	2	3	0.684
11	(0.220, 0.220, -0.000)	4.470	5	4	12	2	3	0.559
12	(0.174, 0.500, 0.174)	4.629	5	4	12	2	5	0.652
13	(0.202, 0.202, -0.000)	4.402	6	5	12	2	3	0.595

Supplementary Table 1361: Nodal ring points along high symmetry lines of mp-12585-CrRh3.

	Coordinates	Frequency	Mode 1	Mode 2	Multi.	Deg.	Path	Ratio
14	(0.123, 0.123, 0.123)	3.989	6	5	8	2	4	0.246
15	(0.133, 0.133, 0.133)	4.061	6	5	8	2	4	0.265
16	(0.143, 0.143, 0.143)	4.135	6	5	8	2	4	0.286
17	(0.330, 0.500, -0.000)	5.755	7	6	12	2	2	0.660
18	(0.375, 0.375, 0.000)	5.790	7	6	12	2	3	0.249
19	(0.284, 0.500, 0.284)	5.431	7	6	12	2	5	0.432
20	(0.349, 0.500, 0.349)	4.909	7	6	12	2	5	0.302
21	(0.075, 0.075, -0.000)	3.899	8	7	12	2	3	0.850
22	(0.282, 0.500, 0.282)	5.449	8	7	12	2	5	0.436
23	(0.209, 0.500, -0.000)	6.198	9	8	12	2	2	0.418
24	(0.010, 0.010, 0.010)	3.777	9	8	8	2	4	0.020
25	(0.020, 0.020, 0.020)	3.793	9	8	8	2	4	0.041
26	(0.031, 0.031, 0.031)	3.820	9	8	8	2	4	0.061
27	(0.041, 0.041, 0.041)	3.856	9	8	8	2	4	0.082
28	(0.051, 0.051, 0.051)	3.901	9	8	8	2	4	0.102
29	(0.061, 0.061, 0.061)	3.955	9	8	8	2	4	0.122
30	(0.139, 0.500, 0.139)	6.254	9	8	12	2	5	0.721
31	(0.312, 0.500, 0.312)	5.461	9	8	12	2	5	0.376
32	(0.327, 0.500, 0.000)	6.411	10	9	12	2	2	0.654
33	(0.272, 0.272, 0.000)	6.371	10	9	12	2	3	0.455
34	(0.177, 0.500, 0.177)	6.423	10	9	12	2	5	0.645
35	(0.000, 0.194, 0.000)	6.871	11	10	6	2	1	0.388
36	(0.000, 0.265, 0.000)	6.903	11	10	6	2	1	0.531
37	(0.192, 0.500, 0.192)	6.472	11	10	12	2	5	0.617

The followings are details for Ir$_3$. It has 9 topological nodal lines and 20 nodal ring points along high symmetry line as shown in Fig 645.

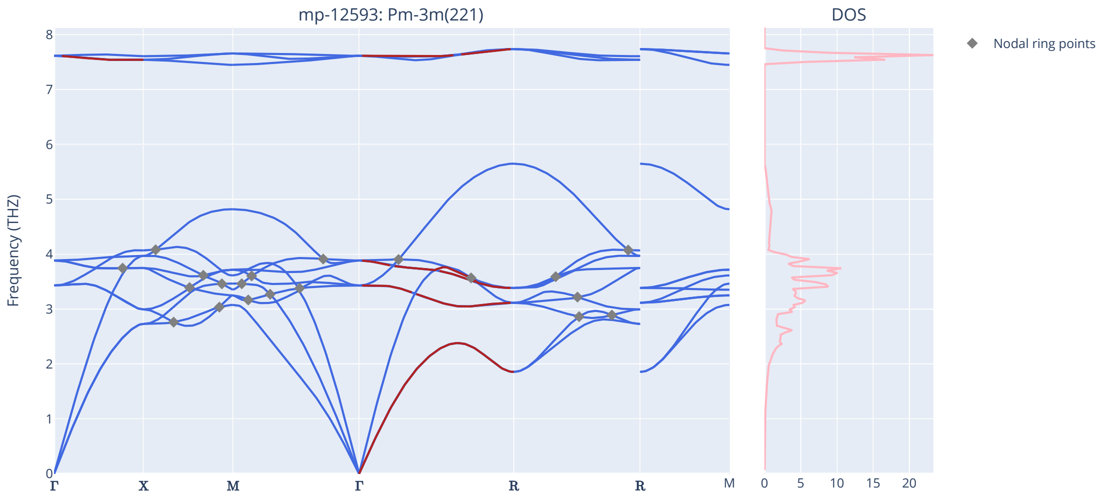

Supplementary Figure 645: The phonon band and DOS of Ir3Sc.

Supplementary Table 1362: High symmetry lines of mp-12593-Ir3Sc

	Start	End
0	Γ (0.000, 0.000, 0.000)	X (0.000, 0.500, 0.000)
1	X (0.000, 0.500, 0.000)	M (0.500, 0.500, 0.000)
2	M (0.500, 0.500, 0.000)	Γ (0.000, 0.000, 0.000)
3	Γ (0.000, 0.000, 0.000)	R (0.500, 0.500, 0.500)
4	R (0.500, 0.500, 0.500)	X (0.000, 0.500, 0.000)
5	R (0.500, 0.500, 0.500)	M (0.500, 0.500, 0.000)

Supplementary Table 1363: Nodal ring points along high symmetry lines of mp-12593-Ir3Sc.

	Coordinates	Frequency	Mode 1	Mode 2	Multi.	Deg.	Path	Ratio
0	(0.169, 0.500, 0.000)	2.761	2	1	12	2	2	0.338
1	(0.425, 0.500, -0.000)	3.035	2	1	12	2	2	0.851
2	(0.439, 0.439, 0.000)	3.166	3	2	12	2	3	0.123
3	(0.241, 0.495, 0.241)	2.861	3	2	24	2	5	0.517
4	(0.112, 0.500, 0.111)	2.890	3	2	12	2	5	0.777
5	(0.352, 0.352, -0.000)	3.269	4	3	12	2	3	0.296
6	(0.439, 0.500, -0.000)	3.460	5	4	12	2	2	0.878
7	(0.258, 0.500, -0.000)	3.392	5	4	12	2	2	0.516
8	(0.235, 0.235, -0.000)	3.379	5	4	12	2	3	0.530
9	(0.464, 0.464, 0.000)	3.461	5	4	12	2	3	0.072
10	(0.248, 0.500, 0.248)	3.218	5	4	12	2	5	0.504
11	(0.425, 0.425, 0.000)	3.604	6	5	12	2	3	0.150
12	(0.000, 0.383, 0.000)	3.745	7	6	6	2	1	0.767
13	(0.335, 0.500, -0.000)	3.614	7	6	12	2	2	0.670

Supplementary Table 1363: Nodal ring points along high symmetry lines of mp-12593-Ir3Sc.

	Coordinates	Frequency	Mode 1	Mode 2	Multi.	Deg.	Path	Ratio
14	(0.362, 0.362, 0.362)	3.567	8	7	8	2	4	0.724
15	(0.334, 0.500, 0.334)	3.590	8	7	12	2	5	0.332
16	(0.069, 0.500, -0.000)	4.080	9	8	12	2	2	0.137
17	(0.142, 0.142, -0.000)	3.911	9	8	12	2	3	0.716
18	(0.128, 0.128, 0.128)	3.903	9	8	8	2	4	0.255
19	(0.047, 0.500, 0.047)	4.074	9	8	12	2	5	0.906

The followings are details for La$_3$. It has 8 topological nodal lines and 28 nodal ring points along high symmetry line as shown in Fig 646.

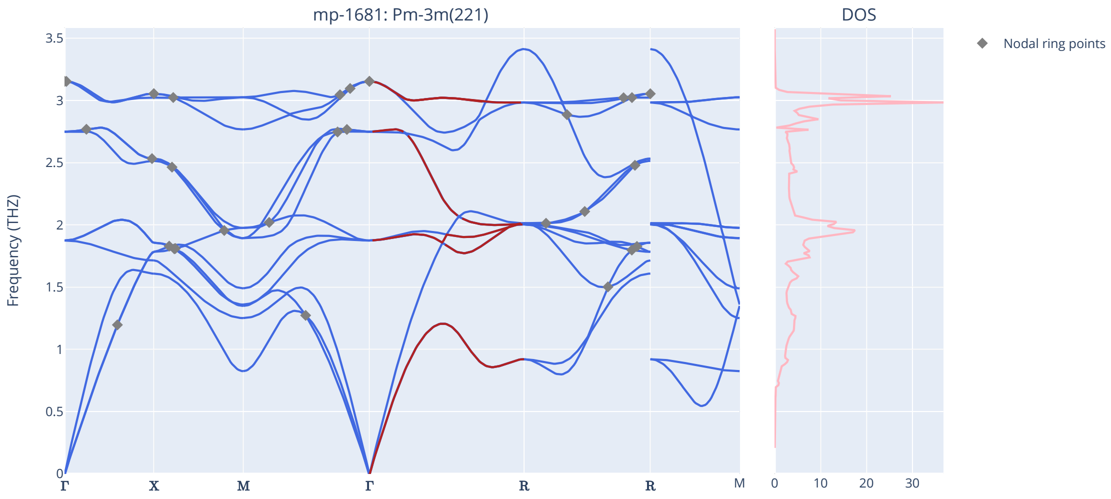

Supplementary Figure 646: The phonon band and DOS of La3Sn.

Supplementary Table 1364: High symmetry lines of mp-1681-La3Sn

	Start	End
0	Γ (0.000, 0.000, 0.000)	X (0.000, 0.500, 0.000)
1	X (0.000, 0.500, 0.000)	M (0.500, 0.500, 0.000)
2	M (0.500, 0.500, 0.000)	Γ (0.000, 0.000, 0.000)
3	Γ (0.000, 0.000, 0.000)	R (0.500, 0.500, 0.500)
4	R (0.500, 0.500, 0.500)	X (0.000, 0.500, 0.000)
5	R (0.500, 0.500, 0.500)	M (0.500, 0.500, 0.000)

Supplementary Table 1365: Nodal ring points along high symmetry lines of mp-1681-La3Sn.

	Coordinates	Frequency	Mode 1	Mode 2	Multi.	Deg.	Path	Ratio
0	(0.000, 0.296, 0.000)	1.196	2	1	6	2	1	0.592
1	(0.252, 0.252, -0.000)	1.273	2	1	12	2	3	0.495
2	(0.168, 0.500, 0.168)	1.502	3	2	12	2	5	0.665
3	(0.115, 0.500, -0.000)	1.808	4	3	12	2	2	0.229
4	(0.073, 0.500, 0.073)	1.798	4	3	12	2	5	0.854
5	(0.086, 0.500, -0.000)	1.829	5	4	12	2	2	0.172
6	(0.120, 0.500, 0.000)	1.810	5	4	12	2	2	0.241
7	(0.053, 0.500, 0.053)	1.827	5	4	12	2	5	0.894
8	(0.394, 0.500, -0.000)	1.955	7	6	12	2	2	0.789
9	(0.102, 0.500, -0.000)	2.464	8	7	12	2	2	0.204
10	(0.397, 0.397, 0.000)	2.020	8	7	12	2	3	0.206
11	(0.126, 0.126, 0.000)	2.747	8	7	12	2	3	0.748
12	(0.413, 0.500, 0.413)	2.013	8	7	12	2	5	0.174
13	(0.260, 0.500, 0.260)	2.108	8	7	12	2	5	0.481

Supplementary Table 1365: Nodal ring points along high symmetry lines of mp-1681-La3Sn.

	Coordinates	Frequency	Mode 1	Mode 2	Multi.	Deg.	Path	Ratio
14	(0.061, 0.500, 0.061)	2.479	8	7	12	2	5	0.878
15	(0.000, 0.122, 0.000)	2.769	9	8	6	2	1	0.245
16	(0.000, 0.490, 0.000)	2.533	9	8	6	2	1	0.980
17	(0.089, 0.089, -0.000)	2.767	9	8	12	2	3	0.822
18	(0.330, 0.500, 0.330)	2.886	10	9	12	2	5	0.340
19	(0.109, 0.500, -0.000)	3.024	11	10	12	2	2	0.217
20	(0.076, 0.076, 0.000)	3.097	11	10	12	2	3	0.847
21	(0.073, 0.500, 0.073)	3.023	11	10	12	2	5	0.855
22	(0.000, 0.000, 0.000)	3.154	12	11	1	3	1	0.000
23	(0.000, 0.010, 0.000)	3.154	12	11	6	2	1	0.020
24	(0.000, 0.500, 0.000)	3.054	12	11	3	2	1	1.000
25	(0.116, 0.116, -0.000)	3.046	12	11	12	2	3	0.767
26	(0.106, 0.500, 0.106)	3.022	12	11	12	2	5	0.788

The followings are details for LuTl3. It has 7 topological nodal lines and 26 nodal ring points along high symmetry line as shown in Fig 647.

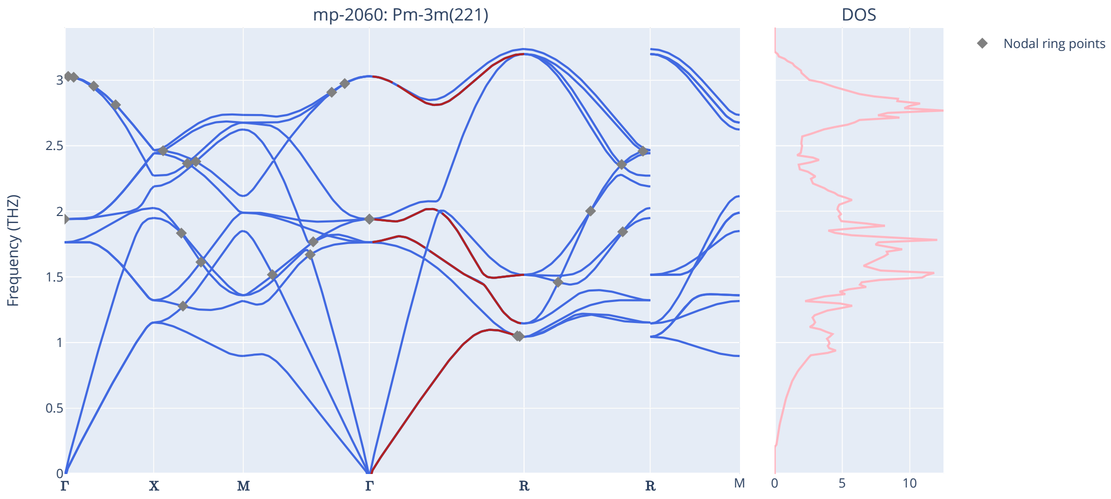

Supplementary Figure 647: The phonon band and DOS of LuTl3.

Supplementary Table 1366: High symmetry lines of mp-2060-LuTl3

	Start	End
0	Γ (0.000, 0.000, 0.000)	X (0.000, 0.500, 0.000)
1	X (0.000, 0.500, 0.000)	M (0.500, 0.500, 0.000)
2	M (0.500, 0.500, 0.000)	Γ (0.000, 0.000, 0.000)
3	Γ (0.000, 0.000, 0.000)	R (0.500, 0.500, 0.500)
4	R (0.500, 0.500, 0.500)	X (0.000, 0.500, 0.000)
5	R (0.500, 0.500, 0.500)	M (0.500, 0.500, 0.000)

Supplementary Table 1367: Nodal ring points along high symmetry lines of mp-2060-LuTl3.

	Coordinates	Frequency	Mode 1	Mode 2	Multi.	Deg.	Path	Ratio
0	(0.163, 0.500, 0.000)	1.278	3	2	12	2	2	0.326
1	(0.486, 0.486, 0.486)	1.047	3	2	8	2	4	0.972
2	(0.478, 0.478, 0.478)	1.051	3	2	8	2	4	0.956
3	(0.234, 0.234, 0.000)	1.670	4	3	12	2	3	0.531
4	(0.264, 0.500, -0.000)	1.613	5	4	12	2	2	0.527
5	(0.384, 0.384, 0.000)	1.517	5	4	12	2	3	0.232
6	(0.154, 0.500, -0.000)	1.834	6	5	12	2	2	0.308
7	(0.222, 0.222, 0.000)	1.768	6	5	12	2	3	0.555
8	(0.365, 0.500, 0.365)	1.460	6	5	12	2	5	0.269
9	(0.109, 0.500, 0.109)	1.844	6	5	12	2	5	0.781
10	(0.000, 0.000, 0.000)	1.941	8	7	1	3	1	0.000
11	(0.237, 0.500, 0.237)	2.003	8	7	12	2	5	0.527
12	(0.235, 0.500, 0.003)	2.380	9	8	24	2	2	0.470
13	(0.188, 0.500, -0.000)	2.365	9	8	12	2	2	0.375

Supplementary Table 1367: Nodal ring points along high symmetry lines of mp-2060-LuTl3.

	Coordinates	Frequency	Mode 1	Mode 2	Multi.	Deg.	Path	Ratio
14	(0.113, 0.497, 0.113)	2.357	10	9	24	2	5	0.773
15	(0.052, 0.500, -0.000)	2.462	11	10	12	2	2	0.104
16	(0.098, 0.098, -0.000)	2.974	11	10	12	2	3	0.805
17	(0.149, 0.149, -0.000)	2.908	11	10	12	2	3	0.702
18	(0.029, 0.500, 0.029)	2.459	11	10	12	2	5	0.942
19	(0.000, 0.020, 0.000)	3.029	12	11	6	2	1	0.041
20	(0.000, 0.051, 0.000)	3.022	12	11	6	2	1	0.102
21	(0.000, 0.163, 0.000)	2.955	12	11	6	2	1	0.327
22	(0.000, 0.286, 0.000)	2.812	12	11	6	2	1	0.571

The followings are details for ThTl$_3$. It has 8 topological nodal lines and 27 nodal ring points along high symmetry line as shown in Fig **648**.

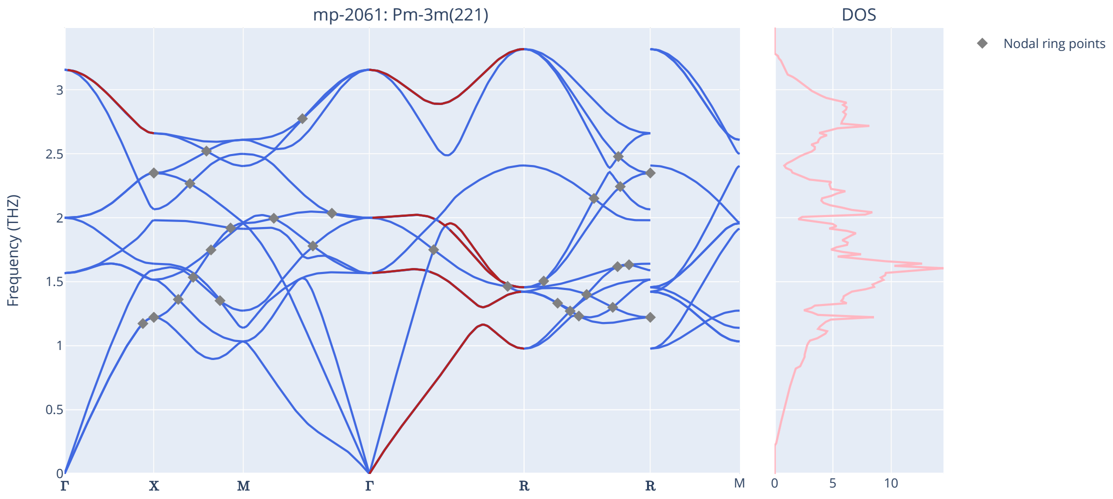

Supplementary Figure 648: The phonon band and DOS of ThTl3.

Supplementary Table 1368: High symmetry lines of mp-2061-ThTl3

	Start	End
0	Γ (0.000, 0.000, 0.000)	X (0.000, 0.500, 0.000)
1	X (0.000, 0.500, 0.000)	M (0.500, 0.500, 0.000)
2	M (0.500, 0.500, 0.000)	Γ (0.000, 0.000, 0.000)
3	Γ (0.000, 0.000, 0.000)	R (0.500, 0.500, 0.500)
4	R (0.500, 0.500, 0.500)	X (0.000, 0.500, 0.000)
5	R (0.500, 0.500, 0.500)	M (0.500, 0.500, 0.000)

Supplementary Table 1369: Nodal ring points along high symmetry lines of mp-2061-ThTl3.

	Coordinates	Frequency	Mode 1	Mode 2	Multi.	Deg.	Path	Ratio
0	(0.000, 0.439, 0.000)	1.174	2	1	6	2	1	0.878
1	(0.000, 0.500, 0.000)	1.222	2	1	3	2	1	1.000
2	(0.283, 0.500, 0.283)	1.232	2	1	12	2	5	0.434
3	(0.137, 0.500, 0.000)	1.362	3	2	12	2	2	0.274
4	(0.317, 0.500, 0.317)	1.271	3	2	12	2	5	0.365
5	(0.149, 0.500, 0.149)	1.300	3	2	12	2	5	0.702
6	(0.371, 0.500, 0.000)	1.352	4	3	12	2	2	0.741
7	(0.220, 0.500, -0.000)	1.535	4	3	12	2	2	0.440
8	(0.367, 0.500, 0.367)	1.333	4	3	12	2	5	0.266
9	(0.252, 0.500, 0.252)	1.400	5	4	12	2	5	0.496
10	(0.320, 0.500, -0.000)	1.749	6	5	12	2	2	0.640
11	(0.208, 0.208, 0.208)	1.750	6	5	8	2	4	0.415
12	(0.447, 0.447, 0.447)	1.464	6	5	8	2	4	0.894
13	(0.130, 0.500, 0.130)	1.618	6	5	12	2	5	0.741

Supplementary Table 1369: Nodal ring points along high symmetry lines of mp-2061-ThTl3.

	Coordinates	Frequency	Mode 1	Mode 2	Multi.	Deg.	Path	Ratio
14	(0.084, 0.500, 0.084)	1.633	6	5	12	2	5	0.831
15	(0.430, 0.500, 0.000)	1.920	7	6	12	2	2	0.860
16	(0.224, 0.224, -0.000)	1.780	7	6	12	2	3	0.553
17	(0.378, 0.378, -0.000)	1.996	8	7	12	2	3	0.243
18	(0.422, 0.500, 0.422)	1.505	8	7	12	2	5	0.156
19	(0.202, 0.500, -0.000)	2.267	9	8	12	2	2	0.405
20	(0.149, 0.149, -0.000)	2.035	9	8	12	2	3	0.703
21	(0.223, 0.500, 0.223)	2.151	9	8	12	2	5	0.554
22	(0.119, 0.500, 0.119)	2.244	9	8	12	2	5	0.762
23	(0.000, 0.500, 0.000)	2.350	10	9	3	2	1	1.000
24	(0.295, 0.500, -0.000)	2.521	11	10	12	2	2	0.589
25	(0.127, 0.500, 0.127)	2.479	11	10	12	2	5	0.747
26	(0.265, 0.265, 0.000)	2.775	12	11	12	2	3	0.470

The followings are details for CePd$_3$. It has 8 topological nodal lines and 27 nodal ring points along high symmetry line as shown in Fig **649**.

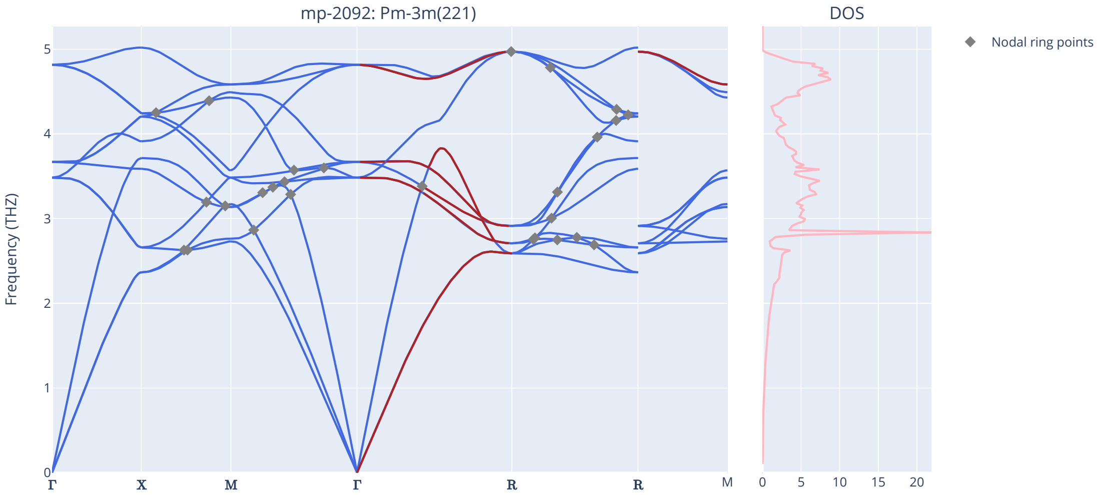

Supplementary Figure 649: The phonon band and DOS of CePd3.

Supplementary Table 1370: High symmetry lines of mp-2092-CePd3

	Start	End
0	Γ (0.000, 0.000, 0.000)	X (0.000, 0.500, 0.000)
1	X (0.000, 0.500, 0.000)	M (0.500, 0.500, 0.000)
2	M (0.500, 0.500, 0.000)	Γ (0.000, 0.000, 0.000)
3	Γ (0.000, 0.000, 0.000)	R (0.500, 0.500, 0.500)
4	R (0.500, 0.500, 0.500)	X (0.000, 0.500, 0.000)
5	R (0.500, 0.500, 0.500)	M (0.500, 0.500, 0.000)

Supplementary Table 1371: Nodal ring points along high symmetry lines of mp-2092-CePd3.

	Coordinates	Frequency	Mode 1	Mode 2	Multi.	Deg.	Path	Ratio
0	(0.258, 0.500, 0.000)	2.629	2	1	12	2	2	0.516
1	(0.238, 0.500, -0.000)	2.627	3	2	12	2	2	0.476
2	(0.409, 0.409, 0.000)	2.865	3	2	12	2	3	0.182
3	(0.320, 0.495, 0.320)	2.749	3	2	24	2	5	0.360
4	(0.174, 0.500, 0.174)	2.690	3	2	12	2	5	0.652
5	(0.263, 0.263, 0.000)	3.286	4	3	12	2	3	0.474
6	(0.242, 0.500, 0.242)	2.778	4	3	12	2	5	0.515
7	(0.415, 0.500, 0.415)	2.754	4	3	12	2	5	0.170
8	(0.469, 0.500, -0.000)	3.149	5	4	12	2	2	0.939
9	(0.364, 0.500, 0.000)	3.194	5	4	12	2	2	0.728
10	(0.374, 0.374, -0.000)	3.306	5	4	12	2	3	0.253
11	(0.333, 0.333, -0.000)	3.370	5	4	12	2	3	0.333
12	(0.408, 0.500, 0.408)	2.772	5	4	12	2	5	0.183
13	(0.287, 0.287, -0.000)	3.435	6	5	12	2	3	0.426

Supplementary Table 1371: Nodal ring points along high symmetry lines of mp-2092-CePd3.

	Coordinates	Frequency	Mode 1	Mode 2	Multi.	Deg.	Path	Ratio
14	(0.210, 0.210, 0.210)	3.382	6	5	8	2	4	0.421
15	(0.343, 0.500, 0.343)	3.005	6	5	12	2	5	0.314
16	(0.131, 0.131, -0.000)	3.598	7	6	12	2	3	0.737
17	(0.250, 0.250, 0.000)	3.572	8	7	12	2	3	0.500
18	(0.162, 0.500, 0.162)	3.962	8	7	12	2	5	0.677
19	(0.319, 0.500, 0.319)	3.313	8	7	12	2	5	0.361
20	(0.087, 0.500, 0.087)	4.158	9	8	12	2	5	0.826
21	(0.379, 0.500, -0.000)	4.391	10	9	12	2	2	0.758
22	(0.347, 0.500, 0.347)	4.783	10	9	12	2	5	0.305
23	(0.039, 0.500, 0.039)	4.225	10	9	12	2	5	0.921
24	(0.082, 0.500, 0.000)	4.250	11	10	12	2	2	0.164
25	(0.085, 0.500, 0.085)	4.289	11	10	12	2	5	0.830
26	(0.498, 0.498, 0.498)	4.972	12	11	8	3	4	0.996

The followings are details for Au$_3$. It has 8 topological nodal lines and 22 nodal ring points along high symmetry line as shown in Fig **650**.

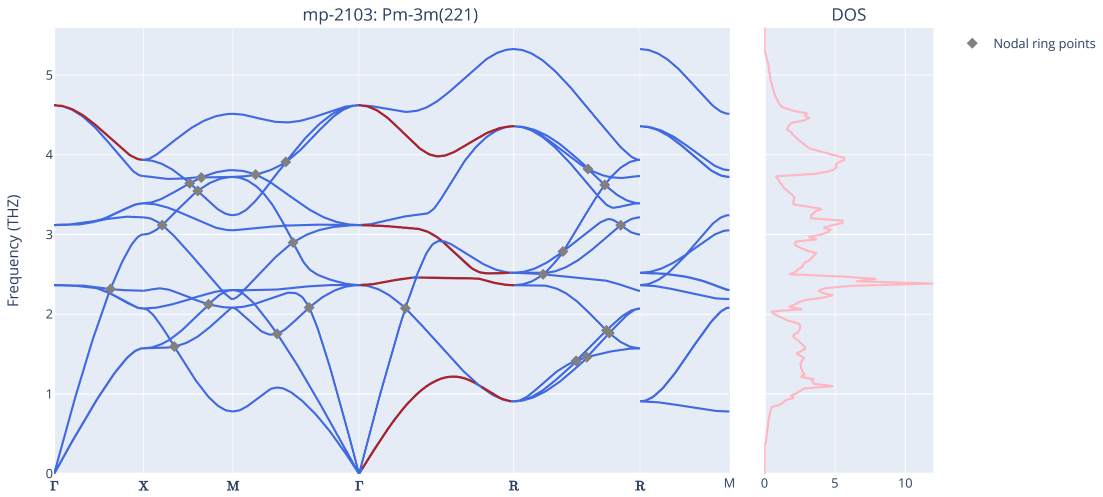

Supplementary Figure 650: The phonon band and DOS of Au3Cu.

Supplementary Table 1372: High symmetry lines of mp-2103-Au3Cu

	Start	End
0	Γ (0.000, 0.000, 0.000)	X (0.000, 0.500, 0.000)
1	X (0.000, 0.500, 0.000)	M (0.500, 0.500, 0.000)
2	M (0.500, 0.500, 0.000)	Γ (0.000, 0.000, 0.000)
3	Γ (0.000, 0.000, 0.000)	R (0.500, 0.500, 0.500)
4	R (0.500, 0.500, 0.500)	X (0.000, 0.500, 0.000)
5	R (0.500, 0.500, 0.500)	M (0.500, 0.500, 0.000)

Supplementary Table 1373: Nodal ring points along high symmetry lines of mp-2103-Au3Cu.

	Coordinates	Frequency	Mode 1	Mode 2	Multi.	Deg.	Path	Ratio
0	(0.173, 0.500, 0.000)	1.594	2	1	12	2	2	0.346
1	(0.211, 0.500, 0.211)	1.461	2	1	12	2	5	0.578
2	(0.324, 0.324, 0.000)	1.754	3	2	12	2	3	0.351
3	(0.253, 0.500, 0.253)	1.415	3	2	12	2	5	0.493
4	(0.122, 0.500, 0.122)	1.764	3	2	12	2	5	0.756
5	(0.365, 0.500, 0.000)	2.124	4	3	12	2	2	0.729
6	(0.197, 0.197, -0.000)	2.084	4	3	12	2	3	0.605
7	(0.150, 0.150, 0.150)	2.074	4	3	8	2	4	0.299
8	(0.133, 0.500, 0.133)	1.797	4	3	12	2	5	0.733
9	(0.001, 0.316, 0.001)	2.316	6	5	24	2	1	0.632
10	(0.384, 0.500, 0.384)	2.500	6	5	12	2	5	0.232
11	(0.079, 0.500, 0.075)	3.115	7	6	24	2	5	0.847
12	(0.105, 0.500, -0.000)	3.117	7	6	12	2	2	0.210
13	(0.261, 0.261, -0.000)	2.898	7	6	12	2	3	0.478

Supplementary Table 1373: Nodal ring points along high symmetry lines of mp-2103-Au3Cu.

	Coordinates	Frequency	Mode 1	Mode 2	Multi.	Deg.	Path	Ratio
14	(0.305, 0.500, 0.305)	2.786	8	7	12	2	5	0.389
15	(0.304, 0.500, -0.000)	3.546	9	8	12	2	2	0.609
16	(0.259, 0.500, -0.000)	3.641	10	9	12	2	2	0.518
17	(0.140, 0.500, 0.140)	3.621	10	9	12	2	5	0.720
18	(0.324, 0.500, -0.000)	3.714	11	10	12	2	2	0.647
19	(0.410, 0.410, 0.000)	3.751	11	10	12	2	3	0.181
20	(0.290, 0.290, 0.000)	3.910	11	10	12	2	3	0.420
21	(0.206, 0.500, 0.206)	3.821	11	10	12	2	5	0.587

The followings are details for $HfIr_3$. It has 7 topological nodal lines and 27 nodal ring points along high symmetry line as shown in Fig **651**.

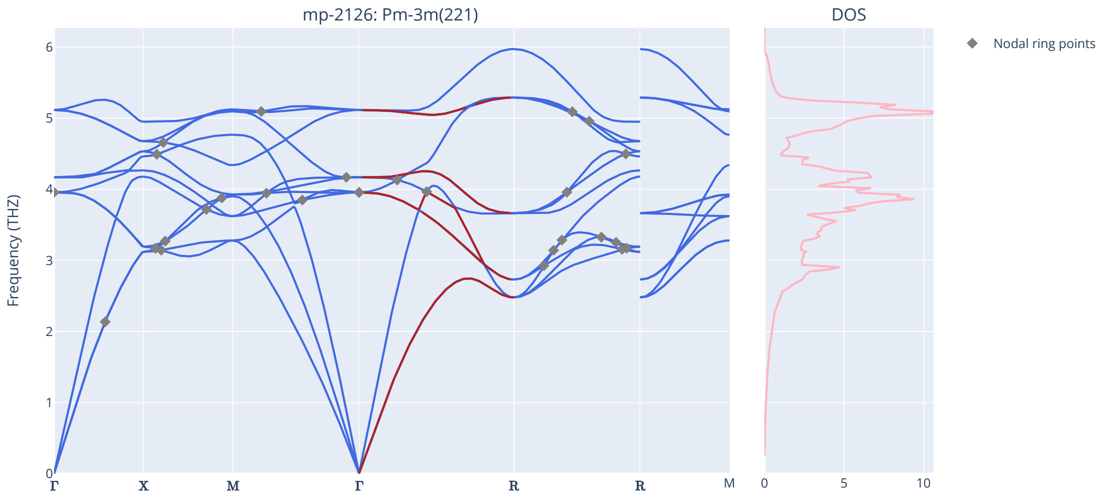

Supplementary Figure 651: The phonon band and DOS of HfIr3.

Supplementary Table 1374: High symmetry lines of mp-2126-HfIr3

	Start	End
0	Γ (0.000, 0.000, 0.000)	X (0.000, 0.500, 0.000)
1	X (0.000, 0.500, 0.000)	M (0.500, 0.500, 0.000)
2	M (0.500, 0.500, 0.000)	Γ (0.000, 0.000, 0.000)
3	Γ (0.000, 0.000, 0.000)	R (0.500, 0.500, 0.500)
4	R (0.500, 0.500, 0.500)	X (0.000, 0.500, 0.000)
5	R (0.500, 0.500, 0.500)	M (0.500, 0.500, 0.000)

Supplementary Table 1375: Nodal ring points along high symmetry lines of mp-2126-HfIr3.

	Coordinates	Frequency	Mode 1	Mode 2	Multi.	Deg.	Path	Ratio
0	(0.000, 0.286, 0.000)	2.135	2	1	6	2	1	0.571
1	(0.100, 0.500, -0.000)	3.144	2	1	12	2	2	0.199
2	(0.071, 0.500, 0.071)	3.155	2	1	12	2	5	0.857
3	(0.067, 0.500, -0.000)	3.168	3	2	12	2	2	0.134
4	(0.053, 0.500, 0.053)	3.170	3	2	12	2	5	0.893
5	(0.122, 0.500, -0.000)	3.271	4	3	12	2	2	0.245
6	(0.353, 0.500, -0.000)	3.716	4	3	12	2	2	0.706
7	(0.095, 0.500, 0.095)	3.253	4	3	12	2	5	0.809
8	(0.153, 0.500, 0.153)	3.326	4	3	12	2	5	0.693
9	(0.381, 0.500, 0.381)	2.925	4	3	12	2	5	0.238
10	(0.000, 0.000, 0.000)	3.955	5	4	1	3	1	0.000
11	(0.224, 0.224, 0.000)	3.848	5	4	12	2	3	0.551
12	(0.342, 0.500, 0.342)	3.141	5	4	12	2	5	0.315
13	(0.309, 0.500, 0.309)	3.285	5	4	12	2	5	0.381

Supplementary Table 1375: Nodal ring points along high symmetry lines of mp-2126-HfIr3.

	Coordinates	Frequency	Mode 1	Mode 2	Multi.	Deg.	Path	Ratio
14	(0.439, 0.500, -0.000)	3.878	6	5	12	2	2	0.878
15	(0.367, 0.367, 0.000)	3.944	6	5	12	2	3	0.267
16	(0.218, 0.218, 0.218)	3.965	6	5	8	2	4	0.437
17	(0.123, 0.123, 0.123)	4.130	7	6	8	2	4	0.246
18	(0.075, 0.500, -0.000)	4.491	8	7	12	2	2	0.150
19	(0.290, 0.500, 0.290)	3.959	8	7	12	2	5	0.420
20	(0.057, 0.500, 0.057)	4.496	8	7	12	2	5	0.887
21	(0.050, 0.050, -0.000)	4.168	9	8	12	2	3	0.899
22	(0.112, 0.500, -0.000)	4.657	10	9	12	2	2	0.223
23	(0.268, 0.500, 0.268)	5.088	11	10	12	2	5	0.463
24	(0.201, 0.500, 0.201)	4.955	11	10	12	2	5	0.598
25	(0.387, 0.387, -0.000)	5.092	12	11	12	2	3	0.226

The followings are details for Bi_4Li_{12}. It has 4 topological nodal lines and 14 nodal ring points along high symmetry line as shown in Fig 652.

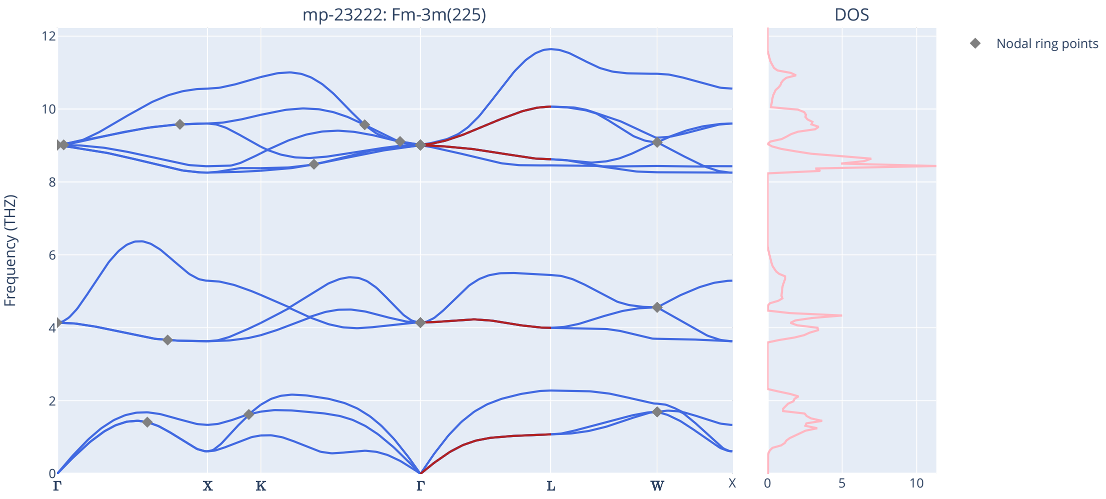

Supplementary Figure 652: The phonon band and DOS of Bi4Li12.

Supplementary Table 1376: High symmetry lines of mp-23222-Bi4Li12

	Start	End
0	Γ (0.000, 0.000, 0.000)	X (0.500, 0.000, 0.500)
1	X (0.500, 0.000, 0.500)	U (0.625, 0.250, 0.625)
2	K (0.375, 0.375, 0.750)	Γ (0.000, 0.000, 0.000)
3	Γ (0.000, 0.000, 0.000)	L (0.500, 0.500, 0.500)
4	L (0.500, 0.500, 0.500)	W (0.500, 0.250, 0.750)
5	W (0.500, 0.250, 0.750)	X (0.500, 0.000, 0.500)

Supplementary Table 1377: Nodal ring points along high symmetry lines of mp-23222-Bi4Li12.

	Coordinates	Frequency	Mode 1	Mode 2	Multi.	Deg.	Path	Ratio
0	(0.305, 0.000, 0.295)	1.414	2	1	24	2	1	0.600
1	(0.500, 0.250, 0.750)	1.696	2	1	6	2	5	1.000
2	(0.597, 0.194, 0.597)	1.625	3	2	12	2	2	0.775
3	(0.000, 0.000, 0.000)	4.143	5	4	1	3	1	0.000
4	(0.367, 0.000, 0.367)	3.667	5	4	6	2	1	0.735
5	(0.500, 0.250, 0.750)	4.563	6	5	6	2	5	1.000
6	(0.000, 0.000, 0.000)	9.007	8	7	1	3	1	0.000
7	(0.253, 0.253, 0.498)	8.485	8	7	24	2	3	0.333
8	(0.022, -0.000, 0.022)	9.018	10	9	6	2	1	0.044
9	(0.500, 0.250, 0.750)	9.088	10	9	6	2	5	1.000
10	(0.408, 0.000, 0.408)	9.579	11	10	6	2	1	0.816
11	(0.046, 0.046, 0.097)	9.110	11	10	24	2	3	0.873
12	(0.000, 0.000, 0.000)	9.019	12	11	1	3	1	0.000
13	(0.130, 0.130, 0.263)	9.568	12	11	24	2	3	0.651

The followings are details for Al3. It has 5 topological nodal lines and 13 nodal ring points along high symmetry line as shown in Fig 653.

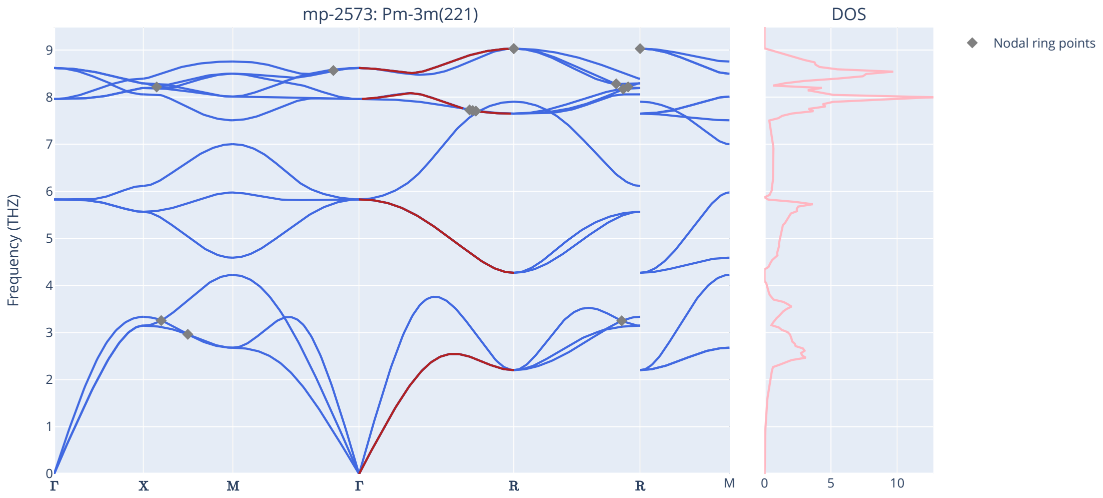

Supplementary Figure 653: The phonon band and DOS of Al3Np.

Supplementary Table 1378: High symmetry lines of mp-2573-Al3Np

	Start	End
0	Γ (0.000, 0.000, 0.000)	X (0.000, 0.500, 0.000)
1	X (0.000, 0.500, 0.000)	M (0.500, 0.500, 0.000)
2	M (0.500, 0.500, 0.000)	Γ (0.000, 0.000, 0.000)
3	Γ (0.000, 0.000, 0.000)	R (0.500, 0.500, 0.500)
4	R (0.500, 0.500, 0.500)	X (0.000, 0.500, 0.000)
5	R (0.500, 0.500, 0.500)	M (0.500, 0.500, 0.000)

Supplementary Table 1379: Nodal ring points along high symmetry lines of mp-2573-Al3Np.

	Coordinates	Frequency	Mode 1	Mode 2	Multi.	Deg.	Path	Ratio
0	(0.249, 0.500, -0.000)	2.961	2	1	12	2	2	0.497
1	(0.100, 0.500, 0.003)	3.254	3	2	24	2	2	0.199
2	(0.073, 0.500, 0.073)	3.248	3	2	12	2	5	0.854
3	(0.357, 0.357, 0.357)	7.728	8	7	8	2	4	0.714
4	(0.367, 0.367, 0.367)	7.714	8	7	8	2	4	0.735
5	(0.378, 0.378, 0.378)	7.702	8	7	8	2	4	0.755
6	(0.067, 0.500, 0.067)	8.172	9	8	12	2	5	0.867
7	(0.047, 0.498, 0.047)	8.216	10	9	24	2	5	0.905
8	(0.074, 0.500, 0.000)	8.215	10	9	12	2	2	0.149
9	(0.102, 0.102, 0.000)	8.564	11	10	12	2	3	0.797
10	(0.500, 0.500, 0.500)	9.029	11	10	1	3	4	1.000
11	(0.094, 0.500, 0.094)	8.282	11	10	12	2	5	0.811

The followings are details for UGe$_3$. It has 6 topological nodal lines and 12 nodal ring points along high symmetry line as shown in Fig **654**.

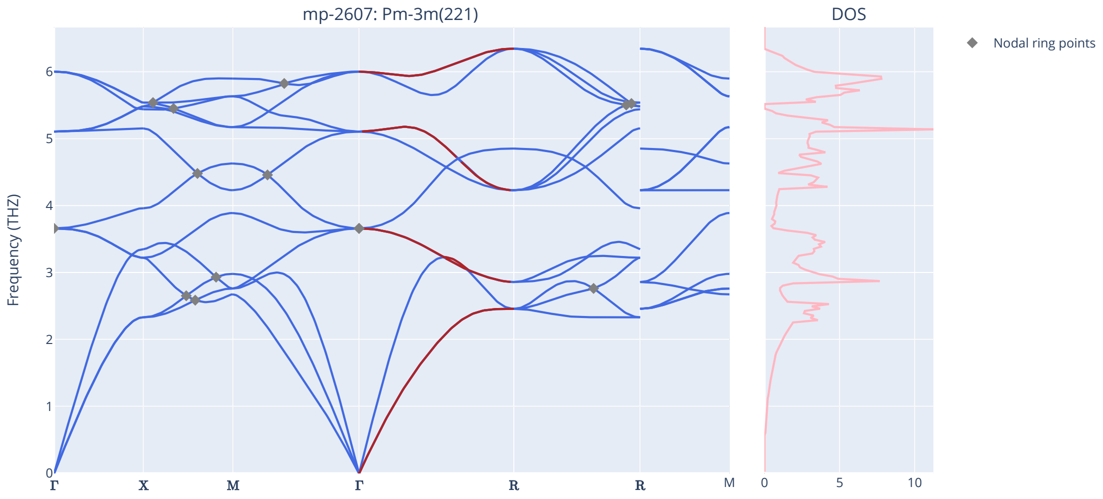

Supplementary Figure 654: The phonon band and DOS of UGe3.

Supplementary Table 1380: High symmetry lines of mp-2607-UGe3

	Start	End
0	Γ (0.000, 0.000, 0.000)	X (0.000, 0.500, 0.000)
1	X (0.000, 0.500, 0.000)	M (0.500, 0.500, 0.000)
2	M (0.500, 0.500, 0.000)	Γ (0.000, 0.000, 0.000)
3	Γ (0.000, 0.000, 0.000)	R (0.500, 0.500, 0.500)
4	R (0.500, 0.500, 0.500)	X (0.000, 0.500, 0.000)
5	R (0.500, 0.500, 0.500)	M (0.500, 0.500, 0.000)

Supplementary Table 1381: Nodal ring points along high symmetry lines of mp-2607-UGe3.

	Coordinates	Frequency	Mode 1	Mode 2	Multi.	Deg.	Path	Ratio
0	(0.290, 0.500, 0.000)	2.586	2	1	12	2	2	0.580
1	(0.240, 0.499, 0.000)	2.652	3	2	24	2	2	0.480
2	(0.184, 0.500, 0.184)	2.762	3	2	12	2	5	0.632
3	(0.407, 0.500, -0.000)	2.930	4	3	12	2	2	0.814
4	(0.000, 0.000, 0.000)	3.659	5	4	1	3	1	0.000
5	(0.303, 0.500, -0.000)	4.479	7	6	12	2	2	0.605
6	(0.362, 0.362, -0.000)	4.458	7	6	12	2	3	0.275
7	(0.169, 0.500, 0.000)	5.451	10	9	12	2	2	0.337
8	(0.055, 0.500, 0.055)	5.502	10	9	12	2	5	0.890
9	(0.034, 0.500, 0.034)	5.525	11	10	12	2	5	0.931
10	(0.054, 0.500, -0.000)	5.538	12	11	12	2	2	0.108
11	(0.296, 0.296, -0.005)	5.824	12	11	24	2	3	0.408

The followings are details for USi_3. It has 4 topological nodal lines and 9 nodal ring points along high symmetry line as shown in Fig **655**.

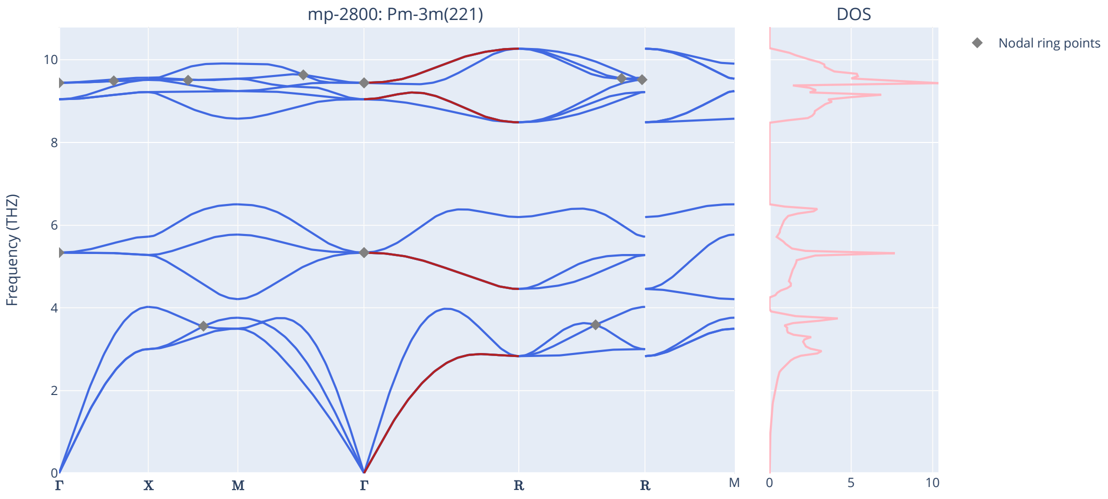

Supplementary Figure 655: The phonon band and DOS of USi3.

Supplementary Table 1382: High symmetry lines of mp-2800-USi3

	Start	End
0	Γ (0.000, 0.000, 0.000)	X (0.000, 0.500, 0.000)
1	X (0.000, 0.500, 0.000)	M (0.500, 0.500, 0.000)
2	M (0.500, 0.500, 0.000)	Γ (0.000, 0.000, 0.000)
3	Γ (0.000, 0.000, 0.000)	R (0.500, 0.500, 0.500)
4	R (0.500, 0.500, 0.500)	X (0.000, 0.500, 0.000)
5	R (0.500, 0.500, 0.500)	M (0.500, 0.500, 0.000)

Supplementary Table 1383: Nodal ring points along high symmetry lines of mp-2800-USi3.

	Coordinates	Frequency	Mode 1	Mode 2	Multi.	Deg.	Path	Ratio
0	(0.308, 0.500, -0.000)	3.558	3	2	12	2	2	0.616
1	(0.196, 0.500, 0.196)	3.590	3	2	12	2	5	0.608
2	(0.000, 0.000, 0.000)	5.336	5	4	1	3	1	0.000
3	(0.000, 0.000, 0.000)	9.442	11	10	1	3	1	0.000
4	(0.000, 0.306, 0.000)	9.490	11	10	6	2	1	0.612
5	(0.222, 0.500, 0.000)	9.505	11	10	12	2	2	0.444
6	(0.093, 0.500, 0.093)	9.550	11	10	12	2	5	0.814
7	(0.012, 0.500, 0.012)	9.518	11	10	12	2	5	0.976
8	(0.240, 0.240, 0.000)	9.635	12	11	12	2	3	0.519

The followings are details for Pt$_3$. It has 7 topological nodal lines and 24 nodal ring points along high symmetry line as shown in Fig **656**.

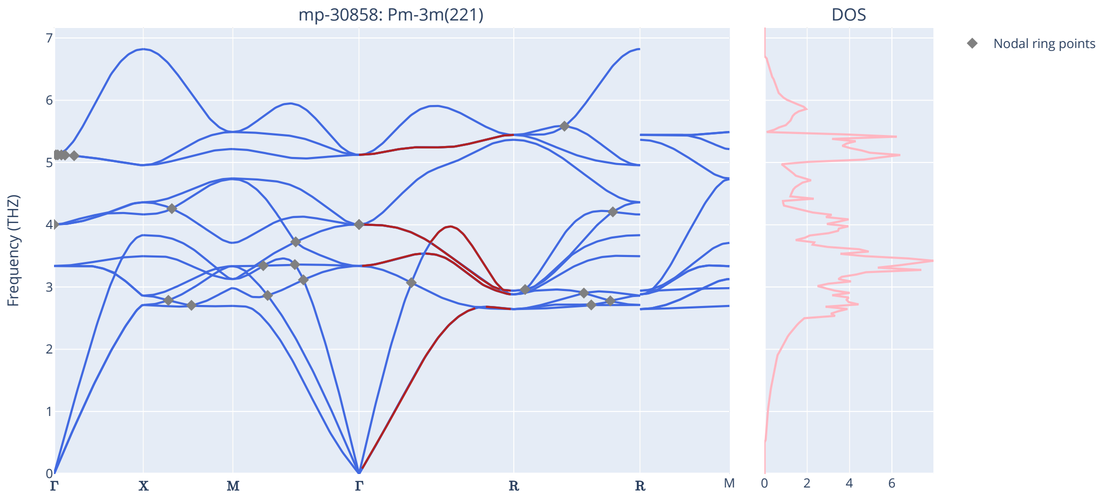

Supplementary Figure 656: The phonon band and DOS of Pt3Zr.

Supplementary Table 1384: High symmetry lines of mp-30858-Pt3Zr

	Start	End
0	Γ (0.000, 0.000, 0.000)	X (0.000, 0.500, 0.000)
1	X (0.000, 0.500, 0.000)	M (0.500, 0.500, 0.000)
2	M (0.500, 0.500, 0.000)	Γ (0.000, 0.000, 0.000)
3	Γ (0.000, 0.000, 0.000)	R (0.500, 0.500, 0.500)
4	R (0.500, 0.500, 0.500)	X (0.000, 0.500, 0.000)
5	R (0.500, 0.500, 0.500)	M (0.500, 0.500, 0.000)

Supplementary Table 1385: Nodal ring points along high symmetry lines of mp-30858-Pt3Zr.

	Coordinates	Frequency	Mode 1	Mode 2	Multi.	Deg.	Path	Ratio
0	(0.269, 0.500, -0.000)	2.705	2	1	12	2	2	0.538
1	(0.193, 0.500, 0.193)	2.711	2	1	12	2	5	0.613
2	(0.140, 0.500, 0.000)	2.786	3	2	12	2	2	0.280
3	(0.362, 0.362, -0.000)	2.864	3	2	12	2	3	0.276
4	(0.118, 0.500, 0.118)	2.777	3	2	12	2	5	0.764
5	(0.220, 0.220, 0.000)	3.115	4	3	12	2	3	0.560
6	(0.223, 0.500, 0.223)	2.902	4	3	12	2	5	0.555
7	(0.169, 0.169, 0.169)	3.069	4	3	8	2	4	0.338
8	(0.379, 0.379, 0.000)	3.343	5	4	12	2	3	0.242
9	(0.254, 0.254, -0.000)	3.360	5	4	12	2	3	0.492
10	(0.251, 0.251, -0.003)	3.725	7	6	24	2	3	0.499
11	(0.159, 0.500, -0.000)	4.259	8	7	12	2	2	0.318
12	(0.455, 0.500, 0.455)	2.959	8	7	12	2	5	0.090
13	(0.108, 0.500, 0.108)	4.206	8	7	12	2	5	0.784

Supplementary Table 1385: Nodal ring points along high symmetry lines of mp-30858-Pt3Zr.

	Coordinates	Frequency	Mode 1	Mode 2	Multi.	Deg.	Path	Ratio
14	(0.000, 0.000, 0.000)	4.004	9	8	1	3	1	0.000
15	(0.000, 0.010, 0.000)	5.122	11	10	6	2	1	0.020
16	(0.000, 0.020, 0.000)	5.122	11	10	6	2	1	0.041
17	(0.000, 0.041, 0.000)	5.121	11	10	6	2	1	0.082
18	(0.000, 0.061, 0.000)	5.118	11	10	6	2	1	0.122
19	(0.000, 0.112, 0.000)	5.109	11	10	6	2	1	0.224
20	(0.299, 0.500, 0.299)	5.583	12	11	12	2	5	0.401

The followings are details for Cu$_3$. It has 6 topological nodal lines and 25 nodal ring points along high symmetry line as shown in Fig **657**.

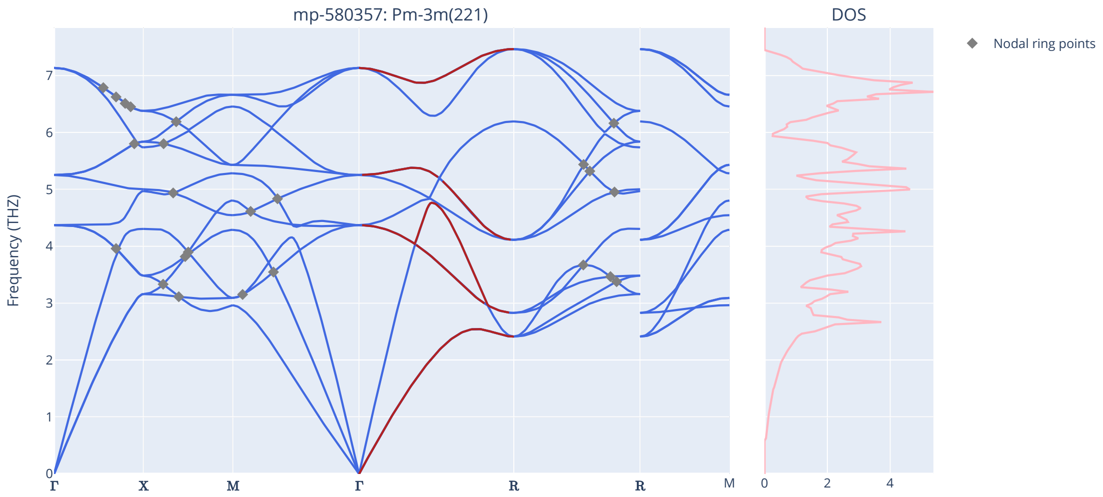

Supplementary Figure 657: The phonon band and DOS of Cu3Pd.

Supplementary Table 1386: High symmetry lines of mp-580357-Cu3Pd

	Start	End
0	Γ (0.000, 0.000, 0.000)	X (0.000, 0.500, 0.000)
1	X (0.000, 0.500, 0.000)	M (0.500, 0.500, 0.000)
2	M (0.500, 0.500, 0.000)	Γ (0.000, 0.000, 0.000)
3	Γ (0.000, 0.000, 0.000)	R (0.500, 0.500, 0.500)
4	R (0.500, 0.500, 0.500)	X (0.000, 0.500, 0.000)
5	R (0.500, 0.500, 0.500)	M (0.500, 0.500, 0.000)

Supplementary Table 1387: Nodal ring points along high symmetry lines of mp-580357-Cu3Pd.

	Coordinates	Frequency	Mode 1	Mode 2	Multi.	Deg.	Path	Ratio
0	(0.198, 0.500, -0.000)	3.112	2	1	12	2	2	0.396
1	(0.110, 0.500, 0.000)	3.331	3	2	12	2	2	0.221
2	(0.461, 0.461, -0.000)	3.151	3	2	12	2	3	0.078
3	(0.339, 0.339, 0.000)	3.546	3	2	12	2	3	0.322
4	(0.093, 0.500, 0.093)	3.376	3	2	12	2	5	0.814
5	(0.234, 0.500, -0.000)	3.817	4	3	12	2	2	0.468
6	(0.117, 0.500, 0.117)	3.466	4	3	12	2	5	0.765
7	(0.000, 0.347, 0.000)	3.959	5	4	6	2	1	0.694
8	(0.251, 0.500, -0.000)	3.896	5	4	12	2	2	0.502
9	(0.225, 0.500, 0.225)	3.670	5	4	12	2	5	0.550
10	(0.429, 0.429, -0.000)	4.612	6	5	12	2	3	0.142
11	(0.167, 0.500, -0.000)	4.936	7	6	12	2	2	0.333
12	(0.323, 0.323, 0.000)	4.834	7	6	12	2	3	0.354
13	(0.101, 0.500, 0.101)	4.953	7	6	12	2	5	0.797

Supplementary Table 1387: Nodal ring points along high symmetry lines of mp-580357-Cu3Pd.

	Coordinates	Frequency	Mode 1	Mode 2	Multi.	Deg.	Path	Ratio
14	(0.199, 0.494, 0.199)	5.320	8	7	24	2	5	0.602
15	(0.000, 0.449, 0.000)	5.801	9	8	6	2	1	0.898
16	(0.113, 0.500, -0.000)	5.802	9	8	12	2	2	0.226
17	(0.224, 0.500, 0.224)	5.437	9	8	12	2	5	0.553
18	(0.184, 0.500, -0.000)	6.189	11	10	12	2	2	0.367
19	(0.104, 0.500, 0.104)	6.162	11	10	12	2	5	0.792
20	(0.000, 0.276, 0.000)	6.787	12	11	6	2	1	0.551
21	(0.000, 0.347, 0.000)	6.624	12	11	6	2	1	0.694
22	(0.000, 0.398, 0.000)	6.511	12	11	6	2	1	0.796
23	(0.000, 0.429, 0.000)	6.451	12	11	6	2	1	0.857

The followings are details for $Ag_3La_3O_6$. It has 5 topological nodal lines and 13 nodal ring points along high symmetry line as shown in Fig **658**.

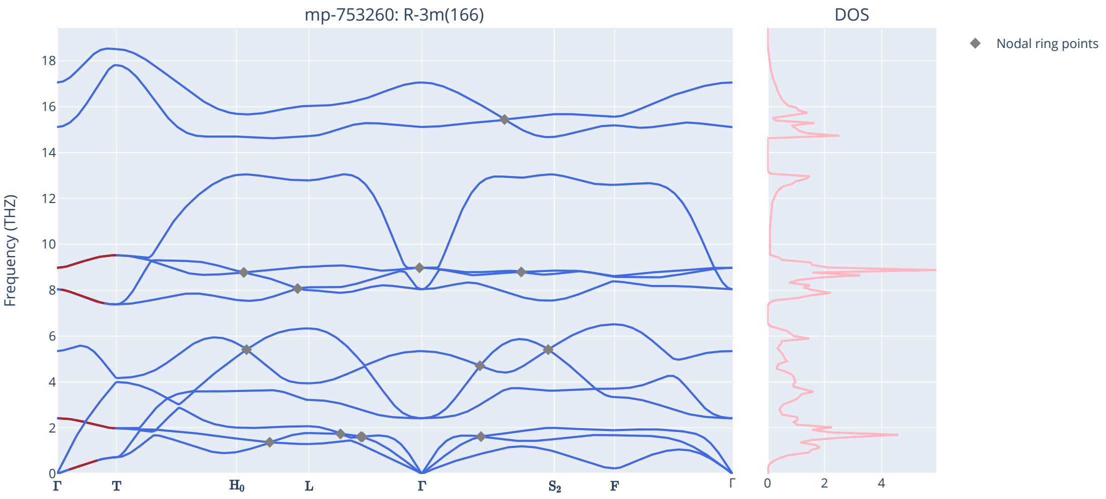

Supplementary Figure 658: The phonon band and DOS of Ag3La3O6.

Supplementary Table 1388: High symmetry lines of mp-753260-Ag3La3O6

	Start	End
0	Γ (0.000, 0.000, 0.000)	T (0.500, 0.500, 0.500)
1	T (0.500, 0.500, 0.500)	H_2 (0.812, 0.188, 0.500)
2	H_0 (0.500, -0.188, 0.188)	L (0.500, 0.000, 0.000)
3	L (0.500, 0.000, 0.000)	Γ (0.000, 0.000, 0.000)
4	Γ (0.000, 0.000, 0.000)	S_0 (0.344, -0.344, 0.000)
5	S_2 (0.656, 0.000, 0.344)	F (0.500, 0.000, 0.500)
6	F (0.500, 0.000, 0.500)	Γ (0.000, 0.000, 0.000)

Supplementary Table 1389: Nodal ring points along high symmetry lines of mp-753260-Ag3La3O6.

	Coordinates	Frequency	Mode 1	Mode 2	Multi.	Deg.	Path	Ratio
0	(0.500, -0.102, 0.102)	1.371	2	1	6	2	3	0.459
1	(0.360, 0.001, 0.001)	1.737	3	2	6	2	4	0.280
2	(0.270, 0.003, 0.003)	1.612	3	2	6	2	4	0.460
3	(0.263, -0.004, -0.004)	1.605	3	2	6	2	4	0.474
4	(0.154, -0.154, -0.000)	1.617	3	2	6	2	5	0.447
5	(0.500, -0.162, 0.162)	5.408	6	5	6	2	3	0.138
6	(0.328, -0.328, -0.000)	5.408	6	5	6	2	5	0.954
7	(0.151, -0.151, -0.000)	4.705	6	5	6	2	5	0.439
8	(0.500, -0.029, 0.029)	8.070	8	7	6	2	3	0.845
9	(0.500, -0.169, 0.169)	8.773	9	8	6	2	3	0.100
10	(0.258, -0.258, 0.000)	8.793	9	8	6	2	5	0.751
11	(0.010, -0.000, -0.000)	8.975	10	9	6	2	4	0.980
12	(0.215, -0.215, 0.000)	15.437	12	11	6	2	5	0.625

The followings are details for AgAu$_3$. It has 7 topological nodal lines and 11 nodal ring points along high symmetry line as shown in Fig 659.

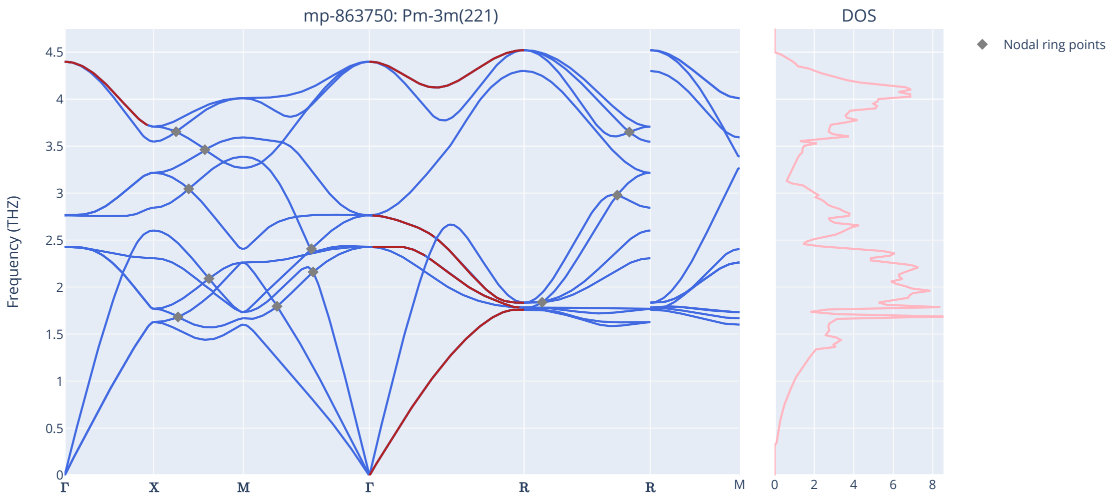

Supplementary Figure 659: The phonon band and DOS of AgAu3.

Supplementary Table 1390: High symmetry lines of mp-863750-AgAu3

	Start	End
0	Γ (0.000, 0.000, 0.000)	X (0.000, 0.500, 0.000)
1	X (0.000, 0.500, 0.000)	M (0.500, 0.500, 0.000)
2	M (0.500, 0.500, 0.000)	Γ (0.000, 0.000, 0.000)
3	Γ (0.000, 0.000, 0.000)	R (0.500, 0.500, 0.500)
4	R (0.500, 0.500, 0.500)	X (0.000, 0.500, 0.000)
5	R (0.500, 0.500, 0.500)	M (0.500, 0.500, 0.000)

Supplementary Table 1391: Nodal ring points along high symmetry lines of mp-863750-AgAu3.

	Coordinates	Frequency	Mode 1	Mode 2	Multi.	Deg.	Path	Ratio
0	(0.135, 0.500, 0.000)	1.683	3	2	12	2	2	0.271
1	(0.366, 0.366, 0.000)	1.796	3	2	12	2	3	0.268
2	(0.223, 0.223, -0.000)	2.161	4	3	12	2	3	0.554
3	(0.309, 0.500, 0.000)	2.091	6	5	12	2	2	0.618
4	(0.428, 0.500, 0.428)	1.839	6	5	12	2	5	0.143
5	(0.230, 0.230, 0.000)	2.407	7	6	12	2	3	0.541
6	(0.195, 0.500, -0.000)	3.045	8	7	12	2	2	0.391
7	(0.131, 0.500, 0.131)	2.979	8	7	12	2	5	0.738
8	(0.286, 0.500, -0.000)	3.462	10	9	12	2	2	0.572
9	(0.124, 0.500, -0.000)	3.653	11	10	12	2	2	0.249
10	(0.083, 0.500, 0.083)	3.649	11	10	12	2	5	0.834

The followings are details for $Li_{12}Pt_4$. It has 5 topological nodal lines and 7 nodal ring points along high symmetry line as shown in Fig **660**.

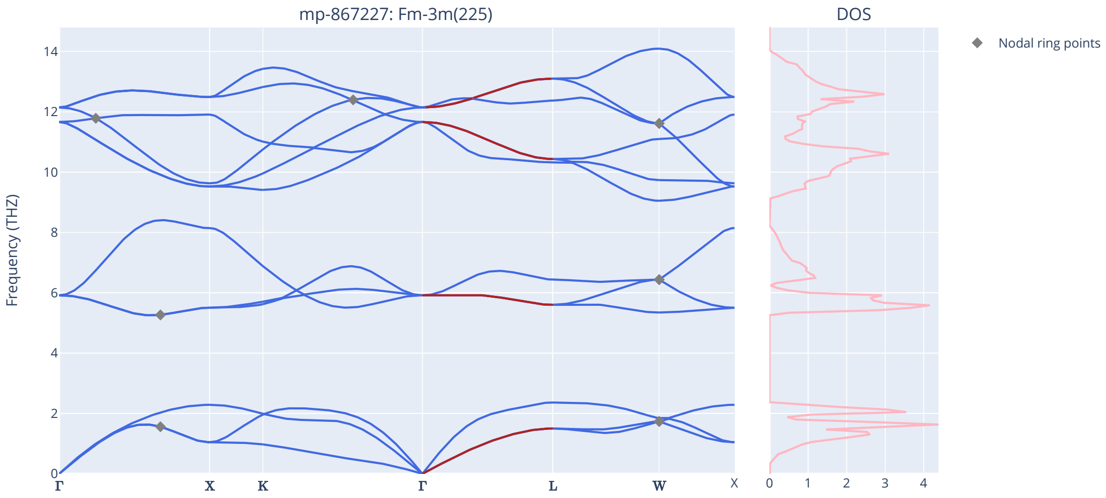

Supplementary Figure 660: The phonon band and DOS of Li12Pt4.

Supplementary Table 1392: High symmetry lines of mp-867227-Li12Pt4

	Start	End
0	Γ (0.000, 0.000, 0.000)	X (0.500, 0.000, 0.500)
1	X (0.500, 0.000, 0.500)	U (0.625, 0.250, 0.625)
2	K (0.375, 0.375, 0.750)	Γ (0.000, 0.000, 0.000)
3	Γ (0.000, 0.000, 0.000)	L (0.500, 0.500, 0.500)
4	L (0.500, 0.500, 0.500)	W (0.500, 0.250, 0.750)
5	W (0.500, 0.250, 0.750)	X (0.500, 0.000, 0.500)

Supplementary Table 1393: Nodal ring points along high symmetry lines of mp-867227-Li12Pt4.

	Coordinates	Frequency	Mode 1	Mode 2	Multi.	Deg.	Path	Ratio
0	(0.337, 0.000, 0.337)	1.559	2	1	6	2	1	0.673
1	(0.500, 0.250, 0.750)	1.733	2	1	6	2	5	1.000
2	(0.337, 0.000, 0.337)	5.266	5	4	6	2	1	0.673
3	(0.500, 0.250, 0.750)	6.436	6	5	6	2	5	1.000
4	(0.122, -0.000, 0.122)	11.785	10	9	6	2	1	0.244
5	(0.159, 0.159, 0.329)	12.395	11	10	24	2	3	0.566
6	(0.500, 0.250, 0.750)	11.614	11	10	6	2	5	1.000

The followings are details for $Li_{12}Mg_4$. It has 5 topological nodal lines and 12 nodal ring points along high symmetry line as shown in Fig **661**.

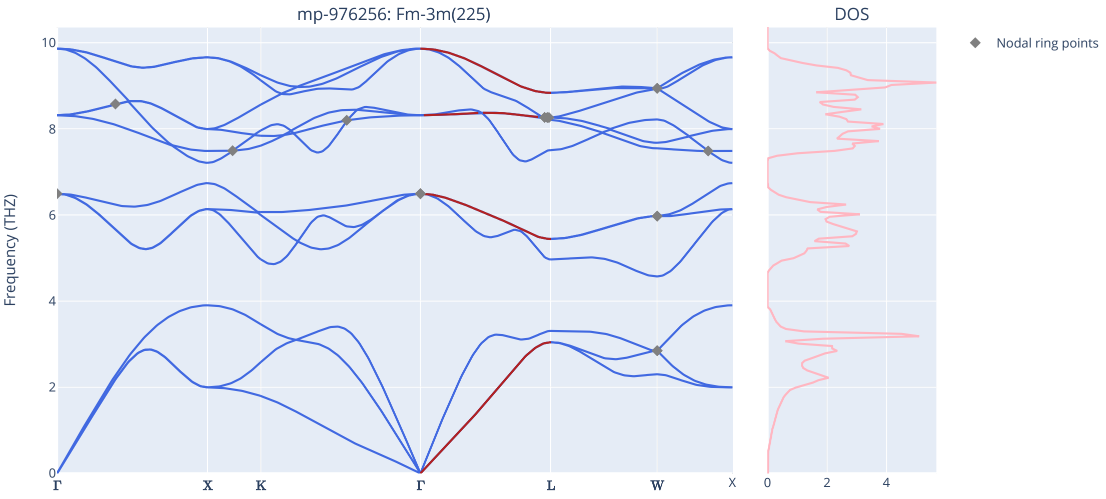

Supplementary Figure 661: The phonon band and DOS of Li12Mg4.

Supplementary Table 1394: High symmetry lines of mp-976256-Li12Mg4

	Start	End
0	Γ (0.000, 0.000, 0.000)	X (0.500, 0.000, 0.500)
1	X (0.500, 0.000, 0.500)	U (0.625, 0.250, 0.625)
2	K (0.375, 0.375, 0.750)	Γ (0.000, 0.000, 0.000)
3	Γ (0.000, 0.000, 0.000)	L (0.500, 0.500, 0.500)
4	L (0.500, 0.500, 0.500)	W (0.500, 0.250, 0.750)
5	W (0.500, 0.250, 0.750)	X (0.500, 0.000, 0.500)

Supplementary Table 1395: Nodal ring points along high symmetry lines of mp-976256-Li12Mg4.

	Coordinates	Frequency	Mode 1	Mode 2	Multi.	Deg.	Path	Ratio
0	(0.500, 0.250, 0.750)	2.847	3	2	6	2	5	1.000
1	(0.000, 0.000, 0.000)	6.494	6	5	1	3	1	0.000
2	(0.500, 0.250, 0.750)	5.973	6	5	6	2	5	1.000
3	(0.559, 0.118, 0.559)	7.493	8	7	12	2	2	0.470
4	(0.176, 0.176, 0.343)	8.199	8	7	24	2	3	0.538
5	(0.499, 0.082, 0.579)	7.486	8	7	48	2	6	0.678
6	(0.194, 0.000, 0.194)	8.578	9	8	6	2	1	0.388
7	(0.493, 0.493, 0.493)	8.263	10	9	8	2	4	0.986
8	(0.487, 0.487, 0.487)	8.263	10	9	8	2	4	0.973
9	(0.476, 0.476, 0.476)	8.266	10	9	8	2	4	0.953
10	(0.500, 0.250, 0.750)	8.942	12	11	6	2	5	1.000

The followings are details for AuPd$_3$. It has 10 topological nodal lines and 18 nodal ring points along high symmetry line as shown in Fig 662.

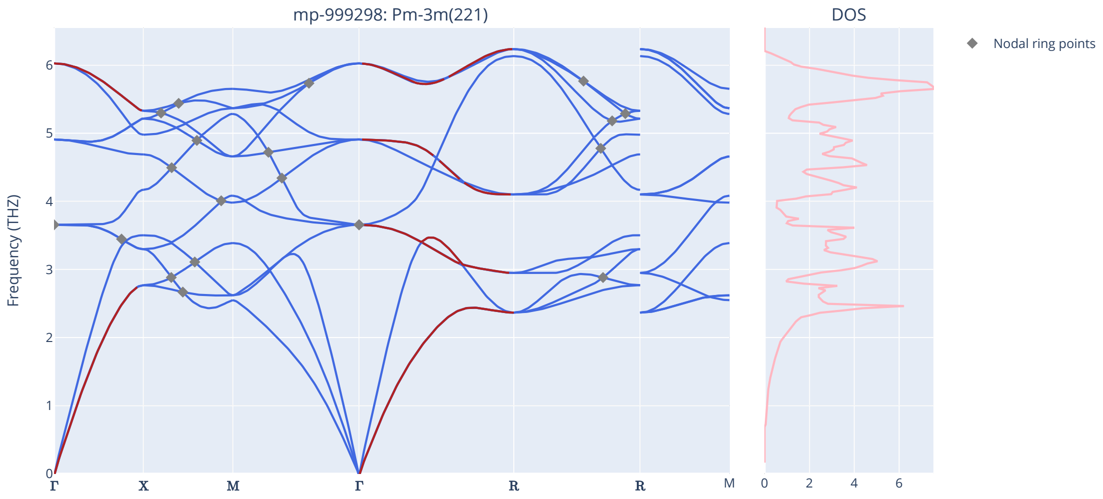

Supplementary Figure 662: The phonon band and DOS of AuPd3.

Supplementary Table 1396: High symmetry lines of mp-999298-AuPd3

	Start	End
0	Γ (0.000, 0.000, 0.000)	X (0.000, 0.500, 0.000)
1	X (0.000, 0.500, 0.000)	M (0.500, 0.500, 0.000)
2	M (0.500, 0.500, 0.000)	Γ (0.000, 0.000, 0.000)
3	Γ (0.000, 0.000, 0.000)	R (0.500, 0.500, 0.500)
4	R (0.500, 0.500, 0.500)	X (0.000, 0.500, 0.000)
5	R (0.500, 0.500, 0.500)	M (0.500, 0.500, 0.000)

Supplementary Table 1397: Nodal ring points along high symmetry lines of mp-999298-AuPd3.

	Coordinates	Frequency	Mode 1	Mode 2	Multi.	Deg.	Path	Ratio
0	(0.221, 0.500, -0.000)	2.668	2	1	12	2	2	0.441
1	(0.156, 0.500, -0.000)	2.882	3	2	12	2	2	0.312
2	(0.147, 0.500, 0.147)	2.881	3	2	12	2	5	0.707
3	(0.288, 0.500, 0.000)	3.109	4	3	12	2	2	0.575
4	(0.000, 0.378, 0.000)	3.446	5	4	6	2	1	0.755
5	(0.000, 0.000, 0.000)	3.655	6	5	1	3	1	0.000
6	(0.436, 0.500, -0.000)	4.008	6	5	12	2	2	0.871
7	(0.158, 0.500, -0.000)	4.494	7	6	12	2	2	0.316
8	(0.305, 0.305, 0.000)	4.342	7	6	12	2	3	0.389
9	(0.299, 0.499, 0.000)	4.894	8	7	24	2	2	0.599
10	(0.359, 0.359, -0.000)	4.721	8	7	12	2	3	0.282
11	(0.156, 0.500, 0.156)	4.778	8	7	12	2	5	0.689
12	(0.111, 0.500, 0.111)	5.182	10	9	12	2	5	0.779
13	(0.099, 0.500, -0.000)	5.297	11	10	12	2	2	0.198

Supplementary Table 1397: Nodal ring points along high symmetry lines of mp-999298-AuPd3.

	Coordinates	Frequency	Mode 1	Mode 2	Multi.	Deg.	Path	Ratio
14	(0.198, 0.198, -0.000)	5.734	11	10	12	2	3	0.603
15	(0.058, 0.500, 0.058)	5.288	11	10	12	2	5	0.883
16	(0.197, 0.500, 0.000)	5.439	12	11	12	2	2	0.395
17	(0.224, 0.500, 0.224)	5.766	12	11	12	2	5	0.552

The followings are details for Bi_2Pt_2. It has 22 topological nodal lines and 34 nodal ring points along high symmetry line as shown in Fig **663**.

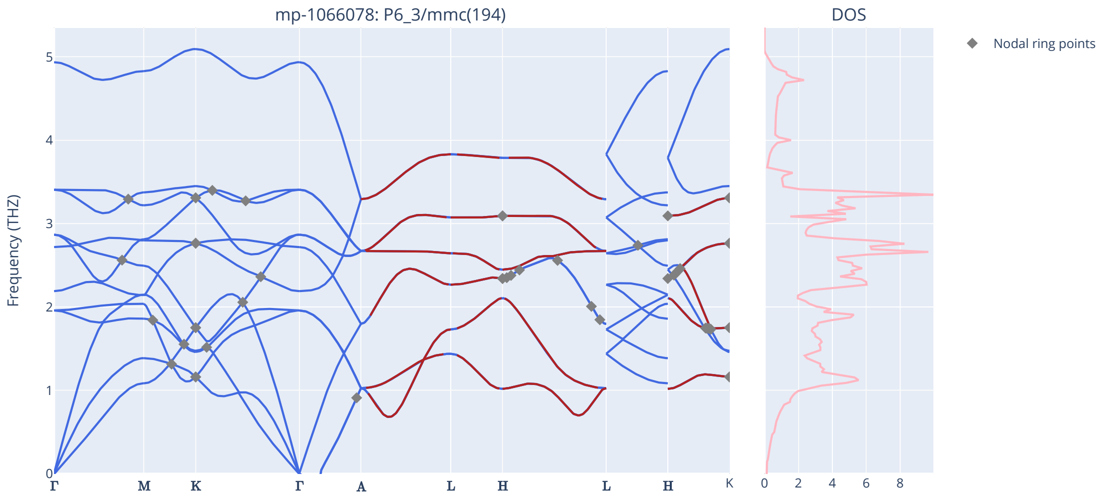

Supplementary Figure 663: The phonon band and DOS of Bi2Pt2.

Supplementary Table 1398: High symmetry lines of mp-1066078-Bi2Pt2

	Start	End
0	Γ (0.000, 0.000, 0.000)	M (0.500, 0.000, 0.000)
1	M (0.500, 0.000, 0.000)	K (0.333, 0.333, 0.000)
2	K (0.333, 0.333, 0.000)	Γ (0.000, 0.000, 0.000)
3	Γ (0.000, 0.000, 0.000)	A (0.000, 0.000, 0.500)
4	A (0.000, 0.000, 0.500)	L (0.500, 0.000, 0.500)
5	L (0.500, 0.000, 0.500)	H (0.333, 0.333, 0.500)
6	H (0.333, 0.333, 0.500)	A (0.000, 0.000, 0.500)
7	L (0.500, 0.000, 0.500)	M (0.500, 0.000, 0.000)
8	H (0.333, 0.333, 0.500)	K (0.333, 0.333, 0.000)

Supplementary Table 1399: Nodal ring points along high symmetry lines of mp-1066078-Bi2Pt2.

	Coordinates	Frequency	Mode 1	Mode 2	Multi.	Deg.	Path	Ratio
0	(0.411, 0.178, 0.000)	1.315	2	1	6	2	2	0.535
1	(0.333, 0.333, 0.000)	1.161	2	1	2	2	2	1.000
2	(-0.000, -0.000, 0.465)	0.910	2	1	2	2	4	0.930
3	(0.471, 0.058, -0.000)	1.842	4	3	6	2	2	0.173
4	(0.298, 0.298, 0.000)	1.516	4	3	6	2	3	0.106
5	(0.371, 0.258, 0.000)	1.553	5	4	6	2	2	0.774
6	(0.332, 0.337, 0.151)	1.733	5	4	12	2	9	0.698
7	(0.333, 0.333, 0.194)	1.747	5	4	4	2	9	0.612
8	(0.333, 0.333, 0.184)	1.745	5	4	4	2	9	0.633
9	(0.333, 0.333, 0.173)	1.743	5	4	4	2	9	0.653
10	(0.333, 0.333, 0.163)	1.742	5	4	4	2	9	0.673

Supplementary Table 1399: Nodal ring points along high symmetry lines of mp-1066078-Bi2Pt2.

	Coordinates	Frequency	Mode 1	Mode 2	Multi.	Deg.	Path	Ratio
11	(0.182, 0.182, -0.000)	2.056	6	5	6	2	3	0.454
12	(0.333, 0.333, 0.000)	1.751	6	5	2	2	2	1.000
13	(0.333, 0.333, 0.500)	2.341	6	5	2	2	6	1.000
14	(0.320, 0.320, 0.500)	2.351	6	5	6	2	7	0.041
15	(0.306, 0.306, 0.500)	2.378	6	5	6	2	7	0.082
16	(0.279, 0.279, 0.500)	2.444	6	5	6	2	7	0.163
17	(0.156, 0.156, 0.500)	2.558	6	5	6	2	7	0.531
18	(0.048, 0.048, 0.500)	2.007	6	5	6	2	7	0.857
19	(0.020, 0.020, 0.500)	1.847	6	5	6	2	7	0.939
20	(0.124, 0.124, -0.000)	2.362	7	6	6	2	3	0.628
21	(0.333, 0.333, 0.400)	2.460	7	6	4	2	9	0.200
22	(0.333, 0.333, 0.420)	2.427	7	6	4	2	9	0.159
23	(0.333, 0.333, 0.450)	2.382	7	6	4	2	9	0.100
24	(0.333, 0.333, 0.439)	2.398	7	6	4	2	9	0.122
25	(0.333, 0.333, 0.000)	2.764	8	7	2	2	2	1.000
26	(0.379, 0.000, -0.000)	2.562	8	7	6	2	1	0.757
27	(0.497, 0.000, 0.243)	2.739	9	8	12	2	8	0.515
28	(0.333, 0.333, 0.000)	3.308	10	9	2	2	2	1.000
29	(0.333, 0.333, 0.500)	3.092	10	9	2	2	6	1.000
30	(0.173, 0.173, -0.000)	3.272	11	10	6	2	3	0.482
31	(0.280, 0.280, 0.000)	3.398	11	10	6	2	3	0.161
32	(0.413, -0.000, -0.000)	3.293	11	10	6	2	1	0.826

The followings are details for Au_4Li_{12}. It has 5 topological nodal lines and 8 nodal ring points along high symmetry line as shown in Fig **664**.

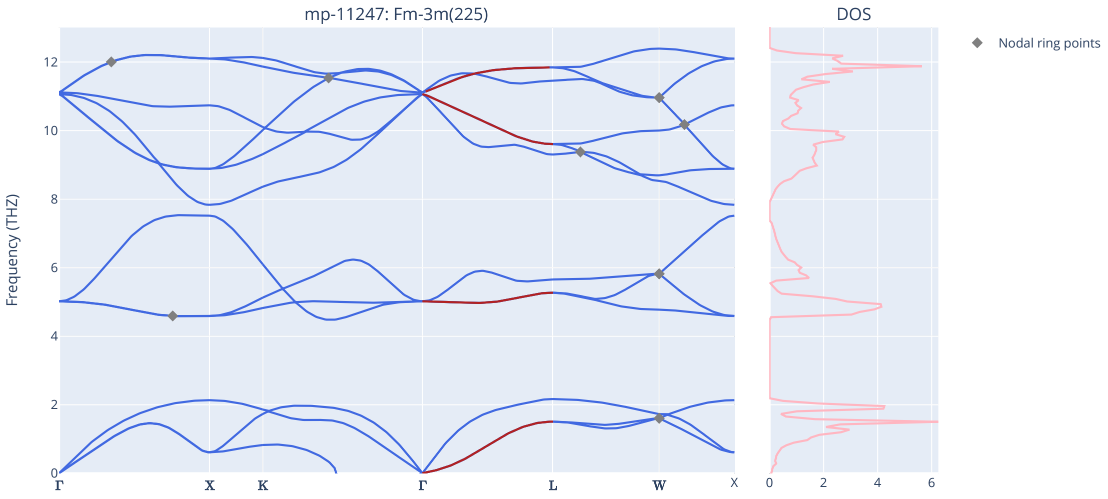

Supplementary Figure 664: The phonon band and DOS of Au4Li12.

Supplementary Table 1400: High symmetry lines of mp-11247-Au4Li12

	Start	End
0	Γ (0.000, 0.000, 0.000)	X (0.500, 0.000, 0.500)
1	X (0.500, 0.000, 0.500)	U (0.625, 0.250, 0.625)
2	K (0.375, 0.375, 0.750)	Γ (0.000, 0.000, 0.000)
3	Γ (0.000, 0.000, 0.000)	L (0.500, 0.500, 0.500)
4	L (0.500, 0.500, 0.500)	W (0.500, 0.250, 0.750)
5	W (0.500, 0.250, 0.750)	X (0.500, 0.000, 0.500)

Supplementary Table 1401: Nodal ring points along high symmetry lines of mp-11247-Au4Li12.

	Coordinates	Frequency	Mode 1	Mode 2	Multi.	Deg.	Path	Ratio
0	(0.500, 0.250, 0.750)	1.605	2	1	6	2	5	1.000
1	(0.378, 0.000, 0.378)	4.590	5	4	6	2	1	0.755
2	(0.500, 0.250, 0.750)	5.819	6	5	6	2	5	1.000
3	(0.500, 0.435, 0.565)	9.376	8	7	24	2	5	0.260
4	(0.499, 0.166, 0.667)	10.174	10	9	24	2	6	0.336
5	(0.222, 0.222, 0.439)	11.536	11	10	24	2	3	0.412
6	(0.500, 0.250, 0.750)	10.960	11	10	6	2	5	1.000
7	(0.173, 0.000, 0.173)	12.003	12	11	6	2	1	0.347

The followings are details for Ba_4Sr_{12}. It has 8 topological nodal lines and 11 nodal ring points along high symmetry line as shown in Fig **665**.

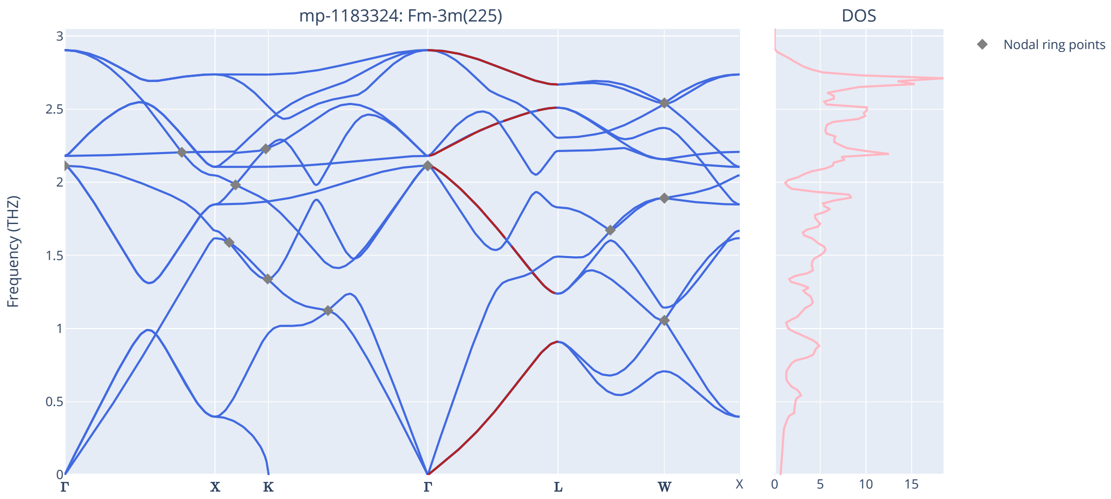

Supplementary Figure 665: The phonon band and DOS of Ba4Sr12.

Supplementary Table 1402: High symmetry lines of mp-1183324-Ba4Sr12

	Start	End
0	Γ (0.000, 0.000, 0.000)	X (0.500, 0.000, 0.500)
1	X (0.500, 0.000, 0.500)	U (0.625, 0.250, 0.625)
2	K (0.375, 0.375, 0.750)	Γ (0.000, 0.000, 0.000)
3	Γ (0.000, 0.000, 0.000)	L (0.500, 0.500, 0.500)
4	L (0.500, 0.500, 0.500)	W (0.500, 0.250, 0.750)
5	W (0.500, 0.250, 0.750)	X (0.500, 0.000, 0.500)

Supplementary Table 1403: Nodal ring points along high symmetry lines of mp-1183324-Ba4Sr12.

	Coordinates	Frequency	Mode 1	Mode 2	Multi.	Deg.	Path	Ratio
0	(0.241, 0.229, 0.469)	1.123	3	2	24	2	3	0.374
1	(0.500, 0.250, 0.750)	1.056	3	2	6	2	5	1.000
2	(0.533, 0.067, 0.533)	1.589	4	3	12	2	2	0.267
3	(0.624, 0.248, 0.624)	1.339	4	3	12	2	2	0.991
4	(0.000, 0.000, 0.000)	2.113	6	5	1	3	1	0.000
5	(0.500, 0.250, 0.750)	1.891	6	5	6	2	5	1.000
6	(0.500, 0.377, 0.623)	1.673	6	5	24	2	5	0.492
7	(0.548, 0.096, 0.548)	1.982	7	6	12	2	2	0.386
8	(0.390, 0.000, 0.390)	2.205	8	7	6	2	1	0.779
9	(0.619, 0.239, 0.619)	2.228	9	8	12	2	2	0.955
10	(0.500, 0.250, 0.750)	2.542	12	11	6	2	5	1.000

The followings are details for $Ba_{12}Sr_4$. It has 8 topological nodal lines and 7 nodal ring points along high symmetry line as shown in Fig **666**.

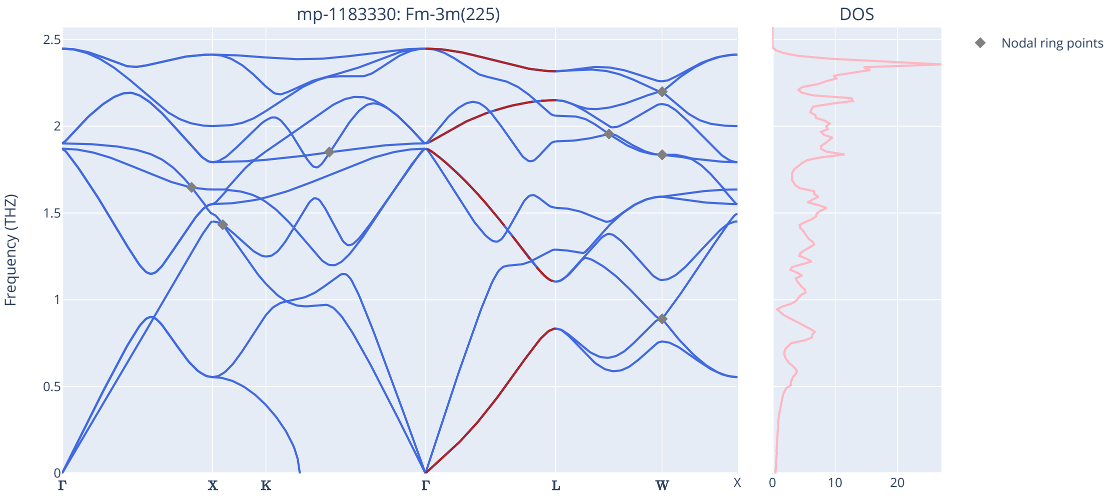

Supplementary Figure 666: The phonon band and DOS of Ba12Sr4.

Supplementary Table 1404: High symmetry lines of mp-1183330-Ba12Sr4

	Start	End
0	Γ (0.000, 0.000, 0.000)	X (0.500, 0.000, 0.500)
1	X (0.500, 0.000, 0.500)	U (0.625, 0.250, 0.625)
2	K (0.375, 0.375, 0.750)	Γ (0.000, 0.000, 0.000)
3	Γ (0.000, 0.000, 0.000)	L (0.500, 0.500, 0.500)
4	L (0.500, 0.500, 0.500)	W (0.500, 0.250, 0.750)
5	W (0.500, 0.250, 0.750)	X (0.500, 0.000, 0.500)

Supplementary Table 1405: Nodal ring points along high symmetry lines of mp-1183330-Ba12Sr4.

	Coordinates	Frequency	Mode 1	Mode 2	Multi.	Deg.	Path	Ratio
0	(0.500, 0.250, 0.750)	0.890	3	2	6	2	5	1.000
1	(0.524, 0.048, 0.524)	1.432	4	3	12	2	2	0.192
2	(0.430, 0.000, 0.430)	1.647	7	6	6	2	1	0.861
3	(0.229, 0.229, 0.448)	1.851	8	7	24	2	3	0.398
4	(0.500, 0.250, 0.750)	1.835	8	7	6	2	5	1.000
5	(0.500, 0.375, 0.625)	1.955	8	7	24	2	5	0.501
6	(0.500, 0.250, 0.750)	2.199	11	10	6	2	5	1.000

The followings are details for Cd_4Na_{12}. It has 6 topological nodal lines and 12 nodal ring points along high symmetry line as shown in Fig **667**.

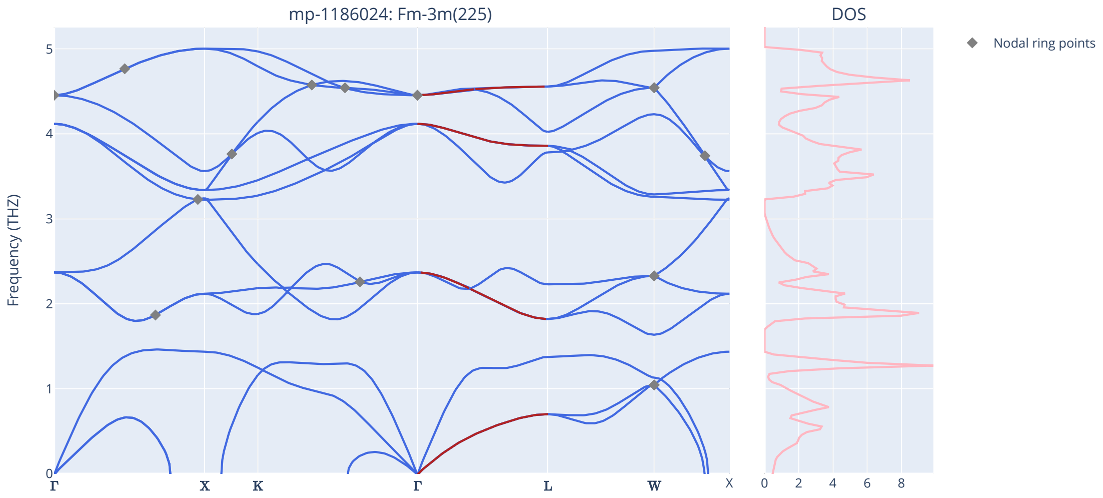

Supplementary Figure 667: The phonon band and DOS of Cd4Na12.

Supplementary Table 1406: High symmetry lines of mp-1186024-Cd4Na12

	Start	End
0	Γ (0.000, 0.000, 0.000)	X (0.500, 0.000, 0.500)
1	X (0.500, 0.000, 0.500)	U (0.625, 0.250, 0.625)
2	K (0.375, 0.375, 0.750)	Γ (0.000, 0.000, 0.000)
3	Γ (0.000, 0.000, 0.000)	L (0.500, 0.500, 0.500)
4	L (0.500, 0.500, 0.500)	W (0.500, 0.250, 0.750)
5	W (0.500, 0.250, 0.750)	X (0.500, 0.000, 0.500)

Supplementary Table 1407: Nodal ring points along high symmetry lines of mp-1186024-Cd4Na12.

	Coordinates	Frequency	Mode 1	Mode 2	Multi.	Deg.	Path	Ratio
0	(0.500, 0.250, 0.750)	1.045	2	1	6	2	5	1.000
1	(0.337, 0.000, 0.337)	1.868	5	4	6	2	1	0.673
2	(0.138, 0.138, 0.267)	2.259	6	5	24	2	3	0.640
3	(0.500, 0.250, 0.750)	2.329	6	5	6	2	5	1.000
4	(0.478, -0.001, 0.478)	3.229	7	6	24	2	1	0.955
5	(0.564, 0.128, 0.564)	3.763	10	9	12	2	2	0.513
6	(0.498, 0.081, 0.582)	3.743	10	9	24	2	6	0.674
7	(0.000, 0.000, 0.000)	4.455	11	10	1	3	1	0.000
8	(0.166, 0.166, 0.345)	4.543	11	10	24	2	3	0.546
9	(0.500, 0.250, 0.750)	4.544	11	10	6	2	5	1.000
10	(0.243, 0.253, 0.496)	4.575	11	10	24	2	3	0.338
11	(0.235, 0.000, 0.235)	4.767	12	11	6	2	1	0.469

The followings are details for $Ta_{12}W_4$. It has 8 topological nodal lines and 17 nodal ring points along high symmetry line as shown in Fig **668**.

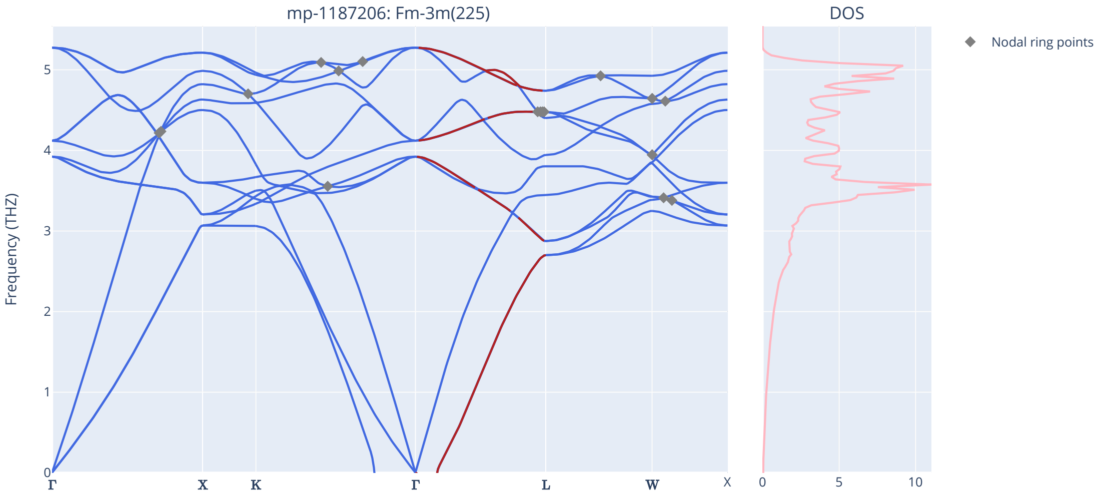

Supplementary Figure 668: The phonon band and DOS of Ta12W4.

Supplementary Table 1408: High symmetry lines of mp-1187206-Ta12W4

	Start	End
0	Γ (0.000, 0.000, 0.000)	X (0.500, 0.000, 0.500)
1	X (0.500, 0.000, 0.500)	U (0.625, 0.250, 0.625)
2	K (0.375, 0.375, 0.750)	Γ (0.000, 0.000, 0.000)
3	Γ (0.000, 0.000, 0.000)	L (0.500, 0.500, 0.500)
4	L (0.500, 0.500, 0.500)	W (0.500, 0.250, 0.750)
5	W (0.500, 0.250, 0.750)	X (0.500, 0.000, 0.500)

Supplementary Table 1409: Nodal ring points along high symmetry lines of mp-1187206-Ta12W4.

	Coordinates	Frequency	Mode 1	Mode 2	Multi.	Deg.	Path	Ratio
0	(0.498, 0.183, 0.685)	3.378	3	2	24	2	6	0.262
1	(0.499, 0.213, 0.712)	3.411	4	3	24	2	6	0.152
2	(0.207, 0.207, 0.412)	3.554	6	5	24	2	3	0.451
3	(0.362, -0.000, 0.362)	4.235	8	7	6	2	1	0.724
4	(0.500, 0.250, 0.750)	3.944	8	7	6	2	5	1.000
5	(0.356, -0.000, 0.356)	4.216	9	8	6	2	1	0.711
6	(0.469, 0.469, 0.469)	4.478	10	9	8	2	4	0.938
7	(0.607, 0.214, 0.607)	4.700	10	9	12	2	2	0.856
8	(0.487, 0.487, 0.487)	4.477	10	9	8	2	4	0.974
9	(0.494, 0.494, 0.494)	4.477	10	9	8	2	4	0.987
10	(0.480, 0.480, 0.480)	4.477	10	9	8	2	4	0.959
11	(0.499, 0.206, 0.707)	4.610	10	9	24	2	6	0.174
12	(0.184, 0.184, 0.357)	4.985	11	10	24	2	3	0.519
13	(0.500, 0.250, 0.750)	4.643	11	10	6	2	5	1.000

Supplementary Table 1409: Nodal ring points along high symmetry lines of mp-1187206-Ta12W4.

	Coordinates	Frequency	Mode 1	Mode 2	Multi.	Deg.	Path	Ratio
14	(0.122, 0.122, 0.249)	5.101	12	11	24	2	3	0.670
15	(0.500, 0.371, 0.629)	4.924	12	11	24	2	5	0.515
16	(0.216, 0.227, 0.443)	5.089	12	11	24	2	3	0.409

The followings are details for Pb_2O_2. It has 24 topological nodal lines and 21 nodal ring points along high symmetry line as shown in Fig **669**.

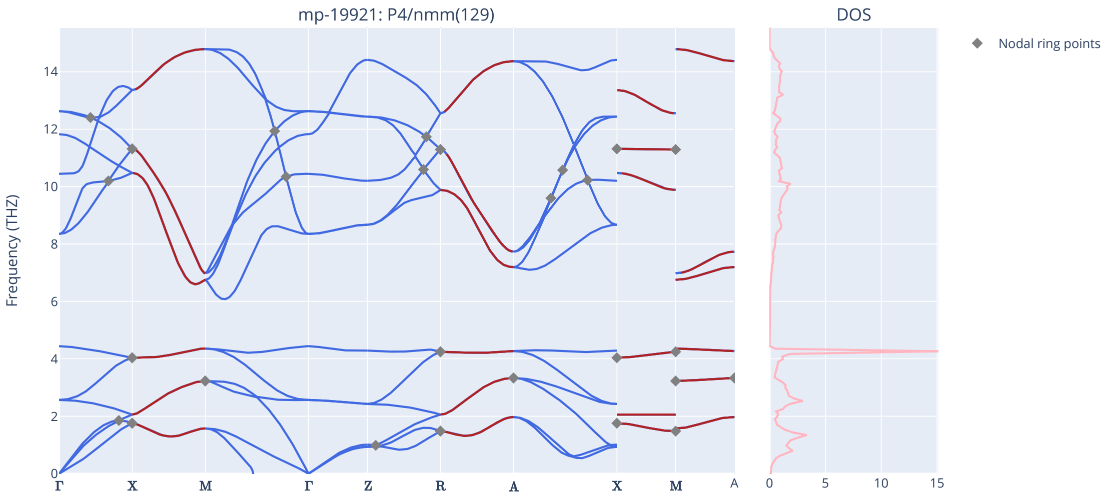

Supplementary Figure 669: The phonon band and DOS of Pb2O2.

Supplementary Table 1410: High symmetry lines of mp-19921-Pb2O2

	Start	End
0	Γ (0.000, 0.000, 0.000)	X (0.000, 0.500, 0.000)
1	X (0.000, 0.500, 0.000)	M (0.500, 0.500, 0.000)
2	M (0.500, 0.500, 0.000)	Γ (0.000, 0.000, 0.000)
3	Γ (0.000, 0.000, 0.000)	Z (0.000, 0.000, 0.500)
4	Z (0.000, 0.000, 0.500)	R (0.000, 0.500, 0.500)
5	R (0.000, 0.500, 0.500)	A (0.500, 0.500, 0.500)
6	A (0.500, 0.500, 0.500)	Z (0.000, 0.000, 0.500)
7	X (0.000, 0.500, 0.000)	R (0.000, 0.500, 0.500)
8	M (0.500, 0.500, 0.000)	A (0.500, 0.500, 0.500)

Supplementary Table 1411: Nodal ring points along high symmetry lines of mp-19921-Pb2O2.

	Coordinates	Frequency	Mode 1	Mode 2	Multi.	Deg.	Path	Ratio
0	(0.000, 0.500, 0.000)	1.758	2	1	2	2	1	1.000
1	(0.000, 0.500, 0.500)	1.483	2	1	2	2	5	1.000
2	(-0.000, 0.409, -0.000)	1.854	3	2	4	2	1	0.818
3	(0.000, 0.057, 0.500)	0.989	3	2	4	2	5	0.114
4	(0.500, 0.500, 0.000)	3.230	4	3	1	2	2	1.000
5	(0.500, 0.500, 0.500)	3.334	4	3	1	2	6	1.000
6	(0.000, 0.500, 0.000)	4.038	6	5	2	2	1	1.000
7	(0.000, 0.500, 0.500)	4.246	6	5	2	2	5	1.000
8	(-0.000, 0.337, -0.000)	10.195	8	7	4	2	1	0.675
9	(0.109, 0.109, 0.000)	10.343	9	8	4	2	3	0.782
10	(-0.000, 0.384, 0.500)	10.593	9	8	4	2	5	0.768

Supplementary Table 1411: Nodal ring points along high symmetry lines of mp-19921-Pb2O2.

	Coordinates	Frequency	Mode 1	Mode 2	Multi.	Deg.	Path	Ratio
11	(0.142, 0.142, 0.500)	10.224	9	8	4	2	7	0.717
12	(0.000, 0.500, 0.000)	11.313	10	9	2	2	1	1.000
13	(0.000, 0.500, 0.500)	11.289	10	9	2	2	5	1.000
14	(0.262, 0.262, 0.500)	10.573	10	9	4	2	7	0.475
15	(0.319, 0.319, 0.500)	9.597	10	9	4	2	7	0.362
16	(0.164, 0.164, 0.000)	11.933	11	10	4	2	3	0.672
17	(0.000, 0.404, 0.500)	11.733	11	10	4	2	5	0.807
18	(-0.000, 0.215, 0.000)	12.403	12	11	4	2	1	0.429

The followings are details for Fe_2Se_2. It has 24 topological nodal lines and 20 nodal ring points along high symmetry line as shown in Fig **670**.

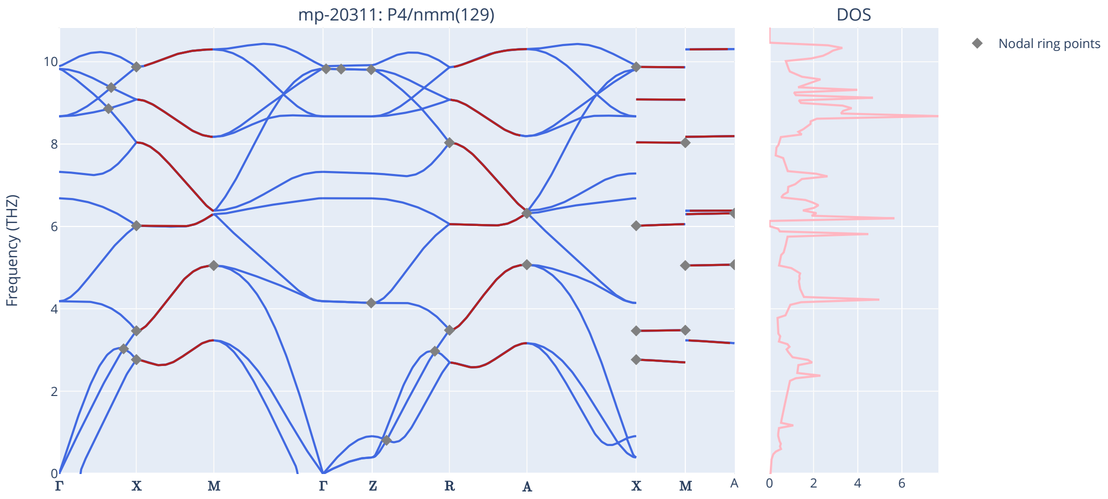

Supplementary Figure 670: The phonon band and DOS of Fe2Se2.

Supplementary Table 1412: High symmetry lines of mp-20311-Fe2Se2

	Start	End
0	Γ (0.000, 0.000, 0.000)	X (0.000, 0.500, 0.000)
1	X (0.000, 0.500, 0.000)	M (0.500, 0.500, 0.000)
2	M (0.500, 0.500, 0.000)	Γ (0.000, 0.000, 0.000)
3	Γ (0.000, 0.000, 0.000)	Z (0.000, 0.000, 0.500)
4	Z (0.000, 0.000, 0.500)	R (0.000, 0.500, 0.500)
5	R (0.000, 0.500, 0.500)	A (0.500, 0.500, 0.500)
6	A (0.500, 0.500, 0.500)	Z (0.000, 0.000, 0.500)
7	X (0.000, 0.500, 0.000)	R (0.000, 0.500, 0.500)
8	M (0.500, 0.500, 0.000)	A (0.500, 0.500, 0.500)

Supplementary Table 1413: Nodal ring points along high symmetry lines of mp-20311-Fe2Se2.

	Coordinates	Frequency	Mode 1	Mode 2	Multi.	Deg.	Path	Ratio
0	(0.000, 0.500, 0.000)	2.769	2	1	2	2	1	1.000
1	(-0.000, 0.093, 0.500)	0.811	2	1	4	2	5	0.185
2	(0.000, 0.417, -0.000)	3.031	3	2	4	2	1	0.834
3	(-0.000, 0.405, 0.500)	2.973	3	2	4	2	5	0.810
4	(0.000, 0.500, 0.000)	3.467	4	3	2	2	1	1.000
5	(0.500, 0.500, 0.000)	5.052	4	3	1	2	2	1.000
6	(0.000, 0.500, 0.500)	3.483	4	3	2	2	5	1.000
7	(0.500, 0.500, 0.500)	5.072	4	3	1	2	6	1.000
8	(0.000, 0.000, 0.490)	4.144	5	4	2	2	4	0.980
9	(0.000, 0.500, 0.000)	6.018	6	5	2	2	1	1.000
10	(0.500, 0.500, 0.500)	6.320	6	5	1	2	6	1.000

Supplementary Table 1413: Nodal ring points along high symmetry lines of mp-20311-Fe2Se2.

	Coordinates	Frequency	Mode 1	Mode 2	Multi.	Deg.	Path	Ratio
11	(0.000, 0.500, 0.500)	8.032	8	7	2	2	5	1.000
12	(0.000, 0.320, 0.000)	8.859	9	8	4	2	1	0.639
13	(-0.000, 0.337, -0.000)	9.371	11	10	4	2	1	0.674
14	(0.000, 0.000, 0.031)	9.825	11	10	2	2	4	0.061
15	(0.000, 0.000, 0.184)	9.819	11	10	2	2	4	0.367
16	(0.000, 0.000, 0.490)	9.805	11	10	2	2	4	0.980
17	(0.000, 0.500, 0.000)	9.871	12	11	2	2	1	1.000

The followings are details for Sn_2O_2. It has 24 topological nodal lines and 23 nodal ring points along high symmetry line as shown in Fig **671**.

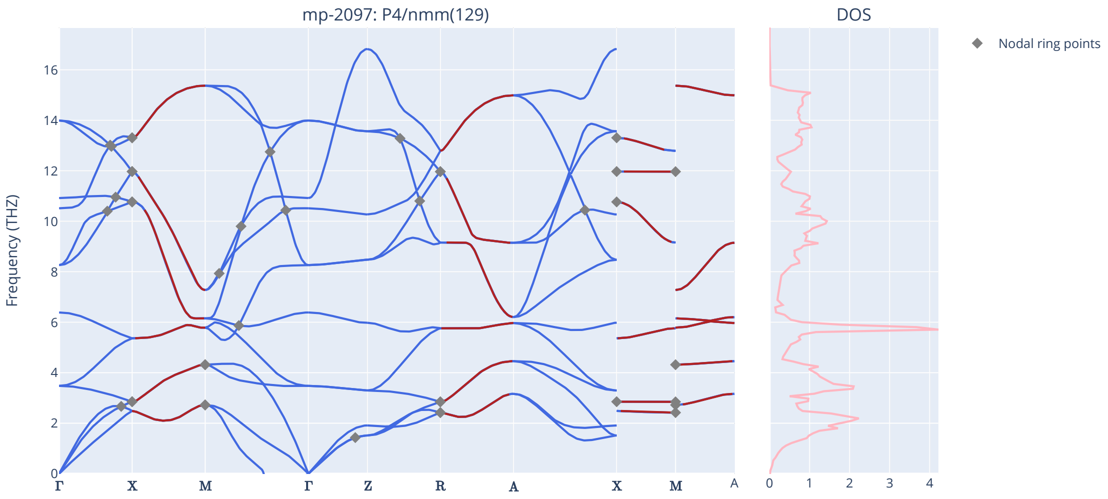

Supplementary Figure 671: The phonon band and DOS of Sn2O2.

Supplementary Table 1414: High symmetry lines of mp-2097-Sn2O2

	Start	End
0	Γ (0.000, 0.000, 0.000)	X (0.000, 0.500, 0.000)
1	X (0.000, 0.500, 0.000)	M (0.500, 0.500, 0.000)
2	M (0.500, 0.500, 0.000)	Γ (0.000, 0.000, 0.000)
3	Γ (0.000, 0.000, 0.000)	Z (0.000, 0.000, 0.500)
4	Z (0.000, 0.000, 0.500)	R (0.000, 0.500, 0.500)
5	R (0.000, 0.500, 0.500)	A (0.500, 0.500, 0.500)
6	A (0.500, 0.500, 0.500)	Z (0.000, 0.000, 0.500)
7	X (0.000, 0.500, 0.000)	R (0.000, 0.500, 0.500)
8	M (0.500, 0.500, 0.000)	A (0.500, 0.500, 0.500)

Supplementary Table 1415: Nodal ring points along high symmetry lines of mp-2097-Sn2O2.

	Coordinates	Frequency	Mode 1	Mode 2	Multi.	Deg.	Path	Ratio
0	(0.500, 0.500, 0.000)	2.725	2	1	1	2	2	1.000
1	(0.000, 0.000, 0.398)	1.432	2	1	2	2	4	0.796
2	(0.000, 0.500, 0.500)	2.422	2	1	2	2	5	1.000
3	(0.000, 0.425, -0.000)	2.669	3	2	4	2	1	0.850
4	(0.000, 0.500, 0.000)	2.854	4	3	2	2	1	1.000
5	(0.500, 0.500, 0.000)	4.320	4	3	1	2	2	1.000
6	(0.000, 0.500, 0.500)	2.848	4	3	2	2	5	1.000
7	(0.337, 0.337, -0.000)	5.871	7	6	4	2	3	0.326
8	(0.000, 0.500, 0.000)	10.770	8	7	2	2	1	1.000
9	(0.000, 0.330, -0.000)	10.404	8	7	4	2	1	0.660
10	(0.000, 0.387, 0.000)	10.960	9	8	4	2	1	0.774

Supplementary Table 1415: Nodal ring points along high symmetry lines of mp-2097-Sn2O2.

	Coordinates	Frequency	Mode 1	Mode 2	Multi.	Deg.	Path	Ratio
11	(0.110, 0.110, -0.000)	10.440	9	8	4	2	3	0.780
12	(0.000, 0.359, 0.500)	10.806	9	8	4	2	5	0.718
13	(0.154, 0.154, 0.500)	10.447	9	8	4	2	7	0.693
14	(0.000, 0.500, 0.000)	11.974	10	9	2	2	1	1.000
15	(0.432, 0.432, 0.000)	7.937	10	9	4	2	3	0.136
16	(0.326, 0.326, 0.000)	9.804	10	9	4	2	3	0.348
17	(0.000, 0.500, 0.500)	11.970	10	9	2	2	5	1.000
18	(-0.000, 0.358, -0.000)	12.970	11	10	4	2	1	0.717
19	(0.185, 0.185, 0.000)	12.755	11	10	4	2	3	0.629
20	(0.000, 0.224, 0.500)	13.287	11	10	4	2	5	0.448
21	(0.000, 0.350, 0.000)	13.020	12	11	4	2	1	0.700
22	(0.000, 0.500, 0.000)	13.311	12	11	2	2	1	1.000

The followings are details for Pt$_3$. It has 8 topological nodal lines and 22 nodal ring points along high symmetry line as shown in Fig **672**.

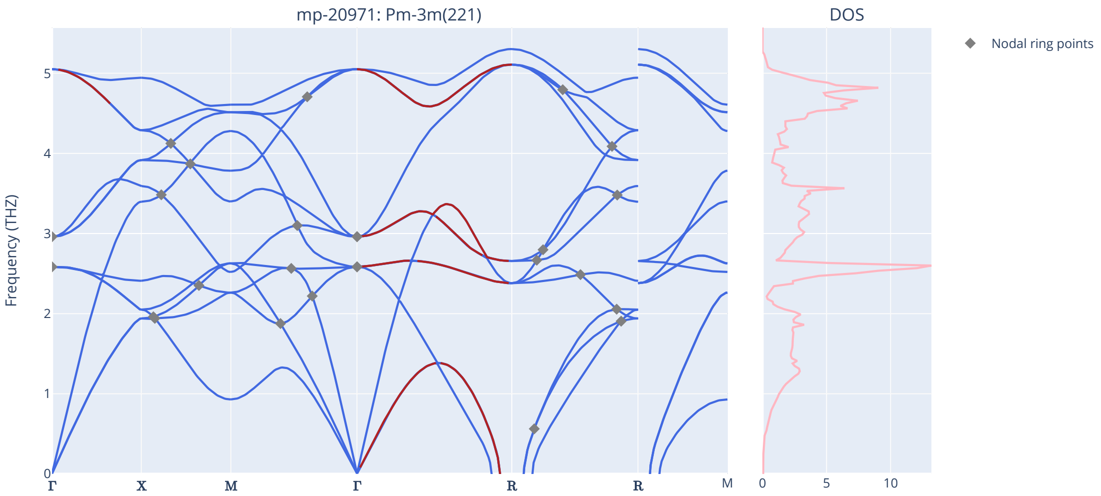

Supplementary Figure 672: The phonon band and DOS of Pt3Sn.

Supplementary Table 1416: High symmetry lines of mp-20971-Pt3Sn

	Start	End
0	Γ (0.000, 0.000, 0.000)	X (0.000, 0.500, 0.000)
1	X (0.000, 0.500, 0.000)	M (0.500, 0.500, 0.000)
2	M (0.500, 0.500, 0.000)	Γ (0.000, 0.000, 0.000)
3	Γ (0.000, 0.000, 0.000)	R (0.500, 0.500, 0.500)
4	R (0.500, 0.500, 0.500)	X (0.000, 0.500, 0.000)
5	R (0.500, 0.500, 0.500)	M (0.500, 0.500, 0.000)

Supplementary Table 1417: Nodal ring points along high symmetry lines of mp-20971-Pt3Sn.

	Coordinates	Frequency	Mode 1	Mode 2	Multi.	Deg.	Path	Ratio
0	(0.075, 0.500, 0.000)	1.940	2	1	12	2	2	0.150
1	(0.067, 0.500, 0.067)	1.905	2	1	12	2	5	0.866
2	(0.067, 0.500, -0.000)	1.960	3	2	12	2	2	0.134
3	(0.303, 0.303, -0.000)	1.876	3	2	12	2	3	0.394
4	(0.411, 0.500, 0.411)	0.561	3	2	12	2	5	0.178
5	(0.322, 0.500, 0.000)	2.352	4	3	12	2	2	0.644
6	(0.177, 0.177, 0.000)	2.219	4	3	12	2	3	0.645
7	(0.085, 0.500, 0.085)	2.055	4	3	12	2	5	0.830
8	(0.260, 0.260, -0.000)	2.564	5	4	12	2	3	0.479
9	(0.228, 0.500, 0.228)	2.486	5	4	12	2	5	0.544
10	(0.000, 0.000, 0.000)	2.584	6	5	1	3	1	0.000
11	(0.083, 0.500, 0.083)	3.480	7	6	12	2	5	0.835
12	(0.111, 0.500, -0.000)	3.484	7	6	12	2	2	0.223
13	(0.237, 0.237, -0.000)	3.100	7	6	12	2	3	0.527

Supplementary Table 1417: Nodal ring points along high symmetry lines of mp-20971-Pt3Sn.

	Coordinates	Frequency	Mode 1	Mode 2	Multi.	Deg.	Path	Ratio
14	(0.401, 0.500, 0.401)	2.667	7	6	12	2	5	0.198
15	(0.274, 0.500, -0.000)	3.869	8	7	12	2	2	0.548
16	(0.376, 0.500, 0.376)	2.798	8	7	12	2	5	0.248
17	(0.000, 0.000, 0.000)	2.960	9	8	1	3	1	0.000
18	(0.104, 0.500, 0.102)	4.089	10	9	24	2	5	0.794
19	(0.165, 0.500, -0.000)	4.125	10	9	12	2	2	0.329
20	(0.197, 0.197, -0.000)	4.707	11	10	12	2	3	0.606
21	(0.298, 0.500, 0.298)	4.796	11	10	12	2	5	0.403

The followings are details for $Au_4Li_8Pb_4$. As shown in Fig 673, it has 7 topological nodal lines and 2 nodal ring points. At the same time, it has 1 single Weyl point and 2 high degenerate Weyl points whose degeneracy are more than 2 and charges are bigger than 1.

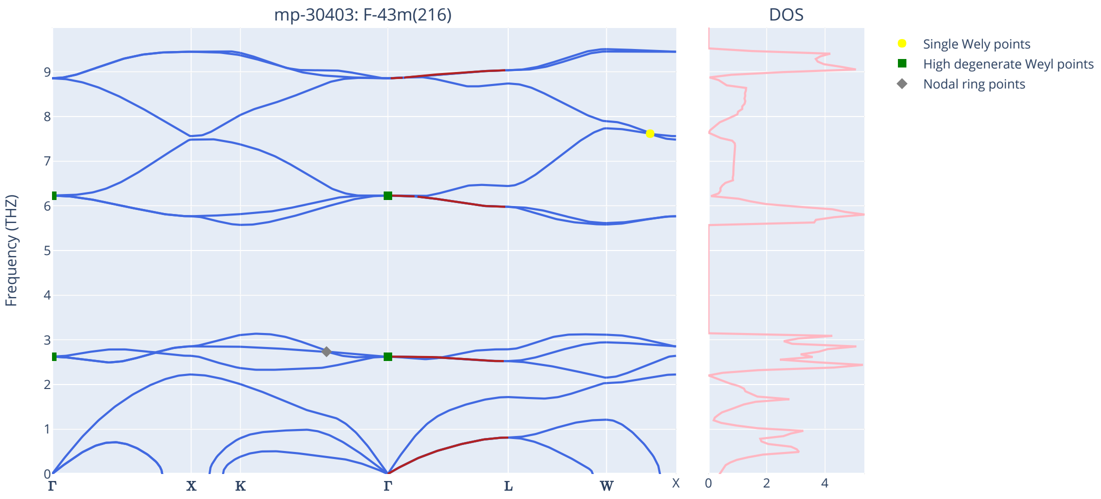

Supplementary Figure 673: The phonon band and DOS of Au4Li8Pb4.

Supplementary Table 1418: High symmetry lines of mp-30403-Au4Li8Pb4

	Start	End
0	Γ (0.000, 0.000, 0.000)	X (0.500, 0.000, 0.500)
1	X (0.500, 0.000, 0.500)	U (0.625, 0.250, 0.625)
2	K (0.375, 0.375, 0.750)	Γ (0.000, 0.000, 0.000)
3	Γ (0.000, 0.000, 0.000)	L (0.500, 0.500, 0.500)
4	L (0.500, 0.500, 0.500)	W (0.500, 0.250, 0.750)
5	W (0.500, 0.250, 0.750)	X (0.500, 0.000, 0.500)

Supplementary Table 1419: Single Weyl points along high symmetry lines of mp-30403-Au4Li8Pb4.

	Coordinates	Frequency	Mode 1	Mode 2	Multi.	Deg.	Path	Ratio
0	(0.500, 0.093, 0.593)	7.617	10	9	12	2	6	0.627

Supplementary Table 1420: High degenerate Weyl points along high symmetry lines of mp-30403-Au4Li8Pb4.

	Coordinates	Frequency	Mode 1	Mode 2	Multi.	Deg.	Path	Ratio
0	(0.000, 0.000, 0.000)	2.624	6	4	1	3	1	0.000
1	(0.000, 0.000, 0.000)	6.231	9	7	1	3	1	0.000

Supplementary Table 1421: Nodal ring points along high symmetry lines of mp-30403-Au4Li8Pb4.

	Coordinates	Frequency	Mode 1	Mode 2	Multi.	Deg.	Path	Ratio
0	(0.161, 0.161, 0.307)	2.735	6	5	24	2	3	0.585

The followings are details for Fe_2S_2. It has 24 topological nodal lines and 32 nodal ring points along high symmetry line as shown in Fig **674**.

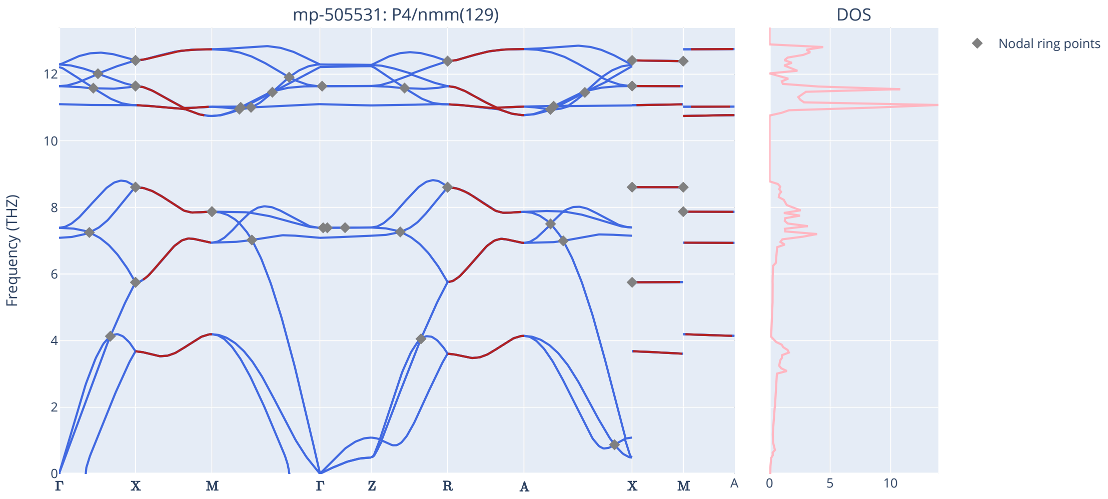

Supplementary Figure 674: The phonon band and DOS of Fe2S2.

Supplementary Table 1422: High symmetry lines of mp-505531-Fe2S2

	Start	End
0	Γ (0.000, 0.000, 0.000)	X (0.000, 0.500, 0.000)
1	X (0.000, 0.500, 0.000)	M (0.500, 0.500, 0.000)
2	M (0.500, 0.500, 0.000)	Γ (0.000, 0.000, 0.000)
3	Γ (0.000, 0.000, 0.000)	Z (0.000, 0.000, 0.500)
4	Z (0.000, 0.000, 0.500)	R (0.000, 0.500, 0.500)
5	R (0.000, 0.500, 0.500)	A (0.500, 0.500, 0.500)
6	A (0.500, 0.500, 0.500)	Z (0.000, 0.000, 0.500)
7	X (0.000, 0.500, 0.000)	R (0.000, 0.500, 0.500)
8	M (0.500, 0.500, 0.000)	A (0.500, 0.500, 0.500)

Supplementary Table 1423: Nodal ring points along high symmetry lines of mp-505531-Fe2S2.

	Coordinates	Frequency	Mode 1	Mode 2	Multi.	Deg.	Path	Ratio
0	(0.081, 0.081, 0.500)	0.872	2	1	4	2	7	0.837
1	(-0.000, 0.335, 0.000)	4.130	3	2	4	2	1	0.671
2	(-0.000, 0.326, 0.500)	4.051	3	2	4	2	5	0.651
3	(0.000, 0.500, 0.000)	5.750	4	3	2	2	1	1.000
4	(0.315, 0.315, -0.000)	7.022	4	3	4	2	3	0.369
5	(0.319, 0.319, 0.500)	6.996	4	3	4	2	7	0.363
6	(-0.000, 0.198, 0.000)	7.248	5	4	4	2	1	0.396
7	(0.000, 0.190, 0.500)	7.264	5	4	4	2	5	0.381
8	(0.377, 0.377, 0.500)	7.505	5	4	4	2	7	0.245
9	(0.000, 0.500, 0.000)	8.609	6	5	2	2	1	1.000
10	(0.500, 0.500, 0.000)	7.873	6	5	1	2	2	1.000

Supplementary Table 1423: Nodal ring points along high symmetry lines of mp-505531-Fe2S2.

	Coordinates	Frequency	Mode 1	Mode 2	Multi.	Deg.	Path	Ratio
11	(0.000, 0.000, 0.031)	7.388	6	5	2	2	4	0.061
12	(0.000, 0.000, 0.071)	7.389	6	5	2	2	4	0.143
13	(0.000, 0.000, 0.245)	7.392	6	5	2	2	4	0.490
14	(0.000, 0.500, 0.500)	8.607	6	5	2	2	5	1.000
15	(0.373, 0.373, 0.000)	10.952	8	7	4	2	3	0.254
16	(0.319, 0.319, -0.000)	11.006	8	7	4	2	3	0.362
17	(0.378, 0.378, 0.500)	10.943	8	7	4	2	7	0.244
18	(-0.000, 0.224, 0.000)	11.583	9	8	4	2	1	0.448
19	(0.220, 0.220, -0.000)	11.460	9	8	4	2	3	0.559
20	(0.000, 0.000, 0.020)	11.642	9	8	2	2	4	0.041
21	(0.000, 0.218, 0.500)	11.586	9	8	4	2	5	0.437
22	(0.219, 0.219, 0.500)	11.455	9	8	4	2	7	0.563
23	(0.000, 0.500, 0.000)	11.647	10	9	2	2	1	1.000
24	(0.367, 0.367, 0.000)	11.007	10	9	4	2	3	0.266
25	(0.363, 0.363, 0.500)	11.040	10	9	4	2	7	0.274
26	(0.000, 0.254, 0.000)	12.017	11	10	4	2	1	0.509
27	(0.143, 0.143, 0.000)	11.912	11	10	4	2	3	0.714
28	(0.000, 0.500, 0.000)	12.416	12	11	2	2	1	1.000
29	(0.000, 0.500, 0.500)	12.395	12	11	2	2	5	1.000

The followings are details for $CaTl_3$. It has 7 topological nodal lines and 18 nodal ring points along high symmetry line as shown in Fig **675**.

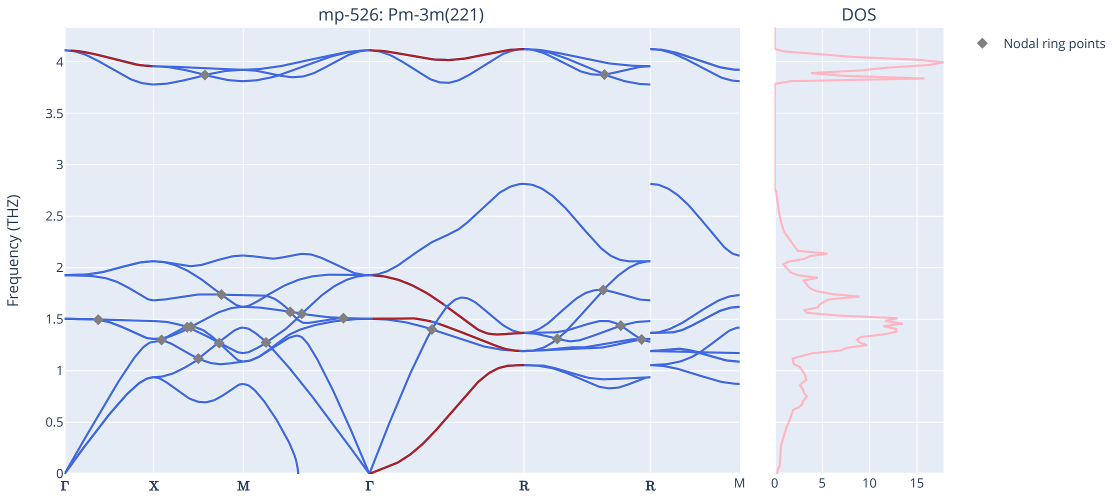

Supplementary Figure 675: The phonon band and DOS of CaTl3.

Supplementary Table 1424: High symmetry lines of mp-526-CaTl3

	Start	End
0	Γ (0.000, 0.000, 0.000)	X (0.000, 0.500, 0.000)
1	X (0.000, 0.500, 0.000)	M (0.500, 0.500, 0.000)
2	M (0.500, 0.500, 0.000)	Γ (0.000, 0.000, 0.000)
3	Γ (0.000, 0.000, 0.000)	R (0.500, 0.500, 0.500)
4	R (0.500, 0.500, 0.500)	X (0.000, 0.500, 0.000)
5	R (0.500, 0.500, 0.500)	M (0.500, 0.500, 0.000)

Supplementary Table 1425: Nodal ring points along high symmetry lines of mp-526-CaTl3.

	Coordinates	Frequency	Mode 1	Mode 2	Multi.	Deg.	Path	Ratio
0	(0.249, 0.500, 0.000)	1.118	3	2	12	2	2	0.499
1	(0.043, 0.500, -0.000)	1.299	4	3	12	2	2	0.086
2	(0.366, 0.500, -0.000)	1.269	4	3	12	2	2	0.732
3	(0.202, 0.202, 0.202)	1.401	4	3	8	2	4	0.404
4	(0.035, 0.500, 0.035)	1.304	4	3	12	2	5	0.931
5	(0.000, 0.189, -0.000)	1.496	5	4	6	2	1	0.377
6	(0.409, 0.409, -0.000)	1.275	5	4	12	2	3	0.181
7	(0.187, 0.500, 0.000)	1.422	6	5	12	2	2	0.374
8	(0.208, 0.500, 0.000)	1.426	6	5	12	2	2	0.416
9	(0.103, 0.103, 0.000)	1.510	6	5	12	2	3	0.794
10	(0.117, 0.500, 0.117)	1.438	6	5	12	2	5	0.766
11	(0.368, 0.500, 0.368)	1.309	6	5	12	2	5	0.263
12	(0.268, 0.268, 0.000)	1.553	7	6	12	2	3	0.464
13	(0.314, 0.314, -0.000)	1.571	7	6	12	2	3	0.373

Supplementary Table 1425: Nodal ring points along high symmetry lines of mp-526-CaTl3.

	Coordinates	Frequency	Mode 1	Mode 2	Multi.	Deg.	Path	Ratio
14	(0.379, 0.500, 0.000)	1.740	8	7	12	2	2	0.757
15	(0.187, 0.500, 0.187)	1.784	8	7	12	2	5	0.627
16	(0.286, 0.500, -0.000)	3.871	11	10	12	2	2	0.572
17	(0.182, 0.500, 0.182)	3.876	11	10	12	2	5	0.637

The followings are details for Hg$_3$. It has 7 topological nodal lines and 22 nodal ring points along high symmetry line as shown in Fig **676**.

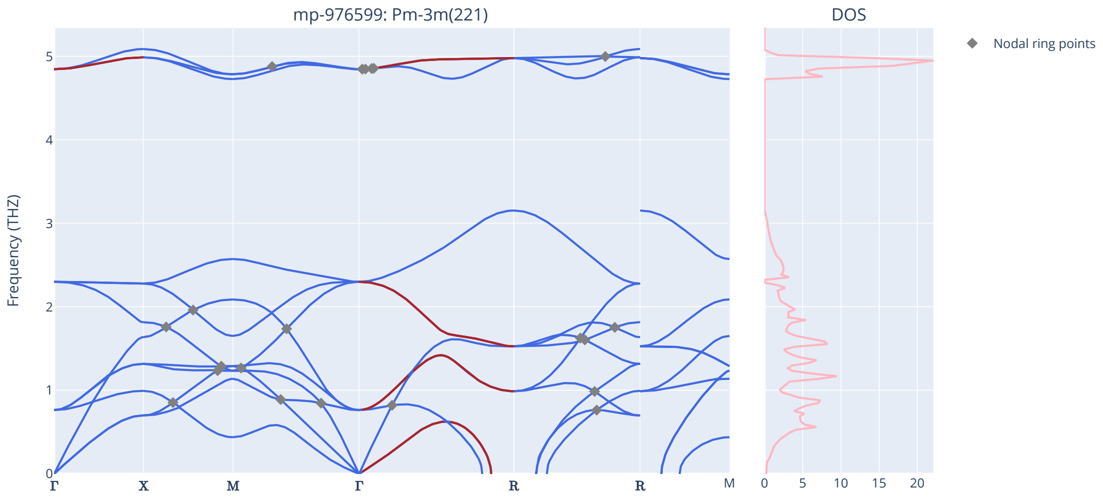

Supplementary Figure 676: The phonon band and DOS of Hg3Li.

Supplementary Table 1426: High symmetry lines of mp-976599-Hg3Li

	Start	End
0	Γ (0.000, 0.000, 0.000)	X (0.000, 0.500, 0.000)
1	X (0.000, 0.500, 0.000)	M (0.500, 0.500, 0.000)
2	M (0.500, 0.500, 0.000)	Γ (0.000, 0.000, 0.000)
3	Γ (0.000, 0.000, 0.000)	R (0.500, 0.500, 0.500)
4	R (0.500, 0.500, 0.500)	X (0.000, 0.500, 0.000)
5	R (0.500, 0.500, 0.500)	M (0.500, 0.500, 0.000)

Supplementary Table 1427: Nodal ring points along high symmetry lines of mp-976599-Hg3Li.

	Coordinates	Frequency	Mode 1	Mode 2	Multi.	Deg.	Path	Ratio
0	(0.172, 0.500, 0.172)	0.762	2	1	12	2	5	0.656
1	(0.166, 0.500, 0.000)	0.852	3	2	12	2	2	0.331
2	(0.310, 0.310, 0.000)	0.889	3	2	12	2	3	0.380
3	(0.175, 0.500, 0.185)	0.986	4	3	24	2	5	0.640
4	(0.415, 0.500, -0.000)	1.235	4	3	12	2	2	0.831
5	(0.150, 0.150, -0.000)	0.845	4	3	12	2	3	0.699
6	(0.107, 0.107, 0.107)	0.820	4	3	8	2	4	0.214
7	(0.467, 0.467, -0.000)	1.267	5	4	12	2	3	0.066
8	(0.436, 0.500, -0.000)	1.289	6	5	12	2	2	0.872
9	(0.219, 0.500, 0.219)	1.604	6	5	12	2	5	0.562
10	(0.102, 0.500, 0.098)	1.754	7	6	24	2	5	0.800
11	(0.127, 0.500, -0.000)	1.759	7	6	12	2	2	0.255
12	(0.287, 0.287, 0.000)	1.736	7	6	12	2	3	0.426
13	(0.236, 0.500, 0.236)	1.625	7	6	12	2	5	0.527

Supplementary Table 1427: Nodal ring points along high symmetry lines of mp-976599-Hg3Li.

	Coordinates	Frequency	Mode 1	Mode 2	Multi.	Deg.	Path	Ratio
14	(0.278, 0.500, -0.000)	1.961	8	7	12	2	2	0.556
15	(0.010, 0.010, 0.010)	4.847	11	10	8	3	4	0.020
16	(0.020, 0.020, 0.020)	4.849	11	10	8	2	4	0.041
17	(0.041, 0.041, 0.041)	4.855	11	10	8	2	4	0.082
18	(0.048, 0.048, 0.048)	4.857	11	10	8	3	4	0.095
19	(0.344, 0.344, 0.000)	4.879	12	11	12	2	3	0.312
20	(0.138, 0.500, 0.138)	4.999	12	11	12	2	5	0.725

The followings are details for Bi_4Na_{12}. It has 5 topological nodal lines and 2 nodal ring points along high symmetry line as shown in Fig **677**.

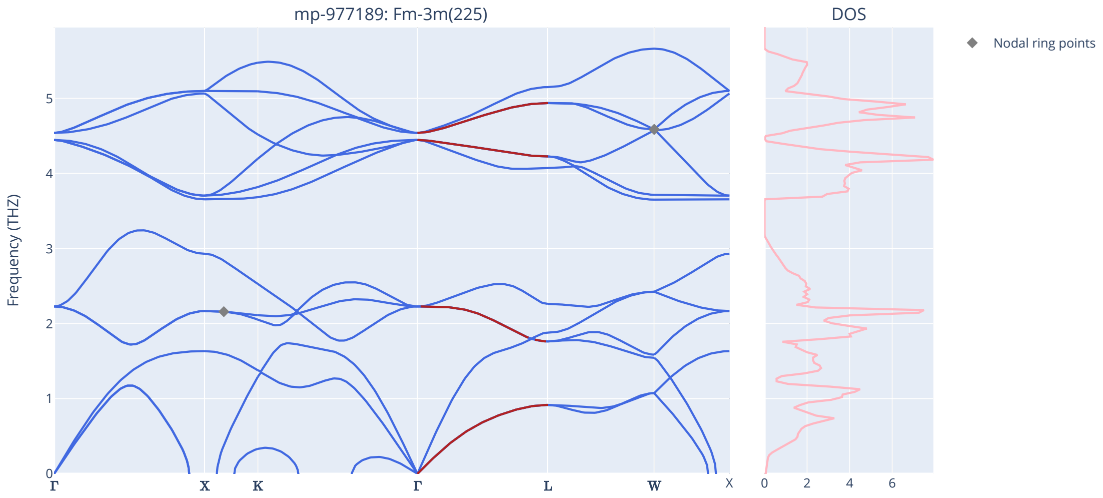

Supplementary Figure 677: The phonon band and DOS of Bi4Na12.

Supplementary Table 1428: High symmetry lines of mp-977189-Bi4Na12

	Start	End
0	Γ (0.000, 0.000, 0.000)	X (0.500, 0.000, 0.500)
1	X (0.500, 0.000, 0.500)	U (0.625, 0.250, 0.625)
2	K (0.375, 0.375, 0.750)	Γ (0.000, 0.000, 0.000)
3	Γ (0.000, 0.000, 0.000)	L (0.500, 0.500, 0.500)
4	L (0.500, 0.500, 0.500)	W (0.500, 0.250, 0.750)
5	W (0.500, 0.250, 0.750)	X (0.500, 0.000, 0.500)

Supplementary Table 1429: Nodal ring points along high symmetry lines of mp-977189-Bi4Na12.

	Coordinates	Frequency	Mode 1	Mode 2	Multi.	Deg.	Path	Ratio
0	(0.546, 0.090, 0.546)	2.159	5	4	24	2	2	0.362
1	(0.500, 0.250, 0.750)	4.587	11	10	6	2	5	1.000

The followings are details for $Na_{12}Tl_4$. It has 5 topological nodal lines and 7 nodal ring points along high symmetry line as shown in Fig **678**.

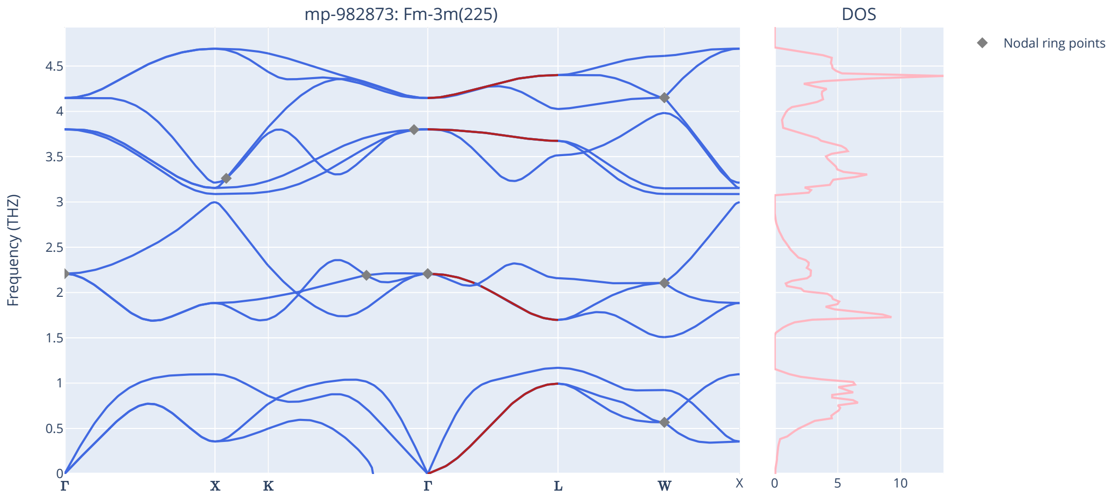

Supplementary Figure 678: The phonon band and DOS of Na12Tl4.

Supplementary Table 1430: High symmetry lines of mp-982873-Na12Tl4

	Start	End
0	Γ (0.000, 0.000, 0.000)	X (0.500, 0.000, 0.500)
1	X (0.500, 0.000, 0.500)	U (0.625, 0.250, 0.625)
2	K (0.375, 0.375, 0.750)	Γ (0.000, 0.000, 0.000)
3	Γ (0.000, 0.000, 0.000)	L (0.500, 0.500, 0.500)
4	L (0.500, 0.500, 0.500)	W (0.500, 0.250, 0.750)
5	W (0.500, 0.250, 0.750)	X (0.500, 0.000, 0.500)

Supplementary Table 1431: Nodal ring points along high symmetry lines of mp-982873-Na12Tl4.

	Coordinates	Frequency	Mode 1	Mode 2	Multi.	Deg.	Path	Ratio
0	(0.500, 0.250, 0.750)	0.567	2	1	6	2	5	1.000
1	(0.000, 0.000, 0.000)	2.208	6	5	1	3	1	0.000
2	(0.142, 0.142, 0.290)	2.190	6	5	24	2	3	0.616
3	(0.500, 0.250, 0.750)	2.105	6	5	6	2	5	1.000
4	(0.031, 0.031, 0.066)	3.798	9	8	24	2	3	0.914
5	(0.527, 0.053, 0.527)	3.261	10	9	12	2	2	0.212
6	(0.500, 0.250, 0.750)	4.151	11	10	6	2	5	1.000

The followings are details for Bi_2Ni_2. It has 24 topological nodal lines and 21 nodal ring points along high symmetry line as shown in Fig 679.

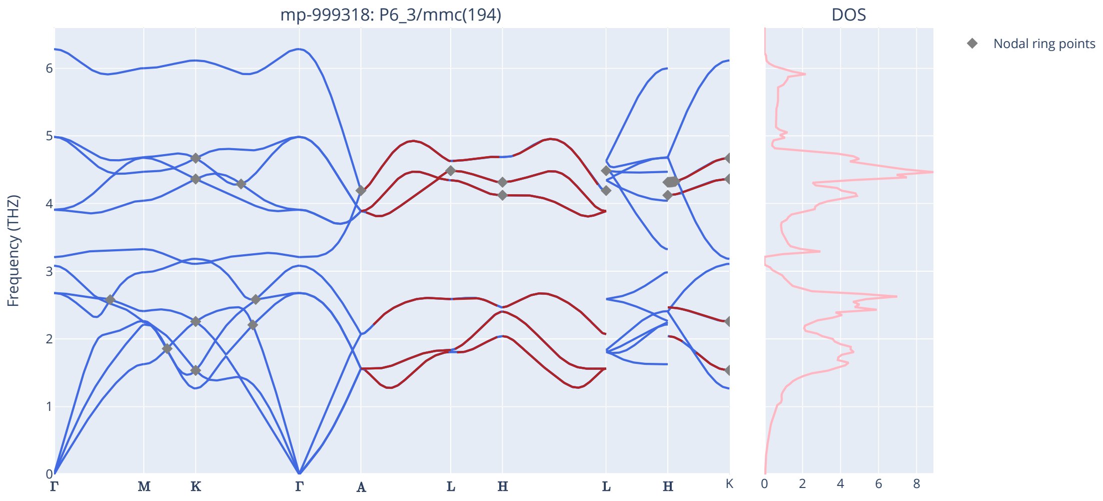

Supplementary Figure 679: The phonon band and DOS of Bi2Ni2.

Supplementary Table 1432: High symmetry lines of mp-999318-Bi2Ni2

	Start	End
0	Γ (0.000, 0.000, 0.000)	M (0.500, 0.000, 0.000)
1	M (0.500, 0.000, 0.000)	K (0.333, 0.333, 0.000)
2	K (0.333, 0.333, 0.000)	Γ (0.000, 0.000, 0.000)
3	Γ (0.000, 0.000, 0.000)	A (0.000, 0.000, 0.500)
4	A (0.000, 0.000, 0.500)	L (0.500, 0.000, 0.500)
5	L (0.500, 0.000, 0.500)	H (0.333, 0.333, 0.500)
6	H (0.333, 0.333, 0.500)	A (0.000, 0.000, 0.500)
7	L (0.500, 0.000, 0.500)	M (0.500, 0.000, 0.000)
8	H (0.333, 0.333, 0.500)	K (0.333, 0.333, 0.000)

Supplementary Table 1433: Nodal ring points along high symmetry lines of mp-999318-Bi2Ni2.

	Coordinates	Frequency	Mode 1	Mode 2	Multi.	Deg.	Path	Ratio
0	(0.425, 0.150, 0.000)	1.856	3	2	6	2	2	0.450
1	(0.333, 0.333, 0.000)	1.534	3	2	2	2	2	1.000
2	(0.150, 0.150, 0.000)	2.207	4	3	6	2	3	0.551
3	(0.333, 0.333, 0.000)	2.257	5	4	2	2	2	1.000
4	(0.312, 0.000, 0.000)	2.580	6	5	6	2	1	0.625
5	(0.140, 0.140, -0.000)	2.583	6	5	6	2	3	0.579
6	(0.333, 0.333, 0.500)	4.123	8	7	2	2	6	1.000
7	(0.333, 0.333, 0.000)	4.363	9	8	2	2	2	1.000
8	(0.188, 0.187, 0.000)	4.289	10	9	12	2	3	0.438
9	(0.500, 0.000, 0.500)	4.486	10	9	3	2	5	1.000
10	(0.333, 0.333, 0.500)	4.315	10	9	2	2	6	1.000

Supplementary Table 1433: Nodal ring points along high symmetry lines of mp-999318-Bi2Ni2.

	Coordinates	Frequency	Mode 1	Mode 2	Multi.	Deg.	Path	Ratio
11	(0.333, 0.333, 0.490)	4.316	10	9	4	2	9	0.020
12	(0.333, 0.333, 0.480)	4.317	10	9	4	2	9	0.041
13	(0.333, 0.333, 0.469)	4.319	10	9	4	2	9	0.061
14	(0.333, 0.333, 0.459)	4.321	10	9	4	2	9	0.082
15	(0.333, 0.333, 0.449)	4.324	10	9	4	2	9	0.102
16	(0.333, 0.333, 0.439)	4.328	10	9	4	2	9	0.122
17	(0.333, 0.333, 0.000)	4.671	11	10	2	2	2	1.000
18	(0.000, 0.000, 0.500)	4.192	12	11	1	2	4	1.000

The followings are details for $NpRh_3$. It has 8 topological nodal lines and 34 nodal ring points along high symmetry line as shown in Fig **680**.

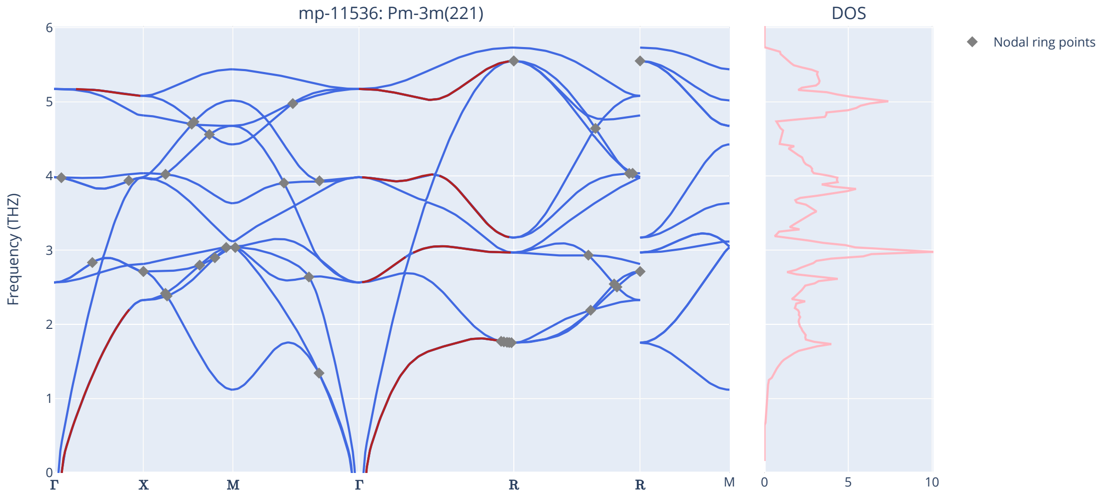

Supplementary Figure 680: The phonon band and DOS of NpRh3.

Supplementary Table 1434: High symmetry lines of mp-11536-NpRh3

	Start	End
0	Γ (0.000, 0.000, 0.000)	X (0.000, 0.500, 0.000)
1	X (0.000, 0.500, 0.000)	M (0.500, 0.500, 0.000)
2	M (0.500, 0.500, 0.000)	Γ (0.000, 0.000, 0.000)
3	Γ (0.000, 0.000, 0.000)	R (0.500, 0.500, 0.500)
4	R (0.500, 0.500, 0.500)	X (0.000, 0.500, 0.000)
5	R (0.500, 0.500, 0.500)	M (0.500, 0.500, 0.000)

Supplementary Table 1435: Nodal ring points along high symmetry lines of mp-11536-NpRh3.

	Coordinates	Frequency	Mode 1	Mode 2	Multi.	Deg.	Path	Ratio
0	(0.133, 0.500, -0.000)	2.382	2	1	12	2	2	0.265
1	(0.158, 0.158, -0.000)	1.343	2	1	12	2	3	0.683
2	(0.400, 0.500, 0.001)	2.898	3	2	24	2	2	0.800
3	(0.123, 0.500, -0.000)	2.417	3	2	12	2	2	0.247
4	(0.490, 0.490, -0.000)	3.035	3	2	12	2	3	0.020
5	(0.493, 0.493, 0.493)	1.753	3	2	8	2	4	0.985
6	(0.459, 0.459, 0.459)	1.770	3	2	8	2	4	0.918
7	(0.468, 0.468, 0.468)	1.764	3	2	8	2	4	0.936
8	(0.478, 0.478, 0.478)	1.758	3	2	8	2	4	0.956
9	(0.485, 0.485, 0.485)	1.755	3	2	8	2	4	0.970
10	(0.196, 0.500, 0.196)	2.190	3	2	12	2	5	0.609
11	(0.092, 0.500, 0.092)	2.503	3	2	12	2	5	0.817
12	(0.000, 0.500, 0.000)	2.712	4	3	3	2	1	1.000
13	(0.315, 0.500, 0.000)	2.798	4	3	12	2	2	0.630

Supplementary Table 1435: Nodal ring points along high symmetry lines of mp-11536-NpRh3.

	Coordinates	Frequency	Mode 1	Mode 2	Multi.	Deg.	Path	Ratio
14	(0.198, 0.198, -0.000)	2.638	4	3	12	2	3	0.603
15	(0.103, 0.500, 0.103)	2.544	4	3	12	2	5	0.795
16	(0.464, 0.500, 0.001)	3.036	5	4	24	2	2	0.927
17	(0.205, 0.500, 0.205)	2.932	5	4	12	2	5	0.590
18	(0.000, 0.214, 0.000)	2.833	6	5	6	2	1	0.429
19	(0.000, 0.041, 0.000)	3.976	8	7	6	2	1	0.082
20	(0.000, 0.418, 0.000)	3.938	8	7	6	2	1	0.837
21	(0.124, 0.500, -0.000)	4.023	8	7	12	2	2	0.247
22	(0.297, 0.297, -0.000)	3.905	8	7	12	2	3	0.406
23	(0.043, 0.500, 0.043)	4.034	8	7	12	2	5	0.914
24	(0.030, 0.500, 0.029)	4.035	9	8	12	2	5	0.941
25	(0.369, 0.500, -0.000)	4.559	9	8	12	2	2	0.739
26	(0.157, 0.157, 0.000)	3.934	9	8	12	2	3	0.686
27	(0.270, 0.500, -0.000)	4.699	10	9	12	2	2	0.541
28	(0.500, 0.500, 0.500)	5.551	10	9	1	3	4	1.000
29	(0.177, 0.500, 0.177)	4.641	10	9	12	2	5	0.646
30	(0.283, 0.500, -0.000)	4.730	11	10	12	2	2	0.566
31	(0.262, 0.262, -0.000)	4.976	11	10	12	2	3	0.476

The followings are details for Pt_3. It has 7 topological nodal lines and 31 nodal ring points along high symmetry line as shown in Fig **681**.

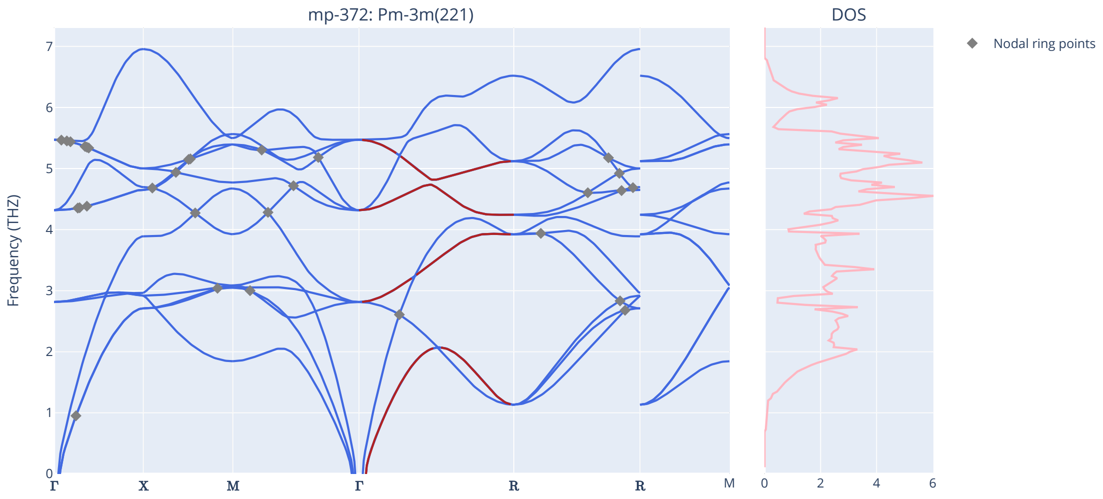

Supplementary Figure 681: The phonon band and DOS of Pt3V.

Supplementary Table 1436: High symmetry lines of mp-372-Pt3V

	Start	End
0	Γ (0.000, 0.000, 0.000)	X (0.000, 0.500, 0.000)
1	X (0.000, 0.500, 0.000)	M (0.500, 0.500, 0.000)
2	M (0.500, 0.500, 0.000)	Γ (0.000, 0.000, 0.000)
3	Γ (0.000, 0.000, 0.000)	R (0.500, 0.500, 0.500)
4	R (0.500, 0.500, 0.500)	X (0.000, 0.500, 0.000)
5	R (0.500, 0.500, 0.500)	M (0.500, 0.500, 0.000)

Supplementary Table 1437: Nodal ring points along high symmetry lines of mp-372-Pt3V.

	Coordinates	Frequency	Mode 1	Mode 2	Multi.	Deg.	Path	Ratio
0	(0.000, 0.122, 0.000)	0.949	2	1	6	2	1	0.245
1	(0.060, 0.500, 0.060)	2.677	2	1	12	2	5	0.881
2	(0.431, 0.431, 0.000)	2.999	3	2	12	2	3	0.137
3	(0.415, 0.500, -0.000)	3.038	4	3	12	2	2	0.830
4	(0.130, 0.130, 0.130)	2.606	4	3	8	2	4	0.260
5	(0.079, 0.500, 0.079)	2.831	4	3	12	2	5	0.842
6	(0.393, 0.500, 0.393)	3.937	5	4	12	2	5	0.214
7	(0.290, 0.500, 0.000)	4.272	7	6	12	2	2	0.580
8	(0.360, 0.360, 0.000)	4.282	7	6	12	2	3	0.279
9	(0.000, 0.133, 0.000)	4.352	8	7	6	2	1	0.265
10	(0.000, 0.143, 0.000)	4.358	8	7	6	2	1	0.286
11	(0.000, 0.184, 0.000)	4.383	8	7	6	2	1	0.367
12	(0.260, 0.260, 0.000)	4.717	8	7	12	2	3	0.481
13	(0.073, 0.500, 0.073)	4.638	8	7	12	2	5	0.853

Supplementary Table 1437: Nodal ring points along high symmetry lines of mp-372-Pt3V.

	Coordinates	Frequency	Mode 1	Mode 2	Multi.	Deg.	Path	Ratio
14	(0.028, 0.500, 0.028)	4.686	9	8	12	2	5	0.943
15	(0.050, 0.500, -0.000)	4.681	9	8	12	2	2	0.100
16	(0.182, 0.500, 0.000)	4.937	9	8	12	2	2	0.364
17	(0.207, 0.500, 0.207)	4.603	9	8	12	2	5	0.586
18	(0.263, 0.500, 0.003)	5.158	10	9	24	2	2	0.526
19	(0.385, 0.385, 0.000)	5.303	10	9	12	2	3	0.229
20	(0.161, 0.161, 0.000)	5.182	10	9	12	2	3	0.677
21	(0.082, 0.500, 0.082)	4.921	10	9	12	2	5	0.836
22	(0.000, 0.041, 0.000)	5.465	11	10	6	2	1	0.082
23	(0.000, 0.071, 0.000)	5.452	11	10	6	2	1	0.143
24	(0.000, 0.092, 0.000)	5.439	11	10	6	2	1	0.184
25	(0.000, 0.173, 0.000)	5.361	11	10	6	2	1	0.347
26	(0.000, 0.184, 0.000)	5.349	11	10	6	2	1	0.367
27	(0.000, 0.194, 0.000)	5.337	11	10	6	2	1	0.388
28	(0.253, 0.500, -0.000)	5.149	11	10	12	2	2	0.506
29	(0.125, 0.500, 0.125)	5.176	11	10	12	2	5	0.750

The followings are details for Co_2Se_2. It has 24 topological nodal lines and 21 nodal ring points along high symmetry line as shown in Fig **682**.

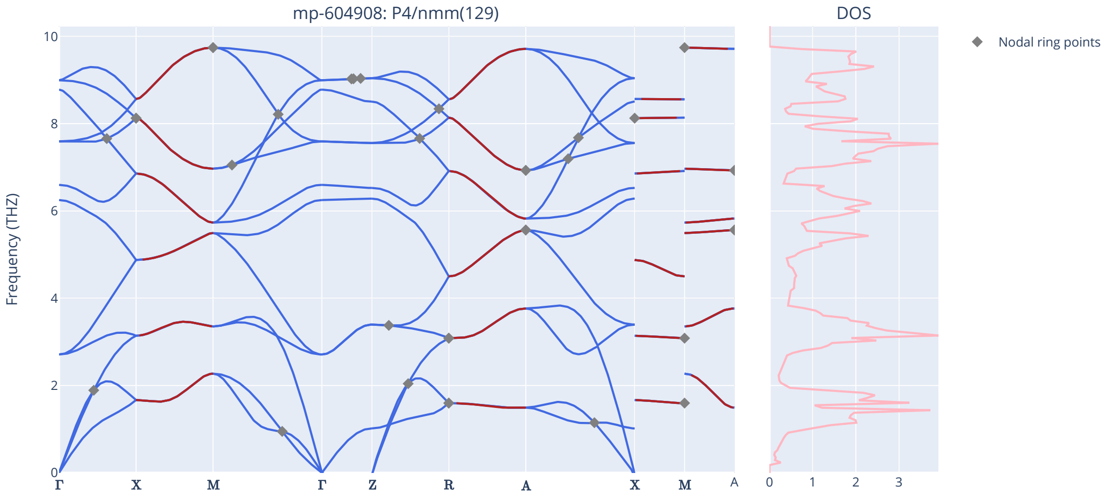

Supplementary Figure 682: The phonon band and DOS of Co2Se2.

Supplementary Table 1438: High symmetry lines of mp-604908-Co2Se2

	Start	End
0	Γ (0.000, 0.000, 0.000)	X (0.000, 0.500, 0.000)
1	X (0.000, 0.500, 0.000)	M (0.500, 0.500, 0.000)
2	M (0.500, 0.500, 0.000)	Γ (0.000, 0.000, 0.000)
3	Γ (0.000, 0.000, 0.000)	Z (0.000, 0.000, 0.500)
4	Z (0.000, 0.000, 0.500)	R (0.000, 0.500, 0.500)
5	R (0.000, 0.500, 0.500)	A (0.500, 0.500, 0.500)
6	A (0.500, 0.500, 0.500)	Z (0.000, 0.000, 0.500)
7	X (0.000, 0.500, 0.000)	R (0.000, 0.500, 0.500)
8	M (0.500, 0.500, 0.000)	A (0.500, 0.500, 0.500)

Supplementary Table 1439: Nodal ring points along high symmetry lines of mp-604908-Co2Se2.

	Coordinates	Frequency	Mode 1	Mode 2	Multi.	Deg.	Path	Ratio
0	(0.182, 0.182, -0.000)	0.947	2	1	4	2	3	0.635
1	(0.000, 0.500, 0.500)	1.596	2	1	2	2	5	1.000
2	(0.185, 0.185, 0.500)	1.146	2	1	4	2	7	0.630
3	(-0.000, 0.225, -0.000)	1.889	3	2	4	2	1	0.450
4	(0.000, 0.236, 0.500)	2.041	3	2	4	2	5	0.472
5	(0.000, 0.500, 0.500)	3.083	4	3	2	2	5	1.000
6	(0.000, 0.110, 0.500)	3.377	5	4	4	2	5	0.221
7	(0.500, 0.500, 0.500)	5.563	6	5	1	2	6	1.000
8	(-0.000, 0.310, -0.000)	7.658	9	8	4	2	1	0.619
9	(-0.000, 0.311, 0.500)	7.661	9	8	4	2	5	0.621
10	(0.305, 0.305, 0.500)	7.196	9	8	4	2	7	0.390

Supplementary Table 1439: Nodal ring points along high symmetry lines of mp-604908-Co2Se2.

	Coordinates	Frequency	Mode 1	Mode 2	Multi.	Deg.	Path	Ratio
11	(0.000, 0.500, 0.000)	8.126	10	9	2	2	1	1.000
12	(0.413, 0.413, 0.000)	7.052	10	9	4	2	3	0.174
13	(0.500, 0.500, 0.500)	6.929	10	9	1	2	6	1.000
14	(0.258, 0.258, 0.500)	7.679	10	9	4	2	7	0.483
15	(0.201, 0.201, 0.000)	8.214	11	10	4	2	3	0.598
16	(0.000, 0.436, 0.500)	8.340	11	10	4	2	5	0.872
17	(0.500, 0.500, 0.000)	9.743	12	11	1	2	2	1.000
18	(0.000, 0.000, 0.296)	9.025	12	11	2	2	4	0.592
19	(0.000, 0.000, 0.316)	9.028	12	11	2	2	4	0.633
20	(0.000, 0.000, 0.388)	9.037	12	11	2	2	4	0.776

The followings are details for LuPt$_3$. It has 8 topological nodal lines and 21 nodal ring points along high symmetry line as shown in Fig 683.

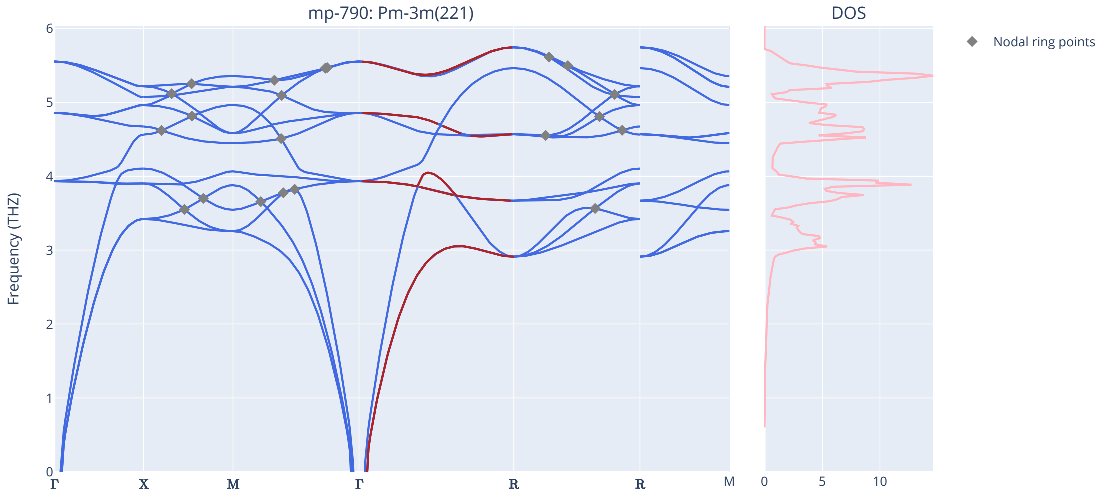

Supplementary Figure 683: The phonon band and DOS of LuPt3.

Supplementary Table 1440: High symmetry lines of mp-790-LuPt3

	Start	End
0	Γ (0.000, 0.000, 0.000)	X (0.000, 0.500, 0.000)
1	X (0.000, 0.500, 0.000)	M (0.500, 0.500, 0.000)
2	M (0.500, 0.500, 0.000)	Γ (0.000, 0.000, 0.000)
3	Γ (0.000, 0.000, 0.000)	R (0.500, 0.500, 0.500)
4	R (0.500, 0.500, 0.500)	X (0.000, 0.500, 0.000)
5	R (0.500, 0.500, 0.500)	M (0.500, 0.500, 0.000)

Supplementary Table 1441: Nodal ring points along high symmetry lines of mp-790-LuPt3.

	Coordinates	Frequency	Mode 1	Mode 2	Multi.	Deg.	Path	Ratio
0	(0.228, 0.499, 0.000)	3.551	3	2	24	2	2	0.456
1	(0.178, 0.500, 0.178)	3.564	3	2	12	2	5	0.644
2	(0.334, 0.500, 0.000)	3.700	4	3	12	2	2	0.668
3	(0.300, 0.300, 0.000)	3.775	4	3	12	2	3	0.400
4	(0.389, 0.389, -0.000)	3.656	4	3	12	2	3	0.221
5	(0.255, 0.255, -0.000)	3.823	4	3	12	2	3	0.490
6	(0.073, 0.500, 0.069)	4.621	7	6	24	2	5	0.858
7	(0.101, 0.500, -0.000)	4.619	7	6	12	2	2	0.202
8	(0.309, 0.309, -0.000)	4.509	7	6	12	2	3	0.382
9	(0.271, 0.500, -0.000)	4.812	8	7	12	2	2	0.542
10	(0.374, 0.500, 0.374)	4.552	8	7	12	2	5	0.253
11	(0.161, 0.500, 0.161)	4.805	8	7	12	2	5	0.679
12	(0.307, 0.307, 0.000)	5.093	10	9	12	2	3	0.387
13	(0.157, 0.500, 0.000)	5.115	11	10	12	2	2	0.314

Supplementary Table 1441: Nodal ring points along high symmetry lines of mp-790-LuPt3.

	Coordinates	Frequency	Mode 1	Mode 2	Multi.	Deg.	Path	Ratio
14	(0.127, 0.127, -0.000)	5.469	11	10	12	2	3	0.747
15	(0.361, 0.500, 0.361)	5.611	11	10	12	2	5	0.279
16	(0.101, 0.500, 0.101)	5.107	11	10	12	2	5	0.798
17	(0.269, 0.500, 0.000)	5.250	12	11	12	2	2	0.537
18	(0.336, 0.336, 0.000)	5.299	12	11	12	2	3	0.329
19	(0.134, 0.134, 0.000)	5.462	12	11	12	2	3	0.732
20	(0.286, 0.500, 0.286)	5.496	12	11	12	2	5	0.428

The followings are details for Li3. It has 8 topological nodal lines and 25 nodal ring points along high symmetry line as shown in Fig 684.

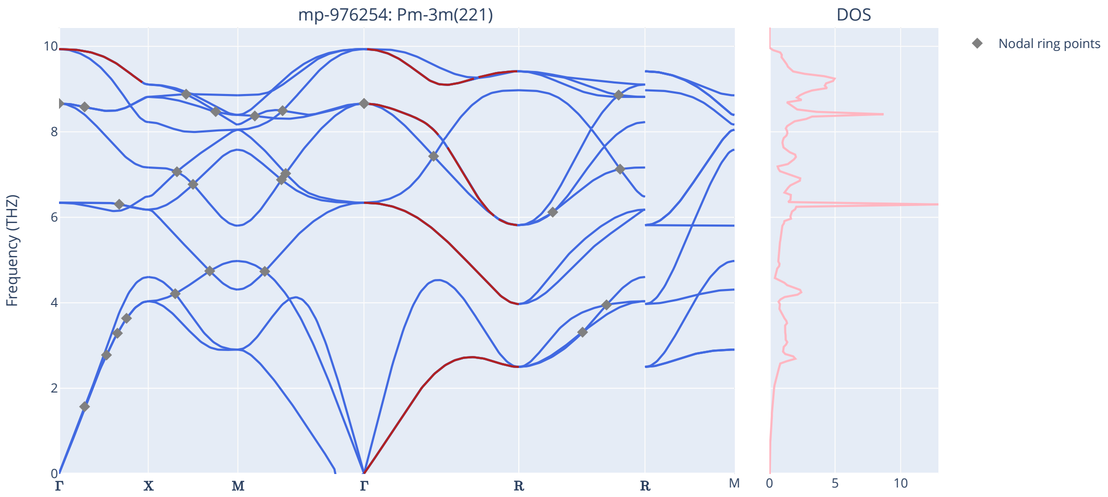

Supplementary Figure 684: The phonon band and DOS of Li3Mg.

Supplementary Table 1442: High symmetry lines of mp-976254-Li3Mg

	Start	End
0	Γ (0.000, 0.000, 0.000)	X (0.000, 0.500, 0.000)
1	X (0.000, 0.500, 0.000)	M (0.500, 0.500, 0.000)
2	M (0.500, 0.500, 0.000)	Γ (0.000, 0.000, 0.000)
3	Γ (0.000, 0.000, 0.000)	R (0.500, 0.500, 0.500)
4	R (0.500, 0.500, 0.500)	X (0.000, 0.500, 0.000)
5	R (0.500, 0.500, 0.500)	M (0.500, 0.500, 0.000)

Supplementary Table 1443: Nodal ring points along high symmetry lines of mp-976254-Li3Mg.

	Coordinates	Frequency	Mode 1	Mode 2	Multi.	Deg.	Path	Ratio
0	(0.000, 0.265, 0.000)	2.777	2	1	6	2	1	0.531
1	(0.000, 0.327, 0.000)	3.286	2	1	6	2	1	0.653
2	(0.000, 0.378, 0.000)	3.636	2	1	6	2	1	0.755
3	(0.248, 0.500, 0.248)	3.311	2	1	12	2	5	0.505
4	(0.000, 0.143, 0.000)	1.569	3	2	6	2	1	0.286
5	(0.150, 0.500, 0.000)	4.210	3	2	12	2	2	0.300
6	(0.153, 0.500, 0.153)	3.949	3	2	12	2	5	0.695
7	(0.343, 0.500, 0.000)	4.742	4	3	12	2	2	0.687
8	(0.393, 0.393, 0.000)	4.732	4	3	12	2	3	0.214
9	(0.000, 0.337, 0.000)	6.303	6	5	6	2	1	0.673
10	(0.250, 0.500, 0.000)	6.769	6	5	12	2	2	0.499
11	(0.327, 0.327, -0.000)	6.876	6	5	12	2	3	0.346
12	(0.101, 0.500, 0.097)	7.124	7	6	24	2	5	0.802
13	(0.160, 0.500, -0.000)	7.062	7	6	12	2	2	0.319

Supplementary Table 1443: Nodal ring points along high symmetry lines of mp-976254-Li3Mg.

	Coordinates	Frequency	Mode 1	Mode 2	Multi.	Deg.	Path	Ratio
14	(0.310, 0.310, -0.002)	7.023	7	6	24	2	3	0.380
15	(0.225, 0.225, 0.225)	7.426	7	6	8	2	4	0.449
16	(0.366, 0.499, 0.366)	6.119	7	6	24	2	5	0.269
17	(0.000, 0.000, 0.000)	8.659	9	8	1	3	1	0.000
18	(0.000, 0.143, 0.000)	8.580	9	8	6	2	1	0.286
19	(0.376, 0.500, -0.000)	8.471	10	9	12	2	2	0.753
20	(0.432, 0.432, -0.000)	8.367	10	9	12	2	3	0.135
21	(0.322, 0.322, -0.000)	8.491	10	9	12	2	3	0.355
22	(0.211, 0.500, -0.000)	8.877	11	10	12	2	2	0.422
23	(0.105, 0.500, 0.105)	8.857	11	10	12	2	5	0.791

The followings are details for Be$_3$. It has 7 topological nodal lines and 15 nodal ring points along high symmetry line as shown in Fig **685**.

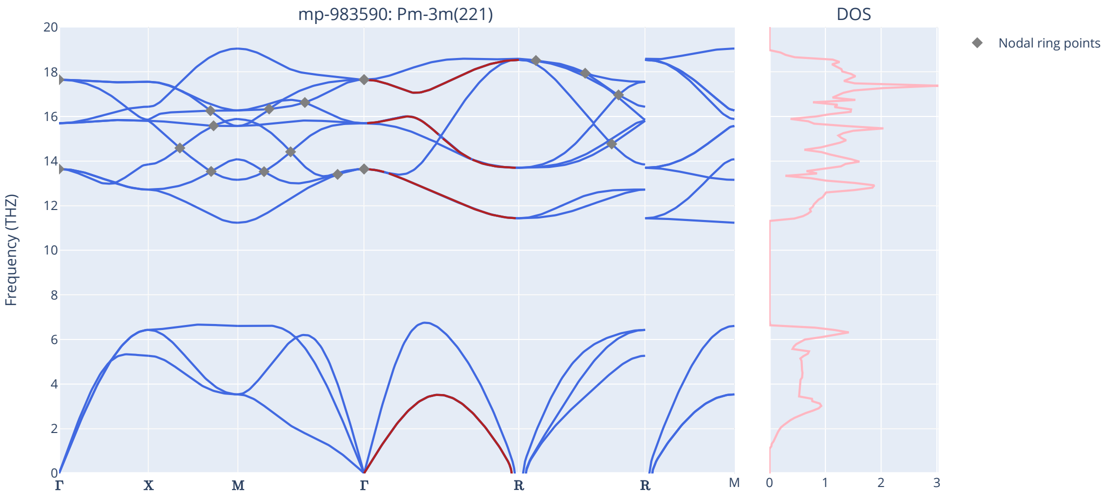

Supplementary Figure 685: The phonon band and DOS of Be3Fe.

Supplementary Table 1444: High symmetry lines of mp-983590-Be3Fe

	Start	End
0	Γ (0.000, 0.000, 0.000)	X (0.000, 0.500, 0.000)
1	X (0.000, 0.500, 0.000)	M (0.500, 0.500, 0.000)
2	M (0.500, 0.500, 0.000)	Γ (0.000, 0.000, 0.000)
3	Γ (0.000, 0.000, 0.000)	R (0.500, 0.500, 0.500)
4	R (0.500, 0.500, 0.500)	X (0.000, 0.500, 0.000)
5	R (0.500, 0.500, 0.500)	M (0.500, 0.500, 0.000)

Supplementary Table 1445: Nodal ring points along high symmetry lines of mp-983590-Be3Fe.

	Coordinates	Frequency	Mode 1	Mode 2	Multi.	Deg.	Path	Ratio
0	(0.105, 0.105, -0.000)	13.403	5	4	12	2	3	0.791
1	(0.000, 0.000, 0.000)	13.645	6	5	1	3	1	0.000
2	(0.351, 0.500, 0.000)	13.535	6	5	12	2	2	0.702
3	(0.396, 0.396, 0.000)	13.526	6	5	12	2	3	0.209
4	(0.176, 0.500, -0.000)	14.586	7	6	12	2	2	0.352
5	(0.291, 0.291, -0.000)	14.415	7	6	12	2	3	0.419
6	(0.132, 0.500, 0.132)	14.767	7	6	12	2	5	0.736
7	(0.365, 0.500, -0.000)	15.581	8	7	12	2	2	0.730
8	(0.347, 0.500, 0.000)	16.257	10	9	12	2	2	0.695
9	(0.234, 0.234, -0.000)	16.627	10	9	12	2	3	0.531
10	(0.376, 0.376, -0.000)	16.336	10	9	12	2	3	0.249
11	(0.105, 0.500, 0.105)	16.967	11	10	12	2	5	0.790
12	(0.000, 0.000, 0.000)	17.652	12	11	1	3	1	0.000
13	(0.432, 0.500, 0.432)	18.511	12	11	12	2	5	0.136
14	(0.237, 0.500, 0.237)	17.941	12	11	12	2	5	0.526

Supplementary Table 1445: Nodal ring points along high symmetry lines of mp-983590-Be3Fe.

Coordinates	Frequency	Mode 1	Mode 2	Multi.	Deg.	Path	Ratio

The followings are details for Ti_2As_2. It has 25 topological nodal lines and 24 nodal ring points along high symmetry line as shown in Fig **686**.

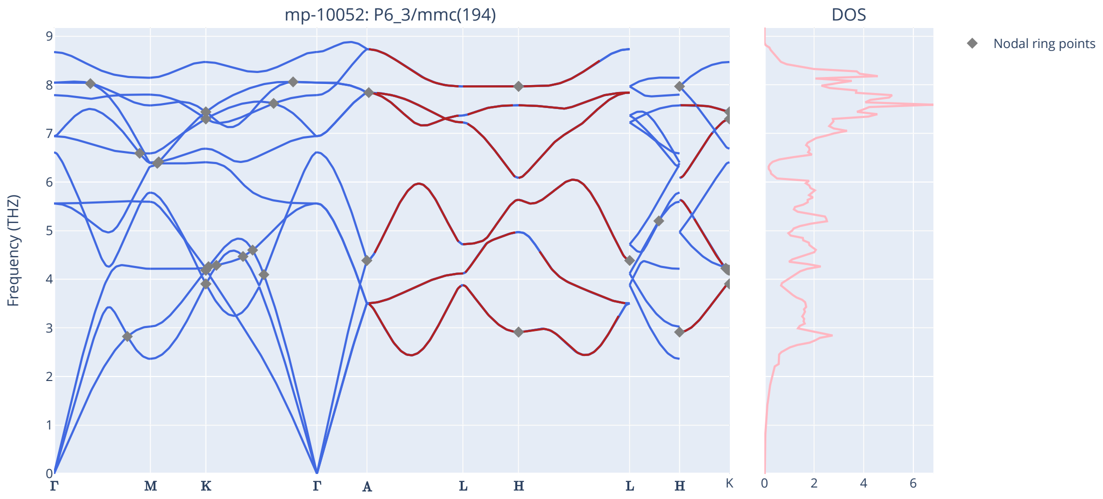

Supplementary Figure 686: The phonon band and DOS of Ti2As2.

Supplementary Table 1446: High symmetry lines of mp-10052-Ti2As2

	Start	End
0	Γ (0.000, 0.000, 0.000)	M (0.500, 0.000, 0.000)
1	M (0.500, 0.000, 0.000)	K (0.333, 0.333, 0.000)
2	K (0.333, 0.333, 0.000)	Γ (0.000, 0.000, 0.000)
3	Γ (0.000, 0.000, 0.000)	A (0.000, 0.000, 0.500)
4	A (0.000, 0.000, 0.500)	L (0.500, 0.000, 0.500)
5	L (0.500, 0.000, 0.500)	H (0.333, 0.333, 0.500)
6	H (0.333, 0.333, 0.500)	A (0.000, 0.000, 0.500)
7	L (0.500, 0.000, 0.500)	M (0.500, 0.000, 0.000)
8	H (0.333, 0.333, 0.500)	K (0.333, 0.333, 0.000)

Supplementary Table 1447: Nodal ring points along high symmetry lines of mp-10052-Ti2As2.

	Coordinates	Frequency	Mode 1	Mode 2	Multi.	Deg.	Path	Ratio
0	(0.381, 0.002, 0.000)	2.823	2	1	12	2	1	0.762
1	(0.333, 0.333, 0.000)	3.905	2	1	2	2	2	1.000
2	(0.333, 0.333, 0.500)	2.914	2	1	2	2	6	1.000
3	(0.333, 0.333, 0.000)	4.186	4	3	2	2	2	1.000
4	(0.302, 0.302, -0.000)	4.287	4	3	6	2	3	0.095
5	(0.222, 0.222, 0.000)	4.469	4	3	6	2	3	0.335
6	(0.159, 0.159, -0.000)	4.095	4	3	6	2	3	0.522
7	(0.333, 0.333, 0.037)	4.227	4	3	4	2	9	0.927
8	(0.333, 0.333, 0.023)	4.203	4	3	4	2	9	0.955
9	(0.333, 0.333, 0.010)	4.189	4	3	4	2	9	0.980
10	(0.325, 0.325, -0.000)	4.259	5	4	6	2	3	0.024

Supplementary Table 1447: Nodal ring points along high symmetry lines of mp-10052-Ti2As2.

	Coordinates	Frequency	Mode 1	Mode 2	Multi.	Deg.	Path	Ratio
11	(0.193, 0.193, -0.001)	4.600	5	4	12	2	3	0.421
12	(0.499, 0.000, 0.208)	5.199	5	4	12	2	8	0.584
13	(0.000, 0.000, 0.500)	4.386	6	5	1	2	4	1.000
14	(0.479, 0.042, 0.000)	6.385	7	6	6	2	2	0.126
15	(0.475, 0.051, 0.000)	6.415	8	7	12	2	2	0.152
16	(0.445, -0.000, -0.000)	6.592	8	7	6	2	1	0.889
17	(0.130, 0.130, -0.000)	7.617	9	8	6	2	3	0.610
18	(0.333, 0.333, 0.000)	7.298	9	8	2	2	2	1.000
19	(0.010, 0.000, 0.500)	7.840	9	8	6	2	5	0.020
20	(0.071, 0.071, -0.000)	8.060	11	10	6	2	3	0.786
21	(0.189, 0.000, -0.000)	8.024	11	10	6	2	1	0.378
22	(0.333, 0.333, 0.000)	7.442	11	10	2	2	2	1.000
23	(0.333, 0.333, 0.500)	7.968	12	11	2	2	6	1.000

The followings are details for In_2As_2. As shown in Fig 687, it has 23 topological nodal lines, 19 nodal ring points and 2 high degenerate Weyl points whose degeneracy are more than 2 and charges are bigger than 1.

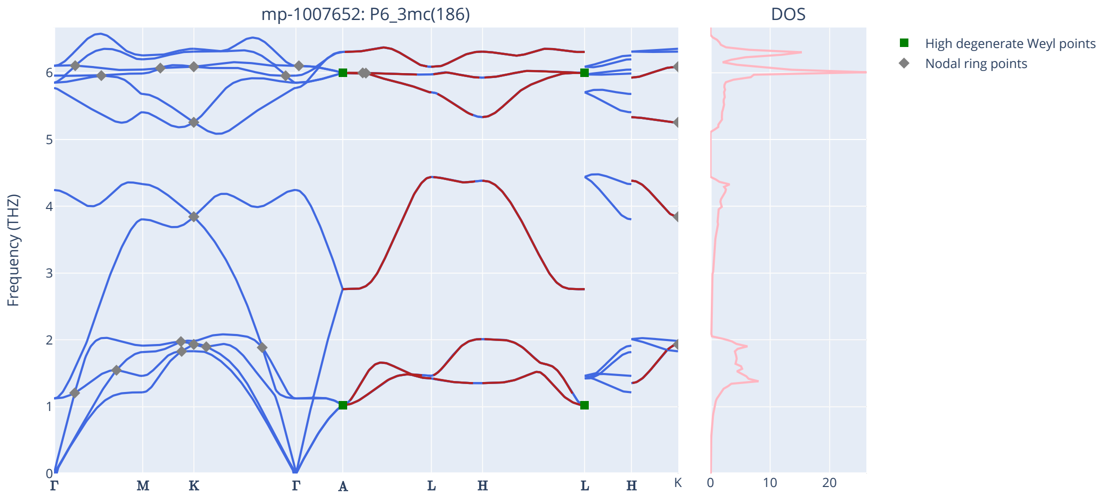

Supplementary Figure 687: The phonon band and DOS of In2As2.

Supplementary Table 1448: High symmetry lines of mp-1007652-In2As2

	Start	End
0	Γ (0.000, 0.000, 0.000)	M (0.500, 0.000, 0.000)
1	M (0.500, 0.000, 0.000)	K (0.333, 0.333, 0.000)
2	K (0.333, 0.333, 0.000)	Γ (0.000, 0.000, 0.000)
3	Γ (0.000, 0.000, 0.000)	A (0.000, 0.000, 0.500)
4	A (0.000, 0.000, 0.500)	L (0.500, 0.000, 0.500)
5	L (0.500, 0.000, 0.500)	H (0.333, 0.333, 0.500)
6	H (0.333, 0.333, 0.500)	A (0.000, 0.000, 0.500)
7	L (0.500, 0.000, 0.500)	M (0.500, 0.000, 0.000)
8	H (0.333, 0.333, 0.500)	K (0.333, 0.333, 0.000)

Supplementary Table 1449: High degenerate Weyl points along high symmetry lines of mp-1007652-In2As2.

	Coordinates	Frequency	Mode 1	Mode 2	Multi.	Deg.	Path	Ratio
0	(0.000, 0.000, 0.500)	1.023	4	1	1	4	4	1.000
1	(0.000, 0.000, 0.500)	5.996	10	7	1	4	4	1.000

Supplementary Table 1450: Nodal ring points along high symmetry lines of mp-1007652-In2As2.

	Coordinates	Frequency	Mode 1	Mode 2	Multi.	Deg.	Path	Ratio
0	(0.373, 0.254, -0.000)	1.828	2	1	6	2	2	0.763

Supplementary Table 1450: Nodal ring points along high symmetry lines of mp-1007652-In2As2.

	Coordinates	Frequency	Mode 1	Mode 2	Multi.	Deg.	Path	Ratio
1	(0.352, 0.000, 0.000)	1.545	3	2	6	2	1	0.704
2	(0.293, 0.293, 0.000)	1.896	3	2	6	2	3	0.122
3	(0.333, 0.333, 0.000)	1.932	3	2	2	2	2	1.000
4	(0.375, 0.250, -0.000)	1.974	4	3	6	2	2	0.749
5	(0.116, -0.000, 0.000)	1.204	4	3	6	2	1	0.233
6	(0.110, 0.110, 0.000)	1.885	5	4	6	2	3	0.669
7	(0.333, 0.333, 0.000)	3.843	6	5	2	2	2	1.000
8	(0.333, 0.333, 0.000)	5.257	8	7	2	2	2	1.000
9	(0.267, 0.000, 0.000)	5.956	9	8	6	2	1	0.534
10	(0.113, 0.000, 0.498)	5.997	9	8	12	2	5	0.226
11	(0.131, 0.000, 0.496)	5.994	9	8	12	2	5	0.262
12	(0.442, 0.115, -0.000)	6.069	10	9	6	2	2	0.346
13	(0.034, 0.034, -0.000)	5.954	10	9	6	2	3	0.898
14	(0.333, 0.333, 0.000)	6.090	10	9	2	2	2	1.000
15	(0.119, 0.000, 0.000)	6.105	11	10	6	2	1	0.239
16	(0.000, 0.000, 0.031)	6.104	12	11	2	2	4	0.061

The followings are details for In_2Sb_2. As shown in Fig **688**, it has 22 topological nodal lines, 23 nodal ring points and 2 high degenerate Weyl points whose degeneracy are more than 2 and charges are bigger than 1.

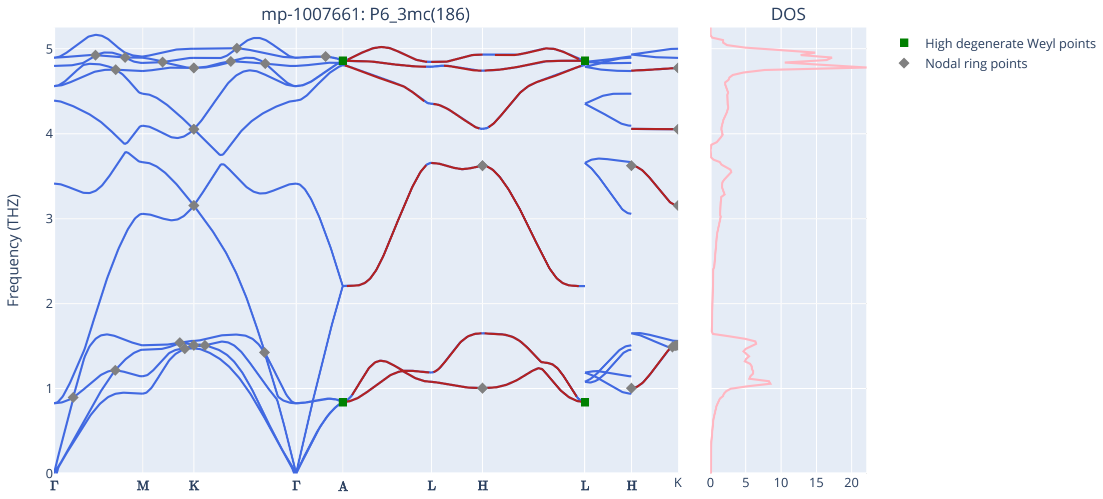

Supplementary Figure 688: The phonon band and DOS of In2Sb2.

Supplementary Table 1451: High symmetry lines of mp-1007661-In2Sb2

	Start	End
0	Γ (0.000, 0.000, 0.000)	M (0.500, 0.000, 0.000)
1	M (0.500, 0.000, 0.000)	K (0.333, 0.333, 0.000)
2	K (0.333, 0.333, 0.000)	Γ (0.000, 0.000, 0.000)
3	Γ (0.000, 0.000, 0.000)	A (0.000, 0.000, 0.500)
4	A (0.000, 0.000, 0.500)	L (0.500, 0.000, 0.500)
5	L (0.500, 0.000, 0.500)	H (0.333, 0.333, 0.500)
6	H (0.333, 0.333, 0.500)	A (0.000, 0.000, 0.500)
7	L (0.500, 0.000, 0.500)	M (0.500, 0.000, 0.000)
8	H (0.333, 0.333, 0.500)	K (0.333, 0.333, 0.000)

Supplementary Table 1452: High degenerate Weyl points along high symmetry lines of mp-1007661-In2Sb2.

	Coordinates	Frequency	Mode 1	Mode 2	Multi.	Deg.	Path	Ratio
0	(0.000, 0.000, 0.500)	0.841	4	1	1	4	4	1.000
1	(0.000, 0.000, 0.500)	4.859	12	9	1	4	4	1.000

Supplementary Table 1453: Nodal ring points along high symmetry lines of mp-1007661-In2Sb2.

	Coordinates	Frequency	Mode 1	Mode 2	Multi.	Deg.	Path	Ratio
0	(0.363, 0.274, 0.000)	1.468	2	1	6	2	2	0.823

Supplementary Table 1453: Nodal ring points along high symmetry lines of mp-1007661-In2Sb2.

	Coordinates	Frequency	Mode 1	Mode 2	Multi.	Deg.	Path	Ratio
1	(0.333, 0.333, 0.500)	1.004	2	1	2	2	6	1.000
2	(0.345, -0.000, 0.000)	1.214	3	2	6	2	1	0.691
3	(0.297, 0.297, -0.000)	1.508	3	2	6	2	3	0.109
4	(0.333, 0.333, 0.000)	1.509	3	2	2	2	2	1.000
5	(0.333, 0.333, 0.020)	1.507	3	2	4	2	9	0.960
6	(0.333, 0.333, 0.032)	1.504	3	2	4	2	9	0.937
7	(0.333, 0.333, 0.060)	1.489	3	2	4	2	9	0.879
8	(0.379, 0.242, 0.000)	1.540	4	3	6	2	2	0.726
9	(0.108, 0.000, 0.000)	0.895	4	3	6	2	1	0.215
10	(0.103, 0.103, 0.000)	1.425	5	4	6	2	3	0.691
11	(0.333, 0.333, 0.000)	3.155	6	5	2	2	2	1.000
12	(0.333, 0.333, 0.500)	3.624	6	5	2	2	6	1.000
13	(0.333, 0.333, 0.000)	4.053	8	7	2	2	2	1.000
14	(0.348, 0.000, -0.000)	4.754	9	8	6	2	1	0.695
15	(0.333, 0.333, 0.000)	4.775	10	9	2	2	2	1.000
16	(0.101, 0.101, 0.000)	4.826	10	9	6	2	3	0.698
17	(0.213, 0.213, 0.000)	4.851	10	9	6	2	3	0.361
18	(0.234, 0.000, -0.000)	4.925	11	10	6	2	1	0.467
19	(0.435, 0.130, 0.000)	4.844	11	10	6	2	2	0.390
20	(0.401, -0.000, -0.000)	4.898	11	10	6	2	1	0.802
21	(0.000, 0.000, 0.316)	4.909	11	10	2	2	4	0.633
22	(0.193, 0.193, -0.000)	5.007	12	11	6	2	3	0.421

The followings are details for Bi_2Pd_2. It has 23 topological nodal lines and 29 nodal ring points along high symmetry line as shown in Fig **689**.

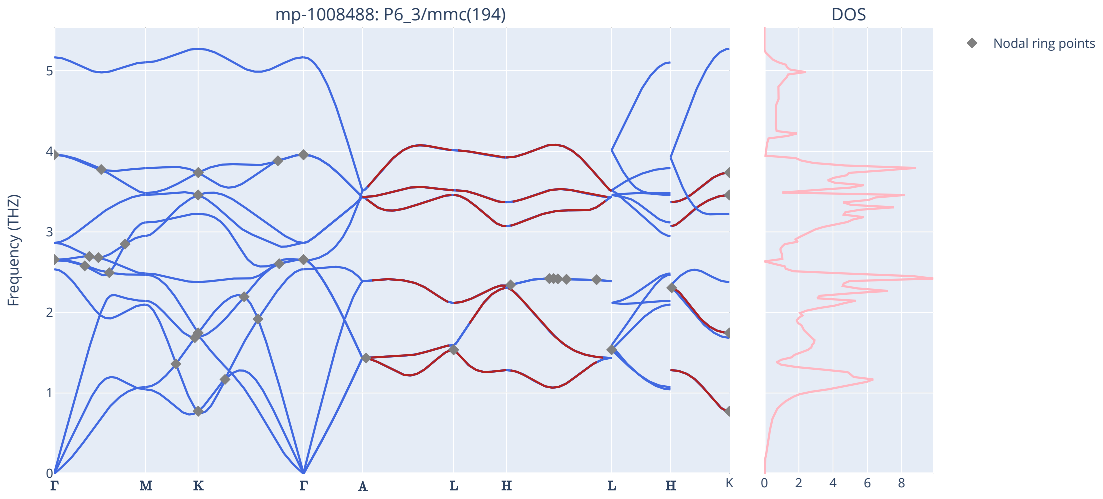

Supplementary Figure 689: The phonon band and DOS of Bi2Pd2.

Supplementary Table 1454: High symmetry lines of mp-1008488-Bi2Pd2

	Start	End
0	Γ (0.000, 0.000, 0.000)	M (0.500, 0.000, 0.000)
1	M (0.500, 0.000, 0.000)	K (0.333, 0.333, 0.000)
2	K (0.333, 0.333, 0.000)	Γ (0.000, 0.000, 0.000)
3	Γ (0.000, 0.000, 0.000)	A (0.000, 0.000, 0.500)
4	A (0.000, 0.000, 0.500)	L (0.500, 0.000, 0.500)
5	L (0.500, 0.000, 0.500)	H (0.333, 0.333, 0.500)
6	H (0.333, 0.333, 0.500)	A (0.000, 0.000, 0.500)
7	L (0.500, 0.000, 0.500)	M (0.500, 0.000, 0.000)
8	H (0.333, 0.333, 0.500)	K (0.333, 0.333, 0.000)

Supplementary Table 1455: Nodal ring points along high symmetry lines of mp-1008488-Bi2Pd2.

	Coordinates	Frequency	Mode 1	Mode 2	Multi.	Deg.	Path	Ratio
0	(0.333, 0.333, 0.000)	0.773	2	1	2	2	2	1.000
1	(0.248, 0.248, -0.000)	1.167	2	1	6	2	3	0.255
2	(0.500, 0.000, 0.500)	1.535	2	1	3	2	5	1.000
3	(0.405, 0.193, -0.000)	1.361	3	2	12	2	2	0.577
4	(0.020, 0.000, 0.499)	1.433	3	2	12	2	5	0.041
5	(0.344, 0.312, 0.000)	1.686	4	3	6	2	2	0.937
6	(0.144, 0.144, -0.000)	1.916	4	3	6	2	3	0.568
7	(0.333, 0.333, 0.490)	2.305	4	3	4	2	9	0.020
8	(0.188, 0.188, -0.000)	2.195	5	4	6	2	3	0.436
9	(0.333, 0.333, 0.000)	1.744	5	4	2	2	2	1.000
10	(0.000, 0.000, 0.000)	2.654	6	5	1	2	1	0.000

Supplementary Table 1455: Nodal ring points along high symmetry lines of mp-1008488-Bi2Pd2.

	Coordinates	Frequency	Mode 1	Mode 2	Multi.	Deg.	Path	Ratio
11	(0.167, 0.000, 0.000)	2.577	6	5	6	2	1	0.333
12	(0.077, 0.077, -0.000)	2.605	6	5	6	2	3	0.769
13	(0.301, 0.000, 0.000)	2.493	6	5	6	2	1	0.602
14	(0.320, 0.320, 0.500)	2.341	6	5	6	2	7	0.041
15	(0.197, 0.197, 0.500)	2.420	6	5	6	2	7	0.408
16	(0.184, 0.184, 0.500)	2.418	6	5	6	2	7	0.449
17	(0.170, 0.170, 0.500)	2.416	6	5	6	2	7	0.490
18	(0.143, 0.143, 0.500)	2.412	6	5	6	2	7	0.571
19	(0.048, 0.048, 0.500)	2.405	6	5	6	2	7	0.857
20	(0.000, 0.000, 0.000)	2.654	7	6	1	2	1	0.000
21	(0.242, -0.000, 0.000)	2.679	8	7	6	2	1	0.483
22	(0.192, -0.000, -0.000)	2.695	8	7	6	2	1	0.383
23	(0.388, 0.000, -0.000)	2.848	8	7	6	2	1	0.776
24	(0.333, 0.333, 0.000)	3.457	9	8	2	2	2	1.000
25	(0.000, 0.000, 0.000)	3.956	11	10	1	2	1	0.000
26	(0.081, 0.081, -0.000)	3.883	11	10	6	2	3	0.757
27	(0.257, -0.000, -0.000)	3.774	11	10	6	2	1	0.514
28	(0.333, 0.333, 0.000)	3.736	11	10	2	2	2	1.000

The followings are details for B_2P_2. As shown in Fig **690**, it has 23 topological nodal lines, 20 nodal ring points and 2 high degenerate Weyl points whose degeneracy are more than 2 and charges are bigger than 1.

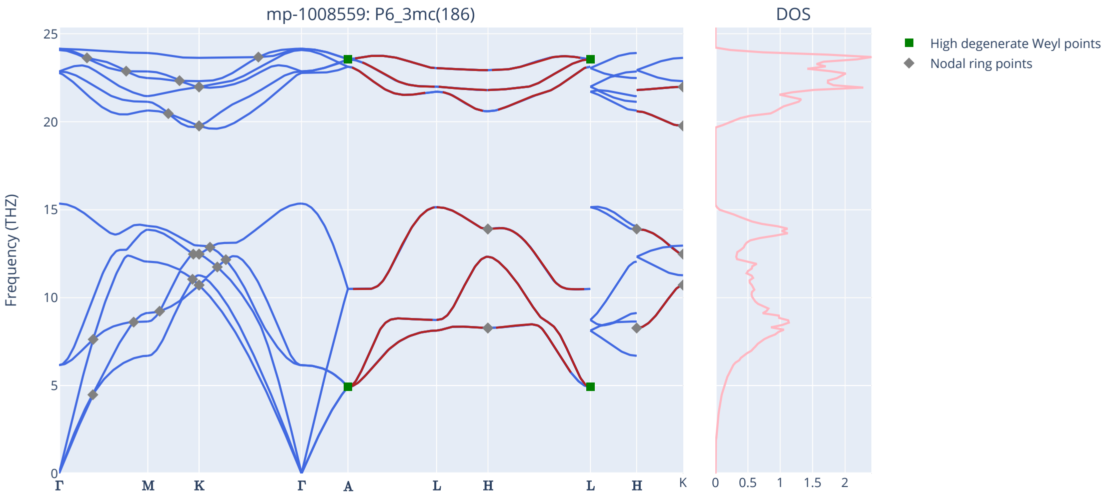

Supplementary Figure 690: The phonon band and DOS of B2P2.

Supplementary Table 1456: High symmetry lines of mp-1008559-B2P2

	Start	End
0	Γ (0.000, 0.000, 0.000)	M (0.500, 0.000, 0.000)
1	M (0.500, 0.000, 0.000)	K (0.333, 0.333, 0.000)
2	K (0.333, 0.333, 0.000)	Γ (0.000, 0.000, 0.000)
3	Γ (0.000, 0.000, 0.000)	A (0.000, 0.000, 0.500)
4	A (0.000, 0.000, 0.500)	L (0.500, 0.000, 0.500)
5	L (0.500, 0.000, 0.500)	H (0.333, 0.333, 0.500)
6	H (0.333, 0.333, 0.500)	A (0.000, 0.000, 0.500)
7	L (0.500, 0.000, 0.500)	M (0.500, 0.000, 0.000)
8	H (0.333, 0.333, 0.500)	K (0.333, 0.333, 0.000)

Supplementary Table 1457: High degenerate Weyl points along high symmetry lines of mp-1008559-B2P2.

	Coordinates	Frequency	Mode 1	Mode 2	Multi.	Deg.	Path	Ratio
0	(0.000, 0.000, 0.500)	4.919	4	1	1	4	4	1.000
1	(0.000, 0.000, 0.500)	23.551	12	9	1	4	4	1.000

Supplementary Table 1458: Nodal ring points along high symmetry lines of mp-1008559-B2P2.

	Coordinates	Frequency	Mode 1	Mode 2	Multi.	Deg.	Path	Ratio
0	(0.190, -0.000, -0.003)	4.476	2	1	12	2	1	0.381

Supplementary Table 1458: Nodal ring points along high symmetry lines of mp-1008559-B2P2.

	Coordinates	Frequency	Mode 1	Mode 2	Multi.	Deg.	Path	Ratio
1	(0.333, 0.333, 0.000)	10.715	2	1	2	2	2	1.000
2	(0.333, 0.333, 0.500)	8.269	2	1	2	2	6	1.000
3	(0.462, 0.077, -0.000)	9.227	3	2	6	2	2	0.230
4	(0.420, -0.000, -0.000)	8.606	3	2	6	2	1	0.840
5	(0.355, 0.291, 0.000)	11.044	3	2	6	2	2	0.873
6	(0.192, -0.000, 0.000)	7.624	4	3	6	2	1	0.384
7	(0.274, 0.274, 0.000)	11.743	4	3	6	2	3	0.178
8	(0.352, 0.296, -0.000)	12.473	5	4	6	2	2	0.888
9	(0.246, 0.246, -0.000)	12.158	5	4	6	2	3	0.261
10	(0.333, 0.333, 0.000)	12.475	5	4	2	2	2	1.000
11	(0.298, 0.298, -0.000)	12.865	6	5	6	2	3	0.106
12	(0.333, 0.333, 0.500)	13.901	6	5	2	2	6	1.000
13	(0.433, 0.133, -0.000)	20.467	8	7	6	2	2	0.400
14	(0.333, 0.333, 0.000)	19.765	8	7	2	2	2	1.000
15	(0.333, 0.333, 0.000)	21.984	10	9	2	2	2	1.000
16	(0.157, 0.000, 0.000)	23.635	11	10	6	2	1	0.314
17	(0.378, 0.000, 0.000)	22.880	11	10	6	2	1	0.756
18	(0.397, 0.206, -0.000)	22.338	11	10	6	2	2	0.619
19	(0.140, 0.140, -0.000)	23.680	12	11	6	2	3	0.579

The followings are details for Co_3. It has 7 topological nodal lines and 36 nodal ring points along high symmetry line as shown in Fig **691**.

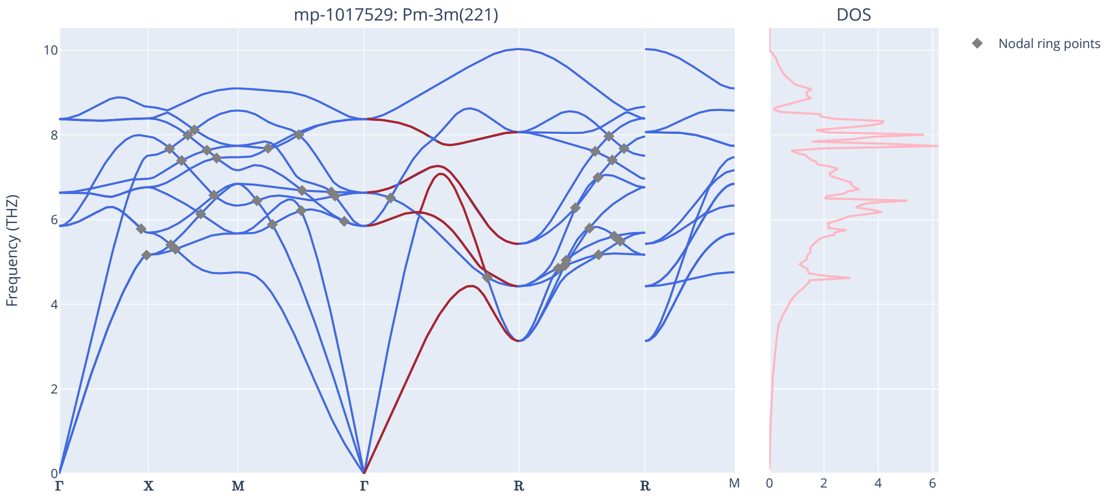

Supplementary Figure 691: The phonon band and DOS of Co3V.

Supplementary Table 1459: High symmetry lines of mp-1017529-Co3V

	Start	End
0	Γ (0.000, 0.000, 0.000)	X (0.000, 0.500, 0.000)
1	X (0.000, 0.500, 0.000)	M (0.500, 0.500, 0.000)
2	M (0.500, 0.500, 0.000)	Γ (0.000, 0.000, 0.000)
3	Γ (0.000, 0.000, 0.000)	R (0.500, 0.500, 0.500)
4	R (0.500, 0.500, 0.500)	X (0.000, 0.500, 0.000)
5	R (0.500, 0.500, 0.500)	M (0.500, 0.500, 0.000)

Supplementary Table 1460: Nodal ring points along high symmetry lines of mp-1017529-Co3V.

	Coordinates	Frequency	Mode 1	Mode 2	Multi.	Deg.	Path	Ratio
0	(0.000, 0.490, 0.000)	5.161	2	1	6	2	1	0.980
1	(0.152, 0.500, -0.000)	5.302	2	1	12	2	2	0.303
2	(0.184, 0.500, 0.184)	5.170	2	1	12	2	5	0.632
3	(0.125, 0.500, -0.000)	5.404	3	2	12	2	2	0.251
4	(0.363, 0.363, 0.000)	5.885	3	2	12	2	3	0.275
5	(0.318, 0.500, 0.318)	4.913	3	2	12	2	5	0.364
6	(0.099, 0.500, 0.099)	5.491	3	2	12	2	5	0.802
7	(0.000, 0.459, 0.000)	5.782	4	3	6	2	1	0.918
8	(0.293, 0.500, 0.000)	6.133	4	3	12	2	2	0.585
9	(0.248, 0.248, -0.000)	6.212	4	3	12	2	3	0.503
10	(0.395, 0.397, 0.403)	4.631	4	3	48	2	4	0.796
11	(0.122, 0.500, 0.122)	5.610	4	3	12	2	5	0.756
12	(0.078, 0.078, 0.000)	5.959	5	4	12	2	3	0.843
13	(0.424, 0.424, -0.000)	6.447	5	4	12	2	3	0.152

Supplementary Table 1460: Nodal ring points along high symmetry lines of mp-1017529-Co3V.

	Coordinates	Frequency	Mode 1	Mode 2	Multi.	Deg.	Path	Ratio
14	(0.313, 0.500, 0.313)	5.038	5	4	12	2	5	0.374
15	(0.344, 0.500, 0.344)	4.850	5	4	12	2	5	0.313
16	(0.220, 0.500, 0.220)	5.798	5	4	12	2	5	0.560
17	(0.366, 0.500, 0.000)	6.576	6	5	12	2	2	0.733
18	(0.245, 0.245, -0.000)	6.687	7	6	12	2	3	0.509
19	(0.115, 0.115, 0.000)	6.557	7	6	12	2	3	0.770
20	(0.086, 0.086, 0.086)	6.511	7	6	8	2	4	0.172
21	(0.187, 0.500, 0.187)	6.996	7	6	12	2	5	0.627
22	(0.276, 0.500, 0.276)	6.278	7	6	12	2	5	0.448
23	(0.382, 0.500, 0.000)	7.453	8	7	12	2	2	0.763
24	(0.186, 0.500, 0.000)	7.395	8	7	12	2	2	0.371
25	(0.130, 0.130, -0.000)	6.652	8	7	12	2	3	0.740
26	(0.130, 0.500, 0.130)	7.407	8	7	12	2	5	0.740
27	(0.327, 0.500, -0.000)	7.637	9	8	12	2	2	0.655
28	(0.118, 0.500, -0.000)	7.673	9	8	12	2	2	0.237
29	(0.379, 0.379, -0.000)	7.686	9	8	12	2	3	0.241
30	(0.083, 0.500, 0.083)	7.678	9	8	12	2	5	0.833
31	(0.198, 0.500, 0.198)	7.610	9	8	12	2	5	0.604
32	(0.221, 0.500, -0.000)	7.994	10	9	12	2	2	0.441
33	(0.258, 0.258, -0.000)	8.003	10	9	12	2	3	0.483
34	(0.143, 0.500, 0.143)	7.969	10	9	12	2	5	0.713
35	(0.258, 0.500, 0.000)	8.119	11	10	12	2	2	0.515

The followings are details for $Li_8Pd_4Sb_4$. As shown in Fig **692**, it has 7 topological nodal lines, 3 nodal ring points and 2 high degenerate Weyl points whose degeneracy are more than 2 and charges are bigger than 1.

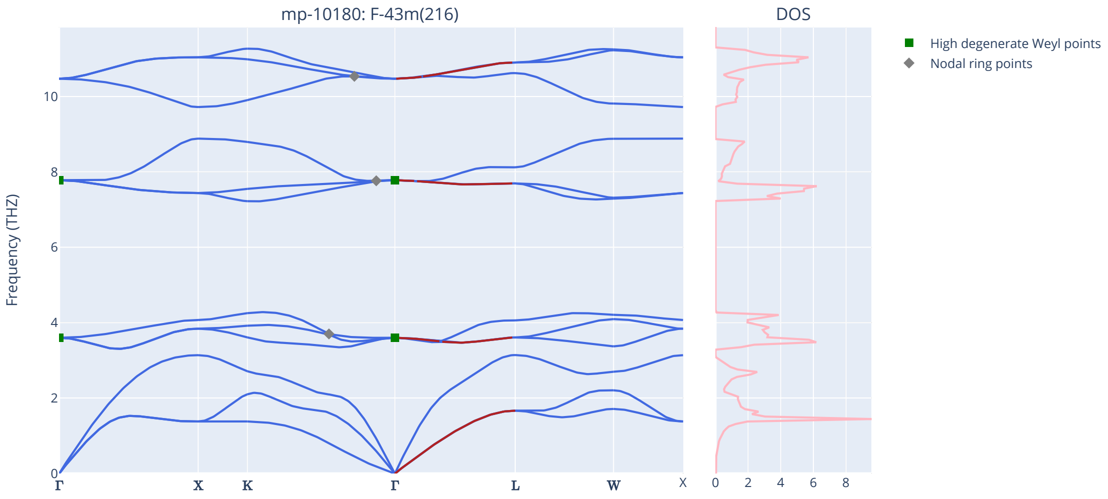

Supplementary Figure 692: The phonon band and DOS of Li8Pd4Sb4.

Supplementary Table 1461: High symmetry lines of mp-10180-Li8Pd4Sb4

	Start	End
0	Γ (0.000, 0.000, 0.000)	X (0.500, 0.000, 0.500)
1	X (0.500, 0.000, 0.500)	U (0.625, 0.250, 0.625)
2	K (0.375, 0.375, 0.750)	Γ (0.000, 0.000, 0.000)
3	Γ (0.000, 0.000, 0.000)	L (0.500, 0.500, 0.500)
4	L (0.500, 0.500, 0.500)	W (0.500, 0.250, 0.750)
5	W (0.500, 0.250, 0.750)	X (0.500, 0.000, 0.500)

Supplementary Table 1462: High degenerate Weyl points along high symmetry lines of mp-10180-Li8Pd4Sb4.

	Coordinates	Frequency	Mode 1	Mode 2	Multi.	Deg.	Path	Ratio
0	(0.000, 0.000, 0.000)	3.598	6	4	1	3	1	0.000
1	(0.000, 0.000, 0.000)	7.779	9	7	1	3	1	0.000

Supplementary Table 1463: Nodal ring points along high symmetry lines of mp-10180-Li8Pd4Sb4.

	Coordinates	Frequency	Mode 1	Mode 2	Multi.	Deg.	Path	Ratio
0	(0.163, 0.163, 0.339)	3.700	6	5	24	2	3	0.554
1	(0.046, 0.046, 0.095)	7.764	9	8	24	2	3	0.874
2	(0.101, 0.101, 0.207)	10.536	11	10	24	2	3	0.726

The followings are details for Mg_2Se_2. As shown in Fig 693, it has 22 topological nodal lines and 19 nodal ring points. At the same time, it has 1 single Weyl point and 2 high degenerate Weyl points whose degeneracy are more than 2 and charges are bigger than 1.

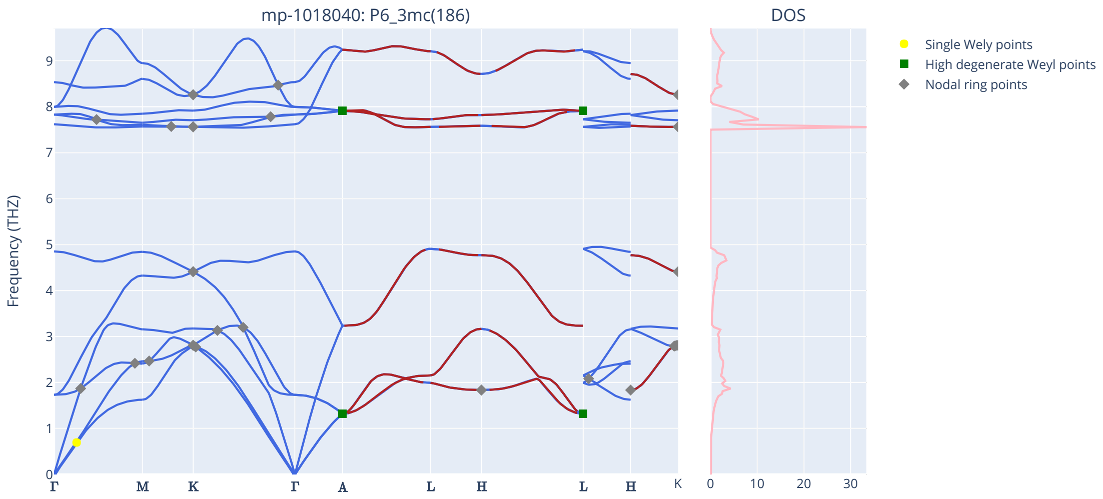

Supplementary Figure 693: The phonon band and DOS of Mg2Se2.

Supplementary Table 1464: High symmetry lines of mp-1018040-Mg2Se2

	Start	End
0	Γ (0.000, 0.000, 0.000)	M (0.500, 0.000, 0.000)
1	M (0.500, 0.000, 0.000)	K (0.333, 0.333, 0.000)
2	K (0.333, 0.333, 0.000)	Γ (0.000, 0.000, 0.000)
3	Γ (0.000, 0.000, 0.000)	A (0.000, 0.000, 0.500)
4	A (0.000, 0.000, 0.500)	L (0.500, 0.000, 0.500)
5	L (0.500, 0.000, 0.500)	H (0.333, 0.333, 0.500)
6	H (0.333, 0.333, 0.500)	A (0.000, 0.000, 0.500)
7	L (0.500, 0.000, 0.500)	M (0.500, 0.000, 0.000)
8	H (0.333, 0.333, 0.500)	K (0.333, 0.333, 0.000)

Supplementary Table 1465: Single Weyl points along high symmetry lines of mp-1018040-Mg2Se2.

	Coordinates	Frequency	Mode 1	Mode 2	Multi.	Deg.	Path	Ratio
0	(0.128, 0.000, -0.000)	0.693	2	1	6	2	1	0.255

Supplementary Table 1466: High degenerate Weyl points along high symmetry lines of mp-1018040-Mg2Se2.

	Coordinates	Frequency	Mode 1	Mode 2	Multi.	Deg.	Path	Ratio
0	(0.000, 0.000, 0.500)	1.318	4	1	1	4	4	1.000
1	(0.000, 0.000, 0.500)	7.911	10	7	1	4	4	1.000

Supplementary Table 1467: Nodal ring points along high symmetry lines of mp-1018040-Mg2Se2.

	Coordinates	Frequency	Mode 1	Mode 2	Multi.	Deg.	Path	Ratio

Supplementary Table 1467: Nodal ring points along high symmetry lines of mp-1018040-Mg2Se2.

	Coordinates	Frequency	Mode 1	Mode 2	Multi.	Deg.	Path	Ratio
0	(0.325, 0.325, -0.000)	2.772	2	1	6	2	3	0.025
1	(0.333, 0.333, 0.500)	1.834	2	1	2	2	6	1.000
2	(0.477, 0.045, 0.000)	2.466	3	2	6	2	2	0.136
3	(0.333, 0.333, 0.000)	2.808	3	2	2	2	2	1.000
4	(0.458, -0.000, 0.000)	2.417	3	2	6	2	1	0.917
5	(0.499, 0.000, 0.442)	2.081	3	2	12	2	8	0.115
6	(0.333, 0.333, 0.012)	2.807	3	2	4	2	9	0.976
7	(0.333, 0.333, 0.041)	2.790	3	2	4	2	9	0.918
8	(0.333, 0.333, 0.031)	2.798	3	2	4	2	9	0.939
9	(0.254, 0.254, -0.000)	3.132	4	3	6	2	3	0.239
10	(0.151, 0.000, 0.000)	1.868	4	3	6	2	1	0.301
11	(0.170, 0.170, 0.000)	3.200	5	4	6	2	3	0.491
12	(0.333, 0.333, 0.000)	4.410	6	5	2	2	2	1.000
13	(0.405, 0.190, 0.000)	7.571	8	7	6	2	2	0.570
14	(0.333, 0.333, 0.000)	7.563	8	7	2	2	2	1.000
15	(0.080, 0.080, 0.000)	7.783	9	8	6	2	3	0.761
16	(0.241, 0.000, -0.000)	7.721	9	8	6	2	1	0.482
17	(0.055, 0.055, -0.000)	8.470	12	11	6	2	3	0.835
18	(0.333, 0.333, 0.000)	8.262	12	11	2	2	2	1.000

The followings are details for Ga_2Sb_2. As shown in Fig **694**, it has 18 topological nodal lines, 4 nodal ring points and 4 single Weyl points.

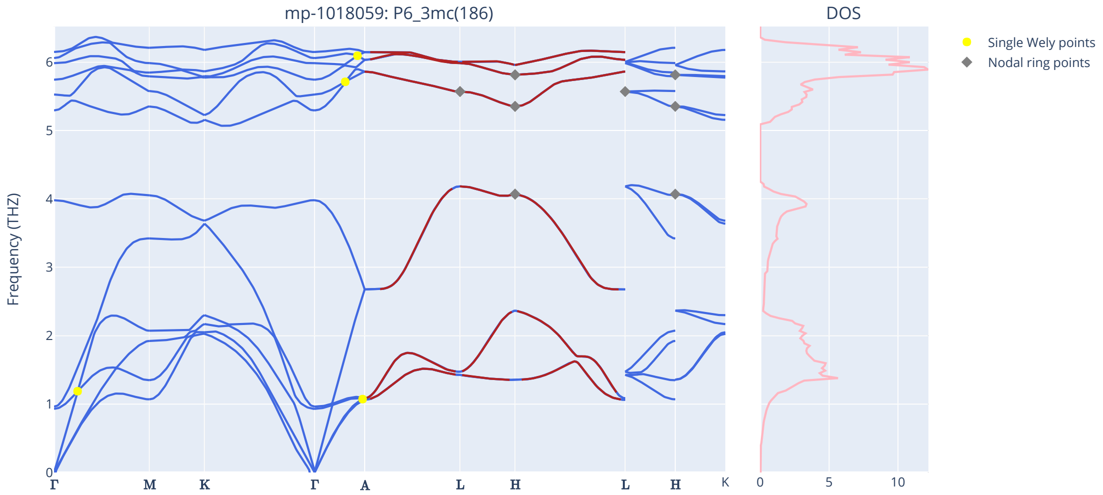

Supplementary Figure 694: The phonon band and DOS of Ga2Sb2.

Supplementary Table 1468: High symmetry lines of mp-1018059-Ga2Sb2

	Start	End
0	Γ (0.000, 0.000, 0.000)	M (0.500, 0.000, 0.000)
1	M (0.500, 0.000, 0.000)	K (0.333, 0.333, 0.000)
2	K (0.333, 0.333, 0.000)	Γ (0.000, 0.000, 0.000)
3	Γ (0.000, 0.000, 0.000)	A (0.000, 0.000, 0.500)
4	A (0.000, 0.000, 0.500)	L (0.500, 0.000, 0.500)
5	L (0.500, 0.000, 0.500)	H (0.333, 0.333, 0.500)
6	H (0.333, 0.333, 0.500)	A (0.000, 0.000, 0.500)
7	L (0.500, 0.000, 0.500)	M (0.500, 0.000, 0.000)
8	H (0.333, 0.333, 0.500)	K (0.333, 0.333, 0.000)

Supplementary Table 1469: Single Weyl points along high symmetry lines of mp-1018059-Ga2Sb2.

	Coordinates	Frequency	Mode 1	Mode 2	Multi.	Deg.	Path	Ratio
0	(-0.000, -0.000, 0.479)	1.072	3	2	2	2	4	0.958
1	(0.123, 0.001, 0.000)	1.188	4	3	12	2	1	0.247
2	(-0.000, 0.000, 0.306)	5.712	8	7	2	2	4	0.612
3	(-0.000, -0.000, 0.428)	6.093	11	10	2	2	4	0.857

Supplementary Table 1470: Nodal ring points along high symmetry lines of mp-1018059-Ga2Sb2.

	Coordinates	Frequency	Mode 1	Mode 2	Multi.	Deg.	Path	Ratio
0	(0.333, 0.333, 0.500)	4.070	6	5	2	2	6	1.000

Supplementary Table 1470: Nodal ring points along high symmetry lines of mp-1018059-Ga2Sb2.

	Coordinates	Frequency	Mode 1	Mode 2	Multi.	Deg.	Path	Ratio
1	(0.500, 0.000, 0.500)	5.569	8	7	3	2	5	1.000
2	(0.333, 0.333, 0.500)	5.353	8	7	2	2	6	1.000
3	(0.333, 0.333, 0.500)	5.813	10	9	2	2	6	1.000

The followings are details for Al_2Sb_2. As shown in Fig 695, it has 18 topological nodal lines, 1 nodal ring point and 6 single Weyl points.

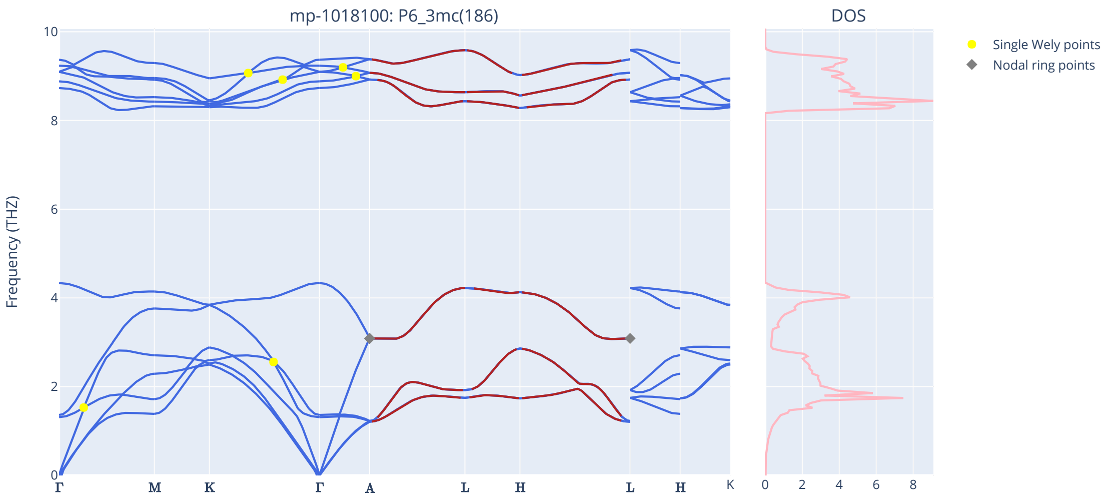

Supplementary Figure 695: The phonon band and DOS of Al2Sb2.

Supplementary Table 1471: High symmetry lines of mp-1018100-Al2Sb2

	Start	End
0	Γ (0.000, 0.000, 0.000)	M (0.500, 0.000, 0.000)
1	M (0.500, 0.000, 0.000)	K (0.333, 0.333, 0.000)
2	K (0.333, 0.333, 0.000)	Γ (0.000, 0.000, 0.000)
3	Γ (0.000, 0.000, 0.000)	A (0.000, 0.000, 0.500)
4	A (0.000, 0.000, 0.500)	L (0.500, 0.000, 0.500)
5	L (0.500, 0.000, 0.500)	H (0.333, 0.333, 0.500)
6	H (0.333, 0.333, 0.500)	A (0.000, 0.000, 0.500)
7	L (0.500, 0.000, 0.500)	M (0.500, 0.000, 0.000)
8	H (0.333, 0.333, 0.500)	K (0.333, 0.333, 0.000)

Supplementary Table 1472: Single Weyl points along high symmetry lines of mp-1018100-Al2Sb2.

	Coordinates	Frequency	Mode 1	Mode 2	Multi.	Deg.	Path	Ratio
0	(0.129, 0.002, -0.000)	1.527	4	3	12	2	1	0.258
1	(0.142, 0.137, -0.000)	2.555	5	4	12	2	3	0.582
2	(-0.000, -0.000, 0.363)	8.999	9	8	2	2	4	0.726
3	(0.113, 0.110, 0.000)	8.921	10	9	12	2	3	0.665
4	(-0.000, -0.000, 0.235)	9.197	11	10	2	2	4	0.469
5	(0.213, 0.219, 0.000)	9.069	12	11	12	2	3	0.352

Supplementary Table 1473: Nodal ring points along high symmetry lines of mp-1018100-Al2Sb2.

	Coordinates	Frequency	Mode 1	Mode 2	Multi.	Deg.	Path	Ratio
0	(0.000, 0.000, 0.500)	3.085	6	5	1	2	4	1.000

The followings are details for Pb_2Pt_2. It has 23 topological nodal lines and 25 nodal ring points along high symmetry line as shown in Fig **696**.

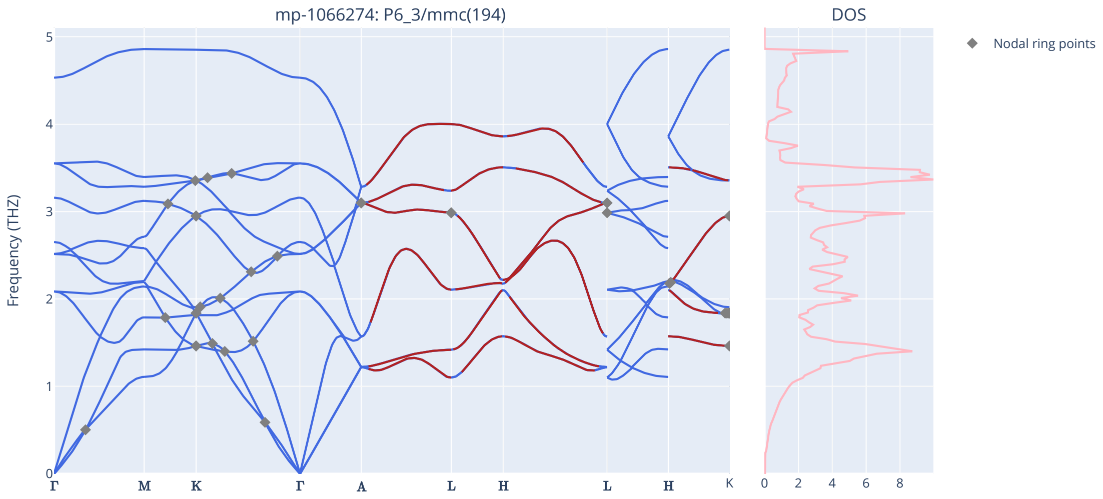

Supplementary Figure 696: The phonon band and DOS of Pb2Pt2.

Supplementary Table 1474: High symmetry lines of mp-1066274-Pb2Pt2

	Start	End
0	Γ (0.000, 0.000, 0.000)	M (0.500, 0.000, 0.000)
1	M (0.500, 0.000, 0.000)	K (0.333, 0.333, 0.000)
2	K (0.333, 0.333, 0.000)	Γ (0.000, 0.000, 0.000)
3	Γ (0.000, 0.000, 0.000)	A (0.000, 0.000, 0.500)
4	A (0.000, 0.000, 0.500)	L (0.500, 0.000, 0.500)
5	L (0.500, 0.000, 0.500)	H (0.333, 0.333, 0.500)
6	H (0.333, 0.333, 0.500)	A (0.000, 0.000, 0.500)
7	L (0.500, 0.000, 0.500)	M (0.500, 0.000, 0.000)
8	H (0.333, 0.333, 0.500)	K (0.333, 0.333, 0.000)

Supplementary Table 1475: Nodal ring points along high symmetry lines of mp-1066274-Pb2Pt2.

	Coordinates	Frequency	Mode 1	Mode 2	Multi.	Deg.	Path	Ratio
0	(0.174, 0.001, 0.000)	0.503	2	1	10	2	1	0.349
1	(0.333, 0.333, 0.000)	1.462	2	1	2	2	2	1.000
2	(0.112, 0.112, -0.000)	0.588	2	1	6	2	3	0.663
3	(0.280, 0.282, 0.000)	1.490	3	2	12	2	3	0.157
4	(0.241, 0.241, 0.000)	1.399	3	2	6	2	3	0.276
5	(0.432, 0.136, -0.000)	1.787	4	3	6	2	2	0.407
6	(0.150, 0.150, 0.000)	1.516	4	3	6	2	3	0.549
7	(0.333, 0.333, 0.000)	1.838	5	4	2	2	2	1.000
8	(0.333, 0.333, 0.035)	1.838	5	4	4	2	9	0.929
9	(0.333, 0.333, 0.021)	1.838	5	4	4	2	9	0.958
10	(0.333, 0.333, 0.010)	1.838	5	4	4	2	9	0.980

Supplementary Table 1475: Nodal ring points along high symmetry lines of mp-1066274-Pb2Pt2.

	Coordinates	Frequency	Mode 1	Mode 2	Multi.	Deg.	Path	Ratio
11	(0.256, 0.256, 0.000)	2.009	6	5	6	2	3	0.233
12	(0.320, 0.320, -0.000)	1.909	6	5	6	2	3	0.040
13	(0.333, 0.333, 0.490)	2.178	6	5	4	2	9	0.020
14	(0.333, 0.333, 0.480)	2.190	6	5	4	2	9	0.041
15	(0.073, 0.073, -0.000)	2.490	7	6	6	2	3	0.782
16	(0.157, 0.157, -0.000)	2.311	7	6	6	2	3	0.530
17	(0.333, 0.333, 0.000)	2.950	8	7	2	2	2	1.000
18	(0.000, 0.000, 0.500)	3.099	8	7	1	4	4	1.000
19	(0.500, 0.000, 0.500)	2.986	8	7	3	2	5	1.000
20	(0.424, 0.153, 0.000)	3.088	9	8	6	2	2	0.459
21	(0.336, 0.328, 0.000)	3.354	10	9	6	2	2	0.985
22	(0.297, 0.297, -0.000)	3.388	11	10	6	2	3	0.110
23	(0.219, 0.219, 0.000)	3.437	11	10	6	2	3	0.342

The followings are details for Li_4Mg_{12}. It has 7 topological nodal lines and 13 nodal ring points along high symmetry line as shown in Fig 697.

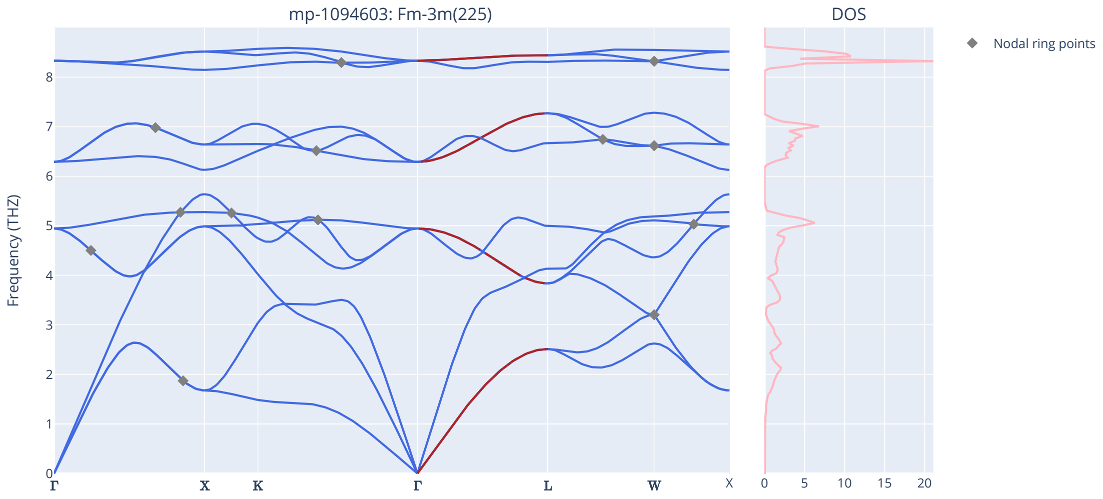

Supplementary Figure 697: The phonon band and DOS of Li4Mg12.

Supplementary Table 1476: High symmetry lines of mp-1094603-Li4Mg12

	Start	End
0	Γ (0.000, 0.000, 0.000)	X (0.500, 0.000, 0.500)
1	X (0.500, 0.000, 0.500)	U (0.625, 0.250, 0.625)
2	K (0.375, 0.375, 0.750)	Γ (0.000, 0.000, 0.000)
3	Γ (0.000, 0.000, 0.000)	L (0.500, 0.500, 0.500)
4	L (0.500, 0.500, 0.500)	W (0.500, 0.250, 0.750)
5	W (0.500, 0.250, 0.750)	X (0.500, 0.000, 0.500)

Supplementary Table 1477: Nodal ring points along high symmetry lines of mp-1094603-Li4Mg12.

	Coordinates	Frequency	Mode 1	Mode 2	Multi.	Deg.	Path	Ratio
0	(0.429, 0.000, 0.429)	1.871	2	1	6	2	1	0.857
1	(0.500, 0.250, 0.750)	3.204	3	2	6	2	5	1.000
2	(0.122, 0.000, 0.122)	4.502	5	4	6	2	1	0.245
3	(0.499, 0.119, 0.618)	5.031	5	4	24	2	6	0.527
4	(0.420, -0.001, 0.420)	5.271	6	5	24	2	1	0.840
5	(0.563, 0.126, 0.563)	5.257	6	5	12	2	2	0.505
6	(0.237, 0.237, 0.464)	5.118	6	5	24	2	3	0.377
7	(0.242, 0.242, 0.470)	6.514	8	7	24	2	3	0.367
8	(0.500, 0.250, 0.750)	6.615	8	7	6	2	5	1.000
9	(0.500, 0.371, 0.629)	6.743	8	7	24	2	5	0.518
10	(0.337, 0.000, 0.337)	6.979	9	8	6	2	1	0.673
11	(0.183, 0.175, 0.357)	8.293	11	10	24	2	3	0.524
12	(0.500, 0.250, 0.750)	8.319	11	10	6	2	5	1.000

The followings are details for V_2P_2. It has 21 topological nodal lines and 25 nodal ring points along high symmetry line as shown in Fig **698**.

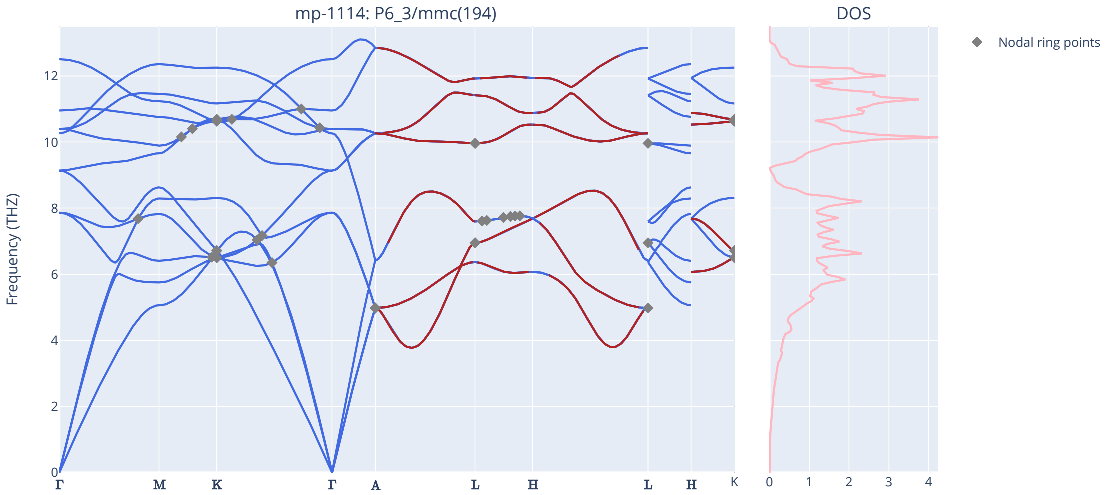

Supplementary Figure 698: The phonon band and DOS of V2P2.

Supplementary Table 1478: High symmetry lines of mp-1114-V2P2

	Start	End
0	Γ (0.000, 0.000, 0.000)	M (0.500, 0.000, 0.000)
1	M (0.500, 0.000, 0.000)	K (0.333, 0.333, 0.000)
2	K (0.333, 0.333, 0.000)	Γ (0.000, 0.000, 0.000)
3	Γ (0.000, 0.000, 0.000)	A (0.000, 0.000, 0.500)
4	A (0.000, 0.000, 0.500)	L (0.500, 0.000, 0.500)
5	L (0.500, 0.000, 0.500)	H (0.333, 0.333, 0.500)
6	H (0.333, 0.333, 0.500)	A (0.000, 0.000, 0.500)
7	L (0.500, 0.000, 0.500)	M (0.500, 0.000, 0.000)
8	H (0.333, 0.333, 0.500)	K (0.333, 0.333, 0.000)

Supplementary Table 1479: Nodal ring points along high symmetry lines of mp-1114-V2P2.

	Coordinates	Frequency	Mode 1	Mode 2	Multi.	Deg.	Path	Ratio
0	(0.333, 0.333, 0.000)	6.501	2	1	2	2	2	1.000
1	(0.348, 0.304, -0.000)	6.517	3	2	6	2	2	0.913
2	(0.000, 0.000, 0.500)	4.977	3	2	1	4	4	1.000
3	(0.174, 0.174, -0.003)	6.353	4	3	12	2	3	0.479
4	(0.000, 0.000, 0.500)	4.977	4	3	1	4	4	1.000
5	(0.500, 0.000, 0.500)	6.952	4	3	3	2	5	1.000
6	(0.395, -0.000, -0.000)	7.683	5	4	6	2	1	0.789
7	(0.217, 0.217, 0.000)	7.040	5	4	6	2	3	0.350
8	(0.333, 0.333, 0.000)	6.716	5	4	2	2	2	1.000
9	(0.202, 0.202, -0.000)	7.170	6	5	6	2	3	0.393
10	(0.480, 0.041, 0.500)	7.609	6	5	6	2	6	0.122

Supplementary Table 1479: Nodal ring points along high symmetry lines of mp-1114-V2P2.

	Coordinates	Frequency	Mode 1	Mode 2	Multi.	Deg.	Path	Ratio
11	(0.466, 0.068, 0.500)	7.628	6	5	6	2	6	0.204
12	(0.418, 0.163, 0.500)	7.717	6	5	6	2	6	0.490
13	(0.398, 0.204, 0.500)	7.748	6	5	6	2	6	0.612
14	(0.384, 0.231, 0.500)	7.762	6	5	6	2	6	0.694
15	(0.371, 0.259, 0.500)	7.766	6	5	6	2	6	0.776
16	(0.436, 0.129, -0.000)	10.156	8	7	6	2	2	0.386
17	(0.404, 0.192, 0.000)	10.403	8	7	6	2	2	0.577
18	(0.333, 0.333, 0.000)	10.631	8	7	2	2	2	1.000
19	(0.500, 0.000, 0.500)	9.960	8	7	3	2	5	1.000
20	(0.290, 0.290, 0.000)	10.687	9	8	6	2	3	0.131
21	(0.036, 0.036, -0.000)	10.432	10	9	6	2	3	0.893
22	(0.333, 0.333, 0.000)	10.686	10	9	2	2	2	1.000
23	(0.089, 0.089, 0.000)	11.001	11	10	6	2	3	0.734

The followings are details for $Li_{12}Pd_4$. It has 5 topological nodal lines and 6 nodal ring points along high symmetry line as shown in Fig **699**.

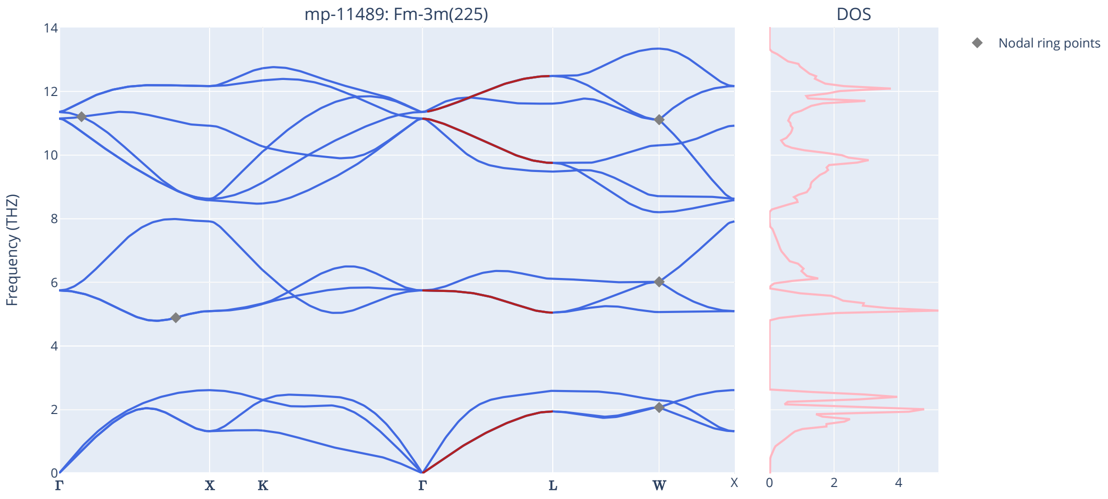

Supplementary Figure 699: The phonon band and DOS of Li12Pd4.

Supplementary Table 1480: High symmetry lines of mp-11489-Li12Pd4

	Start	End
0	Γ (0.000, 0.000, 0.000)	X (0.500, 0.000, 0.500)
1	X (0.500, 0.000, 0.500)	U (0.625, 0.250, 0.625)
2	K (0.375, 0.375, 0.750)	Γ (0.000, 0.000, 0.000)
3	Γ (0.000, 0.000, 0.000)	L (0.500, 0.500, 0.500)
4	L (0.500, 0.500, 0.500)	W (0.500, 0.250, 0.750)
5	W (0.500, 0.250, 0.750)	X (0.500, 0.000, 0.500)

Supplementary Table 1481: Nodal ring points along high symmetry lines of mp-11489-Li12Pd4.

	Coordinates	Frequency	Mode 1	Mode 2	Multi.	Deg.	Path	Ratio
0	(0.500, 0.250, 0.750)	2.067	2	1	6	2	5	1.000
1	(0.388, 0.000, 0.388)	4.889	5	4	6	2	1	0.776
2	(0.500, 0.250, 0.750)	6.018	6	5	6	2	5	1.000
3	(0.075, 0.000, 0.075)	11.206	10	9	6	2	1	0.149
4	(0.500, 0.250, 0.750)	11.113	11	10	6	2	5	1.000

The followings are details for Ba_2O_2. As shown in Fig **700**, it has 23 topological nodal lines, 14 nodal ring points and 2 high degenerate Weyl points whose degeneracy are more than 2 and charges are bigger than 1.

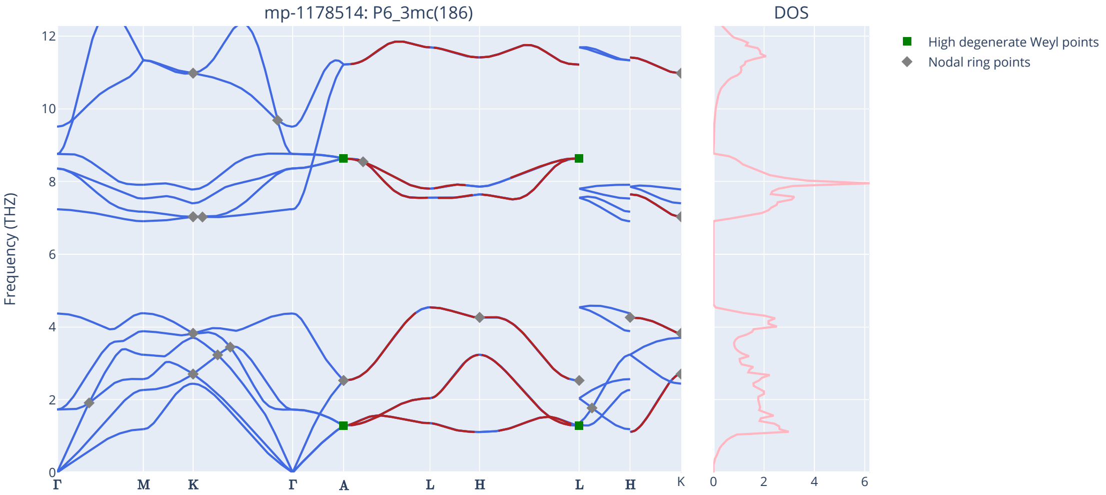

Supplementary Figure 700: The phonon band and DOS of Ba2O2.

Supplementary Table 1482: High symmetry lines of mp-1178514-Ba2O2

	Start	End
0	Γ (0.000, 0.000, 0.000)	M (0.500, 0.000, 0.000)
1	M (0.500, 0.000, 0.000)	K (0.333, 0.333, 0.000)
2	K (0.333, 0.333, 0.000)	Γ (0.000, 0.000, 0.000)
3	Γ (0.000, 0.000, 0.000)	A (0.000, 0.000, 0.500)
4	A (0.000, 0.000, 0.500)	L (0.500, 0.000, 0.500)
5	L (0.500, 0.000, 0.500)	H (0.333, 0.333, 0.500)
6	H (0.333, 0.333, 0.500)	A (0.000, 0.000, 0.500)
7	L (0.500, 0.000, 0.500)	M (0.500, 0.000, 0.000)
8	H (0.333, 0.333, 0.500)	K (0.333, 0.333, 0.000)

Supplementary Table 1483: High degenerate Weyl points along high symmetry lines of mp-1178514-Ba2O2.

	Coordinates	Frequency	Mode 1	Mode 2	Multi.	Deg.	Path	Ratio
0	(0.000, 0.000, 0.500)	1.284	4	1	1	4	4	1.000
1	(0.000, 0.000, 0.500)	8.635	10	7	1	4	4	1.000

Supplementary Table 1484: Nodal ring points along high symmetry lines of mp-1178514-Ba2O2.

	Coordinates	Frequency	Mode 1	Mode 2	Multi.	Deg.	Path	Ratio
0	(0.333, 0.333, 0.000)	2.705	3	2	2	2	2	1.000

Supplementary Table 1484: Nodal ring points along high symmetry lines of mp-1178514-Ba2O2.

	Coordinates	Frequency	Mode 1	Mode 2	Multi.	Deg.	Path	Ratio
1	(0.498, 0.000, 0.374)	1.773	3	2	12	2	8	0.252
2	(0.251, 0.251, 0.000)	3.232	4	3	6	2	3	0.247
3	(0.186, 0.000, 0.000)	1.912	4	3	6	2	1	0.371
4	(0.209, 0.209, -0.000)	3.447	5	4	6	2	3	0.374
5	(0.333, 0.333, 0.000)	3.825	6	5	2	2	2	1.000
6	(0.000, 0.000, 0.500)	2.528	6	5	1	2	4	1.000
7	(0.333, 0.333, 0.500)	4.262	6	5	2	2	6	1.000
8	(0.333, 0.333, 0.000)	7.031	8	7	2	2	2	1.000
9	(0.302, 0.302, 0.000)	7.024	8	7	6	2	3	0.094
10	(0.113, 0.000, 0.497)	8.543	9	8	12	2	5	0.226
11	(0.051, 0.051, -0.000)	9.686	12	11	6	2	3	0.848
12	(0.333, 0.333, 0.000)	10.979	12	11	2	2	2	1.000

The followings are details for $CdPd_3$. It has 8 topological nodal lines and 28 nodal ring points along high symmetry line as shown in Fig **701**.

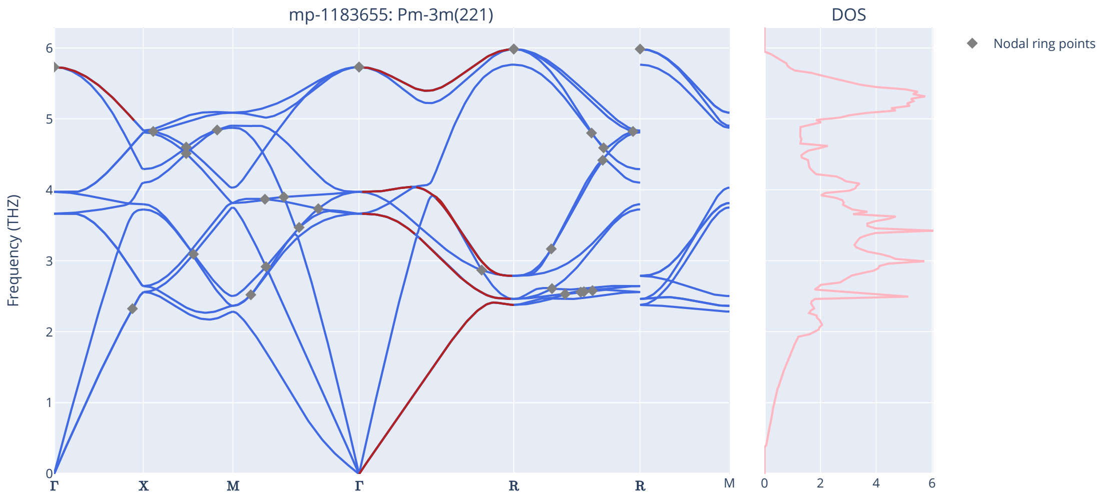

Supplementary Figure 701: The phonon band and DOS of CdPd3.

Supplementary Table 1485: High symmetry lines of mp-1183655-CdPd3

	Start	End
0	Γ (0.000, 0.000, 0.000)	X (0.000, 0.500, 0.000)
1	X (0.000, 0.500, 0.000)	M (0.500, 0.500, 0.000)
2	M (0.500, 0.500, 0.000)	Γ (0.000, 0.000, 0.000)
3	Γ (0.000, 0.000, 0.000)	R (0.500, 0.500, 0.500)
4	R (0.500, 0.500, 0.500)	X (0.000, 0.500, 0.000)
5	R (0.500, 0.500, 0.500)	M (0.500, 0.500, 0.000)

Supplementary Table 1486: Nodal ring points along high symmetry lines of mp-1183655-CdPd3.

	Coordinates	Frequency	Mode 1	Mode 2	Multi.	Deg.	Path	Ratio
0	(0.000, 0.439, 0.000)	2.326	2	1	6	2	1	0.878
1	(0.428, 0.428, 0.000)	2.522	3	2	12	2	3	0.143
2	(0.188, 0.495, 0.188)	2.580	3	2	24	2	5	0.623
3	(0.297, 0.500, 0.297)	2.532	3	2	12	2	5	0.407
4	(0.222, 0.494, 0.222)	2.565	3	2	24	2	5	0.555
5	(0.235, 0.494, 0.235)	2.560	3	2	24	2	5	0.530
6	(0.280, 0.500, -0.000)	3.094	5	4	12	2	2	0.560
7	(0.368, 0.368, -0.000)	2.918	5	4	12	2	3	0.263
8	(0.349, 0.500, 0.349)	2.610	5	4	12	2	5	0.302
9	(0.237, 0.237, 0.000)	3.471	6	5	12	2	3	0.525
10	(0.396, 0.396, 0.396)	2.869	6	5	8	2	4	0.793
11	(0.372, 0.372, 0.000)	3.868	7	6	12	2	3	0.255
12	(0.161, 0.161, -0.000)	3.735	7	6	12	2	3	0.677
13	(0.240, 0.500, -0.000)	4.512	8	7	12	2	2	0.479

Supplementary Table 1486: Nodal ring points along high symmetry lines of mp-1183655-CdPd3.

	Coordinates	Frequency	Mode 1	Mode 2	Multi.	Deg.	Path	Ratio
14	(0.298, 0.298, 0.000)	3.900	8	7	12	2	3	0.405
15	(0.352, 0.500, 0.352)	3.168	8	7	12	2	5	0.297
16	(0.148, 0.500, 0.148)	4.418	8	7	12	2	5	0.704
17	(0.240, 0.500, -0.000)	4.604	10	9	12	2	2	0.479
18	(0.413, 0.500, 0.000)	4.844	10	9	12	2	2	0.826
19	(0.192, 0.500, 0.192)	4.802	10	9	12	2	5	0.616
20	(0.144, 0.500, 0.144)	4.593	10	9	12	2	5	0.711
21	(0.000, 0.000, 0.000)	5.731	11	10	1	3	1	0.000
22	(0.055, 0.500, -0.000)	4.824	11	10	12	2	2	0.110
23	(0.028, 0.500, 0.028)	4.824	11	10	12	2	5	0.944
24	(0.500, 0.500, 0.500)	5.985	12	11	1	3	4	1.000

The followings are details for Cu$_3$. It has 11 topological nodal lines and 29 nodal ring points along high symmetry line as shown in Fig 702.

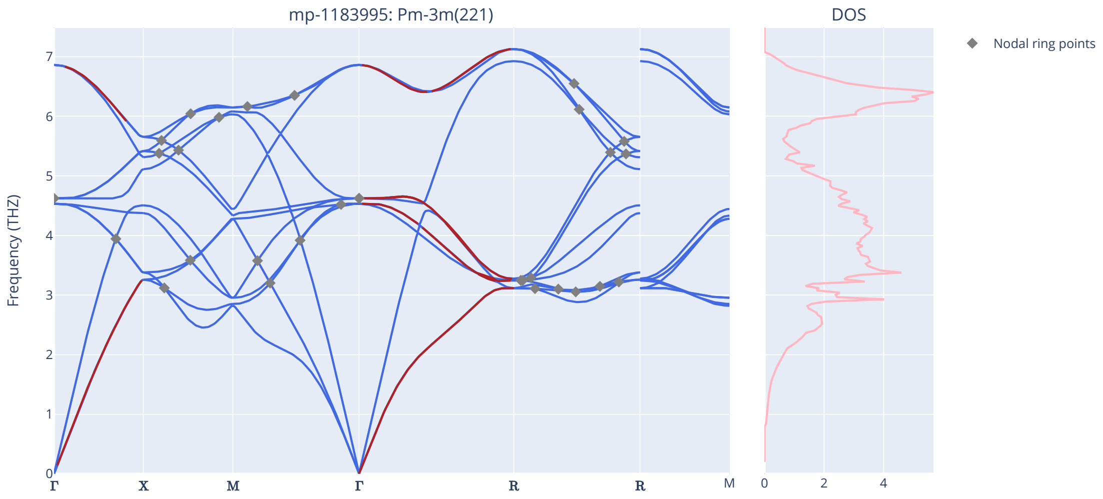

Supplementary Figure 702: The phonon band and DOS of Cu3Ga.

Supplementary Table 1487: High symmetry lines of mp-1183995-Cu3Ga

	Start	End
0	Γ (0.000, 0.000, 0.000)	X (0.000, 0.500, 0.000)
1	X (0.000, 0.500, 0.000)	M (0.500, 0.500, 0.000)
2	M (0.500, 0.500, 0.000)	Γ (0.000, 0.000, 0.000)
3	Γ (0.000, 0.000, 0.000)	R (0.500, 0.500, 0.500)
4	R (0.500, 0.500, 0.500)	X (0.000, 0.500, 0.000)
5	R (0.500, 0.500, 0.500)	M (0.500, 0.500, 0.000)

Supplementary Table 1488: Nodal ring points along high symmetry lines of mp-1183995-Cu3Ga.

	Coordinates	Frequency	Mode 1	Mode 2	Multi.	Deg.	Path	Ratio
0	(0.117, 0.500, 0.000)	3.119	2	1	12	2	2	0.234
1	(0.416, 0.500, 0.416)	3.108	2	1	12	2	5	0.168
2	(0.352, 0.352, -0.000)	3.202	3	2	12	2	3	0.296
3	(0.256, 0.500, 0.254)	3.054	3	2	24	2	5	0.490
4	(0.084, 0.500, 0.084)	3.219	3	2	12	2	5	0.832
5	(0.000, 0.346, 0.000)	3.944	4	3	6	2	1	0.692
6	(0.233, 0.233, 0.000)	3.919	4	3	12	2	3	0.533
7	(0.159, 0.500, 0.159)	3.146	4	3	12	2	5	0.682
8	(0.324, 0.500, 0.324)	3.100	4	3	12	2	5	0.353
9	(0.263, 0.500, -0.000)	3.584	5	4	12	2	2	0.525
10	(0.402, 0.402, 0.000)	3.577	5	4	12	2	3	0.196
11	(0.465, 0.500, 0.465)	3.245	5	4	12	2	5	0.071
12	(0.071, 0.071, -0.000)	4.521	6	5	12	2	3	0.857
13	(0.472, 0.500, 0.472)	3.247	6	5	12	2	5	0.056

Supplementary Table 1488: Nodal ring points along high symmetry lines of mp-1183995-Cu3Ga.

	Coordinates	Frequency	Mode 1	Mode 2	Multi.	Deg.	Path	Ratio
14	(0.432, 0.500, 0.432)	3.291	7	6	12	2	5	0.136
15	(0.000, 0.000, 0.000)	4.624	9	8	1	3	1	0.000
16	(0.088, 0.500, -0.000)	5.379	9	8	12	2	2	0.175
17	(0.197, 0.500, -0.000)	5.432	9	8	12	2	2	0.393
18	(0.056, 0.500, 0.056)	5.369	9	8	12	2	5	0.888
19	(0.118, 0.500, 0.118)	5.396	9	8	12	2	5	0.765
20	(0.423, 0.500, 0.000)	5.984	10	9	12	2	2	0.847
21	(0.241, 0.500, 0.241)	6.117	10	9	12	2	5	0.518
22	(0.101, 0.500, -0.000)	5.595	11	10	12	2	2	0.203
23	(0.063, 0.500, 0.063)	5.580	11	10	12	2	5	0.874
24	(0.265, 0.500, -0.000)	6.043	12	11	12	2	2	0.529
25	(0.442, 0.442, -0.000)	6.164	12	11	12	2	3	0.116
26	(0.255, 0.255, 0.000)	6.352	12	11	12	2	3	0.489
27	(0.261, 0.500, 0.261)	6.551	12	11	12	2	5	0.477

The followings are details for Cu_2Cl_2. As shown in Fig 703, it has 23 topological nodal lines, 29 nodal ring points and 2 high degenerate Weyl points whose degeneracy are more than 2 and charges are bigger than 1.

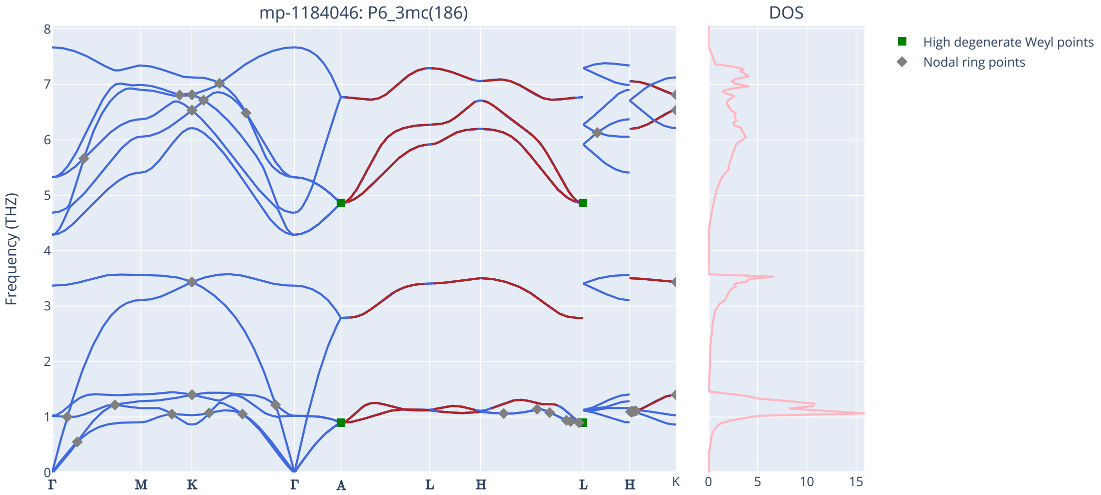

Supplementary Figure 703: The phonon band and DOS of Cu2Cl2.

Supplementary Table 1489: High symmetry lines of mp-1184046-Cu2Cl2

	Start	End
0	Γ (0.000, 0.000, 0.000)	M (0.500, 0.000, 0.000)
1	M (0.500, 0.000, 0.000)	K (0.333, 0.333, 0.000)
2	K (0.333, 0.333, 0.000)	Γ (0.000, 0.000, 0.000)
3	Γ (0.000, 0.000, 0.000)	A (0.000, 0.000, 0.500)
4	A (0.000, 0.000, 0.500)	L (0.500, 0.000, 0.500)
5	L (0.500, 0.000, 0.500)	H (0.333, 0.333, 0.500)
6	H (0.333, 0.333, 0.500)	A (0.000, 0.000, 0.500)
7	L (0.500, 0.000, 0.500)	M (0.500, 0.000, 0.000)
8	H (0.333, 0.333, 0.500)	K (0.333, 0.333, 0.000)

Supplementary Table 1490: High degenerate Weyl points along high symmetry lines of mp-1184046-Cu2Cl2.

	Coordinates	Frequency	Mode 1	Mode 2	Multi.	Deg.	Path	Ratio
0	(0.000, 0.000, 0.500)	0.895	4	1	1	4	4	1.000
1	(0.000, 0.000, 0.500)	4.856	10	7	1	4	4	1.000

Supplementary Table 1491: Nodal ring points along high symmetry lines of mp-1184046-Cu2Cl2.

	Coordinates	Frequency	Mode 1	Mode 2	Multi.	Deg.	Path	Ratio
0	(0.399, 0.203, 0.000)	1.050	2	1	6	2	2	0.608

Supplementary Table 1491: Nodal ring points along high symmetry lines of mp-1184046-Cu2Cl2.

	Coordinates	Frequency	Mode 1	Mode 2	Multi.	Deg.	Path	Ratio
1	(0.277, 0.277, 0.000)	1.073	2	1	6	2	3	0.168
2	(0.142, 0.000, 0.000)	0.551	2	1	6	2	1	0.285
3	(0.169, 0.169, -0.000)	1.052	2	1	6	2	3	0.493
4	(0.259, 0.259, 0.500)	1.062	2	1	6	2	7	0.224
5	(0.150, 0.150, 0.500)	1.141	2	1	6	2	7	0.551
6	(0.109, 0.109, 0.500)	1.079	2	1	6	2	7	0.673
7	(0.054, 0.054, 0.500)	0.940	2	1	6	2	7	0.837
8	(0.041, 0.041, 0.500)	0.920	2	1	6	2	7	0.878
9	(0.014, 0.014, 0.500)	0.897	2	1	6	2	7	0.959
10	(0.333, 0.333, 0.490)	1.094	2	1	4	2	9	0.020
11	(0.333, 0.333, 0.480)	1.095	2	1	4	2	9	0.041
12	(0.333, 0.333, 0.469)	1.097	2	1	4	2	9	0.061
13	(0.354, 0.000, 0.000)	1.217	3	2	6	2	1	0.709
14	(0.333, 0.333, 0.451)	1.102	3	2	4	2	9	0.098
15	(0.333, 0.333, 0.433)	1.109	3	2	4	2	9	0.133
16	(0.333, 0.333, 0.000)	1.400	4	3	2	2	2	1.000
17	(0.085, 0.000, -0.000)	1.001	4	3	6	2	1	0.171
18	(0.061, 0.061, -0.000)	1.218	5	4	6	2	3	0.816
19	(0.333, 0.333, 0.000)	3.434	6	5	2	2	2	1.000
20	(0.333, 0.333, 0.000)	6.533	9	8	2	2	2	1.000
21	(0.498, 0.003, 0.348)	6.129	9	8	12	2	8	0.304
22	(0.179, 0.000, 0.000)	5.662	10	9	6	2	1	0.358
23	(0.296, 0.296, -0.000)	6.714	10	9	6	2	3	0.113
24	(0.158, 0.158, -0.000)	6.483	11	10	6	2	3	0.527
25	(0.333, 0.333, 0.000)	6.812	11	10	2	2	2	1.000
26	(0.373, 0.253, 0.000)	6.806	11	10	6	2	2	0.760
27	(0.243, 0.243, 0.000)	7.017	12	11	6	2	3	0.270

The followings are details for Pd_2H_2. As shown in Fig 704, it has 23 topological nodal lines and 14 nodal ring points. At the same time, it has 2 single Weyl points and 1 high degenerate Weyl point whose degeneracy is more than 2 and charge is bigger than 1.

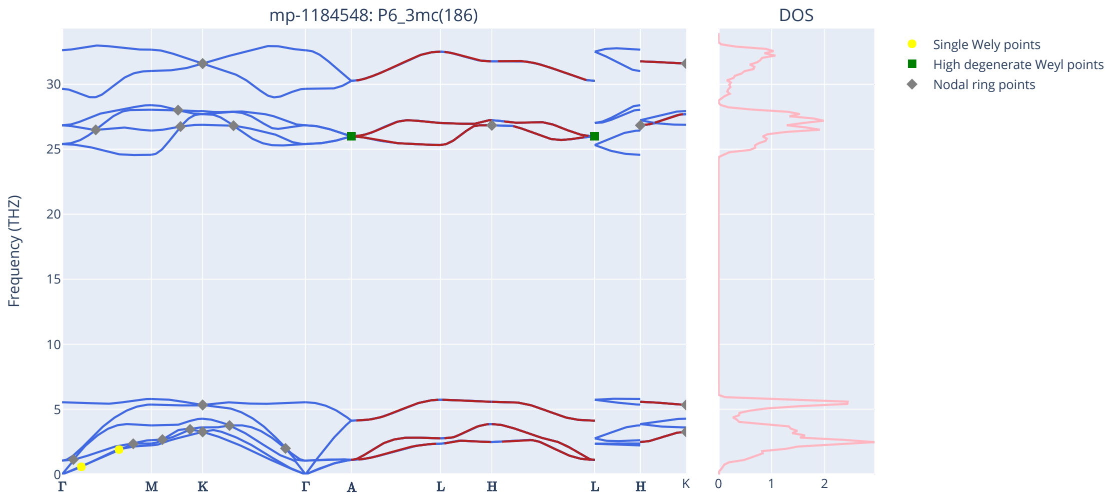

Supplementary Figure 704: The phonon band and DOS of Pd2H2.

Supplementary Table 1492: High symmetry lines of mp-1184548-Pd2H2

	Start	End
0	Γ (0.000, 0.000, 0.000)	M (0.500, 0.000, 0.000)
1	M (0.500, 0.000, 0.000)	K (0.333, 0.333, 0.000)
2	K (0.333, 0.333, 0.000)	Γ (0.000, 0.000, 0.000)
3	Γ (0.000, 0.000, 0.000)	A (0.000, 0.000, 0.500)
4	A (0.000, 0.000, 0.500)	L (0.500, 0.000, 0.500)
5	L (0.500, 0.000, 0.500)	H (0.333, 0.333, 0.500)
6	H (0.333, 0.333, 0.500)	A (0.000, 0.000, 0.500)
7	L (0.500, 0.000, 0.500)	M (0.500, 0.000, 0.000)
8	H (0.333, 0.333, 0.500)	K (0.333, 0.333, 0.000)

Supplementary Table 1493: Single Weyl points along high symmetry lines of mp-1184548-Pd2H2.

	Coordinates	Frequency	Mode 1	Mode 2	Multi.	Deg.	Path	Ratio
0	(0.108, 0.000, -0.000)	0.578	2	1	6	2	1	0.216
1	(0.319, 0.000, -0.000)	1.905	2	1	6	2	1	0.639

Supplementary Table 1494: High degenerate Weyl points along high symmetry lines of mp-1184548-Pd2H2.

	Coordinates	Frequency	Mode 1	Mode 2	Multi.	Deg.	Path	Ratio
0	(0.000, 0.000, 0.500)	25.995	10	7	1	4	4	1.000

Supplementary Table 1495: Nodal ring points along high symmetry lines of mp-1184548-Pd2H2.

	Coordinates	Frequency	Mode 1	Mode 2	Multi.	Deg.	Path	Ratio

Supplementary Table 1495: Nodal ring points along high symmetry lines of mp-1184548-Pd2H2.

	Coordinates	Frequency	Mode 1	Mode 2	Multi.	Deg.	Path	Ratio
0	(0.333, 0.333, 0.000)	3.267	2	1	2	2	2	1.000
1	(0.464, 0.073, 0.000)	2.674	3	2	6	2	2	0.219
2	(0.399, -0.000, 0.000)	2.350	3	2	6	2	1	0.799
3	(0.374, 0.252, 0.000)	3.453	3	2	6	2	2	0.756
4	(0.246, 0.246, 0.000)	3.752	4	3	6	2	3	0.261
5	(0.063, -0.000, -0.000)	1.134	4	3	6	2	1	0.126
6	(0.065, 0.065, -0.000)	1.994	5	4	6	2	3	0.805
7	(0.333, 0.333, 0.000)	5.335	6	5	2	2	2	1.000
8	(0.405, 0.190, -0.000)	26.745	8	7	6	2	2	0.570
9	(0.233, 0.233, 0.000)	26.810	8	7	6	2	3	0.300
10	(0.333, 0.333, 0.500)	26.841	8	7	2	2	6	1.000
11	(0.189, 0.000, 0.000)	26.492	9	8	6	2	1	0.378
12	(0.413, 0.174, 0.000)	28.005	10	9	6	2	2	0.523
13	(0.333, 0.333, 0.000)	31.589	12	11	2	2	2	1.000

The followings are details for Hf$_3$. It has 8 topological nodal lines and 26 nodal ring points along high symmetry line as shown in Fig 705.

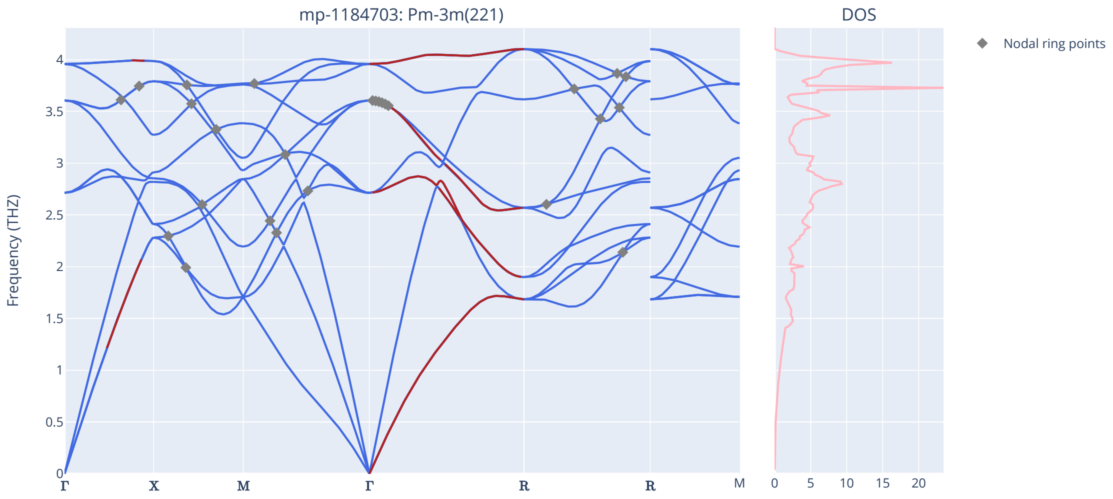

Supplementary Figure 705: The phonon band and DOS of Hf3Pb.

Supplementary Table 1496: High symmetry lines of mp-1184703-Hf3Pb

	Start	End
0	Γ (0.000, 0.000, 0.000)	X (0.000, 0.500, 0.000)
1	X (0.000, 0.500, 0.000)	M (0.500, 0.500, 0.000)
2	M (0.500, 0.500, 0.000)	Γ (0.000, 0.000, 0.000)
3	Γ (0.000, 0.000, 0.000)	R (0.500, 0.500, 0.500)
4	R (0.500, 0.500, 0.500)	X (0.000, 0.500, 0.000)
5	R (0.500, 0.500, 0.500)	M (0.500, 0.500, 0.000)

Supplementary Table 1497: Nodal ring points along high symmetry lines of mp-1184703-Hf3Pb.

	Coordinates	Frequency	Mode 1	Mode 2	Multi.	Deg.	Path	Ratio
0	(0.179, 0.500, 0.000)	1.991	2	1	12	2	2	0.359
1	(0.109, 0.500, 0.109)	2.139	2	1	12	2	5	0.782
2	(0.081, 0.500, 0.000)	2.296	3	2	12	2	2	0.161
3	(0.368, 0.368, 0.000)	2.328	4	3	12	2	3	0.265
4	(0.244, 0.244, -0.000)	2.732	5	4	12	2	3	0.513
5	(0.393, 0.393, 0.000)	2.443	5	4	12	2	3	0.213
6	(0.270, 0.500, 0.000)	2.600	6	5	12	2	2	0.541
7	(0.333, 0.333, 0.000)	3.083	7	6	12	2	3	0.333
8	(0.411, 0.500, 0.411)	2.602	8	7	12	2	5	0.177
9	(0.000, 0.316, 0.000)	3.611	9	8	6	2	1	0.633
10	(0.349, 0.500, -0.000)	3.326	9	8	12	2	2	0.699
11	(0.211, 0.500, -0.000)	3.574	9	8	12	2	2	0.422
12	(0.010, 0.010, 0.010)	3.605	9	8	8	2	4	0.020
13	(0.020, 0.020, 0.020)	3.601	9	8	8	2	4	0.041

Supplementary Table 1497: Nodal ring points along high symmetry lines of mp-1184703-Hf3Pb.

	Coordinates	Frequency	Mode 1	Mode 2	Multi.	Deg.	Path	Ratio
14	(0.031, 0.031, 0.031)	3.594	9	8	8	2	4	0.061
15	(0.041, 0.041, 0.041)	3.584	9	8	8	2	4	0.082
16	(0.051, 0.051, 0.051)	3.571	9	8	8	2	4	0.102
17	(0.061, 0.061, 0.061)	3.555	9	8	8	2	4	0.122
18	(0.123, 0.500, 0.123)	3.537	9	8	12	2	5	0.755
19	(0.198, 0.500, 0.198)	3.428	9	8	12	2	5	0.604
20	(0.000, 0.418, 0.000)	3.745	10	9	6	2	1	0.835
21	(0.301, 0.500, 0.301)	3.717	10	9	12	2	5	0.398
22	(0.185, 0.500, -0.000)	3.755	11	10	12	2	2	0.370
23	(0.097, 0.500, 0.097)	3.835	11	10	12	2	5	0.806
24	(0.456, 0.456, 0.000)	3.769	12	11	12	2	3	0.089
25	(0.131, 0.500, 0.131)	3.867	12	11	12	2	5	0.737

The followings are details for Li_2F_2. As shown in Fig **706**, it has 25 topological nodal lines, 21 nodal ring points and 2 high degenerate Weyl points whose degeneracy are more than 2 and charges are bigger than 1.

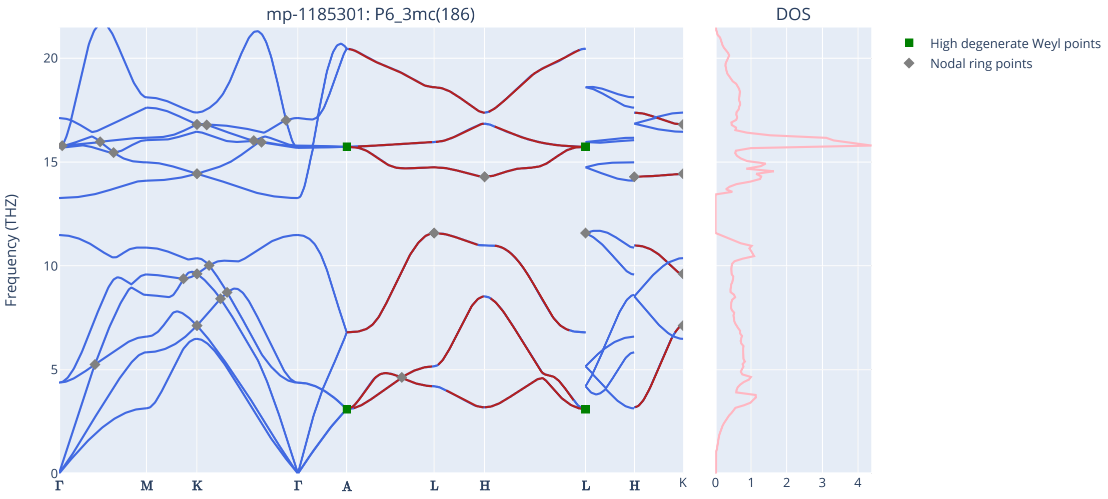

Supplementary Figure 706: The phonon band and DOS of Li2F2.

Supplementary Table 1498: High symmetry lines of mp-1185301-Li2F2

	Start	End
0	Γ (0.000, 0.000, 0.000)	M (0.500, 0.000, 0.000)
1	M (0.500, 0.000, 0.000)	K (0.333, 0.333, 0.000)
2	K (0.333, 0.333, 0.000)	Γ (0.000, 0.000, 0.000)
3	Γ (0.000, 0.000, 0.000)	A (0.000, 0.000, 0.500)
4	A (0.000, 0.000, 0.500)	L (0.500, 0.000, 0.500)
5	L (0.500, 0.000, 0.500)	H (0.333, 0.333, 0.500)
6	H (0.333, 0.333, 0.500)	A (0.000, 0.000, 0.500)
7	L (0.500, 0.000, 0.500)	M (0.500, 0.000, 0.000)
8	H (0.333, 0.333, 0.500)	K (0.333, 0.333, 0.000)

Supplementary Table 1499: High degenerate Weyl points along high symmetry lines of mp-1185301-Li2F2.

	Coordinates	Frequency	Mode 1	Mode 2	Multi.	Deg.	Path	Ratio
0	(0.000, 0.000, 0.500)	3.099	4	1	1	4	4	1.000
1	(0.000, 0.000, 0.500)	15.737	10	7	1	4	4	1.000

Supplementary Table 1500: Nodal ring points along high symmetry lines of mp-1185301-Li2F2.

	Coordinates	Frequency	Mode 1	Mode 2	Multi.	Deg.	Path	Ratio
0	(0.333, 0.333, 0.000)	7.122	3	2	2	2	2	1.000

Supplementary Table 1500: Nodal ring points along high symmetry lines of mp-1185301-Li2F2.

	Coordinates	Frequency	Mode 1	Mode 2	Multi.	Deg.	Path	Ratio
1	(0.315, 0.000, 0.498)	4.627	3	2	12	2	5	0.630
2	(0.205, -0.000, -0.000)	5.248	4	3	6	2	1	0.410
3	(0.256, 0.256, -0.000)	8.415	4	3	6	2	3	0.233
4	(0.234, 0.234, 0.000)	8.723	5	4	6	2	3	0.298
5	(0.378, 0.244, -0.000)	9.379	5	4	6	2	2	0.732
6	(0.333, 0.333, 0.000)	9.614	5	4	2	2	2	1.000
7	(0.293, 0.293, -0.000)	10.019	6	5	6	2	3	0.120
8	(0.500, 0.000, 0.500)	11.580	6	5	3	2	5	1.000
9	(0.333, 0.333, 0.000)	14.437	8	7	2	2	2	1.000
10	(0.333, 0.333, 0.500)	14.290	8	7	2	2	6	1.000
11	(0.120, 0.120, -0.000)	15.954	9	8	6	2	3	0.639
12	(0.312, -0.000, 0.000)	15.456	9	8	6	2	1	0.623
13	(0.235, -0.000, 0.000)	15.976	10	9	6	2	1	0.470
14	(0.018, 0.000, 0.000)	15.787	10	9	6	2	1	0.036
15	(0.146, 0.146, -0.000)	16.033	10	9	6	2	3	0.562
16	(0.301, 0.301, -0.000)	16.788	11	10	6	2	3	0.096
17	(0.333, 0.333, 0.000)	16.810	11	10	2	2	2	1.000
18	(0.039, 0.039, -0.000)	17.010	12	11	6	2	3	0.882

The followings are details for Li_2Cl_2. As shown in Fig **707**, it has 22 topological nodal lines and 21 nodal ring points. At the same time, it has 1 single Weyl point and 2 high degenerate Weyl points whose degeneracy are more than 2 and charges are bigger than 1.

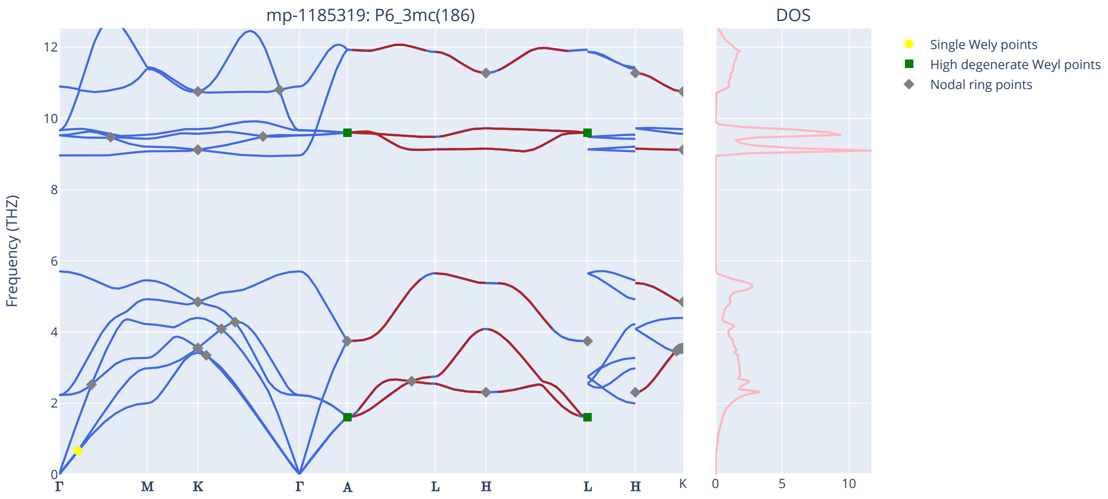

Supplementary Figure 707: The phonon band and DOS of Li2Cl2.

Supplementary Table 1501: High symmetry lines of mp-1185319-Li2Cl2

	Start	End
0	Γ (0.000, 0.000, 0.000)	M (0.500, 0.000, 0.000)
1	M (0.500, 0.000, 0.000)	K (0.333, 0.333, 0.000)
2	K (0.333, 0.333, 0.000)	Γ (0.000, 0.000, 0.000)
3	Γ (0.000, 0.000, 0.000)	A (0.000, 0.000, 0.500)
4	A (0.000, 0.000, 0.500)	L (0.500, 0.000, 0.500)
5	L (0.500, 0.000, 0.500)	H (0.333, 0.333, 0.500)
6	H (0.333, 0.333, 0.500)	A (0.000, 0.000, 0.500)
7	L (0.500, 0.000, 0.500)	M (0.500, 0.000, 0.000)
8	H (0.333, 0.333, 0.500)	K (0.333, 0.333, 0.000)

Supplementary Table 1502: Single Weyl points along high symmetry lines of mp-1185319-Li2Cl2.

	Coordinates	Frequency	Mode 1	Mode 2	Multi.	Deg.	Path	Ratio
0	(0.107, -0.000, 0.000)	0.664	2	1	6	2	1	0.214

Supplementary Table 1503: High degenerate Weyl points along high symmetry lines of mp-1185319-Li2Cl2.

	Coordinates	Frequency	Mode 1	Mode 2	Multi.	Deg.	Path	Ratio
0	(0.000, 0.000, 0.500)	1.609	4	1	1	4	4	1.000
1	(0.000, 0.000, 0.500)	9.600	10	7	1	4	4	1.000

Supplementary Table 1504: Nodal ring points along high symmetry lines of mp-1185319-Li2Cl2.

	Coordinates	Frequency	Mode 1	Mode 2	Multi.	Deg.	Path	Ratio

Supplementary Table 1504: Nodal ring points along high symmetry lines of mp-1185319-Li2Cl2.

	Coordinates	Frequency	Mode 1	Mode 2	Multi.	Deg.	Path	Ratio
0	(0.306, 0.306, -0.000)	3.346	2	1	6	2	3	0.083
1	(0.333, 0.333, 0.500)	2.304	2	1	2	2	6	1.000
2	(0.333, 0.333, 0.000)	3.547	3	2	2	2	2	1.000
3	(0.366, 0.000, 0.498)	2.616	3	2	12	2	5	0.733
4	(0.333, 0.333, 0.016)	3.542	3	2	4	2	9	0.969
5	(0.333, 0.333, 0.071)	3.465	3	2	4	2	9	0.857
6	(0.333, 0.333, 0.061)	3.486	3	2	4	2	9	0.878
7	(0.333, 0.333, 0.051)	3.504	3	2	4	2	9	0.898
8	(0.333, 0.333, 0.041)	3.519	3	2	4	2	9	0.918
9	(0.333, 0.333, 0.031)	3.531	3	2	4	2	9	0.939
10	(0.256, 0.256, -0.000)	4.088	4	3	6	2	3	0.232
11	(0.184, 0.000, 0.000)	2.521	4	3	6	2	1	0.368
12	(0.211, 0.211, 0.000)	4.279	5	4	6	2	3	0.366
13	(0.333, 0.333, 0.000)	4.845	6	5	2	2	2	1.000
14	(0.000, 0.000, 0.500)	3.744	6	5	1	2	4	1.000
15	(0.333, 0.333, 0.000)	9.120	8	7	2	2	2	1.000
16	(0.120, 0.120, -0.000)	9.493	9	8	6	2	3	0.641
17	(0.293, 0.000, 0.000)	9.474	9	8	6	2	1	0.586
18	(0.066, 0.066, -0.000)	10.806	12	11	6	2	3	0.802
19	(0.333, 0.333, 0.000)	10.758	12	11	2	2	2	1.000
20	(0.333, 0.333, 0.500)	11.277	12	11	2	2	6	1.000

The followings are details for Au_3. It has 8 topological nodal lines and 16 nodal ring points along high symmetry line as shown in Fig **708**.

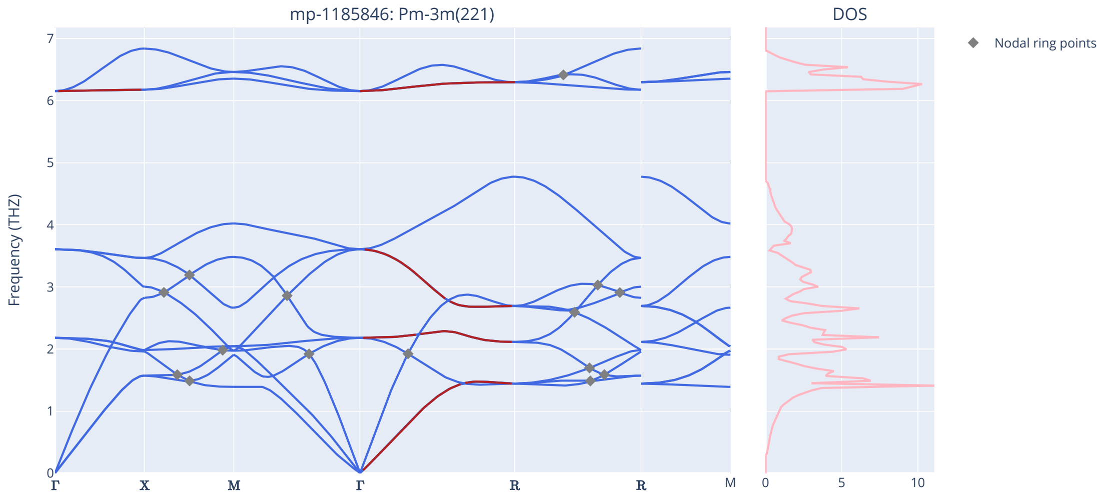

Supplementary Figure 708: The phonon band and DOS of Au3Mg.

Supplementary Table 1505: High symmetry lines of mp-1185846-Au3Mg

	Start	End
0	Γ (0.000, 0.000, 0.000)	X (0.000, 0.500, 0.000)
1	X (0.000, 0.500, 0.000)	M (0.500, 0.500, 0.000)
2	M (0.500, 0.500, 0.000)	Γ (0.000, 0.000, 0.000)
3	Γ (0.000, 0.000, 0.000)	R (0.500, 0.500, 0.500)
4	R (0.500, 0.500, 0.500)	X (0.000, 0.500, 0.000)
5	R (0.500, 0.500, 0.500)	M (0.500, 0.500, 0.000)

Supplementary Table 1506: Nodal ring points along high symmetry lines of mp-1185846-Au3Mg.

	Coordinates	Frequency	Mode 1	Mode 2	Multi.	Deg.	Path	Ratio
0	(0.253, 0.500, 0.000)	1.487	2	1	12	2	2	0.506
1	(0.201, 0.500, 0.201)	1.486	2	1	12	2	5	0.598
2	(0.184, 0.500, -0.000)	1.586	3	2	12	2	2	0.368
3	(0.145, 0.500, 0.145)	1.588	3	2	12	2	5	0.709
4	(0.438, 0.500, -0.000)	1.982	4	3	12	2	2	0.876
5	(0.202, 0.202, -0.000)	1.921	4	3	12	2	3	0.596
6	(0.155, 0.155, 0.155)	1.923	4	3	8	2	4	0.310
7	(0.204, 0.500, 0.204)	1.695	4	3	12	2	5	0.592
8	(0.263, 0.500, 0.263)	2.591	6	5	12	2	5	0.474
9	(0.109, 0.500, -0.000)	2.911	7	6	12	2	2	0.219
10	(0.289, 0.289, -0.000)	2.859	7	6	12	2	3	0.423
11	(0.084, 0.500, 0.084)	2.909	7	6	12	2	5	0.832
12	(0.252, 0.500, -0.000)	3.192	8	7	12	2	2	0.504
13	(0.171, 0.500, 0.171)	3.030	8	7	12	2	5	0.657
14	(0.308, 0.500, 0.308)	6.414	12	11	12	2	5	0.385

Supplementary Table 1506: Nodal ring points along high symmetry lines of mp-1185846-Au3Mg.

Coordinates	Frequency	Mode 1	Mode 2	Multi.	Deg.	Path	Ratio

The followings are details for Mg_2S_2. As shown in Fig 709, it has 18 topological nodal lines, 6 nodal ring points and 7 single Weyl points.

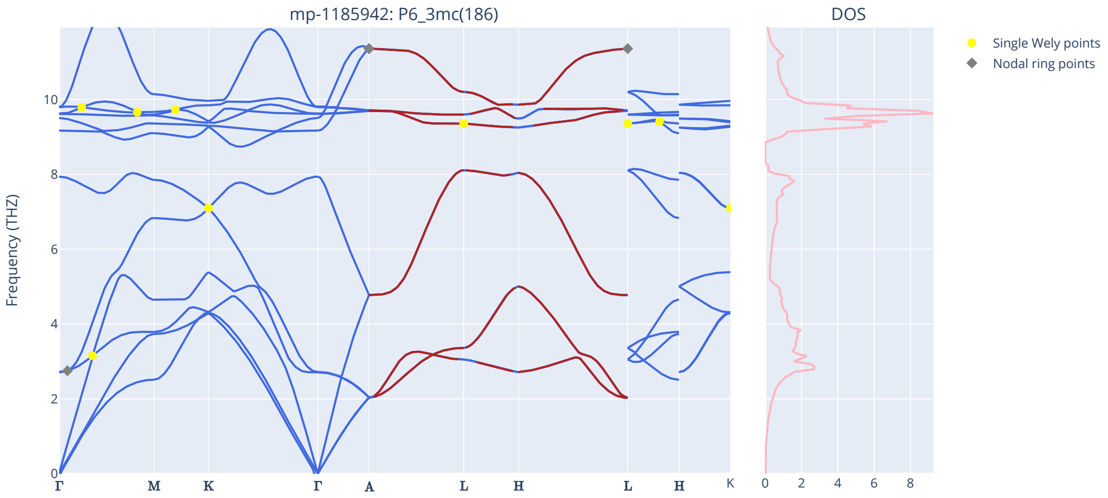

Supplementary Figure 709: The phonon band and DOS of Mg2S2.

Supplementary Table 1507: High symmetry lines of mp-1185942-Mg2S2

	Start	End
0	Γ (0.000, 0.000, 0.000)	M (0.500, 0.000, 0.000)
1	M (0.500, 0.000, 0.000)	K (0.333, 0.333, 0.000)
2	K (0.333, 0.333, 0.000)	Γ (0.000, 0.000, 0.000)
3	Γ (0.000, 0.000, 0.000)	A (0.000, 0.000, 0.500)
4	A (0.000, 0.000, 0.500)	L (0.500, 0.000, 0.500)
5	L (0.500, 0.000, 0.500)	H (0.333, 0.333, 0.500)
6	H (0.333, 0.333, 0.500)	A (0.000, 0.000, 0.500)
7	L (0.500, 0.000, 0.500)	M (0.500, 0.000, 0.000)
8	H (0.333, 0.333, 0.500)	K (0.333, 0.333, 0.000)

Supplementary Table 1508: Single Weyl points along high symmetry lines of mp-1185942-Mg2S2.

	Coordinates	Frequency	Mode 1	Mode 2	Multi.	Deg.	Path	Ratio
0	(0.176, 0.000, 0.000)	3.154	4	3	6	2	1	0.353
1	(0.500, 0.000, 0.500)	9.356	8	7	3	2	5	1.000
2	(0.500, 0.000, 0.189)	9.411	8	7	6	2	8	0.623
3	(0.120, 0.002, 0.000)	9.792	11	10	12	2	1	0.240
4	(0.413, 0.001, 0.000)	9.660	11	10	12	2	1	0.826
5	(0.433, 0.132, -0.000)	9.722	11	10	12	2	2	0.396
6	(0.333, 0.333, 0.000)	7.095	6	5	2	2	2	1.000

Supplementary Table 1509: Nodal ring points along high symmetry lines of mp-1185942-Mg2S2.

	Coordinates	Frequency	Mode 1	Mode 2	Multi.	Deg.	Path	Ratio
0	(0.044, 0.000, 0.000)	2.752	5	4	6	2	1	0.089
1	(0.000, 0.000, 0.500)	11.359	12	11	1	2	4	1.000

The followings are details for Ni$_3$. It has 8 topological nodal lines and 20 nodal ring points along high symmetry line as shown in Fig **710**.

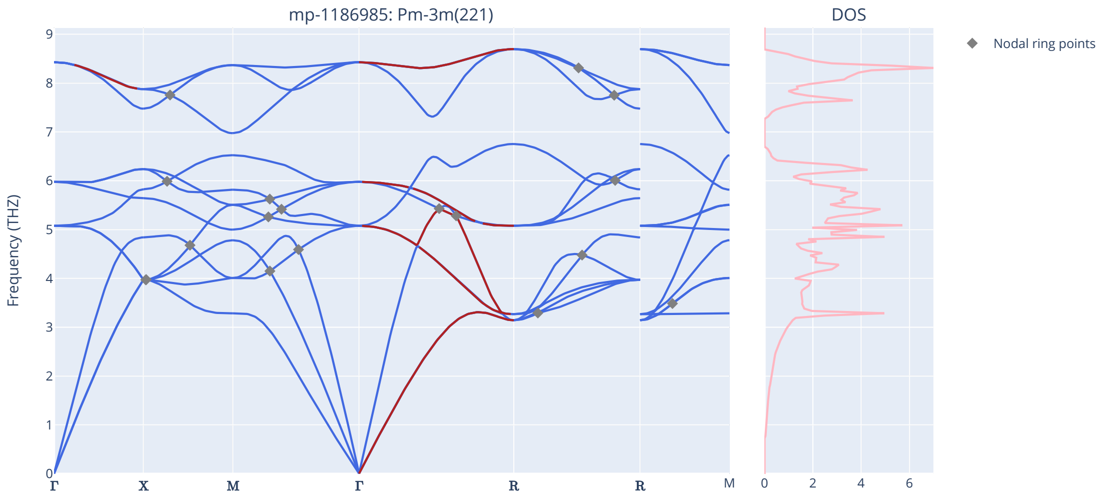

Supplementary Figure 710: The phonon band and DOS of Ni3Sc.

Supplementary Table 1510: High symmetry lines of mp-1186985-Ni3Sc

	Start	End
0	Γ (0.000, 0.000, 0.000)	X (0.000, 0.500, 0.000)
1	X (0.000, 0.500, 0.000)	M (0.500, 0.500, 0.000)
2	M (0.500, 0.500, 0.000)	Γ (0.000, 0.000, 0.000)
3	Γ (0.000, 0.000, 0.000)	R (0.500, 0.500, 0.500)
4	R (0.500, 0.500, 0.500)	X (0.000, 0.500, 0.000)
5	R (0.500, 0.500, 0.500)	M (0.500, 0.500, 0.000)

Supplementary Table 1511: Nodal ring points along high symmetry lines of mp-1186985-Ni3Sc.

	Coordinates	Frequency	Mode 1	Mode 2	Multi.	Deg.	Path	Ratio
0	(0.013, 0.500, -0.000)	3.971	2	1	12	2	2	0.026
1	(0.405, 0.500, 0.405)	3.292	2	1	12	2	5	0.191
2	(0.353, 0.353, 0.000)	4.150	3	2	12	2	3	0.294
3	(0.499, 0.499, 0.319)	3.486	3	2	24	2	6	0.362
4	(0.016, 0.500, -0.000)	3.975	4	3	12	2	2	0.032
5	(0.240, 0.240, -0.000)	4.592	4	3	12	2	3	0.521
6	(0.261, 0.500, 0.000)	4.680	5	4	12	2	2	0.521
7	(0.230, 0.500, 0.230)	4.478	5	4	12	2	5	0.541
8	(0.359, 0.359, 0.000)	5.260	6	5	12	2	3	0.282
9	(0.259, 0.259, 0.259)	5.430	6	5	8	2	4	0.518
10	(0.314, 0.314, 0.314)	5.282	6	5	8	2	4	0.628
11	(0.307, 0.307, 0.000)	5.417	7	6	12	2	3	0.386
12	(0.133, 0.500, 0.000)	5.989	8	7	12	2	2	0.265
13	(0.353, 0.353, 0.000)	5.625	8	7	12	2	3	0.293

Supplementary Table 1511: Nodal ring points along high symmetry lines of mp-1186985-Ni3Sc.

	Coordinates	Frequency	Mode 1	Mode 2	Multi.	Deg.	Path	Ratio
14	(0.099, 0.500, 0.099)	6.008	8	7	12	2	5	0.803
15	(0.149, 0.500, -0.000)	7.756	11	10	12	2	2	0.298
16	(0.102, 0.500, 0.102)	7.752	11	10	12	2	5	0.795
17	(0.244, 0.500, 0.244)	8.311	12	11	12	2	5	0.513

The followings are details for Ca_3. It has 6 topological nodal lines and 17 nodal ring points along high symmetry line as shown in Fig 711.

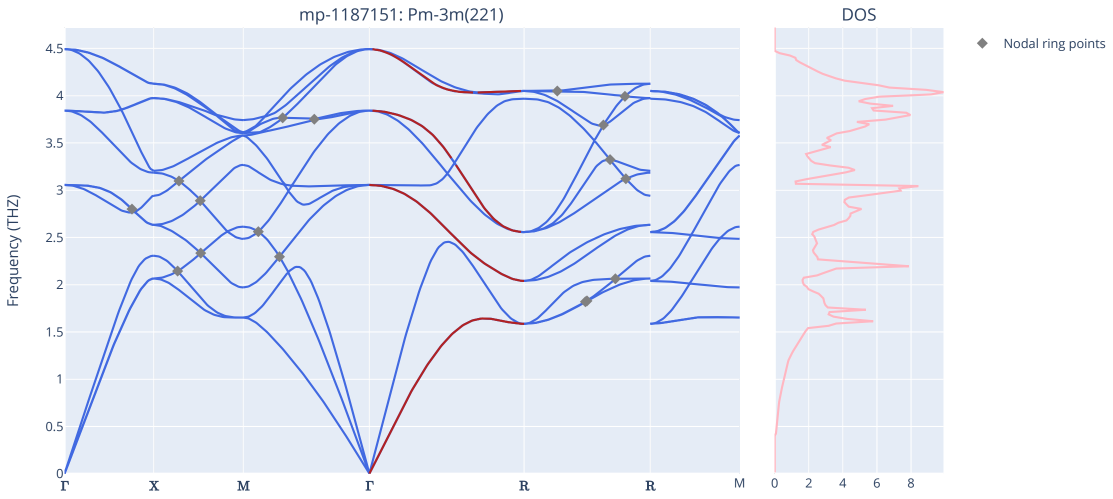

Supplementary Figure 711: The phonon band and DOS of Ca3Sr.

Supplementary Table 1512: High symmetry lines of mp-1187151-Ca3Sr

	Start	End
0	Γ (0.000, 0.000, 0.000)	X (0.000, 0.500, 0.000)
1	X (0.000, 0.500, 0.000)	M (0.500, 0.500, 0.000)
2	M (0.500, 0.500, 0.000)	Γ (0.000, 0.000, 0.000)
3	Γ (0.000, 0.000, 0.000)	R (0.500, 0.500, 0.500)
4	R (0.500, 0.500, 0.500)	X (0.000, 0.500, 0.000)
5	R (0.500, 0.500, 0.500)	M (0.500, 0.500, 0.000)

Supplementary Table 1513: Nodal ring points along high symmetry lines of mp-1187151-Ca3Sr.

	Coordinates	Frequency	Mode 1	Mode 2	Multi.	Deg.	Path	Ratio
0	(0.257, 0.500, 0.257)	1.820	2	1	12	2	5	0.486
1	(0.251, 0.494, 0.251)	1.829	2	1	24	2	5	0.498
2	(0.133, 0.500, 0.000)	2.144	3	2	12	2	2	0.266
3	(0.138, 0.500, 0.138)	2.063	3	2	12	2	5	0.723
4	(0.263, 0.500, 0.000)	2.334	4	3	12	2	2	0.525
5	(0.356, 0.356, -0.000)	2.296	4	3	12	2	3	0.288
6	(0.440, 0.440, 0.000)	2.561	5	4	12	2	3	0.120
7	(0.000, 0.378, 0.000)	2.800	6	5	6	2	1	0.755
8	(0.260, 0.500, 0.000)	2.889	6	5	12	2	2	0.520
9	(0.141, 0.500, -0.000)	3.097	7	6	12	2	2	0.282
10	(0.097, 0.500, 0.097)	3.121	7	6	12	2	5	0.806
11	(0.159, 0.500, 0.159)	3.323	8	7	12	2	5	0.681
12	(0.218, 0.218, -0.000)	3.751	9	8	12	2	3	0.564
13	(0.343, 0.343, 0.000)	3.766	10	9	12	2	3	0.313

Supplementary Table 1513: Nodal ring points along high symmetry lines of mp-1187151-Ca3Sr.

	Coordinates	Frequency	Mode 1	Mode 2	Multi.	Deg.	Path	Ratio
14	(0.186, 0.500, 0.186)	3.689	10	9	12	2	5	0.629
15	(0.100, 0.500, 0.100)	3.993	11	10	12	2	5	0.799
16	(0.368, 0.500, 0.368)	4.049	12	11	12	2	5	0.263

The followings are details for Os_4Ta_{12}. It has 7 topological nodal lines and 18 nodal ring points along high symmetry line as shown in Fig **712**.

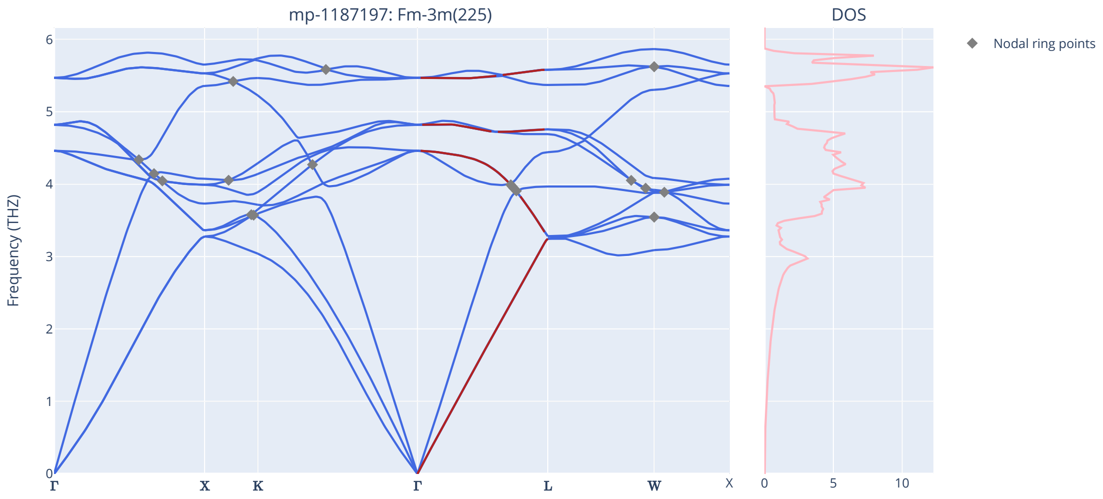

Supplementary Figure 712: The phonon band and DOS of Os4Ta12.

Supplementary Table 1514: High symmetry lines of mp-1187197-Os4Ta12

	Start	End
0	Γ (0.000, 0.000, 0.000)	X (0.500, 0.000, 0.500)
1	X (0.500, 0.000, 0.500)	U (0.625, 0.250, 0.625)
2	K (0.375, 0.375, 0.750)	Γ (0.000, 0.000, 0.000)
3	Γ (0.000, 0.000, 0.000)	L (0.500, 0.500, 0.500)
4	L (0.500, 0.500, 0.500)	W (0.500, 0.250, 0.750)
5	W (0.500, 0.250, 0.750)	X (0.500, 0.000, 0.500)

Supplementary Table 1515: Nodal ring points along high symmetry lines of mp-1187197-Os4Ta12.

	Coordinates	Frequency	Mode 1	Mode 2	Multi.	Deg.	Path	Ratio
0	(0.615, 0.231, 0.615)	3.571	3	2	24	2	2	0.922
1	(0.500, 0.250, 0.750)	3.545	3	2	6	2	5	1.000
2	(0.610, 0.219, 0.610)	3.578	4	3	12	2	2	0.878
3	(0.377, 0.377, 0.384)	3.906	5	4	24	2	4	0.759
4	(0.364, 0.364, 0.364)	3.967	5	4	8	2	4	0.728
5	(0.370, 0.370, 0.370)	3.943	5	4	8	2	4	0.740
6	(0.358, 0.358, 0.358)	3.991	5	4	8	2	4	0.716
7	(0.499, 0.216, 0.717)	3.888	5	4	24	2	6	0.135
8	(0.245, 0.245, 0.494)	4.270	6	5	24	2	3	0.343
9	(0.360, -0.000, 0.360)	4.045	7	6	6	2	1	0.719
10	(0.500, 0.270, 0.730)	3.944	7	6	24	2	5	0.920
11	(0.500, 0.304, 0.696)	4.052	7	6	24	2	5	0.784
12	(0.332, -0.000, 0.332)	4.145	8	7	6	2	1	0.664
13	(0.556, 0.113, 0.556)	4.054	8	7	12	2	2	0.451

Supplementary Table 1515: Nodal ring points along high symmetry lines of mp-1187197-Os4Ta12.

	Coordinates	Frequency	Mode 1	Mode 2	Multi.	Deg.	Path	Ratio
14	(0.282, 0.000, 0.282)	4.337	9	8	6	2	1	0.563
15	(0.567, 0.135, 0.567)	5.419	10	9	12	2	2	0.538
16	(0.500, 0.250, 0.750)	5.622	11	10	6	2	5	1.000
17	(0.220, 0.220, 0.425)	5.584	12	11	24	2	3	0.427

The followings are details for Re_4Ta_{12}. It has 8 topological nodal lines and 14 nodal ring points along high symmetry line as shown in Fig **713**.

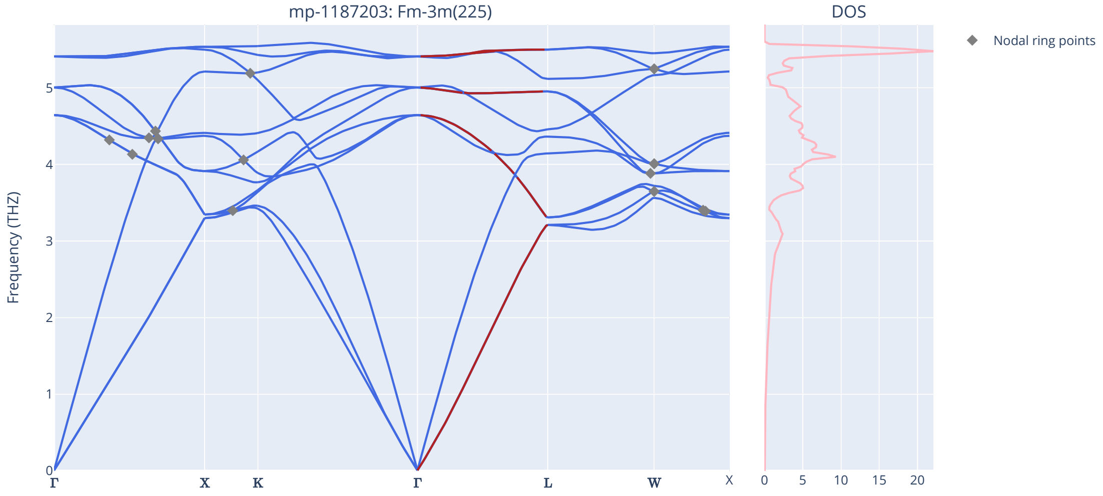

Supplementary Figure 713: The phonon band and DOS of Re4Ta12.

Supplementary Table 1516: High symmetry lines of mp-1187203-Re4Ta12

	Start	End
0	Γ (0.000, 0.000, 0.000)	X (0.500, 0.000, 0.500)
1	X (0.500, 0.000, 0.500)	U (0.625, 0.250, 0.625)
2	K (0.375, 0.375, 0.750)	Γ (0.000, 0.000, 0.000)
3	Γ (0.000, 0.000, 0.000)	L (0.500, 0.500, 0.500)
4	L (0.500, 0.500, 0.500)	W (0.500, 0.250, 0.750)
5	W (0.500, 0.250, 0.750)	X (0.500, 0.000, 0.500)

Supplementary Table 1517: Nodal ring points along high symmetry lines of mp-1187203-Re4Ta12.

	Coordinates	Frequency	Mode 1	Mode 2	Multi.	Deg.	Path	Ratio
0	(0.566, 0.133, 0.566)	3.397	3	2	12	2	2	0.530
1	(0.500, 0.250, 0.750)	3.647	3	2	6	2	5	1.000
2	(0.497, 0.089, 0.586)	3.403	3	2	24	2	6	0.650
3	(0.498, 0.080, 0.578)	3.390	3	2	24	2	6	0.683
4	(0.184, 0.000, 0.184)	4.320	5	4	6	2	1	0.367
5	(0.260, -0.008, 0.260)	4.133	5	4	24	2	1	0.520
6	(0.346, 0.000, 0.346)	4.335	6	5	6	2	1	0.691
7	(0.500, 0.259, 0.741)	3.883	6	5	24	2	5	0.965
8	(0.315, 0.000, 0.315)	4.347	7	6	6	2	1	0.630
9	(0.592, 0.183, 0.592)	4.059	7	6	12	2	2	0.733
10	(0.500, 0.250, 0.750)	4.009	8	7	6	2	5	1.000
11	(0.337, 0.000, 0.337)	4.435	9	8	6	2	1	0.673
12	(0.607, 0.215, 0.607)	5.190	10	9	12	2	2	0.859
13	(0.500, 0.250, 0.750)	5.248	11	10	6	2	5	1.000

The followings are details for GaZr$_3$. It has 7 topological nodal lines and 38 nodal ring points along high symmetry line as shown in Fig 714.

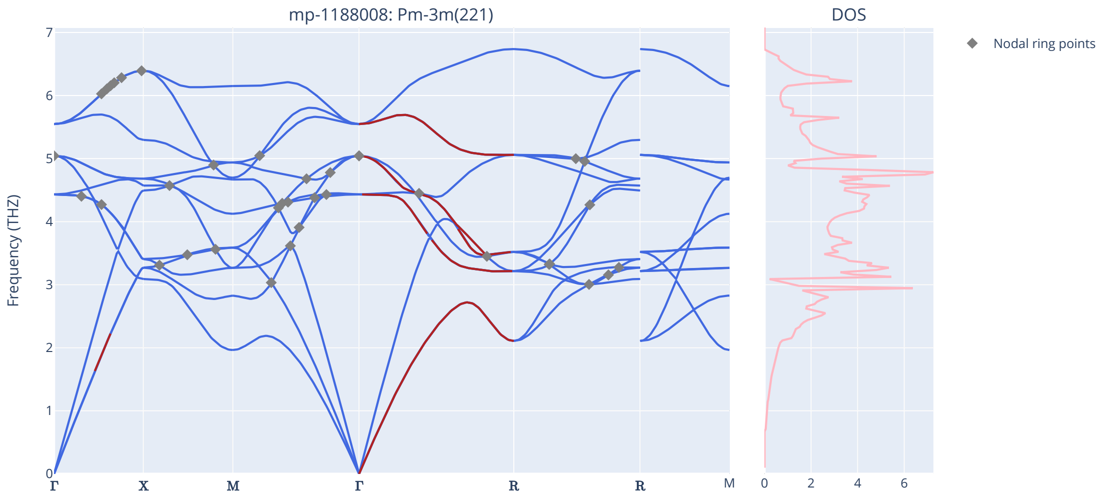

Supplementary Figure 714: The phonon band and DOS of GaZr3.

Supplementary Table 1518: High symmetry lines of mp-1188008-GaZr3

	Start	End
0	Γ (0.000, 0.000, 0.000)	X (0.000, 0.500, 0.000)
1	X (0.000, 0.500, 0.000)	M (0.500, 0.500, 0.000)
2	M (0.500, 0.500, 0.000)	Γ (0.000, 0.000, 0.000)
3	Γ (0.000, 0.000, 0.000)	R (0.500, 0.500, 0.500)
4	R (0.500, 0.500, 0.500)	X (0.000, 0.500, 0.000)
5	R (0.500, 0.500, 0.500)	M (0.500, 0.500, 0.000)

Supplementary Table 1519: Nodal ring points along high symmetry lines of mp-1188008-GaZr3.

	Coordinates	Frequency	Mode 1	Mode 2	Multi.	Deg.	Path	Ratio
0	(0.202, 0.500, 0.202)	3.003	2	1	12	2	5	0.596
1	(0.348, 0.348, 0.000)	3.032	3	2	12	2	3	0.305
2	(0.125, 0.500, 0.125)	3.154	3	2	12	2	5	0.749
3	(0.090, 0.500, -0.000)	3.309	4	3	12	2	2	0.180
4	(0.272, 0.272, -0.000)	3.615	4	3	12	2	3	0.456
5	(0.084, 0.500, 0.084)	3.273	4	3	12	2	5	0.832
6	(0.000, 0.153, 0.000)	4.401	5	4	6	2	1	0.306
7	(0.246, 0.500, 0.000)	3.474	5	4	12	2	2	0.491
8	(0.404, 0.500, 0.000)	3.561	5	4	12	2	2	0.807
9	(0.237, 0.237, -0.000)	3.908	5	4	12	2	3	0.526
10	(0.360, 0.500, 0.360)	3.321	5	4	12	2	5	0.280
11	(0.000, 0.265, 0.000)	4.270	6	5	6	2	1	0.531
12	(0.129, 0.129, -0.000)	4.429	6	5	12	2	3	0.741
13	(0.176, 0.176, 0.000)	4.379	6	5	12	2	3	0.648

Supplementary Table 1519: Nodal ring points along high symmetry lines of mp-1188008-GaZr3.

	Coordinates	Frequency	Mode 1	Mode 2	Multi.	Deg.	Path	Ratio
14	(0.413, 0.413, 0.413)	3.449	6	5	8	2	4	0.826
15	(0.358, 0.500, 0.358)	3.328	6	5	12	2	5	0.284
16	(0.146, 0.500, -0.000)	4.571	7	6	12	2	2	0.292
17	(0.281, 0.281, -0.000)	4.309	7	6	12	2	3	0.437
18	(0.319, 0.319, 0.000)	4.218	7	6	12	2	3	0.362
19	(0.199, 0.500, 0.199)	4.266	7	6	12	2	5	0.602
20	(0.304, 0.304, -0.000)	4.290	8	7	12	2	3	0.392
21	(0.114, 0.114, 0.000)	4.778	8	7	12	2	3	0.772
22	(0.000, 0.000, 0.000)	5.043	9	8	1	3	1	0.000
23	(0.208, 0.208, -0.000)	4.679	9	8	12	2	3	0.584
24	(0.194, 0.194, 0.194)	4.448	9	8	8	2	4	0.388
25	(0.393, 0.499, -0.000)	4.897	10	9	24	2	2	0.786
26	(0.219, 0.500, 0.219)	4.955	10	9	12	2	5	0.562
27	(0.393, 0.393, -0.000)	5.046	11	10	12	2	3	0.213
28	(0.254, 0.500, 0.254)	4.998	11	10	12	2	5	0.491
29	(0.000, 0.265, 0.000)	6.027	12	11	6	2	1	0.531
30	(0.000, 0.296, 0.000)	6.106	12	11	6	2	1	0.592
31	(0.000, 0.316, 0.000)	6.156	12	11	6	2	1	0.633
32	(0.000, 0.337, 0.000)	6.202	12	11	6	2	1	0.673
33	(0.000, 0.378, 0.000)	6.283	12	11	6	2	1	0.755
34	(0.000, 0.490, 0.000)	6.392	12	11	6	2	1	0.980

The followings are details for $SrTl_3$. It has 6 topological nodal lines and 23 nodal ring points along high symmetry line as shown in Fig **715**.

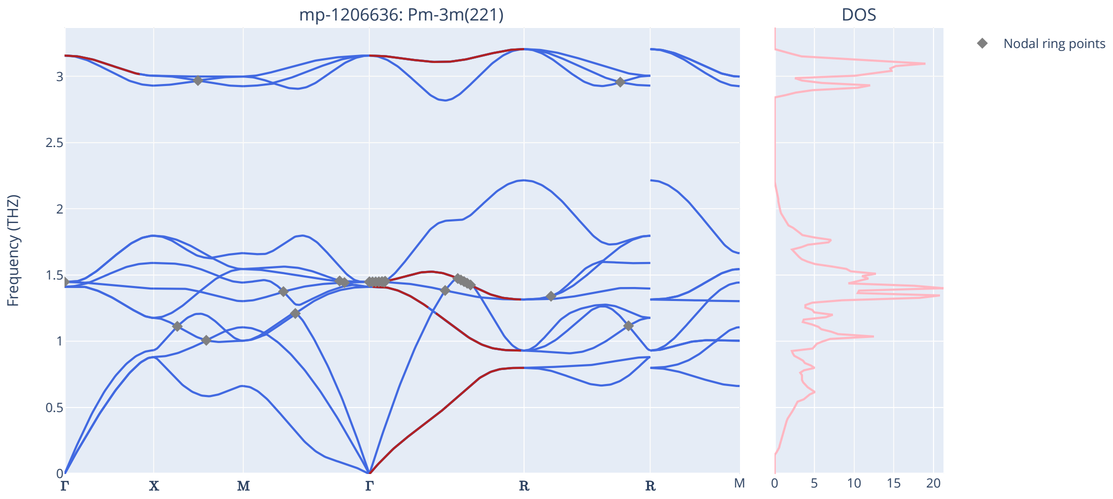

Supplementary Figure 715: The phonon band and DOS of SrTl3.

Supplementary Table 1520: High symmetry lines of mp-1206636-SrTl3

	Start	End
0	Γ (0.000, 0.000, 0.000)	X (0.000, 0.500, 0.000)
1	X (0.000, 0.500, 0.000)	M (0.500, 0.500, 0.000)
2	M (0.500, 0.500, 0.000)	Γ (0.000, 0.000, 0.000)
3	Γ (0.000, 0.000, 0.000)	R (0.500, 0.500, 0.500)
4	R (0.500, 0.500, 0.500)	X (0.000, 0.500, 0.000)
5	R (0.500, 0.500, 0.500)	M (0.500, 0.500, 0.000)

Supplementary Table 1521: Nodal ring points along high symmetry lines of mp-1206636-SrTl3.

	Coordinates	Frequency	Mode 1	Mode 2	Multi.	Deg.	Path	Ratio
0	(0.294, 0.500, -0.000)	1.007	3	2	12	2	2	0.587
1	(0.086, 0.500, 0.086)	1.116	4	3	12	2	5	0.827
2	(0.131, 0.500, 0.000)	1.113	4	3	12	2	2	0.263
3	(0.293, 0.293, -0.000)	1.210	4	3	12	2	3	0.413
4	(0.340, 0.340, 0.000)	1.375	6	5	12	2	3	0.319
5	(0.245, 0.245, 0.245)	1.383	6	5	8	2	4	0.490
6	(0.099, 0.099, 0.000)	1.443	7	6	12	2	3	0.803
7	(0.048, 0.048, 0.048)	1.447	7	6	8	2	4	0.096
8	(0.327, 0.327, 0.327)	1.425	7	6	8	2	4	0.654
9	(0.286, 0.286, 0.286)	1.474	7	6	8	2	4	0.571
10	(0.296, 0.296, 0.296)	1.462	7	6	8	2	4	0.592
11	(0.306, 0.306, 0.306)	1.451	7	6	8	2	4	0.612
12	(0.316, 0.316, 0.316)	1.438	7	6	8	2	4	0.633
13	(0.118, 0.118, 0.000)	1.455	8	7	12	2	3	0.764

Supplementary Table 1521: Nodal ring points along high symmetry lines of mp-1206636-SrTl3.

	Coordinates	Frequency	Mode 1	Mode 2	Multi.	Deg.	Path	Ratio
14	(0.393, 0.500, 0.393)	1.340	8	7	12	2	5	0.214
15	(0.000, 0.000, 0.000)	1.448	9	8	1	3	1	0.000
16	(0.010, 0.010, 0.010)	1.448	9	8	8	2	4	0.020
17	(0.020, 0.020, 0.020)	1.448	9	8	8	2	4	0.041
18	(0.031, 0.031, 0.031)	1.449	9	8	8	2	4	0.061
19	(0.041, 0.041, 0.041)	1.451	9	8	8	2	4	0.082
20	(0.051, 0.051, 0.051)	1.453	9	8	8	2	4	0.102
21	(0.247, 0.500, -0.000)	2.969	11	10	12	2	2	0.494
22	(0.119, 0.500, 0.119)	2.956	11	10	12	2	5	0.762

The followings are details for $Hg_4Na_8Tl_4$. As shown in Fig 716, it has 6 topological nodal lines, 2 nodal ring points and 2 high degenerate Weyl points whose degeneracy are more than 2 and charges are bigger than 1.

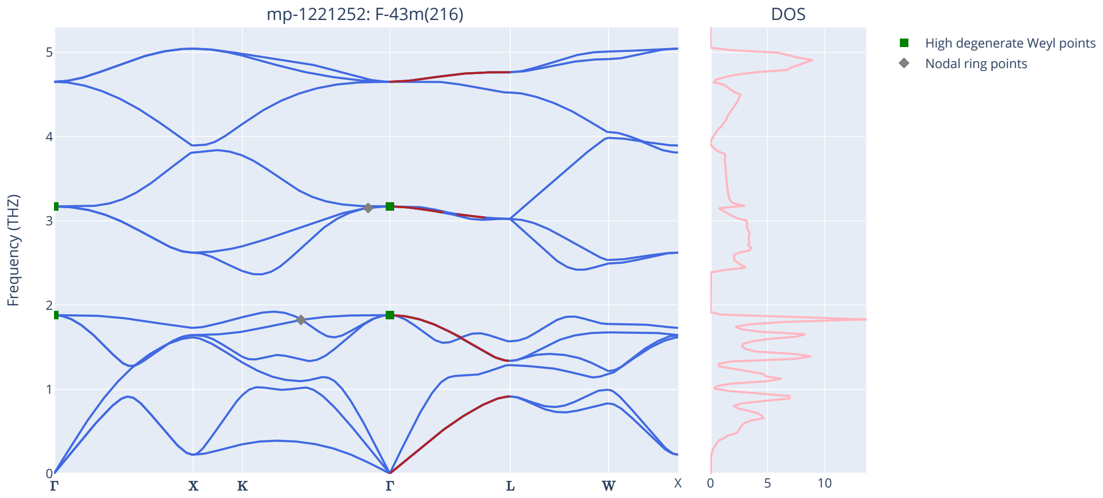

Supplementary Figure 716: The phonon band and DOS of Hg4Na8Tl4.

Supplementary Table 1522: High symmetry lines of mp-1221252-Hg4Na8Tl4

	Start	End
0	Γ (0.000, 0.000, 0.000)	X (0.500, 0.000, 0.500)
1	X (0.500, 0.000, 0.500)	U (0.625, 0.250, 0.625)
2	K (0.375, 0.375, 0.750)	Γ (0.000, 0.000, 0.000)
3	Γ (0.000, 0.000, 0.000)	L (0.500, 0.500, 0.500)
4	L (0.500, 0.500, 0.500)	W (0.500, 0.250, 0.750)
5	W (0.500, 0.250, 0.750)	X (0.500, 0.000, 0.500)

Supplementary Table 1523: High degenerate Weyl points along high symmetry lines of mp-1221252-Hg4Na8Tl4.

	Coordinates	Frequency	Mode 1	Mode 2	Multi.	Deg.	Path	Ratio
0	(0.000, 0.000, 0.000)	1.882	6	4	1	3	1	0.000
1	(0.000, 0.000, 0.000)	3.170	9	7	1	3	1	0.000

Supplementary Table 1524: Nodal ring points along high symmetry lines of mp-1221252-Hg4Na8Tl4.

	Coordinates	Frequency	Mode 1	Mode 2	Multi.	Deg.	Path	Ratio
0	(0.230, 0.230, 0.448)	1.824	6	5	24	2	3	0.398
1	(0.054, 0.054, 0.111)	3.152	8	7	24	2	3	0.853

The followings are details for $Au_4Li_8Si_4$. As shown in Fig **717**, it has 6 topological nodal lines, 2 nodal ring points and 2 high degenerate Weyl points whose degeneracy are more than 2 and charges are bigger than 1.

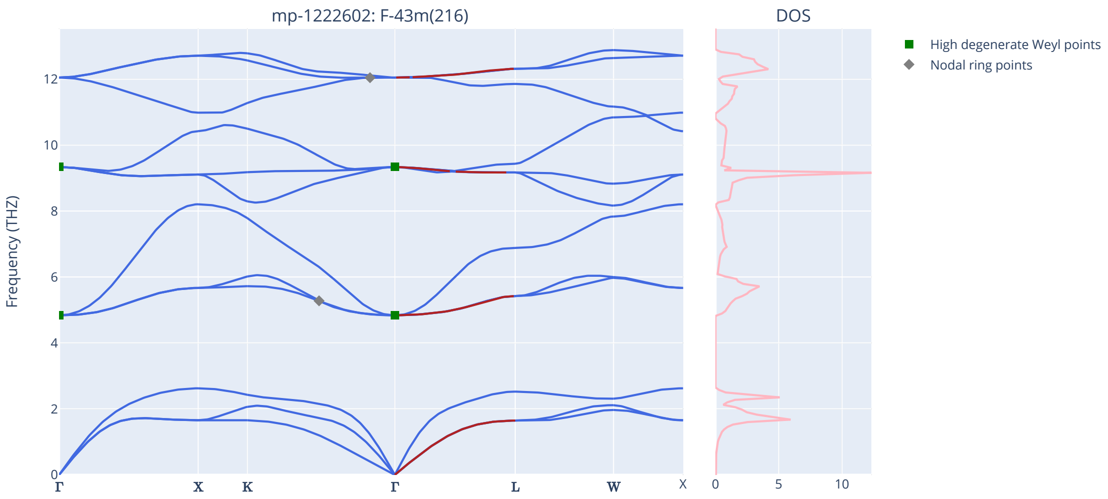

Supplementary Figure 717: The phonon band and DOS of Au4Li8Si4.

Supplementary Table 1525: High symmetry lines of mp-1222602-Au4Li8Si4

	Start	End
0	Γ (0.000, 0.000, 0.000)	X (0.500, 0.000, 0.500)
1	X (0.500, 0.000, 0.500)	U (0.625, 0.250, 0.625)
2	K (0.375, 0.375, 0.750)	Γ (0.000, 0.000, 0.000)
3	Γ (0.000, 0.000, 0.000)	L (0.500, 0.500, 0.500)
4	L (0.500, 0.500, 0.500)	W (0.500, 0.250, 0.750)
5	W (0.500, 0.250, 0.750)	X (0.500, 0.000, 0.500)

Supplementary Table 1526: High degenerate Weyl points along high symmetry lines of mp-1222602-Au4Li8Si4.

	Coordinates	Frequency	Mode 1	Mode 2	Multi.	Deg.	Path	Ratio
0	(0.000, 0.000, 0.000)	4.836	6	4	1	3	1	0.000
1	(0.000, 0.000, 0.000)	9.336	9	7	1	3	1	0.000

Supplementary Table 1527: Nodal ring points along high symmetry lines of mp-1222602-Au4Li8Si4.

	Coordinates	Frequency	Mode 1	Mode 2	Multi.	Deg.	Path	Ratio
0	(0.191, 0.191, 0.386)	5.276	5	4	24	2	3	0.487
1	(0.064, 0.064, 0.125)	12.045	11	10	24	2	3	0.832

The followings are details for $Cd_4Li_8Tl_4$. As shown in Fig 718, it has 5 topological nodal lines, 3 nodal ring points and 2 high degenerate Weyl points whose degeneracy are more than 2 and charges are bigger than 1.

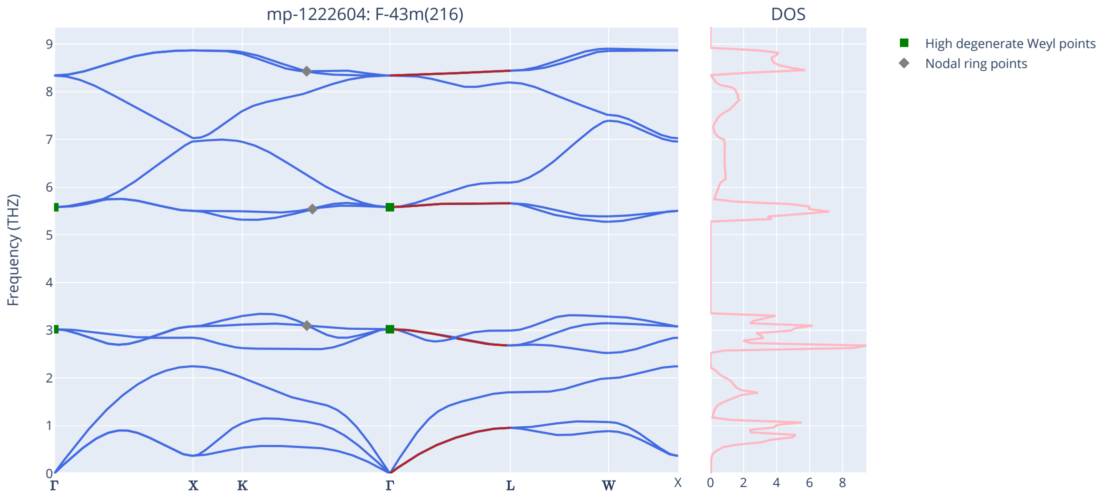

Supplementary Figure 718: The phonon band and DOS of Cd4Li8Tl4.

Supplementary Table 1528: High symmetry lines of mp-1222604-Cd4Li8Tl4

	Start	End
0	Γ (0.000, 0.000, 0.000)	X (0.500, 0.000, 0.500)
1	X (0.500, 0.000, 0.500)	U (0.625, 0.250, 0.625)
2	K (0.375, 0.375, 0.750)	Γ (0.000, 0.000, 0.000)
3	Γ (0.000, 0.000, 0.000)	L (0.500, 0.500, 0.500)
4	L (0.500, 0.500, 0.500)	W (0.500, 0.250, 0.750)
5	W (0.500, 0.250, 0.750)	X (0.500, 0.000, 0.500)

Supplementary Table 1529: High degenerate Weyl points along high symmetry lines of mp-1222604-Cd4Li8Tl4.

	Coordinates	Frequency	Mode 1	Mode 2	Multi.	Deg.	Path	Ratio
0	(0.000, 0.000, 0.000)	3.023	6	4	1	3	1	0.000
1	(0.000, 0.000, 0.000)	5.578	9	7	1	3	1	0.000

Supplementary Table 1530: Nodal ring points along high symmetry lines of mp-1222604-Cd4Li8Tl4.

	Coordinates	Frequency	Mode 1	Mode 2	Multi.	Deg.	Path	Ratio
0	(0.214, 0.214, 0.418)	3.097	6	5	24	2	3	0.438
1	(0.191, 0.191, 0.399)	5.541	8	7	24	2	3	0.475
2	(0.214, 0.214, 0.420)	8.429	12	11	24	2	3	0.437

The followings are details for $Li_8Pt_4Sn_4$. As shown in Fig 719, it has 5 topological nodal lines, 2 nodal ring points and 2 high degenerate Weyl points whose degeneracy are more than 2 and charges are bigger than 1.

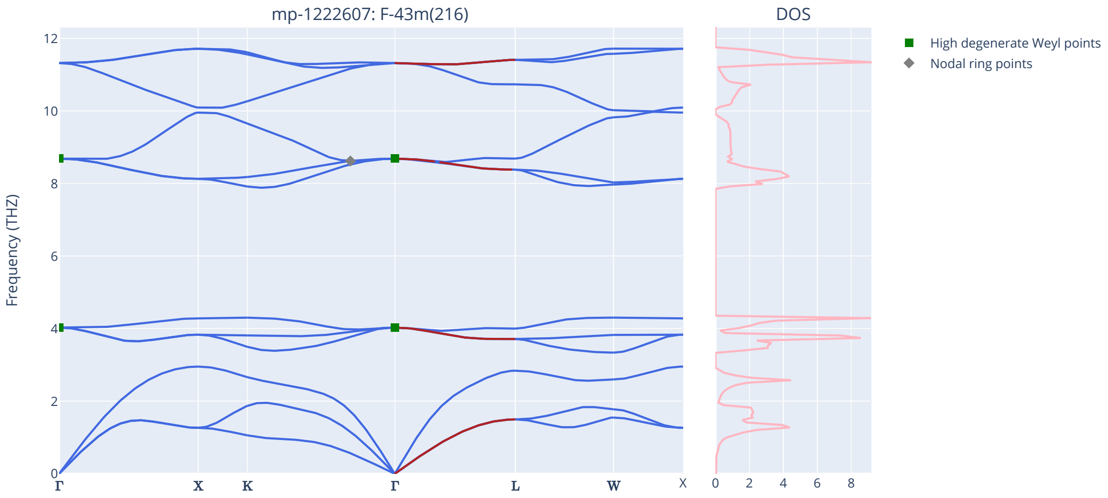

Supplementary Figure 719: The phonon band and DOS of Li8Pt4Sn4.

Supplementary Table 1531: High symmetry lines of mp-1222607-Li8Pt4Sn4

	Start	End
0	Γ (0.000, 0.000, 0.000)	X (0.500, 0.000, 0.500)
1	X (0.500, 0.000, 0.500)	U (0.625, 0.250, 0.625)
2	K (0.375, 0.375, 0.750)	Γ (0.000, 0.000, 0.000)
3	Γ (0.000, 0.000, 0.000)	L (0.500, 0.500, 0.500)
4	L (0.500, 0.500, 0.500)	W (0.500, 0.250, 0.750)
5	W (0.500, 0.250, 0.750)	X (0.500, 0.000, 0.500)

Supplementary Table 1532: High degenerate Weyl points along high symmetry lines of mp-1222607-Li8Pt4Sn4.

	Coordinates	Frequency	Mode 1	Mode 2	Multi.	Deg.	Path	Ratio
0	(0.000, 0.000, 0.000)	4.030	6	4	1	3	1	0.000
1	(0.000, 0.000, 0.000)	8.687	9	7	1	3	1	0.000

Supplementary Table 1533: Nodal ring points along high symmetry lines of mp-1222607-Li8Pt4Sn4.

	Coordinates	Frequency	Mode 1	Mode 2	Multi.	Deg.	Path	Ratio
0	(0.115, 0.115, 0.224)	8.622	9	8	24	2	3	0.699

The followings are details for $Li_8Pd_4Sn_4$. As shown in Fig 720, it has 6 topological nodal lines, 1 single Weyl point and 2 high degenerate Weyl points whose degeneracy are more than 2 and charges are bigger than 1.

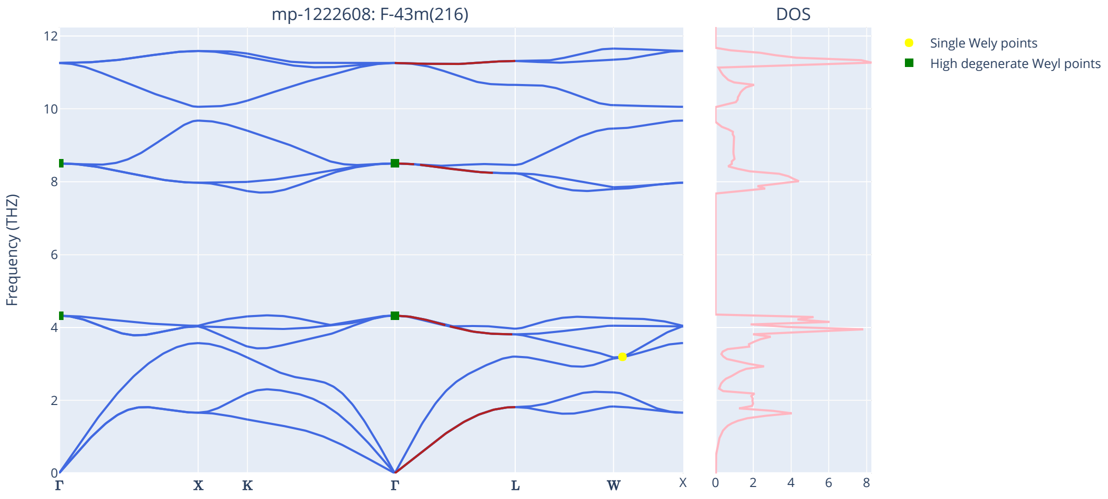

Supplementary Figure 720: The phonon band and DOS of Li8Pd4Sn4.

Supplementary Table 1534: High symmetry lines of mp-1222608-Li8Pd4Sn4

	Start	End
0	Γ (0.000, 0.000, 0.000)	X (0.500, 0.000, 0.500)
1	X (0.500, 0.000, 0.500)	U (0.625, 0.250, 0.625)
2	K (0.375, 0.375, 0.750)	Γ (0.000, 0.000, 0.000)
3	Γ (0.000, 0.000, 0.000)	L (0.500, 0.500, 0.500)
4	L (0.500, 0.500, 0.500)	W (0.500, 0.250, 0.750)
5	W (0.500, 0.250, 0.750)	X (0.500, 0.000, 0.500)

Supplementary Table 1535: Single Weyl points along high symmetry lines of mp-1222608-Li8Pd4Sn4.

	Coordinates	Frequency	Mode 1	Mode 2	Multi.	Deg.	Path	Ratio
0	(0.500, 0.218, 0.718)	3.195	4	3	12	2	6	0.129

Supplementary Table 1536: High degenerate Weyl points along high symmetry lines of mp-1222608-Li8Pd4Sn4.

	Coordinates	Frequency	Mode 1	Mode 2	Multi.	Deg.	Path	Ratio
0	(0.000, 0.000, 0.000)	4.326	6	4	1	3	1	0.000
1	(0.000, 0.000, 0.000)	8.503	9	7	1	3	1	0.000

The followings are details for $Li_8Mg_4Zn_4$. As shown in Fig **721**, it has 5 topological nodal lines, 3 nodal ring points and 2 high degenerate Weyl points whose degeneracy are more than 2 and charges are bigger than 1.

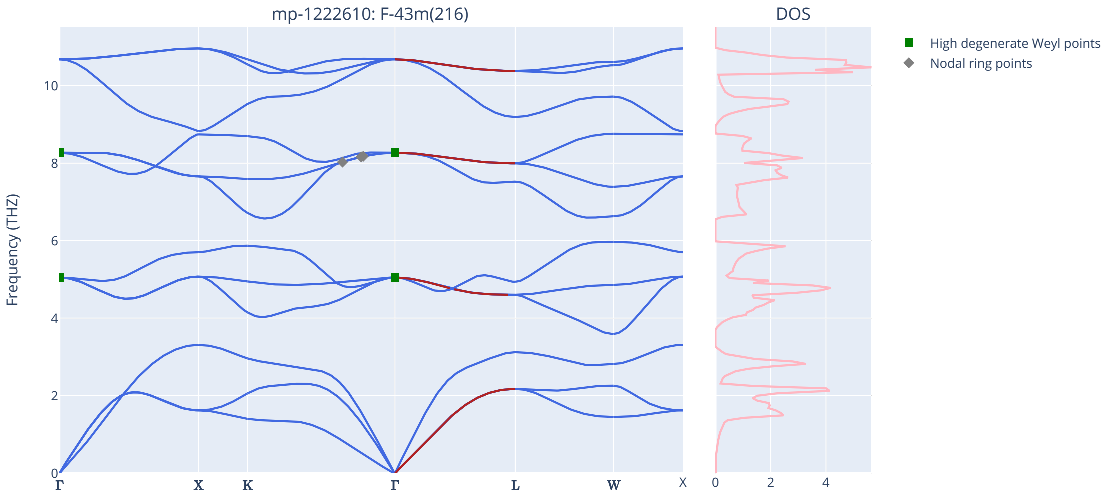

Supplementary Figure 721: The phonon band and DOS of Li8Mg4Zn4.

Supplementary Table 1537: High symmetry lines of mp-1222610-Li8Mg4Zn4

	Start	End
0	Γ (0.000, 0.000, 0.000)	X (0.500, 0.000, 0.500)
1	X (0.500, 0.000, 0.500)	U (0.625, 0.250, 0.625)
2	K (0.375, 0.375, 0.750)	Γ (0.000, 0.000, 0.000)
3	Γ (0.000, 0.000, 0.000)	L (0.500, 0.500, 0.500)
4	L (0.500, 0.500, 0.500)	W (0.500, 0.250, 0.750)
5	W (0.500, 0.250, 0.750)	X (0.500, 0.000, 0.500)

Supplementary Table 1538: High degenerate Weyl points along high symmetry lines of mp-1222610-Li8Mg4Zn4.

	Coordinates	Frequency	Mode 1	Mode 2	Multi.	Deg.	Path	Ratio
0	(0.000, 0.000, 0.000)	5.049	6	4	1	3	1	0.000
1	(0.000, 0.000, 0.000)	8.271	9	7	1	3	1	0.000

Supplementary Table 1539: Nodal ring points along high symmetry lines of mp-1222610-Li8Mg4Zn4.

	Coordinates	Frequency	Mode 1	Mode 2	Multi.	Deg.	Path	Ratio
0	(0.082, 0.082, 0.175)	8.162	8	7	24	2	3	0.772
1	(0.080, 0.080, 0.162)	8.176	8	7	24	2	3	0.785
2	(0.129, 0.129, 0.271)	8.029	8	7	24	2	3	0.645

The followings are details for $Ag_4Li_8Tl_4$. As shown in Fig 722, it has 7 topological nodal lines, 3 nodal ring points and 2 high degenerate Weyl points whose degeneracy are more than 2 and charges are bigger than 1.

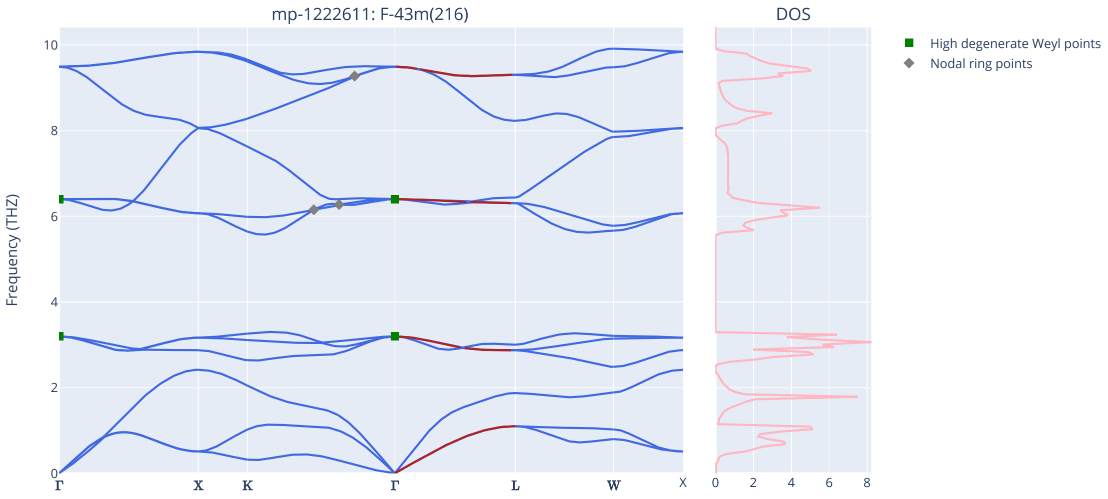

Supplementary Figure 722: The phonon band and DOS of Ag4Li8Tl4.

Supplementary Table 1540: High symmetry lines of mp-1222611-Ag4Li8Tl4

	Start	End
0	Γ (0.000, 0.000, 0.000)	X (0.500, 0.000, 0.500)
1	X (0.500, 0.000, 0.500)	U (0.625, 0.250, 0.625)
2	K (0.375, 0.375, 0.750)	Γ (0.000, 0.000, 0.000)
3	Γ (0.000, 0.000, 0.000)	L (0.500, 0.500, 0.500)
4	L (0.500, 0.500, 0.500)	W (0.500, 0.250, 0.750)
5	W (0.500, 0.250, 0.750)	X (0.500, 0.000, 0.500)

Supplementary Table 1541: High degenerate Weyl points along high symmetry lines of mp-1222611-Ag4Li8Tl4.

	Coordinates	Frequency	Mode 1	Mode 2	Multi.	Deg.	Path	Ratio
0	(0.000, 0.000, 0.000)	3.196	6	4	1	3	1	0.000
1	(0.000, 0.000, 0.000)	6.403	9	7	1	3	1	0.000

Supplementary Table 1542: Nodal ring points along high symmetry lines of mp-1222611-Ag4Li8Tl4.

	Coordinates	Frequency	Mode 1	Mode 2	Multi.	Deg.	Path	Ratio
0	(0.207, 0.207, 0.411)	6.155	8	7	24	2	3	0.451
1	(0.145, 0.145, 0.279)	6.276	8	7	24	2	3	0.622
2	(0.103, 0.103, 0.204)	9.275	11	10	24	2	3	0.728

The followings are details for $Ir_4Li_8Sn_4$. As shown in Fig 723, it has 5 topological nodal lines, 1 nodal ring point and 2 high degenerate Weyl points whose degeneracy are more than 2 and charges are bigger than 1.

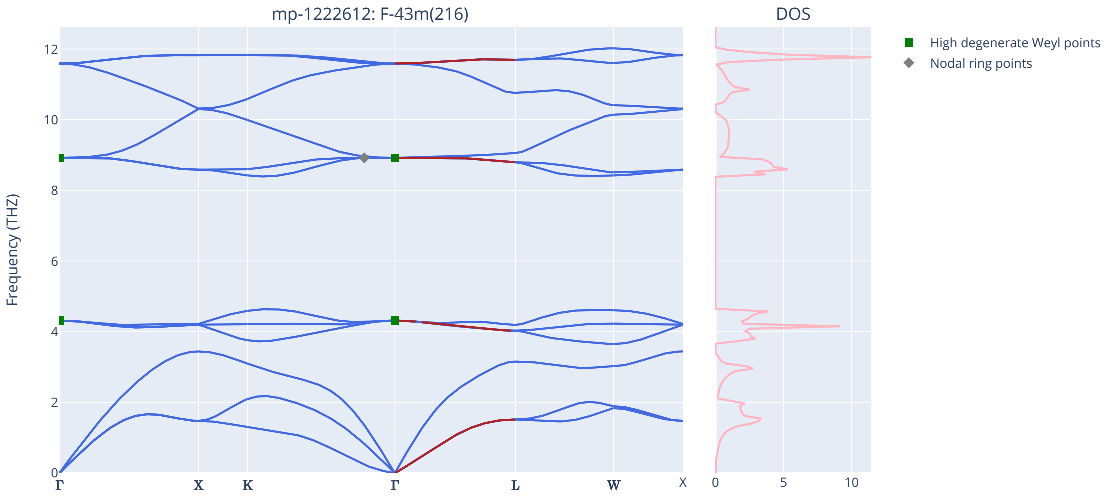

Supplementary Figure 723: The phonon band and DOS of Ir4Li8Sn4.

Supplementary Table 1543: High symmetry lines of mp-1222612-Ir4Li8Sn4

	Start	End
0	Γ (0.000, 0.000, 0.000)	X (0.500, 0.000, 0.500)
1	X (0.500, 0.000, 0.500)	U (0.625, 0.250, 0.625)
2	K (0.375, 0.375, 0.750)	Γ (0.000, 0.000, 0.000)
3	Γ (0.000, 0.000, 0.000)	L (0.500, 0.500, 0.500)
4	L (0.500, 0.500, 0.500)	W (0.500, 0.250, 0.750)
5	W (0.500, 0.250, 0.750)	X (0.500, 0.000, 0.500)

Supplementary Table 1544: High degenerate Weyl points along high symmetry lines of mp-1222612-Ir4Li8Sn4.

	Coordinates	Frequency	Mode 1	Mode 2	Multi.	Deg.	Path	Ratio
0	(0.000, 0.000, 0.000)	4.315	6	4	1	3	1	0.000
1	(0.000, 0.000, 0.000)	8.921	9	7	1	3	1	0.000

Supplementary Table 1545: Nodal ring points along high symmetry lines of mp-1222612-Ir4Li8Sn4.

	Coordinates	Frequency	Mode 1	Mode 2	Multi.	Deg.	Path	Ratio
0	(0.077, 0.077, 0.157)	8.920	8	7	24	2	3	0.793

The followings are details for $Li_8Mg_4Tl_4$. As shown in Fig 724, it has 6 topological nodal lines and 2 nodal ring points. At the same time, it has 1 single Weyl point and 2 high degenerate Weyl points whose degeneracy are more than 2 and charges are bigger than 1.

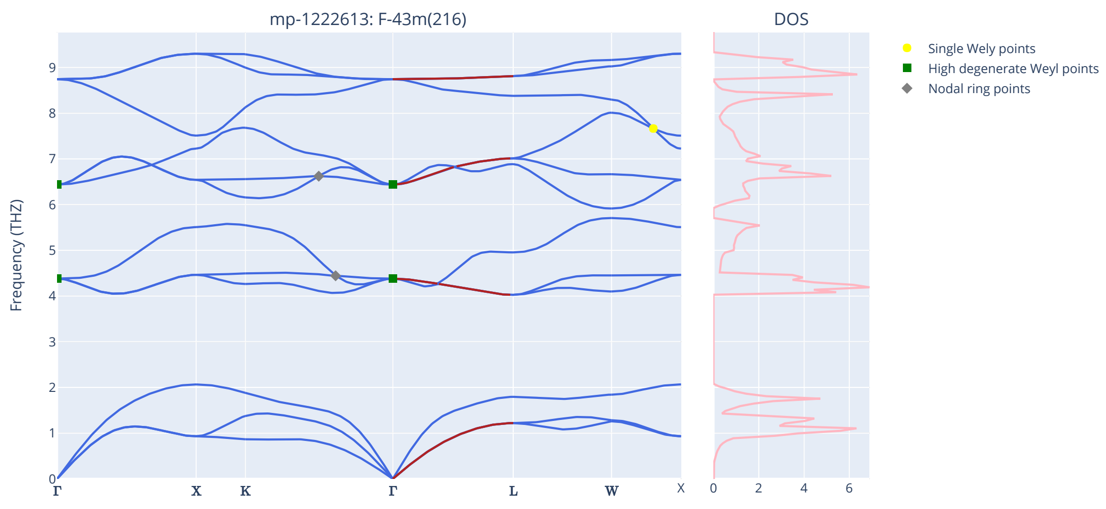

Supplementary Figure 724: The phonon band and DOS of Li8Mg4Tl4.

Supplementary Table 1546: High symmetry lines of mp-1222613-Li8Mg4Tl4

	Start	End
0	Γ (0.000, 0.000, 0.000)	X (0.500, 0.000, 0.500)
1	X (0.500, 0.000, 0.500)	U (0.625, 0.250, 0.625)
2	K (0.375, 0.375, 0.750)	Γ (0.000, 0.000, 0.000)
3	Γ (0.000, 0.000, 0.000)	L (0.500, 0.500, 0.500)
4	L (0.500, 0.500, 0.500)	W (0.500, 0.250, 0.750)
5	W (0.500, 0.250, 0.750)	X (0.500, 0.000, 0.500)

Supplementary Table 1547: Single Weyl points along high symmetry lines of mp-1222613-Li8Mg4Tl4.

	Coordinates	Frequency	Mode 1	Mode 2	Multi.	Deg.	Path	Ratio
0	(0.500, 0.100, 0.600)	7.665	10	9	12	2	6	0.601

Supplementary Table 1548: High degenerate Weyl points along high symmetry lines of mp-1222613-Li8Mg4Tl4.

	Coordinates	Frequency	Mode 1	Mode 2	Multi.	Deg.	Path	Ratio
0	(0.000, 0.000, 0.000)	4.380	6	4	1	3	1	0.000
1	(0.000, 0.000, 0.000)	6.438	9	7	1	3	1	0.000

Supplementary Table 1549: Nodal ring points along high symmetry lines of mp-1222613-Li8Mg4Tl4.

	Coordinates	Frequency	Mode 1	Mode 2	Multi.	Deg.	Path	Ratio
0	(0.149, 0.149, 0.287)	4.443	6	5	24	2	3	0.612
1	(0.187, 0.187, 0.377)	6.622	8	7	24	2	3	0.498

The followings are details for $Li_8Mg_4Sn_4$. As shown in Fig 725, it has 6 topological nodal lines and 4 nodal ring points. At the same time, it has 1 single Weyl point and 2 high degenerate Weyl points whose degeneracy are more than 2 and charges are bigger than 1.

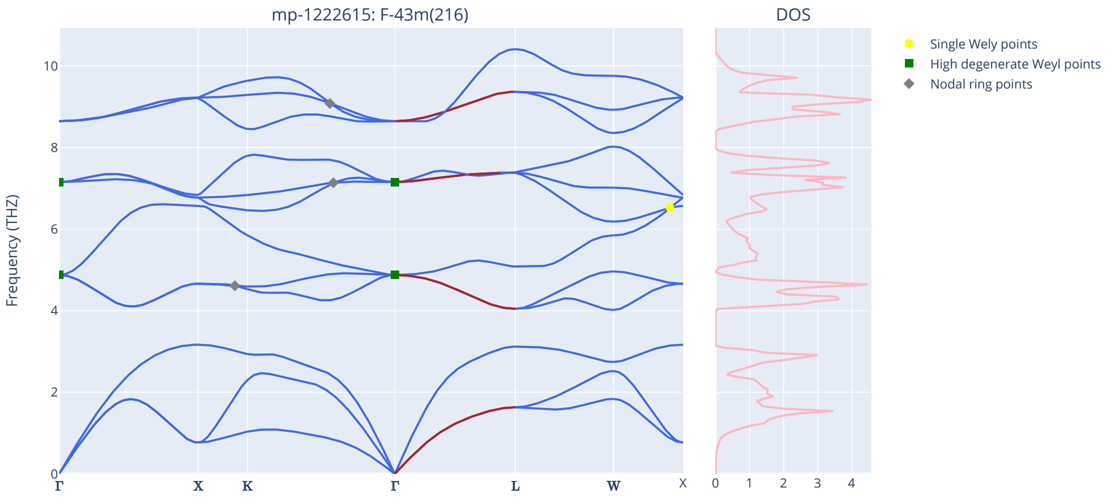

Supplementary Figure 725: The phonon band and DOS of Li8Mg4Sn4.

Supplementary Table 1550: High symmetry lines of mp-1222615-Li8Mg4Sn4

	Start	End
0	Γ (0.000, 0.000, 0.000)	X (0.500, 0.000, 0.500)
1	X (0.500, 0.000, 0.500)	U (0.625, 0.250, 0.625)
2	K (0.375, 0.375, 0.750)	Γ (0.000, 0.000, 0.000)
3	Γ (0.000, 0.000, 0.000)	L (0.500, 0.500, 0.500)
4	L (0.500, 0.500, 0.500)	W (0.500, 0.250, 0.750)
5	W (0.500, 0.250, 0.750)	X (0.500, 0.000, 0.500)

Supplementary Table 1551: Single Weyl points along high symmetry lines of mp-1222615-Li8Mg4Sn4.

	Coordinates	Frequency	Mode 1	Mode 2	Multi.	Deg.	Path	Ratio
0	(0.500, 0.047, 0.547)	6.522	7	6	12	2	6	0.814

Supplementary Table 1552: High degenerate Weyl points along high symmetry lines of mp-1222615-Li8Mg4Sn4.

	Coordinates	Frequency	Mode 1	Mode 2	Multi.	Deg.	Path	Ratio
0	(0.000, 0.000, 0.000)	4.875	6	4	1	3	1	0.000
1	(0.000, 0.000, 0.000)	7.152	9	7	1	3	1	0.000

Supplementary Table 1553: Nodal ring points along high symmetry lines of mp-1222615-Li8Mg4Sn4.

	Coordinates	Frequency	Mode 1	Mode 2	Multi.	Deg.	Path	Ratio
0	(0.593, 0.187, 0.593)	4.611	5	4	24	2	2	0.747
1	(0.161, 0.161, 0.308)	7.136	8	7	24	2	3	0.584
2	(0.168, 0.168, 0.326)	9.080	12	11	24	2	3	0.560

The followings are details for $Hg_4Li_8Mg_4$. As shown in Fig **726**, it has 6 topological nodal lines, 2 nodal ring points and 2 high degenerate Weyl points whose degeneracy are more than 2 and charges are bigger than 1.

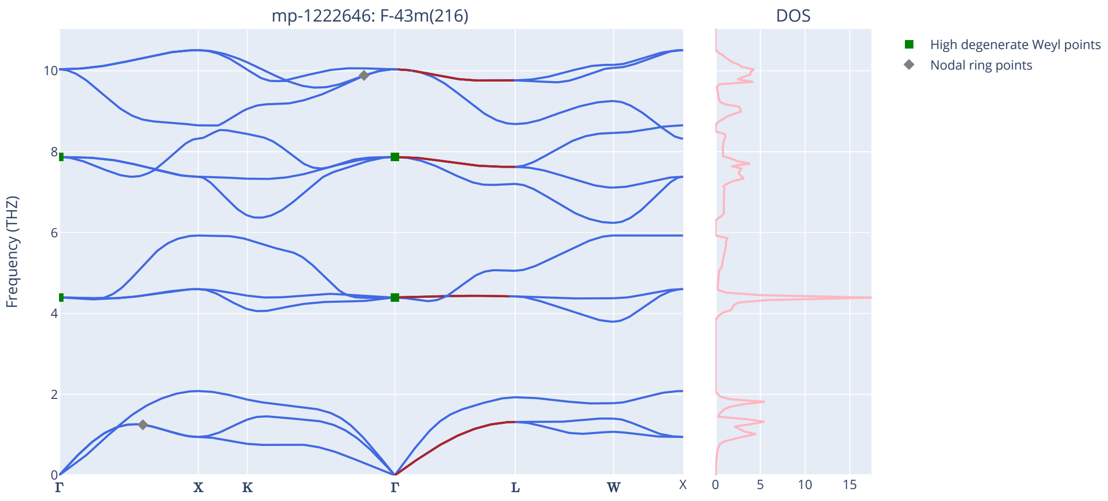

Supplementary Figure 726: The phonon band and DOS of Hg4Li8Mg4.

Supplementary Table 1554: High symmetry lines of mp-1222646-Hg4Li8Mg4

	Start	End
0	Γ (0.000, 0.000, 0.000)	X (0.500, 0.000, 0.500)
1	X (0.500, 0.000, 0.500)	U (0.625, 0.250, 0.625)
2	K (0.375, 0.375, 0.750)	Γ (0.000, 0.000, 0.000)
3	Γ (0.000, 0.000, 0.000)	L (0.500, 0.500, 0.500)
4	L (0.500, 0.500, 0.500)	W (0.500, 0.250, 0.750)
5	W (0.500, 0.250, 0.750)	X (0.500, 0.000, 0.500)

Supplementary Table 1555: High degenerate Weyl points along high symmetry lines of mp-1222646-Hg4Li8Mg4.

	Coordinates	Frequency	Mode 1	Mode 2	Multi.	Deg.	Path	Ratio
0	(0.000, 0.000, 0.000)	4.398	6	4	1	3	1	0.000
1	(0.000, 0.000, 0.000)	7.869	9	7	1	3	1	0.000

Supplementary Table 1556: Nodal ring points along high symmetry lines of mp-1222646-Hg4Li8Mg4.

	Coordinates	Frequency	Mode 1	Mode 2	Multi.	Deg.	Path	Ratio
0	(0.306, 0.000, 0.296)	1.248	2	1	24	2	1	0.603
1	(0.081, 0.081, 0.154)	9.885	11	10	24	2	3	0.791

The followings are details for $Ga_4Li_8Mg_4$. As shown in Fig 727, it has 6 topological nodal lines, 1 nodal ring point and 2 high degenerate Weyl points whose degeneracy are more than 2 and charges are bigger than 1.

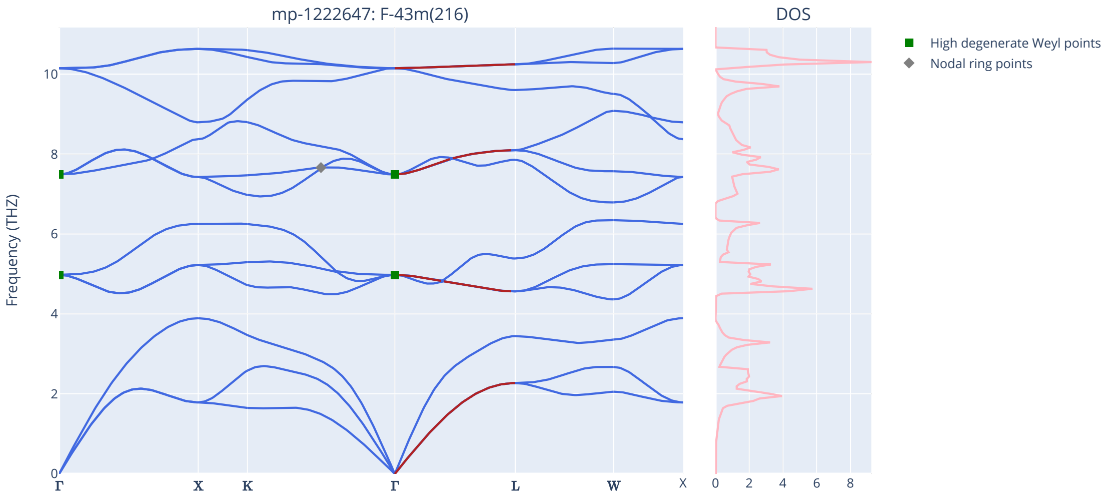

Supplementary Figure 727: The phonon band and DOS of Ga4Li8Mg4.

Supplementary Table 1557: High symmetry lines of mp-1222647-Ga4Li8Mg4

	Start	End
0	Γ (0.000, 0.000, 0.000)	X (0.500, 0.000, 0.500)
1	X (0.500, 0.000, 0.500)	U (0.625, 0.250, 0.625)
2	K (0.375, 0.375, 0.750)	Γ (0.000, 0.000, 0.000)
3	Γ (0.000, 0.000, 0.000)	L (0.500, 0.500, 0.500)
4	L (0.500, 0.500, 0.500)	W (0.500, 0.250, 0.750)
5	W (0.500, 0.250, 0.750)	X (0.500, 0.000, 0.500)

Supplementary Table 1558: High degenerate Weyl points along high symmetry lines of mp-1222647-Ga4Li8Mg4.

	Coordinates	Frequency	Mode 1	Mode 2	Multi.	Deg.	Path	Ratio
0	(0.000, 0.000, 0.000)	4.978	6	4	1	3	1	0.000
1	(0.000, 0.000, 0.000)	7.489	9	7	1	3	1	0.000

Supplementary Table 1559: Nodal ring points along high symmetry lines of mp-1222647-Ga4Li8Mg4.

	Coordinates	Frequency	Mode 1	Mode 2	Multi.	Deg.	Path	Ratio
0	(0.191, 0.191, 0.372)	7.661	8	7	24	2	3	0.499

The followings are details for $Cd_4Li_8Mg_4$. As shown in Fig **728**, it has 6 topological nodal lines, 2 nodal ring points and 2 high degenerate Weyl points whose degeneracy are more than 2 and charges are bigger than 1.

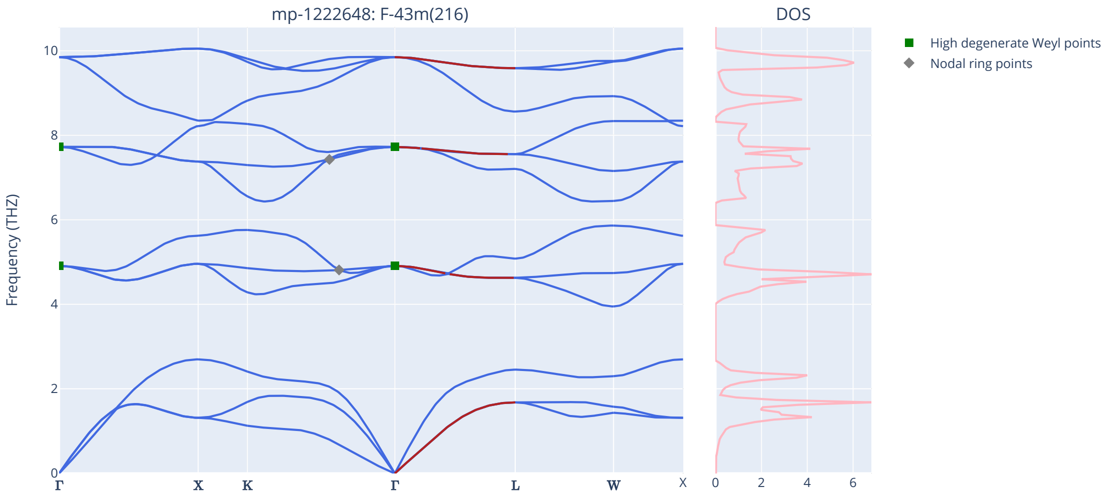

Supplementary Figure 728: The phonon band and DOS of Cd4Li8Mg4.

Supplementary Table 1560: High symmetry lines of mp-1222648-Cd4Li8Mg4

	Start	End
0	Γ (0.000, 0.000, 0.000)	X (0.500, 0.000, 0.500)
1	X (0.500, 0.000, 0.500)	U (0.625, 0.250, 0.625)
2	K (0.375, 0.375, 0.750)	Γ (0.000, 0.000, 0.000)
3	Γ (0.000, 0.000, 0.000)	L (0.500, 0.500, 0.500)
4	L (0.500, 0.500, 0.500)	W (0.500, 0.250, 0.750)
5	W (0.500, 0.250, 0.750)	X (0.500, 0.000, 0.500)

Supplementary Table 1561: High degenerate Weyl points along high symmetry lines of mp-1222648-Cd4Li8Mg4.

	Coordinates	Frequency	Mode 1	Mode 2	Multi.	Deg.	Path	Ratio
0	(0.000, 0.000, 0.000)	4.908	6	4	1	3	1	0.000
1	(0.000, 0.000, 0.000)	7.722	9	7	1	3	1	0.000

Supplementary Table 1562: Nodal ring points along high symmetry lines of mp-1222648-Cd4Li8Mg4.

	Coordinates	Frequency	Mode 1	Mode 2	Multi.	Deg.	Path	Ratio
0	(0.144, 0.144, 0.280)	4.811	6	5	24	2	3	0.622
1	(0.161, 0.161, 0.338)	7.425	8	7	24	2	3	0.557

The followings are details for $Au_4Li_8Mg_4$. As shown in Fig **729**, it has 4 topological nodal lines and 1 nodal ring point. At the same time, it has 1 single Weyl point and 2 high degenerate Weyl points whose degeneracy are more than 2 and charges are bigger than 1.

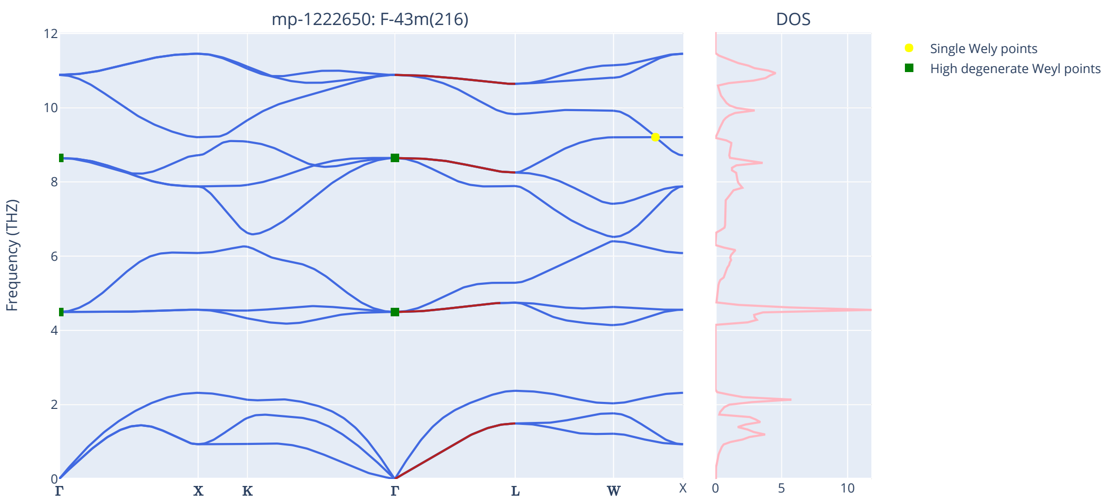

Supplementary Figure 729: The phonon band and DOS of Au4Li8Mg4.

Supplementary Table 1563: High symmetry lines of mp-1222650-Au4Li8Mg4

	Start	End
0	Γ (0.000, 0.000, 0.000)	X (0.500, 0.000, 0.500)
1	X (0.500, 0.000, 0.500)	U (0.625, 0.250, 0.625)
2	K (0.375, 0.375, 0.750)	Γ (0.000, 0.000, 0.000)
3	Γ (0.000, 0.000, 0.000)	L (0.500, 0.500, 0.500)
4	L (0.500, 0.500, 0.500)	W (0.500, 0.250, 0.750)
5	W (0.500, 0.250, 0.750)	X (0.500, 0.000, 0.500)

Supplementary Table 1564: Single Weyl points along high symmetry lines of mp-1222650-Au4Li8Mg4.

	Coordinates	Frequency	Mode 1	Mode 2	Multi.	Deg.	Path	Ratio
0	(0.500, 0.099, 0.599)	9.207	10	9	12	2	6	0.603

Supplementary Table 1565: High degenerate Weyl points along high symmetry lines of mp-1222650-Au4Li8Mg4.

	Coordinates	Frequency	Mode 1	Mode 2	Multi.	Deg.	Path	Ratio
0	(0.000, 0.000, 0.000)	4.496	6	4	1	3	1	0.000
1	(0.000, 0.000, 0.000)	8.646	9	7	1	3	1	0.000

The followings are details for $Li_8Mg_4Ge_4$. As shown in Fig 730, it has 6 topological nodal lines and 2 nodal ring points. At the same time, it has 2 single Weyl points and 2 high degenerate Weyl points whose degeneracy are more than 2 and charges are bigger than 1.

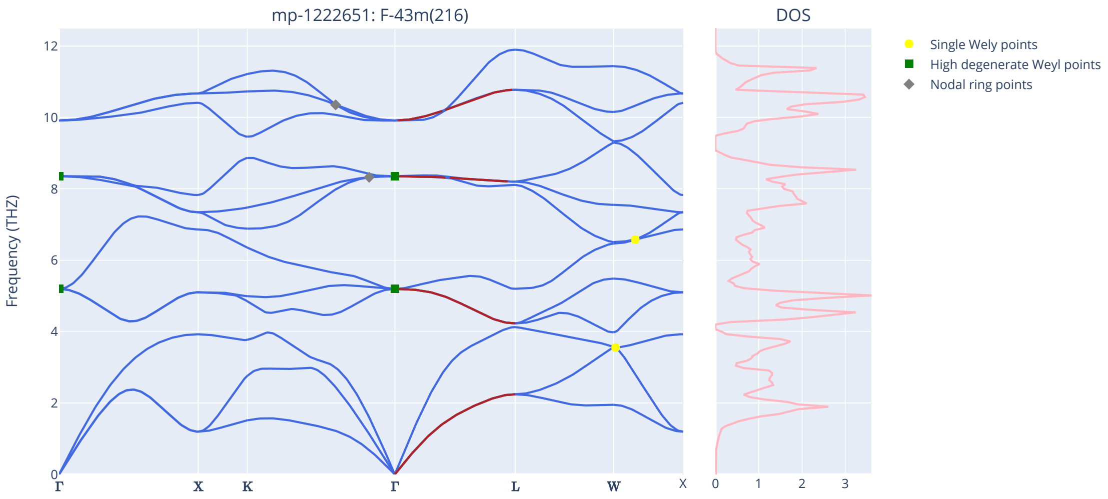

Supplementary Figure 730: The phonon band and DOS of Li8Mg4Ge4.

Supplementary Table 1566: High symmetry lines of mp-1222651-Li8Mg4Ge4

	Start	End
0	Γ (0.000, 0.000, 0.000)	X (0.500, 0.000, 0.500)
1	X (0.500, 0.000, 0.500)	U (0.625, 0.250, 0.625)
2	K (0.375, 0.375, 0.750)	Γ (0.000, 0.000, 0.000)
3	Γ (0.000, 0.000, 0.000)	L (0.500, 0.500, 0.500)
4	L (0.500, 0.500, 0.500)	W (0.500, 0.250, 0.750)
5	W (0.500, 0.250, 0.750)	X (0.500, 0.000, 0.500)

Supplementary Table 1567: Single Weyl points along high symmetry lines of mp-1222651-Li8Mg4Ge4.

	Coordinates	Frequency	Mode 1	Mode 2	Multi.	Deg.	Path	Ratio
0	(0.500, 0.243, 0.743)	3.552	3	2	12	2	6	0.030
1	(0.500, 0.172, 0.672)	6.579	7	6	12	2	6	0.313

Supplementary Table 1568: High degenerate Weyl points along high symmetry lines of mp-1222651-Li8Mg4Ge4.

	Coordinates	Frequency	Mode 1	Mode 2	Multi.	Deg.	Path	Ratio
0	(0.000, 0.000, 0.000)	5.196	6	4	1	3	1	0.000
1	(0.000, 0.000, 0.000)	8.353	9	7	1	3	1	0.000

Supplementary Table 1569: Nodal ring points along high symmetry lines of mp-1222651-Li8Mg4Ge4.

	Coordinates	Frequency	Mode 1	Mode 2	Multi.	Deg.	Path	Ratio

Supplementary Table 1569: Nodal ring points along high symmetry lines of mp-1222651-Li8Mg4Ge4.

	Coordinates	Frequency	Mode 1	Mode 2	Multi.	Deg.	Path	Ratio
0	(0.063, 0.063, 0.132)	8.324	8	7	24	2	3	0.826
1	(0.153, 0.153, 0.298)	10.352	12	11	24	2	3	0.599

The followings are details for $Li_8Mg_4Pb_4$. As shown in Fig **731**, it has 6 topological nodal lines and 3 nodal ring points. At the same time, it has 1 single Weyl point and 2 high degenerate Weyl points whose degeneracy are more than 2 and charges are bigger than 1.

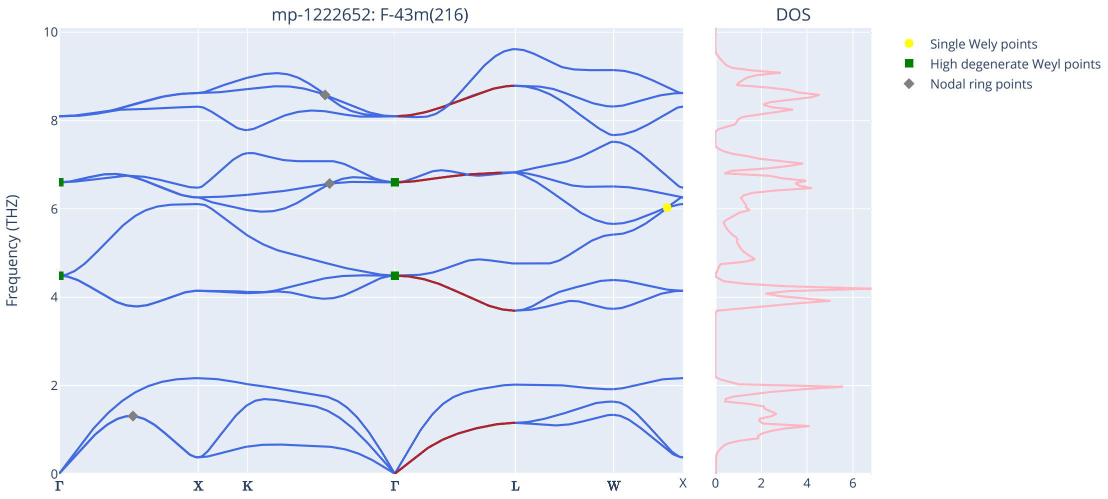

Supplementary Figure 731: The phonon band and DOS of Li8Mg4Pb4.

Supplementary Table 1570: High symmetry lines of mp-1222652-Li8Mg4Pb4

	Start	End
0	Γ (0.000, 0.000, 0.000)	X (0.500, 0.000, 0.500)
1	X (0.500, 0.000, 0.500)	U (0.625, 0.250, 0.625)
2	K (0.375, 0.375, 0.750)	Γ (0.000, 0.000, 0.000)
3	Γ (0.000, 0.000, 0.000)	L (0.500, 0.500, 0.500)
4	L (0.500, 0.500, 0.500)	W (0.500, 0.250, 0.750)
5	W (0.500, 0.250, 0.750)	X (0.500, 0.000, 0.500)

Supplementary Table 1571: Single Weyl points along high symmetry lines of mp-1222652-Li8Mg4Pb4.

	Coordinates	Frequency	Mode 1	Mode 2	Multi.	Deg.	Path	Ratio
0	(0.500, 0.057, 0.557)	6.029	7	6	12	2	6	0.772

Supplementary Table 1572: High degenerate Weyl points along high symmetry lines of mp-1222652-Li8Mg4Pb4.

	Coordinates	Frequency	Mode 1	Mode 2	Multi.	Deg.	Path	Ratio
0	(0.000, 0.000, 0.000)	4.489	6	4	1	3	1	0.000
1	(0.000, 0.000, 0.000)	6.600	9	7	1	3	1	0.000

Supplementary Table 1573: Nodal ring points along high symmetry lines of mp-1222652-Li8Mg4Pb4.

	Coordinates	Frequency	Mode 1	Mode 2	Multi.	Deg.	Path	Ratio
0	(0.261, -0.000, 0.271)	1.308	2	1	24	2	1	0.532
1	(0.168, 0.168, 0.329)	6.574	8	7	24	2	3	0.558
2	(0.176, 0.176, 0.356)	8.581	12	11	24	2	3	0.527

The followings are details for $Li_8Pd_4Ge_4$. As shown in Fig 732, it has 6 topological nodal lines, 2 nodal ring points and 2 high degenerate Weyl points whose degeneracy are more than 2 and charges are bigger than 1.

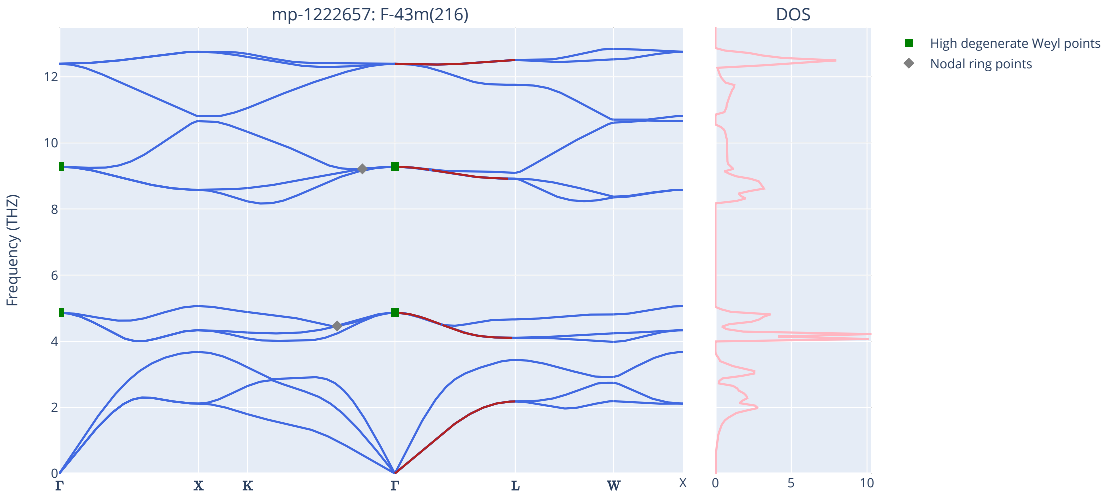

Supplementary Figure 732: The phonon band and DOS of Li8Pd4Ge4.

Supplementary Table 1574: High symmetry lines of mp-1222657-Li8Pd4Ge4

	Start	End
0	Γ (0.000, 0.000, 0.000)	X (0.500, 0.000, 0.500)
1	X (0.500, 0.000, 0.500)	U (0.625, 0.250, 0.625)
2	K (0.375, 0.375, 0.750)	Γ (0.000, 0.000, 0.000)
3	Γ (0.000, 0.000, 0.000)	L (0.500, 0.500, 0.500)
4	L (0.500, 0.500, 0.500)	W (0.500, 0.250, 0.750)
5	W (0.500, 0.250, 0.750)	X (0.500, 0.000, 0.500)

Supplementary Table 1575: High degenerate Weyl points along high symmetry lines of mp-1222657-Li8Pd4Ge4.

	Coordinates	Frequency	Mode 1	Mode 2	Multi.	Deg.	Path	Ratio
0	(0.000, 0.000, 0.000)	4.863	6	4	1	3	1	0.000
1	(0.000, 0.000, 0.000)	9.279	9	7	1	3	1	0.000

Supplementary Table 1576: Nodal ring points along high symmetry lines of mp-1222657-Li8Pd4Ge4.

	Coordinates	Frequency	Mode 1	Mode 2	Multi.	Deg.	Path	Ratio
0	(0.142, 0.142, 0.297)	4.463	6	5	24	2	3	0.609
1	(0.086, 0.086, 0.161)	9.214	9	8	24	2	3	0.780

The followings are details for $Ag_4Ga_4Li_8$. As shown in Fig 733, it has 6 topological nodal lines, 3 nodal ring points and 2 high degenerate Weyl points whose degeneracy are more than 2 and charges are bigger than 1.

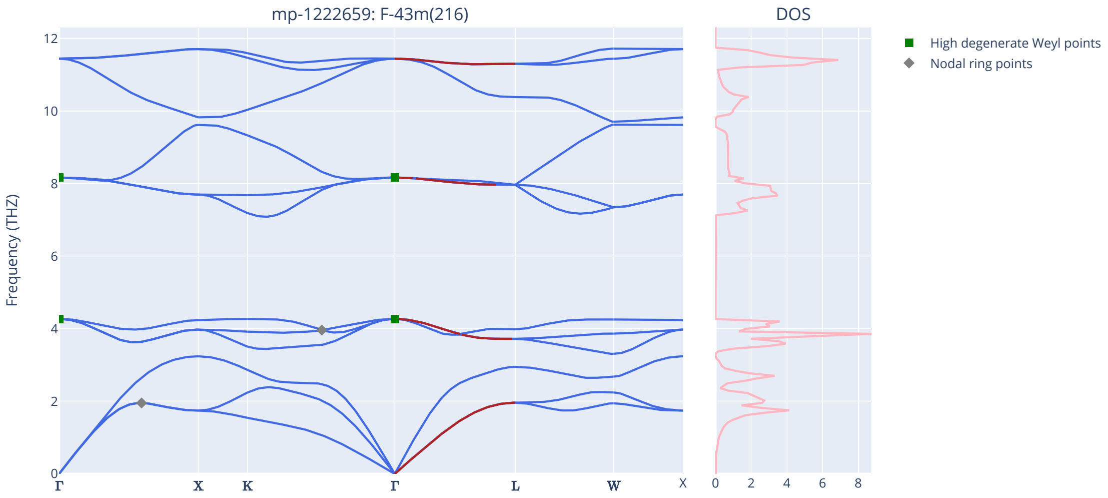

Supplementary Figure 733: The phonon band and DOS of Ag4Ga4Li8.

Supplementary Table 1577: High symmetry lines of mp-1222659-Ag4Ga4Li8

	Start	End
0	Γ (0.000, 0.000, 0.000)	X (0.500, 0.000, 0.500)
1	X (0.500, 0.000, 0.500)	U (0.625, 0.250, 0.625)
2	K (0.375, 0.375, 0.750)	Γ (0.000, 0.000, 0.000)
3	Γ (0.000, 0.000, 0.000)	L (0.500, 0.500, 0.500)
4	L (0.500, 0.500, 0.500)	W (0.500, 0.250, 0.750)
5	W (0.500, 0.250, 0.750)	X (0.500, 0.000, 0.500)

Supplementary Table 1578: High degenerate Weyl points along high symmetry lines of mp-1222659-Ag4Ga4Li8.

	Coordinates	Frequency	Mode 1	Mode 2	Multi.	Deg.	Path	Ratio
0	(0.000, 0.000, 0.000)	4.271	6	4	1	3	1	0.000
1	(0.000, 0.000, 0.000)	8.168	9	7	1	3	1	0.000

Supplementary Table 1579: Nodal ring points along high symmetry lines of mp-1222659-Ag4Ga4Li8.

	Coordinates	Frequency	Mode 1	Mode 2	Multi.	Deg.	Path	Ratio
0	(0.302, -0.000, 0.291)	1.951	2	1	24	2	1	0.593
1	(0.184, 0.184, 0.373)	3.961	6	5	24	2	3	0.506

The followings are details for $Al_4Ga_4Li_8$. As shown in Fig **734**, it has 7 topological nodal lines, 3 nodal ring points and 2 high degenerate Weyl points whose degeneracy are more than 2 and charges are bigger than 1.

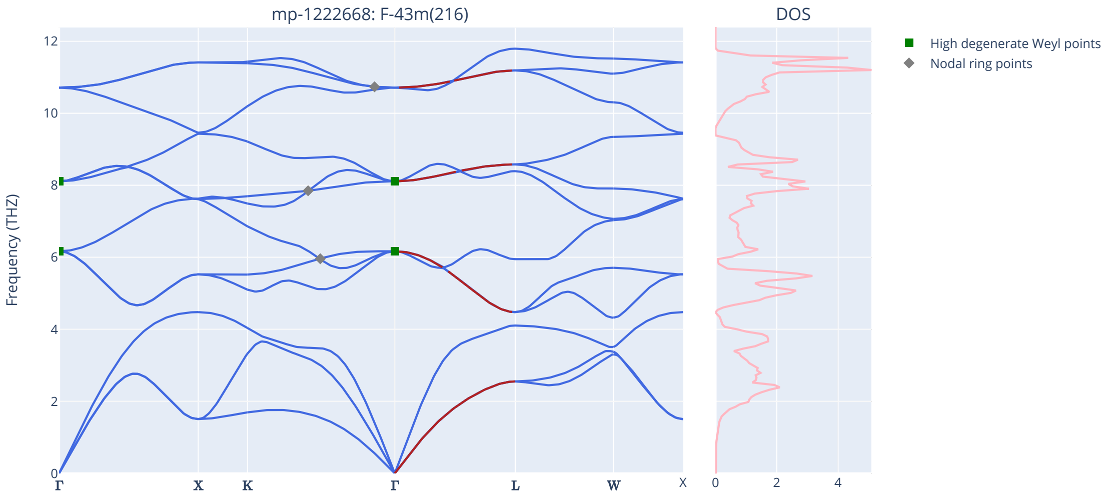

Supplementary Figure 734: The phonon band and DOS of Al4Ga4Li8.

Supplementary Table 1580: High symmetry lines of mp-1222668-Al4Ga4Li8

	Start	End
0	Γ (0.000, 0.000, 0.000)	X (0.500, 0.000, 0.500)
1	X (0.500, 0.000, 0.500)	U (0.625, 0.250, 0.625)
2	K (0.375, 0.375, 0.750)	Γ (0.000, 0.000, 0.000)
3	Γ (0.000, 0.000, 0.000)	L (0.500, 0.500, 0.500)
4	L (0.500, 0.500, 0.500)	W (0.500, 0.250, 0.750)
5	W (0.500, 0.250, 0.750)	X (0.500, 0.000, 0.500)

Supplementary Table 1581: High degenerate Weyl points along high symmetry lines of mp-1222668-Al4Ga4Li8.

	Coordinates	Frequency	Mode 1	Mode 2	Multi.	Deg.	Path	Ratio
0	(0.000, 0.000, 0.000)	6.166	6	4	1	3	1	0.000
1	(0.000, 0.000, 0.000)	8.110	9	7	1	3	1	0.000

Supplementary Table 1582: Nodal ring points along high symmetry lines of mp-1222668-Al4Ga4Li8.

	Coordinates	Frequency	Mode 1	Mode 2	Multi.	Deg.	Path	Ratio
0	(0.185, 0.185, 0.383)	5.959	6	5	24	2	3	0.495
1	(0.222, 0.222, 0.439)	7.847	8	7	24	2	3	0.412
2	(0.054, 0.054, 0.101)	10.734	12	11	24	2	3	0.863

The followings are details for $Cd_4In_4Li_8$. As shown in Fig 735, it has 5 topological nodal lines, 2 nodal ring points and 2 high degenerate Weyl points whose degeneracy are more than 2 and charges are bigger than 1.

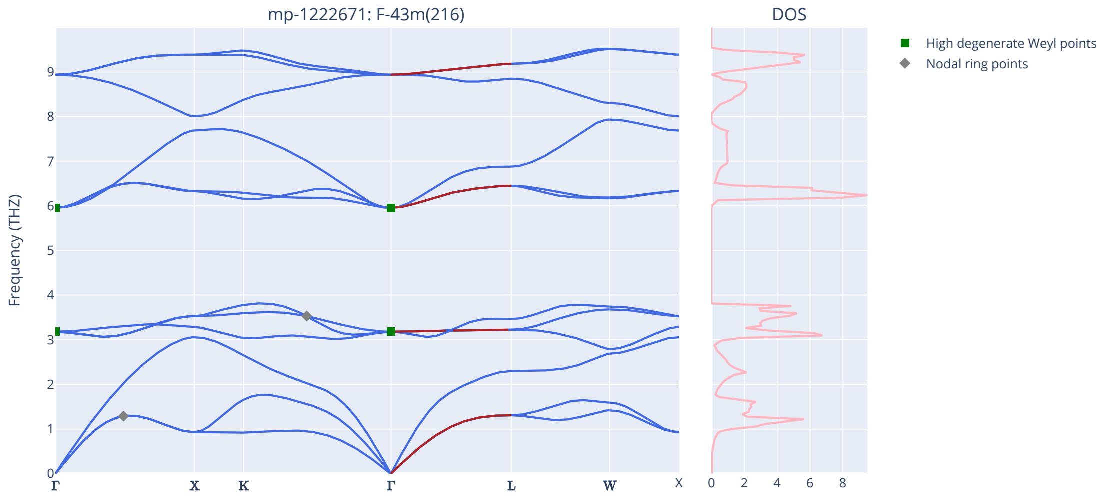

Supplementary Figure 735: The phonon band and DOS of Cd4In4Li8.

Supplementary Table 1583: High symmetry lines of mp-1222671-Cd4In4Li8

	Start	End
0	Γ (0.000, 0.000, 0.000)	X (0.500, 0.000, 0.500)
1	X (0.500, 0.000, 0.500)	U (0.625, 0.250, 0.625)
2	K (0.375, 0.375, 0.750)	Γ (0.000, 0.000, 0.000)
3	Γ (0.000, 0.000, 0.000)	L (0.500, 0.500, 0.500)
4	L (0.500, 0.500, 0.500)	W (0.500, 0.250, 0.750)
5	W (0.500, 0.250, 0.750)	X (0.500, 0.000, 0.500)

Supplementary Table 1584: High degenerate Weyl points along high symmetry lines of mp-1222671-Cd4In4Li8.

	Coordinates	Frequency	Mode 1	Mode 2	Multi.	Deg.	Path	Ratio
0	(0.000, 0.000, 0.000)	3.178	6	4	1	3	1	0.000
1	(0.000, 0.000, 0.000)	5.955	9	7	1	3	1	0.000

Supplementary Table 1585: Nodal ring points along high symmetry lines of mp-1222671-Cd4In4Li8.

	Coordinates	Frequency	Mode 1	Mode 2	Multi.	Deg.	Path	Ratio
0	(0.241, 0.000, 0.250)	1.286	2	1	24	2	1	0.490
1	(0.211, 0.211, 0.431)	3.530	6	5	24	2	3	0.429

The followings are details for Cd_2O_2. As shown in Fig **736**, it has 23 topological nodal lines, 15 nodal ring points and 1 high degenerate Weyl point whose degeneracy is more than 2 and charge is bigger than 1.

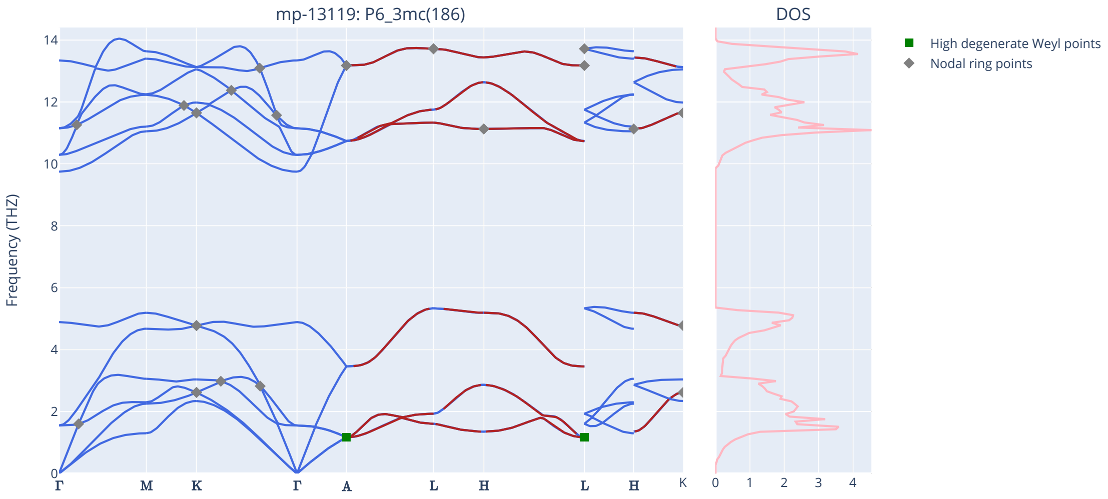

Supplementary Figure 736: The phonon band and DOS of Cd2O2.

Supplementary Table 1586: High symmetry lines of mp-13119-Cd2O2

	Start	End
0	Γ (0.000, 0.000, 0.000)	M (0.500, 0.000, 0.000)
1	M (0.500, 0.000, 0.000)	K (0.333, 0.333, 0.000)
2	K (0.333, 0.333, 0.000)	Γ (0.000, 0.000, 0.000)
3	Γ (0.000, 0.000, 0.000)	A (0.000, 0.000, 0.500)
4	A (0.000, 0.000, 0.500)	L (0.500, 0.000, 0.500)
5	L (0.500, 0.000, 0.500)	H (0.333, 0.333, 0.500)
6	H (0.333, 0.333, 0.500)	A (0.000, 0.000, 0.500)
7	L (0.500, 0.000, 0.500)	M (0.500, 0.000, 0.000)
8	H (0.333, 0.333, 0.500)	K (0.333, 0.333, 0.000)

Supplementary Table 1587: High degenerate Weyl points along high symmetry lines of mp-13119-Cd2O2.

	Coordinates	Frequency	Mode 1	Mode 2	Multi.	Deg.	Path	Ratio
0	(0.000, 0.000, 0.500)	1.165	4	1	1	4	4	1.000

Supplementary Table 1588: Nodal ring points along high symmetry lines of mp-13119-Cd2O2.

	Coordinates	Frequency	Mode 1	Mode 2	Multi.	Deg.	Path	Ratio
0	(0.333, 0.333, 0.000)	2.617	3	2	2	2	2	1.000
1	(0.253, 0.253, 0.000)	2.978	4	3	6	2	3	0.242

Supplementary Table 1588: Nodal ring points along high symmetry lines of mp-13119-Cd2O2.

	Coordinates	Frequency	Mode 1	Mode 2	Multi.	Deg.	Path	Ratio
2	(0.112, 0.000, -0.000)	1.600	4	3	6	2	1	0.224
3	(0.122, 0.122, 0.000)	2.822	5	4	6	2	3	0.633
4	(0.333, 0.333, 0.000)	4.776	6	5	2	2	2	1.000
5	(0.333, 0.333, 0.000)	11.647	8	7	2	2	2	1.000
6	(0.333, 0.333, 0.500)	11.128	8	7	2	2	6	1.000
7	(0.375, 0.250, 0.000)	11.886	9	8	6	2	2	0.751
8	(0.101, 0.000, 0.000)	11.259	10	9	6	2	1	0.203
9	(0.218, 0.218, 0.000)	12.376	10	9	6	2	3	0.346
10	(0.068, 0.068, 0.000)	11.571	11	10	6	2	3	0.796
11	(0.124, 0.124, -0.000)	13.092	12	11	6	2	3	0.629
12	(0.000, 0.000, 0.500)	13.177	12	11	1	2	4	1.000
13	(0.500, 0.000, 0.500)	13.719	12	11	3	2	5	1.000

The followings are details for Ce_4Mg_{12}. It has 6 topological nodal lines and 15 nodal ring points along high symmetry line as shown in Fig 737.

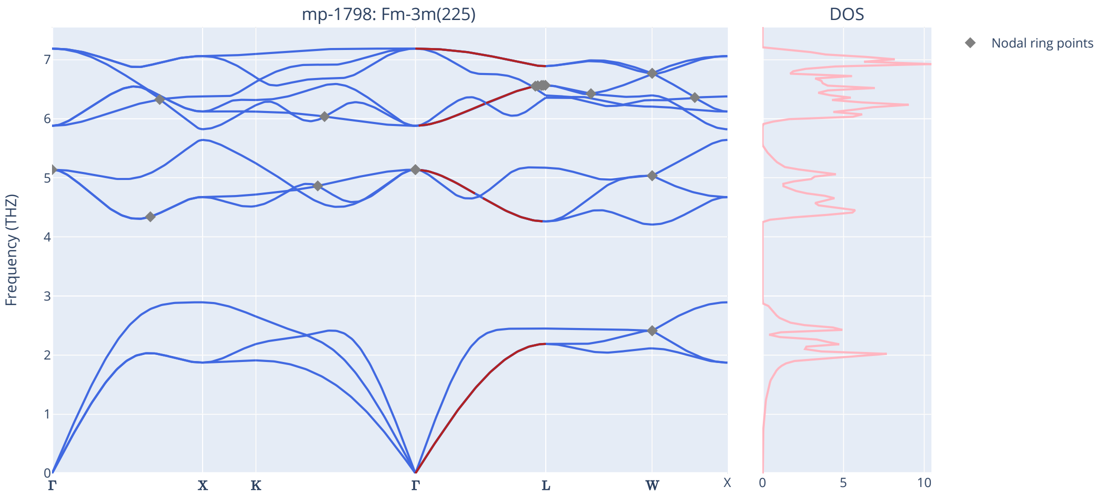

Supplementary Figure 737: The phonon band and DOS of Ce4Mg12.

Supplementary Table 1589: High symmetry lines of mp-1798-Ce4Mg12

	Start	End
0	Γ (0.000, 0.000, 0.000)	X (0.500, 0.000, 0.500)
1	X (0.500, 0.000, 0.500)	U (0.625, 0.250, 0.625)
2	K (0.375, 0.375, 0.750)	Γ (0.000, 0.000, 0.000)
3	Γ (0.000, 0.000, 0.000)	L (0.500, 0.500, 0.500)
4	L (0.500, 0.500, 0.500)	W (0.500, 0.250, 0.750)
5	W (0.500, 0.250, 0.750)	X (0.500, 0.000, 0.500)

Supplementary Table 1590: Nodal ring points along high symmetry lines of mp-1798-Ce4Mg12.

	Coordinates	Frequency	Mode 1	Mode 2	Multi.	Deg.	Path	Ratio
0	(0.500, 0.250, 0.750)	2.412	3	2	6	2	5	1.000
1	(0.000, 0.000, 0.000)	5.138	5	4	1	3	1	0.000
2	(0.327, 0.000, 0.327)	4.341	5	4	6	2	1	0.653
3	(0.228, 0.228, 0.460)	4.863	6	5	24	2	3	0.388
4	(0.500, 0.250, 0.750)	5.035	6	5	6	2	5	1.000
5	(0.357, -0.001, 0.357)	6.326	8	7	24	2	1	0.715
6	(0.214, 0.214, 0.426)	6.036	8	7	24	2	3	0.431
7	(0.498, 0.498, 0.498)	6.567	10	9	8	2	4	0.997
8	(0.483, 0.483, 0.483)	6.564	10	9	8	2	4	0.966
9	(0.460, 0.460, 0.460)	6.551	10	9	8	2	4	0.920
10	(0.469, 0.469, 0.469)	6.557	10	9	8	2	4	0.939
11	(0.490, 0.490, 0.490)	6.566	10	9	8	2	4	0.980
12	(0.500, 0.394, 0.606)	6.425	10	9	24	2	5	0.425
13	(0.498, 0.109, 0.608)	6.359	10	9	24	2	6	0.566
14	(0.500, 0.250, 0.750)	6.769	12	11	6	2	5	1.000

Supplementary Table 1590: Nodal ring points along high symmetry lines of mp-1798-Ce4Mg12.

Coordinates	Frequency	Mode 1	Mode 2	Multi.	Deg.	Path	Ratio

The followings are details for Pt_2Sn_2. It has 22 topological nodal lines and 13 nodal ring points along high symmetry line as shown in Fig **738**.

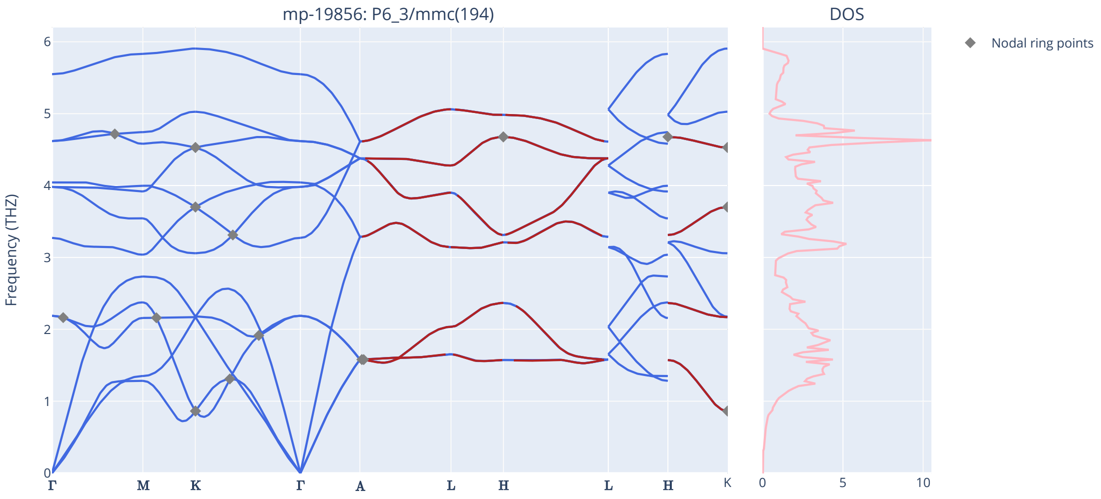

Supplementary Figure 738: The phonon band and DOS of Pt2Sn2.

Supplementary Table 1591: High symmetry lines of mp-19856-Pt2Sn2

	Start	End
0	Γ (0.000, 0.000, 0.000)	M (0.500, 0.000, 0.000)
1	M (0.500, 0.000, 0.000)	K (0.333, 0.333, 0.000)
2	K (0.333, 0.333, 0.000)	Γ (0.000, 0.000, 0.000)
3	Γ (0.000, 0.000, 0.000)	A (0.000, 0.000, 0.500)
4	A (0.000, 0.000, 0.500)	L (0.500, 0.000, 0.500)
5	L (0.500, 0.000, 0.500)	H (0.333, 0.333, 0.500)
6	H (0.333, 0.333, 0.500)	A (0.000, 0.000, 0.500)
7	L (0.500, 0.000, 0.500)	M (0.500, 0.000, 0.000)
8	H (0.333, 0.333, 0.500)	K (0.333, 0.333, 0.000)

Supplementary Table 1592: Nodal ring points along high symmetry lines of mp-19856-Pt2Sn2.

	Coordinates	Frequency	Mode 1	Mode 2	Multi.	Deg.	Path	Ratio
0	(0.224, 0.224, 0.000)	1.311	2	1	6	2	3	0.327
1	(0.333, 0.333, 0.000)	0.865	2	1	2	2	2	1.000
2	(0.010, 0.000, 0.500)	1.581	3	2	6	2	5	0.020
3	(0.020, 0.000, 0.499)	1.580	3	2	12	2	5	0.041
4	(0.457, 0.085, -0.000)	2.161	4	3	6	2	2	0.256
5	(0.132, 0.132, -0.000)	1.916	4	3	6	2	3	0.604
6	(0.061, 0.000, 0.000)	2.164	5	4	6	2	1	0.122
7	(0.215, 0.215, 0.000)	3.312	7	6	6	2	3	0.356
8	(0.333, 0.333, 0.000)	3.701	8	7	2	2	2	1.000
9	(0.333, 0.333, 0.000)	4.529	10	9	2	2	2	1.000
10	(0.333, 0.333, 0.500)	4.676	10	9	2	2	6	1.000
11	(0.345, -0.000, -0.000)	4.717	11	10	6	2	1	0.690

Supplementary Table 1592: Nodal ring points along high symmetry lines of mp-19856-Pt2Sn2.

Coordinates	Frequency	Mode 1	Mode 2	Multi.	Deg.	Path	Ratio

The followings are details for Ir_2Sn_2. It has 25 topological nodal lines and 17 nodal ring points along high symmetry line as shown in Fig 739.

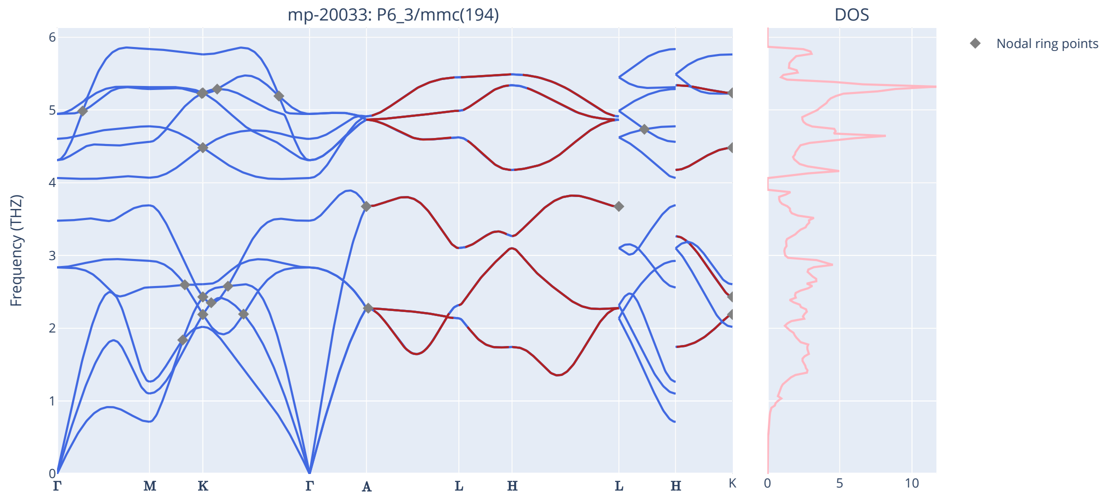

Supplementary Figure 739: The phonon band and DOS of Ir2Sn2.

Supplementary Table 1593: High symmetry lines of mp-20033-Ir2Sn2

	Start	End
0	Γ (0.000, 0.000, 0.000)	M (0.500, 0.000, 0.000)
1	M (0.500, 0.000, 0.000)	K (0.333, 0.333, 0.000)
2	K (0.333, 0.333, 0.000)	Γ (0.000, 0.000, 0.000)
3	Γ (0.000, 0.000, 0.000)	A (0.000, 0.000, 0.500)
4	A (0.000, 0.000, 0.500)	L (0.500, 0.000, 0.500)
5	L (0.500, 0.000, 0.500)	H (0.333, 0.333, 0.500)
6	H (0.333, 0.333, 0.500)	A (0.000, 0.000, 0.500)
7	L (0.500, 0.000, 0.500)	M (0.500, 0.000, 0.000)
8	H (0.333, 0.333, 0.500)	K (0.333, 0.333, 0.000)

Supplementary Table 1594: Nodal ring points along high symmetry lines of mp-20033-Ir2Sn2.

	Coordinates	Frequency	Mode 1	Mode 2	Multi.	Deg.	Path	Ratio
0	(0.396, 0.208, 0.000)	1.838	3	2	6	2	2	0.623
1	(0.206, 0.206, 0.000)	2.194	3	2	6	2	3	0.381
2	(0.333, 0.333, 0.000)	2.189	3	2	2	2	2	1.000
3	(0.010, 0.000, 0.500)	2.275	3	2	6	2	5	0.020
4	(0.306, 0.308, -0.000)	2.350	4	3	12	2	3	0.078
5	(0.390, 0.221, 0.000)	2.596	5	4	6	2	2	0.662
6	(0.255, 0.255, 0.000)	2.577	5	4	6	2	3	0.235
7	(0.333, 0.333, 0.000)	2.430	5	4	2	2	2	1.000
8	(0.000, 0.000, 0.500)	3.674	6	5	1	2	4	1.000
9	(0.333, 0.333, 0.000)	4.481	8	7	2	2	2	1.000
10	(0.499, 0.001, 0.275)	4.734	9	8	12	2	8	0.450

Supplementary Table 1594: Nodal ring points along high symmetry lines of mp-20033-Ir2Sn2.

	Coordinates	Frequency	Mode 1	Mode 2	Multi.	Deg.	Path	Ratio
11	(0.337, 0.326, -0.000)	5.226	10	9	6	2	2	0.979
12	(0.289, 0.289, 0.000)	5.286	11	10	6	2	3	0.134
13	(0.138, -0.000, 0.000)	4.984	11	10	6	2	1	0.276
14	(0.333, 0.333, 0.000)	5.234	11	10	2	2	2	1.000
15	(0.096, 0.096, 0.000)	5.191	12	11	6	2	3	0.712

The followings are details for Al_4Fe_{12}. It has 7 topological nodal lines and 19 nodal ring points along high symmetry line as shown in Fig **740**.

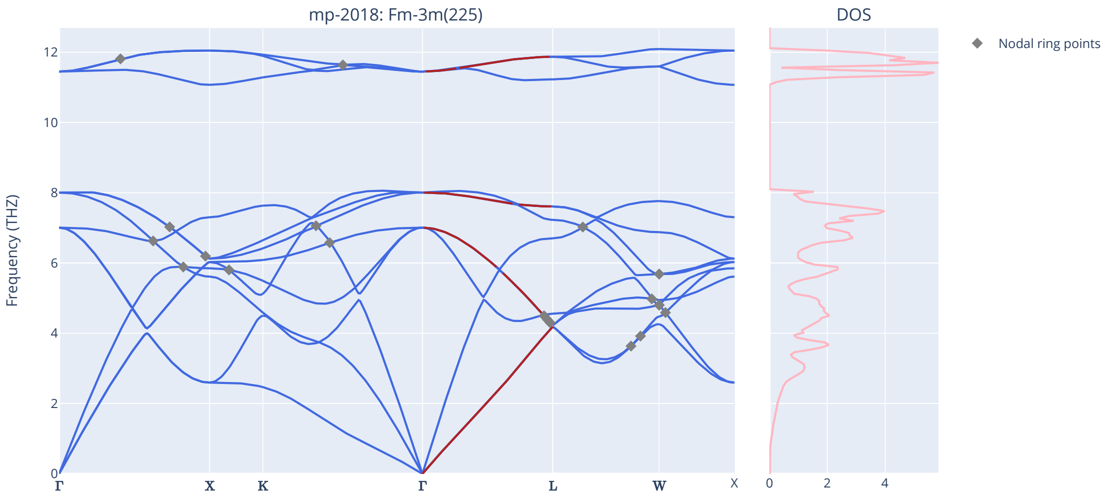

Supplementary Figure 740: The phonon band and DOS of Al4Fe12.

Supplementary Table 1595: High symmetry lines of mp-2018-Al4Fe12

	Start	End
0	Γ (0.000, 0.000, 0.000)	X (0.500, 0.000, 0.500)
1	X (0.500, 0.000, 0.500)	U (0.625, 0.250, 0.625)
2	K (0.375, 0.375, 0.750)	Γ (0.000, 0.000, 0.000)
3	Γ (0.000, 0.000, 0.000)	L (0.500, 0.500, 0.500)
4	L (0.500, 0.500, 0.500)	W (0.500, 0.250, 0.750)
5	W (0.500, 0.250, 0.750)	X (0.500, 0.000, 0.500)

Supplementary Table 1596: Nodal ring points along high symmetry lines of mp-2018-Al4Fe12.

	Coordinates	Frequency	Mode 1	Mode 2	Multi.	Deg.	Path	Ratio
0	(0.500, 0.316, 0.684)	3.635	2	1	24	2	5	0.735
1	(0.500, 0.294, 0.706)	3.918	2	1	24	2	5	0.824
2	(0.500, 0.229, 0.729)	4.588	3	2	12	2	6	0.084
3	(0.467, 0.467, 0.467)	4.497	4	3	8	2	4	0.933
4	(0.480, 0.480, 0.480)	4.386	4	3	8	2	4	0.959
5	(0.490, 0.490, 0.490)	4.300	4	3	8	2	4	0.980
6	(0.500, 0.250, 0.750)	4.801	4	3	6	2	5	1.000
7	(0.546, 0.091, 0.546)	5.802	5	4	12	2	2	0.366
8	(0.500, 0.267, 0.733)	4.978	5	4	24	2	5	0.930
9	(0.413, 0.000, 0.413)	5.887	6	5	6	2	1	0.825
10	(0.222, 0.222, 0.432)	6.577	6	5	24	2	3	0.419
11	(0.312, -0.001, 0.312)	6.628	7	6	10	2	1	0.625
12	(0.253, 0.253, 0.497)	7.059	7	6	24	2	3	0.333
13	(0.500, 0.429, 0.571)	7.024	7	6	24	2	5	0.282

Supplementary Table 1596: Nodal ring points along high symmetry lines of mp-2018-Al4Fe12.

	Coordinates	Frequency	Mode 1	Mode 2	Multi.	Deg.	Path	Ratio
14	(0.500, 0.250, 0.750)	5.684	7	6	6	2	5	1.000
15	(0.486, -0.000, 0.486)	6.194	8	7	6	2	1	0.973
16	(0.367, 0.000, 0.367)	7.026	9	8	6	2	1	0.735
17	(0.204, 0.000, 0.204)	11.812	12	11	6	2	1	0.408
18	(0.191, 0.191, 0.369)	11.636	12	11	24	2	3	0.502

The followings are details for Ni_2Te_2. It has 23 topological nodal lines and 17 nodal ring points along high symmetry line as shown in Fig **741**.

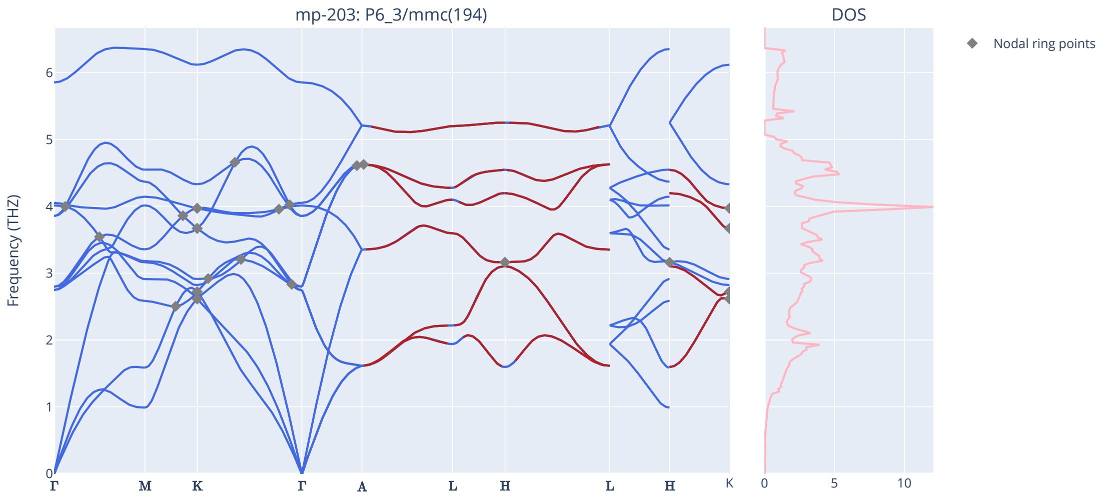

Supplementary Figure 741: The phonon band and DOS of Ni2Te2.

Supplementary Table 1597: High symmetry lines of mp-203-Ni2Te2

	Start	End
0	Γ (0.000, 0.000, 0.000)	M (0.500, 0.000, 0.000)
1	M (0.500, 0.000, 0.000)	K (0.333, 0.333, 0.000)
2	K (0.333, 0.333, 0.000)	Γ (0.000, 0.000, 0.000)
3	Γ (0.000, 0.000, 0.000)	A (0.000, 0.000, 0.500)
4	A (0.000, 0.000, 0.500)	L (0.500, 0.000, 0.500)
5	L (0.500, 0.000, 0.500)	H (0.333, 0.333, 0.500)
6	H (0.333, 0.333, 0.500)	A (0.000, 0.000, 0.500)
7	L (0.500, 0.000, 0.500)	M (0.500, 0.000, 0.000)
8	H (0.333, 0.333, 0.500)	K (0.333, 0.333, 0.000)

Supplementary Table 1598: Nodal ring points along high symmetry lines of mp-203-Ni2Te2.

	Coordinates	Frequency	Mode 1	Mode 2	Multi.	Deg.	Path	Ratio
0	(0.333, 0.333, 0.000)	2.615	2	1	2	2	2	1.000
1	(0.402, 0.196, 0.000)	2.504	3	2	6	2	2	0.589
2	(0.333, 0.333, 0.000)	2.722	4	3	2	2	2	1.000
3	(0.194, 0.194, -0.000)	3.205	5	4	6	2	3	0.418
4	(0.298, 0.298, -0.000)	2.919	6	5	6	2	3	0.105
5	(0.032, 0.032, 0.000)	2.832	6	5	6	2	3	0.903
6	(0.333, 0.333, 0.500)	3.163	6	5	2	2	6	1.000
7	(0.250, 0.000, -0.000)	3.543	8	7	6	2	1	0.500
8	(0.333, 0.333, 0.000)	3.669	8	7	2	2	2	1.000
9	(0.000, 0.000, 0.459)	4.608	8	7	2	2	4	0.918
10	(0.073, 0.073, -0.000)	3.957	9	8	6	2	3	0.780

Supplementary Table 1598: Nodal ring points along high symmetry lines of mp-203-Ni2Te2.

	Coordinates	Frequency	Mode 1	Mode 2	Multi.	Deg.	Path	Ratio
11	(0.379, 0.242, -0.000)	3.859	9	8	6	2	2	0.728
12	(0.010, 0.000, 0.500)	4.626	9	8	6	4	5	0.020
13	(0.061, -0.000, -0.000)	3.997	10	9	6	2	1	0.122
14	(0.333, 0.333, 0.000)	3.970	10	9	2	2	2	1.000
15	(0.213, 0.213, -0.000)	4.658	11	10	6	2	3	0.360
16	(0.039, 0.038, 0.001)	4.023	11	10	24	2	3	0.883

The followings are details for $Cd_4Na_8Pb_4$. As shown in Fig 742, it has 6 topological nodal lines, 11 nodal ring points and 2 high degenerate Weyl points whose degeneracy are more than 2 and charges are bigger than 1.

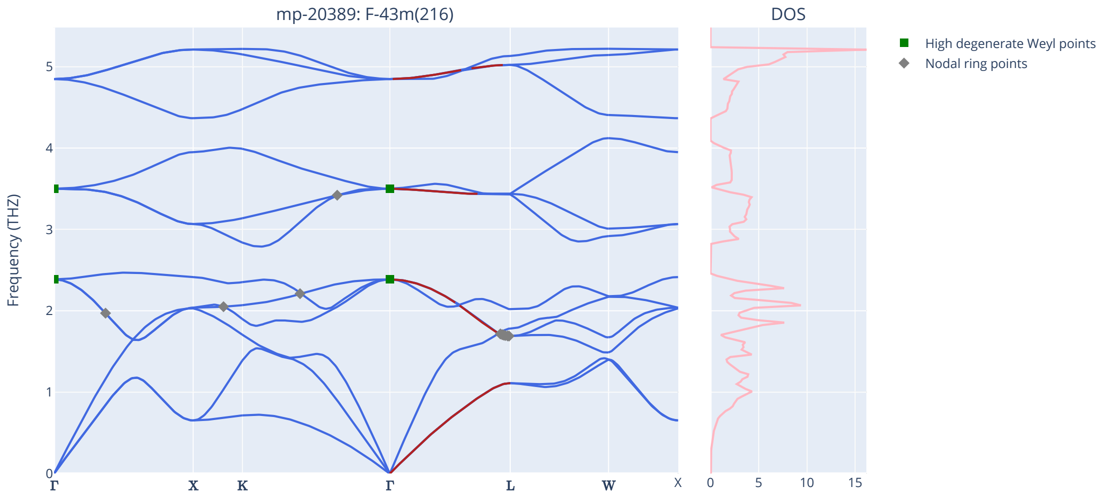

Supplementary Figure 742: The phonon band and DOS of Cd4Na8Pb4.

Supplementary Table 1599: High symmetry lines of mp-20389-Cd4Na8Pb4

	Start	End
0	Γ (0.000, 0.000, 0.000)	X (0.500, 0.000, 0.500)
1	X (0.500, 0.000, 0.500)	U (0.625, 0.250, 0.625)
2	K (0.375, 0.375, 0.750)	Γ (0.000, 0.000, 0.000)
3	Γ (0.000, 0.000, 0.000)	L (0.500, 0.500, 0.500)
4	L (0.500, 0.500, 0.500)	W (0.500, 0.250, 0.750)
5	W (0.500, 0.250, 0.750)	X (0.500, 0.000, 0.500)

Supplementary Table 1600: High degenerate Weyl points along high symmetry lines of mp-20389-Cd4Na8Pb4.

	Coordinates	Frequency	Mode 1	Mode 2	Multi.	Deg.	Path	Ratio
0	(0.000, 0.000, 0.000)	2.386	6	4	1	3	1	0.000
1	(0.000, 0.000, 0.000)	3.500	9	7	1	3	1	0.000

Supplementary Table 1601: Nodal ring points along high symmetry lines of mp-20389-Cd4Na8Pb4.

	Coordinates	Frequency	Mode 1	Mode 2	Multi.	Deg.	Path	Ratio
0	(0.479, 0.479, 0.479)	1.695	4	3	8	2	4	0.959
1	(0.473, 0.473, 0.473)	1.700	4	3	8	2	4	0.946
2	(0.495, 0.495, 0.495)	1.689	4	3	8	2	4	0.991
3	(0.489, 0.489, 0.489)	1.690	4	3	8	2	4	0.978

Supplementary Table 1601: Nodal ring points along high symmetry lines of mp-20389-Cd4Na8Pb4.

	Coordinates	Frequency	Mode 1	Mode 2	Multi.	Deg.	Path	Ratio
4	(0.467, 0.467, 0.467)	1.706	4	3	8	2	4	0.933
5	(0.459, 0.459, 0.459)	1.715	4	3	8	2	4	0.918
6	(0.576, 0.155, 0.576)	2.050	5	4	24	2	2	0.618
7	(0.190, 0.000, 0.180)	1.970	5	4	24	2	1	0.370
8	(0.234, 0.234, 0.451)	2.210	6	5	24	2	3	0.392
9	(0.136, 0.136, 0.265)	3.420	8	7	24	2	3	0.644

The followings are details for $In_4Li_8Pt_4$. As shown in Fig **743**, it has 5 topological nodal lines, 1 nodal ring point and 2 high degenerate Weyl points whose degeneracy are more than 2 and charges are bigger than 1.

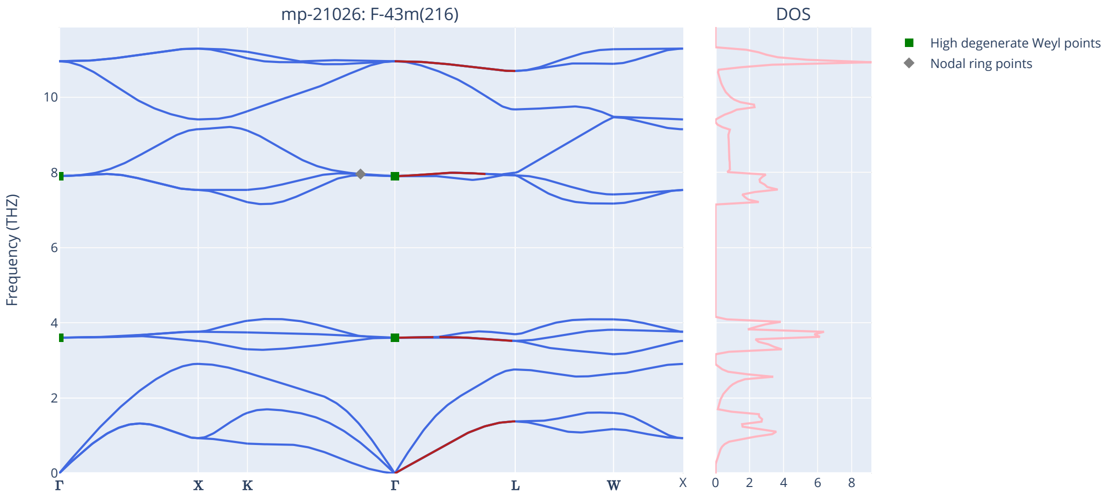

Supplementary Figure 743: The phonon band and DOS of In4Li8Pt4.

Supplementary Table 1602: High symmetry lines of mp-21026-In4Li8Pt4

	Start	End
0	Γ (0.000, 0.000, 0.000)	X (0.500, 0.000, 0.500)
1	X (0.500, 0.000, 0.500)	U (0.625, 0.250, 0.625)
2	K (0.375, 0.375, 0.750)	Γ (0.000, 0.000, 0.000)
3	Γ (0.000, 0.000, 0.000)	L (0.500, 0.500, 0.500)
4	L (0.500, 0.500, 0.500)	W (0.500, 0.250, 0.750)
5	W (0.500, 0.250, 0.750)	X (0.500, 0.000, 0.500)

Supplementary Table 1603: High degenerate Weyl points along high symmetry lines of mp-21026-In4Li8Pt4.

	Coordinates	Frequency	Mode 1	Mode 2	Multi.	Deg.	Path	Ratio
0	(0.000, 0.000, 0.000)	3.605	6	4	1	3	1	0.000
1	(0.000, 0.000, 0.000)	7.899	9	7	1	3	1	0.000

Supplementary Table 1604: Nodal ring points along high symmetry lines of mp-21026-In4Li8Pt4.

	Coordinates	Frequency	Mode 1	Mode 2	Multi.	Deg.	Path	Ratio
0	(0.084, 0.084, 0.177)	7.956	9	8	24	2	3	0.768

The followings are details for $In_4Li_8Pd_4$. As shown in Fig **744**, it has 6 topological nodal lines and 2 nodal ring points. At the same time, it has 1 single Weyl point and 2 high degenerate Weyl points whose degeneracy are more than 2 and charges are bigger than 1.

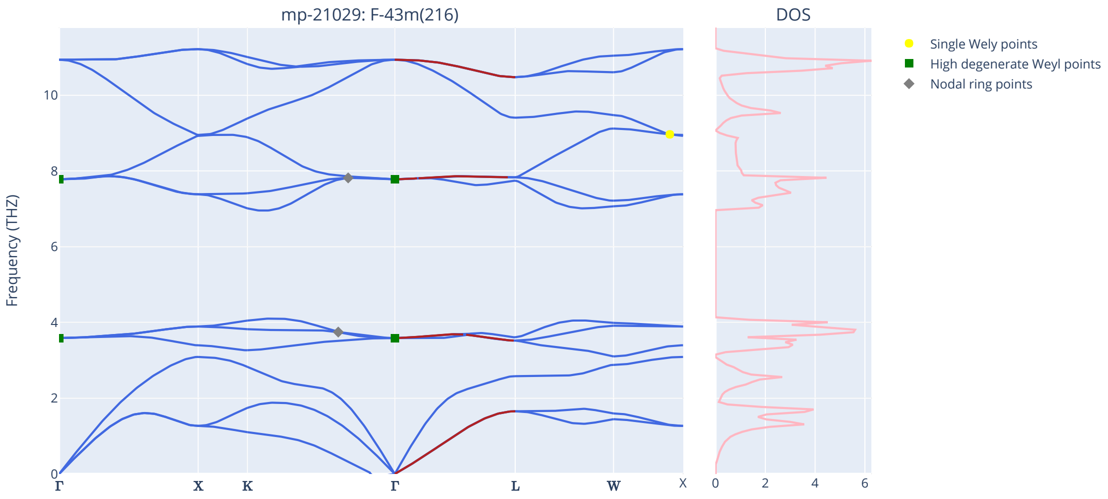

Supplementary Figure 744: The phonon band and DOS of In4Li8Pd4.

Supplementary Table 1605: High symmetry lines of mp-21029-In4Li8Pd4

	Start	End
0	Γ (0.000, 0.000, 0.000)	X (0.500, 0.000, 0.500)
1	X (0.500, 0.000, 0.500)	U (0.625, 0.250, 0.625)
2	K (0.375, 0.375, 0.750)	Γ (0.000, 0.000, 0.000)
3	Γ (0.000, 0.000, 0.000)	L (0.500, 0.500, 0.500)
4	L (0.500, 0.500, 0.500)	W (0.500, 0.250, 0.750)
5	W (0.500, 0.250, 0.750)	X (0.500, 0.000, 0.500)

Supplementary Table 1606: Single Weyl points along high symmetry lines of mp-21029-In4Li8Pd4.

	Coordinates	Frequency	Mode 1	Mode 2	Multi.	Deg.	Path	Ratio
0	(0.500, 0.048, 0.548)	8.966	10	9	12	2	6	0.810

Supplementary Table 1607: High degenerate Weyl points along high symmetry lines of mp-21029-In4Li8Pd4.

	Coordinates	Frequency	Mode 1	Mode 2	Multi.	Deg.	Path	Ratio
0	(0.000, 0.000, 0.000)	3.586	6	4	1	3	1	0.000
1	(0.000, 0.000, 0.000)	7.779	9	7	1	3	1	0.000

Supplementary Table 1608: Nodal ring points along high symmetry lines of mp-21029-In4Li8Pd4.

	Coordinates	Frequency	Mode 1	Mode 2	Multi.	Deg.	Path	Ratio
0	(0.145, 0.145, 0.286)	3.745	6	5	24	2	3	0.617
1	(0.115, 0.115, 0.241)	7.816	8	7	24	2	3	0.684

The followings are details for Ti_2Sb_2. It has 25 topological nodal lines and 19 nodal ring points along high symmetry line as shown in Fig **745**.

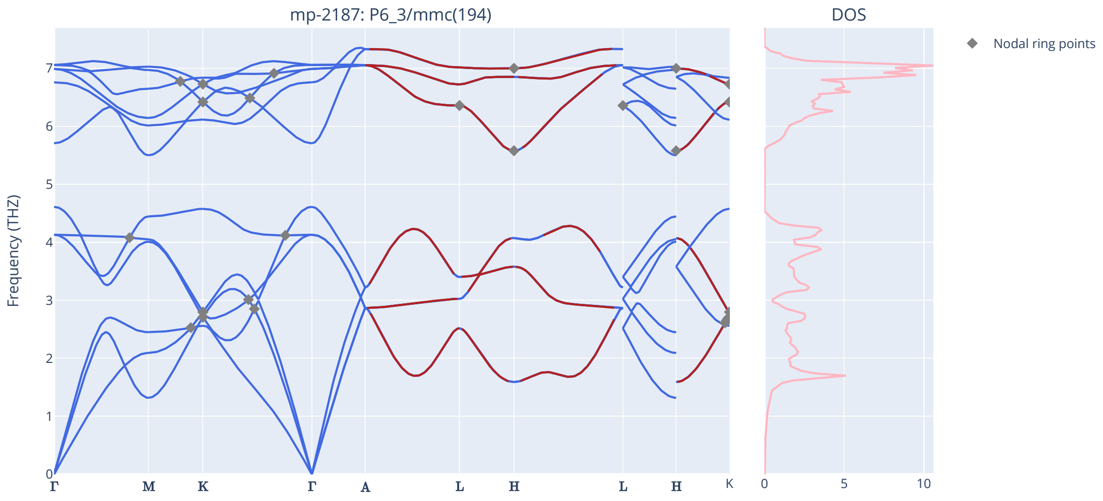

Supplementary Figure 745: The phonon band and DOS of Ti2Sb2.

Supplementary Table 1609: High symmetry lines of mp-2187-Ti2Sb2

	Start	End
0	Γ (0.000, 0.000, 0.000)	M (0.500, 0.000, 0.000)
1	M (0.500, 0.000, 0.000)	K (0.333, 0.333, 0.000)
2	K (0.333, 0.333, 0.000)	Γ (0.000, 0.000, 0.000)
3	Γ (0.000, 0.000, 0.000)	A (0.000, 0.000, 0.500)
4	A (0.000, 0.000, 0.500)	L (0.500, 0.000, 0.500)
5	L (0.500, 0.000, 0.500)	H (0.333, 0.333, 0.500)
6	H (0.333, 0.333, 0.500)	A (0.000, 0.000, 0.500)
7	L (0.500, 0.000, 0.500)	M (0.500, 0.000, 0.000)
8	H (0.333, 0.333, 0.500)	K (0.333, 0.333, 0.000)

Supplementary Table 1610: Nodal ring points along high symmetry lines of mp-2187-Ti2Sb2.

	Coordinates	Frequency	Mode 1	Mode 2	Multi.	Deg.	Path	Ratio
0	(0.370, 0.259, 0.000)	2.527	3	2	6	2	2	0.778
1	(0.333, 0.333, 0.000)	2.708	3	2	2	2	2	1.000
2	(0.176, 0.176, 0.000)	2.850	3	2	6	2	3	0.472
3	(0.333, 0.333, 0.041)	2.609	3	2	4	2	9	0.918
4	(0.333, 0.333, 0.031)	2.641	3	2	4	2	9	0.939
5	(0.333, 0.333, 0.020)	2.671	3	2	4	2	9	0.959
6	(0.333, 0.333, 0.010)	2.696	3	2	4	2	9	0.980
7	(0.194, 0.194, 0.000)	3.011	4	3	6	2	3	0.417
8	(0.333, 0.333, 0.000)	2.796	5	4	2	2	2	1.000
9	(0.400, -0.000, 0.000)	4.079	6	5	6	2	1	0.800
10	(0.081, 0.081, 0.000)	4.118	6	5	6	2	3	0.757

Supplementary Table 1610: Nodal ring points along high symmetry lines of mp-2187-Ti2Sb2.

	Coordinates	Frequency	Mode 1	Mode 2	Multi.	Deg.	Path	Ratio
11	(0.500, 0.000, 0.500)	6.357	8	7	3	2	5	1.000
12	(0.333, 0.333, 0.500)	5.578	8	7	2	2	6	1.000
13	(0.190, 0.190, 0.000)	6.486	9	8	6	2	3	0.431
14	(0.333, 0.333, 0.000)	6.418	9	8	2	2	2	1.000
15	(0.402, 0.195, -0.000)	6.774	11	10	6	2	2	0.585
16	(0.333, 0.333, 0.000)	6.727	11	10	2	2	2	1.000
17	(0.116, 0.116, 0.000)	6.913	11	10	6	2	3	0.652
18	(0.333, 0.333, 0.500)	6.998	12	11	2	2	6	1.000

The followings are details for In_2N_2. As shown in Fig **746**, it has 22 topological nodal lines, 18 nodal ring points and 2 high degenerate Weyl points whose degeneracy are more than 2 and charges are bigger than 1.

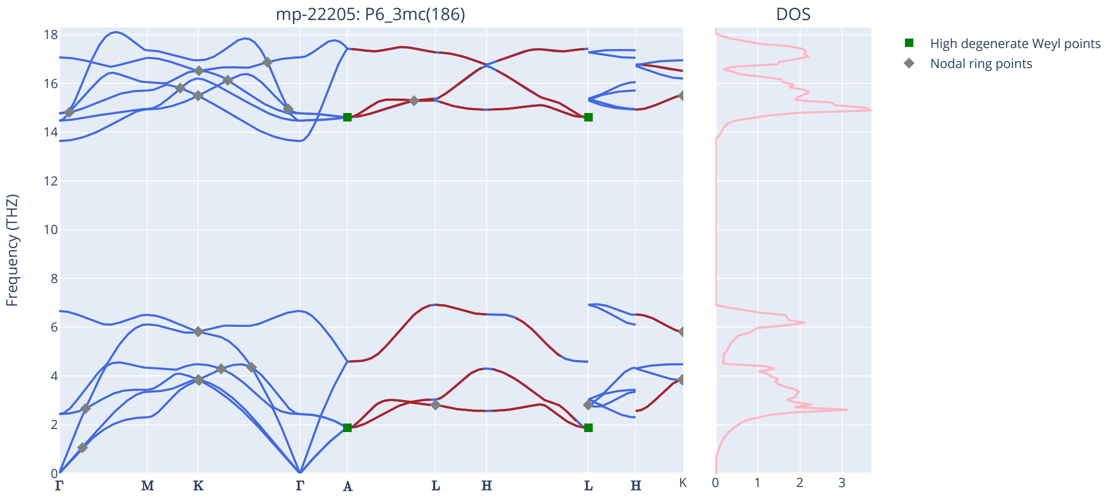

Supplementary Figure 746: The phonon band and DOS of In2N2.

Supplementary Table 1611: High symmetry lines of mp-22205-In2N2

	Start	End
0	Γ (0.000, 0.000, 0.000)	M (0.500, 0.000, 0.000)
1	M (0.500, 0.000, 0.000)	K (0.333, 0.333, 0.000)
2	K (0.333, 0.333, 0.000)	Γ (0.000, 0.000, 0.000)
3	Γ (0.000, 0.000, 0.000)	A (0.000, 0.000, 0.500)
4	A (0.000, 0.000, 0.500)	L (0.500, 0.000, 0.500)
5	L (0.500, 0.000, 0.500)	H (0.333, 0.333, 0.500)
6	H (0.333, 0.333, 0.500)	A (0.000, 0.000, 0.500)
7	L (0.500, 0.000, 0.500)	M (0.500, 0.000, 0.000)
8	H (0.333, 0.333, 0.500)	K (0.333, 0.333, 0.000)

Supplementary Table 1612: High degenerate Weyl points along high symmetry lines of mp-22205-In2N2.

	Coordinates	Frequency	Mode 1	Mode 2	Multi.	Deg.	Path	Ratio
0	(0.000, 0.000, 0.500)	1.869	4	1	1	4	4	1.000
1	(0.000, 0.000, 0.500)	14.605	10	7	1	4	4	1.000

Supplementary Table 1613: Nodal ring points along high symmetry lines of mp-22205-In2N2.

	Coordinates	Frequency	Mode 1	Mode 2	Multi.	Deg.	Path	Ratio
0	(0.133, -0.000, -0.000)	1.057	2	1	6	2	1	0.266

Supplementary Table 1613: Nodal ring points along high symmetry lines of mp-22205-In2N2.

	Coordinates	Frequency	Mode 1	Mode 2	Multi.	Deg.	Path	Ratio
1	(0.330, 0.330, -0.000)	3.822	2	1	6	2	3	0.011
2	(0.500, 0.000, 0.500)	2.809	2	1	3	2	5	1.000
3	(0.333, 0.333, 0.000)	3.844	3	2	2	2	2	1.000
4	(0.333, 0.333, 0.010)	3.842	3	2	4	2	9	0.980
5	(0.258, 0.258, -0.000)	4.292	4	3	6	2	3	0.225
6	(0.150, 0.000, -0.000)	2.660	4	3	6	2	1	0.300
7	(0.159, 0.159, 0.000)	4.352	5	4	6	2	3	0.524
8	(0.333, 0.333, 0.000)	5.816	6	5	2	2	2	1.000
9	(0.333, 0.333, 0.000)	15.498	8	7	2	2	2	1.000
10	(0.392, 0.216, -0.002)	15.808	9	8	12	2	2	0.648
11	(0.378, 0.000, 0.498)	15.290	9	8	12	2	5	0.757
12	(0.058, -0.000, -0.000)	14.813	10	9	6	2	1	0.116
13	(0.236, 0.236, -0.000)	16.130	10	9	6	2	3	0.291
14	(0.039, 0.039, 0.000)	14.961	11	10	6	2	3	0.884
15	(0.331, 0.331, 0.000)	16.521	11	10	6	2	3	0.007
16	(0.106, 0.106, -0.000)	16.874	12	11	6	2	3	0.682

The followings are details for Rh$_3$. It has 8 topological nodal lines and 19 nodal ring points along high symmetry line as shown in Fig **747**.

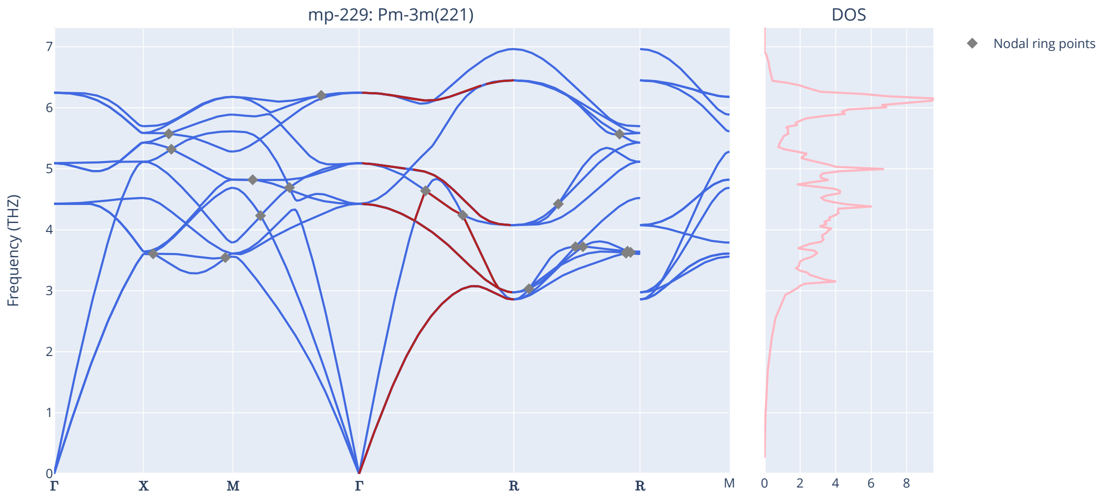

Supplementary Figure 747: The phonon band and DOS of Rh3Zr.

Supplementary Table 1614: High symmetry lines of mp-229-Rh3Zr

	Start	End
0	Γ (0.000, 0.000, 0.000)	X (0.000, 0.500, 0.000)
1	X (0.000, 0.500, 0.000)	M (0.500, 0.500, 0.000)
2	M (0.500, 0.500, 0.000)	Γ (0.000, 0.000, 0.000)
3	Γ (0.000, 0.000, 0.000)	R (0.500, 0.500, 0.500)
4	R (0.500, 0.500, 0.500)	X (0.000, 0.500, 0.000)
5	R (0.500, 0.500, 0.500)	M (0.500, 0.500, 0.000)

Supplementary Table 1615: Nodal ring points along high symmetry lines of mp-229-Rh3Zr.

	Coordinates	Frequency	Mode 1	Mode 2	Multi.	Deg.	Path	Ratio
0	(0.054, 0.500, -0.000)	3.605	2	1	12	2	2	0.109
1	(0.459, 0.500, -0.000)	3.544	2	1	12	2	2	0.917
2	(0.056, 0.500, 0.056)	3.616	2	1	12	2	5	0.888
3	(0.038, 0.500, 0.038)	3.629	3	2	12	2	5	0.925
4	(0.440, 0.500, 0.440)	3.031	4	3	12	2	5	0.120
5	(0.226, 0.500, 0.226)	3.722	4	3	12	2	5	0.547
6	(0.050, 0.500, 0.050)	3.647	4	3	12	2	5	0.900
7	(0.390, 0.390, 0.000)	4.231	5	4	12	2	3	0.220
8	(0.256, 0.500, 0.256)	3.720	5	4	12	2	5	0.488
9	(0.215, 0.215, 0.215)	4.638	6	5	8	2	4	0.430
10	(0.335, 0.335, 0.335)	4.237	6	5	8	2	4	0.670
11	(0.421, 0.421, -0.000)	4.819	7	6	12	2	3	0.158
12	(0.275, 0.275, -0.000)	4.691	7	6	12	2	3	0.449
13	(0.156, 0.500, -0.000)	5.320	8	7	12	2	2	0.313

Supplementary Table 1615: Nodal ring points along high symmetry lines of mp-229-Rh3Zr.

	Coordinates	Frequency	Mode 1	Mode 2	Multi.	Deg.	Path	Ratio
14	(0.324, 0.500, 0.324)	4.422	8	7	12	2	5	0.353
15	(0.142, 0.500, -0.000)	5.573	10	9	12	2	2	0.285
16	(0.081, 0.500, 0.081)	5.568	10	9	12	2	5	0.837
17	(0.150, 0.150, -0.000)	6.201	12	11	12	2	3	0.700

The followings are details for La_4Mg_{12}. It has 6 topological nodal lines and 15 nodal ring points along high symmetry line as shown in Fig **748**.

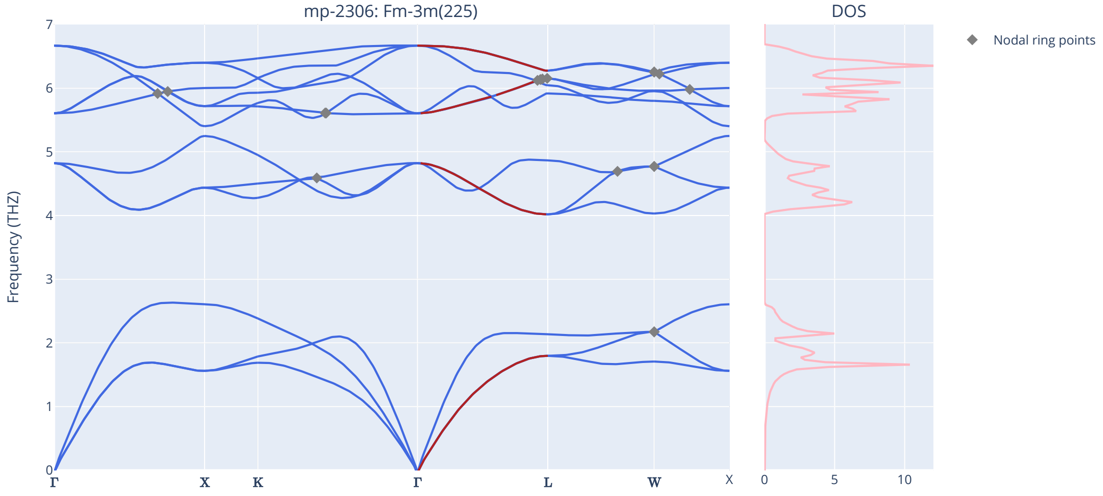

Supplementary Figure 748: The phonon band and DOS of La4Mg12.

Supplementary Table 1616: High symmetry lines of mp-2306-La4Mg12

	Start	End
0	Γ (0.000, 0.000, 0.000)	X (0.500, 0.000, 0.500)
1	X (0.500, 0.000, 0.500)	U (0.625, 0.250, 0.625)
2	K (0.375, 0.375, 0.750)	Γ (0.000, 0.000, 0.000)
3	Γ (0.000, 0.000, 0.000)	L (0.500, 0.500, 0.500)
4	L (0.500, 0.500, 0.500)	W (0.500, 0.250, 0.750)
5	W (0.500, 0.250, 0.750)	X (0.500, 0.000, 0.500)

Supplementary Table 1617: Nodal ring points along high symmetry lines of mp-2306-La4Mg12.

	Coordinates	Frequency	Mode 1	Mode 2	Multi.	Deg.	Path	Ratio
0	(0.500, 0.250, 0.750)	2.173	3	2	6	2	5	1.000
1	(0.231, 0.231, 0.478)	4.590	6	5	24	2	3	0.370
2	(0.500, 0.250, 0.750)	4.771	6	5	6	2	5	1.000
3	(0.500, 0.336, 0.664)	4.693	6	5	24	2	5	0.655
4	(0.344, -0.002, 0.344)	5.916	8	7	24	2	1	0.688
5	(0.222, 0.222, 0.428)	5.610	8	7	24	2	3	0.422
6	(0.209, 0.220, 0.429)	5.609	8	7	24	2	3	0.429
7	(0.378, 0.000, 0.378)	5.947	10	9	6	2	1	0.755
8	(0.498, 0.498, 0.498)	6.154	10	9	8	2	4	0.996
9	(0.472, 0.472, 0.472)	6.136	10	9	8	2	4	0.943
10	(0.461, 0.461, 0.461)	6.122	10	9	8	2	4	0.921
11	(0.480, 0.480, 0.480)	6.144	10	9	8	2	4	0.959
12	(0.500, 0.132, 0.632)	5.980	10	9	12	2	6	0.473
13	(0.499, 0.232, 0.733)	6.223	11	10	24	2	6	0.069
14	(0.500, 0.250, 0.750)	6.253	12	11	6	2	5	1.000

Supplementary Table 1617: Nodal ring points along high symmetry lines of mp-2306-La4Mg12.

Coordinates	Frequency	Mode 1	Mode 2	Multi.	Deg.	Path	Ratio

The followings are details for AlNi$_3$. It has 7 topological nodal lines and 20 nodal ring points along high symmetry line as shown in Fig 749.

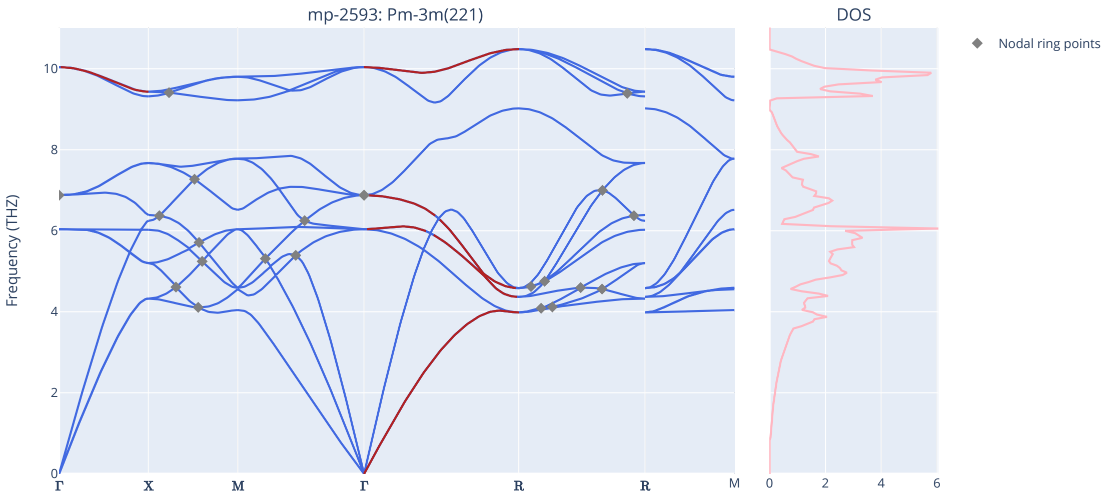

Supplementary Figure 749: The phonon band and DOS of AlNi3.

Supplementary Table 1618: High symmetry lines of mp-2593-AlNi3

	Start	End
0	Γ (0.000, 0.000, 0.000)	X (0.000, 0.500, 0.000)
1	X (0.000, 0.500, 0.000)	M (0.500, 0.500, 0.000)
2	M (0.500, 0.500, 0.000)	Γ (0.000, 0.000, 0.000)
3	Γ (0.000, 0.000, 0.000)	R (0.500, 0.500, 0.500)
4	R (0.500, 0.500, 0.500)	X (0.000, 0.500, 0.000)
5	R (0.500, 0.500, 0.500)	M (0.500, 0.500, 0.000)

Supplementary Table 1619: Nodal ring points along high symmetry lines of mp-2593-AlNi3.

	Coordinates	Frequency	Mode 1	Mode 2	Multi.	Deg.	Path	Ratio
0	(0.279, 0.500, 0.000)	4.108	2	1	12	2	2	0.558
1	(0.367, 0.500, 0.367)	4.118	2	1	12	2	5	0.266
2	(0.154, 0.500, 0.000)	4.609	3	2	12	2	2	0.308
3	(0.412, 0.500, 0.412)	4.086	3	2	12	2	5	0.177
4	(0.170, 0.500, 0.170)	4.561	3	2	12	2	5	0.659
5	(0.301, 0.500, -0.000)	5.244	4	3	12	2	2	0.602
6	(0.270, 0.270, 0.000)	5.393	4	3	12	2	3	0.459
7	(0.255, 0.496, 0.255)	4.597	4	3	24	2	5	0.490
8	(0.391, 0.391, -0.000)	5.311	5	4	12	2	3	0.218
9	(0.284, 0.500, -0.000)	5.711	6	5	12	2	2	0.567
10	(0.401, 0.500, 0.395)	4.753	6	5	24	2	5	0.205
11	(0.044, 0.500, 0.044)	6.371	7	6	12	2	5	0.912
12	(0.061, 0.500, -0.000)	6.371	7	6	12	2	2	0.123
13	(0.235, 0.235, 0.000)	6.249	7	6	12	2	3	0.530

Supplementary Table 1619: Nodal ring points along high symmetry lines of mp-2593-AlNi3.

	Coordinates	Frequency	Mode 1	Mode 2	Multi.	Deg.	Path	Ratio
14	(0.452, 0.500, 0.452)	4.623	7	6	12	2	5	0.097
15	(0.258, 0.500, -0.000)	7.271	8	7	12	2	2	0.516
16	(0.169, 0.500, 0.169)	6.997	8	7	12	2	5	0.663
17	(0.000, 0.000, 0.000)	6.878	9	8	1	3	1	0.000
18	(0.115, 0.500, -0.000)	9.410	11	10	12	2	2	0.231
19	(0.071, 0.500, 0.071)	9.393	11	10	12	2	5	0.859

The followings are details for Co_2Sb_2. It has 21 topological nodal lines and 35 nodal ring points along high symmetry line as shown in Fig **750**.

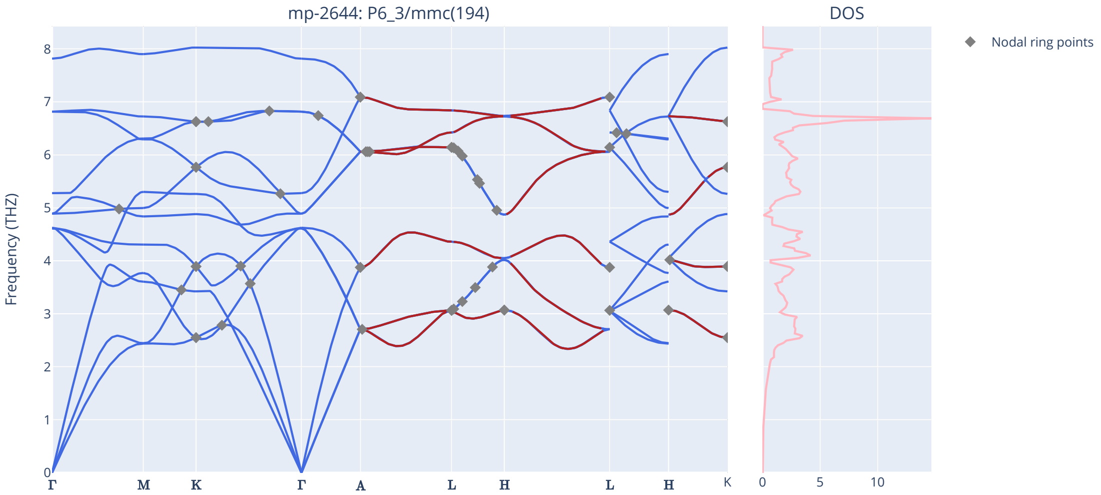

Supplementary Figure 750: The phonon band and DOS of Co2Sb2.

Supplementary Table 1620: High symmetry lines of mp-2644-Co2Sb2

	Start	End
0	Γ (0.000, 0.000, 0.000)	M (0.500, 0.000, 0.000)
1	M (0.500, 0.000, 0.000)	K (0.333, 0.333, 0.000)
2	K (0.333, 0.333, 0.000)	Γ (0.000, 0.000, 0.000)
3	Γ (0.000, 0.000, 0.000)	A (0.000, 0.000, 0.500)
4	A (0.000, 0.000, 0.500)	L (0.500, 0.000, 0.500)
5	L (0.500, 0.000, 0.500)	H (0.333, 0.333, 0.500)
6	H (0.333, 0.333, 0.500)	A (0.000, 0.000, 0.500)
7	L (0.500, 0.000, 0.500)	M (0.500, 0.000, 0.000)
8	H (0.333, 0.333, 0.500)	K (0.333, 0.333, 0.000)

Supplementary Table 1621: Nodal ring points along high symmetry lines of mp-2644-Co2Sb2.

	Coordinates	Frequency	Mode 1	Mode 2	Multi.	Deg.	Path	Ratio
0	(0.333, 0.333, 0.000)	2.550	2	1	2	2	2	1.000
1	(0.251, 0.251, -0.000)	2.784	2	1	6	2	3	0.246
2	(0.333, 0.333, 0.500)	3.070	2	1	2	2	6	1.000
3	(0.010, 0.000, 0.500)	2.707	3	2	6	2	5	0.020
4	(0.380, 0.241, -0.000)	3.451	4	3	6	2	2	0.723
5	(0.162, 0.162, -0.000)	3.569	4	3	6	2	3	0.514
6	(0.500, 0.000, 0.500)	3.064	4	3	3	2	5	1.000
7	(0.493, 0.014, 0.500)	3.090	4	3	6	2	6	0.041
8	(0.466, 0.068, 0.500)	3.232	4	3	6	2	6	0.204
9	(0.425, 0.150, 0.500)	3.495	4	3	6	2	6	0.449
10	(0.371, 0.259, 0.500)	3.883	4	3	6	2	6	0.776

Supplementary Table 1621: Nodal ring points along high symmetry lines of mp-2644-Co2Sb2.

	Coordinates	Frequency	Mode 1	Mode 2	Multi.	Deg.	Path	Ratio
11	(0.333, 0.333, 0.490)	4.018	4	3	4	2	9	0.020
12	(0.192, 0.192, 0.000)	3.901	5	4	6	2	3	0.424
13	(0.333, 0.333, 0.000)	3.893	5	4	2	2	2	1.000
14	(0.000, 0.000, 0.500)	3.876	6	5	1	2	4	1.000
15	(0.367, -0.000, -0.000)	4.978	8	7	6	2	1	0.734
16	(0.500, 0.000, 0.500)	6.142	8	7	3	2	5	1.000
17	(0.493, 0.014, 0.500)	6.133	8	7	6	2	6	0.041
18	(0.480, 0.041, 0.500)	6.072	8	7	6	2	6	0.122
19	(0.466, 0.068, 0.500)	5.977	8	7	6	2	6	0.204
20	(0.418, 0.163, 0.500)	5.533	8	7	6	2	6	0.490
21	(0.412, 0.177, 0.500)	5.462	8	7	6	2	6	0.531
22	(0.357, 0.286, 0.500)	4.954	8	7	6	2	6	0.857
23	(0.067, 0.067, 0.000)	5.266	9	8	6	2	3	0.800
24	(0.333, 0.333, 0.000)	5.767	9	8	2	2	2	1.000
25	(0.031, 0.000, 0.499)	6.060	9	8	12	2	5	0.061
26	(0.041, 0.000, 0.499)	6.059	9	8	12	2	5	0.082
27	(0.051, 0.000, 0.498)	6.059	9	8	12	2	5	0.102
28	(0.499, 0.002, 0.357)	6.399	10	9	12	2	8	0.285
29	(0.499, 0.001, 0.441)	6.418	10	9	12	2	8	0.117
30	(0.102, 0.102, -0.000)	6.828	11	10	6	2	3	0.695
31	(0.294, 0.294, 0.000)	6.627	11	10	6	2	3	0.117
32	(0.333, 0.333, 0.000)	6.626	11	10	2	2	2	1.000
33	(0.000, 0.000, 0.143)	6.741	11	10	2	2	4	0.286
34	(0.000, 0.000, 0.500)	7.090	12	11	1	2	4	1.000

The followings are details for $Au_4Li_8Ge_4$. As shown in Fig **751**, it has 5 topological nodal lines, 1 nodal ring point and 2 high degenerate Weyl points whose degeneracy are more than 2 and charges are bigger than 1.

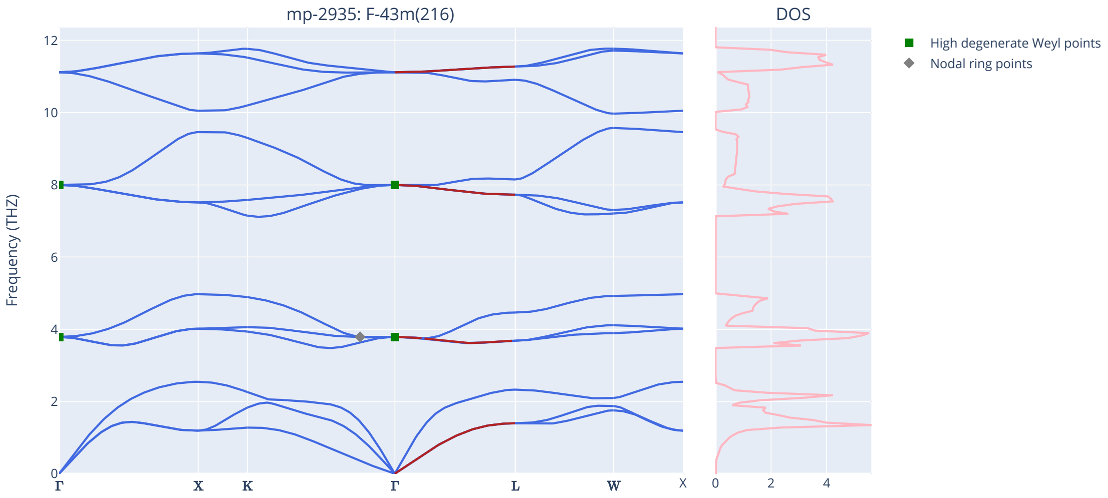

Supplementary Figure 751: The phonon band and DOS of Au4Li8Ge4.

Supplementary Table 1622: High symmetry lines of mp-2935-Au4Li8Ge4

	Start	End
0	Γ (0.000, 0.000, 0.000)	X (0.500, 0.000, 0.500)
1	X (0.500, 0.000, 0.500)	U (0.625, 0.250, 0.625)
2	K (0.375, 0.375, 0.750)	Γ (0.000, 0.000, 0.000)
3	Γ (0.000, 0.000, 0.000)	L (0.500, 0.500, 0.500)
4	L (0.500, 0.500, 0.500)	W (0.500, 0.250, 0.750)
5	W (0.500, 0.250, 0.750)	X (0.500, 0.000, 0.500)

Supplementary Table 1623: High degenerate Weyl points along high symmetry lines of mp-2935-Au4Li8Ge4.

	Coordinates	Frequency	Mode 1	Mode 2	Multi.	Deg.	Path	Ratio
0	(0.000, 0.000, 0.000)	3.788	6	4	1	3	1	0.000
1	(0.000, 0.000, 0.000)	7.998	9	7	1	3	1	0.000

Supplementary Table 1624: Nodal ring points along high symmetry lines of mp-2935-Au4Li8Ge4.

	Coordinates	Frequency	Mode 1	Mode 2	Multi.	Deg.	Path	Ratio
0	(0.090, 0.090, 0.175)	3.789	6	5	24	2	3	0.765

The followings are details for $Ga_4Li_8Rh_4$. As shown in Fig **752**, it has 5 topological nodal lines, 3 nodal ring points and 2 high degenerate Weyl points whose degeneracy are more than 2 and charges are bigger than 1.

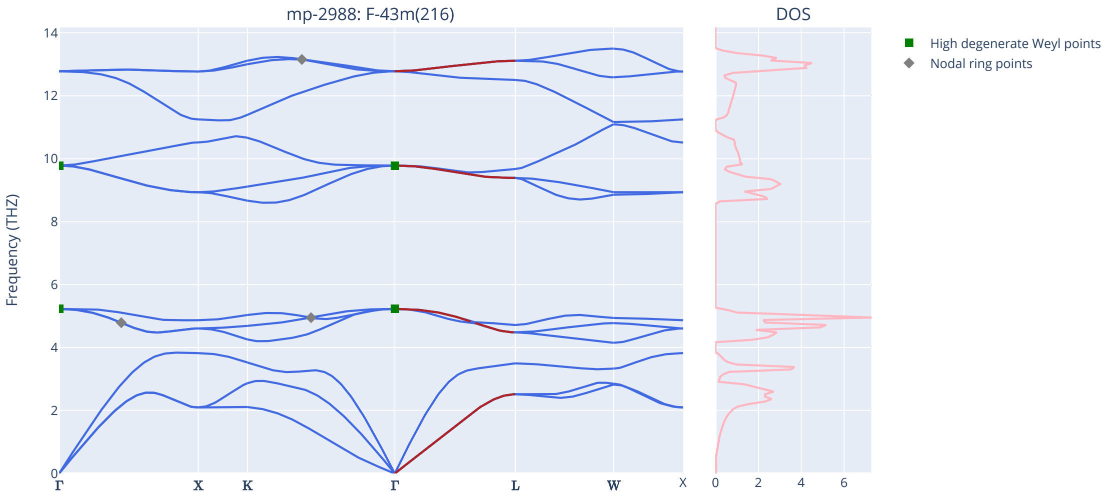

Supplementary Figure 752: The phonon band and DOS of Ga4Li8Rh4.

Supplementary Table 1625: High symmetry lines of mp-2988-Ga4Li8Rh4

	Start	End
0	Γ (0.000, 0.000, 0.000)	X (0.500, 0.000, 0.500)
1	X (0.500, 0.000, 0.500)	U (0.625, 0.250, 0.625)
2	K (0.375, 0.375, 0.750)	Γ (0.000, 0.000, 0.000)
3	Γ (0.000, 0.000, 0.000)	L (0.500, 0.500, 0.500)
4	L (0.500, 0.500, 0.500)	W (0.500, 0.250, 0.750)
5	W (0.500, 0.250, 0.750)	X (0.500, 0.000, 0.500)

Supplementary Table 1626: High degenerate Weyl points along high symmetry lines of mp-2988-Ga4Li8Rh4.

	Coordinates	Frequency	Mode 1	Mode 2	Multi.	Deg.	Path	Ratio
0	(0.000, 0.000, 0.000)	5.226	6	4	1	3	1	0.000
1	(0.000, 0.000, 0.000)	9.782	9	7	1	3	1	0.000

Supplementary Table 1627: Nodal ring points along high symmetry lines of mp-2988-Ga4Li8Rh4.

	Coordinates	Frequency	Mode 1	Mode 2	Multi.	Deg.	Path	Ratio
0	(0.219, -0.000, 0.228)	4.787	5	4	24	2	1	0.447
1	(0.208, 0.208, 0.431)	4.948	6	5	24	2	3	0.432
2	(0.237, 0.237, 0.471)	13.152	12	11	24	2	3	0.370

The followings are details for $Ag_4In_4Li_8$. As shown in Fig **753**, it has 5 topological nodal lines and 5 nodal ring points. At the same time, it has 1 single Weyl point and 2 high degenerate Weyl points whose degeneracy are more than 2 and charges are bigger than 1.

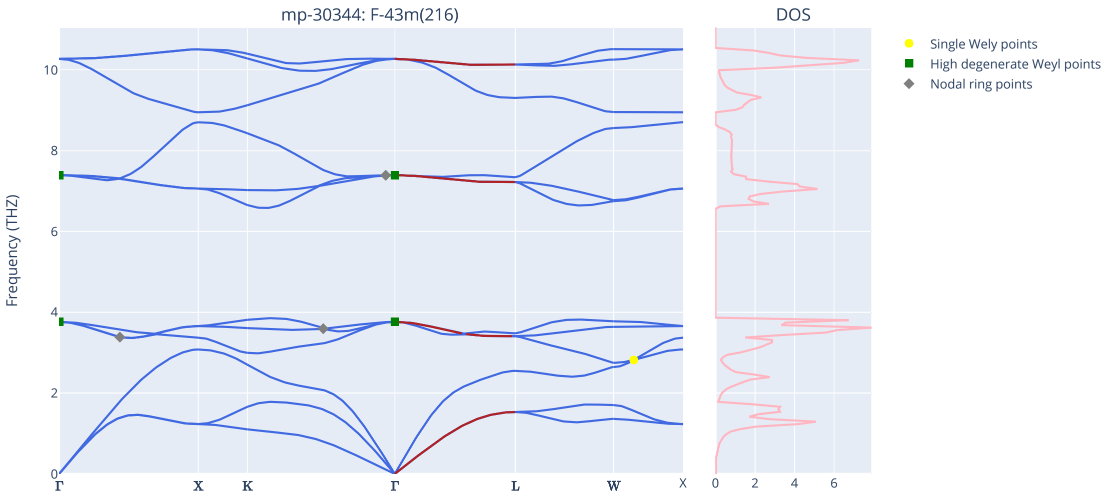

Supplementary Figure 753: The phonon band and DOS of Ag4In4Li8.

Supplementary Table 1628: High symmetry lines of mp-30344-Ag4In4Li8

	Start	End
0	Γ (0.000, 0.000, 0.000)	X (0.500, 0.000, 0.500)
1	X (0.500, 0.000, 0.500)	U (0.625, 0.250, 0.625)
2	K (0.375, 0.375, 0.750)	Γ (0.000, 0.000, 0.000)
3	Γ (0.000, 0.000, 0.000)	L (0.500, 0.500, 0.500)
4	L (0.500, 0.500, 0.500)	W (0.500, 0.250, 0.750)
5	W (0.500, 0.250, 0.750)	X (0.500, 0.000, 0.500)

Supplementary Table 1629: Single Weyl points along high symmetry lines of mp-30344-Ag4In4Li8.

	Coordinates	Frequency	Mode 1	Mode 2	Multi.	Deg.	Path	Ratio
0	(0.500, 0.177, 0.677)	2.819	4	3	12	2	6	0.291

Supplementary Table 1630: High degenerate Weyl points along high symmetry lines of mp-30344-Ag4In4Li8.

	Coordinates	Frequency	Mode 1	Mode 2	Multi.	Deg.	Path	Ratio
0	(0.000, 0.000, 0.000)	3.762	6	4	1	3	1	0.000
1	(0.000, 0.000, 0.000)	7.396	9	7	1	3	1	0.000

Supplementary Table 1631: Nodal ring points along high symmetry lines of mp-30344-Ag4In4Li8.

	Coordinates	Frequency	Mode 1	Mode 2	Multi.	Deg.	Path	Ratio
0	(0.224, -0.000, 0.214)	3.384	5	4	24	2	1	0.437
1	(0.184, 0.184, 0.362)	3.593	6	5	24	2	3	0.515
2	(0.022, 0.022, 0.047)	7.390	8	7	24	2	3	0.939

The followings are details for $Ag_4Li_8Pb_4$. As shown in Fig **754**, it has 5 topological nodal lines, 1 nodal ring point and 2 high degenerate Weyl points whose degeneracy are more than 2 and charges are bigger than 1.

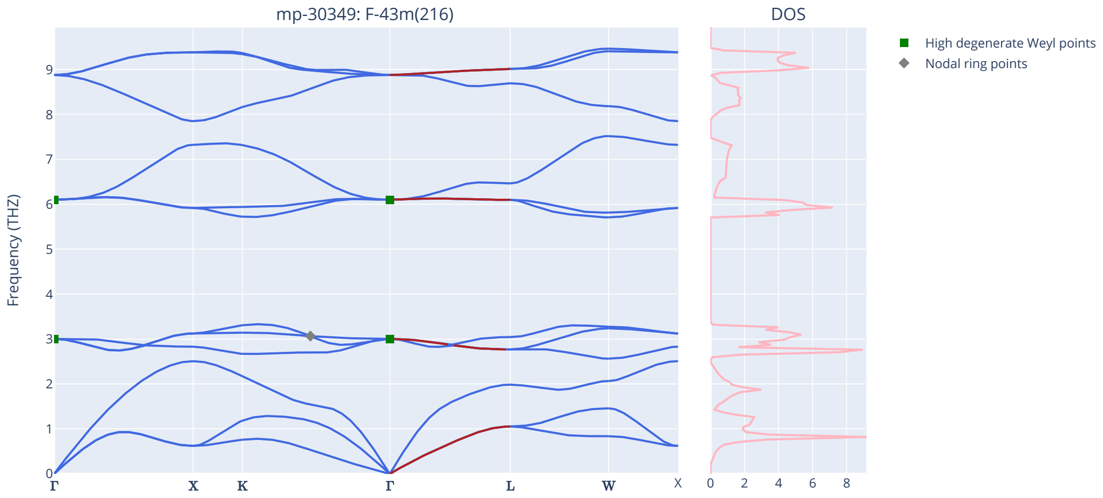

Supplementary Figure 754: The phonon band and DOS of Ag4Li8Pb4.

Supplementary Table 1632: High symmetry lines of mp-30349-Ag4Li8Pb4

	Start	End
0	Γ (0.000, 0.000, 0.000)	X (0.500, 0.000, 0.500)
1	X (0.500, 0.000, 0.500)	U (0.625, 0.250, 0.625)
2	K (0.375, 0.375, 0.750)	Γ (0.000, 0.000, 0.000)
3	Γ (0.000, 0.000, 0.000)	L (0.500, 0.500, 0.500)
4	L (0.500, 0.500, 0.500)	W (0.500, 0.250, 0.750)
5	W (0.500, 0.250, 0.750)	X (0.500, 0.000, 0.500)

Supplementary Table 1633: High degenerate Weyl points along high symmetry lines of mp-30349-Ag4Li8Pb4.

	Coordinates	Frequency	Mode 1	Mode 2	Multi.	Deg.	Path	Ratio
0	(0.000, 0.000, 0.000)	3.002	6	4	1	3	1	0.000
1	(0.000, 0.000, 0.000)	6.100	9	7	1	3	1	0.000

Supplementary Table 1634: Nodal ring points along high symmetry lines of mp-30349-Ag4Li8Pb4.

	Coordinates	Frequency	Mode 1	Mode 2	Multi.	Deg.	Path	Ratio
0	(0.199, 0.199, 0.407)	3.066	6	5	24	2	3	0.462

The followings are details for $Au_4Ga_4Li_8$. As shown in Fig **755**, it has 5 topological nodal lines, 2 nodal ring points and 2 high degenerate Weyl points whose degeneracy are more than 2 and charges are bigger than 1.

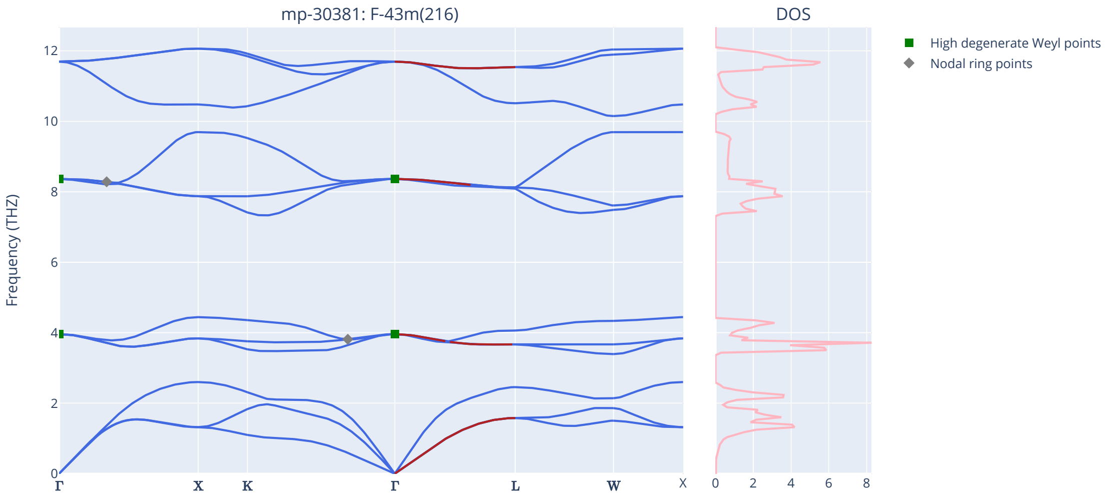

Supplementary Figure 755: The phonon band and DOS of Au4Ga4Li8.

Supplementary Table 1635: High symmetry lines of mp-30381-Au4Ga4Li8

	Start	End
0	Γ (0.000, 0.000, 0.000)	X (0.500, 0.000, 0.500)
1	X (0.500, 0.000, 0.500)	U (0.625, 0.250, 0.625)
2	K (0.375, 0.375, 0.750)	Γ (0.000, 0.000, 0.000)
3	Γ (0.000, 0.000, 0.000)	L (0.500, 0.500, 0.500)
4	L (0.500, 0.500, 0.500)	W (0.500, 0.250, 0.750)
5	W (0.500, 0.250, 0.750)	X (0.500, 0.000, 0.500)

Supplementary Table 1636: High degenerate Weyl points along high symmetry lines of mp-30381-Au4Ga4Li8.

	Coordinates	Frequency	Mode 1	Mode 2	Multi.	Deg.	Path	Ratio
0	(0.000, 0.000, 0.000)	3.960	6	4	1	3	1	0.000
1	(0.000, 0.000, 0.000)	8.363	9	7	1	3	1	0.000

Supplementary Table 1637: Nodal ring points along high symmetry lines of mp-30381-Au4Ga4Li8.

	Coordinates	Frequency	Mode 1	Mode 2	Multi.	Deg.	Path	Ratio
0	(0.118, 0.118, 0.241)	3.815	6	5	24	2	3	0.681
1	(0.166, -0.000, 0.176)	8.283	9	8	24	2	1	0.342

The followings are details for $Au_4In_4Li_8$. As shown in Fig **756**, it has 7 topological nodal lines and 2 nodal ring points. At the same time, it has 1 single Weyl point and 2 high degenerate Weyl points whose degeneracy are more than 2 and charges are bigger than 1.

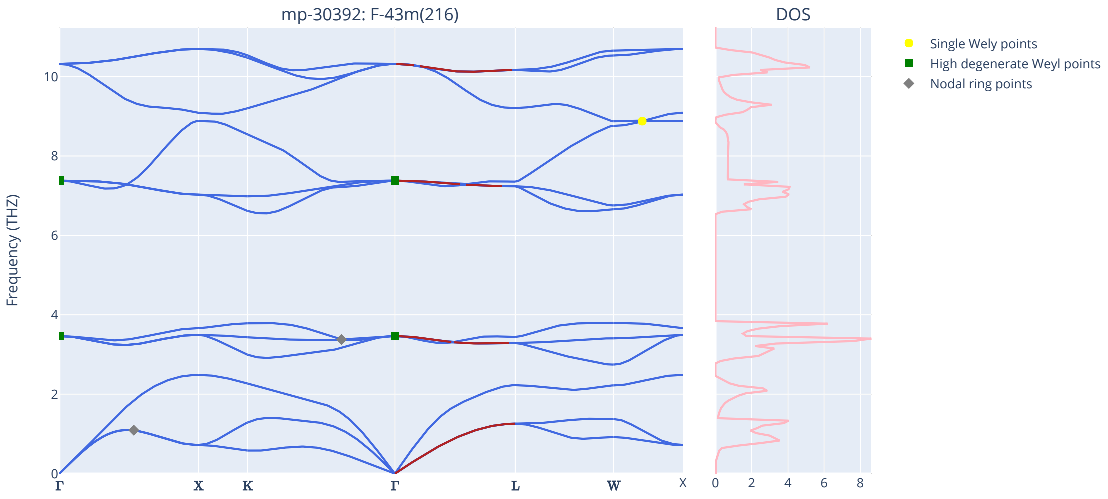

Supplementary Figure 756: The phonon band and DOS of Au4In4Li8.

Supplementary Table 1638: High symmetry lines of mp-30392-Au4In4Li8

	Start	End
0	Γ (0.000, 0.000, 0.000)	X (0.500, 0.000, 0.500)
1	X (0.500, 0.000, 0.500)	U (0.625, 0.250, 0.625)
2	K (0.375, 0.375, 0.750)	Γ (0.000, 0.000, 0.000)
3	Γ (0.000, 0.000, 0.000)	L (0.500, 0.500, 0.500)
4	L (0.500, 0.500, 0.500)	W (0.500, 0.250, 0.750)
5	W (0.500, 0.250, 0.750)	X (0.500, 0.000, 0.500)

Supplementary Table 1639: Single Weyl points along high symmetry lines of mp-30392-Au4In4Li8.

	Coordinates	Frequency	Mode 1	Mode 2	Multi.	Deg.	Path	Ratio
0	(0.500, 0.147, 0.647)	8.877	10	9	12	2	6	0.414

Supplementary Table 1640: High degenerate Weyl points along high symmetry lines of mp-30392-Au4In4Li8.

	Coordinates	Frequency	Mode 1	Mode 2	Multi.	Deg.	Path	Ratio
0	(0.000, 0.000, 0.000)	3.464	6	4	1	3	1	0.000
1	(0.000, 0.000, 0.000)	7.379	9	7	1	3	1	0.000

Supplementary Table 1641: Nodal ring points along high symmetry lines of mp-30392-Au4In4Li8.

	Coordinates	Frequency	Mode 1	Mode 2	Multi.	Deg.	Path	Ratio
0	(0.268, 0.006, 0.268)	1.093	2	1	24	2	1	0.536
1	(0.138, 0.138, 0.270)	3.378	6	5	24	2	3	0.638

The followings are details for $Au_4Li_8Sn_4$. As shown in Fig 757, it has 6 topological nodal lines, 8 nodal ring points and 2 high degenerate Weyl points whose degeneracy are more than 2 and charges are bigger than 1.

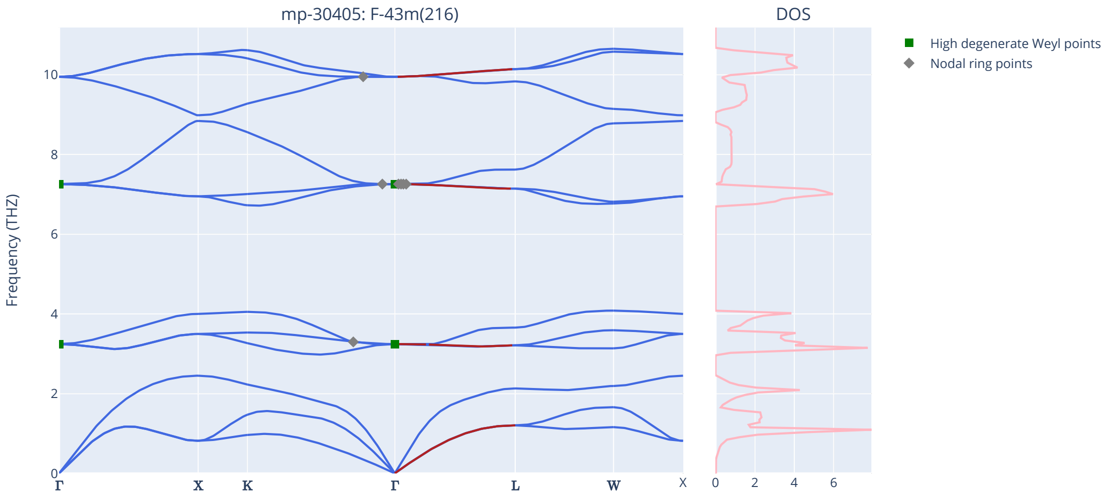

Supplementary Figure 757: The phonon band and DOS of Au4Li8Sn4.

Supplementary Table 1642: High symmetry lines of mp-30405-Au4Li8Sn4

	Start	End
0	Γ (0.000, 0.000, 0.000)	X (0.500, 0.000, 0.500)
1	X (0.500, 0.000, 0.500)	U (0.625, 0.250, 0.625)
2	K (0.375, 0.375, 0.750)	Γ (0.000, 0.000, 0.000)
3	Γ (0.000, 0.000, 0.000)	L (0.500, 0.500, 0.500)
4	L (0.500, 0.500, 0.500)	W (0.500, 0.250, 0.750)
5	W (0.500, 0.250, 0.750)	X (0.500, 0.000, 0.500)

Supplementary Table 1643: High degenerate Weyl points along high symmetry lines of mp-30405-Au4Li8Sn4.

	Coordinates	Frequency	Mode 1	Mode 2	Multi.	Deg.	Path	Ratio
0	(0.000, 0.000, 0.000)	3.245	6	4	1	3	1	0.000
1	(0.000, 0.000, 0.000)	7.257	9	7	1	3	1	0.000

Supplementary Table 1644: Nodal ring points along high symmetry lines of mp-30405-Au4Li8Sn4.

	Coordinates	Frequency	Mode 1	Mode 2	Multi.	Deg.	Path	Ratio
0	(0.107, 0.107, 0.209)	3.299	6	5	24	2	3	0.719
1	(0.026, 0.026, 0.026)	7.257	9	8	8	2	4	0.052
2	(0.015, 0.015, 0.015)	7.257	9	8	8	2	4	0.031
3	(0.033, 0.033, 0.062)	7.257	9	8	24	2	3	0.916

Supplementary Table 1644: Nodal ring points along high symmetry lines of mp-30405-Au4Li8Sn4.

	Coordinates	Frequency	Mode 1	Mode 2	Multi.	Deg.	Path	Ratio
4	(0.036, 0.036, 0.036)	7.256	9	8	8	2	4	0.071
5	(0.047, 0.047, 0.047)	7.255	9	8	8	2	4	0.095
6	(0.082, 0.082, 0.160)	9.949	11	10	24	2	3	0.786

The followings are details for $Cu_4Li_8Sn_4$. As shown in Fig **758**, it has 5 topological nodal lines, 2 nodal ring points and 2 high degenerate Weyl points whose degeneracy are more than 2 and charges are bigger than 1.

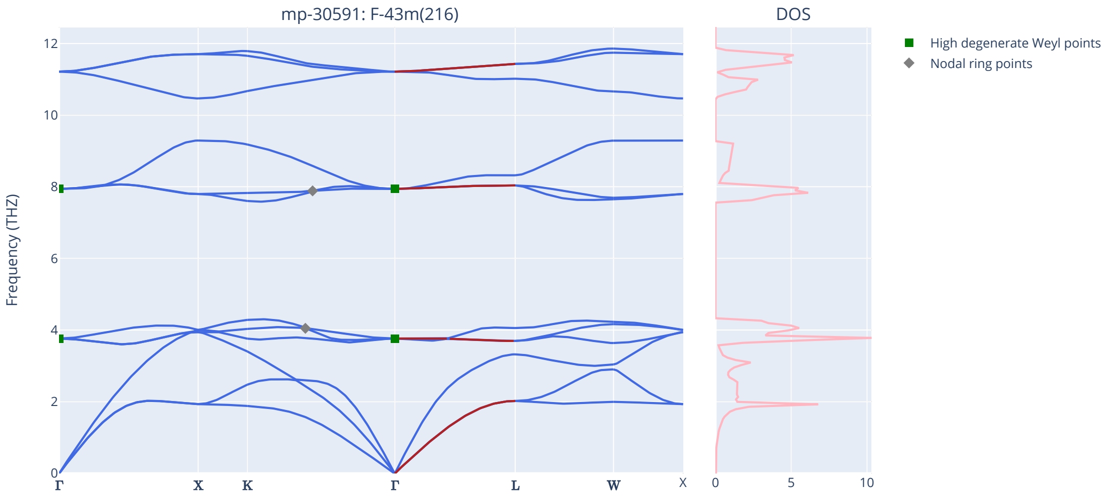

Supplementary Figure 758: The phonon band and DOS of Cu4Li8Sn4.

Supplementary Table 1645: High symmetry lines of mp-30591-Cu4Li8Sn4

	Start	End
0	Γ (0.000, 0.000, 0.000)	X (0.500, 0.000, 0.500)
1	X (0.500, 0.000, 0.500)	U (0.625, 0.250, 0.625)
2	K (0.375, 0.375, 0.750)	Γ (0.000, 0.000, 0.000)
3	Γ (0.000, 0.000, 0.000)	L (0.500, 0.500, 0.500)
4	L (0.500, 0.500, 0.500)	W (0.500, 0.250, 0.750)
5	W (0.500, 0.250, 0.750)	X (0.500, 0.000, 0.500)

Supplementary Table 1646: High degenerate Weyl points along high symmetry lines of mp-30591-Cu4Li8Sn4.

	Coordinates	Frequency	Mode 1	Mode 2	Multi.	Deg.	Path	Ratio
0	(0.000, 0.000, 0.000)	3.762	6	4	1	3	1	0.000
1	(0.000, 0.000, 0.000)	7.940	9	7	1	3	1	0.000

Supplementary Table 1647: Nodal ring points along high symmetry lines of mp-30591-Cu4Li8Sn4.

	Coordinates	Frequency	Mode 1	Mode 2	Multi.	Deg.	Path	Ratio
0	(0.230, 0.230, 0.452)	4.048	6	5	24	2	3	0.394
1	(0.214, 0.214, 0.413)	7.886	8	7	24	2	3	0.443

The followings are details for $Al_4Li_8Pd_4$. As shown in Fig 759, it has 5 topological nodal lines, 2 nodal ring points and 2 high degenerate Weyl points whose degeneracy are more than 2 and charges are bigger than 1.

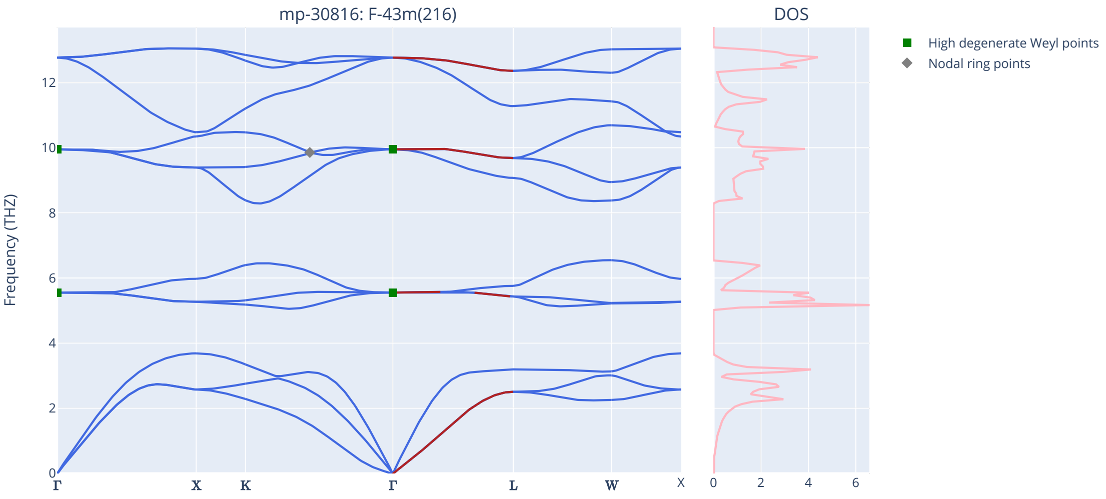

Supplementary Figure 759: The phonon band and DOS of Al4Li8Pd4.

Supplementary Table 1648: High symmetry lines of mp-30816-Al4Li8Pd4

	Start	End
0	Γ (0.000, 0.000, 0.000)	X (0.500, 0.000, 0.500)
1	X (0.500, 0.000, 0.500)	U (0.625, 0.250, 0.625)
2	K (0.375, 0.375, 0.750)	Γ (0.000, 0.000, 0.000)
3	Γ (0.000, 0.000, 0.000)	L (0.500, 0.500, 0.500)
4	L (0.500, 0.500, 0.500)	W (0.500, 0.250, 0.750)
5	W (0.500, 0.250, 0.750)	X (0.500, 0.000, 0.500)

Supplementary Table 1649: High degenerate Weyl points along high symmetry lines of mp-30816-Al4Li8Pd4.

	Coordinates	Frequency	Mode 1	Mode 2	Multi.	Deg.	Path	Ratio
0	(0.000, 0.000, 0.000)	5.552	6	4	1	3	1	0.000
1	(0.000, 0.000, 0.000)	9.951	9	7	1	3	1	0.000

Supplementary Table 1650: Nodal ring points along high symmetry lines of mp-30816-Al4Li8Pd4.

	Coordinates	Frequency	Mode 1	Mode 2	Multi.	Deg.	Path	Ratio
0	(0.214, 0.214, 0.419)	9.856	9	8	24	2	3	0.437

The followings are details for $Al_4Li_8Pt_4$. As shown in Fig **760**, it has 4 topological nodal lines and 3 nodal ring points. At the same time, it has 1 single Weyl point and 2 high degenerate Weyl points whose degeneracy are more than 2 and charges are bigger than 1.

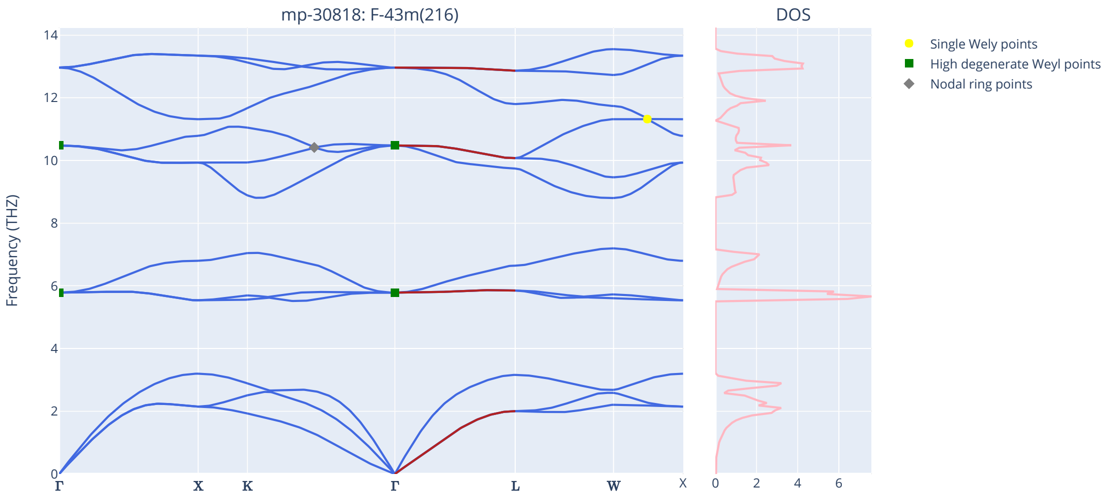

Supplementary Figure 760: The phonon band and DOS of Al4Li8Pt4.

Supplementary Table 1651: High symmetry lines of mp-30818-Al4Li8Pt4

	Start	End
0	Γ (0.000, 0.000, 0.000)	X (0.500, 0.000, 0.500)
1	X (0.500, 0.000, 0.500)	U (0.625, 0.250, 0.625)
2	K (0.375, 0.375, 0.750)	Γ (0.000, 0.000, 0.000)
3	Γ (0.000, 0.000, 0.000)	L (0.500, 0.500, 0.500)
4	L (0.500, 0.500, 0.500)	W (0.500, 0.250, 0.750)
5	W (0.500, 0.250, 0.750)	X (0.500, 0.000, 0.500)

Supplementary Table 1652: Single Weyl points along high symmetry lines of mp-30818-Al4Li8Pt4.

	Coordinates	Frequency	Mode 1	Mode 2	Multi.	Deg.	Path	Ratio
0	(0.500, 0.128, 0.628)	11.321	10	9	12	2	6	0.486

Supplementary Table 1653: High degenerate Weyl points along high symmetry lines of mp-30818-Al4Li8Pt4.

	Coordinates	Frequency	Mode 1	Mode 2	Multi.	Deg.	Path	Ratio
0	(0.000, 0.000, 0.000)	5.782	6	4	1	3	1	0.000
1	(0.000, 0.000, 0.000)	10.479	9	7	1	3	1	0.000

Supplementary Table 1654: Nodal ring points along high symmetry lines of mp-30818-Al4Li8Pt4.

	Coordinates	Frequency	Mode 1	Mode 2	Multi.	Deg.	Path	Ratio
0	(0.207, 0.207, 0.407)	10.415	9	8	24	2	3	0.454

The followings are details for $Ag_4Al_4Li_8$. As shown in Fig **761**, it has 5 topological nodal lines, 2 nodal ring points and 2 high degenerate Weyl points whose degeneracy are more than 2 and charges are bigger than 1.

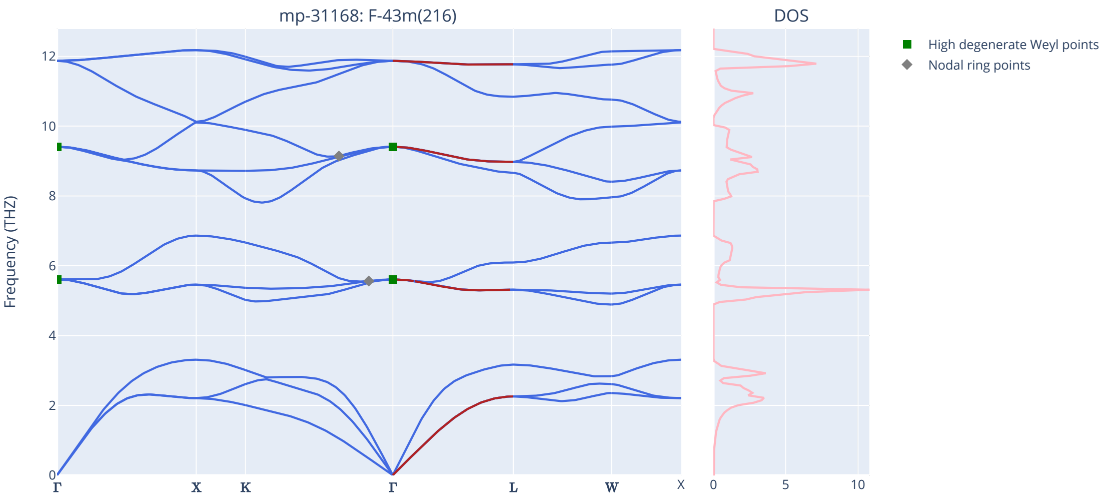

Supplementary Figure 761: The phonon band and DOS of Ag4Al4Li8.

Supplementary Table 1655: High symmetry lines of mp-31168-Ag4Al4Li8

	Start	End
0	Γ (0.000, 0.000, 0.000)	X (0.500, 0.000, 0.500)
1	X (0.500, 0.000, 0.500)	U (0.625, 0.250, 0.625)
2	K (0.375, 0.375, 0.750)	Γ (0.000, 0.000, 0.000)
3	Γ (0.000, 0.000, 0.000)	L (0.500, 0.500, 0.500)
4	L (0.500, 0.500, 0.500)	W (0.500, 0.250, 0.750)
5	W (0.500, 0.250, 0.750)	X (0.500, 0.000, 0.500)

Supplementary Table 1656: High degenerate Weyl points along high symmetry lines of mp-31168-Ag4Al4Li8.

	Coordinates	Frequency	Mode 1	Mode 2	Multi.	Deg.	Path	Ratio
0	(0.000, 0.000, 0.000)	5.610	6	4	1	3	1	0.000
1	(0.000, 0.000, 0.000)	9.408	9	7	1	3	1	0.000

Supplementary Table 1657: Nodal ring points along high symmetry lines of mp-31168-Ag4Al4Li8.

	Coordinates	Frequency	Mode 1	Mode 2	Multi.	Deg.	Path	Ratio
0	(0.060, 0.060, 0.122)	5.562	6	5	24	2	3	0.837
1	(0.138, 0.138, 0.273)	9.140	9	8	24	2	3	0.635

The followings are details for $Ga_4Ir_4Li_8$. As shown in Fig 762, it has 4 topological nodal lines, 1 nodal ring point and 2 high degenerate Weyl points whose degeneracy are more than 2 and charges are bigger than 1.

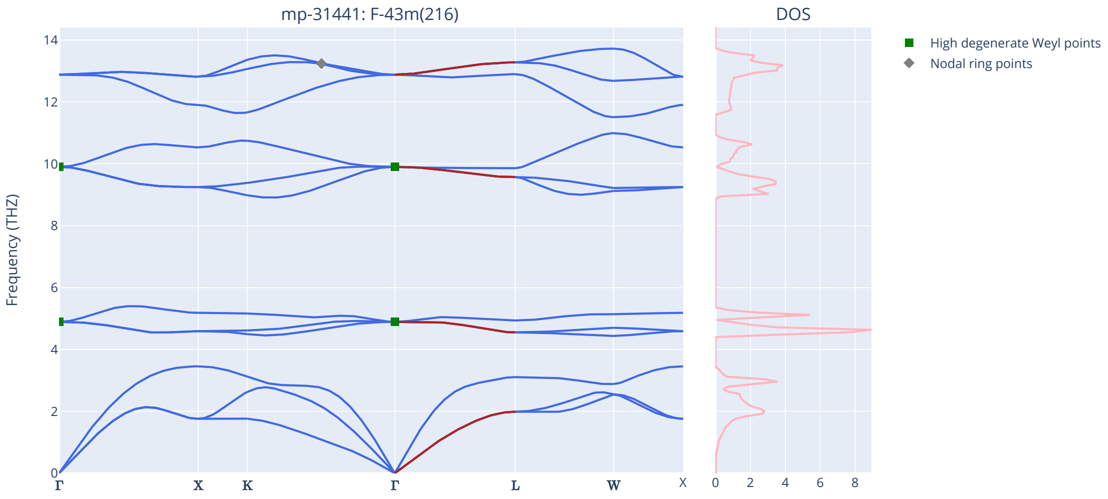

Supplementary Figure 762: The phonon band and DOS of Ga4Ir4Li8.

Supplementary Table 1658: High symmetry lines of mp-31441-Ga4Ir4Li8

	Start	End
0	Γ (0.000, 0.000, 0.000)	X (0.500, 0.000, 0.500)
1	X (0.500, 0.000, 0.500)	U (0.625, 0.250, 0.625)
2	K (0.375, 0.375, 0.750)	Γ (0.000, 0.000, 0.000)
3	Γ (0.000, 0.000, 0.000)	L (0.500, 0.500, 0.500)
4	L (0.500, 0.500, 0.500)	W (0.500, 0.250, 0.750)
5	W (0.500, 0.250, 0.750)	X (0.500, 0.000, 0.500)

Supplementary Table 1659: High degenerate Weyl points along high symmetry lines of mp-31441-Ga4Ir4Li8.

	Coordinates	Frequency	Mode 1	Mode 2	Multi.	Deg.	Path	Ratio
0	(0.000, 0.000, 0.000)	4.892	6	4	1	3	1	0.000
1	(0.000, 0.000, 0.000)	9.902	9	7	1	3	1	0.000

Supplementary Table 1660: Nodal ring points along high symmetry lines of mp-31441-Ga4Ir4Li8.

	Coordinates	Frequency	Mode 1	Mode 2	Multi.	Deg.	Path	Ratio
0	(0.182, 0.182, 0.377)	13.239	12	11	24	2	3	0.503

The followings are details for $In_4Li_8Rh_4$. As shown in Fig 763, it has 5 topological nodal lines and 1 nodal ring point. At the same time, it has 1 single Weyl point and 2 high degenerate Weyl points whose degeneracy are more than 2 and charges are bigger than 1.

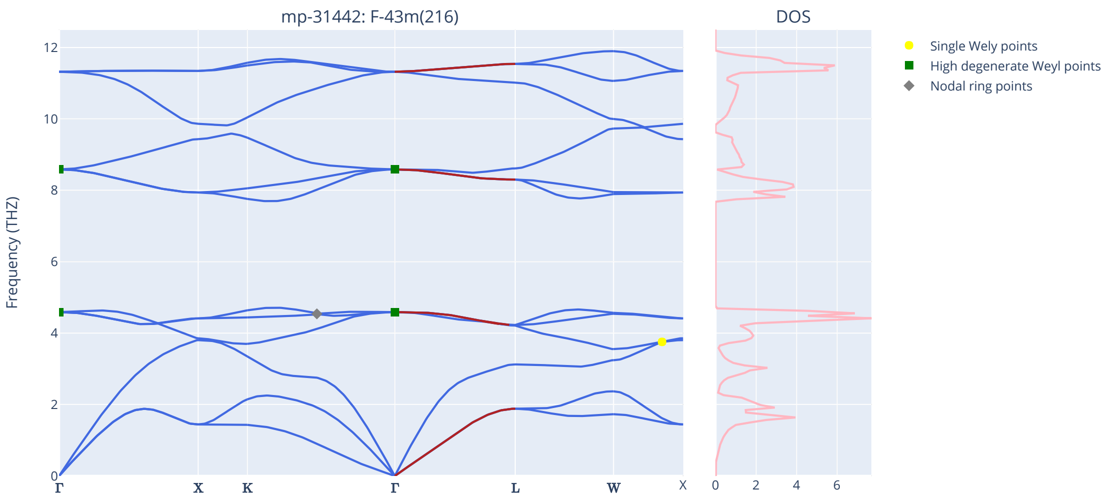

Supplementary Figure 763: The phonon band and DOS of In4Li8Rh4.

Supplementary Table 1661: High symmetry lines of mp-31442-In4Li8Rh4

	Start	End
0	Γ (0.000, 0.000, 0.000)	X (0.500, 0.000, 0.500)
1	X (0.500, 0.000, 0.500)	U (0.625, 0.250, 0.625)
2	K (0.375, 0.375, 0.750)	Γ (0.000, 0.000, 0.000)
3	Γ (0.000, 0.000, 0.000)	L (0.500, 0.500, 0.500)
4	L (0.500, 0.500, 0.500)	W (0.500, 0.250, 0.750)
5	W (0.500, 0.250, 0.750)	X (0.500, 0.000, 0.500)

Supplementary Table 1662: Single Weyl points along high symmetry lines of mp-31442-In4Li8Rh4.

	Coordinates	Frequency	Mode 1	Mode 2	Multi.	Deg.	Path	Ratio
0	(0.500, 0.075, 0.575)	3.753	4	3	12	2	6	0.699

Supplementary Table 1663: High degenerate Weyl points along high symmetry lines of mp-31442-In4Li8Rh4.

	Coordinates	Frequency	Mode 1	Mode 2	Multi.	Deg.	Path	Ratio
0	(0.000, 0.000, 0.000)	4.587	6	4	1	3	1	0.000
1	(0.000, 0.000, 0.000)	8.585	9	7	1	3	1	0.000

Supplementary Table 1664: Nodal ring points along high symmetry lines of mp-31442-In4Li8Rh4.

	Coordinates	Frequency	Mode 1	Mode 2	Multi.	Deg.	Path	Ratio
0	(0.199, 0.199, 0.395)	4.538	6	5	24	2	3	0.472

The followings are details for $In_4Ir_4Li_8$. As shown in Fig **764**, it has 4 topological nodal lines, 2 nodal ring points and 2 high degenerate Weyl points whose degeneracy are more than 2 and charges are bigger than 1.

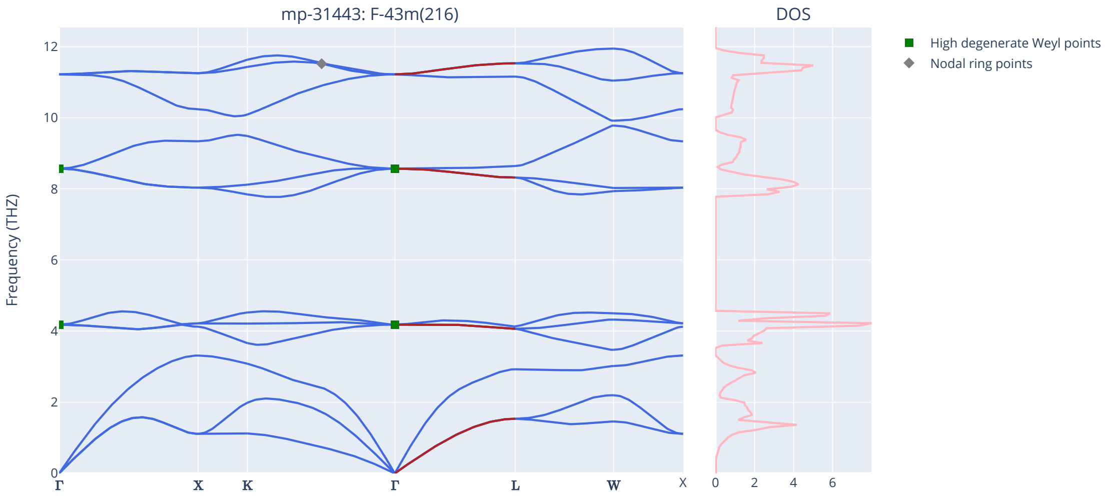

Supplementary Figure 764: The phonon band and DOS of In4Ir4Li8.

Supplementary Table 1665: High symmetry lines of mp-31443-In4Ir4Li8

	Start	End
0	Γ (0.000, 0.000, 0.000)	X (0.500, 0.000, 0.500)
1	X (0.500, 0.000, 0.500)	U (0.625, 0.250, 0.625)
2	K (0.375, 0.375, 0.750)	Γ (0.000, 0.000, 0.000)
3	Γ (0.000, 0.000, 0.000)	L (0.500, 0.500, 0.500)
4	L (0.500, 0.500, 0.500)	W (0.500, 0.250, 0.750)
5	W (0.500, 0.250, 0.750)	X (0.500, 0.000, 0.500)

Supplementary Table 1666: High degenerate Weyl points along high symmetry lines of mp-31443-In4Ir4Li8.

	Coordinates	Frequency	Mode 1	Mode 2	Multi.	Deg.	Path	Ratio
0	(0.000, 0.000, 0.000)	4.181	6	4	1	3	1	0.000
1	(0.000, 0.000, 0.000)	8.569	9	7	1	3	1	0.000

Supplementary Table 1667: Nodal ring points along high symmetry lines of mp-31443-In4Ir4Li8.

	Coordinates	Frequency	Mode 1	Mode 2	Multi.	Deg.	Path	Ratio
0	(0.191, 0.191, 0.366)	11.517	12	11	24	2	3	0.504

The followings are details for $Li_8Pt_4Sb_4$. As shown in Fig **765**, it has 6 topological nodal lines, 2 nodal ring points and 2 high degenerate Weyl points whose degeneracy are more than 2 and charges are bigger than 1.

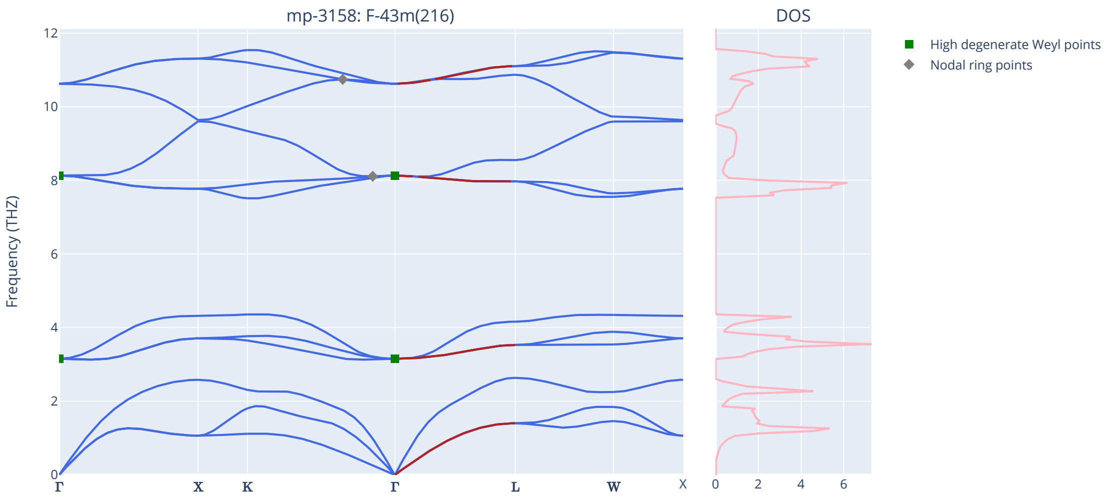

Supplementary Figure 765: The phonon band and DOS of Li8Pt4Sb4.

Supplementary Table 1668: High symmetry lines of mp-3158-Li8Pt4Sb4

	Start	End
0	Γ (0.000, 0.000, 0.000)	X (0.500, 0.000, 0.500)
1	X (0.500, 0.000, 0.500)	U (0.625, 0.250, 0.625)
2	K (0.375, 0.375, 0.750)	Γ (0.000, 0.000, 0.000)
3	Γ (0.000, 0.000, 0.000)	L (0.500, 0.500, 0.500)
4	L (0.500, 0.500, 0.500)	W (0.500, 0.250, 0.750)
5	W (0.500, 0.250, 0.750)	X (0.500, 0.000, 0.500)

Supplementary Table 1669: High degenerate Weyl points along high symmetry lines of mp-3158-Li8Pt4Sb4.

	Coordinates	Frequency	Mode 1	Mode 2	Multi.	Deg.	Path	Ratio
0	(0.000, 0.000, 0.000)	3.152	6	4	1	3	1	0.000
1	(0.000, 0.000, 0.000)	8.129	9	7	1	3	1	0.000

Supplementary Table 1670: Nodal ring points along high symmetry lines of mp-3158-Li8Pt4Sb4.

	Coordinates	Frequency	Mode 1	Mode 2	Multi.	Deg.	Path	Ratio
0	(0.058, 0.058, 0.110)	8.109	9	8	24	2	3	0.851
1	(0.129, 0.129, 0.268)	10.742	11	10	24	2	3	0.647

The followings are details for $Ga_4Li_8Pd_4$. As shown in Fig 766, it has 5 topological nodal lines and 5 nodal ring points. At the same time, it has 1 single Weyl point and 2 high degenerate Weyl points whose degeneracy are more than 2 and charges are bigger than 1.

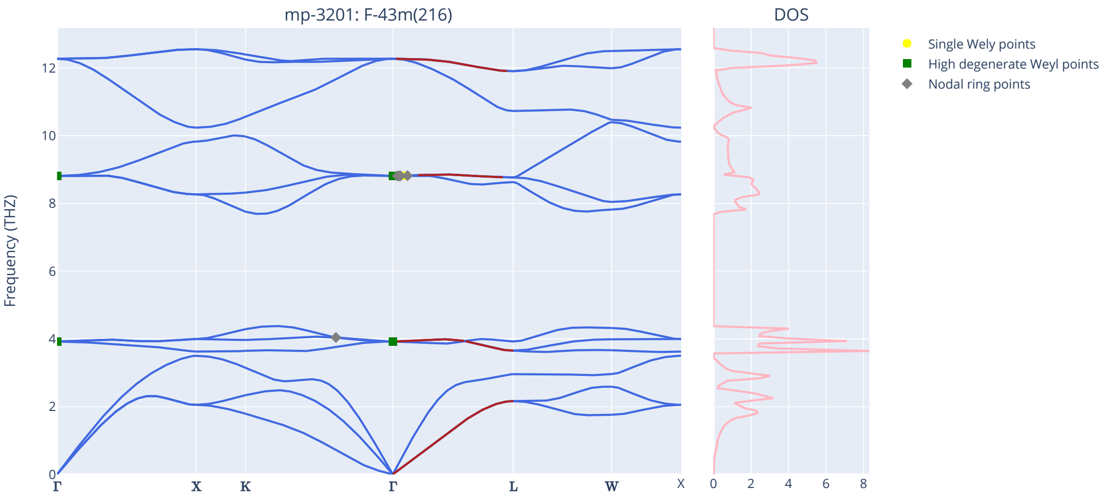

Supplementary Figure 766: The phonon band and DOS of Ga4Li8Pd4.

Supplementary Table 1671: High symmetry lines of mp-3201-Ga4Li8Pd4

	Start	End
0	Γ (0.000, 0.000, 0.000)	X (0.500, 0.000, 0.500)
1	X (0.500, 0.000, 0.500)	U (0.625, 0.250, 0.625)
2	K (0.375, 0.375, 0.750)	Γ (0.000, 0.000, 0.000)
3	Γ (0.000, 0.000, 0.000)	L (0.500, 0.500, 0.500)
4	L (0.500, 0.500, 0.500)	W (0.500, 0.250, 0.750)
5	W (0.500, 0.250, 0.750)	X (0.500, 0.000, 0.500)

Supplementary Table 1672: Single Weyl points along high symmetry lines of mp-3201-Ga4Li8Pd4.

	Coordinates	Frequency	Mode 1	Mode 2	Multi.	Deg.	Path	Ratio
0	(0.051, 0.051, 0.051)	8.817	8	7	8	2	4	0.102

Supplementary Table 1673: High degenerate Weyl points along high symmetry lines of mp-3201-Ga4Li8Pd4.

	Coordinates	Frequency	Mode 1	Mode 2	Multi.	Deg.	Path	Ratio
0	(0.000, 0.000, 0.000)	3.921	6	4	1	3	1	0.000
1	(0.000, 0.000, 0.000)	8.811	9	7	1	3	1	0.000

Supplementary Table 1674: Nodal ring points along high symmetry lines of mp-3201-Ga4Li8Pd4.

	Coordinates	Frequency	Mode 1	Mode 2	Multi.	Deg.	Path	Ratio
0	(0.145, 0.145, 0.289)	4.041	6	5	24	2	3	0.614
1	(0.026, 0.026, 0.026)	8.812	8	7	8	2	4	0.052
2	(0.061, 0.061, 0.061)	8.820	8	7	8	2	4	0.122
3	(0.019, 0.019, 0.019)	8.812	8	7	8	2	4	0.039
4	(0.032, 0.032, 0.032)	8.813	8	7	8	2	4	0.064

The followings are details for $Cu_4Li_8Ge_4$. As shown in Fig 767, it has 6 topological nodal lines, 2 nodal ring points and 2 high degenerate Weyl points whose degeneracy are more than 2 and charges are bigger than 1.

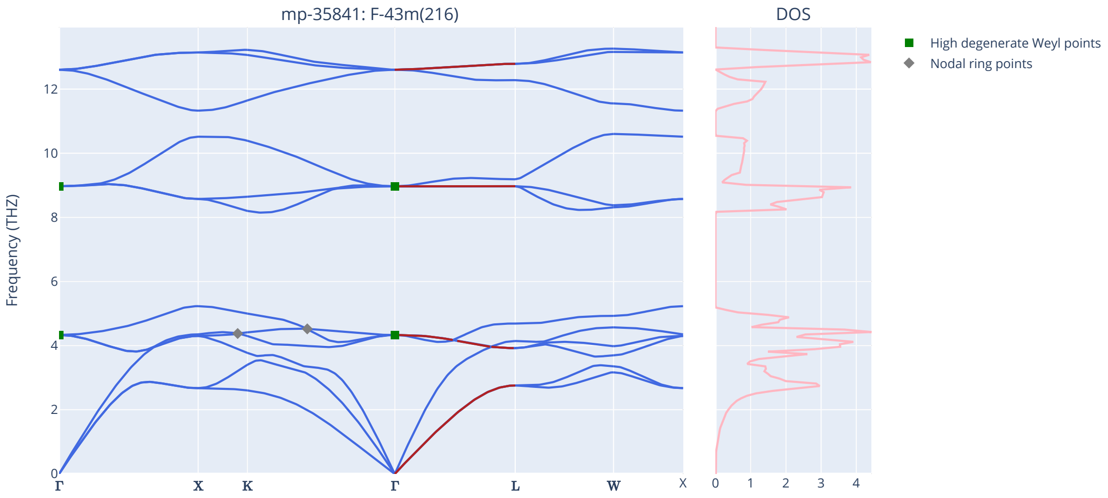

Supplementary Figure 767: The phonon band and DOS of Cu4Li8Ge4.

Supplementary Table 1675: High symmetry lines of mp-35841-Cu4Li8Ge4

	Start	End
0	Γ (0.000, 0.000, 0.000)	X (0.500, 0.000, 0.500)
1	X (0.500, 0.000, 0.500)	U (0.625, 0.250, 0.625)
2	K (0.375, 0.375, 0.750)	Γ (0.000, 0.000, 0.000)
3	Γ (0.000, 0.000, 0.000)	L (0.500, 0.500, 0.500)
4	L (0.500, 0.500, 0.500)	W (0.500, 0.250, 0.750)
5	W (0.500, 0.250, 0.750)	X (0.500, 0.000, 0.500)

Supplementary Table 1676: High degenerate Weyl points along high symmetry lines of mp-35841-Cu4Li8Ge4.

	Coordinates	Frequency	Mode 1	Mode 2	Multi.	Deg.	Path	Ratio
0	(0.000, 0.000, 0.000)	4.329	6	4	1	3	1	0.000
1	(0.000, 0.000, 0.000)	8.967	9	7	1	3	1	0.000

Supplementary Table 1677: Nodal ring points along high symmetry lines of mp-35841-Cu4Li8Ge4.

	Coordinates	Frequency	Mode 1	Mode 2	Multi.	Deg.	Path	Ratio
0	(0.601, 0.201, 0.601)	4.375	5	4	24	2	2	0.804
1	(0.219, 0.219, 0.448)	4.519	6	5	24	2	3	0.407

The followings are details for $Ga_4Li_8Pt_4$. As shown in Fig 768, it has 6 topological nodal lines, 2 nodal ring points and 2 high degenerate Weyl points whose degeneracy are more than 2 and charges are bigger than 1.

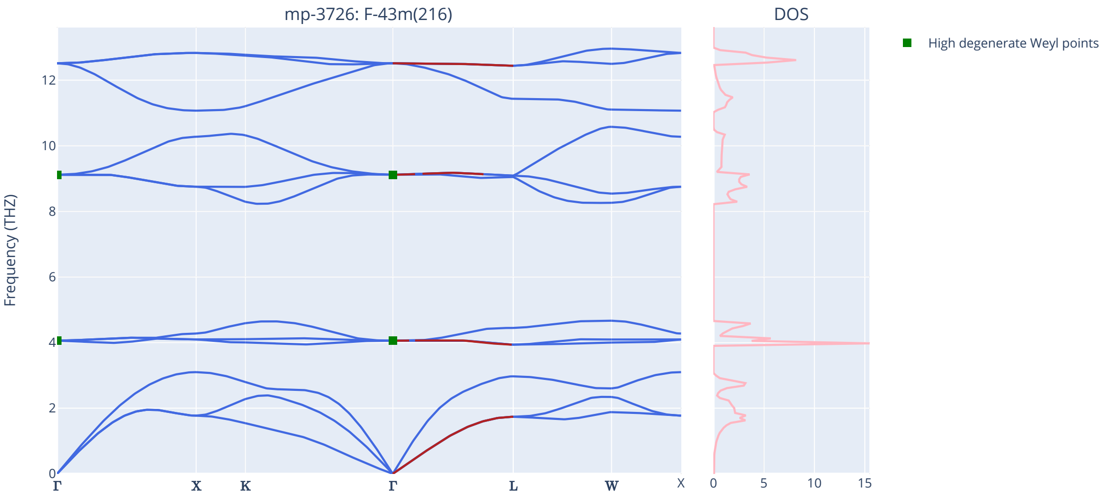

Supplementary Figure 768: The phonon band and DOS of Ga4Li8Pt4.

Supplementary Table 1678: High symmetry lines of mp-3726-Ga4Li8Pt4

	Start	End
0	Γ (0.000, 0.000, 0.000)	X (0.500, 0.000, 0.500)
1	X (0.500, 0.000, 0.500)	U (0.625, 0.250, 0.625)
2	K (0.375, 0.375, 0.750)	Γ (0.000, 0.000, 0.000)
3	Γ (0.000, 0.000, 0.000)	L (0.500, 0.500, 0.500)
4	L (0.500, 0.500, 0.500)	W (0.500, 0.250, 0.750)
5	W (0.500, 0.250, 0.750)	X (0.500, 0.000, 0.500)

Supplementary Table 1679: High degenerate Weyl points along high symmetry lines of mp-3726-Ga4Li8Pt4.

	Coordinates	Frequency	Mode 1	Mode 2	Multi.	Deg.	Path	Ratio
0	(0.000, 0.000, 0.000)	4.056	6	4	1	3	1	0.000
1	(0.000, 0.000, 0.000)	9.115	9	7	1	3	1	0.000

The followings are details for PrSn$_3$. It has 7 topological nodal lines and 25 nodal ring points along high symmetry line as shown in Fig 769.

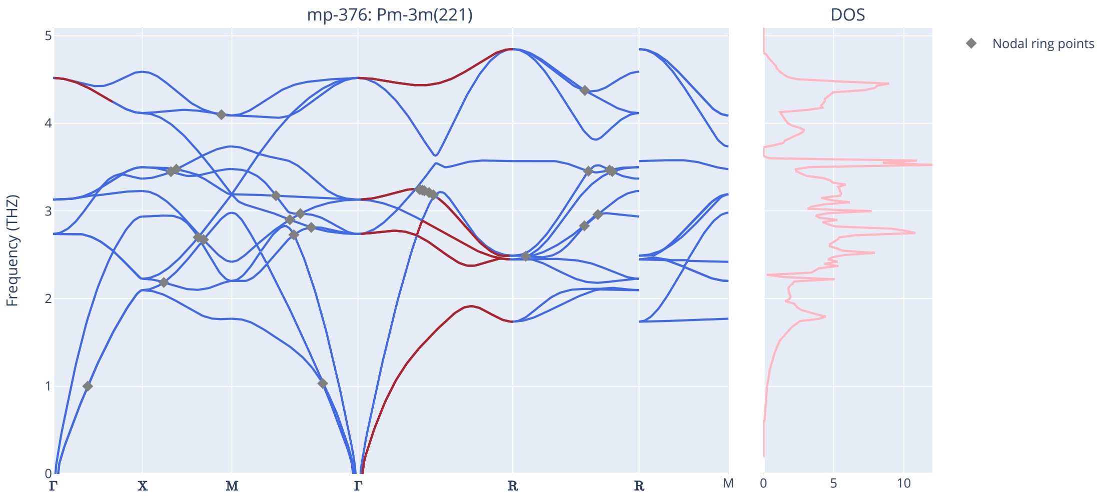

Supplementary Figure 769: The phonon band and DOS of PrSn3.

Supplementary Table 1680: High symmetry lines of mp-376-PrSn3

	Start	End
0	Γ (0.000, 0.000, 0.000)	X (0.000, 0.500, 0.000)
1	X (0.000, 0.500, 0.000)	M (0.500, 0.500, 0.000)
2	M (0.500, 0.500, 0.000)	Γ (0.000, 0.000, 0.000)
3	Γ (0.000, 0.000, 0.000)	R (0.500, 0.500, 0.500)
4	R (0.500, 0.500, 0.500)	X (0.000, 0.500, 0.000)
5	R (0.500, 0.500, 0.500)	M (0.500, 0.500, 0.000)

Supplementary Table 1681: Nodal ring points along high symmetry lines of mp-376-PrSn3.

	Coordinates	Frequency	Mode 1	Mode 2	Multi.	Deg.	Path	Ratio
0	(0.000, 0.194, 0.000)	0.999	2	1	6	2	1	0.388
1	(0.140, 0.140, -0.000)	1.031	2	1	12	2	3	0.720
2	(0.120, 0.498, -0.000)	2.183	3	2	24	2	2	0.239
3	(0.254, 0.254, 0.000)	2.726	4	3	12	2	3	0.492
4	(0.312, 0.500, 0.000)	2.698	5	4	12	2	2	0.625
5	(0.342, 0.500, 0.000)	2.672	5	4	12	2	2	0.684
6	(0.185, 0.185, 0.000)	2.811	5	4	12	2	3	0.630
7	(0.270, 0.270, -0.000)	2.900	6	5	12	2	3	0.460
8	(0.216, 0.495, 0.216)	2.829	6	5	24	2	5	0.567
9	(0.163, 0.500, 0.163)	2.958	6	5	12	2	5	0.674
10	(0.228, 0.228, 0.000)	2.969	7	6	12	2	3	0.543
11	(0.449, 0.500, 0.449)	2.482	7	6	12	2	5	0.102
12	(0.160, 0.500, 0.000)	3.447	8	7	12	2	2	0.320
13	(0.325, 0.325, -0.000)	3.173	8	7	12	2	3	0.350

Supplementary Table 1681: Nodal ring points along high symmetry lines of mp-376-PrSn3.

	Coordinates	Frequency	Mode 1	Mode 2	Multi.	Deg.	Path	Ratio
14	(0.199, 0.199, 0.199)	3.242	8	7	8	2	4	0.399
15	(0.229, 0.229, 0.229)	3.209	8	7	8	2	4	0.459
16	(0.243, 0.243, 0.243)	3.183	8	7	8	2	4	0.487
17	(0.214, 0.214, 0.214)	3.230	8	7	8	2	4	0.429
18	(0.208, 0.208, 0.208)	3.236	8	7	8	2	4	0.415
19	(0.106, 0.500, 0.106)	3.449	8	7	12	2	5	0.788
20	(0.190, 0.500, -0.000)	3.475	9	8	12	2	2	0.380
21	(0.200, 0.500, 0.200)	3.453	9	8	12	2	5	0.599
22	(0.117, 0.500, 0.117)	3.466	9	8	12	2	5	0.767
23	(0.442, 0.500, -0.000)	4.098	12	11	12	2	2	0.885
24	(0.215, 0.500, 0.215)	4.374	12	11	12	2	5	0.571

The followings are details for Pd3. It has 8 topological nodal lines and 26 nodal ring points along high symmetry line as shown in Fig 770.

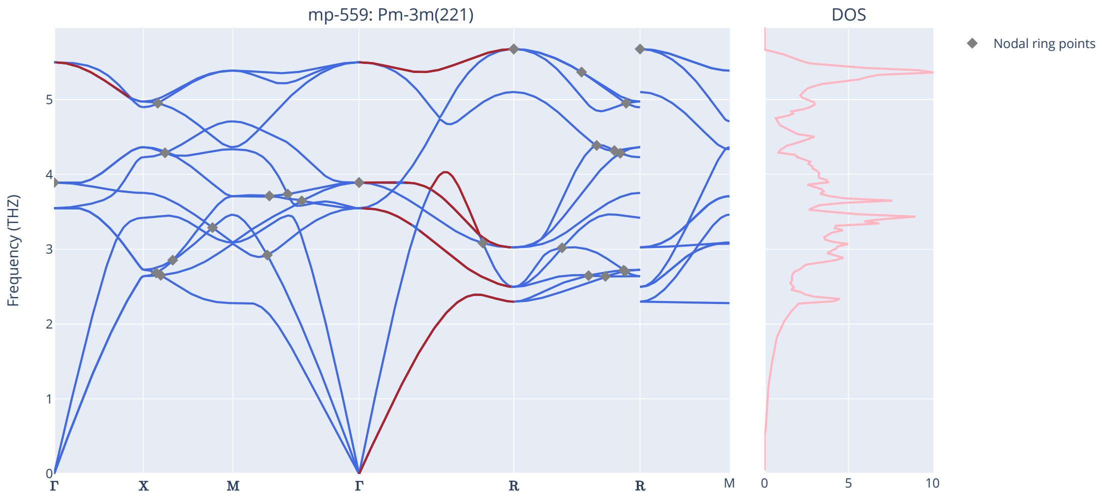

Supplementary Figure 770: The phonon band and DOS of Pd3Y.

Supplementary Table 1682: High symmetry lines of mp-559-Pd3Y

	Start	End
0	Γ (0.000, 0.000, 0.000)	X (0.000, 0.500, 0.000)
1	X (0.000, 0.500, 0.000)	M (0.500, 0.500, 0.000)
2	M (0.500, 0.500, 0.000)	Γ (0.000, 0.000, 0.000)
3	Γ (0.000, 0.000, 0.000)	R (0.500, 0.500, 0.500)
4	R (0.500, 0.500, 0.500)	X (0.000, 0.500, 0.000)
5	R (0.500, 0.500, 0.500)	M (0.500, 0.500, 0.000)

Supplementary Table 1683: Nodal ring points along high symmetry lines of mp-559-Pd3Y.

	Coordinates	Frequency	Mode 1	Mode 2	Multi.	Deg.	Path	Ratio
0	(0.097, 0.500, 0.000)	2.652	2	1	12	2	2	0.195
1	(0.137, 0.500, 0.137)	2.637	2	1	12	2	5	0.726
2	(0.072, 0.500, -0.000)	2.683	3	2	12	2	2	0.143
3	(0.363, 0.363, 0.000)	2.922	3	2	12	2	3	0.274
4	(0.058, 0.500, 0.061)	2.705	3	2	24	2	5	0.881
5	(0.204, 0.500, 0.204)	2.646	3	2	12	2	5	0.592
6	(0.164, 0.500, -0.000)	2.853	4	3	12	2	2	0.328
7	(0.065, 0.500, 0.065)	2.718	4	3	12	2	5	0.870
8	(0.387, 0.500, 0.000)	3.289	5	4	12	2	2	0.774
9	(0.309, 0.500, 0.309)	3.018	5	4	12	2	5	0.382
10	(0.400, 0.400, 0.400)	3.082	6	5	8	2	4	0.800
11	(0.355, 0.355, 0.000)	3.711	7	6	12	2	3	0.290
12	(0.227, 0.227, 0.000)	3.645	7	6	12	2	3	0.547
13	(0.000, 0.000, 0.000)	3.891	8	7	1	3	1	0.000

Supplementary Table 1683: Nodal ring points along high symmetry lines of mp-559-Pd3Y.

	Coordinates	Frequency	Mode 1	Mode 2	Multi.	Deg.	Path	Ratio
14	(0.121, 0.500, -0.000)	4.288	8	7	12	2	2	0.242
15	(0.282, 0.282, -0.000)	3.736	8	7	12	2	3	0.435
16	(0.079, 0.500, 0.079)	4.282	8	7	12	2	5	0.843
17	(0.102, 0.500, 0.102)	4.318	9	8	12	2	5	0.796
18	(0.172, 0.500, 0.172)	4.386	9	8	12	2	5	0.656
19	(0.080, 0.500, -0.000)	4.951	11	10	12	2	2	0.160
20	(0.055, 0.500, 0.055)	4.950	11	10	12	2	5	0.890
21	(0.500, 0.500, 0.500)	5.674	12	11	1	3	4	1.000
22	(0.232, 0.500, 0.232)	5.367	12	11	12	2	5	0.537

The followings are details for $Ag_4Li_8Sn_4$. As shown in Fig **771**, it has 5 topological nodal lines, 2 nodal ring points and 2 high degenerate Weyl points whose degeneracy are more than 2 and charges are bigger than 1.

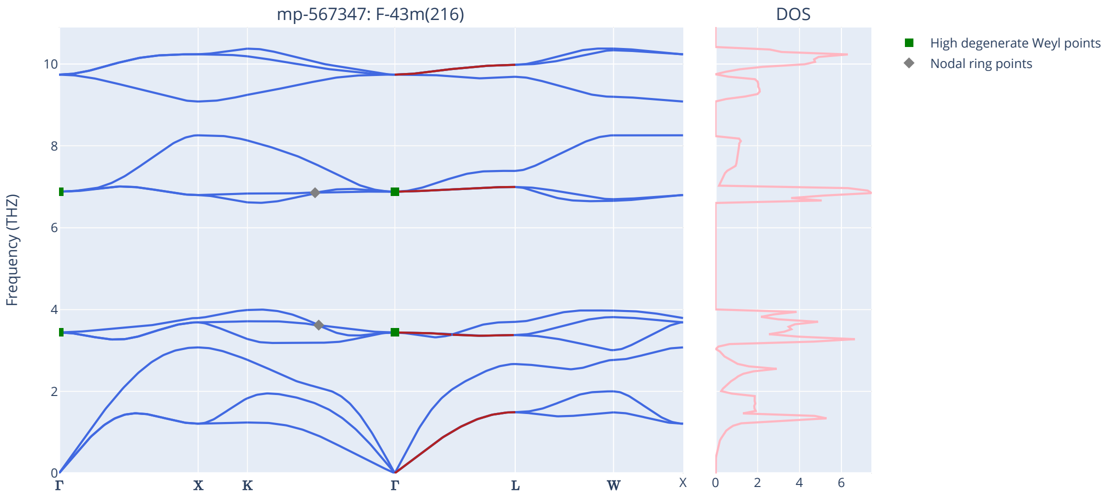

Supplementary Figure 771: The phonon band and DOS of Ag4Li8Sn4.

Supplementary Table 1684: High symmetry lines of mp-567347-Ag4Li8Sn4

	Start	End
0	Γ (0.000, 0.000, 0.000)	X (0.500, 0.000, 0.500)
1	X (0.500, 0.000, 0.500)	U (0.625, 0.250, 0.625)
2	K (0.375, 0.375, 0.750)	Γ (0.000, 0.000, 0.000)
3	Γ (0.000, 0.000, 0.000)	L (0.500, 0.500, 0.500)
4	L (0.500, 0.500, 0.500)	W (0.500, 0.250, 0.750)
5	W (0.500, 0.250, 0.750)	X (0.500, 0.000, 0.500)

Supplementary Table 1685: High degenerate Weyl points along high symmetry lines of mp-567347-Ag4Li8Sn4.

	Coordinates	Frequency	Mode 1	Mode 2	Multi.	Deg.	Path	Ratio
0	(0.000, 0.000, 0.000)	3.441	6	4	1	3	1	0.000
1	(0.000, 0.000, 0.000)	6.879	9	7	1	3	1	0.000

Supplementary Table 1686: Nodal ring points along high symmetry lines of mp-567347-Ag4Li8Sn4.

	Coordinates	Frequency	Mode 1	Mode 2	Multi.	Deg.	Path	Ratio
0	(0.190, 0.190, 0.390)	3.620	6	5	24	2	3	0.484
1	(0.207, 0.207, 0.401)	6.855	8	7	24	2	3	0.459

The followings are details for Cr_2As_2. It has 22 topological nodal lines and 32 nodal ring points along high symmetry line as shown in Fig 772.

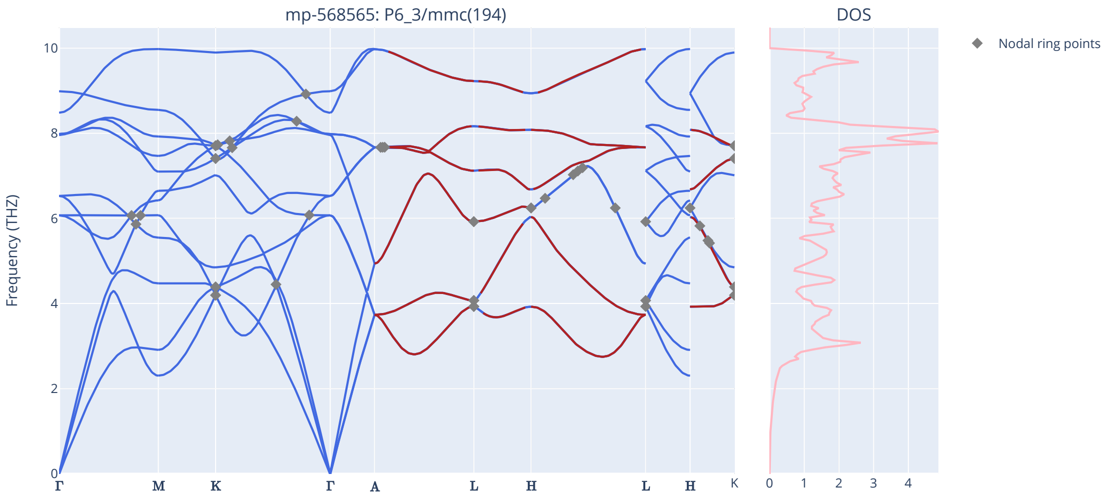

Supplementary Figure 772: The phonon band and DOS of Cr2As2.

Supplementary Table 1687: High symmetry lines of mp-568565-Cr2As2

	Start	End
0	Γ (0.000, 0.000, 0.000)	M (0.500, 0.000, 0.000)
1	M (0.500, 0.000, 0.000)	K (0.333, 0.333, 0.000)
2	K (0.333, 0.333, 0.000)	Γ (0.000, 0.000, 0.000)
3	Γ (0.000, 0.000, 0.000)	A (0.000, 0.000, 0.500)
4	A (0.000, 0.000, 0.500)	L (0.500, 0.000, 0.500)
5	L (0.500, 0.000, 0.500)	H (0.333, 0.333, 0.500)
6	H (0.333, 0.333, 0.500)	A (0.000, 0.000, 0.500)
7	L (0.500, 0.000, 0.500)	M (0.500, 0.000, 0.000)
8	H (0.333, 0.333, 0.500)	K (0.333, 0.333, 0.000)

Supplementary Table 1688: Nodal ring points along high symmetry lines of mp-568565-Cr2As2.

	Coordinates	Frequency	Mode 1	Mode 2	Multi.	Deg.	Path	Ratio
0	(0.333, 0.333, 0.000)	4.195	2	1	2	2	2	1.000
1	(0.500, 0.000, 0.500)	3.930	2	1	3	2	5	1.000
2	(0.158, 0.158, 0.000)	4.448	3	2	6	2	3	0.527
3	(0.333, 0.333, 0.000)	4.388	4	3	2	2	2	1.000
4	(0.500, 0.000, 0.500)	4.070	4	3	3	2	5	1.000
5	(0.387, -0.000, 0.000)	5.863	5	4	6	2	1	0.775
6	(0.332, 0.332, 0.390)	5.823	5	4	12	2	9	0.221
7	(0.329, 0.329, 0.280)	5.419	5	4	12	2	9	0.439
8	(0.331, 0.338, 0.299)	5.479	5	4	12	2	9	0.403
9	(0.410, -0.002, 0.000)	6.068	6	5	12	2	1	0.821
10	(0.060, 0.062, 0.000)	6.077	6	5	12	2	3	0.817

Supplementary Table 1688: Nodal ring points along high symmetry lines of mp-568565-Cr2As2.

	Coordinates	Frequency	Mode 1	Mode 2	Multi.	Deg.	Path	Ratio
11	(0.500, 0.000, 0.500)	5.923	6	5	3	2	5	1.000
12	(0.333, 0.333, 0.500)	6.244	6	5	2	2	6	1.000
13	(0.293, 0.293, 0.500)	6.471	6	5	6	2	7	0.122
14	(0.211, 0.211, 0.500)	7.025	6	5	6	2	7	0.367
15	(0.197, 0.197, 0.500)	7.112	6	5	6	2	7	0.408
16	(0.184, 0.184, 0.500)	7.189	6	5	6	2	7	0.449
17	(0.088, 0.088, 0.500)	6.244	6	5	6	2	7	0.735
18	(0.365, -0.000, 0.000)	6.067	7	6	6	2	1	0.730
19	(0.333, 0.333, 0.000)	7.407	8	7	2	2	2	1.000
20	(0.285, 0.285, -0.000)	7.659	9	8	6	2	3	0.144
21	(0.031, -0.000, 0.499)	7.668	9	8	12	2	5	0.061
22	(0.041, 0.000, 0.499)	7.669	9	8	12	2	5	0.081
23	(0.051, 0.000, 0.498)	7.671	9	8	12	2	5	0.102
24	(0.333, 0.333, 0.000)	7.705	10	9	2	2	2	1.000
25	(0.098, 0.098, -0.000)	8.282	10	9	6	2	3	0.707
26	(0.292, 0.292, 0.000)	7.820	11	10	6	2	3	0.123
27	(0.327, 0.327, 0.000)	7.728	11	10	6	2	3	0.018
28	(0.071, 0.071, 0.000)	8.920	12	11	6	2	3	0.788

The followings are details for Al3. It has 7 topological nodal lines and 14 nodal ring points along high symmetry line as shown in Fig 773.

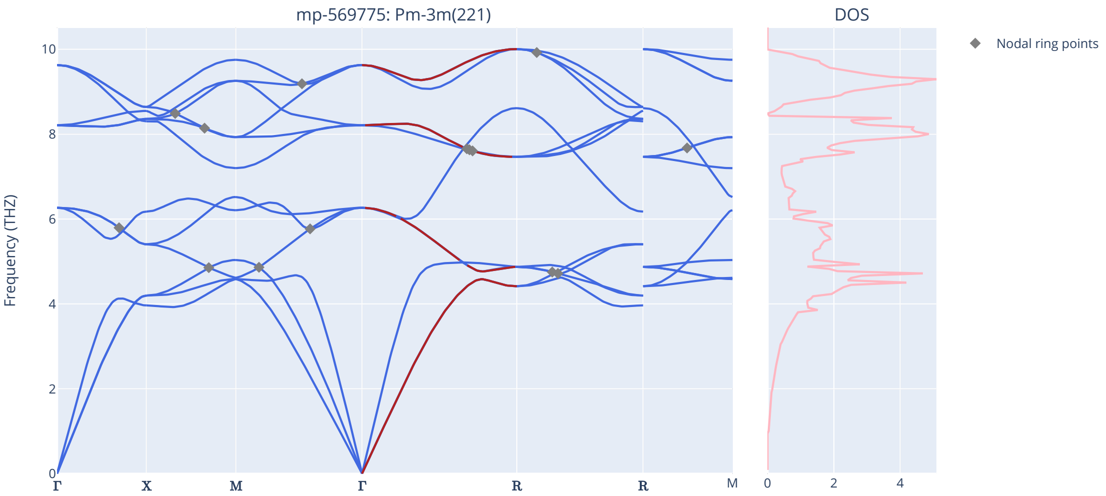

Supplementary Figure 773: The phonon band and DOS of Al3Zr.

Supplementary Table 1689: High symmetry lines of mp-569775-Al3Zr

	Start	End
0	Γ (0.000, 0.000, 0.000)	X (0.000, 0.500, 0.000)
1	X (0.000, 0.500, 0.000)	M (0.500, 0.500, 0.000)
2	M (0.500, 0.500, 0.000)	Γ (0.000, 0.000, 0.000)
3	Γ (0.000, 0.000, 0.000)	R (0.500, 0.500, 0.500)
4	R (0.500, 0.500, 0.500)	X (0.000, 0.500, 0.000)
5	R (0.500, 0.500, 0.500)	M (0.500, 0.500, 0.000)

Supplementary Table 1690: Nodal ring points along high symmetry lines of mp-569775-Al3Zr.

	Coordinates	Frequency	Mode 1	Mode 2	Multi.	Deg.	Path	Ratio
0	(0.337, 0.499, 0.337)	4.719	3	2	24	2	5	0.326
1	(0.349, 0.500, -0.000)	4.855	4	3	12	2	2	0.698
2	(0.408, 0.408, 0.000)	4.863	4	3	12	2	3	0.184
3	(0.360, 0.500, 0.360)	4.752	4	3	12	2	5	0.281
4	(0.206, 0.206, 0.000)	5.768	5	4	12	2	3	0.589
5	(0.000, 0.347, 0.000)	5.794	6	5	6	2	1	0.694
6	(0.339, 0.339, 0.339)	7.655	8	7	8	2	4	0.677
7	(0.498, 0.499, 0.254)	7.674	8	7	24	2	6	0.492
8	(0.347, 0.347, 0.347)	7.631	8	7	8	2	4	0.694
9	(0.357, 0.357, 0.357)	7.605	8	7	8	2	4	0.714
10	(0.325, 0.500, -0.000)	8.141	9	8	12	2	2	0.651
11	(0.161, 0.500, -0.000)	8.487	10	9	12	2	2	0.322
12	(0.421, 0.500, 0.421)	9.923	11	10	12	2	5	0.158
13	(0.238, 0.238, -0.000)	9.187	12	11	12	2	3	0.524

The followings are details for $InSc_3$. It has 7 topological nodal lines and 29 nodal ring points along high symmetry line as shown in Fig 774.

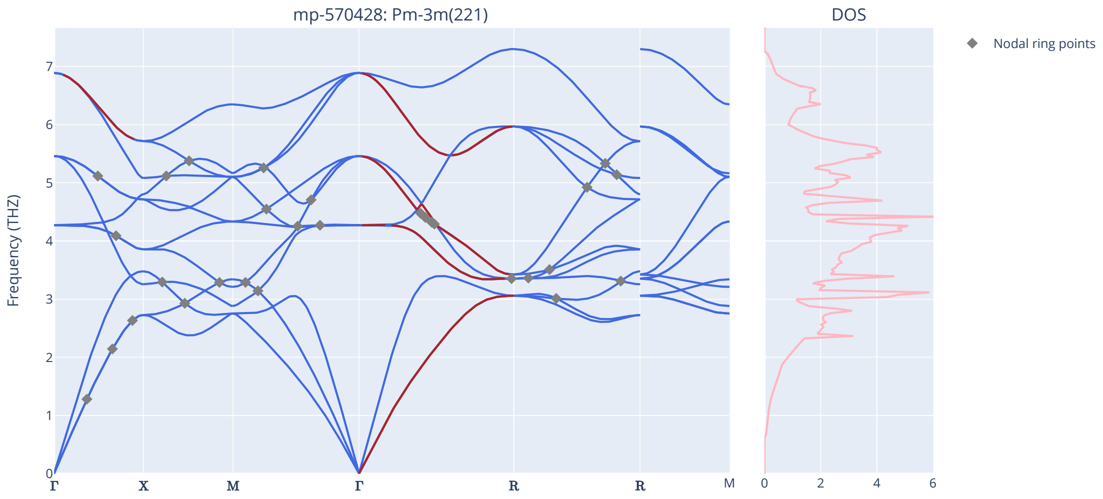

Supplementary Figure 774: The phonon band and DOS of InSc3.

Supplementary Table 1691: High symmetry lines of mp-570428-InSc3

	Start	End
0	Γ (0.000, 0.000, 0.000)	X (0.000, 0.500, 0.000)
1	X (0.000, 0.500, 0.000)	M (0.500, 0.500, 0.000)
2	M (0.500, 0.500, 0.000)	Γ (0.000, 0.000, 0.000)
3	Γ (0.000, 0.000, 0.000)	R (0.500, 0.500, 0.500)
4	R (0.500, 0.500, 0.500)	X (0.000, 0.500, 0.000)
5	R (0.500, 0.500, 0.500)	M (0.500, 0.500, 0.000)

Supplementary Table 1692: Nodal ring points along high symmetry lines of mp-570428-InSc3.

	Coordinates	Frequency	Mode 1	Mode 2	Multi.	Deg.	Path	Ratio
0	(0.000, 0.184, 0.000)	1.282	2	1	6	2	1	0.367
1	(0.000, 0.327, 0.000)	2.145	2	1	6	2	1	0.653
2	(0.000, 0.439, 0.000)	2.633	2	1	6	2	1	0.878
3	(0.232, 0.500, 0.000)	2.929	3	2	12	2	2	0.463
4	(0.332, 0.500, 0.332)	3.013	3	2	12	2	5	0.336
5	(0.106, 0.500, -0.000)	3.294	4	3	12	2	2	0.211
6	(0.400, 0.400, 0.000)	3.143	4	3	12	2	3	0.200
7	(0.076, 0.500, 0.076)	3.310	4	3	12	2	5	0.847
8	(0.426, 0.500, 0.000)	3.287	5	4	12	2	2	0.851
9	(0.450, 0.450, -0.000)	3.289	5	4	12	2	3	0.101
10	(0.000, 0.347, 0.000)	4.090	6	5	6	2	1	0.694
11	(0.155, 0.155, 0.000)	4.270	6	5	12	2	3	0.691
12	(0.493, 0.493, 0.493)	3.353	6	5	8	2	4	0.985
13	(0.442, 0.500, 0.442)	3.363	6	5	12	2	5	0.115

Supplementary Table 1692: Nodal ring points along high symmetry lines of mp-570428-InSc3.

	Coordinates	Frequency	Mode 1	Mode 2	Multi.	Deg.	Path	Ratio
14	(0.243, 0.243, -0.000)	4.254	7	6	12	2	3	0.514
15	(0.214, 0.214, 0.214)	4.404	7	6	8	2	4	0.429
16	(0.199, 0.199, 0.199)	4.475	7	6	8	2	4	0.398
17	(0.243, 0.243, 0.243)	4.291	7	6	8	2	4	0.487
18	(0.235, 0.235, 0.235)	4.323	7	6	8	2	4	0.469
19	(0.358, 0.500, 0.358)	3.508	7	6	12	2	5	0.283
20	(0.190, 0.190, 0.000)	4.706	8	7	12	2	3	0.620
21	(0.367, 0.367, -0.000)	4.548	8	7	12	2	3	0.267
22	(0.000, 0.245, 0.000)	5.117	9	8	6	2	1	0.490
23	(0.210, 0.500, 0.210)	4.923	9	8	12	2	5	0.580
24	(0.093, 0.500, 0.093)	5.139	10	9	12	2	5	0.815
25	(0.128, 0.500, -0.000)	5.118	10	9	12	2	2	0.256
26	(0.378, 0.378, -0.000)	5.256	10	9	12	2	3	0.244
27	(0.256, 0.500, 0.000)	5.377	11	10	12	2	2	0.512
28	(0.138, 0.500, 0.138)	5.333	11	10	12	2	5	0.724

The followings are details for Ni_2As_2. It has 22 topological nodal lines and 24 nodal ring points along high symmetry line as shown in Fig **775**.

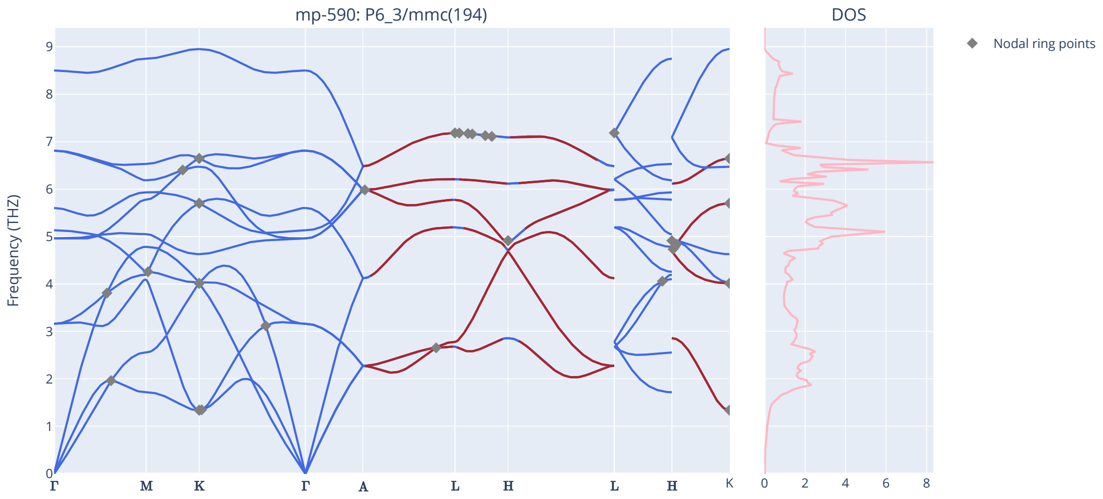

Supplementary Figure 775: The phonon band and DOS of Ni2As2.

Supplementary Table 1693: High symmetry lines of mp-590-Ni2As2

	Start	End
0	Γ (0.000, 0.000, 0.000)	M (0.500, 0.000, 0.000)
1	M (0.500, 0.000, 0.000)	K (0.333, 0.333, 0.000)
2	K (0.333, 0.333, 0.000)	Γ (0.000, 0.000, 0.000)
3	Γ (0.000, 0.000, 0.000)	A (0.000, 0.000, 0.500)
4	A (0.000, 0.000, 0.500)	L (0.500, 0.000, 0.500)
5	L (0.500, 0.000, 0.500)	H (0.333, 0.333, 0.500)
6	H (0.333, 0.333, 0.500)	A (0.000, 0.000, 0.500)
7	L (0.500, 0.000, 0.500)	M (0.500, 0.000, 0.000)
8	H (0.333, 0.333, 0.500)	K (0.333, 0.333, 0.000)

Supplementary Table 1694: Nodal ring points along high symmetry lines of mp-590-Ni2As2.

	Coordinates	Frequency	Mode 1	Mode 2	Multi.	Deg.	Path	Ratio
0	(0.326, 0.326, -0.000)	1.347	2	1	6	2	3	0.023
1	(0.309, 0.002, 0.000)	1.962	2	1	12	2	1	0.619
2	(0.333, 0.333, 0.000)	1.339	2	1	2	2	2	1.000
3	(0.398, -0.000, 0.499)	2.654	3	2	12	2	5	0.795
4	(0.333, 0.333, 0.000)	4.013	4	3	2	2	2	1.000
5	(0.124, 0.124, -0.000)	3.119	4	3	6	2	3	0.627
6	(0.498, 0.000, 0.082)	4.057	4	3	12	2	8	0.835
7	(0.287, 0.001, 0.000)	3.809	5	4	12	2	1	0.575
8	(0.494, 0.012, -0.000)	4.257	5	4	6	2	2	0.036
9	(0.333, 0.333, 0.490)	4.733	6	5	4	2	9	0.020
10	(0.333, 0.333, 0.480)	4.768	6	5	4	2	9	0.041

Supplementary Table 1694: Nodal ring points along high symmetry lines of mp-590-Ni2As2.

	Coordinates	Frequency	Mode 1	Mode 2	Multi.	Deg.	Path	Ratio
11	(0.333, 0.333, 0.469)	4.804	6	5	4	2	9	0.061
12	(0.333, 0.333, 0.459)	4.840	6	5	4	2	9	0.082
13	(0.333, 0.333, 0.000)	5.701	8	7	2	2	2	1.000
14	(0.333, 0.333, 0.500)	4.913	8	7	2	2	6	1.000
15	(0.010, 0.000, 0.500)	5.983	9	8	6	2	5	0.020
16	(0.385, 0.230, 0.000)	6.402	10	9	6	2	2	0.690
17	(0.333, 0.333, 0.000)	6.646	11	10	2	2	2	1.000
18	(0.500, 0.000, 0.500)	7.183	12	11	3	2	5	1.000
19	(0.486, 0.027, 0.500)	7.182	12	11	6	2	6	0.082
20	(0.459, 0.082, 0.500)	7.170	12	11	6	2	6	0.245
21	(0.446, 0.109, 0.500)	7.161	12	11	6	2	6	0.327
22	(0.405, 0.190, 0.500)	7.126	12	11	6	2	6	0.571
23	(0.384, 0.231, 0.500)	7.109	12	11	6	2	6	0.694

The followings are details for Co_3. It has 9 topological nodal lines and 38 nodal ring points along high symmetry line as shown in Fig **776**.

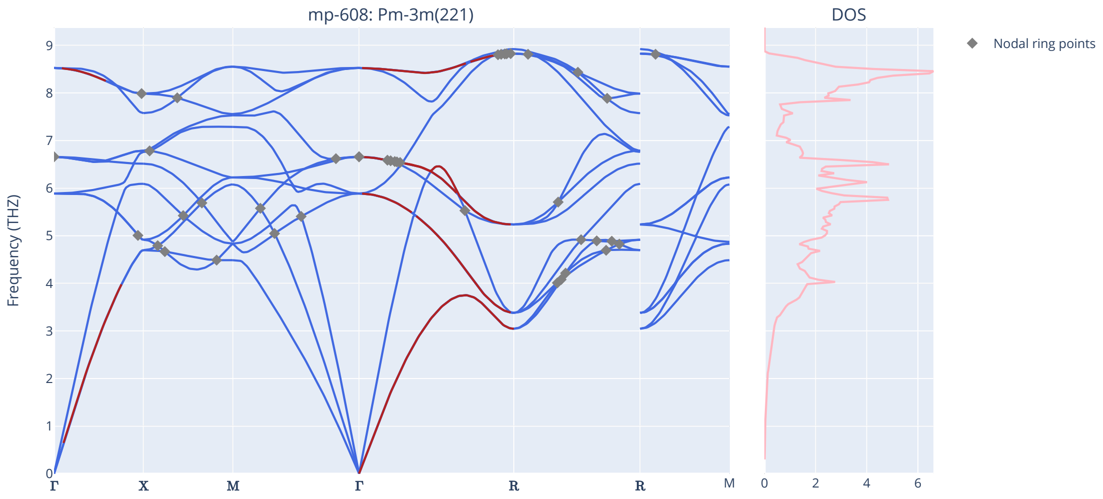

Supplementary Figure 776: The phonon band and DOS of Co3Ti.

Supplementary Table 1695: High symmetry lines of mp-608-Co3Ti

	Start	End
0	Γ (0.000, 0.000, 0.000)	X (0.000, 0.500, 0.000)
1	X (0.000, 0.500, 0.000)	M (0.500, 0.500, 0.000)
2	M (0.500, 0.500, 0.000)	Γ (0.000, 0.000, 0.000)
3	Γ (0.000, 0.000, 0.000)	R (0.500, 0.500, 0.500)
4	R (0.500, 0.500, 0.500)	X (0.000, 0.500, 0.000)
5	R (0.500, 0.500, 0.500)	M (0.500, 0.500, 0.000)

Supplementary Table 1696: Nodal ring points along high symmetry lines of mp-608-Co3Ti.

	Coordinates	Frequency	Mode 1	Mode 2	Multi.	Deg.	Path	Ratio
0	(0.120, 0.500, -0.000)	4.667	2	1	12	2	2	0.240
1	(0.410, 0.500, 0.000)	4.488	2	1	12	2	2	0.820
2	(0.134, 0.500, 0.134)	4.694	2	1	12	2	5	0.731
3	(0.312, 0.500, 0.312)	4.079	2	1	12	2	5	0.375
4	(0.080, 0.500, -0.000)	4.791	3	2	12	2	2	0.160
5	(0.335, 0.335, -0.000)	5.047	3	2	12	2	3	0.331
6	(0.295, 0.493, 0.295)	4.214	3	2	24	2	5	0.410
7	(0.328, 0.500, 0.328)	4.009	3	2	12	2	5	0.345
8	(0.082, 0.500, 0.082)	4.823	3	2	12	2	5	0.835
9	(0.000, 0.469, 0.000)	5.007	4	3	6	2	1	0.939
10	(0.229, 0.229, -0.000)	5.406	4	3	12	2	3	0.542
11	(0.112, 0.500, 0.112)	4.885	4	3	12	2	5	0.776
12	(0.172, 0.500, 0.172)	4.892	4	3	12	2	5	0.657
13	(0.327, 0.500, 0.000)	5.689	5	4	12	2	2	0.655

Supplementary Table 1696: Nodal ring points along high symmetry lines of mp-608-Co3Ti.

	Coordinates	Frequency	Mode 1	Mode 2	Multi.	Deg.	Path	Ratio
14	(0.223, 0.500, 0.000)	5.423	5	4	12	2	2	0.446
15	(0.391, 0.391, 0.000)	5.576	5	4	12	2	3	0.219
16	(0.234, 0.500, 0.234)	4.917	5	4	12	2	5	0.533
17	(0.342, 0.342, 0.342)	5.529	6	5	8	2	4	0.684
18	(0.000, 0.000, 0.000)	6.659	8	7	1	3	1	0.000
19	(0.035, 0.500, -0.000)	6.782	8	7	12	2	2	0.069
20	(0.324, 0.500, 0.324)	5.707	8	7	12	2	5	0.351
21	(0.092, 0.092, -0.000)	6.619	9	8	12	2	3	0.817
22	(0.092, 0.092, 0.092)	6.587	9	8	8	2	4	0.183
23	(0.115, 0.115, 0.115)	6.559	9	8	8	2	4	0.231
24	(0.102, 0.102, 0.102)	6.575	9	8	8	2	4	0.204
25	(0.122, 0.122, 0.122)	6.551	9	8	8	2	4	0.245
26	(0.133, 0.133, 0.133)	6.539	9	8	8	2	4	0.265
27	(0.490, 0.490, 0.490)	8.826	10	9	8	2	4	0.980
28	(0.471, 0.471, 0.471)	8.819	10	9	8	2	4	0.942
29	(0.459, 0.459, 0.459)	8.811	10	9	8	2	4	0.918
30	(0.477, 0.477, 0.477)	8.822	10	9	8	2	4	0.954
31	(0.449, 0.449, 0.449)	8.803	10	9	8	2	4	0.898
32	(0.190, 0.500, -0.000)	7.897	11	10	12	2	2	0.379
33	(0.498, 0.498, 0.414)	8.813	11	10	24	2	6	0.173
34	(0.444, 0.500, 0.444)	8.812	11	10	12	2	5	0.113
35	(0.130, 0.500, 0.130)	7.887	11	10	12	2	5	0.739
36	(0.000, 0.490, 0.000)	7.987	12	11	6	2	1	0.980
37	(0.246, 0.500, 0.246)	8.435	12	11	12	2	5	0.508

The followings are details for LaSn$_3$. It has 6 topological nodal lines and 18 nodal ring points along high symmetry line as shown in Fig 777.

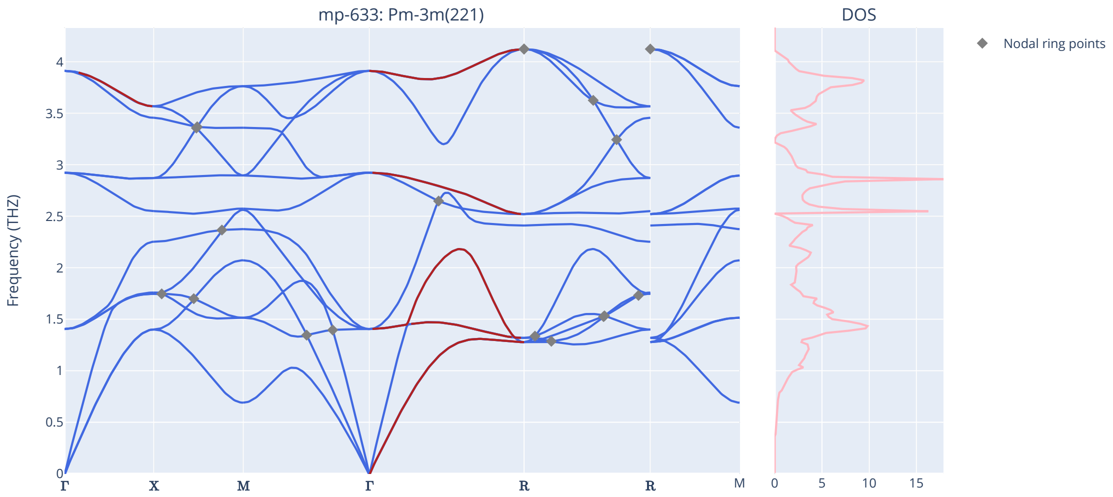

Supplementary Figure 777: The phonon band and DOS of LaSn3.

Supplementary Table 1697: High symmetry lines of mp-633-LaSn3

	Start	End
0	Γ (0.000, 0.000, 0.000)	X (0.000, 0.500, 0.000)
1	X (0.000, 0.500, 0.000)	M (0.500, 0.500, 0.000)
2	M (0.500, 0.500, 0.000)	Γ (0.000, 0.000, 0.000)
3	Γ (0.000, 0.000, 0.000)	R (0.500, 0.500, 0.500)
4	R (0.500, 0.500, 0.500)	X (0.000, 0.500, 0.000)
5	R (0.500, 0.500, 0.500)	M (0.500, 0.500, 0.000)

Supplementary Table 1698: Nodal ring points along high symmetry lines of mp-633-LaSn3.

	Coordinates	Frequency	Mode 1	Mode 2	Multi.	Deg.	Path	Ratio
0	(0.392, 0.500, 0.392)	1.287	2	1	12	2	5	0.216
1	(0.189, 0.500, 0.181)	1.526	3	2	24	2	5	0.630
2	(0.248, 0.248, -0.000)	1.346	3	2	12	2	3	0.503
3	(0.044, 0.500, -0.000)	1.747	4	3	12	2	2	0.088
4	(0.224, 0.500, -0.000)	1.700	4	3	12	2	2	0.447
5	(0.145, 0.145, 0.000)	1.396	4	3	12	2	3	0.710
6	(0.181, 0.500, 0.181)	1.533	4	3	12	2	5	0.637
7	(0.047, 0.500, 0.047)	1.732	4	3	12	2	5	0.906
8	(0.457, 0.500, 0.457)	1.336	5	4	12	2	5	0.086
9	(0.381, 0.500, 0.000)	2.367	6	5	12	2	2	0.762
10	(0.224, 0.224, 0.224)	2.648	7	6	8	2	4	0.447
11	(0.133, 0.500, 0.133)	3.244	10	9	12	2	5	0.733
12	(0.238, 0.500, -0.000)	3.357	10	9	12	2	2	0.477
13	(0.243, 0.500, 0.000)	3.369	11	10	12	2	2	0.486

Supplementary Table 1698: Nodal ring points along high symmetry lines of mp-633-LaSn3.

	Coordinates	Frequency	Mode 1	Mode 2	Multi.	Deg.	Path	Ratio
14	(0.226, 0.500, 0.226)	3.626	11	10	12	2	5	0.548
15	(0.500, 0.500, 0.500)	4.124	12	11	1	3	4	1.000

The followings are details for Rh$_3$. It has 7 topological nodal lines and 23 nodal ring points along high symmetry line as shown in Fig 778.

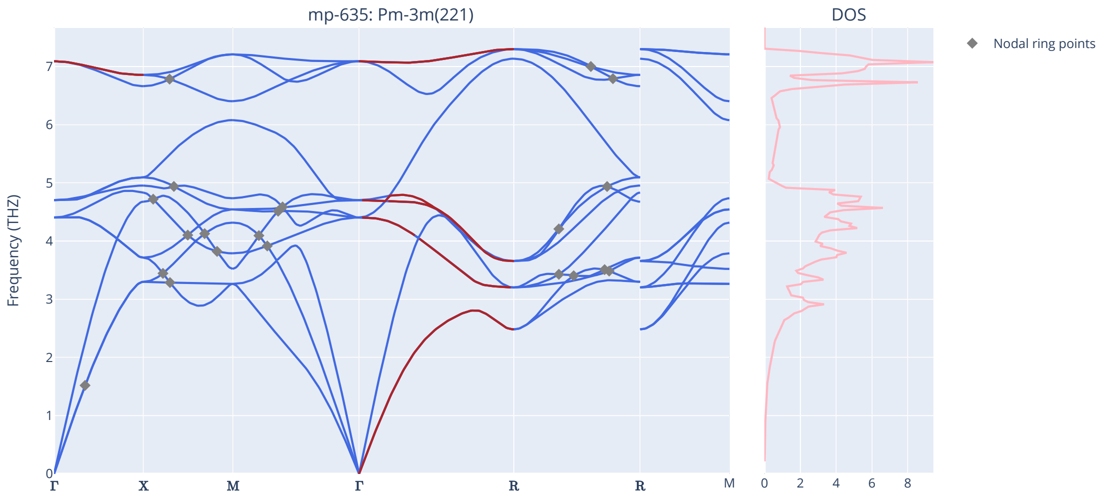

Supplementary Figure 778: The phonon band and DOS of Rh3Sc.

Supplementary Table 1699: High symmetry lines of mp-635-Rh3Sc

	Start	End
0	Γ (0.000, 0.000, 0.000)	X (0.000, 0.500, 0.000)
1	X (0.000, 0.500, 0.000)	M (0.500, 0.500, 0.000)
2	M (0.500, 0.500, 0.000)	Γ (0.000, 0.000, 0.000)
3	Γ (0.000, 0.000, 0.000)	R (0.500, 0.500, 0.500)
4	R (0.500, 0.500, 0.500)	X (0.000, 0.500, 0.000)
5	R (0.500, 0.500, 0.500)	M (0.500, 0.500, 0.000)

Supplementary Table 1700: Nodal ring points along high symmetry lines of mp-635-Rh3Sc.

	Coordinates	Frequency	Mode 1	Mode 2	Multi.	Deg.	Path	Ratio
0	(0.000, 0.173, 0.000)	1.518	2	1	6	2	1	0.347
1	(0.149, 0.500, -0.000)	3.286	2	1	12	2	2	0.298
2	(0.109, 0.500, 0.000)	3.444	3	2	12	2	2	0.219
3	(0.122, 0.500, 0.122)	3.484	3	2	12	2	5	0.755
4	(0.412, 0.500, -0.000)	3.821	4	3	12	2	2	0.825
5	(0.363, 0.363, 0.000)	3.916	4	3	12	2	3	0.273
6	(0.263, 0.500, 0.263)	3.403	4	3	12	2	5	0.474
7	(0.140, 0.500, 0.140)	3.509	4	3	12	2	5	0.719
8	(0.248, 0.500, -0.000)	4.102	5	4	12	2	2	0.497
9	(0.343, 0.500, -0.000)	4.127	5	4	12	2	2	0.686
10	(0.396, 0.396, 0.000)	4.093	5	4	12	2	3	0.207
11	(0.322, 0.500, 0.322)	3.428	5	4	12	2	5	0.357
12	(0.055, 0.500, -0.000)	4.720	6	5	12	2	2	0.110
13	(0.320, 0.320, -0.000)	4.513	6	5	12	2	3	0.360

Supplementary Table 1700: Nodal ring points along high symmetry lines of mp-635-Rh3Sc.

	Coordinates	Frequency	Mode 1	Mode 2	Multi.	Deg.	Path	Ratio
14	(0.171, 0.500, -0.000)	4.938	8	7	12	2	2	0.341
15	(0.303, 0.303, 0.000)	4.586	8	7	12	2	3	0.395
16	(0.322, 0.500, 0.322)	4.208	8	7	12	2	5	0.357
17	(0.131, 0.500, 0.131)	4.937	8	7	12	2	5	0.739
18	(0.146, 0.500, -0.000)	6.783	11	10	12	2	2	0.293
19	(0.107, 0.500, 0.107)	6.791	11	10	12	2	5	0.785
20	(0.195, 0.500, 0.195)	7.000	12	11	12	2	5	0.610

The followings are details for $Ca_3Hg_3O_6$. It has 6 topological nodal lines and 12 nodal ring points along high symmetry line as shown in Fig 779.

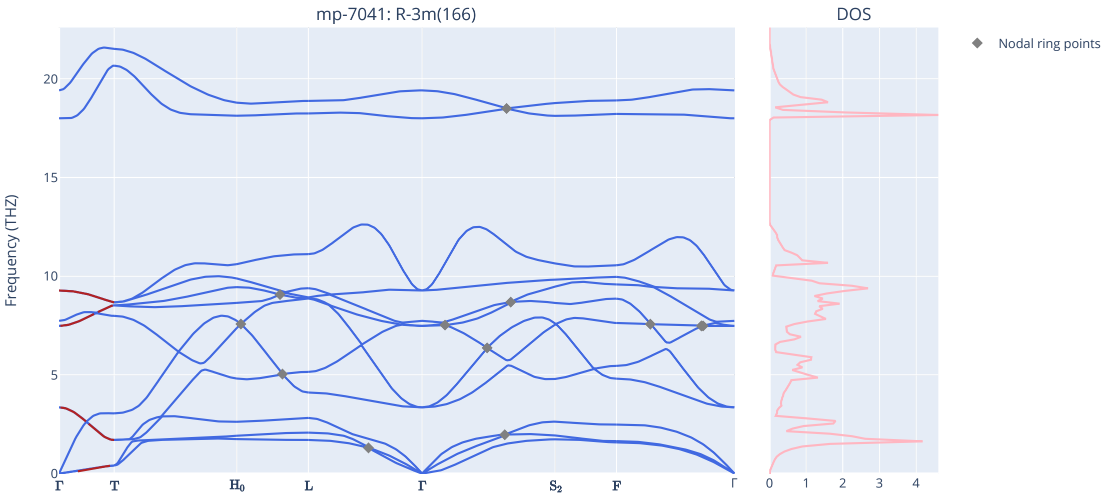

Supplementary Figure 779: The phonon band and DOS of Ca3Hg3O6.

Supplementary Table 1701: High symmetry lines of mp-7041-Ca3Hg3O6

	Start	End
0	Γ (0.000, 0.000, 0.000)	T (0.500, 0.500, 0.500)
1	T (0.500, 0.500, 0.500)	H_2 (0.816, 0.184, 0.500)
2	H_0 (0.500, -0.184, 0.184)	L (0.500, 0.000, 0.000)
3	L (0.500, 0.000, 0.000)	Γ (0.000, 0.000, 0.000)
4	Γ (0.000, 0.000, 0.000)	S_0 (0.342, -0.342, 0.000)
5	S_2 (0.658, 0.000, 0.342)	F (0.500, 0.000, 0.500)
6	F (0.500, 0.000, 0.500)	Γ (0.000, 0.000, 0.000)

Supplementary Table 1702: Nodal ring points along high symmetry lines of mp-7041-Ca3Hg3O6.

	Coordinates	Frequency	Mode 1	Mode 2	Multi.	Deg.	Path	Ratio
0	(0.236, 0.000, 0.000)	1.300	2	1	6	2	4	0.528
1	(0.213, -0.213, 0.000)	1.965	3	2	6	2	5	0.622
2	(0.500, -0.067, 0.067)	5.040	5	4	6	2	3	0.638
3	(0.500, -0.174, 0.174)	7.575	6	5	6	2	3	0.058
4	(0.167, -0.167, 0.000)	6.366	6	5	6	2	5	0.489
5	(0.140, 0.006, 0.140)	7.480	7	6	6	2	7	0.720
6	(0.059, -0.059, 0.000)	7.523	7	6	6	2	5	0.172
7	(0.356, 0.002, 0.356)	7.569	7	6	6	2	7	0.288
8	(0.133, -0.000, 0.133)	7.479	7	6	6	2	7	0.735
9	(0.500, -0.073, 0.073)	9.069	8	7	6	2	3	0.604
10	(0.228, -0.228, 0.000)	8.686	8	7	6	2	5	0.667
11	(0.217, -0.217, -0.000)	18.497	12	11	6	2	5	0.635

The followings are details for Pd$_3$. It has 6 topological nodal lines and 33 nodal ring points along high symmetry line as shown in Fig **780**.

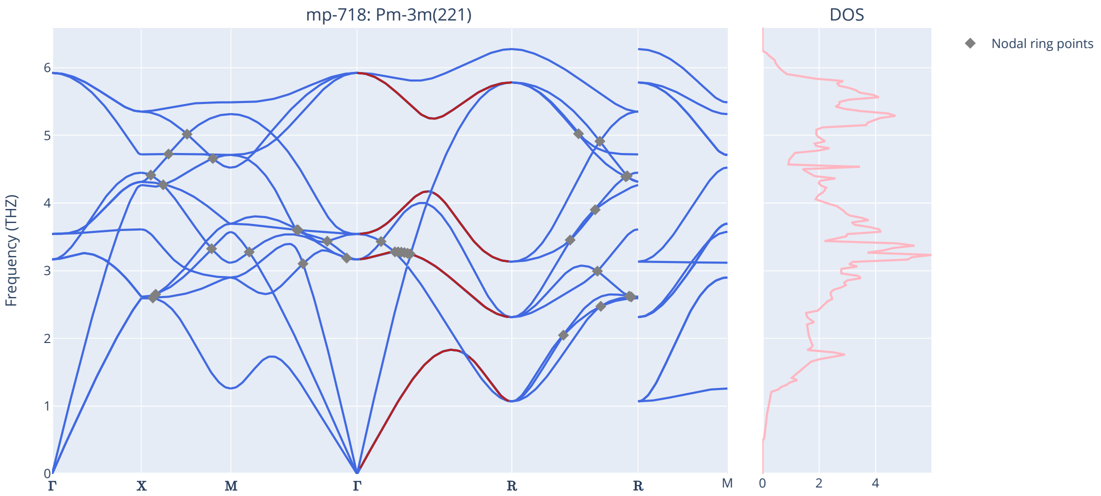

Supplementary Figure 780: The phonon band and DOS of Pd3Sn.

Supplementary Table 1703: High symmetry lines of mp-718-Pd3Sn

	Start	End
0	Γ (0.000, 0.000, 0.000)	X (0.000, 0.500, 0.000)
1	X (0.000, 0.500, 0.000)	M (0.500, 0.500, 0.000)
2	M (0.500, 0.500, 0.000)	Γ (0.000, 0.000, 0.000)
3	Γ (0.000, 0.000, 0.000)	R (0.500, 0.500, 0.500)
4	R (0.500, 0.500, 0.500)	X (0.000, 0.500, 0.000)
5	R (0.500, 0.500, 0.500)	M (0.500, 0.500, 0.000)

Supplementary Table 1704: Nodal ring points along high symmetry lines of mp-718-Pd3Sn.

	Coordinates	Frequency	Mode 1	Mode 2	Multi.	Deg.	Path	Ratio
0	(0.064, 0.500, 0.000)	2.598	2	1	12	2	2	0.128
1	(0.148, 0.500, 0.148)	2.474	2	1	12	2	5	0.705
2	(0.296, 0.500, 0.296)	2.047	3	2	12	2	5	0.409
3	(0.027, 0.500, 0.027)	2.613	3	2	12	2	5	0.945
4	(0.080, 0.500, -0.000)	2.652	4	3	12	2	2	0.159
5	(0.214, 0.214, -0.000)	3.104	4	3	12	2	3	0.572
6	(0.035, 0.500, 0.035)	2.624	4	3	12	2	5	0.930
7	(0.393, 0.500, 0.000)	3.325	5	4	12	2	2	0.786
8	(0.041, 0.041, 0.000)	3.187	5	4	12	2	3	0.918
9	(0.427, 0.427, 0.000)	3.277	5	4	12	2	3	0.146
10	(0.161, 0.500, 0.161)	2.994	5	4	12	2	5	0.678
11	(0.153, 0.153, 0.153)	3.267	6	5	8	2	4	0.306
12	(0.172, 0.172, 0.172)	3.248	6	5	8	2	4	0.343
13	(0.122, 0.122, 0.122)	3.278	6	5	8	2	4	0.245

Supplementary Table 1704: Nodal ring points along high symmetry lines of mp-718-Pd3Sn.

	Coordinates	Frequency	Mode 1	Mode 2	Multi.	Deg.	Path	Ratio
14	(0.133, 0.133, 0.133)	3.278	6	5	8	2	4	0.265
15	(0.143, 0.143, 0.143)	3.274	6	5	8	2	4	0.286
16	(0.163, 0.163, 0.163)	3.258	6	5	8	2	4	0.327
17	(0.122, 0.500, 0.000)	4.269	7	6	12	2	2	0.244
18	(0.238, 0.238, -0.001)	3.603	7	6	24	2	3	0.524
19	(0.118, 0.118, -0.000)	3.436	7	6	12	2	3	0.764
20	(0.078, 0.078, 0.078)	3.431	7	6	8	2	4	0.155
21	(0.170, 0.500, 0.170)	3.900	7	6	12	2	5	0.660
22	(0.269, 0.500, 0.269)	3.453	7	6	12	2	5	0.462
23	(0.233, 0.233, -0.000)	3.601	8	7	12	2	3	0.533
24	(0.049, 0.500, 0.049)	4.388	8	7	12	2	5	0.903
25	(0.053, 0.500, 0.000)	4.413	9	8	12	2	2	0.105
26	(0.400, 0.500, 0.000)	4.658	9	8	12	2	2	0.800
27	(0.043, 0.500, 0.043)	4.401	9	8	12	2	5	0.914
28	(0.152, 0.500, 0.000)	4.724	10	9	12	2	2	0.304
29	(0.236, 0.500, 0.236)	5.024	10	9	12	2	5	0.529
30	(0.256, 0.500, -0.000)	5.017	11	10	12	2	2	0.511
31	(0.151, 0.500, 0.151)	4.913	11	10	12	2	5	0.697

The followings are details for $Li_{12}Tl_4$. It has 7 topological nodal lines and 9 nodal ring points along high symmetry line as shown in Fig **781**.

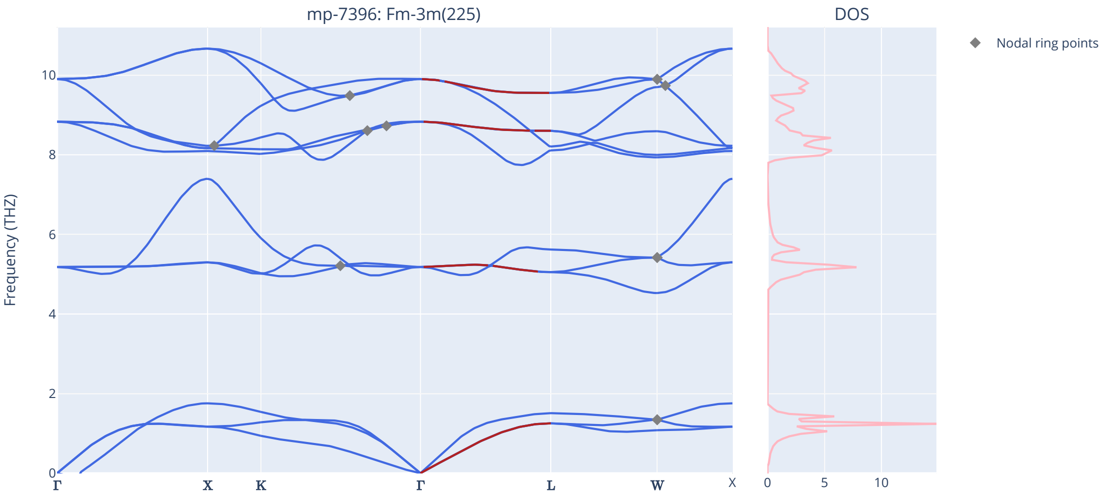

Supplementary Figure 781: The phonon band and DOS of Li12Tl4.

Supplementary Table 1705: High symmetry lines of mp-7396-Li12Tl4

	Start	End
0	Γ (0.000, 0.000, 0.000)	X (0.500, 0.000, 0.500)
1	X (0.500, 0.000, 0.500)	U (0.625, 0.250, 0.625)
2	K (0.375, 0.375, 0.750)	Γ (0.000, 0.000, 0.000)
3	Γ (0.000, 0.000, 0.000)	L (0.500, 0.500, 0.500)
4	L (0.500, 0.500, 0.500)	W (0.500, 0.250, 0.750)
5	W (0.500, 0.250, 0.750)	X (0.500, 0.000, 0.500)

Supplementary Table 1706: Nodal ring points along high symmetry lines of mp-7396-Li12Tl4.

	Coordinates	Frequency	Mode 1	Mode 2	Multi.	Deg.	Path	Ratio
0	(0.500, 0.250, 0.750)	1.345	3	2	6	2	5	1.000
1	(0.191, 0.191, 0.373)	5.212	5	4	24	2	3	0.498
2	(0.500, 0.250, 0.750)	5.418	6	5	6	2	5	1.000
3	(0.076, 0.076, 0.163)	8.726	8	7	24	2	3	0.787
4	(0.128, 0.128, 0.246)	8.605	8	7	24	2	3	0.667
5	(0.516, 0.031, 0.516)	8.227	10	9	12	2	2	0.126
6	(0.161, 0.161, 0.337)	9.484	11	10	24	2	3	0.558
7	(0.499, 0.222, 0.723)	9.737	11	10	24	2	6	0.109
8	(0.500, 0.250, 0.750)	9.895	12	11	6	2	5	1.000

The followings are details for MgPd$_3$. It has 8 topological nodal lines and 20 nodal ring points along high symmetry line as shown in Fig **782**.

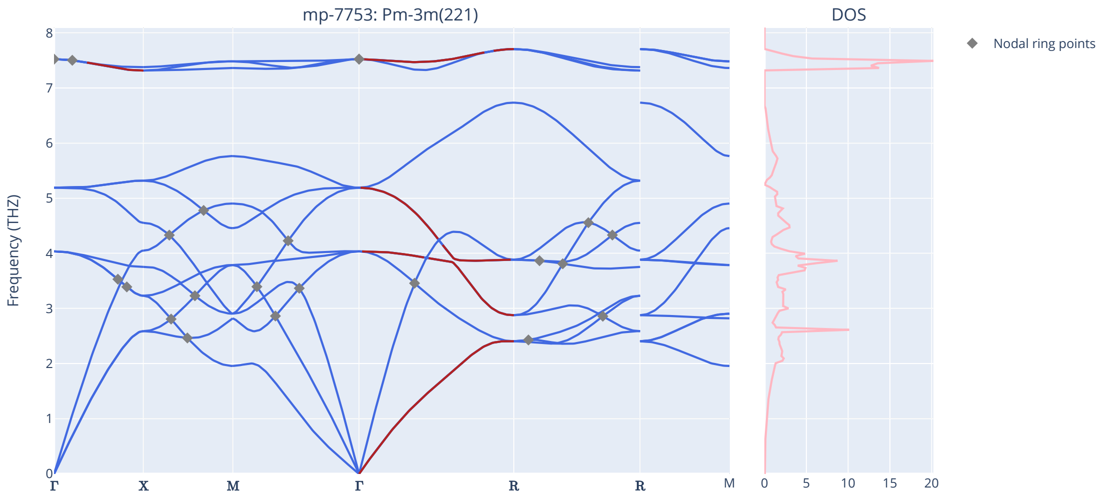

Supplementary Figure 782: The phonon band and DOS of MgPd3.

Supplementary Table 1707: High symmetry lines of mp-7753-MgPd3

	Start	End
0	Γ (0.000, 0.000, 0.000)	X (0.000, 0.500, 0.000)
1	X (0.000, 0.500, 0.000)	M (0.500, 0.500, 0.000)
2	M (0.500, 0.500, 0.000)	Γ (0.000, 0.000, 0.000)
3	Γ (0.000, 0.000, 0.000)	R (0.500, 0.500, 0.500)
4	R (0.500, 0.500, 0.500)	X (0.000, 0.500, 0.000)
5	R (0.500, 0.500, 0.500)	M (0.500, 0.500, 0.000)

Supplementary Table 1708: Nodal ring points along high symmetry lines of mp-7753-MgPd3.

	Coordinates	Frequency	Mode 1	Mode 2	Multi.	Deg.	Path	Ratio
0	(0.246, 0.500, -0.000)	2.464	2	1	12	2	2	0.493
1	(0.155, 0.500, -0.000)	2.807	3	2	12	2	2	0.310
2	(0.331, 0.331, -0.000)	2.862	3	2	12	2	3	0.338
3	(0.442, 0.500, 0.442)	2.428	3	2	12	2	5	0.116
4	(0.148, 0.500, 0.148)	2.858	3	2	12	2	5	0.705
5	(0.000, 0.407, 0.000)	3.390	4	3	6	2	1	0.814
6	(0.288, 0.500, -0.000)	3.230	4	3	12	2	2	0.577
7	(0.237, 0.237, 0.000)	3.366	4	3	12	2	3	0.527
8	(0.179, 0.179, 0.179)	3.457	4	3	8	2	4	0.359
9	(0.000, 0.357, 0.000)	3.530	5	4	6	2	1	0.714
10	(0.404, 0.404, 0.000)	3.394	5	4	12	2	3	0.191
11	(0.306, 0.500, 0.306)	3.810	6	5	12	2	5	0.388
12	(0.145, 0.500, -0.000)	4.330	7	6	12	2	2	0.290
13	(0.281, 0.281, -0.000)	4.227	7	6	12	2	3	0.438

Supplementary Table 1708: Nodal ring points along high symmetry lines of mp-7753-MgPd3.

	Coordinates	Frequency	Mode 1	Mode 2	Multi.	Deg.	Path	Ratio
14	(0.398, 0.500, 0.398)	3.866	7	6	12	2	5	0.204
15	(0.110, 0.500, 0.110)	4.332	7	6	12	2	5	0.781
16	(0.337, 0.500, -0.000)	4.778	8	7	12	2	2	0.674
17	(0.205, 0.500, 0.205)	4.556	8	7	12	2	5	0.591
18	(0.000, 0.000, 0.000)	7.523	12	11	1	3	1	0.000
19	(0.000, 0.102, 0.000)	7.503	12	11	6	2	1	0.204

The followings are details for Ni_2Sb_2. It has 25 topological nodal lines and 16 nodal ring points along high symmetry line as shown in Fig **783**.

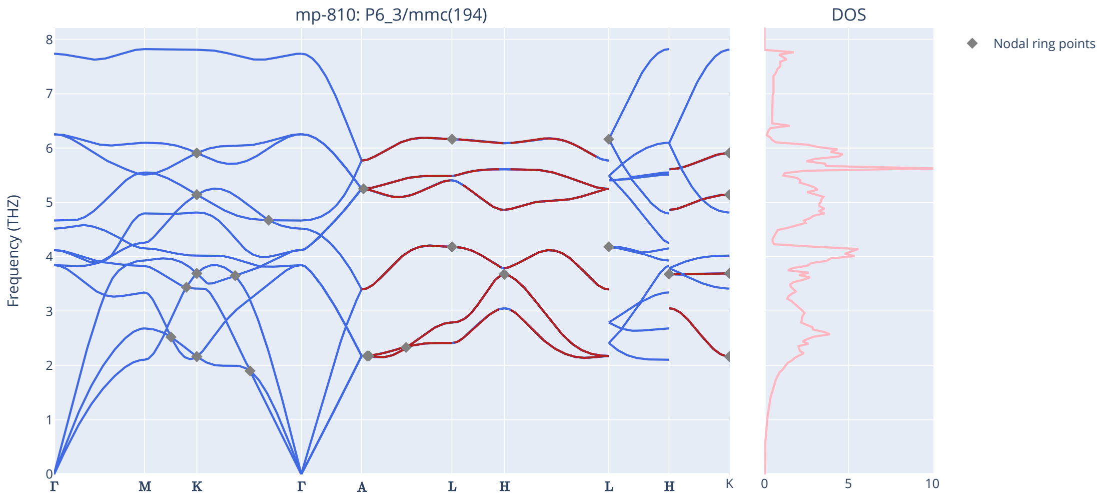

Supplementary Figure 783: The phonon band and DOS of Ni2Sb2.

Supplementary Table 1709: High symmetry lines of mp-810-Ni2Sb2

	Start	End
0	Γ (0.000, 0.000, 0.000)	M (0.500, 0.000, 0.000)
1	M (0.500, 0.000, 0.000)	K (0.333, 0.333, 0.000)
2	K (0.333, 0.333, 0.000)	Γ (0.000, 0.000, 0.000)
3	Γ (0.000, 0.000, 0.000)	A (0.000, 0.000, 0.500)
4	A (0.000, 0.000, 0.500)	L (0.500, 0.000, 0.500)
5	L (0.500, 0.000, 0.500)	H (0.333, 0.333, 0.500)
6	H (0.333, 0.333, 0.500)	A (0.000, 0.000, 0.500)
7	L (0.500, 0.000, 0.500)	M (0.500, 0.000, 0.000)
8	H (0.333, 0.333, 0.500)	K (0.333, 0.333, 0.000)

Supplementary Table 1710: Nodal ring points along high symmetry lines of mp-810-Ni2Sb2.

	Coordinates	Frequency	Mode 1	Mode 2	Multi.	Deg.	Path	Ratio
0	(0.333, 0.333, 0.000)	2.163	2	1	2	2	2	1.000
1	(0.416, 0.169, -0.000)	2.524	2	1	6	2	2	0.506
2	(0.164, 0.164, 0.000)	1.901	2	1	6	2	3	0.509
3	(0.031, -0.000, 0.499)	2.175	3	2	12	2	5	0.061
4	(0.041, -0.000, 0.498)	2.175	3	2	12	2	5	0.081
5	(0.246, 0.000, 0.497)	2.333	3	2	12	2	5	0.492
6	(0.366, 0.267, -0.000)	3.440	4	3	6	2	2	0.801
7	(0.333, 0.333, 0.500)	3.680	4	3	2	2	6	1.000
8	(0.211, 0.211, 0.000)	3.653	5	4	6	2	3	0.367
9	(0.333, 0.333, 0.000)	3.694	5	4	2	2	2	1.000
10	(0.500, 0.000, 0.500)	4.184	6	5	3	2	5	1.000

Supplementary Table 1710: Nodal ring points along high symmetry lines of mp-810-Ni2Sb2.

	Coordinates	Frequency	Mode 1	Mode 2	Multi.	Deg.	Path	Ratio
11	(0.104, 0.104, 0.000)	4.673	9	8	6	2	3	0.688
12	(0.333, 0.333, 0.000)	5.143	9	8	2	2	2	1.000
13	(0.010, 0.000, 0.500)	5.251	9	8	6	2	5	0.020
14	(0.333, 0.333, 0.000)	5.909	11	10	2	2	2	1.000
15	(0.500, 0.000, 0.500)	6.163	12	11	3	2	5	1.000

The followings are details for GaNi$_3$. It has 6 topological nodal lines and 33 nodal ring points along high symmetry line as shown in Fig **784**.

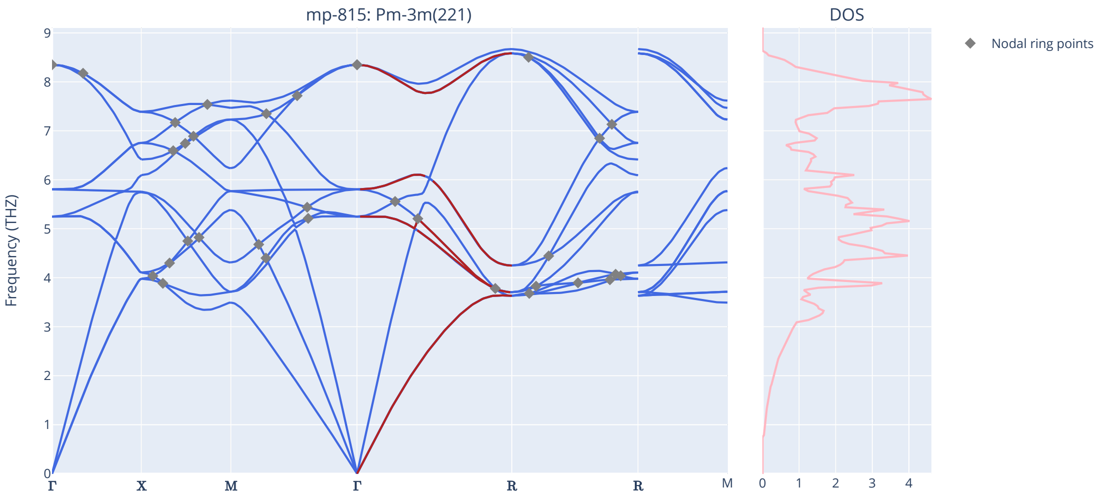

Supplementary Figure 784: The phonon band and DOS of GaNi3.

Supplementary Table 1711: High symmetry lines of mp-815-GaNi3

	Start	End
0	Γ (0.000, 0.000, 0.000)	X (0.000, 0.500, 0.000)
1	X (0.000, 0.500, 0.000)	M (0.500, 0.500, 0.000)
2	M (0.500, 0.500, 0.000)	Γ (0.000, 0.000, 0.000)
3	Γ (0.000, 0.000, 0.000)	R (0.500, 0.500, 0.500)
4	R (0.500, 0.500, 0.500)	X (0.000, 0.500, 0.000)
5	R (0.500, 0.500, 0.500)	M (0.500, 0.500, 0.000)

Supplementary Table 1712: Nodal ring points along high symmetry lines of mp-815-GaNi3.

	Coordinates	Frequency	Mode 1	Mode 2	Multi.	Deg.	Path	Ratio
0	(0.120, 0.500, 0.000)	3.888	2	1	12	2	2	0.240
1	(0.112, 0.500, 0.112)	3.961	2	1	12	2	5	0.777
2	(0.064, 0.500, 0.000)	4.038	3	2	12	2	2	0.128
3	(0.431, 0.500, 0.431)	3.682	3	2	12	2	5	0.138
4	(0.238, 0.500, 0.238)	3.898	3	2	12	2	5	0.524
5	(0.069, 0.500, 0.069)	4.043	3	2	12	2	5	0.862
6	(0.158, 0.500, -0.000)	4.302	4	3	12	2	2	0.315
7	(0.361, 0.361, 0.000)	4.403	4	3	12	2	3	0.279
8	(0.089, 0.500, 0.089)	4.073	4	3	12	2	5	0.822
9	(0.259, 0.500, -0.000)	4.751	5	4	12	2	2	0.519
10	(0.322, 0.500, 0.000)	4.825	5	4	12	2	2	0.645
11	(0.193, 0.193, 0.000)	5.213	5	4	12	2	3	0.613
12	(0.389, 0.389, -0.000)	4.682	5	4	12	2	3	0.222
13	(0.404, 0.500, 0.404)	3.830	5	4	12	2	5	0.192

Supplementary Table 1712: Nodal ring points along high symmetry lines of mp-815-GaNi3.

	Coordinates	Frequency	Mode 1	Mode 2	Multi.	Deg.	Path	Ratio
14	(0.196, 0.196, 0.196)	5.208	6	5	8	2	4	0.393
15	(0.447, 0.447, 0.447)	3.783	6	5	8	2	4	0.894
16	(0.197, 0.197, -0.000)	5.441	7	6	12	2	3	0.605
17	(0.123, 0.123, 0.123)	5.559	7	6	8	2	4	0.246
18	(0.354, 0.500, 0.354)	4.447	7	6	12	2	5	0.292
19	(0.245, 0.500, -0.000)	6.745	9	8	12	2	2	0.491
20	(0.178, 0.500, -0.000)	6.595	9	8	12	2	2	0.356
21	(0.291, 0.500, -0.000)	6.891	10	9	12	2	2	0.582
22	(0.433, 0.500, 0.433)	8.503	10	9	12	2	5	0.133
23	(0.152, 0.500, 0.152)	6.849	10	9	12	2	5	0.695
24	(0.000, 0.000, 0.000)	8.351	11	10	1	3	1	0.000
25	(0.189, 0.500, -0.000)	7.171	11	10	12	2	2	0.378
26	(0.237, 0.237, 0.000)	7.719	11	10	12	2	3	0.526
27	(0.359, 0.359, 0.000)	7.351	11	10	12	2	3	0.282
28	(0.104, 0.500, 0.104)	7.134	11	10	12	2	5	0.792
29	(0.000, 0.173, 0.000)	8.177	12	11	6	2	1	0.347
30	(0.369, 0.500, -0.000)	7.540	12	11	12	2	2	0.738

The followings are details for Ni3. It has 7 topological nodal lines and 30 nodal ring points along high symmetry line as shown in Fig **785**.

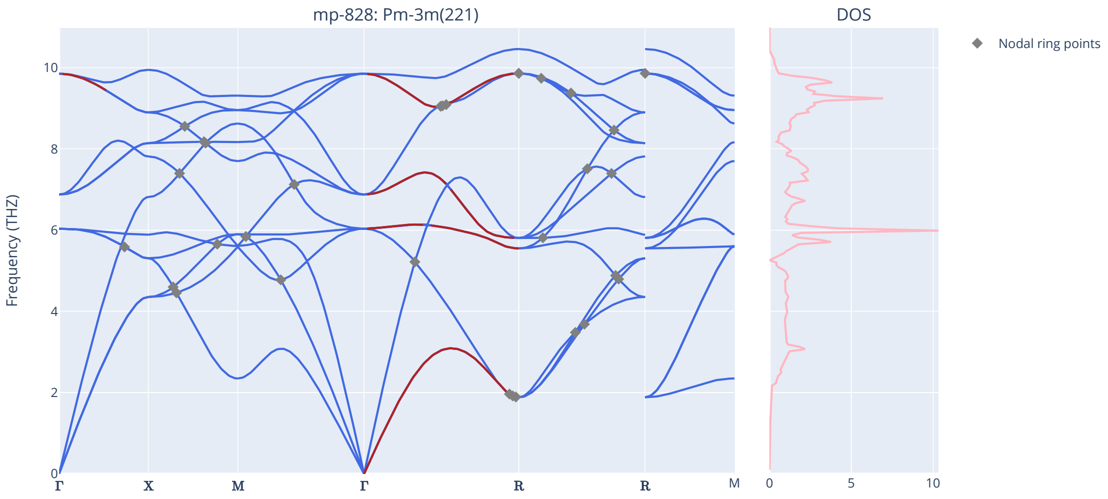

Supplementary Figure 785: The phonon band and DOS of Ni3Si.

Supplementary Table 1713: High symmetry lines of mp-828-Ni3Si

	Start	End
0	Γ (0.000, 0.000, 0.000)	X (0.000, 0.500, 0.000)
1	X (0.000, 0.500, 0.000)	M (0.500, 0.500, 0.000)
2	M (0.500, 0.500, 0.000)	Γ (0.000, 0.000, 0.000)
3	Γ (0.000, 0.000, 0.000)	R (0.500, 0.500, 0.500)
4	R (0.500, 0.500, 0.500)	X (0.000, 0.500, 0.000)
5	R (0.500, 0.500, 0.500)	M (0.500, 0.500, 0.000)

Supplementary Table 1714: Nodal ring points along high symmetry lines of mp-828-Ni3Si.

	Coordinates	Frequency	Mode 1	Mode 2	Multi.	Deg.	Path	Ratio
0	(0.157, 0.500, 0.000)	4.455	2	1	12	2	2	0.314
1	(0.240, 0.500, 0.240)	3.682	2	1	12	2	5	0.519
2	(0.139, 0.500, 0.000)	4.598	3	2	12	2	2	0.277
3	(0.330, 0.330, -0.000)	4.774	3	2	12	2	3	0.341
4	(0.470, 0.470, 0.470)	1.962	3	2	8	2	4	0.940
5	(0.481, 0.481, 0.481)	1.916	3	2	8	2	4	0.961
6	(0.491, 0.491, 0.491)	1.890	3	2	8	3	4	0.981
7	(0.276, 0.500, 0.276)	3.479	3	2	12	2	5	0.448
8	(0.105, 0.500, 0.105)	4.793	3	2	12	2	5	0.791
9	(0.000, 0.366, 0.000)	5.584	4	3	6	2	1	0.733
10	(0.386, 0.500, 0.000)	5.654	4	3	12	2	2	0.771
11	(0.164, 0.164, 0.164)	5.216	4	3	8	2	4	0.328
12	(0.117, 0.500, 0.117)	4.882	4	3	12	2	5	0.766
13	(0.468, 0.468, -0.000)	5.842	5	4	12	2	3	0.065

Supplementary Table 1714: Nodal ring points along high symmetry lines of mp-828-Ni3Si.

	Coordinates	Frequency	Mode 1	Mode 2	Multi.	Deg.	Path	Ratio
14	(0.405, 0.500, 0.405)	5.810	6	5	12	2	5	0.191
15	(0.174, 0.500, 0.000)	7.396	7	6	12	2	2	0.348
16	(0.277, 0.277, 0.000)	7.124	7	6	12	2	3	0.447
17	(0.133, 0.500, 0.133)	7.394	7	6	12	2	5	0.734
18	(0.320, 0.500, 0.000)	8.141	8	7	12	2	2	0.640
19	(0.228, 0.500, 0.228)	7.510	8	7	12	2	5	0.543
20	(0.311, 0.500, 0.000)	8.175	9	8	12	2	2	0.622
21	(0.203, 0.500, -0.000)	8.554	10	9	12	2	2	0.407
22	(0.254, 0.254, 0.254)	9.060	10	9	8	2	4	0.507
23	(0.500, 0.500, 0.500)	9.860	10	9	1	3	4	1.000
24	(0.265, 0.265, 0.265)	9.090	10	9	8	2	4	0.531
25	(0.247, 0.247, 0.247)	9.049	10	9	8	2	4	0.495
26	(0.411, 0.500, 0.411)	9.738	10	9	12	2	5	0.178
27	(0.123, 0.500, 0.123)	8.461	10	9	12	2	5	0.754
28	(0.293, 0.500, 0.293)	9.375	11	10	12	2	5	0.413

The followings are details for Ga$_3$. It has 8 topological nodal lines and 27 nodal ring points along high symmetry line as shown in Fig **786**.

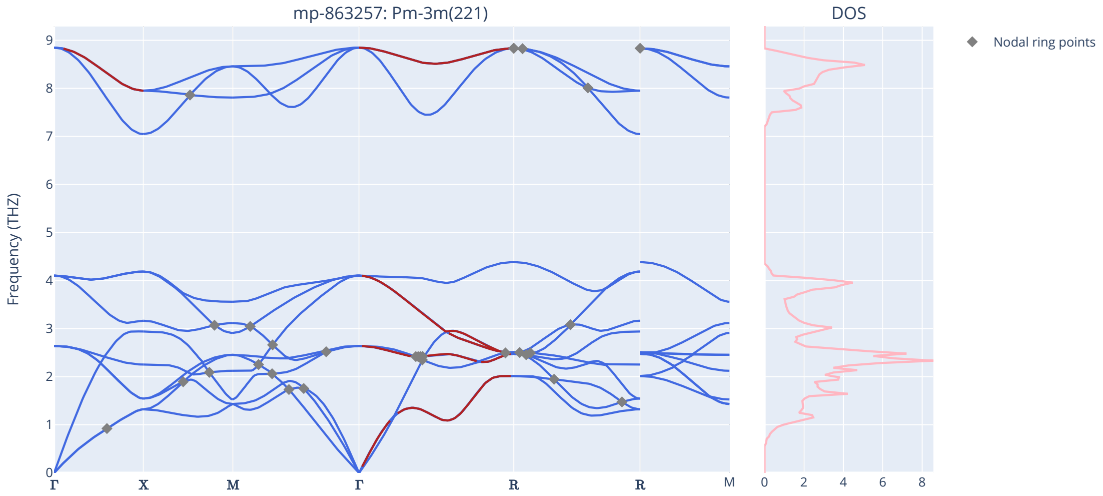

Supplementary Figure 786: The phonon band and DOS of Ga3Li.

Supplementary Table 1715: High symmetry lines of mp-863257-Ga3Li

	Start	End
0	Γ (0.000, 0.000, 0.000)	X (0.000, 0.500, 0.000)
1	X (0.000, 0.500, 0.000)	M (0.500, 0.500, 0.000)
2	M (0.500, 0.500, 0.000)	Γ (0.000, 0.000, 0.000)
3	Γ (0.000, 0.000, 0.000)	R (0.500, 0.500, 0.500)
4	R (0.500, 0.500, 0.500)	X (0.000, 0.500, 0.000)
5	R (0.500, 0.500, 0.500)	M (0.500, 0.500, 0.000)

Supplementary Table 1716: Nodal ring points along high symmetry lines of mp-863257-Ga3Li.

	Coordinates	Frequency	Mode 1	Mode 2	Multi.	Deg.	Path	Ratio
0	(0.000, 0.296, 0.000)	0.919	2	1	6	2	1	0.592
1	(0.278, 0.278, 0.000)	1.731	2	1	12	2	3	0.445
2	(0.223, 0.500, -0.000)	1.892	3	2	12	2	2	0.445
3	(0.219, 0.219, -0.000)	1.753	3	2	12	2	3	0.562
4	(0.340, 0.500, 0.340)	1.946	3	2	12	2	5	0.319
5	(0.072, 0.500, 0.072)	1.473	3	2	12	2	5	0.856
6	(0.369, 0.500, 0.000)	2.089	4	3	12	2	2	0.738
7	(0.344, 0.344, -0.000)	2.060	4	3	12	2	3	0.312
8	(0.208, 0.208, 0.197)	2.348	4	3	24	2	4	0.409
9	(0.130, 0.130, -0.000)	2.517	5	4	12	2	3	0.740
10	(0.398, 0.398, 0.000)	2.251	5	4	12	2	3	0.204
11	(0.451, 0.500, 0.451)	2.456	5	4	12	2	5	0.098
12	(0.183, 0.183, 0.183)	2.418	6	5	8	2	4	0.367
13	(0.205, 0.205, 0.205)	2.420	6	5	8	2	4	0.410

Supplementary Table 1716: Nodal ring points along high symmetry lines of mp-863257-Ga3Li.

	Coordinates	Frequency	Mode 1	Mode 2	Multi.	Deg.	Path	Ratio
14	(0.191, 0.191, 0.191)	2.417	6	5	8	2	4	0.382
15	(0.473, 0.473, 0.473)	2.494	6	5	8	2	4	0.946
16	(0.198, 0.198, 0.198)	2.417	6	5	8	2	4	0.396
17	(0.342, 0.342, 0.000)	2.661	7	6	12	2	3	0.316
18	(0.477, 0.500, 0.477)	2.502	7	6	12	2	5	0.047
19	(0.434, 0.500, 0.434)	2.491	7	6	12	2	5	0.132
20	(0.398, 0.500, 0.000)	3.070	8	7	12	2	2	0.796
21	(0.431, 0.431, 0.000)	3.045	8	7	12	2	3	0.138
22	(0.276, 0.500, 0.276)	3.081	8	7	12	2	5	0.448
23	(0.261, 0.500, -0.000)	7.861	11	10	12	2	2	0.522
24	(0.207, 0.500, 0.207)	8.010	11	10	12	2	5	0.587
25	(0.500, 0.500, 0.500)	8.834	12	11	1	3	4	1.000
26	(0.465, 0.500, 0.465)	8.825	12	11	12	2	5	0.069

The followings are details for Mn_4V_{12}. It has 8 topological nodal lines and 14 nodal ring points along high symmetry line as shown in Fig **787**.

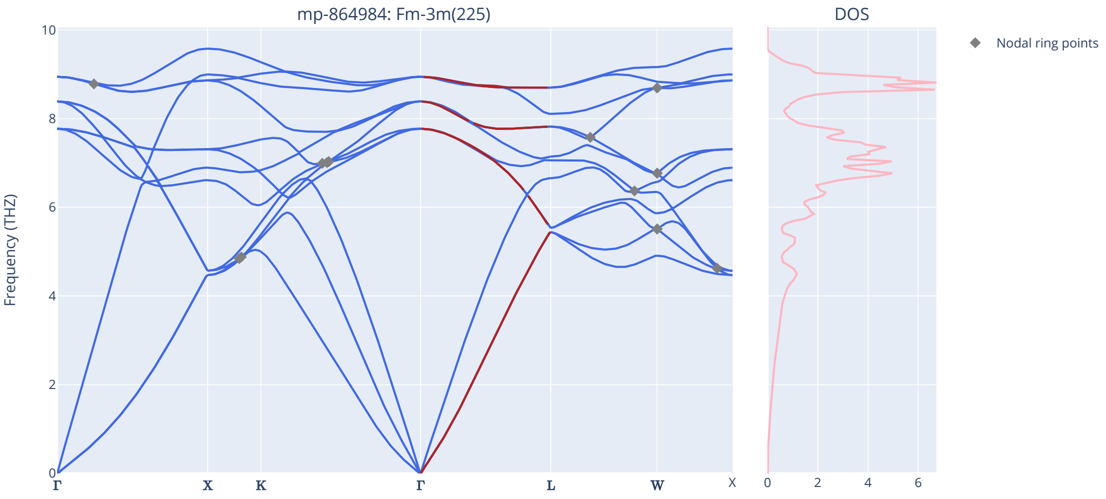

Supplementary Figure 787: The phonon band and DOS of Mn4V12.

Supplementary Table 1717: High symmetry lines of mp-864984-Mn4V12

	Start	End
0	Γ (0.000, 0.000, 0.000)	X (0.500, 0.000, 0.500)
1	X (0.500, 0.000, 0.500)	U (0.625, 0.250, 0.625)
2	K (0.375, 0.375, 0.750)	Γ (0.000, 0.000, 0.000)
3	Γ (0.000, 0.000, 0.000)	L (0.500, 0.500, 0.500)
4	L (0.500, 0.500, 0.500)	W (0.500, 0.250, 0.750)
5	W (0.500, 0.250, 0.750)	X (0.500, 0.000, 0.500)

Supplementary Table 1718: Nodal ring points along high symmetry lines of mp-864984-Mn4V12.

	Coordinates	Frequency	Mode 1	Mode 2	Multi.	Deg.	Path	Ratio
0	(0.578, 0.158, 0.580)	4.879	3	2	24	2	2	0.634
1	(0.573, 0.149, 0.576)	4.842	3	2	24	2	2	0.595
2	(0.500, 0.250, 0.750)	5.510	3	2	6	2	5	1.000
3	(0.498, 0.052, 0.550)	4.621	3	2	24	2	6	0.797
4	(0.221, 0.221, 0.438)	7.007	6	5	24	2	3	0.414
5	(0.500, 0.303, 0.697)	6.368	6	5	24	2	5	0.787
6	(0.214, 0.214, 0.431)	7.031	7	6	24	2	3	0.426
7	(0.225, 0.236, 0.461)	6.991	7	6	24	2	3	0.386
8	(0.500, 0.250, 0.750)	6.768	8	7	6	2	5	1.000
9	(0.500, 0.407, 0.593)	7.578	9	8	24	2	5	0.372
10	(0.500, 0.250, 0.750)	8.692	10	9	6	2	5	1.000
11	(0.122, 0.000, 0.122)	8.782	11	10	6	2	1	0.245

The followings are details for Re_4V_{12}. It has 6 topological nodal lines and 13 nodal ring points along high symmetry line as shown in Fig **788**.

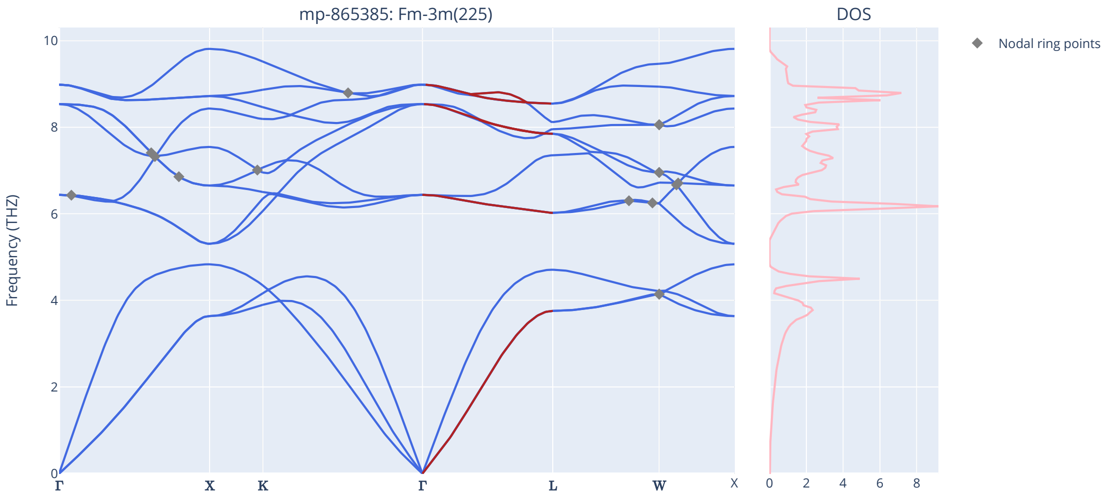

Supplementary Figure 788: The phonon band and DOS of Re4V12.

Supplementary Table 1719: High symmetry lines of mp-865385-Re4V12

	Start	End
0	Γ (0.000, 0.000, 0.000)	X (0.500, 0.000, 0.500)
1	X (0.500, 0.000, 0.500)	U (0.625, 0.250, 0.625)
2	K (0.375, 0.375, 0.750)	Γ (0.000, 0.000, 0.000)
3	Γ (0.000, 0.000, 0.000)	L (0.500, 0.500, 0.500)
4	L (0.500, 0.500, 0.500)	W (0.500, 0.250, 0.750)
5	W (0.500, 0.250, 0.750)	X (0.500, 0.000, 0.500)

Supplementary Table 1720: Nodal ring points along high symmetry lines of mp-865385-Re4V12.

	Coordinates	Frequency	Mode 1	Mode 2	Multi.	Deg.	Path	Ratio
0	(0.500, 0.250, 0.750)	4.143	2	1	6	2	5	1.000
1	(0.500, 0.266, 0.734)	6.251	5	4	24	2	5	0.937
2	(0.500, 0.321, 0.679)	6.301	5	4	24	2	5	0.716
3	(0.041, 0.000, 0.041)	6.430	6	5	6	2	1	0.082
4	(0.499, 0.193, 0.692)	6.668	6	5	24	2	6	0.230
5	(0.398, 0.000, 0.398)	6.857	7	6	6	2	1	0.796
6	(0.500, 0.187, 0.687)	6.711	7	6	12	2	6	0.252
7	(0.322, -0.000, 0.314)	7.322	8	7	24	2	1	0.637
8	(0.612, 0.224, 0.612)	7.011	8	7	12	2	2	0.898
9	(0.500, 0.250, 0.750)	6.954	8	7	6	2	5	1.000
10	(0.306, 0.000, 0.306)	7.409	9	8	6	2	1	0.612
11	(0.500, 0.250, 0.750)	8.057	10	9	6	2	5	1.000
12	(0.174, 0.174, 0.350)	8.793	12	11	24	2	3	0.534

The followings are details for Li_4Zn_{12}. It has 7 topological nodal lines and 6 nodal ring points along high symmetry line as shown in Fig 789.

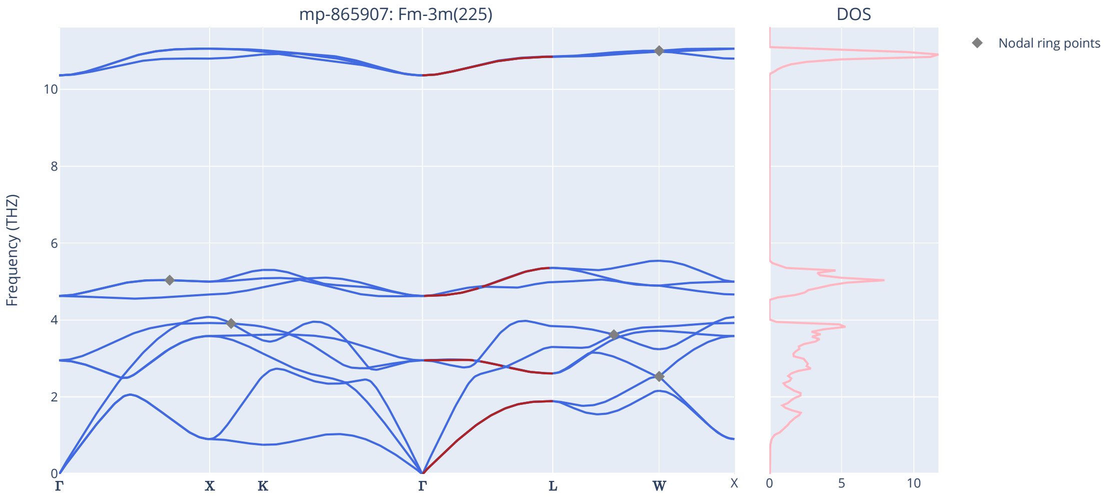

Supplementary Figure 789: The phonon band and DOS of Li4Zn12.

Supplementary Table 1721: High symmetry lines of mp-865907-Li4Zn12

	Start	End
0	Γ (0.000, 0.000, 0.000)	X (0.500, 0.000, 0.500)
1	X (0.500, 0.000, 0.500)	U (0.625, 0.250, 0.625)
2	K (0.375, 0.375, 0.750)	Γ (0.000, 0.000, 0.000)
3	Γ (0.000, 0.000, 0.000)	L (0.500, 0.500, 0.500)
4	L (0.500, 0.500, 0.500)	W (0.500, 0.250, 0.750)
5	W (0.500, 0.250, 0.750)	X (0.500, 0.000, 0.500)

Supplementary Table 1722: Nodal ring points along high symmetry lines of mp-865907-Li4Zn12.

	Coordinates	Frequency	Mode 1	Mode 2	Multi.	Deg.	Path	Ratio
0	(0.500, 0.250, 0.750)	2.531	3	2	6	2	5	1.000
1	(0.551, 0.101, 0.551)	3.909	6	5	12	2	2	0.405
2	(0.500, 0.356, 0.644)	3.616	6	5	24	2	5	0.574
3	(0.367, 0.000, 0.367)	5.035	9	8	6	2	1	0.735
4	(0.500, 0.250, 0.750)	11.003	12	11	6	2	5	1.000

The followings are details for Ac_2N_2. As shown in Fig **790**, it has 24 topological nodal lines, 24 nodal ring points and 1 high degenerate Weyl point whose degeneracy is more than 2 and charge is bigger than 1.

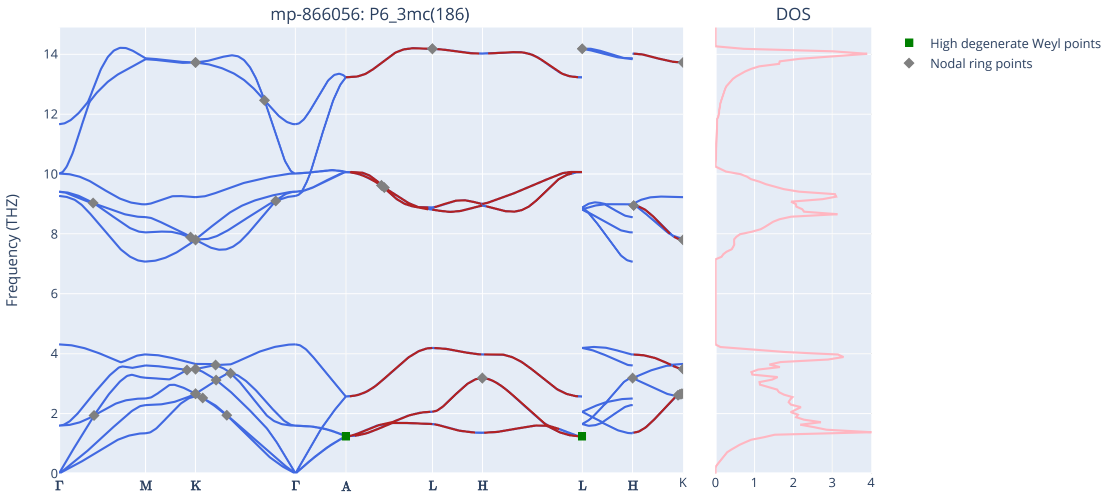

Supplementary Figure 790: The phonon band and DOS of Ac2N2.

Supplementary Table 1723: High symmetry lines of mp-866056-Ac2N2

	Start	End
0	Γ (0.000, 0.000, 0.000)	M (0.500, 0.000, 0.000)
1	M (0.500, 0.000, 0.000)	K (0.333, 0.333, 0.000)
2	K (0.333, 0.333, 0.000)	Γ (0.000, 0.000, 0.000)
3	Γ (0.000, 0.000, 0.000)	A (0.000, 0.000, 0.500)
4	A (0.000, 0.000, 0.500)	L (0.500, 0.000, 0.500)
5	L (0.500, 0.000, 0.500)	H (0.333, 0.333, 0.500)
6	H (0.333, 0.333, 0.500)	A (0.000, 0.000, 0.500)
7	L (0.500, 0.000, 0.500)	M (0.500, 0.000, 0.000)
8	H (0.333, 0.333, 0.500)	K (0.333, 0.333, 0.000)

Supplementary Table 1724: High degenerate Weyl points along high symmetry lines of mp-866056-Ac2N2.

	Coordinates	Frequency	Mode 1	Mode 2	Multi.	Deg.	Path	Ratio
0	(0.000, 0.000, 0.500)	1.238	4	1	1	4	4	1.000

Supplementary Table 1725: Nodal ring points along high symmetry lines of mp-866056-Ac2N2.

	Coordinates	Frequency	Mode 1	Mode 2	Multi.	Deg.	Path	Ratio
0	(0.229, 0.229, -0.000)	1.944	2	1	6	2	3	0.313
1	(0.309, 0.309, 0.000)	2.521	2	1	6	2	3	0.072

Supplementary Table 1725: Nodal ring points along high symmetry lines of mp-866056-Ac2N2.

	Coordinates	Frequency	Mode 1	Mode 2	Multi.	Deg.	Path	Ratio
2	(0.333, 0.333, 0.000)	2.661	3	2	2	2	2	1.000
3	(0.333, 0.333, 0.017)	2.656	3	2	4	2	9	0.967
4	(0.333, 0.333, 0.050)	2.612	3	2	4	2	9	0.901
5	(0.333, 0.333, 0.031)	2.642	3	2	4	2	9	0.939
6	(0.265, 0.265, -0.000)	3.117	4	3	6	2	3	0.206
7	(0.202, 0.000, 0.000)	1.936	4	3	6	2	1	0.404
8	(0.333, 0.333, 0.500)	3.184	4	3	2	2	6	1.000
9	(0.217, 0.217, -0.000)	3.344	5	4	6	2	3	0.350
10	(0.362, 0.276, 0.000)	3.453	5	4	6	2	2	0.829
11	(0.333, 0.333, 0.000)	3.486	5	4	2	2	2	1.000
12	(0.266, 0.266, -0.000)	3.620	6	5	6	2	3	0.201
13	(0.066, 0.066, 0.000)	9.099	8	7	6	2	3	0.803
14	(0.333, 0.333, 0.000)	7.799	8	7	2	2	2	1.000
15	(0.333, 0.333, 0.490)	8.947	8	7	4	2	9	0.020
16	(0.197, -0.000, 0.000)	9.031	9	8	6	2	1	0.393
17	(0.350, 0.300, -0.000)	7.891	9	8	6	2	2	0.901
18	(0.206, -0.000, 0.497)	9.619	9	8	12	2	5	0.411
19	(0.222, -0.000, 0.495)	9.547	9	8	12	2	5	0.445
20	(0.103, 0.103, 0.000)	12.460	12	11	6	2	3	0.690
21	(0.333, 0.333, 0.000)	13.724	12	11	2	2	2	1.000
22	(0.500, 0.000, 0.500)	14.182	12	11	3	2	5	1.000

The followings are details for Os_4V_{12}. It has 7 topological nodal lines and 12 nodal ring points along high symmetry line as shown in Fig **791**.

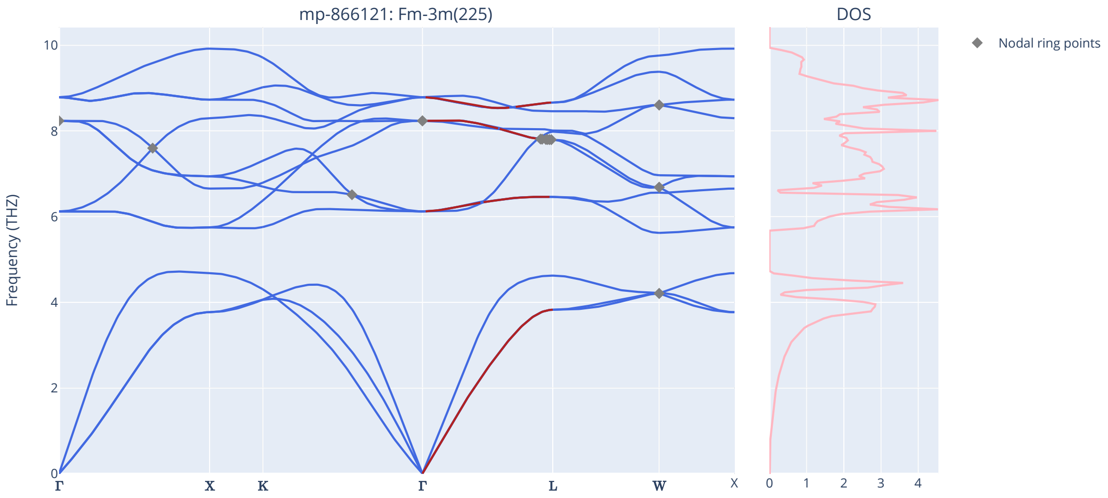

Supplementary Figure 791: The phonon band and DOS of Os4V12.

Supplementary Table 1726: High symmetry lines of mp-866121-Os4V12

	Start	End
0	Γ (0.000, 0.000, 0.000)	X (0.500, 0.000, 0.500)
1	X (0.500, 0.000, 0.500)	U (0.625, 0.250, 0.625)
2	K (0.375, 0.375, 0.750)	Γ (0.000, 0.000, 0.000)
3	Γ (0.000, 0.000, 0.000)	L (0.500, 0.500, 0.500)
4	L (0.500, 0.500, 0.500)	W (0.500, 0.250, 0.750)
5	W (0.500, 0.250, 0.750)	X (0.500, 0.000, 0.500)

Supplementary Table 1727: Nodal ring points along high symmetry lines of mp-866121-Os4V12.

	Coordinates	Frequency	Mode 1	Mode 2	Multi.	Deg.	Path	Ratio
0	(0.500, 0.250, 0.750)	4.208	2	1	6	2	5	1.000
1	(0.168, 0.168, 0.328)	6.511	6	5	24	2	3	0.559
2	(0.495, 0.495, 0.495)	7.797	7	6	8	2	4	0.989
3	(0.453, 0.453, 0.453)	7.811	7	6	8	2	4	0.906
4	(0.487, 0.487, 0.487)	7.797	7	6	8	2	4	0.974
5	(0.479, 0.479, 0.479)	7.799	7	6	8	2	4	0.959
6	(0.474, 0.474, 0.474)	7.801	7	6	8	2	4	0.947
7	(0.459, 0.459, 0.459)	7.807	7	6	8	2	4	0.918
8	(0.500, 0.250, 0.750)	6.686	7	6	6	2	5	1.000
9	(0.000, 0.000, 0.000)	8.235	8	7	1	3	1	0.000
10	(0.311, -0.001, 0.311)	7.599	9	8	24	2	1	0.622
11	(0.500, 0.250, 0.750)	8.605	10	9	6	2	5	1.000

The followings are details for $Li_{12}Ge_4$. It has 6 topological nodal lines and 10 nodal ring points along high symmetry line as shown in Fig 792.

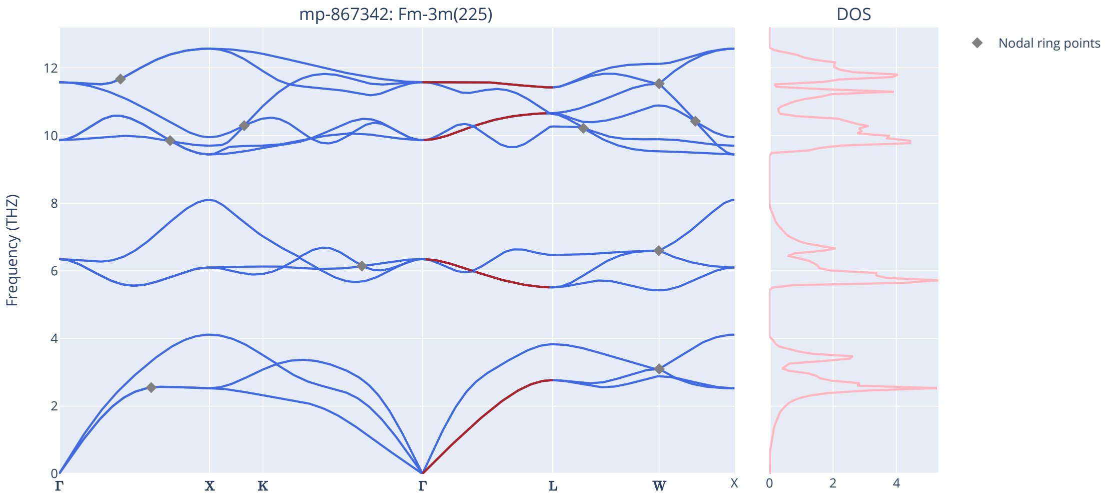

Supplementary Figure 792: The phonon band and DOS of Li12Ge4.

Supplementary Table 1728: High symmetry lines of mp-867342-Li12Ge4

	Start	End
0	Γ (0.000, 0.000, 0.000)	X (0.500, 0.000, 0.500)
1	X (0.500, 0.000, 0.500)	U (0.625, 0.250, 0.625)
2	K (0.375, 0.375, 0.750)	Γ (0.000, 0.000, 0.000)
3	Γ (0.000, 0.000, 0.000)	L (0.500, 0.500, 0.500)
4	L (0.500, 0.500, 0.500)	W (0.500, 0.250, 0.750)
5	W (0.500, 0.250, 0.750)	X (0.500, 0.000, 0.500)

Supplementary Table 1729: Nodal ring points along high symmetry lines of mp-867342-Li12Ge4.

	Coordinates	Frequency	Mode 1	Mode 2	Multi.	Deg.	Path	Ratio
0	(0.306, 0.000, 0.306)	2.549	2	1	6	2	1	0.612
1	(0.500, 0.250, 0.750)	3.098	3	2	6	2	5	1.000
2	(0.145, 0.145, 0.281)	6.142	6	5	24	2	3	0.621
3	(0.500, 0.251, 0.749)	6.596	6	5	24	2	5	0.996
4	(0.500, 0.428, 0.572)	10.216	8	7	24	2	5	0.287
5	(0.365, -0.000, 0.373)	9.851	9	8	24	2	1	0.738
6	(0.581, 0.162, 0.581)	10.292	10	9	12	2	2	0.650
7	(0.499, 0.129, 0.630)	10.422	10	9	24	2	6	0.481
8	(0.500, 0.250, 0.750)	11.534	11	10	6	2	5	1.000
9	(0.204, 0.000, 0.204)	11.671	12	11	6	2	1	0.408

The followings are details for NpSn$_3$. It has 6 topological nodal lines and 15 nodal ring points along high symmetry line as shown in Fig **793**.

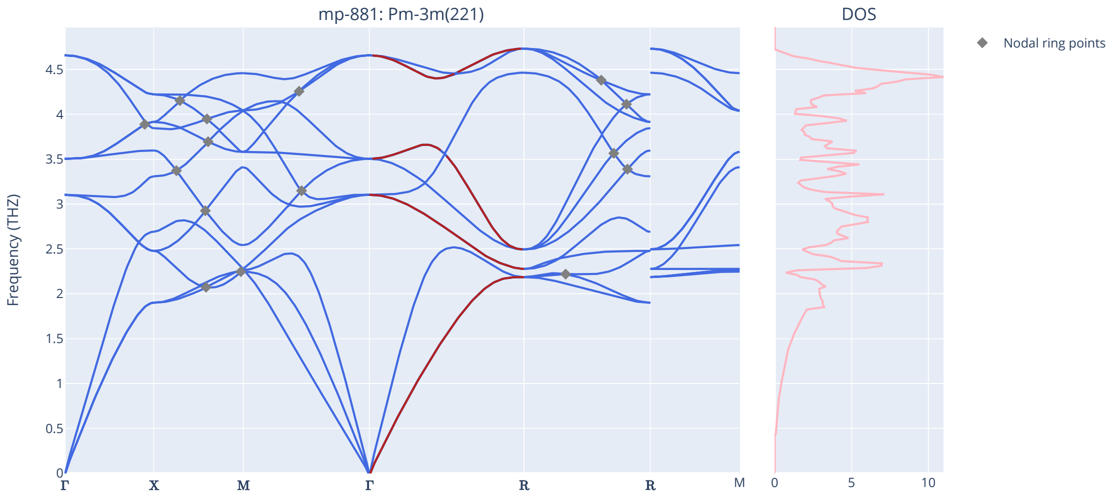

Supplementary Figure 793: The phonon band and DOS of NpSn3.

Supplementary Table 1730: High symmetry lines of mp-881-NpSn3

	Start	End
0	Γ (0.000, 0.000, 0.000)	X (0.000, 0.500, 0.000)
1	X (0.000, 0.500, 0.000)	M (0.500, 0.500, 0.000)
2	M (0.500, 0.500, 0.000)	Γ (0.000, 0.000, 0.000)
3	Γ (0.000, 0.000, 0.000)	R (0.500, 0.500, 0.500)
4	R (0.500, 0.500, 0.500)	X (0.000, 0.500, 0.000)
5	R (0.500, 0.500, 0.500)	M (0.500, 0.500, 0.000)

Supplementary Table 1731: Nodal ring points along high symmetry lines of mp-881-NpSn3.

	Coordinates	Frequency	Mode 1	Mode 2	Multi.	Deg.	Path	Ratio
0	(0.292, 0.500, 0.000)	2.073	2	1	12	2	2	0.585
1	(0.487, 0.500, 0.000)	2.246	2	1	12	2	2	0.974
2	(0.335, 0.500, 0.335)	2.219	3	2	12	2	5	0.329
3	(0.288, 0.500, 0.000)	2.926	6	5	12	2	2	0.577
4	(0.127, 0.500, -0.000)	3.372	7	6	12	2	2	0.253
5	(0.268, 0.268, 0.000)	3.148	7	6	12	2	3	0.463
6	(0.090, 0.500, 0.090)	3.389	7	6	12	2	5	0.819
7	(0.304, 0.500, 0.000)	3.695	8	7	12	2	2	0.608
8	(0.145, 0.500, 0.145)	3.566	8	7	12	2	5	0.711
9	(0.000, 0.449, 0.000)	3.888	9	8	6	2	1	0.898
10	(0.297, 0.500, 0.000)	3.948	10	9	12	2	2	0.595
11	(0.146, 0.500, -0.000)	4.153	11	10	12	2	2	0.292
12	(0.278, 0.278, 0.000)	4.256	11	10	12	2	3	0.443
13	(0.094, 0.500, 0.094)	4.113	11	10	12	2	5	0.811
14	(0.194, 0.500, 0.194)	4.381	12	11	12	2	5	0.611

Supplementary Table 1731: Nodal ring points along high symmetry lines of mp-881-NpSn3.

Coordinates	Frequency	Mode 1	Mode 2	Multi.	Deg.	Path	Ratio

The followings are details for Ga_2As_2. As shown in Fig **794**, it has 22 topological nodal lines, 21 nodal ring points and 2 high degenerate Weyl points whose degeneracy are more than 2 and charges are bigger than 1.

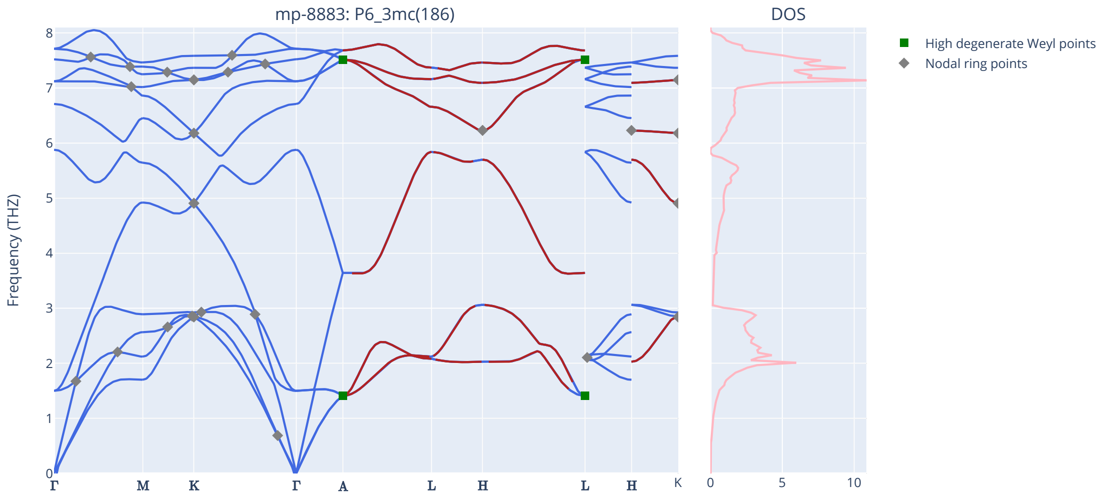

Supplementary Figure 794: The phonon band and DOS of Ga2As2.

Supplementary Table 1732: High symmetry lines of mp-8883-Ga2As2

	Start	End
0	Γ (0.000, 0.000, 0.000)	M (0.500, 0.000, 0.000)
1	M (0.500, 0.000, 0.000)	K (0.333, 0.333, 0.000)
2	K (0.333, 0.333, 0.000)	Γ (0.000, 0.000, 0.000)
3	Γ (0.000, 0.000, 0.000)	A (0.000, 0.000, 0.500)
4	A (0.000, 0.000, 0.500)	L (0.500, 0.000, 0.500)
5	L (0.500, 0.000, 0.500)	H (0.333, 0.333, 0.500)
6	H (0.333, 0.333, 0.500)	A (0.000, 0.000, 0.500)
7	L (0.500, 0.000, 0.500)	M (0.500, 0.000, 0.000)
8	H (0.333, 0.333, 0.500)	K (0.333, 0.333, 0.000)

Supplementary Table 1733: High degenerate Weyl points along high symmetry lines of mp-8883-Ga2As2.

	Coordinates	Frequency	Mode 1	Mode 2	Multi.	Deg.	Path	Ratio
0	(0.000, 0.000, 0.500)	1.409	4	1	1	4	4	1.000
1	(0.000, 0.000, 0.500)	7.516	10	7	1	4	4	1.000

Supplementary Table 1734: Nodal ring points along high symmetry lines of mp-8883-Ga2As2.

	Coordinates	Frequency	Mode 1	Mode 2	Multi.	Deg.	Path	Ratio
0	(0.061, 0.061, 0.000)	0.689	2	1	6	2	3	0.818

Supplementary Table 1734: Nodal ring points along high symmetry lines of mp-8883-Ga2As2.

	Coordinates	Frequency	Mode 1	Mode 2	Multi.	Deg.	Path	Ratio
1	(0.333, 0.333, 0.000)	2.843	2	1	2	2	2	1.000
2	(0.419, 0.162, -0.000)	2.660	3	2	6	2	2	0.487
3	(0.339, 0.322, -0.000)	2.858	3	2	6	2	2	0.966
4	(0.358, 0.000, 0.000)	2.206	3	2	6	2	1	0.716
5	(0.499, -0.000, 0.476)	2.106	3	2	12	2	8	0.049
6	(0.309, 0.309, 0.000)	2.930	4	3	6	2	3	0.073
7	(0.123, -0.000, -0.000)	1.675	4	3	6	2	1	0.246
8	(0.134, 0.134, -0.000)	2.890	5	4	6	2	3	0.598
9	(0.333, 0.333, 0.000)	4.907	6	5	2	2	2	1.000
10	(0.333, 0.333, 0.000)	6.179	8	7	2	2	2	1.000
11	(0.333, 0.333, 0.500)	6.230	8	7	2	2	6	1.000
12	(0.436, 0.000, -0.000)	7.024	9	8	6	2	1	0.871
13	(0.222, 0.222, -0.000)	7.289	10	9	6	2	3	0.334
14	(0.333, 0.333, 0.000)	7.148	10	9	2	2	2	1.000
15	(0.101, 0.101, 0.000)	7.436	10	9	6	2	3	0.697
16	(0.420, 0.160, -0.000)	7.288	11	10	6	2	2	0.479
17	(0.428, -0.000, 0.000)	7.387	11	10	6	2	1	0.856
18	(0.207, -0.000, -0.000)	7.563	11	10	6	2	1	0.413
19	(0.209, 0.209, -0.000)	7.594	12	11	6	2	3	0.373

The followings are details for Ga$_3$. It has 8 topological nodal lines and 13 nodal ring points along high symmetry line as shown in Fig **795**.

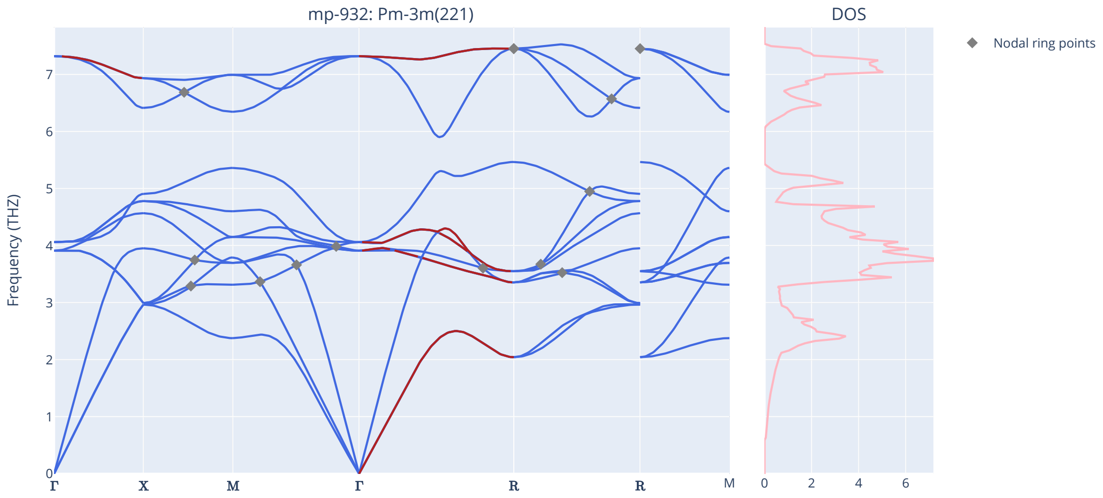

Supplementary Figure 795: The phonon band and DOS of Ga3Sc.

Supplementary Table 1735: High symmetry lines of mp-932-Ga3Sc

	Start	End
0	Γ (0.000, 0.000, 0.000)	X (0.000, 0.500, 0.000)
1	X (0.000, 0.500, 0.000)	M (0.500, 0.500, 0.000)
2	M (0.500, 0.500, 0.000)	Γ (0.000, 0.000, 0.000)
3	Γ (0.000, 0.000, 0.000)	R (0.500, 0.500, 0.500)
4	R (0.500, 0.500, 0.500)	X (0.000, 0.500, 0.000)
5	R (0.500, 0.500, 0.500)	M (0.500, 0.500, 0.000)

Supplementary Table 1736: Nodal ring points along high symmetry lines of mp-932-Ga3Sc.

	Coordinates	Frequency	Mode 1	Mode 2	Multi.	Deg.	Path	Ratio
0	(0.266, 0.500, 0.000)	3.291	3	2	12	2	2	0.532
1	(0.392, 0.392, 0.000)	3.367	3	2	12	2	3	0.216
2	(0.247, 0.247, -0.000)	3.662	4	3	12	2	3	0.505
3	(0.308, 0.500, 0.308)	3.526	4	3	12	2	5	0.383
4	(0.287, 0.500, -0.000)	3.748	5	4	12	2	2	0.574
5	(0.401, 0.401, 0.401)	3.605	6	5	8	2	4	0.801
6	(0.090, 0.090, 0.000)	3.987	7	6	12	2	3	0.819
7	(0.393, 0.500, 0.393)	3.674	8	7	12	2	5	0.214
8	(0.200, 0.500, 0.200)	4.950	9	8	12	2	5	0.601
9	(0.228, 0.500, -0.000)	6.688	11	10	12	2	2	0.456
10	(0.113, 0.500, 0.113)	6.573	11	10	12	2	5	0.774
11	(0.500, 0.500, 0.500)	7.453	12	11	1	3	4	1.000

The followings are details for Rh$_3$. It has 6 topological nodal lines and 21 nodal ring points along high symmetry line as shown in Fig **796**.

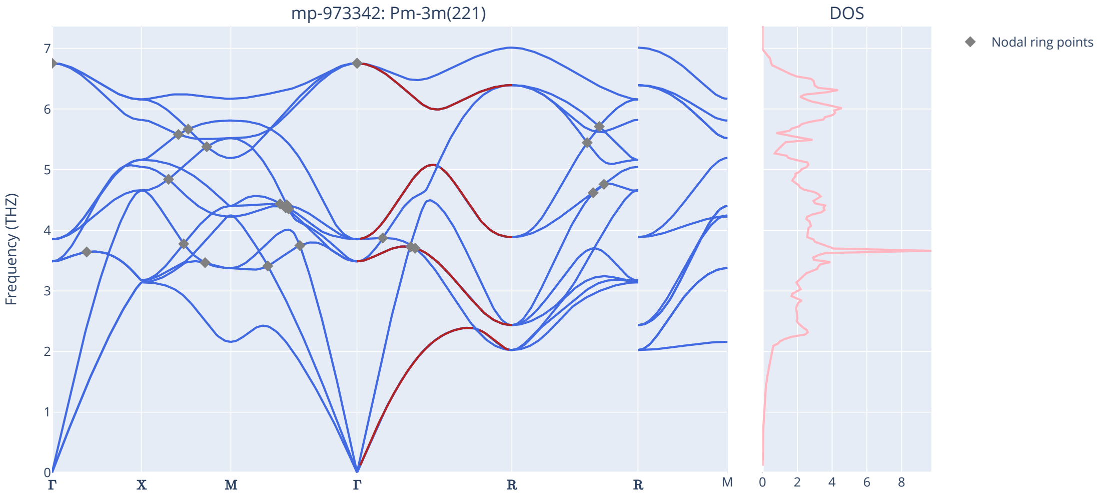

Supplementary Figure 796: The phonon band and DOS of Rh3Sb.

Supplementary Table 1737: High symmetry lines of mp-973342-Rh3Sb

	Start	End
0	Γ (0.000, 0.000, 0.000)	X (0.000, 0.500, 0.000)
1	X (0.000, 0.500, 0.000)	M (0.500, 0.500, 0.000)
2	M (0.500, 0.500, 0.000)	Γ (0.000, 0.000, 0.000)
3	Γ (0.000, 0.000, 0.000)	R (0.500, 0.500, 0.500)
4	R (0.500, 0.500, 0.500)	X (0.000, 0.500, 0.000)
5	R (0.500, 0.500, 0.500)	M (0.500, 0.500, 0.000)

Supplementary Table 1738: Nodal ring points along high symmetry lines of mp-973342-Rh3Sb.

	Coordinates	Frequency	Mode 1	Mode 2	Multi.	Deg.	Path	Ratio
0	(0.357, 0.500, 0.000)	3.465	3	2	12	2	2	0.713
1	(0.353, 0.353, -0.000)	3.410	3	2	12	2	3	0.294
2	(0.226, 0.226, -0.000)	3.747	4	3	12	2	3	0.547
3	(0.173, 0.173, 0.173)	3.726	4	3	8	2	4	0.347
4	(0.191, 0.181, 0.191)	3.703	4	3	24	2	4	0.375
5	(0.000, 0.194, 0.000)	3.642	5	4	6	2	1	0.388
6	(0.237, 0.500, -0.000)	3.775	5	4	12	2	2	0.473
7	(0.282, 0.282, 0.002)	4.370	6	5	24	2	3	0.435
8	(0.152, 0.500, 0.000)	4.841	7	6	12	2	2	0.304
9	(0.271, 0.271, 0.000)	4.353	7	6	12	2	3	0.459
10	(0.305, 0.305, -0.000)	4.434	7	6	12	2	3	0.391
11	(0.083, 0.083, 0.083)	3.872	7	6	8	2	4	0.166
12	(0.135, 0.500, 0.135)	4.759	7	6	12	2	5	0.730
13	(0.178, 0.500, 0.178)	4.618	7	6	12	2	5	0.644

Supplementary Table 1738: Nodal ring points along high symmetry lines of mp-973342-Rh3Sb.

	Coordinates	Frequency	Mode 1	Mode 2	Multi.	Deg.	Path	Ratio
14	(0.278, 0.278, -0.000)	4.419	8	7	12	2	3	0.444
15	(0.366, 0.500, 0.000)	5.378	9	8	12	2	2	0.732
16	(0.201, 0.500, 0.201)	5.445	9	8	12	2	5	0.597
17	(0.207, 0.500, 0.000)	5.580	10	9	12	2	2	0.415
18	(0.262, 0.500, 0.000)	5.668	11	10	12	2	2	0.523
19	(0.154, 0.500, 0.154)	5.711	11	10	12	2	5	0.693
20	(0.000, 0.000, 0.000)	6.755	12	11	1	3	1	0.000

The followings are details for Hg_2S_2. As shown in Fig 797, it has 22 topological nodal lines, 32 nodal ring points and 2 high degenerate Weyl points whose degeneracy are more than 2 and charges are bigger than 1.

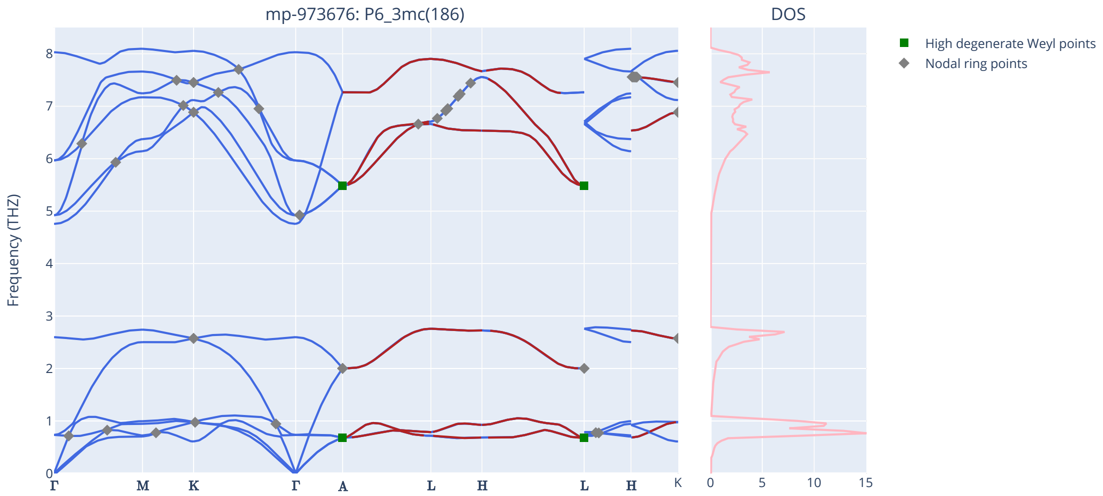

Supplementary Figure 797: The phonon band and DOS of Hg2S2.

Supplementary Table 1739: High symmetry lines of mp-973676-Hg2S2

	Start	End
0	Γ (0.000, 0.000, 0.000)	M (0.500, 0.000, 0.000)
1	M (0.500, 0.000, 0.000)	K (0.333, 0.333, 0.000)
2	K (0.333, 0.333, 0.000)	Γ (0.000, 0.000, 0.000)
3	Γ (0.000, 0.000, 0.000)	A (0.000, 0.000, 0.500)
4	A (0.000, 0.000, 0.500)	L (0.500, 0.000, 0.500)
5	L (0.500, 0.000, 0.500)	H (0.333, 0.333, 0.500)
6	H (0.333, 0.333, 0.500)	A (0.000, 0.000, 0.500)
7	L (0.500, 0.000, 0.500)	M (0.500, 0.000, 0.000)
8	H (0.333, 0.333, 0.500)	K (0.333, 0.333, 0.000)

Supplementary Table 1740: High degenerate Weyl points along high symmetry lines of mp-973676-Hg2S2.

	Coordinates	Frequency	Mode 1	Mode 2	Multi.	Deg.	Path	Ratio
0	(0.000, 0.000, 0.500)	0.679	4	1	1	4	4	1.000
1	(0.000, 0.000, 0.500)	5.487	10	7	1	4	4	1.000

Supplementary Table 1741: Nodal ring points along high symmetry lines of mp-973676-Hg2S2.

	Coordinates	Frequency	Mode 1	Mode 2	Multi.	Deg.	Path	Ratio
0	(0.456, 0.088, -0.000)	0.775	2	1	6	2	2	0.264

Supplementary Table 1741: Nodal ring points along high symmetry lines of mp-973676-Hg2S2.

	Coordinates	Frequency	Mode 1	Mode 2	Multi.	Deg.	Path	Ratio
1	(0.300, 0.000, 0.000)	0.825	3	2	6	2	1	0.601
2	(0.328, 0.328, 0.000)	0.975	3	2	6	2	3	0.015
3	(0.498, 0.004, 0.345)	0.773	3	2	12	2	8	0.311
4	(0.499, 0.003, 0.376)	0.777	3	2	12	2	8	0.247
5	(0.083, 0.000, -0.000)	0.715	4	3	6	2	1	0.165
6	(0.065, 0.065, 0.000)	0.944	5	4	6	2	3	0.806
7	(0.333, 0.333, 0.000)	2.574	6	5	2	2	2	1.000
8	(0.000, 0.000, 0.500)	2.002	6	5	1	2	4	1.000
9	(0.348, -0.000, -0.000)	5.930	8	7	6	2	1	0.697
10	(0.333, 0.333, 0.000)	6.883	8	7	2	2	2	1.000
11	(0.367, 0.266, -0.000)	7.013	9	8	6	2	2	0.799
12	(0.000, 0.000, 0.041)	4.925	9	8	2	2	4	0.082
13	(0.428, 0.000, 0.499)	6.657	9	8	12	2	5	0.856
14	(0.157, -0.000, -0.000)	6.285	10	9	6	2	1	0.313
15	(0.253, 0.253, 0.000)	7.260	10	9	6	2	3	0.242
16	(0.480, 0.041, 0.500)	6.766	10	9	6	2	6	0.122
17	(0.452, 0.095, 0.500)	6.910	10	9	6	2	6	0.286
18	(0.446, 0.109, 0.500)	6.953	10	9	6	2	6	0.327
19	(0.412, 0.177, 0.500)	7.185	10	9	6	2	6	0.531
20	(0.405, 0.190, 0.500)	7.232	10	9	6	2	6	0.571
21	(0.371, 0.259, 0.500)	7.439	10	9	6	2	6	0.776
22	(0.333, 0.333, 0.490)	7.555	10	9	4	2	9	0.020
23	(0.333, 0.333, 0.480)	7.555	10	9	4	2	9	0.041
24	(0.333, 0.333, 0.469)	7.555	10	9	4	2	9	0.061
25	(0.333, 0.333, 0.459)	7.554	10	9	4	2	9	0.082
26	(0.333, 0.333, 0.449)	7.554	10	9	4	2	9	0.102
27	(0.333, 0.333, 0.439)	7.553	10	9	4	2	9	0.122
28	(0.120, 0.120, -0.000)	6.951	11	10	6	2	3	0.639
29	(0.389, 0.223, -0.000)	7.492	11	10	6	2	2	0.668
30	(0.333, 0.333, 0.000)	7.450	11	10	2	2	2	1.000
31	(0.186, 0.186, 0.000)	7.699	12	11	6	2	3	0.441

The followings are details for Au$_3$. It has 8 topological nodal lines and 22 nodal ring points along high symmetry line as shown in Fig **798**.

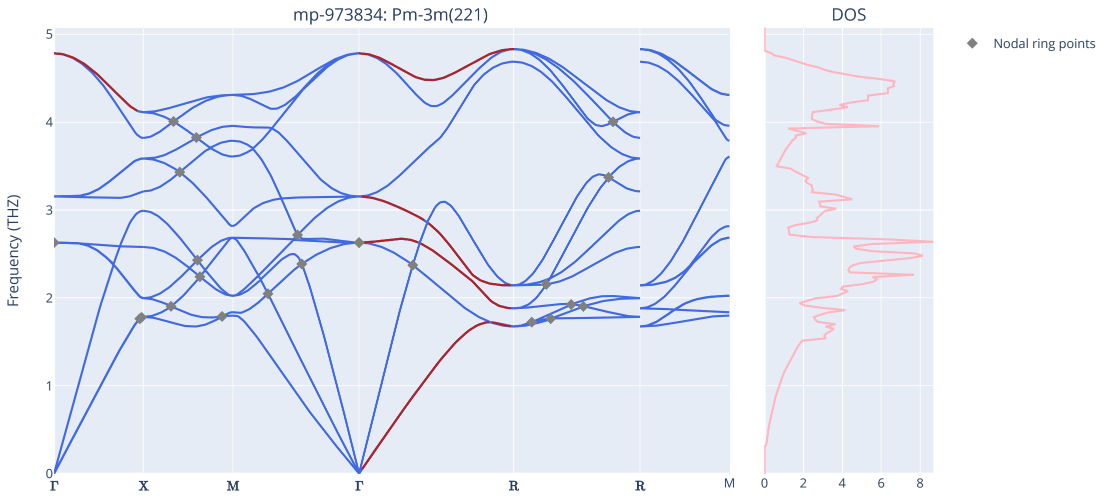

Supplementary Figure 798: The phonon band and DOS of Au3Pd.

Supplementary Table 1742: High symmetry lines of mp-973834-Au3Pd

	Start	End
0	Γ (0.000, 0.000, 0.000)	X (0.000, 0.500, 0.000)
1	X (0.000, 0.500, 0.000)	M (0.500, 0.500, 0.000)
2	M (0.500, 0.500, 0.000)	Γ (0.000, 0.000, 0.000)
3	Γ (0.000, 0.000, 0.000)	R (0.500, 0.500, 0.500)
4	R (0.500, 0.500, 0.500)	X (0.000, 0.500, 0.000)
5	R (0.500, 0.500, 0.500)	M (0.500, 0.500, 0.000)

Supplementary Table 1743: Nodal ring points along high symmetry lines of mp-973834-Au3Pd.

	Coordinates	Frequency	Mode 1	Mode 2	Multi.	Deg.	Path	Ratio
0	(0.000, 0.480, 0.000)	1.764	2	1	6	2	1	0.959
1	(0.000, 0.490, 0.000)	1.779	2	1	6	2	1	0.980
2	(0.440, 0.500, 0.000)	1.789	2	1	12	2	2	0.880
3	(0.354, 0.500, 0.354)	1.764	2	1	12	2	5	0.292
4	(0.155, 0.500, 0.000)	1.904	3	2	12	2	2	0.309
5	(0.360, 0.360, -0.000)	2.047	3	2	12	2	3	0.279
6	(0.429, 0.500, 0.429)	1.724	3	2	12	2	5	0.143
7	(0.225, 0.497, 0.225)	1.903	3	2	24	2	5	0.551
8	(0.316, 0.500, -0.000)	2.241	4	3	12	2	2	0.633
9	(0.227, 0.227, 0.000)	2.386	4	3	12	2	3	0.547
10	(0.174, 0.174, 0.174)	2.373	4	3	8	2	4	0.347
11	(0.273, 0.500, 0.273)	1.925	4	3	12	2	5	0.454
12	(0.000, 0.000, 0.000)	2.629	6	5	1	3	1	0.000
13	(0.302, 0.500, -0.000)	2.430	6	5	12	2	2	0.605

Supplementary Table 1743: Nodal ring points along high symmetry lines of mp-973834-Au3Pd.

	Coordinates	Frequency	Mode 1	Mode 2	Multi.	Deg.	Path	Ratio
14	(0.371, 0.500, 0.371)	2.154	6	5	12	2	5	0.257
15	(0.243, 0.243, -0.006)	2.716	7	6	24	2	3	0.513
16	(0.203, 0.500, -0.000)	3.431	8	7	12	2	2	0.407
17	(0.125, 0.500, 0.125)	3.372	8	7	12	2	5	0.751
18	(0.296, 0.500, -0.000)	3.824	10	9	12	2	2	0.593
19	(0.169, 0.500, 0.000)	4.006	11	10	12	2	2	0.338
20	(0.107, 0.500, 0.107)	4.003	11	10	12	2	5	0.786

The followings are details for Al3. It has 4 topological nodal lines and 15 nodal ring points along high symmetry line as shown in Fig 799.

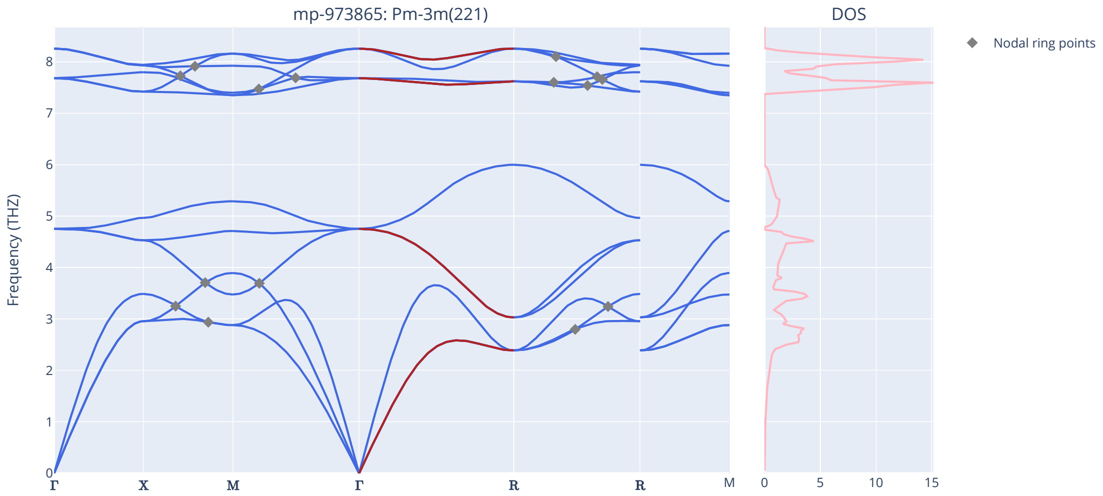

Supplementary Figure 799: The phonon band and DOS of Al3Pa.

Supplementary Table 1744: High symmetry lines of mp-973865-Al3Pa

	Start	End
0	Γ (0.000, 0.000, 0.000)	X (0.000, 0.500, 0.000)
1	X (0.000, 0.500, 0.000)	M (0.500, 0.500, 0.000)
2	M (0.500, 0.500, 0.000)	Γ (0.000, 0.000, 0.000)
3	Γ (0.000, 0.000, 0.000)	R (0.500, 0.500, 0.500)
4	R (0.500, 0.500, 0.500)	X (0.000, 0.500, 0.000)
5	R (0.500, 0.500, 0.500)	M (0.500, 0.500, 0.000)

Supplementary Table 1745: Nodal ring points along high symmetry lines of mp-973865-Al3Pa.

	Coordinates	Frequency	Mode 1	Mode 2	Multi.	Deg.	Path	Ratio
0	(0.363, 0.500, 0.000)	2.937	2	1	12	2	2	0.726
1	(0.257, 0.500, 0.257)	2.797	2	1	12	2	5	0.486
2	(0.180, 0.500, -0.000)	3.248	3	2	12	2	2	0.360
3	(0.127, 0.500, 0.127)	3.241	3	2	12	2	5	0.746
4	(0.346, 0.500, -0.000)	3.705	4	3	12	2	2	0.691
5	(0.395, 0.395, -0.000)	3.693	4	3	12	2	3	0.211
6	(0.208, 0.500, 0.208)	7.538	8	7	12	2	5	0.584
7	(0.397, 0.397, 0.000)	7.469	9	8	12	2	3	0.207
8	(0.251, 0.251, -0.000)	7.687	9	8	12	2	3	0.498
9	(0.345, 0.500, 0.338)	7.600	9	8	24	2	5	0.317
10	(0.149, 0.500, 0.149)	7.655	9	8	12	2	5	0.701
11	(0.207, 0.500, -0.000)	7.725	10	9	12	2	2	0.414
12	(0.170, 0.500, 0.170)	7.709	10	9	12	2	5	0.660
13	(0.289, 0.500, 0.000)	7.912	11	10	12	2	2	0.577
14	(0.333, 0.500, 0.333)	8.100	11	10	12	2	5	0.334

Supplementary Table 1745: Nodal ring points along high symmetry lines of mp-973865-Al3Pa.

Coordinates	Frequency	Mode 1	Mode 2	Multi.	Deg.	Path	Ratio

The followings are details for Fe$_3$. It has 6 topological nodal lines and 25 nodal ring points along high symmetry line as shown in Fig 800.

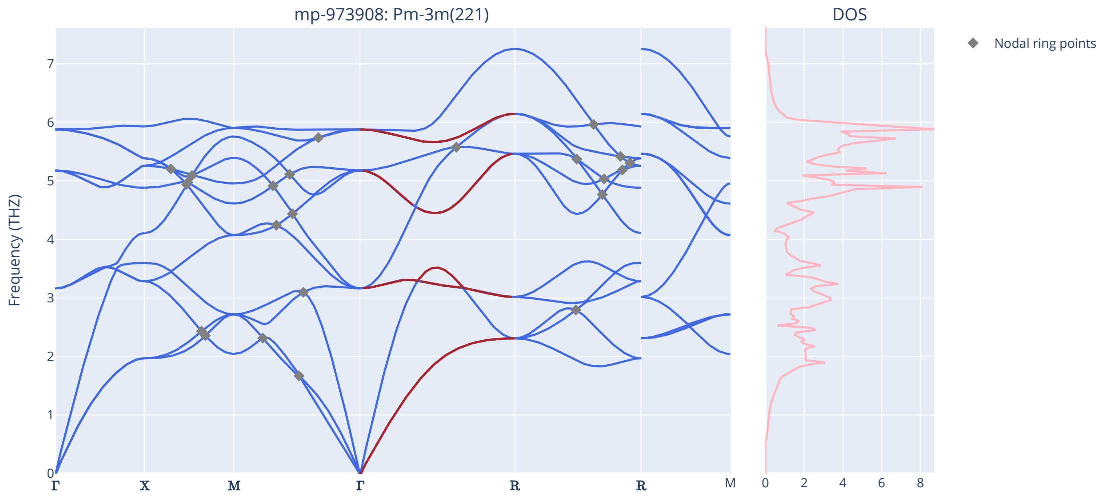

Supplementary Figure 800: The phonon band and DOS of Fe3Pa.

Supplementary Table 1746: High symmetry lines of mp-973908-Fe3Pa

	Start	End
0	Γ (0.000, 0.000, 0.000)	X (0.000, 0.500, 0.000)
1	X (0.000, 0.500, 0.000)	M (0.500, 0.500, 0.000)
2	M (0.500, 0.500, 0.000)	Γ (0.000, 0.000, 0.000)
3	Γ (0.000, 0.000, 0.000)	R (0.500, 0.500, 0.500)
4	R (0.500, 0.500, 0.500)	X (0.000, 0.500, 0.000)
5	R (0.500, 0.500, 0.500)	M (0.500, 0.500, 0.000)

Supplementary Table 1747: Nodal ring points along high symmetry lines of mp-973908-Fe3Pa.

	Coordinates	Frequency	Mode 1	Mode 2	Multi.	Deg.	Path	Ratio
0	(0.340, 0.500, 0.000)	2.356	2	1	12	2	2	0.681
1	(0.242, 0.242, -0.000)	1.667	2	1	12	2	3	0.517
2	(0.385, 0.385, -0.000)	2.312	2	1	12	2	3	0.229
3	(0.319, 0.500, 0.001)	2.430	3	2	24	2	2	0.638
4	(0.258, 0.500, 0.258)	2.794	3	2	12	2	5	0.484
5	(0.225, 0.225, 0.000)	3.095	4	3	12	2	3	0.551
6	(0.332, 0.332, 0.000)	4.241	6	5	12	2	3	0.336
7	(0.235, 0.500, -0.000)	4.938	7	6	12	2	2	0.471
8	(0.268, 0.268, -0.000)	4.436	7	6	12	2	3	0.464
9	(0.153, 0.500, 0.153)	4.766	7	6	12	2	5	0.693
10	(0.242, 0.500, -0.000)	4.972	8	7	12	2	2	0.484
11	(0.345, 0.345, 0.000)	4.915	8	7	12	2	3	0.310
12	(0.147, 0.500, 0.000)	5.203	9	8	12	2	2	0.295
13	(0.266, 0.500, 0.000)	5.091	9	8	12	2	2	0.531

Supplementary Table 1747: Nodal ring points along high symmetry lines of mp-973908-Fe3Pa.

	Coordinates	Frequency	Mode 1	Mode 2	Multi.	Deg.	Path	Ratio
14	(0.279, 0.279, 0.000)	5.111	9	8	12	2	3	0.442
15	(0.311, 0.311, 0.311)	5.575	9	8	8	2	4	0.622
16	(0.074, 0.500, 0.074)	5.186	9	8	12	2	5	0.852
17	(0.146, 0.500, 0.146)	5.032	9	8	12	2	5	0.707
18	(0.254, 0.500, 0.254)	5.370	9	8	12	2	5	0.492
19	(0.044, 0.500, 0.044)	5.307	10	9	12	2	5	0.912
20	(0.165, 0.165, -0.000)	5.737	11	10	12	2	3	0.670
21	(0.082, 0.500, 0.082)	5.417	11	10	12	2	5	0.837
22	(0.190, 0.500, 0.186)	5.964	12	11	24	2	5	0.624

The followings are details for PtRh3. It has 6 topological nodal lines and 17 nodal ring points along high symmetry line as shown in Fig **801**.

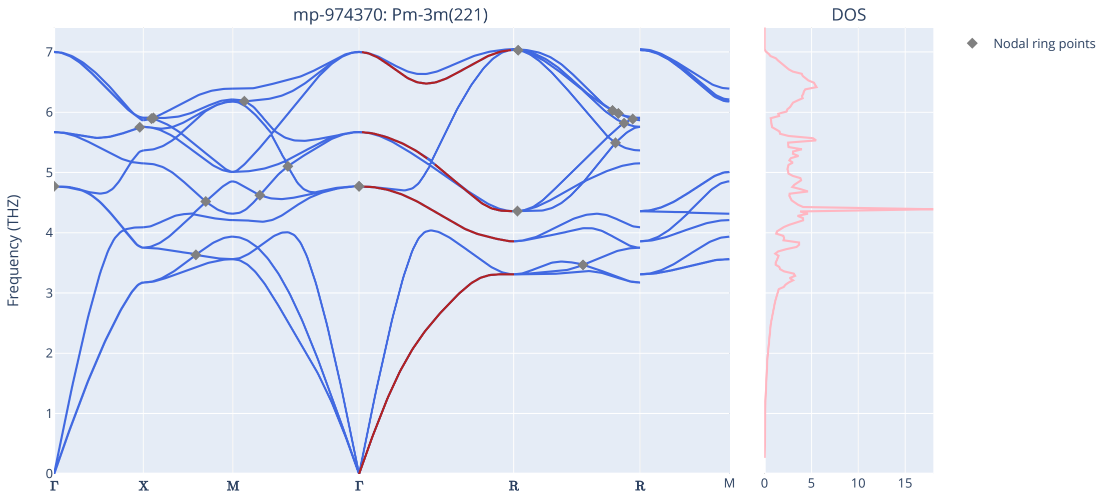

Supplementary Figure 801: The phonon band and DOS of PtRh3.

Supplementary Table 1748: High symmetry lines of mp-974370-PtRh3

	Start	End
0	Γ (0.000, 0.000, 0.000)	X (0.000, 0.500, 0.000)
1	X (0.000, 0.500, 0.000)	M (0.500, 0.500, 0.000)
2	M (0.500, 0.500, 0.000)	Γ (0.000, 0.000, 0.000)
3	Γ (0.000, 0.000, 0.000)	R (0.500, 0.500, 0.500)
4	R (0.500, 0.500, 0.500)	X (0.000, 0.500, 0.000)
5	R (0.500, 0.500, 0.500)	M (0.500, 0.500, 0.000)

Supplementary Table 1749: Nodal ring points along high symmetry lines of mp-974370-PtRh3.

	Coordinates	Frequency	Mode 1	Mode 2	Multi.	Deg.	Path	Ratio
0	(0.294, 0.500, -0.000)	3.633	3	2	12	2	2	0.588
1	(0.226, 0.500, 0.226)	3.466	3	2	12	2	5	0.548
2	(0.000, 0.000, 0.000)	4.770	6	5	1	3	1	0.000
3	(0.349, 0.500, -0.000)	4.518	6	5	12	2	2	0.698
4	(0.393, 0.393, 0.000)	4.622	6	5	12	2	3	0.215
5	(0.282, 0.282, -0.000)	5.102	7	6	12	2	3	0.436
6	(0.486, 0.500, 0.486)	4.359	8	7	12	2	5	0.029
7	(0.097, 0.500, 0.097)	5.492	8	7	12	2	5	0.805
8	(0.000, 0.480, 0.000)	5.750	9	8	6	2	1	0.959
9	(0.483, 0.500, 0.483)	7.029	10	9	12	2	5	0.035
10	(0.063, 0.500, 0.063)	5.817	10	9	12	2	5	0.873
11	(0.454, 0.454, 0.000)	6.182	11	10	12	2	3	0.092
12	(0.045, 0.500, -0.000)	5.893	11	10	12	2	2	0.091
13	(0.029, 0.500, 0.029)	5.887	11	10	12	2	5	0.942

Supplementary Table 1749: Nodal ring points along high symmetry lines of mp-974370-PtRh3.

	Coordinates	Frequency	Mode 1	Mode 2	Multi.	Deg.	Path	Ratio
14	(0.056, 0.500, 0.000)	5.905	12	11	12	2	2	0.112
15	(0.086, 0.500, 0.086)	5.979	12	11	12	2	5	0.828
16	(0.109, 0.500, 0.109)	6.028	12	11	12	2	5	0.782

The followings are details for Ga_4Li_{12}. It has 5 topological nodal lines and 13 nodal ring points along high symmetry line as shown in Fig 802.

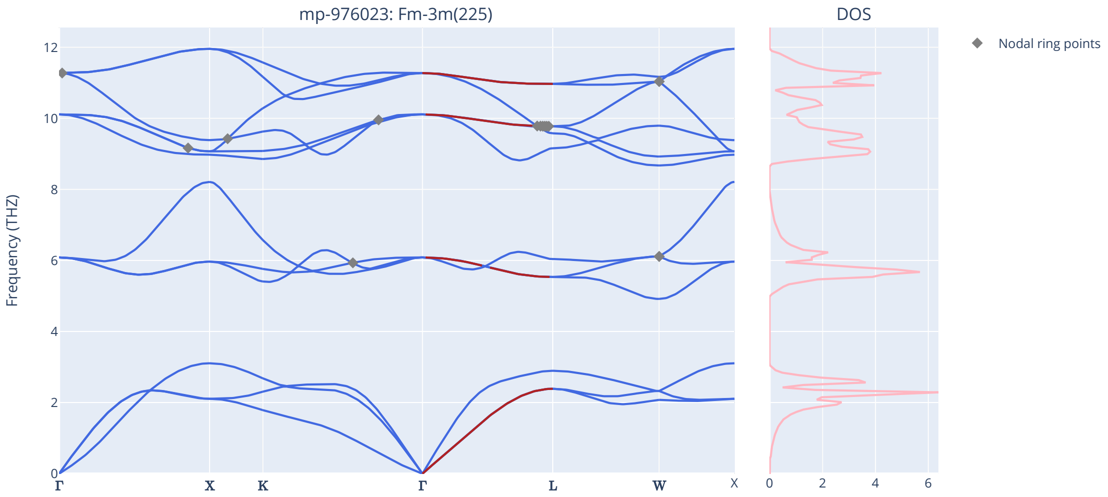

Supplementary Figure 802: The phonon band and DOS of Ga4Li12.

Supplementary Table 1750: High symmetry lines of mp-976023-Ga4Li12

	Start	End
0	Γ (0.000, 0.000, 0.000)	X (0.500, 0.000, 0.500)
1	X (0.500, 0.000, 0.500)	U (0.625, 0.250, 0.625)
2	K (0.375, 0.375, 0.750)	Γ (0.000, 0.000, 0.000)
3	Γ (0.000, 0.000, 0.000)	L (0.500, 0.500, 0.500)
4	L (0.500, 0.500, 0.500)	W (0.500, 0.250, 0.750)
5	W (0.500, 0.250, 0.750)	X (0.500, 0.000, 0.500)

Supplementary Table 1751: Nodal ring points along high symmetry lines of mp-976023-Ga4Li12.

	Coordinates	Frequency	Mode 1	Mode 2	Multi.	Deg.	Path	Ratio
0	(0.160, 0.160, 0.330)	5.933	6	5	24	2	3	0.564
1	(0.500, 0.250, 0.750)	6.113	6	5	6	2	5	1.000
2	(0.429, 0.000, 0.429)	9.167	9	8	6	2	1	0.857
3	(0.107, 0.107, 0.202)	9.958	9	8	24	2	3	0.725
4	(0.542, 0.085, 0.542)	9.428	10	9	12	2	2	0.339
5	(0.469, 0.469, 0.469)	9.780	10	9	8	2	4	0.939
6	(0.477, 0.477, 0.477)	9.779	10	9	8	2	4	0.954
7	(0.453, 0.453, 0.453)	9.782	10	9	8	2	4	0.906
8	(0.441, 0.440, 0.441)	9.785	10	9	24	2	4	0.881
9	(0.461, 0.461, 0.461)	9.781	10	9	8	2	4	0.923
10	(0.485, 0.485, 0.485)	9.779	10	9	8	2	4	0.969
11	(0.500, 0.250, 0.750)	11.039	11	10	6	2	5	1.000
12	(0.010, 0.000, 0.010)	11.278	12	11	6	2	1	0.020

The followings are details for Ni3. It has 8 topological nodal lines and 36 nodal ring points along high symmetry line as shown in Fig 803.

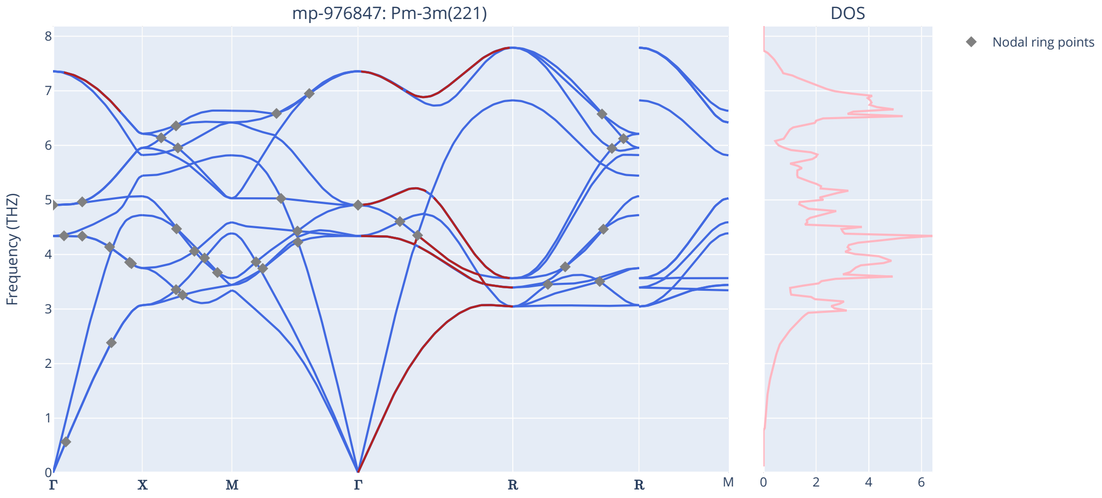

Supplementary Figure 803: The phonon band and DOS of Ni3Sb.

Supplementary Table 1752: High symmetry lines of mp-976847-Ni3Sb

	Start	End
0	Γ (0.000, 0.000, 0.000)	X (0.000, 0.500, 0.000)
1	X (0.000, 0.500, 0.000)	M (0.500, 0.500, 0.000)
2	M (0.500, 0.500, 0.000)	Γ (0.000, 0.000, 0.000)
3	Γ (0.000, 0.000, 0.000)	R (0.500, 0.500, 0.500)
4	R (0.500, 0.500, 0.500)	X (0.000, 0.500, 0.000)
5	R (0.500, 0.500, 0.500)	M (0.500, 0.500, 0.000)

Supplementary Table 1753: Nodal ring points along high symmetry lines of mp-976847-Ni3Sb.

	Coordinates	Frequency	Mode 1	Mode 2	Multi.	Deg.	Path	Ratio
0	(0.000, 0.071, 0.000)	0.565	2	1	6	2	1	0.143
1	(0.000, 0.327, 0.000)	2.384	2	1	6	2	1	0.653
2	(0.225, 0.500, 0.001)	3.257	2	1	24	2	2	0.451
3	(0.188, 0.500, 0.000)	3.355	3	2	12	2	2	0.377
4	(0.160, 0.500, 0.152)	3.507	3	2	24	2	5	0.688
5	(0.000, 0.429, 0.000)	3.858	4	3	6	2	1	0.857
6	(0.000, 0.439, 0.000)	3.834	4	3	6	2	1	0.878
7	(0.420, 0.500, 0.000)	3.670	4	3	12	2	2	0.840
8	(0.377, 0.377, -0.000)	3.744	4	3	12	2	3	0.245
9	(0.238, 0.238, -0.000)	4.228	4	3	12	2	3	0.524
10	(0.361, 0.500, 0.361)	3.453	4	3	12	2	5	0.278
11	(0.000, 0.061, 0.000)	4.343	5	4	6	2	1	0.122
12	(0.000, 0.163, 0.000)	4.337	5	4	6	2	1	0.327
13	(0.000, 0.316, 0.000)	4.138	5	4	6	2	1	0.633

Supplementary Table 1753: Nodal ring points along high symmetry lines of mp-976847-Ni3Sb.

	Coordinates	Frequency	Mode 1	Mode 2	Multi.	Deg.	Path	Ratio
14	(0.348, 0.500, 0.000)	3.935	5	4	12	2	2	0.696
15	(0.291, 0.500, 0.000)	4.060	5	4	12	2	2	0.582
16	(0.404, 0.404, 0.000)	3.865	5	4	12	2	3	0.192
17	(0.191, 0.500, -0.000)	4.471	6	5	12	2	2	0.382
18	(0.192, 0.192, 0.192)	4.352	6	5	8	2	4	0.385
19	(0.141, 0.500, 0.141)	4.464	6	5	12	2	5	0.717
20	(0.293, 0.500, 0.293)	3.775	6	5	12	2	5	0.415
21	(0.239, 0.241, 0.000)	4.429	7	6	24	2	3	0.520
22	(0.135, 0.135, 0.135)	4.605	7	6	8	2	4	0.271
23	(0.000, 0.000, 0.000)	4.906	8	7	1	3	1	0.000
24	(0.305, 0.305, -0.000)	5.027	8	7	12	2	3	0.390
25	(0.000, 0.163, 0.000)	4.965	9	8	6	2	1	0.327
26	(0.199, 0.500, -0.000)	5.954	10	9	12	2	2	0.397
27	(0.108, 0.500, 0.108)	5.946	10	9	12	2	5	0.785
28	(0.105, 0.500, -0.000)	6.139	11	10	12	2	2	0.210
29	(0.193, 0.193, 0.000)	6.950	11	10	12	2	3	0.614
30	(0.062, 0.500, 0.062)	6.124	11	10	12	2	5	0.876
31	(0.188, 0.500, 0.000)	6.358	12	11	12	2	2	0.376
32	(0.323, 0.323, 0.000)	6.586	12	11	12	2	3	0.354
33	(0.147, 0.500, 0.147)	6.575	12	11	12	2	5	0.706

The followings are details for TlZr$_3$. It has 7 topological nodal lines and 20 nodal ring points along high symmetry line as shown in Fig 804.

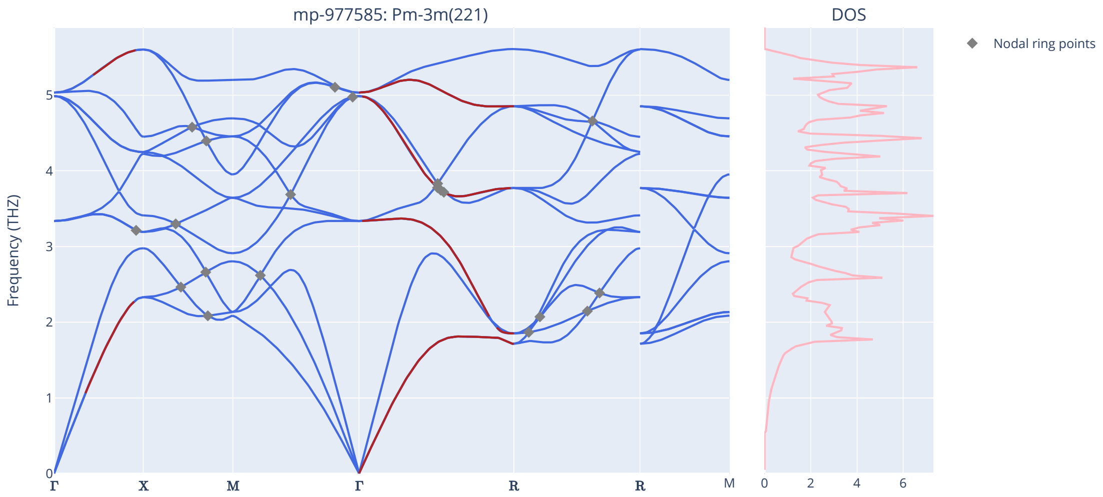

Supplementary Figure 804: The phonon band and DOS of TlZr3.

Supplementary Table 1754: High symmetry lines of mp-977585-TlZr3

	Start	End
0	Γ (0.000, 0.000, 0.000)	X (0.000, 0.500, 0.000)
1	X (0.000, 0.500, 0.000)	M (0.500, 0.500, 0.000)
2	M (0.500, 0.500, 0.000)	Γ (0.000, 0.000, 0.000)
3	Γ (0.000, 0.000, 0.000)	R (0.500, 0.500, 0.500)
4	R (0.500, 0.500, 0.500)	X (0.000, 0.500, 0.000)
5	R (0.500, 0.500, 0.500)	M (0.500, 0.500, 0.000)

Supplementary Table 1755: Nodal ring points along high symmetry lines of mp-977585-TlZr3.

	Coordinates	Frequency	Mode 1	Mode 2	Multi.	Deg.	Path	Ratio
0	(0.361, 0.500, -0.000)	2.086	2	1	12	2	2	0.721
1	(0.209, 0.500, 0.209)	2.147	2	1	12	2	5	0.582
2	(0.210, 0.500, -0.000)	2.465	3	2	12	2	2	0.419
3	(0.441, 0.500, 0.441)	1.867	3	2	12	2	5	0.119
4	(0.161, 0.500, 0.161)	2.387	3	2	12	2	5	0.679
5	(0.349, 0.500, -0.000)	2.662	4	3	12	2	2	0.698
6	(0.391, 0.391, -0.000)	2.620	4	3	12	2	3	0.219
7	(0.397, 0.500, 0.397)	2.072	4	3	12	2	5	0.207
8	(0.000, 0.459, 0.000)	3.214	5	4	6	2	1	0.918
9	(0.181, 0.500, -0.000)	3.299	6	5	12	2	2	0.361
10	(0.271, 0.271, 0.000)	3.687	7	6	12	2	3	0.458
11	(0.274, 0.274, 0.274)	3.717	7	6	8	2	4	0.548
12	(0.264, 0.264, 0.264)	3.745	7	6	8	2	4	0.528
13	(0.254, 0.254, 0.254)	3.777	7	6	8	2	4	0.507

Supplementary Table 1755: Nodal ring points along high symmetry lines of mp-977585-TlZr3.

	Coordinates	Frequency	Mode 1	Mode 2	Multi.	Deg.	Path	Ratio
14	(0.025, 0.025, -0.000)	4.974	8	7	12	2	3	0.949
15	(0.352, 0.500, -0.000)	4.394	9	8	12	2	2	0.705
16	(0.254, 0.254, 0.254)	3.832	9	8	8	2	4	0.508
17	(0.273, 0.500, -0.000)	4.577	10	9	12	2	2	0.545
18	(0.095, 0.095, -0.000)	5.103	11	10	12	2	3	0.809
19	(0.189, 0.500, 0.189)	4.658	11	10	12	2	5	0.623

The followings are details for Mg$_3$. It has 7 topological nodal lines and 21 nodal ring points along high symmetry line as shown in Fig 805.

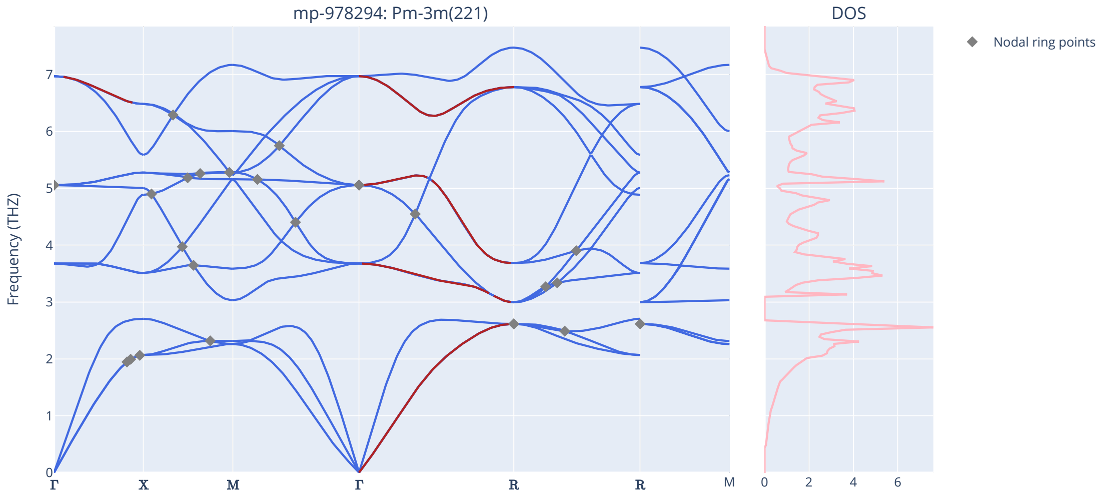

Supplementary Figure 805: The phonon band and DOS of Mg3Sn.

Supplementary Table 1756: High symmetry lines of mp-978294-Mg3Sn

	Start	End
0	Γ (0.000, 0.000, 0.000)	X (0.000, 0.500, 0.000)
1	X (0.000, 0.500, 0.000)	M (0.500, 0.500, 0.000)
2	M (0.500, 0.500, 0.000)	Γ (0.000, 0.000, 0.000)
3	Γ (0.000, 0.000, 0.000)	R (0.500, 0.500, 0.500)
4	R (0.500, 0.500, 0.500)	X (0.000, 0.500, 0.000)
5	R (0.500, 0.500, 0.500)	M (0.500, 0.500, 0.000)

Supplementary Table 1757: Nodal ring points along high symmetry lines of mp-978294-Mg3Sn.

	Coordinates	Frequency	Mode 1	Mode 2	Multi.	Deg.	Path	Ratio
0	(0.000, 0.408, 0.000)	1.947	2	1	6	2	1	0.816
1	(0.000, 0.429, 0.000)	1.994	2	1	6	2	1	0.857
2	(0.000, 0.480, 0.000)	2.063	2	1	6	2	1	0.959
3	(0.500, 0.500, 0.500)	2.616	2	1	1	3	4	1.000
4	(0.374, 0.500, -0.000)	2.317	3	2	12	2	2	0.748
5	(0.298, 0.500, 0.298)	2.488	3	2	12	2	5	0.403
6	(0.280, 0.500, 0.000)	3.646	5	4	12	2	2	0.560
7	(0.328, 0.500, 0.328)	3.338	5	4	12	2	5	0.344
8	(0.218, 0.500, 0.000)	3.972	6	5	12	2	2	0.435
9	(0.374, 0.500, 0.374)	3.268	6	5	12	2	5	0.252
10	(0.045, 0.500, -0.000)	4.899	7	6	12	2	2	0.090
11	(0.252, 0.252, -0.000)	4.403	7	6	12	2	3	0.497
12	(0.182, 0.182, 0.182)	4.547	7	6	8	2	4	0.363
13	(0.253, 0.500, 0.253)	3.902	7	6	12	2	5	0.495

Supplementary Table 1757: Nodal ring points along high symmetry lines of mp-978294-Mg3Sn.

	Coordinates	Frequency	Mode 1	Mode 2	Multi.	Deg.	Path	Ratio
14	(0.248, 0.500, -0.001)	5.184	8	7	24	2	2	0.495
15	(0.402, 0.402, 0.000)	5.155	8	7	12	2	3	0.196
16	(0.000, 0.000, 0.000)	5.055	9	8	1	3	1	0.000
17	(0.318, 0.500, 0.000)	5.258	9	8	12	2	2	0.635
18	(0.481, 0.500, -0.001)	5.279	9	8	24	2	2	0.962
19	(0.315, 0.315, -0.000)	5.748	10	9	12	2	3	0.369
20	(0.166, 0.500, 0.001)	6.288	11	10	24	2	2	0.332

The followings are details for Ga$_3$. It has 7 topological nodal lines and 19 nodal ring points along high symmetry line as shown in Fig **806**.

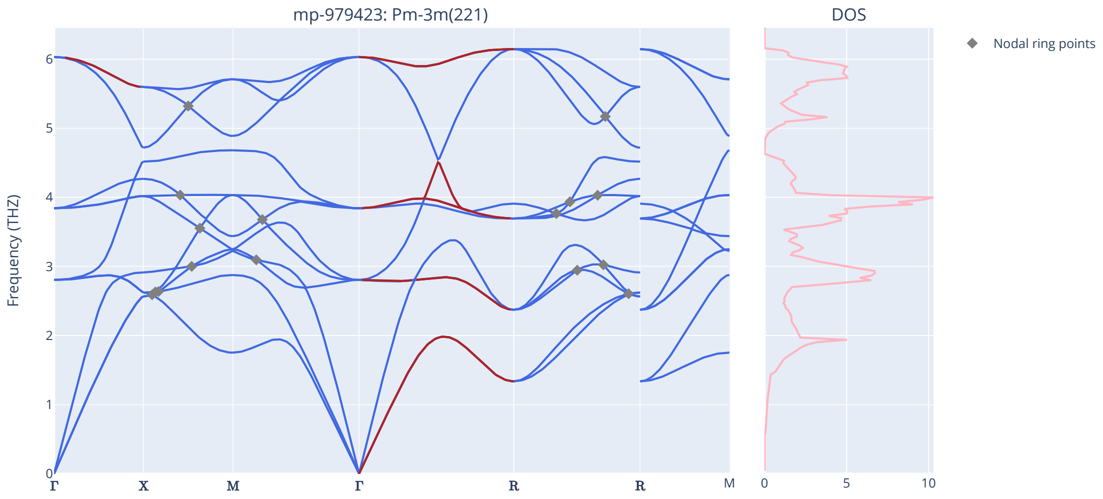

Supplementary Figure 806: The phonon band and DOS of Ga3Y.

Supplementary Table 1758: High symmetry lines of mp-979423-Ga3Y

	Start	End
0	Γ (0.000, 0.000, 0.000)	X (0.000, 0.500, 0.000)
1	X (0.000, 0.500, 0.000)	M (0.500, 0.500, 0.000)
2	M (0.500, 0.500, 0.000)	Γ (0.000, 0.000, 0.000)
3	Γ (0.000, 0.000, 0.000)	R (0.500, 0.500, 0.500)
4	R (0.500, 0.500, 0.500)	X (0.000, 0.500, 0.000)
5	R (0.500, 0.500, 0.500)	M (0.500, 0.500, 0.000)

Supplementary Table 1759: Nodal ring points along high symmetry lines of mp-979423-Ga3Y.

	Coordinates	Frequency	Mode 1	Mode 2	Multi.	Deg.	Path	Ratio
0	(0.049, 0.500, -0.000)	2.590	2	1	12	2	2	0.098
1	(0.085, 0.500, -0.000)	2.636	3	2	12	2	2	0.170
2	(0.045, 0.500, 0.045)	2.604	3	2	12	2	5	0.909
3	(0.067, 0.500, -0.000)	2.631	4	3	12	2	2	0.135
4	(0.270, 0.500, 0.000)	3.000	4	3	12	2	2	0.541
5	(0.407, 0.407, -0.000)	3.095	4	3	12	2	3	0.187
6	(0.249, 0.500, 0.249)	2.944	4	3	12	2	5	0.502
7	(0.145, 0.500, 0.145)	3.023	5	4	12	2	5	0.710
8	(0.316, 0.500, -0.000)	3.553	6	5	12	2	2	0.633
9	(0.383, 0.383, -0.000)	3.678	7	6	12	2	3	0.234
10	(0.206, 0.500, 0.000)	4.032	8	7	12	2	2	0.412
11	(0.332, 0.500, 0.332)	3.764	8	7	12	2	5	0.337
12	(0.168, 0.500, 0.168)	4.029	8	7	12	2	5	0.664
13	(0.278, 0.500, 0.278)	3.935	9	8	12	2	5	0.445

Supplementary Table 1759: Nodal ring points along high symmetry lines of mp-979423-Ga3Y.

	Coordinates	Frequency	Mode 1	Mode 2	Multi.	Deg.	Path	Ratio
14	(0.251, 0.500, -0.000)	5.320	11	10	12	2	2	0.502
15	(0.138, 0.500, 0.138)	5.169	11	10	12	2	5	0.725

The followings are details for Na$_3$. It has 6 topological nodal lines and 20 nodal ring points along high symmetry line as shown in Fig **807**.

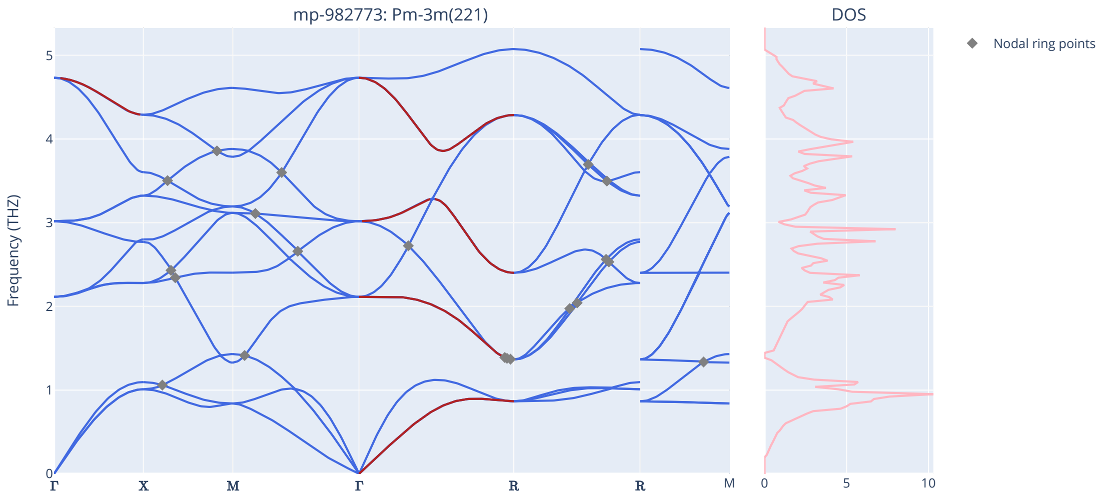

Supplementary Figure 807: The phonon band and DOS of Na3Tl.

Supplementary Table 1760: High symmetry lines of mp-982773-Na3Tl

	Start	End
0	Γ (0.000, 0.000, 0.000)	X (0.000, 0.500, 0.000)
1	X (0.000, 0.500, 0.000)	M (0.500, 0.500, 0.000)
2	M (0.500, 0.500, 0.000)	Γ (0.000, 0.000, 0.000)
3	Γ (0.000, 0.000, 0.000)	R (0.500, 0.500, 0.500)
4	R (0.500, 0.500, 0.500)	X (0.000, 0.500, 0.000)
5	R (0.500, 0.500, 0.500)	M (0.500, 0.500, 0.000)

Supplementary Table 1761: Nodal ring points along high symmetry lines of mp-982773-Na3Tl.

	Coordinates	Frequency	Mode 1	Mode 2	Multi.	Deg.	Path	Ratio
0	(0.106, 0.500, -0.000)	1.059	3	2	12	2	2	0.212
1	(0.453, 0.453, 0.000)	1.413	4	3	12	2	3	0.094
2	(0.498, 0.498, 0.145)	1.335	4	3	24	2	6	0.711
3	(0.178, 0.500, 0.000)	2.342	5	4	12	2	2	0.357
4	(0.249, 0.500, 0.249)	2.042	5	4	12	2	5	0.502
5	(0.154, 0.500, -0.000)	2.430	6	5	12	2	2	0.309
6	(0.489, 0.489, 0.489)	1.369	6	5	8	3	4	0.979
7	(0.478, 0.478, 0.478)	1.379	6	5	8	2	4	0.956
8	(0.471, 0.471, 0.471)	1.389	6	5	8	2	4	0.942
9	(0.279, 0.500, 0.279)	1.974	6	5	12	2	5	0.443
10	(0.124, 0.500, 0.124)	2.531	6	5	12	2	5	0.753
11	(0.243, 0.243, -0.000)	2.658	7	6	12	2	3	0.515
12	(0.159, 0.159, 0.159)	2.724	7	6	8	2	4	0.319
13	(0.135, 0.500, 0.135)	2.562	7	6	12	2	5	0.731

Supplementary Table 1761: Nodal ring points along high symmetry lines of mp-982773-Na3Tl.

	Coordinates	Frequency	Mode 1	Mode 2	Multi.	Deg.	Path	Ratio
14	(0.410, 0.410, -0.000)	3.109	8	7	12	2	3	0.179
15	(0.131, 0.500, 0.131)	3.497	9	8	12	2	5	0.737
16	(0.135, 0.500, 0.000)	3.502	10	9	12	2	2	0.270
17	(0.306, 0.306, -0.000)	3.600	10	9	12	2	3	0.387
18	(0.205, 0.500, 0.205)	3.696	10	9	12	2	5	0.589
19	(0.412, 0.500, -0.001)	3.857	11	10	24	2	2	0.823

The followings are details for $Mn_8Ru_4Si_4$. As shown in Fig **808**, it has 7 topological nodal lines and 4 nodal ring points. At the same time, it has 1 single Weyl point and 2 high degenerate Weyl points whose degeneracy are more than 2 and charges are bigger than 1.

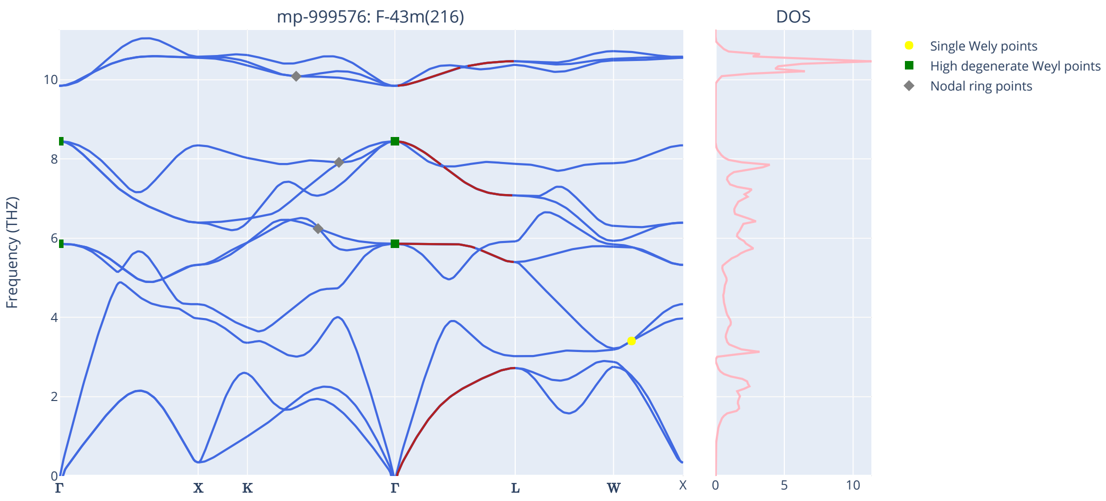

Supplementary Figure 808: The phonon band and DOS of Mn8Ru4Si4.

Supplementary Table 1762: High symmetry lines of mp-999576-Mn8Ru4Si4

	Start	End
0	Γ (0.000, 0.000, 0.000)	X (0.500, 0.000, 0.500)
1	X (0.500, 0.000, 0.500)	U (0.625, 0.250, 0.625)
2	K (0.375, 0.375, 0.750)	Γ (0.000, 0.000, 0.000)
3	Γ (0.000, 0.000, 0.000)	L (0.500, 0.500, 0.500)
4	L (0.500, 0.500, 0.500)	W (0.500, 0.250, 0.750)
5	W (0.500, 0.250, 0.750)	X (0.500, 0.000, 0.500)

Supplementary Table 1763: Single Weyl points along high symmetry lines of mp-999576-Mn8Ru4Si4.

	Coordinates	Frequency	Mode 1	Mode 2	Multi.	Deg.	Path	Ratio
0	(0.500, 0.185, 0.685)	3.404	4	3	12	2	6	0.260

Supplementary Table 1764: High degenerate Weyl points along high symmetry lines of mp-999576-Mn8Ru4Si4.

	Coordinates	Frequency	Mode 1	Mode 2	Multi.	Deg.	Path	Ratio
0	(0.000, 0.000, 0.000)	5.860	6	4	1	3	1	0.000
1	(0.000, 0.000, 0.000)	8.447	9	7	1	3	1	0.000

Supplementary Table 1765: Nodal ring points along high symmetry lines of mp-999576-Mn8Ru4Si4.

	Coordinates	Frequency	Mode 1	Mode 2	Multi.	Deg.	Path	Ratio
0	(0.199, 0.199, 0.386)	6.240	6	5	24	2	3	0.480
1	(0.145, 0.145, 0.281)	7.913	9	8	24	2	3	0.621
2	(0.253, 0.253, 0.500)	10.087	11	10	24	2	3	0.331

The followings are details for Rh$_3$. It has 8 topological nodal lines and 22 nodal ring points along high symmetry line as shown in Fig **809**.

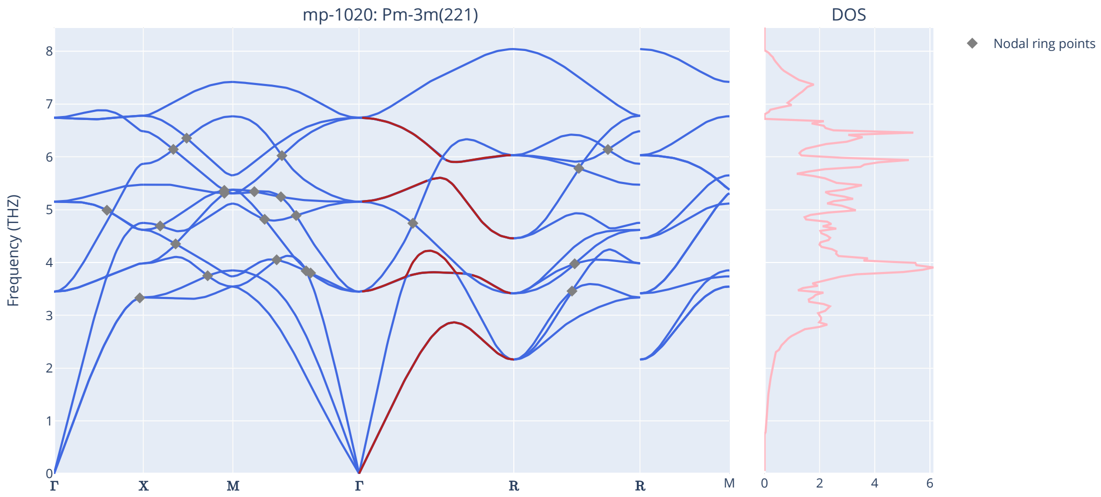

Supplementary Figure 809: The phonon band and DOS of Rh3Ta.

Supplementary Table 1766: High symmetry lines of mp-1020-Rh3Ta

	Start	End
0	Γ (0.000, 0.000, 0.000)	X (0.000, 0.500, 0.000)
1	X (0.000, 0.500, 0.000)	M (0.500, 0.500, 0.000)
2	M (0.500, 0.500, 0.000)	Γ (0.000, 0.000, 0.000)
3	Γ (0.000, 0.000, 0.000)	R (0.500, 0.500, 0.500)
4	R (0.500, 0.500, 0.500)	X (0.000, 0.500, 0.000)
5	R (0.500, 0.500, 0.500)	M (0.500, 0.500, 0.000)

Supplementary Table 1767: Nodal ring points along high symmetry lines of mp-1020-Rh3Ta.

	Coordinates	Frequency	Mode 1	Mode 2	Multi.	Deg.	Path	Ratio
0	(0.000, 0.480, 0.000)	3.329	2	1	6	2	1	0.959
1	(0.360, 0.500, -0.000)	3.749	3	2	12	2	2	0.719
2	(0.270, 0.500, 0.270)	3.459	3	2	12	2	5	0.461
3	(0.326, 0.326, -0.000)	4.051	4	3	12	2	3	0.348
4	(0.209, 0.209, -0.000)	3.841	4	3	12	2	3	0.581
5	(0.180, 0.500, -0.000)	4.352	5	4	12	2	2	0.360
6	(0.192, 0.192, -0.000)	3.801	5	4	12	2	3	0.616
7	(0.259, 0.500, 0.259)	3.976	5	4	12	2	5	0.482
8	(0.374, 0.374, 0.000)	4.818	6	5	12	2	3	0.253
9	(0.094, 0.500, -0.000)	4.691	7	6	12	2	2	0.188
10	(0.453, 0.500, 0.000)	5.311	7	6	12	2	2	0.906
11	(0.249, 0.249, 0.000)	4.889	7	6	12	2	3	0.502
12	(0.174, 0.174, 0.174)	4.743	7	6	8	2	4	0.348
13	(0.000, 0.296, 0.000)	4.990	8	7	6	2	1	0.592

Supplementary Table 1767: Nodal ring points along high symmetry lines of mp-1020-Rh3Ta.

	Coordinates	Frequency	Mode 1	Mode 2	Multi.	Deg.	Path	Ratio
14	(0.414, 0.414, -0.000)	5.342	8	7	12	2	3	0.171
15	(0.309, 0.309, 0.000)	5.243	8	7	12	2	3	0.381
16	(0.453, 0.500, -0.000)	5.366	9	8	12	2	2	0.906
17	(0.242, 0.500, 0.242)	5.782	9	8	12	2	5	0.515
18	(0.168, 0.500, 0.000)	6.143	10	9	12	2	2	0.335
19	(0.305, 0.305, -0.000)	6.023	10	9	12	2	3	0.389
20	(0.127, 0.500, 0.127)	6.139	10	9	12	2	5	0.745
21	(0.242, 0.500, -0.000)	6.354	11	10	12	2	2	0.484

The followings are details for VZn$_3$. It has 9 topological nodal lines and 9 nodal ring points along high symmetry line as shown in Fig **810**.

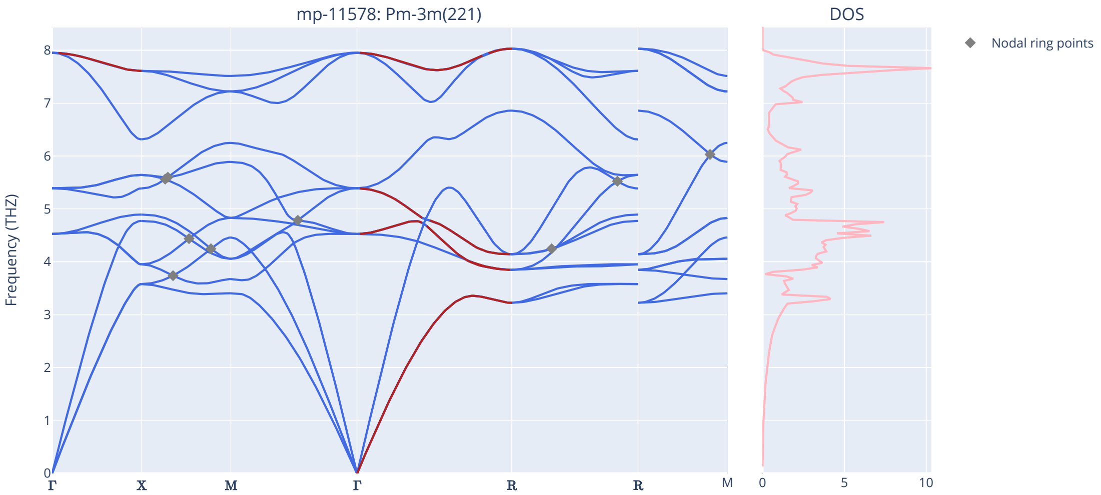

Supplementary Figure 810: The phonon band and DOS of VZn3.

Supplementary Table 1768: High symmetry lines of mp-11578-VZn3

	Start	End
0	Γ (0.000, 0.000, 0.000)	X (0.000, 0.500, 0.000)
1	X (0.000, 0.500, 0.000)	M (0.500, 0.500, 0.000)
2	M (0.500, 0.500, 0.000)	Γ (0.000, 0.000, 0.000)
3	Γ (0.000, 0.000, 0.000)	R (0.500, 0.500, 0.500)
4	R (0.500, 0.500, 0.500)	X (0.000, 0.500, 0.000)
5	R (0.500, 0.500, 0.500)	M (0.500, 0.500, 0.000)

Supplementary Table 1769: Nodal ring points along high symmetry lines of mp-11578-VZn3.

	Coordinates	Frequency	Mode 1	Mode 2	Multi.	Deg.	Path	Ratio
0	(0.177, 0.500, -0.000)	3.737	3	2	12	2	2	0.354
1	(0.389, 0.500, -0.000)	4.246	5	4	12	2	2	0.778
2	(0.267, 0.500, 0.000)	4.438	5	4	12	2	2	0.533
3	(0.234, 0.234, -0.000)	4.784	7	6	12	2	3	0.531
4	(0.342, 0.500, 0.342)	4.246	7	6	12	2	5	0.316
5	(0.133, 0.500, -0.000)	5.563	8	7	12	2	2	0.266
6	(0.082, 0.500, 0.082)	5.522	8	7	12	2	5	0.836
7	(0.148, 0.500, -0.000)	5.599	9	8	12	2	2	0.296
8	(0.500, 0.498, 0.097)	6.029	9	8	24	2	6	0.805

The followings are details for MnPt$_3$. It has 7 topological nodal lines and 25 nodal ring points along high symmetry line as shown in Fig 811.

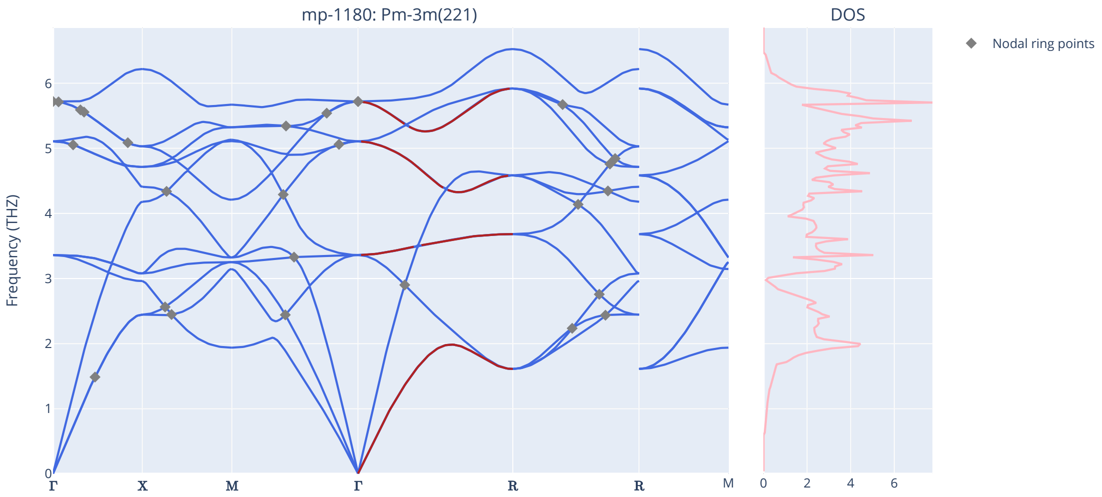

Supplementary Figure 811: The phonon band and DOS of MnPt3.

Supplementary Table 1770: High symmetry lines of mp-1180-MnPt3

	Start	End
0	Γ (0.000, 0.000, 0.000)	X (0.000, 0.500, 0.000)
1	X (0.000, 0.500, 0.000)	M (0.500, 0.500, 0.000)
2	M (0.500, 0.500, 0.000)	Γ (0.000, 0.000, 0.000)
3	Γ (0.000, 0.000, 0.000)	R (0.500, 0.500, 0.500)
4	R (0.500, 0.500, 0.500)	X (0.000, 0.500, 0.000)
5	R (0.500, 0.500, 0.500)	M (0.500, 0.500, 0.000)

Supplementary Table 1771: Nodal ring points along high symmetry lines of mp-1180-MnPt3.

	Coordinates	Frequency	Mode 1	Mode 2	Multi.	Deg.	Path	Ratio
0	(0.000, 0.235, 0.000)	1.486	2	1	6	2	1	0.469
1	(0.163, 0.500, 0.000)	2.447	2	1	12	2	2	0.326
2	(0.133, 0.500, 0.133)	2.436	2	1	12	2	5	0.734
3	(0.127, 0.500, -0.000)	2.563	3	2	12	2	2	0.254
4	(0.288, 0.288, -0.000)	2.441	3	2	12	2	3	0.424
5	(0.265, 0.500, 0.265)	2.235	3	2	12	2	5	0.470
6	(0.151, 0.151, 0.151)	2.901	4	3	8	2	4	0.302
7	(0.158, 0.500, 0.158)	2.757	4	3	12	2	5	0.685
8	(0.253, 0.253, -0.000)	3.329	5	4	12	2	3	0.493
9	(0.241, 0.500, 0.241)	4.139	6	5	12	2	5	0.517
10	(0.135, 0.500, 0.000)	4.339	7	6	12	2	2	0.270
11	(0.296, 0.296, 0.000)	4.291	7	6	12	2	3	0.408
12	(0.123, 0.500, 0.123)	4.346	7	6	12	2	5	0.753
13	(0.000, 0.112, 0.000)	5.054	8	7	6	2	1	0.224

Supplementary Table 1771: Nodal ring points along high symmetry lines of mp-1180-MnPt3.

	Coordinates	Frequency	Mode 1	Mode 2	Multi.	Deg.	Path	Ratio
14	(0.075, 0.075, 0.000)	5.061	8	7	12	2	3	0.849
15	(0.115, 0.500, 0.115)	4.760	9	8	12	2	5	0.769
16	(0.095, 0.500, 0.095)	4.843	10	9	12	2	5	0.810
17	(0.000, 0.000, 0.000)	5.720	11	10	1	3	1	0.000
18	(0.000, 0.031, 0.000)	5.715	11	10	6	2	1	0.061
19	(0.000, 0.153, 0.000)	5.591	11	10	6	2	1	0.306
20	(0.000, 0.173, 0.000)	5.557	11	10	6	2	1	0.347
21	(0.000, 0.418, 0.000)	5.087	11	10	6	2	1	0.837
22	(0.285, 0.285, 0.000)	5.344	11	10	12	2	3	0.430
23	(0.124, 0.124, -0.000)	5.541	11	10	12	2	3	0.752
24	(0.303, 0.500, 0.303)	5.673	11	10	12	2	5	0.395

The followings are details for Ag3. It has 8 topological nodal lines and 29 nodal ring points along high symmetry line as shown in Fig 812.

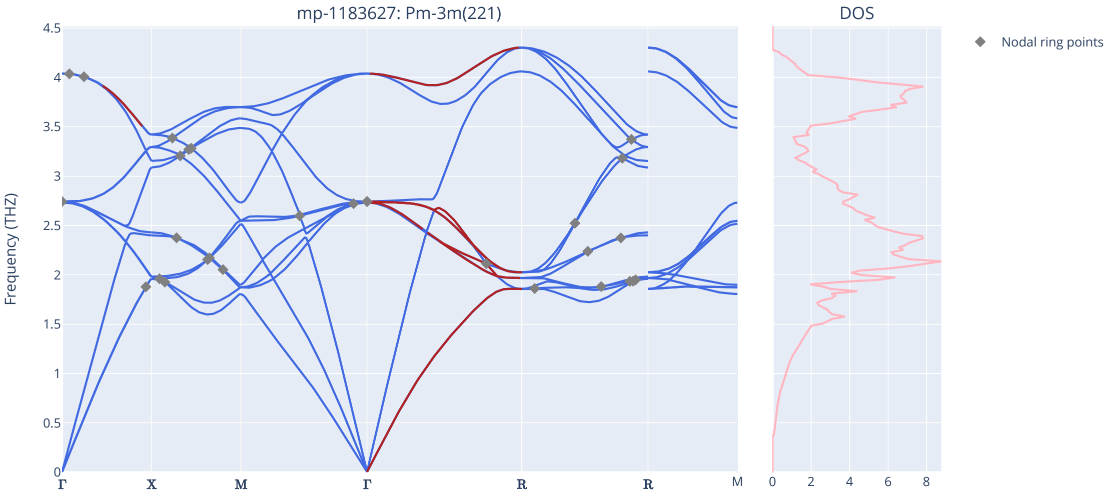

Supplementary Figure 812: The phonon band and DOS of Ag3Cd.

Supplementary Table 1772: High symmetry lines of mp-1183627-Ag3Cd

	Start	End
0	Γ (0.000, 0.000, 0.000)	X (0.000, 0.500, 0.000)
1	X (0.000, 0.500, 0.000)	M (0.500, 0.500, 0.000)
2	M (0.500, 0.500, 0.000)	Γ (0.000, 0.000, 0.000)
3	Γ (0.000, 0.000, 0.000)	R (0.500, 0.500, 0.500)
4	R (0.500, 0.500, 0.500)	X (0.000, 0.500, 0.000)
5	R (0.500, 0.500, 0.500)	M (0.500, 0.500, 0.000)

Supplementary Table 1773: Nodal ring points along high symmetry lines of mp-1183627-Ag3Cd.

	Coordinates	Frequency	Mode 1	Mode 2	Multi.	Deg.	Path	Ratio
0	(0.000, 0.469, 0.000)	1.879	2	1	6	2	1	0.939
1	(0.075, 0.500, 0.000)	1.926	2	1	12	2	2	0.151
2	(0.449, 0.500, 0.449)	1.861	2	1	12	2	5	0.102
3	(0.060, 0.500, 0.060)	1.936	2	1	12	2	5	0.881
4	(0.045, 0.500, -0.000)	1.961	3	2	12	2	2	0.090
5	(0.049, 0.499, 0.049)	1.949	3	2	24	2	5	0.901
6	(0.072, 0.496, 0.072)	1.934	3	2	24	2	5	0.856
7	(0.401, 0.500, 0.000)	2.053	4	3	12	2	2	0.801
8	(0.315, 0.500, -0.000)	2.155	4	3	12	2	2	0.629
9	(0.325, 0.500, 0.000)	2.171	4	3	12	2	2	0.650
10	(0.182, 0.500, 0.189)	1.881	4	3	24	2	5	0.629
11	(0.141, 0.500, -0.000)	2.375	6	5	12	2	2	0.282
12	(0.054, 0.054, -0.000)	2.721	6	5	12	2	3	0.893
13	(0.385, 0.385, 0.385)	2.113	6	5	8	2	4	0.771

Supplementary Table 1773: Nodal ring points along high symmetry lines of mp-1183627-Ag3Cd.

	Coordinates	Frequency	Mode 1	Mode 2	Multi.	Deg.	Path	Ratio
14	(0.108, 0.500, 0.108)	2.374	6	5	12	2	5	0.784
15	(0.239, 0.500, 0.239)	2.238	6	5	12	2	5	0.523
16	(0.267, 0.267, 0.000)	2.598	7	6	12	2	3	0.466
17	(0.000, 0.000, 0.000)	2.743	8	7	1	3	1	0.000
18	(0.161, 0.500, 0.000)	3.206	8	7	12	2	2	0.323
19	(0.289, 0.500, 0.289)	2.523	8	7	12	2	5	0.421
20	(0.102, 0.500, 0.102)	3.181	8	7	12	2	5	0.795
21	(0.209, 0.500, -0.000)	3.270	9	8	12	2	2	0.418
22	(0.223, 0.499, 0.000)	3.282	9	8	24	2	2	0.446
23	(0.000, 0.041, 0.000)	4.037	11	10	6	2	1	0.082
24	(0.000, 0.122, 0.000)	4.007	11	10	6	2	1	0.245
25	(0.117, 0.500, -0.000)	3.386	11	10	12	2	2	0.235
26	(0.066, 0.500, 0.066)	3.372	11	10	12	2	5	0.868

The followings are details for Hf$_3$. It has 6 topological nodal lines and 28 nodal ring points along high symmetry line as shown in Fig 813.

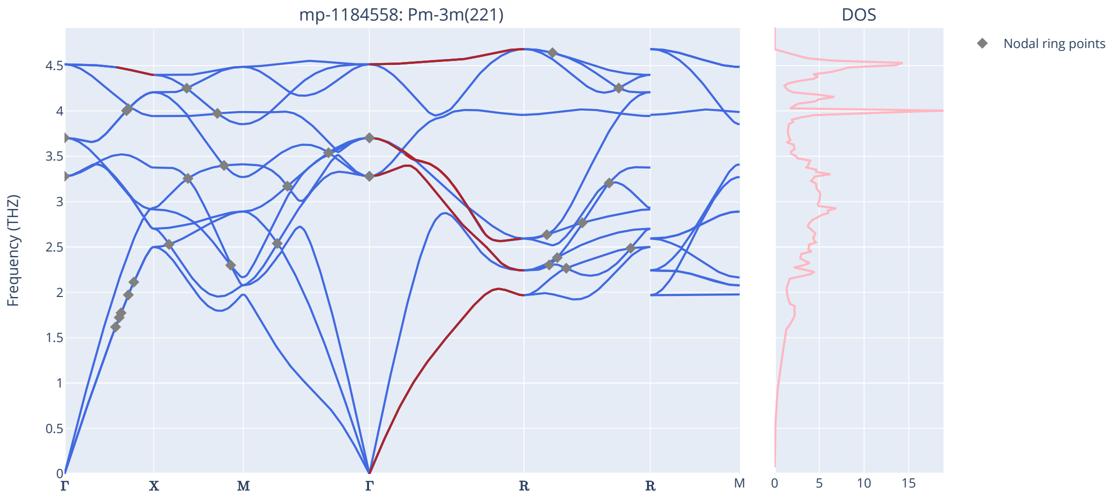

Supplementary Figure 813: The phonon band and DOS of Hf3Sn.

Supplementary Table 1774: High symmetry lines of mp-1184558-Hf3Sn

	Start	End
0	Γ (0.000, 0.000, 0.000)	X (0.000, 0.500, 0.000)
1	X (0.000, 0.500, 0.000)	M (0.500, 0.500, 0.000)
2	M (0.500, 0.500, 0.000)	Γ (0.000, 0.000, 0.000)
3	Γ (0.000, 0.000, 0.000)	R (0.500, 0.500, 0.500)
4	R (0.500, 0.500, 0.500)	X (0.000, 0.500, 0.000)
5	R (0.500, 0.500, 0.500)	M (0.500, 0.500, 0.000)

Supplementary Table 1775: Nodal ring points along high symmetry lines of mp-1184558-Hf3Sn.

	Coordinates	Frequency	Mode 1	Mode 2	Multi.	Deg.	Path	Ratio
0	(0.000, 0.286, 0.000)	1.618	2	1	6	2	1	0.571
1	(0.000, 0.306, 0.000)	1.722	2	1	6	2	1	0.612
2	(0.000, 0.316, 0.000)	1.773	2	1	6	2	1	0.633
3	(0.000, 0.357, 0.000)	1.972	2	1	6	2	1	0.714
4	(0.000, 0.388, 0.000)	2.113	2	1	6	2	1	0.776
5	(0.085, 0.500, -0.000)	2.528	3	2	12	2	2	0.171
6	(0.078, 0.500, 0.078)	2.486	3	2	12	2	5	0.845
7	(0.333, 0.500, 0.333)	2.266	3	2	12	2	5	0.334
8	(0.430, 0.500, 0.000)	2.299	4	3	12	2	2	0.861
9	(0.365, 0.365, 0.000)	2.539	4	3	12	2	3	0.270
10	(0.400, 0.500, 0.400)	2.302	4	3	12	2	5	0.200
11	(0.369, 0.500, 0.369)	2.382	5	4	12	2	5	0.263
12	(0.000, 0.000, 0.000)	3.280	6	5	1	3	1	0.000
13	(0.269, 0.500, 0.269)	2.767	6	5	12	2	5	0.462

Supplementary Table 1775: Nodal ring points along high symmetry lines of mp-1184558-Hf3Sn.

	Coordinates	Frequency	Mode 1	Mode 2	Multi.	Deg.	Path	Ratio
14	(0.191, 0.500, -0.000)	3.255	7	6	12	2	2	0.382
15	(0.324, 0.324, 0.000)	3.170	7	6	12	2	3	0.351
16	(0.163, 0.500, 0.163)	3.205	7	6	12	2	5	0.674
17	(0.393, 0.500, -0.000)	3.399	8	7	12	2	2	0.786
18	(0.162, 0.162, -0.000)	3.539	8	7	12	2	3	0.676
19	(0.410, 0.500, 0.410)	2.635	8	7	12	2	5	0.181
20	(0.000, 0.000, 0.000)	3.703	9	8	1	3	1	0.000
21	(0.000, 0.347, 0.000)	4.003	9	8	6	2	1	0.694
22	(0.000, 0.357, 0.000)	4.024	9	8	6	2	1	0.714
23	(0.356, 0.500, 0.000)	3.973	10	9	12	2	2	0.712
24	(0.184, 0.500, -0.000)	4.250	11	10	12	2	2	0.368
25	(0.125, 0.500, 0.125)	4.252	11	10	12	2	5	0.750
26	(0.387, 0.500, 0.387)	4.641	12	11	12	2	5	0.225

The followings are details for Rh$_3$. It has 8 topological nodal lines and 30 nodal ring points along high symmetry line as shown in Fig **814**.

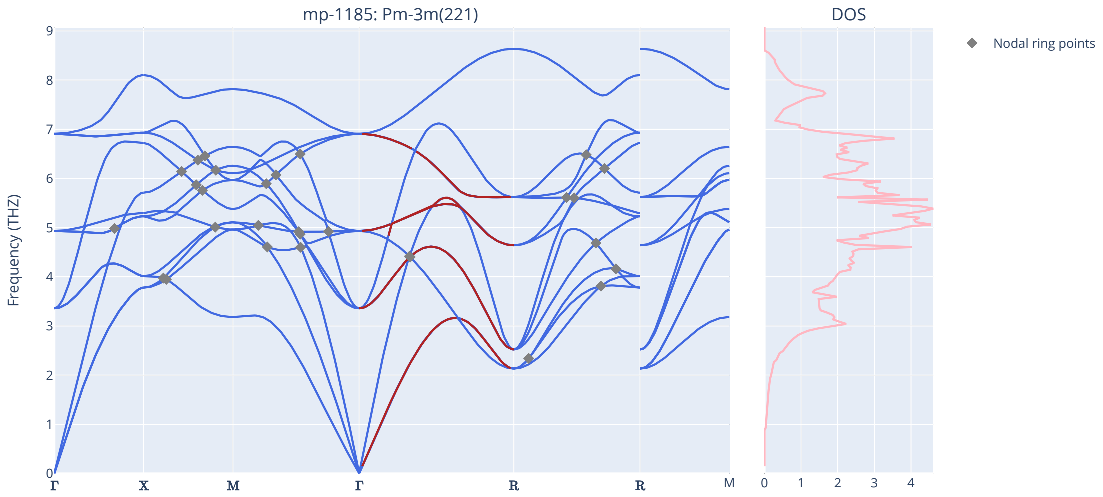

Supplementary Figure 814: The phonon band and DOS of Rh3V.

Supplementary Table 1776: High symmetry lines of mp-1185-Rh3V

	Start	End
0	Γ (0.000, 0.000, 0.000)	X (0.000, 0.500, 0.000)
1	X (0.000, 0.500, 0.000)	M (0.500, 0.500, 0.000)
2	M (0.500, 0.500, 0.000)	Γ (0.000, 0.000, 0.000)
3	Γ (0.000, 0.000, 0.000)	R (0.500, 0.500, 0.500)
4	R (0.500, 0.500, 0.500)	X (0.000, 0.500, 0.000)
5	R (0.500, 0.500, 0.500)	M (0.500, 0.500, 0.000)

Supplementary Table 1777: Nodal ring points along high symmetry lines of mp-1185-Rh3V.

	Coordinates	Frequency	Mode 1	Mode 2	Multi.	Deg.	Path	Ratio
0	(0.127, 0.500, 0.000)	3.947	2	1	12	2	2	0.254
1	(0.155, 0.500, 0.155)	3.810	2	1	12	2	5	0.691
2	(0.111, 0.500, -0.000)	3.973	3	2	12	2	2	0.221
3	(0.364, 0.364, -0.000)	4.606	3	2	12	2	3	0.273
4	(0.441, 0.500, 0.441)	2.340	3	2	12	2	5	0.118
5	(0.402, 0.500, -0.000)	5.009	4	3	12	2	2	0.803
6	(0.232, 0.232, 0.000)	4.600	4	3	12	2	3	0.536
7	(0.167, 0.167, 0.161)	4.402	4	3	24	2	4	0.330
8	(0.096, 0.500, 0.096)	4.161	4	3	12	2	5	0.809
9	(0.399, 0.399, 0.000)	5.045	5	4	12	2	3	0.202
10	(0.175, 0.500, 0.175)	4.685	5	4	12	2	5	0.650
11	(0.234, 0.234, -0.000)	4.866	6	5	12	2	3	0.533
12	(0.163, 0.163, 0.163)	4.417	6	5	8	2	4	0.325
13	(0.000, 0.337, 0.000)	4.981	7	6	6	2	1	0.673

Supplementary Table 1777: Nodal ring points along high symmetry lines of mp-1185-Rh3V.

	Coordinates	Frequency	Mode 1	Mode 2	Multi.	Deg.	Path	Ratio
14	(0.329, 0.500, 0.000)	5.758	7	6	12	2	2	0.659
15	(0.239, 0.239, 0.000)	4.917	7	6	12	2	3	0.522
16	(0.295, 0.500, -0.000)	5.870	8	7	12	2	2	0.589
17	(0.368, 0.368, 0.000)	5.893	8	7	12	2	3	0.264
18	(0.121, 0.121, -0.000)	4.922	8	7	12	2	3	0.757
19	(0.261, 0.500, 0.261)	5.604	8	7	12	2	5	0.478
20	(0.213, 0.500, -0.000)	6.141	9	8	12	2	2	0.426
21	(0.405, 0.500, -0.000)	6.166	9	8	12	2	2	0.809
22	(0.329, 0.329, -0.000)	6.072	9	8	12	2	3	0.341
23	(0.291, 0.500, 0.291)	5.611	9	8	12	2	5	0.418
24	(0.141, 0.500, 0.141)	6.202	9	8	12	2	5	0.718
25	(0.304, 0.500, -0.000)	6.370	10	9	12	2	2	0.609
26	(0.233, 0.233, -0.000)	6.496	10	9	12	2	3	0.534
27	(0.343, 0.500, -0.000)	6.458	11	10	12	2	2	0.686
28	(0.213, 0.500, 0.213)	6.481	11	10	12	2	5	0.573

The followings are details for $Li_{12}Sn_4$. It has 6 topological nodal lines and 14 nodal ring points along high symmetry line as shown in Fig **815**.

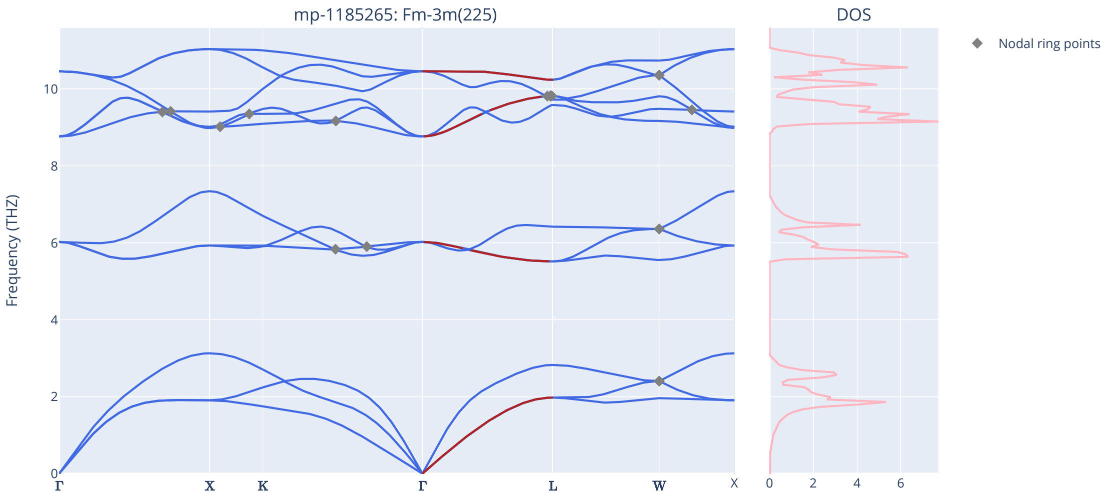

Supplementary Figure 815: The phonon band and DOS of Li12Sn4.

Supplementary Table 1778: High symmetry lines of mp-1185265-Li12Sn4

	Start	End
0	Γ (0.000, 0.000, 0.000)	X (0.500, 0.000, 0.500)
1	X (0.500, 0.000, 0.500)	U (0.625, 0.250, 0.625)
2	K (0.375, 0.375, 0.750)	Γ (0.000, 0.000, 0.000)
3	Γ (0.000, 0.000, 0.000)	L (0.500, 0.500, 0.500)
4	L (0.500, 0.500, 0.500)	W (0.500, 0.250, 0.750)
5	W (0.500, 0.250, 0.750)	X (0.500, 0.000, 0.500)

Supplementary Table 1779: Nodal ring points along high symmetry lines of mp-1185265-Li12Sn4.

	Coordinates	Frequency	Mode 1	Mode 2	Multi.	Deg.	Path	Ratio
0	(0.500, 0.250, 0.750)	2.407	3	2	6	2	5	1.000
1	(0.199, 0.199, 0.414)	5.834	5	4	24	2	3	0.456
2	(0.127, 0.127, 0.265)	5.903	6	5	24	2	3	0.651
3	(0.500, 0.250, 0.750)	6.361	6	5	6	2	5	1.000
4	(0.343, 0.000, 0.343)	9.401	8	7	6	2	1	0.687
5	(0.526, 0.049, 0.526)	9.018	8	7	24	2	2	0.198
6	(0.199, 0.199, 0.411)	9.167	8	7	24	2	3	0.458
7	(0.593, 0.186, 0.593)	9.350	9	8	12	2	2	0.746
8	(0.500, 0.141, 0.641)	9.455	9	8	12	2	6	0.435
9	(0.496, 0.496, 0.496)	9.818	10	9	8	2	4	0.993
10	(0.370, 0.000, 0.370)	9.416	10	9	10	2	1	0.741
11	(0.491, 0.491, 0.491)	9.817	10	9	8	2	4	0.981
12	(0.479, 0.479, 0.479)	9.814	10	9	8	2	4	0.958
13	(0.500, 0.250, 0.750)	10.357	11	10	6	2	5	1.000

The followings are details for Ag3. It has 7 topological nodal lines and 14 nodal ring points along high symmetry line as shown in Fig 816.

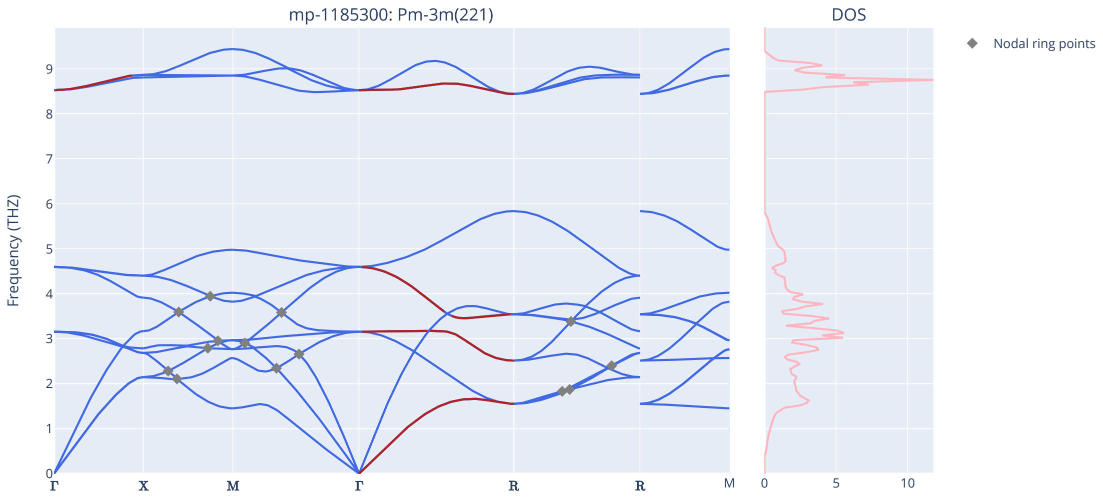

Supplementary Figure 816: The phonon band and DOS of Ag3Li.

Supplementary Table 1780: High symmetry lines of mp-1185300-Ag3Li

	Start	End
0	Γ (0.000, 0.000, 0.000)	X (0.000, 0.500, 0.000)
1	X (0.000, 0.500, 0.000)	M (0.500, 0.500, 0.000)
2	M (0.500, 0.500, 0.000)	Γ (0.000, 0.000, 0.000)
3	Γ (0.000, 0.000, 0.000)	R (0.500, 0.500, 0.500)
4	R (0.500, 0.500, 0.500)	X (0.000, 0.500, 0.000)
5	R (0.500, 0.500, 0.500)	M (0.500, 0.500, 0.000)

Supplementary Table 1781: Nodal ring points along high symmetry lines of mp-1185300-Ag3Li.

	Coordinates	Frequency	Mode 1	Mode 2	Multi.	Deg.	Path	Ratio
0	(0.188, 0.500, 0.000)	2.109	2	1	12	2	2	0.376
1	(0.279, 0.500, 0.279)	1.873	2	1	12	2	5	0.442
2	(0.138, 0.500, -0.000)	2.283	3	2	12	2	2	0.277
3	(0.327, 0.327, -0.000)	2.346	3	2	12	2	3	0.346
4	(0.308, 0.500, 0.308)	1.832	3	2	12	2	5	0.384
5	(0.361, 0.500, -0.000)	2.792	4	3	12	2	2	0.721
6	(0.238, 0.238, -0.000)	2.659	4	3	12	2	3	0.525
7	(0.112, 0.496, 0.112)	2.402	4	3	24	2	5	0.775
8	(0.452, 0.452, 0.002)	2.905	5	4	24	2	3	0.096
9	(0.417, 0.500, 0.000)	2.955	6	5	12	2	2	0.834
10	(0.274, 0.500, 0.274)	3.383	6	5	12	2	5	0.452
11	(0.198, 0.500, -0.000)	3.595	7	6	12	2	2	0.395
12	(0.307, 0.307, -0.000)	3.584	7	6	12	2	3	0.386
13	(0.374, 0.500, -0.000)	3.945	8	7	12	2	2	0.748

The followings are details for Cu$_3$. It has 7 topological nodal lines and 19 nodal ring points along high symmetry line as shown in Fig **817**.

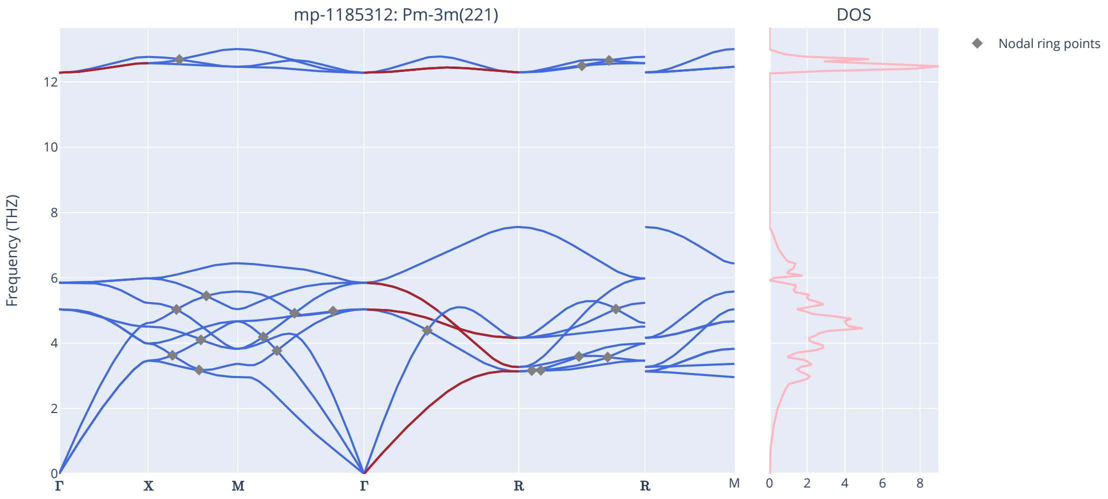

Supplementary Figure 817: The phonon band and DOS of Cu3Li.

Supplementary Table 1782: High symmetry lines of mp-1185312-Cu3Li

	Start	End
0	Γ (0.000, 0.000, 0.000)	X (0.000, 0.500, 0.000)
1	X (0.000, 0.500, 0.000)	M (0.500, 0.500, 0.000)
2	M (0.500, 0.500, 0.000)	Γ (0.000, 0.000, 0.000)
3	Γ (0.000, 0.000, 0.000)	R (0.500, 0.500, 0.500)
4	R (0.500, 0.500, 0.500)	X (0.000, 0.500, 0.000)
5	R (0.500, 0.500, 0.500)	M (0.500, 0.500, 0.000)

Supplementary Table 1783: Nodal ring points along high symmetry lines of mp-1185312-Cu3Li.

	Coordinates	Frequency	Mode 1	Mode 2	Multi.	Deg.	Path	Ratio
0	(0.284, 0.500, -0.000)	3.177	2	1	12	2	2	0.568
1	(0.413, 0.500, 0.413)	3.166	2	1	12	2	5	0.175
2	(0.135, 0.500, 0.000)	3.626	3	2	12	2	2	0.270
3	(0.345, 0.345, -0.000)	3.772	3	2	12	2	3	0.311
4	(0.449, 0.500, 0.449)	3.160	3	2	12	2	5	0.103
5	(0.148, 0.500, 0.148)	3.573	3	2	12	2	5	0.704
6	(0.294, 0.500, 0.000)	4.099	4	3	12	2	2	0.587
7	(0.204, 0.204, 0.204)	4.389	4	3	8	2	4	0.409
8	(0.262, 0.500, 0.262)	3.592	4	3	12	2	5	0.476
9	(0.399, 0.399, 0.000)	4.191	5	4	12	2	3	0.203
10	(0.123, 0.123, 0.000)	4.979	6	5	12	2	3	0.755
11	(0.157, 0.500, 0.000)	5.031	7	6	12	2	2	0.314
12	(0.275, 0.275, -0.000)	4.917	7	6	12	2	3	0.450
13	(0.115, 0.500, 0.115)	5.045	7	6	12	2	5	0.769

Supplementary Table 1783: Nodal ring points along high symmetry lines of mp-1185312-Cu3Li.

	Coordinates	Frequency	Mode 1	Mode 2	Multi.	Deg.	Path	Ratio
14	(0.324, 0.500, 0.000)	5.446	8	7	12	2	2	0.649
15	(0.250, 0.500, 0.250)	12.495	11	10	12	2	5	0.500
16	(0.173, 0.500, -0.000)	12.686	12	11	12	2	2	0.347
17	(0.142, 0.500, 0.142)	12.658	12	11	12	2	5	0.715

The followings are details for AlLu$_3$. It has 8 topological nodal lines and 18 nodal ring points along high symmetry line as shown in Fig 818.

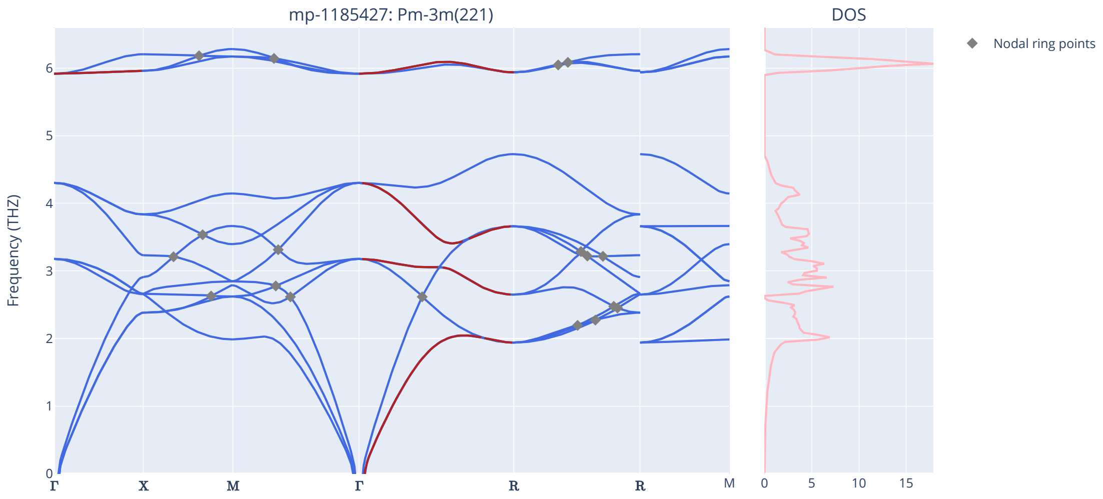

Supplementary Figure 818: The phonon band and DOS of AlLu3.

Supplementary Table 1784: High symmetry lines of mp-1185427-AlLu3

	Start	End
0	Γ (0.000, 0.000, 0.000)	X (0.000, 0.500, 0.000)
1	X (0.000, 0.500, 0.000)	M (0.500, 0.500, 0.000)
2	M (0.500, 0.500, 0.000)	Γ (0.000, 0.000, 0.000)
3	Γ (0.000, 0.000, 0.000)	R (0.500, 0.500, 0.500)
4	R (0.500, 0.500, 0.500)	X (0.000, 0.500, 0.000)
5	R (0.500, 0.500, 0.500)	M (0.500, 0.500, 0.000)

Supplementary Table 1785: Nodal ring points along high symmetry lines of mp-1185427-AlLu3.

	Coordinates	Frequency	Mode 1	Mode 2	Multi.	Deg.	Path	Ratio
0	(0.177, 0.500, 0.177)	2.273	2	1	12	2	5	0.646
1	(0.247, 0.500, 0.247)	2.195	3	2	12	2	5	0.505
2	(0.089, 0.500, 0.089)	2.451	3	2	12	2	5	0.821
3	(0.379, 0.500, -0.003)	2.629	4	3	24	2	2	0.758
4	(0.272, 0.272, 0.000)	2.616	4	3	12	2	3	0.457
5	(0.204, 0.204, 0.204)	2.617	4	3	8	2	4	0.408
6	(0.104, 0.500, 0.104)	2.475	4	3	12	2	5	0.792
7	(0.330, 0.330, 0.000)	2.778	5	4	12	2	3	0.341
8	(0.209, 0.500, 0.209)	3.220	6	5	12	2	5	0.583
9	(0.169, 0.500, -0.000)	3.209	7	6	12	2	2	0.337
10	(0.320, 0.320, 0.000)	3.314	7	6	12	2	3	0.360
11	(0.234, 0.500, 0.234)	3.287	7	6	12	2	5	0.532
12	(0.147, 0.500, 0.147)	3.216	7	6	12	2	5	0.706
13	(0.331, 0.500, -0.000)	3.535	8	7	12	2	2	0.663

Supplementary Table 1785: Nodal ring points along high symmetry lines of mp-1185427-AlLu3.

	Coordinates	Frequency	Mode 1	Mode 2	Multi.	Deg.	Path	Ratio
14	(0.324, 0.500, 0.324)	6.046	11	10	12	2	5	0.352
15	(0.311, 0.500, -0.000)	6.182	12	11	12	2	2	0.623
16	(0.337, 0.337, -0.001)	6.143	12	11	24	2	3	0.327
17	(0.286, 0.500, 0.286)	6.084	12	11	12	2	5	0.429

The followings are details for Mg3. It has 6 topological nodal lines and 24 nodal ring points along high symmetry line as shown in Fig 819.

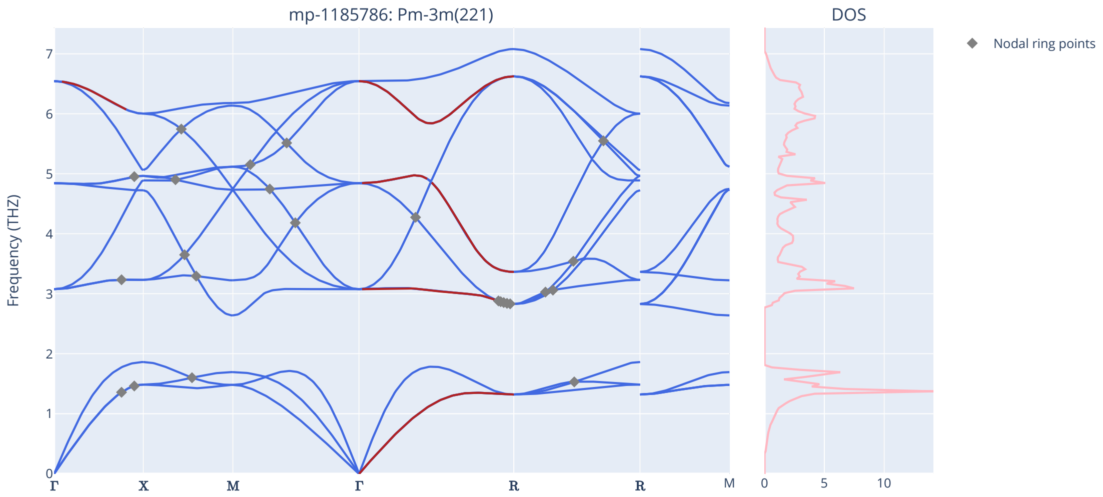

Supplementary Figure 819: The phonon band and DOS of Mg3Tl.

Supplementary Table 1786: High symmetry lines of mp-1185786-Mg3Tl

	Start	End
0	Γ (0.000, 0.000, 0.000)	X (0.000, 0.500, 0.000)
1	X (0.000, 0.500, 0.000)	M (0.500, 0.500, 0.000)
2	M (0.500, 0.500, 0.000)	Γ (0.000, 0.000, 0.000)
3	Γ (0.000, 0.000, 0.000)	R (0.500, 0.500, 0.500)
4	R (0.500, 0.500, 0.500)	X (0.000, 0.500, 0.000)
5	R (0.500, 0.500, 0.500)	M (0.500, 0.500, 0.000)

Supplementary Table 1787: Nodal ring points along high symmetry lines of mp-1185786-Mg3Tl.

	Coordinates	Frequency	Mode 1	Mode 2	Multi.	Deg.	Path	Ratio
0	(0.000, 0.378, 0.000)	1.358	2	1	6	2	1	0.755
1	(0.000, 0.449, 0.000)	1.463	2	1	6	2	1	0.898
2	(0.272, 0.500, 0.000)	1.601	3	2	12	2	2	0.544
3	(0.261, 0.500, 0.261)	1.531	3	2	12	2	5	0.479
4	(0.000, 0.378, 0.000)	3.234	5	4	6	2	1	0.755
5	(0.296, 0.500, 0.000)	3.296	5	4	12	2	2	0.591
6	(0.345, 0.500, 0.345)	3.062	5	4	12	2	5	0.310
7	(0.231, 0.500, 0.000)	3.651	6	5	12	2	2	0.461
8	(0.468, 0.468, 0.468)	2.854	6	5	8	2	4	0.935
9	(0.458, 0.458, 0.458)	2.869	6	5	8	2	4	0.915
10	(0.478, 0.478, 0.478)	2.841	6	5	8	2	4	0.956
11	(0.488, 0.488, 0.488)	2.833	6	5	8	2	4	0.977
12	(0.450, 0.450, 0.450)	2.881	6	5	8	2	4	0.901
13	(0.375, 0.500, 0.375)	3.029	6	5	12	2	5	0.251

Supplementary Table 1787: Nodal ring points along high symmetry lines of mp-1185786-Mg3Tl.

	Coordinates	Frequency	Mode 1	Mode 2	Multi.	Deg.	Path	Ratio
14	(0.252, 0.252, 0.000)	4.184	7	6	12	2	3	0.495
15	(0.183, 0.183, 0.183)	4.275	7	6	8	2	4	0.365
16	(0.264, 0.500, 0.264)	3.543	7	6	12	2	5	0.472
17	(0.180, 0.500, 0.000)	4.903	8	7	12	2	2	0.359
18	(0.353, 0.353, -0.000)	4.747	8	7	12	2	3	0.293
19	(0.000, 0.449, 0.000)	4.954	9	8	6	2	1	0.898
20	(0.430, 0.430, 0.000)	5.154	10	9	12	2	3	0.139
21	(0.287, 0.287, -0.000)	5.515	10	9	12	2	3	0.427
22	(0.145, 0.500, 0.145)	5.550	10	9	12	2	5	0.709
23	(0.212, 0.500, -0.000)	5.747	11	10	12	2	2	0.424

The followings are details for NiPt$_3$. It has 8 topological nodal lines and 27 nodal ring points along high symmetry line as shown in Fig **820**.

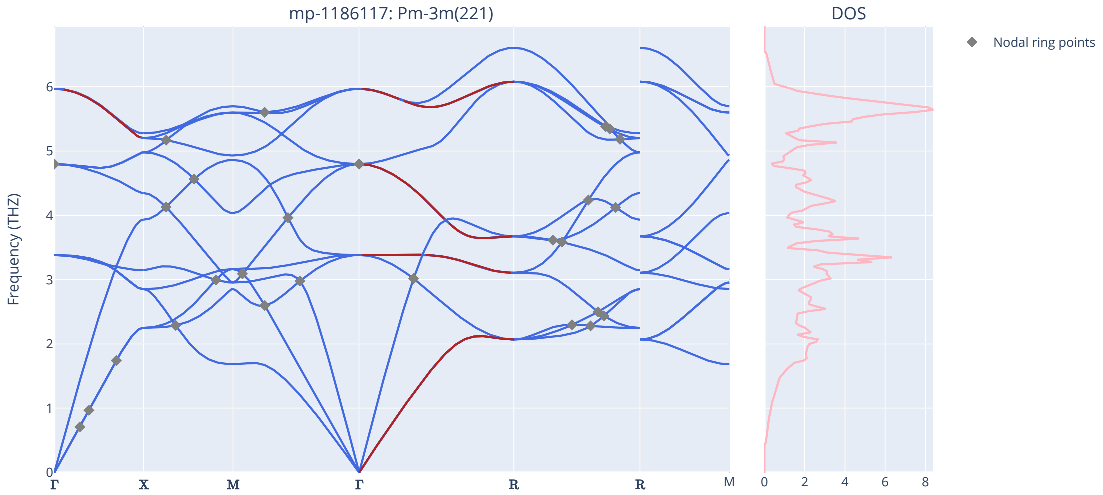

Supplementary Figure 820: The phonon band and DOS of NiPt3.

Supplementary Table 1788: High symmetry lines of mp-1186117-NiPt3

	Start	End
0	Γ (0.000, 0.000, 0.000)	X (0.000, 0.500, 0.000)
1	X (0.000, 0.500, 0.000)	M (0.500, 0.500, 0.000)
2	M (0.500, 0.500, 0.000)	Γ (0.000, 0.000, 0.000)
3	Γ (0.000, 0.000, 0.000)	R (0.500, 0.500, 0.500)
4	R (0.500, 0.500, 0.500)	X (0.000, 0.500, 0.000)
5	R (0.500, 0.500, 0.500)	M (0.500, 0.500, 0.000)

Supplementary Table 1789: Nodal ring points along high symmetry lines of mp-1186117-NiPt3.

	Coordinates	Frequency	Mode 1	Mode 2	Multi.	Deg.	Path	Ratio
0	(0.000, 0.143, 0.000)	0.708	2	1	6	2	1	0.286
1	(0.000, 0.194, 0.000)	0.966	2	1	6	2	1	0.388
2	(0.000, 0.347, 0.000)	1.741	2	1	6	2	1	0.694
3	(0.179, 0.500, -0.000)	2.287	2	1	12	2	2	0.359
4	(0.196, 0.500, 0.196)	2.279	2	1	12	2	5	0.608
5	(0.374, 0.374, 0.000)	2.596	3	2	12	2	3	0.253
6	(0.269, 0.500, 0.269)	2.297	3	2	12	2	5	0.461
7	(0.143, 0.500, 0.143)	2.435	3	2	12	2	5	0.714
8	(0.405, 0.500, -0.000)	2.994	4	3	12	2	2	0.810
9	(0.235, 0.235, -0.000)	2.976	4	3	12	2	3	0.531
10	(0.176, 0.176, 0.176)	3.014	4	3	8	2	4	0.352
11	(0.166, 0.500, 0.166)	2.498	4	3	12	2	5	0.667
12	(0.462, 0.462, -0.000)	3.089	5	4	12	2	3	0.076
13	(0.309, 0.500, 0.309)	3.582	6	5	12	2	5	0.381

Supplementary Table 1789: Nodal ring points along high symmetry lines of mp-1186117-NiPt3.

	Coordinates	Frequency	Mode 1	Mode 2	Multi.	Deg.	Path	Ratio
14	(0.126, 0.500, -0.000)	4.126	7	6	12	2	2	0.251
15	(0.282, 0.282, 0.000)	3.962	7	6	12	2	3	0.436
16	(0.345, 0.500, 0.345)	3.612	7	6	12	2	5	0.310
17	(0.097, 0.500, 0.097)	4.121	7	6	12	2	5	0.807
18	(0.000, 0.000, 0.000)	4.796	8	7	1	3	1	0.000
19	(0.283, 0.498, 0.000)	4.562	8	7	24	2	2	0.566
20	(0.205, 0.500, 0.205)	4.236	8	7	12	2	5	0.591
21	(0.127, 0.500, -0.000)	5.168	10	9	12	2	2	0.254
22	(0.079, 0.500, 0.079)	5.180	10	9	12	2	5	0.841
23	(0.136, 0.500, 0.136)	5.379	11	10	12	2	5	0.728
24	(0.120, 0.500, 0.120)	5.341	11	10	12	2	5	0.761
25	(0.374, 0.374, -0.000)	5.601	12	11	12	2	3	0.251

The followings are details for Hg$_3$. It has 7 topological nodal lines and 18 nodal ring points along high symmetry line as shown in Fig **821**.

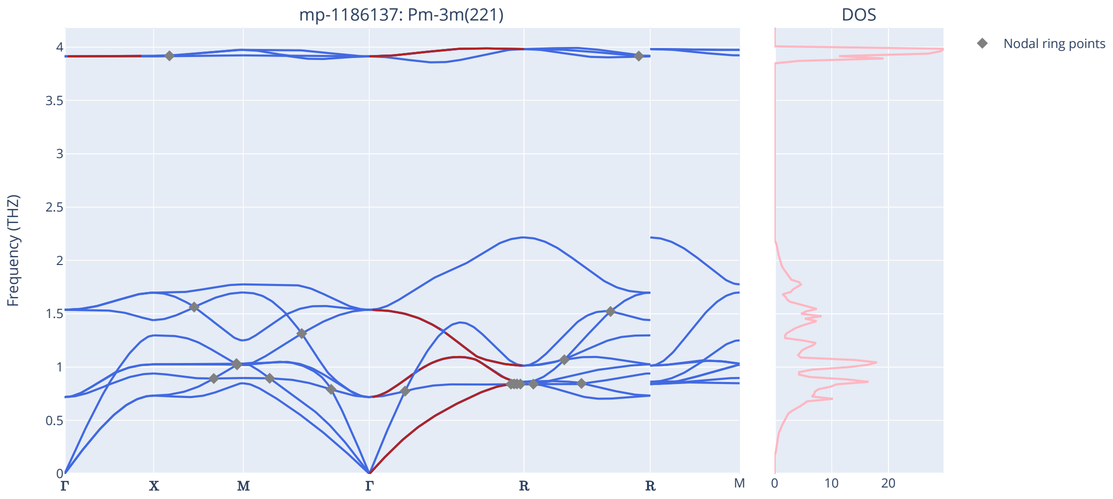

Supplementary Figure 821: The phonon band and DOS of Hg3Na.

Supplementary Table 1790: High symmetry lines of mp-1186137-Hg3Na

	Start	End
0	Γ (0.000, 0.000, 0.000)	X (0.000, 0.500, 0.000)
1	X (0.000, 0.500, 0.000)	M (0.500, 0.500, 0.000)
2	M (0.500, 0.500, 0.000)	Γ (0.000, 0.000, 0.000)
3	Γ (0.000, 0.000, 0.000)	R (0.500, 0.500, 0.500)
4	R (0.500, 0.500, 0.500)	X (0.000, 0.500, 0.000)
5	R (0.500, 0.500, 0.500)	M (0.500, 0.500, 0.000)

Supplementary Table 1791: Nodal ring points along high symmetry lines of mp-1186137-Hg3Na.

	Coordinates	Frequency	Mode 1	Mode 2	Multi.	Deg.	Path	Ratio
0	(0.463, 0.500, 0.463)	0.840	2	1	12	2	5	0.074
1	(0.336, 0.500, 0.000)	0.893	3	2	12	2	2	0.671
2	(0.395, 0.395, 0.000)	0.895	3	2	12	2	3	0.210
3	(0.477, 0.477, 0.477)	0.842	3	2	8	2	4	0.954
4	(0.458, 0.458, 0.458)	0.840	3	2	8	2	4	0.915
5	(0.468, 0.468, 0.468)	0.842	3	2	8	2	4	0.936
6	(0.488, 0.488, 0.488)	0.841	3	2	8	2	4	0.977
7	(0.273, 0.500, 0.273)	0.846	3	2	12	2	5	0.455
8	(0.466, 0.500, -0.000)	1.021	4	3	12	2	2	0.932
9	(0.152, 0.152, 0.000)	0.791	4	3	12	2	3	0.696
10	(0.115, 0.115, 0.115)	0.774	4	3	8	2	4	0.231
11	(0.463, 0.500, 0.000)	1.032	6	5	12	2	2	0.925
12	(0.340, 0.500, 0.340)	1.068	6	5	12	2	5	0.319
13	(0.268, 0.268, -0.000)	1.314	7	6	12	2	3	0.464

Supplementary Table 1791: Nodal ring points along high symmetry lines of mp-1186137-Hg3Na.

	Coordinates	Frequency	Mode 1	Mode 2	Multi.	Deg.	Path	Ratio
14	(0.226, 0.500, -0.000)	1.563	8	7	12	2	2	0.451
15	(0.158, 0.500, 0.158)	1.521	8	7	12	2	5	0.685
16	(0.087, 0.500, -0.000)	3.918	11	10	12	2	2	0.175
17	(0.046, 0.500, 0.046)	3.916	11	10	12	2	5	0.909

The followings are details for $Nb_{12}Os_4$. It has 7 topological nodal lines and 12 nodal ring points along high symmetry line as shown in Fig **822**.

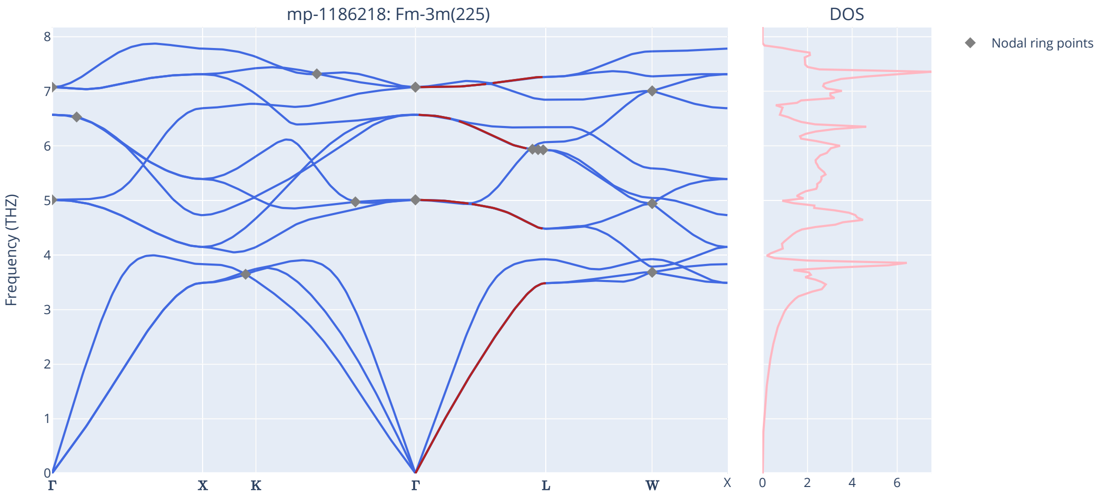

Supplementary Figure 822: The phonon band and DOS of Nb12Os4.

Supplementary Table 1792: High symmetry lines of mp-1186218-Nb12Os4

	Start	End
0	Γ (0.000, 0.000, 0.000)	X (0.500, 0.000, 0.500)
1	X (0.500, 0.000, 0.500)	U (0.625, 0.250, 0.625)
2	K (0.375, 0.375, 0.750)	Γ (0.000, 0.000, 0.000)
3	Γ (0.000, 0.000, 0.000)	L (0.500, 0.500, 0.500)
4	L (0.500, 0.500, 0.500)	W (0.500, 0.250, 0.750)
5	W (0.500, 0.250, 0.750)	X (0.500, 0.000, 0.500)

Supplementary Table 1793: Nodal ring points along high symmetry lines of mp-1186218-Nb12Os4.

	Coordinates	Frequency	Mode 1	Mode 2	Multi.	Deg.	Path	Ratio
0	(0.601, 0.202, 0.601)	3.647	2	1	12	2	2	0.808
1	(0.500, 0.250, 0.750)	3.685	2	1	6	2	5	1.000
2	(0.000, 0.000, 0.000)	5.012	5	4	1	3	1	0.000
3	(0.145, 0.145, 0.278)	4.974	6	5	24	2	3	0.624
4	(0.500, 0.250, 0.750)	4.943	6	5	6	2	5	1.000
5	(0.490, 0.490, 0.490)	5.927	7	6	8	2	4	0.980
6	(0.471, 0.471, 0.471)	5.931	7	6	8	2	4	0.941
7	(0.449, 0.449, 0.449)	5.940	7	6	8	2	4	0.898
8	(0.082, 0.000, 0.082)	6.530	9	8	6	2	1	0.163
9	(0.500, 0.250, 0.750)	7.008	10	9	6	2	5	1.000
10	(0.000, 0.000, 0.000)	7.077	11	10	1	3	1	0.000
11	(0.237, 0.237, 0.458)	7.320	12	11	24	2	3	0.382

The followings are details for $InTh_3$. It has 8 topological nodal lines and 30 nodal ring points along high symmetry line as shown in Fig 823.

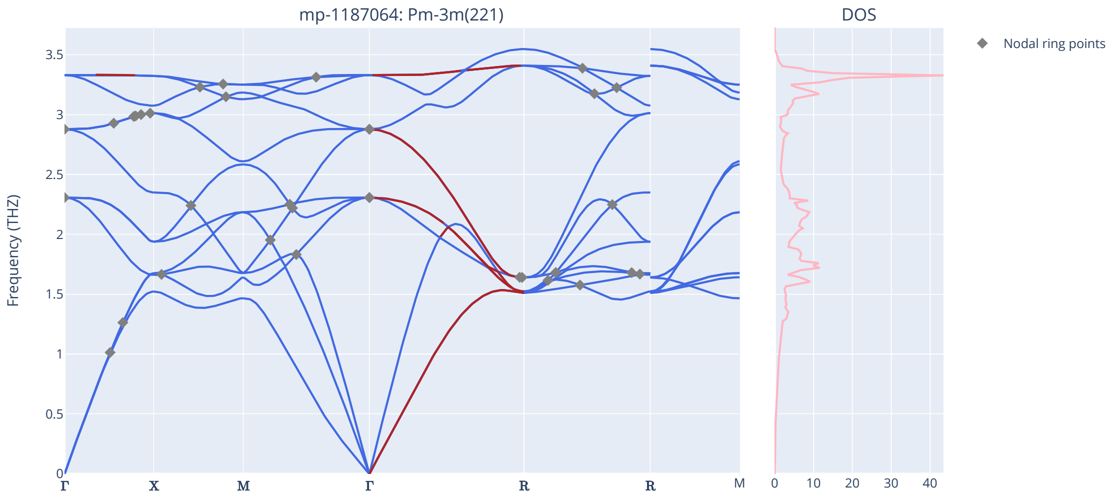

Supplementary Figure 823: The phonon band and DOS of InTh3.

Supplementary Table 1794: High symmetry lines of mp-1187064-InTh3

	Start	End
0	Γ (0.000, 0.000, 0.000)	X (0.000, 0.500, 0.000)
1	X (0.000, 0.500, 0.000)	M (0.500, 0.500, 0.000)
2	M (0.500, 0.500, 0.000)	Γ (0.000, 0.000, 0.000)
3	Γ (0.000, 0.000, 0.000)	R (0.500, 0.500, 0.500)
4	R (0.500, 0.500, 0.500)	X (0.000, 0.500, 0.000)
5	R (0.500, 0.500, 0.500)	M (0.500, 0.500, 0.000)

Supplementary Table 1795: Nodal ring points along high symmetry lines of mp-1187064-InTh3.

	Coordinates	Frequency	Mode 1	Mode 2	Multi.	Deg.	Path	Ratio
0	(0.278, 0.500, 0.278)	1.576	2	1	12	2	5	0.443
1	(0.000, 0.255, 0.000)	1.013	3	2	6	2	1	0.510
2	(0.000, 0.327, 0.000)	1.263	3	2	6	2	1	0.653
3	(0.043, 0.500, -0.000)	1.665	3	2	12	2	2	0.086
4	(0.041, 0.500, 0.041)	1.668	3	2	12	2	5	0.917
5	(0.289, 0.289, 0.000)	1.831	4	3	12	2	3	0.421
6	(0.072, 0.500, 0.075)	1.680	4	3	24	2	5	0.853
7	(0.392, 0.392, -0.000)	1.953	5	4	12	2	3	0.215
8	(0.405, 0.500, 0.405)	1.616	5	4	12	2	5	0.190
9	(0.000, 0.000, 0.000)	2.307	6	5	1	3	1	0.000
10	(0.305, 0.305, 0.000)	2.220	6	5	12	2	3	0.391
11	(0.374, 0.500, 0.374)	1.681	6	5	12	2	5	0.251
12	(0.208, 0.500, 0.000)	2.241	7	6	12	2	2	0.415
13	(0.315, 0.315, 0.000)	2.251	7	6	12	2	3	0.370

Supplementary Table 1795: Nodal ring points along high symmetry lines of mp-1187064-InTh3.

	Coordinates	Frequency	Mode 1	Mode 2	Multi.	Deg.	Path	Ratio
14	(0.494, 0.494, 0.494)	1.640	7	6	8	3	4	0.988
15	(0.486, 0.486, 0.486)	1.641	7	6	8	2	4	0.972
16	(0.151, 0.500, 0.151)	2.248	7	6	12	2	5	0.699
17	(0.000, 0.000, 0.000)	2.878	8	7	1	3	1	0.000
18	(0.000, 0.276, 0.000)	2.928	9	8	6	2	1	0.551
19	(0.000, 0.388, 0.000)	2.985	9	8	6	2	1	0.776
20	(0.000, 0.398, 0.000)	2.989	9	8	6	2	1	0.796
21	(0.000, 0.429, 0.000)	3.001	9	8	6	2	1	0.857
22	(0.000, 0.480, 0.000)	3.012	9	8	6	2	1	0.959
23	(0.404, 0.500, 0.000)	3.149	10	9	12	2	2	0.808
24	(0.221, 0.500, 0.221)	3.175	10	9	12	2	5	0.557
25	(0.258, 0.500, 0.000)	3.230	11	10	12	2	2	0.516
26	(0.133, 0.500, 0.133)	3.225	11	10	12	2	5	0.734
27	(0.388, 0.500, 0.000)	3.255	12	11	12	2	2	0.776
28	(0.211, 0.211, -0.000)	3.313	12	11	12	2	3	0.578
29	(0.269, 0.500, 0.269)	3.388	12	11	12	2	5	0.463

The followings are details for Ir_4Ta_{12}. It has 7 topological nodal lines and 15 nodal ring points along high symmetry line as shown in Fig **824**.

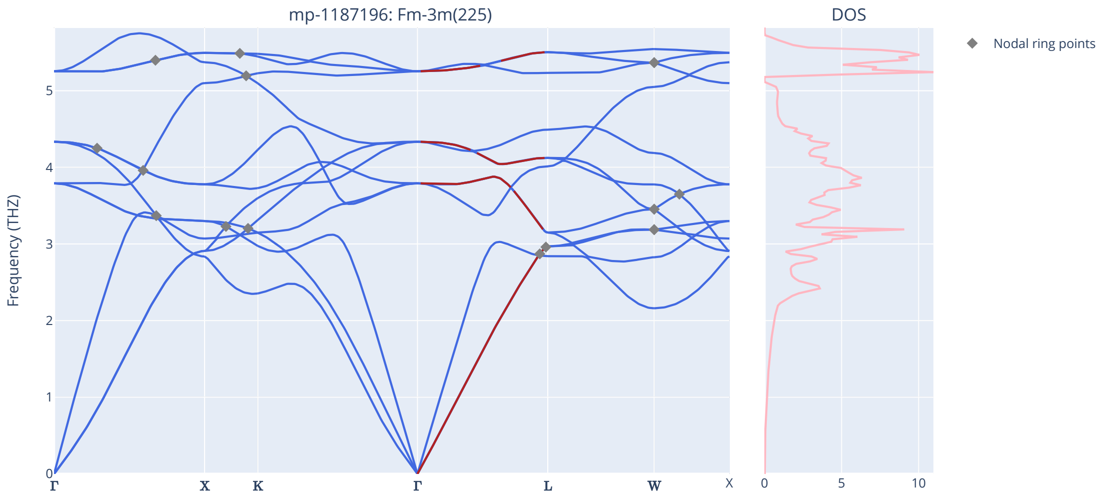

Supplementary Figure 824: The phonon band and DOS of Ir4Ta12.

Supplementary Table 1796: High symmetry lines of mp-1187196-Ir4Ta12

	Start	End
0	Γ (0.000, 0.000, 0.000)	X (0.500, 0.000, 0.500)
1	X (0.500, 0.000, 0.500)	U (0.625, 0.250, 0.625)
2	K (0.375, 0.375, 0.750)	Γ (0.000, 0.000, 0.000)
3	Γ (0.000, 0.000, 0.000)	L (0.500, 0.500, 0.500)
4	L (0.500, 0.500, 0.500)	W (0.500, 0.250, 0.750)
5	W (0.500, 0.250, 0.750)	X (0.500, 0.000, 0.500)

Supplementary Table 1797: Nodal ring points along high symmetry lines of mp-1187196-Ir4Ta12.

	Coordinates	Frequency	Mode 1	Mode 2	Multi.	Deg.	Path	Ratio
0	(0.493, 0.493, 0.493)	2.960	3	2	8	2	4	0.986
1	(0.469, 0.469, 0.469)	2.870	3	2	8	2	4	0.939
2	(0.500, 0.250, 0.750)	3.187	4	3	6	2	5	1.000
3	(0.550, 0.100, 0.550)	3.228	5	4	12	2	2	0.402
4	(0.602, 0.204, 0.602)	3.203	5	4	12	2	2	0.817
5	(0.339, 0.000, 0.339)	3.369	6	5	6	2	1	0.679
6	(0.500, 0.250, 0.750)	3.454	6	5	6	2	5	1.000
7	(0.499, 0.166, 0.667)	3.650	7	6	24	2	6	0.335
8	(0.296, 0.000, 0.296)	3.960	8	7	6	2	1	0.592
9	(0.143, 0.000, 0.143)	4.250	9	8	6	2	1	0.286
10	(0.598, 0.195, 0.598)	5.195	10	9	12	2	2	0.781
11	(0.337, 0.000, 0.337)	5.398	11	10	6	2	1	0.673
12	(0.500, 0.250, 0.750)	5.367	11	10	6	2	5	1.000
13	(0.583, 0.166, 0.583)	5.488	12	11	12	2	2	0.662

The followings are details for $Cr_{12}V_4$. It has 8 topological nodal lines and 18 nodal ring points along high symmetry line as shown in Fig **825**.

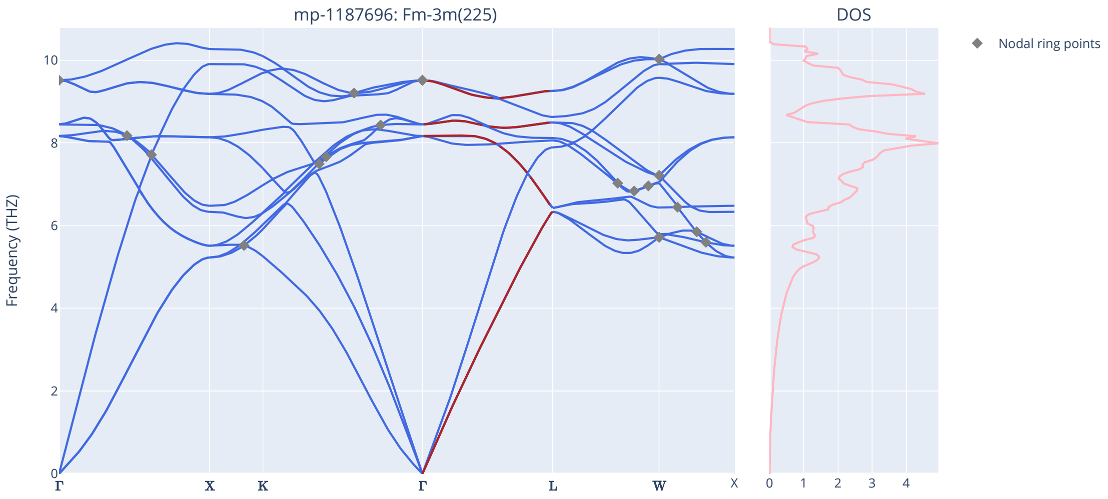

Supplementary Figure 825: The phonon band and DOS of Cr12V4.

Supplementary Table 1798: High symmetry lines of mp-1187696-Cr12V4

	Start	End
0	Γ (0.000, 0.000, 0.000)	X (0.500, 0.000, 0.500)
1	X (0.500, 0.000, 0.500)	U (0.625, 0.250, 0.625)
2	K (0.375, 0.375, 0.750)	Γ (0.000, 0.000, 0.000)
3	Γ (0.000, 0.000, 0.000)	L (0.500, 0.500, 0.500)
4	L (0.500, 0.500, 0.500)	W (0.500, 0.250, 0.750)
5	W (0.500, 0.250, 0.750)	X (0.500, 0.000, 0.500)

Supplementary Table 1799: Nodal ring points along high symmetry lines of mp-1187696-Cr12V4.

	Coordinates	Frequency	Mode 1	Mode 2	Multi.	Deg.	Path	Ratio
0	(0.581, 0.162, 0.581)	5.518	2	1	12	2	2	0.648
1	(0.500, 0.250, 0.750)	5.717	2	1	6	2	5	1.000
2	(0.500, 0.095, 0.595)	5.596	3	2	12	2	6	0.621
3	(0.497, 0.124, 0.627)	5.851	4	3	24	2	6	0.500
4	(0.500, 0.189, 0.689)	6.446	5	4	24	2	6	0.243
5	(0.237, 0.237, 0.490)	7.491	6	5	24	2	3	0.353
6	(0.500, 0.275, 0.725)	6.960	6	5	24	2	5	0.898
7	(0.500, 0.309, 0.691)	6.835	6	5	24	2	5	0.765
8	(0.500, 0.347, 0.653)	7.025	6	5	24	2	5	0.611
9	(0.307, -0.001, 0.307)	7.713	7	6	24	2	1	0.614
10	(0.220, 0.220, 0.458)	7.664	7	6	24	2	3	0.397
11	(0.099, 0.099, 0.195)	8.429	8	7	24	2	3	0.738
12	(0.500, 0.250, 0.750)	7.212	8	7	6	2	5	1.000
13	(0.226, -0.000, 0.226)	8.175	9	8	10	2	1	0.451

Supplementary Table 1799: Nodal ring points along high symmetry lines of mp-1187696-Cr12V4.

	Coordinates	Frequency	Mode 1	Mode 2	Multi.	Deg.	Path	Ratio
14	(0.000, 0.000, 0.000)	9.513	11	10	1	3	1	0.000
15	(0.164, 0.164, 0.318)	9.204	12	11	24	2	3	0.572
16	(0.500, 0.250, 0.750)	10.023	12	11	6	2	5	1.000

The followings are details for V_4W_{12}. It has 7 topological nodal lines and 11 nodal ring points along high symmetry line as shown in Fig **826**.

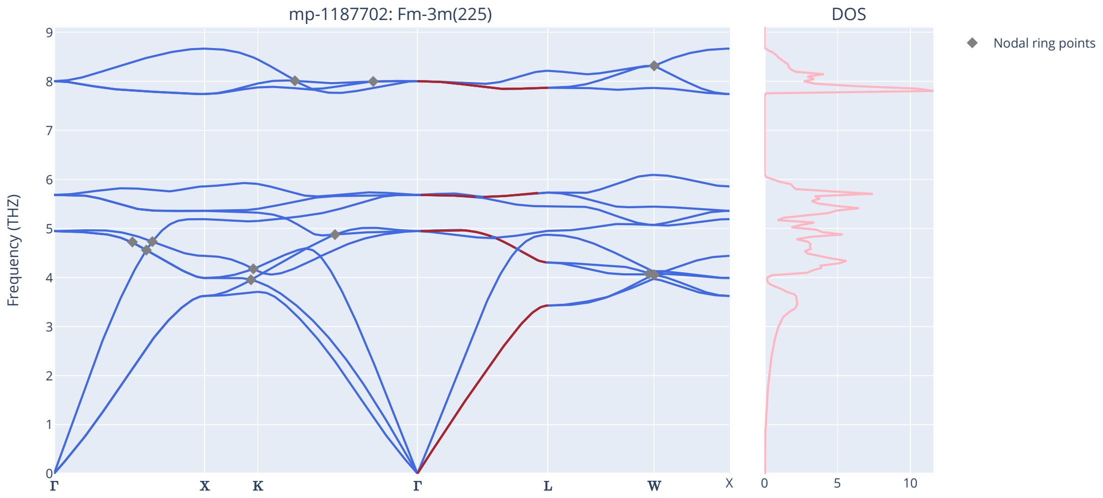

Supplementary Figure 826: The phonon band and DOS of V4W12.

Supplementary Table 1800: High symmetry lines of mp-1187702-V4W12

	Start	End
0	Γ (0.000, 0.000, 0.000)	X (0.500, 0.000, 0.500)
1	X (0.500, 0.000, 0.500)	U (0.625, 0.250, 0.625)
2	K (0.375, 0.375, 0.750)	Γ (0.000, 0.000, 0.000)
3	Γ (0.000, 0.000, 0.000)	L (0.500, 0.500, 0.500)
4	L (0.500, 0.500, 0.500)	W (0.500, 0.250, 0.750)
5	W (0.500, 0.250, 0.750)	X (0.500, 0.000, 0.500)

Supplementary Table 1801: Nodal ring points along high symmetry lines of mp-1187702-V4W12.

	Coordinates	Frequency	Mode 1	Mode 2	Multi.	Deg.	Path	Ratio
0	(0.609, 0.218, 0.609)	3.954	3	2	12	2	2	0.871
1	(0.500, 0.250, 0.750)	4.056	3	2	6	2	5	1.000
2	(0.307, -0.000, 0.307)	4.560	4	3	6	2	1	0.613
3	(0.500, 0.262, 0.738)	4.077	4	3	24	2	5	0.951
4	(0.615, 0.229, 0.615)	4.177	5	4	12	2	2	0.916
5	(0.260, 0.007, 0.260)	4.725	5	4	24	2	1	0.520
6	(0.326, 0.000, 0.326)	4.733	6	5	6	2	1	0.653
7	(0.187, 0.200, 0.387)	4.876	6	5	24	2	3	0.484
8	(0.108, 0.108, 0.203)	7.999	12	11	24	2	3	0.723
9	(0.500, 0.250, 0.750)	8.318	12	11	6	2	5	1.000
10	(0.287, 0.287, 0.576)	8.012	12	11	24	2	3	0.232

The followings are details for Rh$_3$. It has 6 topological nodal lines and 19 nodal ring points along high symmetry line as shown in Fig **827**.

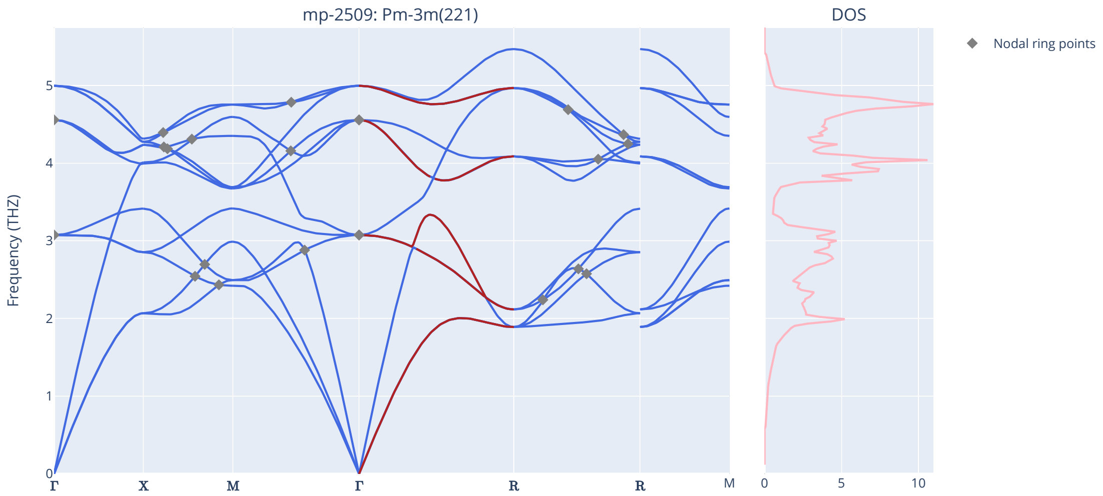

Supplementary Figure 827: The phonon band and DOS of Rh3Th.

Supplementary Table 1802: High symmetry lines of mp-2509-Rh3Th

	Start	End
0	Γ (0.000, 0.000, 0.000)	X (0.000, 0.500, 0.000)
1	X (0.000, 0.500, 0.000)	M (0.500, 0.500, 0.000)
2	M (0.500, 0.500, 0.000)	Γ (0.000, 0.000, 0.000)
3	Γ (0.000, 0.000, 0.000)	R (0.500, 0.500, 0.500)
4	R (0.500, 0.500, 0.500)	X (0.000, 0.500, 0.000)
5	R (0.500, 0.500, 0.500)	M (0.500, 0.500, 0.000)

Supplementary Table 1803: Nodal ring points along high symmetry lines of mp-2509-Rh3Th.

	Coordinates	Frequency	Mode 1	Mode 2	Multi.	Deg.	Path	Ratio
0	(0.423, 0.500, 0.000)	2.433	2	1	12	2	2	0.845
1	(0.289, 0.500, 0.000)	2.543	3	2	12	2	2	0.578
2	(0.212, 0.500, 0.212)	2.575	3	2	12	2	5	0.576
3	(0.343, 0.500, -0.000)	2.696	4	3	12	2	2	0.686
4	(0.215, 0.215, -0.000)	2.880	4	3	12	2	3	0.569
5	(0.244, 0.500, 0.244)	2.638	4	3	12	2	5	0.511
6	(0.385, 0.500, 0.385)	2.240	4	3	12	2	5	0.230
7	(0.000, 0.000, 0.000)	3.075	5	4	1	3	1	0.000
8	(0.166, 0.500, 0.166)	4.051	8	7	12	2	5	0.668
9	(0.000, 0.000, 0.000)	4.557	9	8	1	3	1	0.000
10	(0.135, 0.500, 0.000)	4.185	9	8	12	2	2	0.269
11	(0.270, 0.270, 0.000)	4.158	9	8	12	2	3	0.460
12	(0.271, 0.500, -0.000)	4.309	10	9	12	2	2	0.542
13	(0.114, 0.500, -0.000)	4.209	10	9	12	2	2	0.229

Supplementary Table 1803: Nodal ring points along high symmetry lines of mp-2509-Rh3Th.

	Coordinates	Frequency	Mode 1	Mode 2	Multi.	Deg.	Path	Ratio
14	(0.286, 0.500, 0.284)	4.689	10	9	24	2	5	0.431
15	(0.048, 0.500, 0.048)	4.248	10	9	12	2	5	0.904
16	(0.111, 0.500, -0.000)	4.394	12	11	12	2	2	0.221
17	(0.268, 0.268, -0.000)	4.784	12	11	12	2	3	0.463
18	(0.065, 0.500, 0.065)	4.367	12	11	12	2	5	0.869

The followings are details for $CrPt_3$. It has 9 topological nodal lines and 27 nodal ring points along high symmetry line as shown in Fig **828**.

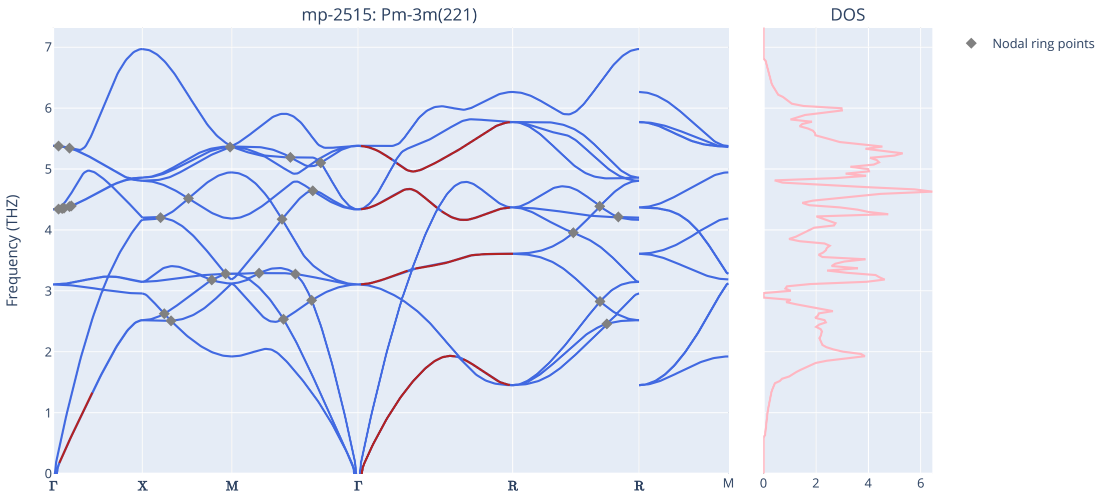

Supplementary Figure 828: The phonon band and DOS of CrPt3.

Supplementary Table 1804: High symmetry lines of mp-2515-CrPt3

	Start	End
0	Γ (0.000, 0.000, 0.000)	X (0.000, 0.500, 0.000)
1	X (0.000, 0.500, 0.000)	M (0.500, 0.500, 0.000)
2	M (0.500, 0.500, 0.000)	Γ (0.000, 0.000, 0.000)
3	Γ (0.000, 0.000, 0.000)	R (0.500, 0.500, 0.500)
4	R (0.500, 0.500, 0.500)	X (0.000, 0.500, 0.000)
5	R (0.500, 0.500, 0.500)	M (0.500, 0.500, 0.000)

Supplementary Table 1805: Nodal ring points along high symmetry lines of mp-2515-CrPt3.

	Coordinates	Frequency	Mode 1	Mode 2	Multi.	Deg.	Path	Ratio
0	(0.160, 0.500, -0.000)	2.509	2	1	12	2	2	0.321
1	(0.128, 0.500, 0.128)	2.459	2	1	12	2	5	0.745
2	(0.123, 0.500, 0.000)	2.625	3	2	12	2	2	0.245
3	(0.295, 0.295, -0.000)	2.535	3	2	12	2	3	0.410
4	(0.388, 0.500, 0.000)	3.180	4	3	12	2	2	0.776
5	(0.184, 0.184, 0.000)	2.846	4	3	12	2	3	0.632
6	(0.155, 0.500, 0.155)	2.827	4	3	12	2	5	0.691
7	(0.392, 0.392, -0.000)	3.290	5	4	12	2	3	0.217
8	(0.248, 0.248, -0.000)	3.276	5	4	12	2	3	0.504
9	(0.466, 0.500, -0.000)	3.281	6	5	12	2	2	0.931
10	(0.260, 0.500, 0.260)	3.954	6	5	12	2	5	0.479
11	(0.102, 0.500, 0.000)	4.202	7	6	12	2	2	0.203
12	(0.301, 0.301, 0.000)	4.176	7	6	12	2	3	0.398
13	(0.082, 0.500, 0.082)	4.214	7	6	12	2	5	0.836

Supplementary Table 1805: Nodal ring points along high symmetry lines of mp-2515-CrPt3.

	Coordinates	Frequency	Mode 1	Mode 2	Multi.	Deg.	Path	Ratio
14	(0.000, 0.031, 0.000)	4.342	8	7	6	2	1	0.061
15	(0.000, 0.051, 0.000)	4.352	8	7	6	2	1	0.102
16	(0.000, 0.061, 0.000)	4.358	8	7	6	2	1	0.122
17	(0.000, 0.092, 0.000)	4.384	8	7	6	2	1	0.184
18	(0.000, 0.102, 0.000)	4.394	8	7	6	2	1	0.204
19	(0.258, 0.500, -0.000)	4.517	8	7	12	2	2	0.515
20	(0.179, 0.179, -0.000)	4.642	8	7	12	2	3	0.642
21	(0.156, 0.500, 0.156)	4.388	8	7	12	2	5	0.689
22	(0.491, 0.500, 0.000)	5.360	10	9	12	2	2	0.982
23	(0.148, 0.148, -0.000)	5.101	10	9	12	2	3	0.705
24	(0.268, 0.268, 0.000)	5.191	11	10	12	2	3	0.464
25	(0.000, 0.031, 0.000)	5.376	12	11	6	2	1	0.061
26	(0.000, 0.092, 0.000)	5.342	12	11	6	2	1	0.184

The followings are details for $Cu_3Y_3O_6$. It has 5 topological nodal lines and 11 nodal ring points along high symmetry line as shown in Fig **829**.

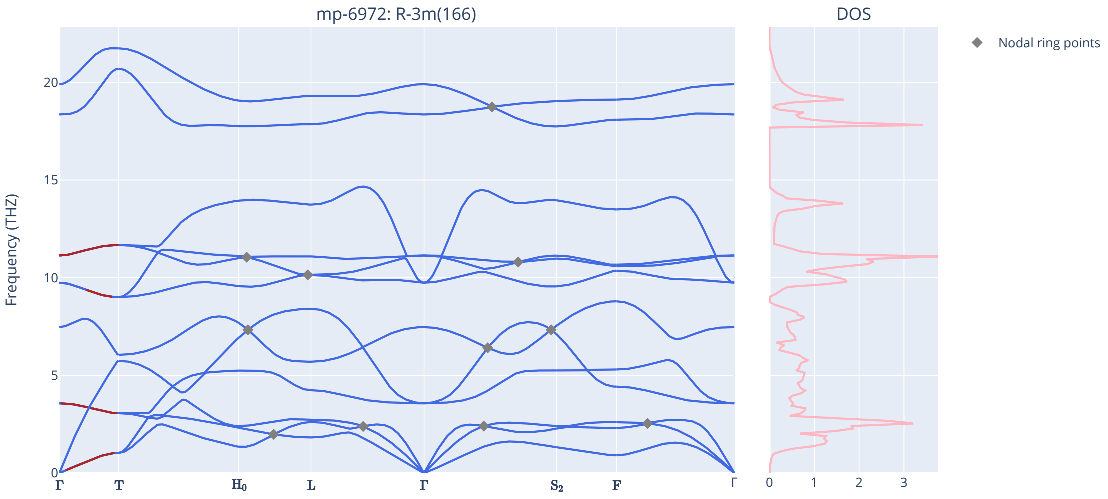

Supplementary Figure 829: The phonon band and DOS of Cu3Y3O6.

Supplementary Table 1806: High symmetry lines of mp-6972-Cu3Y3O6

	Start	End
0	Γ (0.000, 0.000, 0.000)	T (0.500, 0.500, 0.500)
1	T (0.500, 0.500, 0.500)	H_2 (0.812, 0.188, 0.500)
2	H_0 (0.500, -0.188, 0.188)	L (0.500, 0.000, 0.000)
3	L (0.500, 0.000, 0.000)	Γ (0.000, 0.000, 0.000)
4	Γ (0.000, 0.000, 0.000)	S_0 (0.344, -0.344, 0.000)
5	S_2 (0.656, 0.000, 0.344)	F (0.500, 0.000, 0.500)
6	F (0.500, 0.000, 0.500)	Γ (0.000, 0.000, 0.000)

Supplementary Table 1807: Nodal ring points along high symmetry lines of mp-6972-Cu3Y3O6.

	Coordinates	Frequency	Mode 1	Mode 2	Multi.	Deg.	Path	Ratio
0	(0.500, -0.097, 0.097)	1.979	2	1	6	2	3	0.483
1	(0.269, -0.002, -0.002)	2.384	3	2	6	2	4	0.461
2	(0.155, -0.155, -0.000)	2.404	3	2	6	2	5	0.452
3	(0.368, -0.002, 0.368)	2.545	3	2	6	2	7	0.264
4	(0.500, -0.164, 0.164)	7.334	6	5	6	2	3	0.126
5	(0.166, -0.166, 0.000)	6.409	6	5	6	2	5	0.481
6	(0.330, -0.330, 0.000)	7.333	6	5	6	2	5	0.961
7	(0.500, -0.009, 0.009)	10.142	8	7	6	2	3	0.954
8	(0.500, -0.167, 0.167)	11.051	9	8	6	2	3	0.109
9	(0.245, -0.245, 0.000)	10.801	9	8	6	2	5	0.712
10	(0.177, -0.177, 0.000)	18.747	12	11	6	2	5	0.514

The followings are details for In$_3$. It has 7 topological nodal lines and 23 nodal ring points along high symmetry line as shown in Fig 830.

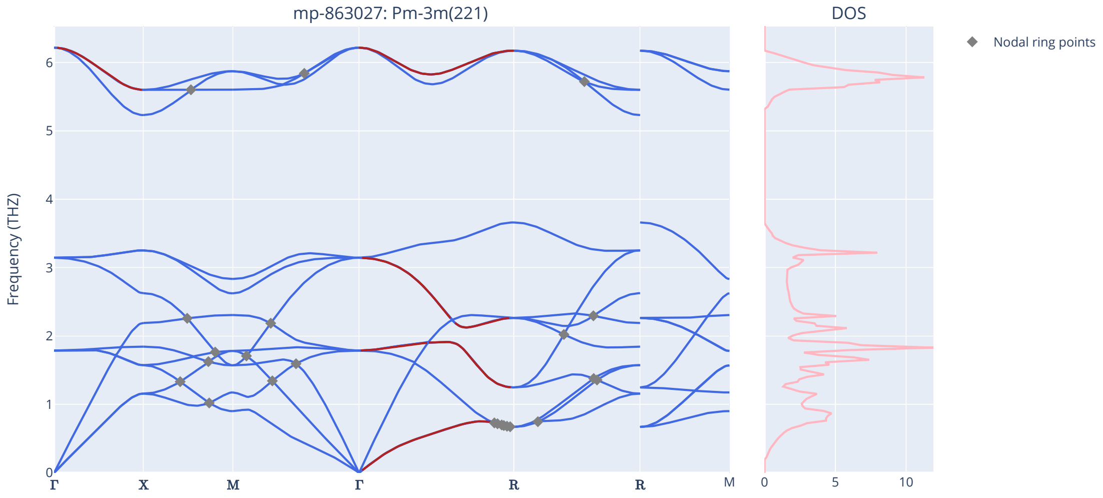

Supplementary Figure 830: The phonon band and DOS of In3Li.

Supplementary Table 1808: High symmetry lines of mp-863027-In3Li

	Start	End
0	Γ (0.000, 0.000, 0.000)	X (0.000, 0.500, 0.000)
1	X (0.000, 0.500, 0.000)	M (0.500, 0.500, 0.000)
2	M (0.500, 0.500, 0.000)	Γ (0.000, 0.000, 0.000)
3	Γ (0.000, 0.000, 0.000)	R (0.500, 0.500, 0.500)
4	R (0.500, 0.500, 0.500)	X (0.000, 0.500, 0.000)
5	R (0.500, 0.500, 0.500)	M (0.500, 0.500, 0.000)

Supplementary Table 1809: Nodal ring points along high symmetry lines of mp-863027-In3Li.

	Coordinates	Frequency	Mode 1	Mode 2	Multi.	Deg.	Path	Ratio
0	(0.368, 0.500, 0.000)	1.020	2	1	12	2	2	0.737
1	(0.405, 0.500, 0.405)	0.746	2	1	12	2	5	0.191
2	(0.206, 0.500, -0.000)	1.331	3	2	12	2	2	0.412
3	(0.343, 0.343, -0.000)	1.343	3	2	12	2	3	0.314
4	(0.461, 0.461, 0.461)	0.698	3	2	8	2	4	0.922
5	(0.437, 0.437, 0.437)	0.728	3	2	8	2	4	0.875
6	(0.447, 0.447, 0.447)	0.716	3	2	8	2	4	0.895
7	(0.468, 0.468, 0.468)	0.690	3	2	8	2	4	0.936
8	(0.478, 0.478, 0.478)	0.679	3	2	8	2	4	0.956
9	(0.488, 0.488, 0.488)	0.672	3	2	8	2	4	0.977
10	(0.171, 0.500, 0.171)	1.357	3	2	12	2	5	0.659
11	(0.364, 0.500, -0.000)	1.624	4	3	12	2	2	0.728
12	(0.249, 0.249, 0.000)	1.594	4	3	12	2	3	0.501
13	(0.184, 0.500, 0.184)	1.377	4	3	12	2	5	0.633

Supplementary Table 1809: Nodal ring points along high symmetry lines of mp-863027-In3Li.

	Coordinates	Frequency	Mode 1	Mode 2	Multi.	Deg.	Path	Ratio
14	(0.446, 0.446, -0.000)	1.708	5	4	12	2	3	0.108
15	(0.402, 0.500, 0.000)	1.764	6	5	12	2	2	0.804
16	(0.302, 0.500, 0.302)	2.022	6	5	12	2	5	0.397
17	(0.245, 0.500, 0.000)	2.258	7	6	12	2	2	0.490
18	(0.350, 0.350, 0.000)	2.186	7	6	12	2	3	0.301
19	(0.185, 0.500, 0.185)	2.295	7	6	12	2	5	0.630
20	(0.266, 0.500, -0.000)	5.601	11	10	12	2	2	0.532
21	(0.221, 0.500, 0.221)	5.721	11	10	12	2	5	0.559
22	(0.217, 0.217, 0.000)	5.841	12	11	12	2	3	0.566

The followings are details for Pt3. It has 8 topological nodal lines and 21 nodal ring points along high symmetry line as shown in Fig **831**.

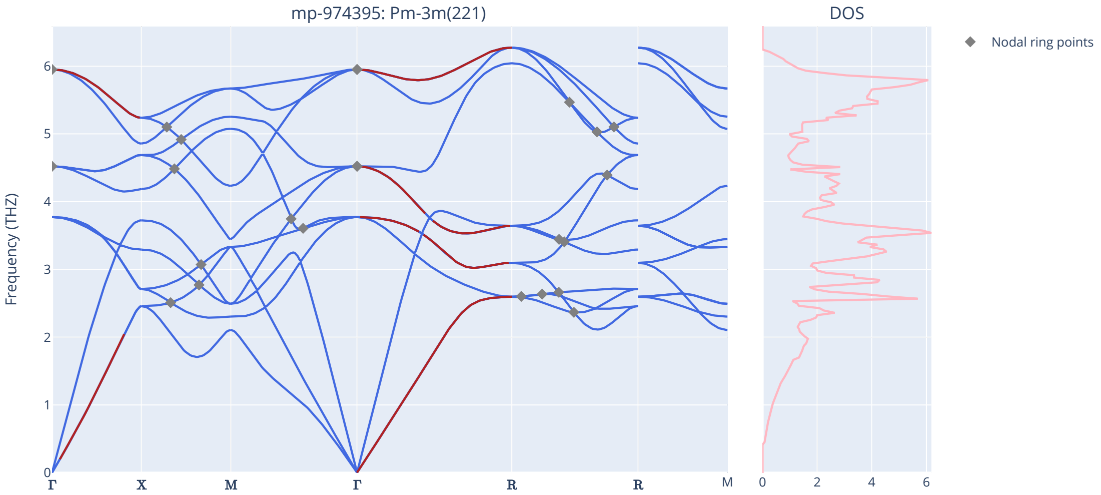

Supplementary Figure 831: The phonon band and DOS of Pt3Rh.

Supplementary Table 1810: High symmetry lines of mp-974395-Pt3Rh

	Start	End
0	Γ (0.000, 0.000, 0.000)	X (0.000, 0.500, 0.000)
1	X (0.000, 0.500, 0.000)	M (0.500, 0.500, 0.000)
2	M (0.500, 0.500, 0.000)	Γ (0.000, 0.000, 0.000)
3	Γ (0.000, 0.000, 0.000)	R (0.500, 0.500, 0.500)
4	R (0.500, 0.500, 0.500)	X (0.000, 0.500, 0.000)
5	R (0.500, 0.500, 0.500)	M (0.500, 0.500, 0.000)

Supplementary Table 1811: Nodal ring points along high symmetry lines of mp-974395-Pt3Rh.

	Coordinates	Frequency	Mode 1	Mode 2	Multi.	Deg.	Path	Ratio
0	(0.254, 0.500, 0.254)	2.367	2	1	12	2	5	0.492
1	(0.164, 0.500, 0.000)	2.510	3	2	12	2	2	0.327
2	(0.462, 0.500, 0.462)	2.602	3	2	12	2	5	0.075
3	(0.380, 0.500, 0.380)	2.636	3	2	12	2	5	0.241
4	(0.323, 0.500, -0.000)	2.771	4	3	12	2	2	0.646
5	(0.314, 0.500, 0.314)	2.662	4	3	12	2	5	0.373
6	(0.334, 0.500, -0.000)	3.072	6	5	12	2	2	0.668
7	(0.214, 0.214, 0.000)	3.606	6	5	12	2	3	0.573
8	(0.292, 0.500, 0.292)	3.406	6	5	12	2	5	0.416
[truncated: 559,085 more chars]
